# Automatic mapping of atoms across both simple and complex chemical reactions

**DOI:** 10.1038/s41467-019-09440-2

**Published:** 2019-03-29

**Authors:** Wojciech Jaworski, Sara Szymkuć, Barbara Mikulak-Klucznik, Krzysztof Piecuch, Tomasz Klucznik, Michał Kaźmierowski, Jan Rydzewski, Anna Gambin, Bartosz A. Grzybowski

**Affiliations:** 1 Faculty of Mathematics, Informatics and Mechanics, University of Warsaw, Ul. Banacha 2, 02-097 Warszawa, Poland; 2 Institute of Organic Chemistry, Polish Academy of Sciences, Ul. Kasprzaka 44/52, Warsaw, 02-224 Poland; 3 IBS Center for Soft and Living Matter, UNIST, 50, UNIST-gil, Eonyang-eup, Ulju-gun, Ulsan, South Korea; 4 Department of Chemistry, UNIST, 50, UNIST-gil, Eonyang-eup, Ulju-gun, Ulsan, South Korea

## Abstract

Mapping atoms across chemical reactions is important for substructure searches, automatic extraction of reaction rules, identification of metabolic pathways, and more. Unfortunately, the existing mapping algorithms can deal adequately only with relatively simple reactions but not those in which expert chemists would benefit from computer’s help. Here we report how a combination of algorithmics and expert chemical knowledge significantly improves the performance of atom mapping, allowing the machine to deal with even the most mechanistically complex chemical and biochemical transformations. The key feature of our approach is the use of few but judiciously chosen reaction templates that are used to generate plausible “intermediate” atom assignments which then guide a graph-theoretical algorithm towards the chemically correct isomorphic mappings. The algorithm performs significantly better than the available state-of-the-art reaction mappers, suggesting its uses in database curation, mechanism assignments, and – above all – machine extraction of reaction rules underlying modern synthesis-planning programs.

## Abstract (web summary)

Mapping atoms across chemical reactions represents a challenging computational task. Here the authors show via a combination of graph theory and combinatorics with expert chemical knowledge the possibility to map very complex organic reactions.

## Introduction

Mapping atoms across chemical reactions—that is, numbering them to indicate which atom of the substrate(s) becomes which atom of the product(s)—is not only one of the classic exercises in organic chemistry textbooks^1,2^ but also of growing importance in classifying reactions^3^ in large databases and facilitating substructure searches^4^, in assigning the roles (reactant, reagent, product) specific molecules play in a given reaction^5^, and in elucidating mechanisms of enzymatic reactions or identifying metabolic pathways^6–9^. Most recently, atom mapping has become a central component of chemical AI^10–13^ as it is required for automatic extraction of reaction cores/rules from literature precedents; such rules are subsequently used as the knowledge base of various synthesis-design programs^10–17^. Unfortunately, the existing algorithms^6–9,18–25^ are capable of correctly mapping relatively simple reactions (sometimes only when full stoichiometry is provided^25^) but not those in which expert chemists would actually benefit from computer’s help. In addition, their purportedly high correctness is often reported based on comparisons to the results of other algorithms (i.e., not to the chemically correct mappings^25^) or to examples from databases in which mappings were never systematically verified^7–9,22–24^.

**Fig. 1:**
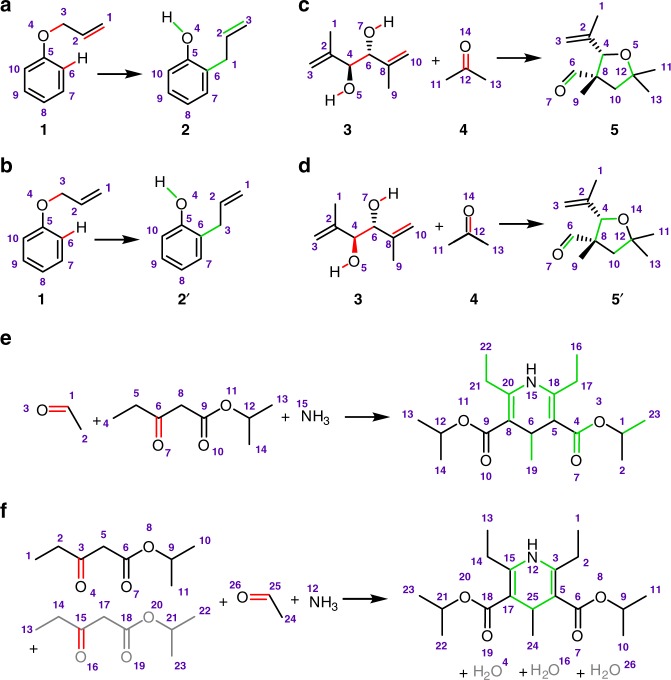
Examples of problems encountered by traditional matching algorithms. **a**, **b** The number of bonds being cut might not necessarily be minimal. The example shown here is for a (sigmatropic) Claisen rearrangement^1^. The mapping in (**a**) is correct, even though the one in (**b**) entails fewer bonds being cut (bonds that are disconnected are colored in red whereas bonds created are marked green). **c**, **d** Several alternative mappings with the same “bonds-cut” score might exist. Shown here are two alternative mappings of Prins rearrangement^39^—in both cases, six bonds are being cut and four are formed. The correct mapping, one determined by our algorithm, is shown in (**c**). **e**, **f** When chemists write organic reactions, they usually do not account for full stoichiometry. The example here is for the Hantzsch dihydropyridine synthesis^40^ in which only one molecule of ketoester would typically be provided in a synthetic scheme. **e** Competitive mappers do not account for the missing substrate and either yield incorrect mapping (as shown here based on Marvin^33^) or find no mapping at all (Baldi’s ReactionMap^32^). **f** Our method considers missing atoms or substrates (here, one with bonds colored gray) and maps the reaction correctly

Computationally, the atom mapping problem is known to be NP hard (as it encloses the subgraph isomorphism problem^26^). The existing algorithms for atom matching^6–9,18–25^—described in comprehensive recent reviews^18,27^—typically fall into two broad and partly overlapping categories. Methods based on the so-called extended connectivity (EC)^19,28^ are often extensions of the Morgan algorithm^29^ (which assigns a unique number to each atom in a molecule on the basis of its chemical neighborhood) and use iterative procedures to establish unique labelling of graph vertices (i.e., of atoms), identify common substructure(s) between substrates and products, and ultimately construct complete atom mapping. Another class of algorithms relies on the so-called principle of minimal chemical distance (MCD)^30^, which is an ansatz stipulating that most chemical reactions follow the shortest path from reactants to products, in the process cutting the minimal possible number of bonds. In order to find the optimal/correct reaction mapping, these algorithms try to solve the subgraph isomorphism problem^7^, use problem solving methods (such as the A*-algorithm)^8^, or introduce integer linear optimization^24^. In all cases, the algorithms face NP-hard problems which are in general intractable without the use of additional domain knowledge that could reduce the problem’s complexity. More significantly, they are incapable of mapping reactions for which the assumptions such as MCD are simply incorrect, as in various types of pericyclic reactions, 1,2-rearrangements, or metathesis reactions for which the number of bonds being cut is not minimal (vs. alternative mappings, see example in Fig. 1a, b). Another problem concerns reactions in which cutting different bonds leads to answers with the same overall algorithm score—one example is the Prins rearrangement in Fig. 1c, d, for which traditional algorithms would not be able to decide whether the oxygen atom in the product’s ring comes from substrate 3 (Fig. 1c) or 4 (Fig. 1d). To overcome such problems, Baldi’s group^25^ has recently combined the substructure and optimization methods with an atom-assigning cost function trained on a large (>250,000) set of atom-mapped reactions from the SPRESI database from ICSynth^31^. The authors claimed that for relatively basic reactions with complete stoichiometry (Fig. 1e, f), this approach and the accompanying ReactionMap software^32^ performed superior to other algorithms (including commercial ChemAxon’s software, MarvinJS^33^). On the other hand, this work showed one of the major flaws of the field—namely, mapping was considered correct if it matched the mapping of another program (in this particular case, ICSynth’s proprietary program used to map SPRESI reactions). Such examples emphasize the need to validate algorithm’s predictions against true mappings performed by human experts and accounting for reaction mechanism.

Here, we describe and extensively validate an algorithm that can map even the most mechanistically complex chemical and biochemical transformations, including those with incomplete stoichiometry. The distinctive feature of our approach is that it supplements graph-theoretical considerations and optimization schemes^6–9,18–23^ with few (20) but judiciously chosen chemical rules/heuristics that allow the algorithm to explore plausible “intermediate” atom assignments, avoid decoy solutions (e.g., those obtained under the assumption of minimal number of bonds being cut^7–9,22,23^), and ultimately be guided towards the correct mappings. The advantages of this approach are most manifest for complex to very complex reactions where it reaches 84% mapping correctness vs. up to ~62% of other, state-of-the-art solutions^25,32,33^ (and ~86% vs. ~71% over reactions provided with full stoichiometry). We also demonstrate similar improvements in the mapping of reactions from which “synthetic rules” were previously extracted (often incorrectly, as it turns out) and were used as the basis for synthetic design algorithms. Accurate and general-scope mapping algorithms like the one we describe are important to ensure that computers can extract, process, annotate, and apply not only simple chemical transforms but also the advanced chemistries without which any synthesis-design programs cannot address realistic synthetic challenges. The analyses and comparisons described below are based on over 1400 expert-mapped reactions of different complexities (cf. Supplementary Notes 2–5 and file Supplementary Data 1).

## Results

### Establishing isomorphic mapping

Our algorithm has two major, interrelated components: a module for isomorphic (i.e., one-to-one) mapping and a module for the application of reaction heuristics guiding correct atom assignments. To simplify the problem to the isomorphism of subgraphs (rather than full molecular graphs), we first label atoms by their environments (represented as subgraphs around each atom, not the routinely used scalar values) and use the so-called bucket sorting^34^ to group together atoms with identical environments. These subsets of atoms within the substrate/product molecules define possible candidates for isomorphic mapping. To reduce solution search space and thus avoid time consuming exhaustive analysis—which is computationally prohibitive for large molecules—we introduce and apply sequentially four combinatorial tests: Test 1: Whether the number of connected components in reactant(s) and product(s) graphs are equal; Test 2: Whether connected components may be matched according to the number of atoms/nodes; Test 3: Whether the connected components may be matched according to node types (i.e., have pairwise identical multisets of nodes’ environments); and Test 4: Whether the connected components are pairwise isomorphic. These tests exclude the majority of possible candidates.

The most computationally-intensive part of the above operations is to find the correct isomorphism. This is done by creating a decision tree^35^ of all possible isomorphisms, with the size of this tree limited to a predefined number of vertices (currently, 1,000,000)—evaluation of a candidate reaction typically requires trees with tens of thousands of vertices but for either very large molecules and/or those with many symmetries, it can approach or even exceed the one-million limit. The procedure is similar to the so-called VF2 algorithm^36^ with an important difference that VF2 adds single nodes (here, atoms) to the matching while our algorithm extends the matching simultaneously to all immediate neighbors of a given atom. Specifically, the isomorphisms are first calculated at the level of the fourth-order environments, and then recursively at the third, second, and first levels. The purpose of this recursion is to match the atoms sequentially from the periphery of the molecules (where atoms agree to higher-order environments) towards the reaction center (where the conserved neighborhoods become smaller). Regarding the VF2 part of the algorithm, it checks the graph isomorphism by constructing the matching between graphs’ nodes. For a given partial matching, it computes the set of candidate pairs for inclusion in the matching. Then, for each pair from the candidate set, it executes itself recursively with the matching extended by the selected candidate as an argument. When the algorithm obtains matching that covers all the nodes of one of the graphs, it terminates. When all candidate pairs fail to establish complete matching or the set of the candidate pairs is empty, the algorithm back-traces. If after such procedure some unmatched atoms still remain, we again resort to combinatorics, sequentially cutting/removing all possible subsets of bonds originating from the unlabeled atoms—first all possible single bonds then, if needed, pairs of bonds, and so on to up to sets of six bonds. By this bond cutting we strive to identify minimal sets of bonds defining the “reaction center” and whose disconnection gives a full isomorphism between reactants and products (for further details and examples, see Supplementary Notes 1.1 and 1.2).

### Addition of reaction heuristics

**Fig. 2:**
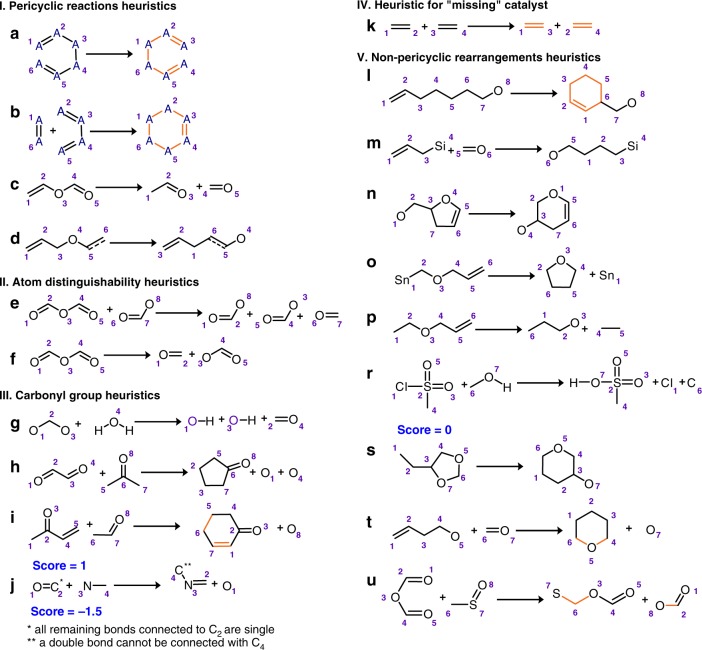
Schemes of reaction heuristics guiding the searches. Specific heuristics span various reaction classes: **a**–**d** pericyclic reactions; **e**, **f** reactions in which two atoms of the same type can be hard to distinguish due to symmetry; **g**–**j** select reactions involving carbonyl group; **k** reactions such as metathesis; **l**–**u** non-pericyclic rearrangements. The algorithm strives to apply these templates to the substrates in all possible ways. The intermediates thus created are then subject to the mapping procedure as described in the main text and illustrated in Figs. 3 and 4. The score for the application of a heuristic is usually +0.5 (vs. +1 for each bond cut without the use of heuristics). Exceptions are heursitics **i**, **j**, and **r**, for which the scores are, respectively, +1, −1.5, and 0. In the templates shown, free valences of all atoms can be filled by atom(s) of any type, unless otherwise stated. Dashed lines specify aromatic bonds; orange color indicates bonds which cannot be broken in any other subsequent operations during mapping

**Fig. 3:**
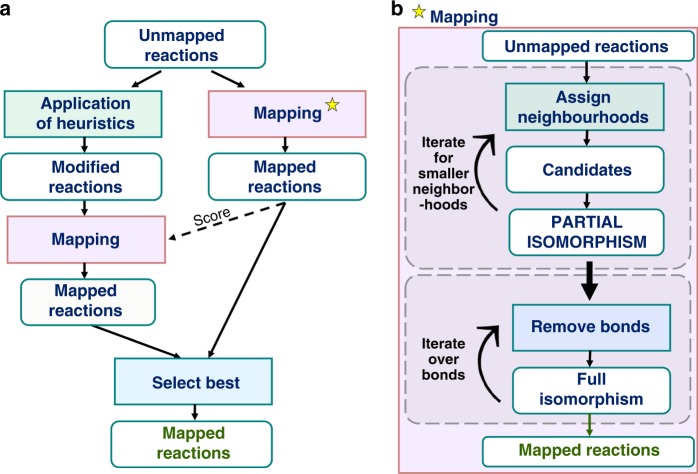
Algorithm scheme. **a** A diagram illustrating the algorithm with (left arm of the flowchart) and without (right arm) the use of heuristics. Both paths are processed simultaneously—in the end, the scores are compared and a lower-scoring solution is selected. The purpose of additionally performing the search without heuristics is that the best solution it produces (with some score S, communicated from the right to the left branches; dotted arrow) allows rapid rejection of any other with-heuristics solutions for which the score is above S. This allows the algorithm to significantly limit the search space and yield results faster. **b** A scheme of the mapping procedure, in which two iterative processes are involved: iteration for smaller neighborhoods and iteration over bonds—see main text for details

Although the above algorithm—using an improved representation of neighborhoods and various original combinatorial tests/procedures—is efficient in mapping simple reactions, it fails, for instance, when the number of disconnected bonds is not minimal or when there are multiple different solutions with the same score (see Fig. 1). To take these and other cases into account, we have augmented the algorithm with 20 reaction heuristics listed in Fig. 2. In addition to generating mapping candidates as described previously, the algorithm now tries to apply these heuristics at all possible sites of the reagent(s). In this way a set—sometimes quite large, up to hundreds—of intermediate candidates is created that is then subject to isomorphic mapping against the product(s) as described above. Importantly, whereas the candidates generated without the use of heuristics are scored based on the number of bonds disconnected (+1 for every bond cut, including bonds involving H atoms), the score for the application of heuristics is defined as lower than the actual number of bonds being cut or changed by each heuristics—that is, heuristics are being preferred to allow the algorithm explore solutions with non-minimal numbers of bonds being cut. In other words, the algorithm still strives to find the mapping with minimal score but the heuristics help “channel” the calculations towards chemically viable solutions. The block diagram of the algorithm is shown in Fig. 3. We make several additional comments about this protocol.

**Fig. 4:**
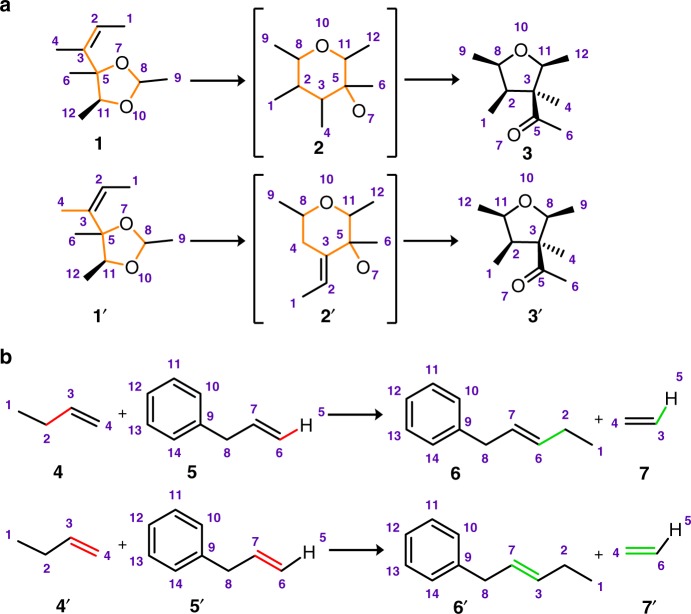
Application of reaction heuristics. The example in **a**, illustrates the consequences of applying reaction heuristic [s] (see Fig. 2) in various places of the same substrate 1 in the Prins-Pinacol rearrangement^39,52^. In the top row, the heuristic is properly applied to the substrate (the substructure of the substrate recognized by the heuristic is marked orange) producing intermediate 2, which is then correctly mapped into product 3 with overall score = 3.5 (3 for disconnecting bonds 5–11, 3-H, and 7-H plus 0.5 for using the heuristic). In contrast, when the same heuristic is applied to the wrong part of the molecule (1′), it yields intermediate 2′, which is incorrectly mapped into product 3′ with score = 4.5 (3 for disconnecting single bonds 4–8, 5–11, 7-H, additional 1 for converting double bond 2–3 into a single bond, and 0.5 for using the heuristic). The second example in **b**, illustrates the performance of the algorithm with and without the application of the metathesis heuristics (k in Fig. 3). (top row) Without the heuristics, the algorithm can identify the lowest-scoring isomorphic mapping by cutting only two single bonds—still, this solution is chemically incorrect. (bottom row) With the heuristics applied, the algorithm is not heavily (score + 4) penalized for cutting as many as four bonds (two σ and two π)—application of the heuristics costs much less (+0.5) and the algorithm can find the chemically correct isomorphic mapping

The heuristics can be divided into five sub-groups (see Fig. 2). The first one includes reaction types, to which the ansatz of the minimal number of bonds being cut does not apply (e.g., pericyclic transformations including cycloadditions, electrocyclic reactions, or sigmatropic rearrangements). In the cyclic transition states of such transformations, breaking several bonds is energetically more favorable than cutting one sigma bond (see Fig. 1a, b). Heuristics in the second group are for reactions in which disconnections of certain chemically distinct bonds are indistinguishable from the algorithm’s point of view. An example here could be a simple reaction between an anhydride and a carboxylic acid (marked as [e] in Fig. 2) in which another anhydride is formed. In the newly formed anhydride, the central (non-carbonyl) oxygen atom might derive either from the initial anhydride or from carboxylic acid’s OH group—both of the solutions are algorithmically equivalent, although only the second variant is chemically correct. The third group covers chemistry of the carbonyl group (e.g., heuristics [h] and [i] in Fig. 2 suggest that two carbonyl compounds might undergo a condensation, such as aldol, accompanied by the loss of a water molecule). The fourth group of heuristics deals with the problem of incomplete chemical information crucial for the reaction mechanism and outcome but not explicitly present in the substrate(s) or product(s). For example, cutting two double bonds in a metathesis reaction might appear illogical from the algorithm’s point of view, because cutting two single bonds instead would result in a lower-score—although chemically incorrect—solution (see Fig. 4b). Chemically, in this case cutting two double bonds makes perfect sense if one accounts for the presence of an organometallic catalyst that coordinates to double bonds and facilitates the bond-breaking step. In other words, this heuristic corrects for the missing catalyst. The fifth and the last group of heuristics comprises important non-pericyclic rearrangements and includes certain reaction motifs popular in chemical transformations of different types.

For the best performance of the algorithm, not all rules are assigned the same score preference: the score for the majority of applied heuristics is +0.5, but +1 for heuristic [i] (Robinson annulation; the value is still lower than if each disconnected bond were scored as +1), −1.5 for [j], and 0 for [r].

Any preferences given to the heuristics are substantial only if this heuristics is applied in a proper way. If it is applied at a wrong locus of a molecule, it transforms the substrate into a decoy form (see examples in Fig. 4) that is significantly less similar to the product. Consequently, more bonds need to be cut to obtain the proper product structure (i.e., finding the full isomorphism is more difficult) and the overall score is high.

Addition of any new heuristics not only increases the number of candidates for isomorphic matching and the computation time but can also create more decoy solutions (see Fig. 4) that are chemically improper—the set of 20 heuristics we use was identified by an iterative protocol in which we aimed to minimize the number of heuristics while, without increasing the computation time, maximizing the applicability of the algorithm to chemistries as diverse as possible. When additional heuristics were added, they could rarely help in mapping some specialized type of chemistry but, as a rule, they concomitantly decreased the efficiently of correct mapping of many other reaction types.

### Correcting for the missing stoichiometry

The last and important aspect of the algorithm is the ability to match reactions with incomplete stoichiometry. The stoichiometry correction is executed before the main mapping process. The algorithm first checks if the reaction could be balanced by adding copies of some of the substrates (if so, such copies are added; see example in Fig. 1f) or by matching against hard-coded, atom-mapped templates of some popular reactions in which the groups, typically unspecified by chemists while writing reactions, are explicitly included (if a template fits the reaction, the core atoms already have atom assignments and no heuristics are needed, which speeds up the algorithm; for templates, see Supplementary Fig. 6). In the next stage, the algorithm tries to balance stoichiometry by adding water molecules to either reactants or products. If the reaction is still unbalanced, individual missing atoms are added to the appropriate side of the reaction. The algorithm then treats them as one-atom-molecules and performs full mapping routine as described earlier. This scheme works well for less than ca. eight missing atoms—for larger numbers, the problem becomes intractable due to combinatorial explosion of mapping options.

## Discussion

During algorithm development and selection of heuristics (cf. above), we scrutinized its results on the total of 548 reactions which can be referred to as a “training set”. This set comprised 241 typical reactions with full stoichiometry and taken from the Organic Syntheses collection^37^; set of 191 randomly selected and typically mostly stoichiometrically unbalanced reactions from Reaxys collection^38^; and 116 mechanistically complex reactions (73 stoichiometrically balanced and 43 unbalanced reactions) taken from various literature sources (e.g., Kurti’s “Strategic Application of Named Reactions in Organic Synthesis”^39^ or Grossman’s “The Art of Writing Reasonable Organic Reaction Mechanisms”^1^)—whose mapping should pose a challenge even to human experts. These reactions were mapped by our algorithm, by ReactionMap^32^, Marvin JS version 16.4.18^33^, and as a benchmark for correctness, by the authors (S.S., B.M.K., T.K., all expert organic chemists with track record as co-developers of the Chematica retrosynthetic software^13,40^). All mapped reactions are provided in the Supplementary Note 2. For the 241 typical reactions with full stoichiometry, our algorithm provides 93.8% correct assignments compared to 92.1% for ReactionMap and 86.7% for MarvinJS methods—that is, it does slightly better than the competing solutions. For the 191 Reaxys reactions in the second collection, the accuracy of our algorithm is 94.2% vs 90.5% for MarvinJS and only 12% for ReactionMap. The poor performance of ReactionMap could be expected since, as its authors admit^25^, the program cannot generally tackle reactions without full stoichiometry. Our algorithm outperforms the competition most decisively in mapping the 116 complex reactions—here, it mapped correctly 85.3% reactions compared to 44.8% for ReactionMap and 59.4% for MarvinJS.

**Fig. 5:**
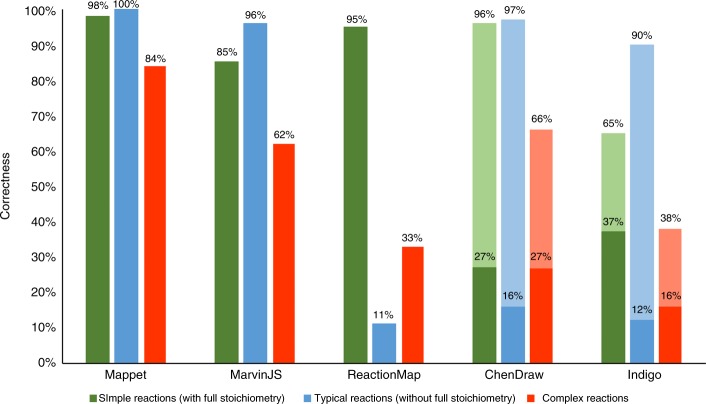
Performance of various mappers quantified on the 401 reactions from the main test set. For ChemDraw and Indigo, solid parts of the bars correspond to the more stringent criterion that all atoms in the molecule must be assigned numbers. Shaded parts of the bars give the percentages of correct answers assuming a more lenient criterion allowing for unmapped but chemically unique atoms (see examples in Supplementary Fig. 6). ReactionMap does significantly better (~71%) on complex reactions with full stoichiometry (see main text)

Next, we performed similar comparisons on a set of 401 reactions that were not considered during training (for reaction miniatures with mappings, see Supplementary Note 3). This “test” set comprised: 100 relatively simple reactions with full stoichiometry taken from total syntheses published in Org. Lett., J. Am. Chem. Soc., and J. Org. Chem.; 100 typical reactions without full stoichiometry taken from patents; and 201 mechanistically complex reactions (92 stoichiometrically balanced and 109 unbalanced reactions) which include rearrangements and multicomponent reactions taken from recent literature (in most cases, after 2010 and from Org. Lett., J. Am. Chem. Soc., and J. Org. Chem.). For all 401 reactions, we compared the performance of our algorithm not only against MarvinJS and ReactionMap but also ChemDraw Prime (version 16.0.0.82) and Indigo (version 1.3.0 beta). The results summarized in Fig. 5 evidence that for simple reactions with full stoichiometry (green bars), all algorithms with exception of Indigo (65% correctness) are performing well—ours has 98% correctness, MarvinJS 85%, ReactionMap 95%, and ChemDraw 96%. For 100 reactions without full stoichiometry (blue bars), our algorithm is slightly more accurate than ChemDraw, MarvinJS, and Indigo (100% vs 97%, 96% and 90% correctness, respectively) and significantly better than ReactionMap (11% correctness), which does not handle missing stoichiometry. As in the training set, the major differences are observed for complex reactions (red bars) for which our algorithm is correct in 84% of cases compared to 66% for ChemDraw, 62% for MarvinJS, 38% for Indigo, and 33% for ReactionMap. We make two comments regarding these results. First, the figures of merit for ChemDraw and Indigo are overestimated, because a sizable fraction of the results these mappers produce come without full mappings (see Supplementary Fig. 5)—though the fragments missing atom assignments are chemically unique and can be unambiguously assigned by a human chemist (on the other hand, such results might not be treated as correct during, say, automatic reaction rule extraction). If a more stringent criterion is applied that all atoms in the molecule must have unique numbers, the statistics for the two mappers are much worse (solid parts of the bars in the figure): for ChemDraw, 27% correctness on simple reactions with full stoichiometry, 16% for typical reactions without full stoichiometry, and 27% for complex reactions; for Indigo, 37% for simple reactions with full stoichiometry, 12% for typical reactions without full stoichiometry, and 16% for complex reactions. Second, it is instructive to compare the performance of all mappers on complex reactions with full stoichiometry (73 such reactions in the training set and 92 in the test set). For such reactions, correctness of ChemDraw, MarvinJS, and Indigo does not change much (respectively, 67%, 59%, 34%) but the performance of ReactionMap improves quite significantly (~71%)—though it is still perceptibly below our mapper (86% correctness for the stoichiometrically-balanced reactions in the training set and 87% for balanced reactions in the test set).

**Fig. 6:**
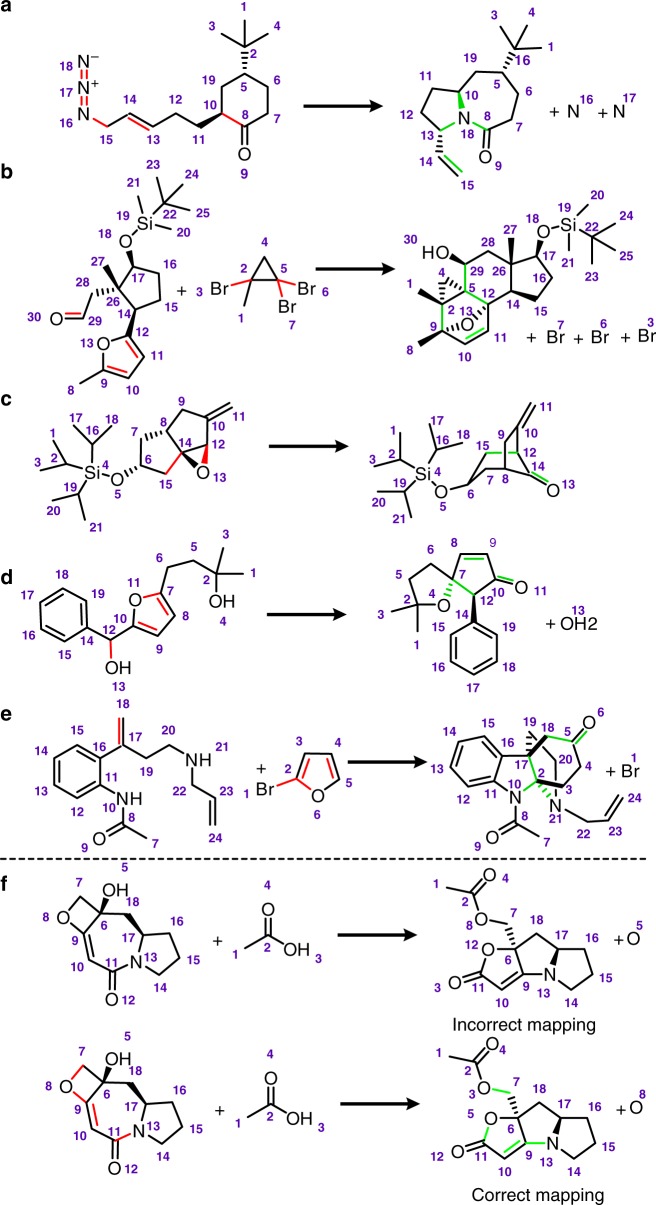
Examples of mapped, complex reactions. **a**–**e** Reactions mapped correctly by our algorithm. **f** An example of a reaction in which the mapping found is incorrect. Correct mapping is shown in the bottom row. Bonds that are disconnected are colored in red whereas bonds created are marked green. For discussion, see main text and refs. ^41–46^ for chemical details

Some of these complex reactions we tested on, involving multiple mechanistic steps and/or rearrangements, are shown in Fig. 6. For example, reaction in Fig. 6a commences with a [3,3]-sigmatropic allylic azide rearrangement with subsequent intramolecular Schmidt-Aubé reaction to form bicyclic amide^41^. In Fig. 6b, cyclopropenyl lithium reagent generated in situ reacts with an aldehyde, followed by an intramolecular Diels-Alder reaction with a furan ring to form bridged bicyclic scaffold^42^. In Fig. 6c, a Lewis acid catalyzed semipinacol rearrangement creates bicyclo[3.2.1]octan-8-one scaffold^43^. In Fig. 6d, a Lewis acid catalyzed oxa-Piancatelli rearrangement of an alcohol creates oxaspirocycle scaffold^44^. A multistep sequence in Fig. 6e involves cross-coupling of 2-bromofuran with an amide, intramolecular Diels-Alder reaction, thermal rearrangement of amidofuran to dihydro-*2H*-carbazolone and, finally, cyclization with allylamine to form the main scaffold of minfiensine alkaloid^45^. On the flipside of the coin, the 16% of incorrectly mapped reactions are usually ones in which key, mechanistically important substrates or by-products are missing, those that comprise sequences of mechanistically complex rearrangements and unusual migrations of functional groups (changing atomic environments in non-trivial ways, cf. Supplementary Fig. 8), or those that are cascades of not necessarily complex but just too many (>4, 5) reactions^46^ that could/should be written as separate transformations (Fig. 6f). As narrated earlier in the text, such corner cases cannot be overcome by simply adding more specialized heuristics since their application creates additional decoy solutions—especially when the heuristics’ atom cores overlap—having comparable scores but ultimately yielding wrong atom assignments.

**Fig. 7:**
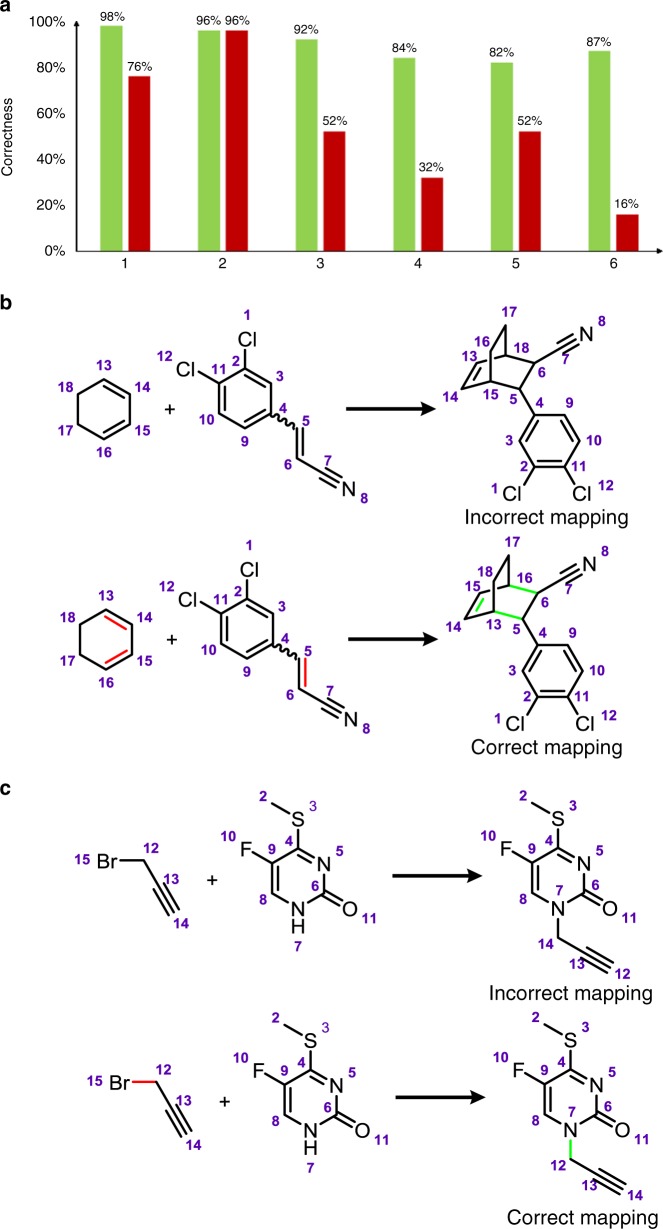
Comparison of our algorithm against USPTO mappings. **a** Percentages of correct USPTO mappings (red bars) and those by our algorithm (green bars) categorized according to the number of bonds broken/created, from one to six. Statistics are based on 50 reactions each for one to five bonds being changed and 31 reactions for six bonds. We note that in addition to chemically meaningful reactions for which we compared the mappings, the USPTO set also contained a large fraction of nonsensical reactions that were likely due to human entry errors in the databases they used (e.g., missing key reaction partners, creating atoms “ex nihilo”, etc., see Supplementary Fig. 9). Such reactions as well as simple deprotections were not included in the statistics shown. **b**, **c** Examples of two reactions—Diels-Alder cycloaddition (**b**) and *N*-alkylation of amide (**c**)—mapped incorrectly in the USPTO set and correctly by our software. For more examples, see Supplementary Note 4

As the second test set, we considered the performance of our algorithm in mapping patent reactions from which reactivity scores for atom pairs^11^ or reaction templates/cores^47^ were previously derived and then used for, respectively, reaction prediction or retrosynthetic planning. Proper atom assignments in this and similar reaction sets are important since machine-extraction of mapped reaction cores is common to most retrosynthetic design programs (Wiley/CAS ChemPlanner^17^, InfoChem’s ICSynth^16^, BenevolentAI/Waller’s^12^, and MIT/Coley’s^47^ programs), though not of our Chematica platform^13,40^. Here, we considered a subset of 50,000 reactions selected by Landrum and co-workers^5,48^ from the United States Patent and Trademark Office (USPTO) database to represent reactions most essential for medicinal chemistry (this collection was later used by the MIT team in the abovementioned studies^11^ and^47^). After further cleaning for chemically nonsensical entries (see Supplementary Fig. 9), we categorized the reactions according to the number of bonds that were altered (from one to six) and selected samples from each class at random to ultimately collect 281 examples (cf. Fig. 7 and Supplementary Note 4)—we note that this method of categorization and selection placed emphasis on the more complicated reactions that would otherwise be infrequent (~72% of the USPTO set alters one or two bonds) but are important in the context of learning non-trivial chemical reaction rules. When the reactions were mapped by human experts, we compared the correctness of mappings provided in the USPTO set (red bars in Fig. 7a) vs. the mappings generated by our algorithm (green bars). As seen, for reactions disconnecting/creating one to two bonds, there are no major performance differences; on the other hand, for more complex reactions changing more than two bonds, the percentage of correctly mapped reactions in the USPTO collection drops rapidly to 16–52% while it remains between 82 and 92% for our mapper. A manifestation of this trend are illustrated in Fig. 7b which shows two useful reactions, Diels-Alder cycloaddition and *N*-alkylation of amides, incorrectly mapped in the USPTO set. We observe that when the authors of ref. ^11^ used such mappings to derive reactivity scores for atom pairs and then—using a deep neural network based on Weisfeiler-Lehman architecture— predicted outcomes of new reactions, they claimed that “the overall model performance does not depend strongly on atom mapping quality”. While this might be the case for some very simple chemistries, we have verified that if incorrect mappings are used to train the neural net, predictions of products of test reactions are, in vast majority, chemically nonsensical (see Supplementary Notes 5.2 vs. 5.3 and more thorough discussion in Section 8 of the Supplementary Information to ref. ^49^). In a wider context, such examples help us understand why synthesis-planning softwares based on machine-extracted rules or reactivity indices that come with faulty atom mappings are generally not applicable to complex targets whose syntheses require the use of more advanced chemistries. We note, however, that other approaches are also being developed that do not require atom mappings but, instead, base reaction predictions on reaction fingerprints^14^ or the so-called sequence to sequence models^50,51^.

We sought additional validation from synthetic chemists outside of our group (three M.Sc. students, two Ph.D. candidates, two postdoctoral fellows, all listed in the Acknowledgments section). First, to benchmark our expert mappings, four of these chemists re-mapped a randomly-chosen sample of our 100 reactions—their mappings agreed with those of our internal experts. Then, to ensure that the reactions we chose for our tests were not in any way biased, all seven external chemists were asked to provide additional samples (25 reactions per person) they considered representative to modern synthetic chemistry. All these reactions—differing in the level of mechanistic complexity, provided to us in a typical synthetic notation (in 38.9% of cases, without full stoichiometry), and all listed in the Supplementary Section 5—were mapped by our algorithm and the results were compared against external mappings (in addition, our internal experts validated the external mappings once more). In the end, the algorithm provided 90% correct mappings for 100 reactions provided by Ph.D. candidates and postdocs, and 95% correct answers for reactions provided by M.Sc. students.

Finally, we considered algorithm’s speed. Because our method uses several heuristics for the NP hard subgraph isomorphism problem, its speed is, in principle, exponential with respect to graph size—however, tests summarized in Supplementary Fig. 7 indicate that typical mapping times remain practical (91.5% of reactions mapped within 1 s, 96.5% in less than 10 s).

In summary, mapping of organic reactions is an example of a NP-hard problem for which prior attempts have been largely restricted to simple reaction types which chemists can typically map without much effort. In our approach, junction of graph theory and combinatorics with domain chemical knowledge (embodied in the minimal set of reaction heuristics) enables mapping of both simple and very complex organic reactions, including those that might challenge human experts. The graphical user interface of our algorithm is made freely available at http://mapper.grzybowskigroup.pl/marvinjs/ (see Supplementary Note 1.3 for a short tutorial) and we hope it will be useful for colleagues working on the applications we touched upon above (especially assignment of atoms in machine-extracted synthetic rules) and also in related fields, notably in the mapping of biochemical pathways (see examples in the Supplementary Note 1.4).

## Supplementary information

Supplementary Information

Description of Additional Supplementary Files

Supplementary Data

## Data Availability

The source code of the program is made available for academic users upon request to the corresponding authors.
