# Supplementary material for: Automatic mapping of atoms across both simple and complex chemical reactions
Source: Nat Commun. 2019 Mar 29;10:1434. doi: 10.1038/s41467-019-09440-2 (PMC6441094; doi:10.1038/s41467-019-09440-2)
Supplement: Supplementary file 1 — Supplementary Information [file 41467_2019_9440_MOESM1_ESM.pdf]

Supplementary Information

Automatic mapping of atoms across both simple and complex chemical reactions

Jaworski et al.

Supplementary Information for Manuscript titled "***Automatic mapping of atoms across both simple and complex chemical reactions***" by Wojciech Jaworski[1†], Sara Szymkuć[2†], Barbara Mikulak-Klucznik[2†], Krzysztof Piecuch[1], Tomasz Klucznik[2], Michał Kaźmierowski[1], Jan Rydzewski[1], Anna Gambin[1]* & Bartosz A. Grzybowski[2,3,4]*

[1] Faculty of Mathematics, Informatics and Mechanics, University of Warsaw, Ul. Banacha 2, 02-097 Warszawa, Poland

[2] Institute of Organic Chemistry, Polish Academy of Sciences, Ul. Kasprzaka 44/52, Warsaw 02-224, Poland

[3] IBS Center for Soft and Living Matter and

[4] Department of Chemistry, UNIST, 50, UNIST-gil, Eonyang-eup, Ulju-gun, Ulsan, South Korea

† Authors contributed equally

* Correspondence to aniag@mimuw.edu.pl or nanogrzybowski@gmail.com

TABLE OF CONTENTS

Supplementary Note 1. Additional algorithmic details and examples………..**pages 3-25**

- **1.1.** Further comments on isomorphic mapping and pseudocodes.............**pages 4-9**
- **1.2.** Illustrative example of isomorphic mapping without heuristics….......**pages 10-12**
- **1.3.** Short tutorial for using mapper's graphical user interface…................**pages 13-14**
- **1.4.** Examples of biosynthetic pathways mapped by our algorithm............**pages 15-17**
- **1.5.** Examples of mappings missing some atom assignments but otherwise correct……………………..…………………………………………..…….**page 18**
- **1.6.** Hard-coded reaction templates for some popular reaction classes………………………………………………………………..…..**pages 19-20**
- **1.7.** Quantification of mapping times…..................…………………………...**page 21**
- **1.8.** Additional examples of algorithm failures……….………..…………..**pages 22-23**
- **1.9.** Examples of chemically nonsensical reactions and mappings in the USPTO database……...……………….....…..**pages 24-25**

Supplementary Note 2. Training Set of 548 reactions with human/correct mappings compared to those of our software (“MAPPET”), ReactionMap, and MarvinJS…………………………………………………………………..….**pages 26-311**

- **p.27 to p.124** – 241 typical reactions with full stoichiometry and taken from the Organic Syntheses collection;
- **p.125 to p.248** – 191 randomly selected and mostly stoichiometrically unbalanced reactions from Reaxys;
- **p.249 to p.311** – 116 mechanistically complex reactions (both stoichiometrically balanced and unbalanced) taken from various literature sources.

Supplementary Note 3. Test Set #1 of 401 reactions with human/correct mappings compared to those of our software (“MAPPET”), ReactionMap, MarvinJS, ChemDraw and Indigo…………………………………………………………..………**pages 312 - 616**

- **p. 312 to p.384** – 100 simple reactions with full stoichiometry taken from total syntheses published in *Org. Lett.*, *J. Am. Chem. Soc.* and *J.Org. Chem.*
- **p.385 to p.451** – 100 randomly selected and mostly stoichiometrically unbalanced reactions from patents;
- **p.452 to p. 616** 201 mechanistically complex reactions (both stoichiometrically balanced and unbalanced) which include rearrangements and multicomponent reactions taken from recent (in most cases published after 2010) literature: *Org. Lett.*, *J. Am. Chem. Soc.* and *J.Org. Chem.*

Supplementary Note 4. Test Set #2 of 281 reactions selected from the USPTO’s set of 50,000 patent reactions accompanying ref. 11 (original USPTO’s mappings, in SMILES format, are compared to those of our algorithm)……………………………......**pages 618 - 727**

- **p.618 to p.637** – reactions in which one bond is being cut/created
- **p.637 to p.657** – reactions in which two bonds are being cut/created
- **p.657 to p.676** – reactions in which three bonds are being cut/created
- **p.676 to p.696** – reactions in which four bonds are being cut/created
- **p.696 to p.715**– reactions in which five bonds are being cut/created
- **p.715 to p.727** – reactions in which six bonds are being cut/created

Supplementary Note 5. Test set of 175 mapped reactions provided by external experts……………………………………………………………………..**pages 728-834**

Supplementary Note 6. Consequences of incorrect atom mapping for the prediction of reaction outcomes (with reference to the algorithm from main-text ref 11)…………………………..………………………………………..….**pages 835 - 872**

6.1. An illustrative example of the correct outcome of a Diels-Alder reaction vs. top predictions (all incorrect) of the MIT’s program …………….…………….. **page 835**

6.2. Examples of 10 pairs of reactions with *correct* mapping in the MIT training set. The first reaction in each pair has correct mapping and was used with such a mapping to train the MIT's deep neural network for reaction prediction. The second reaction is a closely related transformation (with chemically correct product shown) with which we queried/tested the MIT's network. The question asked is whether the MIT network can or cannot predict the correct product based on reaction substrates. On this set of 10 examples, **90% of outputs are correct**……………..**page 836 - 853**
6.3. Examples of 10 pairs of reactions with *incorrect* mapping in the MIT training set. The first reaction in each pair has incorrect mapping and was used with such a mapping to train the MIT's deep neural network for reaction prediction. The second reaction is a closely related transformation (with chemically correct product shown) with which we queried/tested the MIT's network. The question asked is whether the MIT network can or cannot predict the correct product based on reaction substrates. On this set of 10 examples, **only 20% of outputs are correct**………………………………………………………………... **page 854 - 872**

Supplementary Note 1. Additional algorithmic details and examples.

1.1. Further comments on isomorphic mapping and pseudocodes. The atom-atom mapping problem is formulated by means of reaction multi graphs as follows. We represent reactants and products molecules as two undirected and potentially disconnected multi graphs R and P. Vertices (also called nodes) of graphs R and P are labeled with disjoint sets of natural numbers N_R and N_P .

Assume that function

$$n_P : N_P \longrightarrow \mathbb{P} \text{ and } n_R : N_R \longrightarrow \mathbb{P} \tag{1}$$

maps node labels into the set of chemical elements

$$\mathbb{P} = \{\text{H, He, Li, } \ldots\}. \tag{2}$$

Moreover, the function

$$e : \mathbb{N} \times \mathbb{N} \longrightarrow \{0,1,2,3\} \tag{3}$$

corresponds to chemical bonds, i.e., $e\ (i, j)$ equal to 0 means that there is no bond between atoms i and j, $e\ (i, j) = 1$ represents single bond, 2 – double bond and 3 – triple one.

We are looking for a mapping ϕ from N_R to N_P, such that each node in N_R is assigned to the corresponding node in N_P, i.e., the atom-to-atom assignment reflects the underlying chemical changes in the course of reaction.

To reduce the problem's complexity, atom environments are defined recursively. Let us start with trivial observation that atoms do not change their types in the course of chemical reactions. Hence for every

$$p \in \mathbb{P} \tag{4}$$

the following equality holds:

$$\phi\big(n_R{}^{-1}(p)\big) = n_P{}^{-1}(p) \tag{5}$$

Therefore, the problem of finding correct ϕ may be split into several distinct problems for different atom types. The main idea can be viewed as the extension of Morgan's approach to identify other properties of atoms that are invariant in the reaction. These properties are analogues of EC-values and we regard them as hash values from the set $\mathbb{H}$.

Let us introduce two mappings $h_R^n : N_R \longrightarrow \mathbb{H}$ and $h_R^n : N_R \longrightarrow \mathbb{H}$. The hash values are

defined recursively as follows:

$$h_R^0(i) = \{n_R(i)\} \quad (6)$$

$$h_R^{n+1}(i) = \{n_R(i)\} \cup h_R^n(\Gamma(i)) \quad (7)$$

where $\Gamma(i) = \{j : e(i,j) > 0\}$ is a set of neighbors of the node i and $h_R^n(\Gamma(i))$ is a multiset image of the set $(\Gamma(i))$ of the function h_R^n.

Hash values for reaction products are defined analogously:

$$h_P^0(i) = \{n_P(i)\} \quad (8)$$

$$h_P^{n+1}(i) = \{n_P(i)\} \cup h_P^n(\Gamma(i)) \quad (9)$$

The exemplary hash-function labels for reaction from for the first level of recursion (i.e. h_R^0 and h_P^0) and for the second level (i.e., h_R^1 and h_P^1) are illustrated in the example in Supplementary Note 1.2 (Supplementary Figures 1a and 1b, respectively).

For each atom, hash function provides the set of features that enable to distinguish them and, at the same time, remain invariant in the course of reaction. Since hash values of the n-th level describe the neighborhood of a given atom within the radius n, they have much more discriminatory power than simple atom types.

However, the usability of hash values is limited to the stable parts of reaction graphs. When the reaction bonds are broken, the incident atoms are rearranged in novel ways. Fortunately, in a typical reaction, the number of broken bonds is relatively small and most of atoms could be matched using hash values.

Having defined the hash labeling, we generate the candidates for isomorphic subgraphs of reagents and products as follows: We execute *bucket sort* algorithm on sets N_R and N_P with respect to their hash values. Then, we group together the sets corresponding to pairs of nodes having identical hash values. If in a such pair the number of reagent nodes is different than the number of product nodes, we exclude nodes in all possible ways to make these numbers identical, thus obtaining a set of candidates. The following pseudocode formalizes CreateCandidates procedure together with the several auxiliary functions:

```
function CreateCandidates(n)
 | return GetExclusionSchemas(PairBucketGroup(N_P, N_R, (x → h^n(x))))
end
```

```
function Map(s, f)
    l ← {};
    foreach x ∈ s do
        l ← l ∪ f(x)
    end
    return l
end

function Product(s)
    if s = {} then return {{}};
    x ← an element from s;
    t ← Product(s \ {x});
    ret ← {};
    foreach a ∈ x do
        foreach b ∈ t do
            ret ← {{a} ∪ b} ∪ ret
        end
    end
    return ret
end

function GetExclusionSchemas(s)
    l ← {};
    foreach (S, T) ∈ s do
        if |S| > |T| then
            k ← |S| − |T|;
            x ← set of all k−element subsets of S;
            l ← l ∪ {x}
        end
        if |S| < |T| then
            k ← |T| − |S|;
            x ← set of all k−element subsets of T;
            l ← l ∪ {x}
        end
    end
    return Map(Product(l),(s → ⋃ s))
end
```

```
function PairBucketGroup(c_1, c_2, f)
    l ← {};
    foreach k ∈ Range(f) do
        s_1 ← {};
        s_2 ← {};
        foreach x ∈ c_1 do
            if f(x) = k then s_1 ← s_1 ∪ {x};
        end
        foreach x ∈ c_2 do
            if f(x) = k then s_2 ← s_2 ∪ {x};
        end
        if s_1 ≠ {} ∨ s_2 ≠ {} then l ← l ∪ {(s_1, s_2)};
    end
    return l
end
```

Next, for each candidate subgraph, we strive to establish one-to-one mapping (isomorphism) between reagent atoms and product atoms. The isomorphism is constructed separately for each connected component of candidate graphs. The following tests are used to exclude most candidates before checking the actual graph isomorphism (see Supplementary Figure 1 and the related description in Supplementary Note 1.2):

Test 1: The number of connected components in the reagent graph is equal to the number of connected components in the product graph.

Test 2: Connected components may be matched according to the number of nodes.

Test 3: Connected components may be matched according to node types (they have pairwise identical multisets of nodes' hash values).

Test 4: Connected components are pairwise isomorphic.

The following pseudocode formalizes MatchIsomorphicConnectedComponents procedure that implements the abovementioned tests:

function GetConnectedComponents($graph, selection$)
  $g \leftarrow$ subgraph of $graph$ induced by $selection$;
  return *connnected components of g*
end
function MatchIsomorphicConnectedComponents($excluded, graph, n$)
  $c_R \leftarrow$ GetConnectedComponents($graph, N_R \setminus excluded$);
  $c_P \leftarrow$ GetConnectedComponents($graph, N_P \setminus excluded$);
  if $|c_R| \neq |c_P|$ **then return** $failure$;
  $buckets \leftarrow$ PairBucketGroup($c_R, c_P, (c \rightarrow |c|)$);
  if $\exists_{(c_R,c_P)\in buckets}|c_R| \neq |c_P|$ **then return** $failure$;
  $buckets \leftarrow \bigcup$Map($buckets, ((c_R, c_P) \rightarrow$
    PairBucketGroup($c_R, c_P, (c \rightarrow \vec{h}^c)$));
  if $\exists_{(c_R,c_P)\in buckets}|c_R| \neq |c_P|$ **then return** $failure$;
  $part_buckets \leftarrow \bigcup$Map($buckets, ((c_R, c_P) \rightarrow$
    $part_R \leftarrow$ Quotient($c_R, ((x, y) \rightarrow$ AreIsomorphic($graph, n, x, y$));
    $part_P \leftarrow$ Quotient($c_P, ((x, y) \rightarrow$ AreIsomorphic($graph, n, x, y$));
    PairBucketGroup($part_R, part_P, (c \rightarrow |c|)$)));
  if $\exists_{(part_R,part_P)\in part\_buckets}|part_R| \neq |part_P|$ **then return** $failure$;
  return $\bigcup$Map($part\_bucket, ((part_R, part_P) \rightarrow$
    Combine($part_R, part_P, ((c_R, c_P) \rightarrow$
      AreIsomorphic($graph$,n,Elem(x),Elem(y))))))
end

To test whether graphs are isomorphic, we implemented the constrained search over a space of all partial mappings. Appropriately defined hashing values increase the distinctness of vertices which reduces the size of the search space. The nodes with the most unique labels are chosen as starting points for the search procedure. Then, a decision tree describing all possible isomorphisms is created. The size of this tree cannot exceed the predefined threshold (in the current implementation, 1,000,000 vertices). Failure is admitted by the algorithm when the decision tree is too large.

The described algorithm is similar in spirit to the popular VF2 algorithm. The main difference lies in the fact that VF2 adds single nodes to match sequentially while our algorithm extends the matching simultaneously to all neighbors of a given node.

In order to further reduce computational complexity, we treat hydrogen and fluorine atoms in a special way. We consider them as features of atoms to which they are linked. In this way, we substantially reduce the number of graph nodes for which we look for isomorphism and solve the problem of ambiguity caused by the fact that hydrogen (or fluorine) atoms linked to a given carbon atom are indistinguishable.

When mapping for other atoms is found, hydrogen and fluorine atoms are expanded and a simple mapping procedure is executed for them. In this procedure, we take into account that hydrogen and fluorine atoms may have only one bond and we look for any mapping that preserves as many bonds as possible.

With the key concepts of heuristics discussed at length in the main text, we briefly mention two other issues – namely, the treatment of aromaticity and disambiguation.

There are two possible representations for aromatic rings in SMILES format: one can mark atoms belonging to a ring as aromatic or point out a sequence of single and double bonds. However, the internal representation/notation of aromaticity in our algorithm is different and each aromatic bond is counted as one-and-a-half single bonds. We have chosen such notion because the notion of aromatic atoms (rather than bonds) is hard to combine with the concept of minimal chemical distance, whereas the alternating single-double representation requires to apply the algorithm to all isomers of a molecule, which causes a combinatorial explosion for molecules with many aromatic carbon rings.

Disambiguation procedures are another important component of the algorithm. While they do not affect the algorithm's outcome, they make the task tractable. During the whole mapping process, the algorithm operates on collections of candidates for mappings. Disambiguation is a process of elimination of repetitive candidates and is performed after each step of the algorithm: After each execution of the atom mapping procedure, on a set of candidates for which we have found complete matching, and on a set of modified versions of reactions obtained after application of heuristics. Down to technical detail, candidates are redundant if there exists isomorphism between substrates of the first candidate and substrates of the second one and between products of the first candidate and products of the second one. This isomorphism takes into account any partial mapping or labeling that is assigned to candidates. Disambiguation is implemented using the mapping algorithm with the omission of node exclusion step. If the mapping algorithm finds complete isomorphism, we consider candidates identical. Otherwise they are different.

1.2. Illustrative example of isomorphic mapping without heuristics

As discussed above and in the main text, we simplify the mapping problem to the isomorphism of subgraphs (rather than full molecular graphs), and first detect atoms whose environments are not changing during the reaction. The environments are defined not by scalar values used in previous approaches but by molecular subgraphs (i.e., smaller graphs within the graph defining the entire molecule) assigned to each atom, extending to within a given "radius" (measured in terms of bond distances), and defined recursively as illustrated in Supplementary Figures 1a and 1b for the "zero-order" environments (atoms themselves) and the first-order ones (nearest neighbors). In general, our algorithm uses environments up to the fourth order. The algorithm then uses the so-called bucket sorting to group together atoms with identical environment subgraphs. This procedure is illustrated in Supplementary Figure 1c which builds on the example from Supplementary Figures 1a,b (again, for the sake of illustration, restricting the analysis to the first-order environments). As seen, the numbers of atoms within each group are not necessarily equal. For instance, the reactant has one more atom of first-order environment type 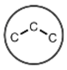

than the product, the product has one more atom of type 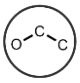

than the reactant, etc. In case of such discrepancies, the algorithm excludes extraneous atoms in all possible ways (see Supplementary Note 1.1 above for pseudocode) to make numbers in each type category equal. In our simple example, there are the following sets of atoms that could be excluded: A{3,5,14,18}, B{3,5,14,22}, C{3,8,14,18}, D{3,8,14,22}, E{3,9,14,18}, F{3,9,14,22}, G{3,11,14,18}, H{3,11,14,22}. These sets define possible candidates for mapping. For each candidate, we try to establish one-to-one mapping (isomorphism) between reagent atoms and product atoms. The isomorphism is constructed separately for each connected component of candidate graphs. To reduce solution search space and thus avoid time consuming exhaustive analysis – which is computationally prohibitive for large molecules – we introduce and apply sequentially four combinatorial tests detailed in the main text and in Supplementary Note 1.1. These tests exclude the majority of possible candidates – in our example, only candidates G and H survive with Supplementary Figure 1d showing their matching connected components colored in yellow and in cyan.

The most computationally-intensive part of the above operations is to find the correct isomorphism. This is done by creating a decision tree of all possible isomorphisms, with the size of this tree limited to a predefined number of vertices (currently, 1,000,000) – evaluation of a candidate reaction typically requires trees with tens of thousands of vertices but for either

very large molecules and/or those with many symmetries, it can approach or even exceed the one-million limit. The procedure is similar to the so-called VF2 algorithm with an important difference that VF2 adds single nodes (here, atoms) to the matching while our algorithm extends the matching simultaneously to all immediate neighbors of a given atom. The isomorphisms are first calculated at the level of the fourth-order environments, and then recursively at the third, second, and first levels. The purpose of this recursion is to match the atoms sequentially from the "periphery" of the molecules (where atoms agree to higher-order environments) towards the reaction center (where the conserved neighborhoods become smaller).

In some, rather rare cases, all atoms in the molecule are matched after this procedure – more generally, there remain unmatched nodes such as the uncolored atoms in Supplementary Figure 1d. For these atoms, mapping is straightforward when their neighbors are already matched and this matching fits environments across the reaction – in our example, candidate G yields correspondence between atoms 3 ~ 14 and 11 ~ 18 while for candidate H, 3 ~ 14 and 11 ~ 22. When, in the next step, these correspondences are extended to complete isomorphisms spanning entire molecules, only candidate H passes the test, yielding a mapping that is, above all, chemically correct. In the most general case, however, not all atoms will already have matched neighbors. If this is so, we again resort to combinatorics, sequentially cutting/removing all possible subsets of bonds originating from the unlabeled atoms – first all possible single bonds then, if needed, pairs of bonds, and so on to up to sets of six bonds. By this bond cutting we strive to identify minimal sets of bonds defining the "reaction center" and whose disconnection gives a full isomorphism between reactants and products. Overall, the above algorithm – using an improved representation of neighborhoods and various original combinatorial tests/procedures is crafted to and solves efficiently sub-graph isomorphism problem for graphs representing chemical molecules, which represent a special class of graphs that are almost always planar and have node degrees of, at most, four.

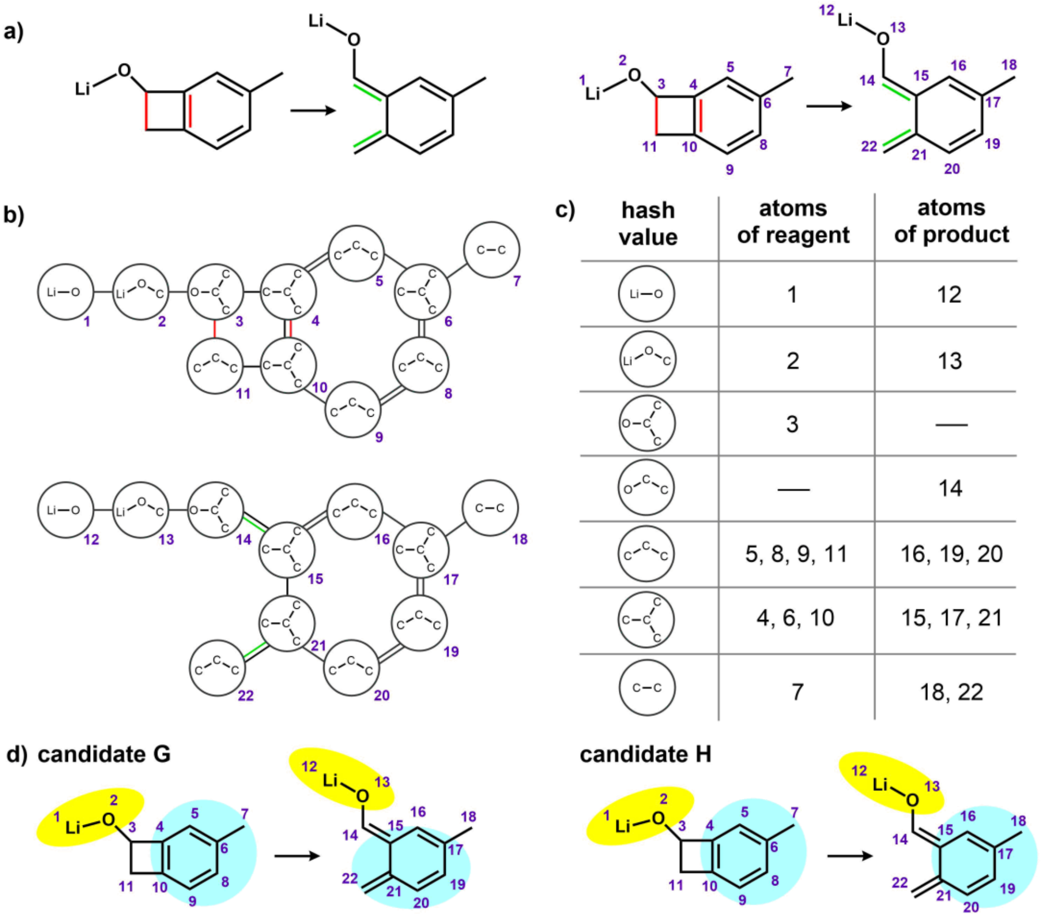

Supplementary Figure 1. Defining atom-environment subgraphs and matching of connected components. (a) A scheme of retro [2+2] cycloaddition. Numbering of atoms is equivalent to zero-level environments. **(b)** First-order environments assign to each atom a graph of its nearest neighbors. Higher-order environments extend these graphs to near-nearest atoms, etc. (in real calculations, up to order four). **(c)** The lists of substrate/product atoms assigned to the same environment types (here, for illustration, of order one). **(d)** Isomorphisms of two candidates surviving all combinatorial tests 1-4 (see main text). The matching connected components are colored yellow and blue. In G, atoms 1,2 correspond to atoms 12,13 and atoms 4,5,6,7,8,9,10 correspond to atoms 15,16,17,19,20,21,22, respectively. In H, atoms 1,2 correspond to atoms 12,13 and atoms 4,5,6,7,8,9,10 correspond to atoms 15,16,17,18,19,20,21, respectively.

1.3. Short tutorial for using mapper's graphical user interface.

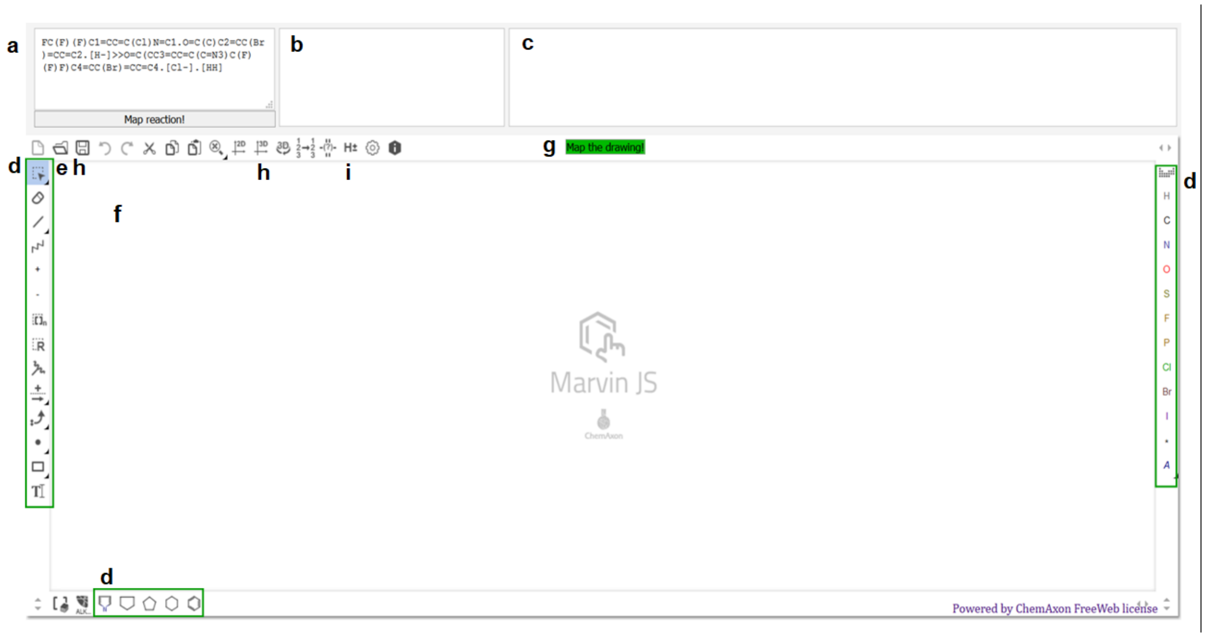

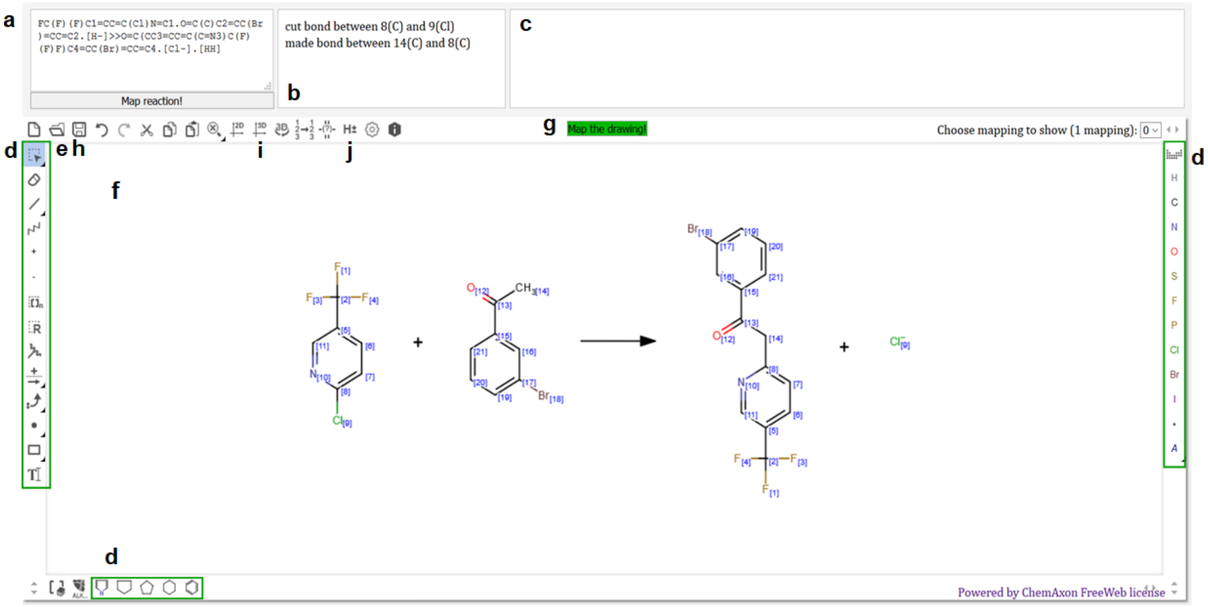

Supplementary Figure 2. Mapper's main page before (top) and after (bottom) reaction mapping.

(a) SMILES input field. User can type in/paste a reaction SMILES and map it with the "Map reaction!" button to start the mapping.

(b) Mapping output field. After mapping, details of the mapping process (numbers of bonds cut, made and changed) are shown here.

(c) If, for any reason, the algorithm is not able to map the reaction (timeout, problems with communication with server etc.) the information will be shown here. In case of unbalanced reactions, the information about incomplete stoichiometry will also be shown.

(d) Instead of entering SMILES of the reaction, the user is free to use drawing toolbars (left, right and bottom edges of mapping window) to draw chemical structures/reactions on his/her own. To confirm the structure and start mapping press "Map the drawing!" button.

(e) Import files button. It is also possible to import data in formats other than SMILES. Use the "import" option to check available file formats.

(f) Drawing and visualization window. Here, the user can draw chemical reaction and confirm it with the "Map the drawing!" button. Mapped reaction will also be displayed in this window.

(g) Map the drawing button. After finishing drawing the reaction choose "Map the drawing!" button to start the mapping.

(h) Export outcome button. Mapped reaction is ready to be saved in a desired format – as a picture, SMARTS, etc.

(i) 3D-visualisation mode. Useful while mapping reactions with complex chemical structures – strained molecules, condensed ring systems etc.

(j) Add/remove explicit H. By default, hydrogen atoms are not shown in mapped reactions. In case you wish to see them choose the "Add explicit H" option.

1.4. Examples of biosynthetic pathways mapped by our algorithm.

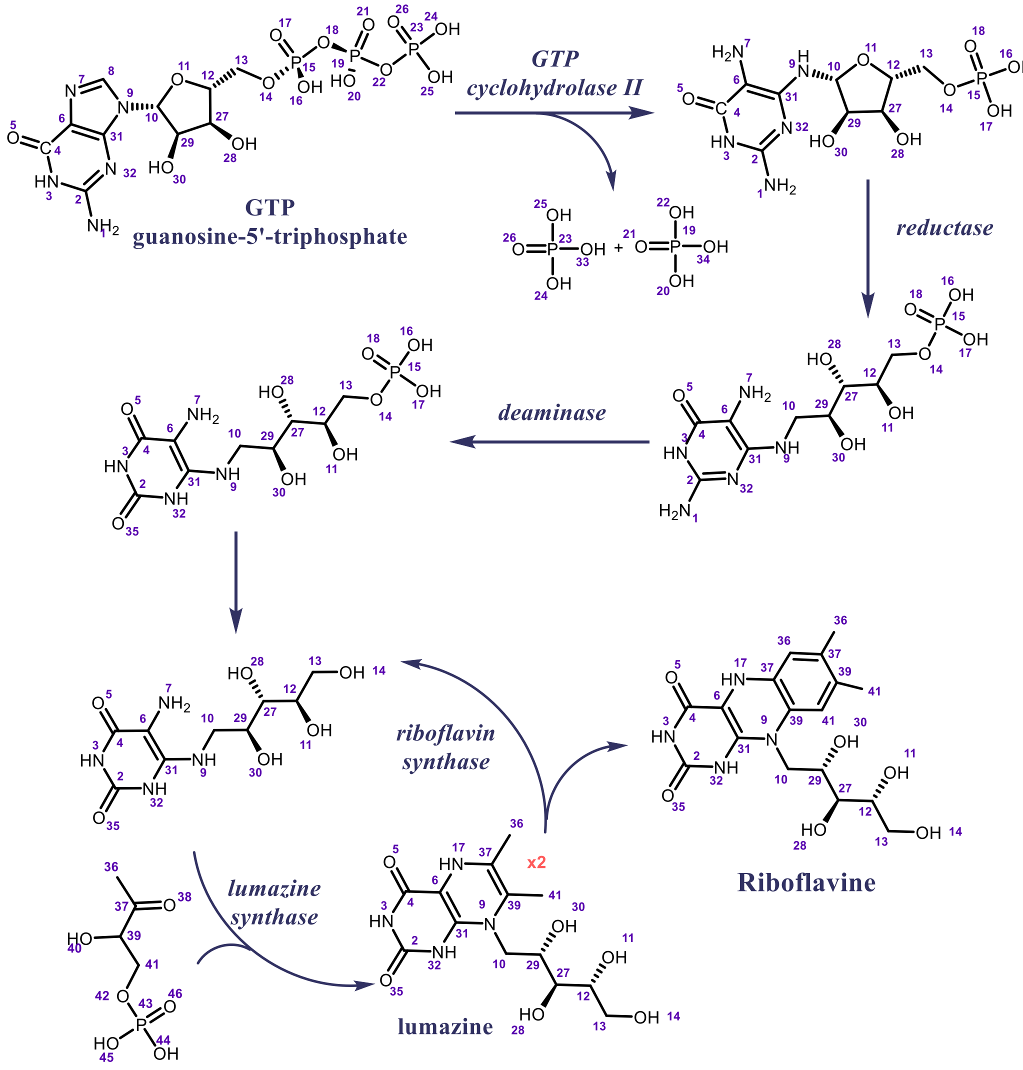

Supplementary Figure 3. Biosynthetic pathway of riboflavine (from *Saccharomyces cerevisiae*) mapped correctly by our algorithm. The first step involves opening of the imidazole ring of GTP accompanied by the loss of a pyrophosphate moiety. Further reduction of the ribosyl moiety results in the formation of the polyol side chain, then the amino group in positon 2 of the ring is hydrolyzed. Dephosphorylation and condensation with 2-hydroxy-3-oxobutyl phosphate yield lumazine. Two molecules of lumazine are then combined in the dismutation reaction yielding riboflavine and recovering one molecule of lumazine precursor (see Bacher, A., Eberhardt, S., Eisenreich, W., Fischer, M., Herz, S., Illarionov, B., Kis K. & Richter, G. Biosynthesis of riboflavin. *Vitam. Horm*, **61**, 1-49 (2001)).

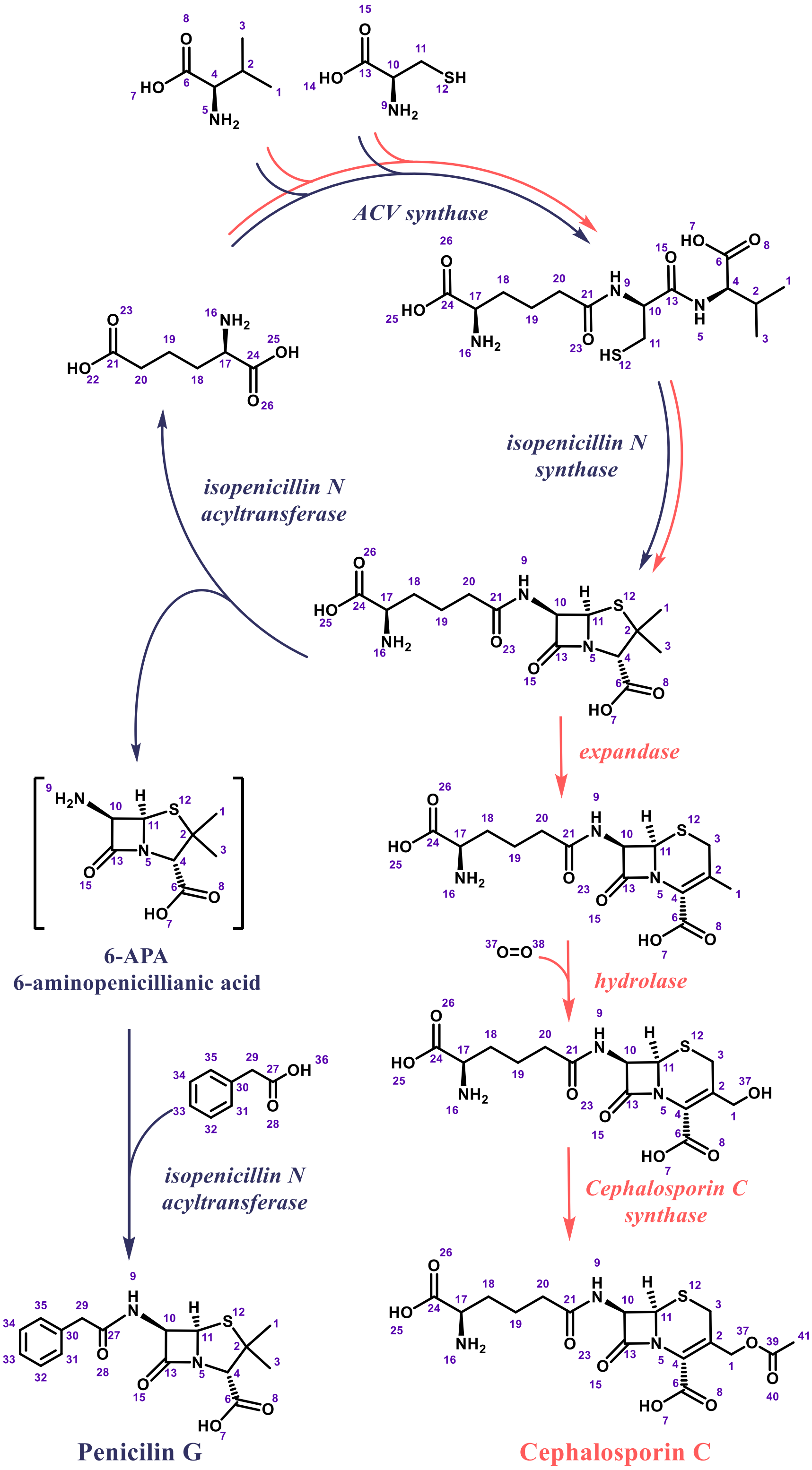

Supplementary Figure 4. Biosynthesis pathways of penicillin G (from *Penicillium chrysogenum*) and cephalosporin C (from *Cephalosporium acremonium*) mapped correctly by our algorithm. In both pathways L-aminoadipic acid, L-cysteine and L-valine are combined by ACV synthase to form ACV tripeptide, which is further converted in isopenicillin N. Isopenicillin N is then converted to penicillin G via 6-aminopenicillanic acid

(in *Penicillium chrysogenum*) or to cephalosporin C (*Cephalosporium acremonium*). For details, see Waites, M. J., Morgan, N. L.; Rockey, J. S. & Higton, G. *Industrial microbiology: an introduction* (John Wiley & Sons, 2009) and Weil, J., Miramonti, J. & Ladisch, M. R. Cephalosporin C: Mode of action and biosynthetic pathway. *Enzyme Microb. Technol* **17**, 85-87 (1995).

1.5. Examples of mappings missing some atom assignments but otherwise correct.

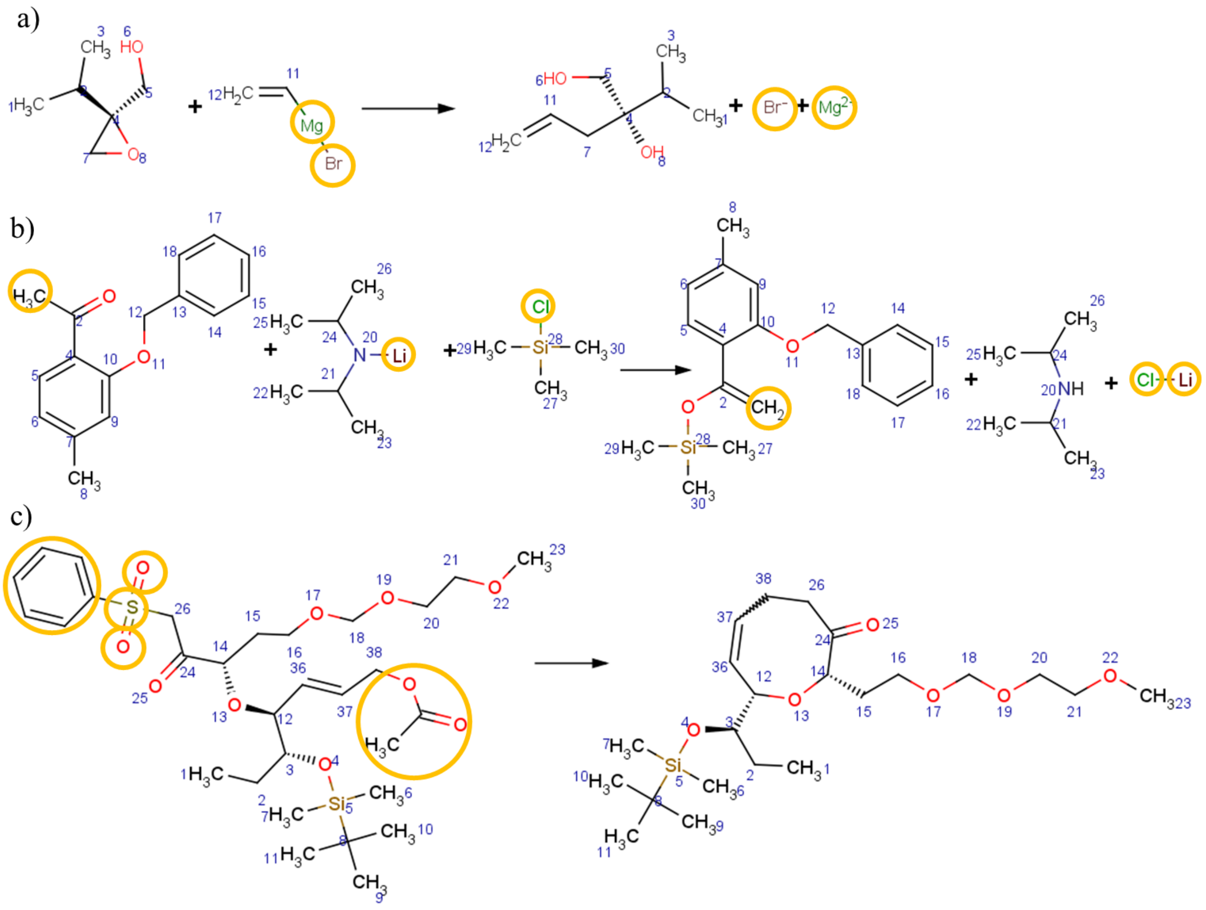

Supplementary Figure 5. Examples of mappings missing some atom assignments but otherwise correct. Examples are mostly from Indigo, but similar problems are common in ChemDraw. Unmapped atoms/groups are denoted by yellow circles. **a,** Opening of an epoxide with methylmagnesium bromide is mapped without assigning numbers to bromine and magnesium atoms. **b,** Mapping of this silyl-enol ether formation is chemically correct with exception that the chlorine, lithium and carbon α to the carbonyl group are left unmapped. **c,** Pd(0)-catalyzed cyclization has correctly assigned atom numbering in the product but has no numbers assigned to atoms within the tosyl and acetate groups present in the substrate.

1.6. Hard-coded reaction templates for some popular reaction classes.

a) Transesterification

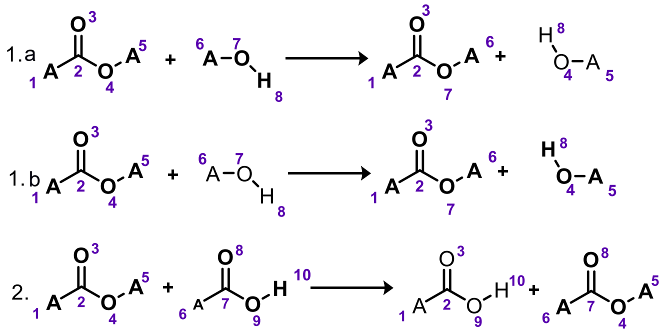

b) Amidation

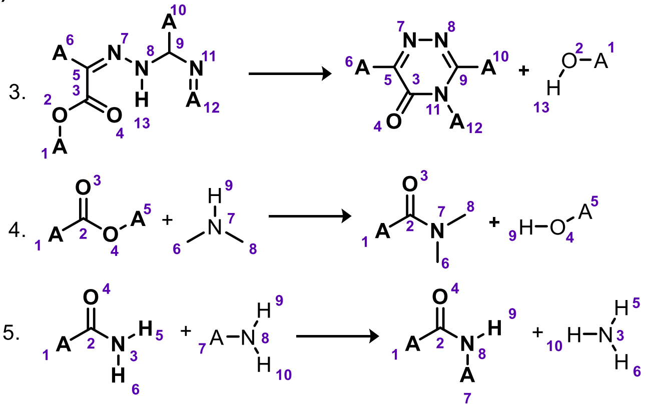

c) Nucleophilic substitution

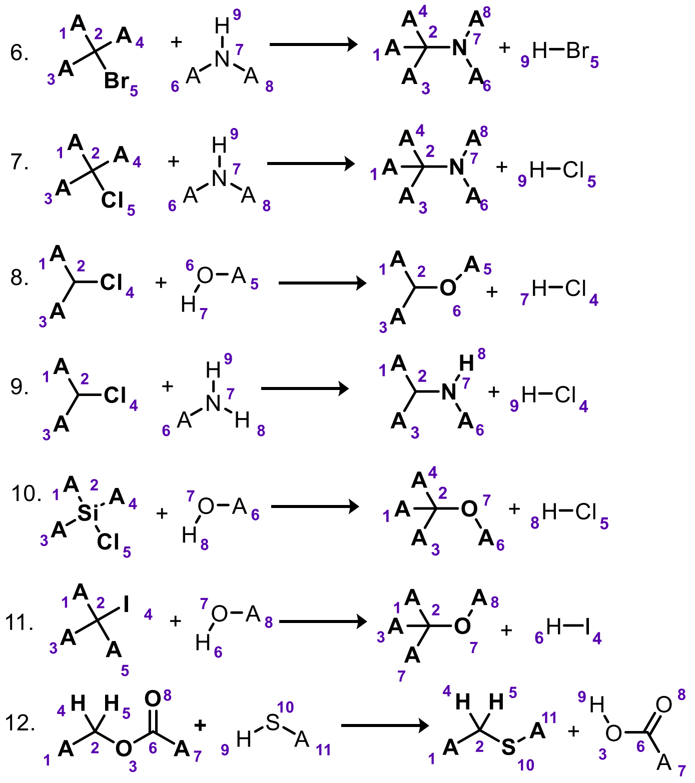

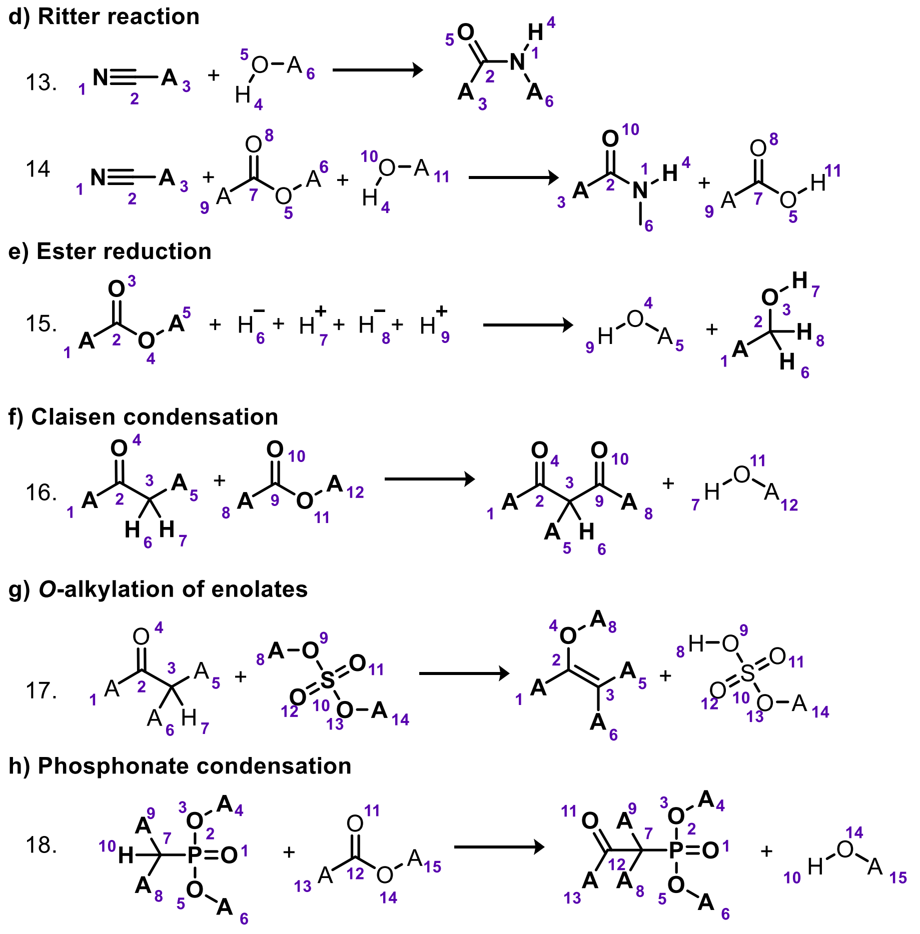

Supplementary Figure 6. Hard-coded reaction templates for some popular reaction classes. If each molecule present in the as-written reaction matches one of the molecules in a template and if the key molecules from the template (drawn in thicker lines) match some molecules in the as-written reaction, then the reaction matches the template. The mapping of such reaction's core is then provided by the template, without application of heuristics.

1.7. Quantification of mapping times

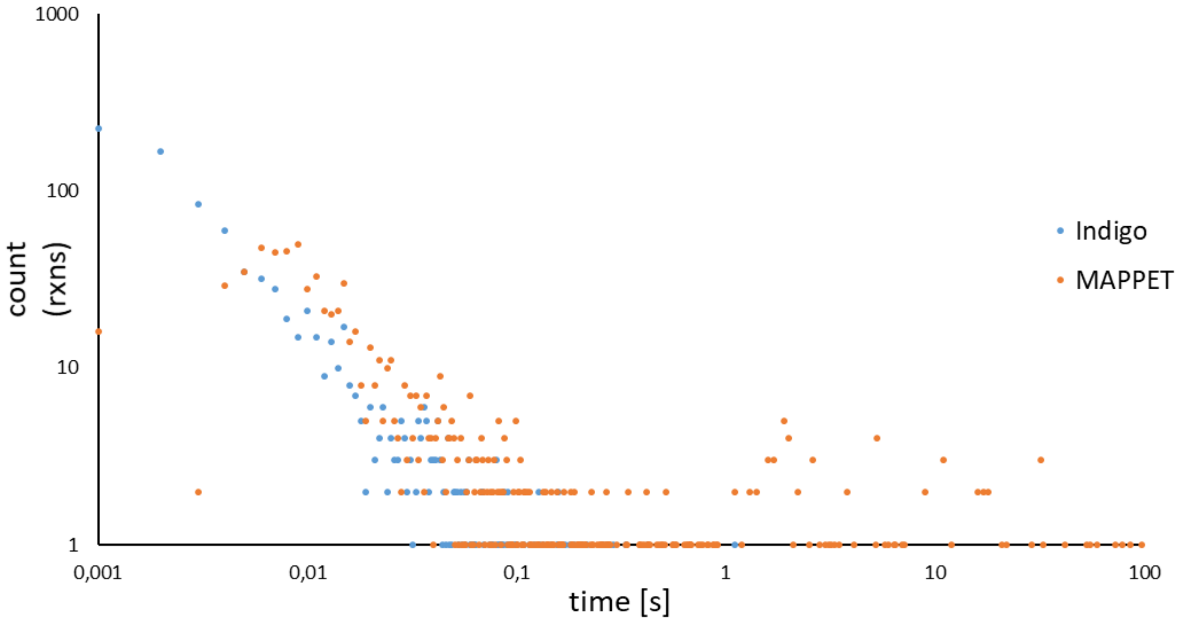

Supplementary Figure 7. Distribution of mapping times plotted on a double-logarithmic scale. Transformations were taken from the training and the main test sets (949 reactions in total). Our algorithm maps 91.5% of reactions in less than 1 sec and 96.8% of reactions in less than 10 sec. For our mapper, five reactions (0.5%) were mapped for times above 100 sec (not shown on the plot) and two were unmapped; on the other hand, for Indigo, the program returned no mapping for six reactions. All calculations were performed on Intel(R) Core(TM) i7-6700K CPU @ 4.00GHz. Blue dots correspond to the mapping times obtained by Indigo (the only other mapper allowing for batch-mode mappings) – Indigo is certainly faster but is also significantly less accurate (cf. Figure 5 in the main text).

1.8. Additional examples of algorithm failures.

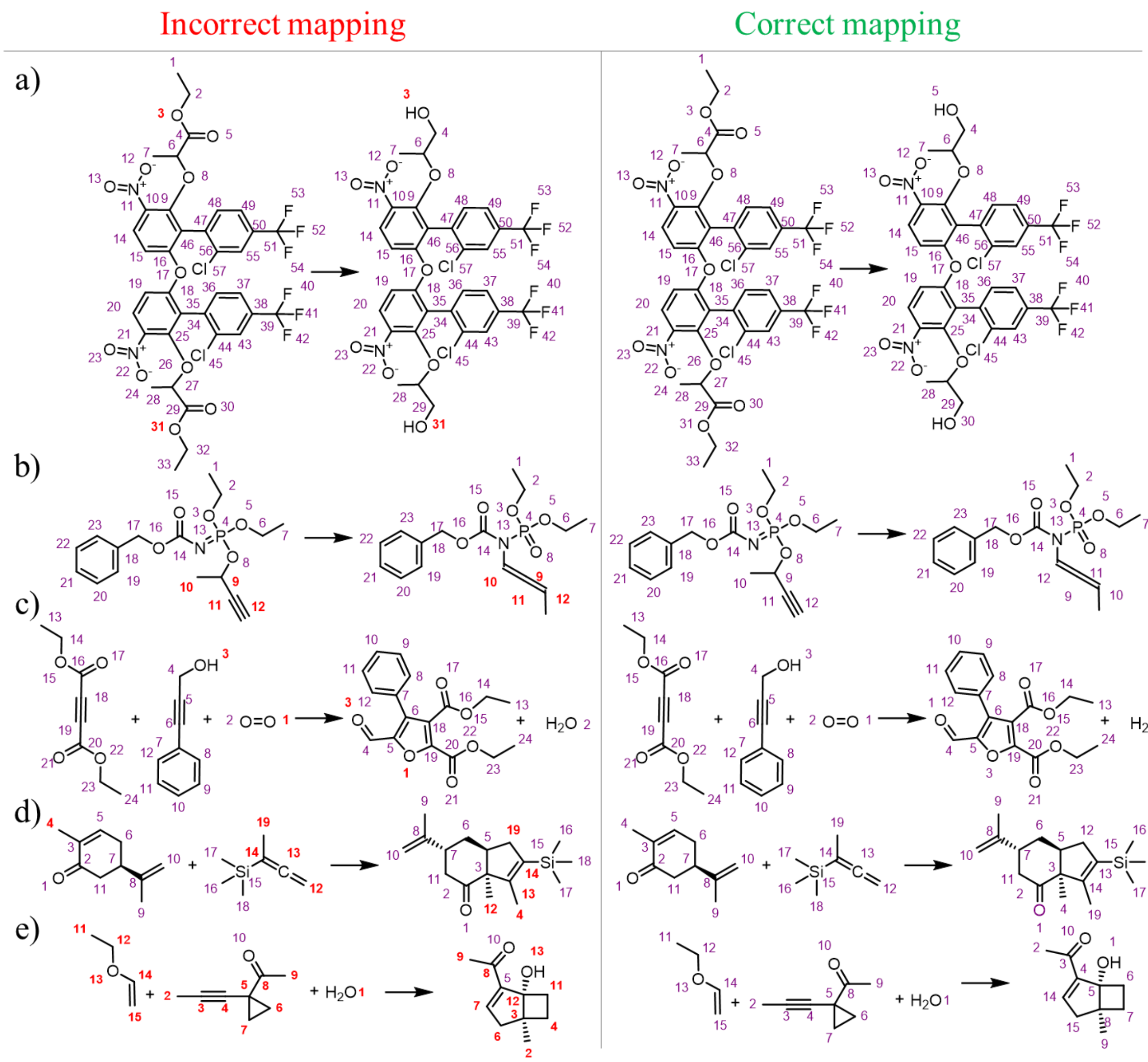

Supplementary Figure 8. Additional examples of algorithm failures. Red numbers in the left panel indicate incorrectly mapped atoms. Chemically correct mappings are shown in the right portion of the figure. **a,** An example of a stoichiometrically imbalanced transformation lacking mechanistically important by-products. This double reduction of an ester is written without specifying the two molecules of the ethanol by-product. As a result, the algorithm incorrectly retains in the product and maps the singly-bonded oxygens #3 and #31 (marked in red) rather than the "dissimilar-looking," doubly-bonded carbonyl oxygens #5 and #30. **b,** A specialized variant of a [3,3]-sigmatropic rearrangement is undetected by the mapper as it is not covered by the appropriate heuristics (Ia in main-text Figure 2). **c,** A mechanistically multistep reaction involving a rearrangement that is not recognized (i.e., not present in Figure 2) resulting in misplacing oxygens #1 and #3. **d,** The algorithm does not properly account for the migration

of the silyl group. **e**, This Au(I)-catalyzed synthesis of bicyclo[3.2.0]heptanes comprises 10 (sic!) mechanistic steps involving 1,3-dipolar cycloaddition and 1,2-alkyl rearrangement (cf. Li, G., Huang X. & Zhang L. Au(I)-Catalyzed Efficient Synthesis of Functionalized Bicyclo[3.2.0]heptanes. *J. Am. Chem. Soc.*, **130**, 6944–6945, 2008).

1.9. Examples of chemically nonsensical reactions and mappings in the USPTO database.

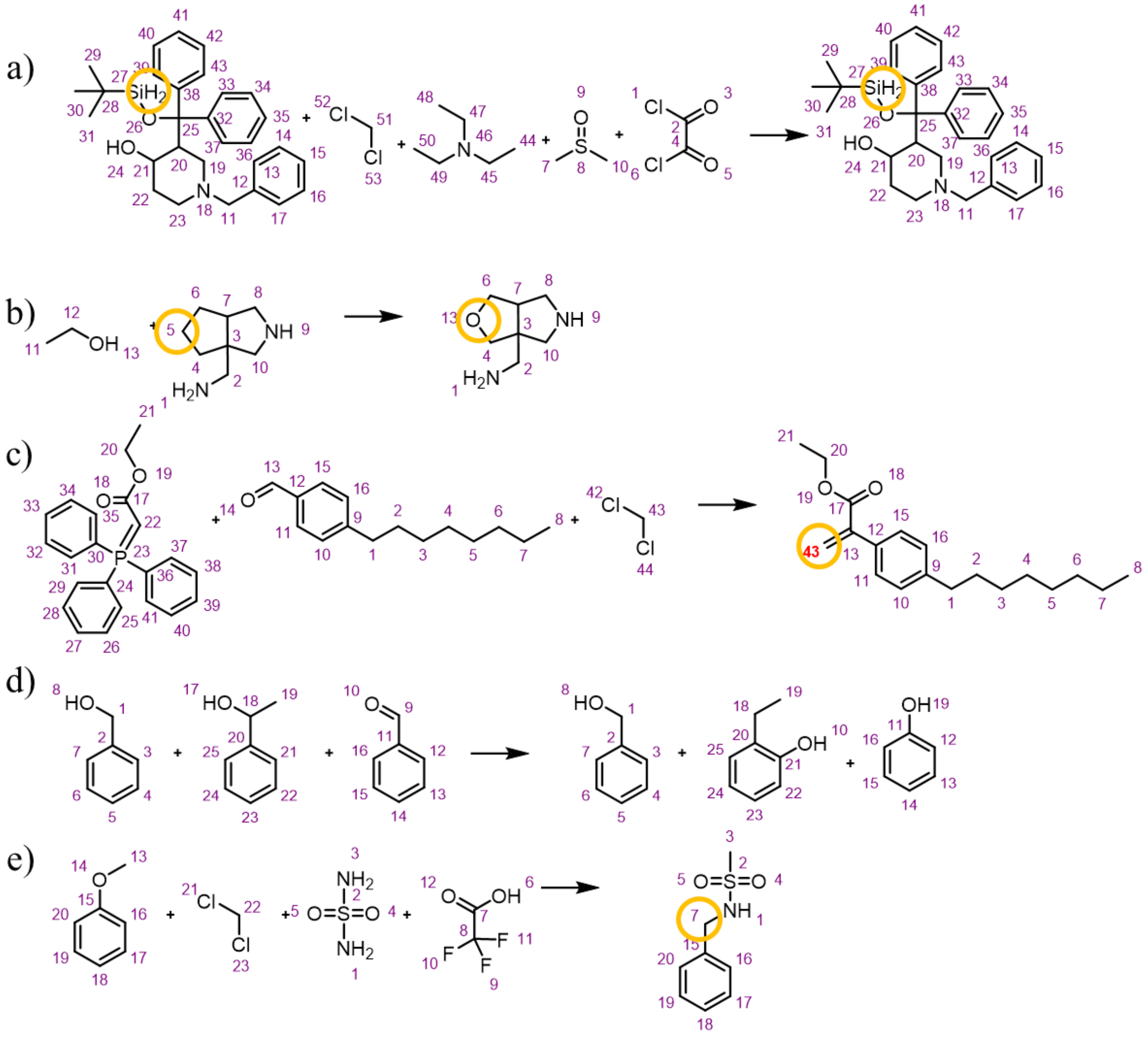

Supplementary Figure 9. Examples of chemically nonsensical reactions and mappings in the USPTO database. a, A chemical "typo" – in one of the substrates, the silyl ether protecting group does not contain all carbon substituents. We suppose that in the real reaction, it was the -OTBS group, but here two methyl groups are missing and replaced by hydrogens. This difference results in a dramatic change in the reactivity of the substrate: the R_2SiH_2 group – compared to the -OTBS – is much more reactive and *incompatible* in the reaction shown. **b,** "Transmutation" – by changing a carbon atom into an oxygen, cyclopentane ring is somehow transformed into the tetrahydrofuran ring. This reaction is certainly not possible. **c,** Wrong structure of the product – in this example of the Wittig reaction, the double bond is created as

expected, but is placed at the wrong part of the product (in exomethylene position which is chemically impossible considering the proposed substrates). **d,** List of chemicals rather than a reaction – there is no known reaction that would convert simultaneously the three substrates on the left side into the three "products" on the right side of the reaction arrow. **e,** It is impossible to obtain this reaction product from anisole in one step. Atom mapping – especially fate of carbon #7 is also mysterious. Based on these and many other USPTO examples we scrutinized, this collection contains the following (approximate) percentages of nonsensical reactions: for reaction in which one bond is altered, ~15%; for two bonds, ~2%; for three bonds, ~ 6%; for four bonds, ~15%; for five bonds, ~34%; for six bonds, ~58%.

Supplementary Note 2. Training Set of 548 reactions with human/correct mappings compared to those of our software (“MAPPET”), ReactionMap, and MarvinJS……………………………………………………………….pages 27-311

- **p.27 to p.124** – 241 typical reactions with full stoichiometry and taken from the Organic Syntheses collection;
- **p.125 to p.248** – 191 randomly selected and mostly stoichiometrically unbalanced reactions from Reaxys;
- **p.249 to p.311** – 116 mechanistically complex reactions (both stoichiometrically balanced and unbalanced) taken from various literature sources.

Reaction no 1

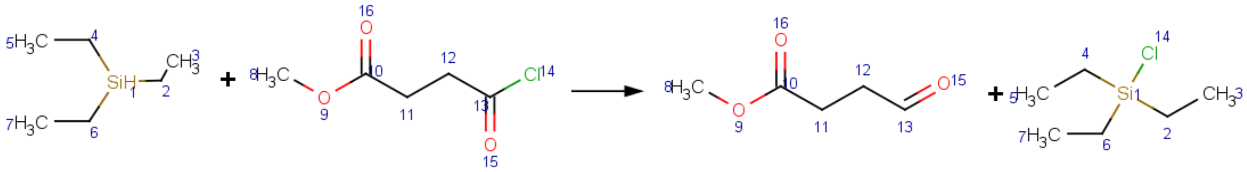

Correct mapped SMILES/SMARTS of the reaction:
[#6:3]-[#6:2]-[#14:1](-[#6:4]-[#6:5])-[#6:6]-[#6:7].[#6:8]-[#8:9]-[#6:10](=[O:16])-[#6:11]-[#6:12]-[#6:13]([Cl:14])=[O:15]>>[#6:8]-[#8:9]-[#6:10](=[O:16])-[#6:11]-[#6:12]-[#6:13]=[O:15].[#6:3]-[#6:2][Si:1]([Cl:14])([#6:4]-[#6:5])[#6:6]-[#6:7]

Correctness of the mapping
MAPPET YES
ReactionMap YES
Marvin YES

Reaction no 2

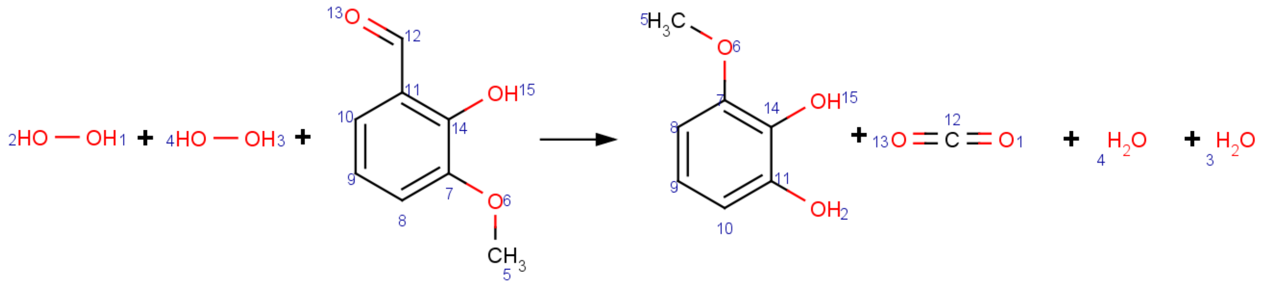

Correct mapped SMILES/SMARTS of the reaction:
[#8:1]-[#8:2].[#8:3]-[#8:4].[#6:5]-[#8:6]-[#6:7]-1=[#6:8]-[#6:9]=[#6:10]-[#6:11](-[#6:12]=[O:13])=[#6:14]-1-[#8:15]>>[#6:5]-[#8:6]-[#6:7]-1=[#6:14](-[#8:15])-[#6:11](-[#8:2])=[#6:10]-[#6:9]=[#6:8]-1.[O:13]=[C:12]=[O:1].[#8:4].[#8:3]

Correctness of the mapping
MAPPET YES
ReactionMap YES
Marvin NO

Reaction no 3

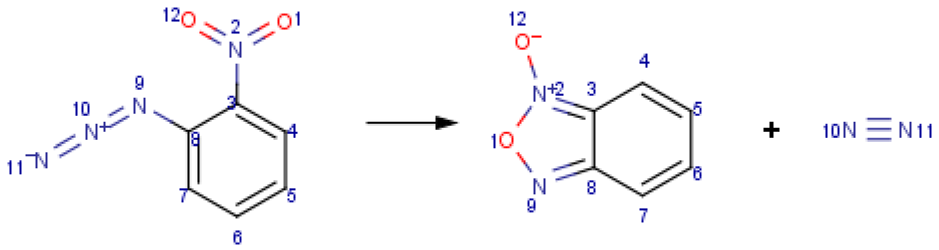

Correct mapped SMILES/SMARTS of the reaction:
[O:1]=[N:2](=[O:12])[#6:3]-1=[#6:4]-[#6:5]=[#6:6]-[#6:7]=[#6:8]-1-[#7:9]=[N+:10]=[N-:11]>>[#8-:12]-[#7+:2]-1=[#6:3]-2-[#6:4]=[#6:5]-[#6:6]=[#6:7]-[#6:8]-2=[#7:9]-[#8:1]-1.[N:11]#[N:10]

Correctness of the mapping
MAPPET YES
ReactionMap YES
Marvin YES

Reaction no 4

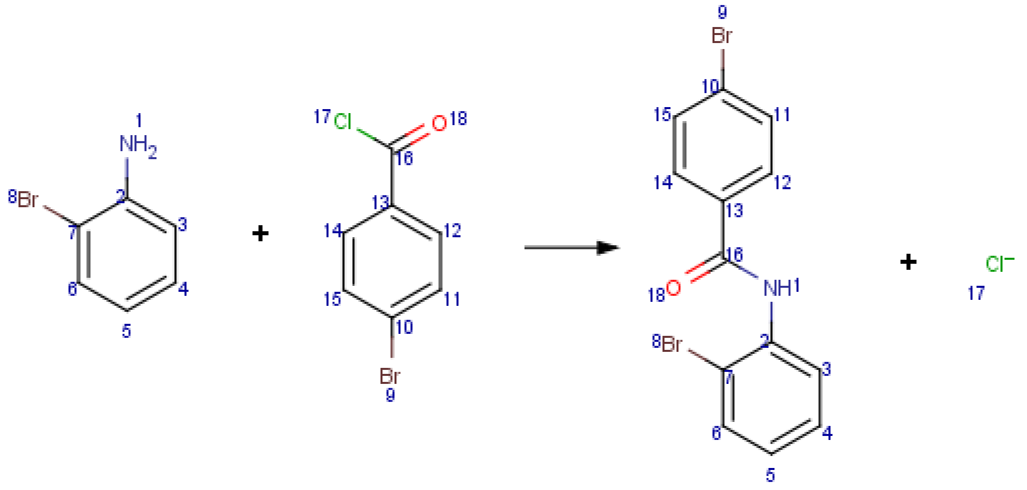

Correct mapped SMILES/SMARTS of the reaction:
[#7:1]-[#6:2]-1=[#6:3]-[#6:4]=[#6:5]-[#6:6]=[#6:7]-1[Br:8].[Cl:17][#6:16](=[O:18])-[#6:13]-1=[#6:12]-[#6:11]=[#6:10]([Br:9])-[#6:15]=[#6:14]-1>>[Br:9][#6:10]-1=[#6:11]-[#6:12]=[#6:13](-[#6:14]=[#6:15]-1)-[#6:16](=[O:18])-[#7:1]-[#6:2]-1=[#6:3]-[#6:4]=[#6:5]-[#6:6]=[#6:7]-1[Br:8].[Cl-:17]

Correctness of the mapping
MAPPET YES
ReactionMap NO
Marvin YES

Reaction no 5

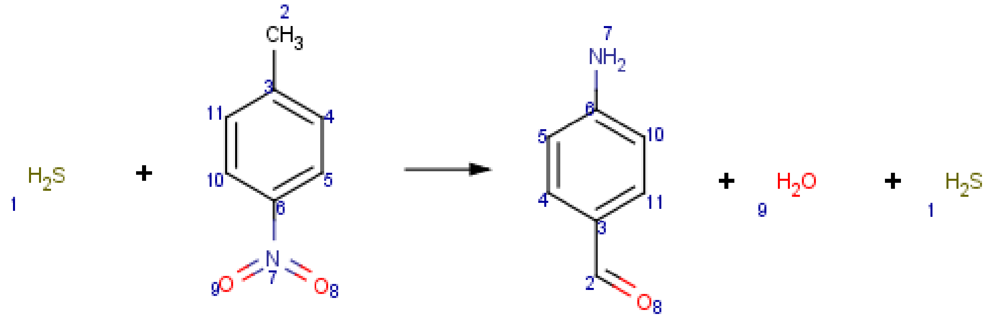

Correct mapped SMILES/SMARTS of the reaction:
[#16:1].[#6:2]-[#6:3]-1=[#6:4]-[#6:5]=[#6:6](-[#6:10]=[#6:11]-1)[N:7](=[O:8])=[O:9]>>[#7:7]-[#6:6]-1=[#6:10]-[#6:11]=[#6:3](-[#6:2]=[O:8])-[#6:4]=[#6:5]-1.[#8:9].[#16:1]

Correctness of the mapping
MAPPET YES
ReactionMap YES
Marvin YES

Reaction no 6

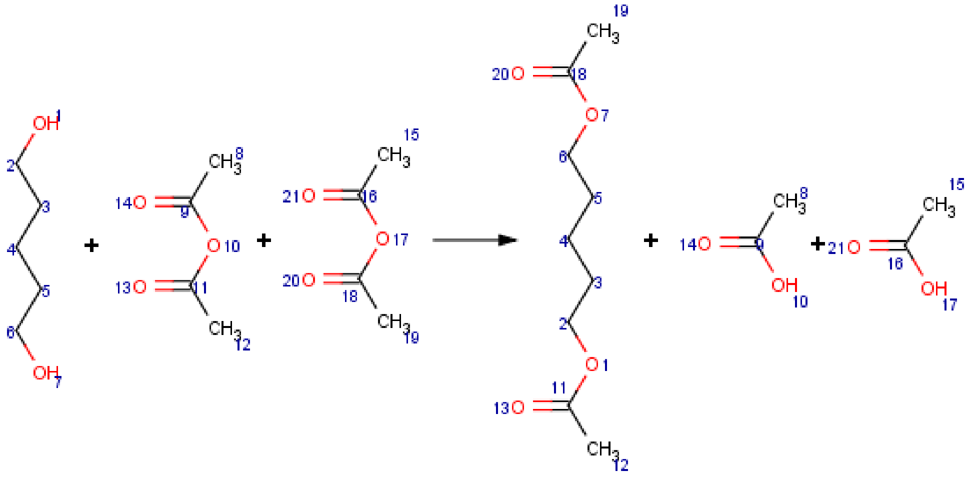

Correct mapped SMILES/SMARTS of the reaction:
[#8:1]-[#6:2]-[#6:3]-[#6:4]-[#6:5]-[#6:6]-[#8:7].[#6:8]-[#6:9](=[O:14])-[#8:10]-[#6:11](-[#6:12])=[O:13].[#6:15]-[#6:16](=[O:21])-[#8:17]-[#6:18](-[#6:19])=[O:20]>>[#6:19]-[#6:18](=[O:20])-[#8:7]-[#6:6]-[#6:5]-[#6:4]-[#6:3]-[#6:2]-[#8:1]-[#6:11](-[#6:12])=[O:13].[#6:8]-[#6:9](-[#8:10])=[O:14].[#6:15]-[#6:16](-[#8:17])=[O:21]

Correctness of the mapping
MAPPET YES
ReactionMap YES
Marvin YES

Reaction no 7

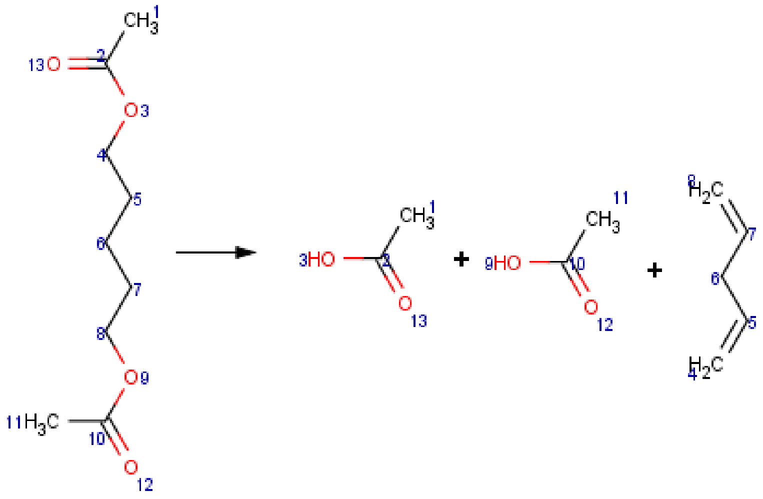

Correct mapped SMILES/SMARTS of the reaction:
[CH3:1][C:2](=[O:13])[O:3][CH2:4][CH2:5][CH2:6][CH2:7][CH2:8][O:9][C:10](=[O:12])[CH3:11]>>[CH3:1][C:2](=[O:13])[OH:3].[CH3:11][C:10](=[O:12])[OH:9].[CH2:4]=[CH:5][CH2:6][CH:7]=[CH2:8]

Correctness of the mapping
MAPPET NO
ReactionMap YES
Marvin NO

Reaction no 8

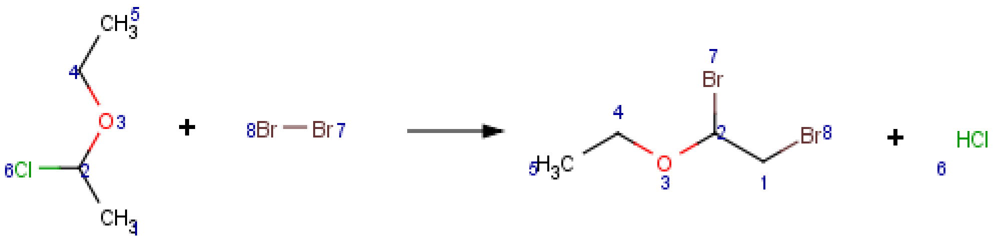

Correct mapped SMILES/SMARTS of the reaction:
[#6:5]-[#6:4]-[#8:3]-[#6:2](-[#6:1])[Cl:6].[Br:7][Br:8]>>[#6:5]-[#6:4]-[#8:3]-[#6:2]([Br:7])-[#6:1][Br:8].[Cl:6]

Correctness of the mapping
MAPPET YES
ReactionMap YES
Marvin YES

Reaction no 9

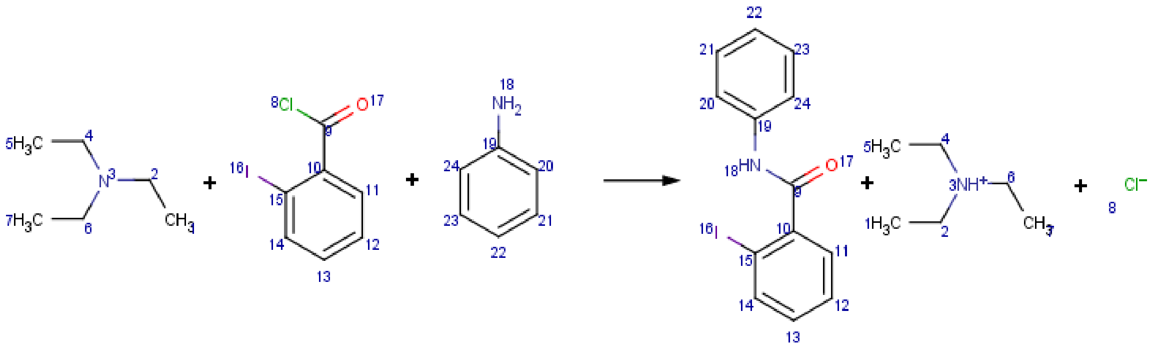

Correct mapped SMILES/SMARTS of the reaction:
[#6:1]-[#6:2]-[#7:3](-[#6:4]-[#6:5])-[#6:6]-[#6:7].[Cl:8][#6:9](=[O:17])-[#6:10]-1=[#6:11]-[#6:12]=[#6:13]-[#6:14]=[#6:15]-1[I:16].[#7:18]-[#6:19]-1=[#6:20]-[#6:21]=[#6:22]-[#6:23]=[#6:24]-1>>[I:16][#6:15]-1=[#6:10](-[#6:11]=[#6:12]-[#6:13]=[#6:14]-1)-[#6:9](=[O:17])-[#7:18]-[#6:19]-1=[#6:20]-[#6:21]=[#6:22]-[#6:23]=[#6:24]-1.[#6:7]-[#6:6]-[#7+:3](-[#6:4]-[#6:5])-[#6:2]-[#6:1].[Cl-:8]

Correctness of the mapping
MAPPET YES
ReactionMap YES
Marvin YES

Reaction no 10

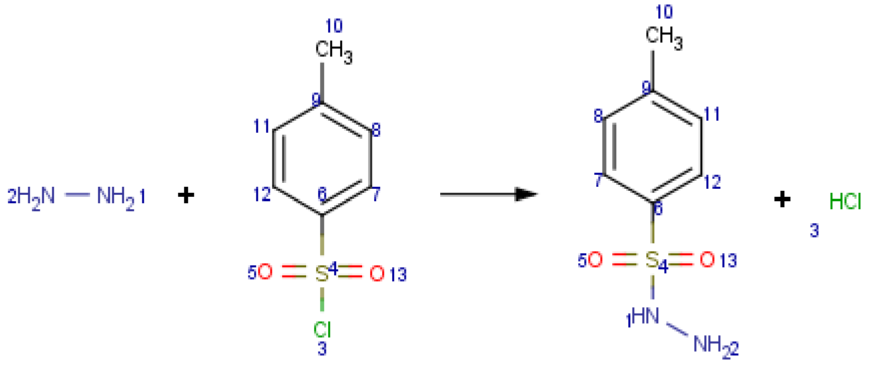

Correct mapped SMILES/SMARTS of the reaction:
[NH2:1][NH2:2].[CH3:10][C:9]1=[CH:8][CH:7]=[C:6]([CH:12]=[CH:11]1)[S:4]([Cl:3])(
=[O:5])=[O:13]>>[CH3:10][C:9]1=[CH:11][CH:12]=[C:6]([CH:7]=[CH:8]1)[S:4](=[O:5])
(=[O:13])[NH:1][NH2:2].[ClH:3]

Correctness of the mapping
MAPPET YES
ReactionMap YES
Marvin YES

Reaction no 11

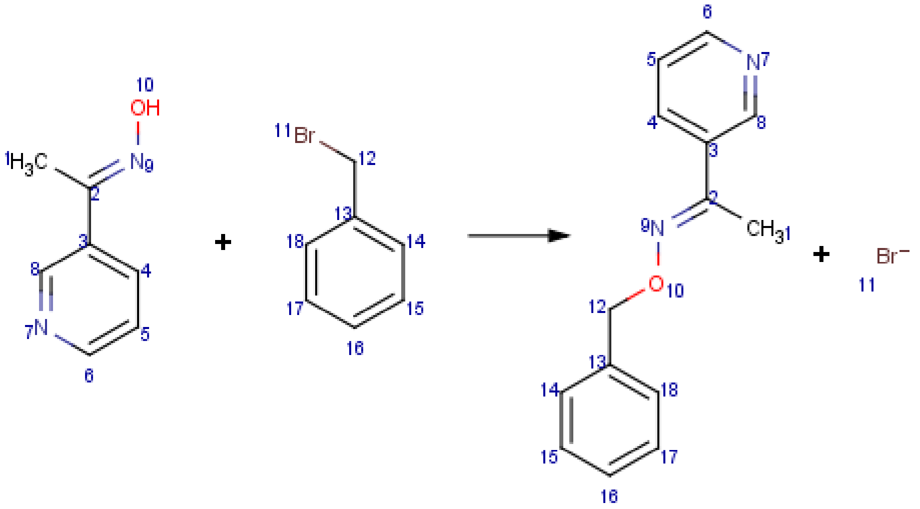

Correct mapped SMILES/SMARTS of the reaction:
[#6:1]\[#6:2](=[#7:9]/[#8:10])-[#6:3]-1=[#6:4]-[#6:5]=[#6:6]-[#7:7]=[#6:8]-
1.[Br:11][#6:12]-[#6:13]-1=[#6:14]-[#6:15]=[#6:16]-[#6:17]=[#6:18]-
1>>[#6:1]\[#6:2](=[#7:9]/[#8:10]-[#6:12]-[#6:13]-1=[#6:18]-[#6:17]=[#6:16]-
[#6:15]=[#6:14]-1)-[#6:3]-1=[#6:4]-[#6:5]=[#6:6]-[#7:7]=[#6:8]-1.[Br-:11]

Correctness of the mapping
MAPPET YES
ReactionMap YES
Marvin YES

Reaction no 12

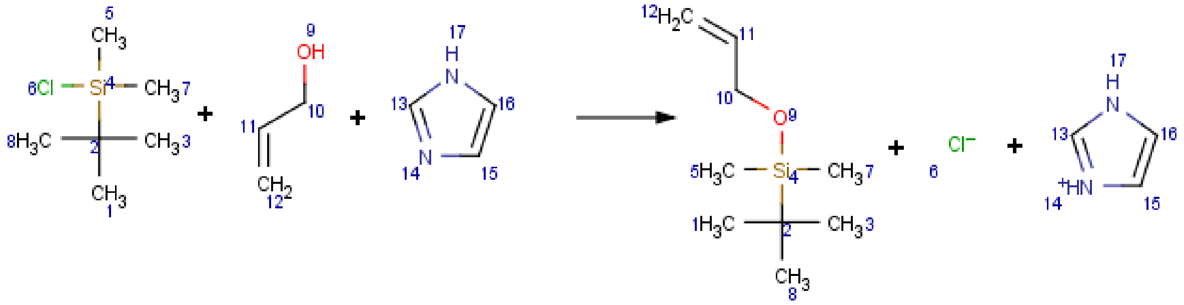

Correct mapped SMILES/SMARTS of the reaction:
[#6:1][C:2]([#6:3])([#6:8])[Si:4]([#6:5])([#6:7])[Cl:6].[#8:9]-[#6:10]-
[#6:11]=[#6:12].[#7:17]-1-[#6:16]=[#6:15]-[#7:14]=[#6:13]-
1>>[#6:8][C:2]([#6:3])([#6:1])[Si:4]([#6:7])([#6:5])[#8:9]-[#6:10]-
[#6:11]=[#6:12].[Cl-:6].[#7:17]-1-[#6:16]=[#6:15]-[#7+:14]=[#6:13]-1

Correctness of the mapping
MAPPET YES
ReactionMap YES
Marvin YES

Reaction no 13

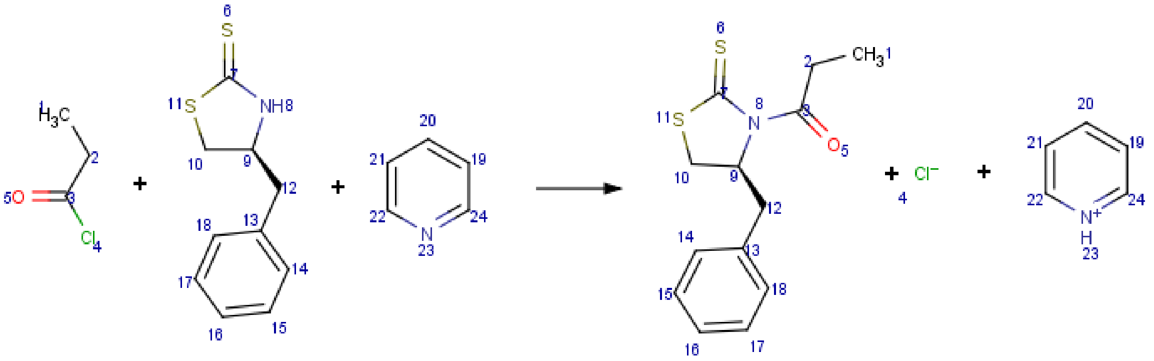

Correct mapped SMILES/SMARTS of the reaction:
[#6:1]-[#6:2]-[#6:3]([Cl:4])=[O:5].[S:6]=[#6:7]-1-[#7:8]-[#6@@H:9](-[#6:12]-
[#6:13]-2=[#6:14]-[#6:15]=[#6:16]-[#6:17]=[#6:18]-2)-[#6:10]-[#16:11]-1.[#6:20]-
1=[#6:19]-[#6:24]=[#7:23]-[#6:22]=[#6:21]-1>>[#6:1]-[#6:2]-[#6:3](=[O:5])-
[#7:8]-1-[#6@@H:9](-[#6:12]-[#6:13]-2=[#6:18]-[#6:17]=[#6:16]-[#6:15]=[#6:14]-
2)-[#6:10]-[#16:11]-[#6:7]-1=[S:6].[Cl-:4].[#6:20]-1=[#6:19]-[#6:24]=[#7+:23]-
[#6:22]=[#6:21]-1

Correctness of the mapping
MAPPET YES
ReactionMap YES
Marvin YES

Reaction no 14

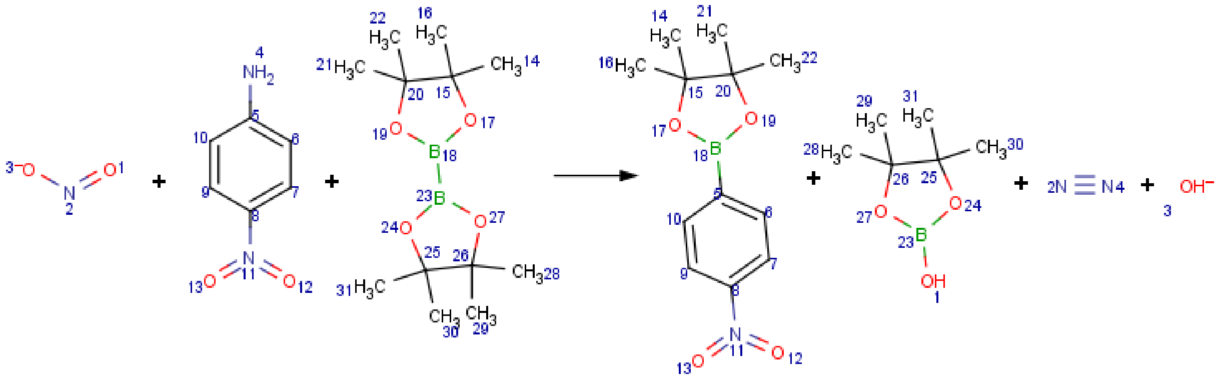

Correct mapped SMILES/SMARTS of the reaction:
[#8-:3]-[#7:2]=[O:1].[#7:4]-[#6:5]-1=[#6:6]-[#6:7]=[#6:8](-[#6:9]=[#6:10]-
1)[N:11](=[O:12])=[O:13].[#6:14][C:15]1([#6:16])[#8:17]-[#5:18](-
[#8:19][C:20]1([#6:21])[#6:22])-[#5:23]-1-
[#8:27][C:26]([#6:28])([#6:29])[C:25]([#6:30])([#6:31])[#8:24]-
1>>[#6:22][C:20]1([#6:21])[#8:19]-[#5:18](-[#8:17][C:15]1([#6:16])[#6:14])-
[#6:5]-1=[#6:6]-[#6:7]=[#6:8](-[#6:9]=[#6:10]-
1)[N:11](=[O:12])=[O:13].[#6:30][C:25]1([#6:31])[#8:24]-[#5:23](-[#8:1])-
[#8:27][C:26]1([#6:28])[#6:29].[N:4]#[N:2].[#8-:3]

Correctness of the mapping
MAPPET YES
ReactionMap YES
Marvin NO

Reaction no 15

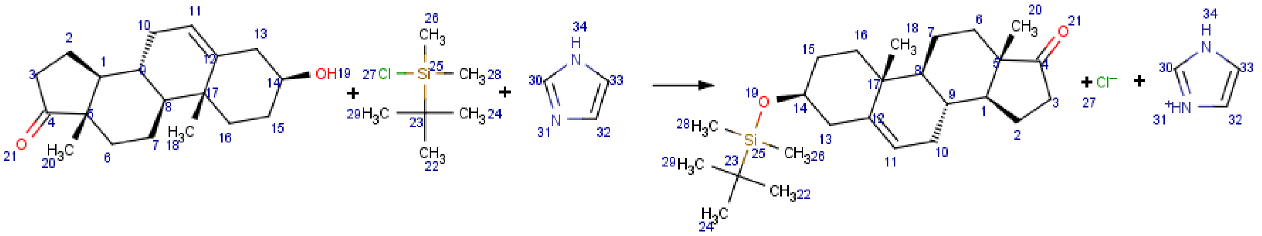

Correct mapped SMILES/SMARTS of the reaction:
[#6:20][C@:5]12[#6:6]-[#6:7]-[#6@H:8]3-[#6@@H:9](-[#6:10]-[#6:11]=[#6:12]4-[#6:13]-[#6@@H:14](-[#8:19])-[#6:15]-[#6:16][C@:17]34[#6:18])-[#6@@H:1]1-[#6:2]-[#6:3]-[#6:4]2=[O:21].[#6:22][C:23]([#6:24])([#6:29])[Si:25]([#6:26])([#6:28])[Cl:27].[#7:34]-1-[#6:33]=[#6:32]-[#7:31]=[#6:30]-1>>[#6:24][C:23]([#6:22])([#6:29])[Si:25]([#6:26])([#6:28])[#8:19]-[#6@H:14]-1-[#6:15]-[#6:16][C@:17]2([#6:18])[#6@H:8]-3-[#6:7]-[#6:6][C@@:5]4([#6:20])[#6@@H:1](-[#6:2]-[#6:3]-[#6:4]4=[O:21])-[#6@@H:9]-3-[#6:10]-[#6:11]=[#6:12]2-[#6:13]-1.[Cl-:27].[#7:34]-1-[#6:33]=[#6:32]-[#7+:31]=[#6:30]-1

Correctness of the mapping
MAPPET YES
ReactionMap YES
Marvin YES

Reaction no 16

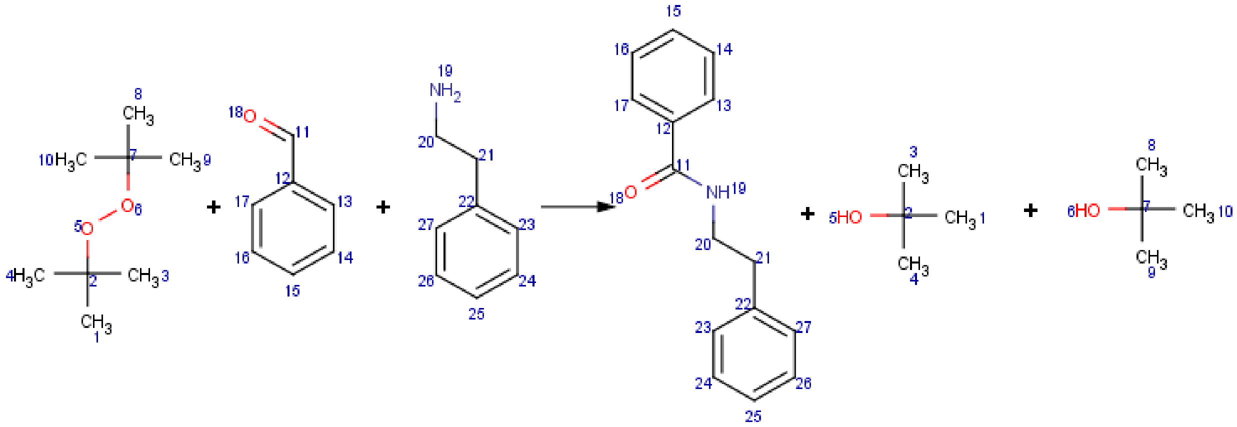

Correct mapped SMILES/SMARTS of the reaction:
[#6:1][C:2]([#6:3])([#6:4])[#8:5]-[#8:6][C:7]([#6:8])([#6:9])[#6:10].[O:18]=[#6:11]-[#6:12]-1=[#6:13]-[#6:14]=[#6:15]-[#6:16]=[#6:17]-1.[#7:19]-[#6:20]-[#6:21]-[#6:22]-1=[#6:23]-[#6:24]=[#6:25]-[#6:26]=[#6:27]-1>>[O:18]=[#6:11](-[#7:19]-[#6:20]-[#6:21]-[#6:22]-1=[#6:27]-[#6:26]=[#6:25]-[#6:24]=[#6:23]-1)-[#6:12]-1=[#6:17]-[#6:16]=[#6:15]-[#6:14]=[#6:13]-1.[#6:4][C:2]([#6:3])([#6:1])[#8:5].[#6:9][C:7]([#6:8])([#6:10])[#8:6]

Correctness of the mapping
MAPPET YES
ReactionMap YES
Marvin YES

Reaction no 17

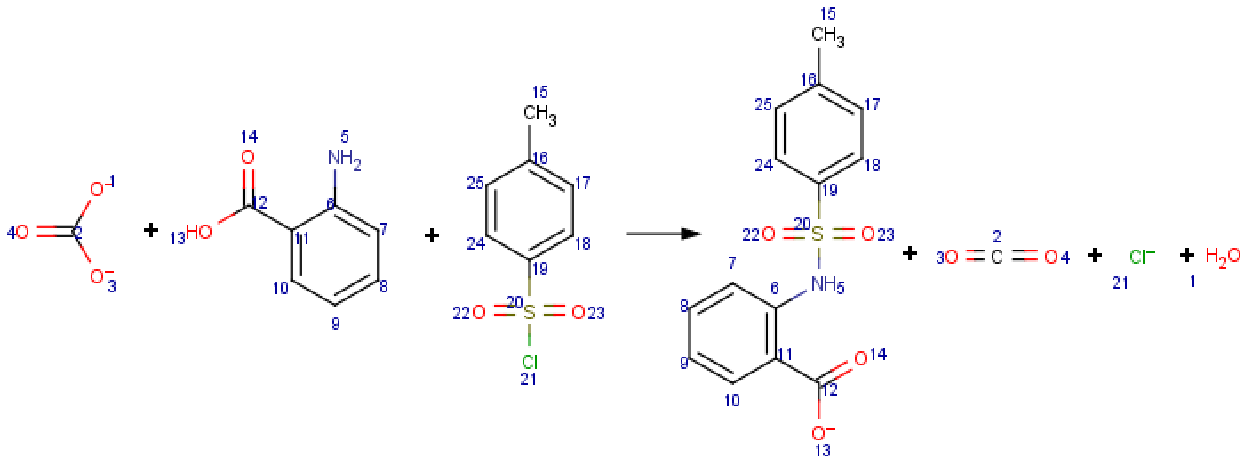

Correct mapped SMILES/SMARTS of the reaction:
[#8-:1]-[#6:2](-[#8-:3])=[O:4].[#7:5]-[#6:6]-1=[#6:7]-[#6:8]=[#6:9]-[#6:10]=[#6:11]-1-[#6:12](-[#8:13])=[O:14].[#6:15]-[#6:16]-1=[#6:17]-[#6:18]=[#6:19](-[#6:24]=[#6:25]-1)[S:20]([Cl:21])(=[O:22])=[O:23]>>[#6:15]-[#6:16]-1=[#6:17]-[#6:18]=[#6:19](-[#6:24]=[#6:25]-1)[S:20](=[O:22])(=[O:23])[#7:5]-[#6:6]-1=[#6:11](-[#6:10]=[#6:9]-[#6:8]=[#6:7]-1)-[#6:12](-[#8-:13])=[O:14].[O:3]=[C:2]=[O:4].[Cl-:21].[#8:1]

Correctness of the mapping
MAPPET YES
ReactionMap YES
Marvin YES

Reaction no 18

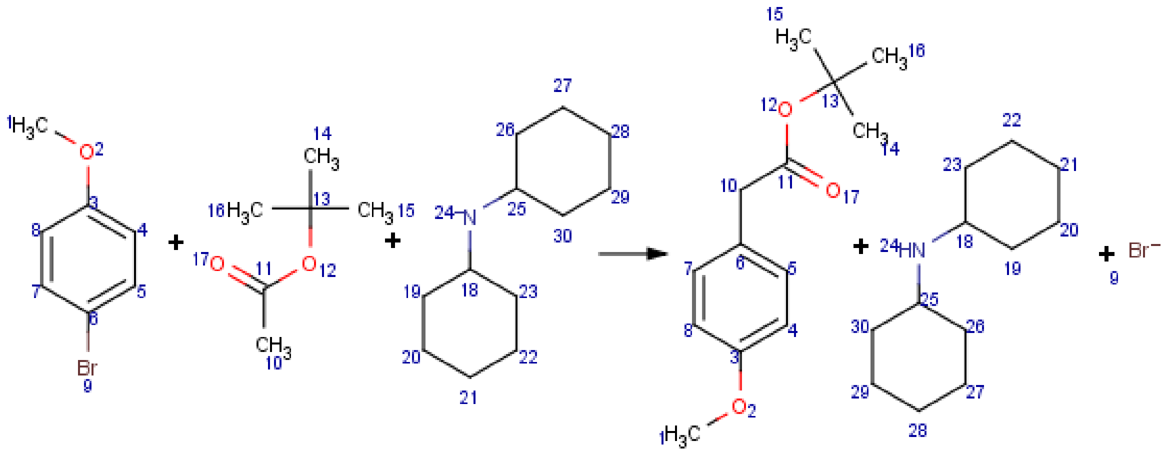

Correct mapped SMILES/SMARTS of the reaction:
[#6:1]-[#8:2]-[#6:3]-1=[#6:4]-[#6:5]=[#6:6]([Br:9])-[#6:7]=[#6:8]-1.[#6:10]-[#6:11](=[O:17])-[#8:12][C:13]([#6:14])([#6:15])[#6:16].[#6:21]-1-[#6:20]-[#6:19]-[#6:18](-[#6:23]-[#6:22]-1)-[#7-:24]-[#6:25]-1-[#6:26]-[#6:27]-[#6:28]-[#6:29]-[#6:30]-1>>[#6:1]-[#8:2]-[#6:3]-1=[#6:4]-[#6:5]=[#6:6](-[#6:10]-[#6:11](=[O:17])-[#8:12][C:13]([#6:16])([#6:15])[#6:14])-[#6:7]=[#6:8]-1.[#6:28]-1-[#6:29]-[#6:30]-[#6:25](-[#6:26]-[#6:27]-1)-[#7:24]-[#6:18]-1-[#6:23]-[#6:22]-[#6:21]-[#6:20]-[#6:19]-1.[Br-:9]

Correctness of the mapping
MAPPET YES
ReactionMap YES
Marvin YES

Reaction no 19

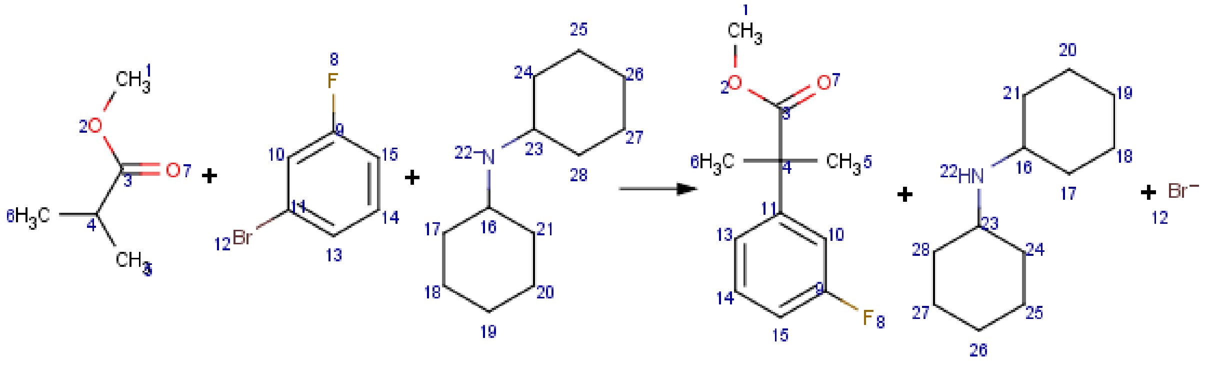

Correct mapped SMILES/SMARTS of the reaction:

[#6:1]-[#8:2]-[#6:3](=[O:7])-[#6:4](-[#6:5])-[#6:6].[F:8][#6:9]-1=[#6:10]-[#6:11]([Br:12])=[#6:13]-[#6:14]=[#6:15]-1.[#6:19]-1-[#6:18]-[#6:17]-[#6:16](-[#6:21]-[#6:20]-1)-[#7-:22]-[#6:23]-1-[#6:24]-[#6:25]-[#6:26]-[#6:27]-[#6:28]-1>>[#6:1]-[#8:2]-[#6:3](=[O:7])[C:4]([#6:6])([#6:5])[#6:11]-1=[#6:10]-[#6:9]([F:8])=[#6:15]-[#6:14]=[#6:13]-1.[#6:26]-1-[#6:27]-[#6:28]-[#6:23](-[#6:24]-[#6:25]-1)-[#7:22]-[#6:16]-1-[#6:21]-[#6:20]-[#6:19]-[#6:18]-[#6:17]-1.[Br-:12]

Correctness of the mapping
MAPPET YES
ReactionMap YES
Marvin YES

Reaction no 20

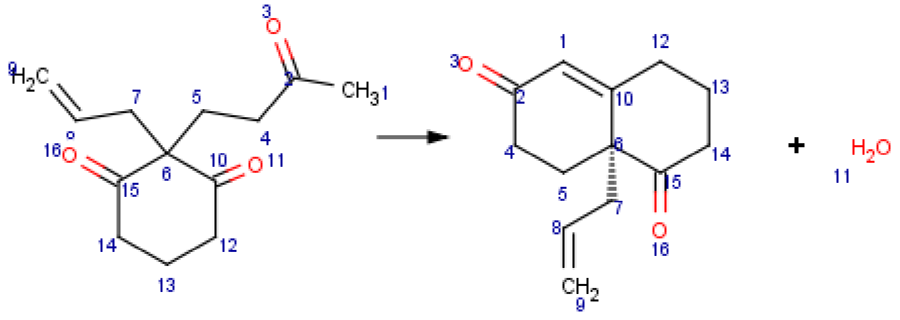

Correct mapped SMILES/SMARTS of the reaction:
[#6:1]-[#6:2](=[O:3])-[#6:4]-[#6:5][C:6]1([#6:7]-[#6:8]=[#6:9])[#6:10](=[O:11])-[#6:12]-[#6:13]-[#6:14]-[#6:15]1=[O:16]>>[#6:9]=[#6:8]-[#6:7][C@:6]12[#6:5]-[#6:4]-[#6:2](=[O:3])-[#6:1]=[#6:10]1-[#6:12]-[#6:13]-[#6:14]-[#6:15]2=[O:16].[#8:11]

Correctness of the mapping
MAPPET YES
ReactionMap YES
Marvin YES

Reaction no 21

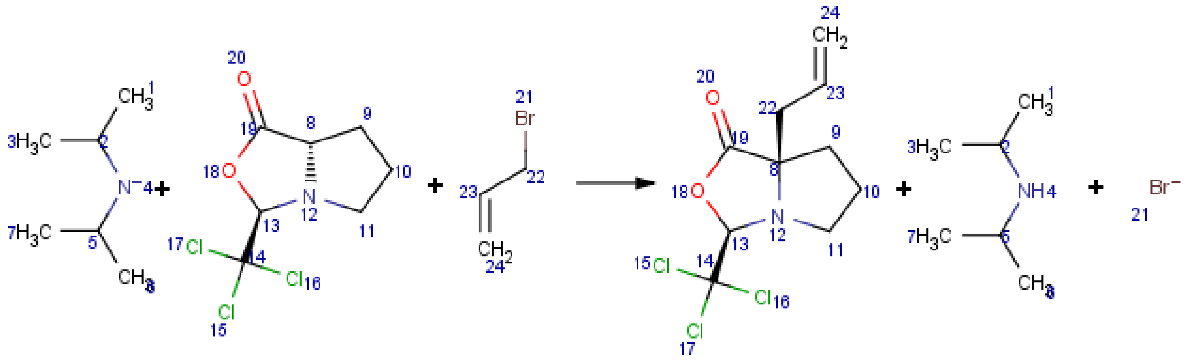

Correct mapped SMILES/SMARTS of the reaction:
[CH3:1][CH:2]([CH3:3])[N-
:4][CH:5]([CH3:6])[CH3:7].[Cl:15][C:14]([Cl:16])([Cl:17])[C@H:13]1[O:18][C:19](=
[O:20])[C@@H:8]2[CH2:9][CH2:10][CH2:11][N:12]12.[Br:21][CH2:22][CH:23]=[CH2:24]>
>[Cl:17][C:14]([Cl:16])([Cl:15])[C@H:13]1[O:18][C:19](=[O:20])[C@:8]2([CH2:22][C
H:23]=[CH2:24])[CH2:9][CH2:10][CH2:11][N:12]12.[CH3:1][CH:2]([CH3:3])[NH:4][CH:5
]([CH3:6])[CH3:7].[Br-:21]

Correctness of the mapping
MAPPET YES
ReactionMap YES
Marvin YES

Reaction no 22

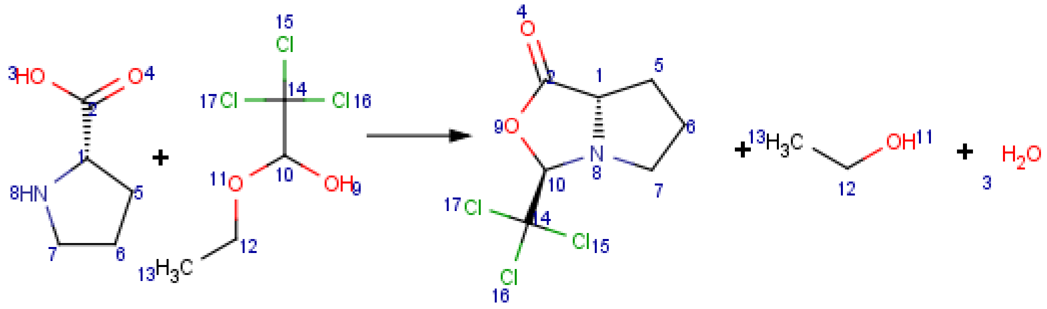

Correct mapped SMILES/SMARTS of the reaction: [#8:3]-[#6:2](=[O:4])-[#6@@H:1]-1-
[#6:5]-[#6:6]-[#6:7]-[#7:8]-1.[#6:13]-[#6:12]-[#8:11]-[#6:10](-
[#8:9])[C:14]([Cl:15])([Cl:16])[Cl:17]>>[Cl:16][C:14]([Cl:15])([Cl:17])[#6@H:10]
-1-[#8:9]-[#6:2](=[O:4])-[#6@@H:1]-2-[#6:5]-[#6:6]-[#6:7]-[#7:8]-1-2.[#6:13]-
[#6:12]-[#8:11].[#8:3]

Correctness of the mapping
MAPPET YES
ReactionMap NO
Marvin YES

Reaction no 23

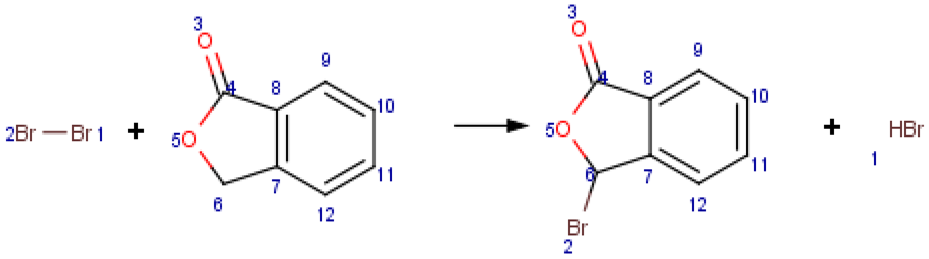

Correct mapped SMILES/SMARTS of the reaction:
[Br:1][Br:2].[O:3]=[#6:4]-1-[#8:5]-[#6:6]-[#6:7]-2=[#6:8]-1-[#6:9]=[#6:10]-
[#6:11]=[#6:12]-2>>[Br:2][#6:6]-1-[#8:5]-[#6:4](=[O:3])-[#6:8]-2=[#6:9]-
[#6:10]=[#6:11]-[#6:12]=[#6:7]-1-2.[Br:1]

Correctness of the mapping
MAPPET YES
ReactionMap YES
Marvin YES

Reaction no 24

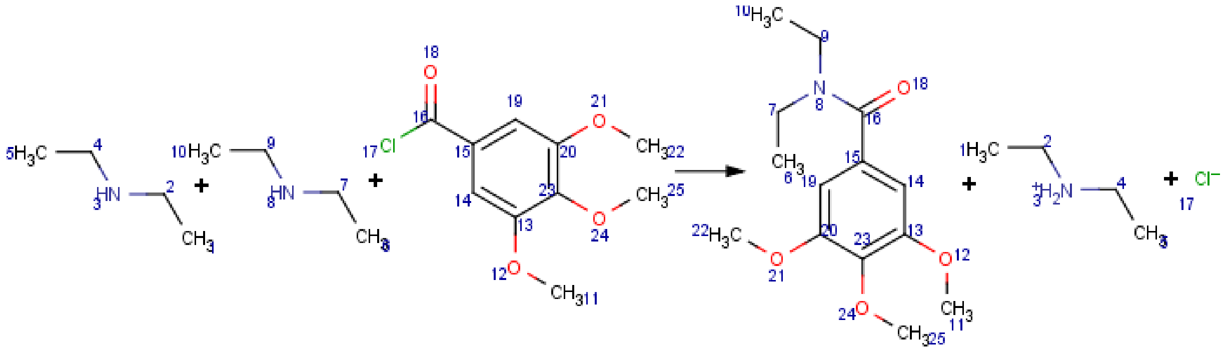

Correct mapped SMILES/SMARTS of the reaction:
[#6:1]-[#6:2]-[#7:3]-[#6:4]-[#6:5].[#6:6]-[#6:7]-[#7:8]-[#6:9]-[#6:10].[#6:11]-
[#8:12]-[#6:13]-1=[#6:14]-[#6:15](=[#6:19]-[#6:20](-[#8:21]-[#6:22])=[#6:23]-1-
[#8:24]-[#6:25])-[#6:16]([Cl:17])=[O:18]>>[#6:10]-[#6:9]-[#7:8](-[#6:7]-[#6:6])-
[#6:16](=[O:18])-[#6:15]-1=[#6:14]-[#6:13](-[#8:12]-[#6:11])=[#6:23](-[#8:24]-
[#6:25])-[#6:20](-[#8:21]-[#6:22])=[#6:19]-1.[#6:5]-[#6:4]-[#7+:3]-[#6:2]-
[#6:1].[Cl-:17]

Correctness of the mapping
MAPPET YES
ReactionMap YES
Marvin YES

Reaction no 25

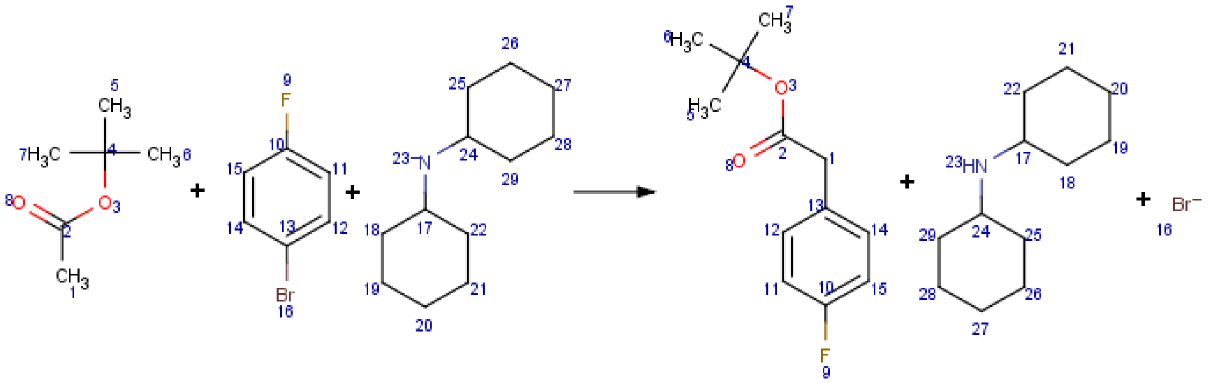

Correct mapped SMILES/SMARTS of the reaction:
[#6:1]-[#6:2](=[O:8])-[#8:3][C:4]([#6:5])([#6:6])[#6:7].[F:9][#6:10]-1=[#6:11]-
[#6:12]=[#6:13]([Br:16])-[#6:14]=[#6:15]-1.[#6:20]-1-[#6:19]-[#6:18]-[#6:17](-
[#6:22]-[#6:21]-1)-[#7-:23]-[#6:24]-1-[#6:25]-[#6:26]-[#6:27]-[#6:28]-[#6:29]-
1>>[#6:6][C:4]([#6:5])([#6:7])[#8:3]-[#6:2](=[O:8])-[#6:1]-[#6:13]-1=[#6:14]-
[#6:15]=[#6:10]([F:9])-[#6:11]=[#6:12]-1.[#6:27]-1-[#6:28]-[#6:29]-[#6:24](-
[#6:25]-[#6:26]-1)-[#7:23]-[#6:17]-1-[#6:22]-[#6:21]-[#6:20]-[#6:19]-[#6:18]-
1.[Br-:16]

Correctness of the mapping
MAPPET YES
ReactionMap YES
Marvin YES

Reaction no 26

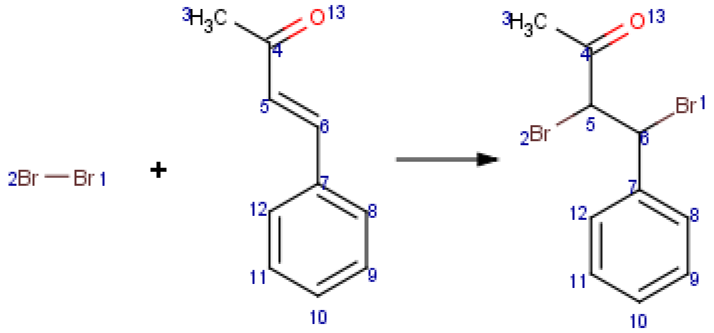

Correct mapped SMILES/SMARTS of the reaction:
[Br:1][Br:2].[#6:3]-[#6:4](=[O:13])\[#6:5]=[#6:6]\[#6:7]-1=[#6:8]-[#6:9]=[#6:10]-[#6:11]=[#6:12]1>>[#6:3]-[#6:4](=[O:13])-[#6:5]([Br:2])-[#6:6]([Br:1])-[#6:7]-1=[#6:8]-[#6:9]=[#6:10]-[#6:11]=[#6:12]-1

Correctness of the mapping
MAPPET YES
ReactionMap YES
Marvin YES

Reaction no 27

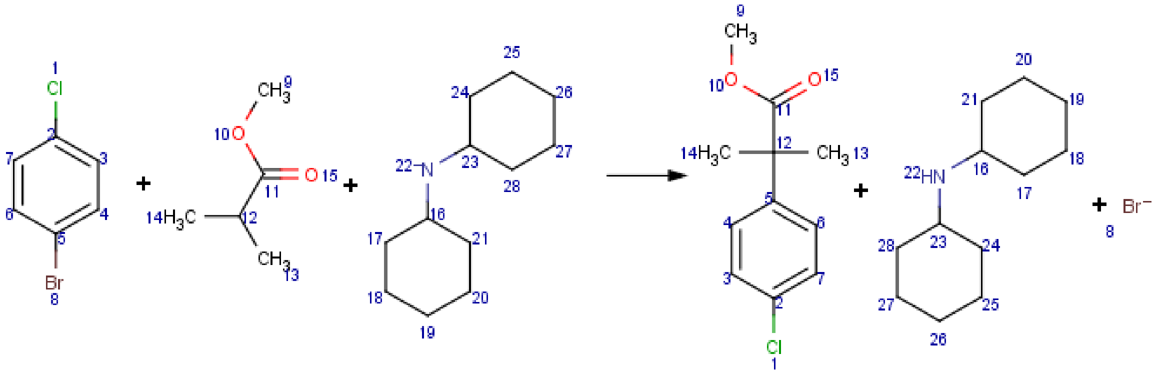

Correct mapped SMILES/SMARTS of the reaction:
[Cl:1][#6:2]-1=[#6:3]-[#6:4]=[#6:5]([Br:8])-[#6:6]=[#6:7]-1.[#6:9]-[#8:10]-[#6:11](=[O:15])-[#6:12](-[#6:13])-[#6:14].[#6:19]-1-[#6:18]-[#6:17]-[#6:16](-[#6:21]-[#6:20]-1)-[#7-:22]-[#6:23]-1-[#6:24]-[#6:25]-[#6:26]-[#6:27]-[#6:28]-1>>[#6:9]-[#8:10]-[#6:11](=[O:15])[C:12]([#6:14])([#6:13])[#6:5]-1=[#6:6]-[#6:7]=[#6:2]([Cl:1])-[#6:3]=[#6:4]-1.[#6:26]-1-[#6:27]-[#6:28]-[#6:23](-[#6:24]-[#6:25]-1)-[#7:22]-[#6:16]-1-[#6:21]-[#6:20]-[#6:19]-[#6:18]-[#6:17]-1.[Br-:8]

Correctness of the mapping
MAPPET YES
ReactionMap YES
Marvin YES

Reaction no 28

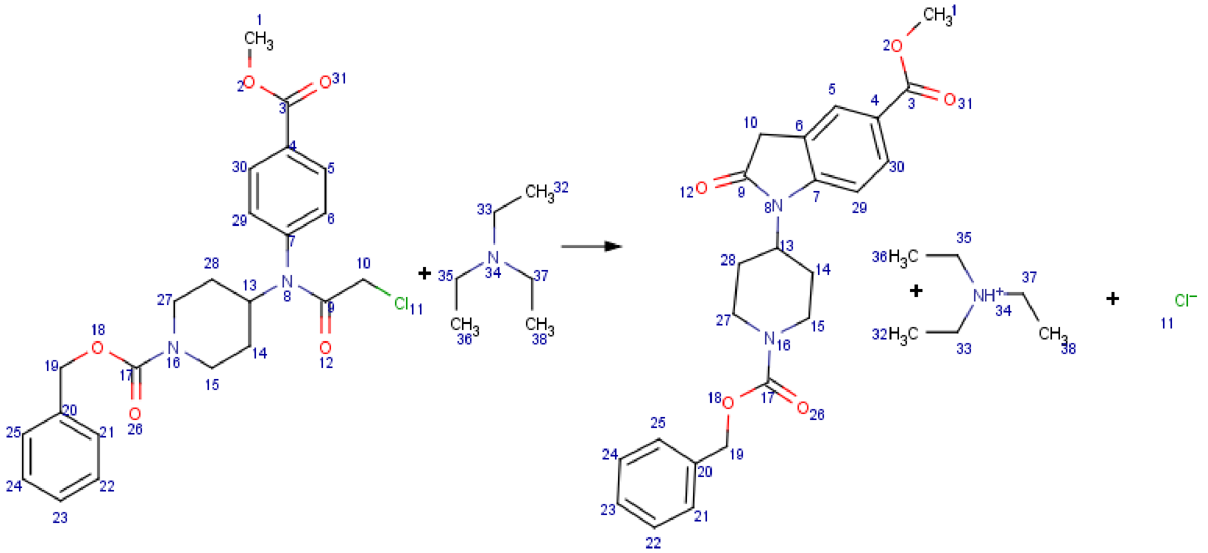

Correct mapped SMILES/SMARTS of the reaction:
[#6:1]-[#8:2]-[#6:3](=[O:31])-[#6:4]-1=[#6:5]-[#6:6]=[#6:7](-[#6:29]=[#6:30]-1)-[#7:8](-[#6:13]-1-[#6:14]-[#6:15]-[#7:16](-[#6:27]-[#6:28]-1)-[#6:17](=[O:26])-[#8:18]-[#6:19]-[#6:20]-1=[#6:21]-[#6:22]=[#6:23]-[#6:24]=[#6:25]-1)-[#6:9](=[O:12])-[#6:10][Cl:11].[#6:32]-[#6:33]-[#7:34](-[#6:35]-[#6:36])-[#6:37]-[#6:38]>>[#6:1]-[#8:2]-[#6:3](=[O:31])-[#6:4]-1=[#6:30]-[#6:29]=[#6:7]-2-[#7:8](-[#6:13]-3-[#6:14]-[#6:15]-[#7:16](-[#6:27]-[#6:28]-3)-[#6:17](=[O:26])-[#8:18]-[#6:19]-[#6:20]-3=[#6:21]-[#6:22]=[#6:23]-[#6:24]=[#6:25]-3)-[#6:9](=[O:12])-[#6:10]-[#6:6]-2=[#6:5]-1.[#6:38]-[#6:37]-[#7+:34](-[#6:35]-[#6:36])-[#6:33]-[#6:32].[Cl-:11]

Correctness of the mapping
MAPPET YES
ReactionMap YES
Marvin YES

Reaction no 29

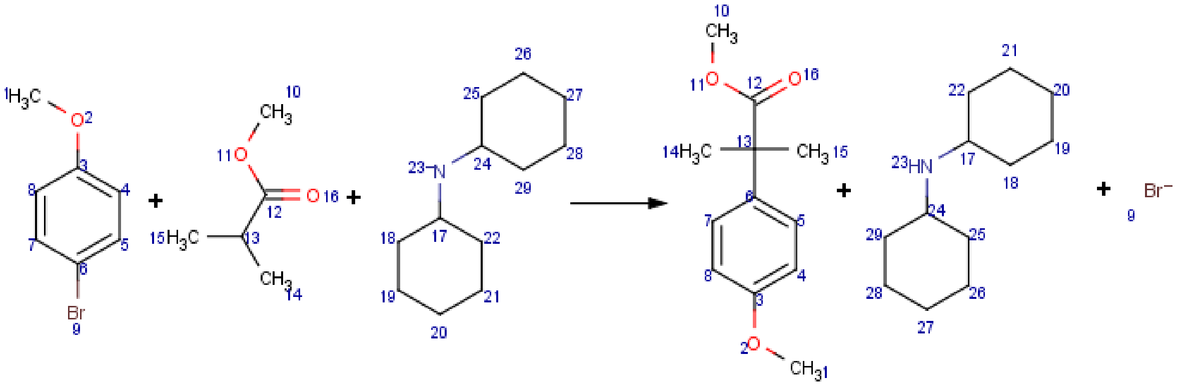

Correct mapped SMILES/SMARTS of the reaction:
[#6:1]-[#8:2]-[#6:3]-1=[#6:4]-[#6:5]=[#6:6]([Br:9])-[#6:7]=[#6:8]-1.[#6:10]-[#8:11]-[#6:12](=[O:16])-[#6:13](-[#6:14])-[#6:15].[#6:20]-1-[#6:19]-[#6:18]-[#6:17](-[#6:22]-[#6:21]-1)-[#7-:23]-[#6:24]-1-[#6:25]-[#6:26]-[#6:27]-[#6:28]-[#6:29]-1>>[#6:10]-[#8:11]-[#6:12](=[O:16])[C:13]([#6:14])([#6:15])[#6:6]-1=[#6:5]-[#6:4]=[#6:3](-[#8:2]-[#6:1])-[#6:8]=[#6:7]-1.[#6:27]-1-[#6:28]-[#6:29]-[#6:24](-[#6:25]-[#6:26]-1)-[#7:23]-[#6:17]-1-[#6:22]-[#6:21]-[#6:20]-[#6:19]-[#6:18]-1.[Br-:9]

Correctness of the mapping
MAPPET YES
ReactionMap YES
Marvin YES

Reaction no 30

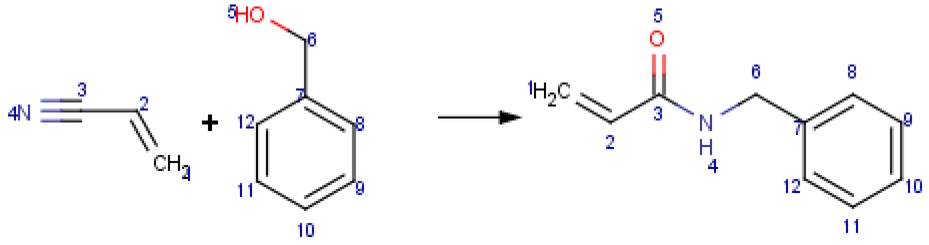

Correct mapped SMILES/SMARTS of the reaction:
[CH2:1]=[CH:2][C:3]#[N:4].[OH:5][CH2:6][C:7]1=[CH:8][CH:9]=[CH:10][CH:11]=[CH:12]1>>[CH2:1]=[CH:2][C:3](=[O:5])[NH:4][CH2:6][C:7]1=[CH:8][CH:9]=[CH:10][CH:11]=[CH:12]1

Correctness of the mapping
MAPPET YES
ReactionMap YES
Marvin YES

Reaction no 31

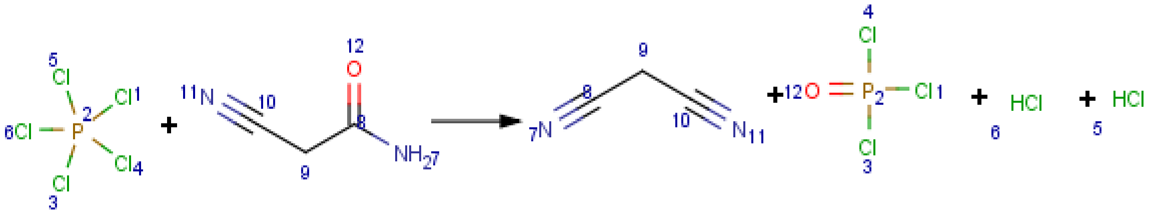

Correct mapped SMILES/SMARTS of the reaction:
[Cl:1][P:2]([Cl:3])([Cl:4])([Cl:5])[Cl:6].[NH2:7][C:8](=[O:12])[CH2:9][C:10]#[N:11]>>[N:11]#[C:10][CH2:9][C:8]#[N:7].[Cl:3][P:2]([Cl:4])([Cl:1])=[O:12].[ClH:6].[ClH:5]

Correctness of the mapping
MAPPET YES
ReactionMap YES
Marvin YES

Reaction no 32

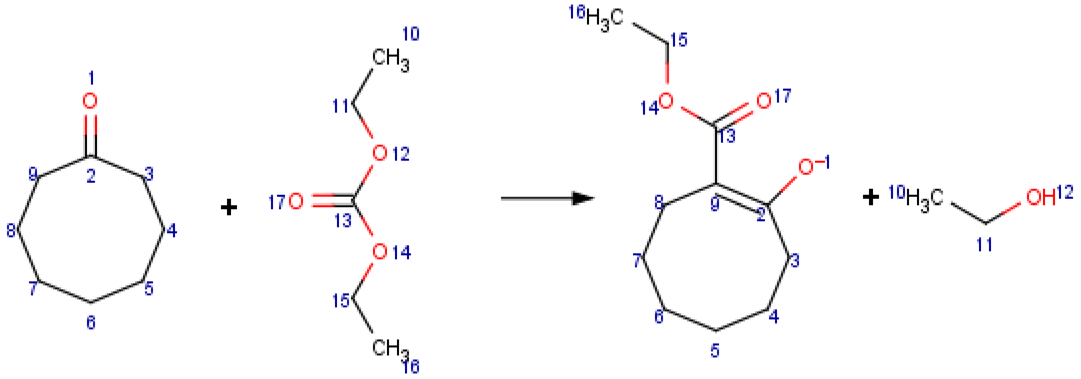

Correct mapped SMILES/SMARTS of the reaction:
[O:1]=[#6:2]-1-[#6:3]-[#6:4]-[#6:5]-[#6:6]-[#6:7]-[#6:8]-[#6:9]-1.[#6:10]-[#6:11]-[#8:12]-[#6:13](=[O:17])-[#8:14]-[#6:15]-[#6:16]>>[#6:16]-[#6:15]-[#8:14]-[#6:13](=[O:17])-[#6:9]-1=[#6:2](-[#8-:1])/[#6:3]-[#6:4]-[#6:5]-[#6:6]-[#6:7]-[#6:8]\1.[#6:10]-[#6:11]-[#8:12]

Correctness of the mapping
MAPPET YES
ReactionMap YES
Marvin NO

Reaction no 33

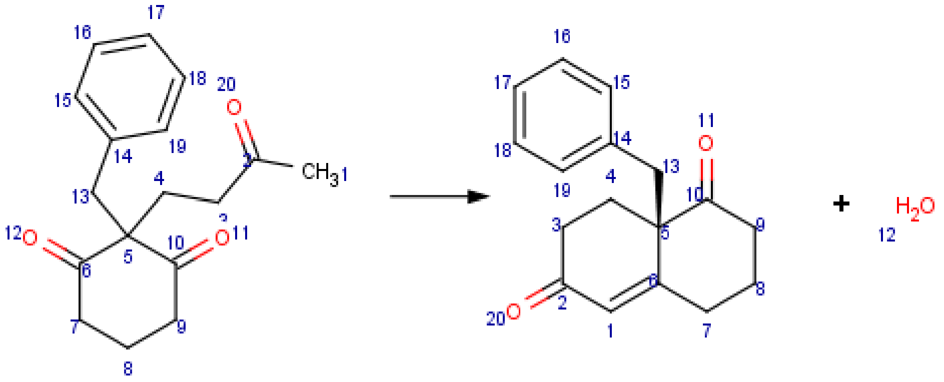

Correct mapped SMILES/SMARTS of the reaction:
[#6:1]-[#6:2](=[O:20])-[#6:3]-[#6:4][C:5]1([#6:13]-[#6:14]-2=[#6:15]-[#6:16]=[#6:17]-[#6:18]=[#6:19]-2)[#6:10](=[O:11])-[#6:9]-[#6:8]-[#6:7]-[#6:6]1=[O:12]>>[O:20]=[#6:2]-1-[#6:3]-[#6:4][C@:5]2([#6:13]-[#6:14]-3=[#6:19]-[#6:18]=[#6:17]-[#6:16]=[#6:15]-3)[#6:10](=[O:11])-[#6:9]-[#6:8]-[#6:7]-[#6:6]2=[#6:1]-1.[#8:12]

Correctness of the mapping
MAPPET YES
ReactionMap YES
Marvin YES

Reaction no 34

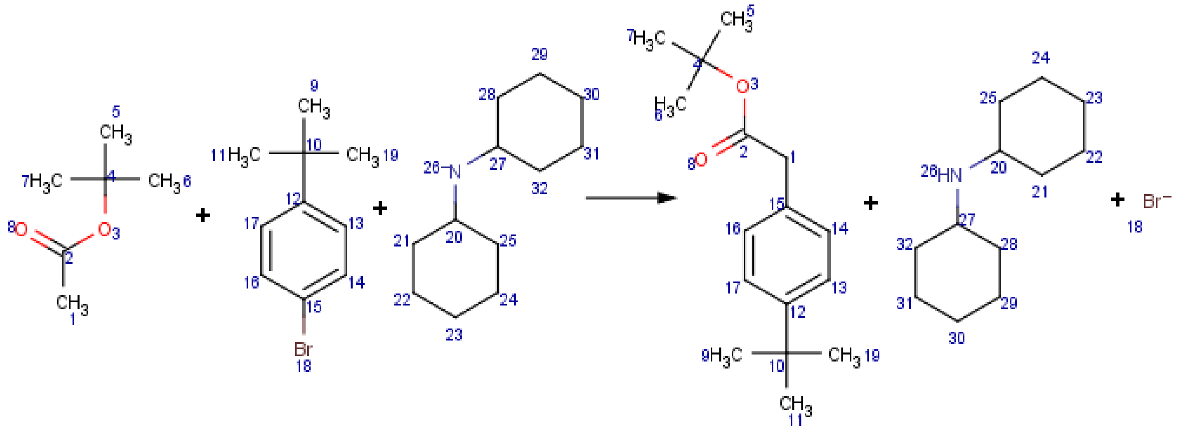

Correct mapped SMILES/SMARTS of the reaction:
[#6:1]-[#6:2](=[O:8])-
[#8:3][C:4]([#6:5])([#6:6])[#6:7].[#6:9][C:10]([#6:11])([#6:19])[#6:12]-
1=[#6:13]-[#6:14]=[#6:15]([Br:18])-[#6:16]=[#6:17]-1.[#6:23]-1-[#6:22]-[#6:21]-
[#6:20](-[#6:25]-[#6:24]-1)-[#7-:26]-[#6:27]-1-[#6:28]-[#6:29]-[#6:30]-[#6:31]-
[#6:32]-1>>[#6:7][C:4]([#6:6])([#6:5])[#8:3]-[#6:2](=[O:8])-[#6:1]-[#6:15]-
1=[#6:14]-[#6:13]=[#6:12](-[#6:17]=[#6:16]-
1)[C:10]([#6:11])([#6:9])[#6:19].[#6:30]-1-[#6:31]-[#6:32]-[#6:27](-[#6:28]-
[#6:29]-1)-[#7:26]-[#6:20]-1-[#6:25]-[#6:24]-[#6:23]-[#6:22]-[#6:21]-1.[Br-:18]

Correctness of the mapping
MAPPET YES
ReactionMap YES
Marvin YES

Reaction no 35

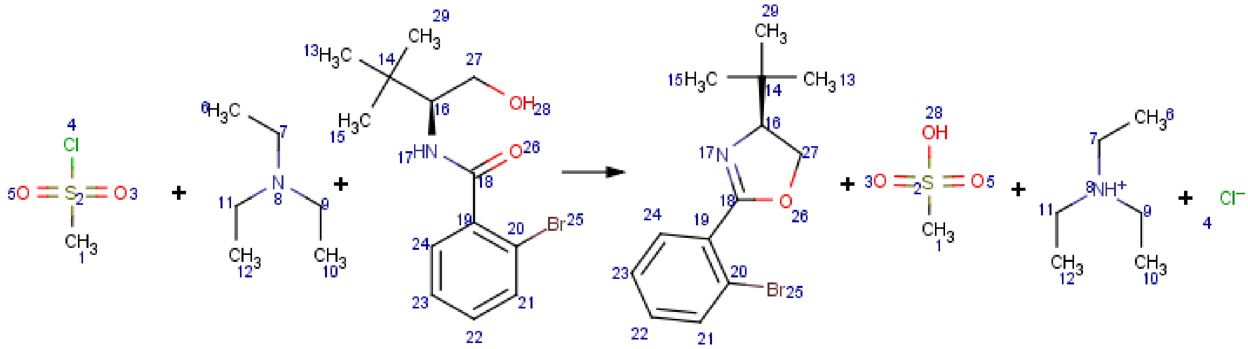

Correct mapped SMILES/SMARTS of the reaction:
[CH3:1][S:2]([Cl:4])(=[O:3])=[O:5].[CH3:6][CH2:7][N:8]([CH2:9][CH3:10])[CH2:11][
CH3:12].[CH3:13][C:14]([CH3:15])([CH3:29])[C@@H:16]([CH2:27][OH:28])[NH:17][C:18
](=[O:26])[C:19]1=[C:20]([Br:25])[CH:21]=[CH:22][CH:23]=[CH:24]1>>[CH3:29][C:14]
([CH3:15])([CH3:13])[C@H:16]1[CH2:27][O:26][C:18](=[N:17]1)[C:19]1=[C:20]([Br:25
])[CH:21]=[CH:22][CH:23]=[CH:24]1.[CH3:1][S:2]([OH:28])(=[O:5])=[O:3].[CH3:6][CH
2:7][NH+:8]([CH2:11][CH3:12])[CH2:9][CH3:10].[Cl-:4]

Correctness of the mapping
MAPPET YES
ReactionMap NO
Marvin NO

Reaction no 36

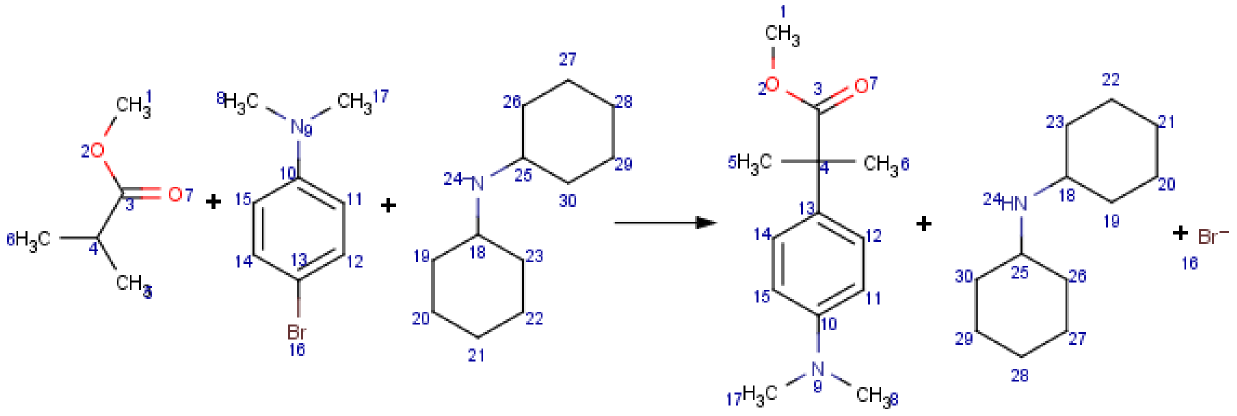

Correct mapped SMILES/SMARTS of the reaction:
[#6:1]-[#8:2]-[#6:3](=[O:7])-[#6:4](-[#6:5])-[#6:6].[#6:8]-[#7:9](-[#6:17])-[#6:10]-1=[#6:11]-[#6:12]=[#6:13]([Br:16])-[#6:14]=[#6:15]-1.[#6:21]-1-[#6:20]-[#6:19]-[#6:18](-[#6:23]-[#6:22]-1)-[#7-:24]-[#6:25]-1-[#6:26]-[#6:27]-[#6:28]-[#6:29]-[#6:30]-1>>[#6:1]-[#8:2]-[#6:3](=[O:7])[C:4]([#6:5])([#6:6])[#6:13]-1=[#6:12]-[#6:11]=[#6:10](-[#6:15]=[#6:14]-1)-[#7:9](-[#6:8])-[#6:17].[#6:28]-1-[#6:29]-[#6:30]-[#6:25](-[#6:26]-[#6:27]-1)-[#7:24]-[#6:18]-1-[#6:23]-[#6:22]-[#6:21]-[#6:20]-[#6:19]-1.[Br-:16]

Correctness of the mapping
MAPPET YES
ReactionMap YES
Marvin YES

Reaction no 37

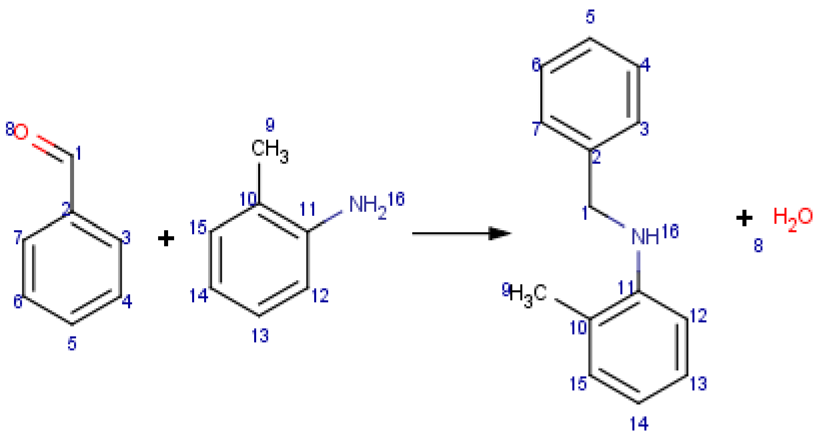

Correct mapped SMILES/SMARTS of the reaction:

[O:8]=[CH:1][C:2]1=[CH:3][CH:4]=[CH:5][CH:6]=[CH:7]1.[CH3:9][C:10]1=[C:11]([NH2:16])[CH:12]=[CH:13][CH:14]=[CH:15]1>>[CH3:9][C:10]1=[C:11]([NH:16][CH2:1][C:2]2=[CH:7][CH:6]=[CH:5][CH:4]=[CH:3]2)[CH:12]=[CH:13][CH:14]=[CH:15]1.[OH2:8]

Correctness of the mapping
MAPPET YES
ReactionMap NO
Marvin YES

Reaction no 38

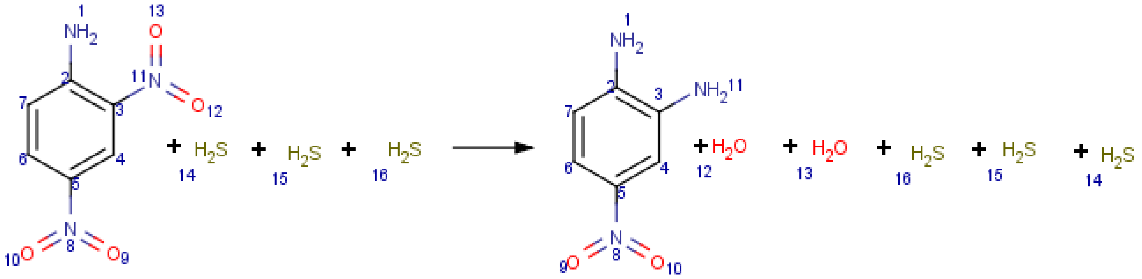

Correct mapped SMILES/SMARTS of the reaction:
[#7:1]-[#6:2]-1=[#6:3](-[#6:4]=[#6:5](-[#6:6]=[#6:7]-
1)[N:8](=[O:9])=[O:10])[N:11](=[O:12])=[O:13].[#16:14].[#16:15].[#16:16]>>[#7:1]
-[#6:2]-1=[#6:3](-[#7:11])-[#6:4]=[#6:5](-[#6:6]=[#6:7]-
1)[N:8](=[O:10])=[O:9].[#8:12].[#8:13].[#16:16].[#16:15].[#16:14]

Correctness of the mapping
MAPPET YES
ReactionMap YES
Marvin YES

Reaction no 39

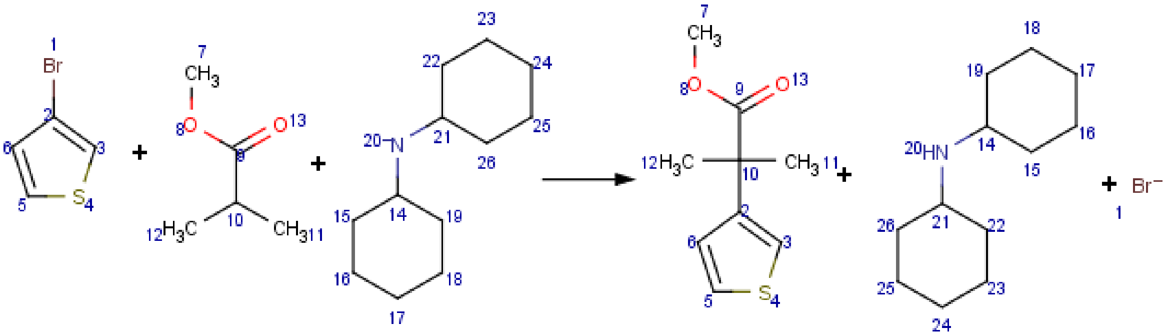

Correct mapped SMILES/SMARTS of the reaction:

[Br:1][#6:2]-1=[#6:3]-[#16:4]-[#6:5]=[#6:6]-1.[#6:7]-[#8:8]-[#6:9](=[O:13])-
[#6:10](-[#6:11])-[#6:12].[#6:17]-1-[#6:16]-[#6:15]-[#6:14](-[#6:19]-[#6:18]-1)-
[#7-:20]-[#6:21]-1-[#6:22]-[#6:23]-[#6:24]-[#6:25]-[#6:26]-1>>[#6:7]-[#8:8]-
[#6:9](=[O:13])[C:10]([#6:12])([#6:11])[#6:2]-1=[#6:3]-[#16:4]-[#6:5]=[#6:6]-
1.[#6:24]-1-[#6:25]-[#6:26]-[#6:21](-[#6:22]-[#6:23]-1)-[#7:20]-[#6:14]-1-
[#6:19]-[#6:18]-[#6:17]-[#6:16]-[#6:15]-1.[Br-:1]

Correctness of the mapping
MAPPET YES
ReactionMap YES
Marvin YES

Reaction no 40

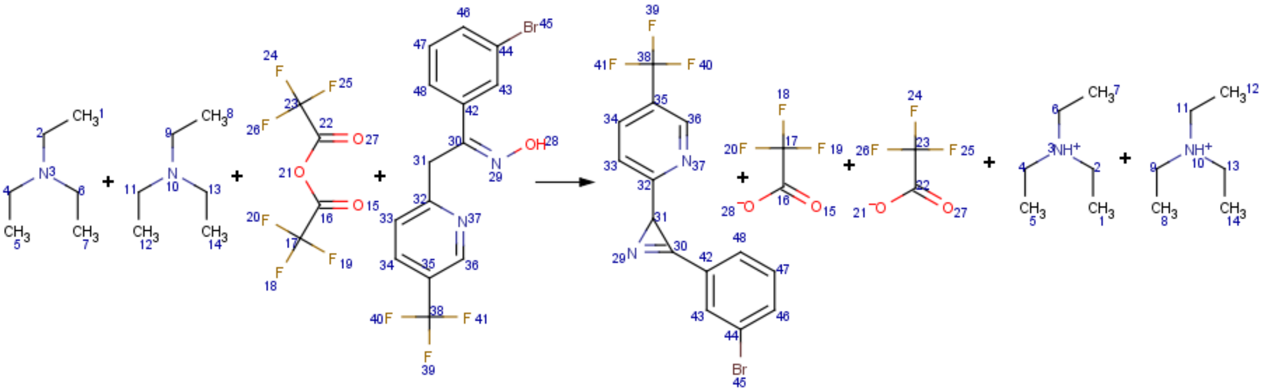

Correct mapped SMILES/SMARTS of the reaction:
[#6:1]-[#6:2]-[#7:3](-[#6:4]-[#6:5])-[#6:6]-[#6:7].[#6:8]-[#6:9]-[#7:10](-
[#6:11]-[#6:12])-[#6:13]-[#6:14].[F:18][C:17]([F:19])([F:20])[#6:16](=[O:15])-
[#8:21]-
[#6:22](=[O:27])[C:23]([F:24])([F:25])[F:26].[#8:28]\[#7:29]=[#6:30](\[#6:31]-
[#6:32]-1=[#6:33]-[#6:34]=[#6:35](-[#6:36]=[#7:37]-
1)[C:38]([F:39])([F:40])[F:41])-[#6:42]-1=[#6:43]-[#6:44]([Br:45])=[#6:46]-
[#6:47]=[#6:48]-1>>[F:39][C:38]([F:40])([F:41])[#6:35]-1=[#6:34]-
[#6:33]=[#6:32](-[#7:37]=[#6:36]-1)-[#6:31]-1-[#7:29]=[#6:30]-1-[#6:42]-
1=[#6:43]-[#6:44]([Br:45])=[#6:46]-[#6:47]=[#6:48]-1.[#8-:28]-
[#6:16](=[O:15])[C:17]([F:18])([F:19])[F:20].[#8-:21]-
[#6:22](=[O:27])[C:23]([F:24])([F:25])[F:26].[#6:7]-[#6:6]-[#7+:3](-[#6:4]-
[#6:5])-[#6:2]-[#6:1].[#6:12]-[#6:11]-[#7+:10](-[#6:9]-[#6:8])-[#6:13]-[#6:14]

Correctness of the mapping
MAPPET YES
ReactionMap YES
Marvin YES

Reaction no 41

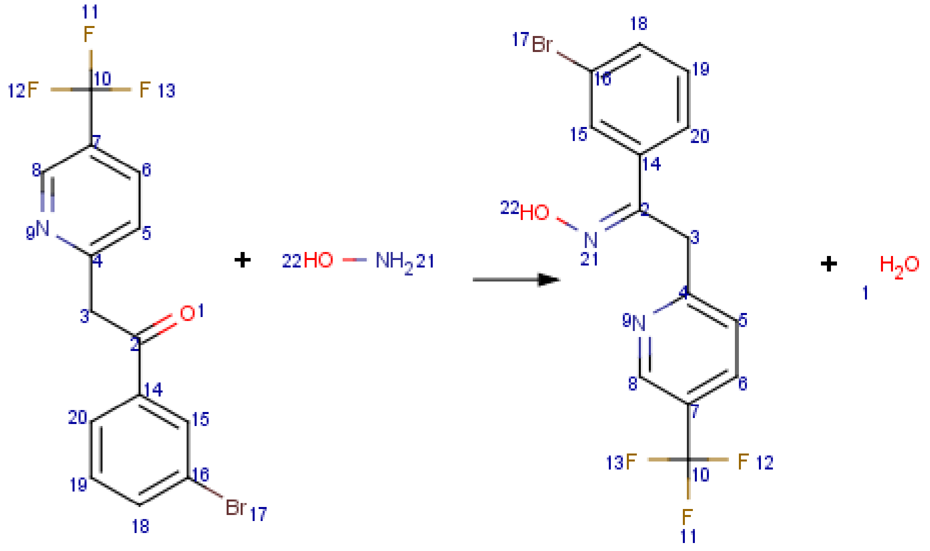

Correct mapped SMILES/SMARTS of the reaction:
[F:11][C:10]([F:12])([F:13])[#6:7]-1=[#6:6]-[#6:5]=[#6:4](-[#6:3]-
[#6:2](=[O:1])-[#6:14]-2=[#6:15]-[#6:16]([Br:17])=[#6:18]-[#6:19]=[#6:20]-2)-
[#7:9]=[#6:8]-1.[#7:21]-[#8:22]>>[#8:22]\[#7:21]=[#6:2](\[#6:3]-[#6:4]-1=[#6:5]-
[#6:6]=[#6:7](-[#6:8]=[#7:9]-1)[C:10]([F:11])([F:12])[F:13])-[#6:14]-1=[#6:15]-
[#6:16]([Br:17])=[#6:18]-[#6:19]=[#6:20]-1.[#8:1]

Correctness of the mapping
MAPPET YES
ReactionMap YES
Marvin YES

Reaction no 42

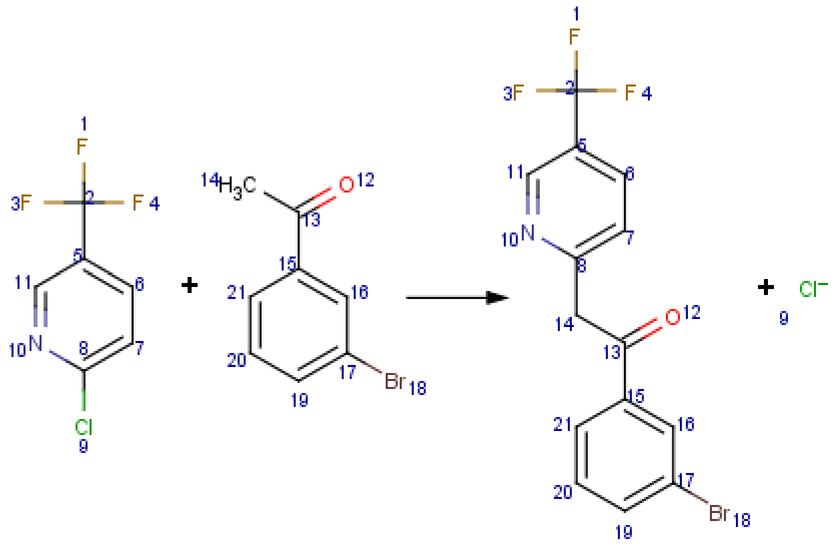

Correct mapped SMILES/SMARTS of the reaction:
[F:1][C:2]([F:3])([F:4])[#6:5]-1=[#6:6]-[#6:7]=[#6:8]([Cl:9])-[#7:10]=[#6:11]-1.[#6:14]-[#6:13](=[O:12])-[#6:15]-1=[#6:16]-[#6:17]([Br:18])=[#6:19]-[#6:20]=[#6:21]-1>>[F:1][C:2]([F:3])([F:4])[#6:5]-1=[#6:6]-[#6:7]=[#6:8](-[#6:14]-[#6:13](=[O:12])-[#6:15]-2=[#6:16]-[#6:17]([Br:18])=[#6:19]-[#6:20]=[#6:21]-2)-[#7:10]=[#6:11]-1.[Cl-:9]

Correctness of the mapping
MAPPET YES
ReactionMap YES
Marvin YES

Reaction no 43

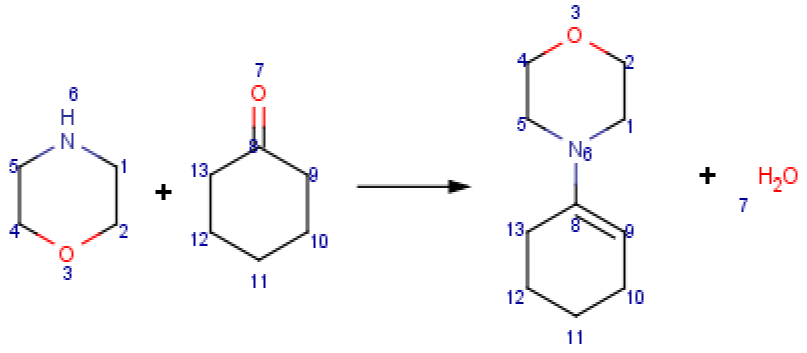

Correct mapped SMILES/SMARTS of the reaction:
[#6:1]-1-[#6:2]-[#8:3]-[#6:4]-[#6:5]-[#7:6]-1.[O:7]=[#6:8]-1-[#6:9]-[#6:10]-[#6:11]-[#6:12]-[#6:13]-1>>[#6:11]-1-[#6:12]-[#6:13]-[#6:8](=[#6:9]-[#6:10]-1)-[#7:6]-1-[#6:5]-[#6:4]-[#8:3]-[#6:2]-[#6:1]-1.[#8:7]

Correctness of the mapping
MAPPET YES
ReactionMap YES
Marvin YES

Reaction no 44

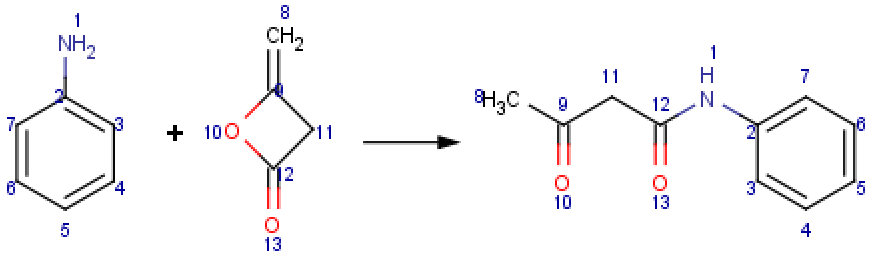

Correct mapped SMILES/SMARTS of the reaction:
[#7:1]-[#6:2]-1=[#6:3]-[#6:4]=[#6:5]-[#6:6]=[#6:7]-1.[#6:8]=[#6:9]-1-[#6:11]-[#6:12](=[O:13])-[#8:10]-1>>[#6:8]-[#6:9](=[O:10])-[#6:11]-[#6:12](=[O:13])-[#7:1]-[#6:2]-1=[#6:7]-[#6:6]=[#6:5]-[#6:4]=[#6:3]-1

Correctness of the mapping
MAPPET YES
ReactionMap YES
Marvin NO

Reaction no 45

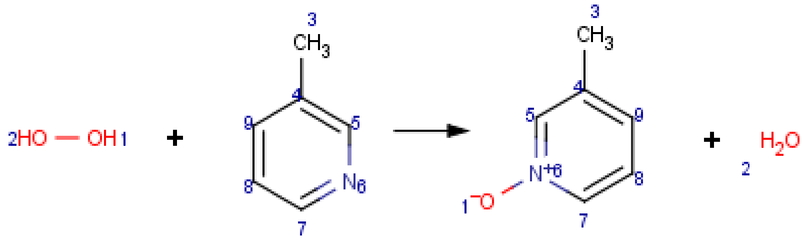

Correct mapped SMILES/SMARTS of the reaction:
[#8:1]-[#8:2].[#6:3]-[#6:4]-1=[#6:5]-[#7:6]=[#6:7]-[#6:8]=[#6:9]-1>>[#6:3]-[#6:4]-1=[#6:9]-[#6:8]=[#6:7]-[#7+:6](-[#8-:1])=[#6:5]-1.[#8:2]

Correctness of the mapping
MAPPET YES
ReactionMap YES
Marvin YES

Reaction no 46

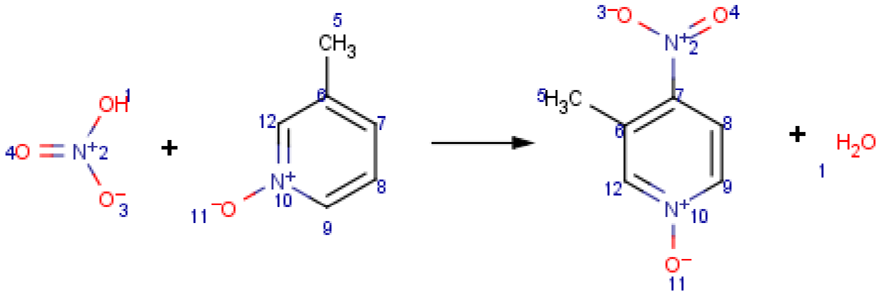

Correct mapped SMILES/SMARTS of the reaction:
[#8:1]-[#7+:2](-[#8-:3])=[O:4].[#6:5]-[#6:6]-1=[#6:7]-[#6:8]=[#6:9]-[#7+:10](-[#8-:11])=[#6:12]-1>>[#6:5]-[#6:6]-1=[#6:7](-[#6:8]=[#6:9]-[#7+:10](-[#8-:11])=[#6:12]-1)-[#7+:2](-[#8-:3])=[O:4].[#8:1]

Correctness of the mapping
MAPPET YES
ReactionMap YES
Marvin YES

Reaction no 47

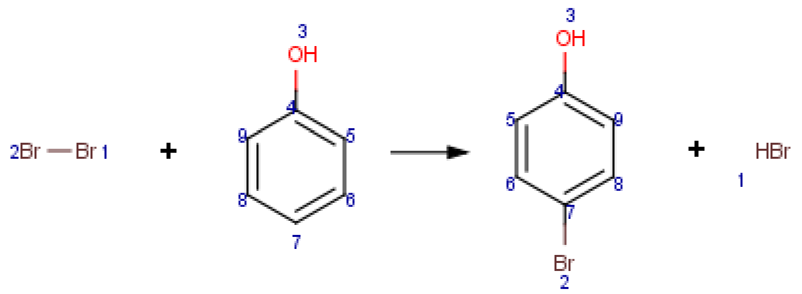

Correct mapped SMILES/SMARTS of the reaction:
[Br:1][Br:2].[#8:3]-[#6:4]-1=[#6:5]-[#6:6]=[#6:7]-[#6:8]=[#6:9]-1>>[#8:3]-[#6:4]-1=[#6:9]-[#6:8]=[#6:7]([Br:2])-[#6:6]=[#6:5]-1.[Br:1]
Correctness of the mapping
MAPPET YES
ReactionMap YES
Marvin YES

Reaction no 48

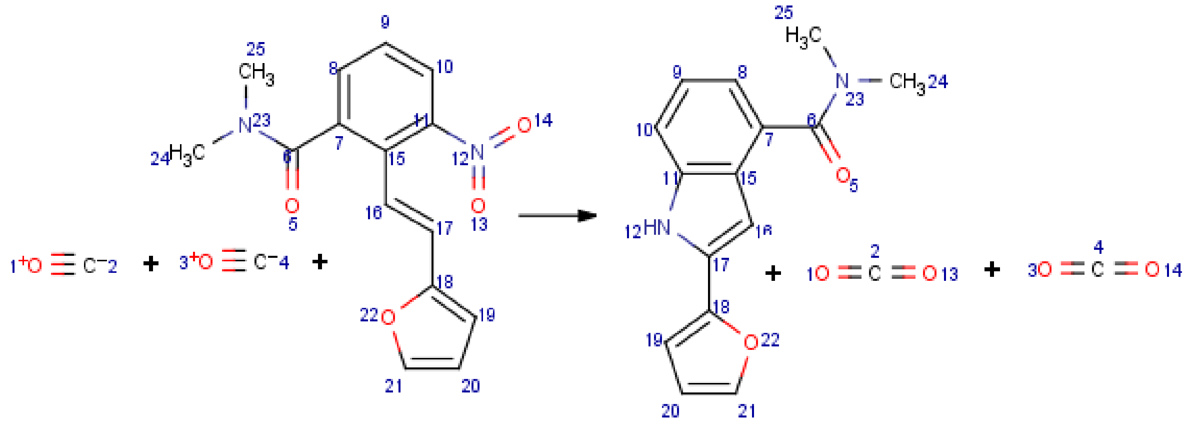

Correct mapped SMILES/SMARTS of the reaction:
[C-:2]#[O+:1].[C-:4]#[O+:3].[#6:24]-[#7:23](-[#6:25])-[#6:6](=[O:5])-[#6:7]-1=[#6:8]-[#6:9]=[#6:10]-[#6:11](=[#6:15]-1\[#6:16]=[#6:17]\[#6:18]-1=[#6:19]-[#6:20]=[#6:21]-[#8:22]1)[N:12](=[O:13])=[O:14]>>[#6:24]-[#7:23](-[#6:25])-[#6:6](=[O:5])-[#6:7]-1=[#6:8]-[#6:9]=[#6:10]-[#6:11]-2=[#6:15]-1-[#6:16]=[#6:17](-[#7:12]-2)-[#6:18]-1=[#6:19]-[#6:20]=[#6:21]-[#8:22]-1.[O:1]=[C:2]=[O:13].[O:3]=[C:4]=[O:14]

Correctness of the mapping
MAPPET YES
ReactionMap YES
Marvin YES

Reaction no 49

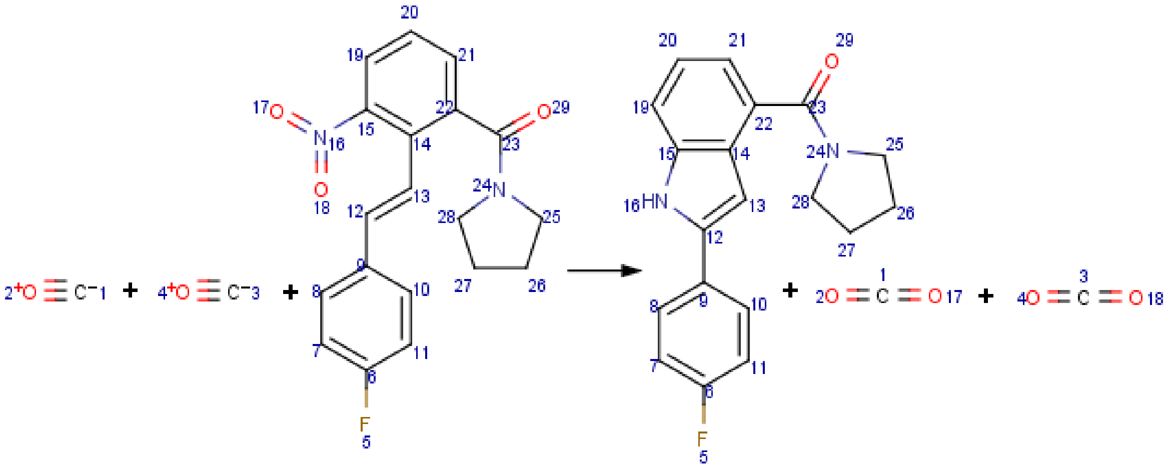

Correct mapped SMILES/SMARTS of the reaction:
[C-:1]#[O+:2].[C-:3]#[O+:4].[F:5][#6:6]-1=[#6:7]-
[#6:8]=[#6:9](\[#6:12]=[#6:13]\[#6:14]-2=[#6:15](-[#6:19]=[#6:20]-
[#6:21]=[#6:22]2-[#6:23](=[O:29])-[#7:24]-2-[#6:25]-[#6:26]-[#6:27]-[#6:28]-
2)[N:16](=[O:17])=[O:18])-[#6:10]=[#6:11]-1>>[F:5][#6:6]-1=[#6:7]-
[#6:8]=[#6:9](-[#6:10]=[#6:11]-1)-[#6:12]-1=[#6:13]-[#6:14]-2=[#6:15](-[#7:16]-
1)-[#6:19]=[#6:20]-[#6:21]=[#6:22]-2-[#6:23](=[O:29])-[#7:24]-1-[#6:25]-[#6:26]-
[#6:27]-[#6:28]-1.[O:2]=[C:1]=[O:17].[O:4]=[C:3]=[O:18]

Correctness of the mapping
MAPPET YES
ReactionMap YES
Marvin YES

Reaction no 50

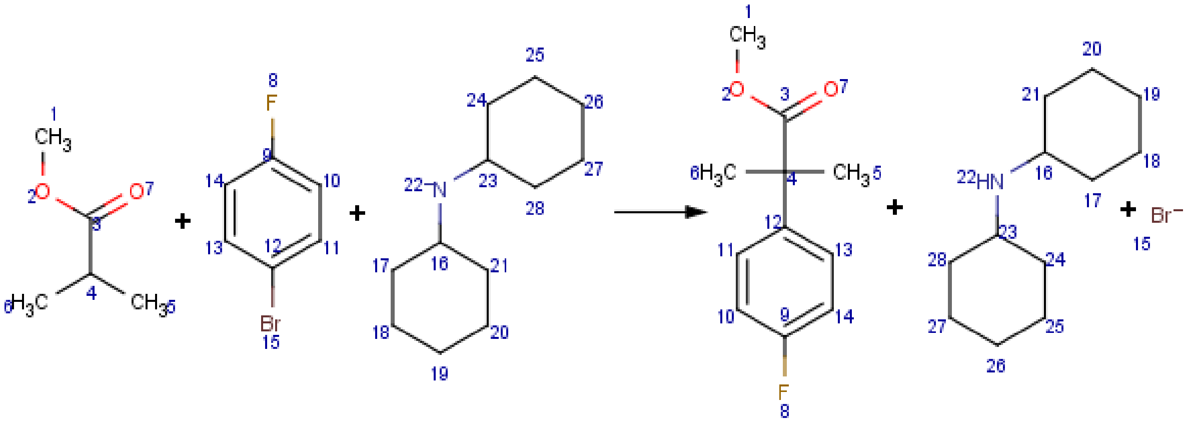

Correct mapped SMILES/SMARTS of the reaction:
[#6:1]-[#8:2]-[#6:3](=[O:7])-[#6:4](-[#6:5])-[#6:6].[F:8][#6:9]-1=[#6:10]-
[#6:11]=[#6:12]([Br:15])-[#6:13]=[#6:14]-1.[#6:19]-1-[#6:18]-[#6:17]-[#6:16](-
[#6:21]-[#6:20]-1)-[#7-:22]-[#6:23]-1-[#6:24]-[#6:25]-[#6:26]-[#6:27]-[#6:28]-
1>>[#6:1]-[#8:2]-[#6:3](=[O:7])[C:4]([#6:6])([#6:5])[#6:12]-1=[#6:13]-
[#6:14]=[#6:9]([F:8])-[#6:10]=[#6:11]-1.[#6:26]-1-[#6:27]-[#6:28]-[#6:23](-
[#6:24]-[#6:25]-1)-[#7:22]-[#6:16]-1-[#6:21]-[#6:20]-[#6:19]-[#6:18]-[#6:17]-
1.[Br-:15]

Correctness of the mapping
MAPPET YES
ReactionMap YES
Marvin YES

Reaction no 51

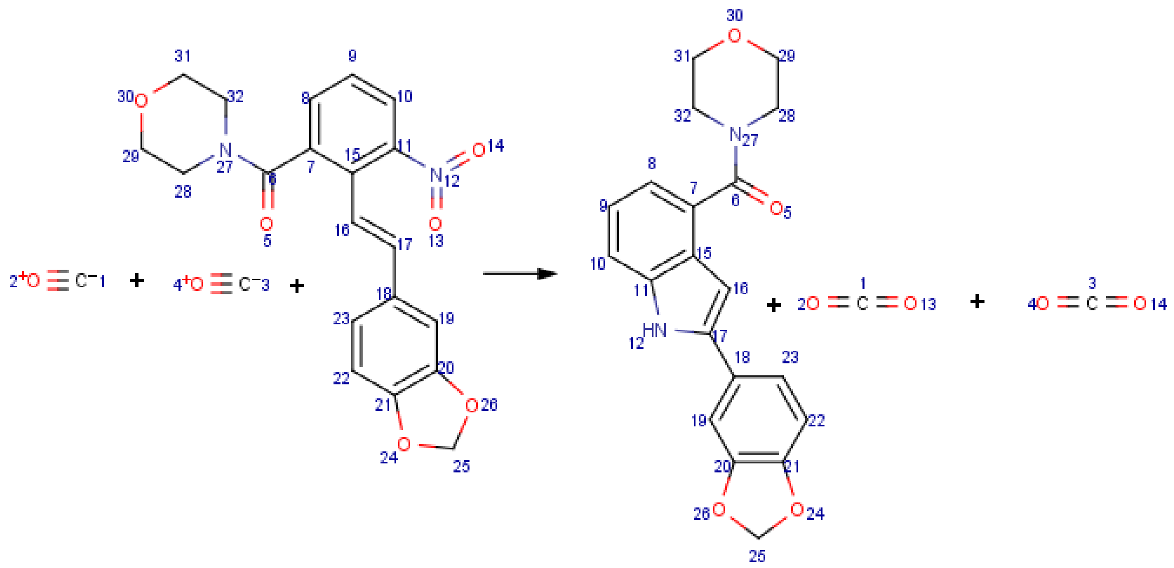

Correct mapped SMILES/SMARTS of the reaction:
[C-:1]#[O+:2].[C-:3]#[O+:4].[O:5]=[#6:6](-[#7:27]-1-[#6:28]-[#6:29]-[#8:30]-
[#6:31]-[#6:32]-1)-[#6:7]-1=[#6:8]-[#6:9]=[#6:10]-[#6:11](=[#6:15]-
1\[#6:16]=[#6:17]\[#6:18]-1=[#6:19]-[#6:20]-2=[#6:21](-[#8:24]-[#6:25]-[#8:26]-
2)-[#6:22]=[#6:23]1)[N:12](=[O:13])=[O:14]>>[O:5]=[#6:6](-[#7:27]-1-[#6:28]-
[#6:29]-[#8:30]-[#6:31]-[#6:32]-1)-[#6:7]-1=[#6:8]-[#6:9]=[#6:10]-[#6:11]-
2=[#6:15]-1-[#6:16]=[#6:17](-[#7:12]-2)-[#6:18]-1=[#6:19]-[#6:20]-2=[#6:21](-
[#8:24]-[#6:25]-[#8:26]-2)-[#6:22]=[#6:23]-
1.[O:2]=[C:1]=[O:13].[O:4]=[C:3]=[O:14]

Correctness of the mapping
MAPPET YES
ReactionMap YES
Marvin YES

Reaction no 52

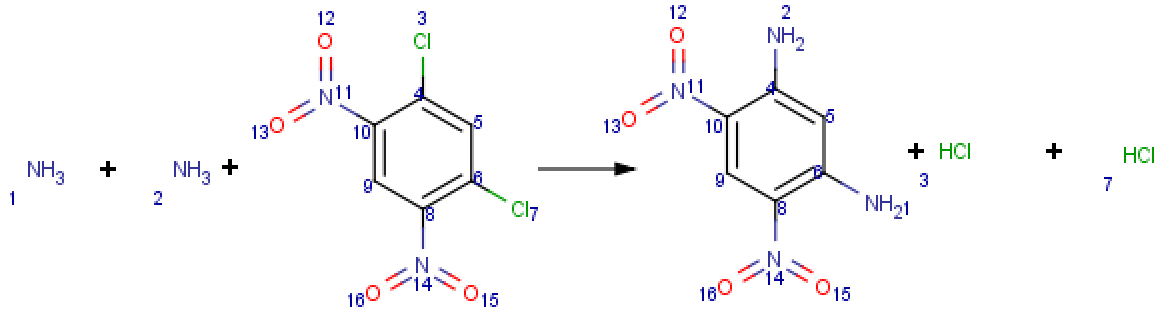

Correct mapped SMILES/SMARTS of the reaction:
[#7:1].[#7:2].[Cl:3][#6:4]-1=[#6:5]-[#6:6]([Cl:7])=[#6:8](-[#6:9]=[#6:10]-
1[N:11](=[O:12])=[O:13])[N:14](=[O:15])=[O:16]>>[#7:2]-[#6:4]-1=[#6:5]-[#6:6](-
[#7:1])=[#6:8](-[#6:9]=[#6:10]-
1[N:11](=[O:12])=[O:13])[N:14](=[O:15])=[O:16].[Cl:3].[Cl:7]

Correctness of the mapping
MAPPET YES
ReactionMap YES
Marvin YES

Reaction no 53

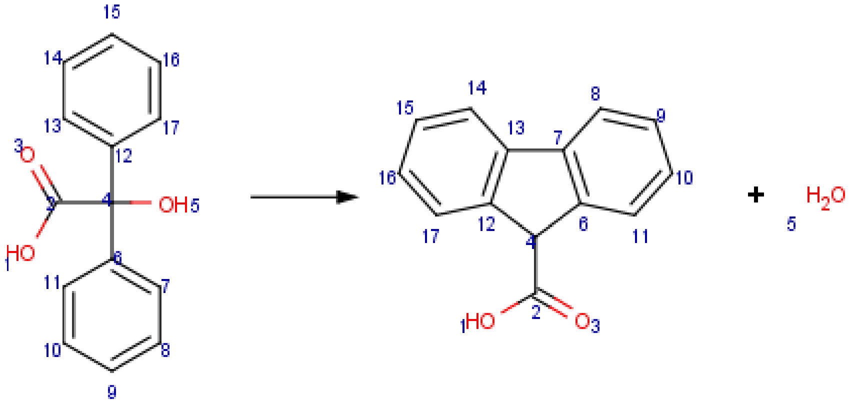

Correct mapped SMILES/SMARTS of the reaction:
[#8:1]-[#6:2](=[O:3])[C:4]([#8:5])([#6:6]-1=[#6:7]-[#6:8]=[#6:9]-[#6:10]=[#6:11]-1)[#6:12]-1=[#6:13]-[#6:14]=[#6:15]-[#6:16]=[#6:17]-1>>[#8:1]-[#6:2](=[O:3])-[#6:4]-1-[#6:6]-2=[#6:7](-[#6:8]=[#6:9]-[#6:10]=[#6:11]-2)-[#6:13]-2=[#6:12]-1-[#6:17]=[#6:16]-[#6:15]=[#6:14]-2.[#8:5]

Correctness of the mapping
MAPPET YES
ReactionMap YES
Marvin YES

Reaction no 54

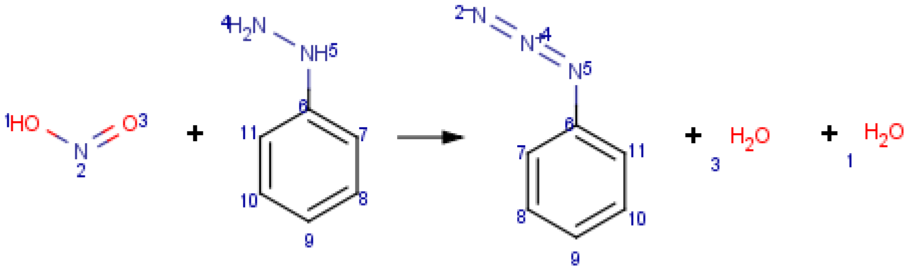

Correct mapped SMILES/SMARTS of the reaction:
[OH:1][N:2]=[O:3].[NH2:4][NH:5][C:6]1=[CH:7][CH:8]=[CH:9][CH:10]=[CH:11]1>>[N-:2]=[N+:4]=[N:5][C:6]1=[CH:11][CH:10]=[CH:9][CH:8]=[CH:7]1.[OH2:3].[OH2:1]

Correctness of the mapping
MAPPET YES
ReactionMap YES
Marvin NO

Reaction no 55

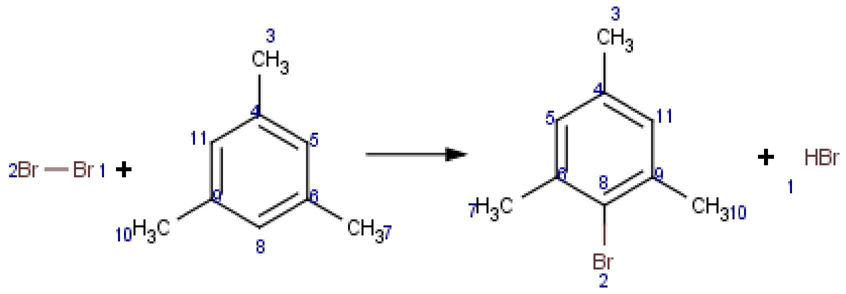

Correct mapped SMILES/SMARTS of the reaction:
[Br:1][Br:2].[#6:3]-[#6:4]-1=[#6:5]-[#6:6](-[#6:7])=[#6:8]-[#6:9](-
[#6:10])=[#6:11]-1>>[#6:3]-[#6:4]-1=[#6:11]-[#6:9](-[#6:10])=[#6:8]([Br:2])-
[#6:6](-[#6:7])=[#6:5]-1.[Br:1]

Correctness of the mapping
MAPPET YES
ReactionMap YES
Marvin YES

Reaction no 56

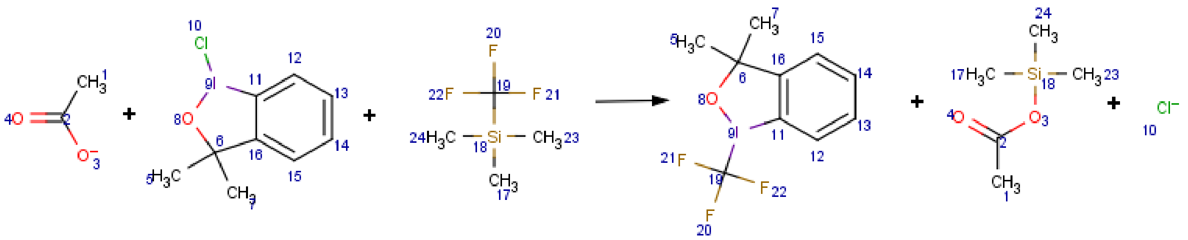

Correct mapped SMILES/SMARTS of the reaction:
[#6:1]-[#6:2](-[#8-:3])=[O:4].[#6:5][C:6]1([#6:7])[#8:8][I:9]([Cl:10])[#6:11]-
2=[#6:12]-[#6:13]=[#6:14]-[#6:15]=[#6:16]1-
2.[#6:17][Si:18]([#6:23])([#6:24])[C:19]([F:20])([F:21])[F:22]>>[#6:5][C:6]1([#6
:7])[#8:8][I:9]([#6:11]-2=[#6:16]1-[#6:15]=[#6:14]-[#6:13]=[#6:12]-
2)[C:19]([F:20])([F:21])[F:22].[#6:1]-[#6:2](=[O:4])-
[#8:3][Si:18]([#6:24])([#6:23])[#6:17].[Cl-:10]

Correctness of the mapping
MAPPET YES
ReactionMap YES
Marvin YES

Reaction no 57

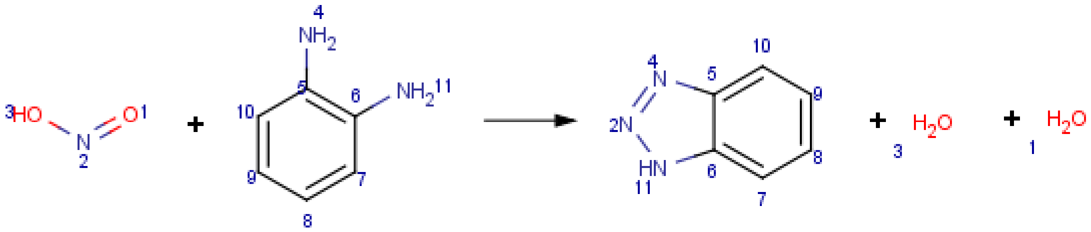

Correct mapped SMILES/SMARTS of the reaction:
[#8:3]-[#7:2]=[O:1].[#7:4]-[#6:5]-1=[#6:6](-[#7:11])-[#6:7]=[#6:8]-
[#6:9]=[#6:10]-1>>[#7:11]-1-[#7:2]=[#7:4]-[#6:5]-2=[#6:10]-[#6:9]=[#6:8]-
[#6:7]=[#6:6]-1-2.[#8:3].[#8:1]

Correctness of the mapping
MAPPET YES
ReactionMap YES
Marvin YES

Reaction no 58

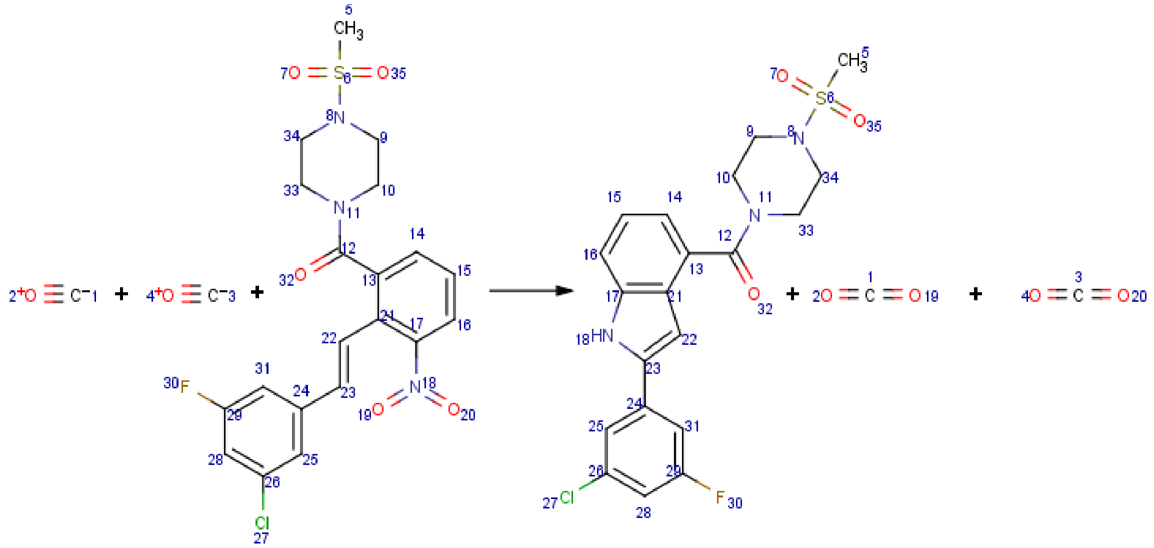

Correct mapped SMILES/SMARTS of the reaction:
[C-:1]#[O+:2].[C-:3]#[O+:4].[#6:5][S:6](=[O:7])(=[O:35])[#7:8]-1-[#6:9]-[#6:10]-[#7:11](-[#6:33]-[#6:34]-1)-[#6:12](=[O:32])-[#6:13]-1=[#6:14]-[#6:15]=[#6:16]-[#6:17](=[#6:21]-1\[#6:22]=[#6:23]\[#6:24]-1=[#6:25]-[#6:26]([Cl:27])=[#6:28]-[#6:29]([F:30])=[#6:31]1)[N:18](=[O:19])=[O:20]>>[#6:5][S:6](=[O:35])(=[O:7])[#7:8]-1-[#6:9]-[#6:10]-[#7:11](-[#6:33]-[#6:34]-1)-[#6:12](=[O:32])-[#6:13]-1=[#6:14]-[#6:15]=[#6:16]-[#6:17]-2=[#6:21]-1-[#6:22]=[#6:23](-[#7:18]-2)-[#6:24]-1=[#6:25]-[#6:26]([Cl:27])=[#6:28]-[#6:29]([F:30])=[#6:31]-1.[O:2]=[C:1]=[O:19].[O:4]=[C:3]=[O:20]

Correctness of the mapping
MAPPET YES
ReactionMap YES
Marvin YES

Reaction no 59

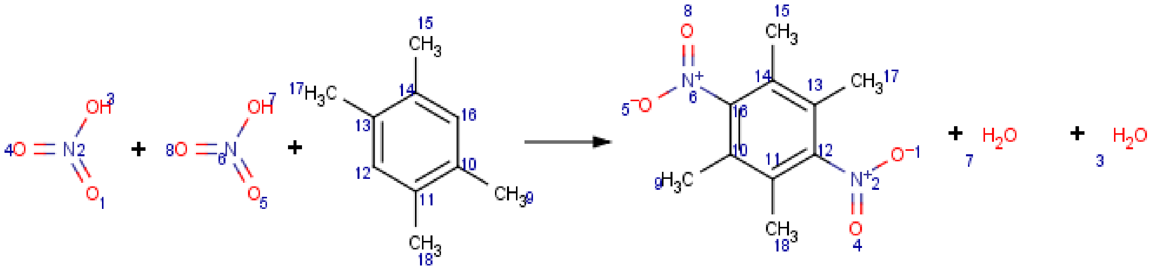

Correct mapped SMILES/SMARTS of the reaction:
[#8:3][N:2](=[O:1])=[O:4].[#8:7][N:6](=[O:5])=[O:8].[#6:15]-[#6:14]-1=[#6:16]-[#6:10](-[#6:9])=[#6:11](-[#6:18])-[#6:12]=[#6:13]-1-[#6:17]>>[#6:15]-[#6:14]-1=[#6:13](-[#6:17])-[#6:12](=[#6:11](-[#6:18])-[#6:10](-[#6:9])=[#6:16]-1-[#7+:6](-[#8-:5])=[O:8])-[#7+:2](-[#8-:1])=[O:4].[#8:7].[#8:3]

Correctness of the mapping
MAPPET YES
ReactionMap YES
Marvin YES

Reaction no 60

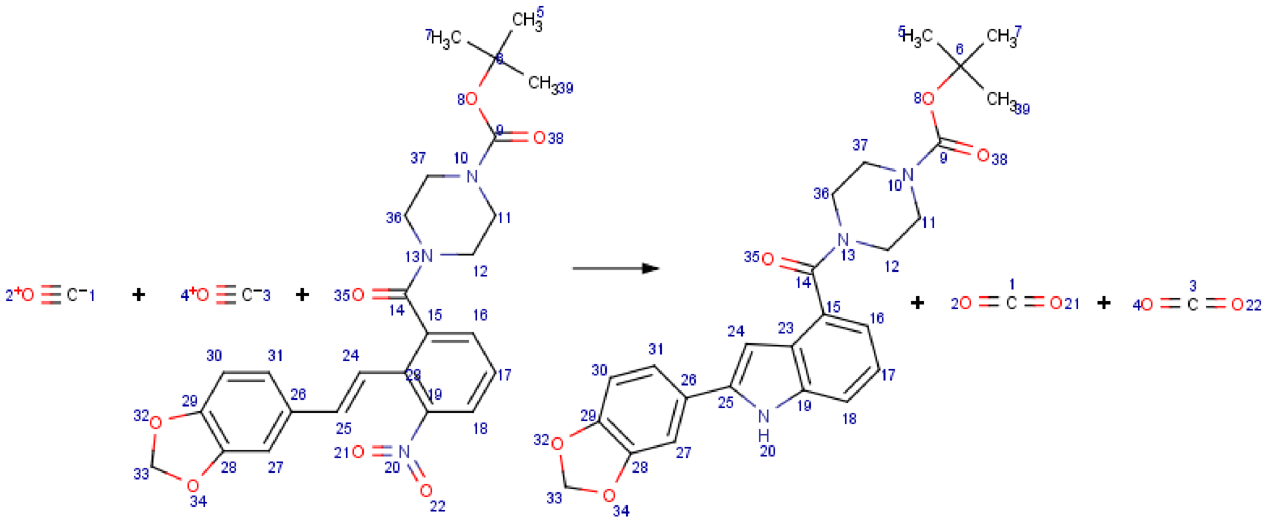

Correct mapped SMILES/SMARTS of the reaction:
[C-:1]#[O+:2].[C-:3]#[O+:4].[#6:5][C:6]([#6:7])([#6:39])[#8:8]-[#6:9](=[O:38])-[#7:10]-1-[#6:11]-[#6:12]-[#7:13](-[#6:36]-[#6:37]-1)-[#6:14](=[O:35])-[#6:15]-1=[#6:16]-[#6:17]=[#6:18]-[#6:19](=[#6:23]-1\[#6:24]=[#6:25]\[#6:26]-1=[#6:27]-[#6:28]-2=[#6:29](-[#8:32]-[#6:33]-[#8:34]-2)-[#6:30]=[#6:31]1)[N:20](=[O:21])=[O:22]>>[#6:7][C:6]([#6:5])([#6:39])[#8:8]-[#6:9](=[O:38])-[#7:10]-1-[#6:11]-[#6:12]-[#7:13](-[#6:36]-[#6:37]-1)-[#6:14](=[O:35])-[#6:15]-1=[#6:16]-[#6:17]=[#6:18]-[#6:19]-2=[#6:23]-1-[#6:24]=[#6:25](-[#7:20]-2)-[#6:26]-1=[#6:27]-[#6:28]-2=[#6:29](-[#8:32]-[#6:33]-[#8:34]-2)-[#6:30]=[#6:31]-1.[O:2]=[C:1]=[O:21].[O:4]=[C:3]=[O:22]

Correctness of the mapping
MAPPET YES
ReactionMap YES
Marvin YES

Reaction no 61

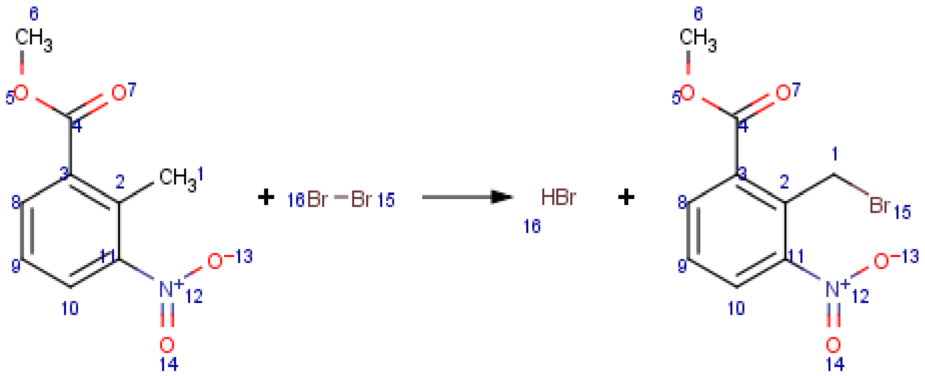

Correct mapped SMILES/SMARTS of the reaction:
[#6:6]-[#8:5]-[#6:4](=[O:7])-[#6:3]-1=[#6:2](-[#6:1])-[#6:11](=[#6:10]-[#6:9]=[#6:8]-1)-[#7+:12](-[#8-:13])=[O:14].[Br:15][Br:16]>>[Br:16].[#6:6]-[#8:5]-[#6:4](=[O:7])-[#6:3]-1=[#6:2](-[#6:1][Br:15])-[#6:11](=[#6:10]-[#6:9]=[#6:8]-1)-[#7+:12](-[#8-:13])=[O:14]

Correctness of the mapping
MAPPET YES
ReactionMap YES
Marvin YES

Reaction no 62

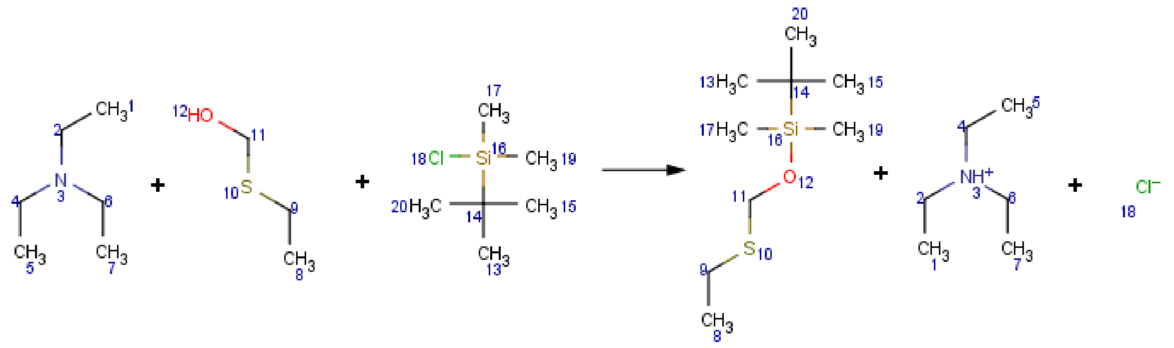

Correct mapped SMILES/SMARTS of the reaction:
[#6:1]-[#6:2]-[#7:3](-[#6:4]-[#6:5])-[#6:6]-[#6:7].[#6:8]-[#6:9]-[#16:10]-[#6:11]-
[#8:12].[#6:13][C:14]([#6:15])([#6:20])[Si:16]([#6:17])([#6:19])[Cl:18]>>[#6:8]-[#6:9]-[#16:10]-[#6:11]-
[#8:12][Si:16]([#6:19])([#6:17])[C:14]([#6:20])([#6:15])[#6:13].[#6:5]-[#6:4]-[#7+:3](-[#6:2]-[#6:1])-[#6:6]-[#6:7].[Cl-:18]

Correctness of the mapping
MAPPET YES
ReactionMap YES
Marvin YES

Reaction no 63

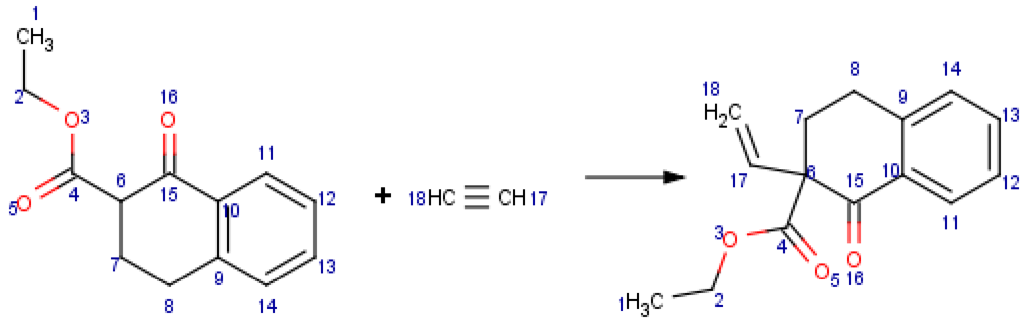

Correct mapped SMILES/SMARTS of the reaction:
[#6:1]-[#6:2]-[#8:3]-[#6:4](=[O:5])-[#6:6]-1-[#6:7]-[#6:8]-[#6:9]-2=[#6:10](-[#6:11]=[#6:12]-[#6:13]=[#6:14]-2)-[#6:15]-1=[O:16].[C:17]#[C:18]>>[#6:1]-[#6:2]-[#8:3]-[#6:4](=[O:5])[C:6]1([#6:7]-[#6:8]-[#6:9]-2=[#6:14]-[#6:13]=[#6:12]-[#6:11]=[#6:10]-2-[#6:15]1=[O:16])[#6:17]=[#6:18]

Correctness of the mapping
MAPPET YES
ReactionMap YES
Marvin YES

Reaction no 64

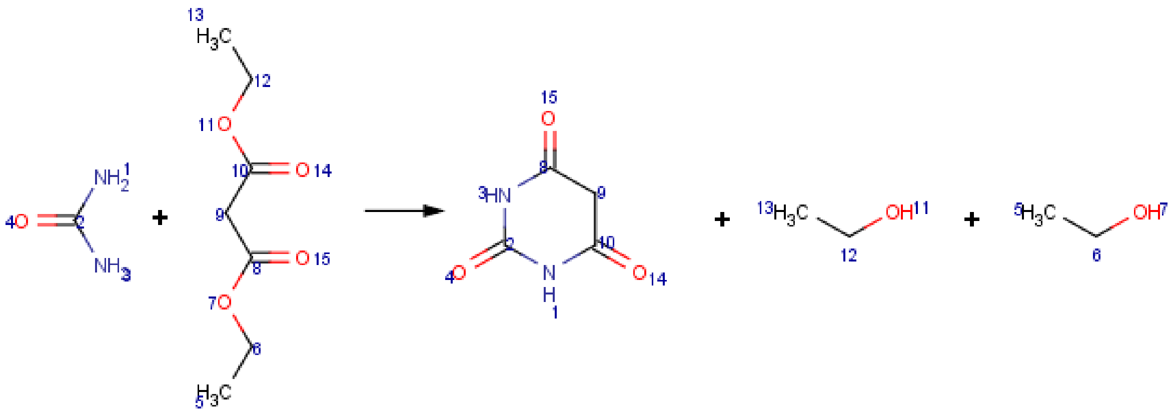

Correct mapped SMILES/SMARTS of the reaction:
[#7:1]-[#6:2](-[#7:3])=[O:4].[#6:5]-[#6:6]-[#8:7]-[#6:8](=[O:15])-[#6:9]-[#6:10](=[O:14])-[#8:11]-[#6:12]-[#6:13]>>[O:15]=[#6:8]-1-[#6:9]-[#6:10](=[O:14])-[#7:1]-[#6:2](=[O:4])-[#7:3]-1.[#6:13]-[#6:12]-[#8:11].[#6:5]-[#6:6]-[#8:7]

Correctness of the mapping
MAPPET YES
ReactionMap YES
Marvin YES

Reaction no 65

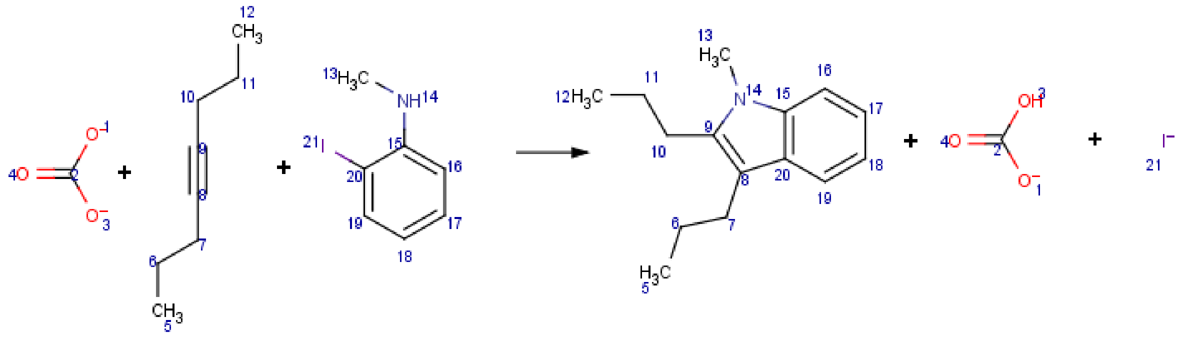

Correct mapped SMILES/SMARTS of the reaction:
[#8-:1]-[#6:2](-[#8-:3])=[O:4].[#6:5]-[#6:6]-[#6:7][C:8]#[C:9][#6:10]-[#6:11]-[#6:12].[#6:13]-[#7:14]-[#6:15]-1=[#6:16]-[#6:17]=[#6:18]-[#6:19]=[#6:20]-1[I:21]>>[#6:12]-[#6:11]-[#6:10]-[#6:9]-1=[#6:8](-[#6:7]-[#6:6]-[#6:5])-[#6:20]-2=[#6:15](-[#6:16]=[#6:17]-[#6:18]=[#6:19]-2)-[#7:14]-1-[#6:13].[#8:3]-[#6:2](-[#8-:1])=[O:4].[I-:21]

Correctness of the mapping
MAPPET YES
ReactionMap YES
Marvin YES

Reaction no 66

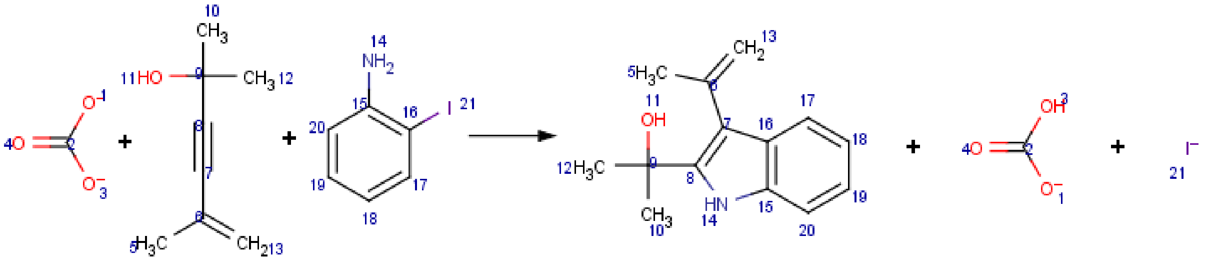

Correct mapped SMILES/SMARTS of the reaction:
[#8-:1]-[#6:2](-[#8-:3])=[O:4].[#6:5]-[#6:6](=[#6:13])[C:7]#[C:8][C:9]([#6:10])([#6:12])[#8:11].[#7:14]-[#6:15]-1=[#6:16]([I:21])-[#6:17]=[#6:18]-[#6:19]=[#6:20]-1>>[#6:5]-[#6:6](=[#6:13])-[#6:7]-1=[#6:8](-[#7:14]-[#6:15]-2=[#6:16]-1-[#6:17]=[#6:18]-[#6:19]=[#6:20]-2)[C:9]([#6:12])([#6:10])[#8:11].[#8:3]-[#6:2](-[#8-:1])=[O:4].[I-:21]

Correctness of the mapping
MAPPET YES
ReactionMap YES
Marvin YES

Reaction no 67

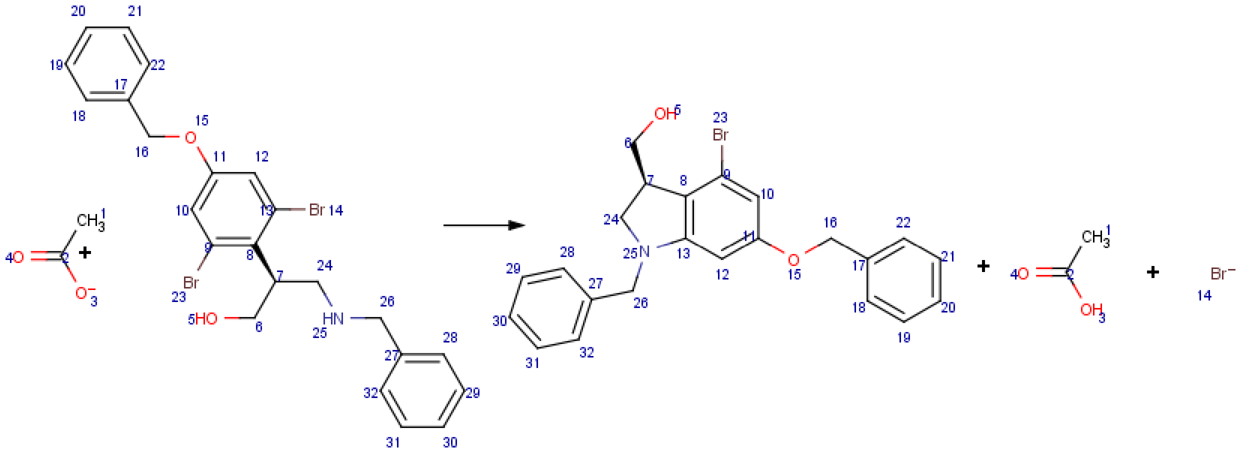

Correct mapped SMILES/SMARTS of the reaction:
[CH3:1][C:2]([O-:3])=[O:4].[OH:5][CH2:6][C@H:7]([CH2:24][NH:25][CH2:26][C:27]1=[CH:28][CH:29]=[CH:30][CH:31]=[CH:32]1)[C:8]1=[C:9]([Br:23])[CH:10]=[C:11]([O:15][CH2:16][C:17]2=[CH:18][CH:19]=[CH:20][CH:21]=[CH:22]2)[CH:12]=[C:13]1[Br:14]>>[OH:5][CH2:6][C@@H:7]1[CH2:24][N:25]([CH2:26][C:27]2=[CH:32][CH:31]=[CH:30][CH:29]=[CH:28]2)[C:13]2=[C:8]1[C:9]([Br:23])=[CH:10][C:11]([O:15][CH2:16][C:17]1=[CH:22][CH:21]=[CH:20][CH:19]=[CH:18]1)=[CH:12]2.[CH3:1][C:2]([OH:3])=[O:4].[Br-:14]

Correctness of the mapping
MAPPET YES
ReactionMap YES
Marvin YES

Reaction no 68

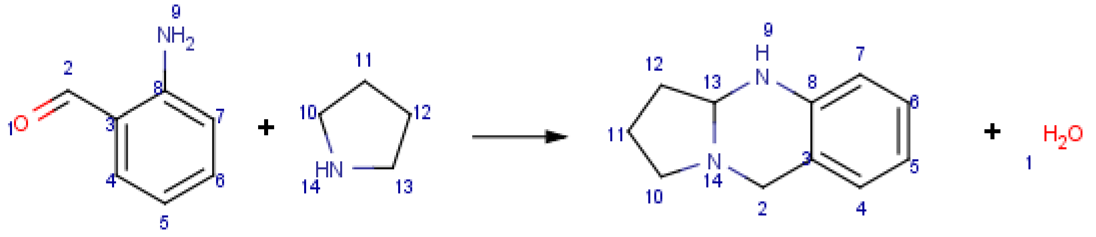

Correct mapped SMILES/SMARTS of the reaction:
[#7:9]-[#6:8]-1=[#6:7]-[#6:6]=[#6:5]-[#6:4]=[#6:3]-1-[#6:2]=[O:1].[#6:11]-1-[#6:12]-[#6:13]-[#7:14]-[#6:10]-1>>[#6:11]-1-[#6:12]-[#6:13]-2-[#7:9]-[#6:8]-3=[#6:7]-[#6:6]=[#6:5]-[#6:4]=[#6:3]-3-[#6:2]-[#7:14]-2-[#6:10]-1.[#8:1]

Correctness of the mapping
MAPPET YES
ReactionMap YES
Marvin YES

Reaction no 69

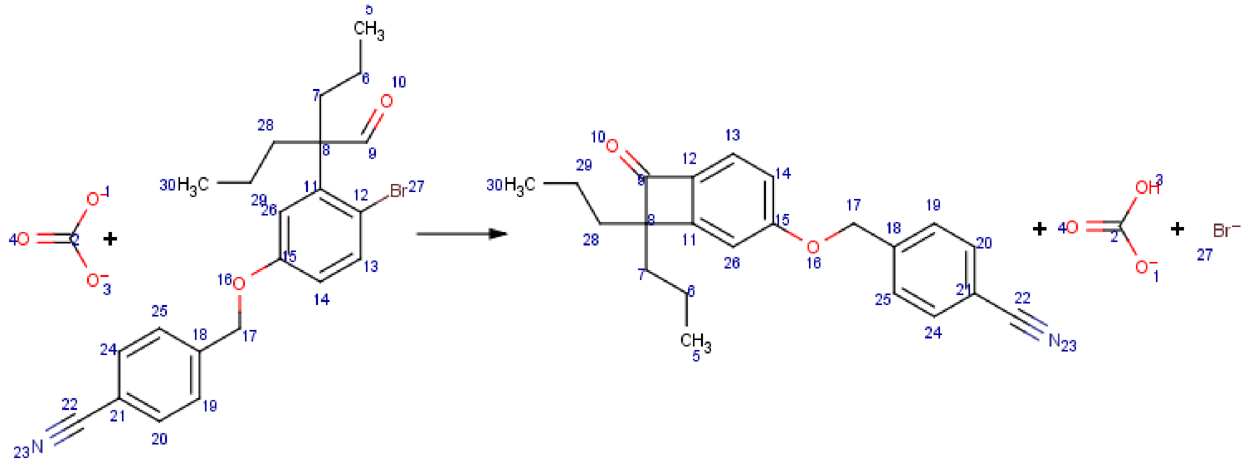

Correct mapped SMILES/SMARTS of the reaction:
[#8-:1]-[#6:2](-[#8-:3])=[O:4].[#6:5]-[#6:6]-[#6:7][C:8]([#6:28]-[#6:29]-[#6:30])([#6:9]=[O:10])[#6:11]-1=[#6:12]([Br:27])-[#6:13]=[#6:14]-[#6:15](-[#8:16]-[#6:17]-[#6:18]-2=[#6:19]-[#6:20]=[#6:21](-[#6:24]=[#6:25]-2)[C:22]#[N:23])=[#6:26]-1>>[#6:30]-[#6:29]-[#6:28][C:8]1([#6:7]-[#6:6]-[#6:5])[#6:9](=[O:10])-[#6:12]-2=[#6:13]-[#6:14]=[#6:15](-[#8:16]-[#6:17]-[#6:18]-3=[#6:19]-[#6:20]=[#6:21](-[#6:24]=[#6:25]-3)[C:22]#[N:23])-[#6:26]=[#6:11]1-2.[#8:3]-[#6:2](-[#8-:1])=[O:4].[Br-:27]

Correctness of the mapping
MAPPET YES
ReactionMap YES
Marvin YES

Reaction no 70

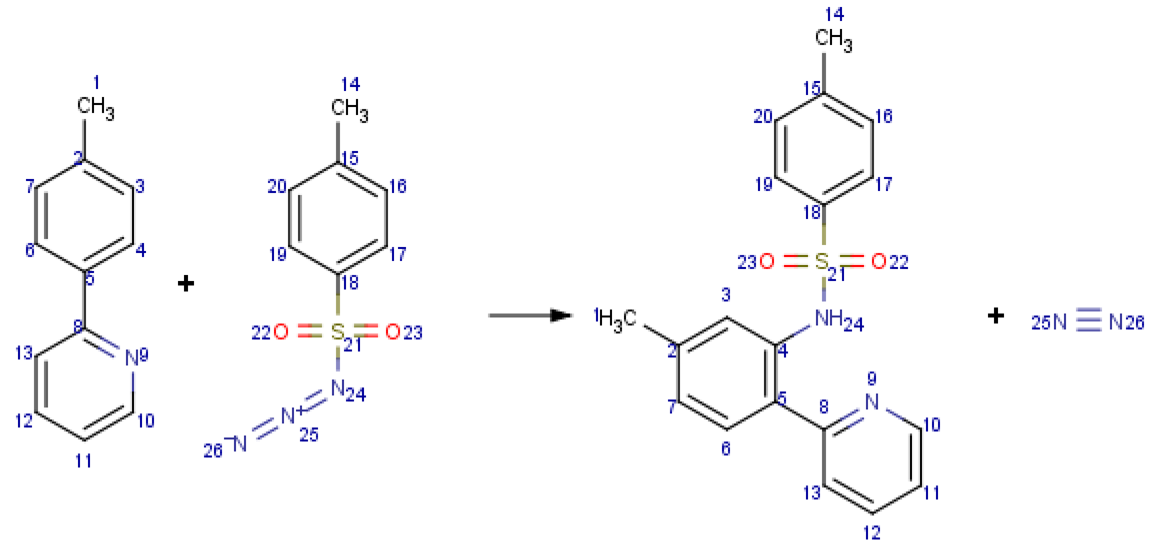

Correct mapped SMILES/SMARTS of the reaction:
[#6:1]-[#6:2]-1=[#6:3]-[#6:4]=[#6:5](-[#6:6]=[#6:7]-1)-[#6:8]-1=[#7:9]-[#6:10]=[#6:11]-[#6:12]=[#6:13]-1.[#6:14]-[#6:15]-1=[#6:16]-[#6:17]=[#6:18](-[#6:19]=[#6:20]-1)[S:21](=[O:22])(=[O:23])[#7:24]=[N:25]#[N:26]>>[#6:14]-[#6:15]-1=[#6:20]-[#6:19]=[#6:18](-[#6:17]=[#6:16]-1)[S:21](=[O:22])(=[O:23])[#7:24]-[#6:4]-1=[#6:5](-[#6:6]=[#6:7]-[#6:2](-[#6:1])=[#6:3]-1)-[#6:8]-1=[#7:9]-[#6:10]=[#6:11]-[#6:12]=[#6:13]-1.[N:26]#[N:25]

Correctness of the mapping
MAPPET YES
ReactionMap YES
Marvin NO

Reaction no 71

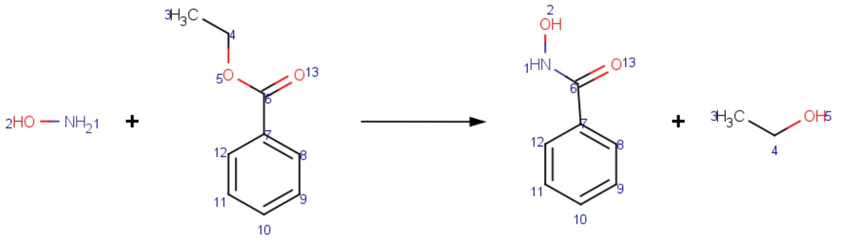

Correct mapped SMILES/SMARTS of the reaction:
[#7:1]-[#8:2].[#6:3]-[#6:4]-[#8:5]-[#6:6](=[O:13])-[#6:7]-1=[#6:8]-[#6:9]=[#6:10]-[#6:11]=[#6:12]-1>>[#8:2]-[#7:1]-[#6:6](=[O:13])-[#6:7]-1=[#6:8]-[#6:9]=[#6:10]-[#6:11]=[#6:12]-1.[#6:3]-[#6:4]-[#8:5]

Correctness of the mapping
MAPPET YES
ReactionMap YES
Marvin YES

Reaction no 72

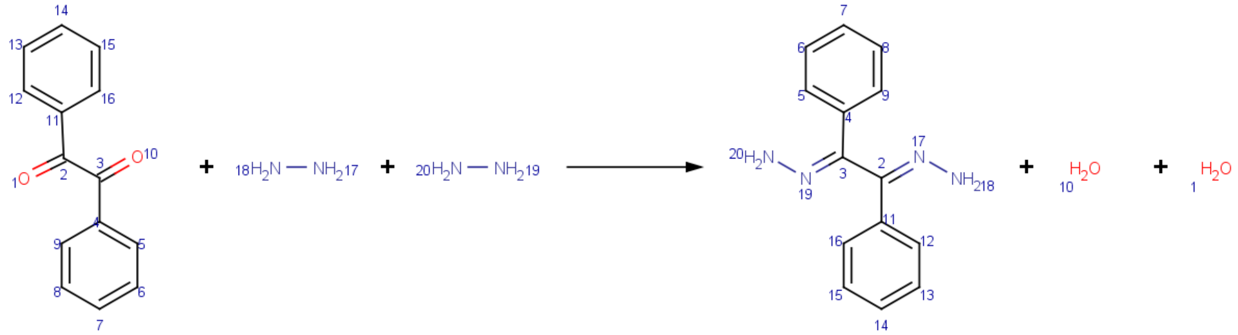

Correct mapped SMILES/SMARTS of the reaction:
[O:1]=[#6:2](-[#6:3](=[O:10])-[#6:4]-1=[#6:5]-[#6:6]=[#6:7]-[#6:8]=[#6:9]-1)-
[#6:11]-1=[#6:12]-[#6:13]=[#6:14]-[#6:15]=[#6:16]-1.[#7:17]-[#7:18].[#7:19]-
[#7:20]>>[#7:20]\[#7:19]=[#6:3](\[#6:2](=[#7:17]\[#7:18])\[#6:11]-1=[#6:12]-
[#6:13]=[#6:14]-[#6:15]=[#6:16]1)/[#6:4]-1=[#6:5]-[#6:6]=[#6:7]-
[#6:8]=[#6:9]1.[#8:10].[#8:1]

Correctness of the mapping
MAPPET YES
ReactionMap YES
Marvin YES

Reaction no 73

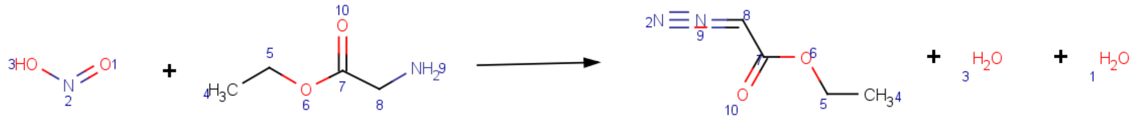

Correct mapped SMILES/SMARTS of the reaction:
[#8:3]-[#7:2]=[O:1].[#6:4]-[#6:5]-[#8:6]-[#6:7](=[O:10])-[#6:8]-[#7:9]>>[#6:4]-
[#6:5]-[#8:6]-[#6:7](=[O:10])-[#6:8]=[N:9]#[N:2].[#8:3].[#8:1]

Correctness of the mapping
MAPPET YES
ReactionMap YES
Marvin NO

Reaction no 74

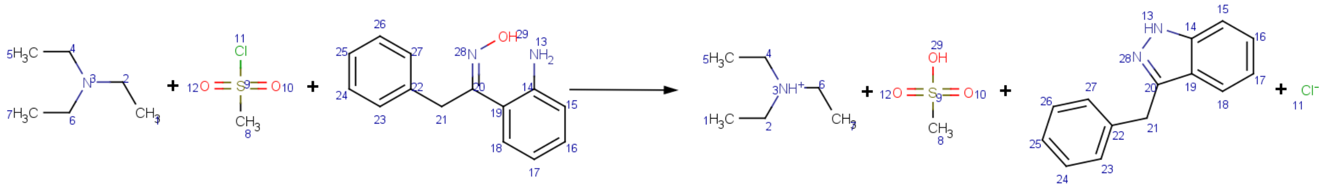

Correct mapped SMILES/SMARTS of the reaction:
[#6:1]-[#6:2]-[#7:3](-[#6:4]-[#6:5])-[#6:6]-
[#6:7].[#6:8][S:9]([Cl:11])(=[O:10])=[O:12].[#7:13]-[#6:14]-1=[#6:15]-
[#6:16]=[#6:17]-[#6:18]=[#6:19]-1\[#6:20](-[#6:21]-[#6:22]-1=[#6:23]-
[#6:24]=[#6:25]-[#6:26]=[#6:27]-1)=[#7:28]/[#8:29]>>[#6:7]-[#6:6]-[#7+:3](-
[#6:4]-[#6:5])-[#6:2]-[#6:1].[#6:8][S:9]([#8:29])(=[O:10])=[O:12].[#6:21](-
[#6:20]-1=[#7:28]-[#7:13]-[#6:14]-2=[#6:15]-[#6:16]=[#6:17]-[#6:18]=[#6:19]-1-
2)-[#6:22]-1=[#6:23]-[#6:24]=[#6:25]-[#6:26]=[#6:27]-1.[Cl-:11]

Correctness of the mapping
MAPPET YES
ReactionMap YES
Marvin YES

Reaction no 75

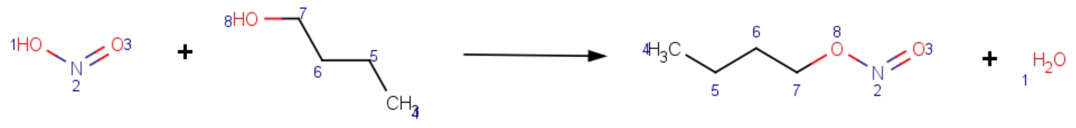

Correct mapped SMILES/SMARTS of the reaction:
[#8:1]-[#7:2]=[O:3].[#6:4]-[#6:5]-[#6:6]-[#6:7]-[#8:8]>>[#6:4]-[#6:5]-[#6:6]-[#6:7]-[#8:8]-[#7:2]=[O:3].[#8:1]

Correctness of the mapping
MAPPET   YES
ReactionMap YES
Marvin   NO

Reaction no 76

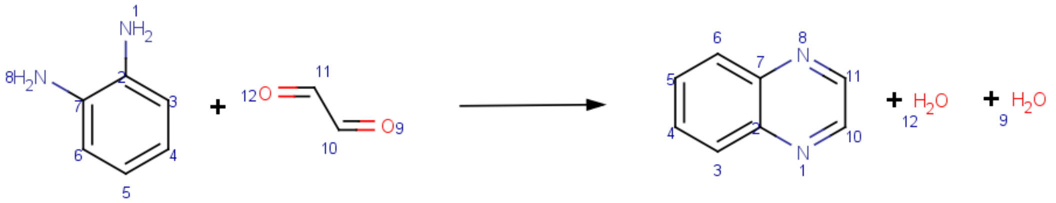

Correct mapped SMILES/SMARTS of the reaction:
[#7:1]-[#6:2]-1=[#6:3]-[#6:4]=[#6:5]-[#6:6]=[#6:7]-1-[#7:8].[O:9]=[#6:10]-[#6:11]=[O:12]>>[#6:4]-1=[#6:5]-[#6:6]=[#6:7]-2-[#7:8]=[#6:11]-[#6:10]=[#7:1]-[#6:2]-2=[#6:3]-1.[#8:12].[#8:9]

Correctness of the mapping
MAPPET   YES
ReactionMap YES
Marvin   YES

Reaction no 77

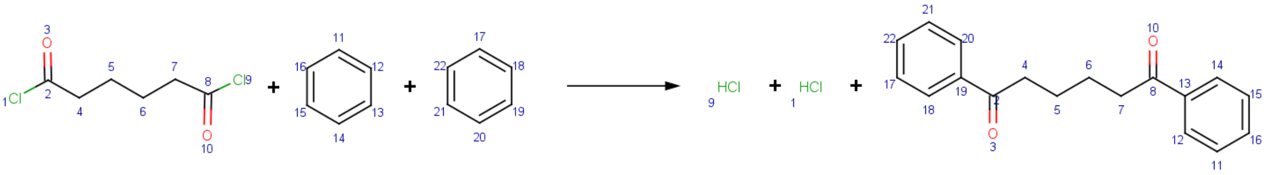

Correct mapped SMILES/SMARTS of the reaction:
[Cl:1][#6:2](=[O:3])-[#6:4]-[#6:5]-[#6:6]-[#6:7]-[#6:8]([Cl:9])=[O:10].[#6:11]-1=[#6:12]-[#6:13]=[#6:14]-[#6:15]=[#6:16]-1.[#6:17]-1=[#6:18]-[#6:19]=[#6:20]-[#6:21]=[#6:22]-1>>[Cl:9].[Cl:1].[O:3]=[#6:2](-[#6:4]-[#6:5]-[#6:6]-[#6:7]-[#6:8](=[O:10])-[#6:13]-1=[#6:14]-[#6:15]=[#6:16]-[#6:11]=[#6:12]-1)-[#6:19]-1=[#6:18]-[#6:17]=[#6:22]-[#6:21]=[#6:20]-1

Correctness of the mapping
MAPPET   YES
ReactionMap YES
Marvin   YES

Reaction no 78

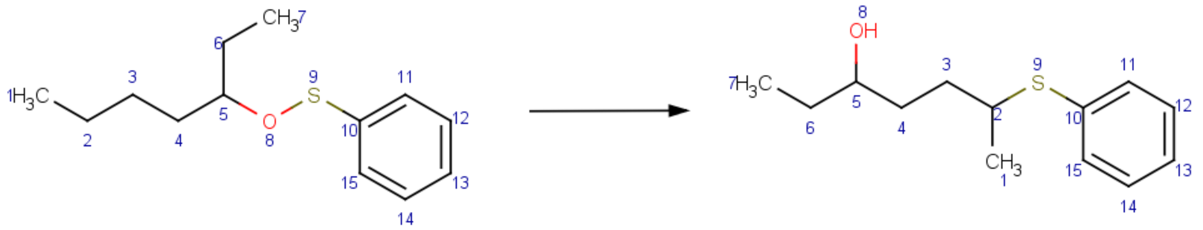

Correct mapped SMILES/SMARTS of the reaction:
[#6:1]-[#6:2]-[#6:3]-[#6:4]-[#6:5](-[#6:6]-[#6:7])-[#8:8]-[#16:9]-[c:10]1[c:11][c:12][c:13][c:14][c:15]1>>[#6:7]-[#6:6]-[#6:5](-[#8:8])-[#6:4]-[#6:3]-[#6:2](-[#6:1])-[#16:9]-[c:10]1[c:11][c:12][c:13][c:14][c:15]1

Correctness of the mapping
MAPPET YES
ReactionMap YES
Marvin YES

Reaction no 79

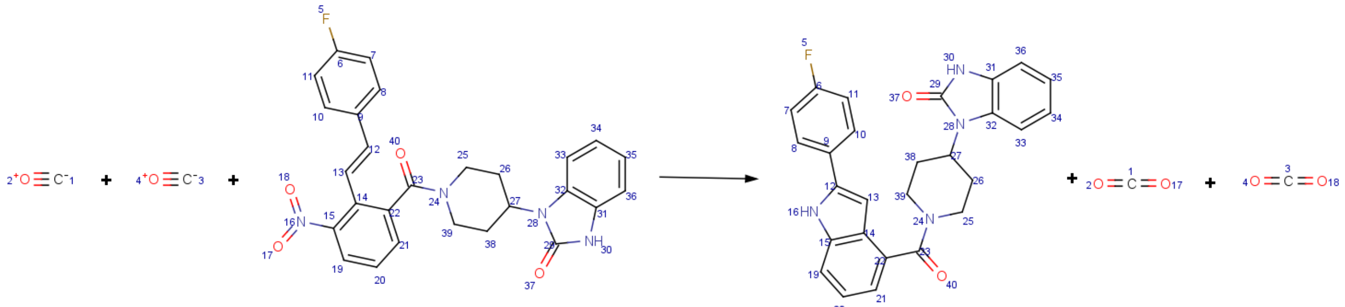

Correct mapped SMILES/SMARTS of the reaction:
[C-:1]#[O+:2].[C-:3]#[O+:4].[F:5][#6:6]-1=[#6:7]-[#6:8]=[#6:9](\[#6:12]=[#6:13]\[#6:14]-2=[#6:15](-[#6:19]=[#6:20]-[#6:21]=[#6:22]2-[#6:23](=[O:40])-[#7:24]-2-[#6:25]-[#6:26]-[#6:27](-[#6:38]-[#6:39]-2)-[#7:28]-2-[#6:29](=[O:37])-[#7:30]-[#6:31]-3=[#6:32]-2-[#6:33]=[#6:34]-[#6:35]=[#6:36]-3)[N:16](=[O:17])=[O:18])-[#6:10]=[#6:11]-1>>[F:5][#6:6]-1=[#6:7]-[#6:8]=[#6:9](-[#6:10]=[#6:11]-1)-[#6:12]-1=[#6:13]-[#6:14]-2=[#6:15](-[#7:16]-1)-[#6:19]=[#6:20]-[#6:21]=[#6:22]-2-[#6:23](=[O:40])-[#7:24]-1-[#6:25]-[#6:26]-[#6:27](-[#6:38]-[#6:39]-1)-[#7:28]-1-[#6:29](=[O:37])-[#7:30]-[#6:31]-2=[#6:32]-1-[#6:33]=[#6:34]-[#6:35]=[#6:36]-2.[O:2]=[C:1]=[O:17].[O:4]=[C:3]=[O:18]

Correctness of the mapping
MAPPET YES
ReactionMap YES
Marvin YES

Reaction no 80

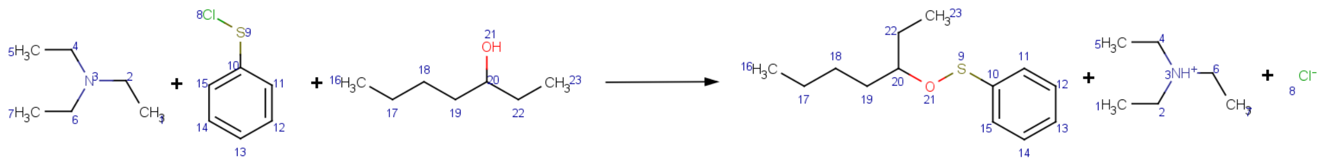

Correct mapped SMILES/SMARTS of the reaction:
[#6:1]-[#6:2]-[#7:3](-[#6:4]-[#6:5])-[#6:6]-[#6:7].[Cl:8][#16:9]-[c:10]1[c:11][c:12][c:13][c:14][c:15]1.[#6:16]-[#6:17]-[#6:18]-[#6:19]-[#6:20](-[#8:21])-[#6:22]-[#6:23]>>[#6:16]-[#6:17]-[#6:18]-[#6:19]-[#6:20](-[#6:22]-

[#6:23])-[#8:21]-[#16:9]-[c:10]1[c:11][c:12][c:13][c:14][c:15]1.[#6:7]-[#6:6]-[#7+:3](-[#6:4]-[#6:5])-[#6:2]-[#6:1].[Cl-:8]

Correctness of the mapping
MAPPET YES
ReactionMap YES
Marvin YES

Reaction no 81

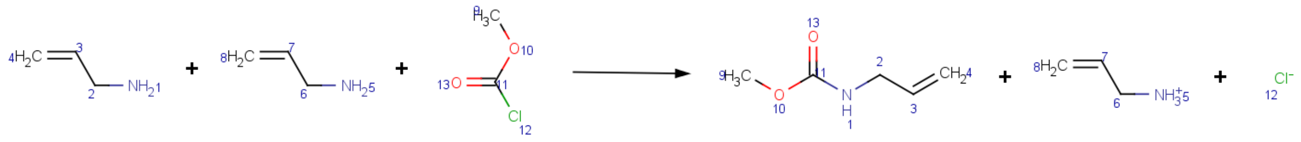

Correct mapped SMILES/SMARTS of the reaction:
[#7:1]-[#6:2]-[#6:3]=[#6:4].[#7:5]-[#6:6]-[#6:7]=[#6:8].[#6:9]-[#8:10]-[#6:11]([Cl:12])=[O:13]>>[#6:9]-[#8:10]-[#6:11](=[O:13])-[#7:1]-[#6:2]-[#6:3]=[#6:4].[#7+:5]-[#6:6]-[#6:7]=[#6:8].[Cl-:12]

Correctness of the mapping
MAPPET YES
ReactionMap YES
Marvin YES

Reaction no 82

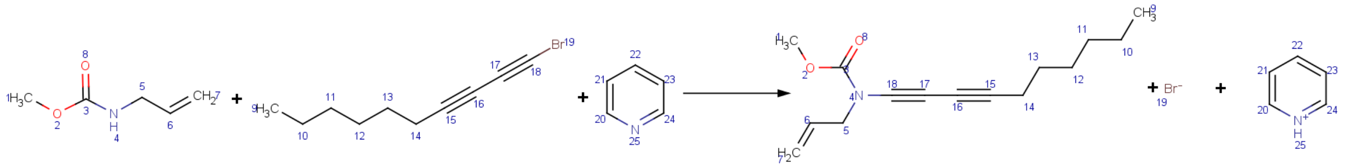

Correct mapped SMILES/SMARTS of the reaction:
[#6:1]-[#8:2]-[#6:3](=[O:8])-[#7:4]-[#6:5]-[#6:6]=[#6:7].[#6:9]-[#6:10]-[#6:11]-[#6:12]-[#6:13]-[#6:14][C:15]#[C:16][C:17]#[C:18][Br:19].[#6:22]-1=[#6:23]-[#6:24]=[#7:25]-[#6:20]=[#6:21]-1>>[#6:9]-[#6:10]-[#6:11]-[#6:12]-[#6:13]-[#6:14][C:15]#[C:16][C:17]#[C:18][#7:4](-[#6:5]-[#6:6]=[#6:7])-[#6:3](=[O:8])-[#8:2]-[#6:1].[Br-:19].[#6:22]-1=[#6:23]-[#6:24]=[#7+:25]-[#6:20]=[#6:21]-1

Correctness of the mapping
MAPPET YES
ReactionMap YES
Marvin YES

Reaction no 83

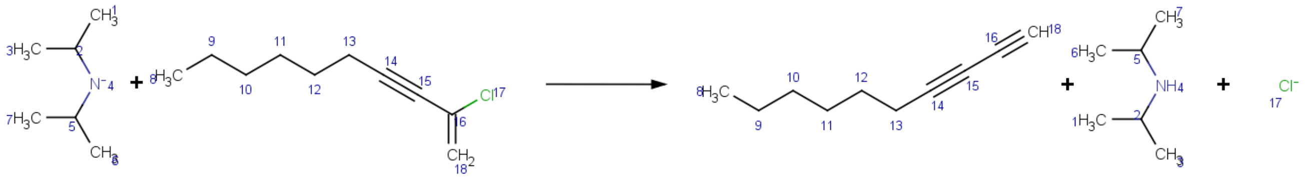

Correct mapped SMILES/SMARTS of the reaction:
[#6:1]-[#6:2](-[#6:3])-[#7-:4]-[#6:5](-[#6:6])-[#6:7].[#6:8]-[#6:9]-[#6:10]-[#6:11]-[#6:12]-[#6:13][C:14]#[C:15][#6:16]([Cl:17])=[#6:18]>>[#6:8]-[#6:9]-[#6:10]-[#6:11]-[#6:12]-[#6:13][C:14]#[C:15][C:16]#[C:18].[#6:7]-[#6:5](-[#6:6])-[#7:4]-[#6:2](-[#6:3])-[#6:1].[Cl-:17]

Correctness of the mapping
MAPPET   YES
ReactionMap YES
Marvin   YES

Reaction no 84

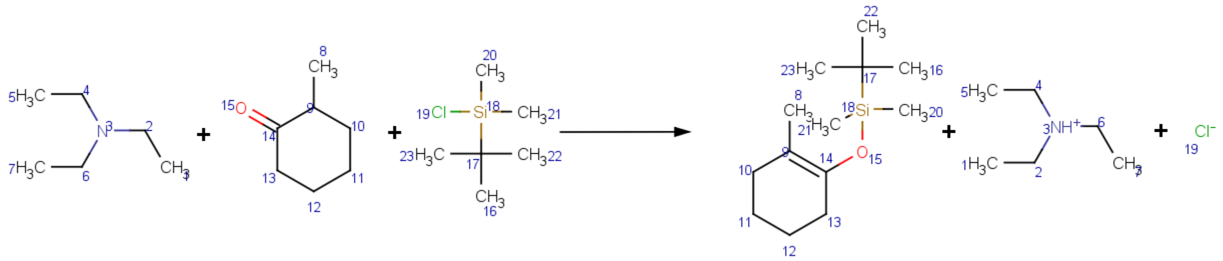

Correct mapped SMILES/SMARTS of the reaction:
[#6:1]-[#6:2]-[#7:3](-[#6:4]-[#6:5])-[#6:6]-[#6:7].[#6:8]-[#6:9]-1-[#6:10]-[#6:11]-[#6:12]-[#6:13]-[#6:14]-1=[O:15].[#6:16][C:17]([#6:22])([#6:23])[Si:18]([#6:20])([#6:21])[Cl:19]>>[#6:8]-[#6:9]-1=[#6:14](-[#6:13]-[#6:12]-[#6:11]-[#6:10]-1)-[#8:15][Si:18]([#6:20])([#6:21])[C:17]([#6:22])([#6:16])[#6:23].[#6:7]-[#6:6]-[#7+:3](-[#6:4]-[#6:5])-[#6:2]-[#6:1].[Cl-:19]

Correctness of the mapping
MAPPET   YES
ReactionMap YES
Marvin   YES

Reaction no 85

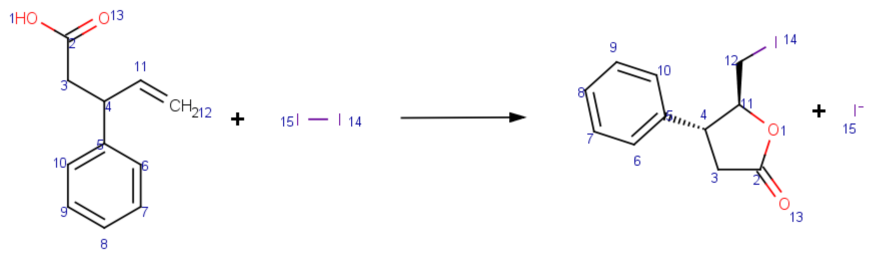

Correct mapped SMILES/SMARTS of the reaction:
[#8:1]-[#6:2](=[O:13])-[#6:3]-[#6:4](-[#6:11]=[#6:12])-[c:5]1[c:6][c:7][c:8][c:9][c:10]1.[I:14][I:15]>>[I:14][#6:12]-[#6@H:11]-1-[#8:1]-[#6:2](=[O:13])-[#6:3]-[#6@@H:4]-1-[c:5]1[c:6][c:7][c:8][c:9][c:10]1.[I-:15]

Correctness of the mapping
MAPPET   YES
ReactionMap NO
Marvin   YES

Reaction no 86

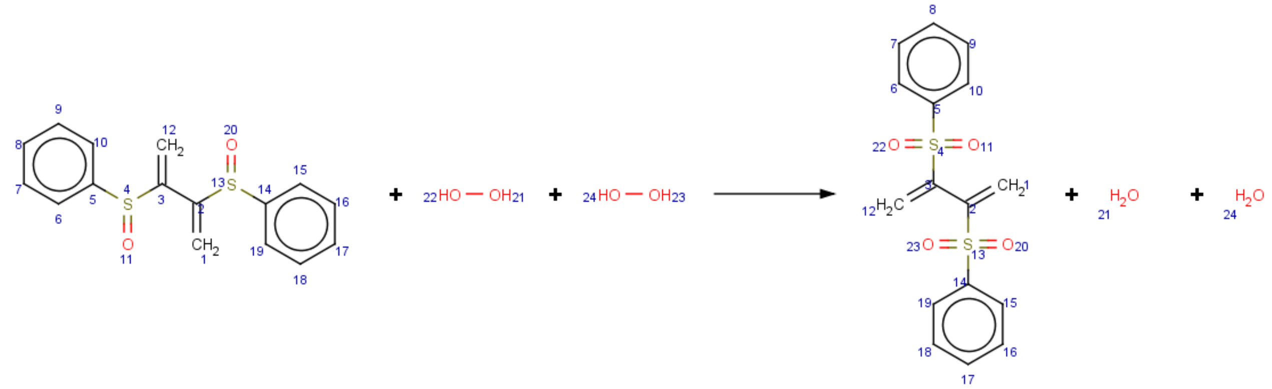

Correct mapped SMILES/SMARTS of the reaction:
 [#6:1]=[#6:2](-
[#6:3](=[#6:12])[S:4](=[O:11])[c:5]1[c:6][c:7][c:8][c:9][c:10]1)[S:13](=[O:20])[
c:14]1[c:15][c:16][c:17][c:18][c:19]1.[#8:21]-[#8:22].[#8:23]-
[#8:24]>>[#6:12]=[#6:3](-
[#6:2](=[#6:1])[S:13](=[O:20])(=[O:23])[c:14]1[c:15][c:16][c:17][c:18][c:19]1)[S
:4](=[O:11])(=[O:22])[c:5]1[c:6][c:7][c:8][c:9][c:10]1.[#8:21].[#8:24]
Correctness of the mapping
MAPPET   NO
ReactionMap NO
Marvin   NO

Reaction no 87

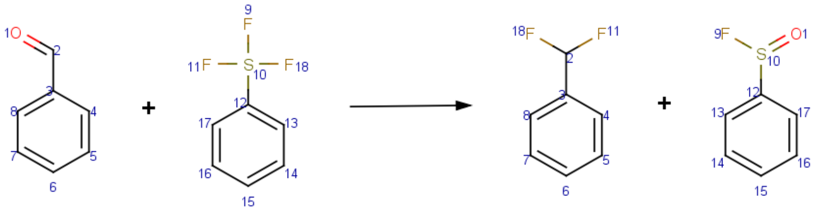

Correct mapped SMILES/SMARTS of the reaction:
[O:1]=[#6:2]-
[c:3]1[c:4][c:5][c:6][c:7][c:8]1.[F:9][S:10]([F:11])([F:18])[c:12]1[c:13][c:14][
c:15][c:16][c:17]1>>[F:18][#6:2]([F:11])-
[c:3]1[c:4][c:5][c:6][c:7][c:8]1.[F:9][S:10](=[O:1])[c:12]1[c:17][c:16][c:15][c:
14][c:13]1

Correctness of the mapping
MAPPET   YES
ReactionMap YES
Marvin   YES

Reaction no 88

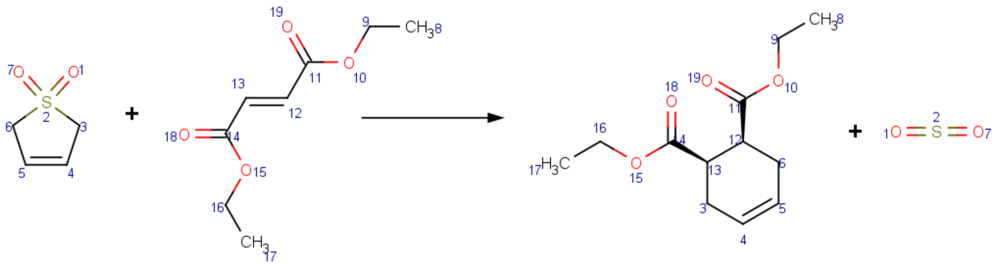

Correct mapped SMILES/SMARTS of the reaction:
[O:1]=[S:2]1(=[O:7])[#6:3]-[#6:4]=[#6:5]-[#6:6]1.[#6:8]-[#6:9]-[#8:10]-
[#6:11](=[O:19])\[#6:12]=[#6:13]\[#6:14](=[O:18])-[#8:15]-[#6:16]-

[#6:17]>>[#6:8]-[#6:9]-[#8:10]-[#6:11](=[O:19])-[#6@H:12]-1-[#6:6]-
[#6:5]=[#6:4]-[#6:3]-[#6@H:13]-1-[#6:14](=[O:18])-[#8:15]-[#6:16]-
[#6:17].[O:1]=[S:2]=[O:7]

Correctness of the mapping
MAPPET YES
ReactionMap YES
Marvin YES

Reaction no 89

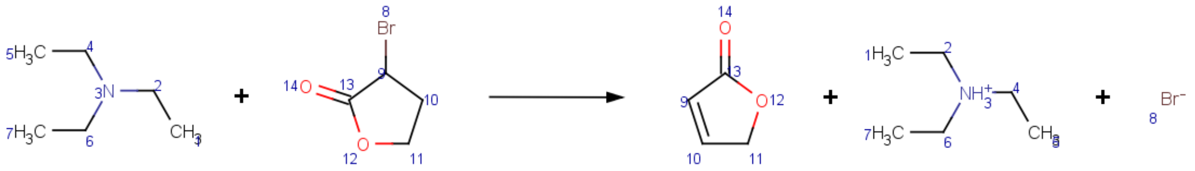

Correct mapped SMILES/SMARTS of the reaction:
[#6:1]-[#6:2]-[#7:3](-[#6:4]-[#6:5])-[#6:6]-[#6:7].[Br:8][#6:9]-1-[#6:10]-
[#6:11]-[#8:12]-[#6:13]-1=[O:14]>>[O:14]=[#6:13]-1-[#8:12]-[#6:11]-
[#6:10]=[#6:9]-1.[#6:5]-[#6:4]-[#7+:3](-[#6:2]-[#6:1])-[#6:6]-[#6:7].[Br-:8]

Correctness of the mapping
MAPPET YES
ReactionMap YES
Marvin YES

Reaction no 90

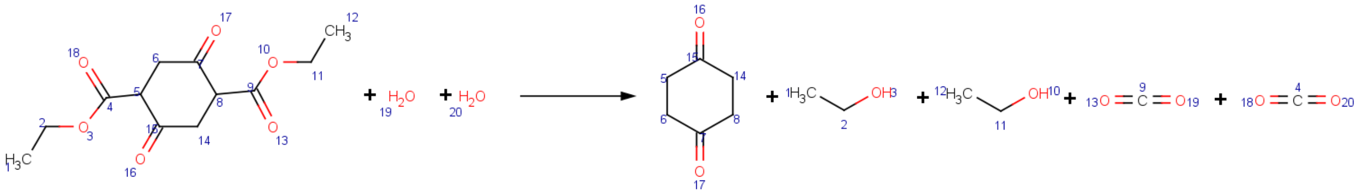

Correct mapped SMILES/SMARTS of the reaction:
[#6:1]-[#6:2]-[#8:3]-[#6:4](=[O:18])-[#6:5]-1-[#6:6]-[#6:7](=[O:17])-[#6:8](-
[#6:14]-[#6:15]-1=[O:16])-[#6:9](=[O:13])-[#8:10]-[#6:11]-
[#6:12].[#8:19].[#8:20]>>[O:16]=[#6:15]-1-[#6:14]-[#6:8]-[#6:7](=[O:17])-[#6:6]-
[#6:5]-1.[#6:1]-[#6:2]-[#8:3].[#6:12]-[#6:11]-
[#8:10].[O:13]=[C:9]=[O:19].[O:18]=[C:4]=[O:20]

Correctness of the mapping
MAPPET YES
ReactionMap YES
Marvin YES

Reaction no 91

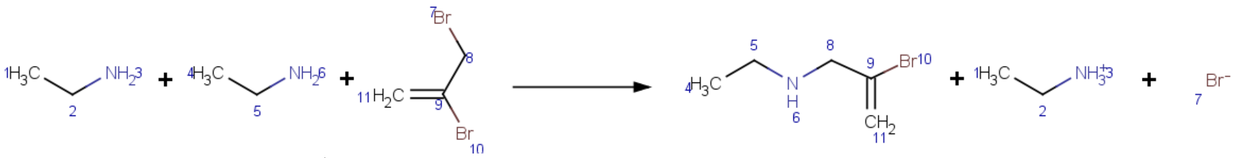

Correct mapped SMILES/SMARTS of the reaction:
[#6:1]-[#6:2]-[#7:3].[#6:4]-[#6:5]-[#7:6].[Br:7][#6:8]-
[#6:9]([Br:10])=[#6:11]>>[#6:4]-[#6:5]-[#7:6]-[#6:8]-
[#6:9]([Br:10])=[#6:11].[#6:1]-[#6:2]-[#7+:3].[Br-:7]

Correctness of the mapping
MAPPET YES
ReactionMap YES
Marvin YES

Reaction no 92

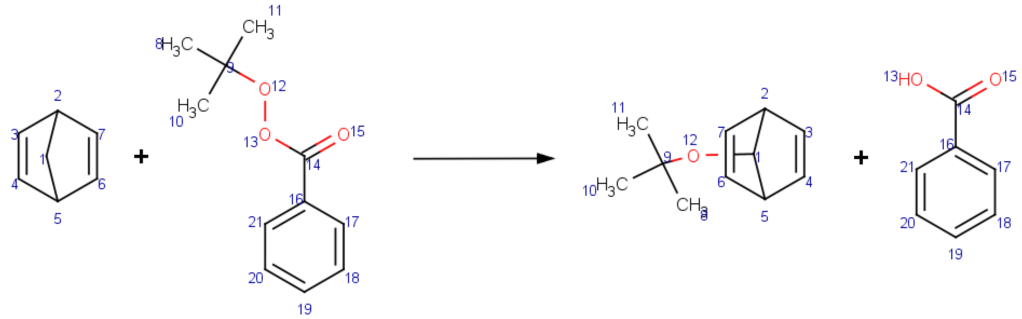

Correct mapped SMILES/SMARTS of the reaction:
[#6:1]-1-[#6:2]-2-[#6:3]=[#6:4]-[#6:5]-1-[#6:6]=[#6:7]-2.[#6:8][C:9]([#6:10])([#6:11])[#8:12]-[#8:13]-[#6:14](=[O:15])-[c:16]1[c:17][c:18][c:19][c:20][c:21]1>>[#6:10][C:9]([#6:8])([#6:11])[#8:12]-[#6:1]-1-[#6:2]-2-[#6:7]=[#6:6]-[#6:5]-1-[#6:4]=[#6:3]-2.[#8:13]-[#6:14](=[O:15])-[c:16]1[c:17][c:18][c:19][c:20][c:21]1

Correctness of the mapping
MAPPET YES
ReactionMap YES
Marvin YES

Reaction no 93

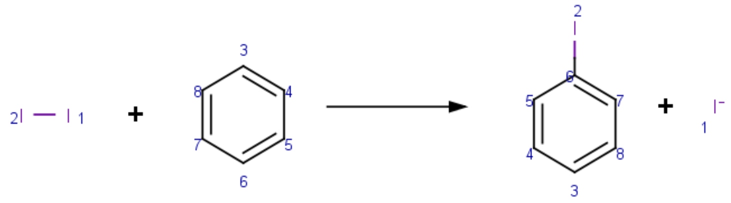

Correct mapped SMILES/SMARTS of the reaction:
[I:1][I:2].[#6:3]-1=[#6:4]-[#6:5]=[#6:6]-[#6:7]=[#6:8]-1>>[I:2][#6:6]-1=[#6:7]-[#6:8]=[#6:3]-[#6:4]=[#6:5]-1.[I-:1]

Correctness of the mapping
MAPPET YES
ReactionMap NO
Marvin YES

Reaction no 94

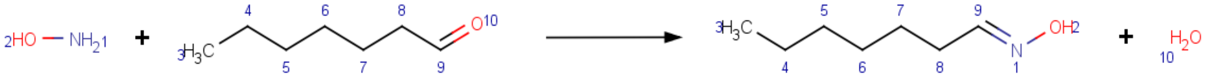

Correct mapped SMILES/SMARTS of the reaction:
[#7:1]-[#8:2].[#6:3]-[#6:4]-[#6:5]-[#6:6]-[#6:7]-[#6:8]-[#6:9]=[O:10]>>[#6:3]-[#6:4]-[#6:5]-[#6:6]-[#6:7]-[#6:8]\[#6:9]=[#7:1]\[#8:2].[#8:10]

Correctness of the mapping
MAPPET YES
ReactionMap YES
Marvin YES

Reaction no 95

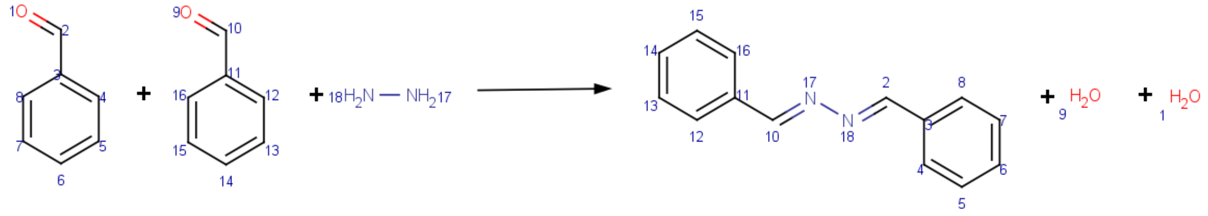

Correct mapped SMILES/SMARTS of the reaction:
[O:1]=[#6:2]-[#6:3]-1=[#6:4]-[#6:5]=[#6:6]-[#6:7]=[#6:8]-1.[O:9]=[#6:10]-[#6:11]-1=[#6:12]-[#6:13]=[#6:14]-[#6:15]=[#6:16]-1.[#7:17]-[#7:18]>>[#6:10](=[#7:17]/[#7:18]=[#6:2]/[#6:3]-1=[#6:8]-[#6:7]=[#6:6]-[#6:5]=[#6:4]1)\[#6:11]-1=[#6:12]-[#6:13]=[#6:14]-[#6:15]=[#6:16]1.[#8:9].[#8:1]

Correctness of the mapping
MAPPET   YES
ReactionMap YES
Marvin   YES

Reaction no 96

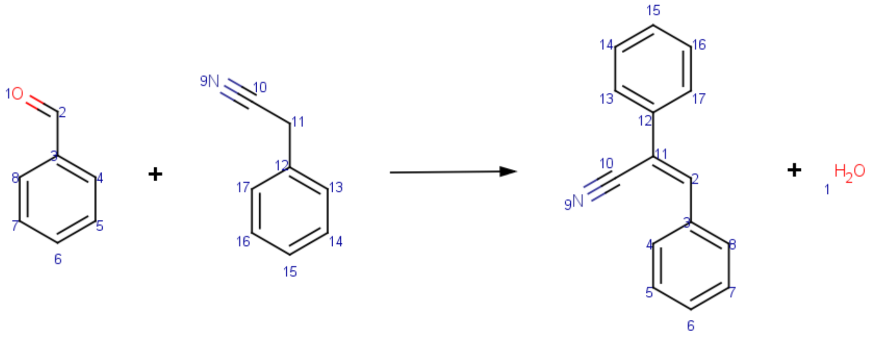

Correct mapped SMILES/SMARTS of the reaction:
[O:1]=[#6:2]-[#6:3]-1=[#6:4]-[#6:5]=[#6:6]-[#6:7]=[#6:8]-1.[N:9]#[C:10][#6:11]-[#6:12]-1=[#6:13]-[#6:14]=[#6:15]-[#6:16]=[#6:17]-1>>[N:9]#[C:10]\[#6:11](=[#6:2]/[#6:3]-1=[#6:8]-[#6:7]=[#6:6]-[#6:5]=[#6:4]1)-[#6:12]-1=[#6:13]-[#6:14]=[#6:15]-[#6:16]=[#6:17]-1.[#8:1]

Correctness of the mapping
MAPPET   NO
ReactionMap YES
Marvin   YES

Reaction no 97

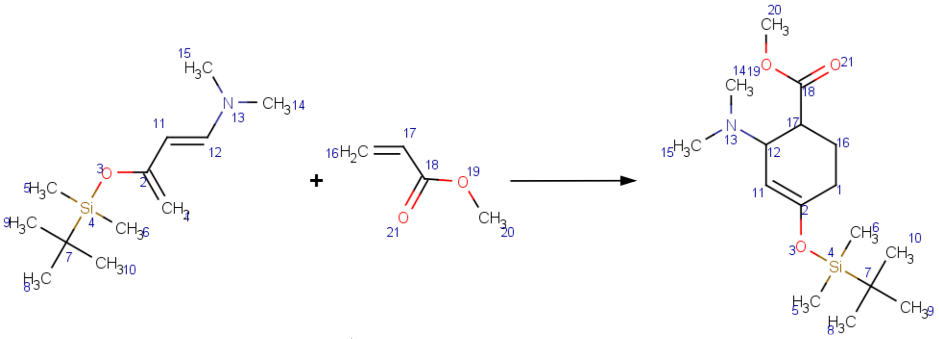

Correct mapped SMILES/SMARTS of the reaction:
[#6:14]-[#7:13](-[#6:15])\[#6:12]=[#6:11]\[#6:2](=[#6:1])-[#8:3][Si:4]([#6:5])([#6:6])[C:7]([#6:8])([#6:9])[#6:10].[#6:20]-[#8:19]-[#6:18](=[O:21])-[#6:17]=[#6:16]>>[#6:20]-[#8:19]-[#6:18](=[O:21])-[#6:17]-1-

[#6:16]-[#6:1]-[#6:2](-
[#8:3][Si:4]([#6:5])([#6:6])[C:7]([#6:9])([#6:8])[#6:10])=[#6:11]-[#6:12]-1-
[#7:13](-[#6:14])-[#6:15]

Correctness of the mapping
MAPPET YES
ReactionMap YES
Marvin NO

Reaction no 98

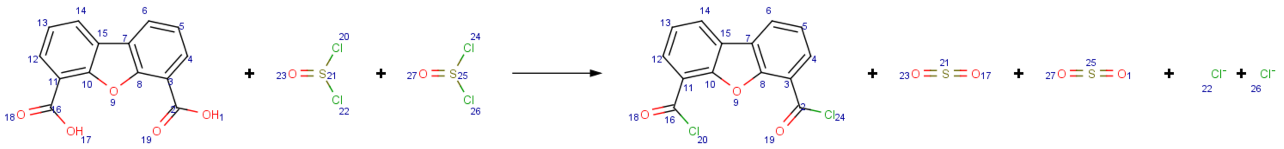

Correct mapped SMILES/SMARTS of the reaction:
[#8:1]-[#6:2](=[O:19])-[#6:3]-1=[#6:4]-[#6:5]=[#6:6]-[#6:7]-2=[#6:8]-1-[#8:9]-
[#6:10]-1=[#6:11](-[#6:12]=[#6:13]-[#6:14]=[#6:15]-2-1)-[#6:16](-
[#8:17])=[O:18].[Cl:20][S:21]([Cl:22])=[O:23].[Cl:24][S:25]([Cl:26])=[O:27]>>[Cl
:24][#6:2](=[O:19])-[#6:3]-1=[#6:4]-[#6:5]=[#6:6]-[#6:7]-2=[#6:8]-1-[#8:9]-
[#6:10]-1=[#6:11](-[#6:12]=[#6:13]-[#6:14]=[#6:15]-2-1)-
[#6:16]([Cl:20])=[O:18].[O:23]=[S:21]=[O:17].[O:27]=[S:25]=[O:1].[Cl-:22].[Cl-
:26]

Correctness of the mapping
MAPPET NO
ReactionMap NO
Marvin NO

Reaction no 99

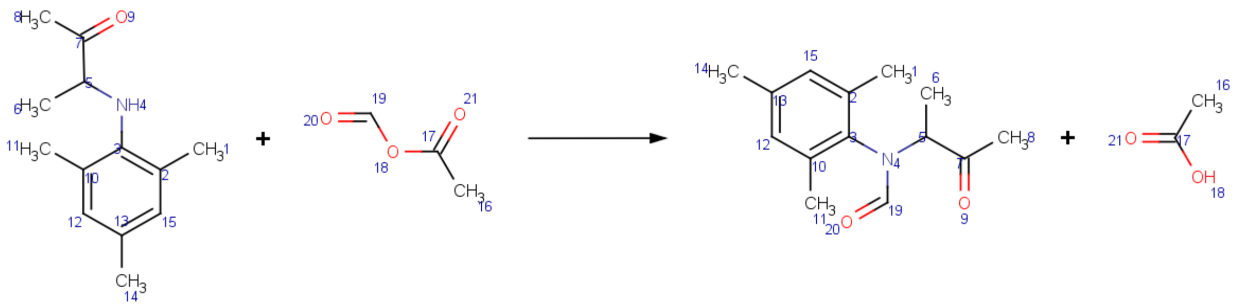

Correct mapped SMILES/SMARTS of the reaction:
[#6:6]-[#6:5](-[#7:4]-[#6:3]-1=[#6:2](-[#6:1])-[#6:15]=[#6:13](-[#6:14])-
[#6:12]=[#6:10]-1-[#6:11])-[#6:7](-[#6:8])=[O:9].[#6:16]-[#6:17](=[O:21])-
[#8:18]-[#6:19]=[O:20]>>[#6:6]-[#6:5](-[#7:4](-[#6:19]=[O:20])-[#6:3]-
1=[#6:10](-[#6:11])-[#6:12]=[#6:13](-[#6:14])-[#6:15]=[#6:2]-1-[#6:1])-[#6:7](-
[#6:8])=[O:9].[#6:16]-[#6:17](-[#8:18])=[O:21]

Correctness of the mapping
MAPPET YES
ReactionMap YES
Marvin YES

Reaction no 100

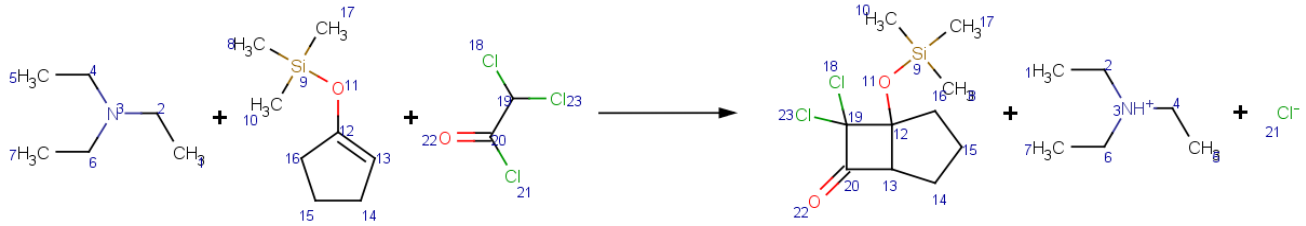

Correct mapped SMILES/SMARTS of the reaction:
[#6:1]-[#6:2]-[#7:3](-[#6:4]-[#6:5])-[#6:6]-[#6:7].[#6:8][Si:9]([#6:10])([#6:17])[#8:11]-[#6:12]-1=[#6:13]-[#6:14]-[#6:15]-[#6:16]-1.[Cl:18][#6:19]([Cl:23])-[#6:20]([Cl:21])=[O:22]>>[#6:17][Si:9]([#6:10])([#6:8])[#8:11][C:12]12[#6:16]-[#6:15]-[#6:14]-[#6:13]1-[#6:20](=[O:22])[C:19]2([Cl:23])[Cl:18].[#6:5]-[#6:4]-[#7+:3](-[#6:2]-[#6:1])-[#6:6]-[#6:7].[Cl-:21]

Correctness of the mapping
MAPPET   YES
ReactionMap YES
Marvin   YES

Reaction no 101

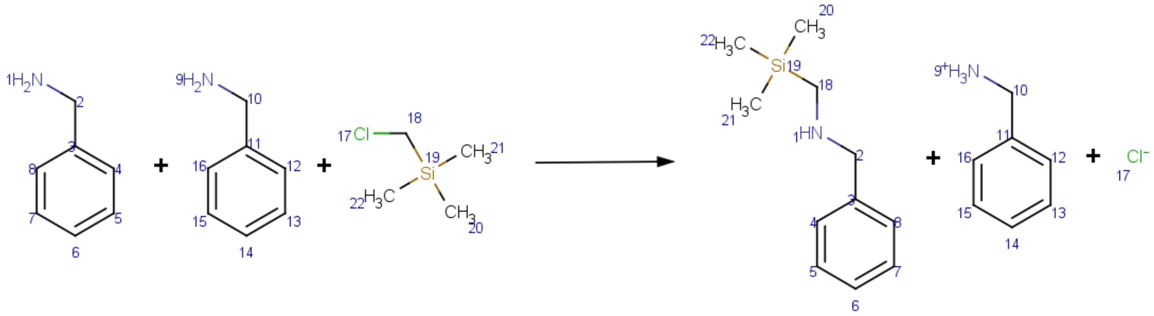

Correct mapped SMILES/SMARTS of the reaction:
[#7:1]-[#6:2]-[#6:3]-1=[#6:4]-[#6:5]=[#6:6]-[#6:7]=[#6:8]-1.[#7:9]-[#6:10]-[#6:11]-1=[#6:12]-[#6:13]=[#6:14]-[#6:15]=[#6:16]-1.[#6:20][Si:19]([#6:21])([#6:22])[#6:18][Cl:17]>>[#6:22][Si:19]([#6:21])([#6:20])[#6:18]-[#7:1]-[#6:2]-[#6:3]-1=[#6:8]-[#6:7]=[#6:6]-[#6:5]=[#6:4]-1.[#7+:9]-[#6:10]-[#6:11]-1=[#6:12]-[#6:13]=[#6:14]-[#6:15]=[#6:16]-1.[Cl-:17]

Correctness of the mapping
MAPPET   YES
ReactionMap YES
Marvin   YES

Reaction no 102

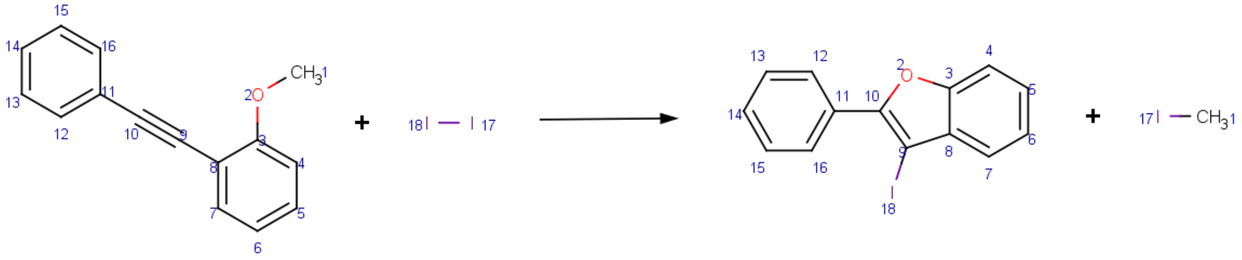

Correct mapped SMILES/SMARTS of the reaction:
[#6:1]-[#8:2]-[#6:3]-1=[#6:4]-[#6:5]=[#6:6]-[#6:7]=[#6:8]-1[C:9]#[C:10][#6:11]-1=[#6:12]-[#6:13]=[#6:14]-[#6:15]=[#6:16]-1.[I:17][I:18]>>[I:18][#6:9]-

1=[#6:10](-[#8:2]-[#6:3]-2=[#6:4]-[#6:5]=[#6:6]-[#6:7]=[#6:8]-1-2)-[#6:11]-1=[#6:16]-[#6:15]=[#6:14]-[#6:13]=[#6:12]-1.[#6:1][I:17]

Correctness of the mapping
MAPPET YES
ReactionMap YES
Marvin NO

Reaction no 103

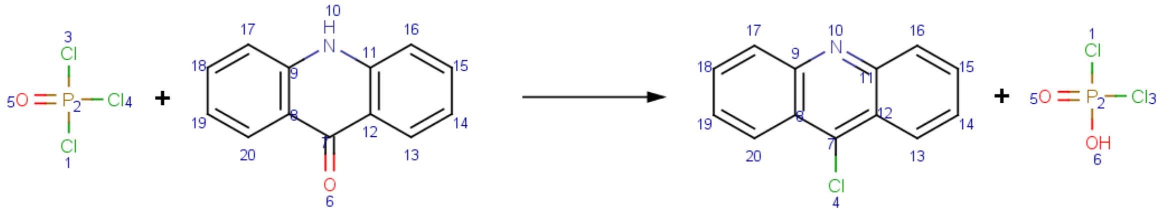

Correct mapped SMILES/SMARTS of the reaction:
[Cl:1][P:2]([Cl:3])([Cl:4])=[O:5].[O:6]=[#6:7]-1-[#6:12]-2=[#6:11](-[#7:10]-[#6:9]-3=[#6:8]-1-[#6:20]=[#6:19]-[#6:18]=[#6:17]-3)-[#6:16]=[#6:15]-[#6:14]=[#6:13]-2>>[Cl:4][#6:7]-1=[#6:12]-2-[#6:13]=[#6:14]-[#6:15]=[#6:16]-[#6:11]-2=[#7:10]-[#6:9]-2=[#6:8]-1-[#6:20]=[#6:19]-[#6:18]=[#6:17]-2.[#8:6][P:2]([Cl:1])([Cl:3])=[O:5]

Correctness of the mapping
MAPPET YES
ReactionMap YES
Marvin YES

Reaction no 104

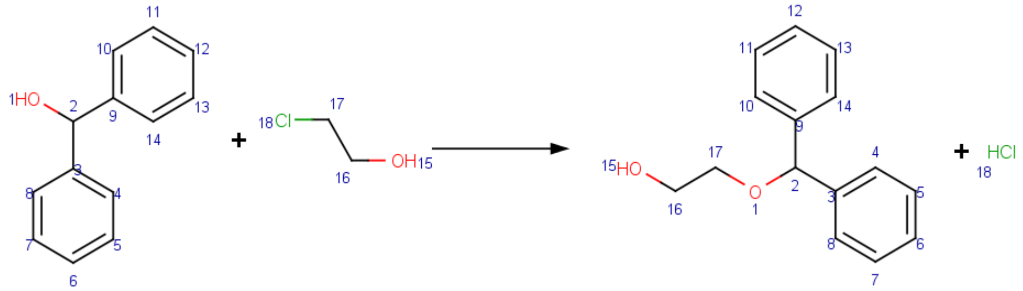

Correct mapped SMILES/SMARTS of the reaction:
[#8:1]-[#6:2](-[#6:3]-1=[#6:4]-[#6:5]=[#6:6]-[#6:7]=[#6:8]-1)-[#6:9]-1=[#6:10]-[#6:11]=[#6:12]-[#6:13]=[#6:14]-1.[#8:15]-[#6:16]-[#6:17][Cl:18]>>[#8:15]-[#6:16]-[#6:17]-[#8:1]-[#6:2](-[#6:3]-1=[#6:4]-[#6:5]=[#6:6]-[#6:7]=[#6:8]-1)-[#6:9]-1=[#6:10]-[#6:11]=[#6:12]-[#6:13]=[#6:14]-1.[Cl:18]

Correctness of the mapping
MAPPET YES
ReactionMap YES
Marvin YES

Reaction no 105

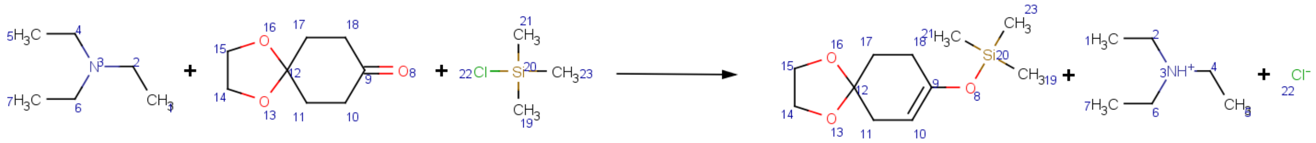

Correct mapped SMILES/SMARTS of the reaction:
[#6:1]-[#6:2]-[#7:3](-[#6:4]-[#6:5])-[#6:6]-[#6:7].[O:8]=[#6:9]-1-[#6:10]-[#6:11][C:12]2([#6:17]-[#6:18]-1)[#8:13]-[#6:14]-[#6:15]-[#8:16]2.[#6:19][Si:20]([#6:21])([#6:23])[Cl:22]>>[#6:23][Si:20]([#6:21])([#6:19])[#8:8]-[#6:9]-1=[#6:10]-[#6:11][C:12]2([#6:17]-[#6:18]-1)[#8:13]-[#6:14]-[#6:15]-[#8:16]2.[#6:5]-[#6:4]-[#7+:3](-[#6:2]-[#6:1])-[#6:6]-[#6:7].[Cl-:22]

Correctness of the mapping
MAPPET YES
ReactionMap YES
Marvin YES

Reaction no 106

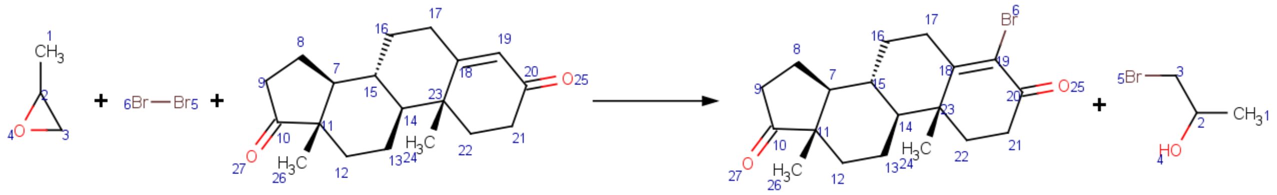

Correct mapped SMILES/SMARTS of the reaction:
[#6:1]-[#6:2]-1-[#6:3]-[#8:4]-1.[Br:5][Br:6].[#6:26][C@:11]12[#6:12]-[#6:13]-[#6@H:14]3-[#6@@H:15](-[#6:16]-[#6:17]-[#6:18]4=[#6:19]-[#6:20](=[O:25])-[#6:21]-[#6:22][C@:23]34[#6:24])-[#6@@H:7]1-[#6:8]-[#6:9]-[#6:10]2=[O:27]>>[#6:26][C@:11]12[#6:12]-[#6:13]-[#6@H:14]3-[#6@@H:15](-[#6:16]-[#6:17]-[#6:18]4=[#6:19]([Br:6])-[#6:20](=[O:25])-[#6:21]-[#6:22][C@:23]34[#6:24])-[#6@@H:7]1-[#6:8]-[#6:9]-[#6:10]2=[O:27].[#6:1]-[#6:2](-[#8:4])-[#6:3][Br:5]

Correctness of the mapping
MAPPET YES
ReactionMap YES
Marvin NO

Reaction no 107

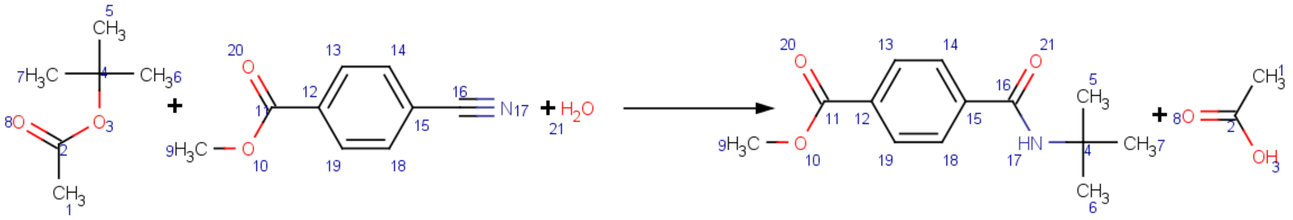

Correct mapped SMILES/SMARTS of the reaction:
[CH3:1][C:2](=[O:8])[O:3][C:4]([CH3:5])([CH3:6])[CH3:7].[CH3:9][O:10][C:11](=[O:20])[C:12]1=[CH:13][CH:14]=[C:15]([CH:18]=[CH:19]1)[C:16]#[N:17].[OH2:21]>>[CH3:9][O:10][C:11](=[O:20])[C:12]1=[CH:13][CH:14]=[C:15]([CH:18]=[CH:19]1)[C:16](=[O:21])[NH:17][C:4]([CH3:7])([CH3:6])[CH3:5].[CH3:1][C:2]([OH:3])=[O:8]

Correctness of the mapping
MAPPET YES

ReactionMap YES
Marvin YES

Reaction no 108

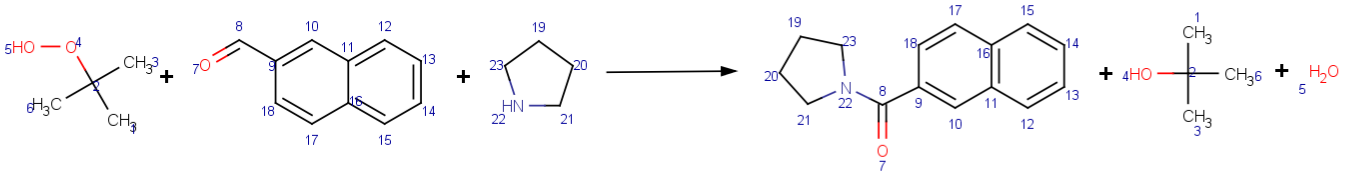

Correct mapped SMILES/SMARTS of the reaction:
[#6:1][C:2]([#6:3])([#6:6])[#8:4]-[#8:5].[O:7]=[#6:8]-[#6:9]-1=[#6:10]-[#6:11]-
2=[#6:12]-[#6:13]=[#6:14]-[#6:15]=[#6:16]-2-[#6:17]=[#6:18]-1.[#6:19]-1-[#6:20]-
[#6:21]-[#7:22]-[#6:23]-1>>[O:7]=[#6:8](-[#7:22]-1-[#6:21]-[#6:20]-[#6:19]-
[#6:23]-1)-[#6:9]-1=[#6:10]-[#6:11]-2=[#6:16](-[#6:15]=[#6:14]-[#6:13]=[#6:12]-
2)-[#6:17]=[#6:18]-1.[#6:3][C:2]([#6:1])([#6:6])[#8:4].[#8:5]

Correctness of the mapping
MAPPET YES
ReactionMap YES
Marvin YES

Reaction no 109

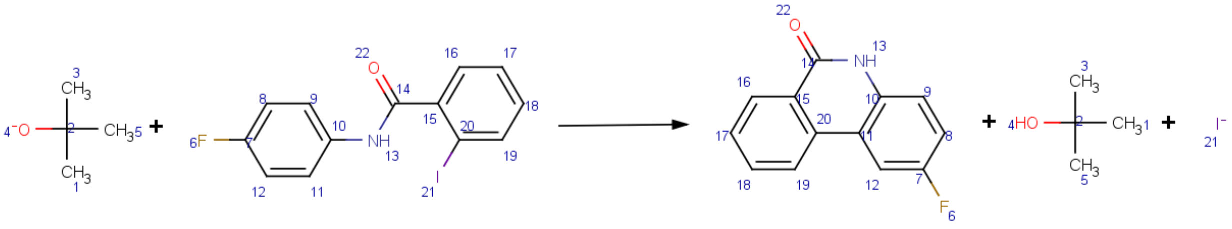

Correct mapped SMILES/SMARTS of the reaction:
[#6:1][C:2]([#6:3])([#6:5])[#8-:4].[F:6][#6:7]-1=[#6:8]-[#6:9]=[#6:10](-[#7:13]-
[#6:14](=[O:22])-[#6:15]-2=[#6:16]-[#6:17]=[#6:18]-[#6:19]=[#6:20]-2[I:21])-
[#6:11]=[#6:12]-1>>[F:6][#6:7]-1=[#6:12]-[#6:11]-2=[#6:10](-[#7:13]-
[#6:14](=[O:22])-[#6:15]-3=[#6:20]-2-[#6:19]=[#6:18]-[#6:17]=[#6:16]-3)-
[#6:9]=[#6:8]-1.[#6:5][C:2]([#6:3])([#6:1])[#8:4].[I-:21]

Correctness of the mapping
MAPPET YES
ReactionMap YES
Marvin YES

Reaction no 110

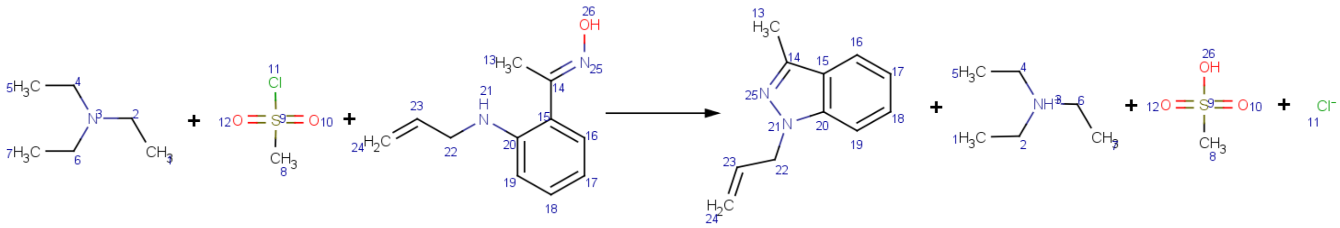

Correct mapped SMILES/SMARTS of the reaction:
[#6:1]-[#6:2]-[#7:3](-[#6:4]-[#6:5])-[#6:6]-
[#6:7].[#6:8][S:9]([Cl:11])(=[O:10])=[O:12].[#6:13]\[#6:14](=[#7:25]/[#8:26])-
[#6:15]-1=[#6:16]-[#6:17]=[#6:18]-[#6:19]=[#6:20]-1-[#7:21]-[#6:22]-
[#6:23]=[#6:24]>>[#6:13]-[#6:14]-1=[#7:25]-[#7:21](-[#6:22]-[#6:23]=[#6:24])-
[#6:20]-2=[#6:15]-1-[#6:16]=[#6:17]-[#6:18]=[#6:19]-2.[#6:7]-[#6:6]-[#7+:3](-
[#6:4]-[#6:5])-[#6:2]-[#6:1].[#6:8][S:9]([#8:26])(=[O:10])=[O:12].[Cl-:11]

Correctness of the mapping
MAPPET YES
ReactionMap YES
Marvin YES

Reaction no 111

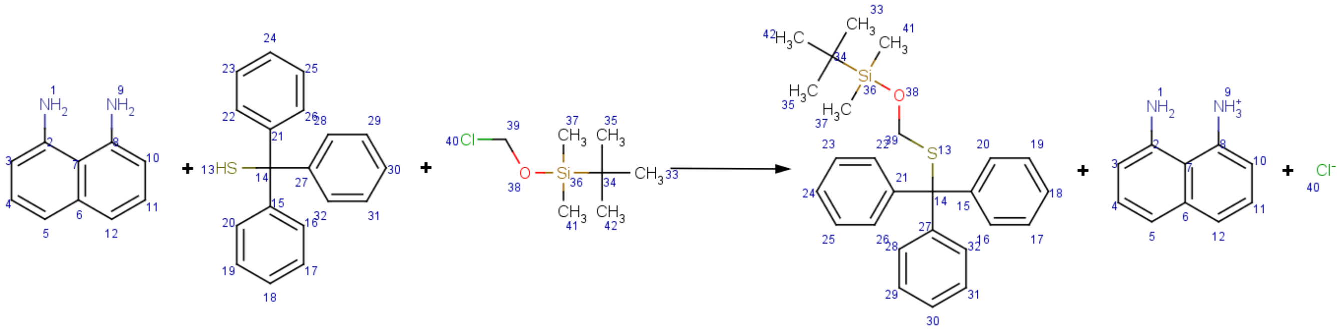

Correct mapped SMILES/SMARTS of the reaction:
[#7:1]-[#6:2]-1=[#6:3]-[#6:4]=[#6:5]-[#6:6]-2=[#6:7]-1-[#6:8](-[#7:9])=[#6:10]-[#6:11]=[#6:12]-2.[#16:13][C:14]([#6:15]-1=[#6:16]-[#6:17]=[#6:18]-[#6:19]=[#6:20]-1)([#6:21]-1=[#6:22]-[#6:23]=[#6:24]-[#6:25]=[#6:26]-1)[#6:27]-1=[#6:28]-[#6:29]=[#6:30]-[#6:31]=[#6:32]-1.[#6:33][C:34]([#6:35])([#6:42])[Si:36]([#6:37])([#6:41])[#8:38]-[#6:39][Cl:40]>>[#6:42][C:34]([#6:35])([#6:33])[Si:36]([#6:37])([#6:41])[#8:38]-[#6:39]-[#16:13][C:14]([#6:27]-1=[#6:32]-[#6:31]=[#6:30]-[#6:29]=[#6:28]-1)([#6:21]-1=[#6:26]-[#6:25]=[#6:24]-[#6:23]=[#6:22]-1)[#6:15]-1=[#6:20]-[#6:19]=[#6:18]-[#6:17]=[#6:16]-1.[#7:1]-[#6:2]-1=[#6:3]-[#6:4]=[#6:5]-[#6:6]-2=[#6:7]-1-[#6:8](-[#7+:9])=[#6:10]-[#6:11]=[#6:12]-2.[Cl-:40]

Correctness of the mapping
MAPPET YES
ReactionMap YES
Marvin YES

Reaction no 112

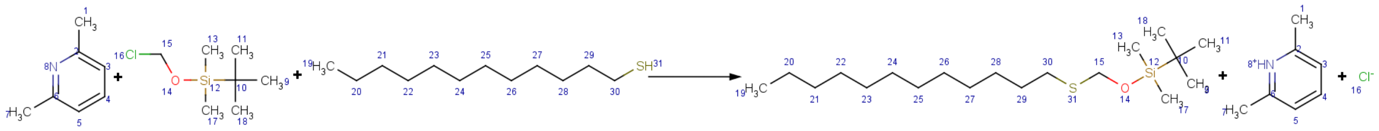

Correct mapped SMILES/SMARTS of the reaction:
[#6:1]-[#6:2]-1=[#6:3]-[#6:4]=[#6:5]-[#6:6](-[#6:7])=[#7:8]-1.[#6:9][C:10]([#6:11])([#6:18])[Si:12]([#6:13])([#6:17])[#8:14]-[#6:15][Cl:16].[#6:19]-[#6:20]-[#6:21]-[#6:22]-[#6:23]-[#6:24]-[#6:25]-[#6:26]-[#6:27]-[#6:28]-[#6:29]-[#6:30]-[#16:31]>>[#6:19]-[#6:20]-[#6:21]-[#6:22]-[#6:23]-[#6:24]-[#6:25]-[#6:26]-[#6:27]-[#6:28]-[#6:29]-[#6:30]-[#16:31]-[#6:15]-[#8:14][Si:12]([#6:17])([#6:13])[C:10]([#6:11])([#6:9])[#6:18].[#6:1]-[#6:2]-1=[#6:3]-[#6:4]=[#6:5]-[#6:6](-[#6:7])=[#7+:8]-1.[Cl-:16]

Correctness of the mapping
MAPPET YES
ReactionMap YES
Marvin YES

Reaction no 113

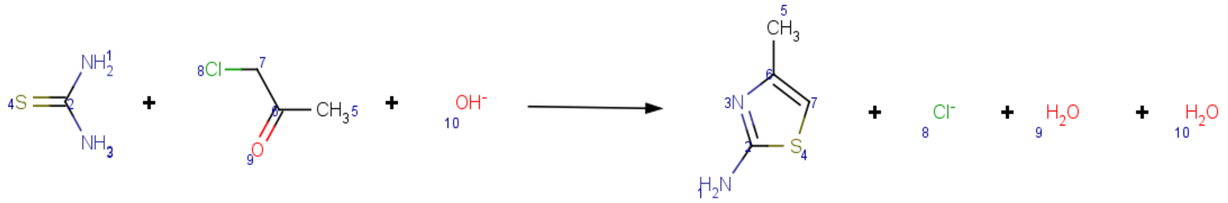

Correct mapped SMILES/SMARTS of the reaction:
[#7:1]-[#6:2](-[#7:3])=[S:4].[#6:5]-[#6:6](=[O:9])-[#6:7][Cl:8].[#8-:10]>>[#6:5]-[#6:6]-1=[#6:7]-[#16:4]-[#6:2](-[#7:1])=[#7:3]-1.[Cl-:8].[#8:9].[#8:10]

Correctness of the mapping
MAPPET   YES
ReactionMap YES
Marvin   NO

Reaction no 114

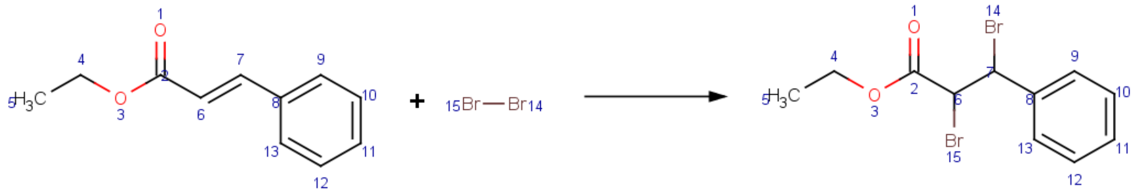

Correct mapped SMILES/SMARTS of the reaction:
[#6:5]-[#6:4]-[#8:3]-[#6:2](=[O:1])\[#6:6]=[#6:7]\[#6:8]-1=[#6:9]-[#6:10]=[#6:11]-[#6:12]=[#6:13]1.[Br:14][Br:15]>>[#6:5]-[#6:4]-[#8:3]-[#6:2](=[O:1])-[#6:6]([Br:15])-[#6:7]([Br:14])-[#6:8]-1=[#6:9]-[#6:10]=[#6:11]-[#6:12]=[#6:13]-1

Correctness of the mapping
MAPPET   YES
ReactionMap YES
Marvin   YES

Reaction no 115

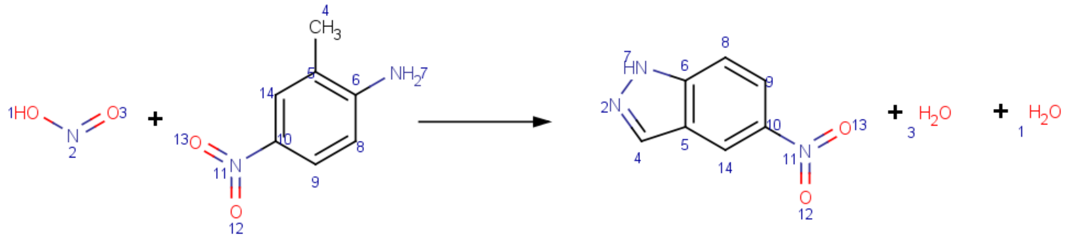

Correct mapped SMILES/SMARTS of the reaction:
[#8:1]-[#7:2]=[O:3].[#6:4]-[#6:5]-1=[#6:6](-[#7:7])-[#6:8]=[#6:9]-[#6:10](=[#6:14]-1)[N:11](=[O:12])=[O:13]>>[O:13]=[N:11](=[O:12])[#6:10]-1=[#6:14]-[#6:5]-2=[#6:6](-[#7:7]-[#7:2]=[#6:4]-2)-[#6:8]=[#6:9]-1.[#8:3].[#8:1]

Correctness of the mapping
MAPPET   YES
ReactionMap YES
Marvin   YES

Reaction no 116

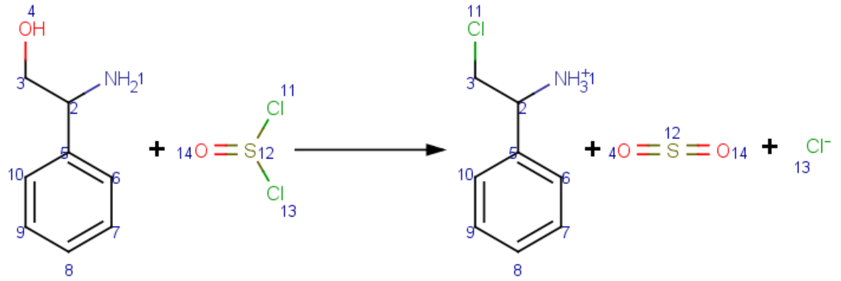

Correct mapped SMILES/SMARTS of the reaction:
[NH2:1][CH:2]([CH2:3][OH:4])[C:5]1=[CH:6][CH:7]=[CH:8][CH:9]=[CH:10]1.[Cl:11][S:12]([Cl:13])=[O:14]>>[NH3+:1][CH:2]([CH2:3][Cl:11])[C:5]1=[CH:6][CH:7]=[CH:8][CH:9]=[CH:10]1.[O:4]=[S:12]=[O:14].[Cl-:13]

Correctness of the mapping
MAPPET   NO
ReactionMap YES
Marvin   YES

Reaction no 117

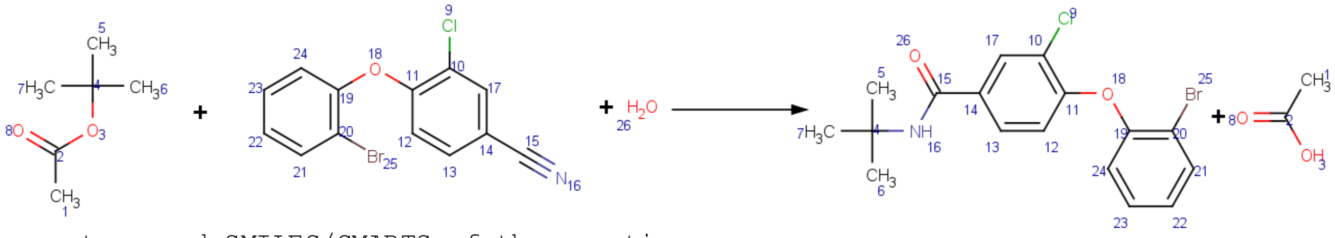

Correct mapped SMILES/SMARTS of the reaction:
[CH3:1][C:2](=[O:8])[O:3][C:4]([CH3:5])([CH3:6])[CH3:7].[Cl:9][C:10]1=[C:11]([O:18][C:19]2=[C:20]([Br:25])[CH:21]=[CH:22][CH:23]=[CH:24]2)[CH:12]=[CH:13][C:14](=[CH:17]1)[C:15]#[N:16].[OH2:26]>>[CH3:7][C:4]([CH3:6])([CH3:5])[NH:16][C:15](=[O:26])[C:14]1=[CH:17][C:10]([Cl:9])=[C:11]([O:18][C:19]2=[C:20]([Br:25])[CH:21]=[CH:22][CH:23]=[CH:24]2)[CH:12]=[CH:13]1.[CH3:1][C:2]([OH:3])=[O:8]

Correctness of the mapping
MAPPET   YES
ReactionMap YES
Marvin   YES

Reaction no 118

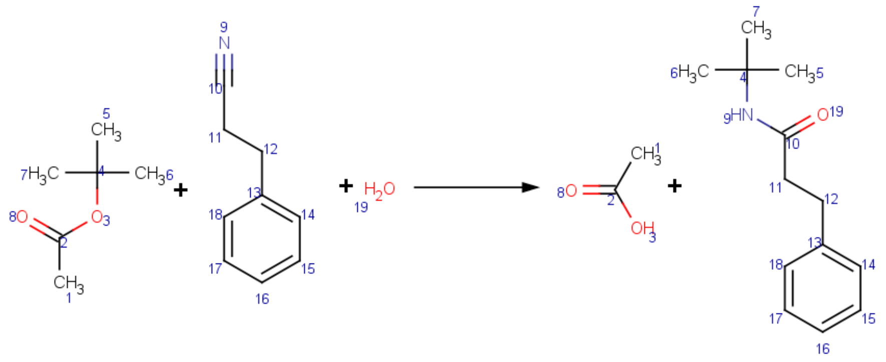

Correct mapped SMILES/SMARTS of the reaction:
[CH3:1][C:2](=[O:8])[O:3][C:4]([CH3:5])([CH3:6])[CH3:7].[N:9]#[C:10][CH2:11][CH2:12][C:13]1=[CH:14][CH:15]=[CH:16][CH:17]=[CH:18]1.[OH2:19]>>[CH3:1][C:2]([OH:3])=[O:8].[CH3:7][C:4]([CH3:6])([CH3:5])[NH:9][C:10](=[O:19])[CH2:11][CH2:12][C:13]1=[CH:14][CH:15]=[CH:16][CH:17]=[CH:18]1

Correctness of the mapping

MAPPET YES
ReactionMap YES
Marvin YES

Reaction no 119

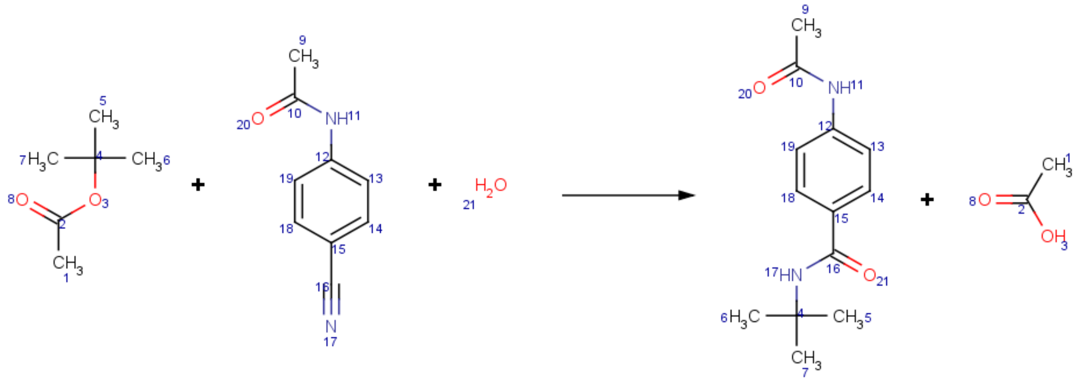

Correct mapped SMILES/SMARTS of the reaction:
[CH3:1][C:2](=[O:8])[O:3][C:4]([CH3:5])([CH3:6])[CH3:7].[CH3:9][C:10](=[O:20])[NH:11][C:12]1=[CH:13][CH:14]=[C:15]([CH:18]=[CH:19]1)[C:16]#[N:17].[OH2:21]>>[CH3:9][C:10](=[O:20])[NH:11][C:12]1=[CH:13][CH:14]=[C:15]([CH:18]=[CH:19]1)[C:16](=[O:21])[NH:17][C:4]([CH3:7])([CH3:6])[CH3:5].[CH3:1][C:2]([OH:3])=[O:8]

Correctness of the mapping
MAPPET YES
ReactionMap YES
Marvin YES

Reaction no 120

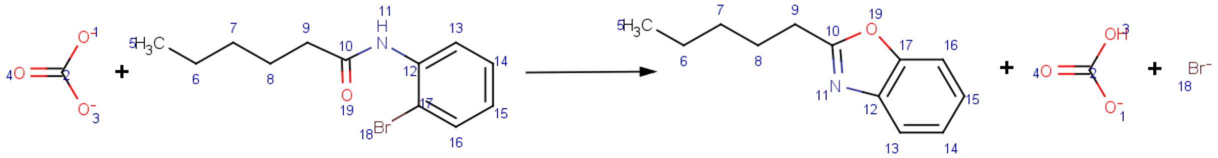

Correct mapped SMILES/SMARTS of the reaction:
[#8-:1]-[#6:2](-[#8-:3])=[O:4].[#6:5]-[#6:6]-[#6:7]-[#6:8]-[#6:9]-[#6:10](=[O:19])-[#7:11]-[#6:12]-1=[#6:13]-[#6:14]=[#6:15]-[#6:16]=[#6:17]-1[Br:18]>>[#6:5]-[#6:6]-[#6:7]-[#6:8]-[#6:9]-[#6:10]-1=[#7:11]-[#6:12]-2=[#6:17](-[#8:19]-1)-[#6:16]=[#6:15]-[#6:14]=[#6:13]-2.[#8:3]-[#6:2](-[#8-:1])=[O:4].[Br-:18]

Correctness of the mapping
MAPPET YES
ReactionMap YES
Marvin YES

Reaction no 121

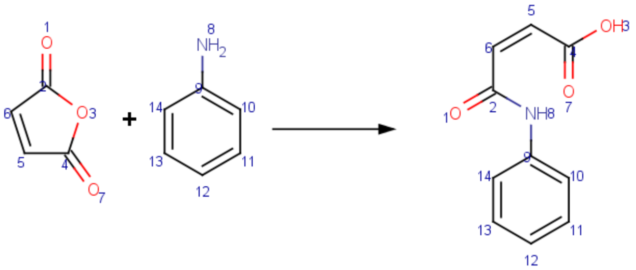

Correct mapped SMILES/SMARTS of the reaction:
[O:1]=[#6:2]-1-[#8:3]-[#6:4](=[O:7])-[#6:5]=[#6:6]-1.[#7:8]-[#6:9]-1=[#6:10]-[#6:11]=[#6:12]-[#6:13]=[#6:14]-1>>[#8:3]-[#6:4](=[O:7])\[#6:5]=[#6:6]/[#6:2](=[O:1])-[#7:8]-[#6:9]-1=[#6:10]-[#6:11]=[#6:12]-[#6:13]=[#6:14]-1

Correctness of the mapping
MAPPET   YES
ReactionMap YES
Marvin   YES

Reaction no 122

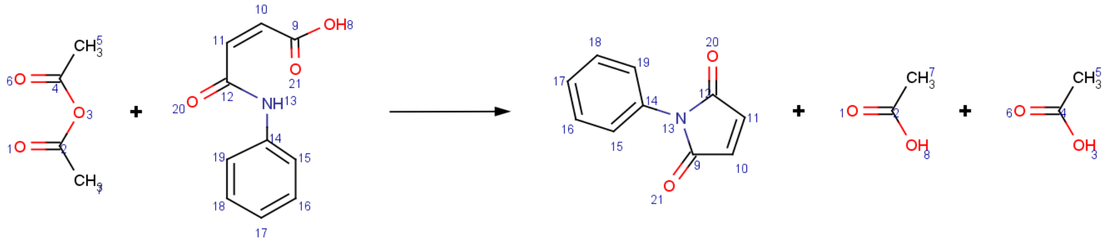

Correct mapped SMILES/SMARTS of the reaction:
[#6:5]-[#6:4](=[O:6])-[#8:3]-[#6:2](-[#6:7])=[O:1].[#8:8]-[#6:9](=[O:21])\[#6:10]=[#6:11]/[#6:12](=[O:20])-[#7:13]-[#6:14]-1=[#6:15]-[#6:16]=[#6:17]-[#6:18]=[#6:19]-1>>[O:20]=[#6:12]-1-[#6:11]=[#6:10]-[#6:9](=[O:21])-[#7:13]-1-[#6:14]-1=[#6:15]-[#6:16]=[#6:17]-[#6:18]=[#6:19]-1.[#6:7]-[#6:2](-[#8:8])=[O:1].[#6:5]-[#6:4](-[#8:3])=[O:6]

Correctness of the mapping
MAPPET   YES
ReactionMap YES
Marvin   YES

Reaction no 123

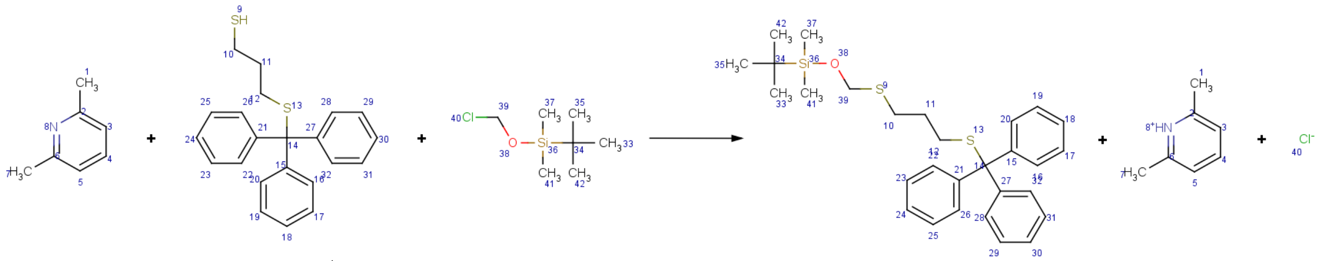

Correct mapped SMILES/SMARTS of the reaction:
[#6:1]-[#6:2]-1=[#6:3]-[#6:4]=[#6:5]-[#6:6](-[#6:7])=[#7:8]-1.[#16:9]-[#6:10]-[#6:11]-[#6:12]-[#16:13][C:14]([#6:15]-1=[#6:16]-[#6:17]=[#6:18]-[#6:19]=[#6:20]-1)([#6:21]-1=[#6:22]-[#6:23]=[#6:24]-[#6:25]=[#6:26]-1)[#6:27]-1=[#6:28]-[#6:29]=[#6:30]-[#6:31]=[#6:32]-1.[#6:33][C:34]([#6:35])([#6:42])[Si:36]([#6:37])([#6:41])[#8:38]-[#6:39][Cl:40]>>[#6:35][C:34]([#6:33])([#6:42])[Si:36]([#6:41])([#6:37])[#8:38]-[#6:39]-[#16:9]-[#6:10]-[#6:11]-[#6:12]-[#16:13][C:14]([#6:27]-1=[#6:32]-[#6:31]=[#6:30]-[#6:29]=[#6:28]-1)([#6:21]-1=[#6:26]-[#6:25]=[#6:24]-

[#6:23]=[#6:22]-1)[#6:15]-1=[#6:20]-[#6:19]=[#6:18]-[#6:17]=[#6:16]-1.[#6:1]-[#6:2]-1=[#6:3]-[#6:4]=[#6:5]-[#6:6](-[#6:7])=[#7+:8]-1.[Cl-:40]

Correctness of the mapping
MAPPET YES
ReactionMap YES
Marvin YES

Reaction no 124

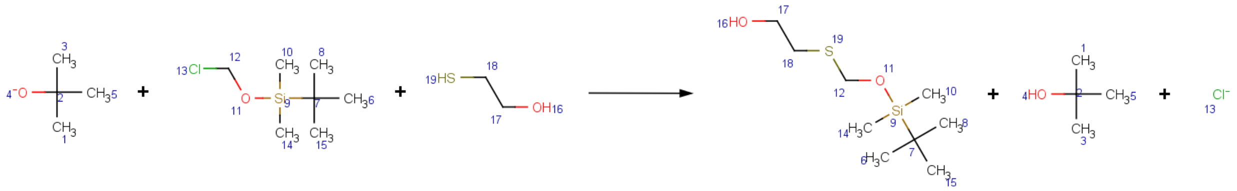

Correct mapped SMILES/SMARTS of the reaction:
[#6:1][C:2]([#6:3])([#6:5])[#8-:4].[#6:6][C:7]([#6:8])([#6:15])[Si:9]([#6:10])([#6:14])[#8:11]-[#6:12][Cl:13].[#8:16]-[#6:17]-[#6:18]-[#16:19]>>[#6:15][C:7]([#6:8])([#6:6])[Si:9]([#6:10])([#6:14])[#8:11]-[#6:12]-[#16:19]-[#6:18]-[#6:17]-[#8:16].[#6:3][C:2]([#6:1])([#6:5])[#8:4].[Cl-:13]

Correctness of the mapping
MAPPET YES
ReactionMap YES
Marvin YES

Reaction no 125

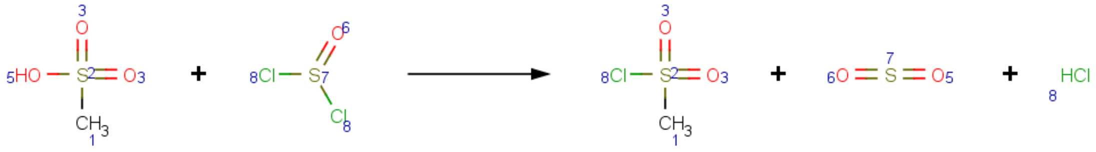

[CH3:1][S:2](=[O:3])(=[O:3])[OH:5].[O:6]=[S:7]([Cl:8])[Cl:8]>>[CH3:1][S:2](=[O:3])(=[O:3])[Cl:8].[O:6]=[S:7]=[O:5].[ClH:8]

Correctness of the mapping
MAPPET NO
ReactionMap YES
Marvin YES

Reaction no 126

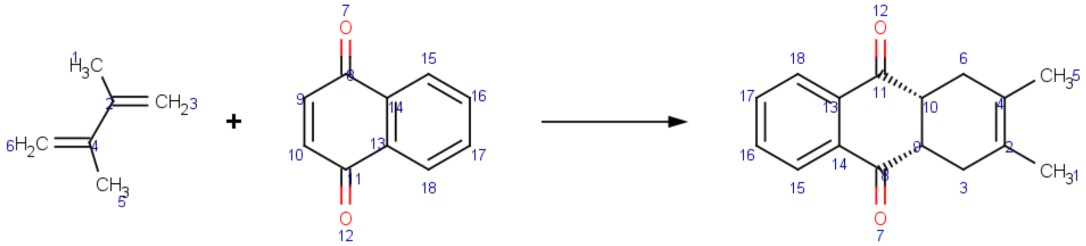

Correct mapped SMILES/SMARTS of the reaction:
[#6:1]-[#6:2](=[#6:3])-[#6:4](-[#6:5])=[#6:6].[O:7]=[#6:8]-1-[#6:9]=[#6:10]-[#6:11](=[O:12])-[#6:13]-2=[#6:14]-1-[#6:15]=[#6:16]-[#6:17]=[#6:18]-2>>[#6:1]-[#6:2]-1=[#6:4](-[#6:5])-[#6:6]-[#6@@H:10]-2-[#6@H:9](-[#6:3]-1)-[#6:8](=[O:7])-[#6:14]-1=[#6:15]-[#6:16]=[#6:17]-[#6:18]=[#6:13]-1-[#6:11]-2=[O:12]

```
Correctness of the mapping
MAPPET    YES
ReactionMap YES
Marvin    NO
```

Reaction no 127

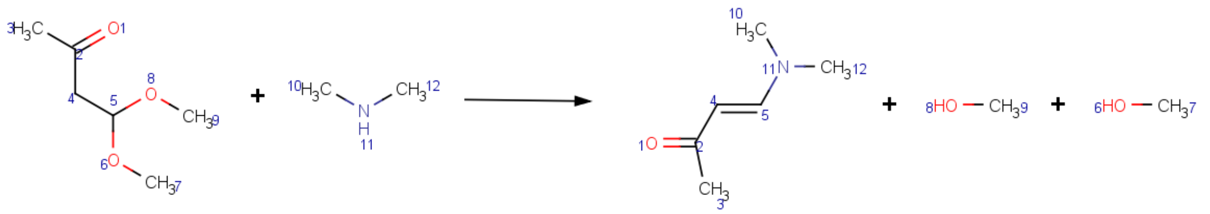

Correct mapped SMILES/SMARTS of the reaction:
[#6:7]-[#8:6]-[#6:5](-[#6:4]-[#6:2](-[#6:3])=[O:1])-[#8:8]-[#6:9].[#6:10]-
[#7:11]-[#6:12]>>[#6:12]-[#7:11](-[#6:10])\[#6:5]=[#6:4]\[#6:2](-
[#6:3])=[O:1].[#6:9]-[#8:8].[#6:7]-[#8:6]

Correctness of the mapping
MAPPET YES
ReactionMap YES
Marvin YES

Reaction no 128

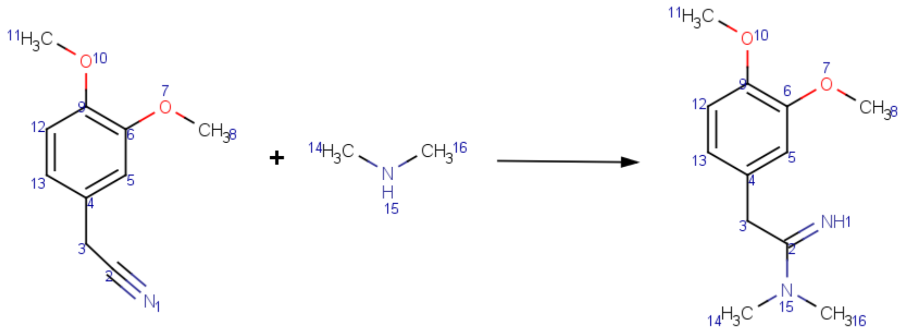

Correct mapped SMILES/SMARTS of the reaction:
[#6:11]-[#8:10]-[#6:9]-1=[#6:6](-[#8:7]-[#6:8])-[#6:5]=[#6:4](-
[#6:3][C:2]#[N:1])-[#6:13]=[#6:12]-1.[#6:14]-[#7:15]-[#6:16]>>[#6:11]-[#8:10]-
[#6:9]-1=[#6:6](-[#8:7]-[#6:8])-[#6:5]=[#6:4](-[#6:3]-[#6:2](=[#7:1])-[#7:15](-
[#6:16])-[#6:14])-[#6:13]=[#6:12]-1

Correctness of the mapping
MAPPET YES
ReactionMap YES
Marvin YES

Reaction no 129

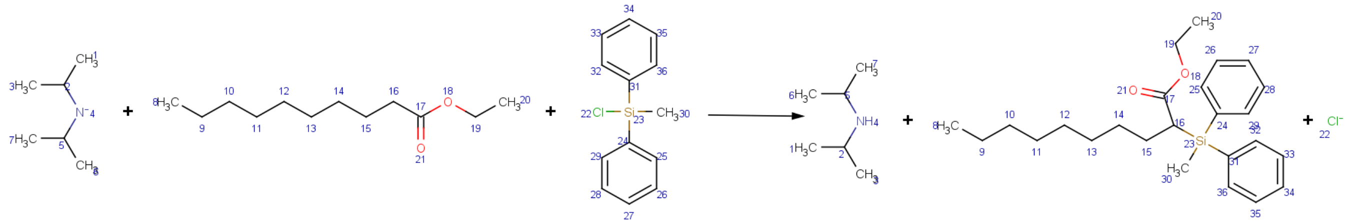

Correct mapped SMILES/SMARTS of the reaction:
[#6:1]-[#6:2](-[#6:3])-[#7-:4]-[#6:5](-[#6:6])-[#6:7].[#6:8]-[#6:9]-[#6:10]-
[#6:11]-[#6:12]-[#6:13]-[#6:14]-[#6:15]-[#6:16]-[#6:17](=[O:21])-[#8:18]-
[#6:19]-[#6:20].[#6:30][Si:23]([Cl:22])([#6:24]-1=[#6:25]-[#6:26]=[#6:27]-
[#6:28]=[#6:29]-1)[#6:31]-1=[#6:32]-[#6:33]=[#6:34]-[#6:35]=[#6:36]-1>>[#6:7]-
[#6:5](-[#6:6])-[#7:4]-[#6:2](-[#6:3])-[#6:1].[#6:8]-[#6:9]-[#6:10]-[#6:11]-
[#6:12]-[#6:13]-[#6:14]-[#6:15]-[#6:16](-[#6:17](=[O:21])-[#8:18]-[#6:19]-
[#6:20])[Si:23]([#6:30])([#6:24]-1=[#6:25]-[#6:26]=[#6:27]-[#6:28]=[#6:29]-
1)[#6:31]-1=[#6:32]-[#6:33]=[#6:34]-[#6:35]=[#6:36]-1.[Cl-:22]

Correctness of the mapping
MAPPET   YES
ReactionMap YES
Marvin   YES

Reaction no 130

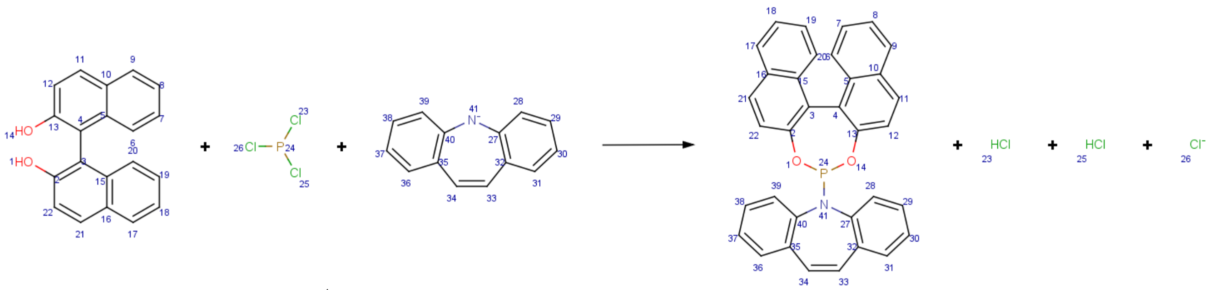

Correct mapped SMILES/SMARTS of the reaction:
[#8:14]-[#6:13]-1=[#6:12]-[#6:11]=[#6:10]-2-[#6:9]=[#6:8]-[#6:7]=[#6:6]-[#6:5]-
2=[#6:4]-1-[#6:3]-1=[#6:2](-[#8:1])-[#6:22]=[#6:21]-[#6:16]-2=[#6:15]-1-
[#6:20]=[#6:19]-[#6:18]=[#6:17]-2.[Cl:23][#15:24]([Cl:25])[Cl:26].[#7-:41]-1-
[#6:27]-2=[#6:32](-[#6:31]=[#6:30]-[#6:29]=[#6:28]-2)-[#6:33]=[#6:34]-[#6:35]-
2=[#6:40]-1-[#6:39]=[#6:38]-[#6:37]=[#6:36]-2>>[#8:14]-1-[#15:24](-[#8:1]-
[#6:2]-2=[#6:3](-[#6:15]-3=[#6:16](-[#6:17]=[#6:18]-[#6:19]=[#6:20]-3)-
[#6:21]=[#6:22]-2)-[#6:4]-2=[#6:5]-3-[#6:6]=[#6:7]-[#6:8]=[#6:9]-[#6:10]-
3=[#6:11]-[#6:12]=[#6:13]-1-2)-[#7:41]-1-[#6:27]-2=[#6:32](-[#6:31]=[#6:30]-
[#6:29]=[#6:28]-2)-[#6:33]=[#6:34]-[#6:35]-2=[#6:40]-1-[#6:39]=[#6:38]-
[#6:37]=[#6:36]-2.[Cl:23].[Cl:25].[Cl-:26]

Correctness of the mapping
MAPPET   YES
ReactionMap YES
Marvin   NO

Reaction no 131

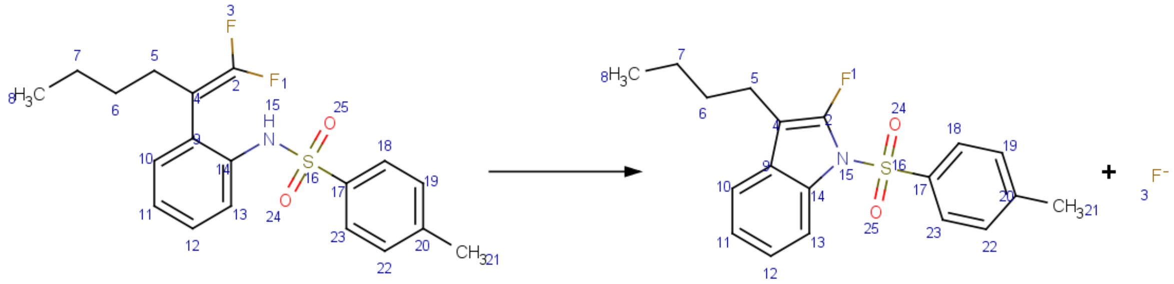

Correct mapped SMILES/SMARTS of the reaction:
[#6:8]-[#6:7]-[#6:6]-[#6:5]\[#6:4](=[#6:2](\[F:1])[F:3])-[#6:9]-1=[#6:10]-
[#6:11]=[#6:12]-[#6:13]=[#6:14]-1-[#7:15][S:16](=[O:24])(=[O:25])[#6:17]-
1=[#6:18]-[#6:19]=[#6:20](-[#6:21])-[#6:22]=[#6:23]-1>>[#6:8]-[#6:7]-[#6:6]-
[#6:5]-[#6:4]-1=[#6:2]([F:1])-[#7:15](-[#6:14]-2=[#6:13]-[#6:12]=[#6:11]-
[#6:10]=[#6:9]-1-2)[S:16](=[O:25])(=[O:24])[#6:17]-1=[#6:18]-[#6:19]=[#6:20](-
[#6:21])-[#6:22]=[#6:23]-1.[F-:3]

Correctness of the mapping
MAPPET   YES
ReactionMap YES
Marvin   YES

Reaction no 132

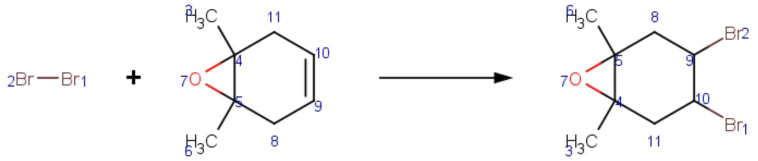

Correct mapped SMILES/SMARTS of the reaction:
[Br:1][Br:2].[#6:3][C:4]12[#6:11]-[#6:10]=[#6:9]-
[#6:8][C:5]1([#6:6])[#8:7]2>>[#6:6][C:5]12[#6:8]-[#6:9]([Br:2])-[#6:10]([Br:1])-
[#6:11][C:4]1([#6:3])[#8:7]2

Correctness of the mapping
MAPPET YES
ReactionMap YES
Marvin YES

Reaction no 133

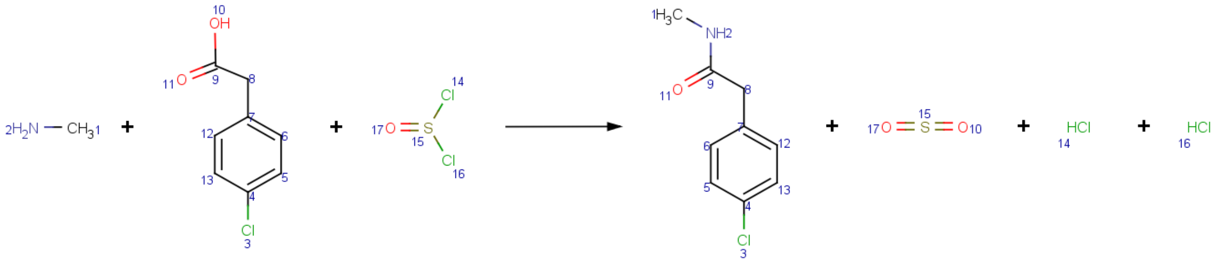

Correct mapped SMILES/SMARTS of the reaction:
[#6:1]-[#7:2].[#8:10]-[#6:9](=[O:11])-[#6:8]-[#6:7]-1=[#6:6]-
[#6:5]=[#6:4]([Cl:3])-[#6:13]=[#6:12]-1.[Cl:14][S:15]([Cl:16])=[O:17]>>[#6:1]-
[#7:2]-[#6:9](=[O:11])-[#6:8]-[#6:7]-1=[#6:12]-[#6:13]=[#6:4]([Cl:3])-
[#6:5]=[#6:6]-1.[O:17]=[S:15]=[O:10].[Cl:14].[Cl:16]

Correctness of the mapping
MAPPET YES
ReactionMap YES
Marvin YES

Reaction no 134

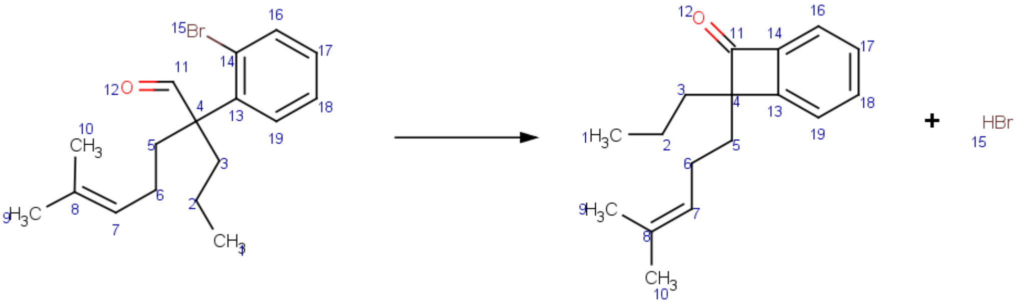

Correct mapped SMILES/SMARTS of the reaction:
[#6:1]-[#6:2]-[#6:3][C:4]([#6:5]-[#6:6]\[#6:7]=[#6:8](\[#6:9])-
[#6:10])([#6:11]=[O:12])[#6:13]-1=[#6:14]([Br:15])-[#6:16]=[#6:17]-
[#6:18]=[#6:19]-1>>[#6:1]-[#6:2]-[#6:3][C:4]1([#6:5]-
[#6:6]\[#6:7]=[#6:8](\[#6:10])-[#6:9])[#6:11](=[O:12])-[#6:14]-2=[#6:16]-
[#6:17]=[#6:18]-[#6:19]=[#6:13]1-2.[Br:15]

```
Correctness of the mapping
MAPPET      YES
ReactionMap YES
Marvin      YES

Reaction no  135
```

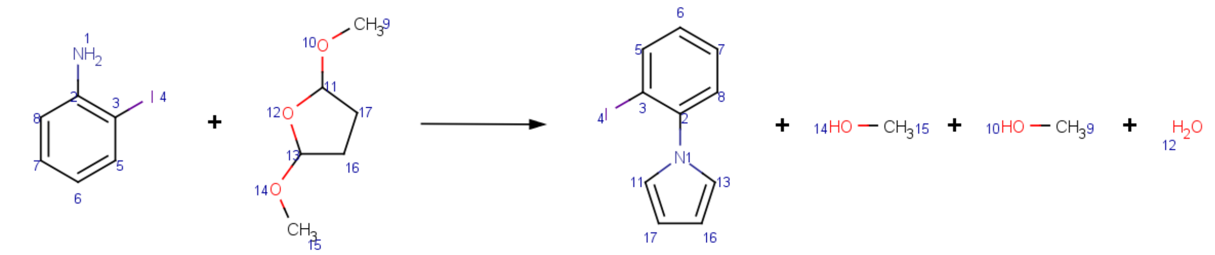

```
Correct mapped SMILES/SMARTS of the reaction:
[#7:1]-[#6:2]-1=[#6:3]([I:4])-[#6:5]=[#6:6]-[#6:7]=[#6:8]-1.[#6:9]-[#8:10]-
[#6:11]-1-[#6:17]-[#6:16]-[#6:13](-[#8:14]-[#6:15])-[#8:12]-1>>[I:4][#6:3]-
1=[#6:5]-[#6:6]=[#6:7]-[#6:8]=[#6:2]-1-[#7:1]-1-[#6:13]=[#6:16]-[#6:17]=[#6:11]-
1.[#6:15]-[#8:14].[#6:9]-[#8:10].[#8:12]

Correctness of the mapping
MAPPET      YES
ReactionMap YES
Marvin      NO

Reaction no  136
```

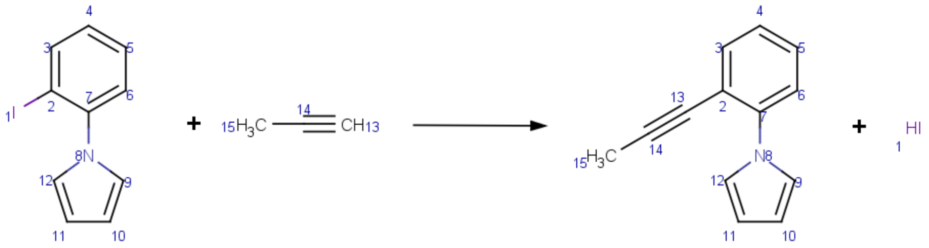

```
Correct mapped SMILES/SMARTS of the reaction:
[I:1][#6:2]-1=[#6:3]-[#6:4]=[#6:5]-[#6:6]=[#6:7]-1-[#7:8]-1-[#6:9]=[#6:10]-
[#6:11]=[#6:12]-1.[#6:15][C:14]#[C:13]>>[#6:15][C:14]#[C:13][#6:2]-1=[#6:3]-
[#6:4]=[#6:5]-[#6:6]=[#6:7]-1-[#7:8]-1-[#6:9]=[#6:10]-[#6:11]=[#6:12]-1.[I:1]

Correctness of the mapping
MAPPET      YES
ReactionMap YES
Marvin      YES

Reaction no  137
```

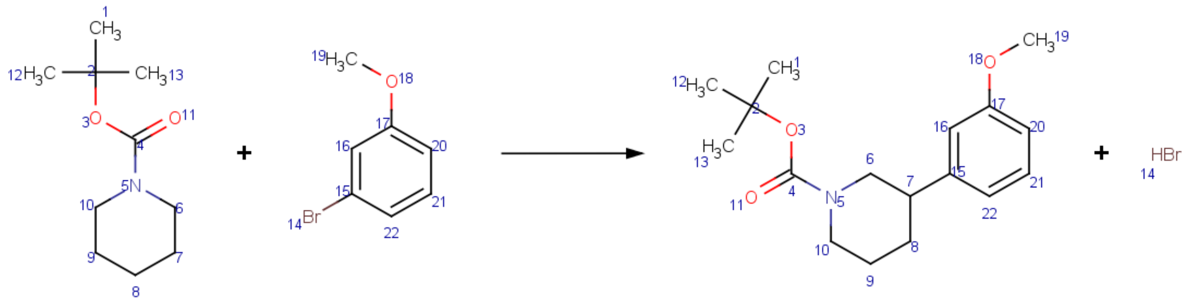

Correct mapped SMILES/SMARTS of the reaction:
[#6:1][C:2]([#6:12])([#6:13])[#8:3]-[#6:4](=[O:11])-[#7:5]-1-[#6:6]-[#6:7]-[#6:8]-[#6:9]-[#6:10]-1.[#6:19]-[#8:18]-[#6:17]-1=[#6:20]-[#6:21]=[#6:22]-[#6:15]([Br:14])=[#6:16]-1>>[#6:19]-[#8:18]-[#6:17]-1=[#6:20]-[#6:21]=[#6:22]-[#6:15](=[#6:16]-1)-[#6:7]-1-[#6:8]-[#6:9]-[#6:10]-[#7:5](-[#6:6]-1)-[#6:4](=[O:11])-[#8:3][C:2]([#6:12])([#6:1])[#6:13].[Br:14]

Correctness of the mapping
MAPPET YES
ReactionMap YES
Marvin YES

Reaction no 138

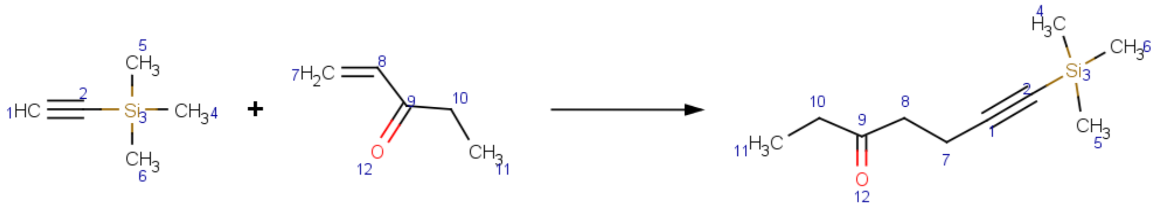

Correct mapped SMILES/SMARTS of the reaction:
[#6:4][Si:3]([#6:5])([#6:6])[C:2]#[C:1].[#6:11]-[#6:10]-[#6:9](=[O:12])-[#6:8]=[#6:7]>>[#6:11]-[#6:10]-[#6:9](=[O:12])-[#6:8]-[#6:7][C:1]#[C:2][Si:3]([#6:6])([#6:5])[#6:4]

Correctness of the mapping
MAPPET YES
ReactionMap YES
Marvin YES

Reaction no 139

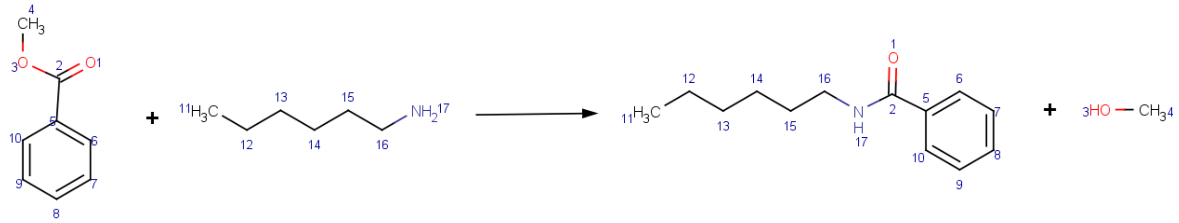

Correct mapped SMILES/SMARTS of the reaction:
[#6:4]-[#8:3]-[#6:2](=[O:1])-[#6:5]-1=[#6:6]-[#6:7]=[#6:8]-[#6:9]=[#6:10]-1.[#6:11]-[#6:12]-[#6:13]-[#6:14]-[#6:15]-[#6:16]-[#7:17]>>[#6:11]-[#6:12]-[#6:13]-[#6:14]-[#6:15]-[#6:16]-[#7:17]-[#6:2](=[O:1])-[#6:5]-1=[#6:6]-[#6:7]=[#6:8]-[#6:9]=[#6:10]-1.[#6:4]-[#8:3]

Correctness of the mapping
MAPPET YES
ReactionMap YES
Marvin YES

Reaction no 140

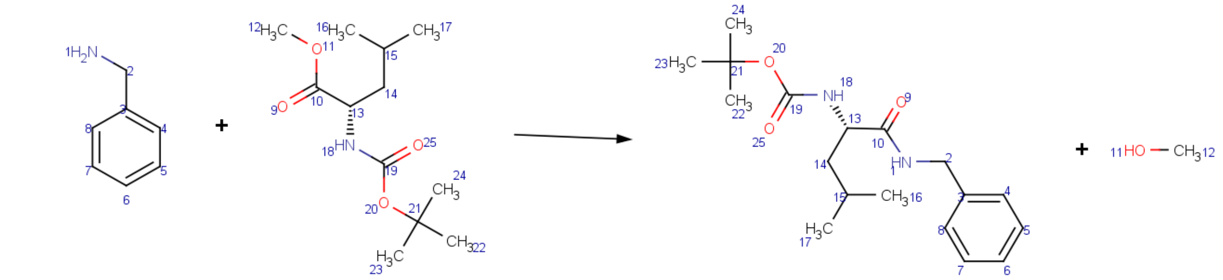

Correct mapped SMILES/SMARTS of the reaction:
[#7:1]-[#6:2]-[#6:3]-1=[#6:4]-[#6:5]=[#6:6]-[#6:7]=[#6:8]-1.[#6:12]-[#8:11]-
[#6:10](=[O:9])-[#6@H:13](-[#6:14]-[#6:15](-[#6:16])-[#6:17])-[#7:18]-
[#6:19](=[O:25])-[#8:20][C:21]([#6:22])([#6:23])[#6:24]>>[#6:16]-[#6:15](-
[#6:17])-[#6:14]-[#6@H:13](-[#7:18]-[#6:19](=[O:25])-
[#8:20][C:21]([#6:23])([#6:22])[#6:24])-[#6:10](=[O:9])-[#7:1]-[#6:2]-[#6:3]-
1=[#6:4]-[#6:5]=[#6:6]-[#6:7]=[#6:8]-1.[#6:12]-[#8:11]

Correctness of the mapping
MAPPET   YES
ReactionMap YES
Marvin   YES

Reaction no 141

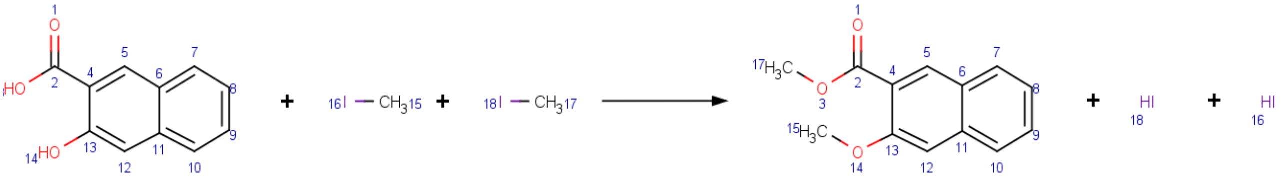

Correct mapped SMILES/SMARTS of the reaction:
[#8:3]-[#6:2](=[O:1])-[#6:4]-1=[#6:5]-[#6:6]-2=[#6:7]-[#6:8]=[#6:9]-
[#6:10]=[#6:11]-2-[#6:12]=[#6:13]-1-
[#8:14].[#6:15][I:16].[#6:17][I:18]>>[#6:17]-[#8:3]-[#6:2](=[O:1])-[#6:4]-
1=[#6:5]-[#6:6]-2=[#6:7]-[#6:8]=[#6:9]-[#6:10]=[#6:11]-2-[#6:12]=[#6:13]-1-
[#8:14]-[#6:15].[I:18].[I:16]

Correctness of the mapping
MAPPET   YES
ReactionMap YES
Marvin   YES

Reaction no 142

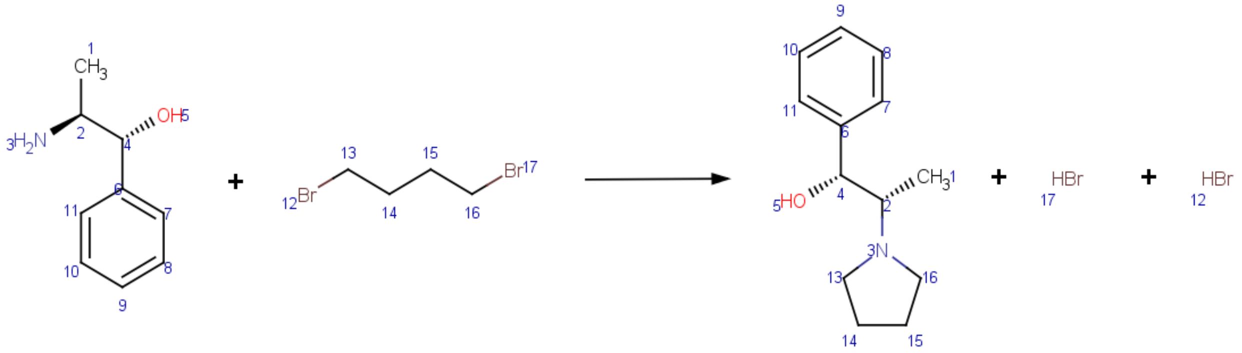

Correct mapped SMILES/SMARTS of the reaction:
[#6:1]-[#6@H:2](-[#7:3])-[#6@H:4](-[#8:5])-[#6:6]-1=[#6:7]-[#6:8]=[#6:9]-[#6:10]=[#6:11]-1.[Br:12][#6:13]-[#6:14]-[#6:15]-[#6:16][Br:17]>>[#6:1]-[#6@@H:2](-[#6@H:4](-[#8:5])-[#6:6]-1=[#6:11]-[#6:10]=[#6:9]-[#6:8]=[#6:7]-1)-[#7:3]-1-[#6:16]-[#6:15]-[#6:14]-[#6:13]-1.[Br:17].[Br:12]

Correctness of the mapping
MAPPET   YES
ReactionMap YES
Marvin   YES

Reaction no 143

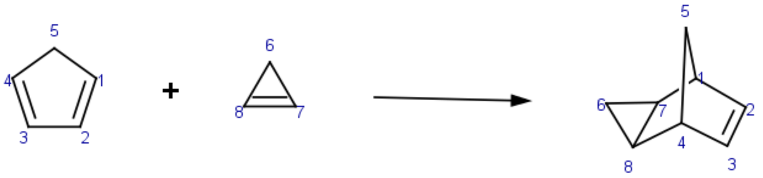

Correct mapped SMILES/SMARTS of the reaction:
[#6:5]-1-[#6:1]=[#6:2]-[#6:3]=[#6:4]-1.[#6:6]-1-[#6:7]=[#6:8]-1>>[#6:6]-1-[#6:8]-2-[#6:7]-1-[#6:1]-1-[#6:5]-[#6:4]-2-[#6:3]=[#6:2]-1

Correctness of the mapping
MAPPET   YES
ReactionMap YES
Marvin   NO

Reaction no 144

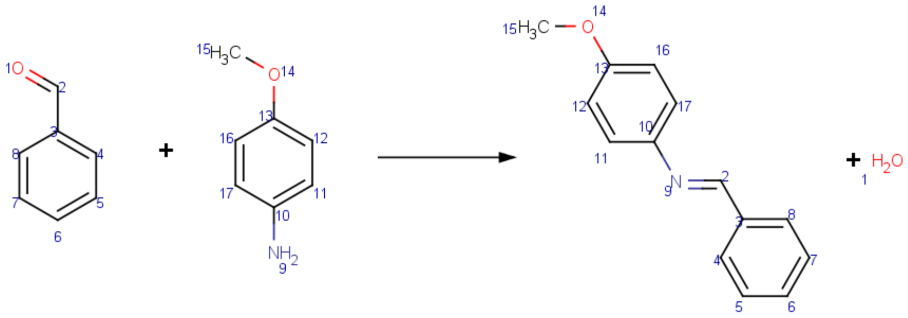

Correct mapped SMILES/SMARTS of the reaction:
[O:1]=[#6:2]-[#6:3]-1=[#6:4]-[#6:5]=[#6:6]-[#6:7]=[#6:8]-1.[#6:15]-[#8:14]-[#6:13]-1=[#6:12]-[#6:11]=[#6:10](-[#7:9])-[#6:17]=[#6:16]-1>>[#6:15]-[#8:14]-[#6:13]-1=[#6:16]-[#6:17]=[#6:10](-[#6:11]=[#6:12]-1)\[#7:9]=[#6:2]\[#6:3]-1=[#6:8]-[#6:7]=[#6:6]-[#6:5]=[#6:4]1.[#8:1]

Correctness of the mapping
MAPPET   YES
ReactionMap YES
Marvin   YES

Reaction no 145

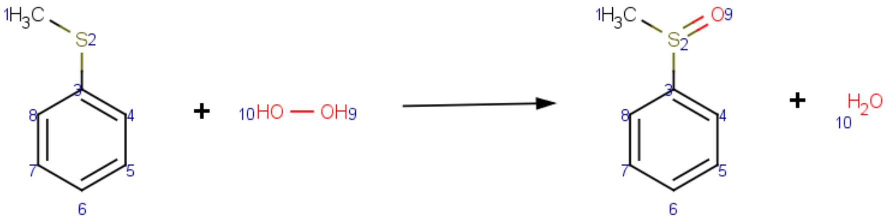

Correct mapped SMILES/SMARTS of the reaction:
[#6:1]-[#16:2]-[#6:3]-1=[#6:4]-[#6:5]=[#6:6]-[#6:7]=[#6:8]-1.[#8:9]-[#8:10]>>[#6:1][S:2](=[O:9])[#6:3]-1=[#6:4]-[#6:5]=[#6:6]-[#6:7]=[#6:8]-1.[#8:10]

Correctness of the mapping
MAPPET YES
ReactionMap YES
Marvin YES

Reaction no 146

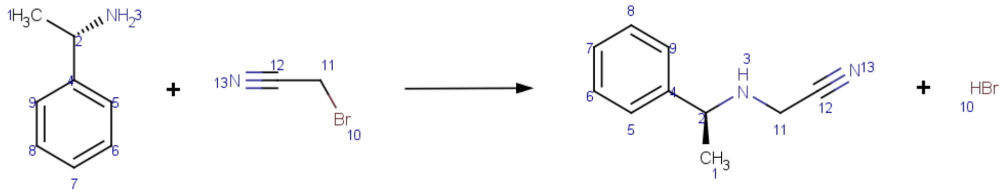

Correct mapped SMILES/SMARTS of the reaction:
[#6:1]-[#6@H:2](-[#7:3])-[#6:4]-1=[#6:5]-[#6:6]=[#6:7]-[#6:8]=[#6:9]-1.[Br:10][#6:11][C:12]#[N:13]>>[#6:1]-[#6@H:2](-[#7:3]-[#6:11][C:12]#[N:13])-[#6:4]-1=[#6:5]-[#6:6]=[#6:7]-[#6:8]=[#6:9]-1.[Br:10]

Correctness of the mapping
MAPPET YES
ReactionMap YES
Marvin YES

Reaction no 147

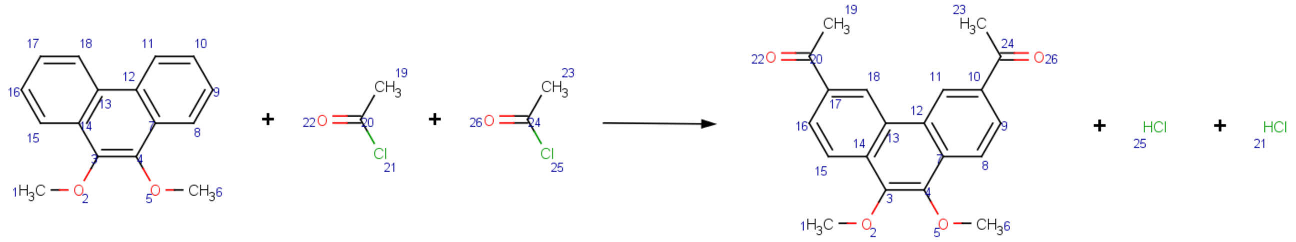

Correct mapped SMILES/SMARTS of the reaction:
[#6:1]-[#8:2]-[#6:3]-1=[#6:4](-[#8:5]-[#6:6])-[#6:7]-2=[#6:12](-[#6:11]=[#6:10]-[#6:9]=[#6:8]-2)-[#6:13]-2=[#6:14]-1-[#6:15]=[#6:16]-[#6:17]=[#6:18]-2.[#6:19]-[#6:20]([Cl:21])=[O:22].[#6:23]-[#6:24]([Cl:25])=[O:26]>>[#6:1]-[#8:2]-[#6:3]-1=[#6:4](-[#8:5]-[#6:6])-[#6:7]-2=[#6:12](-[#6:11]=[#6:10](-[#6:9]=[#6:8]-2)-[#6:24](-[#6:23])=[O:26])-[#6:13]-2=[#6:14]-1-[#6:15]=[#6:16]-[#6:17](=[#6:18]-2)-[#6:20](-[#6:19])=[O:22].[Cl:25].[Cl:21]

Correctness of the mapping
MAPPET YES
ReactionMap YES
Marvin YES

Reaction no 148

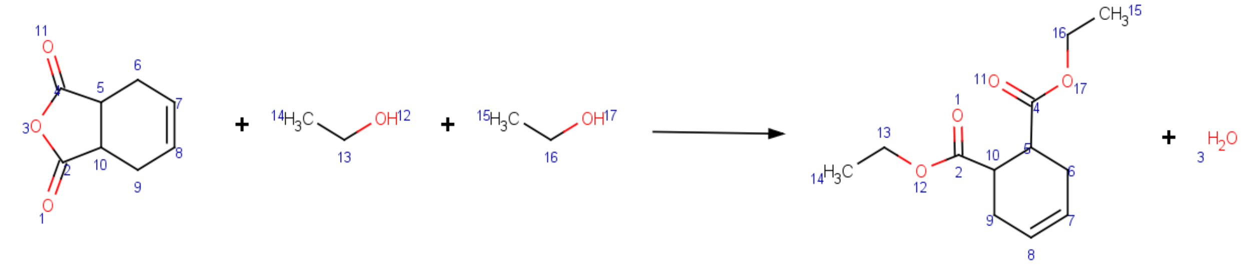

Correct mapped SMILES/SMARTS of the reaction:
[O:1]=[#6:2]-1-[#8:3]-[#6:4](=[O:11])-[#6:5]-2-[#6:6]-[#6:7]=[#6:8]-[#6:9]-[#6:10]-1-2.[#6:14]-[#6:13]-[#8:12].[#6:15]-[#6:16]-[#8:17]>>[#6:15]-[#6:16]-[#8:17]-[#6:4](=[O:11])-[#6:5]-1-[#6:6]-[#6:7]=[#6:8]-[#6:9]-[#6:10]-1-[#6:2](=[O:1])-[#8:12]-[#6:13]-[#6:14].[#8:3]

Correctness of the mapping
MAPPET   YES
ReactionMap NO
Marvin   NO

Reaction no 149

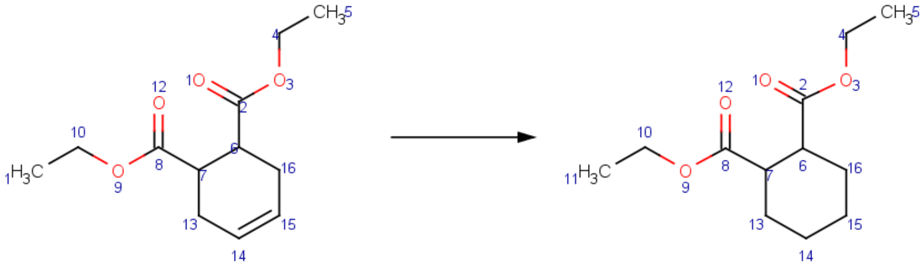

Correct mapped SMILES/SMARTS of the reaction:
[#6:5]-[#6:4]-[#8:3]-[#6:2](=[O:1])-[#6:6]-1-[#6:16]-[#6:15]=[#6:14]-[#6:13]-[#6:7]-1-[#6:8](=[O:12])-[#8:9]-[#6:10]-[#6:11]>>[#6:5]-[#6:4]-[#8:3]-[#6:2](=[O:1])-[#6:6]-1-[#6:16]-[#6:15]-[#6:14]-[#6:13]-[#6:7]-1-[#6:8](=[O:12])-[#8:9]-[#6:10]-[#6:11]

Correctness of the mapping
MAPPET   YES
ReactionMap NO
Marvin   YES

Reaction no 150

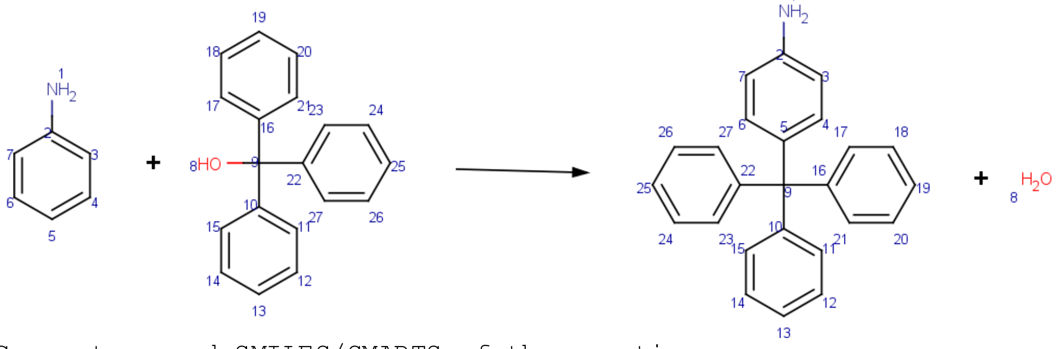

Correct mapped SMILES/SMARTS of the reaction:

[#7:1]-[#6:2]-1=[#6:3]-[#6:4]=[#6:5]-[#6:6]=[#6:7]-1.[#8:8][C:9]([#6:10]-1=[#6:11]-[#6:12]=[#6:13]-[#6:14]=[#6:15]-1)([#6:16]-1=[#6:17]-[#6:18]=[#6:19]-[#6:20]=[#6:21]-1)[#6:22]-1=[#6:23]-[#6:24]=[#6:25]-[#6:26]=[#6:27]-1>>[#7:1]-[#6:2]-1=[#6:3]-[#6:4]=[#6:5](-[#6:6]=[#6:7]-1)[C:9]([#6:10]-1=[#6:11]-[#6:12]=[#6:13]-[#6:14]=[#6:15]-1)([#6:22]-1=[#6:23]-[#6:24]=[#6:25]-[#6:26]=[#6:27]-1)[#6:16]-1=[#6:17]-[#6:18]=[#6:19]-[#6:20]=[#6:21]-1.[#8:8]

Correctness of the mapping
MAPPET YES
ReactionMap YES
Marvin YES

Reaction no 151

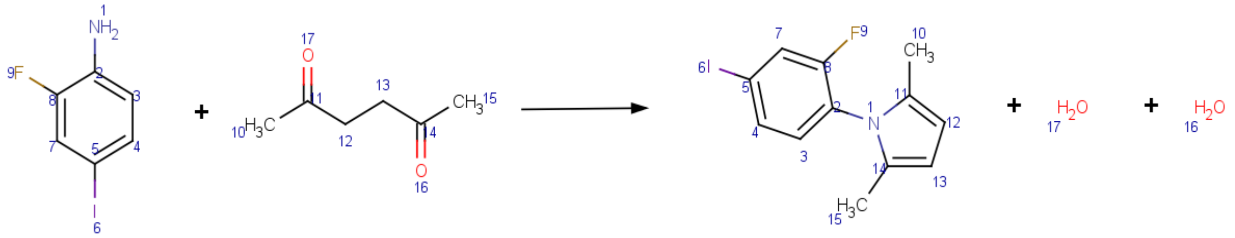

Correct mapped SMILES/SMARTS of the reaction:
[#7:1]-[#6:2]-1=[#6:3]-[#6:4]=[#6:5]([I:6])-[#6:7]=[#6:8]-1[F:9].[#6:10]-[#6:11](=[O:17])-[#6:12]-[#6:13]-[#6:14](-[#6:15])=[O:16]>>[#6:10]-[#6:11]-1=[#6:12]-[#6:13]=[#6:14](-[#6:15])-[#7:1]-1-[#6:2]-1=[#6:3]-[#6:4]=[#6:5]([I:6])-[#6:7]=[#6:8]-1[F:9].[#8:17].[#8:16]

Correctness of the mapping
MAPPET YES
ReactionMap YES
Marvin NO

Reaction no 152

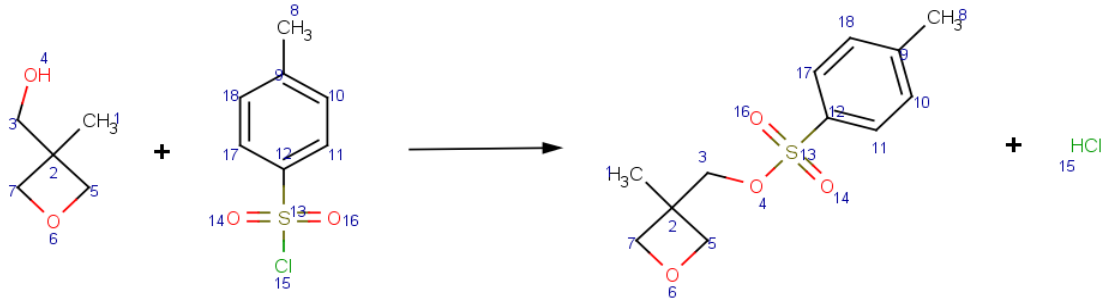

Correct mapped SMILES/SMARTS of the reaction:
[#6:1][C:2]1([#6:3]-[#8:4])[#6:5]-[#8:6]-[#6:7]1.[#6:8]-[#6:9]-1=[#6:10]-[#6:11]=[#6:12](-[#6:17]=[#6:18]-1)[S:13]([Cl:15])(=[O:14])=[O:16]>>[#6:8]-[#6:9]-1=[#6:10]-[#6:11]=[#6:12](-[#6:17]=[#6:18]-1)[S:13](=[O:16])(=[O:14])[#8:4]-[#6:3][C:2]1([#6:1])[#6:5]-[#8:6]-[#6:7]1.[Cl:15]

Correctness of the mapping
MAPPET YES
ReactionMap YES
Marvin YES

Reaction no 153

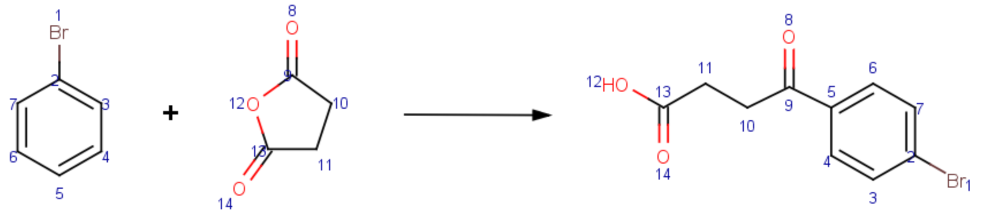

Correct mapped SMILES/SMARTS of the reaction:
[Br:1][#6:2]-1=[#6:3]-[#6:4]=[#6:5]-[#6:6]=[#6:7]-1.[O:8]=[#6:9]-1-[#6:10]-[#6:11]-[#6:13](=[O:14])-[#8:12]-1>>[#8:12]-[#6:13](=[O:14])-[#6:11]-[#6:10]-[#6:9](=[O:8])-[#6:5]-1=[#6:6]-[#6:7]=[#6:2]([Br:1])-[#6:3]=[#6:4]-1

Correctness of the mapping
MAPPET   YES
ReactionMap YES
Marvin   YES

Reaction no 154

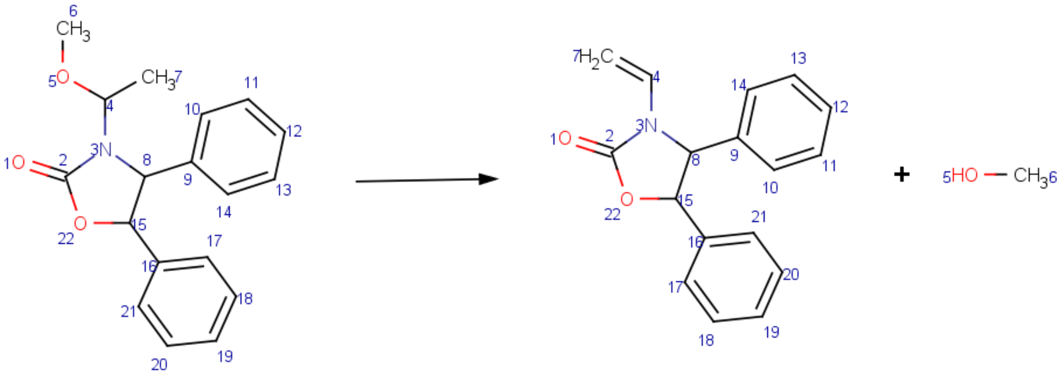

Correct mapped SMILES/SMARTS of the reaction:
[#6:6]-[#8:5]-[#6:4](-[#6:7])-[#7:3]-1-[#6:8](-[#6:15](-[#8:22]-[#6:2]-1=[O:1])-[#6:16]-1=[#6:17]-[#6:18]=[#6:19]-[#6:20]=[#6:21]-1)-[#6:9]-1=[#6:10]-[#6:11]=[#6:12]-[#6:13]=[#6:14]-1>>[#6:7]=[#6:4]-[#7:3]-1-[#6:8](-[#6:15](-[#8:22]-[#6:2]-1=[O:1])-[#6:16]-1=[#6:21]-[#6:20]=[#6:19]-[#6:18]=[#6:17]-1)-[#6:9]-1=[#6:14]-[#6:13]=[#6:12]-[#6:11]=[#6:10]-1.[#6:6]-[#8:5]

Correctness of the mapping
MAPPET   YES
ReactionMap YES
Marvin   YES

Reaction no 155

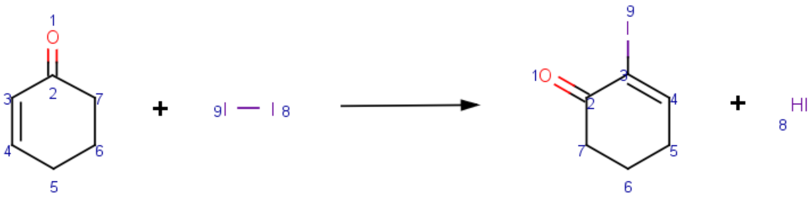

Correct mapped SMILES/SMARTS of the reaction:
[O:1]=[#6:2]-1-[#6:7]-[#6:6]-[#6:5]-[#6:4]=[#6:3]-1.[I:8][I:9]>>[I:9][#6:3]-1=[#6:4]-[#6:5]-[#6:6]-[#6:7]-[#6:2]-1=[O:1].[I:8]

Correctness of the mapping
MAPPET   YES
ReactionMap YES
Marvin   YES

Reaction no 156

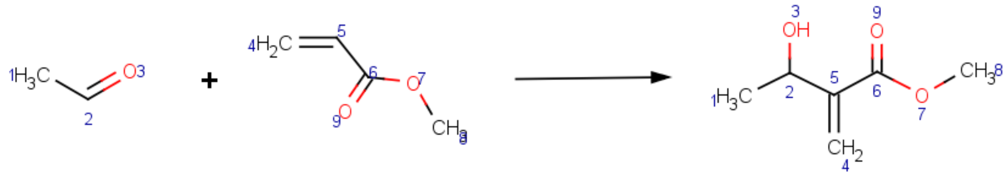

Correct mapped SMILES/SMARTS of the reaction:
[CH3:1][CH:2]=[O:3].[CH3:8][O:7][C:6](=[O:9])[CH:5]=[CH2:4]>>[CH3:1][CH:2]([C:5](=[CH2:4])[C:6](=[O:9])[O:7][CH3:8])[OH:3]

Correctness of the mapping
MAPPET   YES
ReactionMap YES
Marvin   YES

Reaction no 157

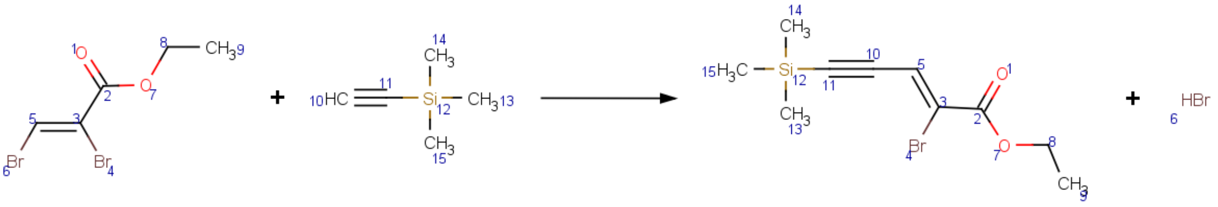

Correct mapped SMILES/SMARTS of the reaction:
[#6:9]-[#6:8]-[#8:7]-[#6:2](=[O:1])-[#6:3](\[Br:4])=[#6:5]\[Br:6].[#6:13][Si:12]([#6:14])([#6:15])[C:11]#[C:10]>>[#6:9]-[#6:8]-[#8:7]-[#6:2](=[O:1])-[#6:3](\[Br:4])=[#6:5]\[C:10]#[C:11][Si:12]([#6:15])([#6:14])[#6:13].[Br:6]

Correctness of the mapping
MAPPET   YES
ReactionMap YES
Marvin   YES

Reaction no 158

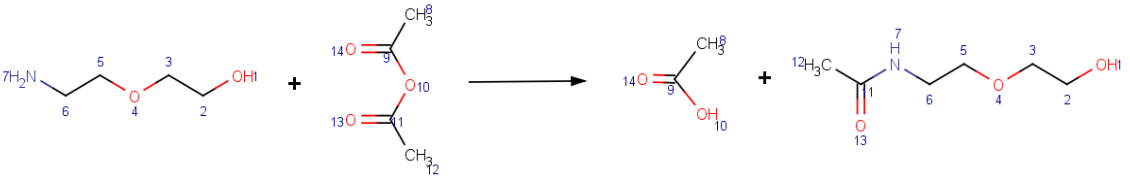

Correct mapped SMILES/SMARTS of the reaction:
[#7:7]-[#6:6]-[#6:5]-[#8:4]-[#6:3]-[#6:2]-[#8:1].[#6:8]-[#6:9](=[O:14])-[#8:10]-[#6:11](-[#6:12])=[O:13]>>[#6:8]-[#6:9](-[#8:10])=[O:14].[#6:12]-[#6:11](=[O:13])-[#7:7]-[#6:6]-[#6:5]-[#8:4]-[#6:3]-[#6:2]-[#8:1]

Correctness of the mapping
MAPPET   YES
ReactionMap YES
Marvin   YES

Reaction no 159

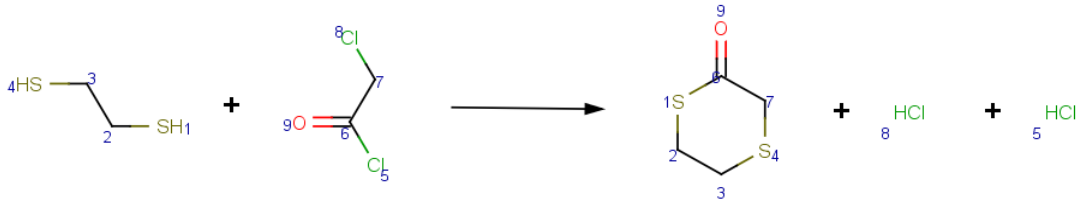

Correct mapped SMILES/SMARTS of the reaction:
[#16:1]-[#6:2]-[#6:3]-[#16:4].[Cl:8][#6:7]-[#6:6]([Cl:5])=[O:9]>>[O:9]=[#6:6]-1-[#6:7]-[#16:4]-[#6:3]-[#6:2]-[#16:1]-1.[Cl:8].[Cl:5]

Correctness of the mapping
MAPPET   YES
ReactionMap YES
Marvin   YES

Reaction no 160

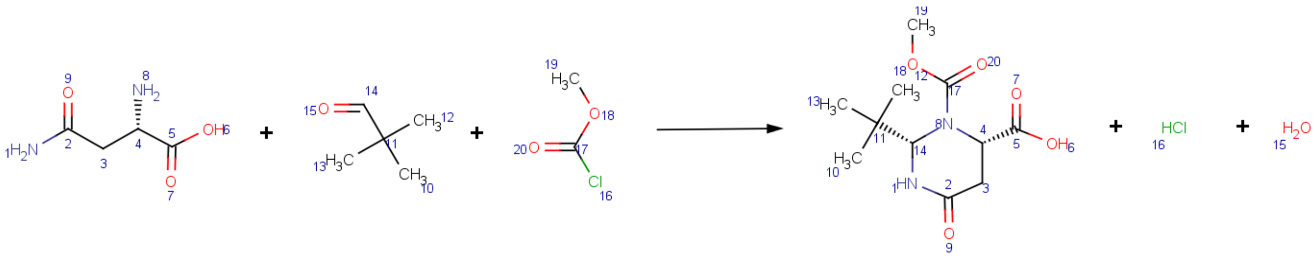

Correct mapped SMILES/SMARTS of the reaction:
[#7:8]-[#6@@H:4](-[#6:3]-[#6:2](-[#7:1])=[O:9])-[#6:5](-[#8:6])=[O:7].[#6:10][C:11]([#6:12])([#6:13])[#6:14]=[O:15].[#6:19]-[#8:18]-[#6:17]([Cl:16])=[O:20]>>[#6:19]-[#8:18]-[#6:17](=[O:20])-[#7:8]-1-[#6@@H:4](-[#6:3]-[#6:2](=[O:9])-[#7:1]-[#6@@H:14]-1[C:11]([#6:13])([#6:12])[#6:10])-[#6:5](-[#8:6])=[O:7].[Cl:16].[#8:15]

Correctness of the mapping
MAPPET   YES
ReactionMap YES
Marvin   YES

Reaction no 161

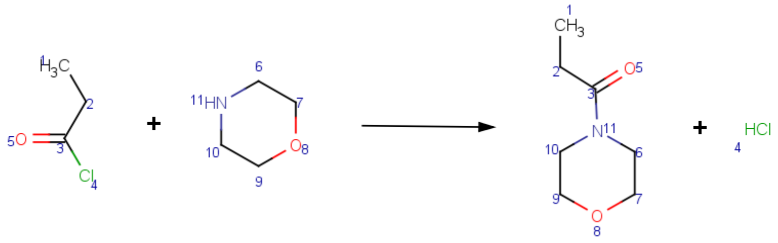

Correct mapped SMILES/SMARTS of the reaction:
[#6:1]-[#6:2]-[#6:3]([Cl:4])=[O:5].[#6:6]-1-[#6:7]-[#8:8]-[#6:9]-[#6:10]-[#7:11]-1>>[#6:1]-[#6:2]-[#6:3](=[O:5])-[#7:11]-1-[#6:6]-[#6:7]-[#8:8]-[#6:9]-[#6:10]-1.[Cl:4]

Correctness of the mapping
MAPPET YES
ReactionMap YES
Marvin YES

Reaction no 162

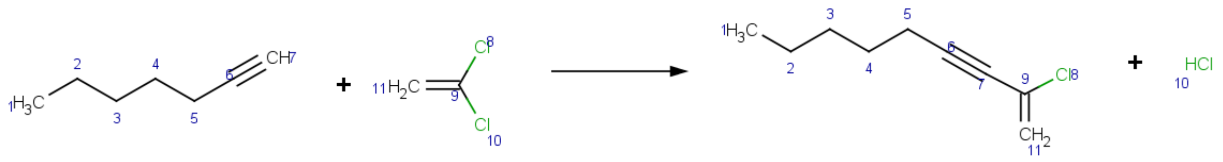

Correct mapped SMILES/SMARTS of the reaction:
[#6:1]-[#6:2]-[#6:3]-[#6:4]-
[#6:5][C:6]#[C:7].[Cl:8][#6:9]([Cl:10])=[#6:11]>>[#6:1]-[#6:2]-[#6:3]-[#6:4]-
[#6:5][C:6]#[C:7][#6:9]([Cl:8])=[#6:11].[Cl:10]

Correctness of the mapping
MAPPET YES
ReactionMap YES
Marvin YES

Reaction no 163

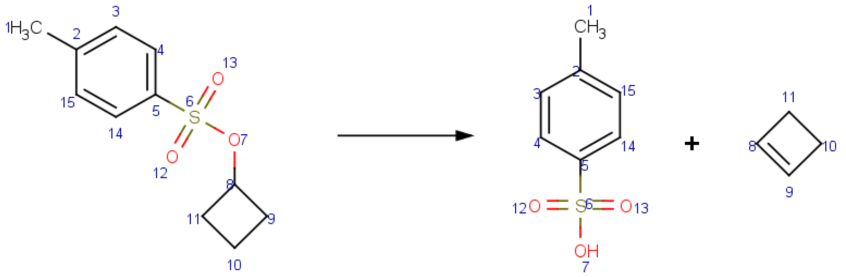

Correct mapped SMILES/SMARTS of the reaction:
[#6:1]-[#6:2]-1=[#6:3]-[#6:4]=[#6:5](-[#6:14]=[#6:15]-
1)[S:6](=[O:12])(=[O:13])[#8:7]-[#6:8]-1-[#6:9]-[#6:10]-[#6:11]-1>>[#6:1]-
[#6:2]-1=[#6:15]-[#6:14]=[#6:5](-[#6:4]=[#6:3]-
1)[S:6]([#8:7])(=[O:12])=[O:13].[#6:11]-1-[#6:10]-[#6:9]=[#6:8]-1

Correctness of the mapping
MAPPET YES
ReactionMap YES
Marvin YES

Reaction no 164

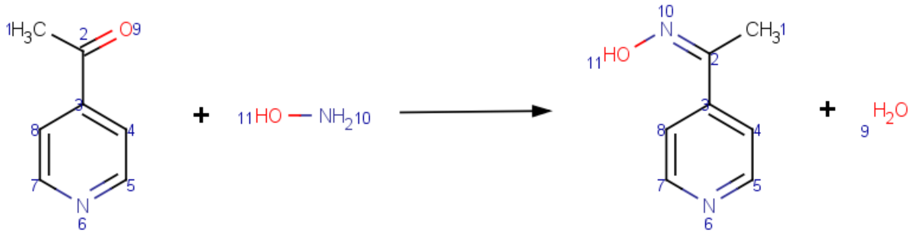

Correct mapped SMILES/SMARTS of the reaction:
[#6:1]-[#6:2](=[O:9])-[#6:3]-1=[#6:4]-[#6:5]=[#7:6]-[#6:7]=[#6:8]-1.[#7:10]-[#8:11]>>[#6:1]\[#6:2](=[#7:10]\[#8:11])-[#6:3]-1=[#6:4]-[#6:5]=[#7:6]-[#6:7]=[#6:8]-1.[#8:9]

Correctness of the mapping
MAPPET YES
ReactionMap YES
Marvin YES

Reaction no 165

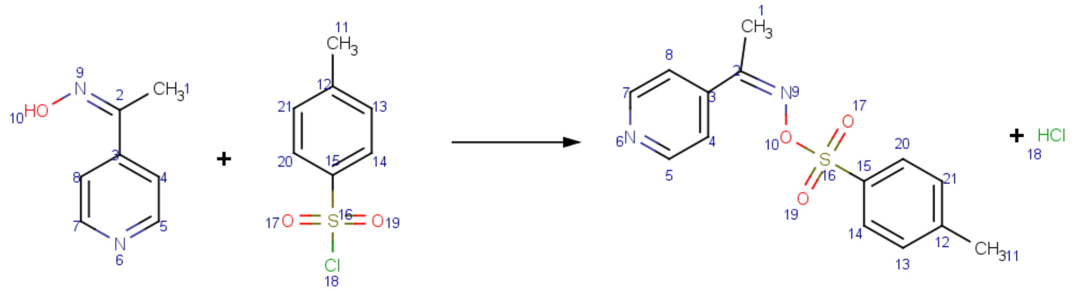

Correct mapped SMILES/SMARTS of the reaction:
[#6:1]\[#6:2](=[#7:9]\[#8:10])-[#6:3]-1=[#6:4]-[#6:5]=[#7:6]-[#6:7]=[#6:8]-1.[#6:11]-[#6:12]-1=[#6:13]-[#6:14]=[#6:15](-[#6:20]=[#6:21]-1)[S:16]([Cl:18])(=[O:17])=[O:19]>>[#6:1]\[#6:2](=[#7:9]\[#8:10][S:16](=[O:17])(=[O:19])[#6:15]-1=[#6:20]-[#6:21]=[#6:12](-[#6:11])-[#6:13]=[#6:14]-1)-[#6:3]-1=[#6:4]-[#6:5]=[#7:6]-[#6:7]=[#6:8]-1.[Cl:18]

Correctness of the mapping
MAPPET YES
ReactionMap YES
Marvin YES

Reaction no 166

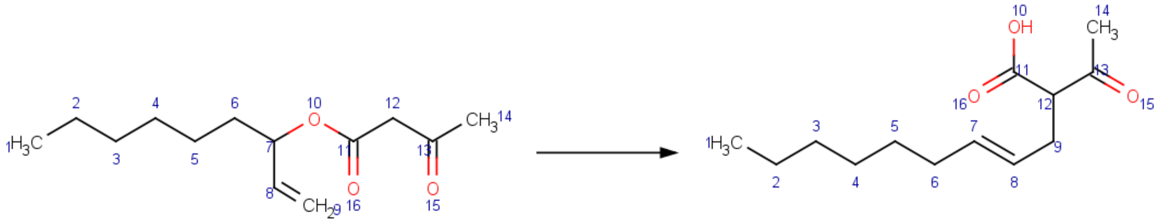

Correct mapped SMILES/SMARTS of the reaction:
[#6:1]-[#6:2]-[#6:3]-[#6:4]-[#6:5]-[#6:6]-[#6:7](-[#8:10]-[#6:11](=[O:16])-[#6:12]-[#6:13](-[#6:14])=[O:15])-[#6:8]=[#6:9]>>[#6:1]-[#6:2]-[#6:3]-[#6:4]-[#6:5]-[#6:6]\[#6:7]=[#6:8]\[#6:9]-[#6:12](-[#6:13](-[#6:14])=[O:15])-[#6:11](-[#8:10])=[O:16]

Correctness of the mapping
MAPPET YES
ReactionMap YES
Marvin YES

Reaction no 167

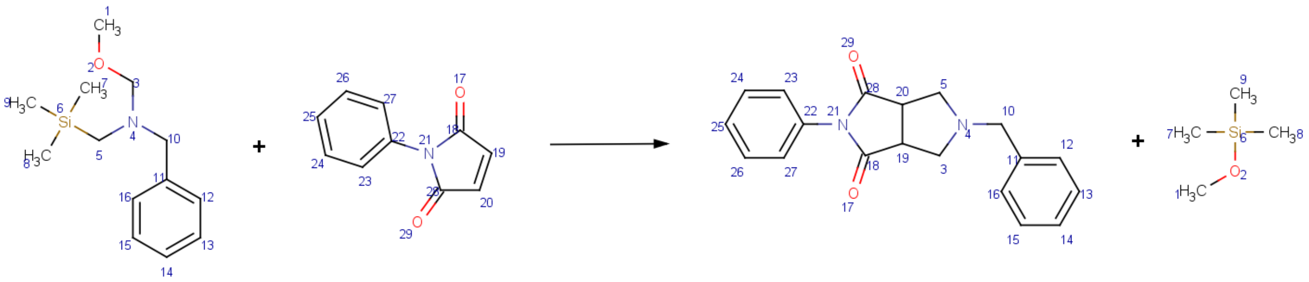

Correct mapped SMILES/SMARTS of the reaction:
[#6:1]-[#8:2]-[#6:3]-[#7:4](-[#6:10]-[#6:11]-1=[#6:12]-[#6:13]=[#6:14]-
[#6:15]=[#6:16]-1)-[#6:5][Si:6]([#6:7])([#6:8])[#6:9].[O:17]=[#6:18]-1-
[#6:19]=[#6:20]-[#6:28](=[O:29])-[#7:21]-1-[#6:22]-1=[#6:23]-[#6:24]=[#6:25]-
[#6:26]=[#6:27]-1>>[O:29]=[#6:28]-1-[#6:20]-2-[#6:5]-[#7:4](-[#6:10]-[#6:11]-
3=[#6:12]-[#6:13]=[#6:14]-[#6:15]=[#6:16]-3)-[#6:3]-[#6:19]-2-[#6:18](=[O:17])-
[#7:21]-1-[#6:22]-1=[#6:27]-[#6:26]=[#6:25]-[#6:24]=[#6:23]-1.[#6:1]-
[#8:2][Si:6]([#6:9])([#6:8])[#6:7]

Correctness of the mapping
MAPPET YES
ReactionMap YES
Marvin YES

Reaction no 168

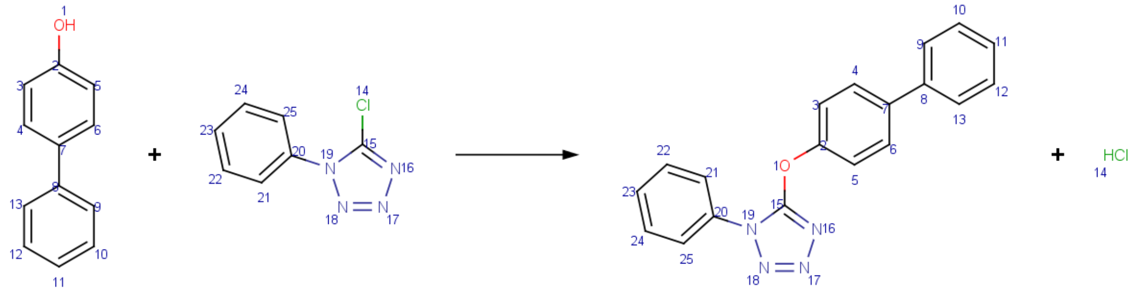

Correct mapped SMILES/SMARTS of the reaction:
[#8:1]-[#6:2]-1=[#6:5]-[#6:6]=[#6:7](-[#6:4]=[#6:3]-1)-[#6:8]-1=[#6:9]-
[#6:10]=[#6:11]-[#6:12]=[#6:13]-1.[Cl:14][#6:15]-1=[#7:16]-[#7:17]=[#7:18]-
[#7:19]-1-[#6:20]-1=[#6:21]-[#6:22]=[#6:23]-[#6:24]=[#6:25]-1>>[#8:1](-[#6:15]-
1=[#7:16]-[#7:17]=[#7:18]-[#7:19]-1-[#6:20]-1=[#6:25]-[#6:24]=[#6:23]-
[#6:22]=[#6:21]-1)-[#6:2]-1=[#6:3]-[#6:4]=[#6:7](-[#6:6]=[#6:5]-1)-[#6:8]-
1=[#6:9]-[#6:10]=[#6:11]-[#6:12]=[#6:13]-1.[Cl:14]

Correctness of the mapping
MAPPET YES
ReactionMap YES
Marvin YES

Reaction no 169

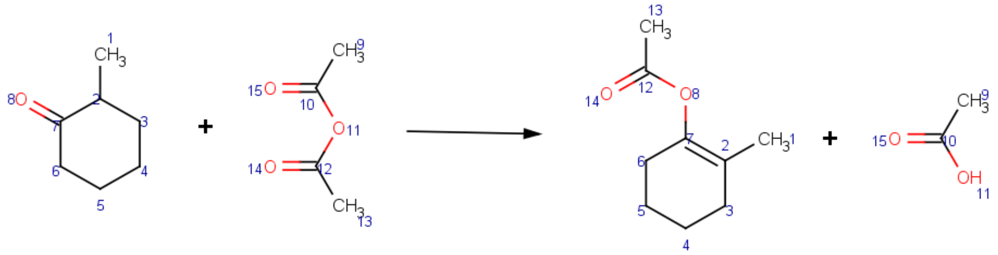

Correct mapped SMILES/SMARTS of the reaction:
[#6:1]-[#6:2]-1-[#6:3]-[#6:4]-[#6:5]-[#6:6]-[#6:7]-1=[O:8].[#6:9]-
[#6:10](=[O:15])-[#8:11]-[#6:12](-[#6:13])=[O:14]>>[#6:13]-[#6:12](=[O:14])-
[#8:8]-[#6:7]-1=[#6:2](-[#6:1])-[#6:3]-[#6:4]-[#6:5]-[#6:6]-1.[#6:9]-[#6:10](-
[#8:11])=[O:15]

Correctness of the mapping
MAPPET YES
ReactionMap YES
Marvin YES

Reaction no 170

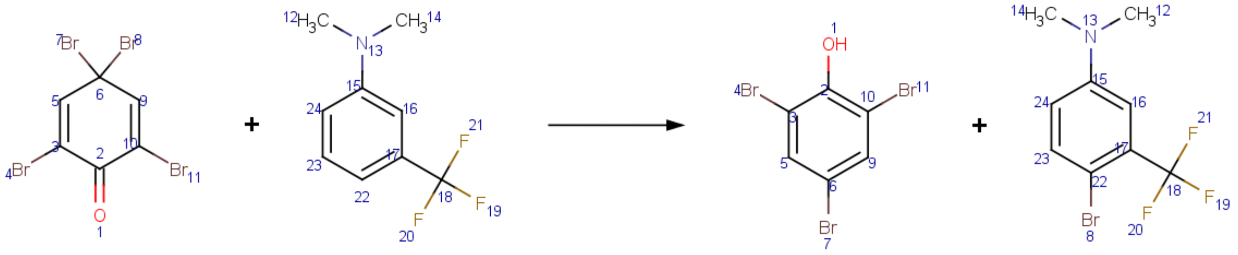

Correct mapped SMILES/SMARTS of the reaction:
[Br:4][#6:3]-1=[#6:5][C:6]([Br:7])([Br:8])[#6:9]=[#6:10]([Br:11])-[#6:2]-
1=[O:1].[#6:12]-[#7:13](-[#6:14])-[#6:15]-1=[#6:16]-[#6:17](=[#6:22]-
[#6:23]=[#6:24]-1)[C:18]([F:19])([F:20])[F:21]>>[#8:1]-[#6:2]-
1=[#6:10]([Br:11])-[#6:9]=[#6:6]([Br:7])-[#6:5]=[#6:3]-1[Br:4].[#6:14]-[#7:13](-
[#6:12])-[#6:15]-1=[#6:16]-[#6:17](=[#6:22]([Br:8])-[#6:23]=[#6:24]-
1)[C:18]([F:19])([F:20])[F:21]

Correctness of the mapping
MAPPET YES
ReactionMap YES
Marvin YES

Reaction no 171

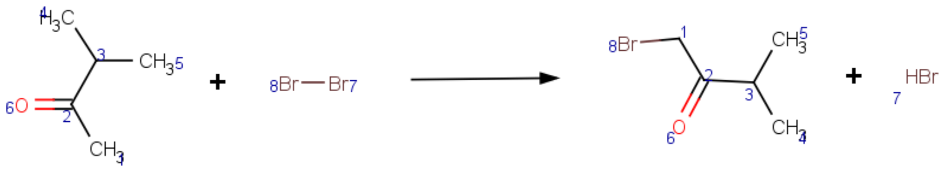

Correct mapped SMILES/SMARTS of the reaction:
[#6:4]-[#6:3](-[#6:5])-[#6:2](-[#6:1])=[O:6].[Br:7][Br:8]>>[#6:4]-[#6:3](-
[#6:5])-[#6:2](=[O:6])-[#6:1][Br:8].[Br:7]

Correctness of the mapping
MAPPET YES
ReactionMap YES
Marvin YES

Reaction no 172

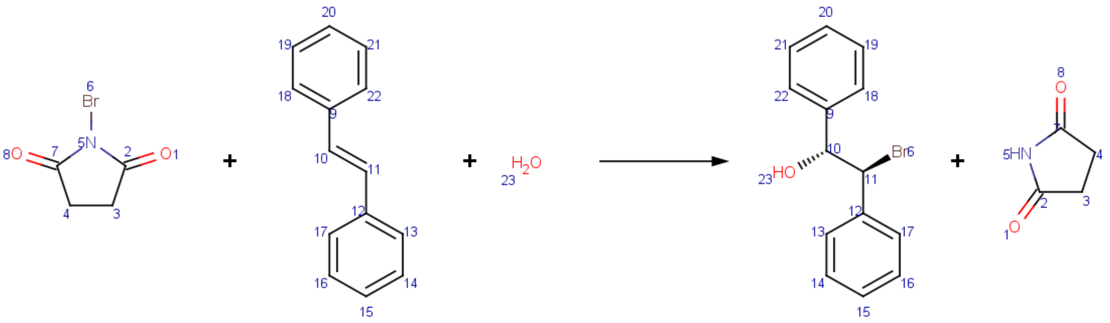

Correct mapped SMILES/SMARTS of the reaction:
[Br:6][#7:5]-1-[#6:2](=[O:1])-[#6:3]-[#6:4]-[#6:7]-
1=[O:8].[#6:10](=[#6:11]/[#6:12]-1=[#6:13]-[#6:14]=[#6:15]-
[#6:16]=[#6:17]1)\[#6:9]-1=[#6:18]-[#6:19]=[#6:20]-
[#6:21]=[#6:22]1.[#8:23]>>[#8:23]-[#6@@H:10](-[#6@@H:11]([Br:6])-[#6:12]-
1=[#6:17]-[#6:16]=[#6:15]-[#6:14]=[#6:13]-1)-[#6:9]-1=[#6:22]-[#6:21]=[#6:20]-
[#6:19]=[#6:18]-1.[O:8]=[#6:7]-1-[#6:4]-[#6:3]-[#6:2](=[O:1])-[#7:5]-1

Correctness of the mapping
MAPPET YES
ReactionMap YES
Marvin YES

Reaction no 173

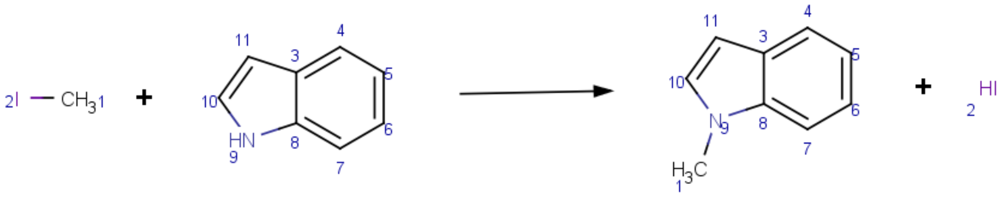

Correct mapped SMILES/SMARTS of the reaction:
[#6:1][I:2].[#7:9]-1-[#6:10]=[#6:11]-[#6:3]-2=[#6:4]-[#6:5]=[#6:6]-
[#6:7]=[#6:8]-1-2>>[#6:1]-[#7:9]-1-[#6:10]=[#6:11]-[#6:3]-2=[#6:4]-
[#6:5]=[#6:6]-[#6:7]=[#6:8]-1-2.[I:2]

Correctness of the mapping
MAPPET YES
ReactionMap YES
Marvin YES

Reaction no 174

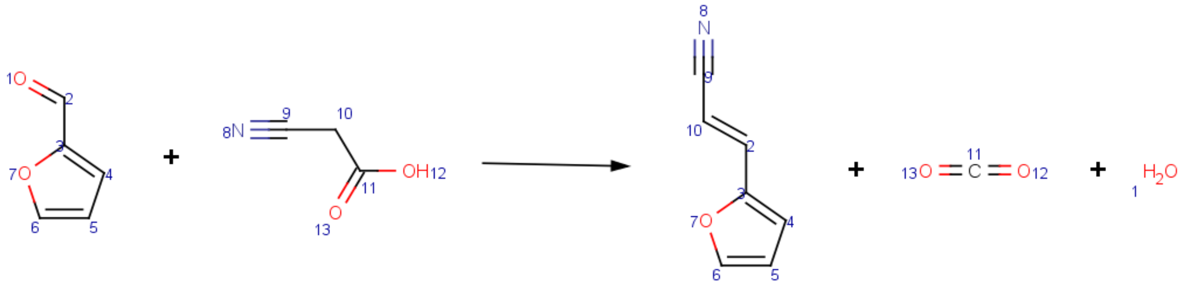

Correct mapped SMILES/SMARTS of the reaction:
[O:1]=[#6:2]-[#6:3]-1=[#6:4]-[#6:5]=[#6:6]-[#8:7]-1.[#8:12]-[#6:11](=[O:13])-
[#6:10][C:9]#[N:8]>>[N:8]#[C:9]\[#6:10]=[#6:2]\[#6:3]-1=[#6:4]-[#6:5]=[#6:6]-
[#8:7]1.[O:13]=[C:11]=[O:12].[#8:1]

Correctness of the mapping
MAPPET   YES
ReactionMap YES
Marvin   YES

Reaction no 175

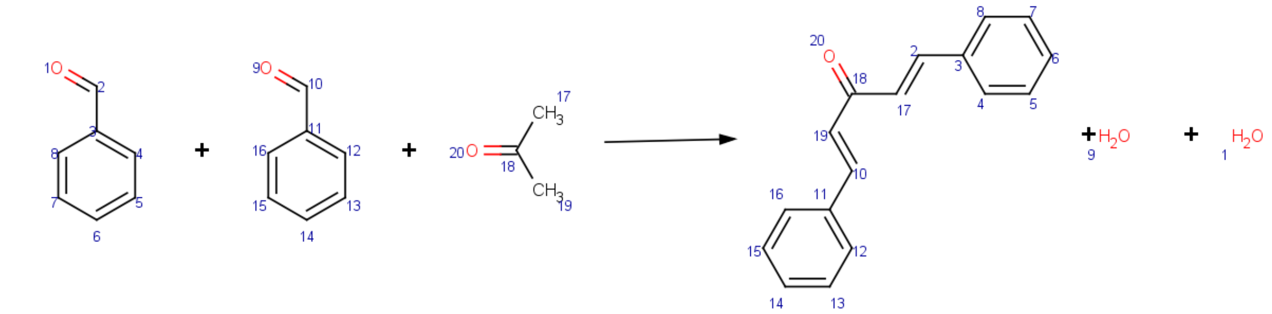

Correct mapped SMILES/SMARTS of the reaction:
[O:1]=[#6:2]-[#6:3]-1=[#6:4]-[#6:5]=[#6:6]-[#6:7]=[#6:8]-1.[O:9]=[#6:10]-[#6:11]-1=[#6:12]-[#6:13]=[#6:14]-[#6:15]=[#6:16]-1.[#6:17]-[#6:18](-[#6:19])=[O:20]>>[O:20]=[#6:18](\[#6:19]=[#6:10]\[#6:11]-1=[#6:12]-[#6:13]=[#6:14]-[#6:15]=[#6:16]1)/[#6:17]=[#6:2]/[#6:3]-1=[#6:8]-[#6:7]=[#6:6]-[#6:5]=[#6:4]1.[#8:9].[#8:1]

Correctness of the mapping
MAPPET   YES
ReactionMap YES
Marvin   YES

Reaction no 176

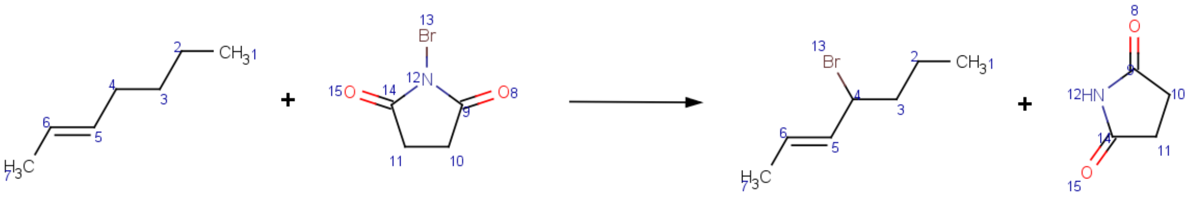

Correct mapped SMILES/SMARTS of the reaction:
[#6:1]-[#6:2]-[#6:3]-[#6:4]\[#6:5]=[#6:6]\[#6:7].[Br:13][#7:12]-1-[#6:9](=[O:8])-[#6:10]-[#6:11]-[#6:14]-1=[O:15]>>[#6:1]-[#6:2]-[#6:3]-[#6:4]([Br:13])\[#6:5]=[#6:6]\[#6:7].[O:8]=[#6:9]-1-[#6:10]-[#6:11]-[#6:14](=[O:15])-[#7:12]-1

Correctness of the mapping
MAPPET   YES
ReactionMap YES
Marvin   YES

Reaction no 177

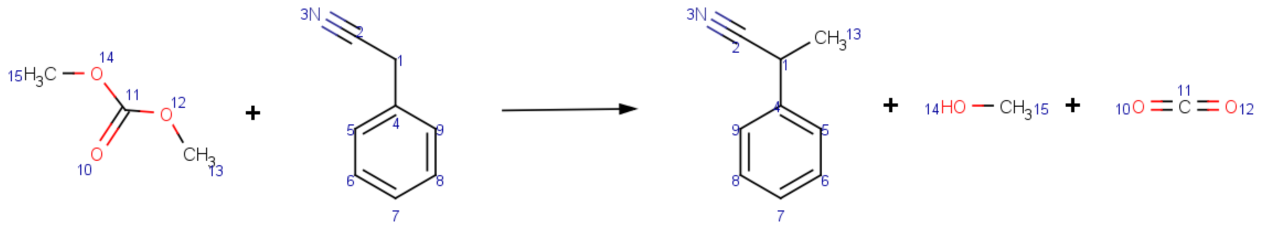

[CH3:13][O:12][C:11](=[O:10])[O:14][CH3:15].[CH:7]1=[CH:6][CH:5]=[C:4]([CH:9]=[CH:8]1)[CH2:1][C:2]#[N:3]>>[CH3:13][CH:1]([C:2]#[N:3])[C:4]1=[CH:5][CH:6]=[CH:7][CH:8]=[CH:9]1.[CH3:15][OH:14].[C:11](=[O:10])=[O:12]

Correctness of the mapping
MAPPET   NO
ReactionMap YES
Marvin   YES

Reaction no 178

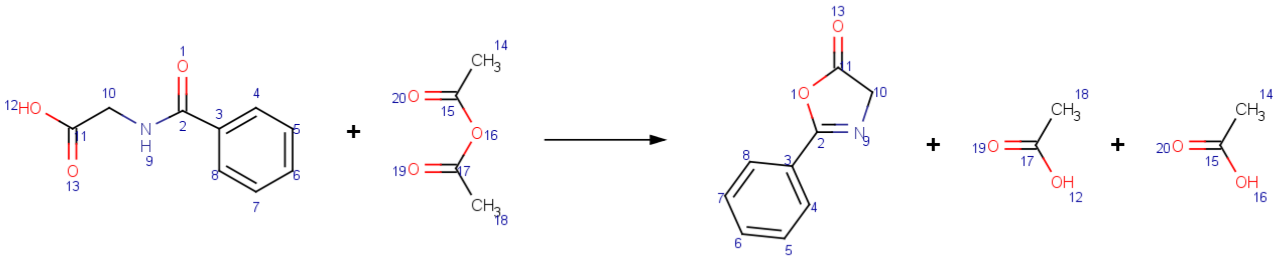

Correct mapped SMILES/SMARTS of the reaction:
[#8:12]-[#6:11](=[O:13])-[#6:10]-[#7:9]-[#6:2](=[O:1])-[#6:3]-1=[#6:4]-[#6:5]=[#6:6]-[#6:7]=[#6:8]-1.[#6:14]-[#6:15](=[O:20])-[#8:16]-[#6:17](-[#6:18])=[O:19]>>[O:13]=[#6:11]-1-[#6:10]-[#7:9]=[#6:2](-[#8:1]-1)-[#6:3]-1=[#6:4]-[#6:5]=[#6:6]-[#6:7]=[#6:8]-1.[#6:18]-[#6:17](-[#8:12])=[O:19].[#6:14]-[#6:15](-[#8:16])=[O:20]

Correctness of the mapping
MAPPET   YES
ReactionMap YES
Marvin   NO

Reaction no 179

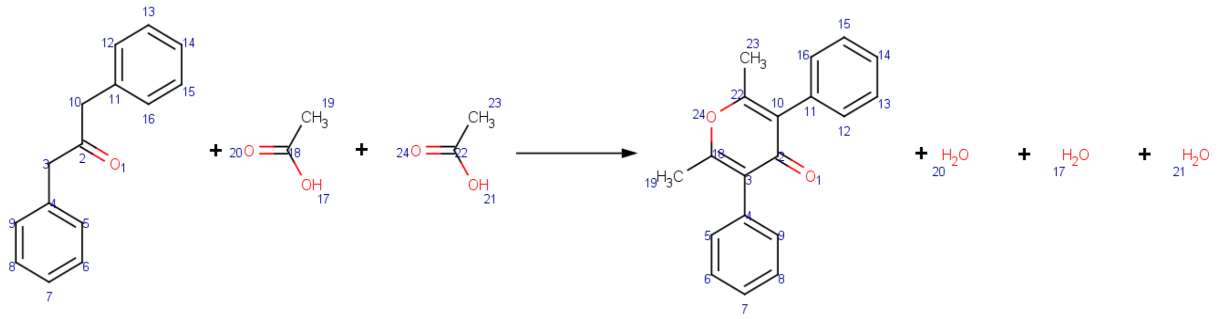

Correct mapped SMILES/SMARTS of the reaction:
[Cl:1][#6:2](=[O:9])-[#6:3]-1-[#6:4]-[#6:5]-[#6:6]-[#6:7]-[#6:8]-1.[Cl:10][#6:11](=[O:18])-[#6:12]-1-[#6:13]-[#6:14]-[#6:15]-[#6:16]-[#6:17]-1>>[O:18]=[#6:11]1[C:12]2([#6:17]-[#6:16]-[#6:15]-[#6:14]-[#6:13]2)[#6:2](=[O:9])[C:3]11[#6:4]-[#6:5]-[#6:6]-[#6:7]-[#6:8]1.[Cl:1].[Cl:10]

Correctness of the mapping
MAPPET   YES
ReactionMap YES
Marvin   YES

Reaction no 180

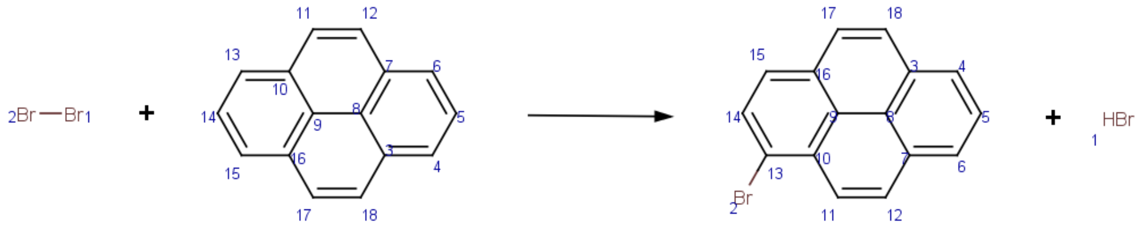

Correct mapped SMILES/SMARTS of the reaction:
[Br:1][Br:2].[#6:5]-1=[#6:6]-[#6:7]-2=[#6:8]-3-[#6:3](-[#6:18]=[#6:17]-[#6:16]-
4=[#6:9]-3-[#6:10](-[#6:11]=[#6:12]-2)=[#6:13]-[#6:14]=[#6:15]-4)=[#6:4]-
1>>[Br:2][#6:13]-1=[#6:14]-[#6:15]=[#6:16]-2-[#6:17]=[#6:18]-[#6:3]-3=[#6:8]-4-
[#6:7](-[#6:12]=[#6:11]-[#6:10]-1=[#6:9]-2-4)=[#6:6]-[#6:5]=[#6:4]-3.[Br:1]

Correctness of the mapping
MAPPET YES
ReactionMap YES
Marvin YES

Reaction no 181

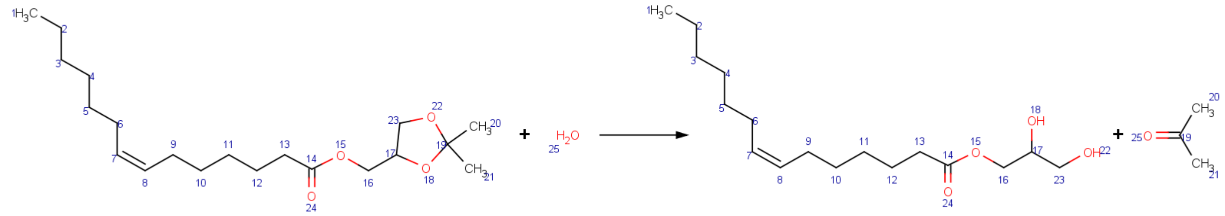

Correct mapped SMILES/SMARTS of the reaction:
[#6:1]-[#6:2]-[#6:3]-[#6:4]-[#6:5]-[#6:6]\[#6:7]=[#6:8]/[#6:9]-[#6:10]-[#6:11]-
[#6:12]-[#6:13]-[#6:14](=[O:24])-[#8:15]-[#6:16]-[#6:17]-1-[#6:23]-
[#8:22][C:19]([#6:20])([#6:21])[#8:18]-1.[#8:25]>>[#6:1]-[#6:2]-[#6:3]-[#6:4]-
[#6:5]-[#6:6]\[#6:7]=[#6:8]/[#6:9]-[#6:10]-[#6:11]-[#6:12]-[#6:13]-
[#6:14](=[O:24])-[#8:15]-[#6:16]-[#6:17](-[#8:18])-[#6:23]-[#8:22].[#6:20]-
[#6:19](-[#6:21])=[O:25]

Correctness of the mapping
MAPPET YES
ReactionMap YES
Marvin YES

Reaction no 182

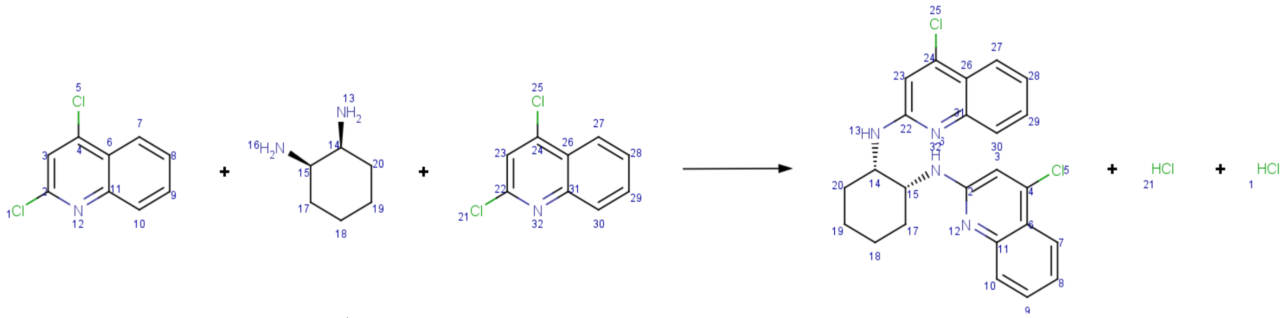

Correct mapped SMILES/SMARTS of the reaction:
[Cl:1][#6:2]-1=[#6:3]-[#6:4]([Cl:5])=[#6:6]-2-[#6:7]=[#6:8]-[#6:9]=[#6:10]-
[#6:11]-2=[#7:12]-1.[#7:13]-[#6@H:14]-1-[#6:20]-[#6:19]-[#6:18]-[#6:17]-
[#6@H:15]-1-[#7:16].[Cl:21][#6:22]-1=[#6:23]-[#6:24]([Cl:25])=[#6:26]-2-
[#6:27]=[#6:28]-[#6:29]=[#6:30]-[#6:31]-2=[#7:32]-1>>[Cl:5][#6:4]-1=[#6:6]-2-
[#6:7]=[#6:8]-[#6:9]=[#6:10]-[#6:11]-2=[#7:12]-[#6:2](-[#7:16]-[#6@@H:15]-2-
[#6:17]-[#6:18]-[#6:19]-[#6:20]-[#6@@H:14]-2-[#7:13]-[#6:22]-2=[#6:23]-

[#6:24]([Cl:25])=[#6:26]-3-[#6:27]=[#6:28]-[#6:29]=[#6:30]-[#6:31]-3=[#7:32]-2)=[#6:3]-1.[Cl:21].[Cl:1]

Correctness of the mapping
MAPPET   YES
ReactionMap YES
Marvin   YES

Reaction no 183

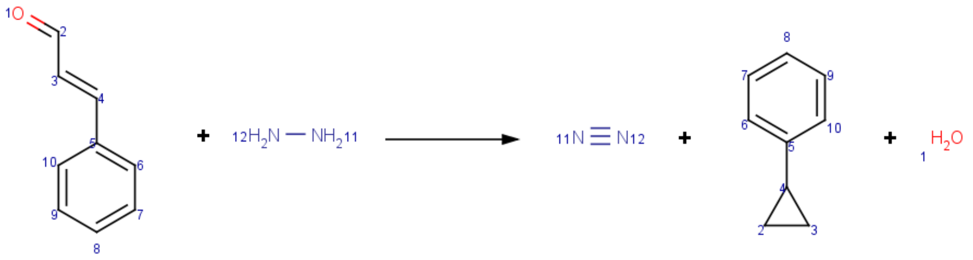

Correct mapped SMILES/SMARTS of the reaction:
[O:1]=[#6:2]\[#6:3]=[#6:4]\[#6:5]-1=[#6:6]-[#6:7]=[#6:8]-[#6:9]=[#6:10]1.[#7:11]-[#7:12]>>[N:12]#[N:11].[#6:3]-1-[#6:2]-[#6:4]-1-[#6:5]-1=[#6:6]-[#6:7]=[#6:8]-[#6:9]=[#6:10]-1.[#8:1]

Correctness of the mapping
MAPPET   YES
ReactionMap YES
Marvin   YES

Reaction no 184

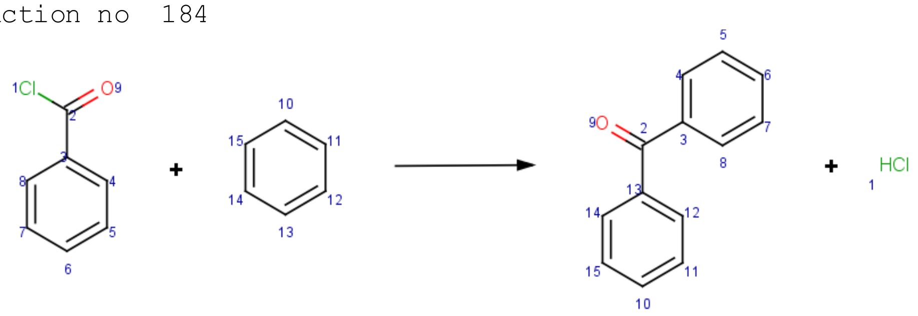

Correct mapped SMILES/SMARTS of the reaction:
[Cl:1][#6:2](=[O:9])-[#6:3]-1=[#6:4]-[#6:5]=[#6:6]-[#6:7]=[#6:8]-1.[#6:10]-1=[#6:11]-[#6:12]=[#6:13]-[#6:14]=[#6:15]-1>>[O:9]=[#6:2](-[#6:13]-1=[#6:12]-[#6:11]=[#6:10]-[#6:15]=[#6:14]-1)-[#6:3]-1=[#6:4]-[#6:5]=[#6:6]-[#6:7]=[#6:8]-1.[Cl:1]

Correctness of the mapping
MAPPET   YES
ReactionMap YES
Marvin   YES

Reaction no 185

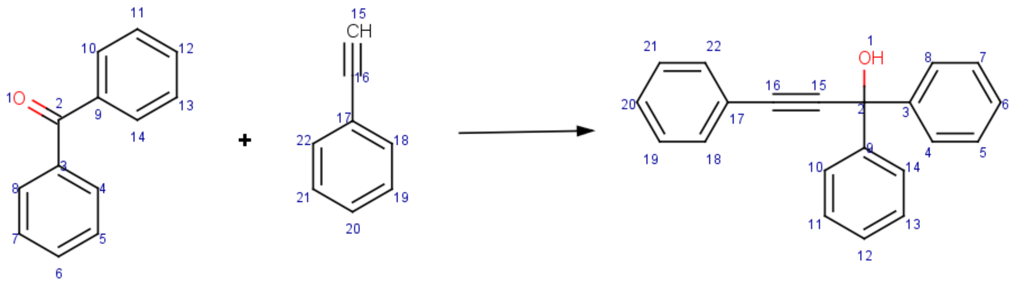

Correct mapped SMILES/SMARTS of the reaction:
[O:1]=[#6:2](-[#6:3]-1=[#6:4]-[#6:5]=[#6:6]-[#6:7]=[#6:8]-1)-[#6:9]-1=[#6:10]-[#6:11]=[#6:12]-[#6:13]=[#6:14]-1.[C:15]#[C:16][#6:17]-1=[#6:18]-[#6:19]=[#6:20]-[#6:21]=[#6:22]-1>>[#8:1][C:2]([C:15]#[C:16][#6:17]-1=[#6:18]-[#6:19]=[#6:20]-[#6:21]=[#6:22]-1)([#6:3]-1=[#6:8]-[#6:7]=[#6:6]-[#6:5]=[#6:4]-1)[#6:9]-1=[#6:14]-[#6:13]=[#6:12]-[#6:11]=[#6:10]-1

Correctness of the mapping
MAPPET   YES
ReactionMap YES
Marvin   YES

Reaction no 186

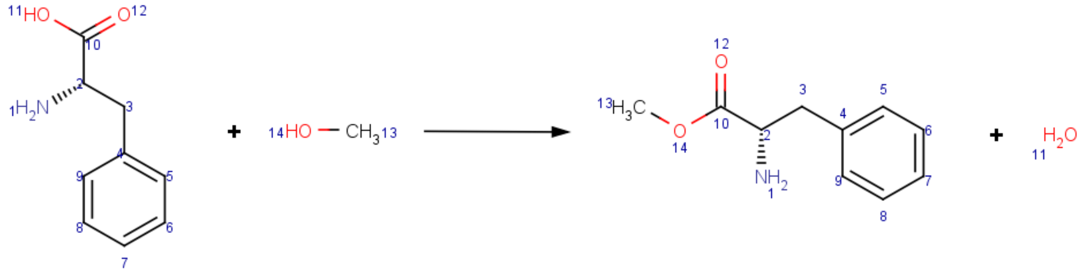

Correct mapped SMILES/SMARTS of the reaction:
[#7:1]-[#6@@H:2](-[#6:3]-[#6:4]-1=[#6:5]-[#6:6]=[#6:7]-[#6:8]=[#6:9]-1)-[#6:10](-[#8:11])=[O:12].[#6:13]-[#8:14]>>[#6:13]-[#8:14]-[#6:10](=[O:12])-[#6@@H:2](-[#7:1])-[#6:3]-[#6:4]-1=[#6:5]-[#6:6]=[#6:7]-[#6:8]=[#6:9]-1.[#8:11]

Correctness of the mapping
MAPPET   YES
ReactionMap NO
Marvin   NO

Reaction no 187

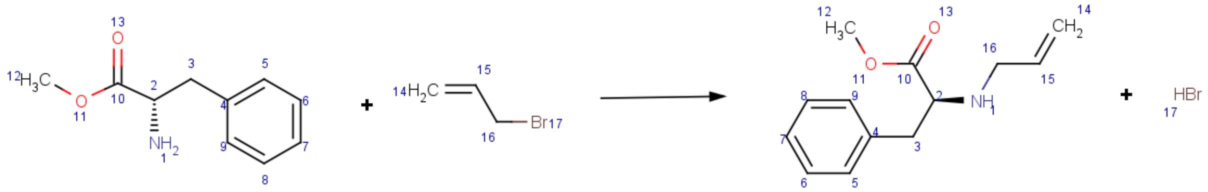

Correct mapped SMILES/SMARTS of the reaction:
[#6:12]-[#8:11]-[#6:10](=[O:13])-[#6@@H:2](-[#7:1])-[#6:3]-[#6:4]-1=[#6:5]-[#6:6]=[#6:7]-[#6:8]=[#6:9]-1.[Br:17][#6:16]-[#6:15]=[#6:14]>>[#6:12]-[#8:11]-

[#6:10](=[O:13])-[#6@H:2](-[#6:3]-[#6:4]-1=[#6:5]-[#6:6]=[#6:7]-[#6:8]=[#6:9]-1)-[#7:1]-[#6:16]-[#6:15]=[#6:14].[Br:17]

Correctness of the mapping
MAPPET YES
ReactionMap YES
Marvin YES

Reaction no 188

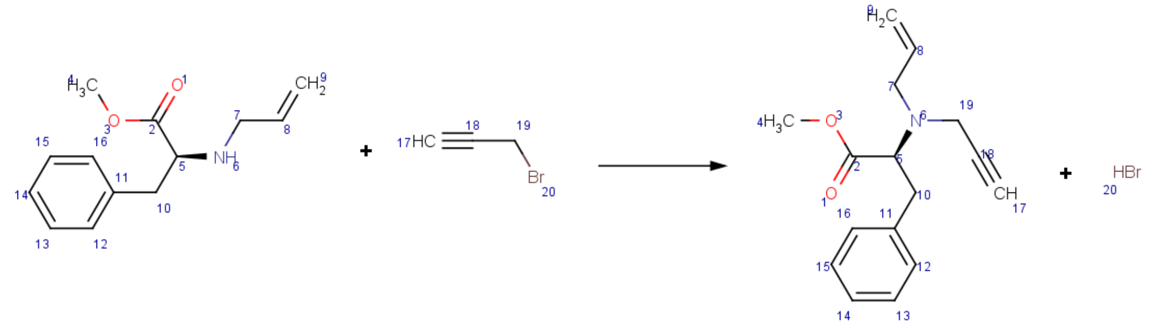

Correct mapped SMILES/SMARTS of the reaction:
[#6:4]-[#8:3]-[#6:2](=[O:1])-[#6@H:5](-[#6:10]-[#6:11]-1=[#6:12]-[#6:13]=[#6:14]-[#6:15]=[#6:16]-1)-[#7:6]-[#6:7]-[#6:8]=[#6:9].[Br:20][#6:19][C:18]#[C:17]>>[#6:4]-[#8:3]-[#6:2](=[O:1])-[#6@H:5](-[#6:10]-[#6:11]-1=[#6:12]-[#6:13]=[#6:14]-[#6:15]=[#6:16]-1)-[#7:6](-[#6:7]-[#6:8]=[#6:9])-[#6:19][C:18]#[C:17].[Br:20]

Correctness of the mapping
MAPPET YES
ReactionMap YES
Marvin YES

Reaction no 189

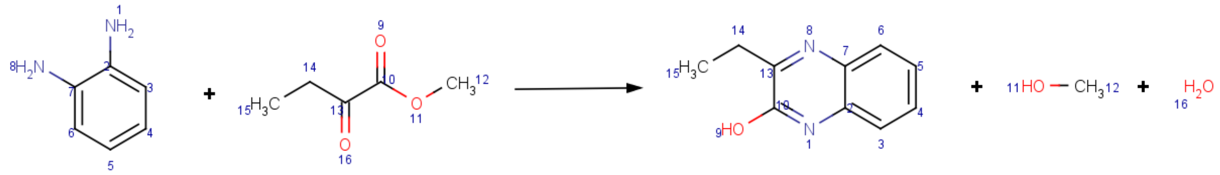

Correct mapped SMILES/SMARTS of the reaction:
[#7:1]-[#6:2]-1=[#6:3]-[#6:4]=[#6:5]-[#6:6]=[#6:7]-1-[#7:8].[#6:15]-[#6:14]-[#6:13](=[O:16])-[#6:10](=[O:9])-[#8:11]-[#6:12]>>[#6:15]-[#6:14]-[#6:13]-1=[#7:8]-[#6:7]-2=[#6:6]-[#6:5]=[#6:4]-[#6:3]=[#6:2]-2-[#7:1]=[#6:10]-1-[#8:9].[#6:12]-[#8:11].[#8:16]

Correctness of the mapping
MAPPET YES
ReactionMap YES
Marvin NO

Reaction no 190

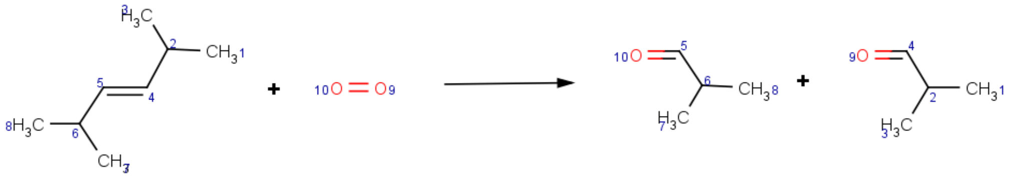

```
Correct mapped SMILES/SMARTS of the reaction:
[#6:1]-[#6:2](-[#6:3])\[#6:4]=[#6:5]\[#6:6](-[#6:7])-
[#6:8].[O:9]=[O:10]>>[#6:8]-[#6:6](-[#6:7])-[#6:5]=[O:10].[#6:1]-[#6:2](-
[#6:3])-[#6:4]=[O:9]

Correctness of the mapping
MAPPET     YES
ReactionMap YES
Marvin     YES

Reaction no  191
```

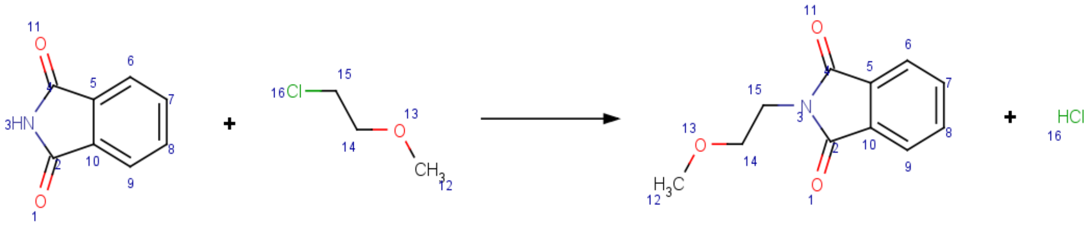

```
Correct mapped SMILES/SMARTS of the reaction:
[O:1]=[#6:2]-1-[#7:3]-[#6:4](=[O:11])-[#6:5]-2=[#6:6]-[#6:7]=[#6:8]-
[#6:9]=[#6:10]-1-2.[#6:12]-[#8:13]-[#6:14]-[#6:15][Cl:16]>>[#6:12]-[#8:13]-
[#6:14]-[#6:15]-[#7:3]-1-[#6:4](=[O:11])-[#6:5]-2=[#6:6]-[#6:7]=[#6:8]-
[#6:9]=[#6:10]-2-[#6:2]-1=[O:1].[Cl:16]

Correctness of the mapping
MAPPET     YES
ReactionMap YES
Marvin     YES

Reaction no  192
```

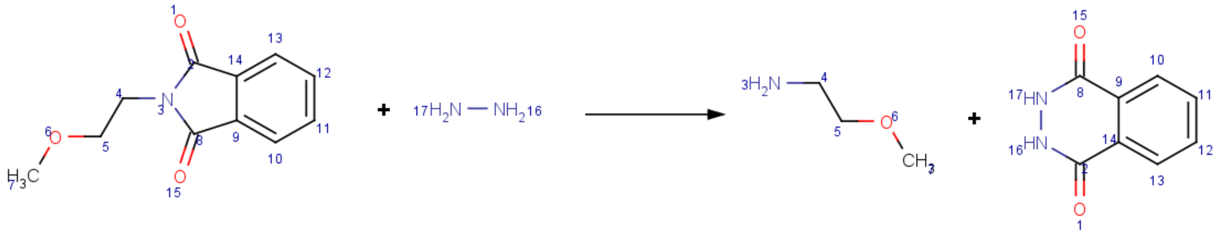

```
Correct mapped SMILES/SMARTS of the reaction:
[#6:7]-[#8:6]-[#6:5]-[#6:4]-[#7:3]-1-[#6:2](=[O:1])-[#6:14]-2=[#6:13]-
[#6:12]=[#6:11]-[#6:10]=[#6:9]-2-[#6:8]-1=[O:15].[#7:16]-[#7:17]>>[#6:7]-[#8:6]-
[#6:5]-[#6:4]-[#7:3].[O:1]=[#6:2]-1-[#7:16]-[#7:17]-[#6:8](=[O:15])-[#6:9]-
2=[#6:10]-[#6:11]=[#6:12]-[#6:13]=[#6:14]-1-2

Correctness of the mapping
MAPPET     YES
ReactionMap NO
Marvin     YES

Reaction no  193
```

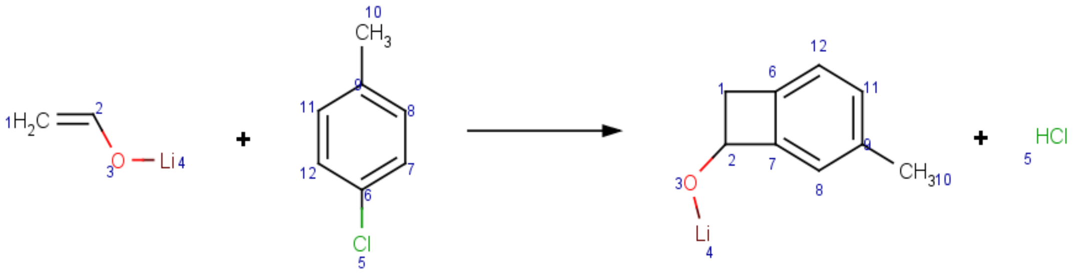

Correct mapped SMILES/SMARTS of the reaction:
[Li:4][#8:3]-[#6:2]=[#6:1].[#6:10]-[#6:9]-1=[#6:8]-[#6:7]=[#6:6]([Cl:5])-[#6:12]=[#6:11]-1>>[Li:4][#8:3]-[#6:2]-1-[#6:1]-[#6:6]-2=[#6:12]-[#6:11]=[#6:9](-[#6:10])-[#6:8]=[#6:7]-1-2.[Cl:5]

Correctness of the mapping
MAPPET YES
ReactionMap YES
Marvin YES

Reaction no 194

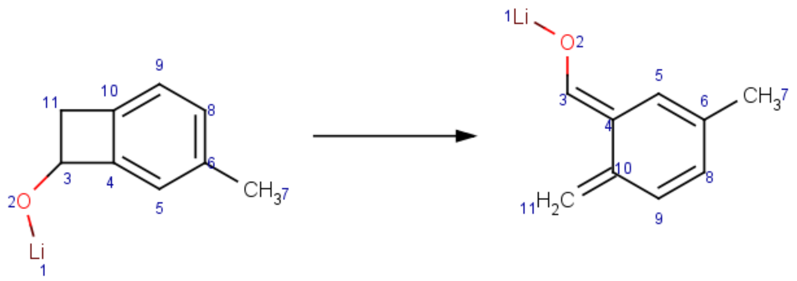

Correct mapped SMILES/SMARTS of the reaction:
[Li:1][#8:2]-[#6:3]-1-[#6:11]-[#6:10]-2=[#6:9]-[#6:8]=[#6:6](-[#6:7])-[#6:5]=[#6:4]-1-2>>[Li:1][#8:2]\[#6:3]=[#6:4]-1/[#6:5]=[#6:6](-[#6:7])-[#6:8]=[#6:9]-[#6:10]-1=[#6:11]

Correctness of the mapping
MAPPET YES
ReactionMap YES
Marvin YES

Reaction no 195

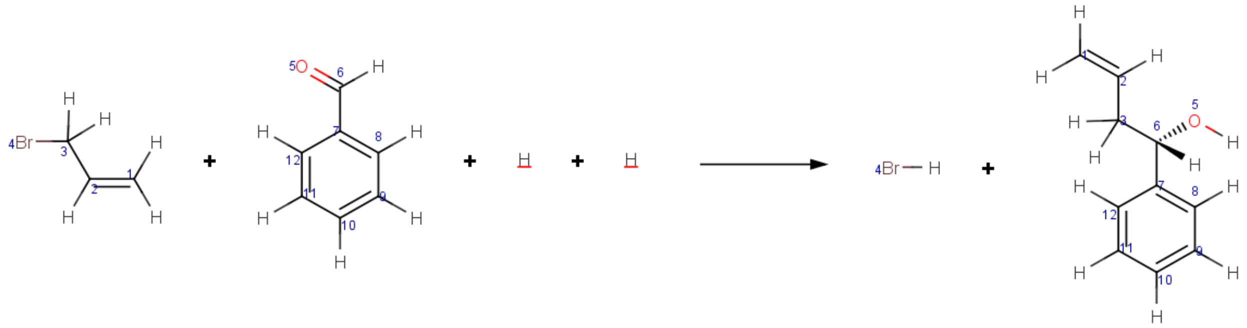

Correct mapped SMILES/SMARTS of the reaction:
[H]\[#6:1]([H])=[#6:2](/[H])[C:3]([H])([H])[Br:4].[H][#6:6](=[O:5])-[#6:7]-1=[#6:8]([H])-[#6:9]([H])=[#6:10]([H])-[#6:11]([H])=[#6:12]-1[H].[H].[H]>>[Br:4][H].[H][#8:5][C@:6]([H])([#6:7]-1=[#6:8]([H])-[#6:9]([H])=[#6:10]([H])-[#6:11]([H])=[#6:12]-1[H])[C:3]([H])([H])[#6:2](\[H])=[#6:1](\[H])[H]

Correctness of the mapping
MAPPET YES
ReactionMap YES
Marvin YES

Reaction no 196

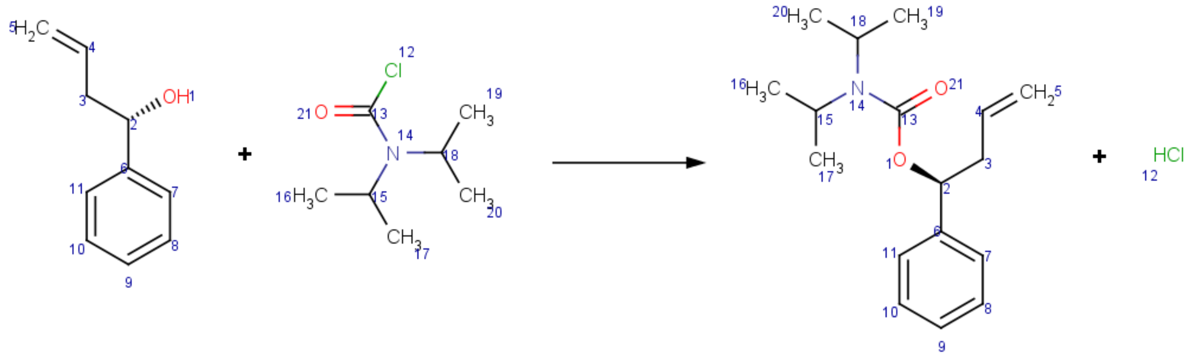

Correct mapped SMILES/SMARTS of the reaction:
[#8:1]-[#6@@H:2](-[#6:3]-[#6:4]=[#6:5])-[#6:6]-1=[#6:7]-[#6:8]=[#6:9]-[#6:10]=[#6:11]-1.[#6:16]-[#6:15](-[#6:17])-[#7:14](-[#6:18](-[#6:19])-[#6:20])-[#6:13]([Cl:12])=[O:21]>>[#6:20]-[#6:18](-[#6:19])-[#7:14](-[#6:15](-[#6:17])-[#6:16])-[#6:13](=[O:21])-[#8:1]-[#6@@H:2](-[#6:3]-[#6:4]=[#6:5])-[#6:6]-1=[#6:7]-[#6:8]=[#6:9]-[#6:10]=[#6:11]-1.[Cl:12]

Correctness of the mapping
MAPPET   YES
ReactionMap YES
Marvin   YES

Reaction no 197

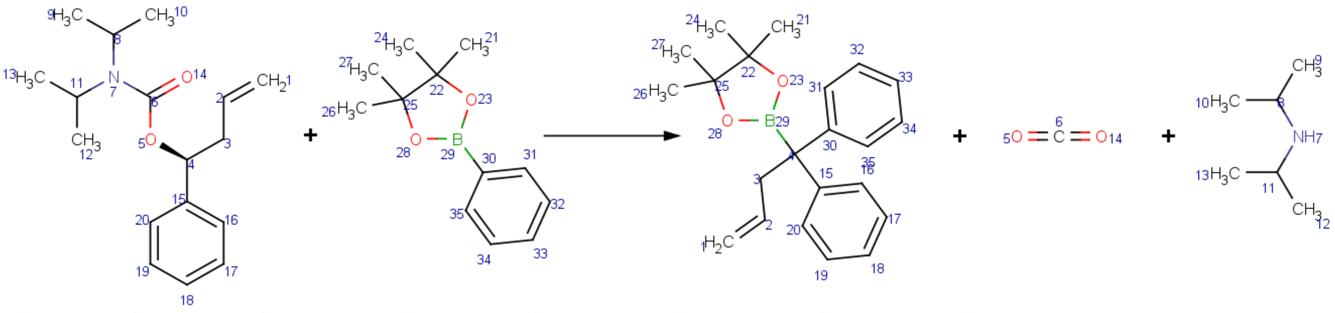

[#6:9]-[#6:8](-[#6:10])-[#7:7](-[#6:11](-[#6:12])-[#6:13])-[#6:6](=[O:14])-[#8:5]-[#6@@H:4](-[#6:3]-[#6:2]=[#6:1])-[#6:15]-1=[#6:16]-[#6:17]=[#6:18]-[#6:19]=[#6:20]-1.[#6:21][C:22]1([#6:24])[#8:23]-[#5:29](-[#8:28][C:25]1([#6:26])[#6:27])-[#6:30]-1=[#6:31]-[#6:32]=[#6:33]-[#6:34]=[#6:35]-1>>[#6:21][C:22]1([#6:24])[#8:23]-[#5:29](-[#8:28][C:25]1([#6:26])[#6:27])[C:4]([#6:3]-[#6:2]=[#6:1])([#6:15]-1=[#6:16]-[#6:17]=[#6:18]-[#6:19]=[#6:20]-1)[#6:30]-1=[#6:31]-[#6:32]=[#6:33]-[#6:34]=[#6:35]-1.[O:5]=[C:6]=[O:14].[#6:9]-[#6:8](-[#6:10])-[#7:7]-[#6:11](-[#6:12])-[#6:13]

Correctness of the mapping
MAPPET   NO
ReactionMap NO
Marvin   YES

Reaction no 198

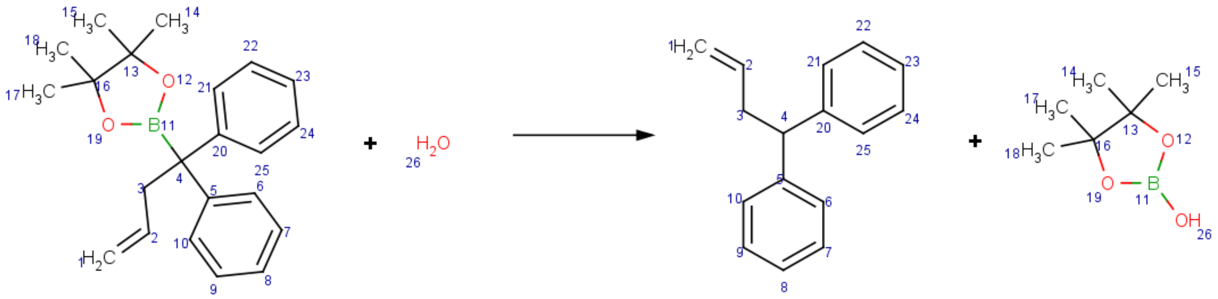

Correct mapped SMILES/SMARTS of the reaction:
[#6:14][C:13]1([#6:15])[#8:12]-[#5:11](-
[#8:19][C:16]1([#6:17])[#6:18])[C:4]([#6:3]-[#6:2]=[#6:1])([#6:5]-1=[#6:6]-
[#6:7]=[#6:8]-[#6:9]=[#6:10]-1)[#6:20]-1=[#6:21]-[#6:22]=[#6:23]-
[#6:24]=[#6:25]-1.[#8:26]>>[#6:1]=[#6:2]-[#6:3]-[#6:4](-[#6:5]-1=[#6:6]-
[#6:7]=[#6:8]-[#6:9]=[#6:10]-1)-[#6:20]-1=[#6:21]-[#6:22]=[#6:23]-
[#6:24]=[#6:25]-1.[#6:15][C:13]1([#6:14])[#8:12]-[#5:11](-[#8:26])-
[#8:19][C:16]1([#6:18])[#6:17]

Correctness of the mapping
MAPPET   YES
ReactionMap YES
Marvin   YES

Reaction no 199

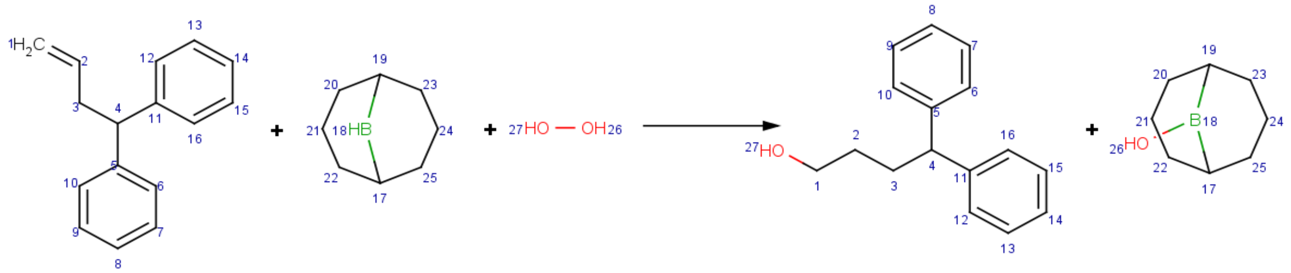

Correct mapped SMILES/SMARTS of the reaction:
[#6:1]=[#6:2]-[#6:3]-[#6:4](-[#6:5]-1=[#6:6]-[#6:7]=[#6:8]-[#6:9]=[#6:10]-1)-
[#6:11]-1=[#6:12]-[#6:13]=[#6:14]-[#6:15]=[#6:16]-1.[#5:18]-1-[#6:19]-2-[#6:20]-
[#6:21]-[#6:22]-[#6:17]-1-[#6:25]-[#6:24]-[#6:23]-2.[#8:26]-[#8:27]>>[#8:27]-
[#6:1]-[#6:2]-[#6:3]-[#6:4](-[#6:11]-1=[#6:16]-[#6:15]=[#6:14]-[#6:13]=[#6:12]-
1)-[#6:5]-1=[#6:10]-[#6:9]=[#6:8]-[#6:7]=[#6:6]-1.[#8:26]-[#5:18]-1-[#6:19]-2-
[#6:20]-[#6:21]-[#6:22]-[#6:17]-1-[#6:25]-[#6:24]-[#6:23]-2

Correctness of the mapping
MAPPET   YES
ReactionMap YES
Marvin   NO

Reaction no 200

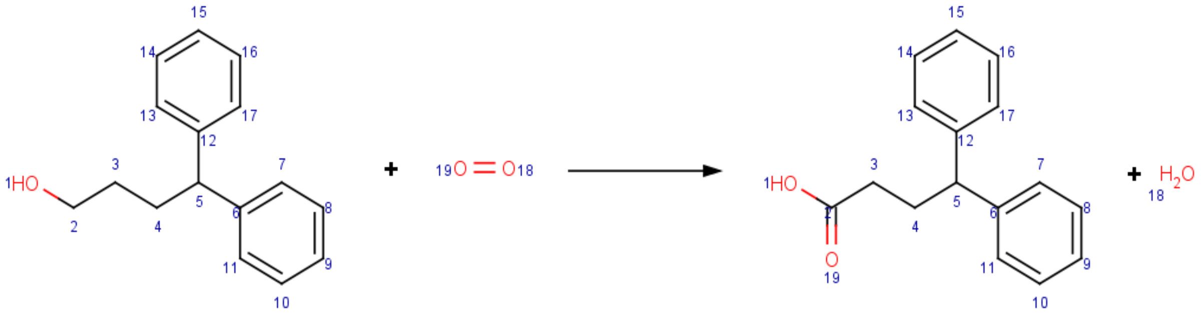

Correct mapped SMILES/SMARTS of the reaction:
[#8:1]-[#6:2]-[#6:3]-[#6:4]-[#6:5](-[#6:6]-1=[#6:7]-[#6:8]=[#6:9]-[#6:10]=[#6:11]-1)-[#6:12]-1=[#6:13]-[#6:14]=[#6:15]-[#6:16]=[#6:17]-1.[O:18]=[O:19]>>[#8:1]-[#6:2](=[O:19])-[#6:3]-[#6:4]-[#6:5](-[#6:6]-1=[#6:7]-[#6:8]=[#6:9]-[#6:10]=[#6:11]-1)-[#6:12]-1=[#6:13]-[#6:14]=[#6:15]-[#6:16]=[#6:17]-1.[#8:18]

Correctness of the mapping
MAPPET YES
ReactionMap YES
Marvin YES

Reaction no 201

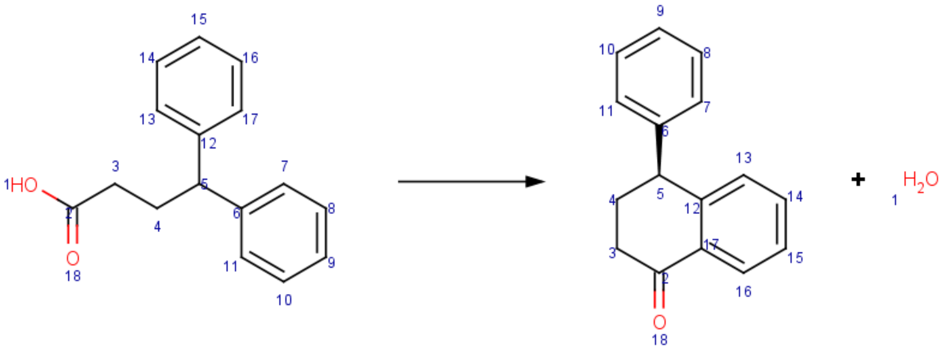

Correct mapped SMILES/SMARTS of the reaction:
[#8:1]-[#6:2](=[O:18])-[#6:3]-[#6:4]-[#6:5](-[#6:6]-1=[#6:7]-[#6:8]=[#6:9]-[#6:10]=[#6:11]-1)-[#6:12]-1=[#6:13]-[#6:14]=[#6:15]-[#6:16]=[#6:17]-1>>[O:18]=[#6:2]-1-[#6:3]-[#6:4]-[#6@@H:5](-[#6:6]-2=[#6:11]-[#6:10]=[#6:9]-[#6:8]=[#6:7]-2)-[#6:12]-2=[#6:13]-[#6:14]=[#6:15]-[#6:16]=[#6:17]-1-2.[#8:1]

Correctness of the mapping
MAPPET YES
ReactionMap YES
Marvin YES

Reaction no 202

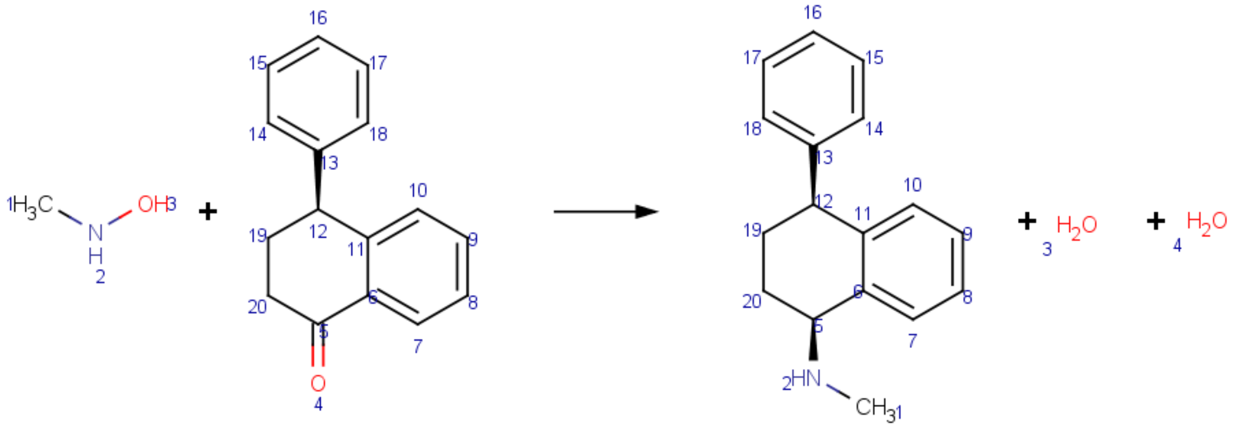

Correct mapped SMILES/SMARTS of the reaction:
[CH3:1][NH:2][OH:3].[O:4]=[C:5]1[CH2:20][CH2:19][C@@H:12]([C:13]2=[CH:14][CH:15]=[CH:16][CH:17]=[CH:18]2)[C:11]2=[CH:10][CH:9]=[CH:8][CH:7]=[C:6]12>>[OH2:4].[OH2:3].[CH3:1][NH:2][C@H:5]1[CH2:20][CH2:19][C@@H:12]([C:13]2=[CH:18][CH:17]=[CH:16][CH:15]=[CH:14]2)[C:11]2=[CH:10][CH:9]=[CH:8][CH:7]=[C:6]12

Correctness of the mapping
MAPPET   YES
ReactionMap YES
Marvin   YES

Reaction no 203

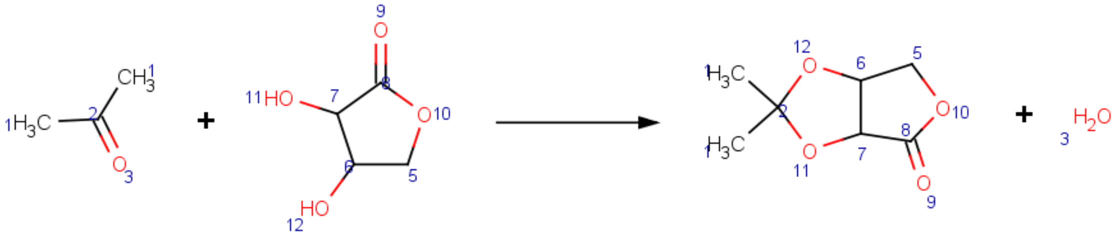

[CH3:1][C:2](=[O:3])[CH3:1].[CH2:5]1[CH:6]([CH:7]([C:8](=[O:9])[O:10]1)[OH:11])[OH:12]>>[CH3:1][C:2]1([O:12][CH:6]2[CH2:5][O:10][C:8](=[O:9])[CH:7]2[O:11]1)[CH3:1].[OH2:3]

Correctness of the mapping
MAPPET   NO
ReactionMap YES
Marvin   YES

Reaction no 204

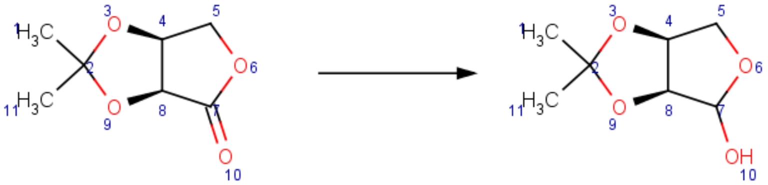

Correct mapped SMILES/SMARTS of the reaction:
[#6:1][C:2]1([#6:11])[#8:3]-[#6@H:4]-2-[#6:5]-[#8:6]-[#6:7](=[O:10])-[#6@H:8]-2-[#8:9]1>>[#6:1][C:2]1([#6:11])[#8:3]-[#6@H:4]-2-[#6:5]-[#8:6]-[#6:7](-[#8:10])-[#6@H:8]-2-[#8:9]1

Correctness of the mapping
MAPPET YES
ReactionMap YES
Marvin YES

Reaction no 205

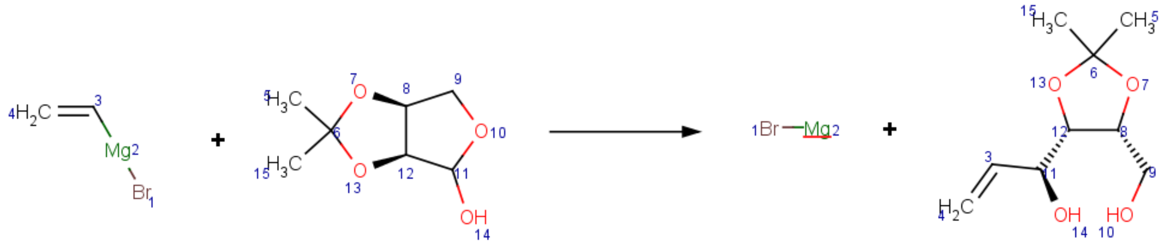

Correct mapped SMILES/SMARTS of the reaction:
[Br:1][Mg:2][#6:3]=[#6:4].[#6:5][C:6]1([#6:15])[#8:7]-[#6@H:8]-2-[#6:9]-[#8:10]-[#6:11](-[#8:14])-[#6@H:12]-2-[#8:13]1>>[Mg:2][Br:1].[#6:5][C:6]1([#6:15])[#8:7]-[#6@H:8](-[#6:9]-[#8:10])-[#6@@H:12](-[#8:13]1)-[#6@@H:11](-[#8:14])-[#6:3]=[#6:4]

Correctness of the mapping
MAPPET YES
ReactionMap YES
Marvin YES

Reaction no 206

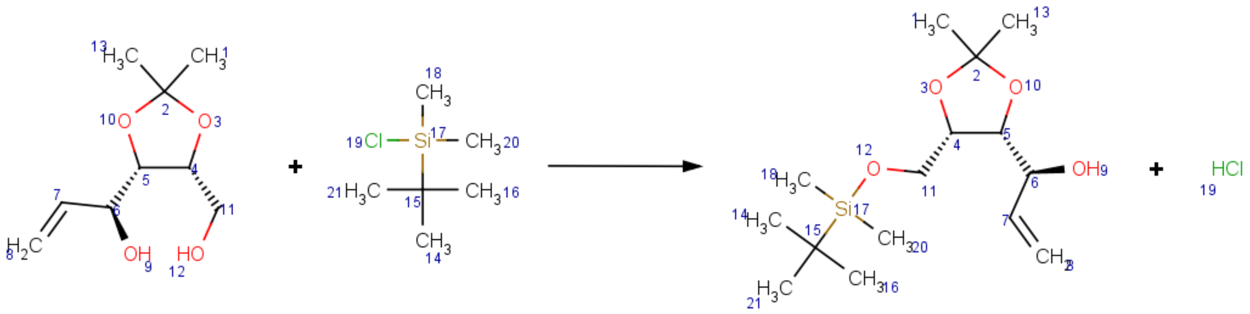

Correct mapped SMILES/SMARTS of the reaction:
[#6:1][C:2]1([#6:13])[#8:3]-[#6@H:4](-[#6:11]-[#8:12])-[#6@@H:5](-[#8:10]1)-[#6@@H:6](-[#8:9])-[#6:7]=[#6:8].[#6:14][C:15]([#6:16])([#6:21])[Si:17]([#6:18])([#6:20])[Cl:19]>>[#6:21][C:15]([#6:16])([#6:14])[Si:17]([#6:20])([#6:18])[#8:12]-[#6:11]-[#6@@H:4]-1-[#8:3][C:2]([#6:1])([#6:13])[#8:10]-[#6@@H:5]-1-[#6@@H:6](-[#8:9])-[#6:7]=[#6:8].[Cl:19]

Correctness of the mapping
MAPPET YES
ReactionMap YES
Marvin YES

Reaction no 207

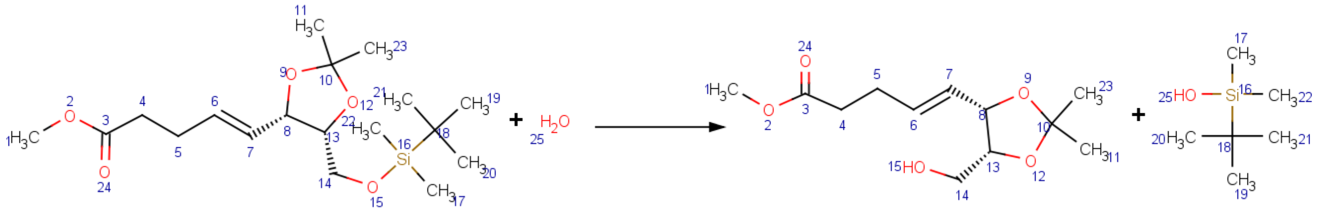

[#6:1]-[#8:2]-[#6:3](=[O:24])-[#6:4]-[#6:5]\[#6:6]=[#6:7]\[#6@@H:8]-1-
[#8:9][C:10]([#6:11])([#6:23])[#8:12]-[#6@@H:13]1-[#6:14]-
[#8:15][Si:16]([#6:17])([#6:22])[C:18]([#6:19])([#6:20])[#6:21].[#8:25]>>[#6:1]-
[#8:2]-[#6:3](=[O:24])-[#6:4]-[#6:5]\[#6:6]=[#6:7]\[#6@@H:8]-1-
[#8:9][C:10]([#6:23])([#6:11])[#8:12]-[#6@@H:13]1-[#6:14]-
[#8:15].[#6:19][C:18]([#6:21])([#6:20])[Si:16]([#6:17])([#6:22])[#8:25]

Correctness of the mapping
MAPPET   NO
ReactionMap YES
Marvin   YES

Reaction no 208

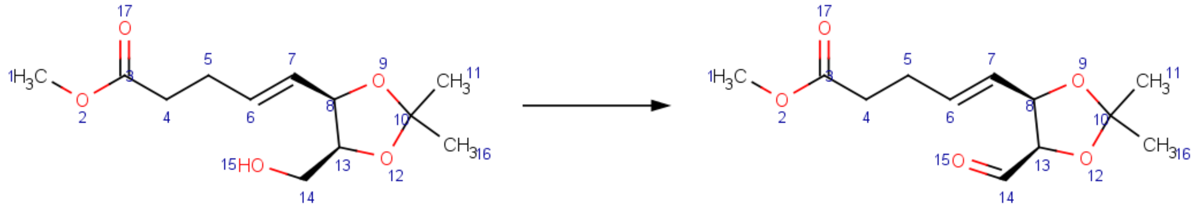

Correct mapped SMILES/SMARTS of the reaction:
[#6:1]-[#8:2]-[#6:3](=[O:17])-[#6:4]-[#6:5]\[#6:6]=[#6:7]\[#6@H:8]-1-
[#8:9][C:10]([#6:11])([#6:16])[#8:12]-[#6@H:13]1-[#6:14]-[#8:15]>>[#6:1]-[#8:2]-
[#6:3](=[O:17])-[#6:4]-[#6:5]\[#6:6]=[#6:7]\[#6@H:8]-1-
[#8:9][C:10]([#6:11])([#6:16])[#8:12]-[#6@H:13]1-[#6:14]=[O:15]

Correctness of the mapping
MAPPET   YES
ReactionMap YES
Marvin   YES

Reaction no 209

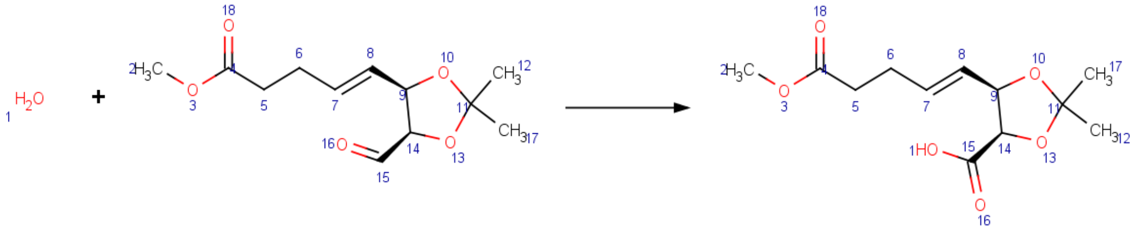

Correct mapped SMILES/SMARTS of the reaction:
[#8:1].[#6:2]-[#8:3]-[#6:4](=[O:18])-[#6:5]-[#6:6]\[#6:7]=[#6:8]\[#6@H:9]-1-
[#8:10][C:11]([#6:12])([#6:17])[#8:13]-[#6@H:14]1-[#6:15]=[O:16]>>[#6:2]-[#8:3]-
[#6:4](=[O:18])-[#6:5]-[#6:6]\[#6:7]=[#6:8]\[#6@H:9]-1-
[#8:10][C:11]([#6:17])([#6:12])[#8:13]-[#6@H:14]1-[#6:15](-[#8:1])=[O:16]

Correctness of the mapping
MAPPET   YES
ReactionMap YES
Marvin   YES

Reaction no 210

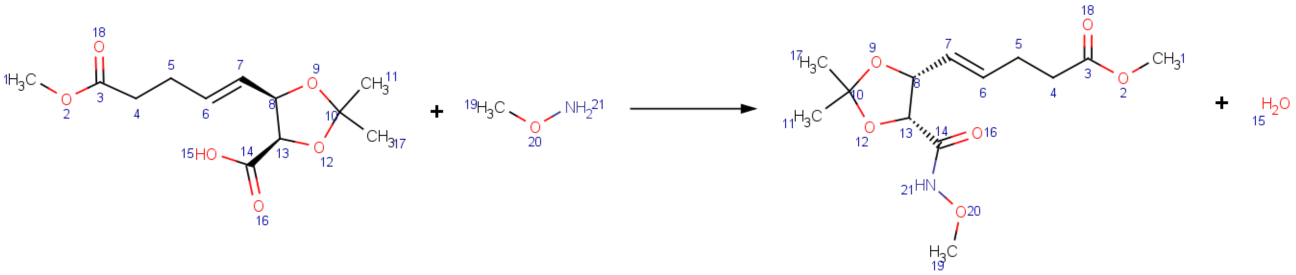

Correct mapped SMILES/SMARTS of the reaction:
[#6:1]-[#8:2]-[#6:3](=[O:18])-[#6:4]-[#6:5]\[#6:6]=[#6:7]\[#6@H:8]-1-[#8:9][C:10]([#6:11])([#6:17])[#8:12]-[#6@H:13]1-[#6:14](-[#8:15])=[O:16].[#6:19]-[#8:20]-[#7:21]>>[#6:19]-[#8:20]-[#7:21]-[#6:14](=[O:16])-[#6@@H:13]-1-[#8:12][C:10]([#6:11])([#6:17])[#8:9]-[#6@@H:8]-1\[#6:7]=[#6:6]\[#6:5]-[#6:4]-[#6:3](=[O:18])-[#8:2]-[#6:1].[#8:15]

Correctness of the mapping
MAPPET YES
ReactionMap YES
Marvin YES

Reaction no 211

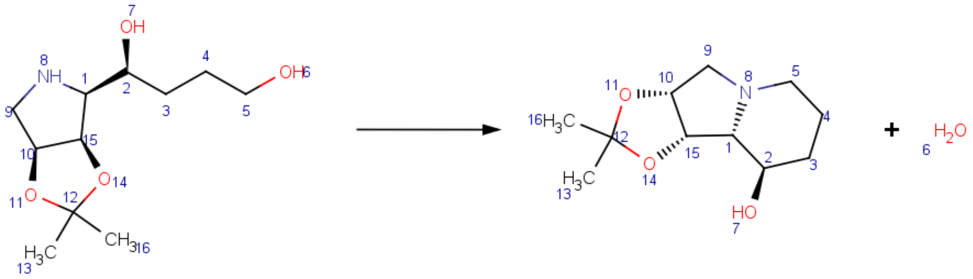

Correct mapped SMILES/SMARTS of the reaction:
[#6:13][C:12]1([#6:16])[#8:11]-[#6@H:10]-2-[#6:9]-[#7:8]-[#6@@H:1](-[#6@@H:2](-[#8:7])-[#6:3]-[#6:4]-[#6:5]-[#8:6])-[#6@H:15]-2-[#8:14]1>>[#6:16][C:12]1([#6:13])[#8:11]-[#6@@H:10]-2-[#6:9]-[#7:8]-3-[#6:5]-[#6:4]-[#6:3]-[#6@@H:2](-[#8:7])-[#6@@H:1]-3-[#6@@H:15]-2-[#8:14]1.[#8:6]

Correctness of the mapping
MAPPET NO
ReactionMap YES
Marvin YES

Reaction no 212

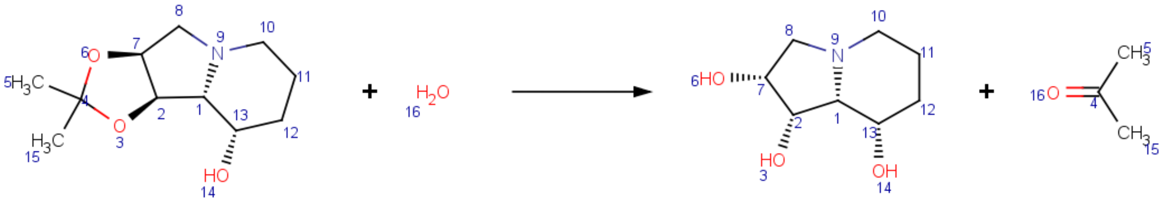

Correct mapped SMILES/SMARTS of the reaction:
[#6:5][C:4]1([#6:15])[#8:6]-[#6@H:7]-2-[#6:8]-[#7:9]-3-[#6:10]-[#6:11]-[#6:12]-[#6@H:13](-[#8:14])-[#6@@H:1]-3-[#6@H:2]-2-[#8:3]1.[#8:16]>>[#8:6]-[#6@@H:7]-1-[#6:8]-[#7:9]-2-[#6:10]-[#6:11]-[#6:12]-[#6@H:13](-[#8:14])-[#6@@H:1]-2-[#6@@H:2]-1-[#8:3].[#6:5]-[#6:4](-[#6:15])=[O:16]

Correctness of the mapping
MAPPET YES
ReactionMap YES
Marvin YES

Reaction no 213

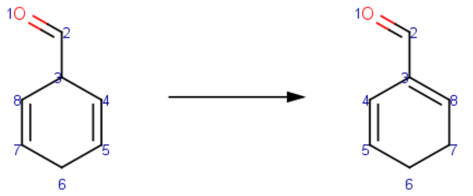

Correct mapped SMILES/SMARTS of the reaction:
[O:1]=[#6:2]-[#6:3]-1-[#6:4]=[#6:5]-[#6:6]-[#6:7]=[#6:8]-1>>[O:1]=[#6:2]-[#6:3]-1=[#6:8]-[#6:7]-[#6:6]-[#6:5]=[#6:4]-1

Correctness of the mapping
MAPPET YES
ReactionMap YES
Marvin YES

Reaction no 214

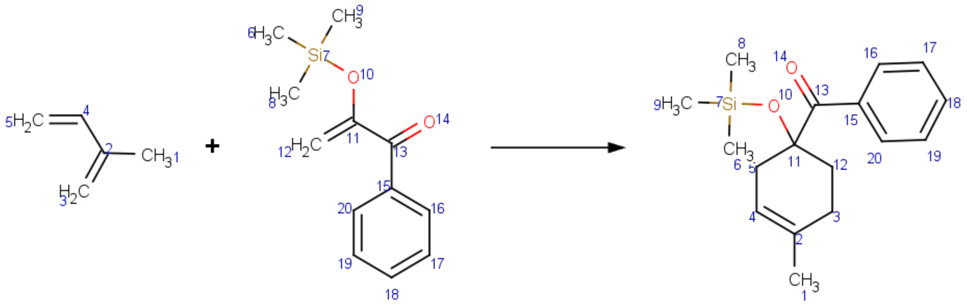

Correct mapped SMILES/SMARTS of the reaction:
[#6:1]-[#6:2](=[#6:3])-[#6:4]=[#6:5].[#6:6][Si:7]([#6:8])([#6:9])[#8:10]-[#6:11](=[#6:12])-[#6:13](=[O:14])-[c:15]1[c:16][c:17][c:18][c:19][c:20]1>>[#6:1]-[#6:2]-1=[#6:4]-[#6:5][C:11]([#6:12]-[#6:3]-1)([#8:10][Si:7]([#6:8])([#6:6])[#6:9])[#6:13](=[O:14])-[c:15]1[c:16][c:17][c:18][c:19][c:20]1

Correctness of the mapping
MAPPET YES
ReactionMap YES
Marvin NO

Reaction no 215

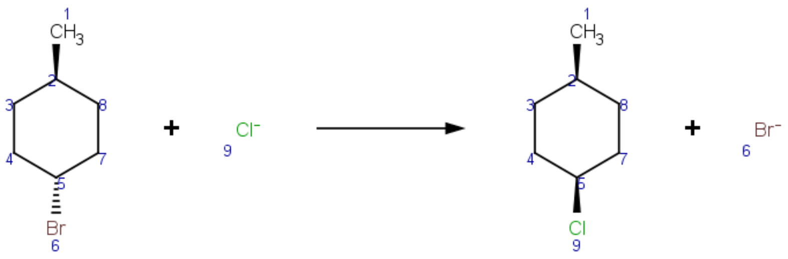

Correct mapped SMILES/SMARTS of the reaction:
[#6:1]-[#6@H:2]-1-[#6:8]-[#6:7]-[#6@H:5]([Br:6])-[#6:4]-[#6:3]-1.[Cl-:9]>>[#6:1]-[#6@H:2]-1-[#6:8]-[#6:7]-[#6@@H:5]([Cl:9])-[#6:4]-[#6:3]-1.[Br-:6]

Correctness of the mapping
MAPPET YES
ReactionMap YES
Marvin YES

Reaction no 216

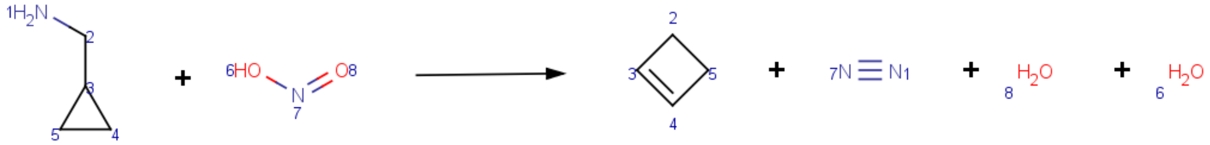

Correct mapped SMILES/SMARTS of the reaction:
[#7:1]-[#6:2]-[#6:3]-1-[#6:4]-[#6:5]-1.[#8:6]-[#7:7]=[O:8]>>[#6:2]-1-[#6:5]-[#6:4]=[#6:3]-1.[N:1]#[N:7].[#8:8].[#8:6]

Correctness of the mapping
MAPPET YES
ReactionMap YES
Marvin YES

Reaction no 217

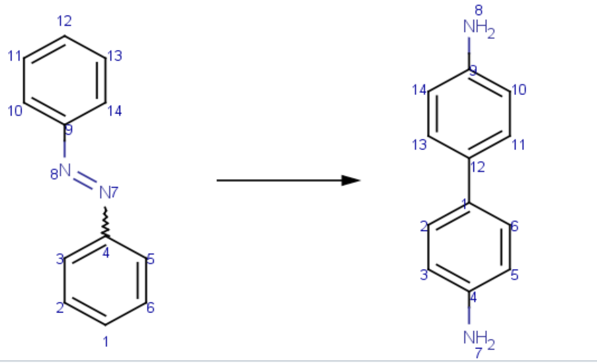

Correct mapped SMILES/SMARTS of the reaction:
[c:1]1[c:2][c:3][c:4]([c:5][c:6]1)-[#7:7]=[#7:8]-[c:9]1[c:10][c:11][c:12][c:13][c:14]1>>[#7:8]-[c:9]1[c:10][c:11][c:12]([c:13][c:14]1)-[c:1]1[c:6][c:5][c:4](-[#7:7])[c:3][c:2]1

Correctness of the mapping
MAPPET YES
ReactionMap YES
Marvin YES

Reaction no 218

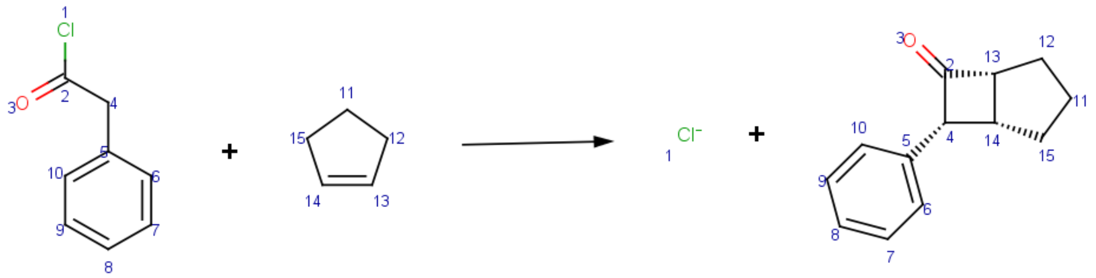

Correct mapped SMILES/SMARTS of the reaction:
[Cl:1][#6:2](=[O:3])-[#6:4]-[c:5]1[c:6][c:7][c:8][c:9][c:10]1.[#6:11]-1-[#6:12]-[#6:13]=[#6:14]-[#6:15]-1>>[Cl-:1].[O:3]=[#6:2]-1-[#6@@H:13]-2-[#6:12]-[#6:11]-[#6:15]-[#6@@H:14]-2-[#6@@H:4]-1-[c:5]1[c:6][c:7][c:8][c:9][c:10]1

Correctness of the mapping
MAPPET YES
ReactionMap YES
Marvin YES

Reaction no 219

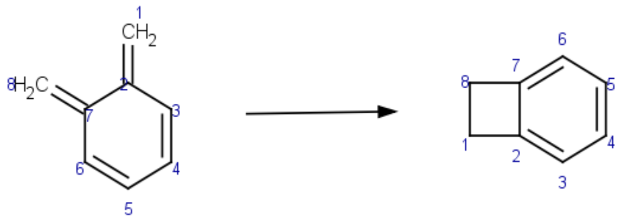

Correct mapped SMILES/SMARTS of the reaction:
[#6:1]=[#6:2]-1-[#6:3]=[#6:4]-[#6:5]=[#6:6]-[#6:7]-1=[#6:8]>>[#6:1]-1-[#6:8]-[c:7]2[c:6][c:5][c:4][c:3][c:2]-12

Correctness of the mapping
MAPPET YES
ReactionMap YES
Marvin YES

Reaction no 220

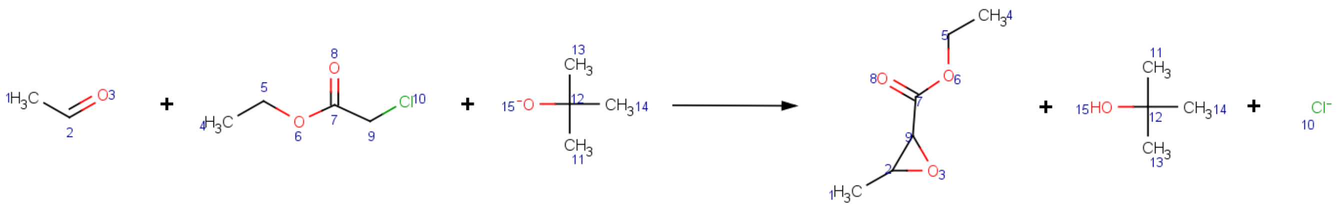

Correct mapped SMILES/SMARTS of the reaction:
[#6:1]-[#6:2]=[O:3].[#6:4]-[#6:5]-[#8:6]-[#6:7](=[O:8])-[#6:9][Cl:10].[#6:11][C:12]([#6:13])([#6:14])[#8-:15]>>[#6:4]-[#6:5]-[#8:6]-[#6:7](=[O:8])-[#6:9]-1-[#8:3]-[#6:2]-1-[#6:1].[#6:13][C:12]([#6:11])([#6:14])[#8:15].[Cl-:10]

Correctness of the mapping
MAPPET YES
ReactionMap YES
Marvin YES

Reaction no 221

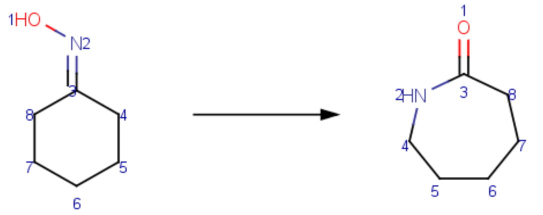

Correct mapped SMILES/SMARTS of the reaction:
[#8:1]\[#7:2]=[#6:3]-1\[#6:4]-[#6:5]-[#6:6]-[#6:7]-[#6:8]-1>>[O:1]=[#6:3]-1-[#6:8]-[#6:7]-[#6:6]-[#6:5]-[#6:4]-[#7:2]-1

Correctness of the mapping
MAPPET   YES
ReactionMap YES
Marvin   YES

Reaction no 222

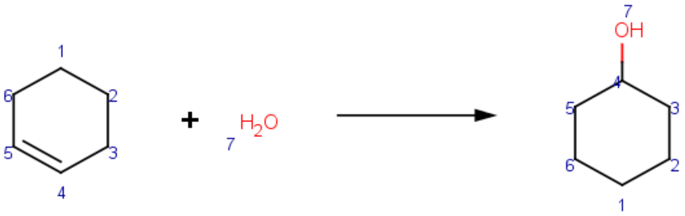

Correct mapped SMILES/SMARTS of the reaction:
[#6:1]-1-[#6:2]-[#6:3]-[#6:4]=[#6:5]-[#6:6]-1.[#8:7]>>[#8:7]-[#6:4]-1-[#6:3]-[#6:2]-[#6:1]-[#6:6]-[#6:5]-1

Correctness of the mapping
MAPPET   YES
ReactionMap YES
Marvin   YES

Reaction no 223

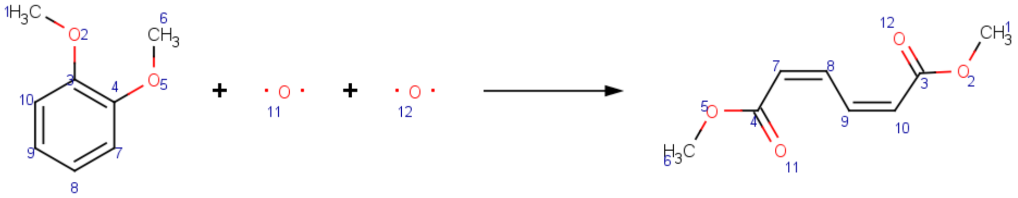

Correct mapped SMILES/SMARTS of the reaction:
[CH3:1][O:2][C:3]1=[C:4]([O:5][CH3:6])[CH:7]=[CH:8][CH:9]=[CH:10]1.[O:11].[O:12]>>[CH3:1][O:2][C:3](=[O:12])\[CH:10]=[CH:9]/[CH:8]=[CH:7]\[C:4](=[O:11])[O:5][CH3:6]

Correctness of the mapping
MAPPET   YES
ReactionMap NO
Marvin   YES

Reaction no 224

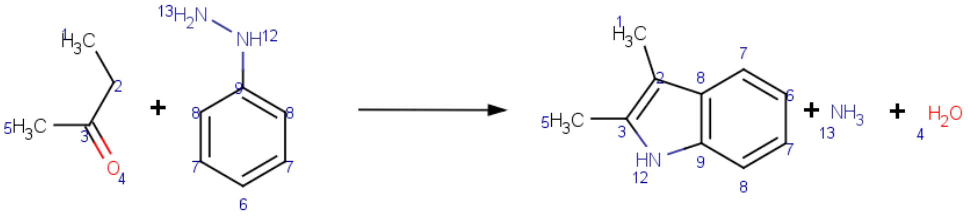

Correct mapped SMILES/SMARTS of the reaction:
[CH3:1][CH2:2][C:3](=[O:4])[CH3:5].[cH:6]1[cH:7][cH:8][c:9]([cH:8][cH:7]1)[NH:12][NH2:13]>>[CH3:1][C:2]1=[C:3]([NH:12][C:9]2=[C:8]1[CH:7]=[CH:6][CH:7]=[CH:8]2)[CH3:5].[NH3:13].[OH2:4]

Correctness of the mapping
MAPPET   NO
ReactionMap YES
Marvin   NO

Reaction no 225

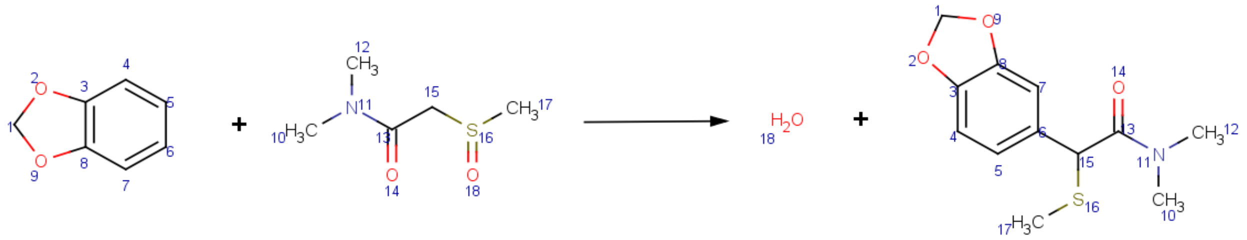

Correct mapped SMILES/SMARTS of the reaction:
[CH2:1]1[O:2][c:3]2[cH:4][cH:5][cH:6][cH:7][c:8]2[O:9]1.[CH3:10][N:11]([CH3:12])[C:13](=[O:14])[CH2:15][S:16]([CH3:17])=[O:18]>>[OH2:18].[CH3:17][S:16][CH:15]([C:13](=[O:14])[N:11]([CH3:12])[CH3:10])[c:6]1[cH:5][cH:4][c:3]2[O:2][CH2:1][O:9][c:8]2[cH:7]1

Correctness of the mapping
MAPPET   YES
ReactionMap YES
Marvin   YES

Reaction no 226

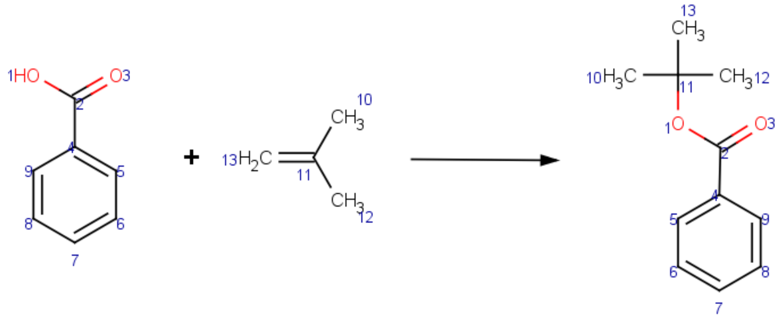

Correct mapped SMILES/SMARTS of the reaction:
[OH:1][C:2](=[O:3])[c:4]1[cH:5][cH:6][cH:7][cH:8][cH:9]1.[CH3:10][C:11]([CH3:12])=[CH2:13]>>[CH3:13][C:11]([CH3:10])([CH3:12])[O:1][C:2](=[O:3])[c:4]1[cH:9][cH:8][cH:7][cH:6][cH:5]1

Correctness of the mapping
MAPPET   YES
ReactionMap YES
Marvin   YES

Reaction no 227

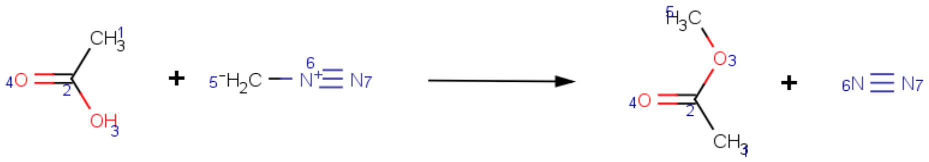

Correct mapped SMILES/SMARTS of the reaction:
[CH3:1][C:2]([OH:3])=[O:4].[CH2-
:5][N+:6]#[N:7]>>[CH3:5][O:3][C:2]([CH3:1])=[O:4].[N:7]#[N:6]

Correctness of the mapping
MAPPET YES
ReactionMap YES
Marvin YES

Reaction no 228

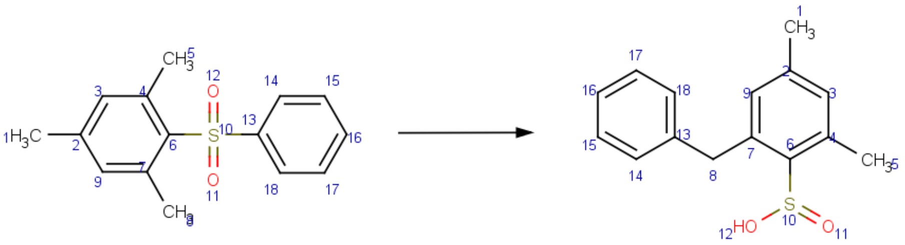

Correct mapped SMILES/SMARTS of the reaction:
[CH3:1][c:2]1[cH:3][c:4]([CH3:5])[c:6]([c:7]([CH3:8])[cH:9]1)[S:10](=[O:11])(=[O
:12])[c:13]1[cH:14][cH:15][cH:16][cH:17][cH:18]1>>[CH3:1][c:2]1[cH:3][c:4]([CH3:
5])[c:6]([c:7]([CH2:8][c:13]2[cH:14][cH:15][cH:16][cH:17][cH:18]2)[cH:9]1)[S:10]
([OH:12])=[O:11]

Correctness of the mapping
MAPPET YES
ReactionMap YES
Marvin YES

Reaction no 229

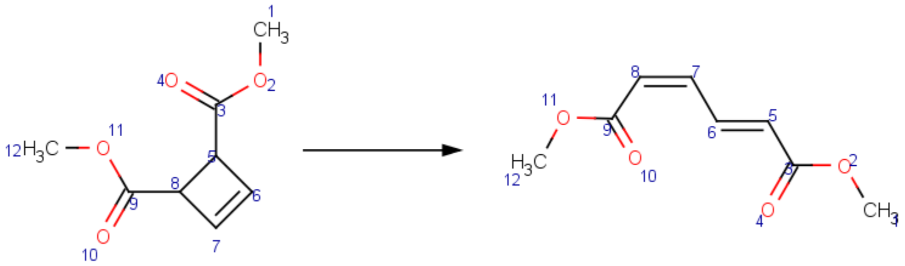

Correct mapped SMILES/SMARTS of the reaction:
[#6:1]-[#8:2]-[#6:3](=[O:4])-[#6:5]-1-[#6:6]=[#6:7]-[#6:8]-1-[#6:9](=[O:10])-
[#8:11]-[#6:12]>>[#6:1]-[#8:2]-
[#6:3](=[O:4])\[#6:5]=[#6:6]\[#6:7]=[#6:8]/[#6:9](=[O:10])-[#8:11]-[#6:12]

Correctness of the mapping
MAPPET YES
ReactionMap YES
Marvin YES

Reaction no 230

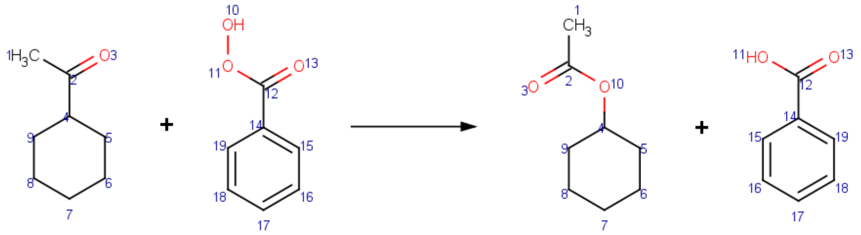

Correct mapped SMILES/SMARTS of the reaction:
[#6:1]-[#6:2](=[O:3])-[#6:4]-1-[#6:5]-[#6:6]-[#6:7]-[#6:8]-[#6:9]-1.[#8:10]-[#8:11]-[#6:12](=[O:13])-[#6:14]-1=[#6:15]-[#6:16]=[#6:17]-[#6:18]=[#6:19]-1>>[#6:1]-[#6:2](=[O:3])-[#8:10]-[#6:4]-1-[#6:5]-[#6:6]-[#6:7]-[#6:8]-[#6:9]-1.[#8:11]-[#6:12](=[O:13])-[#6:14]-1=[#6:19]-[#6:18]=[#6:17]-[#6:16]=[#6:15]-1

Correctness of the mapping
MAPPET   YES
ReactionMap YES
Marvin   YES

Reaction no 231

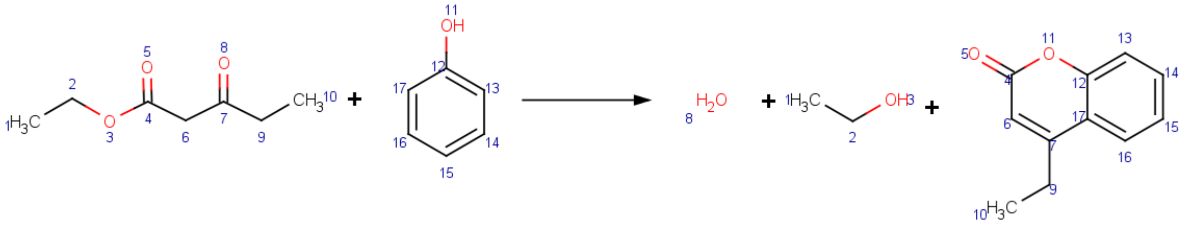

Correct mapped SMILES/SMARTS of the reaction:
[CH3:1][CH2:2][O:3][C:4](=[O:5])[CH2:6][C:7](=[O:8])[CH2:9][CH3:10].[OH:11][c:12]1[cH:13][cH:14][cH:15][cH:16][cH:17]1>>[OH2:8].[CH3:1][CH2:2][OH:3].[CH3:10][CH2:9][C:7]1=[CH:6][C:4](=[O:5])[O:11][c:12]2[cH:13][cH:14][cH:15][cH:16][c:17]12

Correctness of the mapping
MAPPET   YES
ReactionMap NO
Marvin   NO

Reaction no 232

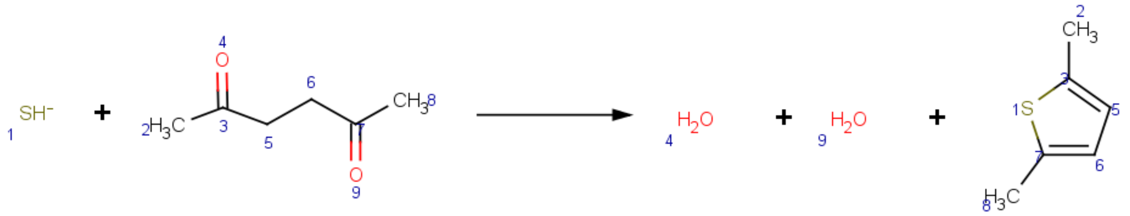

Correct mapped SMILES/SMARTS of the reaction:
[#16-:1].[#6:2]-[#6:3](=[O:4])-[#6:5]-[#6:6]-[#6:7](-[#6:8])=[O:9]>>[#8:4].[#8:9].[#6:2]-[#6:3]-1=[#6:5]-[#6:6]=[#6:7](-[#6:8])-[#16:1]-1

Correctness of the mapping
MAPPET   YES
ReactionMap YES
Marvin   YES

Reaction no 233

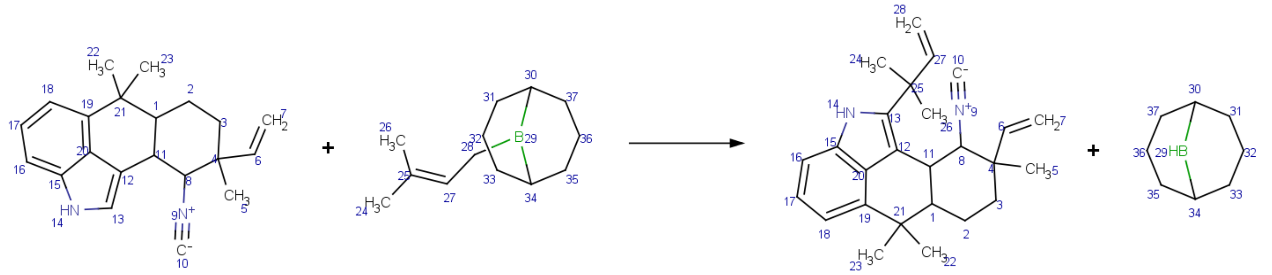

Correct mapped SMILES/SMARTS of the reaction:
[CH3:5][C:4]1([CH2:3][CH2:2][CH:1]2[CH:11]([CH:8]1[N+:9]#[C-
:10])[C:12]1=[CH:13][NH:14][c:15]3[cH:16][cH:17][cH:18][c:19]([c:20]13)[C:21]2([
CH3:22])[CH3:23])[CH:6]=[CH2:7].[CH3:24][C:25]([CH3:26])=[CH:27][CH2:28][B:29]1[
CH:30]2[CH2:31][CH2:32][CH2:33][CH:34]1[CH2:35][CH2:36][CH2:37]2>>[CH3:24][C:25]
([CH3:26])([CH:27]=[CH2:28])[C:13]1=[C:12]2[CH:11]3[CH:1]([CH2:2][CH2:3][C:4]([C
H3:5])([CH:6]=[CH2:7])[CH:8]3[N+:9]#[C-
:10])[C:21]([CH3:22])([CH3:23])[c:19]3[cH:18][cH:17][cH:16][c:15]([NH:14]1)[c:20
]23.[BH:29]1[CH:30]2[CH2:37][CH2:36][CH2:35][CH:34]1[CH2:33][CH2:32][CH2:31]2

Correctness of the mapping
MAPPET   YES
ReactionMap YES
Marvin   NO

Reaction no 234

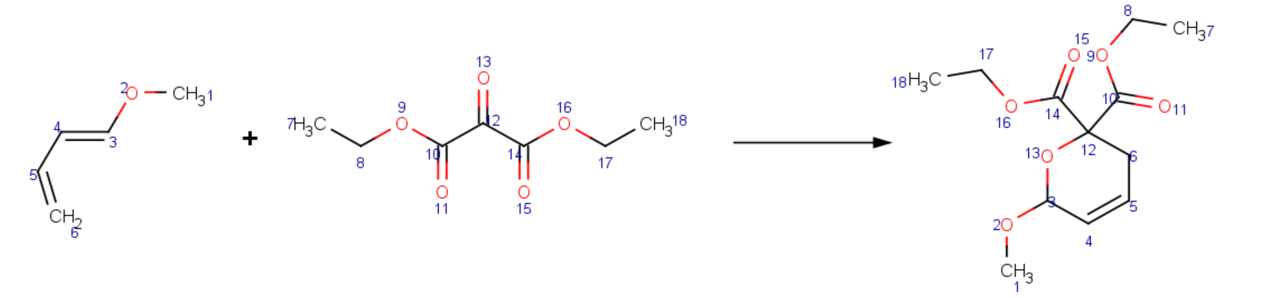

Correct mapped SMILES/SMARTS of the reaction:
[CH3:1][O:2]\[CH:3]=[CH:4]\[CH:5]=[CH2:6].[CH3:7][CH2:8][O:9][C:10](=[O:11])[C:1
2](=[O:13])[C:14](=[O:15])[O:16][CH2:17][CH3:18]>>[CH3:18][CH2:17][O:16][C:14](=
[O:15])[C:12]1([CH2:6][CH:5]=[CH:4][CH:3]([O:2][CH3:1])[O:13]1)[C:10](=[O:11])[O
:9][CH2:8][CH3:7]

Correctness of the mapping
MAPPET   YES
ReactionMap YES
Marvin   YES

Reaction no 235

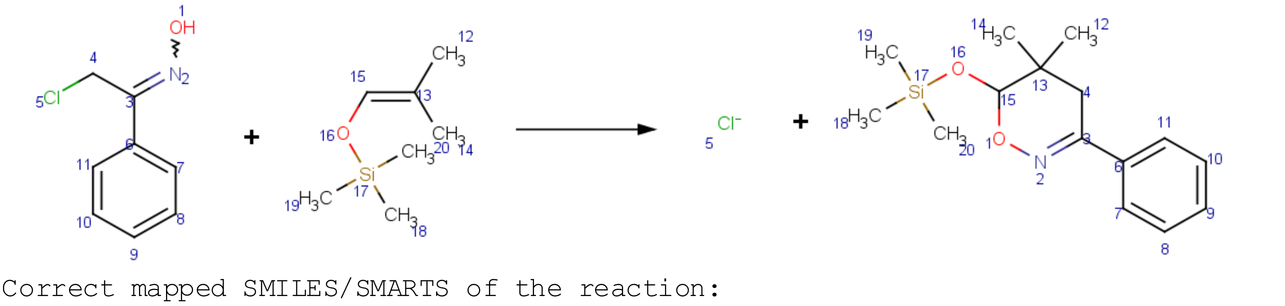

Correct mapped SMILES/SMARTS of the reaction:
[OH:1][N:2]=[C:3]([CH2:4][Cl:5])[c:6]1[cH:7][cH:8][cH:9][cH:10][cH:11]1.[CH3:12]
[C:13]([CH3:14])=[CH:15][O:16][Si:17]([CH3:18])([CH3:19])[CH3:20]>>[Cl-

:5].[CH3:12][C:13]1([CH3:14])[CH2:4][C:3](=[N:2][O:1][CH:15]1[O:16][Si:17]([CH3:18])([CH3:20])[CH3:19])[c:6]1[cH:11][cH:10][cH:9][cH:8][cH:7]1

Correctness of the mapping
MAPPET YES
ReactionMap YES
Marvin YES

Reaction no 236

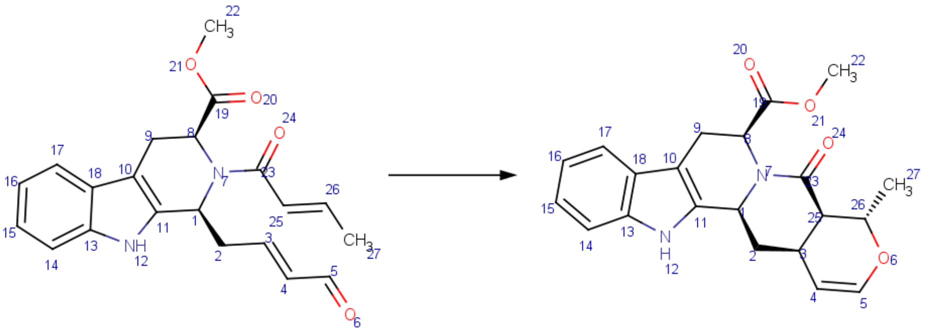

Correct mapped SMILES/SMARTS of the reaction:
[CH3:22][O:21][C:19](=[O:20])[C@@H:8]1[CH2:9][C:10]2=[C:11]([NH:12][c:13]3[cH:14][cH:15][cH:16][cH:17][c:18]23)[C@H:1]([CH2:2]\[CH:3]=[CH:4]\[CH:5]=[O:6])[N:7]1[C:23](=[O:24])\[CH:25]=[CH:26]\[CH3:27]>>[CH3:22][O:21][C:19](=[O:20])[C@@H:8]1[CH2:9][C:10]2=[C:11]([NH:12][c:13]3[cH:14][cH:15][cH:16][cH:17][c:18]23)[C@@H:1]2[CH2:2][C@@H:3]3[CH:4]=[CH:5][O:6][C@@H:26]([CH3:27])[C@@H:25]3[C:23](=[O:24])[N:7]12

Correctness of the mapping
MAPPET YES
ReactionMap YES
Marvin YES

Reaction no 237

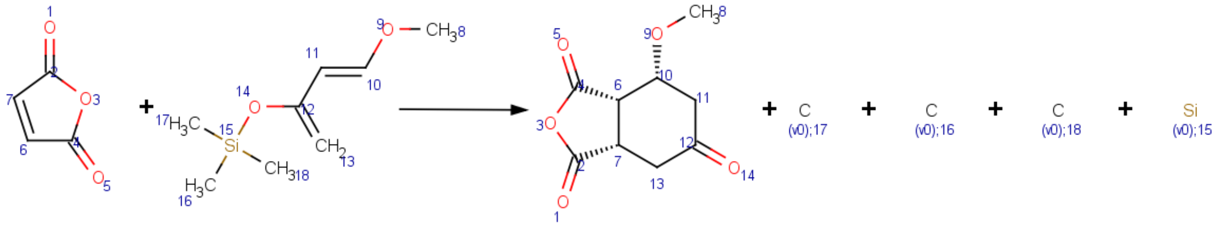

Correct mapped SMILES/SMARTS of the reaction:
[O:1]=[C:2]1[O:3][C:4](=[O:5])[CH:6]=[CH:7]1.[CH3:8][O:9]\[CH:10]=[CH:11]\[C:12](=[CH2:13])[O:14][Si:15]([CH3:16])([CH3:17])[CH3:18]>>[CH3:8][O:9][C@@H:10]1[CH2:11][C:12](=[O:14])[CH2:13][C@H:7]2[C@@H:6]1[C:4](=[O:5])[O:3][C:2]2=[O:1].[C:17].[C:16].[C:18].[Si:15]

Correctness of the mapping
MAPPET YES
ReactionMap NO
Marvin NO

Reaction no 238

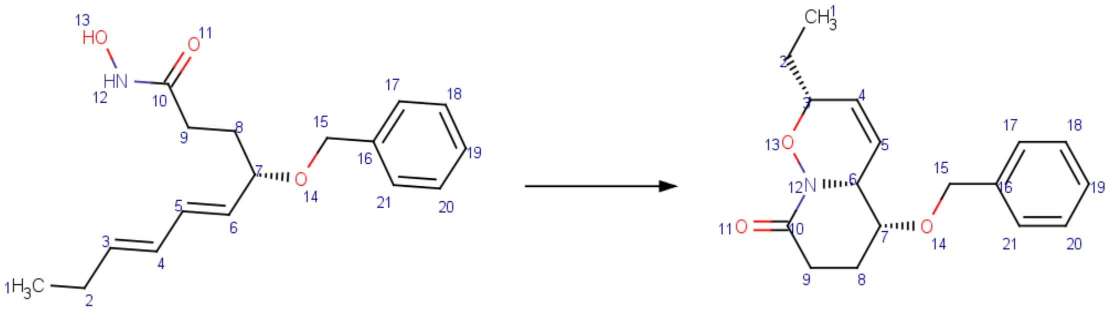

Correct mapped SMILES/SMARTS of the reaction:
[CH3:1][CH2:2]\[CH:3]=[CH:4]\[CH:5]=[CH:6]\[C@H:7]([CH2:8][CH2:9][C:10](=[O:11])[NH:12][OH:13])[O:14][CH2:15][c:16]1[cH:17][cH:18][cH:19][cH:20][cH:21]1>>[CH3:1][CH2:2][C@H:3]1[O:13][N:12]2[C@H:6]([CH:5]=[CH:4]1)[C@@H:7]([CH2:8][CH2:9][C:10]2=[O:11])[O:14][CH2:15][C:16]1=[CH:17][CH:18]=[CH:19][CH:20]=[CH:21]1

Correctness of the mapping
MAPPET   YES
ReactionMap YES
Marvin   YES

Reaction no 239

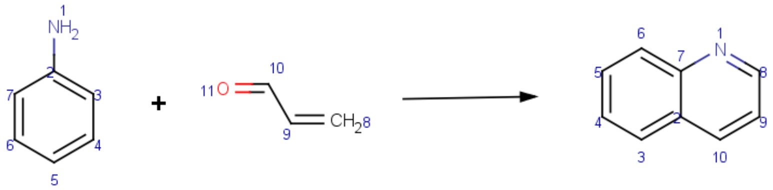

Correct mapped SMILES/SMARTS of the reaction:
[#7:1]-[c:2]1[c:3][c:4][c:5][c:6][c:7]1.[#6:8]=[#6:9]-[#6:10]=[O:11]>>[c:4]1[c:5][c:6][c:7]2[n:1][c:8][c:9][c:10][c:2]2[c:3]1

Correctness of the mapping
MAPPET   NO
ReactionMap NO
Marvin   YES

Reaction no 240

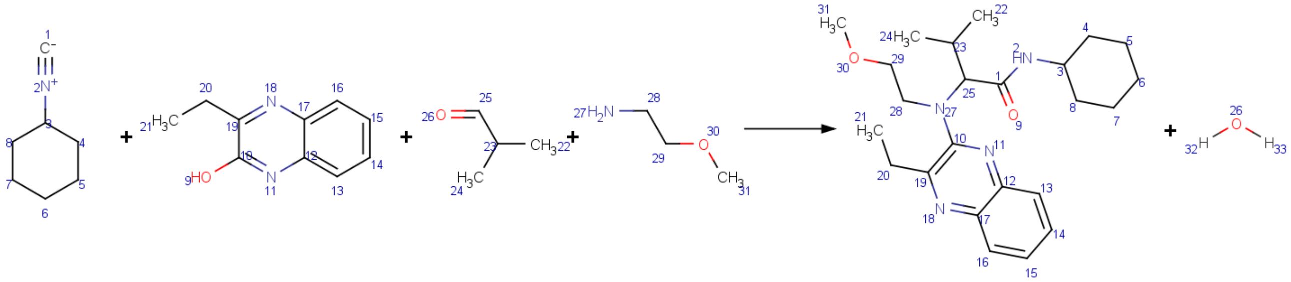

Correct mapped SMILES/SMARTS of the reaction:
[C-:1]#[N+:2][#6:3]-1-[#6:4]-[#6:5]-[#6:6]-[#6:7]-[#6:8]-1.[#6:21]-[#6:20]-[#6:19]-1=[#7:18]-[#6:17]-2=[#6:16]-[#6:15]=[#6:14]-[#6:13]=[#6:12]-2-[#7:11]=[#6:10]-1-[#8:9].[#6:22]-[#6:23](-[#6:24])-[#6:25]=[O:26].[#6:31]-[#8:30]-[#6:29]-[#6:28]-[#7:27]>>[#6:21]-[#6:20]-[#6:19]-1=[#6:10](-[#7:11]=[#6:12]-2-[#6:13]=[#6:14]-[#6:15]=[#6:16]-[#6:17]-2=[#7:18]-1)-[#7:27](-[#6:28]-[#6:29]-[#8:30]-[#6:31])-[#6:25](-[#6:23](-[#6:22])-[#6:24])-[#6:1](=[O:9])-[#7:2]-[#6:3]-1-[#6:4]-[#6:5]-[#6:6]-[#6:7]-[#6:8]-1.[H:32][#8:26][H:33]

Correctness of the mapping
MAPPET NO
ReactionMap NO
Marvin YES

Reaction no 241

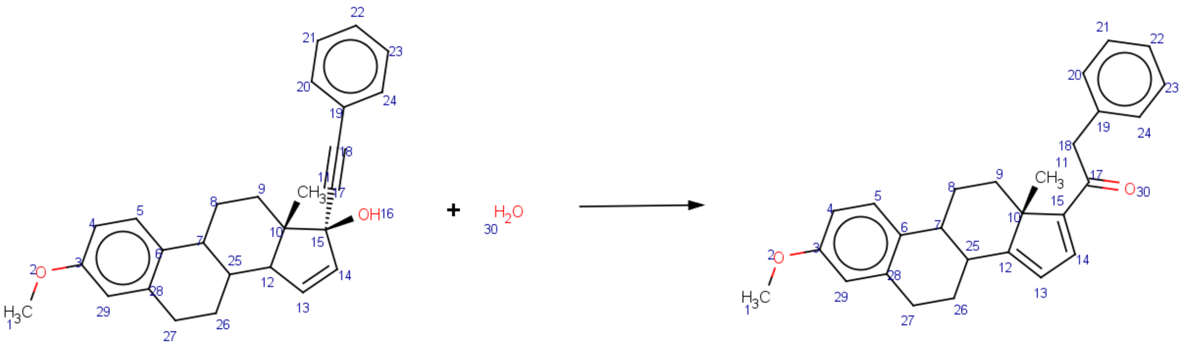

[CH3:1][O:2][c:3]1[cH:4][cH:5][c:6]2[CH:7]3[CH2:8][CH2:9][C@@:10]4([CH3:11])[CH:12]([CH:13]=[CH:14][C@@:15]4([OH:16])[C:17]#[C:18][c:19]4[cH:20][cH:21][cH:22][cH:23][cH:24]4)[CH:25]3[CH2:26][CH2:27][c:28]2[cH:29]1.[OH2:30]>>[CH3:1][O:2][c:3]1[cH:4][cH:5][c:6]2[CH:7]3[CH2:8][CH2:9][C@@:10]4([CH3:11])[C:12](=[CH:13][CH:14]=[C:15]4[C:17](=[O:30])[CH2:18][c:19]4[cH:20][cH:21][cH:22][cH:23][cH:24]4)[CH:25]3[CH2:26][CH2:27][c:28]2[cH:29]1

Correctness of the mapping
MAPPET NO
ReactionMap NO
Marvin NO

Reaction no 1

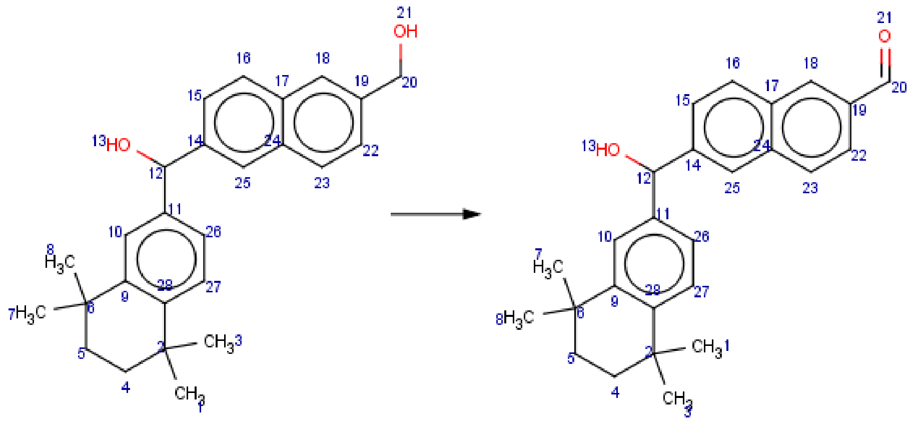

Correct mapped SMILES/SMARTS of the reaction:
[CH3:1][C:2]1([CH3:3])[CH2:4][CH2:5][C:6]([CH3:7])([CH3:8])[c:9]2[cH:10][
c:11]([cH:26][cH:27][c:28]12)[CH:12]([OH:13])[c:14]1[cH:15][cH:16][c:17]2
[cH:18][c:19]([CH2:20][OH:21])[cH:22][cH:23][c:24]2[cH:25]1>>[CH3:3][C:2]
1([CH3:1])[CH2:4][CH2:5][C:6]([CH3:8])([CH3:7])[c:9]2[cH:10][c:11]([cH:26
][cH:27][c:28]12)[CH:12]([OH:13])[c:14]1[cH:15][cH:16][c:17]2[cH:18][c:19
]([CH:20]=[O:21])[cH:22][cH:23][c:24]2[cH:25]1

Correctness of the mapping

MAPPET	YES
ReactionMap	YES
Marvin	YES

Reaction no 2

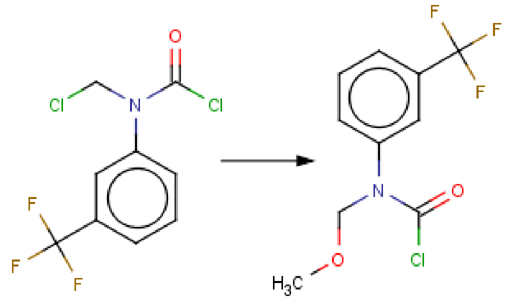

SMILES of the input:
O=C(Cl)N(CCl)c1cccc(C(F)(F)F)c1>>COCN(C(=O)Cl)c1cccc(C(F)(F)F)c1

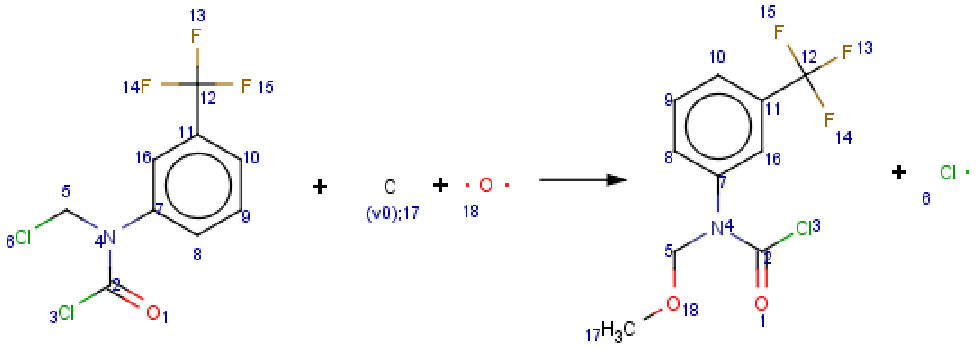

Correct mapped SMILES/SMARTS of the reaction:
[F:13][C:12]([F:14])([F:15])[c:11]1[cH:10][cH:9][cH:8][c:7]([cH:16]1)[N:4
]([CH2:5][Cl:6])[C:2]([Cl:3])=[O:1].[C:17].[O:18]>>[CH3:17][O:18][CH2:5][

N:4]([C:2]([Cl:3])=[O:1])[c:7]1[cH:8][cH:9][cH:10][c:11]([cH:16]1)[C:12]([F:13])([F:14])[F:15].[Cl:6]

Correctness of the mapping
MAPPET YES
ReactionMap NO
Marvin YES

Reaction no 3

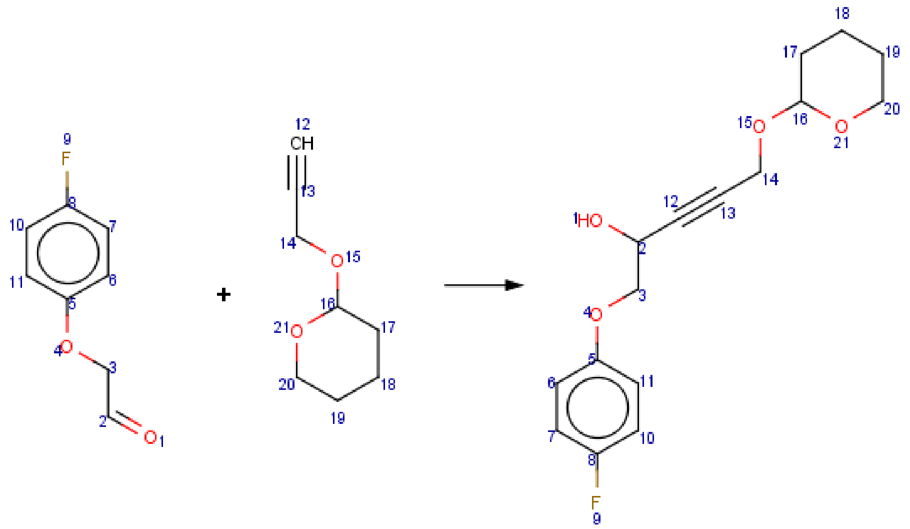

Correct mapped SMILES/SMARTS of the reaction:
[F:9][c:8]1[cH:7][cH:6][c:5]([O:4][CH2:3][CH:2]=[O:1])[cH:11][cH:10]1.[CH:12]#[C:13][CH2:14][O:15][CH:16]1[CH2:17][CH2:18][CH2:19][CH2:20][O:21]1>>[OH:1][CH:2]([CH2:3][O:4][c:5]1[cH:11][cH:10][c:8]([F:9])[cH:7][cH:6]1)[C:12]#[C:13][CH2:14][O:15][CH:16]1[CH2:17][CH2:18][CH2:19][CH2:20][O:21]1

Correctness of the mapping
MAPPET YES
ReactionMap YES
Marvin YES

Reaction no 4

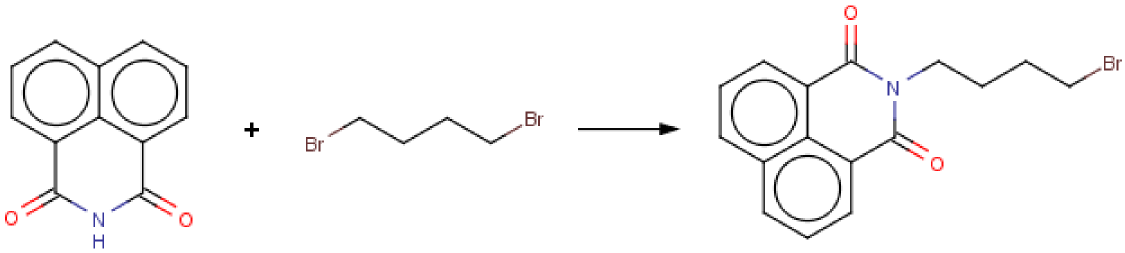

SMILES of the input:
O=C1NC(=O)c2cccc3cccc1c32.BrCCCCBr>>O=C1c2cccc3cccc(c32)C(=O)N1CCCCBr

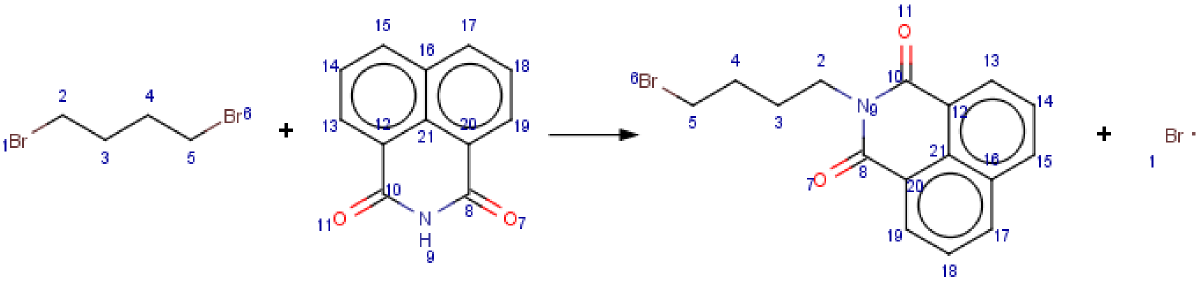

Correct mapped SMILES/SMARTS of the reaction:
[Br:1][CH2:2][CH2:3][CH2:4][CH2:5][Br:6].[O:7]=[C:8]1[NH:9][C:10](=[O:11]
)[c:12]2[cH:13][cH:14][cH:15][c:16]3[cH:17][cH:18][cH:19][c:20]1[c:21]23>
>[Br:6][CH2:5][CH2:4][CH2:3][CH2:2][N:9]1[C:10](=[O:11])[c:12]2[cH:13][cH
:14][cH:15][c:16]3[cH:17][cH:18][cH:19][c:20]([C:8]1=[O:7])[c:21]23.[Br:1
]

Correctness of the mapping
MAPPET YES
ReactionMap NO
Marvin YES

Reaction no 5

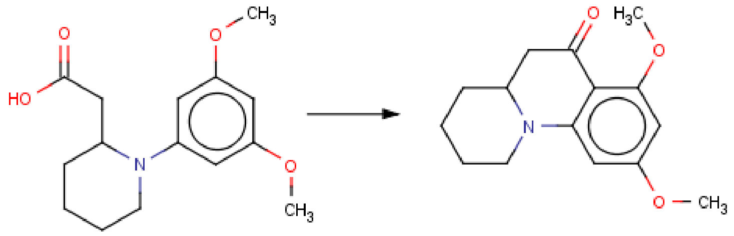

SMILES of the input:
COc1cc(OC)cc(N2CCCCC2CC(=O)O)c1>>COc1cc(OC)c2c(c1)N1CCCCC1CC2=O

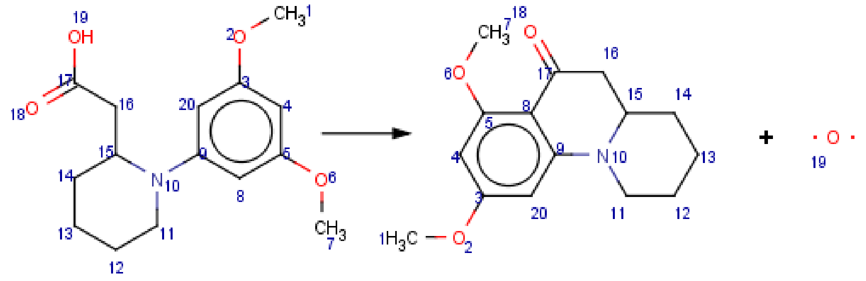

Correct mapped SMILES/SMARTS of the reaction:
[CH3:1][O:2][c:3]1[cH:4][c:5]([O:6][CH3:7])[cH:8][c:9]([cH:20]1)[N:10]1[C
H2:11][CH2:12][CH2:13][CH2:14][CH:15]1[CH2:16][C:17]([OH:19])=[O:18]>>[CH
3:1][O:2][c:3]1[cH:4][c:5]([O:6][CH3:7])[c:8]2[C:17](=[O:18])[CH2:16][CH:
15]3[CH2:14][CH2:13][CH2:12][CH2:11][N:10]3[c:9]2[cH:20]1.[O:19]

Correctness of the mapping
MAPPET YES
ReactionMap NO
Marvin YES

Reaction no 6

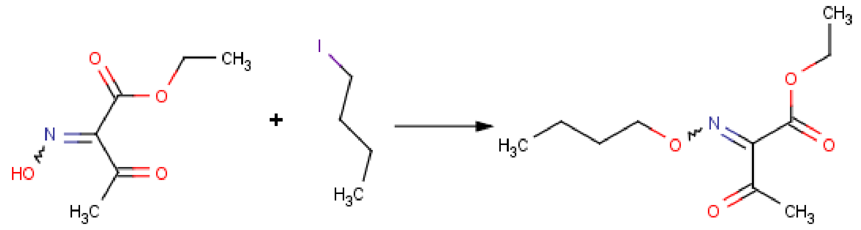

SMILES of the input:
CCOC(=O)C(=NO)C(C)=O.CCCCI>>CCCCON=C(C(C)=O)C(=O)OCC

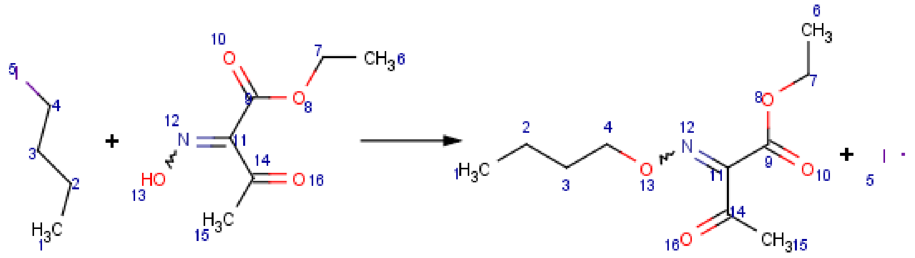

Correct mapped SMILES/SMARTS of the reaction:
[CH3:1][CH2:2][CH2:3][CH2:4][I:5].[CH3:6][CH2:7][O:8][C:9](=[O:10])[C:11](=[N:12][OH:13])[C:14]([CH3:15])=[O:16]>>[CH3:1][CH2:2][CH2:3][CH2:4][O:13][N:12]=[C:11]([C:14]([CH3:15])=[O:16])[C:9](=[O:10])[O:8][CH2:7][CH3:6].[I:5]

Correctness of the mapping

MAPPET	YES
ReactionMap	NO
Marvin	YES

Reaction no 7

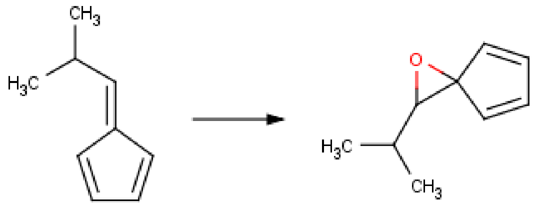

SMILES of the input:
CC(C)C=C1C=CC=C1>>CC(C)C1OC12C=CC=C2

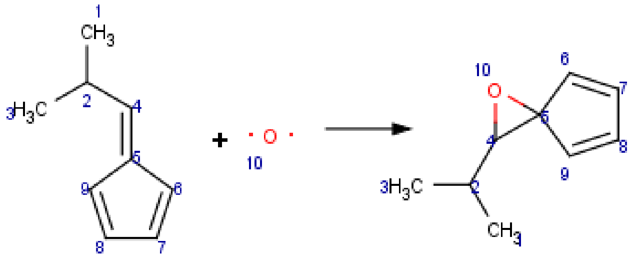

Correct mapped SMILES/SMARTS of the reaction:
[CH3:1][CH:2]([CH3:3])[CH:4]=[C:5]1[CH:6]=[CH:7][CH:8]=[CH:9]1.[O:10]>>[CH3:3][CH:2]([CH3:1])[CH:4]1[O:10][C:5]11[CH:6]=[CH:7][CH:8]=[CH:9]1

Correctness of the mapping

MAPPET	YES
ReactionMap	NO
Marvin	YES

Reaction no 8

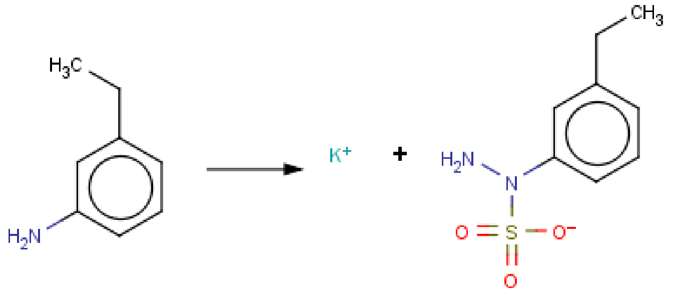

SMILES of the input:
CCc1cccc(N)c1>>[K+].CCc1cccc(N(N)S(=O)(=O)[O-])c1

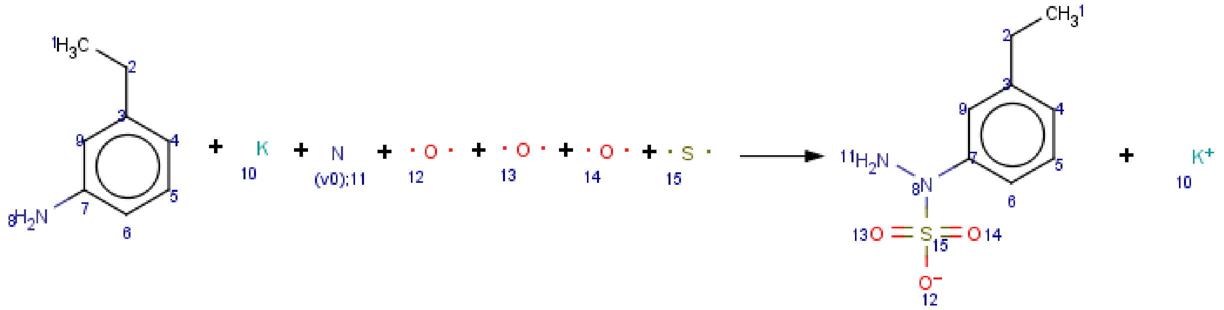

Correct mapped SMILES/SMARTS of the reaction:
[CH3:1][CH2:2][c:3]1[cH:4][cH:5][cH:6][c:7]([NH2:8])[cH:9]1.[K:10].[N:11]
.[O:12].[O:13].[O:14].[S:15]>>[CH3:1][CH2:2][c:3]1[cH:4][cH:5][cH:6][c:7]
([cH:9]1)[N:8]([NH2:11])[S:15]([O-:12])(=[O:13])=[O:14].[K+:10]

Correctness of the mapping

MAPPET	YES
ReactionMap	NO
Marvin	YES

Reaction no 9

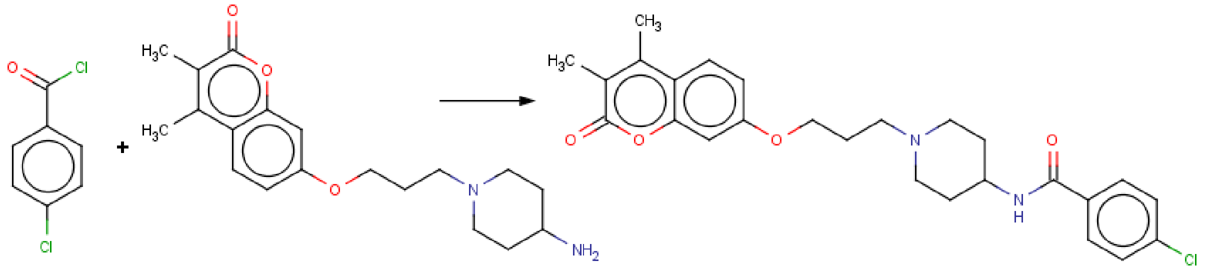

SMILES of the input:
O=C(Cl)c1ccc(Cl)cc1.Cc1c(C)c(=O)oc2cc(OCCCN3CCC(N)CC3)ccc12>>Cc1c(C)c2ccc
(OCCCN3CCC(NC(=O)c4ccc(Cl)cc4)CC3)cc2oc1=O

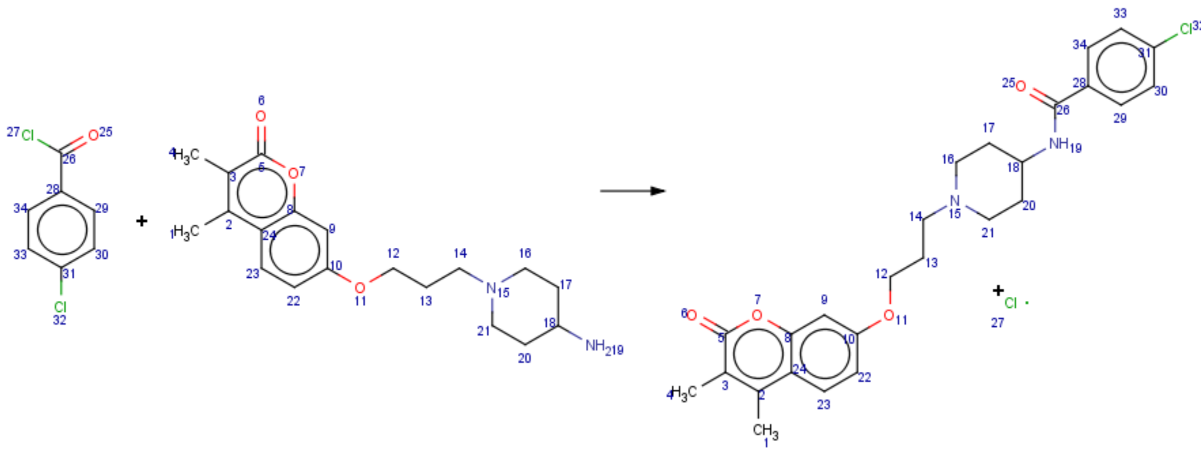

Correct mapped SMILES/SMARTS of the reaction:
[CH3:1][c:2]1[c:3]([CH3:4])[c:5](=[O:6])[o:7][c:8]2[cH:9][c:10]([O:11][CH2:12][CH2:13][CH2:14][N:15]3[CH2:16][CH2:17][CH:18]([NH2:19])[CH2:20][CH2:21]3)[cH:22][cH:23][c:24]12.[Cl:27][C:26](=[O:25])[c:28]1[cH:29][cH:30][c:31]([Cl:32])[cH:33][cH:34]1>>[CH3:1][c:2]1[c:3]([CH3:4])[c:5](=[O:6])[o:7][c:8]2[cH:9][c:10]([O:11][CH2:12][CH2:13][CH2:14][N:15]3[CH2:16][CH2:17][CH:18]([CH2:20][CH2:21]3)[NH:19][C:26](=[O:25])[c:28]3[cH:34][cH:33][c:31]([Cl:32])[cH:30][cH:29]3)[cH:22][cH:23][c:24]12.[Cl:27]

Correctness of the mapping

MAPPET	YES
ReactionMap	NO
Marvin	YES

Reaction no 10

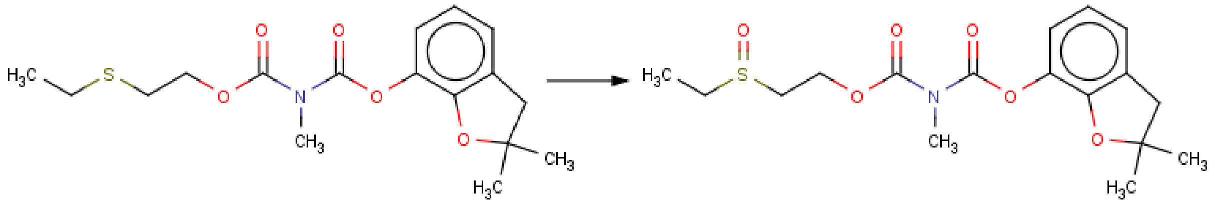

SMILES of the input:
CCSCCOC(=O)N(C)C(=O)Oc1cccc2c1OC(C)(C)C2>>CCS(=O)CCOC(=O)N(C)C(=O)Oc1cccc2c1OC(C)(C)C2

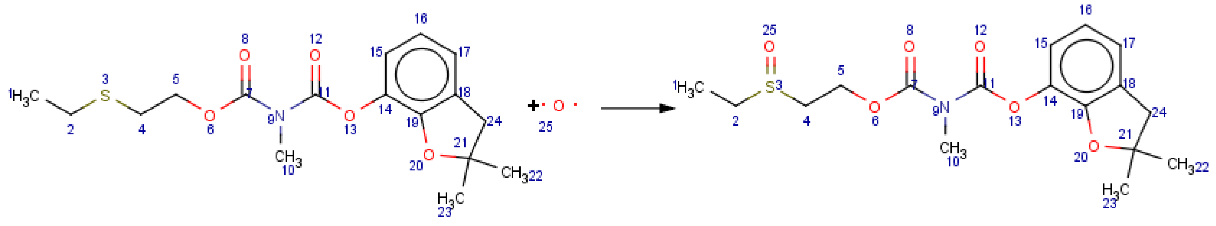

Correct mapped SMILES/SMARTS of the reaction:
[CH3:1][CH2:2][S:3][CH2:4][CH2:5][O:6][C:7](=[O:8])[N:9]([CH3:10])[C:11](=[O:12])[O:13][c:14]1[cH:15][cH:16][cH:17][c:18]2[CH2:24][C:21]([CH3:22])([CH3:23])[O:20][c:19]12.[O:25]>>[CH3:1][CH2:2][S:3](=[O:25])[CH2:4][CH2:5][O:6][C:7](=[O:8])[N:9]([CH3:10])[C:11](=[O:12])[O:13][c:14]1[cH:15][cH:16][cH:17][c:18]2[CH2:24][C:21]([CH3:22])([CH3:23])[O:20][c:19]12

Correctness of the mapping
MAPPET YES
ReactionMap NO
Marvin YES

Reaction no 11

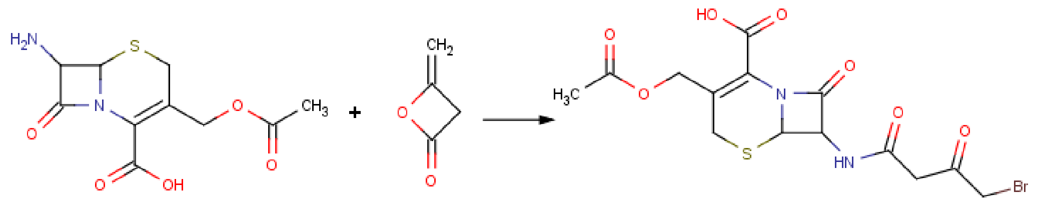

SMILES of the input:
CC(=O)OCC1=C(C(=O)O)N2C(=O)C(N)C2SC1.C=C1CC(=O)O1>>CC(=O)OCC1=C(C(=O)O)N2
C(=O)C(NC(=O)CC(=O)CBr)C2SC1

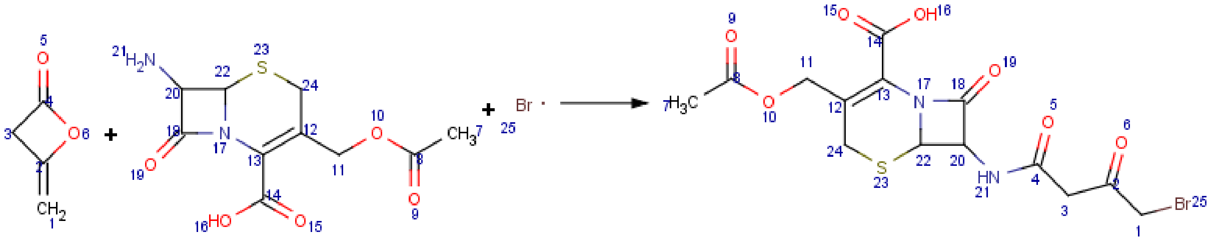

Correct mapped SMILES/SMARTS of the reaction:
[CH2:1]=[C:2]1[CH2:3][C:4](=[O:5])[O:6]1.[CH3:7][C:8](=[O:9])[O:10][CH2:1
1][C:12]1=[C:13]([N:17]2[CH:22]([S:23][CH2:24]1)[CH:20]([NH2:21])[C:18]2=
[O:19])[C:14]([OH:16])=[O:15].[Br:25]>>[CH3:7][C:8](=[O:9])[O:10][CH2:11]
[C:12]1=[C:13]([N:17]2[CH:22]([S:23][CH2:24]1)[CH:20]([NH:21][C:4](=[O:5]
)[CH2:3][C:2](=[O:6])[CH2:1][Br:25])[C:18]2=[O:19])[C:14]([OH:16])=[O:15]

Correctness of the mapping
MAPPET YES
ReactionMap NO
Marvin NO

Reaction no 12

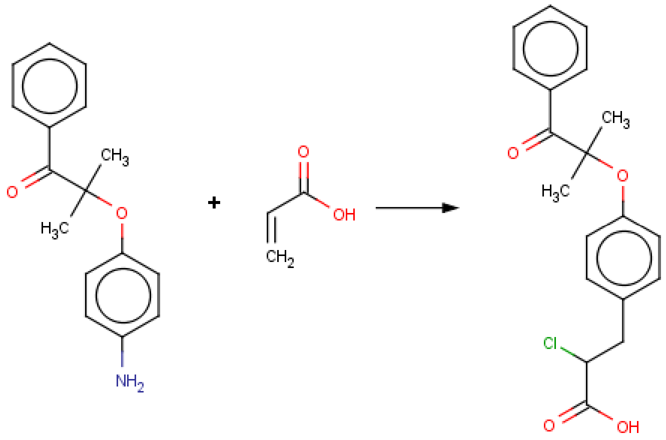

SMILES of the input:
CC(C)(Oc1ccc(N)cc1)C(=O)c1ccccc1.C=CC(=O)O>>CC(C)(Oc1ccc(CC(Cl)C(=O)O)cc1
)C(=O)c1ccccc1

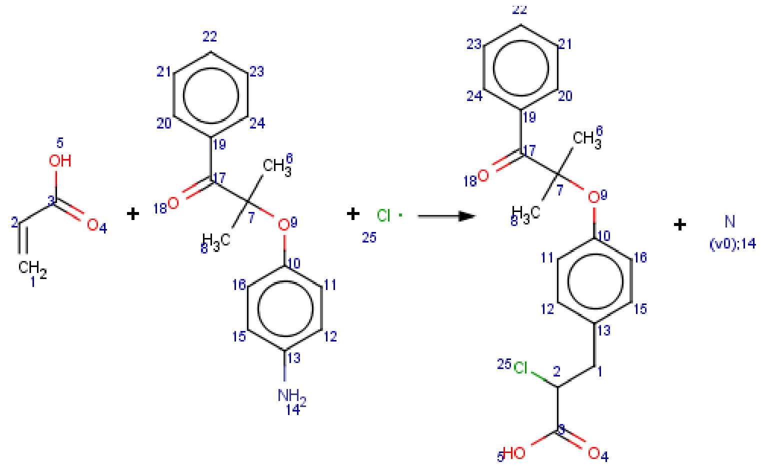

Correct mapped SMILES/SMARTS of the reaction:
[OH:5][C:3](=[O:4])[CH:2]=[CH2:1].[CH3:6][C:7]([CH3:8])([O:9][c:10]1[cH:1
1][cH:12][c:13]([NH2:14])[cH:15][cH:16]1)[C:17](=[O:18])[c:19]1[cH:20][cH
:21][cH:22][cH:23][cH:24]1.[Cl:25]>>[CH3:6][C:7]([CH3:8])([O:9][c:10]1[cH
:16][cH:15][c:13]([CH2:1][CH:2]([Cl:25])[C:3]([OH:5])=[O:4])[cH:12][cH:11
]1)[C:17](=[O:18])[c:19]1[cH:24][cH:23][cH:22][cH:21][cH:20]1.[N:14]

Correctness of the mapping

MAPPET	YES
ReactionMap	NO
Marvin	YES

Reaction no 13

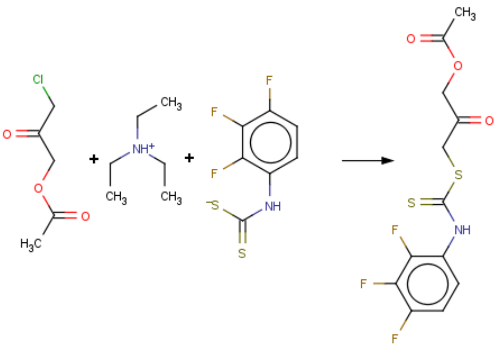

SMILES of the input:
CC(=O)OCC(=O)CCl.CC[NH+](CC)CC.Fc1ccc(NC(=S)[S-
])c(F)c1F>>CC(=O)OCC(=O)CSC(=S)Nc1ccc(F)c(F)c1F

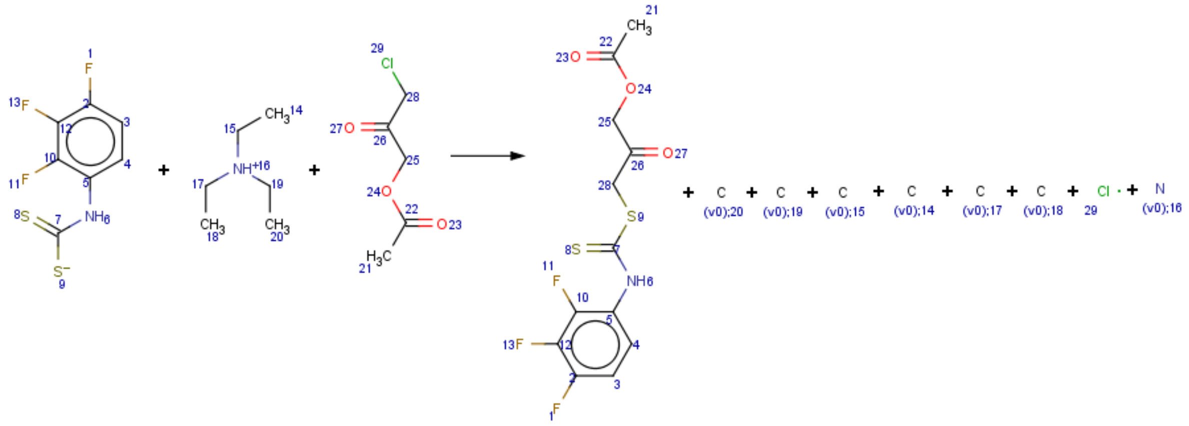

Correct mapped SMILES/SMARTS of the reaction:
[F:1][c:2]1[cH:3][cH:4][c:5]([NH:6][C:7]([S-
:9])=[S:8])[c:10]([F:11])[c:12]1[F:13].[CH3:14][CH2:15][NH+:16]([CH2:17][
CH3:18])[CH2:19][CH3:20].[CH3:21][C:22](=[O:23])[O:24][CH2:25][C:26](=[O:
27])[CH2:28][Cl:29]>>[CH3:21][C:22](=[O:23])[O:24][CH2:25][C:26](=[O:27])
[CH2:28][S:9][C:7](=[S:8])[NH:6][c:5]1[cH:4][cH:3][c:2]([F:1])[c:12]([F:1
3])[c:10]1[F:11].[C:20].[C:19].[C:15].[C:14].[C:17].[C:18].[Cl:29].[N:16]

Correctness of the mapping

MAPPET	YES
ReactionMap	NO
Marvin	YES

Reaction no 14

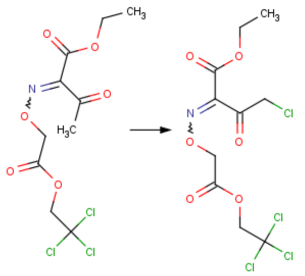

SMILES of the input:
CCOC(=O)C(=NOCC(=O)OCC(Cl)(Cl)Cl)C(C)=O>>CCOC(=O)C(=NOCC(=O)OCC(Cl)(Cl)Cl
)C(=O)CCl

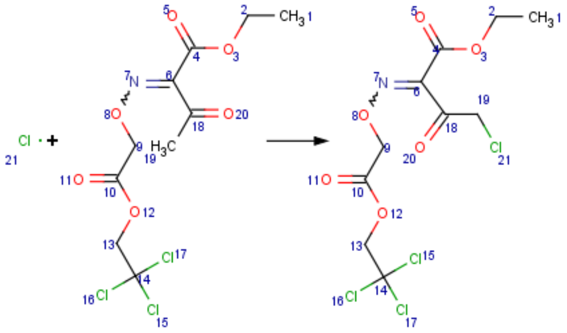

Correct mapped SMILES/SMARTS of the reaction:
[CH3:1][CH2:2][O:3][C:4](=[O:5])[C:6](=[N:7][O:8][CH2:9][C:10](=[O:11])[O
:12][CH2:13][C:14]([Cl:15])([Cl:16])[Cl:17])[C:18]([CH3:19])=[O:20].[Cl:2
1]>>[CH3:1][CH2:2][O:3][C:4](=[O:5])[C:6](=[N:7][O:8][CH2:9][C:10](=[O:11
])[O:12][CH2:13][C:14]([Cl:17])([Cl:16])[Cl:15])[C:18](=[O:20])[CH2:19][C
l:21]

Correctness of the mapping

MAPPET	YES
ReactionMap	NO
Marvin	YES

Reaction no 15

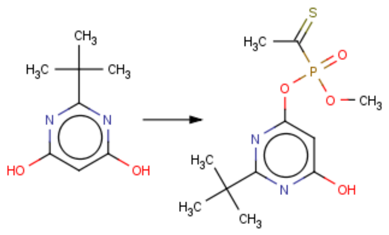

SMILES of the input:
CC(C)(C)c1nc(O)cc(O)n1>>COP(=O)(Oc1cc(O)nc(C(C)(C)C)n1)C(C)=S

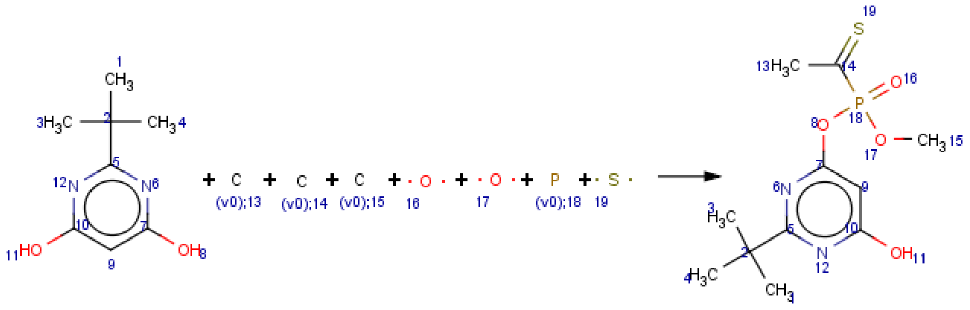

Correct mapped SMILES/SMARTS of the reaction:
[CH3:1][C:2]([CH3:3])([CH3:4])[c:5]1[n:6][c:7]([OH:8])[cH:9][c:10]([OH:11
])[n:12]1.[C:13].[C:14].[C:15].[O:16].[O:17].[P:18].[S:19]>>[CH3:15][O:17
][P:18](=[O:16])([O:8][c:7]1[cH:9][c:10]([OH:11])[n:12][c:5]([n:6]1)[C:2]
([CH3:4])([CH3:3])[CH3:1])[C:14]([CH3:13])=[S:19]

Correctness of the mapping
MAPPET YES
ReactionMap NO
Marvin YES

Reaction no 16

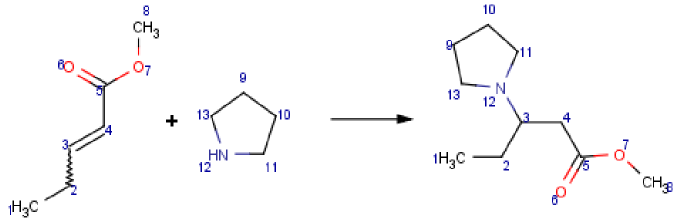

Correct mapped SMILES/SMARTS of the reaction:
[CH3:1][CH2:2][CH:3]=[CH:4][C:5](=[O:6])[O:7][CH3:8].[CH2:9]1[CH2:10][CH2
:11][NH:12][CH2:13]1>>[CH3:1][CH2:2][CH:3]([CH2:4][C:5](=[O:6])[O:7][CH3:
8])[N:12]1[CH2:13][CH2:9][CH2:10][CH2:11]1

Correctness of the mapping
MAPPET YES
ReactionMap YES
Marvin YES

Reaction no 17

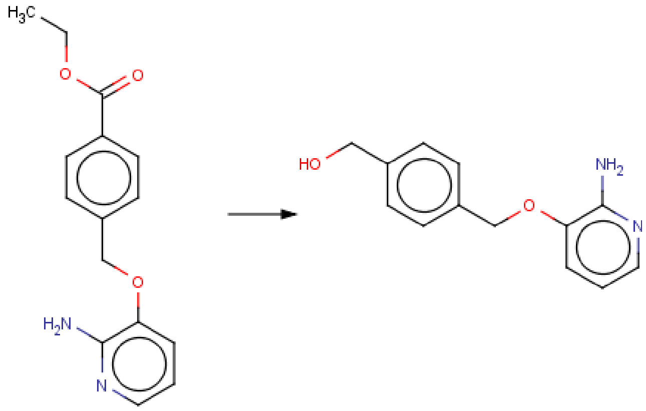

SMILES of the input:
CCOC(=O)c1ccc(COc2cccnc2N)cc1>>Nc1ncccc1OCc1ccc(CO)cc1

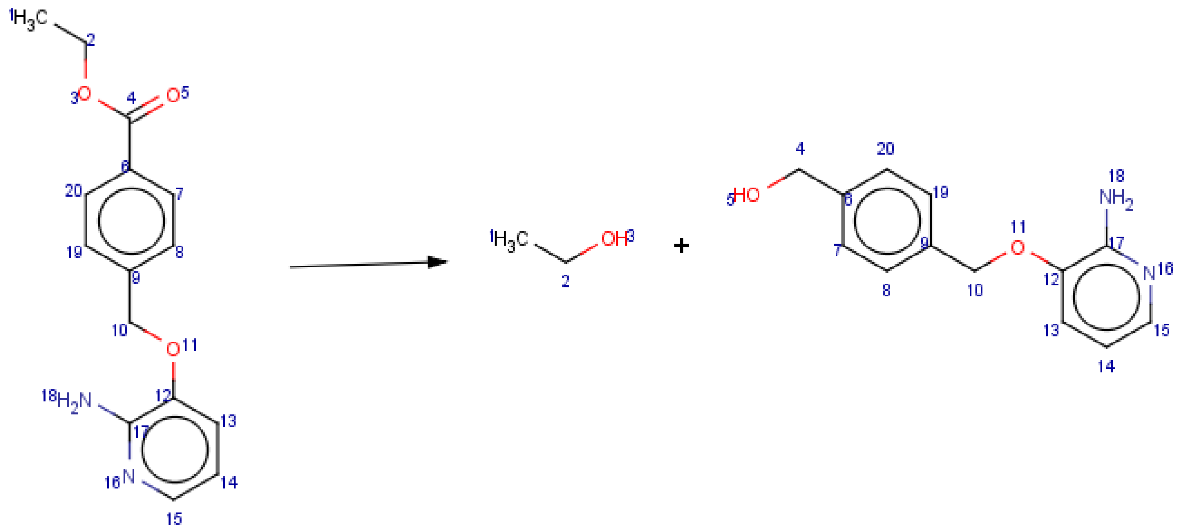

Correct mapped SMILES/SMARTS of the reaction:
[CH3:1][CH2:2][O:3][C:4](=[O:5])[c:6]1[cH:7][cH:8][c:9]([CH2:10][O:11][c:12]2[cH:13][cH:14][cH:15][n:16][c:17]2[NH2:18])[cH:19][cH:20]1>>[CH3:1][CH2:2][OH:3].[NH2:18][c:17]1[n:16][cH:15][cH:14][cH:13][c:12]1[O:11][CH2:10][c:9]1[cH:8][cH:7][c:6]([CH2:4][OH:5])[cH:20][cH:19]1

Correctness of the mapping

MAPPET	YES
ReactionMap	NO
Marvin	NO

Reaction no 18

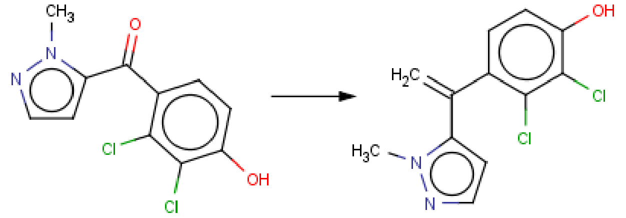

SMILES of the input:
Cn1nccc1C(=O)c1ccc(O)c(Cl)c1Cl>>C=C(c1ccnn1C)c1ccc(O)c(Cl)c1Cl

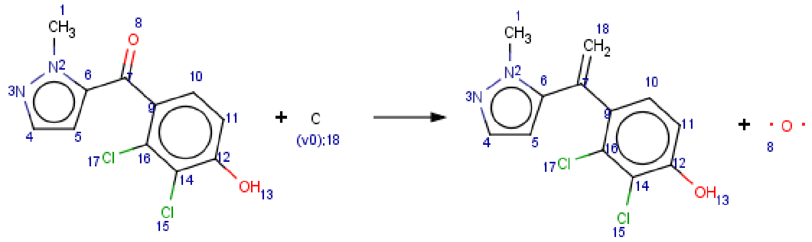

Correct mapped SMILES/SMARTS of the reaction:
[CH3:1][n:2]1[n:3][cH:4][cH:5][c:6]1[C:7](=[O:8])[c:9]1[cH:10][cH:11][c:12]([OH:13])[c:14]([Cl:15])[c:16]1[Cl:17].[C:18]>>[CH3:1][n:2]1[n:3][cH:4][cH:5][c:6]1[C:7](=[CH2:18])[c:9]1[cH:10][cH:11][c:12]([OH:13])[c:14]([Cl:15])[c:16]1[Cl:17].[O:8]

Correctness of the mapping

```
MAPPET            YES
ReactionMap       NO
Marvin            YES
```

Reaction no 19

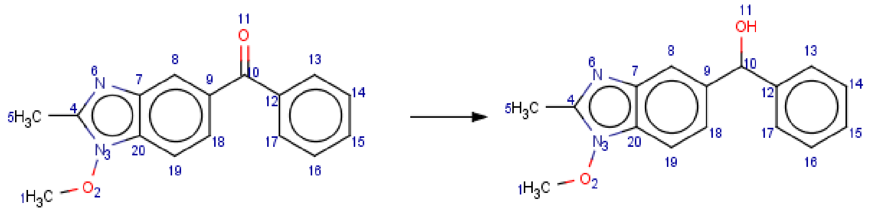

Correct mapped SMILES/SMARTS of the reaction:
[CH3:1][O:2][n:3]1[c:4]([CH3:5])[n:6][c:7]2[cH:8][c:9]([cH:18][cH:19][c:2
0]12)[C:10](=[O:11])[c:12]1[cH:13][cH:14][cH:15][cH:16][cH:17]1>>[CH3:1][
O:2][n:3]1[c:4]([CH3:5])[n:6][c:7]2[cH:8][c:9]([cH:18][cH:19][c:20]12)[CH
:10]([OH:11])[c:12]1[cH:13][cH:14][cH:15][cH:16][cH:17]1

```
Correctness of the mapping
MAPPET            YES
ReactionMap       YES
Marvin            YES
```

Reaction no 20

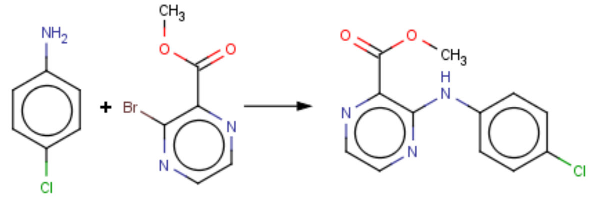

SMILES of the input:
Nc1ccc(Cl)cc1.COC(=O)c1nccnc1Br>>COC(=O)c1nccnc1Nc1ccc(Cl)cc1

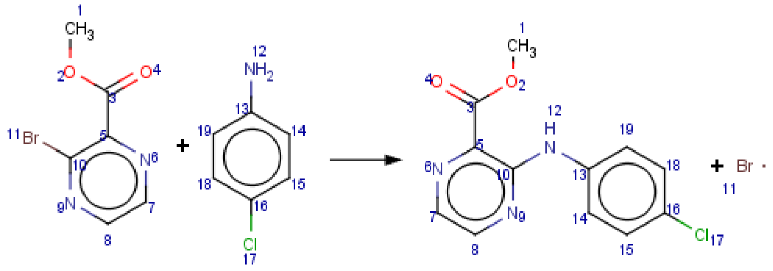

Correct mapped SMILES/SMARTS of the reaction:
[CH3:1][O:2][C:3](=[O:4])[c:5]1[n:6][cH:7][cH:8][n:9][c:10]1[Br:11].[NH2:
12][c:13]1[cH:14][cH:15][c:16]([Cl:17])[cH:18][cH:19]1>>[CH3:1][O:2][C:3]
(=[O:4])[c:5]1[n:6][cH:7][cH:8][n:9][c:10]1[NH:12][c:13]1[cH:19][cH:18][c
:16]([Cl:17])[cH:15][cH:14]1.[Br:11]

```
Correctness of the mapping
MAPPET            YES
ReactionMap       NO
Marvin            YES
```

Reaction no 21

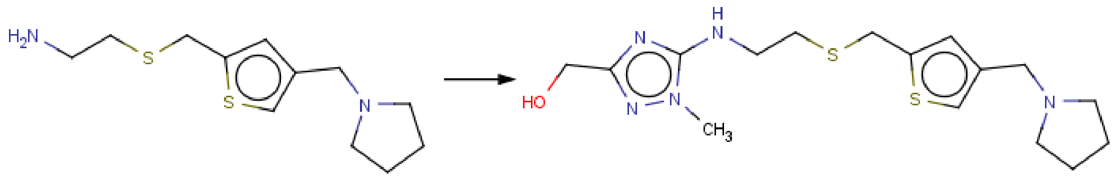

SMILES of the input:
NCCSCc1cc(CN2CCCC2)cs1>>Cn1nc(CO)nc1NCCSCc1cc(CN2CCCC2)cs1

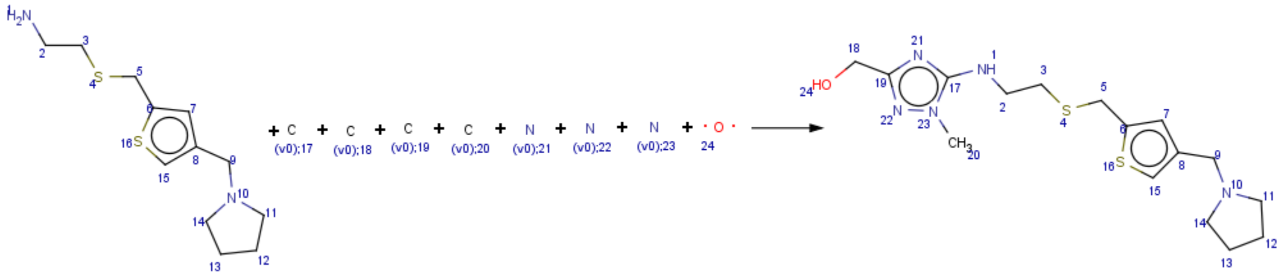

Correct mapped SMILES/SMARTS of the reaction:
[NH2:1][CH2:2][CH2:3][S:4][CH2:5][c:6]1[cH:7][c:8]([CH2:9][N:10]2[CH2:11][CH2:12][CH2:13][CH2:14]2)[cH:15][s:16]1.[C:17].[C:18].[C:19].[C:20].[N:21].[N:22].[N:23].[O:24]>>[CH3:20][n:23]1[n:22][c:19]([CH2:18][OH:24])[n:21][c:17]1[NH:1][CH2:2][CH2:3][S:4][CH2:5][c:6]1[cH:7][c:8]([CH2:9][N:10]2[CH2:11][CH2:12][CH2:13][CH2:14]2)[cH:15][s:16]1

Correctness of the mapping
MAPPET YES
ReactionMap NO
Marvin YES

Reaction no 22

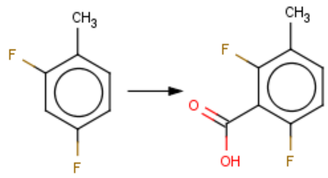

SMILES of the input:
Cc1ccc(F)cc1F>>Cc1ccc(F)c(C(=O)O)c1F

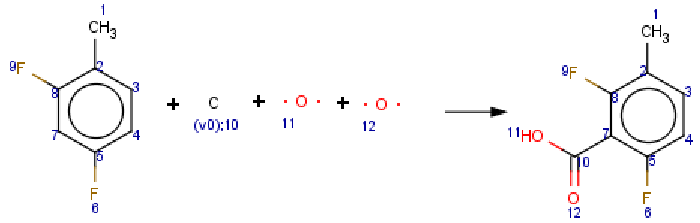

Correct mapped SMILES/SMARTS of the reaction:
[CH3:1][c:2]1[cH:3][cH:4][c:5]([F:6])[cH:7][c:8]1[F:9].[C:10].[O:11].[O:12]>>[CH3:1][c:2]1[cH:3][cH:4][c:5]([F:6])[c:7]([C:10]([OH:11])=[O:12])[c:8]1[F:9]

Correctness of the mapping
MAPPET YES
ReactionMap NO
Marvin YES

Reaction no 23

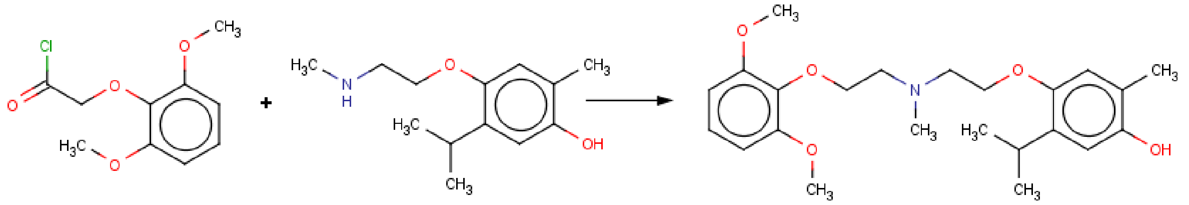

SMILES of the input:
COc1cccc(OC)c1OCC(=O)Cl.CNCCOc1cc(C)c(O)cc1C(C)C>>COc1cccc(OC)c1OCCN(C)CCOc1cc(C)c(O)cc1C(C)C

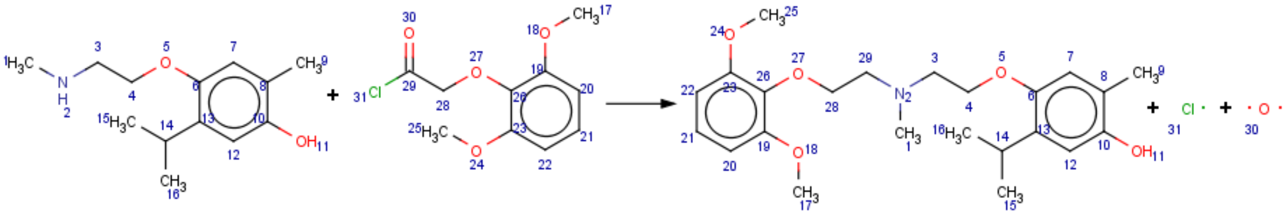

Correct mapped SMILES/SMARTS of the reaction:
[CH3:1][NH:2][CH2:3][CH2:4][O:5][c:6]1[cH:7][c:8]([CH3:9])[c:10]([OH:11])[cH:12][c:13]1[CH:14]([CH3:15])[CH3:16].[CH3:17][O:18][c:19]1[cH:20][cH:21][cH:22][c:23]([O:24][CH3:25])[c:26]1[O:27][CH2:28][C:29]([Cl:31])=[O:30]>>[CH3:17][O:18][c:19]1[cH:20][cH:21][cH:22][c:23]([O:24][CH3:25])[c:26]1[O:27][CH2:28][CH2:29][N:2]([CH3:1])[CH2:3][CH2:4][O:5][c:6]1[cH:7][c:8]([CH3:9])[c:10]([OH:11])[cH:12][c:13]1[CH:14]([CH3:16])[CH3:15].[Cl:31].[O:30]

Correctness of the mapping
MAPPET YES
ReactionMap NO
Marvin YES

Reaction no 24

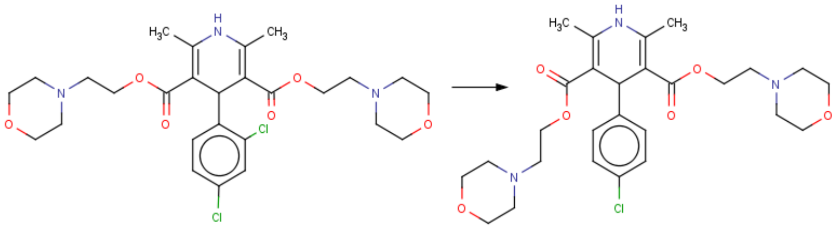

SMILES of the input:
CC1=C(C(=O)OCCN2CCOCC2)C(c2ccc(Cl)cc2Cl)C(C(=O)OCCN2CCOCC2)=C(C)N1>>CC1=C(C(=O)OCCN2CCOCC2)C(c2ccc(Cl)cc2)C(C(=O)OCCN2CCOCC2)=C(C)N1

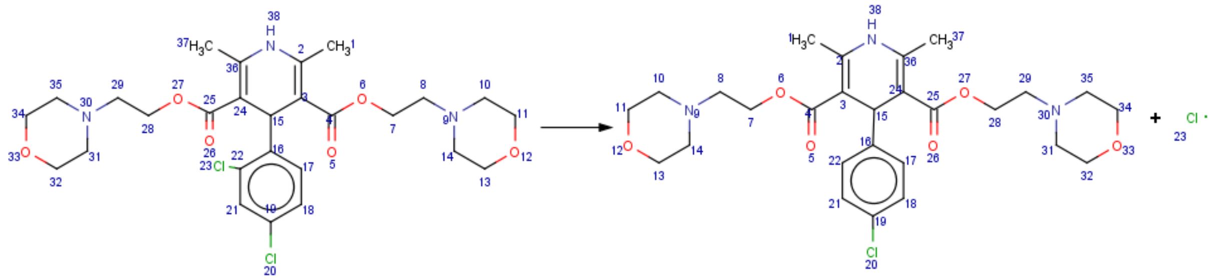

Correct mapped SMILES/SMARTS of the reaction:
[CH3:1][C:2]1=[C:3]([CH:15]([C:24]([C:25](=[O:26])[O:27][CH2:28][CH2:29][
N:30]2[CH2:31][CH2:32][O:33][CH2:34][CH2:35]2)=[C:36]([CH3:37])[NH:38]1)[
c:16]1[cH:17][cH:18][c:19]([Cl:20])[cH:21][c:22]1[Cl:23])[C:4](=[O:5])[O:
6][CH2:7][CH2:8][N:9]1[CH2:10][CH2:11][O:12][CH2:13][CH2:14]1>>[CH3:37][C
:36]1=[C:24]([CH:15]([C:3]([C:4](=[O:5])[O:6][CH2:7][CH2:8][N:9]2[CH2:14]
[CH2:13][O:12][CH2:11][CH2:10]2)=[C:2]([CH3:1])[NH:38]1)[c:16]1[cH:17][cH
:18][c:19]([Cl:20])[cH:21][cH:22]1)[C:25](=[O:26])[O:27][CH2:28][CH2:29][
N:30]1[CH2:35][CH2:34][O:33][CH2:32][CH2:31]1.[Cl:23]

Correctness of the mapping
MAPPET YES
ReactionMap NO
Marvin YES

Reaction no 25

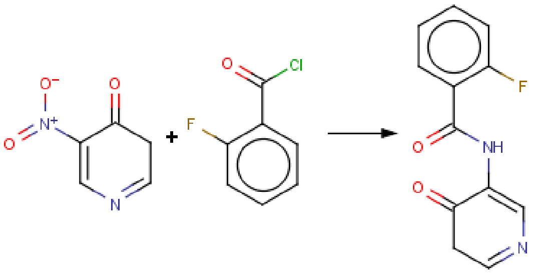

SMILES of the input:
O=C1CC=NC=C1[N+](=O)[O-].O=C(Cl)c1ccccc1F>>O=C(NC1=CN=CCC1=O)c1ccccc1F

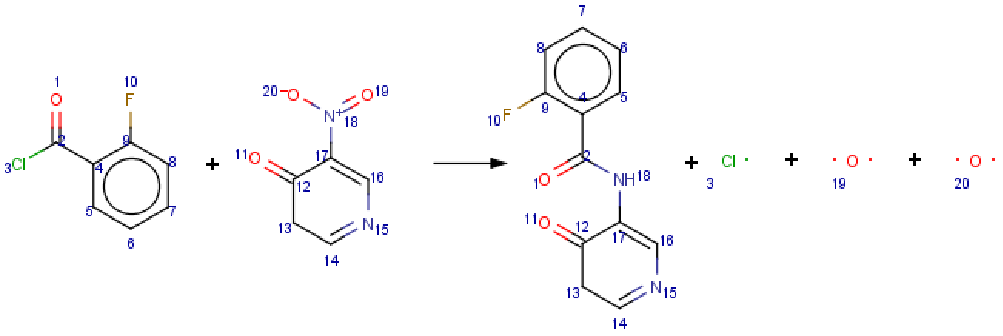

Correct mapped SMILES/SMARTS of the reaction:
[F:10][c:9]1[cH:8][cH:7][cH:6][cH:5][c:4]1[C:2]([Cl:3])=[O:1].[O-
:20][N+:18](=[O:19])[C:17]1=[CH:16][N:15]=[CH:14][CH2:13][C:12]1=[O:11]>>
[F:10][c:9]1[cH:8][cH:7][cH:6][cH:5][c:4]1[C:2](=[O:1])[NH:18][C:17]1=[CH
:16][N:15]=[CH:14][CH2:13][C:12]1=[O:11].[Cl:3].[O:19].[O:20]

Correctness of the mapping
MAPPET YES
ReactionMap NO
Marvin YES

Reaction no 26

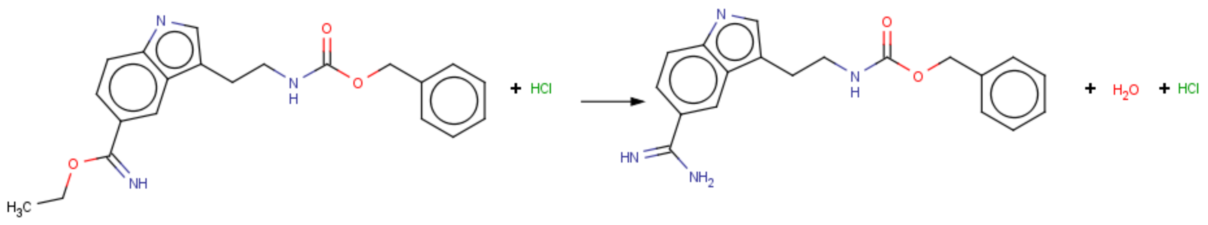

SMILES of the input:
Cl.CCOC(=N)c1ccc2ncc(CCNC(=O)OCc3ccccc3)c2c1>>O.Cl.N=C(N)c1ccc2ncc(CCNC(=
O)OCc3ccccc3)c2c1

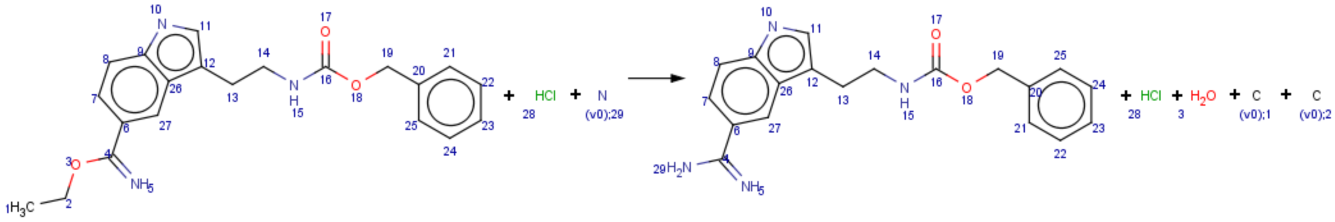

Correct mapped SMILES/SMARTS of the reaction:
[CH3:1][CH2:2][O:3][C:4](=[NH:5])[c:6]1[cH:7][cH:8][c:9]2[n:10][cH:11][c:
12]([CH2:13][CH2:14][NH:15][C:16](=[O:17])[O:18][CH2:19][c:20]3[cH:21][cH
:22][cH:23][cH:24][cH:25]3)[c:26]2[cH:27]1.[ClH:28].[N:29]>>[NH2:29][C:4]
(=[NH:5])[c:6]1[cH:7][cH:8][c:9]2[n:10][cH:11][c:12]([CH2:13][CH2:14][NH:
15][C:16](=[O:17])[O:18][CH2:19][c:20]3[cH:25][cH:24][cH:23][cH:22][cH:21
]3)[c:26]2[cH:27]1.[ClH:28].[OH2:3].[C:1].[C:2]

Correctness of the mapping

MAPPET	YES
ReactionMap	NO
Marvin	YES

Reaction no 27

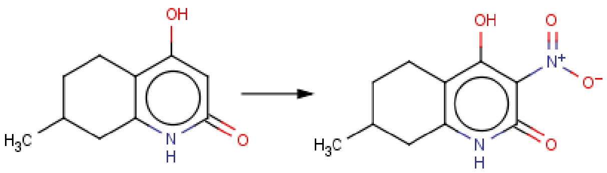

SMILES of the input:
CC1CCc2c(O)cc(=O)[nH]c2C1>>CC1CCc2c(O)c([N+](=O)[O-])c(=O)[nH]c2C1

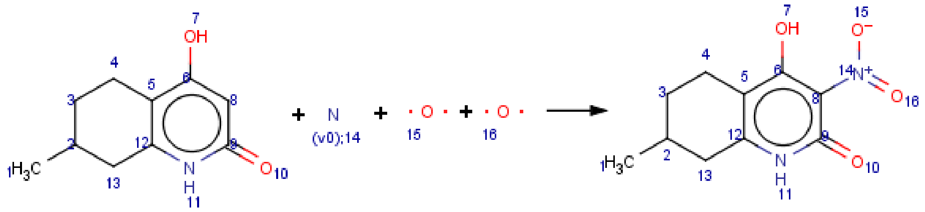

Correct mapped SMILES/SMARTS of the reaction:
[CH3:1][CH:2]1[CH2:3][CH2:4][c:5]2[c:6]([OH:7])[cH:8][c:9](=[O:10])[nH:11
][c:12]2[CH2:13]1.[N:14].[O:15].[O:16]>>[CH3:1][CH:2]1[CH2:3][CH2:4][c:5]
2[c:6]([OH:7])[c:8]([N+:14]([O-
:15])=[O:16])[c:9](=[O:10])[nH:11][c:12]2[CH2:13]1

Correctness of the mapping
MAPPET YES
ReactionMap NO
Marvin YES

Reaction no 28

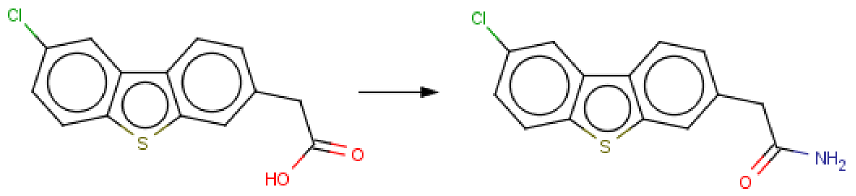

SMILES of the input:
O=C(O)Cc1ccc2c(c1)sc1ccc(Cl)cc12>>NC(=O)Cc1ccc2c(c1)sc1ccc(Cl)cc12

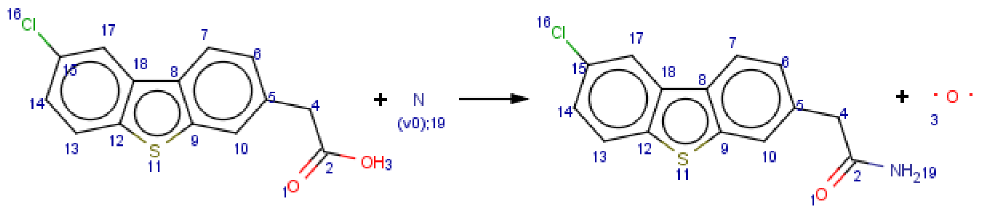

Correct mapped SMILES/SMARTS of the reaction:
[OH:3][C:2](=[O:1])[CH2:4][c:5]1[cH:6][cH:7][c:8]2[c:9]([cH:10]1)[s:11][c
:12]1[cH:13][cH:14][c:15]([Cl:16])[cH:17][c:18]21.[N:19]>>[NH2:19][C:2](=
[O:1])[CH2:4][c:5]1[cH:6][cH:7][c:8]2[c:9]([cH:10]1)[s:11][c:12]1[cH:13][
cH:14][c:15]([Cl:16])[cH:17][c:18]21.[O:3]

Correctness of the mapping
MAPPET YES
ReactionMap NO
Marvin YES

Reaction no 29

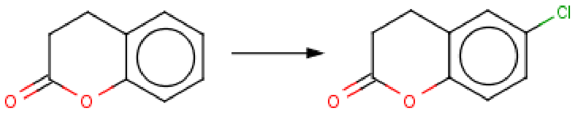

SMILES of the input:
O=C1CCc2ccccc2O1>>O=C1CCc2cc(Cl)ccc2O1

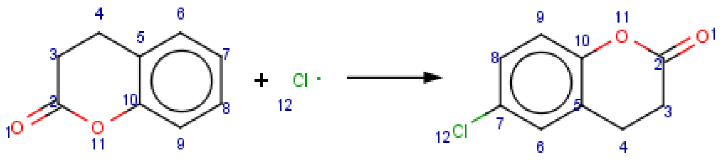

Correct mapped SMILES/SMARTS of the reaction:
[O:1]=[C:2]1[CH2:3][CH2:4][c:5]2[cH:6][cH:7][cH:8][cH:9][c:10]2[O:11]1.[C
l:12]>>[Cl:12][c:7]1[cH:8][cH:9][c:10]2[O:11][C:2](=[O:1])[CH2:3][CH2:4][
c:5]2[cH:6]1

Correctness of the mapping
MAPPET YES
ReactionMap NO
Marvin YES

Reaction no 30

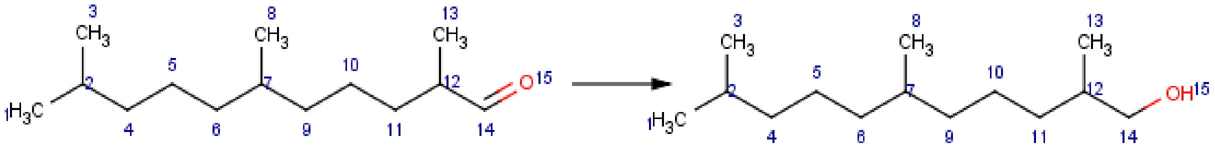

Correct mapped SMILES/SMARTS of the reaction:
[CH3:1][CH:2]([CH3:3])[CH2:4][CH2:5][CH2:6][CH:7]([CH3:8])[CH2:9][CH2:10][CH2:11][CH:12]([CH3:13])[CH:14]=[O:15]>>[CH3:1][CH:2]([CH3:3])[CH2:4][CH2:5][CH2:6][CH:7]([CH3:8])[CH2:9][CH2:10][CH2:11][CH:12]([CH3:13])[CH2:14][OH:15]

Correctness of the mapping
MAPPET YES
ReactionMap YES
Marvin YES

Reaction no 31

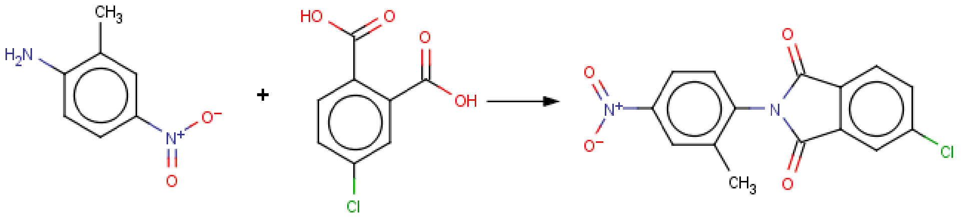

SMILES of the input:
Cc1cc([N+](=O)[O-])ccc1N.O=C(O)c1ccc(Cl)cc1C(=O)O>>Cc1cc([N+](=O)[O-])ccc1N1C(=O)c2ccc(Cl)cc2C1=O

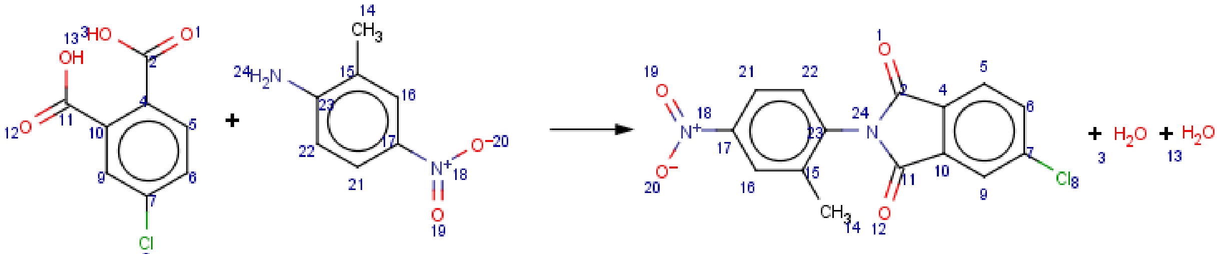

Correct mapped SMILES/SMARTS of the reaction:
[OH:3][C:2](=[O:1])[c:4]1[cH:5][cH:6][c:7]([Cl:8])[cH:9][c:10]1[C:11]([OH:13])=[O:12].[CH3:14][c:15]1[cH:16][c:17]([cH:21][cH:22][c:23]1[NH2:24])[N+:18]([O-:20])=[O:19]>>[OH2:13].[OH2:3].[CH3:14][c:15]1[cH:16][c:17]([cH:21][cH:22][c:23]1[N:24]1[C:2](=[O:1])[c:4]2[cH:5][cH:6][c:7]([Cl:8])[cH:9][c:10]2[C:11]1=[O:12])[N+:18]([O-:20])=[O:19]

Correctness of the mapping
MAPPET YES
ReactionMap NO
Marvin YES

Reaction no 32

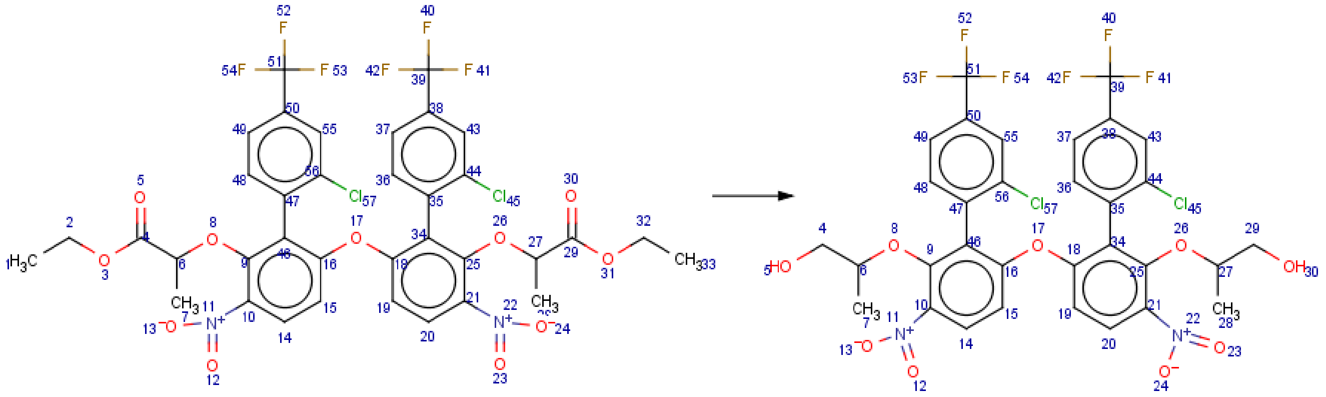

Correct mapped SMILES/SMARTS of the reaction:
[CH3:1][CH2:2][O:3][C:4](=[O:5])[CH:6]([CH3:7])[O:8][c:9]1[c:10]([cH:14][cH:15][c:16]([O:17][c:18]2[cH:19][cH:20][c:21]([c:25]([O:26][CH:27]([CH3:28])[C:29](=[O:30])[O:31][CH2:32][CH3:33])[c:34]2-[c:35]2[cH:36][cH:37][c:38]([cH:43][c:44]2[Cl:45])[C:39]([F:40])([F:41])[F:42])[N+:22]([O-:24])=[O:23])[c:46]1-[c:47]1[cH:48][cH:49][c:50]([cH:55][c:56]1[Cl:57])[C:51]([F:52])([F:53])[F:54])[N+:11]([O:13])=[O:12]>>[CH3:28][CH:27]([CH2:29][OH:30])[O:26][c:25]1[c:21]([cH:20][cH:19][c:18]([O:17][c:16]2[cH:15][cH:14][c:10]([c:9]([O:8][CH:6]([CH3:7])[CH2:4][OH:5])[c:46]2-[c:47]2[cH:48][cH:49][c:50]([cH:55][c:56]2[Cl:57])[C:51]([F:52])([F:53])[F:54])[N+:11]([O-:13])=[O:12])[c:34]1-[c:35]1[cH:36][cH:37][c:38]([cH:43][c:44]1[Cl:45])[C:39]([F:40])([F:41])[F:42])[N+:22]([O:24])=[O:23]

Correctness of the mapping

MAPPET	NO
ReactionMap	NO
Marvin	NO

Reaction no 33

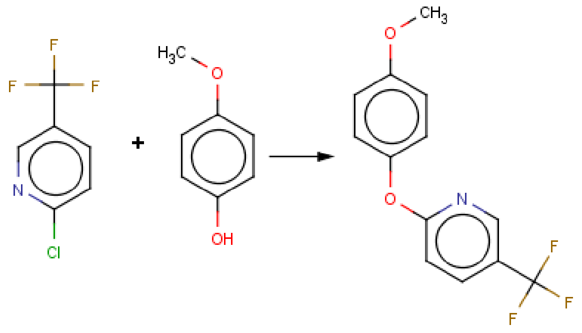

SMILES of the input:
FC(F)(F)c1ccc(Cl)nc1.COc1ccc(O)cc1>>COc1ccc(Oc2ccc(C(F)(F)F)cn2)cc1

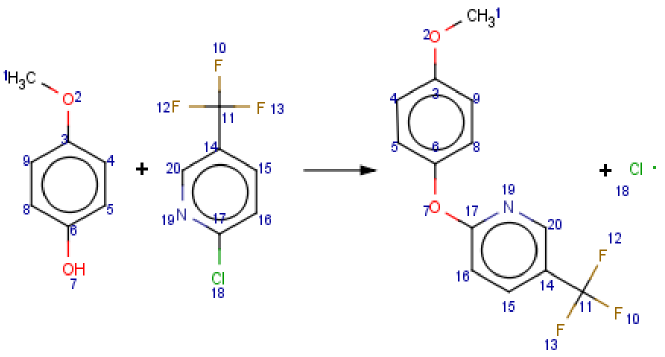

Correct mapped SMILES/SMARTS of the reaction:
[CH3:1][O:2][c:3]1[cH:4][cH:5][c:6]([OH:7])[cH:8][cH:9]1.[F:10][C:11]([F:
12])([F:13])[c:14]1[cH:15][cH:16][c:17]([Cl:18])[n:19][cH:20]1>>[CH3:1][O
:2][c:3]1[cH:4][cH:5][c:6]([O:7][c:17]2[cH:16][cH:15][c:14]([cH:20][n:19]
2)[C:11]([F:10])([F:12])[F:13])[cH:8][cH:9]1.[Cl:18]

Correctness of the mapping

MAPPET	YES
ReactionMap	NO
Marvin	YES

Reaction no 34

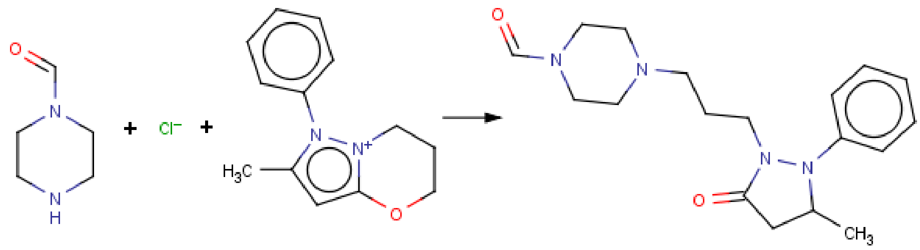

SMILES of the input:
O=CN1CCNCC1.[Cl-].Cc1cc2[n+](n1-
c1ccccc1)CCCO2>>CC1CC(=O)N(CCCN2CCN(C=O)CC2)N1c1ccccc1

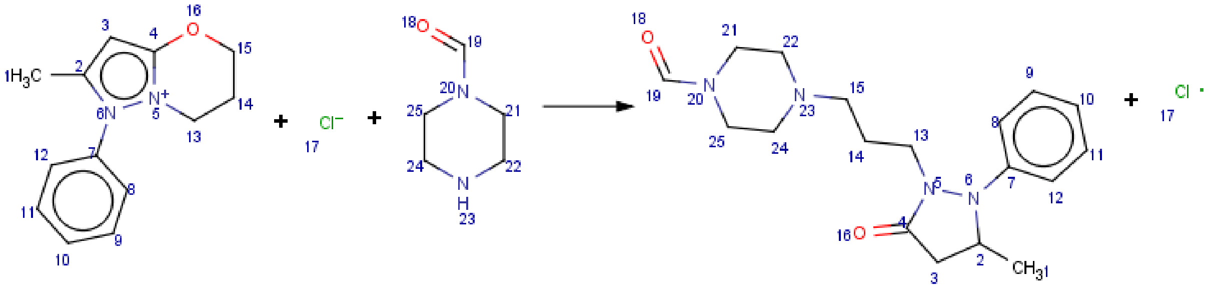

Correct mapped SMILES/SMARTS of the reaction:
[CH3:1][c:2]1[cH:3][c:4]2[O:16][CH2:15][CH2:14][CH2:13][n+:5]2[n:6]1-
[c:7]1[cH:8][cH:9][cH:10][cH:11][cH:12]1.[Cl-
:17].[O:18]=[CH:19][N:20]1[CH2:21][CH2:22][NH:23][CH2:24][CH2:25]1>>[CH3:
1][CH:2]1[CH2:3][C:4](=[O:16])[N:5]([CH2:13][CH2:14][CH2:15][N:23]2[CH2:2
4][CH2:25][N:20]([CH2:21][CH2:22]2)[CH:19]=[O:18])[N:6]1[c:7]1[cH:8][cH:9
][cH:10][cH:11][cH:12]1.[Cl:17]

Correctness of the mapping

MAPPET	YES
ReactionMap	NO
Marvin	YES

Reaction no 35

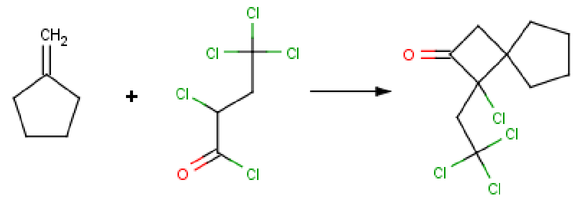

SMILES of the input:
C=C1CCCC1.O=C(Cl)C(Cl)CC(Cl)(Cl)Cl>>O=C1CC2(CCCC2)C1(Cl)CC(Cl)(Cl)Cl

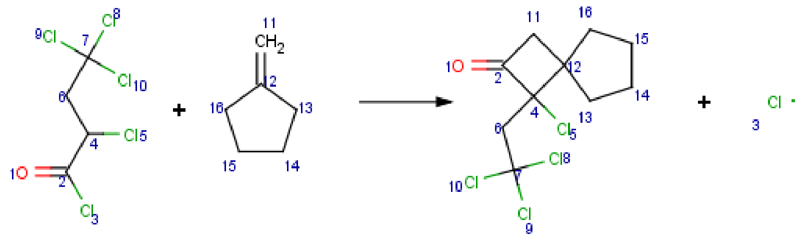

Correct mapped SMILES/SMARTS of the reaction:
[Cl:5][CH:4]([CH2:6][C:7]([Cl:8])([Cl:9])[Cl:10])[C:2]([Cl:3])=[O:1].[CH2:11]=[C:12]1[CH2:13][CH2:14][CH2:15][CH2:16]1>>[Cl:9][C:7]([Cl:8])([Cl:10])[CH2:6][C:4]1([Cl:5])[C:2](=[O:1])[CH2:11][C:12]11[CH2:16][CH2:15][CH2:14][CH2:13]1.[Cl:3]

Correctness of the mapping

MAPPET	YES
ReactionMap	NO
Marvin	YES

Reaction no 36

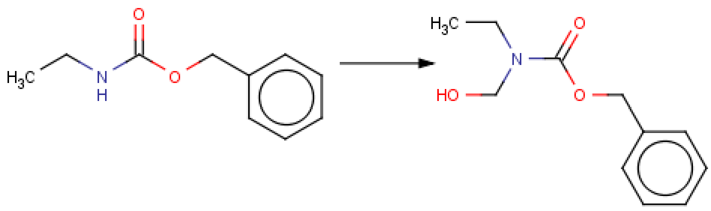

SMILES of the input:
CCNC(=O)OCc1ccccc1>>CCN(CO)C(=O)OCc1ccccc1

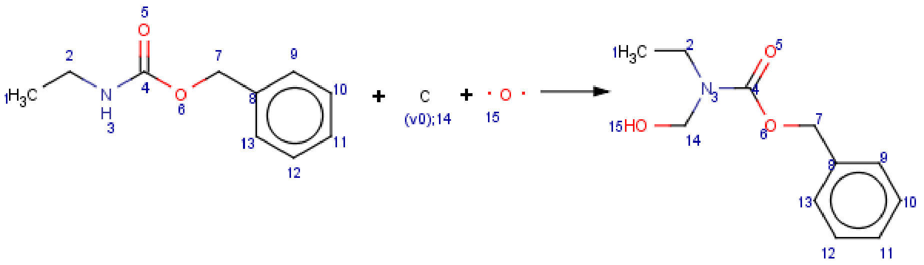

Correct mapped SMILES/SMARTS of the reaction:
[CH3:1][CH2:2][NH:3][C:4](=[O:5])[O:6][CH2:7][c:8]1[cH:9][cH:10][cH:11][cH:12][cH:13]1.[C:14].[O:15]>>[CH3:1][CH2:2][N:3]([CH2:14][OH:15])[C:4](=[O:5])[O:6][CH2:7][c:8]1[cH:9][cH:10][cH:11][cH:12][cH:13]1

Correctness of the mapping

MAPPET	YES
ReactionMap	NO
Marvin	YES

Reaction no 37

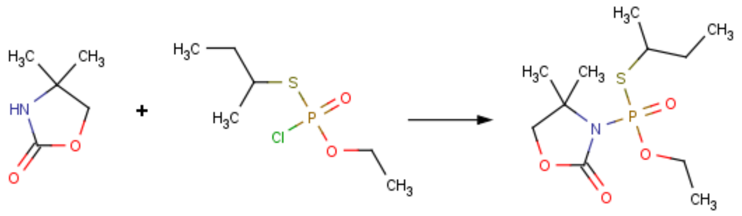

SMILES of the input:
CC1(C)COC(=O)N1.CCOP(=O)(Cl)SC(C)CC>>CCOP(=O)(SC(C)CC)N1C(=O)OCC1(C)C

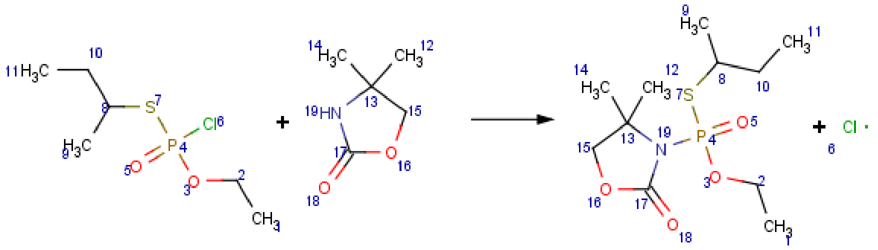

Correct mapped SMILES/SMARTS of the reaction:
[CH3:1][CH2:2][O:3][P:4]([Cl:6])(=[O:5])[S:7][CH:8]([CH3:9])[CH2:10][CH3:11].[CH3:12][C:13]1([CH3:14])[CH2:15][O:16][C:17](=[O:18])[NH:19]1>>[CH3:1][CH2:2][O:3][P:4](=[O:5])([S:7][CH:8]([CH3:9])[CH2:10][CH3:11])[N:19]1[C:17](=[O:18])[O:16][CH2:15][C:13]1([CH3:14])[CH3:12].[Cl:6]

Correctness of the mapping

MAPPET	YES
ReactionMap	NO
Marvin	YES

Reaction no 38

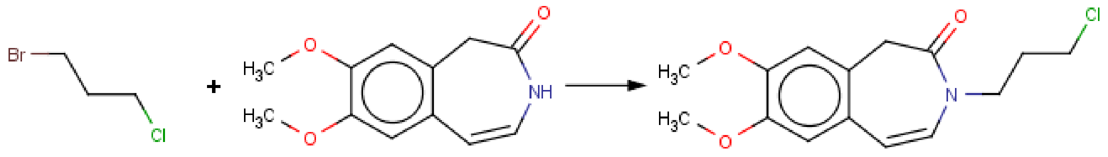

SMILES of the input:
ClCCCBr.COc1cc2c(cc1OC)CC(=O)NC=C2>>COc1cc2c(cc1OC)CC(=O)N(CCCCl)C=C2

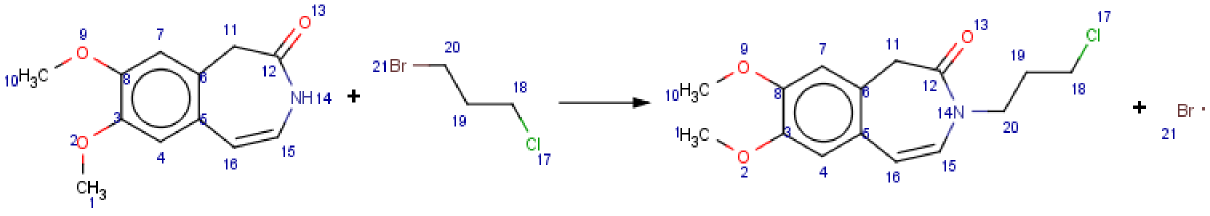

Correct mapped SMILES/SMARTS of the reaction:
[CH3:10][O:9][c:8]1[cH:7][c:6]2[CH2:11][C:12](=[O:13])[NH:14][CH:15]=[CH:16][c:5]2[cH:4][c:3]1[O:2][CH3:1].[Cl:17][CH2:18][CH2:19][CH2:20][Br:21]>>[CH3:10][O:9][c:8]1[cH:7][c:6]2[CH2:11][C:12](=[O:13])[N:14]([CH2:20][CH2:19][CH2:18][Cl:17])[CH:15]=[CH:16][c:5]2[cH:4][c:3]1[O:2][CH3:1].[Br:21]

Correctness of the mapping
MAPPET YES
ReactionMap NO
Marvin YES

Reaction no 39

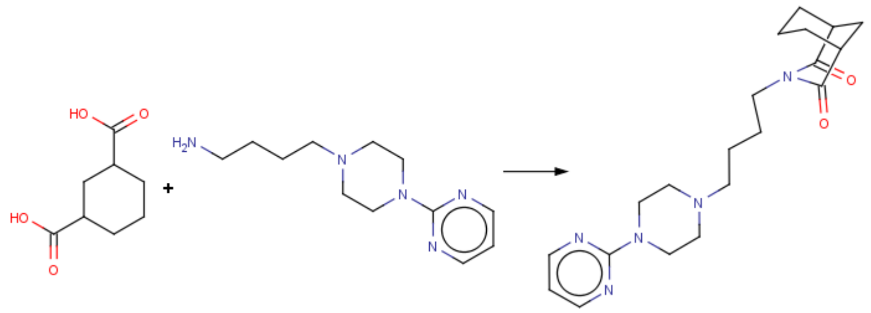

SMILES of the input:
O=C(O)C1CCCC(C(=O)O)C1.NCCCCN1CCN(c2ncccn2)CC1>>O=C1C2CCCC(C2)C(=O)N1CCCCN1CCN(c2ncccn2)CC1

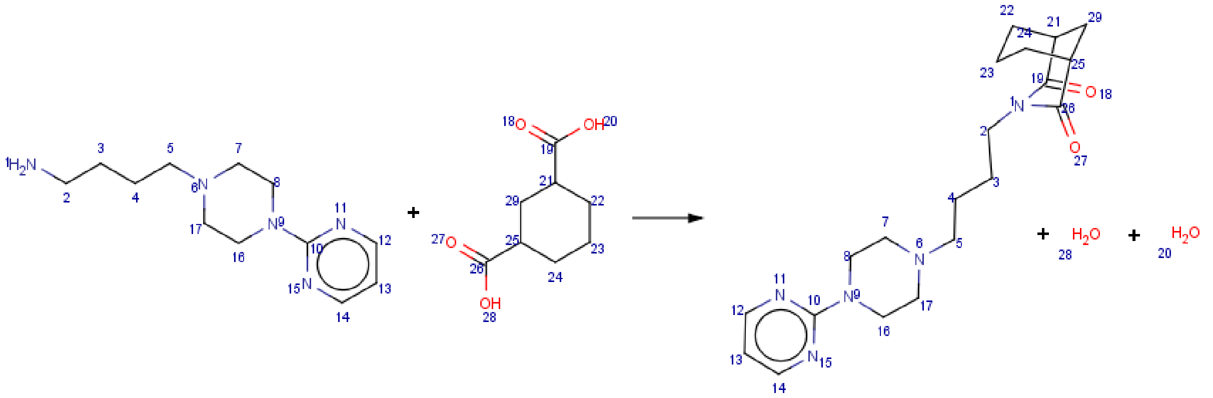

Correct mapped SMILES/SMARTS of the reaction:
[NH2:1][CH2:2][CH2:3][CH2:4][CH2:5][N:6]1[CH2:7][CH2:8][N:9]([CH2:16][CH2:17]1)[c:10]1[n:11][cH:12][cH:13][cH:14][n:15]1.[OH:20][C:19](=[O:18])[CH:21]1[CH2:22][CH2:23][CH2:24][CH:25]([CH2:29]1)[C:26]([OH:28])=[O:27]>>[OH2:28].[OH2:20].[O:18]=[C:19]1[CH:21]2[CH2:22][CH2:23][CH2:24][CH:25]([CH2:29]2)[C:26](=[O:27])[N:1]1[CH2:2][CH2:3][CH2:4][CH2:5][N:6]1[CH2:17][CH2:16][N:9]([CH2:8][CH2:7]1)[c:10]1[n:15][cH:14][cH:13][cH:12][n:11]1

Correctness of the mapping
MAPPET YES
ReactionMap NO
Marvin YES

Reaction no 40

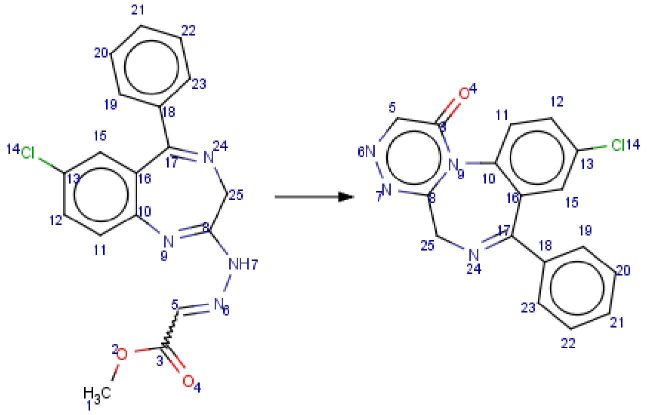

Correct mapped SMILES/SMARTS of the reaction:
[CH3:1][O:2][C:3](=[O:4])[CH:5]=[N:6][NH:7][C:8]1=[N:9][c:10]2[cH:11][cH:
12][c:13]([Cl:14])[cH:15][c:16]2[C:17](=[N:24][CH2:25]1)[c:18]1[cH:19][cH
:20][cH:21][cH:22][cH:23]1>>[Cl:14][c:13]1[cH:12][cH:11][c:10]-
2[c:16]([cH:15]1)[C:17](=[N:24][CH2:25][c:8]1[n:7][n:6][cH:5][c:3](=[O:4]
)[n:9]-21)[c:18]1[cH:19][cH:20][cH:21][cH:22][cH:23]1

Correctness of the mapping
MAPPET NO
ReactionMap NO
Marvin YES

Reaction no 41

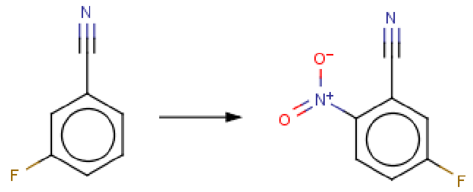

SMILES of the input:
N#Cc1cccc(F)c1>>N#Cc1cc(F)ccc1[N+](=O)[O-]

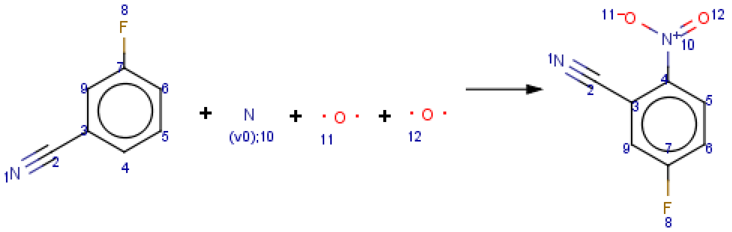

Correct mapped SMILES/SMARTS of the reaction:
[F:8][c:7]1[cH:6][cH:5][cH:4][c:3]([cH:9]1)[C:2]#[N:1].[N:10].[O:11].[O:1
2]>>[O-
:11][N+:10](=[O:12])[c:4]1[cH:5][cH:6][c:7]([F:8])[cH:9][c:3]1[C:2]#[N:1]

Correctness of the mapping
MAPPET YES
ReactionMap NO
Marvin YES

Reaction no 42

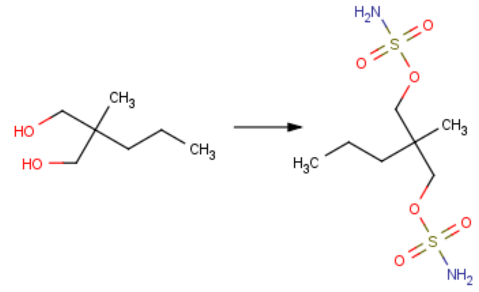

SMILES of the input:
CCCC(C)(CO)CO>>CCCC(C)(COS(N)(=O)=O)COS(N)(=O)=O

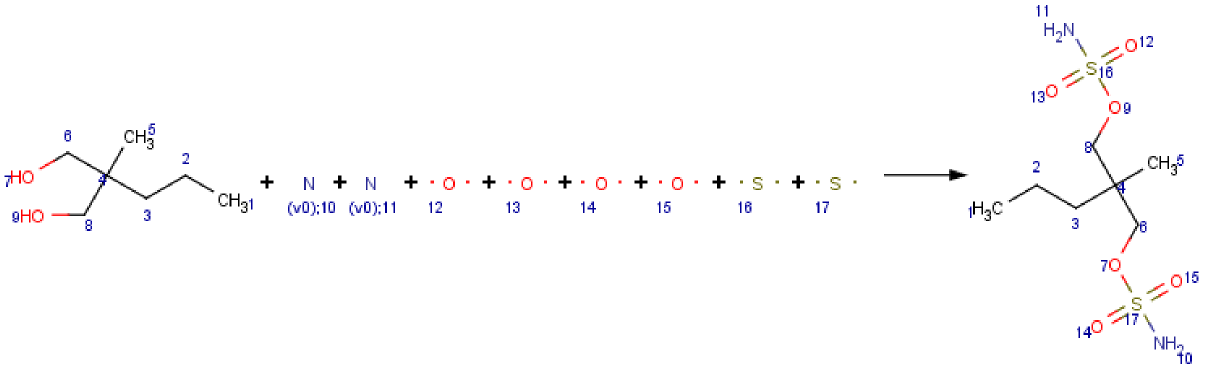

Correct mapped SMILES/SMARTS of the reaction:
[CH3:1][CH2:2][CH2:3][C:4]([CH3:5])([CH2:6][OH:7])[CH2:8][OH:9].[N:10].[N
:11].[O:12].[O:13].[O:14].[O:15].[S:16].[S:17]>>[CH3:1][CH2:2][CH2:3][C:4
]([CH3:5])([CH2:6][O:7][S:17]([NH2:10])(=[O:15])=[O:14])[CH2:8][O:9][S:16
]([NH2:11])(=[O:12])=[O:13]

Correctness of the mapping

MAPPET	YES
ReactionMap	NO
Marvin	YES

Reaction no 43

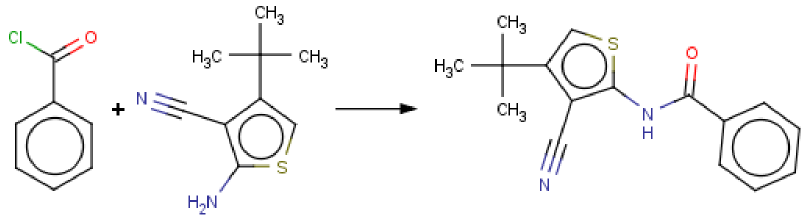

SMILES of the input:
O=C(Cl)c1ccccc1.CC(C)(C)c1csc(N)c1C#N>>CC(C)(C)c1csc(NC(=O)c2ccccc2)c1C#N

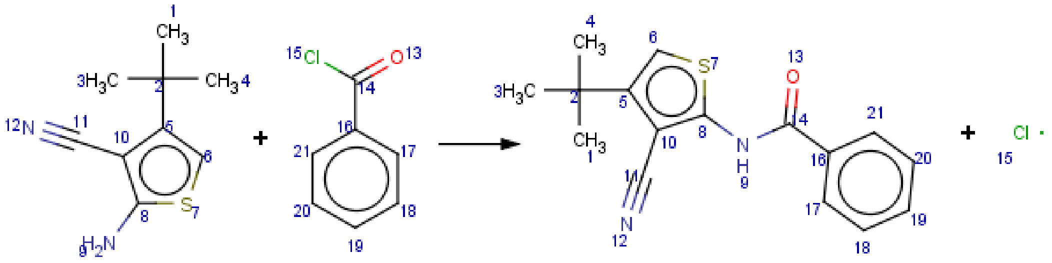

Correct mapped SMILES/SMARTS of the reaction:
[CH3:1][C:2]([CH3:3])([CH3:4])[c:5]1[cH:6][s:7][c:8]([NH2:9])[c:10]1[C:11
]#[N:12].[Cl:15][C:14](=[O:13])[c:16]1[cH:17][cH:18][cH:19][cH:20][cH:21]
1>>[CH3:3][C:2]([CH3:1])([CH3:4])[c:5]1[cH:6][s:7][c:8]([NH:9][C:14](=[O:
13])[c:16]2[cH:21][cH:20][cH:19][cH:18][cH:17]2)[c:10]1[C:11]#[N:12].[Cl:
15]

Correctness of the mapping

MAPPET	YES
ReactionMap	NO
Marvin	YES

Reaction no 44

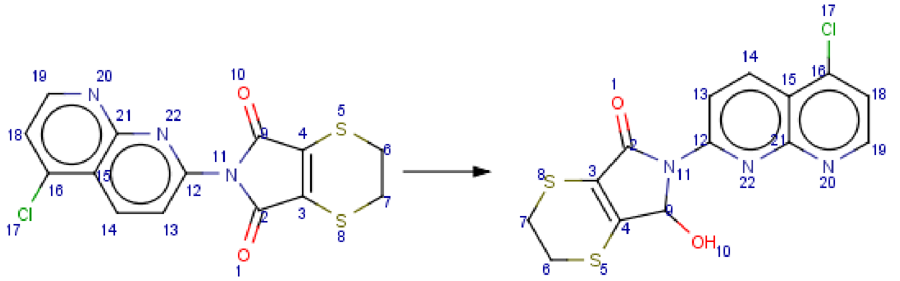

Correct mapped SMILES/SMARTS of the reaction:
[Cl:17][c:16]1[cH:18][cH:19][n:20][c:21]2[n:22][c:12]([cH:13][cH:14][c:15
]12)[N:11]1[C:2](=[O:1])[C:3]2=[C:4]([S:5][CH2:6][CH2:7][S:8]2)[C:9]1=[O:
10]>>[OH:10][CH:9]1[N:11]([C:2](=[O:1])[C:3]2=[C:4]1[S:5][CH2:6][CH2:7][S
:8]2)[c:12]1[cH:13][cH:14][c:15]2[c:16]([Cl:17])[cH:18][cH:19][n:20][c:21
]2[n:22]1

Correctness of the mapping

MAPPET	YES
ReactionMap	YES
Marvin	YES

Reaction no 45

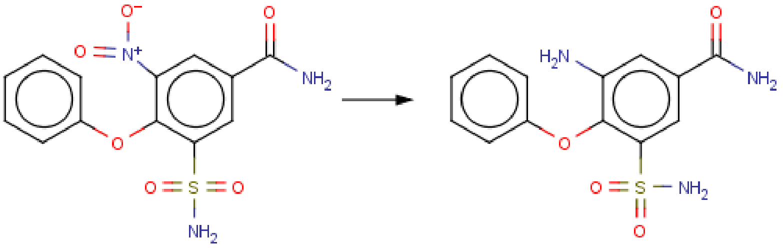

SMILES of the input:
NC(=O)c1cc([N+](=O)[O-
])c(Oc2ccccc2)c(S(N)(=O)=O)c1>>NC(=O)c1cc(N)c(Oc2ccccc2)c(S(N)(=O)=O)c1

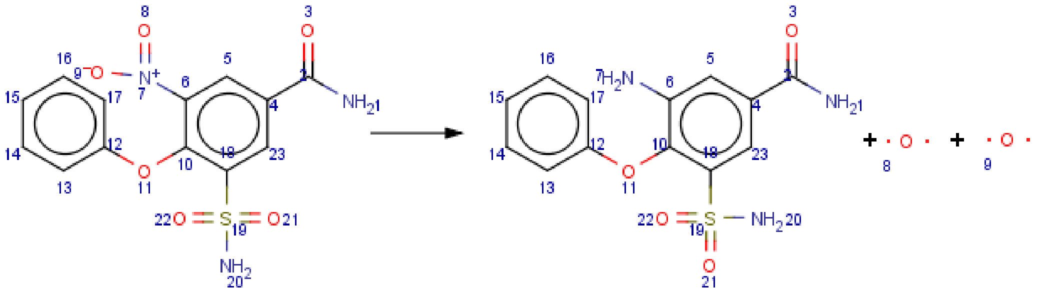

Correct mapped SMILES/SMARTS of the reaction:
[NH2:1][C:2](=[O:3])[c:4]1[cH:5][c:6]([c:10]([O:11][c:12]2[cH:13][cH:14][
cH:15][cH:16][cH:17]2)[c:18]([cH:23]1)[S:19]([NH2:20])(=[O:21])=[O:22])[N
+:7]([O-
:9])=[O:8]>>[NH2:1][C:2](=[O:3])[c:4]1[cH:5][c:6]([NH2:7])[c:10]([O:11][c
:12]2[cH:13][cH:14][cH:15][cH:16][cH:17]2)[c:18]([cH:23]1)[S:19]([NH2:20]
)(=[O:22])=[O:21].[O:8].[O:9]

Correctness of the mapping
MAPPET YES
ReactionMap NO
Marvin YES

Reaction no 46

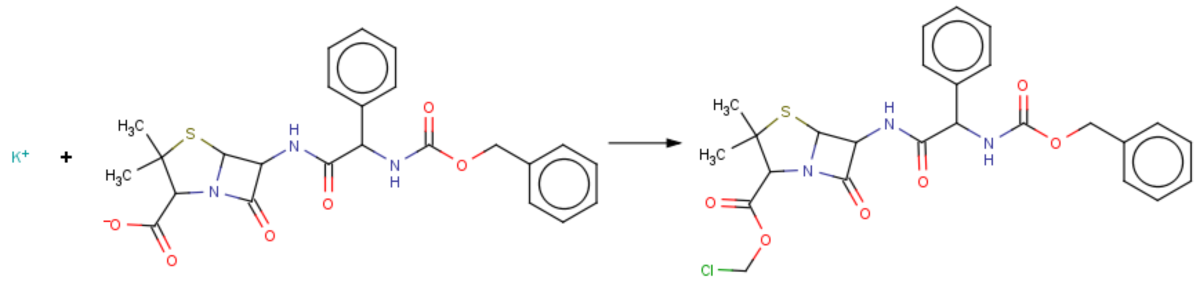

SMILES of the input:
[K+].CC1(C)SC2C(NC(=O)C(NC(=O)OCc3ccccc3)c3ccccc3)C(=O)N2C1C(=O)[O-
]>>CC1(C)SC2C(NC(=O)C(NC(=O)OCc3ccccc3)c3ccccc3)C(=O)N2C1C(=O)OCCl

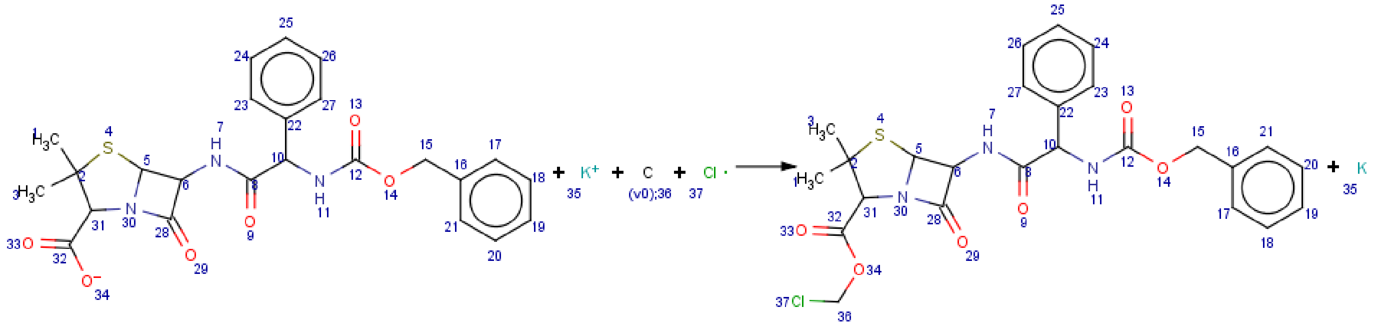

Correct mapped SMILES/SMARTS of the reaction:
[CH3:1][C:2]1([CH3:3])[S:4][CH:5]2[CH:6]([NH:7][C:8](=[O:9])[CH:10]([NH:1
1][C:12](=[O:13])[O:14][CH2:15][c:16]3[cH:17][cH:18][cH:19][cH:20][cH:21]
3)[c:22]3[cH:23][cH:24][cH:25][cH:26][cH:27]3)[C:28](=[O:29])[N:30]2[CH:3
1]1[C:32]([O-
:34])=[O:33].[K+:35].[C:36].[Cl:37]>>[CH3:3][C:2]1([CH3:1])[S:4][CH:5]2[C
H:6]([NH:7][C:8](=[O:9])[CH:10]([NH:11][C:12](=[O:13])[O:14][CH2:15][c:16
]3[cH:21][cH:20][cH:19][cH:18][cH:17]3)[c:22]3[cH:27][cH:26][cH:25][cH:24
][cH:23]3)[C:28](=[O:29])[N:30]2[CH:31]1[C:32](=[O:33])[O:34][CH2:36][Cl:
37].[K:35]

Correctness of the mapping
MAPPET YES
ReactionMap NO
Marvin YES

Reaction no 47

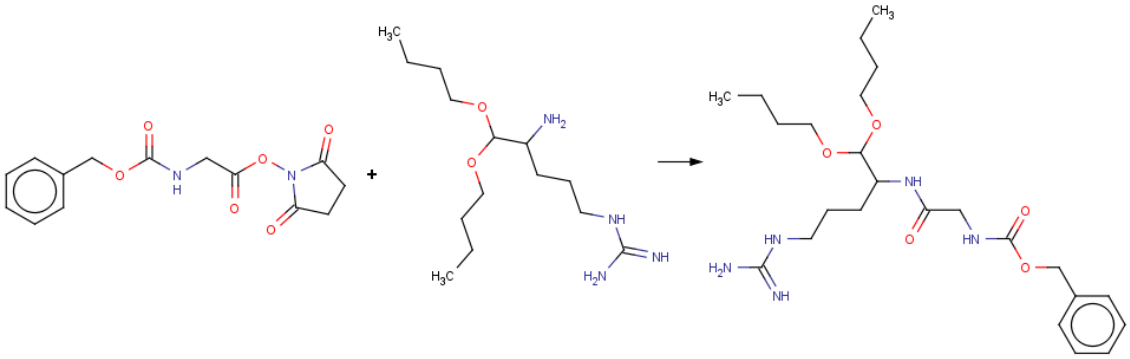

SMILES of the input:
O=C(CNC(=O)OCc1ccccc1)ON1C(=O)CCC1=O.CCCCOC(OCCCC)C(N)CCCNC(=N)N>>CCCCOC(OCCCC)C(CCCNC(=N)N)NC(=O)CNC(=O)OCc1ccccc1

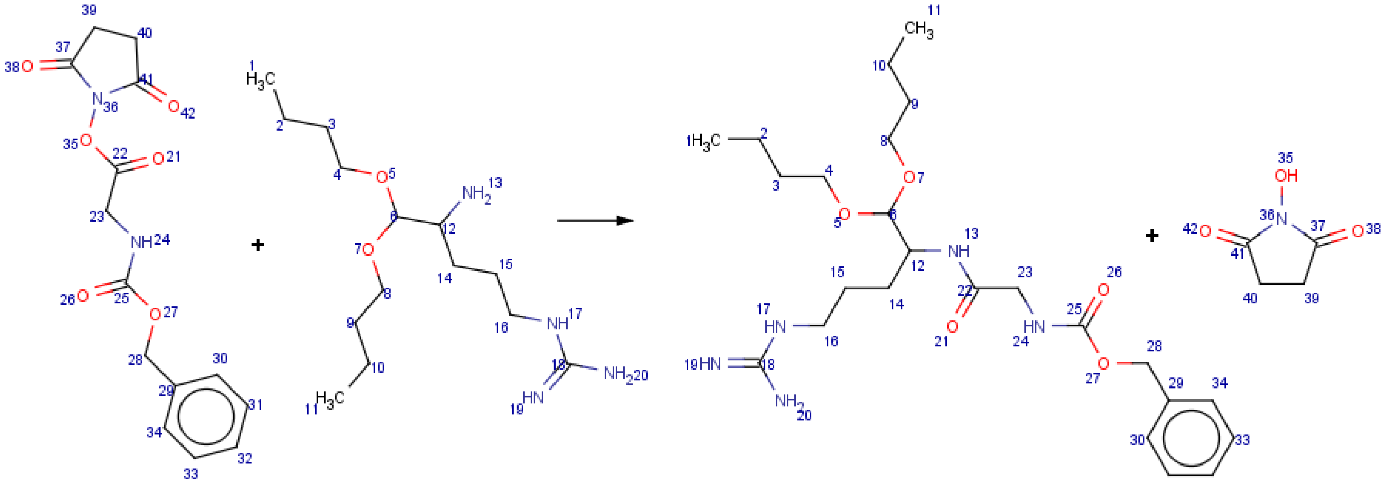

Correct mapped SMILES/SMARTS of the reaction:
[O:21]=[C:22]([CH2:23][NH:24][C:25](=[O:26])[O:27][CH2:28][c:29]1[cH:30][cH:31][cH:32][cH:33][cH:34]1)[O:35][N:36]1[C:37](=[O:38])[CH2:39][CH2:40][C:41]1=[O:42].[CH3:1][CH2:2][CH2:3][CH2:4][O:5][CH:6]([O:7][CH2:8][CH2:9][CH2:10][CH3:11])[CH:12]([NH2:13])[CH2:14][CH2:15][CH2:16][NH:17][C:18]([NH2:20])=[NH:19]>>[CH3:1][CH2:2][CH2:3][CH2:4][O:5][CH:6]([O:7][CH2:8][CH2:9][CH2:10][CH3:11])[CH:12]([CH2:14][CH2:15][CH2:16][NH:17][C:18]([NH2:20])=[NH:19])[NH:13][C:22](=[O:21])[CH2:23][NH:24][C:25](=[O:26])[O:27][CH2:28][c:29]1[cH:34][cH:33][cH:32][cH:31][cH:30]1.[OH:35][N:36]1[C:37](=[O:38])[CH2:39][CH2:40][C:41]1=[O:42]

Correctness of the mapping

MAPPET	YES
ReactionMap	NO
Marvin	YES

Reaction no 48

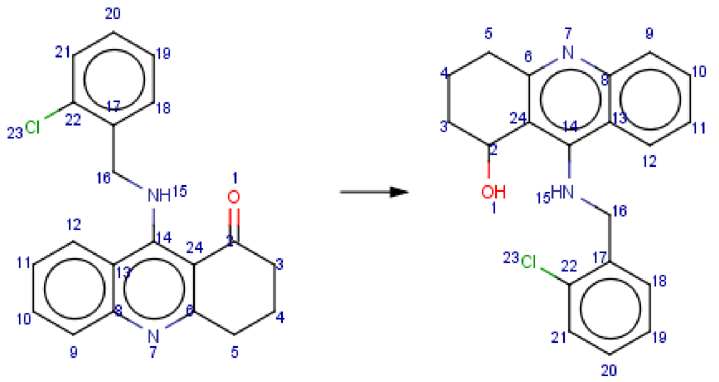

Correct mapped SMILES/SMARTS of the reaction:
[Cl:23][c:22]1[cH:21][cH:20][cH:19][cH:18][c:17]1[CH2:16][NH:15][c:14]1[c
:24]2[C:2](=[O:1])[CH2:3][CH2:4][CH2:5][c:6]2[n:7][c:8]2[cH:9][cH:10][cH:
11][cH:12][c:13]12>>[OH:1][CH:2]1[CH2:3][CH2:4][CH2:5][c:6]2[n:7][c:8]3[c
H:9][cH:10][cH:11][cH:12][c:13]3[c:14]([NH:15][CH2:16][c:17]3[cH:18][cH:1
9][cH:20][cH:21][c:22]3[Cl:23])[c:24]12

Correctness of the mapping

MAPPET	YES
ReactionMap	YES
Marvin	YES

Reaction no 49

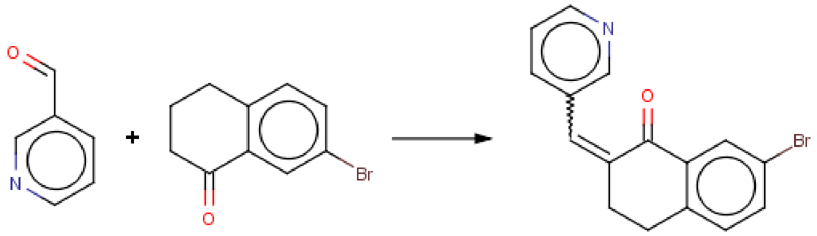

SMILES of the input:
O=Cc1cccnc1.O=C1CCCc2ccc(Br)cc21>>O=C1c2cc(Br)ccc2CCC1=Cc1cccnc1

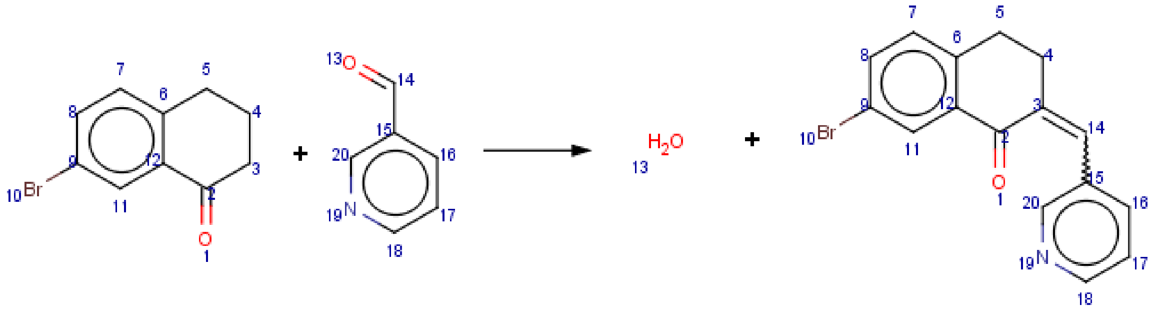

Correct mapped SMILES/SMARTS of the reaction:
[Br:10][c:9]1[cH:8][cH:7][c:6]2[CH2:5][CH2:4][CH2:3][C:2](=[O:1])[c:12]2[
cH:11]1.[O:13]=[CH:14][c:15]1[cH:16][cH:17][cH:18][n:19][cH:20]1>>[OH2:13
].[Br:10][c:9]1[cH:8][cH:7][c:6]2[CH2:5][CH2:4][C:3](=[CH:14][c:15]3[cH:1
6][cH:17][cH:18][n:19][cH:20]3)[C:2](=[O:1])[c:12]2[cH:11]1

Correctness of the mapping
MAPPET YES
ReactionMap NO
Marvin YES

Reaction no 50

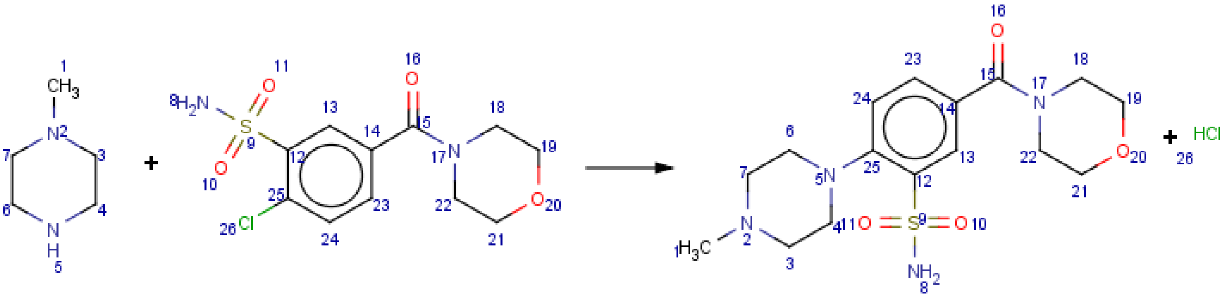

Correct mapped SMILES/SMARTS of the reaction:
[CH3:1][N:2]1[CH2:3][CH2:4][NH:5][CH2:6][CH2:7]1.[NH2:8][S:9](=[O:10])(=[O:11])[c:12]1[cH:13][c:14]([cH:23][cH:24][c:25]1[Cl:26])[C:15](=[O:16])[N:17]1[CH2:18][CH2:19][O:20][CH2:21][CH2:22]1>>[ClH:26].[CH3:1][N:2]1[CH2:7][CH2:6][N:5]([CH2:4][CH2:3]1)[c:25]1[cH:24][cH:23][c:14]([cH:13][c:12]1[S:9]([NH2:8])(=[O:11])=[O:10])[C:15](=[O:16])[N:17]1[CH2:18][CH2:19][O:20][CH2:21][CH2:22]1

Correctness of the mapping
MAPPET YES
ReactionMap YES
Marvin YES

Reaction no 51

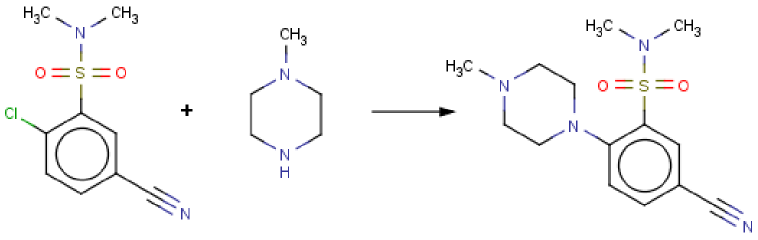

SMILES of the input:
CN(C)S(=O)(=O)c1cc(C#N)ccc1Cl.CN1CCNCC1>>CN(C)S(=O)(=O)c1cc(C#N)ccc1N1CCN(C)CC1

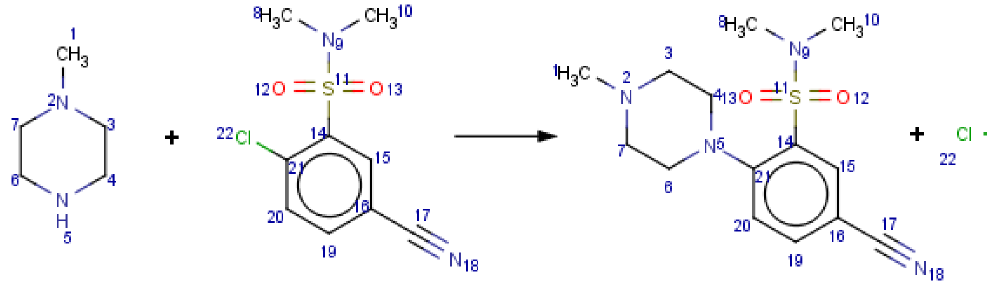

Correct mapped SMILES/SMARTS of the reaction:
[CH3:1][N:2]1[CH2:3][CH2:4][NH:5][CH2:6][CH2:7]1.[CH3:8][N:9]([CH3:10])[S:11](=[O:12])(=[O:13])[c:14]1[cH:15][c:16]([cH:19][cH:20][c:21]1[Cl:22])[C:17]#[N:18]>>[CH3:8][N:9]([CH3:10])[S:11](=[O:13])(=[O:12])[c:14]1[cH:15][c:16]([cH:19][cH:20][c:21]1[N:5]1[CH2:6][CH2:7][N:2]([CH3:1])[CH2:3][CH2:4]1)[C:17]#[N:18].[Cl:22]

```
Correctness of the mapping
MAPPET             YES
ReactionMap        NO
Marvin             YES

Reaction no  52
```

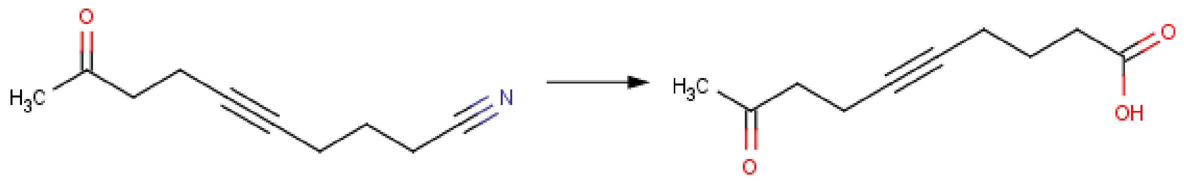


```
SMILES of the input:
CC(=O)CCC#CCCCC#N>>CC(=O)CCC#CCCCC(=O)O
```

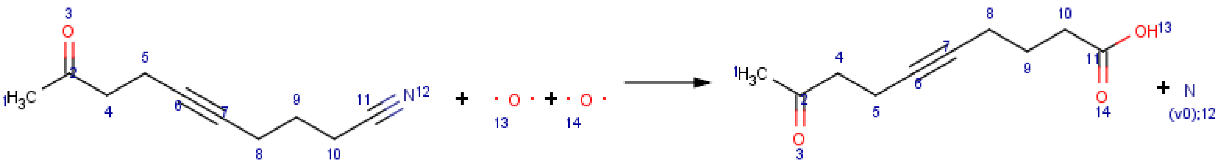

```
Correct mapped SMILES/SMARTS of the reaction:
[CH3:1][C:2](=[O:3])[CH2:4][CH2:5][C:6]#[C:7][CH2:8][CH2:9][CH2:10][C:11]
#[N:12].[O:13].[O:14]>>[CH3:1][C:2](=[O:3])[CH2:4][CH2:5][C:6]#[C:7][CH2:
8][CH2:9][CH2:10][C:11]([OH:13])=[O:14].[N:12]

Correctness of the mapping
MAPPET             YES
ReactionMap        NO
Marvin             YES

Reaction no  53
```

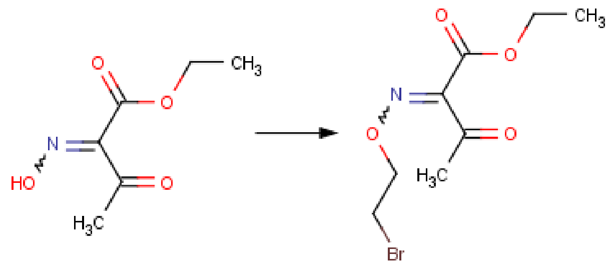


```
SMILES of the input:
CCOC(=O)C(=NO)C(C)=O>>CCOC(=O)C(=NOCCBr)C(C)=O
```

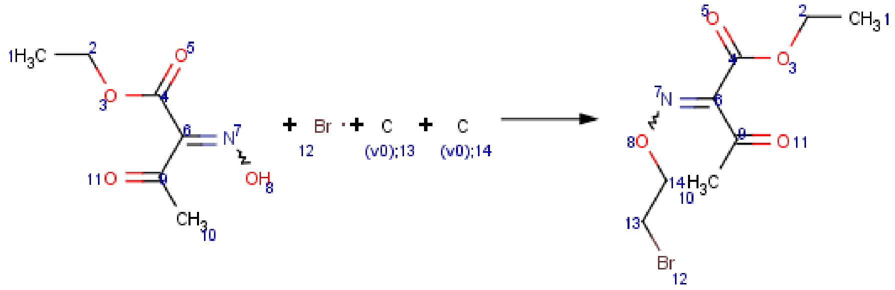

```
Correct mapped SMILES/SMARTS of the reaction:
[CH3:1][CH2:2][O:3][C:4](=[O:5])[C:6](=[N:7][OH:8])[C:9]([CH3:10])=[O:11]
.[Br:12].[C:13].[C:14]>>[CH3:1][CH2:2][O:3][C:4](=[O:5])[C:6](=[N:7][O:8]
[CH2:14][CH2:13][Br:12])[C:9]([CH3:10])=[O:11]
```

Correctness of the mapping
MAPPET YES
ReactionMap NO
Marvin YES

Reaction no 54

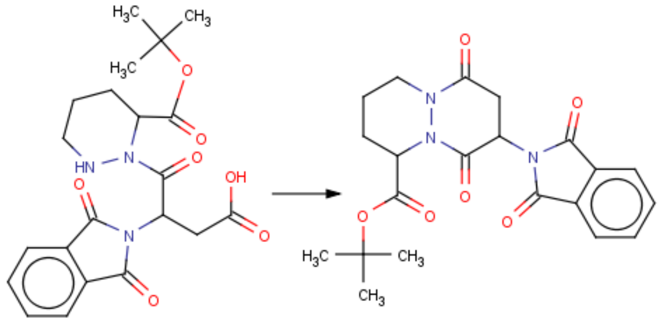

SMILES of the input:
CC(C)(C)OC(=O)C1CCCNN1C(=O)C(CC(=O)O)N1C(=O)c2ccccc2C1=O>>CC(C)(C)OC(=O)C
1CCCN2C(=O)CC(N3C(=O)c4ccccc4C3=O)C(=O)N12

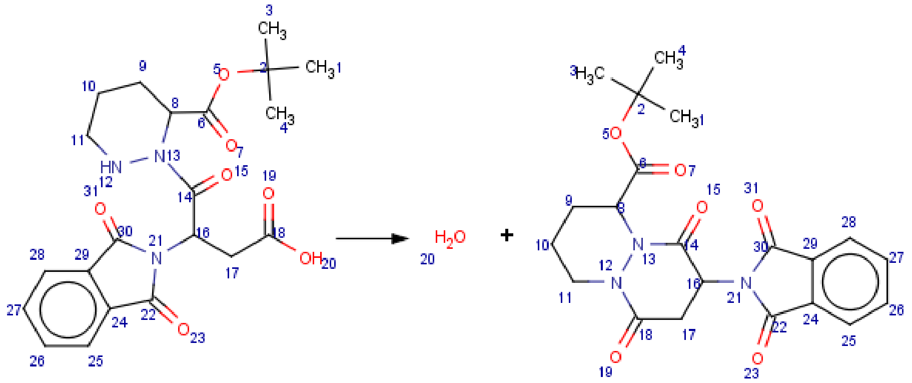

Correct mapped SMILES/SMARTS of the reaction:
[CH3:1][C:2]([CH3:3])([CH3:4])[O:5][C:6](=[O:7])[CH:8]1[CH2:9][CH2:10][CH
2:11][NH:12][N:13]1[C:14](=[O:15])[CH:16]([CH2:17][C:18]([OH:20])=[O:19])
[N:21]1[C:22](=[O:23])[c:24]2[cH:25][cH:26][cH:27][cH:28][c:29]2[C:30]1=[
O:31]>>[OH2:20].[CH3:4][C:2]([CH3:3])([CH3:1])[O:5][C:6](=[O:7])[CH:8]1[C
H2:9][CH2:10][CH2:11][N:12]2[N:13]1[C:14](=[O:15])[CH:16]([CH2:17][C:18]2
=[O:19])[N:21]1[C:30](=[O:31])[c:29]2[cH:28][cH:27][cH:26][cH:25][c:24]2[
C:22]1=[O:23]

Correctness of the mapping
MAPPET YES
ReactionMap NO
Marvin YES

Reaction no 55

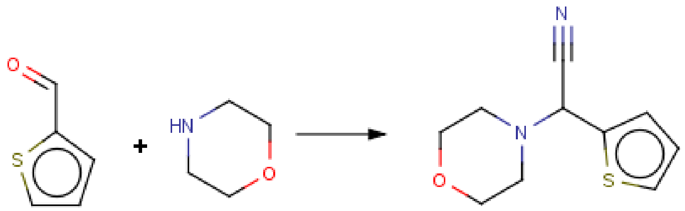

SMILES of the input:
O=Cc1cccs1.C1COCCN1>>N#CC(c1cccs1)N1CCOCC1

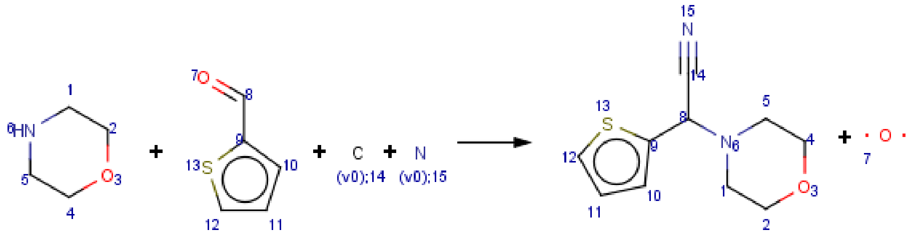

Correct mapped SMILES/SMARTS of the reaction:
[CH2:1]1[CH2:2][O:3][CH2:4][CH2:5][NH:6]1.[O:7]=[CH:8][c:9]1[cH:10][cH:11][cH:12][s:13]1.[C:14].[N:15]>>[N:15]#[C:14][CH:8]([N:6]1[CH2:5][CH2:4][O:3][CH2:2][CH2:1]1)[c:9]1[cH:10][cH:11][cH:12][s:13]1.[O:7]

Correctness of the mapping
MAPPET YES
ReactionMap NO
Marvin YES

Reaction no 56

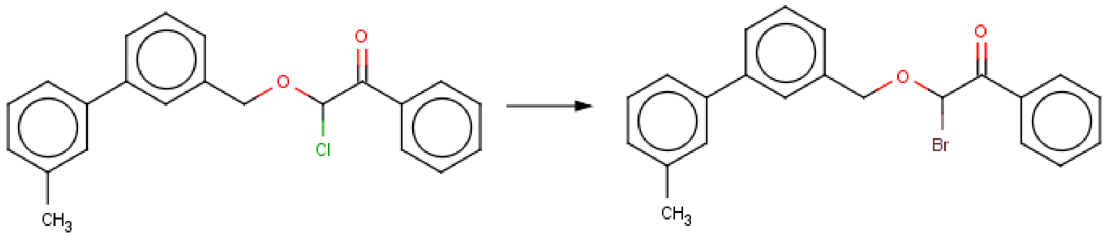

SMILES of the input:
Cc1cccc(-c2cccc(COC(Cl)C(=O)c3ccccc3)c2)c1>>Cc1cccc(-c2cccc(COC(Br)C(=O)c3ccccc3)c2)c1

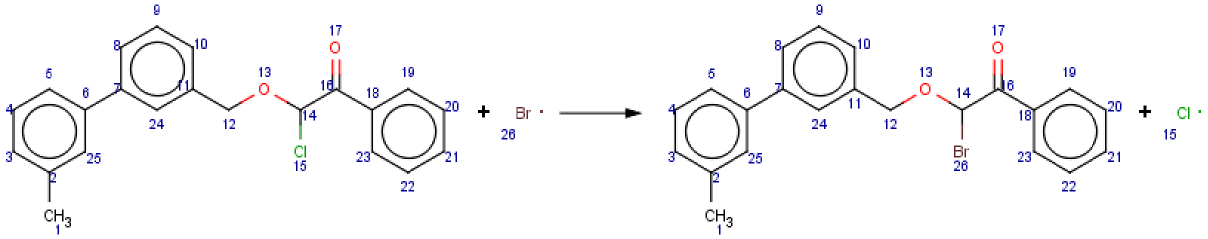

Correct mapped SMILES/SMARTS of the reaction:
[CH3:1][c:2]1[cH:3][cH:4][cH:5][c:6]([cH:25]1)-[c:7]1[cH:8][cH:9][cH:10][c:11]([CH2:12][O:13][CH:14]([Cl:15])[C:16](=[O:17])[c:18]2[cH:19][cH:20][cH:21][cH:22][cH:23]2)[cH:24]1.[Br:26]>>[CH3:1][c:2]1[cH:3][cH:4][cH:5][c:6]([cH:25]1)-[c:7]1[cH:8][cH:9][cH:10][c:11]([CH2:12][O:13][CH:14]([Br:26])[C:16](=[O:17])[c:18]2[cH:19][cH:20][cH:21][cH:22][cH:23]2)[cH:24]1.[Cl:15]

Correctness of the mapping
MAPPET YES
ReactionMap NO
Marvin YES

Reaction no 57

SMILES of the input:

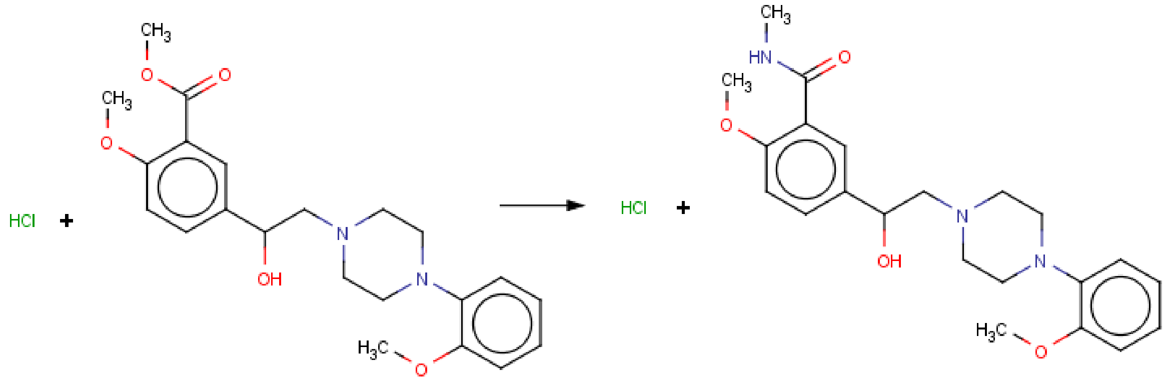

SMILES of the input:
Cl.COC(=O)c1cc(C(O)CN2CCN(c3ccccc3OC)CC2)ccc1OC>>Cl.CNC(=O)c1cc(C(O)CN2CC
N(c3ccccc3OC)CC2)ccc1OC

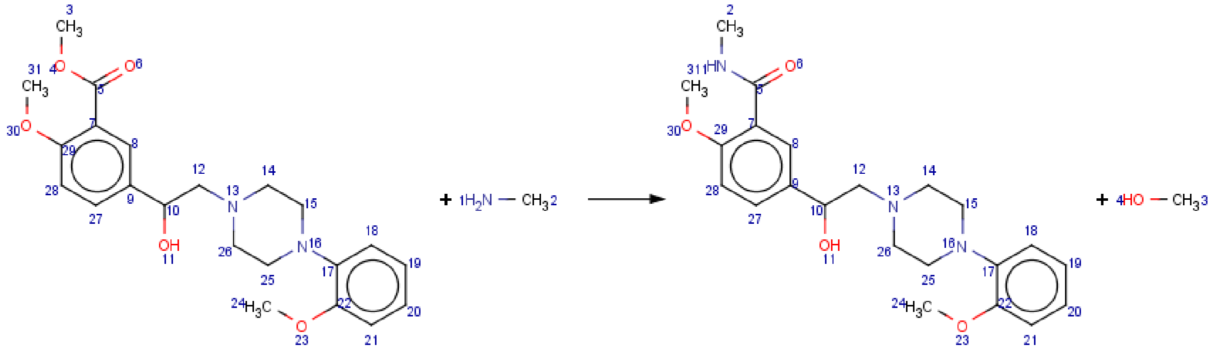

Correct mapped SMILES/SMARTS of the reaction:
[CH3:3][O:4][C:5](=[O:6])[c:7]1[cH:8][c:9]([cH:27][cH:28][c:29]1[O:30][CH
3:31])[CH:10]([OH:11])[CH2:12][N:13]1[CH2:14][CH2:15][N:16]([CH2:25][CH2:
26]1)[c:17]1[cH:18][cH:19][cH:20][cH:21][c:22]1[O:23][CH3:24].[CH3:2][NH2
:1]>>[CH3:2][NH:1][C:5](=[O:6])[c:7]1[cH:8][c:9]([cH:27][cH:28][c:29]1[O:
30][CH3:31])[CH:10]([OH:11])[CH2:12][N:13]1[CH2:14][CH2:15][N:16]([CH2:25
][CH2:26]1)[c:17]1[cH:18][cH:19][cH:20][cH:21][c:22]1[O:23][CH3:24].[CH3:
3][OH:4]

Correctness of the mapping

MAPPET	YES
ReactionMap	NO
Marvin	NO

Reaction no 58

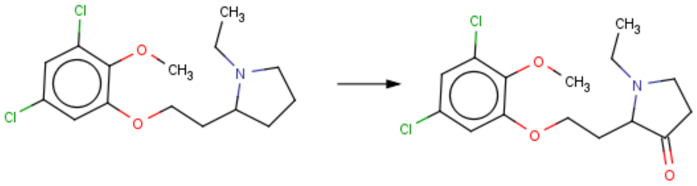

SMILES of the input:
CCN1CCCC1CCOc1cc(Cl)cc(Cl)c1OC>>CCN1CCC(=O)C1CCOc1cc(Cl)cc(Cl)c1OC

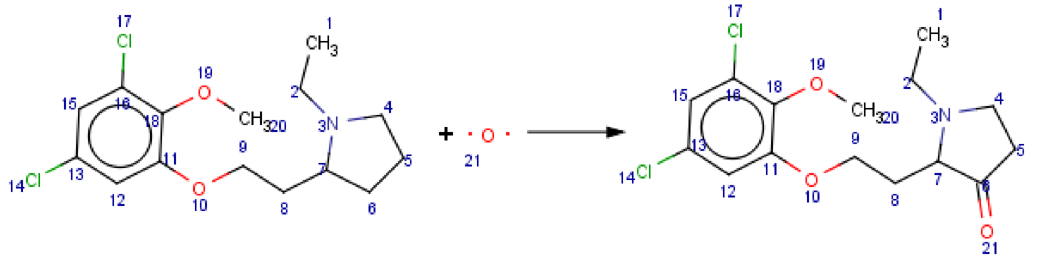

Correct mapped SMILES/SMARTS of the reaction:
[CH3:1][CH2:2][N:3]1[CH2:4][CH2:5][CH2:6][CH:7]1[CH2:8][CH2:9][O:10][c:11
]1[cH:12][c:13]([Cl:14])[cH:15][c:16]([Cl:17])[c:18]1[O:19][CH3:20].[O:21
]>>[CH3:1][CH2:2][N:3]1[CH2:4][CH2:5][C:6](=[O:21])[CH:7]1[CH2:8][CH2:9][
O:10][c:11]1[cH:12][c:13]([Cl:14])[cH:15][c:16]([Cl:17])[c:18]1[O:19][CH3
:20]

Correctness of the mapping

MAPPET	YES
ReactionMap	NO
Marvin	YES

Reaction no 59

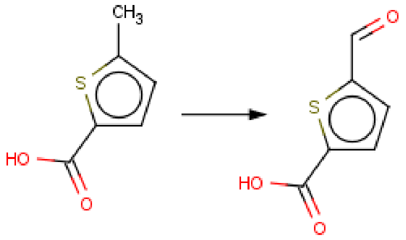

SMILES of the input:
Cc1ccc(C(=O)O)s1>>O=Cc1ccc(C(=O)O)s1

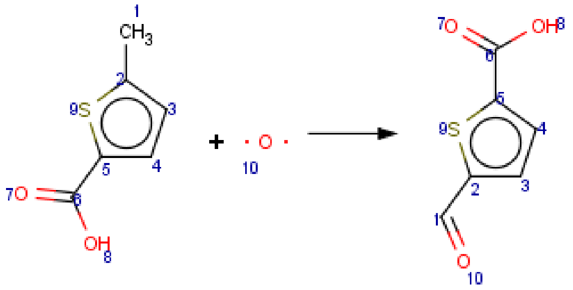

Correct mapped SMILES/SMARTS of the reaction:
[CH3:1][c:2]1[cH:3][cH:4][c:5]([s:9]1)[C:6]([OH:8])=[O:7].[O:10]>>[OH:8][
C:6](=[O:7])[c:5]1[cH:4][cH:3][c:2]([CH:1]=[O:10])[s:9]1

Correctness of the mapping

MAPPET	YES
ReactionMap	NO
Marvin	YES

Reaction no 60

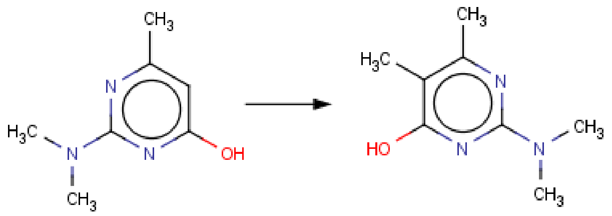

SMILES of the input:
Cc1cc(O)nc(N(C)C)n1>>Cc1nc(N(C)C)nc(O)c1C

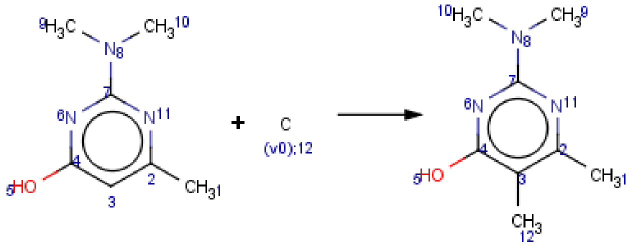

Correct mapped SMILES/SMARTS of the reaction:
[CH3:9][N:8]([CH3:10])[c:7]1[n:11][c:2]([CH3:1])[cH:3][c:4]([OH:5])[n:6]1
.[C:12]>>[CH3:10][N:8]([CH3:9])[c:7]1[n:11][c:2]([CH3:1])[c:3]([CH3:12])[
c:4]([OH:5])[n:6]1

Correctness of the mapping
MAPPET YES
ReactionMap NO
Marvin YES

Reaction no 61

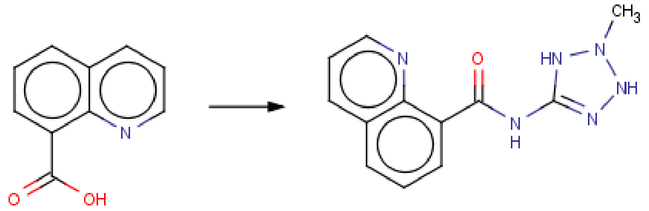

SMILES of the input:
O=C(O)c1cccc2cccnc21>>CN1NN=C(NC(=O)c2cccc3cccnc32)N1

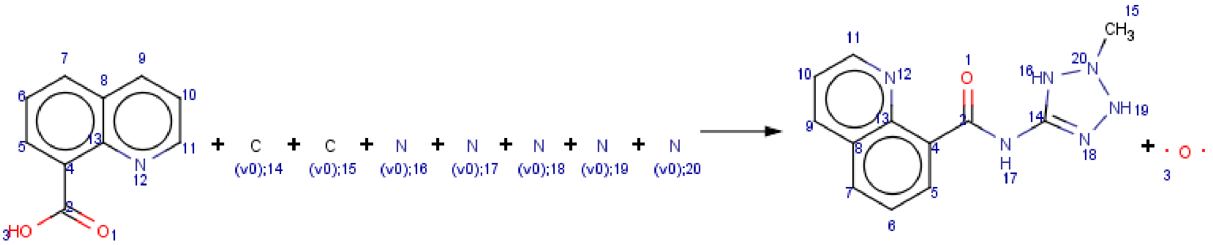

Correct mapped SMILES/SMARTS of the reaction:
[OH:3][C:2](=[O:1])[c:4]1[cH:5][cH:6][cH:7][c:8]2[cH:9][cH:10][cH:11][n:1
2][c:13]12.[C:14].[C:15].[N:16].[N:17].[N:18].[N:19].[N:20]>>[CH3:15][N:2
0]1[NH:19][N:18]=[C:14]([NH:17][C:2](=[O:1])[c:4]2[cH:5][cH:6][cH:7][c:8]
3[cH:9][cH:10][cH:11][n:12][c:13]23)[NH:16]1.[O:3]

Correctness of the mapping
MAPPET YES
ReactionMap NO
Marvin YES

Reaction no 62

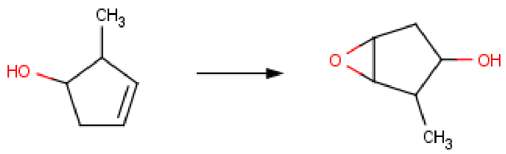

SMILES of the input:
CC1C=CCC1O>>CC1C2OC2CC1O

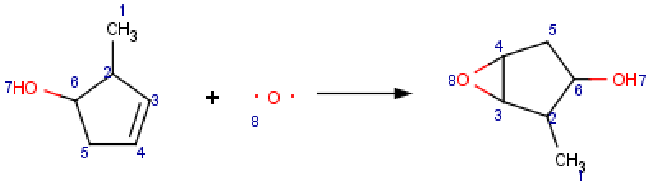

Correct mapped SMILES/SMARTS of the reaction:
[CH3:1][CH:2]1[CH:3]=[CH:4][CH2:5][CH:6]1[OH:7].[O:8]>>[CH3:1][CH:2]1[CH:
3]2[O:8][CH:4]2[CH2:5][CH:6]1[OH:7]

Correctness of the mapping
MAPPET YES
ReactionMap NO
Marvin YES

Reaction no 63

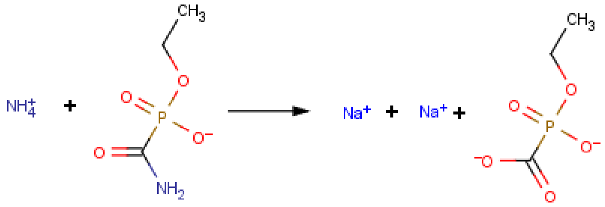

SMILES of the input:
[NH4+].CCOP(=O)([O-])C(N)=O>>[Na+].[Na+].CCOP(=O)([O-])C(=O)[O-]

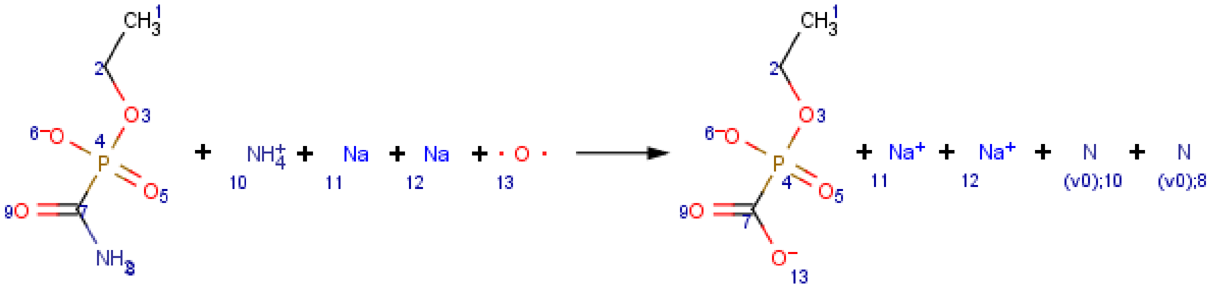

Correct mapped SMILES/SMARTS of the reaction:
[CH3:1][CH2:2][O:3][P:4]([O-
:6])(=[O:5])[C:7]([NH2:8])=[O:9].[NH4+:10].[Na:11].[Na:12].[O:13]>>[CH3:1
][CH2:2][O:3][P:4]([O-:6])(=[O:5])[C:7]([O-
:13])=[O:9].[Na+:11].[Na+:12].[N:10].[N:8]

Correctness of the mapping
MAPPET YES
ReactionMap NO
Marvin YES

Reaction no 64

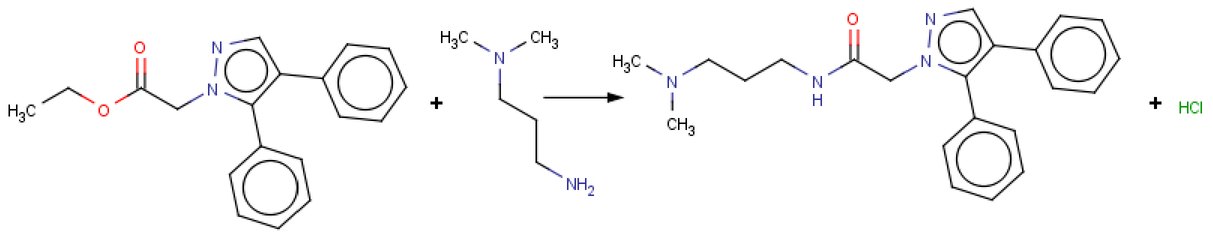

SMILES of the input:
CCOC(=O)Cn1ncc(-c2ccccc2)c1-c1ccccc1.CN(C)CCCN>>Cl.CN(C)CCCNC(=O)Cn1ncc(-c2ccccc2)c1-c1ccccc1

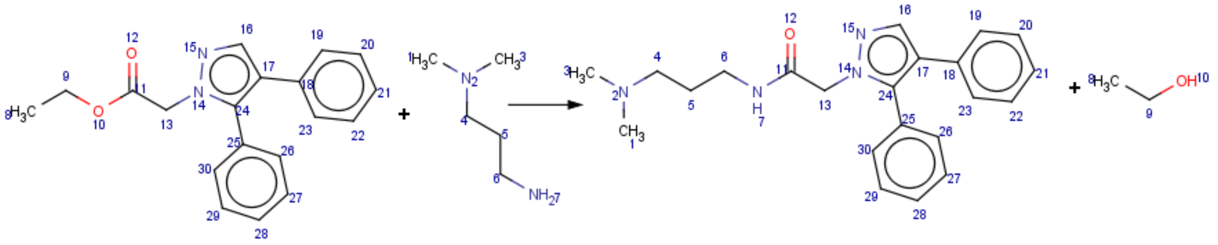

Correct mapped SMILES/SMARTS of the reaction:
[CH3:8][CH2:9][O:10][C:11](=[O:12])[CH2:13][n:14]1[n:15][cH:16][c:17]([c:24]1-[c:25]1[cH:26][cH:27][cH:28][cH:29][cH:30]1)-[c:18]1[cH:19][cH:20][cH:21][cH:22][cH:23]1.[CH3:1][N:2]([CH3:3])[CH2:4][CH2:5][CH2:6][NH2:7]>>[CH3:3][N:2]([CH3:1])[CH2:4][CH2:5][CH2:6][NH:7][C:11](=[O:12])[CH2:13][n:14]1[n:15][cH:16][c:17]([c:24]1-[c:25]1[cH:26][cH:27][cH:28][cH:29][cH:30]1)-[c:18]1[cH:19][cH:20][cH:21][cH:22][cH:23]1.[CH3:8][CH2:9][OH:10]

Correctness of the mapping

MAPPET	YES
ReactionMap	NO
Marvin	YES

Reaction no 65

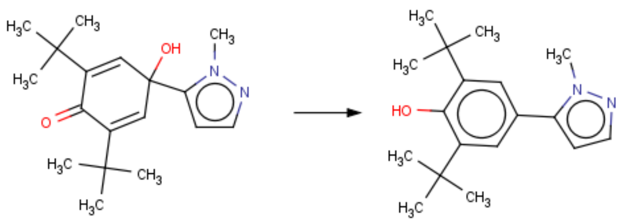

SMILES of the input:
Cn1nccc1C1(O)C=C(C(C)(C)C)C(=O)C(C(C)(C)C)=C1>>Cn1nccc1-c1cc(C(C)(C)C)c(O)c(C(C)(C)C)c1

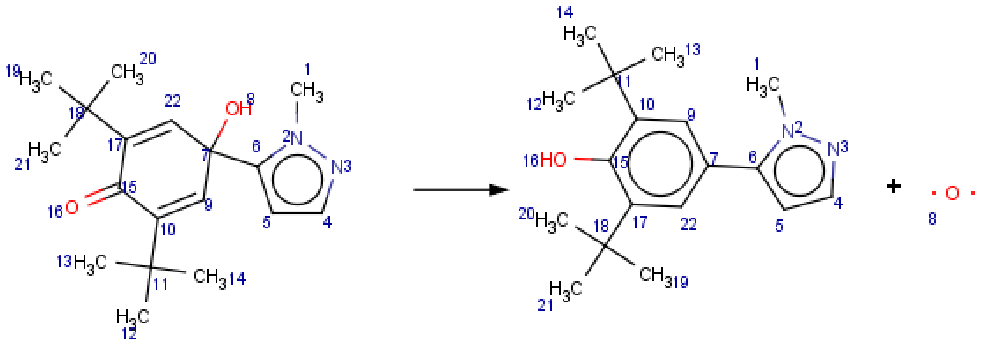

Correct mapped SMILES/SMARTS of the reaction:
[CH3:1][n:2]1[n:3][cH:4][cH:5][c:6]1[C:7]1([OH:8])[CH:9]=[C:10]([C:15](=[
O:16])[C:17](=[CH:22]1)[C:18]([CH3:19])([CH3:20])[CH3:21])[C:11]([CH3:12]
)([CH3:13])[CH3:14]>>[CH3:1][n:2]1[n:3][cH:4][cH:5][c:6]1-
[c:7]1[cH:22][c:17]([c:15]([OH:16])[c:10]([cH:9]1)[C:11]([CH3:14])([CH3:1
3])[CH3:12])[C:18]([CH3:21])([CH3:20])[CH3:19].[O:8]

Correctness of the mapping

MAPPET	YES
ReactionMap	NO
Marvin	YES

Reaction no 66

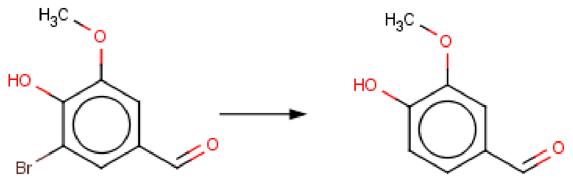

SMILES of the input:
COc1cc(C=O)cc(Br)c1O>>COc1cc(C=O)ccc1O

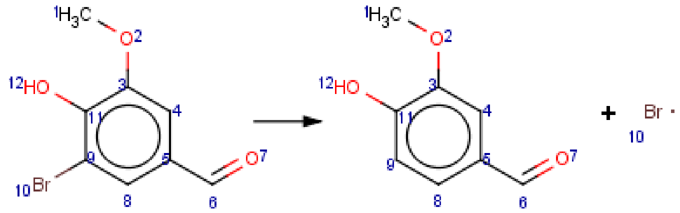

Correct mapped SMILES/SMARTS of the reaction:
[CH3:1][O:2][c:3]1[cH:4][c:5]([CH:6]=[O:7])[cH:8][c:9]([Br:10])[c:11]1[OH
:12]>>[CH3:1][O:2][c:3]1[cH:4][c:5]([CH:6]=[O:7])[cH:8][cH:9][c:11]1[OH:1
2].[Br:10]

Correctness of the mapping

MAPPET	YES
ReactionMap	NO
Marvin	YES

Reaction no 67

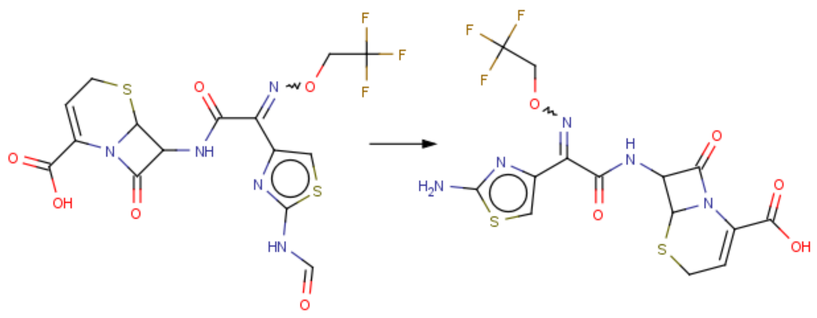

SMILES of the input:
O=CNc1nc(C(=NOCC(F)(F)F)C(=O)NC2C(=O)N3C(C(=O)O)=CCSC23)cs1>>Nc1nc(C(=NOCC(F)(F)F)C(=O)NC2C(=O)N3C(C(=O)O)=CCSC23)cs1

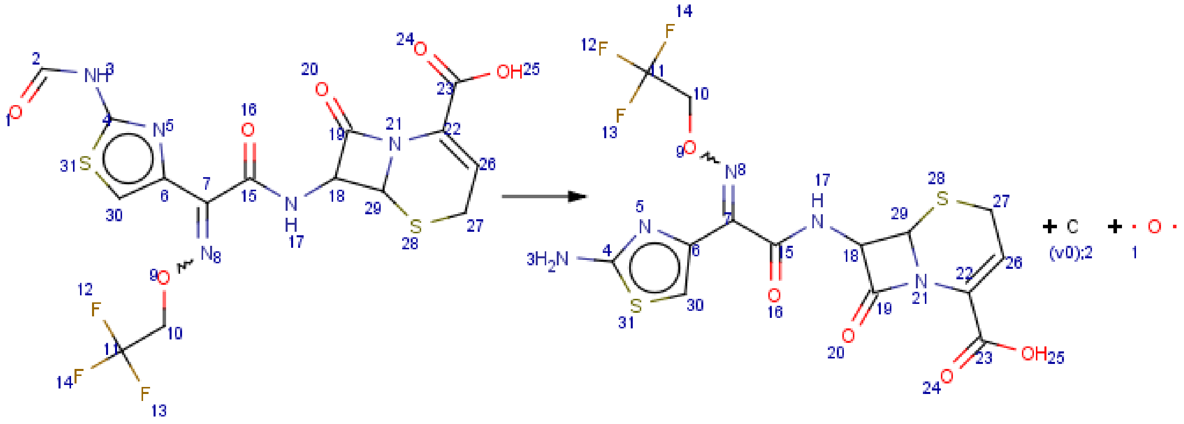

Correct mapped SMILES/SMARTS of the reaction:
[OH:25][C:23](=[O:24])[C:22]1=[CH:26][CH2:27][S:28][CH:29]2[CH:18]([NH:17][C:15](=[O:16])[C:7](=[N:8][O:9][CH2:10][C:11]([F:12])([F:13])[F:14])[c:6]3[cH:30][s:31][c:4]([NH:3][CH:2]=[O:1])[n:5]3)[C:19](=[O:20])[N:21]12>>[NH2:3][c:4]1[n:5][c:6]([cH:30][s:31]1)[C:7](=[N:8][O:9][CH2:10][C:11]([F:12])([F:13])[F:14])[C:15](=[O:16])[NH:17][CH:18]1[CH:29]2[S:28][CH2:27][CH:26]=[C:22]([N:21]2[C:19]1=[O:20])[C:23]([OH:25])=[O:24].[C:2].[O:1]

Correctness of the mapping

MAPPET	YES
ReactionMap	NO
Marvin	YES

Reaction no 68

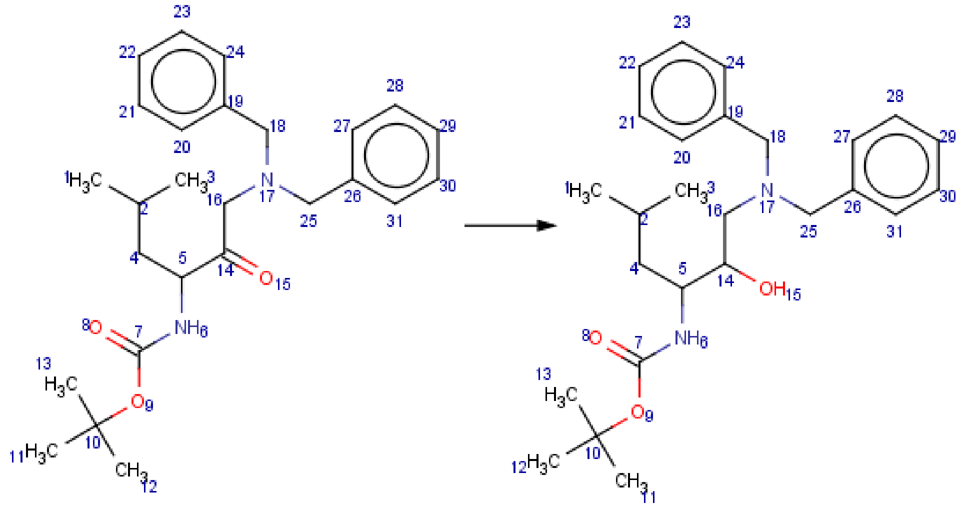

Correct mapped SMILES/SMARTS of the reaction:
[CH3:1][CH:2]([CH3:3])[CH2:4][CH:5]([NH:6][C:7](=[O:8])[O:9][C:10]([CH3:1
1])([CH3:12])[CH3:13])[C:14](=[O:15])[CH2:16][N:17]([CH2:18][c:19]1[cH:20
][cH:21][cH:22][cH:23][cH:24]1)[CH2:25][c:26]1[cH:27][cH:28][cH:29][cH:30
][cH:31]1>>[CH3:1][CH:2]([CH3:3])[CH2:4][CH:5]([NH:6][C:7](=[O:8])[O:9][C
:10]([CH3:12])([CH3:11])[CH3:13])[CH:14]([OH:15])[CH2:16][N:17]([CH2:18][
c:19]1[cH:20][cH:21][cH:22][cH:23][cH:24]1)[CH2:25][c:26]1[cH:27][cH:28][
cH:29][cH:30][cH:31]1

Correctness of the mapping
MAPPET YES
ReactionMap YES
Marvin YES

Reaction no 69

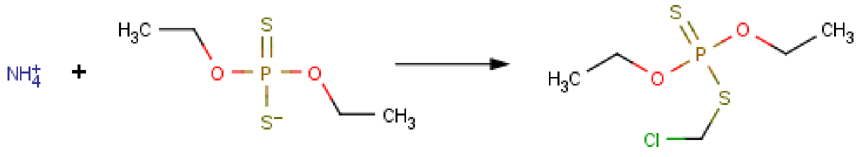

SMILES of the input:
[NH4+].CCOP(=S)([S-])OCC>>CCOP(=S)(OCC)SCCl

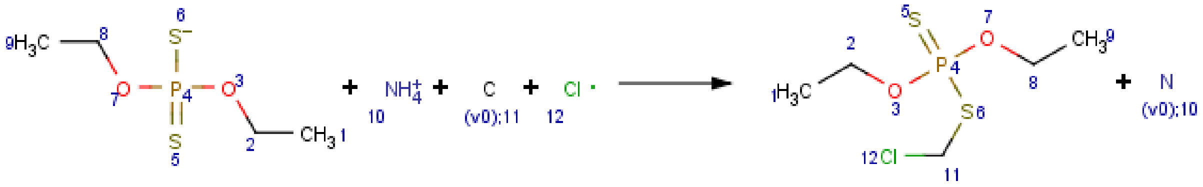

Correct mapped SMILES/SMARTS of the reaction:
[CH3:1][CH2:2][O:3][P:4]([S-
:6])(=[S:5])[O:7][CH2:8][CH3:9].[NH4+:10].[C:11].[Cl:12]>>[CH3:9][CH2:8][
O:7][P:4](=[S:5])([O:3][CH2:2][CH3:1])[S:6][CH2:11][Cl:12].[N:10]

Correctness of the mapping
MAPPET YES
ReactionMap NO
Marvin YES

Reaction no 70

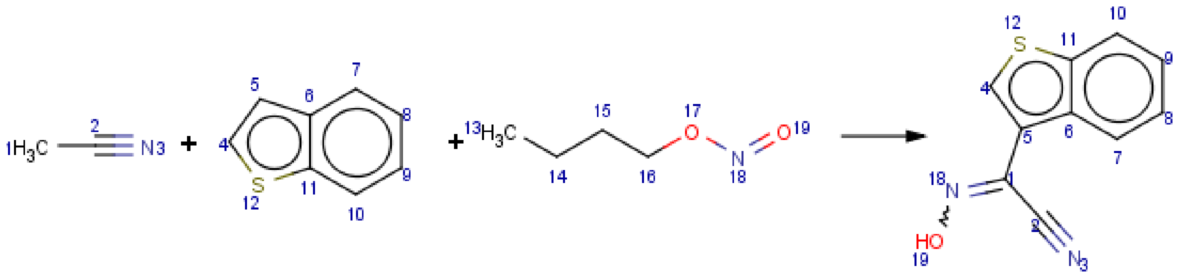

Correct mapped SMILES/SMARTS of the reaction:
[CH3:1][C:2]#[N:3].[cH:4]1[cH:5][c:6]2[cH:7][cH:8][cH:9][cH:10][c:11]2[s:12]1.[CH3:13][CH2:14][CH2:15][CH2:16][O:17][N:18]=[O:19]>>[OH:19][N:18]=[C:1]([C:2]#[N:3])[c:5]1[cH:4][s:12][c:11]2[cH:10][cH:9][cH:8][cH:7][c:6]12

Correctness of the mapping

MAPPET	NO
ReactionMap	NO
Marvin	NO

Reaction no 71

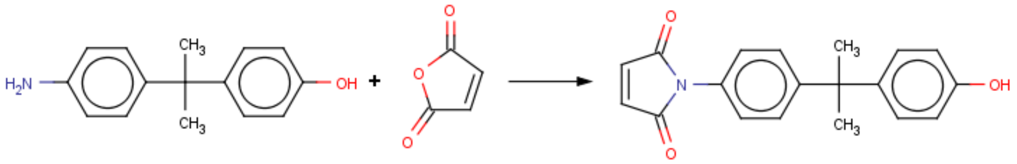

SMILES of the input:
CC(C)(c1ccc(N)cc1)c1ccc(O)cc1.O=C1C=CC(=O)O1>>CC(C)(c1ccc(O)cc1)c1ccc(N2C(=O)C=CC2=O)cc1

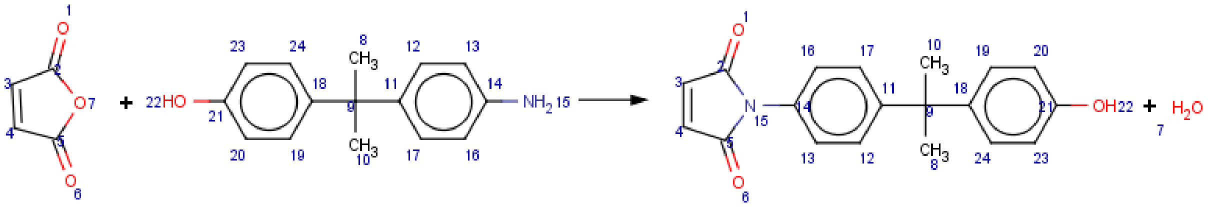

Correct mapped SMILES/SMARTS of the reaction:
[O:1]=[C:2]1[O:7][C:5](=[O:6])[CH:4]=[CH:3]1.[CH3:8][C:9]([CH3:10])([c:11]1[cH:12][cH:13][c:14]([NH2:15])[cH:16][cH:17]1)[c:18]1[cH:19][cH:20][c:21]([OH:22])[cH:23][cH:24]1>>[OH2:7].[CH3:10][C:9]([CH3:8])([c:18]1[cH:19][cH:20][c:21]([OH:22])[cH:23][cH:24]1)[c:11]1[cH:12][cH:13][c:14]([cH:16][cH:17]1)[N:15]1[C:5](=[O:6])[CH:4]=[CH:3][C:2]1=[O:1]

Correctness of the mapping

MAPPET	YES
ReactionMap	NO
Marvin	YES

Reaction no 72

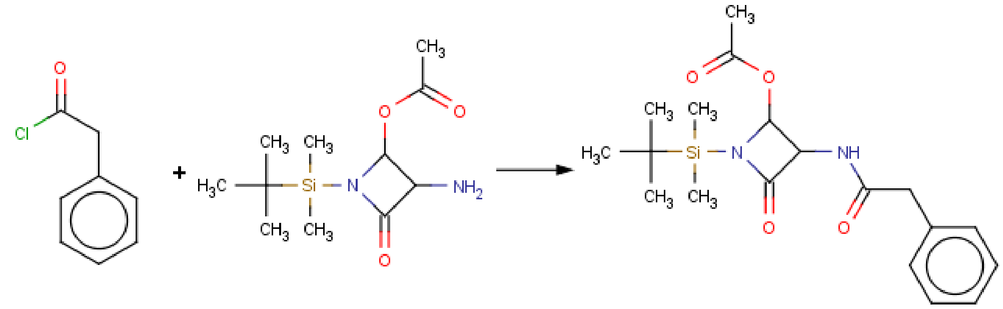

SMILES of the input:
O=C(Cl)Cc1ccccc1.CC(=O)OC1C(N)C(=O)N1[Si](C)(C)C(C)(C)C>>CC(=O)OC1C(NC(=O
)Cc2ccccc2)C(=O)N1[Si](C)(C)C(C)(C)C

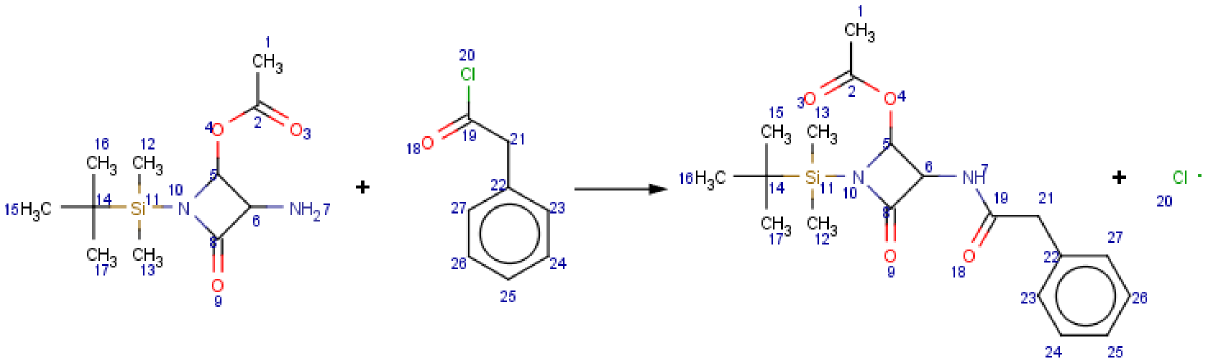

Correct mapped SMILES/SMARTS of the reaction:
[CH3:1][C:2](=[O:3])[O:4][CH:5]1[CH:6]([NH2:7])[C:8](=[O:9])[N:10]1[Si:11
]([CH3:12])([CH3:13])[C:14]([CH3:15])([CH3:16])[CH3:17].[Cl:20][C:19](=[O
:18])[CH2:21][c:22]1[cH:23][cH:24][cH:25][cH:26][cH:27]1>>[CH3:1][C:2](=[
O:3])[O:4][CH:5]1[CH:6]([NH:7][C:19](=[O:18])[CH2:21][c:22]2[cH:27][cH:26
][cH:25][cH:24][cH:23]2)[C:8](=[O:9])[N:10]1[Si:11]([CH3:13])([CH3:12])[C
:14]([CH3:16])([CH3:15])[CH3:17].[Cl:20]

Correctness of the mapping

MAPPET	YES
ReactionMap	NO
Marvin	YES

Reaction no 73

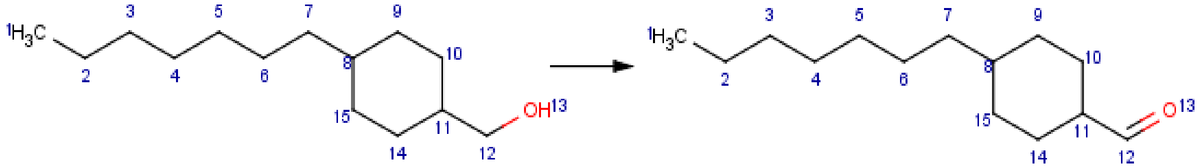

Correct mapped SMILES/SMARTS of the reaction:
[CH3:1][CH2:2][CH2:3][CH2:4][CH2:5][CH2:6][CH2:7][CH:8]1[CH2:9][CH2:10][C
H:11]([CH2:12][OH:13])[CH2:14][CH2:15]1>>[CH3:1][CH2:2][CH2:3][CH2:4][CH2
:5][CH2:6][CH2:7][CH:8]1[CH2:9][CH2:10][CH:11]([CH2:14][CH2:15]1)[CH:12]=
[O:13]

Correctness of the mapping

MAPPET	YES
ReactionMap	YES
Marvin	YES

Reaction no 74

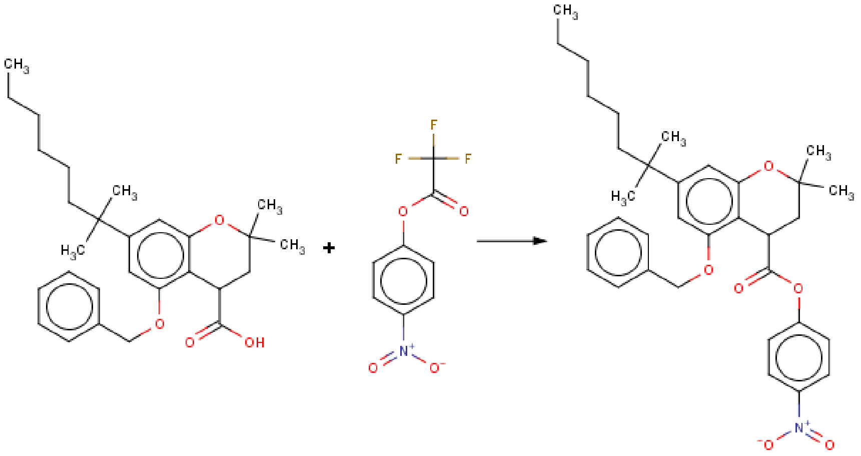

SMILES of the input:
CCCCCCC(C)(C)c1cc(OCc2ccccc2)c2c(c1)OC(C)(C)CC2C(=O)O.O=C(Oc1ccc([N+](=O)[O-])cc1)C(F)(F)F>>CCCCCCC(C)(C)c1cc(OCc2ccccc2)c2c(c1)OC(C)(C)CC2C(=O)Oc1ccc([N+](=O)[O-])cc1

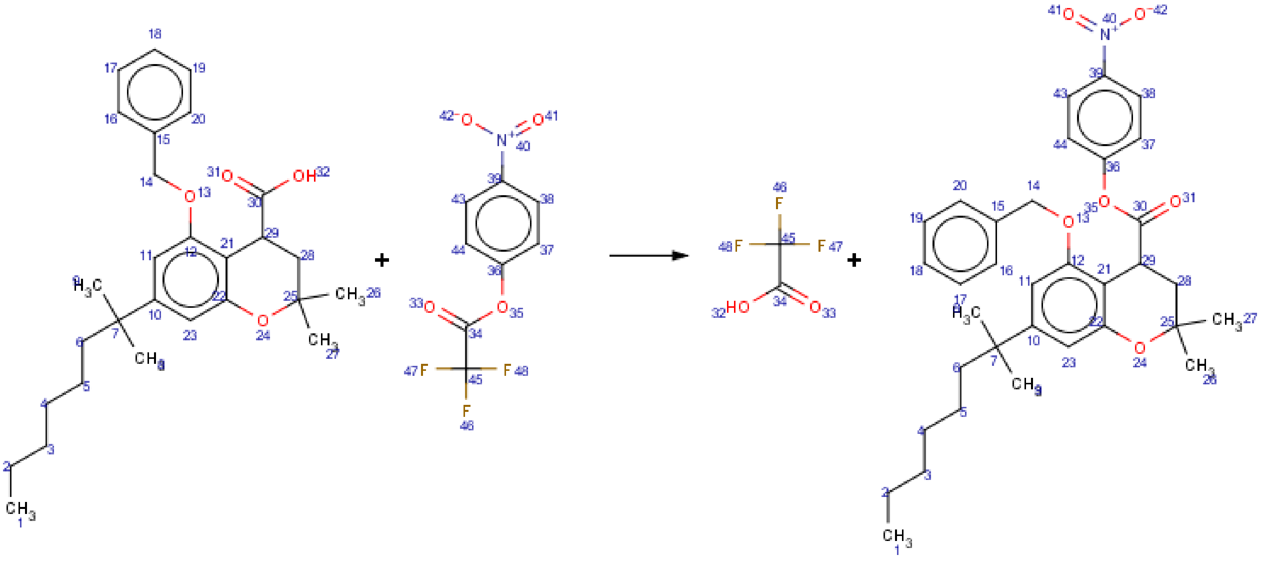

Correct mapped SMILES/SMARTS of the reaction:
[O-:42][N+:40](=[O:41])[c:39]1[cH:38][cH:37][c:36]([O:35][C:34](=[O:33])[C:45]([F:46])([F:47])[F:48])[cH:44][cH:43]1.[CH3:1][CH2:2][CH2:3][CH2:4][CH2:5][CH2:6][C:7]([CH3:8])([CH3:9])[c:10]1[cH:11][c:12]([O:13][CH2:14][c:15]2[cH:16][cH:17][cH:18][cH:19][cH:20]2)[c:21]2[CH:29]([CH2:28][C:25]([CH3:26])([CH3:27])[O:24][c:22]2[cH:23]1)[C:30]([OH:32])=[O:31]>>[OH:32][C:34](=[O:33])[C:45]([F:46])([F:47])[F:48].[CH3:1][CH2:2][CH2:3][CH2:4][CH2:5][CH2:6][C:7]([CH3:9])([CH3:8])[c:10]1[cH:11][c:12]([O:13][CH2:14][c:15]2[cH:16][cH:17][cH:18][cH:19][cH:20]2)[c:21]2[CH:29]([CH2:28][C:25]([CH3:27])([CH3:26])[O:24][c:22]2[cH:23]1)[C:30](=[O:31])[O:35][c:36]1[cH:44][cH:43][c:39]([cH:38][cH:37]1)[N+:40]([O-:42])=[O:41]

Correctness of the mapping
MAPPET YES
ReactionMap NO
Marvin NO

Reaction no 75

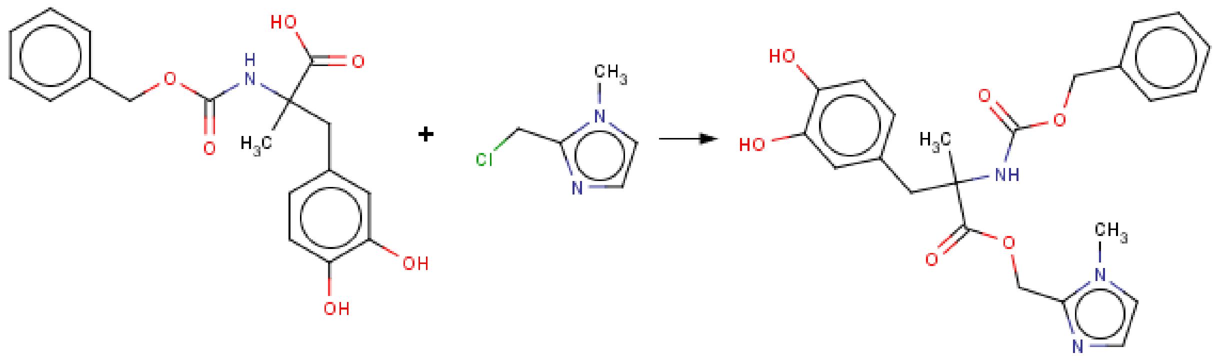

SMILES of the input:
CC(Cc1ccc(O)c(O)c1)(NC(=O)OCc1ccccc1)C(=O)O.Cn1ccnc1CCl>>Cn1ccnc1COC(=O)C(C)(Cc1ccc(O)c(O)c1)NC(=O)OCc1ccccc1

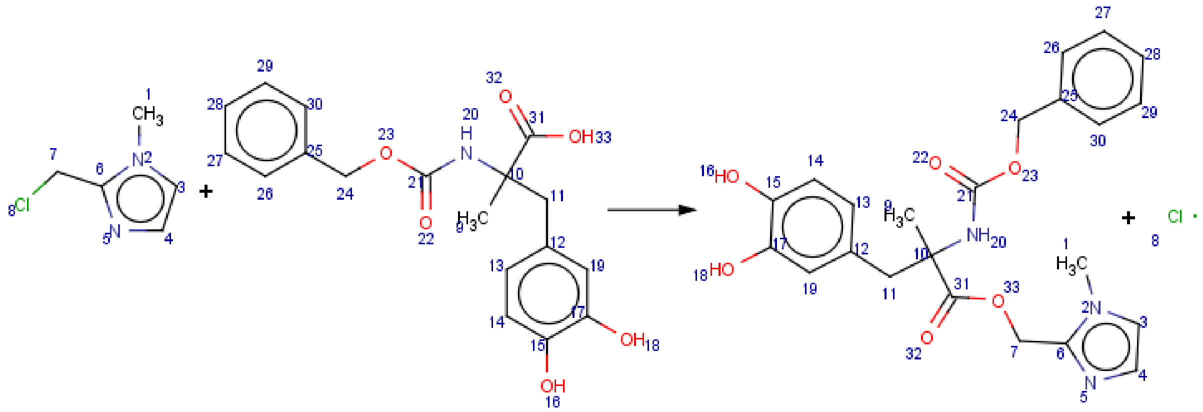

Correct mapped SMILES/SMARTS of the reaction:
[CH3:1][n:2]1[cH:3][cH:4][n:5][c:6]1[CH2:7][Cl:8].[CH3:9][C:10]([CH2:11][c:12]1[cH:13][cH:14][c:15]([OH:16])[c:17]([OH:18])[cH:19]1)([NH:20][C:21](=[O:22])[O:23][CH2:24][c:25]1[cH:26][cH:27][cH:28][cH:29][cH:30]1)[C:31]([OH:33])=[O:32]>>[CH3:1][n:2]1[cH:3][cH:4][n:5][c:6]1[CH2:7][O:33][C:31](=[O:32])[C:10]([CH3:9])([CH2:11][c:12]1[cH:13][cH:14][c:15]([OH:16])[c:17]([OH:18])[cH:19]1)[NH:20][C:21](=[O:22])[O:23][CH2:24][c:25]1[cH:26][cH:27][cH:28][cH:29][cH:30]1.[Cl:8]

Correctness of the mapping
MAPPET YES
ReactionMap NO
Marvin YES

Reaction no 76

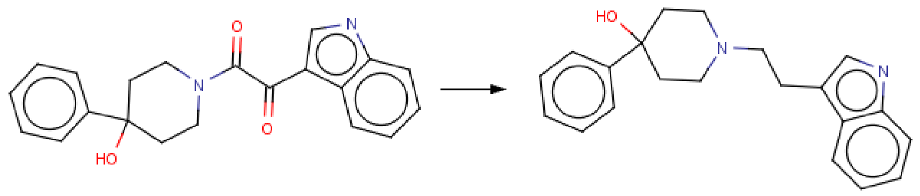

SMILES of the input:
O=C(C(=O)N1CCC(O)(c2ccccc2)CC1)c1cnc2ccccc12>>OC1(c2ccccc2)CCN(CCc2cnc3ccccc23)CC1

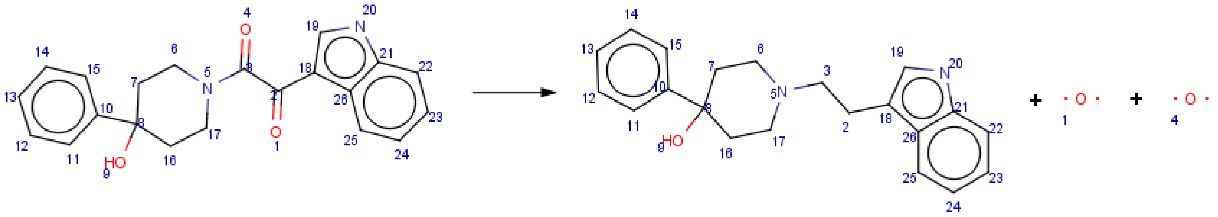

Correct mapped SMILES/SMARTS of the reaction:
[OH:9][C:8]1([CH2:7][CH2:6][N:5]([CH2:17][CH2:16]1)[C:3](=[O:4])[C:2](=[O
:1])[c:18]1[cH:19][n:20][c:21]2[cH:22][cH:23][cH:24][cH:25][c:26]12)[c:10
]1[cH:11][cH:12][cH:13][cH:14][cH:15]1>>[OH:9][C:8]1([CH2:7][CH2:6][N:5](
[CH2:3][CH2:2][c:18]2[cH:19][n:20][c:21]3[cH:22][cH:23][cH:24][cH:25][c:2
6]23)[CH2:17][CH2:16]1)[c:10]1[cH:11][cH:12][cH:13][cH:14][cH:15]1.[O:1].
[O:4]

Correctness of the mapping

MAPPET	YES
ReactionMap	NO
Marvin	YES

Reaction no 77

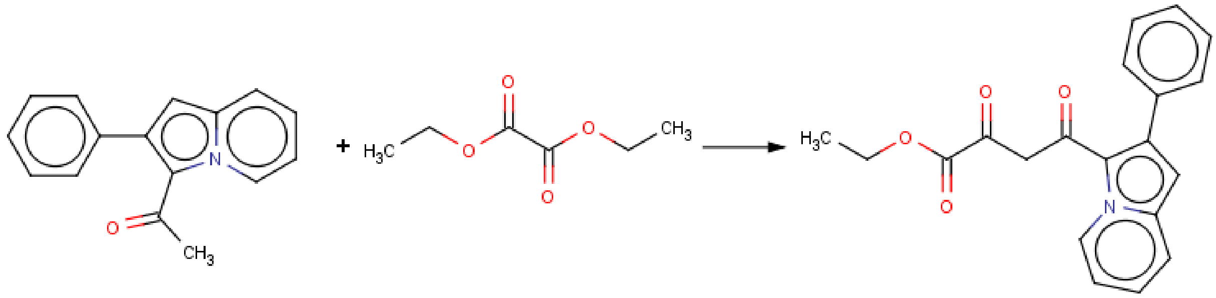

SMILES of the input:
CC(=O)c1c(-c2ccccc2)cc2ccccn21.CCOC(=O)C(=O)OCC>>CCOC(=O)C(=O)CC(=O)c1c(-
c2ccccc2)cc2ccccn21

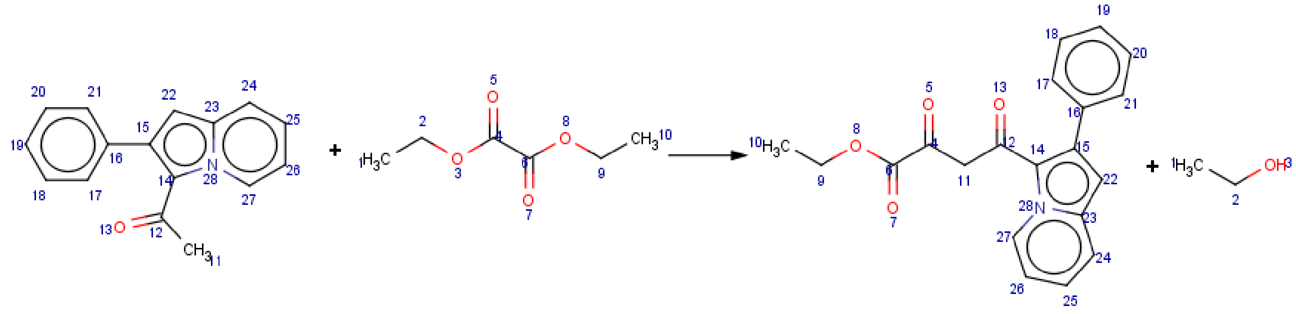

Correct mapped SMILES/SMARTS of the reaction:
[CH3:11][C:12](=[O:13])[c:14]1[c:15]([cH:22][c:23]2[cH:24][cH:25][cH:26][
cH:27][n:28]12)-
[c:16]1[cH:17][cH:18][cH:19][cH:20][cH:21]1.[CH3:1][CH2:2][O:3][C:4](=[O:
5])[C:6](=[O:7])[O:8][CH2:9][CH3:10]>>[CH3:10][CH2:9][O:8][C:6](=[O:7])[C
:4](=[O:5])[CH2:11][C:12](=[O:13])[c:14]1[c:15]([cH:22][c:23]2[cH:24][cH:
25][cH:26][cH:27][n:28]12)-
[c:16]1[cH:17][cH:18][cH:19][cH:20][cH:21]1.[CH3:1][CH2:2][OH:3]

Correctness of the mapping
MAPPET YES
ReactionMap NO
Marvin YES

Reaction no 78

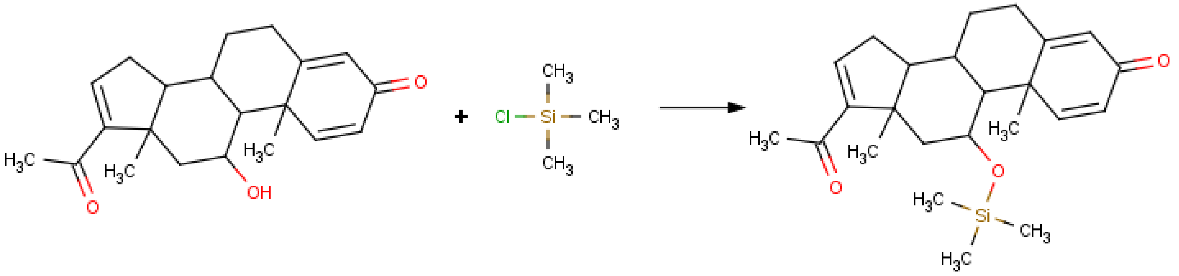

SMILES of the input:
CC(=O)C1=CCC2C3CCC4=CC(=O)C=CC4(C)C3C(O)CC12C.C[Si](C)(C)Cl>>CC(=O)C1=CCC2C3CCC4=CC(=O)C=CC4(C)C3C(O[Si](C)(C)C)CC12C

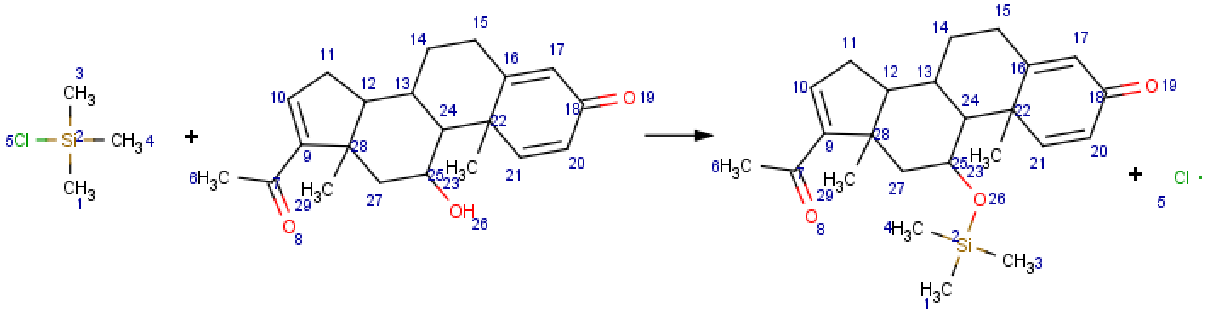

Correct mapped SMILES/SMARTS of the reaction:
[CH3:1][Si:2]([CH3:3])([CH3:4])[Cl:5].[CH3:6][C:7](=[O:8])[C:9]1=[CH:10][CH2:11][CH:12]2[CH:13]3[CH2:14][CH2:15][C:16]4=[CH:17][C:18](=[O:19])[CH:20]=[CH:21][C:22]4([CH3:23])[CH:24]3[CH:25]([OH:26])[CH2:27][C:28]12[CH3:29]>>[CH3:6][C:7](=[O:8])[C:9]1=[CH:10][CH2:11][CH:12]2[CH:13]3[CH2:14][CH2:15][C:16]4=[CH:17][C:18](=[O:19])[CH:20]=[CH:21][C:22]4([CH3:23])[CH:24]3[CH:25]([CH2:27][C:28]12[CH3:29])[O:26][Si:2]([CH3:4])([CH3:3])[CH3:1].[Cl:5]

Correctness of the mapping
MAPPET YES
ReactionMap NO
Marvin YES

Reaction no 79

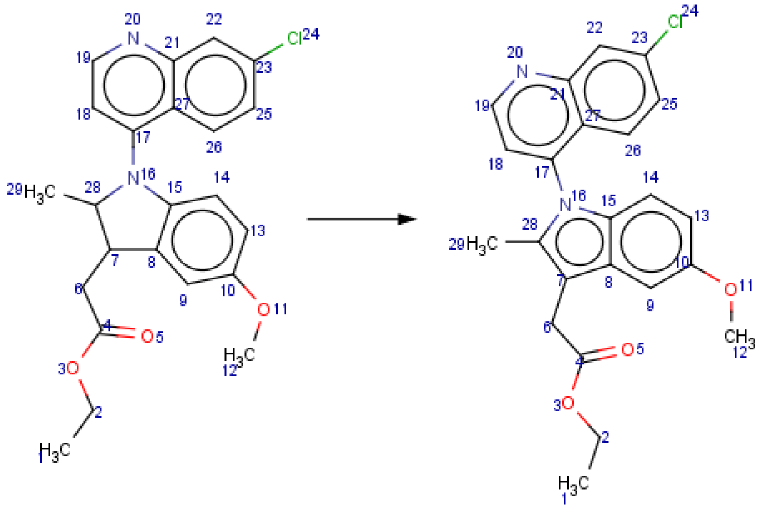

Correct mapped SMILES/SMARTS of the reaction:
[CH3:1][CH2:2][O:3][C:4](=[O:5])[CH2:6][CH:7]1[CH:28]([CH3:29])[N:16]([c:15]2[cH:14][cH:13][c:10]([O:11][CH3:12])[cH:9][c:8]12)[c:17]1[cH:18][cH:19][n:20][c:21]2[cH:22][c:23]([Cl:24])[cH:25][cH:26][c:27]12>>[CH3:1][CH2:2][O:3][C:4](=[O:5])[CH2:6][c:7]1[c:28]([CH3:29])[n:16](-[c:17]2[cH:18][cH:19][n:20][c:21]3[cH:22][c:23]([Cl:24])[cH:25][cH:26][c:27]23)[c:15]2[cH:14][cH:13][c:10]([O:11][CH3:12])[cH:9][c:8]12

Correctness of the mapping

MAPPET	YES
ReactionMap	YES
Marvin	YES

Reaction no 80

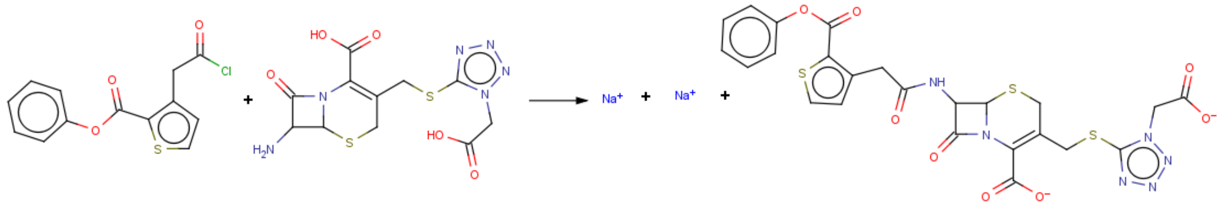

SMILES of the input:
O=C(Cl)Cc1ccsc1C(=O)Oc1ccccc1.NC1C(=O)N2C(C(=O)O)=C(CSc3nnnn3CC(=O)O)CSC12>>[Na+].[Na+].O=C([O-])Cn1nnnc1SCC1=C(C(=O)[O-])N2C(=O)C(NC(=O)Cc3ccsc3C(=O)Oc3ccccc3)C2SC1

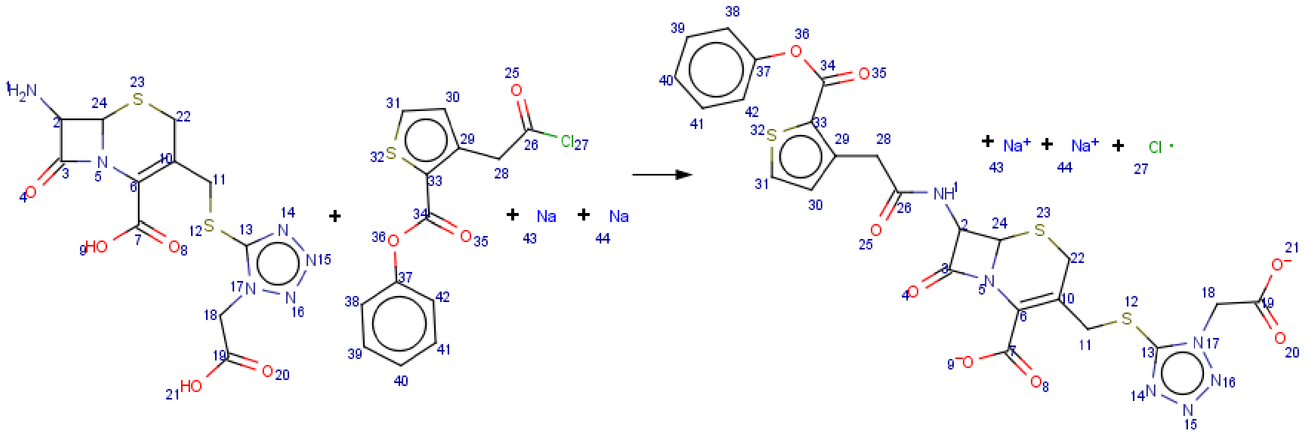

Correct mapped SMILES/SMARTS of the reaction:
[NH2:1][CH:2]1[CH:24]2[S:23][CH2:22][C:10]([CH2:11][S:12][c:13]3[n:14][n:15][n:16][n:17]3[CH2:18][C:19]([OH:21])=[O:20])=[C:6]([N:5]2[C:3]1=[O:4])[C:7]([OH:9])=[O:8].[Cl:27][C:26](=[O:25])[CH2:28][c:29]1[cH:30][cH:31][s:32][c:33]1[C:34](=[O:35])[O:36][c:37]1[cH:38][cH:39][cH:40][cH:41][cH:42]1.[Na:43].[Na:44]>>[O-:21][C:19](=[O:20])[CH2:18][n:17]1[n:16][n:15][n:14][c:13]1[S:12][CH2:11][C:10]1=[C:6]([N:5]2[CH:24]([S:23][CH2:22]1)[CH:2]([NH:1][C:26](=[O:25])[CH2:28][c:29]1[cH:30][cH:31][s:32][c:33]1[C:34](=[O:35])[O:36][c:37]1[cH:42][cH:41][cH:40][cH:39][cH:38]1)[C:3]2=[O:4])[C:7]([O-:9])=[O:8].[Na+:43].[Na+:44].[Cl:27]

Correctness of the mapping

MAPPET	YES
ReactionMap	NO
Marvin	YES

Reaction no 81

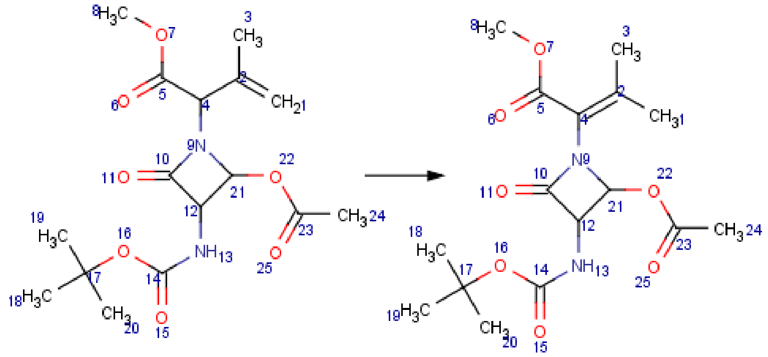

Correct mapped SMILES/SMARTS of the reaction:
[CH3:8][O:7][C:5](=[O:6])[CH:4]([N:9]1[CH:21]([O:22][C:23]([CH3:24])=[O:25])[CH:12]([NH:13][C:14](=[O:15])[O:16][C:17]([CH3:18])([CH3:19])[CH3:20])[C:10]1=[O:11])[C:2]([CH3:3])=[CH2:1]>>[CH3:8][O:7][C:5](=[O:6])[C:4]([N:9]1[CH:21]([O:22][C:23]([CH3:24])=[O:25])[CH:12]([NH:13][C:14](=[O:15])[O:16][C:17]([CH3:19])([CH3:18])[CH3:20])[C:10]1=[O:11])=[C:2]([CH3:1])[CH3:3]

Correctness of the mapping

MAPPET	YES
ReactionMap	YES
Marvin	YES

Reaction no 82

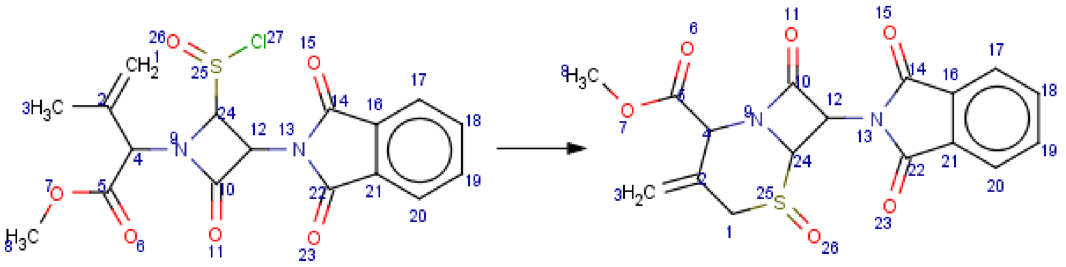

Correct mapped SMILES/SMARTS of the reaction:
[CH3:8][O:7][C:5](=[O:6])[CH:4]([N:9]1[CH:24]([CH:12]([N:13]2[C:14](=[O:15])[c:16]3[cH:17][cH:18][cH:19][cH:20][c:21]3[C:22]2=[O:23])[C:10]1=[O:11])[S:25]([Cl:27])=[O:26])[C:2]([CH3:3])=[CH2:1]>>[CH3:8][O:7][C:5](=[O:6])[CH:4]1[N:9]2[CH:24]([CH:12]([N:13]3[C:22](=[O:23])[c:21]4[cH:20][cH:19][cH:18][cH:17][c:16]4[C:14]3=[O:15])[C:10]2=[O:11])[S:25](=[O:26])[CH2:1][C:2]1=[CH2:3].[Cl:27]

Correctness of the mapping

MAPPET	NO
ReactionMap	NO
Marvin	NO

Reaction no 83

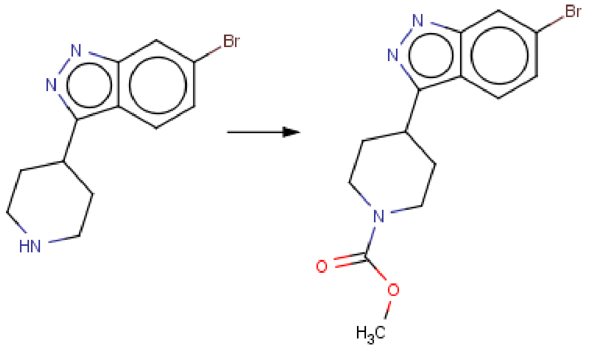

SMILES of the input:
Brc1ccc2c(c1)nnc2C1CCNCC1>>COC(=O)N1CCC(c2nnc3cc(Br)ccc32)CC1

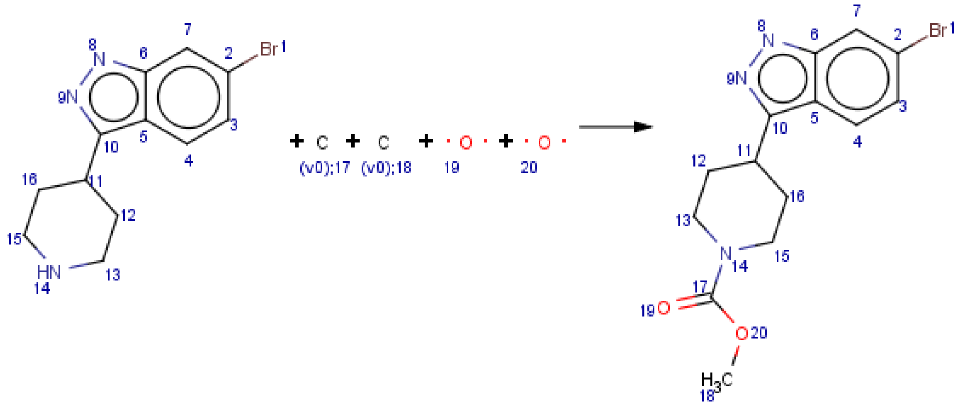

Correct mapped SMILES/SMARTS of the reaction:
[Br:1][c:2]1[cH:3][cH:4][c:5]2[c:10]([n:9][n:8][c:6]2[cH:7]1)[CH:11]1[CH2:12][CH2:13][NH:14][CH2:15][CH2:16]1.[C:17].[C:18].[O:19].[O:20]>>[CH3:18][O:20][C:17](=[O:19])[N:14]1[CH2:13][CH2:12][CH:11]([CH2:16][CH2:15]1)[c:10]1[n:9][n:8][c:6]2[cH:7][c:2]([Br:1])[cH:3][cH:4][c:5]12

Correctness of the mapping

MAPPET	YES
ReactionMap	NO
Marvin	YES

Reaction no 84

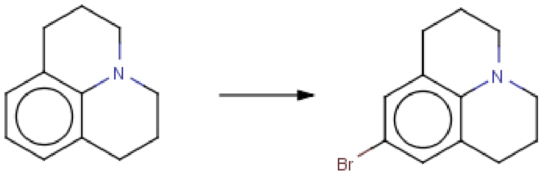

SMILES of the input:
c1cc2c3c(c1)CCCN3CCC2>>Brc1cc2c3c(c1)CCCN3CCC2

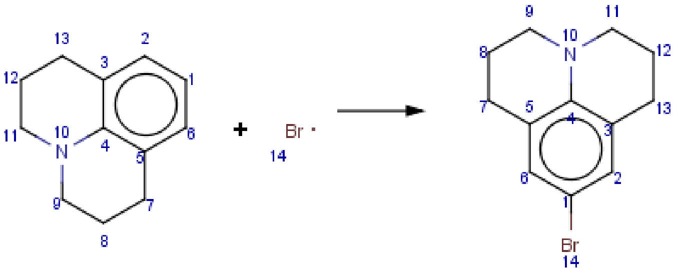

Correct mapped SMILES/SMARTS of the reaction:
[CH2:8]1[CH2:9][N:10]2[CH2:11][CH2:12][CH2:13][c:3]3[cH:2][cH:1][cH:6][c:
5]([CH2:7]1)[c:4]23.[Br:14]>>[Br:14][c:1]1[cH:6][c:5]2[CH2:7][CH2:8][CH2:
9][N:10]3[CH2:11][CH2:12][CH2:13][c:3]([cH:2]1)[c:4]23

Correctness of the mapping
MAPPET YES
ReactionMap NO
Marvin YES

Reaction no 85

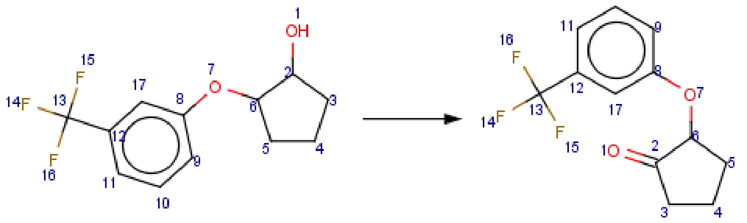

Correct mapped SMILES/SMARTS of the reaction:
[OH:1][CH:2]1[CH2:3][CH2:4][CH2:5][CH:6]1[O:7][c:8]1[cH:9][cH:10][cH:11][
c:12]([cH:17]1)[C:13]([F:14])([F:15])[F:16]>>[F:14][C:13]([F:15])([F:16])
[c:12]1[cH:11][cH:10][cH:9][c:8]([O:7][CH:6]2[CH2:5][CH2:4][CH2:3][C:2]2=
[O:1])[cH:17]1

Correctness of the mapping
MAPPET YES
ReactionMap YES
Marvin YES
Reaction no 86

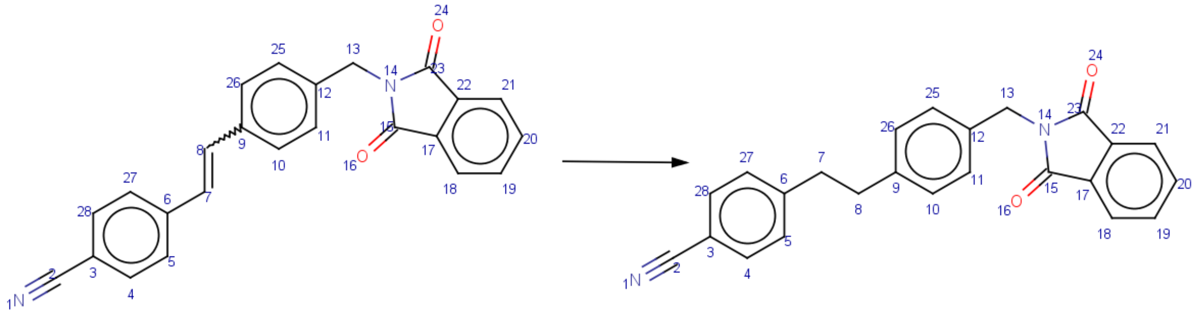

Correct mapped SMILES/SMARTS of the reaction:
[O:16]=[C:15]1[N:14]([CH2:13][c:12]2[cH:11][cH:10][c:9]([CH:8]=[CH:7][c:6
]3[cH:5][cH:4][c:3]([cH:28][cH:27]3)[C:2]#[N:1])[cH:26][cH:25]2)[C:23](=[
O:24])[c:22]2[cH:21][cH:20][cH:19][cH:18][c:17]12>>[O:16]=[C:15]1[N:14]([
CH2:13][c:12]2[cH:11][cH:10][c:9]([CH2:8][CH2:7][c:6]3[cH:5][cH:4][c:3]([
cH:28][cH:27]3)[C:2]#[N:1])[cH:26][cH:25]2)[C:23](=[O:24])[c:22]2[cH:21][
cH:20][cH:19][cH:18][c:17]12

Correctness of the mapping
MAPPET YES
ReactionMap YES
Marvin YES

Reaction no 87

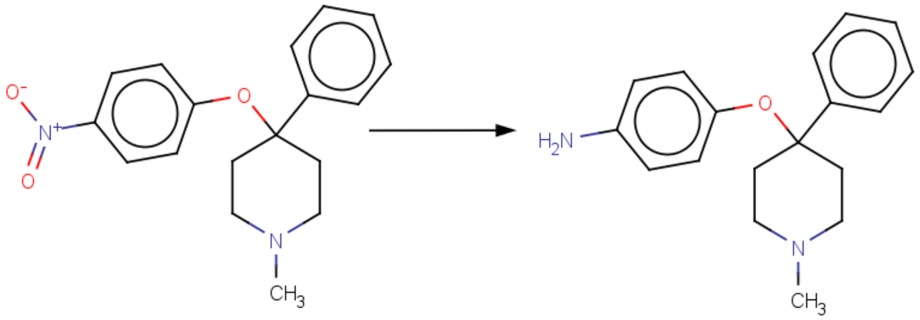

SMILES of the input:
CN1CCC(Oc2ccc([N+](=O)[O-
])cc2)(c2ccccc2)CC1>>CN1CCC(Oc2ccc(N)cc2)(c2ccccc2)CC1

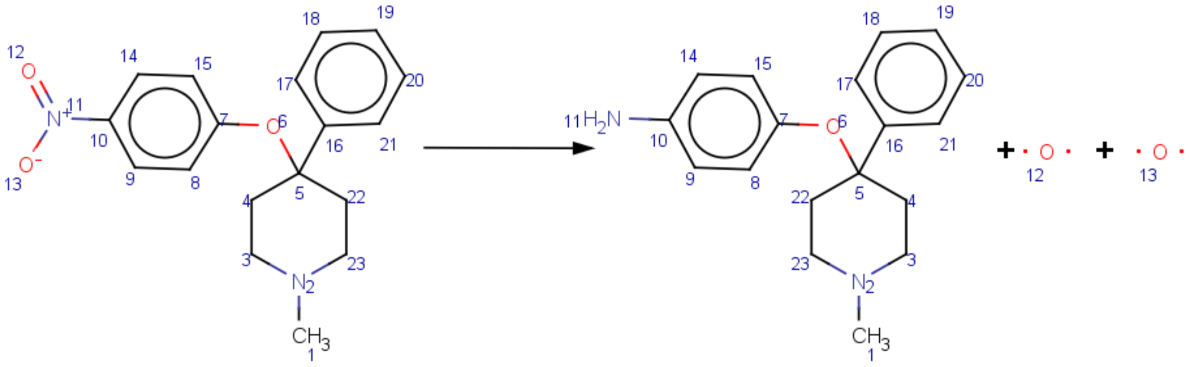

Correct mapped SMILES/SMARTS of the reaction:
[CH3:1][N:2]1[CH2:3][CH2:4][C:5]([CH2:22][CH2:23]1)([O:6][c:7]1[cH:8][cH:
9][c:10]([cH:14][cH:15]1)[N+:11]([O-
:13])=[O:12])[c:16]1[cH:17][cH:18][cH:19][cH:20][cH:21]1>>[CH3:1][N:2]1[C
H2:23][CH2:22][C:5]([CH2:4][CH2:3]1)([O:6][c:7]1[cH:8][cH:9][c:10]([NH2:1
1])[cH:14][cH:15]1)[c:16]1[cH:17][cH:18][cH:19][cH:20][cH:21]1.[O:12].[O:
13]

Correctness of the mapping

MAPPET	YES
ReactionMap	NO
Marvin	YES

Reaction no 88

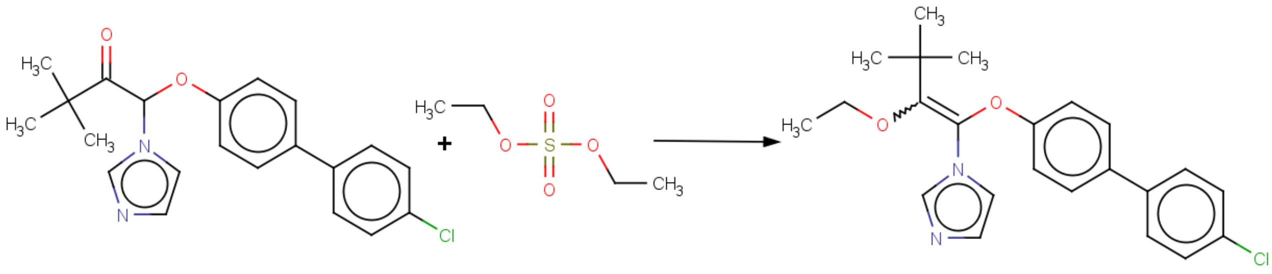

SMILES of the input:
CC(C)(C)C(=O)C(Oc1ccc(-
c2ccc(Cl)cc2)cc1)n1ccnc1.CCOS(=O)(=O)OCC>>CCOC(=C(Oc1ccc(-
c2ccc(Cl)cc2)cc1)n1ccnc1)C(C)(C)C

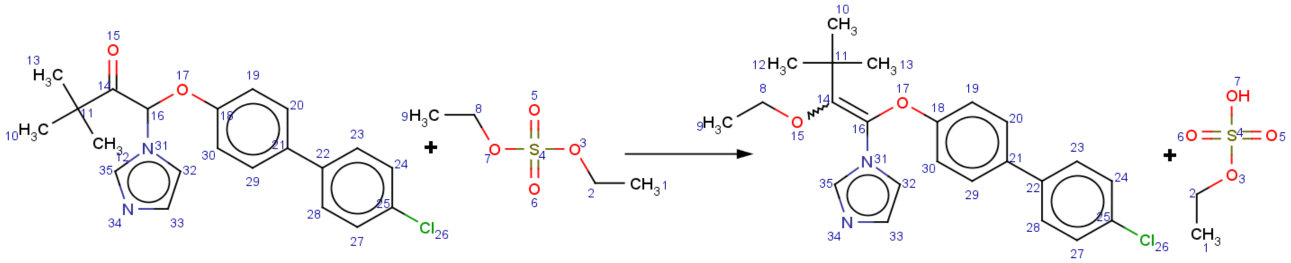

Correct mapped SMILES/SMARTS of the reaction:
[CH3:10][C:11]([CH3:12])([CH3:13])[C:14](=[O:15])[CH:16]([O:17][c:18]1[cH:19][cH:20][c:21]([cH:29][cH:30]1)-
[c:22]1[cH:23][cH:24][c:25]([Cl:26])[cH:27][cH:28]1)[n:31]1[cH:32][cH:33][n:34][cH:35]1.[CH3:1][CH2:2][O:3][S:4](=[O:5])(=[O:6])[O:7][CH2:8][CH3:9]>>[CH3:9][CH2:8][O:15][C:14](=[C:16]([O:17][c:18]1[cH:19][cH:20][c:21]([cH:29][cH:30]1)-
[c:22]1[cH:23][cH:24][c:25]([Cl:26])[cH:27][cH:28]1)[n:31]1[cH:32][cH:33][n:34][cH:35]1)[C:11]([CH3:10])([CH3:13])[CH3:12].[CH3:1][CH2:2][O:3][S:4]([OH:7])(=[O:5])=[O:6]

Correctness of the mapping

MAPPET	YES
ReactionMap	NO
Marvin	NO

Reaction no 89

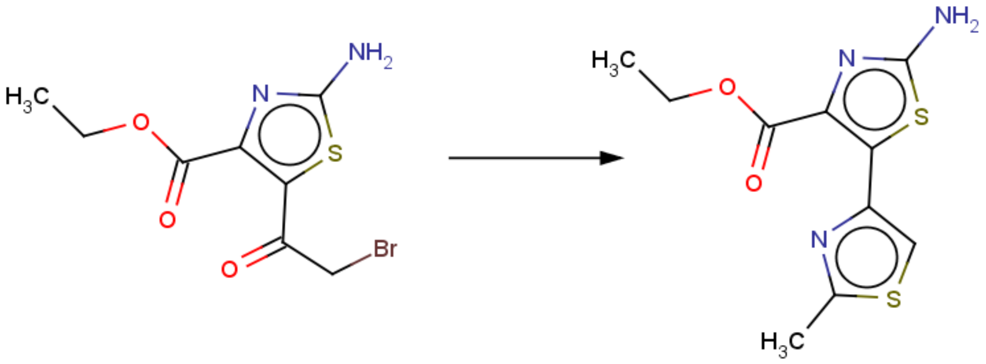

SMILES of the input:
CCOC(=O)c1nc(N)sc1C(=O)CBr>>CCOC(=O)c1nc(N)sc1-c1csc(C)n1

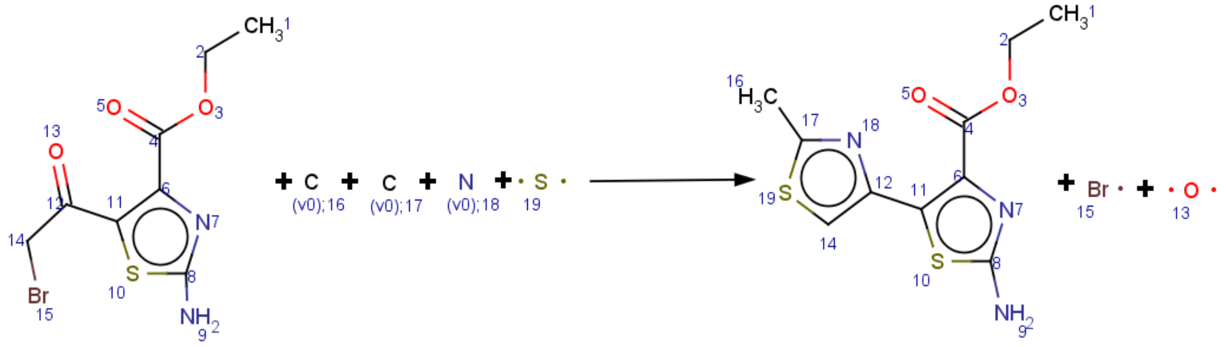

Correct mapped SMILES/SMARTS of the reaction:
[CH3:1][CH2:2][O:3][C:4](=[O:5])[c:6]1[n:7][c:8]([NH2:9])[s:10][c:11]1[C:12](=[O:13])[CH2:14][Br:15].[C:16].[C:17].[N:18].[S:19]>>[CH3:1][CH2:2][O:3][C:4](=[O:5])[c:6]1[n:7][c:8]([NH2:9])[s:10][c:11]1-
[c:12]1[cH:14][s:19][c:17]([CH3:16])[n:18]1.[Br:15].[O:13]

Correctness of the mapping
MAPPET YES
ReactionMap NO
Marvin YES

Reaction no 90

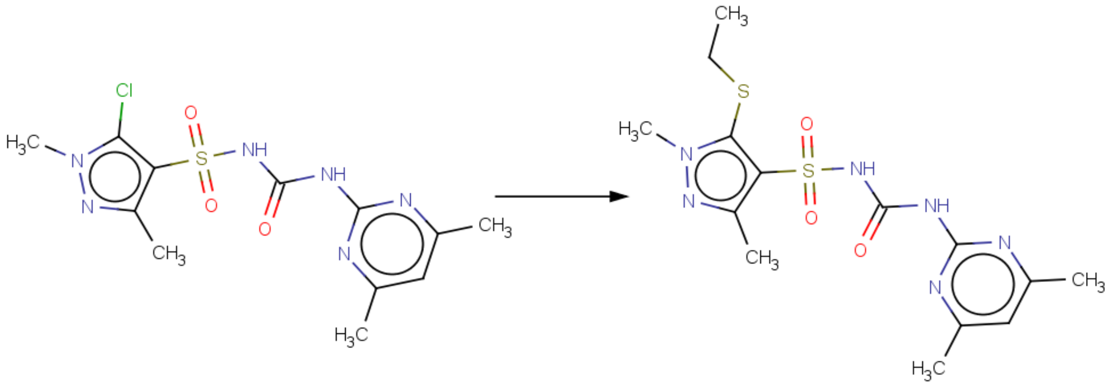

SMILES of the input:
Cc1nn(C)c(Cl)c1S(=O)(=O)NC(=O)Nc1nc(C)cc(C)n1>>CCSc1c(S(=O)(=O)NC(=O)Nc2nc(C)cc(C)n2)c(C)nn1C

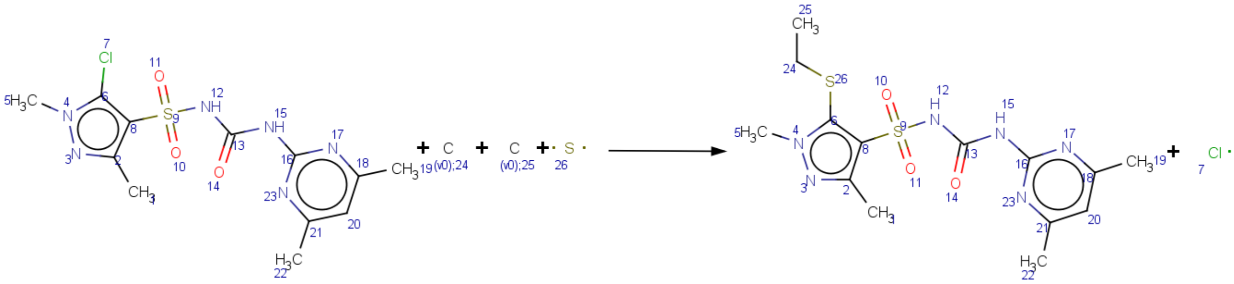

Correct mapped SMILES/SMARTS of the reaction:
[CH3:1][c:2]1[n:3][n:4]([CH3:5])[c:6]([Cl:7])[c:8]1[S:9](=[O:10])(=[O:11])[NH:12][C:13](=[O:14])[NH:15][c:16]1[n:17][c:18]([CH3:19])[cH:20][c:21]([CH3:22])[n:23]1.[C:24].[C:25].[S:26]>>[CH3:25][CH2:24][S:26][c:6]1[c:8]([c:2]([CH3:1])[n:3][n:4]1[CH3:5])[S:9](=[O:11])(=[O:10])[NH:12][C:13](=[O:14])[NH:15][c:16]1[n:17][c:18]([CH3:19])[cH:20][c:21]([CH3:22])[n:23]1.[Cl:7]

Correctness of the mapping
MAPPET YES
ReactionMap NO
Marvin YES

Reaction no 91

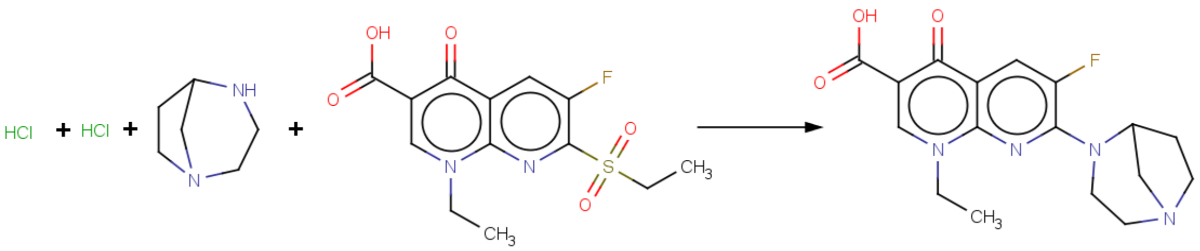

SMILES of the input:
Cl.Cl.C1CN2CC1NCC2.CCn1cc(C(=O)O)c(=O)c2cc(F)c(S(=O)(=O)CC)nc21>>CCn1cc(C(=O)O)c(=O)c2cc(F)c(N3CCN4CCC3C4)nc21

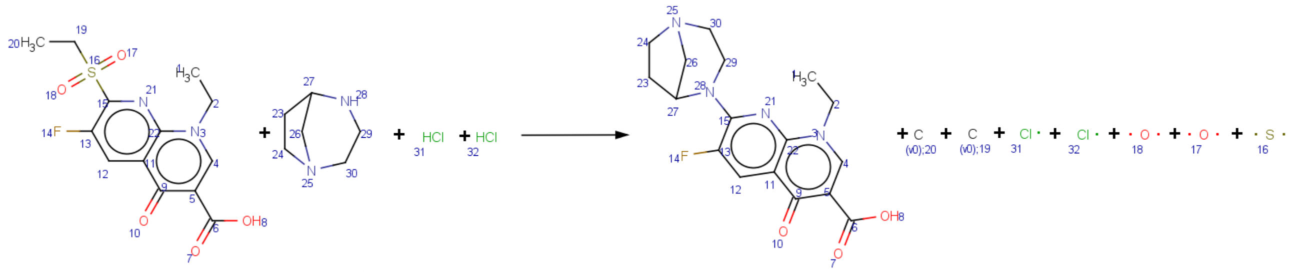

Correct mapped SMILES/SMARTS of the reaction:
[CH3:1][CH2:2][n:3]1[cH:4][c:5]([C:6]([OH:8])=[O:7])[c:9](=[O:10])[c:11]2
[cH:12][c:13]([F:14])[c:15]([n:21][c:22]12)[S:16](=[O:17])(=[O:18])[CH2:1
9][CH3:20].[CH2:23]1[CH2:24][N:25]2[CH2:26][CH:27]1[NH:28][CH2:29][CH2:30
]2.[ClH:31].[ClH:32]>>[CH3:1][CH2:2][n:3]1[cH:4][c:5]([C:6]([OH:8])=[O:7]
)[c:9](=[O:10])[c:11]2[cH:12][c:13]([F:14])[c:15]([n:21][c:22]12)[N:28]1[
CH2:29][CH2:30][N:25]2[CH2:24][CH2:23][CH:27]1[CH2:26]2.[C:20].[C:19].[Cl
:31].[Cl:32].[O:18].[O:17].[S:16]

Correctness of the mapping

MAPPET	YES
ReactionMap	NO
Marvin	YES

Reaction no 92

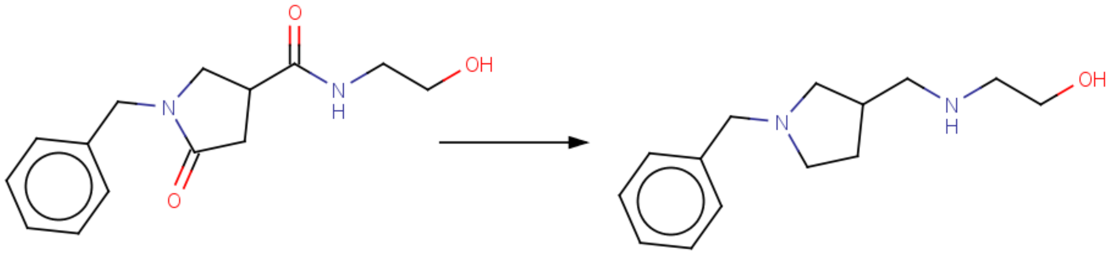

SMILES of the input:
O=C(NCCO)C1CC(=O)N(Cc2ccccc2)C1>>OCCNCC1CCN(Cc2ccccc2)C1

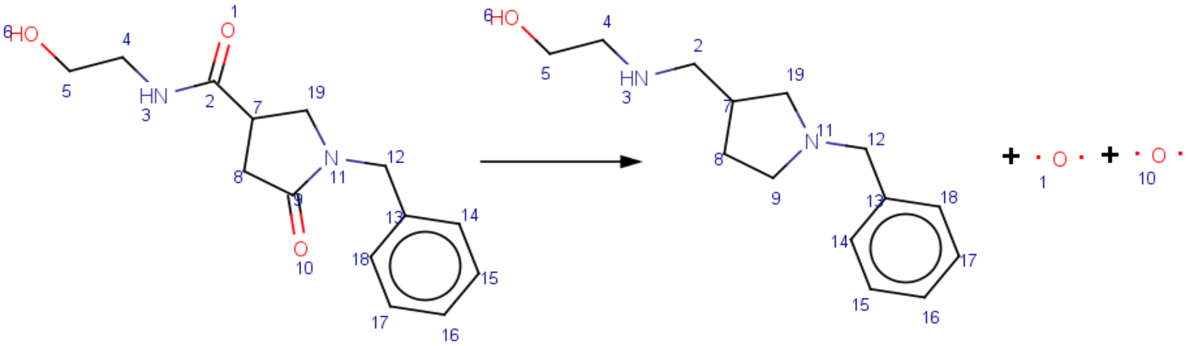

Correct mapped SMILES/SMARTS of the reaction:
[OH:6][CH2:5][CH2:4][NH:3][C:2](=[O:1])[CH:7]1[CH2:19][N:11]([CH2:12][c:1
3]2[cH:14][cH:15][cH:16][cH:17][cH:18]2)[C:9](=[O:10])[CH2:8]1>>[OH:6][CH
2:5][CH2:4][NH:3][CH2:2][CH:7]1[CH2:8][CH2:9][N:11]([CH2:12][c:13]2[cH:14
][cH:15][cH:16][cH:17][cH:18]2)[CH2:19]1.[O:1].[O:10]

Correctness of the mapping

MAPPET	YES
ReactionMap	NO
Marvin	YES

Reaction no 93

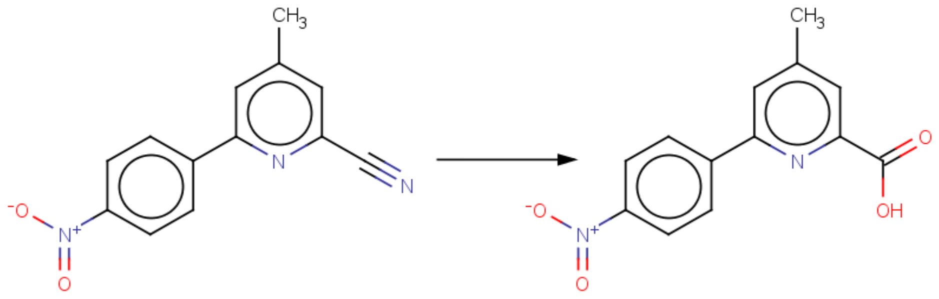

SMILES of the input:
Cc1cc(C#N)nc(-c2ccc([N+](=O)[O-])cc2)c1>>Cc1cc(C(=O)O)nc(-c2ccc([N+](=O)[O-])cc2)c1

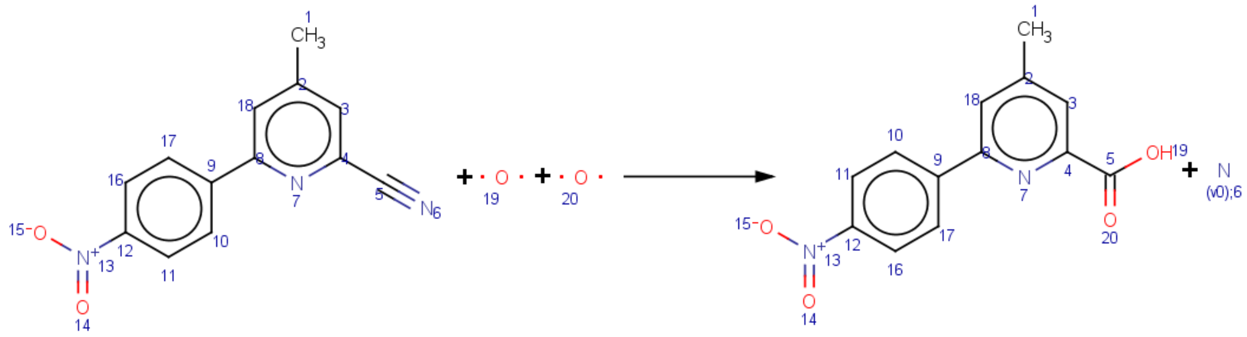

Correct mapped SMILES/SMARTS of the reaction:
[CH3:1][c:2]1[cH:3][c:4]([n:7][c:8]([cH:18]1)-[c:9]1[cH:10][cH:11][c:12]([cH:16][cH:17]1)[N+:13]([O-:15])=[O:14])[C:5]#[N:6].[O:19].[O:20]>>[CH3:1][c:2]1[cH:3][c:4]([n:7][c:8]([cH:18]1)-[c:9]1[cH:17][cH:16][c:12]([cH:11][cH:10]1)[N+:13]([O-:15])=[O:14])[C:5]([OH:19])=[O:20].[N:6]

Correctness of the mapping

MAPPET	YES
ReactionMap	NO
Marvin	YES

Reaction no 94

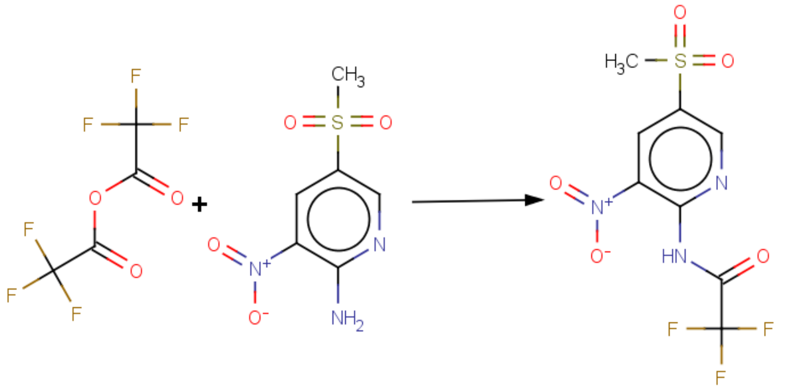

SMILES of the input:
O=C(OC(=O)C(F)(F)F)C(F)(F)F.CS(=O)(=O)c1cnc(N)c([N+](=O)[O-])c1>>CS(=O)(=O)c1cnc(NC(=O)C(F)(F)F)c([N+](=O)[O-])c1

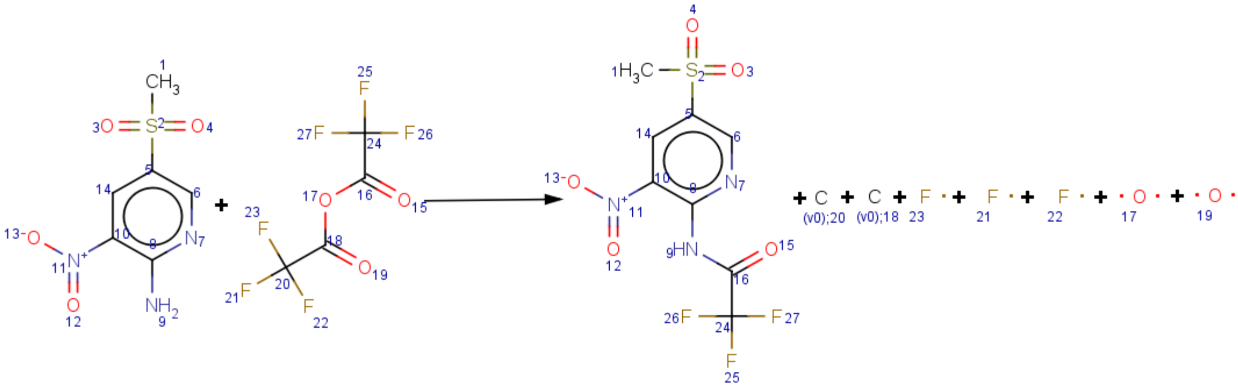

Correct mapped SMILES/SMARTS of the reaction:
[CH3:1][S:2](=[O:3])(=[O:4])[c:5]1[cH:6][n:7][c:8]([NH2:9])[c:10]([cH:14]1)[N+:11]([O-:13])=[O:12].[F:21][C:20]([F:22])([F:23])[C:18](=[O:19])[O:17][C:16](=[O:15])[C:24]([F:25])([F:26])[F:27]>>[CH3:1][S:2](=[O:3])(=[O:4])[c:5]1[cH:6][n:7][c:8]([NH:9][C:16](=[O:15])[C:24]([F:25])([F:26])[F:27])[c:10]([cH:14]1)[N+:11]([O-:13])=[O:12].[C:20].[C:18].[F:23].[F:21].[F:22].[O:17].[O:19]

Correctness of the mapping

MAPPET	YES
ReactionMap	NO
Marvin	YES

Reaction no 95

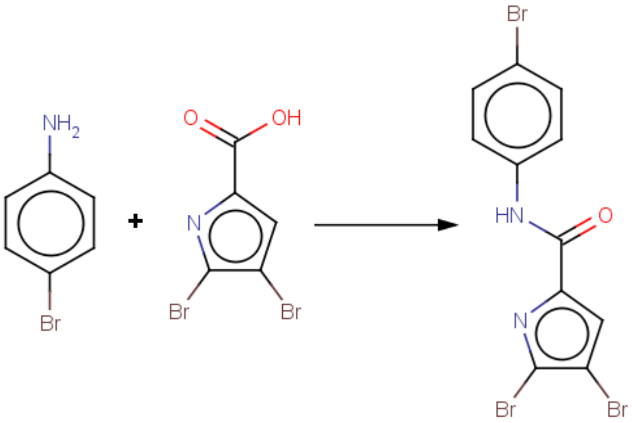

SMILES of the input:
Nc1ccc(Br)cc1.O=C(O)c1cc(Br)c(Br)n1>>O=C(Nc1ccc(Br)cc1)c1cc(Br)c(Br)n1

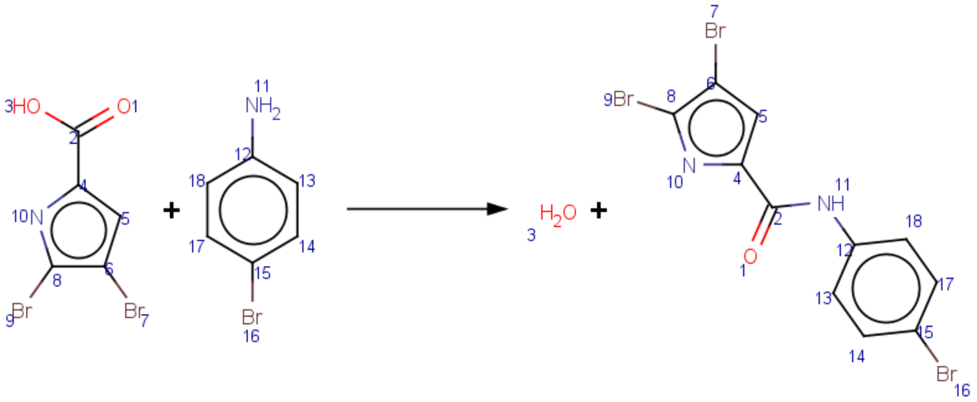

Correct mapped SMILES/SMARTS of the reaction:

[OH:3][C:2](=[O:1])[c:4]1[cH:5][c:6]([Br:7])[c:8]([Br:9])[n:10]1.[NH2:11][c:12]1[cH:13][cH:14][c:15]([Br:16])[cH:17][cH:18]1>>[OH2:3].[Br:7][c:6]1[cH:5][c:4]([n:10][c:8]1[Br:9])[C:2](=[O:1])[NH:11][c:12]1[cH:18][cH:17][c:15]([Br:16])[cH:14][cH:13]1

Correctness of the mapping

MAPPET	YES
ReactionMap	NO
Marvin	YES

Reaction no 96

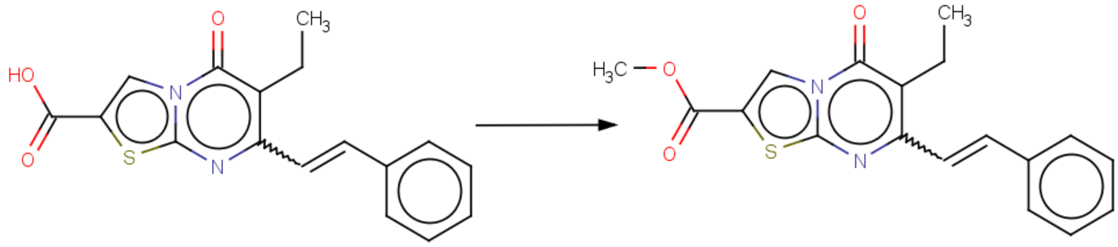

SMILES of the input:
CCc1c(C=Cc2ccccc2)nc2sc(C(=O)O)cn2c1=O>>CCc1c(C=Cc2ccccc2)nc2sc(C(=O)OC)cn2c1=O

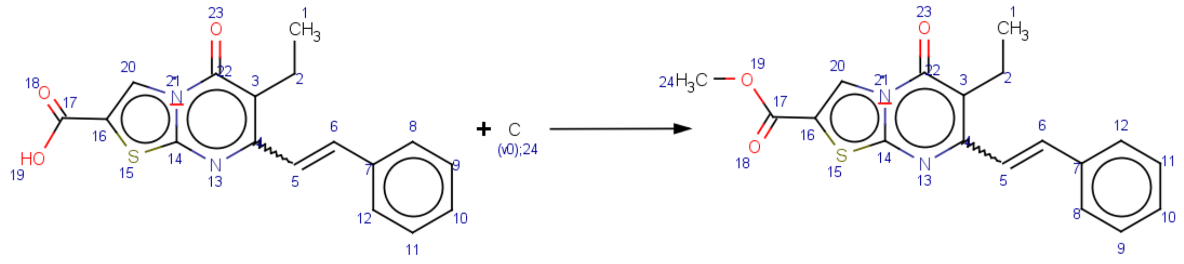

Correct mapped SMILES/SMARTS of the reaction:
[CH3:1][CH2:2][c:3]1[c:4]([CH:5]=[CH:6][c:7]2[cH:8][cH:9][cH:10][cH:11][cH:12]2)[n:13][c:14]2[s:15][c:16]([cH:20][n:21]2[c:22]1=[O:23])[C:17]([OH:19])=[O:18].[C:24]>>[CH3:1][CH2:2][c:3]1[c:4]([CH:5]=[CH:6][c:7]2[cH:12][cH:11][cH:10][cH:9][cH:8]2)[n:13][c:14]2[s:15][c:16]([cH:20][n:21]2[c:22]1=[O:23])[C:17](=[O:18])[O:19][CH3:24]

Correctness of the mapping

MAPPET	YES
ReactionMap	NO
Marvin	YES

Reaction no 97

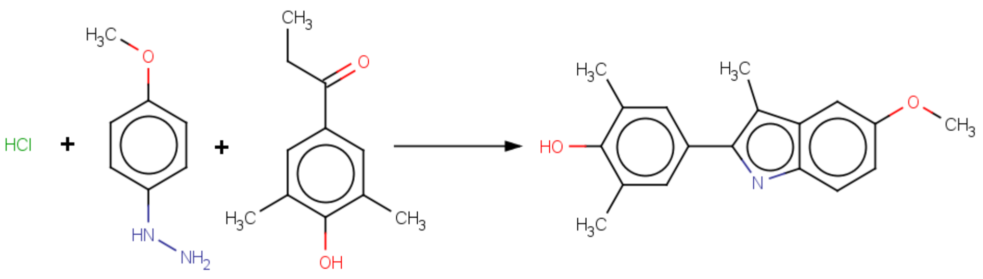

SMILES of the input:
Cl.COc1ccc(NN)cc1.CCC(=O)c1cc(C)c(O)c(C)c1>>COc1ccc2nc(-c3cc(C)c(O)c(C)c3)c(C)c2c1

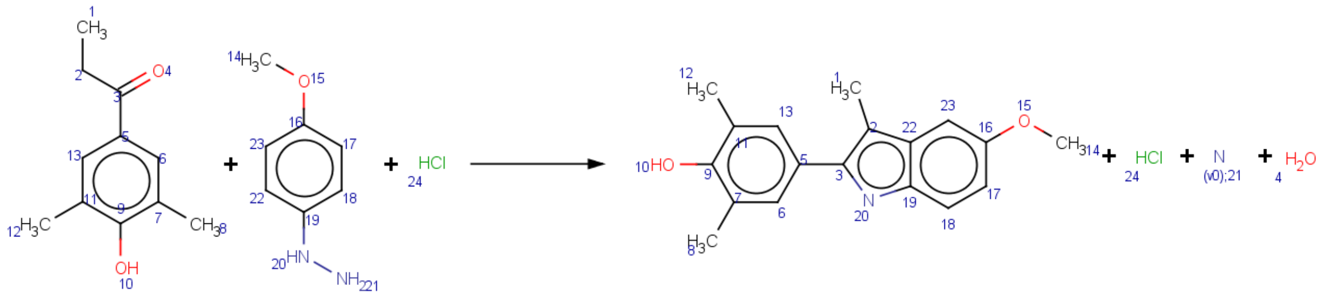

Correct mapped SMILES/SMARTS of the reaction:
[#6:1]-[#6:2]-[#6:3](=[O:4])-[c:5]1[c:6][c:7](-[#6:8])[c:9](-[#8:10])[c:11](-[#6:12])[c:13]1.[#6:14]-[#8:15]-[c:16]1[c:17][c:18][c:19](-[#7:20]-[#7:21])[c:22][c:23]1.[Cl:24]>>[#6:14]-[#8:15]-[c:16]1[c:17][c:18][c:19]2[n:20][c:3]([c:2](-[#6:1])[c:22]2[c:23]1)-[c:5]1[c:6][c:7](-[#6:8])[c:9](-[#8:10])[c:11](-[#6:12])[c:13]1.[Cl:24].[#7;v0:21].[#8:4]

Correctness of the mapping

MAPPET	YES
ReactionMap	NO
Marvin	YES

Reaction no 98

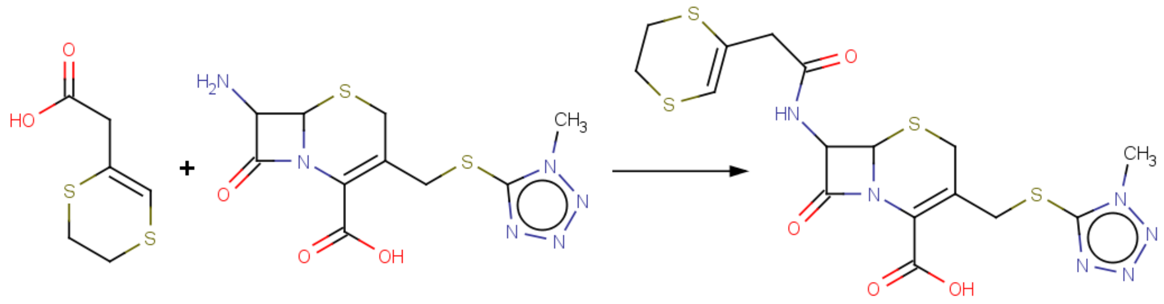

SMILES of the input:
O=C(O)CC1=CSCCS1.Cn1nnnc1SCC1=C(C(=O)O)N2C(=O)C(N)C2SC1>>Cn1nnnc1SCC1=C(C(=O)O)N2C(=O)C(NC(=O)CC3=CSCCS3)C2SC1

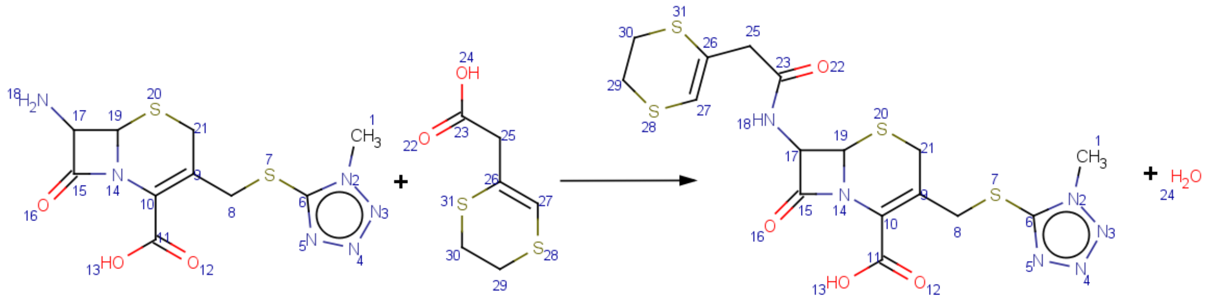

Correct mapped SMILES/SMARTS of the reaction:
[#6:1]-[n:2]1[n:3][n:4][n:5][c:6]1-[#16:7]-[#6:8]-[#6:9]-1=[#6:10](-[#7:14]-2-[#6:19](-[#16:20]-[#6:21]-1)-[#6:17](-[#7:18])-[#6:15]-2=[O:16])-[#6:11](-[#8:13])=[O:12].[#8:24]-[#6:23](=[O:22])-[#6:25]-[#6:26]-1=[#6:27]-[#16:28]-[#6:29]-[#6:30]-[#16:31]-1>>[#8:24].[#6:1]-

[n:2]1[n:3][n:4][n:5][c:6]1-[#16:7]-[#6:8]-[#6:9]-1=[#6:10](-[#7:14]-2-[#6:19](-[#16:20]-[#6:21]-1)-[#6:17](-[#7:18]-[#6:23](=[O:22])-[#6:25]-[#6:26]-1=[#6:27]-[#16:28]-[#6:29]-[#6:30]-[#16:31]-1)-[#6:15]-2=[O:16])-[#6:11](-[#8:13])=[O:12]

Correctness of the mapping
MAPPET YES
ReactionMap NO
Marvin YES

Reaction no 99

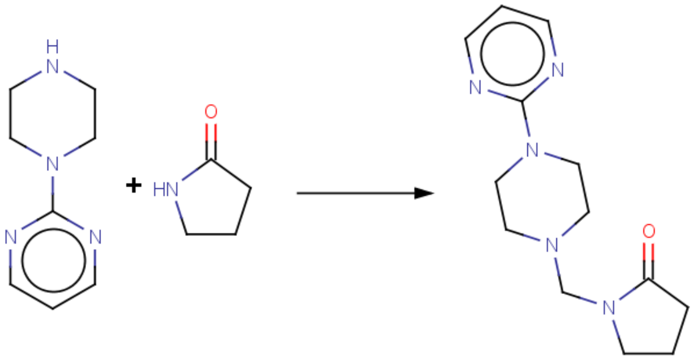

SMILES of the input:
c1cnc(N2CCNCC2)nc1.O=C1CCCN1>>O=C1CCCN1CN1CCN(c2ncccn2)CC1

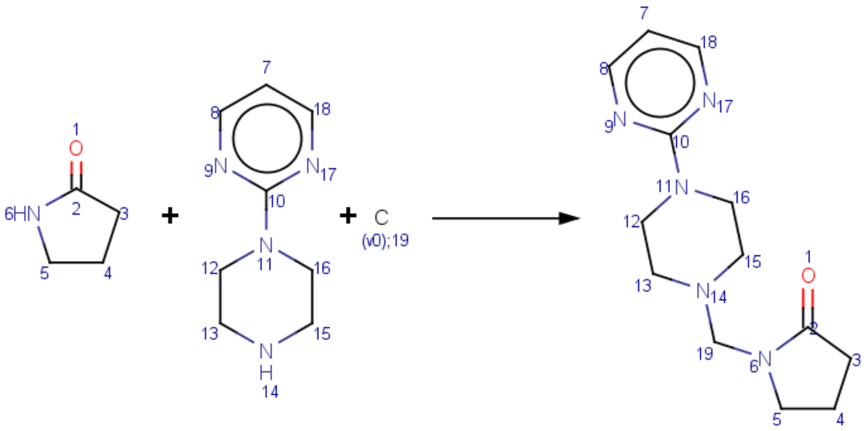

Correct mapped SMILES/SMARTS of the reaction:
[O:1]=[#6:2]-1-[#6:3]-[#6:4]-[#6:5]-[#7:6]-1.[#6:13]-1-[#6:12]-[#7:11](-[#6:16]-[#6:15]-[#7:14]-1)-[c:10]1[n:9][c:8][c:7][c:18][n:17]1.[#6;v0:19]>>[O:1]=[#6:2]-1-[#6:3]-[#6:4]-[#6:5]-[#7:6]-1-[#6:19]-[#7:14]-1-[#6:13]-[#6:12]-[#7:11](-[#6:16]-[#6:15]-1)-[c:10]1[n:9][c:8][c:7][c:18][n:17]1

Correctness of the mapping
MAPPET YES
ReactionMap NO
Marvin YES

Reaction no 100

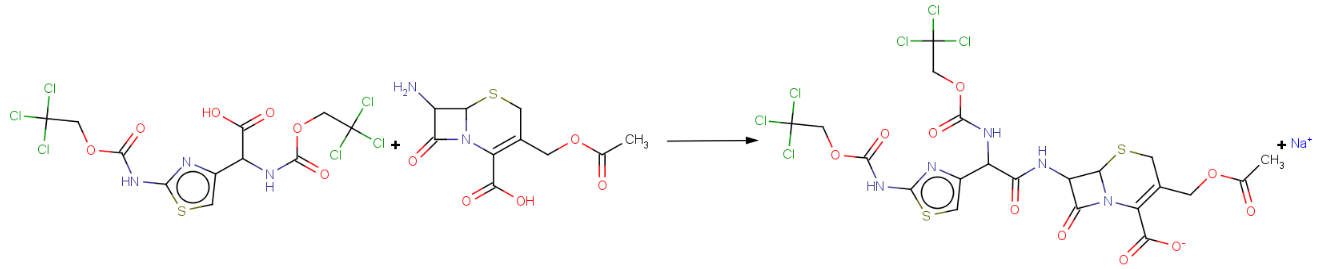

SMILES of the input:
O=C(O)C(NC(=O)OCC(Cl)(Cl)Cl)c1csc(NC(=O)OCC(Cl)(Cl)Cl)n1.CC(=O)OCC1=C(C(=
O)O)N2C(=O)C(N)C2SC1>>[Na+].CC(=O)OCC1=C(C(=O)[O-
])N2C(=O)C(NC(=O)C(NC(=O)OCC(Cl)(Cl)Cl)c3csc(NC(=O)OCC(Cl)(Cl)Cl)n3)C2SC1

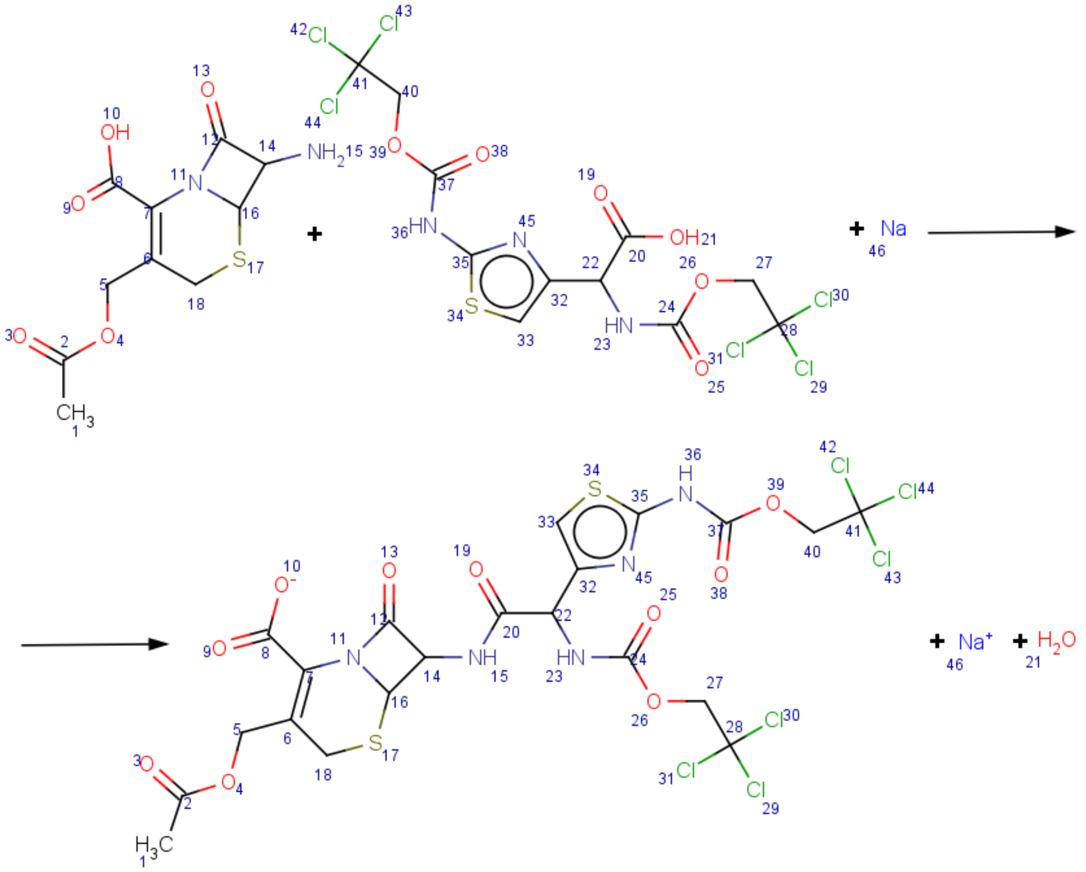

Correct mapped SMILES/SMARTS of the reaction:
[#6:1]-[#6:2](=[O:3])-[#8:4]-[#6:5]-[#6:6]-1=[#6:7](-[#7:11]-2-[#6:16](-
[#16:17]-[#6:18]-1)-[#6:14](-[#7:15])-[#6:12]-2=[O:13])-[#6:8](-
[#8:10])=[O:9].[#8:21]-[#6:20](=[O:19])-[#6:22](-[#7:23]-
[#6:24](=[O:25])-[#8:26]-[#6:27][C:28]([Cl:29])([Cl:30])[Cl:31])-
[c:32]1[c:33][s:34][c:35](-[#7:36]-[#6:37](=[O:38])-[#8:39]-
[#6:40][C:41]([Cl:42])([Cl:43])[Cl:44])[n:45]1.[Na:46]>>[#6:1]-
[#6:2](=[O:3])-[#8:4]-[#6:5]-[#6:6]-1=[#6:7](-[#7:11]-2-[#6:16](-
[#16:17]-[#6:18]-1)-[#6:14](-[#7:15]-[#6:20](=[O:19])-[#6:22](-[#7:23]-
[#6:24](=[O:25])-[#8:26]-[#6:27][C:28]([Cl:31])([Cl:30])[Cl:29])-
[c:32]1[c:33][s:34][c:35](-[#7:36]-[#6:37](=[O:38])-[#8:39]-
[#6:40][C:41]([Cl:44])([Cl:43])[Cl:42])[n:45]1)-[#6:12]-2=[O:13])-
[#6:8](-[#8-:10])=[O:9].[Na+:46].[#8:21]

Correctness of the mapping

MAPPET YES
ReactionMap NO
Marvin YES

Reaction no 101

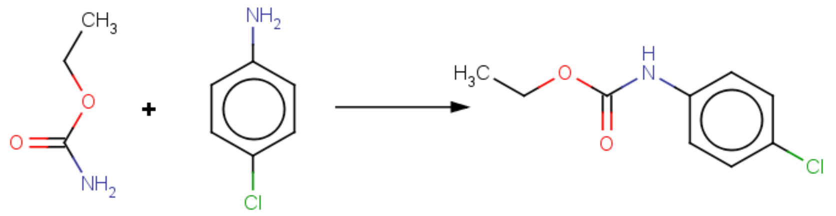

SMILES of the input:
CCOC(N)=O.Nc1ccc(Cl)cc1>>CCOC(=O)Nc1ccc(Cl)cc1

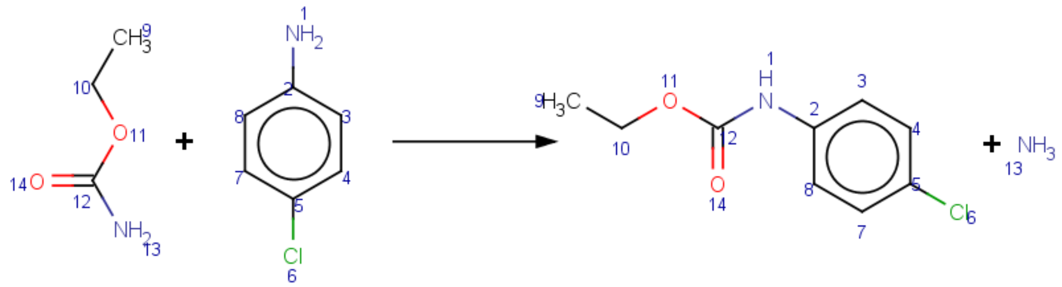

Correct mapped SMILES/SMARTS of the reaction:
[CH3:9][CH2:10][O:11][C:12]([NH2:13])=[O:14].[NH2:1][c:2]1[cH:3][cH:4][c:5]([Cl:6])[cH:7][cH:8]1>>[CH3:9][CH2:10][O:11][C:12](=[O:14])[NH:1][c:2]1[cH:3][cH:4][c:5]([Cl:6])[cH:7][cH:8]1.[NH3:13]

Correctness of the mapping
MAPPET YES
ReactionMap NO
Marvin NO

Reaction no 102

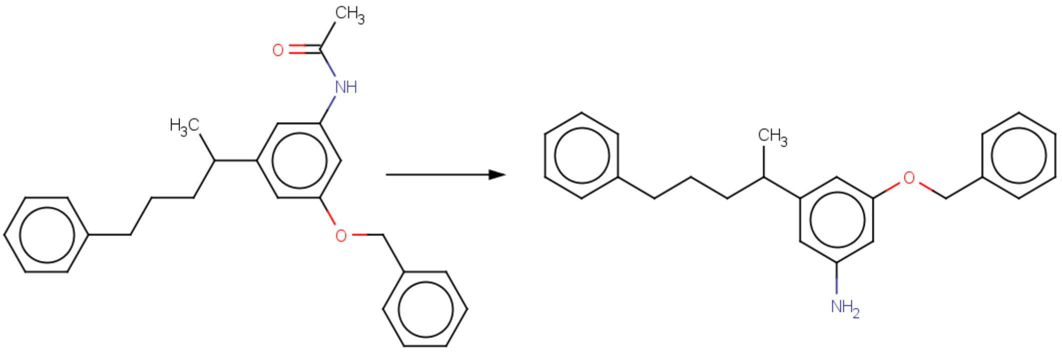

SMILES of the input:
CC(=O)Nc1cc(OCc2ccccc2)cc(C(C)CCCc2ccccc2)c1>>CC(CCCc1ccccc1)c1cc(N)cc(OCc2ccccc2)c1

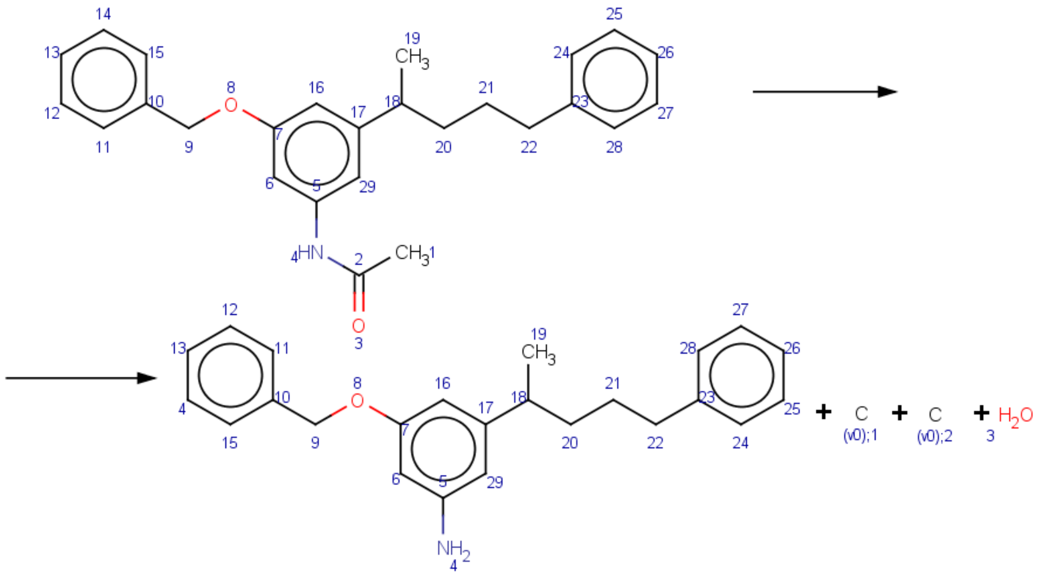

Correct mapped SMILES/SMARTS of the reaction:
[#6:19]-[#6:18](-[#6:20]-[#6:21]-[#6:22]-
[c:23]1[c:24][c:25][c:26][c:27][c:28]1)-[c:17]1[c:29][c:5](-[#7:4]-
[#6:2](-[#6:1])=[O:3])[c:6][c:7](-[#8:8]-[#6:9]-
[c:10]2[c:11][c:12][c:13][c:14][c:15]2)[c:16]1>>[#6:19]-[#6:18](-[#6:20]-
[#6:21]-[#6:22]-[c:23]1[c:28][c:27][c:26][c:25][c:24]1)-
[c:17]1[c:29][c:5](-[#7:4])[c:6][c:7](-[#8:8]-[#6:9]-
[c:10]2[c:15][c:14][c:13][c:12][c:11]2)[c:16]1.[#6;v0:1].[#6;v0:2].[#8:3]

Correctness of the mapping

MAPPET	YES
ReactionMap	NO
Marvin	YES

Reaction no 103

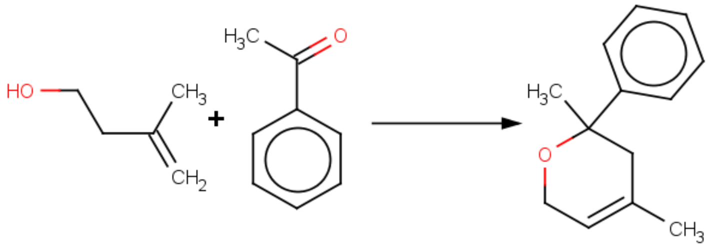

SMILES of the input:
C=C(C)CCO.CC(=O)c1ccccc1>>CC1=CCOC(C)(c2ccccc2)C1

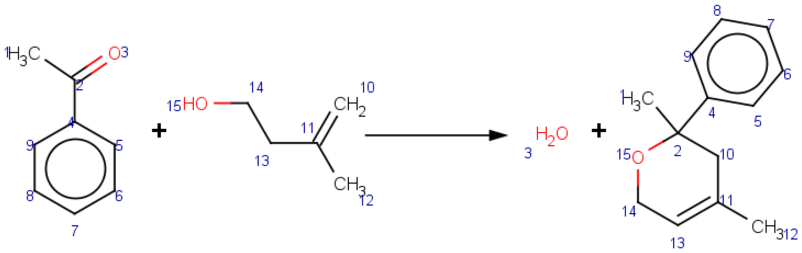

Correct mapped SMILES/SMARTS of the reaction:
[#6:1]-[#6:2](=[O:3])-[c:4]1[c:5][c:6][c:7][c:8][c:9]1.[#6:12]-
[#6:11](=[#6:10])-[#6:13]-[#6:14]-[#8:15]>>[#8:3].[#6:12]-[#6:11]-

1=[#6:13]-[#6:14]-[#8:15][C:2]([#6:1])([#6:10]-
1)[c:4]1[c:9][c:8][c:7][c:6][c:5]1

Correctness of the mapping

MAPPET	YES
ReactionMap	NO
Marvin	NO

Reaction no 104

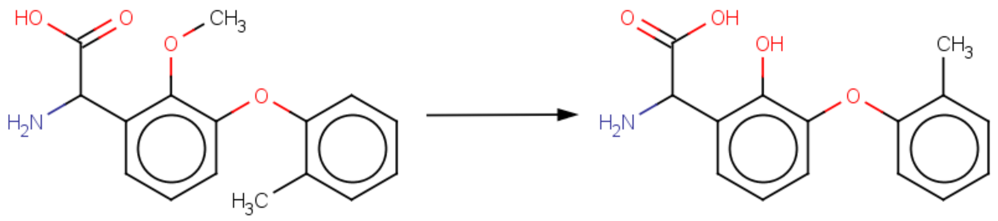

SMILES of the input:
COc1c(Oc2ccccc2C)cccc1C(N)C(=O)O>>Cc1ccccc1Oc1cccc(C(N)C(=O)O)c1O

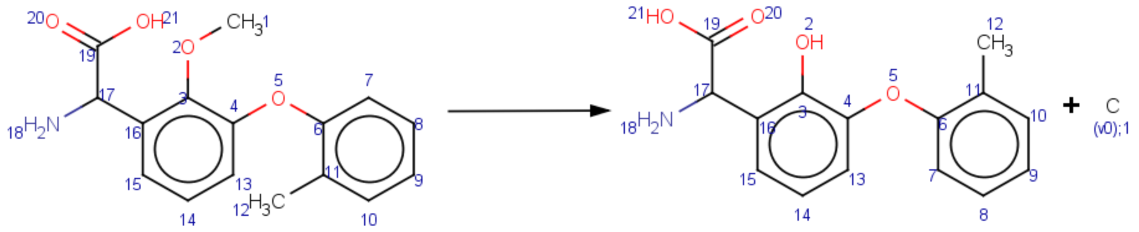

Correct mapped SMILES/SMARTS of the reaction:
[#6:1]-[#8:2]-[c:3]1[c:4](-[#8:5]-[c:6]2[c:7][c:8][c:9][c:10][c:11]2-[#6:12])[c:13][c:14][c:15][c:16]1-[#6:17](-[#7:18])-[#6:19](-[#8:21])=[O:20]>>[#6:12]-[c:11]1[c:10][c:9][c:8][c:7][c:6]1-[#8:5]-[c:4]1[c:13][c:14][c:15][c:16](-[#6:17](-[#7:18])-[#6:19](-[#8:21])=[O:20])[c:3]1-[#8:2].[#6;v0:1]

Correctness of the mapping

MAPPET	YES
ReactionMap	NO
Marvin	YES

Reaction no 105

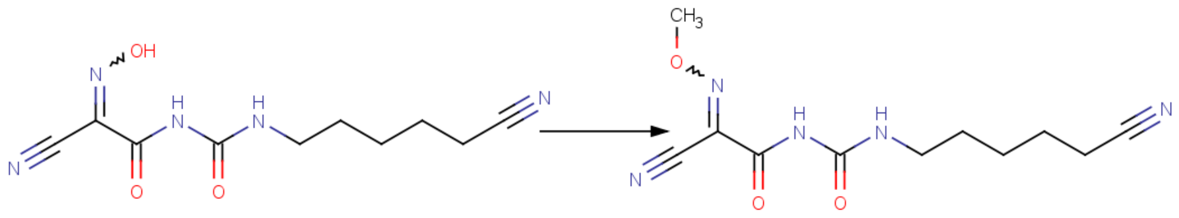

SMILES of the input:
N#CCCCCCNC(=O)NC(=O)C(C#N)=NO>>CON=C(C#N)C(=O)NC(=O)NCCCCCC#N

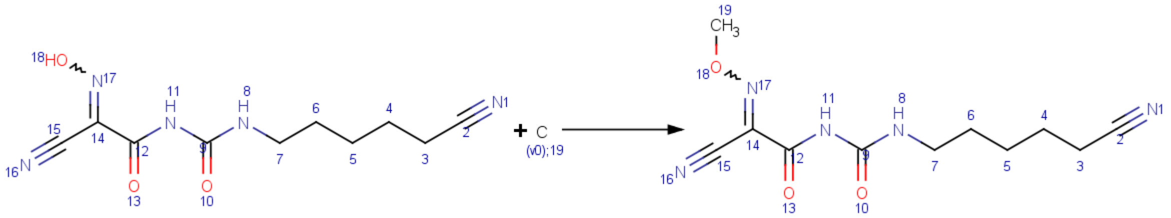

Correct mapped SMILES/SMARTS of the reaction:
[#8:18]-[#7:17]=[#6:14]([C:15]#[N:16])-[#6:12](=[O:13])-[#7:11]-
[#6:9](=[O:10])-[#7:8]-[#6:7]-[#6:6]-[#6:5]-[#6:4]-
[#6:3][C:2]#[N:1].[#6;v0:19]>>[#6:19]-[#8:18]-
[#7:17]=[#6:14]([C:15]#[N:16])-[#6:12](=[O:13])-[#7:11]-[#6:9](=[O:10])-
[#7:8]-[#6:7]-[#6:6]-[#6:5]-[#6:4]-[#6:3][C:2]#[N:1]

Correctness of the mapping

MAPPET	YES
ReactionMap	NO
Marvin	YES

Reaction no 106

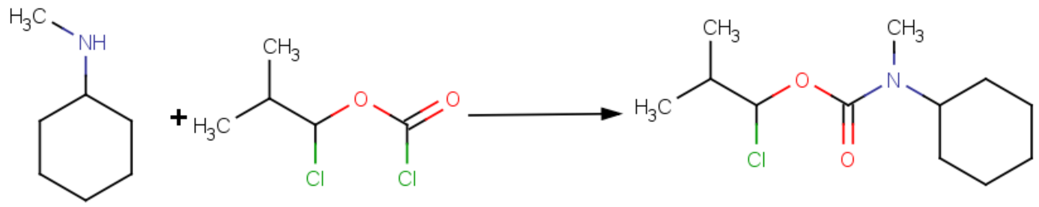

SMILES of the input:
CNC1CCCCC1.CC(C)C(Cl)OC(=O)Cl>>CC(C)C(Cl)OC(=O)N(C)C1CCCCC1

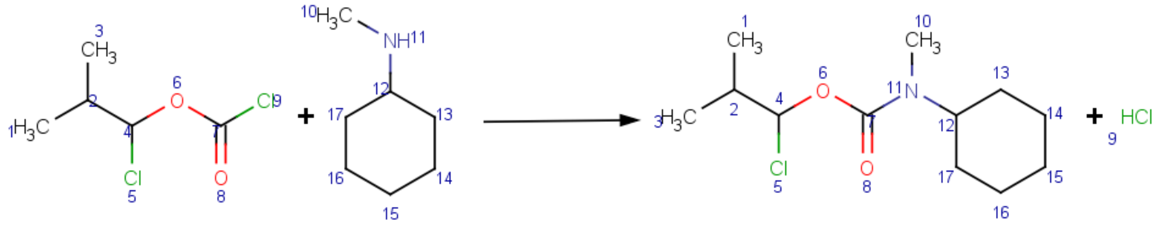

Correct mapped SMILES/SMARTS of the reaction:
[#6:1]-[#6:2](-[#6:3])-[#6:4]([Cl:5])-[#8:6]-
[#6:7]([Cl:9])=[O:8].[#6:10]-[#7:11]-[#6:12]-1-[#6:13]-[#6:14]-[#6:15]-
[#6:16]-[#6:17]-1>>[#6:3]-[#6:2](-[#6:1])-[#6:4]([Cl:5])-[#8:6]-
[#6:7](=[O:8])-[#7:11](-[#6:10])-[#6:12]-1-[#6:13]-[#6:14]-[#6:15]-
[#6:16]-[#6:17]-1.[Cl:9]

Correctness of the mapping

MAPPET	YES
ReactionMap	NO
Marvin	YES

Reaction no 107

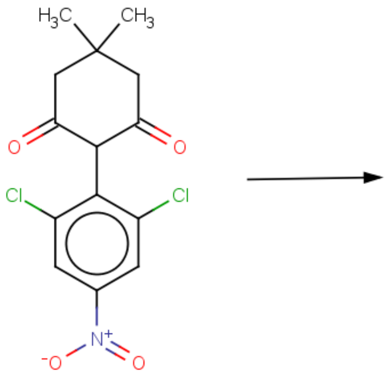

SMILES of the input:
CC1(C)CC(=O)C(c2c(Cl)cc([N+](=O)[O-
])cc2Cl)C(=O)C1>>CC1(C)CC(=O)C(c2c(Cl)cc(N)cc2Cl)C(=O)C1

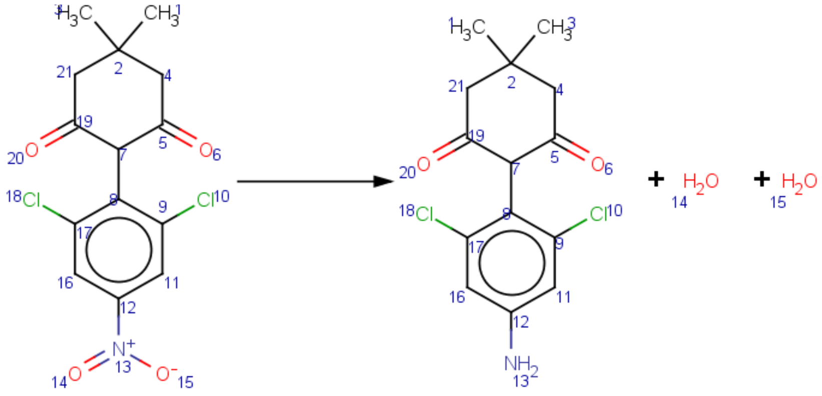

Correct mapped SMILES/SMARTS of the reaction:
[#6:1][C:2]1([#6:3])[#6:4]-[#6:5](=[O:6])-[#6:7](-[#6:19](=[O:20])-
[#6:21]1)-[c:8]1[c:9]([Cl:10])[c:11][c:12]([c:16][c:17]1[Cl:18])-
[#7+:13](-[#8-:15])=[O:14]>>[#6:3][C:2]1([#6:1])[#6:4]-[#6:5](=[O:6])-
[#6:7](-[#6:19](=[O:20])-[#6:21]1)-[c:8]1[c:9]([Cl:10])[c:11][c:12](-
[#7:13])[c:16][c:17]1[Cl:18].[#8:14].[#8:15]

Correctness of the mapping

MAPPET	YES
ReactionMap	NO
Marvin	YES

Reaction no 108

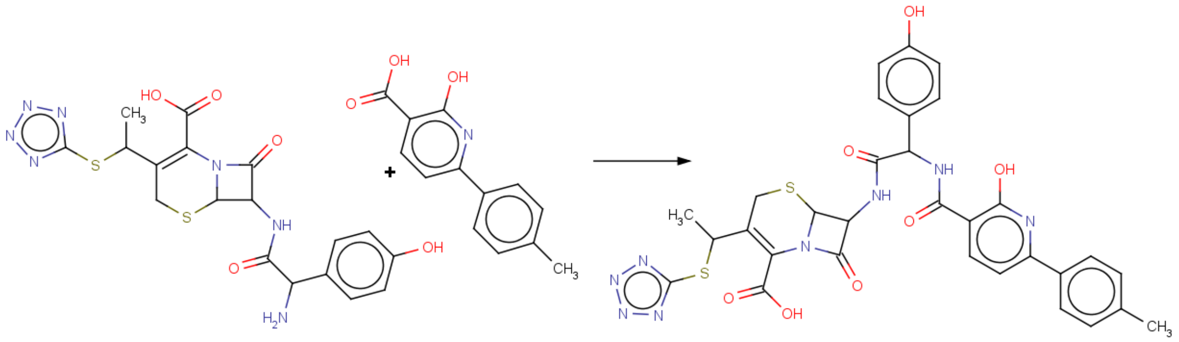

SMILES of the input:
CC(Sc1nnnn1)C1=C(C(=O)O)N2C(=O)C(NC(=O)C(N)c3ccc(O)cc3)C2SC1.Cc1ccc(-
c2ccc(C(=O)O)c(O)n2)cc1>>Cc1ccc(-
c2ccc(C(=O)NC(C(=O)NC3C(=O)N4C(C(=O)O)=C(C(C)Sc5nnnn5)CSC34)c3ccc(O)cc3)c
(O)n2)cc1

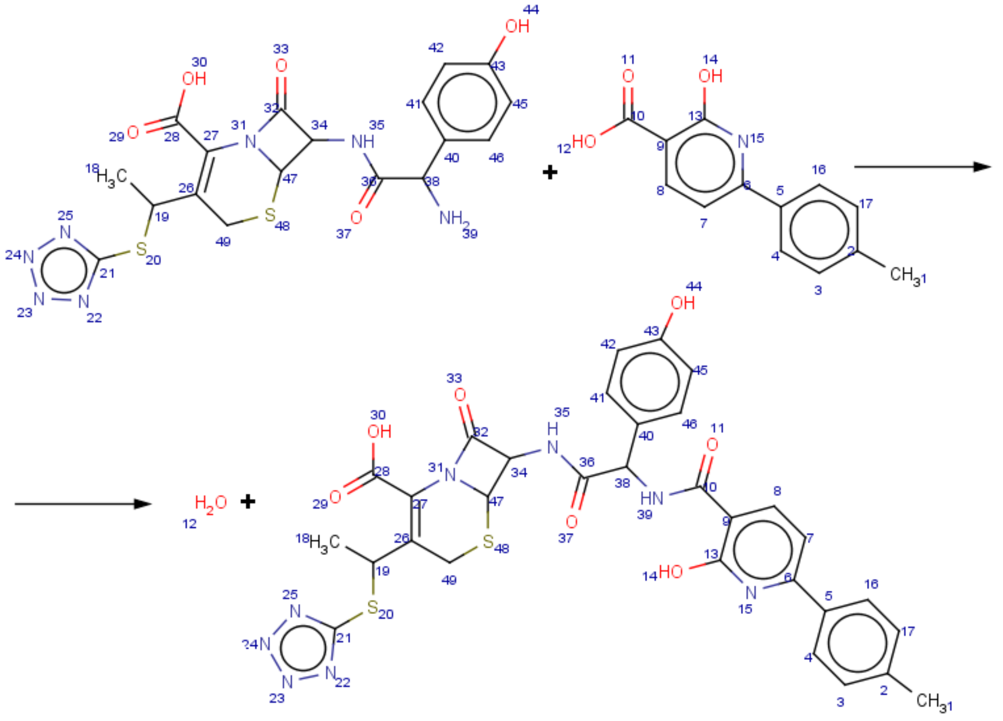

Correct mapped SMILES/SMARTS of the reaction:
[#6:1]-[c:2]1[c:3][c:4][c:5]([c:16][c:17]1)-[c:6]1[c:7][c:8][c:9](-
[#6:10](-[#8:12])=[O:11])[c:13](-[#8:14])[n:15]1.[#6:18]-[#6:19](-
[#16:20]-[c:21]1[n:22][n:23][n:24][n:25]1)-[#6:26]-1=[#6:27](-[#7:31]-2-
[#6:47](-[#16:48]-[#6:49]-1)-[#6:34](-[#7:35]-[#6:36](=[O:37])-[#6:38](-
[#7:39])-[c:40]1[c:41][c:42][c:43](-[#8:44])[c:45][c:46]1)-[#6:32]-
2=[O:33])-[#6:28](-[#8:30])=[O:29]>>[#8:12].[#6:18]-[#6:19](-[#16:20]-
[c:21]1[n:22][n:23][n:24][n:25]1)-[#6:26]-1=[#6:27](-[#7:31]-2-[#6:47](-
[#16:48]-[#6:49]-1)-[#6:34](-[#7:35]-[#6:36](=[O:37])-[#6:38](-[#7:39]-
[#6:10](=[O:11])-[c:9]1[c:8][c:7][c:6]([n:15][c:13]1-[#8:14])-
[c:5]1[c:16][c:17][c:2](-[#6:1])[c:3][c:4]1)-[c:40]1[c:41][c:42][c:43](-
[#8:44])[c:45][c:46]1)-[#6:32]-2=[O:33])-[#6:28](-[#8:30])=[O:29]

Correctness of the mapping

MAPPET	YES
ReactionMap	NO
Marvin	YES

Reaction no 109

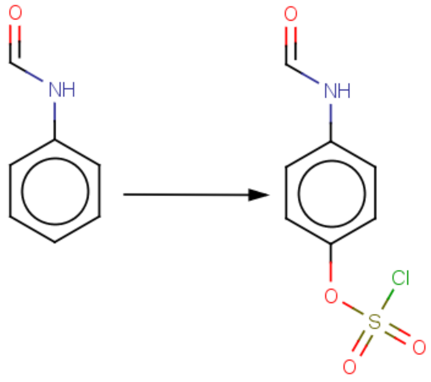

SMILES of the input:
O=CNc1ccccc1>>O=CNc1ccc(OS(=O)(=O)Cl)cc1

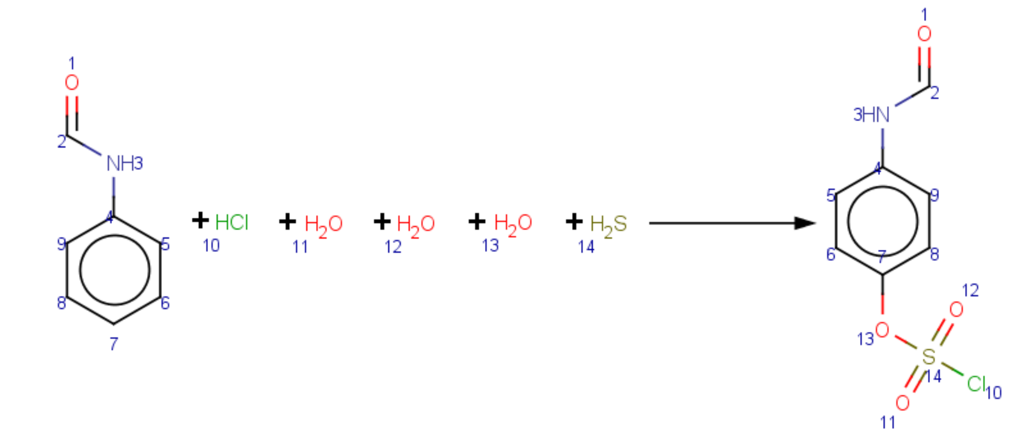

Correct mapped SMILES/SMARTS of the reaction:
[O:1]=[#6:2]-[#7:3]-[c:4]1[c:5][c:6][c:7][c:8][c:9]1.[Cl:10].[#8:11].[#8:12].[#8:13].[#16:14]>>[Cl:10][S:14](=[O:12])(=[O:11])[#8:13]-[c:7]1[c:6][c:5][c:4](-[#7:3]-[#6:2]=[O:1])[c:9][c:8]1

Correctness of the mapping
MAPPET YES
ReactionMap NO
Marvin YES

Reaction no 110

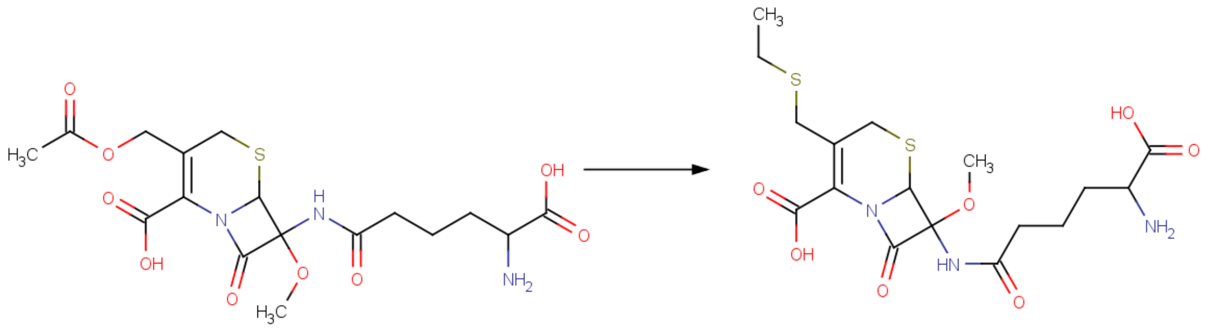

SMILES of the input:
COC1(NC(=O)CCCC(N)C(=O)O)C(=O)N2C(C(=O)O)=C(COC(C)=O)CSC21>>CCSCC1=C(C(=O)O)N2C(=O)C(NC(=O)CCCC(N)C(=O)O)(OC)C2SC1

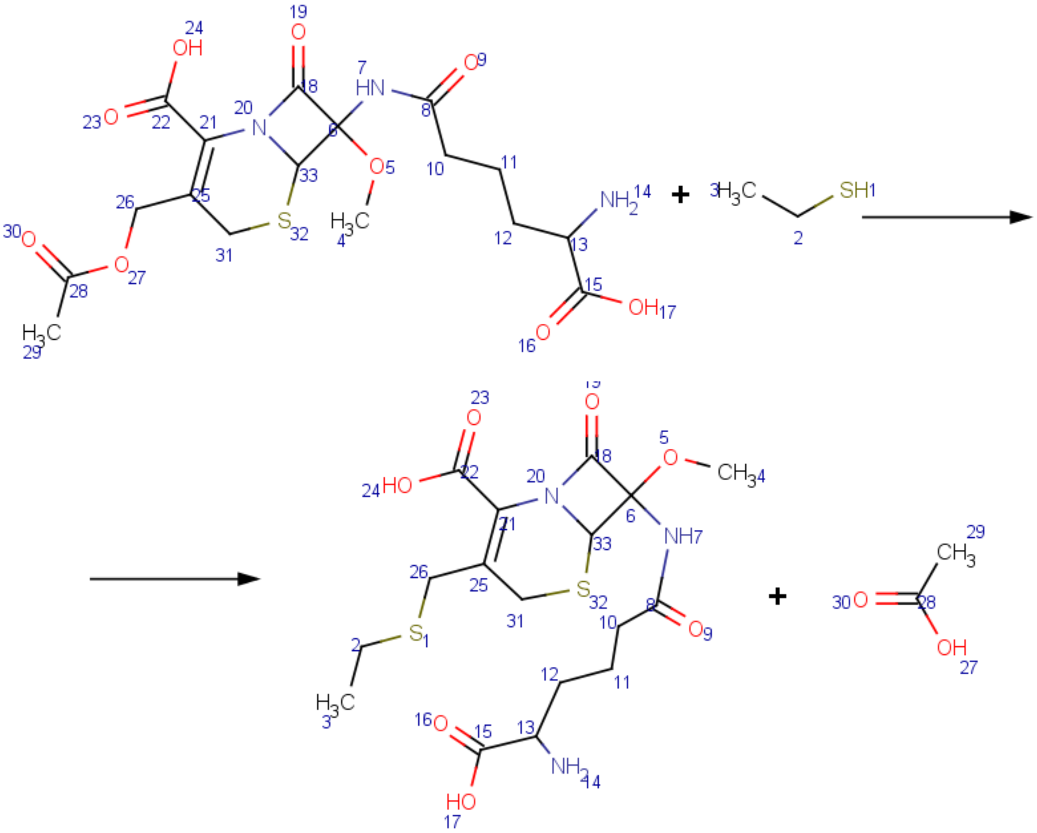

Correct mapped SMILES/SMARTS of the reaction:
[CH3:4][O:5][C:6]1([NH:7][C:8](=[O:9])[CH2:10][CH2:11][CH2:12][CH:13]([NH2:14])[C:15]([OH:17])=[O:16])[CH:33]2[S:32][CH2:31][C:25]([CH2:26][O:27][C:28]([CH3:29])=[O:30])=[C:21]([N:20]2[C:18]1=[O:19])[C:22]([OH:24])=[O:23].[CH3:3][CH2:2][SH:1]>>[CH3:3][CH2:2][S:1][CH2:26][C:25]1=[C:21]([N:20]2[CH:33]([S:32][CH2:31]1)[C:6]([NH:7][C:8](=[O:9])[CH2:10][CH2:11][CH2:12][CH:13]([NH2:14])[C:15]([OH:17])=[O:16])([O:5][CH3:4])[C:18]2=[O:19])[C:22]([OH:24])=[O:23].[CH3:29][C:28]([OH:27])=[O:30]

Correctness of the mapping
MAPPET YES
ReactionMap NO
Marvin YES

Reaction no 111

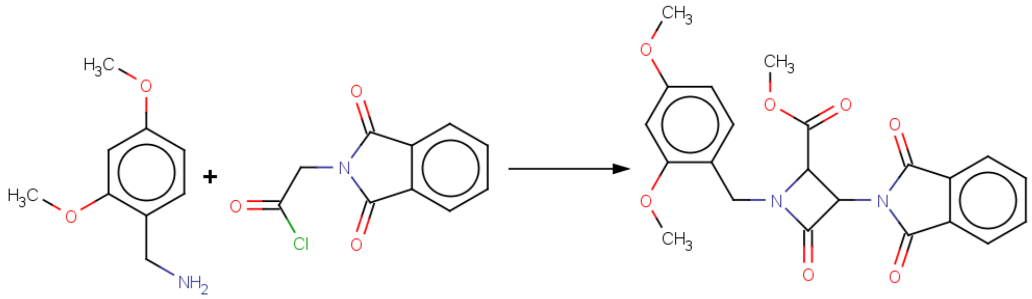

SMILES of the input:
COc1ccc(CN)c(OC)c1.O=C(Cl)CN1C(=O)c2ccccc2C1=O>>COC(=O)C1C(N2C(=O)c3ccccc3C2=O)C(=O)N1Cc1ccc(OC)cc1OC

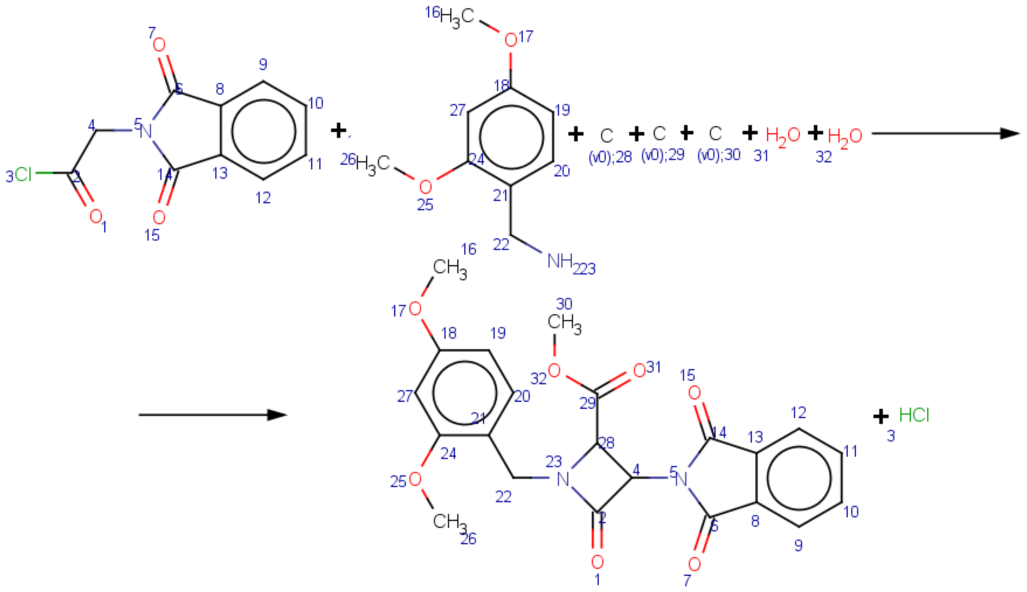

Correct mapped SMILES/SMARTS of the reaction:
[Cl:3][#6:2](=[O:1])-[#6:4]-[#7:5]-1-[#6:6](=[O:7])-
[c:8]2[c:9][c:10][c:11][c:12][c:13]2-[#6:14]-1=[O:15].[#6:16]-[#8:17]-
[c:18]1[c:19][c:20][c:21](-[#6:22]-[#7:23])[c:24](-[#8:25]-
[#6:26])[c:27]1.[#6;v0:28].[#6;v0:29].[#6;v0:30].[#8:31].[#8:32]>>[#6:30]
-[#8:32]-[#6:29](=[O:31])-[#6:28]-1-[#6:4](-[#7:5]-2-[#6:14](=[O:15])-
[c:13]3[c:12][c:11][c:10][c:9][c:8]3-[#6:6]-2=[O:7])-[#6:2](=[O:1])-
[#7:23]-1-[#6:22]-[c:21]1[c:20][c:19][c:18](-[#8:17]-
[#6:16])[c:27][c:24]1-[#8:25]-[#6:26].[Cl:3]

Correctness of the mapping

MAPPET	YES
ReactionMap	NO
Marvin	YES

Reaction no 112

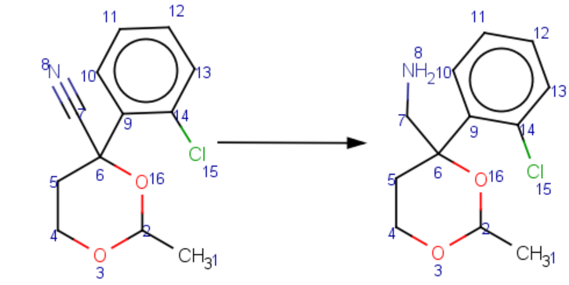

Correct mapped SMILES/SMARTS of the reaction:
[#6:1]-[#6:2]-1-[#8:3]-[#6:4]-[#6:5][C:6]([#8:16]-
1)([C:7]#[N:8])[c:9]1[c:10][c:11][c:12][c:13][c:14]1[Cl:15]>>[#6:1]-
[#6:2]-1-[#8:3]-[#6:4]-[#6:5][C:6]([#6:7]-[#7:8])([#8:16]-
1)[c:9]1[c:10][c:11][c:12][c:13][c:14]1[Cl:15]

Correctness of the mapping

MAPPET	YES
ReactionMap	YES
Marvin	YES

Reaction no 113

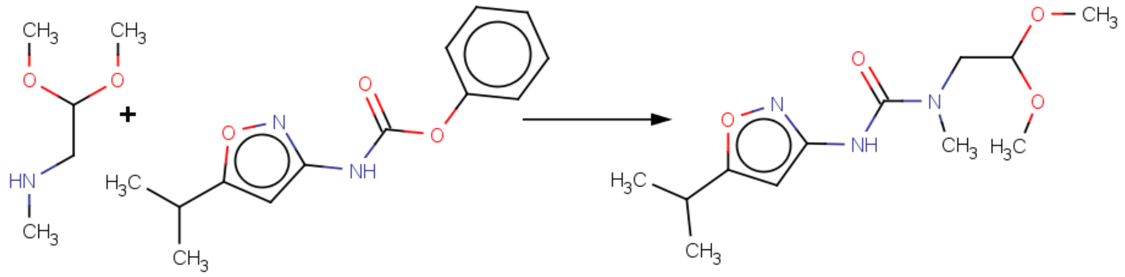

SMILES of the input:
CNCC(OC)OC.CC(C)c1cc(NC(=O)Oc2ccccc2)no1>>COC(CN(C)C(=O)Nc1cc(C(C)C)on1)O
C

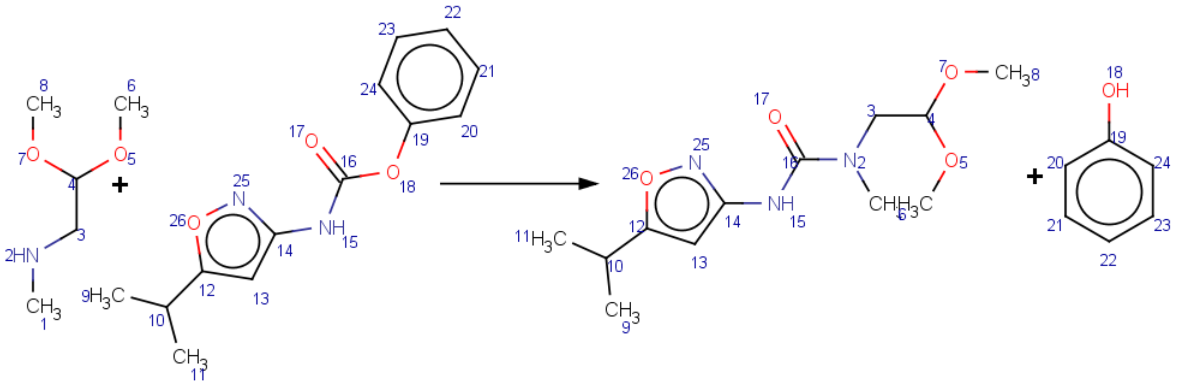

Correct mapped SMILES/SMARTS of the reaction:
[CH3:9][CH:10]([CH3:11])[c:12]1[cH:13][c:14]([NH:15][C:16](=[O:17])[O:18][c:19]2[cH:20][cH:21][cH:22][cH:23][cH:24]2)[n:25][o:26]1.[CH3:1][NH:2][CH2:3][CH:4]([O:5][CH3:6])[O:7][CH3:8]>>[CH3:8][O:7][CH:4]([CH2:3][N:2]([CH3:1])[C:16](=[O:17])[NH:15][c:14]1[cH:13][c:12]([o:26][n:25]1)[CH:10]([CH3:9])[CH3:11])[O:5][CH3:6].[OH:18][c:19]1[cH:24][cH:23][cH:22][cH:21][cH:20]1

Correctness of the mapping

MAPPET	YES
ReactionMap	NO
Marvin	YES

Reaction no 114

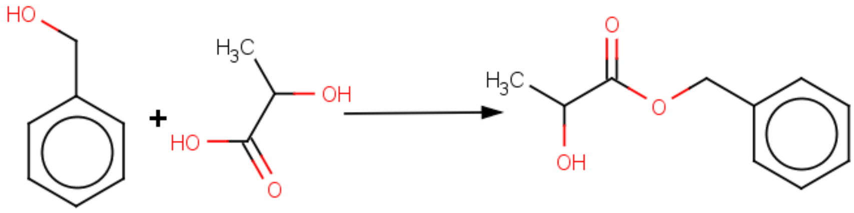

SMILES of the input:
OCc1ccccc1.CC(O)C(=O)O>>CC(O)C(=O)OCc1ccccc1

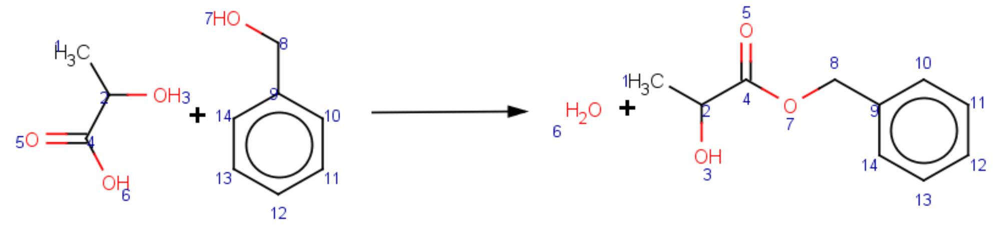

Correct mapped SMILES/SMARTS of the reaction:
[#6:1]-[#6:2](-[#8:3])-[#6:4](-[#8:6])=[O:5].[#8:7]-[#6:8]-
[c:9]1[c:10][c:11][c:12][c:13][c:14]1>>[#8:6].[#6:1]-[#6:2](-[#8:3])-
[#6:4](=[O:5])-[#8:7]-[#6:8]-[c:9]1[c:10][c:11][c:12][c:13][c:14]1

Correctness of the mapping

MAPPET	YES
ReactionMap	NO
Marvin	YES

Reaction no 115

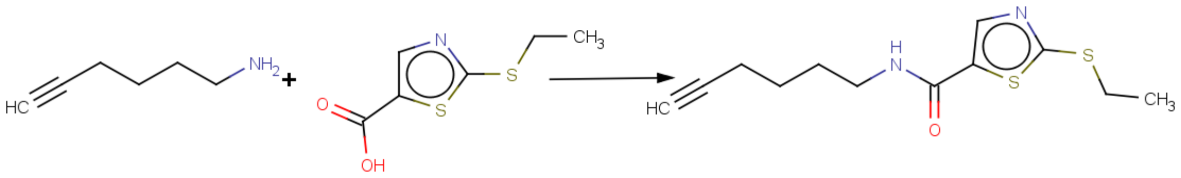

SMILES of the input:
C#CCCCCN.CCSc1ncc(C(=O)O)s1>>C#CCCCCNC(=O)c1cnc(SCC)s1

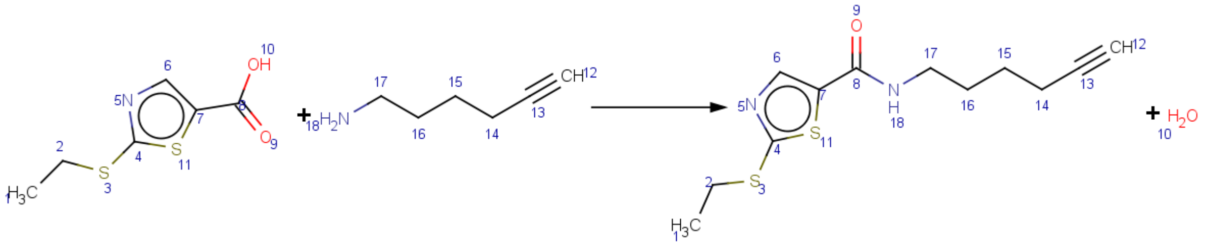

Correct mapped SMILES/SMARTS of the reaction:
[#6:1]-[#6:2]-[#16:3]-[c:4]1[n:5][c:6][c:7]([s:11]1)-[#6:8](-
[#8:10])=[O:9].[#7:18]-[#6:17]-[#6:16]-[#6:15]-
[#6:14][C:13]#[C:12]>>[#8:10].[#6:1]-[#6:2]-[#16:3]-
[c:4]1[n:5][c:6][c:7]([s:11]1)-[#6:8](=[O:9])-[#7:18]-[#6:17]-[#6:16]-
[#6:15]-[#6:14][C:13]#[C:12]

Correctness of the mapping

MAPPET	YES
ReactionMap	NO
Marvin	YES

Reaction no 116

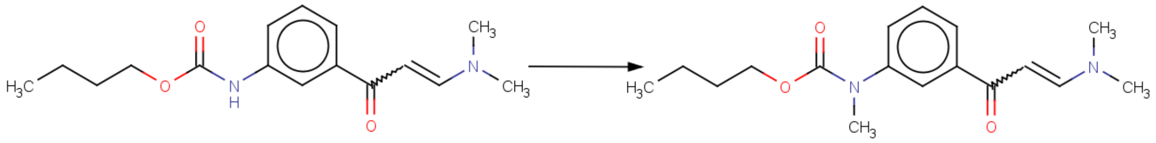

SMILES of the input:
CCCCOC(=O)Nc1cccc(C(=O)C=CN(C)C)c1>>CCCCOC(=O)N(C)c1cccc(C(=O)C=CN(C)C)c1

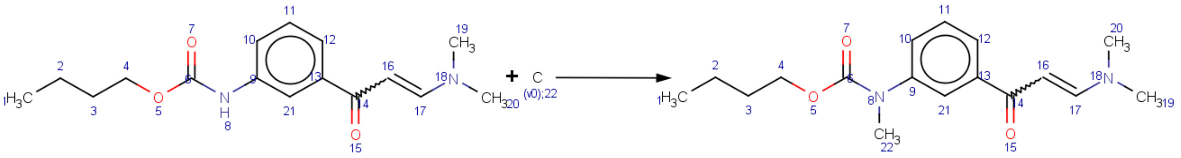

Correct mapped SMILES/SMARTS of the reaction:
[#6:1]-[#6:2]-[#6:3]-[#6:4]-[#8:5]-[#6:6](=[O:7])-[#7:8]-[c:9]1[c:10][c:11][c:12][c:13]([c:21]1)-[#6:14](=[O:15])-[#6:16]=[#6:17]-[#7:18](-[#6:19])-[#6:20].[#6;v0:22]>>[#6:1]-[#6:2]-[#6:3]-[#6:4]-[#8:5]-[#6:6](=[O:7])-[#7:8](-[#6:22])-[c:9]1[c:10][c:11][c:12][c:13]([c:21]1)-[#6:14](=[O:15])-[#6:16]=[#6:17]-[#7:18](-[#6:20])-[#6:19]

Correctness of the mapping

MAPPET	YES
ReactionMap	NO
Marvin	YES

Reaction no 117

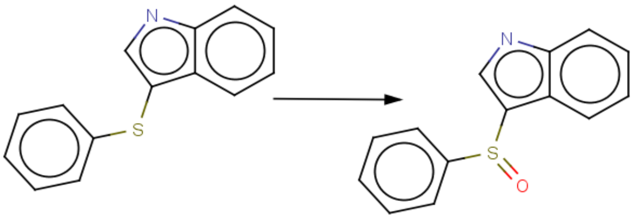

SMILES of the input:
c1nc2ccccc2c1Sc1ccccc1>>O=S(c1cnc2ccccc21)c1ccccc1

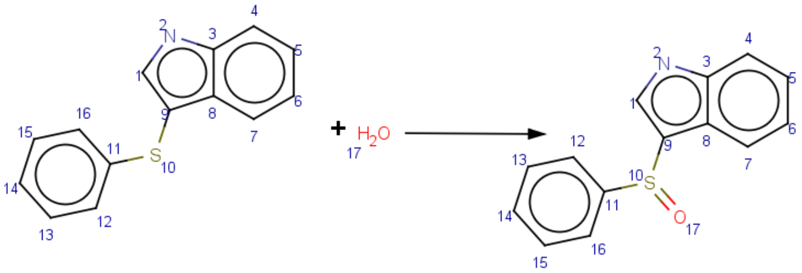

Correct mapped SMILES/SMARTS of the reaction:
[#16:10](-[c:9]1[c:1][n:2][c:3]2[c:4][c:5][c:6][c:7][c:8]12)-[c:11]1[c:12][c:13][c:14][c:15][c:16]1.[#8:17]>>[O:17]=[S:10]([c:9]1[c:1][n:2][c:3]2[c:4][c:5][c:6][c:7][c:8]12)[c:11]1[c:16][c:15][c:14][c:13][c:12]1

Correctness of the mapping

MAPPET	YES
ReactionMap	NO
Marvin	YES

Reaction no 118

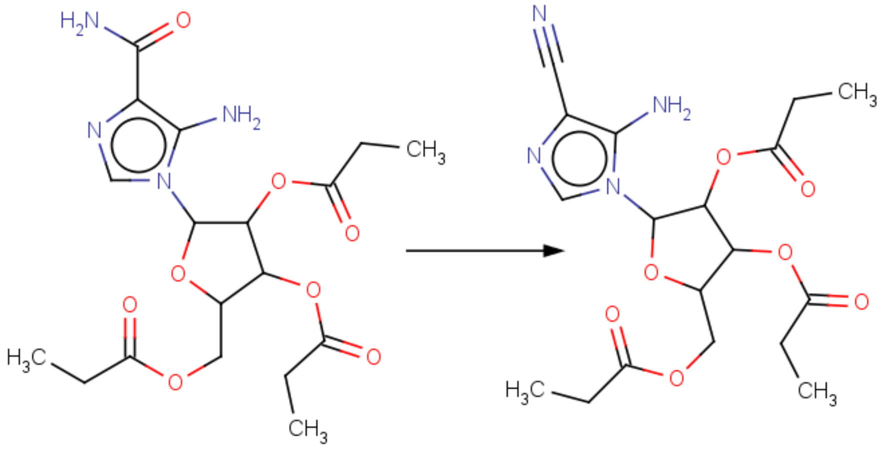

SMILES of the input:
CCC(=O)OCC1OC(n2cnc(C(N)=O)c2N)C(OC(=O)CC)C1OC(=O)CC>>CCC(=O)OCC1OC(n2cnc(C#N)c2N)C(OC(=O)CC)C1OC(=O)CC

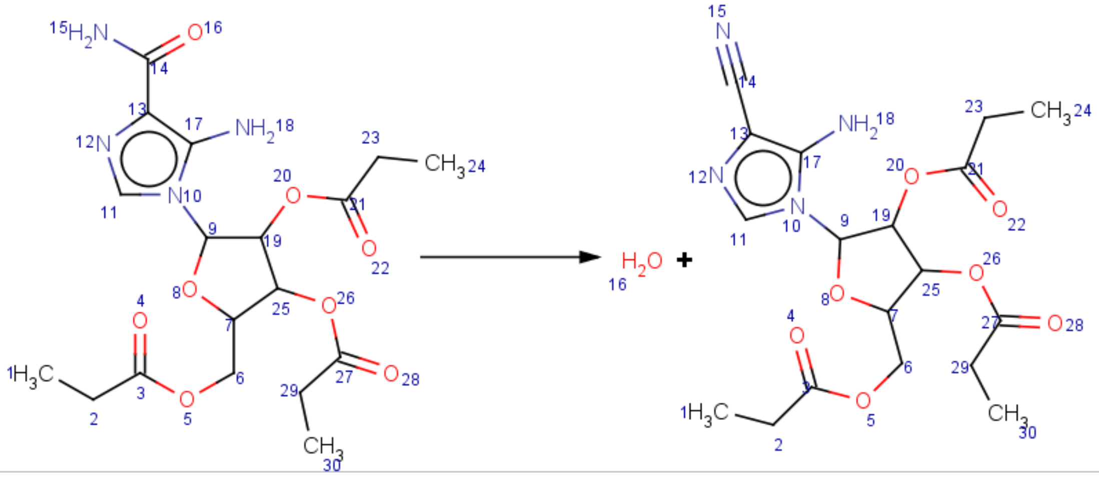

Correct mapped SMILES/SMARTS of the reaction:
[#6:1]-[#6:2]-[#6:3](=[O:4])-[#8:5]-[#6:6]-[#6:7]-1-[#8:8]-[#6:9](-[#6:19](-[#8:20]-[#6:21](=[O:22])-[#6:23]-[#6:24])-[#6:25]-1-[#8:26]-[#6:27](=[O:28])-[#6:29]-[#6:30])-[n:10]1[c:11][n:12][c:13](-[#6:14](-[#7:15])=[O:16])[c:17]1-[#7:18]>>[#8:16].[#6:1]-[#6:2]-[#6:3](=[O:4])-[#8:5]-[#6:6]-[#6:7]-1-[#8:8]-[#6:9](-[#6:19](-[#8:20]-[#6:21](=[O:22])-[#6:23]-[#6:24])-[#6:25]-1-[#8:26]-[#6:27](=[O:28])-[#6:29]-[#6:30])-[n:10]1[c:11][n:12][c:13]([C:14]#[N:15])[c:17]1-[#7:18]

Correctness of the mapping

MAPPET	YES
ReactionMap	NO
Marvin	YES

Reaction no 119

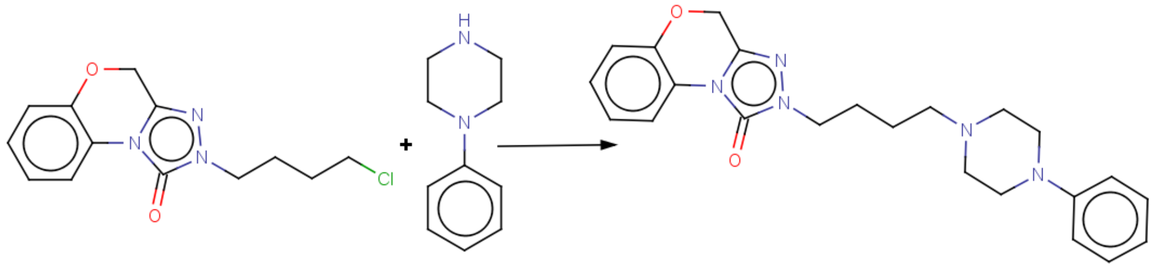

SMILES of the input:
O=c1n(CCCCCl)nc2n1-
c1ccccc1OC2.c1ccc(N2CCNCC2)cc1>>O=c1n(CCCCN2CCN(c3ccccc3)CC2)nc2n1-
c1ccccc1OC2

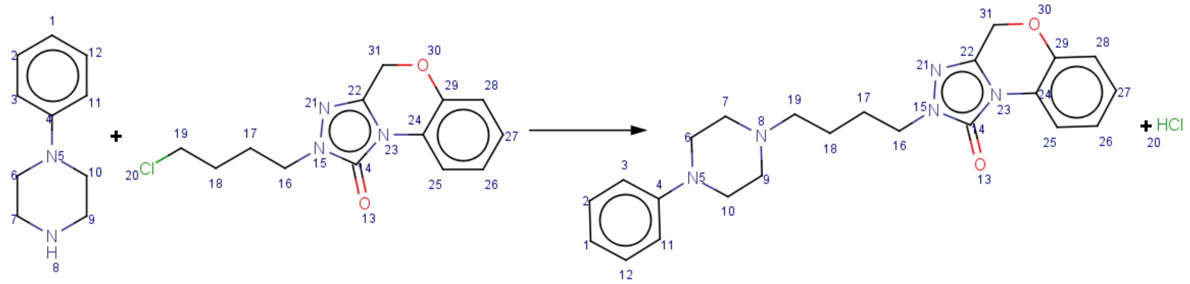

Correct mapped SMILES/SMARTS of the reaction:
[#6:7]-1-[#6:6]-[#7:5](-[#6:10]-[#6:9]-[#7:8]-1)-
[c:4]1[c:3][c:2][c:1][c:12][c:11]1.[Cl:20][#6:19]-[#6:18]-[#6:17]-
[#6:16]-[n:15]1[n:21][c:22]2-[#6:31]-[#8:30]-
[c:29]3[c:28][c:27][c:26][c:25][c:24]3-
[n:23]2[c:14]1=[O:13]>>[O:13]=[c:14]1[n:15](-[#6:16]-[#6:17]-[#6:18]-
[#6:19]-[#7:8]-2-[#6:9]-[#6:10]-[#7:5](-[#6:6]-[#6:7]-2)-
[c:4]2[c:11][c:12][c:1][c:2][c:3]2)[n:21][c:22]2-[#6:31]-[#8:30]-
[c:29]3[c:28][c:27][c:26][c:25][c:24]3-[n:23]12.[Cl:20]

Correctness of the mapping

MAPPET	YES
ReactionMap	NO
Marvin	YES

Reaction no 120

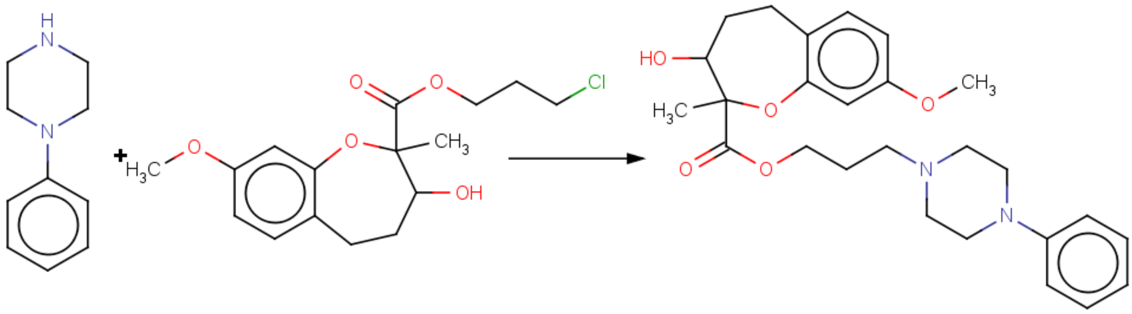

SMILES of the input:
c1ccc(N2CCNCC2)cc1.COc1ccc2c(c1)OC(C)(C(=O)OCCCCl)C(O)CC2>>COc1ccc2c(c1)O
C(C)(C(=O)OCCCN1CCN(c3ccccc3)CC1)C(O)CC2

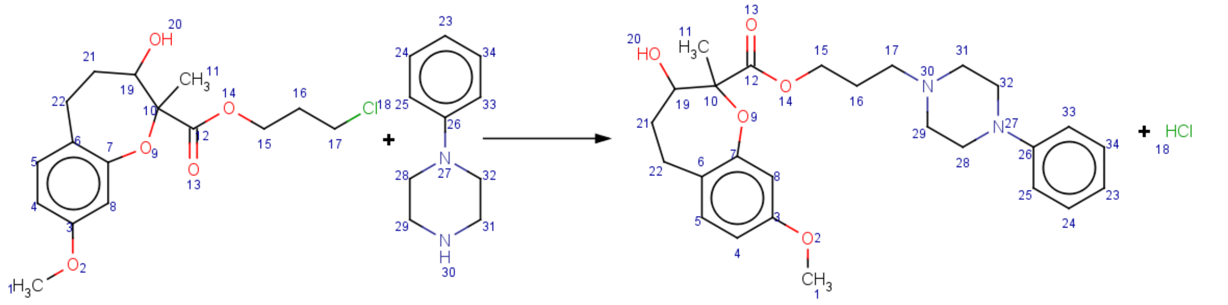

Correct mapped SMILES/SMARTS of the reaction:
[#6:1]-[#8:2]-[c:3]1[c:4][c:5][c:6]2-[#6:22]-[#6:21]-[#6:19](-[#8:20])[C:10]([#6:11])([#8:9]-[c:7]2[c:8]1)[#6:12](=[O:13])-[#8:14]-[#6:15]-[#6:16]-[#6:17][Cl:18].[#6:29]-1-[#6:28]-[#7:27](-[#6:32]-[#6:31]-[#7:30]-1)-[c:26]1[c:25][c:24][c:23][c:34][c:33]1>>[#6:1]-[#8:2]-[c:3]1[c:4][c:5][c:6]2-[#6:22]-[#6:21]-[#6:19](-[#8:20])[C:10]([#6:11])([#8:9]-[c:7]2[c:8]1)[#6:12](=[O:13])-[#8:14]-[#6:15]-[#6:16]-[#6:17]-[#7:30]-1-[#6:31]-[#6:32]-[#7:27](-[#6:28]-[#6:29]-1)-[c:26]1[c:33][c:34][c:23][c:24][c:25]1.[Cl:18]

Correctness of the mapping

MAPPET	YES
ReactionMap	NO
Marvin	YES

Reaction no 121

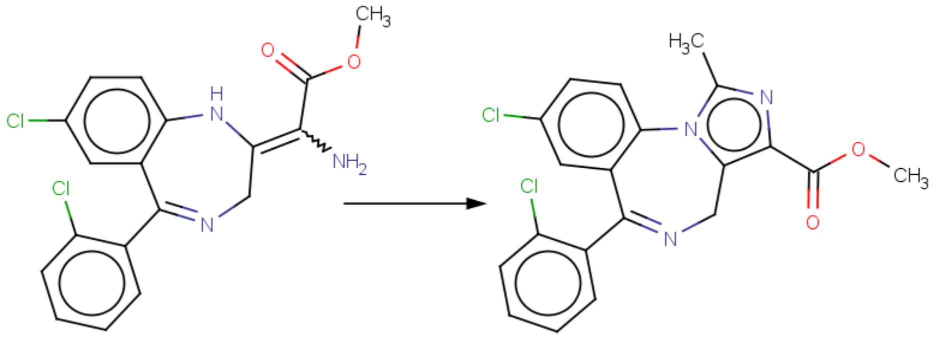

SMILES of the input:
COC(=O)C(N)=C1CN=C(c2ccccc2Cl)c2cc(Cl)ccc2N1>>COC(=O)c1nc(C)n2c1CN=C(c1cc ccc1Cl)c1cc(Cl)ccc1-2

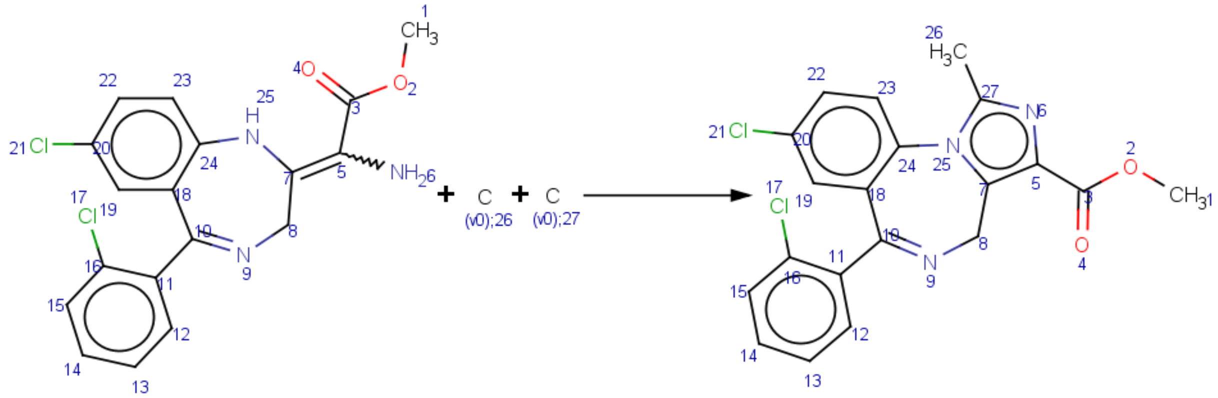

Correct mapped SMILES/SMARTS of the reaction:
[#6:1]-[#8:2]-[#6:3](=[O:4])-[#6:5](-[#7:6])=[#6:7]-1-[#6:8]-[#7:9]=[#6:10](-[c:11]2[c:12][c:13][c:14][c:15][c:16]2[Cl:17])-[c:18]2[c:19][c:20]([Cl:21])[c:22][c:23][c:24]2-[#7:25]-

1.[#6;v0:26].[#6;v0:27]>>[#6:1]-[#8:2]-[#6:3](=[O:4])-[c:5]1[n:6][c:27](-[#6:26])[n:25]-2[c:7]1-[#6:8]-[#7:9]=[#6:10](-[c:11]1[c:12][c:13][c:14][c:15][c:16]1[Cl:17])-[c:18]1[c:19][c:20]([Cl:21])[c:22][c:23][c:24]-21

Correctness of the mapping
MAPPET YES
ReactionMap NO
Marvin YES

Reaction no 122

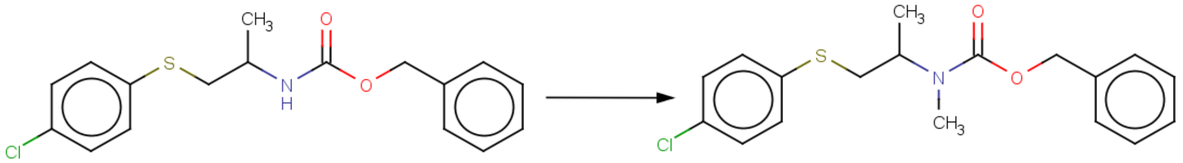

SMILES of the input:
CC(CSc1ccc(Cl)cc1)NC(=O)OCc1ccccc1>>CC(CSc1ccc(Cl)cc1)N(C)C(=O)OCc1ccccc1

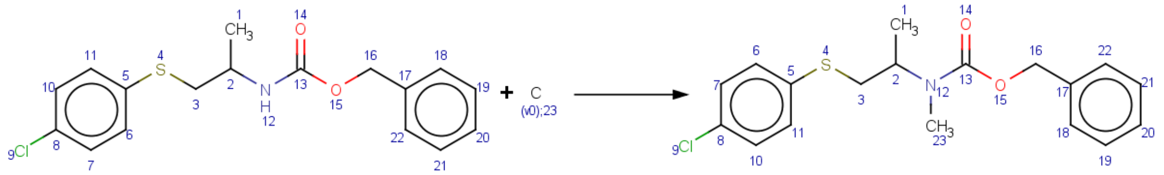

Correct mapped SMILES/SMARTS of the reaction:
[#6:1]-[#6:2](-[#6:3]-[#16:4]-[c:5]1[c:6][c:7][c:8]([Cl:9])[c:10][c:11]1)-[#7:12]-[#6:13](=[O:14])-[#8:15]-[#6:16]-[c:17]1[c:18][c:19][c:20][c:21][c:22]1.[#6;v0:23]>>[#6:1]-[#6:2](-[#6:3]-[#16:4]-[c:5]1[c:11][c:10][c:8]([Cl:9])[c:7][c:6]1)-[#7:12](-[#6:23])-[#6:13](=[O:14])-[#8:15]-[#6:16]-[c:17]1[c:22][c:21][c:20][c:19][c:18]1

Correctness of the mapping
MAPPET YES
ReactionMap NO
Marvin YES

Reaction no 123

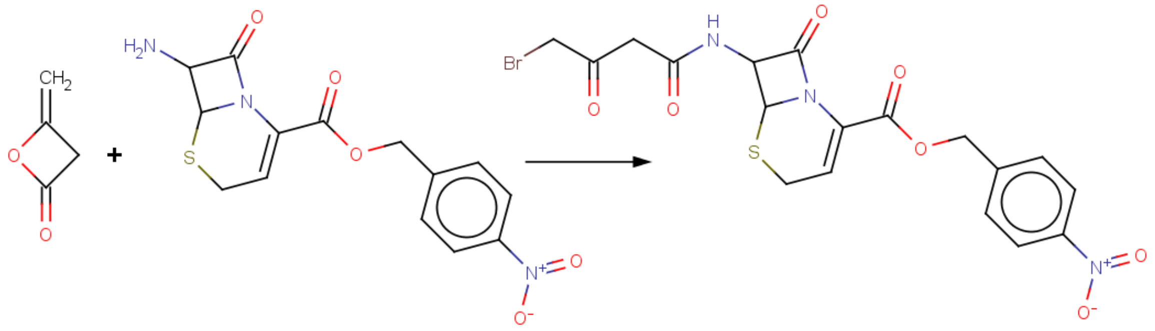

SMILES of the input:
NC1C(=O)N2C(C(=O)OCc3ccc([N+](=O)[O-])cc3)=CCSC12.C=C1CC(=O)O1>>O=C(CBr)CC(=O)NC1C(=O)N2C(C(=O)OCc3ccc([N+](=O)[O-])cc3)=CCSC12

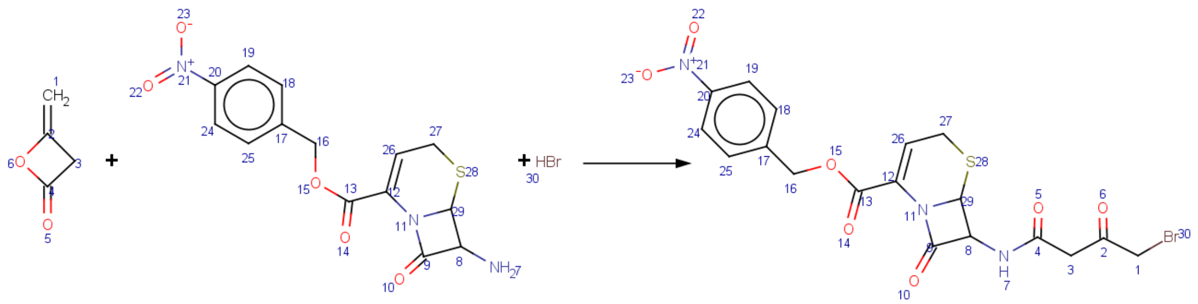

Correct mapped SMILES/SMARTS of the reaction:
[#6:1]=[#6:2]-1-[#6:3]-[#6:4](=[O:5])-[#8:6]-1.[#7:7]-[#6:8]-1-[#6:29]-2-
[#16:28]-[#6:27]-[#6:26]=[#6:12](-[#7:11]-2-[#6:9]-1=[O:10])-
[#6:13](=[O:14])-[#8:15]-[#6:16]-
[c:17]1[c:18][c:19][c:20]([c:24][c:25]1)-[#7+:21](-[#8-
:23])=[O:22].[Br:30]>>[#8-:23]-[#7+:21](=[O:22])-
[c:20]1[c:19][c:18][c:17](-[#6:16]-[#8:15]-[#6:13](=[O:14])-[#6:12]-
2=[#6:26]-[#6:27]-[#16:28]-[#6:29]-3-[#6:8](-[#7:7]-[#6:4](=[O:5])-
[#6:3]-[#6:2](=[O:6])-[#6:1][Br:30])-[#6:9](=[O:10])-[#7:11]-2-
3)[c:25][c:24]1

Correctness of the mapping

MAPPET	YES
ReactionMap	NO
Marvin	NO

Reaction no 124

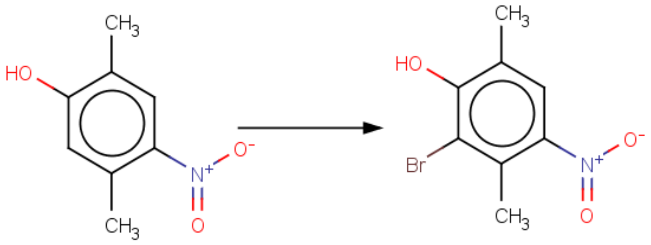

SMILES of the input:
Cc1cc([N+](=O)[O-])c(C)cc1O>>Cc1cc([N+](=O)[O-])c(C)c(Br)c1O

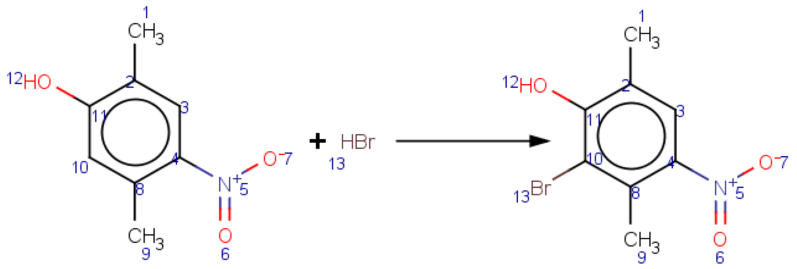

Correct mapped SMILES/SMARTS of the reaction:
[#6:1]-[c:2]1[c:3][c:4]([c:8](-[#6:9])[c:10][c:11]1-[#8:12])-[#7+:5](-
[#8-:7])=[O:6].[Br:13]>>[#6:1]-[c:2]1[c:3][c:4]([c:8](-
[#6:9])[c:10]([Br:13])[c:11]1-[#8:12])-[#7+:5](-[#8-:7])=[O:6]

Correctness of the mapping

MAPPET	YES
ReactionMap	NO
Marvin	YES

Reaction no 125

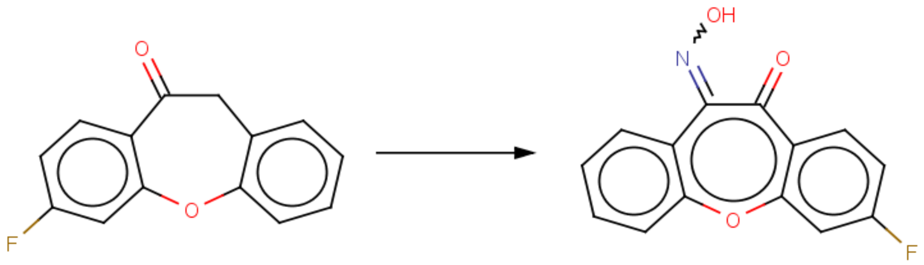

SMILES of the input:
O=C1Cc2ccccc2Oc2cc(F)ccc21>>O=c1c2ccc(F)cc2oc2ccccc2c1=NO

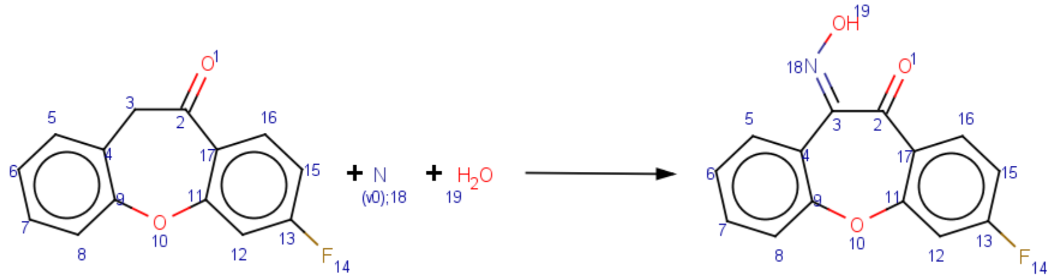

Correct mapped SMILES/SMARTS of the reaction:
[F:14][c:13]1[c:15][c:16][c:17]-2[c:11](-[#8:10]-[c:9]3[c:8][c:7][c:6][c:5][c:4]3-[#6:3]-[#6:2]-2=[O:1])[c:12]1.[#7;v0:18].[#8:19]>>[#8:19]\[#7:18]=[c:3]1\[c:4]2[c:5][c:6][c:7][c:8][c:9]2[o:10][c:11]2[c:12][c:13]([F:14])[c:15][c:16][c:17]2[c:2]1=[O:1]

Correctness of the mapping
MAPPET YES
ReactionMap NO
Marvin YES

Reaction no 126

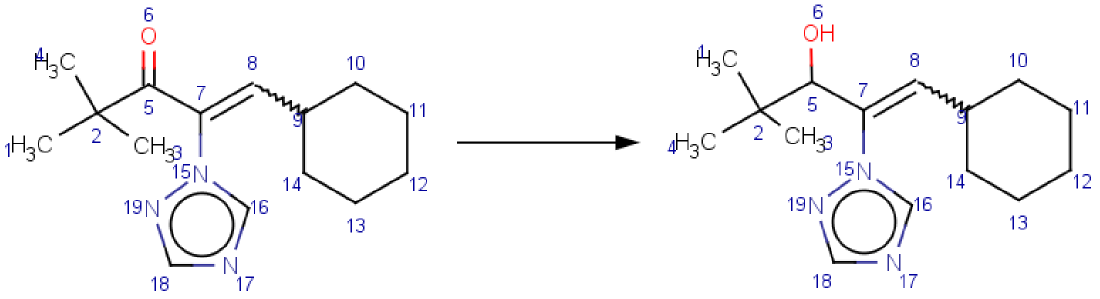

Correct mapped SMILES/SMARTS of the reaction:
[#6:1][C:2]([#6:3])([#6:4])[#6:5](=[O:6])-[#6:7](=[#6:8]-[#6:9]-1-[#6:10]-[#6:11]-[#6:12]-[#6:13]-[#6:14]-1)-[n:15]1[c:16][n:17][c:18][n:19]1>>[#6:4][C:2]([#6:3])([#6:1])[#6:5](-[#8:6])-[#6:7](=[#6:8]-[#6:9]-1-[#6:10]-[#6:11]-[#6:12]-[#6:13]-[#6:14]-1)-[n:15]1[c:16][n:17][c:18][n:19]1

Correctness of the mapping
MAPPET YES
ReactionMap YES
Marvin YES

Reaction no 127

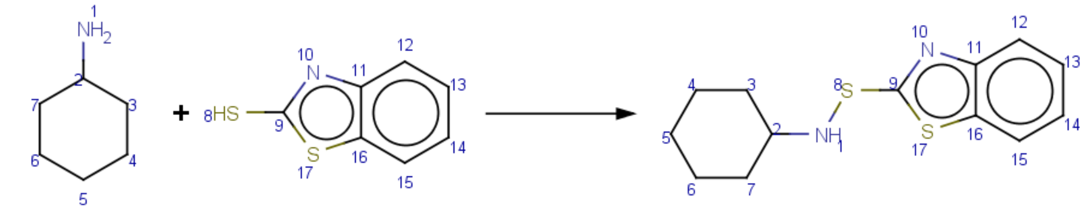

Correct mapped SMILES/SMARTS of the reaction:
[#7:1]-[#6:2]-1-[#6:3]-[#6:4]-[#6:5]-[#6:6]-[#6:7]-1.[#16:8]-[c:9]1[n:10][c:11]2[c:12][c:13][c:14][c:15][c:16]2[s:17]1>>[#6:5]-1-[#6:4]-[#6:3]-[#6:2](-[#6:7]-[#6:6]-1)-[#7:1]-[#16:8]-[c:9]1[n:10][c:11]2[c:12][c:13][c:14][c:15][c:16]2[s:17]1

Correctness of the mapping
MAPPET YES
ReactionMap YES
Marvin YES

Reaction no 128

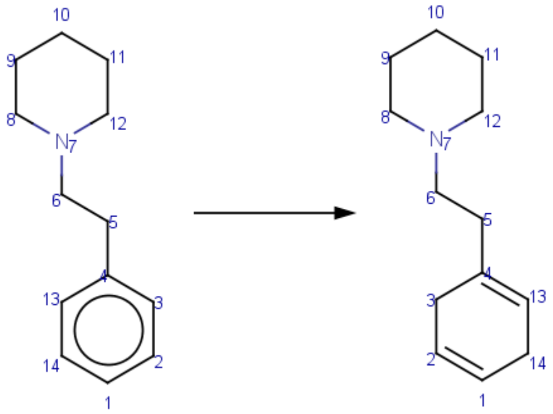

Correct mapped SMILES/SMARTS of the reaction:
[#6:6](-[#6:5]-[c:4]1[c:3][c:2][c:1][c:14][c:13]1)-[#7:7]-1-[#6:8]-[#6:9]-[#6:10]-[#6:11]-[#6:12]-1>>[#6:6](-[#6:5]-[#6:4]-1=[#6:13]-[#6:14]-[#6:1]=[#6:2]-[#6:3]-1)-[#7:7]-1-[#6:8]-[#6:9]-[#6:10]-[#6:11]-[#6:12]-1

Correctness of the mapping
MAPPET YES
ReactionMap YES
Marvin YES

Reaction no 129

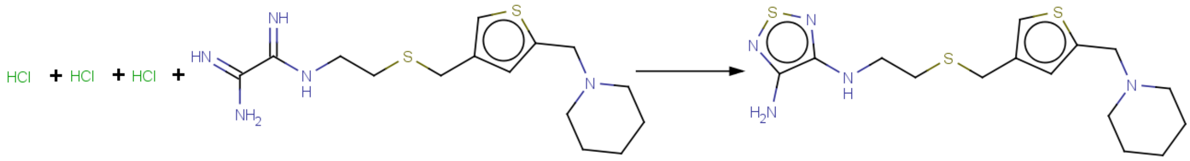

SMILES of the input:
Cl.Cl.Cl.N=C(N)C(=N)NCCSCc1csc(CN2CCCCC2)c1>>Nc1nsnc1NCCSCc1csc(CN2CCCCC2)c1

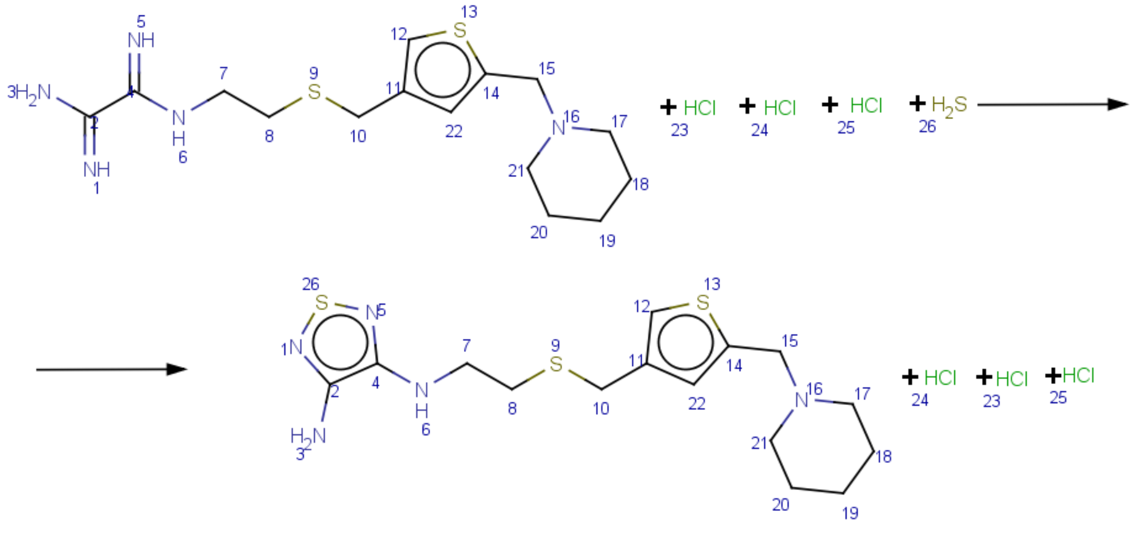

Correct mapped SMILES/SMARTS of the reaction:
[#7:3]-[#6:2](=[#7:1])-[#6:4](=[#7:5])-[#7:6]-[#6:7]-[#6:8]-[#16:9]-[#6:10]-[c:11]1[c:12][s:13][c:14](-[#6:15]-[#7:16]-2-[#6:17]-[#6:18]-[#6:19]-[#6:20]-[#6:21]-2)[c:22]1.[Cl:23].[Cl:24].[Cl:25].[#16:26]>>[#7:3]-[c:2]1[n:1][s:26][n:5][c:4]1-[#7:6]-[#6:7]-[#6:8]-[#16:9]-[#6:10]-[c:11]1[c:12][s:13][c:14](-[#6:15]-[#7:16]-2-[#6:17]-[#6:18]-[#6:19]-[#6:20]-[#6:21]-2)[c:22]1.[Cl:24].[Cl:23].[Cl:25]

Correctness of the mapping

MAPPET	YES
ReactionMap	NO
Marvin	YES

Reaction no 130

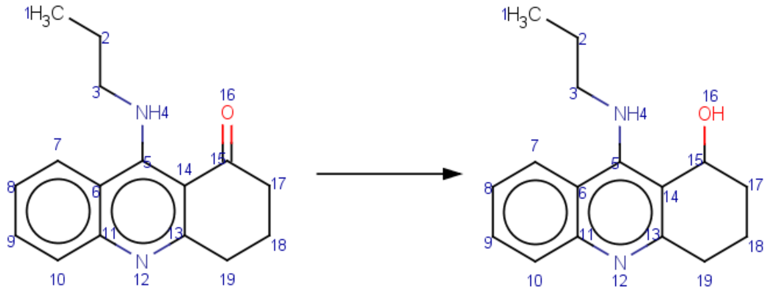

Correct mapped SMILES/SMARTS of the reaction:
[#6:1]-[#6:2]-[#6:3]-[#7:4]-[c:5]1[c:14]2-[#6:15](=[O:16])-[#6:17]-[#6:18]-[#6:19]-[c:13]2[n:12][c:11]2[c:10][c:9][c:8][c:7][c:6]12>>[#6:1]-[#6:2]-[#6:3]-[#7:4]-[c:5]1[c:14]2-[#6:15](-[#8:16])-[#6:17]-[#6:18]-[#6:19]-[c:13]2[n:12][c:11]2[c:10][c:9][c:8][c:7][c:6]12

Correctness of the mapping

MAPPET	YES
ReactionMap	YES
Marvin	YES

Reaction no 131

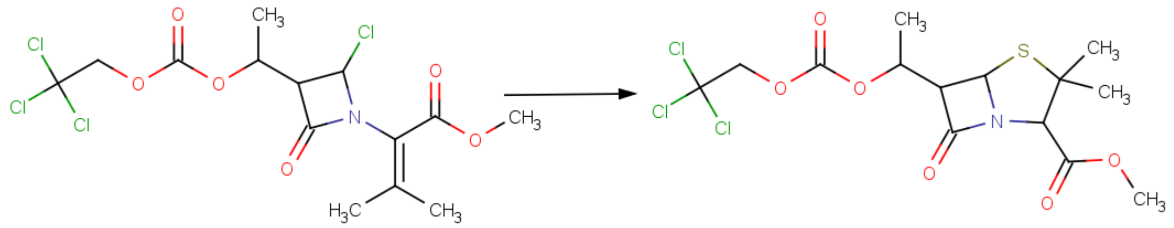

SMILES of the input:
COC(=O)C(=C(C)C)N1C(=O)C(C(C)OC(=O)OCC(Cl)(Cl)Cl)C1Cl>>COC(=O)C1N2C(=O)C(C(C)OC(=O)OCC(Cl)(Cl)Cl)C2SC1(C)C

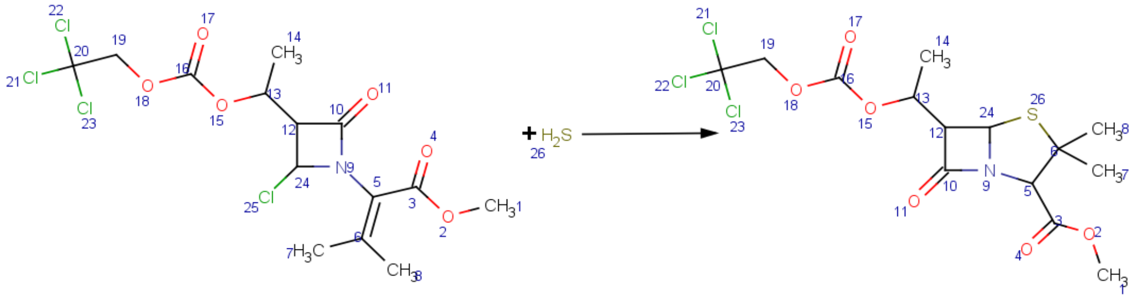

Correct mapped SMILES/SMARTS of the reaction:
[#6:1]-[#8:2]-[#6:3](=[O:4])-[#6:5](-[#7:9]-1-[#6:24]([Cl:25])-[#6:12](-[#6:13](-[#6:14])-[#8:15]-[#6:16](=[O:17])-[#8:18]-[#6:19][C:20]([Cl:21])([Cl:22])[Cl:23])-[#6:10]-1=[O:11])=[#6:6](-[#6:7])-[#6:8].[#16:26]>>[#6:1]-[#8:2]-[#6:3](=[O:4])-[#6:5]1-[#7:9]-2-[#6:24](-[#16:26][C:6]1([#6:8])[#6:7])-[#6:12](-[#6:13](-[#6:14])-[#8:15]-[#6:16](=[O:17])-[#8:18]-[#6:19][C:20]([Cl:22])([Cl:21])[Cl:23])-[#6:10]-2=[O:11].[Cl:25]

Correctness of the mapping

MAPPET	YES
ReactionMap	NO
Marvin	YES

Reaction no 132

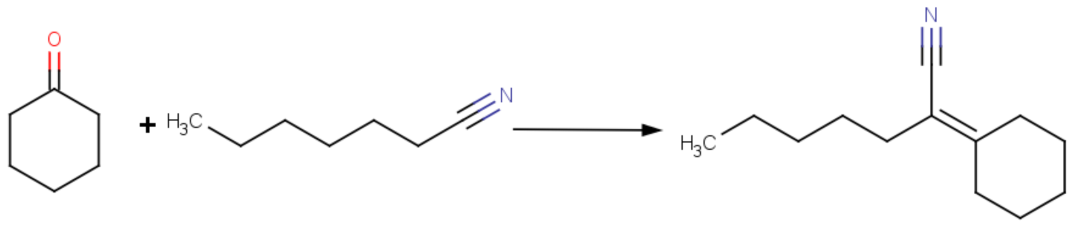

SMILES of the input:
O=C1CCCCC1.CCCCCCC#N>>CCCCCC(C#N)=C1CCCCC1

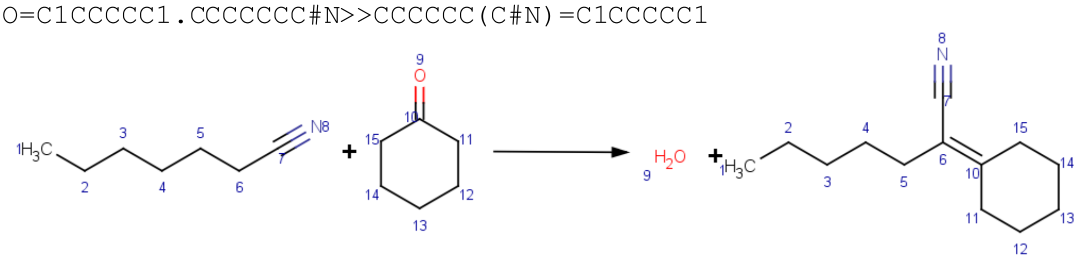

Correct mapped SMILES/SMARTS of the reaction:

[#6:1]-[#6:2]-[#6:3]-[#6:4]-[#6:5]-[#6:6][C:7]#[N:8].[O:9]=[#6:10]-1-[#6:11]-[#6:12]-[#6:13]-[#6:14]-[#6:15]-1>>[#8:9].[#6:1]-[#6:2]-[#6:3]-[#6:4]-[#6:5]\[#6:6]([C:7]#[N:8])=[#6:10]-1\[#6:15]-[#6:14]-[#6:13]-[#6:12]-[#6:11]-1

Correctness of the mapping
MAPPET YES
ReactionMap NO
Marvin YES

Reaction no 133

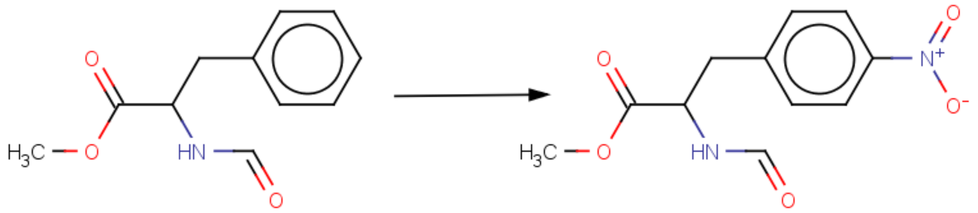

SMILES of the input:
COC(=O)C(Cc1ccccc1)NC=O>>COC(=O)C(Cc1ccc([N+](=O)[O-])cc1)NC=O

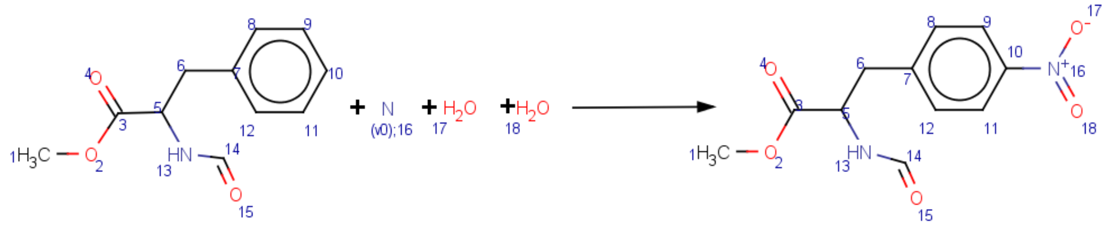

Correct mapped SMILES/SMARTS of the reaction:
[#6:1]-[#8:2]-[#6:3](=[O:4])-[#6:5](-[#6:6]-[c:7]1[c:8][c:9][c:10][c:11][c:12]1)-[#7:13]-[#6:14]=[O:15].[#7;v0:16].[#8:17].[#8:18]>>[#6:1]-[#8:2]-[#6:3](=[O:4])-[#6:5](-[#6:6]-[c:7]1[c:8][c:9][c:10]([c:11][c:12]1)-[#7+:16](-[#8-:17])=[O:18])-[#7:13]-[#6:14]=[O:15]

Correctness of the mapping
MAPPET YES
ReactionMap NO
Marvin YES

Reaction no 134

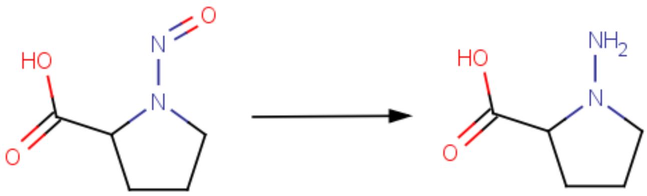

SMILES of the input:
O=NN1CCCC1C(=O)O>>NN1CCCC1C(=O)O

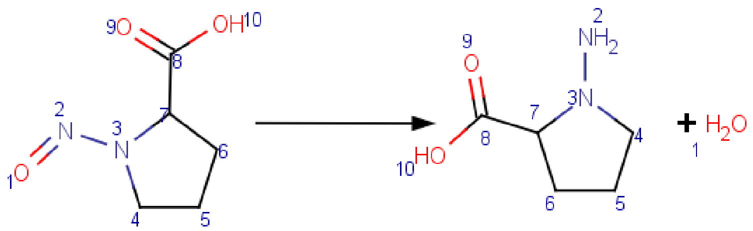

Correct mapped SMILES/SMARTS of the reaction:
[#8:10]-[#6:8](=[O:9])-[#6:7]-1-[#6:6]-[#6:5]-[#6:4]-[#7:3]-1-[#7:2]=[O:1]>>[#7:2]-[#7:3]-1-[#6:4]-[#6:5]-[#6:6]-[#6:7]-1-[#6:8](-[#8:10])=[O:9].[#8:1]

Correctness of the mapping

MAPPET	YES
ReactionMap	NO
Marvin	YES

Reaction no 135

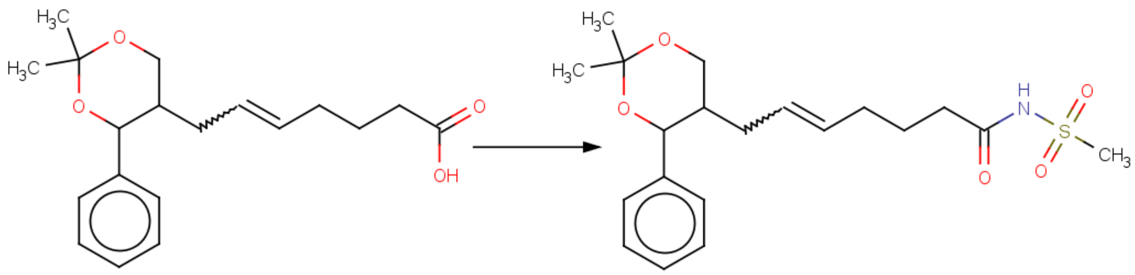

SMILES of the input:
CC1(C)OCC(CC=CCCCC(=O)O)C(c2ccccc2)O1>>CC1(C)OCC(CC=CCCCC(=O)NS(C)(=O)=O)C(c2ccccc2)O1

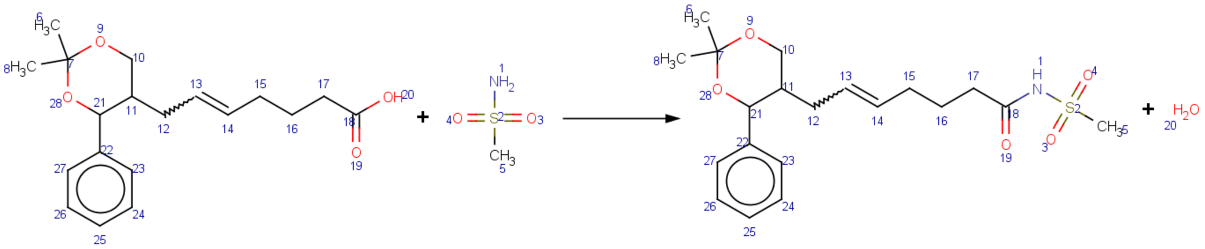

Correct mapped SMILES/SMARTS of the reaction:
[CH3:6][C:7]1([CH3:8])[O:9][CH2:10][CH:11]([CH2:12][CH:13]=[CH:14][CH2:15][CH2:16][CH2:17][C:18]([OH:20])=[O:19])[CH:21]([O:28]1)[c:22]1[cH:23][cH:24][cH:25][cH:26][cH:27]1.[CH3:5][S:2]([NH2:1])(=[O:3])=[O:4]>>[CH3:6][C:7]1([CH3:8])[O:9][CH2:10][CH:11]([CH2:12][CH:13]=[CH:14][CH2:15][CH2:16][CH2:17][C:18](=[O:19])[NH:1][S:2]([CH3:5])(=[O:4])=[O:3])[CH:21]([O:28]1)[c:22]1[cH:23][cH:24][cH:25][cH:26][cH:27]1.[OH2:20]

Correctness of the mapping

MAPPET	YES
ReactionMap	NO
Marvin	YES

Reaction no 136

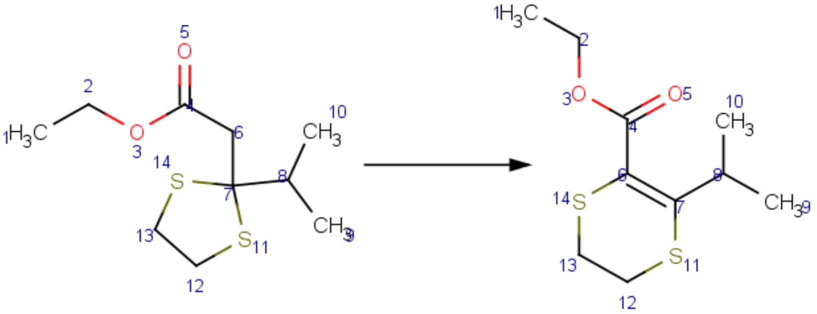

Correct mapped SMILES/SMARTS of the reaction:
[#6:1]-[#6:2]-[#8:3]-[#6:4](=[O:5])-[#6:6][C:7]1([#16:11]-[#6:12]-[#6:13]-[#16:14]1)[#6:8](-[#6:9])-[#6:10]>>[#6:1]-[#6:2]-[#8:3]-[#6:4](=[O:5])-[#6:6]-1=[#6:7](-[#16:11]-[#6:12]-[#6:13]-[#16:14]-1)-[#6:8](-[#6:9])-[#6:10]

Correctness of the mapping

MAPPET	YES
ReactionMap	YES
Marvin	YES

Reaction no 137

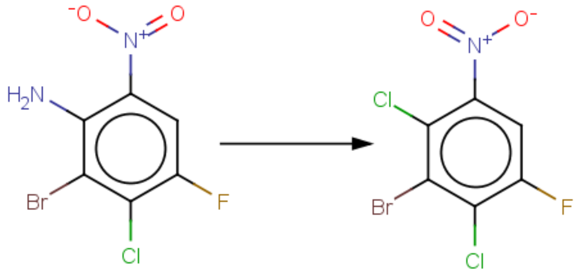

SMILES of the input:
Nc1c(Br)c(Cl)c(F)cc1[N+](=O)[O-]>>O=[N+]([O-])c1cc(F)c(Cl)c(Br)c1Cl

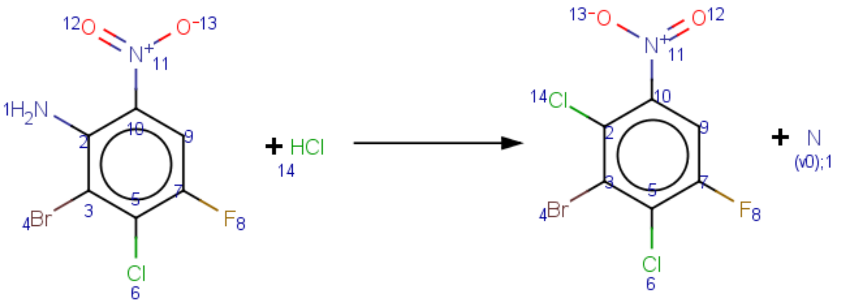

Correct mapped SMILES/SMARTS of the reaction:
[#7:1]-[c:2]1[c:3]([Br:4])[c:5]([Cl:6])[c:7]([F:8])[c:9][c:10]1-[#7+:11](-[#8-:13])=[O:12].[Cl:14]>>[#8-:13]-[#7+:11](=[O:12])-[c:10]1[c:9][c:7]([F:8])[c:5]([Cl:6])[c:3]([Br:4])[c:2]1[Cl:14].[#7;v0:1]

Correctness of the mapping

MAPPET	YES
ReactionMap	NO
Marvin	YES

Reaction no 138

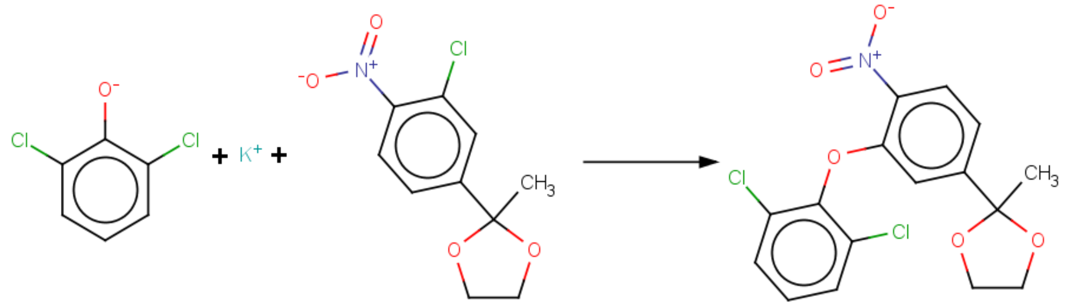

SMILES of the input:
CC1(c2ccc([N+](=O)[O-])c(Cl)c2)OCCO1.[K+].[O-]c1c(Cl)cccc1Cl>>CC1(c2ccc([N+](=O)[O-])c(Oc3c(Cl)cccc3Cl)c2)OCCO1

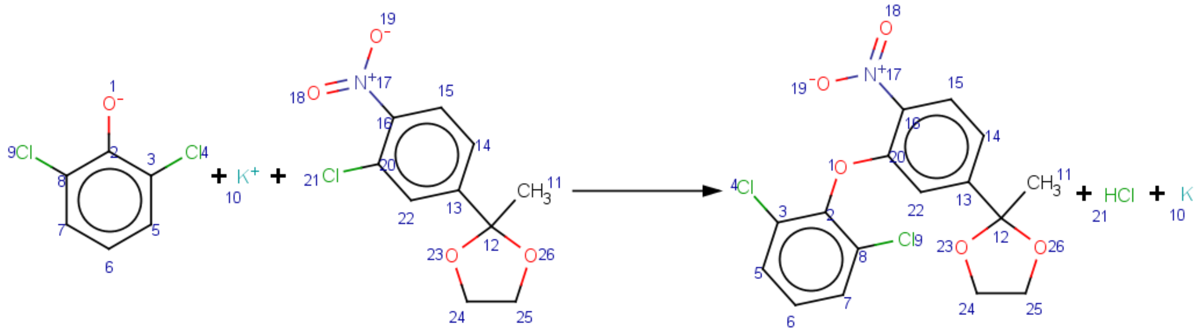

Correct mapped SMILES/SMARTS of the reaction:
[#8-:1]-[c:2]1[c:3]([Cl:4])[c:5][c:6][c:7][c:8]1[Cl:9].[K+:10].[#6:11][C:12]1([#8:23]-[#6:24]-[#6:25]-[#8:26]1)[c:13]1[c:14][c:15][c:16]([c:20]([Cl:21])[c:22]1)-[#7+:17](-[#8-:19])=[O:18]>>[#6:11][C:12]1([#8:23]-[#6:24]-[#6:25]-[#8:26]1)[c:13]1[c:14][c:15][c:16]([c:20](-[#8:1]-[c:2]2[c:3]([Cl:4])[c:5][c:6][c:7][c:8]2[Cl:9])[c:22]1)-[#7+:17](-[#8-:19])=[O:18].[Cl:21].[K:10]

Correctness of the mapping

MAPPET	YES
ReactionMap	NO
Marvin	YES

Reaction no 139

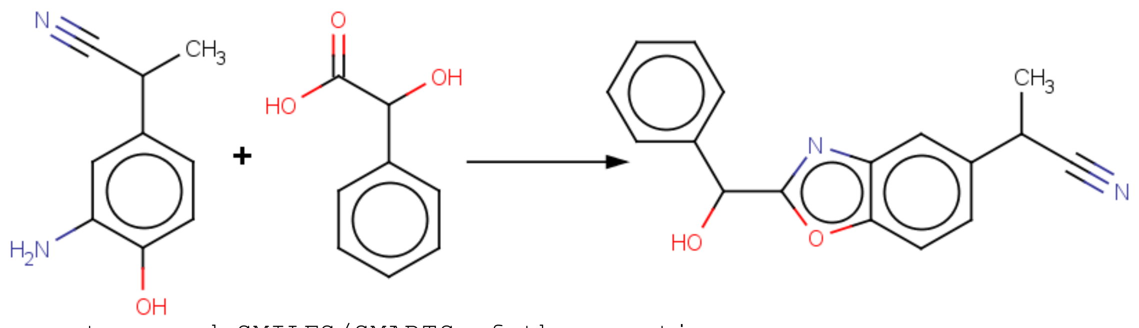

Correct mapped SMILES/SMARTS of the reaction:
CC(C#N)c1ccc(O)c(N)c1.O=C(O)C(O)c1ccccc1>>CC(C#N)c1ccc2oc(C(O)c3ccccc3)nc2c1

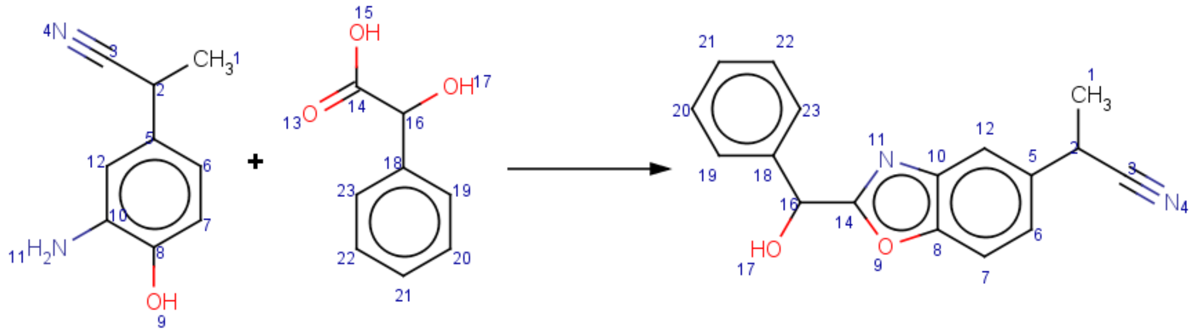

[#6:1]-[#6:2]([C:3]#[N:4])-[c:5]1[c:6][c:7][c:8](-[#8:9])[c:10](-[#7:11])[c:12]1.[#8:17]-[#6:16](-[#6:14](-[#8:15])=[O:13])-[c:18]1[c:19][c:20][c:21][c:22][c:23]1>>[#6:1]-[#6:2]([C:3]#[N:4])-[c:5]1[c:6][c:7][c:8]2[o:9][c:14]([n:11][c:10]2[c:12]1)-[#6:16](-[#8:17])-[c:18]1[c:19][c:20][c:21][c:22][c:23]1

Correctness of the mapping
MAPPET NO
ReactionMap NO
Marvin NO

Reaction no 140

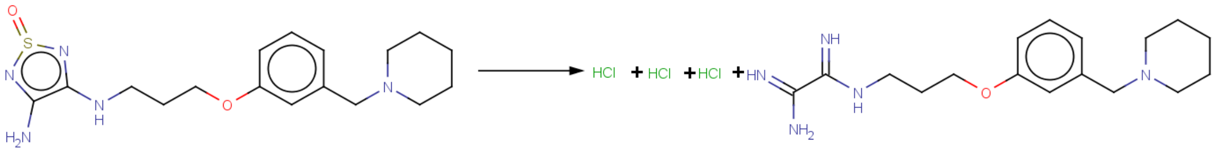

SMILES of the input:
Nc1ns(=O)nc1NCCCOc1cccc(CN2CCCCC2)c1>>Cl.Cl.Cl.N=C(N)C(=N)NCCCOc1cccc(CN2CCCCC2)c1

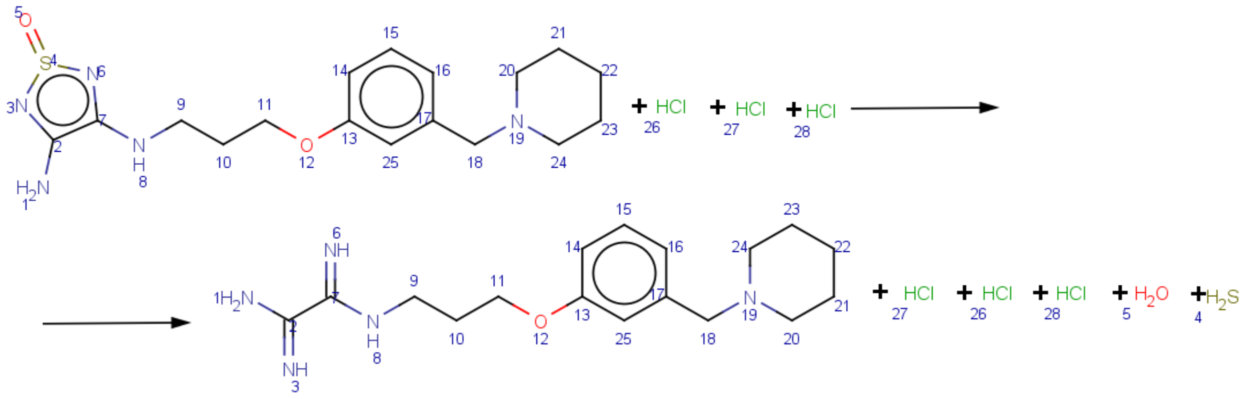

Correct mapped SMILES/SMARTS of the reaction:
[#7:1]-[c:2]1[n:3][s:4](=[O:5])[n:6][c:7]1-[#7:8]-[#6:9]-[#6:10]-[#6:11]-[#8:12]-[c:13]1[c:14][c:15][c:16][c:17](-[#6:18]-[#7:19]-2-[#6:20]-[#6:21]-[#6:22]-[#6:23]-[#6:24]-2)[c:25]1.[Cl:26].[Cl:27].[Cl:28]>>[#7:1]-[#6:2](=[#7:3])-[#6:7](=[#7:6])-[#7:8]-[#6:9]-[#6:10]-[#6:11]-[#8:12]-[c:13]1[c:14][c:15][c:16][c:17](-[#6:18]-[#7:19]-2-[#6:24]-[#6:23]-[#6:22]-[#6:21]-[#6:20]-2)[c:25]1.[Cl:27].[Cl:26].[Cl:28].[#8:5].[#16:4]

Correctness of the mapping
MAPPET YES

ReactionMap NO
Marvin YES

Reaction no 141

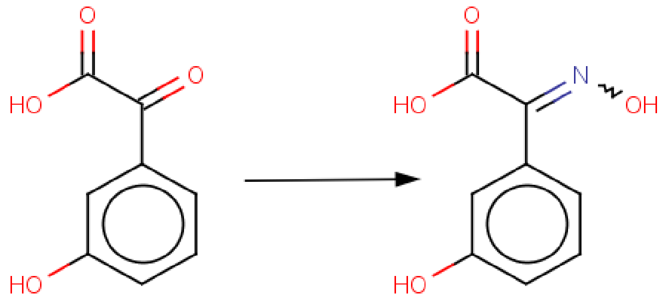

Correct mapped SMILES/SMARTS of the reaction:
O=C(O)C(=O)c1cccc(O)c1>>O=C(O)C(=NO)c1cccc(O)c1

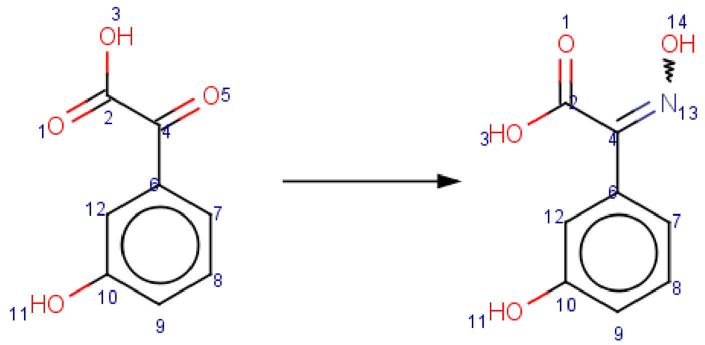

[#8:3]-[#6:2](=[O:1])-[#6:4](=[O:5])-[c:6]1[c:7][c:8][c:9][c:10](-[#8:11])[c:12]1>>[#8:14]-[#7:13]=[#6:4](-[#6:2](-[#8:3])=[O:1])-[c:6]1[c:7][c:8][c:9][c:10](-[#8:11])[c:12]1
Correctness of the mapping
MAPPET NO
ReactionMap NO
Marvin NO

Reaction no 142

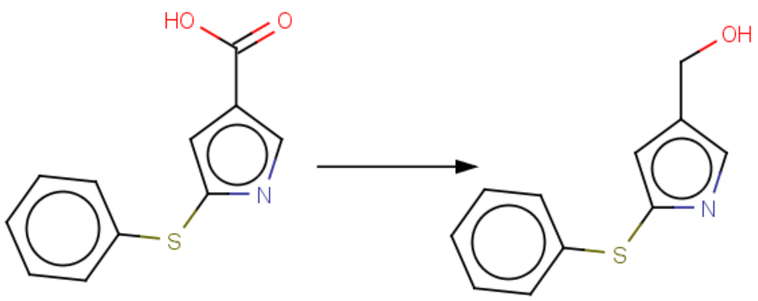

SMILES of the input:
O=C(O)c1cnc(Sc2ccccc2)c1>>OCc1cnc(Sc2ccccc2)c1

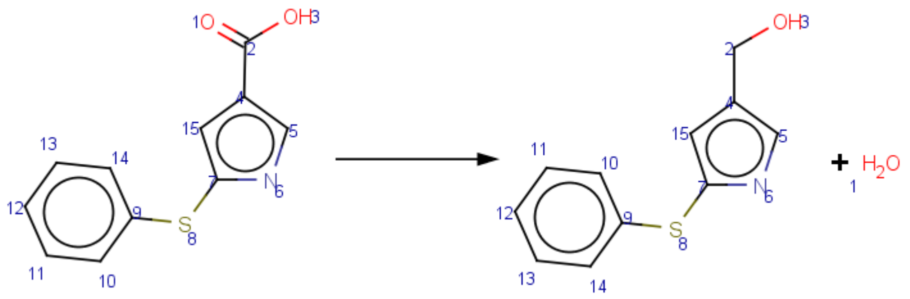

Correct mapped SMILES/SMARTS of the reaction:
[#8:3]-[#6:2](=[O:1])-[c:4]1[c:5][n:6][c:7](-[#16:8]-[c:9]2[c:10][c:11][c:12][c:13][c:14]2)[c:15]1>>[#8:3]-[#6:2]-[c:4]1[c:5][n:6][c:7](-[#16:8]-[c:9]2[c:14][c:13][c:12][c:11][c:10]2)[c:15]1.[#8:1]

Correctness of the mapping

MAPPET	YES
ReactionMap	NO
Marvin	YES

Reaction no 143

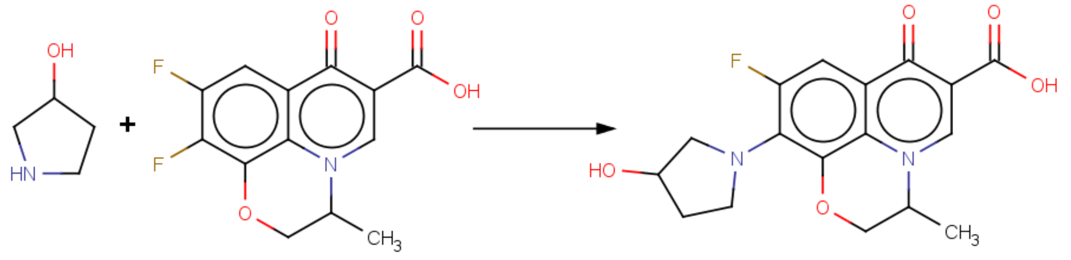

SMILES of the input:
OC1CCNC1.CC1COc2c(F)c(F)cc3c(=O)c(C(=O)O)cn1c32>>CC1COc2c(N3CCC(O)C3)c(F)cc3c(=O)c(C(=O)O)cn1c32

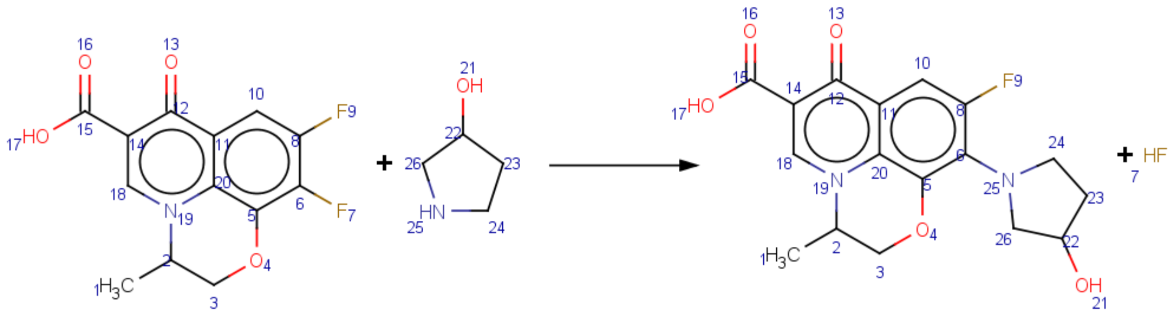

Correct mapped SMILES/SMARTS of the reaction:
[#6:1]-[#6:2]-1-[#6:3]-[#8:4]-[c:5]2[c:6]([F:7])[c:8]([F:9])[c:10][c:11]3[c:20]2[n:19]-1[c:18][c:14](-[#6:15](-[#8:17])=[O:16])[c:12]3=[O:13].[#8:21]-[#6:22]-1-[#6:23]-[#6:24]-[#7:25]-[#6:26]-1>>[#6:1]-[#6:2]-1-[#6:3]-[#8:4]-[c:5]2[c:6](-[#7:25]-3-[#6:24]-[#6:23]-[#6:22](-[#8:21])-[#6:26]-3)[c:8]([F:9])[c:10][c:11]3[c:20]2[n:19]-1[c:18][c:14](-[#6:15](-[#8:17])=[O:16])[c:12]3=[O:13].[F:7]

Correctness of the mapping

MAPPET	YES
ReactionMap	NO

Marvin YES

Reaction no 144

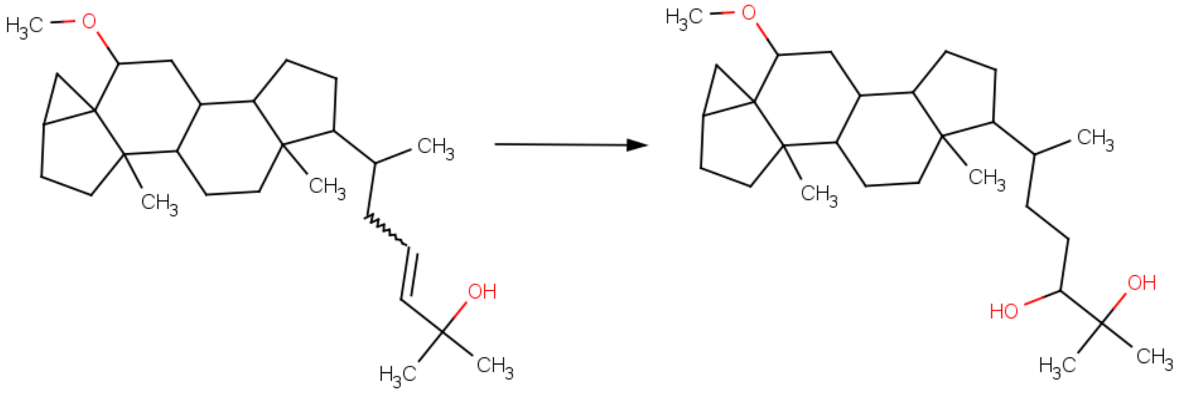

SMILES of the input:
COC1CC2C3CCC(C(C)CC=CC(C)(C)O)C3(C)CCC2C2(C)CCC3CC312>>COC1CC2C3CCC(C(C)CCC(O)C(C)(C)O)C3(C)CCC2C2(C)CCC3CC312

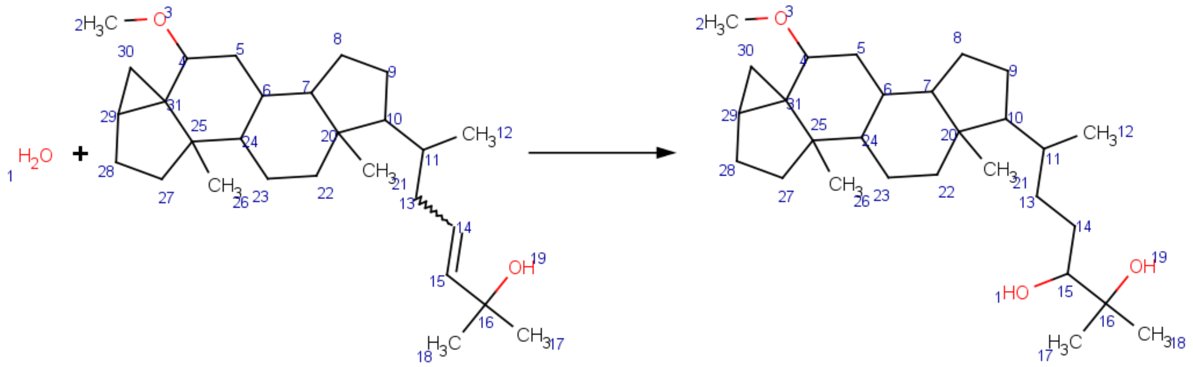

Correct mapped SMILES/SMARTS of the reaction:
[#8:1].[#6:2]-[#8:3]-[#6:4]1-[#6:5]-[#6:6]-2-[#6:7]3-[#6:8]-[#6:9]-[#6:10](-[#6:11](-[#6:12])-[#6:13]-[#6:14]=[#6:15][C:16]([#6:17])([#6:18])[#8:19])[C:20]3([#6:21])[#6:22]-[#6:23]-[#6:24]-2[C:25]2([#6:26])[#6:27]-[#6:28]-[#6:29]3-[#6:30][C:31]123>>[#6:2]-[#8:3]-[#6:4]1-[#6:5]-[#6:6]-2-[#6:7]3-[#6:8]-[#6:9]-[#6:10](-[#6:11](-[#6:12])-[#6:13]-[#6:14]-[#6:15](-[#8:1])[C:16]([#6:18])([#6:17])[#8:19])[C:20]3([#6:21])[#6:22]-[#6:23]-[#6:24]-2[C:25]2([#6:26])[#6:27]-[#6:28]-[#6:29]3-[#6:30][C:31]123

Correctness of the mapping
MAPPET YES
ReactionMap NO
Marvin YES

Reaction no 145

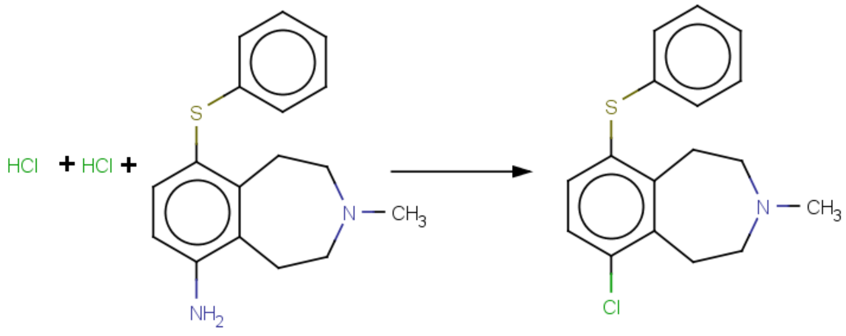

SMILES of the input:
Cl.Cl.CN1CCc2c(N)ccc(Sc3ccccc3)c2CC1>>CN1CCc2c(Cl)ccc(Sc3ccccc3)c2CC1

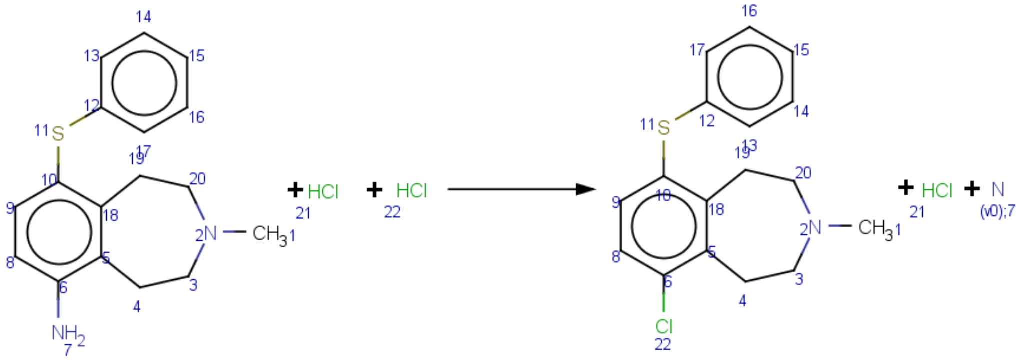

Correct mapped SMILES/SMARTS of the reaction:
[#6:1]-[#7:2]-1-[#6:3]-[#6:4]-[c:5]2[c:6](-[#7:7])[c:8][c:9][c:10](-
[#16:11]-[c:12]3[c:13][c:14][c:15][c:16][c:17]3)[c:18]2-[#6:19]-[#6:20]-
1.[Cl:21].[Cl:22]>>[#6:1]-[#7:2]-1-[#6:3]-[#6:4]-
[c:5]2[c:6]([Cl:22])[c:8][c:9][c:10](-[#16:11]-
[c:12]3[c:17][c:16][c:15][c:14][c:13]3)[c:18]2-[#6:19]-[#6:20]-
1.[Cl:21].[#7;v0:7]

Correctness of the mapping
MAPPET YES
ReactionMap NO
Marvin YES

Reaction no 146

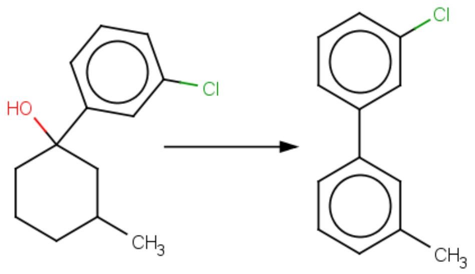

SMILES of the input:
CC1CCCC(O)(c2cccc(Cl)c2)C1>>Cc1cccc(-c2cccc(Cl)c2)c1

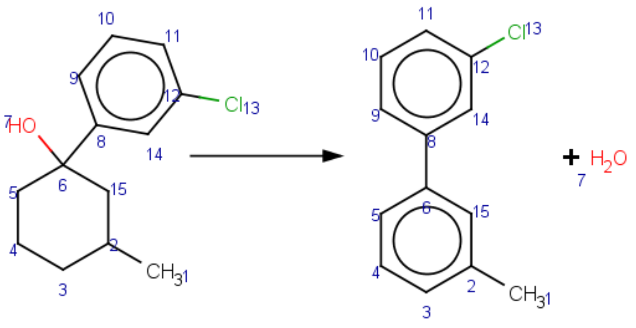

Correct mapped SMILES/SMARTS of the reaction:
[#6:1]-[#6:2]-1-[#6:3]-[#6:4]-[#6:5][C:6]([#8:7])([#6:15]-
1)[c:8]1[c:9][c:10][c:11][c:12]([Cl:13])[c:14]1>>[#6:1]-
[c:2]1[c:3][c:4][c:5][c:6]([c:15]1)-
[c:8]1[c:9][c:10][c:11][c:12]([Cl:13])[c:14]1.[#8:7]

Correctness of the mapping
MAPPET YES

ReactionMap NO
Marvin YES

Reaction no 147

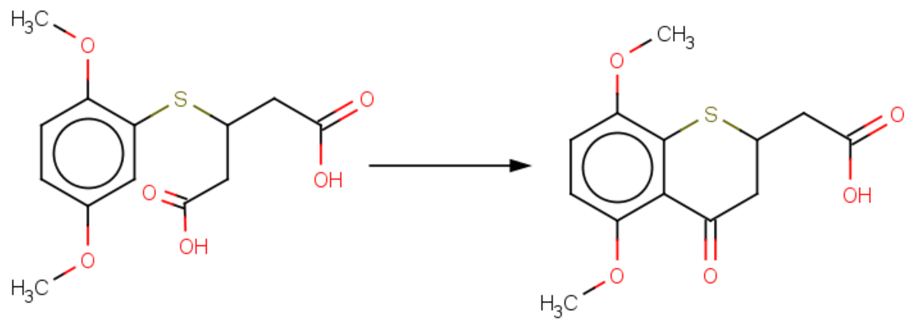

SMILES of the input:
COc1ccc(OC)c(SC(CC(=O)O)CC(=O)O)c1>>COc1ccc(OC)c2c1SC(CC(=O)O)CC2=O

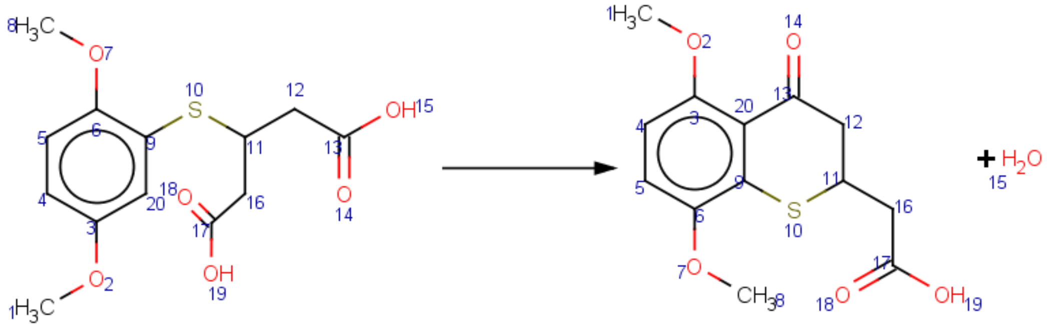

Correct mapped SMILES/SMARTS of the reaction:
[#6:1]-[#8:2]-[c:3]1[c:4][c:5][c:6](-[#8:7]-[#6:8])[c:9](-[#16:10]-[#6:11](-[#6:12]-[#6:13](-[#8:15])=[O:14])-[#6:16]-[#6:17](-[#8:19])=[O:18])[c:20]1>>[#6:8]-[#8:7]-[c:6]1[c:5][c:4][c:3](-[#8:2]-[#6:1])[c:20]2-[#6:13](=[O:14])-[#6:12]-[#6:11](-[#6:16]-[#6:17](-[#8:19])=[O:18])-[#16:10]-[c:9]12.[#8:15]

Correctness of the mapping
MAPPET YES
ReactionMap NO
Marvin YES

Reaction no 148

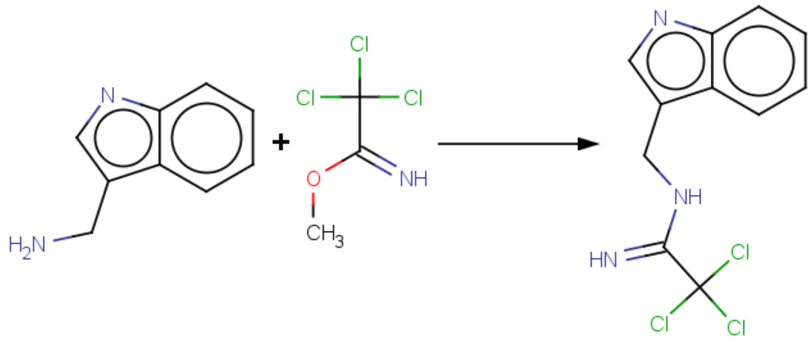

SMILES of the input:
NCc1cnc2ccccc12.COC(=N)C(Cl)(Cl)Cl>>N=C(NCc1cnc2ccccc12)C(Cl)(Cl)Cl

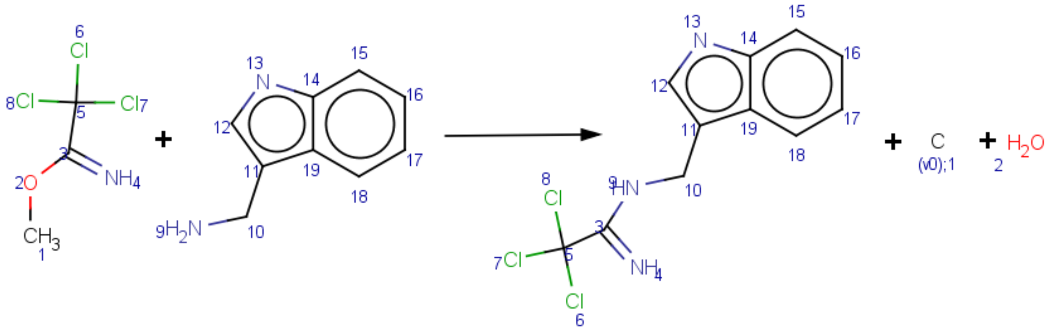

Correct mapped SMILES/SMARTS of the reaction:
[#6:1]-[#8:2]-[#6:3](=[#7:4])[C:5]([Cl:6])([Cl:7])[Cl:8].[#7:9]-[#6:10]-[c:11]1[c:12][n:13][c:14]2[c:15][c:16][c:17][c:18][c:19]12>>[Cl:7][C:5]([Cl:6])([Cl:8])[#6:3](=[#7:4])-[#7:9]-[#6:10]-[c:11]1[c:12][n:13][c:14]2[c:15][c:16][c:17][c:18][c:19]12.[#6;v0:1].[#8:2]

Correctness of the mapping

MAPPET	YES
ReactionMap	NO
Marvin	YES

Reaction no 149

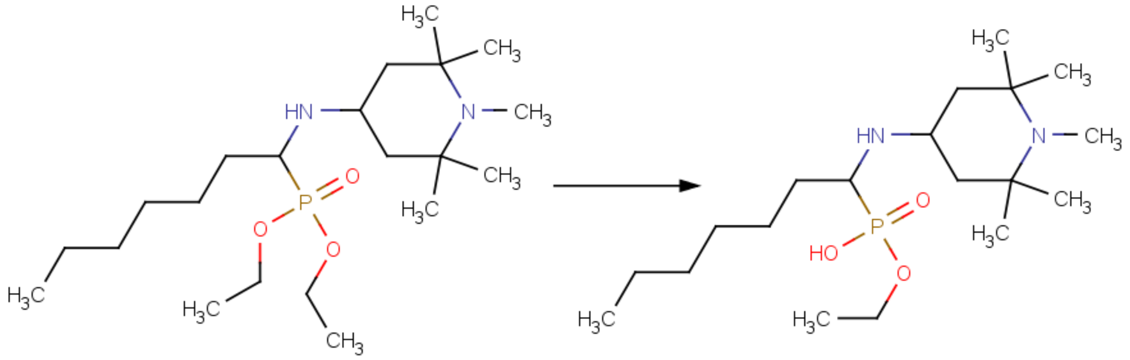

Correct mapped SMILES/SMARTS of the reaction:
CCCCCCC(NC1CC(C)(C)N(C)C(C)(C)C1)P(=O)(OCC)OCC>>CCCCCCC(NC1CC(C)(C)N(C)C(C)(C)C1)P(=O)(O)OCC

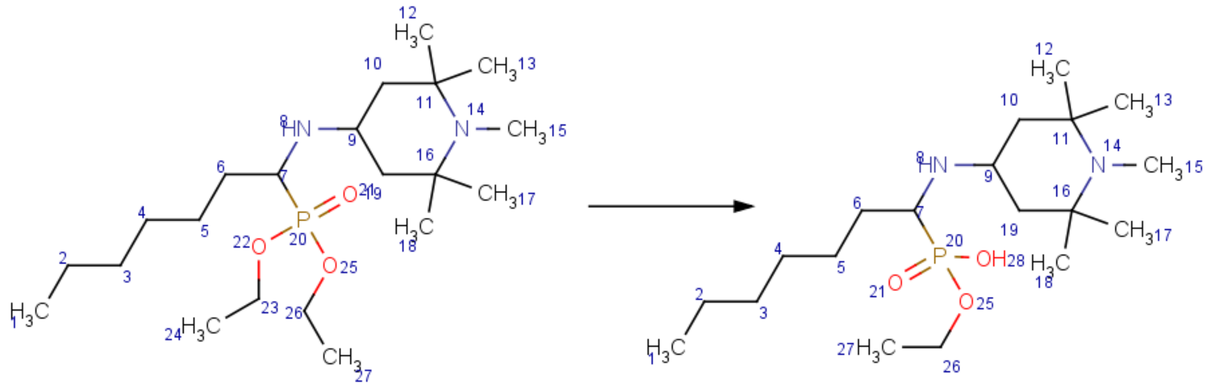

[#6:1]-[#6:2]-[#6:3]-[#6:4]-[#6:5]-[#6:6]-[#6:7](-[#7:8]-[#6:9]-1-[#6:10][C:11]([#6:12])([#6:13])[#7:14](-[#6:15])[C:16]([#6:17])([#6:18])[#6:19]-1)[P:20](=[O:21])([#8:22]-[#6:23]-[#6:24])[#8:25]-[#6:26]-[#6:27]>>[#6:1]-[#6:2]-[#6:3]-[#6:4]-[#6:5]-[#6:6]-[#6:7](-[#7:8]-[#6:9]-1-

[#6:10][C:11]([#6:12])([#6:13])[#7:14](-
[#6:15])[C:16]([#6:17])([#6:18])[#6:19]-
1)[P:20]([#8:28])(=[O:21])[#8:25]-[#6:26]-[#6:27]

Correctness of the mapping
MAPPET NO
ReactionMap NO
Marvin NO

Reaction no 150

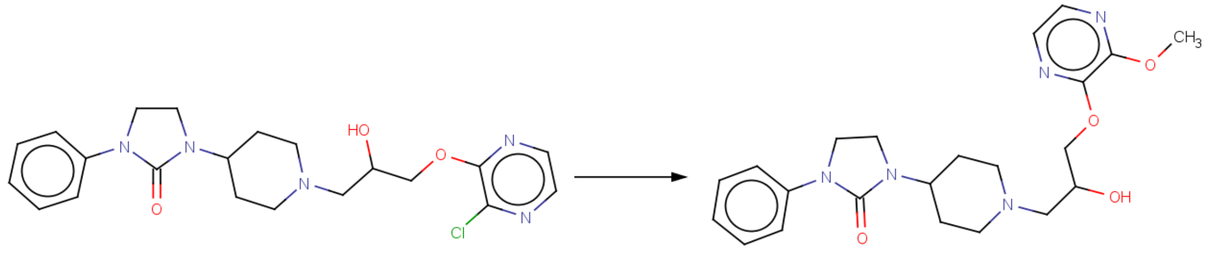

SMILES of the input:
O=C1N(c2ccccc2)CCN1C1CCN(CC(O)COc2nccnc2Cl)CC1>>COc1nccnc1OCC(O)CN1CCC(N2
CCN(c3ccccc3)C2=O)CC1

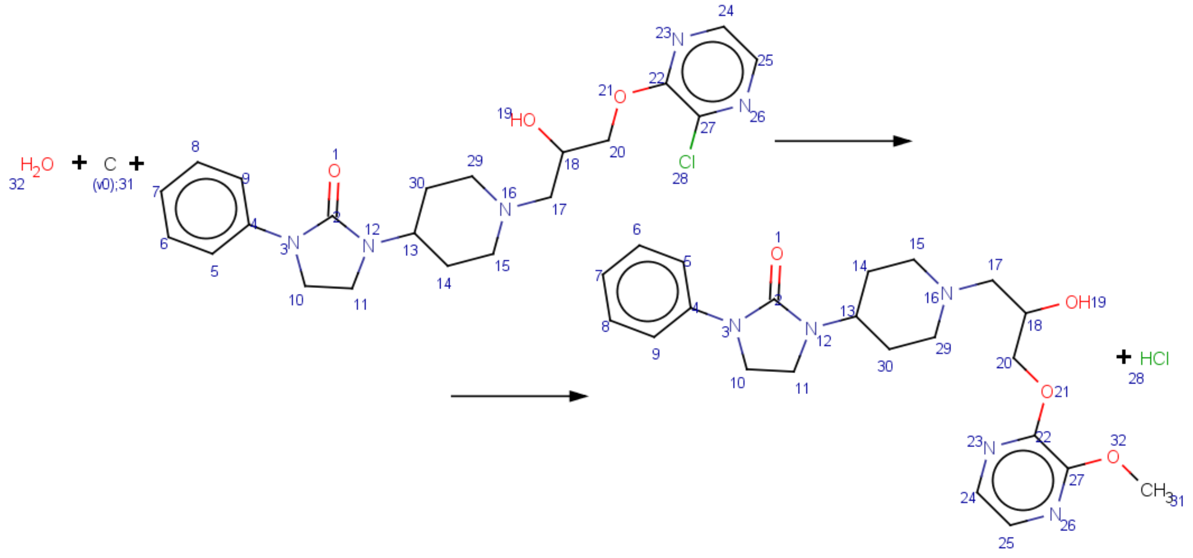

Correct mapped SMILES/SMARTS of the reaction:
[#8:19]-[#6:18](-[#6:20]-[#8:21]-
[c:22]1[n:23][c:24][c:25][n:26][c:27]1[Cl:28])-[#6:17]-[#7:16]-1-[#6:15]-
[#6:14]-[#6:13](-[#6:30]-[#6:29]-1)-[#7:12]-1-[#6:11]-[#6:10]-[#7:3](-
[#6:2]-1=[O:1])-
[c:4]1[c:5][c:6][c:7][c:8][c:9]1.[#6;v0:31].[#8:32]>>[#6:31]-[#8:32]-
[c:27]1[n:26][c:25][c:24][n:23][c:22]1-[#8:21]-[#6:20]-[#6:18](-[#8:19])-
[#6:17]-[#7:16]-1-[#6:29]-[#6:30]-[#6:13](-[#6:14]-[#6:15]-1)-[#7:12]-1-
[#6:11]-[#6:10]-[#7:3](-[#6:2]-1=[O:1])-
[c:4]1[c:9][c:8][c:7][c:6][c:5]1.[Cl:28]

Correctness of the mapping
MAPPET YES
ReactionMap NO
Marvin YES

Reaction no 151

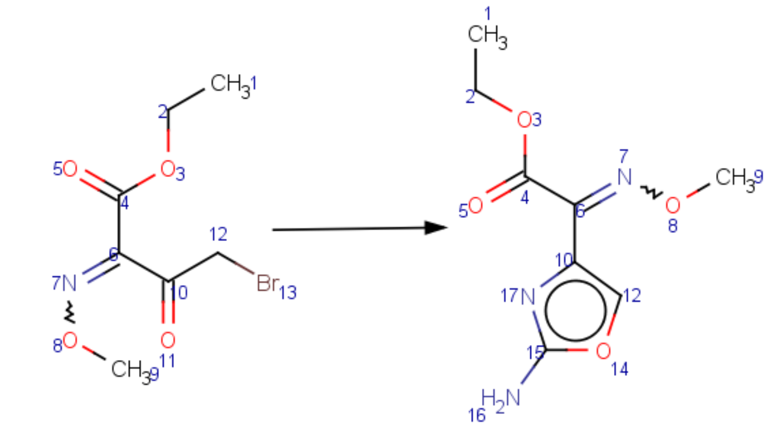

Correct mapped SMILES/SMARTS of the reaction:
[#6:1]-[#6:2]-[#8:3]-[#6:4](=[O:5])-[#6:6](=[#7:7]-[#8:8]-[#6:9])-[#6:10](=[O:11])-[#6:12][Br:13]>>[#6:1]-[#6:2]-[#8:3]-[#6:4](=[O:5])-[#6:6](=[#7:7]-[#8:8]-[#6:9])-[c:10]1[c:12][o:14][c:15](-[#7:16])[n:17]1

Correctness of the mapping

MAPPET	NO
ReactionMap	NO
Marvin	YES

Reaction no 152

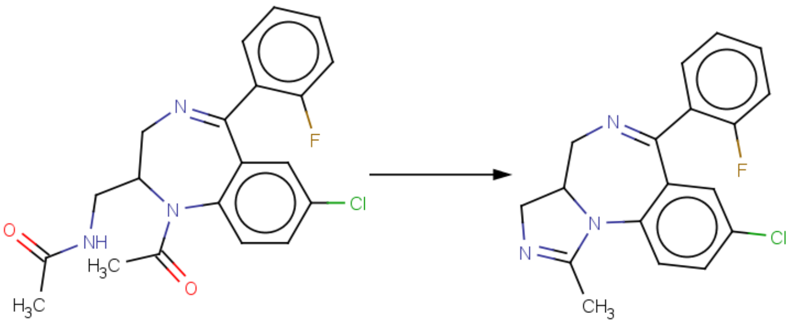

SMILES of the input:
CC(=O)NCC1CN=C(c2ccccc2F)c2cc(Cl)ccc2N1C(C)=O>>CC1=NCC2CN=C(c3ccccc3F)c3cc(Cl)ccc3N12

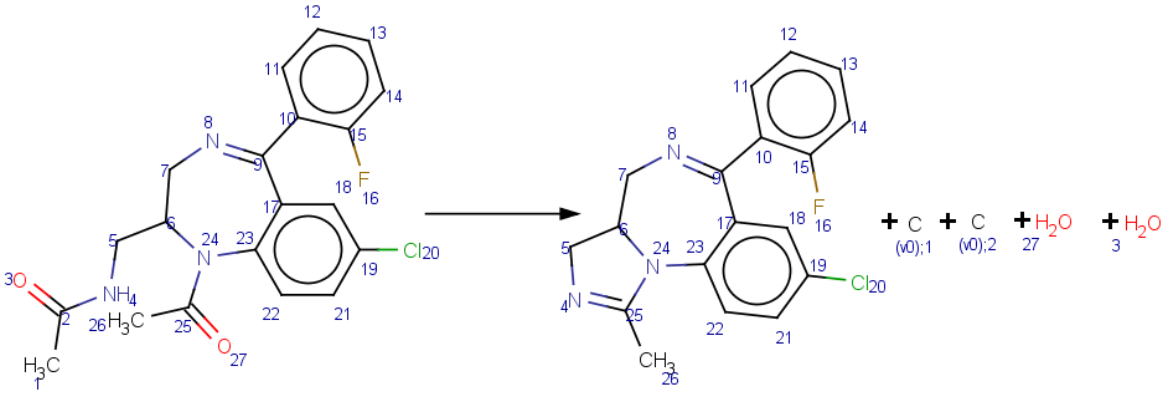

Correct mapped SMILES/SMARTS of the reaction:
[#6:1]-[#6:2](=[O:3])-[#7:4]-[#6:5]-[#6:6]-1-[#6:7]-[#7:8]=[#6:9](-[c:10]2[c:11][c:12][c:13][c:14][c:15]2[F:16])-[c:17]2[c:18][c:19]([Cl:20])[c:21][c:22][c:23]2-[#7:24]-1-[#6:25](-[#6:26])=[O:27]>>[#6:26]-[#6:25]-1=[#7:4]-[#6:5]-[#6:6]-2-[#6:7]-[#7:8]=[#6:9](-[c:10]3[c:11][c:12][c:13][c:14][c:15]3[F:16])-

[c:17]3[c:18][c:19]([Cl:20])[c:21][c:22][c:23]3-[#7:24]-1-2.[#6;v0:1].[#6;v0:2].[#8:27].[#8:3]

Correctness of the mapping
MAPPET YES
ReactionMap NO
Marvin YES

Reaction no 153

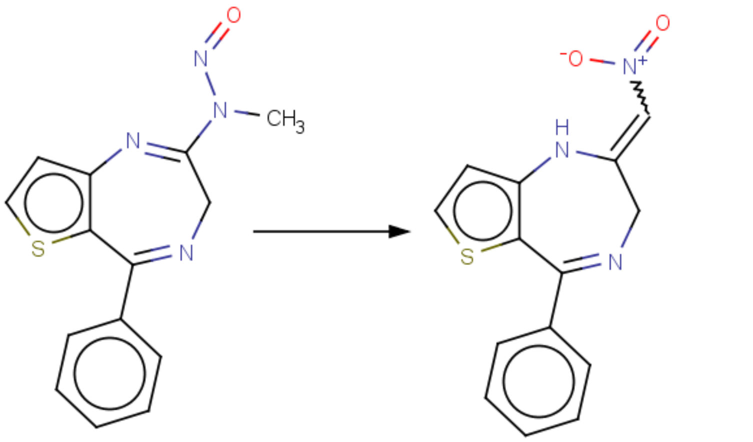

Correct mapped SMILES/SMARTS of the reaction:
CN(N=O)C1=Nc2ccsc2C(c2ccccc2)=NC1>>O=[N+]([O-])C=C1CN=C(c2ccccc2)c2sccc2N1

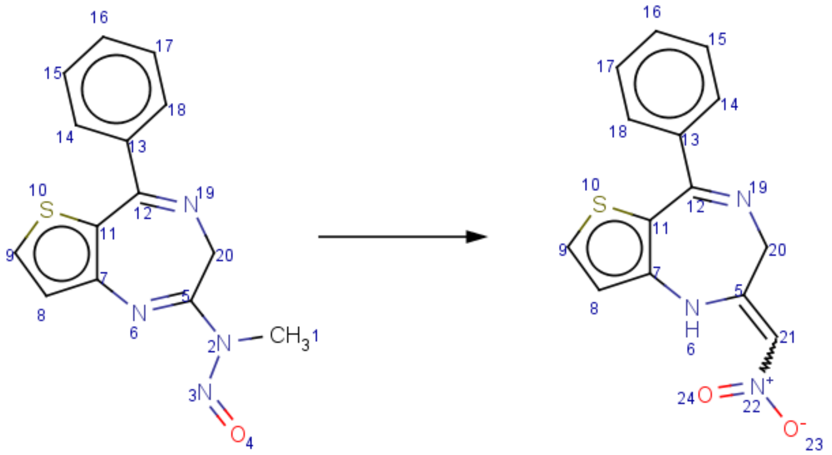

[#6:1]-[#7:2](-[#7:3]=[O:4])-[#6:5]-1=[#7:6]-[c:7]2[c:8][c:9][s:10][c:11]2-[#6:12](=[#7:19]-[#6:20]-1)-[c:13]1[c:14][c:15][c:16][c:17][c:18]1>>[#8-:23]-[#7+:22](=[O:24])-[#6:21]=[#6:5]-1-[#6:20]-[#7:19]=[#6:12](-[c:11]2[s:10][c:9][c:8][c:7]2-[#7:6]-1)-[c:13]1[c:14][c:15][c:16][c:17][c:18]1
Correctness of the mapping
MAPPET NO
ReactionMap NO
Marvin NO

Reaction no 154

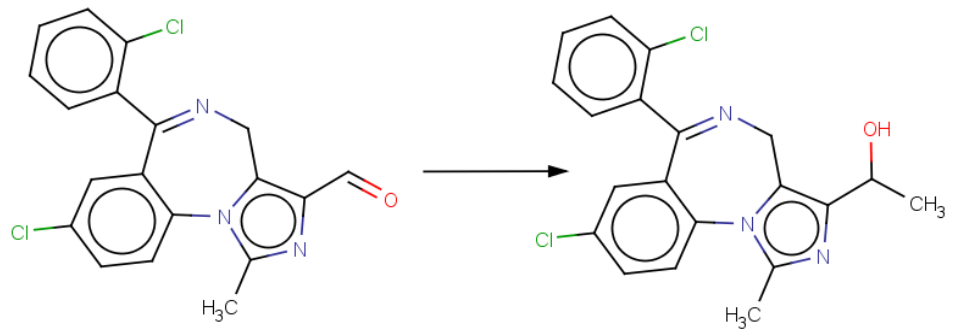

SMILES of the input:
Cc1nc(C=O)c2n1-c1ccc(Cl)cc1C(c1ccccc1Cl)=NC2>>Cc1nc(C(C)O)c2n1-c1ccc(Cl)cc1C(c1ccccc1Cl)=NC2

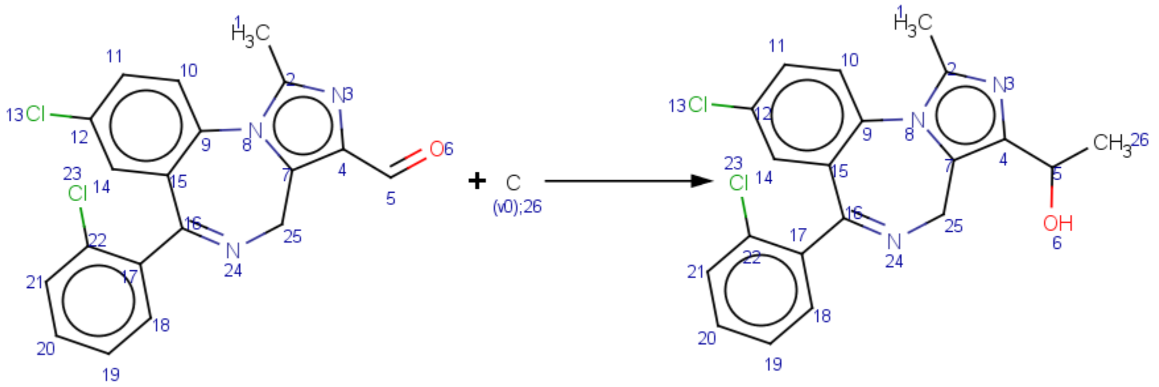

Correct mapped SMILES/SMARTS of the reaction:
[#6:1]-[c:2]1[n:3][c:4](-[#6:5]=[O:6])[c:7]2-[#6:25]-[#7:24]=[#6:16](-[c:17]3[c:18][c:19][c:20][c:21][c:22]3[Cl:23])-[c:15]3[c:14][c:12]([Cl:13])[c:11][c:10][c:9]3-[n:8]12.[#6;v0:26]>>[#6:26]-[#6:5](-[#8:6])-[c:4]1[n:3][c:2](-[#6:1])[n:8]-2[c:7]1-[#6:25]-[#7:24]=[#6:16](-[c:17]1[c:18][c:19][c:20][c:21][c:22]1[Cl:23])-[c:15]1[c:14][c:12]([Cl:13])[c:11][c:10][c:9]-21

Correctness of the mapping

MAPPET	YES
ReactionMap	NO
Marvin	YES

Reaction no 155

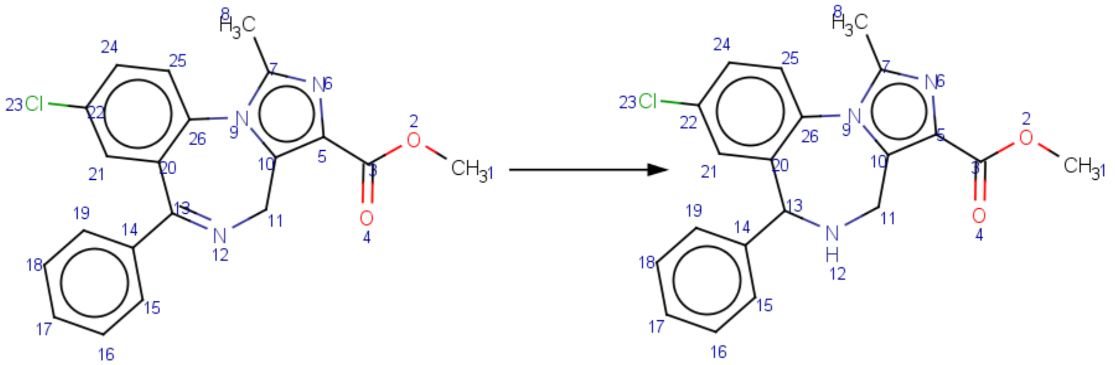

Correct mapped SMILES/SMARTS of the reaction:
[#6:1]-[#8:2]-[#6:3](=[O:4])-[c:5]1[n:6][c:7](-[#6:8])[n:9]-2[c:10]1-[#6:11]-[#7:12]=[#6:13](-[c:14]1[c:15][c:16][c:17][c:18][c:19]1)-[c:20]1[c:21][c:22]([Cl:23])[c:24][c:25][c:26]-21>>[#6:1]-[#8:2]-[#6:3](=[O:4])-[c:5]1[n:6][c:7](-[#6:8])[n:9]-2[c:10]1-[#6:11]-[#7:12]-[#6:13](-[c:14]1[c:15][c:16][c:17][c:18][c:19]1)-[c:20]1[c:21][c:22]([Cl:23])[c:24][c:25][c:26]-21

Correctness of the mapping
MAPPET YES
ReactionMap YES
Marvin YES

Reaction no 156

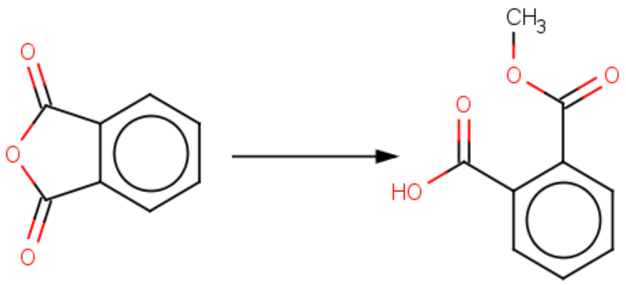

SMILES of the input:
O=C1OC(=O)c2ccccc21>>COC(=O)c1ccccc1C(=O)O

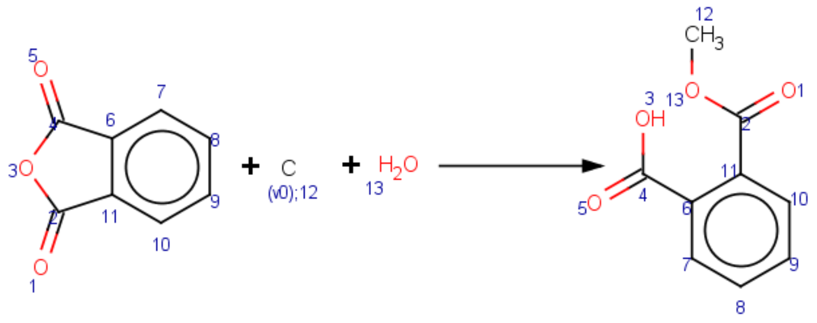

Correct mapped SMILES/SMARTS of the reaction:
[O:1]=[#6:2]-1-[#8:3]-[#6:4](=[O:5])-[c:6]2[c:7][c:8][c:9][c:10][c:11]-12.[#6;v0:12].[#8:13]>>[#6:12]-[#8:13]-[#6:2](=[O:1])-[c:11]1[c:10][c:9][c:8][c:7][c:6]1-[#6:4](-[#8:3])=[O:5]

Correctness of the mapping
MAPPET YES
ReactionMap NO
Marvin YES

Reaction no 157

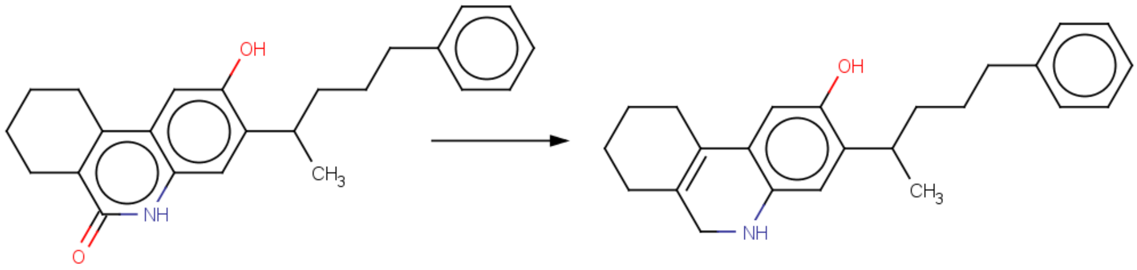

SMILES of the input:
CC(CCCc1ccccc1)c1cc2[nH]c(=O)c3c(c2cc1O)CCCC3>>CC(CCCc1ccccc1)c1cc2c(cc1O
)C1=C(CCCC1)CN2

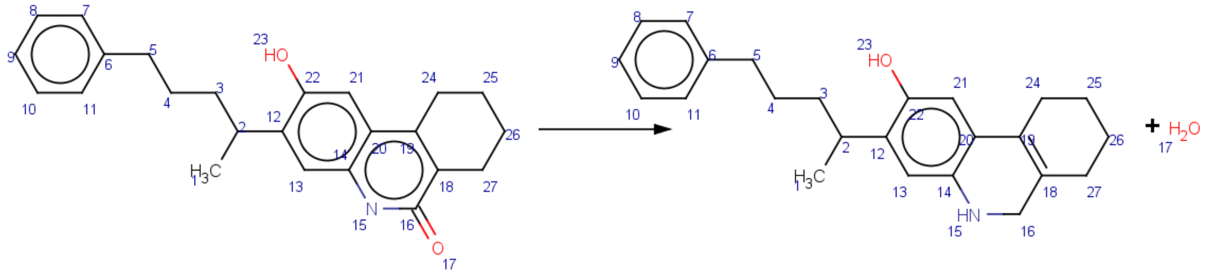

Correct mapped SMILES/SMARTS of the reaction:
[#6:1]-[#6:2](-[#6:3]-[#6:4]-[#6:5]-[c:6]1[c:7][c:8][c:9][c:10][c:11]1)-
[c:12]1[c:13][c:14]2[n:15][c:16](=[O:17])[c:18]3-[#6:27]-[#6:26]-[#6:25]-
[#6:24]-[c:19]3[c:20]2[c:21][c:22]1-[#8:23]>>[#6:1]-[#6:2](-[#6:3]-
[#6:4]-[#6:5]-[c:6]1[c:11][c:10][c:9][c:8][c:7]1)-[c:12]1[c:13][c:14]2-
[#7:15]-[#6:16]-[#6:18]-3=[#6:19](-[#6:24]-[#6:25]-[#6:26]-[#6:27]-3)-
[c:20]2[c:21][c:22]1-[#8:23].[#8:17]

Correctness of the mapping

MAPPET	YES
ReactionMap	NO
Marvin	YES

Reaction no 158

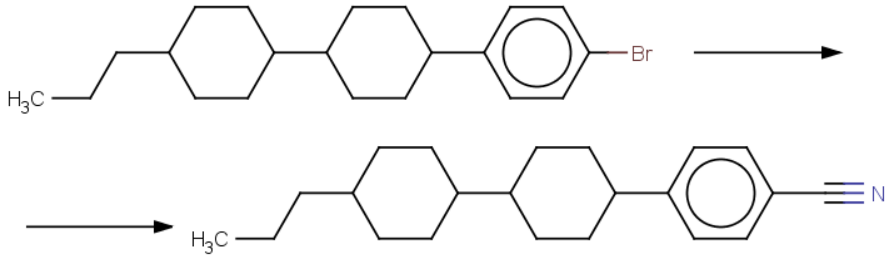

SMILES of the input:
CCCC1CCC(C2CCC(c3ccc(Br)cc3)CC2)CC1>>CCCC1CCC(C2CCC(c3ccc(C#N)cc3)CC2)CC1

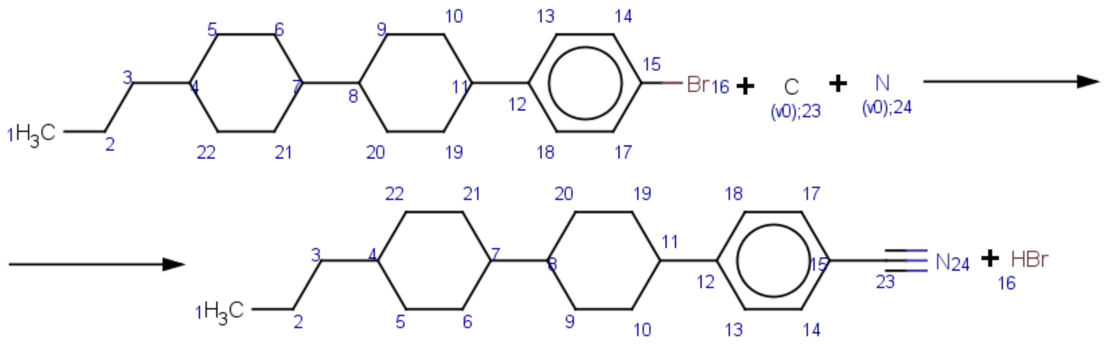

Correct mapped SMILES/SMARTS of the reaction:
[#6:1]-[#6:2]-[#6:3]-[#6:4]-1-[#6:5]-[#6:6]-[#6:7](-[#6:21]-[#6:22]-1)-[#6:8]-1-[#6:9]-[#6:10]-[#6:11](-[#6:19]-[#6:20]-1)-[c:12]1[c:13][c:14][c:15]([Br:16])[c:17][c:18]1.[#6;v0:23].[#7;v0:24]>>[#6:1]-[#6:2]-[#6:3]-[#6:4]-1-[#6:22]-[#6:21]-[#6:7](-[#6:6]-[#6:5]-1)-[#6:8]-1-[#6:20]-[#6:19]-[#6:11](-[#6:10]-[#6:9]-1)-[c:12]1[c:18][c:17][c:15]([c:14][c:13]1)[C:23]#[N:24].[Br:16]

Correctness of the mapping

MAPPET	YES
ReactionMap	NO
Marvin	NO

Reaction no 159

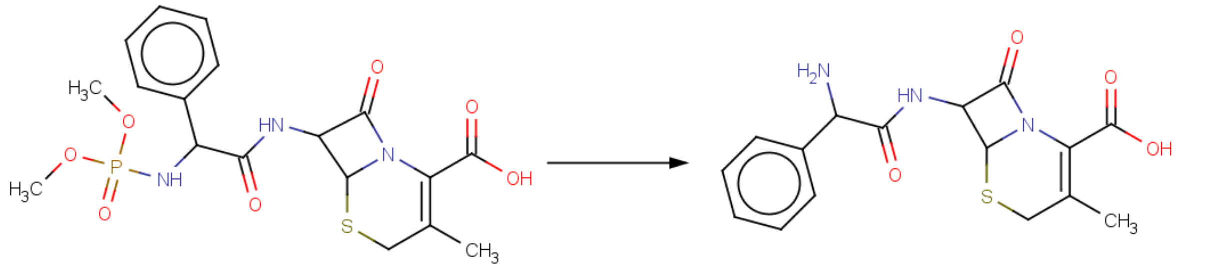

SMILES of the input:
COP(=O)(NC(C(=O)NC1C(=O)N2C(C(=O)O)=C(C)CSC12)c1ccccc1)OC>>CC1=C(C(=O)O)N2C(=O)C(NC(=O)C(N)c3ccccc3)C2SC1

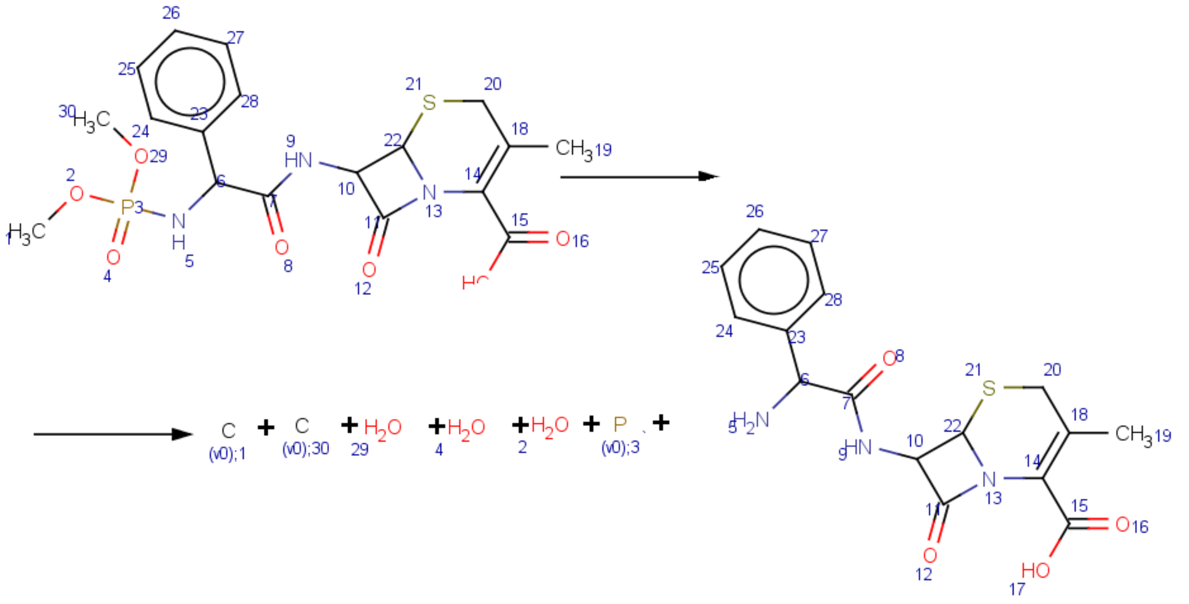

Correct mapped SMILES/SMARTS of the reaction:
[#6:1]-[#8:2][P:3](=[O:4])([#7:5]-[#6:6](-[#6:7](=[O:8])-[#7:9]-[#6:10]-1-[#6:22]-2-[#16:21]-[#6:20]-[#6:18](-[#6:19])=[#6:14](-[#7:13]-2-[#6:11]-1=[O:12])-[#6:15](-[#8:17])=[O:16])-[c:23]1[c:24][c:25][c:26][c:27][c:28]1)[#8:29]-[#6:30]>>[#6:19]-[#6:18]-1=[#6:14](-[#7:13]-2-[#6:22](-[#16:21]-[#6:20]-1)-[#6:10](-[#7:9]-[#6:7](=[O:8])-[#6:6](-[#7:5])-[c:23]1[c:24][c:25][c:26][c:27][c:28]1)-[#6:11]-2=[O:12])-[#6:15](-[#8:17])=[O:16].[#6;v0:1].[#6;v0:30].[#8:29].[#8:4].[#8:2].[#15;v0:3]

Correctness of the mapping
MAPPET YES
ReactionMap NO
Marvin YES

Reaction no 160

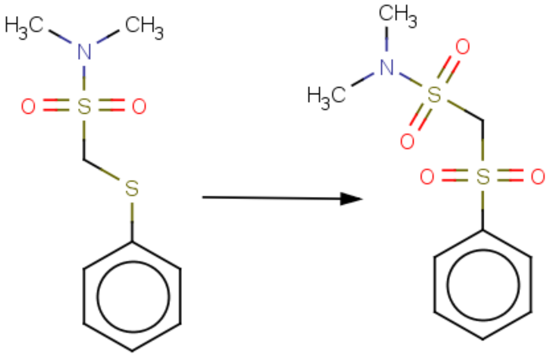

SMILES of the input:
CN(C)S(=O)(=O)CSc1ccccc1>>CN(C)S(=O)(=O)CS(=O)(=O)c1ccccc1

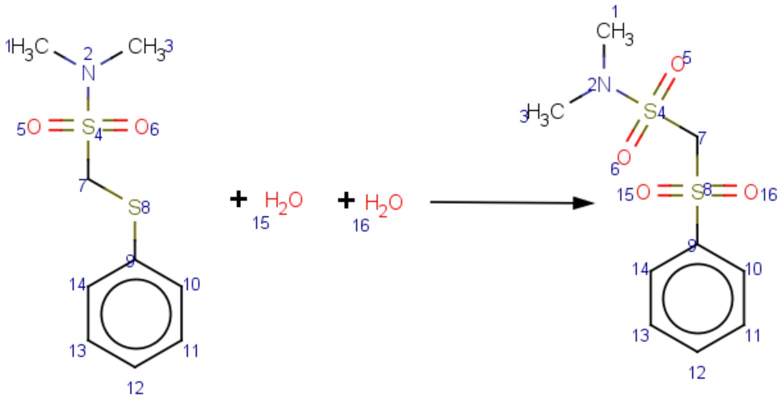

Correct mapped SMILES/SMARTS of the reaction:
[#6:1]-[#7:2](-[#6:3])[S:4](=[O:5])(=[O:6])[#6:7]-[#16:8]-[c:9]1[c:10][c:11][c:12][c:13][c:14]1.[#8:15].[#8:16]>>[#6:1]-[#7:2](-[#6:3])[S:4](=[O:6])(=[O:5])[#6:7][S:8](=[O:15])(=[O:16])[c:9]1[c:10][c:11][c:12][c:13][c:14]1

Correctness of the mapping
MAPPET YES
ReactionMap NO
Marvin YES

Reaction no 161

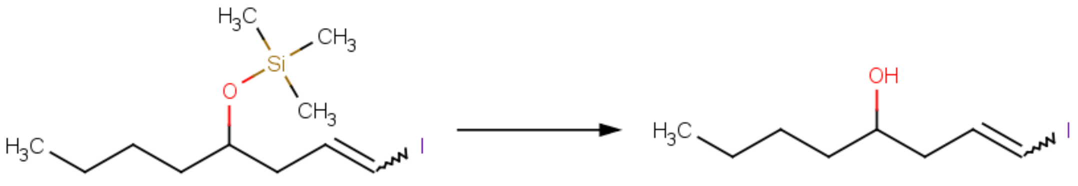

SMILES of the input:
CCCCC(CC=CI)O[Si](C)(C)C>>CCCCC(O)CC=CI

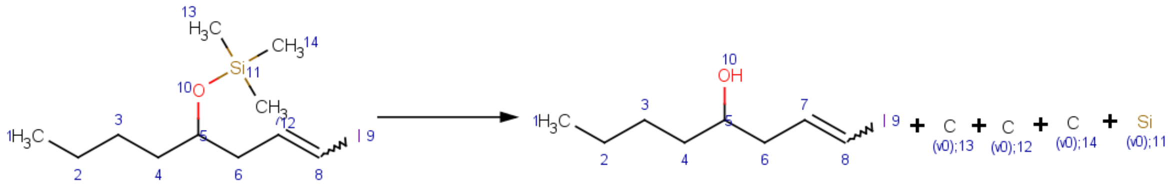

Correct mapped SMILES/SMARTS of the reaction:
[#6:1]-[#6:2]-[#6:3]-[#6:4]-[#6:5](-[#6:6]-[#6:7]=[#6:8][I:9])-[#8:10][Si:11]([#6:12])([#6:13])[#6:14]>>[#6:1]-[#6:2]-[#6:3]-[#6:4]-[#6:5](-[#8:10])-[#6:6]-[#6:7]=[#6:8][I:9].[#6;v0:13].[#6;v0:12].[#6;v0:14].[#14;v0:11]

Correctness of the mapping
MAPPET YES
ReactionMap NO
Marvin YES

Reaction no 162

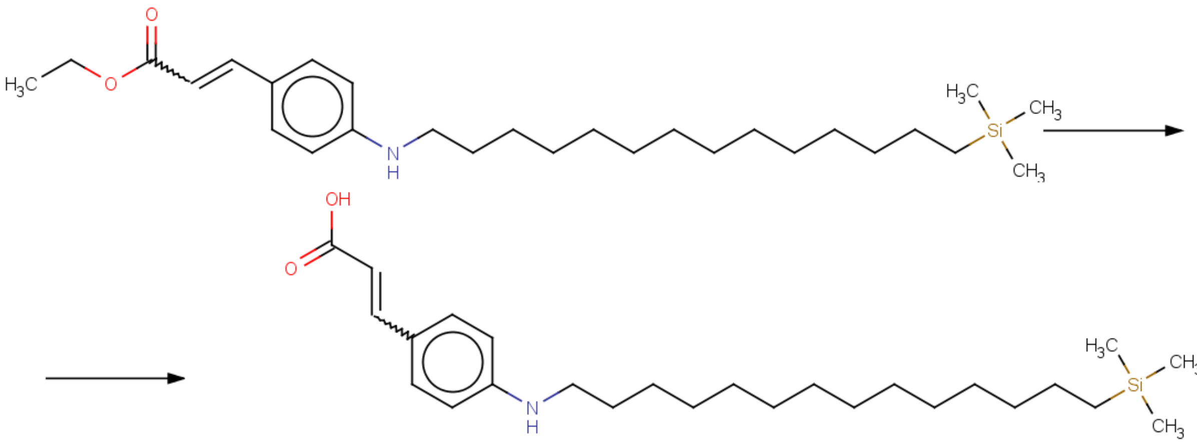

Correct mapped SMILES/SMARTS of the reaction:
CCOC(=O)C=Cc1ccc(NCCCCCCCCCCCCC[Si](C)(C)C)cc1>>C[Si](C)(C)CCCCCCCCCCCCCNc1ccc(C=CC(=O)O)cc1

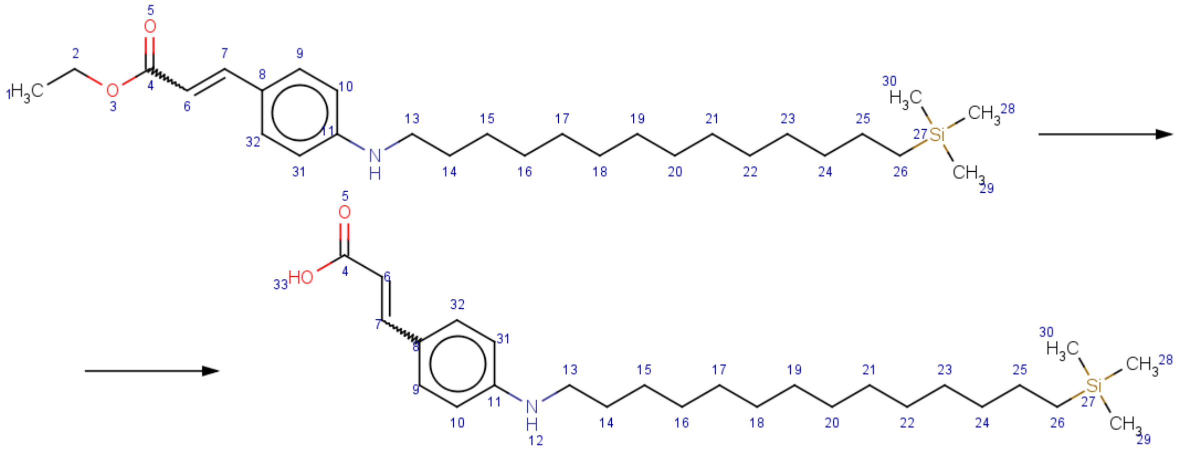

[#6:1]-[#6:2]-[#8:3]-[#6:4](=[O:5])-[#6:6]=[#6:7]-
[c:8]1[c:9][c:10][c:11](-[#7:12]-[#6:13]-[#6:14]-[#6:15]-[#6:16]-[#6:17]-
[#6:18]-[#6:19]-[#6:20]-[#6:21]-[#6:22]-[#6:23]-[#6:24]-[#6:25]-
[#6:26][Si:27]([#6:28])([#6:29])[#6:30])[c:31][c:32]1>>[#6:28][Si:27]([#6
:30])([#6:29])[#6:26]-[#6:25]-[#6:24]-[#6:23]-[#6:22]-[#6:21]-[#6:20]-
[#6:19]-[#6:18]-[#6:17]-[#6:16]-[#6:15]-[#6:14]-[#6:13]-[#7:12]-
[c:11]1[c:10][c:9][c:8](-[#6:7]=[#6:6]-[#6:4](-
[#8:33])=[O:5])[c:32][c:31]1

Correctness of the mapping

MAPPET	NO
ReactionMap	NO
Marvin	NO

Reaction no 163

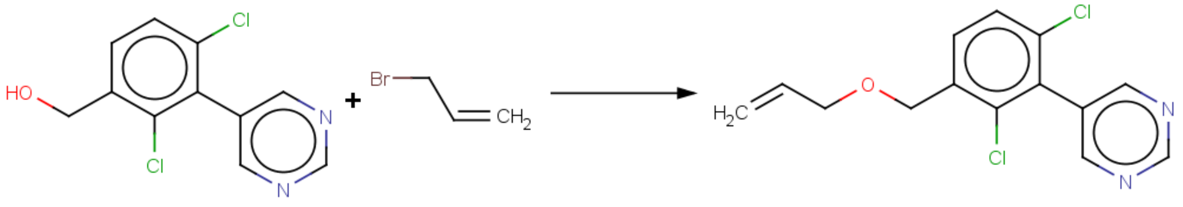

SMILES of the input:
OCc1ccc(Cl)c(-c2cncnc2)c1Cl.C=CCBr>>C=CCOCc1ccc(Cl)c(-c2cncnc2)c1Cl

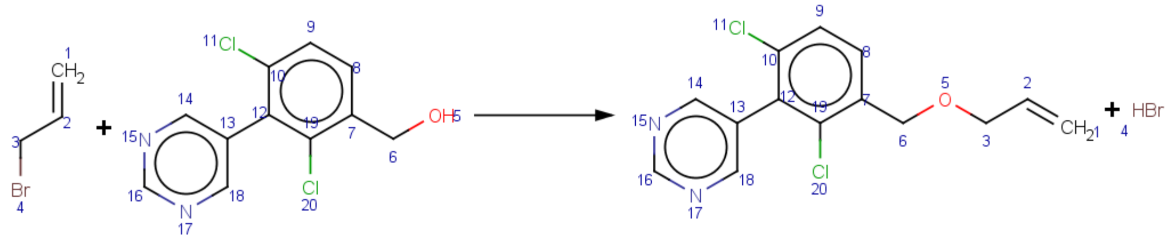

Correct mapped SMILES/SMARTS of the reaction:
[Br:4][#6:3]-[#6:2]=[#6:1].[#8:5]-[#6:6]-
[c:7]1[c:8][c:9][c:10]([Cl:11])[c:12]([c:19]1[Cl:20])-
[c:13]1[c:14][n:15][c:16][n:17][c:18]1>>[Cl:11][c:10]1[c:9][c:8][c:7](-

[#6:6]-[#8:5]-[#6:3]-[#6:2]=[#6:1])[c:19]([Cl:20])[c:12]1-
[c:13]1[c:18][n:17][c:16][n:15][c:14]1.[Br:4]

Correctness of the mapping
MAPPET YES
ReactionMap NO
Marvin YES

Reaction no 164

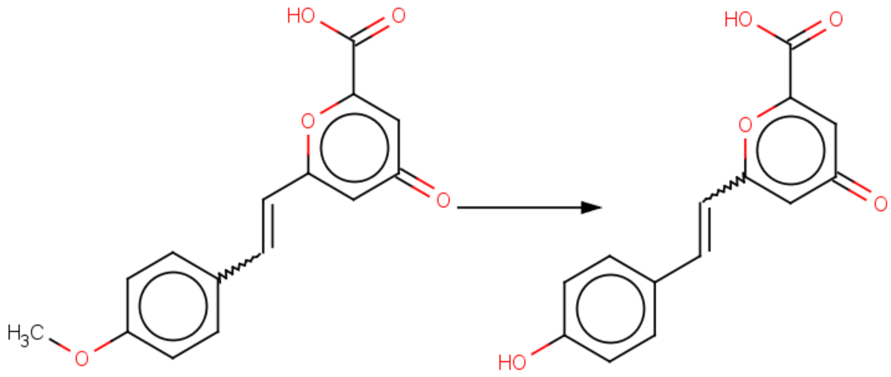

SMILES of the input:
COc1ccc(C=Cc2cc(=O)cc(C(=O)O)o2)cc1>>O=C(O)c1cc(=O)cc(C=Cc2ccc(O)cc2)o1

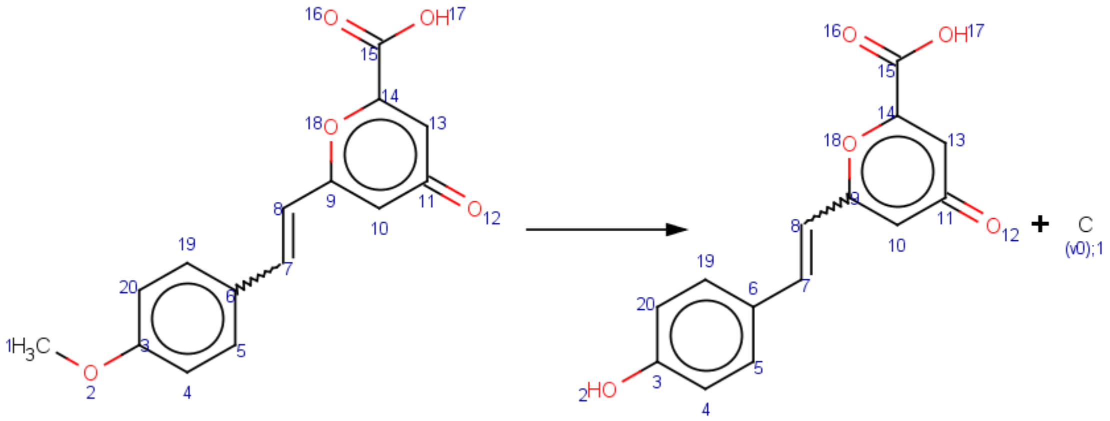

Correct mapped SMILES/SMARTS of the reaction:
[#6:1]-[#8:2]-[c:3]1[c:4][c:5][c:6](-[#6:7]=[#6:8]-
[c:9]2[c:10][c:11](=[O:12])[c:13][c:14]([o:18]2)-[#6:15](-
[#8:17])=[O:16])[c:19][c:20]1>>[#8:17]-[#6:15](=[O:16])-
[c:14]1[c:13][c:11](=[O:12])[c:10][c:9](-[#6:8]=[#6:7]-
[c:6]2[c:5][c:4][c:3](-[#8:2])[c:20][c:19]2)[o:18]1.[#6;v0:1]

Correctness of the mapping
MAPPET YES
ReactionMap NO
Marvin YES

Reaction no 165

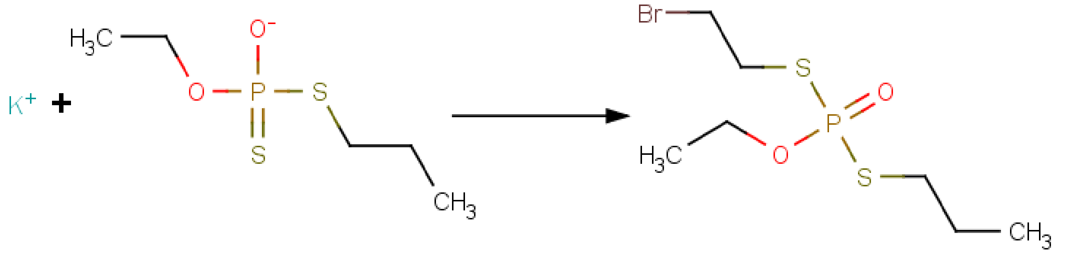

SMILES of the input:
[K+].CCCSP([O-])(=S)OCC>>CCCSP(=O)(OCC)SCCBr

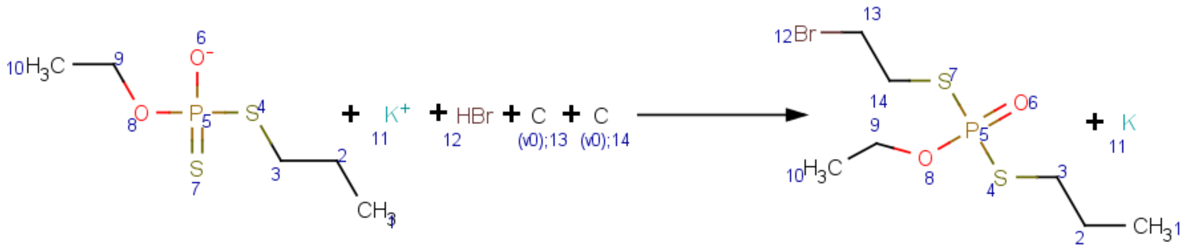

Correct mapped SMILES/SMARTS of the reaction:
[#6:1]-[#6:2]-[#6:3]-[#16:4][P:5]([#8-:6])(=[S:7])[#8:8]-[#6:9]-[#6:10].[K+:11].[Br:12].[#6;v0:13].[#6;v0:14]>>[#6:1]-[#6:2]-[#6:3]-[#16:4][P:5](=[O:6])([#8:8]-[#6:9]-[#6:10])[#16:7]-[#6:14]-[#6:13][Br:12].[K:11]

Correctness of the mapping
MAPPET YES
ReactionMap NO
Marvin YES

Reaction no 166

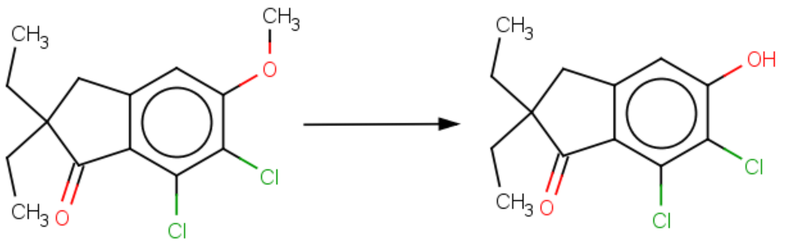

SMILES of the input:
CCC1(CC)Cc2cc(OC)c(Cl)c(Cl)c2C1=O>>CCC1(CC)Cc2cc(O)c(Cl)c(Cl)c2C1=O

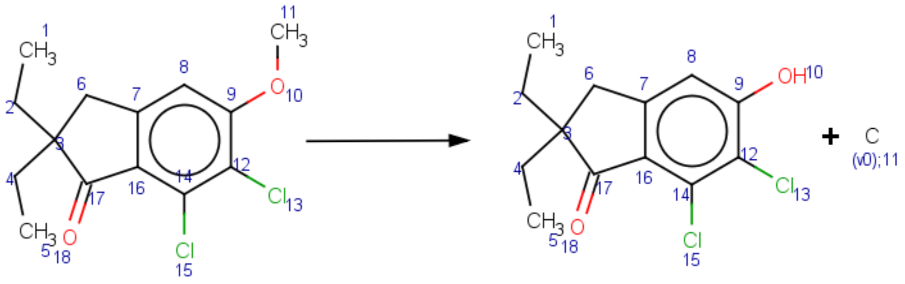

Correct mapped SMILES/SMARTS of the reaction:
[#6:1]-[#6:2][C:3]1([#6:4]-[#6:5])[#6:6]-[c:7]2[c:8][c:9](-[#8:10]-[#6:11])[c:12]([Cl:13])[c:14]([Cl:15])[c:16]2-[#6:17]1=[O:18]>>[#6:1]-

[#6:2][C:3]1([#6:4]-[#6:5])[#6:6]-[c:7]2[c:8][c:9](-
[#8:10])[c:12]([Cl:13])[c:14]([Cl:15])[c:16]2-[#6:17]1=[O:18].[#6;v0:11]

Correctness of the mapping
MAPPET YES
ReactionMap NO
Marvin YES

Reaction no 167

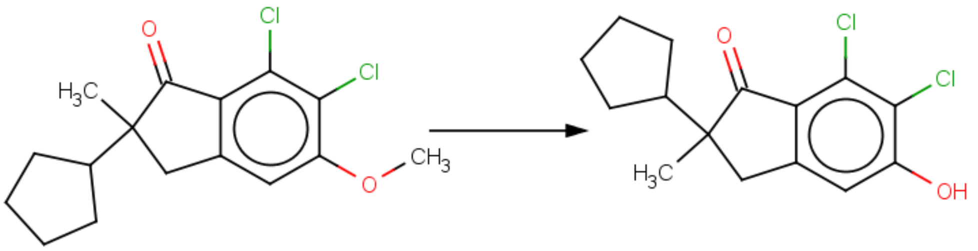

SMILES of the input:
COc1cc2c(c(Cl)c1Cl)C(=O)C(C)(C1CCCC1)C2>>CC1(C2CCCC2)Cc2cc(O)c(Cl)c(Cl)c2
C1=O

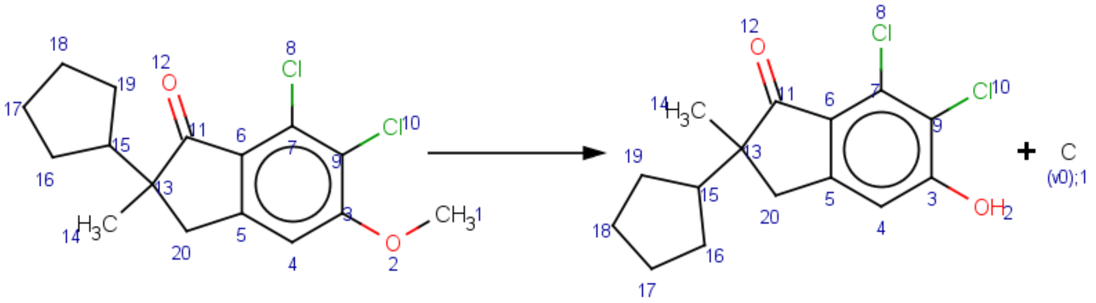

Correct mapped SMILES/SMARTS of the reaction:
[#6:1]-[#8:2]-[c:3]1[c:4][c:5]2-[#6:20][C:13]([#6:14])([#6:15]-3-[#6:16]-
[#6:17]-[#6:18]-[#6:19]-3)[#6:11](=[O:12])-
[c:6]2[c:7]([Cl:8])[c:9]1[Cl:10]>>[#6:14][C:13]1([#6:20]-
[c:5]2[c:4][c:3](-[#8:2])[c:9]([Cl:10])[c:7]([Cl:8])[c:6]2-
[#6:11]1=[O:12])[#6:15]-1-[#6:19]-[#6:18]-[#6:17]-[#6:16]-1.[#6;v0:1]

Correctness of the mapping
MAPPET YES
ReactionMap NO
Marvin YES

Reaction no 168

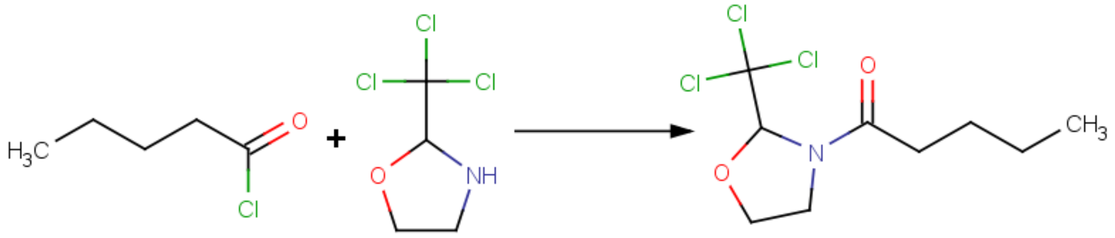

SMILES of the input:
CCCCC(=O)Cl.ClC(Cl)(Cl)C1NCCO1>>CCCCC(=O)N1CCOC1C(Cl)(Cl)Cl

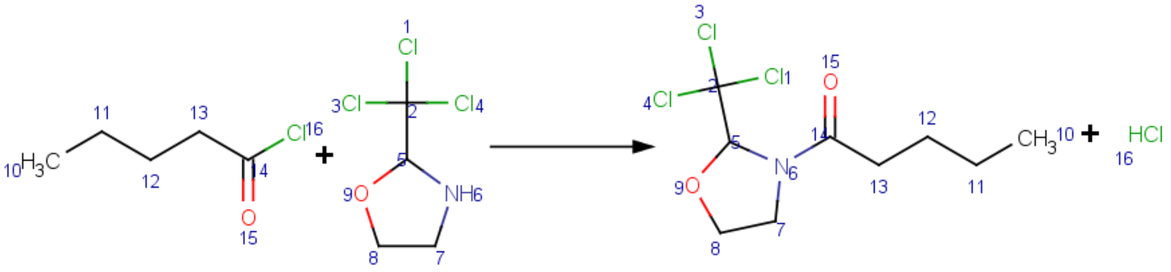

Correct mapped SMILES/SMARTS of the reaction:
[#6:10]-[#6:11]-[#6:12]-[#6:13]-
[#6:14]([Cl:16])=[O:15].[Cl:1][C:2]([Cl:3])([Cl:4])[#6:5]-1-[#7:6]-
[#6:7]-[#6:8]-[#8:9]-1>>[#6:10]-[#6:11]-[#6:12]-[#6:13]-[#6:14](=[O:15])-
[#7:6]-1-[#6:7]-[#6:8]-[#8:9]-[#6:5]-1[C:2]([Cl:3])([Cl:1])[Cl:4].[Cl:16]

Correctness of the mapping

MAPPET	YES
ReactionMap	NO
Marvin	YES

Reaction no 169

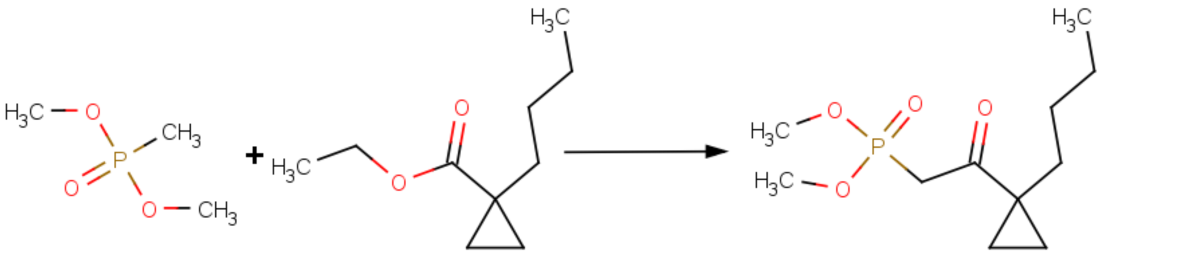

SMILES of the input:
COP(C)(=O)OC.CCCCC1(C(=O)OCC)CC1>>CCCCC1(C(=O)CP(=O)(OC)OC)CC1

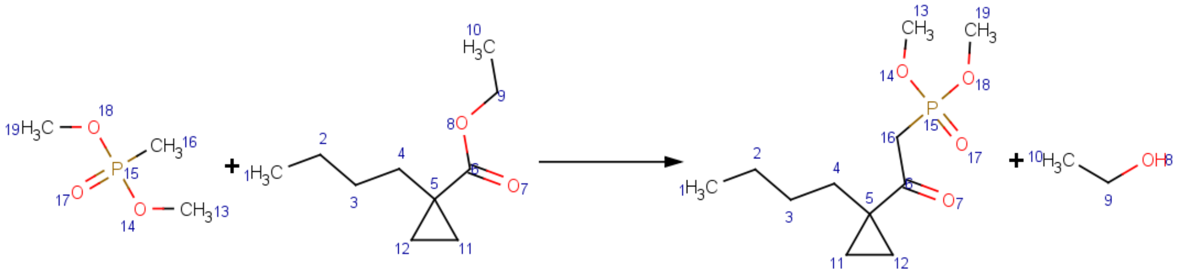

Correct mapped SMILES/SMARTS of the reaction:
[CH3:13][O:14][P:15]([CH3:16])(=[O:17])[O:18][CH3:19].[CH3:1][CH2:2][CH2:
3][CH2:4][C:5]1([CH2:11][CH2:12]1)[C:6](=[O:7])[O:8][CH2:9][CH3:10]>>[CH3
:1][CH2:2][CH2:3][CH2:4][C:5]1([CH2:12][CH2:11]1)[C:6](=[O:7])[CH2:16][P:
15](=[O:17])([O:18][CH3:19])[O:14][CH3:13].[CH3:10][CH2:9][OH:8]

Correctness of the mapping

MAPPET	YES
ReactionMap	NO
Marvin	YES

Reaction no 170

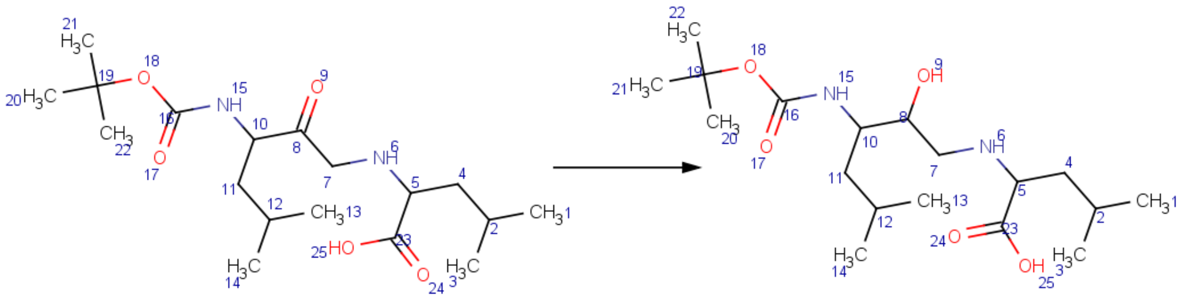

Correct mapped SMILES/SMARTS of the reaction:
[#6:1]-[#6:2](-[#6:3])-[#6:4]-[#6:5](-[#7:6]-[#6:7]-[#6:8](=[O:9])-
[#6:10](-[#6:11]-[#6:12](-[#6:13])-[#6:14])-[#7:15]-[#6:16](=[O:17])-
[#8:18][C:19]([#6:20])([#6:21])[#6:22])-[#6:23](-
[#8:25])=[O:24]>>[#6:13]-[#6:12](-[#6:14])-[#6:11]-[#6:10](-[#7:15]-
[#6:16](=[O:17])-[#8:18][C:19]([#6:21])([#6:20])[#6:22])-[#6:8](-[#8:9])-
[#6:7]-[#7:6]-[#6:5](-[#6:4]-[#6:2](-[#6:1])-[#6:3])-[#6:23](-
[#8:25])=[O:24]

Correctness of the mapping

MAPPET	YES
ReactionMap	YES
Marvin	YES

Reaction no 171

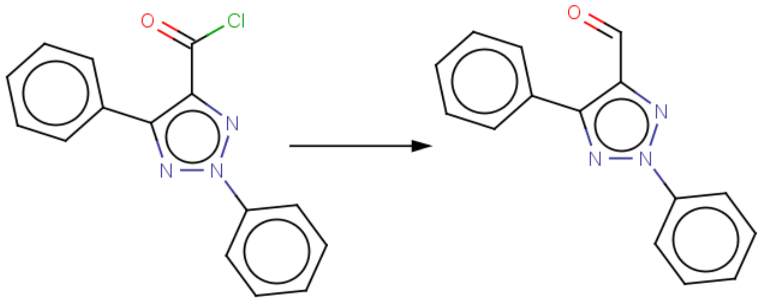

SMILES of the input:
O=C(Cl)c1nn(-c2ccccc2)nc1-c1ccccc1>>O=Cc1nn(-c2ccccc2)nc1-c1ccccc1

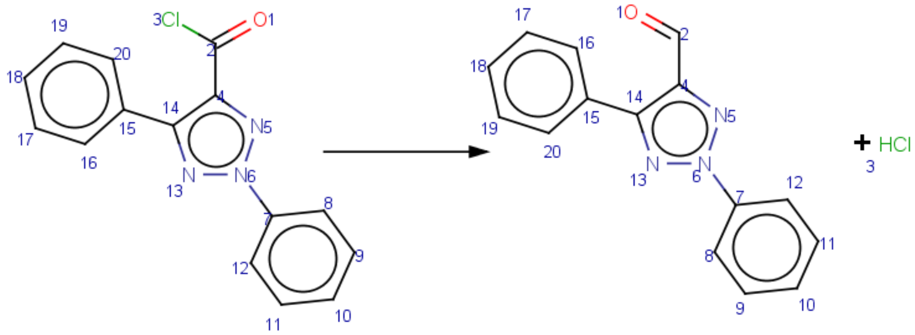

Correct mapped SMILES/SMARTS of the reaction:
[Cl:3][#6:2](=[O:1])-[c:4]1[n:5][n:6]([n:13][c:14]1-
[c:15]1[c:16][c:17][c:18][c:19][c:20]1)-
[c:7]1[c:8][c:9][c:10][c:11][c:12]1>>[O:1]=[#6:2]-
[c:4]1[n:5][n:6]([n:13][c:14]1-[c:15]1[c:20][c:19][c:18][c:17][c:16]1)-
[c:7]1[c:12][c:11][c:10][c:9][c:8]1.[Cl:3]

Correctness of the mapping
MAPPET YES
ReactionMap NO
Marvin YES

Reaction no 172

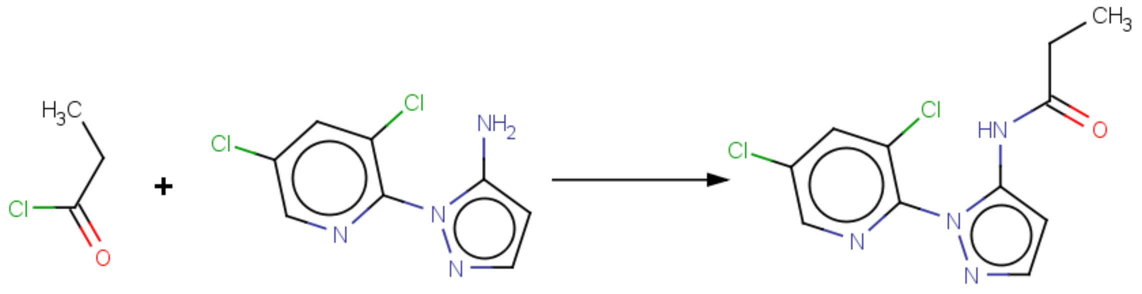

SMILES of the input:
CCC(=O)Cl.Nc1ccnn1-c1ncc(Cl)cc1Cl>>CCC(=O)Nc1ccnn1-c1ncc(Cl)cc1Cl

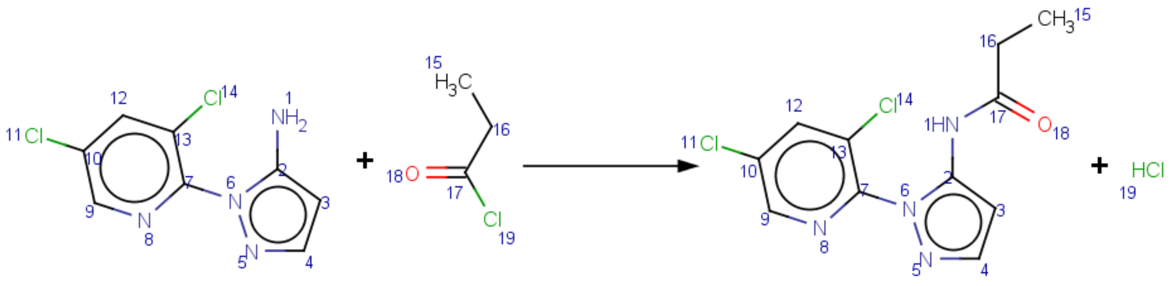

Correct mapped SMILES/SMARTS of the reaction:
[#7:1]-[c:2]1[c:3][c:4][n:5][n:6]1-
[c:7]1[n:8][c:9][c:10]([Cl:11])[c:12][c:13]1[Cl:14].[#6:15]-[#6:16]-
[#6:17]([Cl:19])=[O:18]>>[#6:15]-[#6:16]-[#6:17](=[O:18])-[#7:1]-
[c:2]1[c:3][c:4][n:5][n:6]1-
[c:7]1[n:8][c:9][c:10]([Cl:11])[c:12][c:13]1[Cl:14].[Cl:19]

Correctness of the mapping
MAPPET YES
ReactionMap NO
Marvin YES

Reaction no 173

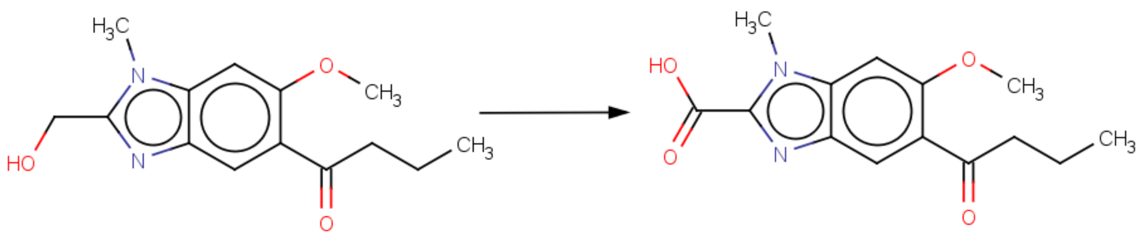

SMILES of the input:
CCCC(=O)c1cc2nc(CO)n(C)c2cc1OC>>CCCC(=O)c1cc2nc(C(=O)O)n(C)c2cc1OC

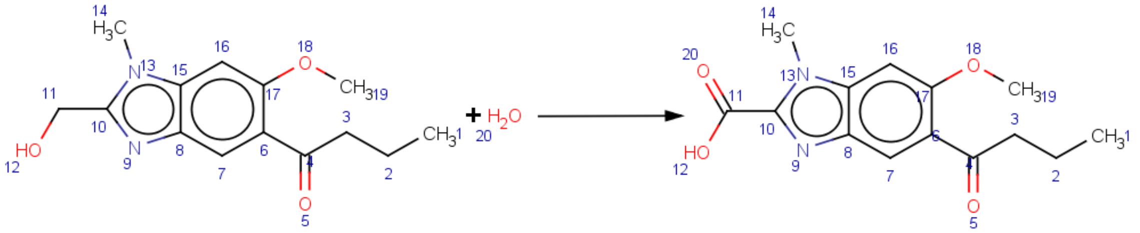

Correct mapped SMILES/SMARTS of the reaction:
[#6:1]-[#6:2]-[#6:3]-[#6:4](=[O:5])-[c:6]1[c:7][c:8]2[n:9][c:10](-[#6:11]-[#8:12])[n:13](-[#6:14])[c:15]2[c:16][c:17]1-[#8:18]-[#6:19].[#8:20]>>[#6:1]-[#6:2]-[#6:3]-[#6:4](=[O:5])-[c:6]1[c:7][c:8]2[n:9][c:10](-[#6:11](-[#8:12])=[O:20])[n:13](-[#6:14])[c:15]2[c:16][c:17]1-[#8:18]-[#6:19]

Correctness of the mapping

MAPPET	YES
ReactionMap	NO
Marvin	YES

Reaction no 174

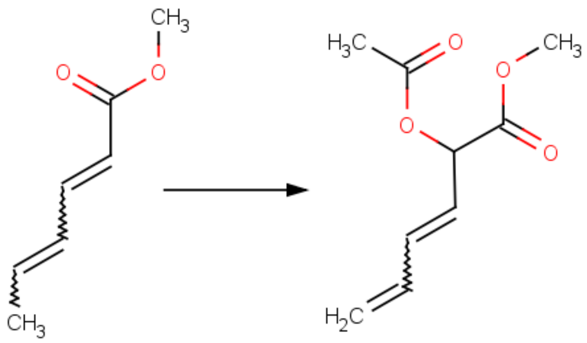

SMILES of the input:
CC=CC=CC(=O)OC>>C=CC=CC(OC(C)=O)C(=O)OC

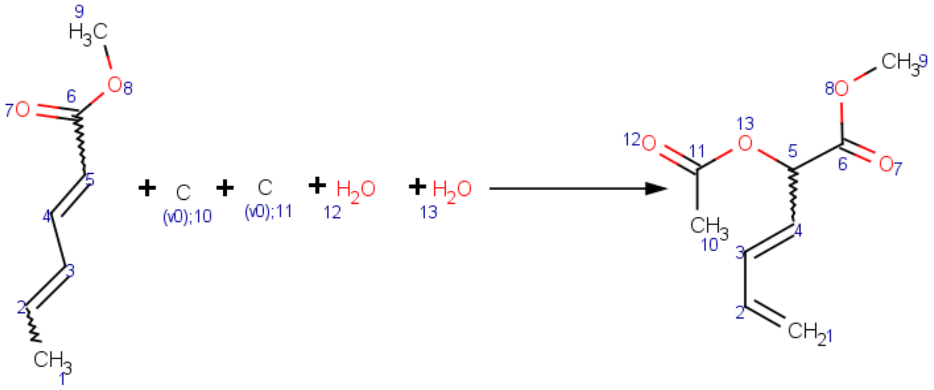

Correct mapped SMILES/SMARTS of the reaction:
[#6:9]-[#8:8]-[#6:6](=[O:7])-[#6:5]=[#6:4]-[#6:3]=[#6:2]-[#6:1].[#6;v0:10].[#6;v0:11].[#8:12].[#8:13]>>[#6:9]-[#8:8]-[#6:6](=[O:7])-[#6:5](-[#8:13]-[#6:11](-[#6:10])=[O:12])-[#6:4]=[#6:3]-[#6:2]=[#6:1]

```
Correctness of the mapping
MAPPET          YES
ReactionMap     NO
Marvin          NO
```

Reaction no 175

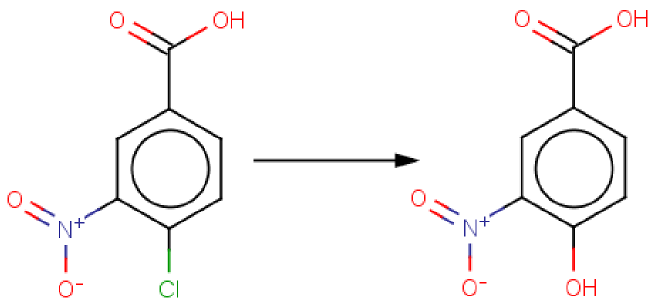

SMILES of the input:
O=C(O)c1ccc(Cl)c([N+](=O)[O-])c1>>O=C(O)c1ccc(O)c([N+](=O)[O-])c1

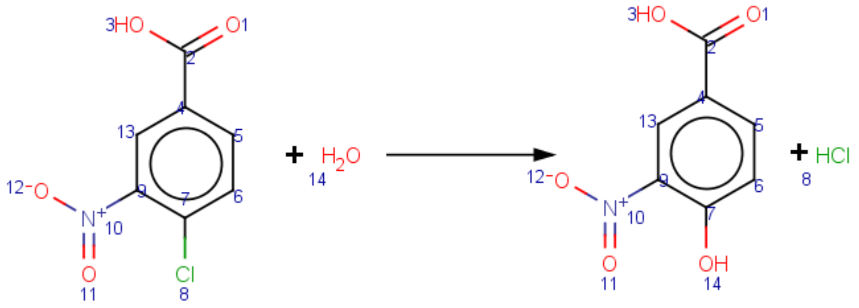

Correct mapped SMILES/SMARTS of the reaction:
[#8:3]-[#6:2](=[O:1])-[c:4]1[c:5][c:6][c:7]([Cl:8])[c:9]([c:13]1)-[#7+:10](-[#8-:12])=[O:11].[#8:14]>>[#8:3]-[#6:2](=[O:1])-[c:4]1[c:5][c:6][c:7](-[#8:14])[c:9]([c:13]1)-[#7+:10](-[#8-:12])=[O:11].[Cl:8]

```
Correctness of the mapping
MAPPET          YES
ReactionMap     NO
Marvin          YES
```

Reaction no 176

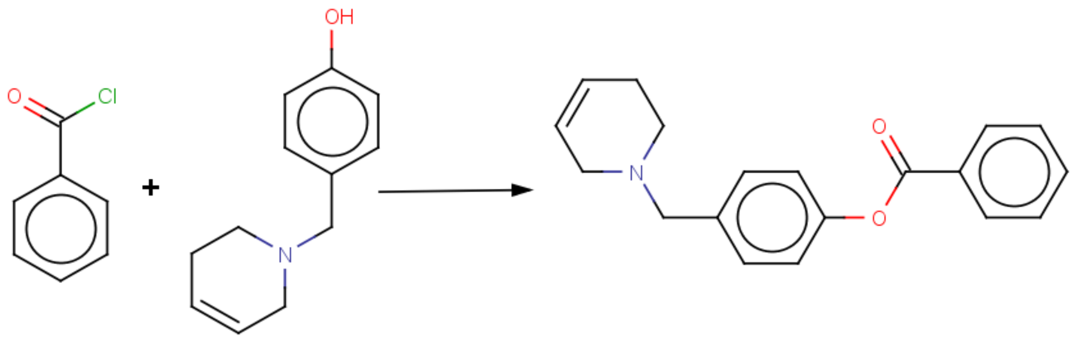

SMILES of the input:
O=C(Cl)c1ccccc1.Oc1ccc(CN2CC=CCC2)cc1>>O=C(Oc1ccc(CN2CC=CCC2)cc1)c1ccccc1

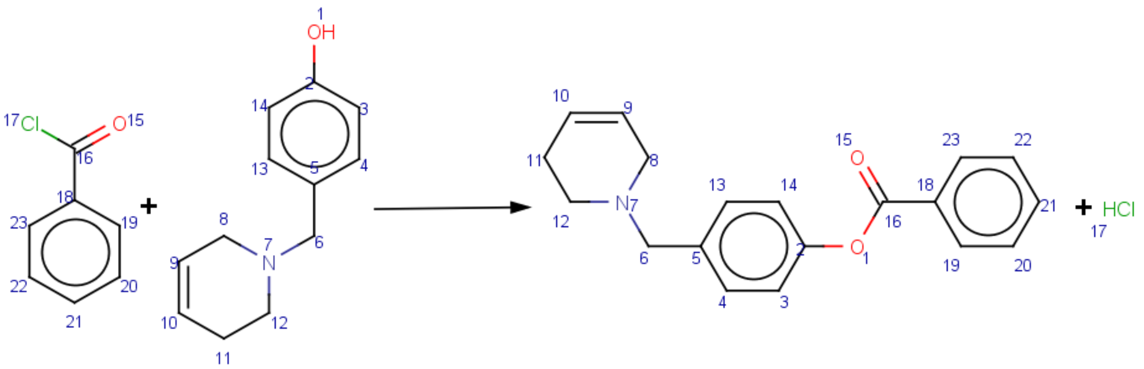

Correct mapped SMILES/SMARTS of the reaction:
[#8:1]-[c:2]1[c:3][c:4][c:5](-[#6:6]-[#7:7]-2-[#6:12]-[#6:11]-[#6:10]=[#6:9]-[#6:8]-2)[c:13][c:14]1.[Cl:17][#6:16](=[O:15])-[c:18]1[c:19][c:20][c:21][c:22][c:23]1>>[O:15]=[#6:16](-[#8:1]-[c:2]1[c:3][c:4][c:5](-[#6:6]-[#7:7]-2-[#6:12]-[#6:11]-[#6:10]=[#6:9]-[#6:8]-2)[c:13][c:14]1)-[c:18]1[c:23][c:22][c:21][c:20][c:19]1.[Cl:17]

Correctness of the mapping

MAPPET	YES
ReactionMap	NO
Marvin	YES

Reaction no 177

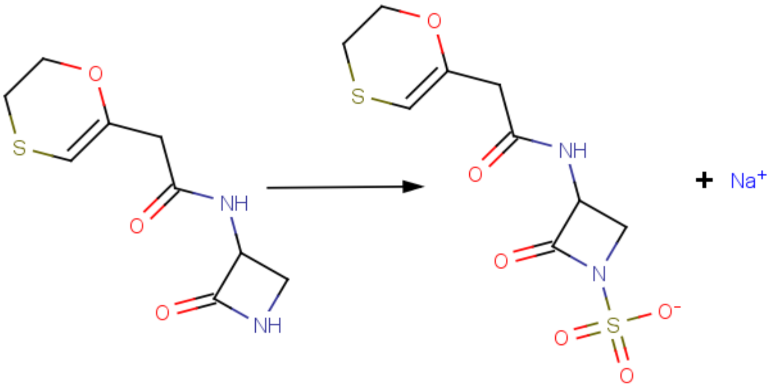

SMILES of the input:
O=C(CC1=CSCCO1)NC1CNC1=O>>[Na+].O=C(CC1=CSCCO1)NC1CN(S(=O)(=O)[O-])C1=O

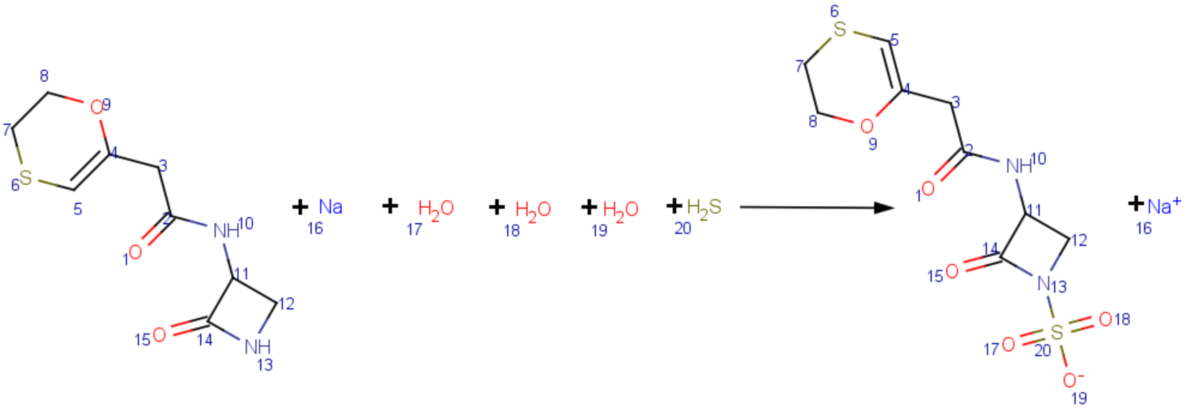

Correct mapped SMILES/SMARTS of the reaction:
[O:1]=[#6:2](-[#6:3]-[#6:4]-1=[#6:5]-[#16:6]-[#6:7]-[#6:8]-[#8:9]-1)-[#7:10]-[#6:11]-1-[#6:12]-[#7:13]-[#6:14]-1=[O:15].[Na:16].[#8:17].[#8:18].[#8:19].[#16:20]>>[#8-:19][S:20](=[O:17])(=[O:18])[#7:13]-1-[#6:12]-[#6:11](-[#7:10]-

[#6:2](=[O:1])-[#6:3]-[#6:4]-2=[#6:5]-[#16:6]-[#6:7]-[#6:8]-[#8:9]-2)-[#6:14]-1=[O:15].[Na+:16]

Correctness of the mapping
MAPPET YES
ReactionMap NO
Marvin YES

Reaction no 178

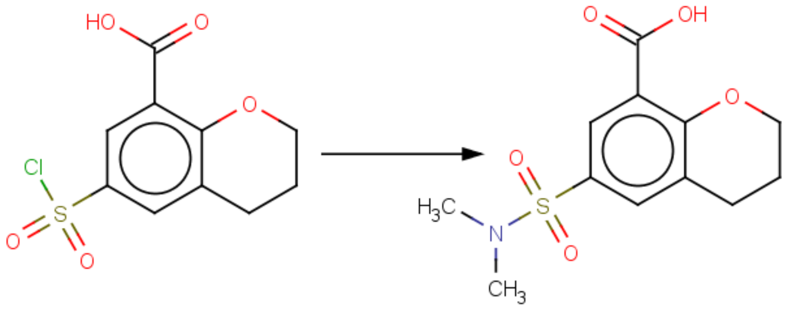

SMILES of the input:
O=C(O)c1cc(S(=O)(=O)Cl)cc2c1OCCC2>>CN(C)S(=O)(=O)c1cc2c(c(C(=O)O)c1)OCCC2

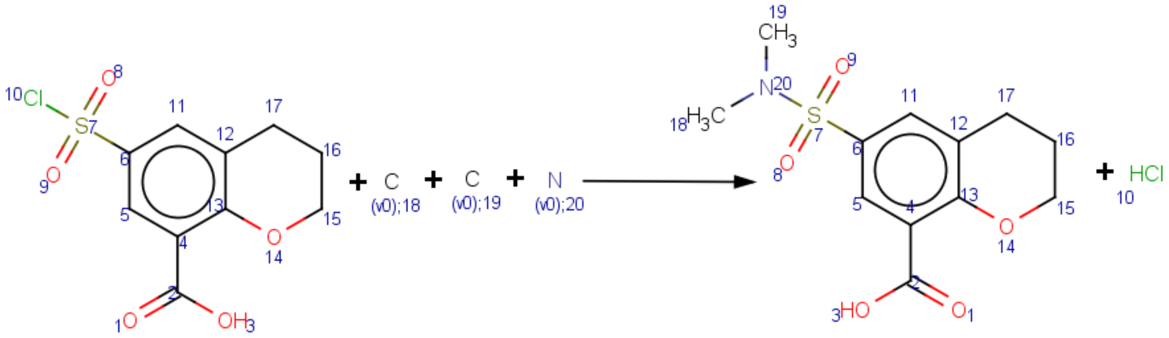

Correct mapped SMILES/SMARTS of the reaction:
[#8:3]-[#6:2](=[O:1])-[c:4]1[c:5][c:6]([c:11][c:12]2-[#6:17]-[#6:16]-[#6:15]-[#8:14]-[c:13]12)[S:7]([Cl:10])(=[O:8])=[O:9].[#6;v0:18].[#6;v0:19].[#7;v0:20]>>[#6:18]-[#7:20](-[#6:19])[S:7](=[O:8])(=[O:9])[c:6]1[c:11][c:12]2-[#6:17]-[#6:16]-[#6:15]-[#8:14]-[c:13]2[c:4]([c:5]1)-[#6:2](-[#8:3])=[O:1].[Cl:10]

Correctness of the mapping
MAPPET YES
ReactionMap NO
Marvin YES

Reaction no 179

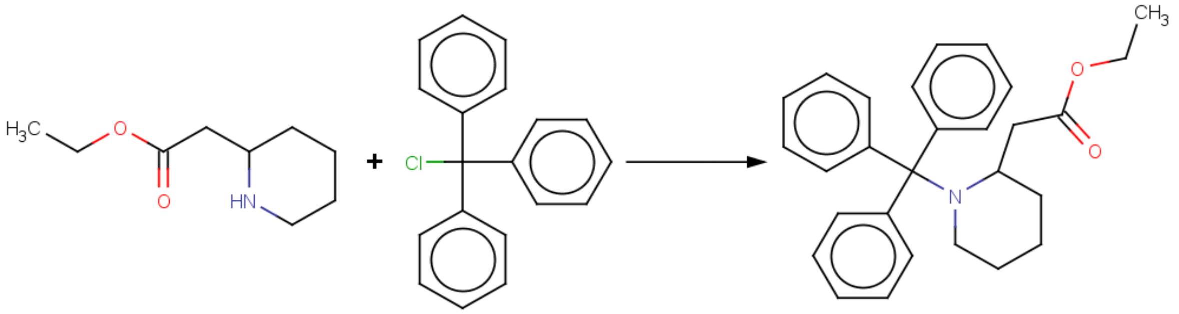

SMILES of the input:
CCOC(=O)CC1CCCCN1.ClC(c1ccccc1)(c1ccccc1)c1ccccc1>>CCOC(=O)CC1CCCCN1C(c1ccccc1)(c1ccccc1)c1ccccc1

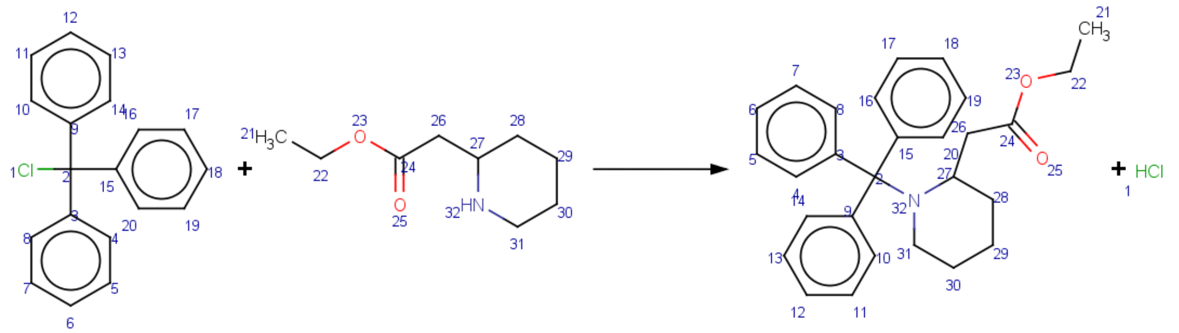

Correct mapped SMILES/SMARTS of the reaction:
[Cl:1][C:2]([c:3]1[c:4][c:5][c:6][c:7][c:8]1)([c:9]1[c:10][c:11][c:12][c:13][c:14]1)[c:15]1[c:16][c:17][c:18][c:19][c:20]1.[#6:21]-[#6:22]-[#8:23]-[#6:24](=[O:25])-[#6:26]-[#6:27]-1-[#6:28]-[#6:29]-[#6:30]-[#6:31]-[#7:32]-1>>[#6:21]-[#6:22]-[#8:23]-[#6:24](=[O:25])-[#6:26]-[#6:27]-1-[#6:28]-[#6:29]-[#6:30]-[#6:31]-[#7:32]-1[C:2]([c:3]1[c:4][c:5][c:6][c:7][c:8]1)([c:15]1[c:16][c:17][c:18][c:19][c:20]1)[c:9]1[c:10][c:11][c:12][c:13][c:14]1.[Cl:1]

Correctness of the mapping

MAPPET	YES
ReactionMap	NO
Marvin	YES

Reaction no 180

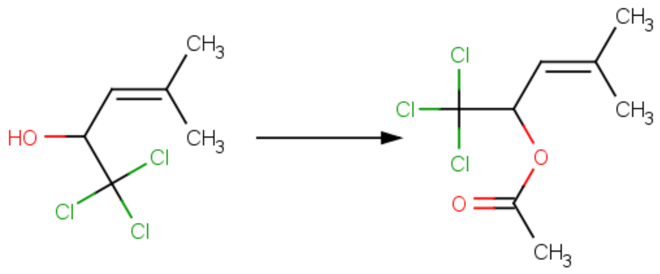

SMILES of the input:
CC(C)=CC(O)C(Cl)(Cl)Cl>>CC(C)=CC(OC(C)=O)C(Cl)(Cl)Cl

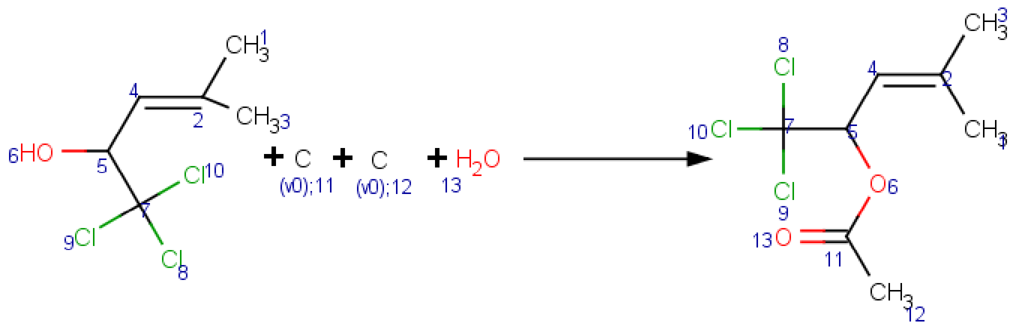

Correct mapped SMILES/SMARTS of the reaction:
[#6:1]\[#6:2](-[#6:3])=[#6:4]\[#6:5](-
[#8:6])[C:7]([Cl:8])([Cl:9])[Cl:10].[#6;v0:11].[#6;v0:12].[#8:13]>>[#6:3]
\[#6:2](-[#6:1])=[#6:4]\[#6:5](-[#8:6]-[#6:11](-
[#6:12])=[O:13])[C:7]([Cl:10])([Cl:9])[Cl:8]

Correctness of the mapping

MAPPET	YES
ReactionMap	NO
Marvin	YES

Reaction no 181

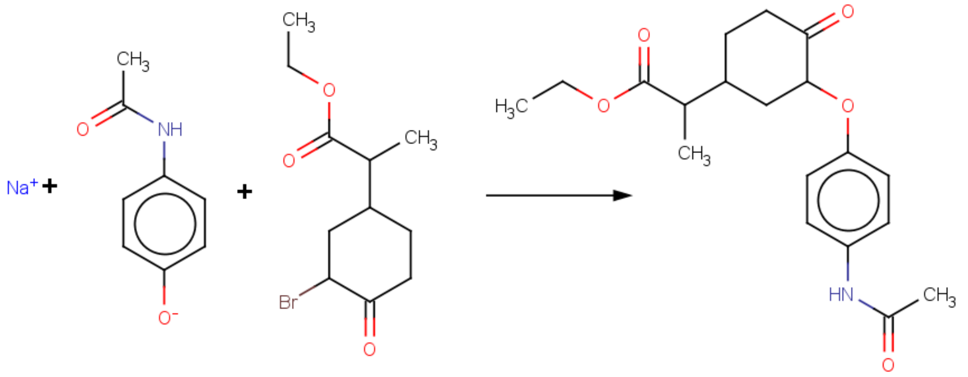

SMILES of the input:
[Na+].CC(=O)Nc1ccc([O-
])cc1.CCOC(=O)C(C)C1CCC(=O)C(Br)C1>>CCOC(=O)C(C)C1CCC(=O)C(Oc2ccc(NC(C)=O
)cc2)C1

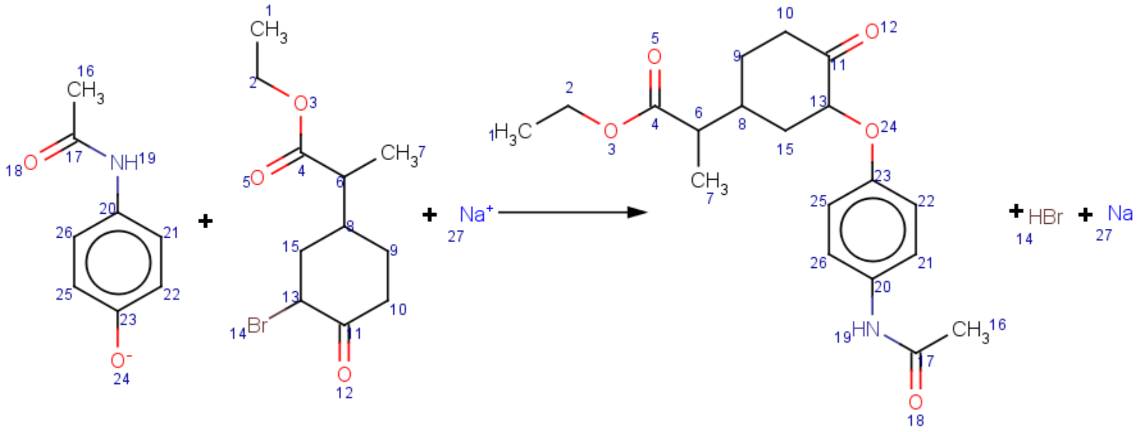

Correct mapped SMILES/SMARTS of the reaction:
[#6:1]-[#6:2]-[#8:3]-[#6:4](=[O:5])-[#6:6](-[#6:7])-[#6:8]-1-[#6:9]-
[#6:10]-[#6:11](=[O:12])-[#6:13]([Br:14])-[#6:15]-1.[#6:16]-
[#6:17](=[O:18])-[#7:19]-[c:20]1[c:21][c:22][c:23](-[#8-
:24])[c:25][c:26]1.[Na+:27]>>[#6:1]-[#6:2]-[#8:3]-[#6:4](=[O:5])-[#6:6](-

[#6:7])-[#6:8]-1-[#6:9]-[#6:10]-[#6:11](=[O:12])-[#6:13](-[#6:15]-1)-[#8:24]-[c:23]1[c:22][c:21][c:20](-[#7:19]-[#6:17](-[#6:16])=[O:18])[c:26][c:25]1.[Br:14].[Na:27]

Correctness of the mapping
MAPPET YES
ReactionMap NO
Marvin YES

Reaction no 182

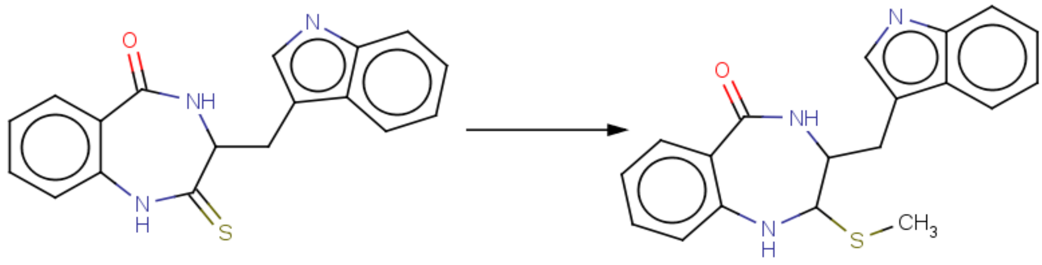

SMILES of the input:
O=C1NC(Cc2cnc3ccccc23)C(=S)Nc2ccccc21>>CSC1Nc2ccccc2C(=O)NC1Cc1cnc2ccccc1
2

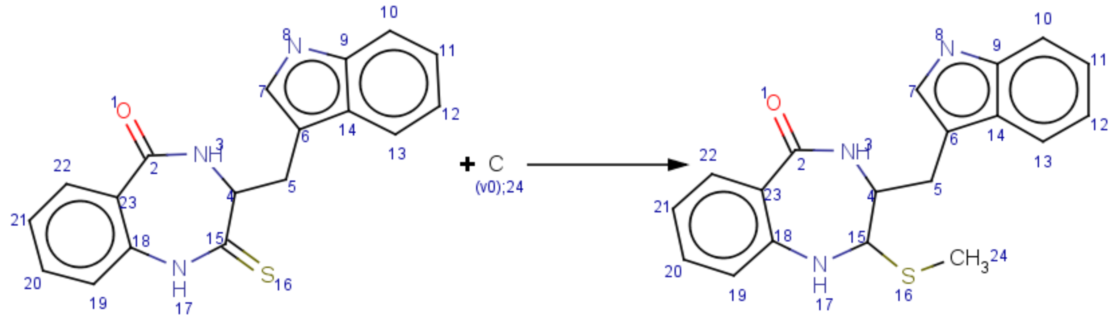

Correct mapped SMILES/SMARTS of the reaction:
[O:1]=[#6:2]-1-[#7:3]-[#6:4](-[#6:5]-
[c:6]2[c:7][n:8][c:9]3[c:10][c:11][c:12][c:13][c:14]23)-[#6:15](=[S:16])-
[#7:17]-[c:18]2[c:19][c:20][c:21][c:22][c:23]-12.[#6;v0:24]>>[#6:24]-
[#16:16]-[#6:15]-1-[#7:17]-[c:18]2[c:19][c:20][c:21][c:22][c:23]2-
[#6:2](=[O:1])-[#7:3]-[#6:4]-1-[#6:5]-
[c:6]1[c:7][n:8][c:9]2[c:10][c:11][c:12][c:13][c:14]12

Correctness of the mapping
MAPPET YES
ReactionMap NO
Marvin YES

Reaction no 183

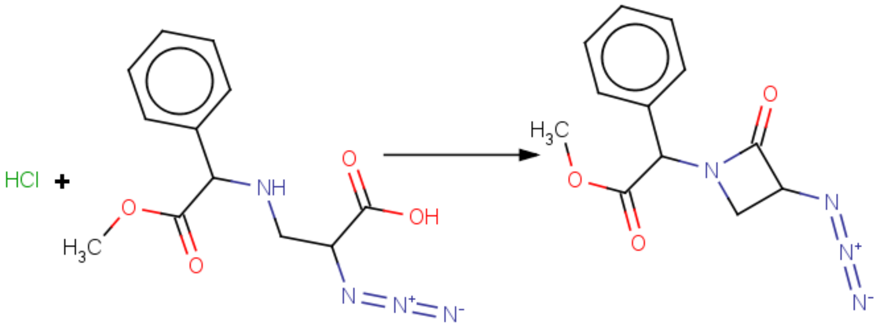

SMILES of the input:
Cl.COC(=O)C(NCC(N=[N+]=[N-
])C(=O)O)c1ccccc1>>COC(=O)C(c1ccccc1)N1CC(N=[N+]=[N-])C1=O

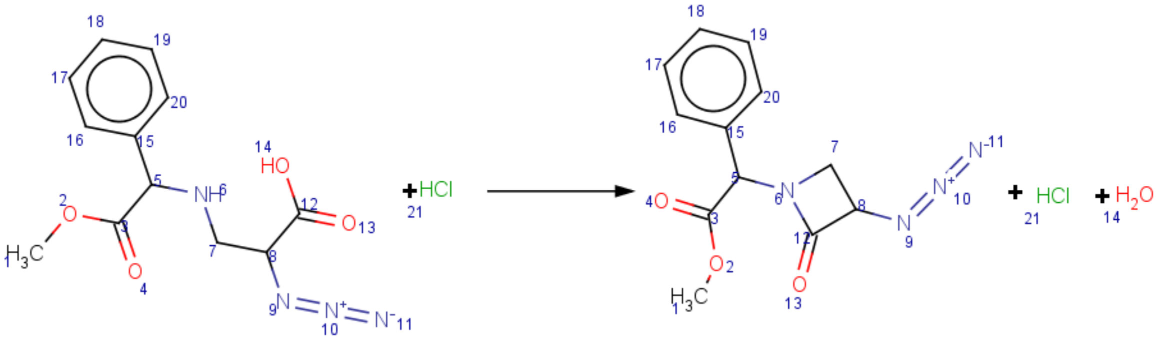

Correct mapped SMILES/SMARTS of the reaction:
[#6:1]-[#8:2]-[#6:3](=[O:4])-[#6:5](-[#7:6]-[#6:7]-[#6:8](-
[#7:9]=[N+:10]=[#7-:11])-[#6:12](-[#8:14])=[O:13])-
[c:15]1[c:16][c:17][c:18][c:19][c:20]1.[Cl:21]>>[#6:1]-[#8:2]-
[#6:3](=[O:4])-[#6:5](-[#7:6]-1-[#6:7]-[#6:8](-[#7:9]=[N+:10]=[#7-:11])-
[#6:12]-1=[O:13])-[c:15]1[c:16][c:17][c:18][c:19][c:20]1.[Cl:21].[#8:14]

Correctness of the mapping
MAPPET YES
ReactionMap NO
Marvin YES

Reaction no 184

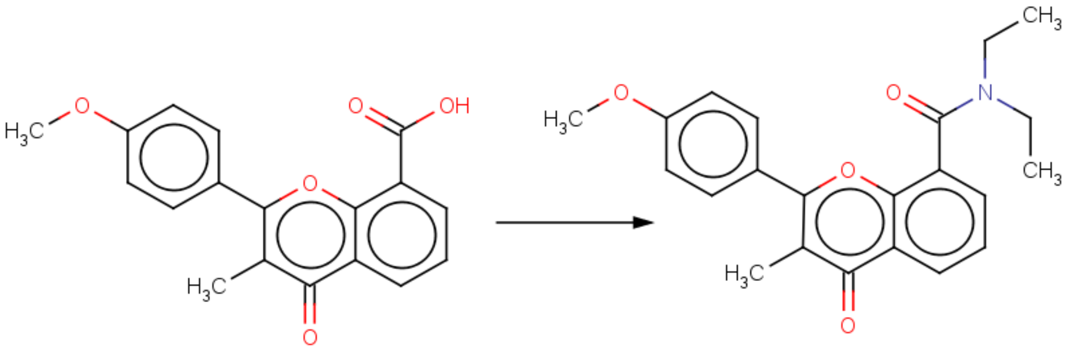

SMILES of the input:
COc1ccc(-c2oc3c(C(=O)O)cccc3c(=O)c2C)cc1>>CCN(CC)C(=O)c1cccc2c(=O)c(C)c(-
c3ccc(OC)cc3)oc12

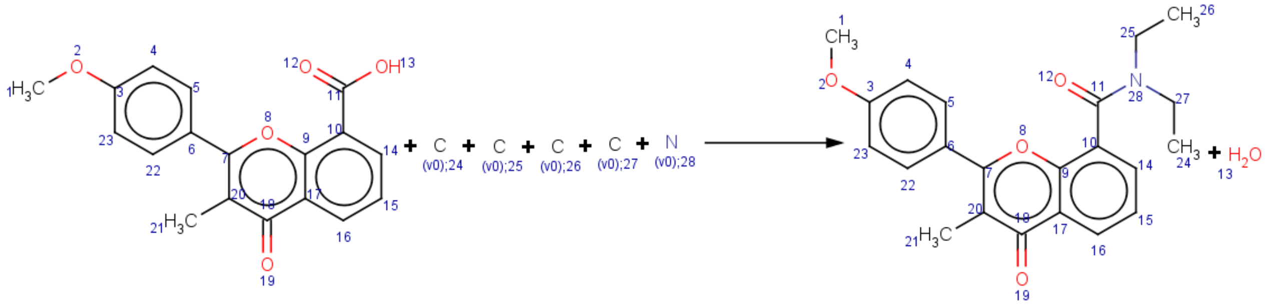

Correct mapped SMILES/SMARTS of the reaction:
[#6:1]-[#8:2]-[c:3]1[c:4][c:5][c:6]([c:22][c:23]1)-
[c:7]1[o:8][c:9]2[c:10]([c:14][c:15][c:16][c:17]2[c:18](=[O:19])[c:20]1-
[#6:21])-[#6:11](-
[#8:13])=[O:12].[#6;v0:24].[#6;v0:25].[#6;v0:26].[#6;v0:27].[#7;v0:28]>>[
#6:26]-[#6:25]-[#7:28](-[#6:27]-[#6:24])-[#6:11](=[O:12])-
[c:10]1[c:14][c:15][c:16][c:17]2[c:9]1[o:8][c:7](-[c:6]1[c:5][c:4][c:3](-
[#8:2]-[#6:1])[c:23][c:22]1)[c:20](-[#6:21])[c:18]2=[O:19].[#8:13]

Correctness of the mapping

MAPPET	YES
ReactionMap	NO
Marvin	YES

Reaction no 185

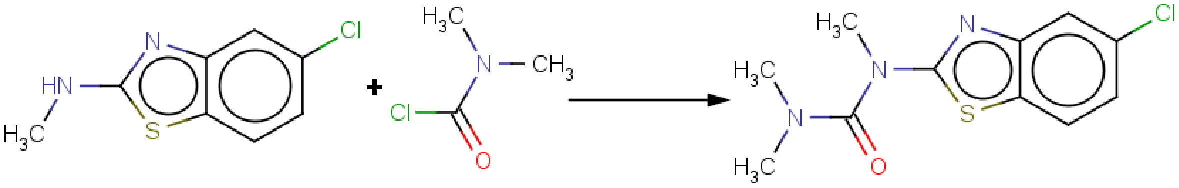

SMILES of the input:
CNc1nc2cc(Cl)ccc2s1.CN(C)C(=O)Cl>>CN(C)C(=O)N(C)c1nc2cc(Cl)ccc2s1

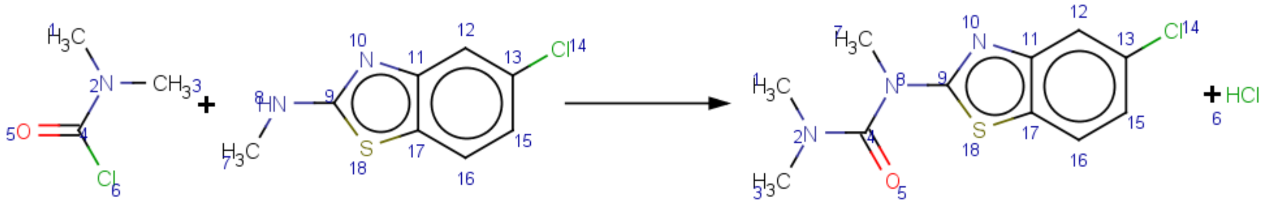

Correct mapped SMILES/SMARTS of the reaction:
[#6:1]-[#7:2](-[#6:3])-[#6:4]([Cl:6])=[O:5].[#6:7]-[#7:8]-
[c:9]1[n:10][c:11]2[c:12][c:13]([Cl:14])[c:15][c:16][c:17]2[s:18]1>>[#6:3
]-[#7:2](-[#6:1])-[#6:4](=[O:5])-[#7:8](-[#6:7])-
[c:9]1[n:10][c:11]2[c:12][c:13]([Cl:14])[c:15][c:16][c:17]2[s:18]1.[Cl:6]

Correctness of the mapping

MAPPET	YES
ReactionMap	NO
Marvin	YES

Reaction no 186

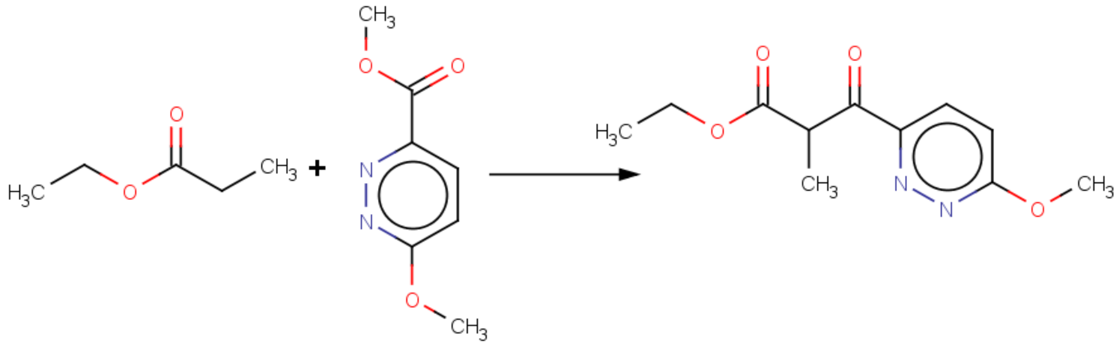

SMILES of the input:
CCOC(=O)CC.COC(=O)c1ccc(OC)nn1>>CCOC(=O)C(C)C(=O)c1ccc(OC)nn1

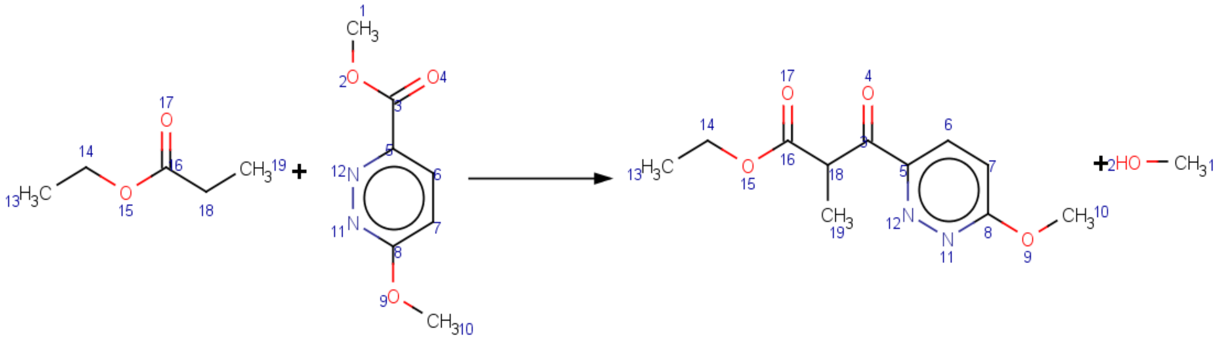

Correct mapped SMILES/SMARTS of the reaction:
[CH3:13][CH2:14][O:15][C:16](=[O:17])[CH2:18][CH3:19].[CH3:1][O:2][C:3](=
[O:4])[c:5]1[cH:6][cH:7][c:8]([O:9][CH3:10])[n:11][n:12]1>>[CH3:13][CH2:1
4][O:15][C:16](=[O:17])[CH:18]([CH3:19])[C:3](=[O:4])[c:5]1[cH:6][cH:7][c
:8]([O:9][CH3:10])[n:11][n:12]1.[CH3:1][OH:2]

Correctness of the mapping
MAPPET YES
ReactionMap NO
Marvin YES

Reaction no 187

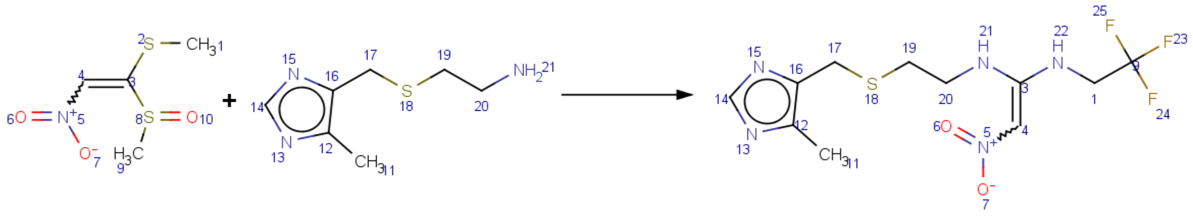

Correct mapped SMILES/SMARTS of the reaction:
[#6:1]-[#16:2]-[#6:3](=[#6:4]-[#7+:5](-[#8-
:7])=[O:6])[S:8]([#6:9])=[O:10].[#6:11]-[c:12]1[n:13][c:14][n:15][c:16]1-
[#6:17]-[#16:18]-[#6:19]-[#6:20]-[#7:21]>>[#6:11]-
[c:12]1[n:13][c:14][n:15][c:16]1-[#6:17]-[#16:18]-[#6:19]-[#6:20]-
[#7:21]-[#6:3](-[#7:22]-[#6:1][C:9]([F:23])([F:24])[F:25])=[#6:4]-
[#7+:5](-[#8-:7])=[O:6]

Correctness of the mapping
MAPPET NO
ReactionMap NO
Marvin YES

Reaction no 188

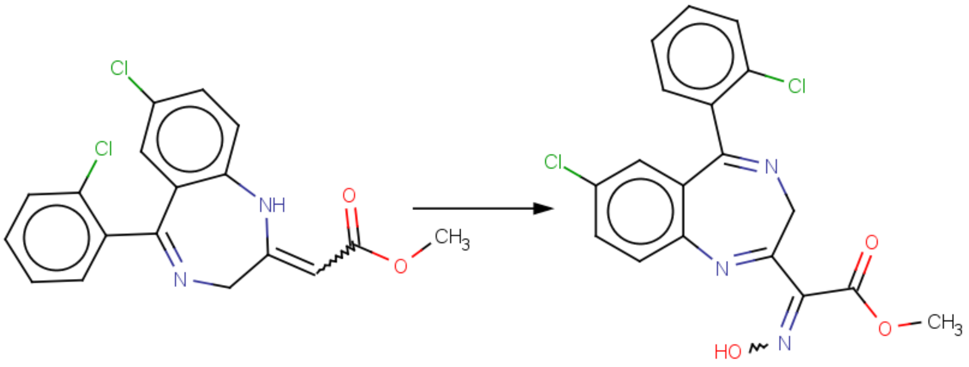

SMILES of the input:
COC(=O)C=C1CN=C(c2ccccc2Cl)c2cc(Cl)ccc2N1>>COC(=O)C(=NO)C1=Nc2ccc(Cl)cc2C(c2ccccc2Cl)=NC1

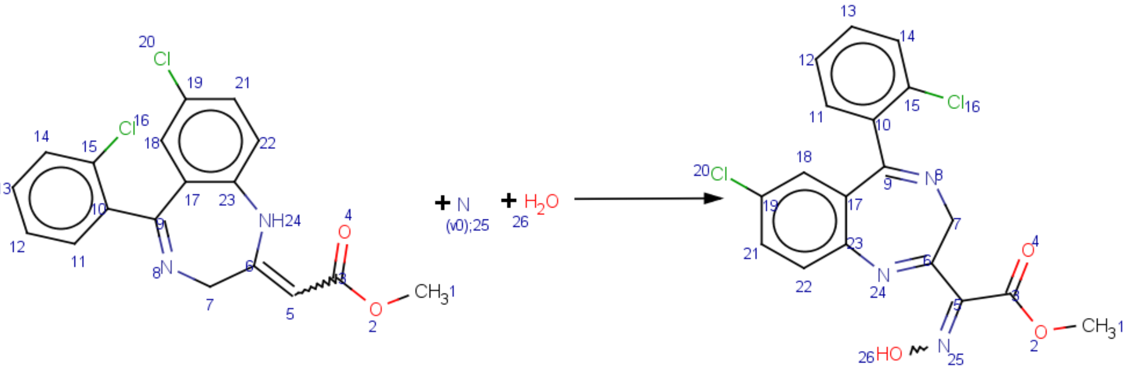

Correct mapped SMILES/SMARTS of the reaction:
[#6:1]-[#8:2]-[#6:3](=[O:4])-[#6:5]=[#6:6]-1-[#6:7]-[#7:8]=[#6:9](-[c:10]2[c:11][c:12][c:13][c:14][c:15]2[Cl:16])-[c:17]2[c:18][c:19]([Cl:20])[c:21][c:22][c:23]2-[#7:24]-1.[#7;v0:25].[#8:26]>>[#6:1]-[#8:2]-[#6:3](=[O:4])-[#6:5](=[#7:25]-[#8:26])-[#6:6]-1=[#7:24]-[c:23]2[c:22][c:21][c:19]([Cl:20])[c:18][c:17]2-[#6:9](=[#7:8]-[#6:7]-1)-[c:10]1[c:11][c:12][c:13][c:14][c:15]1[Cl:16]

Correctness of the mapping

MAPPET	YES
ReactionMap	NO
Marvin	YES

Reaction no 189

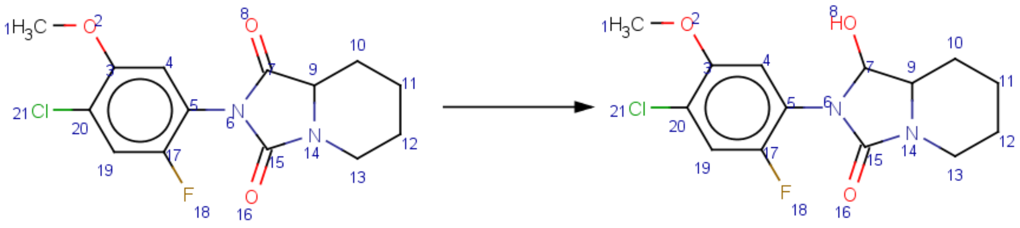

Correct mapped SMILES/SMARTS of the reaction:
[#6:1]-[#8:2]-[c:3]1[c:4][c:5](-[#7:6]-2-[#6:7](=[O:8])-[#6:9]-3-[#6:10]-[#6:11]-[#6:12]-[#6:13]-[#7:14]-3-[#6:15]-

2=[O:16])[c:17]([F:18])[c:19][c:20]1[Cl:21]>>[#6:1]-[#8:2]-[c:3]1[c:4][c:5](-[#7:6]-2-[#6:7](-[#8:8])-[#6:9]-3-[#6:10]-[#6:11]-[#6:12]-[#6:13]-[#7:14]-3-[#6:15]-2=[O:16])[c:17]([F:18])[c:19][c:20]1[Cl:21]

Correctness of the mapping

MAPPET	YES
ReactionMap	YES
Marvin	YES

Reaction no 190

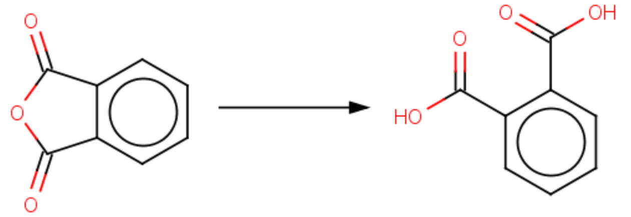

SMILES of the input:
O=C1OC(=O)c2ccccc21>>O=C(O)c1ccccc1C(=O)O

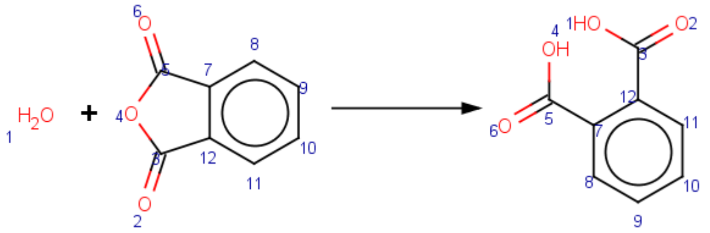

Correct mapped SMILES/SMARTS of the reaction:
[#8:1].[O:2]=[#6:3]-1-[#8:4]-[#6:5](=[O:6])-[c:7]2[c:8][c:9][c:10][c:11][c:12]-12>>[#8:1]-[#6:3](=[O:2])-[c:12]1[c:11][c:10][c:9][c:8][c:7]1-[#6:5](-[#8:4])=[O:6]

Correctness of the mapping

MAPPET	YES
ReactionMap	NO
Marvin	YES

Reaction no 191

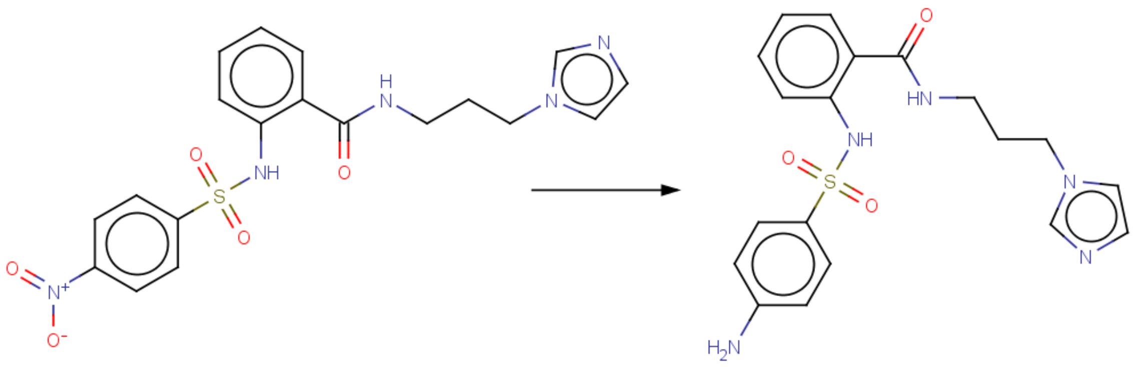

SMILES of the input:
O=C(NCCCn1ccnc1)c1ccccc1NS(=O)(=O)c1ccc([N+](=O)[O-])cc1>>Nc1ccc(S(=O)(=O)Nc2ccccc2C(=O)NCCCn2ccnc2)cc1

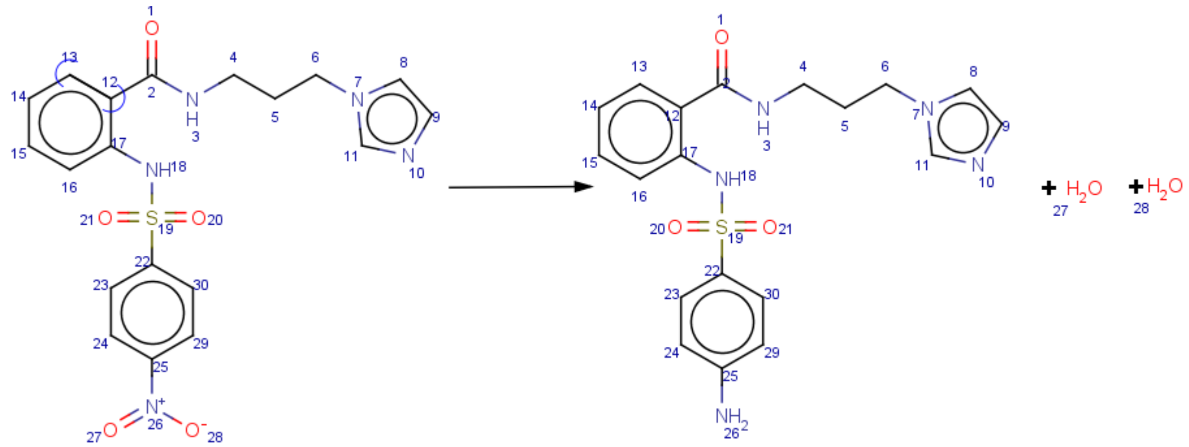

Correct mapped SMILES/SMARTS of the reaction:

```
[#8-:28]-[#7+:26](=[O:27])-
[c:25]1[c:24][c:23][c:22]([c:30][c:29]1)[S:19](=[O:20])(=[O:21])[#7:18]-
[c:17]1[c:16][c:15][c:14][c:13][c:12]1-[#6:2](=[O:1])-[#7:3]-[#6:4]-
[#6:5]-[#6:6]-[n:7]1[c:8][c:9][n:10][c:11]1>>[#7:26]-
[c:25]1[c:24][c:23][c:22]([c:30][c:29]1)[S:19](=[O:21])(=[O:20])[#7:18]-
[c:17]1[c:16][c:15][c:14][c:13][c:12]1-[#6:2](=[O:1])-[#7:3]-[#6:4]-
[#6:5]-[#6:6]-[n:7]1[c:8][c:9][n:10][c:11]1.[#8:27].[#8:28]
```

Correctness of the mapping

MAPPET	YES
ReactionMap	NO
Marvin	YES

Reaction no 1

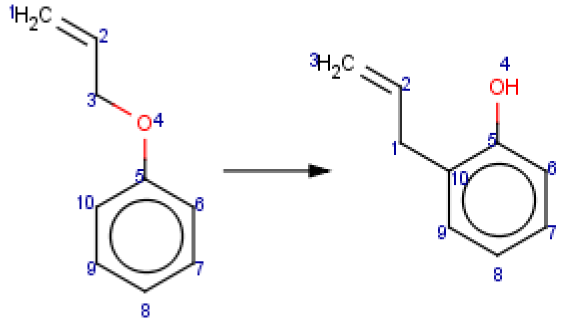

Correct mapped SMILES/SMARTS of the reaction:
[CH2:1]=[CH:2][CH2:3][O:4][c:5]1[cH:6][cH:7][cH:8][cH:9][cH:10]1>>[OH:4][c:5]1[cH:6][cH:7][cH:8][cH:9][c:10]1[CH2:1][CH:2]=[CH2:3]

Correctness of the mapping

MAPPET	YES
ReactionMap	NO
Marvin	NO

Reaction no 2

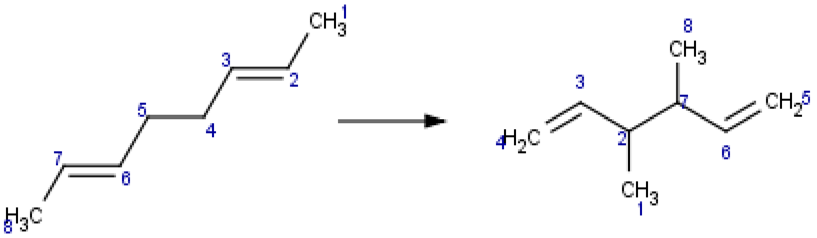

Correct mapped SMILES/SMARTS of the reaction:
[CH3:1]\[CH:2]=[CH:3]\[CH2:4][CH2:5]\[CH:6]=[CH:7]\[CH3:8]>>[CH3:1][CH:2]([CH:3]=[CH2:4])[CH:7]([CH3:8])[CH:6]=[CH2:5]

Correctness of the mapping

MAPPET	YES
ReactionMap	NO
Marvin	NO

Reaction no 3

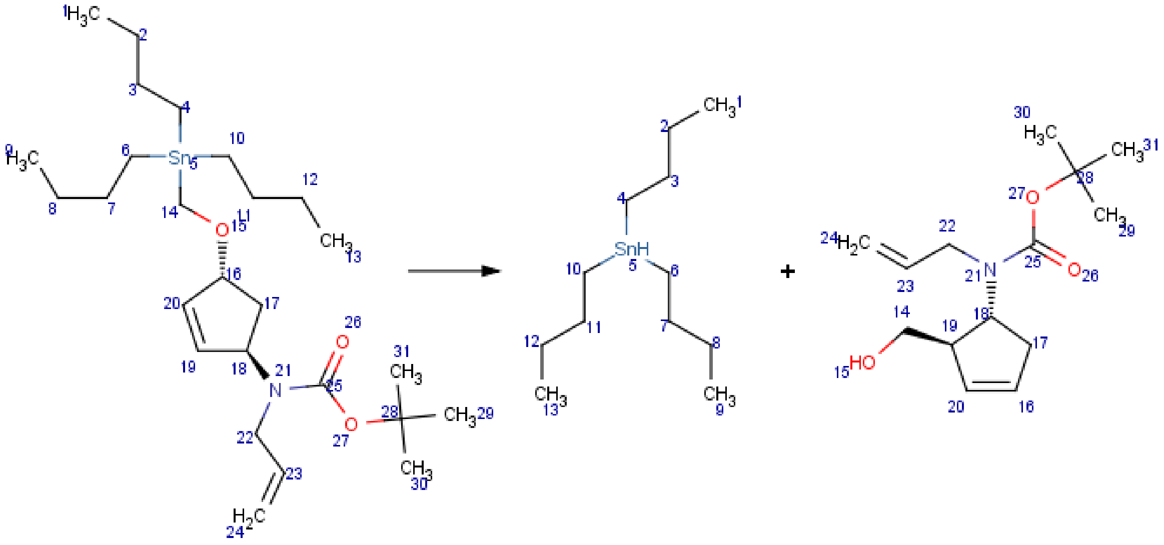

Correct mapped SMILES/SMARTS of the reaction:
[CH3:1][CH2:2][CH2:3][CH2:4][Sn:5]([CH2:6][CH2:7][CH2:8][CH3:9])([CH2:10][CH2:11
][CH2:12][CH3:13])[CH2:14][O:15][C@@H:16]1[CH2:17][C@H:18]([CH:19]=[CH:20]1)[N:2
1]([CH2:22][CH:23]=[CH2:24])[C:25](=[O:26])[O:27][C:28]([CH3:29])([CH3:30])[CH3:
31]>>[CH3:1][CH2:2][CH2:3][CH2:4][SnH:5]([CH2:10][CH2:11][CH2:12][CH3:13])[CH2:6
][CH2:7][CH2:8][CH3:9].[CH3:31][C:28]([CH3:30])([CH3:29])[O:27][C:25](=[O:26])[N
:21]([CH2:22][CH:23]=[CH2:24])[C@@H:18]1[CH2:17][CH:16]=[CH:20][C@H:19]1[CH2:14]
[OH:15]

Correctness of the mapping

MAPPET	YES
ReactionMap	YES
Marvin	NO

Reaction no 4

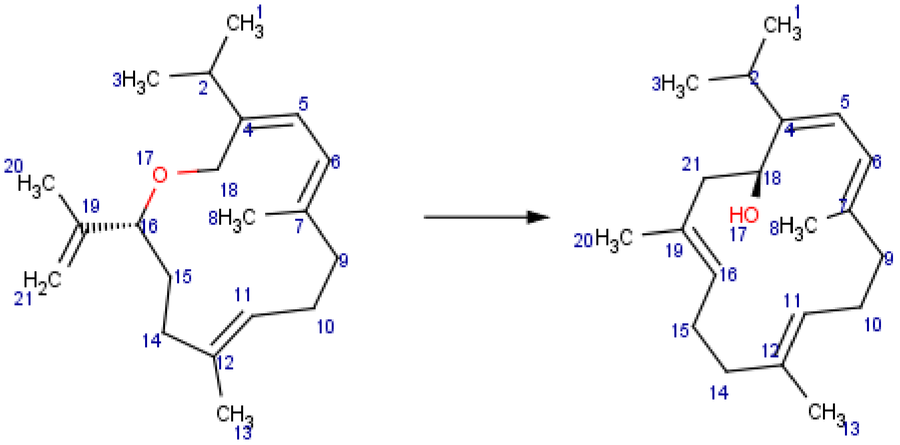

Correct mapped SMILES/SMARTS of the reaction:
[CH3:1][CH:2]([CH3:3])[C:4]1=[CH:5]/[CH:6]=[C:7]([CH3:8])/[CH2:9][CH2:10]\[CH:11
]=[C:12]([CH3:13])\[CH2:14][CH2:15][C@@H:16]([O:17][CH2:18]\1)[C:19]([CH3:20])=[
CH2:21]>>[CH3:3][CH:2]([CH3:1])[C:4]1=[CH:5]/[CH:6]=[C:7]([CH3:8])/[CH2:9][CH2:1
0]\[CH:11]=[C:12]([CH3:13])\[CH2:14][CH2:15]\[CH:16]=[C:19]([CH3:20])\[CH2:21][C
@@H:18]\1[OH:17]

Correctness of the mapping

MAPPET	YES
ReactionMap	YES
Marvin	YES

Reaction no 5

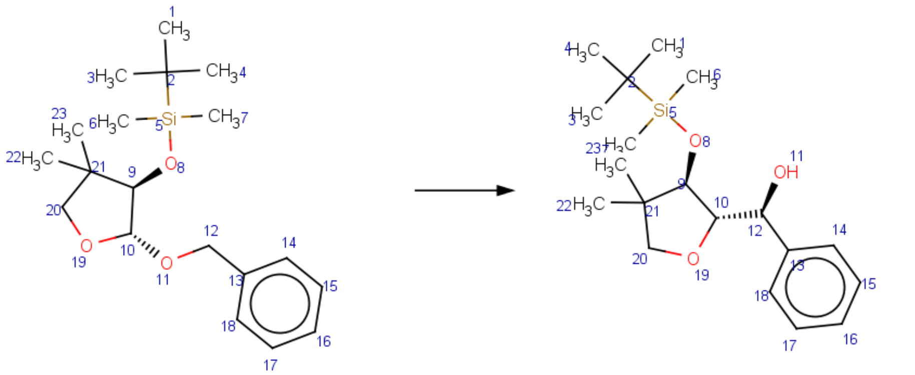

Correct mapped SMILES/SMARTS of the reaction:
[CH3:1][C:2]([CH3:3])([CH3:4])[Si:5]([CH3:6])([CH3:7])[O:8][C@H:9]1[C@H:10]([O:1
1][CH2:12][c:13]2[cH:14][cH:15][cH:16][cH:17][cH:18]2)[O:19][CH2:20][C:21]1([CH3
:22])[CH3:23]>>[CH3:4][C:2]([CH3:3])([CH3:1])[Si:5]([CH3:7])([CH3:6])[O:8][C@H:9

]1[C@@H:10]([O:19][CH2:20][C:21]1([CH3:22])[CH3:23])[C@@H:12]([OH:11])[c:13]1[cH:14][cH:15][cH:16][cH:17][cH:18]1

Correctness of the mapping
MAPPET YES
ReactionMap YES
Marvin YES

Reaction no 6

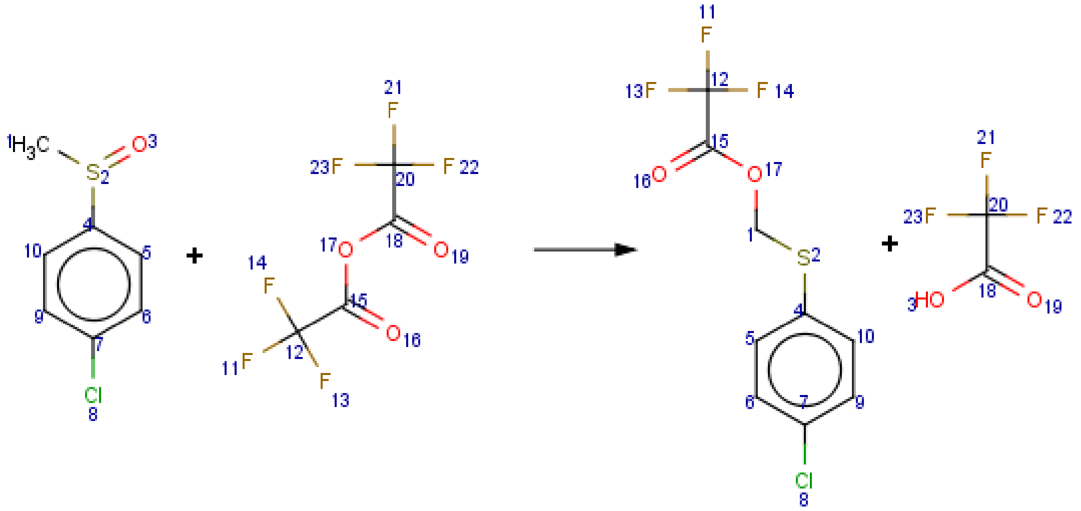

Correct mapped SMILES/SMARTS of the reaction:
[CH3:1][S:2](=[O:3])[c:4]1[cH:5][cH:6][c:7]([Cl:8])[cH:9][cH:10]1.[F:11][C:12]([F:13])([F:14])[C:15](=[O:16])[O:17][C:18](=[O:19])[C:20]([F:21])([F:22])[F:23]>>[F:11][C:12]([F:13])([F:14])[C:15](=[O:16])[O:17][CH2:1][S:2][c:4]1[cH:10][cH:9][c:7]([Cl:8])[cH:6][cH:5]1.[OH:3][C:18](=[O:19])[C:20]([F:21])([F:22])[F:23]

Correctness of the mapping
MAPPET YES
ReactionMap YES
Marvin NO

Reaction no 7

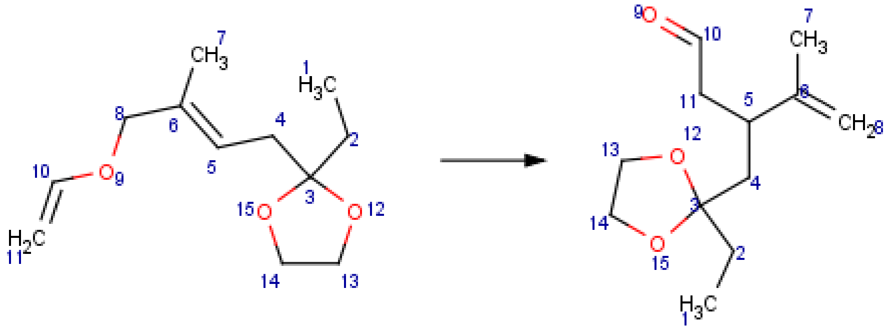

Correct mapped SMILES/SMARTS of the reaction:
[CH3:1][CH2:2][C:3]1([CH2:4]\[CH:5]=[C:6](/[CH3:7])[CH2:8][O:9][CH:10]=[CH2:11])[O:12][CH2:13][CH2:14][O:15]1>>[CH3:1][CH2:2][C:3]1([CH2:4][CH:5]([CH2:11][CH:10]=[O:9])[C:6]([CH3:7])=[CH2:8])[O:15][CH2:14][CH2:13][O:12]1

Correctness of the mapping
MAPPET YES
ReactionMap YES
Marvin NO

Reaction no 8

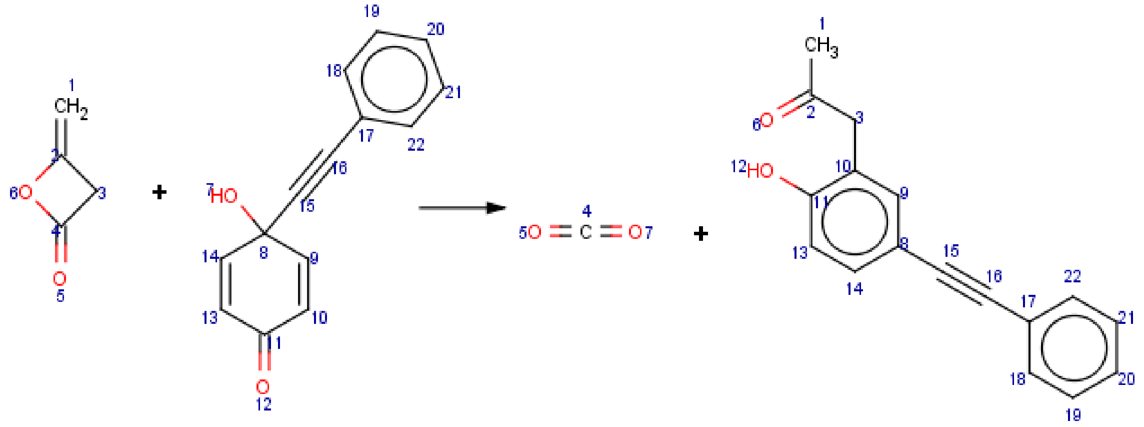

Correct mapped SMILES/SMARTS of the reaction:
[CH2:1]=[C:2]1[CH2:3][C:4](=[O:5])[O:6]1.[OH:7][C:8]1([CH:9]=[CH:10][C:11](=[O:1
2])[CH:13]=[CH:14]1)[C:15]#[C:16][c:17]1[cH:18][cH:19][cH:20][cH:21][cH:22]1>>[O
:5]=[C:4]=[O:7].[CH3:1][C:2](=[O:6])[CH2:3][c:10]1[cH:9][c:8]([cH:14][cH:13][c:1
1]1[OH:12])[C:15]#[C:16][c:17]1[cH:22][cH:21][cH:20][cH:19][cH:18]1

Correctness of the mapping
MAPPET YES
ReactionMap YES
Marvin NO

Reaction no 9

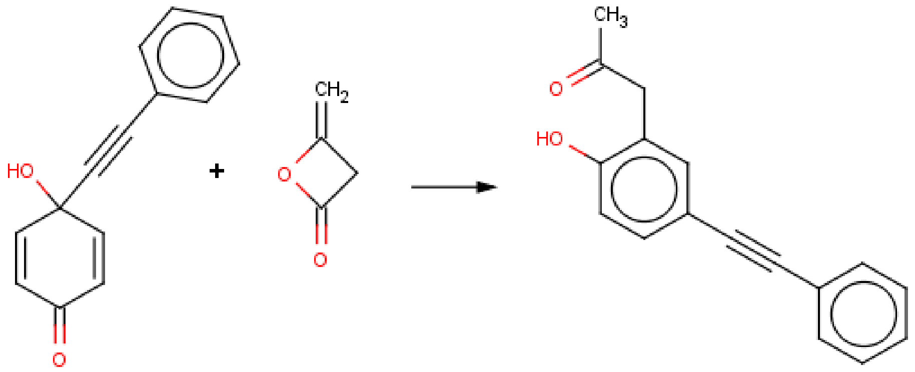

SMILES of the input:
OC1(C=CC(=O)C=C1)C#Cc1ccccc1.C=C1CC(=O)O1>>CC(=O)Cc1cc(ccc1O)C#Cc1ccccc1

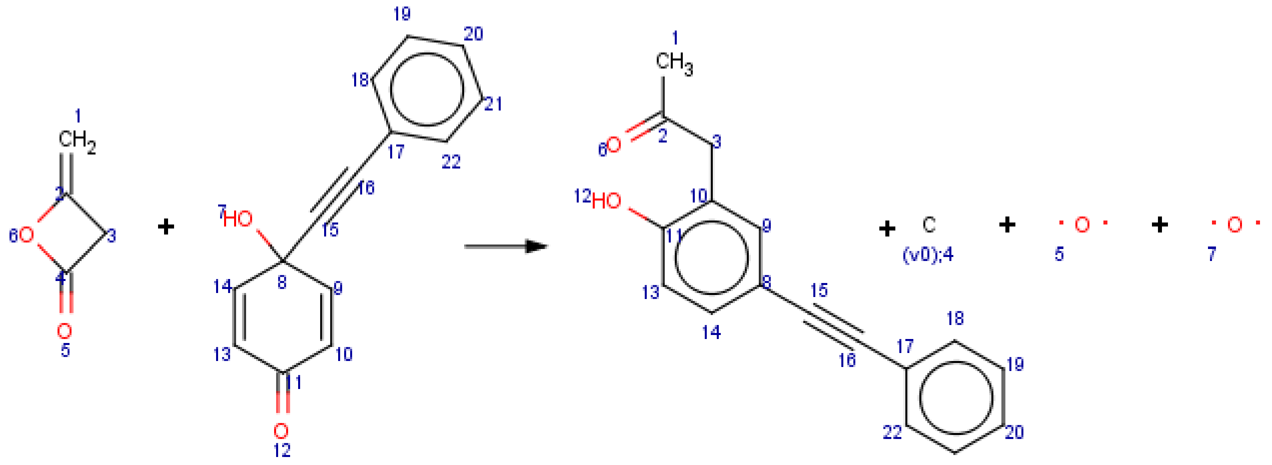

Correct mapped SMILES/SMARTS of the reaction:
[CH2:1]=[C:2]1[CH2:3][C:4](=[O:5])[O:6]1.[OH:7][C:8]1([CH:9]=[CH:10][C:11](=[O:12])[CH:13]=[CH:14]1)[C:15]#[C:16][c:17]1[cH:18][cH:19][cH:20][cH:21][cH:22]1>>[CH3:1][C:2](=[O:6])[CH2:3][c:10]1[cH:9][c:8]([cH:14][cH:13][c:11]1[OH:12])[C:15]#[C:16][c:17]1[cH:18][cH:19][cH:20][cH:21][cH:22]1.[C:4].[O:5].[O:7]

Correctness of the mapping

MAPPET	YES
ReactionMap	NO
Marvin	NO

Reaction no 10

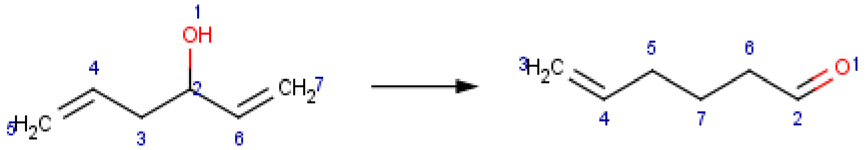

Correct mapped SMILES/SMARTS of the reaction:
[OH:1][CH:2]([CH2:3][CH:4]=[CH2:5])[CH:6]=[CH2:7]>>[CH2:3]=[CH:4][CH2:5][CH2:7][CH2:6][CH:2]=[O:1]

Correctness of the mapping

MAPPET	YES
ReactionMap	NO
Marvin	NO

Reaction no 11

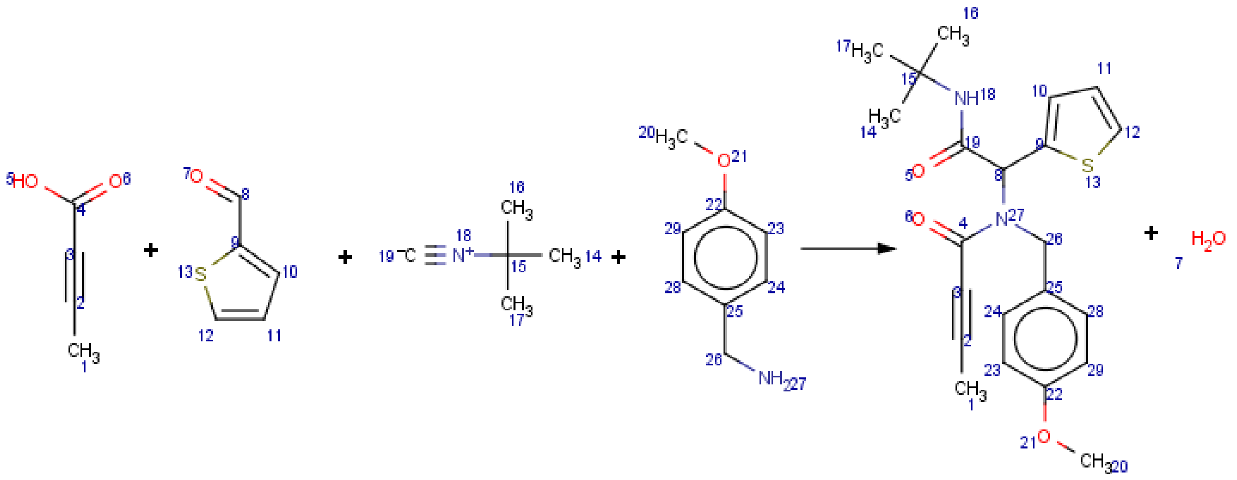

Correct mapped SMILES/SMARTS of the reaction:
[CH3:1][C:2]#[C:3][C:4]([OH:5])=[O:6].[O:7]=[CH:8][C:9]1=[CH:10][CH:11]=[CH:12][S:13]1.[CH3:14][C:15]([CH3:16])([CH3:17])[N+:18]#[C-:19].[CH3:20][O:21][c:22]1[cH:23][cH:24][c:25]([CH2:26][NH2:27])[cH:28][cH:29]1>>[CH3:20][O:21][c:22]1[cH:23][cH:24][c:25]([CH2:26][N:27]([CH:8]([C:19](=[O:5])[NH:18][C:15]([CH3:17])([CH3:16])[CH3:14])[C:9]2=[CH:10][CH:11]=[CH:12][S:13]2)[C:4](=[O:6])[C:3]#[C:2][CH3:1])[cH:28][cH:29]1.[OH2:7]

Correctness of the mapping

MAPPET	YES
ReactionMap	YES
Marvin	NO

Reaction no 12

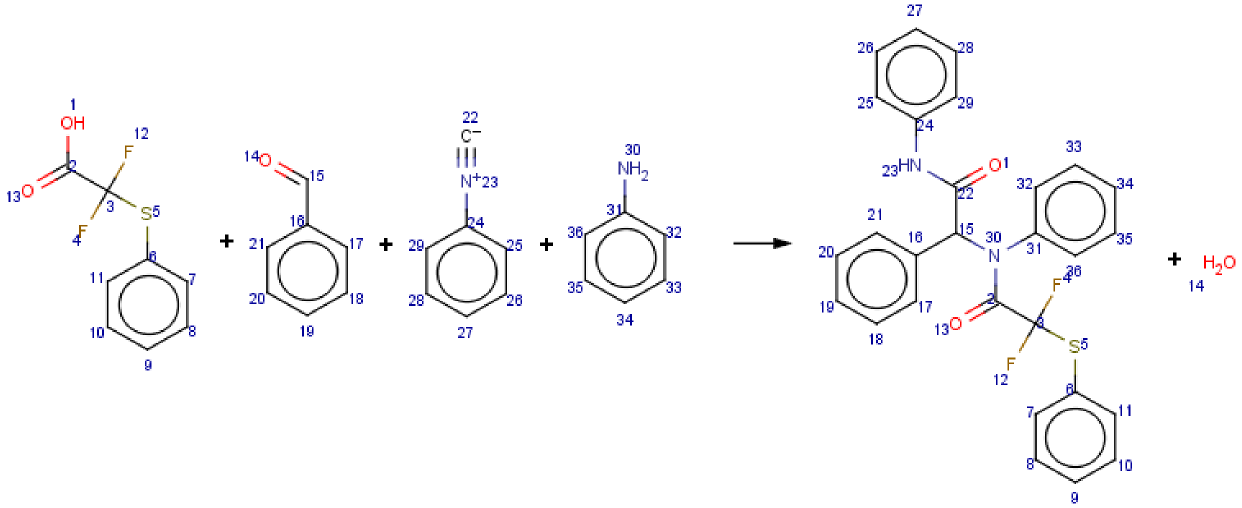

Correct mapped SMILES/SMARTS of the reaction:
[OH:1][C:2](=[O:13])[C:3]([F:4])([F:12])[S:5][c:6]1[cH:7][cH:8][cH:9][cH:10][cH:11]1.[O:14]=[CH:15][c:16]1[cH:17][cH:18][cH:19][cH:20][cH:21]1.[C-:22]#[N+:23][c:24]1[cH:25][cH:26][cH:27][cH:28][cH:29]1.[NH2:30][c:31]1[cH:32][cH:33][cH:34][cH:35][cH:36]1>>[F:4][C:3]([F:12])([S:5][c:6]1[cH:11][cH:10][cH:9][cH:8][cH:7]1)[C:2](=[O:13])[N:30]([CH:15]([C:22](=[O:1])[NH:23][c:24]1[cH:25][cH:26][cH:27][cH:28][cH:29]1)[c:16]1[cH:17][cH:18][cH:19][cH:20][cH:21]1)[c:31]1[cH:32][cH:33][cH:34][cH:35][cH:36]1.[OH2:14]

```
Correctness of the mapping
MAPPET              YES
ReactionMap         YES
Marvin              NO

Reaction no  13
```

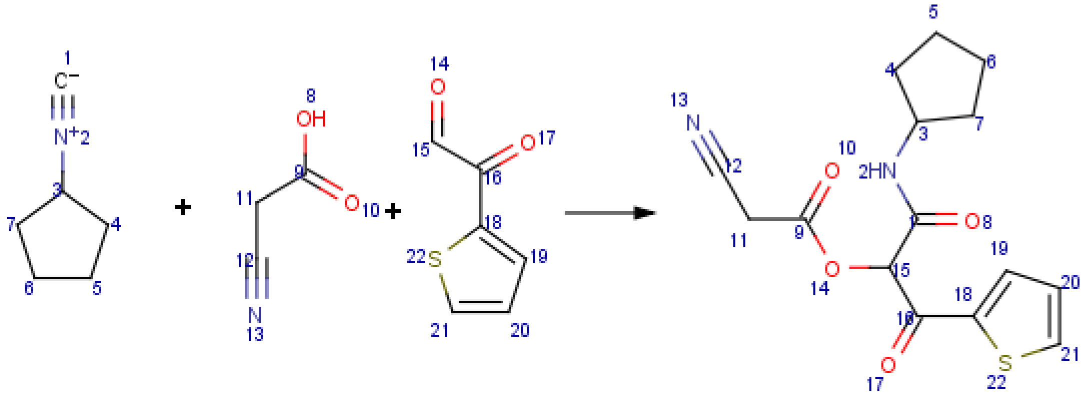

```
Correct mapped SMILES/SMARTS of the reaction:
[C-
:1]#[N+:2][CH:3]1[CH2:4][CH2:5][CH2:6][CH2:7]1.[OH:8][C:9](=[O:10])[CH2:11][C:12
]#[N:13].[O:14]=[CH:15][C:16](=[O:17])[C:18]1=[CH:19][CH:20]=[CH:21][S:22]1>>[O:
10]=[C:9]([CH2:11][C:12]#[N:13])[O:14][CH:15]([C:1](=[O:8])[NH:2][CH:3]1[CH2:4][
CH2:5][CH2:6][CH2:7]1)[C:16](=[O:17])[C:18]1=[CH:19][CH:20]=[CH:21][S:22]1

Correctness of the mapping
MAPPET              YES
ReactionMap         YES
Marvin              NO

Reaction no  14
```

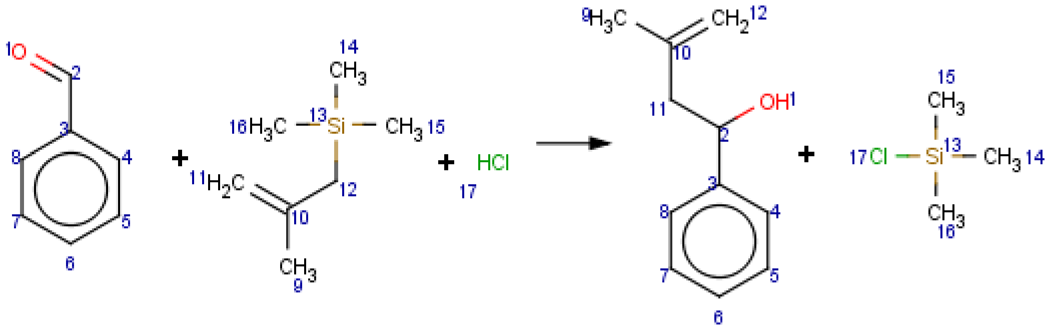

```
Correct mapped SMILES/SMARTS of the reaction:
[O:1]=[CH:2][c:3]1[cH:4][cH:5][cH:6][cH:7][cH:8]1.[CH3:9][C:10](=[CH2:11])[CH2:1
2][Si:13]([CH3:14])([CH3:15])[CH3:16].[ClH:17]>>[CH3:9][C:10](=[CH2:12])[CH2:11]
[CH:2]([OH:1])[c:3]1[cH:4][cH:5][cH:6][cH:7][cH:8]1.[CH3:16][Si:13]([CH3:15])([C
H3:14])[Cl:17]

Correctness of the mapping
MAPPET              YES
ReactionMap         NO
Marvin              NO
```

Reaction no 15

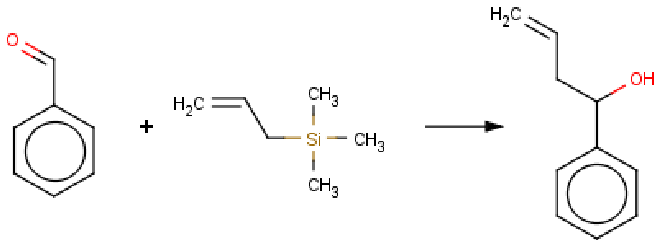

SMILES of the input:
O=Cc1ccccc1.C[Si](C)(C)CC=C>>OC(CC=C)c1ccccc1

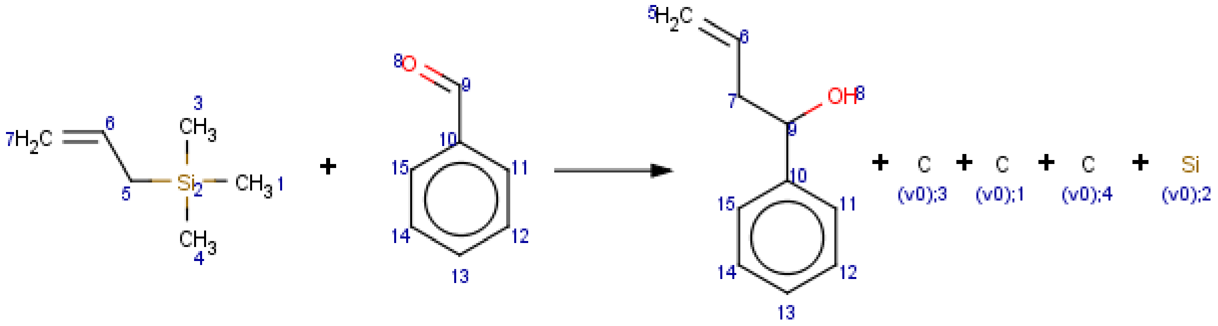

Correct mapped SMILES/SMARTS of the reaction:
[CH3:1][Si:2]([CH3:3])([CH3:4])[CH2:5][CH:6]=[CH2:7].[O:8]=[CH:9][c:10]1[cH:11][cH:12][cH:13][cH:14][cH:15]1>>[OH:8][CH:9]([CH2:7][CH:6]=[CH2:5])[c:10]1[cH:11][cH:12][cH:13][cH:14][cH:15]1.[C:3].[C:1].[C:4].[Si:2]

Correctness of the mapping
MAPPET   YES
ReactionMap   NO
Marvin   NO

Reaction no 16

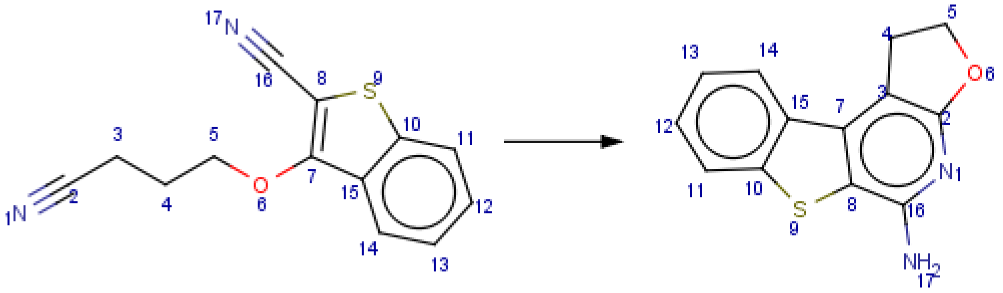

Correct mapped SMILES/SMARTS of the reaction:
[N:1]#[C:2][CH2:3][CH2:4][CH2:5][O:6][C:7]1=[C:8]([S:9][c:10]2[cH:11][cH:12][cH:13][cH:14][c:15]12)[C:16]#[N:17]>>[NH2:17][c:16]1[n:1][c:2]2[O:6][CH2:5][CH2:4][c:3]2[c:7]-2[c:8]1[S:9][c:10]1[cH:11][cH:12][cH:13][cH:14][c:15]-21

Correctness of the mapping
MAPPET   YES
ReactionMap   NO
Marvin   YES

Reaction no 17

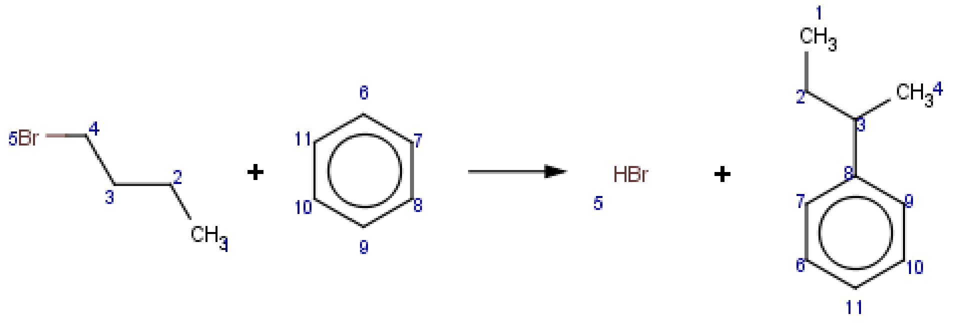

Correct mapped SMILES/SMARTS of the reaction:
[CH3:1][CH2:2][CH2:3][CH2:4][Br:5].[cH:6]1[cH:7][cH:8][cH:9][cH:10][cH:11]1>>[Br
H:5].[CH3:1][CH2:2][CH:3]([CH3:4])[c:8]1[cH:9][cH:10][cH:11][cH:6][cH:7]1

Correctness of the mapping

MAPPET	YES
ReactionMap	YES
Marvin	NO

Reaction no 18

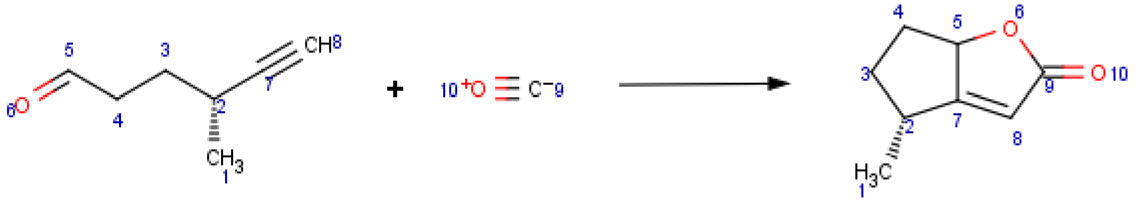

Correct mapped SMILES/SMARTS of the reaction:
[CH3:1][C@H:2]([CH2:3][CH2:4][CH:5]=[O:6])[C:7]#[CH:8].[C-
:9]#[O+:10]>>[CH3:1][C@@H:2]1[CH2:3][CH2:4][CH:5]2[O:6][C:9](=[O:10])[CH:8]=[C:7
]12

Correctness of the mapping

MAPPET	YES
ReactionMap	YES
Marvin	NO

Reaction no 19

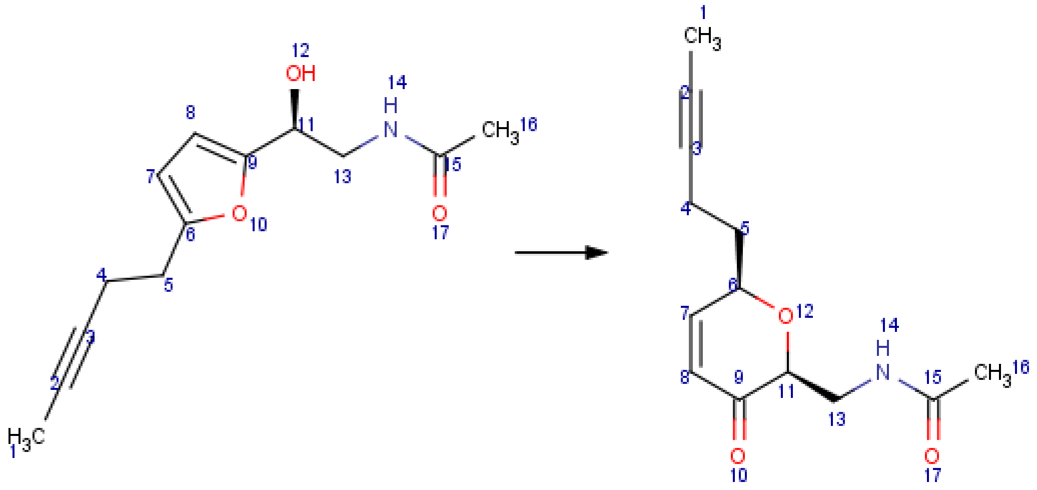

Correct mapped SMILES/SMARTS of the reaction:
[CH3:1][C:2]#[C:3][CH2:4][CH2:5][C:6]1=[CH:7][CH:8]=[C:9]([O:10]1)[C@@H:11]([OH:12])[CH2:13][NH:14][C:15]([CH3:16])=[O:17]>>[CH3:1][C:2]#[C:3][CH2:4][CH2:5][C@H:6]1[O:12][C@@H:11]([CH2:13][NH:14][C:15]([CH3:16])=[O:17])[C:9](=[O:10])[CH:8]=[CH:7]1

Correctness of the mapping
MAPPET YES
ReactionMap YES
Marvin NO

Reaction no 20

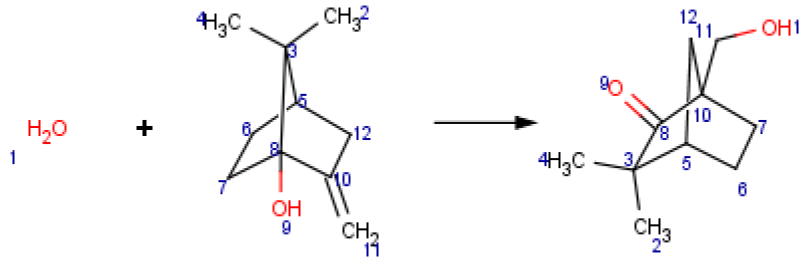

Correct mapped SMILES/SMARTS of the reaction:
[OH2:1].[CH3:2][C:3]1([CH3:4])[CH:5]2[CH2:6][CH2:7][C:8]1([OH:9])[C:10](=[CH2:11])[CH2:12]2>>[CH3:4][C:3]1([CH3:2])[CH:5]2[CH2:6][CH2:7][C:10]([CH2:11][OH:1])([CH2:12]2)[C:8]1=[O:9]

Correctness of the mapping
MAPPET YES
ReactionMap YES
Marvin YES

Reaction no 21

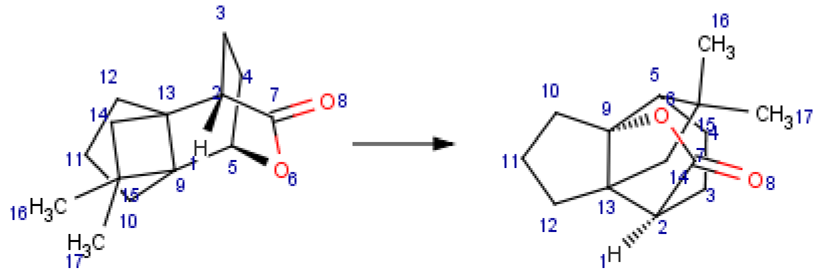

Correct mapped SMILES/SMARTS of the reaction:
[H:1][C@@:2]12[CH2:3][CH2:4][C@@H:5]([O:6][C:7]1=[O:8])[C:9]13[CH2:10][CH2:11][CH2:12][C:13]21[CH2:14][C:15]3([CH3:16])[CH3:17]>>[H:1][C@:2]12[CH2:3][CH2:4][CH:5]3[C:15]([CH3:16])([CH3:17])[CH2:14][C:13]11[CH2:12][CH2:11][CH2:10][C@@:9]31[O:6][C:7]2=[O:8]

Correctness of the mapping
MAPPET NO
ReactionMap NO
Marvin YES

Reaction no 22

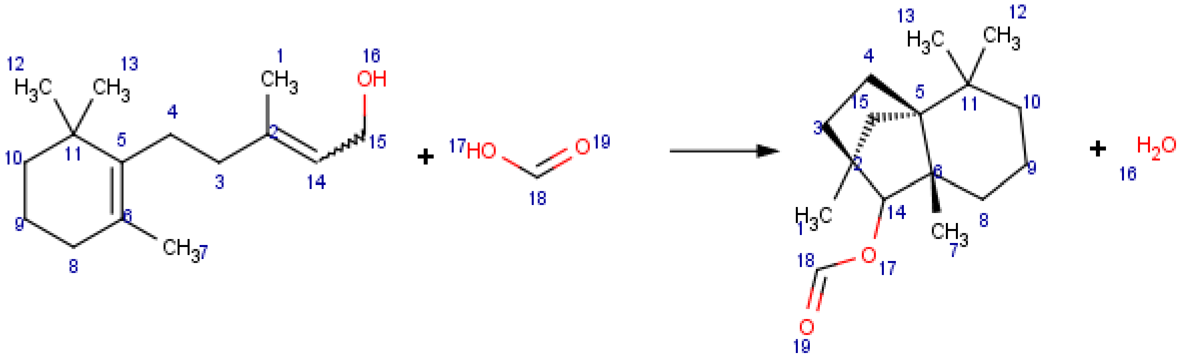

Correct mapped SMILES/SMARTS of the reaction:
[CH3:1][C:2]([CH2:3][CH2:4][C:5]1=[C:6]([CH3:7])[CH2:8][CH2:9][CH2:10][C:11]1([C
H3:12])[CH3:13])=[CH:14][CH2:15][OH:16].[OH:17][CH:18]=[O:19]>>[CH3:1][C@:2]12[C
H2:3][CH2:4][C@:5]3([CH2:15]1)[C@:6]([CH3:7])([CH2:8][CH2:9][CH2:10][C:11]3([CH3
:12])[CH3:13])[CH:14]2[O:17][CH:18]=[O:19].[OH2:16]

Correctness of the mapping

MAPPET	YES
ReactionMap	NO
Marvin	YES

Reaction no 23

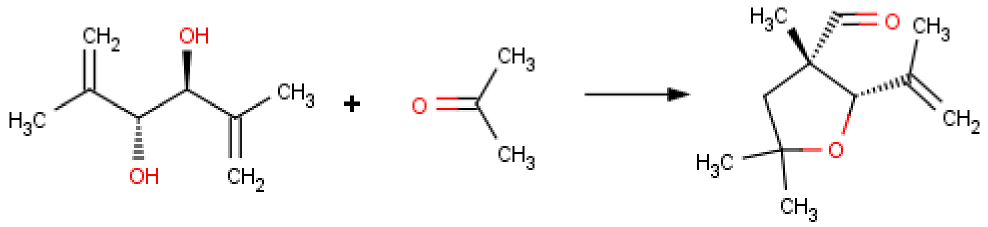

SMILES of the input:
CC(=C)[C@@H](O)[C@@H](O)C(C)=C.CC(C)=O>>CC(=C)[C@@H]1OC(C)(C)C[C@]1(C)C=O

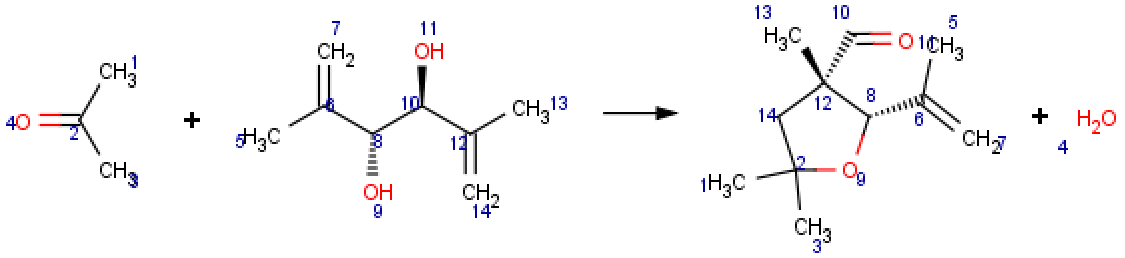

Correct mapped SMILES/SMARTS of the reaction:
[CH3:1][C:2]([CH3:3])=[O:4].[CH3:5][C:6](=[CH2:7])[C@@H:8]([OH:9])[C@@H:10]([OH:
11])[C:12]([CH3:13])=[CH2:14]>>[OH2:4].[CH3:5][C:6](=[CH2:7])[C@@H:8]1[O:9][C:2]
([CH3:3])([CH3:1])[CH2:14][C@:12]1([CH3:13])[CH:10]=[O:11]

Correctness of the mapping

MAPPET	YES
ReactionMap	NO
Marvin	NO

Reaction no 24

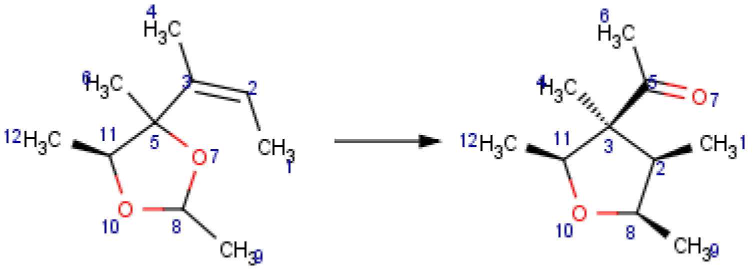

Correct mapped SMILES/SMARTS of the reaction:
[CH3:1]\[CH:2]=[C:3](\[CH3:4])[C:5]1([CH3:6])[O:7][CH:8]([CH3:9])[O:10][C@H:11]1[CH3:12]>>[CH3:9][C@H:8]1[O:10][C@@H:11]([CH3:12])[C@:3]([CH3:4])([C@H:2]1[CH3:1])[C:5]([CH3:6])=[O:7]

Correctness of the mapping
MAPPET YES
ReactionMap NO
Marvin NO

Reaction no 25

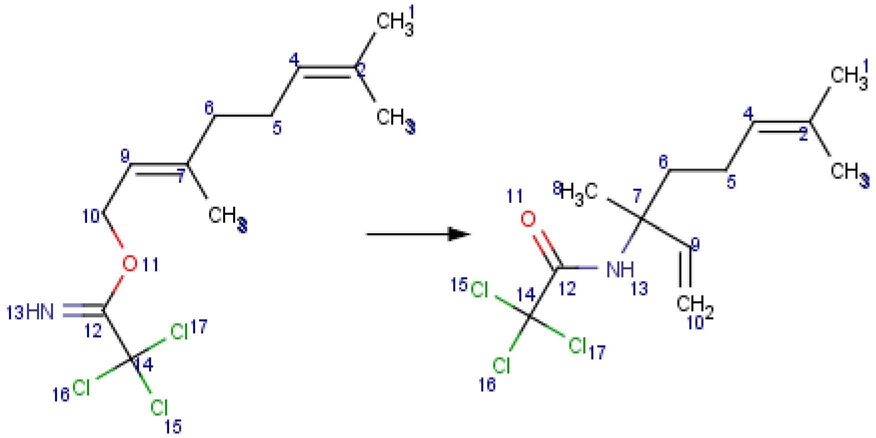

Correct mapped SMILES/SMARTS of the reaction:
[CH3:1][C:2]([CH3:3])=[CH:4][CH2:5][CH2:6]\[C:7]([CH3:8])=[CH:9]\[CH2:10][O:11][C:12](=[NH:13])[C:14]([Cl:15])([Cl:16])[Cl:17]>>[CH3:1][C:2]([CH3:3])=[CH:4][CH2:5][CH2:6][C:7]([CH3:8])([NH:13][C:12](=[O:11])[C:14]([Cl:16])([Cl:15])[Cl:17])[CH:9]=[CH2:10]

Correctness of the mapping
MAPPET YES
ReactionMap YES
Marvin YES

Reaction no 26

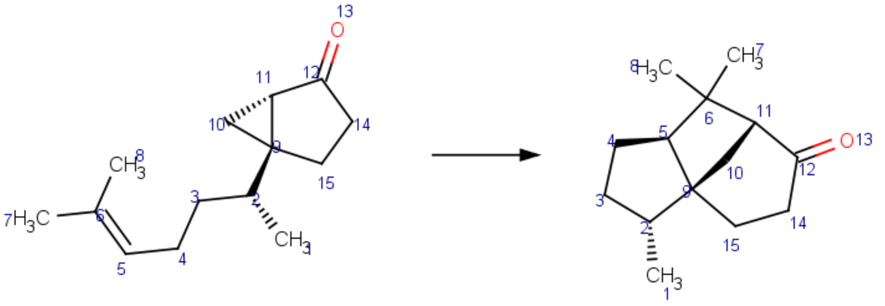

Correct mapped SMILES/SMARTS of the reaction:
[CH3:1][C@H:2]([CH2:3][CH2:4][CH:5]=[C:6]([CH3:7])[CH3:8])[C@@:9]12[CH2:10][C@@H
:11]1[C:12](=[O:13])[CH2:14][CH2:15]2>>[CH3:1][C@@H:2]1[CH2:3][CH2:4][C@H:5]2[C:
6]([CH3:8])([CH3:7])[C@H:11]3[CH2:10][C@@:9]12[CH2:15][CH2:14][C:12]3=[O:13]

Correctness of the mapping
MAPPET YES
ReactionMap YES
Marvin YES

Reaction no 27

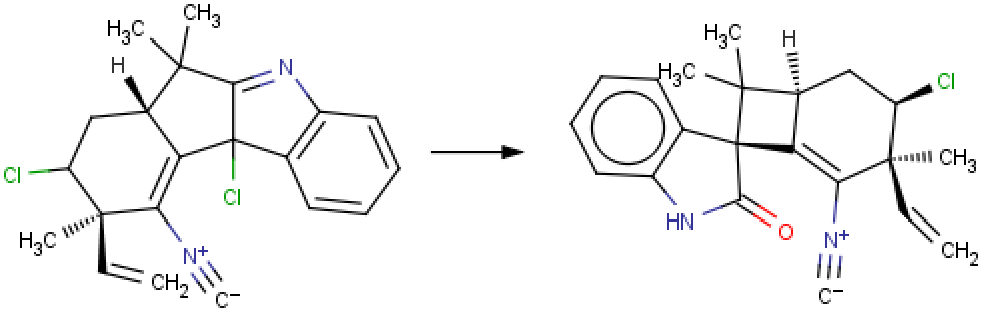

SMILES of the input:
[H][C@]12CC(Cl)[C@](C)(C=C)C([N+]#[C-
])=C1C1(Cl)C3=C(C=CC=C3)N=C1C2(C)C>>[H][C@]12C[C@@H](Cl)[C@@](C)(C=C)C([N+]#[C-
])=C1[C@]1(C(=O)Nc3ccccc13)C2(C)C

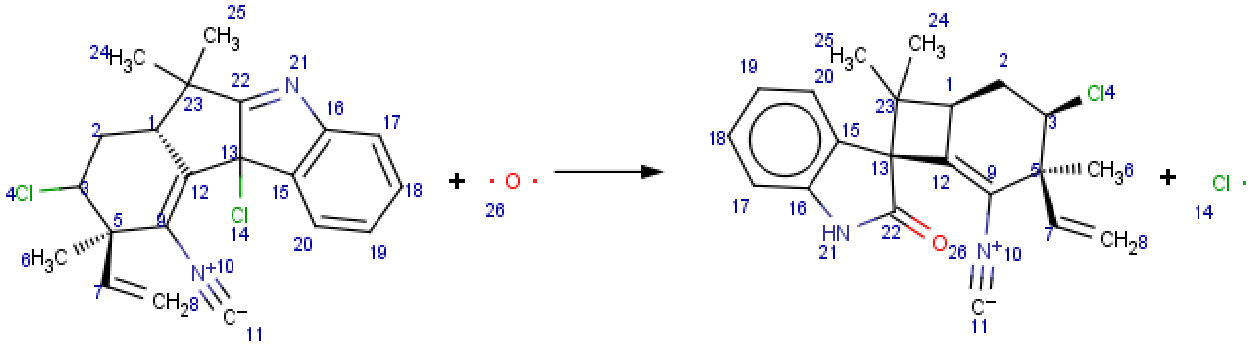

Correct mapped SMILES/SMARTS of the reaction:
[CH3:24][C:23]1([CH3:25])[C@H:1]2[CH2:2][CH:3]([Cl:4])[C@:5]([CH3:6])([CH:7]=[CH
2:8])[C:9]([N+:10]#[C-
:11])=[C:12]2[C:13]2([Cl:14])[C:15]3=[C:16]([CH:17]=[CH:18][CH:19]=[CH:20]3)[N:2
1]=[C:22]12.[O:26]>>[CH3:24][C:23]1([CH3:25])[C@H:1]2[CH2:2][C@@H:3]([Cl:4])[C@@
:5]([CH3:6])([CH:7]=[CH2:8])[C:9]([N+:10]#[C-
:11])=[C:12]2[C@:13]11[C:22](=[O:26])[NH:21][c:16]2[cH:17][cH:18][cH:19][cH:20][
c:15]12.[Cl:14]

```
Correctness of the mapping
MAPPET              YES
ReactionMap         NO
Marvin              YES

Reaction no  28
```

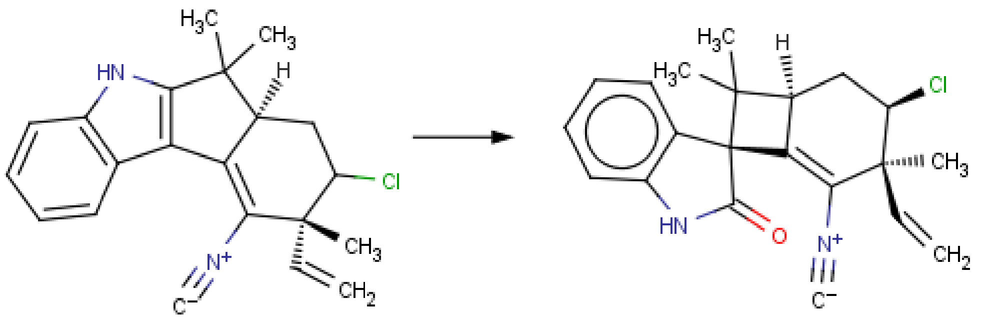

```
SMILES of the input:
[H][C@]12CC(Cl)[C@](C)(C=C)C([N+]#[C-
])=C1C1=C(NC3=C1C=CC=C3)C2(C)C>>[H][C@]12C[C@@H](Cl)[C@@](C)(C=C)C([N+]#[C-
])=C1[C@]1(C(=O)Nc3ccccc13)C2(C)C
```

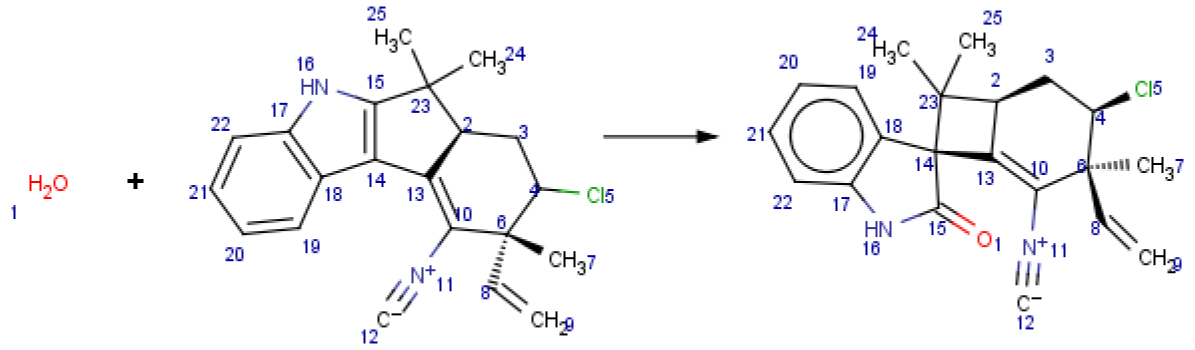

```
Correct mapped SMILES/SMARTS of the reaction:
[OH2:1].[CH3:24][C:23]1([CH3:25])[C@H:2]2[CH2:3][CH:4]([Cl:5])[C@:6]([CH3:7])([C
H:8]=[CH2:9])[C:10]([N+:11]#[C-
:12])=[C:13]2[C:14]2=[C:15]1[NH:16][C:17]1=[C:18]2[CH:19]=[CH:20][CH:21]=[CH:22]
1>>[CH3:25][C:23]1([CH3:24])[C@H:2]2[CH2:3][C@@H:4]([Cl:5])[C@@:6]([CH3:7])([CH:
8]=[CH2:9])[C:10]([N+:11]#[C-
:12])=[C:13]2[C@:14]11[C:15](=[O:1])[NH:16][c:17]2[cH:22][cH:21][cH:20][cH:19][c
:18]12

Correctness of the mapping
MAPPET              YES
ReactionMap         NO
Marvin              YES
```

Reaction no 29

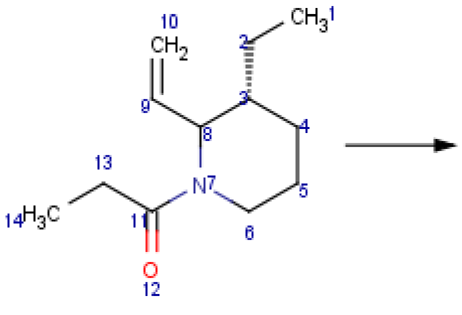

Correct mapped SMILES/SMARTS of the reaction:
[CH3:1][CH2:2][C@@H:3]1[CH2:4][CH2:5][CH2:6][N:7]([CH:8]1[CH:9]=[CH2:10])[C:11](=[O:12])[CH2:13][CH3:14]>>[CH3:1][CH2:2][C@@H:3]1[CH2:4][CH2:5][CH2:6][NH:7][C:11](=[O:12])[C@H:13]([CH3:14])[CH2:10]\[CH:9]=[CH:8]\1

Correctness of the mapping

MAPPET	YES
ReactionMap	YES
Marvin	YES

Reaction no 30

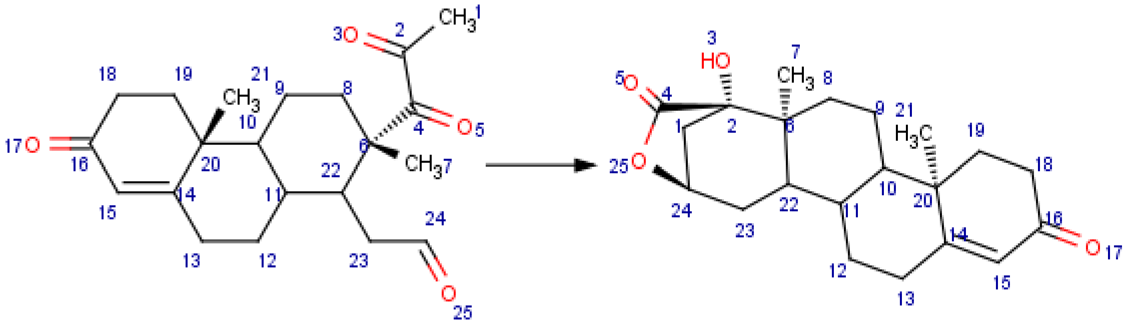

Correct mapped SMILES/SMARTS of the reaction:
[CH3:1][C:2](=[O:3])[C:4](=[O:5])[C@@:6]1([CH3:7])[CH2:8][CH2:9][CH:10]2[CH:11]([CH2:12][CH2:13][C:14]3=[CH:15][C:16](=[O:17])[CH2:18][CH2:19][C@:20]23[CH3:21])[CH:22]1[CH2:23][CH:24]=[O:25]>>[CH3:21][C@:20]12[CH2:19][CH2:18][C:16](=[O:17])[CH:15]=[C:14]1[CH2:13][CH2:12][CH:11]1[CH:10]2[CH2:9][CH2:8][C@@:6]2([CH3:7])[CH:22]1[CH2:23][C@@H:24]1[CH2:1][C@@:2]2([OH:3])[C:4](=[O:5])[O:25]1

Correctness of the mapping

MAPPET	YES
ReactionMap	NO
Marvin	NO

Reaction no 31

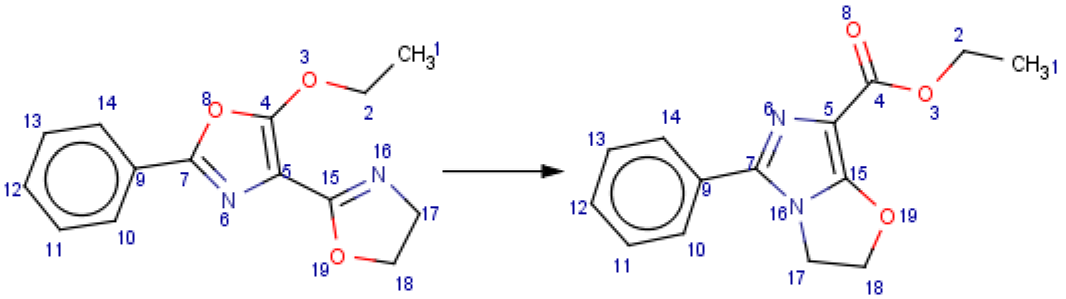

Correct mapped SMILES/SMARTS of the reaction:
[CH3:1][CH2:2][O:3][C:4]1=[C:5]([N:6]=[C:7]([O:8]1)[c:9]1[cH:10][cH:11][cH:12][c
H:13][cH:14]1)[C:15]1=[N:16][CH2:17][CH2:18][O:19]1>>[CH3:1][CH2:2][O:3][C:4](=[
O:8])[C:5]1=[C:15]2[O:19][CH2:18][CH2:17][N:16]2[C:7](=[N:6]1)[c:9]1[cH:10][cH:1
1][cH:12][cH:13][cH:14]1

Correctness of the mapping

MAPPET	YES
ReactionMap	YES
Marvin	NO

Reaction no 32

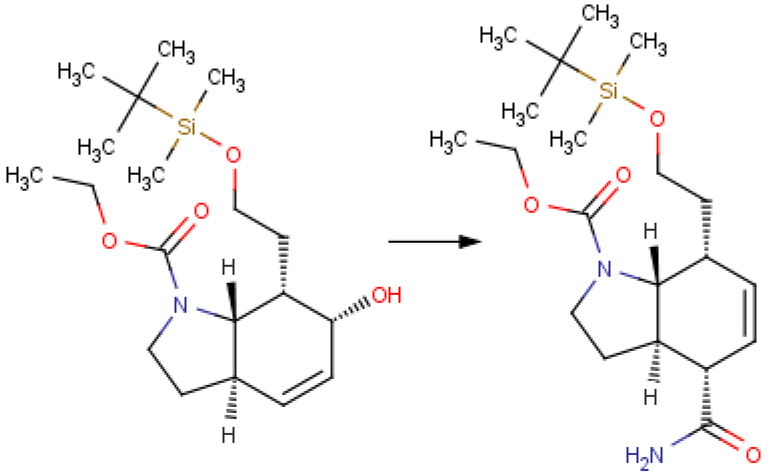

SMILES of the input:
[H][C@]12CCN(C(=O)OCC)[C@]1([H])[C@H](CCO[Si](C)(C)C(C)(C)C)[C@H](O)C=C2>>[H][C@
]12CCN(C(=O)OCC)[C@]1([H])[C@H](CCO[Si](C)(C)C(C)(C)C)C=C[C@@H]2C(N)=O

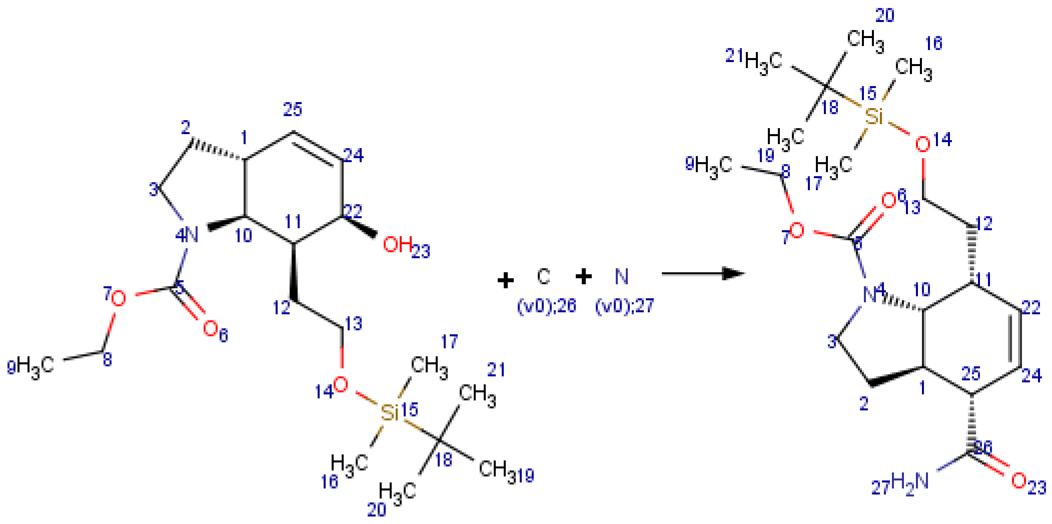

Correct mapped SMILES/SMARTS of the reaction:
[CH3:9][CH2:8][O:7][C:5](=[O:6])[N:4]1[CH2:3][CH2:2][C@@H:1]2[CH:25]=[CH:24][C@@
H:22]([OH:23])[C@@H:11]([CH2:12][CH2:13][O:14][Si:15]([CH3:16])([CH3:17])[C:18](
[CH3:19])([CH3:20])[CH3:21])[C@@H:10]12.[C:26].[N:27]>>[CH3:9][CH2:8][O:7][C:5](
=[O:6])[N:4]1[CH2:3][CH2:2][C@H:1]2[C@H:10]1[C@H:11]([CH2:12][CH2:13][O:14][Si:1
5]([CH3:16])([CH3:17])[C:18]([CH3:21])([CH3:20])[CH3:19])[CH:22]=[CH:24][C@@H:25
]2[C:26]([NH2:27])=[O:23]

Correctness of the mapping
MAPPET YES
ReactionMap NO
Marvin YES

Reaction no 33

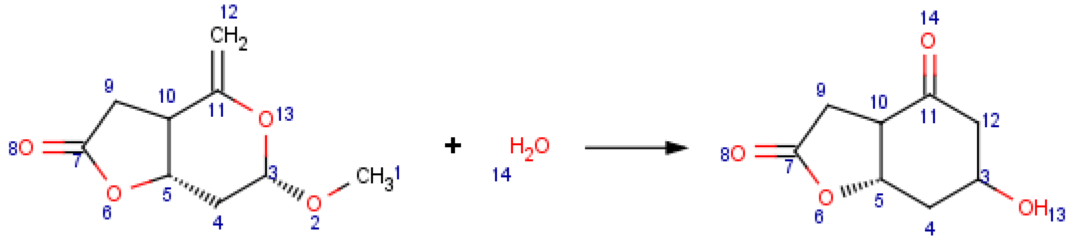

Correct mapped SMILES/SMARTS of the reaction:
[CH3:1][O:2][C@@H:3]1[CH2:4][C@@H:5]2[O:6][C:7](=[O:8])[CH2:9][CH:10]2[C:11](=[C
H2:12])[O:13]1.[OH2:14]>>[OH:13][CH:3]1[CH2:4][C@@H:5]2[O:6][C:7](=[O:8])[CH2:9]
[CH:10]2[C:11](=[O:14])[CH2:12]1

Correctness of the mapping
MAPPET NO
ReactionMap NO
Marvin NO

Reaction no 34

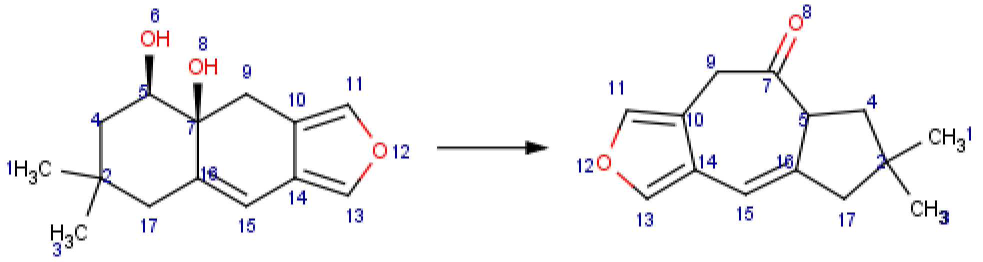

Correct mapped SMILES/SMARTS of the reaction:
[CH3:1][C:2]1([CH3:3])[CH2:4][C@@H:5]([OH:6])[C@:7]2([OH:8])[CH2:9][C:10]3=[CH:1
1][O:12][CH:13]=[C:14]3[CH:15]=[C:16]2[CH2:17]1>>[CH3:1][C:2]1([CH3:3])[CH2:4][C
H:5]2[C:16]([CH2:17]1)=[CH:15][C:14]1=[CH:13][O:12][CH:11]=[C:10]1[CH2:9][C:7]2=
[O:8]

Correctness of the mapping
MAPPET NO
ReactionMap NO
Marvin YES

Reaction no 35

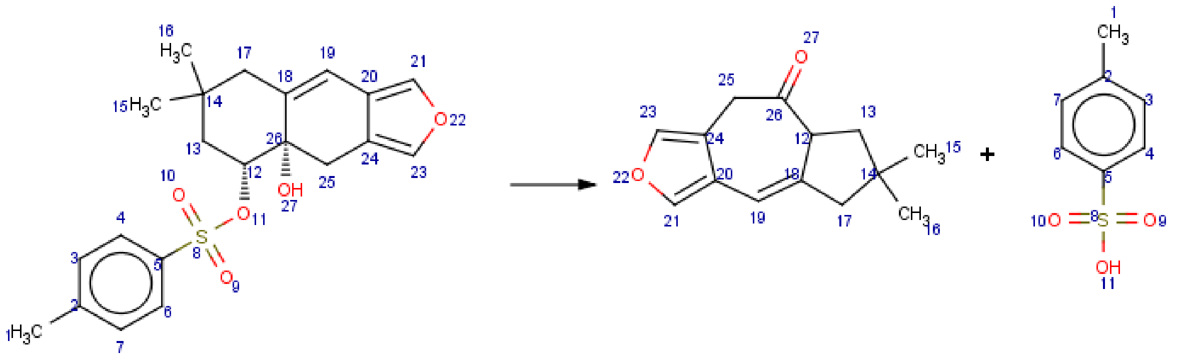

Correct mapped SMILES/SMARTS of the reaction:
[CH3:1][c:2]1[cH:3][cH:4][c:5]([cH:6][cH:7]1)[S:8](=[O:9])(=[O:10])[O:11][C@@H:1
2]1[CH2:13][C:14]([CH3:15])([CH3:16])[CH2:17][C:18]2=[CH:19][C:20]3=[CH:21][O:22
][CH:23]=[C:24]3[CH2:25][C@@:26]12[OH:27]>>[CH3:15][C:14]1([CH3:16])[CH2:13][CH:
12]2[C:18]([CH2:17]1)=[CH:19][C:20]1=[CH:21][O:22][CH:23]=[C:24]1[CH2:25][C:26]2
=[O:27].[CH3:1][c:2]1[cH:3][cH:4][c:5]([cH:6][cH:7]1)[S:8]([OH:11])(=[O:10])=[O:
9]

Correctness of the mapping

MAPPET	YES
ReactionMap	YES
Marvin	YES

Reaction no 36

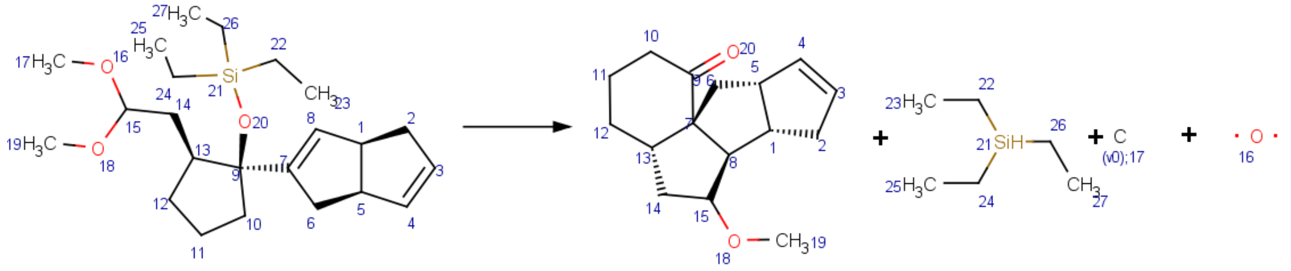

Correct mapped SMILES/SMARTS of the reaction:
[CH3:23][CH2:22][Si:21]([CH2:24][CH3:25])([CH2:26][CH3:27])[O:20][C@:9]1([CH2:10
][CH2:11][CH2:12][C@H:13]1[CH2:14][CH:15]([O:16][CH3:17])[O:18][CH3:19])[C:7]1=[
CH:8][C@H:1]2[CH2:2][CH:3]=[CH:4][C@H:5]2[CH2:6]1>>[CH3:27][CH2:26][SiH:21]([CH2
:22][CH3:23])[CH2:24][CH3:25].[CH3:19][O:18][CH:15]1[CH2:14][C@@H:13]2[CH2:12][C
H2:11][CH2:10][C:9](=[O:20])[C@@:7]22[CH2:6][C@@H:5]3[CH:4]=[CH:3][CH2:2][C@@H:1
]3[C@@H:8]12.[C:17].[O:16]

Correctness of the mapping

MAPPET	YES
ReactionMap	NO
Marvin	NO

Reaction no 37

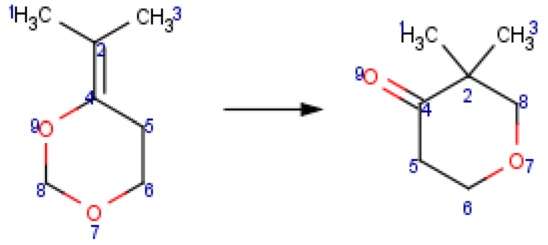

Correct mapped SMILES/SMARTS of the reaction:
[CH3:1][C:2]([CH3:3])=[C:4]1[CH2:5][CH2:6][O:7][CH2:8][O:9]1>>[CH3:3][C:2]1([CH3
:1])[CH2:8][O:7][CH2:6][CH2:5][C:4]1=[O:9]

Correctness of the mapping

MAPPET	YES
ReactionMap	YES
Marvin	YES

Reaction no 38

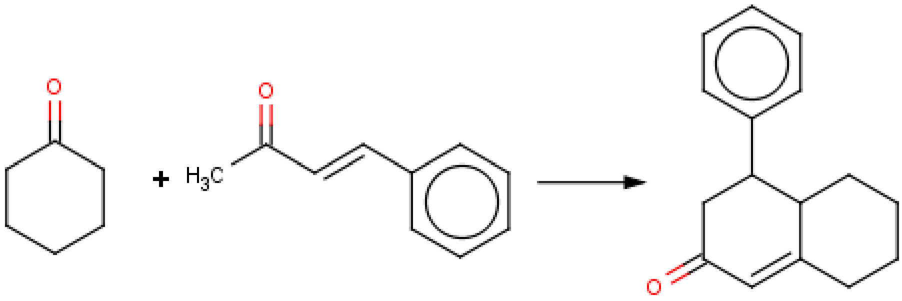

SMILES of the input:
O=C1CCCCC1.CC(=O)\C=C\c1ccccc1>>O=C1CC(C2CCCCC2=C1)c1ccccc1

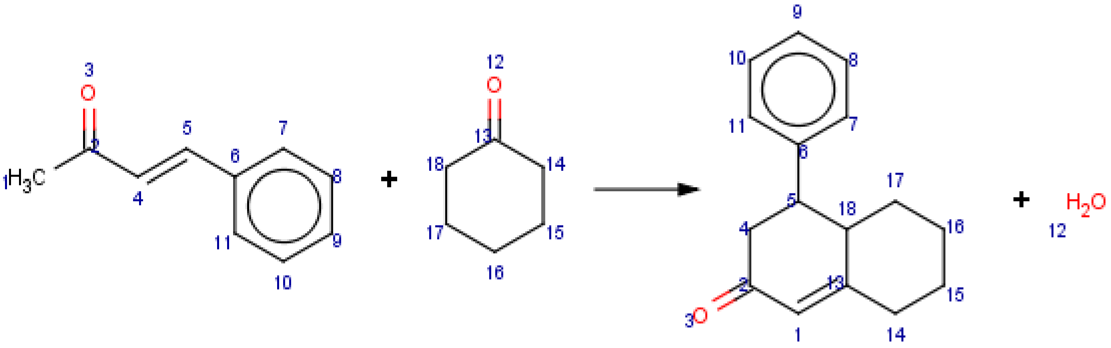

Correct mapped SMILES/SMARTS of the reaction:
[CH3:1][C:2](=[O:3])\[CH:4]=[CH:5]\[c:6]1[cH:7][cH:8][cH:9][cH:10][cH:11]1.[O:12
]=[C:13]1[CH2:14][CH2:15][CH2:16][CH2:17][CH2:18]1>>[OH2:12].[O:3]=[C:2]1[CH2:4]
[CH:5]([CH:18]2[CH2:17][CH2:16][CH2:15][CH2:14][C:13]2=[CH:1]1)[c:6]1[cH:11][cH:
10][cH:9][cH:8][cH:7]1

Correctness of the mapping

MAPPET	YES
ReactionMap	NO
Marvin	NO

Reaction no 39

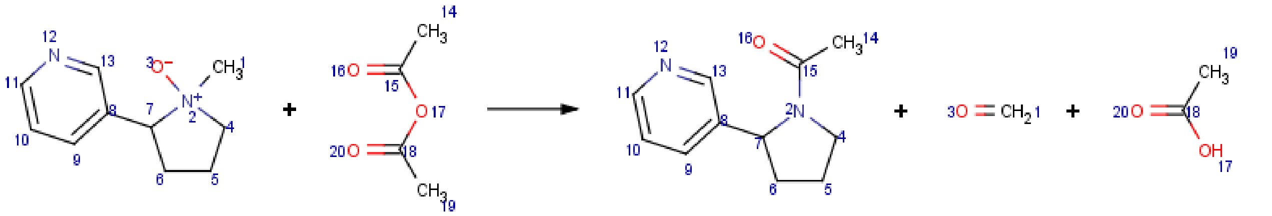

Correct mapped SMILES/SMARTS of the reaction:
[CH3:1][N+:2]1([O-:3])[CH2:4][CH2:5][CH2:6][CH:7]1[C:8]1=[CH:9][CH:10]=[CH:11][N:12]=[CH:13]1.[CH3:14][C:15](=[O:16])[O:17][C:18]([CH3:19])=[O:20]>>[CH3:14][C:15](=[O:16])[N:2]1[CH2:4][CH2:5][CH2:6][CH:7]1[C:8]1=[CH:9][CH:10]=[CH:11][N:12]=[CH:13]1.[CH2:1]=[O:3].[CH3:19][C:18]([OH:17])=[O:20]

Correctness of the mapping
MAPPET YES
ReactionMap NO
Marvin NO

Reaction no 40

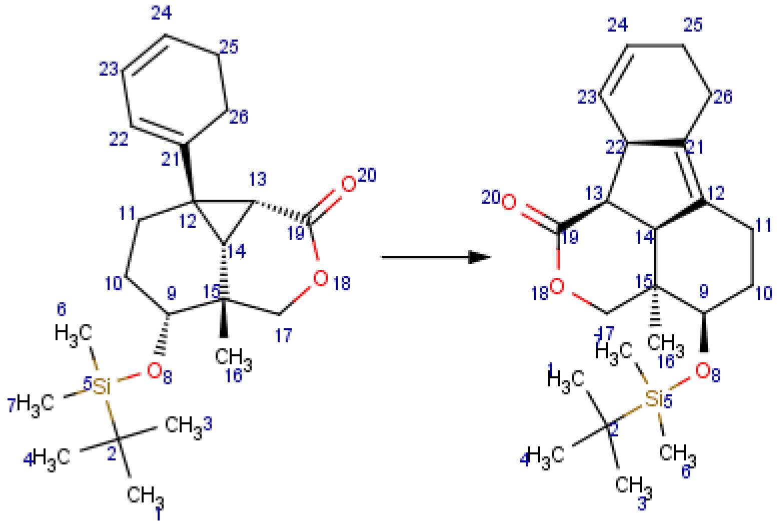

Correct mapped SMILES/SMARTS of the reaction:
[CH3:1][C:2]([CH3:3])([CH3:4])[Si:5]([CH3:6])([CH3:7])[O:8][C@@H:9]1[CH2:10][CH2:11][C@@:12]2([C@H:13]3[C@@H:14]2[C@:15]1([CH3:16])[CH2:17][O:18][C:19]3=[O:20])[C:21]1=[CH:22][CH:23]=[CH:24][CH2:25][CH2:26]1>>[CH3:4][C:2]([CH3:3])([CH3:1])[Si:5]([CH3:6])([CH3:7])[O:8][C@@H:9]1[CH2:10][CH2:11][C:12]2=[C:21]3[CH2:26][CH2:25][CH:24]=[CH:23][C@@H:22]3[C@H:13]3[C@@H:14]2[C@:15]1([CH3:16])[CH2:17][O:18][C:19]3=[O:20]

Correctness of the mapping
MAPPET YES
ReactionMap NO
Marvin YES

Reaction no 41

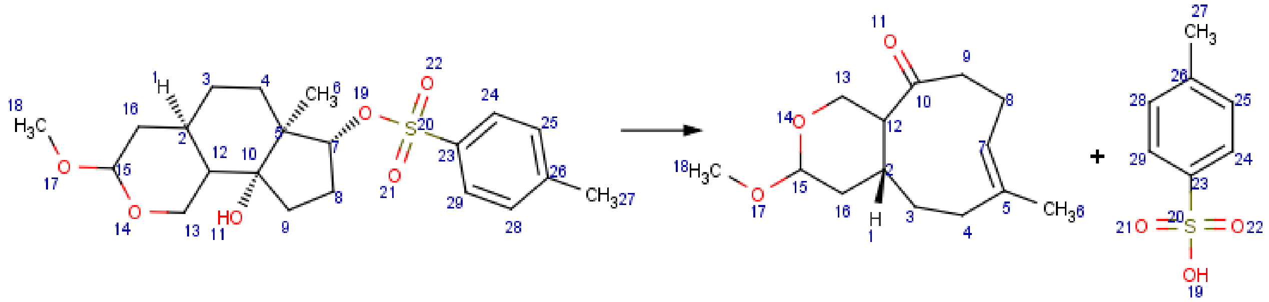

Correct mapped SMILES/SMARTS of the reaction:
[CH3:1][C:2]1=[CH:3][CH:4]=[C:5]([CH:6]=[CH:7]1)[S:8](=[O:9])(=[O:9])[O:11][CH:12]2[CH2:13][CH2:14][C:15]3([C:16]2([CH2:17][CH2:18][CH:19]4[CH:20]3[CH2:21][O:22][CH:23]([CH2:24]4)[O:25][CH3:26])[CH3:27])[OH:28]>>[CH3:27][C:16]1=[CH:12][CH2:13][CH2:14][C:15](=[O:28])[CH:20]2[CH2:21][O:22][CH:23]([CH2:24][CH:19]2[CH2:18][CH2:17]1)[O:25][CH3:26].[CH3:1][C:2]1=[CH:3][CH:4]=[C:5]([CH:6]=[CH:7]1)[S:8](=[O:9])(=[O:9])[OH:11]

Correctness of the mapping

MAPPET	NO
ReactionMap	YES
Marvin	YES

Reaction no 42

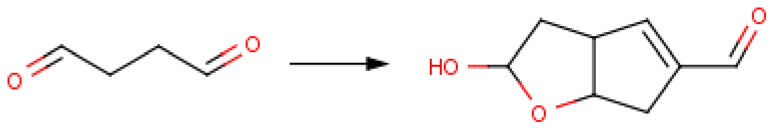

SMILES of the input:
O=CCCC=O>>OC1CC2C=C(CC2O1)C=O

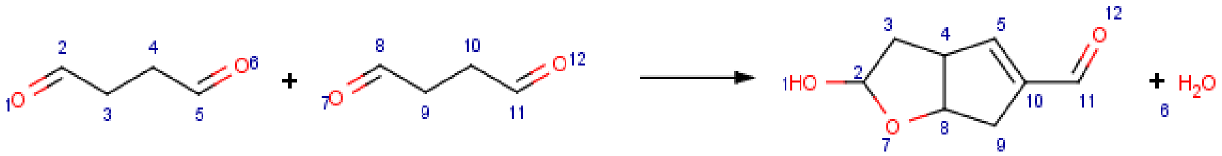

Correct mapped SMILES/SMARTS of the reaction:
[O:1]=[CH:2][CH2:3][CH2:4][CH:5]=[O:6].[O:7]=[CH:8][CH2:9][CH2:10][CH:11]=[O:12]>>[OH2:6].[OH:1][CH:2]1[CH2:3][CH:4]2[CH:5]=[C:10]([CH2:9][CH:8]2[O:7]1)[CH:11]=[O:12]

Correctness of the mapping

MAPPET	YES
ReactionMap	NO
Marvin	YES

Reaction no 43

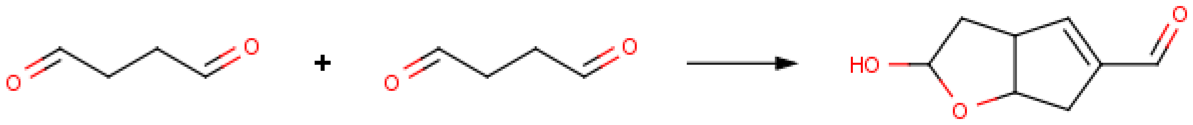

SMILES of the input:
O=CCCC=O.O=CCCC=O>>OC1CC2C=C(CC2O1)C=O

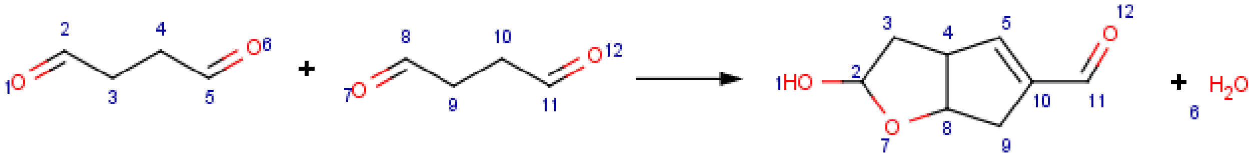

Correct mapped SMILES/SMARTS of the reaction:
[O:1]=[CH:2][CH2:3][CH2:4][CH:5]=[O:6].[O:7]=[CH:8][CH2:9][CH2:10][CH:11]=[O:12]>>[OH2:6].[OH:1][CH:2]1[CH2:3][CH:4]2[CH:5]=[C:10]([CH2:9][CH:8]2[O:7]1)[CH:11]=[O:12]

Correctness of the mapping

MAPPET	YES
ReactionMap	NO
Marvin	YES

Reaction no 44

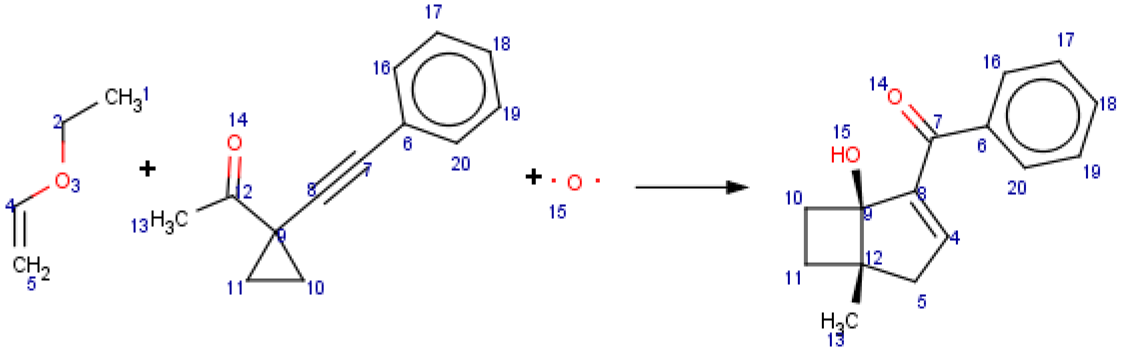

Correct mapped SMILES/SMARTS of the reaction:
[CH3:1][CH2:2][O:3][CH:4]=[CH2:5].[CH3:13][C:12](=[O:14])[C:9]1([CH2:10][CH2:11]1)[C:8]#[C:7][cH:6]1[cH:16][cH:17][cH:18][cH:19][cH:20]1.[O:15]>>[CH3:13][C@@:12]12[CH2:11][CH2:10][C@:9]1([OH:15])[C:8](=[CH:4][CH2:5]2)[C:7](=[O:14])[c:6]1[cH:16][cH:17][cH:18][cH:19][cH:20]1

Correctness of the mapping

MAPPET	NO
ReactionMap	NO
Marvin	NO

Reaction no 45

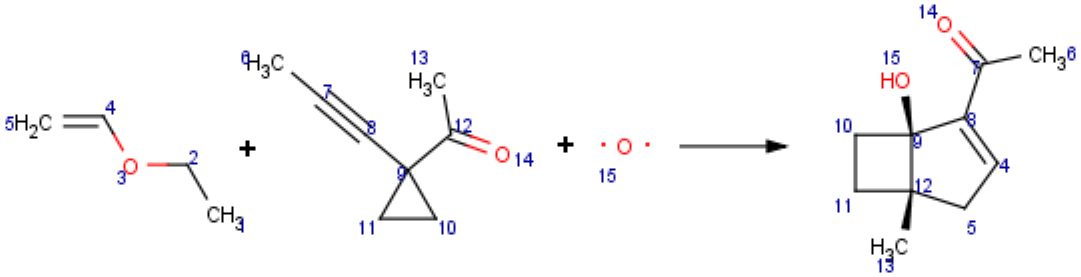

Correct mapped SMILES/SMARTS of the reaction:
[CH3:1][CH2:2][O:3][CH:4]=[CH2:5].[CH3:6][C:7]#[C:8][C:9]1([CH2:10][CH2:11]1)[C:12]([CH3:13])=[O:14].[O:15]>>[CH3:6][C:7](=[O:14])[C:8]1=[CH:4][CH2:5][C@:12]2([CH3:13])[CH2:11][CH2:10][C@:9]12[OH:15]

Correctness of the mapping
MAPPET NO
ReactionMap NO
Marvin NO

Reaction no 46

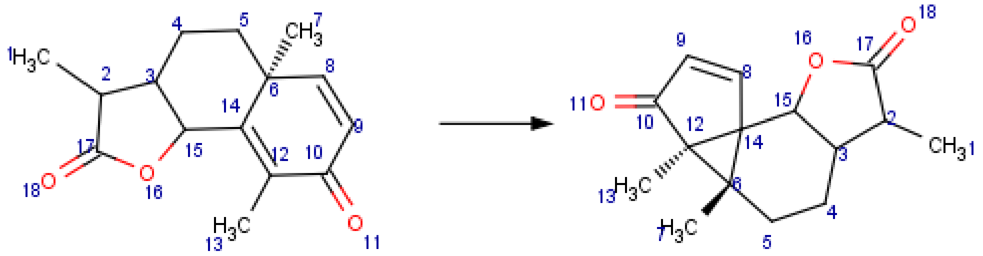

Correct mapped SMILES/SMARTS of the reaction:
[CH3:1][CH:2]1[CH:3]2[CH2:4][CH2:5][C@@:6]3([CH3:7])[CH:8]=[CH:9][C:10](=[O:11])[C:12]([CH3:13])=[C:14]3[CH:15]2[O:16][C:17]1=[O:18]>>[CH3:1][CH:2]1[CH:3]2[CH2:4][CH2:5][C@:6]3([CH3:7])[C@@:12]4([CH3:13])[C:10](=[O:11])[CH:9]=[CH:8][C:14]34[CH:15]2[O:16][C:17]1=[O:18]

Correctness of the mapping
MAPPET YES
ReactionMap YES
Marvin YES

Reaction no 47

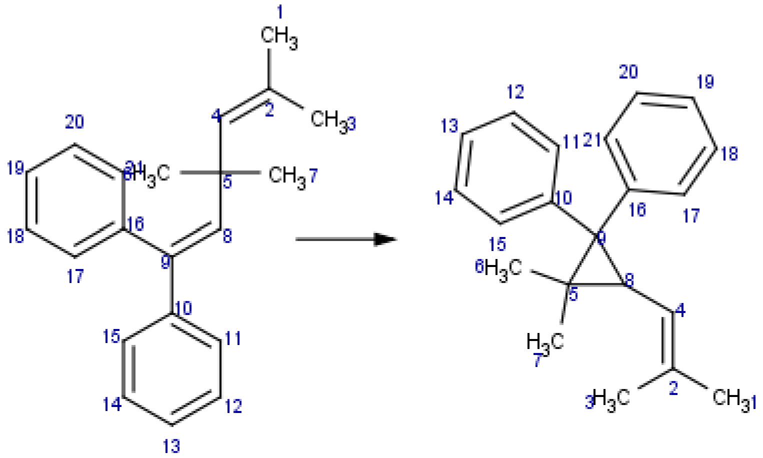

Correct mapped SMILES/SMARTS of the reaction:
[CH3:1][C:2]([CH3:3])=[CH:4][C:5]([CH3:6])([CH3:7])[CH:8]=[C:9]([C:10]1=[CH:11][CH:12]=[CH:13][CH:14]=[CH:15]1)[C:16]1=[CH:17][CH:18]=[CH:19][CH:20]=[CH:21]1>>[CH3:1][C:2]([CH3:3])=[CH:4][CH:8]1[C:5]([CH3:7])([CH3:6])[C:9]1([C:10]1=[CH:15][CH:14]=[CH:13][CH:12]=[CH:11]1)[C:16]1=[CH:21][CH:20]=[CH:19][CH:18]=[CH:17]1

Correctness of the mapping
MAPPET YES
ReactionMap NO
Marvin YES

Reaction no 48

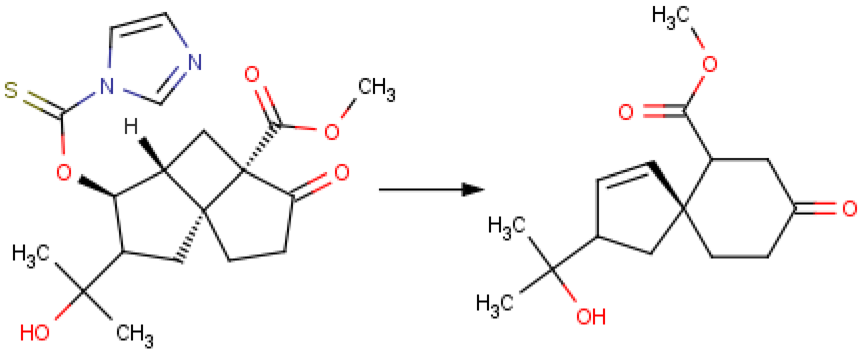

SMILES of the input:
[H][C@@]12C[C@@]3(C(=O)OC)C(=O)CC[C@@]13CC([C@H]2OC(=S)N1C=CN=C1)C(C)(C)O>>COC(=O)C1CC(=O)CC[C@]11CC(C=C1)C(C)(C)O

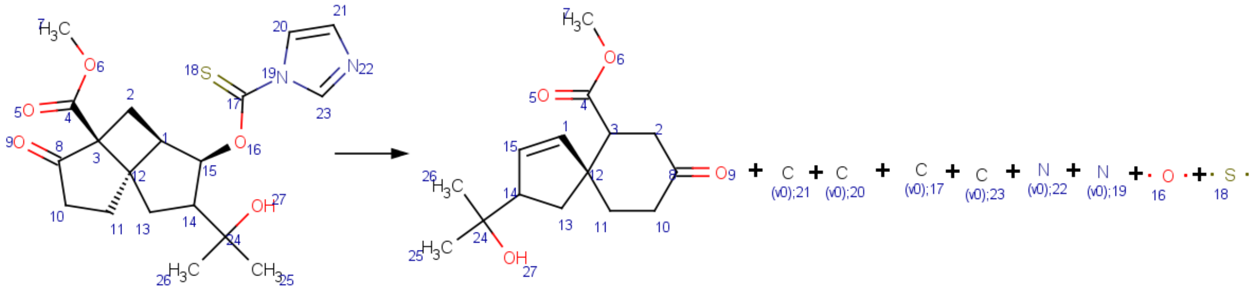

Correct mapped SMILES/SMARTS of the reaction:
[CH3:7][O:6][C:4](=[O:5])[C@@:3]12[CH2:2][C@H:1]3[C@@H:15]([O:16][C:17](=[S:18])[N:19]4[CH:20]=[CH:21][N:22]=[CH:23]4)[CH:14]([CH2:13][C@@:12]13[CH2:11][CH2:10][C:8]2=[O:9])[C:24]([CH3:25])([CH3:26])[OH:27]>>[CH3:7][O:6][C:4](=[O:5])[CH:3]1[CH2:2][C:8](=[O:9])[CH2:10][CH2:11][C@:12]11[CH2:13][CH:14]([CH:15]=[CH:1]1)[C:24]([CH3:25])([CH3:26])[OH:27].[C:21].[C:20].[C:17].[C:23].[N:22].[N:19].[O:16].[S:18]

Correctness of the mapping

MAPPET	YES
ReactionMap	NO
Marvin	YES

Reaction no 49

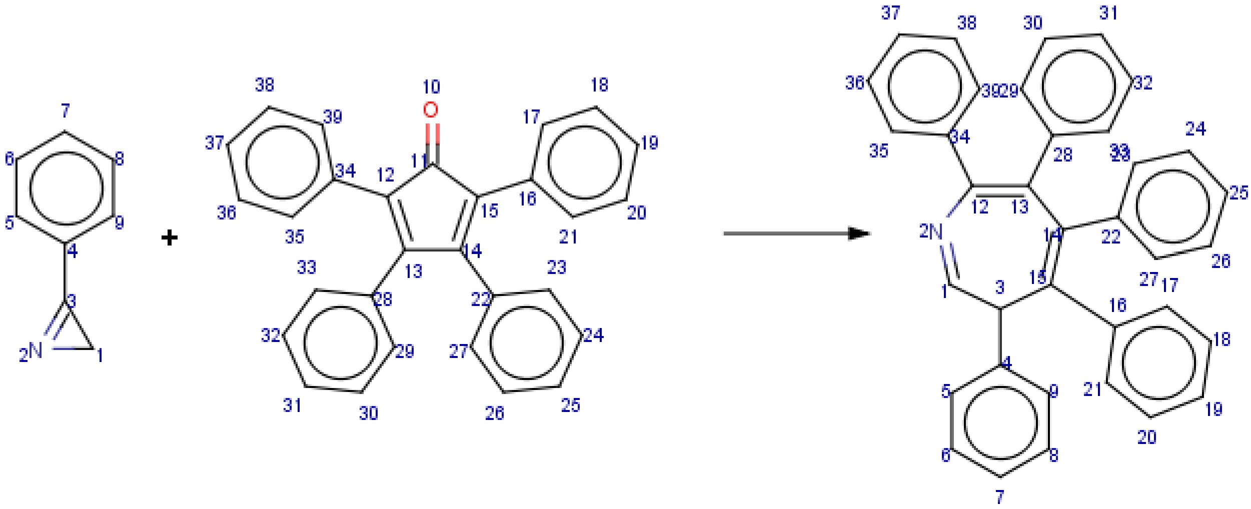

Correct mapped SMILES/SMARTS of the reaction:
[O:1]=[C:2]1[C:6](=[C:5]([C:4](=[C:3]1[c:25]1[cH:26][cH:27][cH:28][cH:29][cH:30]1)[c:19]1[cH:20][cH:21][cH:22][cH:23][cH:24]1)[c:13]1[cH:14][cH:15][cH:16][cH:17][cH:18]1)[c:7]1[cH:8][cH:9][cH:10][cH:11][cH:12]1.[CH:39]1=[N:38][CH:37]1[c:34]1[cH:33][cH:32][cH:31][cH:36][cH:35]1>>[cH:10]1[cH:11][cH:12][c:7]([cH:8][cH:9]1)[CH:6]1[CH:39]=[N:38][C:37]([c:34]2[cH:33][cH:32][cH:31][cH:36][cH:35]2)=[C:3]([c:25]2[cH:30][cH:29][cH:28][cH:27][cH:26]2)[C:4]([c:19]2[cH:24][cH:23][cH:22][cH:21][cH:20]2)=[C:5]1[c:13]1[cH:18][cH:17][cH:16][cH:15][cH:14]1.[C:2].[O:1]

Correctness of the mapping

MAPPET	NO
ReactionMap	NO
Marvin	YES

Reaction no 50

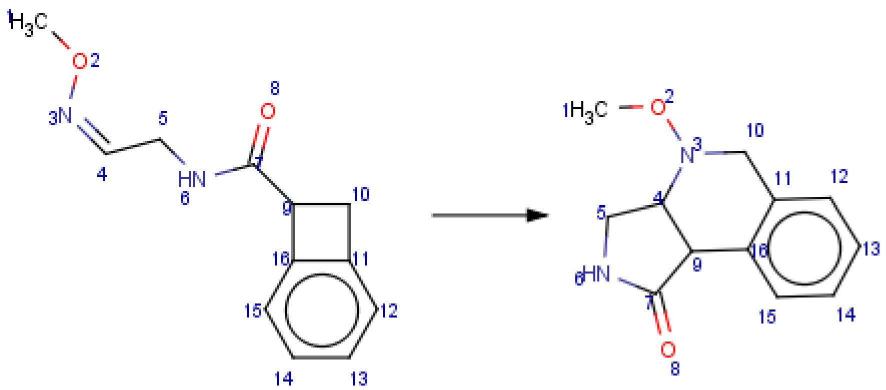

Correct mapped SMILES/SMARTS of the reaction:
[CH3:1][O:2]\[N:3]=[CH:4]/[CH2:5][NH:6][C:7](=[O:8])[CH:9]1[CH2:10][c:11]2[cH:12][cH:13][cH:14][cH:15][c:16]12>>[CH3:1][O:2][N:3]1[CH2:10][c:11]2[cH:12][cH:13][cH:14][cH:15][c:16]2[CH:9]2[CH:4]1[CH2:5][NH:6][C:7]2=[O:8]

Correctness of the mapping

MAPPET	YES
ReactionMap	YES
Marvin	YES

Reaction no 51

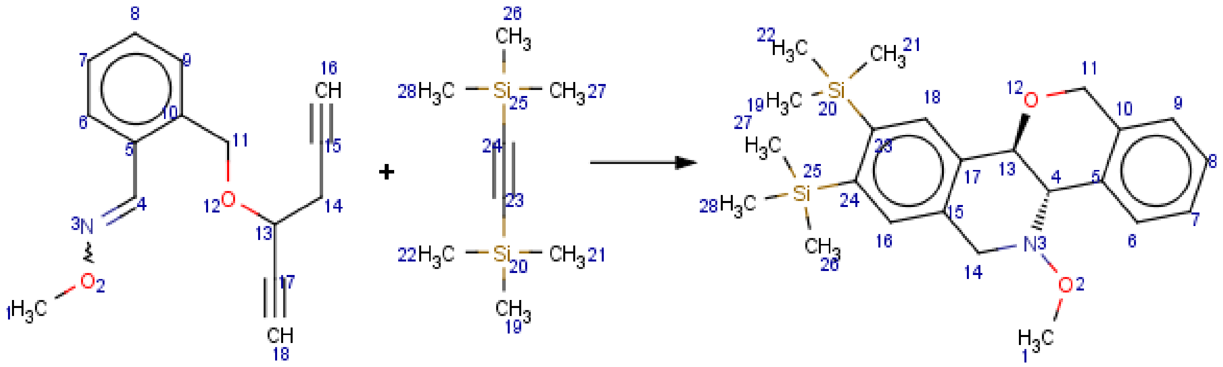

Correct mapped SMILES/SMARTS of the reaction:
[CH3:1][O:2][N:3]=[CH:4][c:5]1[cH:6][cH:7][cH:8][cH:9][c:10]1[CH2:11][O:12][CH:13]([CH2:14][C:15]#[CH:16])[C:17]#[CH:18].[CH3:19][Si:20]([CH3:21])([CH3:22])[C:23]#[C:24][Si:25]([CH3:26])([CH3:27])[CH3:28]>>[CH3:1][O:2][N:3]1[CH2:14][c:15]2[cH:16][c:24]([c:23]([cH:18][c:17]2[C@H:13]2[O:12][CH2:11][c:10]3[cH:9][cH:8][cH:

7][cH:6][c:5]3[C@H:4]12)[Si:20]([CH3:22])([CH3:21])[CH3:19])[Si:25]([CH3:28])([CH3:27])[CH3:26]

Correctness of the mapping

MAPPET	YES
ReactionMap	NO
Marvin	YES

Reaction no 52

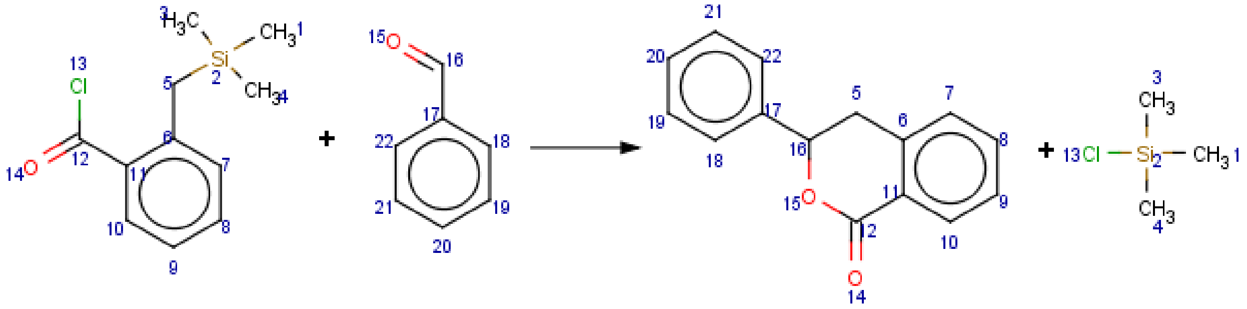

Correct mapped SMILES/SMARTS of the reaction:
[CH3:1][Si:2]([CH3:3])([CH3:4])[CH2:5][c:6]1[cH:7][cH:8][cH:9][cH:10][c:11]1[C:12]([Cl:13])=[O:14].[O:15]=[CH:16][c:17]1[cH:18][cH:19][cH:20][cH:21][cH:22]1>>[O:14]=[C:12]1[O:15][CH:16]([CH2:5][c:6]2[cH:7][cH:8][cH:9][cH:10][c:11]12)[c:17]1[cH:18][cH:19][cH:20][cH:21][cH:22]1.[CH3:4][Si:2]([CH3:3])([CH3:1])[Cl:13]

Correctness of the mapping

MAPPET	YES
ReactionMap	YES
Marvin	YES

Reaction no 53

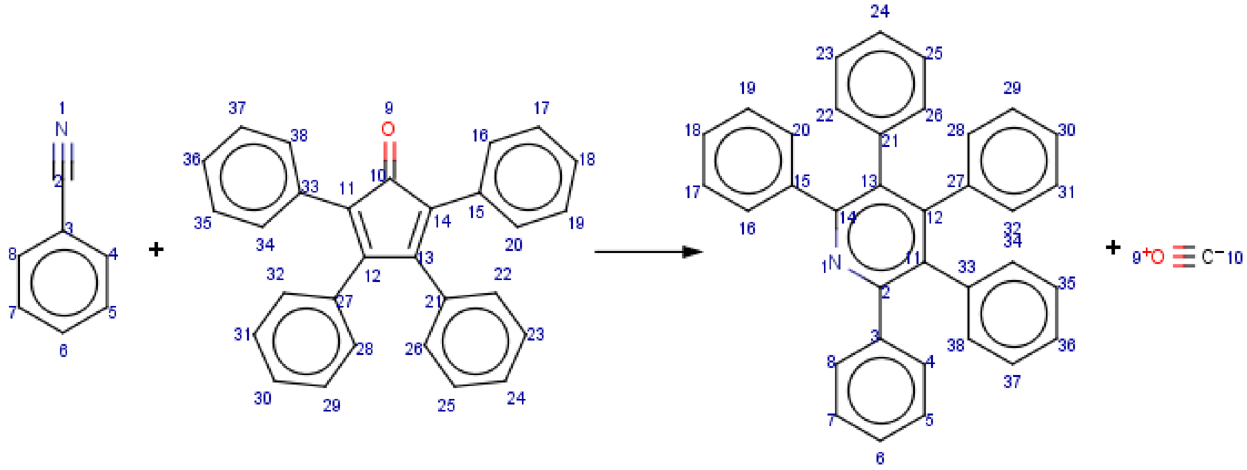

Correct mapped SMILES/SMARTS of the reaction:
[N:1]#[C:2][c:3]1[cH:4][cH:5][cH:6][cH:7][cH:8]1.[O:9]=[C:10]1[C:14](=[C:13]([C:12](=[C:11]1[c:33]1[cH:34][cH:35][cH:36][cH:37][cH:38]1)[c:27]1[cH:28][cH:29][cH:30][cH:31][cH:32]1)[c:21]1[cH:22][cH:23][cH:24][cH:25][cH:26]1)[c:15]1[cH:16][cH:17][cH:18][cH:19][cH:20]1>>[C-:10]#[O+:9].[cH:6]1[cH:7][cH:8][c:3]([cH:4][cH:5]1)-[c:2]1[n:1][c:14](-[c:15]2[cH:16][cH:17][cH:18][cH:19][cH:20]2)[c:13](-[c:21]2[cH:22][cH:23][cH:24][cH:25][cH:26]2)[c:12](-[c:27]2[cH:28][cH:29][cH:30][cH:31][cH:32]2)[c:11]1-[c:33]1[cH:34][cH:35][cH:36][cH:37][cH:38]1

Correctness of the mapping
MAPPET YES
ReactionMap NO
Marvin NO

Reaction no 54

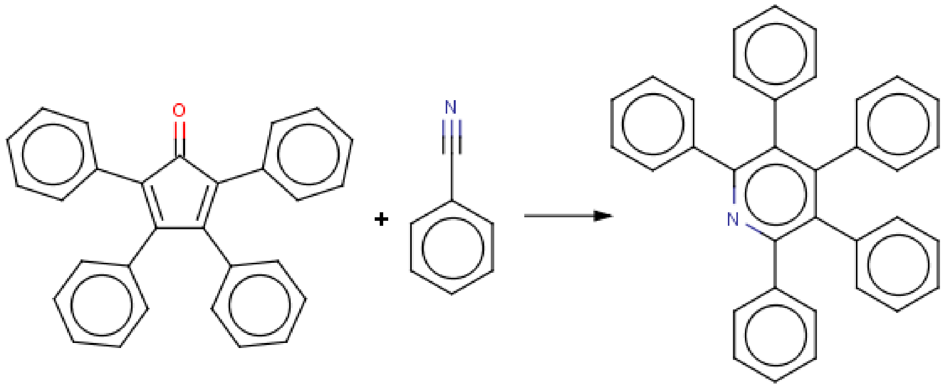

SMILES of the input:
O=C1C(=C(C(=C1c1ccccc1)c1ccccc1)c1ccccc1)c1ccccc1.N#Cc1ccccc1>>c1ccc(cc1)-c1nc(-c2ccccc2)c(-c2ccccc2)c(-c2ccccc2)c1-c1ccccc1

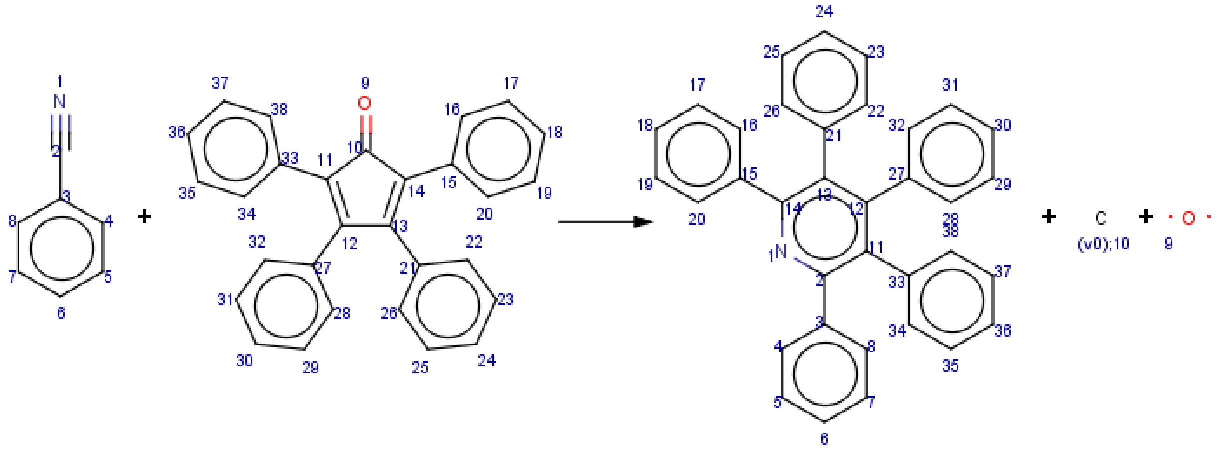

Correct mapped SMILES/SMARTS of the reaction:
[N:1]#[C:2][c:3]1[cH:4][cH:5][cH:6][cH:7][cH:8]1.[O:9]=[C:10]1[C:14](=[C:13]([C:12](=[C:11]1[c:33]1[cH:34][cH:35][cH:36][cH:37][cH:38]1)[c:27]1[cH:28][cH:29][cH:30][cH:31][cH:32]1)[c:21]1[cH:22][cH:23][cH:24][cH:25][cH:26]1)[c:15]1[cH:16][cH:17][cH:18][cH:19][cH:20]1>>[cH:6]1[cH:5][cH:4][c:3]([cH:8][cH:7]1)-[c:2]1[n:1][c:14](-[c:15]2[cH:20][cH:19][cH:18][cH:17][cH:16]2)[c:13](-[c:21]2[cH:26][cH:25][cH:24][cH:23][cH:22]2)[c:12](-[c:27]2[cH:32][cH:31][cH:30][cH:29][cH:28]2)[c:11]1-[c:33]1[cH:38][cH:37][cH:36][cH:35][cH:34]1.[C:10].[O:9]

Correctness of the mapping
MAPPET YES
ReactionMap NO
Marvin NO

Reaction no 55

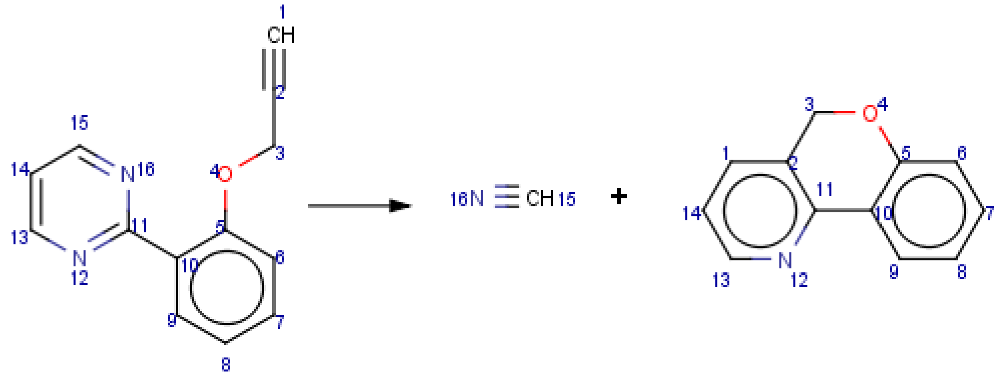

Correct mapped SMILES/SMARTS of the reaction:
[CH:1]#[C:2][CH2:3][O:4][c:5]1[cH:6][cH:7][cH:8][cH:9][c:10]1[C:11]1=[N:12][CH:1
3]=[CH:14][CH:15]=[N:16]1>>[CH:15]#[N:16].[CH2:3]1[O:4][c:5]2[cH:6][cH:7][cH:8][
cH:9][c:10]2-[c:11]2[n:12][cH:13][cH:14][cH:1][c:2]12

Correctness of the mapping

MAPPET	YES
ReactionMap	YES
Marvin	NO

Reaction no 56

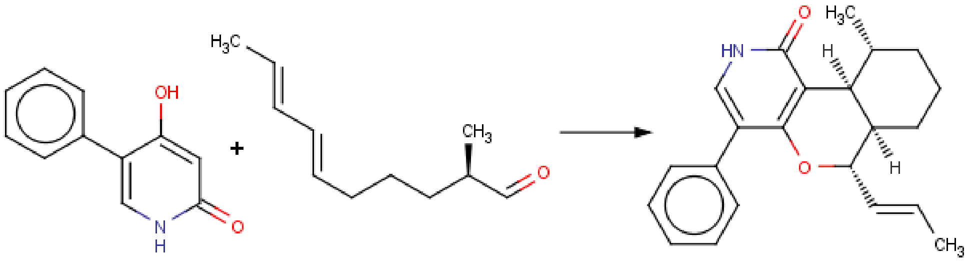

SMILES of the input:
OC1=CC(=O)NC=C1c1ccccc1.C\C=C\C=C\CCC[C@@H](C)C=O>>[H][C@@]12CCC[C@@H](C)[C@]1([
H])C1=C(O[C@H]2\C=C\C)C(=CNC1=O)c1ccccc1

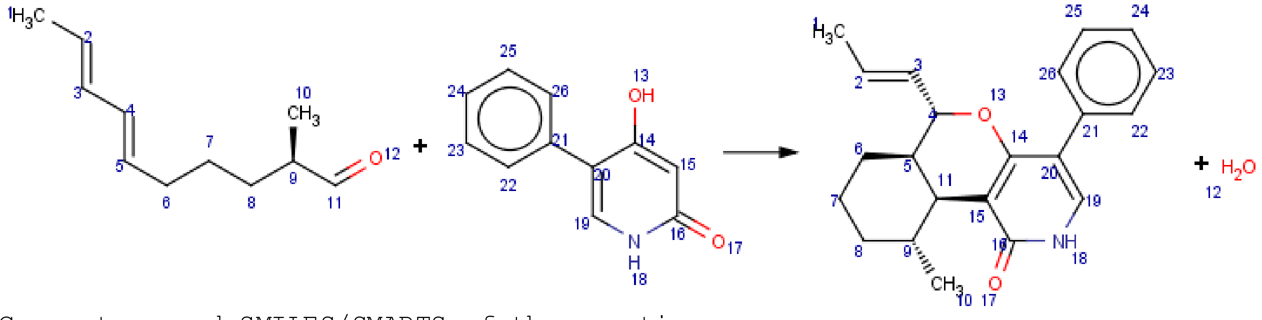

Correct mapped SMILES/SMARTS of the reaction:
[CH3:1]\[CH:2]=[CH:3]\[CH:4]=[CH:5]\[CH2:6][CH2:7][CH2:8][C@@H:9]([CH3:10])[CH:1
1]=[O:12].[OH:13][C:14]1=[CH:15][C:16](=[O:17])[NH:18][CH:19]=[C:20]1[c:21]1[cH:
22][cH:23][cH:24][cH:25][cH:26]1>>[OH2:12].[CH3:1]\[CH:2]=[CH:3]\[C@@H:4]1[O:13]
[C:14]2=[C:15]([C@H:11]3[C@H:9]([CH3:10])[CH2:8][CH2:7][CH2:6][C@@H:5]13)[C:16](
=[O:17])[NH:18][CH:19]=[C:20]2[c:21]1[cH:26][cH:25][cH:24][cH:23][cH:22]1

Correctness of the mapping

MAPPET	YES
ReactionMap	NO
Marvin	YES

Reaction no 57

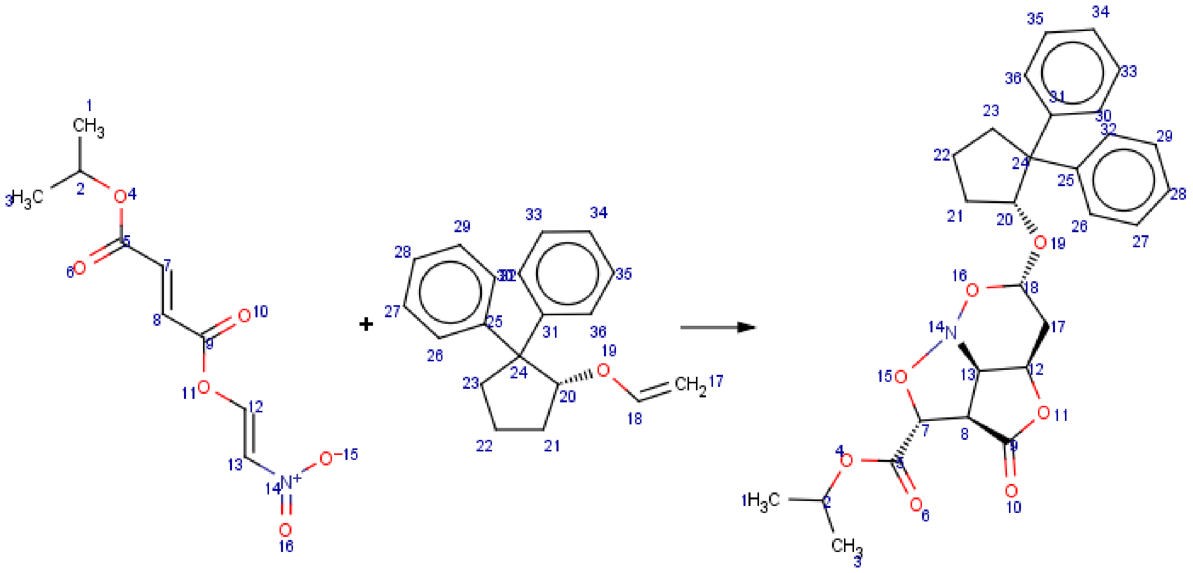

Correct mapped SMILES/SMARTS of the reaction:

```
[CH3:1][CH:2]([CH3:3])[O:4][C:5](=[O:6])\[CH:7]=[CH:8]\[C:9](=[O:10])[O:11]\[CH:
12]=[CH:13]\[N+:14]([O-
:15])=[O:16].[CH2:17]=[CH:18][O:19][C@@H:20]1[CH2:21][CH2:22][CH2:23][C:24]1([c:
25]1[cH:26][cH:27][cH:28][cH:29][cH:30]1)[c:31]1[cH:32][cH:33][cH:34][cH:35][cH:
36]1>>[CH3:3][CH:2]([CH3:1])[O:4][C:5](=[O:6])[C@@H:7]1[O:15][N:14]2[O:16][C@@H:
18]([CH2:17][C@H:12]3[O:11][C:9](=[O:10])[C@@H:8]1[C@@H:13]23)[O:19][C@@H:20]1[C
H2:21][CH2:22][CH2:23][C:24]1([c:31]1[cH:36][cH:35][cH:34][cH:33][cH:32]1)[c:25]
1[cH:30][cH:29][cH:28][cH:27][cH:26]1
```

Correctness of the mapping

MAPPET	YES
ReactionMap	YES
Marvin	NO

Reaction no 58

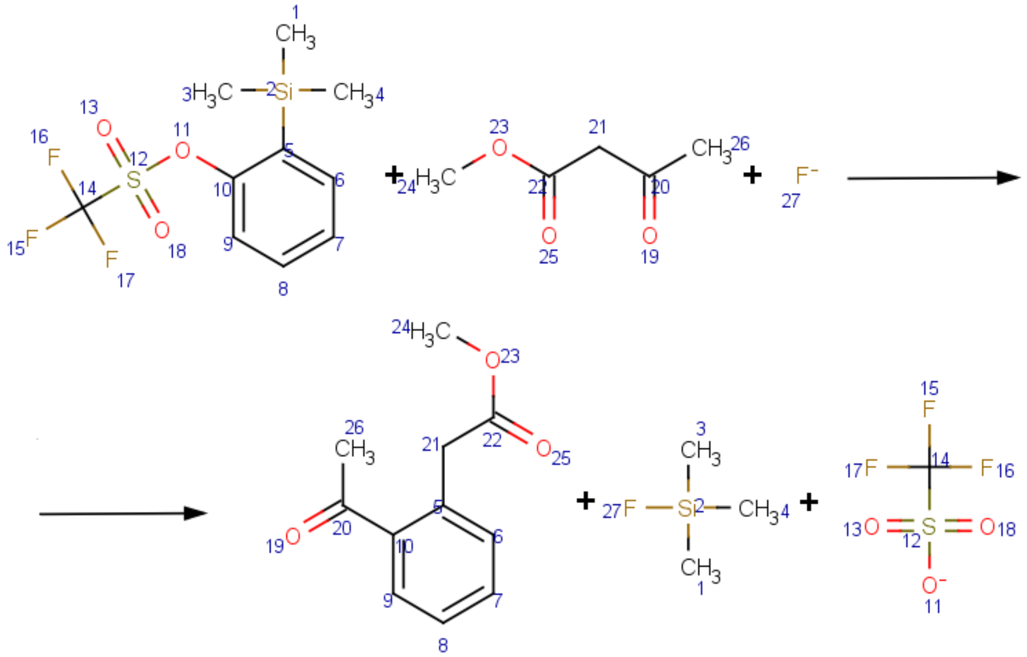

Correct mapped SMILES/SMARTS of the reaction:
[#6:1][Si:2]([#6:3])([#6:4])[#6:5]-1=[#6:6]-[#6:7]=[#6:8]-[#6:9]=[#6:10]-1-[#8:11][S:12](=[O:13])(=[O:18])[C:14]([F:15])([F:16])[F:17].[#6:24]-[#8:23]-[#6:22](=[O:25])-[#6:21]-[#6:20](-[#6:26])=[O:19].[F-:27]>>[#6:24]-[#8:23]-[#6:22](=[O:25])-[#6:21]-[#6:5]-1=[#6:6]-[#6:7]=[#6:8]-[#6:9]=[#6:10]-1-[#6:20](-[#6:26])=[O:19].[#6:1][Si:2]([#6:3])([#6:4])[F:27].[#8-:11][S:12](=[O:18])(=[O:13])[C:14]([F:15])([F:16])[F:17]

Correctness of the mapping
MAPPET YES
ReactionMap NO
Marvin YES

Reaction no 59

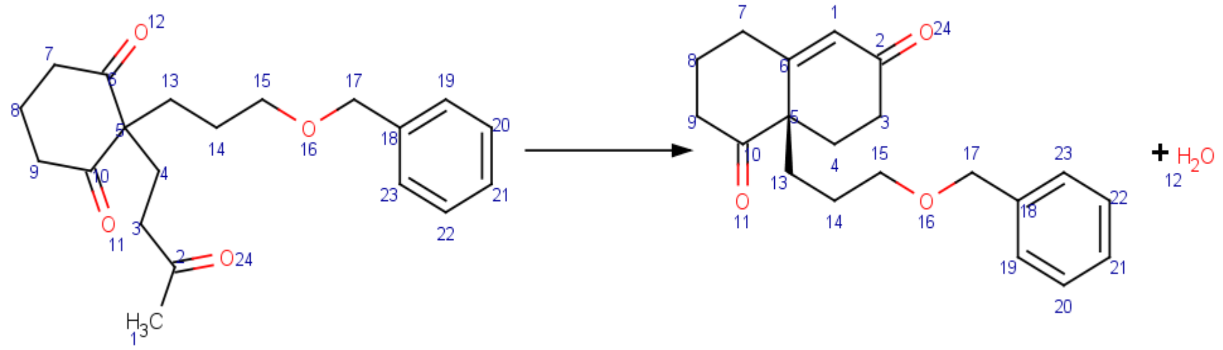

Correct mapped SMILES/SMARTS of the reaction:
[#6:1]-[#6:2](=[O:24])-[#6:3]-[#6:4][C:5]1([#6:13]-[#6:14]-[#6:15]-[#8:16]-[#6:17]-[#6:18]-2=[#6:19]-[#6:20]=[#6:21]-[#6:22]=[#6:23]-2)[#6:10](=[O:11])-[#6:9]-[#6:8]-[#6:7]-[#6:6]1=[O:12]>>[O:24]=[#6:2]-1-[#6:3]-[#6:4][C@:5]2([#6:13]-[#6:14]-[#6:15]-[#8:16]-[#6:17]-[#6:18]-3=[#6:23]-[#6:22]=[#6:21]-[#6:20]=[#6:19]-3)[#6:10](=[O:11])-[#6:9]-[#6:8]-[#6:7]-[#6:6]2=[#6:1]-1.[#8:12]

Correctness of the mapping
MAPPET YES
ReactionMap YES
Marvin YES

Reaction no 60

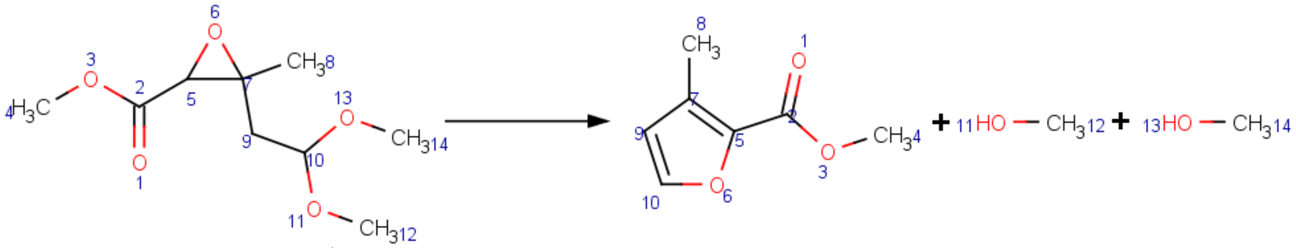

Correct mapped SMILES/SMARTS of the reaction:
[CH3:8][C:7]1([CH:5]([O:6]1)[C:2](=[O:1])[O:3][CH3:4])[CH2:9][CH:10]([O:11][CH3:12])[O:13][CH3:14]>>[CH3:8][C:7]1=[C:5]([O:6][CH:10]=[CH:9]1)[C:2](=[O:1])[O:3][CH3:4].[CH3:12][OH:11].[CH3:14][OH:13]

Correctness of the mapping
MAPPET NO
ReactionMap YES
Marvin NO

Reaction no 61

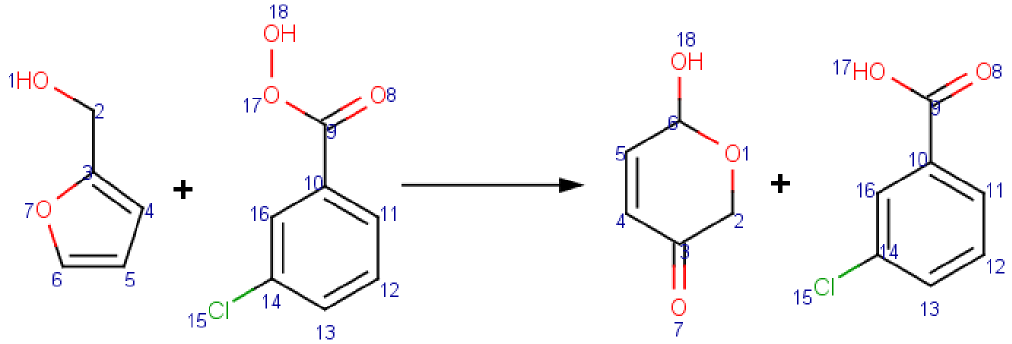

Correct mapped SMILES/SMARTS of the reaction:
[#8:1]-[#6:2]-[#6:3]-1=[#6:4]-[#6:5]=[#6:6]-[#8:7]-1.[#8:18]-[#8:17]-[#6:9](=[O:8])-[#6:10]-1=[#6:11]-[#6:12]=[#6:13]-[#6:14]([Cl:15])=[#6:16]-1>>[#8:18]-[#6:6]-1-[#8:1]-[#6:2]-[#6:3](=[O:7])-[#6:4]=[#6:5]-1.[#8:17]-[#6:9](=[O:8])-[#6:10]-1=[#6:11]-[#6:12]=[#6:13]-[#6:14]([Cl:15])=[#6:16]-1

Correctness of the mapping

MAPPET YES

ReactionMap YES

Marvin YES

Reaction no 62

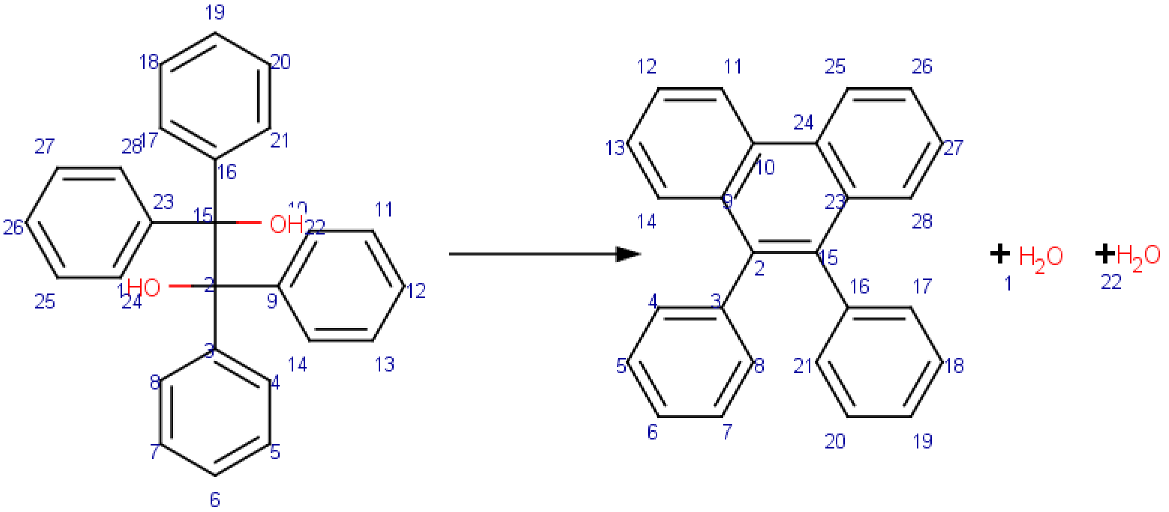

Correct mapped SMILES/SMARTS of the reaction:
[#8:1][C:2]([#6:3]-1=[#6:4]-[#6:5]=[#6:6]-[#6:7]=[#6:8]-1)([#6:9]-1=[#6:10]-[#6:11]=[#6:12]-[#6:13]=[#6:14]-1)[C:15]([#8:22])([#6:16]-1=[#6:17]-[#6:18]=[#6:19]-[#6:20]=[#6:21]-1)[#6:23]-1=[#6:24]-[#6:25]=[#6:26]-[#6:27]=[#6:28]-1>>[#6:6]-1=[#6:5]-[#6:4]=[#6:3](-[#6:8]=[#6:7]-1)-[#6:2]-1=[#6:15](-[#6:16]-2=[#6:17]-[#6:18]=[#6:19]-[#6:20]=[#6:21]-2)-[#6:23]-2=[#6:24](-[#6:25]=[#6:26]-[#6:27]=[#6:28]-2)-[#6:10]-2=[#6:9]-1-[#6:14]=[#6:13]-[#6:12]=[#6:11]-2.[#8:1].[#8:22]

Correctness of the mapping

MAPPET YES

ReactionMap YES

Marvin YES

Reaction no   63

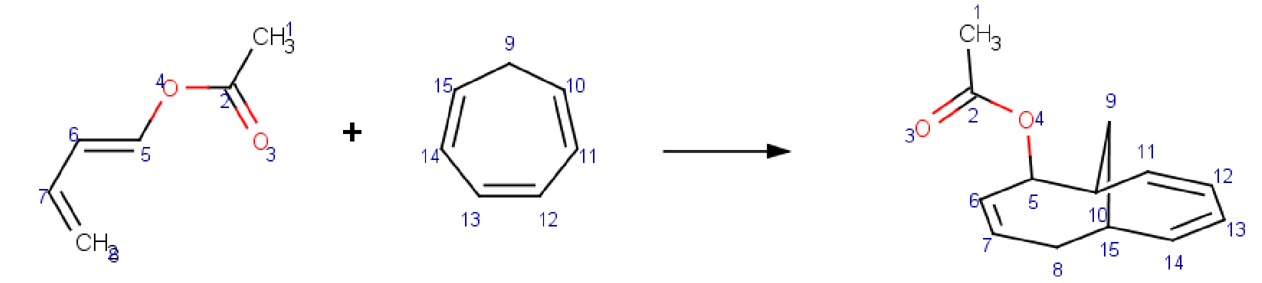

Correct mapped SMILES/SMARTS of the reaction:
[CH3:1][C:2](=[O:3])[O:4]/[CH:5]=[CH:6]/[CH:7]=[CH2:8].[CH2:9]1[CH:10]=[CH:11][C
H:12]=[CH:13][CH:14]=[CH:15]1>>[CH3:1][C:2](=[O:3])[O:4][CH:5]1[CH:6]=[CH:7][CH2
:8][CH:15]2[CH2:9][CH:10]1[CH:11]=[CH:12][CH:13]=[CH:14]2

Correctness of the mapping

MAPPET	NO
ReactionMap	YES
Marvin	NO

Reaction no   64

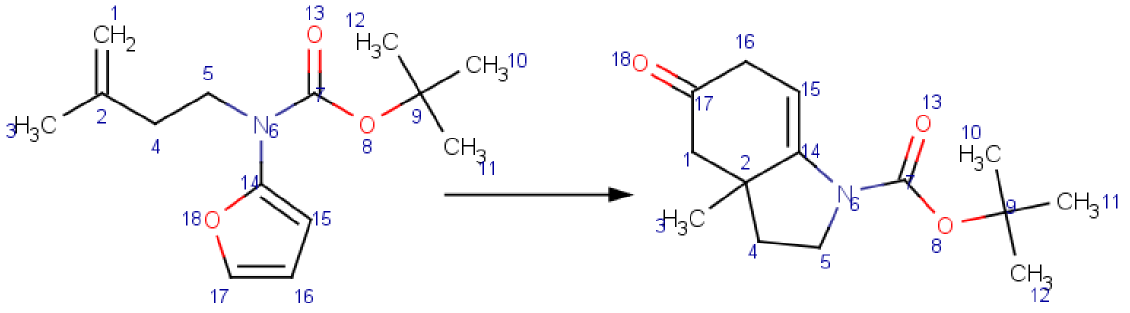

Correct mapped SMILES/SMARTS of the reaction:
[#6:3]-[#6:2](=[#6:1])-[#6:4]-[#6:5]-[#7:6](-[#6:7](=[O:13])-
[#8:8][C:9]([#6:10])([#6:11])[#6:12])-[#6:14]-1=[#6:15]-[#6:16]=[#6:17]-[#8:18]-
1>>[#6:11][C:9]([#6:10])([#6:12])[#8:8]-[#6:7](=[O:13])-[#7:6]-1-[#6:5]-
[#6:4][C:2]2([#6:3])[#6:1]-[#6:17](=[O:18])-[#6:16]-[#6:15]=[#6:14]-12

Correctness of the mapping

MAPPET	YES
ReactionMap	YES
Marvin	YES

Reaction no 65

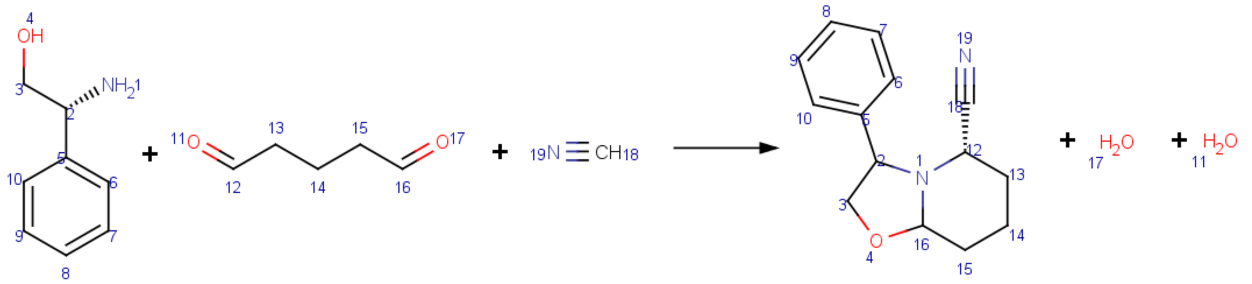

Correct mapped SMILES/SMARTS of the reaction:
[NH2:1][C@@H:2]([CH2:3][OH:4])[C:5]1=[CH:6][CH:7]=[CH:8][CH:9]=[CH:10]1.[O:11]=[CH:12][CH2:13][CH2:14][CH2:15][CH:16]=[O:17].[CH:18]#[N:19]>>[N:19]#[C:18][C@@H:12]1[CH2:13][CH2:14][CH2:15][CH:16]2[O:4][CH2:3][CH:2]([N:1]12)[C:5]1=[CH:10][CH:9]=[CH:8][CH:7]=[CH:6]1.[OH2:17].[OH2:11]

Correctness of the mapping
MAPPET YES
ReactionMap YES
Marvin YES

Reaction no 66

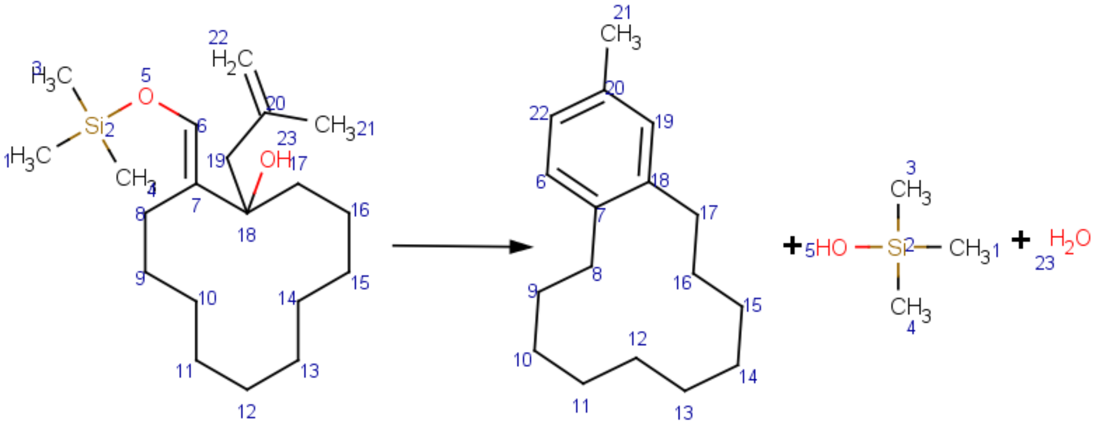

Correct mapped SMILES/SMARTS of the reaction:
[#6:21]-[#6:20](=[#6:22])-[#6:19][C:18]1([#8:23])[#6:17]-[#6:16]-[#6:15]-[#6:14]-[#6:13]-[#6:12]-[#6:11]-[#6:10]-[#6:9]-[#6:8]\[#6:7]1=[#6:6]/[#8:5][Si:2]([#6:1])([#6:3])[#6:4]>>[#6:21]-[#6:20]-1=[#6:22]-[#6:6]=[#6:7]-2-[#6:8]-[#6:9]-[#6:10]-[#6:11]-[#6:12]-[#6:13]-[#6:14]-[#6:15]-[#6:16]-[#6:17]-[#6:18]-2=[#6:19]-1.[#6:4][Si:2]([#6:3])([#6:1])[#8:5].[#8:23]

Correctness of the mapping
MAPPET YES
ReactionMap YES
Marvin NO

Reaction no 67

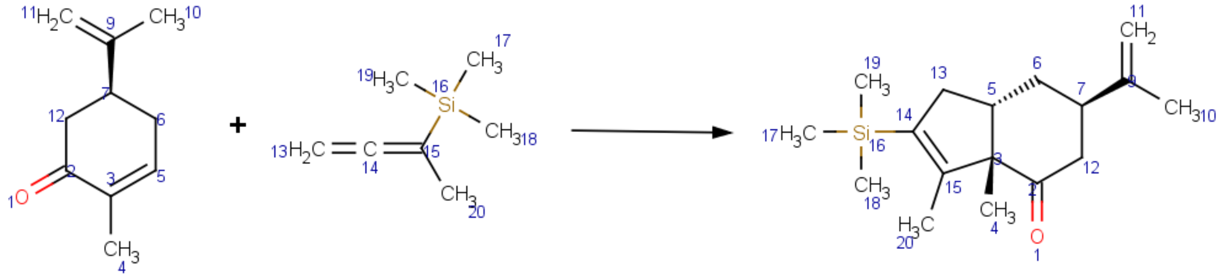

Correct mapped SMILES/SMARTS of the reaction:
[C@@H:7]1([CH2:6][CH:5]=[C:3]([C:2](=[O:1])[CH2:12]1)[CH3:4])[C:9](=[CH2:11])[CH3:10].[CH3:20][C:15](=[C:14]=[CH2:13])[Si:16]([CH3:17])([CH3:18])[CH3:19]>>[C@@H:5]12[CH2:6][C@H:7]([CH2:12][C:2](=[O:1])[C@@:3]1([C:15](=[C:14]([CH2:13]2)[Si:16]([CH3:17])([CH3:18])[CH3:19])[CH3:20])[CH3:4])[C:9](=[CH2:11])[CH3:10]

Correctness of the mapping

MAPPET	NO
ReactionMap	NO
Marvin	NO

Reaction no 68

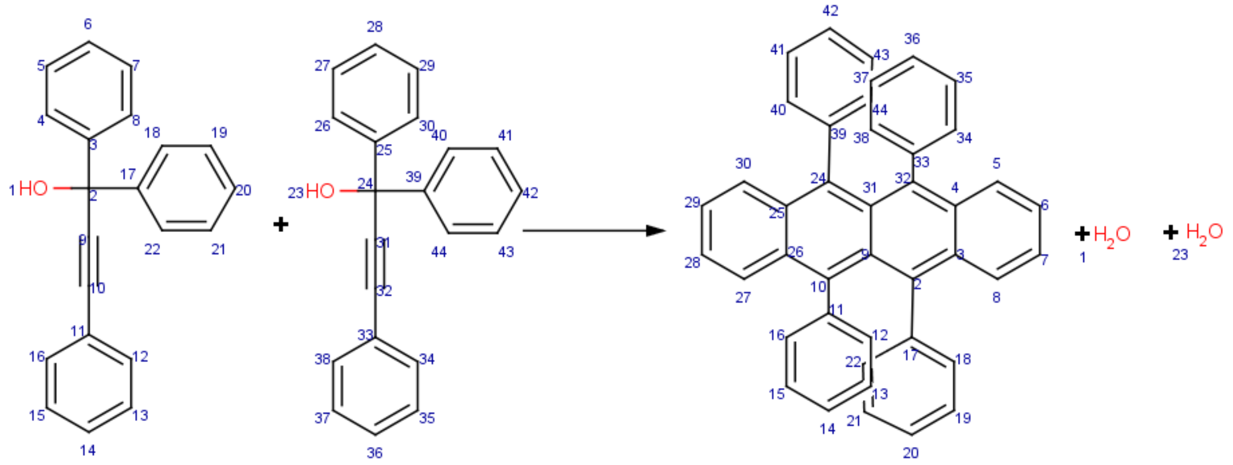

Correct mapped SMILES/SMARTS of the reaction:
[#8:1][C:2]([C:9]#[C:10][#6:11]-1=[#6:12]-[#6:13]=[#6:14]-[#6:15]=[#6:16]-1)([#6:3]-1=[#6:4]-[#6:5]=[#6:6]-[#6:7]=[#6:8]-1)[#6:17]-1=[#6:18]-[#6:19]=[#6:20]-[#6:21]=[#6:22]-1.[#8:23][C:24]([C:31]#[C:32][#6:33]-1=[#6:34]-[#6:35]=[#6:36]-[#6:37]=[#6:38]-1)([#6:25]-1=[#6:26]-[#6:27]=[#6:28]-[#6:29]=[#6:30]-1)[#6:39]-1=[#6:40]-[#6:41]=[#6:42]-[#6:43]=[#6:44]-1>>[#6:42]-1=[#6:43]-[#6:44]=[#6:39](-[#6:40]=[#6:41]-1)-[#6:24]-1=[#6:31]-2-[#6:32](-[#6:33]-3=[#6:38]-[#6:37]=[#6:36]-[#6:35]=[#6:34]-3)=[#6:4]-3-[#6:5]=[#6:6]-[#6:7]=[#6:8]-[#6:3]-3=[#6:2](-[#6:17]-3=[#6:18]-[#6:19]=[#6:20]-[#6:21]=[#6:22]-3)-[#6:9]-2=[#6:10](-[#6:11]-2=[#6:12]-[#6:13]=[#6:14]-[#6:15]=[#6:16]-2)-[#6:26]-2=[#6:27]-[#6:28]=[#6:29]-[#6:30]=[#6:25]-1-2.[#8:1].[#8:23]

Correctness of the mapping

MAPPET	YES
ReactionMap	YES
Marvin	NO

Reaction no 69

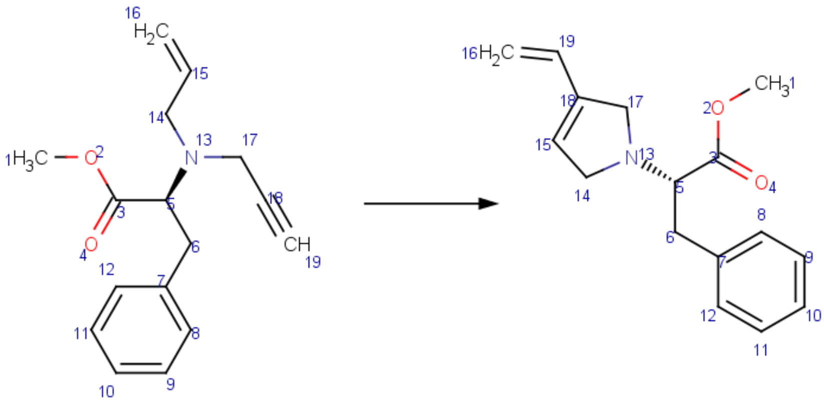

Correct mapped SMILES/SMARTS of the reaction:
[CH3:1][O:2][C:3](=[O:4])[C@H:5]([CH2:6][C:7]1=[CH:8][CH:9]=[CH:10][CH:11]=[CH:12]1)[N:13]([CH2:14][CH:15]=[CH2:16])[CH2:17][C:18]#[CH:19]>>[CH3:1][O:2][C:3](=[O:4])[C@H:5]([CH2:6][C:7]1=[CH:8][CH:9]=[CH:10][CH:11]=[CH:12]1)[N:13]2[CH2:14][CH:15]=[C:18]([CH2:17]2)[CH:19]=[CH2:16]

Correctness of the mapping
MAPPET NO
ReactionMap YES
Marvin YES

Reaction no 70

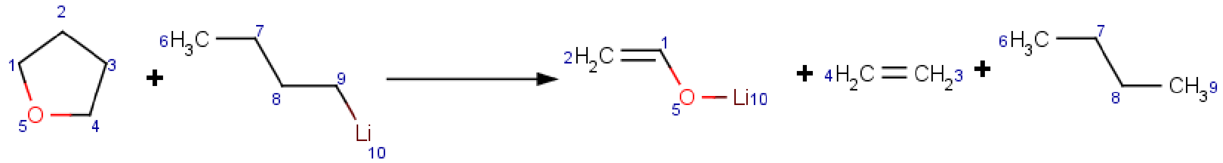

Correct mapped SMILES/SMARTS of the reaction:
[#6:2]-1-[#6:3]-[#6:4]-[#8:5]-[#6:1]-1.[Li:10][#6:9]-[#6:8]-[#6:7]-[#6:6]>>[Li:10][#8:5]-[#6:1]=[#6:2].[#6:3]=[#6:4].[#6:9]-[#6:8]-[#6:7]-[#6:6]

Correctness of the mapping
MAPPET YES
ReactionMap YES
Marvin YES

Reaction no 71

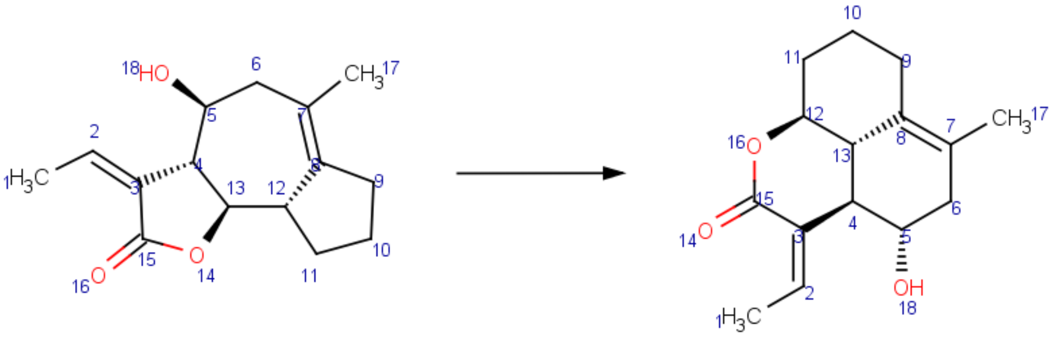

Correct mapped SMILES/SMARTS of the reaction:
[CH3:1]/[CH:2]=[C:3]\1[C@@H:4]2[C@H:5]([CH2:6][C:7](=[C:8]3[CH2:9][CH2:10][CH2:11][C@@H:12]3[C@H:13]2[O:14][C:15]1=[O:16])[CH3:17])[OH:18]>>[CH3:1]/[CH:2]=[C:3]\1[C@@H:4]2[C@H:5]([CH2:6][C:7](=[C:8]3[C@H:13]2[C@H:12]([CH2:11][CH2:10][CH2:9]3)[O:16][C:15]1=[O:14])[CH3:17])[OH:18]

Correctness of the mapping
MAPPET NO
ReactionMap YES
Marvin YES

Reaction no 72

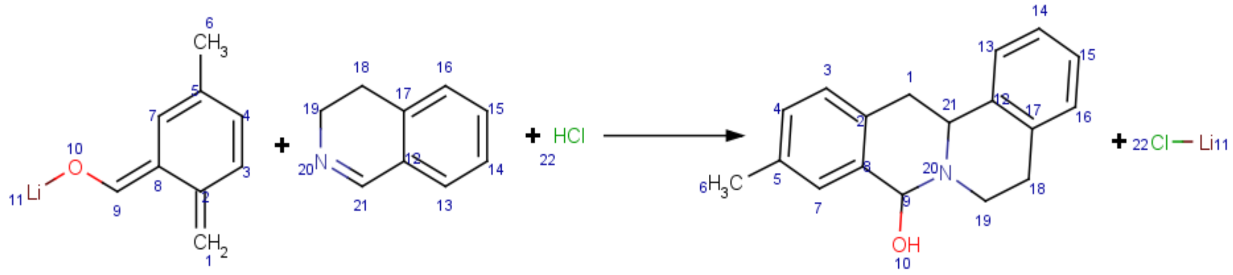

Correct mapped SMILES/SMARTS of the reaction:
[Li:11][#8:10]\[#6:9]=[#6:8]-1/[#6:7]=[#6:5](-[#6:6])-[#6:4]=[#6:3]-[#6:2]-
1=[#6:1].[#6:19]-1-[#6:18]-[#6:17]-2=[#6:16]-[#6:15]=[#6:14]-[#6:13]=[#6:12]-2-
[#6:21]=[#7:20]-1.[Cl:22]>>[#6:6]-[#6:5]-1=[#6:4]-[#6:3]=[#6:2]-2-[#6:1]-
[#6:21]-3-[#7:20](-[#6:19]-[#6:18]-[#6:17]-4=[#6:12]-3-[#6:13]=[#6:14]-
[#6:15]=[#6:16]-4)-[#6:9](-[#8:10])-[#6:8]-2=[#6:7]-1.[Li:11][Cl:22]

Correctness of the mapping
MAPPET YES
ReactionMap YES
Marvin YES

Reaction no 73

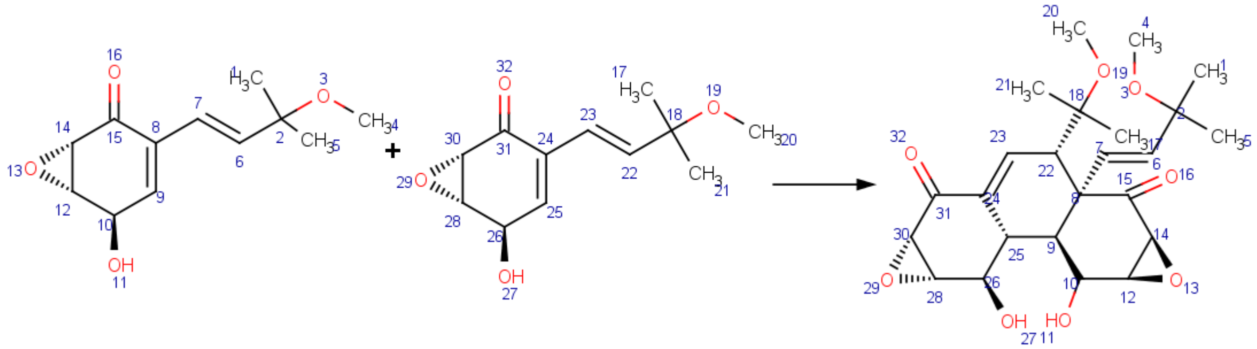

Correct mapped SMILES/SMARTS of the reaction:
[CH3:4][O:3][C:2]([CH3:1])([CH3:5])\[CH:6]=[CH:7]\[C:8]1=[CH:9][C@@H:10]([OH:11]
)[C@@H:12]2[O:13][C@@H:14]2[C:15]1=[O:16].[CH3:20][O:19][C:18]([CH3:17])([CH3:21
])\[CH:22]=[CH:23]\[C:24]1=[CH:25][C@@H:26]([OH:27])[C@@H:28]2[O:29][C@@H:30]2[C
:31]1=[O:32]>>[CH3:4][O:3][C:2]([CH3:5])([CH3:1])\[CH:6]=[CH:7]\[C@@:8]12[C@@H:9
]([CH:10]([OH:11])[C@@H:12]3[O:13][C@@H:14]3[C:15]1=[O:16])[C@@H:25]1[C@@H:26]([
OH:27])[C@@H:28]3[O:29][C@@H:30]3[C:31](=[O:32])[C:24]1=[CH:23][C@@H:22]2[C:18](
[CH3:21])([CH3:17])[O:19][CH3:20]

Correctness of the mapping
MAPPET YES
ReactionMap YES
Marvin YES

Reaction no 74

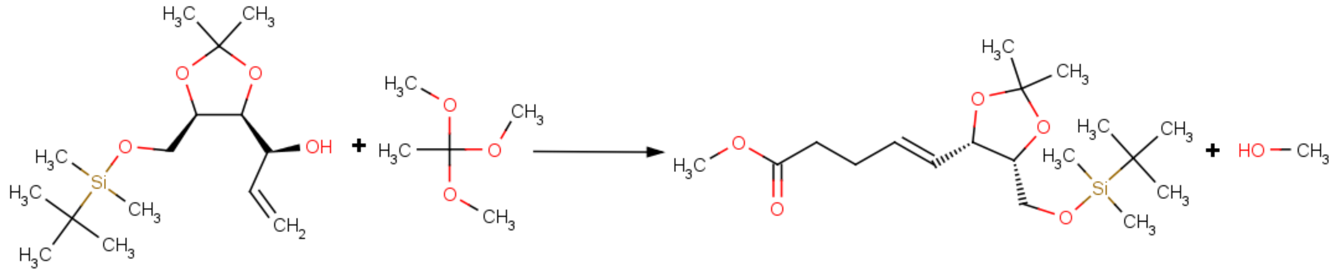

SMILES of the input:
CC(C)([Si](C)(OC[C@H]1OC(C)(O[C@H]1[C@H](C=C)O)C)C)C.COC(OC)(OC)C>>COC(CC/C=C/[C@@H]1OC(C)(O[C@@H]1CO[Si](C)(C(C)(C)C)C)C)=O.CO

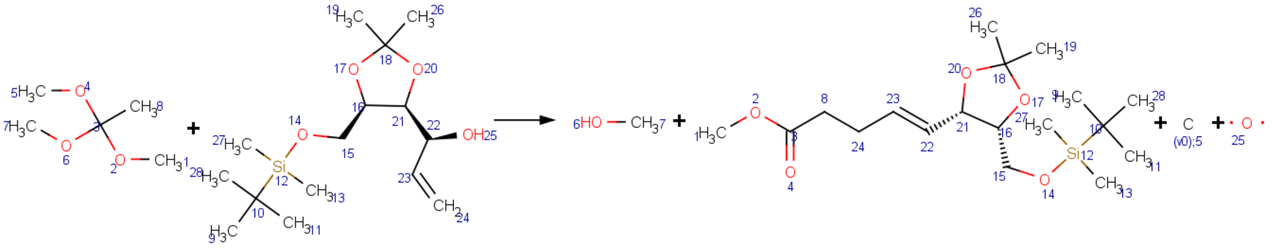

Correct mapped SMILES/SMARTS of the reaction:
[CH3:1][O:2][C:3]([CH3:8])([O:4][CH3:5])[O:6][CH3:7].[CH3:9][C:10]([CH3:11])([CH3:28])[Si:12]([CH3:13])([CH3:27])[O:14][CH2:15][C@H:16]1[O:17][C:18]([CH3:19])([CH3:26])[O:20][C@H:21]1[C@@H:22]([OH:25])[CH:23]=[CH2:24]>>[CH3:7][OH:6].[CH3:1][O:2][C:3](=[O:4])[CH2:8][CH2:24]\[CH:23]=[CH:22]\[C@@H:21]1[O:20][C:18]([CH3:26])([CH3:19])[O:17][C@@H:16]1[CH2:15][O:14][Si:12]([CH3:13])([CH3:27])[C:10]([CH3:28])([CH3:11])[CH3:9].[C:5].[O:25]

Correctness of the mapping

MAPPET	YES
ReactionMap	NO
Marvin	NO

Reaction no 75

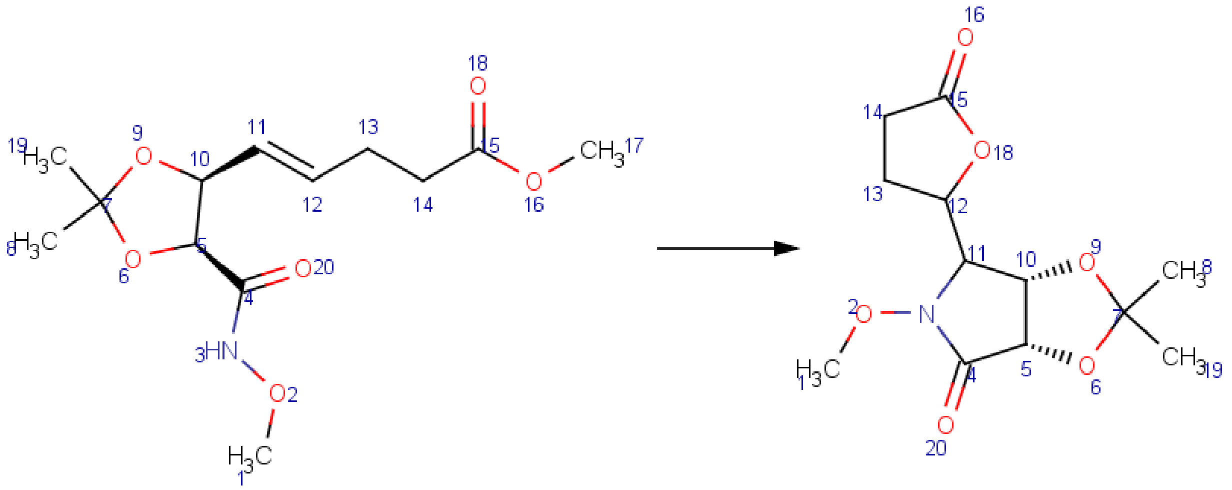

Correct mapped SMILES/SMARTS of the reaction:
[CH3:1][O:2][NH:3][C:4](=[O:20])[C@H:5]1[O:6][C:7]([CH3:8])([CH3:19])[O:9][C@H:1
0]1\[CH:11]=[CH:12]\[CH2:13][CH2:14][C:15](=[O:18])[O:16][CH3:17]>>[CH3:1][O:2]N
1[CH:11]([CH:12]2[CH2:13][CH2:14][C:15](=[O:16])[O:18]2)[C@@H:10]2[O:9][C:7]([CH
3:8])([CH3:19])[O:6][C@@H:5]2[C:4]1=[O:20]

Correctness of the mapping

MAPPET	NO
ReactionMap	NO
Marvin	NO

Reaction no 76

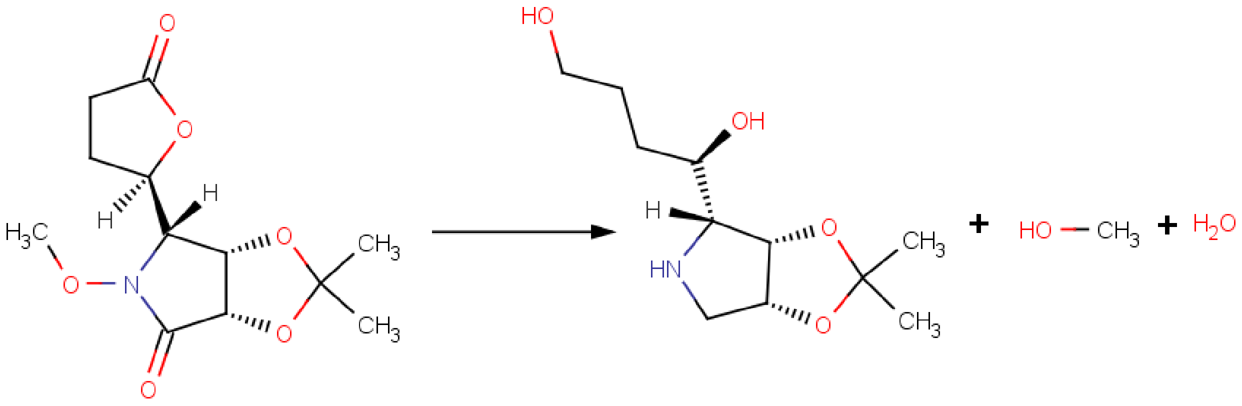

SMILES of the input:
[H][C@]1([C@@]2([C@@H]3OC(C)(O[C@@H]3C(N2OC)=O)C)[H])CCC(O1)=O>>[H][C@]1([C@@H](
CCCO)O)NC[C@H]2OC(C)(O[C@@H]12)C.CO.O

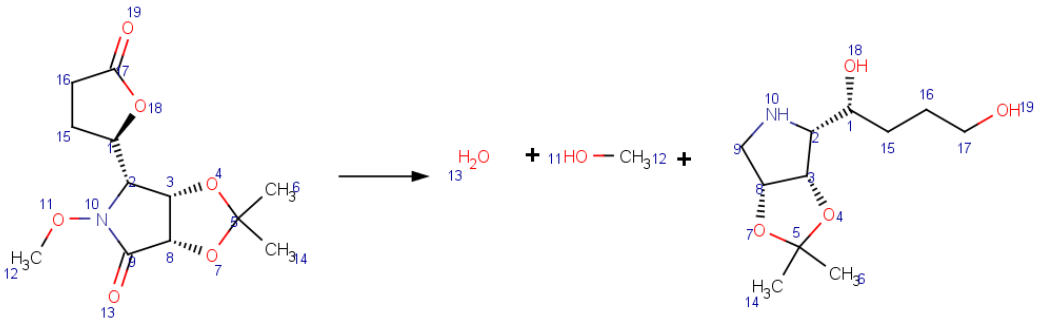

Correct mapped SMILES/SMARTS of the reaction:
[CH3:12][O:11][N:10]1[C@H:2]([C@H:1]2[CH2:15][CH2:16][C:17](=[O:19])[O:18]2)[C@@
H:3]2[O:4][C:5]([CH3:6])([CH3:14])[O:7][C@@H:8]2[C:9]1=[O:13]>>[OH2:13].[CH3:12]

[OH:11].[CH3:14][C:5]1([CH3:6])[O:7][C@@H:8]2[CH2:9][NH:10][C@H:2]([C@H:1]([OH:1
8])[CH2:15][CH2:16][CH2:17][OH:19])[C@@H:3]2[O:4]1

Correctness of the mapping
MAPPET YES
ReactionMap YES
Marvin YES

Reaction no 77

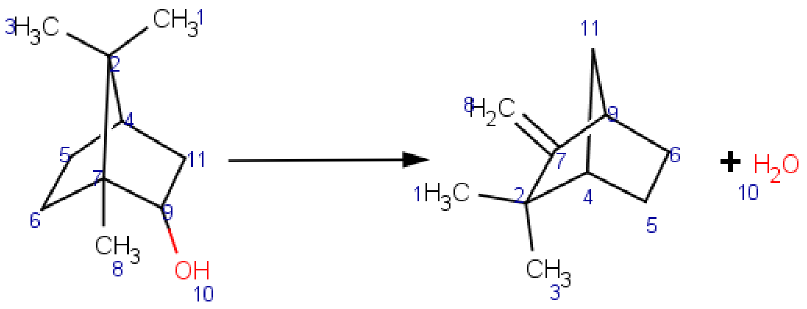

Correct mapped SMILES/SMARTS of the reaction:
[#6:1][C:2]1([#6:3])[#6:4]-2-[#6:5]-[#6:6][C:7]1([#6:8])[#6:9](-[#8:10])-
[#6:11]-2>>[#6:3][C:2]1([#6:1])[#6:4]-2-[#6:5]-[#6:6]-[#6:9](-[#6:11]-2)-
[#6:7]1=[#6:8].[#8:10]

Correctness of the mapping
MAPPET YES
ReactionMap YES
Marvin YES

Reaction no 78

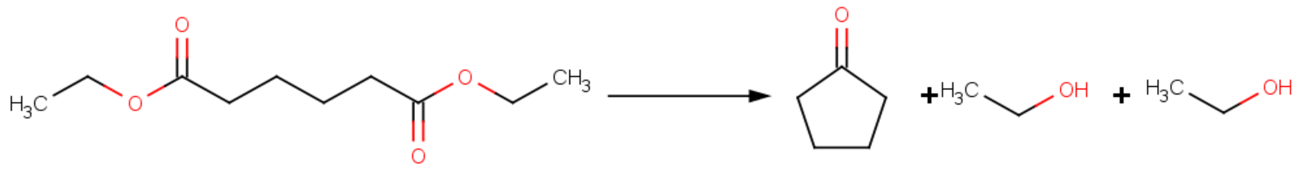

SMILES of the input:
CCOC(=O)CCCCC(=O)OCC>>O=C1CCCC1.CCO.CCO

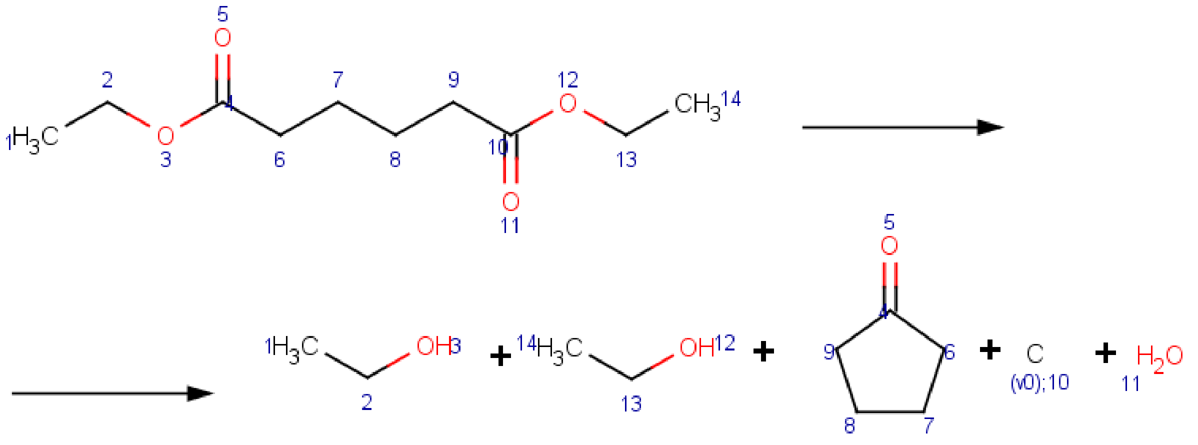

Correct mapped SMILES/SMARTS of the reaction:
[#6:1]-[#6:2]-[#8:3]-[#6:4](=[O:5])-[#6:6]-[#6:7]-[#6:8]-[#6:9]-
[#6:10](=[O:11])-[#8:12]-[#6:13]-[#6:14]>>[#6:1]-[#6:2]-[#8:3].[#6:14]-[#6:13]-
[#8:12].[O:5]=[#6:4]-1-[#6:6]-[#6:7]-[#6:8]-[#6:9]-1.[#6;v0:10].[#8:11]

Correctness of the mapping
MAPPET YES
ReactionMap NO
Marvin YES

Reaction no 79

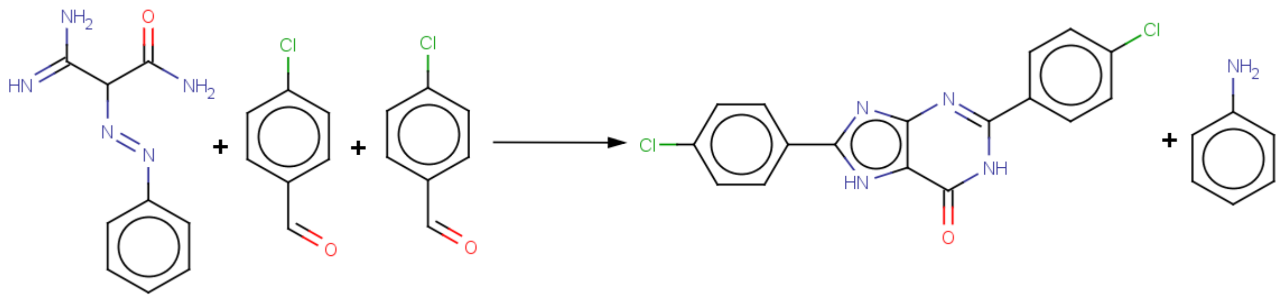

SMILES of the input:
NC(=N)C(\N=N\c1ccccc1)C(N)=O.Clc1ccc(C=O)cc1.Clc1ccc(C=O)cc1>>Clc1ccc(cc1)-c1nc2N=C(NC(=O)c2[nH]1)c1ccc(Cl)cc1.Nc1ccccc1

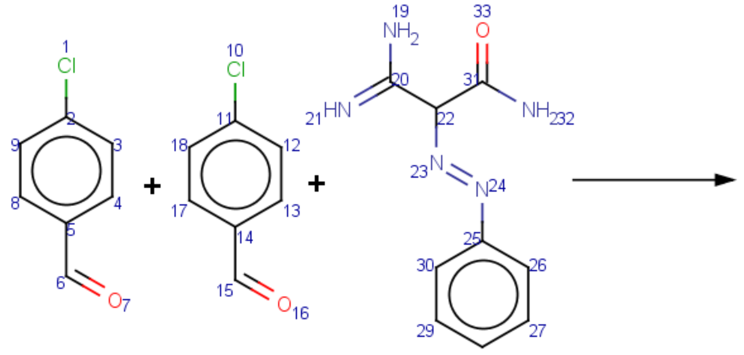

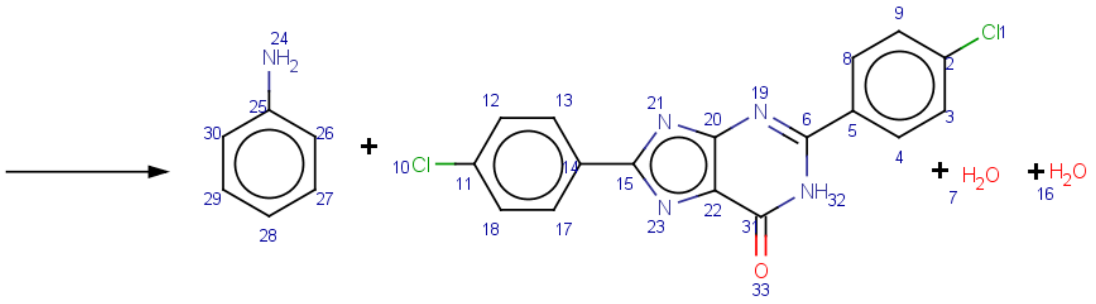

Correct mapped SMILES/SMARTS of the reaction:
[Cl:1][c:2]1[c:3][c:4][c:5](-
[#6:6]=[O:7])[c:8][c:9]1.[Cl:10][c:11]1[c:12][c:13][c:14](-
[#6:15]=[O:16])[c:17][c:18]1.[#7:19]-[#6:20](=[#7:21])-
[#6:22](\[#7:23]=[#7:24]\[c:25]1[c:26][c:27][c:28][c:29][c:30]1)-[#6:31](-
[#7:32])=[O:33]>>[#7:24]-
[c:25]1[c:26][c:27][c:28][c:29][c:30]1.[Cl:10][c:11]1[c:12][c:13][c:14]([c:17][c:18]1)-[c:15]1[n:21][c:20]2-[#7:19]=[#6:6](-[#7:32]-[#6:31](=[O:33])-[c:22]2[n:23]1)-[c:5]1[c:8][c:9][c:2]([Cl:1])[c:3][c:4]1.[#8:7].[#8:16]

Correctness of the mapping
MAPPET YES
ReactionMap NO
Marvin YES

Reaction no 80

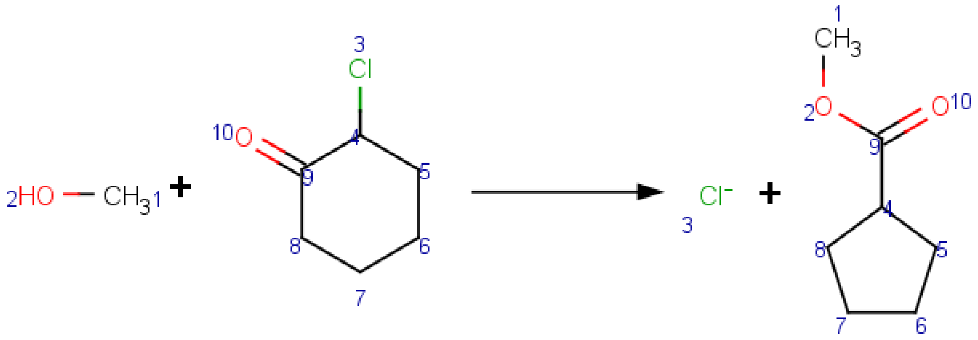

Correct mapped SMILES/SMARTS of the reaction:
[#6:1]-[#8:2].[Cl:3][#6:4]-1-[#6:5]-[#6:6]-[#6:7]-[#6:8]-[#6:9]-1=[O:10]>>[Cl-:3].[#6:1]-[#8:2]-[#6:9](=[O:10])-[#6:4]-1-[#6:5]-[#6:6]-[#6:7]-[#6:8]-1

Correctness of the mapping
MAPPET YES
ReactionMap YES
Marvin YES

Reaction no 81

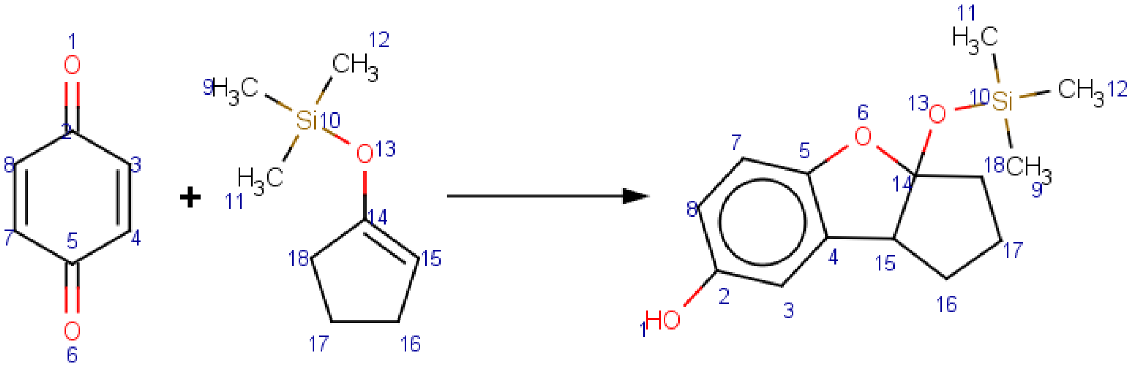

Correct mapped SMILES/SMARTS of the reaction:
[O:1]=[#6:2]-1-[#6:3]=[#6:4]-[#6:5](=[O:6])-[#6:7]=[#6:8]-1.[#6:9][Si:10]([#6:11])([#6:12])[#8:13]-[#6:14]-1=[#6:15]-[#6:16]-[#6:17]-[#6:18]-1>>[#6:12][Si:10]([#6:11])([#6:9])[#8:13][C:14]12[#6:18]-[#6:17]-[#6:16]-[#6:15]1-[c:4]1[c:3][c:2](-[#8:1])[c:8][c:7][c:5]1-[#8:6]2

Correctness of the mapping
MAPPET YES
ReactionMap YES
Marvin YES

Reaction no 82

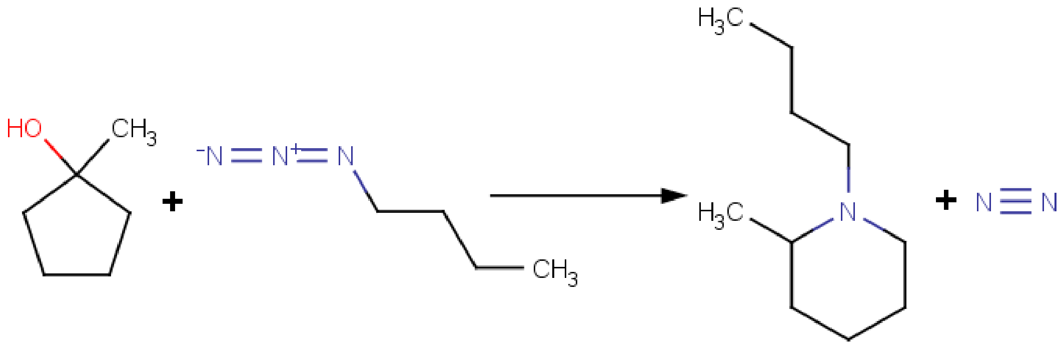

SMILES of the input:
CC1(O)CCCC1.CCCCN=[N+]=[N-]>>CCCCN1CCCCC1C.N#N

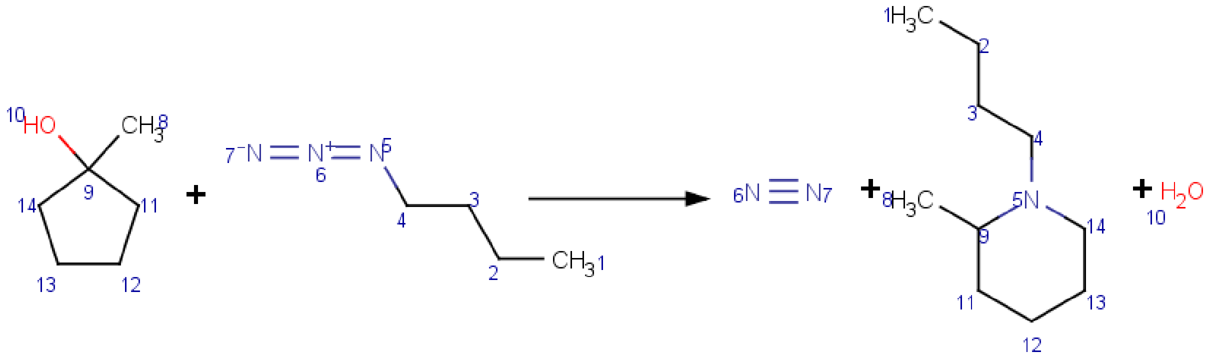

Correct mapped SMILES/SMARTS of the reaction:
[#6:1]-[#6:2]-[#6:3]-[#6:4]-[#7:5]=[N+:6]=[#7-:7].[#6:8][C:9]1([#8:10])[#6:11]-[#6:12]-[#6:13]-[#6:14]1>>[N:7]#[N:6].[#6:1]-[#6:2]-[#6:3]-[#6:4]-[#7:5]-1-[#6:14]-[#6:13]-[#6:12]-[#6:11]-[#6:9]-1-[#6:8].[#8:10]

Correctness of the mapping
MAPPET YES
ReactionMap NO
Marvin YES

Reaction no 83

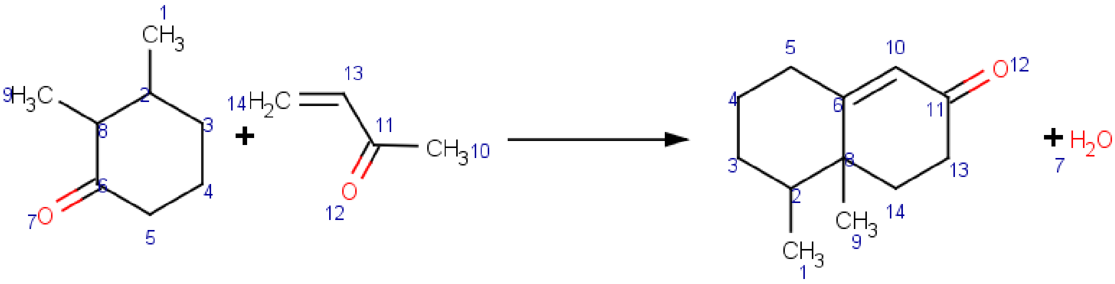

Correct mapped SMILES/SMARTS of the reaction:
[#6:1]-[#6:2]-1-[#6:3]-[#6:4]-[#6:5]-[#6:6](=[O:7])-[#6:8]-1-[#6:9].[#6:10]-[#6:11](=[O:12])-[#6:13]=[#6:14]>>[#6:1]-[#6:2]1-[#6:3]-[#6:4]-[#6:5]-[#6:6]2=[#6:10]-[#6:11](=[O:12])-[#6:13]-[#6:14][C:8]12[#6:9].[#8:7]

Correctness of the mapping
MAPPET YES
ReactionMap YES
Marvin NO

Reaction no 84

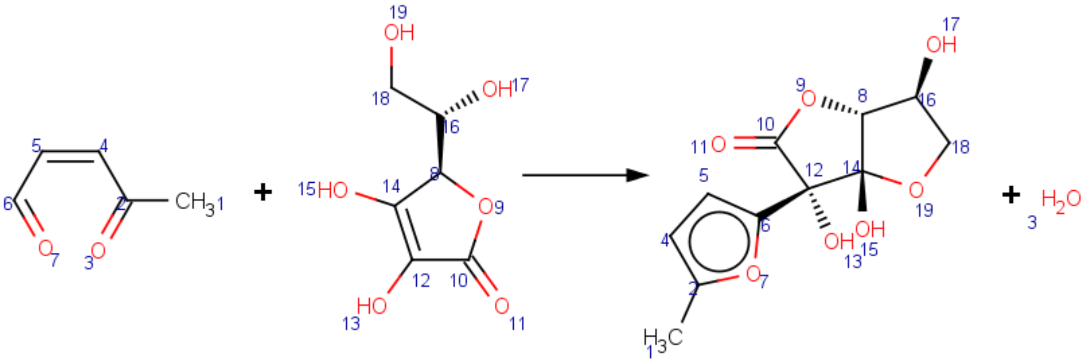

Correct mapped SMILES/SMARTS of the reaction:
[CH3:1][C:2](=[O:3])\[CH:4]=[CH:5]/[CH:6]=[O:7].[OH:19][CH2:18][C@H:16]([OH:17])
[C@H:8]1[O:9][C:10](=[O:11])[C:12]([OH:13])=[C:14]1[OH:15]>>[OH2:3].[CH3:1][c:2]
1[cH:4][cH:5][c:6]([o:7]1)[C@@:12]1([OH:13])[C:10](=[O:11])[O:9][C@@H:8]2[C@@H:1
6]([OH:17])[CH2:18][O:19][C@:14]12[OH:15]

Correctness of the mapping

MAPPET	YES
ReactionMap	YES
Marvin	YES

Reaction no 85

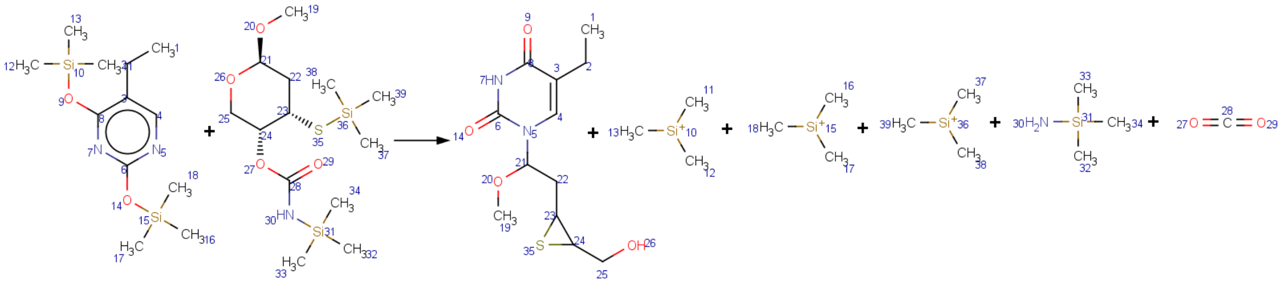

Correct mapped SMILES/SMARTS of the reaction:
[CH3:1][CH2:2][c:3]1[cH:4][n:5][c:6]([n:7][c:8]1[O:9][Si:10]([CH3:11])([CH3:12])
[CH3:13])[O:14][Si:15]([CH3:16])([CH3:17])[CH3:18].[CH3:19][O:20][C@H:21]1[CH2:2
2][C@@H:23]([C@@H:24]([CH2:25][O:26]1)[O:27][C:28](=[O:29])[NH:30][Si:31]([CH3:3
2])([CH3:33])[CH3:34])[S:35][Si:36]([CH3:37])([CH3:38])[CH3:39]>>[CH3:1][CH2:2][
C:3]1=[CH:4][N:5]([C:6](=[O:14])[NH:7][C:8]1=[O:9])[CH:21]([CH2:22][CH:23]2[CH:2
4]([S:35]2)[CH2:25][OH:26])[O:20][CH3:19].[CH3:11][Si+:10]([CH3:12])[CH3:13].[CH
3:16][Si+:15]([CH3:17])[CH3:18].[CH3:37][Si+:36]([CH3:38])[CH3:39].[CH3:32][Si:3
1]([CH3:33])([CH3:34])[NH2:30].[C:28](=[O:27])=[O:29]

Correctness of the mapping

MAPPET	NO
ReactionMap	YES
Marvin	YES

Reaction no 86

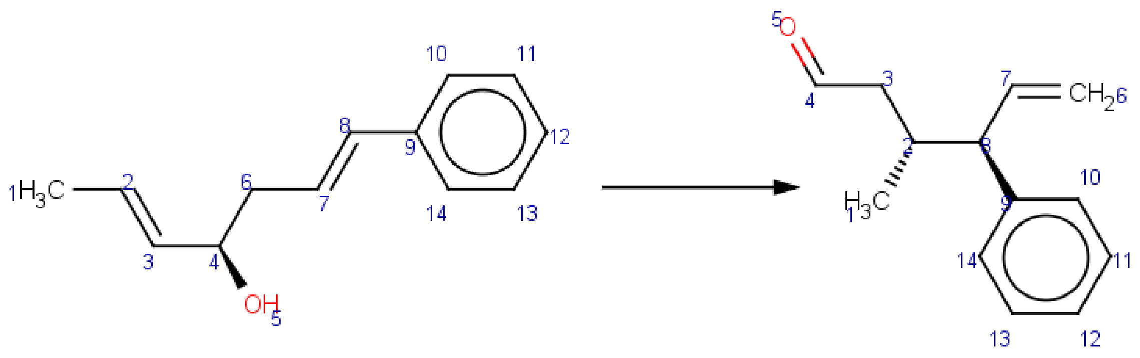

Correct mapped SMILES/SMARTS of the reaction:
[#6:1]\[#6:2]=[#6:3]\[#6@H:4](-[#8:5])-
[#6:6]\[#6:7]=[#6:8]\[c:9]1[c:10][c:11][c:12][c:13][c:14]1>>[#6:1]-[#6@H:2](-
[#6:3]-[#6:4]=[O:5])-[#6@@H:8](-[#6:7]=[#6:6])-
[c:9]1[c:10][c:11][c:12][c:13][c:14]1

Correctness of the mapping

MAPPET	YES
ReactionMap	NO
Marvin	NO

Reaction no 87

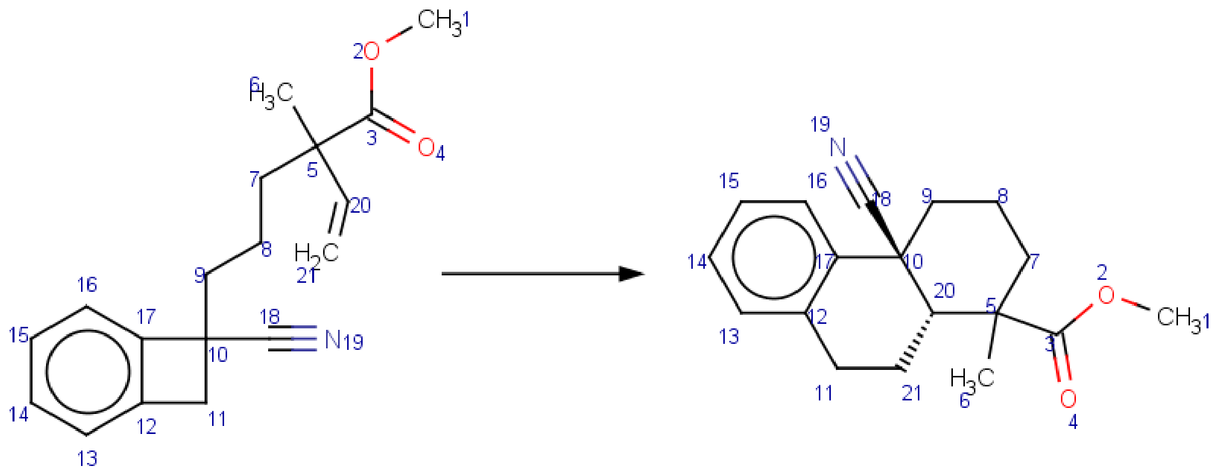

Correct mapped SMILES/SMARTS of the reaction:
[#6:1]-[#8:2]-[#6:3](=[O:4])[C:5]([#6:6])([#6:7]-[#6:8]-[#6:9][C:10]1([#6:11]-
[c:12]2[c:13][c:14][c:15][c:16][c:17]12)[C:18]#[N:19])[#6:20]=[#6:21]>>[#6:1]-
[#8:2]-[#6:3](=[O:4])[C:5]1([#6:6])[#6:7]-[#6:8]-
[#6:9][C@:10]2([C:18]#[N:19])[#6@H:20]1-[#6:21]-[#6:11]-
[c:12]1[c:13][c:14][c:15][c:16][c:17]21

Correctness of the mapping

MAPPET	YES
ReactionMap	YES
Marvin	YES

Reaction no 88

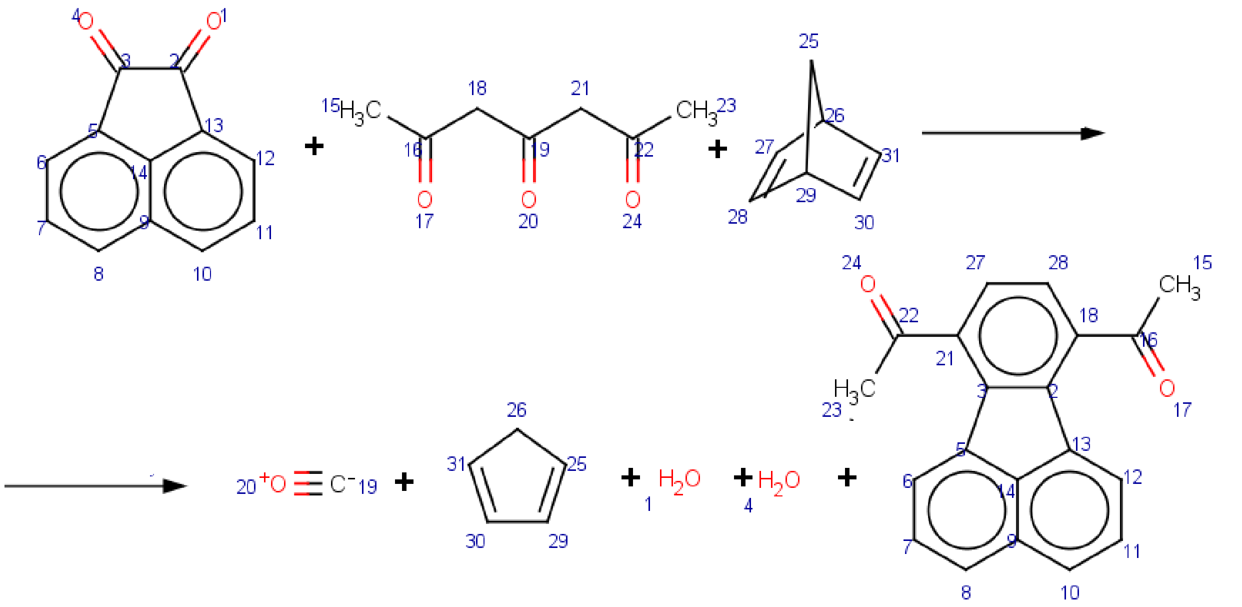

Correct mapped SMILES/SMARTS of the reaction:
[O:1]=[#6:2]-1-[#6:3](=[O:4])-[c:5]2[c:6][c:7][c:8][c:9]3[c:10][c:11][c:12][c:13]-1[c:14]23.[#6:15]-[#6:16](=[O:17])-[#6:18]-[#6:19](=[O:20])-[#6:21]-[#6:22](-[#6:23])=[O:24].[#6:25]-1-[#6:26]-2-[#6:27]=[#6:28]-[#6:29]-1-[#6:30]=[#6:31]-2>>[#6:23]-[#6:22](=[O:24])-[c:21]1[c:27][c:28][c:18](-[#6:16](-[#6:15])=[O:17])[c:2]2-[c:13]3[c:12][c:11][c:10][c:9]4[c:8][c:7][c:6][c:5](-[c:3]12)[c:14]34.[C-:19]#[O+:20].[#6:26]-1-[#6:25]=[#6:29]-[#6:30]=[#6:31]-1.[#8:1].[#8:4]

Correctness of the mapping

MAPPET	NO
ReactionMap	NO
Marvin	YES

Reaction no 89

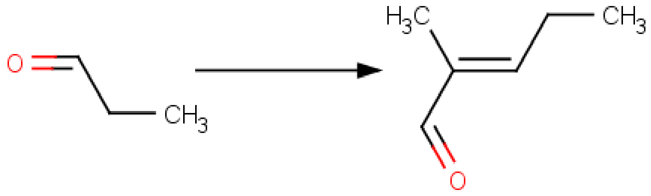

SMILES of the input:
CCC=O>>CC\C=C(/C)C=O

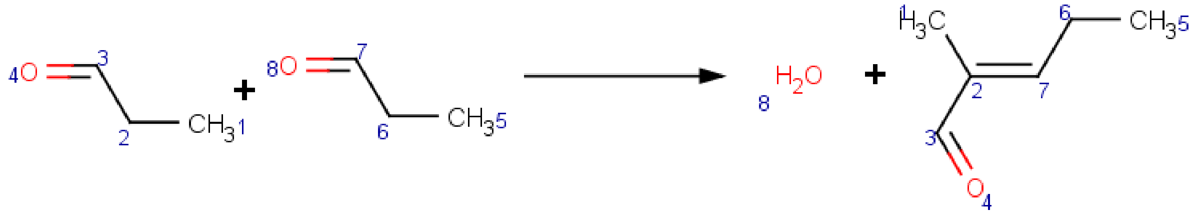

Correct mapped SMILES/SMARTS of the reaction:
[#6:1]-[#6:2]-[#6:3]=[O:4].[#6:5]-[#6:6]-[#6:7]=[O:8]>>[#8:8].[#6:5]-[#6:6]\[#6:7]=[#6:2](/[#6:1])-[#6:3]=[O:4]

Correctness of the mapping
MAPPET YES
ReactionMap NO
Marvin YES

Reaction no 90

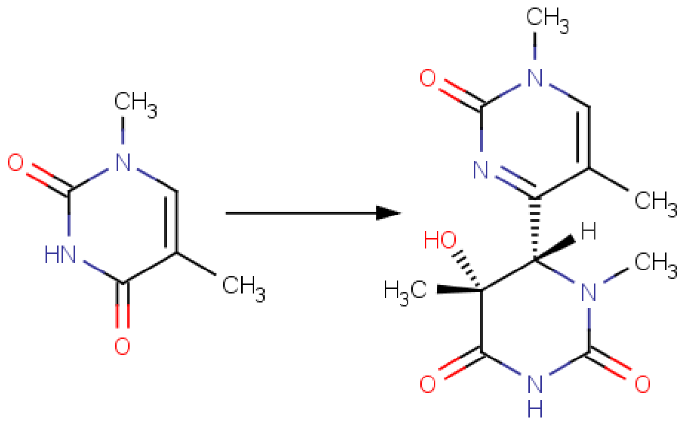

SMILES of the input:
CN1C=C(C)C(=O)NC1=O>>[H][C@]1(N(C)C(=O)NC(=O)[C@]1(C)O)C1=NC(=O)N(C)C=C1C

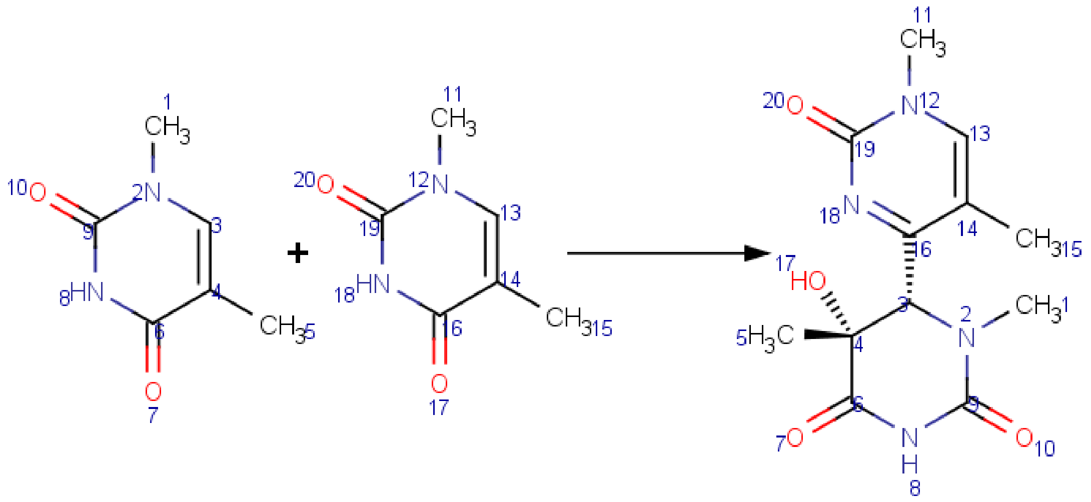

Correct mapped SMILES/SMARTS of the reaction:
[#6:1]-[#7:2]-1-[#6:3]=[#6:4](-[#6:5])-[#6:6](=[O:7])-[#7:8]-[#6:9]-1=[O:10].[#6:11]-[#7:12]-1-[#6:13]=[#6:14](-[#6:15])-[#6:16](=[O:17])-[#7:18]-[#6:19]-1=[O:20]>>[#6:11]-[#7:12]-1-[#6:13]=[#6:14](-[#6:15])-[#6:16](=[#7:18]-[#6:19]-1=[O:20])-[#6@@H:3]1-[#7:2](-[#6:1])-[#6:9](=[O:10])-[#7:8]-[#6:6](=[O:7])[C@:4]1([#6:5])[#8:17]

Correctness of the mapping
MAPPET YES
ReactionMap NO
Marvin YES

Reaction no 91

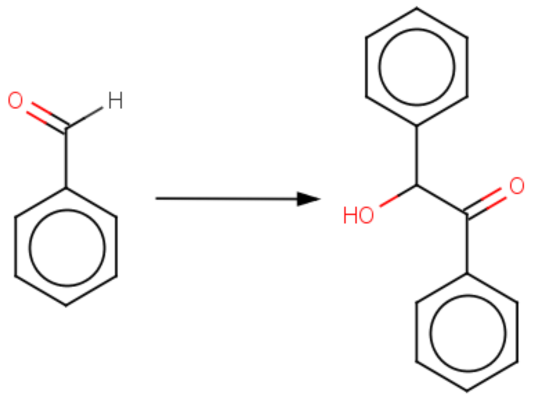

SMILES of the input:
[H]C(=O)c1ccccc1>>OC(C(=O)c1ccccc1)c1ccccc1

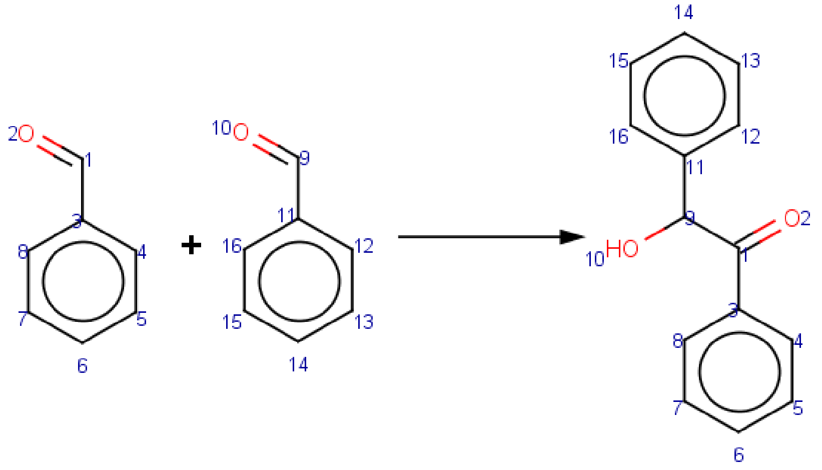

Correct mapped SMILES/SMARTS of the reaction:
[O:2]=[#6:1]-[c:3]1[c:4][c:5][c:6][c:7][c:8]1.[O:10]=[#6:9]-
[c:11]1[c:12][c:13][c:14][c:15][c:16]1>>[#8:10]-[#6:9](-[#6:1](=[O:2])-
[c:3]1[c:4][c:5][c:6][c:7][c:8]1)-[c:11]1[c:16][c:15][c:14][c:13][c:12]1

Correctness of the mapping

MAPPET	YES
ReactionMap	NO
Marvin	YES

Reaction no 92

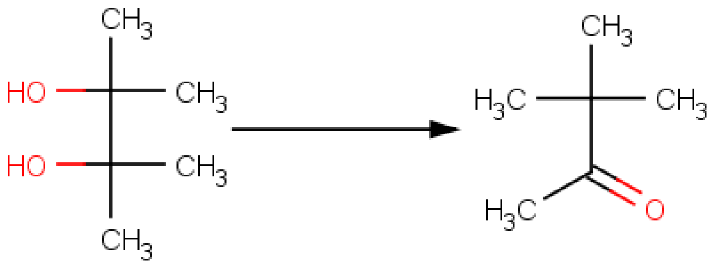

SMILES of the input:
CC(C)(O)C(C)(C)O>>CC(=O)C(C)(C)C

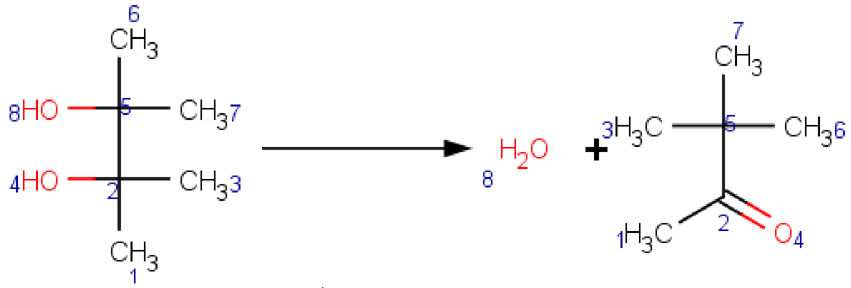

Correct mapped SMILES/SMARTS of the reaction:
[#6:1][C:2]([#6:3])([#8:4])[C:5]([#6:6])([#6:7])[#8:8]>>[#8:8].[#6:1]-[#6:2](=[O:4])[C:5]([#6:7])([#6:6])[#6:3]

Correctness of the mapping

MAPPET	YES
ReactionMap	NO
Marvin	YES

Reaction no 93

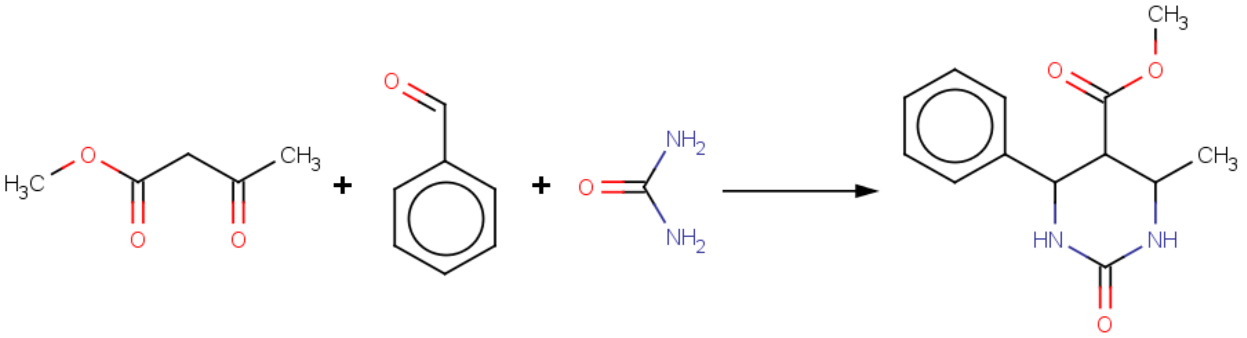

SMILES of the input:
COC(=O)CC(C)=O.O=Cc1ccccc1.NC(N)=O>>COC(=O)C1C(C)NC(=O)NC1c1ccccc1

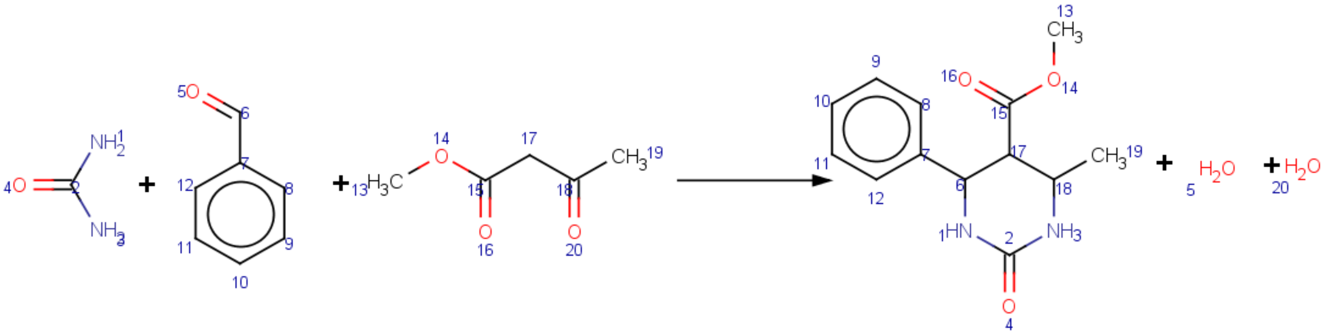

Correct mapped SMILES/SMARTS of the reaction:
[#7:1]-[#6:2](-[#7:3])=[O:4].[O:5]=[#6:6]-[c:7]1[c:8][c:9][c:10][c:11][c:12]1.[#6:13]-[#8:14]-[#6:15](=[O:16])-[#6:17]-[#6:18](-[#6:19])=[O:20]>>[#6:13]-[#8:14]-[#6:15](=[O:16])-[#6:17]-1-[#6:18](-[#6:19])-[#7:3]-[#6:2](=[O:4])-[#7:1]-[#6:6]-1-[c:7]1[c:12][c:11][c:10][c:9][c:8]1.[#8:5].[#8:20]

Correctness of the mapping

MAPPET	YES
ReactionMap	NO
Marvin	YES

Reaction no 94

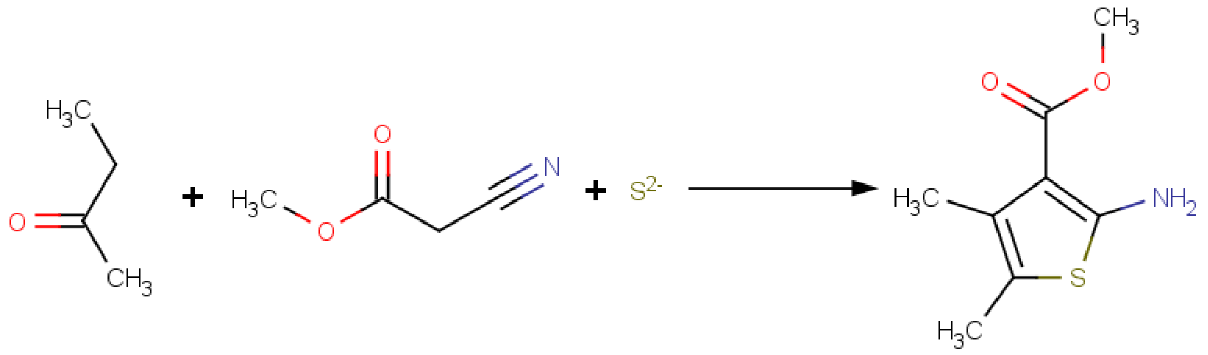

SMILES of the input:
CCC(C)=O.COC(=O)CC#N.[S--]>>COC(=O)C1=C(N)SC(C)=C1C

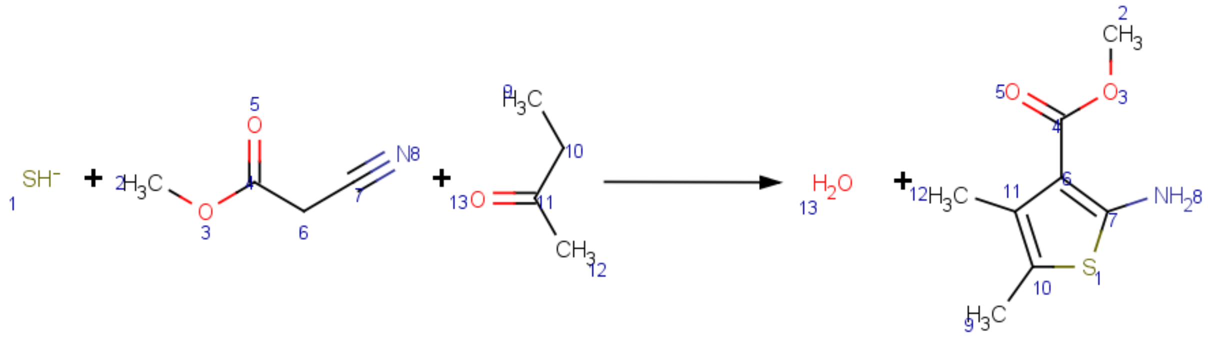

Correct mapped SMILES/SMARTS of the reaction:
[#16-:1].[#6:2]-[#8:3]-[#6:4](=[O:5])-[#6:6][C:7]#[N:8].[#6:9]-[#6:10]-[#6:11](-[#6:12])=[O:13]>>[#8:13].[#6:2]-[#8:3]-[#6:4](=[O:5])-[#6:6]-1=[#6:7](-[#7:8])-[#16:1]-[#6:10](-[#6:9])=[#6:11]-1-[#6:12]

Correctness of the mapping
MAPPET YES
ReactionMap NO
Marvin YES

Reaction no 95

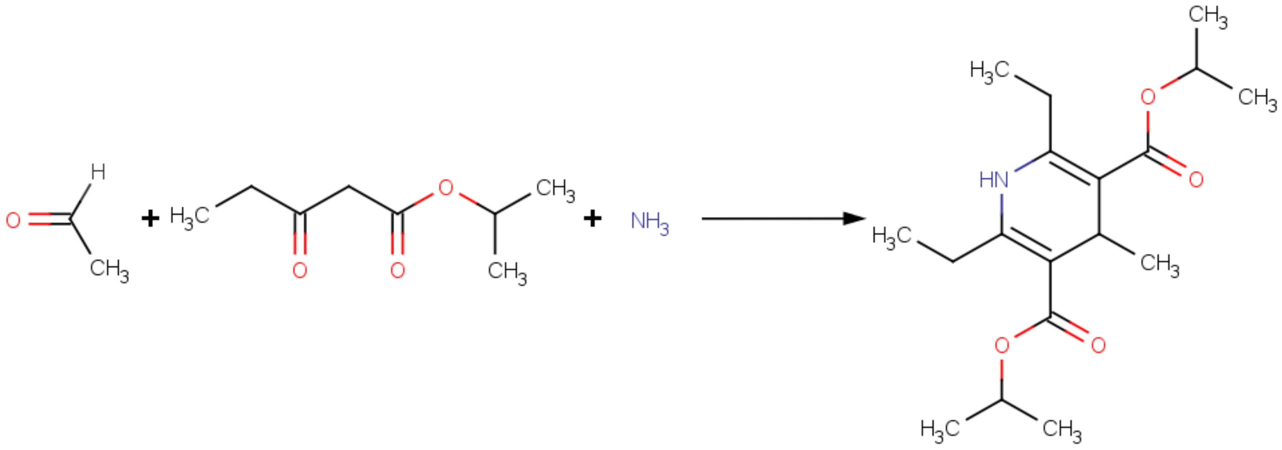

SMILES of the input:
[H]C(C)=O.CCC(=O)CC(=O)OC(C)C.N>>CCC1=C(C(C)C(C(=O)OC(C)C)=C(CC)N1)C(=O)OC(C)C

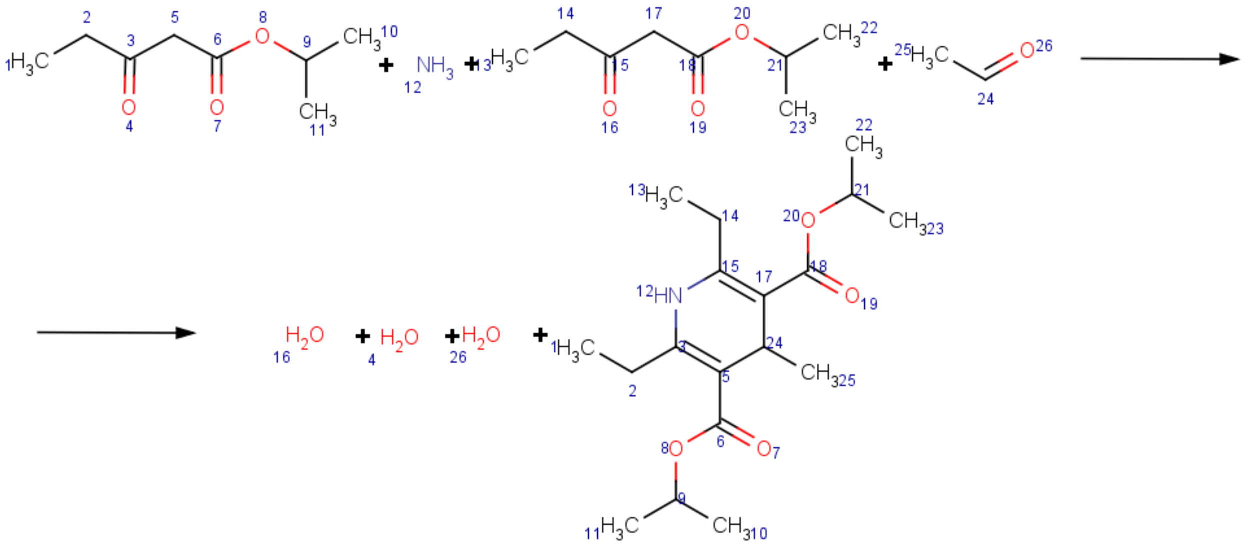

Correct mapped SMILES/SMARTS of the reaction:
[#6:1]-[#6:2]-[#6:3](=[O:4])-[#6:5]-[#6:6](=[O:7])-[#8:8]-[#6:9](-[#6:10])-
[#6:11].[#7:12].[#6:13]-[#6:14]-[#6:15](=[O:16])-[#6:17]-[#6:18](=[O:19])-
[#8:20]-[#6:21](-[#6:22])-[#6:23].[#6:25]-
[#6:24]=[O:26]>>[#8:16].[#8:4].[#8:26].[#6:13]-[#6:14]-[#6:15]-1=[#6:17](-
[#6:24](-[#6:25])-[#6:5](-[#6:6](=[O:7])-[#8:8]-[#6:9](-[#6:11])-
[#6:10])=[#6:3](-[#6:2]-[#6:1])-[#7:12]-1)-[#6:18](=[O:19])-[#8:20]-[#6:21](-
[#6:22])-[#6:23]

Correctness of the mapping

MAPPET	YES
ReactionMap	NO
Marvin	NO

Reaction no 96

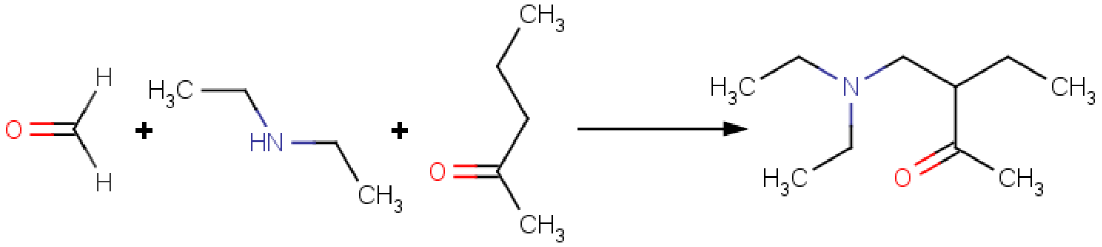

SMILES of the input:
[H]C([H])=O.CCNCC.CCCC(C)=O>>CCC(CN(CC)CC)C(C)=O

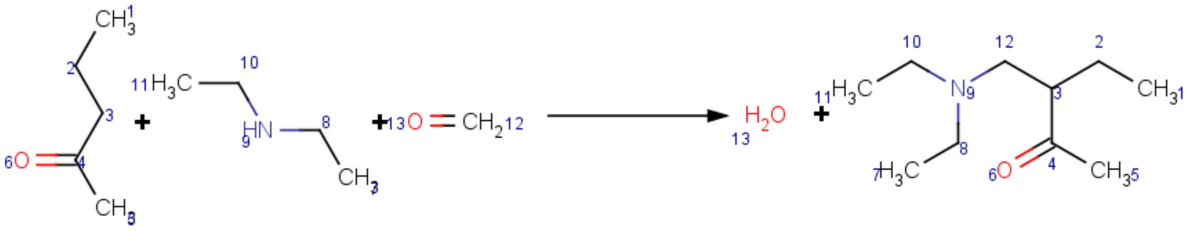

Correct mapped SMILES/SMARTS of the reaction:
[#6:1]-[#6:2]-[#6:3]-[#6:4](-[#6:5])=[O:6].[#6:7]-[#6:8]-[#7:9]-[#6:10]-[#6:11].[#6:12]=[O:13]>>[#8:13].[#6:1]-[#6:2]-[#6:3](-[#6:12]-[#7:9](-[#6:10]-[#6:11])-[#6:8]-[#6:7])-[#6:4](-[#6:5])=[O:6]

Correctness of the mapping

MAPPET	YES
ReactionMap	NO
Marvin	YES

Reaction no 97

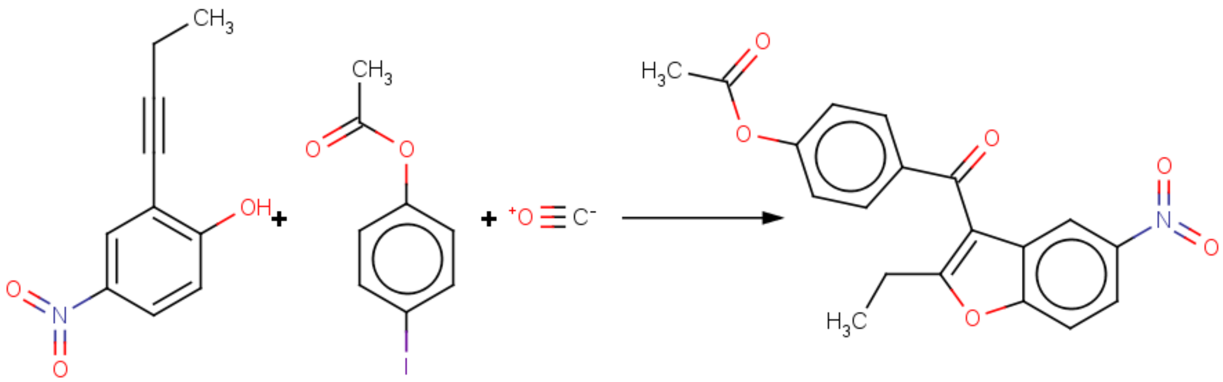

SMILES of the input:
CCC#CC1=C(O)C=CC(=C1)N(=O)=O.CC(=O)Oc1ccc(I)cc1.[C-]#[O+]>>CCC1=C(C(=O)c2ccc(OC(C)=O)cc2)c2cc(ccc2O1)N(=O)=O

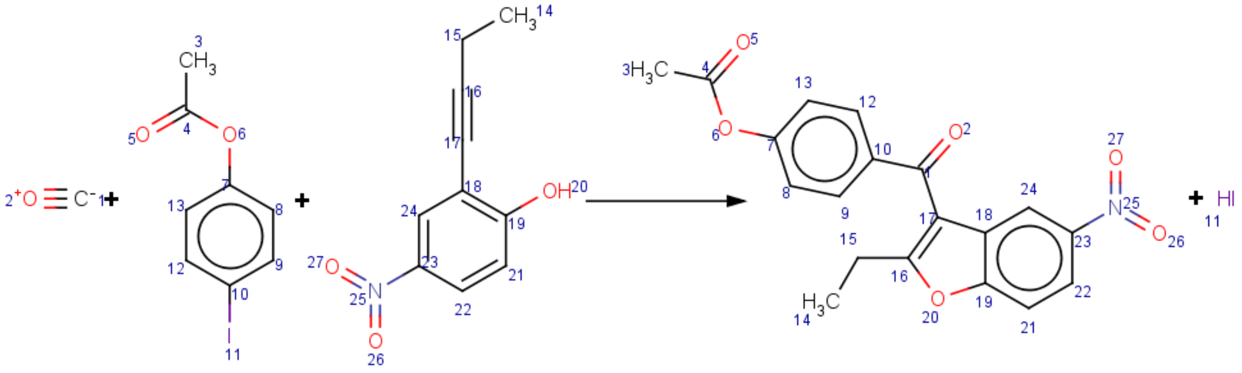

Correct mapped SMILES/SMARTS of the reaction:
[C-:1]#[O+:2].[#6:3]-[#6:4](=[O:5])-[#8:6]-[c:7]1[c:8][c:9][c:10]([I:11])[c:12][c:13]1.[#6:14]-[#6:15][C:16]#[C:17][#6:18]-1=[#6:19](-[#8:20])-[#6:21]=[#6:22]-[#6:23](=[#6:24]-1)[N:25](=[O:26])=[O:27]>>[#6:14]-[#6:15]-[#6:16]-1=[#6:17](-[#6:1](=[O:2])-[c:10]2[c:9][c:8][c:7](-[#8:6]-[#6:4](-[#6:3])=[O:5])[c:13][c:12]2)-[c:18]2[c:24][c:23]([c:22][c:21][c:19]2-[#8:20]-1)[N:25](=[O:27])=[O:26].[I:11]

Correctness of the mapping
MAPPET YES
ReactionMap NO
Marvin YES

Reaction no 98

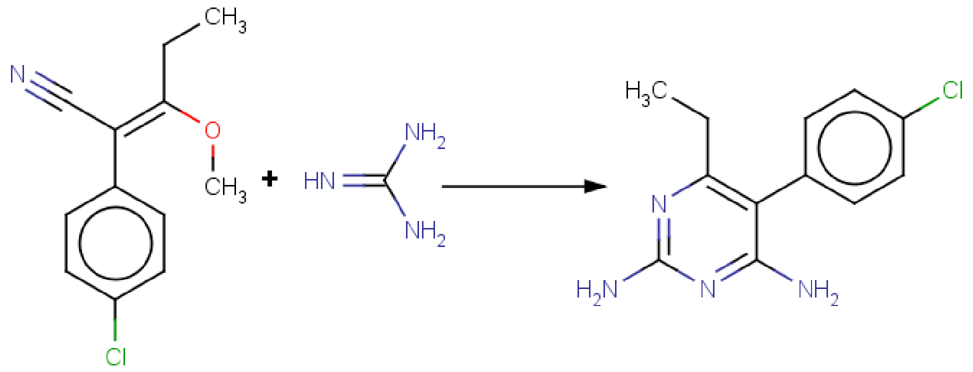

SMILES of the input:
CC\C(OC)=C(/C#N)c1ccc(Cl)cc1.NC(N)=N>>CCC1=C(C(N)=NC(N)=N1)c1ccc(Cl)cc1

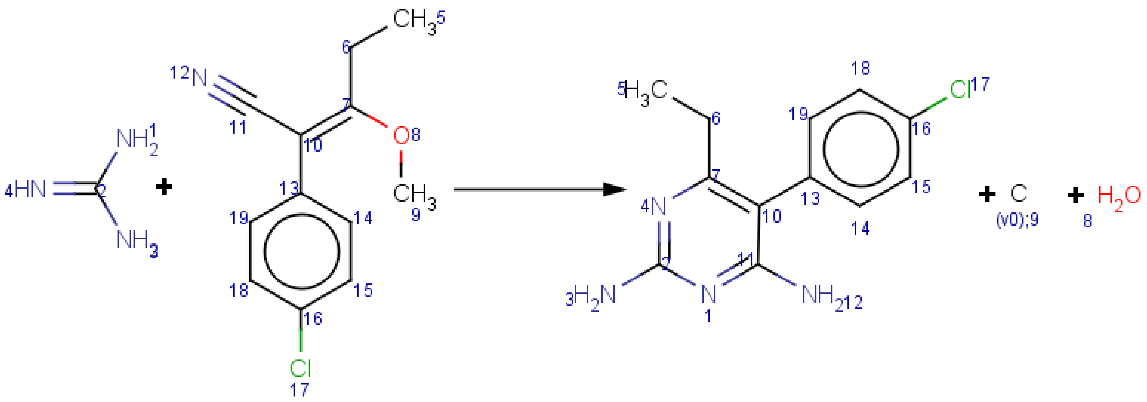

Correct mapped SMILES/SMARTS of the reaction:
[#7:1]-[#6:2](-[#7:3])=[#7:4].[#6:5]-[#6:6]\[#6:7](-[#8:8]-[#6:9])=[#6:10](/[C:11]#[N:12])-[c:13]1[c:14][c:15][c:16]([Cl:17])[c:18][c:19]1>>[#6:5]-[#6:6]-[#6:7]-1=[#6:10](-[#6:11](-[#7:12])=[#7:1]-[#6:2](-[#7:3])=[#7:4]-1)-[c:13]1[c:19][c:18][c:16]([Cl:17])[c:15][c:14]1.[#6;v0:9].[#8:8]

Correctness of the mapping
MAPPET YES
ReactionMap NO
Marvin YES

Reaction no 99

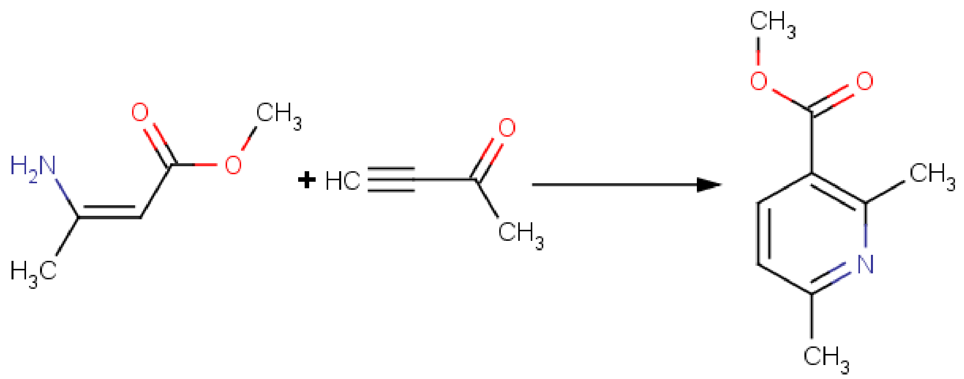

SMILES of the input:
COC(=O)\C=C(\C)N.CC(=O)C#C>>COC(=O)C1=C(C)N=C(C)C=C1

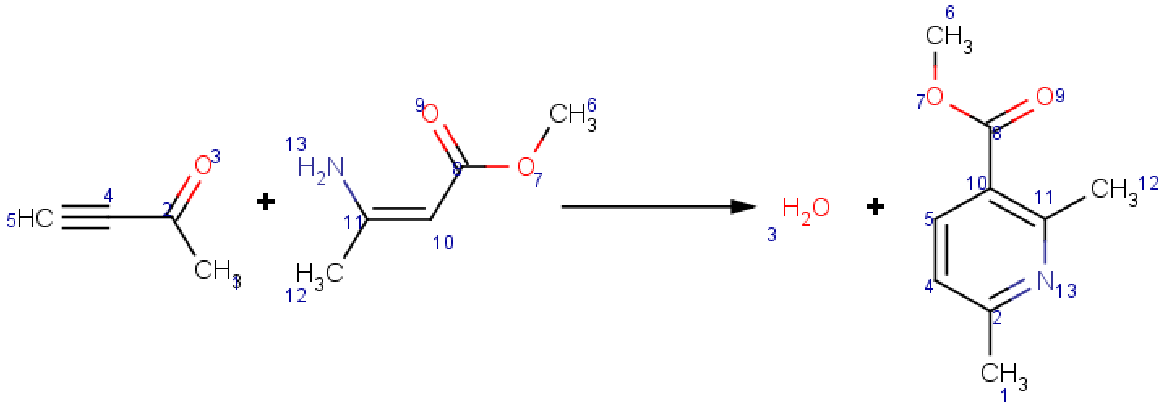

Correct mapped SMILES/SMARTS of the reaction:
[#6:1]-[#6:2](=[O:3])[C:4]#[C:5].[#6:6]-[#8:7]-
[#6:8](=[O:9])\[#6:10]=[#6:11](\[#6:12])-[#7:13]>>[#8:3].[#6:6]-[#8:7]-
[#6:8](=[O:9])-[#6:10]-1=[#6:11](-[#6:12])-[#7:13]=[#6:2](-[#6:1])-
[#6:4]=[#6:5]-1

Correctness of the mapping

MAPPET	YES
ReactionMap	NO
Marvin	YES

Reaction no 100

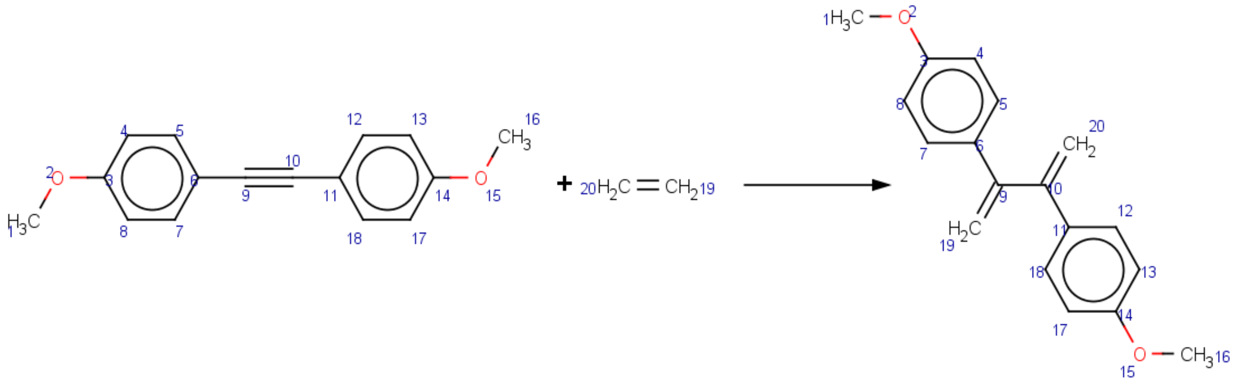

Correct mapped SMILES/SMARTS of the reaction:
[#6:1]-[#8:2]-
[c:3]1[c:4][c:5][c:6]([c:7][c:8]1)[C:9]#[C:10][c:11]1[c:12][c:13][c:14](-
[#8:15]-[#6:16])[c:17][c:18]1.[#6:19]=[#6:20]>>[#6:1]-[#8:2]-
[c:3]1[c:4][c:5][c:6]([c:7][c:8]1)-[#6:9](=[#6:19])-[#6:10](=[#6:20])-
[c:11]1[c:12][c:13][c:14](-[#8:15]-[#6:16])[c:17][c:18]1

```
Correctness of the mapping
MAPPET              YES
ReactionMap         YES
Marvin              NO
```

Reaction no 101

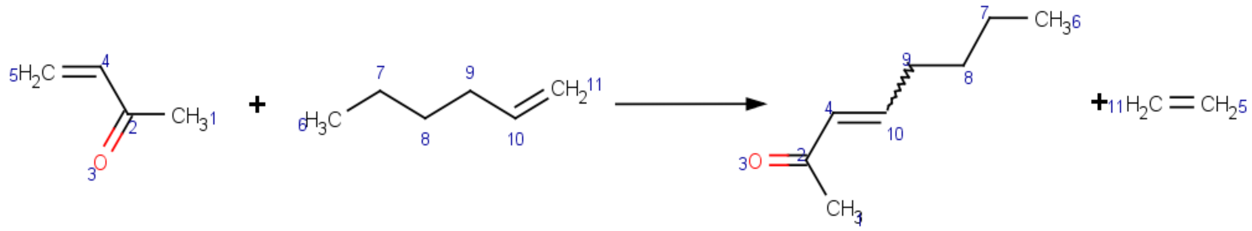

Correct mapped SMILES/SMARTS of the reaction:
[#6:1]-[#6:2](=[O:3])-[#6:4]=[#6:5].[#6:6]-[#6:7]-[#6:8]-[#6:9]-[#6:10]=[#6:11]>>[#6:6]-[#6:7]-[#6:8]-[#6:9]-[#6:10]=[#6:4]-[#6:2](-[#6:1])=[O:3].[#6:5]=[#6:11]

```
Correctness of the mapping
MAPPET              YES
ReactionMap         NO
Marvin              NO
```

Reaction no 102

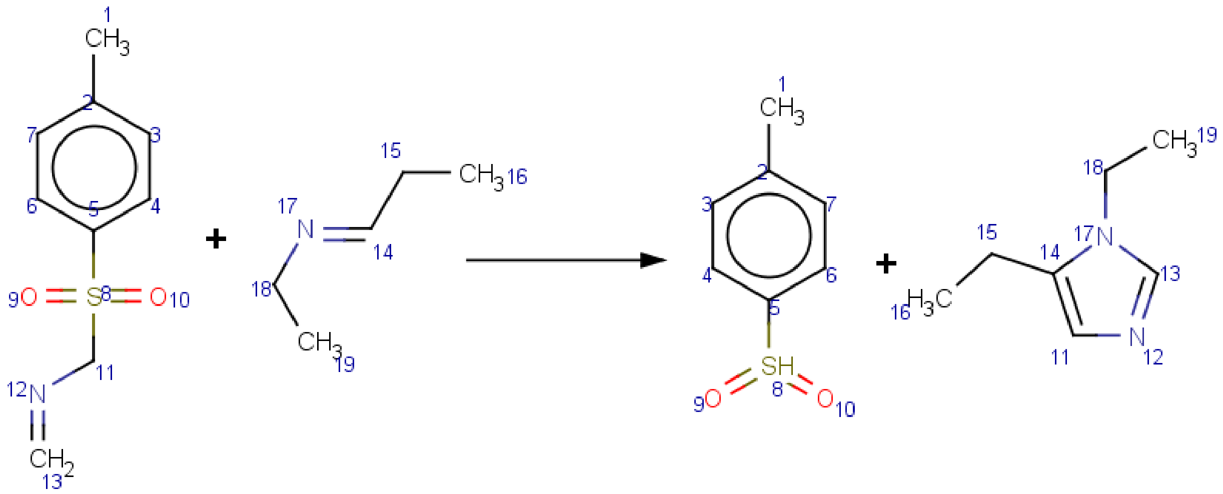

Correct mapped SMILES/SMARTS of the reaction:
[#6:1]-[c:2]1[c:3][c:4][c:5]([c:6][c:7]1)[S:8](=[O:9])(=[O:10])[#6:11]-[#7:12]=[#6:13].[#6:16]-[#6:15]\[#6:14]=[#7:17]\[#6:18]-[#6:19]>>[#6:1]-[c:2]1[c:7][c:6][c:5]([c:4][c:3]1)[S:8](=[O:10])=[O:9].[#6:19]-[#6:18]-[#7:17]-1-[#6:13]=[#7:12]-[#6:11]=[#6:14]-1-[#6:15]-[#6:16]

```
Correctness of the mapping
MAPPET              YES
ReactionMap         YES
Marvin              YES
```

Reaction no 103

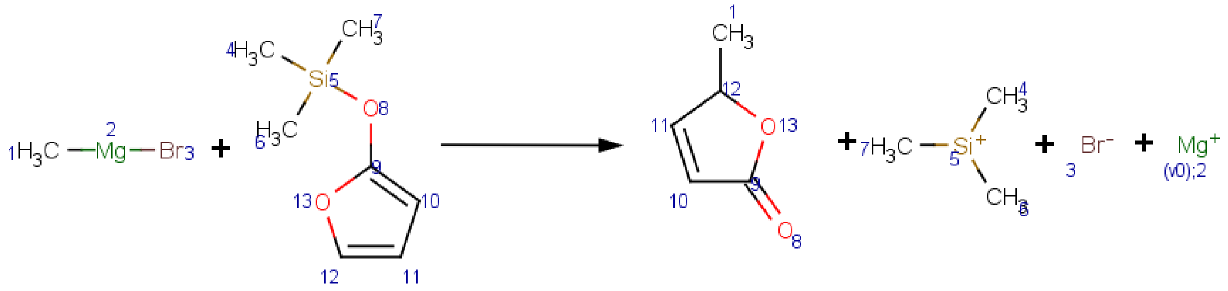

Correct mapped SMILES/SMARTS of the reaction:
[#6:1][Mg:2][Br:3].[#6:4][Si:5]([#6:6])([#6:7])[#8:8]-[#6:9]-1=[#6:10]-
[#6:11]=[#6:12]-[#8:13]-1>>[#6:1]-[#6:12]-1-[#8:13]-[#6:9](=[O:8])-
[#6:10]=[#6:11]-1.[#6:4]-[#14+:5](-[#6:6])-[#6:7].[Br-:3].[Mg;v0+:2]

Correctness of the mapping

MAPPET	YES
ReactionMap	YES
Marvin	NO

Reaction no 104

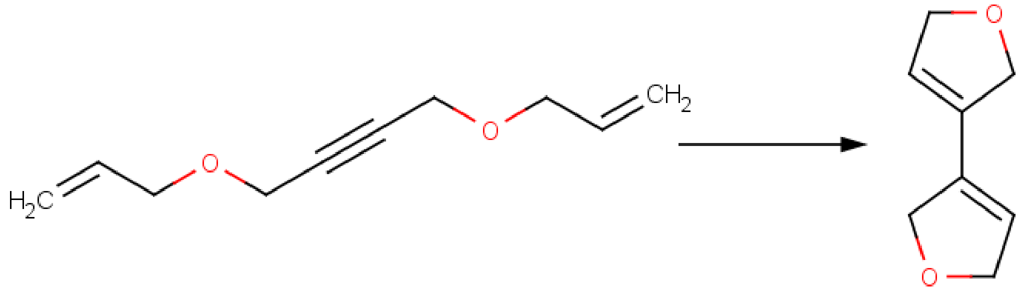

SMILES of the input:
C=CCOCC#CCOCC=C>>C1OCC(=C1)C1=CCOC1

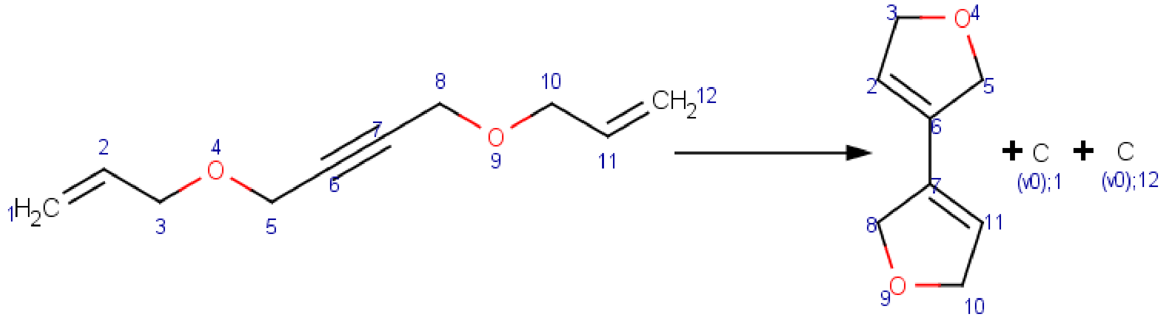

Correct mapped SMILES/SMARTS of the reaction:
[#6:1]=[#6:2]-[#6:3]-[#8:4]-[#6:5][C:6]#[C:7][#6:8]-[#8:9]-[#6:10]-
[#6:11]=[#6:12]>>[#6:10]-1-[#8:9]-[#6:8]-[#6:7](=[#6:11]-1)-[#6:6]-1=[#6:2]-
[#6:3]-[#8:4]-[#6:5]-1.[#6;v0:1].[#6;v0:12]

```
Correctness of the mapping
MAPPET              YES
ReactionMap         NO
Marvin              YES

Reaction no  105
```

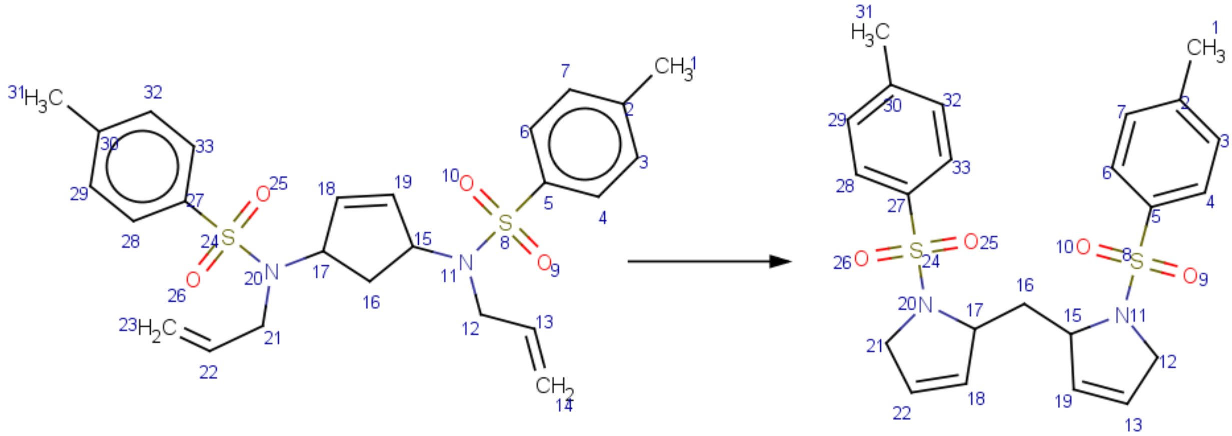

```
Correct mapped SMILES/SMARTS of the reaction:
[#6:1]-[c:2]1[c:3][c:4][c:5]([c:6][c:7]1)[S:8](=[O:9])(=[O:10])[#7:11](-[#6:12]-
[#6:13]=[#6:14])-[#6:15]-1-[#6:16]-[#6:17](-[#6:18]=[#6:19]-1)-[#7:20](-[#6:21]-
[#6:22]=[#6:23])[S:24](=[O:25])(=[O:26])[c:27]1[c:28][c:29][c:30](-
[#6:31])[c:32][c:33]1>>[#6:1]-[#6:2]-1=[#6:3]-[#6:4]=[#6:5](-[#6:6]=[#6:7]-
1)[S:8](=[O:10])(=[O:9])[#7:11]-1-[#6:12]-[#6:13]=[#6:19]-[#6:15]-1-[#6:16]-
[#6:17]-1-[#6:18]=[#6:22]-[#6:21]-[#7:20]-1[S:24](=[O:25])(=[O:26])[#6:27]-
1=[#6:28]-[#6:29]=[#6:30](-[#6:31])-[#6:32]=[#6:33]-1

Correctness of the mapping
MAPPET              NO
ReactionMap         NO
Marvin              YES

Reaction no  106
```

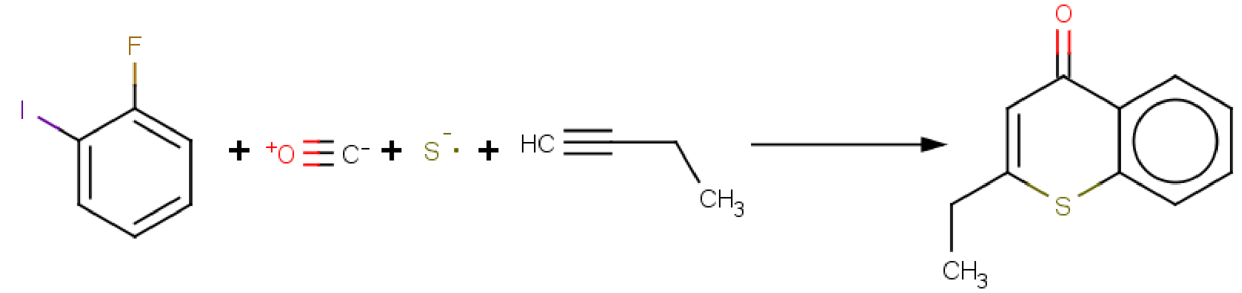

```
SMILES of the input:
FC1=CC=CC=C1I.[C-]#[O+].[S-].CCC#C>>CCC1=CC(=O)c2ccccc2S1
```

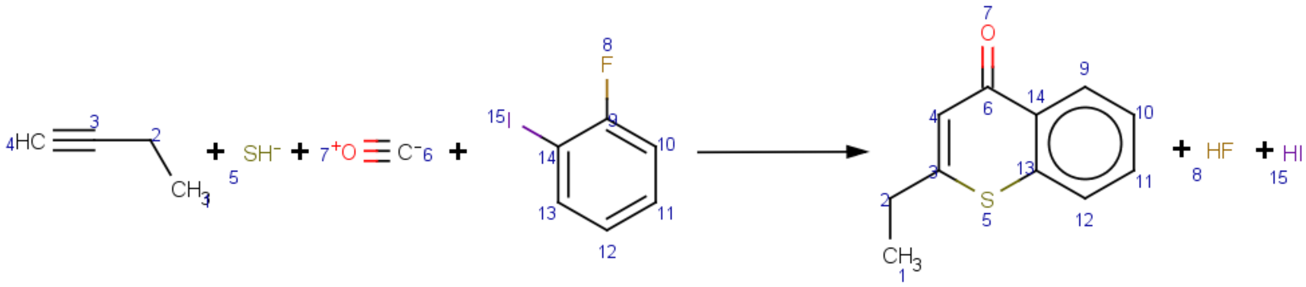

Correct mapped SMILES/SMARTS of the reaction:
[#6:1]-[#6:2][C:3]#[C:4].[#16-:5].[C-:6]#[O+:7].[F:8][#6:9]-1=[#6:10]-[#6:11]=[#6:12]-[#6:13]=[#6:14]-1[I:15]>>[#6:1]-[#6:2]-[#6:3]-1=[#6:4]-[#6:6](=[O:7])-[c:14]2[c:9][c:10][c:11][c:12][c:13]2-[#16:5]-1.[F:8].[I:15]

Correctness of the mapping
MAPPET YES
ReactionMap NO
Marvin YES

Reaction no 107

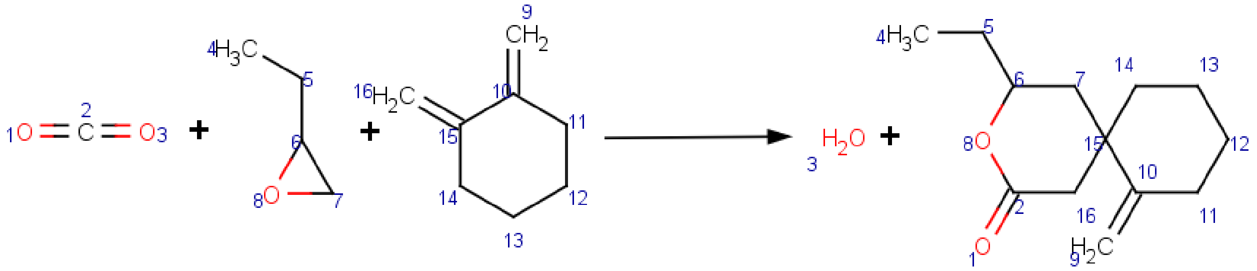

Correct mapped SMILES/SMARTS of the reaction:
[O:1]=[C:2]=[O:3].[#6:4]-[#6:5]-[#6:6]-1-[#6:7]-[#8:8]-1.[#6:9]=[#6:10]-1-[#6:11]-[#6:12]-[#6:13]-[#6:14]-[#6:15]-1=[#6:16]>>[#8:3].[#6:4]-[#6:5]-[#6:6]-1-[#6:7][C:15]2([#6:14]-[#6:13]-[#6:12]-[#6:11]-[#6:10]2=[#6:9])[#6:16]-[#6:2](=[O:1])-[#8:8]-1

Correctness of the mapping
MAPPET YES
ReactionMap NO
Marvin YES

Reaction no 108

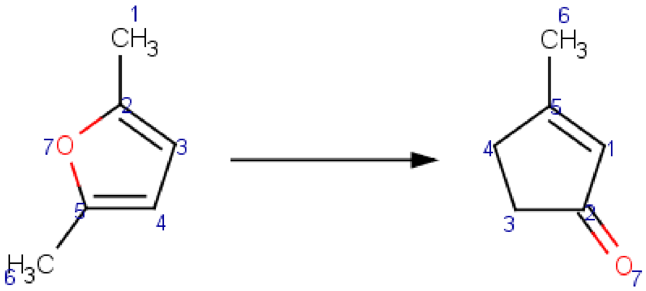

Correct mapped SMILES/SMARTS of the reaction:
[#6:1]-[#6:2]-1=[#6:3]-[#6:4]=[#6:5](-[#6:6])-[#8:7]-1>>[#6:6]-[#6:5]-1=[#6:1]-[#6:2](=[O:7])-[#6:3]-[#6:4]-1

Correctness of the mapping
MAPPET YES
ReactionMap YES
Marvin NO

Reaction no 109

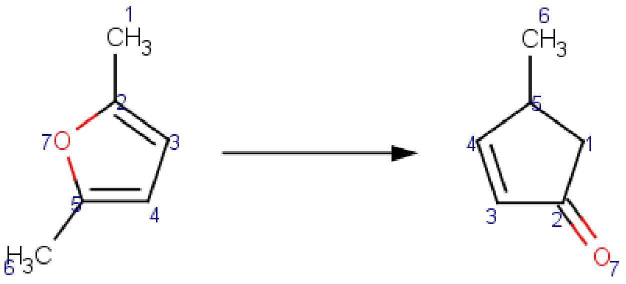

Correct mapped SMILES/SMARTS of the reaction:
[#6:1]-[#6:2]-1=[#6:3]-[#6:4]=[#6:5](-[#6:6])-[#8:7]-1>>[#6:6]-[#6:5]-1-[#6:1]-[#6:2](=[O:7])-[#6:3]=[#6:4]-1

Correctness of the mapping
MAPPET   YES
ReactionMap   YES
Marvin   YES

Reaction no 110

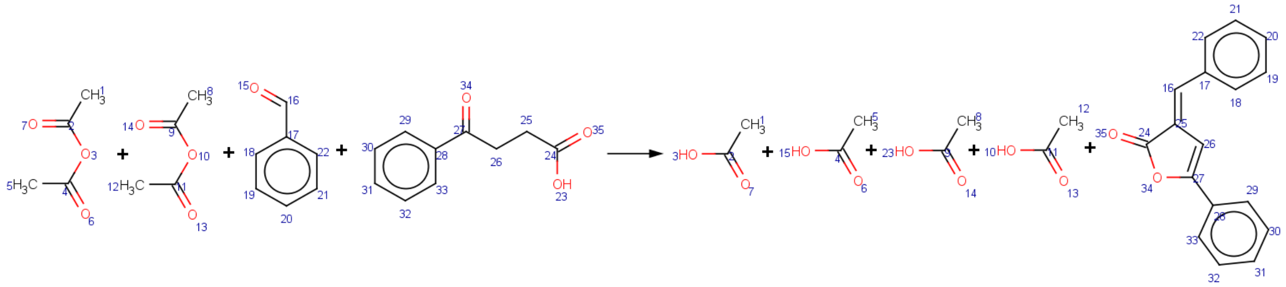

Correct mapped SMILES/SMARTS of the reaction:
[CH3:1][C:2](=[O:7])[O:3][C:4](=[O:6])[CH3:5].[CH3:8][C:9](=[O:14])[O:10][C:11](=[O:13])[CH3:12].[cH:20]1[cH:19][cH:18][c:17]([cH:22][cH:21]1)[CH:16]=[O:15].[cH:31]1[cH:30][cH:29][c:28]([cH:33][cH:32]1)[C:27](=[O:34])[CH2:26][CH2:25][C:24](=[O:35])[OH:23]>>[CH3:1][C:2](=[O:7])[OH:3].[CH3:5][C:4](=[O:6])[OH:15].[CH3:8][C:9](=[O:14])[OH:23].[CH3:12][C:11](=[O:13])[OH:10].[cH:20]1[cH:19][cH:18][c:17]([cH:22][cH:21]1)/[CH:16]=[C:25]/2[CH:26]=[C:27]([O:34][C:24]2=[O:35])[c:28]3[cH:29][cH:30][cH:31][cH:32][cH:33]3

Correctness of the mapping
MAPPET   NO
ReactionMap   NO
Marvin   NO

Reaction no 111

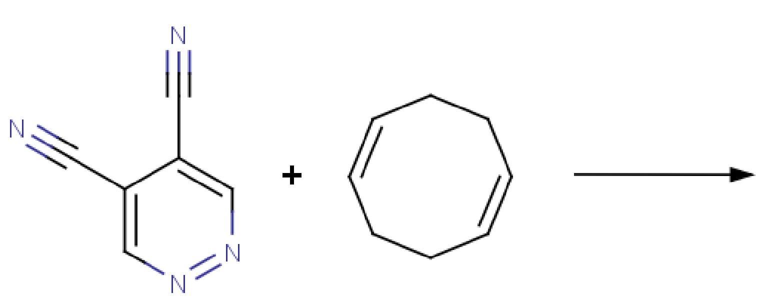

SMILES of the input:
N#CC1=CN=NC=C1C#N.C1C\C=C/CC\C=C/1>>N#CC1=C(C#N)C2C3CCC2C2CCC3C12

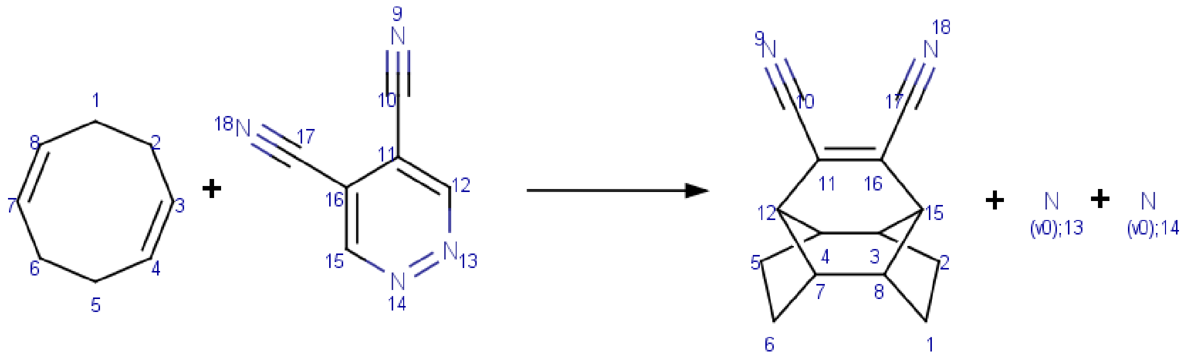

Correct mapped SMILES/SMARTS of the reaction:
[#6:1]-1-[#6:2]\[#6:3]=[#6:4]/[#6:5]-[#6:6]\[#6:7]=[#6:8]/1.[N:9]#[C:10][#6:11]-1=[#6:12]-[#7:13]=[#7:14]-[#6:15]=[#6:16]-1[C:17]#[N:18]>>[N:9]#[C:10][#6:11]-1=[#6:16]([C:17]#[N:18])-[#6:15]-2-[#6:3]-3-[#6:2]-[#6:1]-[#6:8]-2-[#6:7]-2-[#6:6]-[#6:5]-[#6:4]-3-[#6:12]-1-2.[#7;v0:13].[#7;v0:14]

Correctness of the mapping
MAPPET YES
ReactionMap NO
Marvin YES

Reaction no 112

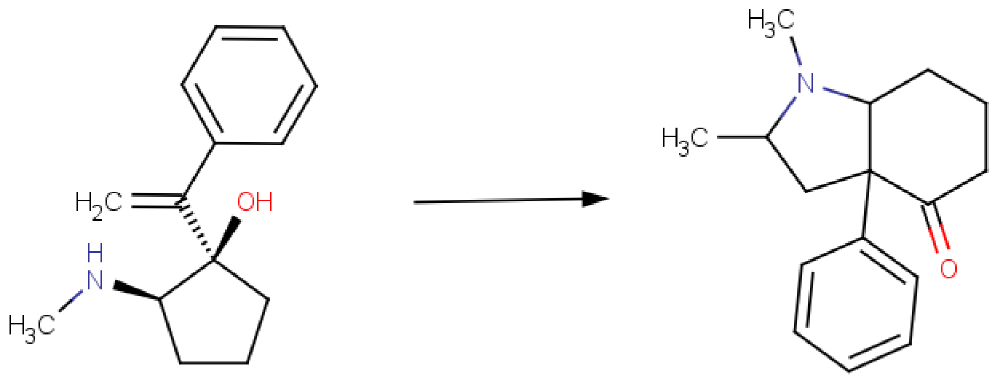

SMILES of the input:
N(C)[C@@H]1CCC[C@@]1(O)C(=C)C1=CC=CC=C1>>CC1CC2(C(CCCC2=O)N1C)C1=CC=CC=C1

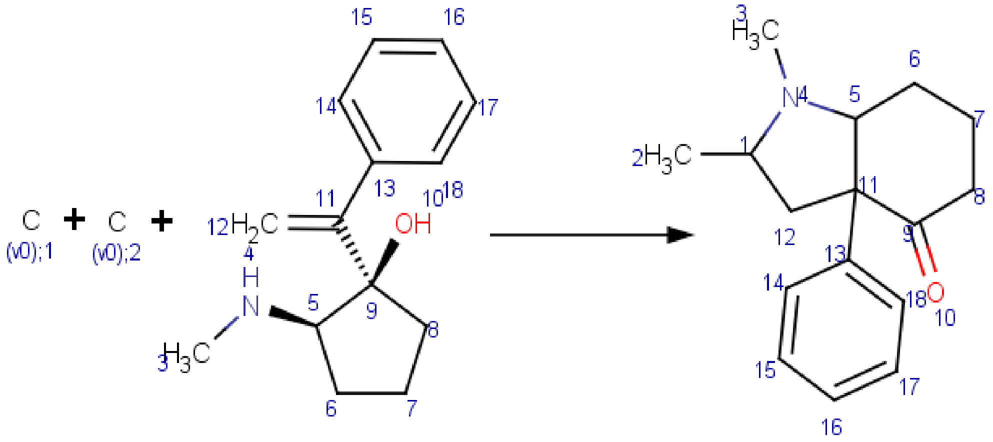

Correct mapped SMILES/SMARTS of the reaction:
[#6;v0:1].[#6;v0:2].[#6:3]-[#7:4]-[#6@@H:5]1-[#6:6]-[#6:7]-
[#6:8][C@@:9]1([#8:10])[#6:11](=[#6:12])-[#6:13]-1=[#6:14]-[#6:15]=[#6:16]-
[#6:17]=[#6:18]-1>>[#6:2]-[#6:1]-1-[#6:12][C:11]2([#6:5](-[#6:6]-[#6:7]-[#6:8]-
[#6:9]2=[O:10])-[#7:4]-1-[#6:3])[#6:13]-1=[#6:18]-[#6:17]=[#6:16]-
[#6:15]=[#6:14]-1

Correctness of the mapping

MAPPET	YES
ReactionMap	NO
Marvin	YES

Reaction no 113

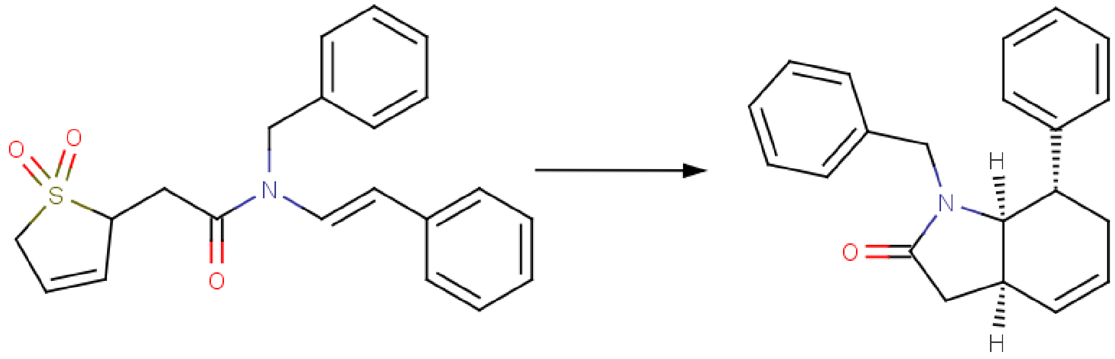

SMILES of the input:
O=C(CC1C=CCS1(=O)=O)N(CC1=CC=CC=C1)\C=C\C1=CC=CC=C1>>[H][C@]12CC(=O)N(CC3=CC=CC=C3)[C@@]1([H])[C@@H](CC=C2)C1=CC=CC=C1

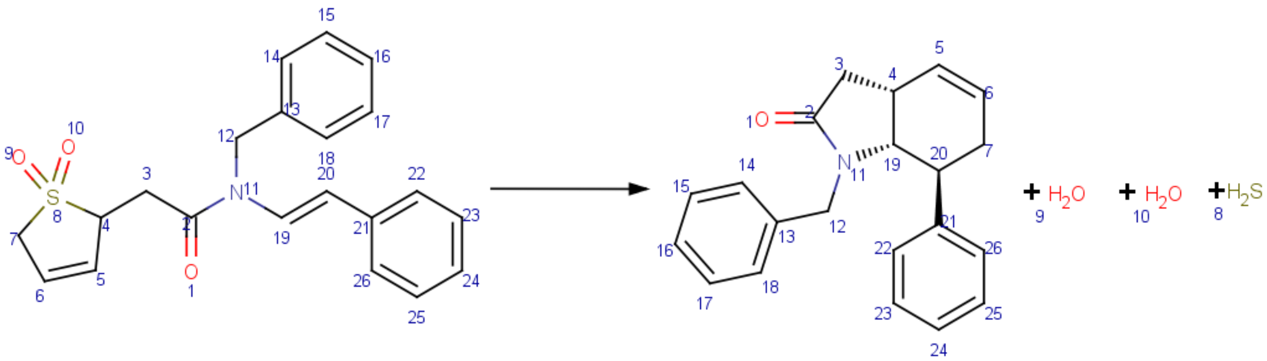

Correct mapped SMILES/SMARTS of the reaction:
[O:1]=[#6:2](-[#6:3]-[#6:4]1-[#6:5]=[#6:6]-[#6:7][S:8]1(=[O:9])=[O:10])-
[#7:11](-[#6:12]-[#6:13]-1=[#6:14]-[#6:15]=[#6:16]-[#6:17]=[#6:18]-

1)\[#6:19]=[#6:20]\[#6:21]-1=[#6:22]-[#6:23]=[#6:24]-[#6:25]=[#6:26]1>>[O:1]=[#6:2]-1-[#6:3]-[#6@@H:4]-2-[#6:5]=[#6:6]-[#6:7]-[#6@H:20](-[#6@@H:19]-2-[#7:11]-1-[#6:12]-[#6:13]-1=[#6:18]-[#6:17]=[#6:16]-[#6:15]=[#6:14]-1)-[#6:21]-1=[#6:26]-[#6:25]=[#6:24]-[#6:23]=[#6:22]-1.[#8:9].[#8:10].[#16:8]

Correctness of the mapping

MAPPET	YES
ReactionMap	NO
Marvin	YES

Reaction no 114

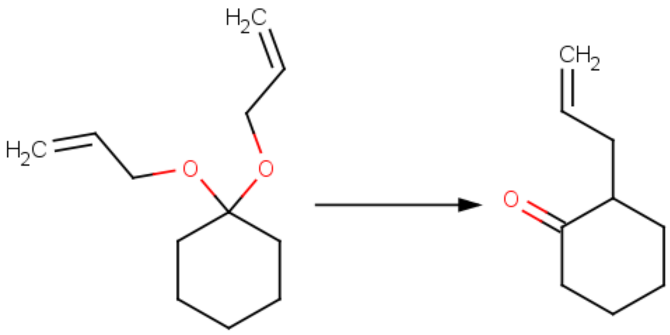

SMILES of the input:
C=CCOC1(CCCCC1)OCC=C>>C=CCC1CCCCC1=O

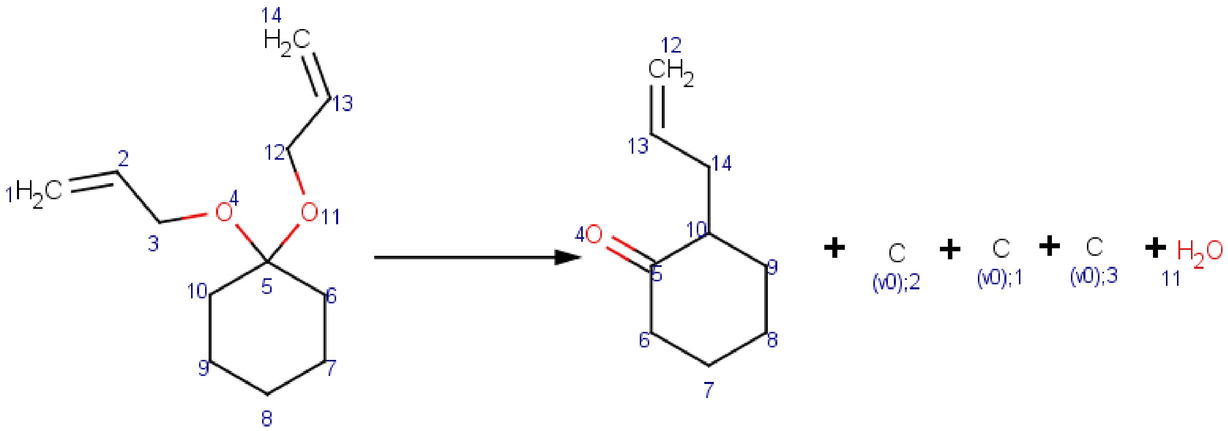

Correct mapped SMILES/SMARTS of the reaction:
[#6:1]=[#6:2]-[#6:3]-[#8:4][C:5]1([#6:6]-[#6:7]-[#6:8]-[#6:9]-[#6:10]1)[#8:11]-[#6:12]-[#6:13]=[#6:14]>>[#6:12]=[#6:13]-[#6:14]-[#6:10]-1-[#6:9]-[#6:8]-[#6:7]-[#6:6]-[#6:5]-1=[O:4].[#6;v0:2].[#6;v0:1].[#6;v0:3].[#8:11]

Correctness of the mapping

MAPPET	YES
ReactionMap	NO
Marvin	NO

Reaction no 115

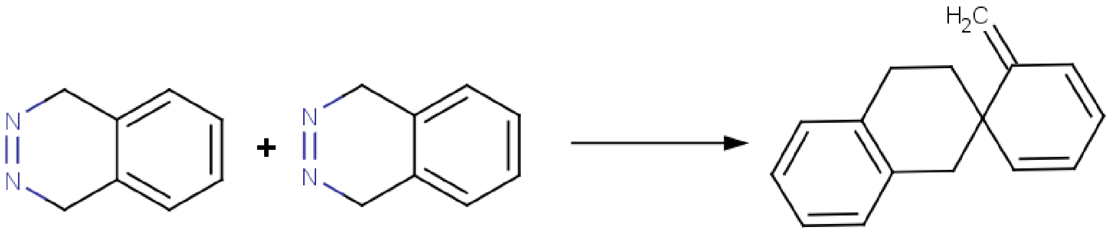

SMILES of the input:
C1N=NCC2=CC=CC=C12.C1N=NCC2=CC=CC=C12>>C=C1C=CC=CC11CCC2=C(C1)C=CC=C2

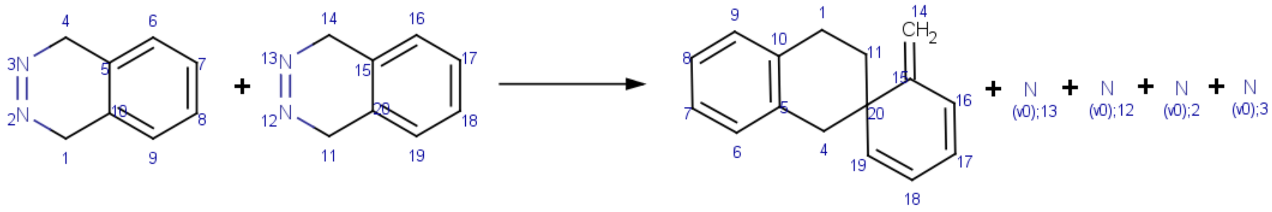

Correct mapped SMILES/SMARTS of the reaction:
[#6:1]-1-[#7:2]=[#7:3]-[#6:4]-[#6:5]-2=[#6:6]-[#6:7]=[#6:8]-[#6:9]=[#6:10]-1-2.[#6:11]-1-[#7:12]=[#7:13]-[#6:14]-[#6:15]-2=[#6:16]-[#6:17]=[#6:18]-[#6:19]=[#6:20]-1-2>>[#6:14]=[#6:15]1-[#6:16]=[#6:17]-[#6:18]=[#6:19][C:20]11[#6:11]-[#6:1]-[#6:10]-2=[#6:5](-[#6:4]1)-[#6:6]=[#6:7]-[#6:8]=[#6:9]-2.[#7;v0:13].[#7;v0:12].[#7;v0:2].[#7;v0:3]

Correctness of the mapping
MAPPET YES
ReactionMap NO
Marvin YES

Reaction no 116

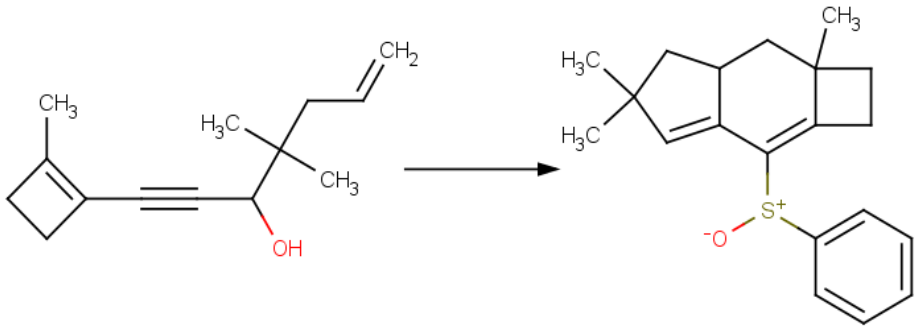

SMILES of the input:
CC1=C(CC1)C#CC(O)C(C)(C)CC=C>>CC12CCC1=C([S+]([O-])C1=CC=CC=C1)C1=CC(C)(C)CC1C2

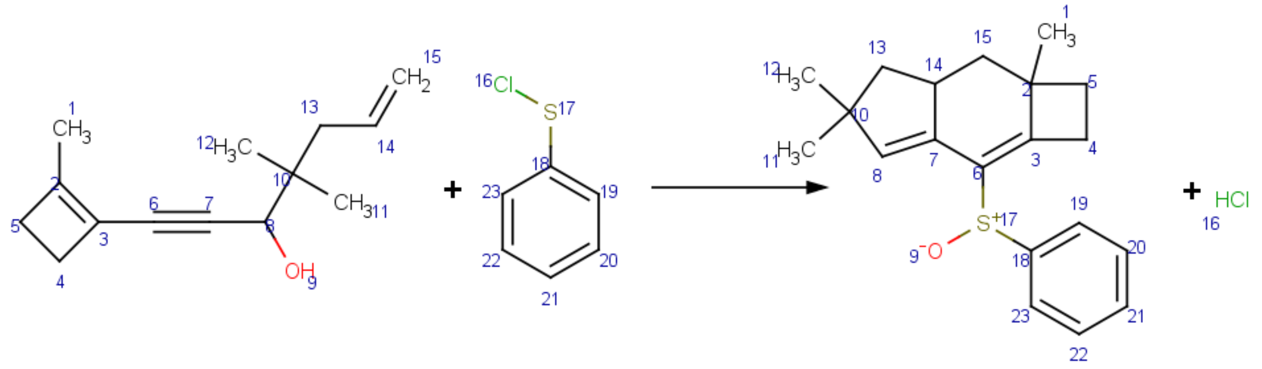

```
Correct mapped SMILES/SMARTS of the reaction:
[#6:1]-[#6:2]-1=[#6:3](-[#6:4]-[#6:5]-1)[C:6]#[C:7][#6:8](-
[#8:9])[C:10]([#6:11])([#6:12])[#6:13]-[#6:14]=[#6:15].[Cl:16][#16:17]-[#6:18]-
1=[#6:19]-[#6:20]=[#6:21]-[#6:22]=[#6:23]-1>>[#6:1][C:2]12[#6:5]-[#6:4]-
[#6:3]1=[#6:6]([S+:17]([#8-:9])[#6:18]-1=[#6:19]-[#6:20]=[#6:21]-
[#6:22]=[#6:23]-1)-[#6:7]-1=[#6:8][C:10]([#6:11])([#6:12])[#6:13]-[#6:14]-1-
[#6:15]2.[Cl:16]

Correctness of the mapping
MAPPET              YES
ReactionMap         NO
Marvin              NO
```

Supplementary Note S3. Test Set #1 of 401 reactions with human/correct mappings compared to those of our software ("MAPPET"), ReactionMap, MarvinJS, ChemDraw and Indigo ....pages 312-616

- **p.312 to p.384** – 100 simple reactions with full stoichiometry taken from total syntheses published in *Org. Lett.*, *J. Am. Chem. Soc.* and *J.Org. Chem.*
- **p.385 to p.451** – 100 randomly selected and mostly stoichiometrically unbalanced reactions from patents;
- **p.452 to p.616** 201 mechanistically complex reactions (both stoichiometrically balanced and unbalanced) which include rearrangements and multicomponent reactions taken from recent (in most cases published after 2010) literature: *Org. Lett.*, *J. Am. Chem. Soc.* and *J.Org. Chem.*

Reaction no 1

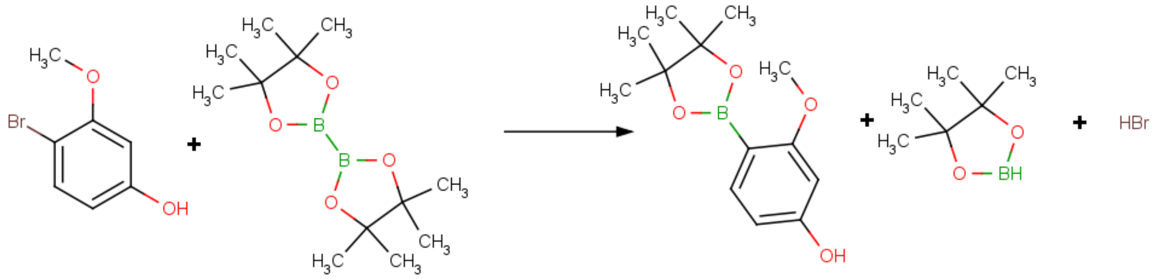

SMILES of the input:
COC1=CC(O)=CC=C1Br.CC1(C)OB(OC1(C)C)B1OC(C)(C)C(C)(C)O1>>COC1=CC(O)=CC=C1
B1OC(C)(C)C(C)(C)O1.CC1(C)OBOC1(C)C.Br

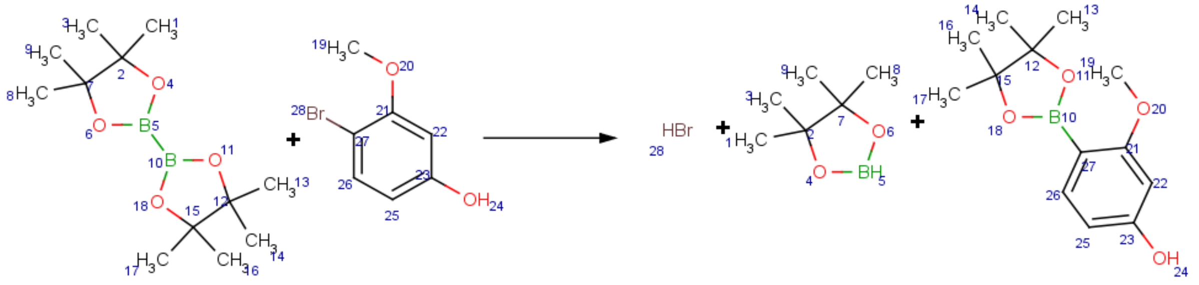

Correct mapped SMILES/SMARTS of the reaction:
[CH3:1][C:2]1([CH3:3])[O:4][B:5]([O:6][C:7]1([CH3:8])[CH3:9])[B:10]1[O:11
][C:12]([CH3:13])([CH3:14])[C:15]([CH3:16])([CH3:17])[O:18]1.[CH3:19][O:2
0][C:21]1=[CH:22][C:23]([OH:24])=[CH:25][CH:26]=[C:27]1[Br:28]>>[BrH:28].
[CH3:8][C:7]1([CH3:9])[O:6][BH:5][O:4][C:2]1([CH3:1])[CH3:3].[CH3:19][O:2
0][C:21]1=[CH:22][C:23]([OH:24])=[CH:25][CH:26]=[C:27]1[B:10]1[O:18][C:15
]([CH3:17])([CH3:16])[C:12]([CH3:14])([CH3:13])[O:11]1

Correctness of the mapping

MAPPET	YES
ReactionMap	NO
Marvin	YES
ChemDraw	YES
Indigo	YES

Reaction no 2

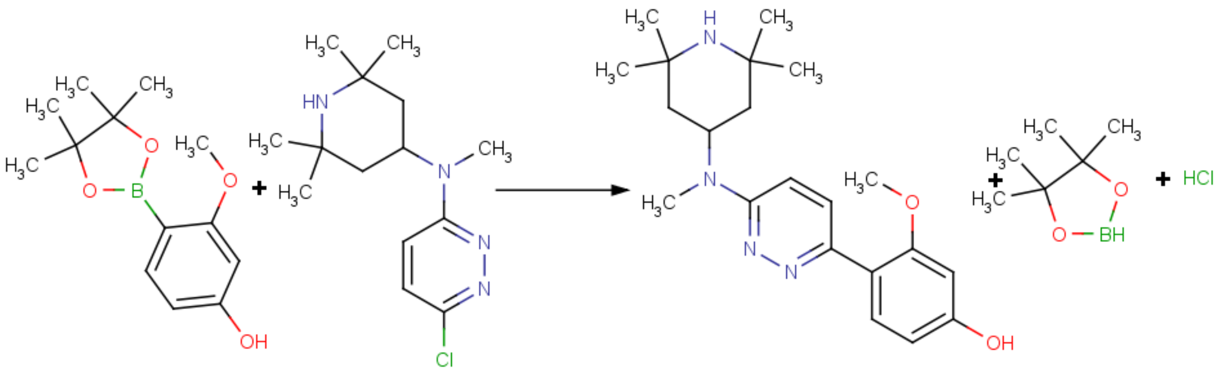

SMILES of the input:
COC1=CC(O)=CC=C1B1OC(C)(C)C(C)(C)O1.CN(C1CC(C)(C)NC(C)(C)C1)C1=NN=C(Cl)C=C1>>COC1=CC(O)=CC=C1C1=NN=C(C=C1)N(C)C1CC(C)(C)NC(C)(C)C1.CC1(C)OBOC1(C)C.Cl

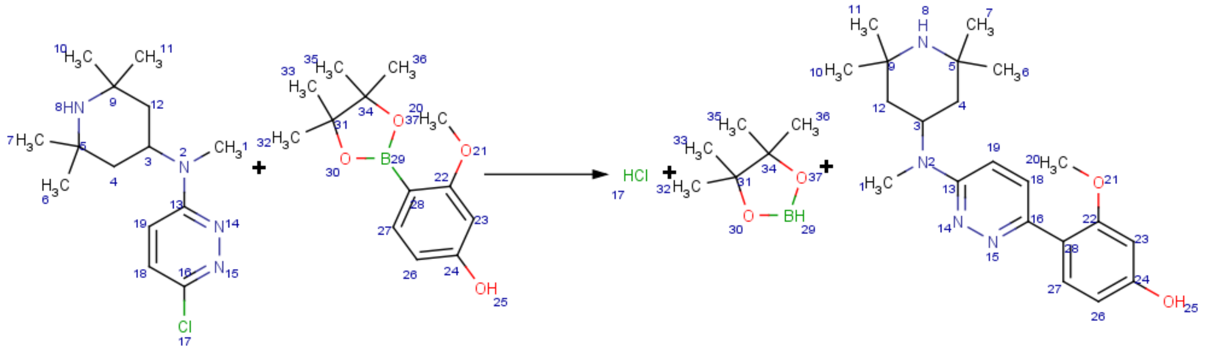

Correct mapped SMILES/SMARTS of the reaction:
[CH3:1][N:2]([CH:3]1[CH2:4][C:5]([CH3:6])([CH3:7])[NH:8][C:9]([CH3:10])([CH3:11])[CH2:12]1)[C:13]1=[N:14][N:15]=[C:16]([Cl:17])[CH:18]=[CH:19]1.[CH3:20][O:21][C:22]1=[CH:23][C:24]([OH:25])=[CH:26][CH:27]=[C:28]1[B:29]1[O:30][C:31]([CH3:32])([CH3:33])[C:34]([CH3:35])([CH3:36])[O:37]1>>[ClH:17].[CH3:36][C:34]1([CH3:35])[O:37][BH:29][O:30][C:31]1([CH3:32])[CH3:33].[CH3:20][O:21][C:22]1=[CH:23][C:24]([OH:25])=[CH:26][CH:27]=[C:28]1[C:16]1=[N:15][N:14]=[C:13]([CH:19]=[CH:18]1)[N:2]([CH3:1])[CH:3]1[CH2:12][C:9]([CH3:10])([CH3:11])[NH:8][C:5]([CH3:7])([CH3:6])[CH2:4]1

Correctness of the mapping

MAPPET	YES
ReactionMap	YES
Marvin	YES
ChemDraw	YES
Indigo	YES

Reaction no 3

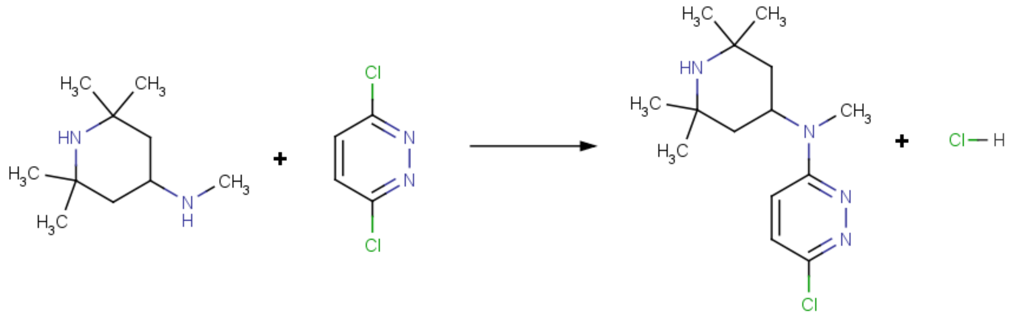

SMILES of the input:
CNC1CC(C)(C)NC(C)(C)C1.ClC1=NN=C(Cl)C=C1>>CN(C1CC(C)(C)NC(C)(C)C1)C1=NN=C(Cl)C=C1.Cl[H]

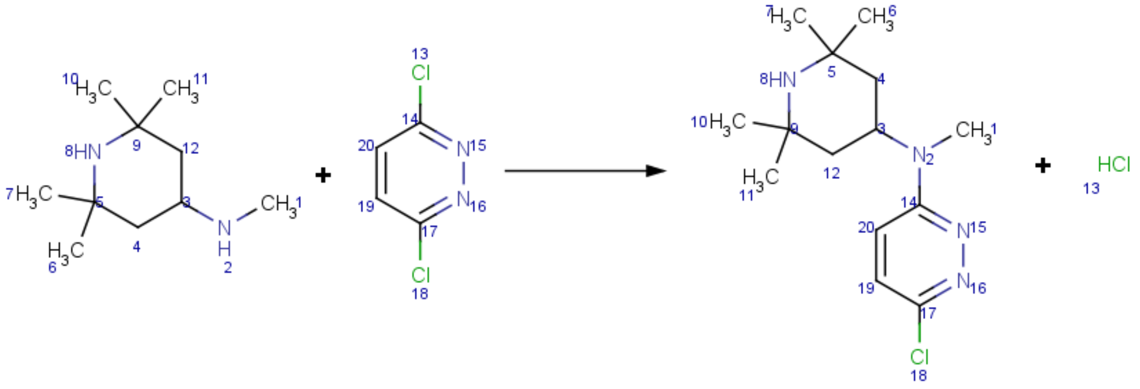

Correct mapped SMILES/SMARTS of the reaction:
[CH3:1][NH:2][CH:3]1[CH2:4][C:5]([CH3:6])([CH3:7])[NH:8][C:9]([CH3:10])([CH3:11])[CH2:12]1.[Cl:13][C:14]1=[N:15][N:16]=[C:17]([Cl:18])[CH:19]=[CH:20]1>>[CH3:1][N:2]([CH:3]1[CH2:12][C:9]([CH3:11])([CH3:10])[NH:8][C:5]([CH3:7])([CH3:6])[CH2:4]1)[C:14]1=[N:15][N:16]=[C:17]([Cl:18])[CH:19]=[CH:20]1.[ClH:13]

Correctness of the mapping

MAPPET	YES
ReactionMap	YES
Marvin	YES
ChemDraw	YES
Indigo	YES

Reaction no 4

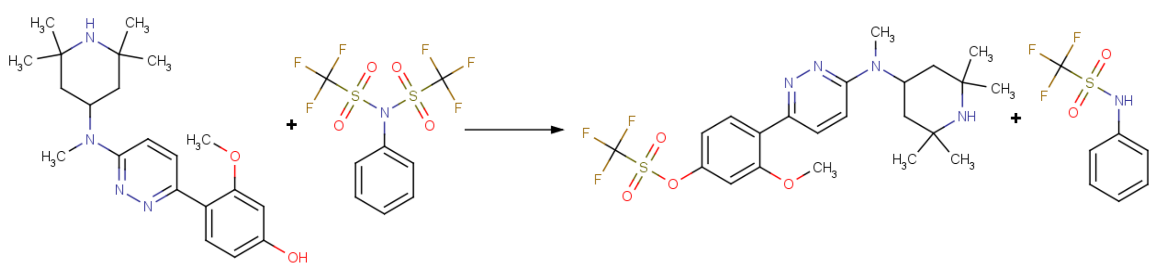

SMILES of the input:
COC1=CC(O)=CC=C1C1=NN=C(C=C1)N(C)C1CC(C)(C)NC(C)(C)C1.FC(F)(F)S(=O)(=O)N(C1=CC=CC=C1)S(=O)(=O)C(F)(F)F>>COC1=CC(OS(=O)(=O)C(F)(F)F)=CC=C1C1=NN=C(C=C1)N(C)C1CC(C)(C)NC(C)(C)C1.FC(F)(F)S(=O)(=O)NC1=CC=CC=C1

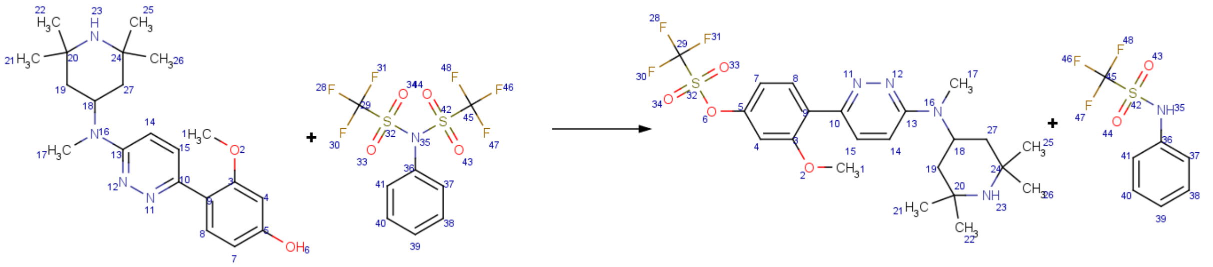

Correct mapped SMILES/SMARTS of the reaction:
[CH3:1][O:2][C:3]1=[CH:4][C:5]([OH:6])=[CH:7][CH:8]=[C:9]1[C:10]1=[N:11][
N:12]=[C:13]([CH:14]=[CH:15]1)[N:16]([CH3:17])[CH:18]1[CH2:19][C:20]([CH3
:21])([CH3:22])[NH:23][C:24]([CH3:25])([CH3:26])[CH2:27]1.[F:28][C:29]([F
:30])([F:31])[S:32](=[O:33])(=[O:34])[N:35]([C:36]1=[CH:37][CH:38]=[CH:39
][CH:40]=[CH:41]1)[S:42](=[O:43])(=[O:44])[C:45]([F:46])([F:47])[F:48]>>[
CH3:1][O:2][C:3]1=[CH:4][C:5]([O:6][S:32](=[O:34])(=[O:33])[C:29]([F:28])
([F:30])[F:31])=[CH:7][CH:8]=[C:9]1[C:10]1=[N:11][N:12]=[C:13]([CH:14]=[C
H:15]1)[N:16]([CH3:17])[CH:18]1[CH2:27][C:24]([CH3:25])([CH3:26])[NH:23][
C:20]([CH3:22])([CH3:21])[CH2:19]1.[F:46][C:45]([F:47])([F:48])[S:42](=[O
:44])(=[O:43])[NH:35][C:36]1=[CH:37][CH:38]=[CH:39][CH:40]=[CH:41]1

Correctness of the mapping

MAPPET	YES
ReactionMap	YES
Marvin	YES
ChemDraw	YES
Indigo	NO

Reaction no 5

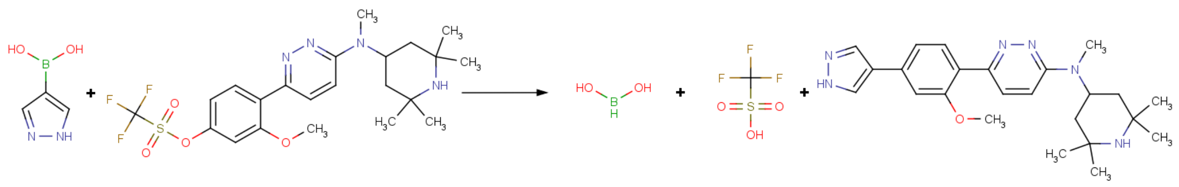

SMILES of the input:
OB(O)C1=CNN=C1.COC1=CC(OS(=O)(=O)C(F)(F)F)=CC=C1C1=NN=C(C=C1)N(C)C1CC(C)(
C)NC(C)(C)C1>>OBO.OS(=O)(=O)C(F)(F)F.COC1=CC(=CC=C1C1=NN=C(C=C1)N(C)C1CC(
C)(C)NC(C)(C)C1)C1=CNN=C1

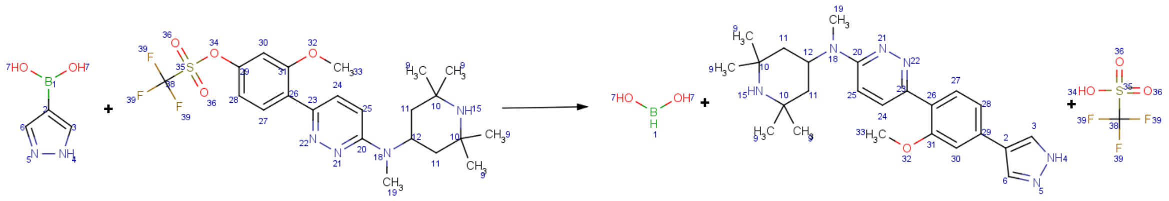

Correct mapped SMILES/SMARTS of the reaction:
[OH:1][B:2]([OH:3])[C:4]1=[CH:5][NH:6][N:7]=[CH:8]1.[CH3:9][O:10][C:11]1=
[CH:12][C:13]([O:14][S:15](=[O:16])(=[O:17])[C:18]([F:19])([F:20])[F:21])
=[CH:22][CH:23]=[C:24]1[C:25]1=[N:26][N:27]=[C:28]([CH:29]=[CH:30]1)[N:31
]([CH3:32])[CH:33]1[CH2:34][C:35]([CH3:36])([CH3:37])[NH:38][C:39]([CH3:4
0])([CH3:41])[CH2:42]1>>[CH3:9][O:10][C:11]1=[CH:12][C:13](=[CH:22][CH:23
]=[C:24]1[C:25]1=[N:26][N:27]=[C:28]([CH:29]=[CH:30]1)[N:31]([CH3:32])[CH
:33]1[CH2:42][C:39]([CH3:40])([CH3:41])[NH:38][C:35]([CH3:37])([CH3:36])[

CH2:34]1)[C:4]1=[CH:5][NH:6][N:7]=[CH:8]1.[B:2].[C:18].[F:21].[F:19].[F:2
0].[O:17].[O:16].[O:1].[O:3].[O:14].[S:15]

Correctness of the mapping

MAPPET	YES
ReactionMap	YES
Marvin	YES
ChemDraw	YES
Indigo	YES

Reaction no 6

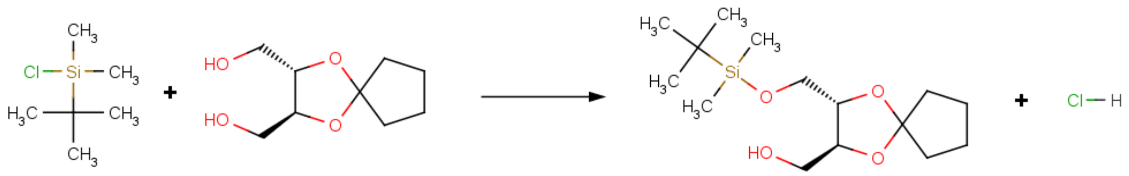

SMILES of the input:
CC(C)(C)[Si](C)(C)Cl.OC[C@@H]1OC2(CCCC2)O[C@H]1CO>>CC(C)(C)[Si](C)(C)OC[C
@@H]1OC2(CCCC2)O[C@H]1CO.Cl[H]

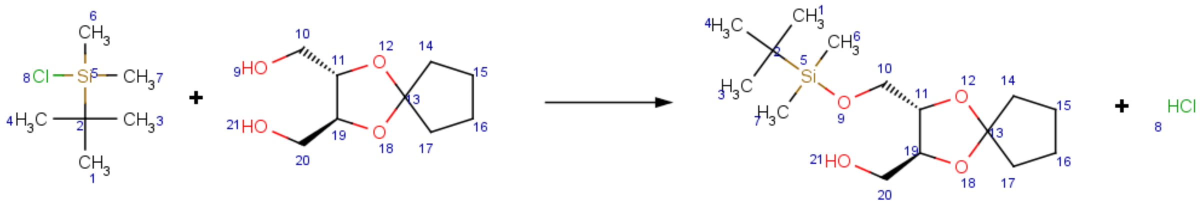

Correct mapped SMILES/SMARTS of the reaction:
[CH3:1][C:2]([CH3:3])([CH3:4])[Si:5]([CH3:6])([CH3:7])[Cl:8].[OH:9][CH2:1
0][C@@H:11]1[O:12][C:13]2([CH2:14][CH2:15][CH2:16][CH2:17]2)[O:18][C@H:19
]1[CH2:20][OH:21]>>[CH3:4][C:2]([CH3:3])([CH3:1])[Si:5]([CH3:7])([CH3:6])
[O:9][CH2:10][C@@H:11]1[O:12][C:13]2([CH2:14][CH2:15][CH2:16][CH2:17]2)[O
:18][C@H:19]1[CH2:20][OH:21].[ClH:8]

Correctness of the mapping

MAPPET	YES
ReactionMap	YES
Marvin	YES
ChemDraw	YES
Indigo	YES

Reaction no 7

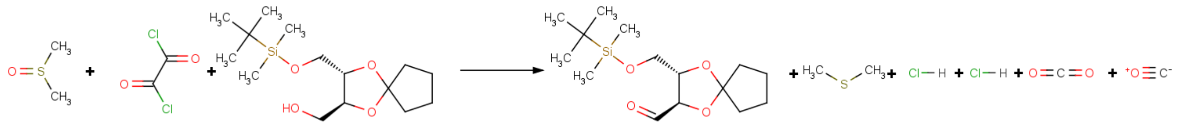

SMILES of the input:
CS(C)=O.ClC(=O)C(Cl)=O.CC(C)(C)[Si](C)(C)OC[C@@H]1OC2(CCCC2)O[C@H]1CO>>CC
(C)(C)[Si](C)(C)OC[C@@H]1OC2(CCCC2)O[C@H]1C=O.CSC.Cl[H].Cl[H].O=C=O.[C-
]#[O+]

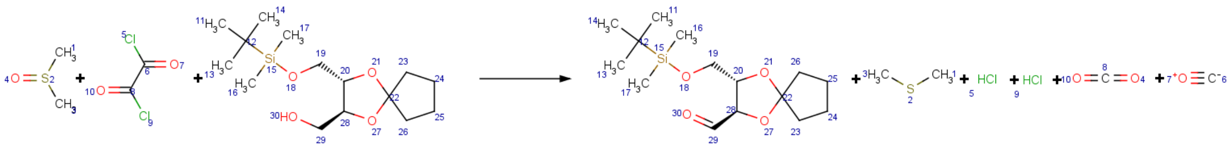

Correct mapped SMILES/SMARTS of the reaction:
[CH3:1][S:2]([CH3:3])=[O:4].[Cl:5][C:6](=[O:7])[C:8]([Cl:9])=[O:10].[CH3:11][C:12]([CH3:13])([CH3:14])[Si:15]([CH3:16])([CH3:17])[O:18][CH2:19][C@@H:20]1[O:21][C:22]2([CH2:23][CH2:24][CH2:25][CH2:26]2)[O:27][C@H:28]1[CH2:29][OH:30]>>[CH3:14][C:12]([CH3:13])([CH3:11])[Si:15]([CH3:17])([CH3:16])[O:18][CH2:19][C@@H:20]1[O:21][C:22]2([CH2:26][CH2:25][CH2:24][CH2:23]2)[O:27][C@H:28]1[CH:29]=[O:30].[CH3:3][S:2][CH3:1].[ClH:5].[ClH:9].[O:10]=[C:8]=[O:4].[C-:6]#[O+:7]

Correctness of the mapping

MAPPET	YES
ReactionMap	YES
Marvin	YES
ChemDraw	YES
Indigo	NO

Reaction no 8

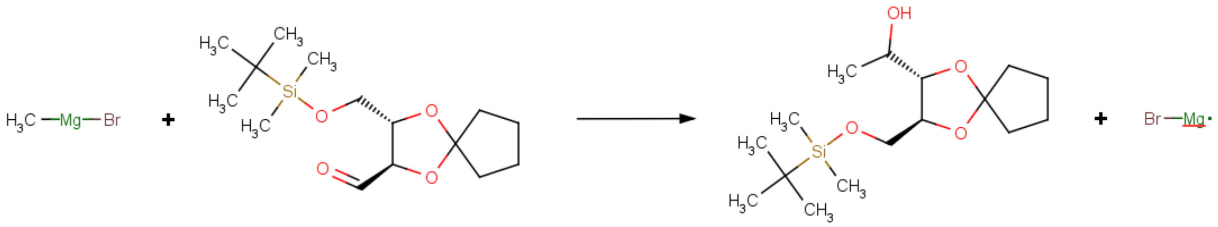

SMILES of the input:
C[Mg]Br.CC(C)(C)[Si](C)(C)OC[C@@H]1OC2(CCCC2)O[C@H]1C=O>>CC(O)[C@@H]1OC2(CCCC2)O[C@H]1CO[Si](C)(C)C(C)(C)C.[Mg]Br

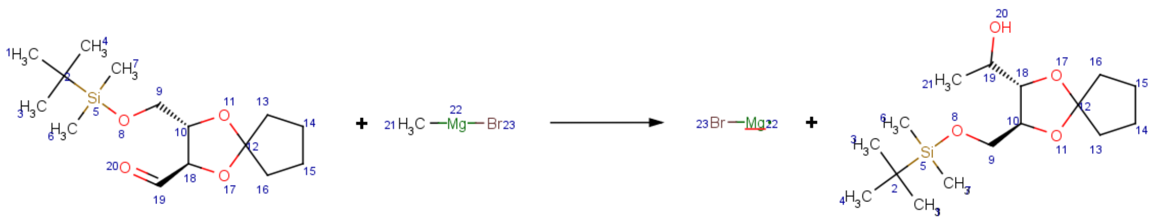

Correct mapped SMILES/SMARTS of the reaction:
[CH3:1][C:2]([CH3:3])([CH3:4])[Si:5]([CH3:6])([CH3:7])[O:8][CH2:9][C@@H:10]1[O:11][C:12]2([CH2:13][CH2:14][CH2:15][CH2:16]2)[O:17][C@H:18]1[CH:19]=[O:20].[CH3:21][Mg:22][Br:23]>>[Mg:22][Br:23].[CH3:21][CH:19]([OH:20])[C@@H:18]1[O:17][C:12]2([CH2:16][CH2:15][CH2:14][CH2:13]2)[O:11][C@H:10]1[CH2:9][O:8][Si:5]([CH3:6])([CH3:7])[C:2]([CH3:4])([CH3:3])[CH3:1]

Correctness of the mapping

MAPPET	YES
ReactionMap	YES
Marvin	YES
ChemDraw	YES
Indigo	YES

Reaction no 9

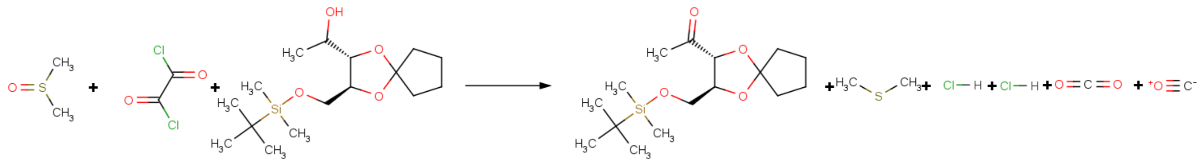

SMILES of the input:
CS(C)=O.ClC(=O)C(Cl)=O.CC(O)[C@@H]1OC2(CCCC2)O[C@H]1CO[Si](C)(C)C(C)(C)C>>CC(=O)[C@@H]1OC2(CCCC2)O[C@H]1CO[Si](C)(C)C(C)(C)C.CSC.Cl[H].Cl[H].O=C=O.[C-]#[O+]

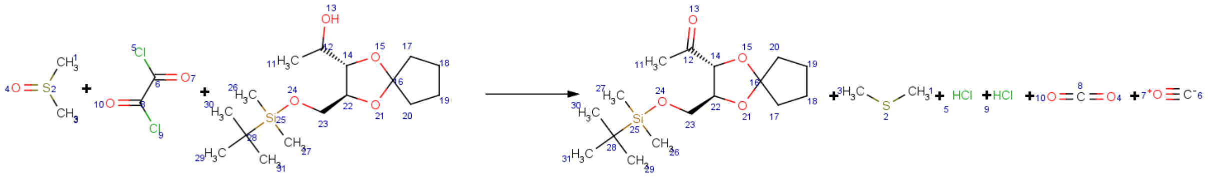

Correct mapped SMILES/SMARTS of the reaction:
[CH3:1][S:2]([CH3:3])=[O:4].[Cl:5][C:6](=[O:7])[C:8]([Cl:9])=[O:10].[CH3:11][CH:12]([OH:13])[C@@H:14]1[O:15][C:16]2([CH2:17][CH2:18][CH2:19][CH2:20]2)[O:21][C@H:22]1[CH2:23][O:24][Si:25]([CH3:26])([CH3:27])[C:28]([CH3:29])([CH3:30])[CH3:31]>>[CH3:11][C:12](=[O:13])[C@@H:14]1[O:15][C:16]2([CH2:20][CH2:19][CH2:18][CH2:17]2)[O:21][C@H:22]1[CH2:23][O:24][Si:25]([CH3:27])([CH3:26])[C:28]([CH3:31])([CH3:30])[CH3:29].[CH3:3][S:2][CH3:1].[ClH:5].[ClH:9].[O:10]=[C:8]=[O:4].[C-:6]#[O+:7]

Correctness of the mapping

MAPPET	YES
ReactionMap	YES
Marvin	YES
ChemDraw	YES
Indigo	NO

Reaction no 10

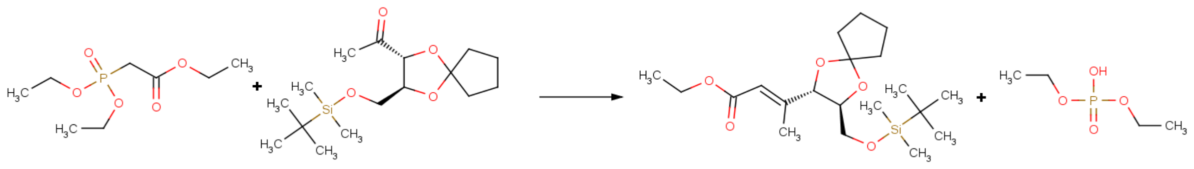

SMILES of the input:
CCOC(=O)CP(=O)(OCC)OCC.CC(=O)[C@@H]1OC2(CCCC2)O[C@H]1CO[Si](C)(C)C(C)(C)C>>CCOC(=O)\C=C(/C)[C@@H]1OC2(CCCC2)O[C@H]1CO[Si](C)(C)C(C)(C)C.CCOP(O)(=O)OCC

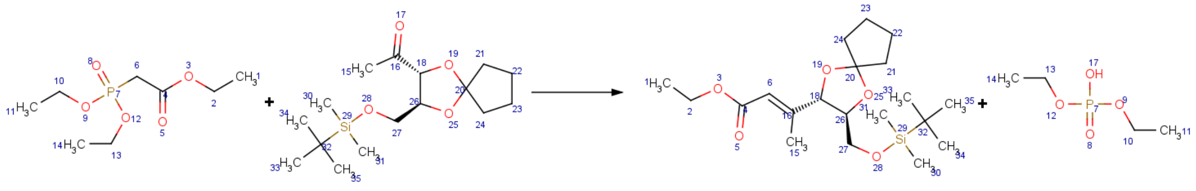

Correct mapped SMILES/SMARTS of the reaction:

[CH3:1][CH2:2][O:3][C:4](=[O:5])[CH2:6][P:7](=[O:8])([O:9][CH2:10][CH3:11
])[O:12][CH2:13][CH3:14].[CH3:15][C:16](=[O:17])[C@@H:18]1[O:19][C:20]2([
CH2:21][CH2:22][CH2:23][CH2:24]2)[O:25][C@H:26]1[CH2:27][O:28][Si:29]([CH
3:30])([CH3:31])[C:32]([CH3:33])([CH3:34])[CH3:35]>>[CH3:1][CH2:2][O:3][C
:4](=[O:5])\[CH:6]=[C:16](/[CH3:15])[C@@H:18]1[O:19][C:20]2([CH2:24][CH2:
23][CH2:22][CH2:21]2)[O:25][C@H:26]1[CH2:27][O:28][Si:29]([CH3:30])([CH3:
31])[C:32]([CH3:35])([CH3:34])[CH3:33].[CH3:11][CH2:10][O:9][P:7]([OH:17]
)(=[O:8])[O:12][CH2:13][CH3:14]

Correctness of the mapping

MAPPET	YES
ReactionMap	YES
Marvin	YES
ChemDraw	YES
Indigo	YES

Reaction no 11

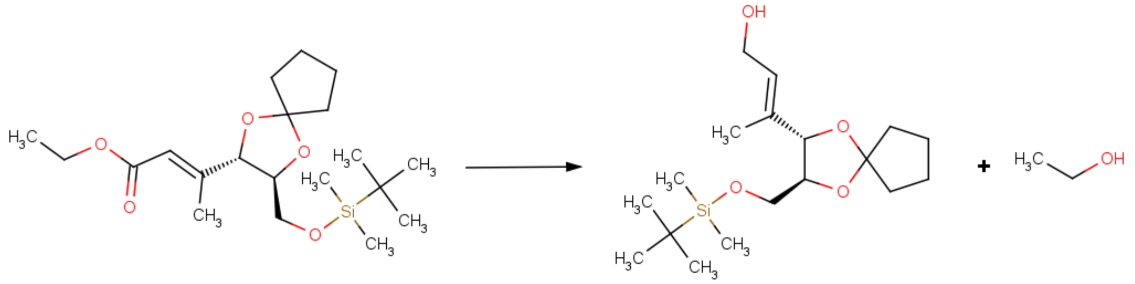

SMILES of the input:
CCOC(=O)\C=C(/C)[C@@H]1OC2(CCCC2)O[C@H]1CO[Si](C)(C)C(C)(C)C>>C\C(=C/CO)[
C@@H]1OC2(CCCC2)O[C@H]1CO[Si](C)(C)C(C)(C)C.CCO

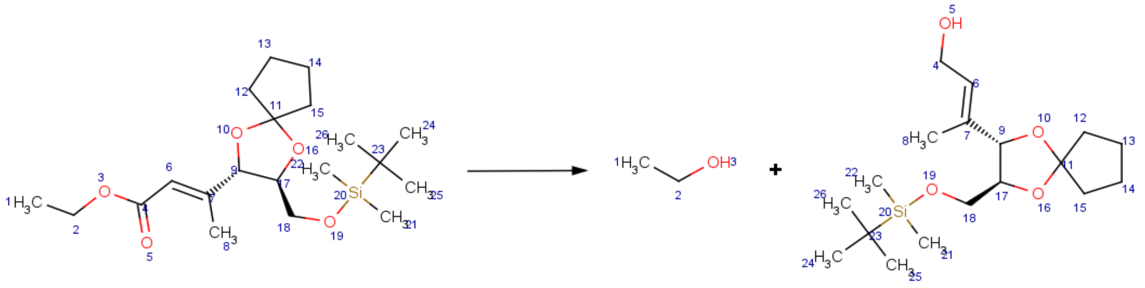

Correct mapped SMILES/SMARTS of the reaction:
[CH3:1][CH2:2][O:3][C:4](=[O:5])\[CH:6]=[C:7](/[CH3:8])[C@@H:9]1[O:10][C:
11]2([CH2:12][CH2:13][CH2:14][CH2:15]2)[O:16][C@H:17]1[CH2:18][O:19][Si:2
0]([CH3:21])([CH3:22])[C:23]([CH3:24])([CH3:25])[CH3:26]>>[CH3:1][CH2:2][
OH:3].[CH3:8]\[C:7](=[CH:6]/[CH2:4][OH:5])[C@@H:9]1[O:10][C:11]2([CH2:12]
[CH2:13][CH2:14][CH2:15]2)[O:16][C@H:17]1[CH2:18][O:19][Si:20]([CH3:22])(
[CH3:21])[C:23]([CH3:24])([CH3:26])[CH3:25]

Correctness of the mapping

MAPPET	YES
ReactionMap	YES
Marvin	YES
ChemDraw	YES
Indigo	YES

Reaction no 12

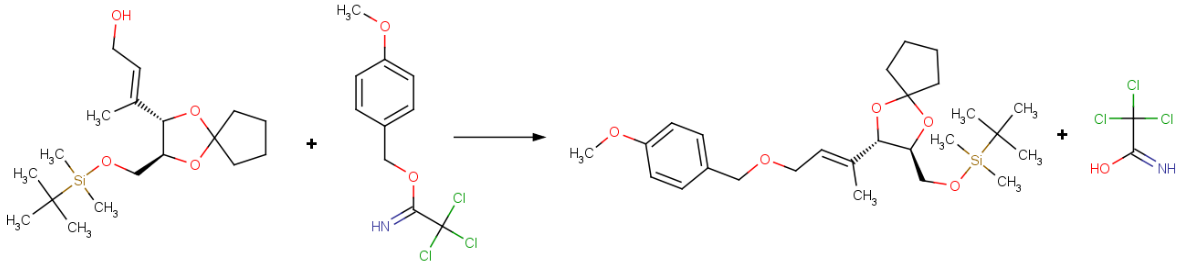

SMILES of the input:

C\C(=C/CO)[C@@H]1OC2(CCCC2)O[C@H]1CO[Si](C)(C)C(C)(C)C.COC1=CC=C(COC(=N)C(Cl)(Cl)Cl)C=C1>>COC1=CC=C(COC\C=C(/C)[C@@H]2OC3(CCCC3)O[C@H]2CO[Si](C)(C)C(C)(C)C)C=C1.OC(=N)C(Cl)(Cl)Cl

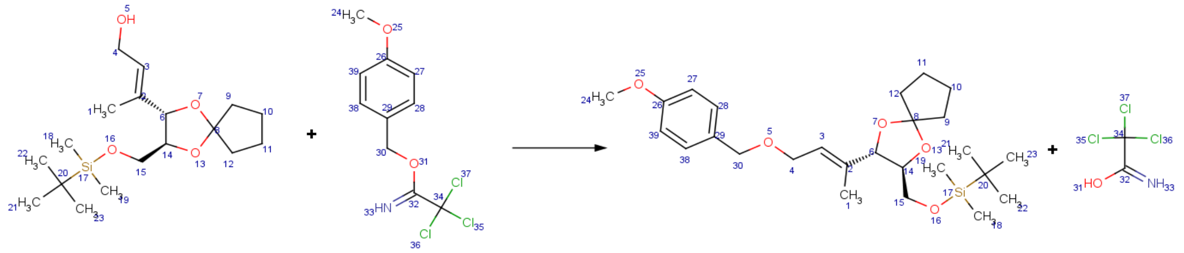

Correct mapped SMILES/SMARTS of the reaction:

[CH3:1]\[C:2](=[CH:3]/[CH2:4][OH:5])[C@@H:6]1[O:7][C:8]2([CH2:9][CH2:10][CH2:11][CH2:12]2)[O:13][C@H:14]1[CH2:15][O:16][Si:17]([CH3:18])([CH3:19])[C:20]([CH3:21])([CH3:22])[CH3:23].[CH3:24][O:25][C:26]1=[CH:27][CH:28]=[C:29]([CH2:30][O:31][C:32](=[NH:33])[C:34]([Cl:35])([Cl:36])[Cl:37])[CH:38]=[CH:39]1>>[CH3:24][O:25][C:26]1=[CH:27][CH:28]=[C:29]([CH2:30][O:5][CH2:4]\[CH:3]=[C:2](/[CH3:1])[C@@H:6]2[O:7][C:8]3([CH2:12][CH2:11][CH2:10][CH2:9]3)[O:13][C@H:14]2[CH2:15][O:16][Si:17]([CH3:18])([CH3:19])[C:20]([CH3:23])([CH3:22])[CH3:21])[CH:38]=[CH:39]1.[OH:31][C:32](=[NH:33])[C:34]([Cl:37])([Cl:36])[Cl:35]

Correctness of the mapping

MAPPET	YES
ReactionMap	YES
Marvin	NO
ChemDraw	YES
Indigo	YES

Reaction no 13

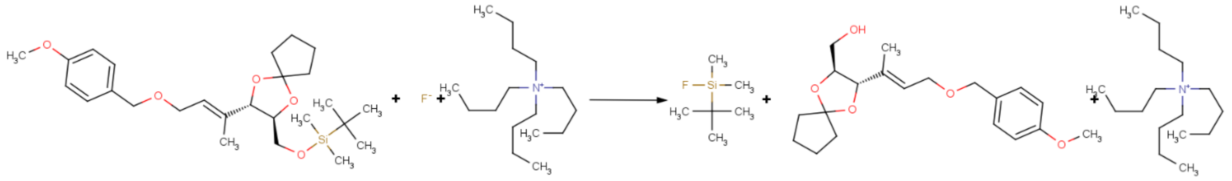

SMILES of the input:

COC1=CC=C(COC\C=C(/C)[C@@H]2OC3(CCCC3)O[C@H]2CO[Si](C)(C)C(C)(C)C)C=C1.[F].CCCC[N+](CCCC)(CCCC)CCCC>>CC(C)(C)[Si](C)(C)F.COC1=CC=C(COC\C=C(/C)[C@@H]2OC3(CCCC3)O[C@H]2CO)C=C1.CCCC[N](CCCC)(CCCC)CCCC

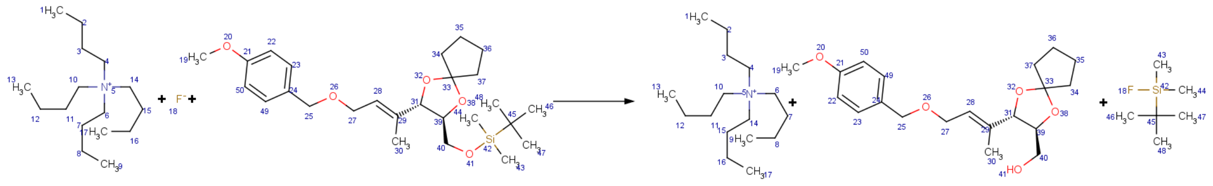

Correct mapped SMILES/SMARTS of the reaction:
[CH3:1][CH2:2][CH2:3][CH2:4][N+:5]([CH2:6][CH2:7][CH2:8][CH3:9])([CH2:10]
[CH2:11][CH2:12][CH3:13])[CH2:14][CH2:15][CH2:16][CH3:17].[F-
:18].[CH3:19][O:20][C:21]1=[CH:22][CH:23]=[C:24]([CH2:25][O:26][CH2:27]\[
CH:28]=[C:29](/[CH3:30])[C@@H:31]2[O:32][C:33]3([CH2:34][CH2:35][CH2:36][
CH2:37]3)[O:38][C@H:39]2[CH2:40][O:41][Si:42]([CH3:43])([CH3:44])[C:45]([
CH3:46])([CH3:47])[CH3:48])[CH:49]=[CH:50]1>>[CH3:1][CH2:2][CH2:3][CH2:4]
[N+:5]([CH2:14][CH2:15][CH2:16][CH3:17])([CH2:10][CH2:11][CH2:12][CH3:13]
)[CH2:6][CH2:7][CH2:8][CH3:9].[CH3:19][O:20][C:21]1=[CH:50][CH:49]=[C:24]
([CH2:25][O:26][CH2:27]\[CH:28]=[C:29](/[CH3:30])[C@@H:31]2[O:32][C:33]3(
[CH2:37][CH2:36][CH2:35][CH2:34]3)[O:38][C@H:39]2[CH2:40][OH:41])[CH:23]=
[CH:22]1.[CH3:48][C:45]([CH3:47])([CH3:46])[Si:42]([CH3:43])([CH3:44])[F:
18]

Correctness of the mapping

MAPPET	YES
ReactionMap	YES
Marvin	YES
ChemDraw	YES
Indigo	YES

Reaction no 14

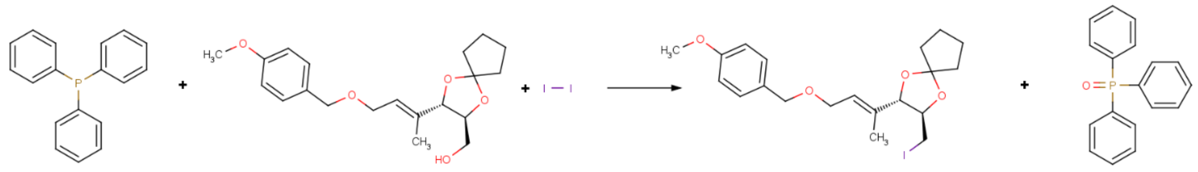

SMILES of the input:
C1=CC=C(C=C1)P(C1=CC=CC=C1)C1=CC=CC=C1.COC1=CC=C(COC\C=C(/C)[C@@H]2OC3(CC
CC3)O[C@H]2CO)C=C1.II>>COC1=CC=C(COC\C=C(/C)[C@@H]2OC3(CCCC3)O[C@H]2CI)C=
C1.O=P(C1=CC=CC=C1)(C1=CC=CC=C1)C1=CC=CC=C1

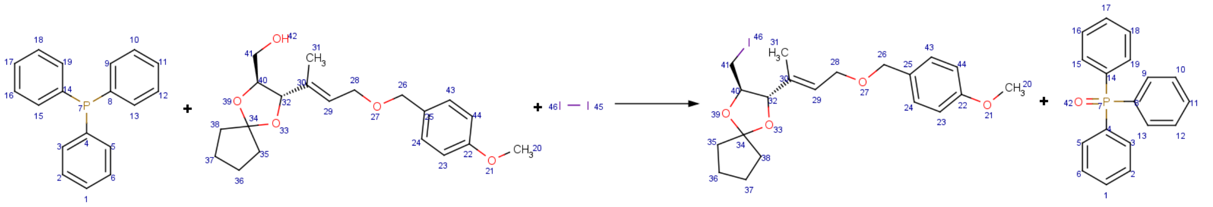

Correct mapped SMILES/SMARTS of the reaction:
[I:1][I:2].[CH3:3][O:4][C:5]1=[CH:6][CH:7]=[C:8]([CH2:9][O:10][CH2:11]\[C
H:12]=[C:13](/[CH3:14])[C@@H:15]2[O:16][C:17]3([CH2:18][CH2:19][CH2:20][C
H2:21]3)[O:22][C@H:23]2[CH2:24][OH:25])[CH:26]=[CH:27]1.[CH:28]1=[CH:29][
CH:30]=[C:31]([CH:32]=[CH:33]1)[P:34]([C:35]1=[CH:36][CH:37]=[CH:38][CH:3
9]=[CH:40]1)[C:41]1=[CH:42][CH:43]=[CH:44][CH:45]=[CH:46]1>>[O:25]=[P:34]
([C:35]1=[CH:36][CH:37]=[CH:38][CH:39]=[CH:40]1)([C:41]1=[CH:42][CH:43]=[
CH:44][CH:45]=[CH:46]1)[C:31]1=[CH:30][CH:29]=[CH:28][CH:33]=[CH:32]1.[CH
3:3][O:4][C:5]1=[CH:27][CH:26]=[C:8]([CH2:9][O:10][CH2:11]\[CH:12]=[C:13]
(/[CH3:14])[C@@H:15]2[O:16][C:17]3([CH2:21][CH2:20][CH2:19][CH2:18]3)[O:2
2][C@H:23]2[CH2:24][I:2])[CH:7]=[CH:6]1.[I:1]

Correctness of the mapping

MAPPET	YES
ReactionMap	NO
Marvin	NO
ChemDraw	YES
Indigo	NO

Reaction no 15

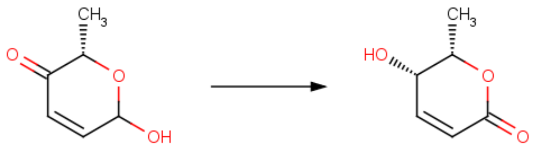

SMILES of the input:
C[C@@H]1OC(O)C=CC1=O>>C[C@@H]1OC(=O)C=C[C@@H]1O

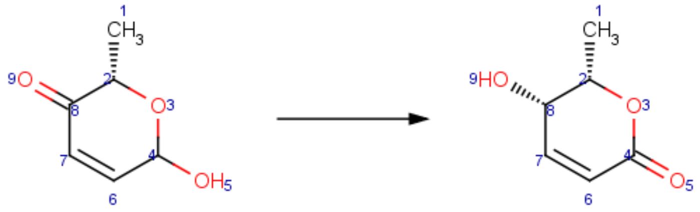

Correct mapped SMILES/SMARTS of the reaction:
[CH3:1][C@@H:2]1[O:3][CH:4]([OH:5])[CH:6]=[CH:7][C:8]1=[O:9]>>[CH3:1][C@@H:2]1[O:3][C:4](=[O:5])[CH:6]=[CH:7][C@@H:8]1[OH:9]

Correctness of the mapping

MAPPET	YES
ReactionMap	YES
Marvin	YES
ChemDraw	YES
Indigo	NO

Reaction no 16

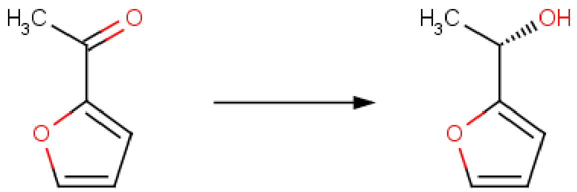

SMILES of the input:
CC(=O)C1=CC=CO1>>C[C@H](O)C1=CC=CO1

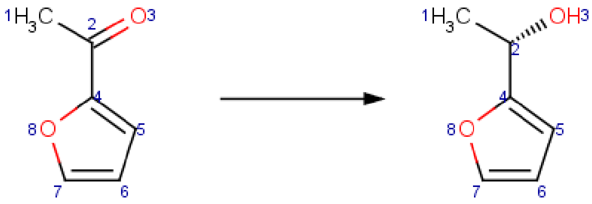

Correct mapped SMILES/SMARTS of the reaction:
[CH3:1][C:2](=[O:3])[C:4]1=[CH:5][CH:6]=[CH:7][O:8]1>>[CH3:1][C@H:2]([OH:3])[C:4]1=[CH:5][CH:6]=[CH:7][O:8]1

Correctness of the mapping

MAPPET	YES
ReactionMap	YES
Marvin	YES
ChemDraw	YES
Indigo	YES

Reaction no 17

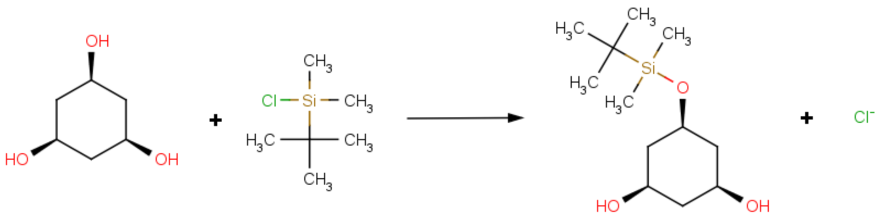

SMILES of the input:
O[C@H]1C[C@@H](O)C[C@@H](O)C1.CC(C)(C)[Si](C)(C)Cl>>CC(C)(C)[Si](C)(C)O[C
@H]1C[C@@H](O)C[C@@H](O)C1.[Cl-]

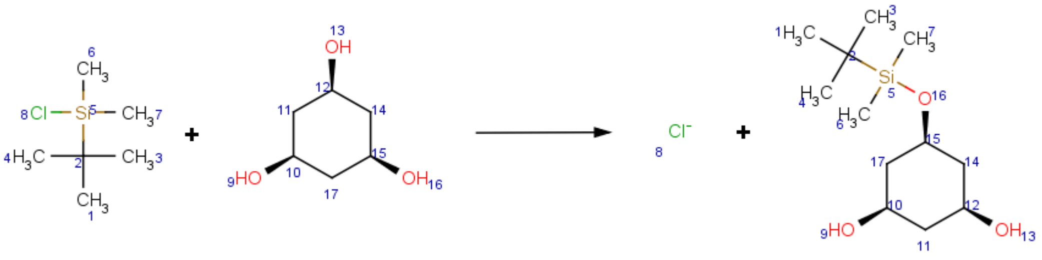

Correct mapped SMILES/SMARTS of the reaction:
[CH3:1][C:2]([CH3:3])([CH3:4])[Si:5]([CH3:6])([CH3:7])[Cl:8].[OH:13][C@H:
12]1[CH2:14][C@@H:15]([OH:16])[CH2:17][C@@H:10]([OH:9])[CH2:11]1>>[Cl-
:8].[CH3:1][C:2]([CH3:4])([CH3:3])[Si:5]([CH3:6])([CH3:7])[O:16][C@H:15]1
[CH2:14][C@@H:12]([OH:13])[CH2:11][C@@H:10]([OH:9])[CH2:17]1

Correctness of the mapping

MAPPET	YES
ReactionMap	YES
Marvin	YES
ChemDraw	YES
Indigo	YES

Reaction no 18

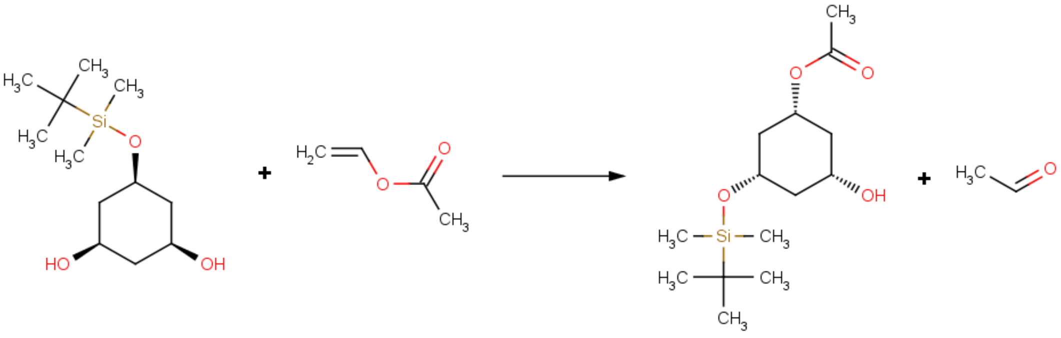

SMILES of the input:

CC(C)(C)[Si](C)(C)O[C@H]1C[C@@H](O)C[C@@H](O)C1.CC(=O)OC=C>>CC(=O)O[C@@H]1C[C@H](O)C[C@@H](C1)O[Si](C)(C)C(C)(C)C.CC=O

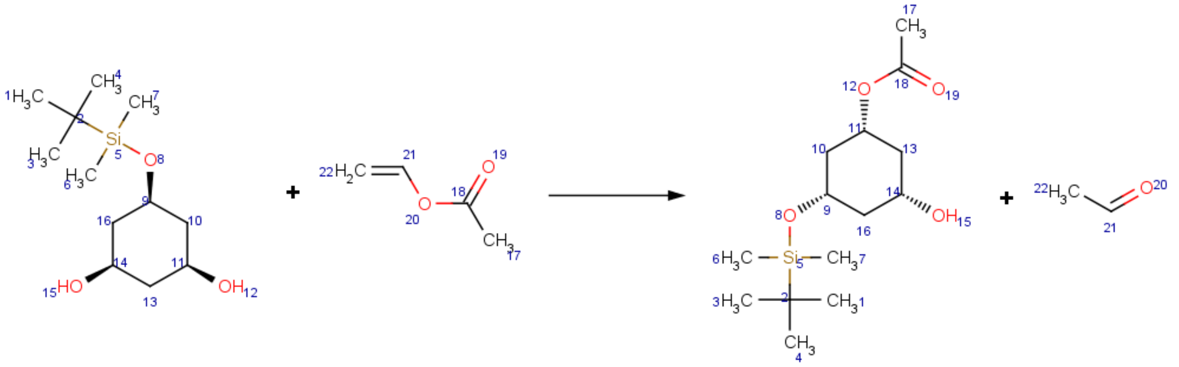

Correct mapped SMILES/SMARTS of the reaction:
[CH3:1][C:2]([CH3:3])([CH3:4])[Si:5]([CH3:6])([CH3:7])[O:8][C@H:9]1[CH2:10][C@@H:11]([OH:12])[CH2:13][C@@H:14]([OH:15])[CH2:16]1.[CH3:17][C:18](=[O:19])[O:20][CH:21]=[CH2:22]>>[CH3:17][C:18](=[O:19])[O:12][C@@H:11]1[CH2:13][C@H:14]([OH:15])[CH2:16][C@@H:9]([CH2:10]1)[O:8][Si:5]([CH3:6])([CH3:7])[C:2]([CH3:4])([CH3:3])[CH3:1].[CH3:22][CH:21]=[O:20]

Correctness of the mapping

MAPPET	YES
ReactionMap	YES
Marvin	NO
ChemDraw	YES
Indigo	NO

Reaction no 19

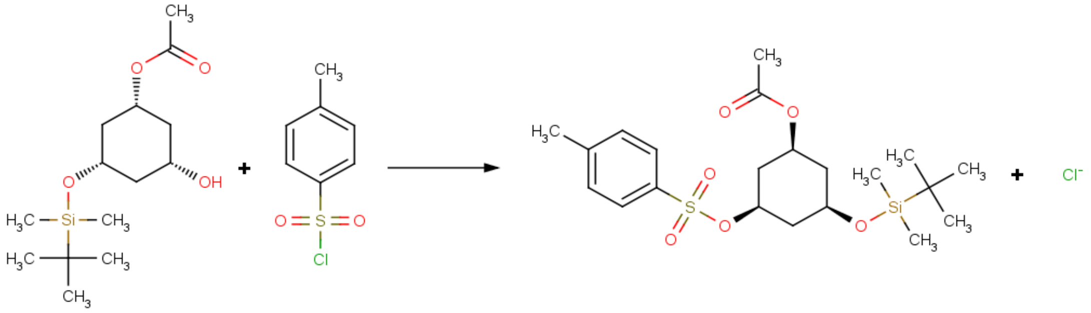

SMILES of the input:
CC(=O)O[C@@H]1C[C@H](O)C[C@@H](C1)O[Si](C)(C)C(C)(C)C.CC1=CC=C(C=C1)S(Cl)(=O)=O>>CC(=O)O[C@H]1C[C@H](C[C@H](C1)OS(=O)(=O)C1=CC=C(C)C=C1)O[Si](C)(C)C(C)(C)C.[Cl-]

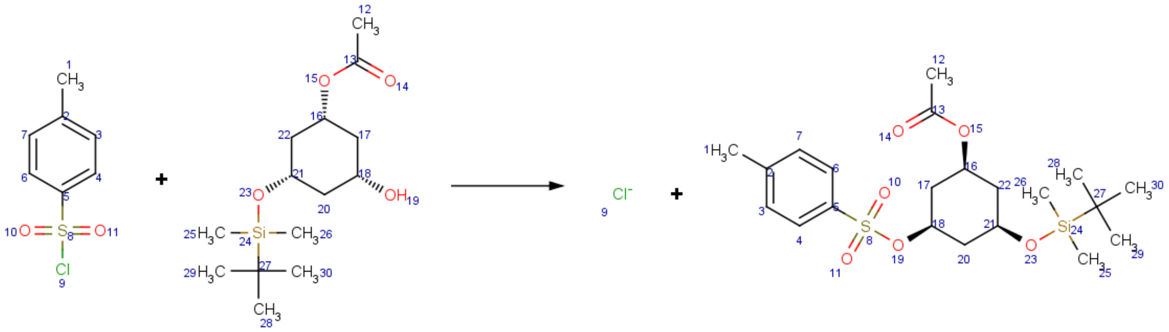

Correct mapped SMILES/SMARTS of the reaction:
[CH3:1][C:2]1=[CH:3][CH:4]=[C:5]([CH:6]=[CH:7]1)[S:8]([Cl:9])(=[O:10])=[O:11].[CH3:12][C:13](=[O:14])[O:15][C@@H:16]1[CH2:17][C@H:18]([OH:19])[CH2

:20][C@@H:21]([CH2:22]1)[O:23][Si:24]([CH3:25])([CH3:26])[C:27]([CH3:28])([CH3:29])[CH3:30]>>[Cl-
:9].[CH3:12][C:13](=[O:14])[O:15][C@H:16]1[CH2:22][C@H:21]([CH2:20][C@H:18]([CH2:17]1)[O:19][S:8](=[O:11])(=[O:10])[C:5]1=[CH:4][CH:3]=[C:2]([CH3:1])[CH:7]=[CH:6]1)[O:23][Si:24]([CH3:25])([CH3:26])[C:27]([CH3:30])([CH3:29])[CH3:28]

Correctness of the mapping
MAPPET YES
ReactionMap YES
Marvin YES
ChemDraw YES
Indigo YES

Reaction no 20

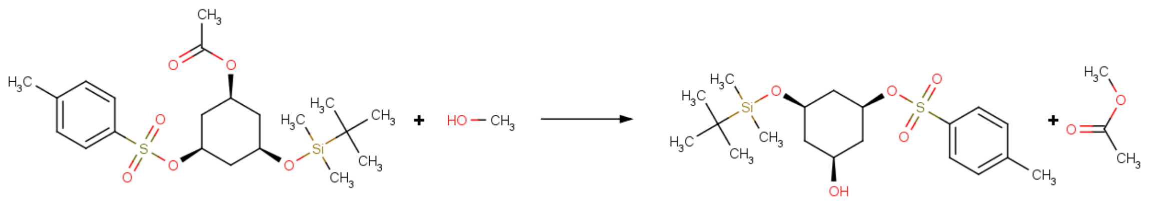

SMILES of the input:
CC(=O)O[C@H]1C[C@H](C[C@H](C1)OS(=O)(=O)C1=CC=C(C)C=C1)O[Si](C)(C)C(C)(C)C.CO>>CC1=CC=C(C=C1)S(=O)(=O)O[C@H]1C[C@@H](O)C[C@H](C1)O[Si](C)(C)C(C)(C)C.COC(C)=O

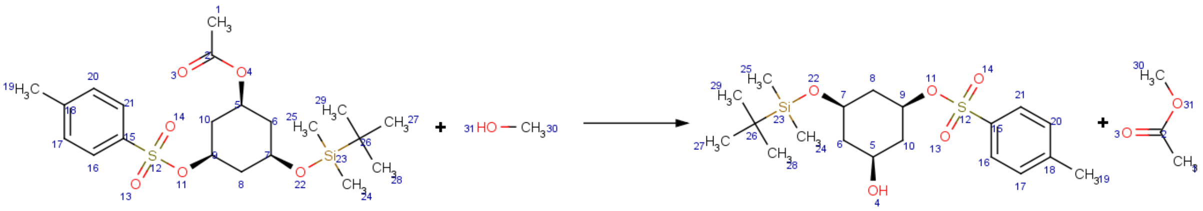

Correct mapped SMILES/SMARTS of the reaction:
[CH3:1][C:2](=[O:3])[O:4][C@H:5]1[CH2:6][C@H:7]([CH2:8][C@H:9]([CH2:10]1)[O:11][S:12](=[O:13])(=[O:14])[C:15]1=[CH:16][CH:17]=[C:18]([CH3:19])[CH:20]=[CH:21]1)[O:22][Si:23]([CH3:24])([CH3:25])[C:26]([CH3:27])([CH3:28])[CH3:29].[CH3:30][OH:31]>>[CH3:19][C:18]1=[CH:17][CH:16]=[C:15]([CH:21]=[CH:20]1)[S:12](=[O:14])(=[O:13])[O:11][C@H:9]1[CH2:10][C@@H:5]([OH:4])[CH2:6][C@H:7]([CH2:8]1)[O:22][Si:23]([CH3:25])([CH3:24])[C:26]([CH3:27])([CH3:29])[CH3:28].[CH3:30][O:31][C:2]([CH3:1])=[O:3]

Correctness of the mapping
MAPPET YES
ReactionMap YES
Marvin YES
ChemDraw YES
Indigo YES

Reaction no 21

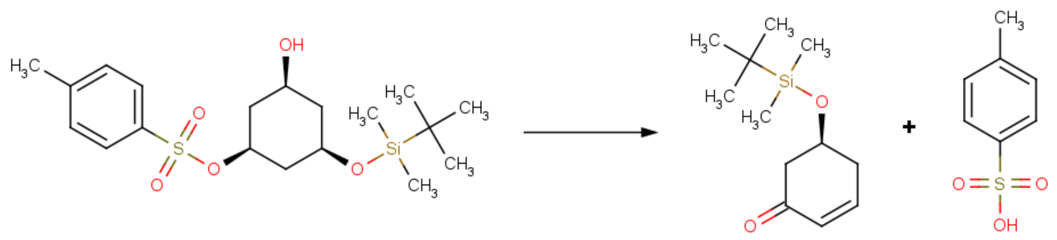

SMILES of the input:
CC1=CC=C(C=C1)S(=O)(=O)O[C@H]1C[C@@H](O)C[C@H](C1)O[Si](C)(C)C(C)(C)C>>CC(C)(C)[Si](C)(C)O[C@H]1CC=CC(=O)C1.CC1=CC=C(C=C1)S(O)(=O)=O

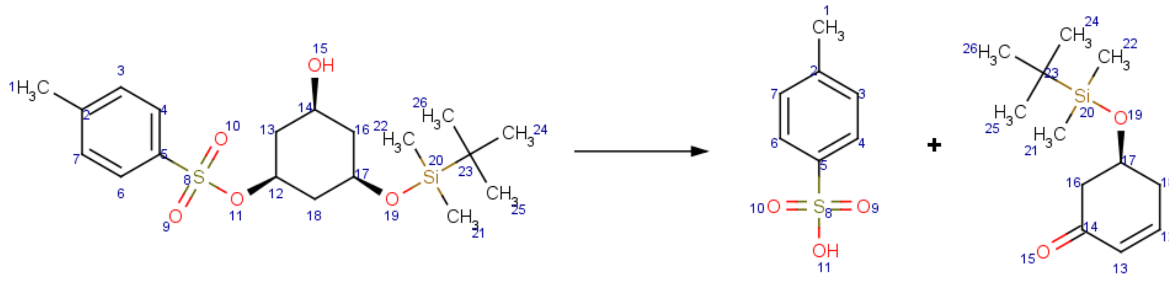

Correct mapped SMILES/SMARTS of the reaction:
[CH3:1][C:2]1=[CH:3][CH:4]=[C:5]([CH:6]=[CH:7]1)[S:8](=[O:9])(=[O:10])[O:11][C@H:12]1[CH2:13][C@@H:14]([OH:15])[CH2:16][C@H:17]([CH2:18]1)[O:19][Si:20]([CH3:21])([CH3:22])[C:23]([CH3:24])([CH3:25])[CH3:26]>>[CH3:1][C:2]1=[CH:3][CH:4]=[C:5]([CH:6]=[CH:7]1)[S:8]([OH:11])(=[O:10])=[O:9].[CH3:26][C:23]([CH3:25])([CH3:24])[Si:20]([CH3:21])([CH3:22])[O:19][C@H:17]1[CH2:18][CH:12]=[CH:13][C:14](=[O:15])[CH2:16]1

Correctness of the mapping

MAPPET	YES
ReactionMap	YES
Marvin	YES
ChemDraw	NO
Indigo	NO

Reaction no 22

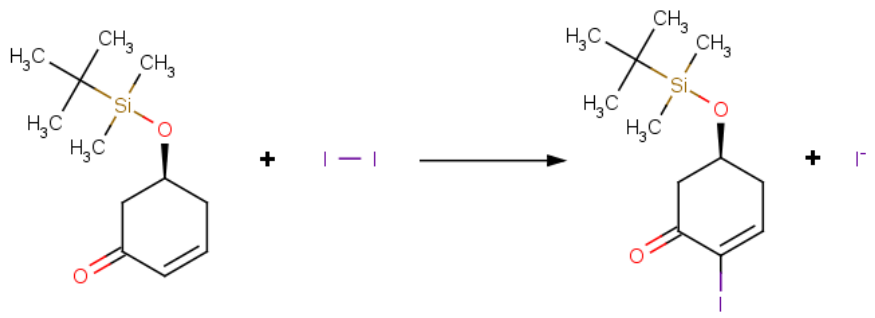

SMILES of the input:
CC(C)(C)[Si](C)(C)O[C@H]1CC=CC(=O)C1.II>>CC(C)(C)[Si](C)(C)O[C@H]1CC=C(I)C(=O)C1.[I-]

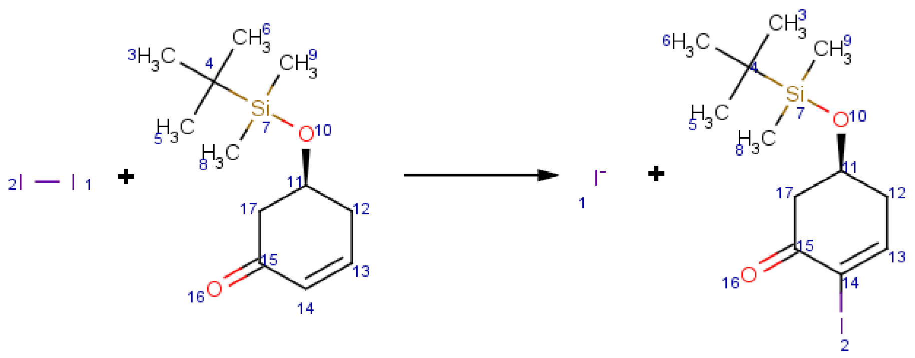

Correct mapped SMILES/SMARTS of the reaction:
[I:1][I:2].[CH3:3][C:4]([CH3:5])([CH3:6])[Si:7]([CH3:8])([CH3:9])[O:10][C@H:11]1[CH2:12][CH:13]=[CH:14][C:15](=[O:16])[CH2:17]1>>[I-:1].[CH3:6][C:4]([CH3:5])([CH3:3])[Si:7]([CH3:8])([CH3:9])[O:10][C@H:11]1[CH2:12][CH:13]=[C:14]([I:2])[C:15](=[O:16])[CH2:17]1

Correctness of the mapping

MAPPET	YES
ReactionMap	YES
Marvin	YES
ChemDraw	YES
Indigo	YES

Reaction no 23

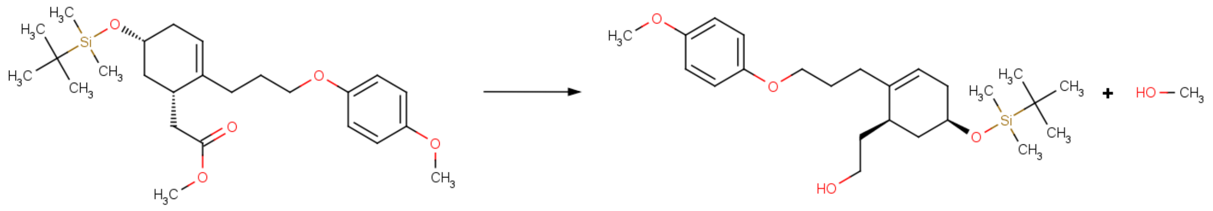

SMILES of the input:
COC(=O)C[C@@H]1C[C@@H](CC=C1CCCOC1=CC=C(OC)C=C1)O[Si](C)(C)C(C)(C)C>>COC1=CC=C(OCCCC2=CC[C@H](C[C@H]2CCO)O[Si](C)(C)C(C)(C)C)C=C1.CO

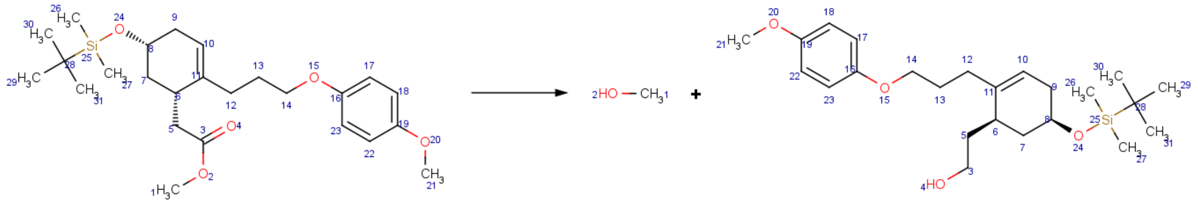

Correct mapped SMILES/SMARTS of the reaction:
[CH3:1][O:2][C:3](=[O:4])[CH2:5][C@@H:6]1[CH2:7][C@@H:8]([CH2:9][CH:10]=[C:11]1[CH2:12][CH2:13][CH2:14][O:15][C:16]1=[CH:17][CH:18]=[C:19]([O:20][CH3:21])[CH:22]=[CH:23]1)[O:24][Si:25]([CH3:26])([CH3:27])[C:28]([CH3:29])([CH3:30])[CH3:31]>>[CH3:1][OH:2].[CH3:21][O:20][C:19]1=[CH:18][CH:17]=[C:16]([O:15][CH2:14][CH2:13][CH2:12][C:11]2=[CH:10][CH2:9][C@H:8]([CH2:7][C@H:6]2[CH2:5][CH2:3][OH:4])[O:24][Si:25]([CH3:27])([CH3:26])[C:28]([CH3:29])([CH3:31])[CH3:30])[CH:23]=[CH:22]1

Correctness of the mapping

MAPPET	YES
ReactionMap	YES

Marvin YES
ChemDraw YES
Indigo NO

Reaction no 24

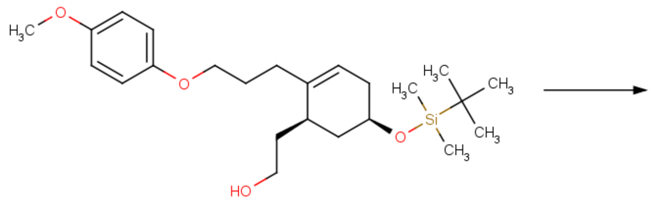

SMILES of the input:
COC1=CC=C(OCCCC2=CC[C@H](C[C@H]2CCO)O[Si](C)(C)C(C)(C)C)C=C1>>COC1=CC=C(OCCCC2=CC[C@H](C[C@H]2CC=O)O[Si](C)(C)C(C)(C)C)C=C1

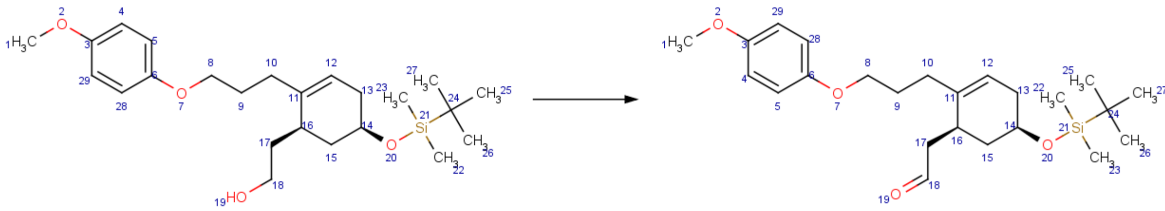

Correct mapped SMILES/SMARTS of the reaction:
[CH3:1][O:2][C:3]1=[CH:4][CH:5]=[C:6]([O:7][CH2:8][CH2:9][CH2:10][C:11]2=[CH:12][CH2:13][C@H:14]([CH2:15][C@H:16]2[CH2:17][CH2:18][OH:19])[O:20][Si:21]([CH3:22])([CH3:23])[C:24]([CH3:25])([CH3:26])[CH3:27])[CH:28]=[CH:29]1>>[CH3:1][O:2][C:3]1=[CH:29][CH:28]=[C:6]([O:7][CH2:8][CH2:9][CH2:10][C:11]2=[CH:12][CH2:13][C@H:14]([CH2:15][C@H:16]2[CH2:17][CH:18]=[O:19])[O:20][Si:21]([CH3:23])([CH3:22])[C:24]([CH3:27])([CH3:26])[CH3:25])[CH:5]=[CH:4]1

Correctness of the mapping
MAPPET YES
ReactionMap YES
Marvin YES
ChemDraw YES
Indigo NO

Reaction no 25

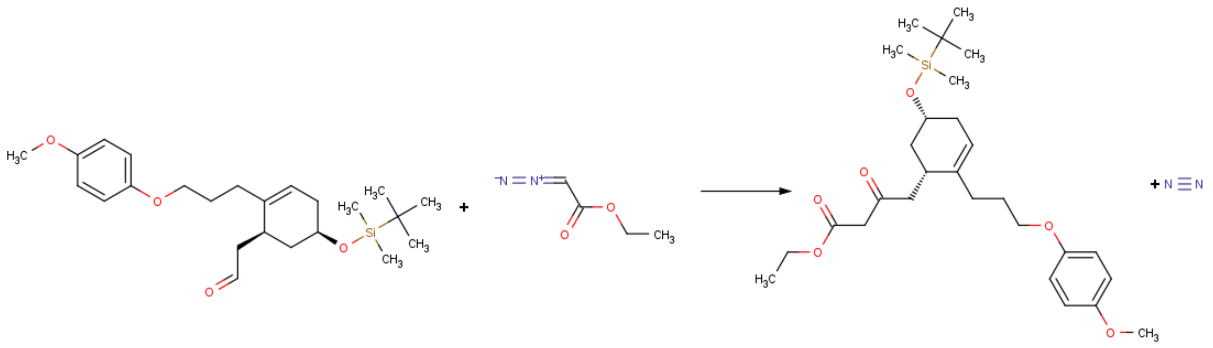

SMILES of the input:
COC1=CC=C(OCCCC2=CC[C@H](C[C@H]2CC=O)O[Si](C)(C)C(C)(C)C)C=C1.CCOC(=O)C=[N+]=[N-

]>>CCOC(=O)CC(=O)C[C@@H]1C[C@@H](CC=C1CCCOC1=CC=C(OC)C=C1)O[Si](C)(C)C(C)(C)C.N#N

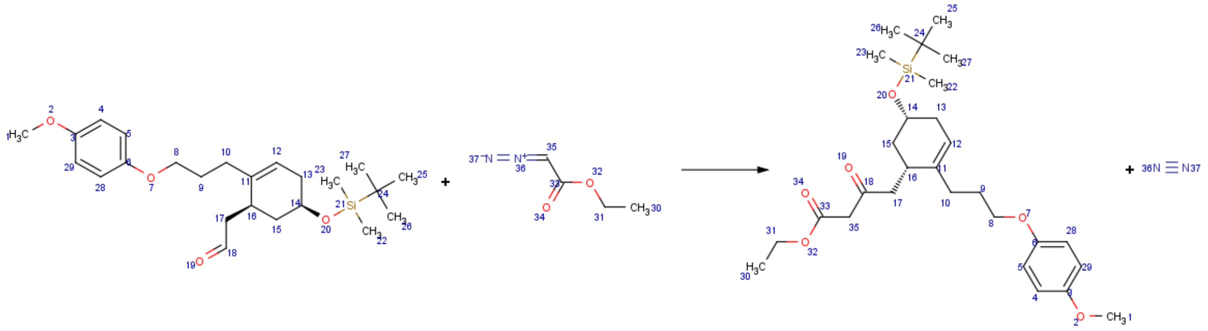

Correct mapped SMILES/SMARTS of the reaction:
[CH3:1][O:2][C:3]1=[CH:4][CH:5]=[C:6]([O:7][CH2:8][CH2:9][CH2:10][C:11]2=[CH:12][CH2:13][C@H:14]([CH2:15][C@H:16]2[CH2:17][CH:18]=[O:19])[O:20][Si:21]([CH3:22])([CH3:23])[C:24]([CH3:25])([CH3:26])[CH3:27])[CH:28]=[CH:29]1.[CH3:30][CH2:31][O:32][C:33](=[O:34])[CH:35]=[N+:36]=[N-:37]>>[CH3:30][CH2:31][O:32][C:33](=[O:34])[CH2:35][C:18](=[O:19])[CH2:17][C@@H:16]1[CH2:15][C@@H:14]([CH2:13][CH:12]=[C:11]1[CH2:10][CH2:9][CH2:8][O:7][C:6]1=[CH:28][CH:29]=[C:3]([O:2][CH3:1])[CH:4]=[CH:5]1)[O:20][Si:21]([CH3:22])([CH3:23])[C:24]([CH3:27])([CH3:26])[CH3:25].[N:37]#[N:36]

Correctness of the mapping

MAPPET	YES
ReactionMap	YES
Marvin	YES
ChemDraw	YES
Indigo	YES

Reaction no 26

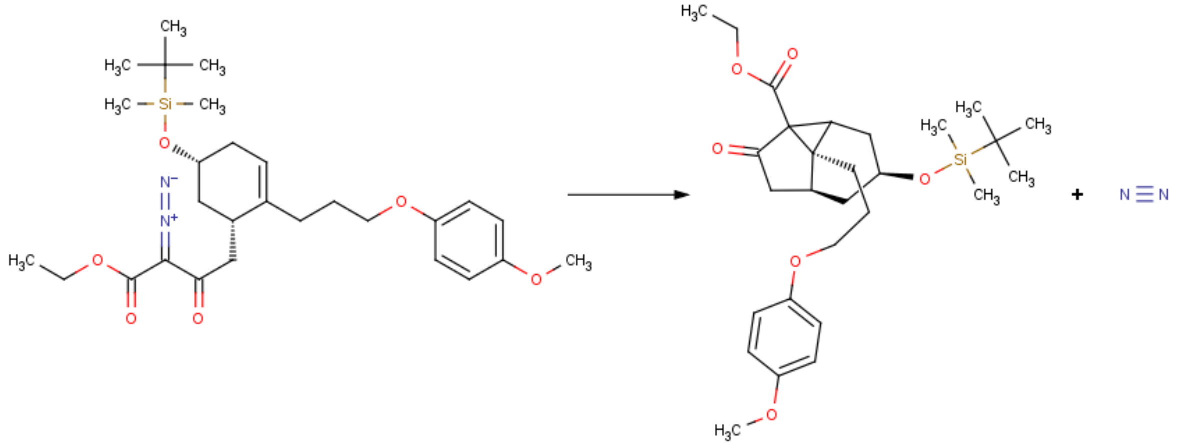

SMILES of the input:
CCOC(=O)C(=[N+]=[N-])C(=O)C[C@@H]1C[C@@H](CC=C1CCCOC1=CC=C(OC)C=C1)O[Si](C)(C)C(C)(C)C>>CCOC(=O)C12C3C[C@H](C[C@@H](CC1=O)[C@@]23CCCOC1=CC=C(OC)C=C1)O[Si](C)(C)C(C)(C)C.N#N

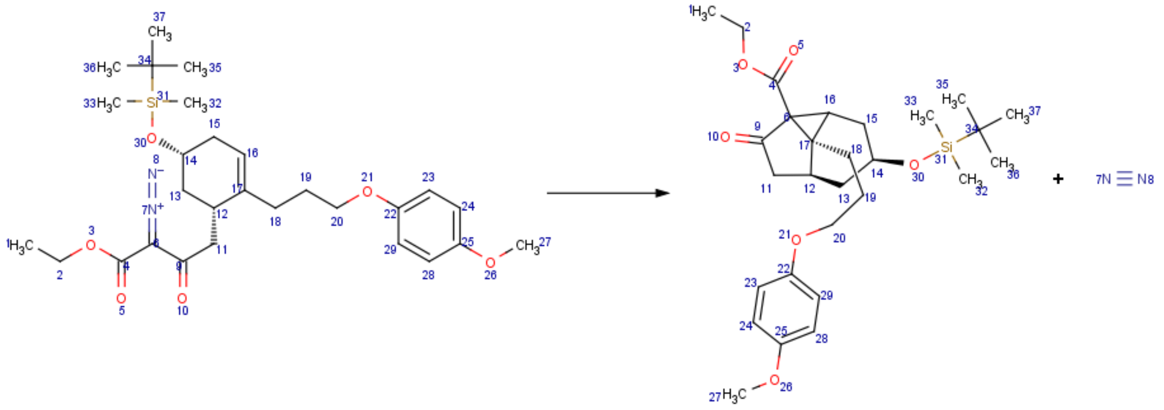

Correct mapped SMILES/SMARTS of the reaction:
[CH3:1][CH2:2][O:3][C:4](=[O:5])[C:6](=[N+:7]=[N-
:8])[C:9](=[O:10])[CH2:11][C@@H:12]1[CH2:13][C@@H:14]([CH2:15][CH:16]=[C:
17]1[CH2:18][CH2:19][CH2:20][O:21][C:22]1=[CH:23][CH:24]=[C:25]([O:26][CH
3:27])[CH:28]=[CH:29]1)[O:30][Si:31]([CH3:32])([CH3:33])[C:34]([CH3:35])(
[CH3:36])[CH3:37]>>[CH3:1][CH2:2][O:3][C:4](=[O:5])[C:6]12[CH:16]3[CH2:15
][C@H:14]([CH2:13][C@@H:12]([CH2:11][C:9]1=[O:10])[C@@:17]23[CH2:18][CH2:
19][CH2:20][O:21][C:22]1=[CH:29][CH:28]=[C:25]([O:26][CH3:27])[CH:24]=[CH
:23]1)[O:30][Si:31]([CH3:32])([CH3:33])[C:34]([CH3:37])([CH3:36])[CH3:35]
.[N:8]#[N:7]

Correctness of the mapping

MAPPET	YES
ReactionMap	YES
Marvin	YES
ChemDraw	YES
Indigo	YES

Reaction no 27

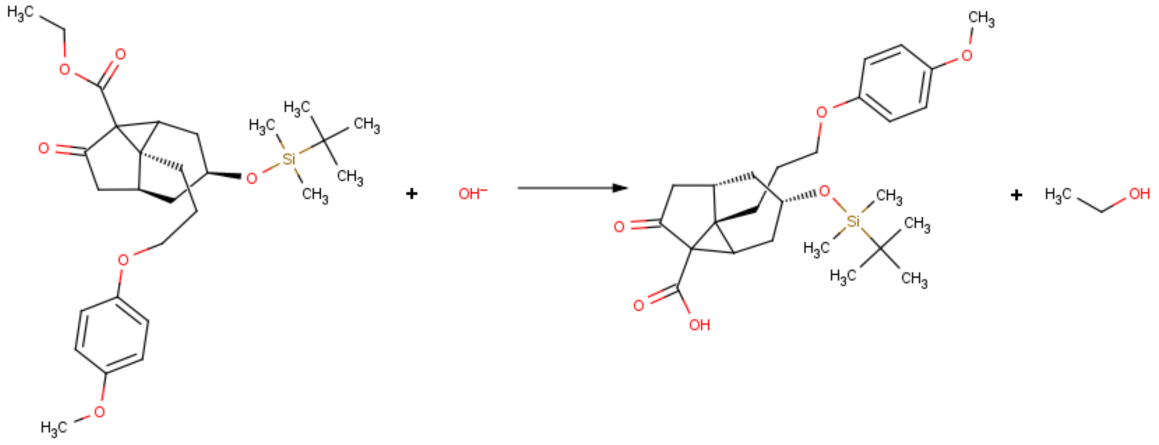

SMILES of the input:
CCOC(=O)C12C3C[C@H](C[C@@H](CC1=O)[C@@]23CCCOC1=CC=C(OC)C=C1)O[Si](C)(C)C
(C)(C)C.[OH-
]>>COC1=CC=C(OCCC[C@]23C4C[C@H](C[C@H]2CC(=O)C34C(O)=O)O[Si](C)(C)C(C)(C)
C)C=C1.CCO

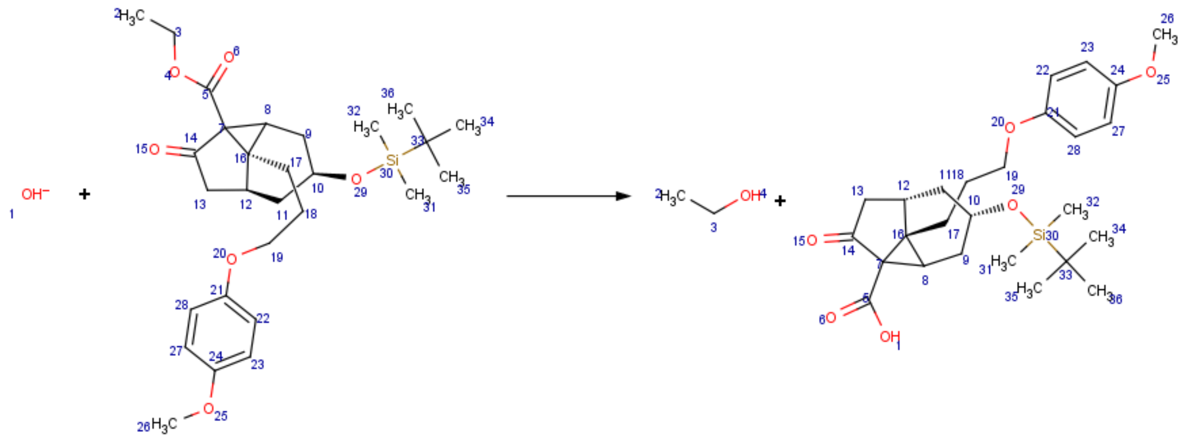

Correct mapped SMILES/SMARTS of the reaction:
[OH-:1].[CH3:2][CH2:3][O:4][C:5](=[O:6])[C:7]12[CH:8]3[CH2:9][C@H:10]([CH2:11][C@@H:12]([CH2:13][C:14]1=[O:15])[C@@:16]23[CH2:17][CH2:18][CH2:19][O:20][C:21]1=[CH:22][CH:23]=[C:24]([O:25][CH3:26])[CH:27]=[CH:28]1)[O:29][Si:30]([CH3:31])([CH3:32])[C:33]([CH3:34])([CH3:35])[CH3:36]>>[CH3:2][CH2:3][OH:4].[CH3:26][O:25][C:24]1=[CH:27][CH:28]=[C:21]([O:20][CH2:19][CH2:18][CH2:17][C@:16]23[CH:8]4[CH2:9][C@H:10]([CH2:11][C@H:12]2[CH2:13][C:14](=[O:15])[C:7]34[C:5]([OH:1])=[O:6])[O:29][Si:30]([CH3:31])([CH3:32])[C:33]([CH3:36])([CH3:35])[CH3:34])[CH:22]=[CH:23]1

Correctness of the mapping

MAPPET	YES
ReactionMap	NO
Marvin	NO
ChemDraw	NO
Indigo	NO

Reaction no 28

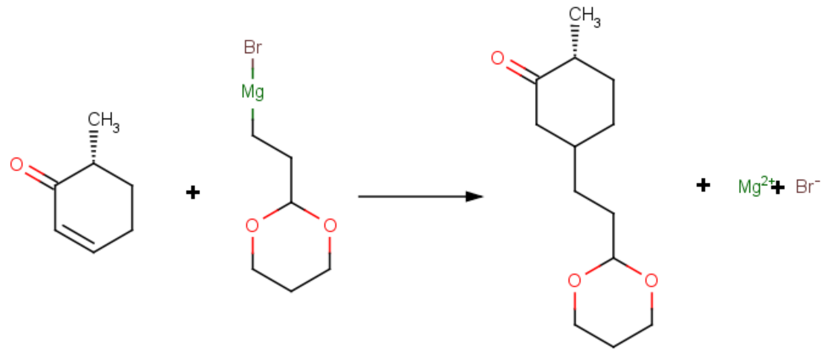

SMILES of the input:
C[C@@H]1CCC=CC1=O.Br[Mg]CCC1OCCCO1>>C[C@@H]1CCC(CCC2OCCCO2)CC1=O.[Mg++].[Br-]

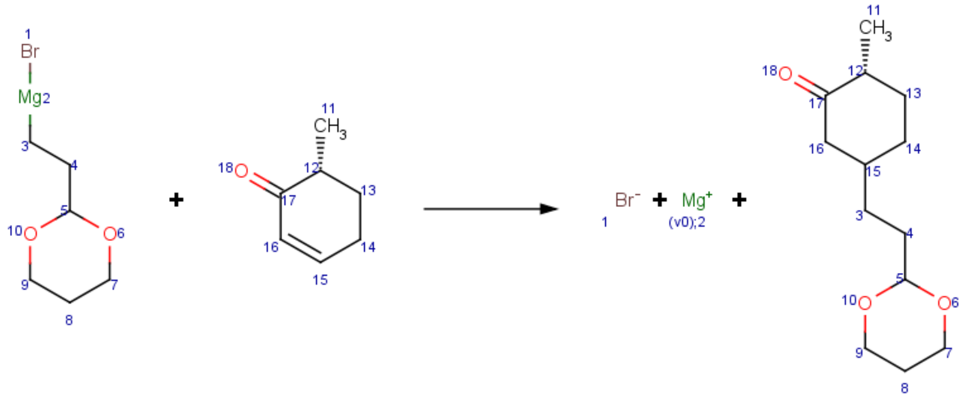

Correct mapped SMILES/SMARTS of the reaction:
[Br:1][Mg:2][CH2:3][CH2:4][CH:5]1[O:6][CH2:7][CH2:8][CH2:9][O:10]1.[CH3:11][C@@H:12]1[CH2:13][CH2:14][CH:15]=[CH:16][C:17]1=[O:18]>>[Br-:1].[Mg+:2].[CH3:11][C@@H:12]1[CH2:13][CH2:14][CH:15]([CH2:3][CH2:4][CH:5]2[O:6][CH2:7][CH2:8][CH2:9][O:10]2)[CH2:16][C:17]1=[O:18]

Correctness of the mapping

MAPPET	YES
ReactionMap	YES
Marvin	YES
ChemDraw	YES
Indigo	YES

Reaction no 29

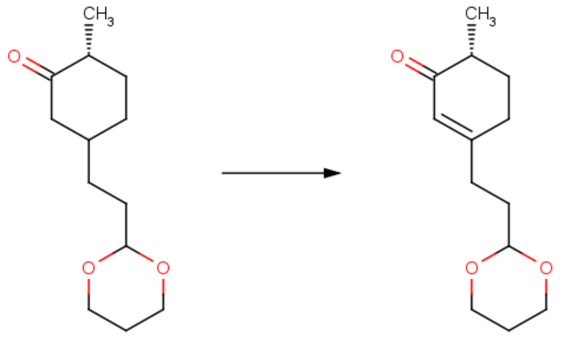

SMILES of the input:
C[C@@H]1CCC(CCC2OCCCO2)CC1=O>>C[C@@H]1CCC(CCC2OCCCO2)=CC1=O

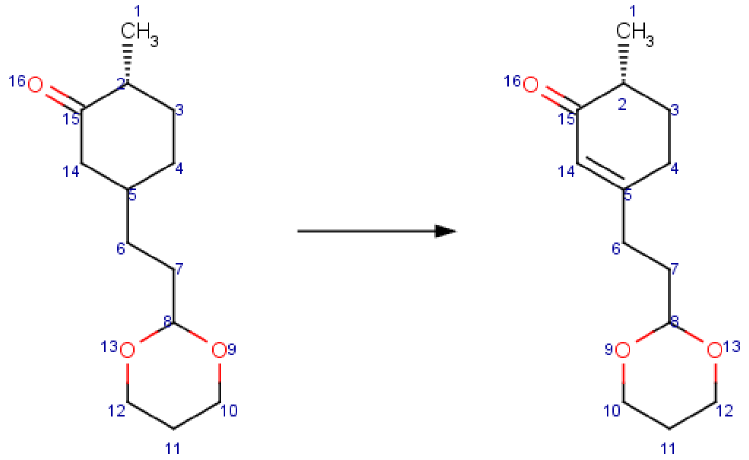

Correct mapped SMILES/SMARTS of the reaction:

[CH3:1][C@@H:2]1[CH2:3][CH2:4][CH:5]([CH2:6][CH2:7][CH:8]2[O:9][CH2:10][CH2:11][CH2:12][O:13]2)[CH2:14][C:15]1=[O:16]>>[CH3:1][C@@H:2]1[CH2:3][CH2:4][C:5]([CH2:6][CH2:7][CH:8]2[O:13][CH2:12][CH2:11][CH2:10][O:9]2)=[CH:14][C:15]1=[O:16]

Correctness of the mapping

MAPPET YES
ReactionMap YES
Marvin YES
ChemDraw YES
Indigo YES

Reaction no 30

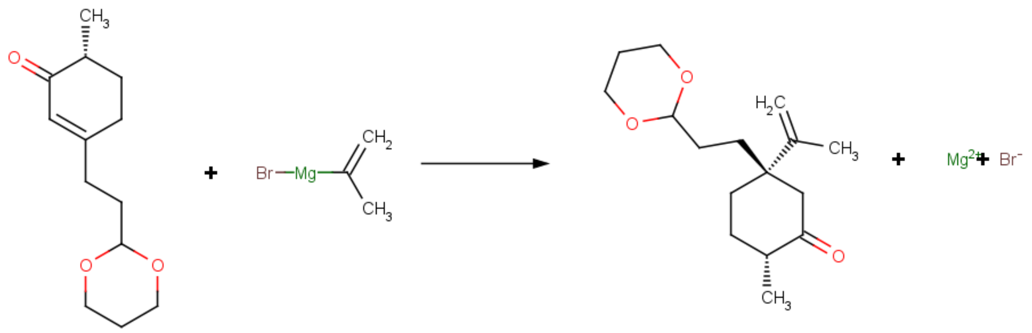

SMILES of the input:
C[C@@H]1CCC(CCC2OCCCO2)=CC1=O.CC(=C)[Mg]Br>>C[C@@H]1CC[C@@](CCC2OCCCO2)(CC1=O)C(C)=C.[Mg++].[Br-]

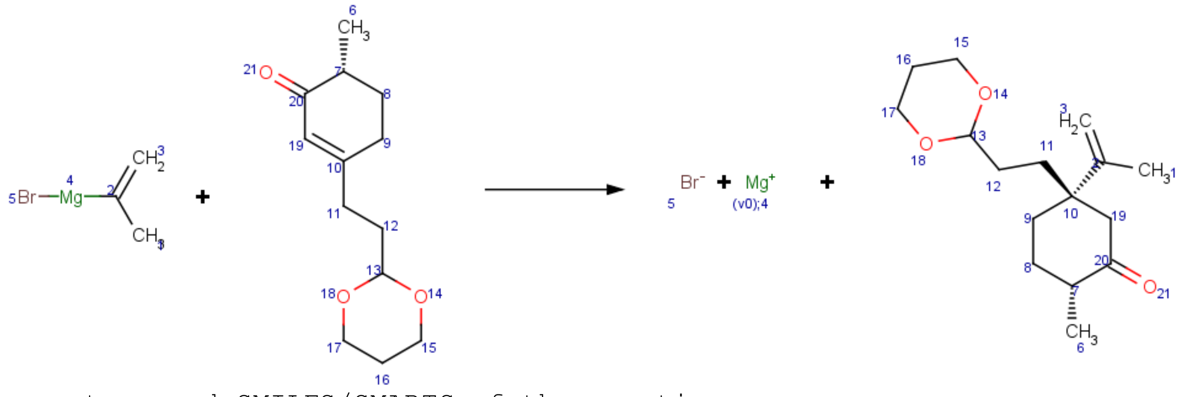

Correct mapped SMILES/SMARTS of the reaction:
[CH3:1][C:2](=[CH2:3])[Mg:4][Br:5].[CH3:6][C@@H:7]1[CH2:8][CH2:9][C:10]([CH2:11][CH2:12][CH:13]2[O:14][CH2:15][CH2:16][CH2:17][O:18]2)=[CH:19][C:20]1=[O:21]>>[Br-:5].[Mg+:4].[CH3:6][C@@H:7]1[CH2:8][CH2:9][C@@:10]([CH2:11][CH2:12][CH:13]2[O:18][CH2:17][CH2:16][CH2:15][O:14]2)([CH2:19][C:20]1=[O:21])[C:2]([CH3:1])=[CH2:3]

Correctness of the mapping

MAPPET YES
ReactionMap YES
Marvin YES
ChemDraw YES
Indigo YES

Reaction no 31

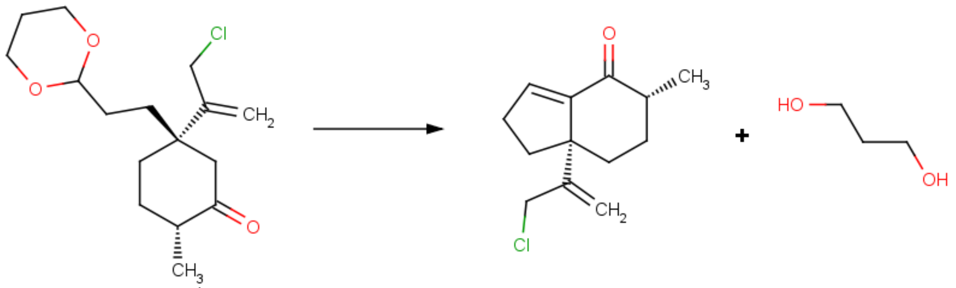

SMILES of the input:
C[C@@H]1CC[C@@](CCC2OCCCO2)(CC1=O)C(=C)CCl>>C[C@@H]1CC[C@]2(CCC=C2C1=O)C(=C)CCl.OCCCO

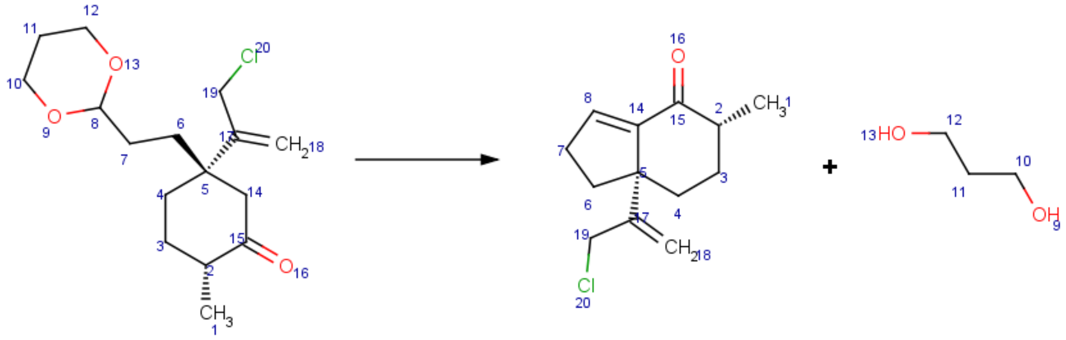

Correct mapped SMILES/SMARTS of the reaction:
[CH3:1][C@@H:2]1[CH2:3][CH2:4][C@@:5]([CH2:6][CH2:7][CH:8]2[O:9][CH2:10][CH2:11][CH2:12][O:13]2)([CH2:14][C:15]1=[O:16])[C:17](=[CH2:18])[CH2:19][Cl:20]>>[CH3:1][C@@H:2]1[CH2:3][CH2:4][C@:5]2([CH2:6][CH2:7][CH:8]=[C:14]2[C:15]1=[O:16])[C:17](=[CH2:18])[CH2:19][Cl:20].[OH:9][CH2:10][CH2:11][CH2:12][OH:13]

Correctness of the mapping

MAPPET	YES
ReactionMap	YES
Marvin	YES
ChemDraw	YES
Indigo	YES

Reaction no 32

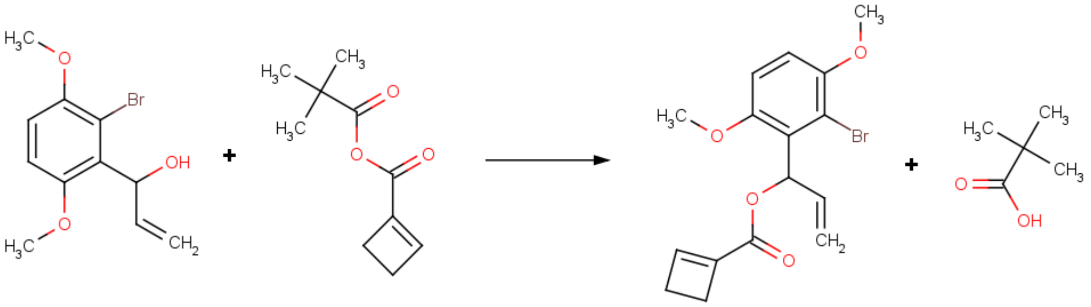

SMILES of the input:
COC1=C(Br)C(C(O)C=C)=C(OC)C=C1.CC(C)(C)C(=O)OC(=O)C1=CCC1>>COC1=C(Br)C(C(OC(=O)C2=CCC2)C=C)=C(OC)C=C1.CC(C)(C)C(O)=O

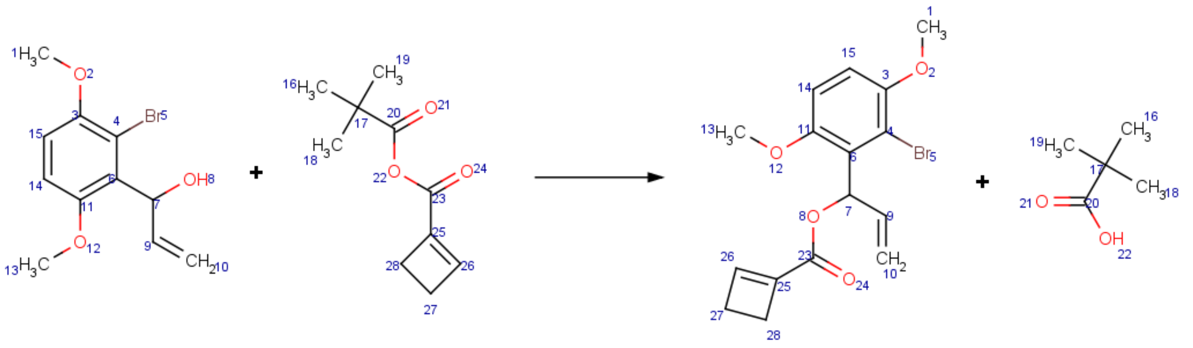

Correct mapped SMILES/SMARTS of the reaction:
[CH3:1][O:2][C:3]1=[C:4]([Br:5])[C:6]([CH:7]([OH:8])[CH:9]=[CH2:10])=[C:1
1]([O:12][CH3:13])[CH:14]=[CH:15]1.[CH3:16][C:17]([CH3:18])([CH3:19])[C:2
0](=[O:21])[O:22][C:23](=[O:24])[C:25]1=[CH:26][CH2:27][CH2:28]1>>[CH3:1]
[O:2][C:3]1=[C:4]([Br:5])[C:6]([CH:7]([O:8][C:23](=[O:24])[C:25]2=[CH:26]
[CH2:27][CH2:28]2)[CH:9]=[CH2:10])=[C:11]([O:12][CH3:13])[CH:14]=[CH:15]1
.[CH3:16][C:17]([CH3:19])([CH3:18])[C:20]([OH:22])=[O:21]

Correctness of the mapping

MAPPET	YES
ReactionMap	YES
Marvin	NO
ChemDraw	YES
Indigo	YES

Reaction no 33

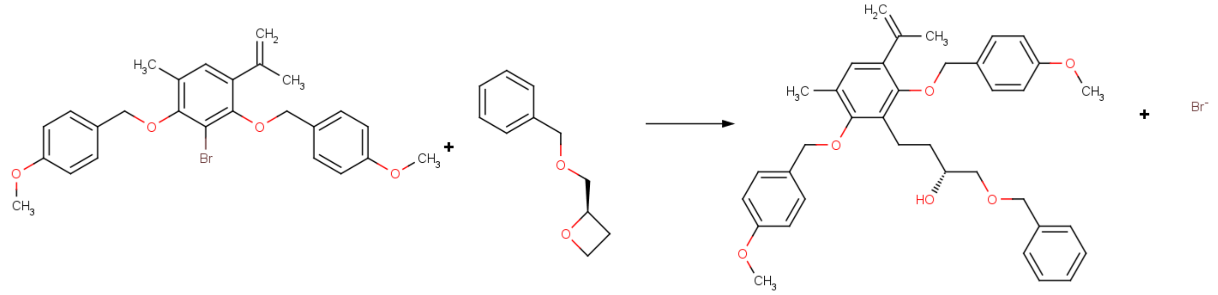

SMILES of the input:
COC1=CC=C(COC2=C(C)C=C(C(C)=C)C(OCC3=CC=C(OC)C=C3)=C2Br)C=C1.C(OCC1=CC=CC
=C1)[C@H]1CCO1>>COC1=CC=C(COC2=C(C)C=C(C(C)=C)C(OCC3=CC=C(OC)C=C3)=C2CC[C
@@H](O)COCC2=CC=CC=C2)C=C1.[Br-]

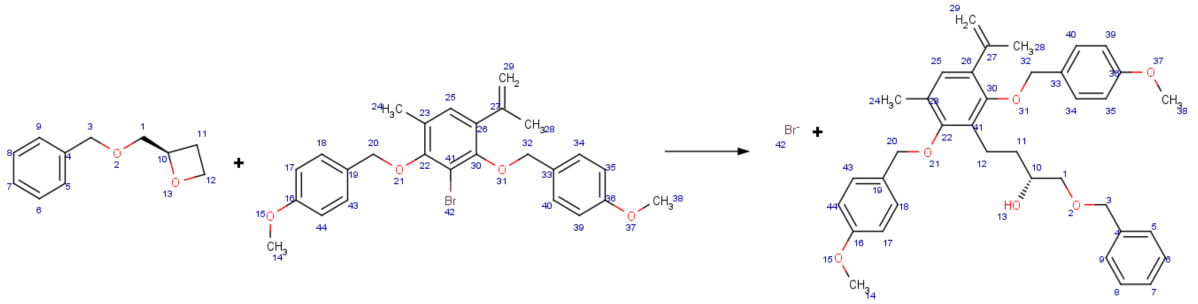

Correct mapped SMILES/SMARTS of the reaction:
[CH2:1]([O:2][CH2:3][C:4]1=[CH:5][CH:6]=[CH:7][CH:8]=[CH:9]1)[C@H:10]1[CH
2:11][CH2:12][O:13]1.[CH3:14][O:15][C:16]1=[CH:17][CH:18]=[C:19]([CH2:20]
[O:21][C:22]2=[C:23]([CH3:24])[CH:25]=[C:26]([C:27]([CH3:28])=[CH2:29])[C
:30]([O:31][CH2:32][C:33]3=[CH:34][CH:35]=[C:36]([O:37][CH3:38])[CH:39]=[
CH:40]3)=[C:41]2[Br:42])[CH:43]=[CH:44]1>>[Br-
:42].[CH3:14][O:15][C:16]1=[CH:44][CH:43]=[C:19]([CH2:20][O:21][C:22]2=[C
:23]([CH3:24])[CH:25]=[C:26]([C:27]([CH3:28])=[CH2:29])[C:30]([O:31][CH2:

32][C:33]3=[CH:40][CH:39]=[C:36]([O:37][CH3:38])[CH:35]=[CH:34]3)=[C:41]2[CH2:12][CH2:11][C@@H:10]([OH:13])[CH2:1][O:2][CH2:3][C:4]2=[CH:5][CH:6]=[CH:7][CH:8]=[CH:9]2)[CH:18]=[CH:17]1

Correctness of the mapping

MAPPET	YES
ReactionMap	YES
Marvin	YES
ChemDraw	YES
Indigo	YES

Reaction no 34

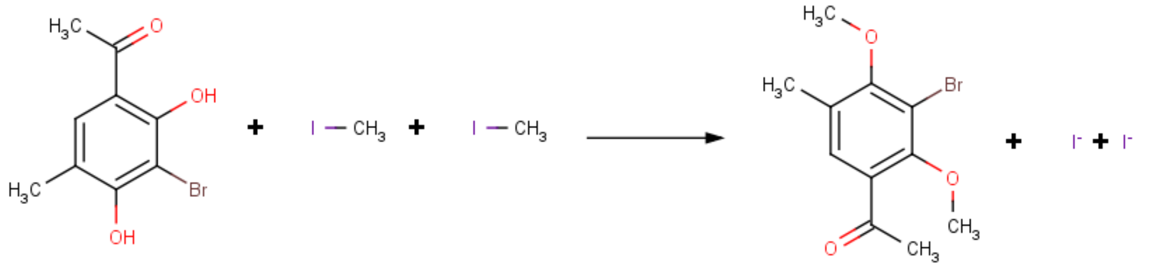

SMILES of the input:
CC(=O)C1=C(O)C(Br)=C(O)C(C)=C1.CI.CI>>COC1=C(Br)C(OC)=C(C=C1C)C(C)=O.[I-].[I-]

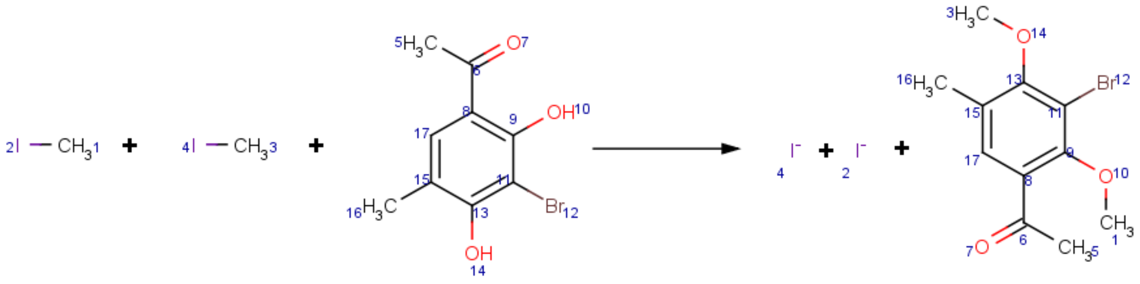

Correct mapped SMILES/SMARTS of the reaction:
[CH3:1][I:2].[CH3:3][I:4].[CH3:5][C:6](=[O:7])[C:8]1=[C:9]([OH:10])[C:11]([Br:12])=[C:13]([OH:14])[C:15]([CH3:16])=[CH:17]1>>[I-:4].[I-:2].[CH3:3][O:14][C:13]1=[C:11]([Br:12])[C:9]([O:10][CH3:1])=[C:8]([CH:17]=[C:15]1[CH3:16])[C:6]([CH3:5])=[O:7]

Correctness of the mapping

MAPPET	YES
ReactionMap	YES
Marvin	YES
ChemDraw	YES
Indigo	YES

Reaction no 35

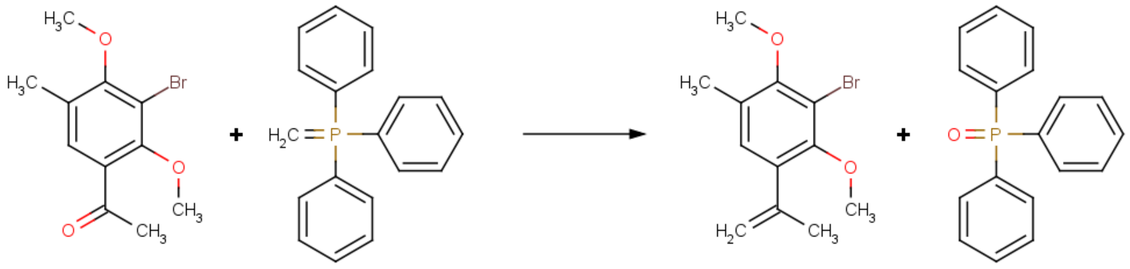

SMILES of the input:
COC1=C(Br)C(OC)=C(C=C1C)C(C)=O.C=P(C1=CC=CC=C1)(C1=CC=CC=C1)C1=CC=CC=C1>>
COC1=C(Br)C(OC)=C(C=C1C)C(C)=C.O=P(C1=CC=CC=C1)(C1=CC=CC=C1)C1=CC=CC=C1

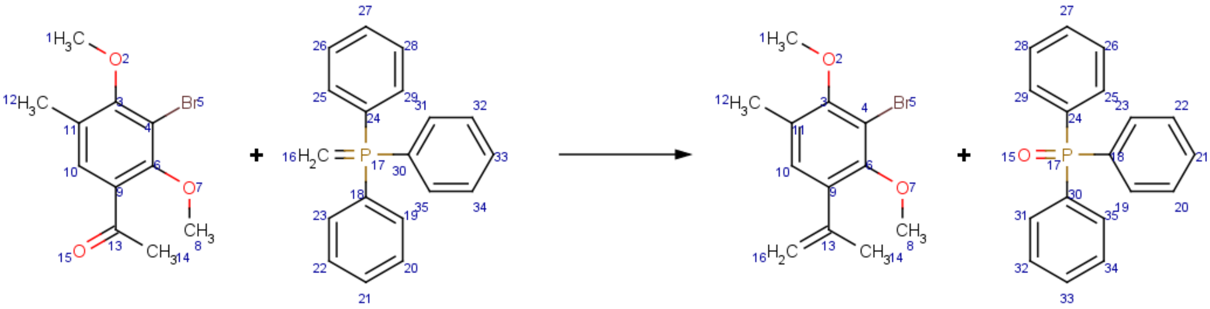

Correct mapped SMILES/SMARTS of the reaction:
[CH3:1][O:2][C:3]1=[C:4]([Br:5])[C:6]([O:7][CH3:8])=[C:9]([CH:10]=[C:11]1[CH3:12])[C:13]([CH3:14])=[O:15].[CH2:16]=[P:17]([C:18]1=[CH:19][CH:20]=[CH:21][CH:22]=[CH:23]1)([C:24]1=[CH:25][CH:26]=[CH:27][CH:28]=[CH:29]1)[C:30]1=[CH:31][CH:32]=[CH:33][CH:34]=[CH:35]1>>[CH3:1][O:2][C:3]1=[C:4]([Br:5])[C:6]([O:7][CH3:8])=[C:9]([CH:10]=[C:11]1[CH3:12])[C:13]([CH3:14])=[CH2:16].[O:15]=[P:17]([C:30]1=[CH:35][CH:34]=[CH:33][CH:32]=[CH:31]1)([C:24]1=[CH:29][CH:28]=[CH:27][CH:26]=[CH:25]1)[C:18]1=[CH:23][CH:22]=[CH:21][CH:20]=[CH:19]1

Correctness of the mapping

MAPPET	YES
ReactionMap	YES
Marvin	YES
ChemDraw	YES
Indigo	NO

Reaction no 36

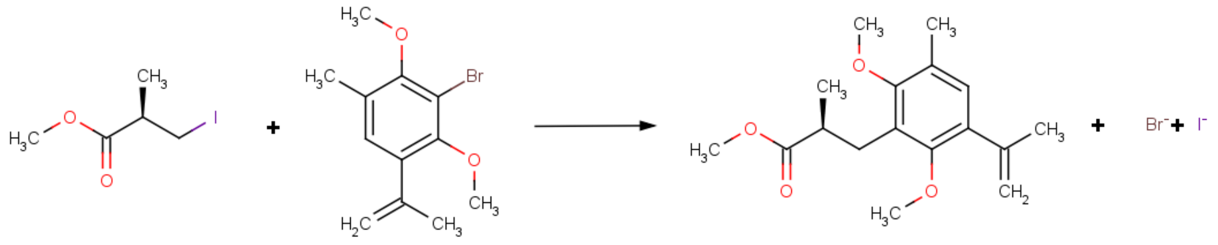

SMILES of the input:
COC(=O)[C@@H](C)CI.COC1=C(Br)C(OC)=C(C=C1C)C(C)=C>>COC(=O)[C@@H](C)CC1=C(OC)C(C)=CC(C(C)=C)=C1OC.[Br-].[I-]

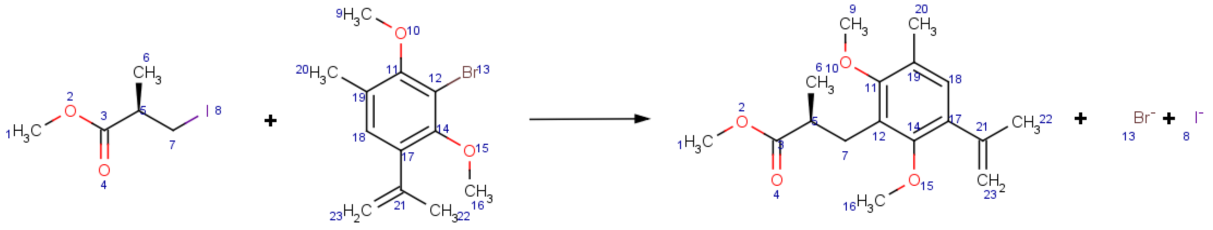

Correct mapped SMILES/SMARTS of the reaction:
[CH3:1][O:2][C:3](=[O:4])[C@@H:5]([CH3:6])[CH2:7][I:8].[CH3:9][O:10][C:11]1=[C:12]([Br:13])[C:14]([O:15][CH3:16])=[C:17]([CH:18]=[C:19]1[CH3:20])[C:21]([CH3:22])=[CH2:23]>>[CH3:1][O:2][C:3](=[O:4])[C@@H:5]([CH3:6])[CH2:7][C:12]1=[C:11]([O:10][CH3:9])[C:19]([CH3:20])=[CH:18][C:17]([C:21]([CH3:22])=[CH2:23])=[C:14]1[O:15][CH3:16].[Br-:13].[I-:8]

Correctness of the mapping

MAPPET	YES
ReactionMap	YES
Marvin	YES
ChemDraw	YES
Indigo	YES

Reaction no 37

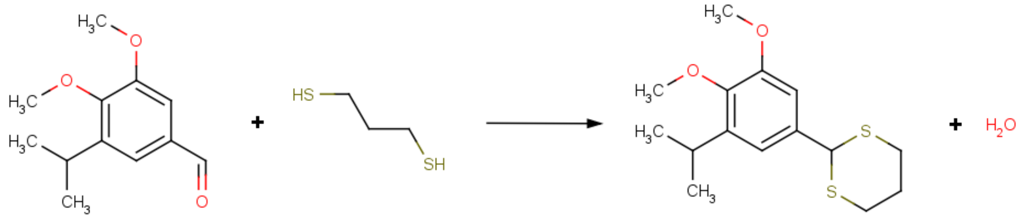

SMILES of the input:
COC1=CC(C=O)=CC(C(C)C)=C1OC.SCCCS>>COC1=CC(=CC(C(C)C)=C1OC)C1SCCCS1.O

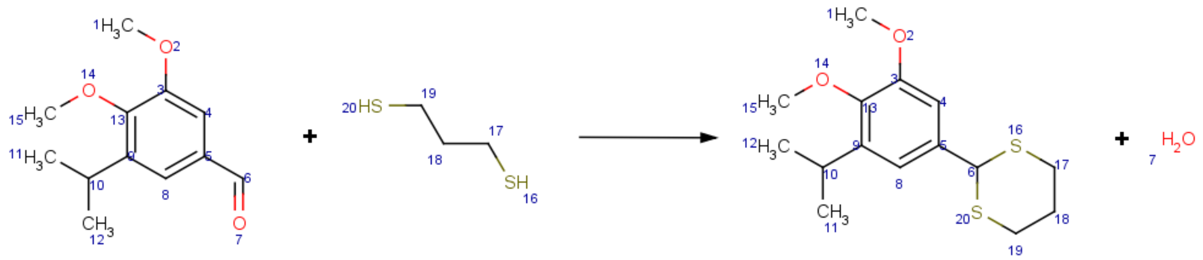

Correct mapped SMILES/SMARTS of the reaction:
[CH3:1][O:2][C:3]1=[CH:4][C:5]([CH:6]=[O:7])=[CH:8][C:9]([CH:10]([CH3:11])[CH3:12])=[C:13]1[O:14][CH3:15].[SH:16][CH2:17][CH2:18][CH2:19][SH:20]>>[CH3:1][O:2][C:3]1=[CH:4][C:5](=[CH:8][C:9]([CH:10]([CH3:12])[CH3:11])=[C:13]1[O:14][CH3:15])[CH:6]1[S:16][CH2:17][CH2:18][CH2:19][S:20]1.[OH2:7]

Correctness of the mapping

MAPPET	YES
ReactionMap	YES
Marvin	YES
ChemDraw	YES
Indigo	NO

Reaction no 38

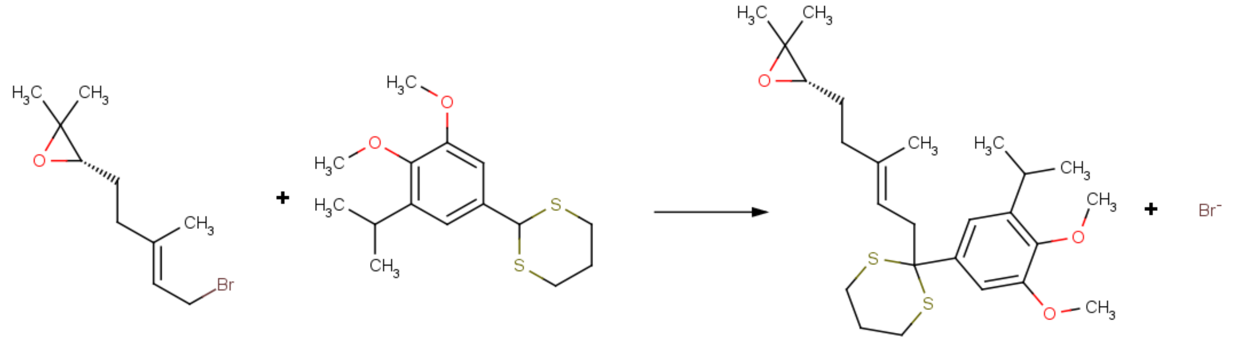

SMILES of the input:
C\C(CC[C@@H]1OC1(C)C)=C/CBr.COC1=CC(=CC(C(C)C)=C1OC)C1SCCCS1>>COC1=CC(=CC
(C(C)C)=C1OC)C1(C\C=C(/C)CC[C@@H]2OC2(C)C)SCCCS1.[Br-]

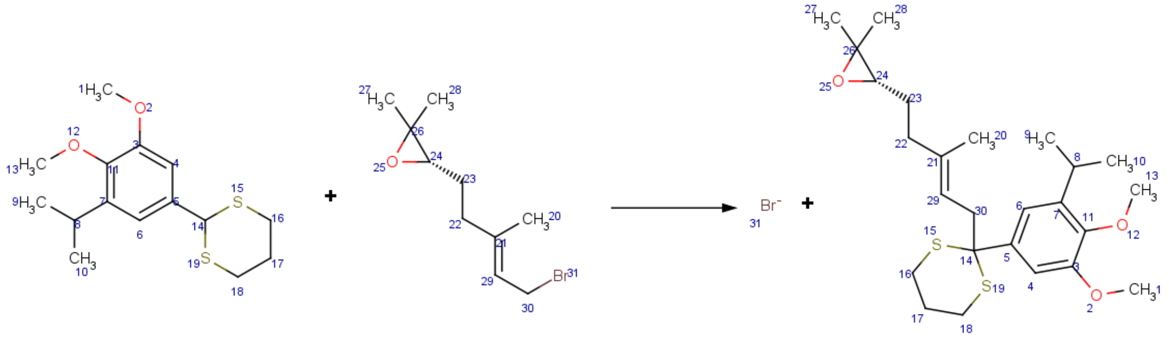

Correct mapped SMILES/SMARTS of the reaction:
[CH3:1][O:2][C:3]1=[CH:4][C:5](=[CH:6][C:7]([CH:8]([CH3:9])[CH3:10])=[C:1
1]1[O:12][CH3:13])[CH:14]1[S:15][CH2:16][CH2:17][CH2:18][S:19]1.[CH3:20]\
[C:21]([CH2:22][CH2:23][C@@H:24]1[O:25][C:26]1([CH3:27])[CH3:28])=[CH:29]
/[CH2:30][Br:31]>>[Br-
:31].[CH3:1][O:2][C:3]1=[CH:4][C:5](=[CH:6][C:7]([CH:8]([CH3:10])[CH3:9])
=[C:11]1[O:12][CH3:13])[C:14]1([CH2:30]\[CH:29]=[C:21](/[CH3:20])[CH2:22]
[CH2:23][C@@H:24]2[O:25][C:26]2([CH3:27])[CH3:28])[S:19][CH2:18][CH2:17][
CH2:16][S:15]1

Correctness of the mapping

MAPPET	YES
ReactionMap	YES
Marvin	YES
ChemDraw	YES
Indigo	YES

Reaction no 39

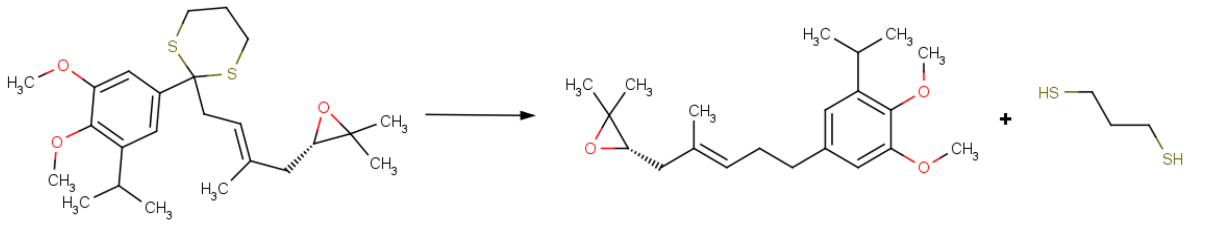

SMILES of the input:
COC1=CC(=CC(C(C)C)=C1OC)C1(C\C=C(/C)C[C@@H]2OC2(C)C)SCCCS1>>COC1=CC(CC\C=
C(/C)C[C@@H]2OC2(C)C)=CC(C(C)C)=C1OC.SCCCS

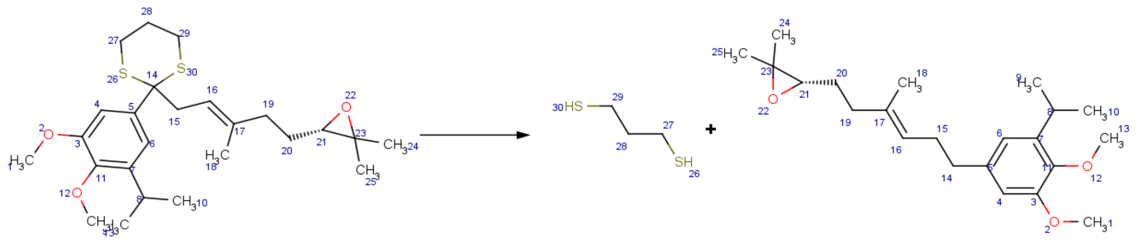

Correct mapped SMILES/SMARTS of the reaction:
[CH3:1][O:2][C:3]1=[CH:4][C:5](=[CH:6][C:7]([CH:8]([CH3:9])[CH3:10])=[C:1
1]1[O:12][CH3:13])[C:14]1([CH2:15]\[CH:16]=[C:17](/[CH3:18])[CH2:19][CH2:
20][C@@H:21]2[O:22][C:23]2([CH3:24])[CH3:25])[S:26][CH2:27][CH2:28][CH2:2
9][S:30]1>>[SH:26][CH2:27][CH2:28][CH2:29][SH:30].[CH3:1][O:2][C:3]1=[CH:
4][C:5]([CH2:14][CH2:15]\[CH:16]=[C:17](/[CH3:18])[CH2:19][CH2:20][C@@H:2
1]2[O:22][C:23]2([CH3:25])[CH3:24])=[CH:6][C:7]([CH:8]([CH3:10])[CH3:9])=
[C:11]1[O:12][CH3:13]

Correctness of the mapping

MAPPET	YES
ReactionMap	YES
Marvin	YES
ChemDraw	YES
Indigo	YES

Reaction no 40

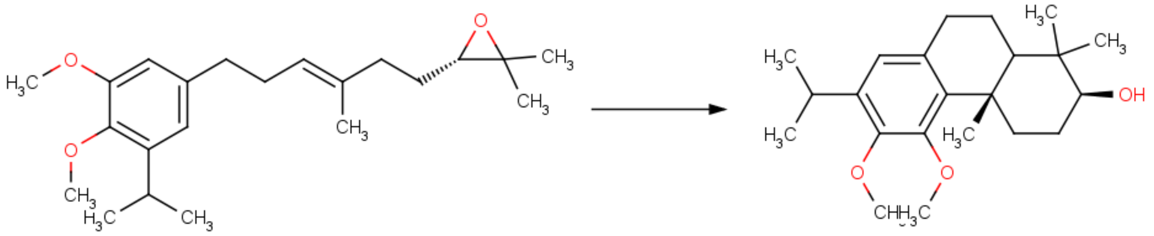

SMILES of the input:
COC1=CC(CC\C=C(/C)CC[C@@H]2OC2(C)C)=CC(C(C)C)=C1OC>>COC1=C(C=C2CCC3C(C)(C
)[C@@H](O)CC[C@]3(C)C2=C1OC)C(C)C

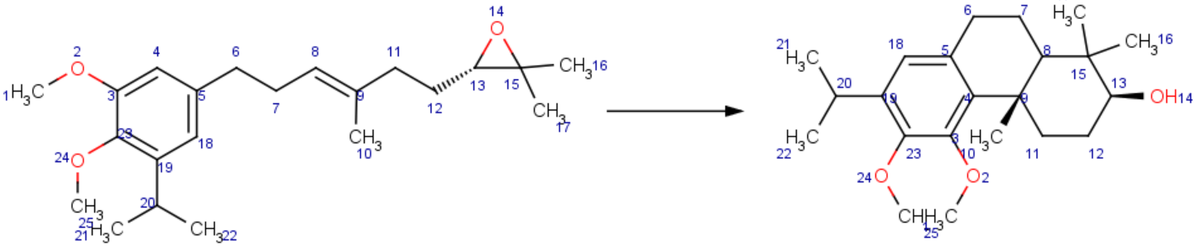

Correct mapped SMILES/SMARTS of the reaction:
[CH3:1][O:2][C:3]1=[CH:4][C:5]([CH2:6][CH2:7]\[CH:8]=[C:9](/[CH3:10])[CH2
:11][CH2:12][C@@H:13]2[O:14][C:15]2([CH3:16])[CH3:17])=[CH:18][C:19]([CH:
20]([CH3:21])[CH3:22])=[C:23]1[O:24][CH3:25]>>[CH3:25][O:24][C:23]1=[C:19
]([CH:18]=[C:5]2[CH2:6][CH2:7][CH:8]3[C:15]([CH3:17])([CH3:16])[C@@H:13](
[OH:14])[CH2:12][CH2:11][C@:9]3([CH3:10])[C:4]2=[C:3]1[O:2][CH3:1])[CH:20
]([CH3:22])[CH3:21]

Correctness of the mapping

MAPPET	YES
ReactionMap	YES
Marvin	YES
ChemDraw	YES
Indigo	NO

Reaction no 41

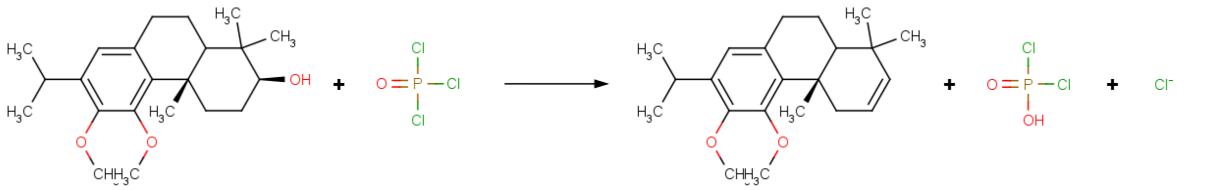

SMILES of the input:
COC1=C(C=C2CCC3C(C)(C)[C@@H](O)CC[C@]3(C)C2=C1OC)C(C)C.ClP(Cl)(Cl)=O>>COC1=C(C=C2CCC3C(C)(C)C=CC[C@]3(C)C2=C1OC)C(C)C.OP(Cl)(Cl)=O.[Cl-]

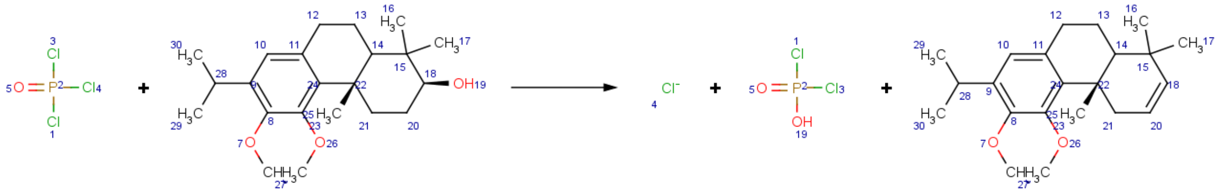

Correct mapped SMILES/SMARTS of the reaction:
[Cl:1][P:2]([Cl:3])([Cl:4])=[O:5].[CH3:6][O:7][C:8]1=[C:9]([CH:10]=[C:11]2[CH2:12][CH2:13][CH:14]3[C:15]([CH3:16])([CH3:17])[C@@H:18]([OH:19])[CH2:20][CH2:21][C@:22]3([CH3:23])[C:24]2=[C:25]1[O:26][CH3:27])[CH:28]([CH3:29])[CH3:30]>>[Cl-:4].[OH:19][P:2]([Cl:1])([Cl:3])=[O:5].[CH3:6][O:7][C:8]1=[C:9]([CH:10]=[C:11]2[CH2:12][CH2:13][CH:14]3[C:15]([CH3:16])([CH3:17])[CH:18]=[CH:20][CH2:21][C@:22]3([CH3:23])[C:24]2=[C:25]1[O:26][CH3:27])[CH:28]([CH3:30])[CH3:29]

Correctness of the mapping

MAPPET	YES
ReactionMap	YES
Marvin	YES
ChemDraw	YES
Indigo	NO

Reaction no 42

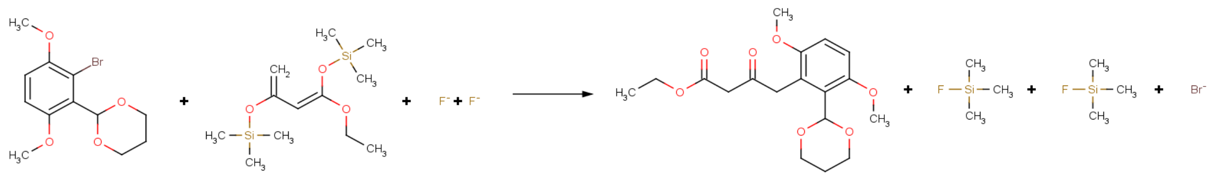

SMILES of the input:
COC1=C(Br)C(C2OCCCO2)=C(OC)C=C1.CCO\C(O[Si](C)(C)C)=C\C(=C)O[Si](C)(C)C.[F-].[F-]>>CCOC(=O)CC(=O)CC1=C(OC)C=CC(OC)=C1C1OCCCO1.C[Si](C)(C)F.C[Si](C)(C)F.[Br-]

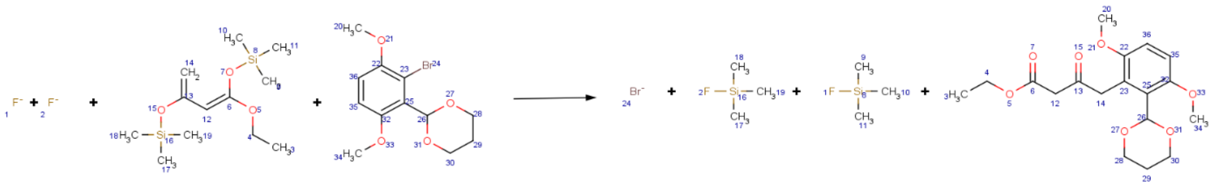

Correct mapped SMILES/SMARTS of the reaction:
[F-:1].[F-
:2].[CH3:3][CH2:4][O:5]\[C:6]([O:7][Si:8]([CH3:9])([CH3:10])[CH3:11])=[CH
:12]\[C:13](=[CH2:14])[O:15][Si:16]([CH3:17])([CH3:18])[CH3:19].[CH3:20][
O:21][C:22]1=[C:23]([Br:24])[C:25]([CH:26]2[O:27][CH2:28][CH2:29][CH2:30]
[O:31]2)=[C:32]([O:33][CH3:34])[CH:35]=[CH:36]1>>[Br-
:24].[CH3:17][Si:16]([CH3:18])([CH3:19])[F:2].[CH3:11][Si:8]([CH3:9])([CH
3:10])[F:1].[CH3:3][CH2:4][O:5][C:6](=[O:7])[CH2:12][C:13](=[O:15])[CH2:1
4][C:23]1=[C:22]([O:21][CH3:20])[CH:36]=[CH:35][C:32]([O:33][CH3:34])=[C:
25]1[CH:26]1[O:31][CH2:30][CH2:29][CH2:28][O:27]1

Correctness of the mapping

MAPPET	YES
ReactionMap	YES
Marvin	NO
ChemDraw	YES
Indigo	NO

Reaction no 43

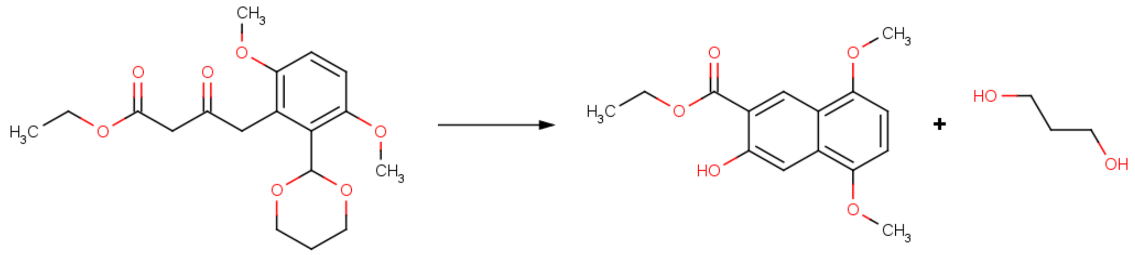

SMILES of the input:
CCOC(=O)CC(=O)CC1=C(OC)C=CC(OC)=C1C1OCCCO1>>CCOC(=O)C1=CC2=C(OC)C=CC(OC)=
C2C=C1O.OCCCO

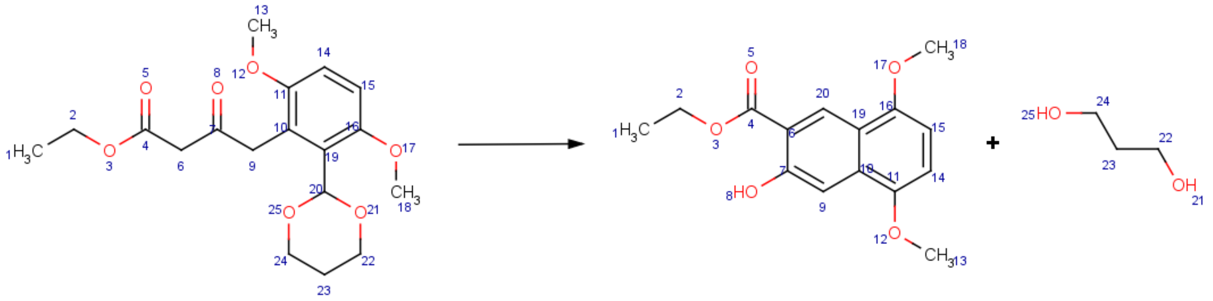

Correct mapped SMILES/SMARTS of the reaction:
[CH3:1][CH2:2][O:3][C:4](=[O:5])[CH2:6][C:7](=[O:8])[CH2:9][C:10]1=[C:11]
([O:12][CH3:13])[CH:14]=[CH:15][C:16]([O:17][CH3:18])=[C:19]1[CH:20]1[O:2
1][CH2:22][CH2:23][CH2:24][O:25]1>>[CH3:1][CH2:2][O:3][C:4](=[O:5])[C:6]1
=[CH:20][C:19]2=[C:16]([O:17][CH3:18])[CH:15]=[CH:14][C:11]([O:12][CH3:13
])=[C:10]2[CH:9]=[C:7]1[OH:8].[OH:21][CH2:22][CH2:23][CH2:24][OH:25]

Correctness of the mapping

MAPPET	YES
ReactionMap	YES

Marvin NO
ChemDraw YES
Indigo NO

Reaction no 44

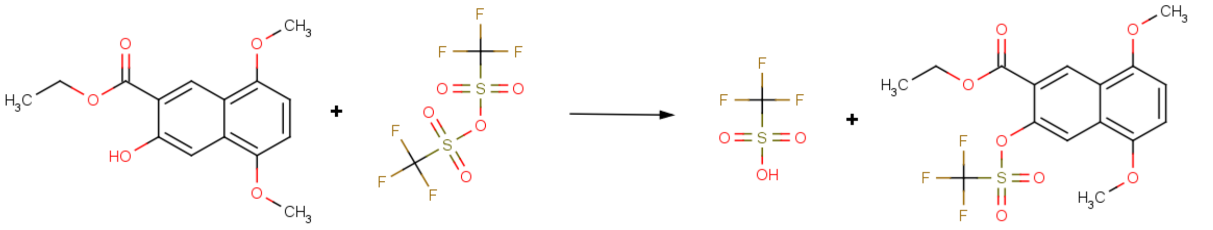

SMILES of the input:
CCOC(=O)C1=CC2=C(OC)C=CC(OC)=C2C=C1O.FC(F)(F)S(=O)(=O)OS(=O)(=O)C(F)(F)F>>OS(=O)(=O)C(F)(F)F.CCOC(=O)C1=CC2=C(OC)C=CC(OC)=C2C=C1OS(=O)(=O)C(F)(F)F

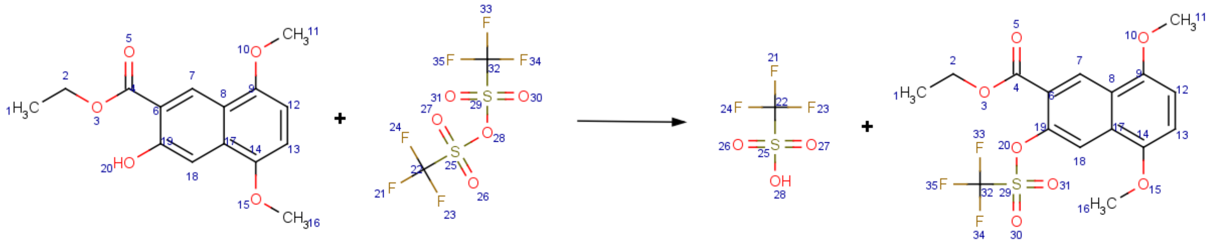

Correct mapped SMILES/SMARTS of the reaction:
[CH3:1][CH2:2][O:3][C:4](=[O:5])[C:6]1=[CH:7][C:8]2=[C:9]([O:10][CH3:11])[CH:12]=[CH:13][C:14]([O:15][CH3:16])=[C:17]2[CH:18]=[C:19]1[OH:20].[F:21][C:22]([F:23])([F:24])[S:25](=[O:26])(=[O:27])[O:28][S:29](=[O:30])(=[O:31])[C:32]([F:33])([F:34])[F:35]>>[OH:28][S:25](=[O:27])(=[O:26])[C:22]([F:21])([F:23])[F:24].[CH3:1][CH2:2][O:3][C:4](=[O:5])[C:6]1=[CH:7][C:8]2=[C:9]([O:10][CH3:11])[CH:12]=[CH:13][C:14]([O:15][CH3:16])=[C:17]2[CH:18]=[C:19]1[O:20][S:29](=[O:31])(=[O:30])[C:32]([F:33])([F:34])[F:35]

Correctness of the mapping
MAPPET YES
ReactionMap YES
Marvin YES
ChemDraw YES
Indigo YES

Reaction no 45

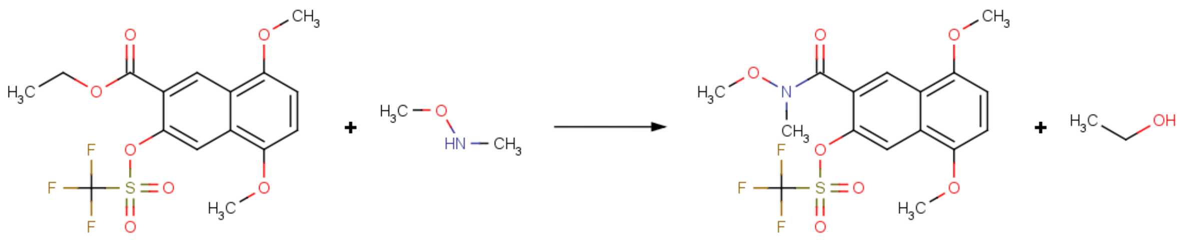

SMILES of the input:
CCOC(=O)C1=CC2=C(OC)C=CC(OC)=C2C=C1OS(=O)(=O)C(F)(F)F.CNOC>>CON(C)C(=O)C1=CC2=C(OC)C=CC(OC)=C2C=C1OS(=O)(=O)C(F)(F)F.CCO

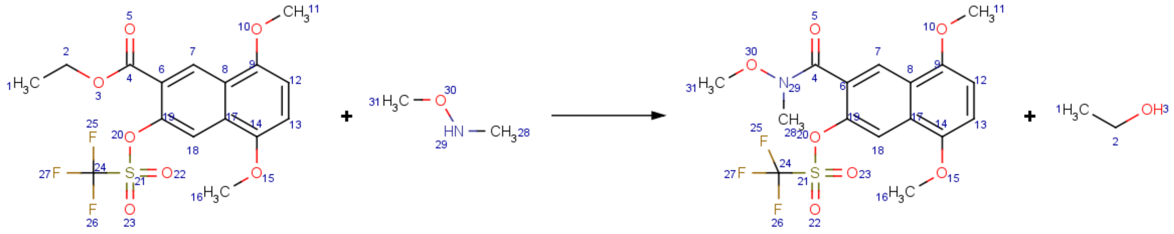

Correct mapped SMILES/SMARTS of the reaction:
[CH3:1][CH2:2][O:3][C:4](=[O:5])[C:6]1=[CH:7][C:8]2=[C:9]([O:10][CH3:11])
[CH:12]=[CH:13][C:14]([O:15][CH3:16])=[C:17]2[CH:18]=[C:19]1[O:20][S:21](
=[O:22])(=[O:23])[C:24]([F:25])([F:26])[F:27].[CH3:28][NH:29][O:30][CH3:3
1]>>[CH3:31][O:30][N:29]([CH3:28])[C:4](=[O:5])[C:6]1=[CH:7][C:8]2=[C:9](
[O:10][CH3:11])[CH:12]=[CH:13][C:14]([O:15][CH3:16])=[C:17]2[CH:18]=[C:19
]1[O:20][S:21](=[O:23])(=[O:22])[C:24]([F:25])([F:26])[F:27].[CH3:1][CH2:
2][OH:3]

Correctness of the mapping

MAPPET	YES
ReactionMap	YES
Marvin	YES
ChemDraw	YES
Indigo	YES

Reaction no 46

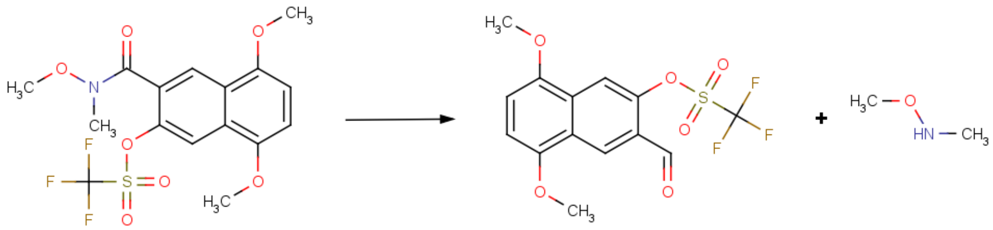

SMILES of the input:
CON(C)C(=O)C1=CC2=C(OC)C=CC(OC)=C2C=C1OS(=O)(=O)C(F)(F)F>>COC1=C2C=C(OS(=
O)(=O)C(F)(F)F)C(C=O)=CC2=C(OC)C=C1.CNOC

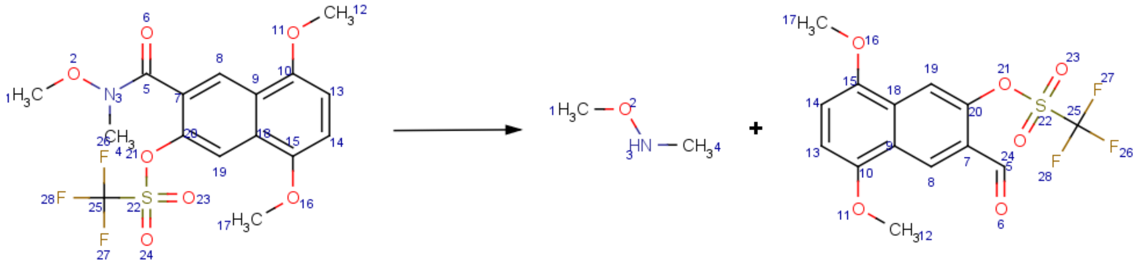

Correct mapped SMILES/SMARTS of the reaction:
[CH3:1][O:2][N:3]([CH3:4])[C:5](=[O:6])[C:7]1=[CH:8][C:9]2=[C:10]([O:11][
CH3:12])[CH:13]=[CH:14][C:15]([O:16][CH3:17])=[C:18]2[CH:19]=[C:20]1[O:21
][S:22](=[O:23])(=[O:24])[C:25]([F:26])([F:27])[F:28]>>[CH3:4][NH:3][O:2]
[CH3:1].[CH3:17][O:16][C:15]1=[C:18]2[CH:19]=[C:20]([O:21][S:22](=[O:23])
(=[O:24])[C:25]([F:26])([F:27])[F:28])[C:7]([CH:5]=[O:6])=[CH:8][C:9]2=[C
:10]([O:11][CH3:12])[CH:13]=[CH:14]1

Correctness of the mapping

MAPPET	YES
ReactionMap	YES

Marvin YES
ChemDraw YES
Indigo YES

Reaction no 47

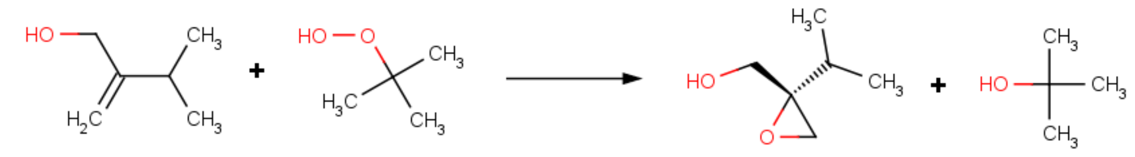

SMILES of the input:
CC(C)C(=C)CO.CC(C)(C)OO>>CC(C)[C@]1(CO)CO1.CC(C)(C)O

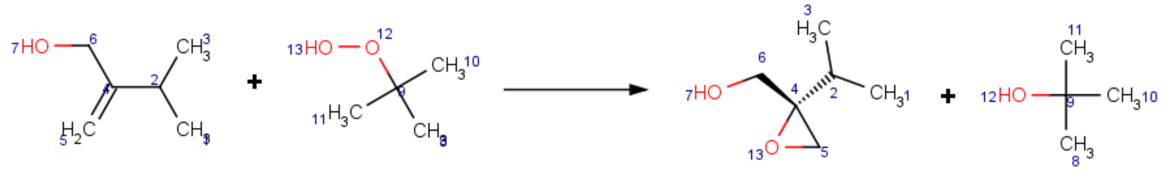

Correct mapped SMILES/SMARTS of the reaction:
[CH3:1][CH:2]([CH3:3])[C:4](=[CH2:5])[CH2:6][OH:7].[CH3:8][C:9]([CH3:10])([CH3:11])[O:12][OH:13]>>[CH3:3][CH:2]([CH3:1])[C@:4]1([CH2:6][OH:7])[CH2:5][O:13]1.[CH3:8][C:9]([CH3:11])([CH3:10])[OH:12]

Correctness of the mapping
MAPPET YES
ReactionMap YES
Marvin YES
ChemDraw YES
Indigo NO

Reaction no 48

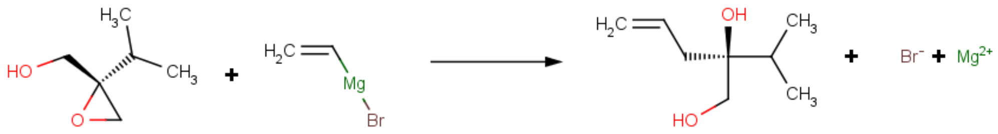

SMILES of the input:
CC(C)[C@]1(CO)CO1.Br[Mg]C=C>>CC(C)[C@](O)(CO)CC=C.[Br-].[Mg++]

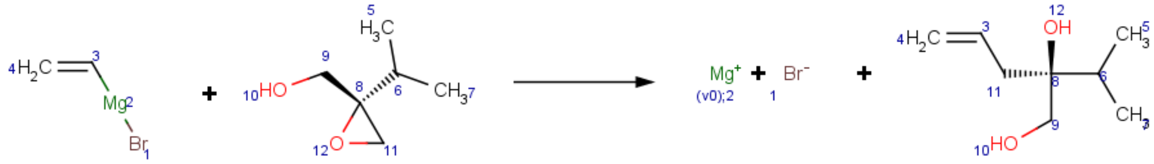

Correct mapped SMILES/SMARTS of the reaction:
[Br:1][Mg:2][CH:3]=[CH2:4].[CH3:5][CH:6]([CH3:7])[C@:8]1([CH2:9][OH:10])[CH2:11][O:12]1>>[Mg+:2].[Br-:1].[CH3:7][CH:6]([CH3:5])[C@:8]([OH:12])([CH2:9][OH:10])[CH2:11][CH:3]=[CH2:4]

Correctness of the mapping
MAPPET YES
ReactionMap YES
Marvin YES
ChemDraw YES
Indigo YES

Reaction no 49

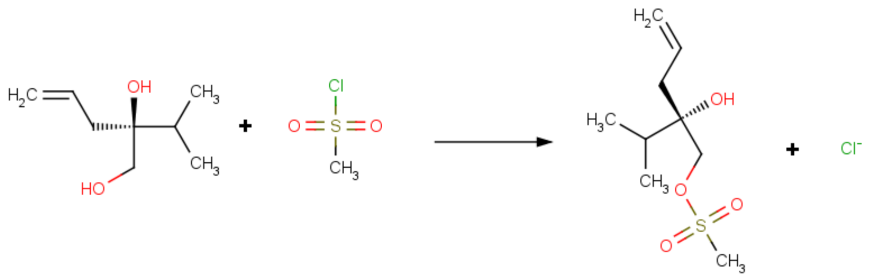

SMILES of the input:
CC(C)[C@](O)(CO)CC=C.CS(Cl)(=O)=O>>CC(C)[C@](O)(COS(C)(=O)=O)CC=C.[Cl-]

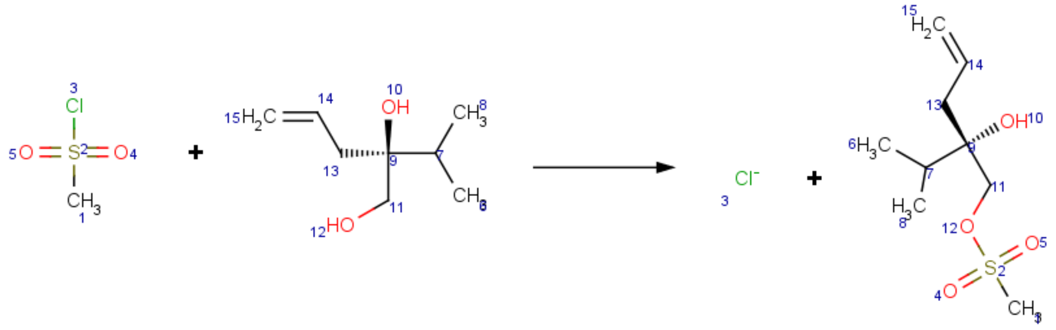

Correct mapped SMILES/SMARTS of the reaction:
[CH3:1][S:2]([Cl:3])(=[O:4])=[O:5].[CH3:6][CH:7]([CH3:8])[C@:9]([OH:10])([CH2:11][OH:12])[CH2:13][CH:14]=[CH2:15]>>[Cl-:3].[CH3:8][CH:7]([CH3:6])[C@:9]([OH:10])([CH2:11][O:12][S:2]([CH3:1])(=[O:5])=[O:4])[CH2:13][CH:14]=[CH2:15]

Correctness of the mapping
MAPPET YES
ReactionMap YES
Marvin YES
ChemDraw YES
Indigo YES

Reaction no 50

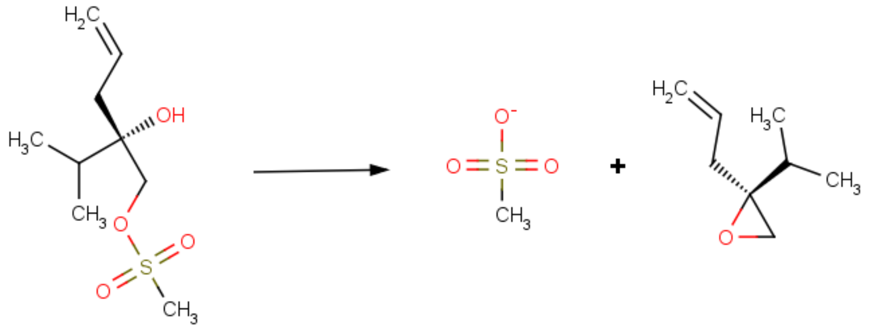

SMILES of the input:
CC(C)[C@](O)(COS(C)(=O)=O)CC=C>>CS([O-])(=O)=O.CC(C)[C@@]1(CC=C)CO1

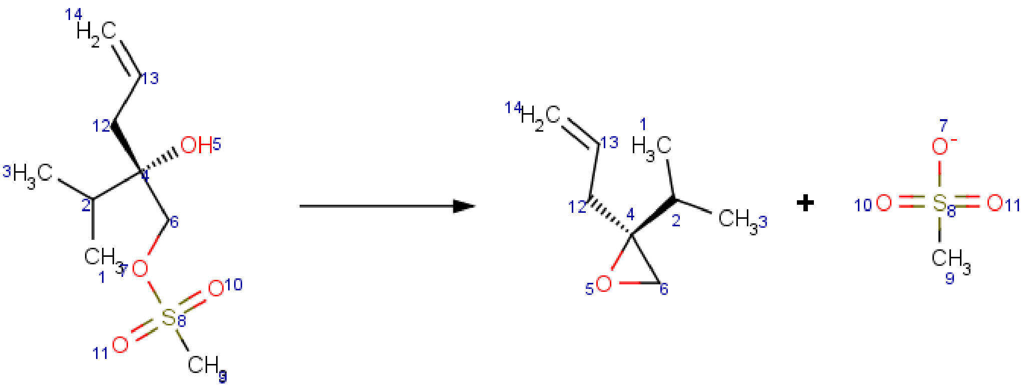

Correct mapped SMILES/SMARTS of the reaction:
[CH3:1][CH:2]([CH3:3])[C@:4]([OH:5])([CH2:6][O:7][S:8]([CH3:9])(=[O:10])=[O:11])[CH2:12][CH:13]=[CH2:14]>>[CH3:3][CH:2]([CH3:1])[C@@:4]1([CH2:12][CH:13]=[CH2:14])[CH2:6][O:5]1.[CH3:9][S:8]([O-:7])(=[O:11])=[O:10]

Correctness of the mapping

MAPPET	YES
ReactionMap	NO
Marvin	YES
ChemDraw	YES
Indigo	NO

Reaction no 51

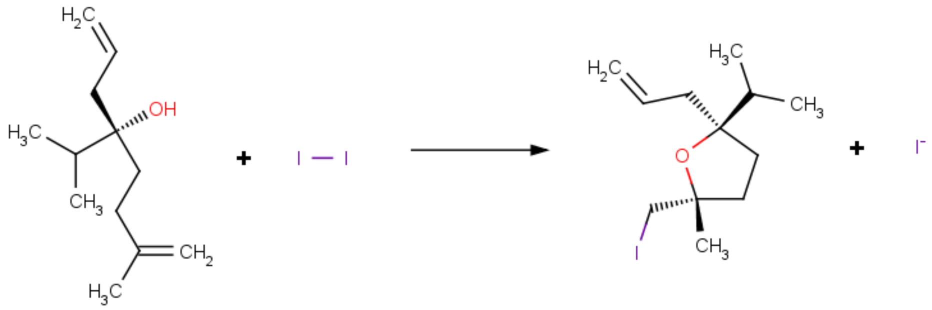

SMILES of the input:
CC(C)[C@](O)(CCC(C)=C)CC=C.II>>CC(C)[C@@]1(CC=C)CC[C@](C)(CI)O1.[I-]

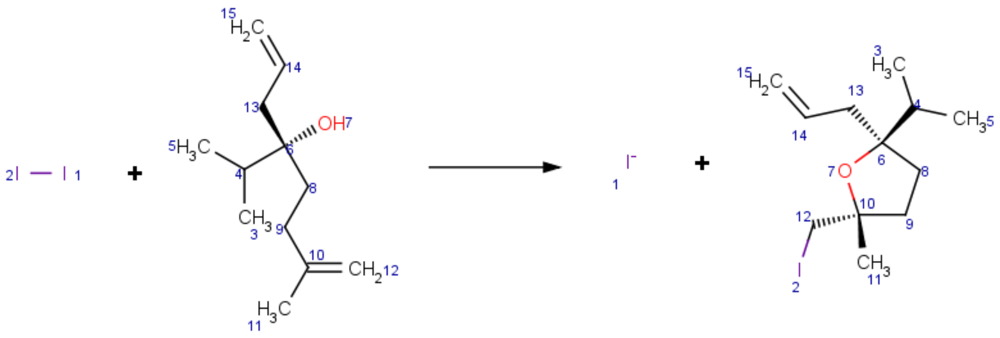

Correct mapped SMILES/SMARTS of the reaction:
[I:1][I:2].[CH3:3][CH:4]([CH3:5])[C@:6]([OH:7])([CH2:8][CH2:9][C:10]([CH3:11])=[CH2:12])[CH2:13][CH:14]=[CH2:15]>>[I-:1].[CH3:5][CH:4]([CH3:3])[C@@:6]1([CH2:13][CH:14]=[CH2:15])[CH2:8][CH2:9][C@:10]([CH3:11])([CH2:12][I:2])[O:7]1

Correctness of the mapping

MAPPET	YES
ReactionMap	YES
Marvin	NO
ChemDraw	YES
Indigo	YES

Reaction no 52

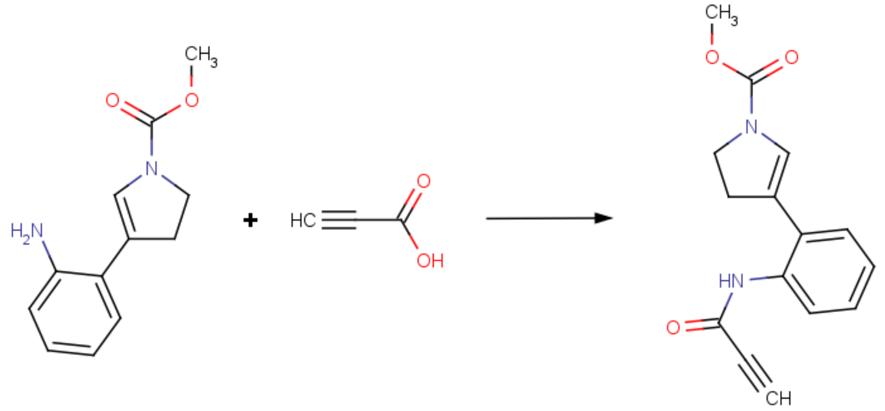

SMILES of the input:
COC(=O)N1CCC(=C1)C1=CC=CC=C1N.OC(=O)C#C>>COC(=O)N1CCC(=C1)C1=CC=CC=C1NC(=O)C#C

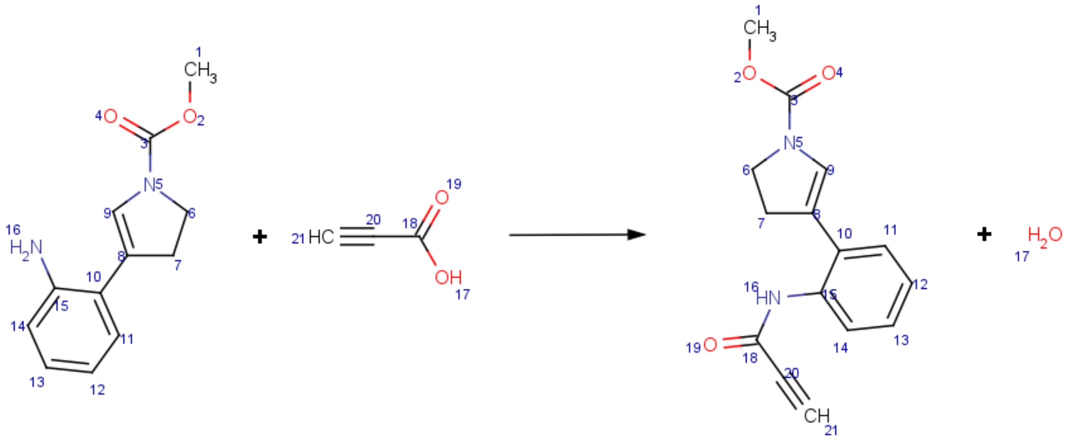

Correct mapped SMILES/SMARTS of the reaction:
[CH3:1][O:2][C:3](=[O:4])[N:5]1[CH2:6][CH2:7][C:8](=[CH:9]1)[C:10]1=[CH:11][CH:12]=[CH:13][CH:14]=[C:15]1[NH2:16].[OH:17][C:18](=[O:19])[C:20]#[CH:21]>>[CH3:1][O:2][C:3](=[O:4])[N:5]1[CH2:6][CH2:7][C:8](=[CH:9]1)[C:10]1=[CH:11][CH:12]=[CH:13][CH:14]=[C:15]1[NH:16][C:18](=[O:19])[C:20]#[CH:21].[OH2:17]

Correctness of the mapping

MAPPET	YES
ReactionMap	YES
Marvin	YES
ChemDraw	YES
Indigo	YES

Reaction no 53

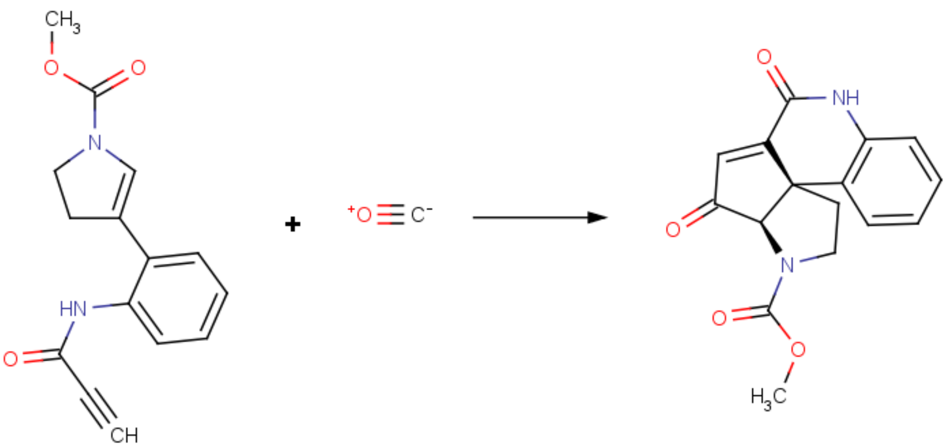

SMILES of the input:
COC(=O)N1CCC(=C1)C1=CC=CC=C1NC(=O)C#C.[C-
]#[O+]>>COC(=O)N1CC[C@@]23[C@@H]1C(=O)C=C2C(=O)NC1=C3C=CC=C1

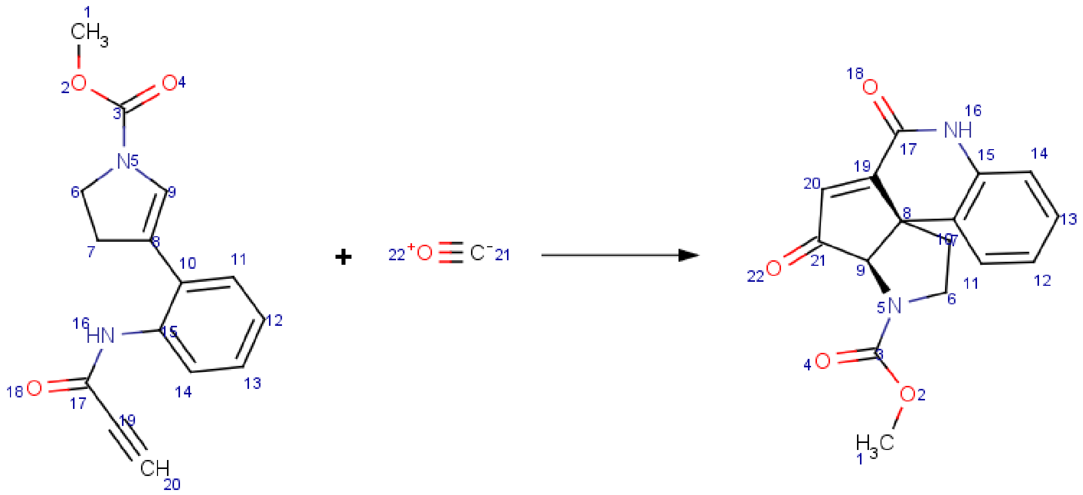

Correct mapped SMILES/SMARTS of the reaction:
[CH3:1][O:2][C:3](=[O:4])[N:5]1[CH2:6][CH2:7][C:8](=[CH:9]1)[C:10]1=[CH:1
1][CH:12]=[CH:13][CH:14]=[C:15]1[NH:16][C:17](=[O:18])[C:19]#[CH:20].[C-
:21]#[O+:22]>>[CH3:1][O:2][C:3](=[O:4])[N:5]1[CH2:6][CH2:7][C@@:8]23[C@@H
:9]1[C:21](=[O:22])[CH:20]=[C:19]2[C:17](=[O:18])[NH:16][C:15]1=[C:10]3[C
H:11]=[CH:12][CH:13]=[CH:14]1

Correctness of the mapping

MAPPET	YES
ReactionMap	YES
Marvin	YES
ChemDraw	YES
Indigo	NO

Reaction no 54

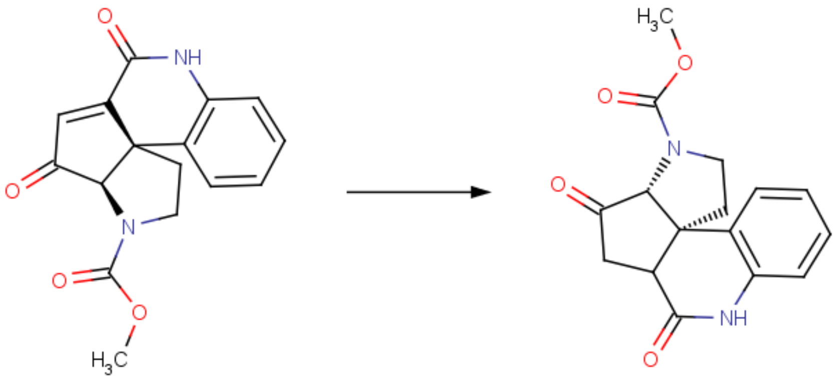

SMILES of the input:
COC(=O)N1CC[C@@]23[C@@H]1C(=O)C=C2C(=O)NC1=C3C=CC=C1>>COC(=O)N1CC[C@]23C(CC(=O)[C@H]12)C(=O)NC1=C3C=CC=C1

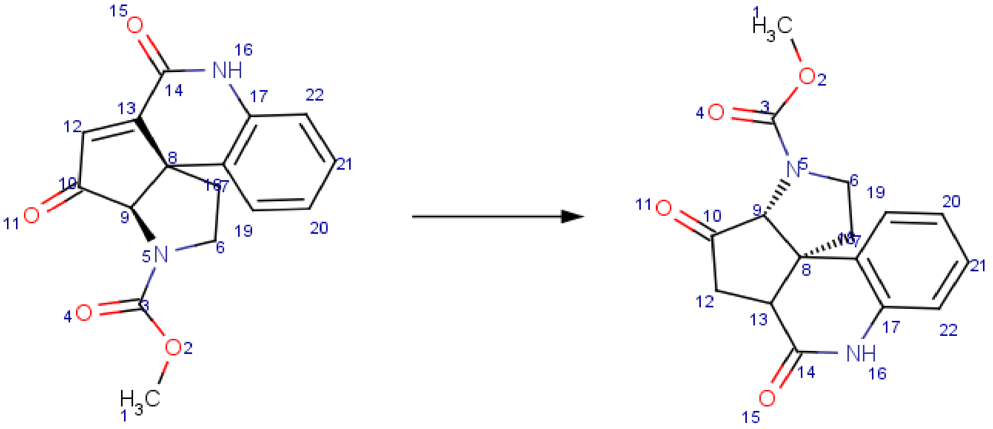

Correct mapped SMILES/SMARTS of the reaction:
[CH3:1][O:2][C:3](=[O:4])[N:5]1[CH2:6][CH2:7][C@@:8]23[C@@H:9]1[C:10](=[O:11])[CH:12]=[C:13]2[C:14](=[O:15])[NH:16][C:17]1=[C:18]3[CH:19]=[CH:20][CH:21]=[CH:22]1>>[CH3:1][O:2][C:3](=[O:4])[N:5]1[CH2:6][CH2:7][C@:8]23[CH:13]([CH2:12][C:10](=[O:11])[C@H:9]12)[C:14](=[O:15])[NH:16][C:17]1=[C:18]3[CH:19]=[CH:20][CH:21]=[CH:22]1

Correctness of the mapping

MAPPET	YES
ReactionMap	YES
Marvin	YES
ChemDraw	YES
Indigo	YES

Reaction no 55

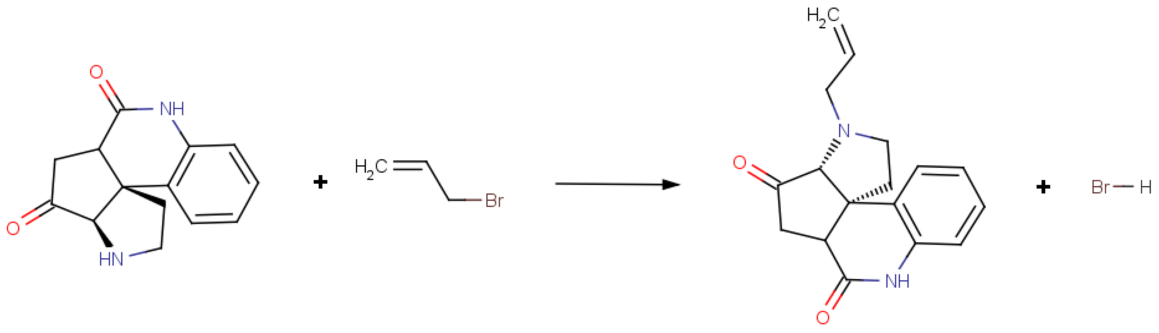

SMILES of the input:
O=C1CC2C(=O)NC3=C(C=CC=C3)[C@@]22CCN[C@@H]12.BrCC=C>>C=CCN1CC[C@]23C(CC(=
O)[C@H]12)C(=O)NC1=C3C=CC=C1.Br[H]

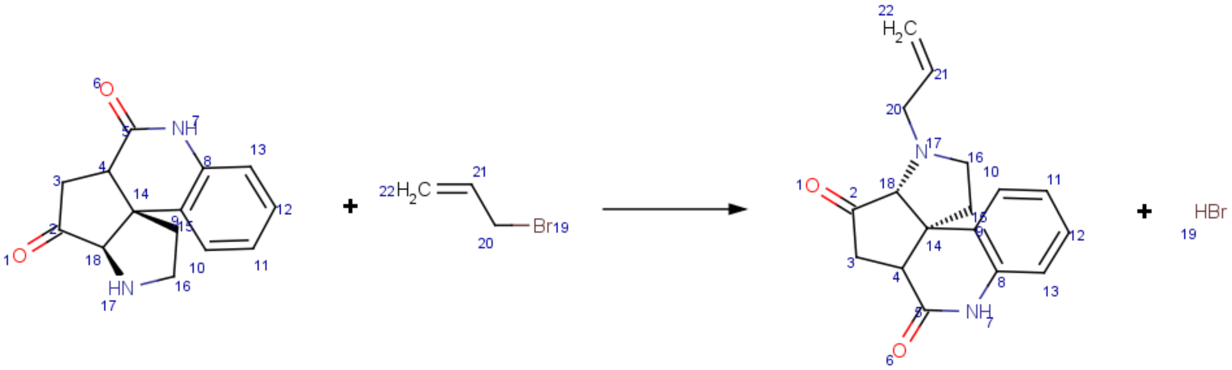

Correct mapped SMILES/SMARTS of the reaction:
[O:1]=[C:2]1[CH2:3][CH:4]2[C:5](=[O:6])[NH:7][C:8]3=[C:9]([CH:10]=[CH:11]
[CH:12]=[CH:13]3)[C@@:14]22[CH2:15][CH2:16][NH:17][C@@H:18]12.[Br:19][CH2
:20][CH:21]=[CH2:22]>>[CH2:22]=[CH:21][CH2:20][N:17]1[CH2:16][CH2:15][C@:
14]23[CH:4]([CH2:3][C:2](=[O:1])[C@H:18]12)[C:5](=[O:6])[NH:7][C:8]1=[C:9
]3[CH:10]=[CH:11][CH:12]=[CH:13]1.[BrH:19]

Correctness of the mapping
MAPPET YES
ReactionMap YES
Marvin YES
ChemDraw YES
Indigo YES

Reaction no 56

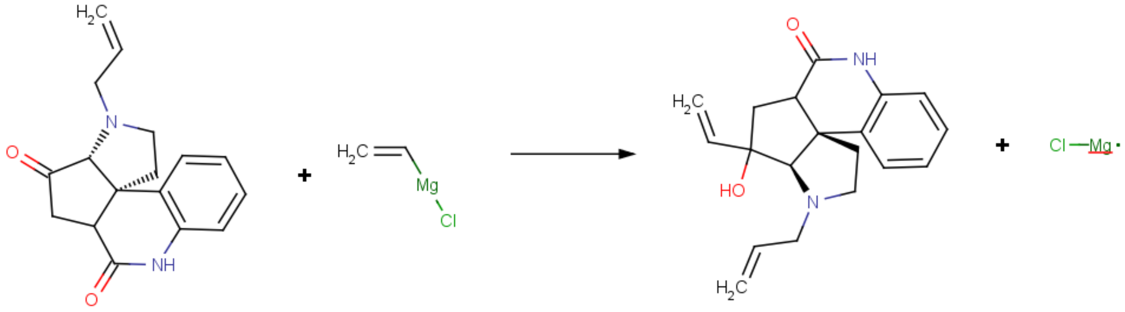

SMILES of the input:
C=CCN1CC[C@]23C(CC(=O)[C@H]12)C(=O)NC1=C3C=CC=C1.Cl[Mg]C=C>>OC1(CC2C(=O)N
C3=C(C=CC=C3)[C@@]22CCN(CC=C)[C@@H]12)C=C.[Mg]Cl

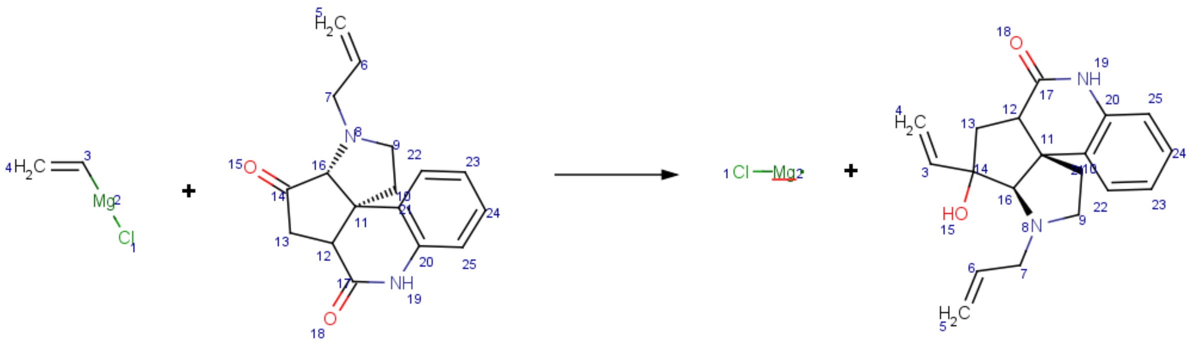

Correct mapped SMILES/SMARTS of the reaction:
[Cl:1][Mg:2][CH:3]=[CH2:4].[CH2:5]=[CH:6][CH2:7][N:8]1[CH2:9][CH2:10][C@:11]23[CH:12]([CH2:13][C:14](=[O:15])[C@H:16]12)[C:17](=[O:18])[NH:19][C:20]1=[C:21]3[CH:22]=[CH:23][CH:24]=[CH:25]1>>[Mg:2][Cl:1].[OH:15][C:14]1([CH2:13][CH:12]2[C:17](=[O:18])[NH:19][C:20]3=[C:21]([CH:22]=[CH:23][CH:24]=[CH:25]3)[C@@:11]22[CH2:10][CH2:9][N:8]([CH2:7][CH:6]=[CH2:5])[C@@H:16]12)[CH:3]=[CH2:4]

Correctness of the mapping

MAPPET	YES
ReactionMap	YES
Marvin	YES
ChemDraw	YES
Indigo	YES

Reaction no 57

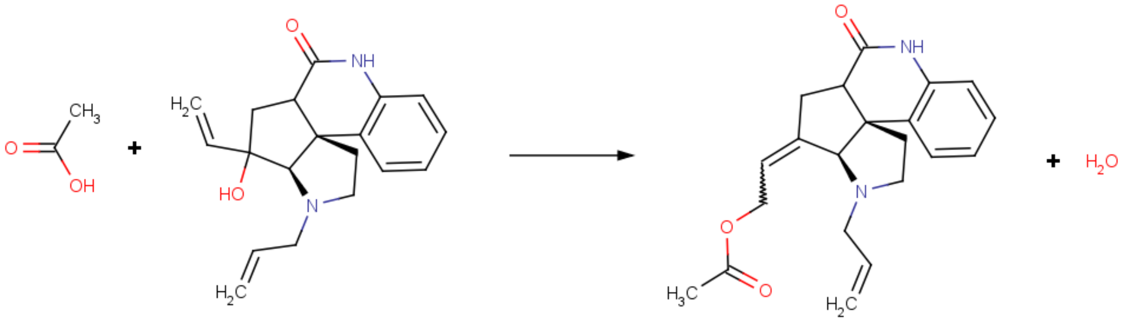

SMILES of the input:
CC(O)=O.OC1(CC2C(=O)NC3=C(C=CC=C3)[C@@]22CCN(CC=C)[C@@H]12)C=C>>CC(=O)OCC=C1CC2C(=O)NC3=C(C=CC=C3)[C@@]22CCN(CC=C)[C@@H]12.O

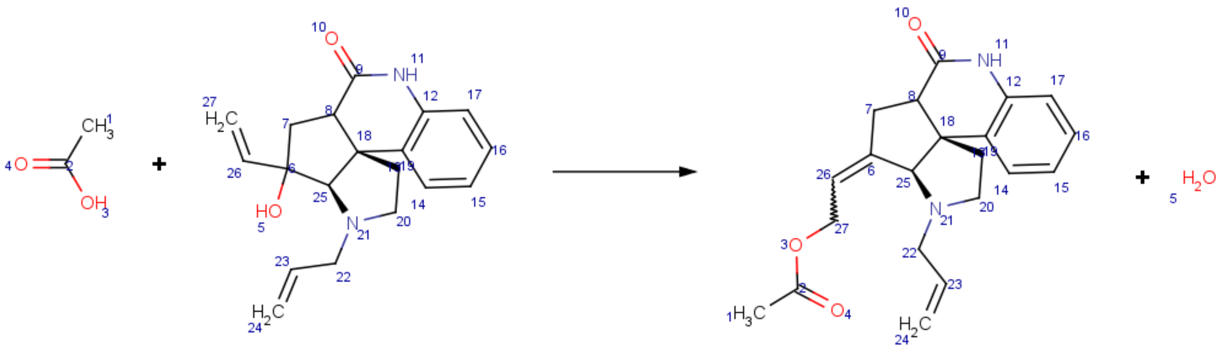

Correct mapped SMILES/SMARTS of the reaction:
[CH3:1][C:2]([OH:3])=[O:4].[OH:5][C:6]1([CH2:7][CH:8]2[C:9](=[O:10])[NH:11][C:12]3=[C:13]([CH:14]=[CH:15][CH:16]=[CH:17]3)[C@@:18]22[CH2:19][CH2:20][N:21]([CH2:22][CH:23]=[CH2:24])[C@@H:25]12)[CH:26]=[CH2:27]>>[CH3:1][C:2](=[O:4])[O:3][CH2:27][CH:26]=[C:6]1[CH2:7][CH:8]2[C:9](=[O:10])[NH:11][C:12]3=[C:13]([CH:14]=[CH:15][CH:16]=[CH:17]3)[C@@:18]22[CH2:19][CH2:20][N:21]([CH2:22][CH:23]=[CH2:24])[C@@H:25]12.[OH2:5]

Correctness of the mapping

MAPPET	YES
ReactionMap	YES
Marvin	NO
ChemDraw	YES
Indigo	NO

Reaction no 58

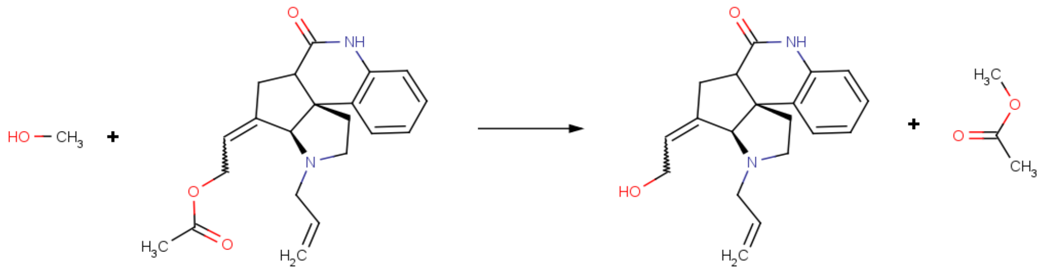

SMILES of the input:
CO.CC(=O)OCC=C1CC2C(=O)NC3=C(C=CC=C3)[C@@]22CCN(CC=C)[C@@H]12>>OCC=C1CC2C(=O)NC3=C(C=CC=C3)[C@@]22CCN(CC=C)[C@@H]12.COC(C)=O

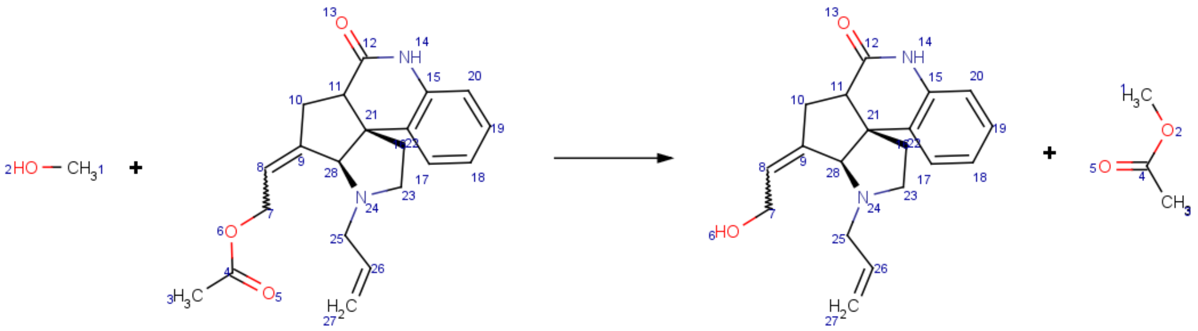

Correct mapped SMILES/SMARTS of the reaction:
[CH3:1][OH:2].[CH3:3][C:4](=[O:5])[O:6][CH2:7][CH:8]=[C:9]1[CH2:10][CH:11]2[C:12](=[O:13])[NH:14][C:15]3=[C:16]([CH:17]=[CH:18][CH:19]=[CH:20]3)[C@@:21]22[CH2:22][CH2:23][N:24]([CH2:25][CH:26]=[CH2:27])[C@@H:28]12>>[OH:6][CH2:7][CH:8]=[C:9]1[CH2:10][CH:11]2[C:12](=[O:13])[NH:14][C:15]3=[C:16]([CH:17]=[CH:18][CH:19]=[CH:20]3)[C@@:21]22[CH2:22][CH2:23][N:24]([CH2:25][CH:26]=[CH2:27])[C@@H:28]12.[CH3:1][O:2][C:4]([CH3:3])=[O:5]

Correctness of the mapping

MAPPET	YES
ReactionMap	YES
Marvin	YES
ChemDraw	YES
Indigo	YES

Reaction no 59

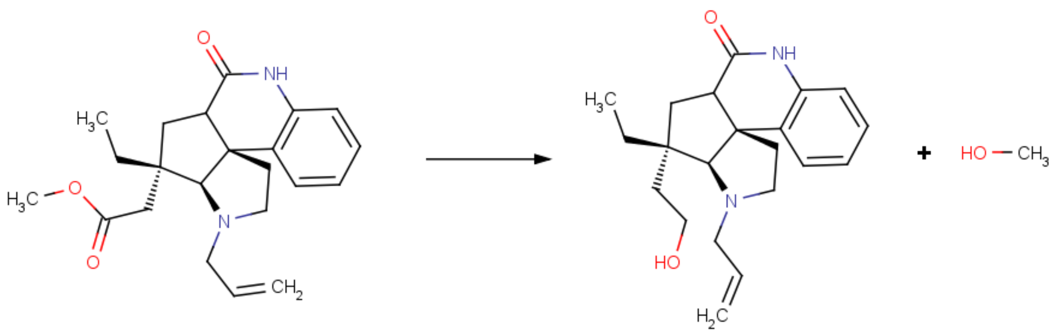

SMILES of the input:
CC[C@@]1(CC(=O)OC)CC2C(=O)NC3=C(C=CC=C3)[C@@]22CCN(CC=C)[C@@H]12>>CC[C@@]1(CCO)CC2C(=O)NC3=C(C=CC=C3)[C@@]22CCN(CC=C)[C@@H]12.CO

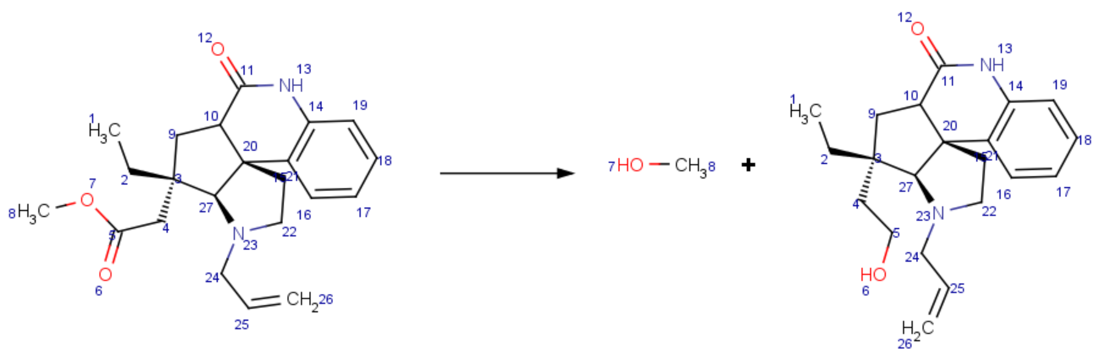

Correct mapped SMILES/SMARTS of the reaction:
[CH3:1][CH2:2][C@@:3]1([CH2:4][C:5](=[O:6])[O:7][CH3:8])[CH2:9][CH:10]2[C
:11](=[O:12])[NH:13][C:14]3=[C:15]([CH:16]=[CH:17][CH:18]=[CH:19]3)[C@@:2
0]22[CH2:21][CH2:22][N:23]([CH2:24][CH:25]=[CH2:26])[C@@H:27]12>>[CH3:8][
OH:7].[CH3:1][CH2:2][C@@:3]1([CH2:4][CH2:5][OH:6])[CH2:9][CH:10]2[C:11](=
[O:12])[NH:13][C:14]3=[C:15]([CH:16]=[CH:17][CH:18]=[CH:19]3)[C@@:20]22[C
H2:21][CH2:22][N:23]([CH2:24][CH:25]=[CH2:26])[C@@H:27]12

Correctness of the mapping

MAPPET	YES
ReactionMap	YES
Marvin	YES
ChemDraw	NO
Indigo	NO

Reaction no 60

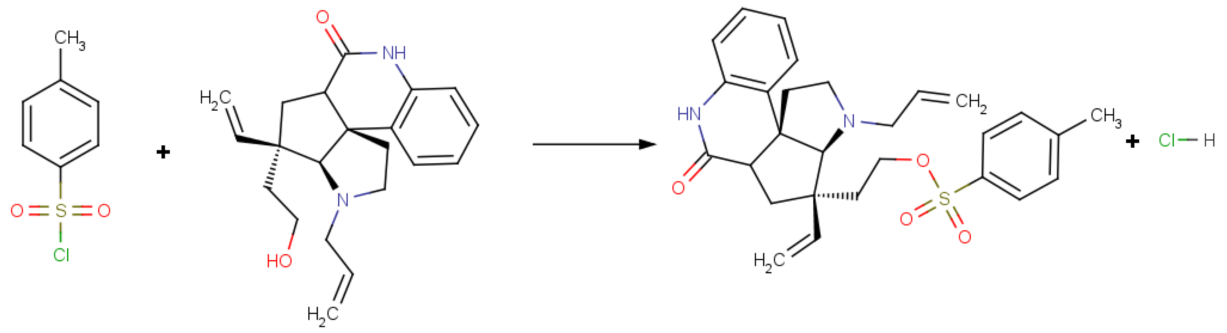

SMILES of the input:
CC1=CC=C(C=C1)S(Cl)(=O)=O.OCC[C@@]1(CC2C(=O)NC3=C(C=CC=C3)[C@@]22CCN(CC=C
)[C@@H]12)C=C>>CC1=CC=C(C=C1)S(=O)(=O)OCC[C@@]1(CC2C(=O)NC3=C(C=CC=C3)[C@
@]22CCN(CC=C)[C@@H]12)C=C.Cl[H]

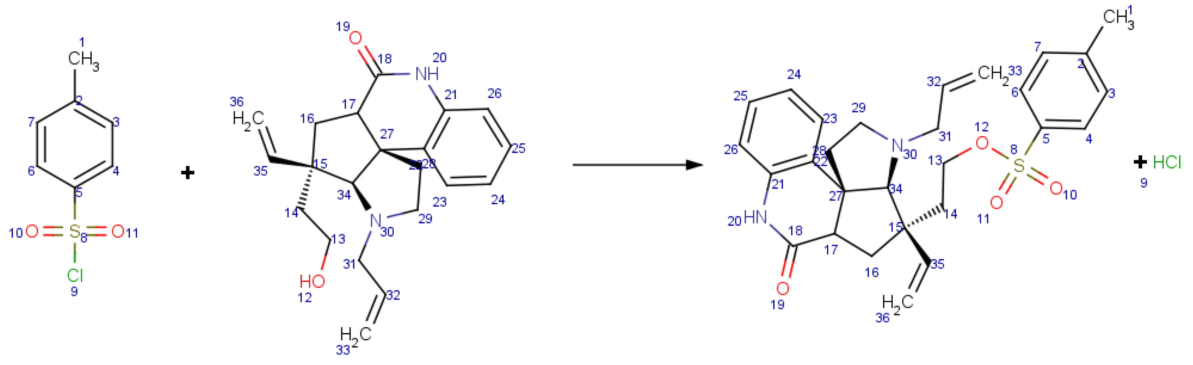

Correct mapped SMILES/SMARTS of the reaction:
[CH3:1][C:2]1=[CH:3][CH:4]=[C:5]([CH:6]=[CH:7]1)[S:8]([Cl:9])(=[O:10])=[O
:11].[OH:12][CH2:13][CH2:14][C@@:15]1([CH2:16][CH:17]2[C:18](=[O:19])[NH:

20][C:21]3=[C:22]([CH:23]=[CH:24][CH:25]=[CH:26]3)[C@@:27]22[CH2:28][CH2:29][N:30]([CH2:31][CH:32]=[CH2:33])[C@@H:34]12)[CH:35]=[CH2:36]>>[CH3:1][C:2]1=[CH:3][CH:4]=[C:5]([CH:6]=[CH:7]1)[S:8](=[O:11])(=[O:10])[O:12][CH2:13][CH2:14][C@@:15]1([CH2:16][CH:17]2[C:18](=[O:19])[NH:20][C:21]3=[C:22]([CH:23]=[CH:24][CH:25]=[CH:26]3)[C@@:27]22[CH2:28][CH2:29][N:30]([CH2:31][CH:32]=[CH2:33])[C@@H:34]12)[CH:35]=[CH2:36].[ClH:9]

Correctness of the mapping

MAPPET	YES
ReactionMap	YES
Marvin	YES
ChemDraw	YES
Indigo	YES

Reaction no 61

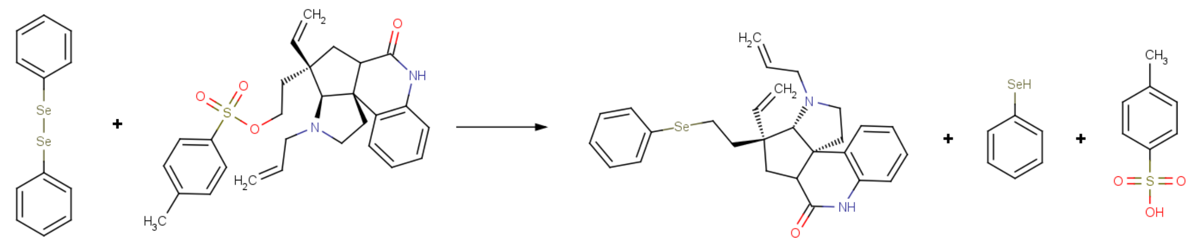

SMILES of the input:
[Se]([Se]C1=CC=CC=C1)C1=CC=CC=C1.CC1=CC=C(C=C1)S(=O)(=O)OCC[C@@]1(CC2C(=O)NC3=C(C=CC=C3)[C@@]22CCN(CC=C)[C@@H]12)C=C>>C=CCN1CC[C@]23C(C[C@](CC[Se]C4=CC=CC=C4)(C=C)[C@H]12)C(=O)NC1=C3C=CC=C1.[SeH]C1=CC=CC=C1.CC1=CC=C(C=C1)S(O)(=O)=O

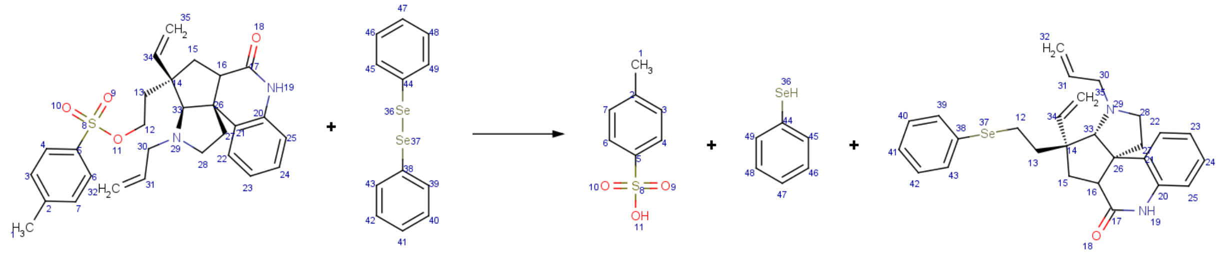

Correct mapped SMILES/SMARTS of the reaction:
[CH3:1][C:2]1=[CH:3][CH:4]=[C:5]([CH:6]=[CH:7]1)[S:8](=[O:9])(=[O:10])[O:11][CH2:12][CH2:13][C@@:14]1([CH2:15][CH:16]2[C:17](=[O:18])[NH:19][C:20]3=[C:21]([CH:22]=[CH:23][CH:24]=[CH:25]3)[C@@:26]22[CH2:27][CH2:28][N:29]([CH2:30][CH:31]=[CH2:32])[C@@H:33]12)[CH:34]=[CH2:35].[Se:36]([Se:37][C:38]1=[CH:39][CH:40]=[CH:41][CH:42]=[CH:43]1)[C:44]1=[CH:45][CH:46]=[CH:47][CH:48]=[CH:49]1>>[CH3:1][C:2]1=[CH:3][CH:4]=[C:5]([CH:6]=[CH:7]1)[S:8]([OH:11])(=[O:10])=[O:9].[SeH:36][C:44]1=[CH:45][CH:46]=[CH:47][CH:48]=[CH:49]1.[CH2:32]=[CH:31][CH2:30][N:29]1[CH2:28][CH2:27][C@:26]23[CH:16]([CH2:15][C@:14]([CH2:13][CH2:12][Se:37][C:38]4=[CH:43][CH:42]=[CH:41][CH:40]=[CH:39]4)([CH:34]=[CH2:35])[C@H:33]12)[C:17](=[O:18])[NH:19][C:20]1=[C:21]3[CH:22]=[CH:23][CH:24]=[CH:25]1

Correctness of the mapping

MAPPET	YES
ReactionMap	YES
Marvin	YES
ChemDraw	YES
Indigo	NO

Reaction no 62

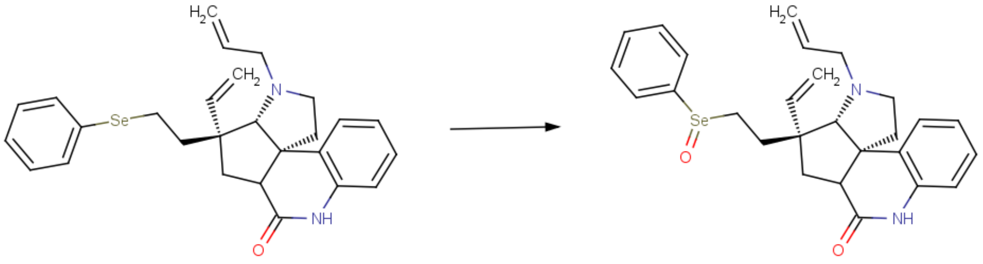

SMILES of the input:
C=CCN1CC[C@]23C(C[C@](CC[Se]C4=CC=CC=C4)(C=C)[C@H]12)C(=O)NC1=C3C=CC=C1>>C=CCN1CC[C@]23C(C[C@](CC[Se](=O)C4=CC=CC=C4)(C=C)[C@H]12)C(=O)NC1=C3C=CC=C1

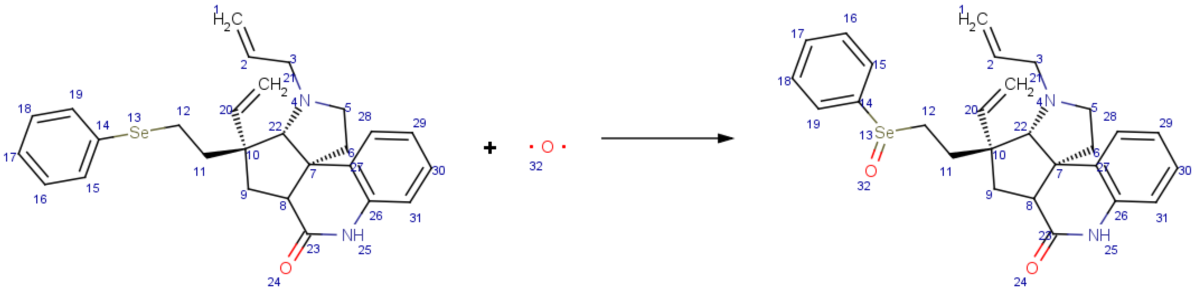

Correct mapped SMILES/SMARTS of the reaction:
[CH2:1]=[CH:2][CH2:3][N:4]1[CH2:5][CH2:6][C@:7]23[CH:8]([CH2:9][C@:10]([CH2:11][CH2:12][Se:13][C:14]4=[CH:15][CH:16]=[CH:17][CH:18]=[CH:19]4)([CH:20]=[CH2:21])[C@H:22]12)[C:23](=[O:24])[NH:25][C:26]1=[C:27]3[CH:28]=[CH:29][CH:30]=[CH:31]1.[O:32]>>[CH2:1]=[CH:2][CH2:3][N:4]1[CH2:5][CH2:6][C@:7]23[CH:8]([CH2:9][C@:10]([CH2:11][CH2:12][Se:13](=[O:32])[C:14]4=[CH:19][CH:18]=[CH:17][CH:16]=[CH:15]4)([CH:20]=[CH2:21])[C@H:22]12)[C:23](=[O:24])[NH:25][C:26]1=[C:27]3[CH:28]=[CH:29][CH:30]=[CH:31]1

Correctness of the mapping

MAPPET	YES
ReactionMap	NO
Marvin	YES
ChemDraw	YES
Indigo	YES

Reaction no 63

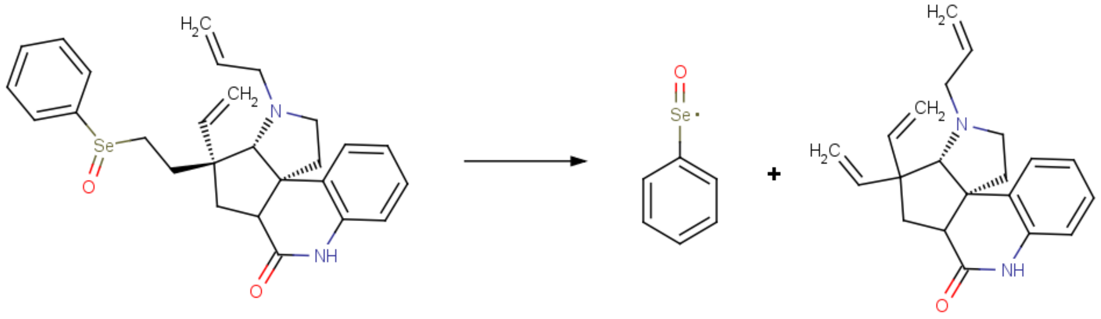

SMILES of the input:
C=CCN1CC[C@]23C(C[C@](CC[Se](=O)C4=CC=CC=C4)(C=C)[C@H]12)C(=O)NC1=C3C=CC=C1>>O=[Se]C1=CC=CC=C1.C=CCN1CC[C@]23C(CC(C=C)(C=C)[C@H]12)C(=O)NC1=C3C=CC=C1

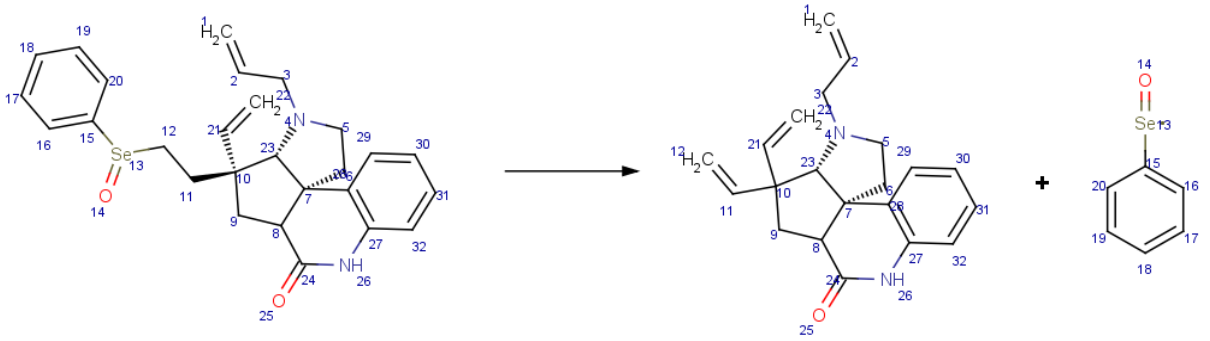

Correct mapped SMILES/SMARTS of the reaction:
[CH2:1]=[CH:2][CH2:3][N:4]1[CH2:5][CH2:6][C@:7]23[CH:8]([CH2:9][C@:10]([C
H2:11][CH2:12][Se:13](=[O:14])[C:15]4=[CH:16][CH:17]=[CH:18][CH:19]=[CH:2
0]4)([CH:21]=[CH2:22])[C@H:23]12)[C:24](=[O:25])[NH:26][C:27]1=[C:28]3[CH
:29]=[CH:30][CH:31]=[CH:32]1>>[CH2:1]=[CH:2][CH2:3][N:4]1[CH2:5][CH2:6][C
@:7]23[CH:8]([CH2:9][C:10]([CH:11]=[CH2:12])([CH:21]=[CH2:22])[C@H:23]12)
[C:24](=[O:25])[NH:26][C:27]1=[C:28]3[CH:29]=[CH:30][CH:31]=[CH:32]1.[O:1
4]=[Se:13][C:15]1=[CH:16][CH:17]=[CH:18][CH:19]=[CH:20]1

Correctness of the mapping

MAPPET	YES
ReactionMap	YES
Marvin	YES
ChemDraw	YES
Indigo	YES

Reaction no 64

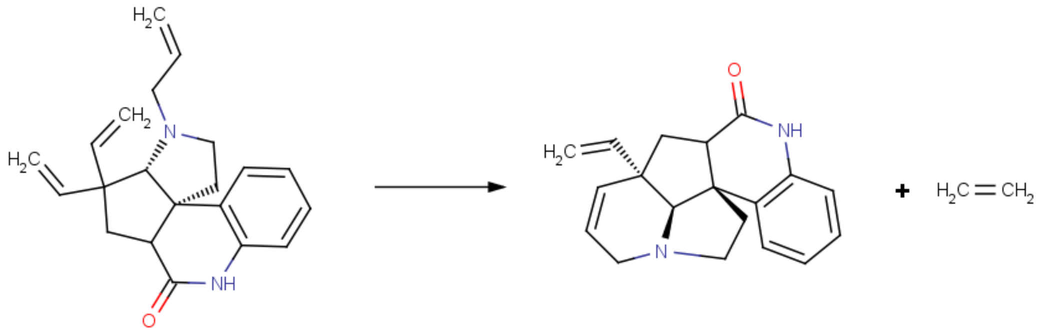

SMILES of the input:
C=CCN1CC[C@]23C(CC(C=C)(C=C)[C@H]12)C(=O)NC1=C3C=CC=C1>>C=C[C@@]12CC3C(=O
)NC4=C(C=CC=C4)[C@@]33CCN(CC=C1)[C@@H]23.C=C

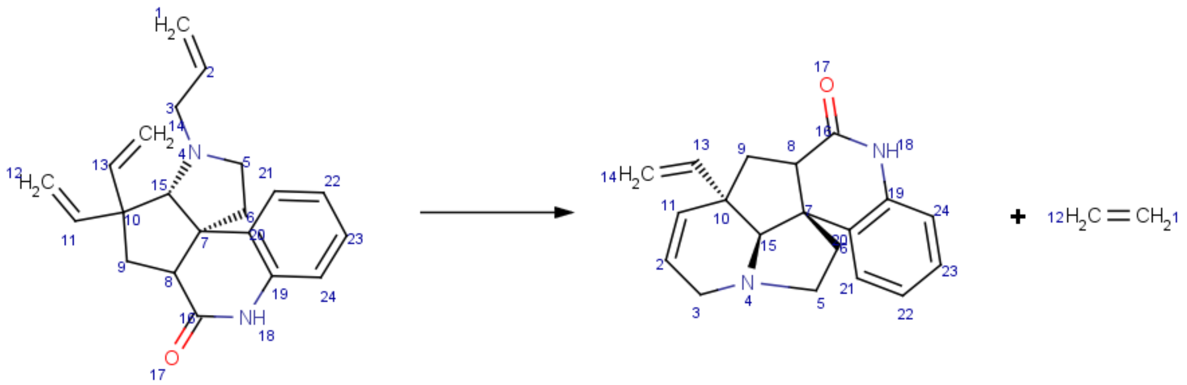

Correct mapped SMILES/SMARTS of the reaction:
[CH2:1]=[CH:2][CH2:3][N:4]1[CH2:5][CH2:6][C@:7]23[CH:8]([CH2:9][C:10]([CH
:11]=[CH2:12])([CH:13]=[CH2:14])[C@H:15]12)[C:16](=[O:17])[NH:18][C:19]1=

[C:20]3[CH:21]=[CH:22][CH:23]=[CH:24]1>>[CH2:14]=[CH:13][C@@:10]12[CH2:9]
[CH:8]3[C:16](=[O:17])[NH:18][C:19]4=[C:20]([CH:21]=[CH:22][CH:23]=[CH:24
]4)[C@@:7]33[CH2:6][CH2:5][N:4]([CH2:3][CH:2]=[CH:11]1)[C@@H:15]23.[CH2:1
]=[CH2:12]

Correctness of the mapping

MAPPET	YES
ReactionMap	YES
Marvin	NO
ChemDraw	YES
Indigo	NO

Reaction no 65

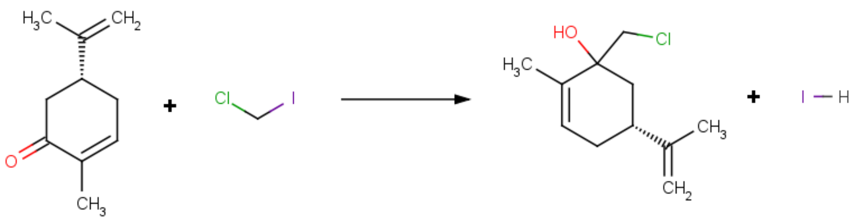

SMILES of the input:
CC(=C)[C@@H]1CC=C(C)C(=O)C1.ClCI>>CC(=C)[C@@H]1CC=C(C)C(O)(CCl)C1.I[H]

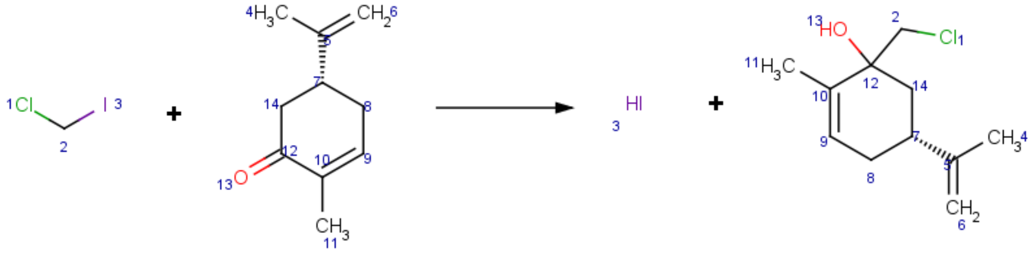

Correct mapped SMILES/SMARTS of the reaction:
[Cl:1][CH2:2][I:3].[CH3:4][C:5](=[CH2:6])[C@@H:7]1[CH2:8][CH:9]=[C:10]([C
H3:11])[C:12](=[O:13])[CH2:14]1>>[IH:3].[CH3:4][C:5](=[CH2:6])[C@@H:7]1[C
H2:8][CH:9]=[C:10]([CH3:11])[C:12]([OH:13])([CH2:2][Cl:1])[CH2:14]1

Correctness of the mapping

MAPPET	YES
ReactionMap	YES
Marvin	YES
ChemDraw	YES
Indigo	YES

Reaction no 66

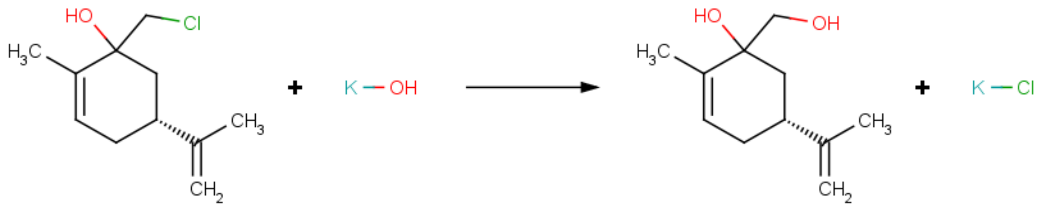

SMILES of the input:

CC(=C)[C@@H]1CC=C(C)C(O)(CCl)C1.O[K]>>CC(=C)[C@@H]1CC=C(C)C(O)(CO)C1.Cl[K
]

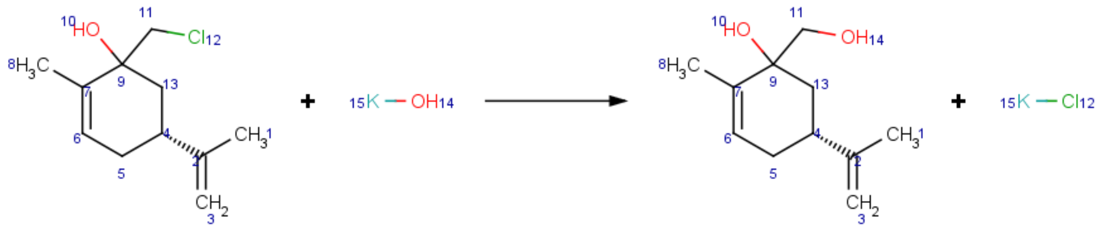

Correct mapped SMILES/SMARTS of the reaction:
[CH3:1][C:2](=[CH2:3])[C@@H:4]1[CH2:5][CH:6]=[C:7]([CH3:8])[C:9]([OH:10])
([CH2:11][Cl:12])[CH2:13]1.[OH:14][K:15]>>[CH3:1][C:2](=[CH2:3])[C@@H:4]1
[CH2:5][CH:6]=[C:7]([CH3:8])[C:9]([OH:10])([CH2:11][OH:14])[CH2:13]1.[Cl:
12][K:15]

Correctness of the mapping

MAPPET	YES
ReactionMap	YES
Marvin	YES
ChemDraw	YES
Indigo	YES

Reaction no 67

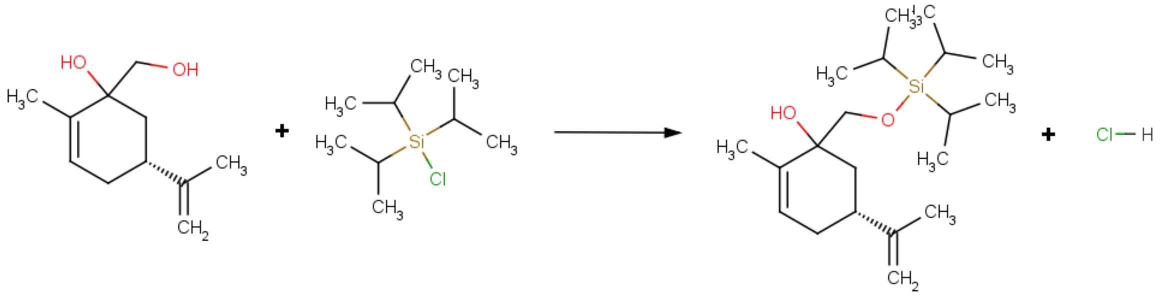

SMILES of the input:
CC(=C)[C@@H]1CC=C(C)C(O)(CO)C1.CC(C)[Si](Cl)(C(C)C)C(C)C>>CC(C)[Si](OCC1(
O)C[C@@H](CC=C1C)C(C)=C)(C(C)C)C(C)C.Cl[H]

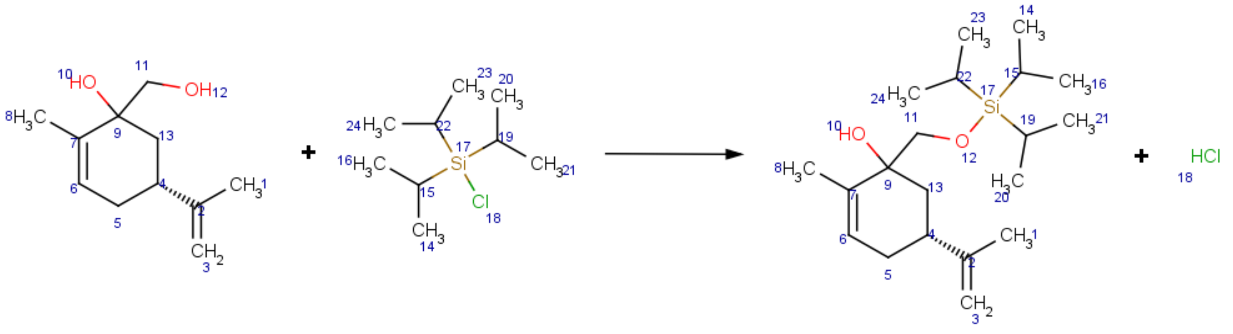

Correct mapped SMILES/SMARTS of the reaction:
[CH3:1][C:2](=[CH2:3])[C@@H:4]1[CH2:5][CH:6]=[C:7]([CH3:8])[C:9]([OH:10])
([CH2:11][OH:12])[CH2:13]1.[CH3:14][CH:15]([CH3:16])[Si:17]([Cl:18])([CH:
19]([CH3:20])[CH3:21])[CH:22]([CH3:23])[CH3:24]>>[CH3:16][CH:15]([CH3:14]
)[Si:17]([O:12][CH2:11][C:9]1([OH:10])[CH2:13][C@@H:4]([CH2:5][CH:6]=[C:7
]1[CH3:8])[C:2]([CH3:1])=[CH2:3])([CH:22]([CH3:24])[CH3:23])[CH:19]([CH3:
20])[CH3:21].[ClH:18]

Correctness of the mapping
MAPPET YES
ReactionMap YES
Marvin YES
ChemDraw YES
Indigo YES

Reaction no 68

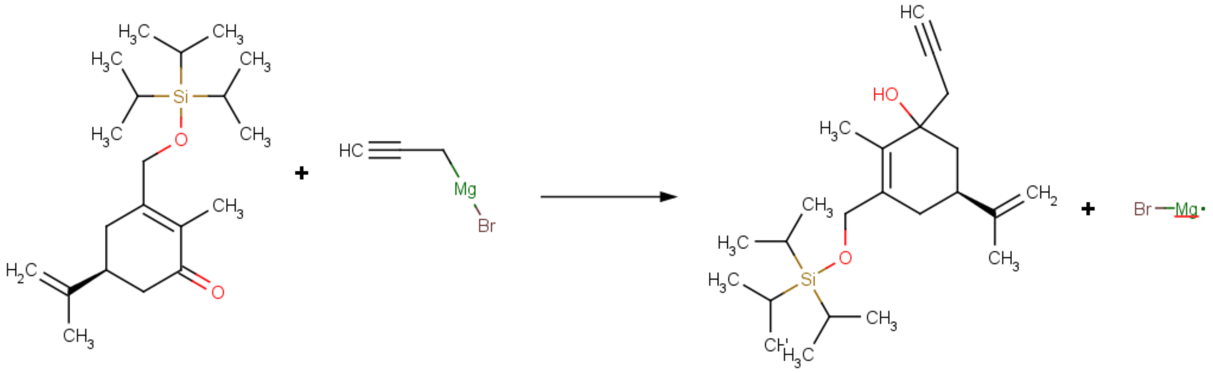

SMILES of the input:
CC(C)[Si](OCC1=C(C)C(=O)C[C@H](C1)C(C)=C)(C(C)C)C(C)C.Br[Mg]CC#C>>CC(C)[Si](OCC1=C(C)C(O)(CC#C)C[C@H](C1)C(C)=C)(C(C)C)C(C)C.[Mg]Br

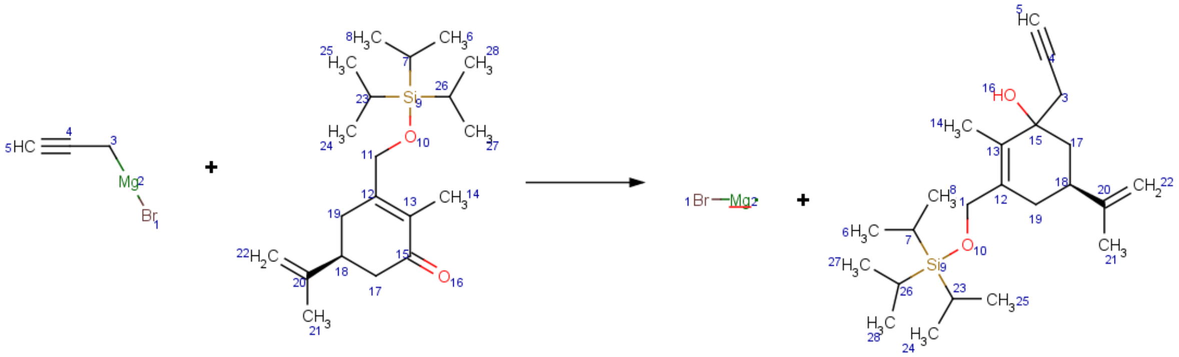

Correct mapped SMILES/SMARTS of the reaction:
[Br:1][Mg:2][CH2:3][C:4]#[CH:5].[CH3:6][CH:7]([CH3:8])[Si:9]([O:10][CH2:11][C:12]1=[C:13]([CH3:14])[C:15](=[O:16])[CH2:17][C@H:18]([CH2:19]1)[C:20]([CH3:21])=[CH2:22])([CH:23]([CH3:24])[CH3:25])[CH:26]([CH3:27])[CH3:28]>>[Mg:2][Br:1].[CH3:28][CH:26]([CH3:27])[Si:9]([O:10][CH2:11][C:12]1=[C:13]([CH3:14])[C:15]([OH:16])([CH2:3][C:4]#[CH:5])[CH2:17][C@H:18]([CH2:19]1)[C:20]([CH3:21])=[CH2:22])([CH:23]([CH3:25])[CH3:24])[CH:7]([CH3:8])[CH3:6]

Correctness of the mapping
MAPPET YES
ReactionMap YES
Marvin YES
ChemDraw YES
Indigo YES

Reaction no 69

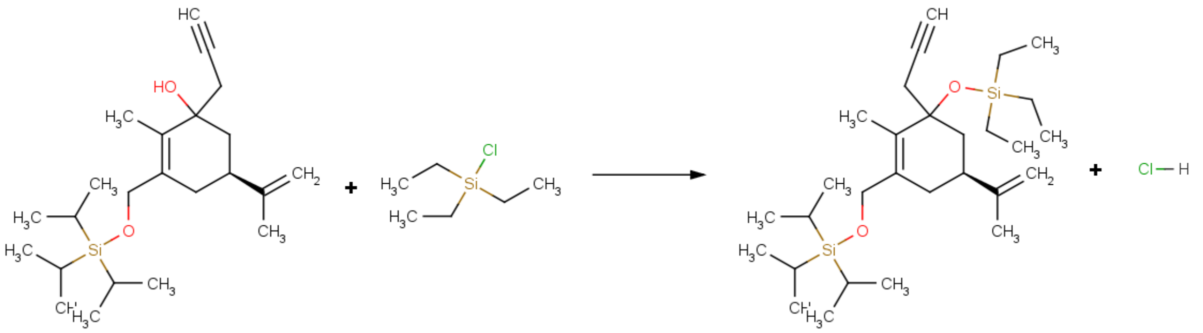

SMILES of the input:
CC(C)[Si](OCC1=C(C)C(O)(CC#C)C[C@H](C1)C(C)=C)(C(C)C)C(C)C.CC[Si](Cl)(CC)CC>>CC[Si](CC)(CC)OC1(CC#C)C[C@H](CC(CO[Si](C(C)C)(C(C)C)C(C)C)=C1C)C(C)=C.Cl[H]

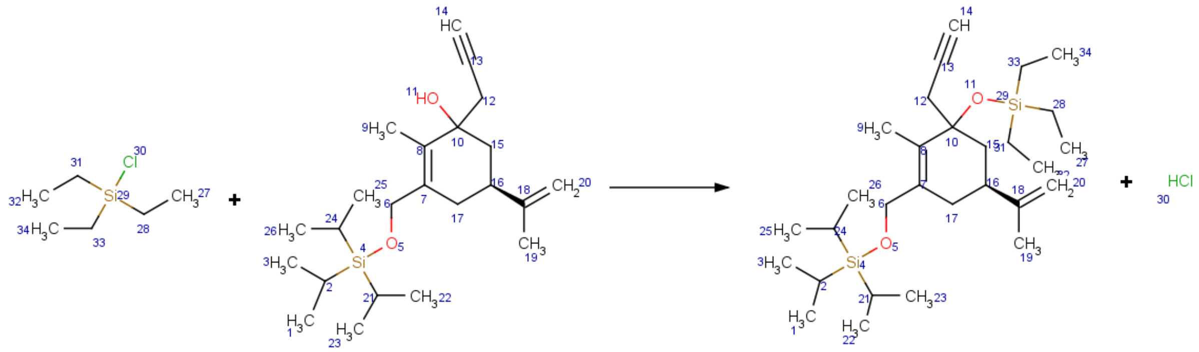

Correct mapped SMILES/SMARTS of the reaction:
[CH3:27][CH2:28][Si:29]([Cl:30])([CH2:31][CH3:32])[CH2:33][CH3:34].[CH3:1][CH:2]([CH3:3])[Si:4]([O:5][CH2:6][C:7]1=[C:8]([CH3:9])[C:10]([OH:11])([CH2:12][C:13]#[CH:14])[CH2:15][C@H:16]([CH2:17]1)[C:18]([CH3:19])=[CH2:20])([CH:21]([CH3:22])[CH3:23])[CH:24]([CH3:25])[CH3:26]>>[CH3:27][CH2:28][Si:29]([CH2:33][CH3:34])([CH2:31][CH3:32])[O:11][C:10]1([CH2:12][C:13]#[CH:14])[CH2:15][C@H:16]([CH2:17][C:7]([CH2:6][O:5][Si:4]([CH:2]([CH3:1])[CH3:3])([CH:24]([CH3:25])[CH3:26])[CH:21]([CH3:22])[CH3:23])=[C:8]1[CH3:9])[C:18]([CH3:19])=[CH2:20].[ClH:30]

Correctness of the mapping

MAPPET	YES
ReactionMap	YES
Marvin	YES
ChemDraw	YES
Indigo	YES

Reaction no 70

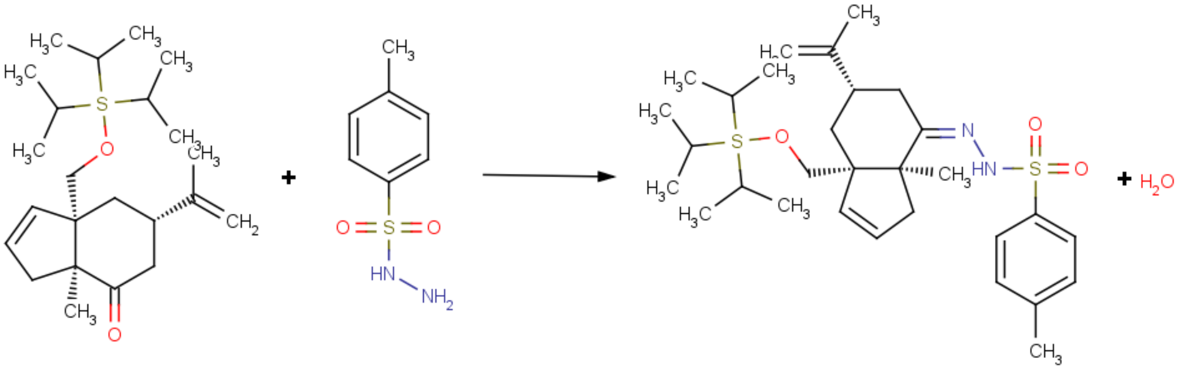

SMILES of the input:
CC(C)S(OC[C@]12C[C@@H](CC(=O)[C@@]1(C)CC=C2)C(C)=C)(C(C)C)C(C)C.CC1=CC=C(C=C1)S(=O)(=O)NN>>CC(C)S(OC[C@]12C[C@@H](C\C(=N\NS(=O)(=O)C3=CC=C(C)C=C3)[C@@]1(C)CC=C2)C(C)=C)(C(C)C)C(C)C.O

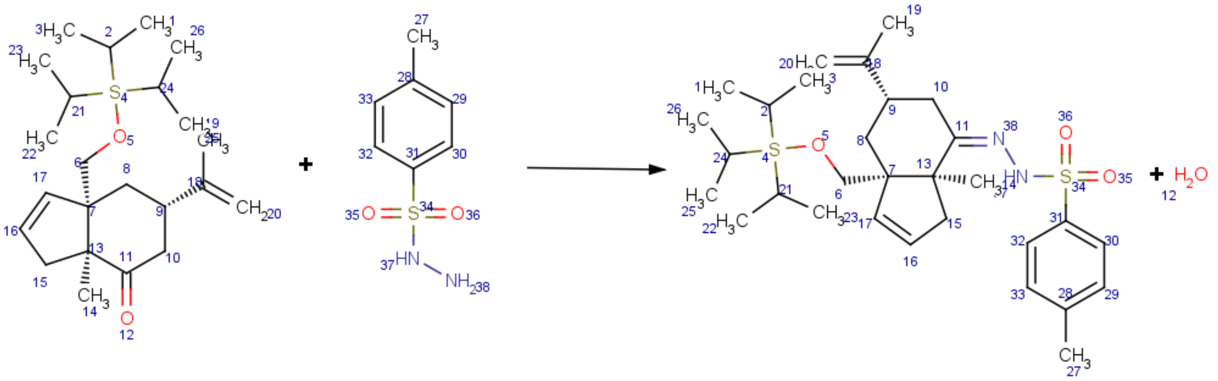

Correct mapped SMILES/SMARTS of the reaction:
[CH3:1][CH:2]([CH3:3])[S:4]([O:5][CH2:6][C@:7]12[CH2:8][C@@H:9]([CH2:10][C:11](=[O:12])[C@@:13]1([CH3:14])[CH2:15][CH:16]=[CH:17]2)[C:18]([CH3:19])=[CH2:20])([CH:21]([CH3:22])[CH3:23])[CH:24]([CH3:25])[CH3:26].[CH3:27][C:28]1=[CH:29][CH:30]=[C:31]([CH:32]=[CH:33]1)[S:34](=[O:35])(=[O:36])[NH:37][NH2:38]>>[CH3:26][CH:24]([CH3:25])[S:4]([O:5][CH2:6][C@:7]12[CH2:8][C@@H:9]([CH2:10]\[C:11](=[N:38]\[NH:37][S:34](=[O:36])(=[O:35])[C:31]3=[CH:30][CH:29]=[C:28]([CH3:27])[CH:33]=[CH:32]3)[C@@:13]1([CH3:14])[CH2:15][CH:16]=[CH:17]2)[C:18]([CH3:19])=[CH2:20])([CH:21]([CH3:23])[CH3:22])[CH:2]([CH3:3])[CH3:1].[OH2:12]

Correctness of the mapping

MAPPET	YES
ReactionMap	YES
Marvin	YES
ChemDraw	YES
Indigo	NO

Reaction no 71

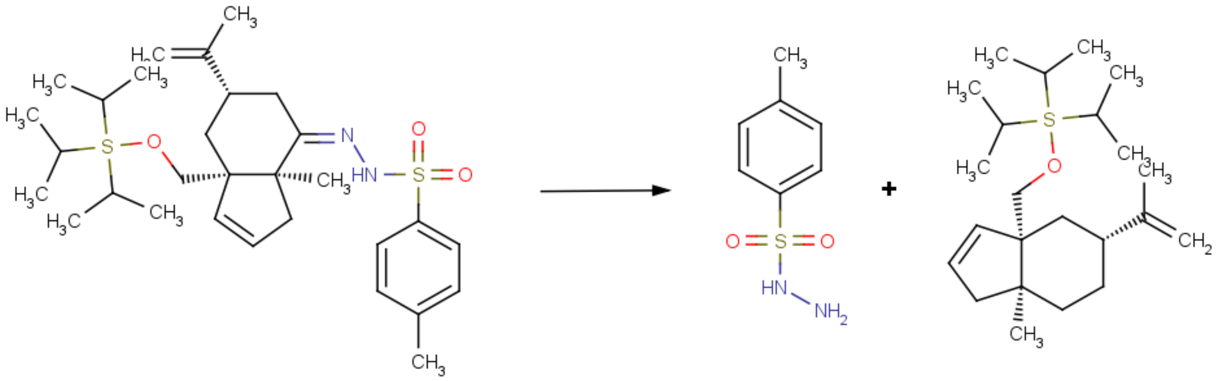

SMILES of the input:
CC(C)S(OC[C@]12C[C@@H](C\C(=N\NS(=O)(=O)C3=CC=C(C)C=C3)[C@@]1(C)CC=C2)C(C)=C)(C(C)C)C(C)C>>CC1=CC=C(C=C1)S(=O)(=O)NN.CC(C)S(OC[C@]12C[C@@H](CC[C@@]1(C)CC=C2)C(C)=C)(C(C)C)C(C)C

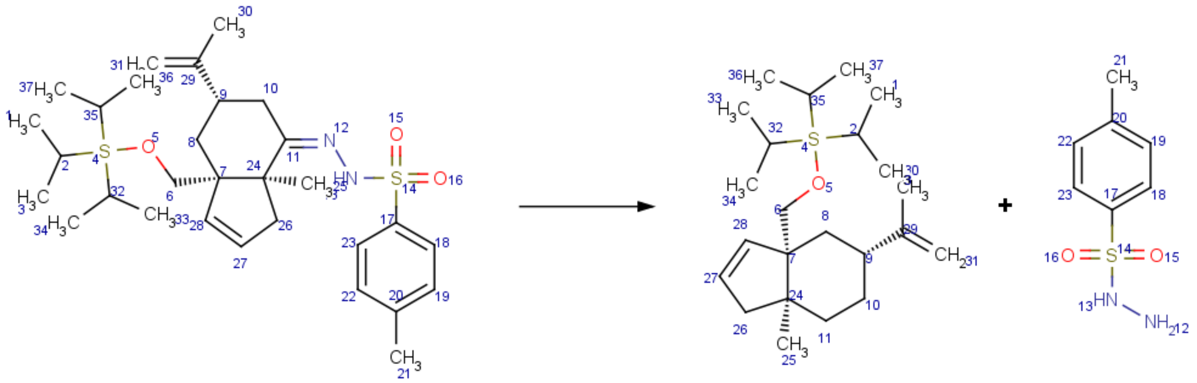

Correct mapped SMILES/SMARTS of the reaction:
[CH3:1][CH:2]([CH3:3])[S:4]([O:5][CH2:6][C@:7]12[CH2:8][C@@H:9]([CH2:10]\
[C:11](=[N:12]\[NH:13][S:14](=[O:15])(=[O:16])[C:17]3=[CH:18][CH:19]=[C:2
0]([CH3:21])[CH:22]=[CH:23]3)[C@@:24]1([CH3:25])[CH2:26][CH:27]=[CH:28]2)
[C:29]([CH3:30])=[CH2:31])([CH:32]([CH3:33])[CH3:34])[CH:35]([CH3:36])[CH
3:37]>>[CH3:37][CH:35]([CH3:36])[S:4]([O:5][CH2:6][C@:7]12[CH2:8][C@@H:9]
([CH2:10][CH2:11][C@@:24]1([CH3:25])[CH2:26][CH:27]=[CH:28]2)[C:29]([CH3:
30])=[CH2:31])([CH:32]([CH3:34])[CH3:33])[CH:2]([CH3:3])[CH3:1].[CH3:21][
C:20]1=[CH:19][CH:18]=[C:17]([CH:23]=[CH:22]1)[S:14](=[O:16])(=[O:15])[NH
:13][NH2:12]

Correctness of the mapping

MAPPET	YES
ReactionMap	YES
Marvin	YES
ChemDraw	NO
Indigo	YES

Reaction no 72

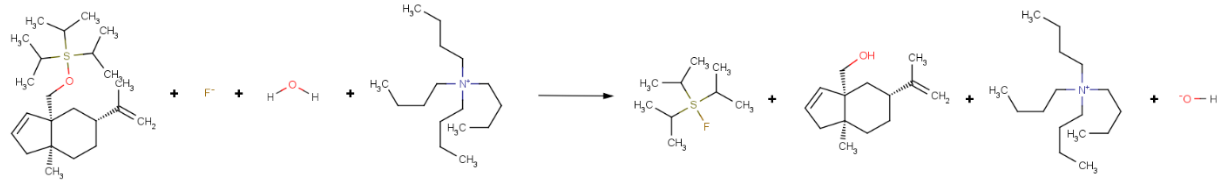

SMILES of the input:
CC(C)S(OC[C@]12C[C@@H](CC[C@@]1(C)CC=C2)C(C)=C)(C(C)C)C(C)C.[F-
].[H]O[H].CCCC[N+](CCCC)(CCCC)CCCC>>CC(C)S(F)(C(C)C)C(C)C.CC(=C)[C@@H]1CC
[C@@]2(C)CC=C[C@@]2(CO)C1.CCCC[N+](CCCC)(CCCC)CCCC.[O-][H]

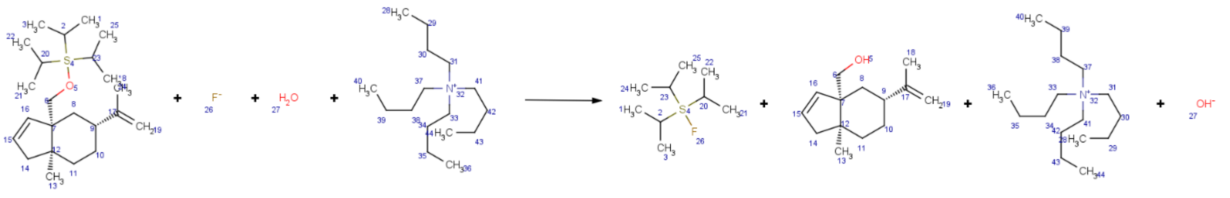

Correct mapped SMILES/SMARTS of the reaction:
[CH3:1][CH:2]([CH3:3])[S:4]([O:5][CH2:6][C@:7]12[CH2:8][C@@H:9]([CH2:10][
CH2:11][C@@:12]1([CH3:13])[CH2:14][CH:15]=[CH:16]2)[C:17]([CH3:18])=[CH2:
19])([CH:20]([CH3:21])[CH3:22])[CH:23]([CH3:24])[CH3:25].[F-
:26].[OH2:27].[CH3:28][CH2:29][CH2:30][CH2:31][N+:32]([CH2:33][CH2:34][CH
2:35][CH3:36])([CH2:37][CH2:38][CH2:39][CH3:40])[CH2:41][CH2:42][CH2:43][
CH3:44]>>[CH3:3][CH:2]([CH3:1])[S:4]([F:26])([CH:20]([CH3:22])[CH3:21])[C
H:23]([CH3:25])[CH3:24].[CH3:18][C:17](=[CH2:19])[C@@H:9]1[CH2:10][CH2:11
][C@@:12]2([CH3:13])[CH2:14][CH:15]=[CH:16][C@@:7]2([CH2:6][OH:5])[CH2:8]

1.[CH3:40][CH2:39][CH2:38][CH2:37][N+:32]([CH2:41][CH2:42][CH2:43][CH3:44])([CH2:33][CH2:34][CH2:35][CH3:36])[CH2:31][CH2:30][CH2:29][CH3:28].[OH-:27]

Correctness of the mapping
MAPPET YES
ReactionMap YES
Marvin YES
ChemDraw YES
Indigo YES

Reaction no 73

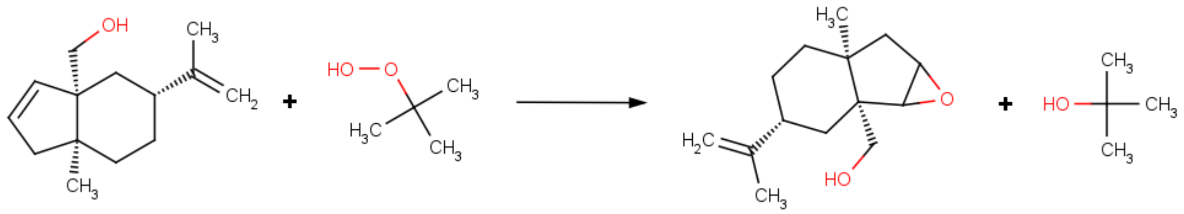

SMILES of the input:
CC(=C)[C@@H]1CC[C@@]2(C)CC=C[C@@]2(CO)C1.CC(C)(C)OO>>CC(=C)[C@@H]1CC[C@@]2(C)CC3OC3[C@@]2(CO)C1.CC(C)(C)O

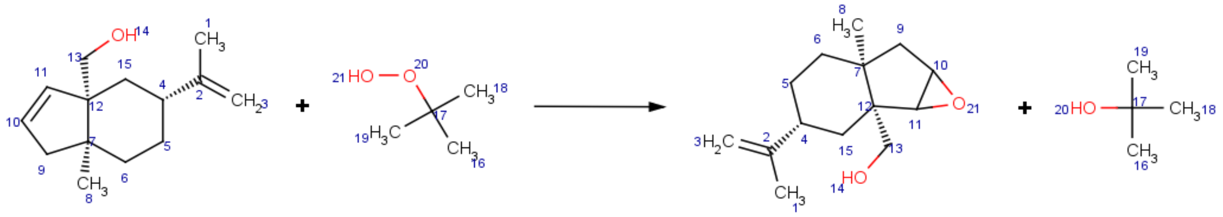

Correct mapped SMILES/SMARTS of the reaction:
[CH3:1][C:2](=[CH2:3])[C@@H:4]1[CH2:5][CH2:6][C@@:7]2([CH3:8])[CH2:9][CH:10]=[CH:11][C@@:12]2([CH2:13][OH:14])[CH2:15]1.[CH3:16][C:17]([CH3:18])([CH3:19])[O:20][OH:21]>>[CH3:1][C:2](=[CH2:3])[C@@H:4]1[CH2:5][CH2:6][C@@:7]2([CH3:8])[CH2:9][CH:10]3[O:21][CH:11]3[C@@:12]2([CH2:13][OH:14])[CH2:15]1.[CH3:16][C:17]([CH3:19])([CH3:18])[OH:20]

Correctness of the mapping
MAPPET YES
ReactionMap YES
Marvin YES
ChemDraw YES
Indigo NO

Reaction no 74

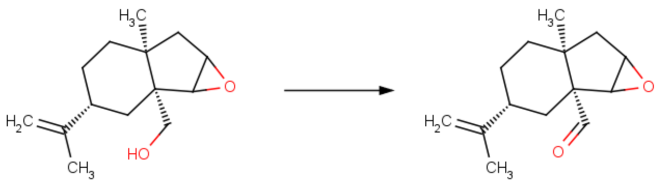

SMILES of the input:
CC(=C)[C@@H]1CC[C@@]2(C)CC3OC3[C@@]2(CO)C1>>CC(=C)[C@@H]1CC[C@@]2(C)CC3OC3[C@]2(C1)C=O

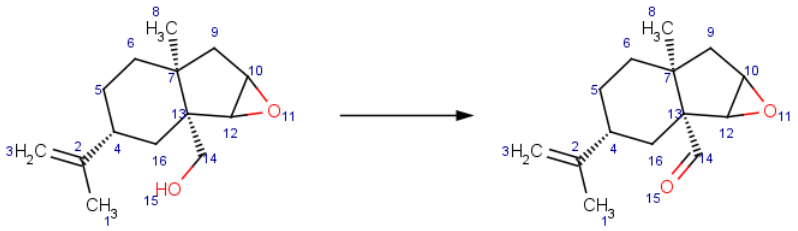

Correct mapped SMILES/SMARTS of the reaction:
[CH3:1][C:2](=[CH2:3])[C@@H:4]1[CH2:5][CH2:6][C@@:7]2([CH3:8])[CH2:9][CH:10]3[O:11][CH:12]3[C@@:13]2([CH2:14][OH:15])[CH2:16]1>>[CH3:1][C:2](=[CH2:3])[C@@H:4]1[CH2:5][CH2:6][C@@:7]2([CH3:8])[CH2:9][CH:10]3[O:11][CH:12]3[C@:13]2([CH2:16]1)[CH:14]=[O:15]

Correctness of the mapping

MAPPET	YES
ReactionMap	YES
Marvin	YES
ChemDraw	YES
Indigo	NO

Reaction no 75

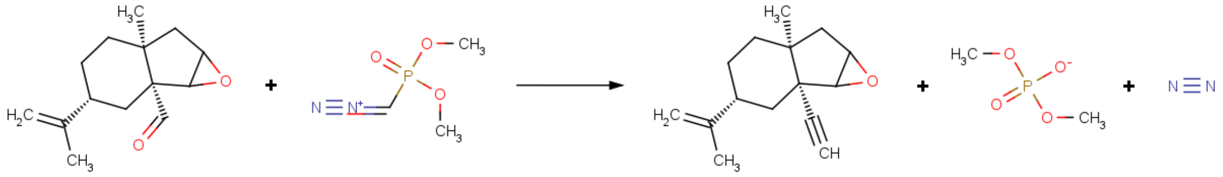

SMILES of the input:
CC(=C)[C@@H]1CC[C@@]2(C)CC3OC3[C@]2(C1)C=O.COP(=O)(OC)C=[N+]#N>>CC(=C)[C@@H]1CC[C@@]2(C)CC3OC3[C@]2(C1)C#C.COP([O-])(=O)OC.N#N

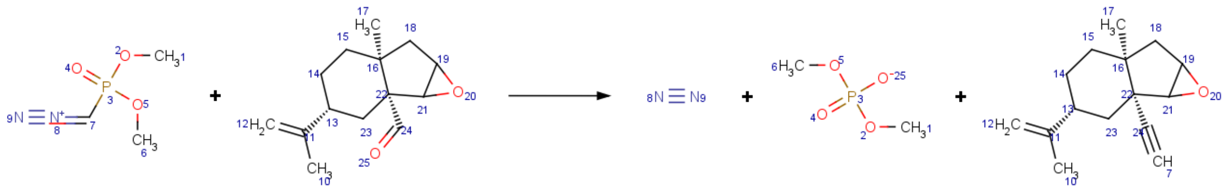

Correct mapped SMILES/SMARTS of the reaction:
[CH3:1][O:2][P:3](=[O:4])([O:5][CH3:6])[CH:7]=[N+:8]#[N:9].[CH3:10][C:11](=[CH2:12])[C@@H:13]1[CH2:14][CH2:15][C@@:16]2([CH3:17])[CH2:18][CH:19]3[O:20][CH:21]3[C@:22]2([CH2:23]1)[CH:24]=[O:25]>>[N:9]#[N:8].[CH3:1][O:2][P:3]([O-:25])(=[O:4])[O:5][CH3:6].[CH3:10][C:11](=[CH2:12])[C@@H:13]1[CH2:14][CH2:15][C@@:16]2([CH3:17])[CH2:18][CH:19]3[O:20][CH:21]3[C@:22]2([CH2:23]1)[C:24]#[CH:7]

Correctness of the mapping

MAPPET	YES
ReactionMap	YES
Marvin	NO
ChemDraw	YES
Indigo	YES

Reaction no 76

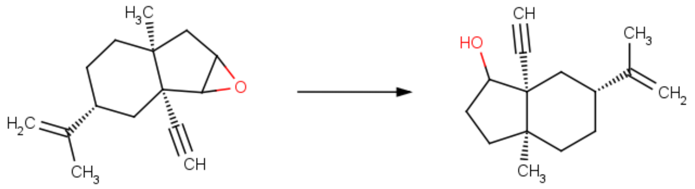

SMILES of the input:
CC(=C)[C@@H]1CC[C@@]2(C)CC3OC3[C@]2(C1)C#C>>CC(=C)[C@@H]1CC[C@@]2(C)CCC(O)[C@]2(C1)C#C

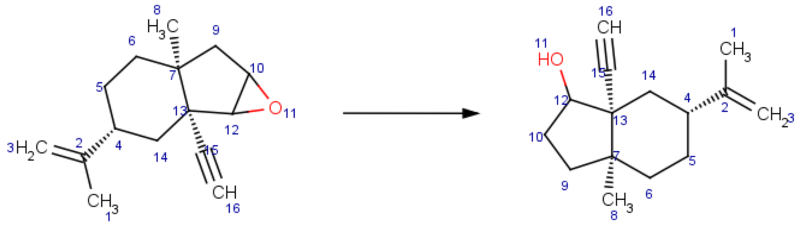

Correct mapped SMILES/SMARTS of the reaction:
[CH3:1][C:2](=[CH2:3])[C@@H:4]1[CH2:5][CH2:6][C@@:7]2([CH3:8])[CH2:9][CH:10]3[O:11][CH:12]3[C@:13]2([CH2:14]1)[C:15]#[CH:16]>>[CH3:1][C:2](=[CH2:3])[C@@H:4]1[CH2:5][CH2:6][C@@:7]2([CH3:8])[CH2:9][CH2:10][CH:12]([OH:11])[C@:13]2([CH2:14]1)[C:15]#[CH:16]

Correctness of the mapping

MAPPET	YES
ReactionMap	YES
Marvin	YES
ChemDraw	YES
Indigo	YES

Reaction no 77

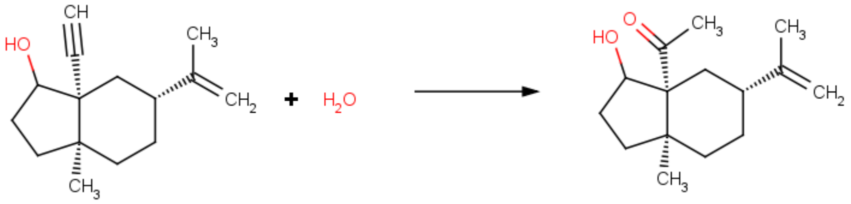

SMILES of the input:
CC(=C)[C@@H]1CC[C@@]2(C)CCC(O)[C@]2(C1)C#C.O>>CC(=C)[C@@H]1CC[C@@]2(C)CCC(O)[C@]2(C1)C(C)=O

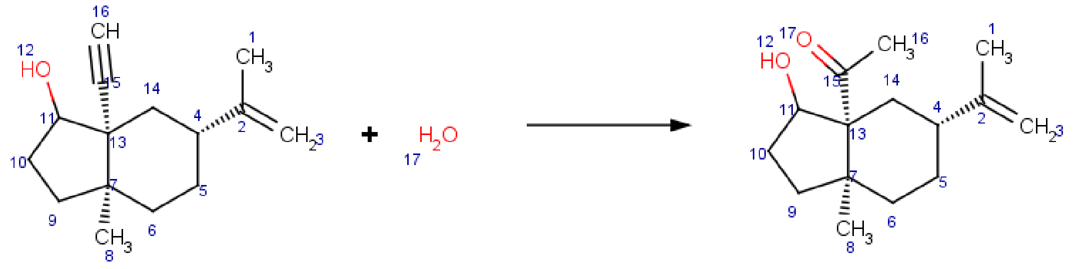

Correct mapped SMILES/SMARTS of the reaction:
[CH3:1][C:2](=[CH2:3])[C@@H:4]1[CH2:5][CH2:6][C@@:7]2([CH3:8])[CH2:9][CH2:10][CH:11]([OH:12])[C@:13]2([CH2:14]1)[C:15]#[CH:16].[OH2:17]>>[CH3:1][C:2](=[CH2:3])[C@@H:4]1[CH2:5][CH2:6][C@@:7]2([CH3:8])[CH2:9][CH2:10][CH:11]([OH:12])[C@:13]2([CH2:14]1)[C:15]([CH3:16])=[O:17]

Correctness of the mapping

MAPPET	YES
ReactionMap	YES
Marvin	YES
ChemDraw	YES
Indigo	YES

Reaction no 78

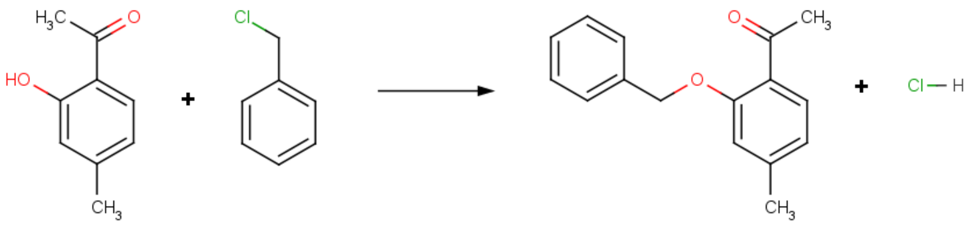

SMILES of the input:
CC(=O)C1=CC=C(C)C=C1O.ClCC1=CC=CC=C1>>CC(=O)C1=CC=C(C)C=C1OCC1=CC=CC=C1.Cl[H]

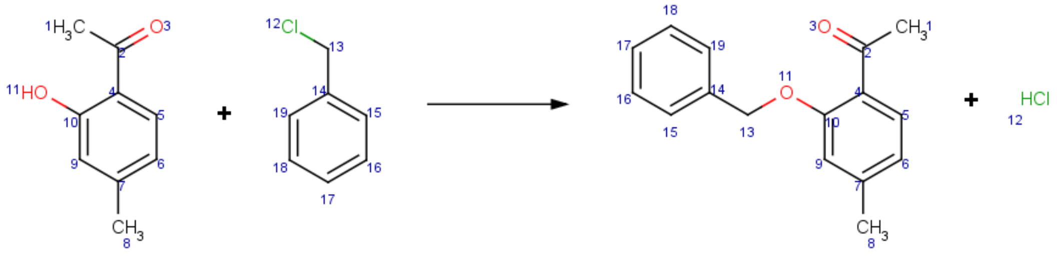

Correct mapped SMILES/SMARTS of the reaction:
[CH3:1][C:2](=[O:3])[C:4]1=[CH:5][CH:6]=[C:7]([CH3:8])[CH:9]=[C:10]1[OH:11].[Cl:12][CH2:13][C:14]1=[CH:15][CH:16]=[CH:17][CH:18]=[CH:19]1>>[CH3:1][C:2](=[O:3])[C:4]1=[CH:5][CH:6]=[C:7]([CH3:8])[CH:9]=[C:10]1[O:11][CH2:13][C:14]1=[CH:15][CH:16]=[CH:17][CH:18]=[CH:19]1.[ClH:12]

Correctness of the mapping

MAPPET	YES
ReactionMap	YES

Marvin YES
ChemDraw YES
Indigo YES

Reaction no 79

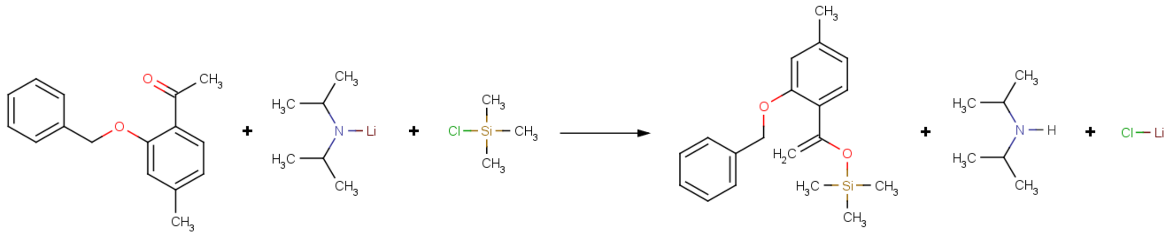

SMILES of the input:
CC(=O)C1=CC=C(C)C=C1OCC1=CC=CC=C1.[Li]N(C(C)C)C(C)C.C[Si](C)(C)Cl>>CC1=CC=C(C(=C)O[Si](C)(C)C)C(OCC2=CC=CC=C2)=C1.[H]N(C(C)C)C(C)C.[Li]Cl

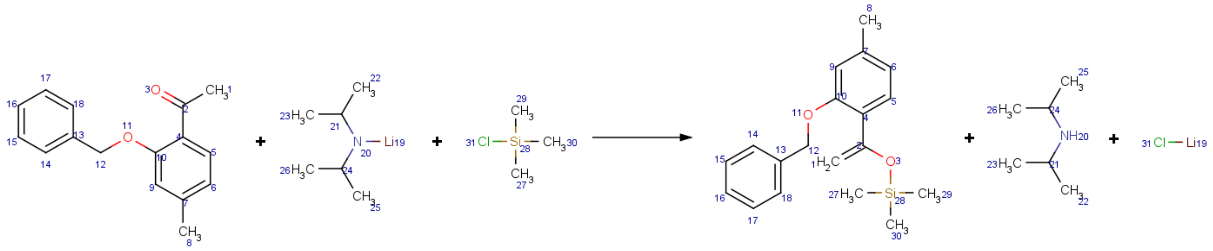

Correct mapped SMILES/SMARTS of the reaction:
[CH3:1][C:2](=[O:3])[C:4]1=[CH:5][CH:6]=[C:7]([CH3:8])[CH:9]=[C:10]1[O:11][CH2:12][C:13]1=[CH:14][CH:15]=[CH:16][CH:17]=[CH:18]1.[Li:19][N:20]([CH:21]([CH3:22])[CH3:23])[CH:24]([CH3:25])[CH3:26].[CH3:27][Si:28]([CH3:29])([CH3:30])[Cl:31]>>[CH3:8][C:7]1=[CH:6][CH:5]=[C:4]([C:2](=[CH2:1])[O:3][Si:28]([CH3:30])([CH3:29])[CH3:27])[C:10]([O:11][CH2:12][C:13]2=[CH:18][CH:17]=[CH:16][CH:15]=[CH:14]2)=[CH:9]1.[CH3:25][CH:24]([CH3:26])[NH:20][CH:21]([CH3:22])[CH3:23].[Li:19][Cl:31]

Correctness of the mapping
MAPPET YES
ReactionMap YES
Marvin YES
ChemDraw YES
Indigo YES

Reaction no 80

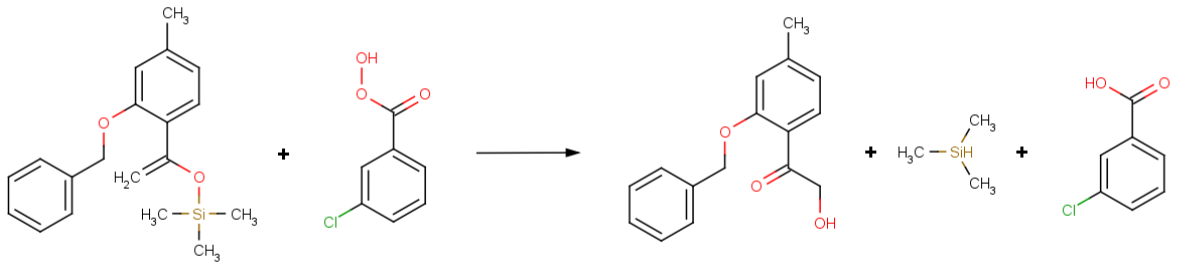

SMILES of the input:
CC1=CC=C(C(=C)O[Si](C)(C)C)C(OCC2=CC=CC=C2)=C1.OOC(=O)C1=CC=CC(Cl)=C1>>CC1=CC=C(C(=O)CO)C(OCC2=CC=CC=C2)=C1.C[SiH](C)C.OC(=O)C1=CC=CC(Cl)=C1

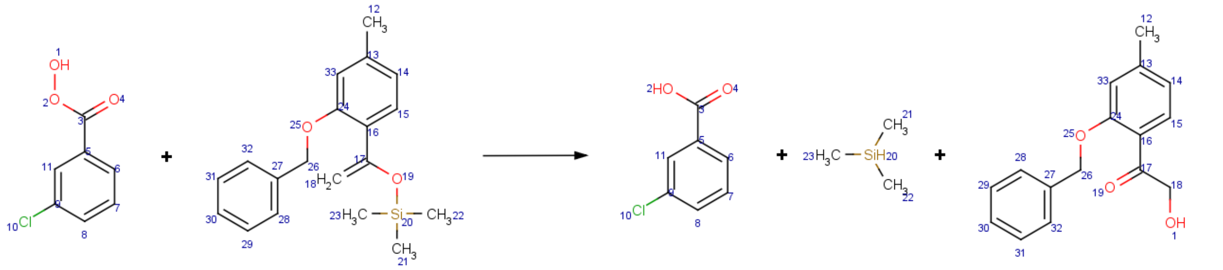

Correct mapped SMILES/SMARTS of the reaction:
[OH:1][O:2][C:3](=[O:4])[C:5]1=[CH:6][CH:7]=[CH:8][C:9]([Cl:10])=[CH:11]1.[CH3:12][C:13]1=[CH:14][CH:15]=[C:16]([C:17](=[CH2:18])[O:19][Si:20]([CH3:21])([CH3:22])[CH3:23])[C:24]([O:25][CH2:26][C:27]2=[CH:28][CH:29]=[CH:30][CH:31]=[CH:32]2)=[CH:33]1>>[OH:2][C:3](=[O:4])[C:5]1=[CH:6][CH:7]=[CH:8][C:9]([Cl:10])=[CH:11]1.[CH3:21][SiH:20]([CH3:22])[CH3:23].[CH3:12][C:13]1=[CH:14][CH:15]=[C:16]([C:17](=[O:19])[CH2:18][OH:1])[C:24]([O:25][CH2:26][C:27]2=[CH:32][CH:31]=[CH:30][CH:29]=[CH:28]2)=[CH:33]1

Correctness of the mapping

MAPPET	YES
ReactionMap	YES
Marvin	YES
ChemDraw	YES
Indigo	NO

Reaction no 81

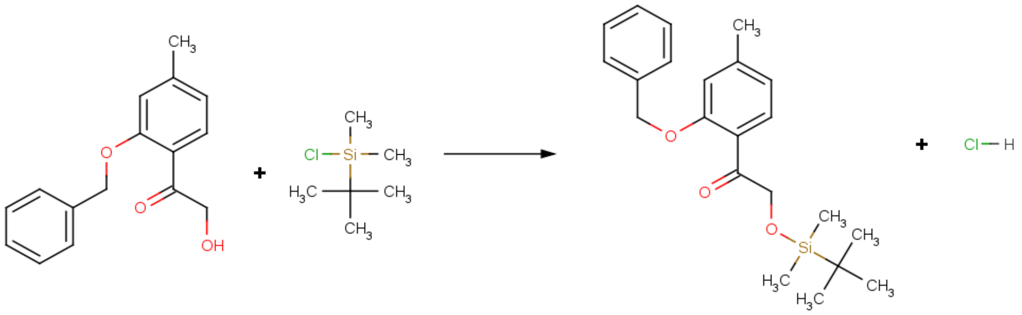

SMILES of the input:
CC1=CC=C(C(=O)CO)C(OCC2=CC=CC=C2)=C1.CC(C)(C)[Si](C)(C)Cl>>CC1=CC=C(C(=O)CO[Si](C)(C)C(C)(C)C)C(OCC2=CC=CC=C2)=C1.Cl[H]

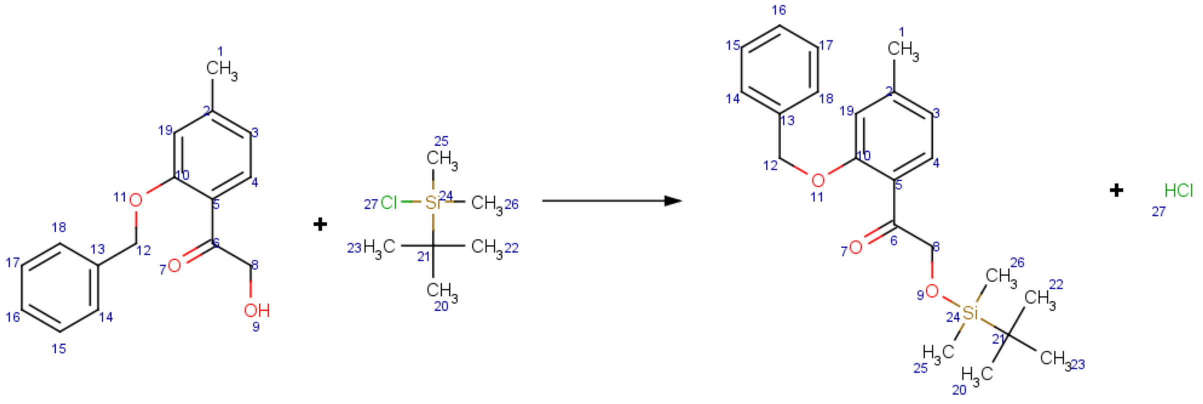

Correct mapped SMILES/SMARTS of the reaction:
[CH3:1][C:2]1=[CH:3][CH:4]=[C:5]([C:6](=[O:7])[CH2:8][OH:9])[C:10]([O:11][CH2:12][C:13]2=[CH:14][CH:15]=[CH:16][CH:17]=[CH:18]2)=[CH:19]1.[CH3:20][C:21]([CH3:22])([CH3:23])[Si:24]([CH3:25])([CH3:26])[Cl:27]>>[CH3:1][C:2]1=[CH:3][CH:4]=[C:5]([C:6](=[O:7])[CH2:8][O:9][Si:24]([CH3:26])([CH3:25]

)[C:21]([CH3:23])([CH3:22])[CH3:20])[C:10]([O:11][CH2:12][C:13]2=[CH:14][CH:15]=[CH:16][CH:17]=[CH:18]2)=[CH:19]1.[ClH:27]

Correctness of the mapping

MAPPET	YES
ReactionMap	YES
Marvin	YES
ChemDraw	YES
Indigo	YES

Reaction no 82

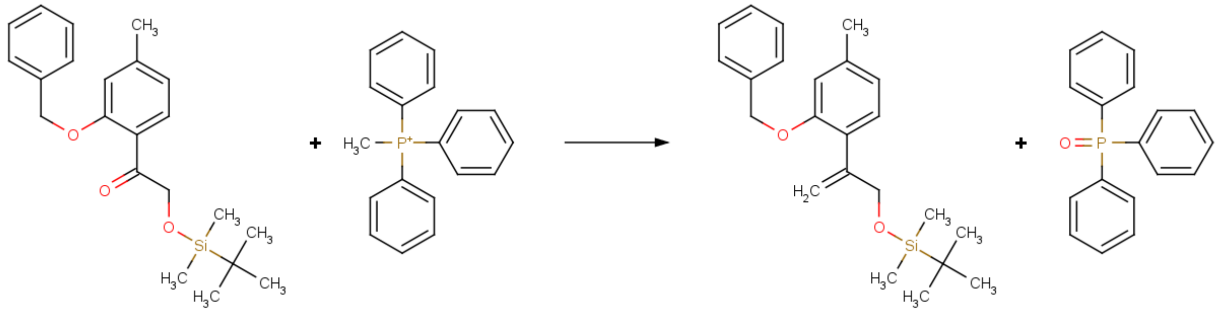

SMILES of the input:
CC1=CC=C(C(=O)CO[Si](C)(C)C(C)(C)C)C(OCC2=CC=CC=C2)=C1.C[P+](C1=CC=CC=C1)(C1=CC=CC=C1)C1=CC=CC=C1>>CC1=CC=C(C(=C)CO[Si](C)(C)C(C)(C)C)C(OCC2=CC=CC=C2)=C1.O=P(C1=CC=CC=C1)(C1=CC=CC=C1)C1=CC=CC=C1

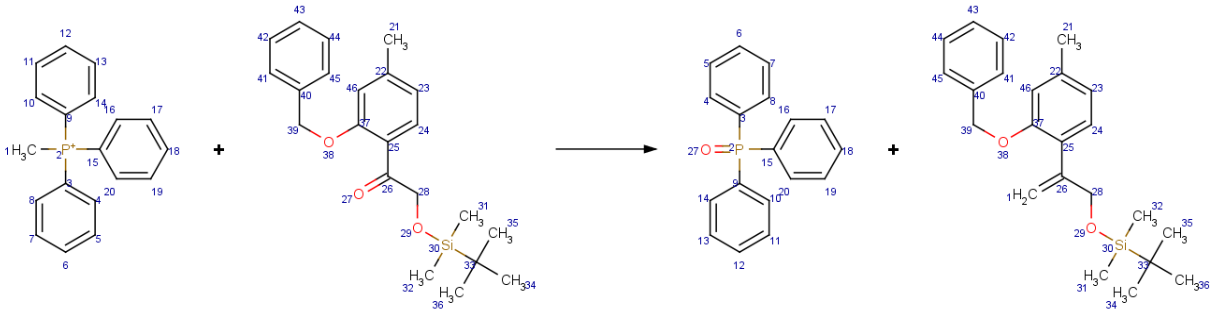

Correct mapped SMILES/SMARTS of the reaction:
[CH3:1][P+:2]([C:3]1=[CH:4][CH:5]=[CH:6][CH:7]=[CH:8]1)([C:9]1=[CH:10][CH:11]=[CH:12][CH:13]=[CH:14]1)[C:15]1=[CH:16][CH:17]=[CH:18][CH:19]=[CH:20]1.[CH3:21][C:22]1=[CH:23][CH:24]=[C:25]([C:26](=[O:27])[CH2:28][O:29][Si:30]([CH3:31])([CH3:32])[C:33]([CH3:34])([CH3:35])[CH3:36])[C:37]([O:38][CH2:39][C:40]2=[CH:41][CH:42]=[CH:43][CH:44]=[CH:45]2)=[CH:46]1>>[O:27]=[P:2]([C:9]1=[CH:10][CH:11]=[CH:12][CH:13]=[CH:14]1)([C:3]1=[CH:4][CH:5]=[CH:6][CH:7]=[CH:8]1)[C:15]1=[CH:16][CH:17]=[CH:18][CH:19]=[CH:20]1.[CH3:21][C:22]1=[CH:23][CH:24]=[C:25]([C:26](=[CH2:1])[CH2:28][O:29][Si:30]([CH3:32])([CH3:31])[C:33]([CH3:36])([CH3:35])[CH3:34])[C:37]([O:38][CH2:39][C:40]2=[CH:45][CH:44]=[CH:43][CH:42]=[CH:41]2)=[CH:46]1

Correctness of the mapping

MAPPET	YES
ReactionMap	YES
Marvin	YES
ChemDraw	YES
Indigo	YES

Reaction no 83

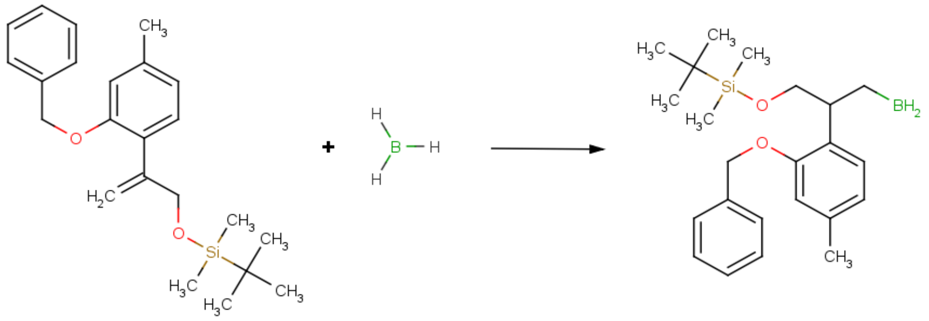

SMILES of the input:
CC1=CC=C(C(=C)CO[Si](C)(C)C(C)(C)C)C(OCC2=CC=CC=C2)=C1.[H]B([H])[H]>>BCC(
CO[Si](C)(C)C(C)(C)C)C1=CC=C(C)C=C1OCC1=CC=CC=C1

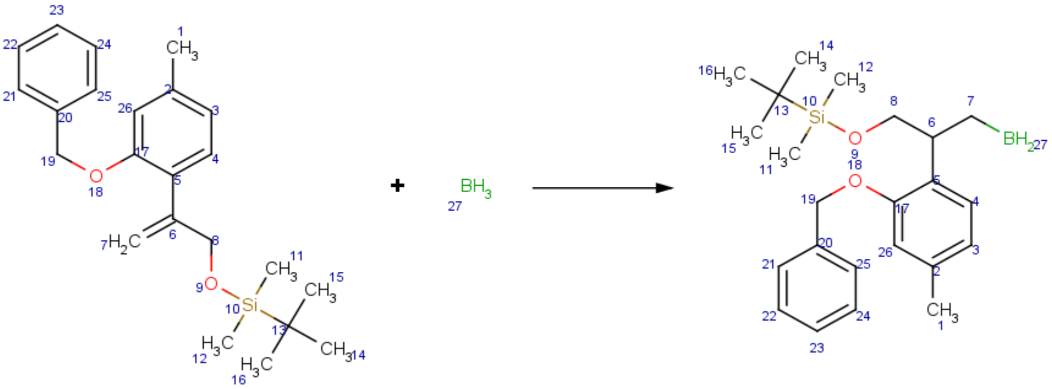

Correct mapped SMILES/SMARTS of the reaction:
[CH3:1][C:2]1=[CH:3][CH:4]=[C:5]([C:6](=[CH2:7])[CH2:8][O:9][Si:10]([CH3:
11])([CH3:12])[C:13]([CH3:14])([CH3:15])[CH3:16])[C:17]([O:18][CH2:19][C:
20]2=[CH:21][CH:22]=[CH:23][CH:24]=[CH:25]2)=[CH:26]1.[BH3:27]>>[BH2:27][
CH2:7][CH:6]([CH2:8][O:9][Si:10]([CH3:11])([CH3:12])[C:13]([CH3:16])([CH3
:15])[CH3:14])[C:5]1=[CH:4][CH:3]=[C:2]([CH3:1])[CH:26]=[C:17]1[O:18][CH2
:19][C:20]1=[CH:25][CH:24]=[CH:23][CH:22]=[CH:21]1

Correctness of the mapping

MAPPET	YES
ReactionMap	YES
Marvin	YES
ChemDraw	YES
Indigo	YES

Reaction no 84

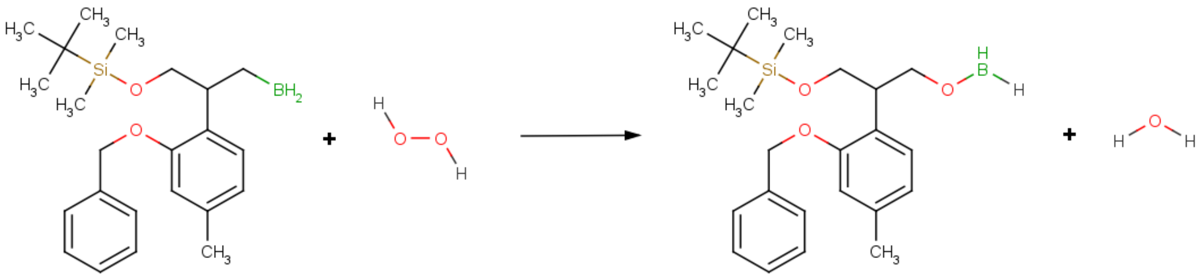

SMILES of the input:
BCC(CO[Si](C)(C)C(C)(C)C)C1=CC=C(C)C=C1OCC1=CC=CC=C1.[H]OO[H]>>[H]BOCC(CO
[Si](C)(C)C(C)(C)C)C1=CC=C(C)C=C1OCC1=CC=CC=C1.[H]O[H]

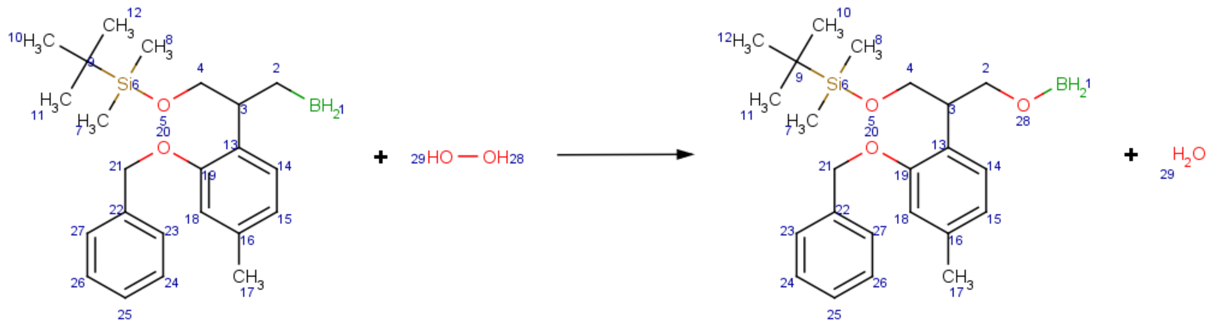

Correct mapped SMILES/SMARTS of the reaction:
[BH2:1][CH2:2][CH:3]([CH2:4][O:5][Si:6]([CH3:7])([CH3:8])[C:9]([CH3:10])(
[CH3:11])[CH3:12])[C:13]1=[CH:14][CH:15]=[C:16]([CH3:17])[CH:18]=[C:19]1[
O:20][CH2:21][C:22]1=[CH:23][CH:24]=[CH:25][CH:26]=[CH:27]1.[OH:28][OH:29
]>>[BH2:1][O:28][CH2:2][CH:3]([CH2:4][O:5][Si:6]([CH3:7])([CH3:8])[C:9]([
CH3:12])([CH3:11])[CH3:10])[C:13]1=[CH:14][CH:15]=[C:16]([CH3:17])[CH:18]
=[C:19]1[O:20][CH2:21][C:22]1=[CH:27][CH:26]=[CH:25][CH:24]=[CH:23]1.[OH2
:29]

Correctness of the mapping

MAPPET	YES
ReactionMap	YES
Marvin	NO
ChemDraw	YES
Indigo	NO

Reaction no 85

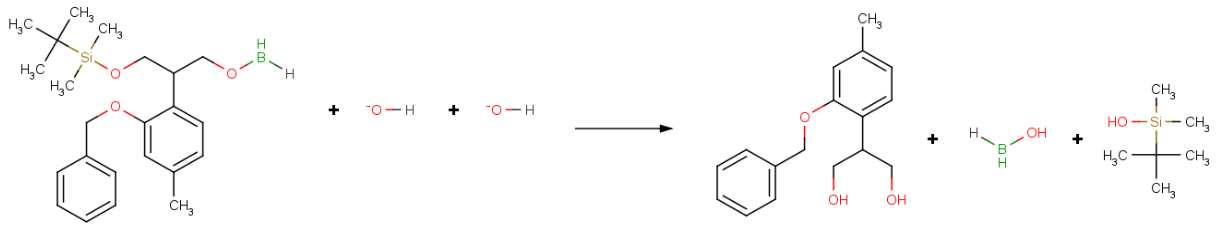

SMILES of the input:
[H]BOCC(CO[Si](C)(C)C(C)(C)C)C1=CC=C(C)C=C1OCC1=CC=CC=C1.[O-][H].[O-
][H]>>CC1=CC=C(C(CO)CO)C(OCC2=CC=CC=C2)=C1.[H]BO.CC(C)(C)[Si](C)(C)O

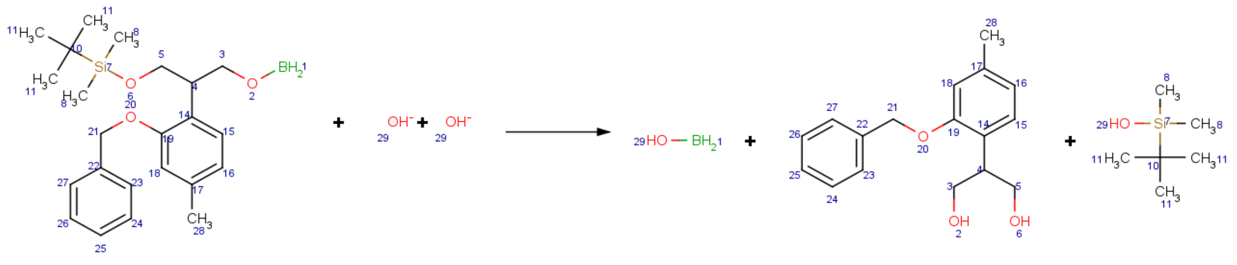

Correct mapped SMILES/SMARTS of the reaction:
[BH2:1][O:2][CH2:3][CH:4]([CH2:5][O:6][Si:7]([CH3:8])([CH3:8])[C:10]([CH3
:11])([CH3:11])[CH3:11])[C:14]1=[CH:15][CH:16]=[C:17]([CH:18]=[C:19]1[O:2
0][CH2:21][C:22]2=[CH:23][CH:24]=[CH:25][CH:26]=[CH:27]2)[CH3:28].[OH-
:29].[OH-
:29]>>[BH2:1][OH:29].[CH3:28][C:17]1=[CH:16][CH:15]=[C:14]([C:19](=[CH:18
]1)[O:20][CH2:21][C:22]2=[CH:23][CH:24]=[CH:25][CH:26]=[CH:27]2)[CH:4]([C
H2:3][OH:2])[CH2:5][OH:6].[CH3:11][C:10]([CH3:11])([CH3:11])[Si:7]([CH3:8
])([CH3:8])[OH:29]

Correctness of the mapping
MAPPET NO
ReactionMap YES
Marvin NO
ChemDraw YES
Indigo NO

Reaction no 86

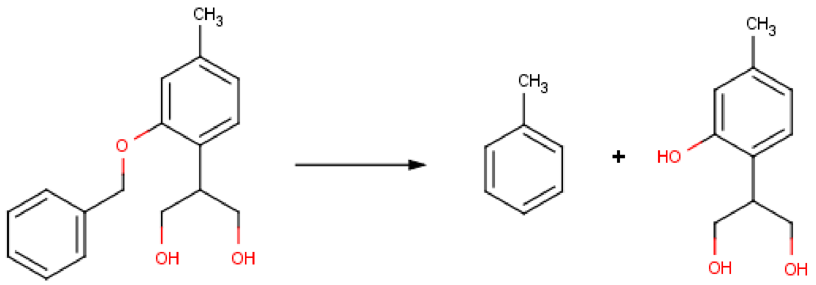

SMILES of the input:
CC1=CC=C(C(CO)CO)C(OCC2=CC=CC=C2)=C1>>CC1=CC=CC=C1.CC1=CC=C(C(CO)CO)C(O)=C1

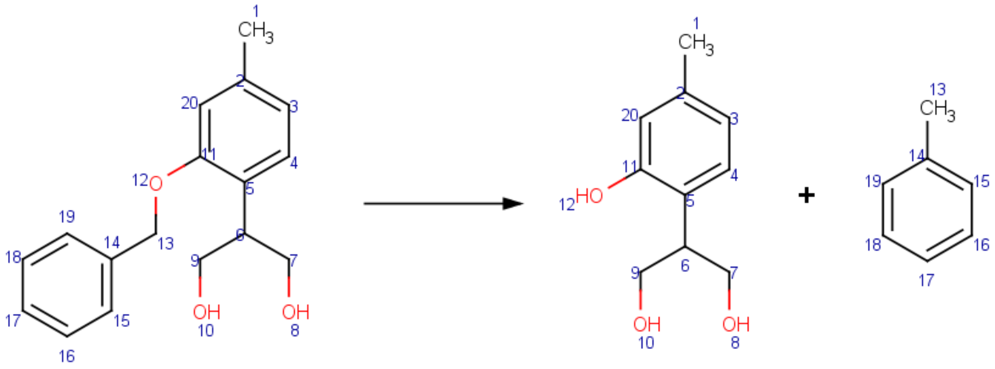

Correct mapped SMILES/SMARTS of the reaction:
[CH3:1][C:2]1=[CH:3][CH:4]=[C:5]([CH:6]([CH2:7][OH:8])[CH2:9][OH:10])[C:11]([O:12][CH2:13][C:14]2=[CH:15][CH:16]=[CH:17][CH:18]=[CH:19]2)=[CH:20]1>>[CH3:1][C:2]1=[CH:3][CH:4]=[C:5]([CH:6]([CH2:9][OH:10])[CH2:7][OH:8])[C:11]([OH:12])=[CH:20]1.[CH3:13][C:14]1=[CH:15][CH:16]=[CH:17][CH:18]=[CH:19]1

Correctness of the mapping
MAPPET YES
ReactionMap YES
Marvin YES
ChemDraw YES
Indigo NO

Reaction no 87

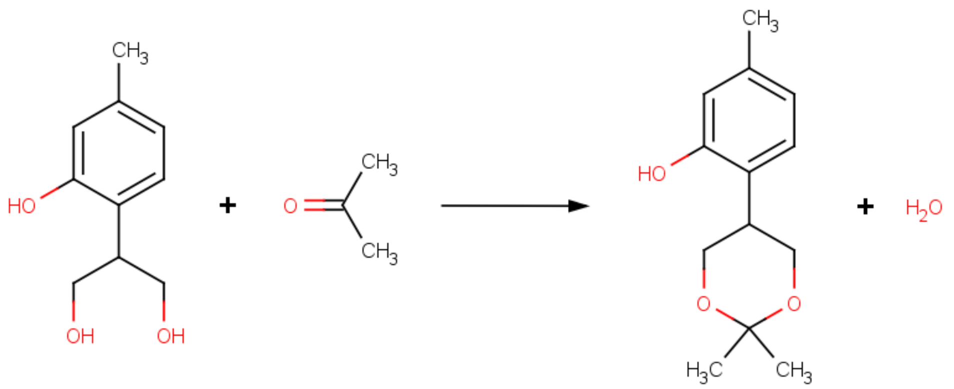

SMILES of the input:
CC1=CC=C(C(CO)CO)C(O)=C1.CC(C)=O>>CC1=CC=C(C2COC(C)(C)OC2)C(O)=C1.O

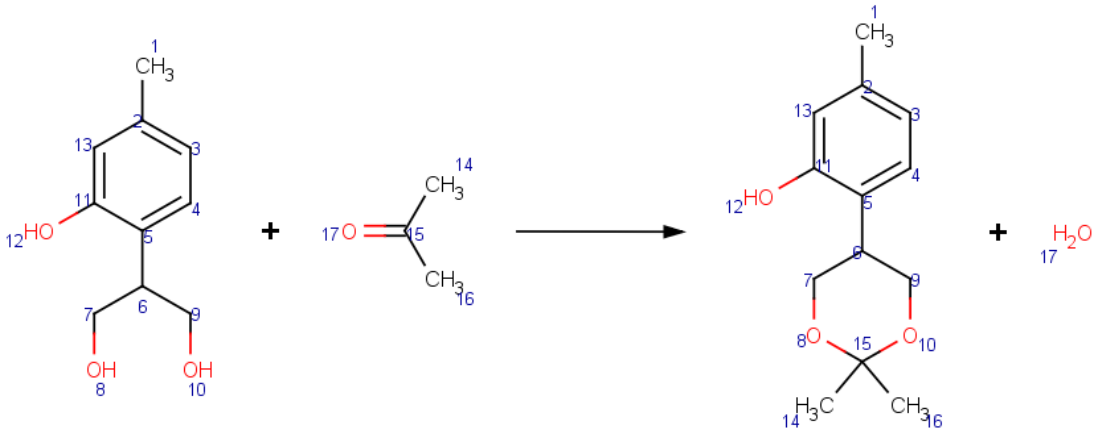

Correct mapped SMILES/SMARTS of the reaction:
[CH3:1][C:2]1=[CH:3][CH:4]=[C:5]([CH:6]([CH2:7][OH:8])[CH2:9][OH:10])[C:11]([OH:12])=[CH:13]1.[CH3:14][C:15]([CH3:16])=[O:17]>>[CH3:1][C:2]1=[CH:3][CH:4]=[C:5]([CH:6]2[CH2:9][O:10][C:15]([CH3:16])([CH3:14])[O:8][CH2:7]2)[C:11]([OH:12])=[CH:13]1.[OH2:17]

Correctness of the mapping

MAPPET	YES
ReactionMap	YES
Marvin	YES
ChemDraw	YES
Indigo	YES

Reaction no 88

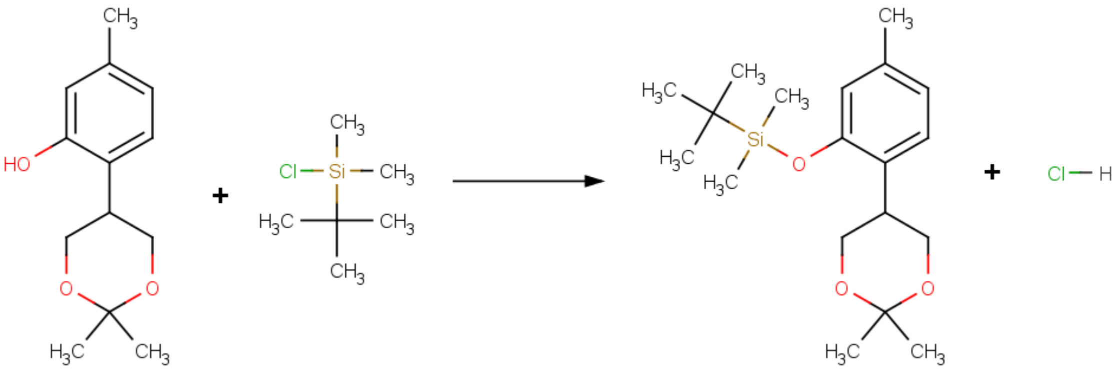

SMILES of the input:
CC1=CC=C(C2COC(C)(C)OC2)C(O)=C1.CC(C)(C)[Si](C)(C)Cl>>CC1=CC=C(C2COC(C)(C)OC2)C(O[Si](C)(C)C(C)(C)C)=C1.Cl[H]

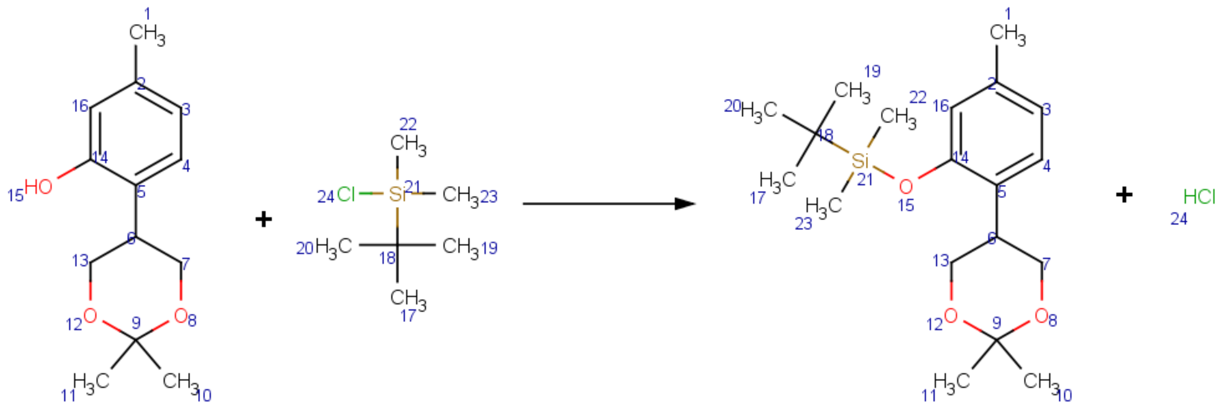

Correct mapped SMILES/SMARTS of the reaction:
[CH3:1][C:2]1=[CH:3][CH:4]=[C:5]([CH:6]2[CH2:7][O:8][C:9]([CH3:10])([CH3:11])[O:12][CH2:13]2)[C:14]([OH:15])=[CH:16]1.[CH3:17][C:18]([CH3:19])([CH3:20])[Si:21]([CH3:22])([CH3:23])[Cl:24]>>[CH3:1][C:2]1=[CH:3][CH:4]=[C:5]([CH:6]2[CH2:7][O:8][C:9]([CH3:10])([CH3:11])[O:12][CH2:13]2)[C:14]([O:15][Si:21]([CH3:22])([CH3:23])[C:18]([CH3:20])([CH3:19])[CH3:17])=[CH:16]1.[ClH:24]

Correctness of the mapping

MAPPET	YES
ReactionMap	YES
Marvin	YES
ChemDraw	YES
Indigo	YES

Reaction no 89

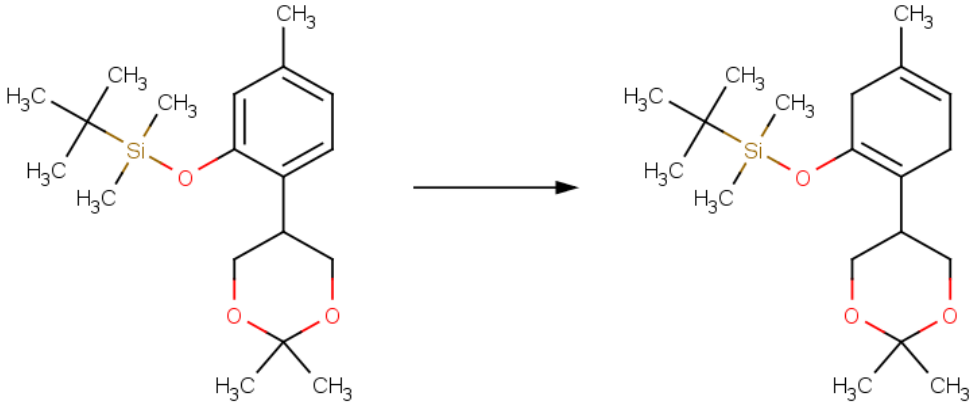

SMILES of the input:
CC1=CC=C(C2COC(C)(C)OC2)C(O[Si](C)(C)C(C)(C)C)=C1>>CC1=CCC(C2COC(C)(C)OC2)=C(C1)O[Si](C)(C)C(C)(C)C

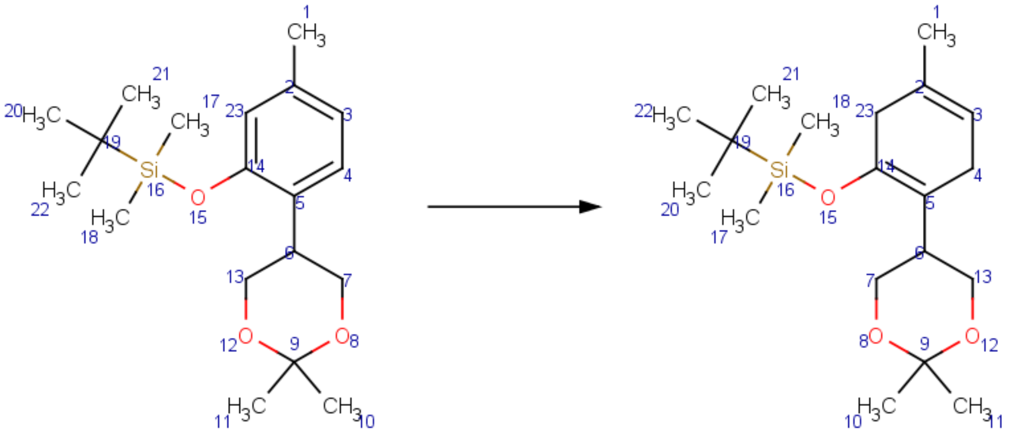

Correct mapped SMILES/SMARTS of the reaction:
[CH3:1][C:2]1=[CH:3][CH:4]=[C:5]([CH:6]2[CH2:7][O:8][C:9]([CH3:10])([CH3:
11])[O:12][CH2:13]2)[C:14]([O:15][Si:16]([CH3:17])([CH3:18])[C:19]([CH3:2
0])([CH3:21])[CH3:22])=[CH:23]1>>[CH3:1][C:2]1=[CH:3][CH2:4][C:5]([CH:6]2
[CH2:13][O:12][C:9]([CH3:11])([CH3:10])[O:8][CH2:7]2)=[C:14]([CH2:23]1)[O
:15][Si:16]([CH3:18])([CH3:17])[C:19]([CH3:22])([CH3:21])[CH3:20]

Correctness of the mapping

MAPPET	YES
ReactionMap	YES
Marvin	YES
ChemDraw	YES
Indigo	YES

Reaction no 90

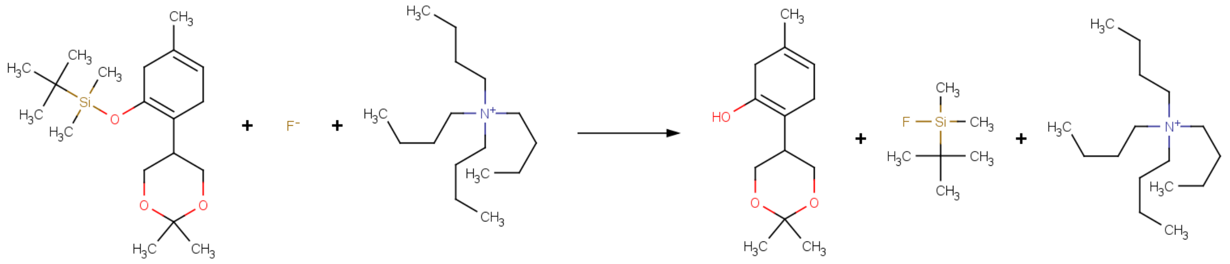

SMILES of the input:
CC1=CCC(C2COC(C)(C)OC2)=C(C1)O[Si](C)(C)C(C)(C)C.[F-
].CCCC[N+](CCCC)(CCCC)CCCC>>CC1=CCC(C2COC(C)(C)OC2)=C(O)C1.CC(C)(C)[Si](C
)(C)F.CCCC[N+](CCCC)(CCCC)CCCC

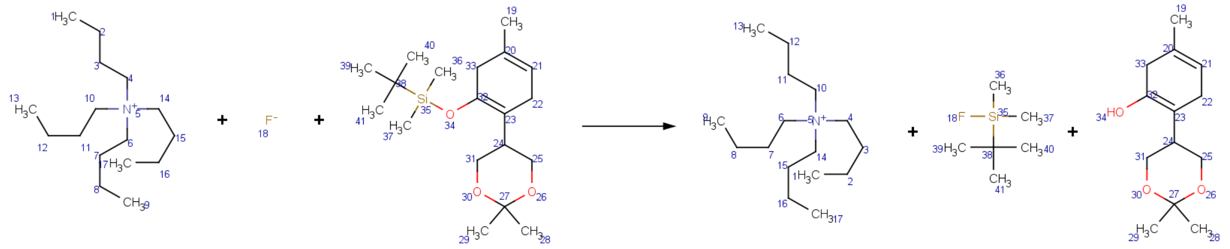

Correct mapped SMILES/SMARTS of the reaction:
[CH3:1][CH2:2][CH2:3][CH2:4][N+:5]([CH2:6][CH2:7][CH2:8][CH3:9])([CH2:10]
[CH2:11][CH2:12][CH3:13])[CH2:14][CH2:15][CH2:16][CH3:17].[F-
:18].[CH3:19][C:20]1=[CH:21][CH2:22][C:23]([CH:24]2[CH2:25][O:26][C:27]([
CH3:28])([CH3:29])[O:30][CH2:31]2)=[C:32]([CH2:33]1)[O:34][Si:35]([CH3:36
])([CH3:37])[C:38]([CH3:39])([CH3:40])[CH3:41]>>[CH3:13][CH2:12][CH2:11][
CH2:10][N+:5]([CH2:14][CH2:15][CH2:16][CH3:17])([CH2:6][CH2:7][CH2:8][CH3
:9])[CH2:4][CH2:3][CH2:2][CH3:1].[CH3:41][C:38]([CH3:40])([CH3:39])[Si:35
]([CH3:36])([CH3:37])[F:18].[CH3:19][C:20]1=[CH:21][CH2:22][C:23]([CH:24]

2[CH2:25][O:26][C:27]([CH3:28])([CH3:29])[O:30][CH2:31]2)=[C:32]([OH:34])
[CH2:33]1

Correctness of the mapping

MAPPET	YES
ReactionMap	YES
Marvin	YES
ChemDraw	YES
Indigo	YES

Reaction no 91

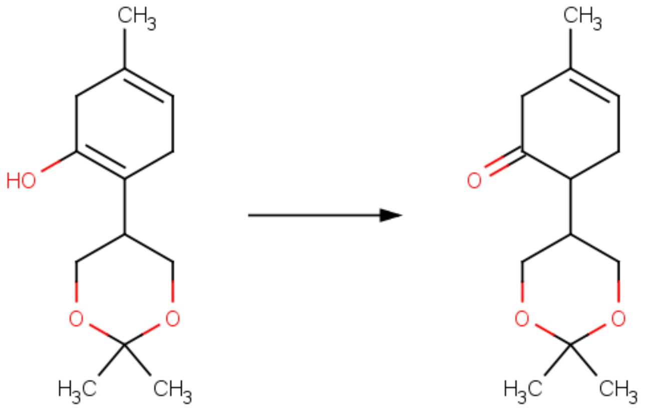

SMILES of the input:
CC1=CCC(C2COC(C)(C)OC2)=C(O)C1>>CC1=CCC(C2COC(C)(C)OC2)C(=O)C1

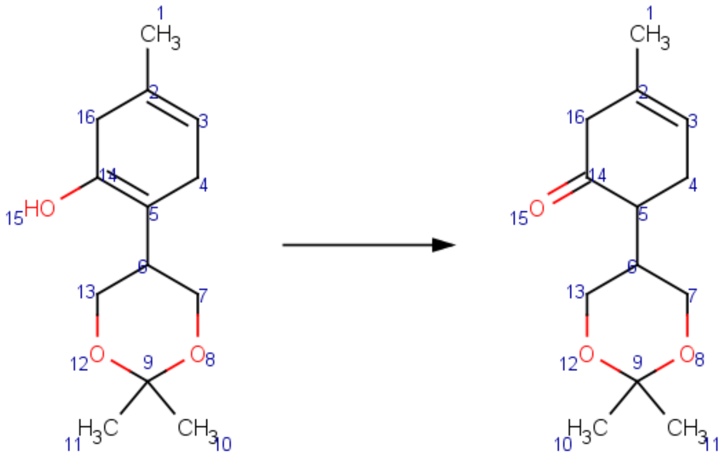

Correct mapped SMILES/SMARTS of the reaction:
[CH3:1][C:2]1=[CH:3][CH2:4][C:5]([CH:6]2[CH2:7][O:8][C:9]([CH3:10])([CH3:
11])[O:12][CH2:13]2)=[C:14]([OH:15])[CH2:16]1>>[CH3:1][C:2]1=[CH:3][CH2:4
][CH:5]([CH:6]2[CH2:7][O:8][C:9]([CH3:11])([CH3:10])[O:12][CH2:13]2)[C:14
](=[O:15])[CH2:16]1

Correctness of the mapping

MAPPET	YES
ReactionMap	YES
Marvin	YES
ChemDraw	YES
Indigo	NO

Reaction no 92

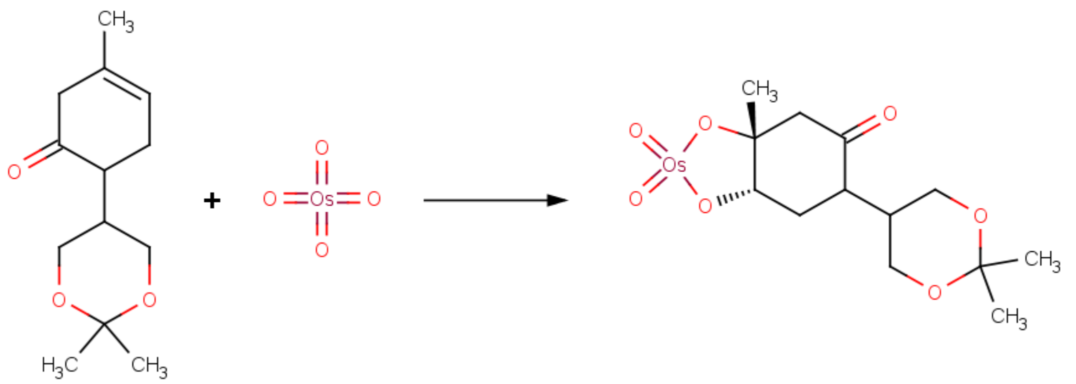

SMILES of the input:
CC1=CCC(C2COC(C)(C)OC2)C(=O)C1.O=[Os](=O)(=O)=O>>C[C@@]12CC(=O)C(C[C@@H]1
O[Os](=O)(=O)O2)C1COC(C)(C)OC1

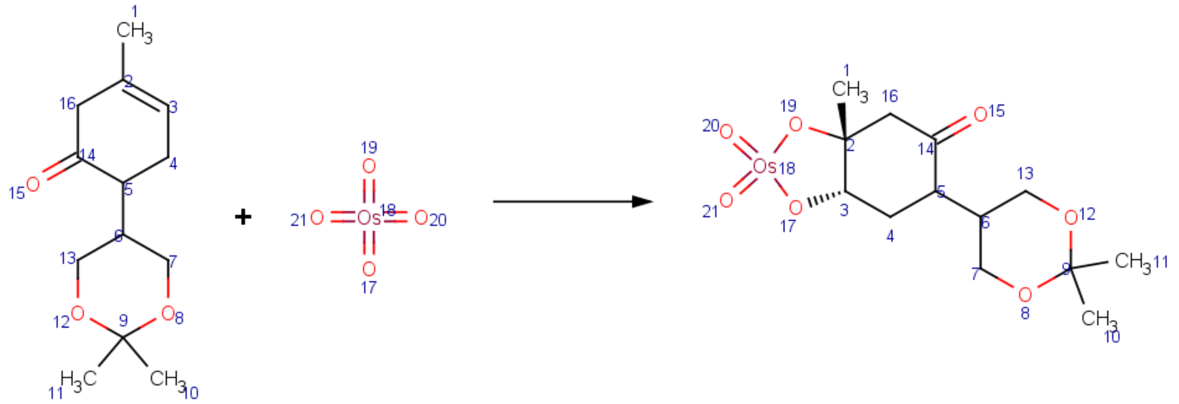

Correct mapped SMILES/SMARTS of the reaction:
[CH3:1][C:2]1=[CH:3][CH2:4][CH:5]([CH:6]2[CH2:7][O:8][C:9]([CH3:10])([CH3
:11])[O:12][CH2:13]2)[C:14](=[O:15])[CH2:16]1.[O:17]=[Os:18](=[O:19])(=[O
:20])=[O:21]>>[CH3:1][C@@:2]12[CH2:16][C:14](=[O:15])[CH:5]([CH2:4][C@@H:
3]1[O:17][Os:18](=[O:21])(=[O:20])[O:19]2)[CH:6]1[CH2:13][O:12][C:9]([CH3
:11])([CH3:10])[O:8][CH2:7]1

Correctness of the mapping

MAPPET	YES
ReactionMap	YES
Marvin	YES
ChemDraw	YES
Indigo	NO

Reaction no 93

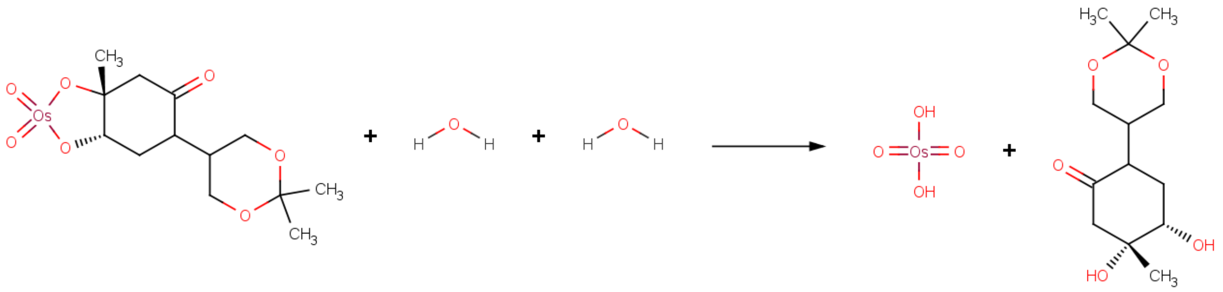

SMILES of the input:
C[C@@]12CC(=O)C(C[C@@H]1O[Os](=O)(=O)O2)C1COC(C)(C)OC1.[H]O[H].[H]O[H]>>O
[Os](O)(=O)=O.CC1(C)OCC(CO1)C1C[C@H](O)[C@](C)(O)CC1=O

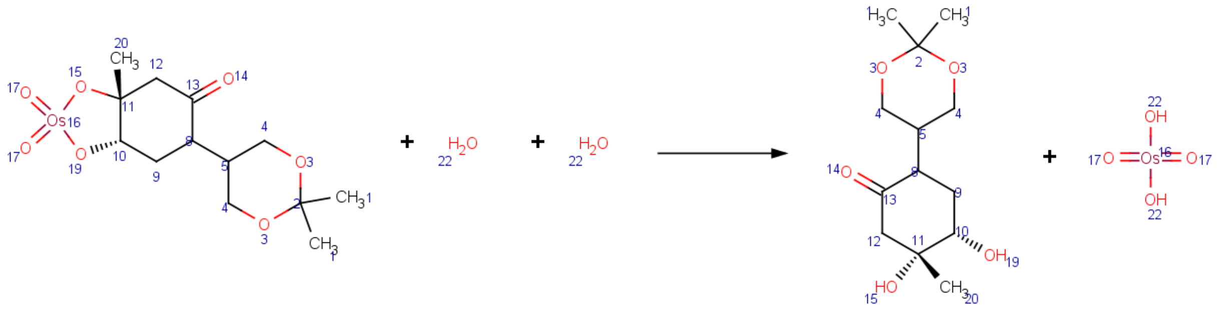

Correct mapped SMILES/SMARTS of the reaction:

[CH3:20][C@@:11]12[CH2:12][C:13](=[O:14])[CH:8]([CH2:9][C@@H:10]1[O:19][Os:16](=[O:17])(=[O:17])[O:15]2)[CH:5]1[CH2:4][O:3][C:2]([CH3:1])([CH3:1])[O:3][CH2:4]1.[OH2:22].[OH2:22]>>[CH3:1][C:2]1([CH3:1])[O:3][CH2:4][CH:5]([CH2:4][O:3]1)[CH:8]1[CH2:9][C@H:10]([OH:19])[C@:11]([CH3:20])([OH:15])[CH2:12][C:13]1=[O:14].[OH:22][Os:16]([OH:22])(=[O:17])=[O:17]

Correctness of the mapping

MAPPET	NO
ReactionMap	YES
Marvin	NO
ChemDraw	YES
Indigo	NO

Reaction no 94

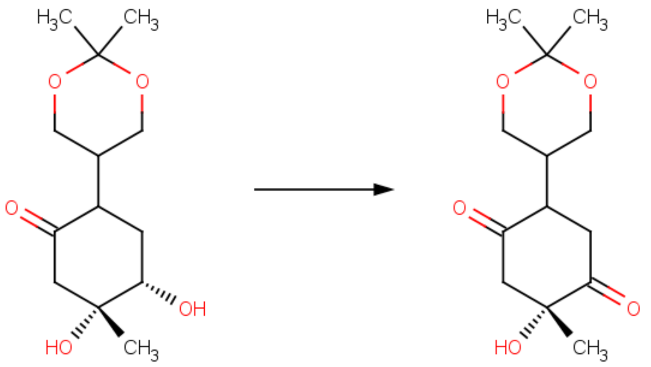

SMILES of the input:

CC1(C)OCC(CO1)C1C[C@H](O)[C@](C)(O)CC1=O>>CC1(C)OCC(CO1)C1CC(=O)[C@](C)(O)CC1=O

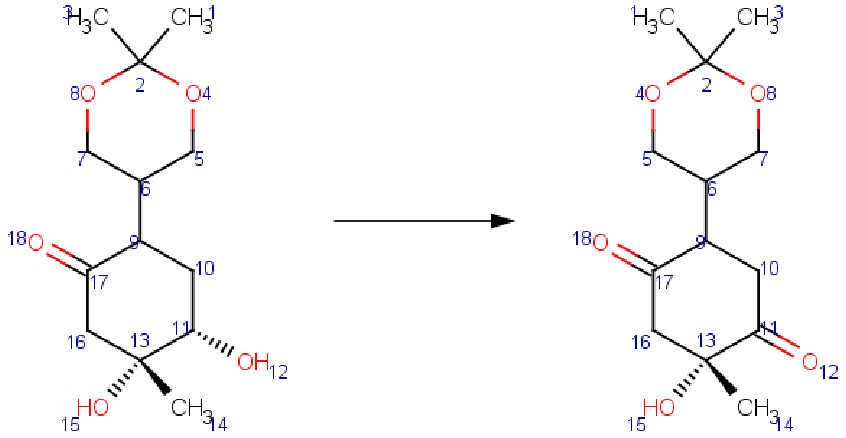

Correct mapped SMILES/SMARTS of the reaction:

[CH3:1][C:2]1([CH3:3])[O:4][CH2:5][CH:6]([CH2:7][O:8]1)[CH:9]1[CH2:10][C@H:11]([OH:12])[C@:13]([CH3:14])([OH:15])[CH2:16][C:17]1=[O:18]>>[CH3:3][C

:2]1([CH3:1])[O:8][CH2:7][CH:6]([CH2:5][O:4]1)[CH:9]1[CH2:10][C:11](=[O:12])[C@:13]([CH3:14])([OH:15])[CH2:16][C:17]1=[O:18]

Correctness of the mapping
MAPPET YES
ReactionMap YES
Marvin YES
ChemDraw YES
Indigo NO

Reaction no 95

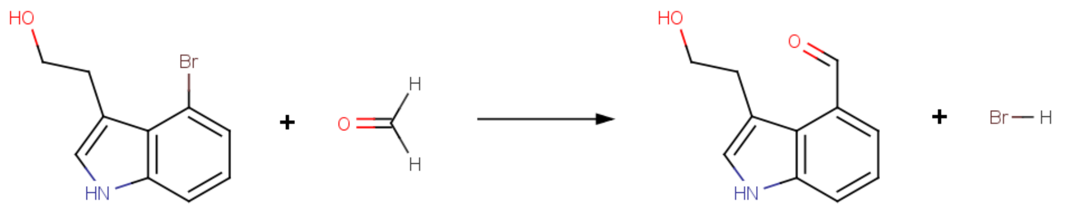

SMILES of the input:
OCCC1=CNC2=C1C(Br)=CC=C2.[H]C([H])=O>>OCCC1=CNC2=C1C(C=O)=CC=C2.Br[H]

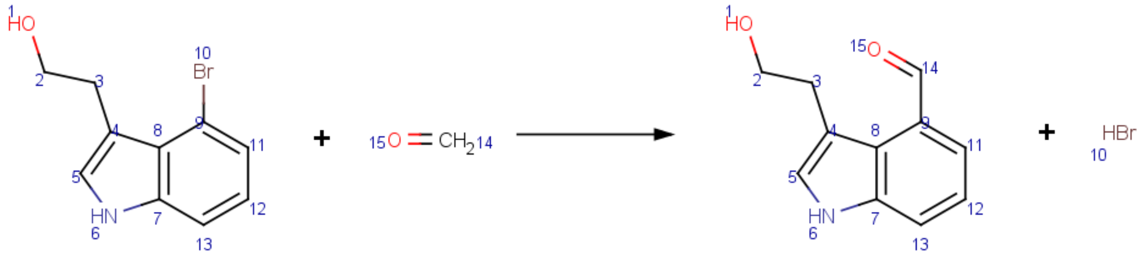

Correct mapped SMILES/SMARTS of the reaction:
[OH:1][CH2:2][CH2:3][C:4]1=[CH:5][NH:6][C:7]2=[C:8]1[C:9]([Br:10])=[CH:11][CH:12]=[CH:13]2.[CH2:14]=[O:15]>>[OH:1][CH2:2][CH2:3][C:4]1=[CH:5][NH:6][C:7]2=[C:8]1[C:9]([CH:14]=[O:15])=[CH:11][CH:12]=[CH:13]2.[BrH:10]

Correctness of the mapping
MAPPET YES
ReactionMap YES
Marvin YES
ChemDraw YES
Indigo YES

Reaction no 96

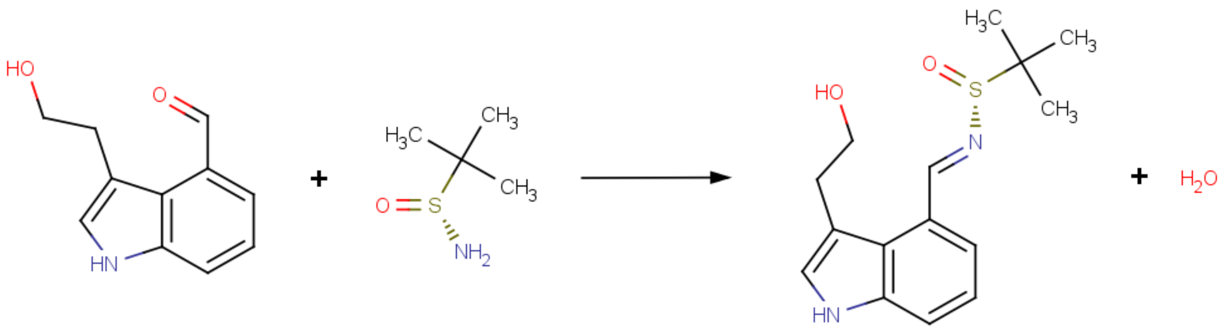

SMILES of the input:
OCCC1=CNC2=C1C(C=O)=CC=C2.CC(C)(C)[S@](N)=O>>CC(C)(C)[S@@](=O)\N=C\C1=CC=CC2=C1C(CCO)=CN2.O

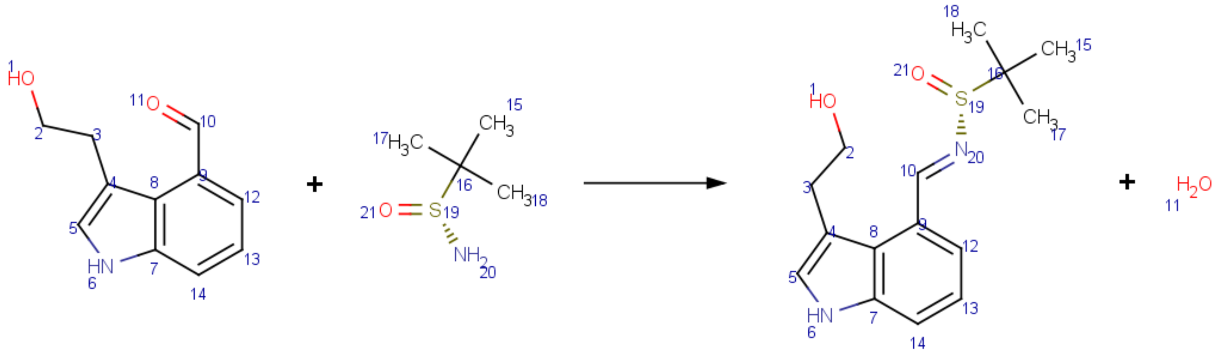

Correct mapped SMILES/SMARTS of the reaction:
[OH:1][CH2:2][CH2:3][C:4]1=[CH:5][NH:6][C:7]2=[C:8]1[C:9]([CH:10]=[O:11])=[CH:12][CH:13]=[CH:14]2.[CH3:15][C:16]([CH3:17])([CH3:18])[S@:19]([NH2:20])=[O:21]>>[CH3:15][C:16]([CH3:18])([CH3:17])[S@@:19](=[O:21])\[N:20]=[CH:10]\[C:9]1=[CH:12][CH:13]=[CH:14][C:7]2=[C:8]1[C:4]([CH2:3][CH2:2][OH:1])=[CH:5][NH:6]2.[OH2:11]

Correctness of the mapping

MAPPET	YES
ReactionMap	YES
Marvin	YES
ChemDraw	YES
Indigo	NO

Reaction no 97

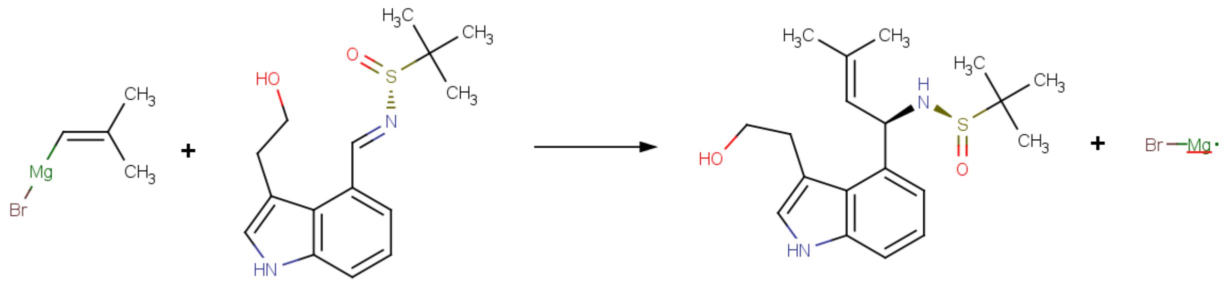

SMILES of the input:
CC(C)=C[Mg]Br.CC(C)(C)[S@@](=O)\N=C\C1=CC=CC2=C1C(CCO)=CN2>>CC(C)=C[C@@H](N[S@](=O)C(C)(C)C)C1=CC=CC2=C1C(CCO)=CN2.[Mg]Br

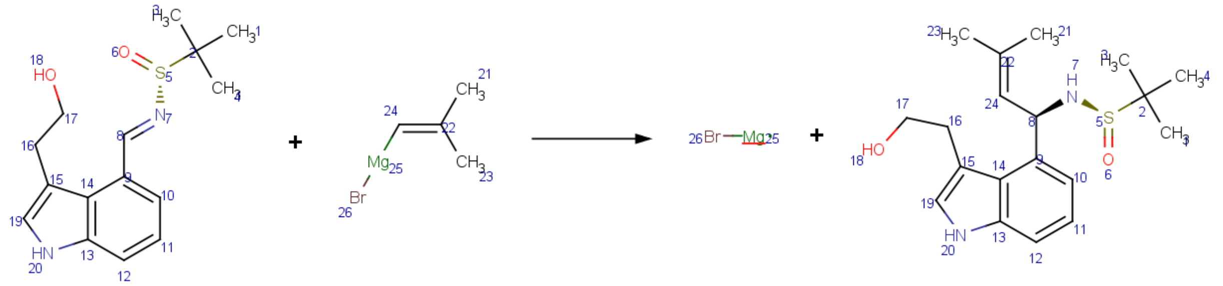

Correct mapped SMILES/SMARTS of the reaction:
[CH3:1][C:2]([CH3:3])([CH3:4])[S@@:5](=[O:6])\[N:7]=[CH:8]\[C:9]1=[CH:10][CH:11]=[CH:12][C:13]2=[C:14]1[C:15]([CH2:16][CH2:17][OH:18])=[CH:19][NH:20]2.[CH3:21][C:22]([CH3:23])=[CH:24][Mg:25][Br:26]>>[Mg:25][Br:26].[CH3:

21][C:22]([CH3:23])=[CH:24][C@@H:8]([NH:7][S@:5](=[O:6])[C:2]([CH3:4])([CH3:3])[CH3:1])[C:9]1=[CH:10][CH:11]=[CH:12][C:13]2=[C:14]1[C:15]([CH2:16][CH2:17][OH:18])=[CH:19][NH:20]2

Correctness of the mapping

MAPPET	YES
ReactionMap	YES
Marvin	YES
ChemDraw	YES
Indigo	YES

Reaction no 98

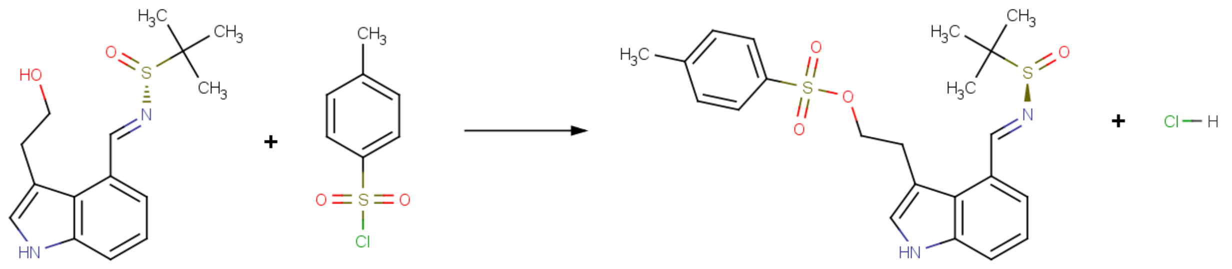

SMILES of the input:
CC(C)(C)[S@@](=O)\N=C\C1=CC=CC2=C1C(CCO)=CN2.CC1=CC=C(C=C1)S(Cl)(=O)=O>>CC1=CC=C(C=C1)S(=O)(=O)OCCC1=CNC2=C1C(\C=N\[S@](=O)C(C)(C)C)=CC=C2.Cl[H]

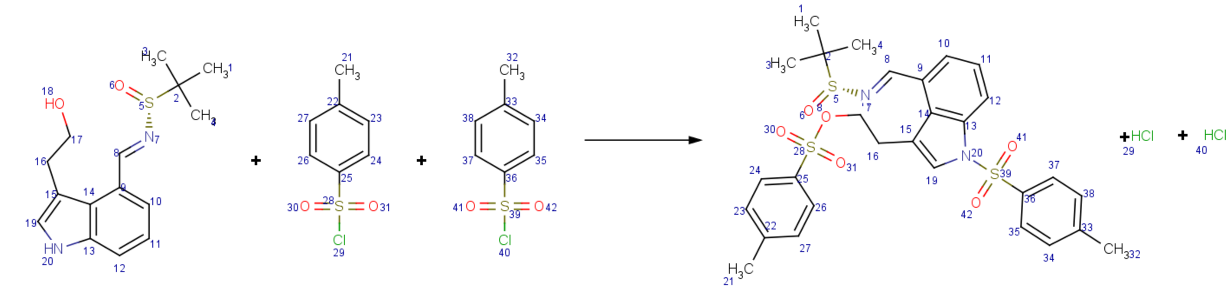

Correct mapped SMILES/SMARTS of the reaction:
[CH3:1][C:2]([CH3:3])([CH3:4])[S@@:5](=[O:6])\[N:7]=[CH:8]\[C:9]1=[CH:10][CH:11]=[CH:12][C:13]2=[C:14]1[C:15]([CH2:16][CH2:17][OH:18])=[CH:19][NH:20]2.[CH3:21][C:22]1=[CH:23][CH:24]=[C:25]([CH:26]=[CH:27]1)[S:28]([Cl:29])(=[O:30])=[O:31].[CH3:32][C:33]1=[CH:34][CH:35]=[C:36]([CH:37]=[CH:38]1)[S:39]([Cl:40])(=[O:41])=[O:42]>>[CH3:21][C:22]1=[CH:23][CH:24]=[C:25]([CH:26]=[CH:27]1)[S:28](=[O:31])(=[O:30])[O:18][CH2:17][CH2:16][C:15]1=[CH:19][N:20]([C:13]2=[C:14]1[C:9](\[CH:8]=[N:7]\[S@:5](=[O:6])[C:2]([CH3:4])([CH3:3])[CH3:1])=[CH:10][CH:11]=[CH:12]2)[S:39](=[O:42])(=[O:41])[C:36]1=[CH:37][CH:38]=[C:33]([CH3:32])[CH:34]=[CH:35]1.[ClH:29].[ClH:40]

Correctness of the mapping

MAPPET	YES
ReactionMap	YES
Marvin	YES
ChemDraw	YES
Indigo	YES

Reaction no 99

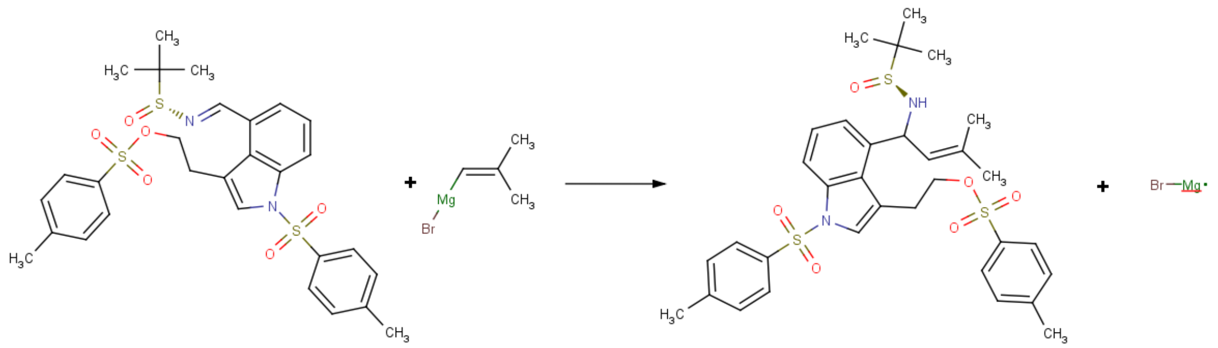

SMILES of the input:
CC1=CC=C(C=C1)S(=O)(=O)OCCC1=CN(C2=C1C(\C=N\[S@](=O)C(C)(C)C)=CC=C2)S(=O)(=O)C1=CC=C(C)C=C1.CC(C)=C[Mg]Br>>CC(C)=CC(N[S@](=O)C(C)(C)C)C1=CC=CC2=C1C(CCOS(=O)(=O)C1=CC=C(C)C=C1)=CN2S(=O)(=O)C1=CC=C(C)C=C1.[Mg]Br

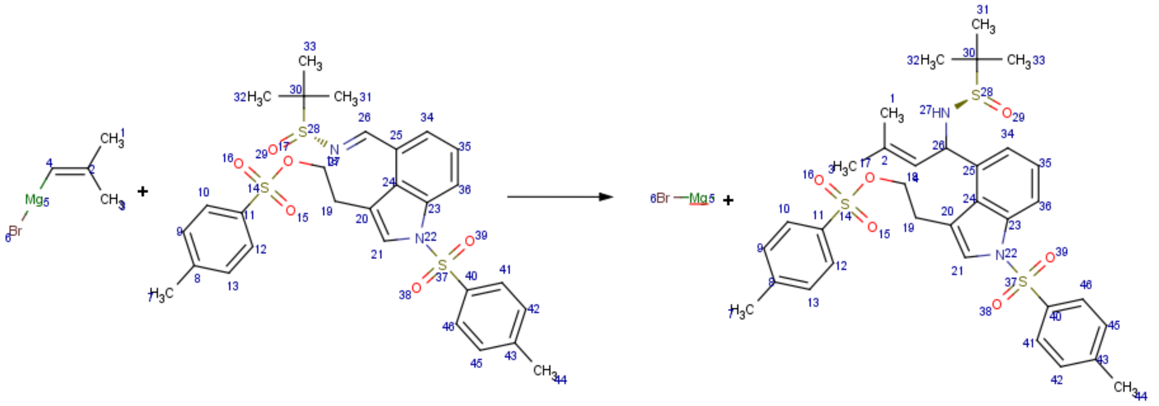

Correct mapped SMILES/SMARTS of the reaction:
[CH3:1][C:2]([CH3:3])=[CH:4][Mg:5][Br:6].[CH3:7][C:8]1=[CH:9][CH:10]=[C:11]([CH:12]=[CH:13]1)[S:14](=[O:15])(=[O:16])[O:17][CH2:18][CH2:19][C:20]1=[CH:21][N:22]([C:23]2=[C:24]1[C:25](\[CH:26]=[N:27]\[S@:28](=[O:29])[C:30]([CH3:31])([CH3:32])[CH3:33])=[CH:34][CH:35]=[CH:36]2)[S:37](=[O:38])(=[O:39])[C:40]1=[CH:41][CH:42]=[C:43]([CH3:44])[CH:45]=[CH:46]1>>[Mg:5][Br:6].[CH3:1][C:2]([CH3:3])=[CH:4][CH:26]([NH:27][S@:28](=[O:29])[C:30]([CH3:33])([CH3:32])[CH3:31])[C:25]1=[CH:34][CH:35]=[CH:36][C:23]2=[C:24]1[C:20]([CH2:19][CH2:18][O:17][S:14](=[O:15])(=[O:16])[C:11]1=[CH:12][CH:13]=[C:8]([CH3:7])[CH:9]=[CH:10]1)=[CH:21][N:22]2[S:37](=[O:38])(=[O:39])[C:40]1=[CH:46][CH:45]=[C:43]([CH3:44])[CH:42]=[CH:41]1

Correctness of the mapping

MAPPET	YES
ReactionMap	YES
Marvin	NO
ChemDraw	YES
Indigo	YES

Reaction no 100

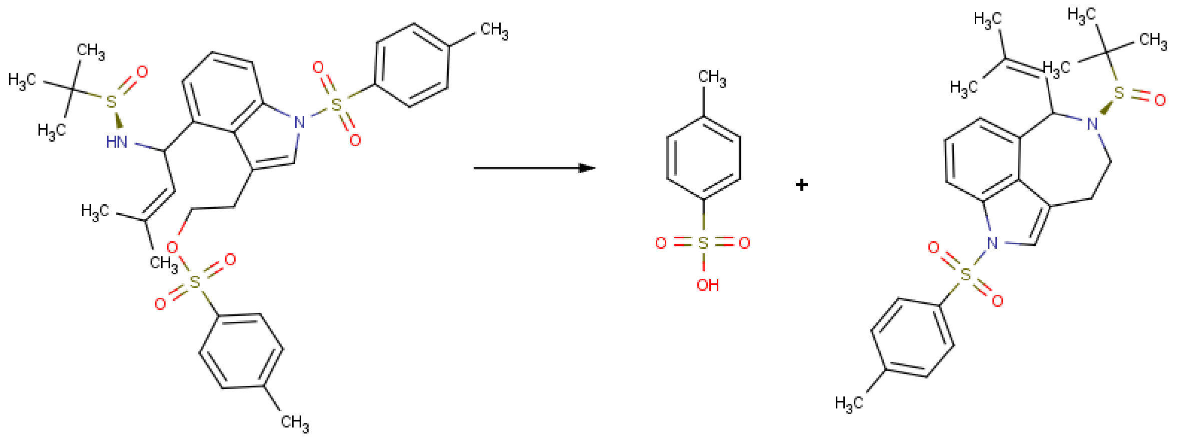

SMILES of the input:
CC(C)=CC(N[S@](=O)C(C)(C)C)C1=CC=CC2=C1C(CCOS(=O)(=O)C1=CC=C(C)C=C1)=CN2S
(=O)(=O)C1=CC=C(C)C=C1>>CC1=CC=C(C=C1)S(O)(=O)=O.CC(C)=CC1N(CCC2=CN(C3=C2
C1=CC=C3)S(=O)(=O)C1=CC=C(C)C=C1)[S@](=O)C(C)(C)C

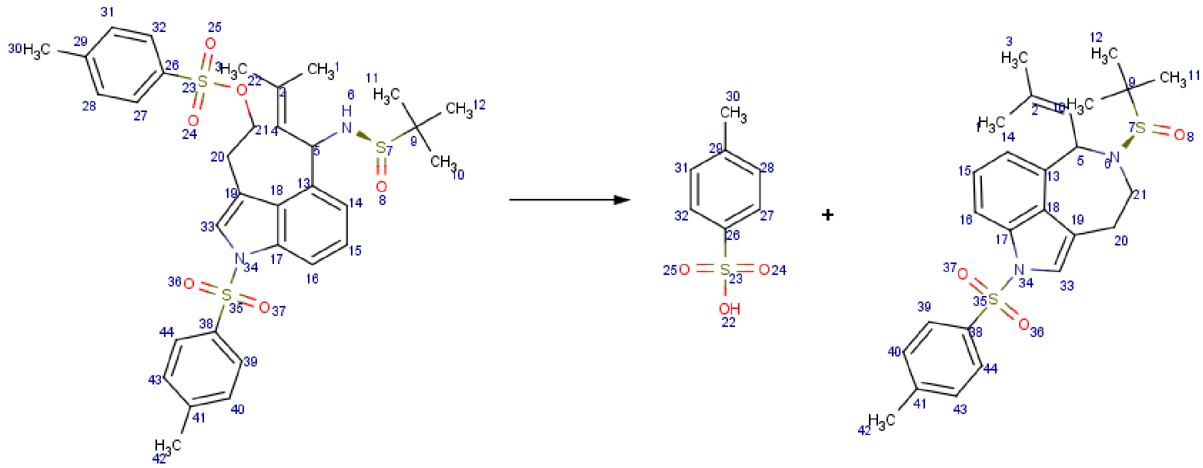

Correct mapped SMILES/SMARTS of the reaction:
[CH3:1][C:2]([CH3:3])=[CH:4][CH:5]([NH:6][S@:7](=[O:8])[C:9]([CH3:10])([C
H3:11])[CH3:12])[C:13]1=[CH:14][CH:15]=[CH:16][C:17]2=[C:18]1[C:19]([CH2:
20][CH2:21][O:22][S:23](=[O:24])(=[O:25])[C:26]1=[CH:27][CH:28]=[C:29]([C
H3:30])[CH:31]=[CH:32]1)=[CH:33][N:34]2[S:35](=[O:36])(=[O:37])[C:38]1=[C
H:39][CH:40]=[C:41]([CH3:42])[CH:43]=[CH:44]1>>[CH3:30][C:29]1=[CH:28][CH
:27]=[C:26]([CH:32]=[CH:31]1)[S:23]([OH:22])(=[O:25])=[O:24].[CH3:3][C:2]
([CH3:1])=[CH:4][CH:5]1[N:6]([CH2:21][CH2:20][C:19]2=[CH:33][N:34]([C:17]
3=[C:18]2[C:13]1=[CH:14][CH:15]=[CH:16]3)[S:35](=[O:36])(=[O:37])[C:38]1=
[CH:44][CH:43]=[C:41]([CH3:42])[CH:40]=[CH:39]1)[S@:7](=[O:8])[C:9]([CH3:
12])([CH3:11])[CH3:10]

Correctness of the mapping

MAPPET	YES
ReactionMap	YES
Marvin	YES
ChemDraw	YES
Indigo	YES

Reaction no 101

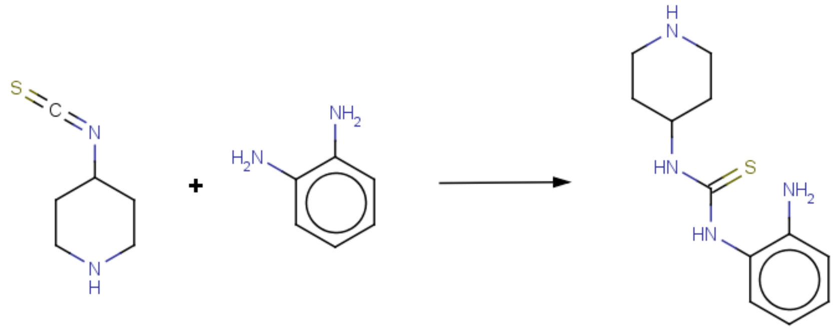

SMILES of the input:
S=C=NC1CCNCC1.Nc1ccccc1N>>Nc1ccccc1NC(=S)NC1CCNCC1

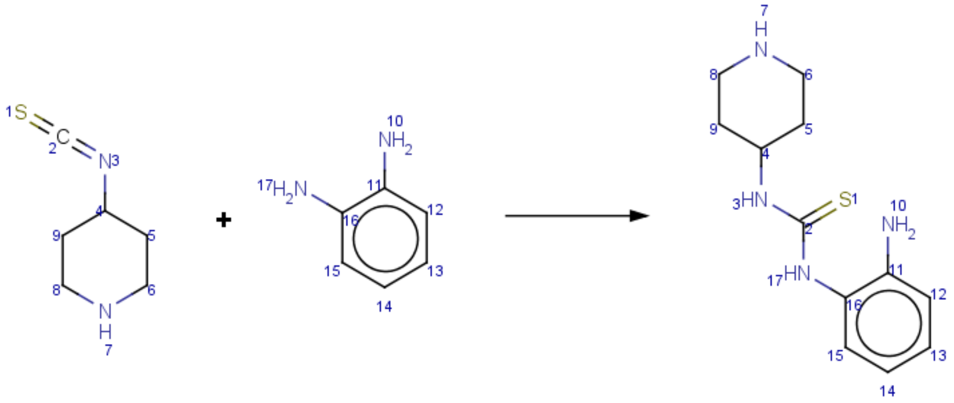

Correct mapped SMILES/SMARTS of the reaction:
[S:1]=[C:2]=[N:3][CH:4]1[CH2:5][CH2:6][NH:7][CH2:8][CH2:9]1.[NH2:10][c:11]1[cH:12][cH:13][cH:14][cH:15][c:16]1[NH2:17]>>[NH2:10][c:11]1[cH:12][cH:13][cH:14][cH:15][c:16]1[NH:17][C:2](=[S:1])[NH:3][CH:4]1[CH2:9][CH2:8][NH:7][CH2:6][CH2:5]1

Correctness of the mapping

MAPPET	YES
ReactionMap	YES
Marvin	YES
ChemDraw	YES
Indigo	YES

Reaction no 102

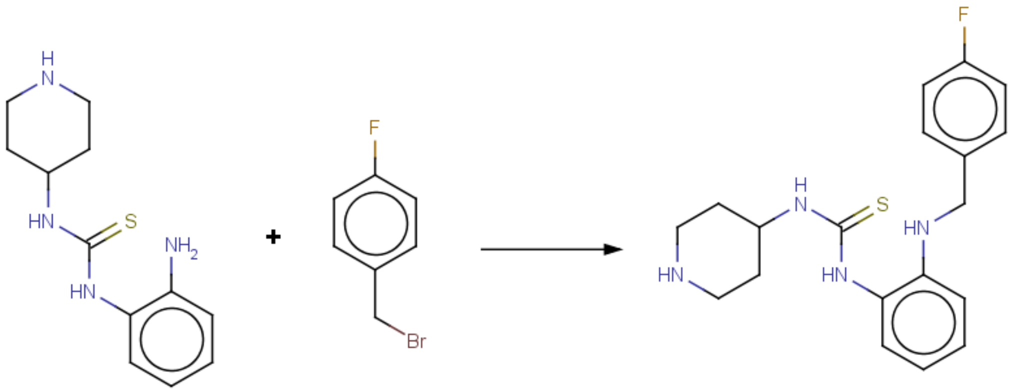

SMILES of the input:

Nc1ccccc1NC(=S)NC1CCNCC1.Fc1ccc(CBr)cc1>>Fc1ccc(CNc2ccccc2NC(=S)NC2CCNCC2)cc1

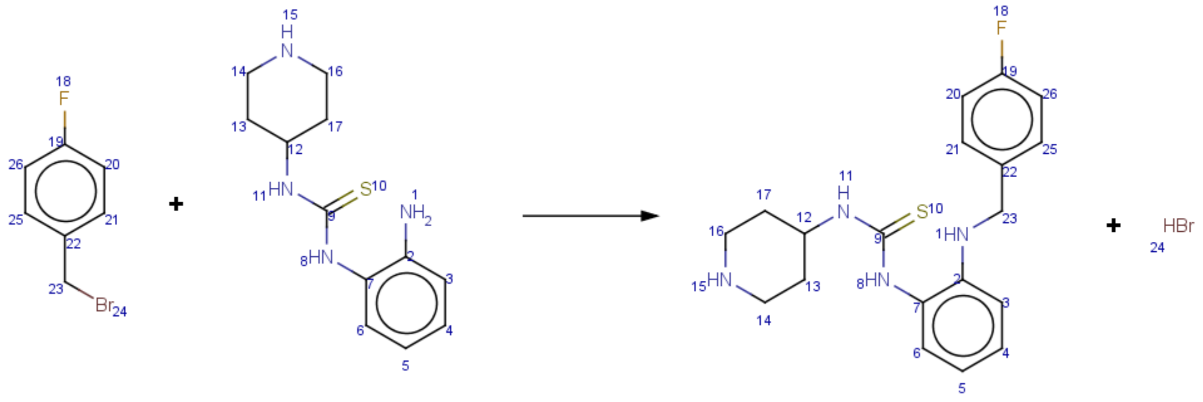

Correct mapped SMILES/SMARTS of the reaction:
[F:18][c:19]1[cH:20][cH:21][c:22]([CH2:23][Br:24])[cH:25][cH:26]1.[NH2:1][c:2]1[cH:3][cH:4][cH:5][cH:6][c:7]1[NH:8][C:9](=[S:10])[NH:11][CH:12]1[CH2:13][CH2:14][NH:15][CH2:16][CH2:17]1>>[F:18][c:19]1[cH:26][cH:25][c:22]([CH2:23][NH:1][c:2]2[cH:3][cH:4][cH:5][cH:6][c:7]2[NH:8][C:9](=[S:10])[NH:11][CH:12]2[CH2:13][CH2:14][NH:15][CH2:16][CH2:17]2)[cH:21][cH:20]1.[BrH:24]

Correctness of the mapping

MAPPET	YES
ReactionMap	NO
Marvin	YES
ChemDraw	YES
Indigo	YES

Reaction no 103

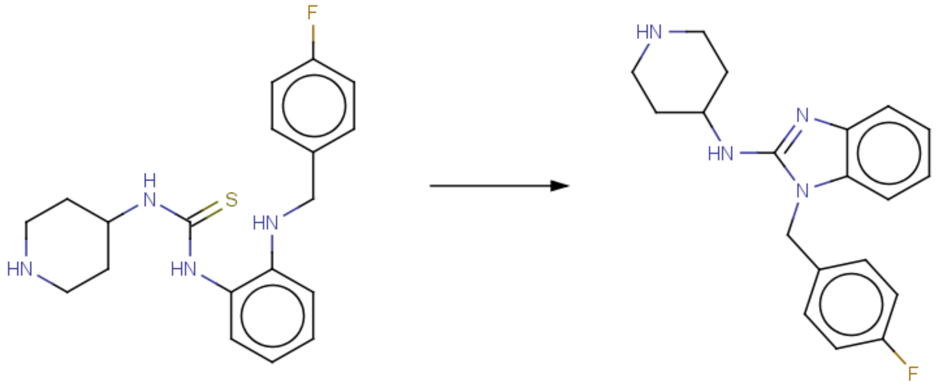

SMILES of the input:
Fc1ccc(CNc2ccccc2NC(=S)NC2CCNCC2)cc1>>Fc1ccc(CN2C(NC3CCNCC3)=Nc3ccccc23)cc1

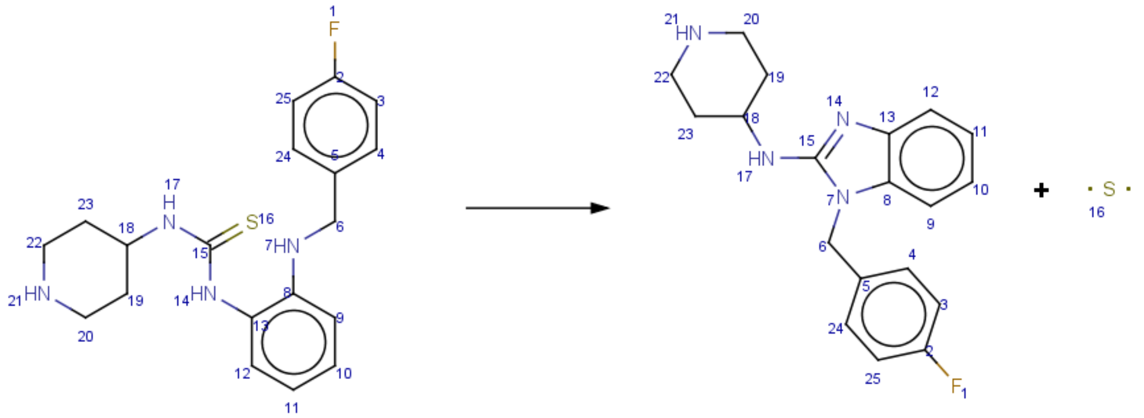

Correct mapped SMILES/SMARTS of the reaction:
[F:1][c:2]1[cH:3][cH:4][c:5]([CH2:6][NH:7][c:8]2[cH:9][cH:10][cH:11][cH:12][c:13]2[NH:14][C:15](=[S:16])[NH:17][CH:18]2[CH2:19][CH2:20][NH:21][CH2:22][CH2:23]2)[cH:24][cH:25]1>>[F:1][c:2]1[cH:25][cH:24][c:5]([CH2:6][N:7]2[C:15]([NH:17][CH:18]3[CH2:23][CH2:22][NH:21][CH2:20][CH2:19]3)=[N:14][c:13]3[cH:12][cH:11][cH:10][cH:9][c:8]23)[cH:4][cH:3]1.[S:16]

Correctness of the mapping

MAPPET	YES
ReactionMap	NO
Marvin	YES
ChemDraw	YES
Indigo	YES

Reaction no 104

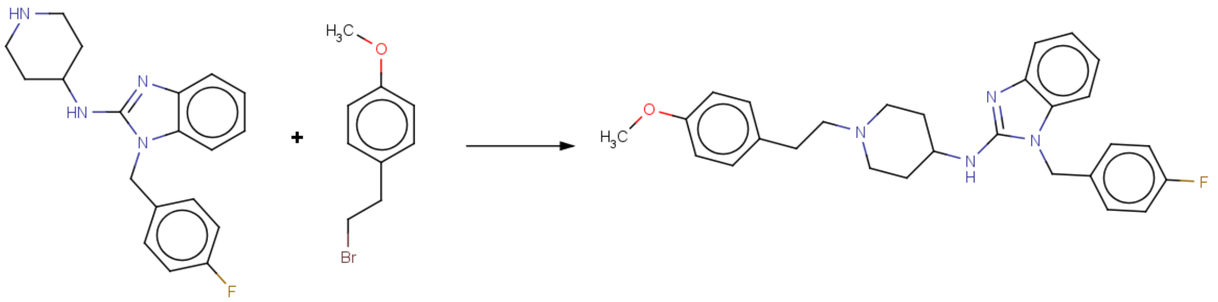

SMILES of the input:
Fc1ccc(CN2C(NC3CCNCC3)=Nc3ccccc23)cc1.COc1ccc(CCBr)cc1>>COc1ccc(CCN2CCC(CC2)NC2=Nc3ccccc3N2Cc2ccc(F)cc2)cc1

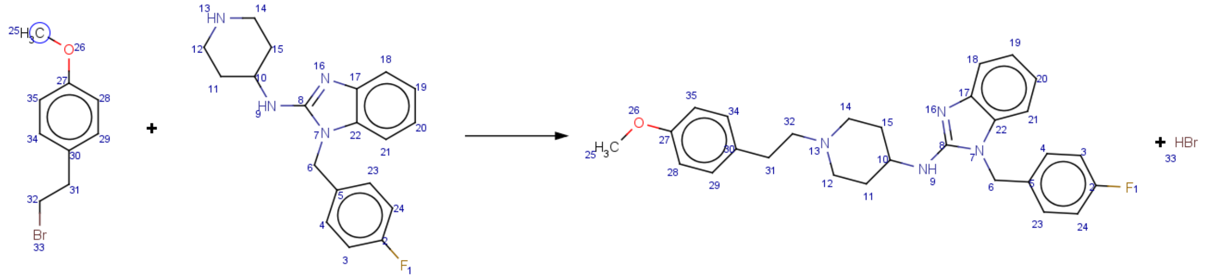

Correct mapped SMILES/SMARTS of the reaction:
[CH3:25][O:26][c:27]1[cH:28][cH:29][c:30]([CH2:31][CH2:32][Br:33])[cH:34][cH:35]1.[F:1][c:2]1[cH:3][cH:4][c:5]([CH2:6][N:7]2[C:8]([NH:9][CH:10]3[CH2:11][CH2:12][NH:13][CH2:14][CH2:15]3)=[N:16][c:17]3[cH:18][cH:19][cH:20][cH:21][c:22]23)[cH:23][cH:24]1>>[CH3:25][O:26][c:27]1[cH:35][cH:34][c:30]([CH2:31][CH2:32][N:13]2[CH2:14][CH2:15][CH:10]([CH2:11][CH2:12]2)[NH:9

][C:8]2=[N:16][c:17]3[cH:18][cH:19][cH:20][cH:21][c:22]3[N:7]2[CH2:6][c:5]2[cH:4][cH:3][c:2]([F:1])[cH:24][cH:23]2)[cH:29][cH:28]1.[BrH:33]

Correctness of the mapping

MAPPET	YES
ReactionMap	NO
Marvin	YES
ChemDraw	YES
Indigo	YES

Reaction no 105

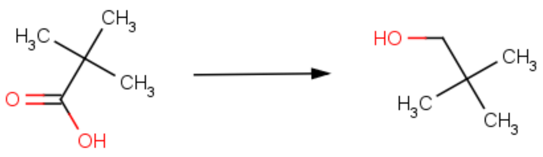

SMILES of the input:
CC(C)(C)C(O)=O>>CC(C)(C)CO

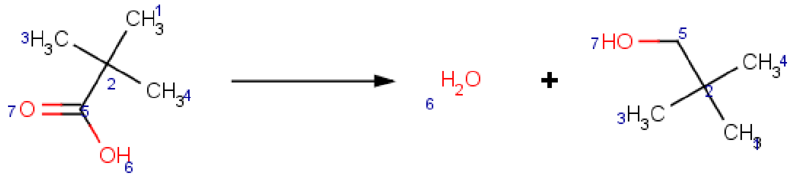

Correct mapped SMILES/SMARTS of the reaction:
[CH3:1][C:2]([CH3:3])([CH3:4])[C:5]([OH:6])=[O:7]>>[OH2:6].[CH3:1][C:2]([CH3:4])([CH3:3])[CH2:5][OH:7]

Correctness of the mapping

MAPPET	YES
ReactionMap	NO
Marvin	YES
ChemDraw	YES
Indigo	YES

Reaction no 106

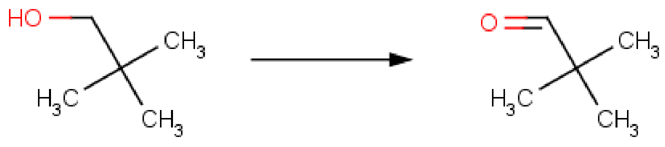

SMILES of the input:
CC(C)(C)CO>>CC(C)(C)C=O

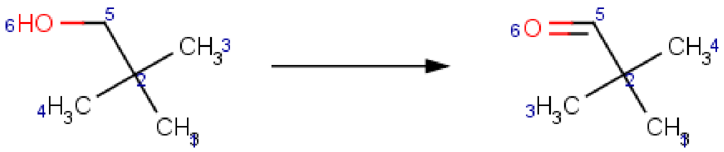

Correct mapped SMILES/SMARTS of the reaction:

[CH3:1][C:2]([CH3:3])([CH3:4])[CH2:5][OH:6]>>[CH3:1][C:2]([CH3:4])([CH3:3
])[CH:5]=[O:6]

Correctness of the mapping

MAPPET	YES
ReactionMap	YES
Marvin	YES
ChemDraw	YES
Indigo	YES

Reaction no 107

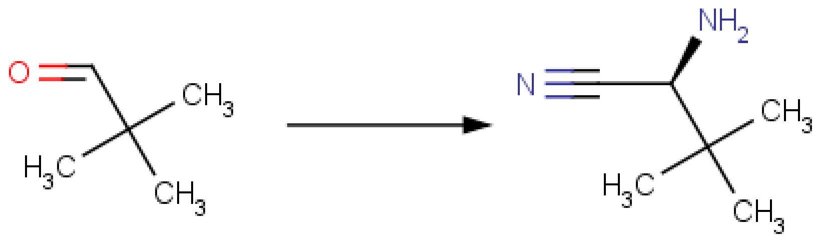

SMILES of the input:
CC(C)(C)C=O>>CC(C)(C)[C@H](N)C#N

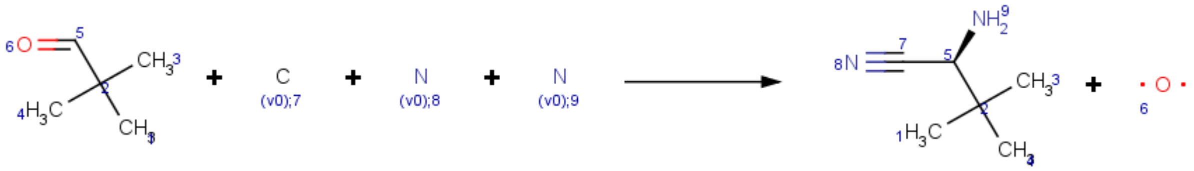

Correct mapped SMILES/SMARTS of the reaction:
[CH3:1][C:2]([CH3:3])([CH3:4])[CH:5]=[O:6].[C:7].[N:8].[N:9]>>[CH3:4][C:2
]([CH3:3])([CH3:1])[C@H:5]([NH2:9])[C:7]#[N:8].[O:6]

Correctness of the mapping

MAPPET	YES
ReactionMap	NO
Marvin	YES
ChemDraw	YES
Indigo	YES

Reaction no 108

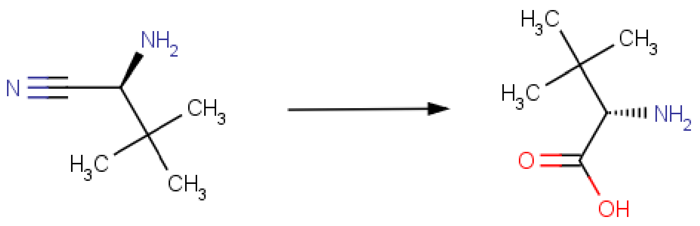

SMILES of the input:
CC(C)(C)[C@H](N)C#N>>CC(C)(C)[C@H](N)C(O)=O

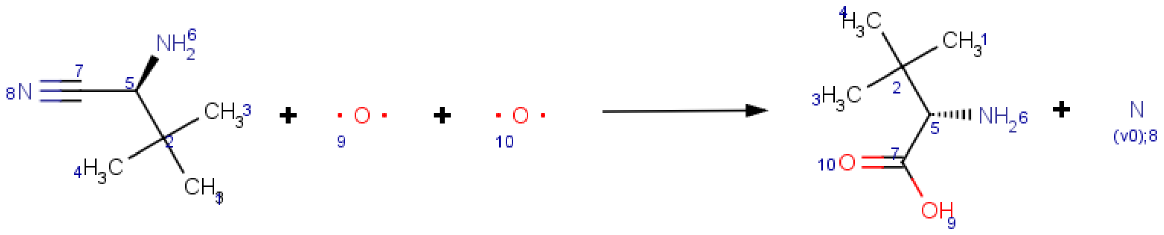

Correct mapped SMILES/SMARTS of the reaction:
[CH3:1][C:2]([CH3:3])([CH3:4])[C@H:5]([NH2:6])[C:7]#[N:8].[O:9].[O:10]>>[CH3:4][C:2]([CH3:3])([CH3:1])[C@H:5]([NH2:6])[C:7]([OH:9])=[O:10].[N:8]

Correctness of the mapping

MAPPET	YES
ReactionMap	NO
Marvin	YES
ChemDraw	YES
Indigo	YES

Reaction no 109

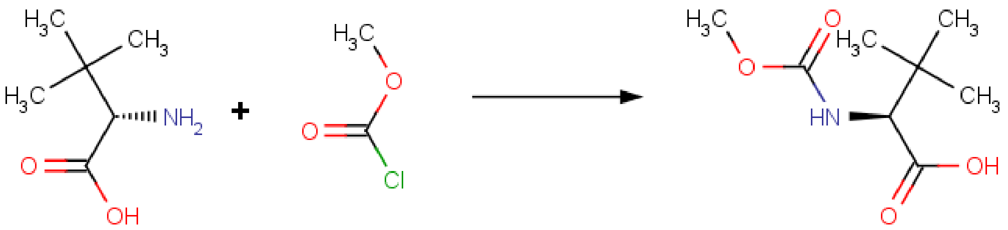

SMILES of the input:
CC(C)(C)[C@H](N)C(O)=O.COC(Cl)=O>>COC(=O)N[C@H](C(O)=O)C(C)(C)C

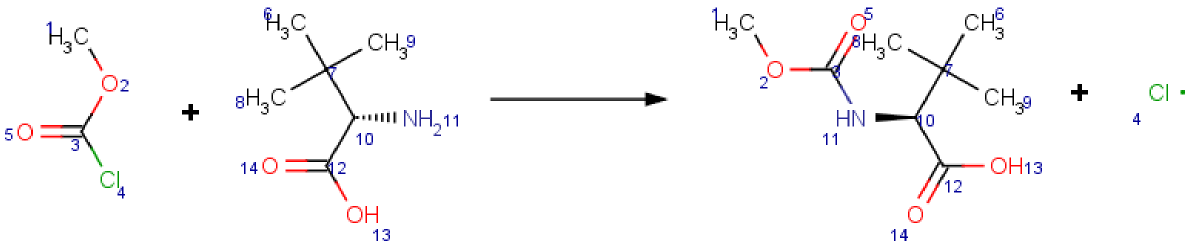

Correct mapped SMILES/SMARTS of the reaction:
[CH3:1][O:2][C:3]([Cl:4])=[O:5].[CH3:6][C:7]([CH3:8])([CH3:9])[C@H:10]([NH2:11])[C:12]([OH:13])=[O:14]>>[CH3:1][O:2][C:3](=[O:5])[NH:11][C@H:10]([C:12]([OH:13])=[O:14])[C:7]([CH3:9])([CH3:8])[CH3:6].[Cl:4]

Correctness of the mapping

MAPPET	YES
ReactionMap	NO
Marvin	YES
ChemDraw	YES
Indigo	YES

Reaction no 110

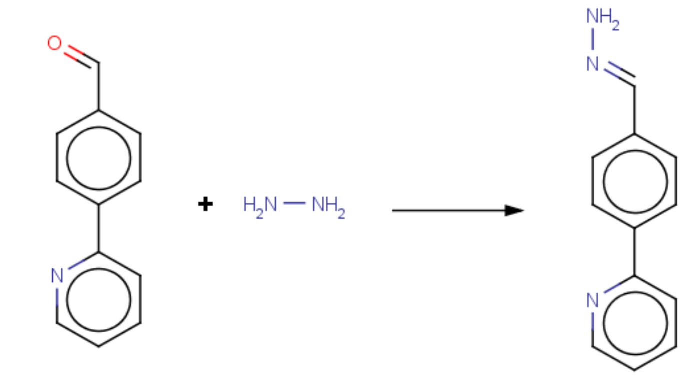

SMILES of the input:
O=Cc1ccc(cc1)-c1ccccn1.NN>>N\N=C\c1ccc(cc1)-c1ccccn1

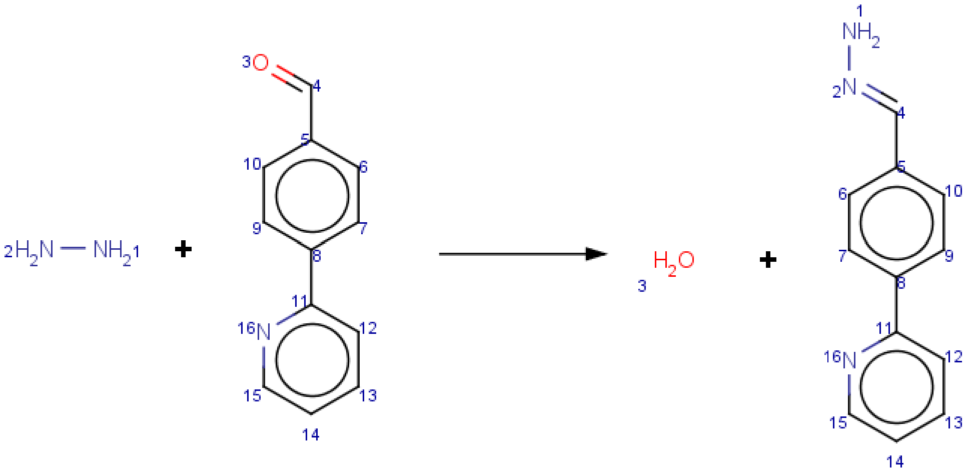

Correct mapped SMILES/SMARTS of the reaction:
[NH2:1][NH2:2].[O:3]=[CH:4][c:5]1[cH:6][cH:7][c:8]([cH:9][cH:10]1)-
[c:11]1[cH:12][cH:13][cH:14][cH:15][n:16]1>>[OH2:3].[NH2:1]\[N:2]=[CH:4]\
[c:5]1[cH:10][cH:9][c:8]([cH:7][cH:6]1)-
[c:11]1[cH:12][cH:13][cH:14][cH:15][n:16]1

Correctness of the mapping

MAPPET	YES
ReactionMap	NO
Marvin	YES
ChemDraw	YES
Indigo	YES

Reaction no 111

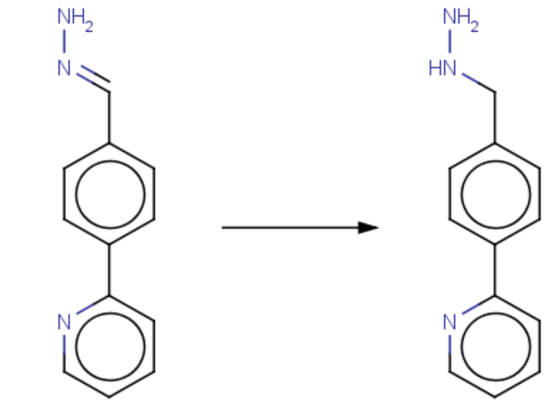

SMILES of the input:
N\N=C\c1ccc(cc1)-c1ccccn1>>NNCc1ccc(cc1)-c1ccccn1

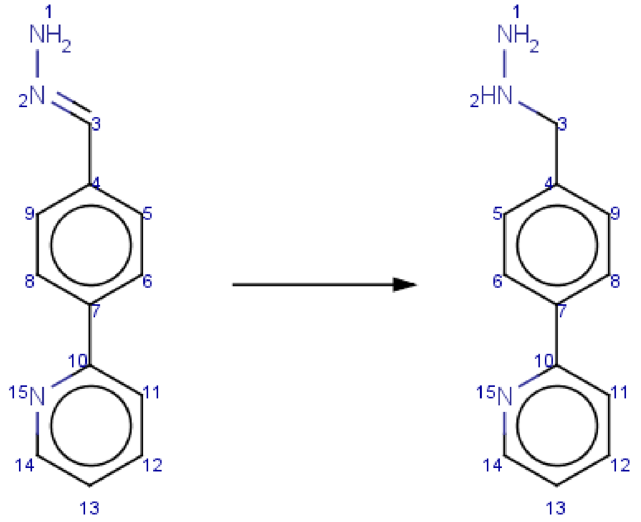

Correct mapped SMILES/SMARTS of the reaction:
[NH2:1]\[N:2]=[CH:3]\[c:4]1[cH:5][cH:6][c:7]([cH:8][cH:9]1)-[c:10]1[cH:11][cH:12][cH:13][cH:14][n:15]1>>[NH2:1][NH:2][CH2:3][c:4]1[cH:9][cH:8][c:7]([cH:6][cH:5]1)-[c:10]1[cH:11][cH:12][cH:13][cH:14][n:15]1

Correctness of the mapping

MAPPET	YES
ReactionMap	YES
Marvin	YES
ChemDraw	YES
Indigo	YES

Reaction no 112

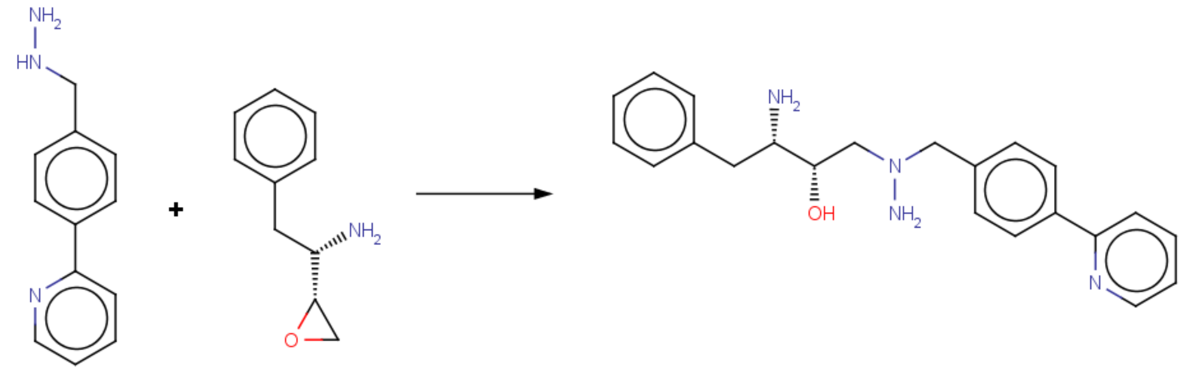

SMILES of the input:
NNCc1ccc(cc1)-c1ccccn1.N[C@@H](Cc1ccccc1)[C@@H]1CO1>>N[C@@H](Cc1ccccc1)[C@@H](O)CN(N)Cc1ccc(cc1)-c1ccccn1

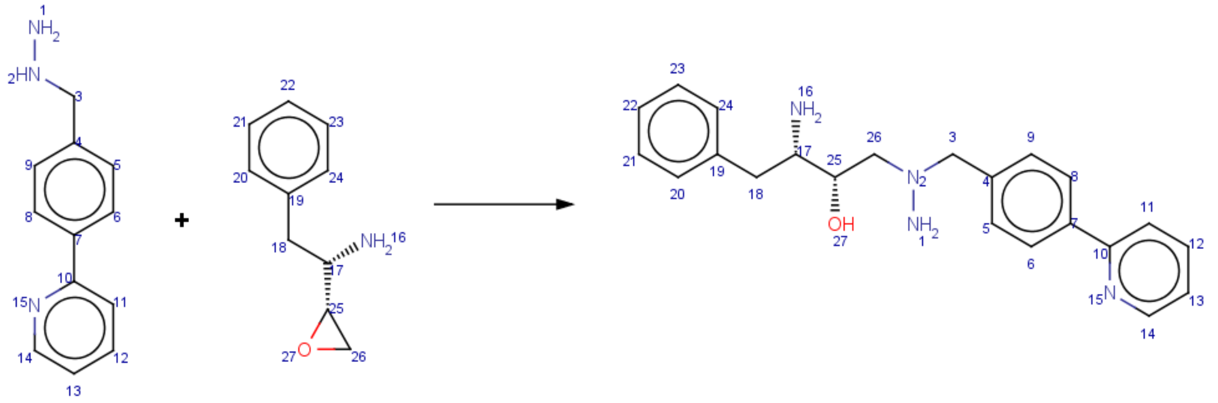

```
Correct mapped SMILES/SMARTS of the reaction:
[NH2:1][NH:2][CH2:3][c:4]1[cH:5][cH:6][c:7]([cH:8][cH:9]1)-
[c:10]1[cH:11][cH:12][cH:13][cH:14][n:15]1.[NH2:16][C@@H:17]([CH2:18][c:1
9]1[cH:20][cH:21][cH:22][cH:23][cH:24]1)[C@@H:25]1[CH2:26][O:27]1>>[NH2:1
6][C@@H:17]([CH2:18][c:19]1[cH:20][cH:21][cH:22][cH:23][cH:24]1)[C@@H:25]
([OH:27])[CH2:26][N:2]([NH2:1])[CH2:3][c:4]1[cH:9][cH:8][c:7]([cH:6][cH:5
]1)-[c:10]1[cH:11][cH:12][cH:13][cH:14][n:15]1

Correctness of the mapping
MAPPET           YES
ReactionMap      YES
Marvin           YES
ChemDraw         YES
Indigo           YES

Reaction no  113
```

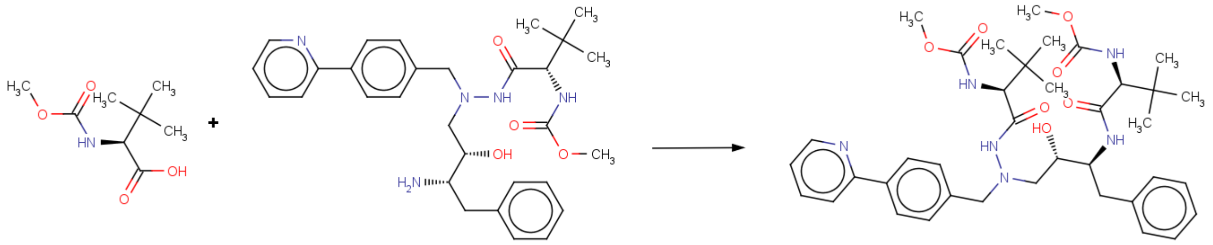

```
SMILES of the input:
COC(=O)N[C@H](C(O)=O)C(C)(C)C.COC(=O)N[C@H](C(=O)NN(C[C@H](O)[C@@H](N)Cc1
ccccc1)Cc1ccc(cc1)-
c1ccccn1)C(C)(C)C>>COC(=O)N[C@H](C(=O)N[C@@H](Cc1ccccc1)[C@@H](O)CN(Cc1cc
c(cc1)-c1ccccn1)NC(=O)[C@@H](NC(=O)OC)C(C)(C)C)C(C)(C)C
```

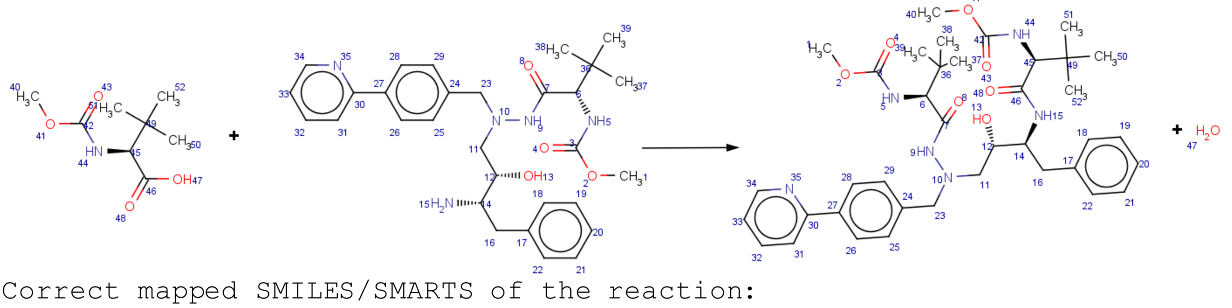

```
Correct mapped SMILES/SMARTS of the reaction:
[CH3:40][O:41][C:42](=[O:43])[NH:44][C@H:45]([C:46]([OH:47])=[O:48])[C:49
]([CH3:50])([CH3:51])[CH3:52].[CH3:1][O:2][C:3](=[O:4])[NH:5][C@H:6]([C:7
](=[O:8])[NH:9][N:10]([CH2:11][C@H:12]([OH:13])[C@@H:14]([NH2:15])[CH2:16
][c:17]1[cH:18][cH:19][cH:20][cH:21][cH:22]1)[CH2:23][c:24]1[cH:25][cH:26
```

][c:27]([cH:28][cH:29]1)-
[c:30]1[cH:31][cH:32][cH:33][cH:34][n:35]1)[C:36]([CH3:37])([CH3:38])[CH3:39]>>[CH3:40][O:41][C:42](=[O:43])[NH:44][C@H:45]([C:46](=[O:48])[NH:15][C@@H:14]([CH2:16][c:17]1[cH:18][cH:19][cH:20][cH:21][cH:22]1)[C@@H:12]([OH:13])[CH2:11][N:10]([CH2:23][c:24]1[cH:25][cH:26][c:27]([cH:28][cH:29]1)-
[c:30]1[cH:31][cH:32][cH:33][cH:34][n:35]1)[NH:9][C:7](=[O:8])[C@@H:6]([NH:5][C:3](=[O:4])[O:2][CH3:1])[C:36]([CH3:37])([CH3:39])[CH3:38])[C:49]([CH3:52])([CH3:51])[CH3:50].[OH2:47]

Correctness of the mapping

MAPPET	YES
ReactionMap	NO
Marvin	YES
ChemDraw	YES
Indigo	YES

Reaction no 114

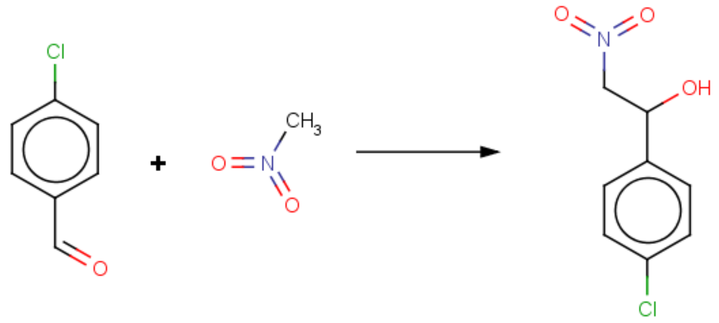

SMILES of the input:
Clc1ccc(C=O)cc1.CN(=O)=O>>OC(CN(=O)=O)c1ccc(Cl)cc1

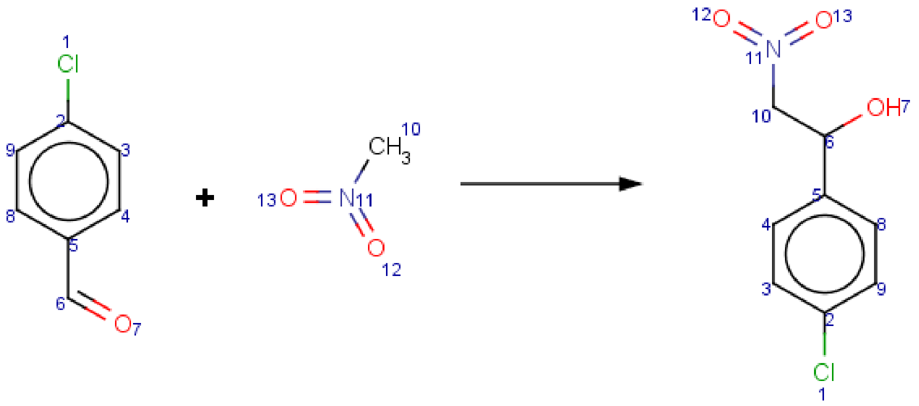

Correct mapped SMILES/SMARTS of the reaction:
[Cl:1][c:2]1[cH:3][cH:4][c:5]([CH:6]=[O:7])[cH:8][cH:9]1.[CH3:10][N:11](=[O:12])=[O:13]>>[OH:7][CH:6]([CH2:10][N:11](=[O:12])=[O:13])[c:5]1[cH:8][cH:9][c:2]([Cl:1])[cH:3][cH:4]1

Correctness of the mapping

MAPPET	YES
ReactionMap	YES
Marvin	YES
ChemDraw	YES
Indigo	YES

Reaction no 115

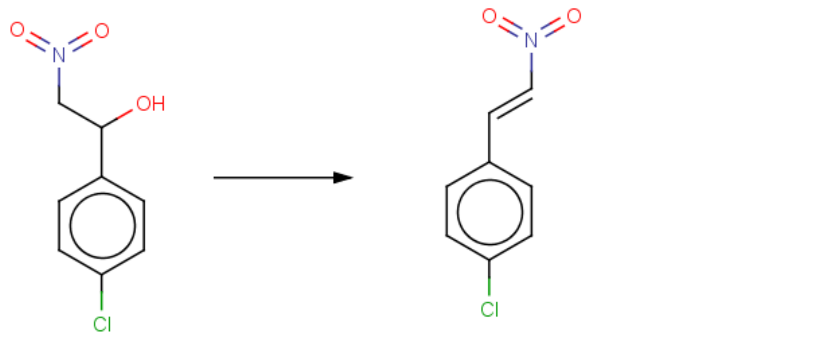

SMILES of the input:
OC(CN(=O)=O)c1ccc(Cl)cc1>>Clc1ccc(\C=C\N(=O)=O)cc1

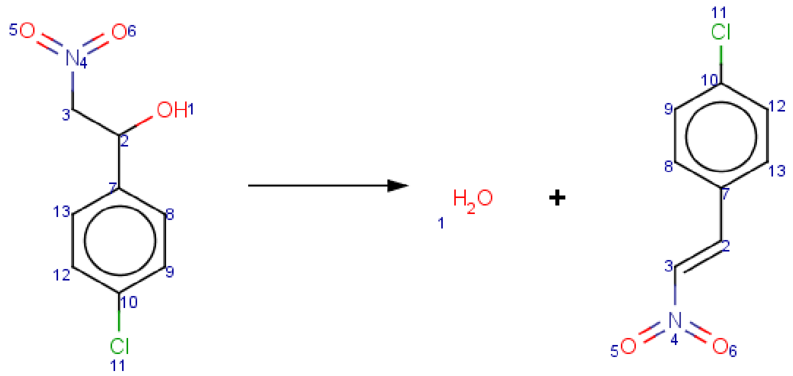

Correct mapped SMILES/SMARTS of the reaction:
[OH:1][CH:2]([CH2:3][N:4](=[O:5])=[O:6])[c:7]1[cH:8][cH:9][c:10]([Cl:11])
[cH:12][cH:13]1>>[OH2:1].[Cl:11][c:10]1[cH:12][cH:13][c:7](\[CH:2]=[CH:3]
\[N:4](=[O:6])=[O:5])[cH:8][cH:9]1

Correctness of the mapping

MAPPET	YES
ReactionMap	NO
Marvin	YES
ChemDraw	YES
Indigo	YES

Reaction no 116

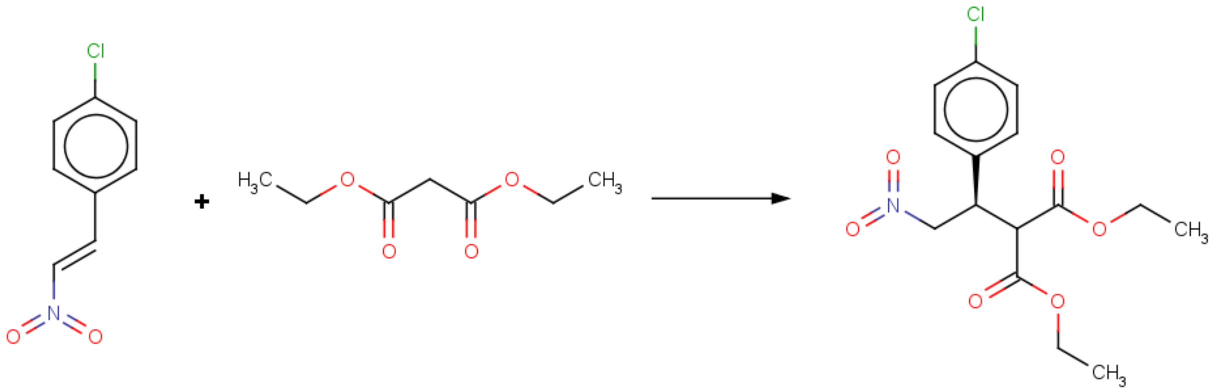

SMILES of the input:
Clc1ccc(\C=C\N(=O)=O)cc1.CCOC(=O)CC(=O)OCC>>CCOC(=O)C([C@@H](CN(=O)=O)c1c
cc(Cl)cc1)C(=O)OCC

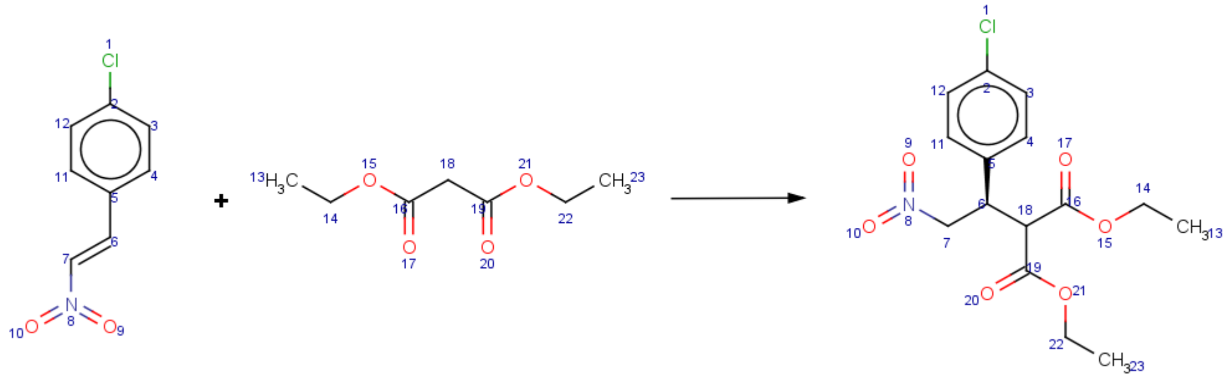

Correct mapped SMILES/SMARTS of the reaction:
[Cl:1][c:2]1[cH:3][cH:4][c:5](\[CH:6]=[CH:7]\[N:8](=[O:9])=[O:10])[cH:11][cH:12]1.[CH3:13][CH2:14][O:15][C:16](=[O:17])[CH2:18][C:19](=[O:20])[O:21][CH2:22][CH3:23]>>[CH3:23][CH2:22][O:21][C:19](=[O:20])[CH:18]([C@@H:6]([CH2:7][N:8](=[O:10])=[O:9])[c:5]1[cH:11][cH:12][c:2]([Cl:1])[cH:3][cH:4]1)[C:16](=[O:17])[O:15][CH2:14][CH3:13]

Correctness of the mapping

MAPPET	YES
ReactionMap	YES
Marvin	YES
ChemDraw	YES
Indigo	YES

Reaction no 117

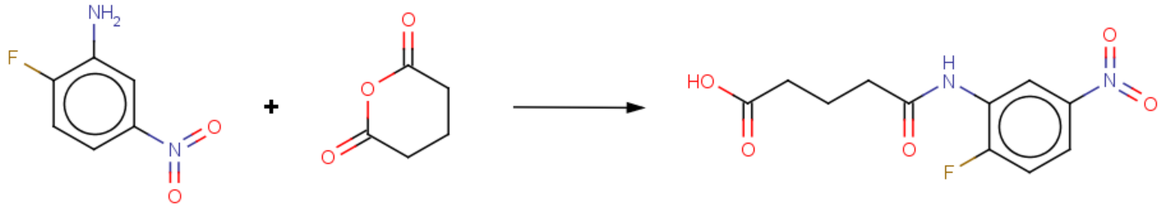

SMILES of the input:
Nc1cc(ccc1F)N(=O)=O.O=C1CCCC(=O)O1>>OC(=O)CCCC(=O)Nc1cc(ccc1F)N(=O)=O

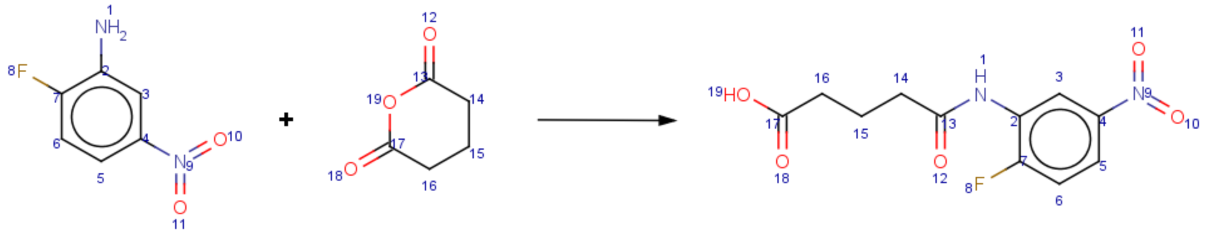

Correct mapped SMILES/SMARTS of the reaction:
[NH2:1][c:2]1[cH:3][c:4]([cH:5][cH:6][c:7]1[F:8])[N:9](=[O:10])=[O:11].[O:12]=[C:13]1[CH2:14][CH2:15][CH2:16][C:17](=[O:18])[O:19]1>>[OH:19][C:17](=[O:18])[CH2:16][CH2:15][CH2:14][C:13](=[O:12])[NH:1][c:2]1[cH:3][c:4]([cH:5][cH:6][c:7]1[F:8])[N:9](=[O:11])=[O:10]

Correctness of the mapping

MAPPET	YES
ReactionMap	YES
Marvin	YES
ChemDraw	YES

Indigo YES

Reaction no 118

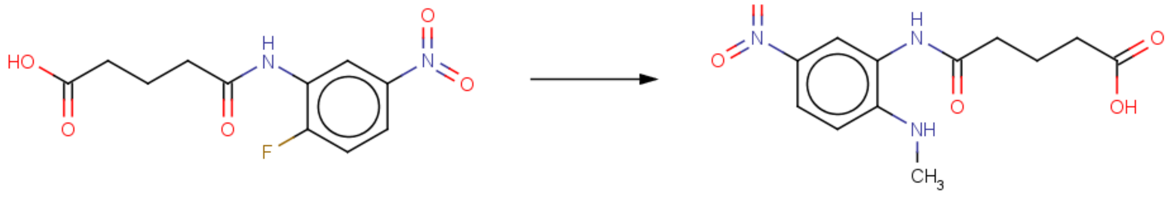

SMILES of the input:
OC(=O)CCCC(=O)Nc1cc(ccc1F)N(=O)=O>>CNc1ccc(cc1NC(=O)CCCC(O)=O)N(=O)=O

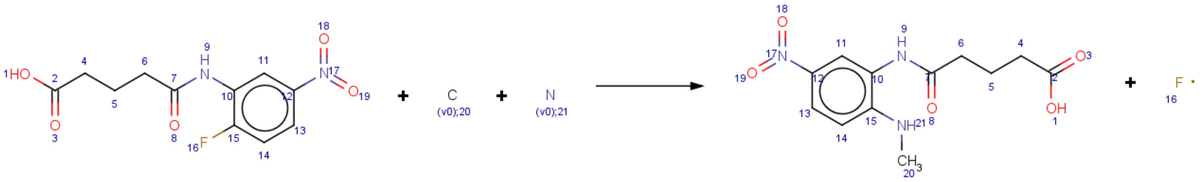

Correct mapped SMILES/SMARTS of the reaction:
[OH:1][C:2](=[O:3])[CH2:4][CH2:5][CH2:6][C:7](=[O:8])[NH:9][c:10]1[cH:11][c:12]([cH:13][cH:14][c:15]1[F:16])[N:17](=[O:18])=[O:19].[C:20].[N:21]>>[CH3:20][NH:21][c:15]1[cH:14][cH:13][c:12]([cH:11][c:10]1[NH:9][C:7](=[O:8])[CH2:6][CH2:5][CH2:4][C:2]([OH:1])=[O:3])[N:17](=[O:19])=[O:18].[F:16]

Correctness of the mapping
MAPPET YES
ReactionMap NO
Marvin YES
ChemDraw YES
Indigo YES

Reaction no 119

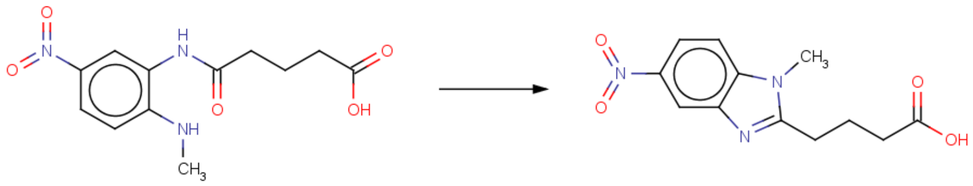

SMILES of the input:
CNc1ccc(cc1NC(=O)CCCC(O)=O)N(=O)=O>>CN1C(CCCC(O)=O)=Nc2cc(ccc12)N(=O)=O

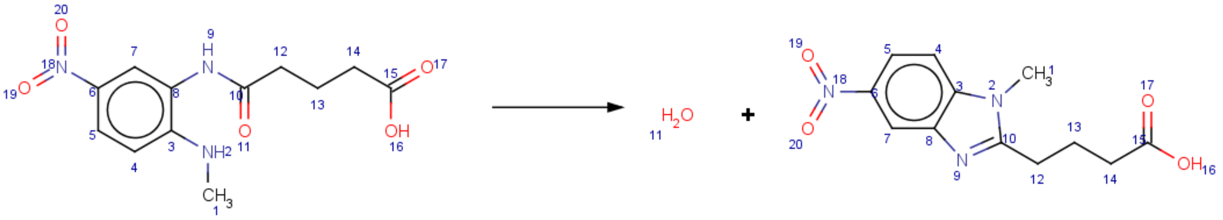

Correct mapped SMILES/SMARTS of the reaction:
[CH3:1][NH:2][c:3]1[cH:4][cH:5][c:6]([cH:7][c:8]1[NH:9][C:10](=[O:11])[CH2:12][CH2:13][CH2:14][C:15]([OH:16])=[O:17])[N:18](=[O:19])=[O:20]>>[OH2:

11].[CH3:1][N:2]1[C:10]([CH2:12][CH2:13][CH2:14][C:15]([OH:16])=[O:17])=[N:9][c:8]2[cH:7][c:6]([cH:5][cH:4][c:3]12)[N:18](=[O:20])=[O:19]

Correctness of the mapping
MAPPET YES
ReactionMap NO
Marvin YES
ChemDraw YES
Indigo NO

Reaction no 120

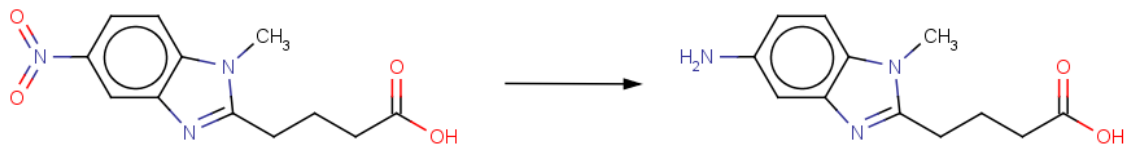

SMILES of the input:
CN1C(CCCC(O)=O)=Nc2cc(ccc12)N(=O)=O>>CN1C(CCCC(O)=O)=Nc2cc(N)ccc12

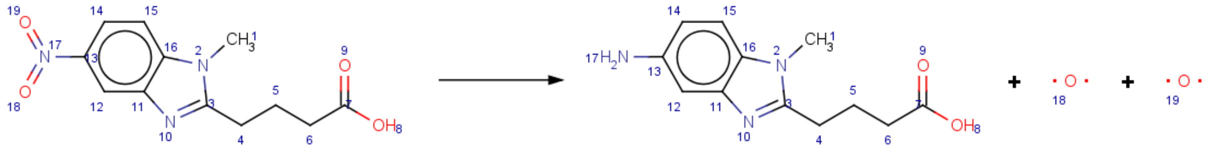

Correct mapped SMILES/SMARTS of the reaction:
[CH3:1][N:2]1[C:3]([CH2:4][CH2:5][CH2:6][C:7]([OH:8])=[O:9])=[N:10][c:11]2[cH:12][c:13]([cH:14][cH:15][c:16]12)[N:17](=[O:18])=[O:19]>>[CH3:1][N:2]1[C:3]([CH2:4][CH2:5][CH2:6][C:7]([OH:8])=[O:9])=[N:10][c:11]2[cH:12][c:13]([NH2:17])[cH:14][cH:15][c:16]12.[O:18].[O:19]

Correctness of the mapping
MAPPET YES
ReactionMap NO
Marvin YES
ChemDraw YES
Indigo YES

Reaction no 121

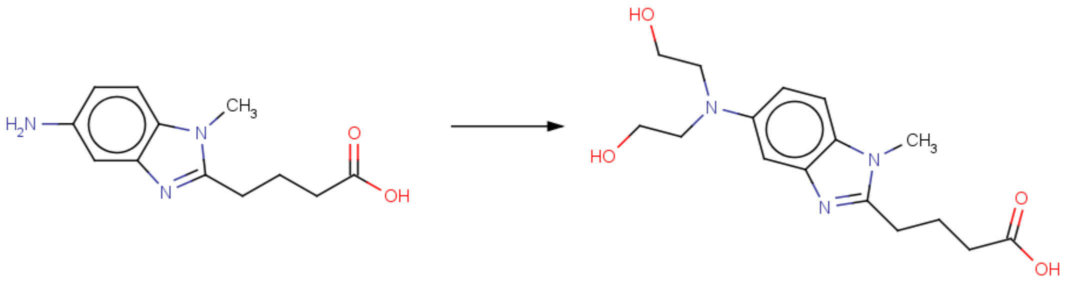

SMILES of the input:
CN1C(CCCC(O)=O)=Nc2cc(N)ccc12>>CN1C(CCCC(O)=O)=Nc2cc(ccc12)N(CCO)CCO

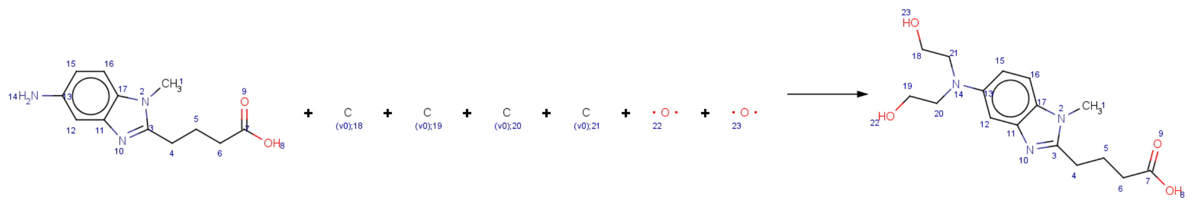

Correct mapped SMILES/SMARTS of the reaction:
[CH3:1][N:2]1[C:3]([CH2:4][CH2:5][CH2:6][C:7]([OH:8])=[O:9])=[N:10][c:11]
2[cH:12][c:13]([NH2:14])[cH:15][cH:16][c:17]12.[C:18].[C:19].[C:20].[C:21
].[O:22].[O:23]>>[CH3:1][N:2]1[C:3]([CH2:4][CH2:5][CH2:6][C:7]([OH:8])=[O
:9])=[N:10][c:11]2[cH:12][c:13]([cH:15][cH:16][c:17]12)[N:14]([CH2:20][CH
2:19][OH:22])[CH2:21][CH2:18][OH:23]

Correctness of the mapping

MAPPET	YES
ReactionMap	NO
Marvin	YES
ChemDraw	YES
Indigo	YES

Reaction no 122

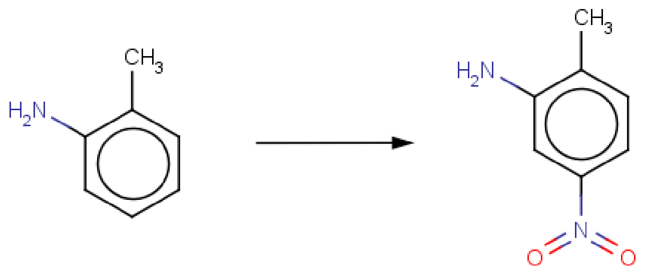

SMILES of the input:
Cc1ccccc1N>>Cc1ccc(cc1N)N(=O)=O

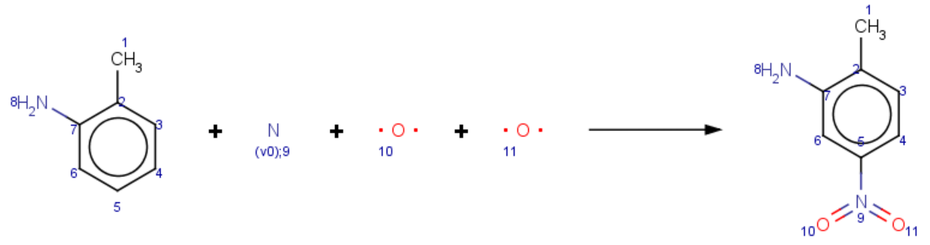

Correct mapped SMILES/SMARTS of the reaction:
[CH3:1][c:2]1[cH:3][cH:4][cH:5][cH:6][c:7]1[NH2:8].[N:9].[O:10].[O:11]>>[
CH3:1][c:2]1[cH:3][cH:4][c:5]([cH:6][c:7]1[NH2:8])[N:9](=[O:11])=[O:10]

Correctness of the mapping

MAPPET	YES
ReactionMap	NO
Marvin	YES
ChemDraw	YES
Indigo	YES

Reaction no 123

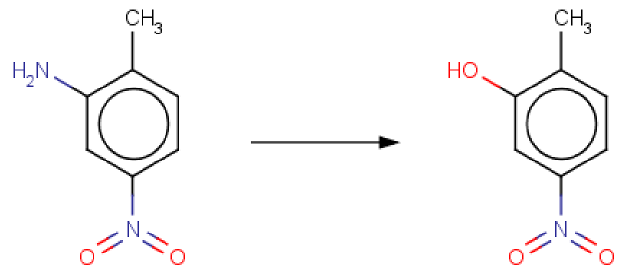

SMILES of the input:
Cc1ccc(cc1N)N(=O)=O>>Cc1ccc(cc1O)N(=O)=O

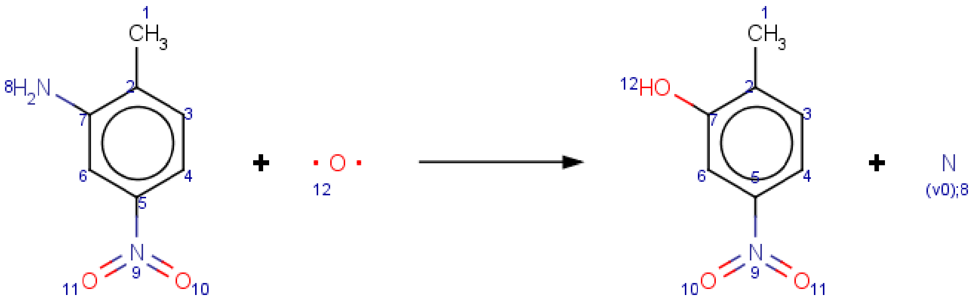

Correct mapped SMILES/SMARTS of the reaction:
[CH3:1][c:2]1[cH:3][cH:4][c:5]([cH:6][c:7]1[NH2:8])[N:9](=[O:10])=[O:11].
[O:12]>>[CH3:1][c:2]1[cH:3][cH:4][c:5]([cH:6][c:7]1[OH:12])[N:9](=[O:11])
=[O:10].[N:8]

Correctness of the mapping

MAPPET	YES
ReactionMap	NO
Marvin	YES
ChemDraw	YES
Indigo	NO

Reaction no 124

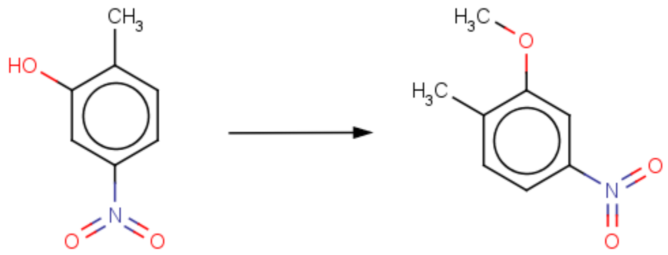

SMILES of the input:
Cc1ccc(cc1O)N(=O)=O>>COc1cc(ccc1C)N(=O)=O

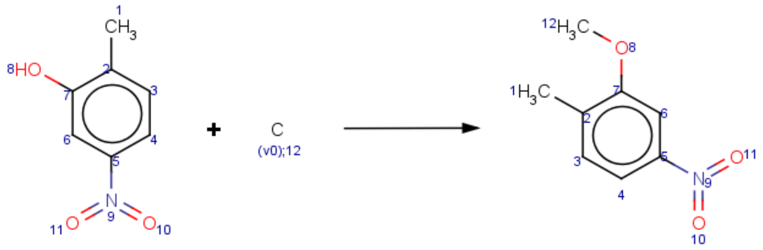

Correct mapped SMILES/SMARTS of the reaction:
[CH3:1][c:2]1[cH:3][cH:4][c:5]([cH:6][c:7]1[OH:8])[N:9](=[O:10])=[O:11].[C:12]>>[CH3:12][O:8][c:7]1[cH:6][c:5]([cH:4][cH:3][c:2]1[CH3:1])[N:9](=[O:11])=[O:10]

Correctness of the mapping

MAPPET	YES
ReactionMap	NO
Marvin	YES
ChemDraw	YES
Indigo	YES

Reaction no 125

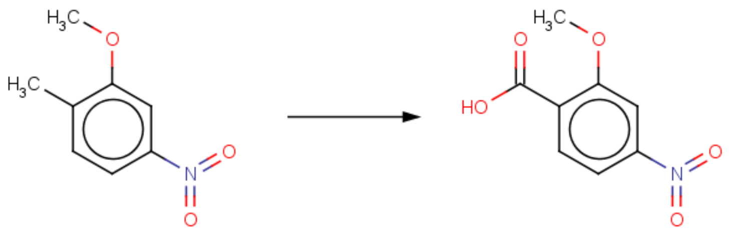

SMILES of the input:
COc1cc(ccc1C)N(=O)=O>>COc1cc(ccc1C(O)=O)N(=O)=O

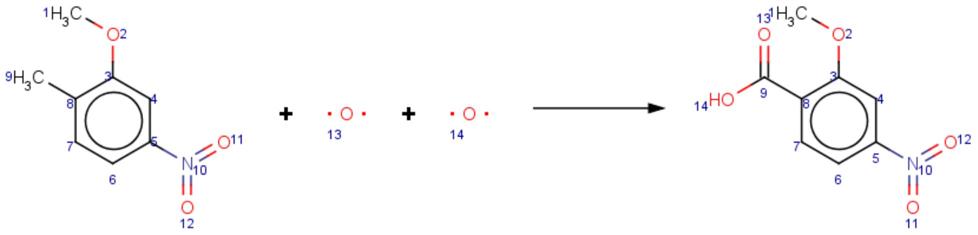

Correct mapped SMILES/SMARTS of the reaction:
[CH3:1][O:2][c:3]1[cH:4][c:5]([cH:6][cH:7][c:8]1[CH3:9])[N:10](=[O:11])=[O:12].[O:13].[O:14]>>[CH3:1][O:2][c:3]1[cH:4][c:5]([cH:6][cH:7][c:8]1[C:9]([OH:14])=[O:13])[N:10](=[O:12])=[O:11]

Correctness of the mapping

MAPPET	YES
ReactionMap	NO
Marvin	YES
ChemDraw	YES
Indigo	YES

Reaction no 126

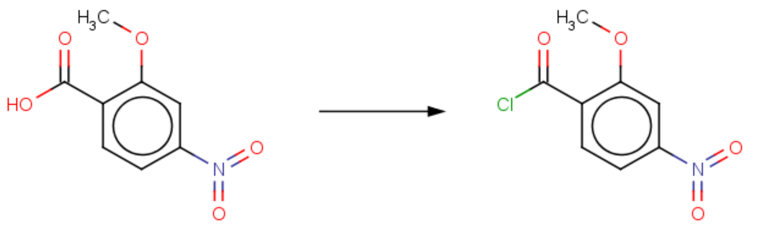

SMILES of the input:
COc1cc(ccc1C(O)=O)N(=O)=O>>COc1cc(ccc1C(Cl)=O)N(=O)=O

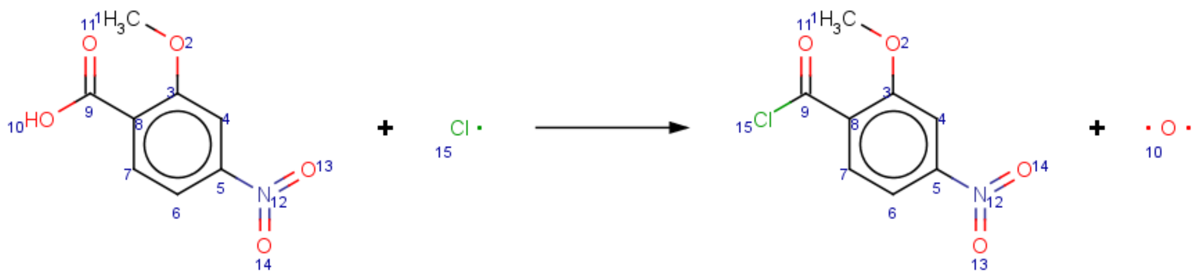

Correct mapped SMILES/SMARTS of the reaction:
[CH3:1][O:2][c:3]1[cH:4][c:5]([cH:6][cH:7][c:8]1[C:9]([OH:10])=[O:11])[N:12](=[O:13])=[O:14].[Cl:15]>>[CH3:1][O:2][c:3]1[cH:4][c:5]([cH:6][cH:7][c:8]1[C:9]([Cl:15])=[O:11])[N:12](=[O:14])=[O:13].[O:10]

Correctness of the mapping

MAPPET	YES
ReactionMap	NO
Marvin	YES
ChemDraw	YES
Indigo	YES

Reaction no 127

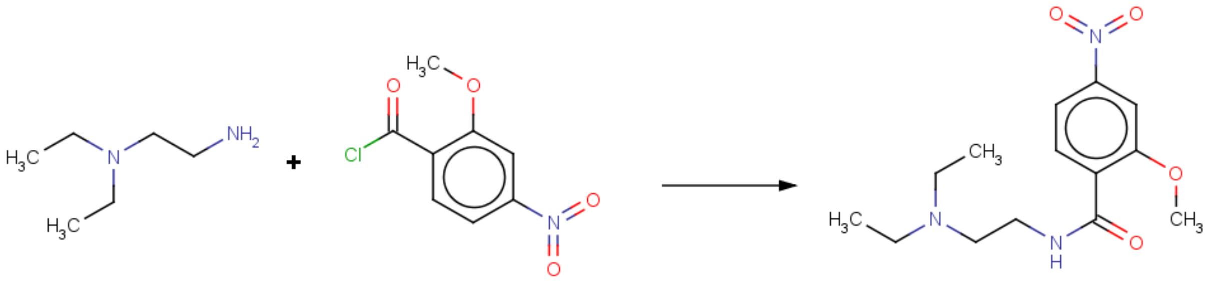

SMILES of the input:
CCN(CC)CCN.COc1cc(ccc1C(Cl)=O)N(=O)=O>>CCN(CC)CCNC(=O)c1ccc(cc1OC)N(=O)=O

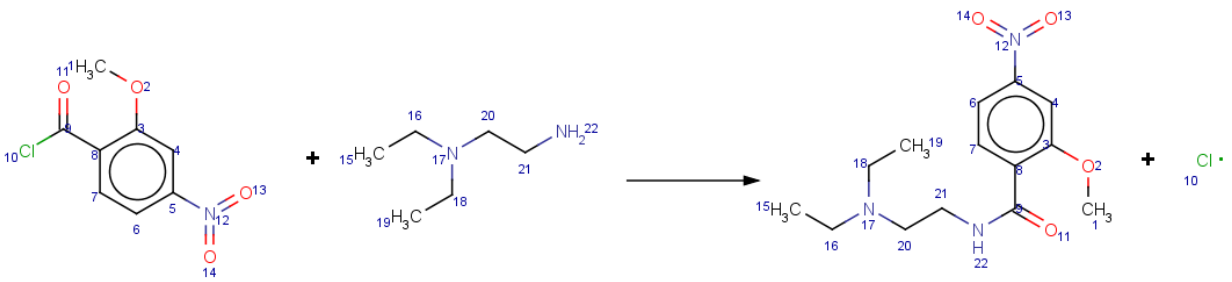

Correct mapped SMILES/SMARTS of the reaction:
[CH3:1][O:2][c:3]1[cH:4][c:5]([cH:6][cH:7][c:8]1[C:9]([Cl:10])=[O:11])[N:12](=[O:13])=[O:14].[CH3:15][CH2:16][N:17]([CH2:18][CH3:19])[CH2:20][CH2:21][NH2:22]>>[CH3:15][CH2:16][N:17]([CH2:18][CH3:19])[CH2:20][CH2:21][NH:22][C:9](=[O:11])[c:8]1[cH:7][cH:6][c:5]([cH:4][c:3]1[O:2][CH3:1])[N:12](=[O:14])=[O:13].[Cl:10]

Correctness of the mapping

MAPPET	YES
ReactionMap	NO
Marvin	YES
ChemDraw	YES
Indigo	YES

Reaction no 128

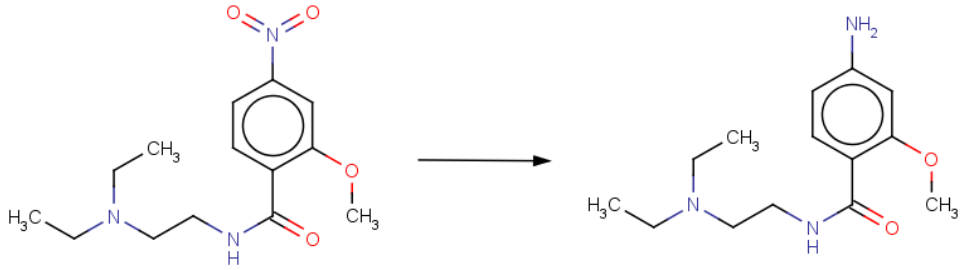

SMILES of the input:
CCN(CC)CCNC(=O)c1ccc(cc1OC)N(=O)=O>>CCN(CC)CCNC(=O)c1ccc(N)cc1OC

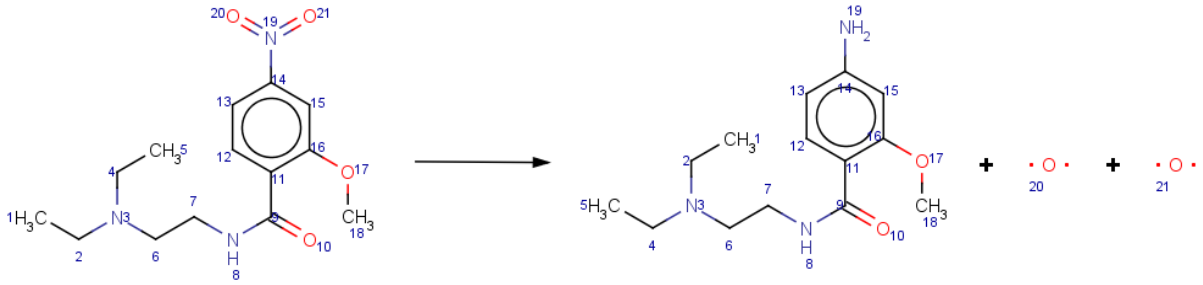

Correct mapped SMILES/SMARTS of the reaction:
[CH3:1][CH2:2][N:3]([CH2:4][CH3:5])[CH2:6][CH2:7][NH:8][C:9](=[O:10])[c:11]1[cH:12][cH:13][c:14]([cH:15][c:16]1[O:17][CH3:18])[N:19](=[O:20])=[O:21]>>[CH3:5][CH2:4][N:3]([CH2:2][CH3:1])[CH2:6][CH2:7][NH:8][C:9](=[O:10])[c:11]1[cH:12][cH:13][c:14]([NH2:19])[cH:15][c:16]1[O:17][CH3:18].[O:20].[O:21]

Correctness of the mapping

MAPPET	YES
ReactionMap	NO

Marvin YES
ChemDraw YES
Indigo YES

Reaction no 129

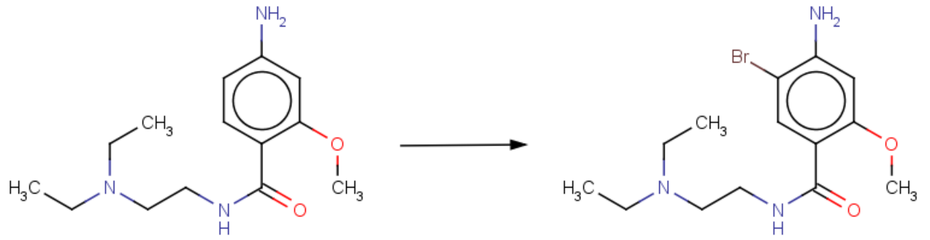

SMILES of the input:
CCN(CC)CCNC(=O)c1ccc(N)cc1OC>>CCN(CC)CCNC(=O)c1cc(Br)c(N)cc1OC

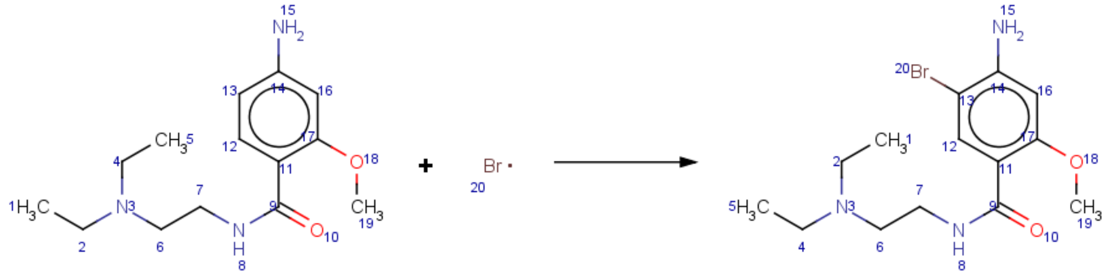

Correct mapped SMILES/SMARTS of the reaction:
[CH3:1][CH2:2][N:3]([CH2:4][CH3:5])[CH2:6][CH2:7][NH:8][C:9](=[O:10])[c:11]1[cH:12][cH:13][c:14]([NH2:15])[cH:16][c:17]1[O:18][CH3:19].[Br:20]>>[CH3:5][CH2:4][N:3]([CH2:2][CH3:1])[CH2:6][CH2:7][NH:8][C:9](=[O:10])[c:11]1[cH:12][c:13]([Br:20])[c:14]([NH2:15])[cH:16][c:17]1[O:18][CH3:19]

Correctness of the mapping
MAPPET YES
ReactionMap NO
Marvin YES
ChemDraw YES
Indigo YES

Reaction no 130

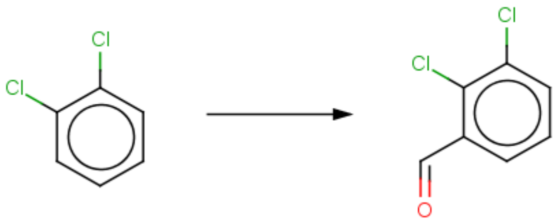

SMILES of the input:
Clc1ccccc1Cl>>Clc1cccc(C=O)c1Cl

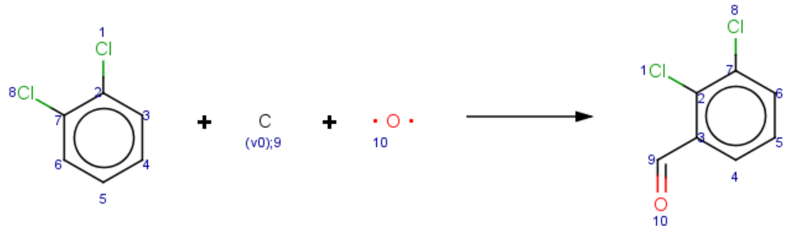

Correct mapped SMILES/SMARTS of the reaction:
[Cl:1][c:2]1[cH:3][cH:4][cH:5][cH:6][c:7]1[Cl:8].[C:9].[O:10]>>[Cl:8][c:7
]1[cH:6][cH:5][cH:4][c:3]([CH:9]=[O:10])[c:2]1[Cl:1]

Correctness of the mapping

MAPPET	YES
ReactionMap	NO
Marvin	YES
ChemDraw	YES
Indigo	YES

Reaction no 131

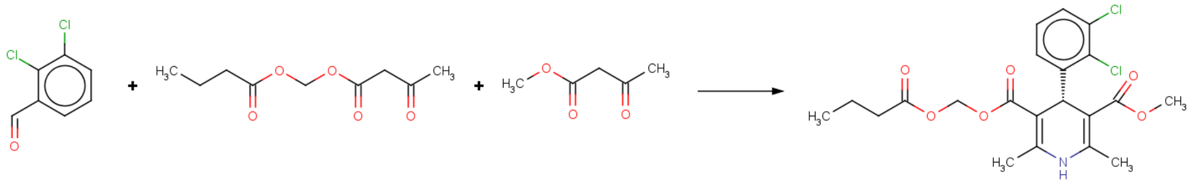

SMILES of the input:
Clc1cccc(C=O)c1Cl.CCCC(=O)OCOC(=O)CC(C)=O.COC(=O)CC(C)=O>>CCCC(=O)OCOC(=O
)C1=C(C)NC(C)=C([C@H]1c1cccc(Cl)c1Cl)C(=O)OC

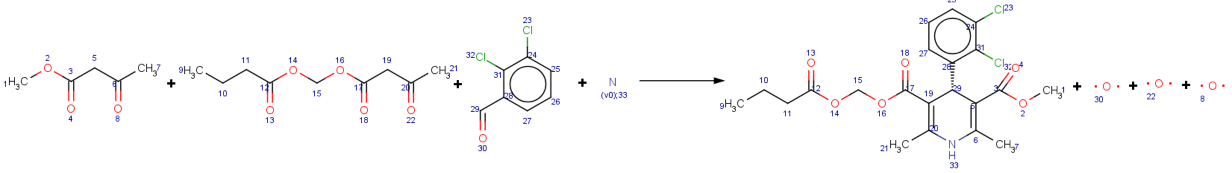

Correct mapped SMILES/SMARTS of the reaction:
[CH3:1][O:2][C:3](=[O:4])[CH2:5][C:6]([CH3:7])=[O:8].[CH3:9][CH2:10][CH2:
11][C:12](=[O:13])[O:14][CH2:15][O:16][C:17](=[O:18])[CH2:19][C:20]([CH3:
21])=[O:22].[Cl:23][c:24]1[cH:25][cH:26][cH:27][c:28]([CH:29]=[O:30])[c:3
1]1[Cl:32].[N:33]>>[CH3:9][CH2:10][CH2:11][C:12](=[O:13])[O:14][CH2:15][O
:16][C:17](=[O:18])[C:19]1=[C:20]([CH3:21])[NH:33][C:6]([CH3:7])=[C:5]([C
@H:29]1[c:28]1[cH:27][cH:26][cH:25][c:24]([Cl:23])[c:31]1[Cl:32])[C:3](=[
O:4])[O:2][CH3:1].[O:30].[O:22].[O:8]

Correctness of the mapping

MAPPET	YES
ReactionMap	NO
Marvin	YES
ChemDraw	YES
Indigo	NO

Reaction no 132

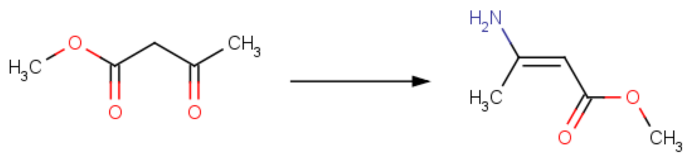

SMILES of the input:
COC(=O)CC(C)=O>>COC(=O)\C=C(/C)N

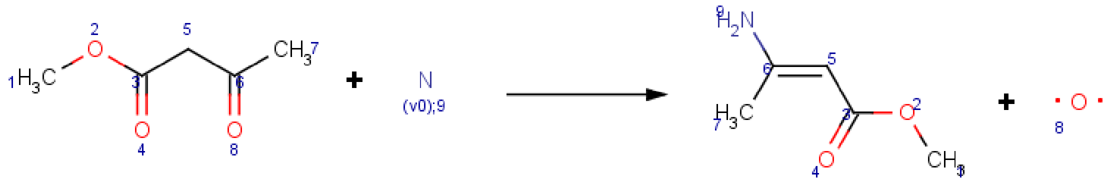

Correct mapped SMILES/SMARTS of the reaction:
[CH3:1][O:2][C:3](=[O:4])[CH2:5][C:6]([CH3:7])=[O:8].[N:9]>>[CH3:1][O:2][C:3](=[O:4])\[CH:5]=[C:6](/[CH3:7])[NH2:9].[O:8]

Correctness of the mapping

MAPPET	YES
ReactionMap	NO
Marvin	YES
ChemDraw	YES
Indigo	YES

Reaction no 133

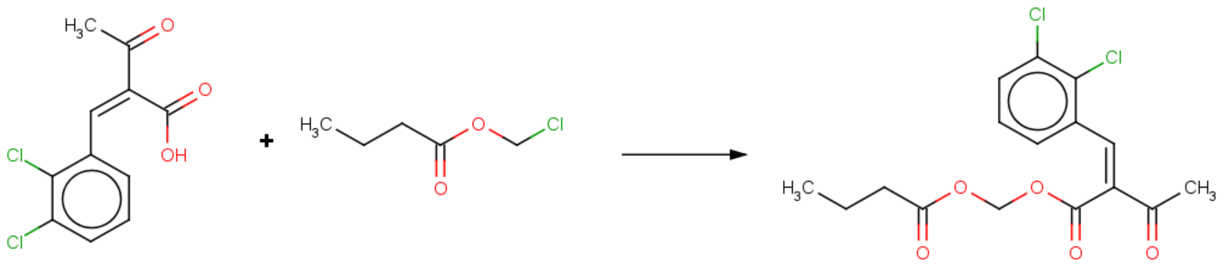

SMILES of the input:
CC(=O)C(=C\c1cccc(Cl)c1Cl)\C(O)=O.CCCC(=O)OCCl>>CCCC(=O)OCOC(=O)C(=C/c1cccc(Cl)c1Cl)\C(C)=O

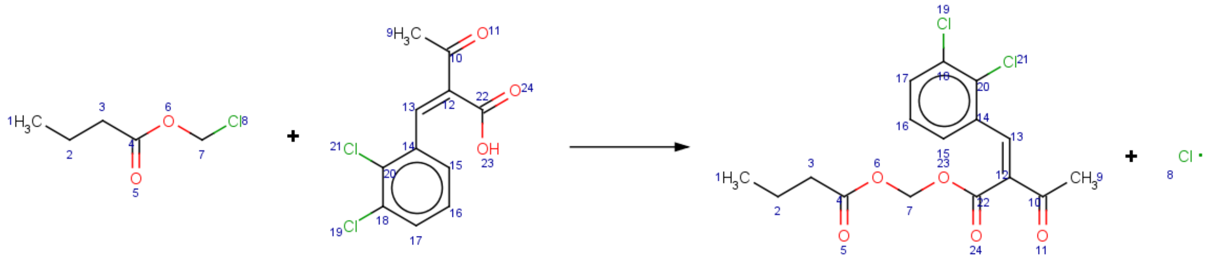

Correct mapped SMILES/SMARTS of the reaction:
[CH3:1][CH2:2][CH2:3][C:4](=[O:5])[O:6][CH2:7][Cl:8].[CH3:9][C:10](=[O:11])[C:12](=[CH:13]\[c:14]1[cH:15][cH:16][cH:17][c:18]([Cl:19])[c:20]1[Cl:2

1])\[C:22]([OH:23])=[O:24]>>[CH3:1][CH2:2][CH2:3][C:4](=[O:5])[O:6][CH2:7][O:23][C:22](=[O:24])[C:12](=[CH:13]/[c:14]1[cH:15][cH:16][cH:17][c:18]([Cl:19])[c:20]1[Cl:21])\[C:10]([CH3:9])=[O:11].[Cl:8]

Correctness of the mapping

MAPPET	YES
ReactionMap	NO
Marvin	YES
ChemDraw	YES
Indigo	YES

Reaction no 134

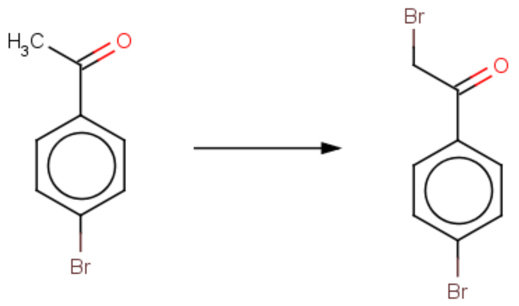

SMILES of the input:
CC(=O)c1ccc(Br)cc1>>BrCC(=O)c1ccc(Br)cc1

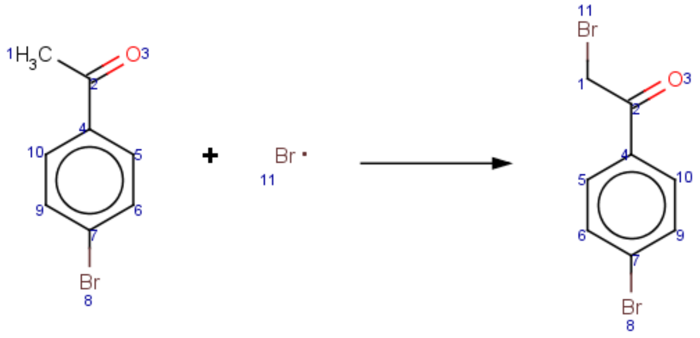

Correct mapped SMILES/SMARTS of the reaction:
[CH3:1][C:2](=[O:3])[c:4]1[cH:5][cH:6][c:7]([Br:8])[cH:9][cH:10]1.[Br:11]>>[Br:11][CH2:1][C:2](=[O:3])[c:4]1[cH:10][cH:9][c:7]([Br:8])[cH:6][cH:5]1

Correctness of the mapping

MAPPET	YES
ReactionMap	NO
Marvin	YES
ChemDraw	YES
Indigo	YES

Reaction no 135

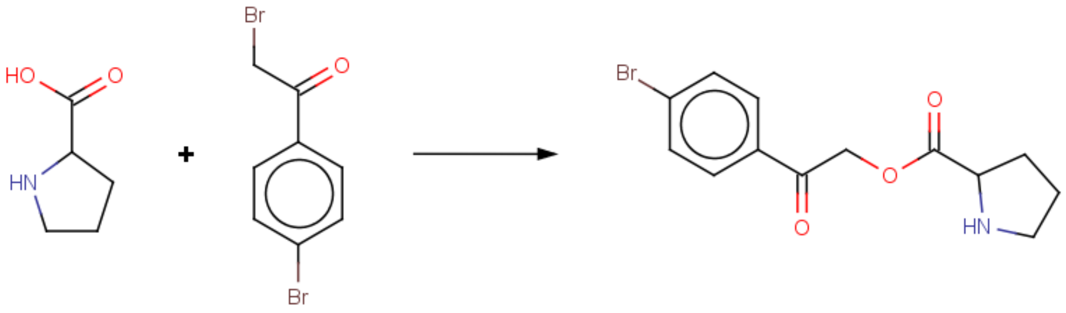

SMILES of the input:
OC(=O)C1CCCN1.BrCC(=O)c1ccc(Br)cc1>>Brc1ccc(cc1)C(=O)COC(=O)C1CCCN1

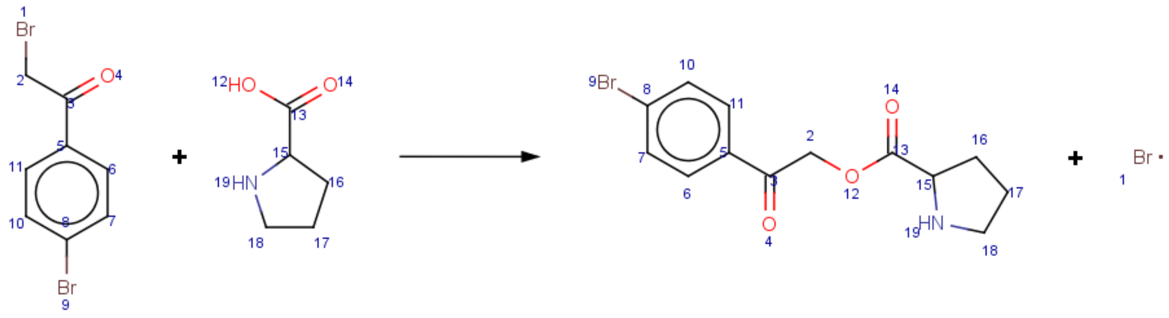

Correct mapped SMILES/SMARTS of the reaction:
[Br:1][CH2:2][C:3](=[O:4])[c:5]1[cH:6][cH:7][c:8]([Br:9])[cH:10][cH:11]1.[OH:12][C:13](=[O:14])[CH:15]1[CH2:16][CH2:17][CH2:18][NH:19]1>>[Br:9][c:8]1[cH:10][cH:11][c:5]([cH:6][cH:7]1)[C:3](=[O:4])[CH2:2][O:12][C:13](=[O:14])[CH:15]1[CH2:16][CH2:17][CH2:18][NH:19]1.[Br:1]

Correctness of the mapping

MAPPET	YES
ReactionMap	NO
Marvin	YES
ChemDraw	YES
Indigo	YES

Reaction no 136

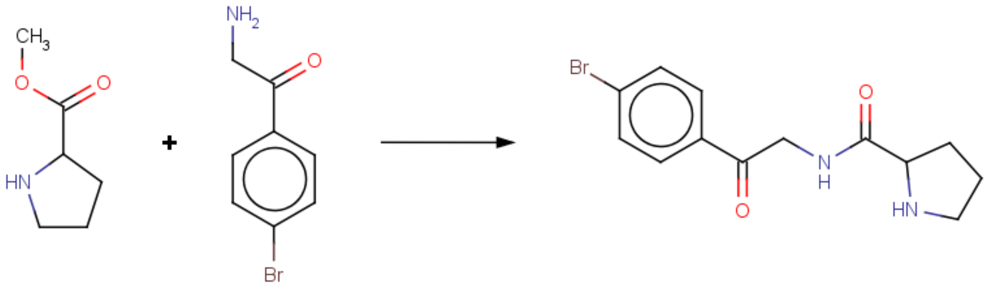

SMILES of the input:
COC(=O)C1CCCN1.NCC(=O)c1ccc(Br)cc1>>Brc1ccc(cc1)C(=O)CNC(=O)C1CCCN1

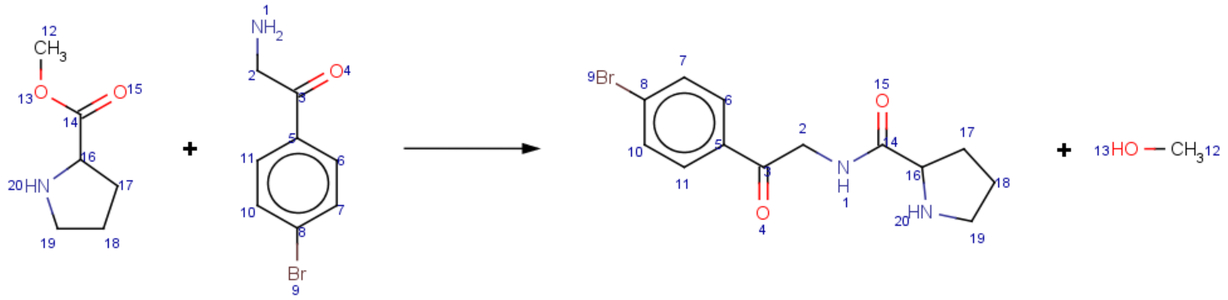

Correct mapped SMILES/SMARTS of the reaction:
[CH3:12][O:13][C:14](=[O:15])[CH:16]1[CH2:17][CH2:18][CH2:19][NH:20]1.[NH2:1][CH2:2][C:3](=[O:4])[c:5]1[cH:6][cH:7][c:8]([Br:9])[cH:10][cH:11]1>>[Br:9][c:8]1[cH:7][cH:6][c:5]([cH:11][cH:10]1)[C:3](=[O:4])[CH2:2][NH:1][C:14](=[O:15])[CH:16]1[CH2:17][CH2:18][CH2:19][NH:20]1.[CH3:12][OH:13]

Correctness of the mapping

MAPPET	YES
ReactionMap	NO
Marvin	YES
ChemDraw	YES
Indigo	YES

Reaction no 137

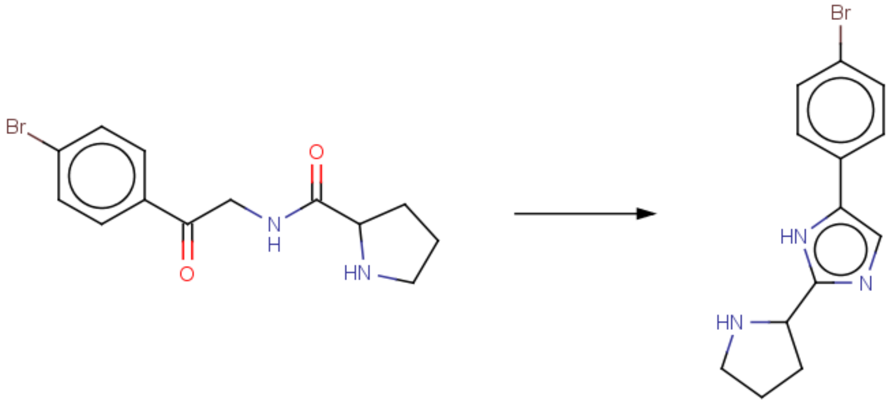

SMILES of the input:
Brc1ccc(cc1)C(=O)CNC(=O)C1CCCN1>>Brc1ccc(cc1)-c1cnc([nH]1)C1CCCN1

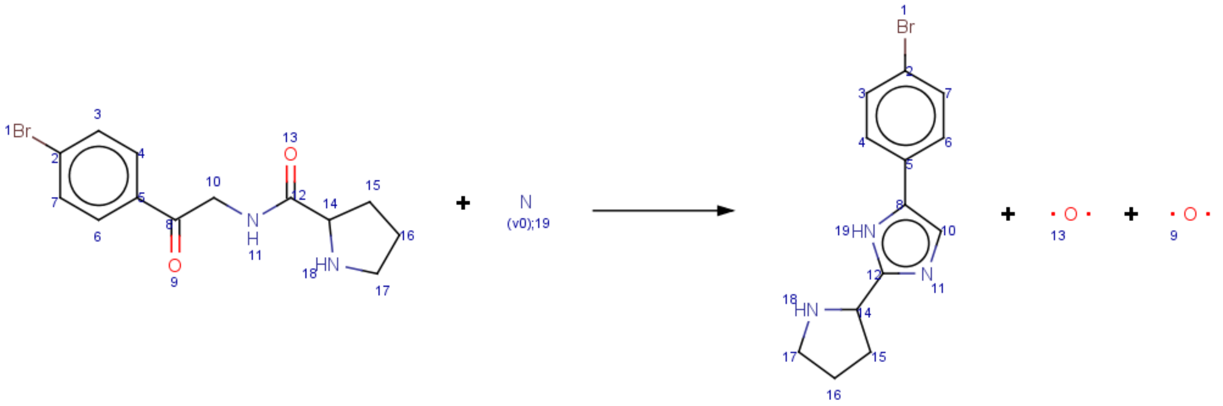

Correct mapped SMILES/SMARTS of the reaction:
[Br:1][c:2]1[cH:3][cH:4][c:5]([cH:6][cH:7]1)[C:8](=[O:9])[CH2:10][NH:11][C:12](=[O:13])[CH:14]1[CH2:15][CH2:16][CH2:17][NH:18]1.[N:19]>>[Br:1][c:2]1[cH:7][cH:6][c:5]([cH:4][cH:3]1)-

[c:8]1[cH:10][n:11][c:12]([nH:19]1)[CH:14]1[CH2:15][CH2:16][CH2:17][NH:18]1.[O:13].[O:9]

Correctness of the mapping

MAPPET	YES
ReactionMap	NO
Marvin	NO
ChemDraw	YES
Indigo	YES

Reaction no 138

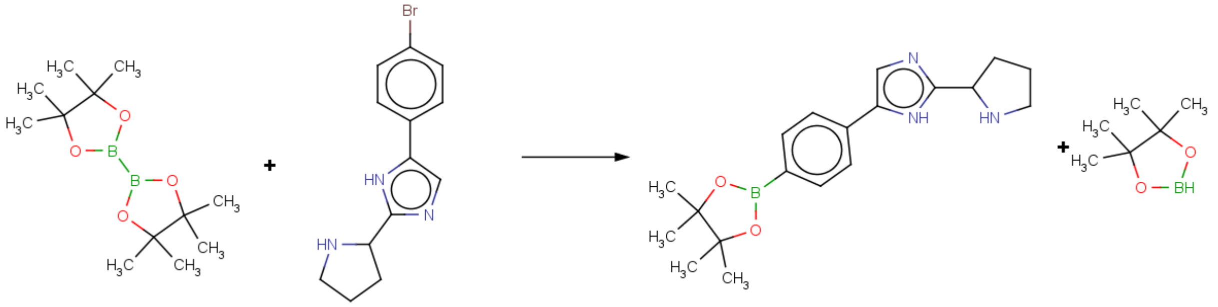

SMILES of the input:
CC1(C)OB(OC1(C)C)B1OC(C)(C)C(C)(C)O1.Brc1ccc(cc1)-c1cnc([nH]1)C1CCCN1>>CC1(C)OB(OC1(C)C)c1ccc(cc1)-c1cnc([nH]1)C1CCCN1.CC1(C)OBOC1(C)C

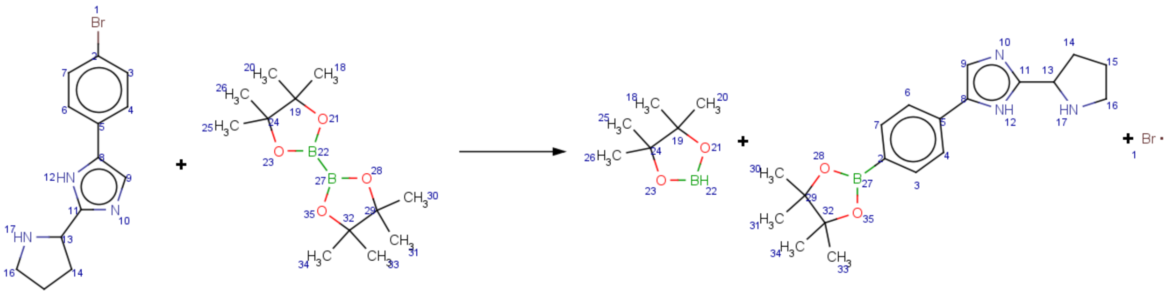

Correct mapped SMILES/SMARTS of the reaction:
[Br:1][c:2]1[cH:3][cH:4][c:5]([cH:6][cH:7]1)-[c:8]1[cH:9][n:10][c:11]([nH:12]1)[CH:13]1[CH2:14][CH2:15][CH2:16][NH:17]1.[CH3:18][C:19]1([CH3:20])[O:21][B:22]([O:23][C:24]1([CH3:25])[CH3:26])[B:27]1[O:28][C:29]([CH3:30])([CH3:31])[C:32]([CH3:33])([CH3:34])[O:35]1>>[CH3:20][C:19]1([CH3:18])[O:21][BH:22][O:23][C:24]1([CH3:26])[CH3:25].[CH3:30][C:29]1([CH3:31])[O:28][B:27]([O:35][C:32]1([CH3:33])[CH3:34])[c:2]1[cH:7][cH:6][c:5]([cH:4][cH:3]1)-[c:8]1[cH:9][n:10][c:11]([nH:12]1)[CH:13]1[CH2:14][CH2:15][CH2:16][NH:17]1.[Br:1]

Correctness of the mapping

MAPPET	YES
ReactionMap	NO
Marvin	YES
ChemDraw	YES
Indigo	YES

Reaction no 139

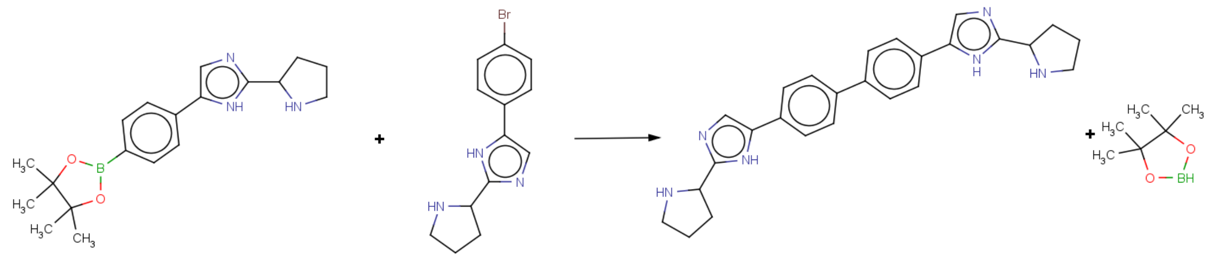

SMILES of the input:
CC1(C)OB(OC1(C)C)c1ccc(cc1)-c1cnc([nH]1)C1CCCN1.Brc1ccc(cc1)-c1cnc([nH]1)C1CCCN1>>C1CNC(C1)c1ncc([nH]1)-c1ccc(cc1)-c1ccc(cc1)-c1cnc([nH]1)C1CCCN1.CC1(C)OBOC1(C)C

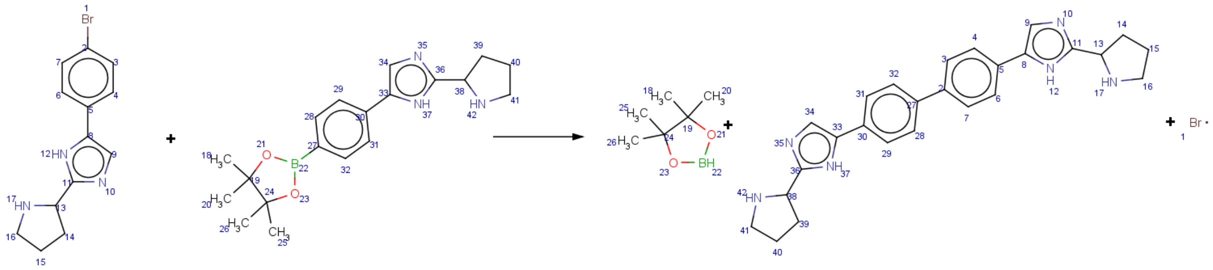

Correct mapped SMILES/SMARTS of the reaction:
[Br:1][c:2]1[cH:3][cH:4][c:5]([cH:6][cH:7]1)-[c:8]1[cH:9][n:10][c:11]([nH:12]1)[CH:13]1[CH2:14][CH2:15][CH2:16][NH:17]1.[CH3:18][C:19]1([CH3:20])[O:21][B:22]([O:23][C:24]1([CH3:25])[CH3:26])[c:27]1[cH:28][cH:29][c:30]([cH:31][cH:32]1)-[c:33]1[cH:34][n:35][c:36]([nH:37]1)[CH:38]1[CH2:39][CH2:40][CH2:41][NH:42]1>>[CH3:20][C:19]1([CH3:18])[O:21][BH:22][O:23][C:24]1([CH3:26])[CH3:25].[CH2:40]1[CH2:41][NH:42][CH:38]([CH2:39]1)[c:36]1[n:35][cH:34][c:33]([nH:37]1)-[c:30]1[cH:31][cH:32][c:27]([cH:28][cH:29]1)-[c:2]1[cH:3][cH:4][c:5]([cH:6][cH:7]1)-[c:8]1[cH:9][n:10][c:11]([nH:12]1)[CH:13]1[CH2:14][CH2:15][CH2:16][NH:17]1.[Br:1]

Correctness of the mapping

MAPPET	YES
ReactionMap	NO
Marvin	YES
ChemDraw	YES
Indigo	YES

Reaction no 140

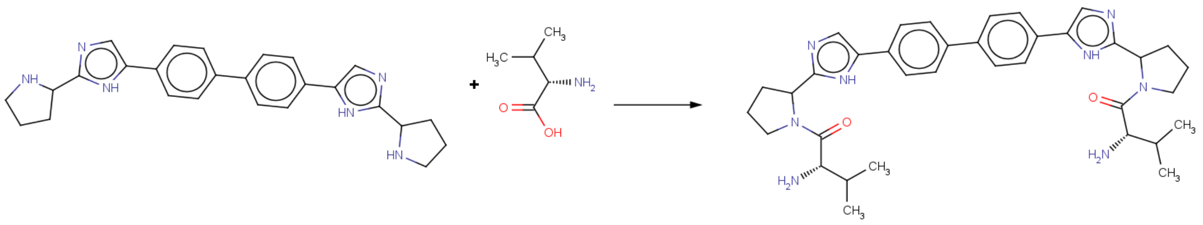

SMILES of the input:
C1CNC(C1)c1ncc([nH]1)-c1ccc(cc1)-c1ccc(cc1)-c1cnc([nH]1)C1CCCN1.CC(C)[C@H](N)C(O)=O>>CC(C)[C@H](N)C(=O)N1CCCC1c1ncc([nH]1)-c1ccc(cc1)-c1ccc(cc1)-c1cnc([nH]1)C1CCCN1C(=O)[C@@H](N)C(C)C

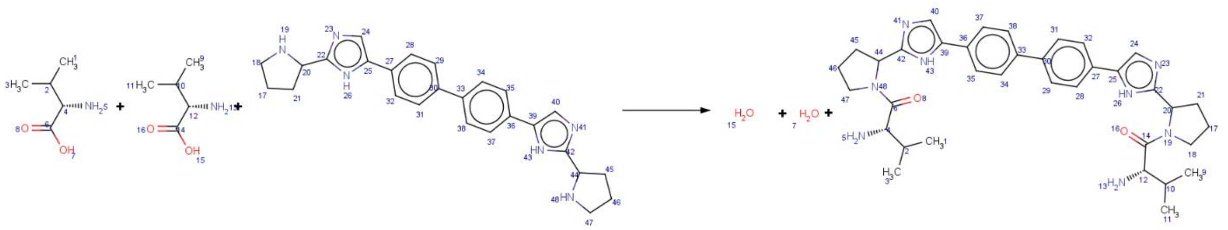

Correct mapped SMILES/SMARTS of the reaction:
[CH3:1][CH:2]([CH3:3])[C@H:4]([NH2:5])[C:6]([OH:7])=[O:8].[CH3:9][CH:10](
[CH3:11])[C@H:12]([NH2:13])[C:14]([OH:15])=[O:16].[CH2:17]1[CH2:18][NH:19
][CH:20]([CH2:21]1)[c:22]1[n:23][cH:24][c:25]([nH:26]1)-
[c:27]1[cH:28][cH:29][c:30]([cH:31][cH:32]1)-
[c:33]1[cH:34][cH:35][c:36]([cH:37][cH:38]1)-
[c:39]1[cH:40][n:41][c:42]([nH:43]1)[CH:44]1[CH2:45][CH2:46][CH2:47][NH:4
8]1>>[OH2:15].[OH2:7].[CH3:3][CH:2]([CH3:1])[C@H:4]([NH2:5])[C:6](=[O:8])
[N:48]1[CH2:47][CH2:46][CH2:45][CH:44]1[c:42]1[n:41][cH:40][c:39]([nH:43]
1)-[c:36]1[cH:37][cH:38][c:33]([cH:34][cH:35]1)-
[c:30]1[cH:31][cH:32][c:27]([cH:28][cH:29]1)-
[c:25]1[cH:24][n:23][c:22]([nH:26]1)[CH:20]1[CH2:21][CH2:17][CH2:18][N:19
]1[C:14](=[O:16])[C@@H:12]([NH2:13])[CH:10]([CH3:9])[CH3:11]

Correctness of the mapping

MAPPET	YES
ReactionMap	NO
Marvin	NO
ChemDraw	YES
Indigo	NO

Reaction no 141

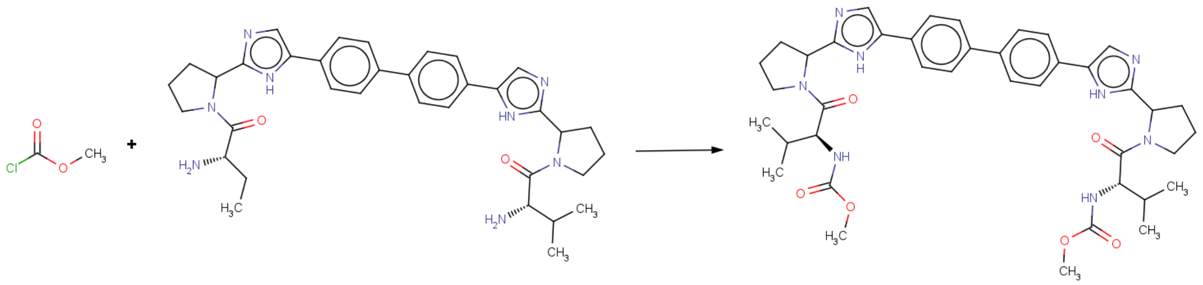

SMILES of the input:
COC(Cl)=O.C(C)[C@H](N)C(=O)N1CCCC1c1ncc([nH]1)-c1ccc(cc1)-c1ccc(cc1)-
c1cnc([nH]1)C1CCCN1C(=O)[C@@H](N)C(C)C>>COC(=O)N[C@@H](C(C)C)C(=O)N1CCCC1
c1ncc([nH]1)-c1ccc(cc1)-c1ccc(cc1)-
c1cnc([nH]1)C1CCCN1C(=O)[C@@H](NC(=O)OC)C(C)C

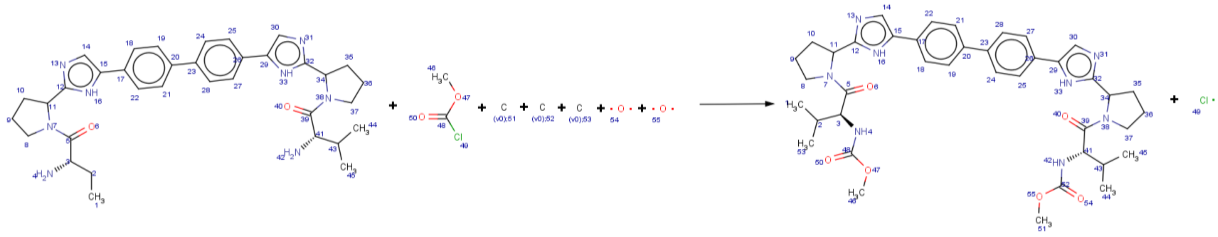

Correct mapped SMILES/SMARTS of the reaction:
[CH3:1][CH2:2][C@H:3]([NH2:4])[C:5](=[O:6])[N:7]1[CH2:8][CH2:9][CH2:10][C
H:11]1[c:12]1[n:13][cH:14][c:15]([nH:16]1)-
[c:17]1[cH:18][cH:19][c:20]([cH:21][cH:22]1)-
[c:23]1[cH:24][cH:25][c:26]([cH:27][cH:28]1)-
[c:29]1[cH:30][n:31][c:32]([nH:33]1)[CH:34]1[CH2:35][CH2:36][CH2:37][N:38
]1[C:39](=[O:40])[C@@H:41]([NH2:42])[CH:43]([CH3:44])[CH3:45].[CH3:46][O:

47][C:48]([Cl:49])=[O:50].[C:51].[C:52].[C:53].[O:54].[O:55]>>[CH3:46][O:47][C:48](=[O:50])[NH:4][C@@H:3]([CH:2]([CH3:1])[CH3:53])[C:5](=[O:6])[N:7]1[CH2:8][CH2:9][CH2:10][CH:11]1[c:12]1[n:13][cH:14][c:15]([nH:16]1)-[c:17]1[cH:22][cH:21][c:20]([cH:19][cH:18]1)-[c:23]1[cH:28][cH:27][c:26]([cH:25][cH:24]1)-[c:29]1[cH:30][n:31][c:32]([nH:33]1)[CH:34]1[CH2:35][CH2:36][CH2:37][N:38]1[C:39](=[O:40])[C@@H:41]([NH:42][C:52](=[O:54])[O:55][CH3:51])[CH:43]([CH3:45])[CH3:44].[Cl:49]

Correctness of the mapping

MAPPET	YES
ReactionMap	NO
Marvin	NO
ChemDraw	YES
Indigo	YES

Reaction no 142

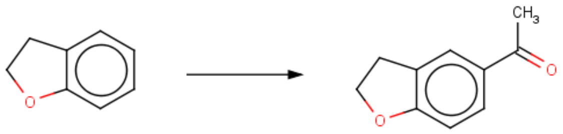

SMILES of the input:
C1Cc2ccccc2O1>>CC(=O)c1ccc2OCCc2c1

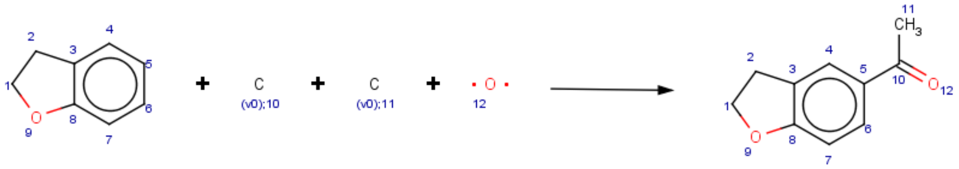

Correct mapped SMILES/SMARTS of the reaction:
[CH2:1]1[CH2:2][c:3]2[cH:4][cH:5][cH:6][cH:7][c:8]2[O:9]1.[C:10].[C:11].[O:12]>>[CH3:11][C:10](=[O:12])[c:5]1[cH:6][cH:7][c:8]2[O:9][CH2:1][CH2:2][c:3]2[cH:4]1

Correctness of the mapping

MAPPET	YES
ReactionMap	NO
Marvin	YES
ChemDraw	YES
Indigo	YES

Reaction no 143

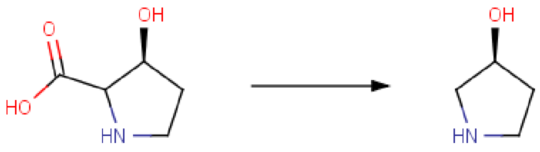

SMILES of the input:
O[C@H]1CCNC1C(O)=O>>O[C@H]1CCNC1

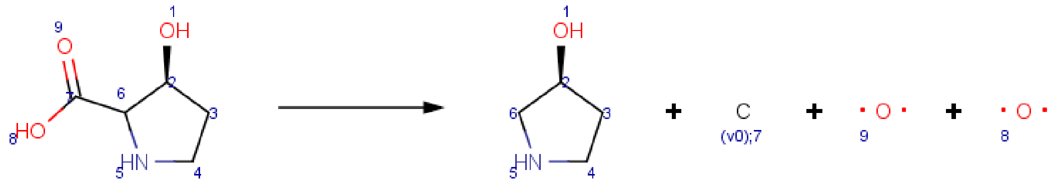

Correct mapped SMILES/SMARTS of the reaction:
[OH:1][C@H:2]1[CH2:3][CH2:4][NH:5][CH:6]1[C:7]([OH:8])=[O:9]>>[OH:1][C@H:2]1[CH2:3][CH2:4][NH:5][CH2:6]1.[C:7].[O:9].[O:8]

Correctness of the mapping

MAPPET	YES
ReactionMap	NO
Marvin	YES
ChemDraw	YES
Indigo	YES

Reaction no 144

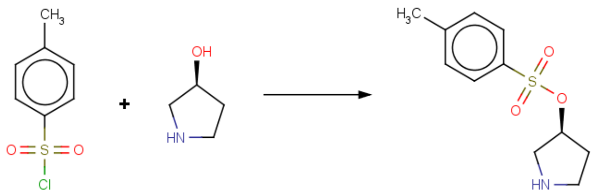

SMILES of the input:
Cc1ccc(cc1)S(Cl)(=O)=O.O[C@H]1CCNC1>>Cc1ccc(cc1)S(=O)(=O)O[C@H]1CCNC1

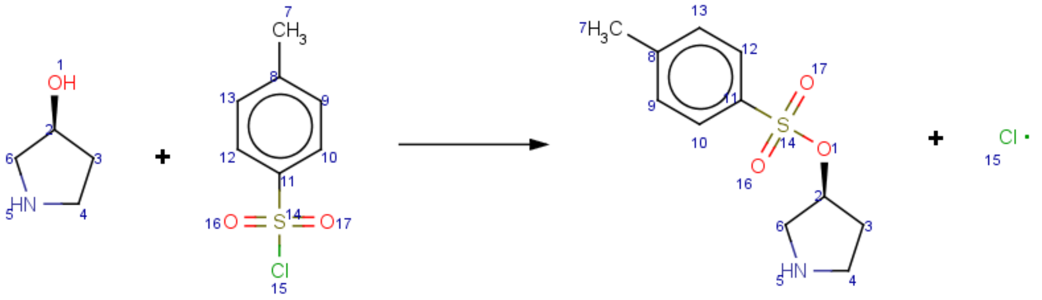

Correct mapped SMILES/SMARTS of the reaction:
[OH:1][C@H:2]1[CH2:3][CH2:4][NH:5][CH2:6]1.[CH3:7][c:8]1[cH:9][cH:10][c:11]([cH:12][cH:13]1)[S:14]([Cl:15])(=[O:16])=[O:17]>>[CH3:7][c:8]1[cH:13][cH:12][c:11]([cH:10][cH:9]1)[S:14](=[O:16])(=[O:17])[O:1][C@H:2]1[CH2:3][CH2:4][NH:5][CH2:6]1.[Cl:15]

Correctness of the mapping

MAPPET	YES
ReactionMap	NO
Marvin	YES
ChemDraw	YES
Indigo	YES

Reaction no 145

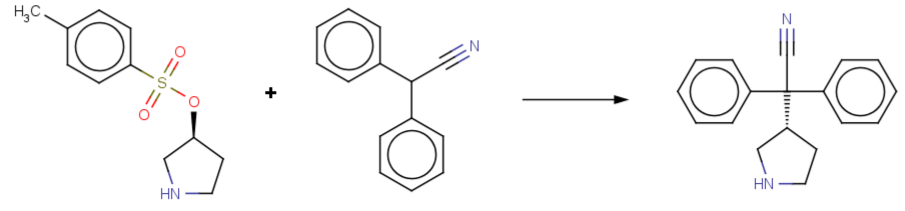

SMILES of the input:
Cc1ccc(cc1)S(=O)(=O)O[C@H]1CCNC1.N#CC(c1ccccc1)c1ccccc1>>N#CC([C@@H]1CCNC1)(c1ccccc1)c1ccccc1

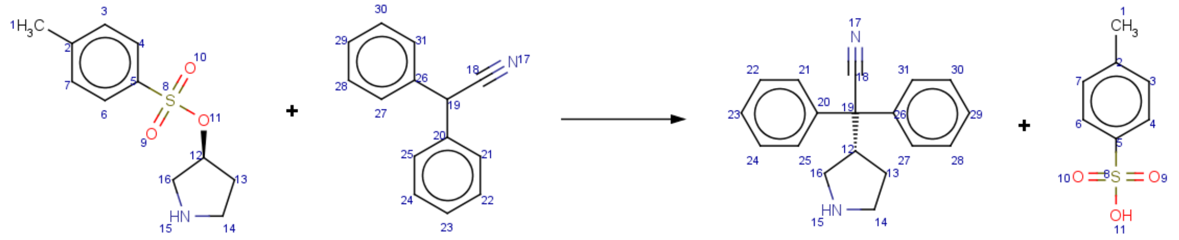

Correct mapped SMILES/SMARTS of the reaction:
[CH3:1][c:2]1[cH:3][cH:4][c:5]([cH:6][cH:7]1)[S:8](=[O:9])(=[O:10])[O:11][C@H:12]1[CH2:13][CH2:14][NH:15][CH2:16]1.[N:17]#[C:18][CH:19]([c:20]1[cH:21][cH:22][cH:23][cH:24][cH:25]1)[c:26]1[cH:27][cH:28][cH:29][cH:30][cH:31]1>>[N:17]#[C:18][C:19]([C@@H:12]1[CH2:13][CH2:14][NH:15][CH2:16]1)([c:26]1[cH:31][cH:30][cH:29][cH:28][cH:27]1)[c:20]1[cH:25][cH:24][cH:23][cH:22][cH:21]1.[CH3:1][c:2]1[cH:3][cH:4][c:5]([cH:6][cH:7]1)[S:8]([OH:11])(=[O:10])=[O:9]

Correctness of the mapping

MAPPET	YES
ReactionMap	NO
Marvin	YES
ChemDraw	YES
Indigo	NO

Reaction no 146

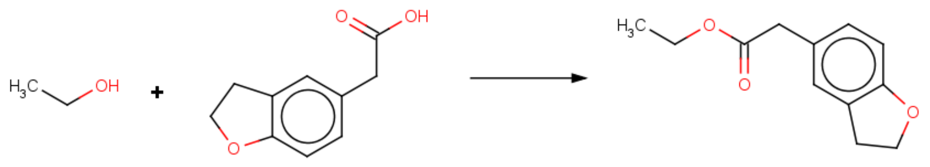

SMILES of the input:
CCO.OC(=O)Cc1ccc2OCCc2c1>>CCOC(=O)Cc1ccc2OCCc2c1

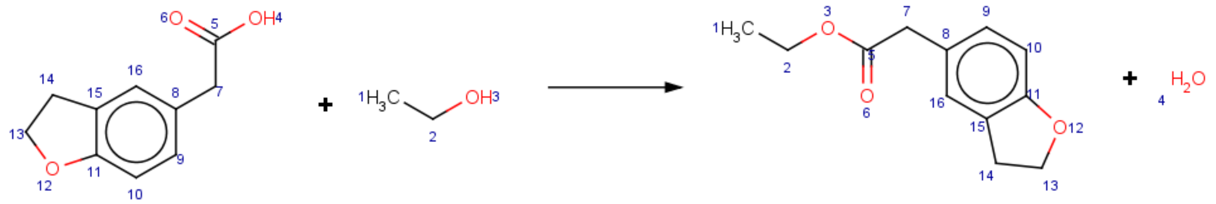

Correct mapped SMILES/SMARTS of the reaction:
[OH:4][C:5](=[O:6])[CH2:7][c:8]1[cH:9][cH:10][c:11]2[O:12][CH2:13][CH2:14
][c:15]2[cH:16]1.[CH3:1][CH2:2][OH:3]>>[CH3:1][CH2:2][O:3][C:5](=[O:6])[C
H2:7][c:8]1[cH:9][cH:10][c:11]2[O:12][CH2:13][CH2:14][c:15]2[cH:16]1.[OH2
:4]

Correctness of the mapping

MAPPET	YES
ReactionMap	NO
Marvin	YES
ChemDraw	NO
Indigo	NO

Reaction no 147

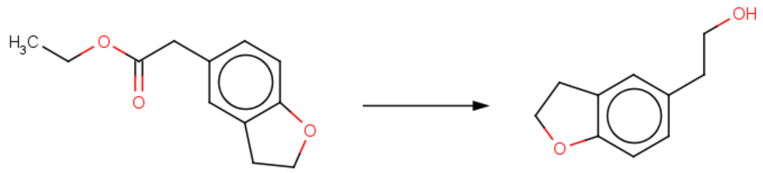

SMILES of the input:
CCOC(=O)Cc1ccc2OCCc2c1>>OCCc1ccc2OCCc2c1

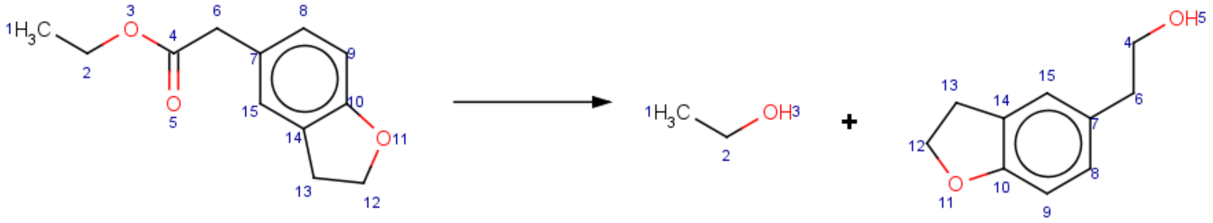

Correct mapped SMILES/SMARTS of the reaction:
[CH3:1][CH2:2][O:3][C:4](=[O:5])[CH2:6][c:7]1[cH:8][cH:9][c:10]2[O:11][CH
2:12][CH2:13][c:14]2[cH:15]1>>[CH3:1][CH2:2][OH:3].[OH:5][CH2:4][CH2:6][c
:7]1[cH:8][cH:9][c:10]2[O:11][CH2:12][CH2:13][c:14]2[cH:15]1

Correctness of the mapping

MAPPET	YES
ReactionMap	NO
Marvin	YES
ChemDraw	YES
Indigo	YES

Reaction no 148

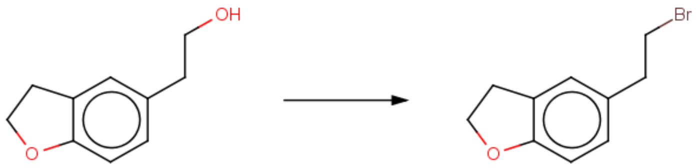

SMILES of the input:
OCCc1ccc2OCCc2c1>>BrCCc1ccc2OCCc2c1

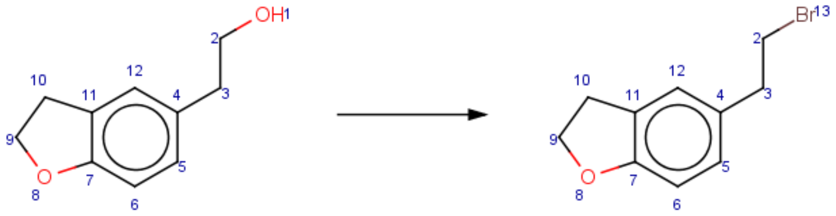

Correct mapped SMILES/SMARTS of the reaction:
[OH:1][CH2:2][CH2:3][c:4]1[cH:5][cH:6][c:7]2[O:8][CH2:9][CH2:10][c:11]2[cH:12]1.[Br:13]>>[Br:13][CH2:2][CH2:3][c:4]1[cH:5][cH:6][c:7]2[O:8][CH2:9][CH2:10][c:11]2[cH:12]1.[O:1]

Correctness of the mapping

MAPPET	YES
ReactionMap	NO
Marvin	YES
ChemDraw	YES
Indigo	YES

Reaction no 149

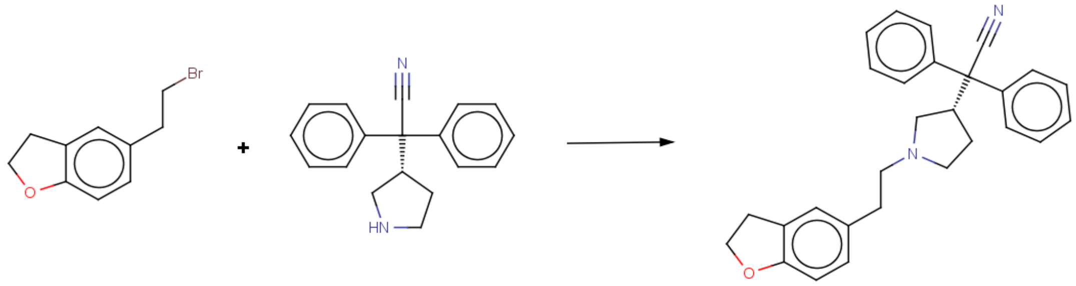

SMILES of the input:
BrCCc1ccc2OCCc2c1.N#CC([C@@H]1CCNC1)(c1ccccc1)c1ccccc1>>N#CC([C@@H]1CCN(CCc2ccc3OCCc3c2)C1)(c1ccccc1)c1ccccc1

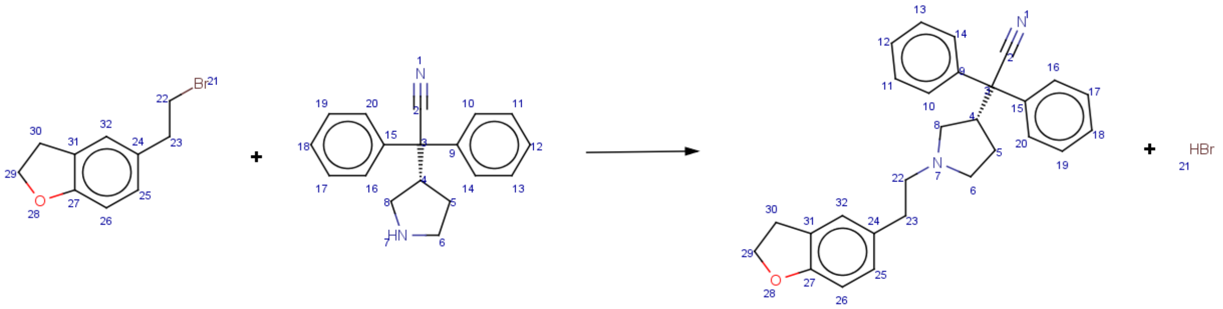

Correct mapped SMILES/SMARTS of the reaction:
[Br:21][CH2:22][CH2:23][c:24]1[cH:25][cH:26][c:27]2[O:28][CH2:29][CH2:30][c:31]2[cH:32]1.[N:1]#[C:2][C:3]([C@@H:4]1[CH2:5][CH2:6][NH:7][CH2:8]1)([

c:9]1[cH:10][cH:11][cH:12][cH:13][cH:14]1)[c:15]1[cH:16][cH:17][cH:18][cH:19][cH:20]1>>[N:1]#[C:2][C:3]([C@@H:4]1[CH2:5][CH2:6][N:7]([CH2:22][CH2:23][c:24]2[cH:25][cH:26][c:27]3[O:28][CH2:29][CH2:30][c:31]3[cH:32]2)[CH2:8]1)([c:15]1[cH:16][cH:17][cH:18][cH:19][cH:20]1)[c:9]1[cH:10][cH:11][cH:12][cH:13][cH:14]1.[BrH:21]

Correctness of the mapping

MAPPET	YES
ReactionMap	NO
Marvin	YES
ChemDraw	YES
Indigo	YES

Reaction no 150

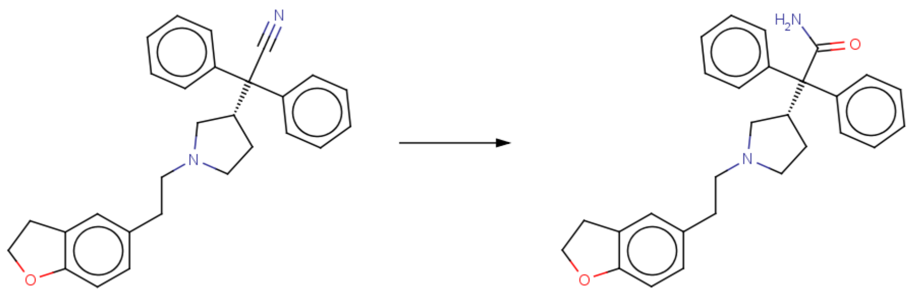

SMILES of the input:
N#CC([C@@H]1CCN(CCc2ccc3OCCc3c2)C1)(c1ccccc1)c1ccccc1>>NC(=O)C([C@@H]1CCN(CCc2ccc3OCCc3c2)C1)(c1ccccc1)c1ccccc1

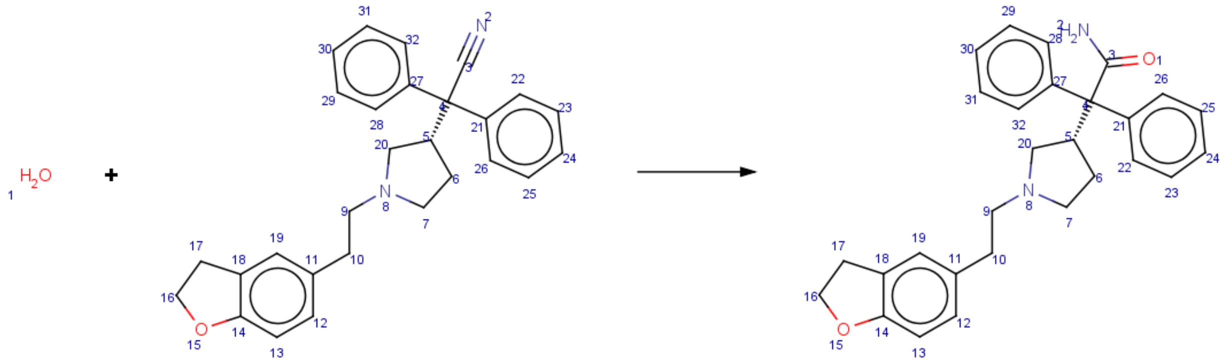

Correct mapped SMILES/SMARTS of the reaction:
[OH2:1].[N:2]#[C:3][C:4]([C@@H:5]1[CH2:6][CH2:7][N:8]([CH2:9][CH2:10][c:11]2[cH:12][cH:13][c:14]3[O:15][CH2:16][CH2:17][c:18]3[cH:19]2)[CH2:20]1)([c:21]1[cH:22][cH:23][cH:24][cH:25][cH:26]1)[c:27]1[cH:28][cH:29][cH:30][cH:31][cH:32]1>>[NH2:2][C:3](=[O:1])[C:4]([C@@H:5]1[CH2:6][CH2:7][N:8]([CH2:9][CH2:10][c:11]2[cH:12][cH:13][c:14]3[O:15][CH2:16][CH2:17][c:18]3[cH:19]2)[CH2:20]1)([c:27]1[cH:32][cH:31][cH:30][cH:29][cH:28]1)[c:21]1[cH:26][cH:25][cH:24][cH:23][cH:22]1

Correctness of the mapping

MAPPET	YES
ReactionMap	NO
Marvin	YES
ChemDraw	YES
Indigo	YES

Reaction no 151

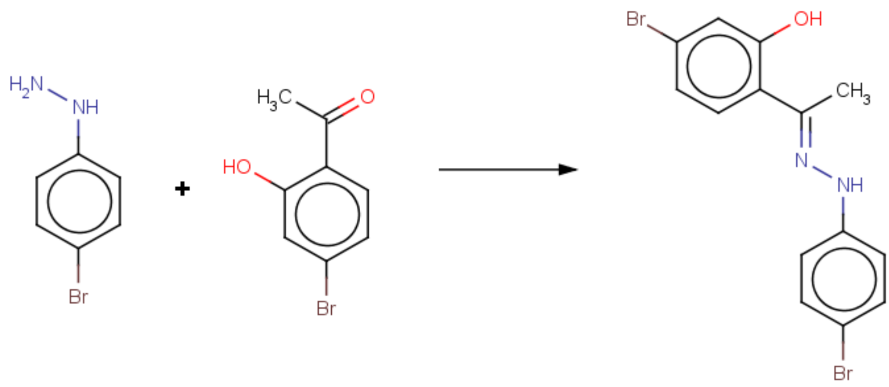

SMILES of the input:
NNc1ccc(Br)cc1.CC(=O)c1ccc(Br)cc1O>>C\C(=N/Nc1ccc(Br)cc1)c1ccc(Br)cc1O

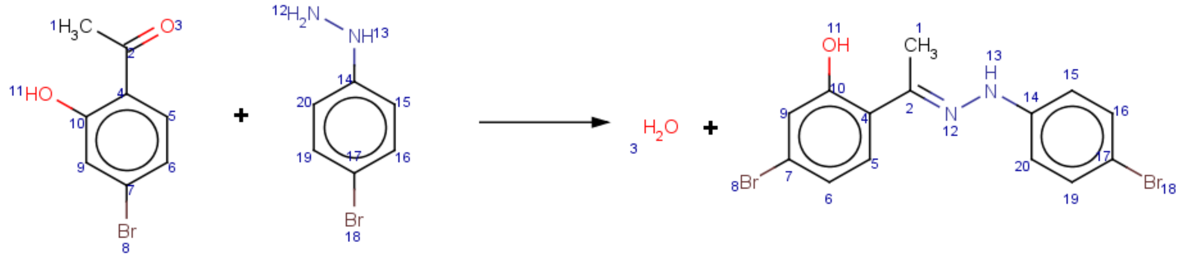

Correct mapped SMILES/SMARTS of the reaction:
[CH3:1][C:2](=[O:3])[c:4]1[cH:5][cH:6][c:7]([Br:8])[cH:9][c:10]1[OH:11].[
NH2:12][NH:13][c:14]1[cH:15][cH:16][c:17]([Br:18])[cH:19][cH:20]1>>[OH2:3
].[CH3:1]\[C:2](=[N:12]/[NH:13][c:14]1[cH:15][cH:16][c:17]([Br:18])[cH:19
][cH:20]1)[c:4]1[cH:5][cH:6][c:7]([Br:8])[cH:9][c:10]1[OH:11]

Correctness of the mapping

MAPPET	YES
ReactionMap	NO
Marvin	YES
ChemDraw	YES
Indigo	YES

Reaction no 152

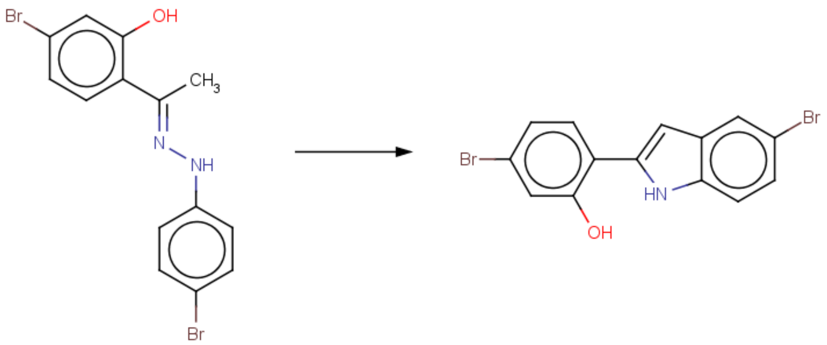

SMILES of the input:
C\C(=N/Nc1ccc(Br)cc1)c1ccc(Br)cc1O>>Oc1cc(Br)ccc1C1=Cc2cc(Br)ccc2N1

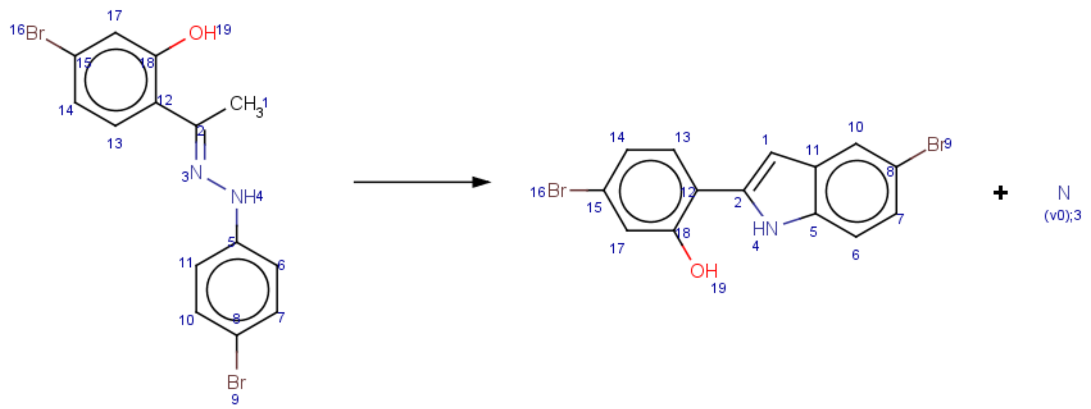

```
Correct mapped SMILES/SMARTS of the reaction:
[CH3:1]\[C:2](=[N:3]/[NH:4][c:5]1[cH:6][cH:7][c:8]([Br:9])[cH:10][cH:11]1
)[c:12]1[cH:13][cH:14][c:15]([Br:16])[cH:17][c:18]1[OH:19]>>[OH:19][c:18]
1[cH:17][c:15]([Br:16])[cH:14][cH:13][c:12]1[C:2]1=[CH:1][c:11]2[cH:10][c
:8]([Br:9])[cH:7][cH:6][c:5]2[NH:4]1.[N:3]

Correctness of the mapping
MAPPET          YES
ReactionMap     NO
Marvin          NO
ChemDraw        NO
Indigo          YES

Reaction no  153
```

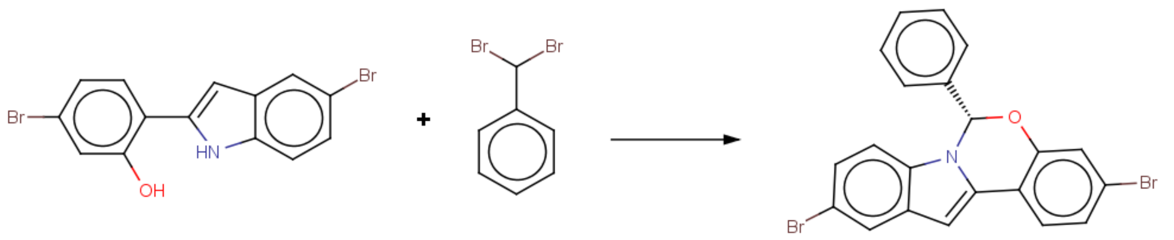

```
SMILES of the input:
Oc1cc(Br)ccc1C1=Cc2cc(Br)ccc2N1.BrC(Br)c1ccccc1>>Brc1ccc2N3[C@@H](Oc4cc(B
r)ccc4C3=Cc2c1)c1ccccc1
```

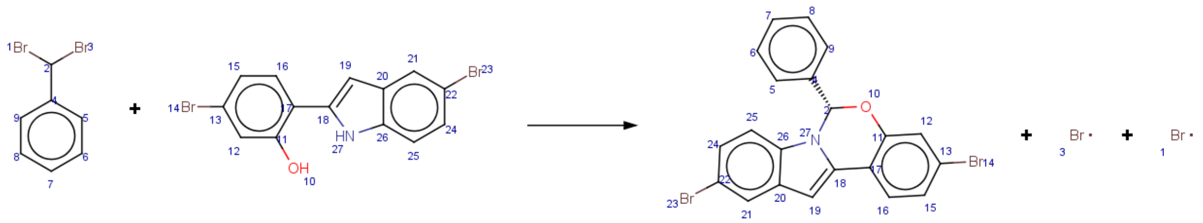

```
Correct mapped SMILES/SMARTS of the reaction:
[Br:1][CH:2]([Br:3])[c:4]1[cH:5][cH:6][cH:7][cH:8][cH:9]1.[OH:10][c:11]1[
cH:12][c:13]([Br:14])[cH:15][cH:16][c:17]1[C:18]1=[CH:19][c:20]2[cH:21][c
:22]([Br:23])[cH:24][cH:25][c:26]2[NH:27]1>>[Br:23][c:22]1[cH:24][cH:25][
c:26]2[N:27]3[C@@H:2]([O:10][c:11]4[cH:12][c:13]([Br:14])[cH:15][cH:16][c
:17]4[C:18]3=[CH:19][c:20]2[cH:21]1)[c:4]1[cH:5][cH:6][cH:7][cH:8][cH:9]1
.[Br:3].[Br:1]

Correctness of the mapping
MAPPET          YES
ReactionMap     NO
```

Marvin YES
ChemDraw YES
Indigo YES

Reaction no 154

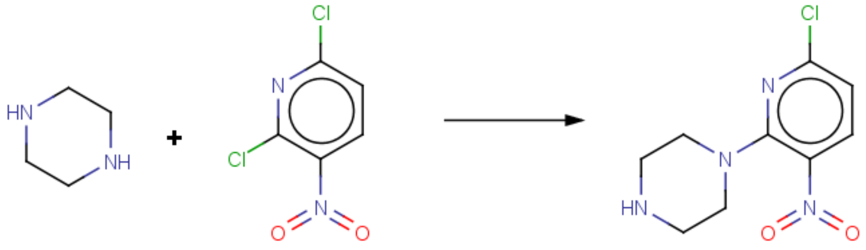

SMILES of the input:
C1CNCCN1.Clc1ccc(c(Cl)n1)N(=O)=O>>Clc1ccc(c(n1)N1CCNCC1)N(=O)=O

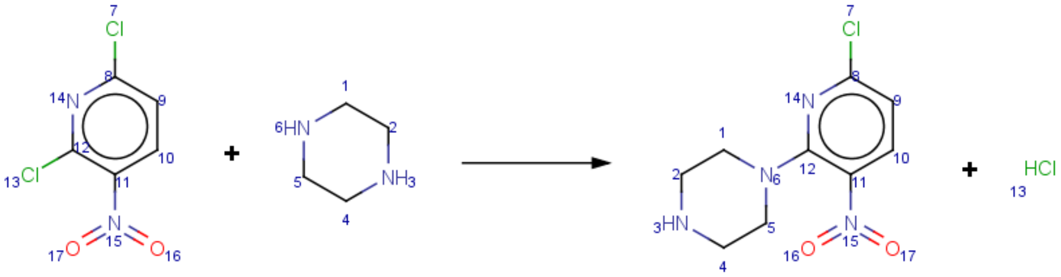

Correct mapped SMILES/SMARTS of the reaction:
[Cl:7][c:8]1[cH:9][cH:10][c:11]([c:12]([Cl:13])[n:14]1)[N:15](=[O:16])=[O:17].[CH2:1]1[CH2:2][NH:3][CH2:4][CH2:5][NH:6]1>>[Cl:7][c:8]1[cH:9][cH:10][c:11]([c:12]([n:14]1)[N:6]1[CH2:5][CH2:4][NH:3][CH2:2][CH2:1]1)[N:15](=[O:16])=[O:17].[ClH:13]

Correctness of the mapping
MAPPET YES
ReactionMap NO
Marvin YES
ChemDraw YES
Indigo YES

Reaction no 155

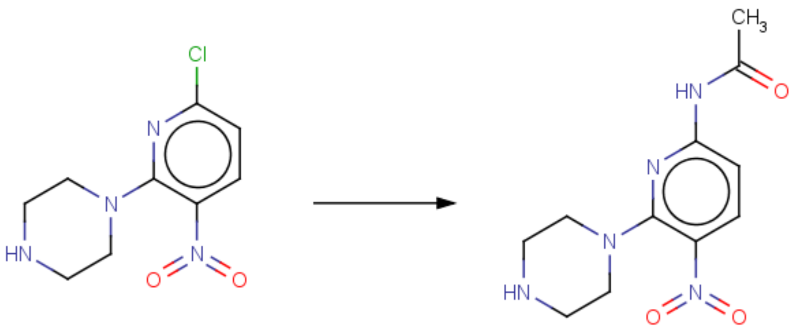

SMILES of the input:
Clc1ccc(c(n1)N1CCNCC1)N(=O)=O>>CC(=O)Nc1ccc(c(n1)N1CCNCC1)N(=O)=O

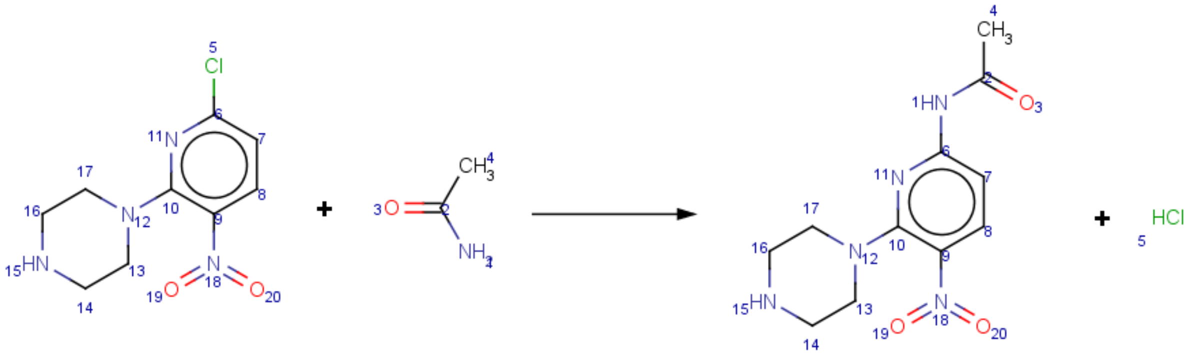

Correct mapped SMILES/SMARTS of the reaction:
[Cl:5][c:6]1[cH:7][cH:8][c:9]([c:10]([n:11]1)[N:12]1[CH2:13][CH2:14][NH:15][CH2:16][CH2:17]1)[N:18](=[O:19])=[O:20].[CH3:4][C:2]([NH2:1])=[O:3]>>[CH3:4][C:2](=[O:3])[NH:1][c:6]1[cH:7][cH:8][c:9]([c:10]([n:11]1)[N:12]1[CH2:13][CH2:14][NH:15][CH2:16][CH2:17]1)[N:18](=[O:19])=[O:20].[ClH:5]

Correctness of the mapping

MAPPET	YES
ReactionMap	NO
Marvin	YES
ChemDraw	YES
Indigo	YES

Reaction no 156

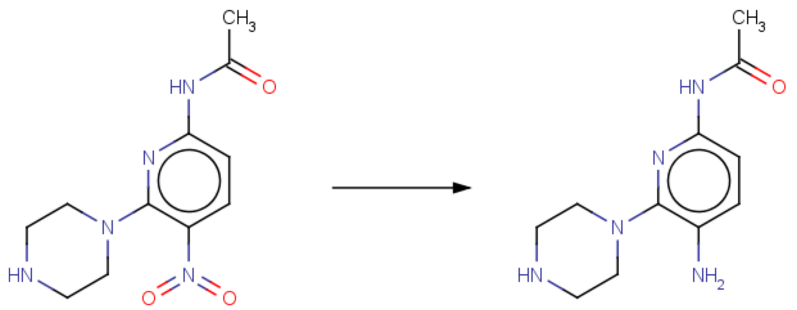

SMILES of the input:
CC(=O)Nc1ccc(c(n1)N1CCNCC1)N(=O)=O>>CC(=O)Nc1ccc(N)c(n1)N1CCNCC1

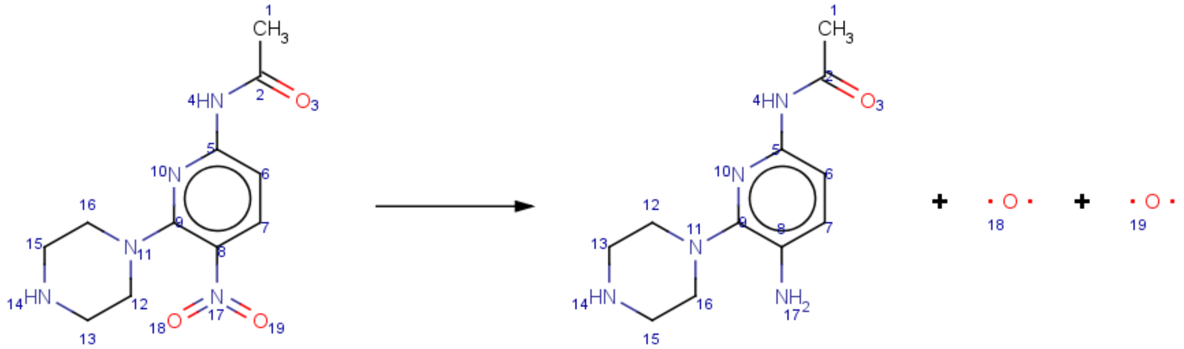

Correct mapped SMILES/SMARTS of the reaction:
[CH3:1][C:2](=[O:3])[NH:4][c:5]1[cH:6][cH:7][c:8]([c:9]([n:10]1)[N:11]1[CH2:12][CH2:13][NH:14][CH2:15][CH2:16]1)[N:17](=[O:18])=[O:19]>>[CH3:1][C:2](=[O:3])[NH:4][c:5]1[cH:6][cH:7][c:8]([NH2:17])[c:9]([n:10]1)[N:11]1[CH2:16][CH2:15][NH:14][CH2:13][CH2:12]1.[O:18].[O:19]

Correctness of the mapping

MAPPET	YES
ReactionMap	NO
Marvin	YES
ChemDraw	YES
Indigo	YES

Reaction no 157

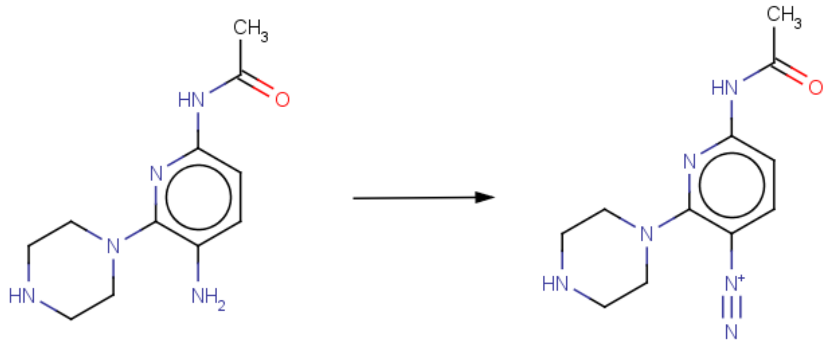

SMILES of the input:
CC(=O)Nc1ccc(N)c(n1)N1CCNCC1>>CC(=O)Nc1ccc([N+]#N)c(n1)N1CCNCC1

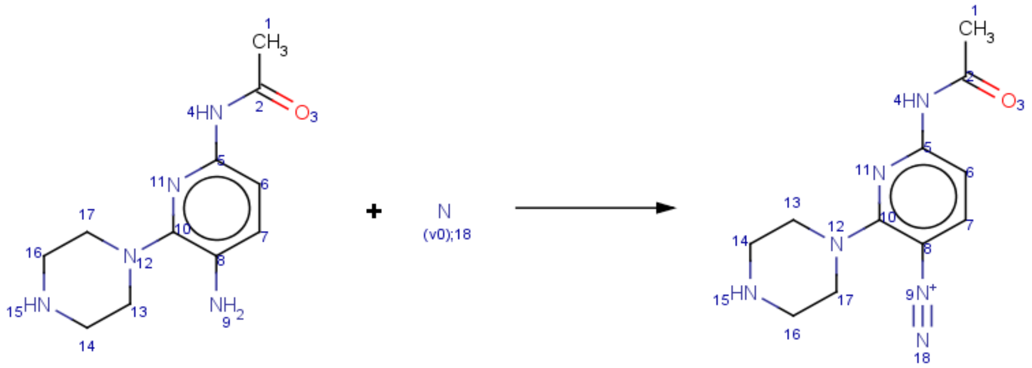

Correct mapped SMILES/SMARTS of the reaction:
[CH3:1][C:2](=[O:3])[NH:4][c:5]1[cH:6][cH:7][c:8]([NH2:9])[c:10]([n:11]1)[N:12]1[CH2:13][CH2:14][NH:15][CH2:16][CH2:17]1.[N:18]>>[CH3:1][C:2](=[O:3])[NH:4][c:5]1[cH:6][cH:7][c:8]([N+:9]#[N:18])[c:10]([n:11]1)[N:12]1[CH2:17][CH2:16][NH:15][CH2:14][CH2:13]1

Correctness of the mapping

MAPPET	YES
ReactionMap	NO
Marvin	YES
ChemDraw	YES
Indigo	NO

Reaction no 158

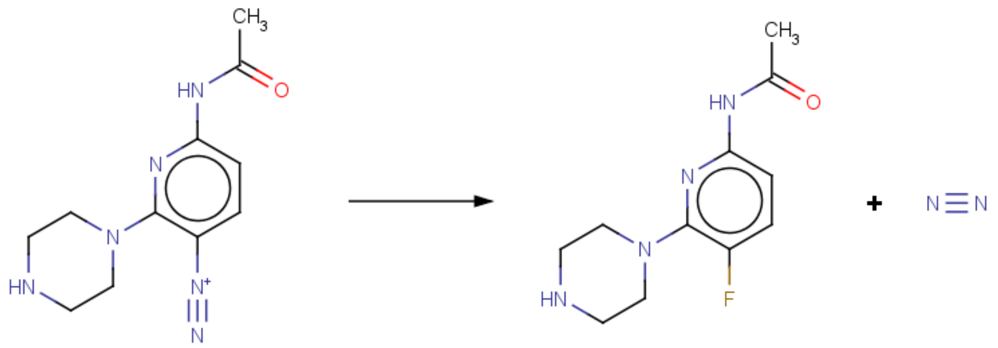

SMILES of the input:
CC(=O)Nc1ccc([N+]#N)c(n1)N1CCNCC1>>CC(=O)Nc1ccc(F)c(n1)N1CCNCC1.N#N

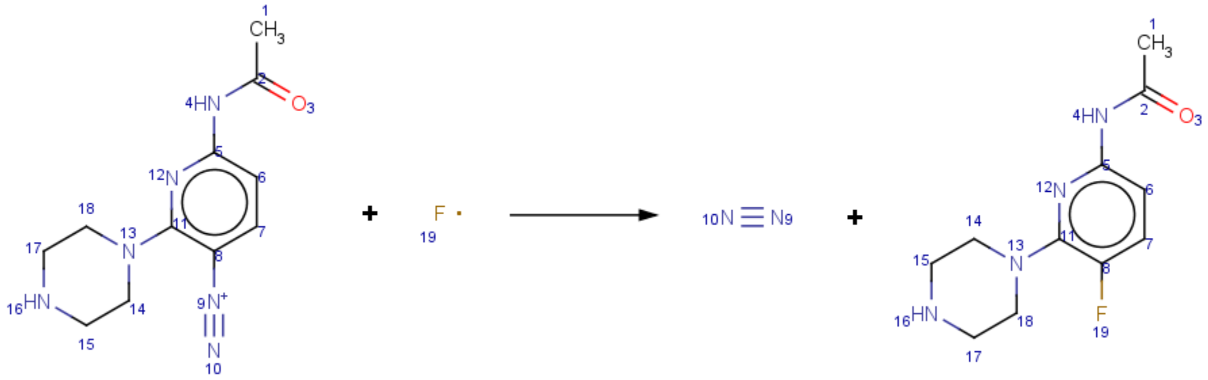

Correct mapped SMILES/SMARTS of the reaction:
[CH3:1][C:2](=[O:3])[NH:4][c:5]1[cH:6][cH:7][c:8]([N+:9]#[N:10])[c:11]([n:12]1)[N:13]1[CH2:14][CH2:15][NH:16][CH2:17][CH2:18]1.[F:19]>>[N:9]#[N:10].[CH3:1][C:2](=[O:3])[NH:4][c:5]1[cH:6][cH:7][c:8]([F:19])[c:11]([n:12]1)[N:13]1[CH2:18][CH2:17][NH:16][CH2:15][CH2:14]1

Correctness of the mapping

MAPPET	YES
ReactionMap	NO
Marvin	YES
ChemDraw	YES
Indigo	YES

Reaction no 159

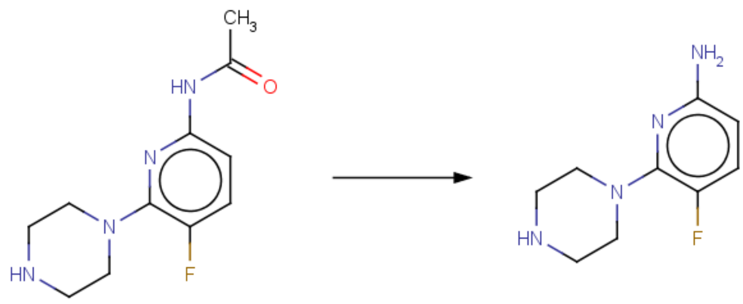

SMILES of the input:
CC(=O)Nc1ccc(F)c(n1)N1CCNCC1>>Nc1ccc(F)c(n1)N1CCNCC1

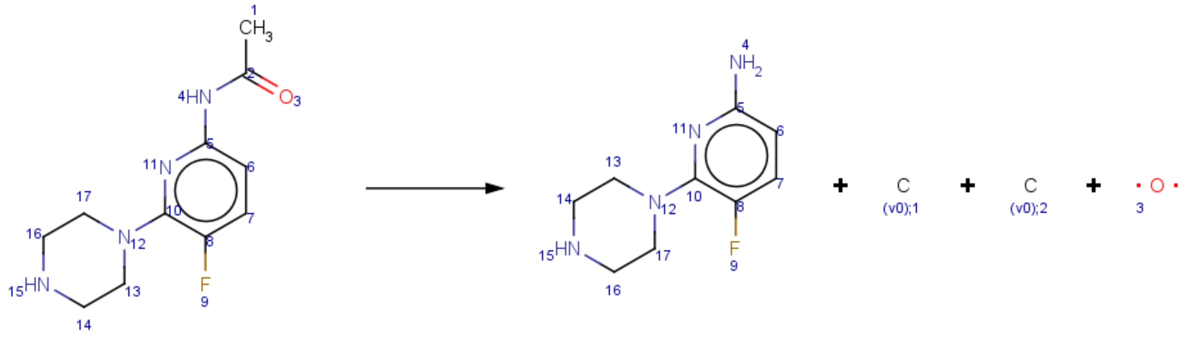

Correct mapped SMILES/SMARTS of the reaction:
[CH3:1][C:2](=[O:3])[NH:4][c:5]1[cH:6][cH:7][c:8]([F:9])[c:10]([n:11]1)[N:12]1[CH2:13][CH2:14][NH:15][CH2:16][CH2:17]1>>[NH2:4][c:5]1[cH:6][cH:7][c:8]([F:9])[c:10]([n:11]1)[N:12]1[CH2:17][CH2:16][NH:15][CH2:14][CH2:13]1.[C:1].[C:2].[O:3]

Correctness of the mapping

MAPPET	YES
ReactionMap	NO
Marvin	YES
ChemDraw	YES
Indigo	YES

Reaction no 160

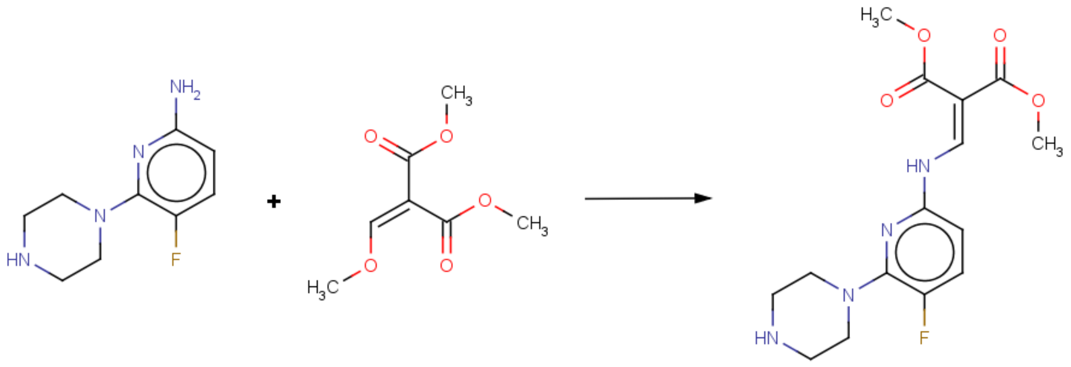

SMILES of the input:
Nc1ccc(F)c(n1)N1CCNCC1.COC=C(C(=O)OC)C(=O)OC>>COC(=O)C(=CNc1ccc(F)c(n1)N1CCNCC1)C(=O)OC

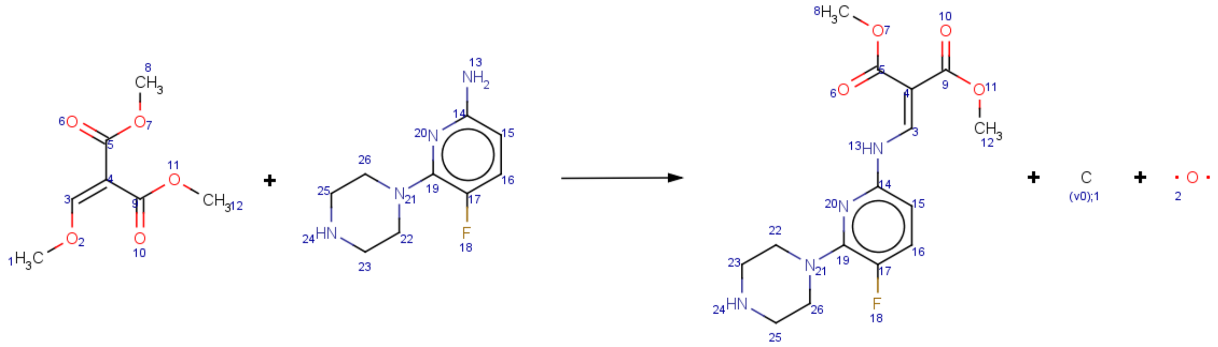

Correct mapped SMILES/SMARTS of the reaction:
[CH3:1][O:2][CH:3]=[C:4]([C:5](=[O:6])[O:7][CH3:8])[C:9](=[O:10])[O:11][CH3:12].[NH2:13][c:14]1[cH:15][cH:16][c:17]([F:18])[c:19]([n:20]1)[N:21]1[CH2:22][CH2:23][NH:24][CH2:25][CH2:26]1>>[CH3:8][O:7][C:5](=[O:6])[C:4](=[CH:3][NH:13][c:14]1[cH:15][cH:16][c:17]([F:18])[c:19]([n:20]1)[N:21]1[CH

2:26][CH2:25][NH:24][CH2:23][CH2:22]1)[C:9](=[O:10])[O:11][CH3:12].[C:1].[O:2]

Correctness of the mapping
MAPPET YES
ReactionMap NO
Marvin YES
ChemDraw YES
Indigo YES

Reaction no 161

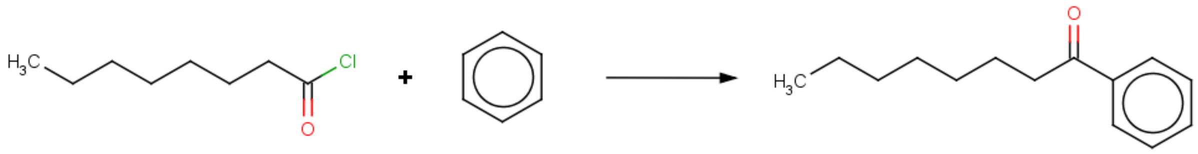

SMILES of the input:
CCCCCCCC(Cl)=O.c1ccccc1>>CCCCCCCC(=O)c1ccccc1

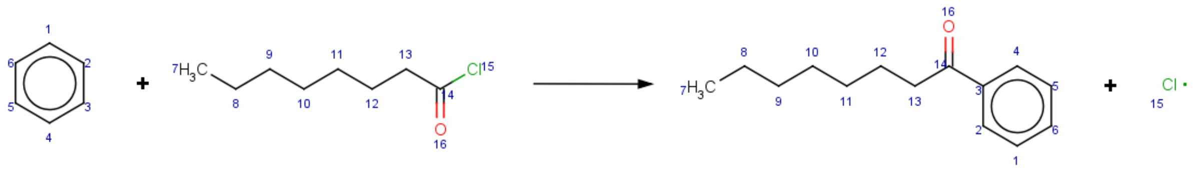

Correct mapped SMILES/SMARTS of the reaction:
[cH:1]1[cH:2][cH:3][cH:4][cH:5][cH:6]1.[CH3:7][CH2:8][CH2:9][CH2:10][CH2:11][CH2:12][CH2:13][C:14]([Cl:15])=[O:16]>>[CH3:7][CH2:8][CH2:9][CH2:10][CH2:11][CH2:12][CH2:13][C:14](=[O:16])[c:3]1[cH:4][cH:5][cH:6][cH:1][cH:2]1.[Cl:15]

Correctness of the mapping
MAPPET YES
ReactionMap NO
Marvin YES
ChemDraw YES
Indigo YES

Reaction no 162

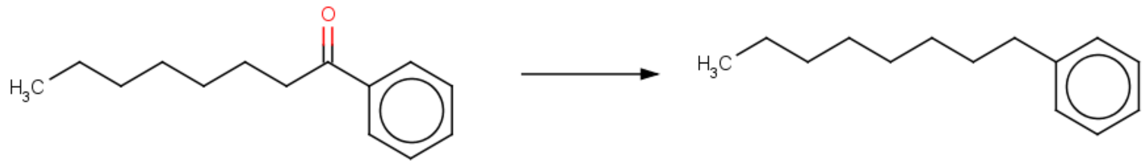

SMILES of the input:
CCCCCCCC(=O)c1ccccc1>>CCCCCCCCc1ccccc1

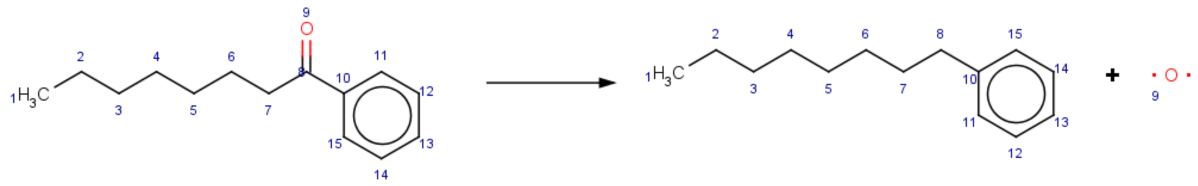

Correct mapped SMILES/SMARTS of the reaction:
[CH3:1][CH2:2][CH2:3][CH2:4][CH2:5][CH2:6][CH2:7][C:8](=[O:9])[c:10]1[cH:11][cH:12][cH:13][cH:14][cH:15]1>>[CH3:1][CH2:2][CH2:3][CH2:4][CH2:5][CH2:6][CH2:7][CH2:8][c:10]1[cH:15][cH:14][cH:13][cH:12][cH:11]1.[O:9]

Correctness of the mapping

MAPPET	YES
ReactionMap	NO
Marvin	YES
ChemDraw	YES
Indigo	YES

Reaction no 163

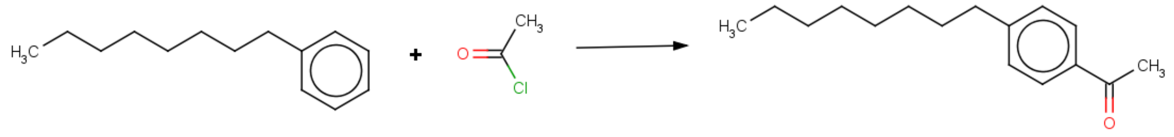

SMILES of the input:
CCCCCCCCc1ccccc1.CC(Cl)=O>>CCCCCCCCc1ccc(cc1)C(C)=O

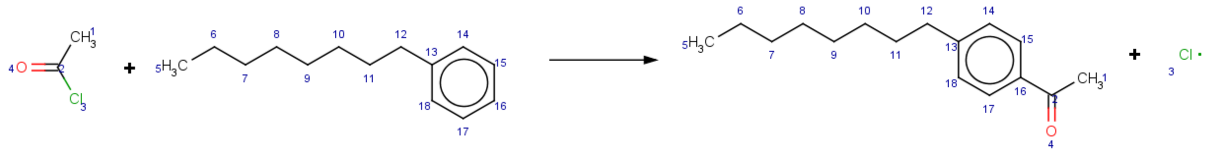

Correct mapped SMILES/SMARTS of the reaction:
[CH3:1][C:2]([Cl:3])=[O:4].[CH3:5][CH2:6][CH2:7][CH2:8][CH2:9][CH2:10][CH2:11][CH2:12][c:13]1[cH:14][cH:15][cH:16][cH:17][cH:18]1>>[CH3:5][CH2:6][CH2:7][CH2:8][CH2:9][CH2:10][CH2:11][CH2:12][c:13]1[cH:14][cH:15][c:16]([cH:17][cH:18]1)[C:2]([CH3:1])=[O:4].[Cl:3]

Correctness of the mapping

MAPPET	YES
ReactionMap	NO
Marvin	YES
ChemDraw	YES
Indigo	YES

Reaction no 164

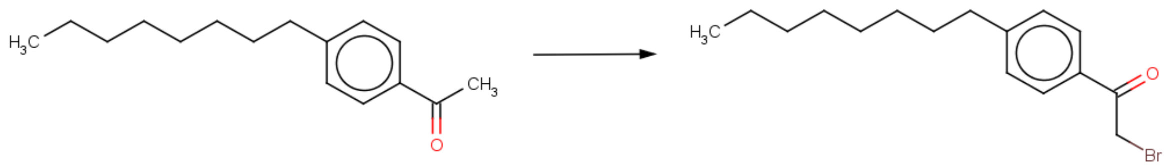

SMILES of the input:
CCCCCCCCc1ccc(cc1)C(C)=O>>CCCCCCCCc1ccc(cc1)C(=O)CBr

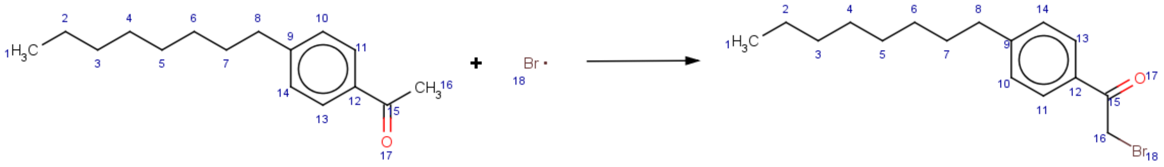

Correct mapped SMILES/SMARTS of the reaction:
[CH3:1][CH2:2][CH2:3][CH2:4][CH2:5][CH2:6][CH2:7][CH2:8][c:9]1[cH:10][cH:
11][c:12]([cH:13][cH:14]1)[C:15]([CH3:16])=[O:17].[Br:18]>>[CH3:1][CH2:2]
[CH2:3][CH2:4][CH2:5][CH2:6][CH2:7][CH2:8][c:9]1[cH:14][cH:13][c:12]([cH:
11][cH:10]1)[C:15](=[O:17])[CH2:16][Br:18]

Correctness of the mapping

MAPPET	YES
ReactionMap	NO
Marvin	YES
ChemDraw	YES
Indigo	YES

Reaction no 165

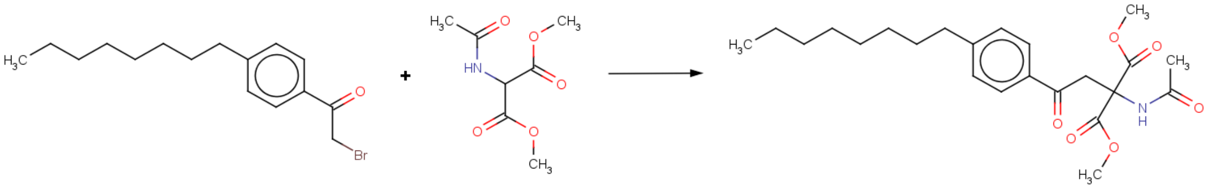

SMILES of the input:
CCCCCCCCc1ccc(cc1)C(=O)CBr.COC(=O)C(NC(C)=O)C(=O)OC>>CCCCCCCCc1ccc(cc1)C(
=O)CC(NC(C)=O)(C(=O)OC)C(=O)OC

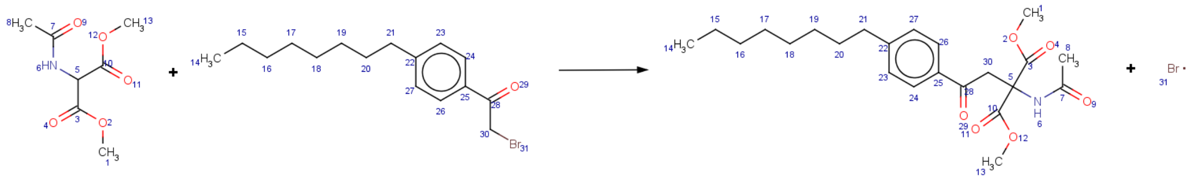

Correct mapped SMILES/SMARTS of the reaction:
[CH3:1][O:2][C:3](=[O:4])[CH:5]([NH:6][C:7]([CH3:8])=[O:9])[C:10](=[O:11]
)[O:12][CH3:13].[CH3:14][CH2:15][CH2:16][CH2:17][CH2:18][CH2:19][CH2:20][
CH2:21][c:22]1[cH:23][cH:24][c:25]([cH:26][cH:27]1)[C:28](=[O:29])[CH2:30
][Br:31]>>[CH3:14][CH2:15][CH2:16][CH2:17][CH2:18][CH2:19][CH2:20][CH2:21
][c:22]1[cH:27][cH:26][c:25]([cH:24][cH:23]1)[C:28](=[O:29])[CH2:30][C:5]
([NH:6][C:7]([CH3:8])=[O:9])([C:10](=[O:11])[O:12][CH3:13])[C:3](=[O:4])[
O:2][CH3:1].[Br:31]

Correctness of the mapping

MAPPET	YES
ReactionMap	NO
Marvin	YES
ChemDraw	YES
Indigo	YES

Reaction no 166

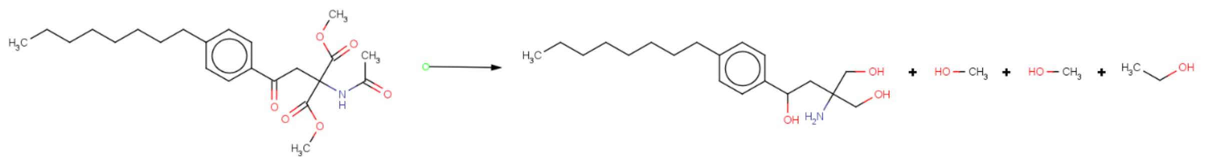

SMILES of the input:
CCCCCCCCc1ccc(cc1)C(=O)CC(NC(C)=O)(C(=O)OC)C(=O)OC>>CCCCCCCCc1ccc(cc1)C(O)CC(N)(CO)CO.CO.CO.CCO

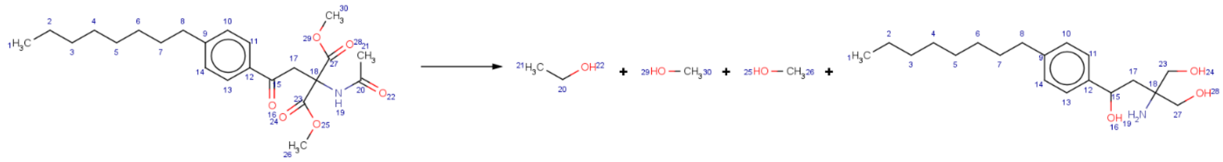

Correct mapped SMILES/SMARTS of the reaction:
[CH3:1][CH2:2][CH2:3][CH2:4][CH2:5][CH2:6][CH2:7][CH2:8][c:9]1[cH:10][cH:11][c:12]([cH:13][cH:14]1)[C:15](=[O:16])[CH2:17][C:18]([NH:19][C:20]([CH3:21])=[O:22])([C:23](=[O:24])[O:25][CH3:26])[C:27](=[O:28])[O:29][CH3:30]>>[CH3:21][CH2:20][OH:22].[CH3:30][OH:29].[CH3:26][OH:25].[CH3:1][CH2:2][CH2:3][CH2:4][CH2:5][CH2:6][CH2:7][CH2:8][c:9]1[cH:10][cH:11][c:12]([cH:13][cH:14]1)[CH:15]([OH:16])[CH2:17][C:18]([NH2:19])([CH2:27][OH:28])[CH2:23][OH:24]

Correctness of the mapping

MAPPET	YES
ReactionMap	YES
Marvin	YES
ChemDraw	NO
Indigo	NO

Reaction no 167

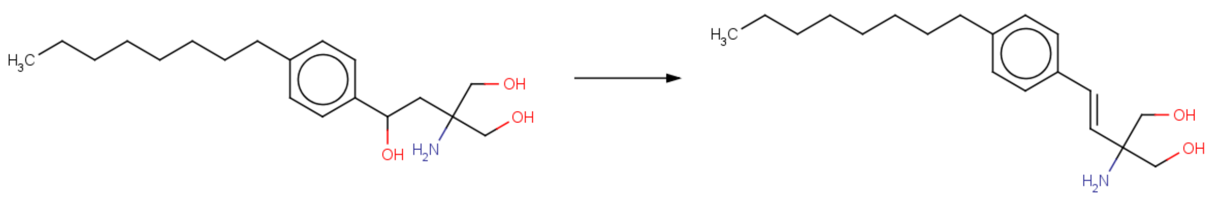

SMILES of the input:
CCCCCCCCc1ccc(cc1)C(O)CC(N)(CO)CO>>CCCCCCCCc1ccc(\C=C\C(N)(CO)CO)cc1

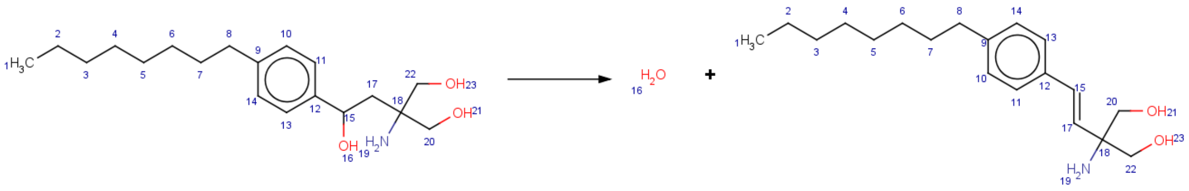

Correct mapped SMILES/SMARTS of the reaction:
[CH3:1][CH2:2][CH2:3][CH2:4][CH2:5][CH2:6][CH2:7][CH2:8][c:9]1[cH:10][cH:
11][c:12]([cH:13][cH:14]1)[CH:15]([OH:16])[CH2:17][C:18]([NH2:19])([CH2:2
0][OH:21])[CH2:22][OH:23]>>[OH2:16].[CH3:1][CH2:2][CH2:3][CH2:4][CH2:5][C
H2:6][CH2:7][CH2:8][c:9]1[cH:14][cH:13][c:12](\[CH:15]=[CH:17]\[C:18]([NH
2:19])([CH2:22][OH:23])[CH2:20][OH:21])[cH:11][cH:10]1

Correctness of the mapping

MAPPET	YES
ReactionMap	NO
Marvin	YES
ChemDraw	YES
Indigo	YES

Reaction no 168

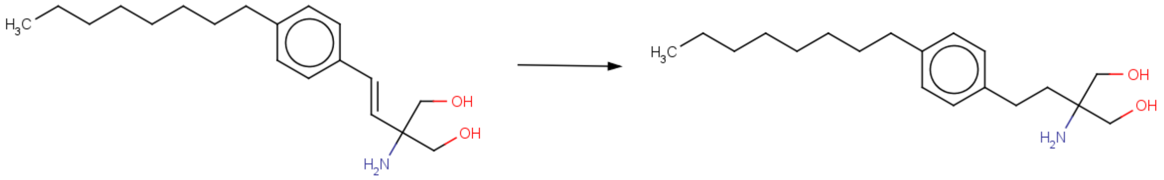

SMILES of the input:
CCCCCCCCc1ccc(\C=C\C(N)(CO)CO)cc1>>CCCCCCCCc1ccc(CCC(N)(CO)CO)cc1

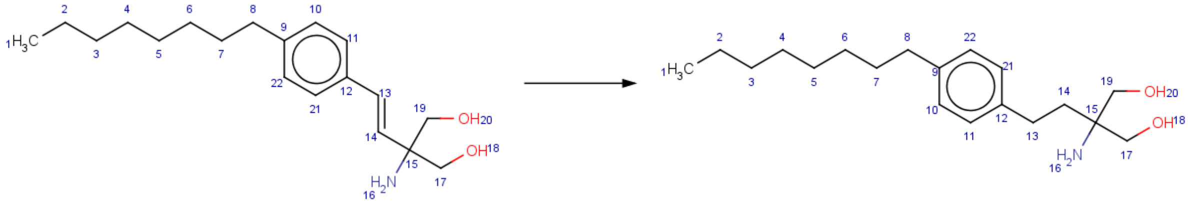

Correct mapped SMILES/SMARTS of the reaction:
[CH3:1][CH2:2][CH2:3][CH2:4][CH2:5][CH2:6][CH2:7][CH2:8][c:9]1[cH:10][cH:
11][c:12](\[CH:13]=[CH:14]\[C:15]([NH2:16])([CH2:17][OH:18])[CH2:19][OH:2
0])[cH:21][cH:22]1>>[CH3:1][CH2:2][CH2:3][CH2:4][CH2:5][CH2:6][CH2:7][CH2
:8][c:9]1[cH:22][cH:21][c:12]([CH2:13][CH2:14][C:15]([NH2:16])([CH2:17][O
H:18])[CH2:19][OH:20])[cH:11][cH:10]1

Correctness of the mapping

MAPPET	YES
ReactionMap	YES
Marvin	YES
ChemDraw	YES
Indigo	YES

Reaction no 169

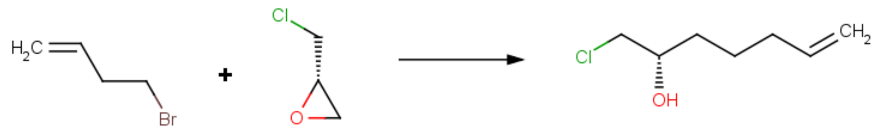

SMILES of the input:
BrCCC=C.ClC[C@@H]1CO1>>O[C@H](CCl)CCCC=C

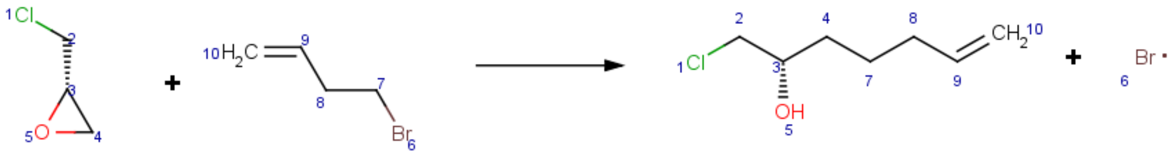

Correct mapped SMILES/SMARTS of the reaction:
[Cl:1][CH2:2][C@@H:3]1[CH2:4][O:5]1.[Br:6][CH2:7][CH2:8][CH:9]=[CH2:10]>>
[OH:5][C@H:3]([CH2:2][Cl:1])[CH2:4][CH2:7][CH2:8][CH:9]=[CH2:10].[Br:6]

Correctness of the mapping

MAPPET	YES
ReactionMap	NO
Marvin	YES
ChemDraw	YES
Indigo	YES

Reaction no 170

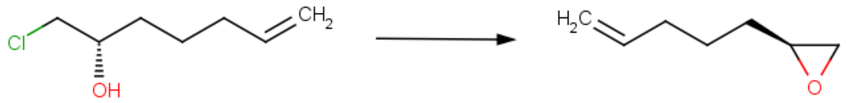

SMILES of the input:
O[C@H](CCl)CCCC=C>>C=CCCC[C@H]1CO1

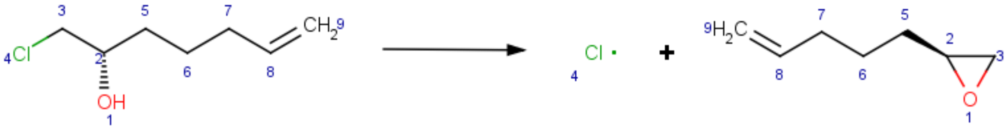

Correct mapped SMILES/SMARTS of the reaction:
[OH:1][C@H:2]([CH2:3][Cl:4])[CH2:5][CH2:6][CH2:7][CH:8]=[CH2:9]>>[Cl:4].[
CH2:9]=[CH:8][CH2:7][CH2:6][CH2:5][C@H:2]1[CH2:3][O:1]1

Correctness of the mapping

MAPPET	YES
ReactionMap	NO
Marvin	YES
ChemDraw	YES
Indigo	YES

Reaction no 171

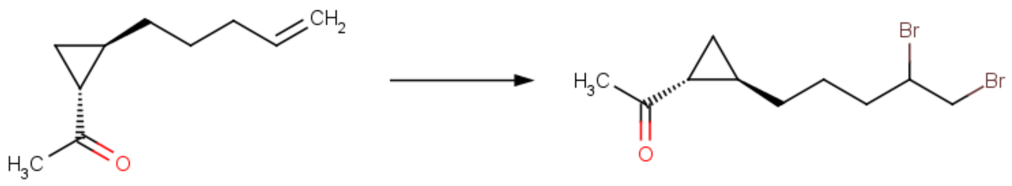

SMILES of the input:
CC(=O)[C@@H]1C[C@H]1CCCC=C>>CC(=O)[C@@H]1C[C@H]1CCCC(Br)CBr

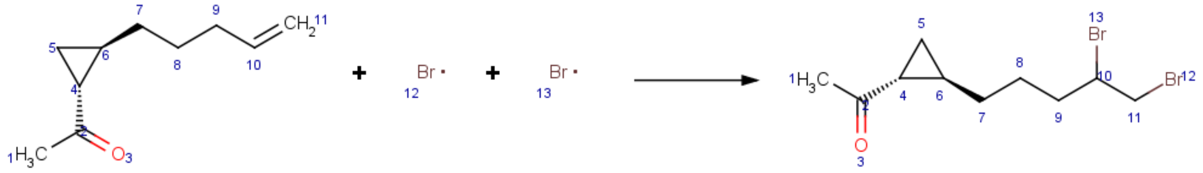

Correct mapped SMILES/SMARTS of the reaction:
[CH3:1][C:2](=[O:3])[C@@H:4]1[CH2:5][C@H:6]1[CH2:7][CH2:8][CH2:9][CH:10]=[CH2:11].[Br:12].[Br:13]>>[CH3:1][C:2](=[O:3])[C@@H:4]1[CH2:5][C@H:6]1[CH2:7][CH2:8][CH2:9][CH:10]([Br:13])[CH2:11][Br:12]

Correctness of the mapping

MAPPET	YES
ReactionMap	NO
Marvin	YES
ChemDraw	YES
Indigo	YES

Reaction no 172

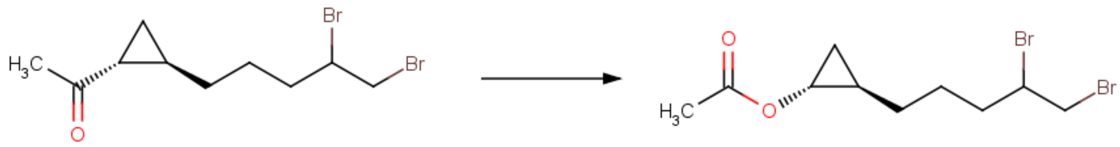

SMILES of the input:
CC(=O)[C@@H]1C[C@H]1CCCC(Br)CBr>>CC(=O)O[C@@H]1C[C@H]1CCCC(Br)CBr

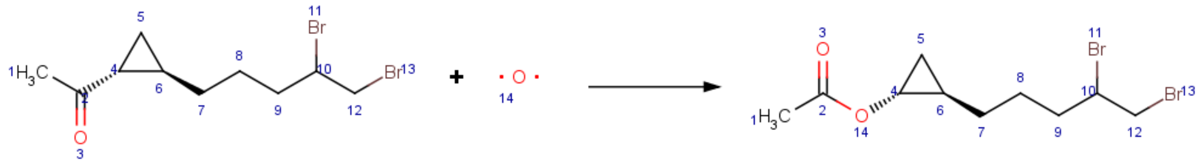

Correct mapped SMILES/SMARTS of the reaction:
[CH3:1][C:2](=[O:3])[C@@H:4]1[CH2:5][C@H:6]1[CH2:7][CH2:8][CH2:9][CH:10]([Br:11])[CH2:12][Br:13].[O:14]>>[CH3:1][C:2](=[O:3])[O:14][C@@H:4]1[CH2:5][C@H:6]1[CH2:7][CH2:8][CH2:9][CH:10]([Br:11])[CH2:12][Br:13]

Correctness of the mapping

MAPPET	YES
ReactionMap	NO
Marvin	YES
ChemDraw	YES
Indigo	YES

Reaction no 173

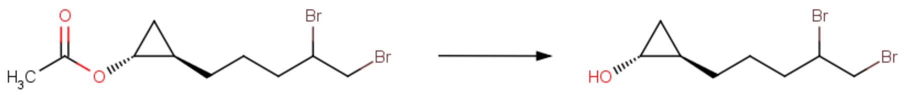

SMILES of the input:
CC(=O)O[C@@H]1C[C@H]1CCCC(Br)CBr>>O[C@@H]1C[C@H]1CCCC(Br)CBr

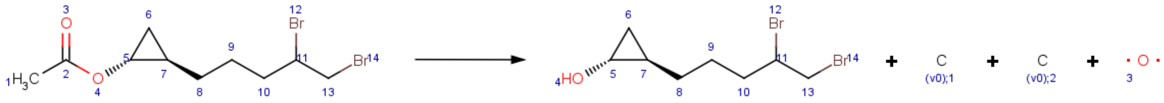

Correct mapped SMILES/SMARTS of the reaction:
[CH3:1][C:2](=[O:3])[O:4][C@@H:5]1[CH2:6][C@H:7]1[CH2:8][CH2:9][CH2:10][CH:11]([Br:12])[CH2:13][Br:14]>>[OH:4][C@@H:5]1[CH2:6][C@H:7]1[CH2:8][CH2:9][CH2:10][CH:11]([Br:12])[CH2:13][Br:14].[C:1].[C:2].[O:3]

Correctness of the mapping

MAPPET	YES
ReactionMap	NO
Marvin	YES
ChemDraw	YES
Indigo	YES

Reaction no 174

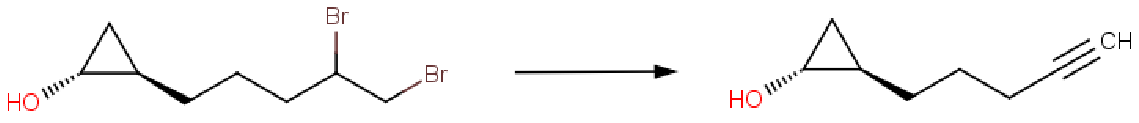

SMILES of the input:
O[C@@H]1C[C@H]1CCCC(Br)CBr>>O[C@@H]1C[C@H]1CCCC#C

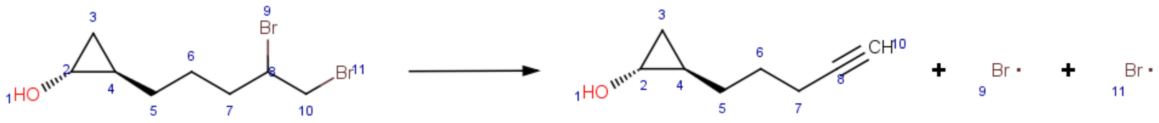

Correct mapped SMILES/SMARTS of the reaction:
[OH:1][C@@H:2]1[CH2:3][C@H:4]1[CH2:5][CH2:6][CH2:7][CH:8]([Br:9])[CH2:10][Br:11]>>[OH:1][C@@H:2]1[CH2:3][C@H:4]1[CH2:5][CH2:6][CH2:7][C:8]#[CH:10].[Br:9].[Br:11]

Correctness of the mapping

MAPPET	YES
ReactionMap	NO
Marvin	YES
ChemDraw	YES
Indigo	YES

Reaction no 175

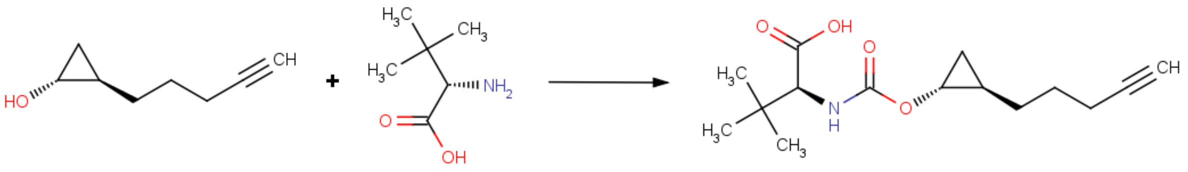

SMILES of the input:
O[C@@H]1C[C@H]1CCCC#C.CC(C)(C)[C@H](N)C(O)=O>>CC(C)(C)[C@H](NC(=O)O[C@@H]
1C[C@H]1CCCC#C)C(O)=O

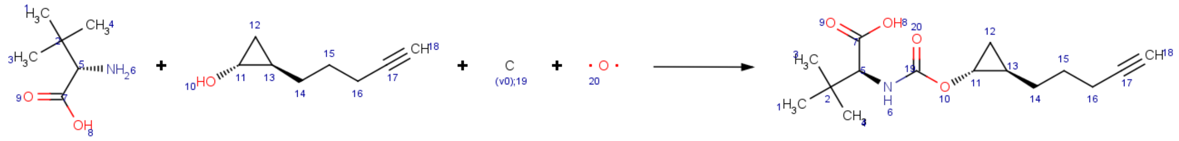

Correct mapped SMILES/SMARTS of the reaction:
[CH3:1][C:2]([CH3:3])([CH3:4])[C@H:5]([NH2:6])[C:7]([OH:8])=[O:9].[OH:10]
[C@@H:11]1[CH2:12][C@H:13]1[CH2:14][CH2:15][CH2:16][C:17]#[CH:18].[C:19].
[O:20]>>[CH3:1][C:2]([CH3:4])([CH3:3])[C@H:5]([NH:6][C:19](=[O:20])[O:10]
[C@@H:11]1[CH2:12][C@H:13]1[CH2:14][CH2:15][CH2:16][C:17]#[CH:18])[C:7]([
OH:8])=[O:9]

Correctness of the mapping

MAPPET	YES
ReactionMap	NO
Marvin	YES
ChemDraw	YES
Indigo	YES

Reaction no 176

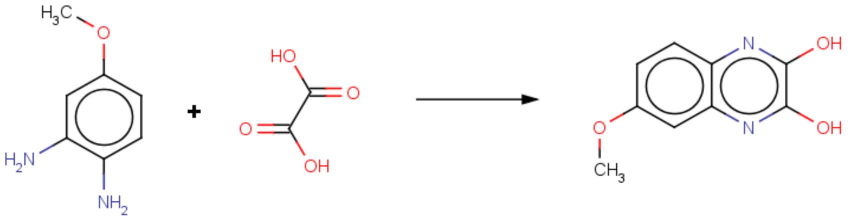

SMILES of the input:
COc1ccc(N)c(N)c1.OC(=O)C(O)=O>>COc1ccc2nc(O)c(O)nc2c1

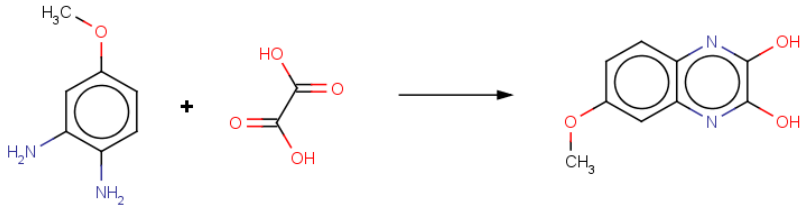

Correct mapped SMILES/SMARTS of the reaction:
[OH:1][C:2](=[O:3])[C:4]([OH:5])=[O:6].[CH3:7][O:8][c:9]1[cH:10][cH:11][c
:12]([NH2:13])[c:14]([NH2:15])[cH:16]1>>[CH3:7][O:8][c:9]1[cH:10][cH:11][
c:12]2[n:13][c:4]([OH:5])[c:2]([OH:1])[n:15][c:14]2[cH:16]1.[O:6].[O:3]

Correctness of the mapping

MAPPET	YES
ReactionMap	NO

Marvin YES
ChemDraw YES
Indigo YES

Reaction no 177

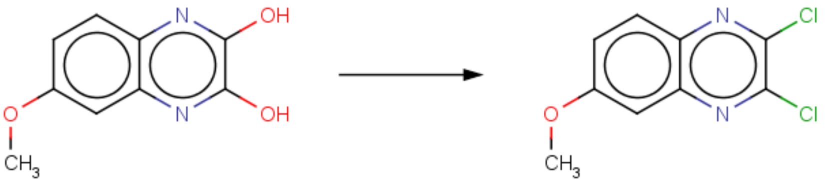

SMILES of the input:
COc1ccc2nc(O)c(O)nc2c1>>COc1ccc2nc(Cl)c(Cl)nc2c1

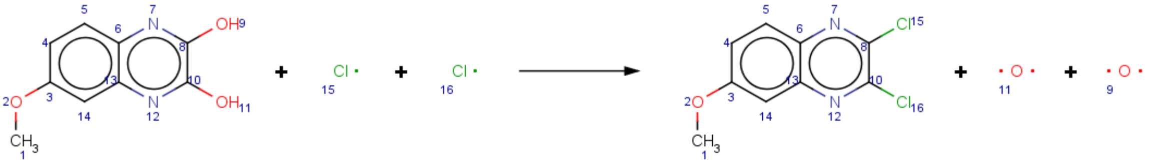

Correct mapped SMILES/SMARTS of the reaction:
[CH3:1][O:2][c:3]1[cH:4][cH:5][c:6]2[n:7][c:8]([OH:9])[c:10]([OH:11])[n:12][c:13]2[cH:14]1.[Cl:15].[Cl:16]>>[CH3:1][O:2][c:3]1[cH:4][cH:5][c:6]2[n:7][c:8]([Cl:15])[c:10]([Cl:16])[n:12][c:13]2[cH:14]1.[O:11].[O:9]

Correctness of the mapping
MAPPET YES
ReactionMap NO
Marvin YES
ChemDraw YES
Indigo YES

Reaction no 178

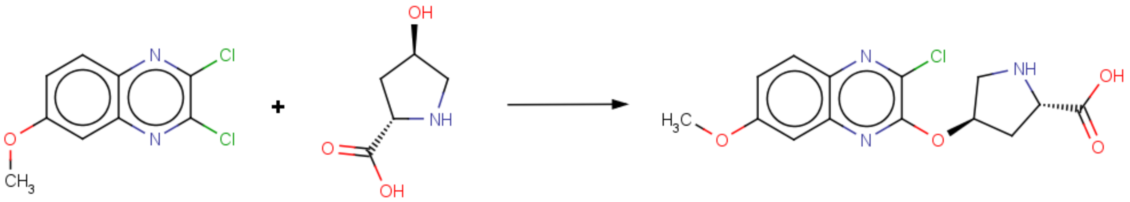

SMILES of the input:
COc1ccc2nc(Cl)c(Cl)nc2c1.O[C@H]1CN[C@@H](C1)C(O)=O>>COc1ccc2nc(Cl)c(O[C@H]3CN[C@@H](C3)C(O)=O)nc2c1

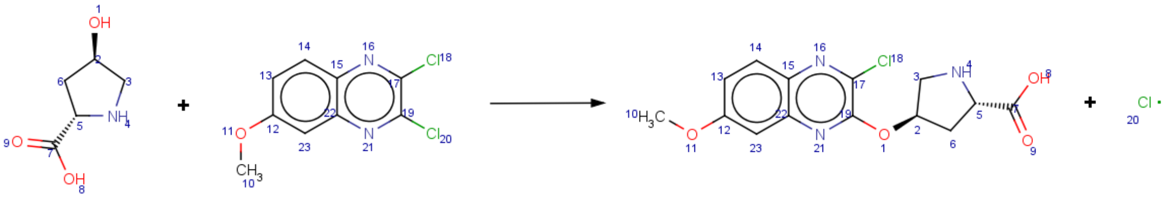

Correct mapped SMILES/SMARTS of the reaction:
[OH:1][C@H:2]1[CH2:3][NH:4][C@@H:5]([CH2:6]1)[C:7]([OH:8])=[O:9].[CH3:10][O:11][c:12]1[cH:13][cH:14][c:15]2[n:16][c:17]([Cl:18])[c:19]([Cl:20])[n:21][c:22]2[cH:23]1>>[CH3:10][O:11][c:12]1[cH:13][cH:14][c:15]2[n:16][c:17]([Cl:18])[c:19]([O:1][C@H:2]3[CH2:3][NH:4][C@@H:5]([CH2:6]3)[C:7]([OH:8])=[O:9])[n:21][c:22]2[cH:23]1.[Cl:20]

Correctness of the mapping

MAPPET	YES
ReactionMap	NO
Marvin	YES
ChemDraw	YES
Indigo	YES

Reaction no 179

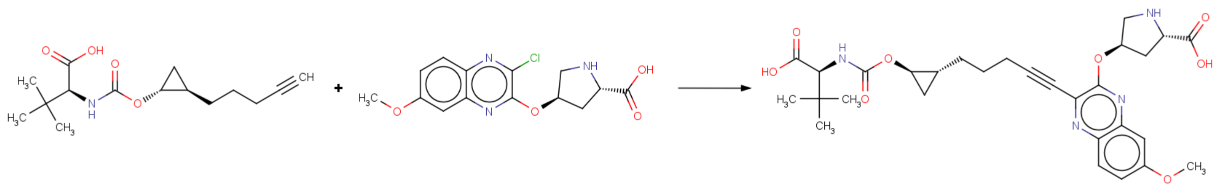

SMILES of the input:
CC(C)(C)[C@H](NC(=O)O[C@@H]1C[C@H]1CCCC#C)C(O)=O.COc1ccc2nc(Cl)c(O[C@H]3CN[C@@H](C3)C(O)=O)nc2c1>>COc1ccc2nc(C#CCCC[C@@H]3C[C@H]3OC(=O)N[C@H](C(O)=O)C(C)(C)C)c(O[C@H]3CN[C@@H](C3)C(O)=O)nc2c1

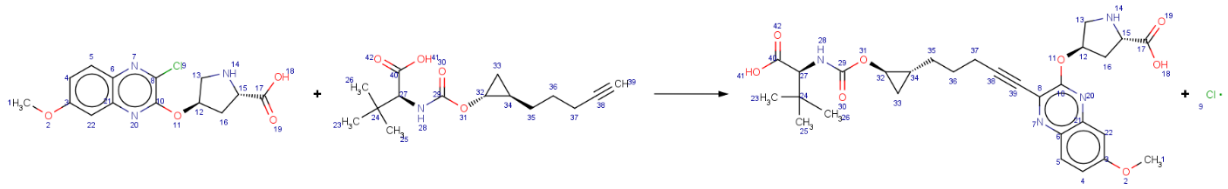

Correct mapped SMILES/SMARTS of the reaction:
[CH3:1][O:2][c:3]1[cH:4][cH:5][c:6]2[n:7][c:8]([Cl:9])[c:10]([O:11][C@H:12]3[CH2:13][NH:14][C@@H:15]([CH2:16]3)[C:17]([OH:18])=[O:19])[n:20][c:21]2[cH:22]1.[CH3:23][C:24]([CH3:25])([CH3:26])[C@H:27]([NH:28][C:29](=[O:30])[O:31][C@@H:32]1[CH2:33][C@H:34]1[CH2:35][CH2:36][CH2:37][C:38]#[CH:39])[C:40]([OH:41])=[O:42]>>[CH3:1][O:2][c:3]1[cH:4][cH:5][c:6]2[n:7][c:8]([C:39]#[C:38][CH2:37][CH2:36][CH2:35][C@@H:34]3[CH2:33][C@H:32]3[O:31][C:29](=[O:30])[NH:28][C@H:27]([C:40]([OH:41])=[O:42])[C:24]([CH3:23])([CH3:26])[CH3:25])[c:10]([O:11][C@H:12]3[CH2:13][NH:14][C@@H:15]([CH2:16]3)[C:17]([OH:18])=[O:19])[n:20][c:21]2[cH:22]1.[Cl:9]

Correctness of the mapping

MAPPET	YES
ReactionMap	NO
Marvin	YES
ChemDraw	YES
Indigo	YES

Reaction no 180

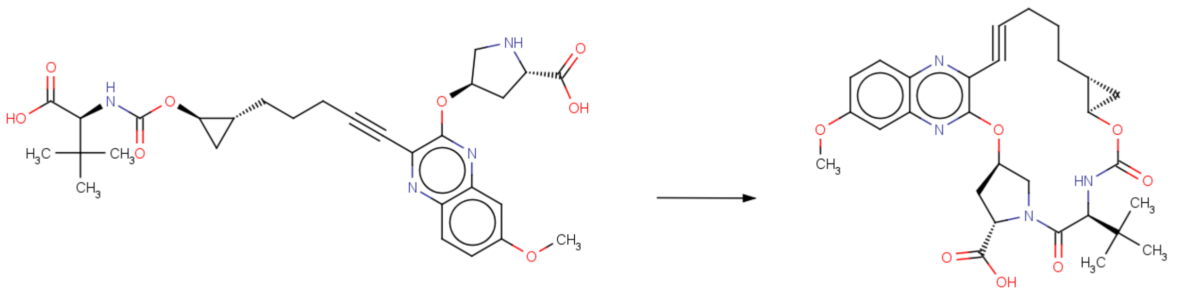

SMILES of the input:

COc1ccc2nc(C#CCCC[C@@H]3C[C@H]3OC(=O)N[C@H](C(O)=O)C(C)(C)C)c(O[C@H]3CN[C@@H](C3)C(O)=O)nc2c1>>COc1ccc2nc3C#CCCC[C@@H]4C[C@H]4OC(=O)N[C@H](C(=O)N4C[C@@H](C[C@H]4C(O)=O)Oc3nc2c1)C(C)(C)C

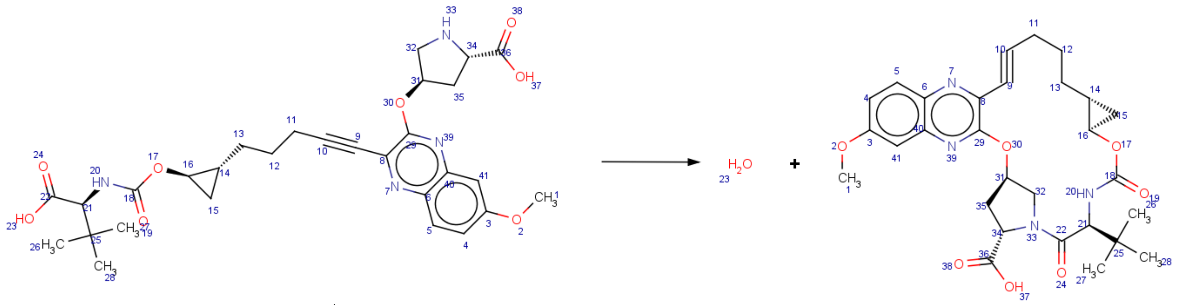

Correct mapped SMILES/SMARTS of the reaction:
[CH3:1][O:2][c:3]1[cH:4][cH:5][c:6]2[n:7][c:8]([C:9]#[C:10][CH2:11][CH2:12][CH2:13][C@@H:14]3[CH2:15][C@H:16]3[O:17][C:18](=[O:19])[NH:20][C@H:21]([C:22]([OH:23])=[O:24])[C:25]([CH3:26])([CH3:27])[CH3:28])[c:29]([O:30][C@H:31]3[CH2:32][NH:33][C@@H:34]([CH2:35]3)[C:36]([OH:37])=[O:38])[n:39][c:40]2[cH:41]1>>[OH2:23].[CH3:1][O:2][c:3]1[cH:4][cH:5][c:6]2[n:7][c:8]3[C:9]#[C:10][CH2:11][CH2:12][CH2:13][C@@H:14]4[CH2:15][C@H:16]4[O:17][C:18](=[O:19])[NH:20][C@H:21]([C:22](=[O:24])[N:33]4[CH2:32][C@@H:31]([CH2:35][C@H:34]4[C:36]([OH:37])=[O:38])[O:30][c:29]3[n:39][c:40]2[cH:41]1)[C:25]([CH3:28])([CH3:27])[CH3:26]

Correctness of the mapping

MAPPET	YES
ReactionMap	NO
Marvin	YES
ChemDraw	YES
Indigo	YES

Reaction no 181

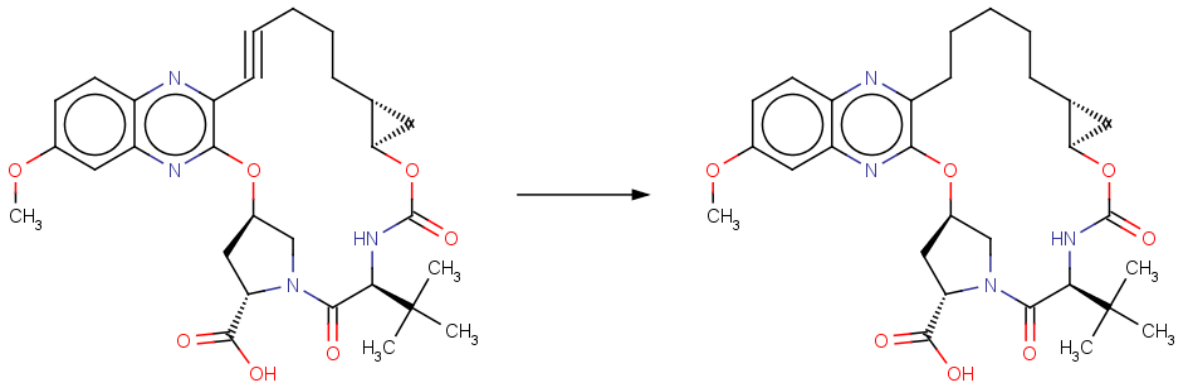

SMILES of the input:
COc1ccc2nc3C#CCCC[C@@H]4C[C@H]4OC(=O)N[C@H](C(=O)N4C[C@@H](C[C@H]4C(O)=O)Oc3nc2c1)C(C)(C)C>>COc1ccc2nc3CCCCC[C@@H]4C[C@H]4OC(=O)N[C@H](C(=O)N4C[C@@H](C[C@H]4C(O)=O)Oc3nc2c1)C(C)(C)C

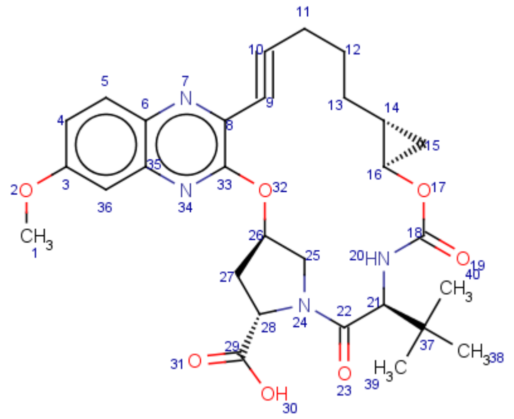

```
Correct mapped SMILES/SMARTS of the reaction:
[CH3:1][O:2][c:3]1[cH:4][cH:5][c:6]2[n:7][c:8]3[C:9]#[C:10][CH2:11][CH2:1
2][CH2:13][C@@H:14]4[CH2:15][C@H:16]4[O:17][C:18](=[O:19])[NH:20][C@H:21]
([C:22](=[O:23])[N:24]4[CH2:25][C@@H:26]([CH2:27][C@H:28]4[C:29]([OH:30])
=[O:31])[O:32][c:33]3[n:34][c:35]2[cH:36]1)[C:37]([CH3:38])([CH3:39])[CH3
:40]>>[CH3:1][O:2][c:3]1[cH:4][cH:5][c:6]2[n:7][c:8]3[CH2:9][CH2:10][CH2:
11][CH2:12][CH2:13][C@@H:14]4[CH2:15][C@H:16]4[O:17][C:18](=[O:19])[NH:20
][C@H:21]([C:22](=[O:23])[N:24]4[CH2:25][C@@H:26]([CH2:27][C@H:28]4[C:29]
([OH:30])=[O:31])[O:32][c:33]3[n:34][c:35]2[cH:36]1)[C:37]([CH3:38])([CH3
:40])[CH3:39]
```

Correctness of the mapping

MAPPET	YES
ReactionMap	YES
Marvin	YES
ChemDraw	YES
Indigo	YES

Reaction no 182

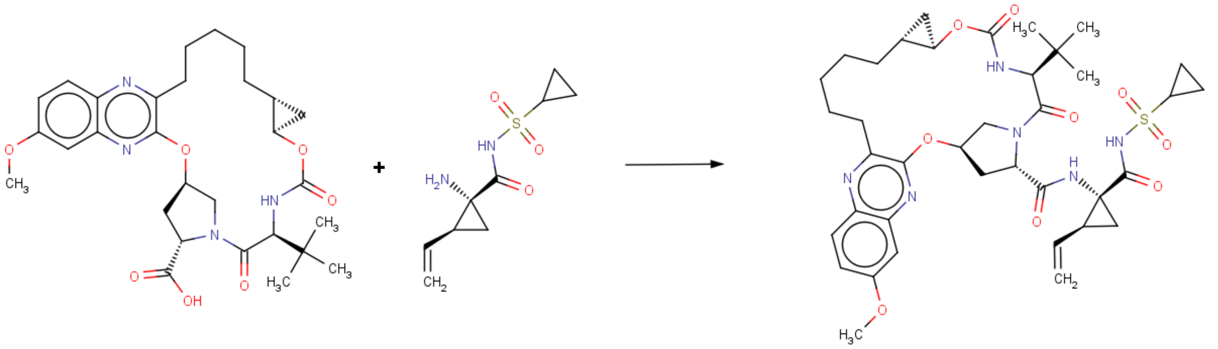

```
SMILES of the input:
COc1ccc2nc3CCCCC[C@@H]4C[C@H]4OC(=O)N[C@H](C(=O)N4C[C@@H](C[C@H]4C(O)=O)O
c3nc2c1)C(C)(C)C.N[C@@]1(C[C@H]1C=C)C(=O)NS(=O)(=O)C1CC1>>COc1ccc2nc3CCCC
C[C@@H]4C[C@H]4OC(=O)N[C@H](C(=O)N4C[C@@H](C[C@H]4C(=O)N[C@@]4(C[C@H]4C=C
)C(=O)NS(=O)(=O)C4CC4)Oc3nc2c1)C(C)(C)C
```

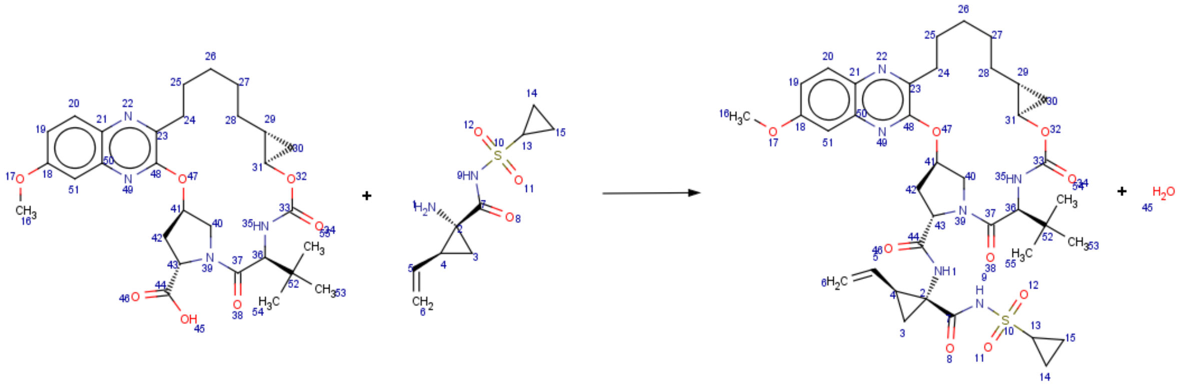

Correct mapped SMILES/SMARTS of the reaction:
[CH3:16][O:17][c:18]1[cH:19][cH:20][c:21]2[n:22][c:23]3[CH2:24][CH2:25][C
H2:26][CH2:27][CH2:28][C@@H:29]4[CH2:30][C@H:31]4[O:32][C:33](=[O:34])[NH
:35][C@H:36]([C:37](=[O:38])[N:39]4[CH2:40][C@@H:41]([CH2:42][C@H:43]4[C:
44]([OH:45])=[O:46])[O:47][c:48]3[n:49][c:50]2[cH:51]1)[C:52]([CH3:53])([
CH3:54])[CH3:55].[NH2:1][C@@:2]1([CH2:3][C@H:4]1[CH:5]=[CH2:6])[C:7](=[O:
8])[NH:9][S:10](=[O:11])(=[O:12])[CH:13]1[CH2:14][CH2:15]1>>[CH3:16][O:17
][c:18]1[cH:19][cH:20][c:21]2[n:22][c:23]3[CH2:24][CH2:25][CH2:26][CH2:27
][CH2:28][C@@H:29]4[CH2:30][C@H:31]4[O:32][C:33](=[O:34])[NH:35][C@H:36](
[C:37](=[O:38])[N:39]4[CH2:40][C@@H:41]([CH2:42][C@H:43]4[C:44](=[O:46])[
NH:1][C@@:2]4([CH2:3][C@H:4]4[CH:5]=[CH2:6])[C:7](=[O:8])[NH:9][S:10](=[O
:11])(=[O:12])[CH:13]4[CH2:15][CH2:14]4)[O:47][c:48]3[n:49][c:50]2[cH:51]
1)[C:52]([CH3:53])([CH3:55])[CH3:54].[OH2:45]

Correctness of the mapping

MAPPET	YES
ReactionMap	NO
Marvin	YES
ChemDraw	YES
Indigo	YES

Reaction no 183

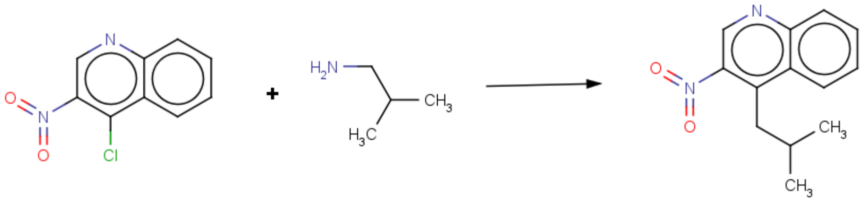

SMILES of the input:
Clc1c(cnc2ccccc12)N(=O)=O.CC(C)CN>>CC(C)Cc1c(cnc2ccccc12)N(=O)=O

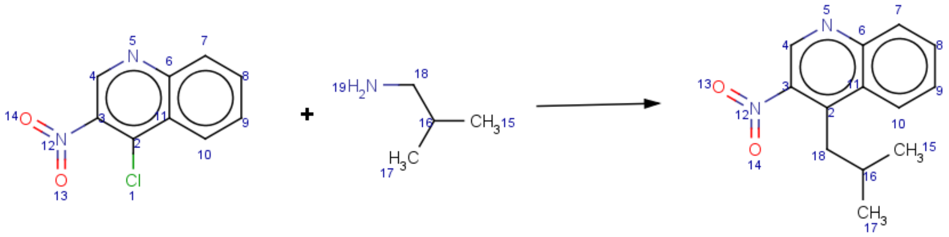

Correct mapped SMILES/SMARTS of the reaction:
[CH3:1][CH:2]([CH3:3])[CH2:4][NH2:5].[Cl:6][c:7]1[c:8]([cH:9][n:10][c:11]
2[cH:12][cH:13][cH:14][cH:15][c:16]12)[N:17](=[O:18])=[O:19]>>[CH3:1][CH:

2]([CH3:3])[CH2:4][c:7]1[c:8]([cH:9][n:10][c:11]2[cH:12][cH:13][cH:14][cH:15][c:16]12)[N:17](=[O:19])=[O:18].[Cl:6].[N:5]

Correctness of the mapping

MAPPET	YES
ReactionMap	NO
Marvin	YES
ChemDraw	YES
Indigo	NO

Reaction no 184

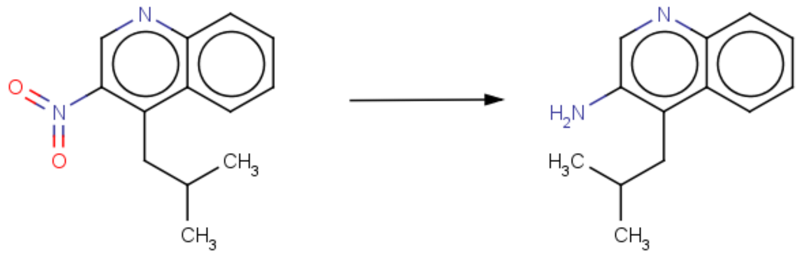

SMILES of the input:
CC(Cc(c(N(=O)=O)cn1)c2c1cccc2)C>>CC(Cc(c(N)cn3)c4c3cccc4)C

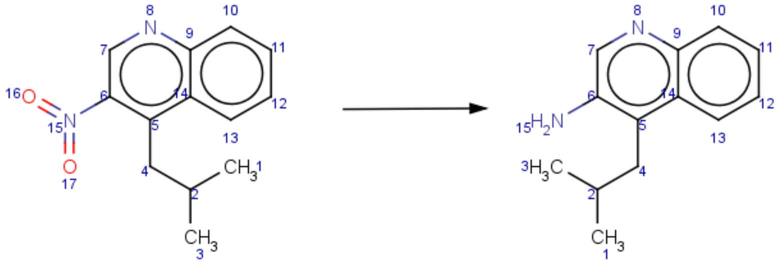

Correct mapped SMILES/SMARTS of the reaction:
[CH3:1][CH:2]([CH3:3])[CH2:4][c:5]1[c:6]([cH:7][n:8][c:9]2[cH:10][cH:11][cH:12][cH:13][c:14]12)[N:15](=[O:16])=[O:17]>>[CH3:3][CH:2]([CH3:1])[CH2:4][c:5]1[c:6]([NH2:15])[cH:7][n:8][c:9]2[cH:10][cH:11][cH:12][cH:13][c:14]12.[O:16].[O:17]

Correctness of the mapping

MAPPET	YES
ReactionMap	NO
Marvin	YES
ChemDraw	YES
Indigo	YES

Reaction no 185

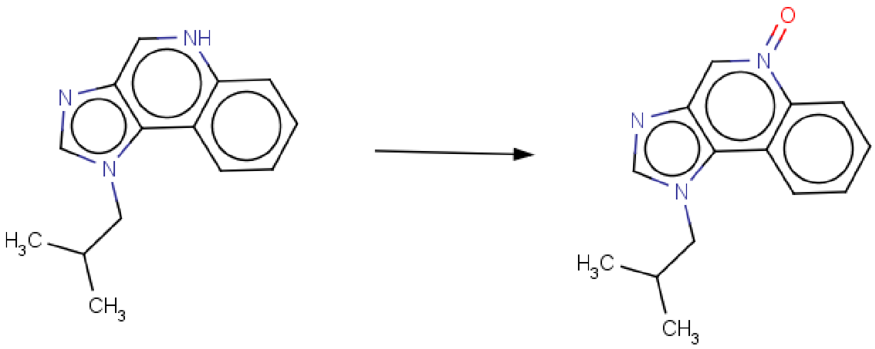

SMILES of the input:
CC(Cn(cn1)c2c1c[nH]c3c2cccc3)C>>CC(Cn(cn4)c5c4cn(=O)c6c5cccc6)C

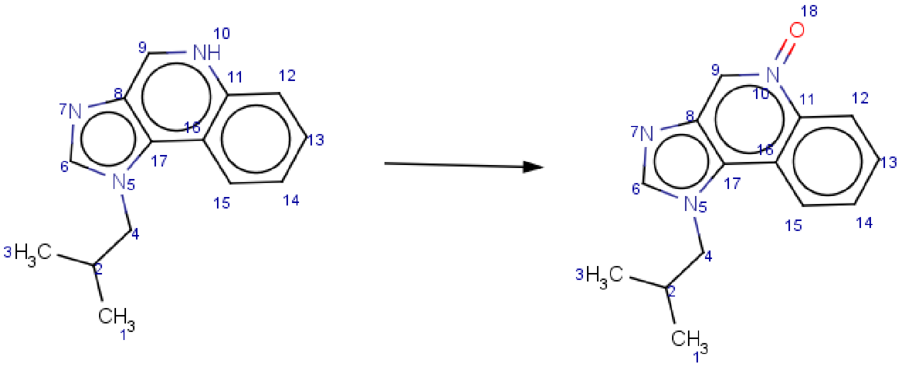

Correct mapped SMILES/SMARTS of the reaction:
[CH3:1][CH:2]([CH3:3])[CH2:4][n:5]1[cH:6][n:7][c:8]2[cH:9][nH:10][c:11]3[cH:12][cH:13][cH:14][cH:15][c:16]3[c:17]12.[O:18]>>[CH3:3][CH:2]([CH3:1])[CH2:4][n:5]1[cH:6][n:7][c:8]2[cH:9][n:10](=[O:18])[c:11]3[cH:12][cH:13][cH:14][cH:15][c:16]3[c:17]12

Correctness of the mapping

MAPPET	YES
ReactionMap	NO
Marvin	YES
ChemDraw	YES
Indigo	YES

Reaction no 186

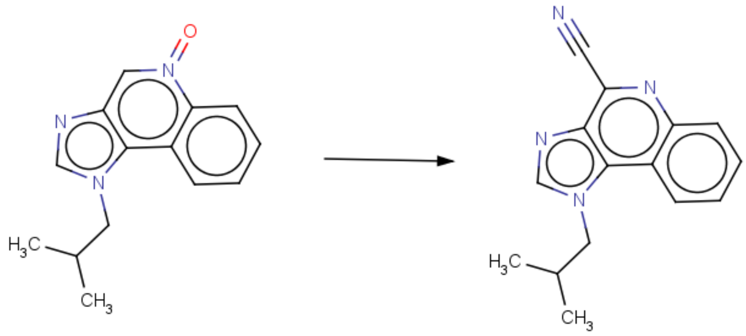

SMILES of the input:
CC(C)Cn1cnc2cn(=O)c3ccccc3c12>>CC(C)Cn1cnc2c(nc3ccccc3c12)C#N

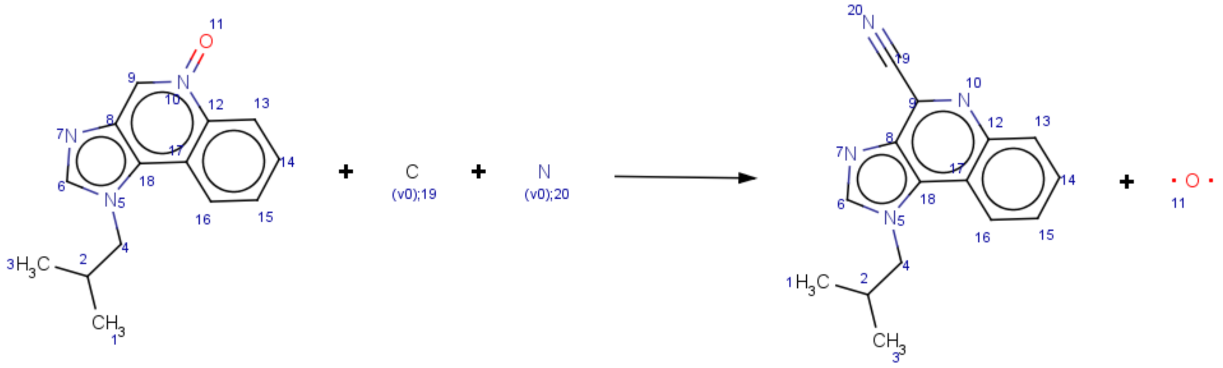

```
Correct mapped SMILES/SMARTS of the reaction:
[CH3:1][CH:2]([CH3:3])[CH2:4][n:5]1[cH:6][n:7][c:8]2[cH:9][n:10](=[O:11])
[c:12]3[cH:13][cH:14][cH:15][cH:16][c:17]3[c:18]12.[C:19].[N:20]>>[CH3:3]
[CH:2]([CH3:1])[CH2:4][n:5]1[cH:6][n:7][c:8]2[c:9]([n:10][c:12]3[cH:13][c
H:14][cH:15][cH:16][c:17]3[c:18]12)[C:19]#[N:20].[O:11]

Correctness of the mapping
MAPPET          YES
ReactionMap     NO
Marvin          YES
ChemDraw        YES
Indigo          YES

Reaction no  187
```

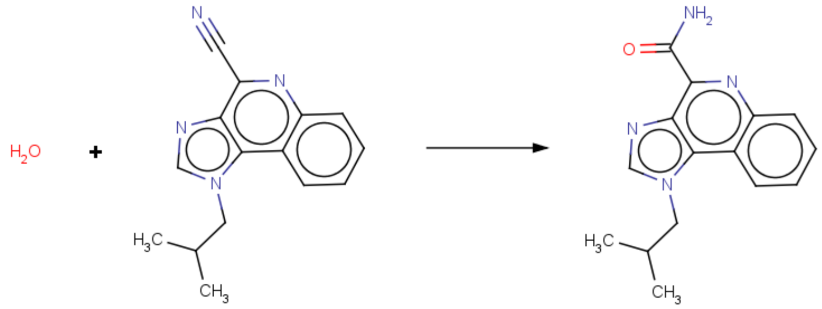

```
SMILES of the input:
CC(Cn(cn1)c2c1c(C#N)nc3c2cccc3)C>>CC(Cn(cn4)c5c4c(C(N)=O)nc6c5cccc6)C
```

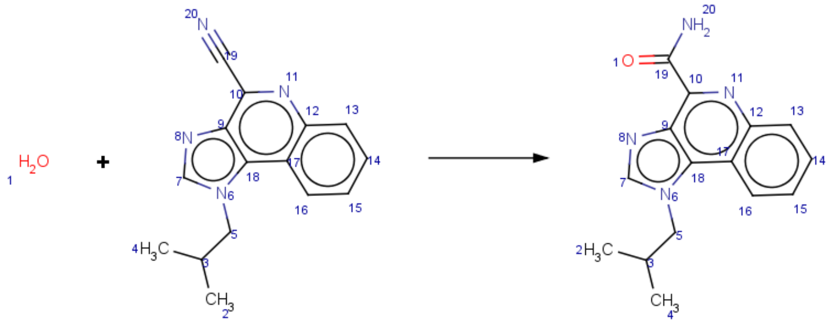

```
Correct mapped SMILES/SMARTS of the reaction:
[OH2:1].[CH3:2][CH:3]([CH3:4])[CH2:5][n:6]1[cH:7][n:8][c:9]2[c:10]([n:11]
[c:12]3[cH:13][cH:14][cH:15][cH:16][c:17]3[c:18]12)[C:19]#[N:20]>>[CH3:4]
[CH:3]([CH3:2])[CH2:5][n:6]1[cH:7][n:8][c:9]2[c:10]([n:11][c:12]3[cH:13][
cH:14][cH:15][cH:16][c:17]3[c:18]12)[C:19]([NH2:20])=[O:1]

Correctness of the mapping
MAPPET          YES
ReactionMap     NO
```

Marvin YES
ChemDraw YES
Indigo YES

Reaction no 188

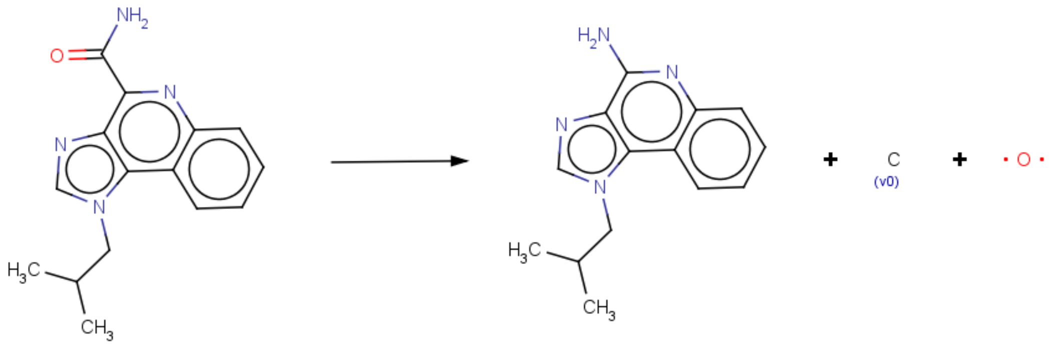

SMILES of the input:
CC(Cn(cn1)c2c1c(C(N)=O)nc3c2cccc3)C>>CC(Cn(cn4)c5c4c(N)nc6c5cccc6)C

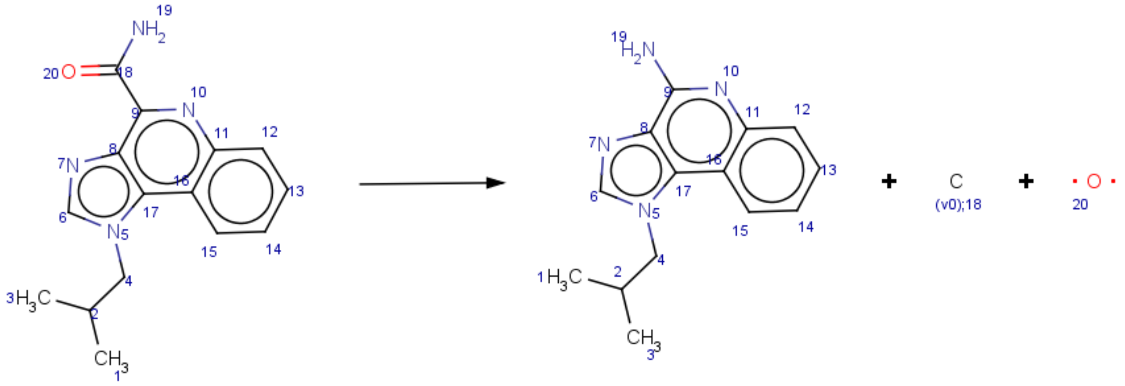

Correct mapped SMILES/SMARTS of the reaction:
[CH3:1][CH:2]([CH3:3])[CH2:4][n:5]1[cH:6][n:7][c:8]2[c:9]([n:10][c:11]3[cH:12][cH:13][cH:14][cH:15][c:16]3[c:17]12)[C:18]([NH2:19])=[O:20]>>[CH3:3][CH:2]([CH3:1])[CH2:4][n:5]1[cH:6][n:7][c:8]2[c:9]([NH2:19])[n:10][c:11]3[cH:12][cH:13][cH:14][cH:15][c:16]3[c:17]12.[C:18].[O:20]

Correctness of the mapping
MAPPET YES
ReactionMap NO
Marvin YES
ChemDraw YES
Indigo YES

Reaction no 189

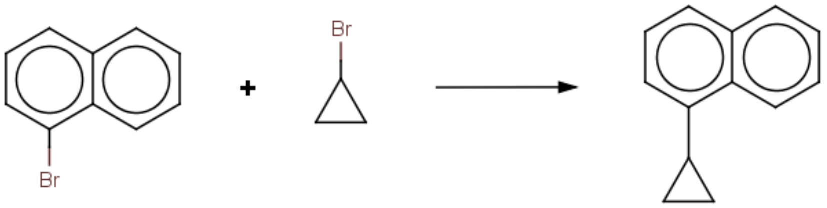

SMILES of the input:
Brc1cccc2ccccc12.BrC1CC1>>C1CC1c1cccc2ccccc12

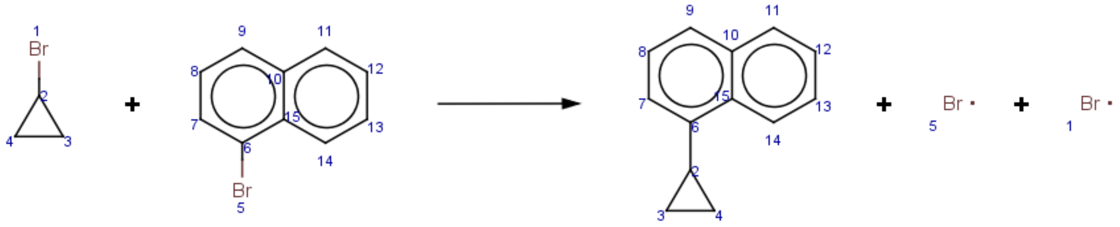

Correct mapped SMILES/SMARTS of the reaction:
[Br:1][CH:2]1[CH2:3][CH2:4]1.[Br:5][c:6]1[cH:7][cH:8][cH:9][c:10]2[cH:11][cH:12][cH:13][cH:14][c:15]12>>[CH2:4]1[CH2:3][CH:2]1[c:6]1[cH:7][cH:8][cH:9][c:10]2[cH:11][cH:12][cH:13][cH:14][c:15]12.[Br:5].[Br:1]

Correctness of the mapping

MAPPET	YES
ReactionMap	NO
Marvin	YES
ChemDraw	YES
Indigo	YES

Reaction no 190

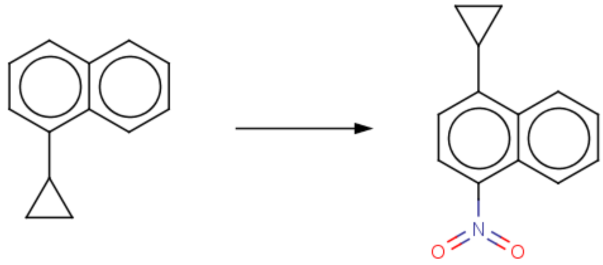

SMILES of the input:
C1(c(ccc2)c3c2cccc3)CC1>>O=N(c(ccc4C5CC5)c6c4cccc6)=O

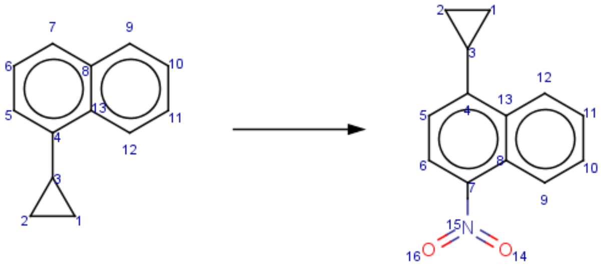

Correct mapped SMILES/SMARTS of the reaction:
[CH2:1]1[CH2:2][CH:3]1[c:4]1[cH:5][cH:6][cH:7][c:8]2[cH:9][cH:10][cH:11][cH:12][c:13]12.[N:14].[O:15].[O:16]>>[O:16]=[N:14](=[O:15])[c:7]1[cH:6][cH:5][c:4]([CH:3]2[CH2:1][CH2:2]2)[c:13]2[cH:12][cH:11][cH:10][cH:9][c:8]12

Correctness of the mapping

MAPPET	YES
ReactionMap	NO
Marvin	YES
ChemDraw	YES
Indigo	YES

Reaction no 191

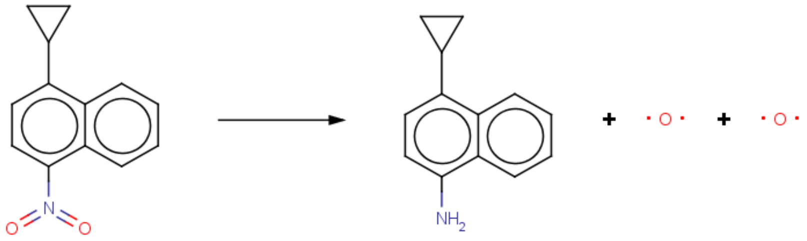

SMILES of the input:
O=N(c(ccc1C2CC2)c3c1cccc3)=O>>Nc(ccc4C5CC5)c6c4cccc6

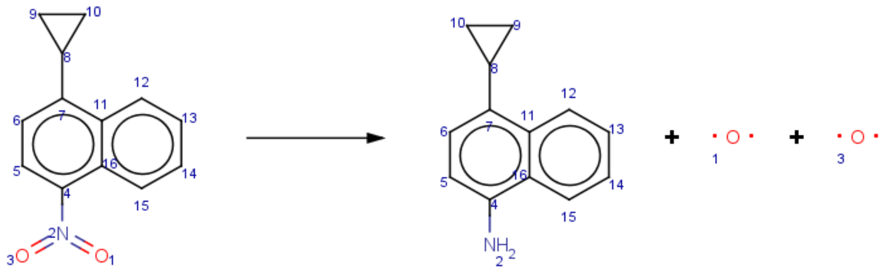

Correct mapped SMILES/SMARTS of the reaction:
[O:1]=[N:2](=[O:3])[c:4]1[cH:5][cH:6][c:7]([CH:8]2[CH2:9][CH2:10]2)[c:11]2[cH:12][cH:13][cH:14][cH:15][c:16]12>>[NH2:2][c:4]1[cH:5][cH:6][c:7]([CH:8]2[CH2:10][CH2:9]2)[c:11]2[cH:12][cH:13][cH:14][cH:15][c:16]12.[O:1].[O:3]

Correctness of the mapping

MAPPET	YES
ReactionMap	NO
Marvin	YES
ChemDraw	YES
Indigo	YES

Reaction no 192

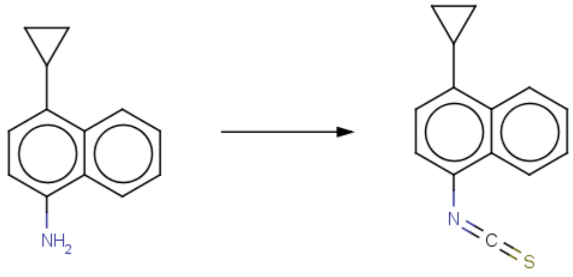

SMILES of the input:
Nc(ccc1C2CC2)c3c1cccc3>>S=C=Nc(ccc4C5CC5)c6c4cccc6

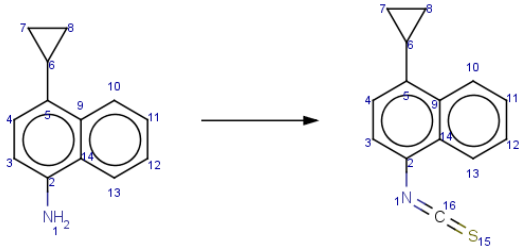

Correct mapped SMILES/SMARTS of the reaction:
[NH2:1][c:2]1[cH:3][cH:4][c:5]([CH:6]2[CH2:7][CH2:8]2)[c:9]2[cH:10][cH:11][cH:12][cH:13][c:14]12.[C:15].[S:16]>>[S:16]=[C:15]=[N:1][c:2]1[cH:3][cH:4][c:5]([CH:6]2[CH2:8][CH2:7]2)[c:9]2[cH:10][cH:11][cH:12][cH:13][c:14]12

Correctness of the mapping
MAPPET YES
ReactionMap NO
Marvin YES
ChemDraw YES
Indigo YES

Reaction no 193

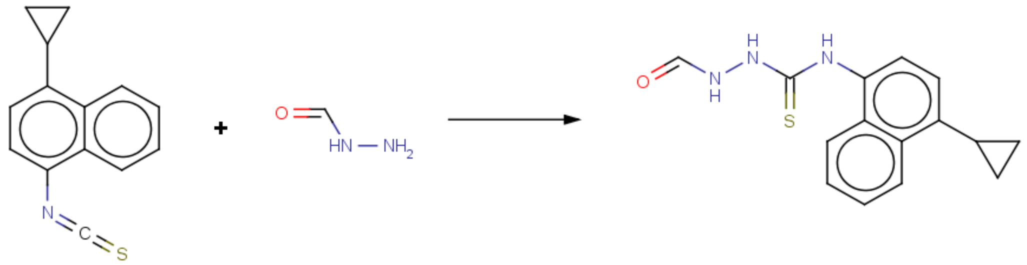

SMILES of the input:
S=C=Nc1ccc(C2CC2)c2ccccc12.NNC=O>>O=CNNC(=S)Nc1ccc(C2CC2)c2ccccc12

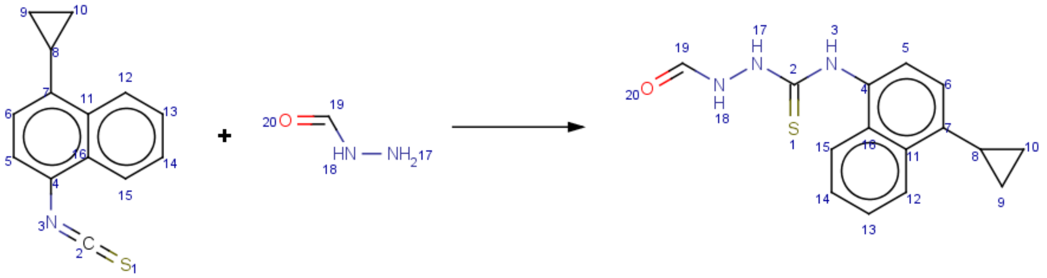

Correct mapped SMILES/SMARTS of the reaction:
[S:1]=[C:2]=[N:3][c:4]1[cH:5][cH:6][c:7]([CH:8]2[CH2:9][CH2:10]2)[c:11]2[cH:12][cH:13][cH:14][cH:15][c:16]12.[NH2:17][NH:18][CH:19]=[O:20]>>[O:20]=[CH:19][NH:18][NH:17][C:2](=[S:1])[NH:3][c:4]1[cH:5][cH:6][c:7]([CH:8]2[CH2:10][CH2:9]2)[c:11]2[cH:12][cH:13][cH:14][cH:15][c:16]12

Correctness of the mapping
MAPPET YES
ReactionMap YES
Marvin YES
ChemDraw YES
Indigo YES

Reaction no 194

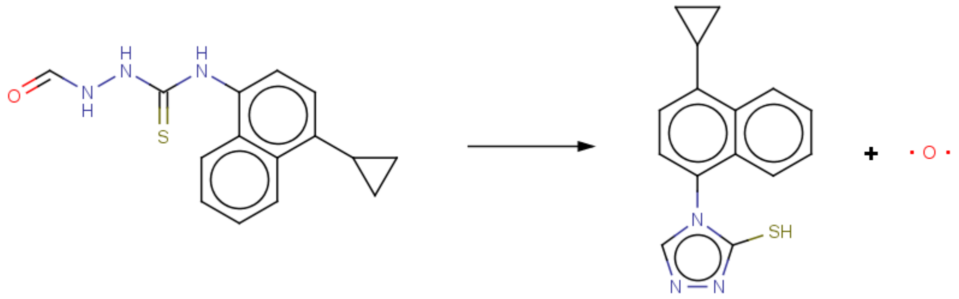

SMILES of the input:
O=CNNC(Nc(ccc1C2CC2)c3c1cccc3)=S>>Sc4n(c(ccc5C6CC6)c7c5cccc7)cnn4

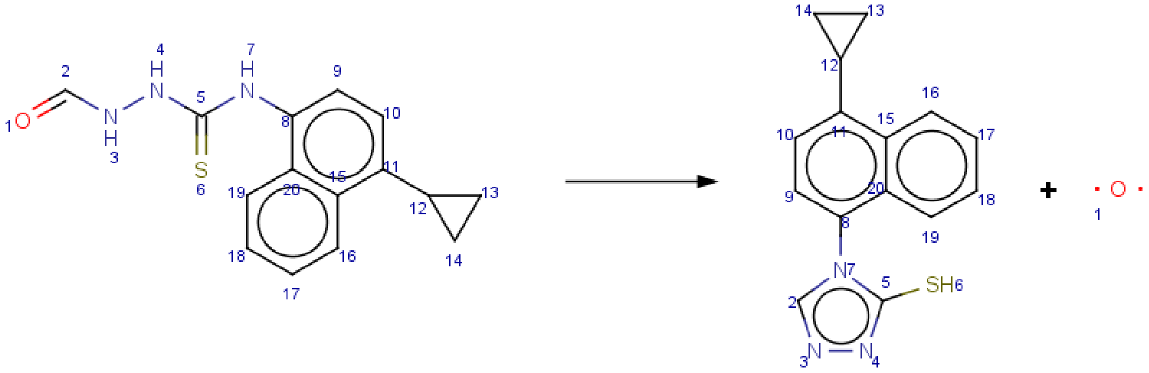

Correct mapped SMILES/SMARTS of the reaction:
[O:1]=[CH:2][NH:3][NH:4][C:5](=[S:6])[NH:7][c:8]1[cH:9][cH:10][c:11]([CH:12]2[CH2:13][CH2:14]2)[c:15]2[cH:16][cH:17][cH:18][cH:19][c:20]12>>[SH:6][c:5]1[n:4][n:3][cH:2][n:7]1-[c:8]1[cH:9][cH:10][c:11]([CH:12]2[CH2:14][CH2:13]2)[c:15]2[cH:16][cH:17][cH:18][cH:19][c:20]12.[O:1]

Correctness of the mapping

MAPPET	YES
ReactionMap	NO
Marvin	YES
ChemDraw	YES
Indigo	YES

Reaction no 195

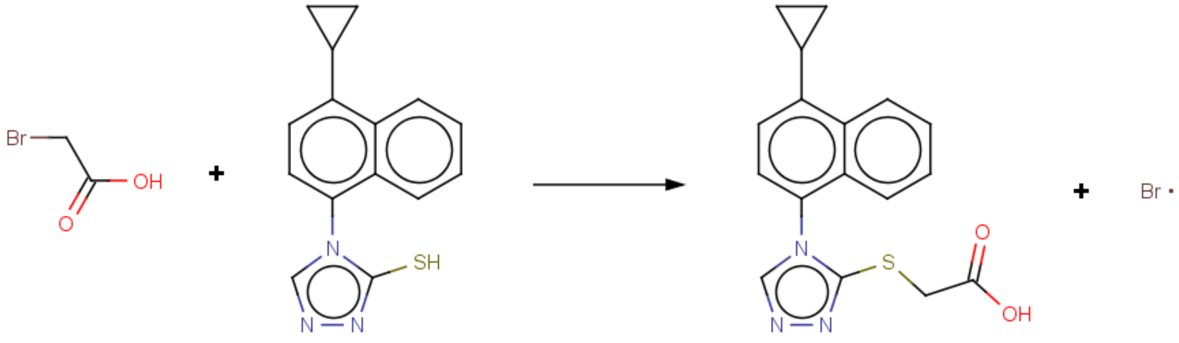

SMILES of the input:
OC(=O)CBr.Sc1nncn1-c1ccc(C2CC2)c2ccccc12>>OC(=O)CSc1nncn1-c1ccc(C2CC2)c2ccccc12.[Br]

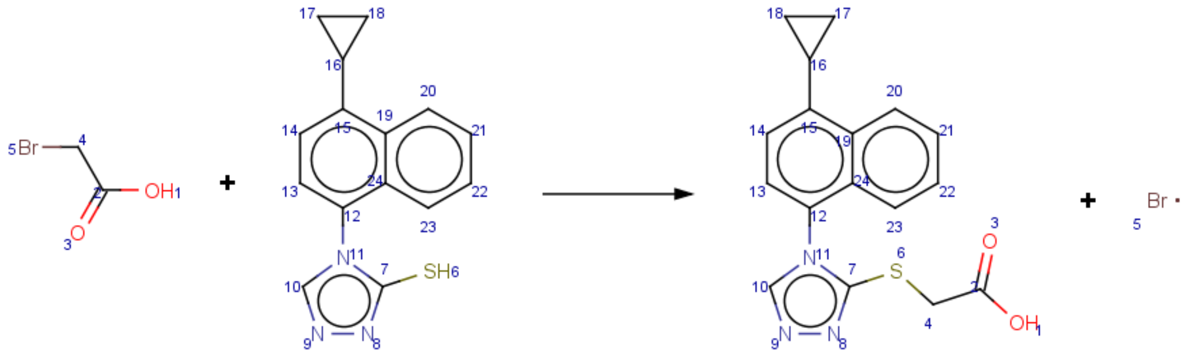

```
Correct mapped SMILES/SMARTS of the reaction:
[OH:1][C:2](=[O:3])[CH2:4][Br:5].[SH:6][c:7]1[n:8][n:9][cH:10][n:11]1-
[c:12]1[cH:13][cH:14][c:15]([CH:16]2[CH2:17][CH2:18]2)[c:19]2[cH:20][cH:2
1][cH:22][cH:23][c:24]12>>[OH:1][C:2](=[O:3])[CH2:4][S:6][c:7]1[n:8][n:9]
[cH:10][n:11]1-
[c:12]1[cH:13][cH:14][c:15]([CH:16]2[CH2:18][CH2:17]2)[c:19]2[cH:20][cH:2
1][cH:22][cH:23][c:24]12.[Br:5]
```

Correctness of the mapping

MAPPET	YES
ReactionMap	NO
Marvin	YES
ChemDraw	YES
Indigo	YES

Reaction no 196

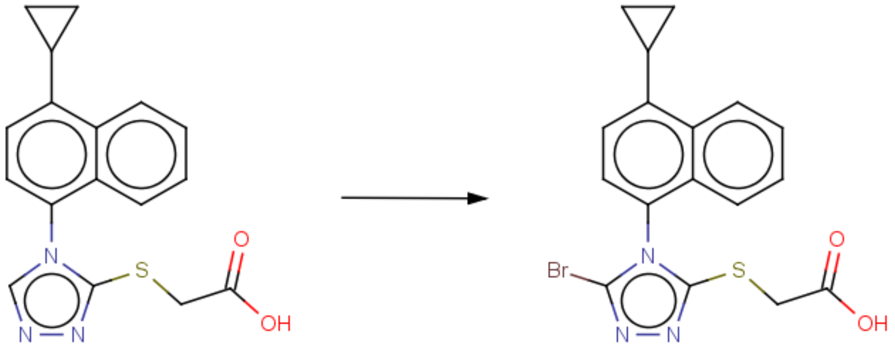

```
SMILES of the input:
OC(=O)CSc1nncn1-c1ccc(C2CC2)c2ccccc12>>OC(=O)CSc1nnc(Br)n1-
c1ccc(C2CC2)c2ccccc12
```

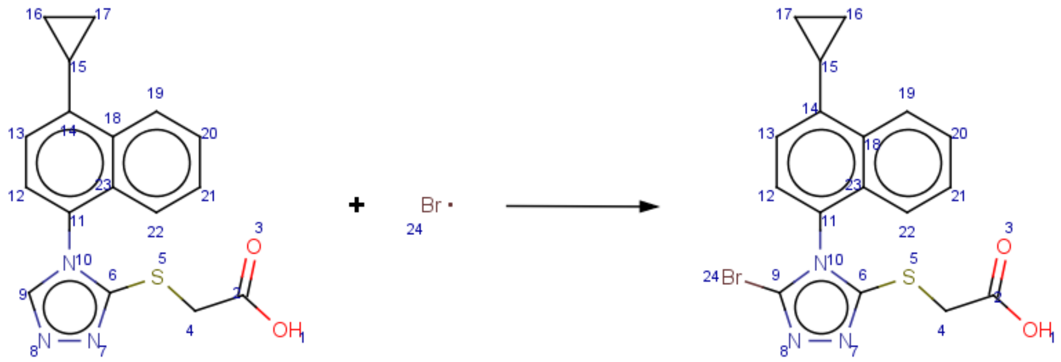

Correct mapped SMILES/SMARTS of the reaction:
[OH:1][C:2](=[O:3])[CH2:4][S:5][c:6]1[n:7][n:8][cH:9][n:10]1-
[c:11]1[cH:12][cH:13][c:14]([CH:15]2[CH2:16][CH2:17]2)[c:18]2[cH:19][cH:2
0][cH:21][cH:22][c:23]12.[Br:24]>>[OH:1][C:2](=[O:3])[CH2:4][S:5][c:6]1[n
:7][n:8][c:9]([Br:24])[n:10]1-
[c:11]1[cH:12][cH:13][c:14]([CH:15]2[CH2:17][CH2:16]2)[c:18]2[cH:19][cH:2
0][cH:21][cH:22][c:23]12

Correctness of the mapping

MAPPET	YES
ReactionMap	NO
Marvin	YES
ChemDraw	YES
Indigo	YES

Reaction no 197

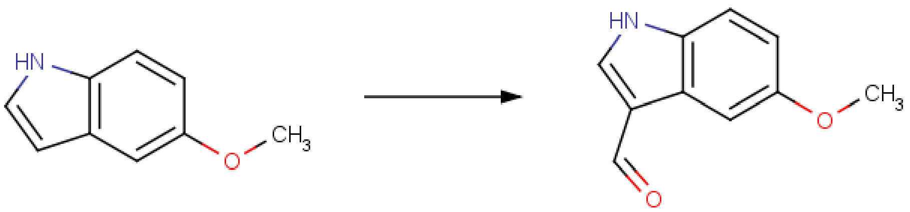

SMILES of the input:
COC1=CC2=C(C=C1)NC=C2>>COC3=CC4=C(C=C3)NC=C4C=O

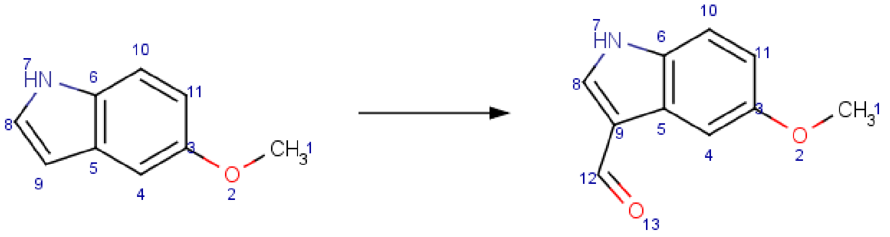

Correct mapped SMILES/SMARTS of the reaction:
[CH3:1][O:2][C:3]1=[CH:4][C:5]2=[C:6]([NH:7][CH:8]=[CH:9]2)[CH:10]=[CH:11
]1.[C:12].[O:13]>>[CH3:1][O:2][C:3]1=[CH:4][C:5]2=[C:6]([NH:7][CH:8]=[C:9
]2[CH:12]=[O:13])[CH:10]=[CH:11]1

Correctness of the mapping

MAPPET	YES

ReactionMap NO
Marvin YES
ChemDraw YES
Indigo YES

Reaction no 198

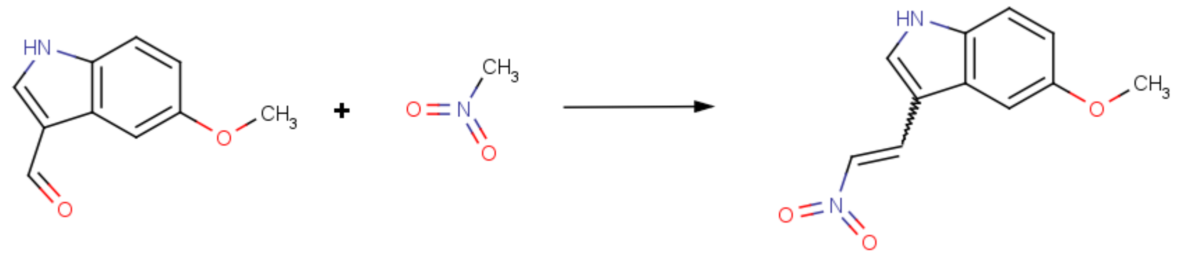

SMILES of the input:
COC1=CC2=C(C=C1)NC=C2C=O.CN(=O)=O>>COC3=CC4=C(C=C3)NC=C4/C=C/N(=O)=O

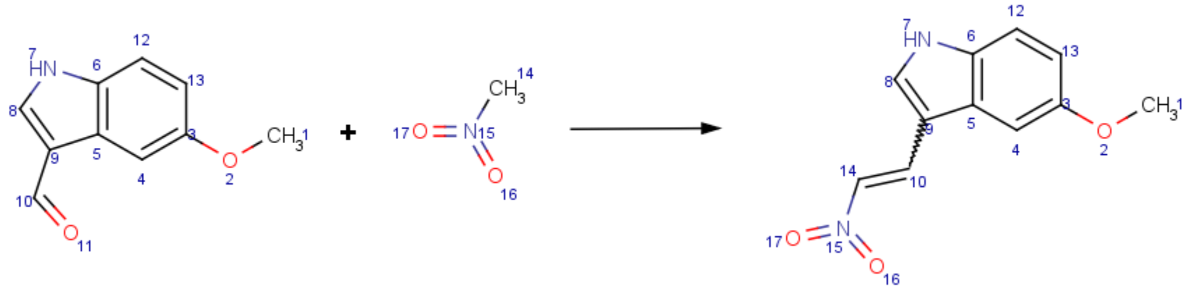

Correct mapped SMILES/SMARTS of the reaction:
[CH3:1][N:2](=[O:3])=[O:4].[CH3:5][O:6][C:7]1=[CH:8][C:9]2=[C:10]([NH:11]
[CH:12]=[C:13]2[CH:14]=[O:15])[CH:16]=[CH:17]1>>[OH2:15].[CH3:5][O:6][C:7
]1=[CH:8][C:9]2=[C:10]([NH:11][CH:12]=[C:13]2\[CH:14]=[CH:1]\[N:2](=[O:3]
)=[O:4])[CH:16]=[CH:17]1

Correctness of the mapping
MAPPET YES
ReactionMap NO
Marvin YES
ChemDraw YES
Indigo YES

Reaction no 199

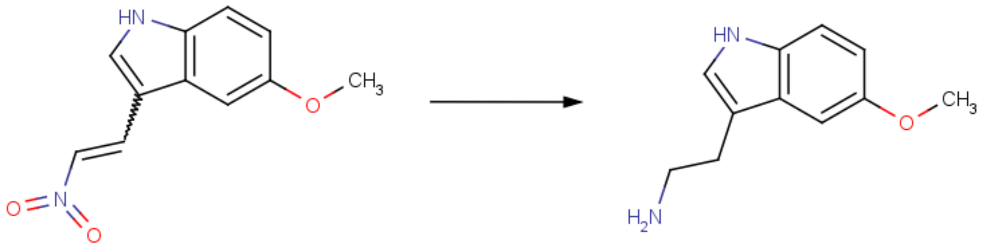

SMILES of the input:
COC1=CC2=C(C=C1)NC=C2/C=C/N(=O)=O>>COC3=CC4=C(C=C3)NC=C4CCN

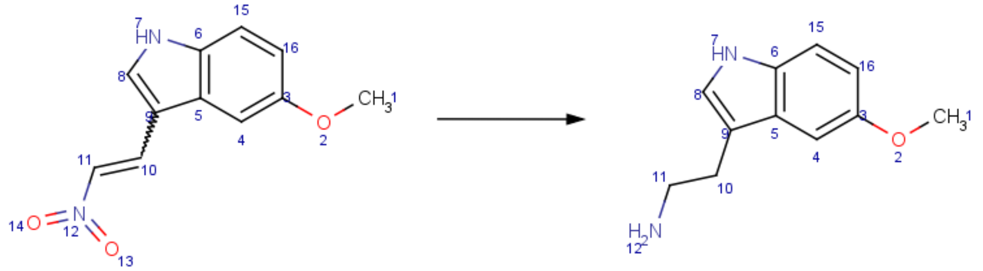

Correct mapped SMILES/SMARTS of the reaction:
[CH3:1][O:2][C:3]1=[CH:4][C:5]2=[C:6]([NH:7][CH:8]=[C:9]2\[CH:10]=[CH:11]\[N:12](=[O:13])=[O:14])[CH:15]=[CH:16]1>>[CH3:1][O:2][C:3]1=[CH:4][C:5]2=[C:6]([NH:7][CH:8]=[C:9]2[CH2:10][CH2:11][NH2:12])[CH:15]=[CH:16]1.[O:13].[O:14]

Correctness of the mapping

MAPPET	YES
ReactionMap	NO
Marvin	YES
ChemDraw	YES
Indigo	NO

Reaction no 200

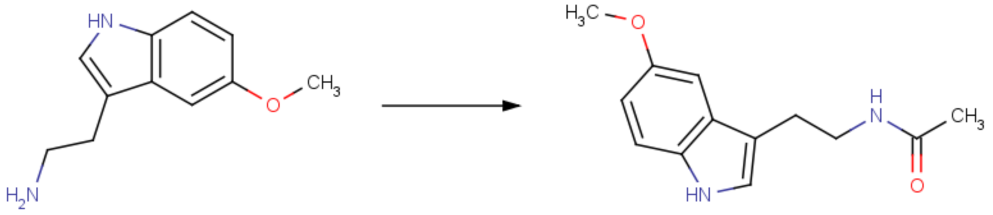

SMILES of the input:
COC1=CC2=C(C=C1)NC=C2CCN>>COC3=CC4=C(C=C3)NC=C4CCNC(C)=O

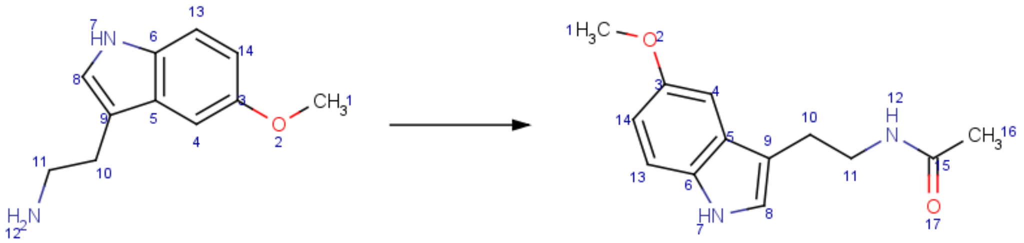

Correct mapped SMILES/SMARTS of the reaction:
[CH3:1][O:2][C:3]1=[CH:4][C:5]2=[C:6]([NH:7][CH:8]=[C:9]2[CH2:10][CH2:11][NH2:12])[CH:13]=[CH:14]1.[C:15].[C:16].[O:17]>>[CH3:1][O:2][C:3]1=[CH:4][C:5]2=[C:6]([NH:7][CH:8]=[C:9]2[CH2:10][CH2:11][NH:12][C:16]([CH3:15])=[O:17])[CH:13]=[CH:14]1

Correctness of the mapping

MAPPET	YES
ReactionMap	NO
Marvin	YES
ChemDraw	YES
Indigo	YES

Reaction no 1

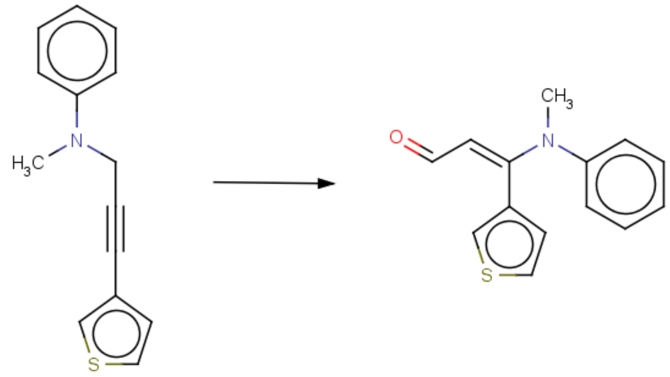

SMILES of the input:
CN(CC#Cc1ccsc1)c1ccccc1>>CN(\C(=C\C=O)c1ccsc1)c1ccccc1

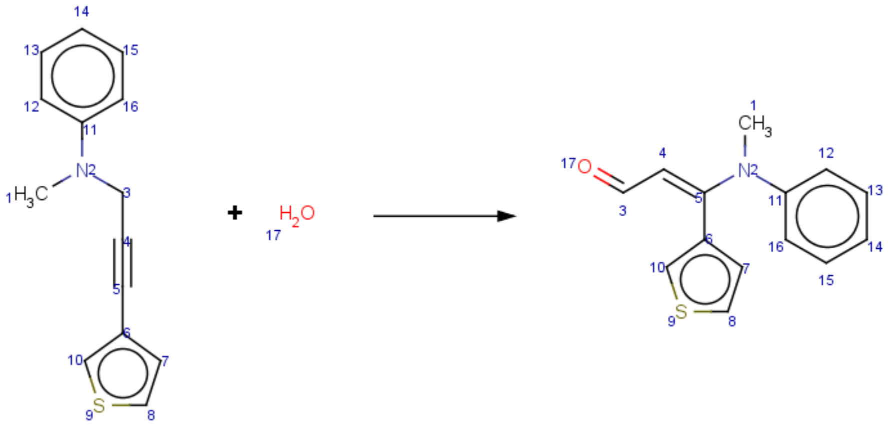

Correct mapped

SMILES/SMARTS of the reaction:
[#6:1]-[#7:2](-[#6:3][C:4]#[C:5][c:6]1[c:7][c:8][s:9][c:10]1)-[c:11]1[c:12][c:13][c:14][c:15][c:16]1.[#8:17]>>[#6:1]-[#7:2](\[#6:5](=[#6:4]\[#6:3]=[O:17])-[c:6]1[c:7][c:8][s:9][c:10]1)-[c:11]1[c:12][c:13][c:14][c:15][c:16]1

Correctness of the mapping

MAPPET	YES
ReactionMap	NO
Marvin	YES
ChemDraw	NO
Indigo	NO

Reaction no 2

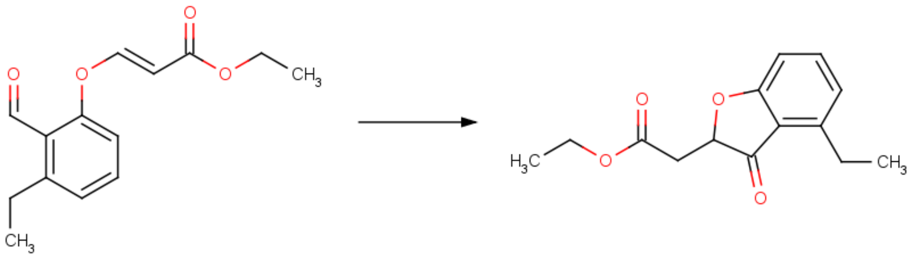

SMILES of the input:
CCOC(=O)\C=C\OC1=CC=CC(CC)=C1C=O>>CCOC(=O)CC1OC2=CC=CC(CC)=C2C1=O

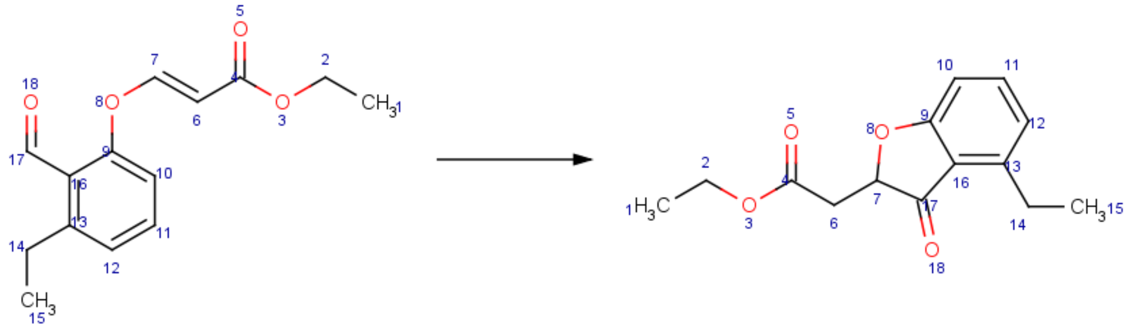

Correct mapped SMILES/SMARTS of the reaction:
[#6:1]-[#6:2]-[#8:3]-[#6:4](=[O:5])\[#6:6]=[#6:7]\[#8:8]-[#6:9]-1=[#6:10]-[#6:11]=[#6:12]-[#6:13](-[#6:14]-[#6:15])=[#6:16]-1-[#6:17]=[O:18]>>[#6:1]-[#6:2]-[#8:3]-[#6:4](=[O:5])-[#6:6]-[#6:7]-1-[#8:8]-[#6:9]-2=[#6:10]-[#6:11]=[#6:12]-[#6:13](-[#6:14]-[#6:15])=[#6:16]-2-[#6:17]-1=[O:18]

Correctness of the mapping

MAPPET	YES
ReactionMap	YES
Marvin	YES
ChemDraw	YES
Indigo	YES

Reaction no 3

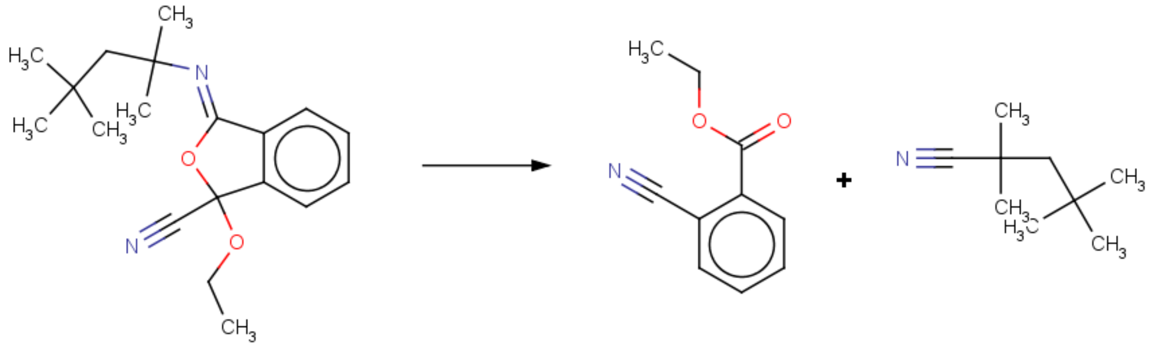

SMILES of the input:
CCOC1(O\C(=N/C(C)(C)CC(C)(C)C)c2ccccc12)C#N>>CCOC(=O)c1ccccc1C#N.CC(C)(C)CC(C)(C)C#N

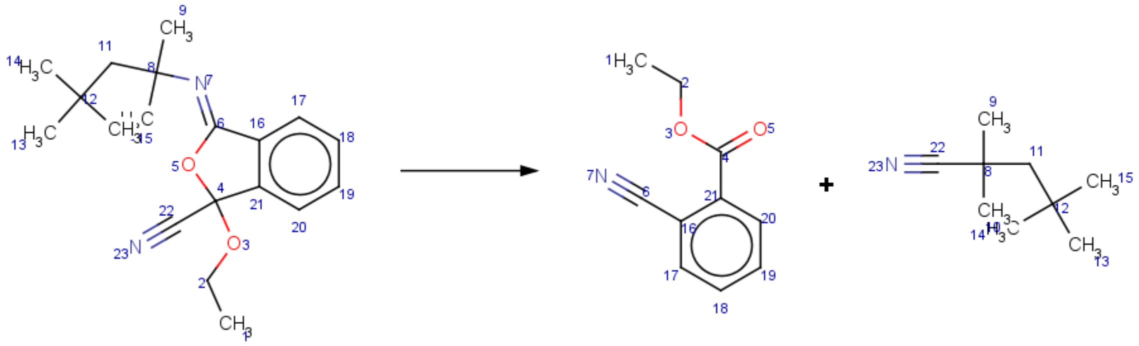

Correct mapped SMILES/SMARTS of the reaction:
[#6:1]-[#6:2]-[#8:3][C:4]1([#8:5]\[#6:6](=[#7:7]/[C:8]([#6:9])([#6:10])[#6:11][C:12]([#

6:13])([#6:14])[#6:15])-
[c:16]2[c:17][c:18][c:19][c:20][c:21]12)[C:22]#[N:23]>>[#6:1]-[#6:2]-
[#8:3]-[#6:4](=[O:5])-
[c:21]1[c:20][c:19][c:18][c:17][c:16]1[C:6]#[N:7].[#6:13][C:12]([#6:15])(
[#6:14])[#6:11][C:8]([#6:9])([#6:10])[C:22]#[N:23]

Correctness of the mapping

MAPPET	YES
ReactionMap	YES
Marvin	YES
ChemDraw	NO
Indigo	NO

Reaction no 4

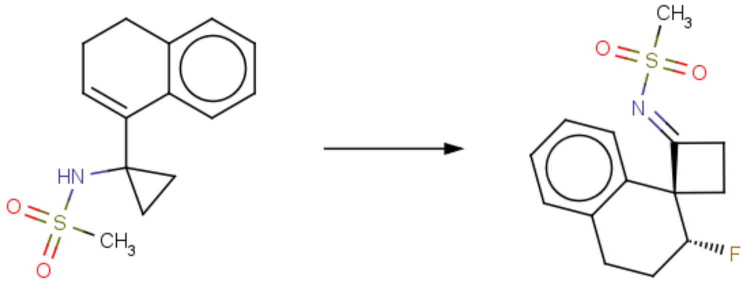

SMILES of the input:
CS(=O)(=O)NC1(CC1)C1=CCCc2ccccc12>>CS(=O)(=O)\N=C1/CC[C@@]11[C@H](F)CCc2c
cccc12

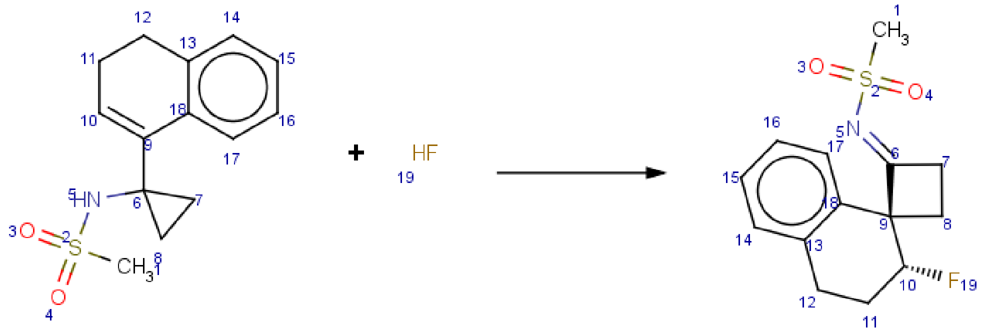

Correct mapped SMILES/SMARTS of the reaction:
[#6:1][S:2](=[O:3])(=[O:4])[#7:5][C:6]1([#6:7]-[#6:8]1)[#6:9]-1=[#6:10]-
[#6:11]-[#6:12]-[c:13]2[c:14][c:15][c:16][c:17][c:18]-
12.[F:19]>>[#6:1][S:2](=[O:3])(=[O:4])\[#7:5]=[#6:6]1/[#6:7]-
[#6:8][C@@:9]11[#6@H:10]([F:19])-[#6:11]-[#6:12]-
[c:13]2[c:14][c:15][c:16][c:17][c:18]12

Correctness of the mapping

MAPPET	YES
ReactionMap	NO
Marvin	YES
ChemDraw	YES
Indigo	YES

Reaction no 5

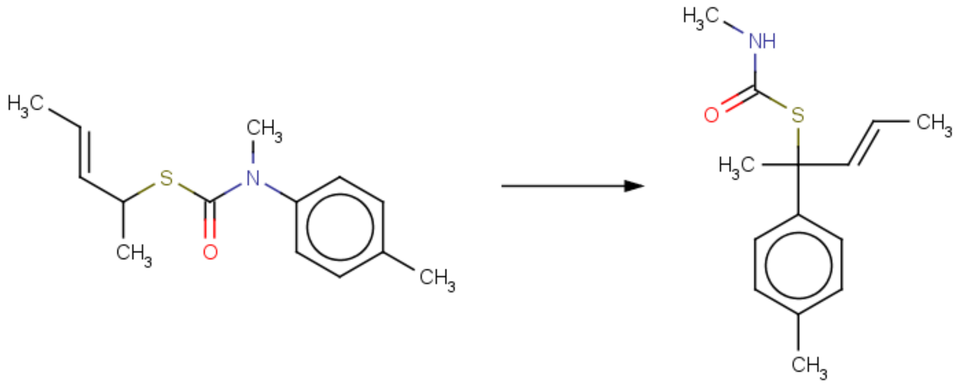

SMILES of the input:
C\C=C\C(C)SC(=O)N(C)c1ccc(C)cc1>>CNC(=O)SC(C)(\C=C\C)c1ccc(C)cc1

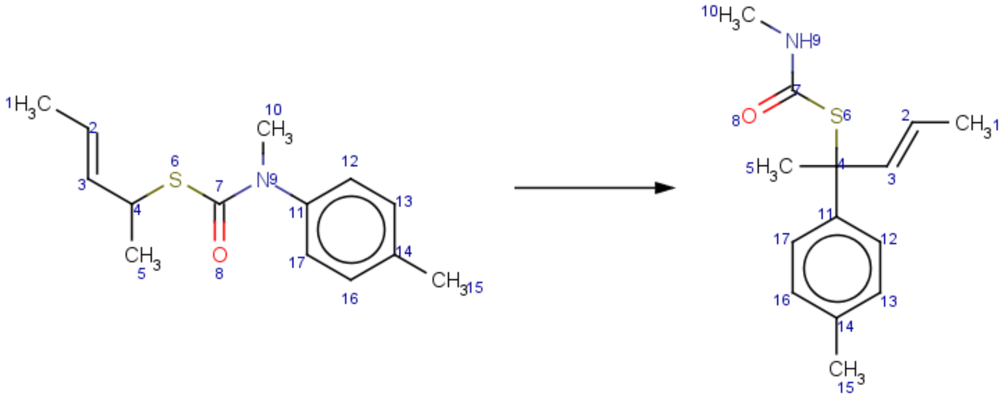

Correct mapped SMILES/SMARTS of the reaction:
[#6:1]\[#6:2]=[#6:3]\[#6:4](-[#6:5])-[#16:6]-[#6:7](=[O:8])-[#7:9](-[#6:10])-[c:11]1[c:12][c:13][c:14](-[#6:15])[c:16][c:17]1>>[#6:10]-[#7:9]-[#6:7](=[O:8])-[#16:6][C:4]([#6:5])(\[#6:3]=[#6:2]\[#6:1])[c:11]1[c:12][c:13][c:14](-[#6:15])[c:16][c:17]1

Correctness of the mapping

MAPPET	YES
ReactionMap	YES
Marvin	YES
ChemDraw	NO
Indigo	YES

Reaction no 6

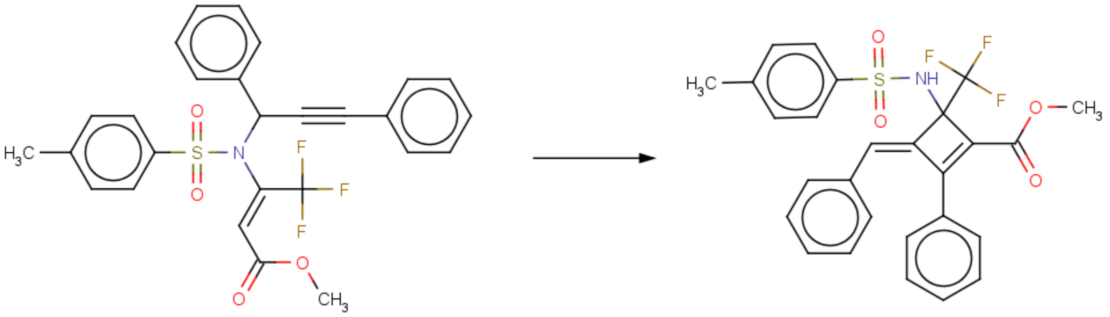

SMILES of the input:

COC(=O)\C=C(\N(C(C#Cc1ccccc1)c1ccccc1)S(=O)(=O)c1ccc(C)cc1)C(F)(F)F>>COC(=O)C1=C(\C(=C/c2ccccc2)C1(NS(=O)(=O)c1ccc(C)cc1)C(F)(F)F)c1ccccc1

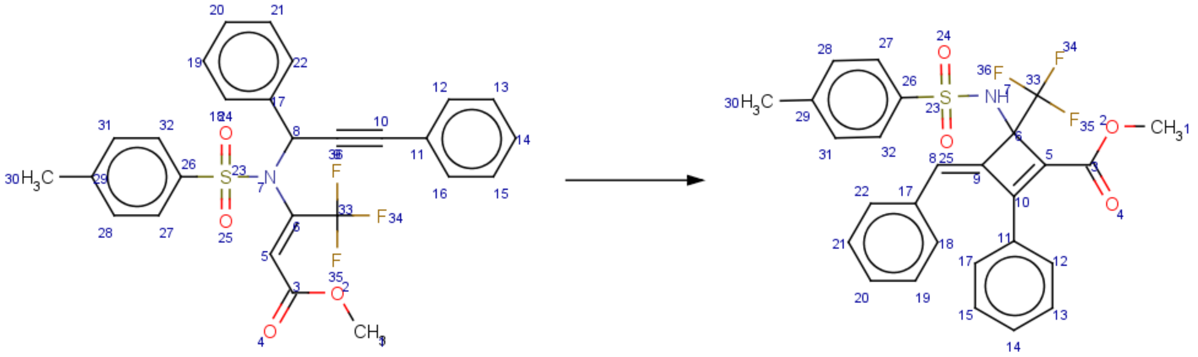

Correct mapped SMILES/SMARTS of the reaction:
[CH3:1][O:2][C:3](/[CH:5]=[C:6](\[C:33]([F:36])([F:35])[F:34])/[N:7]([S:23]([c:26]1[cH:32][cH:31][c:29]([CH3:30])[cH:28][cH:27]1)(=[O:25])=[O:24])[CH:8]([c:17]1[cH:22][cH:21][cH:20][cH:19][cH:18]1)[C:9]#[C:10][c:11]1[cH:16][cH:15][cH:14][cH:13][cH:12]1)=[O:4]>>[CH3:1][O:2][C:3]([C:5]1[C:6]([C:33]([F:35])([F:34])[F:36])([NH:7][S:23]([c:26]2[cH:32][cH:31][c:29]([CH3:30])[cH:28][cH:27]2)(=[O:24])=[O:25])/[C:9](=[CH:10]/[c:11]2[cH:16][cH:15][cH:14][cH:13][cH:12]2)/[C:8]=1[c:17]1[cH:18][cH:19][cH:20][cH:21][cH:22]1)=[O:4]

Correctness of the mapping

MAPPET	NO
ReactionMap	NO
Marvin	NO
ChemDraw	NO
Indigo	YES

Reaction no 7

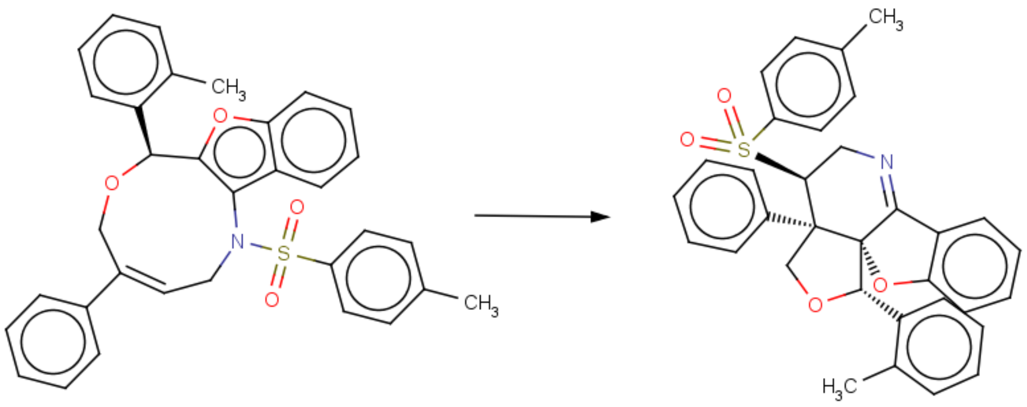

SMILES of the input:
Cc1ccc(cc1)S(=O)(=O)N1C\C=C(/CO[C@H](c2oc3ccccc3c12)c1ccccc1C)c1ccccc1>>Cc1ccc(cc1)S(=O)(=O)[C@H]1CN=C2c3ccccc3O[C@@]22[C@@H](OC[C@@]12c1ccccc1)c1ccccc1C

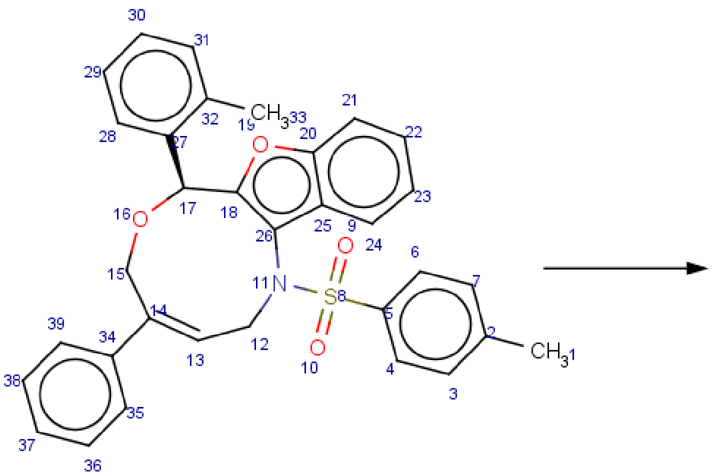

Correct mapped SMILES/SMARTS of the reaction:
[#6:1]-[c:2]1[c:3][c:4][c:5]([c:6][c:7]1)[S:8](=[O:9])(=[O:10])[#7:11]-1-
[#6:12]\[#6:13]=[#6:14](/[#6:15]-[#8:16]-[#6@H:17](-
[c:18]2[o:19][c:20]3[c:21][c:22][c:23][c:24][c:25]3[c:26]-12)-
[c:27]1[c:28][c:29][c:30][c:31][c:32]1-[#6:33])-
[c:34]1[c:35][c:36][c:37][c:38][c:39]1>>[#6:1]-
[c:2]1[c:3][c:4][c:5]([c:6][c:7]1)[S:8](=[O:9])(=[O:10])[#6@H:13]1-
[#6:12]-[#7:11]=[#6:26]2-[c:25]3[c:24][c:23][c:22][c:21][c:20]3-
[#8:19][C@@:18]22[#6@@H:17](-[#8:16]-
[#6:15][C@@:14]12[c:34]1[c:39][c:38][c:37][c:36][c:35]1)-
[c:27]1[c:28][c:29][c:30][c:31][c:32]1-[#6:33]

Correctness of the mapping

MAPPET	YES
ReactionMap	NO
Marvin	YES
ChemDraw	YES
Indigo	YES

Reaction no 8

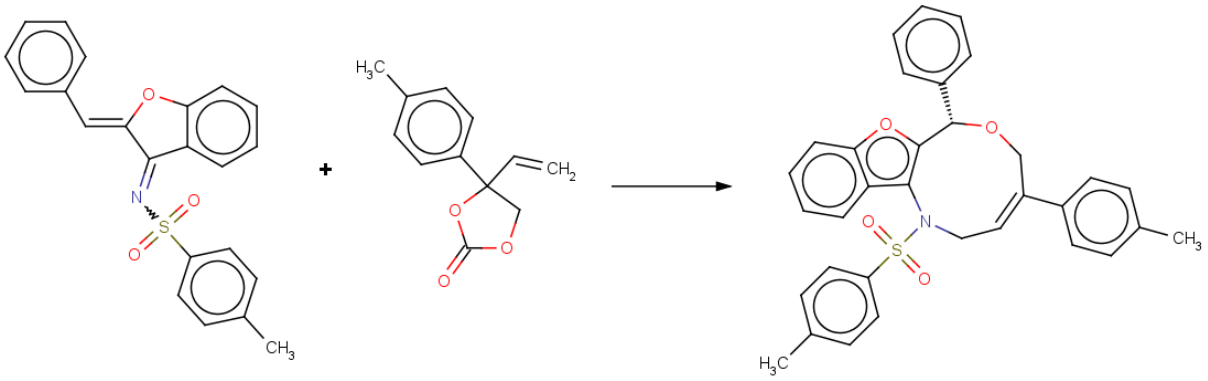

SMILES of the input:
Cc1ccc(cc1)S(=O)(=O)N=C1\C(Oc2ccccc12)=C\c1ccccc1.Cc1ccc(cc1)C1(COC(=O)O1
)C=C>>Cc1ccc(cc1)C1=C/CN(c2c(oc3ccccc23)[C@@H](OC\1)c1ccccc1)S(=O)(=O)c1c
cc(C)cc1

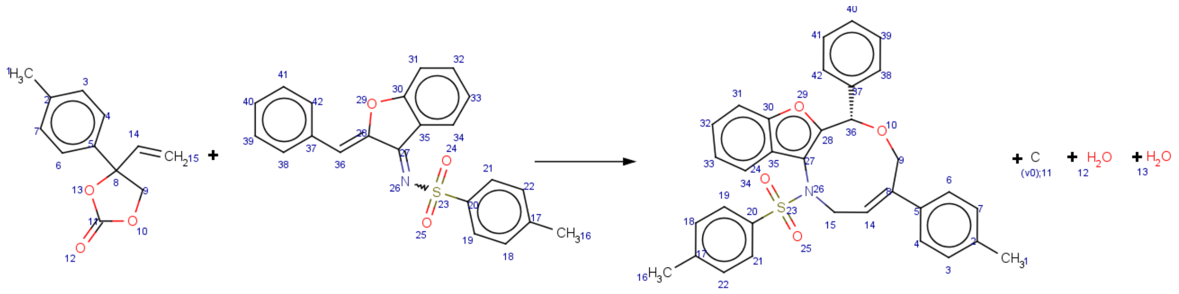

Correct mapped SMILES/SMARTS of the reaction:
[#6:1]-[c:2]1[c:3][c:4][c:5]([c:6][c:7]1)[C:8]1([#6:9]-[#8:10]-
[#6:11](=[O:12])-[#8:13]1)[#6:14]=[#6:15].[#6:16]-
[c:17]1[c:18][c:19][c:20]([c:21][c:22]1)[S:23](=[O:24])(=[O:25])[#7:26]=[
#6:27]-1\[#6:28](-[#8:29]-[c:30]2[c:31][c:32][c:33][c:34][c:35]-
12)=[#6:36]\[c:37]1[c:38][c:39][c:40][c:41][c:42]1>>[#6:1]-
[c:2]1[c:3][c:4][c:5]([c:6][c:7]1)-[#6:8]-1=[#6:14]/[#6:15]-[#7:26](-
[c:27]2[c:28]([o:29][c:30]3[c:31][c:32][c:33][c:34][c:35]23)-[#6@@H:36](-
[#8:10]-[#6:9]\1)-
[c:37]1[c:42][c:41][c:40][c:39][c:38]1)[S:23](=[O:24])(=[O:25])[c:20]1[c:
21][c:22][c:17](-[#6:16])[c:18][c:19]1.[#6;v0:11].[#8:12].[#8:13]

Correctness of the mapping

MAPPET	YES
ReactionMap	NO
Marvin	NO
ChemDraw	YES
Indigo	NO

Reaction no 9

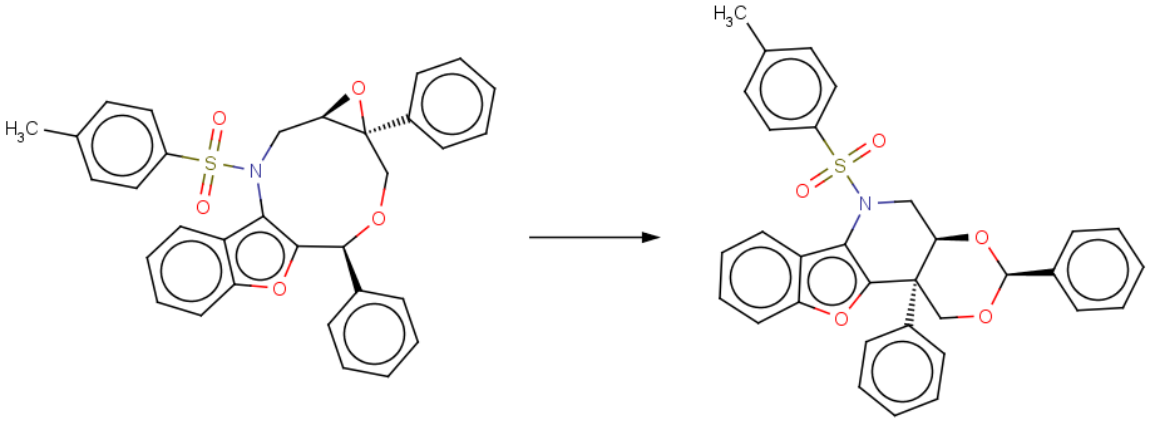

SMILES of the input:
Cc1ccc(cc1)S(=O)(=O)N1C[C@H]2O[C@]2(CO[C@H](c2oc3ccccc3c12)c1ccccc1)c1ccc
cc1>>Cc1ccc(cc1)S(=O)(=O)N1C[C@H]2O[C@H](OC[C@]2(c2oc3ccccc3c12)c1ccccc1)
c1ccccc1

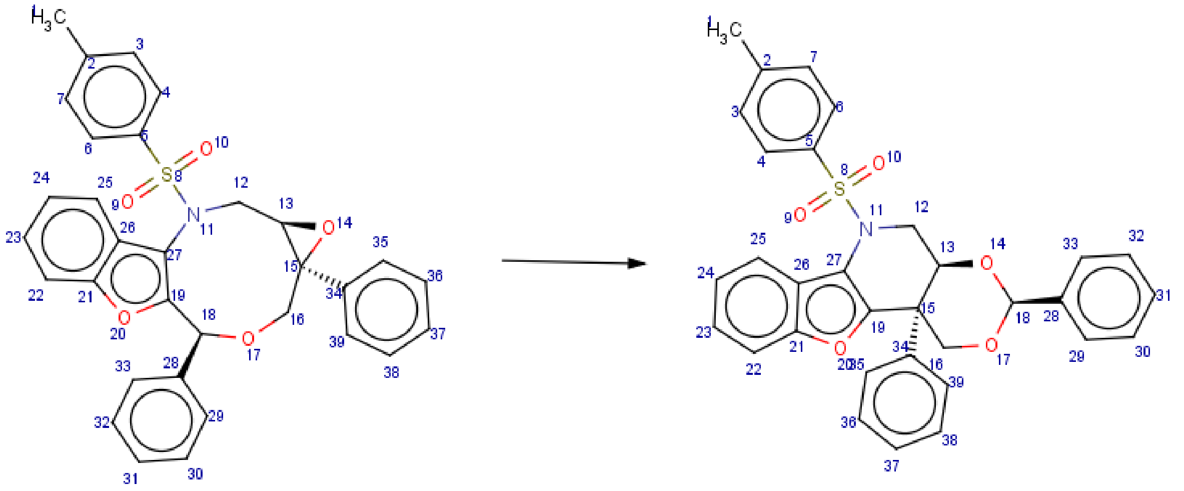

Correct mapped SMILES/SMARTS of the reaction:
[#6:1]-[c:2]1[c:3][c:4][c:5]([c:6][c:7]1)[S:8](=[O:9])(=[O:10])[#7:11]-1-[#6:12]-[#6@H:13]2-[#8:14][C@:15]2([#6:16]-[#8:17]-[#6@H:18](-[c:19]2[o:20][c:21]3[c:22][c:23][c:24][c:25][c:26]3[c:27]-12)-[c:28]1[c:29][c:30][c:31][c:32][c:33]1)[c:34]1[c:35][c:36][c:37][c:38][c:39]1>>[#6:1]-[c:2]1[c:7][c:6][c:5]([c:4][c:3]1)[S:8](=[O:9])(=[O:10])[#7:11]-1-[#6:12]-[#6@H:13]2-[#8:14]-[#6@H:18](-[#8:17]-[#6:16][C@:15]2([c:19]2[o:20][c:21]3[c:22][c:23][c:24][c:25][c:26]3[c:27]-12)[c:34]1[c:39][c:38][c:37][c:36][c:35]1)-[c:28]1[c:33][c:32][c:31][c:30][c:29]1

Correctness of the mapping

MAPPET	YES
ReactionMap	NO
Marvin	NO
ChemDraw	YES
Indigo	YES

Reaction no 10

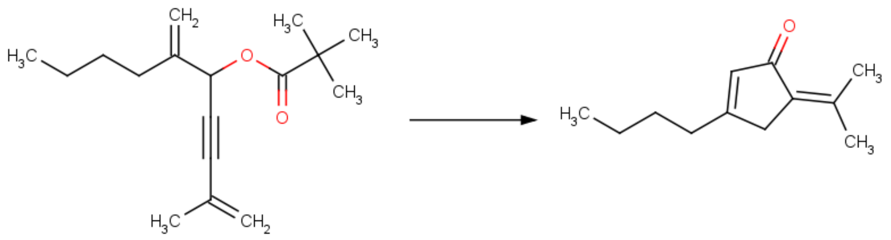

SMILES of the input:
CCCCC(=C)C(OC(=O)C(C)(C)C)C#CC(C)=C>>CCCCC1=CC(=O)C(C1)=C(C)C

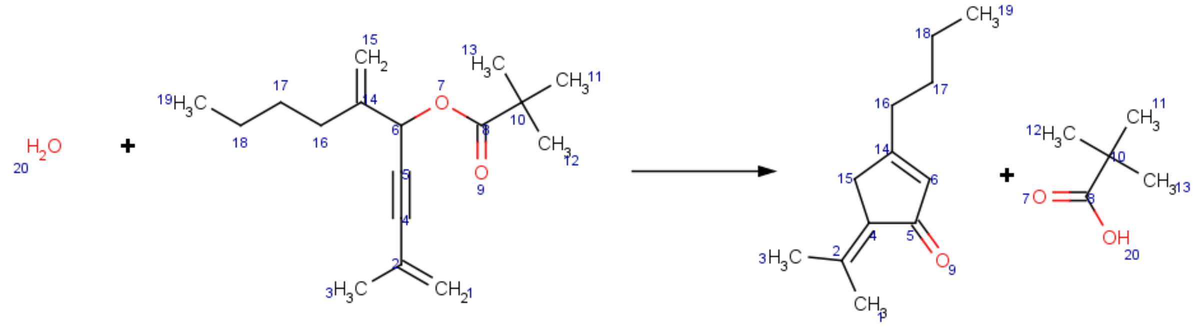

Correct mapped SMILES/SMARTS of the reaction:
C[CH2:1][CH2:2][CH2:3][C:4](=C)[CH:5](OC(=O)C(C)(C)C)[C:6]#[C:7][C:8]([CH3:9])=[CH2:10]>>[CH3:1][CH2:2][CH2:3][CH2:4][C:5]1=CC(=O)[C:7]([CH2:6]1)=[C:8]([CH3:9])[CH3:10]

Correctness of the mapping

MAPPET	NO
ReactionMap	NO
Marvin	NO
ChemDraw	NO
Indigo	NO

Reaction no 11

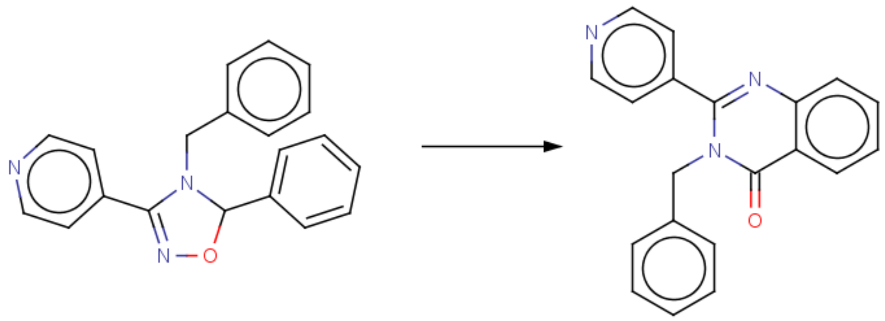

SMILES of the input:
C(N1C(ON=C1c1ccncc1)C1=CC=CC=C1)c1ccccc1>>O=C1N(Cc2ccccc2)C(=Nc2ccccc12)c1ccncc1

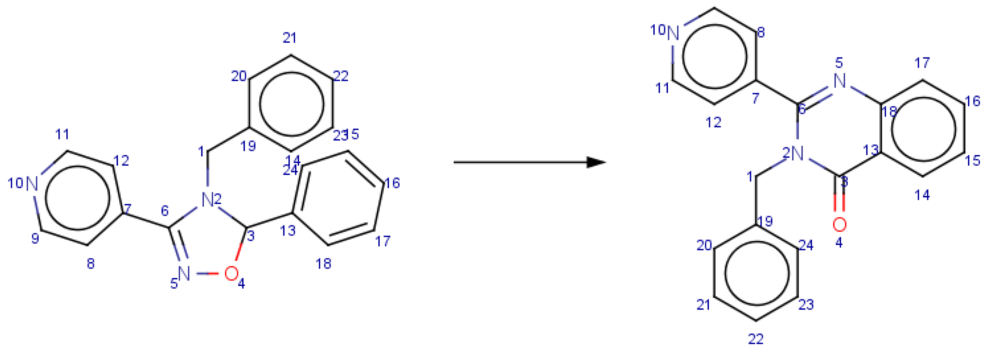

Correct mapped SMILES/SMARTS of the reaction:
[#6:1](-[#7:2]-1-[#6:3](-[#8:4]-[#7:5]=[#6:6]-1-[c:7]1[c:8][c:9][n:10][c:11][c:12]1)-[#6:13]-1=[#6:14]-[#6:15]=[#6:16]-

[#6:17]=[#6:18]-1)-[c:19]1[c:20][c:21][c:22][c:23][c:24]1>>[O:4]=[#6:3]-1-[#7:2](-[#6:1]-[c:19]2[c:24][c:23][c:22][c:21][c:20]2)-[#6:6](=[#7:5]-[c:18]2[c:17][c:16][c:15][c:14][c:13]-12)-[c:7]1[c:12][c:11][n:10][c:9][c:8]1

Correctness of the mapping
MAPPET YES
ReactionMap YES
Marvin YES
ChemDraw YES
Indigo NO

Reaction no 12

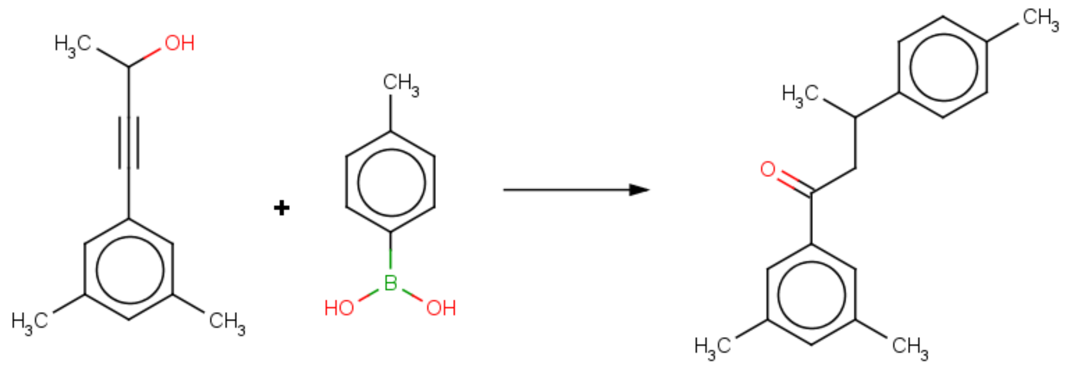

SMILES of the input:
CC(O)C#Cc1cc(C)cc(C)c1.Cc1ccc(cc1)B(O)O>>CC(CC(=O)c1cc(C)cc(C)c1)c1ccc(C)cc1

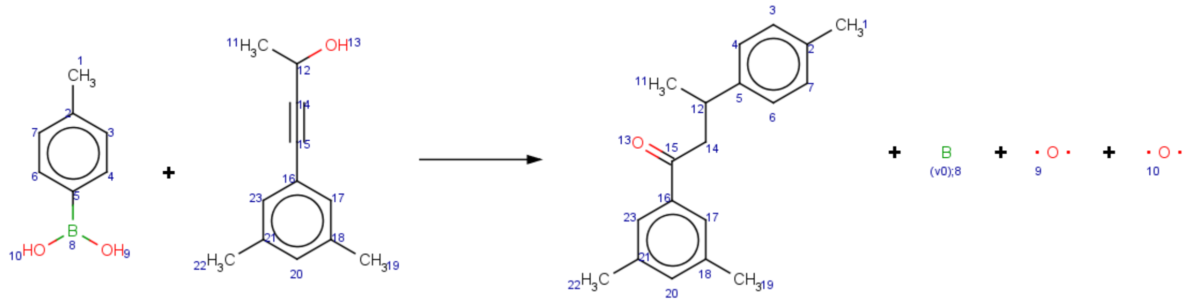

Correct mapped SMILES/SMARTS of the reaction:
[CH3:1][c:2]1[cH:3][cH:4][c:5]([cH:6][cH:7]1)[B:8]([OH:9])[OH:10].[CH3:11][CH:12]([OH:13])[C:14]#[C:15][c:16]1[cH:17][c:18]([CH3:19])[cH:20][c:21]([CH3:22])[cH:23]1>>[CH3:11][CH:12]([CH2:14][C:15](=[O:13])[c:16]1[cH:17][c:18]([CH3:19])[cH:20][c:21]([CH3:22])[cH:23]1)[c:5]1[cH:4][cH:3][c:2]([CH3:1])[cH:7][cH:6]1.[B:8].[O:9].[O:10]

Correctness of the mapping
MAPPET YES
ReactionMap NO
Marvin NO
ChemDraw YES
Indigo NO

Reaction no 13

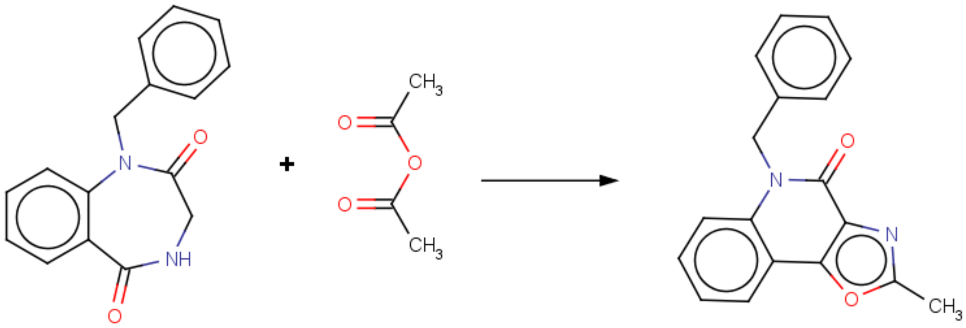

SMILES of the input:
O=C1CNC(=O)c2ccccc2N1Cc1ccccc1.CC(=O)OC(C)=O>>Cc1nc2c(o1)-
c1ccccc1N(Cc1ccccc1)C2=O

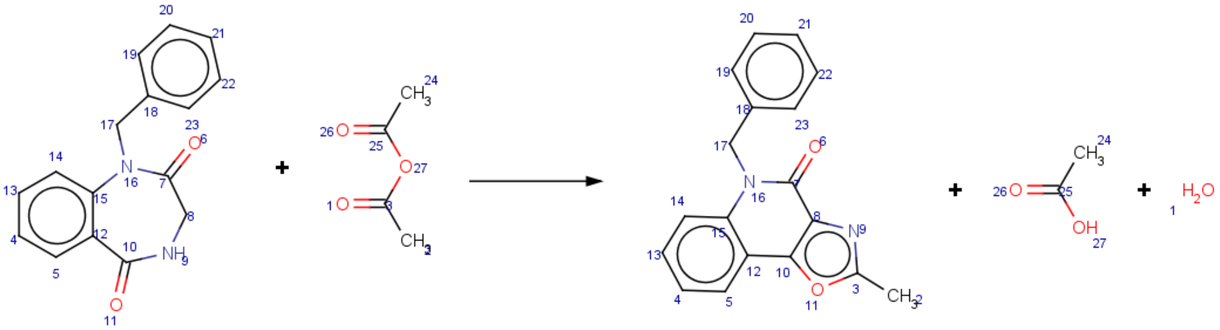

Correct mapped SMILES/SMARTS of the reaction:
[O:1]=[C:2]1[CH2:3][NH:4][C:5](=[O:6])[c:7]2[cH:8][cH:9][cH:10][cH:11][c:
12]2[N:13]1[CH2:14][c:15]1[cH:16][cH:17][cH:18][cH:19][cH:20]1.CC(=O)O[C:
21]([CH3:22])=O>>[CH3:22][c:21]1[n:4][c:3]2[c:5]([o:6]1)-
[c:7]1[cH:8][cH:9][cH:10][cH:11][c:12]1[N:13]([CH2:14][c:15]1[cH:20][cH:1
9][cH:18][cH:17][cH:16]1)[C:2]2=[O:1]

Correctness of the mapping

MAPPET	NO
ReactionMap	NO
Marvin	NO
ChemDraw	YES
Indigo	YES

Reaction no 14

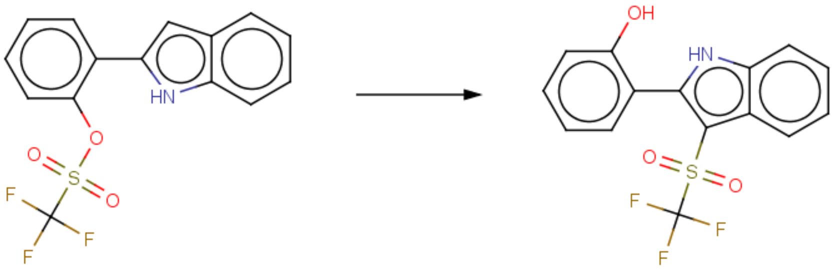

SMILES of the input:
FC(F)(F)S(=O)(=O)Oc1ccccc1-c1cc2ccccc2[nH]1>>Oc1ccccc1-
c1[nH]c2ccccc2c1S(=O)(=O)C(F)(F)F

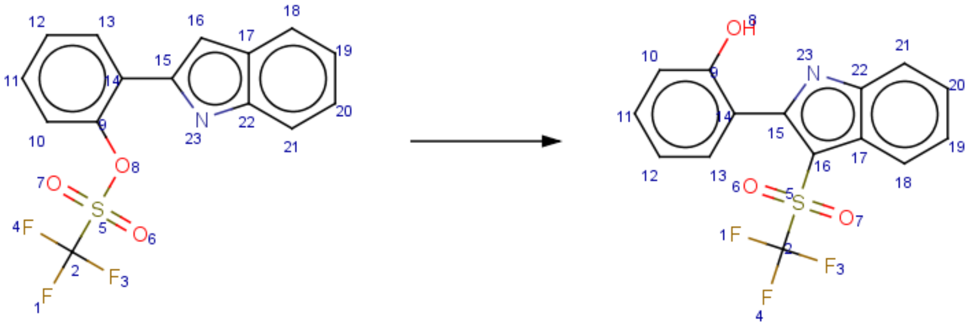

Correct mapped SMILES/SMARTS of the reaction:
[F:1][C:2]([F:3])([F:4])[S:5](=[O:6])(=[O:7])[#8:8]-[c:9]1[c:10][c:11][c:12][c:13][c:14]1-[c:15]1[c:16][c:17]2[c:18][c:19][c:20][c:21][c:22]2[n:23]1>>[#8:8]-[c:9]1[c:10][c:11][c:12][c:13][c:14]1-[c:15]1[n:23][c:22]2[c:21][c:20][c:19][c:18][c:17]2[c:16]1[S:5](=[O:6])(=[O:7])[C:2]([F:1])([F:3])[F:4]

Correctness of the mapping

MAPPET	YES
ReactionMap	YES
Marvin	YES
ChemDraw	YES
Indigo	YES

Reaction no 15

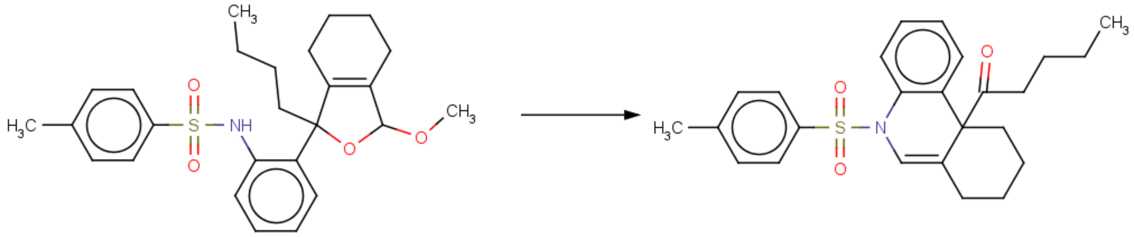

SMILES of the input:
CCCCC1(OC(OC)C2=C1CCCC2)c1ccccc1NS(=O)(=O)c1ccc(C)cc1>>CCCCC(=O)C12CCCCC1=CN(c1ccccc21)S(=O)(=O)c1ccc(C)cc1

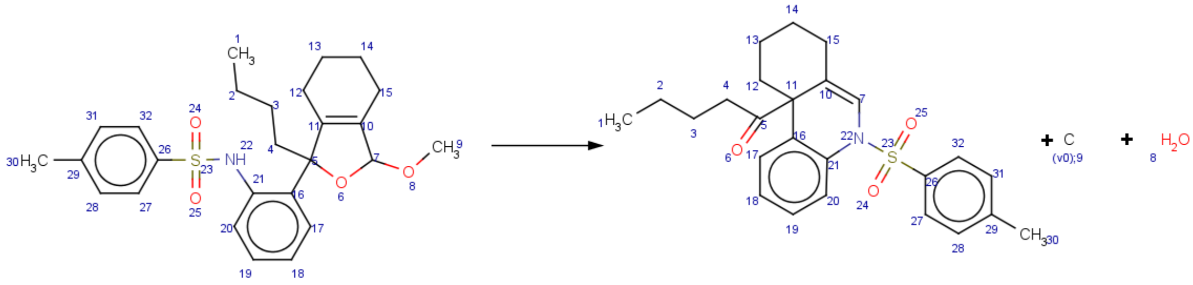

Correct mapped SMILES/SMARTS of the reaction:
[#6:1]-[#6:2]-[#6:3]-[#6:4][C:5]1([#8:6]-[#6:7](-[#8:8]-[#6:9])-[#6:10]-2=[#6:11]1-[#6:12]-[#6:13]-[#6:14]-[#6:15]-2)[c:16]1[c:17][c:18][c:19][c:20][c:21]1-[#7:22][S:23](=[O:24])(=[O:25])[c:26]1[c:27][c:28][c:29](-[#6:30])[c:31][c:32]1>>[#6:1]-[#6:2]-[#6:3]-[#6:4]-

[#6:5](=[O:6])[C:11]12[#6:12]-[#6:13]-[#6:14]-[#6:15]-[#6:10]1=[#6:7]-[#7:22](-[c:21]1[c:20][c:19][c:18][c:17][c:16]21)[S:23](=[O:24])(=[O:25])[c:26]1[c:32][c:31][c:29](-[#6:30])[c:28][c:27]1.[#6;v0:9].[#8:8]

Correctness of the mapping

MAPPET	YES
ReactionMap	NO
Marvin	NO
ChemDraw	NO
Indigo	NO

Reaction no 16

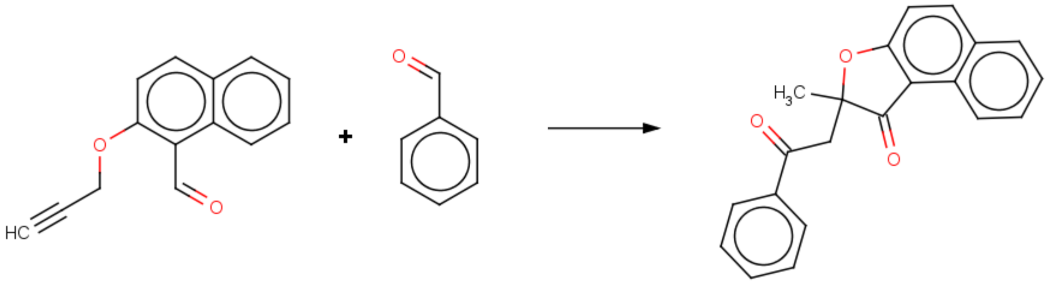

SMILES of the input:
O=Cc1c(OCC#C)ccc2ccccc12.O=Cc1ccccc1>>CC1(CC(=O)c2ccccc2)Oc2ccc3ccccc3c2C1=O

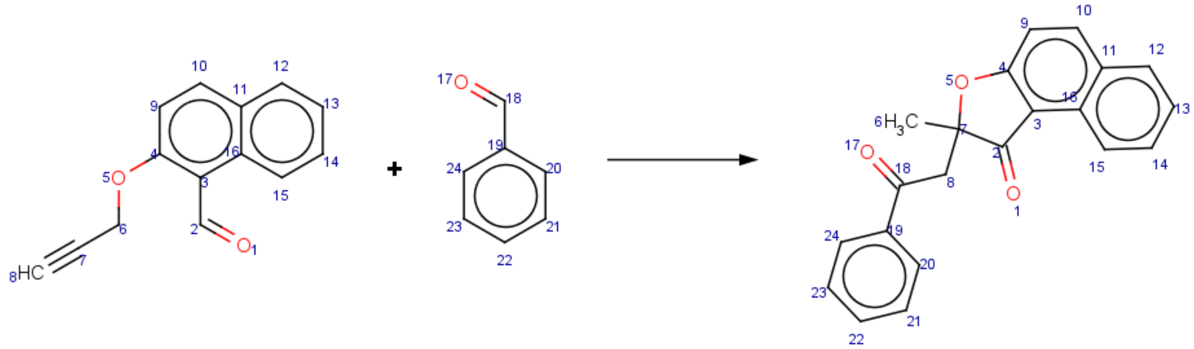

Correct mapped SMILES/SMARTS of the reaction:
[O:1]=[#6:2]-[c:3]1[c:4](-[#8:5]-[#6:6][C:7]#[C:8])[c:9][c:10][c:11]2[c:12][c:13][c:14][c:15][c:16]12.[O:17]=[#6:18]-[c:19]1[c:20][c:21][c:22][c:23][c:24]1>>[#6:6][C:7]1([#6:8]-[#6:18](=[O:17])-[c:19]2[c:20][c:21][c:22][c:23][c:24]2)[#8:5]-[c:4]2[c:9][c:10][c:11]3[c:12][c:13][c:14][c:15][c:16]3[c:3]2-[#6:2]1=[O:1]

Correctness of the mapping

MAPPET	YES
ReactionMap	NO
Marvin	NO
ChemDraw	NO
Indigo	NO

Reaction no 17

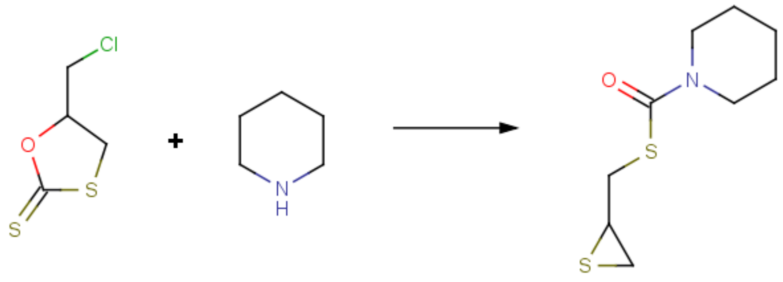

SMILES of the input:
ClCC1CSC(=S)O1.C1CCNCC1>>O=C(SCC1CS1)N1CCCCC1

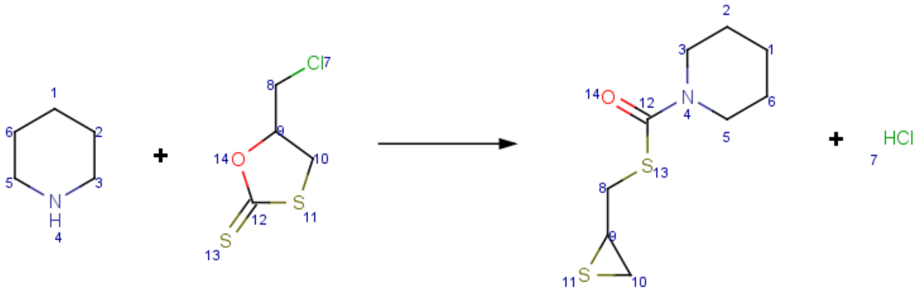

Correct mapped
SMILES/SMARTS of the reaction:
Cl[CH2:1][CH:2]1[CH2:3][S:4]C(=S)O1.[CH2:5]1[CH2:6][CH2:7][NH:8][CH2:9][CH2:10]1>>O=C(S[CH2:1][CH:2]1[CH2:3][S:4]1)[N:8]1[CH2:9][CH2:10][CH2:5][CH2:6][CH2:7]1

Correctness of the mapping

MAPPET	NO
ReactionMap	NO
Marvin	NO
ChemDraw	YES
Indigo	NO

Reaction no 18

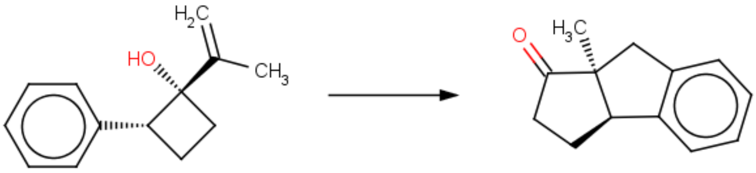

SMILES of the input:
CC(=C)[C@@]1(O)CC[C@@H]1c1ccccc1>>C[C@]12Cc3ccccc3[C@H]1CCC2=O

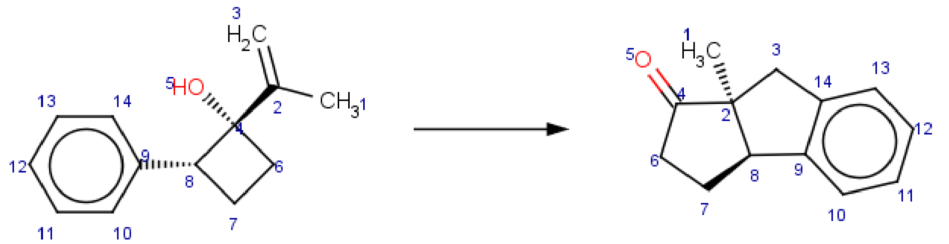

Correct

mapped SMILES/SMARTS of the reaction:
[#6:1]-[#6:2](=[#6:3])[C@@:4]1([#8:5])[#6:6]-[#6:7]-[#6@@H:8]1-[c:9]1[c:10][c:11][c:12][c:13][c:14]1>>[#6:1][C@:2]12[#6:3]-[c:14]3[c:13][c:12][c:11][c:10][c:9]3-[#6@H:8]1-[#6:7]-[#6:6]-[#6:4]2=[O:5]

Correctness of the mapping

MAPPET	YES
ReactionMap	NO
Marvin	NO
ChemDraw	NO
Indigo	NO

Reaction no 19

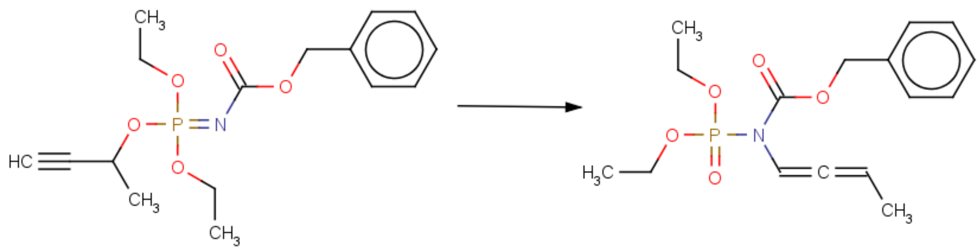

SMILES of the input:
CCOP(OCC)(OC(C)C#C)=NC(=O)OCc1ccccc1>>CCOP(=O)(OCC)N(C=C=CC)C(=O)OCc1cccc c1

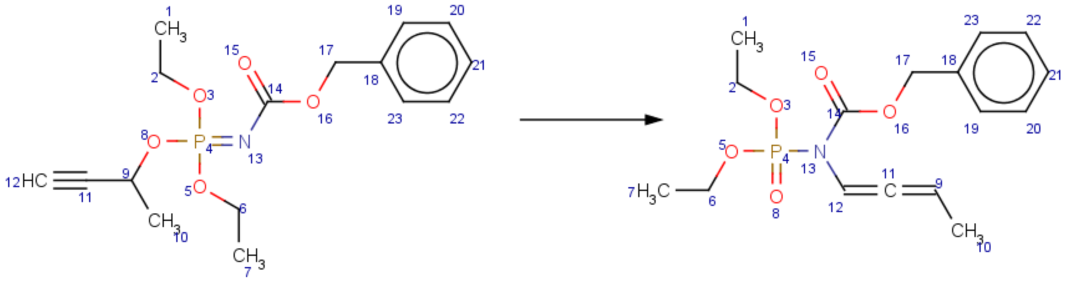

Correct

mapped SMILES/SMARTS of the reaction:
[CH3:1][CH2:2][O:3][P:4](=[N:13][C:14](=[O:15])[O:16][CH2:17][c:18]1[cH:19][cH:20][cH:21][cH:22][cH:23]1)([O:5][CH2:6][CH3:7])[O:8][CH:9]([CH3:10])[C:11]#[CH:12]>>[CH3:7][CH2:6][O:5][P:4](=[O:8])([N:13]([CH:12]=[C:11]=[CH:9][CH3:10])[C:14](=[O:15])[O:16][CH2:17][c:18]1[cH:23][cH:22][cH:21][cH:20][cH:19]1)[O:3][CH2:2][CH3:1]

Correctness of the mapping

MAPPET NO
ReactionMap YES
Marvin NO
ChemDraw YES
Indigo NO

Reaction no 20

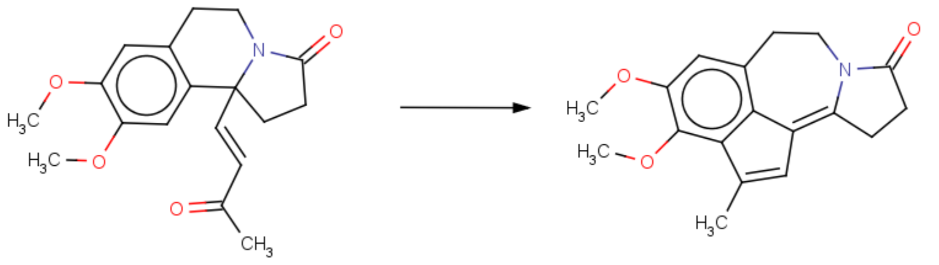

SMILES of the input:
COc1cc2CCN3C(=O)CCC3(\C=C\C(C)=O)c2cc1OC>>COc1cc2CCN3C(=O)CCC3=C3C=C(C)c(c23)c1OC

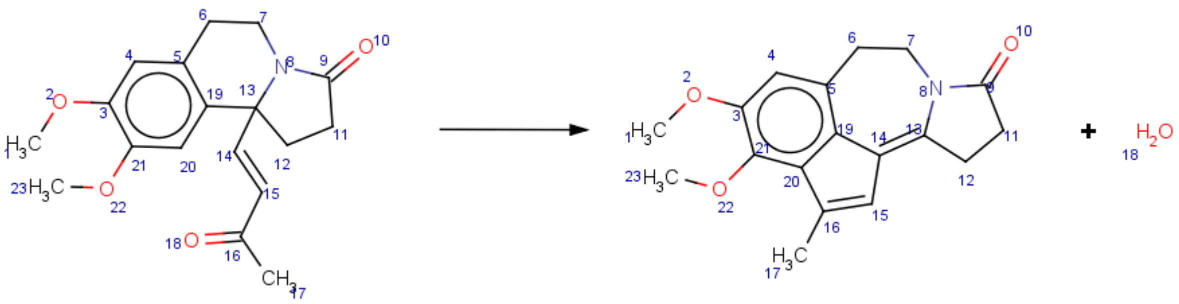

Correct mapped SMILES/SMARTS of the reaction:
[#6:1]-[#8:2]-[c:3]1[c:4][c:5]2-[#6:6]-[#6:7]-[#7:8]3-[#6:9](=[O:10])-[#6:11]-[#6:12][C:13]3(\[#6:14]=[#6:15]\[#6:16](-[#6:17])=[O:18])[c:19]2[c:20][c:21]1-[#8:22]-[#6:23]>>[#6:1]-[#8:2]-[c:3]1[c:4][c:5]2-[#6:6]-[#6:7]-[#7:8]-3-[#6:9](=[O:10])-[#6:11]-[#6:12]-[#6:13]-3=[#6:14]-3-[#6:15]=[#6:16](-[#6:17])-[c:20]([c:19]2-3)[c:21]1-[#8:22]-[#6:23].[#8:18]

Correctness of the mapping
MAPPET YES
ReactionMap NO
Marvin YES
ChemDraw YES
Indigo NO

Reaction no 21

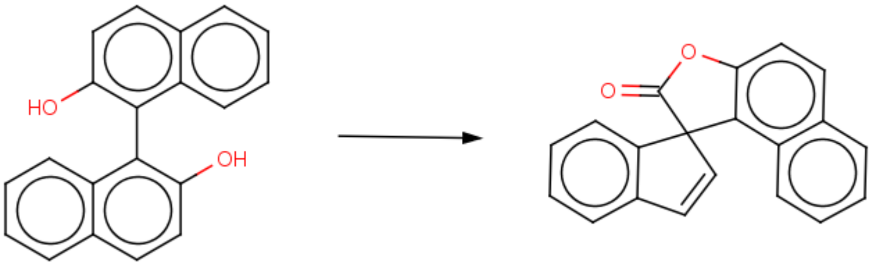

SMILES of the input:
Oc1ccc2ccccc2c1-c1c(O)ccc2ccccc12>>O=C1Oc2ccc3ccccc3c2C11C=Cc2ccccc12

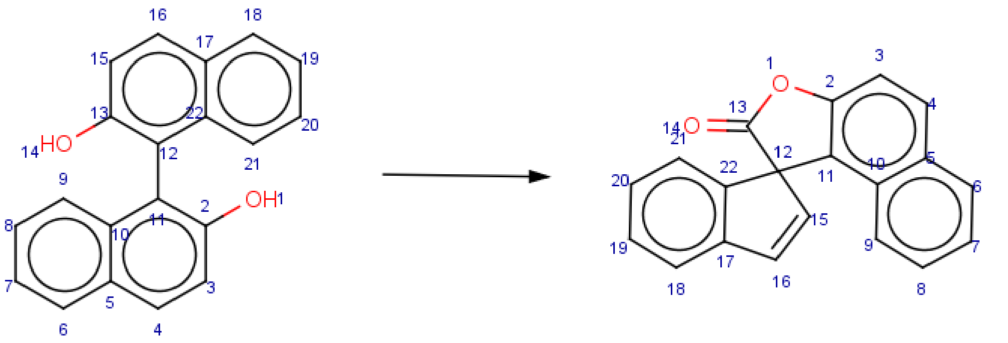

Correct mapped SMILES/SMARTS of the reaction:
[#8:1]-[c:2]1[c:3][c:4][c:5]2[c:6][c:7][c:8][c:9][c:10]2[c:11]1-[c:12]1[c:13](-[#8:14])[c:15][c:16][c:17]2[c:18][c:19][c:20][c:21][c:22]12>>[O:14]=[#6:13]1-[#8:1]-[c:2]2[c:3][c:4][c:5]3[c:6][c:7][c:8][c:9][c:10]3[c:11]2[C:12]11[#6:15]=[#6:16]-[c:17]2[c:18][c:19][c:20][c:21][c:22]12

Correctness of the mapping

MAPPET	YES
ReactionMap	YES
Marvin	YES
ChemDraw	YES
Indigo	NO

Reaction no 22

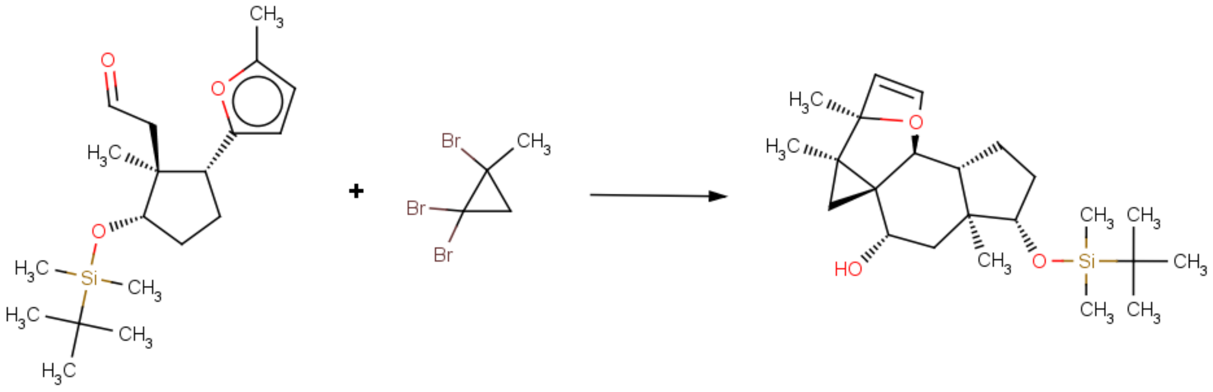

SMILES of the input:

Cc1ccc(o1)[C@@H]1CC[C@H](O[Si](C)(C)C(C)(C)C)[C@@]1(C)CC=O.CC1(Br)CC1(Br)Br>>CC(C)(C)[Si](C)(C)O[C@H]1CC[C@@H]2[C@]1(C)C[C@H](O)[C@]13C[C@@]1(C)[C@]1(C)O[C@@]23C=C1

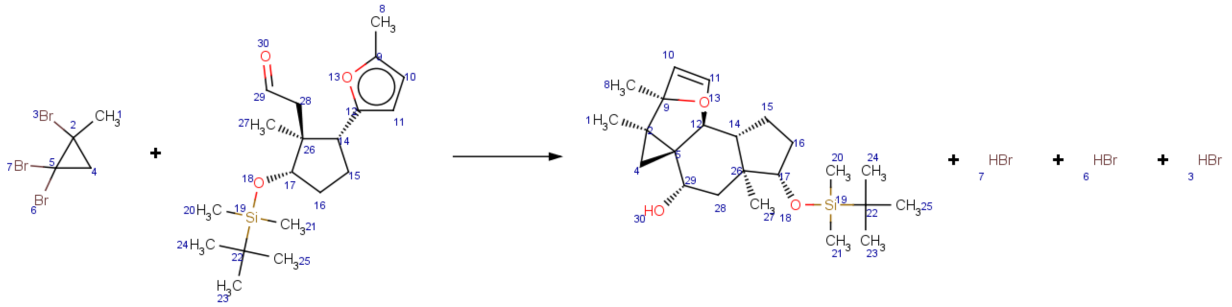

Correct mapped SMILES/SMARTS of the reaction:
[#6:1][C:2]1([Br:3])[#6:4][C:5]1([Br:6])[Br:7].[#6:8]-[c:9]1[c:10][c:11][c:12]([o:13]1)-[#6@@H:14]1-[#6:15]-[#6:16]-[#6@H:17](-[#8:18][Si:19]([#6:20])([#6:21])[C:22]([#6:23])([#6:24])[#6:25])[C@@:26]1([#6:27])[#6:28]-[#6:29]=[O:30]>>[#6:25][C:22]([#6:24])([#6:23])[Si:19]([#6:20])([#6:21])[#8:18]-[#6@H:17]1-[#6:16]-[#6:15]-[#6@@H:14]2[C@:26]1([#6:27])[#6:28]-[#6@H:29](-[#8:30])[C@:5]13[#6:4][C@@:2]1([#6:1])[C@:9]1([#6:8])[#8:13][C@@:12]23[#6:11]=[#6:10]1.[Br:7].[Br:6].[Br:3]

Correctness of the mapping

MAPPET	YES
ReactionMap	NO
Marvin	YES
ChemDraw	NO
Indigo	NO

Reaction no 23

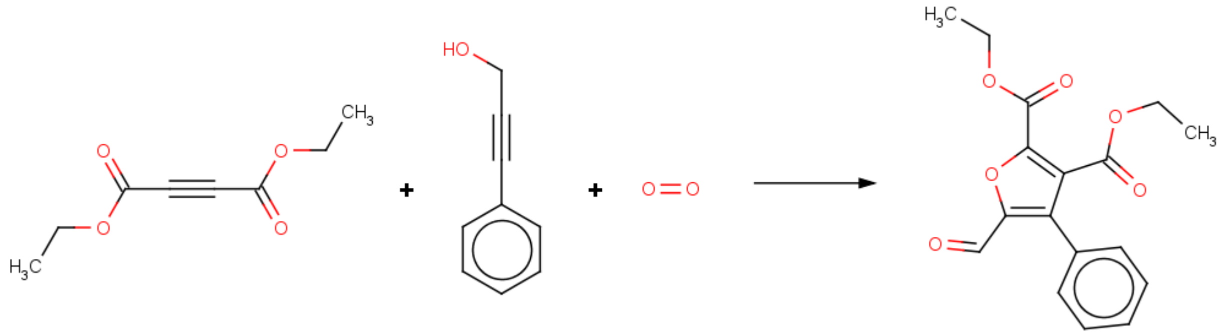

SMILES of the input:
CCOC(=O)C#CC(=O)OCC.OCC#Cc1ccccc1.O=O>>CCOC(=O)C1=C(C(=O)OCC)C(=C(O1)C=O)c1ccccc1

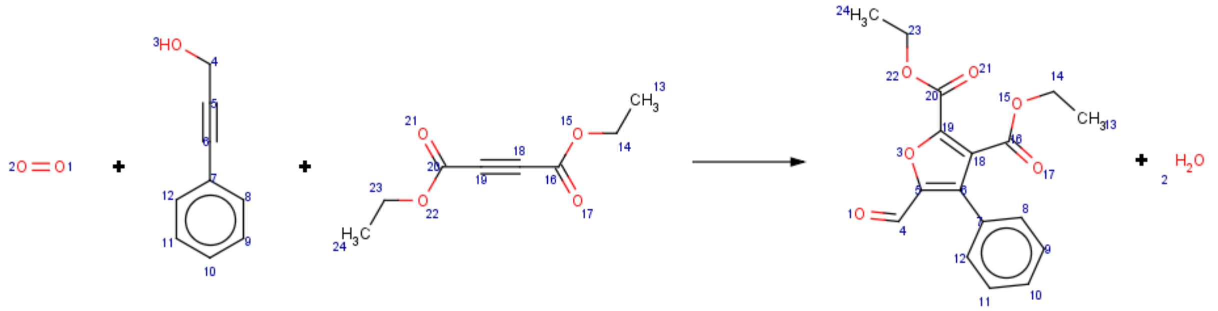

Correct mapped SMILES/SMARTS of the reaction:

[O:1]=[O:2].[OH:3][CH2:4][C:5]#[C:6][c:7]1[cH:8][cH:9][cH:10][cH:11][cH:12]1.[CH3:13][CH2:14][O:15][C:16](=[O:17])[C:18]#[C:19][C:20](=[O:21])[O:22][CH2:23][CH3:24]>>[CH3:24][CH2:23][O:22][C:20](=[O:21])[C:19]1=[C:18]([C:16](=[O:17])[O:15][CH2:14][CH3:13])[C:6](=[C:5]([O:3]1)[CH:4]=[O:1])[c:7]1[cH:8][cH:9][cH:10][cH:11][cH:12]1.[OH2:2]

Correctness of the mapping

MAPPET	NO
ReactionMap	NO
Marvin	NO
ChemDraw	NO
Indigo	YES

Reaction no 24

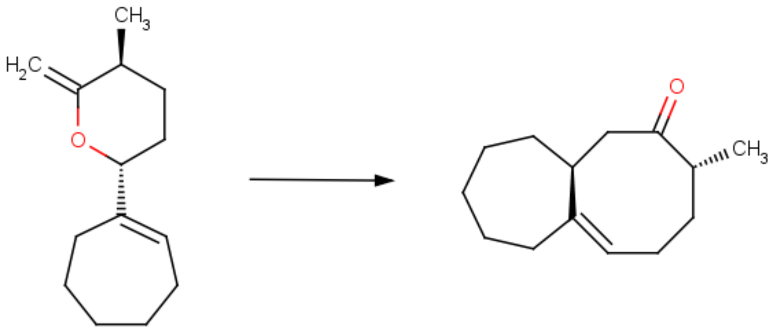

SMILES of the input:
C[C@H]1CC[C@@H](OC1=C)C1=CCCCCC1>>C[C@@H]1CC\C=C2\CCCCC[C@H]2CC1=O

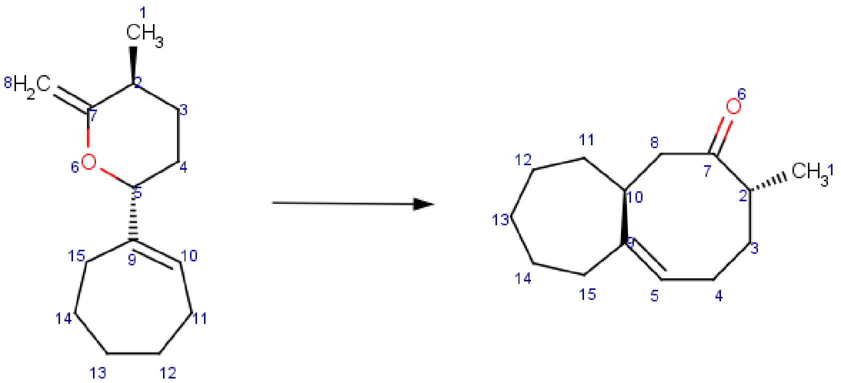

Correct mapped SMILES/SMARTS of the reaction:
[#6:1]-[#6@H:2]-1-[#6:3]-[#6:4]-[#6@@H:5](-[#8:6]-[#6:7]-1=[#6:8])-[#6:9]-1=[#6:10]-[#6:11]-[#6:12]-[#6:13]-[#6:14]-[#6:15]-1>>[#6:1]-[#6@@H:2]-1-[#6:3]-[#6:4]\[#6:5]=[#6:9]-2\[#6:15]-[#6:14]-[#6:13]-[#6:12]-[#6:11]-[#6@H:10]-2-[#6:8]-[#6:7]-1=[O:6]

Correctness of the mapping

MAPPET	YES
ReactionMap	NO
Marvin	NO
ChemDraw	NO
Indigo	NO

Reaction no 25

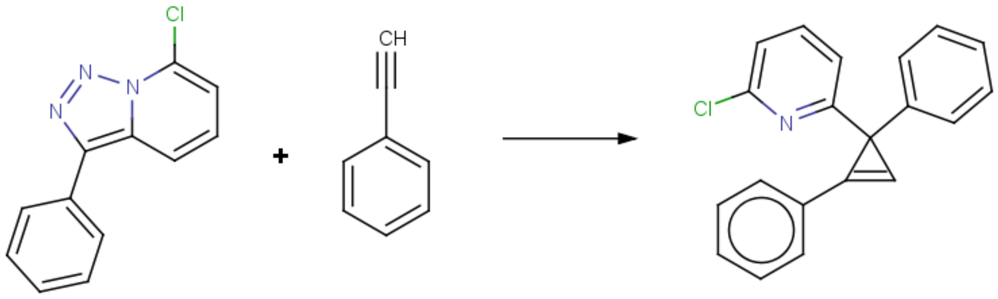

SMILES of the input:
ClC1=CC=CC2=C(N=NN12)C1=CC=CC=C1.C#CC1=CC=CC=C1>>ClC1=CC=CC(=N1)C1(C=C1c1ccccc1)C1=CC=CC=C1

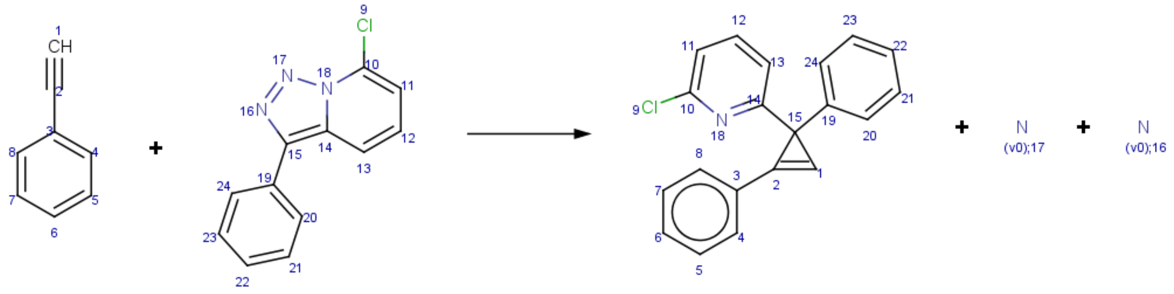

Correct mapped SMILES/SMARTS of the reaction:
[C:1]#[C:2][#6:3]-1=[#6:4]-[#6:5]=[#6:6]-[#6:7]=[#6:8]-1.[Cl:9][#6:10]-1=[#6:11]-[#6:12]=[#6:13]-[#6:14]-2=[#6:15](-[#7:16]=[#7:17]-[#7:18]-1-2)-[#6:19]-1=[#6:20]-[#6:21]=[#6:22]-[#6:23]=[#6:24]-1>>[Cl:9][#6:10]-1=[#6:11]-[#6:12]=[#6:13]-[#6:14](=[#7:18]-1)[C:15]1([#6:1]=[#6:2]1-[c:3]1[c:4][c:5][c:6][c:7][c:8]1)[#6:19]-1=[#6:24]-[#6:23]=[#6:22]-[#6:21]=[#6:20]-1.[#7;v0:17].[#7;v0:16]

Correctness of the mapping

MAPPET	YES
ReactionMap	NO
Marvin	YES
ChemDraw	YES
Indigo	YES

Reaction no 26

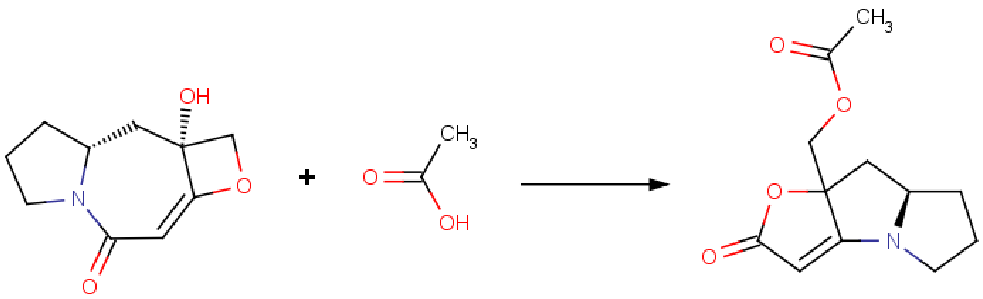

SMILES of the input:
O[C@]12COC1=CC(=O)N1CCC[C@@H]1C2.CC(O)=O>>CC(=O)OCC12C[C@H]3CCCN3C1=CC(=O)O2

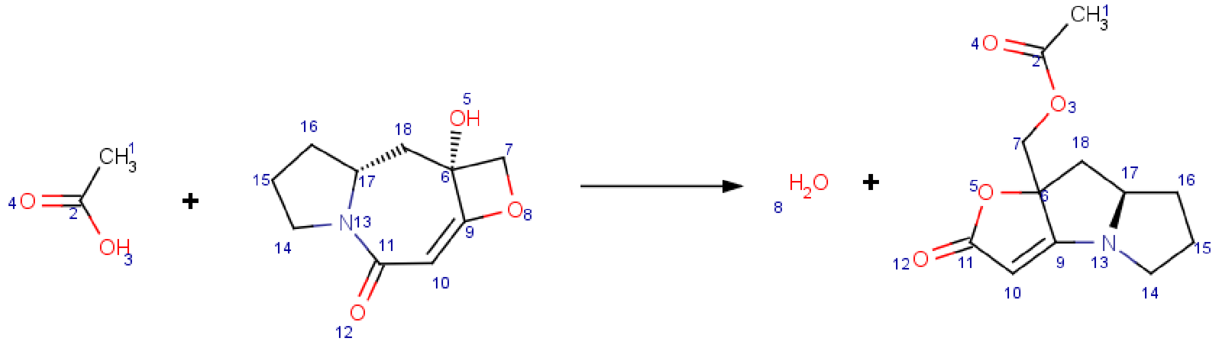

Correct mapped SMILES/SMARTS of the reaction:
[OH:1][C@@:2]12[CH2:14][C@@H:13]3[N:9]([CH2:10][CH2:11][CH2:12]3)[C:7](=[O:8])[CH:6]=[C:5]1O[CH2:3]2.[CH3:15][C:16](=[O:18])[OH:17]>>[CH3:15][C:16]([O:17][CH2:3][C:2]12[O:1][C:7](=[O:8])[CH:6]=[C:5]1[N:9]1[C@H:13]([CH2:12][CH2:11][CH2:10]1)[CH2:14]2)=[O:18]

Correctness of the mapping

MAPPET	NO
ReactionMap	NO
Marvin	NO
ChemDraw	NO
Indigo	YES

Reaction no 27

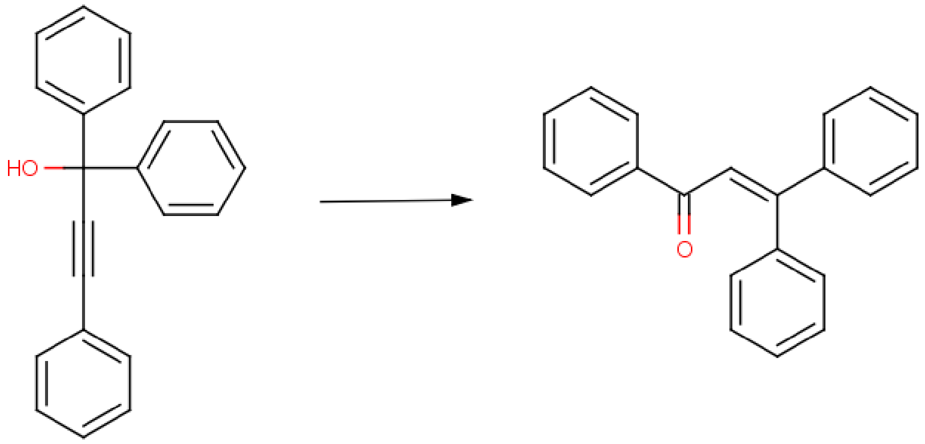

SMILES of the input:
OC(C#CC1=CC=CC=C1)(C1=CC=CC=C1)C1=CC=CC=C1>>O=C(C=C(C1=CC=CC=C1)C1=CC=CC=C1)C1=CC=CC=C1

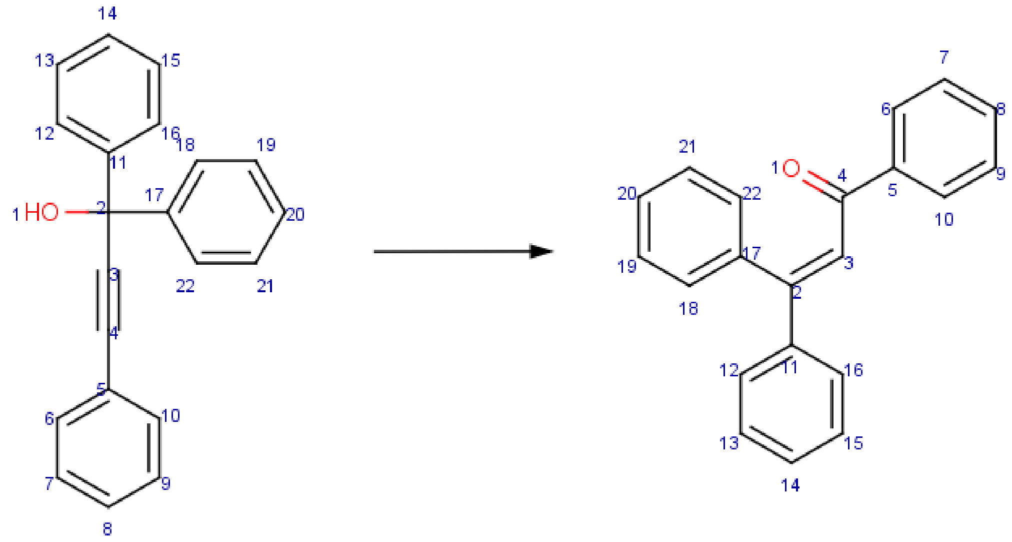

Correct

mapped SMILES/SMARTS of the reaction:
[CH:8]1=[CH:7][CH:6]=[C:5]([CH:10]=[CH:9]1)[C:4]#[C:3][C:2]([C:11]2=[CH:12][CH:13]=[CH:14][CH:15]=[CH:16]2)([C:17]3=[CH:18][CH:19]=[CH:20][CH:21]=[CH:22]3)[OH:1]>>[CH:14]1=[CH:13][CH:12]=[C:11]([CH:16]=[CH:15]1)[C:2](=[CH:3][C:4](=[O:1])[C:5]2=[CH:6][CH:7]=[CH:8][CH:9]=[CH:10]2)[C:17]3=[CH:18][CH:19]=[CH:20][CH:21]=[CH:22]3

Correctness of the mapping

MAPPET	NO
ReactionMap	YES
Marvin	YES
ChemDraw	YES
Indigo	YES

Reaction no 28

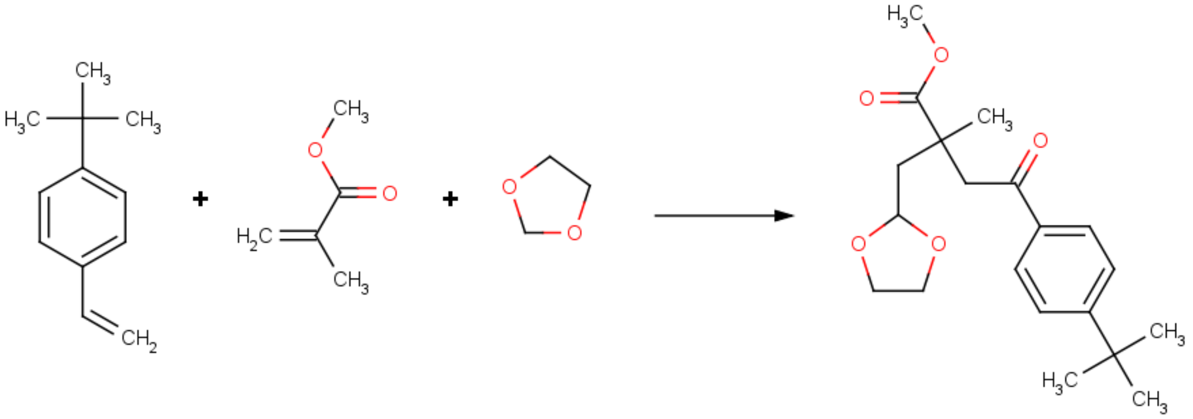

SMILES of the input:
CC(C)(C)C1=CC=C(C=C)C=C1.COC(=O)C(C)=C.C1COCO1>>COC(=O)C(C)(CC1OCCO1)CC(=O)C1=CC=C(C=C1)C(C)(C)C

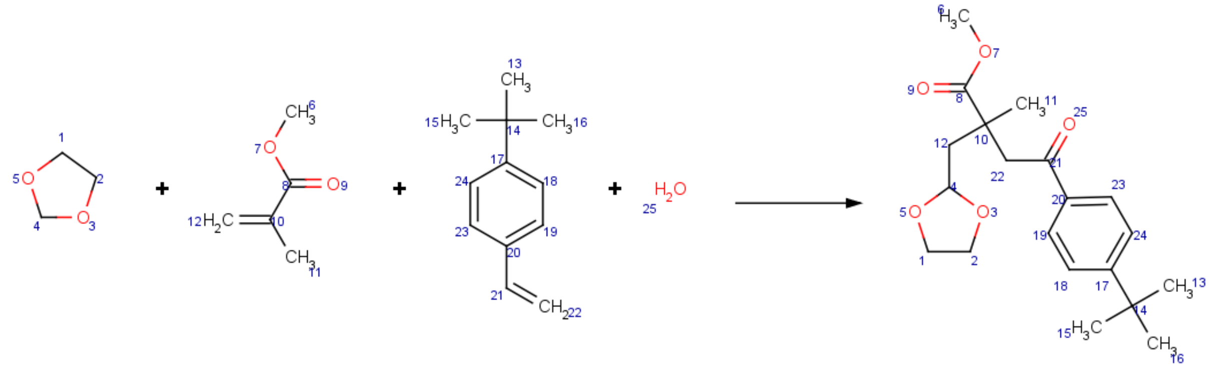

Correct mapped SMILES/SMARTS of the reaction:
[#6:1]-1-[#6:2]-[#8:3]-[#6:4]-[#8:5]-1.[#6:6]-[#8:7]-[#6:8](=[O:9])-
[#6:10](-[#6:11])=[#6:12].[#6:13][C:14]([#6:15])([#6:16])[#6:17]-
1=[#6:18]-[#6:19]=[#6:20](-[#6:21]=[#6:22])-[#6:23]=[#6:24]-
1.[#8:25]>>[#6:6]-[#8:7]-[#6:8](=[O:9])[C:10]([#6:11])([#6:12]-[#6:4]-1-
[#8:3]-[#6:2]-[#6:1]-[#8:5]-1)[#6:22]-[#6:21](=[O:25])-[#6:20]-1=[#6:23]-
[#6:24]=[#6:17](-[#6:18]=[#6:19]-1)[C:14]([#6:16])([#6:15])[#6:13]

Correctness of the mapping

MAPPET	YES
ReactionMap	NO
Marvin	NO
ChemDraw	YES
Indigo	NO

Reaction no 29

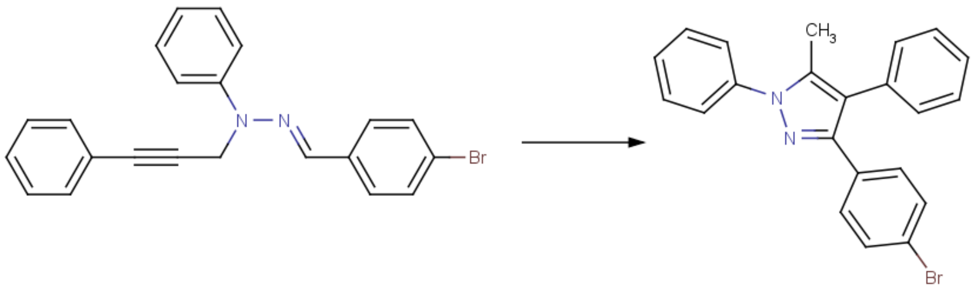

SMILES of the input:
BrC1=CC=C(\C=N\N(CC#CC2=CC=CC=C2)C2=CC=CC=C2)C=C1>>CC1=C(C(=NN1C1=CC=CC=C
1)C1=CC=C(Br)C=C1)C1=CC=CC=C1

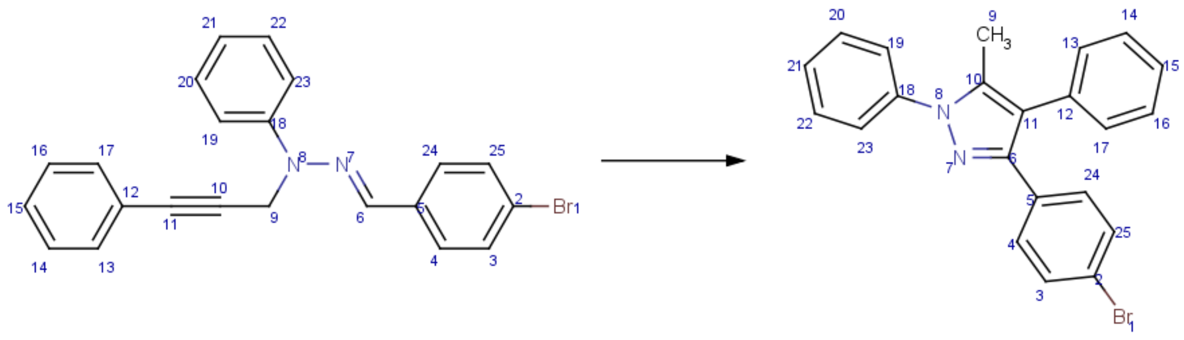

Correct mapped SMILES/SMARTS of the reaction:
[Br:1][#6:2]-1=[#6:3]-[#6:4]=[#6:5](\[#6:6]=[#7:7]\[#7:8](-
[#6:9][C:10]#[C:11][#6:12]-2=[#6:13]-[#6:14]=[#6:15]-[#6:16]=[#6:17]-2)-

[#6:18]-2=[#6:19]-[#6:20]=[#6:21]-[#6:22]=[#6:23]-2)-[#6:24]=[#6:25]-1>>[#6:9]-[#6:10]-1=[#6:11](-[#6:6](=[#7:7]-[#7:8]-1-[#6:18]-1=[#6:23]-[#6:22]=[#6:21]-[#6:20]=[#6:19]-1)-[#6:5]-1=[#6:24]-[#6:25]=[#6:2]([Br:1])-[#6:3]=[#6:4]-1)-[#6:12]-1=[#6:13]-[#6:14]=[#6:15]-[#6:16]=[#6:17]-1

Correctness of the mapping

MAPPET	YES
ReactionMap	NO
Marvin	NO
ChemDraw	NO
Indigo	NO

Reaction no 30

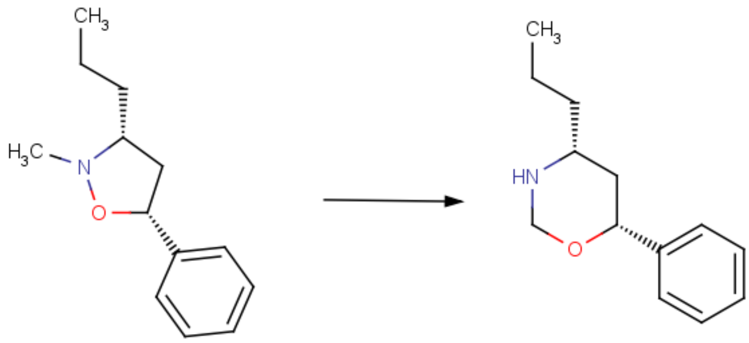

SMILES of the input:
CCC[C@@H]1C[C@@H](ON1C)C1=CC=CC=C1>>CCC[C@@H]1C[C@@H](OCN1)C1=CC=CC=C1

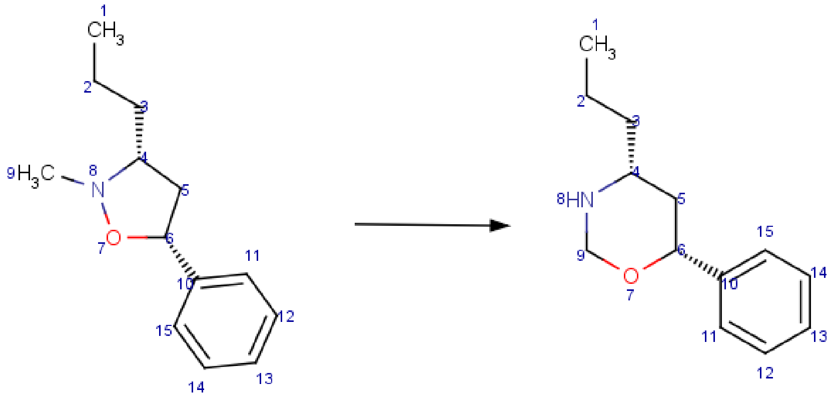

Correct mapped

SMILES/SMARTS of the reaction:
[#6:1]-[#6:2]-[#6:3]-[#6@@H:4]-1-[#6:5]-[#6@@H:6](-[#8:7]-[#7:8]-1-[#6:9])-[#6:10]-1=[#6:11]-[#6:12]=[#6:13]-[#6:14]=[#6:15]-1>>[#6:1]-[#6:2]-[#6:3]-[#6@@H:4]-1-[#6:5]-[#6@@H:6](-[#8:7]-[#6:9]-[#7:8]-1)-[#6:10]-1=[#6:15]-[#6:14]=[#6:13]-[#6:12]=[#6:11]-1

Correctness of the mapping

MAPPET	YES
ReactionMap	NO
Marvin	YES
ChemDraw	YES
Indigo	YES

Reaction no 31

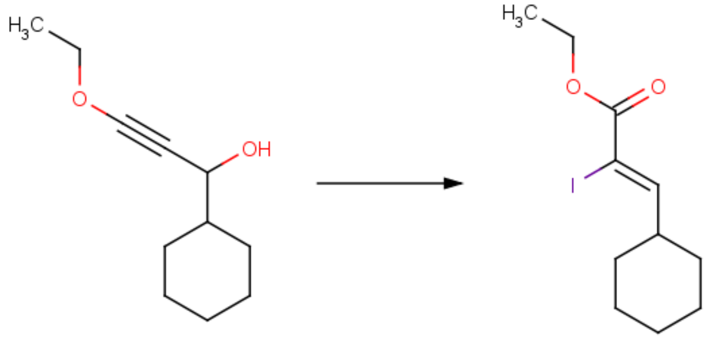

SMILES of the input:
CCOC#CC(O)C1CCCCC1>>CCOC(=O)C(\I)=C\C1CCCCC1

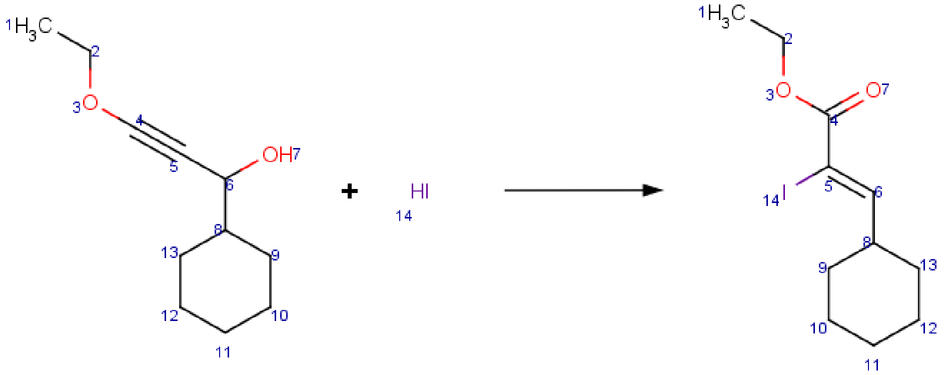

Correct mapped SMILES/SMARTS of the reaction:
[#6:1]-[#6:2]-[#8:3][C:4]#[C:5][#6:6](-[#8:7])-[#6:8]-1-[#6:9]-[#6:10]-[#6:11]-[#6:12]-[#6:13]-1.[I:14]>>[#6:1]-[#6:2]-[#8:3]-[#6:4](=[O:7])-[#6:5](\[I:14])=[#6:6]\[#6:8]-1-[#6:13]-[#6:12]-[#6:11]-[#6:10]-[#6:9]1

Correctness of the mapping

MAPPET	YES
ReactionMap	NO
Marvin	YES
ChemDraw	YES
Indigo	YES

Reaction no 32

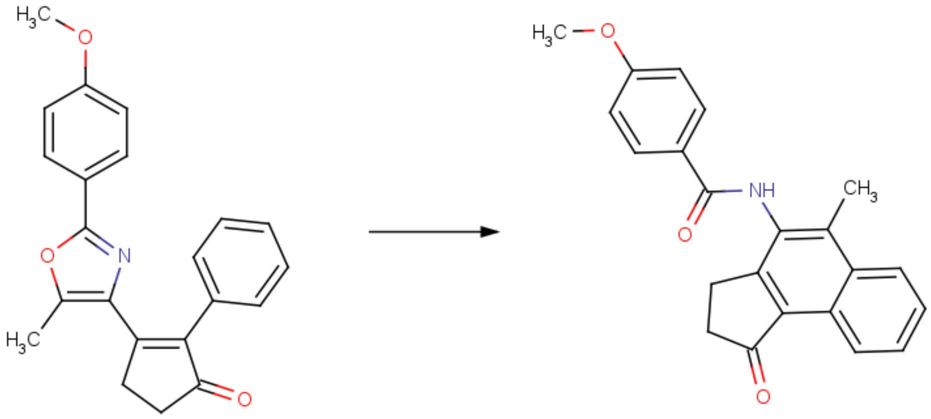

SMILES of the input:
COC1=CC=C(C=C1)C1=NC(=C(C)O1)C1=C(C(=O)CC1)C1=CC=CC=C1>>COC1=CC=C(C=C1)C(=O)NC1=C(C)C2=CC=CC=C2C2=C1CCC2=O

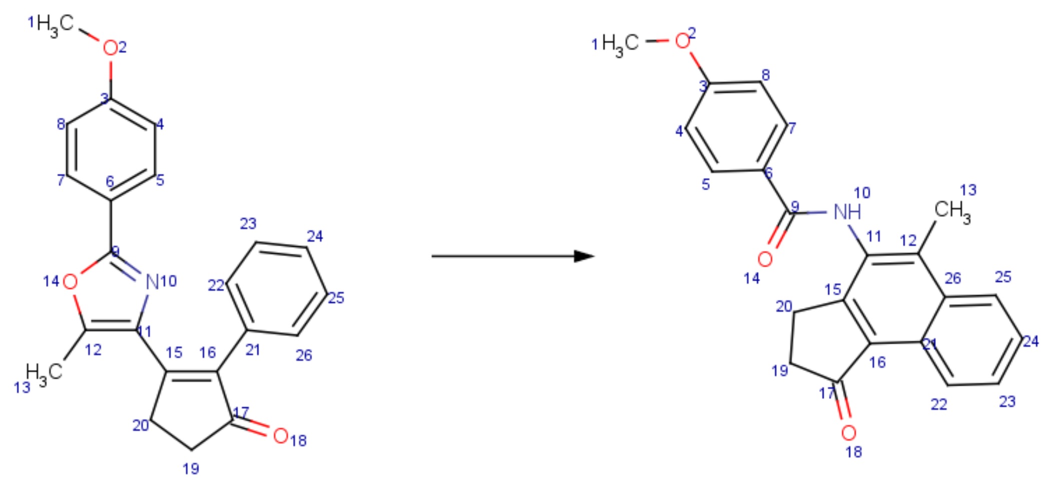

Correct mapped SMILES/SMARTS of the reaction:
[#6:1]-[#8:2]-[#6:3]-1=[#6:4]-[#6:5]=[#6:6](-[#6:7]=[#6:8]-1)-[#6:9]-1=[#7:10]-[#6:11](=[#6:12](-[#6:13])-[#8:14]-1)-[#6:15]-1=[#6:16](-[#6:17](=[O:18])-[#6:19]-[#6:20]-1)-[#6:21]-1=[#6:22]-[#6:23]=[#6:24]-[#6:25]=[#6:26]-1>>[#6:1]-[#8:2]-[#6:3]-1=[#6:8]-[#6:7]=[#6:6](-[#6:5]=[#6:4]-1)-[#6:9](=[O:14])-[#7:10]-[#6:11]-1=[#6:12](-[#6:13])-[#6:26]-2=[#6:25]-[#6:24]=[#6:23]-[#6:22]=[#6:21]-2-[#6:16]-2=[#6:15]-1-[#6:20]-[#6:19]-[#6:17]-2=[O:18]

Correctness of the mapping

MAPPET	YES
ReactionMap	YES
Marvin	NO
ChemDraw	YES
Indigo	YES

Reaction no 33

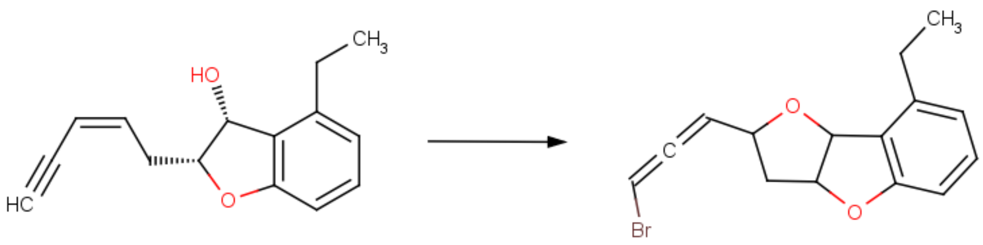

SMILES of the input:
CCC1=CC=CC2=C1[C@@H](O)[C@@H](C\C=C/C#C)O2>>CCC1=CC=CC2=C1[C@H]1O[C@@H](C[C@H]1O2)C=[C@@]=CBr

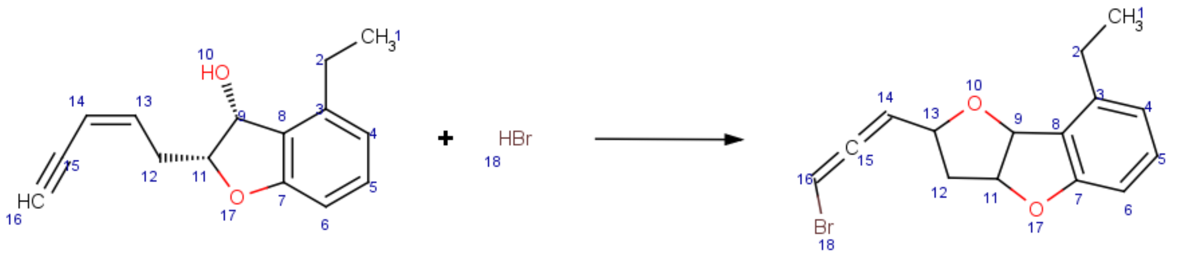

Correct mapped SMILES/SMARTS of the reaction:
[#6:1]-[#6:2]-[#6:3]-1=[#6:4]-[#6:5]=[#6:6]-[#6:7]-2=[#6:8]-1-[#6@@H:9](-[#8:10])-[#6@@H:11](-[#6:12]\[#6:13]=[#6:14]/[C:15]#[C:16])-[#8:17]-2.[Br:18]>>[#6:1]-[#6:2]-[#6:3]-1=[#6:4]-[#6:5]=[#6:6]-[#6:7]-2=[#6:8]-1-[#6:9]-1-[#8:10]-[#6:13](-[#6:12]-[#6:11]-1-[#8:17]-2)-[#6:14]=[C:15]=[#6:16][Br:18]

Correctness of the mapping

MAPPET	YES
ReactionMap	NO
Marvin	YES
ChemDraw	YES
Indigo	NO

Reaction no 34

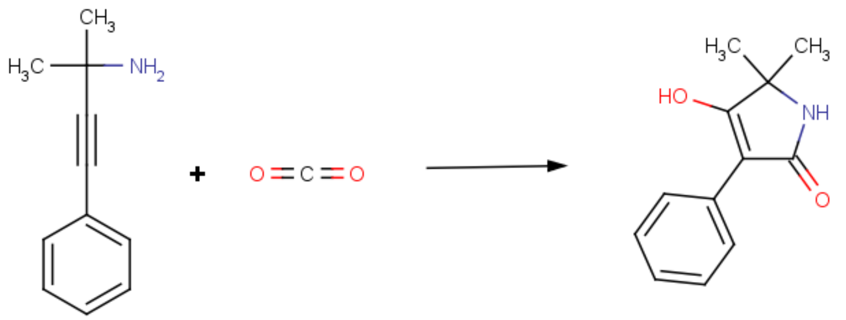

SMILES of the input:
CC(C)(N)C#CC1=CC=CC=C1.O=C=O>>CC1(C)NC(=O)C(=C1O)C1=CC=CC=C1

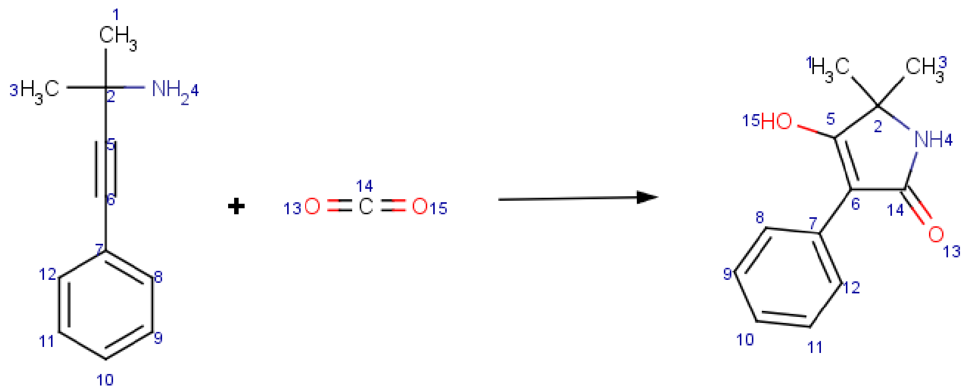

Correct mapped SMILES/SMARTS of the reaction:
[#6:1][C:2]([#6:3])([#7:4])[C:5]#[C:6][#6:7]-1=[#6:8]-[#6:9]=[#6:10]-[#6:11]=[#6:12]-1.[O:13]=[C:14]=[O:15]>>[#6:3][C:2]1([#6:1])[#7:4]-

[#6:14](=[O:13])-[#6:6](=[#6:5]1-[#8:15])-[#6:7]-1=[#6:12]-[#6:11]=[#6:10]-[#6:9]=[#6:8]-1

Correctness of the mapping
MAPPET YES
ReactionMap YES
Marvin YES
ChemDraw YES
Indigo YES

Reaction no 35

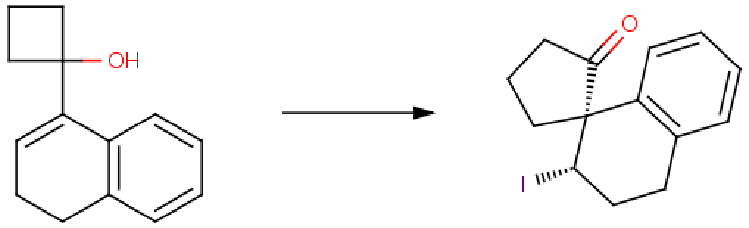

SMILES of the input:
OC1(CCC1)C1=CCCC2=C1C=CC=C2>>I[C@H

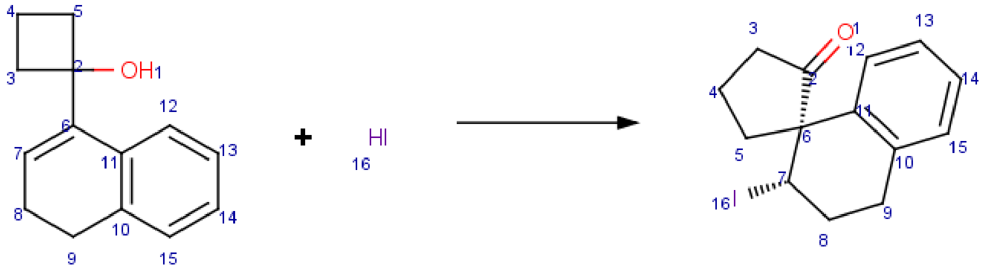

]1CCC2=C(C=CC=C2)[C@]11CCCC1=O

Correct mapped SMILES/SMARTS of the reaction:
[#8:1][C:2]1([#6:3]-[#6:4]-[#6:5]1)[#6:6]-1=[#6:7]-[#6:8]-[#6:9]-[#6:10]-2=[#6:11]-1-[#6:12]=[#6:13]-[#6:14]=[#6:15]-2.[I:16]>>[I:16][#6@H:7]1-[#6:8]-[#6:9]-[#6:10]-2=[#6:11](-[#6:12]=[#6:13]-[#6:14]=[#6:15]-2)[C@:6]11[#6:5]-[#6:4]-[#6:3]-[#6:2]1=[O:1]

Correctness of the mapping
MAPPET YES
ReactionMap NO
Marvin YES
ChemDraw YES
Indigo NO

Reaction no 36

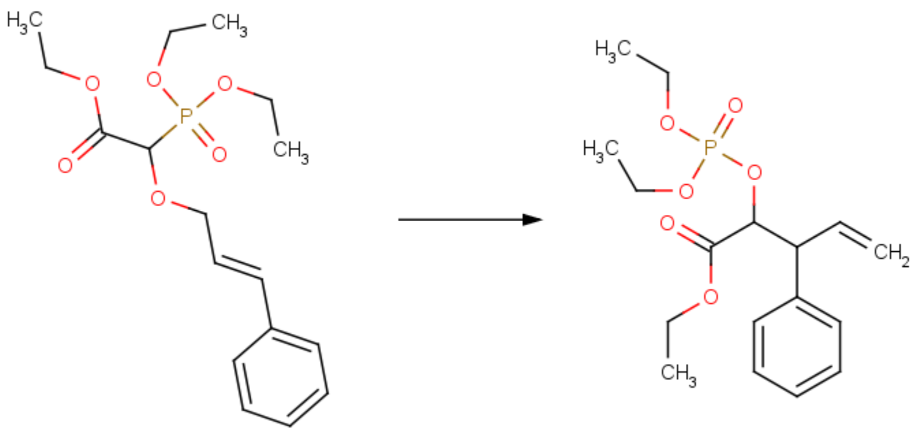

SMILES of the input:

CCOC(=O)C(OC\C=C\C1=CC=CC=C1)P(=O)(OCC)OCC>>CCOC(=O)C(OP(=O)(OCC)OCC)C(C=C)C1=CC=CC=C1

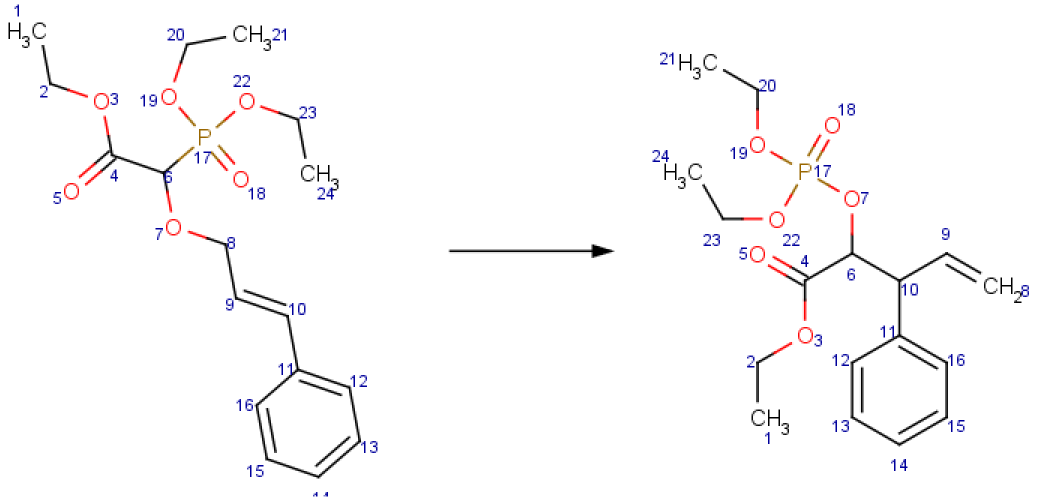

Correct mapped SMILES/SMARTS of the reaction:

[#6:1]-[#6:2]-[#8:3]-[#6:4](=[O:5])-[#6:6](-[#8:7]-[#6:8]\[#6:9]=[#6:10]\[#6:11]-1=[#6:12]-[#6:13]=[#6:14]-[#6:15]=[#6:16]1)[P:17](=[O:18])([#8:19]-[#6:20]-[#6:21])[#8:22]-[#6:23]-[#6:24]>>[#6:1]-[#6:2]-[#8:3]-[#6:4](=[O:5])-[#6:6](-[#8:7][P:17](=[O:18])([#8:19]-[#6:20]-[#6:21])[#8:22]-[#6:23]-[#6:24])-[#6:10](-[#6:9]=[#6:8])-[#6:11]-1=[#6:16]-[#6:15]=[#6:14]-[#6:13]=[#6:12]-1

Correctness of the mapping

MAPPET	YES
ReactionMap	NO
Marvin	NO
ChemDraw	NO
Indigo	NO

Reaction no 37

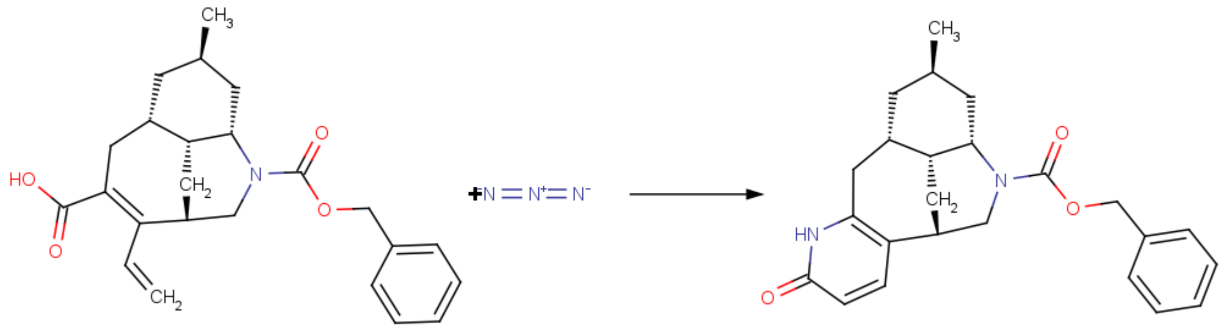

SMILES of the input:
C[C@@H]1C[C@H]2CC(C(O)=O)=C(C=C)[C@@H]3C[C@H]2[C@H](C1)N(C3)C(=O)OCC1=CC=CC=C1.[N-]=[N+]=[N-]>>C[C@@H]1C[C@H]2CC3=C(C=CC(=O)N3)[C@@H]3C[C@H]2[C@H](C1)N(C3)C(=O)OCC1=CC=CC=C1

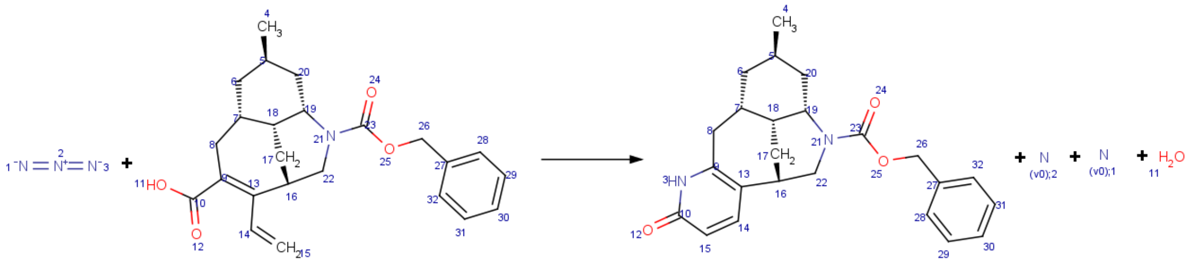

Correct mapped SMILES/SMARTS of the reaction:
[#7-:1]=[N+:2]=[#7-:3].[#6:4]-[#6@@H:5]-1-[#6:6]-[#6@H:7]-2-[#6:8]-[#6:9](-[#6:10](-[#8:11])=[O:12])=[#6:13](-[#6:14]=[#6:15])-[#6@@H:16]-3-[#6:17]-[#6@H:18]-2-[#6@H:19](-[#6:20]-1)-[#7:21](-[#6:22]-3)-[#6:23](=[O:24])-[#8:25]-[#6:26]-[#6:27]-1=[#6:28]-[#6:29]=[#6:30]-[#6:31]=[#6:32]-1>>[#6:4]-[#6@@H:5]-1-[#6:6]-[#6@H:7]-2-[#6:8]-[#6:9]-3=[#6:13](-[#6:14]=[#6:15]-[#6:10](=[O:12])-[#7:3]-3)-[#6@@H:16]-3-[#6:17]-[#6@H:18]-2-[#6@H:19](-[#6:20]-1)-[#7:21](-[#6:22]-3)-[#6:23](=[O:24])-[#8:25]-[#6:26]-[#6:27]-1=[#6:32]-[#6:31]=[#6:30]-[#6:29]=[#6:28]-1.[#7;v0:2].[#7;v0:1].[#8:11]

Correctness of the mapping

MAPPET	YES
ReactionMap	NO
Marvin	NO
ChemDraw	NO
Indigo	NO

Reaction no 38

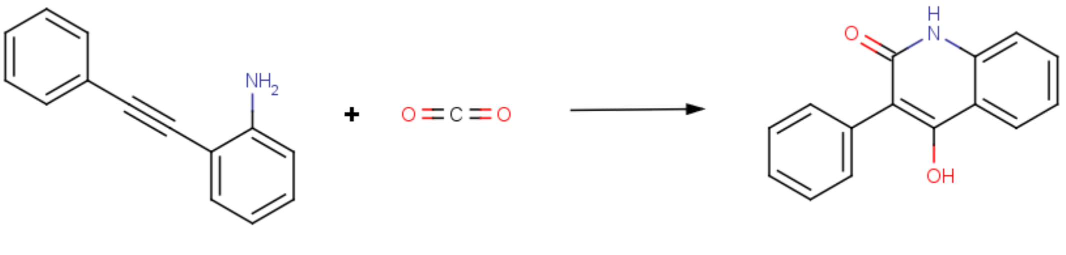

SMILES of the input:
NC1=CC=CC=C1C#CC1=CC=CC=C1.O=C=O>>OC1=C(C(=O)NC2=CC=CC=C12)C1=CC=CC=C1

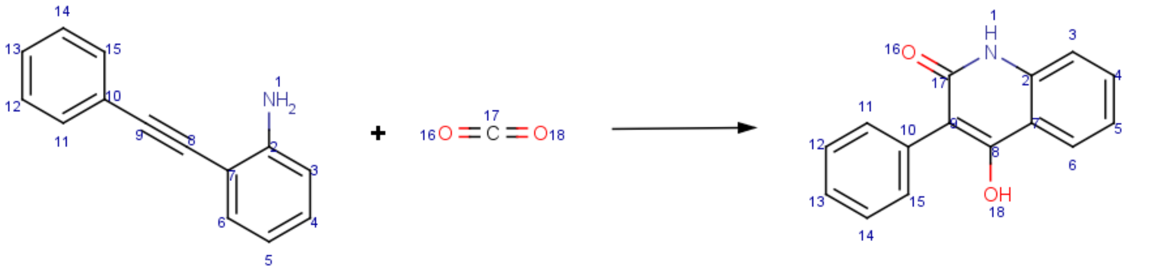

Correct mapped SMILES/SMARTS of the reaction:
[#7:1]-[#6:2]-1=[#6:3]-[#6:4]=[#6:5]-[#6:6]=[#6:7]-1[C:8]#[C:9][#6:10]-1=[#6:11]-[#6:12]=[#6:13]-[#6:14]=[#6:15]-1.[O:16]=[C:17]=[O:18]>>[#8:18]-[#6:8]-1=[#6:9](-[#6:17](=[O:16])-[#7:1]-[#6:2]-2=[#6:3]-[#6:4]=[#6:5]-[#6:6]=[#6:7]-1-2)-[#6:10]-1=[#6:15]-[#6:14]=[#6:13]-[#6:12]=[#6:11]-1

Correctness of the mapping

MAPPET	YES
ReactionMap	YES
Marvin	YES
ChemDraw	YES
Indigo	YES

Reaction no 39

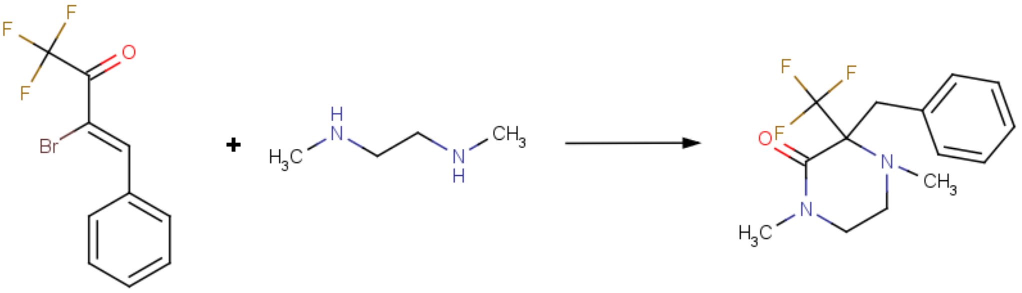

SMILES of the input:
FC(F)(F)C(=O)C(\Br)=C\C1=CC=CC=C1.CNCCNC>>CN1CCN(C)C(CC2=CC=CC=C2)(C1=O)C(F)(F)F

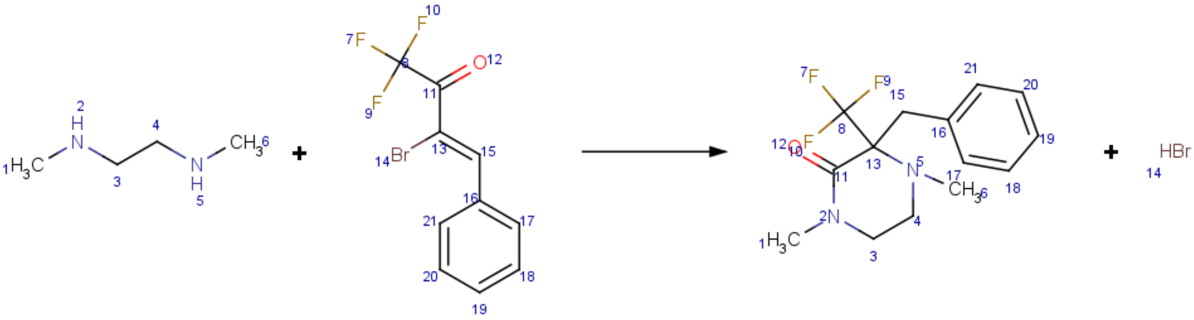

Correct mapped SMILES/SMARTS of the reaction:
[#6:1]-[#7:2]-[#6:3]-[#6:4]-[#7:5]-[#6:6].[F:7][C:8]([F:9])([F:10])[#6:11](=[O:12])-[#6:13](\[Br:14])=[#6:15]\[#6:16]-1=[#6:17]-[#6:18]=[#6:19]-[#6:20]=[#6:21]1>>[#6:1]-[#7:2]-1-[#6:3]-[#6:4]-[#7:5](-

[#6:6])[C:13]([#6:15]-[#6:16]-2=[#6:21]-[#6:20]=[#6:19]-[#6:18]=[#6:17]-2)([#6:11]-1=[O:12])[C:8]([F:7])([F:9])[F:10].[Br:14]

Correctness of the mapping

MAPPET	YES
ReactionMap	NO
Marvin	YES
ChemDraw	NO
Indigo	NO

Reaction no 40

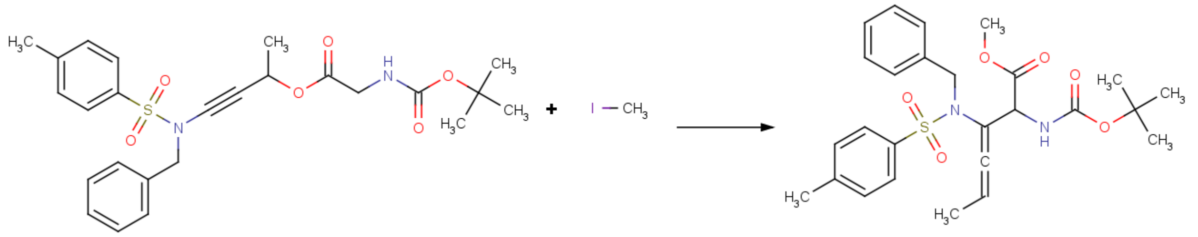

SMILES of the input:
CC(OC(=O)CNC(=O)OC(C)(C)C)C#CN(CC1=CC=CC=C1)S(=O)(=O)C1=CC=C(C)C=C1.CI>>COC(=O)[C@H](NC(=O)OC(C)(C)C)C(=[C@]=CC)N(CC1=CC=CC=C1)S(=O)(=O)C1=CC=C(C)C=C1

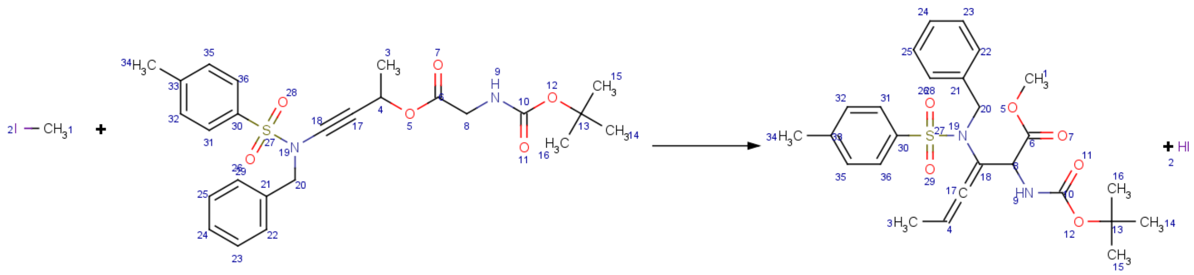

Correct mapped SMILES/SMARTS of the reaction:
C[CH:1]([O:2][C:3](=O)CNC(=O)OC(C)(C)C)[C:4]#[C:5][N:6]([CH2:7][C:8]1=[CH:9][CH:10]=[CH:11][CH:12]=[CH:13]1)[S:14](=[O:15])(=[O:16])[C:17]1=[CH:18][CH:19]=[C:20]([CH3:21])[CH:22]=[CH:23]1.[CH3:24]I>>[CH3:3][O:2][C:1](=O)[C@H:4](NC(=O)OC([CH3:24])(C)C)[C:5](=[C@@]=CC)[N:6]([CH2:7][C:8]1=[CH:9][CH:10]=[CH:11][CH:12]=[CH:13]1)[S:14](=[O:15])(=[O:16])[C:17]1=[CH:18][CH:19]=[C:20]([CH3:21])[CH:22]=[CH:23]1

Correctness of the mapping

MAPPET	NO
ReactionMap	NO
Marvin	NO
ChemDraw	NO
Indigo	NO

Reaction no 41

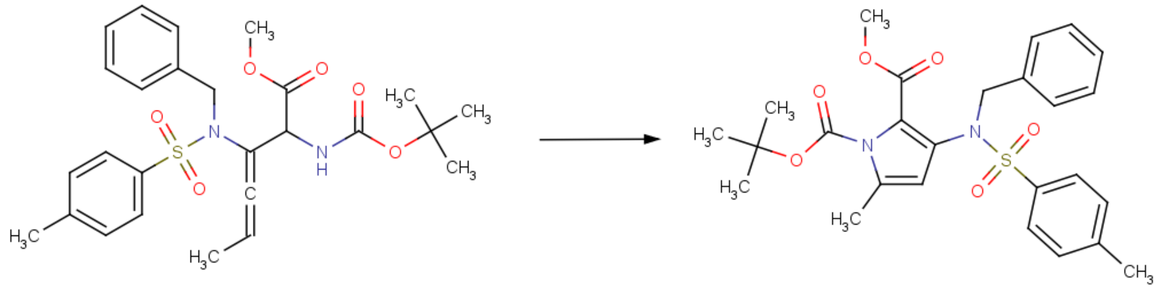

SMILES of the input:
COC(=O)[C@H](NC(=O)OC(C)(C)C)C(=[C@]=CC)N(CC1=CC=CC=C1)S(=O)(=O)C1=CC=C(C)C=C1>>COC(=O)C1=C(C=C(C)N1C(=O)OC(C)(C)C)N(CC1=CC=CC=C1)S(=O)(=O)C1=CC=C(C)C=C1

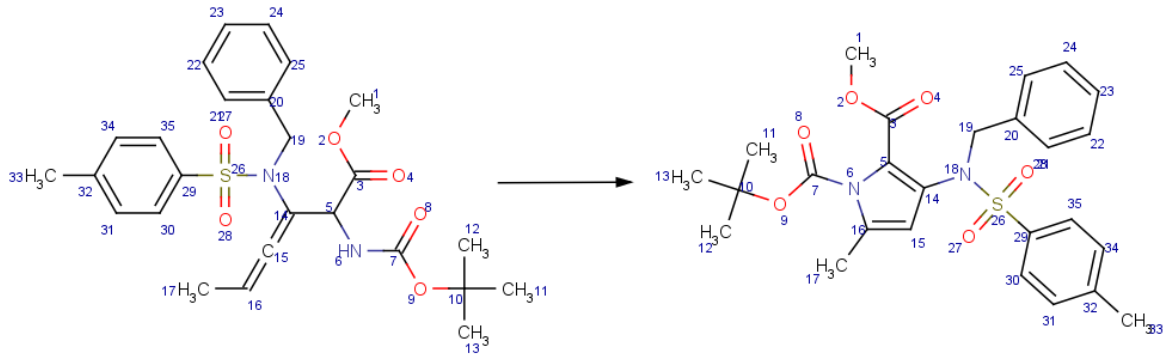

Correct mapped SMILES/SMARTS of the reaction:
[#6:1]-[#8:2]-[#6:3](=[O:4])-[#6:5](-[#7:6]-[#6:7](=[O:8])-[#8:9][C:10]([#6:11])([#6:12])[#6:13])-[#6:14](=[C:15]=[#6:16]-[#6:17])-[#7:18](-[#6:19]-[#6:20]-1=[#6:21]-[#6:22]=[#6:23]-[#6:24]=[#6:25]-1)[S:26](=[O:27])(=[O:28])[#6:29]-1=[#6:30]-[#6:31]=[#6:32](-[#6:33])-[#6:34]=[#6:35]-1>>[#6:1]-[#8:2]-[#6:3](=[O:4])-[#6:5]-1=[#6:14](-[#6:15]=[#6:16](-[#6:17])-[#7:6]-1-[#6:7](=[O:8])-[#8:9][C:10]([#6:13])([#6:12])[#6:11])-[#7:18](-[#6:19]-[#6:20]-1=[#6:25]-[#6:24]=[#6:23]-[#6:22]=[#6:21]-1)[S:26](=[O:27])(=[O:28])[#6:29]-1=[#6:35]-[#6:34]=[#6:32](-[#6:33])-[#6:31]=[#6:30]-1

Correctness of the mapping

MAPPET	YES
ReactionMap	NO
Marvin	YES
ChemDraw	YES
Indigo	YES

Reaction no 42

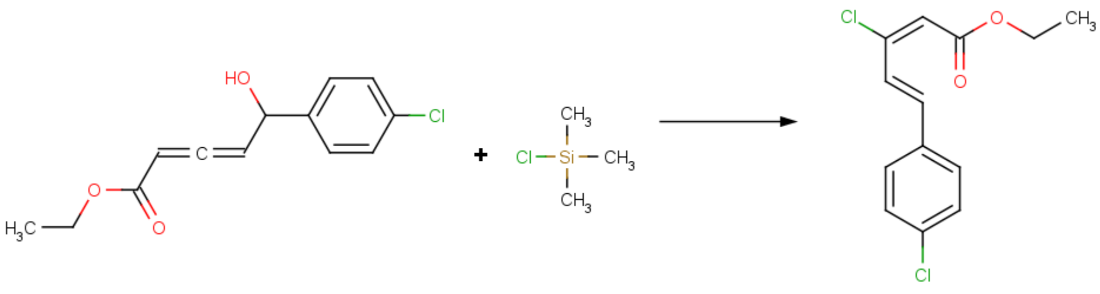

SMILES of the input:
CCOC(=O)C=C=CC(O)C1=CC=C(Cl)C=C1.C[Si](C)(C)Cl>>CCOC(=O)\C=C(\Cl)/C=C/C1=CC=C(Cl)C=C1

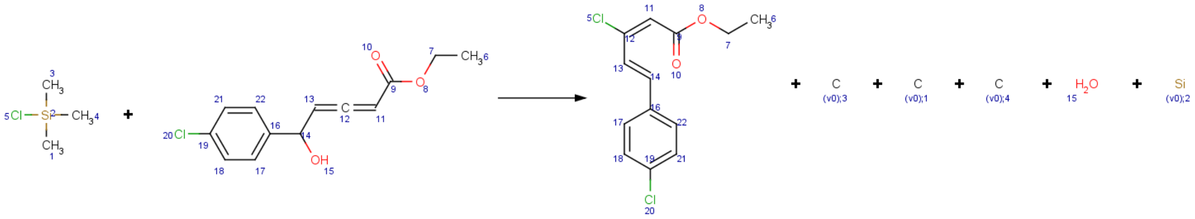

Correct mapped SMILES/SMARTS of the reaction:
[#6:1][Si:2]([#6:3])([#6:4])[Cl:5].[#6:6]-[#6:7]-[#8:8]-[#6:9](=[O:10])-[#6:11]=[C:12]=[#6:13]-[#6:14](-[#8:15])-[#6:16]-1=[#6:17]-[#6:18]=[#6:19]([Cl:20])-[#6:21]=[#6:22]-1>>[#6:6]-[#6:7]-[#8:8]-[#6:9](=[O:10])\[#6:11]=[#6:12](\[Cl:5])/[#6:13]=[#6:14]/[#6:16]-1=[#6:22]-[#6:21]=[#6:19]([Cl:20])-[#6:18]=[#6:17]1.[#6;v0:3].[#6;v0:1].[#6;v0:4].[#8:15].[#14;v0:2]

Correctness of the mapping

MAPPET	YES
ReactionMap	NO
Marvin	YES
ChemDraw	YES
Indigo	NO

Reaction no 43

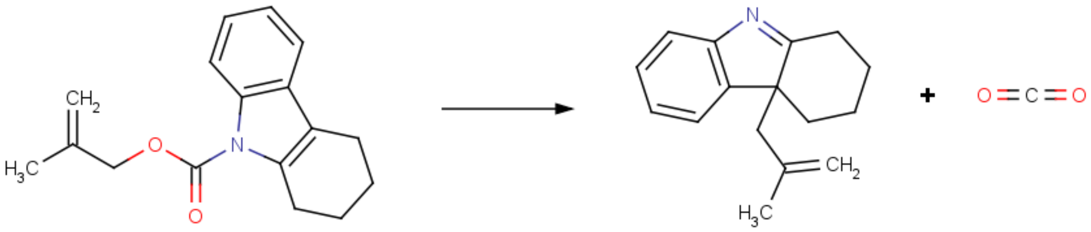

SMILES of the input:
CC(=C)COC(=O)N1C2=C(CCCC2)C2=C1C=CC=C2>>CC(=C)CC12CCCCC1=NC1=C2C=CC=C1.O=C=O

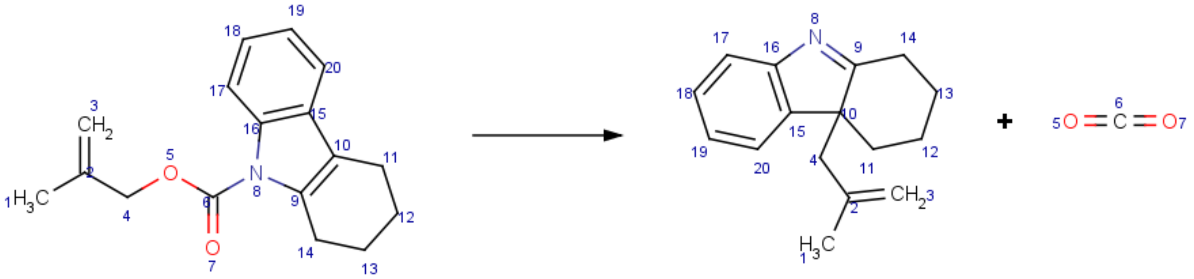

Correct mapped SMILES/SMARTS of the reaction:
[CH3:1][C:2](=[CH2:3])[CH2:4][O:5][C:6](=[O:7])[N:8]1[C:9]2=[C:10]([CH2:11][CH2:12][CH2:13][CH2:14]2)[C:15]3=[C:16]1[CH:17]=[CH:18][CH:19]=[CH:20]3>>[CH3:1][C:2](=[CH2:3])[CH2:4][C:10]12[CH2:11][CH2:12][CH2:13][CH2:14][C:9]1=[N:8][C:16]3=[C:15]2[CH:20]=[CH:19][CH:18]=[CH:17]3.[C:6](=[O:5])=[O:7]

Correctness of the mapping

MAPPET	NO
ReactionMap	YES
Marvin	YES
ChemDraw	YES
Indigo	YES

Reaction no 44

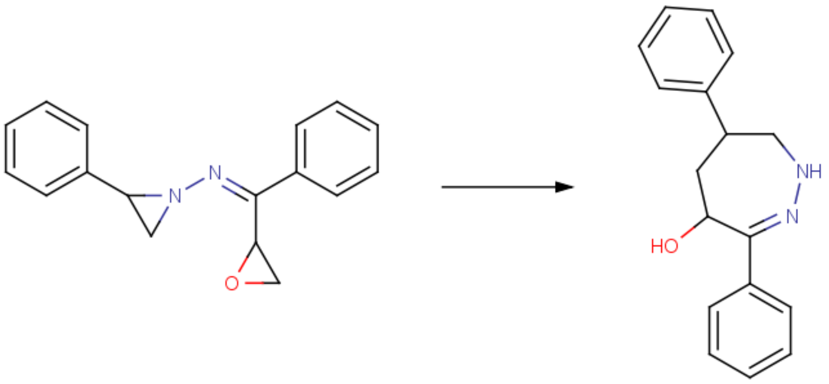

SMILES of the input:
C1OC1\C(=N/N1CC1C1=CC=CC=C1)C1=CC=CC=C1>>OC1CC(CNN=C1C1=CC=CC=C1)C1=CC=CC=C1

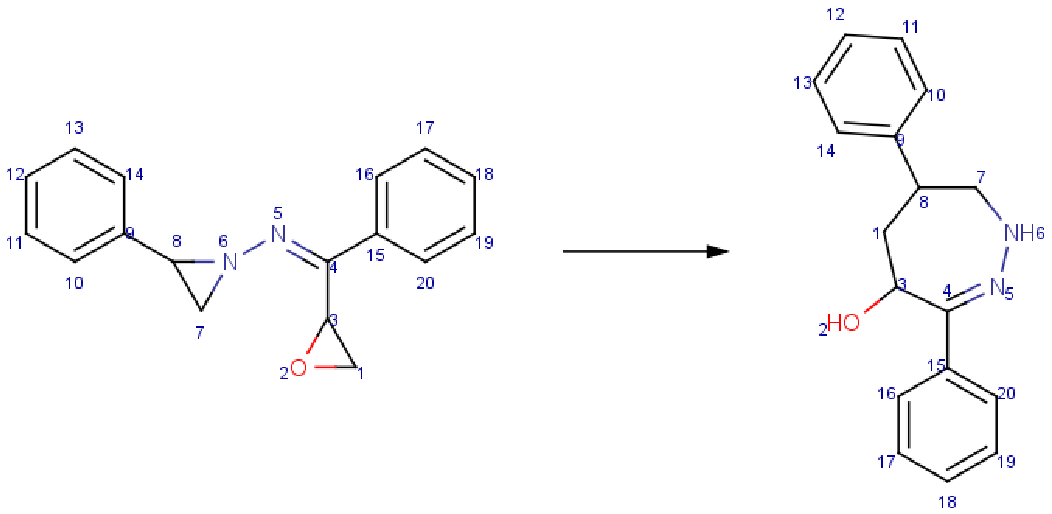

Correct

mapped SMILES/SMARTS of the reaction:
[#6:1]-1-[#8:2]-[#6:3]-1\[#6:4](=[#7:5]/[#7:6]-1-[#6:7]-[#6:8]1-[#6:9]-1=[#6:10]-[#6:11]=[#6:12]-[#6:13]=[#6:14]-1)-[#6:15]-1=[#6:16]-[#6:17]=[#6:18]-[#6:19]=[#6:20]-1>>[#8:2]-[#6:3]-1-[#6:1]-[#6:8](-[#6:7]-[#7:6]-[#7:5]=[#6:4]-1-[#6:15]-1=[#6:20]-[#6:19]=[#6:18]-[#6:17]=[#6:16]-1)-[#6:9]-1=[#6:14]-[#6:13]=[#6:12]-[#6:11]=[#6:10]-1

Correctness of the mapping

MAPPET	YES
ReactionMap	YES
Marvin	YES
ChemDraw	YES
Indigo	YES

Reaction no 45

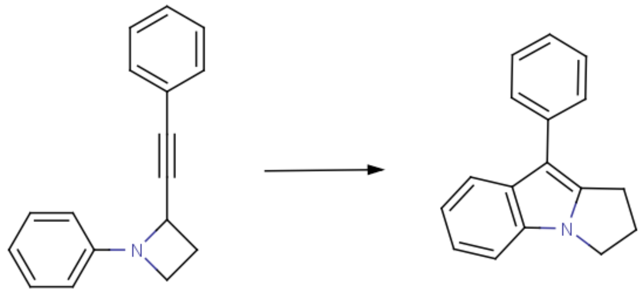

SMILES of the input:
C1CN(C1C#CC1=CC=CC=C1)C1=CC=CC=C1>>C1CN2C(C1)=C(C1=C2C=CC=C1)C1=CC=CC=C1

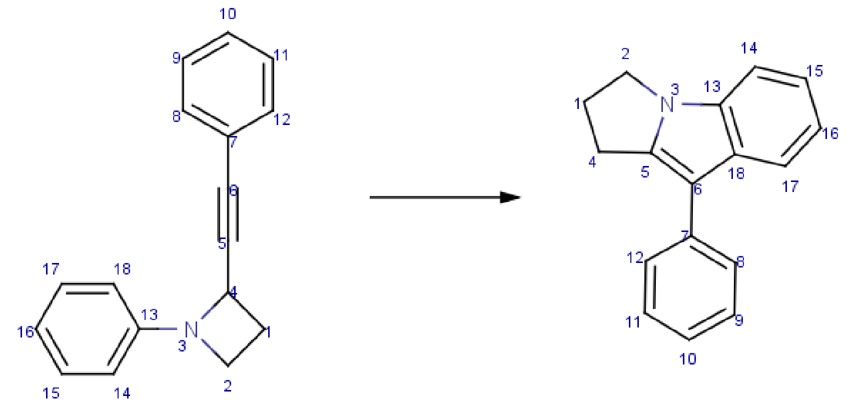

Correct mapped

SMILES/SMARTS of the reaction:
[CH2:1]1[CH2:2][N:3]([CH:4]1[C:5]#[C:6][C:7]2=[CH:8][CH:9]=[CH:10][CH:11]=[CH:12]2)[C:13]3=[CH:14][CH:15]=[CH:16][CH:17]=[CH:18]3>>[CH2:1]1[CH2:4][C:5]2=[C:6]([C:18]3=[C:13]([N:3]2[CH2:2]1)[CH:14]=[CH:15][CH:16]=[CH:17]3)[C:7]4=[CH:8][CH:9]=[CH:10][CH:11]=[CH:12]4

Correctness of the mapping

MAPPET	NO
ReactionMap	YES
Marvin	YES
ChemDraw	NO
Indigo	YES

Reaction no 46

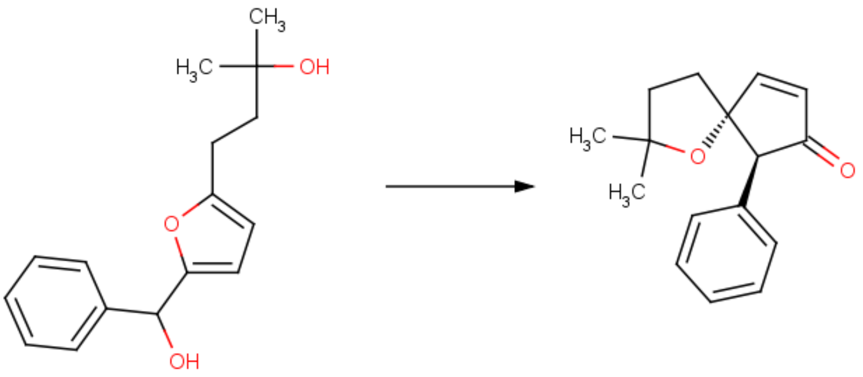

SMILES of the input:
CC(C)(O)CCC1=CC=C(O1)C(O)C1=CC=CC=C1>>CC1(C)CC[C@@]2(O1)C=CC(=O)[C@H]2C1=CC=CC=C1

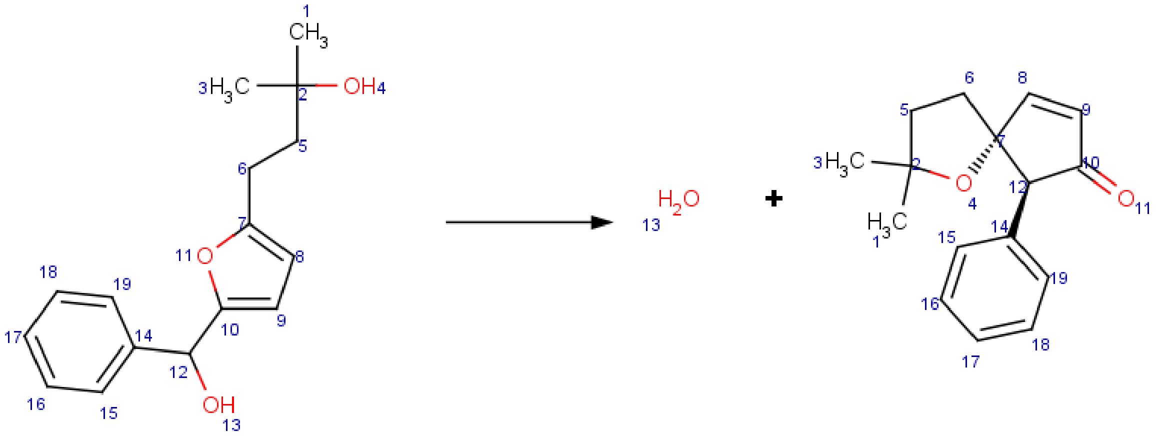

Correct mapped SMILES/SMARTS of the reaction:
[#6:1][C:2]([#6:3])([#8:4])[#6:5]-[#6:6]-[#6:7]-1=[#6:8]-[#6:9]=[#6:10](-[#8:11]-1)-[#6:12](-[#8:13])-[#6:14]-1=[#6:15]-[#6:16]=[#6:17]-[#6:18]=[#6:19]-1>>[#8:13].[#6:3][C:2]1([#6:1])[#6:5]-[#6:6][C@@:7]2([#8:4]1)[#6:8]=[#6:9]-[#6:10](=[O:11])-[#6@H:12]2-[#6:14]-1=[#6:19]-[#6:18]=[#6:17]-[#6:16]=[#6:15]-1

Correctness of the mapping

MAPPET	YES
ReactionMap	NO
Marvin	NO
ChemDraw	NO
Indigo	NO

Reaction no 47

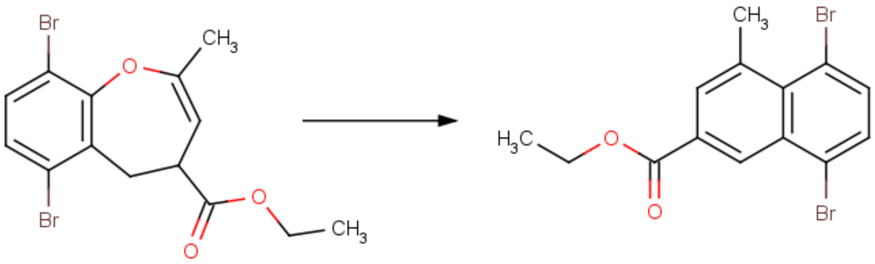

SMILES of the input:
CCOC(=O)C1CC2=C(OC(C)=C1)C(Br)=CC=C2Br>>CCOC(=O)C1=CC2=C(C(C)=C1)C(Br)=CC=C2Br

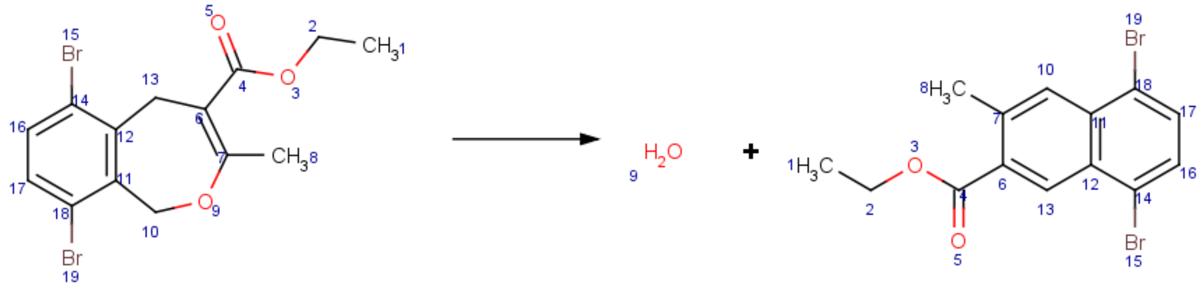

Correct mapped SMILES/SMARTS of the reaction:
[#6:1]-[#6:2]-[#8:3]-[#6:4](=[O:5])-[#6:6]-1=[#6:7](-[#6:8])-[#8:9]-
[#6:10]-[#6:11]-2=[#6:12](-[#6:13]-1)-[#6:14]([Br:15])=[#6:16]-
[#6:17]=[#6:18]-2[Br:19]>>[#8:9].[#6:1]-[#6:2]-[#8:3]-[#6:4](=[O:5])-
[#6:6]-1=[#6:13]-[#6:12]-2=[#6:11](-[#6:10]=[#6:7]-1-[#6:8])-
[#6:18]([Br:19])=[#6:17]-[#6:16]=[#6:14]-2[Br:15]

Correctness of the mapping

MAPPET	YES
ReactionMap	NO
Marvin	YES
ChemDraw	YES
Indigo	YES

Reaction no 48

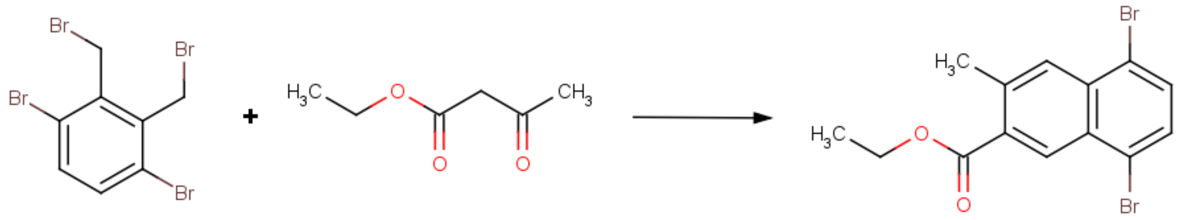

SMILES of the input:
BrCC1=C(CBr)C(Br)=CC=C1Br.CCOC(=O)CC(C)=O>>CCOC(=O)C1=CC2=C(C=C1C)C(Br)=C
C=C2Br

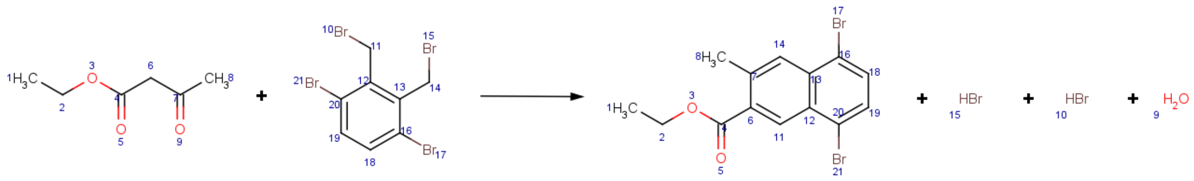

Correct mapped SMILES/SMARTS of the reaction:
[#6:1]-[#6:2]-[#8:3]-[#6:4](=[O:5])-[#6:6]-[#6:7](-
[#6:8])=[O:9].[Br:10][#6:11]-[#6:12]-1=[#6:13](-[#6:14][Br:15])-
[#6:16]([Br:17])=[#6:18]-[#6:19]=[#6:20]-1[Br:21]>>[#6:1]-[#6:2]-[#8:3]-
[#6:4](=[O:5])-[#6:6]-1=[#6:11]-[#6:12]-2=[#6:13](-[#6:14]=[#6:7]-1-
[#6:8])-[#6:16]([Br:17])=[#6:18]-[#6:19]=[#6:20]-
2[Br:21].[Br:15].[Br:10].[#8:9]

Correctness of the mapping

MAPPET	YES
ReactionMap	NO

Marvin YES
ChemDraw YES
Indigo YES

Reaction no 49

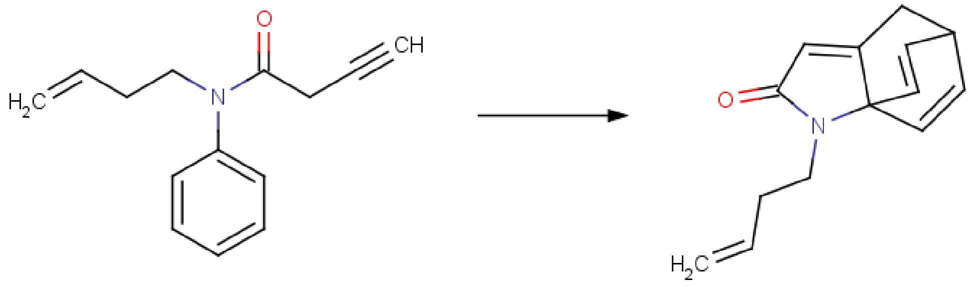

SMILES of the input:
C=CCCN(C(=O)CC#C)C1=CC=CC=C1>>C=CCCN1C(=O)C=C2CC3C=CC12C=C3

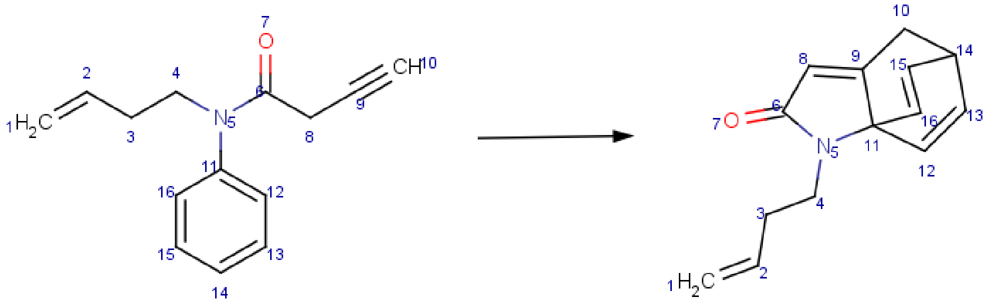

Correct

mapped SMILES/SMARTS of the reaction:
[#6:1]=[#6:2]-[#6:3]-[#6:4]-[#7:5](-[#6:6](=[O:7])-[#6:8][C:9]#[C:10])-[#6:11]-1=[#6:12]-[#6:13]=[#6:14]-[#6:15]=[#6:16]-1>>[#6:1]=[#6:2]-[#6:3]-[#6:4]-[#7:5]1-[#6:6](=[O:7])-[#6:8]=[#6:9]2-[#6:10]-[#6:14]-3-[#6:13]=[#6:12][C:11]12[#6:16]=[#6:15]-3

Correctness of the mapping
MAPPET YES
ReactionMap YES
Marvin YES
ChemDraw YES
Indigo NO

Reaction no 50

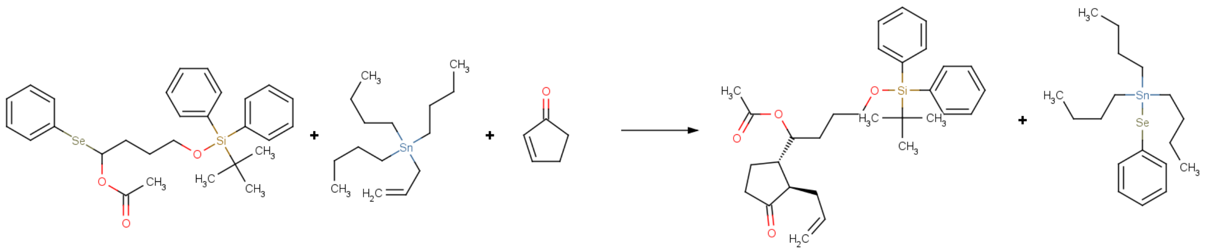

SMILES of the input:
CC(=O)OC(CCCO[Si](C1=CC=CC=C1)(C1=CC=CC=C1)C(C)(C)C)[Se]C1=CC=CC=C1.CCCC[Sn](CCCC)(CCCC)CC=C.O=C1CCC=C1>>CC(=O)OC(CCCO[Si](C1=CC=CC=C1)(C1=CC=CC=C1)C(C)(C)C)[C@H]1CCC(=O)[C@@H]1CC=C.CCCC[Sn](CCCC)(CCCC)[Se]C1=CC=CC=C1

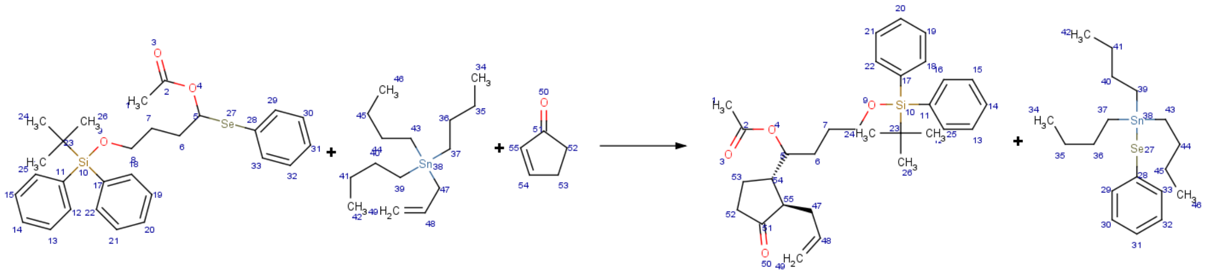

Correct mapped SMILES/SMARTS of the reaction:
[#6:1]-[#6:2](=[O:3])-[#8:4]-[#6:5](-[#6:6]-[#6:7]-[#6:8]-[#8:9][Si:10]([#6:11]-1=[#6:12]-[#6:13]=[#6:14]-[#6:15]=[#6:16]-1)([#6:17]-1=[#6:18]-[#6:19]=[#6:20]-[#6:21]=[#6:22]-1)[C:23]([#6:24])([#6:25])[#6:26])[Se:27][#6:28]-1=[#6:29]-[#6:30]=[#6:31]-[#6:32]=[#6:33]-1.[#6:34]-[#6:35]-[#6:36]-[#6:37][Sn:38]([#6:39]-[#6:40]-[#6:41]-[#6:42])([#6:43]-[#6:44]-[#6:45]-[#6:46])[#6:47]-[#6:48]=[#6:49].[O:50]=[#6:51]-1-[#6:52]-[#6:53]-[#6:54]=[#6:55]-1>>[#6:1]-[#6:2](=[O:3])-[#8:4]-[#6:5](-[#6:6]-[#6:7]-[#6:8]-[#8:9][Si:10]([#6:11]-1=[#6:16]-[#6:15]=[#6:14]-[#6:13]=[#6:12]-1)([#6:17]-1=[#6:22]-[#6:21]=[#6:20]-[#6:19]=[#6:18]-1)[C:23]([#6:26])([#6:25])[#6:24])-[#6@H:54]-1-[#6:53]-[#6:52]-[#6:51](=[O:50])-[#6@@H:55]-1-[#6:47]-[#6:48]=[#6:49].[#6:42]-[#6:41]-[#6:40]-[#6:39][Sn:38]([#6:37]-[#6:36]-[#6:35]-[#6:34])([#6:43]-[#6:44]-[#6:45]-[#6:46])[Se:27][#6:28]-1=[#6:33]-[#6:32]=[#6:31]-[#6:30]=[#6:29]-1

Correctness of the mapping

MAPPET	YES
ReactionMap	YES
Marvin	YES
ChemDraw	NO
Indigo	NO

Reaction no 51

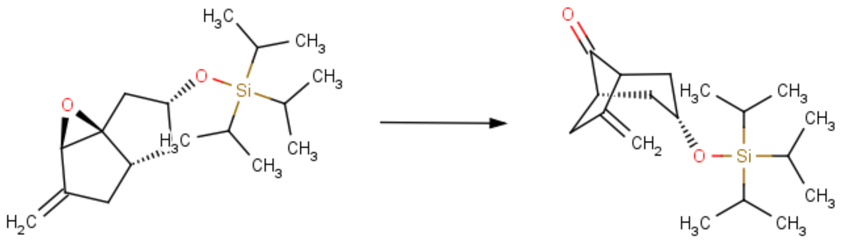

SMILES of the input:
CC(C)[Si](O[C@@H]1C[C@@H]2CC(=C)[C@H]3O[C@@]23C1)(C(C)C)C(C)C>>CC(C)[Si](O[C@@H]1C[C@@H]2CC(=C)C(C1)C2=O)(C(C)C)C(C)C

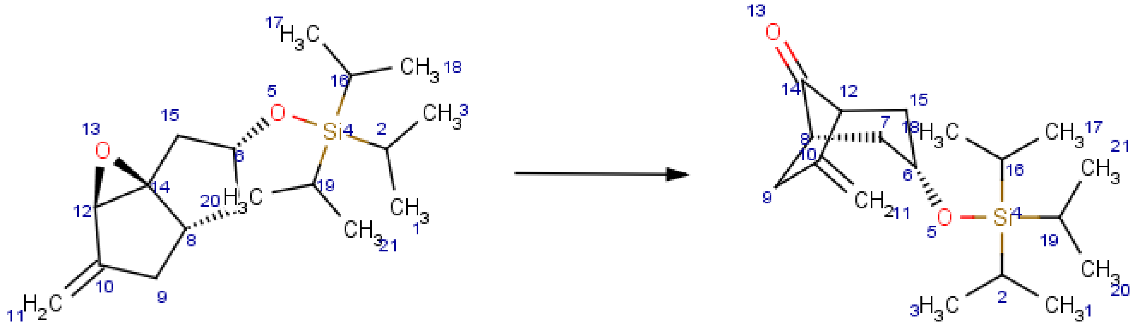

Correct mapped SMILES/SMARTS of the reaction:
[#6:1]-[#6:2](-[#6:3])[Si:4]([#8:5]-[#6@@H:6]-1-[#6:7]-[#6@@H:8]2-[#6:9]-[#6:10](=[#6:11])-[#6@H:12]3-[#8:13][C@@:14]23[#6:15]-1)([#6:16](-[#6:17])-[#6:18])[#6:19](-[#6:20])-[#6:21]>>[#6:21]-[#6:19](-[#6:20])[Si:4]([#8:5]-[#6@@H:6]-1-[#6:7]-[#6@@H:8]-2-[#6:9]-[#6:10](=[#6:11])-[#6:12](-[#6:15]-1)-[#6:14]-2=[O:13])([#6:16](-[#6:18])-[#6:17])[#6:2](-[#6:3])-[#6:1]

Correctness of the mapping

MAPPET	YES
ReactionMap	NO
Marvin	YES
ChemDraw	NO
Indigo	NO

Reaction no 52

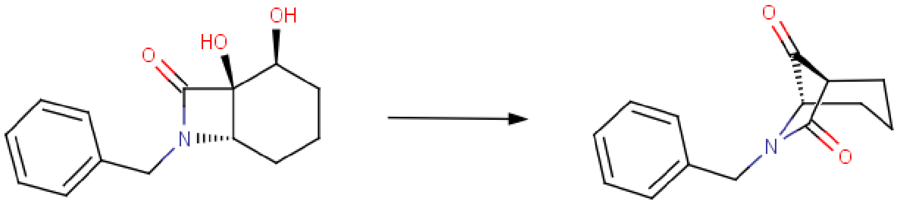

SMILES of the input:
O[C@H]1CCC[C@@H]2N(CC3=CC=CC=C3)C(=O)[C@]12O>>O=C1[C@@H]2CCC[C@H]1C(=O)N2CC1=CC=CC=C1

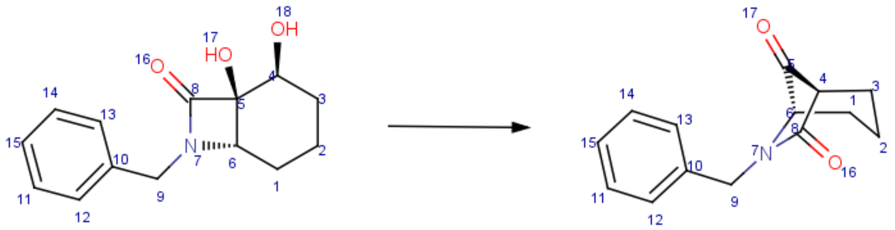

Correct mapped SMILES/SMARTS of the reaction:
[#8:18]-[#6@H:4]1-[#6:3]-[#6:2]-[#6:1]-[#6@@H:6]2-[#7:7](-[#6:9]-[#6:10]-3=[#6:12]-[#6:11]=[#6:15]-[#6:14]=[#6:13]-3)-[#6:8](=[O:16])[C@:5]12[#8:17]>>[O:17]=[#6:5]-1-[#6@@H:6]-2-[#6:1]-

[#6:2]-[#6:3]-[#6@H:4]-1-[#6:8](=[O:16])-[#7:7]-2-[#6:9]-[#6:10]-1=[#6:12]-[#6:11]=[#6:15]-[#6:14]=[#6:13]-1

Correctness of the mapping
MAPPET NO
ReactionMap NO
Marvin YES
ChemDraw YES
Indigo NO

Reaction no 53

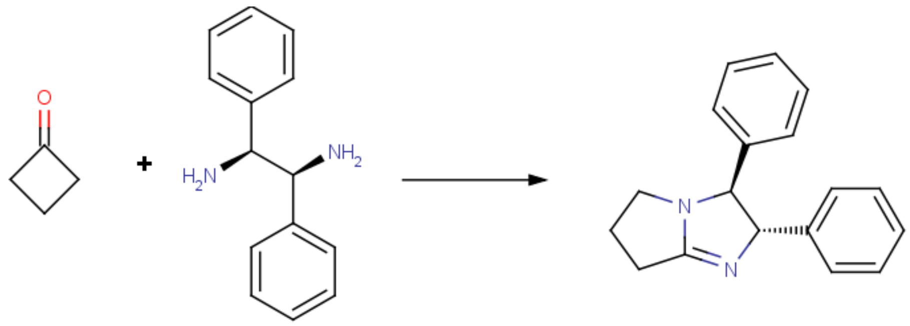

SMILES of the input:
O=C1CCC1.N[C@H]([C@@H](N)C1=CC=CC=C1)C1=CC=CC=C1>>C1CN2[C@H]([C@@H](N=C2C1)C1=CC=CC=C1)C1=CC=CC=C1

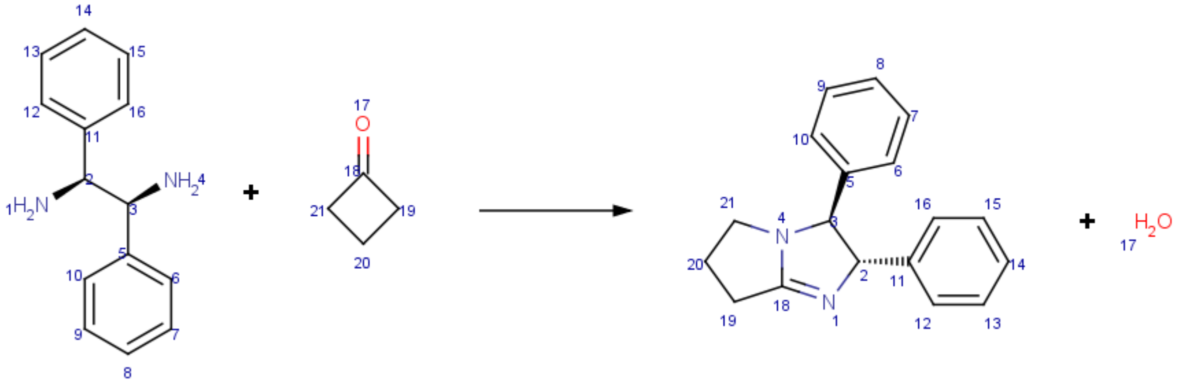

Correct mapped SMILES/SMARTS of the reaction:
[#7:1]-[#6@H:2](-[#6@@H:3](-[#7:4])-[#6:5]-1=[#6:6]-[#6:7]=[#6:8]-[#6:9]=[#6:10]-1)-[#6:11]-1=[#6:12]-[#6:13]=[#6:14]-[#6:15]=[#6:16]-1.[O:17]=[#6:18]-1-[#6:19]-[#6:20]-[#6:21]-1>>[#6:20]-1-[#6:21]-[#7:4]-2-[#6@H:3](-[#6@@H:2](-[#7:1]=[#6:18]-2-[#6:19]-1)-[#6:11]-1=[#6:16]-[#6:15]=[#6:14]-[#6:13]=[#6:12]-1)-[#6:5]-1=[#6:10]-[#6:9]=[#6:8]-[#6:7]=[#6:6]-1.[#8:17]

Correctness of the mapping
MAPPET YES
ReactionMap NO
Marvin YES
ChemDraw YES
Indigo NO

Reaction no 54

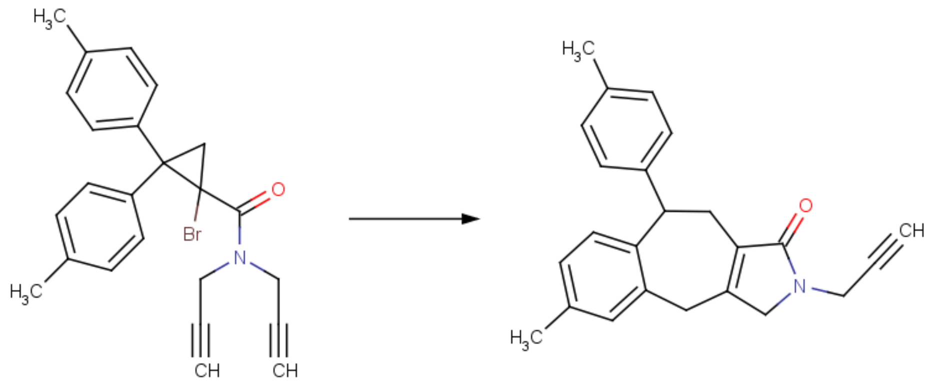

SMILES of the input:
CC1=CC=C(C=C1)C1(CC1(Br)C(=O)N(CC#C)CC#C)C1=CC=C(C)C=C1>>CC1=CC=C(C=C1)C1CC2=C(CN(CC#C)C2=O)CC2=CC(C)=CC=C12

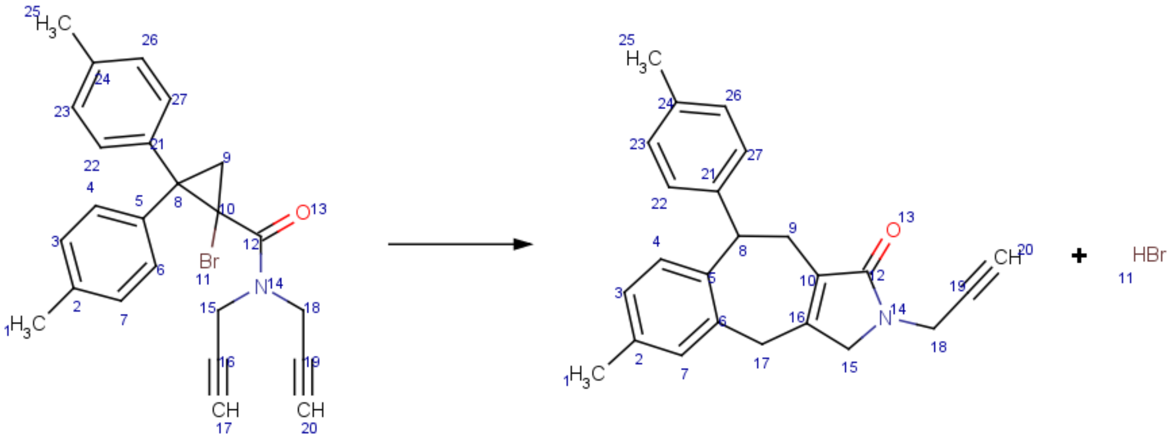

Correct mapped SMILES/SMARTS of the reaction:
[#6:1]-[#6:2]-1=[#6:3]-[#6:4]=[#6:5](-[#6:6]=[#6:7]-1)[C:8]1([#6:9][C:10]1([Br:11])[#6:12](=[O:13])-[#7:14](-[#6:15][C:16]#[C:17])-[#6:18][C:19]#[C:20])[#6:21]-1=[#6:22]-[#6:23]=[#6:24](-[#6:25])-[#6:26]=[#6:27]-1>>[#6:25]-[#6:24]-1=[#6:26]-[#6:27]=[#6:21](-[#6:22]=[#6:23]-1)-[#6:8]-1-[#6:9]-[#6:10]-2=[#6:16](-[#6:15]-[#7:14](-[#6:18][C:19]#[C:20])-[#6:12]-2=[O:13])-[#6:17]-[#6:6]-2=[#6:7]-[#6:2](-[#6:1])=[#6:3]-[#6:4]=[#6:5]-1-2.[Br:11]

Correctness of the mapping

MAPPET	YES
ReactionMap	NO
Marvin	YES
ChemDraw	YES
Indigo	YES

Reaction no 55

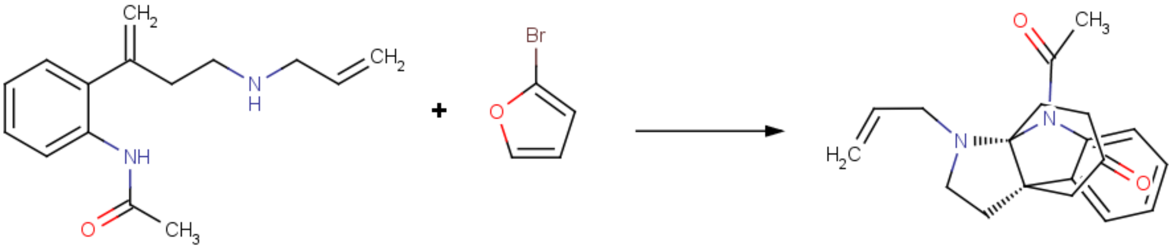

SMILES of the input:

CC(=O)NC1=CC=CC=C1C(=C)CCNCC=C.BrC1=CC=CO1>>CC(=O)N1C2=CC=CC=C2[C@]23CCN(CC=C)[C@]12CCC(=O)C3

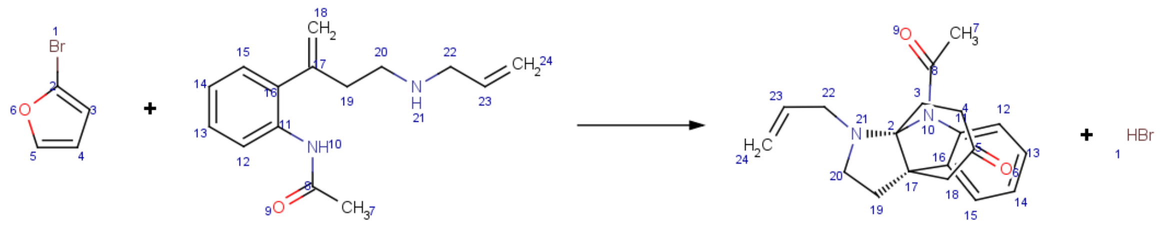

Correct mapped SMILES/SMARTS of the reaction:
[Br:1][#6:2]-1=[#6:3]-[#6:4]=[#6:5]-[#8:6]-1.[#6:7]-[#6:8](=[O:9])-[#7:10]-[#6:11]-1=[#6:12]-[#6:13]=[#6:14]-[#6:15]=[#6:16]-1-[#6:17](=[#6:18])-[#6:19]-[#6:20]-[#7:21]-[#6:22]-[#6:23]=[#6:24]>>[#6:7]-[#6:8](=[O:9])-[#7:10]1-[#6:11]-2=[#6:12]-[#6:13]=[#6:14]-[#6:15]=[#6:16]-2[C@:17]23[#6:19]-[#6:20]-[#7:21](-[#6:22]-[#6:23]=[#6:24])[C@:2]12[#6:3]-[#6:4]-[#6:5](=[O:6])-[#6:18]3.[Br:1]

Correctness of the mapping

MAPPET	YES
ReactionMap	NO
Marvin	NO
ChemDraw	NO
Indigo	NO

Reaction no 56

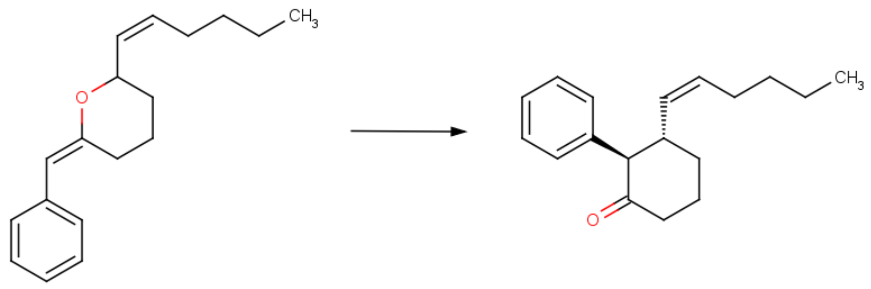

SMILES of the input:
CCCC\C=C/C1CCC\C(O1)=C/C1=CC=CC=C1>>CCCC\C=C/[C@@H]1CCCC(=O)[C@H]1C1=CC=CC=C1

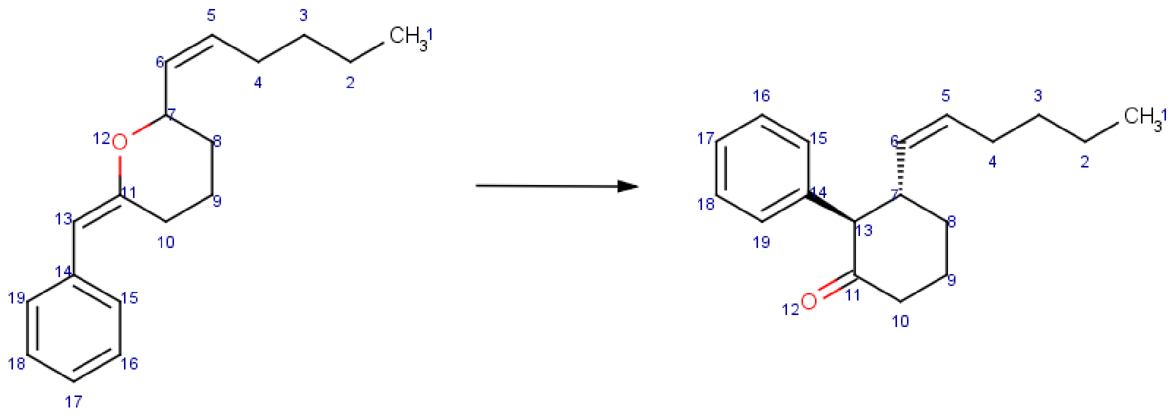

Correct mapped SMILES/SMARTS of the reaction:

[#6:1]-[#6:2]-[#6:3]-[#6:4]\[#6:5]=[#6:6]/[#6:7]-1-[#6:8]-[#6:9]-
[#6:10]\[#6:11](-[#8:12]1)=[#6:13]/[#6:14]-1=[#6:15]-[#6:16]=[#6:17]-
[#6:18]=[#6:19]1>>[#6:1]-[#6:2]-[#6:3]-[#6:4]\[#6:5]=[#6:6]/[#6@@H:7]-1-
[#6:8]-[#6:9]-[#6:10]-[#6:11](=[O:12])-[#6@H:13]1-[#6:14]-1=[#6:19]-
[#6:18]=[#6:17]-[#6:16]=[#6:15]-1

Correctness of the mapping
MAPPET YES
ReactionMap YES
Marvin YES
ChemDraw YES
Indigo NO

Reaction no 57

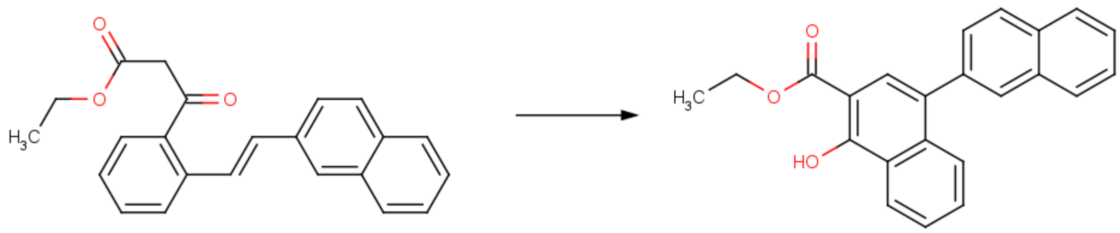

SMILES of the input:
CCOC(=O)CC(=O)C1=C(\C=C\C2=CC=C3C=CC=CC3=C2)C=CC=C1>>CCOC(=O)C1=C(O)C2=C(
C=CC=C2)C(=C1)C1=CC=C2C=CC=CC2=C1

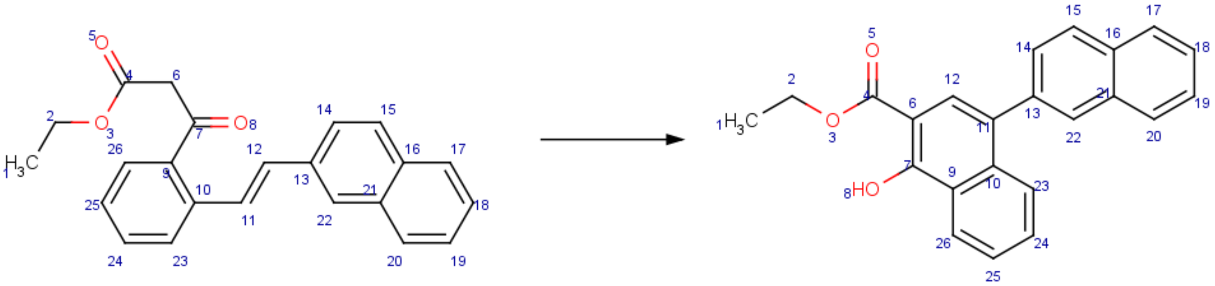

Correct mapped SMILES/SMARTS of the reaction:
CCOC(=O)[CH2:1][C:2](=O)[C:3]1=[C:4](\[CH:5]=[CH:6]\[C:7]2=[CH:8][CH:9]=[
C:10]3[CH:11]=[CH:12][CH:13]=[CH:14][C:15]3=[CH:16]2)[CH:17]=[CH:18][CH:1
9]=[CH:20]1>>CCO[C:1](=O)[C:2]1=[C:3](O)[C:4]2=[C:5]([CH:20]=[CH:19][CH:1
8]=[CH:17]2)[C:6](=C1)[C:7]1=[CH:8][CH:9]=[C:10]2[CH:11]=[CH:12][CH:13]=[
CH:14][C:15]2=[CH:16]1

Correctness of the mapping
MAPPET NO
ReactionMap NO
Marvin NO
ChemDraw NO
Indigo NO

Reaction no 58

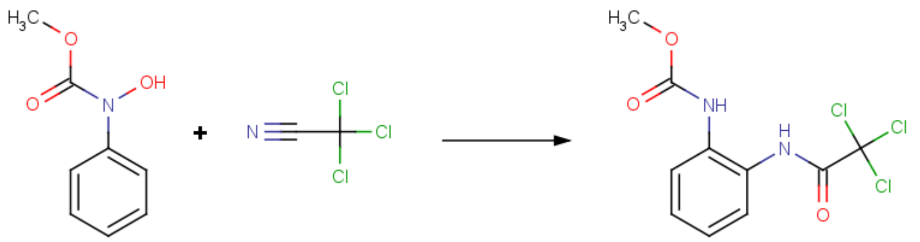

SMILES of the input:
COC(=O)N(O)C1=CC=CC=C1.ClC(Cl)(Cl)C#N>>COC(=O)NC1=C(NC(=O)C(Cl)(Cl)Cl)C=CC=C1

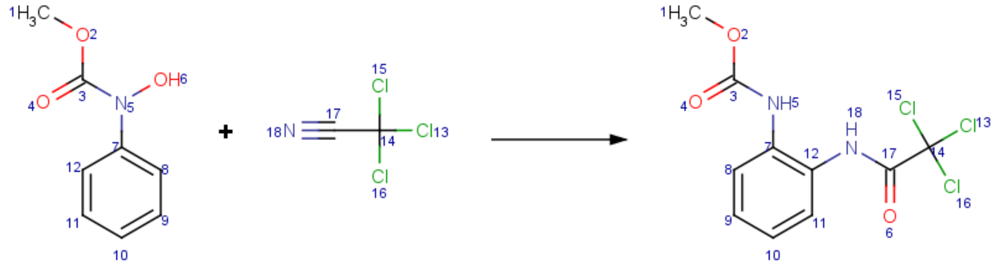

Correct mapped SMILES/SMARTS of the reaction:
[#6:1]-[#8:2]-[#6:3](=[O:4])-[#7:5](-[#8:6])-[#6:7]-1=[#6:8]-[#6:9]=[#6:10]-[#6:11]=[#6:12]-1.[Cl:13][C:14]([Cl:15])([Cl:16])[C:17]#[N:18]>>[#6:1]-[#8:2]-[#6:3](=[O:4])-[#7:5]-[#6:7]-1=[#6:12](-[#7:18]-[#6:17](=[O:6])[C:14]([Cl:13])([Cl:16])[Cl:15])-[#6:11]=[#6:10]-[#6:9]=[#6:8]-1

Correctness of the mapping

MAPPET	YES
ReactionMap	YES
Marvin	YES
ChemDraw	YES
Indigo	YES

Reaction no 59

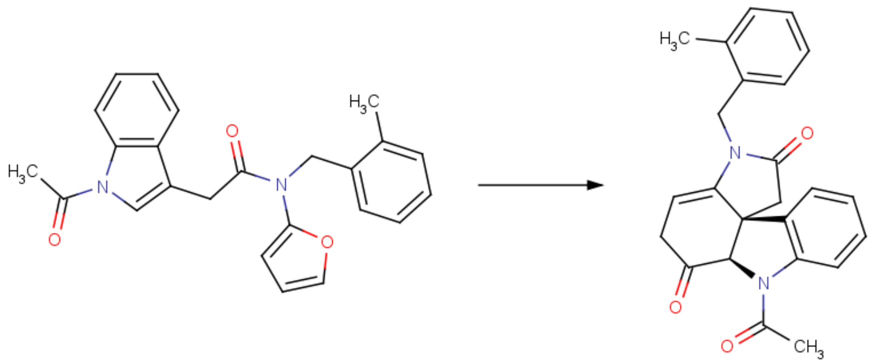

SMILES of the input:
CC(=O)N1C=C(CC(=O)N(CC2=CC=CC=C2C)C2=CC=CO2)C2=C1C=CC=C2>>CC(=O)N1[C@H]2C(=O)CC=C3N(CC4=CC=CC=C4C)C(=O)C[C@]23C2=C1C=CC=C2

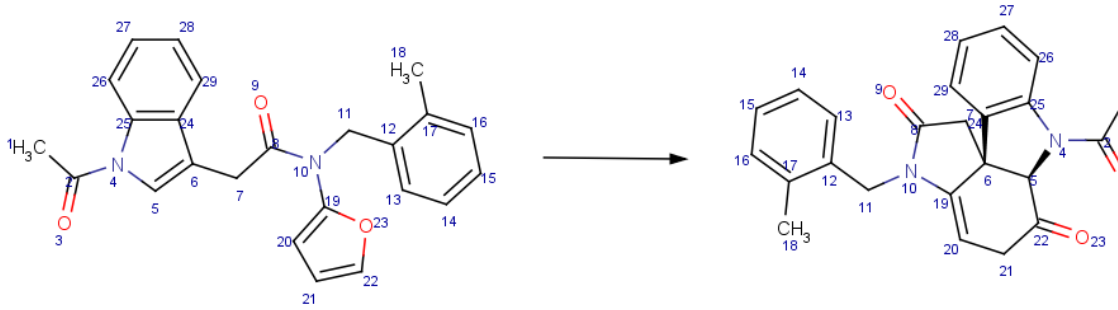

Correct mapped SMILES/SMARTS of the reaction:
[#6:1]-[#6:2](=[O:3])-[#7:4]-1-[#6:5]=[#6:6](-[#6:7]-[#6:8](=[O:9])-[#7:10](-[#6:11]-[#6:12]-2=[#6:13]-[#6:14]=[#6:15]-[#6:16]=[#6:17]-2-[#6:18])-[#6:19]-2=[#6:20]-[#6:21]=[#6:22]-[#8:23]-2)-[#6:24]-2=[#6:25]-1-[#6:26]=[#6:27]-[#6:28]=[#6:29]-2>>[#6:1]-[#6:2](=[O:3])-[#7:4]-1-[#6@H:5]2-[#6:22](=[O:23])-[#6:21]-[#6:20]=[#6:19]3-[#7:10](-[#6:11]-[#6:12]-4=[#6:13]-[#6:14]=[#6:15]-[#6:16]=[#6:17]-4-[#6:18])-[#6:8](=[O:9])-[#6:7][C@:6]23[#6:24]-2=[#6:25]-1-[#6:26]=[#6:27]-[#6:28]=[#6:29]-2

Correctness of the mapping

MAPPET	YES
ReactionMap	YES
Marvin	YES
ChemDraw	YES
Indigo	YES

Reaction no 60

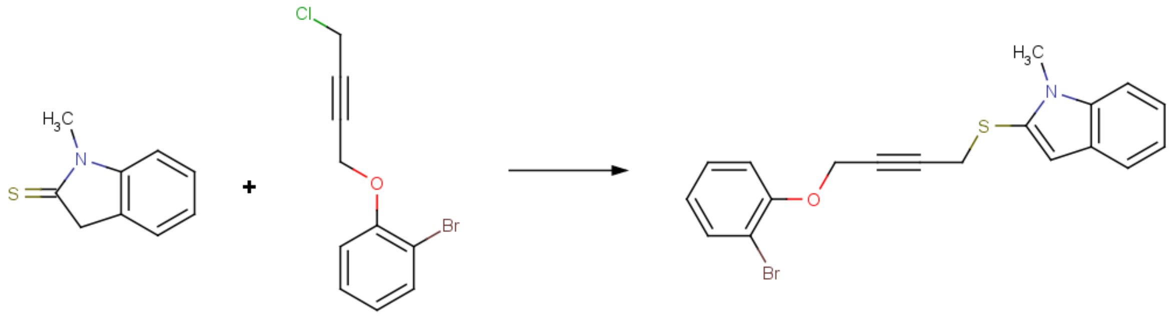

SMILES of the input:
CN1C(=S)CC2=C1C=CC=C2.ClCC#CCOC1=C(Br)C=CC=C1>>CN1C(SCC#CCOC2=C(Br)C=CC=C2)=CC2=C1C=CC=C2

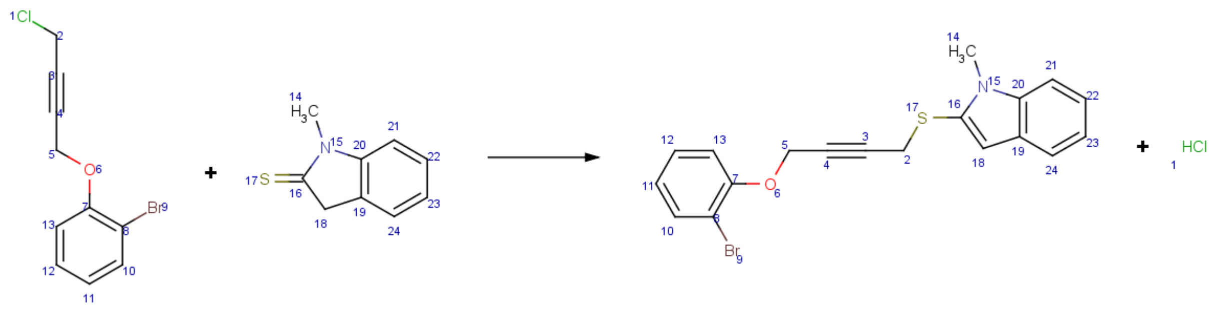

Correct mapped SMILES/SMARTS of the reaction:
[Cl:1][#6:2][C:3]#[C:4][#6:5]-[#8:6]-[#6:7]-1=[#6:8]([Br:9])-[#6:10]=[#6:11]-[#6:12]=[#6:13]-1.[#6:14]-[#7:15]-1-[#6:16](=[S:17])-

[#6:18]-[#6:19]-2=[#6:20]-1-[#6:21]=[#6:22]-[#6:23]=[#6:24]-2>>[#6:14]-
[#7:15]-1-[#6:16](-[#16:17]-[#6:2][C:3]#[C:4][#6:5]-[#8:6]-[#6:7]-
2=[#6:8]([Br:9])-[#6:10]=[#6:11]-[#6:12]=[#6:13]-2)=[#6:18]-[#6:19]-
2=[#6:20]-1-[#6:21]=[#6:22]-[#6:23]=[#6:24]-2.[Cl:1]

Correctness of the mapping

MAPPET	YES
ReactionMap	NO
Marvin	YES
ChemDraw	YES
Indigo	YES

Reaction no 61

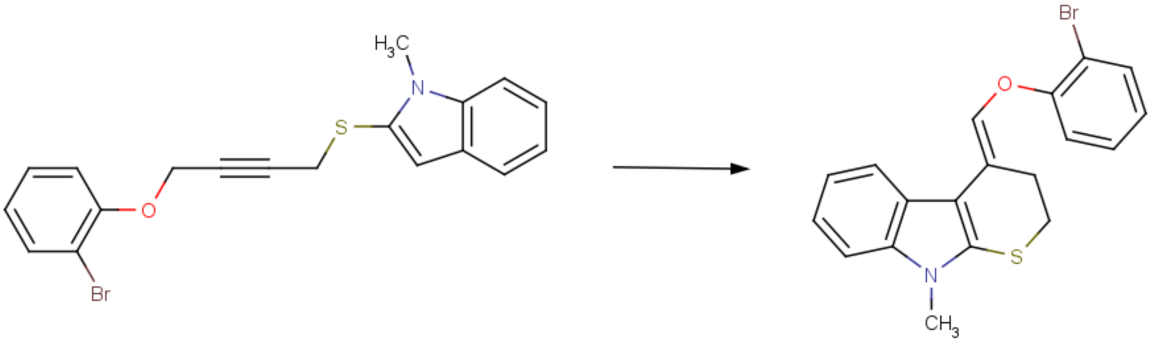

SMILES of the input:
CN1C(SCC#CCOC2=C(Br)C=CC=C2)=CC2=C1C=CC=C2>>CN1C2=C(C3=C1C=CC=C3)\C(CCS2)
=C\OC1=C(Br)C=CC=C1

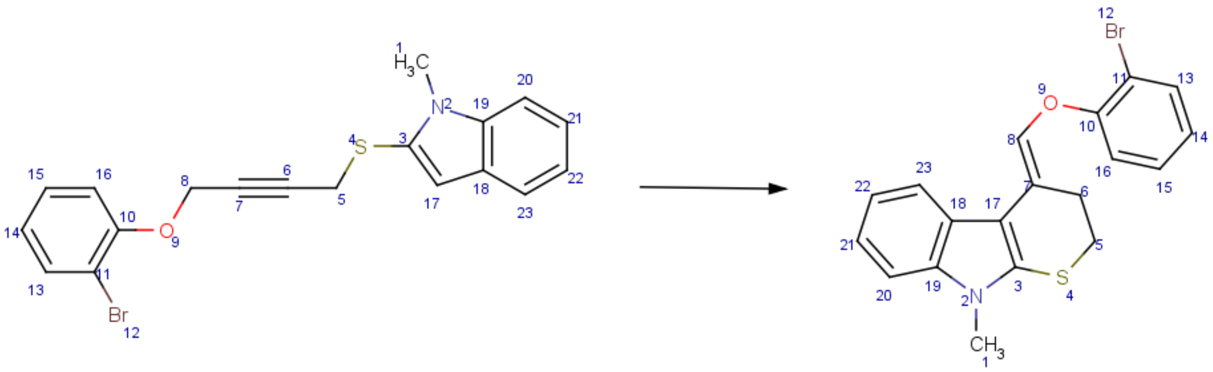

Correct mapped SMILES/SMARTS of the reaction:
[#6:1]-[#7:2]-1-[#6:3](-[#16:4]-[#6:5][C:6]#[C:7][#6:8]-[#8:9]-[#6:10]-
2=[#6:11]([Br:12])-[#6:13]=[#6:14]-[#6:15]=[#6:16]-2)=[#6:17]-[#6:18]-
2=[#6:19]-1-[#6:20]=[#6:21]-[#6:22]=[#6:23]-2>>[#6:1]-[#7:2]-1-[#6:3]-
2=[#6:17](-[#6:18]-3=[#6:19]-1-[#6:20]=[#6:21]-[#6:22]=[#6:23]-
3)\[#6:7](-[#6:6]-[#6:5]-[#16:4]-2)=[#6:8]\[#8:9]-[#6:10]-
1=[#6:11]([Br:12])-[#6:13]=[#6:14]-[#6:15]=[#6:16]-1

Correctness of the mapping

MAPPET	YES
ReactionMap	YES
Marvin	YES
ChemDraw	YES
Indigo	NO

Reaction no 62

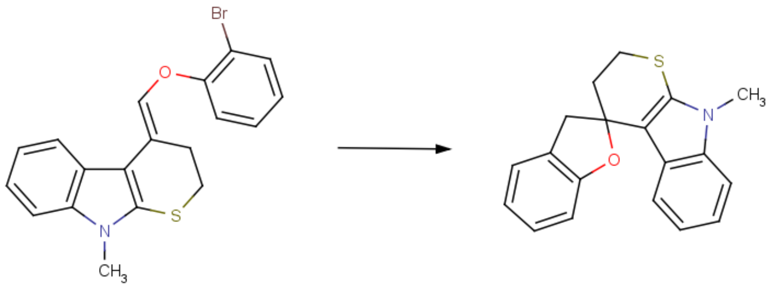

SMILES of the input:
CN1C2=C(C3=C1C=CC=C3)\C(CCS2)=C\OC1=C(Br)C=CC=C1>>CN1C2=C(C3=C1C=CC=C3)C1
(CC3=C(O1)C=CC=C3)CCS2

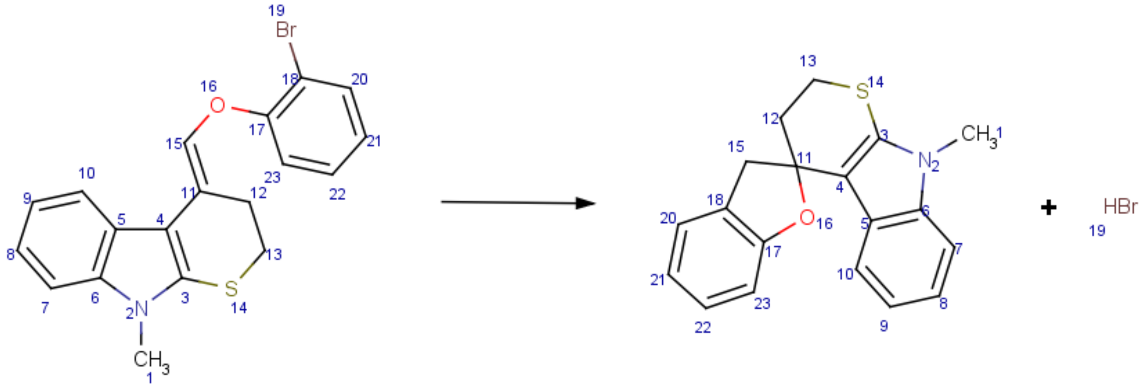

Correct mapped SMILES/SMARTS of the reaction:
[#6:1]-[#7:2]-1-[#6:3]-2=[#6:4](-[#6:5]-3=[#6:6]-1-[#6:7]=[#6:8]-
[#6:9]=[#6:10]-3)\[#6:11](-[#6:12]-[#6:13]-[#16:14]-2)=[#6:15]\[#8:16]-
[#6:17]-1=[#6:18]([Br:19])-[#6:20]=[#6:21]-[#6:22]=[#6:23]-1>>[#6:1]-
[#7:2]-1-[#6:3]-2=[#6:4](-[#6:5]-3=[#6:6]-1-[#6:7]=[#6:8]-[#6:9]=[#6:10]-
3)[C:11]1([#6:15]-[#6:18]-3=[#6:17](-[#8:16]1)-[#6:23]=[#6:22]-
[#6:21]=[#6:20]-3)[#6:12]-[#6:13]-[#16:14]-2.[Br:19]

Correctness of the mapping

MAPPET	YES
ReactionMap	NO
Marvin	YES
ChemDraw	NO
Indigo	YES

Reaction no 63

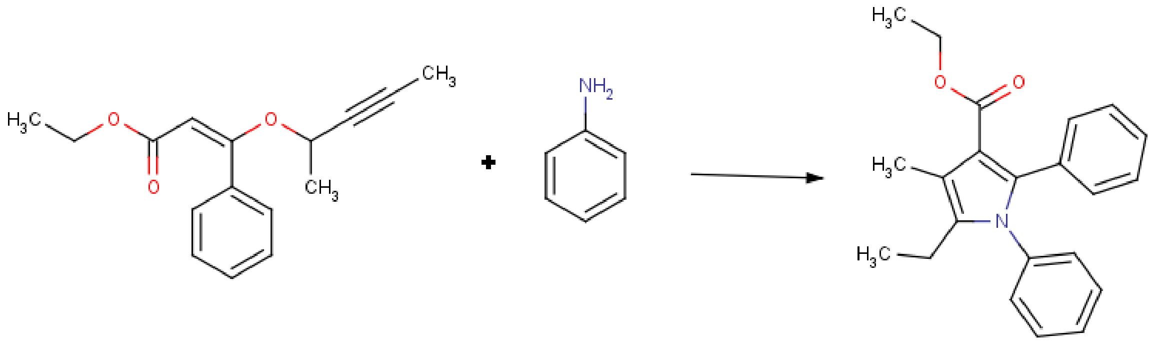

SMILES of the input:
CCOC(=O)\C=C(\OC(C)C#CC)C1=CC=CC=C1.NC1=CC=CC=C1>>CCOC(=O)C1=C(N(C(CC)=C1
C)C1=CC=CC=C1)C1=CC=CC=C1

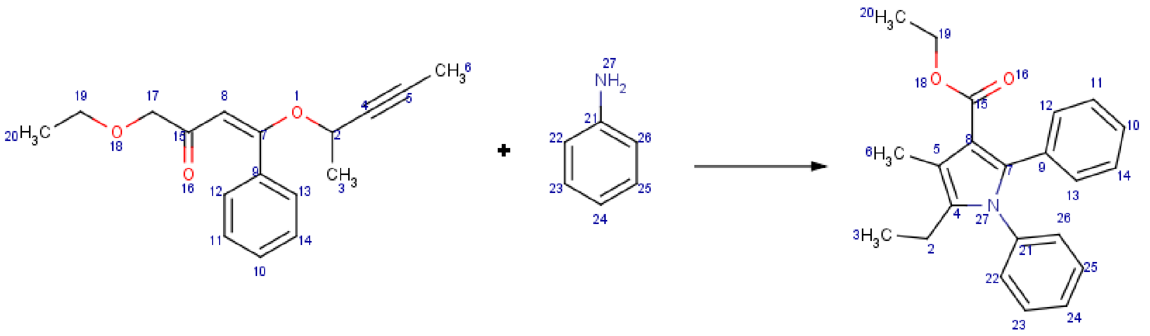

Correct mapped SMILES/SMARTS of the reaction:
[#6:20]-[#6:19]-[#8:18]-[#6:17]-[#6:15](=[O:16])\[#6:8]=[#6:7](\[#8:1]-
[#6:2](-[#6:3])[C:4]#[C:5][#6:6])-[#6:9]-1=[#6:13]-[#6:14]=[#6:10]-
[#6:11]=[#6:12]-1.[#7:27]-[#6:21]-1=[#6:26]-[#6:25]=[#6:24]-
[#6:23]=[#6:22]-1>>[#6:20]-[#6:19]-[#8:18]-[#6:15](=[O:16])-[#6:8]-
1=[#6:7](-[#7:27](-[#6:4](-[#6:2]-[#6:3])=[#6:5]-1-[#6:6])-[#6:21]-
1=[#6:26]-[#6:25]=[#6:24]-[#6:23]=[#6:22]-1)-[#6:9]-1=[#6:12]-
[#6:11]=[#6:10]-[#6:14]=[#6:13]-1

Correctness of the mapping

MAPPET	NO
ReactionMap	NO
Marvin	YES
ChemDraw	YES
Indigo	YES

Reaction no 64

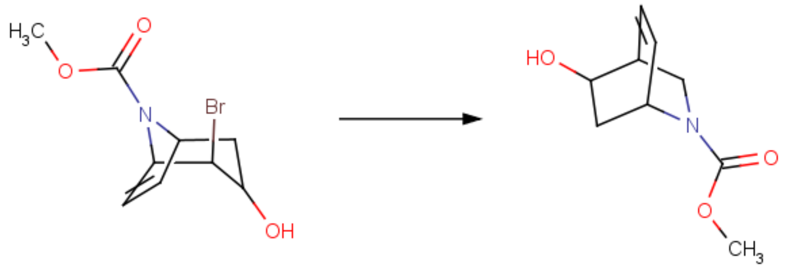

SMILES of the input:
COC(=O)N1C2CC(O)C(Br)C1C=C2>>COC(=O)N1CC2C=CC1CC2O

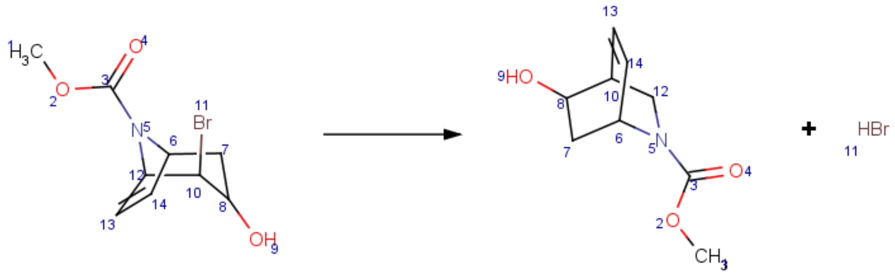

Correct mapped SMILES/SMARTS of the reaction:
[#6:1]-[#8:2]-[#6:3](=[O:4])-[#7:5]-1-[#6:6]-2-[#6:7]-[#6:8](-[#8:9])-
[#6:10]([Br:11])-[#6:12]-1-[#6:13]=[#6:14]-2>>[#6:1]-[#8:2]-
[#6:3](=[O:4])-[#7:5]-1-[#6:12]-[#6:10]-2-[#6:13]=[#6:14]-[#6:6]-1-
[#6:7]-[#6:8]-2-[#8:9].[Br:11]

Correctness of the mapping

MAPPET	YES

```
ReactionMap      NO
Marvin           YES
ChemDraw         YES
Indigo           YES
```

Reaction no 65

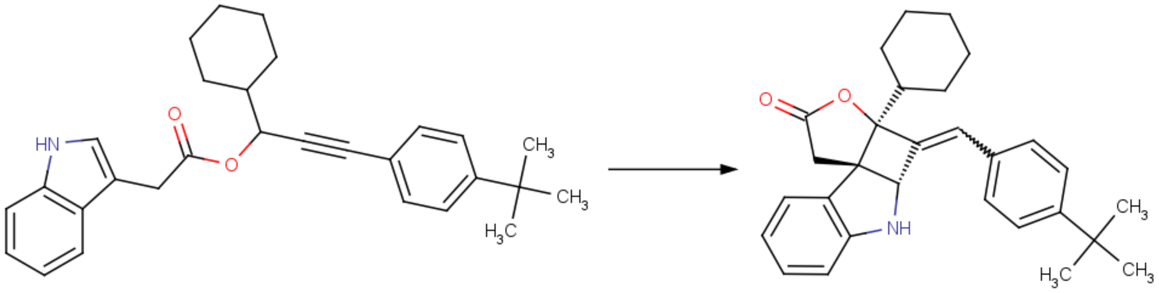

SMILES of the input:
CC(C)(C)C1=CC=C(C=C1)C#CC(OC(=O)CC1=CNC2=C1C=CC=C2)C1CCCCC1>>CC(C)(C)C1=CC=C(C=C2[C@@H]3NC4=C(C=CC=C4)[C@@]33CC(=O)O[C@@]23C2CCCCC2)C=C1

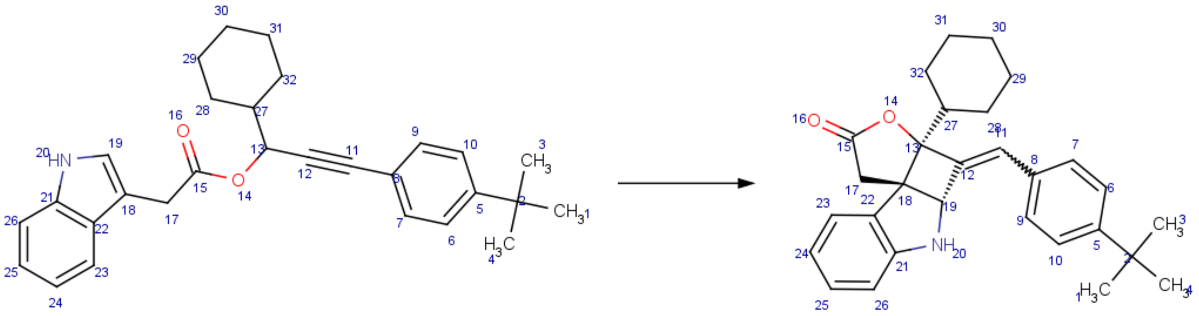

Correct mapped SMILES/SMARTS of the reaction:
[#6:1][C:2]([#6:3])([#6:4])[#6:5]-1=[#6:6]-[#6:7]=[#6:8](-[#6:9]=[#6:10]-1)[C:11]#[C:12][#6:13](-[#8:14]-[#6:15](=[O:16])-[#6:17]-[#6:18]-1=[#6:19]-[#7:20]-[#6:21]-2=[#6:22]-1-[#6:23]=[#6:24]-[#6:25]=[#6:26]-2)-[#6:27]-1-[#6:28]-[#6:29]-[#6:30]-[#6:31]-[#6:32]-1>>[#6:4][C:2]([#6:3])([#6:1])[#6:5]-1=[#6:10]-[#6:9]=[#6:8](-[#6:11]=[#6:12]2-[#6@@H:19]3-[#7:20]-[#6:21]-4=[#6:22](-[#6:23]=[#6:24]-[#6:25]=[#6:26]-4)[C@@:18]33[#6:17]-[#6:15](=[O:16])-[#8:14][C@@:13]23[#6:27]-2-[#6:32]-[#6:31]-[#6:30]-[#6:29]-[#6:28]-2)-[#6:7]=[#6:6]-1

Correctness of the mapping

```
MAPPET           YES
ReactionMap      YES
Marvin           YES
ChemDraw         YES
Indigo           YES
```

Reaction no 66

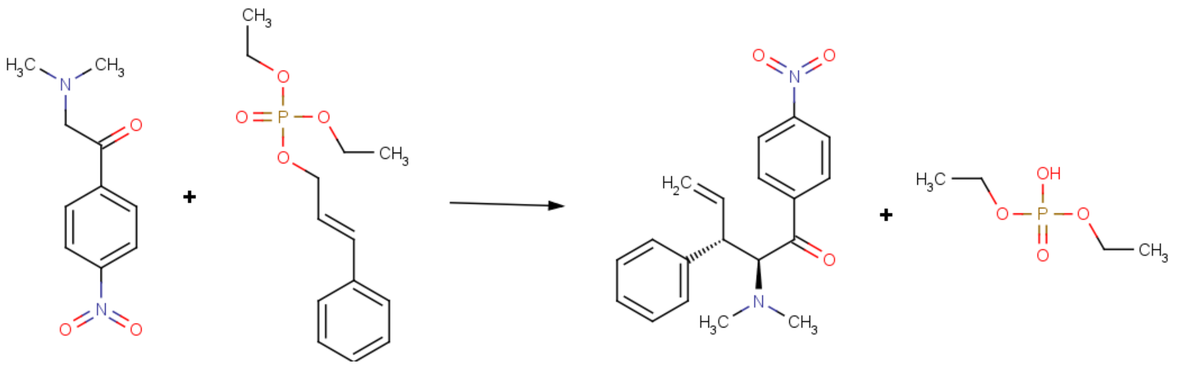

SMILES of the input:
CN(C)CC(=O)C1=CC=C(C=C1)N(=O)=O.CCOP(=O)(OCC)OC\C=C\C1=CC=CC=C1>>CN(C)[C@
@H]([C@@H](C=C)C1=CC=CC=C1)C(=O)C1=CC=C(C=C1)N(=O)=O.CCOP(O)(=O)OCC

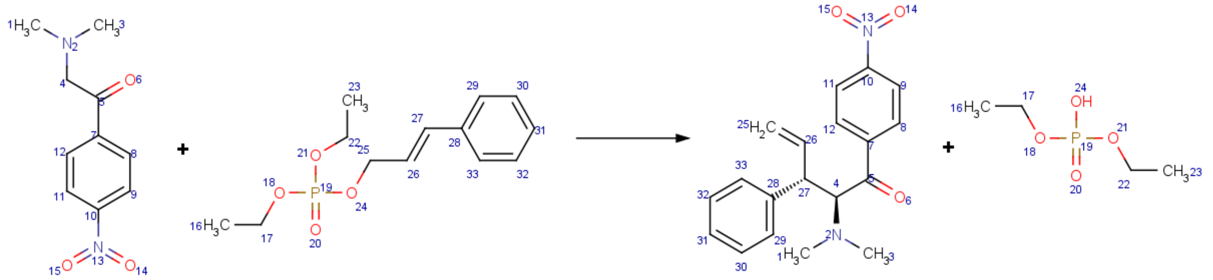

Correct mapped SMILES/SMARTS of the reaction:
[#6:1]-[#7:2](-[#6:3])-[#6:4]-[#6:5](=[O:6])-[#6:7]-1=[#6:8]-
[#6:9]=[#6:10](-[#6:11]=[#6:12]-1)[N:13](=[O:14])=[O:15].[#6:16]-[#6:17]-
[#8:18][P:19](=[O:20])([#8:21]-[#6:22]-[#6:23])[#8:24]-
[#6:25]\[#6:26]=[#6:27]\[#6:28]-1=[#6:29]-[#6:30]=[#6:31]-
[#6:32]=[#6:33]1>>[#6:3]-[#7:2](-[#6:1])-[#6@@H:4](-[#6@@H:27](-
[#6:26]=[#6:25])-[#6:28]-1=[#6:29]-[#6:30]=[#6:31]-[#6:32]=[#6:33]-1)-
[#6:5](=[O:6])-[#6:7]-1=[#6:12]-[#6:11]=[#6:10](-[#6:9]=[#6:8]-
1)[N:13](=[O:15])=[O:14].[#6:23]-[#6:22]-
[#8:21][P:19]([#8:24])(=[O:20])[#8:18]-[#6:17]-[#6:16]

Correctness of the mapping

MAPPET	YES
ReactionMap	YES
Marvin	NO
ChemDraw	NO
Indigo	NO

Reaction no 67

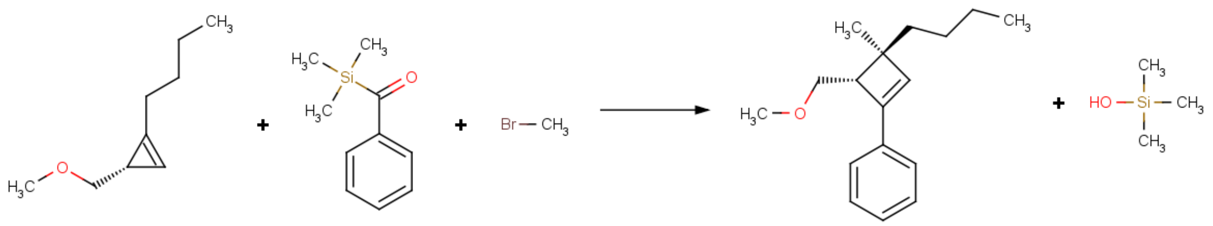

SMILES of the input:
CCCCC1=C[C@@H]1COC.C[Si](C)(C)C(=O)C1=CC=CC=C1.CBr>>CCCC[C@@]1(C)C=C([C@@
H]1COC)C1=CC=CC=C1.C[Si](C)(C)O

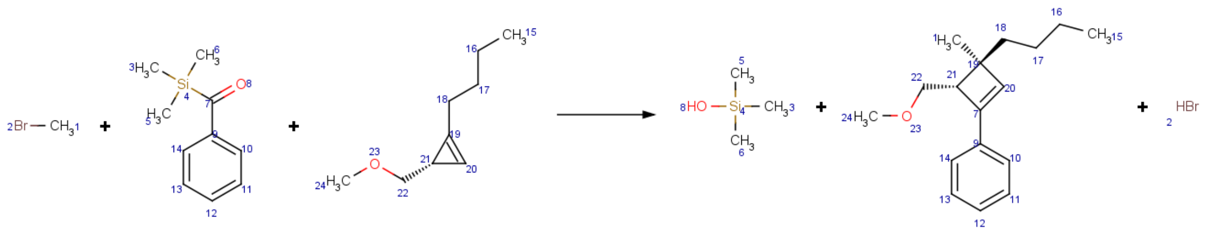

Correct mapped SMILES/SMARTS of the reaction:
[#6:1][Br:2].[#6:3][Si:4]([#6:5])([#6:6])[#6:7](=[O:8])-[#6:9]-1=[#6:10]-[#6:11]=[#6:12]-[#6:13]=[#6:14]-1.[#6:15]-[#6:16]-[#6:17]-[#6:18]-[#6:19]-1=[#6:20]-[#6@@H:21]-1-[#6:22]-[#8:23]-[#6:24]>>[#6:6][Si:4]([#6:5])([#6:3])[#8:8].[#6:15]-[#6:16]-[#6:17]-[#6:18][C@@:19]1([#6:1])[#6:20]=[#6:7](-[#6@@H:21]1-[#6:22]-[#8:23]-[#6:24])-[#6:9]-1=[#6:10]-[#6:11]=[#6:12]-[#6:13]=[#6:14]-1.[Br:2]

Correctness of the mapping

MAPPET	YES
ReactionMap	NO
Marvin	YES
ChemDraw	NO
Indigo	NO

Reaction no 68

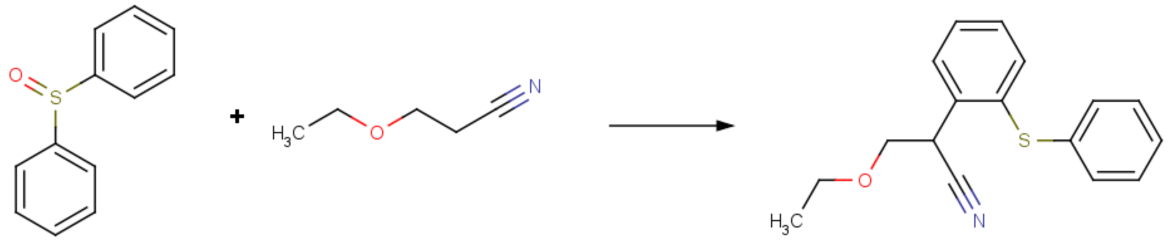

SMILES of the input:
O=S(C1=CC=CC=C1)C1=CC=CC=C1.CCOCCC#N>>CCOCC(C#N)C1=CC=CC=C1SC1=CC=CC=C1

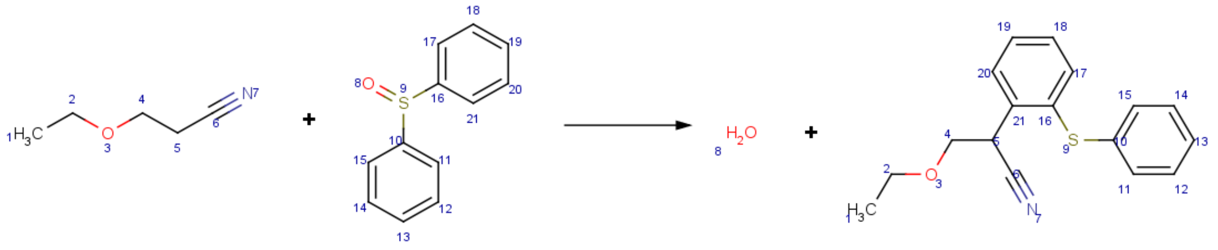

Correct mapped SMILES/SMARTS of the reaction:
[#6:1]-[#6:2]-[#8:3]-[#6:4]-[#6:5][C:6]#[N:7].[O:8]=[S:9]([#6:10]-1=[#6:11]-[#6:12]=[#6:13]-[#6:14]=[#6:15]-1)[#6:16]-1=[#6:17]-[#6:18]=[#6:19]-[#6:20]=[#6:21]-1>>[#8:8].[#6:1]-[#6:2]-[#8:3]-[#6:4]-[#6:5]([C:6]#[N:7])-[#6:21]-1=[#6:20]-[#6:19]=[#6:18]-[#6:17]=[#6:16]-1-[#16:9]-[#6:10]-1=[#6:15]-[#6:14]=[#6:13]-[#6:12]=[#6:11]-1

Correctness of the mapping

MAPPET	YES
ReactionMap	NO
Marvin	YES

ChemDraw YES
Indigo YES

Reaction no 69

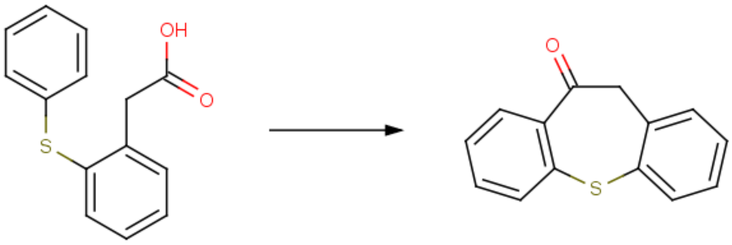

SMILES of the input:
OC(=O)CC1=CC=CC=C1SC1=CC=CC=C1>>O=C1CC2=CC=CC=C2SC2=C1C=CC=C2

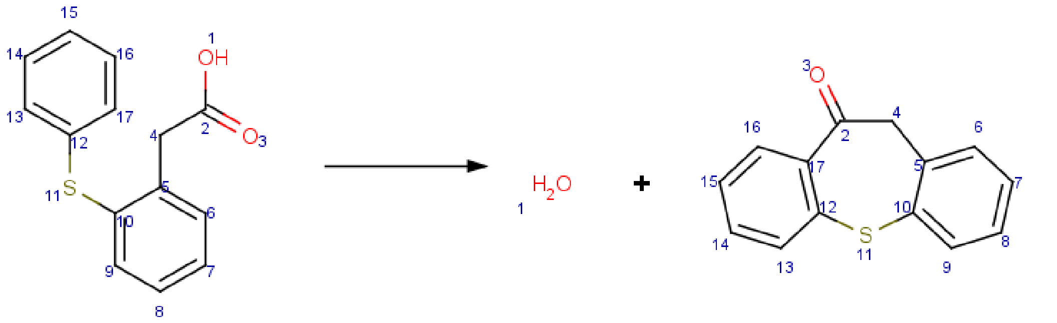

Correct mapped SMILES/SMARTS of the reaction:
[#8:1]-[#6:2](=[O:3])-[#6:4]-[#6:5]-1=[#6:6]-[#6:7]=[#6:8]-[#6:9]=[#6:10]-1-[#16:11]-[#6:12]-1=[#6:13]-[#6:14]=[#6:15]-[#6:16]=[#6:17]-1>>[#8:1].[O:3]=[#6:2]-1-[#6:4]-[#6:5]-2=[#6:6]-[#6:7]=[#6:8]-[#6:9]=[#6:10]-2-[#16:11]-[#6:12]-2=[#6:17]-1-[#6:16]=[#6:15]-[#6:14]=[#6:13]-2

Correctness of the mapping
MAPPET YES
ReactionMap NO
Marvin YES
ChemDraw YES
Indigo YES

Reaction no 70

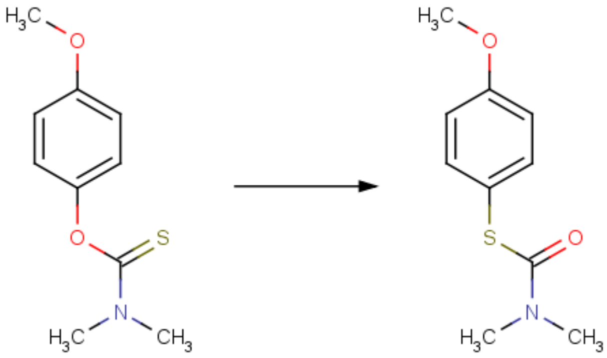

SMILES of the input:
COC1=CC=C(OC(=S)N(C)C)C=C1>>COC1=CC=C(SC(=O)N(C)C)C=C1

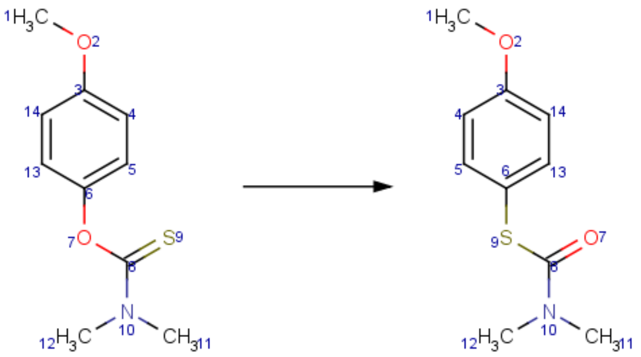

Correct mapped SMILES/SMARTS of the reaction:
[#6:1]-[#8:2]-[#6:3]-1=[#6:4]-[#6:5]=[#6:6](-[#8:7]-[#6:8](=[S:9])-[#7:10](-[#6:11])-[#6:12])-[#6:13]=[#6:14]-1>>[#6:1]-[#8:2]-[#6:3]-1=[#6:14]-[#6:13]=[#6:6](-[#16:9]-[#6:8](=[O:7])-[#7:10](-[#6:11])-[#6:12])-[#6:5]=[#6:4]-1

Correctness of the mapping

MAPPET	YES
ReactionMap	YES
Marvin	YES
ChemDraw	YES
Indigo	NO

Reaction no 71

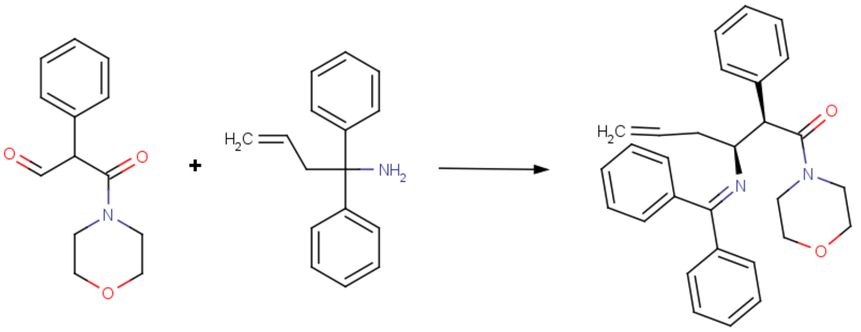

SMILES of the input:
O=CC(C(=O)N1CCOCC1)C1=CC=CC=C1.NC(CC=C)(C1=CC=CC=C1)C1=CC=CC=C1>>C=CC[C@H](N=C(C1=CC=CC=C1)C1=CC=CC=C1)[C@H](C(=O)N1CCOCC1)C1=CC=CC=C1

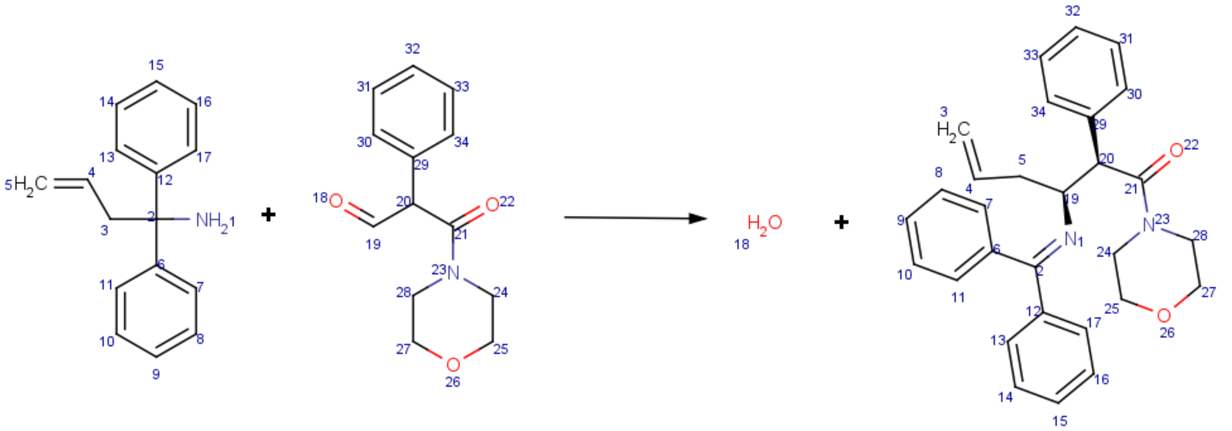

Correct mapped SMILES/SMARTS of the reaction:
O=[CH:1][CH:2]([C:3](=[O:4])[N:5]1[CH2:6][CH2:7][O:8][CH2:9][CH2:10]1)[C:11]1=[CH:12][CH:13]=[CH:14][CH:15]=[CH:16]1.[NH2:17][C:18](CC=C)([C:19]1=

[CH:20][CH:21]=[CH:22][CH:23]=[CH:24]1)[C:25]1=[CH:26][CH:27]=[CH:28][CH:29]=[CH:30]1>>C=CC[C@H:1]([N:17]=[C:18]([C:19]1=[CH:24][CH:23]=[CH:22][CH:21]=[CH:20]1)[C:25]1=[CH:30][CH:29]=[CH:28][CH:27]=[CH:26]1)[C@H:2]([C:3](=[O:4])[N:5]1[CH2:10][CH2:9][O:8][CH2:7][CH2:6]1)[C:11]1=[CH:16][CH:15]=[CH:14][CH:13]=[CH:12]1

Correctness of the mapping

MAPPET	NO
ReactionMap	NO
Marvin	NO
ChemDraw	NO
Indigo	NO

Reaction no 72

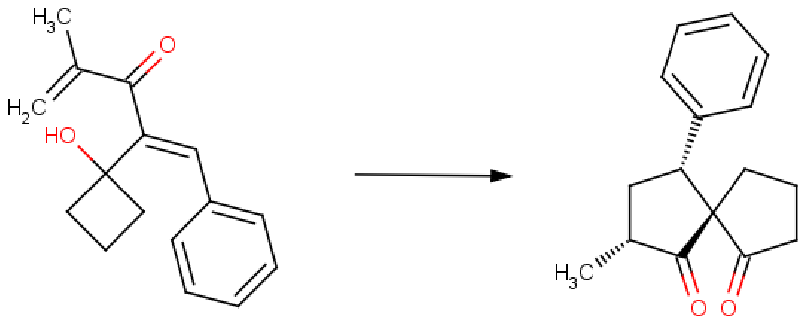

SMILES of the input:
CC(=C)C(=O)C(=C\C1=CC=CC=C1)\C1(O)CCC1>>C[C@@H]1C[C@H](C2=CC=CC=C2)[C@@]2(CCCC2=O)C1=O

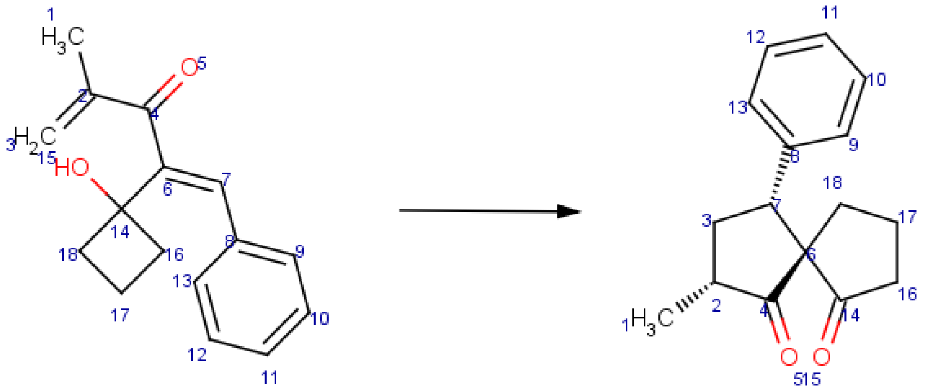

Correct mapped SMILES/SMARTS of the reaction:
[#6:1]-[#6:2](=[#6:3])-[#6:4](=[O:5])-[#6:6](=[#6:7]\[#6:8]-1=[#6:9]-[#6:10]=[#6:11]-[#6:12]=[#6:13]1)\[C:14]1([#8:15])[#6:16]-[#6:17]-[#6:18]1>>[#6:1]-[#6@@H:2]-1-[#6:3]-[#6@H:7](-[#6:8]-2=[#6:13]-[#6:12]=[#6:11]-[#6:10]=[#6:9]-2)[C@@:6]2([#6:18]-[#6:17]-[#6:16]-[#6:14]2=[O:15])[#6:4]-1=[O:5]

Correctness of the mapping

MAPPET	YES
ReactionMap	YES
Marvin	NO
ChemDraw	NO
Indigo	NO

Reaction no 73

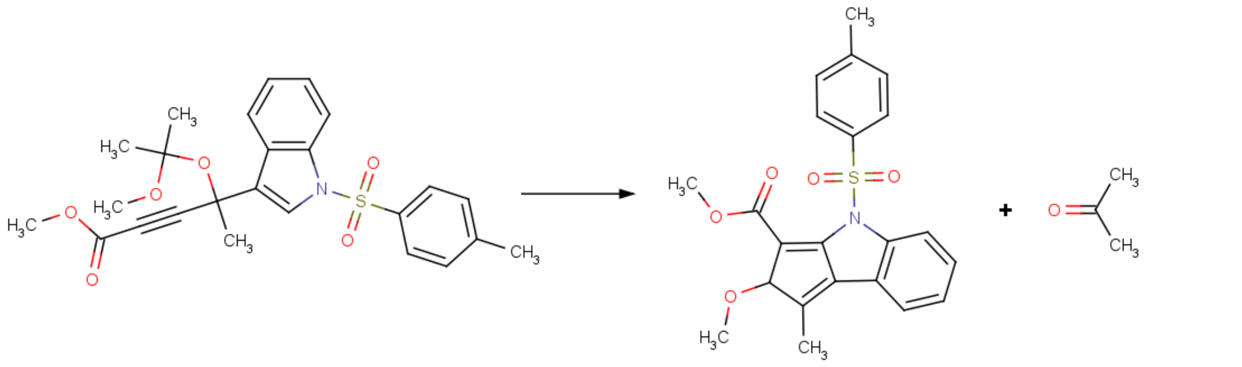

SMILES of the input:
COC(=O)C#CC(C)(OC(C)(C)OC)C1=CN(C2=C1C=CC=C2)S(=O)(=O)C1=CC=C(C)C=C1>>COC1C(C)=C2C3=C(C=CC=C3)N(C2=C1C(=O)OC)S(=O)(=O)C1=CC=C(C)C=C1.CC(C)=O

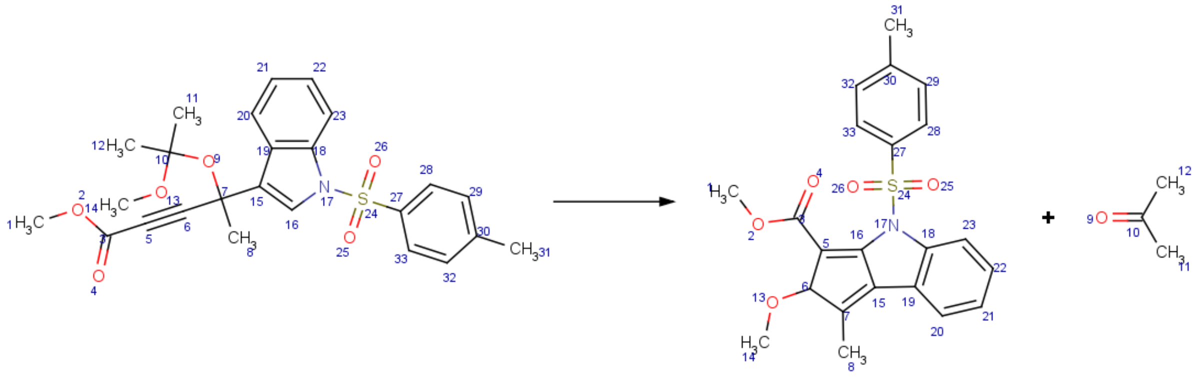

Correct mapped SMILES/SMARTS of the reaction:
[#6:1]-[#8:2]-[#6:3](=[O:4])[C:5]#[C:6][C:7]([#6:8])([#8:9][C:10]([#6:11])([#6:12])[#8:13]-[#6:14])[#6:15]-1=[#6:16]-[#7:17](-[#6:18]-2=[#6:19]-1-[#6:20]=[#6:21]-[#6:22]=[#6:23]-2)[S:24](=[O:25])(=[O:26])[#6:27]-1=[#6:28]-[#6:29]=[#6:30](-[#6:31])-[#6:32]=[#6:33]-1>>[#6:14]-[#8:13]-[#6:6]-1-[#6:7](-[#6:8])=[#6:15]-2-[#6:19]-3=[#6:18](-[#6:23]=[#6:22]-[#6:21]=[#6:20]-3)-[#7:17](-[#6:16]-2=[#6:5]-1-[#6:3](=[O:4])-[#8:2]-[#6:1])[S:24](=[O:25])(=[O:26])[#6:27]-1=[#6:33]-[#6:32]=[#6:30](-[#6:31])-[#6:29]=[#6:28]-1.[#6:12]-[#6:10](-[#6:11])=[O:9]

Correctness of the mapping

MAPPET	YES
ReactionMap	YES
Marvin	YES
ChemDraw	NO
Indigo	NO

Reaction no 74

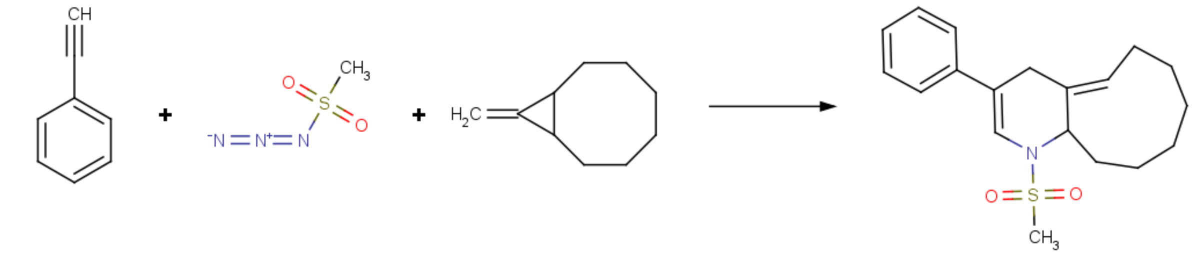

SMILES of the input:
C#CC1=CC=CC=C1.CS(=O)(=O)N=[N+]=[N-].C=C1C2CCCCCCC12>>CS(=O)(=O)N1C=C(C\C2=C/CCCCCCC12)C1=CC=CC=C1

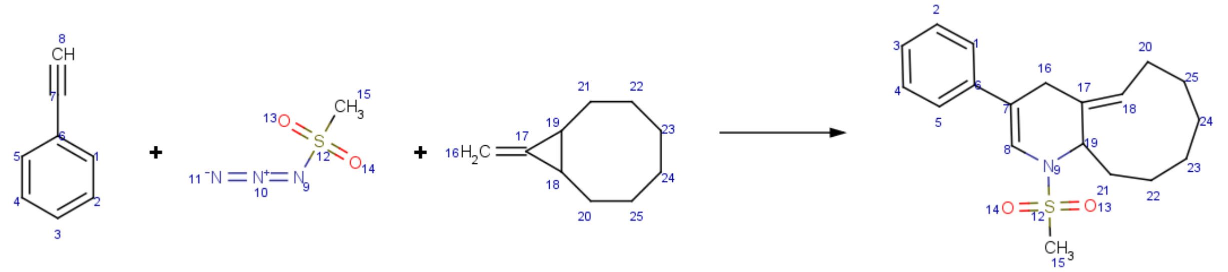

```
Correct mapped SMILES/SMARTS of the reaction:
[C:8]#[C:7][#6:6]-1=[#6:1]-[#6:2]=[#6:3]-[#6:4]=[#6:5]-
1.[#6:15][S:12](=[O:13])(=[O:14])[#7:9]=[N+:10]=[#7-:11].[#6:16]=[#6:17]-
1-[#6:19]-2-[#6:21]-[#6:22]-[#6:23]-[#6:24]-[#6:25]-[#6:20]-[#6:18]-1-
2>>[#6:15][S:12](=[O:13])(=[O:14])[#7:9]-1-[#6:8]=[#6:7](-
[#6:16]\[#6:17]-2=[#6:18]/[#6:20]-[#6:25]-[#6:24]-[#6:23]-[#6:22]-
[#6:21]-[#6:19]-12)-[#6:6]-1=[#6:5]-[#6:4]=[#6:3]-[#6:2]=[#6:1]-1
```

```
Correctness of the mapping
MAPPET          NO
ReactionMap     NO
Marvin          YES
ChemDraw        YES
Indigo          NO
```

Reaction no 75

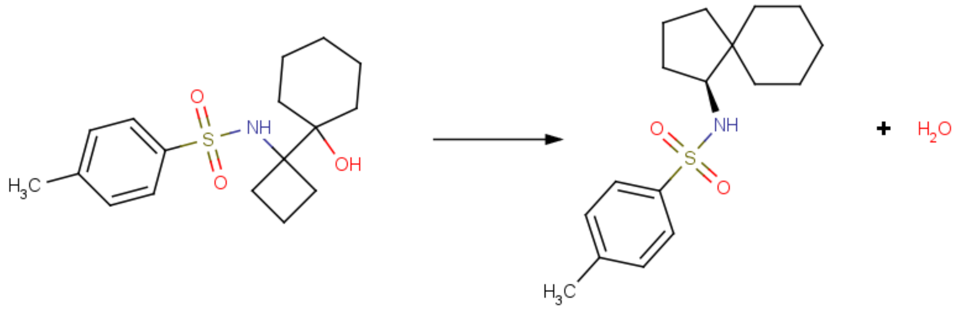

```
SMILES of the input:
CC1=CC=C(C=C1)S(=O)(=O)NC1(CCC1)C1(O)CCCCC1>>CC1=CC=C(C=C1)S(=O)(=O)N[C@H
]1CCCC11CCCCC1.O
```

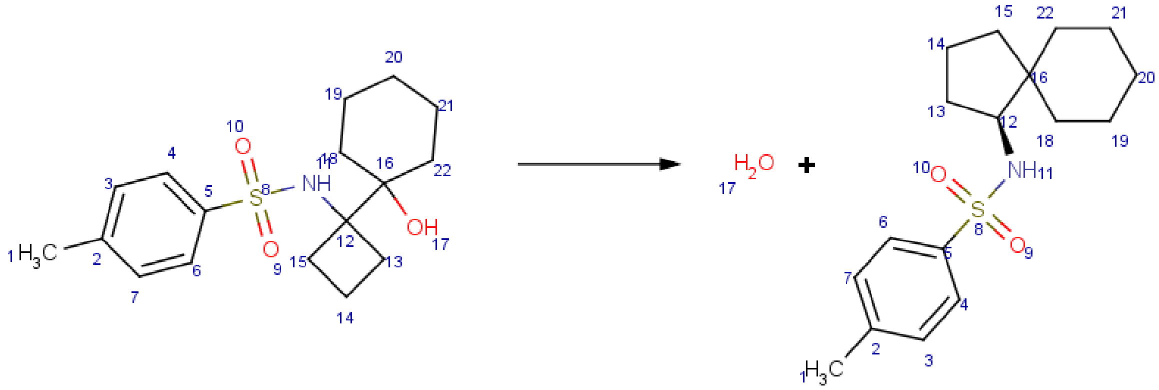

```
Correct mapped SMILES/SMARTS of the reaction:
[#6:1]-[#6:2]-1=[#6:3]-[#6:4]=[#6:5](-[#6:6]=[#6:7]-
1)[S:8](=[O:9])(=[O:10])[#7:11][C:12]1([#6:13]-[#6:14]-
[#6:15]1)[C:16]1([#8:17])[#6:18]-[#6:19]-[#6:20]-[#6:21]-
```

[#6:22]1>>[#8:17].[#6:1]-[#6:2]-1=[#6:7]-[#6:6]=[#6:5](-[#6:4]=[#6:3]-1)[S:8](=[O:9])(=[O:10])[#7:11]-[#6@H:12]1-[#6:13]-[#6:14]-[#6:15][C:16]11[#6:22]-[#6:21]-[#6:20]-[#6:19]-[#6:18]1

Correctness of the mapping

MAPPET	YES
ReactionMap	YES
Marvin	YES
ChemDraw	YES
Indigo	NO

Reaction no 76

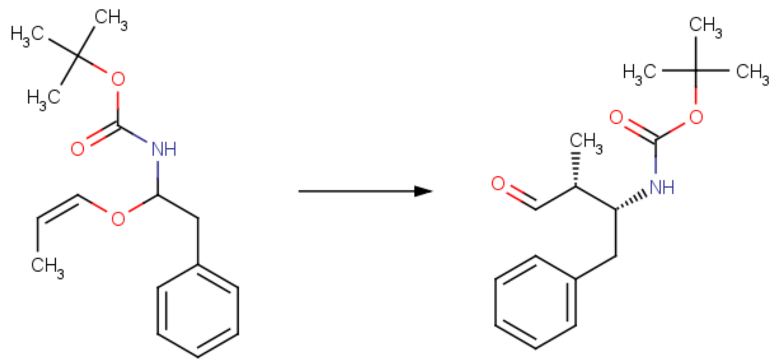

SMILES of the input:
C\C=C/OC(CC1=CC=CC=C1)NC(=O)OC(C)(C)C>>C[C@@H](C=O)[C@@H](CC1=CC=CC=C1)NC(=O)OC(C)(C)C

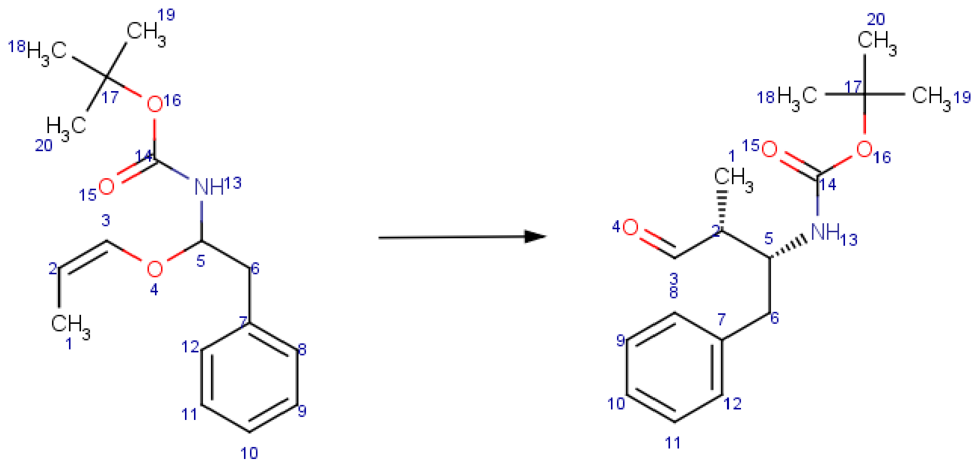

Correct mapped SMILES/SMARTS of the reaction:
[#6:1]\[#6:2]=[#6:3]/[#8:4]-[#6:5](-[#6:6]-[#6:7]-1=[#6:8]-[#6:9]=[#6:10]-[#6:11]=[#6:12]-1)-[#7:13]-[#6:14](=[O:15])-[#8:16][C:17]([#6:18])([#6:19])[#6:20]>>[#6:1]-[#6@@H:2](-[#6:3]=[O:4])-[#6@@H:5](-[#6:6]-[#6:7]-1=[#6:12]-[#6:11]=[#6:10]-[#6:9]=[#6:8]-1)-[#7:13]-[#6:14](=[O:15])-[#8:16][C:17]([#6:20])([#6:19])[#6:18]

Correctness of the mapping

MAPPET	YES
ReactionMap	YES
Marvin	YES
ChemDraw	NO
Indigo	NO

Reaction no 77

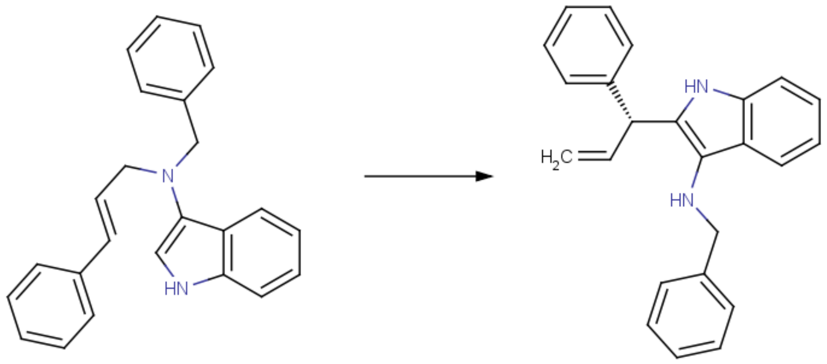

SMILES of the input:
C(\C=C\C1=CC=CC=C1)N(CC1=CC=CC=C1)C1=CNC2=C1C=CC=C2>>C=C[C@@H](C1=C(NCC2=CC=CC=C2)C2=C(N1)C=CC=C2)C1=CC=CC=C1

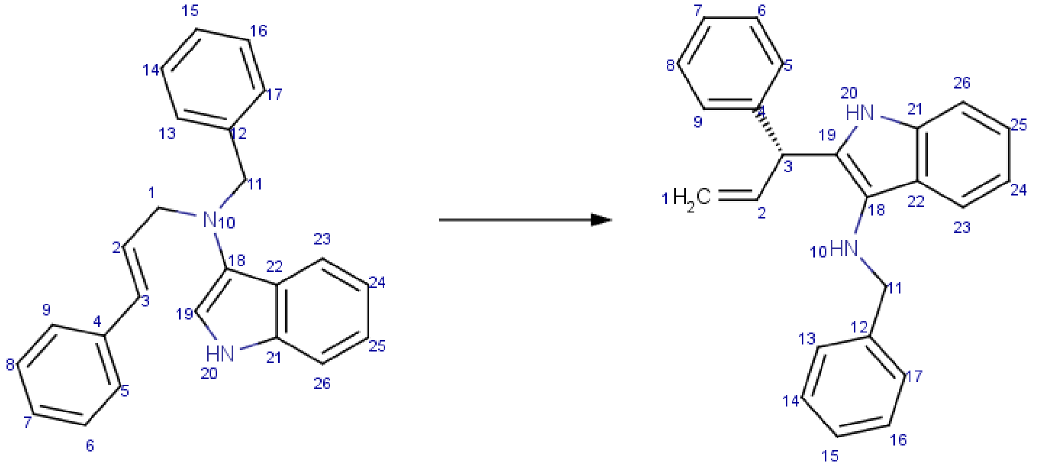

Correct mapped SMILES/SMARTS of the reaction:
[#6:1](\[#6:2]=[#6:3]\[#6:4]-1=[#6:5]-[#6:6]=[#6:7]-[#6:8]=[#6:9]1)-[#7:10](-[#6:11]-[#6:12]-1=[#6:13]-[#6:14]=[#6:15]-[#6:16]=[#6:17]-1)-[#6:18]-1=[#6:19]-[#7:20]-[#6:21]-2=[#6:22]-1-[#6:23]=[#6:24]-[#6:25]=[#6:26]-2>>[#6:1]=[#6:2]-[#6@@H:3](-[#6:19]-1=[#6:18](-[#7:10]-[#6:11]-[#6:12]-2=[#6:17]-[#6:16]=[#6:15]-[#6:14]=[#6:13]-2)-[#6:22]-2=[#6:21](-[#7:20]-1)-[#6:26]=[#6:25]-[#6:24]=[#6:23]-2)-[#6:4]-1=[#6:9]-[#6:8]=[#6:7]-[#6:6]=[#6:5]-1

Correctness of the mapping

MAPPET	YES
ReactionMap	YES
Marvin	NO
ChemDraw	NO
Indigo	NO

Reaction no 78

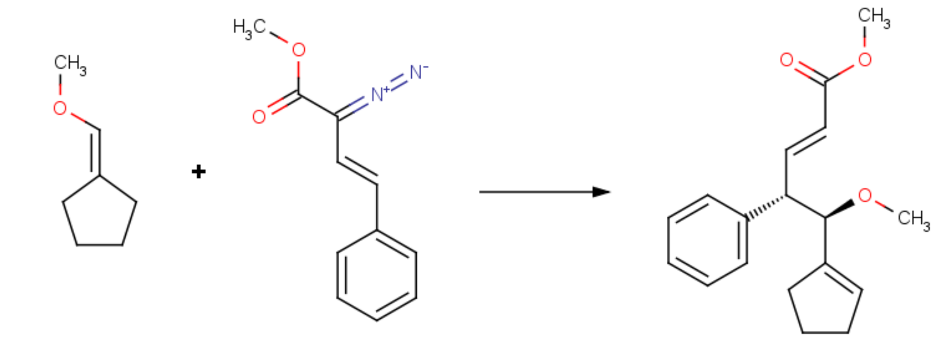

SMILES of the input:
COC=C1CCCC1.COC(=O)C(\C=C\C1=CC=CC=C1)=[N+]=[N-
]>>CO[C@H]([C@@H](\C=C\C(=O)OC)C1=CC=CC=C1)C1=CCCC1

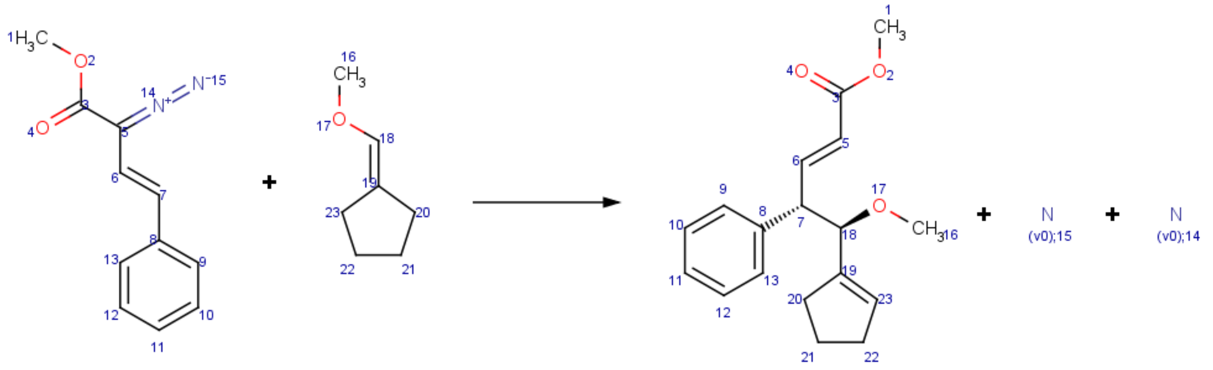

Correct mapped SMILES/SMARTS of the reaction:
[#6:1]-[#8:2]-[#6:3](=[O:4])-[#6:5](\[#6:6]=[#6:7]\[#6:8]-1=[#6:9]-
[#6:10]=[#6:11]-[#6:12]=[#6:13]1)=[N+:14]=[#7-:15].[#6:16]-
[#8:17]\[#6:18]=[#6:19]-1\[#6:20]-[#6:21]-[#6:22]-[#6:23]-1>>[#6:16]-
[#8:17]-[#6@H:18](-[#6@@H:7](\[#6:6]=[#6:5]\[#6:3](=[O:4])-[#8:2]-
[#6:1])-[#6:8]-1=[#6:13]-[#6:12]=[#6:11]-[#6:10]=[#6:9]-1)-[#6:19]-
1=[#6:23]-[#6:22]-[#6:21]-[#6:20]-1.[#7;v0:15].[#7;v0:14]

Correctness of the mapping

MAPPET	YES
ReactionMap	NO
Marvin	YES
ChemDraw	YES
Indigo	NO

Reaction no 79

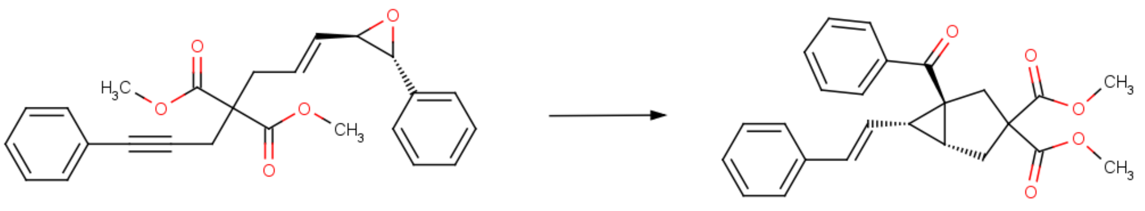

SMILES of the input:
COC(=O)C(C\C=C\[C@H]1O[C@@H]1C1=CC=CC=C1)(CC#CC1=CC=CC=C1)C(=O)OC>>COC(=O
)C1(C[C@@H]2[C@H](\C=C\C3=CC=CC=C3)[C@@]2(C1)C(=O)C1=CC=CC=C1)C(=O)OC

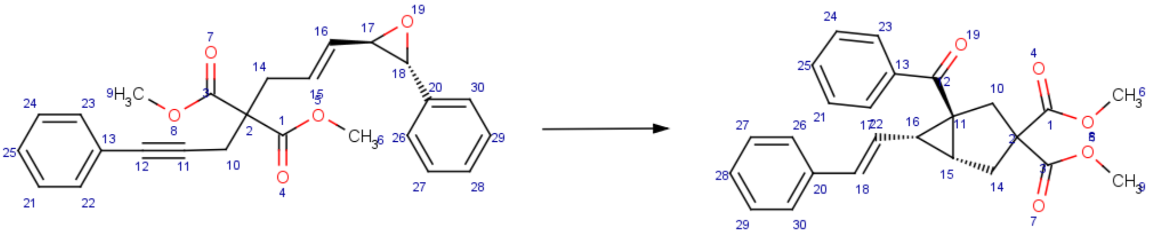

Correct mapped SMILES/SMARTS of the reaction:
[#6:6]-[#8:5]-[#6:1](=[O:4])[C:2]([#6:14]\[#6:15]=[#6:16]\[#6@H:17]-1-
[#8:19]-[#6@@H:18]1-[#6:20]-1=[#6:30]-[#6:29]=[#6:28]-[#6:27]=[#6:26]-
1)([#6:10][C:11]#[C:12][#6:13]-1=[#6:22]-[#6:21]=[#6:25]-[#6:24]=[#6:23]-
1)[#6:3](=[O:7])-[#8:8]-[#6:9]>>[#6:6]-[#8:5]-
[#6:1](=[O:4])[C:2]1([#6:14]-[#6@@H:15]2-
[#6@H:16](\[#6:17]=[#6:18]\[#6:20]-3=[#6:30]-[#6:29]=[#6:28]-
[#6:27]=[#6:26]3)[C@@:11]2([#6:10]1)[#6:12](=[O:19])-[#6:13]-1=[#6:22]-
[#6:21]=[#6:25]-[#6:24]=[#6:23]-1)[#6:3](=[O:7])-[#8:8]-[#6:9]

Correctness of the mapping

MAPPET	NO
ReactionMap	NO
Marvin	YES
ChemDraw	NO
Indigo	NO

Reaction no 80

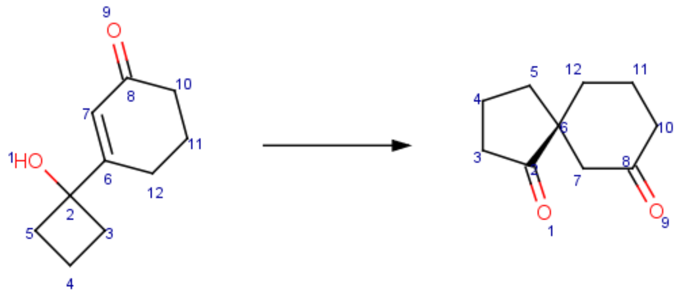

SMILES of the input:
OC1(CCC1)C1=CC(=O)CCC1>>O=C1CCC[C@@]11CCCC(=O)C1

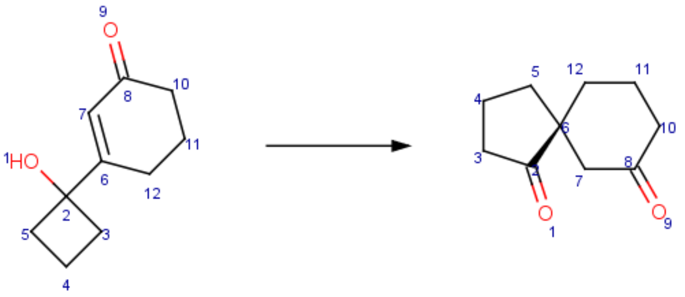

Correct mapped SMILES/SMARTS of the reaction:
[#8:1][C:2]1([#6:3]-[#6:4]-[#6:5]1)[#6:6]-1=[#6:7]-[#6:8](=[O:9])-
[#6:10]-[#6:11]-[#6:12]-1>>[O:1]=[#6:2]1-[#6:3]-[#6:4]-
[#6:5][C@@:6]11[#6:12]-[#6:11]-[#6:10]-[#6:8](=[O:9])-[#6:7]1

Correctness of the mapping

MAPPET YES
ReactionMap YES
Marvin YES
ChemDraw YES
Indigo YES

Reaction no 81

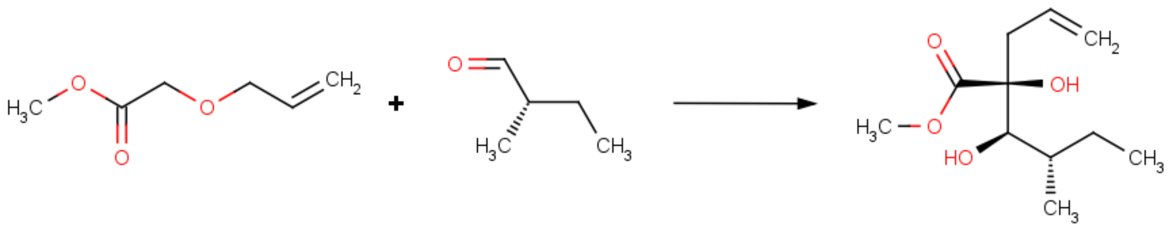

SMILES of the input:
COC(=O)COCC=C.CC[C@H](C)C=O>>CC[C@H](C)[C@@H](O)[C@@](O)(CC=C)C(=O)OC

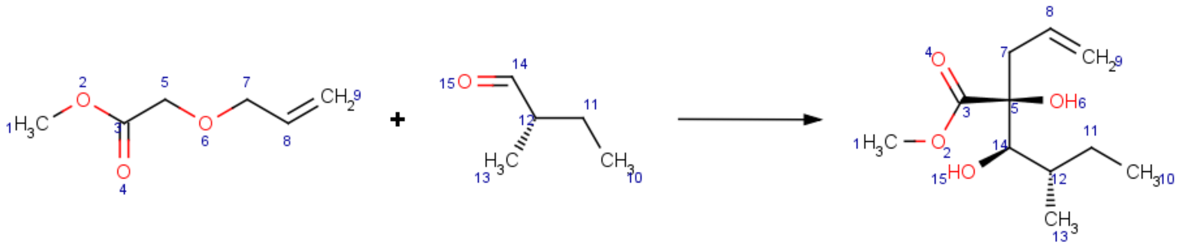

Correct mapped SMILES/SMARTS of the reaction:
[#6:1]-[#8:2]-[#6:3](=[O:4])-[#6:5]-[#8:6]-[#6:7]-[#6:8]=[#6:9].[#6:10]-[#6:11]-[#6@H:12](-[#6:13])-[#6:14]=[O:15]>>[#6:10]-[#6:11]-[#6@H:12](-[#6:13])-[#6@@H:14](-[#8:15])[C@@:5]([#8:6])([#6:7]-[#6:8]=[#6:9])[#6:3](=[O:4])-[#8:2]-[#6:1]

Correctness of the mapping
MAPPET YES
ReactionMap NO
Marvin YES
ChemDraw NO
Indigo YES

Reaction no 82

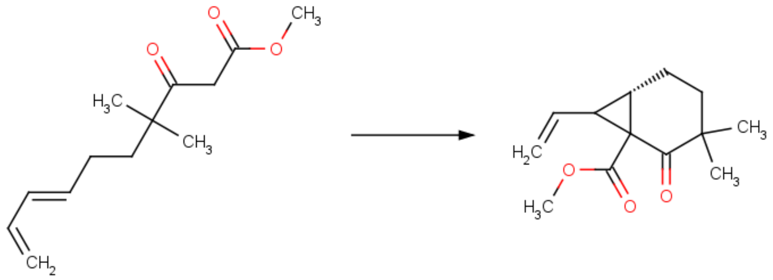

SMILES of the input:
COC(=O)CC(=O)C(C)(C)CC\C=C\C=C>>COC(=O)C12[C@@H](CCC(C)(C)C1=O)C2C=C

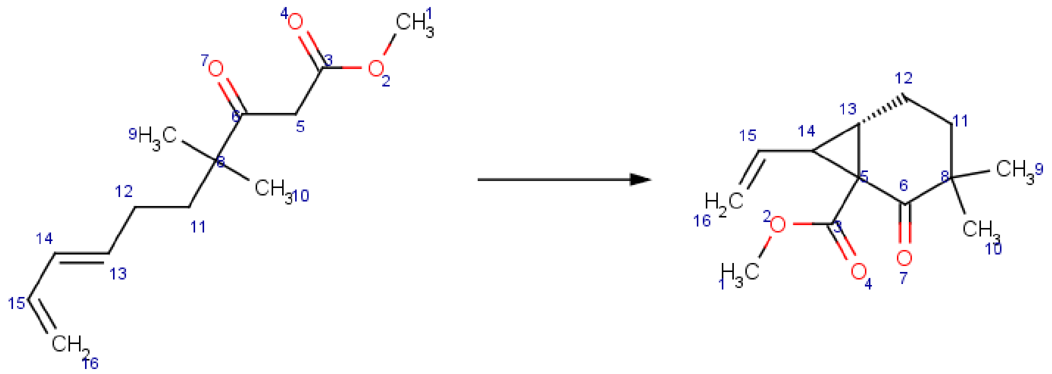

Correct mapped SMILES/SMARTS of the reaction:
[#6:1]-[#8:2]-[#6:3](=[O:4])-[#6:5]-
[#6:6](=[O:7])[C:8]([#6:9])([#6:10])[#6:11]-
[#6:12]\[#6:13]=[#6:14]\[#6:15]=[#6:16]>>[#6:1]-[#8:2]-
[#6:3](=[O:4])[C:5]12[#6@@H:13](-[#6:12]-
[#6:11][C:8]([#6:9])([#6:10])[#6:6]1=[O:7])-[#6:14]2-[#6:15]=[#6:16]

Correctness of the mapping

MAPPET	YES
ReactionMap	YES
Marvin	YES
ChemDraw	YES
Indigo	YES

Reaction no 83

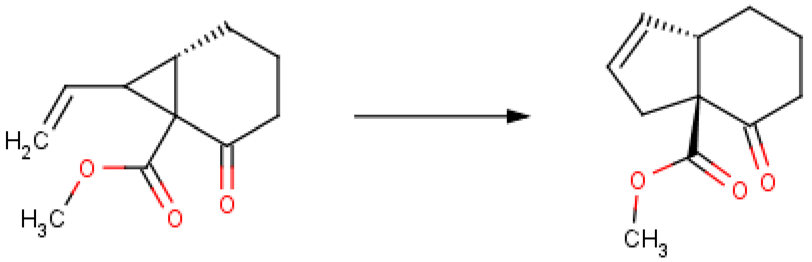

SMILES of the input:
COC(=O)C12[C@@H](CCCC1=O)C2C=C>>COC(=O)[C@@]12CC=C[C@@H]1CCCC2=O

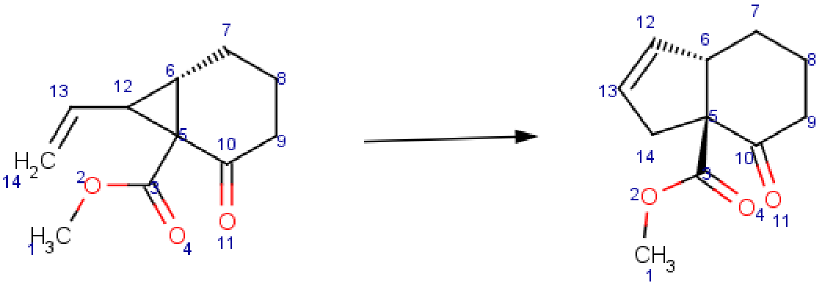

Correct mapped SMILES/SMARTS of the reaction:
[CH3:1][O:2][C:3](=[O:4])[C:5]12[C@@H:6]([CH2:7][CH2:8][CH2:9][C:10]1=[O:11])[CH:12]2[CH:13]=[CH2:14]>>[CH3:1][O:2][C:3](=[O:4])[C@@:5]12[CH2:14][CH:13]=[CH:12][C@@H:6]1[CH2:7][CH2:8][CH2:9][C:10]2=[O:11]

Correctness of the mapping

MAPPET YES
ReactionMap NO
Marvin NO
ChemDraw NO
Indigo NO

Reaction no 84

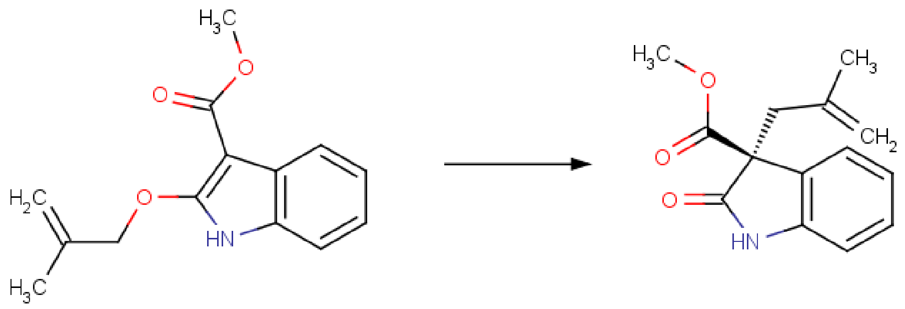

SMILES of the input:
COC(=O)C1=C(NC2=C1C=CC=C2)OCC(C)=C>>COC(=O)[C@]1(CC(C)=C)C(=O)NC2=C1C=CC=C2

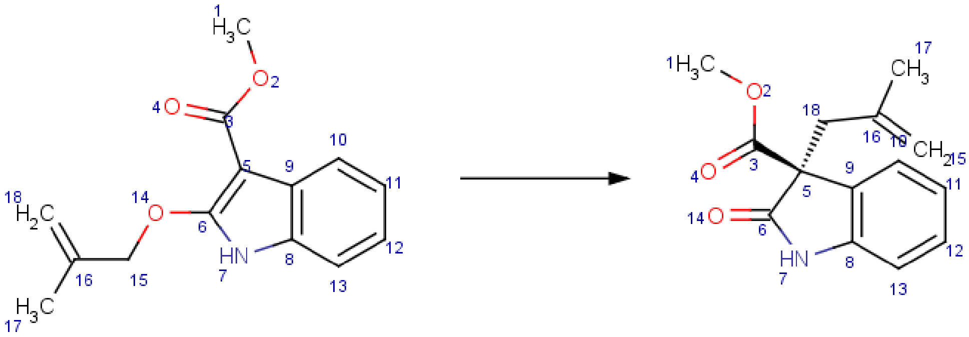

Correct mapped SMILES/SMARTS of the reaction:
[#6:1]-[#8:2]-[#6:3](=[O:4])-[#6:5]-1=[#6:6](-[#7:7]-[#6:8]-2=[#6:9]-1-[#6:10]=[#6:11]-[#6:12]=[#6:13]-2)-[#8:14]-[#6:15]-[#6:16](-[#6:17])=[#6:18]>>[#6:1]-[#8:2]-[#6:3](=[O:4])[C@:5]1([#6:18]-[#6:16](-[#6:17])=[#6:15])[#6:6](=[O:14])-[#7:7]-[#6:8]-2=[#6:9]1-[#6:10]=[#6:11]-[#6:12]=[#6:13]-2

Correctness of the mapping
MAPPET YES
ReactionMap NO
Marvin NO
ChemDraw NO
Indigo NO

Reaction no 85

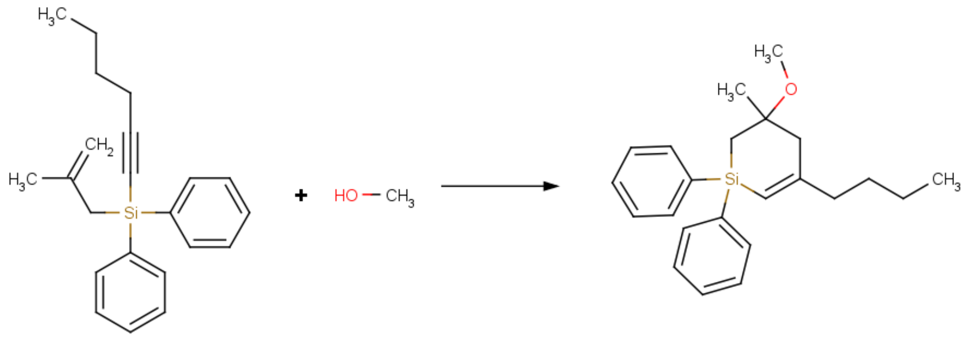

SMILES of the input:
CCCCC#C[Si](CC(C)=C)(C1=CC=CC=C1)C1=CC=CC=C1.CO>>CCCCC1=C[Si](CC(C)(C1)OC
)(C1=CC=CC=C1)C1=CC=CC=C1

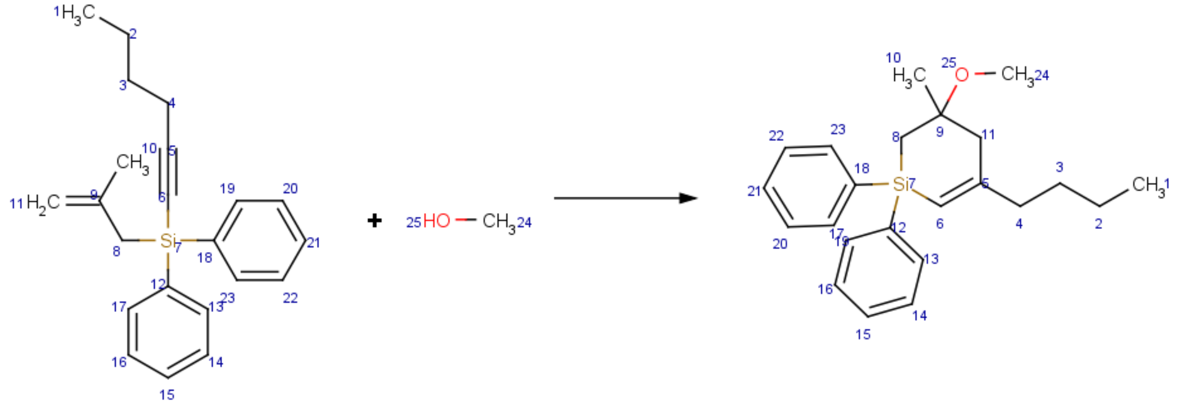

Correct mapped SMILES/SMARTS of the reaction:
[CH3:1][CH2:2][CH2:3][CH2:4][C:5]#[C:6][Si:7]([CH2:8][C:9](=[CH2:11])[CH3
:10])([C:12]1=[CH:13][CH:14]=[CH:15][CH:16]=[CH:17]1)[C:18]2=[CH:19][CH:2
0]=[CH:21][CH:22]=[CH:23]2.[CH3:24][OH:25]>>[CH3:1][CH2:2][CH2:3][CH2:4][
C:5]1=[CH:6][Si:7]([CH2:8][C:9]([CH2:11]1)([CH3:10])[O:25][CH3:24])([C:12
]2=[CH:13][CH:14]=[CH:15][CH:16]=[CH:17]2)[C:18]3=[CH:19][CH:20]=[CH:21][
CH:22]=[CH:23]3

Correctness of the mapping

MAPPET	NO
ReactionMap	YES
Marvin	YES
ChemDraw	YES
Indigo	NO

Reaction no 86

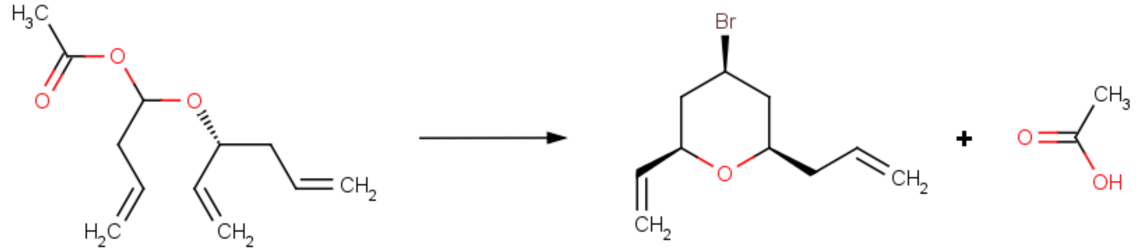

SMILES of the input:
CC(=O)OC(CC=C)O[C@H](CC=C)C=C>>Br[C@H]1C[C@@H](CC=C)O[C@H](C1)C=C.CC(O)=O

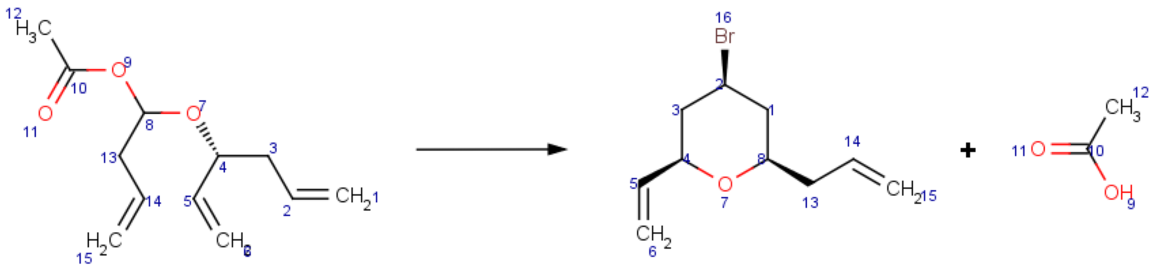

Correct mapped SMILES/SMARTS of the reaction:
[#6:12]-[#6:10](=[O:11])-[#8:9]-[#6:8](-[#6:13]-[#6:14]=[#6:15])-[#8:7]-
[#6@H:4](-[#6:3]-[#6:2]=[#6:1])-[#6:5]=[#6:6]>>[Br:16][#6@H:2]-1-[#6:1]-
[#6@@H:8](-[#6:13]-[#6:14]=[#6:15])-[#8:7]-[#6@H:4](-[#6:3]-1)-
[#6:5]=[#6:6].[#6:12]-[#6:10](-[#8:9])=[O:11]

Correctness of the mapping

MAPPET	NO
ReactionMap	NO
Marvin	YES
ChemDraw	YES
Indigo	YES

Reaction no 87

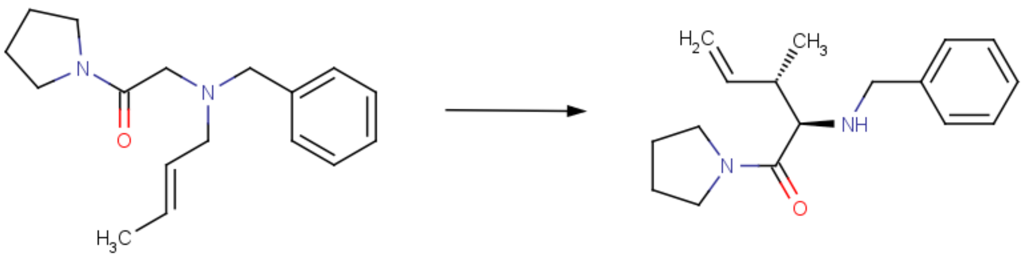

SMILES of the input:
C\C=C\CN(CC(=O)N1CCCC1)CC1=CC=CC=C1>>C[C@@H](C=C)[C@@H](NCC1=CC=CC=C1)C(=
O)N1CCCC1

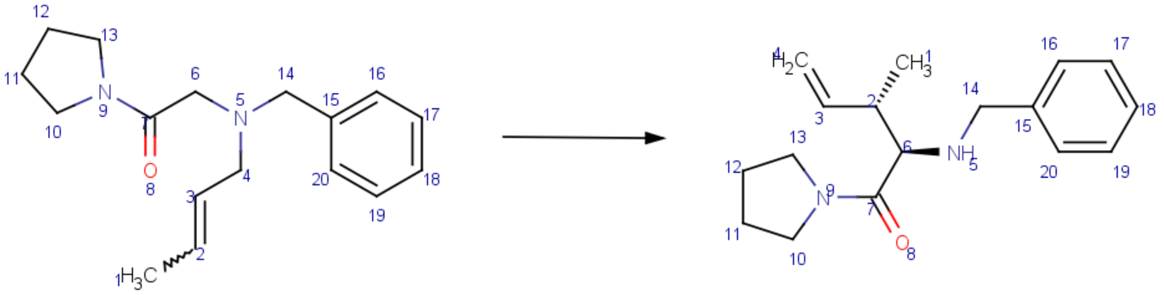

Correct mapped SMILES/SMARTS of the reaction:
[CH3:1][CH:2]=[CH:3][CH2:4][N:5]([CH2:6][C:7](=[O:8])[N:9]1[CH2:10][CH2:1
1][CH2:12][CH2:13]1)[CH2:14][C:15]1=[CH:16][CH:17]=[CH:18][CH:19]=[CH:20]
1>>[CH3:1][C@@H:2]([CH:3]=[CH2:4])[C@@H:6]([NH:5][CH2:14][C:15]1=[CH:16][
CH:17]=[CH:18][CH:19]=[CH:20]1)[C:7](=[O:8])[N:9]1[CH2:10][CH2:11][CH2:12
][CH2:13]1
Correctness of the mapping

```
MAPPET              NO
ReactionMap         YES
Marvin              NO
ChemDraw            NO
Indigo              NO
```

Reaction no 88

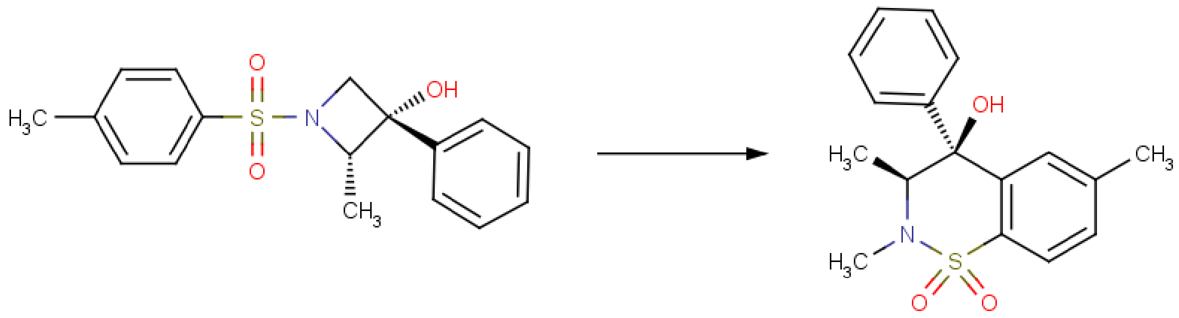

SMILES of the input:
C[C@@H]1N(C[C@@]1(O)C1=CC=CC=C1)S(=O)(=O)C1=CC=C(C)C=C1>>C[C@@H]1N(C)S(=O)(=O)C2=C(C=C(C)C=C2)[C@@]1(O)C1=CC=CC=C1

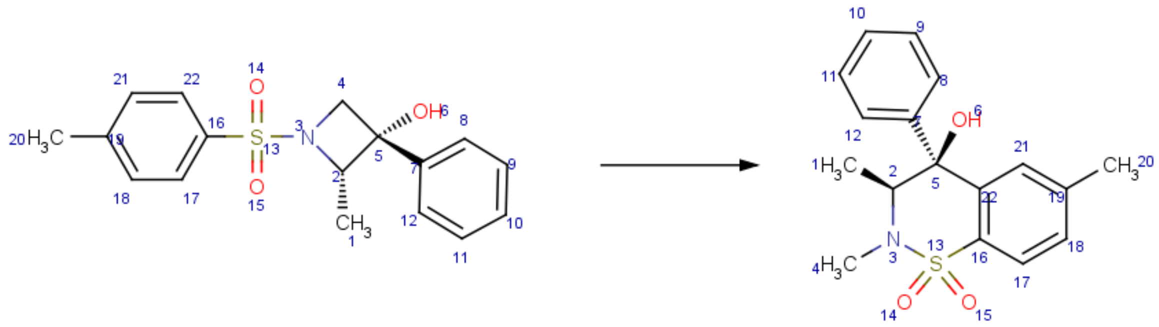

Correct mapped SMILES/SMARTS of the reaction:
[#6:1]-[#6@@H:2]1-[#7:3](-[#6:4][C@@:5]1([#8:6])[#6:7]-1=[#6:8]-[#6:9]=[#6:10]-[#6:11]=[#6:12]-1)[S:13](=[O:14])(=[O:15])[#6:16]-1=[#6:17]-[#6:18]=[#6:19](-[#6:20])-[#6:21]=[#6:22]-1>>[#6:1]-[#6@@H:2]1-[#7:3](-[#6:4])[S:13](=[O:14])(=[O:15])[#6:16]-2=[#6:22](-[#6:21]=[#6:19](-[#6:20])-[#6:18]=[#6:17]-2)[C@@:5]1([#8:6])[#6:7]-1=[#6:12]-[#6:11]=[#6:10]-[#6:9]=[#6:8]-1

Correctness of the mapping

```
MAPPET              YES
ReactionMap         NO
Marvin              YES
ChemDraw            YES
Indigo              YES
```

Reaction no 89

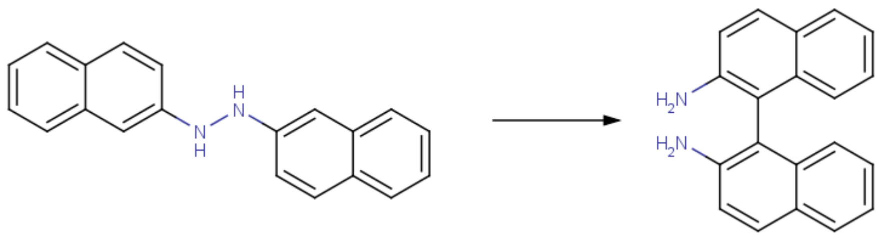

SMILES of the input:
N(NC1=CC2=CC=CC=C2C=C1)C1=CC2=CC=CC=C2C=C1>>NC1=C(C2=CC=CC=C2C=C1)C1=C(N)C=CC2=CC=CC=C12

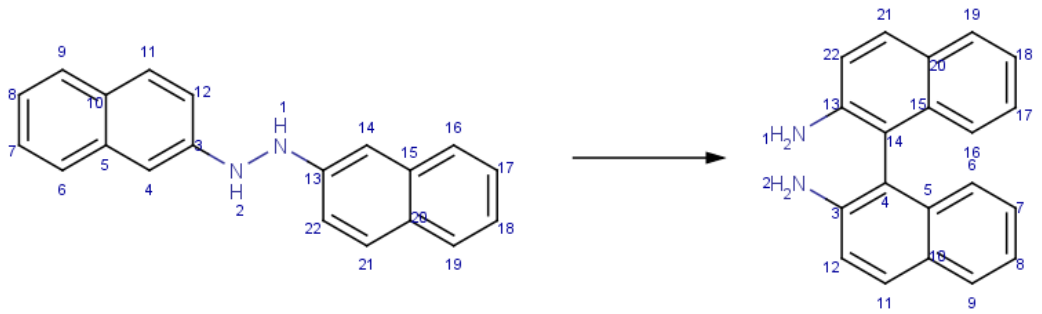

Correct mapped SMILES/SMARTS of the reaction:
[#7:1](-[#7:2]-[#6:3]-1=[#6:4]-[#6:5]-2=[#6:6]-[#6:7]=[#6:8]-[#6:9]=[#6:10]-2-[#6:11]=[#6:12]-1)-[#6:13]-1=[#6:14]-[#6:15]-2=[#6:16]-[#6:17]=[#6:18]-[#6:19]=[#6:20]-2-[#6:21]=[#6:22]-1>>[#7:2]-[#6:3]-1=[#6:4](-[#6:5]-2=[#6:6]-[#6:7]=[#6:8]-[#6:9]=[#6:10]-2-[#6:11]=[#6:12]-1)-[#6:14]-1=[#6:13](-[#7:1])-[#6:22]=[#6:21]-[#6:20]-2=[#6:19]-[#6:18]=[#6:17]-[#6:16]=[#6:15]-1-2

Correctness of the mapping

MAPPET	YES
ReactionMap	YES
Marvin	YES
ChemDraw	YES
Indigo	YES

Reaction no 90

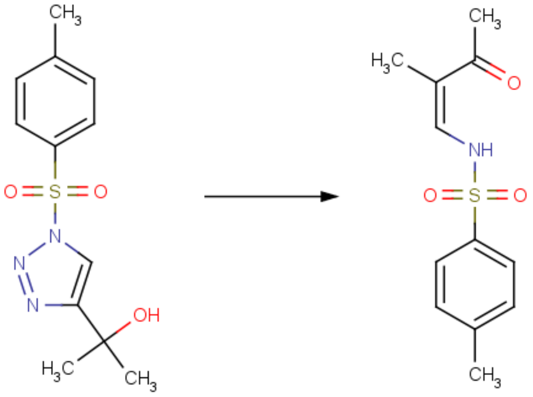

SMILES of the input:
CC1=CC=C(C=C1)S(=O)(=O)N1C=C(N=N1)C(C)(C)O>>CC(=O)C(\C)=C/NS(=O)(=O)C1=CC=C(C)C=C1

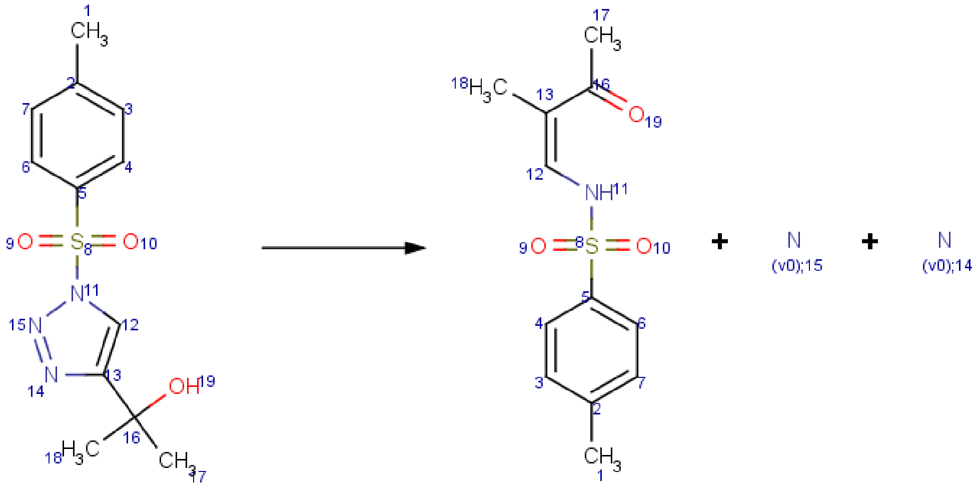

Correct mapped SMILES/SMARTS of the reaction:
[#6:1]-[#6:2]-1=[#6:3]-[#6:4]=[#6:5](-[#6:6]=[#6:7]-1)[S:8](=[O:9])(=[O:10])[#7:11]-1-[#6:12]=[#6:13](-[#7:14]=[#7:15]-1)[C:16]([#6:17])([#6:18])[#8:19]>>[#6:17]-[#6:16](=[O:19])-[#6:13](\[#6:18])=[#6:12]/[#7:11][S:8](=[O:9])(=[O:10])[#6:5]-1=[#6:6]-[#6:7]=[#6:2](-[#6:1])-[#6:3]=[#6:4]-1.[#7;v0:15].[#7;v0:14]

Correctness of the mapping

MAPPET	YES
ReactionMap	NO
Marvin	YES
ChemDraw	NO
Indigo	YES

Reaction no 91

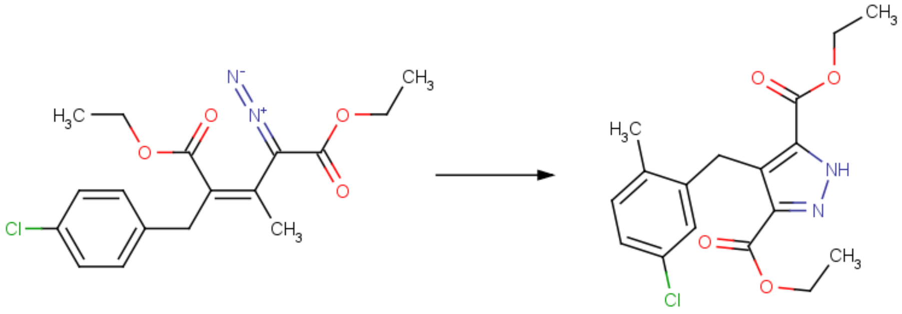

SMILES of the input:
CCOC(=O)C(\CC1=CC=C(Cl)C=C1)=C(\C)C(=[N+]=[N-])C(=O)OCC>>CCOC(=O)C1=C(CC2=CC(Cl)=CC=C2C)C(=NN1)C(=O)OCC

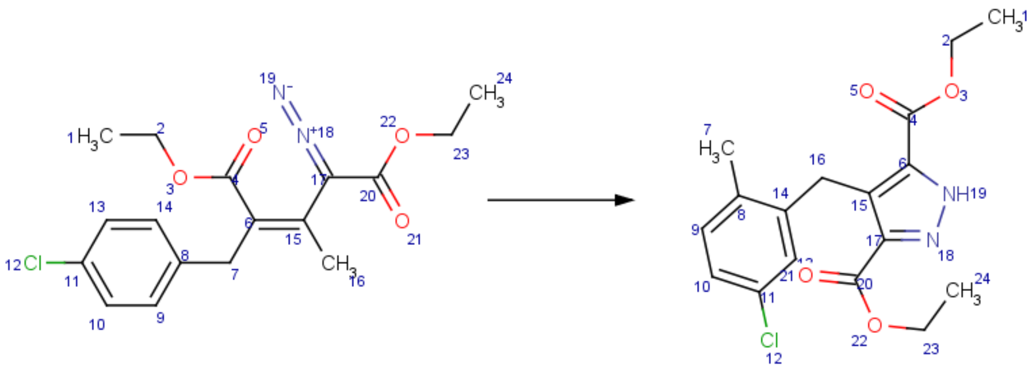

Correct mapped SMILES/SMARTS of the reaction:

[#6:1]-[#6:2]-[#8:3]-[#6:4](=[O:5])-[#6:6](\[#6:7]-[#6:8]-1=[#6:9]-[#6:10]=[#6:11]([Cl:12])-[#6:13]=[#6:14]-1)=[#6:15](\[#6:16])-[#6:17](=[N+:18]=[#7-:19])-[#6:20](=[O:21])-[#8:22]-[#6:23]-[#6:24]>>[#6:1]-[#6:2]-[#8:3]-[#6:4](=[O:5])-[#6:6]-1=[#6:15](-[#6:16]-[#6:14]-2=[#6:13]-[#6:11]([Cl:12])=[#6:10]-[#6:9]=[#6:8]-2-[#6:7])-[#6:17](=[#7:18]-[#7:19]-1)-[#6:20](=[O:21])-[#8:22]-[#6:23]-[#6:24]

Correctness of the mapping

MAPPET	YES
ReactionMap	NO
Marvin	YES
ChemDraw	YES
Indigo	NO

Reaction no 92

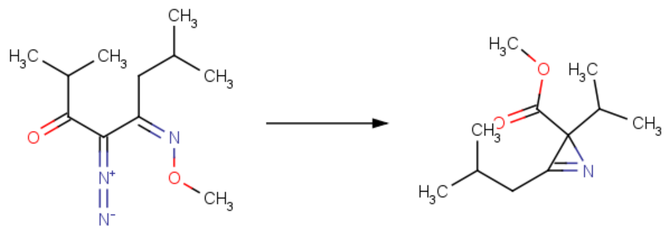

SMILES of the input:
CO\N=C(\CC(C)C)C(=[N+]=[N-])C(=O)C(C)C>>COC(=O)C1(N=C1CC(C)C)C(C)C

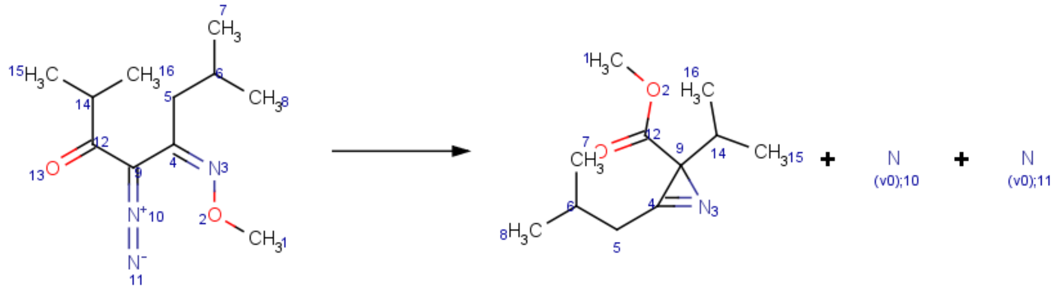

Correct mapped SMILES/SMARTS of the reaction:
[#6:1]-[#8:2]\[#7:3]=[#6:4](\[#6:5]-[#6:6](-[#6:7])-[#6:8])-[#6:9](=[N+:10]=[#7-:11])-[#6:12](=[O:13])-[#6:14](-[#6:15])-[#6:16]>>[#6:1]-[#8:2]-[#6:12](=[O:13])[C:9]1([#7:3]=[#6:4]1-[#6:5]-[#6:6](-[#6:8])-[#6:7])[#6:14](-[#6:15])-[#6:16].[#7;v0:10].[#7;v0:11]

Correctness of the mapping

MAPPET	YES
ReactionMap	NO
Marvin	YES
ChemDraw	NO
Indigo	NO

Reaction no 93

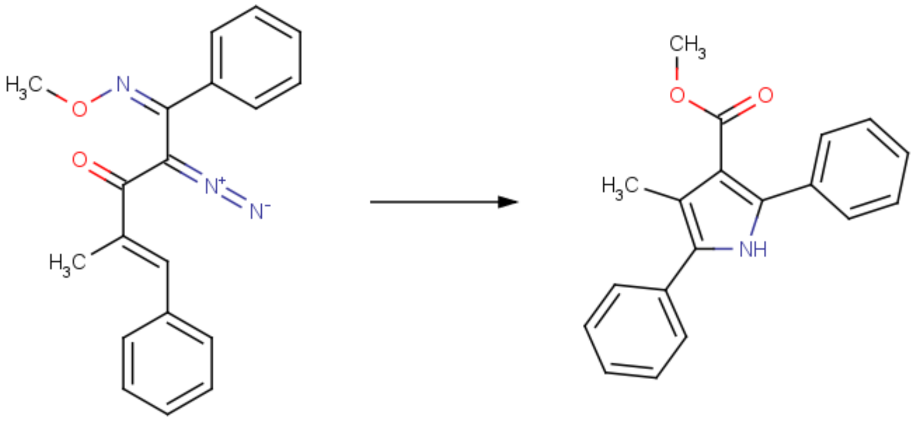

SMILES of the input:
CO\N=C(/C(=[N+]=[N-
])C(=O)C(\C)=C\C1=CC=CC=C1)C1=CC=CC=C1>>COC(=O)C1=C(NC(=C1C)C1=CC=CC=C1)C
1=CC=CC=C1

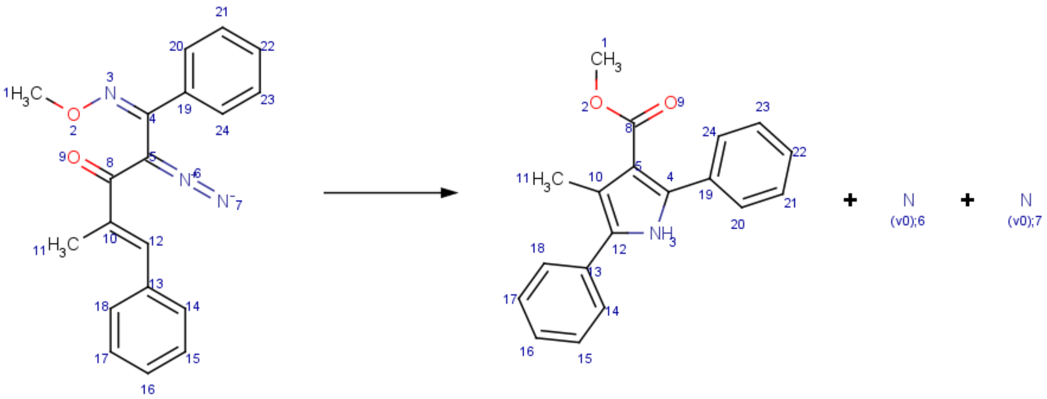

Correct mapped SMILES/SMARTS of the reaction:
[#6:1]-[#8:2]\[#7:3]=[#6:4](/[#6:5](=[N+:6]=[#7-:7])-[#6:8](=[O:9])-
[#6:10](\[#6:11])=[#6:12]\[#6:13]-1=[#6:14]-[#6:15]=[#6:16]-
[#6:17]=[#6:18]1)-[#6:19]-1=[#6:20]-[#6:21]=[#6:22]-[#6:23]=[#6:24]-
1>>[#6:1]-[#8:2]-[#6:8](=[O:9])-[#6:5]-1=[#6:4](-[#7:3]-[#6:12](=[#6:10]-
1-[#6:11])-[#6:13]-1=[#6:14]-[#6:15]=[#6:16]-[#6:17]=[#6:18]-1)-[#6:19]-
1=[#6:24]-[#6:23]=[#6:22]-[#6:21]=[#6:20]-1.[#7;v0:6].[#7;v0:7]

Correctness of the mapping

MAPPET	YES
ReactionMap	NO
Marvin	NO
ChemDraw	NO
Indigo	NO

Reaction no 94

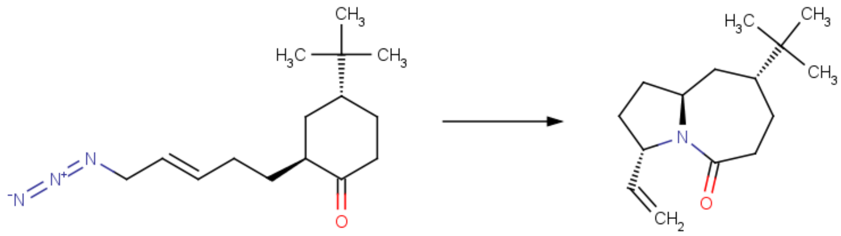

SMILES of the input:
CC(C)(C)[C@@H]1CCC(=O)[C@@H](CC\C=C\CN=[N+]=[N-
])C1>>CC(C)(C)[C@@H]1CCC(=O)N2[C@@H](CC[C@H]2C=C)C1

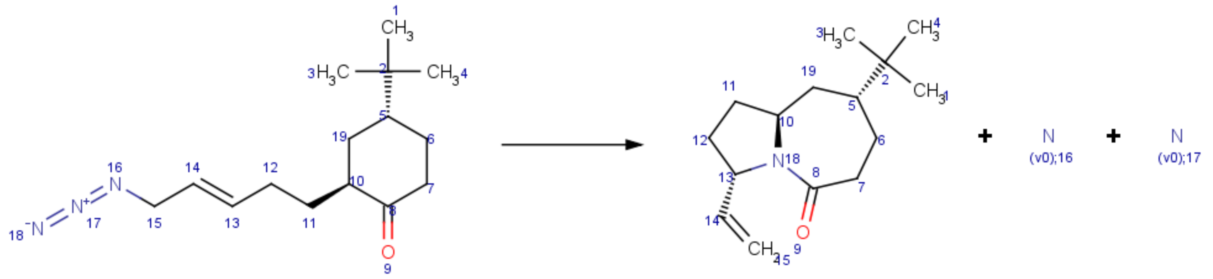

Correct mapped SMILES/SMARTS of the reaction:
[#6:1][C:2]([#6:3])([#6:4])[#6@@H:5]-1-[#6:6]-[#6:7]-[#6:8](=[O:9])-
[#6@@H:10](-[#6:11]-[#6:12]\[#6:13]=[#6:14]\[#6:15]-[#7:16]=[N+:17]=[#7-
:18])-[#6:19]-1>>[#6:4][C:2]([#6:3])([#6:1])[#6@@H:5]-1-[#6:6]-[#6:7]-
[#6:8](=[O:9])-[#7:18]-2-[#6@@H:10](-[#6:11]-[#6:12]-[#6@H:13]-2-
[#6:14]=[#6:15])-[#6:19]-1.[#7;v0:16].[#7;v0:17]

Correctness of the mapping

MAPPET	YES
ReactionMap	NO
Marvin	NO
ChemDraw	NO
Indigo	NO

Reaction no 95

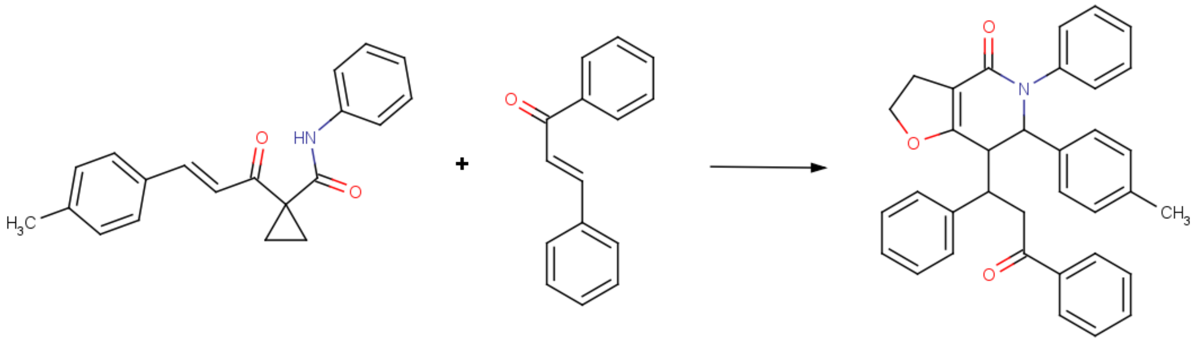

SMILES of the input:
CC1=CC=C(\C=C\C(=O)C2(CC2)C(=O)NC2=CC=CC=C2)C=C1.O=C(\C=C\C1=CC=CC=C1)C1=
CC=CC=C1>>CC1=CC=C(C=C1)C1C(C(CC(=O)C2=CC=CC=C2)C2=CC=CC=C2)C2=C(CCO2)C(=
O)N1C1=CC=CC=C1

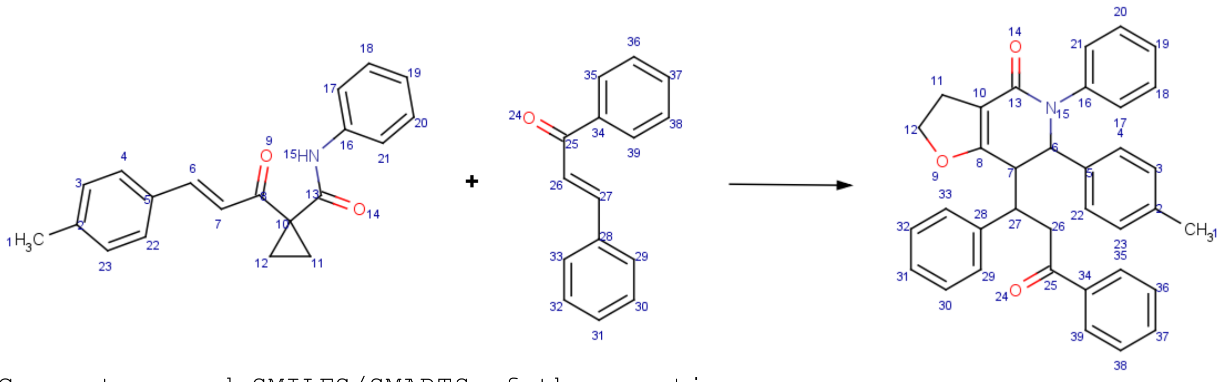

Correct mapped SMILES/SMARTS of the reaction:

[#6:1]-[#6:2]-1=[#6:3]-
[#6:4]=[#6:5](\[#6:6]=[#6:7]\[#6:8](=[O:9])[C:10]2([#6:11]-
[#6:12]2)[#6:13](=[O:14])-[#7:15]-[#6:16]-2=[#6:17]-[#6:18]=[#6:19]-
[#6:20]=[#6:21]-2)-[#6:22]=[#6:23]-
1.[O:24]=[#6:25](\[#6:26]=[#6:27]\[#6:28]-1=[#6:29]-[#6:30]=[#6:31]-
[#6:32]=[#6:33]1)-[#6:34]-1=[#6:35]-[#6:36]=[#6:37]-[#6:38]=[#6:39]-
1>>[#6:1]-[#6:2]-1=[#6:23]-[#6:22]=[#6:5](-[#6:4]=[#6:3]-1)-[#6:6]-1-
[#6:7](-[#6:27](-[#6:26]-[#6:25](=[O:24])-[#6:34]-2=[#6:35]-
[#6:36]=[#6:37]-[#6:38]=[#6:39]-2)-[#6:28]-2=[#6:29]-[#6:30]=[#6:31]-
[#6:32]=[#6:33]-2)-[#6:8]-2=[#6:10](-[#6:11]-[#6:12]-[#8:9]-2)-
[#6:13](=[O:14])-[#7:15]-1-[#6:16]-1=[#6:21]-[#6:20]=[#6:19]-
[#6:18]=[#6:17]-1

Correctness of the mapping

MAPPET	YES
ReactionMap	YES
Marvin	NO
ChemDraw	YES
Indigo	NO

Reaction no 96

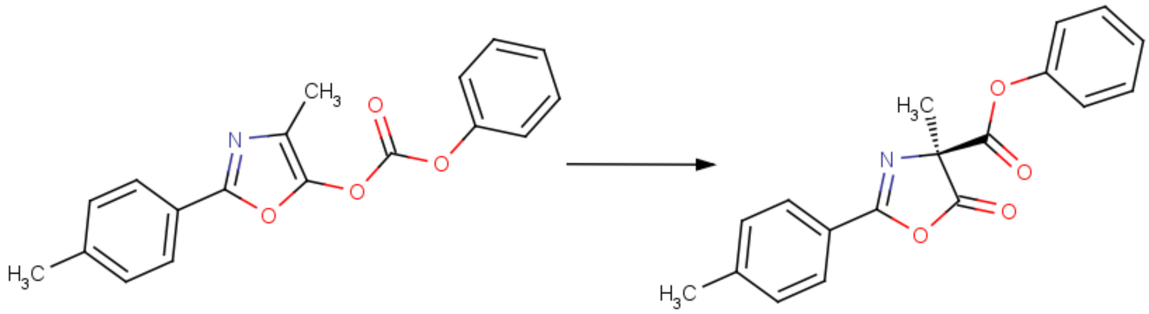

SMILES of the input:
CC1=C(OC(=O)OC2=CC=CC=C2)OC(=N1)C1=CC=C(C)C=C1>>CC1=CC=C(C=C1)C1=N[C@@](C)(C(=O)OC2=CC=CC=C2)C(=O)O1

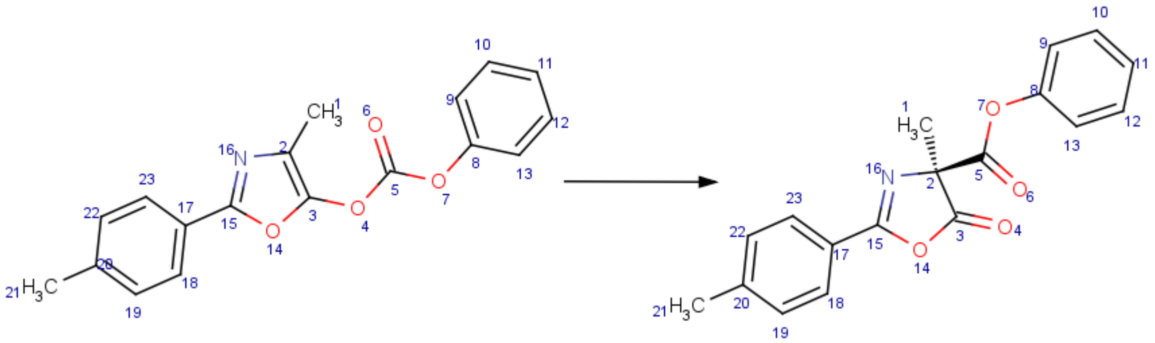

Correct mapped SMILES/SMARTS of the reaction:
[#6:1]-[#6:2]-1=[#6:3](-[#8:4]-[#6:5](=[O:6])-[#8:7]-[#6:8]-2=[#6:9]-
[#6:10]=[#6:11]-[#6:12]=[#6:13]-2)-[#8:14]-[#6:15](=[#7:16]-1)-[#6:17]-
1=[#6:18]-[#6:19]=[#6:20](-[#6:21])-[#6:22]=[#6:23]-1>>[#6:21]-[#6:20]-
1=[#6:22]-[#6:23]=[#6:17](-[#6:18]=[#6:19]-1)-[#6:15]-
1=[#7:16][C@@:2]([#6:1])([#6:5](=[O:6])-[#8:7]-[#6:8]-2=[#6:9]-
[#6:10]=[#6:11]-[#6:12]=[#6:13]-2)[#6:3](=[O:4])-[#8:14]-1

Correctness of the mapping

MAPPET	YES

ReactionMap YES
Marvin YES
ChemDraw YES
Indigo NO

Reaction no 97

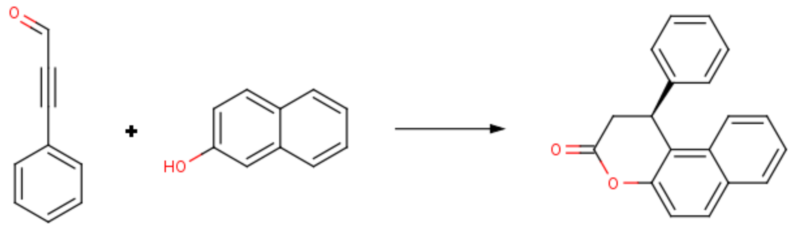

SMILES of the input:
O=CC#CC1=CC=CC=C1.OC1=CC=C2C=CC=CC2=C1>>O=C1C[C@@H](C2=CC=CC=C2)C2=C3C=CC=CC3=CC=C2O1

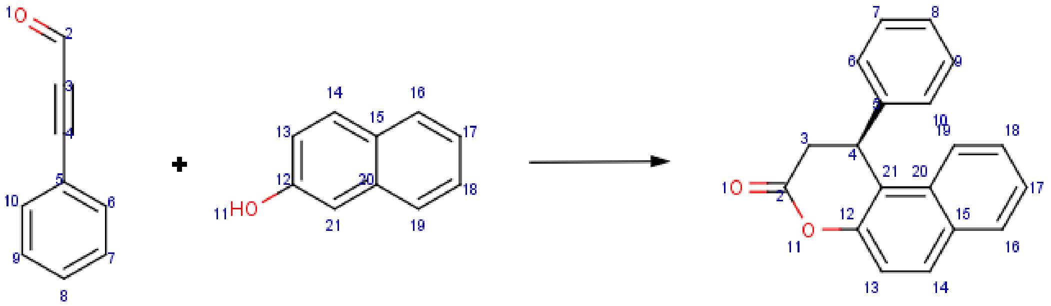

Correct mapped SMILES/SMARTS of the reaction:
[O:1]=[#6:2][C:3]#[C:4][#6:5]-1=[#6:6]-[#6:7]=[#6:8]-[#6:9]=[#6:10]-1.[#8:11]-[#6:12]-1=[#6:13]-[#6:14]=[#6:15]-2-[#6:16]=[#6:17]-[#6:18]=[#6:19]-[#6:20]-2=[#6:21]-1>>[O:1]=[#6:2]-1-[#6:3]-[#6@@H:4](-[#6:5]-2=[#6:6]-[#6:7]=[#6:8]-[#6:9]=[#6:10]-2)-[#6:21]-2=[#6:20]-3-[#6:19]=[#6:18]-[#6:17]=[#6:16]-[#6:15]-3=[#6:14]-[#6:13]=[#6:12]-2-[#8:11]-1

Correctness of the mapping
MAPPET YES
ReactionMap YES
Marvin YES
ChemDraw YES
Indigo NO

Reaction no 98

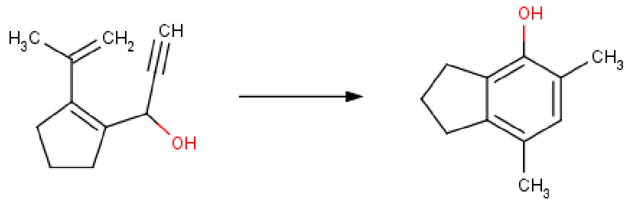

SMILES of the input:
CC(=C)C1=C(CCC1)C(O)C#C>>CC1=C2CCCC2=C(O)C(C)=C1

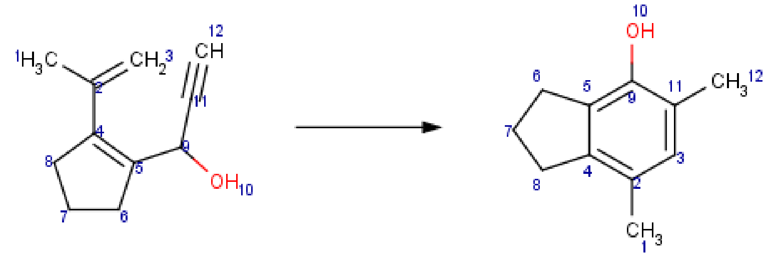

Correct mapped SMILES/SMARTS of the reaction:
[#6:1]-[#6:2](=[#6:3])-[#6:4]-1=[#6:5](-[#6:6]-[#6:7]-[#6:8]-1)-[#6:9](-[#8:10])[C:11]#[C:12]>>[#6:1]-[#6:2]-1=[#6:4]-2-[#6:8]-[#6:7]-[#6:6]-[#6:5]-2=[#6:9](-[#8:10])-[#6:11](-[#6:12])=[#6:3]-1

Correctness of the mapping

MAPPET	YES
ReactionMap	YES
Marvin	YES
ChemDraw	YES
Indigo	NO

Reaction no 99

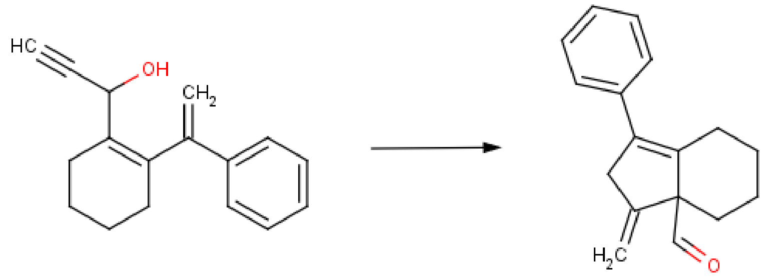

SMILES of the input:
OC(C#C)C1=C(CCCC1)C(=C)C1=CC=CC=C1>>C=C1CC(=C2CCCCC12C=O)C1=CC=CC=C1

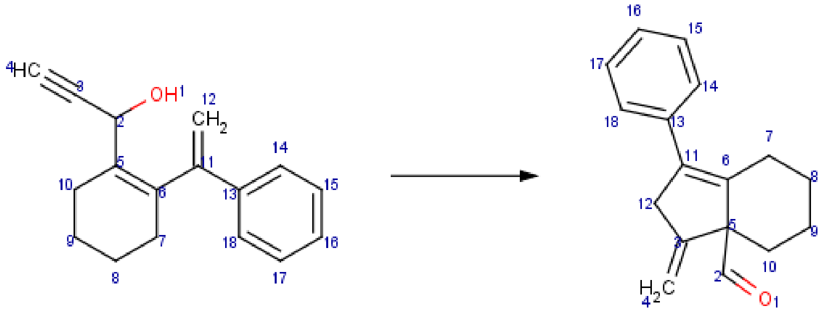

Correct mapped SMILES/SMARTS of the reaction:
[#8:1]-[#6:2]([C:3]#[C:4])-[#6:5]-1=[#6:6](-[#6:7]-[#6:8]-[#6:9]-[#6:10]-1)-[#6:11](=[#6:12])-[#6:13]-1=[#6:14]-[#6:15]=[#6:16]-[#6:17]=[#6:18]-1>>[#6:4]=[#6:3]1-[#6:12]-[#6:11](=[#6:6]2-[#6:7]-[#6:8]-[#6:9]-[#6:10][C:5]12[#6:2]=[O:1])-[#6:13]-1=[#6:18]-[#6:17]=[#6:16]-[#6:15]=[#6:14]-1

Correctness of the mapping

MAPPET YES
ReactionMap YES
Marvin NO
ChemDraw NO
Indigo NO

Reaction no 100

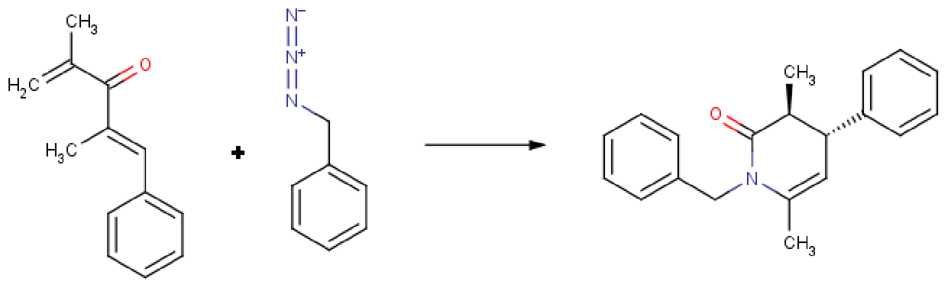

SMILES of the input:
CC(=C)C(=O)C(\C)=C\C1=CC=CC=C1.[N-
]=[N+]=NCC1=CC=CC=C1>>C[C@H]1[C@@H](C=C(C)N(CC2=CC=CC=C2)C1=O)C1=CC=CC=C1

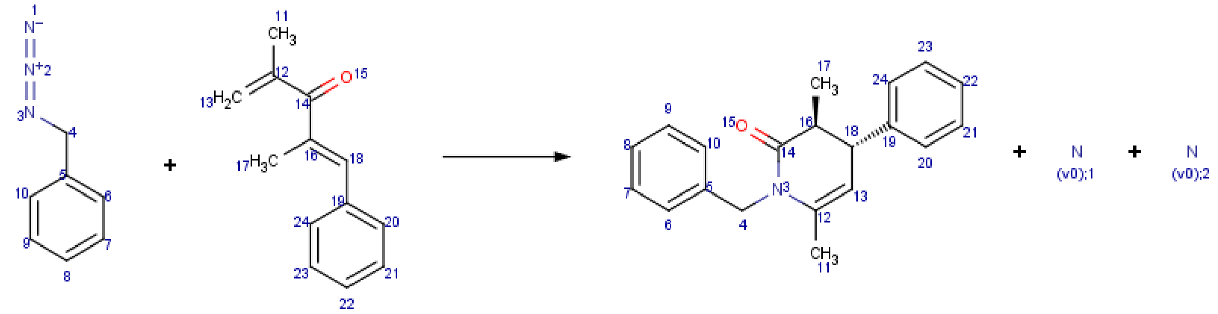

Correct mapped SMILES/SMARTS of the reaction:
[#7-:1]=[N+:2]=[#7:3]-[#6:4]-[#6:5]-1=[#6:6]-[#6:7]=[#6:8]-
[#6:9]=[#6:10]-1.[#6:11]-[#6:12](=[#6:13])-[#6:14](=[O:15])-
[#6:16](\[#6:17])=[#6:18]\[#6:19]-1=[#6:20]-[#6:21]=[#6:22]-
[#6:23]=[#6:24]1>>[#6:17]-[#6@H:16]-1-[#6@@H:18](-[#6:13]=[#6:12](-
[#6:11])-[#7:3](-[#6:4]-[#6:5]-2=[#6:6]-[#6:7]=[#6:8]-[#6:9]=[#6:10]-2)-
[#6:14]-1=[O:15])-[#6:19]-1=[#6:24]-[#6:23]=[#6:22]-[#6:21]=[#6:20]-
1.[#7;v0:1].[#7;v0:2]

Correctness of the mapping
MAPPET YES
ReactionMap NO
Marvin YES
ChemDraw NO
Indigo YES

Reaction no 101

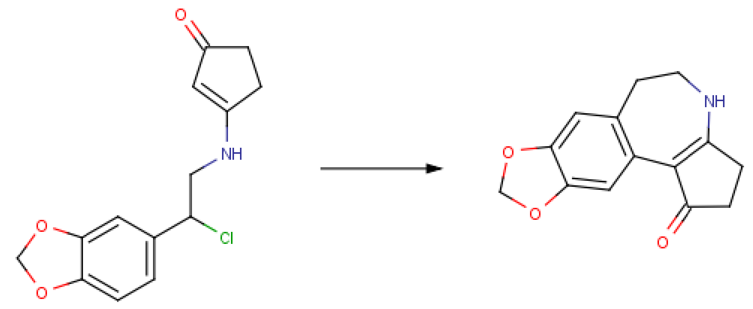

SMILES of the input:
ClC(CNC1=CC(=O)CC1)C1=CC=C2OCOC2=C1>>O=C1CCC2=C1C1=C(CCN2)C=C2OCOC2=C1

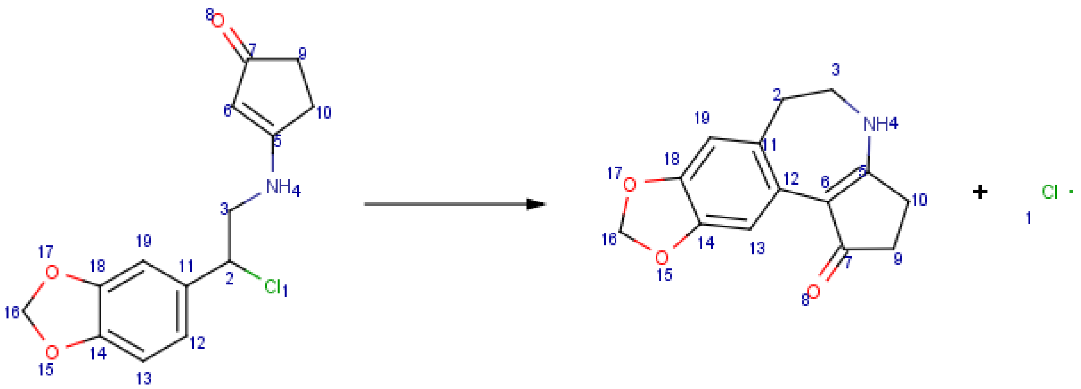

Correct mapped SMILES/SMARTS of the reaction:
[Cl:1][CH:2]([CH2:3][NH:4][C:5]1=[CH:6][C:7](=[O:8])[CH2:9][CH2:10]1)[C:1
1]1=[CH:12][CH:13]=[C:14]2[O:15][CH2:16][O:17][C:18]2=[CH:19]1>>[O:8]=[C:
7]1[CH2:9][CH2:10][C:5]2=[C:6]1[C:12]1=[C:11]([CH2:2][CH2:3][NH:4]2)[CH:1
9]=[C:18]2[O:17][CH2:16][O:15][C:14]2=[CH:13]1.[Cl:1]

Correctness of the mapping

MAPPET	YES
ReactionMap	NO
Marvin	YES
ChemDraw	YES
Indigo	YES

Reaction no 102

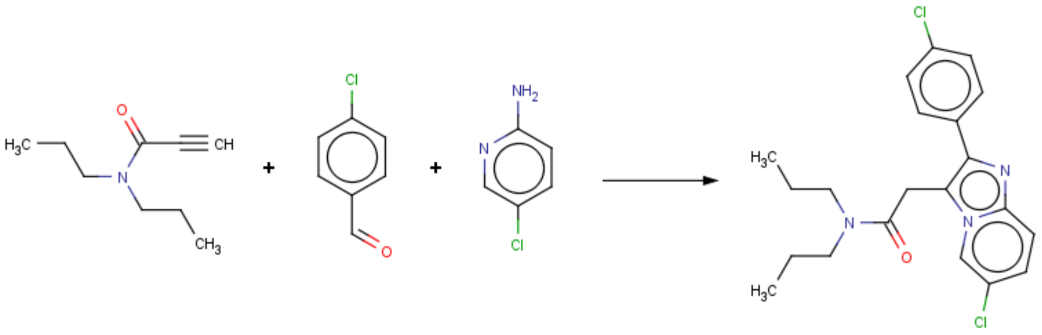

SMILES of the input:
CCCN(CCC)C(=O)C#C.Clc1ccc(C=O)cc1.Nc1ccc(Cl)cn1>>CCCN(CCC)C(=O)Cc1c(nc2cc
c(Cl)cn12)-c1ccc(Cl)cc1

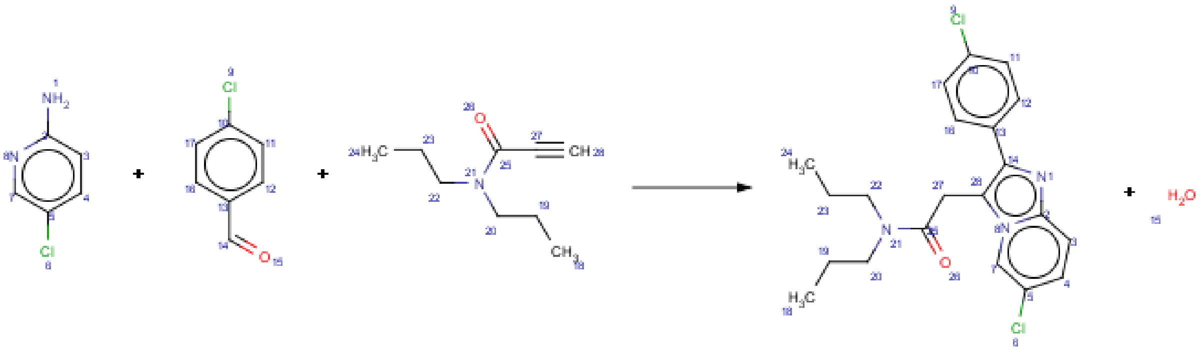

Correct mapped SMILES/SMARTS of the reaction:
[#7:1]-
[c:2]1[c:3][c:4][c:5]([Cl:6])[c:7][n:8]1.[Cl:9][c:10]1[c:11][c:12][c:13](
-[#6:14]=[O:15])[c:16][c:17]1.[#6:18]-[#6:19]-[#6:20]-[#7:21](-[#6:22]-
[#6:23]-[#6:24])-[#6:25](=[O:26])[C:27]#[C:28]>>[#6:18]-[#6:19]-[#6:20]-
[#7:21](-[#6:22]-[#6:23]-[#6:24])-[#6:25](=[O:26])-[#6:27]-
[c:28]1[c:14]([n:1][c:2]2[c:3][c:4][c:5]([Cl:6])[c:7][n:8]12)-
[c:13]1[c:16][c:17][c:10]([Cl:9])[c:11][c:12]1.[#8:15]

Correctness of the mapping

MAPPET	YES
ReactionMap	NO
Marvin	NO
ChemDraw	YES
Indigo	NO

Reaction no 103

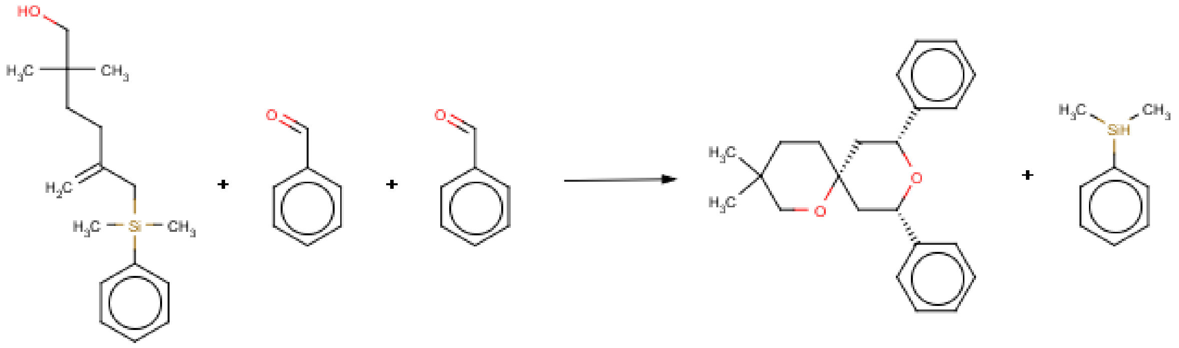

SMILES of the input:
CC(C)(CO)CCC(=C)C[Si](C)(C)c1ccccc1.O=Cc1ccccc1.O=Cc1ccccc1>>CC1(C)CC[C@]
2(C[C@@H](O[C@@H](C2)c2ccccc2)c2ccccc2)OC1.C[SiH](C)c1ccccc1

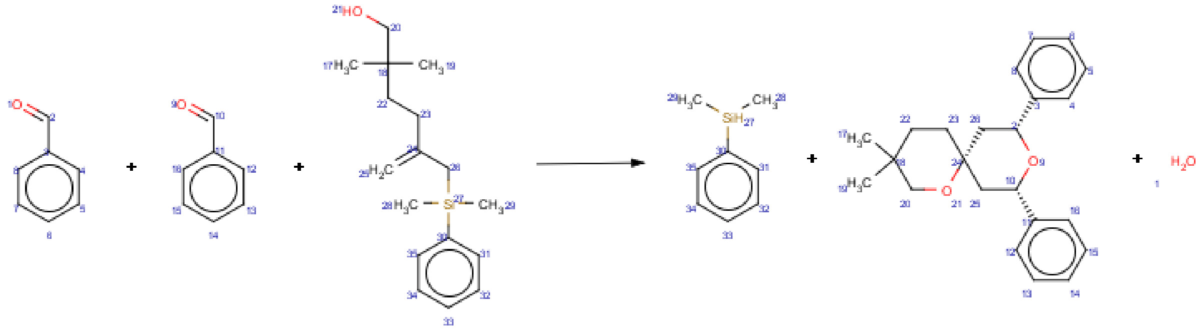

Correct mapped SMILES/SMARTS of the reaction:
[O:1]=[#6:2]-[c:3]1[c:4][c:5][c:6][c:7][c:8]1.[O:9]=[#6:10]-
[c:11]1[c:12][c:13][c:14][c:15][c:16]1.[#6:17][C:18]([#6:19])([#6:20]-
[#8:21])[#6:22]-[#6:23]-[#6:24](=[#6:25])-
[#6:26][Si:27]([#6:28])([#6:29])[c:30]1[c:31][c:32][c:33][c:34][c:35]1>>[
#6:29]-[#14:27](-[#6:28])-
[c:30]1[c:31][c:32][c:33][c:34][c:35]1.[#6:17][C:18]1([#6:19])[#6:22]-
[#6:23][C@:24]2([#6:26]-[#6@@H:2](-[#8:9]-[#6@@H:10](-[#6:25]2)-
[c:11]2[c:16][c:15][c:14][c:13][c:12]2)-
[c:3]2[c:8][c:7][c:6][c:5][c:4]2)[#8:21]-[#6:20]1.[#8:1]

Correctness of the mapping

MAPPET	YES
ReactionMap	NO
Marvin	YES
ChemDraw	NO
Indigo	NO

Reaction no 104

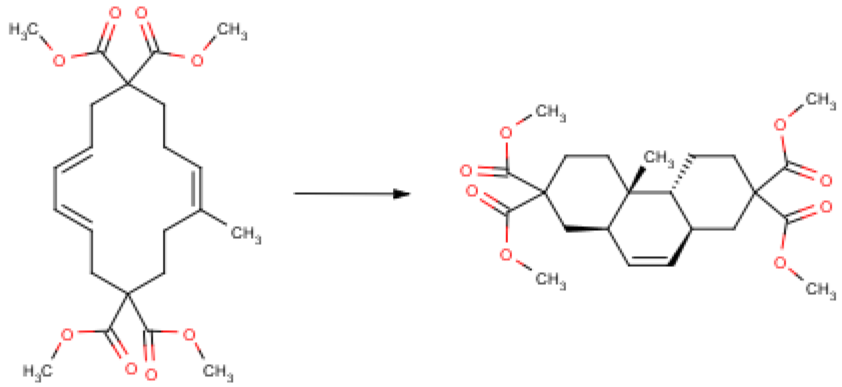

SMILES of the input:
COC(=O)C1(CC\C=C(C)/CCC(C\C=C\C=C\C1)(C(=O)OC)C(=O)OC)C(=O)OC>>COC(=O)C1(
CC[C@@H]2[C@@H](C1)C=C[C@H]1CC(CC[C@]21C)(C(=O)OC)C(=O)OC)C(=O)OC

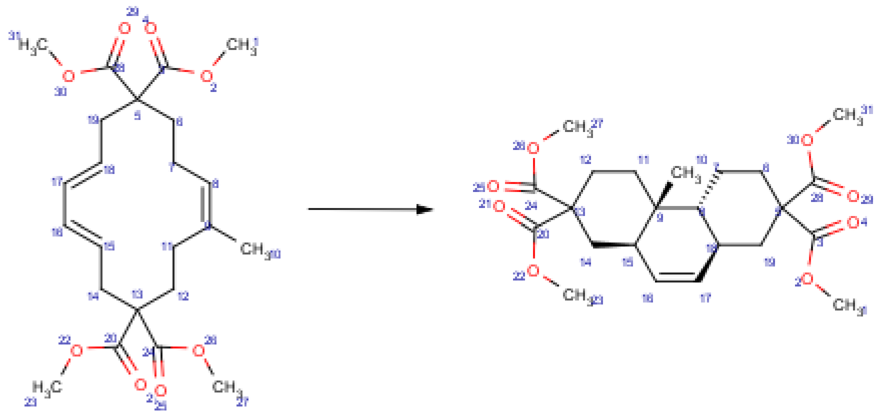

Correct mapped SMILES/SMARTS of the reaction:
[#6:1]-[#8:2]-[#6:3](=[O:4])[C:5]1([#6:6]-[#6:7]\[#6:8]=[#6:9](-
[#6:10])/[#6:11]-
[#6:12][C:13]([#6:14]\[#6:15]=[#6:16]\[#6:17]=[#6:18]\[#6:19]1)([#6:20](=
[O:21])-[#8:22]-[#6:23])[#6:24](=[O:25])-[#8:26]-
[#6:27])[#6:28](=[O:29])-[#8:30]-[#6:31]>>[#6:1]-[#8:2]-
[#6:3](=[O:4])[C:5]1([#6:6]-[#6:7]-[#6@@H:8]2-[#6@@H:18](-[#6:19]1)-
[#6:17]=[#6:16]-[#6@H:15]1-[#6:14][C:13]([#6:12]-
[#6:11][C@:9]21[#6:10])([#6:20](=[O:21])-[#8:22]-
[#6:23])[#6:24](=[O:25])-[#8:26]-[#6:27])[#6:28](=[O:29])-[#8:30]-[#6:31]

Correctness of the mapping

MAPPET	YES
ReactionMap	YES
Marvin	YES
ChemDraw	YES
Indigo	NO

Reaction no 105

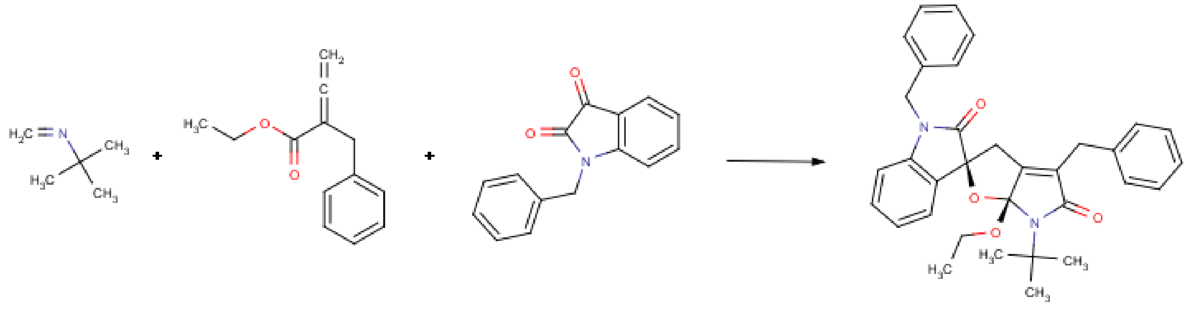

SMILES of the input:
CC(C)(C)N=C.CCOC(=O)C(CC1=CC=CC=C1)=C=C.O=C1N(CC2=CC=CC=C2)C2=C(C=CC=C2)C
1=O>>CCO[C@@]12O[C@]3(CC1=C(CC1=CC=CC=C1)C(=O)N2C(C)(C)C)C(=O)N(CC1=CC=CC
=C1)C1=C3C=CC=C1

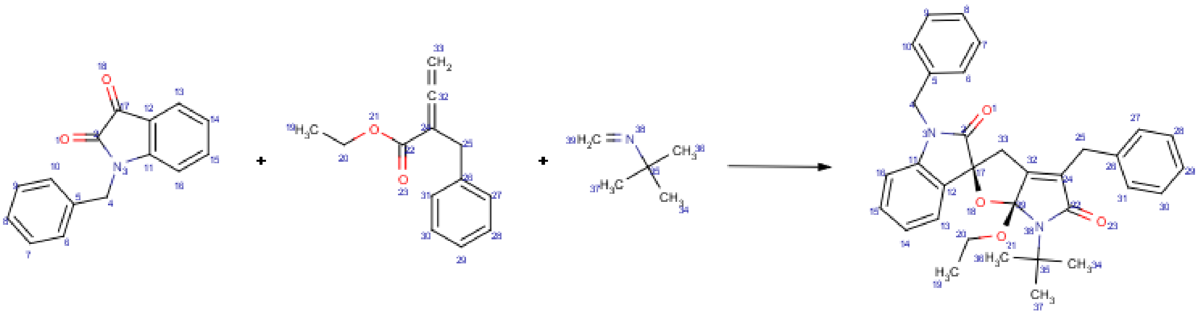

Correct mapped SMILES/SMARTS of the reaction:
[O:1]=[#6:2]-1-[#7:3](-[#6:4]-[#6:5]-2=[#6:6]-[#6:7]=[#6:8]-
[#6:9]=[#6:10]-2)-[#6:11]-2=[#6:12](-[#6:13]=[#6:14]-[#6:15]=[#6:16]-2)-
[#6:17]-1=[O:18].[#6:19]-[#6:20]-[#8:21]-[#6:22](=[O:23])-[#6:24](-
[#6:25]-[#6:26]-1=[#6:27]-[#6:28]=[#6:29]-[#6:30]=[#6:31]-
1)=[C:32]=[#6:33].[#6:34][C:35]([#6:36])([#6:37])[#7:38]=[#6:39]>>[#6:19]
-[#6:20]-[#8:21][C@@:39]12[#8:18][C@:17]3([#6:33]-[#6:32]1=[#6:24](-
[#6:25]-[#6:26]-1=[#6:27]-[#6:28]=[#6:29]-[#6:30]=[#6:31]-1)-
[#6:22](=[O:23])-[#7:38]2[C:35]([#6:37])([#6:36])[#6:34])[#6:2](=[O:1])-
[#7:3](-[#6:4]-[#6:5]-1=[#6:10]-[#6:9]=[#6:8]-[#6:7]=[#6:6]-1)-[#6:11]-
1=[#6:12]3-[#6:13]=[#6:14]-[#6:15]=[#6:16]-1

Correctness of the mapping

MAPPET	YES
ReactionMap	YES
Marvin	YES
ChemDraw	NO
Indigo	NO

Reaction no 106

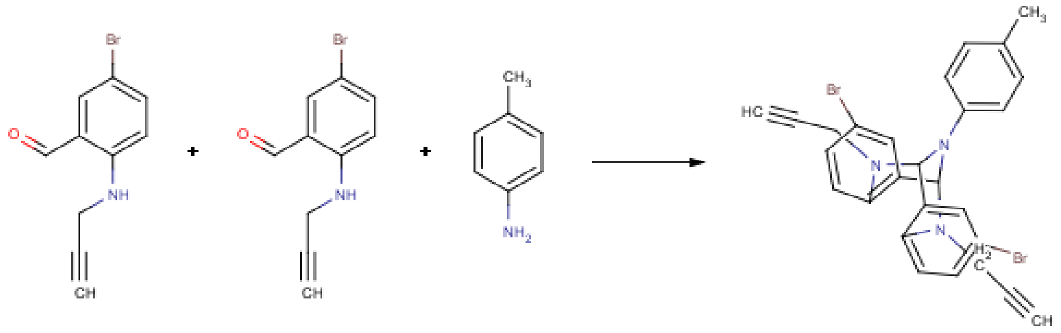

SMILES of the input:
BrC1=CC=C(NCC#C)C(C=O)=C1.BrC1=CC=C(NCC#C)C(C=O)=C1.CC1=CC=C(N)C=C1>>CC1=
CC=C(C=C1)N1C2N(CC#C)C3=C(C=C(Br)C=C3)C1N(CC#C)C1=CC=C(Br)C=C21

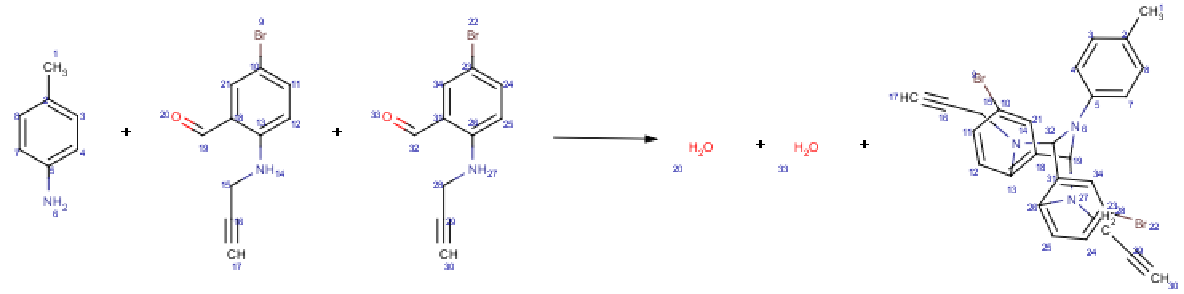

Correct mapped SMILES/SMARTS of the reaction:
[#6:1]-[#6:2]-1=[#6:3]-[#6:4]=[#6:5](-[#7:6])-[#6:7]=[#6:8]-1.[Br:9][#6:10]-1=[#6:11]-[#6:12]=[#6:13](-[#7:14]-[#6:15][C:16]#[C:17])-[#6:18](-[#6:19]=[O:20])=[#6:21]-1.[Br:22][#6:23]-1=[#6:24]-[#6:25]=[#6:26](-[#7:27]-[#6:28][C:29]#[C:30])-[#6:31](-[#6:32]=[O:33])=[#6:34]-1>>[#8:20].[#8:33].[#6:1]-[#6:2]-1=[#6:8]-[#6:7]=[#6:5](-[#6:4]=[#6:3]-1)-[#7:6]-1-[#6:32]-2-[#7:14](-[#6:15][C:16]#[C:17])-[#6:13]-3=[#6:18](-[#6:21]=[#6:10]([Br:9])-[#6:11]=[#6:12]-3)-[#6:19]-1-[#7:27](-[#6:28][C:29]#[C:30])-[#6:26]-1=[#6:25]-[#6:24]=[#6:23]([Br:22])-[#6:34]=[#6:31]-2-1

Correctness of the mapping

MAPPET	YES
ReactionMap	NO
Marvin	YES
ChemDraw	YES
Indigo	NO

Reaction no 107

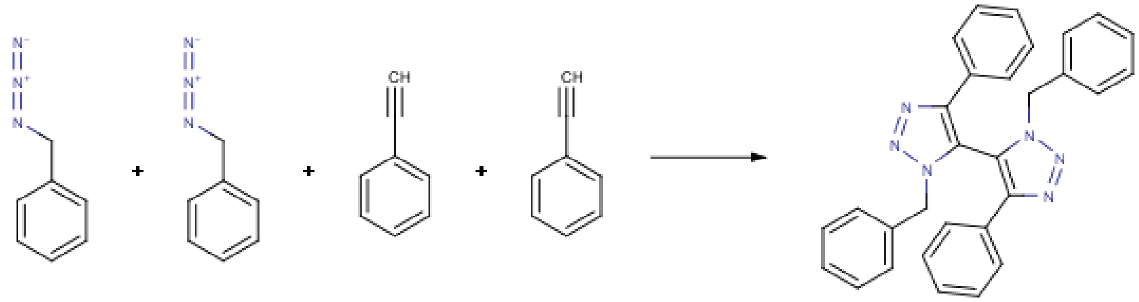

SMILES of the input:
[N-]=[N+]=NCC1=CC=CC=C1.[N-]=[N+]=NCC1=CC=CC=C1.C#CC1=CC=CC=C1.C#CC1=CC=CC=C1>>C(N1N=NC(=C1C1=C(N=NN1CC1=CC=CC=C1)C1=CC=CC=C1)C1=CC=CC=C1)C1=CC=CC=C1

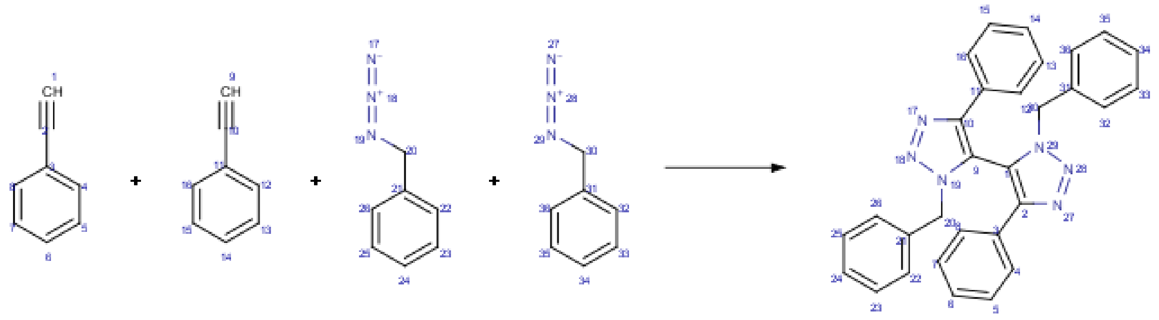

Correct mapped SMILES/SMARTS of the reaction:
[C:1]#[C:2][#6:3]-1=[#6:4]-[#6:5]=[#6:6]-[#6:7]=[#6:8]-1.[C:9]#[C:10][#6:11]-1=[#6:12]-[#6:13]=[#6:14]-[#6:15]=[#6:16]-1.[#7-:17]=[N+:18]=[#7:19]-[#6:20]-[#6:21]-1=[#6:22]-[#6:23]=[#6:24]-[#6:25]=[#6:26]-1.[#7-:27]=[N+:28]=[#7:29]-[#6:30]-[#6:31]-1=[#6:32]-[#6:33]=[#6:34]-[#6:35]=[#6:36]-1>>[#6:30](-[#7:29]-1-[#7:28]=[#7:27]-[#6:2](=[#6:1]-1-[#6:9]-1=[#6:10](-[#7:17]=[#7:18]-[#7:19]-1-[#6:20]-[#6:21]-1=[#6:22]-[#6:23]=[#6:24]-[#6:25]=[#6:26]-1)-[#6:11]-1=[#6:16]-[#6:15]=[#6:14]-[#6:13]=[#6:12]-1)-[#6:3]-1=[#6:4]-[#6:5]=[#6:6]-[#6:7]=[#6:8]-1)-[#6:31]-1=[#6:36]-[#6:35]=[#6:34]-[#6:33]=[#6:32]-1

Correctness of the mapping

MAPPET	YES
ReactionMap	YES
Marvin	NO
ChemDraw	YES
Indigo	NO

Reaction no 108

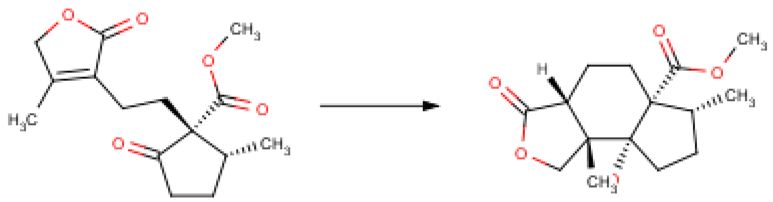

SMILES of the input:
COC(=O)[C@]1(CCC2=C(C)COC2=O)[C@H](C)CCC1=O>>[H][C@@]12CC[C@]3([C@H](C)CC[C@@]3(O)[C@]1(C)COC2=O)C(=O)OC

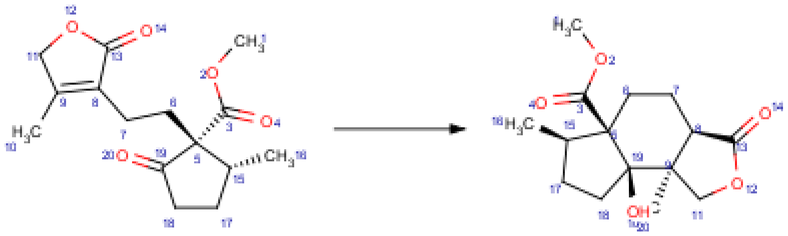

Correct mapped SMILES/SMARTS of the reaction:

[#6:1]-[#8:2]-[#6:3](=[O:4])[C@:5]1([#6:6]-[#6:7]-[#6:8]-2=[#6:9](-[#6:10])-[#6:11]-[#8:12]-[#6:13]-2=[O:14])[#6@H:15](-[#6:16])-[#6:17]-[#6:18]-[#6:19]1=[O:20]>>[#6:1]-[#8:2]-[#6:3](=[O:4])[C@@:5]12[#6:6]-[#6:7]-[#6@H:8]3-[#6:13](=[O:14])-[#8:12]-[#6:11][C@@:9]3([#6:10])[C@:19]1([#8:20])[#6:18]-[#6:17]-[#6@H:15]2-[#6:16]

Correctness of the mapping

MAPPET	YES
ReactionMap	YES
Marvin	YES
ChemDraw	YES
Indigo	NO

Reaction no 109

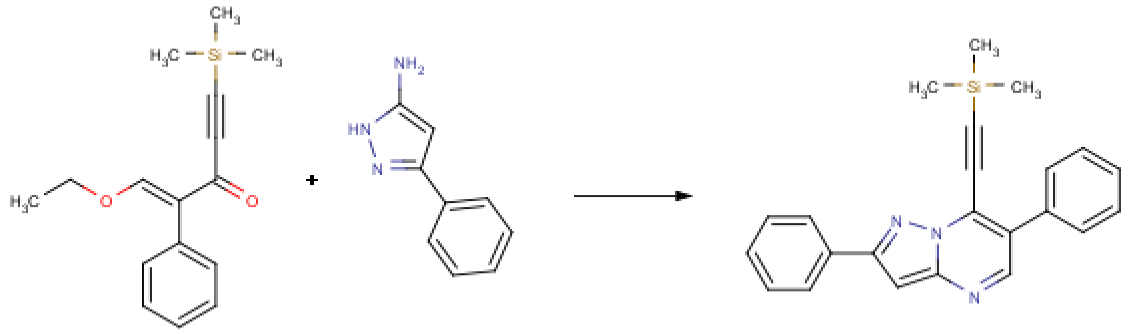

SMILES of the input:
CCO\C=C(\C(=O)C#C[Si](C)(C)C)C1=CC=CC=C1.NC1=CC(=NN1)C1=CC=CC=C1>>C[Si](C)(C)C#CC1=C(C=NC2=CC(=NN12)C1=CC=CC=C1)C1=CC=CC=C1

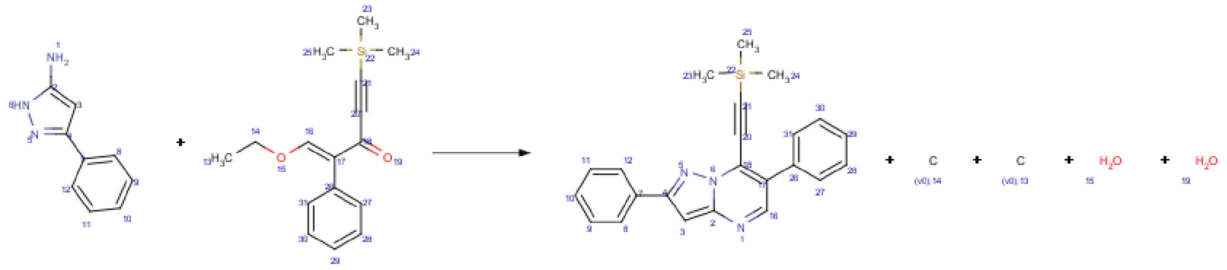

Correct mapped SMILES/SMARTS of the reaction:
[#7:1]-[#6:2]-1=[#6:3]-[#6:4](=[#7:5]-[#7:6]-1)-[#6:7]-1=[#6:8]-[#6:9]=[#6:10]-[#6:11]=[#6:12]-1.[#6:13]-[#6:14]-[#8:15]\[#6:16]=[#6:17](\[#6:18](=[O:19])[C:20]#[C:21][Si:22]([#6:23])([#6:24])[#6:25])-[#6:26]-1=[#6:27]-[#6:28]=[#6:29]-[#6:30]=[#6:31]-1>>[#6:25][Si:22]([#6:24])([#6:23])[C:21]#[C:20][#6:18]-1=[#6:17](-[#6:16]=[#7:1]-[#6:2]-2=[#6:3]-[#6:4](=[#7:5]-[#7:6]-1-2)-[#6:7]-1=[#6:8]-[#6:9]=[#6:10]-[#6:11]=[#6:12]-1)-[#6:26]-1=[#6:31]-[#6:30]=[#6:29]-[#6:28]=[#6:27]-1.[#6;v0:14].[#6;v0:13].[#8:15].[#8:19]

Correctness of the mapping

MAPPET	YES
ReactionMap	NO
Marvin	NO

ChemDraw YES
Indigo YES

Reaction no 110

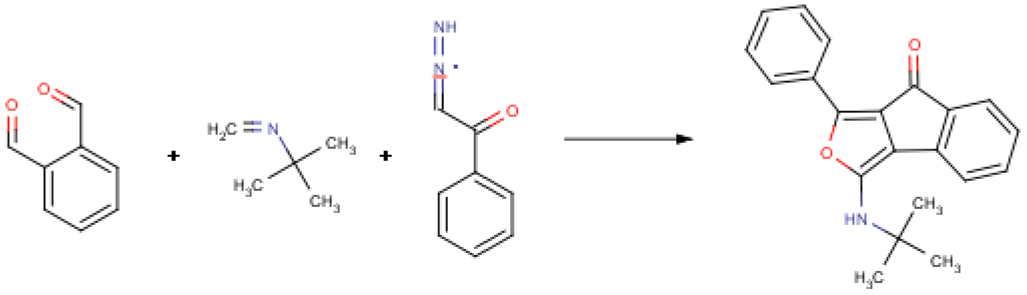

SMILES of the input:
O=CC1=CC=CC=C1C=O.CC(C)(C)N=C.N=[N]=CC(=O)C1=CC=CC=C1>>CC(C)(C)NC1=C2C3=C
(C=CC=C3)C(=O)C2=C(O1)C1=CC=CC=C1

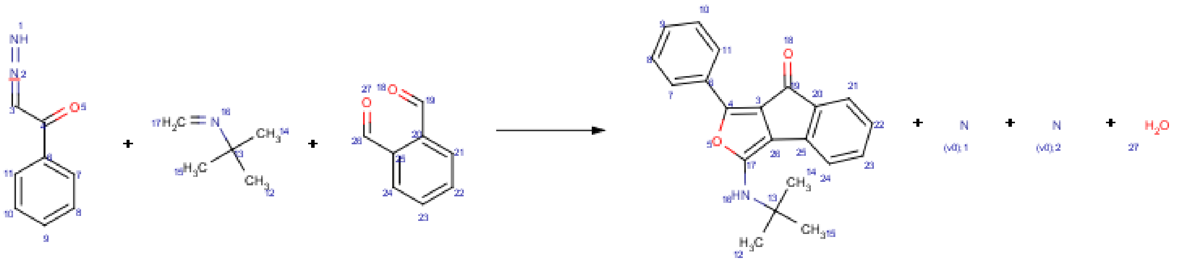

Correct mapped SMILES/SMARTS of the reaction:
[#7:1]=[N:2]=[#6:3]-[#6:4](=[O:5])-[#6:6]-1=[#6:7]-[#6:8]=[#6:9]-
[#6:10]=[#6:11]-
1.[#6:12][C:13]([#6:14])([#6:15])[#7:16]=[#6:17].[O:18]=[#6:19]-[#6:20]-
1=[#6:21]-[#6:22]=[#6:23]-[#6:24]=[#6:25]-1-
[#6:26]=[O:27]>>[#6:15][C:13]([#6:14])([#6:12])[#7:16]-[#6:17]-1=[#6:26]-
2-[#6:25]-3=[#6:20](-[#6:21]=[#6:22]-[#6:23]=[#6:24]-3)-[#6:19](=[O:18])-
[#6:3]-2=[#6:4](-[#8:5]-1)-[#6:6]-1=[#6:7]-[#6:8]=[#6:9]-[#6:10]=[#6:11]-
1.[#7;v0:1].[#7;v0:2].[#8:27]

Correctness of the mapping
MAPPET YES
ReactionMap NO
Marvin YES
ChemDraw YES
Indigo NO

Reaction no 111

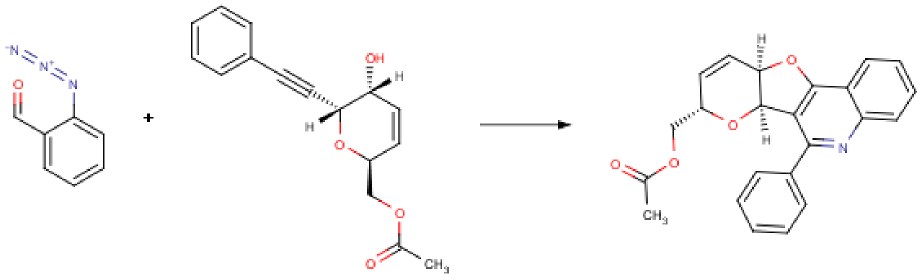

SMILES of the input:
[N-
]=[N+]=NC1=CC=CC=C1C=O.[H][C@@]1(O)C=C[C@@H](COC(C)=O)O[C@]1([H])C#CC1=CC
=CC=C1>>[H][C@]12OC3=C(C(=NC4=CC=CC=C34)C3=CC=CC=C3)[C@@]1([H])O[C@H](COC
(C)=O)C=C2

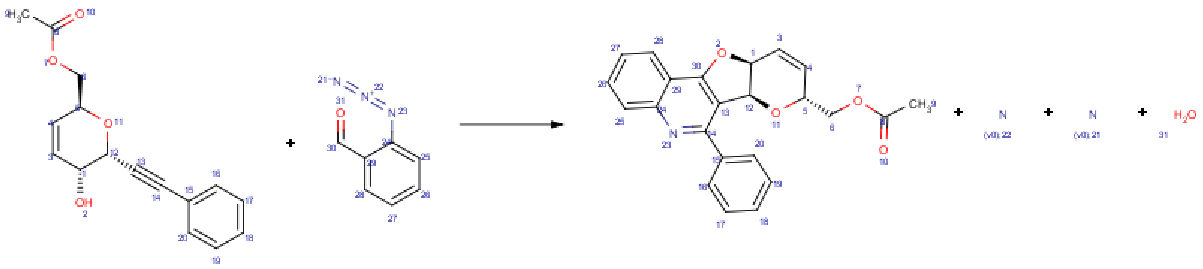

Correct mapped SMILES/SMARTS of the reaction:
[#6:9]-[#6:8](=[O:10])-[#8:7]-[#6:6]-[#6@H:5]-1-[#8:11]-
[#6@H:12]([C:13]#[C:14][#6:15]-2=[#6:16]-[#6:17]=[#6:18]-[#6:19]=[#6:20]-
2)-[#6@H:1](-[#8:2])-[#6:3]=[#6:4]-1.[#7-:21]=[N+:22]=[#7:23]-[#6:24]-
1=[#6:25]-[#6:26]=[#6:27]-[#6:28]=[#6:29]-1-[#6:30]=[O:31]>>[#6:9]-
[#6:8](=[O:10])-[#8:7]-[#6:6]-[#6@@H:5]-1-[#8:11]-[#6@@H:12]-2-
[#6@@H:1](-[#8:2]-[#6:30]-3=[#6:13]-2-[#6:14](=[#7:23]-[#6:24]-2=[#6:25]-
[#6:26]=[#6:27]-[#6:28]=[#6:29]-3-2)-[#6:15]-2=[#6:20]-[#6:19]=[#6:18]-
[#6:17]=[#6:16]-2)-[#6:3]=[#6:4]-1.[#7;v0:22].[#7;v0:21].[#8:31]

Correctness of the mapping
MAPPET YES
ReactionMap NO
Marvin NO
ChemDraw YES
Indigo YES

Reaction no 112

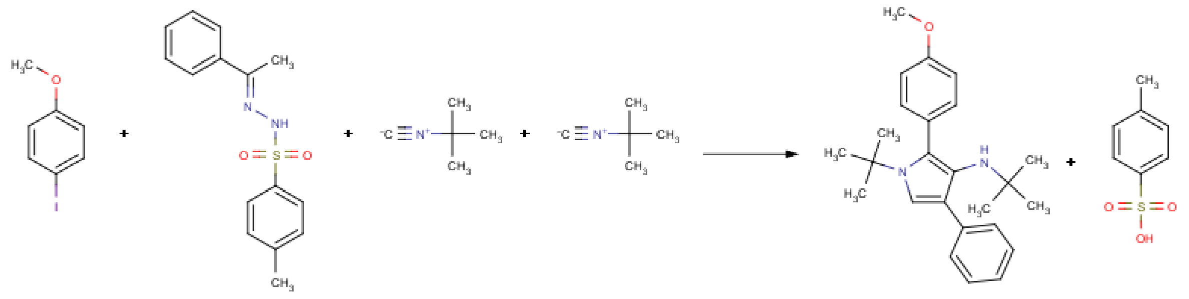

SMILES of the input:
COC1=CC=C(I)C=C1.C\C(=N/NS(=O)(=O)C1=CC=C(C)C=C1)C1=CC=CC=C1.CC(C)(C)[N+]#[C-].CC(C)(C)[N+]#[C-]>>COC1=CC=C(C=C1)C1=C(NC(C)(C)C)C(=CN1C(C)(C)C)C1=CC=CC=C1.CC1=CC=C(C=C1)S(O)(=O)=O

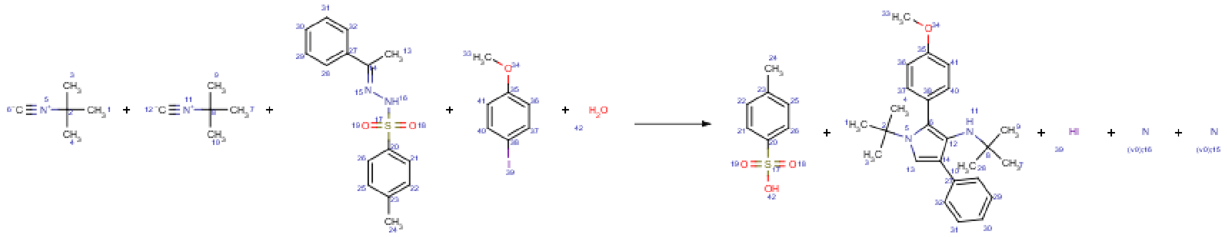

Correct mapped SMILES/SMARTS of the reaction:
[#6:1][C:2]([#6:3])([#6:4])[N+:5]#[C-:6].[#6:7][C:8]([#6:9])([#6:10])[N+:11]#[C-:12].[#6:13]\[#6:14](=[#7:15]/[#7:16][S:17](=[O:18])(=[O:19])[#6:20]-1=[#6:21]-[#6:22]=[#6:23](-[#6:24])-[#6:25]=[#6:26]-1)-[#6:27]-1=[#6:28]-[#6:29]=[#6:30]-[#6:31]=[#6:32]-1.[#6:33]-[#8:34]-[#6:35]-1=[#6:36]-[#6:37]=[#6:38]([I:39])-[#6:40]=[#6:41]-1.[#8:42]>>[#6:24]-[#6:23]-1=[#6:25]-[#6:26]=[#6:20](-[#6:21]=[#6:22]-1)[S:17]([#8:42])(=[O:19])=[O:18].[#6:33]-[#8:34]-[#6:35]-1=[#6:41]-[#6:40]=[#6:38](-[#6:37]=[#6:36]-1)-[#6:6]-1=[#6:12](-[#7:11][C:8]([#6:10])([#6:9])[#6:7])-[#6:14](=[#6:13]-[#7:5]-1[C:2]([#6:1])([#6:4])[#6:3])-[#6:27]-1=[#6:28]-[#6:29]=[#6:30]-[#6:31]=[#6:32]-1.[I:39].[#7;v0:16].[#7;v0:15]

Correctness of the mapping

MAPPET	YES
ReactionMap	NO
Marvin	NO
ChemDraw	NO
Indigo	NO

Reaction no 113

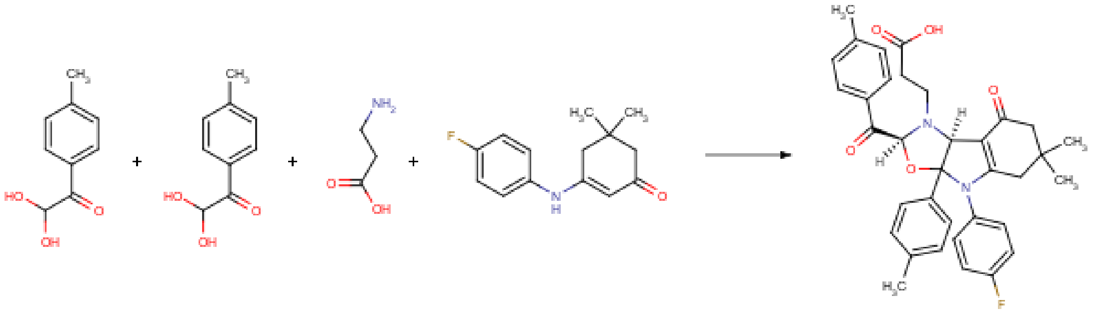

SMILES of the input:
CC1=CC=C(C=C1)C(=O)C(O)O.CC1=CC=C(C=C1)C(=O)C(O)O.NCCC(O)=O.CC1(C)CC(=O)C=C(C1)NC1=CC=C(F)C=C1>>[H][C@]1(OC2(N(C3=C(C(=O)CC(C)(C)C3)[C@]2([H])N1CCC(O)=O)C1=CC=C(F)C=C1)C1=CC=C(C)C=C1)C(=O)C1=CC=C(C)C=C1

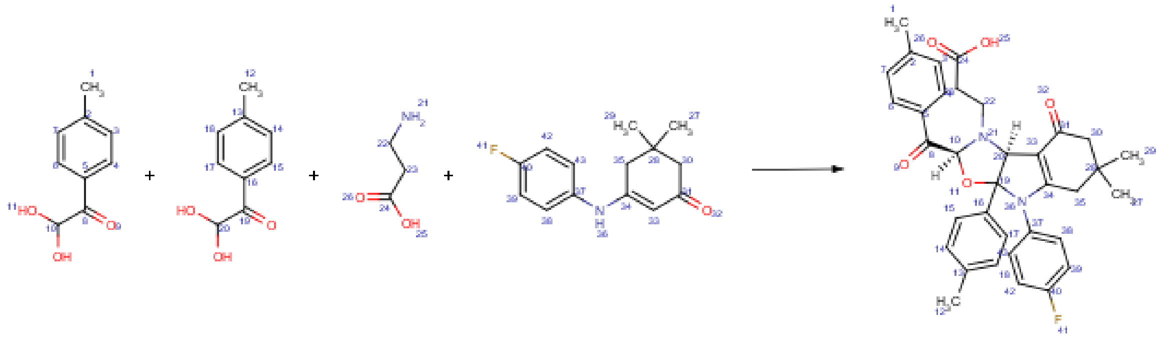

Correct mapped SMILES/SMARTS of the reaction:
[CH3:1][C:2]1=[CH:3][CH:4]=[C:5]([CH:6]=[CH:7]1)[C:8](=[O:9])[CH:10](O)[OH:11].[CH3:12][C:13]1=[CH:14][CH:15]=[C:16]([CH:17]=[CH:18]1)[C:19](=O)[CH:20](O)O.[NH2:21][CH2:22][CH2:23][C:24]([OH:25])=[O:26].[CH3:27][C:28]1([CH3:29])[CH2:30][C:31](=[O:32])[CH:33]=[C:34]([CH2:35]1)[NH:36][C:37]1=[CH:38][CH:39]=[C:40]([F:41])[CH:42]=[CH:43]1>>[H][C@:10]1([O:11][C:19]2([N:36]([C:34]3=[C:33]([C:31](=[O:32])[CH2:30][C:28]([CH3:29])([CH3:27])[CH2:35]3)[C@:20]2([H])[N:21]1[CH2:22][CH2:23][C:24]([OH:25])=[O:26])[C:37]1=[CH:38][CH:39]=[C:40]([F:41])[CH:42]=[CH:43]1)[C:16]1=[CH:17][CH:18]=[C:13]([CH3:12])[CH:14]=[CH:15]1)[C:8](=[O:9])[C:5]1=[CH:6][CH:7]=[C:2]([CH3:1])[CH:3]=[CH:4]1

Correctness of the mapping

MAPPET	NO
ReactionMap	NO
Marvin	NO
ChemDraw	NO
Indigo	NO

Reaction no 114

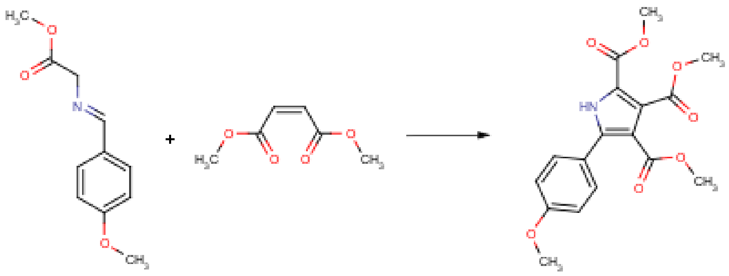

SMILES of the input:
COC(=O)C\N=C\C1=CC=C(OC)C=C1.COC(=O)\C=C/C(=O)OC>>COC(=O)C1=C(C(=O)OC)C(C(=O)OC)=C(N1)C1=CC=C(OC)C=C1

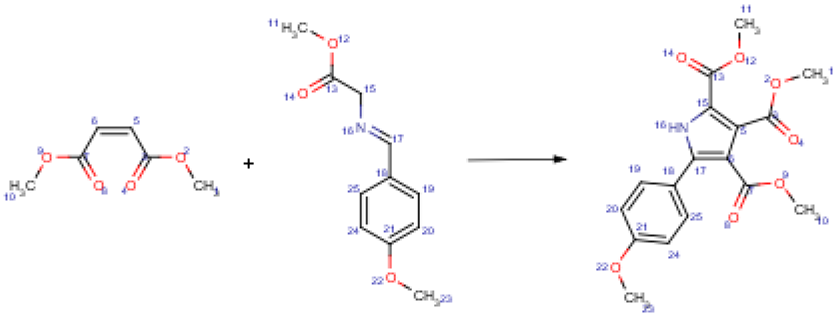

Correct mapped SMILES/SMARTS of the reaction:
[#6:1]-[#8:2]-[#6:3](=[O:4])\[#6:5]=[#6:6]/[#6:7](=[O:8])-[#8:9]-[#6:10].[#6:11]-[#8:12]-[#6:13](=[O:14])-[#6:15]\[#7:16]=[#6:17]\[#6:18]-1=[#6:19]-[#6:20]=[#6:21](-[#8:22]-[#6:23])-[#6:24]=[#6:25]1>>[#6:11]-[#8:12]-[#6:13](=[O:14])-[#6:15]-1=[#6:5](-[#6:3](=[O:4])-[#8:2]-[#6:1])-[#6:6](-[#6:7](=[O:8])-[#8:9]-[#6:10])=[#6:17](-[#7:16]-1)-[#6:18]-1=[#6:25]-[#6:24]=[#6:21](-[#8:22]-[#6:23])-[#6:20]=[#6:19]-1

Correctness of the mapping

MAPPET	YES
ReactionMap	YES
Marvin	YES
ChemDraw	YES
Indigo	YES

Reaction no 115

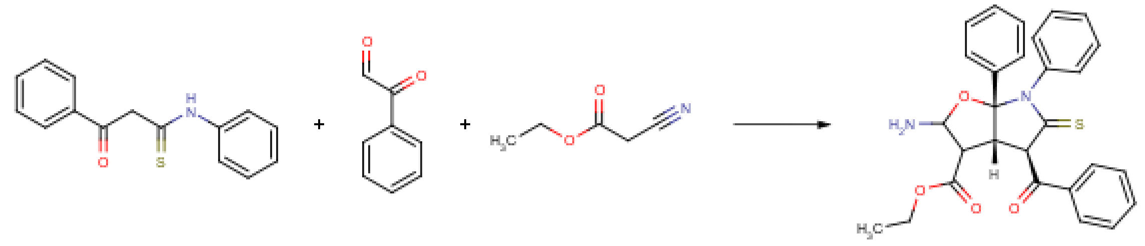

SMILES of the input:
O=C(CC(=S)NC1=CC=CC=C1)C1=CC=CC=C1.O=CC(=O)C1=CC=CC=C1.CCOC(=O)CC#N>>[H][C@@]12C(C(N)O[C@@]1(N(C(=S)[C@H]2C(=O)C1=CC=CC=C1)C1=CC=CC=C1)C1=CC=CC=C1)C(=O)OCC

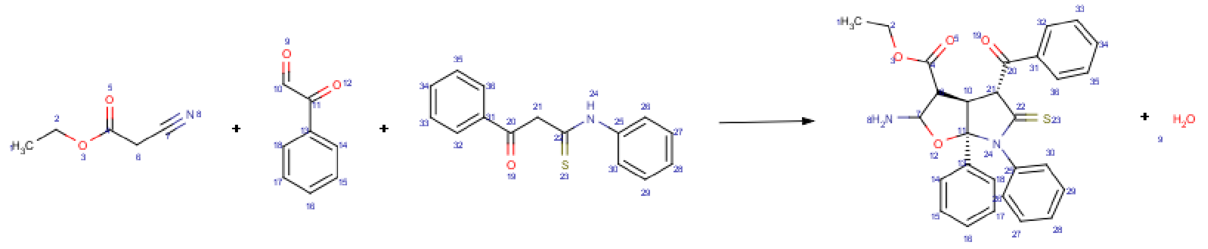

Correct mapped SMILES/SMARTS of the reaction:
[#6:1]-[#6:2]-[#8:3]-[#6:4](=[O:5])-[#6:6][C:7]#[N:8].[O:9]=[#6:10]-[#6:11](=[O:12])-[#6:13]-1=[#6:14]-[#6:15]=[#6:16]-[#6:17]=[#6:18]-1.[O:19]=[#6:20](-[#6:21]-[#6:22](=[S:23])-[#7:24]-[#6:25]-1=[#6:26]-[#6:27]=[#6:28]-[#6:29]=[#6:30]-1)-[#6:31]-1=[#6:32]-[#6:33]=[#6:34]-[#6:35]=[#6:36]-1>>[#6:1]-[#6:2]-[#8:3]-[#6:4](=[O:5])-[#6:6]-1-[#6:7](-[#7:8])-[#8:12][C@:11]2([#6@@H:10]-1-[#6@H:21](-[#6:20](=[O:19])-[#6:31]-1=[#6:32]-[#6:33]=[#6:34]-[#6:35]=[#6:36]-1)-[#6:22](=[S:23])-[#7:24]2-[#6:25]-1=[#6:30]-[#6:29]=[#6:28]-[#6:27]=[#6:26]-1)[#6:13]-1=[#6:18]-[#6:17]=[#6:16]-[#6:15]=[#6:14]-1.[#8:9]

Correctness of the mapping

MAPPET	YES
ReactionMap	NO
Marvin	NO
ChemDraw	YES
Indigo	NO

Reaction no 116

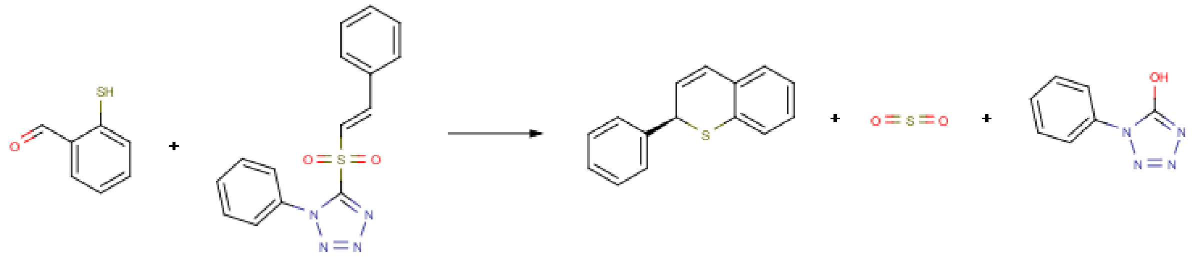

SMILES of the input:
SC1=CC=CC=C1C=O.O=S(=O)(\C=C\C1=CC=CC=C1)C1=NN=NN1C1=CC=CC=C1>>S1[C@H](C=CC2=CC=CC=C12)C1=CC=CC=C1.O=S=O.OC1=NN=NN1C1=CC=CC=C1

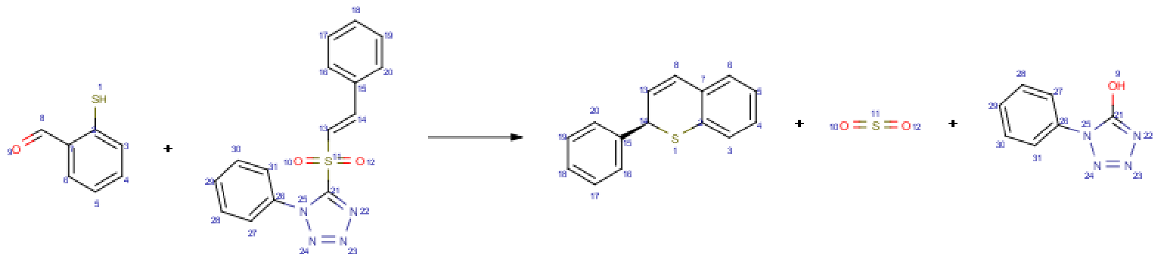

Correct mapped SMILES/SMARTS of the reaction:
[#16:1]-[#6:2]-1=[#6:3]-[#6:4]=[#6:5]-[#6:6]=[#6:7]-1-[#6:8]=[O:9].[O:10]=[S:11](=[O:12])(\[#6:13]=[#6:14]\[#6:15]-1=[#6:16]-[#6:17]=[#6:18]-[#6:19]=[#6:20]1)[#6:21]-1=[#7:22]-[#7:23]=[#7:24]-[#7:25]-1-[#6:26]-1=[#6:27]-[#6:28]=[#6:29]-[#6:30]=[#6:31]-1>>[#16:1]-1-[#6@H:14](-[#6:13]=[#6:8]-[#6:7]-2=[#6:6]-[#6:5]=[#6:4]-[#6:3]=[#6:2]-1-2)-[#6:15]-1=[#6:16]-[#6:17]=[#6:18]-[#6:19]=[#6:20]-1.[O:10]=[S:11]=[O:12].[#8:9]-[#6:21]-1=[#7:22]-[#7:23]=[#7:24]-[#7:25]-1-[#6:26]-1=[#6:31]-[#6:30]=[#6:29]-[#6:28]=[#6:27]-1

Correctness of the mapping

MAPPET	YES
ReactionMap	YES
Marvin	NO
ChemDraw	YES
Indigo	NO

Reaction no 117

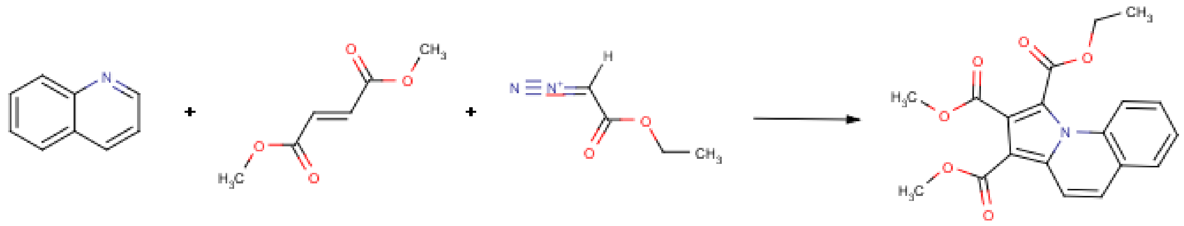

SMILES of the input:

C1=CC=C2N=CC=CC2=C1.COC(=O)\C=C\C(=O)OC.[H]C(=[N+]#N)C(=O)OCC>>CCOC(=O)C1=C(C(=O)OC)C(C(=O)OC)=C2C=CC3=C(C=CC=C3)N12

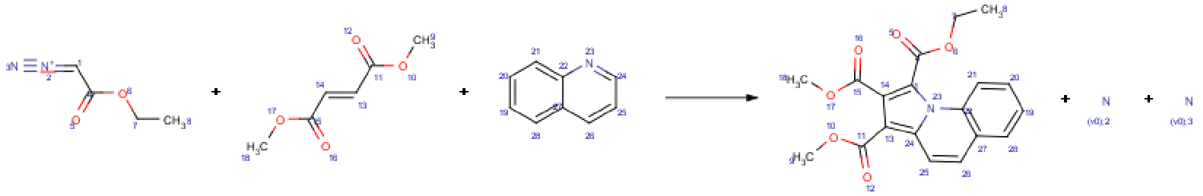

Correct mapped SMILES/SMARTS of the reaction:
[#6:8]-[#6:7]-[#8:6]-[#6:4](=[O:5])-[#6:1]=[N+:2]#[N:3].[#6:9]-[#8:10]-[#6:11](=[O:12])\[#6:13]=[#6:14]\[#6:15](=[O:16])-[#8:17]-[#6:18].[#6:19]-1=[#6:20]-[#6:21]=[#6:22]-2-[#7:23]=[#6:24]-[#6:25]=[#6:26]-[#6:27]-2=[#6:28]-1>>[#6:8]-[#6:7]-[#8:6]-[#6:4](=[O:5])-[#6:1]-1=[#6:14](-[#6:15](=[O:16])-[#8:17]-[#6:18])-[#6:13](-[#6:11](=[O:12])-[#8:10]-[#6:9])=[#6:24]-2-[#6:25]=[#6:26]-[#6:27]-3=[#6:22](-[#6:21]=[#6:20]-[#6:19]=[#6:28]-3)-[#7:23]-1-2.[#7;v0:2].[#7;v0:3]

Correctness of the mapping

MAPPET	YES
ReactionMap	NO
Marvin	YES
ChemDraw	YES
Indigo	NO

Reaction no 118

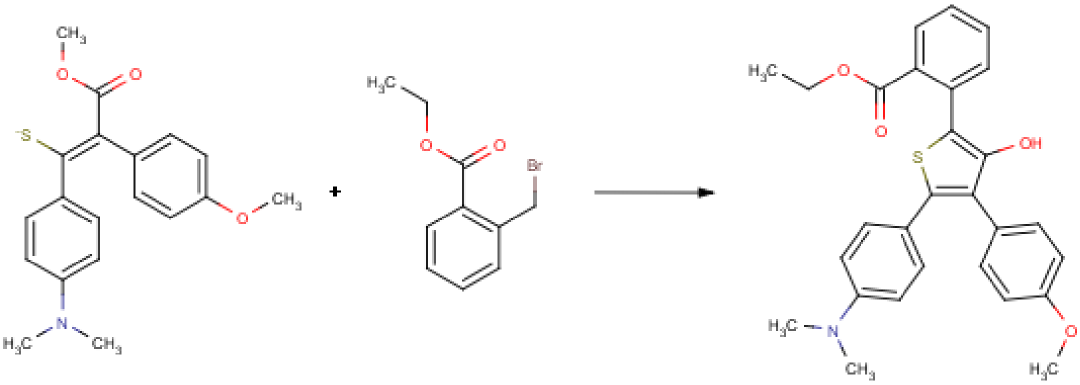

SMILES of the input:
COC(=O)C(=C(/[S-])C1=CC=C(C=C1)N(C)C)\C1=CC=C(OC)C=C1.CCOC(=O)C1=C(CBr)C=CC=C1>>CCOC(=O)C1=CC=CC=C1C1=C(O)C(=C(S1)C1=CC=C(C=C1)N(C)C)C1=CC=C(OC)C=C1

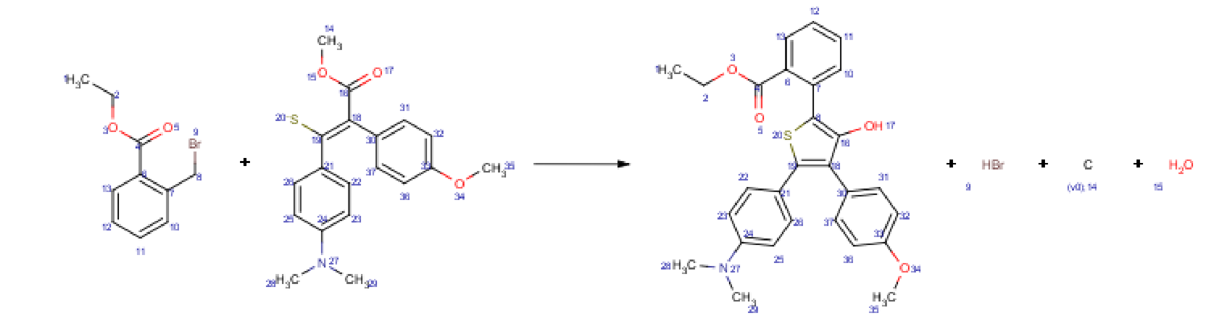

Correct mapped SMILES/SMARTS of the reaction:
[CH3:1][CH2:2][O:3][C:4](=[O:5])[C:6]1=[C:7]([CH2:8][Br:9])[CH:10]=[CH:11
][CH:12]=[CH:13]1.[CH3:14][O:15][C:16](=[O:17])[C:18](=[C:19](/[S-
:20])[C:21]1=[CH:22][CH:23]=[C:24]([CH:25]=[CH:26]1)[N:27]([CH3:28])[CH3:
29])\[C:30]1=[CH:31][CH:32]=[C:33]([O:34][CH3:35])[CH:36]=[CH:37]1>>[CH3:
1][CH2:2][O:3][C:4](=[O:5])[C:6]1=[CH:13][CH:12]=[CH:11][CH:10]=[C:7]1[C:
8]1=[C:16]([OH:17])[C:18](=[C:19]([S:20]1)[C:21]1=[CH:26][CH:25]=[C:24]([
CH:23]=[CH:22]1)[N:27]([CH3:29])[CH3:28])[C:30]1=[CH:31][CH:32]=[C:33]([O
:34][CH3:35])[CH:36]=[CH:37]1.[BrH:9].[C:14].[OH2:15]

Correctness of the mapping

MAPPET	NO
ReactionMap	NO
Marvin	NO
ChemDraw	NO
Indigo	NO

Reaction no 119

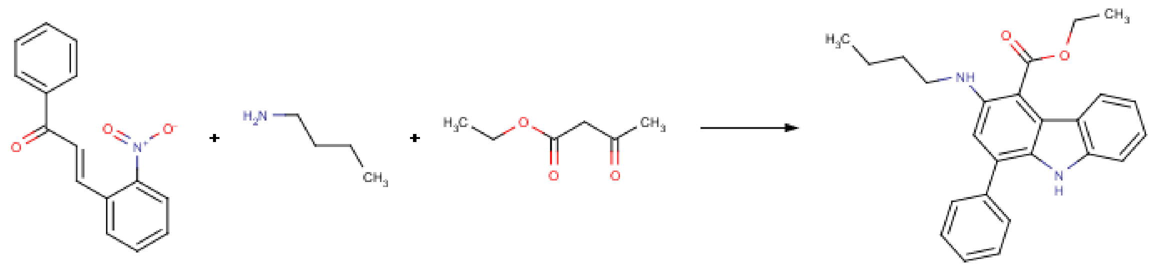

SMILES of the input:
[O-
][N+](=O)C1=CC=CC=C1\C=C\C(=O)C1=CC=CC=C1.CCCCN.CCOC(=O)CC(C)=O>>CCCCNC1=
CC(=C2NC3=C(C=CC=C3)C2=C1C(=O)OCC)C1=CC=CC=C1

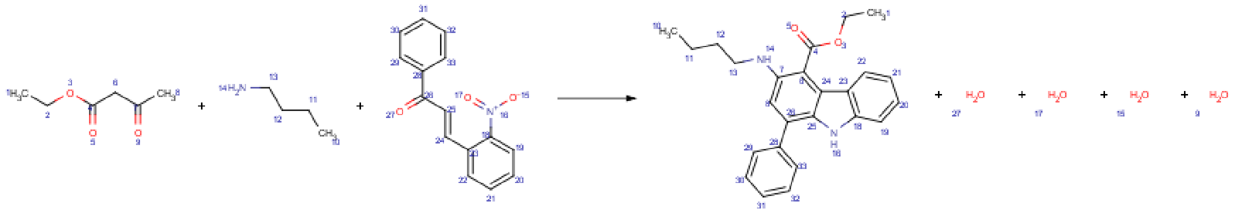

Correct mapped SMILES/SMARTS of the reaction:
[#6:1]-[#6:2]-[#8:3]-[#6:4](=[O:5])-[#6:6]-[#6:7](-[#6:8])=[O:9].[#6:10]-
[#6:11]-[#6:12]-[#6:13]-[#7:14].[#8-:15]-[#7+:16](=[O:17])-[#6:18]-

1=[#6:19]-[#6:20]=[#6:21]-[#6:22]=[#6:23]-
1\[#6:24]=[#6:25]\[#6:26](=[O:27])-[#6:28]-1=[#6:29]-[#6:30]=[#6:31]-
[#6:32]=[#6:33]-1>>[#6:10]-[#6:11]-[#6:12]-[#6:13]-[#7:14]-[#6:7]-
1=[#6:8]-[#6:26](=[#6:25]-2-[#7:16]-[#6:18]-3=[#6:23](-[#6:22]=[#6:21]-
[#6:20]=[#6:19]-3)-[#6:24]-2=[#6:6]-1-[#6:4](=[O:5])-[#8:3]-[#6:2]-
[#6:1])-[#6:28]-1=[#6:33]-[#6:32]=[#6:31]-[#6:30]=[#6:29]-
1.[#8:27].[#8:17].[#8:15].[#8:9]

Correctness of the mapping

MAPPET	YES
ReactionMap	NO
Marvin	NO
ChemDraw	YES
Indigo	YES

Reaction no 120

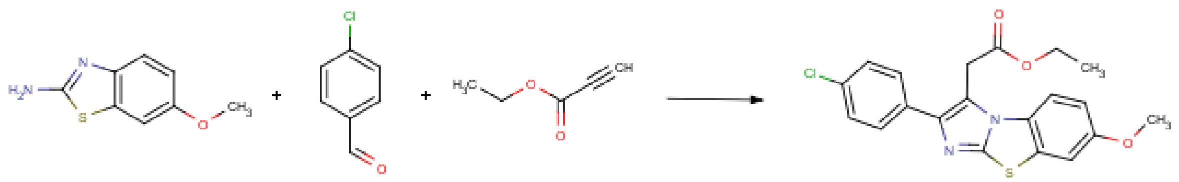

SMILES of the input:
COC1=CC2=C(C=C1)N=C(N)S2.ClC1=CC=C(C=O)C=C1.CCOC(=O)C#C>>CCOC(=O)CC1=C(N=
C2SC3=C(C=CC(OC)=C3)N12)C1=CC=C(Cl)C=C1

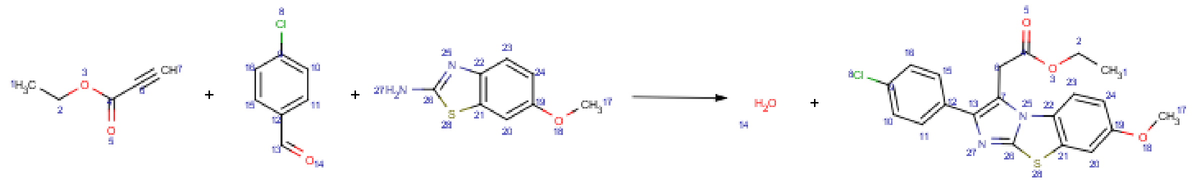

Correct mapped SMILES/SMARTS of the reaction:
[#6:1]-[#6:2]-[#8:3]-[#6:4](=[O:5])[C:6]#[C:7].[Cl:8][#6:9]-1=[#6:10]-
[#6:11]=[#6:12](-[#6:13]=[O:14])-[#6:15]=[#6:16]-1.[#6:17]-[#8:18]-
[#6:19]-1=[#6:20]-[#6:21]-2=[#6:22](-[#6:23]=[#6:24]-1)-[#7:25]=[#6:26](-
[#7:27])-[#16:28]-2>>[#8:14].[#6:1]-[#6:2]-[#8:3]-[#6:4](=[O:5])-[#6:6]-
[#6:7]-1=[#6:13](-[#7:27]=[#6:26]-2-[#16:28]-[#6:21]-3=[#6:22](-
[#6:23]=[#6:24]-[#6:19](-[#8:18]-[#6:17])=[#6:20]-3)-[#7:25]-1-2)-
[#6:12]-1=[#6:11]-[#6:10]=[#6:9]([Cl:8])-[#6:16]=[#6:15]-1

Correctness of the mapping

MAPPET	YES
ReactionMap	NO
Marvin	YES
ChemDraw	YES
Indigo	NO

Reaction no 121

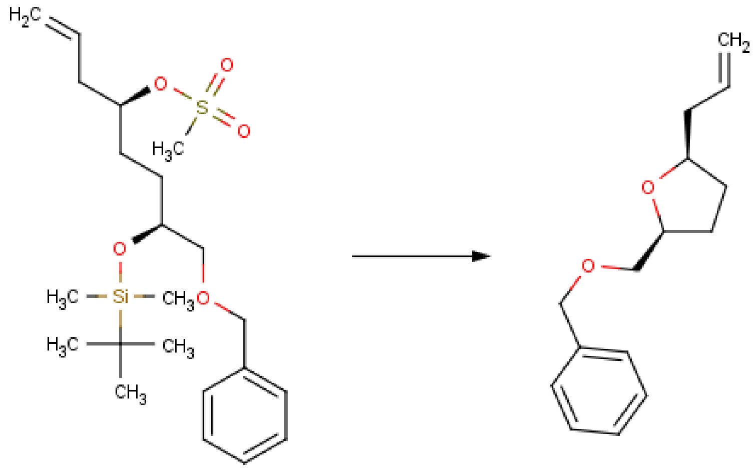

SMILES of the input:
CC(C)(C)[Si](C)(C)O[C@@H](CC[C@@H](CC=C)OS(C)(=O)=O)COCC1=CC=CC=C1>>C=CC[C@H]1CC[C@@H](COCC2=CC=CC=C2)O1

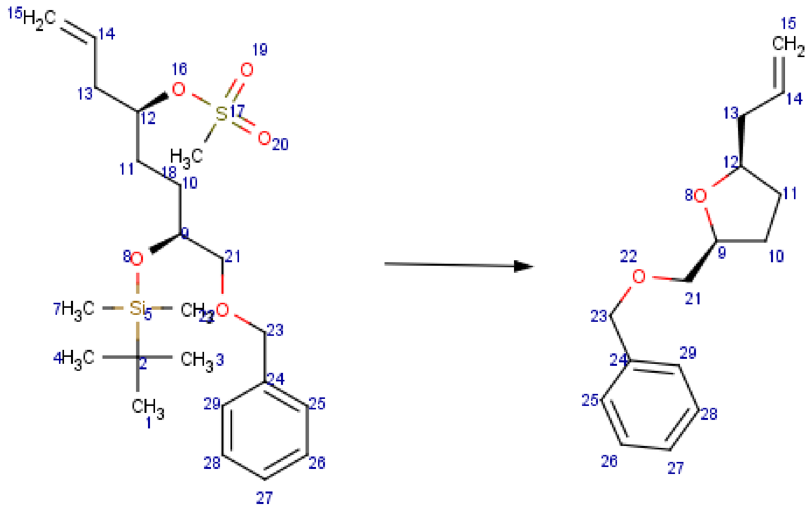

Correct mapped SMILES/SMARTS of the reaction:
[#6:1][C:2]([#6:3])([#6:4])[Si:5]([#6:6])([#6:7])[#8:8]-[#6@@H:9](-[#6:10]-[#6:11]-[#6@@H:12](-[#6:13]-[#6:14]=[#6:15])-[#8:16][S:17]([#6:18])(=[O:19])=[O:20])-[#6:21]-[#8:22]-[#6:23]-[#6:24]-1=[#6:25]-[#6:26]=[#6:27]-[#6:28]=[#6:29]-1>>[#6:15]=[#6:14]-[#6:13]-[#6@H:12]-1-[#6:11]-[#6:10]-[#6@@H:9](-[#6:21]-[#8:22]-[#6:23]-[#6:24]-2=[#6:29]-[#6:28]=[#6:27]-[#6:26]=[#6:25]-2)-[#8:8]-1

Correctness of the mapping

MAPPET	NO
ReactionMap	NO
Marvin	YES
ChemDraw	YES
Indigo	NO

Reaction no 122

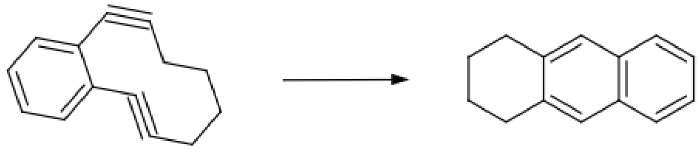

SMILES of the input:
C1CCC#CC2=CC=CC=C2C#CC1>>C1CCC2=CC3=C(C=CC=C3)C=C2C1

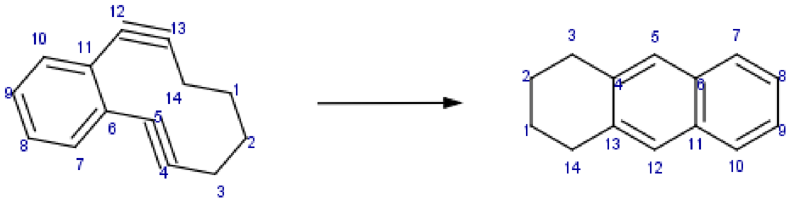

Correct mapped SMILES/SMARTS of the reaction:
[#6:1]-1-[#6:2]-[#6:3][C:4]#[C:5][#6:6]-2=[#6:7]-[#6:8]=[#6:9]-[#6:10]=[#6:11]-2[C:12]#[C:13][#6:14]-1>>[#6:2]-1-[#6:1]-[#6:14]-[#6:13]-2=[#6:12]-[#6:11]-3=[#6:6](-[#6:7]=[#6:8]-[#6:9]=[#6:10]-3)-[#6:5]=[#6:4]-2-[#6:3]-1

Correctness of the mapping

MAPPET	YES
ReactionMap	YES
Marvin	YES
ChemDraw	YES
Indigo	NO

Reaction no 123

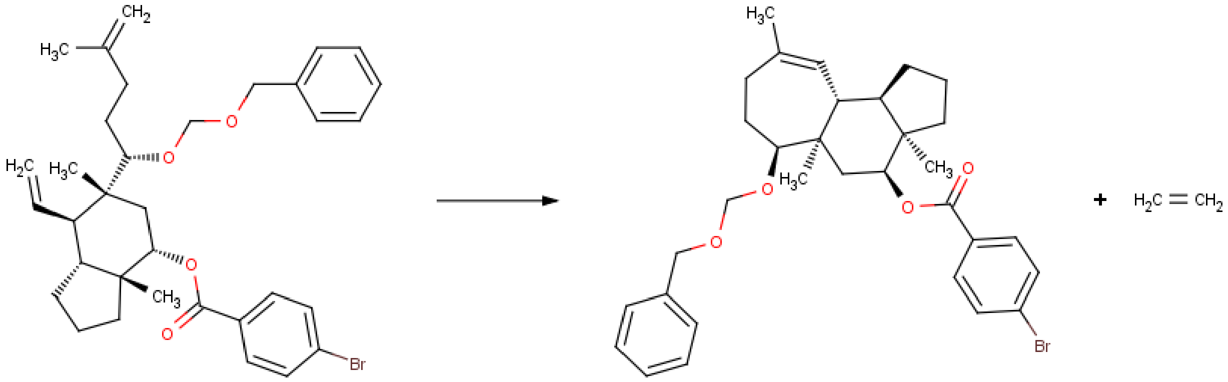

SMILES of the input:
CC(=C)CC[C@H](OCOCC1=CC=CC=C1)[C@]1(C)C[C@H](OC(=O)C2=CC=C(Br)C=C2)[C@]2(C)CCC[C@@H]2[C@H]1C=C>>CC1=C[C@@H]2[C@H]3CCC[C@@]3(C)[C@H](C[C@@]2(C)[C@H](CC1)OCOCC1=CC=CC=C1)OC(=O)C1=CC=C(Br)C=C1.C=C

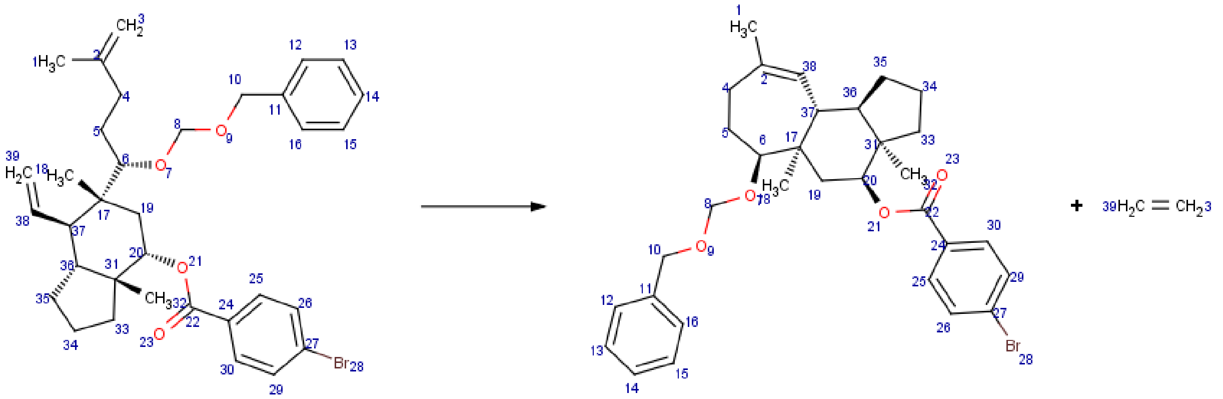

Correct mapped SMILES/SMARTS of the reaction:
[#6:1]-[#6:2](=[#6:3])-[#6:4]-[#6:5]-[#6@H:6](-[#8:7]-[#6:8]-[#8:9]-
[#6:10]-[#6:11]-1=[#6:12]-[#6:13]=[#6:14]-[#6:15]=[#6:16]-
1)[C@:17]1([#6:18])[#6:19]-[#6@H:20](-[#8:21]-[#6:22](=[O:23])-[#6:24]-
2=[#6:25]-[#6:26]=[#6:27]([Br:28])-[#6:29]=[#6:30]-
2)[C@:31]2([#6:32])[#6:33]-[#6:34]-[#6:35]-[#6@@H:36]2-[#6@H:37]1-
[#6:38]=[#6:39]>>[#6:1]-[#6:2]-1=[#6:38]-[#6@@H:37]2-[#6@H:36]3-[#6:35]-
[#6:34]-[#6:33][C@@:31]3([#6:32])[#6@H:20](-
[#6:19][C@@:17]2([#6:18])[#6@H:6](-[#6:5]-[#6:4]-1)-[#8:7]-[#6:8]-[#8:9]-
[#6:10]-[#6:11]-1=[#6:16]-[#6:15]=[#6:14]-[#6:13]=[#6:12]-1)-[#8:21]-
[#6:22](=[O:23])-[#6:24]-1=[#6:30]-[#6:29]=[#6:27]([Br:28])-
[#6:26]=[#6:25]-1.[#6:3]=[#6:39]

Correctness of the mapping

MAPPET	YES
ReactionMap	YES
Marvin	NO
ChemDraw	YES
Indigo	NO

Reaction no 124

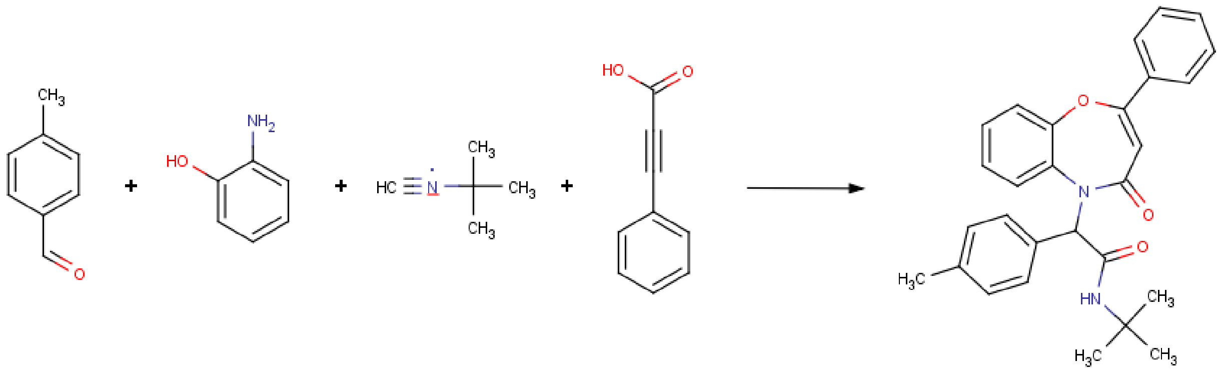

SMILES of the input:
CC1=CC=C(C=O)C=C1.NC1=CC=CC=C1O.CC(C)(C)[N]#C.OC(=O)C#CC1=CC=CC=C1>>CC1=C
C=C(C=C1)C(N1C2=CC=CC=C2OC(=CC1=O)C1=CC=CC=C1)C(=O)NC(C)(C)C

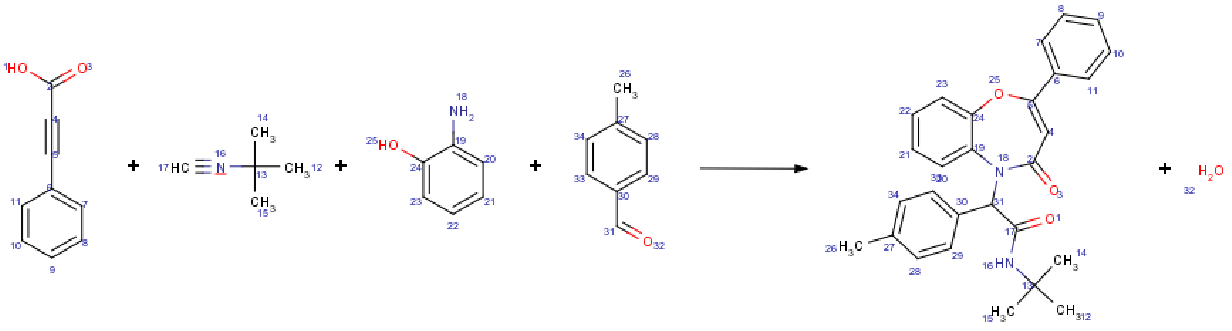

Correct mapped SMILES/SMARTS of the reaction:
[#8:1]-[#6:2](=[O:3])[C:4]#[C:5][#6:6]-1=[#6:7]-[#6:8]=[#6:9]-
[#6:10]=[#6:11]-1.[#6:12][C:13]([#6:14])([#6:15])[N:16]#[C:17].[#7:18]-
[#6:19]-1=[#6:20]-[#6:21]=[#6:22]-[#6:23]=[#6:24]-1-[#8:25].[#6:26]-
[#6:27]-1=[#6:28]-[#6:29]=[#6:30](-[#6:31]=[O:32])-[#6:33]=[#6:34]-
1>>[#6:26]-[#6:27]-1=[#6:34]-[#6:33]=[#6:30](-[#6:29]=[#6:28]-1)-
[#6:31](-[#7:18]-1-[#6:19]-2=[#6:20]-[#6:21]=[#6:22]-[#6:23]=[#6:24]-2-
[#8:25]-[#6:5](=[#6:4]-[#6:2]-1=[O:3])-[#6:6]-1=[#6:7]-[#6:8]=[#6:9]-
[#6:10]=[#6:11]-1)-[#6:17](=[O:1])-
[#7:16][C:13]([#6:15])([#6:14])[#6:12].[#8:32]

Correctness of the mapping

MAPPET	YES
ReactionMap	NO
Marvin	NO
ChemDraw	NO
Indigo	NO

Reaction no 125

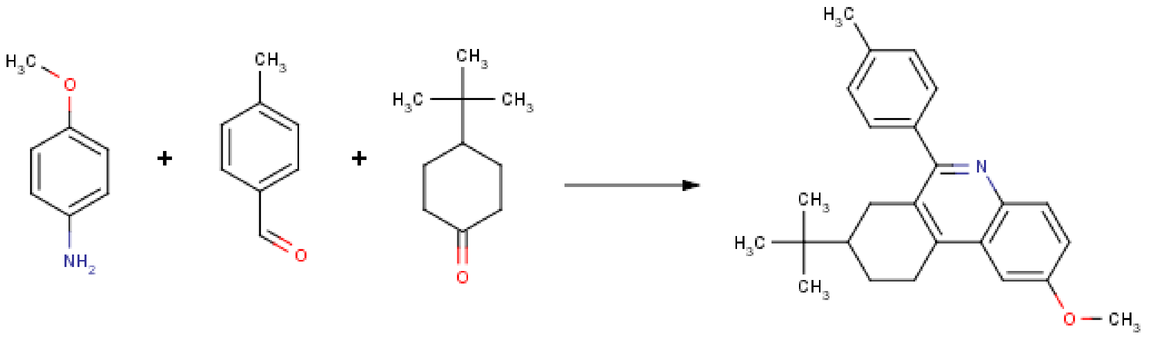

SMILES of the input:
COC1=CC=C(N)C=C1.CC1=CC=C(C=O)C=C1.CC(C)(C)C1CCC(=O)CC1>>COC1=CC2=C(C=C1)
N=C(C1=CC=C(C)C=C1)C1=C2CCC(C1)C(C)(C)C

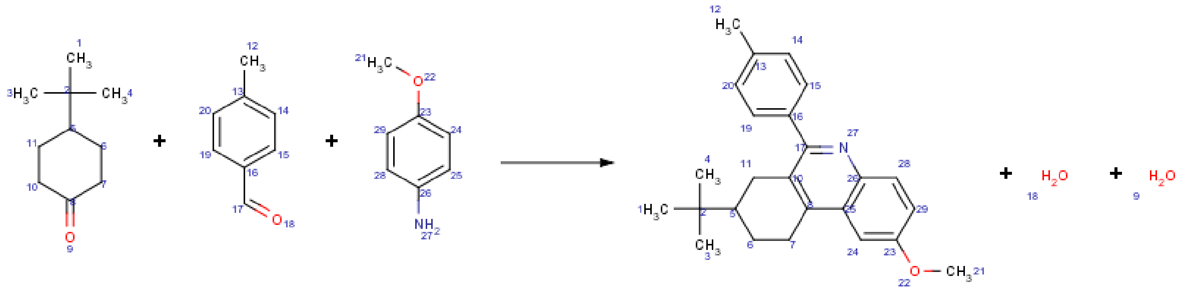

Correct mapped SMILES/SMARTS of the reaction:
[#6:1][C:2]([#6:3])([#6:4])[#6:5]-1-[#6:6]-[#6:7]-[#6:8](=[O:9])-[#6:10]-
[#6:11]-1.[#6:12]-[#6:13]-1=[#6:14]-[#6:15]=[#6:16](-[#6:17]=[O:18])-
[#6:19]=[#6:20]-1.[#6:21]-[#8:22]-[#6:23]-1=[#6:24]-[#6:25]=[#6:26](-
[#7:27])-[#6:28]=[#6:29]-1>>[#6:21]-[#8:22]-[#6:23]-1=[#6:24]-[#6:25]-
2=[#6:26](-[#6:28]=[#6:29]-1)-[#7:27]=[#6:17](-[#6:16]-1=[#6:19]-
[#6:20]=[#6:13](-[#6:12])-[#6:14]=[#6:15]-1)-[#6:10]-1=[#6:8]-2-[#6:7]-
[#6:6]-[#6:5](-[#6:11]-1)[C:2]([#6:1])([#6:4])[#6:3].[#8:18].[#8:9]

Correctness of the mapping

MAPPET	YES
ReactionMap	NO
Marvin	YES
ChemDraw	YES
Indigo	YES

Reaction no 126

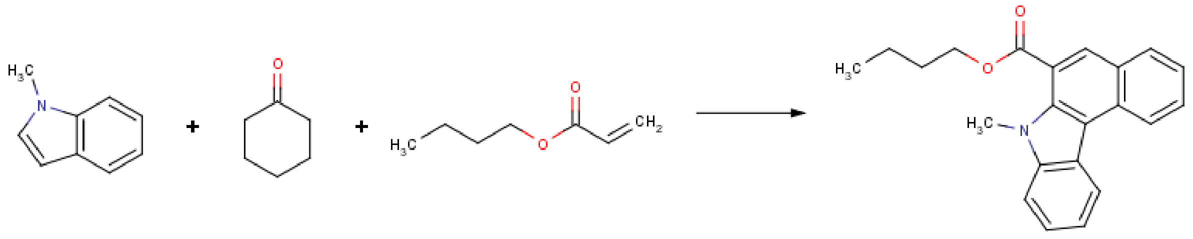

SMILES of the input:
CN1C=CC2=C1C=CC=C2.O=C1CCCCC1.CCCCOC(=O)C=C>>CCCCOC(=O)C1=CC2=C(C=CC=C2)C
2=C1N(C)C1=C2C=CC=C1

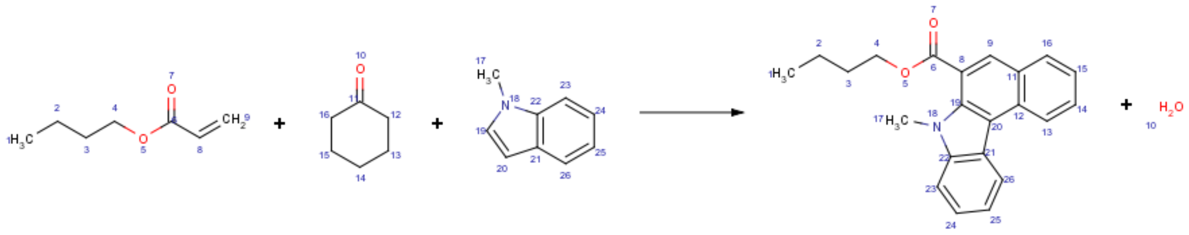

Correct mapped SMILES/SMARTS of the reaction:
[#6:1]-[#6:2]-[#6:3]-[#6:4]-[#8:5]-[#6:6](=[O:7])-
[#6:8]=[#6:9].[O:10]=[#6:11]-1-[#6:12]-[#6:13]-[#6:14]-[#6:15]-[#6:16]-
1.[#6:17]-[#7:18]-1-[#6:19]=[#6:20]-[#6:21]-2=[#6:22]-1-[#6:23]=[#6:24]-
[#6:25]=[#6:26]-2>>[#6:1]-[#6:2]-[#6:3]-[#6:4]-[#8:5]-[#6:6](=[O:7])-

[#6:8]-1=[#6:9]-[#6:11]-2=[#6:12](-[#6:13]=[#6:14]-[#6:15]=[#6:16]-2)-[#6:20]-2=[#6:19]-1-[#7:18](-[#6:17])-[#6:22]-1=[#6:21]-2-[#6:26]=[#6:25]-[#6:24]=[#6:23]-1.[#8:10]

Correctness of the mapping

MAPPET	YES
ReactionMap	NO
Marvin	YES
ChemDraw	YES
Indigo	NO

Reaction no 127

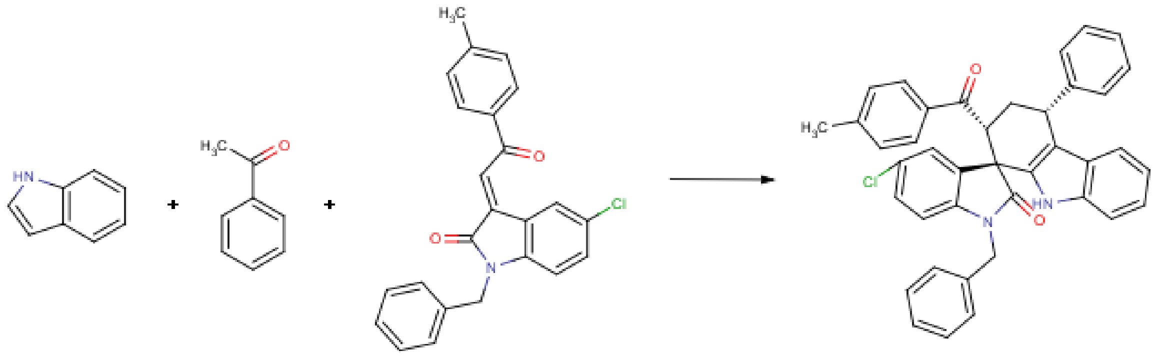

SMILES of the input:
N1C=CC2=C1C=CC=C2.CC(=O)C1=CC=CC=C1.CC1=CC=C(C=C1)C(=O)\C=C1\C(=O)N(CC2=CC=CC=C2)C2=C1C=C(Cl)C=C2>>CC1=CC=C(C=C1)C(=O)[C@@H]1C[C@@H](C2=C(NC3=C2C=CC=C3)[C@]11C(=O)N(CC2=CC=CC=C2)C2=CC=C(Cl)C=C12)C1=CC=CC=C1

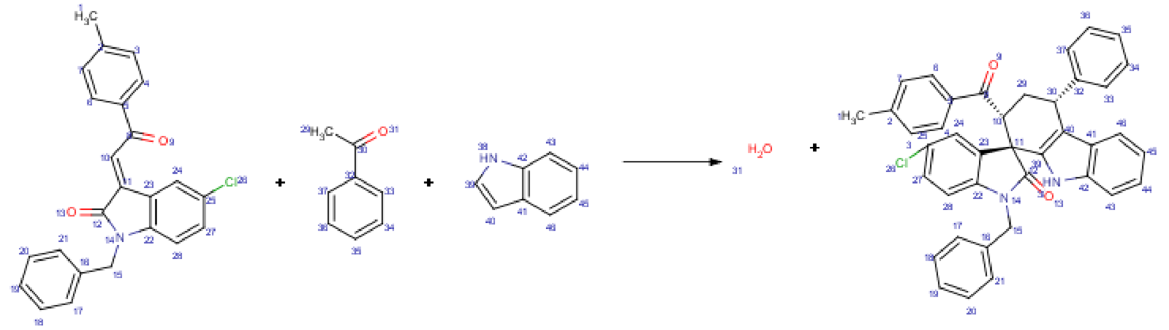

Correct mapped SMILES/SMARTS of the reaction:
[CH3:1][C:2]1=[CH:3][CH:4]=[C:5]([CH:6]=[CH:7]1)[C:8](=[O:9])\[CH:10]=[C:11]1\[C:12](=[O:13])[N:14]([CH2:15][C:16]2=[CH:17][CH:18]=[CH:19][CH:20]=[CH:21]2)[C:22]2=[C:23]1[CH:24]=[C:25]([Cl:26])[CH:27]=[CH:28]2.[CH3:29][C:30](=[O:31])[C:32]1=[CH:33][CH:34]=[CH:35][CH:36]=[CH:37]1.[NH:38]1[CH:39]=[CH:40][C:41]2=[C:42]1[CH:43]=[CH:44][CH:45]=[CH:46]2>>[OH2:31].[CH3:1][C:2]1=[CH:7][CH:6]=[C:5]([CH:4]=[CH:3]1)[C:8](=[O:9])[C@@H:10]1[CH2:29][C@@H:30]([C:40]2=[C:39]([NH:38][C:42]3=[C:41]2[CH:46]=[CH:45][CH:44]=[CH:43]3)[C@:11]11[C:12](=[O:13])[N:14]([CH2:15][C:16]2=[CH:21][CH:20]=[CH:19][CH:18]=[CH:17]2)[C:22]2=[CH:28][CH:27]=[C:25]([Cl:26])[CH:24]=[C:23]12)[C:32]1=[CH:37][CH:36]=[CH:35][CH:34]=[CH:33]1

Correctness of the mapping

MAPPET	YES

```
ReactionMap      NO
Marvin           YES
ChemDraw         YES
Indigo           NO
```

Reaction no 128

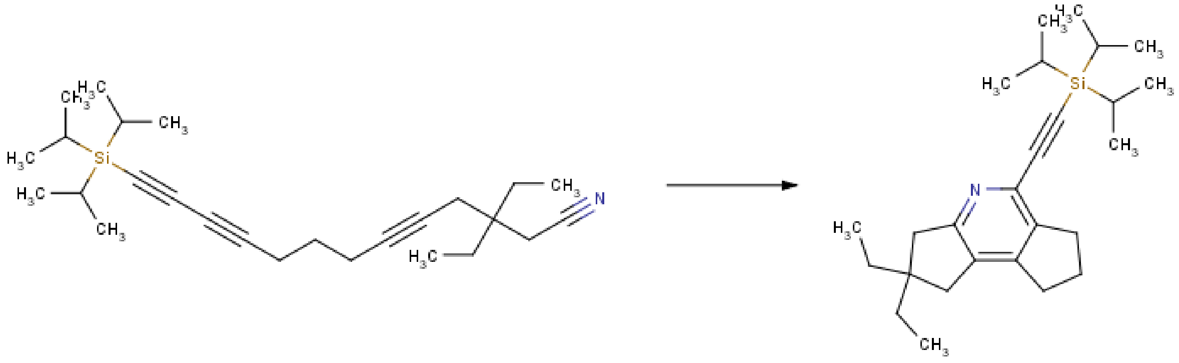

SMILES of the input:
CCC(CC)(CC#N)CC#CCCCC#CC#C[Si](C(C)C)(C(C)C)C(C)C>>CCC1(CC)CC2=NC(C#C[Si](C(C)C)(C(C)C)C(C)C)=C3CCCC3=C2C1

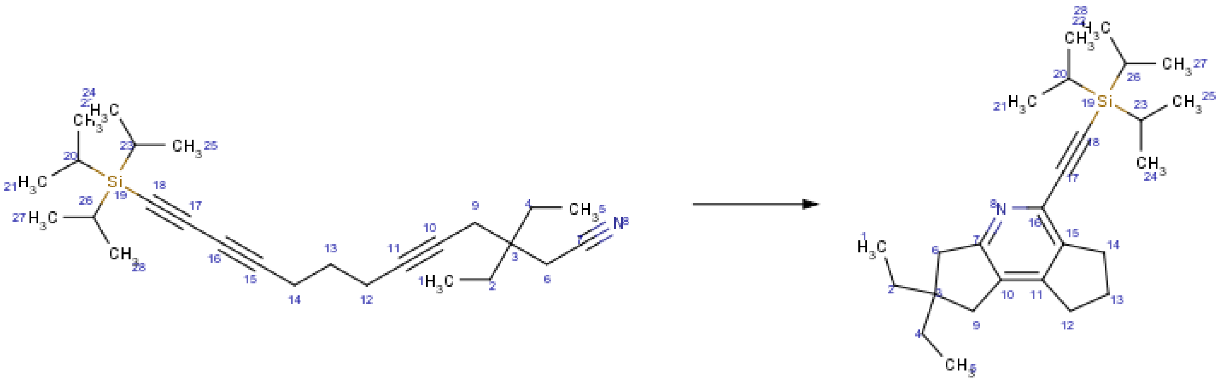

Correct mapped SMILES/SMARTS of the reaction:
[#6:1]-[#6:2][C:3]([#6:4]-[#6:5])([#6:6][C:7]#[N:8])[#6:9][C:10]#[C:11][#6:12]-[#6:13]-[#6:14][C:15]#[C:16][C:17]#[C:18][Si:19]([#6:20](-[#6:21])-[#6:22])([#6:23](-[#6:24])-[#6:25])[#6:26](-[#6:27])-[#6:28]>>[#6:1]-[#6:2][C:3]1([#6:4]-[#6:5])[#6:6]-[#6:7]-2=[#7:8]-[#6:16]([C:17]#[C:18][Si:19]([#6:26](-[#6:28])-[#6:27])([#6:23](-[#6:25])-[#6:24])[#6:20](-[#6:22])-[#6:21])=[#6:15]-3-[#6:14]-[#6:13]-[#6:12]-[#6:11]-3=[#6:10]-2-[#6:9]1

Correctness of the mapping

```
MAPPET           YES
ReactionMap      YES
Marvin           NO
ChemDraw         YES
Indigo           YES
```

Reaction no 129

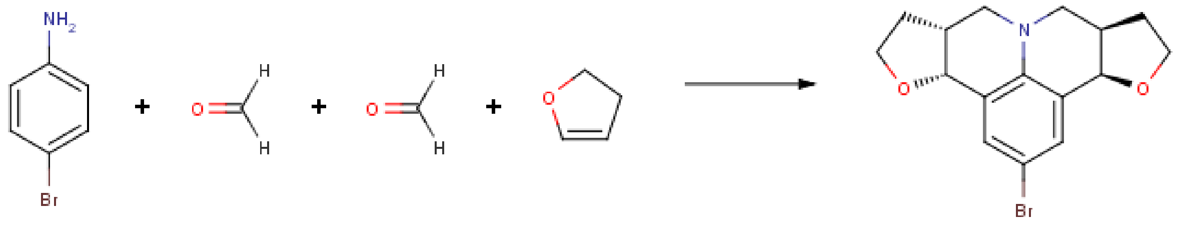

SMILES of the input:
NC1=CC=C(Br)C=C1.[H]C([H])=O.[H]C([H])=O.C1CC=CO1>>BrC1=CC2=C3N(C[C@H]4CCO[C@H]4C3=C1)C[C@H]1CCO[C@@H]21

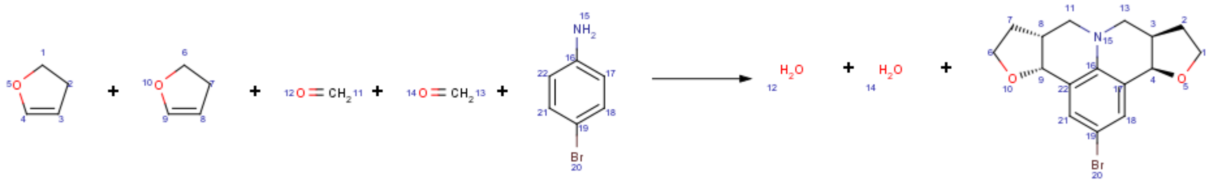

Correct mapped SMILES/SMARTS of the reaction:
[#6:1]-1-[#6:2]-[#6:3]=[#6:4]-[#8:5]-1.[#6:6]-1-[#6:7]-[#6:8]=[#6:9]-[#8:10]-1.[#6:11]=[O:12].[#6:13]=[O:14].[#7:15]-[#6:16]-1=[#6:17]-[#6:18]=[#6:19]([Br:20])-[#6:21]=[#6:22]-1>>[#8:12].[#8:14].[Br:20][#6:19]-1=[#6:21]-[#6:22]-2=[#6:16]-3-[#7:15](-[#6:13]-[#6@H:3]-4-[#6:2]-[#6:1]-[#8:5]-[#6@H:4]-4-[#6:17]-3=[#6:18]-1)-[#6:11]-[#6@H:8]-1-[#6:7]-[#6:6]-[#8:10]-[#6@@H:9]-2-1

Correctness of the mapping

MAPPET	YES
ReactionMap	NO
Marvin	NO
ChemDraw	NO
Indigo	NO

Reaction no 130

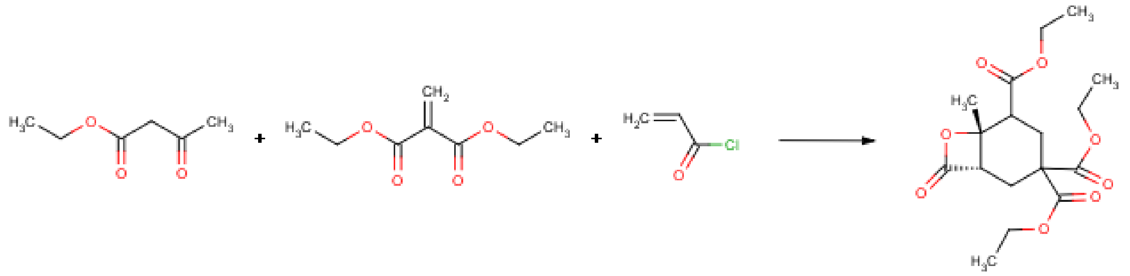

SMILES of the input:
CCOC(=O)CC(C)=O.CCOC(=O)C(=C)C(=O)OCC.ClC(=O)C=C>>CCOC(=O)C1CC(C[C@@H]2C(=O)O[C@]12C)(C(=O)OCC)C(=O)OCC

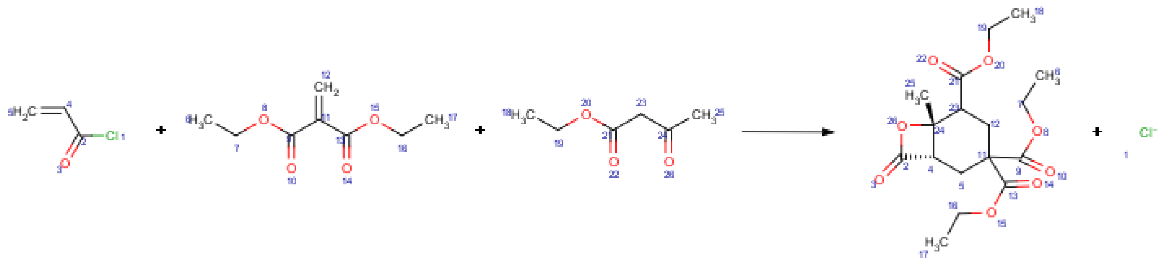

Correct mapped SMILES/SMARTS of the reaction:
[Cl:1][C:2](=[O:3])[CH:4]=[CH2:5].[CH3:6][CH2:7][O:8][C:9](=[O:10])[C:11]
(=[CH2:12])[C:13](=[O:14])[O:15][CH2:16][CH3:17].[CH3:18][CH2:19][O:20][C
:21](=[O:22])[CH2:23][C:24]([CH3:25])=[O:26]>>[CH3:18][CH2:19][O:20][C:21
](=[O:22])[CH:23]1[CH2:12][C:11]([CH2:5][C@@H:4]2[C:2](=[O:3])[O:26][C@:2
4]12[CH3:25])([C:13](=[O:14])[O:15][CH2:16][CH3:17])[C:9](=[O:10])[O:8][C
H2:7][CH3:6].[Cl-:1]

Correctness of the mapping

MAPPET	NO
ReactionMap	NO
Marvin	YES
ChemDraw	NO
Indigo	YES

Reaction no 131

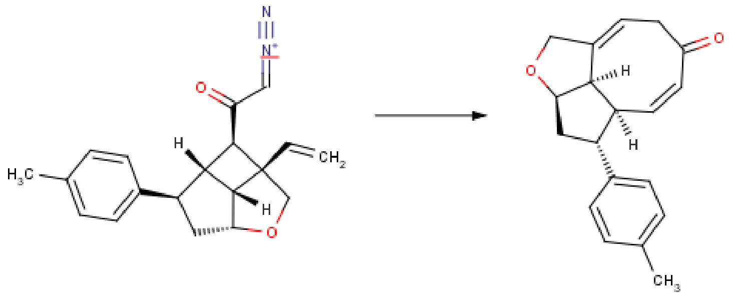

SMILES of the input:
[H][C@]12[C@@H](C(=O)C=[N+]#N)[C@@]3(CO[C@H](C[C@H]1C1=CC=C(C)C=C1)[C@]23
[H])C=C>>[H][C@]12\C=C/C(=O)C\C=C3\CO[C@H](C[C@H]1C1=CC=C(C)C=C1)[C@]23[H
]

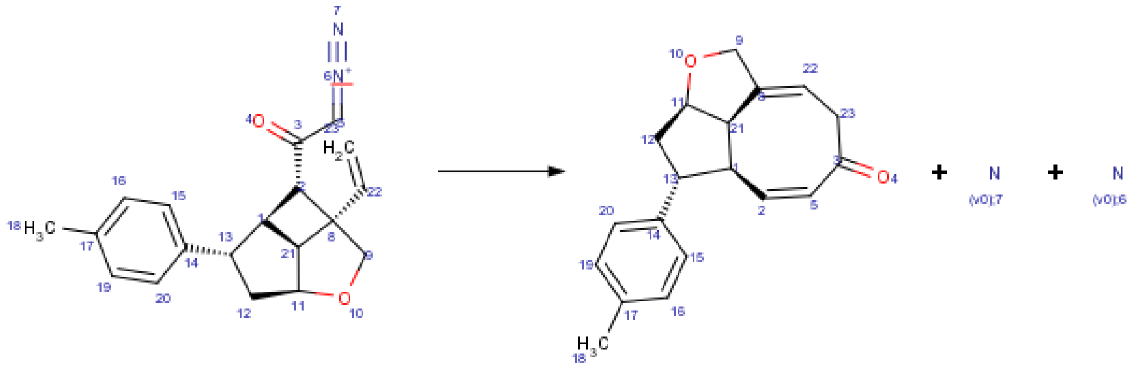

Correct mapped SMILES/SMARTS of the reaction:

[#6:18]-[#6:17]-1=[#6:16]-[#6:15]=[#6:14](-[#6:20]=[#6:19]-1)-[#6@H:13]-1-[#6:12]-[#6@@H:11]-2-[#8:10]-[#6:9][C@@:8]3([#6:22]=[#6:23])[#6@@H:21]-2-[#6@@H:1]-1-[#6@@H:2]3-[#6:3](=[O:4])-[#6:5]=[N+:6]#[N:7]>>[#6:18]-[#6:17]-1=[#6:19]-[#6:20]=[#6:14](-[#6:15]=[#6:16]-1)-[#6@@H:13]1-[#6:12]-[#6@H:11]-2-[#8:10]-[#6:9]\[#6:8]-3=[#6:22]\[#6:23]-[#6:3](=[O:4])\[#6:5]=[#6:2]/[#6@@H:1]-1-[#6@@H:21]-23.[#7;v0:7].[#7;v0:6]

Correctness of the mapping

MAPPET	YES
ReactionMap	NO
Marvin	YES
ChemDraw	NO
Indigo	NO

Reaction no 132

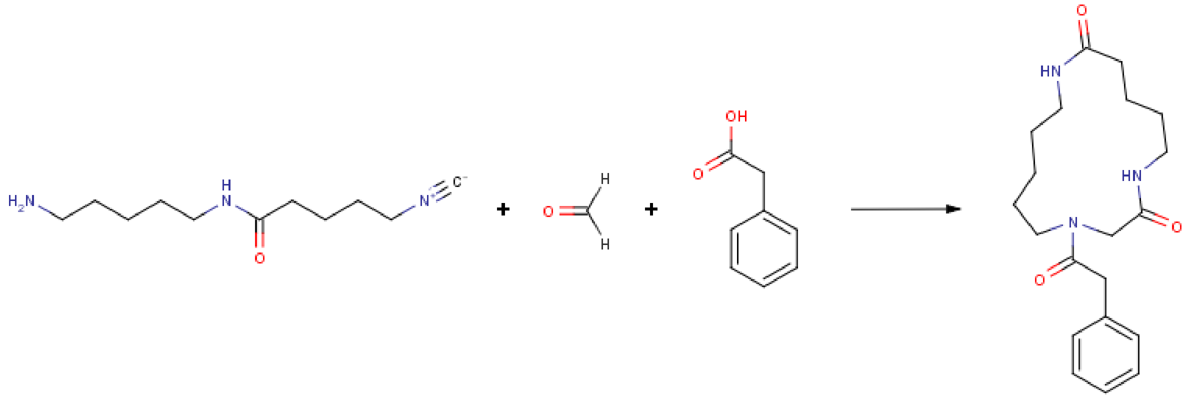

SMILES of the input:
NCCCCCNC(=O)CCCC[N+]#[C-].[H]C([H])=O.OC(=O)CC1=CC=CC=C1>>O=C(CC1=CC=CC=C1)N1CCCCCNC(=O)CCCCNC(=O)C1

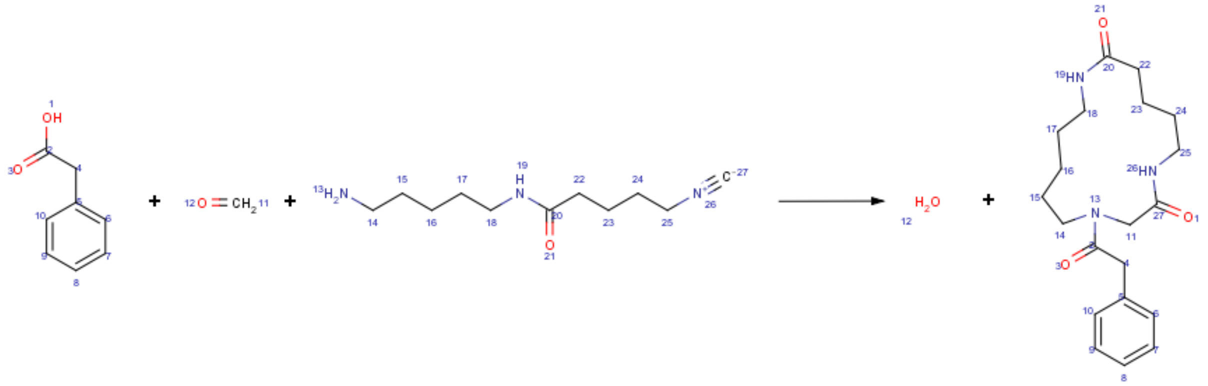

Correct mapped SMILES/SMARTS of the reaction:
[#8:1]-[#6:2](=[O:3])-[#6:4]-[#6:5]-1=[#6:6]-[#6:7]=[#6:8]-[#6:9]=[#6:10]-1.[#6:11]=[O:12].[#7:13]-[#6:14]-[#6:15]-[#6:16]-[#6:17]-[#6:18]-[#7:19]-[#6:20](=[O:21])-[#6:22]-[#6:23]-[#6:24]-[#6:25][N+:26]#[C-:27]>>[#8:12].[O:3]=[#6:2](-[#6:4]-[#6:5]-1=[#6:6]-[#6:7]=[#6:8]-[#6:9]=[#6:10]-1)-[#7:13]-1-[#6:14]-[#6:15]-[#6:16]-[#6:17]-[#6:18]-[#7:19]-[#6:20](=[O:21])-[#6:22]-[#6:23]-[#6:24]-[#6:25]-[#7:26]-[#6:27](=[O:1])-[#6:11]-1

Correctness of the mapping

MAPPET	YES

ReactionMap NO
Marvin NO
ChemDraw NO
Indigo NO

Reaction no 133

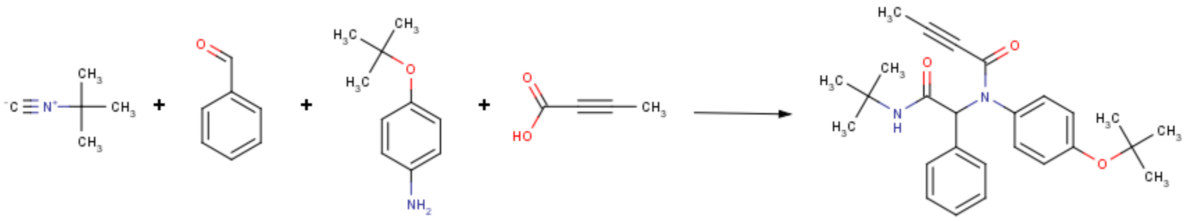

SMILES of the input:
CC(C)(C)[N+]#[C-
].O=CC1=CC=CC=C1.CC(C)(C)OC1=CC=C(N)C=C1.CC#CC(O)=O>>CC#CC(=O)N(C(C(=O)NC
(C)(C)C)C1=CC=CC=C1)C1=CC=C(OC(C)(C)C)C=C1

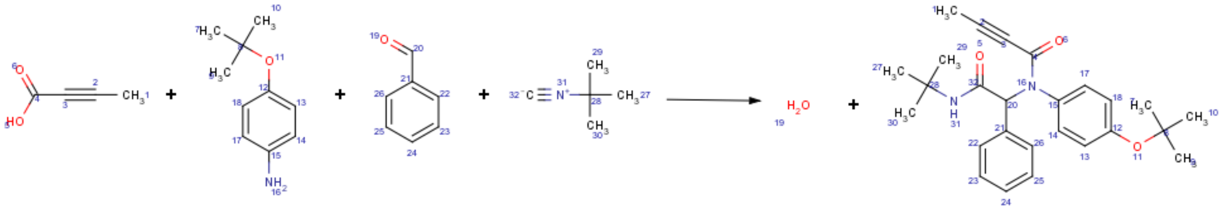

Correct mapped SMILES/SMARTS of the reaction:
[#6:1][C:2]#[C:3][#6:4](-
[#8:5])=[O:6].[#6:7][C:8]([#6:9])([#6:10])[#8:11]-[#6:12]-1=[#6:13]-
[#6:14]=[#6:15](-[#7:16])-[#6:17]=[#6:18]-1.[O:19]=[#6:20]-[#6:21]-
1=[#6:22]-[#6:23]=[#6:24]-[#6:25]=[#6:26]-
1.[#6:27][C:28]([#6:29])([#6:30])[N+:31]#[C-
:32]>>[#8:19].[#6:1][C:2]#[C:3][#6:4](=[O:6])-[#7:16](-[#6:20](-
[#6:32](=[O:5])-[#7:31][C:28]([#6:27])([#6:30])[#6:29])-[#6:21]-
1=[#6:26]-[#6:25]=[#6:24]-[#6:23]=[#6:22]-1)-[#6:15]-1=[#6:17]-
[#6:18]=[#6:12](-[#8:11][C:8]([#6:10])([#6:9])[#6:7])-[#6:13]=[#6:14]-1

Correctness of the mapping
MAPPET YES
ReactionMap NO
Marvin NO
ChemDraw NO
Indigo NO

Reaction no 134

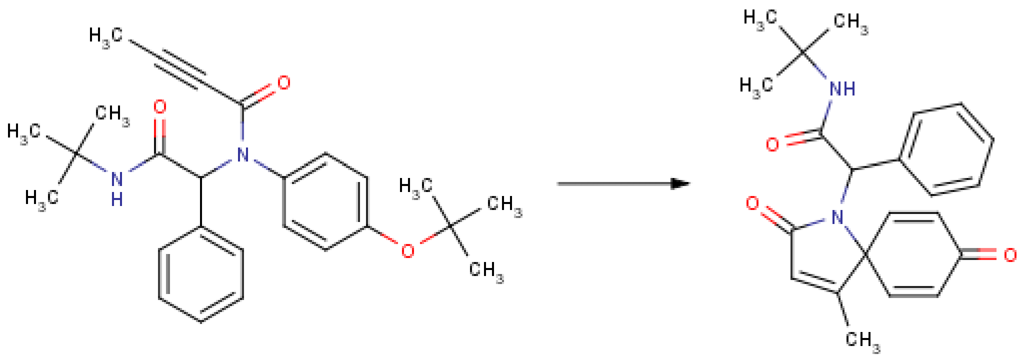

SMILES of the input:
CC#CC(=O)N(C(C(=O)NC(C)(C)C)C1=CC=CC=C1)C1=CC=C(OC(C)(C)C)C=C1>>CC1=CC(=O)N(C(C(=O)NC(C)(C)C)C2=CC=CC=C2)C11C=CC(=O)C=C1

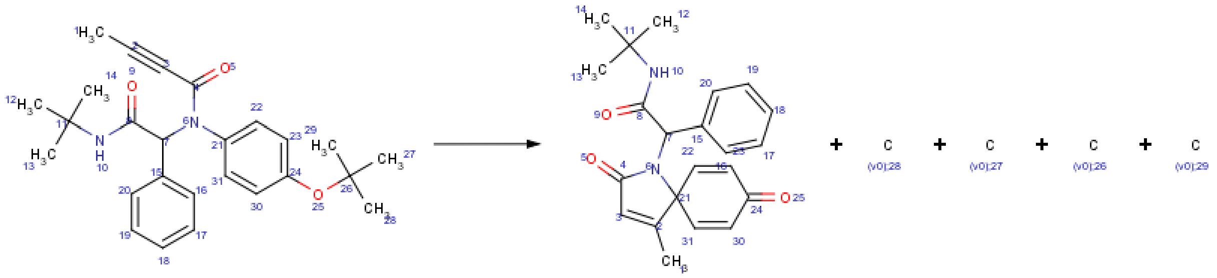

Correct mapped SMILES/SMARTS of the reaction:
[#6:1][C:2]#[C:3][#6:4](=[O:5])-[#7:6](-[#6:7](-[#6:8](=[O:9])-[#7:10][C:11]([#6:12])([#6:13])[#6:14])-[#6:15]-1=[#6:16]-[#6:17]=[#6:18]-[#6:19]=[#6:20]-1)-[#6:21]-1=[#6:22]-[#6:23]=[#6:24](-[#8:25][C:26]([#6:27])([#6:28])[#6:29])-[#6:30]=[#6:31]-1>>[#6:1]-[#6:2]1=[#6:3]-[#6:4](=[O:5])-[#7:6](-[#6:7](-[#6:8](=[O:9])-[#7:10][C:11]([#6:14])([#6:13])[#6:12])-[#6:15]-2=[#6:20]-[#6:19]=[#6:18]-[#6:17]=[#6:16]-2)[C:21]11[#6:22]=[#6:23]-[#6:24](=[O:25])-[#6:30]=[#6:31]1.[#6;v0:28].[#6;v0:27].[#6;v0:26].[#6;v0:29]

Correctness of the mapping

MAPPET	YES
ReactionMap	NO
Marvin	YES
ChemDraw	YES
Indigo	YES

Reaction no 135

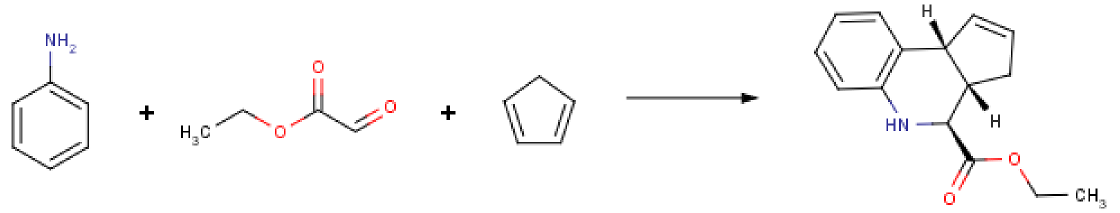

SMILES of the input:
NC1=CC=CC=C1.CCOC(=O)C=O.C1C=CC=C1>>[H][C@]12CC=C[C@@]1([H])C1=C(N[C@@H]2C(=O)OCC)C=CC=C1

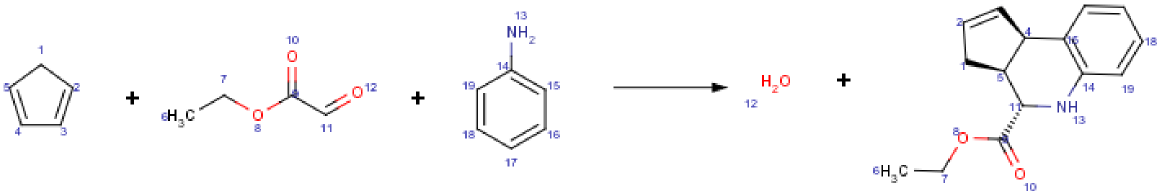

Correct mapped SMILES/SMARTS of the reaction:
[#6:1]-1-[#6:2]=[#6:3]-[#6:4]=[#6:5]-1.[#6:6]-[#6:7]-[#8:8]-[#6:9](=[O:10])-[#6:11]=[O:12].[#7:13]-[#6:14]-1=[#6:15]-[#6:16]=[#6:17]-[#6:18]=[#6:19]-1>>[#8:12].[#6:6]-[#6:7]-[#8:8]-[#6:9](=[O:10])-[#6@H:11]-1-[#7:13]-[#6:14]-2=[#6:15](-[#6:16]=[#6:17]-[#6:18]=[#6:19]-2)-[#6@@H:4]-2-[#6:3]=[#6:2]-[#6:1]-[#6@H:5]-1-2

Correctness of the mapping

MAPPET	YES
ReactionMap	NO
Marvin	YES
ChemDraw	YES
Indigo	NO

Reaction no 136

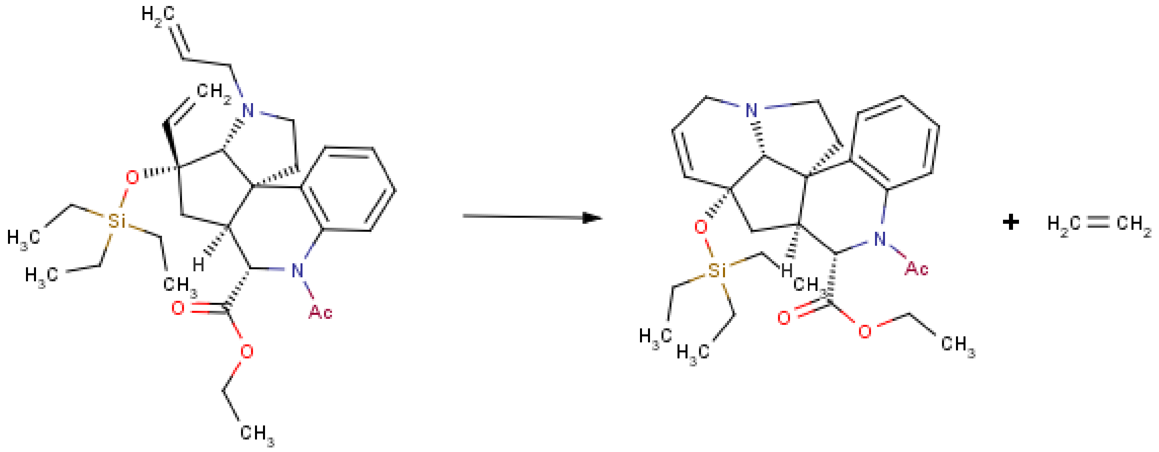

SMILES of the input:
[H][C@]12C[C@@](O[Si](CC)(CC)CC)(C=C)[C@@H]3N(CC=C)CC[C@]13C1=C(C=CC=C1)N([Ac])[C@@H]2C(=O)OCC>>[H][C@]12C[C@@]3(O[Si](CC)(CC)CC)C=CCN4CC[C@]1([C@H]34)C1=C(C=CC=C1)N([Ac])[C@@H]2C(=O)OCC.C=C

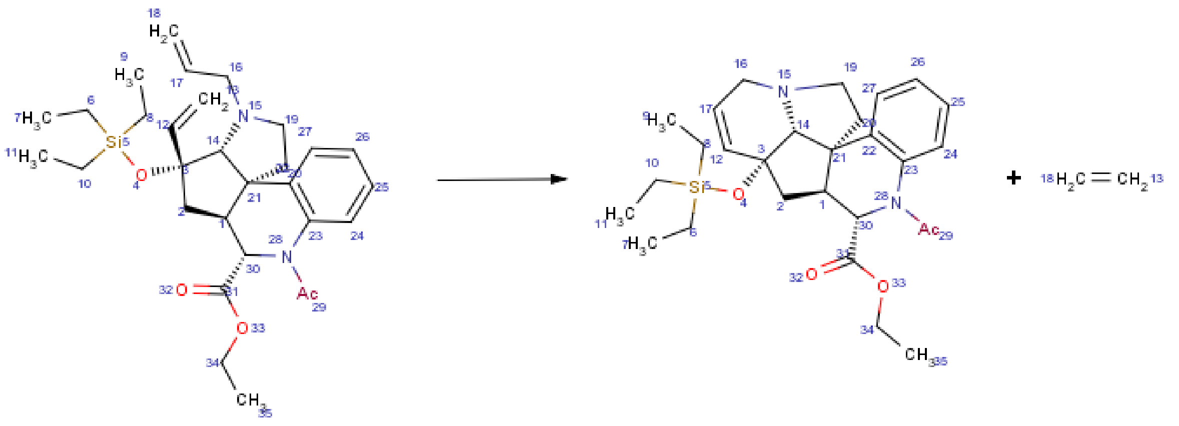

Correct mapped SMILES/SMARTS of the reaction:
[#6:35]-[#6:34]-[#8:33]-[#6:31](=[O:32])-[#6@@H:30]-1-[#6@H:1]2-[#6:2][C@@:3]([#8:4][Si:5]([#6:6]-[#6:7])([#6:8]-[#6:9])[#6:10]-[#6:11])([#6:12]=[#6:13])[#6@@H:14]3-[#7:15](-[#6:16]-[#6:17]=[#6:18])-[#6:19]-[#6:20][C@:21]23[#6:22]-2=[#6:23](-[#6:24]=[#6:25]-[#6:26]=[#6:27]-2)-[#7:28]-1[Ac:29]>>[#6:35]-[#6:34]-[#8:33]-[#6:31](=[O:32])-[#6@@H:30]-1-[#6@H:1]2-[#6:2][C@@:3]3([#8:4][Si:5]([#6:10]-[#6:11])([#6:8]-[#6:9])[#6:6]-[#6:7])[#6:12]=[#6:17]-[#6:16]-[#7:15]-4-[#6:19]-[#6:20][C@:21]2([#6@H:14]3-4)[#6:22]-2=[#6:23](-[#6:24]=[#6:25]-[#6:26]=[#6:27]-2)-[#7:28]-1[Ac:29].[#6:13]=[#6:18]

Correctness of the mapping

MAPPET	YES
ReactionMap	NO
Marvin	NO
ChemDraw	YES
Indigo	NO

Reaction no 137

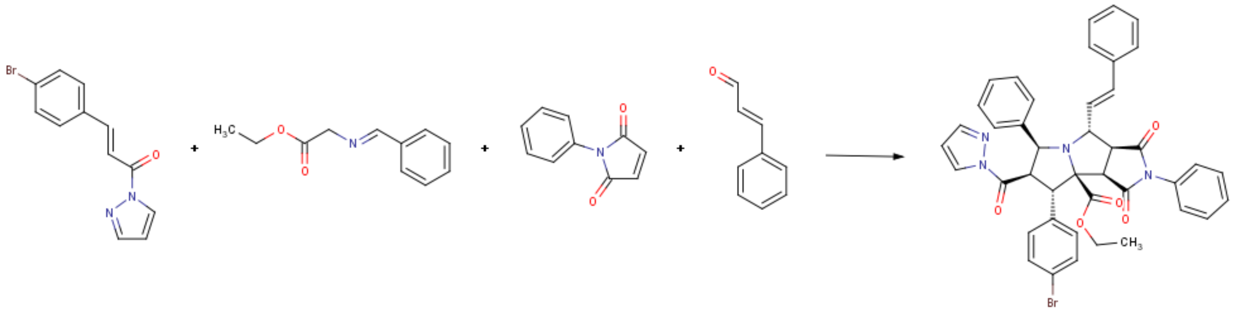

SMILES of the input:
BrC1=CC=C(\C=C\C(=O)N2C=CC=N2)C=C1.CCOC(=O)C\N=C\C1=CC=CC=C1.O=C1C=CC(=O)N1C1=CC=CC=C1.O=C\C=C\C1=CC=CC=C1>>CCOC(=O)[C@]12[C@H]3[C@@H]([C@@H](\C=C\C4=CC=CC=C4)N1[C@@H]([C@@H]([C@H]2C1=CC=C(Br)C=C1)C(=O)N1C=CC=N1)C1=CC=CC=C1)C(=O)N(C3=O)C1=CC=CC=C1

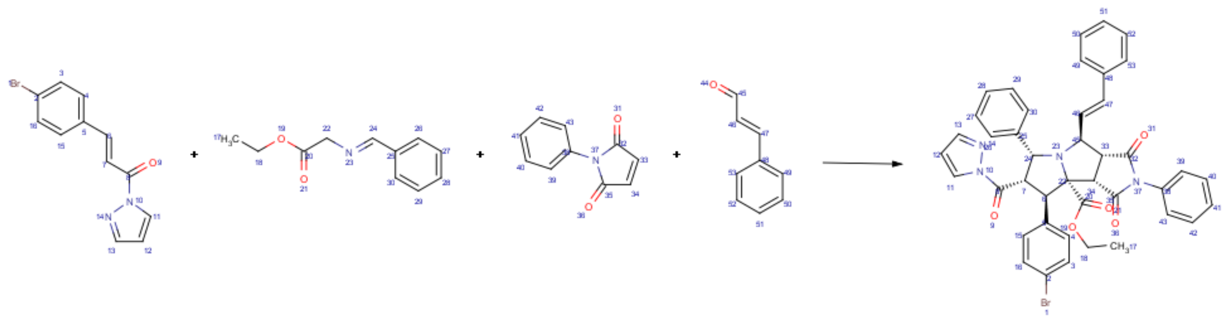

```
Correct mapped SMILES/SMARTS of the reaction:
[Br:1][#6:2]-1=[#6:3]-[#6:4]=[#6:5](\[#6:6]=[#6:7]\[#6:8](=[O:9])-
[#7:10]-2-[#6:11]=[#6:12]-[#6:13]=[#7:14]-2)-[#6:15]=[#6:16]-1.[#6:17]-
[#6:18]-[#8:19]-[#6:20](=[O:21])-[#6:22]\[#7:23]=[#6:24]\[#6:25]-
1=[#6:26]-[#6:27]=[#6:28]-[#6:29]=[#6:30]1.[O:31]=[#6:32]-1-
[#6:33]=[#6:34]-[#6:35](=[O:36])-[#7:37]-1-[#6:38]-1=[#6:39]-
[#6:40]=[#6:41]-[#6:42]=[#6:43]-1.[O:44]=[#6:45]\[#6:46]=[#6:47]\[#6:48]-
1=[#6:49]-[#6:50]=[#6:51]-[#6:52]=[#6:53]1>>[#6:17]-[#6:18]-[#8:19]-
[#6:20](=[O:21])[C@@:22]12[#6@@H:34]-3-[#6@H:33](-
[#6@H:45](\[#6:46]=[#6:47]\[#6:48]-4=[#6:49]-[#6:50]=[#6:51]-
[#6:52]=[#6:53]4)-[#7:23]1-[#6@H:24](-[#6@H:7](-[#6@@H:6]2-[#6:5]-
1=[#6:4]-[#6:3]=[#6:2]([Br:1])-[#6:16]=[#6:15]-1)-[#6:8](=[O:9])-[#7:10]-
1-[#6:11]=[#6:12]-[#6:13]=[#7:14]-1)-[#6:25]-1=[#6:26]-[#6:27]=[#6:28]-
[#6:29]=[#6:30]-1)-[#6:32](=[O:31])-[#7:37](-[#6:35]-3=[O:36])-[#6:38]-
1=[#6:39]-[#6:40]=[#6:41]-[#6:42]=[#6:43]-1
```

Correctness of the mapping

MAPPET	NO
ReactionMap	NO
Marvin	YES
ChemDraw	YES
Indigo	NO

Reaction no 138

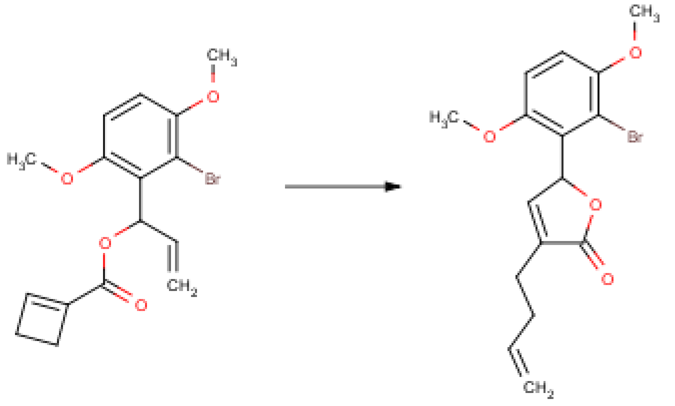

```
SMILES of the input:
COC1=C(Br)C(C(OC(=O)C2=CCC2)C=C)=C(OC)C=C1>>COC1=C(Br)C(C2OC(=O)C(CCC=C)=
C2)=C(OC)C=C1
```

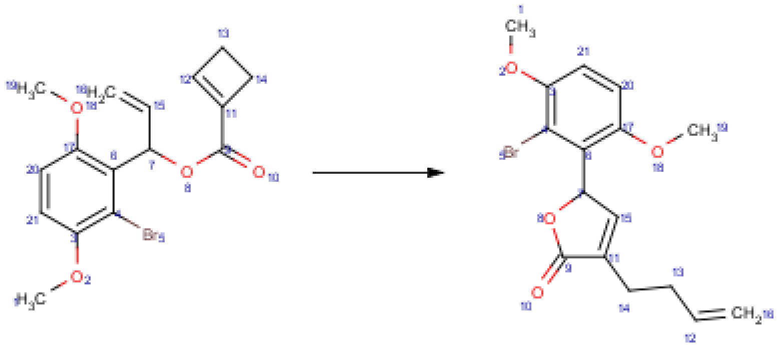

Correct mapped SMILES/SMARTS of the reaction:
[CH3:19][O:18][C:17]1=[C:6]([C:4](=[C:3]([CH:21]=[CH:20]1)[O:2][CH3:1])[Br:5])[CH:7]([CH:15]=[CH2:16])[O:8][C:9](=[O:10])[C:11]2=[CH:12][CH2:13][CH2:14]2>>[CH3:19][O:18][C:17]1=[C:6]([C:4](=[C:3]([CH:21]=[CH:20]1)[O:2][CH3:1])[Br:5])[CH:7]2[CH:15]=[C:11]([C:9](=[O:10])[O:8]2)[CH2:14][CH2:13][CH:12]=[CH2:16]

Correctness of the mapping

MAPPET	NO
ReactionMap	YES
Marvin	YES
ChemDraw	NO
Indigo	NO

Reaction no 139

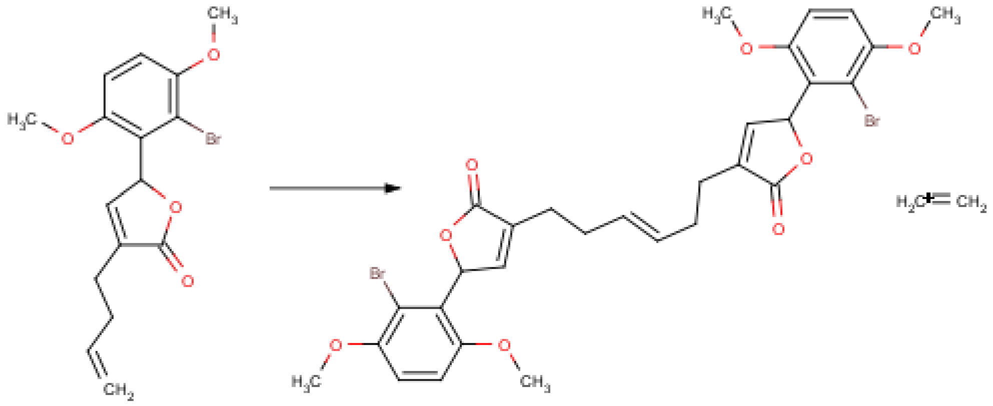

SMILES of the input:
COC1=C(Br)C(C2OC(=O)C(CCC=C)=C2)=C(OC)C=C1>>COC1=C(Br)C(C2OC(=O)C(CC\C=C\CCC3=CC(OC3=O)C3=C(OC)C=CC(OC)=C3Br)=C2)=C(OC)C=C1.C=C

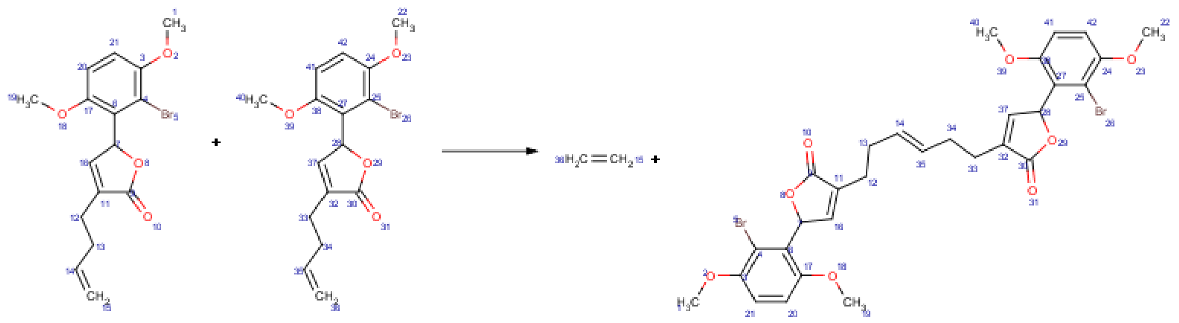

Correct mapped SMILES/SMARTS of the reaction:
[#6:1]-[#8:2]-[#6:3]-1=[#6:4]([Br:5])-[#6:6](-[#6:7]-2-[#8:8]-[#6:9](=[O:10])-[#6:11](-[#6:12]-[#6:13]-[#6:14]=[#6:15])=[#6:16]-2)=[#6:17](-[#8:18]-[#6:19])-[#6:20]=[#6:21]-1.[#6:22]-[#8:23]-[#6:24]-1=[#6:25]([Br:26])-[#6:27](-[#6:28]-2-[#8:29]-[#6:30](=[O:31])-[#6:32](-[#6:33]-[#6:34]-[#6:35]=[#6:36])=[#6:37]-2)=[#6:38](-[#8:39]-[#6:40])-[#6:41]=[#6:42]-1>>[#6:15]=[#6:36].[#6:22]-[#8:23]-[#6:24]-1=[#6:25]([Br:26])-[#6:27](-[#6:28]-2-[#8:29]-[#6:30](=[O:31])-[#6:32](-[#6:33]-[#6:34]\[#6:35]=[#6:14]\[#6:13]-[#6:12]-[#6:11]-3=[#6:16]-[#6:7](-[#8:8]-[#6:9]-3=[O:10])-[#6:6]-3=[#6:17](-[#8:18]-[#6:19])-[#6:20]=[#6:21]-[#6:3](-[#8:2]-[#6:1])=[#6:4]-3[Br:5])=[#6:37]-2)=[#6:38](-[#8:39]-[#6:40])-[#6:41]=[#6:42]-1

Correctness of the mapping

MAPPET	YES
ReactionMap	NO
Marvin	NO
ChemDraw	NO
Indigo	NO

Reaction no 140

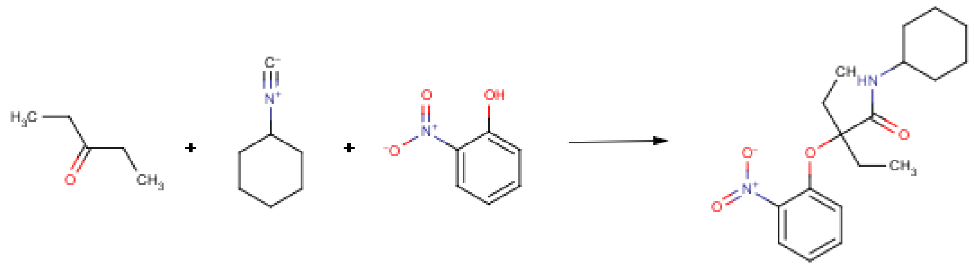

SMILES of the input:
CCC(=O)CC.[C-]#[N+]C1CCCCC1.OC1=CC=CC=C1[N+]([O-])=O>>CCC(CC)(OC1=CC=CC=C1[N+]([O-])=O)C(=O)NC1CCCCC1

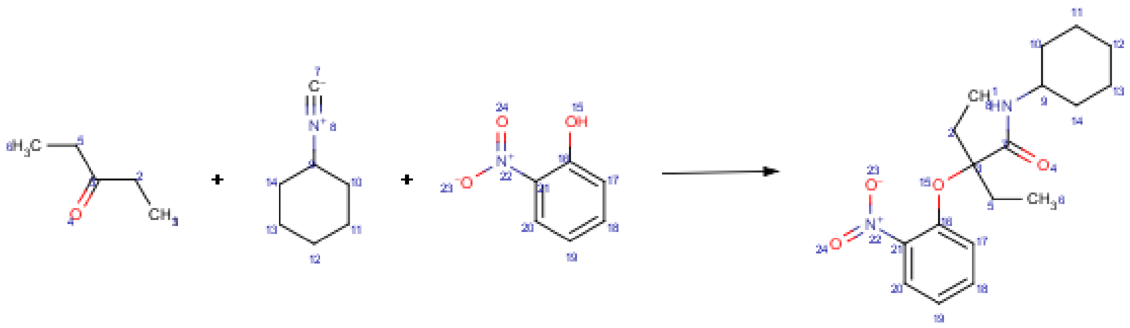

Correct mapped SMILES/SMARTS of the reaction:
[#6:1]-[#6:2]-[#6:3](=[O:4])-[#6:5]-[#6:6].[C-:7]#[N+:8][#6:9]-1-[#6:10]-[#6:11]-[#6:12]-[#6:13]-[#6:14]-1.[#8:15]-[#6:16]-1=[#6:17]-[#6:18]=[#6:19]-[#6:20]=[#6:21]-1-[#7+:22](-[#8-:23])=[O:24]>>[#6:1]-[#6:2][C:3]([#6:5]-[#6:6])([#8:15]-[#6:16]-1=[#6:17]-[#6:18]=[#6:19]-[#6:20]=[#6:21]-1-[#7+:22](-[#8-:23])=[O:24])[#6:7](=[O:4])-[#7:8]-[#6:9]-1-[#6:10]-[#6:11]-[#6:12]-[#6:13]-[#6:14]-1

Correctness of the mapping

MAPPET	YES
ReactionMap	YES
Marvin	YES
ChemDraw	YES
Indigo	YES

Reaction no 141

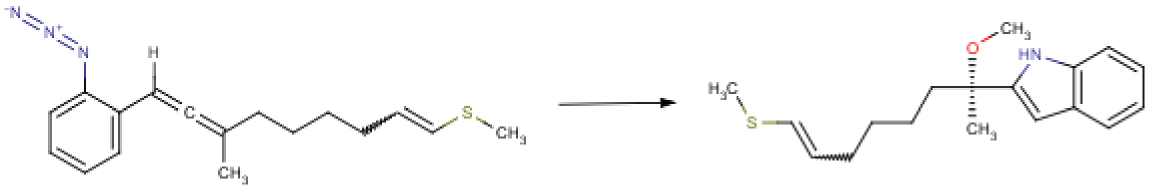

SMILES of the input:
[H]C(=[C@]=C(C)CCCCC=CSC)C1=CC=CC=C1N=[N+]=[N-]>>CO[C@](C)(CCCCC=CSC)C1=CC2=C(N1)C=CC=C2

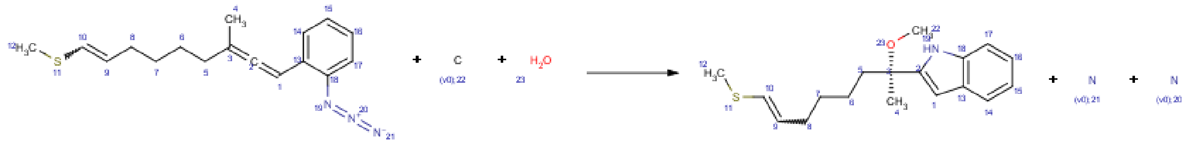

Correct mapped SMILES/SMARTS of the reaction:
[#6:12]-[#16:11]-[#6:10]=[#6:9]-[#6:8]-[#6:7]-[#6:6]-[#6:5]-[#6:3](-[#6:4])=[C:2]=[#6:1]-[#6:13]-1=[#6:14]-[#6:15]=[#6:16]-[#6:17]=[#6:18]-1-[#7:19]=[N+:20]=[#7-:21].[#6;v0:22].[#8:23]>>[#6:22]-[#8:23][C@:3]([#6:4])([#6:5]-[#6:6]-[#6:7]-[#6:8]-[#6:9]=[#6:10]-

[#16:11]-[#6:12])[#6:2]-1=[#6:1]-[#6:13]-2=[#6:18](-[#7:19]-1)-[#6:17]=[#6:16]-[#6:15]=[#6:14]-2.[#7;v0:21].[#7;v0:20]

Correctness of the mapping

MAPPET	YES
ReactionMap	NO
Marvin	NO
ChemDraw	YES
Indigo	YES

Reaction no 142

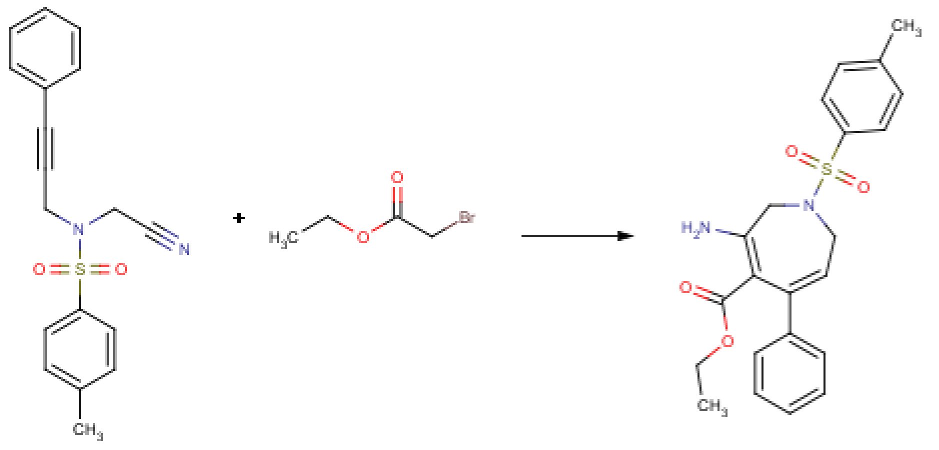

SMILES of the input:
CC1=CC=C(C=C1)S(=O)(=O)N(CC#N)CC#CC1=CC=CC=C1.CCOC(=O)CBr>>CCOC(=O)C1=C(N)CN(CC=C1C1=CC=CC=C1)S(=O)(=O)C1=CC=C(C)C=C1

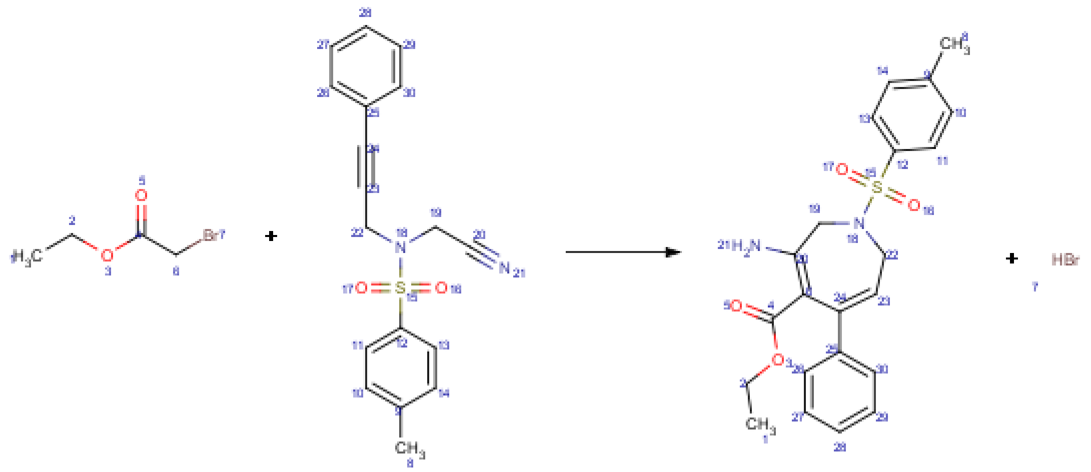

Correct mapped SMILES/SMARTS of the reaction:
[#6:1]-[#6:2]-[#8:3]-[#6:4](=[O:5])-[#6:6][Br:7].[#6:8]-[#6:9]-1=[#6:10]-[#6:11]=[#6:12](-[#6:13]=[#6:14]-1)[S:15](=[O:16])(=[O:17])[#7:18](-[#6:19][C:20]#[N:21])-[#6:22][C:23]#[C:24][#6:25]-1=[#6:26]-[#6:27]=[#6:28]-[#6:29]=[#6:30]-1>>[#6:1]-[#6:2]-[#8:3]-[#6:4](=[O:5])-[#6:6]-1=[#6:20](-[#7:21])-[#6:19]-[#7:18](-[#6:22]-[#6:23]=[#6:24]-1-[#6:25]-1=[#6:30]-[#6:29]=[#6:28]-[#6:27]=[#6:26]-

1) [S:15](=[O:16])(=[O:17])[#6:12]-1=[#6:13]-[#6:14]=[#6:9](-[#6:8])-[#6:10]=[#6:11]-1.[Br:7]

Correctness of the mapping
MAPPET YES
ReactionMap NO
Marvin YES
ChemDraw YES
Indigo NO

Reaction no 143

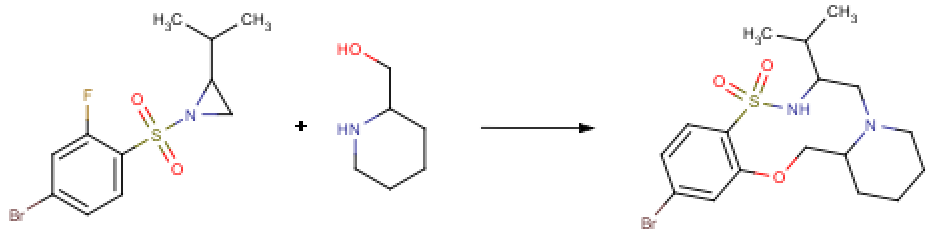

SMILES of the input:
CC(C)C1CN1S(=O)(=O)C1=CC=C(Br)C=C1F.OCC1CCCCN1>>CC(C)C1CN2CCCCC2COC2=C(C=CC(Br)=C2)S(=O)(=O)N1

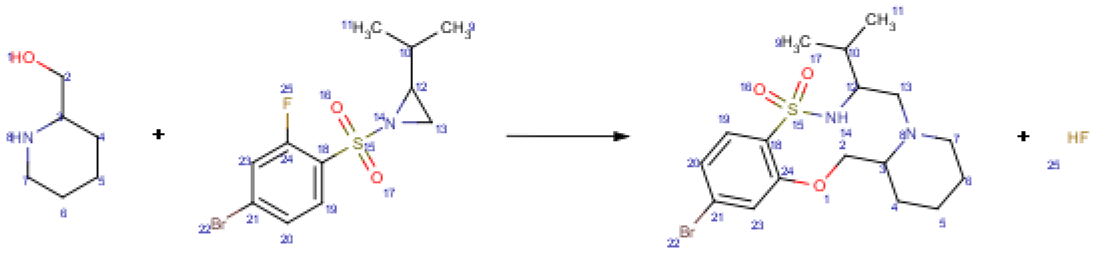

Correct mapped SMILES/SMARTS of the reaction:
[#8:1]-[#6:2]-[#6:3]-1-[#6:4]-[#6:5]-[#6:6]-[#6:7]-[#7:8]-1.[#6:9]-[#6:10](-[#6:11])-[#6:12]-1-[#6:13]-[#7:14]-1[S:15](=[O:16])(=[O:17])[#6:18]-1=[#6:19]-[#6:20]=[#6:21]([Br:22])-[#6:23]=[#6:24]-1[F:25]>>[#6:11]-[#6:10](-[#6:9])-[#6:12]-1-[#6:13]-[#7:8]-2-[#6:7]-[#6:6]-[#6:5]-[#6:4]-[#6:3]-2-[#6:2]-[#8:1]-[#6:24]-2=[#6:18](-[#6:19]=[#6:20]-[#6:21]([Br:22])=[#6:23]-2)[S:15](=[O:16])(=[O:17])[#7:14]-1.[F:25]

Correctness of the mapping
MAPPET YES
ReactionMap NO
Marvin YES
ChemDraw YES
Indigo YES

Reaction no 144

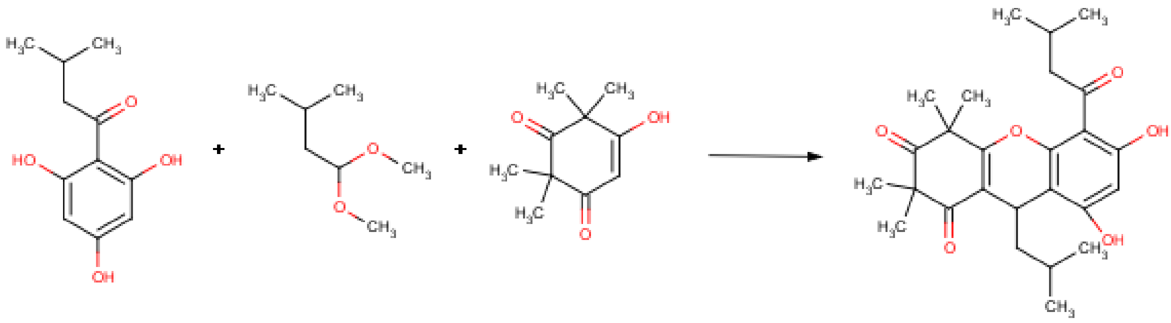

SMILES of the input:
CC(C)CC(=O)C1=C(O)C=C(O)C=C1O.COC(CC(C)C)OC.CC1(C)C(O)=CC(=O)C(C)(C)C1=O>
>CC(C)CC1C2=C(OC3=C1C(=O)C(C)(C)C(=O)C3(C)C)C(C(=O)CC(C)C)=C(O)C=C2O

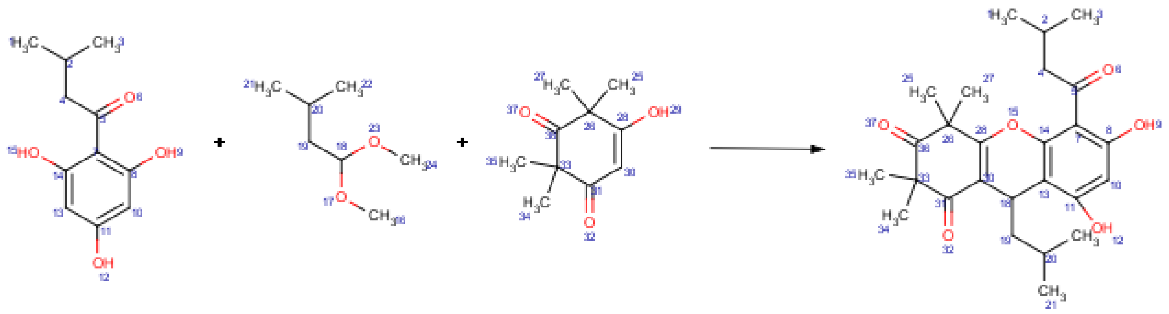

Correct mapped SMILES/SMARTS of the reaction:
[#6:1]-[#6:2](-[#6:3])-[#6:4]-[#6:5](=[O:6])-[#6:7]-1=[#6:8](-[#8:9])-
[#6:10]=[#6:11](-[#8:12])-[#6:13]=[#6:14]-1-[#8:15].[#6:16]-[#8:17]-
[#6:18](-[#6:19]-[#6:20](-[#6:21])-[#6:22])-[#8:23]-
[#6:24].[#6:25][C:26]1([#6:27])[#6:28](-[#8:29])=[#6:30]-
[#6:31](=[O:32])[C:33]([#6:34])([#6:35])[#6:36]1=[O:37]>>[#6:21]-
[#6:20](-[#6:22])-[#6:19]-[#6:18]-1-[#6:13]-2=[#6:14](-[#8:15]-
[#6:28]3=[#6:30]-1-
[#6:31](=[O:32])[C:33]([#6:34])([#6:35])[#6:36](=[O:37])[C:26]3([#6:25])[
#6:27])-[#6:7](-[#6:5](=[O:6])-[#6:4]-[#6:2](-[#6:1])-[#6:3])=[#6:8](-
[#8:9])-[#6:10]=[#6:11]-2-[#8:12]

Correctness of the mapping

MAPPET	NO
ReactionMap	NO
Marvin	YES
ChemDraw	YES
Indigo	YES

Reaction no 145

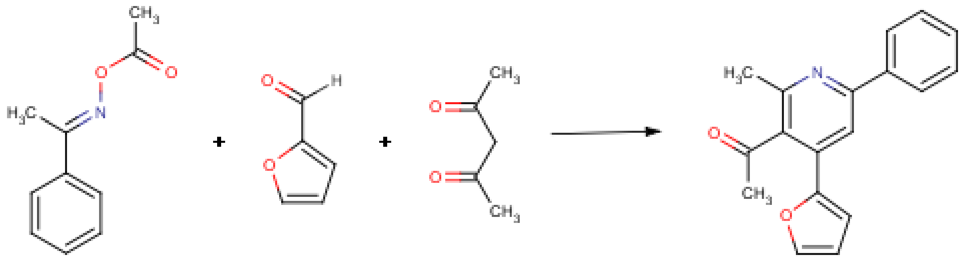

SMILES of the input:
CC(=O)O\N=C(/C)C1=CC=CC=C1.[H]C(=O)C1=CC=CO1.CC(=O)CC(C)=O>>CC(=O)C1=C(C)N=C(C=C1C1=CC=CO1)C1=CC=CC=C1

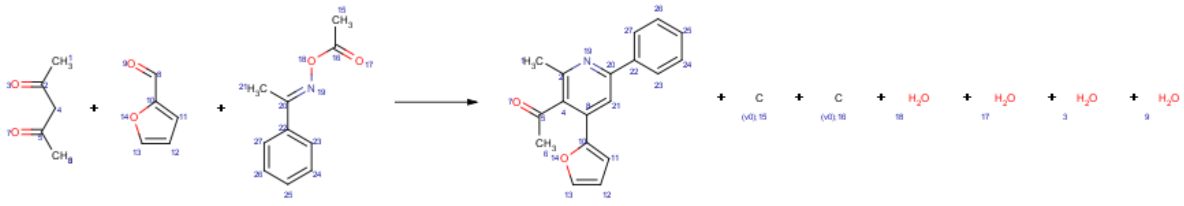

Correct mapped SMILES/SMARTS of the reaction:
[#6:1]-[#6:2](=[O:3])-[#6:4]-[#6:5](-[#6:6])=[O:7].[O:9]=[#6:8]-[#6:10]-1=[#6:11]-[#6:12]=[#6:13]-[#8:14]-1.[#6:15]-[#6:16](=[O:17])-[#8:18]\[#7:19]=[#6:20](/[#6:21])-[#6:22]-1=[#6:23]-[#6:24]=[#6:25]-[#6:26]=[#6:27]-1>>[#6:6]-[#6:5](=[O:7])-[#6:4]-1=[#6:2](-[#6:1])-[#7:19]=[#6:20](-[#6:21]=[#6:8]-1-[#6:10]-1=[#6:11]-[#6:12]=[#6:13]-[#8:14]-1)-[#6:22]-1=[#6:27]-[#6:26]=[#6:25]-[#6:24]=[#6:23]-1.[#6;v0:15].[#6;v0:16].[#8:18].[#8:17].[#8:3].[#8:9]

Correctness of the mapping

MAPPET	YES
ReactionMap	NO
Marvin	YES
ChemDraw	YES
Indigo	YES

Reaction no 146

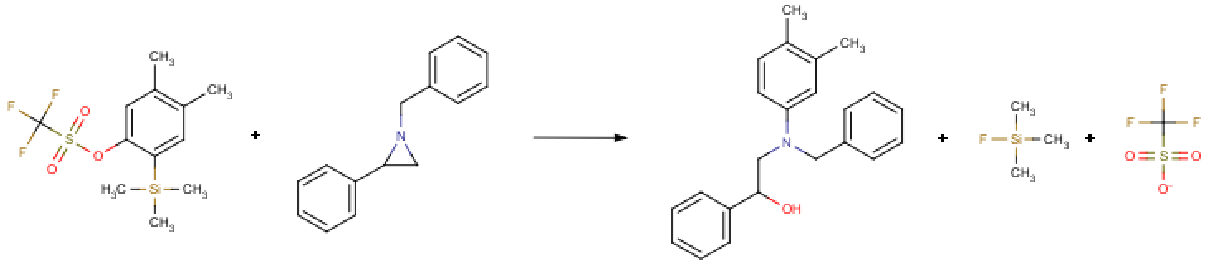

SMILES of the input:
CC1=C(C)C=C(C(OS(=O)(=O)C(F)(F)F)=C1)[Si](C)(C)C.C(N1CC1C1=CC=CC=C1)C1=CC=CC=C1>>CC1=C(C)C=C(C=C1)N(CC(O)C1=CC=CC=C1)CC1=CC=CC=C1.C[Si](C)(C)F.[O-]S(=O)(=O)C(F)(F)F

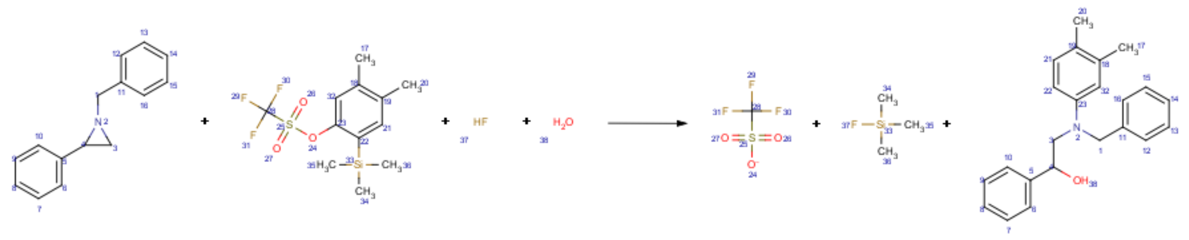

Correct mapped SMILES/SMARTS of the reaction:
[#6:1](-[#7:2]-1-[#6:3]-[#6:4]-1-[#6:5]-1=[#6:6]-[#6:7]=[#6:8]-
[#6:9]=[#6:10]-1)-[#6:11]-1=[#6:12]-[#6:13]=[#6:14]-[#6:15]=[#6:16]-
1.[#6:17]-[#6:18]-1=[#6:19](-[#6:20])-[#6:21]=[#6:22](-[#6:23](-
[#8:24][S:25](=[O:26])(=[O:27])[C:28]([F:29])([F:30])[F:31])=[#6:32]-
1)[Si:33]([#6:34])([#6:35])[#6:36].[F:37].[#8:38]>>[#8-
:24][S:25](=[O:26])(=[O:27])[C:28]([F:29])([F:30])[F:31].[#6:36][Si:33]([
#6:34])([#6:35])[F:37].[#6:20]-[#6:19]-1=[#6:18](-[#6:17])-
[#6:32]=[#6:23](-[#6:22]=[#6:21]-1)-[#7:2](-[#6:3]-[#6:4](-[#8:38])-
[#6:5]-1=[#6:6]-[#6:7]=[#6:8]-[#6:9]=[#6:10]-1)-[#6:1]-[#6:11]-1=[#6:16]-
[#6:15]=[#6:14]-[#6:13]=[#6:12]-1

Correctness of the mapping

MAPPET	YES
ReactionMap	NO
Marvin	YES
ChemDraw	YES
Indigo	NO

Reaction no 147

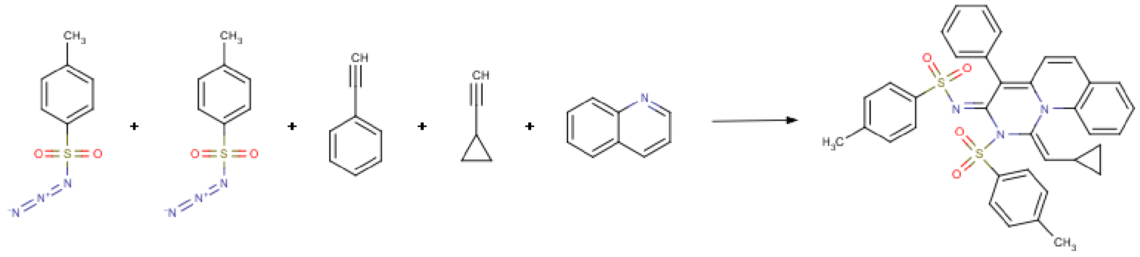

SMILES of the input:
CC1=CC=C(C=C1)S(=O)(=O)N=[N+]=[N-].CC1=CC=C(C=C1)S(=O)(=O)N=[N+]=[N-
].C#CC1=CC=CC=C1.C#CC1CC1.C1=CC=C2N=CC=CC2=C1>>CC1=CC=C(C=C1)S(=O)(=O)\N=
C1\N(\C(=C\C2CC2)N2C3=C(C=CC=C3)C=CC2=C1C1=CC=CC=C1)S(=O)(=O)C1=CC=C(C)C=
C1

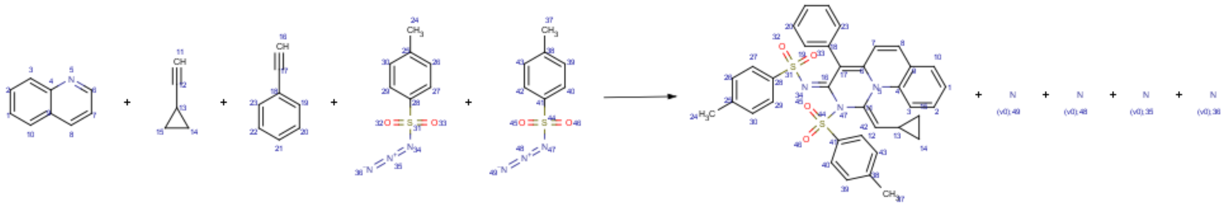

Correct mapped SMILES/SMARTS of the reaction:
[#6:1]-1=[#6:2]-[#6:3]=[#6:4]-2-[#7:5]=[#6:6]-[#6:7]=[#6:8]-[#6:9]-2=[#6:10]-1.[C:11]#[C:12][#6:13]-1-[#6:14]-[#6:15]-1.[C:16]#[C:17][#6:18]-1=[#6:19]-[#6:20]=[#6:21]-[#6:22]=[#6:23]-1.[#6:24]-[#6:25]-1=[#6:26]-[#6:27]=[#6:28](-[#6:29]=[#6:30]-1)[S:31](=[O:32])(=[O:33])[#7:34]=[N+:35]=[#7-:36].[#6:37]-[#6:38]-1=[#6:39]-[#6:40]=[#6:41](-[#6:42]=[#6:43]-1)[S:44](=[O:45])(=[O:46])[#7:47]=[N+:48]=[#7-:49]>>[#6:24]-[#6:25]-1=[#6:26]-[#6:27]=[#6:28](-[#6:29]=[#6:30]-1)[S:31](=[O:33])(=[O:32])\[#7:34]=[#6:16]-1\[#7:47](\[#6:11](=[#6:12]\[#6:13]-2-[#6:15]-[#6:14]2)-[#7:5]-2-[#6:4]-3=[#6:9](-[#6:10]=[#6:1]-[#6:2]=[#6:3]-3)-[#6:8]=[#6:7]-[#6:6]-2=[#6:17]-1-[#6:18]-1=[#6:19]-[#6:20]=[#6:21]-[#6:22]=[#6:23]-1)[S:44](=[O:45])(=[O:46])[#6:41]-1=[#6:42]-[#6:43]=[#6:38](-[#6:37])-[#6:39]=[#6:40]-1.[#7;v0:49].[#7;v0:48].[#7;v0:35].[#7;v0:36]

Correctness of the mapping

MAPPET	YES
ReactionMap	NO
Marvin	NO
ChemDraw	YES
Indigo	YES

Reaction no 148

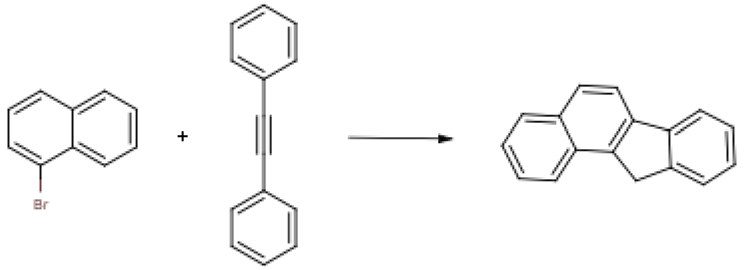

SMILES of the input:
BrC1=CC=CC2=CC=CC=C12.C1=CC=C(C=C1)C#CC1=CC=CC=C1>>C1C2=CC=CC=C2C2=C1C1=CC=CC=C1C=C2

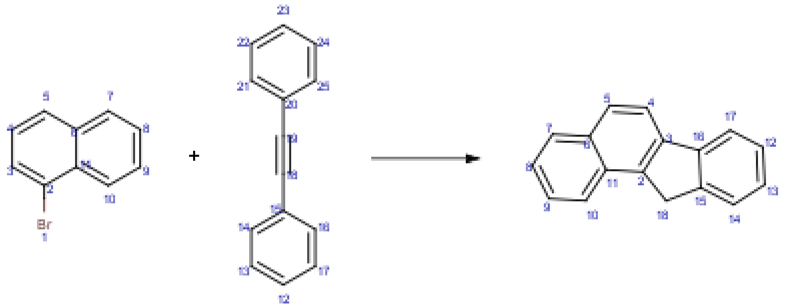

Correct mapped SMILES/SMARTS of the reaction:
[Br:1][#6:2]-1=[#6:3]-[#6:4]=[#6:5]-[#6:6]-2=[#6:7]-[#6:8]=[#6:9]-[#6:10]=[#6:11]-1-2.[#6:12]-1=[#6:13]-[#6:14]=[#6:15](-[#6:16]=[#6:17]-1)[C:18]#[C:19][#6:20]-1=[#6:21]-[#6:22]=[#6:23]-[#6:24]=[#6:25]-1>>[#6:18]-1-[#6:15]-2=[#6:14]-[#6:13]=[#6:12]-[#6:17]=[#6:16]-2-[#6:3]-2=[#6:2]-1-[#6:11]-1=[#6:10]-[#6:9]=[#6:8]-[#6:7]=[#6:6]-1-[#6:5]=[#6:4]-2

Correctness of the mapping
MAPPET NO
ReactionMap NO
Marvin YES
ChemDraw YES
Indigo YES

Reaction no 149

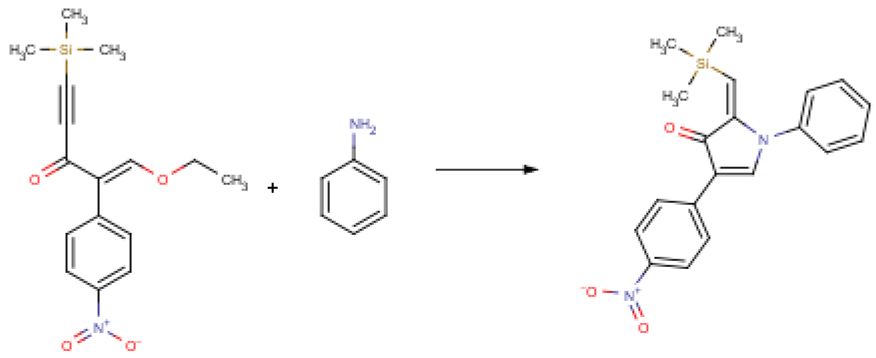

SMILES of the input:
CCO\C=C(\C(=O)C#C[Si](C)(C)C)C1=CC=C(C=C1)[N+]([O-
])=O.NC1=CC=CC=C1>>C[Si](C)(C)\C=C1\N(C=C(C1=O)C1=CC=C(C=C1)[N+]([O-
])=O)C1=CC=CC=C1

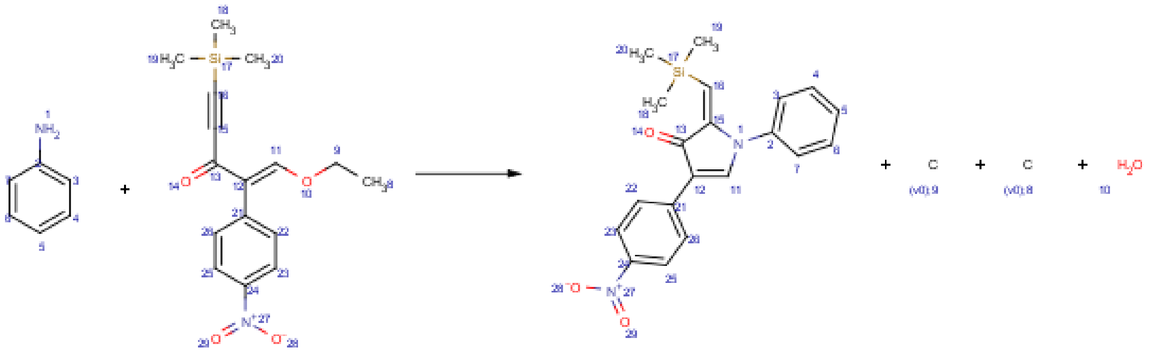

Correct mapped SMILES/SMARTS of the reaction:
[#7:1]-[#6:2]-1=[#6:3]-[#6:4]=[#6:5]-[#6:6]=[#6:7]-1.[#6:8]-[#6:9]-
[#8:10]\[#6:11]=[#6:12](\[#6:13](=[O:14])[C:15]#[C:16][Si:17]([#6:18])([#
6:19])[#6:20])-[#6:21]-1=[#6:22]-[#6:23]=[#6:24](-[#6:25]=[#6:26]-1)-
[#7+:27](-[#8-
:28])=[O:29]>>[#6:20][Si:17]([#6:19])([#6:18])\[#6:16]=[#6:15]-1\[#7:1](-
[#6:11]=[#6:12](-[#6:13]-1=[O:14])-[#6:21]-1=[#6:26]-[#6:25]=[#6:24](-
[#6:23]=[#6:22]-1)-[#7+:27](-[#8-:28])=[O:29])-[#6:2]-1=[#6:3]-
[#6:4]=[#6:5]-[#6:6]=[#6:7]-1.[#6;v0:9].[#6;v0:8].[#8:10]

Correctness of the mapping
MAPPET YES
ReactionMap NO
Marvin YES

ChemDraw YES
Indigo NO

Reaction no 150

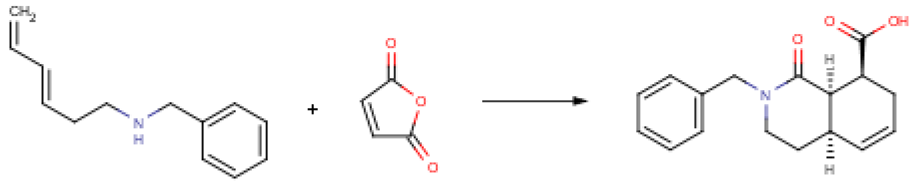

SMILES of the input:
C=C\C=C\CCNCC1=CC=CC=C1.O=C1OC(=O)C=C1>>[H][C@]12CCN(CC3=CC=CC=C3)C(=O)[C@@]1([H])[C@H](CC=C2)C(O)=O

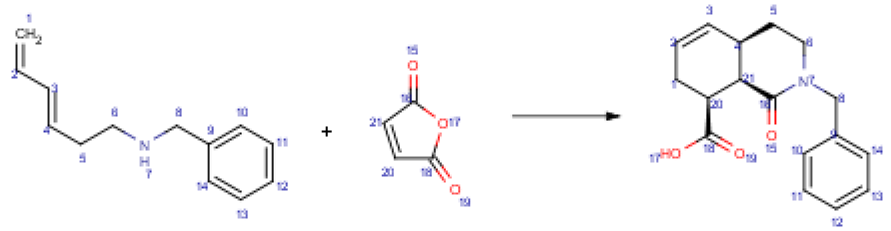

Correct mapped SMILES/SMARTS of the reaction:
[#6:1]=[#6:2]\[#6:3]=[#6:4]\[#6:5]-[#6:6]-[#7:7]-[#6:8]-[#6:9]-1=[#6:10]-[#6:11]=[#6:12]-[#6:13]=[#6:14]-1.[O:15]=[#6:16]-1-[#8:17]-[#6:18](=[O:19])-[#6:20]=[#6:21]-1>>[#8:17]-[#6:18](=[O:19])-[#6@H:20]-1-[#6:1]-[#6:2]=[#6:3]-[#6@H:4]-2-[#6:5]-[#6:6]-[#7:7](-[#6:8]-[#6:9]-3=[#6:14]-[#6:13]=[#6:12]-[#6:11]=[#6:10]-3)-[#6:16](=[O:15])-[#6@@H:21]-1-2

Correctness of the mapping

MAPPET YES
ReactionMap YES
Marvin YES
ChemDraw YES
Indigo NO

Reaction no 151

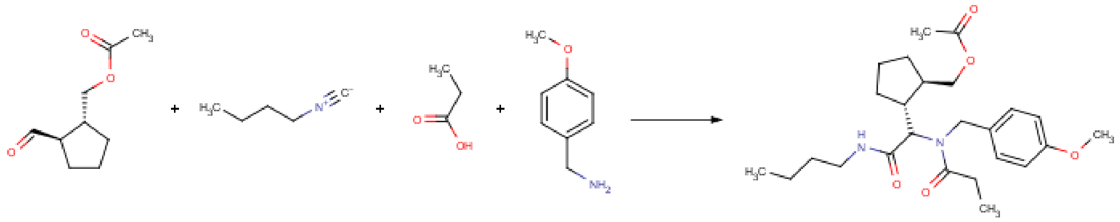

SMILES of the input:
CC(=O)OC[C@@H]1CCC[C@H]1C=O.CCCC[N+]#[C-
].CCC(O)=O.COC1=CC=C(CN)C=C1>>CCCCNC(=O)C([C@@H]1CCC[C@H]1COC(C)=O)N(CC1=
CC=C(OC)C=C1)C(=O)CC

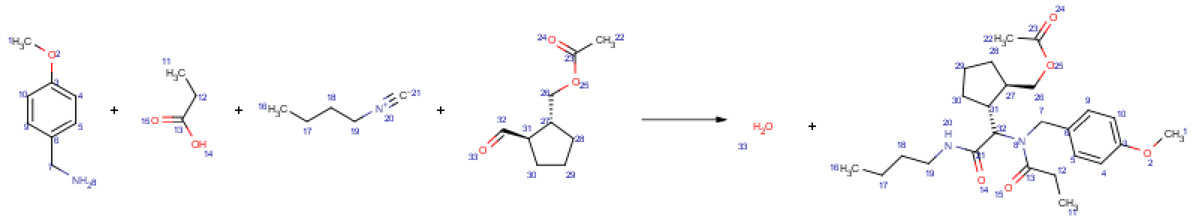

Correct mapped SMILES/SMARTS of the reaction:
[#6:1]-[#8:2]-[#6:3]-1=[#6:4]-[#6:5]=[#6:6](-[#6:7]-[#7:8])-
[#6:9]=[#6:10]-1.[#6:11]-[#6:12]-[#6:13](-[#8:14])=[O:15].[#6:16]-
[#6:17]-[#6:18]-[#6:19][N+:20]#[C-:21].[#6:22]-[#6:23](=[O:24])-[#8:25]-
[#6:26]-[#6@@H:27]-1-[#6:28]-[#6:29]-[#6:30]-[#6@H:31]-1-
[#6:32]=[O:33]>>[#8:33].[#6:16]-[#6:17]-[#6:18]-[#6:19]-[#7:20]-
[#6:21](=[O:14])-[#6:32](-[#6@@H:31]-1-[#6:30]-[#6:29]-[#6:28]-[#6@H:27]-
1-[#6:26]-[#8:25]-[#6:23](-[#6:22])=[O:24])-[#7:8](-[#6:7]-[#6:6]-
1=[#6:9]-[#6:10]=[#6:3](-[#8:2]-[#6:1])-[#6:4]=[#6:5]-1)-
[#6:13](=[O:15])-[#6:12]-[#6:11]

Correctness of the mapping

MAPPET	YES
ReactionMap	NO
Marvin	NO
ChemDraw	NO
Indigo	NO

Reaction no 152

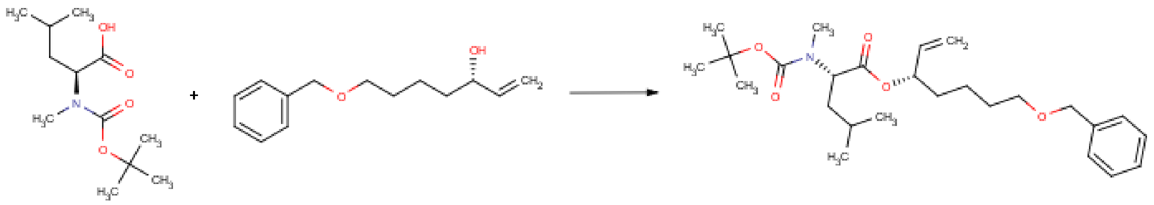

SMILES of the input:
CC(C)C[C@H](N(C)C(=O)OC(C)(C)C)C(O)=O.O[C@@H](CCCCOCC1=CC=CC=C1)C=C>>CC(C
)C[C@H](N(C)C(=O)OC(C)(C)C)C(=O)O[C@@H](CCCCOCC1=CC=CC=C1)C=C

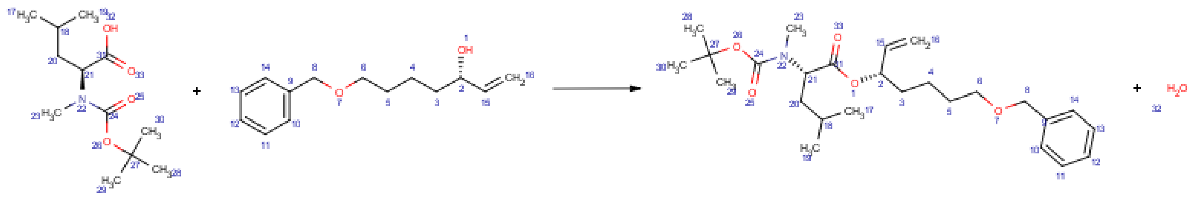

Correct mapped SMILES/SMARTS of the reaction:
[#6:17]-[#6:18](-[#6:19])-[#6:20]-[#6@H:21](-[#7:22](-[#6:23])-[#6:24](=[O:25])-[#8:26][C:27]([#6:28])([#6:29])[#6:30])-[#6:31](-[#8:32])=[O:33].[#8:1]-[#6@@H:2](-[#6:3]-[#6:4]-[#6:5]-[#6:6]-[#8:7]-[#6:8]-[#6:9]-1=[#6:10]-[#6:11]=[#6:12]-[#6:13]=[#6:14]-1)-[#6:15]=[#6:16]>>[#6:17]-[#6:18](-[#6:19])-[#6:20]-[#6@H:21](-[#7:22](-[#6:23])-[#6:24](=[O:25])-[#8:26][C:27]([#6:30])([#6:29])[#6:28])-[#6:31](=[O:33])-[#8:1]-[#6@@H:2](-[#6:3]-[#6:4]-[#6:5]-[#6:6]-[#8:7]-[#6:8]-[#6:9]-1=[#6:14]-[#6:13]=[#6:12]-[#6:11]=[#6:10]-1)-[#6:15]=[#6:16].[#8:32]

Correctness of the mapping

MAPPET	YES
ReactionMap	NO
Marvin	NO
ChemDraw	NO
Indigo	YES

Reaction no 153

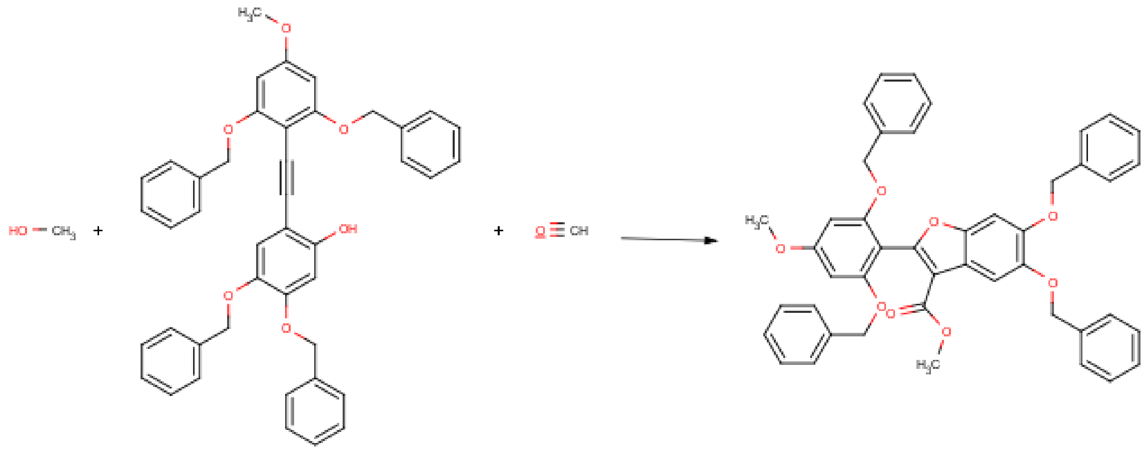

SMILES of the input:
CO.COC1=CC(OCC2=CC=CC=C2)=C(C#CC2=C(O)C=C(OCC3=CC=CC=C3)C(OCC3=CC=CC=C3)=C2)C(OCC2=CC=CC=C2)=C1.C#[O]>>COC(=O)C1=C(OC2=CC(OCC3=CC=CC=C3)=C(OCC3=CC=CC=C3)C=C12)C1=C(OCC2=CC=CC=C2)C=C(OC)C=C1OCC1=CC=CC=C1

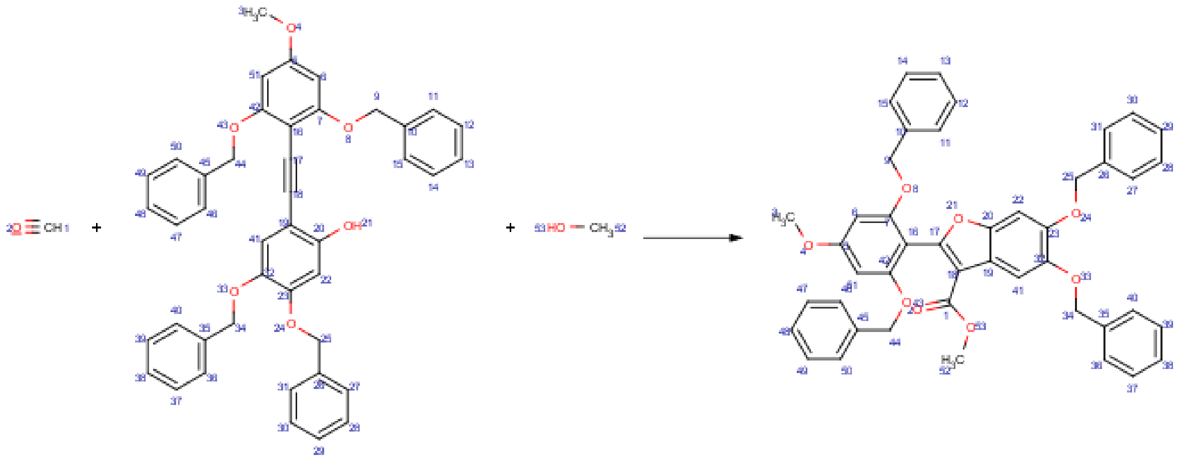

Correct mapped SMILES/SMARTS of the reaction:
[C:1]#[O:2].[#6:3]-[#8:4]-[#6:5]-1=[#6:6]-[#6:7](-[#8:8]-[#6:9]-[#6:10]-2=[#6:11]-[#6:12]=[#6:13]-[#6:14]=[#6:15]-2)=[#6:16]([C:17]#[C:18][#6:19]-2=[#6:20](-[#8:21])-[#6:22]=[#6:23](-[#8:24]-[#6:25]-[#6:26]-3=[#6:27]-[#6:28]=[#6:29]-[#6:30]=[#6:31]-3)-[#6:32](-[#8:33]-[#6:34]-[#6:35]-3=[#6:36]-[#6:37]=[#6:38]-[#6:39]=[#6:40]-3)=[#6:41]-2)-[#6:42](-[#8:43]-[#6:44]-[#6:45]-2=[#6:46]-[#6:47]=[#6:48]-[#6:49]=[#6:50]-2)=[#6:51]-1.[#6:52]-[#8:53]>>[#6:52]-[#8:53]-[#6:1](=[O:2])-[#6:18]-1=[#6:17](-[#8:21]-[#6:20]-2=[#6:22]-[#6:23](-[#8:24]-[#6:25]-[#6:26]-3=[#6:31]-[#6:30]=[#6:29]-[#6:28]=[#6:27]-3)=[#6:32](-[#8:33]-[#6:34]-[#6:35]-3=[#6:40]-[#6:39]=[#6:38]-[#6:37]=[#6:36]-3)-[#6:41]=[#6:19]-1-2)-[#6:16]-1=[#6:42](-[#8:43]-[#6:44]-[#6:45]-2=[#6:50]-[#6:49]=[#6:48]-[#6:47]=[#6:46]-2)-[#6:51]=[#6:5](-[#8:4]-[#6:3])-[#6:6]=[#6:7]-1-[#8:8]-[#6:9]-[#6:10]-1=[#6:15]-[#6:14]=[#6:13]-[#6:12]=[#6:11]-1

Correctness of the mapping

MAPPET	YES
ReactionMap	YES
Marvin	NO
ChemDraw	YES
Indigo	YES

Reaction no 154

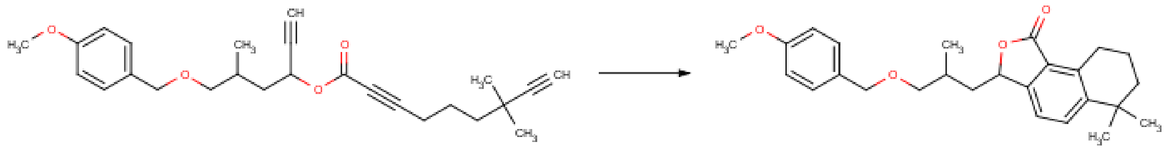

SMILES of the input:
COC1=CC=C(COCC(C)CC(OC(=O)C#CCCCC(C)(C)C#C)C#C)C=C1>>COC1=CC=C(COCC(C)CC2OC(=O)C3=C4CCCC(C)(C)C4=CC=C23)C=C1

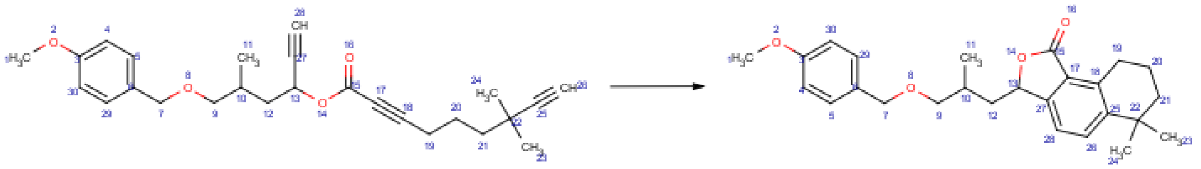

Correct mapped SMILES/SMARTS of the reaction:
[#6:1]-[#8:2]-[#6:3]-1=[#6:4]-[#6:5]=[#6:6](-[#6:7]-[#8:8]-[#6:9]-[#6:10](-[#6:11])-[#6:12]-[#6:13](-[#8:14]-[#6:15](=[O:16])[C:17]#[C:18][#6:19]-[#6:20]-[#6:21][C:22]([#6:23])([#6:24])[C:25]#[C:26])[C:27]#[C:28])-[#6:29]=[#6:30]-1>>[#6:1]-[#8:2]-[#6:3]-1=[#6:30]-[#6:29]=[#6:6](-[#6:7]-[#8:8]-[#6:9]-[#6:10](-[#6:11])-[#6:12]-[#6:13]-2-[#8:14]-[#6:15](=[O:16])-[#6:17]-3=[#6:18]-4-[#6:19]-[#6:20]-[#6:21][C:22]([#6:23])([#6:24])[#6:25]-4=[#6:26]-[#6:28]=[#6:27]-2-3)-[#6:5]=[#6:4]-1

Correctness of the mapping

MAPPET	YES
ReactionMap	YES
Marvin	YES
ChemDraw	YES
Indigo	YES

Reaction no 155

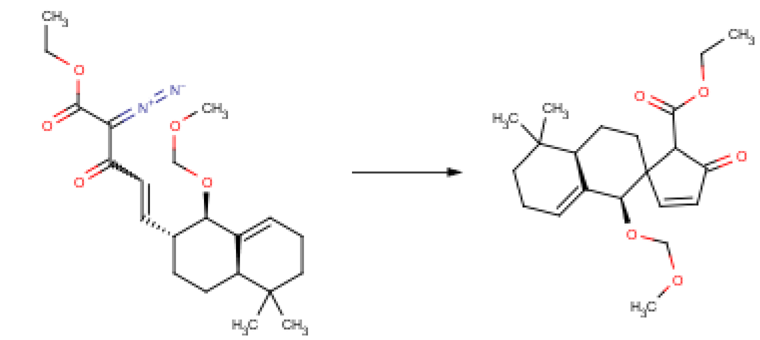

SMILES of the input:
CCOC(=O)C(=[N+]=[N-])C(=O)C=C[C@H]1CC[C@H]2C(=CCCC2(C)C)[C@@H]1OCOC>>CCOC(=O)C1C(=O)C=CC11CC[C@H]2C(=CCCC2(C)C)[C@@H]1OCOC

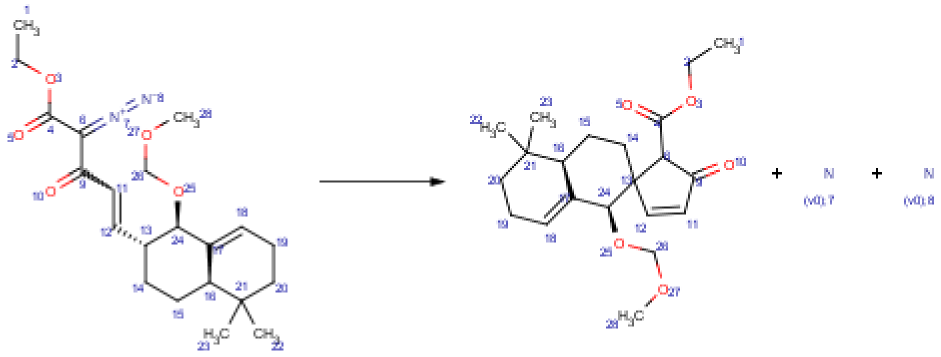

Correct mapped SMILES/SMARTS of the reaction:
[CH3:1][CH2:2][O:3][C:4](=[O:5])[C:6](=[N+:7]=[N-
:8])[C:9](=[O:10])[CH:11]=[CH:12][C@H:13]1[CH2:14][CH2:15][C@H:16]2[C:17]
(=[CH:18][CH2:19][CH2:20][C:21]2([CH3:22])[CH3:23])[C@@H:24]1[O:25][CH2:2
6][O:27][CH3:28]>>[CH3:1][CH2:2][O:3][C:4](=[O:5])[CH:6]1[C:9](=[O:10])[C
H:11]=[CH:12][C:13]11[CH2:14][CH2:15][C@H:16]2[C:17](=[CH:18][CH2:19][CH2
:20][C:21]2([CH3:22])[CH3:23])[C@@H:24]1[O:25][CH2:26][O:27][CH3:28].[N:7
].[N:8]

Correctness of the mapping

MAPPET	YES
ReactionMap	NO
Marvin	YES
ChemDraw	YES
Indigo	YES

Reaction no 156

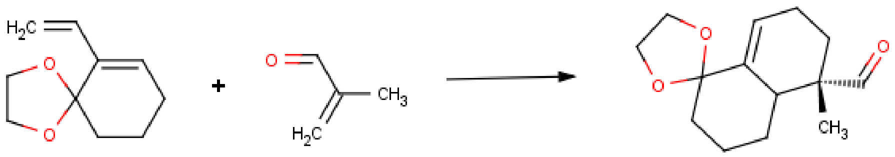

SMILES of the input:
C=CC1=CCCCC11OCCO1.CC(=C)C=O>>C[C@@]1(CCC=C2C1CCCC21OCCO1)C=O

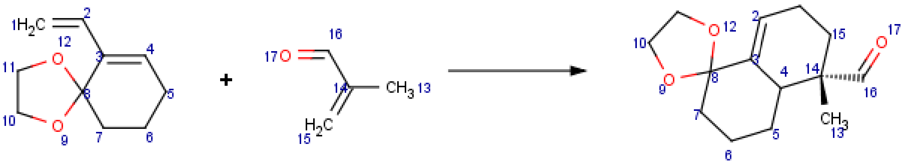

Correct mapped SMILES/SMARTS of the reaction:
[#6:1]=[#6:2]-[#6:3]1=[#6:4]-[#6:5]-[#6:6]-[#6:7][C:8]11[#8:9]-[#6:10]-
[#6:11]-[#8:12]1.[#6:13]-[#6:14](=[#6:15])-
[#6:16]=[O:17]>>[#6:13][C@@:14]1([#6:15]-[#6:1]-[#6:2]=[#6:3]2-[#6:4]1-
[#5:5]-[#6:6]-[#6:7][C:8]21[#8:9]-[#6:10]-[#6:11]-[#8:12]1)[#6:16]=[O:17]

Correctness of the mapping
MAPPET YES
ReactionMap YES
Marvin NO
ChemDraw YES
Indigo NO

Reaction no 157

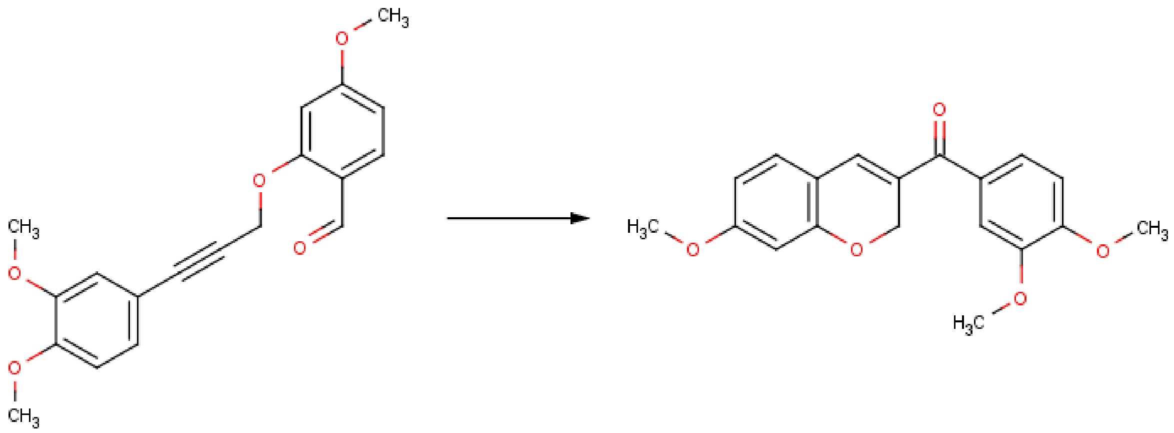

SMILES of the input:
COC1=CC=C(C=O)C(OCC#CC2=CC=C(OC)C(OC)=C2)=C1>>COC1=CC2=C(C=C1)C=C(CO2)C(=O)C1=CC=C(OC)C(OC)=C1

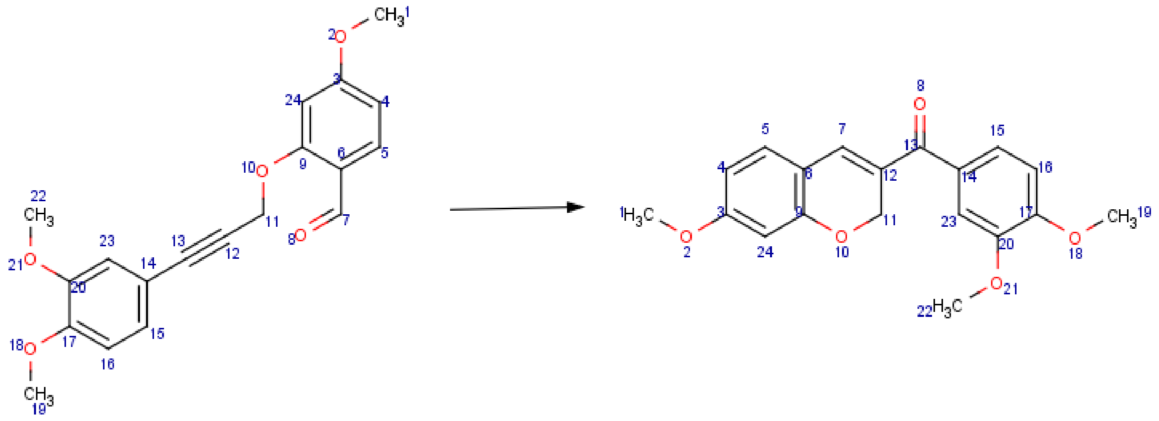

Correct mapped SMILES/SMARTS of the reaction:
[#6:1]-[#8:2]-[#6:3]-1=[#6:4]-[#6:5]=[#6:6](-[#6:7]=[O:8])-[#6:9](-[#8:10]-[#6:11][C:12]#[C:13][#6:14]-2=[#6:15]-[#6:16]=[#6:17](-[#8:18]-[#6:19])-[#6:20](-[#8:21]-[#6:22])=[#6:23]-2)=[#6:24]-1>>[#6:1]-[#8:2]-[#6:3]-1=[#6:24]-[#6:9]-2=[#6:6](-[#6:5]=[#6:4]-1)-[#6:7]=[#6:12](-[#6:11]-[#8:10]-2)-[#6:13](=[O:8])-[#6:14]-1=[#6:15]-[#6:16]=[#6:17](-[#8:18]-[#6:19])-[#6:20](-[#8:21]-[#6:22])=[#6:23]-1

Correctness of the mapping
MAPPET YES
ReactionMap YES
Marvin YES
ChemDraw YES
Indigo NO

Reaction no 158

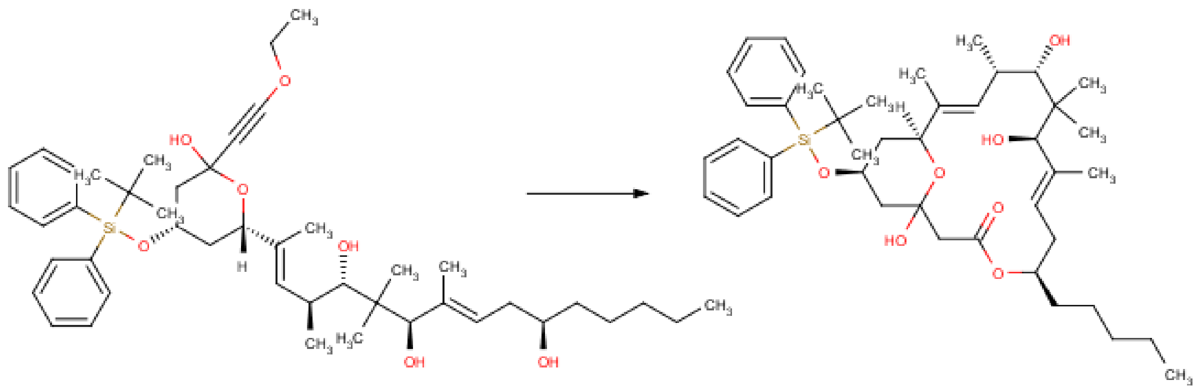

SMILES of the input:
[H][C@]1(C[C@@H](CC(O)(O1)C#COCC)O[Si](C1=CC=CC=C1)(C1=CC=CC=C1)C(C)(C)C)
C(\C)=C\[C@H](C)[C@H](O)C(C)(C)[C@H](O)C(\C)=C\C[C@H](O)CCCCC>>[H][C@]12C
[C@@H](CC(O)(CC(=O)O[C@H](CCCCC)C\C=C(C)\[C@@H](O)C(C)(C)[C@@H](O)[C@@H](
C)\C=C1/C)O2)O[Si](C1=CC=CC=C1)(C1=CC=CC=C1)C(C)(C)C

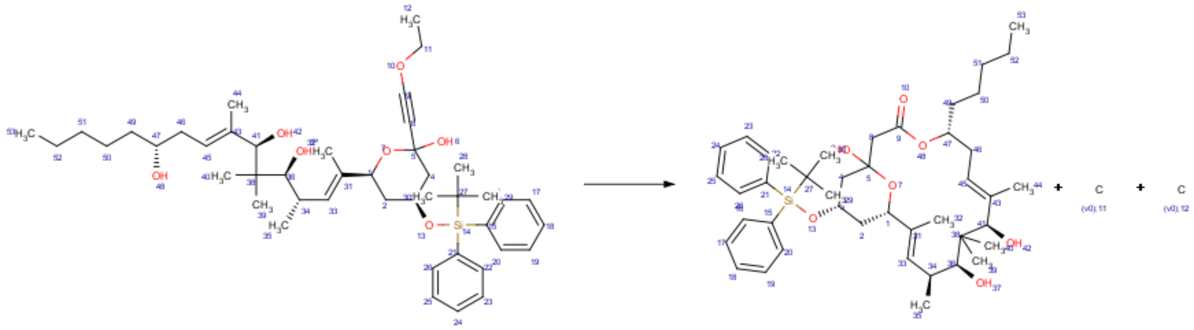

Correct mapped SMILES/SMARTS of the reaction:
[#6:53]-[#6:52]-[#6:51]-[#6:50]-[#6:49]-[#6@@H:47](-[#8:48])-
[#6:46]\[#6:45]=[#6:43](/[#6:44])-[#6@@H:41](-
[#8:42])[C:38]([#6:39])([#6:40])[#6@@H:36](-[#8:37])-[#6@@H:34](-
[#6:35])\[#6:33]=[#6:31](/[#6:32])-[#6@@H:1]-1-[#6:2]-[#6@@H:3](-
[#6:4][C:5]([#8:6])([#8:7]-1)[C:8]#[C:9][#8:10]-[#6:11]-[#6:12])-
[#8:13][Si:14]([#6:15]-1=[#6:16]-[#6:17]=[#6:18]-[#6:19]=[#6:20]-
1)([#6:21]-1=[#6:22]-[#6:23]=[#6:24]-[#6:25]=[#6:26]-
1)[C:27]([#6:28])([#6:29])[#6:30]>>[#6:53]-[#6:52]-[#6:51]-[#6:50]-
[#6:49]-[#6@@H:47]-1-[#6:46]\[#6:45]=[#6:43](-[#6:44])\[#6@@H:41](-
[#8:42])[C:38]([#6:39])([#6:40])[#6@@H:36](-[#8:37])-[#6@@H:34](-
[#6:35])\[#6:33]=[#6:31](-[#6:32])\[#6@@H:1]-2-[#6:2]-[#6@@H:3](-
[#6:4][C:5]([#8:6])([#6:8]-[#6:9](=[O:10])-[#8:48]-1)[#8:7]2)-
[#8:13][Si:14]([#6:15]-1=[#6:20]-[#6:19]=[#6:18]-[#6:17]=[#6:16]-
1)([#6:21]-1=[#6:26]-[#6:25]=[#6:24]-[#6:23]=[#6:22]-
1)[C:27]([#6:30])([#6:29])[#6:28].[#6;v0:11].[#6;v0:12]

Correctness of the mapping

MAPPET	YES
ReactionMap	NO
Marvin	NO
ChemDraw	YES
Indigo	NO

Reaction no 159

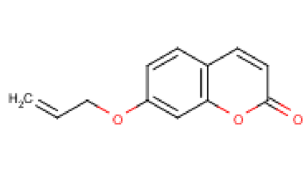

SMILES of the input:
C=CCOC1=CC2=C(C=CC(=O)O2)C=C1>>OC1=C(CC=C)C2=C(C=CC(=O)O2)C=C1

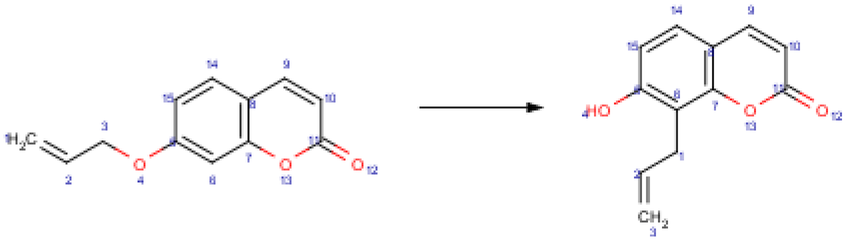

Correct mapped SMILES/SMARTS of the reaction:
[#6:1]=[#6:2]-[#6:3]-[#8:4]-[#6:5]-1=[#6:6]-[#6:7]-2=[#6:8](-[#6:9]=[#6:10]-[#6:11](=[O:12])-[#8:13]-2)-[#6:14]=[#6:15]-1>>[#8:4]-[#6:5]-1=[#6:6](-[#6:1]-[#6:2]=[#6:3])-[#6:7]-2=[#6:8](-[#6:9]=[#6:10]-[#6:11](=[O:12])-[#8:13]-2)-[#6:14]=[#6:15]-1

Correctness of the mapping

MAPPET	YES
ReactionMap	NO
Marvin	NO
ChemDraw	NO
Indigo	NO

Reaction no 160

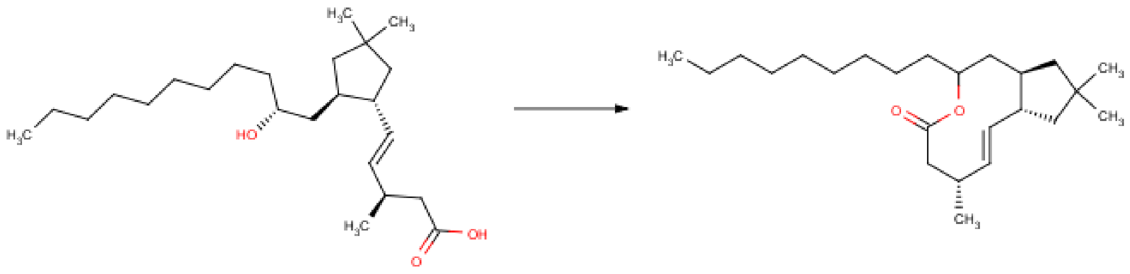

SMILES of the input:
CCCCCCCCC[C@@H](O)C[C@H]1CC(C)(C)C[C@@H]1\C=C\[C@H](C)CC(O)=O>>CCCCCCCCCC1C[C@H]2CC(C)(C)C[C@@H]2\C=C\[C@H](C)CC(=O)O1

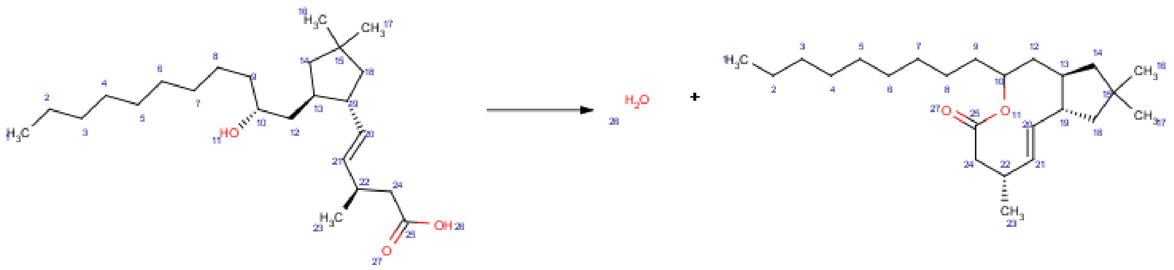

Correct mapped SMILES/SMARTS of the reaction:
[#6:1]-[#6:2]-[#6:3]-[#6:4]-[#6:5]-[#6:6]-[#6:7]-[#6:8]-[#6:9]-
[#6@@H:10](-[#8:11])-[#6:12]-[#6@H:13]-1-
[#6:14][C:15]([#6:16])([#6:17])[#6:18]-[#6@@H:19]-
1\[#6:20]=[#6:21]\[#6@H:22](-[#6:23])-[#6:24]-[#6:25](-
[#8:26])=[O:27]>>[#8:26].[#6:1]-[#6:2]-[#6:3]-[#6:4]-[#6:5]-[#6:6]-
[#6:7]-[#6:8]-[#6:9]-[#6:10]-1-[#6:12]-[#6@H:13]-2-
[#6:14][C:15]([#6:16])([#6:17])[#6:18]-[#6@@H:19]-
2\[#6:20]=[#6:21]\[#6@H:22](-[#6:23])-[#6:24]-[#6:25](=[O:27])-[#8:11]-1

Correctness of the mapping

MAPPET	YES
ReactionMap	NO
Marvin	YES
ChemDraw	NO
Indigo	NO

Reaction no 161

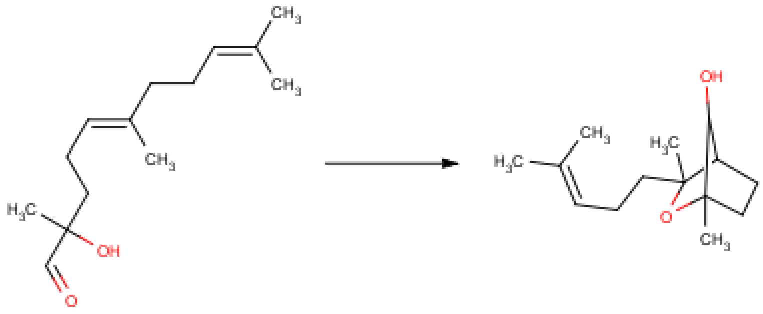

SMILES of the input:
CC(C)=CCC\C(C)=C\CCC(C)(O)C=O>>CC(C)=CCCC1(C)OC2(C)CCC1C2O

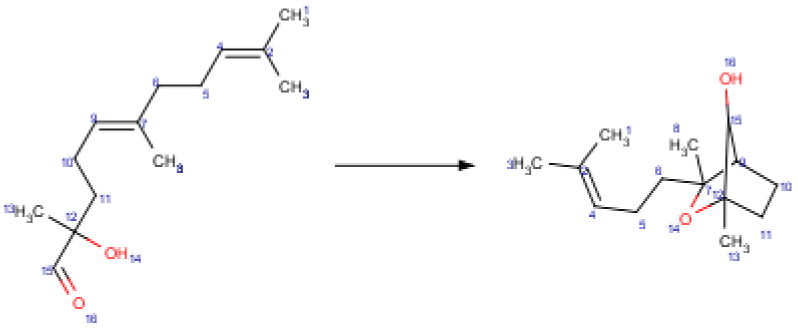

Correct mapped SMILES/SMARTS of the reaction:
[#6:1]\[#6:2](-[#6:3])=[#6:4]\[#6:5]-[#6:6]\[#6:7](-
[#6:8])=[#6:9]\[#6:10]-
[#6:11][C:12]([#6:13])([#8:14])[#6:15]=[O:16]>>[#6:3]\[#6:2](-
[#6:1])=[#6:4]\[#6:5]-[#6:6][C:7]1([#6:8])[#8:14][C:12]2([#6:13])[#6:11]-
[#6:10]-[#6:9]1-[#6:15]2-[#8:16]

Correctness of the mapping

MAPPET	YES
ReactionMap	YES
Marvin	YES
ChemDraw	YES
Indigo	NO

Reaction no 162

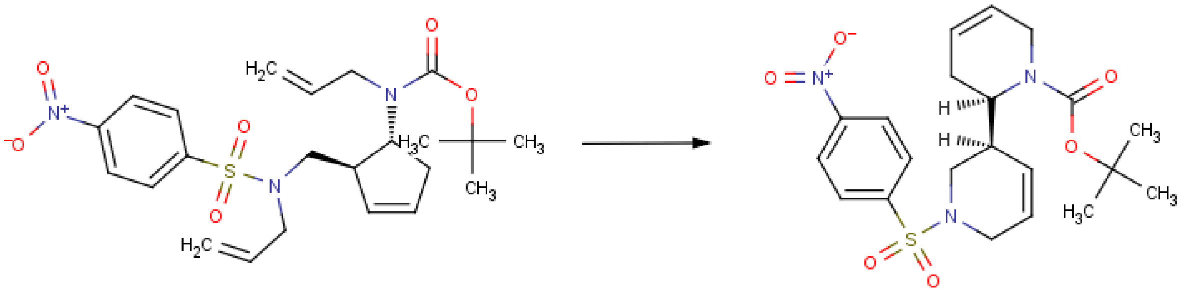

SMILES of the input:
CC(C)(C)OC(=O)N(CC=C)[C@@H]1CC=C[C@H]1CN(CC=C)S(=O)(=O)C1=CC=C(C=C1)[N+](
[O-])=O>>[H][C@]1(CN(CC=C1)S(=O)(=O)C1=CC=C(C=C1)[N+]([O-
])=O)[C@@]1([H])CC=CCN1C(=O)OC(C)(C)C

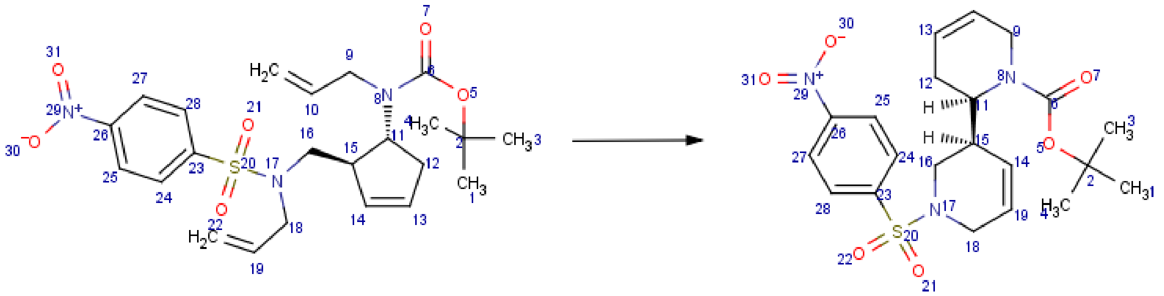

Correct mapped SMILES/SMARTS of the reaction:

[CH3:1][C:2]([CH3:3])([CH3:4])[O:5][C:6](=[O:7])[N:8]([CH2:9][CH:10]=C)[C@@H:11]1[CH2:12][CH:13]=[CH:14][C@H:15]1[CH2:16][N:17]([CH2:18][CH:19]=C)[S:20](=[O:21])(=[O:22])[C:23]1=[CH:24][CH:25]=[C:26]([CH:27]=[CH:28]1)[N+:29]([O-:30])=[O:31]>>[H][C@:15]1([CH2:16][N:17]([CH2:18][CH:19]=[CH:14]1)[S:20](=[O:21])(=[O:22])[C:23]1=[CH:28][CH:27]=[C:26]([CH:25]=[CH:24]1)[N+:29]([O-:30])=[O:31])[C@@:11]1([H])[CH2:12][CH:13]=[CH:10][CH2:9][N:8]1[C:6](=[O:7])[O:5][C:2]([CH3:1])([CH3:4])[CH3:3]

Correctness of the mapping
MAPPET NO
ReactionMap NO
Marvin NO
ChemDraw YES
Indigo NO

Reaction no 163

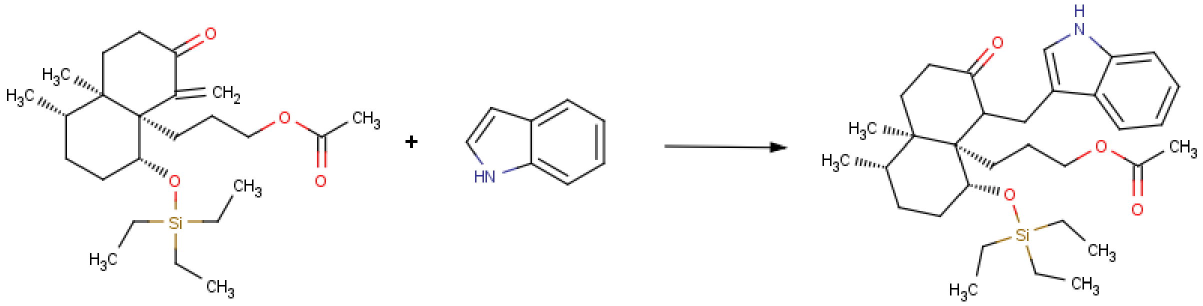

SMILES of the input:
CC[Si](CC)(CC)O[C@@H]1CC[C@H](C)[C@@]2(C)CCC(=O)C(=C)[C@@]12CCCOC(C)=O.N1C=CC2=CC=CC=C12>>CC[Si](CC)(CC)O[C@@H]1CC[C@H](C)[C@@]2(C)CCC(=O)C(CC3=CNC4=CC=CC=C34)[C@@]12CCCOC(C)=O

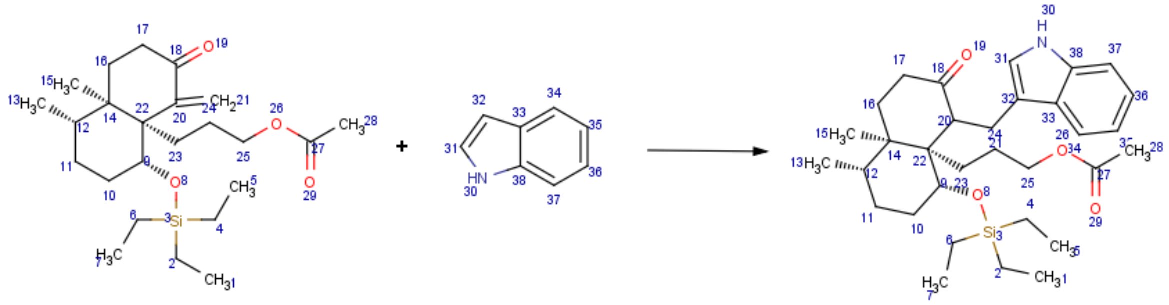

Correct mapped SMILES/SMARTS of the reaction:
[#6:1]-[#6:2][Si:3]([#6:4]-[#6:5])([#6:6]-[#6:7])[#8:8]-[#6@@H:9]1-[#6:10]-[#6:11]-[#6@H:12](-[#6:13])[C@@:14]2([#6:15])[#6:16]-[#6:17]-[#6:18](=[O:19])-[#6:20](=[#6:21])[C@@:22]12[#6:23]-[#6:24]-[#6:25]-[#8:26]-[#6:27](-[#6:28])=[O:29].[#7:30]-1-[#6:31]=[#6:32]-[#6:33]-2=[#6:34]-[#6:35]=[#6:36]-[#6:37]=[#6:38]-1-2>>[#6:7]-[#6:6][Si:3]([#6:4]-[#6:5])([#6:2]-[#6:1])[#8:8]-[#6@@H:9]1-[#6:10]-[#6:11]-[#6@H:12](-[#6:13])[C@@:14]2([#6:15])[#6:16]-[#6:17]-[#6:18](=[O:19])-[#6:20](-[#6:21]-[#6:32]-3=[#6:31]-[#7:30]-[#6:38]-4=[#6:37]-[#6:36]=[#6:35]-[#6:34]=[#6:33]-3-4)[C@@:22]12[#6:23]-[#6:24]-[#6:25]-[#8:26]-[#6:27](-[#6:28])=[O:29]

Correctness of the mapping

MAPPET	YES
ReactionMap	YES
Marvin	YES
ChemDraw	YES
Indigo	NO

Reaction no 164

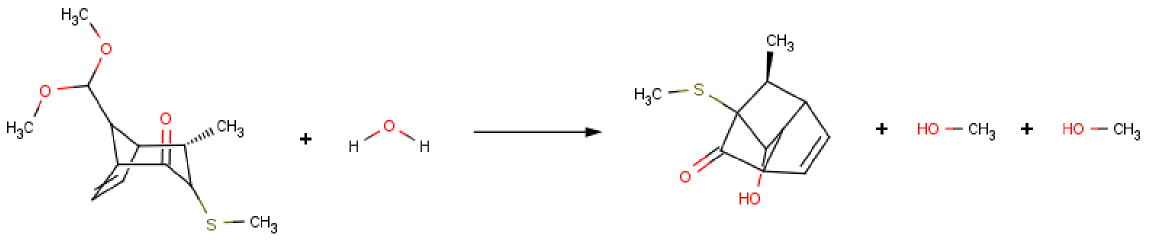

SMILES of the input:
COC(OC)C1C2C=CC1C(=O)C(SC)[C@H]2C.[H]O[H]>>CSC12[C@@H](C)C3C=CC(C3C1O)C2=O.CO.CO

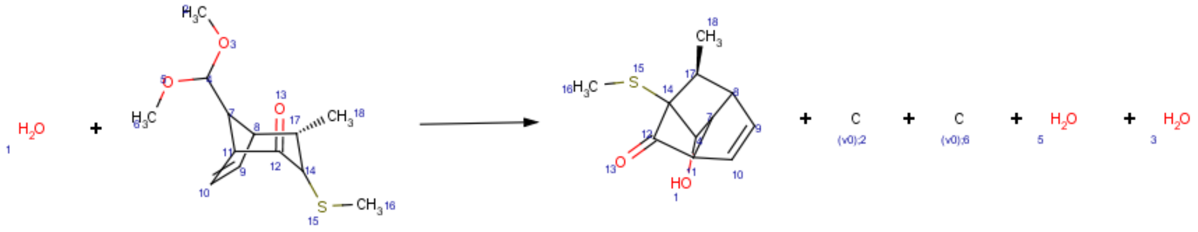

Correct mapped SMILES/SMARTS of the reaction:
[#8:1].[#6:2]-[#8:3]-[#6:4](-[#8:5]-[#6:6])-[#6:7]-1-[#6:8]-2-[#6:9]=[#6:10]-[#6:11]-1-[#6:12](=[O:13])-[#6:14](-[#16:15]-[#6:16])-[#6@H:17]-2-[#6:18]>>[#6:16]-[#16:15][C:14]12[#6@@H:17](-[#6:18])-[#6:8]-3-[#6:9]=[#6:10]-[#6:11](-[#6:7]-3-[#6:4]1-[#8:1])-[#6:12]2=[O:13].[#6;v0:2].[#6;v0:6].[#8:5].[#8:3]

Correctness of the mapping

MAPPET	YES
ReactionMap	NO
Marvin	NO
ChemDraw	NO
Indigo	NO

Reaction no 165

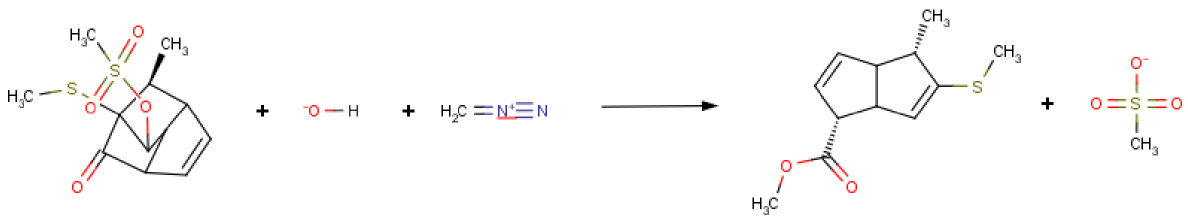

SMILES of the input:

CSC12[C@@H](C)C3C=CC(C3C1OS(C)(=O)=O)C2=O.[O-
][H].C=[N+]#N>>COC(=O)[C@@H]1C=CC2[C@H](C)C(SC)=CC12.CS([O-])(=O)=O

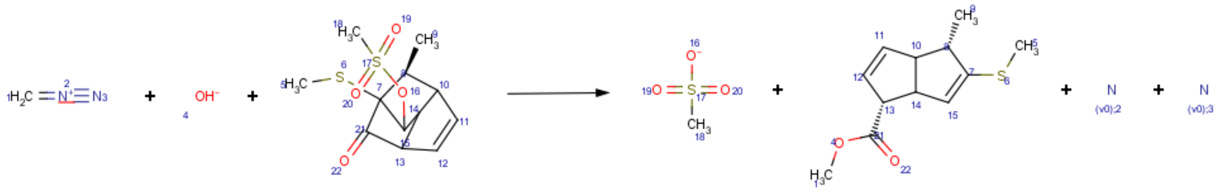

Correct mapped SMILES/SMARTS of the reaction:
[#6:1]=[N+:2]#[N:3].[#8-:4].[#6:5]-[#16:6][C:7]12[#6@@H:8](-[#6:9])-
[#6:10]-3-[#6:11]=[#6:12]-[#6:13](-[#6:14]-3-[#6:15]1-
[#8:16][S:17]([#6:18])(=[O:19])=[O:20])-
[#6:21]2=[O:22]>>[#6:18][S:17]([#8-:16])(=[O:20])=[O:19].[#6:1]-[#8:4]-
[#6:21](=[O:22])-[#6@@H:13]-1-[#6:12]=[#6:11]-[#6:10]-2-[#6@H:8](-
[#6:9])-[#6:7](-[#16:6]-[#6:5])=[#6:15]-[#6:14]-1-2.[#7;v0:2].[#7;v0:3]

Correctness of the mapping

MAPPET	YES
ReactionMap	NO
Marvin	YES
ChemDraw	YES
Indigo	NO

Reaction no 166

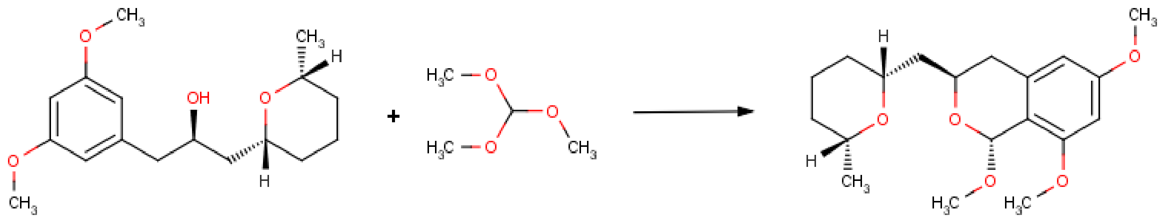

SMILES of the input:
[H][C@@]1(C)CCC[C@]([H])(C[C@H](O)CC2=CC(OC)=CC(OC)=C2)O1.COC(OC)OC>>[H][
C@@]1(C)CCC[C@]([H])(C[C@H]2CC3=CC(OC)=CC(OC)=C3[C@H](OC)O2)O1

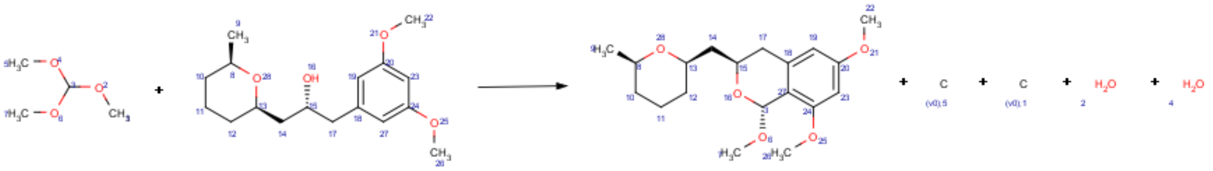

Correct mapped SMILES/SMARTS of the reaction:
[#6:1]-[#8:2]-[#6:3](-[#8:4]-[#6:5])-[#8:6]-[#6:7].[#6:22]-[#8:21]-
[#6:20]-1=[#6:23]-[#6:24](-[#8:25]-[#6:26])=[#6:27]-[#6:18](-[#6:17]-
[#6@@H:15](-[#8:16])-[#6:14]-[#6@H:13]-2-[#6:12]-[#6:11]-[#6:10]-
[#6@@H:8](-[#6:9])-[#8:28]-2)=[#6:19]-1>>[#6:7]-[#8:6]-[#6@@H:3]-1-
[#8:16]-[#6@@H:15](-[#6:14]-[#6@H:13]-2-[#6:12]-[#6:11]-[#6:10]-
[#6@@H:8](-[#6:9])-[#8:28]-2)-[#6:17]-[#6:18]-2=[#6:19]-[#6:20](-[#8:21]-

[#6:22])=[#6:23]-[#6:24](-[#8:25]-[#6:26])=[#6:27]-1-
2.[#6;v0:5].[#6;v0:1].[#8:2].[#8:4]

Correctness of the mapping

MAPPET	YES
ReactionMap	NO
Marvin	YES
ChemDraw	YES
Indigo	YES

Reaction no 167

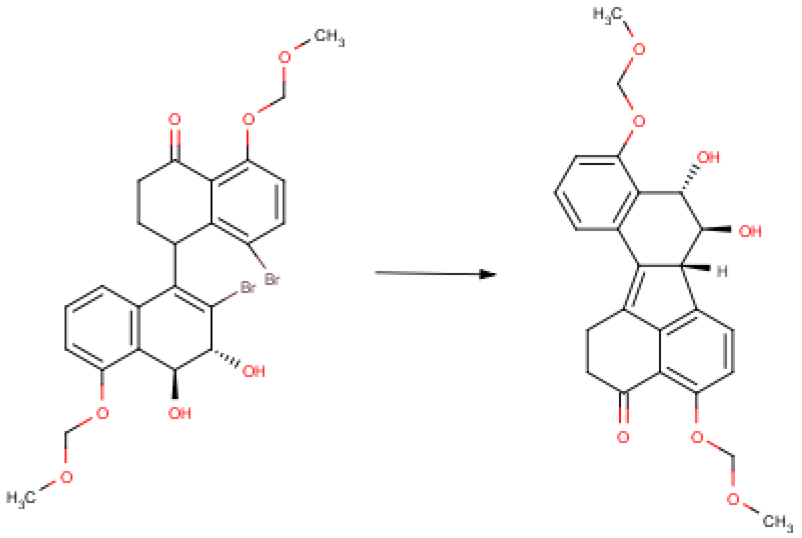

SMILES of the input:
COCOC1=CC=CC2=C1[C@H](O)[C@@H](O)C(Br)=C2C1CCC(=O)C2=C(OCOC)C=CC(Br)=C12>>[H][C@]12[C@H](O)[C@@H](O)C3=C(C=CC=C3OCOC)C1=C1CCC(=O)C3=C(OCOC)C=CC2=C13

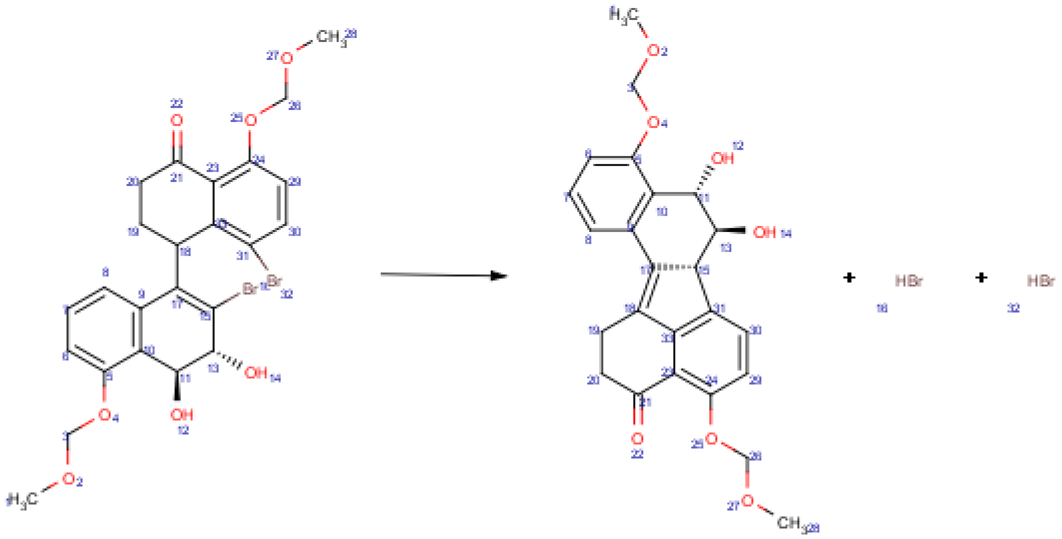

Correct mapped SMILES/SMARTS of the reaction:
[#6:1]-[#8:2]-[#6:3]-[#8:4]-[#6:5]-1=[#6:6]-[#6:7]=[#6:8]-[#6:9]-2=[#6:10]-1-[#6@H:11](-[#8:12])-[#6@@H:13](-[#8:14])-[#6:15]([Br:16])=[#6:17]-2-[#6:18]-1-[#6:19]-[#6:20]-[#6:21](=[O:22])-[#6:23]-2=[#6:24](-[#8:25]-[#6:26]-[#8:27]-[#6:28])-[#6:29]=[#6:30]-

[#6:31]([Br:32])=[#6:33]-1-2>>[#6:1]-[#8:2]-[#6:3]-[#8:4]-[#6:5]-1=[#6:6]-[#6:7]=[#6:8]-[#6:9]-2=[#6:10]-1-[#6@H:11](-[#8:12])-[#6@@H:13](-[#8:14])-[#6@@H:15]-1-[#6:31]-3=[#6:33]-4-[#6:18](-[#6:19]-[#6:20]-[#6:21](=[O:22])-[#6:23]-4=[#6:24](-[#8:25]-[#6:26]-[#8:27]-[#6:28])-[#6:29]=[#6:30]-3)=[#6:17]-2-1.[Br:16].[Br:32]

Correctness of the mapping

MAPPET YES
ReactionMap NO
Marvin YES
ChemDraw YES
Indigo NO

Reaction no 168

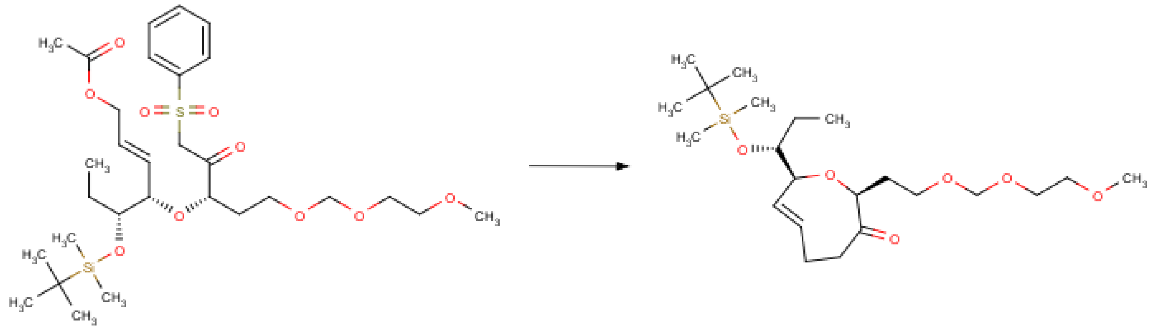

SMILES of the input:
CC[C@@H](O[Si](C)(C)C(C)(C)C)[C@@H](O[C@@H](CCOCOCCOC)C(=O)CS(=O)(=O)C1=CC=CC=C1)\C=C\COC(C)=O>>CC[C@@H](O[Si](C)(C)C(C)(C)C)[C@H]1O[C@@H](CCOCOCCOC)C(=O)CC\C=C\1

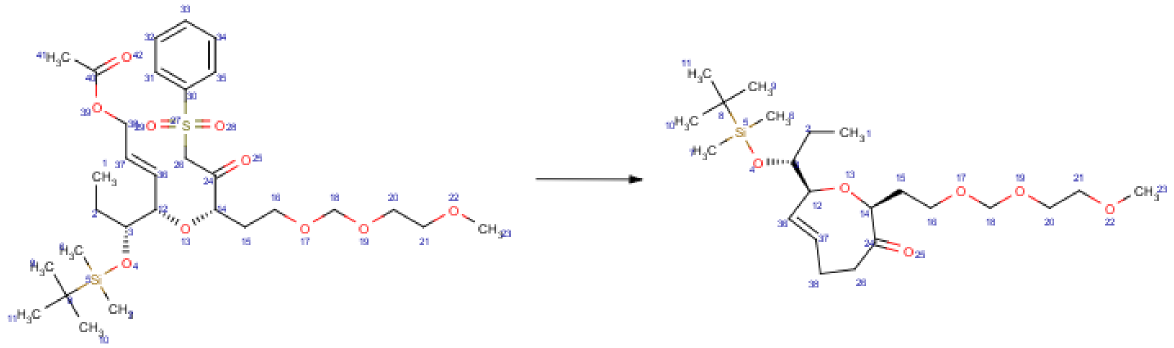

Correct mapped SMILES/SMARTS of the reaction:
[CH3:1][CH2:2][C@@H:3]([O:4][Si:5]([CH3:6])([CH3:7])[C:8]([CH3:9])([CH3:10])[CH3:11])[C@@H:12]([O:13][C@@H:14]([CH2:15][CH2:16][O:17][CH2:18][O:19][CH2:20][CH2:21][O:22][CH3:23])[C:24](=[O:25])[CH2:26][S:27](=[O:28])(=[O:29])[C:30]1=[CH:31][CH:32]=[CH:33][CH:34]=[CH:35]1)\[CH:36]=[CH:37]\[CH2:38][O:39][C:40]([CH3:41])=[O:42]>>[CH3:1][CH2:2][C@@H:3]([O:4][Si:5]([CH3:7])([CH3:6])[C:8]([CH3:11])([CH3:10])[CH3:9])[C@H:12]1[O:13][C@@H:14]([CH2:15][CH2:16][O:17][CH2:18][O:19][CH2:20][CH2:21][O:22][CH3:23])[C:24](=[O:25])[CH2:26][CH2:38]\[CH:37]=[CH:36]\1

Correctness of the mapping

MAPPET NO
ReactionMap NO

Marvin YES
ChemDraw YES
Indigo YES

Reaction no 169

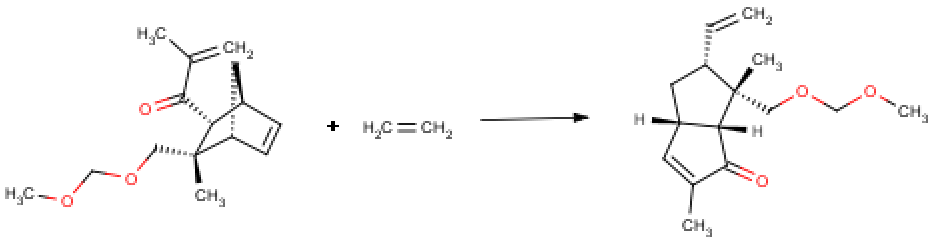

SMILES of the input:
COCOC[C@@]1(C)[C@@H]2C[C@@H](C=C2)[C@H]1C(=O)C(C)=C.C=C>>[H][C@@]12C[C@H](C=C)[C@](C)(COCOC)[C@]1([H])C(=O)C(C)=C2

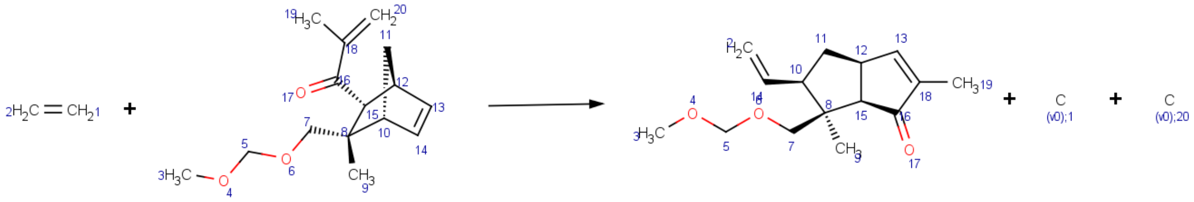

Correct mapped SMILES/SMARTS of the reaction:
[CH2:1]=[CH2:2].[CH3:3][O:4][CH2:5][O:6][CH2:7][C@@:8]1([CH3:9])[C@@H:10]2[CH2:11][C@@H:12]([CH:13]=[CH:14]2)[C@H:15]1[C:16](=[O:17])[C:18]([CH3:19])=[CH2:20]>>[CH3:3][O:4][CH2:5][O:6][CH2:7][C@@:8]1([CH3:9])[C@H:10]([CH2:11][C@H:12]2[CH:13]=[C:18]([CH3:19])[C:16](=[O:17])[C@@H:15]12)[CH:14]=[CH2:2].[C:1].[C:20]

Correctness of the mapping
MAPPET NO
ReactionMap NO
Marvin NO
ChemDraw YES
Indigo NO

Reaction no 170

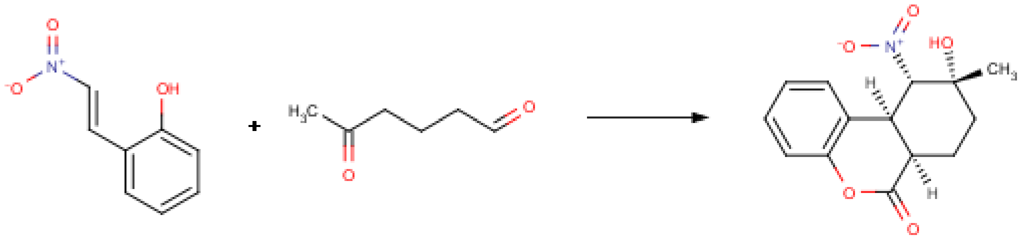

SMILES of the input:
OC1=CC=CC=C1\C=C\[N+]([O-
])=O.CC(=O)CCCC=O>>[H][C@@]12CC[C@@](C)(O)[C@@H]([N+]([O-
])=O)[C@]1([H])C1=C(OC2=O)C=CC=C1

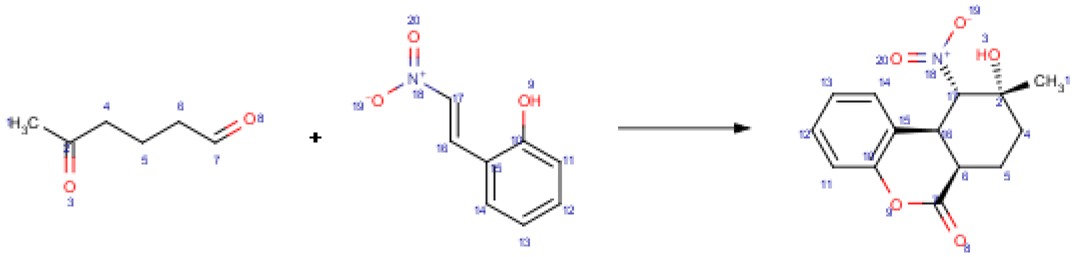

Correct mapped SMILES/SMARTS of the reaction:
[CH3:1][C:2](=[O:3])[CH2:4][CH2:5][CH2:6][CH:7]=[O:8].[OH:9][C:10]1=[CH:1
1][CH:12]=[CH:13][CH:14]=[C:15]1\[CH:16]=[CH:17]\[N+:18]([O-
:19])=[O:20]>>[CH3:1][C@@:2]1([OH:3])[CH2:4][CH2:5][C@@H:6]2[C@H:16]([C@@
H:17]1[N+:18]([O-
:19])=[O:20])[C:15]1=[C:10]([O:9][C:7]2=[O:8])[CH:11]=[CH:12][CH:13]=[CH:
14]1

Correctness of the mapping

MAPPET	YES
ReactionMap	YES
Marvin	YES
ChemDraw	YES
Indigo	NO

Reaction no 171

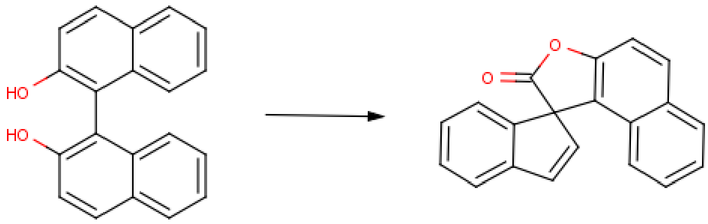

SMILES of the input:
OC1=C(C2=CC=CC=C2C=C1)C1=C(O)C=CC2=CC=CC=C12>>O=C1OC2=C(C3=CC=CC=C3C=C2)C
11C=CC2=CC=CC=C12

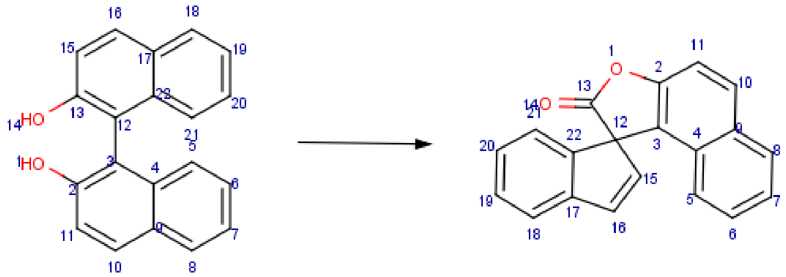

Correct mapped SMILES/SMARTS of the reaction:
[OH:1][C:2]1=[C:3]([C:4]2=[CH:5][CH:6]=[CH:7][CH:8]=[C:9]2[CH:10]=[CH:11]
1)[C:12]1=[C:13]([OH:14])[CH:15]=[CH:16][C:17]2=[CH:18][CH:19]=[CH:20][CH
:21]=[C:22]12>>[O:14]=[C:13]1[O:1][C:2]2=[C:3]([C:4]3=[CH:5][CH:6]=[CH:7]

[CH:8]=[C:9]3[CH:10]=[CH:11]2)[C:12]11[CH:15]=[CH:16][C:17]2=[CH:18][CH:19]=[CH:20][CH:21]=[C:22]12

Correctness of the mapping
MAPPET YES
ReactionMap NO
Marvin YES
ChemDraw YES
Indigo NO

Reaction no 172

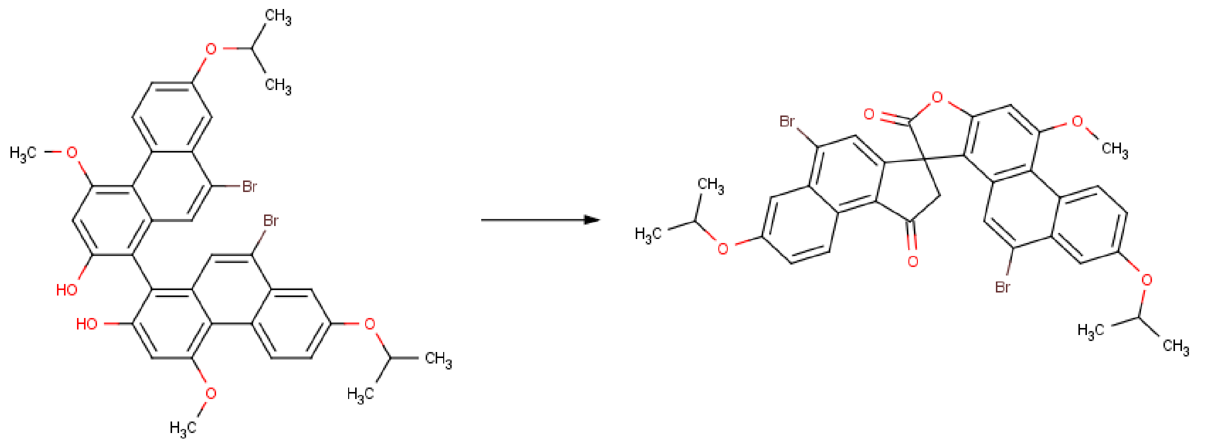

SMILES of the input:
COC1=C2C3=CC=C(OC(C)C)C=C3C(Br)=CC2=C(C(O)=C1)C1=C2C=C(Br)C3=CC(OC(C)C)=CC=C3C2=C(OC)C=C1O>>COC1=CC2=C(C3=C1C1=CC=C(OC(C)C)C=C1C(Br)=C3)C1(CC(=O)C3=C4C=CC(OC(C)C)=CC4=C(Br)C=C13)C(=O)O2

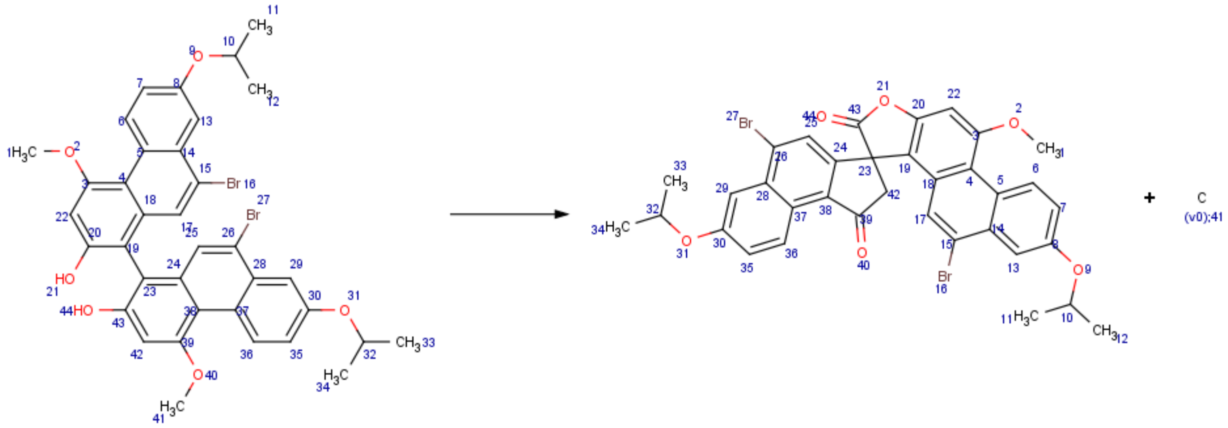

Correct mapped SMILES/SMARTS of the reaction:
[CH3:1][O:2][C:3]1=[C:4]2[C:5]3=[CH:6][CH:7]=[C:8]([O:9][CH:10]([CH3:11])[CH3:12])[CH:13]=[C:14]3[C:15]([Br:16])=[CH:17][C:18]2=[C:19]([C:20]([OH:21])=[CH:22]1)[C:23]1=[C:24]2[CH:25]=[C:26]([Br:27])[C:28]3=[CH:29][C:30]([O:31][CH:32]([CH3:33])[CH3:34])=[CH:35][CH:36]=[C:37]3[C:38]2=[C:39]([O:40][CH3:41])[CH:42]=[C:43]1[OH:44]>>[CH3:1][O:2][C:3]1=[CH:22][C:20]2=[C:19]([C:18]3=[C:4]1[C:5]1=[CH:6][CH:7]=[C:8]([O:9][CH:10]([CH3:12])[CH3:11])[CH:13]=[C:14]1[C:15]([Br:16])=[CH:17]3)[C:23]1([CH2:42][C:39](=[O:40])[C:38]3=[C:37]4[CH:36]=[CH:35][C:30]([O:31][CH:32]([CH3:34])[CH3:33])=[CH:29][C:28]4=[C:26]([Br:27])[CH:25]=[C:24]13)[C:43](=[O:44])[O:21]2.[C:41]

Correctness of the mapping
MAPPET YES
ReactionMap NO

Marvin NO
ChemDraw NO
Indigo NO

Reaction no 173

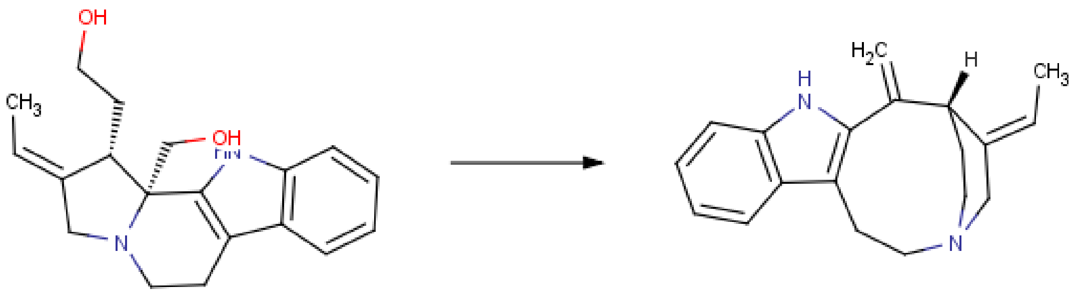

SMILES of the input:
C\C=C1\CN2CCC3=C(NC4=C3C=CC=C4)[C@]2(CO)[C@H]1CCO>>[H][C@]12CCN(C\C1=C\C)CCC1=C(NC3=C1C=CC=C3)C2=C

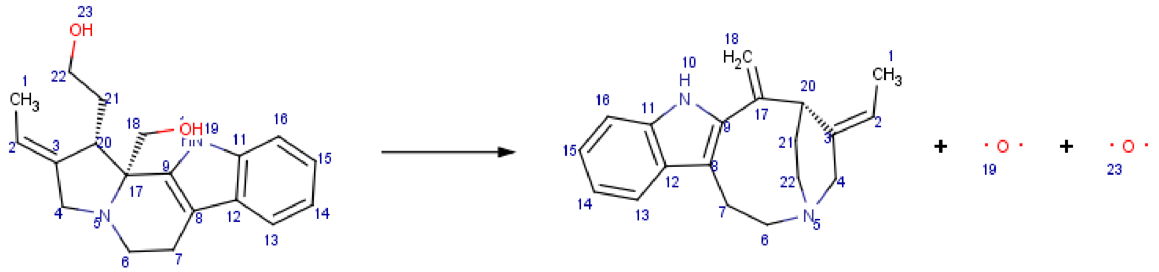

Correct mapped SMILES/SMARTS of the reaction:
[CH3:1]\[CH:2]=[C:3]1\[CH2:4][N:5]2[CH2:6][CH2:7][C:8]3=[C:9]([NH:10][C:11]4=[C:12]3[CH:13]=[CH:14][CH:15]=[CH:16]4)[C@:17]2([CH2:18][OH:19])[C@H:20]1[CH2:21][CH2:22][OH:23]>>[CH3:1]\[CH:2]=[C:3]1\[CH2:4][N:5]2[CH2:22][CH2:21][C@@H:20]1[C:17](=[CH2:18])[C:9]1=[C:8]([CH2:7][CH2:6]2)[C:12]2=[C:11]([NH:10]1)[CH:16]=[CH:15][CH:14]=[CH:13]2.[O:19].[O:23]

Correctness of the mapping
MAPPET YES
ReactionMap NO
Marvin YES
ChemDraw YES
Indigo YES

Reaction no 174

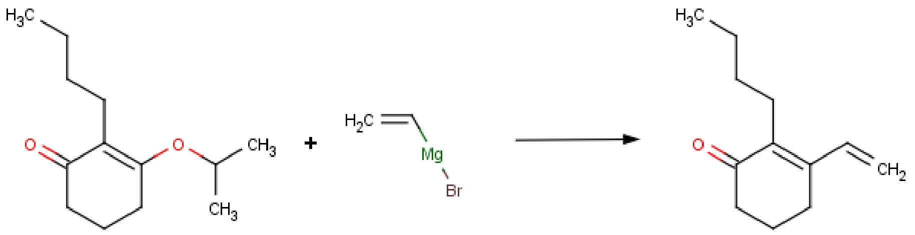

SMILES of the input:
CCCCC1=C(CCCC1=O)OC(C)C.Br[Mg]C=C>>CCCCC1=C(CCCC1=O)C=C

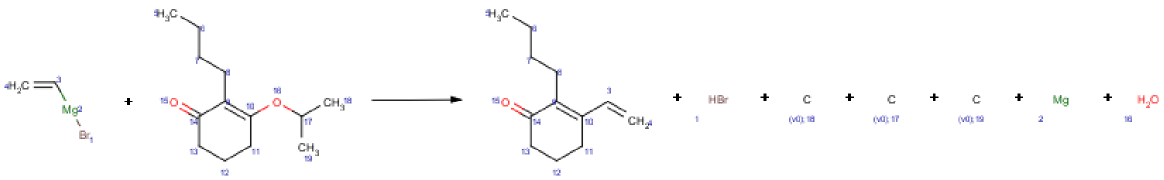

Correct mapped SMILES/SMARTS of the reaction:
[Br:1][Mg:2][#6:3]=[#6:4].[#6:5]-[#6:6]-[#6:7]-[#6:8]-[#6:9]-1=[#6:10](-[#6:11]-[#6:12]-[#6:13]-[#6:14]-1=[O:15])-[#8:16]-[#6:17](-[#6:18])-[#6:19]>>[#6:5]-[#6:6]-[#6:7]-[#6:8]-[#6:9]-1=[#6:10](-[#6:11]-[#6:12]-[#6:13]-[#6:14]-1=[O:15])-[#6:3]=[#6:4].[Br:1].[#6;v0:18].[#6;v0:17].[#6;v0:19].[Mg:2].[#8:16]

Correctness of the mapping

MAPPET	YES
ReactionMap	NO
Marvin	YES
ChemDraw	YES
Indigo	YES

Reaction no 175

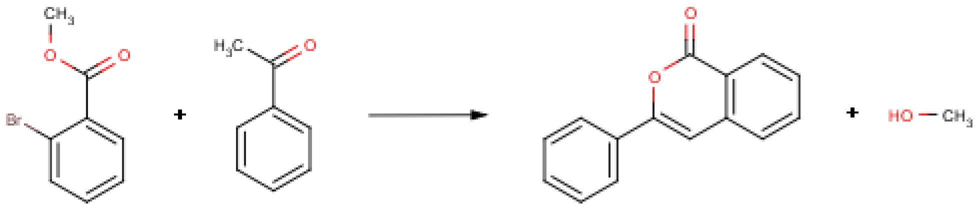

SMILES of the input:
COC(=O)C1=CC=CC=C1Br.CC(=O)C1=CC=CC=C1>>O=C1OC(=CC2=C1C=CC=C2)C1=CC=CC=C1.CO

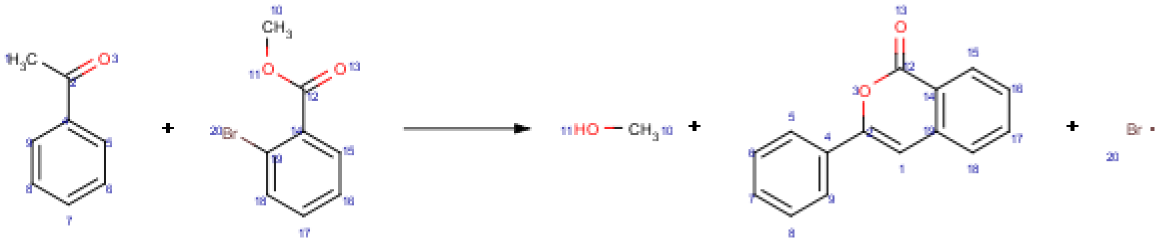

Correct mapped SMILES/SMARTS of the reaction:
[CH3:1][C:2](=[O:3])[C:4]1=[CH:5][CH:6]=[CH:7][CH:8]=[CH:9]1.[CH3:10][O:11][C:12](=[O:13])[C:14]1=[CH:15][CH:16]=[CH:17][CH:18]=[C:19]1[Br:20]>>[CH3:10][OH:11].[O:13]=[C:12]1[O:3][C:2](=[CH:1][C:19]2=[C:14]1[CH:15]=[CH:16][CH:17]=[CH:18]2)[C:4]1=[CH:9][CH:8]=[CH:7][CH:6]=[CH:5]1.[Br:20]

Correctness of the mapping

MAPPET	YES
ReactionMap	NO

Marvin NO
ChemDraw NO
Indigo NO

Reaction no 176

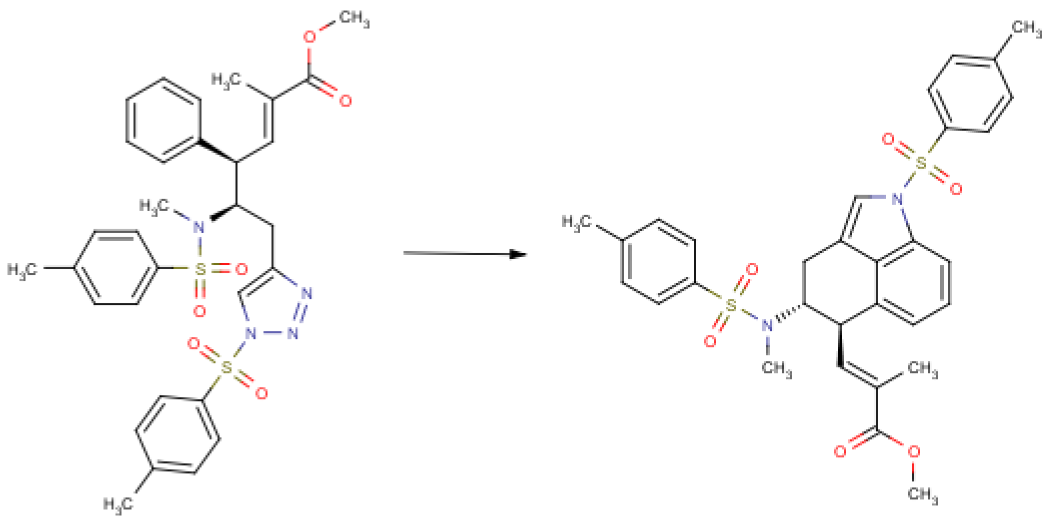

SMILES of the input:
COC(=O)C(\C)=C\[C@@H]([C@@H](CC1=CN(N=N1)S(=O)(=O)C1=CC=C(C)C=C1)N(C)S(=O)(=O)C1=CC=C(C)C=C1)C1=CC=CC=C1>>COC(=O)C(\C)=C\[C@H]1[C@@H](CC2=CN(C3=C2C1=CC=C3)S(=O)(=O)C1=CC=C(C)C=C1)N(C)S(=O)(=O)C1=CC=C(C)C=C1

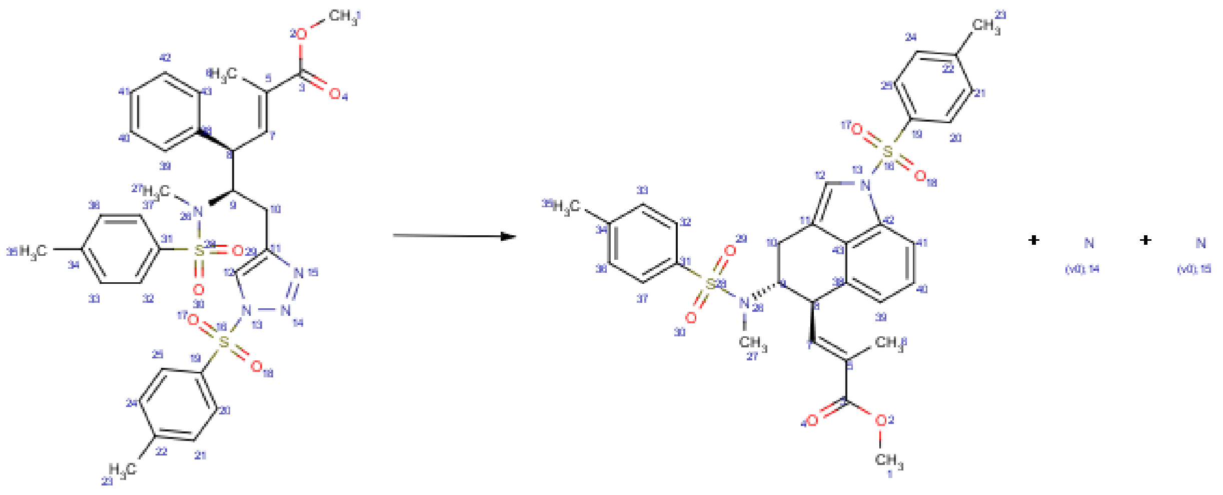

Correct mapped SMILES/SMARTS of the reaction:
[CH3:1][O:2][C:3](=[O:4])[C:5](\[CH3:6])=[CH:7]\[C@@H:8]([C@@H:9]([CH2:10][C:11]1=[CH:12][N:13]([N:14]=[N:15]1)[S:16](=[O:17])(=[O:18])[C:19]1=[CH:20][CH:21]=[C:22]([CH3:23])[CH:24]=[CH:25]1)[N:26]([CH3:27])[S:28](=[O:29])(=[O:30])[C:31]1=[CH:32][CH:33]=[C:34]([CH3:35])[CH:36]=[CH:37]1)[C:38]1=[CH:39][CH:40]=[CH:41][CH:42]=[CH:43]1>>[CH3:1][O:2][C:3](=[O:4])[C:5](\[CH3:6])=[CH:7]\[C@H:8]1[C@@H:9]([CH2:10][C:11]2=[CH:12][N:13]([C:42]3=[C:43]2[C:38]1=[CH:39][CH:40]=[CH:41]3)[S:16](=[O:17])(=[O:18])[C:19]1=[CH:25][CH:24]=[C:22]([CH3:23])[CH:21]=[CH:20]1)[N:26]([CH3:27])[S:28](=[O:29])(=[O:30])[C:31]1=[CH:37][CH:36]=[C:34]([CH3:35])[CH:33]=[CH:32]1.[N:14].[N:15]

Correctness of the mapping
MAPPET YES
ReactionMap NO
Marvin YES
ChemDraw YES
Indigo YES

Reaction no 177

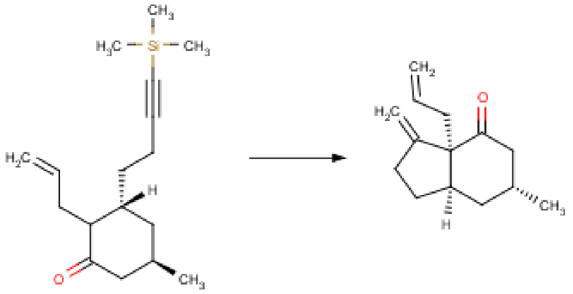

SMILES of the input:
[H][C@@]1(CCC#C[Si](C)(C)C)C[C@@H](C)CC(=O)C1CC=C>>[H][C@]12CCC(=C)[C@@]1(CC=C)C(=O)C[C@H](C)C2

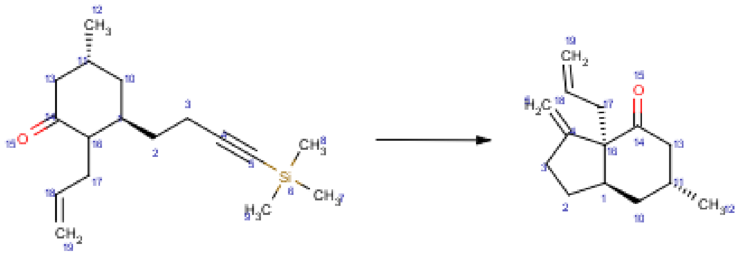

Correct mapped SMILES/SMARTS of the reaction:
[CH3:12][C@@H:11]1[CH2:10][C@@H:1]([CH2:2][CH2:3][C:4]#[C:5][Si:6]([CH3:7])([CH3:8])[CH3:9])[CH:16]([CH2:17][CH:18]=[CH2:19])[C:14](=[O:15])[CH2:13]1>>[CH3:12][C@@H:11]1[CH2:10][C@H:1]2[CH2:2][CH2:3][C:4](=[CH2:5])[C@@:16]2([CH2:17][CH:18]=[CH2:19])[C:14](=[O:15])[CH2:13]1.[C:8].[C:7].[C:9].[Si:6]

Correctness of the mapping
MAPPET YES
ReactionMap NO
Marvin YES
ChemDraw NO
Indigo YES

Reaction no 178

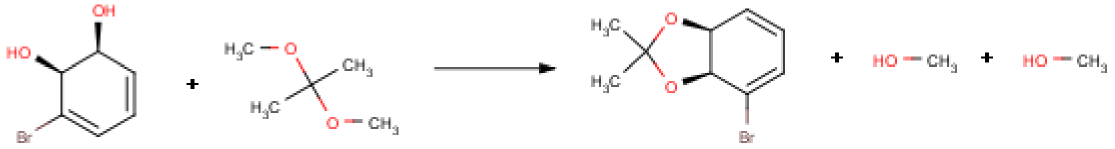

SMILES of the input:

O[C@H]1C=CC=C(Br)[C@H]1O.COC(C)(C)OC>>CC1(C)O[C@H]2C=CC=C(Br)[C@H]2O1.CO.
CO

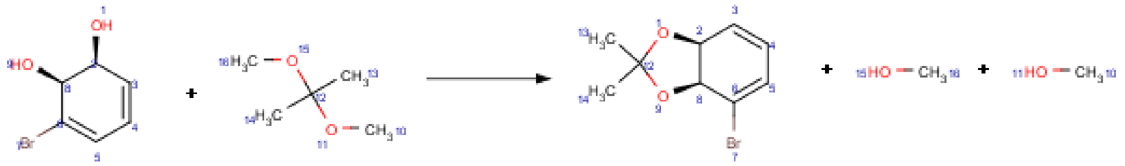

Correct mapped SMILES/SMARTS of the reaction:
[OH:1][C@H:2]1[CH:3]=[CH:4][CH:5]=[C:6]([Br:7])[C@H:8]1[OH:9].[CH3:10][O:
11][C:12]([CH3:13])([CH3:14])[O:15][CH3:16]>>[CH3:13][C:12]1([CH3:14])[O:
1][C@H:2]2[CH:3]=[CH:4][CH:5]=[C:6]([Br:7])[C@H:8]2[O:9]1.[CH3:16][OH:15]
.[CH3:10][OH:11]

Correctness of the mapping

MAPPET	YES
ReactionMap	YES
Marvin	YES
ChemDraw	YES
Indigo	NO

Reaction no 179

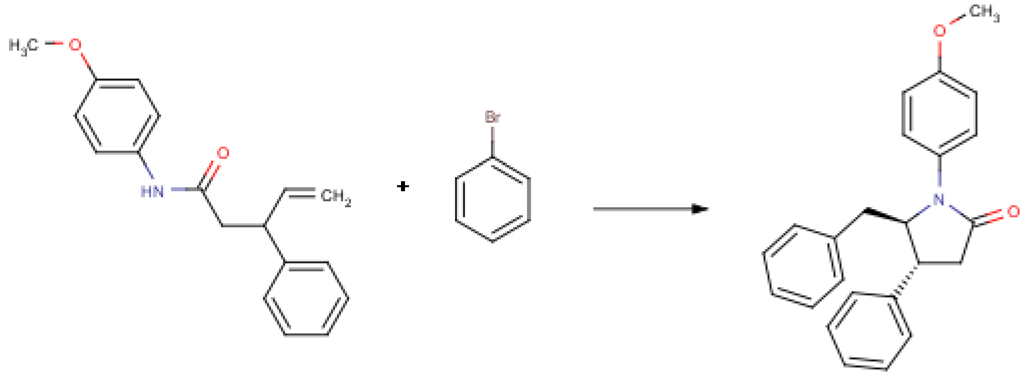

SMILES of the input:
COC1=CC=C(NC(=O)CC(C=C)C2=CC=CC=C2)C=C1.BrC1=CC=CC=C1>>COC1=CC=C(C=C1)N1[
C@H](CC2=CC=CC=C2)[C@H](CC1=O)C1=CC=CC=C1

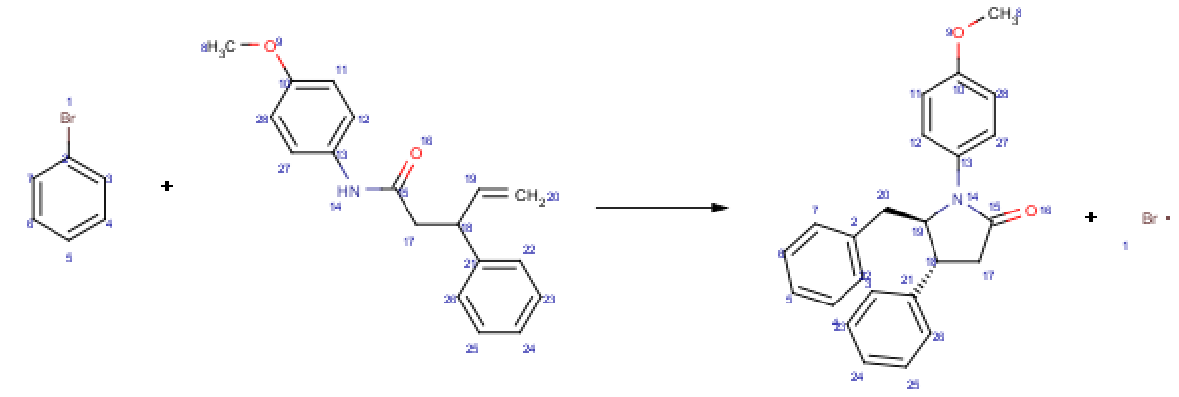

Correct mapped SMILES/SMARTS of the reaction:
[Br:1][C:2]1=[CH:3][CH:4]=[CH:5][CH:6]=[CH:7]1.[CH3:8][O:9][C:10]1=[CH:11
][CH:12]=[C:13]([NH:14][C:15](=[O:16])[CH2:17][CH:18]([CH:19]=[CH2:20])[C
:21]2=[CH:22][CH:23]=[CH:24][CH:25]=[CH:26]2)[CH:27]=[CH:28]1>>[CH3:8][O:
9][C:10]1=[CH:28][CH:27]=[C:13]([CH:12]=[CH:11]1)[N:14]1[C@H:19]([CH2:20]
[C:2]2=[CH:3][CH:4]=[CH:5][CH:6]=[CH:7]2)[C@H:18]([CH2:17][C:15]1=[O:16])
[C:21]1=[CH:26][CH:25]=[CH:24][CH:23]=[CH:22]1.[Br:1]

Correctness of the mapping

MAPPET	YES
ReactionMap	NO
Marvin	YES
ChemDraw	YES
Indigo	NO

Reaction no 180

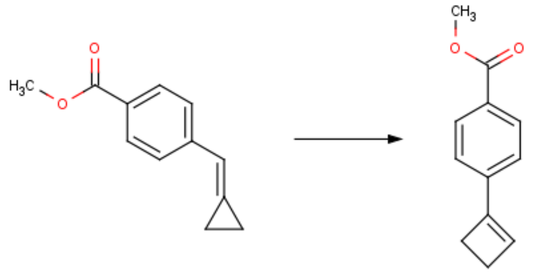

SMILES of the input:
COC(=O)C1=CC=C(C=C2CC2)C=C1>>COC(=O)C1=CC=C(C=C1)C1=CCC1

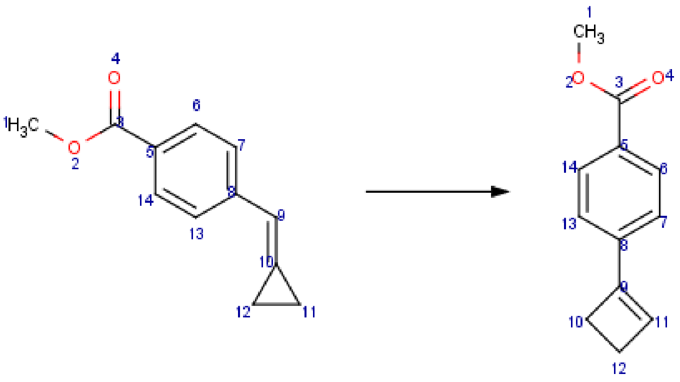

Correct mapped SMILES/SMARTS of the reaction:

[CH3:1][O:2][C:3](=[O:4])[C:5]1=[CH:6][CH:7]=[C:8]([CH:9]=[C:10]2[CH2:11][CH2:12]2)[CH:13]=[CH:14]1>>[CH3:1][O:2][C:3](=[O:4])[C:5]1=[CH:6][CH:7]=[C:8]([CH:13]=[CH:14]1)[C:9]1=[CH:11][CH2:12][CH2:10]1

Correctness of the mapping

MAPPET	NO
ReactionMap	NO
Marvin	NO
ChemDraw	YES
Indigo	NO

Reaction no 181

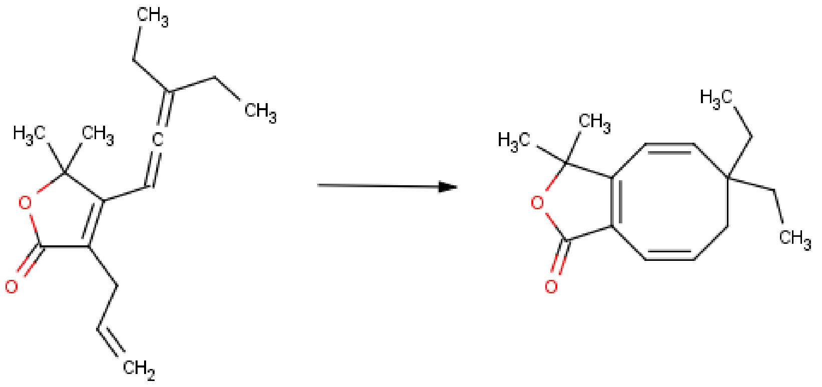

SMILES of the input:
CCC(CC)=C=CC1=C(CC=C)C(=O)OC1(C)C>>CCC1(CC)C\C=C/C2=C(\C=C/1)C(C)(C)OC2=O

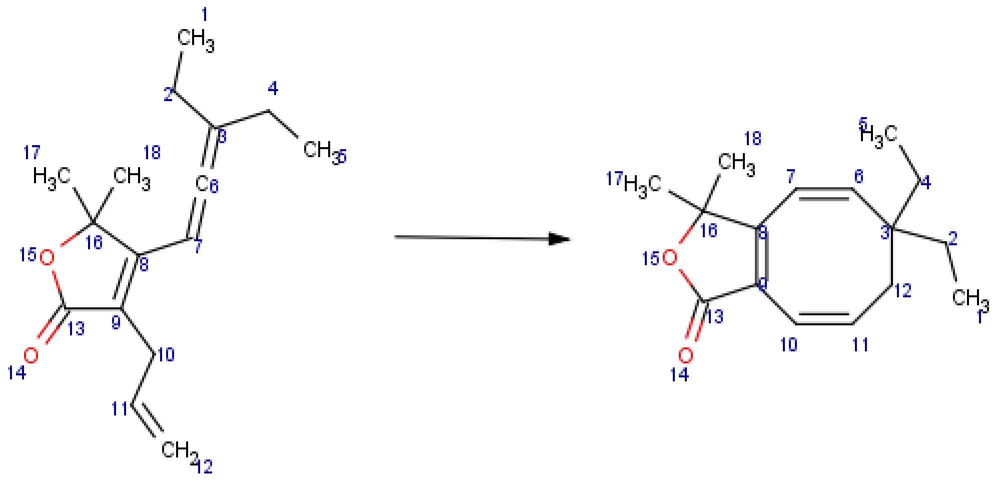

Correct mapped SMILES/SMARTS of the reaction:
[#6:1]-[#6:2]-[#6:3](-[#6:4]-[#6:5])=[C:6]=[#6:7]-[#6:8]1=[#6:9](-[#6:10]-[#6:11]=[#6:12])-[#6:13](=[O:14])-[#8:15][C:16]1([#6:17])[#6:18]>>[#6:1]-[#6:2][C:3]1([#6:4]-[#6:5])[#6:12]\[#6:11]=[#6:10]/[#6:9]-2=[#6:8](\[#6:7]=[#6:6]/1)[C:16]([#6:18])([#6:17])[#8:15]-[#6:13]2=[O:14]

Correctness of the mapping

MAPPET	YES
ReactionMap	YES
Marvin	NO
ChemDraw	YES
Indigo	NO

Reaction no 182

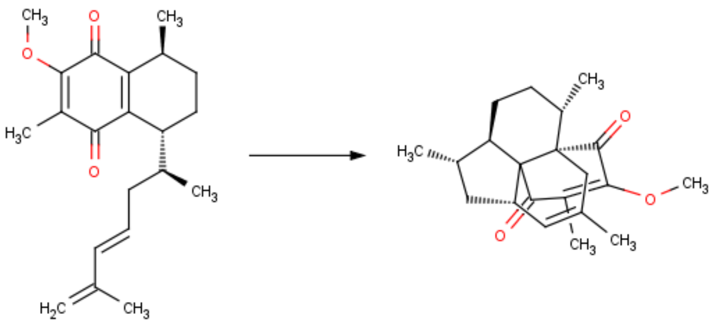

SMILES of the input:
COC1=C(C)C(=O)C2=C([C@@H](C)CC[C@@H]2[C@@H](C)C\C=C\C(C)=C)C1=O>>COC1=C(C)C(=O)C23[C@H]4C[C@H](C)[C@H]2CC[C@H](C)[C@]3(CC(C)=C4)C1=O

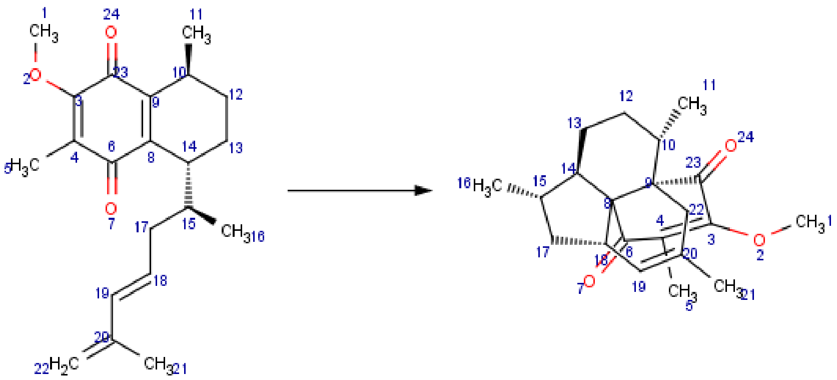

Correct mapped SMILES/SMARTS of the reaction:
[#6:1]-[#8:2]-[#6:3]-1=[#6:4](-[#6:5])-[#6:6](=[O:7])-[#6:8]-2=[#6:9](-[#6@@H:10](-[#6:11])-[#6:12]-[#6:13]-[#6@@H:14]-2-[#6@@H:15](-[#6:16])-[#6:17]\[#6:18]=[#6:19]\[#6:20](-[#6:21])=[#6:22])-[#6:23]-1=[O:24]>>[#6:1]-[#8:2]-[#6:3]-1=[#6:4](-[#6:5])-[#6:6](=[O:7])[C:8]23[#6@H:18]-4-[#6:17]-[#6@H:15](-[#6:16])-[#6@H:14]2-[#6:13]-[#6:12]-[#6@H:10](-[#6:11])[C@:9]3([#6:22]-[#6:20](-[#6:21])=[#6:19]-4)[#6:23]-1=[O:24]

Correctness of the mapping

MAPPET	YES
ReactionMap	NO
Marvin	YES
ChemDraw	YES
Indigo	NO

Reaction no 183

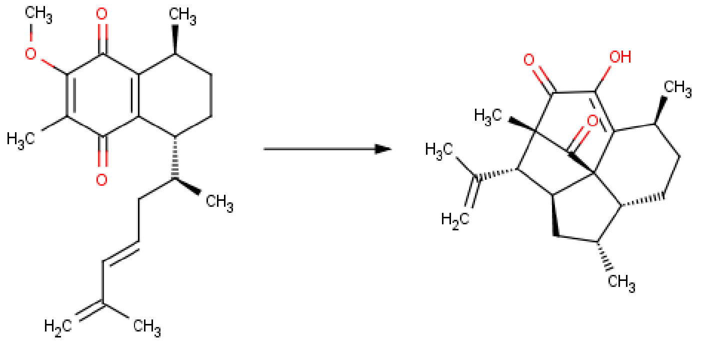

SMILES of the input:
COC1=C(C)C(=O)C2=C([C@@H](C)CC[C@@H]2[C@@H](C)C\C=C\C(C)=C)C1=O>>C[C@@H]1C[C@H]2[C@H](C(C)=C)[C@@]3(C)C(=O)[C@@]22[C@@H]1CC[C@H](C)C2=C(O)C3=O

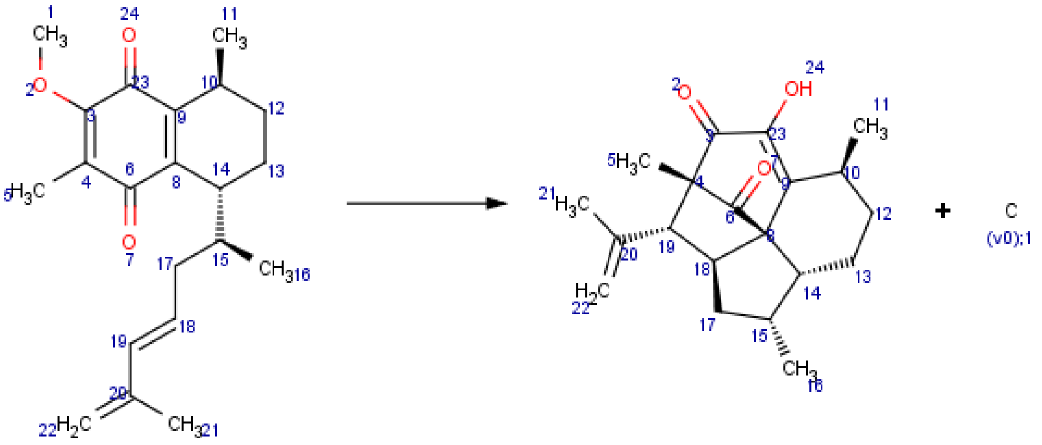

Correct mapped SMILES/SMARTS of the reaction:
[#6:1]-[#8:2]-[#6:3]-1=[#6:4](-[#6:5])-[#6:6](=[O:7])-[#6:8]-2=[#6:9](-[#6@@H:10](-[#6:11])-[#6:12]-[#6:13]-[#6@@H:14]-2-[#6@@H:15](-[#6:16])-[#6:17]\[#6:18]=[#6:19]\[#6:20](-[#6:21])=[#6:22])-[#6:23]-1=[O:24]>>[#6:16]-[#6@@H:15]-1-[#6:17]-[#6@H:18]2-[#6@H:19](-[#6:20](-[#6:21])=[#6:22])[C@@:4]3([#6:5])[#6:6](=[O:7])[C@@:8]22[#6@@H:14]-1-[#6:13]-[#6:12]-[#6@H:10](-[#6:11])-[#6:9]2=[#6:23](-[#8:24])-[#6:3]3=[O:2].[#6;v0:1]

Correctness of the mapping

MAPPET	YES
ReactionMap	NO
Marvin	NO
ChemDraw	YES
Indigo	NO

Reaction no 184

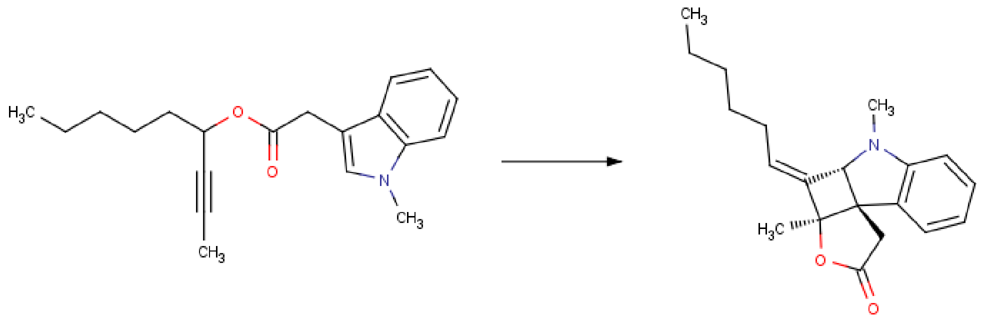

SMILES of the input:
CCCCCC(OC(=O)CC1=CN(C)C2=C1C=CC=C2)C#CC>>CCCCC\C=C1/[C@@H]2N(C)C3=C(C=CC=C3)[C@@]22CC(=O)O[C@@]12C

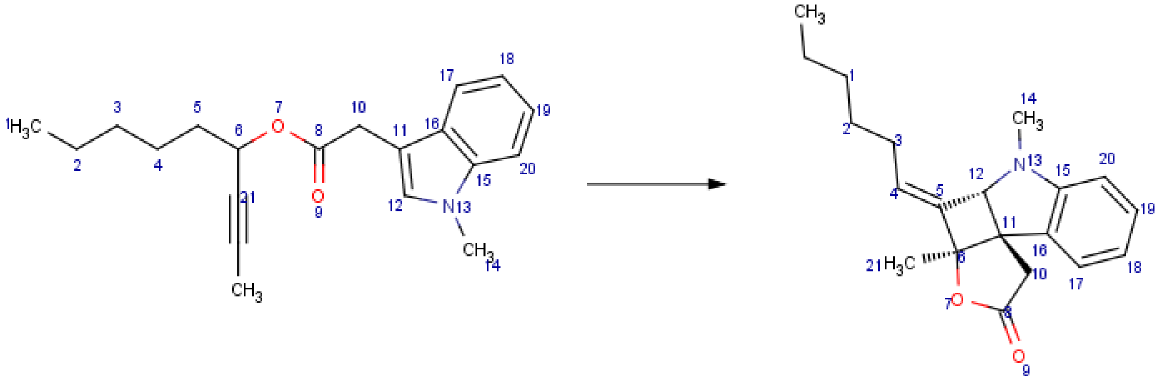

Correct mapped SMILES/SMARTS of the reaction:
[CH3:1][CH2:2][CH2:3][CH2:4][CH2:5][CH:6]([O:7][C:8](=[O:9])[CH2:10][C:11]1=[CH:12][N:13]([CH3:14])[C:15]2=[C:16]1[CH:17]=[CH:18][CH:19]=[CH:20]2)[C:21]#CC>>CC[CH2:1][CH2:2][CH2:3]\[CH:4]=[C:5]1/[C@@H:12]2[N:13]([CH3:14])[C:15]3=[C:16]([CH:17]=[CH:18][CH:19]=[CH:20]3)[C@@:11]22[CH2:10][C:8](=[O:9])[O:7][C@@:6]12[CH3:21]

Correctness of the mapping

MAPPET	NO
ReactionMap	NO
Marvin	NO
ChemDraw	NO
Indigo	NO

Reaction no 185

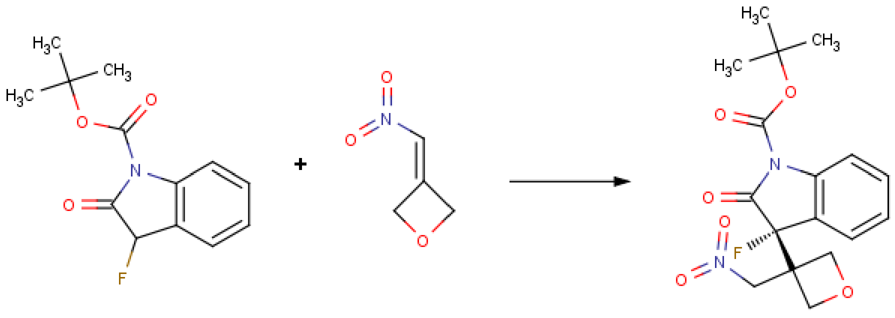

SMILES of the input:

CC(C)(C)OC(=O)N1C(=O)C(F)C2=C1C=CC=C2.O=N(=O)C=C1COC1>>CC(C)(C)OC(=O)N1C(=O)[C@@](F)(C2=C1C=CC=C2)C1(CN(=O)=O)COC1

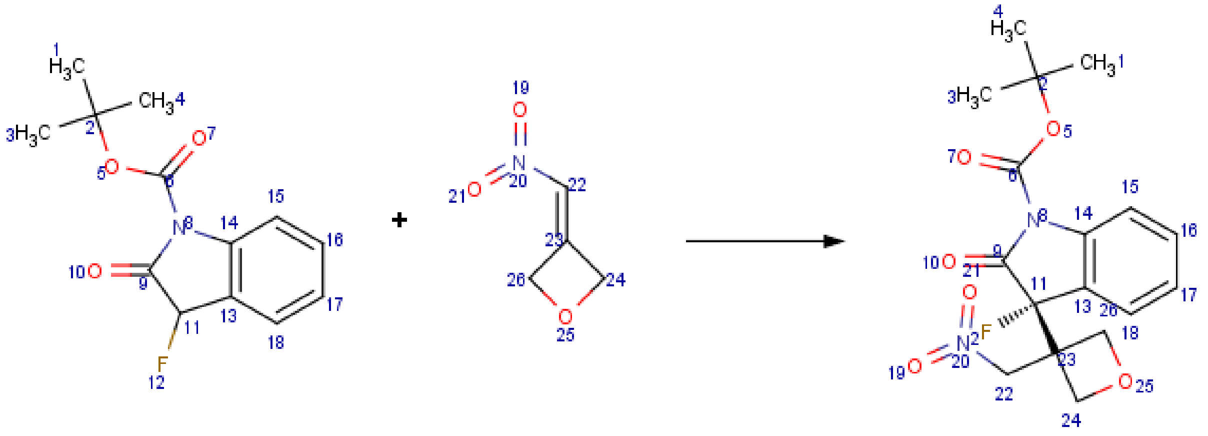

Correct mapped SMILES/SMARTS of the reaction:
[#6:1][C:2]([#6:3])([#6:4])[#8:5]-[#6:6](=[O:7])-[#7:8]-1-[#6:9](=[O:10])-[#6:11]([F:12])-[#6:13]-2=[#6:14]-1-[#6:15]=[#6:16]-[#6:17]=[#6:18]-2.[O:19]=[N:20](=[O:21])\[#6:22]=[#6:23]-1\[#6:24]-[#8:25]-[#6:26]-1>>[#6:4][C:2]([#6:3])([#6:1])[#8:5]-[#6:6](=[O:7])-[#7:8]-1-[#6:9](=[O:10])[C@@:11]([F:12])([#6:13]-2=[#6:14]-1-[#6:15]=[#6:16]-[#6:17]=[#6:18]-2)[C:23]1([#6:22][N:20](=[O:19])=[O:21])[#6:26]-[#8:25]-[#6:24]1

Correctness of the mapping

MAPPET	YES
ReactionMap	YES
Marvin	YES
ChemDraw	YES
Indigo	YES

Reaction no 186

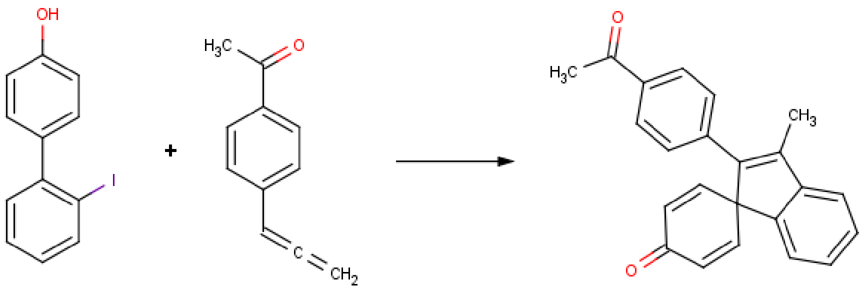

SMILES of the input:
OC1=CC=C(C=C1)C1=C(I)C=CC=C1.CC(=O)C1=CC=C(C=C=C)C=C1>>CC(=O)C1=CC=C(C=C1)C1=C(C)C2=CC=CC=C2C11C=CC(=O)C=C1

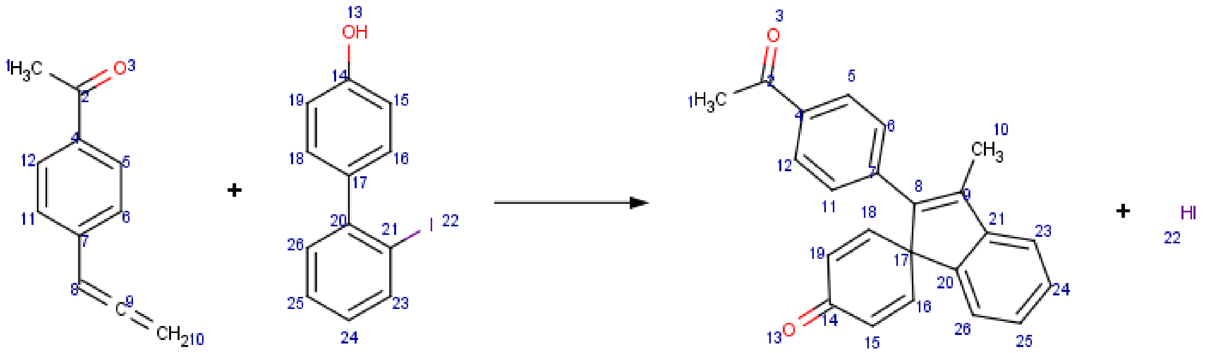

Correct mapped SMILES/SMARTS of the reaction:
[#6:1]-[#6:2](=[O:3])-[#6:4]-1=[#6:5]-[#6:6]=[#6:7](-
[#6:8]=[C:9]=[#6:10])-[#6:11]=[#6:12]-1.[#8:13]-[#6:14]-1=[#6:15]-
[#6:16]=[#6:17](-[#6:18]=[#6:19]-1)-[#6:20]-1=[#6:21]([I:22])-
[#6:23]=[#6:24]-[#6:25]=[#6:26]-1>>[#6:1]-[#6:2](=[O:3])-[#6:4]-1=[#6:5]-
[#6:6]=[#6:7](-[#6:11]=[#6:12]-1)-[#6:8]1=[#6:9](-[#6:10])-[#6:21]-
2=[#6:23]-[#6:24]=[#6:25]-[#6:26]=[#6:20]-2[C:17]11[#6:16]=[#6:15]-
[#6:14](=[O:13])-[#6:19]=[#6:18]1.[I:22]

Correctness of the mapping
MAPPET YES
ReactionMap NO
Marvin YES
ChemDraw YES
Indigo YES

Reaction no 187

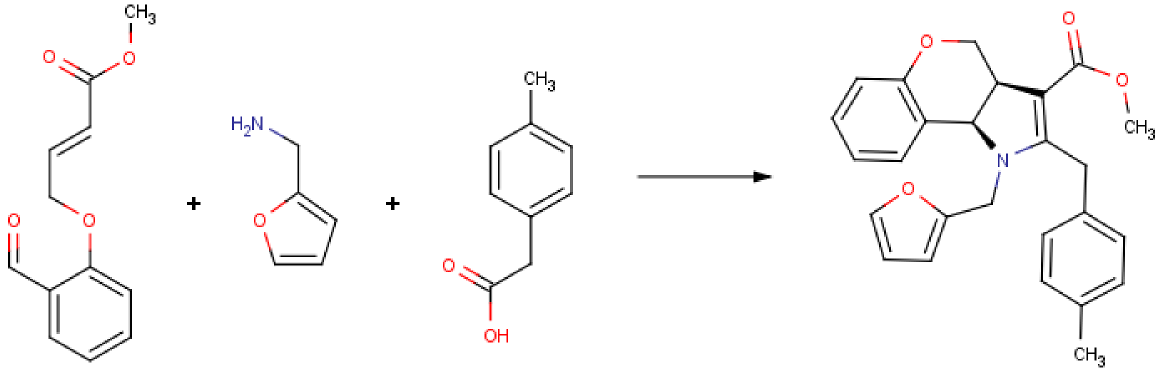

SMILES of the input:
COC(=O)\C=C\COC1=CC=CC=C1C=O.NCC1=CC=CO1.CC1=CC=C(CC(O)=O)C=C1>>COC(=O)C1
=C(CC2=CC=C(C)C=C2)N(CC2=CC=CO2)[C@@H]2[C@H]1COC1=C2C=CC=C1

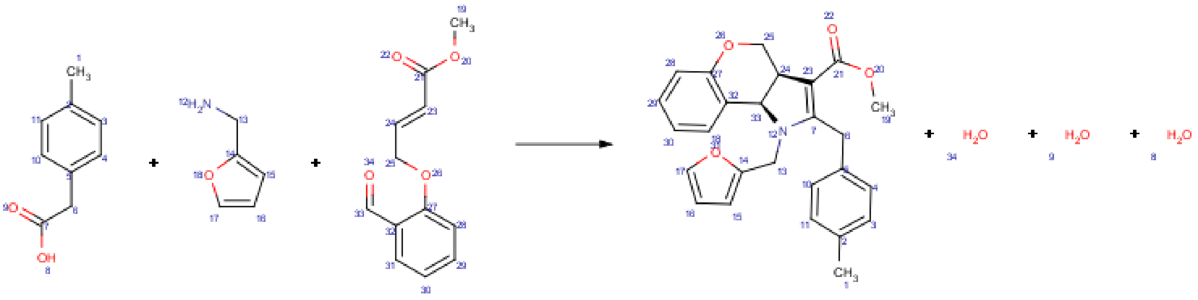

Correct mapped SMILES/SMARTS of the reaction:
[#6:1]-[#6:2]-1=[#6:3]-[#6:4]=[#6:5](-[#6:6]-[#6:7](-[#8:8])=[O:9])-
[#6:10]=[#6:11]-1.[#7:12]-[#6:13]-[#6:14]-1=[#6:15]-[#6:16]=[#6:17]-
[#8:18]-1.[#6:19]-[#8:20]-[#6:21](=[O:22])\[#6:23]=[#6:24]\[#6:25]-
[#8:26]-[#6:27]-1=[#6:28]-[#6:29]=[#6:30]-[#6:31]=[#6:32]-1-
[#6:33]=[O:34]>>[#6:19]-[#8:20]-[#6:21](=[O:22])-[#6:23]-1=[#6:7](-
[#6:6]-[#6:5]-2=[#6:4]-[#6:3]=[#6:2](-[#6:1])-[#6:11]=[#6:10]-2)-
[#7:12](-[#6:13]-[#6:14]-2=[#6:15]-[#6:16]=[#6:17]-[#8:18]-2)-[#6@@H:33]-
2-[#6@H:24]-1-[#6:25]-[#8:26]-[#6:27]-1=[#6:32]-2-[#6:31]=[#6:30]-
[#6:29]=[#6:28]-1.[#8:34].[#8:9].[#8:8]

Correctness of the mapping

MAPPET	YES
ReactionMap	NO
Marvin	YES
ChemDraw	YES
Indigo	YES

Reaction no 188

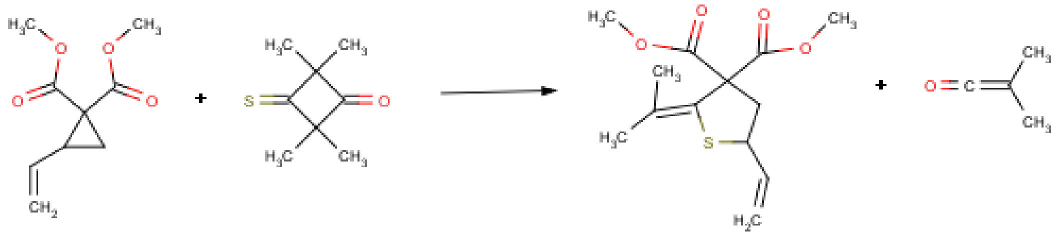

SMILES of the input:
COC(=O)C1(CC1C=C)C(=O)OC.CC1(C)C(=O)C(C)(C)C1=S>>COC(=O)C1(CC(SC1=C(C)C)C
=C)C(=O)OC.CC(C)=C=O

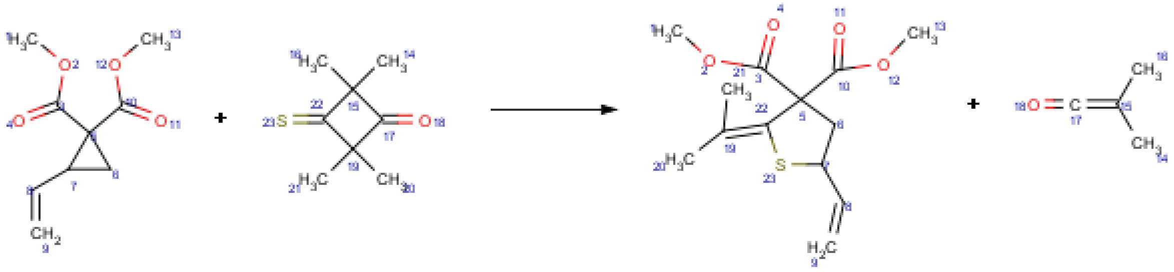

Correct mapped SMILES/SMARTS of the reaction:

[#6:1]-[#8:2]-[#6:3](=[O:4])[C:5]1([#6:6]-[#6:7]1-
[#6:8]=[#6:9])[#6:10](=[O:11])-[#8:12]-
[#6:13].[#6:14][C:15]1([#6:16])[#6:17](=[O:18])[C:19]([#6:20])([#6:21])[#
6:22]1=[S:23]>>[#6:13]-[#8:12]-[#6:10](=[O:11])[C:5]1([#6:6]-[#6:7](-
[#16:23]\[#6:22]1=[#6:19](\[#6:21])-[#6:20])-
[#6:8]=[#6:9])[#6:3](=[O:4])-[#8:2]-[#6:1].[#6:14]-[#6:15](-
[#6:16])=[C:17]=[O:18]

Correctness of the mapping

MAPPET	YES
ReactionMap	YES
Marvin	NO
ChemDraw	YES
Indigo	NO

Reaction no 189

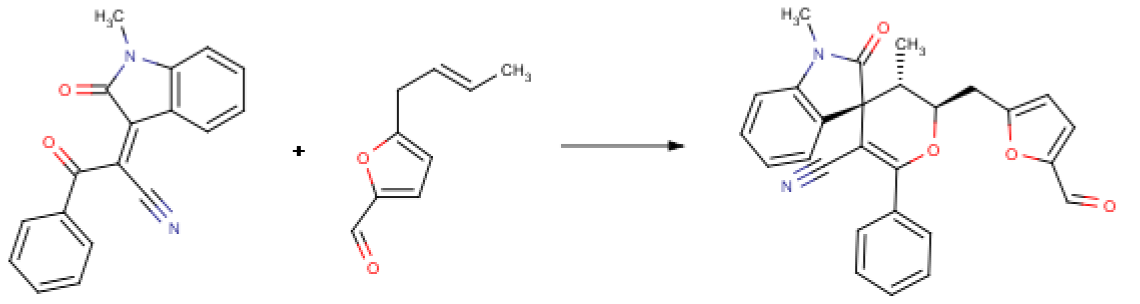

SMILES of the input:
CN1C(=O)\C(=C(\C#N)C(=O)C2=CC=CC=C2)C2=C1C=CC=C2.C\C=C\CC1=CC=C(O1)C=O>>C[C@@H]1[C@@H](CC2=CC=C(O2)C=O)OC(C2=CC=CC=C2)=C(C#N)[C@]11C(=O)N(C)C2=C1C=CC=C2

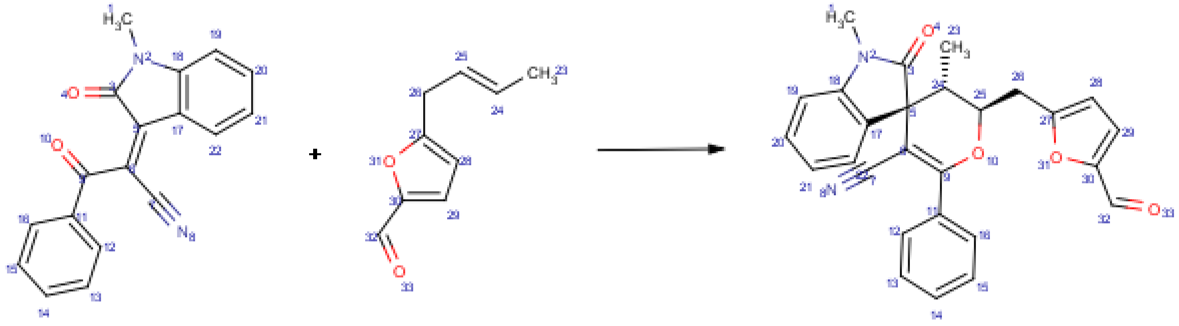

Correct mapped SMILES/SMARTS of the reaction:
[#6:1]-[#7:2]-1-[#6:3](=[O:4])\[#6:5](=[#6:6](\[C:7]#[N:8])-
[#6:9](=[O:10])-[#6:11]-2=[#6:12]-[#6:13]=[#6:14]-[#6:15]=[#6:16]-2)-
[#6:17]-2=[#6:18]-1-[#6:19]=[#6:20]-[#6:21]=[#6:22]-
2.[#6:23]\[#6:24]=[#6:25]\[#6:26]-[#6:27]-1=[#6:28]-[#6:29]=[#6:30](-
[#8:31]-1)-[#6:32]=[O:33]>>[#6:23]-[#6@@H:24]1-[#6@@H:25](-[#6:26]-
[#6:27]-2=[#6:28]-[#6:29]=[#6:30](-[#8:31]-2)-[#6:32]=[O:33])-[#8:10]-
[#6:9](-[#6:11]-2=[#6:16]-[#6:15]=[#6:14]-[#6:13]=[#6:12]-
2)=[#6:6]([C:7]#[N:8])[C@:5]11[#6:3](=[O:4])-[#7:2](-[#6:1])-[#6:18]-
2=[#6:17]1-[#6:22]=[#6:21]-[#6:20]=[#6:19]-2

Correctness of the mapping

MAPPET	YES

ReactionMap YES
Marvin YES
ChemDraw YES
Indigo NO

Reaction no 190

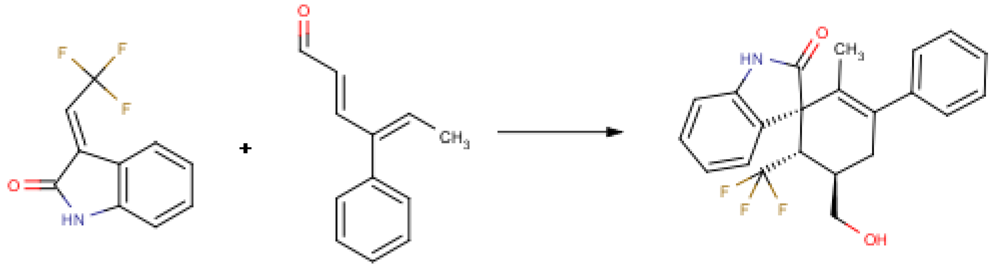

SMILES of the input:
FC(F)(F)\C=C1\C(=O)NC2=C1C=CC=C2.C\C=C(\C=C\C=O)/C1=CC=CC=C1>>CC1=C(C[C@@H](CO)[C@H](C(F)(F)F)[C@@]11C(=O)NC2=C1C=CC=C2)C1=CC=CC=C1

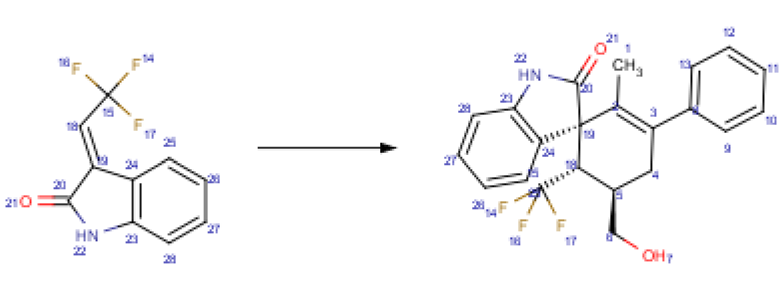

Correct mapped SMILES/SMARTS of the reaction:
[#6:1]\[#6:2]=[#6:3](\[#6:4]=[#6:5]\[#6:6]=[O:7])/[#6:8]-1=[#6:9]-[#6:10]=[#6:11]-[#6:12]=[#6:13]1.[F:14][C:15]([F:16])([F:17])\[#6:18]=[#6:19]-1\[#6:20](=[O:21])-[#7:22]-[#6:23]-2=[#6:24]-1-[#6:25]=[#6:26]-[#6:27]=[#6:28]-2>>[#6:1]-[#6:2]1=[#6:3](-[#6:4]-[#6@@H:5](-[#6:6]-[#8:7])-[#6@H:18]([C:15]([F:14])([F:16])[F:17])[C@@:19]11[#6:20](=[O:21])-[#7:22]-[#6:23]-2=[#6:24]1-[#6:25]=[#6:26]-[#6:27]=[#6:28]-2)-[#6:8]-1=[#6:13]-[#6:12]=[#6:11]-[#6:10]=[#6:9]-1

Correctness of the mapping
MAPPET YES
ReactionMap YES
Marvin YES
ChemDraw YES
Indigo NO

Reaction no 191

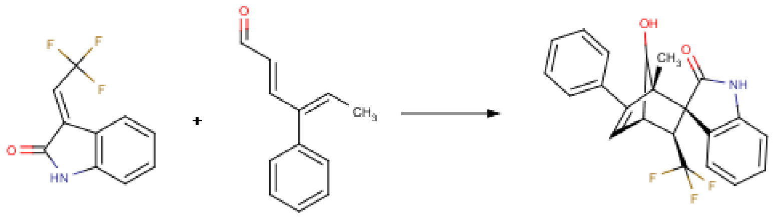

SMILES of the input:
FC(F)(F)\C=C1\C(=O)NC2=C1C=CC=C2.C\C=C(\C=C\C=O)/C1=CC=CC=C1>>C[C@]12C(O)
[C@H](C=C1C1=CC=CC=C1)[C@H](C(F)(F)F)[C@@]21C(=O)NC2=C1C=CC=C2

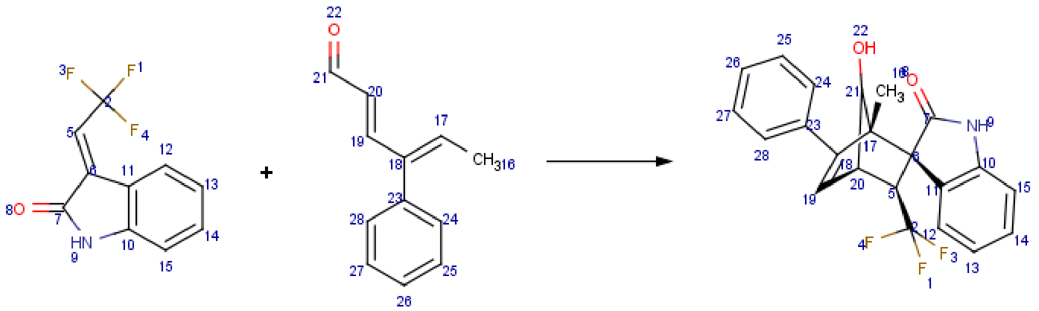

Correct mapped SMILES/SMARTS of the reaction:
[F:1][C:2]([F:3])([F:4])\[#6:5]=[#6:6]-1\[#6:7](=[O:8])-[#7:9]-[#6:10]-
2=[#6:11]-1-[#6:12]=[#6:13]-[#6:14]=[#6:15]-
2.[#6:16]\[#6:17]=[#6:18](\[#6:19]=[#6:20]\[#6:21]=[O:22])/[#6:23]-
1=[#6:24]-[#6:25]=[#6:26]-[#6:27]=[#6:28]1>>[#6:16][C@:17]12[#6:21](-
[#8:22])-[#6@H:20](-[#6:19]=[#6:18]1-[#6:23]-1=[#6:28]-[#6:27]=[#6:26]-
[#6:25]=[#6:24]-1)-
[#6@H:5]([C:2]([F:1])([F:3])[F:4])[C@@:6]21[#6:7](=[O:8])-[#7:9]-[#6:10]-
2=[#6:11]1-[#6:12]=[#6:13]-[#6:14]=[#6:15]-2

Correctness of the mapping

MAPPET	YES
ReactionMap	YES
Marvin	NO
ChemDraw	YES
Indigo	NO

Reaction no 192

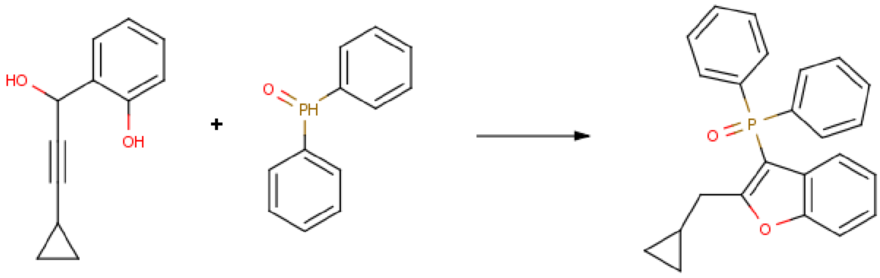

SMILES of the input:
OC(C#CC1CC1)C1=CC=CC=C1O.O=P(C1=CC=CC=C1)C1=CC=CC=C1>>O=P(C1=C(CC2CC2)OC2
=C1C=CC=C2)(C1=CC=CC=C1)C1=CC=CC=C1

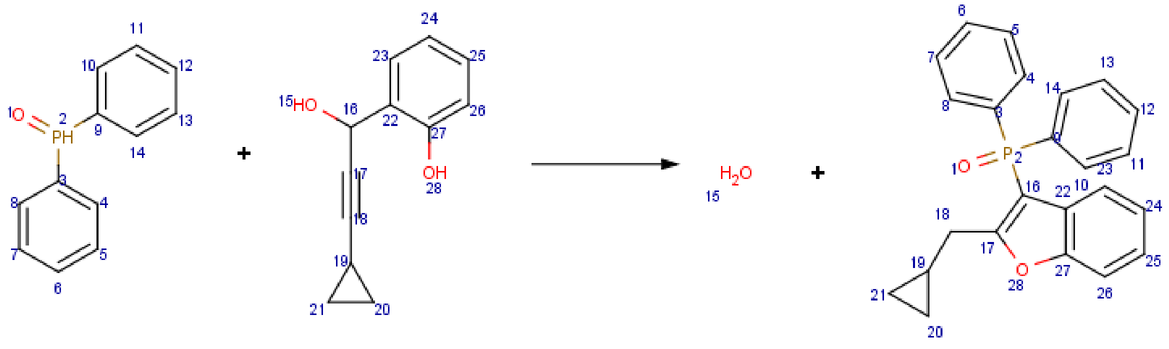

Correct mapped SMILES/SMARTS of the reaction:
[O:1]=[#15:2](-[#6:3]-1=[#6:4]-[#6:5]=[#6:6]-[#6:7]=[#6:8]-1)-[#6:9]-
1=[#6:10]-[#6:11]=[#6:12]-[#6:13]=[#6:14]-1.[#8:15]-
[#6:16]([C:17]#[C:18][#6:19]-1-[#6:20]-[#6:21]-1)-[#6:22]-1=[#6:23]-
[#6:24]=[#6:25]-[#6:26]=[#6:27]-1-[#8:28]>>[#8:15].[O:1]=[P:2]([#6:16]-
1=[#6:17](-[#6:18]-[#6:19]-2-[#6:20]-[#6:21]-2)-[#8:28]-[#6:27]-
2=[#6:22]-1-[#6:23]=[#6:24]-[#6:25]=[#6:26]-2)([#6:3]-1=[#6:8]-
[#6:7]=[#6:6]-[#6:5]=[#6:4]-1)[#6:9]-1=[#6:14]-[#6:13]=[#6:12]-
[#6:11]=[#6:10]-1

Correctness of the mapping

MAPPET	YES
ReactionMap	NO
Marvin	YES
ChemDraw	YES
Indigo	YES

Reaction no 193

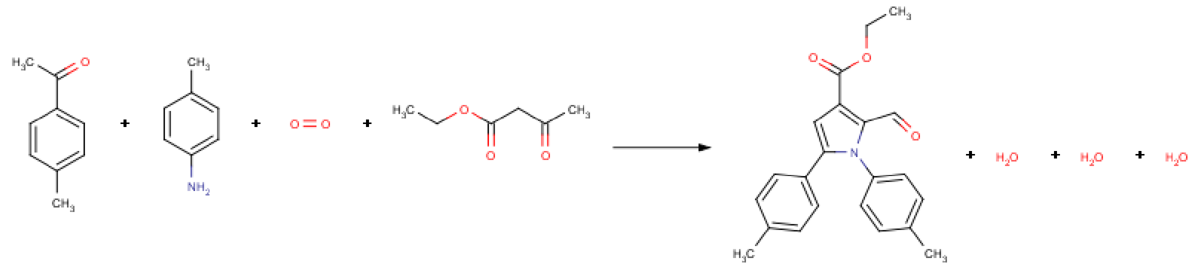

SMILES of the input:
CC(=O)C1=CC=C(C)C=C1.CC1=CC=C(N)C=C1.O=O.CCOC(=O)CC(C)=O>>CCOC(=O)C1=C(C=
O)N(C(=C1)C1=CC=C(C)C=C1)C1=CC=C(C)C=C1.O.O.O

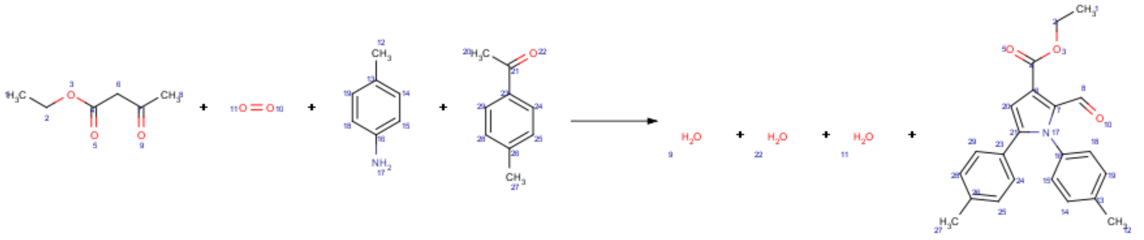

Correct mapped SMILES/SMARTS of the reaction:
[#6:1]-[#6:2]-[#8:3]-[#6:4](=[O:5])-[#6:6]-[#6:7](-
[#6:8])=[O:9].[O:10]=[O:11].[#6:12]-[#6:13]-1=[#6:14]-[#6:15]=[#6:16](-

[#7:17])-[#6:18]=[#6:19]-1.[#6:20]-[#6:21](=[O:22])-[#6:23]-1=[#6:24]-[#6:25]=[#6:26](-[#6:27])-[#6:28]=[#6:29]-1>>[#8:9].[#8:22].[#8:11].[#6:1]-[#6:2]-[#8:3]-[#6:4](=[O:5])-[#6:6]-1=[#6:7](-[#6:8]=[O:10])-[#7:17](-[#6:21](=[#6:20]-1)-[#6:23]-1=[#6:24]-[#6:25]=[#6:26](-[#6:27])-[#6:28]=[#6:29]-1)-[#6:16]-1=[#6:18]-[#6:19]=[#6:13](-[#6:12])-[#6:14]=[#6:15]-1

Correctness of the mapping

MAPPET	YES
ReactionMap	NO
Marvin	NO
ChemDraw	YES
Indigo	NO

Reaction no 194

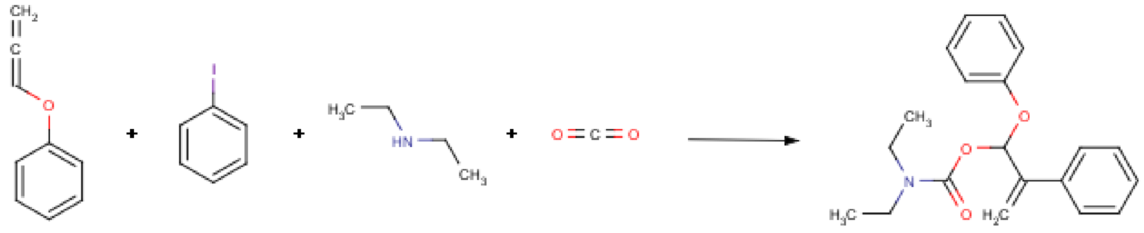

SMILES of the input:
C=C=COC1=CC=CC=C1.IC1=CC=CC=C1.CCNCC.O=C=O>>CCN(CC)C(=O)OC(OC1=CC=CC=C1)C(=C)C1=CC=CC=C1

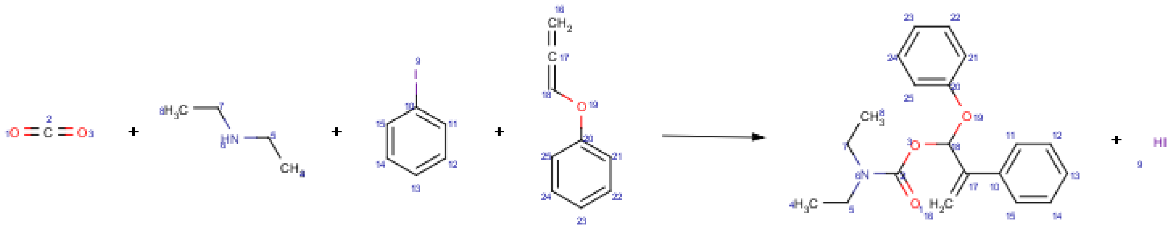

Correct mapped SMILES/SMARTS of the reaction:
[O:1]=[C:2]=[O:3].[#6:4]-[#6:5]-[#7:6]-[#6:7]-[#6:8].[I:9][#6:10]-1=[#6:11]-[#6:12]=[#6:13]-[#6:14]=[#6:15]-1.[#6:16]=[C:17]=[#6:18]-[#8:19]-[#6:20]-1=[#6:21]-[#6:22]=[#6:23]-[#6:24]=[#6:25]-1>>[#6:4]-[#6:5]-[#7:6](-[#6:7]-[#6:8])-[#6:2](=[O:1])-[#8:3]-[#6:18](-[#8:19]-[#6:20]-1=[#6:25]-[#6:24]=[#6:23]-[#6:22]=[#6:21]-1)-[#6:17](=[#6:16])-[#6:10]-1=[#6:11]-[#6:12]=[#6:13]-[#6:14]=[#6:15]-1.[I:9]

Correctness of the mapping

MAPPET	YES
ReactionMap	NO
Marvin	YES
ChemDraw	YES
Indigo	NO

Reaction no 195

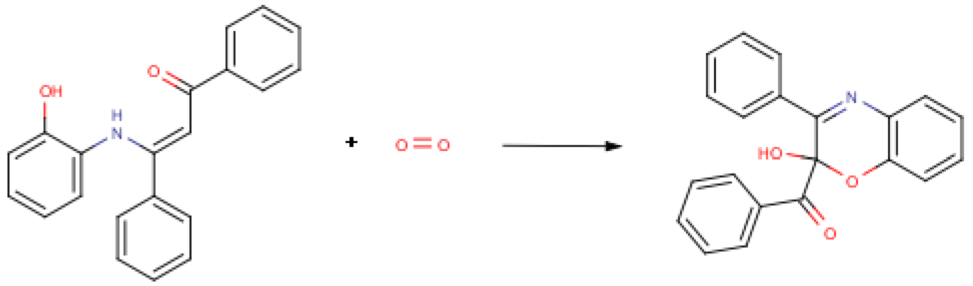

SMILES of the input:
OC1=CC=CC=C1N\C(=C/C(=O)C1=CC=CC=C1)C1=CC=CC=C1.O=O>>OC1(OC2=C(C=CC=C2)N=
C1C1=CC=CC=C1)C(=O)C1=CC=CC=C1

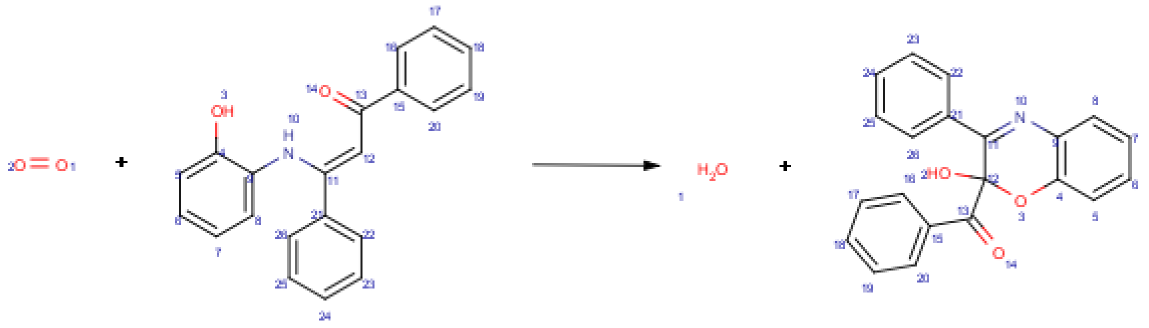

Correct mapped SMILES/SMARTS of the reaction:
[O:1]=[O:2].[#8:3]-[#6:4]-1=[#6:5]-[#6:6]=[#6:7]-[#6:8]=[#6:9]-1-
[#7:10]\[#6:11](=[#6:12]/[#6:13](=[O:14])-[#6:15]-1=[#6:16]-
[#6:17]=[#6:18]-[#6:19]=[#6:20]-1)-[#6:21]-1=[#6:22]-[#6:23]=[#6:24]-
[#6:25]=[#6:26]-1>>[#8:1].[#8:2][C:12]1([#8:3]-[#6:4]-2=[#6:9](-
[#6:8]=[#6:7]-[#6:6]=[#6:5]-2)-[#7:10]=[#6:11]1-[#6:21]-1=[#6:26]-
[#6:25]=[#6:24]-[#6:23]=[#6:22]-1)[#6:13](=[O:14])-[#6:15]-1=[#6:20]-
[#6:19]=[#6:18]-[#6:17]=[#6:16]-1

Correctness of the mapping

MAPPET	YES
ReactionMap	NO
Marvin	NO
ChemDraw	YES
Indigo	YES

Reaction no 196

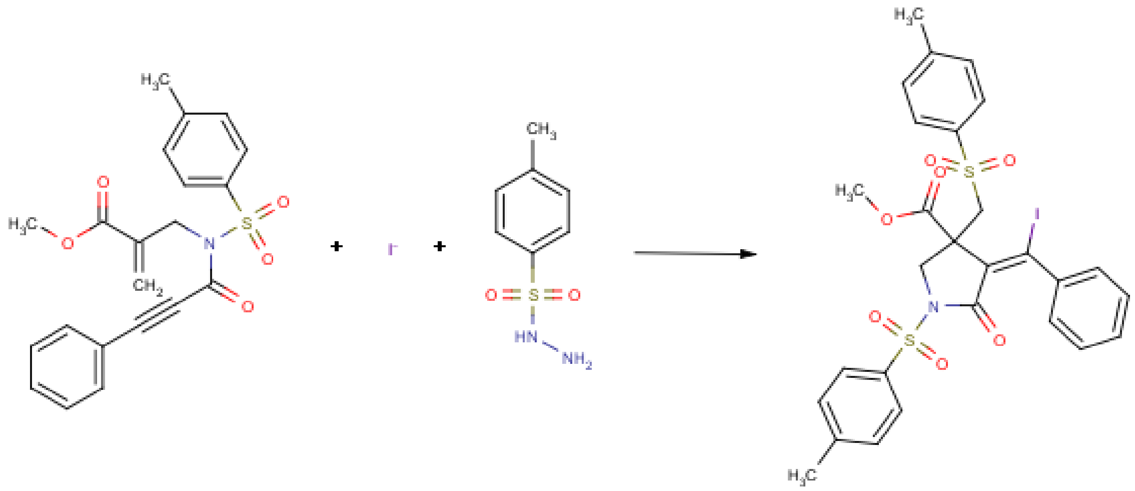

SMILES of the input:
COC(=O)C(=C)CN(C(=O)C#CC1=CC=CC=C1)S(=O)(=O)C1=CC=C(C)C=C1.[I-
].CC1=CC=C(C=C1)S(=O)(=O)NN>>COC(=O)C1(CS(=O)(=O)C2=CC=C(C)C=C2)CN(C(=O)\
C1=C(\I)C1=CC=CC=C1)S(=O)(=O)C1=CC=C(C)C=C1

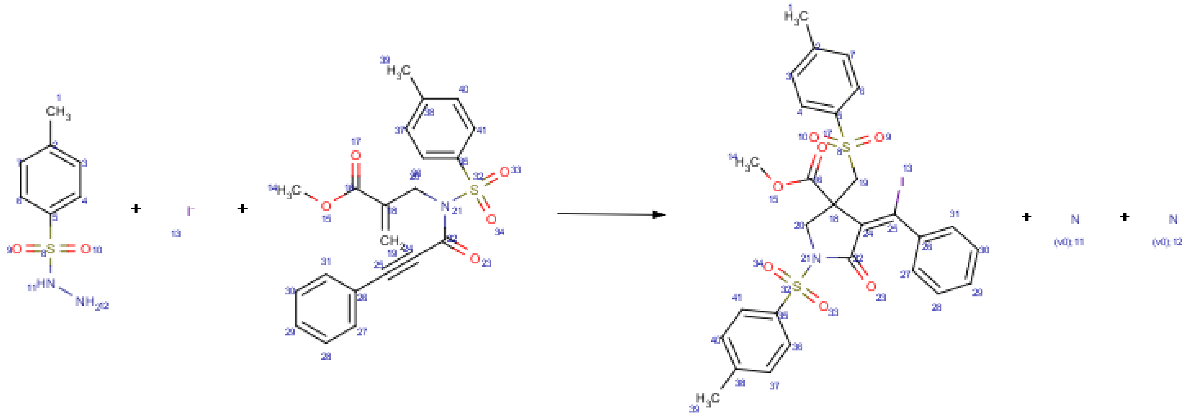

Correct mapped SMILES/SMARTS of the reaction:
[#6:1]-[#6:2]-1=[#6:3]-[#6:4]=[#6:5](-[#6:6]=[#6:7]-
1)[S:8](=[O:9])(=[O:10])[#7:11]-[#7:12].[I-:13].[#6:14]-[#8:15]-
[#6:16](=[O:17])-[#6:18](=[#6:19])-[#6:20]-[#7:21](-
[#6:22](=[O:23])[C:24]#[C:25][#6:26]-1=[#6:27]-[#6:28]=[#6:29]-
[#6:30]=[#6:31]-1)[S:32](=[O:33])(=[O:34])[#6:35]-1=[#6:36]-
[#6:37]=[#6:38](-[#6:39])-[#6:40]=[#6:41]-1>>[#6:14]-[#8:15]-
[#6:16](=[O:17])[C:18]1([#6:19][S:8](=[O:9])(=[O:10])[#6:5]-2=[#6:4]-
[#6:3]=[#6:2](-[#6:1])-[#6:7]=[#6:6]-2)[#6:20]-[#7:21](-
[#6:22](=[O:23])\[#6:24]1=[#6:25](\[I:13])-[#6:26]-1=[#6:31]-
[#6:30]=[#6:29]-[#6:28]=[#6:27]-1)[S:32](=[O:33])(=[O:34])[#6:35]-
1=[#6:41]-[#6:40]=[#6:38](-[#6:39])-[#6:37]=[#6:36]-
1.[#7;v0:11].[#7;v0:12]

Correctness of the mapping

MAPPET	YES
ReactionMap	NO
Marvin	YES
ChemDraw	YES
Indigo	YES

Reaction no 197

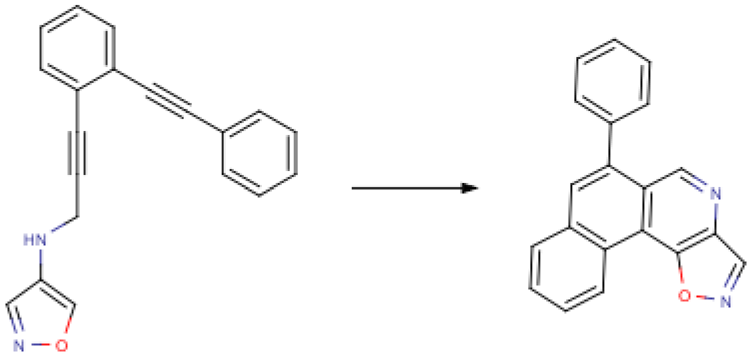

SMILES of the input:
C(NC1=CON=C1)C#CC1=CC=CC=C1C#CC1=CC=CC=C1>>O1N=CC2=C1C1=C(C=N2)C(=CC2=C1C=CC=C2)C1=CC=CC=C1

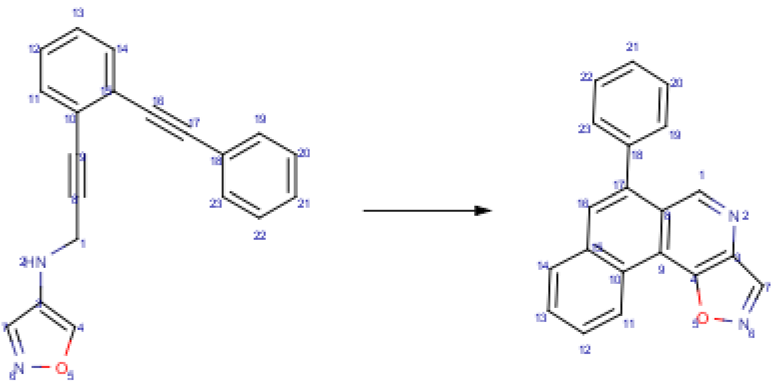

Correct mapped SMILES/SMARTS of the reaction:
[#6:1](-[#7:2]-[#6:3]-1=[#6:4]-[#8:5]-[#7:6]=[#6:7]-1)[C:8]#[C:9][#6:10]-1=[#6:11]-[#6:12]=[#6:13]-[#6:14]=[#6:15]-1[C:16]#[C:17][#6:18]-1=[#6:19]-[#6:20]=[#6:21]-[#6:22]=[#6:23]-1>>[#8:5]-1-[#7:6]=[#6:7]-[#6:3]-2=[#6:4]-1-[#6:9]-1=[#6:8](-[#6:1]=[#7:2]-2)-[#6:17](=[#6:16]-[#6:15]-2=[#6:10]-1-[#6:11]=[#6:12]-[#6:13]=[#6:14]-2)-[#6:18]-1=[#6:23]-[#6:22]=[#6:21]-[#6:20]=[#6:19]-1

Correctness of the mapping

MAPPET	YES
ReactionMap	YES
Marvin	YES
ChemDraw	NO
Indigo	YES

Reaction no 198

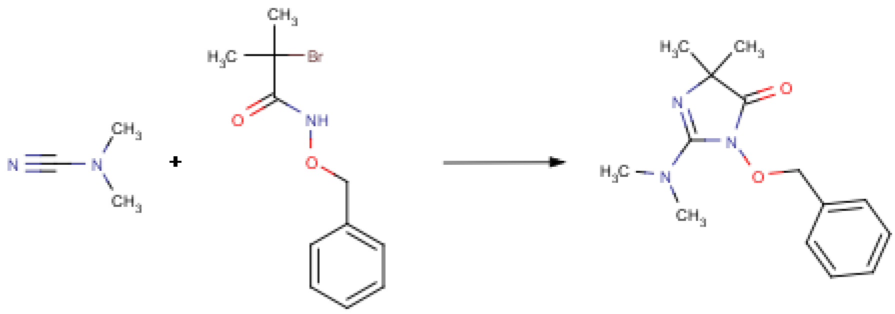

SMILES of the input:
CN(C)C#N.CC(C)(Br)C(=O)NOCC1=CC=CC=C1>>CN(C)C1=NC(C)(C)C(=O)N1OCC1=CC=CC=
C1

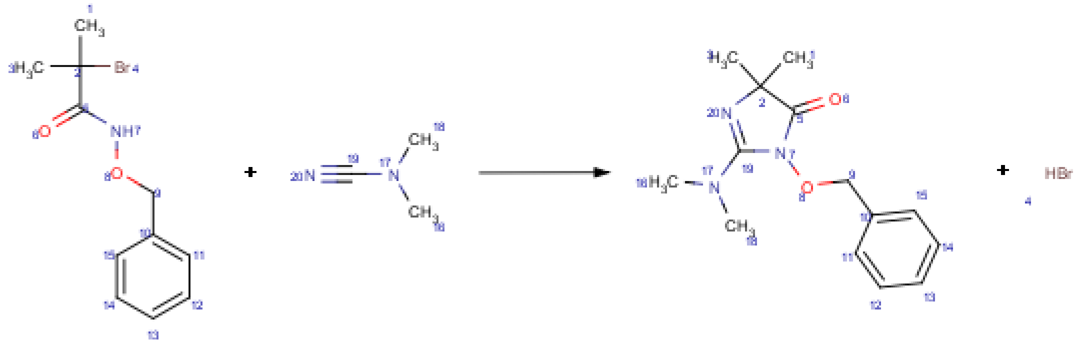

Correct mapped SMILES/SMARTS of the reaction:
[#6:1][C:2]([#6:3])([Br:4])[#6:5](=[O:6])-[#7:7]-[#8:8]-[#6:9]-[#6:10]-
1=[#6:11]-[#6:12]=[#6:13]-[#6:14]=[#6:15]-1.[#6:16]-[#7:17](-
[#6:18])[C:19]#[N:20]>>[#6:16]-[#7:17](-[#6:18])-[#6:19]-
1=[#7:20][C:2]([#6:3])([#6:1])[#6:5](=[O:6])-[#7:7]-1-[#8:8]-[#6:9]-
[#6:10]-1=[#6:15]-[#6:14]=[#6:13]-[#6:12]=[#6:11]-1.[Br:4]

Correctness of the mapping

MAPPET	YES
ReactionMap	NO
Marvin	YES
ChemDraw	YES
Indigo	NO

Reaction no 199

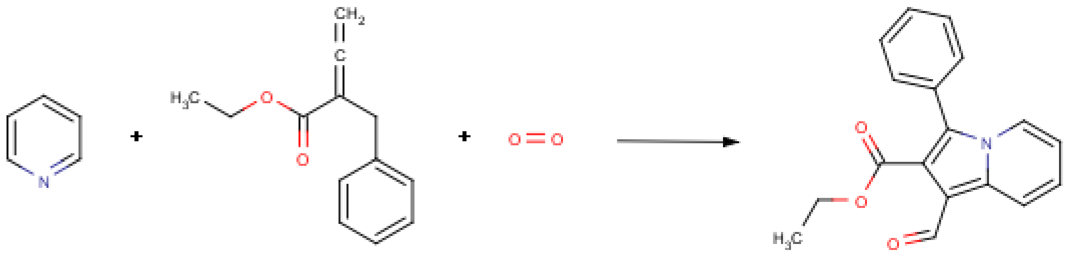

SMILES of the input:
C1=CC=NC=C1.CCOC(=O)C(CC1=CC=CC=C1)=C=C.O=O>>CCOC(=O)C1=C(N2C=CC=CC2=C1C=O)C1=CC=CC=C1

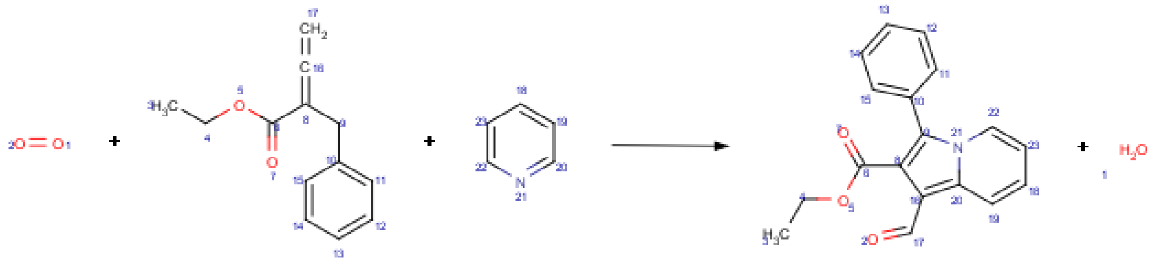

Correct mapped SMILES/SMARTS of the reaction:
[O:1]=[O:2].[#6:3]-[#6:4]-[#8:5]-[#6:6](=[O:7])-[#6:8](-[#6:9]-[#6:10]-1=[#6:11]-[#6:12]=[#6:13]-[#6:14]=[#6:15]-1)=[C:16]=[#6:17].[#6:18]-1=[#6:19]-[#6:20]=[#7:21]-[#6:22]=[#6:23]-1>>[#6:3]-[#6:4]-[#8:5]-[#6:6](=[O:7])-[#6:8]-1=[#6:9](-[#7:21]-2-[#6:22]=[#6:23]-[#6:18]=[#6:19]-[#6:20]-2=[#6:16]-1-[#6:17]=[O:2])-[#6:10]-1=[#6:15]-[#6:14]=[#6:13]-[#6:12]=[#6:11]-1.[#8:1]

Correctness of the mapping

MAPPET	YES
ReactionMap	NO
Marvin	NO
ChemDraw	YES
Indigo	YES

Reaction no 200

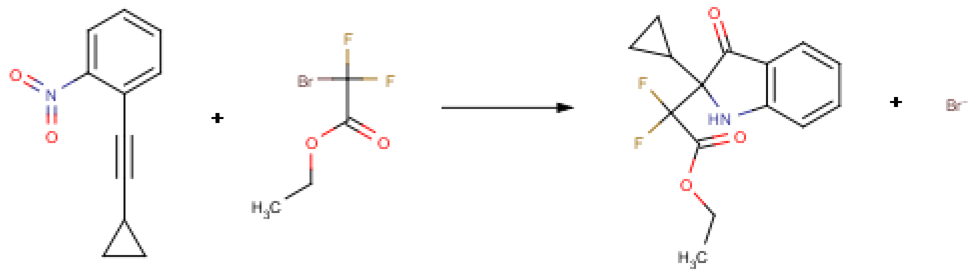

SMILES of the input:
O=N(=O)C1=CC=CC=C1C#CC1CC1.CCOC(=O)C(F)(F)Br>>CCOC(=O)C(F)(F)C1(NC2=C(C=CC=C2)C1=O)C1CC1.[Br-]

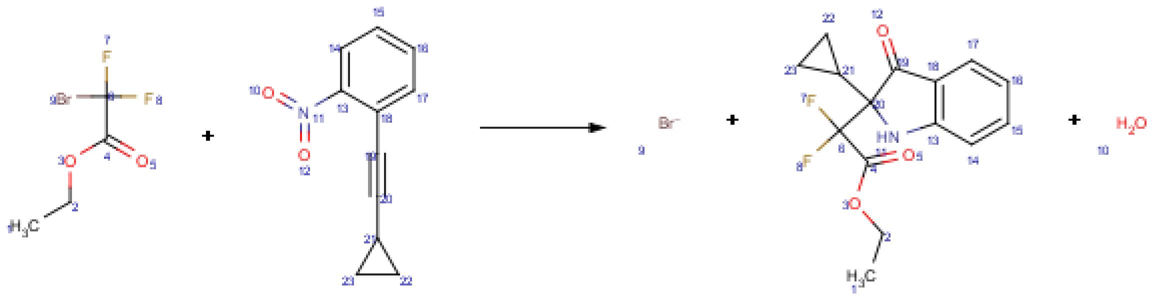

Correct mapped SMILES/SMARTS of the reaction:
[#6:1]-[#6:2]-[#8:3]-
[#6:4](=[O:5])[C:6]([F:7])([F:8])[Br:9].[O:10]=[N:11](=[O:12])[#6:13]-
1=[#6:14]-[#6:15]=[#6:16]-[#6:17]=[#6:18]-1[C:19]#[C:20][#6:21]-1-
[#6:22]-[#6:23]-1>>[Br-:9].[#6:1]-[#6:2]-[#8:3]-
[#6:4](=[O:5])[C:6]([F:7])([F:8])[C:20]1([#7:11]-[#6:13]-2=[#6:18](-
[#6:17]=[#6:16]-[#6:15]=[#6:14]-2)-[#6:19]1=[O:12])[#6:21]-1-[#6:23]-
[#6:22]-1.[#8:10]

Correctness of the mapping

MAPPET	YES
ReactionMap	NO
Marvin	NO
ChemDraw	NO
Indigo	YES

Reaction no 201

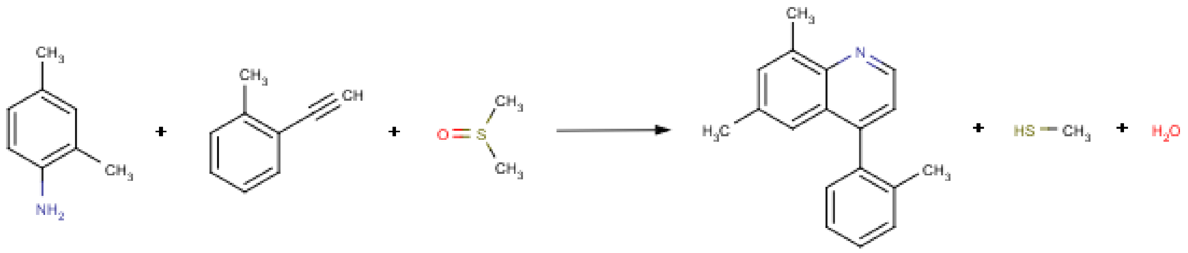

SMILES of the input:
CC1=CC(C)=C(N)C=C1.CC1=C(C=CC=C1)C#C.CS(C)=O>>CC1=CC(C)=C2N=CC=C(C2=C1)C1=C(C)C=CC=C1.CS.O

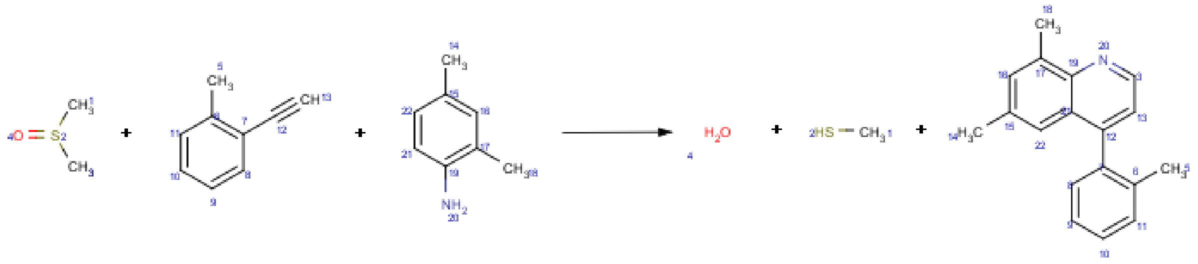

Correct mapped SMILES/SMARTS of the reaction:

```
[#6:1][S:2]([#6:3])=[O:4].[#6:5]-[#6:6]-1=[#6:7](-[#6:8]=[#6:9]-[#6:10]=[#6:11]-1)[C:12]#[C:13].[#6:14]-[#6:15]-1=[#6:16]-[#6:17](-[#6:18])=[#6:19](-[#7:20])-[#6:21]=[#6:22]-1>>[#8:4].[#6:1]-[#16:2].[#6:14]-[#6:15]-1=[#6:16]-[#6:17](-[#6:18])=[#6:19]-2-[#7:20]=[#6:3]-[#6:13]=[#6:12](-[#6:21]-2=[#6:22]-1)-[#6:7]-1=[#6:6](-[#6:5])-[#6:11]=[#6:10]-[#6:9]=[#6:8]-1
```

Correctness of the mapping

MAPPET	YES
ReactionMap	YES
Marvin	NO
ChemDraw	NO
Indigo	YES

Supplementary Note 4. Test Set #2 of 281 reactions selected from the MIT's set of 50,000 patent reactions accompanying ref. 11 (original MIT's mappings, in SMARTS format, are compared to those of our algorithm)……………………..………………………………**pages 618-727**

- **p.618 to p.637** – reactions in which one bond is being cut/created
- **p.637 to p.657** – reactions in which two bonds are being cut/created
- **p.657 to p.676** – reactions in which three bonds are being cut/created
- **p.676 to p.696** – reactions in which four bonds are being cut/created
- **p.696 to p.715** – reactions in which five bonds are being cut/created
- **p.715 to p.727** – reactions in which six bonds are being cut/created

Reaction no 1 (1 bond changed)

SMILES/SMARTS of the USPTO output:
[Cl:28][CH2:29][Cl:30].[F:1][c:2]1[cH:3][c:4]([CH:8]([OH:9])[c:10]2[cH:11][c:12]
3[cH:13][cH:14][c:15]([CH2:20][CH2:21][N:22]4[CH:23]([CH3:27])[CH2:24][CH2:25][C
H2:26]4)[cH:16][c:17]3[cH:18][cH:19]2)[cH:5][cH:6][cH:7]1>>[F:1][c:2]1[cH:3][c:4
]([C:8](=[O:9])[c:10]2[cH:11][c:12]3[cH:13][cH:14][c:15]([CH2:20][CH2: 21][N:22]4
[CH:23]([CH3:27])[CH2:24][CH2:25][CH2:26]4)[cH:16][c:17]3[cH:18][cH:19]2)[cH:5][
cH:6][cH:7]1

SMILES/SMARTS of the MAPPET output:
[CH3:1][CH:2]1[CH2:3][CH2:4][CH2:5][N:6]1[CH2:7][CH2:8][c:9]1[cH:10][cH:11][c:12
]2[cH:13][c:14]([cH:15][cH:16][c:17]2[cH:18]1)[CH:19]([OH:20])[c:21]1[cH:22][cH:
23][cH:24][c:25]([F:26])[cH:27]1>>[CH3:1][CH:2]1[CH2:3][CH2:4][CH2:5][N:6]1[CH2:
7][CH2:8][c:9]1[cH:10][cH:11][c:12]2[cH:13][c:14]([cH:15][cH:16][c:17]2[cH:18]1)
[C:19](=[O:20])[c:21]1[cH:22][cH:23][cH:24][c:25]([F:26])[cH:27]1

Correctness of the mapping
MAPPET YES
USPTO YES

Reaction no 2 (1 bond changed)

SMILES/SMARTS of the USPTO output:
[CH3:1][c:2]1[cH:3][cH:4][c:5]([O:8][C:9]([C:10](=[O:11])[O:12][CH2:13][CH3:14])
([CH3:15])[CH3:16])[cH:6][n:7]1.[CH3:22][S:23]([CH3:24])=[O:25].[CH:19]([OH:20])
=[O:21].[K+:18].[OH-
:17].[OH2:26]>>[CH3:1][c:2]1[cH:3][cH:4][c:5]([O:8][C:9]([C:10](=[O:11])[OH:12])
([CH3:15])[CH3:16])[cH:6][n:7]1

SMILES/SMARTS of the MAPPET output:
[CH3:2][CH2:3][O:4][C:5](=[O:6])[C:7]([CH3 :8])([CH3:9])[O:10][c:11]1[cH:12][cH:1
3][c:14]([CH3:15])[n:16][cH:17]1.[OH2:1]>>[CH3:15][c:14]1[cH:13][cH:12][c:11]([O
:10][C:7]([CH3:8])([CH3:9])[C:5]([OH:1])=[O:6])[cH:17][n:16]1.[CH3:2][CH2:3][OH:
4]

Correctness of the mapping
MAPPET YES
USPTO NO

Reaction no 3 (1 bond changed)

SMILES/SMARTS of the USPTO output:
[CH3:44][OH:45].[CH:1]([CH3:2])([CH3:3])[c:4]1[cH:5][cH:6][c:7]([S:10](=[O:11])(
=[O:12])[NH:13][c:14]2[cH:15][c:16]([C:17](=[O:18])[O:19][CH3:20])[cH:21][c:22](
[O:33][CH2:34][CH2:35][O:36][CH:37]3[CH2:38][CH2:39][CH2:40][CH2:41][O:42]3)[c:2
3]2[O:24][c:25]2[c:26]([O:31][CH3:32])[cH:27][cH:28][cH:29][cH:30]2)[n:8][cH:9]1
.[ClH:43]>>[CH:1]([CH3:2])([CH3:3])[c:4]1[cH:5][cH:6][c:7]([S:10](=[O:11])(=[O:1
2])[NH:13][c:14]2[cH:15][c:16]([C:17](=[O:18])[O:19][CH3:20])[cH:21][c:22]([O:33
][CH2:34][CH2:35][OH:36])[c:23]2[O:24][c:25]2[c:26]([O:31][CH3:32])[cH:27][cH:28
][cH:29][cH:30]2)[n:8][cH:9]1

SMILES/SMARTS of the MAPPET output:
[CH3:1][O:2][C:3](=[O:4])[c:5]1[cH:6][c:7]([NH:8][S:9](=[O:10])(=[O:11])[c:12]2[
cH:13][cH:14][c:15]([cH:16][n:17]2)[CH:18]([CH3:19])[CH3:20])[c:21]([O:22][c:23]
2[cH:24][cH:25][cH:26][cH:27][c:28]2[O:29][CH3:30])[c:31]([O:32][CH2:33][CH2:34]
[O:35][CH:36]2[CH2:37][CH2:38][CH2:39][CH2:40][O:41]2)[cH:42]1>>[CH3:1][O:2][C:3
](=[O:4])[c:5]1[cH:6][c:7]([NH:8][S:9](=[O:10])(=[O:11])[c:12]2[cH:13][cH:14][c:
15]([cH:16][n:17]2)[CH:18]([CH3:20])[CH3:19])[c:21]([O:22][c:23]2[cH:24][cH:25][

cH:26][cH:27][c:28]2[O:29][CH3:30])[c:31]([O:32][CH2:33][CH2:34][OH:35])[cH:42]1
.[C:39].[C:38].[C:36].[C:37].[C:40].[O:41]

Correctness of the mapping
MAPPET YES
USPTO YES

Reaction no 4 (1 bond changed)

SMILES/SMARTS of the USPTO output:
[CH2:1]([CH3:2])[CH:3]([CH:4]([c:5]1[o:6][c:7]2[c:8]([c:9]1[CH3:10])[cH:11][c:12
]([F:15])[cH:13][cH:14]2)[NH:16][c:17]1[cH:18][cH:19][c:20]([C:23](=[O:24])[N:25
]([CH2:26][CH2:27][C:28](=[O:29])[O:30][CH2:31][CH3:32])[CH3:33])[cH:21][cH:22]1
)[CH2:34][CH3:35].[CH2:38]([OH:39])[CH3:40].[CH3:41][CH2:42][CH2:43][CH2:44][CH2
:45][CH3:46].[CH3:47][CH2:48][OH:49].[Na+:37].[O:50]1[CH2:51][CH2:52][CH2:53][CH
2:54]1.[OH-
:36]>>[CH2:1]([CH3:2])[CH:3]([CH:4]([c:5]1[o:6][c:7]2[c:8]([c:9]1[CH3:10])[cH:11
][c:12]([F:15])[cH:13][cH:14]2)[NH:16][c:17]1[cH:18][cH:19][c:20]([C:23](=[O:24]
)[N:25]([CH2:26][CH2:27][C:28](=[O:29])[OH:30])[CH3:33])[cH:21][cH:22]1)[CH2:34]
[CH3:35]

SMILES/SMARTS of the MAPPET output:
[CH3:2][CH2:3][O:4][C:5](=[O:6])[CH2:7][CH2:8][N:9]([CH3:10])[C:11](=[O:12])[c:1
3]1[cH:14][cH:15][c:16]([NH:17][CH:18]([CH:19]([CH2:20][CH3:21])[CH2:22][CH3:23]
)[c:24]2[o:25][c:26]3[cH:27][cH:28][c:29]([F:30])[cH:31][c:32]3[c:33]2[CH3:34])[
cH:35][cH:36]1.[OH2:1]>>[CH3:21][CH2:20][CH:19]([CH2:22][CH3:23])[CH:18]([NH:17]
[c:16]1[cH:15][cH:14][c:13]([cH:36][cH:35]1)[C:11](=[O:12])[N:9]([CH3:10])[CH2:8
][CH2:7][C:5]([OH:1])=[O:6])[c:24]1[o:25][c:26]2[cH:27][cH:28][c:29]([F:30])[cH:
31][c:32]2[c:33]1[CH3:34].[CH3:2][CH2:3][OH:4]

Correctness of the mapping
MAPPET YES
USPTO NO

Reaction no 5 (1 bond changed)

SMILES/SMARTS of the USPTO output:
[Br:1][c:2]1[cH:3][cH:4][c:5]([CH2:6][NH:7][CH:8]([C:9](=[O:10])[O:11][CH3:12])[
CH2:13][CH:14]([CH3:15])[CH3:16])[cH:17][cH:18]1.[CH3:22][OH:23].[ClH:21].[Li+:2
0].[OH-
:19]>>[Br:1][c:2]1[cH:3][cH:4][c:5]([CH2:6][NH:7][CH:8]([C:9](=[O:10])[OH:11])[C
H2:13][CH:14]([CH3:15])[CH3:16])[cH:17][cH:18]1

SMILES/SMARTS of the MAPPET output:
[CH3:2][O:3][C:4](=[O:5])[CH:6]([CH2:7][CH:8]([CH3:9])[CH3:10])[NH:11][CH2:12][c
:13]1[cH:14][cH:15][c:16]([Br:17])[cH:18][cH:19]1.[OH2:1]>>[CH3:9][CH:8]([CH3:10
])[CH2:7][CH:6]([NH:11][CH2:12][c:13]1[cH:14][cH:15][c:16]([Br:17])[cH:18][cH:19
]1)[C:4]([OH:1])=[O:5].[CH3:2][OH:3]

Correctness of the mapping
MAPPET YES
USPTO NO

Reaction no 6 (1 bond changed)

SMILES/SMARTS of the USPTO output:

[CH2:31]1[O:32][CH2:33][CH2:34][CH2:35]1.[O:1]1[CH:2]([c:7]2[cH:8][cH:9][c:10](=
[O:28])[n:11]([CH2:13][CH2:14][O:15][c:16]3[cH:17][cH:18][n:19][c:20]4[cH:21][c:
22]([O:26][CH3:27])[cH:23][cH:24][c:25]34)[cH:12]2)[CH:3]=[CH:4][CH2:5][CH2:6]1.
[Pt:29]=[O:30]>>[O:1]1[CH:2]([c:7]2[cH:8][cH:9][c:10](=[O:28])[n:11]([CH2:13][CH
2:14][O:15][c:16]3[cH:17][cH:18][n:19][c:20]4[cH:21][c:22]([O:26][CH3:27])[cH:23
][cH:24][c:25]34)[cH:12]2)[CH2:3][CH2:4][CH2:5][CH2:6]1

SMILES/SMARTS of the MAPPET output:
[CH3:1][O:2][c:3]1[cH:4][cH:5][c:6]2[c:7]([O:8][CH2:9][CH2:10][n:11]3[cH:12][c:1
3]([cH:14][cH:15][c:16]3=[O:17])[CH:18]3[O:19][CH2:20][CH2:21][CH:22]=[CH:23]3)[
cH:24][cH:25][n:26][c:27]2[cH:28]1>>[CH3:1][O:2][c:3]1[cH:4][cH:5][c:6]2[c:7]([O
:8][CH2:9][CH2:10][n:11]3[cH:12][c:13]([cH:14][cH:15][c:16]3=[O:17])[CH:18]3[CH2
:23][CH2:22][CH2:21][CH2:20][O:19]3)[cH:24][cH:25][n:26][c:27]2[cH:28]1

Correctness of the mapping
MAPPET YES
USPTO YES

Reaction no 7 (1 bond changed)

SMILES/SMARTS of the USPTO output:
[C:1]([CH3:2])([CH3:3])([CH3:4])[O:5][C:6](=[O:7])[NH:8][CH:9]1[C:10](=[O:27])[N
:11]([N:14]=[C:15]2[CH2:16][CH2:17][N:18]([c:21]3[cH:22][cH:23][n:24][cH:25][cH:
26]3)[CH2:19][CH2:20]2)[CH2:12][CH2:13]1.[C:32]([BH3-
:33])#[N:34].[CH3:28][C:29](=[O:30])[OH:31].[CH3:36][OH:37].[Na+:35]>>[C:1]([CH3
:2])([CH3:3])([CH3:4])[O:5][C:6](=[O:7])[NH:8][CH:9]1[C:10](=[O:27])[N:11]([NH:1
4][CH:15]2[CH2:16][CH2:17][N:18]([c:21]3[cH:22][cH:23][n:24][cH:25][cH:26]3)[CH2
:19][CH2:20]2)[CH2:12][CH2:13]1

SMILES/SMARTS of the MAPPET output:
[CH3:1][C:2]([CH3:3])([CH3:4])[O:5][C:6](=[O:7])[NH:8][CH:9]1[CH2:10][CH2:11][N:
12]([N:13]=[C:14]2[CH2:15][CH2:16][N:17]([CH2:18][CH2:19]2)[c:20]2[cH:21][cH:22]
[n:23][cH:24][cH:25]2)[C:26]1=[O:27]>>[CH3:4][C:2]([CH3:3])([CH3:1])[O:5][C:6](=
[O:7])[NH:8][CH:9]1[CH2:10][CH2:11][N:12]([NH:13][CH:14]2[CH2:19][CH2:18][N:17](
[CH2:16][CH2:15]2)[c:20]2[cH:25][cH:24][n:23][cH:22][cH:21]2)[C:26]1=[O:27]

Correctness of the mapping
MAPPET YES
USPTO YES

Reaction no 8 (1 bond changed)

SMILES/SMARTS of the USPTO output:
[CH2:34]1[O:35][CH2:36][CH2:37][CH2:38]1.[CH3:39][OH:40].[Na+:2].[OH-
:1].[nH:3]1[c:4]([CH2:12][NH:13][C:14](=[O:15])[c:16]2[cH:17][cH:18][c:19]3[c:20
]([cH:33]2)[CH2:21][N:22]([CH3:32])[C:23](=[O:31])[CH:24]([CH2:26][C:27](=[O:28]
)[O:29][CH3:30])[NH:25]3)[cH:5][c:6]2[cH:7][cH:8][cH:9][cH:10][c:11]12>>[nH:3]1[
c:4]([CH2:12][NH:13][C:14](=[O:15])[c:16]2[cH:17][cH:18][c:19]3[c:20]([cH:33]2)[
CH2:21][N:22]([CH3:32])[C:23](=[O:31])[CH:24]([CH2:26][C:27](=[O:28])[OH:29])[NH
:25]3)[cH:5][c:6]2[cH:7][cH:8][cH:9][cH:10][c:11]12

SMILES/SMARTS of the MAPPET output:
[CH3:2][O:3][C:4](=[O:5])[CH2:6][CH:7]1[NH:8][c:9]2[cH:10][cH:11][c:12]([cH:13][
c:14]2[CH2:15][N:16]([CH3:17])[C:18]1=[O:19])[C:20](=[O:21])[NH:22][CH2:23][c:24
]1[cH:25][c:26]2[cH:27][cH:28][cH:29][cH:30][c:31]2[nH:32]1.[OH2:1]>>[CH3:17][N:
16]1[CH2:15][c:14]2[cH:13][c:12]([cH:11][cH:10][c:9]2[NH:8][CH:7]([CH2:6][C:4]([

OH:1])=[O:5])[C:18]1=[O:19])[C:20](=[O:21])[NH:22][CH2:23][c:24]1[cH:25][c:26]2[cH:27][cH:28][cH:29][cH:30][c:31]2[nH:32]1.[CH3:2][OH:3]

Correctness of the mapping
MAPPET YES
USPTO NO

Reaction no 9 (1 bond changed)

SMILES/SMARTS of the USPTO output:
[CH:1]1([n:4]2[cH:5][c:6]([C:19](=[O:20])[O:21][CH2:22][CH3:23])[c:7](=[O:18])[c:8]3[c:9]([CH3:17])[c:10]([F:16])[c:11]([F:15])[c:12]([F:14])[c:13]23)[CH2:2][CH2:3]1.[ClH:24]>>[CH:1]1([n:4]2[cH:5][c:6]([C:19](=[O:20])[OH:21])[c:7](=[O:18])[c:8]3[c:9]([CH3:17])[c:10]([F:16])[c:11]([F:15])[c:12]([F:14])[c:13]23)[CH2:2][CH2:3]1

SMILES/SMARTS of the MAPPET output:
[CH3:2][CH2:3][O:4][C:5](=[O:6])[c:7]1[cH:8][n:9]([CH:10]2[CH2:11][CH2:12]2)[c:13]2[c:14]([F:15])[c:16]([F:17])[c:18]([F:19])[c:20]([CH3:21])[c:22]2[c:23]1=[O:24].[OH2:1]>>[CH3:21][c:20]1[c:18]([F:19])[c:16]([F:17])[c:14]([F:15])[c:13]2[n:9]([cH:8][c:7]([C:5]([OH:1])=[O:6])[c:23](=[O:24])[c:22]12)[CH:10]1[CH2:11][CH2:12]1.[CH3:2][CH2:3][OH:4]

Correctness of the mapping
MAPPET YES
USPTO NO

Reaction no 10 (1 bond changed)

SMILES/SMARTS of the USPTO output:
[B:39]([Br:40])([Br:41])[Br:42].[C:43](=[O:44])([OH:45])[O-:46].[CH2:48]([Cl:49])[Cl:50].[CH3:51][C:52]#[N:53].[ClH:38].[F:1][c:2]1[c:3](-[c:9]2[c:10]([C:21](=[O:22])[c:23]3[cH:24][cH:25][c:26]([O:29][CH2:30][CH2:31][N:32]4[CH2:33][CH2:34][CH2:35][CH2:36][CH2:37]4)[cH:27][cH:28]3)[c:11]3[cH:12][cH:13][c:14]([O:19][CH3:20])[cH:15][c:16]3[cH:17][cH:18]2)[cH:4][c:5]([F:8])[cH:6][cH:7]1.[Na+:47]>>[F:1][c:2]1[c:3](-[c:9]2[c:10]([C:21](=[O:22])[c:23]3[cH:24][cH:25][c:26]([O:29][CH2:30][CH2:31][N:32]4[CH2:33][CH2:34][CH2:35][CH2:36][CH2:37]4)[cH:27][cH:28]3)[c:11]3[cH:12][cH:13][c:14]([OH:19])[cH:15][c:16]3[cH:17][cH:18]2)[cH:4][c:5]([F:8])[cH:6][cH:7]1

SMILES/SMARTS of the MAPPET output:
[CH3:1][O:2][c:3]1[cH:4][cH:5][c:6]2[c:7]([C:8](=[O:9])[c:10]3[cH:11][cH:12][c:13]([O:14][CH2:15][CH2:16][N:17]4[CH2:18][CH2:19][CH2:20][CH2:21][CH2:22]4)[cH:23][cH:24]3)[c:25]([cH:26][cH:27][c:28]2[cH:29]1)-[c:30]1[cH:31][c:32]([F:33])[cH:34][cH:35][c:36]1[F:37]>>[OH:2][c:3]1[cH:4][cH:5][c:6]2[c:7]([C:8](=[O:9])[c:10]3[cH:24][cH:23][c:13]([O:14][CH2:15][CH2:16][N:17]4[CH2:22][CH2:21][CH2:20][CH2:19][CH2:18]4)[cH:12][cH:11]3)[c:25]([cH:26][cH:27][c:28]2[cH:29]1)-[c:30]1[cH:31][c:32]([F:33])[cH:34][cH:35][c:36]1[F:37].[C:1]

Correctness of the mapping
MAPPET YES
USPTO NO

Reaction no 11 (1 bond changed)

SMILES/SMARTS of the USPTO output:
[Al+3:22].[H-:21].[H-:23].[H-
:24].[NH2:1][CH2:2][CH2:3][S:4][CH2:5][c:6]1[cH:7][c:8](-
[c:15]2[cH:16][cH:17][cH:18][cH:19][cH:20]2)[c:9]([C:11](=[O:12])[NH:13][CH3:14]
)[o:10]1.[O:26]1[CH2:27][CH2:28][CH2:29][CH2:30]1.[OH2:25]>>[NH2:1][CH2:2][CH2:3
][S:4][CH2:5][c:6]1[cH:7][c:8](-
[c:15]2[cH:16][cH:17][cH:18][cH:19][cH:20]2)[c:9]([CH2:11][NH:13][CH3:14])[o:10]
1

SMILES/SMARTS of the MAPPET output:
[CH3:1][NH:2][C:3](=[O:4])[c:5]1[o:6][c:7]([CH2:8][S:9][CH2:10][CH2:11][NH2:12])
[cH:13][c:14]1-
[c:15]1[cH:16][cH:17][cH:18][cH:19][cH:20]1>>[CH3:1][NH:2][CH2:3][c:5]1[o:6][c:7
]([CH2:8][S:9][CH2:10][CH2:11][NH2:12])[cH:13][c:14]1-
[c:15]1[cH:20][cH:19][cH:18][cH:17][cH:16]1.[O:4]

Correctness of the mapping
MAPPET YES
USPTO YES

Reaction no 12 (1 bond changed)

SMILES/SMARTS of the USPTO output:
[BH4-:14].[CH3:16][CH2:17][OH:18].[CH3:1][c:2]1[cH:3][c:4]([N+:11](=[O:12])[O-
:13])[n:5][n:6]1[CH2:7][C:8]([CH3:9])=[O:10].[Na+:15]>>[CH3:1][c:2]1[cH:3][c:4](
[N+:11](=[O:12])[O-:13])[n:5][n:6]1[CH2:7][CH:8]([CH3:9])[OH:10]

SMILES/SMARTS of the MAPPET output:
[CH3:1][C:2](=[O:3])[CH2:4][n:5]1[n:6][c:7]([cH:8][c:9]1[CH3:10])[N+:11]([O-
:12])=[O:13]>>[CH3:1][CH:2]([OH:3])[CH2:4][n:5]1[n:6][c:7]([cH:8][c:9]1[CH3:10])
[N+:11]([O-:12])=[O:13]

Correctness of the mapping
MAPPET YES
USPTO YES

Reaction no 13 (1 bond changed)

SMILES/SMARTS of the USPTO output:
[CH2:1]([CH3:2])[O:3][c:4]1[cH:5][c:6]([CH:12]([OH:13])[c:14]2[cH:15][c:16]([CH3
:22])[c:17]([O:20][CH3:21])[cH:18][cH:19]2)[cH:7][cH:8][c:9]1[O:10][CH3:11].[CH2
:23]([Cl:24])[Cl:25].[O:26]=[Mn:27]=[O:28]>>[CH2:1]([CH3:2])[O:3][c:4]1[cH:5][c:
6]([C:12](=[O:13])[c:14]2[cH:15][c:16]([CH3:22])[c:17]([O:20][CH3:21])[cH:18][cH
:19]2)[cH:7][cH:8][c:9]1[O:10][CH3:11]

SMILES/SMARTS of the MAPPET output:
[CH3:1][CH2:2][O:3][c:4]1[cH:5][c:6]([cH:7][cH:8][c:9]1[O:10][CH3:11])[CH:12]([O
H:13])[c:14]1[cH:15][cH:16][c:17]([O:18][CH3:19])[c:20]([CH3:21])[cH:22]1>>[CH3:
1][CH2:2][O:3][c:4]1[cH:5][c:6]([cH:7][cH:8][c:9]1[O:10][CH3:11])[C:12](=[O:13])
[c:14]1[cH:15][cH:16][c:17]([O:18][CH3:19])[c:20]([CH3:21])[cH:22]1

Correctness of the mapping
MAPPET YES
USPTO YES

Reaction no 14 (1 bond changed)

SMILES/SMARTS of the USPTO output:
[C:1](=[O:2])([CH3:3])[O:4][CH:5]([CH3:6])[c:7]1[n:8][cH:9][cH:10][c:11]([N:13]2[CH2:14][c:15]3[cH:16][c:17]([S:23]([N:24]([CH3:25])[CH3:26])(=[O:27])=[O:28])[cH:18][cH:19][c:20]3[CH2:21][CH2:22]2)[n:12]1.[CH3:33][OH:34].[Li+:31].[OH-:30].[OH2:29].[OH2:32]>>[OH:4][CH:5]([CH3:6])[c:7]1[n:8][cH:9][cH:10][c:11]([N:13]2[CH2:14][c:15]3[cH:16][c:17]([S:23]([N:24]([CH3:25])[CH3:26])(=[O:27])=[O:28])[cH:18][cH:19][c:20]3[CH2:21][CH2:22]2)[n:12]1

SMILES/SMARTS of the MAPPET output:
[CH3:1][CH:2]([O:3][C:4]([CH3:5])=[O:6])[c:7]1[n:8][cH:9][cH:10][c:11]([n:12]1)[N:13]1[CH2:14][CH2:15][c:16]2[cH:17][cH:18][c:19]([cH:20][c:21]2[CH2:22]1)[S:23](=[O:24])(=[O:25])[N:26]([CH3:27])[CH3:28]>>[CH3:1][CH:2]([OH:3])[c:7]1[n:8][cH:9][cH:10][c:11]([n:12]1)[N:13]1[CH2:14][CH2:15][c:16]2[cH:17][cH:18][c:19]([cH:20][c:21]2[CH2:22]1)[S:23](=[O:24])(=[O:25])[N:26]([CH3:28])[CH3:27].[C:4].[C:5].[O:6]

Correctness of the mapping
MAPPET YES
USPTO YES

Reaction no 15 (1 bond changed)

SMILES/SMARTS of the USPTO output:
[Cl:32][CH2:33][CH2:34][Cl:35].[Cl:6][c:7]1[c:8]([CH:14]=[C:15]([C:16]([C:17]([CH3:18])([CH3:19])[CH3:20])=[O:21])[n:22]2[n:23][cH:24][n:25][cH:26]2)[cH:9][cH:10][c:11]([Cl:13])[cH:12]1.[OH2:31].[OH:27][N+:28](=[O:29])[O-:30].[P:1](=[O:2])([OH:3])([OH:4])[OH:5]>>[Cl:6][c:7]1[c:8]([CH:14]=[C:15]([CH:16]([C:17]([CH3:18])([CH3:19])[CH3:20])[OH:21])[n:22]2[n:23][cH:24][n:25][cH:26]2)[cH:9][cH:10][c:11]([Cl:13])[cH:12]1

SMILES/SMARTS of the MAPPET output:
[CH3:1][C:2]([CH3:3])([CH3:4])[C:5](=[O:6])[C:7](=[CH:8][c:9]1[cH:10][cH:11][c:12]([Cl:13])[cH:14][c:15]1[Cl:16])[n:17]1[cH:18][n:19][cH:20][n:21]1>>[CH3:1][C:2]([CH3:4])([CH3:3])[CH:5]([OH:6])[C:7](=[CH:8][c:9]1[cH:10][cH:11][c:12]([Cl:13])[cH:14][c:15]1[Cl:16])[n:17]1[cH:18][n:19][cH:20][n:21]1

Correctness of the mapping
MAPPET YES
USPTO YES

Reaction no 16 (1 bond changed)

SMILES/SMARTS of the USPTO output:
[C:1]([CH3:2])([CH3:3])([CH3:4])[c:5]1[n:6][c:7](-[c:10]2[o:11][c:12]3[c:13]([cH:14]2)[cH:15][c:16]([CH2:19][CH2:20][c:21]2[c:22]([C:27](=[O:28])[O:29][CH2:30][CH3:31])[cH:23][cH:24][cH:25][cH:26]2)[cH:17][cH:18]3)[s:8][cH:9]1.[CH3:33][OH:34].[ClH:32].[Na+:36].[OH-:35]>>[C:1]([CH3:2])([CH3:3])([CH3:4])[c:5]1[n:6][c:7](-[c:10]2[o:11][c:12]3[c:13]([cH:14]2)[cH:15][c:16]([CH2:19][CH2:20][c:21]2[c:22]([C:27](=[O:28])[OH:29])[cH:23][cH:24][cH:25][cH:26]2)[cH:17][cH:18]3)[s:8][cH:9]1

SMILES/SMARTS of the MAPPET output:
[CH3:2][CH2:3][O:4][C:5](=[O:6])[c:7]1[cH:8][cH:9][cH:10][cH:11][c:12]1[CH2:13][CH2:14][c:15]1[cH:16][cH:17][c:18]2[o:19][c:20]([cH:21][c:22]2[cH:23]1)-

[c:24]1[n:25][c:26]([cH:27][s:28]1)[C:29]([CH3:30])([CH3:31])[CH3:32].[OH2:1]>>[
CH3:32][C:29]([CH3:31])([CH3:30])[c:26]1[cH:27][s:28][c:24]([n:25]1)-
[c:20]1[cH:21][c:22]2[cH:23][c:15]([CH2:14][CH2:13][c:12]3[cH:11][cH:10][cH:9][c
H:8][c:7]3[C:5]([OH:1])=[O:6])[cH:16][cH:17][c:18]2[o:19]1.[CH3:2][CH2:3][OH:4]

Correctness of the mapping
MAPPET YES
USPTO NO

Reaction no 17 (1 bond changed)

SMILES/SMARTS of the USPTO output:
[CH3:11][OH:12].[O:1]1[CH2:2][C:3](=[CH:7][CH2:8][CH2:9][OH:10])[CH2:4][CH2:5][C
H2:6]1>>[O:1]1[CH2:2][CH:3]([CH2:7][CH2:8][CH2:9][OH:10])[CH2:4][CH2:5][CH2:6]1

SMILES/SMARTS of the MAPPET output:
[OH:1][CH2:2][CH2:3][CH:4]=[C:5]1[CH2:6][CH2:7][CH2:8][O:9][CH2:10]1>>[OH:1][CH2
:2][CH2:3][CH2:4][CH:5]1[CH2:6][CH2:7][CH2:8][O:9][CH2:10]1

Correctness of the mapping
MAPPET YES
USPTO YES

Reaction no 18 (1 bond changed)

SMILES/SMARTS of the USPTO output:
[CH2:1]([CH3:2])[O:3][P:4](=[O:5])([O:6][CH2:7][CH3:8])[CH:9]=[CH:10][c:11]1[c:1
2]([O:22][CH2:23][c:24]2[cH:25][c:26]([O:45][CH3:46])[c:27]([O:28][CH2:29][c:30]
3[n:31][c:32]([CH:36]=[CH:37][C:38](=[O:39])[O:40][CH2:41][CH3:42])[o:33][c:34]3
[CH3:35])[cH:43][cH:44]2)[n:13][n:14](-
[c:16]2[cH:17][cH:18][cH:19][cH:20][cH:21]2)[cH:15]1.[CH3:56][CH2:57][OH:58].[Cl
H:54].[Na+:53].[O:47]1[CH2:48][CH2:49][CH2:50][CH2:51]1.[OH-
:52].[OH2:55]>>[CH2:1]([CH3:2])[O:3][P:4](=[O:5])([O:6][CH2:7][CH3:8])[CH:9]=[CH
:10][c:11]1[c:12]([O:22][CH2:23][c:24]2[cH:25][c:26]([O:45][CH3:46])[c:27]([O:28
][CH2:29][c:30]3[n:31][c:32]([CH:36]=[CH:37][C:38](=[O:39])[OH:40])[o:33][c:34]3
[CH3:35])[cH:43][cH:44]2)[n:13][n:14](-
[c:16]2[cH:17][cH:18][cH:19][cH:20][cH:21]2)[cH:15]1

SMILES/SMARTS of the MAPPET output:
[CH3:2][CH2:3][O:4][C:5](=[O:6])[CH:7]=[CH:8][c:9]1[n:10][c:11]([CH2:12][O:13][c
:14]2[cH:15][cH:16][c:17]([CH2:18][O:19][c:20]3[n:21][n:22]([cH:23][c:24]3[CH:25
]=[CH:26][P:27](=[O:28])([O:29][CH2:30][CH3:31])[O:32][CH2:33][CH3:34])-
[c:35]3[cH:36][cH:37][cH:38][cH:39][cH:40]3)[cH:41][c:42]2[O:43][CH3:44])[c:45](
[CH3:46])[o:47]1.[OH2:1]>>[CH3:34][CH2:33][O:32][P:27](=[O:28])([O:29][CH2:30][C
H3:31])[CH:26]=[CH:25][c:24]1[cH:23][n:22]([n:21][c:20]1[O:19][CH2:18][c:17]1[cH
:16][cH:15][c:14]([O:13][CH2:12][c:11]2[n:10][c:9]([CH:8]=[CH:7][C:5]([OH:1])=[O
:6])[o:47][c:45]2[CH3:46])[c:42]([O:43][CH3:44])[cH:41]1)-
[c:35]1[cH:36][cH:37][cH:38][cH:39][cH:40]1.[CH3:2][CH2:3][OH:4]

Correctness of the mapping
MAPPET YES
USPTO NO

Reaction no 19 (1 bond changed)

SMILES/SMARTS of the USPTO output:
[CH2:29]1[O:30][CH2:31][CH2:32][CH2:33]1.[CH3:26][CH2:27][OH:28].[O:1]=[C:2]([CH:3]=[CH:4][c:5]1[cH:6][cH:7][c:8]([C:9](=[O:10])[OH:11])[cH:12][cH:13]1)[c:14]1[c:15]2[c:19]([c:20]([CH3:22])[s:21]1)[CH:18]1[CH:17]([CH2:16]2)[C:23]1([CH3:24])[CH3:25]>>[O:1]=[C:2]([CH2:3][CH2:4][c:5]1[cH:6][cH:7][c:8]([C:9](=[O:10])[OH:11])[cH:12][cH:13]1)[c:14]1[c:15]2[c:19]([c:20]([CH3:22])[s:21]1)[CH:18]1[CH:17]([CH2:16]2)[C:23]1([CH3:24])[CH3:25]

SMILES/SMARTS of the MAPPET output:
[CH3:1][c:2]1[s:3][c:4]([C:5](=[O:6])[CH:7]=[CH:8][c:9]2[cH:10][cH:11][c:12]([cH:13][cH:14]2)[C:15]([OH:16])=[O:17])[c:18]2[CH2:19][CH:20]3[CH:21]([c:22]12)[C:23]3([CH3:24])[CH3:25]>>[CH3:1][c:2]1[s:3][c:4]([C:5](=[O:6])[CH2:7][CH2:8][c:9]2[cH:14][cH:13][c:12]([cH:11][cH:10]2)[C:15]([OH:16])=[O:17])[c:18]2[CH2:19][CH:20]3[CH:21]([c:22]12)[C:23]3([CH3:24])[CH3:25]

Correctness of the mapping
MAPPET YES
USPTO YES

Reaction no 20 (1 bond changed)

SMILES/SMARTS of the USPTO output:
[CH3:1][O:2][c:3]1[cH:4][c:5]2[c:6]([n:7]3[c:8]([s:9]2)[n:10][c:11]([CH:13]([OH:14])[CH:15]2[CH2:16][CH2:17]2)[cH:12]3)[cH:18][cH:19]1.[CH3:20][CH2:21][O:22][CH2:23][CH3:24].[CH3:25][CH2:26][OH:27].[CH:28]([Cl:29])([Cl:30])[Cl:31]>>[CH3:1][O:2][c:3]1[cH:4][c:5]2[c:6]([n:7]3[c:8]([s:9]2)[n:10][c:11]([C:13](=[O:14])[CH:15]2[CH2:16][CH2:17]2)[cH:12]3)[cH:18][cH:19]1

SMILES/SMARTS of the MAPPET output:
[CH3:1][O:2][c:3]1[cH:4][cH:5][c:6]2[c:7]([cH:8]1)[s:9][c:10]1[n:11][c:12]([cH:13][n:14]21)[CH:15]([OH:16])[CH:17]1[CH2:18][CH2:19]1>>[CH3:1][O:2][c:3]1[cH:4][cH:5][c:6]2[c:7]([cH:8]1)[s:9][c:10]1[n:11][c:12]([cH:13][n:14]21)[C:15](=[O:16])[CH:17]1[CH2:18][CH2:19]1

Correctness of the mapping
MAPPET YES
USPTO YES

Reaction no 21 (1 bond changed)

SMILES/SMARTS of the USPTO output:
[CH3:1][O:2][c:3]1[cH:4][cH:5][c:6]([N+:13](=[O:14])[O-:15])[c:7]2[n:8][c:9]([CH3:12])[s:10][c:11]12.[ClH:16].[n:17]1[cH:18][cH:19][cH:20][cH:21][cH:22]1>>[OH:2][c:3]1[cH:4][cH:5][c:6]([N+:13](=[O:14])[O-:15])[c:7]2[n:8][c:9]([CH3:12])[s:10][c:11]12

SMILES/SMARTS of the MAPPET output:
[CH3:1][O:2][c:3]1[cH:4][cH:5][c:6]([N+:7]([O-:8])=[O:9])[c:10]2[n:11][c:12]([CH3:13])[s:14][c:15]12>>[CH3:13][c:12]1[n:11][c:10]2[c:6]([cH:5][cH:4][c:3]([OH:2])[c:15]2[s:14]1)[N+:7]([O-:8])=[O:9].[C:1]

Correctness of the mapping
MAPPET YES
USPTO YES

Reaction no 22 (1 bond changed)

SMILES/SMARTS of the USPTO output:
[CH2:1]([c:2]1[cH:3][cH:4][cH:5][cH:6][cH:7]1)[O:8][c:9]1[cH:10][c:11]([CH:15]([
OH:16])[c:17]2[cH:18][c:19]([O:25][CH3:26])[cH:20][c:21]([O:23][CH3:24])[cH:22]2
)[cH:12][cH:13][cH:14]1.[Cl:27][CH2:28][Cl:29].[O:30]=[Mn:31]=[O:32]>>[CH2:1]([c
:2]1[cH:3][cH:4][cH:5][cH:6][cH:7]1)[O:8][c:9]1[cH:10][c:11]([C:15](=[O:16])[c:1
7]2[cH:18][c:19]([O:25][CH3:26])[cH:20][c:21]([O:23][CH3:24])[cH:22]2)[cH:12][cH
:13][cH:14]1

SMILES/SMARTS of the MAPPET output:
[CH3:1][O:2][c:3]1[cH:4][c:5]([O:6][CH3:7])[cH:8][c:9]([cH:10]1)[CH:11]([OH:12])
[c:13]1[cH:14][cH:15][cH:16][c:17]([O:18][CH2:19][c:20]2[cH:21][cH:22][cH:23][cH
:24][cH:25]2)[cH:26]1>>[CH3:7][O:6][c:5]1[cH:4][c:3]([O:2][CH3:1])[cH:10][c:9]([
cH:8]1)[C:11](=[O:12])[c:13]1[cH:14][cH:15][cH:16][c:17]([O:18][CH2:19][c:20]2[c
H:25][cH:24][cH:23][cH:22][cH:21]2)[cH:26]1

Correctness of the mapping
MAPPET YES
USPTO YES

Reaction no 23 (1 bond changed)

SMILES/SMARTS of the USPTO output:
[C:1]([CH3:2])([CH3:3])([CH3:4])[N:5]([CH2:6][C:7](=[O:8])[O-
:9])[C:10](=[O:11])[C:12]1=[C:13]([OH:29])[C:14]2([c:15]3[c:16]([Cl:23])[cH:17][
cH:18][cH:19][c:20]3[C:21]1=[O:22])[CH2:24][CH2:25][O:26][CH2:27][CH2:28]2.[F:30
][C:31]([F:32])([F:33])[C:34]([OH:35])=[O:36].[OH2:37]>>[NH:5]([CH2:6][C:7](=[O:
8])[OH:9])[C:10](=[O:11])[C:12]1=[C:13]([OH:29])[C:14]2([c:15]3[c:16]([Cl:23])[c
H:17][cH:18][cH:19][c:20]3[C:21]1=[O:22])[CH2:24][CH2:25][O:26][CH2:27][CH2:28]2

SMILES/SMARTS of the MAPPET output:
[CH3:1][C:2]([CH3:3])([CH3:4])[N:5]([CH2:6][C:7]([O-
:8])=[O:9])[C:10](=[O:11])[C:12]1=[C:13]([OH:14])[C:15]2([CH2:16][CH2:17][O:18][
CH2:19][CH2:20]2)[c:21]2[c:22]([Cl:23])[cH:24][cH:25][cH:26][c:27]2[C:28]1=[O:29
]>>[OH:8][C:7](=[O:9])[CH2:6][NH:5][C:10](=[O:11])[C:12]1=[C:13]([OH:14])[C:15]2
([CH2:20][CH2:19][O:18][CH2:17][CH2:16]2)[c:21]2[c:22]([Cl:23])[cH:24][cH:25][cH
:26][c:27]2[C:28]1=[O:29].[C:3].[C:2].[C:1].[C:4]

Correctness of the mapping
MAPPET YES
USPTO YES

Reaction no 24 (1 bond changed)

SMILES/SMARTS of the USPTO output:
[Al+3:34].[CH2:37]([Cl:38])[Cl:39].[Cl-:33].[Cl-:35].[Cl-
:36].[NH2:1][c:2]1[n:3][c:4]([NH:22][CH:23]2[CH2:24][CH2:25][N:26]([S:29](=[O:30
])(=[O:31])[CH3:32])[CH2:27][CH2:28]2)[n:5][cH:6][c:7]1[C:8](=[O:9])[c:10]1[c:11
]([F:21])[c:12]([F:20])[cH:13][c:14]([O:18][CH3:19])[c:15]1[O:16][CH3:17]>>[NH2:
1][c:2]1[n:3][c:4]([NH:22][CH:23]2[CH2:24][CH2:25][N:26]([S:29](=[O:30])(=[O:31]
)[CH3:32])[CH2:27][CH2:28]2)[n:5][cH:6][c:7]1[C:8](=[O:9])[c:10]1[c:11]([F:21])[
c:12]([F:20])[cH:13][c:14]([O:18][CH3:19])[c:15]1[OH:16]

SMILES/SMARTS of the MAPPET output:
[CH3:1][O:2][c:3]1[cH:4][c:5]([F:6])[c:7]([F:8])[c:9]([C:10](=[O:11])[c:12]2[cH:
13][n:14][c:15]([NH:16][CH:17]3[CH2:18][CH2:19][N:20]([CH2:21][CH2:22]3)[S:23]([

CH3:24])(=[O:25])=[O:26])[n:27][c:28]2[NH2:29])[c:30]1[O:31][CH3:32]>>[CH3:1][O:2][c:3]1[cH:4][c:5]([F:6])[c:7]([F:8])[c:9]([C:10](=[O:11])[c:12]2[cH:13][n:14][c:15]([NH:16][CH:17]3[CH2:22][CH2:21][N:20]([CH2:19][CH2:18]3)[S:23]([CH3:24])(=[O:25])=[O:26])[n:27][c:28]2[NH2:29])[c:30]1[OH:31].[C:32]

Correctness of the mapping
MAPPET YES
USPTO YES

Reaction no 25 (1 bond changed)

SMILES/SMARTS of the USPTO output:
[CH2:1]([CH3:2])[n:3]1[c:4](-[c:13]2[cH:14][cH:15][cH:16][c:17]3[cH:18][cH:19][cH:20][cH:21][c:22]23)[n:5][c:6]([F:12])[c:7]1[Si:8]([CH3:9])([CH3:10])[CH3:11].[CH2:25]([N+:26]([CH2:27][CH2:28][CH2:29][CH3:30])([CH2:31][CH2:32][CH2:33][CH3:34])[CH2:35][CH2:36][CH2:37][CH3:38])[CH2:39][CH2:40][CH3:41].[CH2:42]1[O:43][CH2:44][CH2:45][CH2:46]1.[F-:24].[OH2:23]>>[CH2:1]([CH3:2])[n:3]1[c:4](-[c:13]2[cH:14][cH:15][cH:16][c:17]3[cH:18][cH:19][cH:20][cH:21][c:22]23)[n:5][c:6]([F:12])[cH:7]1

SMILES/SMARTS of the MAPPET output:
[CH3:1][CH2:2][n:3]1[c:4]([n:5][c:6]([F:7])[c:8]1[Si:9]([CH3:10])([CH3:11])[CH3:12])-[c:13]1[cH:14][cH:15][cH:16][c:17]2[cH:18][cH:19][cH:20][cH:21][c:22]12>>[CH3:1][CH2:2][n:3]1[cH:8][c:6]([F:7])[n:5][c:4]1-[c:13]1[cH:14][cH:15][cH:16][c:17]2[cH:18][cH:19][cH:20][cH:21][c:22]12.[C:11].[C:10].[C:12].[Si:9]

Correctness of the mapping
MAPPET YES
USPTO YES

Reaction no 26 (1 bond changed)

SMILES/SMARTS of the USPTO output:
[CH2:1]([CH:2]([CH3:3])[CH3:4])[CH:5]([C:6](=[O:7])[O:8][CH3:9])[N:10]1[C:11](=[O:34])[CH:12]([N:22]2[C:23](=[O:33])[O:24][CH2:25][CH:26]2[c:27]2[cH:28][cH:29][cH:30][cH:31][cH:32]2)[CH:13]1[c:14]1[c:15]([CH:16]=[CH2:17])[cH:18][cH:19][cH:20][cH:21]1.[Na+:36].[O:37]1[CH2:38][CH2:39][CH2:40][CH2:41]1.[OH-:35].[OH2:42]>>[CH2:1]([CH:2]([CH3:3])[CH3:4])[CH:5]([C:6](=[O:7])[OH:8])[N:10]1[C:11](=[O:34])[CH:12]([N:22]2[C:23](=[O:33])[O:24][CH2:25][CH:26]2[c:27]2[cH:28][cH:29][cH:30][cH:31][cH:32]2)[CH:13]1[c:14]1[c:15]([CH:16]=[CH2:17])[cH:18][cH:19][cH:20][cH:21]1

SMILES/SMARTS of the MAPPET output:
[CH3:2][O:3][C:4](=[O:5])[CH:6]([CH2:7][CH:8]([CH3:9])[CH3:10])[N:11]1[CH:12]([CH:13]([N:14]2[CH:15]([CH2:16][O:17][C:18]2=[O:19])[c:20]2[cH:21][cH:22][cH:23][cH:24][cH:25]2)[C:26]1=[O:27])[c:28]1[cH:29][cH:30][cH:31][cH:32][c:33]1[CH:34]=[CH2:35].[OH2:1]>>[CH3:9][CH:8]([CH3:10])[CH2:7][CH:6]([N:11]1[CH:12]([CH:13]([N:14]2[CH:15]([CH2:16][O:17][C:18]2=[O:19])[c:20]2[cH:21][cH:22][cH:23][cH:24][cH:25]2)[C:26]1=[O:27])[c:28]1[cH:29][cH:30][cH:31][cH:32][c:33]1[CH:34]=[CH2:35])[C:4]([OH:1])=[O:5].[CH3:2][OH:3]

Correctness of the mapping
MAPPET YES
USPTO NO

Reaction no 27 (1 bond changed)

SMILES/SMARTS of the USPTO output:
[Cl:25][CH2:26][Cl:27].[Na+:24].[O-
:20][C:21]([OH:22])=[O:23].[n:1]1[c:2]2[c:3]([n:4][o:5]1)[cH:6][c:7](-
[c:10]1[c:11]([O:18][CH3:19])[cH:12][c:13]([NH:16][CH3:17])[cH:14][cH:15]1)[cH:8
][cH:9]2>>[n:1]1[c:2]2[c:3]([n:4][o:5]1)[cH:6][c:7](-
[c:10]1[c:11]([OH:18])[cH:12][c:13]([NH:16][CH3:17])[cH:14][cH:15]1)[cH:8][cH:9]
2

SMILES/SMARTS of the MAPPET output:
[CH3:1][NH:2][c:3]1[cH:4][cH:5][c:6]([c:7]([O:8][CH3:9])[cH:10]1)-
[c:11]1[cH:12][cH:13][c:14]2[n:15][o:16][n:17][c:18]2[cH:19]1>>[CH3:1][NH:2][c:3
]1[cH:4][cH:5][c:6]([c:7]([OH:8])[cH:10]1)-
[c:11]1[cH:12][cH:13][c:14]2[n:15][o:16][n:17][c:18]2[cH:19]1.[C:9]

Correctness of the mapping
MAPPET YES
USPTO YES

Reaction no 28 (1 bond changed)

SMILES/SMARTS of the USPTO output:
[C:3](=[O:4])([O:5][CH2:6][CH3:7])[c:8]1[cH:9][cH:10][c:11]([O:12][CH2:13][CH2:1
4][CH2:15][CH2:16][CH2:17][CH2:18][P:19]([O:20][CH2:21][CH3:22])([O:23][CH2:24][
CH3:25])=[O:26])[cH:27][cH:28]1.[CH3:29][CH2:30][OH:31].[Na+:2].[OH-
:1]>>[C:3](=[O:4])([OH:5])[c:8]1[cH:9][cH:10][c:11]([O:12][CH2:13][CH2:14][CH2:1
5][CH2:16][CH2:17][CH2:18][P:19]([O:20][CH2:21][CH3:22])([O:23][CH2:24][CH3:25])
=[O:26])[cH:27][cH:28]1

SMILES/SMARTS of the MAPPET output:
[CH3:2][CH2:3][O:4][C:5](=[O:6])[c:7]1[cH:8][cH:9][c:10]([O:11][CH2:12][CH2:13][
CH2:14][CH2:15][CH2:16][CH2:17][P:18](=[O:19])([O:20][CH2:21][CH3:22])[O:23][CH2
:24][CH3:25])[cH:26][cH:27]1.[OH2:1]>>[CH3:25][CH2:24][O:23][P:18](=[O:19])([CH2
:17][CH2:16][CH2:15][CH2:14][CH2:13][CH2:12][O:11][c:10]1[cH:9][cH:8][c:7]([cH:2
7][cH:26]1)[C:5]([OH:1])=[O:6])[O:20][CH2:21][CH3:22].[CH3:2][CH2:3][OH:4]

Correctness of the mapping
MAPPET YES
USPTO NO

Reaction no 29 (1 bond changed)

SMILES/SMARTS of the USPTO output:
[CH3:24][OH:25].[F:1][c:2]1[cH:3][cH:4][c:5]([CH:6]=[C:7]2[CH2:8][N:9]([C:13](=[
O:14])[O:15][C:16]([CH3:17])([CH3:18])[CH3:19])[CH2:10][CH2:11][CH2:12]2)[cH:20]
[cH:21]1.[H:22][H:23]>>[F:1][c:2]1[cH:3][cH:4][c:5]([CH2:6][CH:7]2[CH2:8][N:9]([
C:13](=[O:14])[O:15][C:16]([CH3:17])([CH3:18])[CH3:19])[CH2:10][CH2:11][CH2:12]2
)[cH:20][cH:21]1

SMILES/SMARTS of the MAPPET output:
[CH3:1][C:2]([CH3:3])([CH3:4])[O:5][C:6](=[O:7])[N:8]1[CH2:9][CH2:10][CH2:11][C:
12]([CH2:13]1)=[CH:14][c:15]1[cH:16][cH:17][c:18]([F:19])[cH:20][cH:21]1>>[CH3:4

][C:2]([CH3:3])([CH3:1])[O:5][C:6](=[O:7])[N:8]1[CH2:9][CH2:10][CH2:11][CH:12]([CH2:14][c:15]2[cH:21][cH:20][c:18]([F:19])[cH:17][cH:16]2)[CH2:13]1

Correctness of the mapping
MAPPET YES
USPTO YES

Reaction no 30 (1 bond changed)

SMILES/SMARTS of the USPTO output:
[Br:1][c:2]1[cH:3][cH:4][c:5]([C:8]2=[C:9]([CH2:13][OH:14])[CH2:10][CH2:11][CH2:12]2)[cH:6][cH:7]1.[CH:15]([Cl:16])([Cl:17])[Cl:18]>>[Br:1][c:2]1[cH:3][cH:4][c:5]([C:8]2=[C:9]([CH:13]=[O:14])[CH2:10][CH2:11][CH2:12]2)[cH:6][cH:7]1

SMILES/SMARTS of the MAPPET output:
[OH:1][CH2:2][C:3]1=[C:4]([CH2:5][CH2:6][CH2:7]1)[c:8]1[cH:9][cH:10][c:11]([Br:12])[cH:13][cH:14]1>>[Br:12][c:11]1[cH:13][cH:14][c:8]([cH:9][cH:10]1)[C:4]1=[C:3]([CH2:7][CH2:6][CH2:5]1)[CH:2]=[O:1]

Correctness of the mapping
MAPPET YES
USPTO YES

Reaction no 31 (1 bond changed)

SMILES/SMARTS of the USPTO output:
[CH3:46][S:47](=[O:48])[CH3:49].[CH:50]([N:51]([CH2:52][CH3:53])[CH:54]([CH3:55])[CH3:56])([CH3:57])[CH3:58].[Cl:69][CH2:70][Cl:71].[OH2:72].[OH:1][CH2:2][c:3]1[cH:4][c:5]([CH3:45])[c:6]([NH:10][C:11](=[O:12])[CH2:13][CH2:14][CH2:15][CH2:16][N:17]([C:18](=[O:19])[CH2:20][CH2:21][N:22]2[CH2:23][CH2:24][CH:25]([O:28][C:29]([NH:30][c:31]3[c:32](-
[c:37]4[cH:38][cH:39][cH:40][cH:41][cH:42]4)[cH:33][cH:34][cH:35][cH:36]3)=[O:43])[CH2:26][CH2:27]2)[CH3:44])[cH:7][c:8]1[CH3:9].[S:65](=[O:66])(=[O:67])=[O:68].[n:59]1[cH:60][cH:61][cH:62][cH:63][cH:64]1>>[O:1]=[CH:2][c:3]1[cH:4][c:5]([CH3:45])[c:6]([NH:10][C:11](=[O:12])[CH2:13][CH2:14][CH2:15][CH2:16][N:17]([C:18](=[O:19])[CH2:20][CH2:21][N:22]2[CH2:23][CH2:24][CH:25]([O:28][C:29]([NH:30][c:31]3[c:32](-
[c:37]4[cH:38][cH:39][cH:40][cH:41][cH:42]4)[cH:33][cH:34][cH:35][cH:36]3)=[O:43])[CH2:26][CH2:27]2)[CH3:44])[cH:7][c:8]1[CH3:9]

SMILES/SMARTS of the MAPPET output:
[CH3:1][N:2]([CH2:3][CH2:4][CH2:5][CH2:6][C:7](=[O:8])[NH:9][c:10]1[cH:11][c:12]([CH3:13])[c:14]([CH2:15][OH:16])[cH:17][c:18]1[CH3:19])[C:20](=[O:21])[CH2:22][CH2:23][N:24]1[CH2:25][CH2:26][CH:27]([CH2:28][CH2:29]1)[O:30][C:31](=[O:32])[NH:33][c:34]1[cH:35][cH:36][cH:37][cH:38][c:39]1-
[c:40]1[cH:41][cH:42][cH:43][cH:44][cH:45]1>>[CH3:1][N:2]([CH2:3][CH2:4][CH2:5][CH2:6][C:7](=[O:8])[NH:9][c:10]1[cH:11][c:12]([CH3:13])[c:14]([CH:15]=[O:16])[cH:17][c:18]1[CH3:19])[C:20](=[O:21])[CH2:22][CH2:23][N:24]1[CH2:29][CH2:28][CH:27]([CH2:26][CH2:25]1)[O:30][C:31](=[O:32])[NH:33][c:34]1[cH:35][cH:36][cH:37][cH:38][c:39]1-[c:40]1[cH:45][cH:44][cH:43][cH:42][cH:41]1

Correctness of the mapping
MAPPET YES
USPTO YES

Reaction no 32 (1 bond changed)

SMILES/SMARTS of the USPTO output:
[CH3:18][CH2:19][O:20][C:21](=[O:22])[CH3:23].[CH3:24][N:25]([CH3:26])[CH:27]=[O:28].[F:1][C:2]([C:3](=[O:4])[NH:5][c:6]1[cH:7][cH:8][c:9]2[c:13]([cH:14]1)[C:12](=[O:15])[CH2:11][CH2:10]2)([F:16])[F:17]>>[F:1][C:2]([C:3](=[O:4])[NH:5][c:6]1[cH:7][cH:8][c:9]2[c:13]([cH:14]1)[CH:12]([OH:15])[CH2:11][CH2:10]2)([F:16])[F:17]

SMILES/SMARTS of the MAPPET output:
[F:1][C:2]([F:3])([F:4])[C:5](=[O:6])[NH:7][c:8]1[cH:9][cH:10][c:11]2[CH2:12][CH2:13][C:14](=[O:15])[c:16]2[cH:17]1>>[OH:15][CH:14]1[CH2:13][CH2:12][c:11]2[cH:10][cH:9][c:8]([NH:7][C:5](=[O:6])[C:2]([F:1])([F:3])[F:4])[cH:17][c:16]12

Correctness of the mapping
MAPPET YES
USPTO YES

Reaction no 33 (1 bond changed)

SMILES/SMARTS of the USPTO output:
[BH4-:37].[C:1]([O:2][N:3]=[O:4])([CH3:5])([CH3:6])[CH3:7].[CH2:45]1[O:46][CH2:47][CH2:48][CH2:49]1.[CH3:39][CH2:40][O:41][C:42]([CH3:43])=[O:44].[Cl:50][CH2:51][Cl:52].[NH2:8][CH:9]1[O:10][C:11]2=[c:20]3[c:15]([cH:16][c:17]([CH3:22])[c:18](=[O:21])[o:19]3)=[CH:14][CH:13]([c:23]3[cH:24][c:25]([Br:33])[c:26]([O:31][CH3:32])[c:27]([O:29][CH3:30])[cH:28]3)[CH:12]2[N:34]1[C:35]#[N:36].[Na+:38]>>[CH2:9]1[O:10][C:11]2=[c:20]3[c:15]([cH:16][c:17]([CH3:22])[c:18](=[O:21])[o:19]3)=[CH:14][CH:13]([c:23]3[cH:24][c:25]([Br:33])[c:26]([O:31][CH3:32])[c:27]([O:29][CH3:30])[cH:28]3)[CH:12]2[N:34]1[C:35]#[N:36]

SMILES/SMARTS of the MAPPET output:
-

Correctness of the mapping
MAPPET NO
USPTO YES

Reaction no 34 (1 bond changed)

SMILES/SMARTS of the USPTO output:
[CH3:21][CH2:22][N:23]([CH2:24][CH3:25])[CH2:26][CH3:27].[CH3:7][S:8]([CH3:9])=[O:10].[Cl:11][c:12]1[c:13]([CH2:19][OH:20])[c:14]([I:18])[cH:15][cH:16][cH:17]1.[Cl:1][C:2]([C:3]([Cl:4])=[O:5])=[O:6].[Cl:28][CH2:29][Cl:30].[OH2:31]>>[Cl:11][c:12]1[c:13]([CH:19]=[O:20])[c:14]([I:18])[cH:15][cH:16][cH:17]1

SMILES/SMARTS of the MAPPET output:
[OH:1][CH2:2][c:3]1[c:4]([Cl:5])[cH:6][cH:7][cH:8][c:9]1[I:10]>>[Cl:5][c:4]1[cH:6][cH:7][cH:8][c:9]([I:10])[c:3]1[CH:2]=[O:1]

Correctness of the mapping
MAPPET YES
USPTO YES

Reaction no 35 (1 bond changed)

SMILES/SMARTS of the USPTO output:
[CH3:1][Si:2]([Br:3])([CH3:4])[CH3:5].[CH3:6][O:7][c:8]1[c:9]([O:40][CH2:41][O:4
2][CH3:43])[c:10]([C:11](=[O:12])[O:13][C:14]([CH3:15])([CH3:16])[CH3:17])[c:18]
([CH2:21][O:22][c:23]2[cH:24][cH:25][c:26](-
[c:29]3[cH:30][cH:31][c:32]([CH2:35][C:36](=[O:37])[O:38][CH3:39])[cH:33][cH:34]
3)[cH:27][cH:28]2)[cH:19][cH:20]1.[Cl:49][CH2:50][Cl:51].[Na+:44].[OH:45][C:46](
=[O:47])[O-
:48]>>[CH3:6][O:7][c:8]1[c:9]([OH:40])[c:10]([C:11](=[O:12])[O:13][C:14]([CH3:15
])([CH3:16])[CH3:17])[c:18]([CH2:21][O:22][c:23]2[cH:24][cH:25][c:26](-
[c:29]3[cH:30][cH:31][c:32]([CH2:35][C:36](=[O:37])[O:38][CH3:39])[cH:33][cH:34]
3)[cH:27][cH:28]2)[cH:19][cH:20]1

SMILES/SMARTS of the MAPPET output:
[CH3:1][O:2][CH2:3][O:4][c:5]1[c:6]([O:7][CH3:8])[cH:9][cH:10][c:11]([CH2:12][O:
13][c:14]2[cH:15][cH:16][c:17]([cH:18][cH:19]2)-
[c:20]2[cH:21][cH:22][c:23]([CH2:24][C:25](=[O:26])[O:27][CH3:28])[cH:29][cH:30]
2)[c:31]1[C:32](=[O:33])[O:34][C:35]([CH3:36])([CH3:37])[CH3:38]>>[CH3:28][O:27]
[C:25](=[O:26])[CH2:24][c:23]1[cH:29][cH:30][c:20]([cH:21][cH:22]1)-
[c:17]1[cH:18][cH:19][c:14]([O:13][CH2:12][c:11]2[cH:10][cH:9][c:6]([O:7][CH3:8]
)[c:5]([OH:4])[c:31]2[C:32](=[O:33])[O:34][C:35]([CH3:38])([CH3:37])[CH3:36])[cH
:15][cH:16]1.[C:1].[C:3].[O:2]

Correctness of the mapping
MAPPET YES
USPTO YES

Reaction no 36 (1 bond changed)

SMILES/SMARTS of the USPTO output:
[CH3:1][O:2][c:3]1[cH:4][c:5]2[c:6]3[c:7]([CH3:17])[cH:8][cH:9][c:10]([CH3:16])[
c:11]3[nH:12][c:13]2[cH:14][cH:15]1.[ClH:18].[n:19]1[cH:20][cH:21][cH:22][cH:23]
[cH:24]1>>[OH:2][c:3]1[cH:4][c:5]2[c:6]3[c:7]([CH3:17])[cH:8][cH:9][c:10]([CH3:1
6])[c:11]3[nH:12][c:13]2[cH:14][cH:15]1

SMILES/SMARTS of the MAPPET output:
[CH3:1][O:2][c:3]1[cH:4][cH:5][c:6]2[nH:7][c:8]3[c:9]([CH3:10])[cH:11][cH:12][c:
13]([CH3:14])[c:15]3[c:16]2[cH:17]1>>[CH3:10][c:9]1[cH:11][cH:12][c:13]([CH3:14]
)[c:15]2[c:16]3[cH:17][c:3]([OH:2])[cH:4][cH:5][c:6]3[nH:7][c:8]12.[C:1]

Correctness of the mapping
MAPPET YES
USPTO YES

Reaction no 37 (1 bond changed)

SMILES/SMARTS of the USPTO output:
[C:1]([CH3:2])([CH3:3])([CH3:4])[O:5][C:6](=[O:7])[CH2:8][O:9][c:10]1[cH:11][c:1
2]([C:16]([CH2:17][CH2:18][c:19]2[cH:20][c:21]([O:27][CH3:28])[c:22]([O:25][CH3:
26])[cH:23][cH:24]2)=[O:29])[cH:13][cH:14][cH:15]1.[CH2:30]1[O:31][CH2:32][CH2:3
3][CH2:34]1>>[C:1]([CH3:2])([CH3:3])([CH3:4])[O:5][C:6](=[O:7])[CH2:8][O:9][c:10
]1[cH:11][c:12]([CH:16]([CH2:17][CH2:18][c:19]2[cH:20][c:21]([O:27][CH3:28])[c:2
2]([O:25][CH3:26])[cH:23][cH:24]2)[OH:29])[cH:13][cH:14][cH:15]1

SMILES/SMARTS of the MAPPET output:
[CH3:1][O:2][c:3]1[cH:4][cH:5][c:6]([CH2:7][CH2:8][C:9](=[O:10])[c:11]2[cH:12][c
H:13][cH:14][c:15]([O:16][CH2:17][C:18](=[O:19])[O:20][C:21]([CH3:22])([CH3:23])

[CH3:24])[cH:25]2)[cH:26][c:27]1[O:28][CH3:29]>>[CH3:1][O:2][c:3]1[cH:4][cH:5][c:6]([CH2:7][CH2:8][CH:9]([OH:10])[c:11]2[cH:12][cH:13][cH:14][c:15]([O:16][CH2:17][C:18](=[O:19])[O:20][C:21]([CH3:24])([CH3:23])[CH3:22])[cH:25]2)[cH:26][c:27]1[O:28][CH3:29]

Correctness of the mapping
MAPPET YES
USPTO YES

Reaction no 38 (1 bond changed)

SMILES/SMARTS of the USPTO output:
[Br:1][B:2]([Br:3])[Br:4].[CH3:22][OH:23].[Cl:24][CH2:25][Cl:26].[F:5][c:6]1[cH:7][c:8]([C:15]2([C:18](=[O:19])[O:20][CH3:21])[CH2:16][CH2:17]2)[cH:9][c:10]([F:14])[c:11]1[O:12][CH3:13]>>[F:5][c:6]1[cH:7][c:8]([C:15]2([C:18](=[O:19])[O:20][CH3:21])[CH2:16][CH2:17]2)[cH:9][c:10]([F:14])[c:11]1[OH:12]

SMILES/SMARTS of the MAPPET output:
[CH3:1][O:2][C:3](=[O:4])[C:5]1([CH2:6][CH2:7]1)[c:8]1[cH:9][c:10]([F:11])[c:12]([O:13][CH3:14])[c:15]([F:16])[cH:17]1>>[CH3:1][O:2][C:3](=[O:4])[C:5]1([CH2:7][CH2:6]1)[c:8]1[cH:17][c:15]([F:16])[c:12]([OH:13])[c:10]([F:11])[cH:9]1.[C:14]

Correctness of the mapping
MAPPET YES
USPTO YES

Reaction no 39 (1 bond changed)

SMILES/SMARTS of the USPTO output:
[BrH:21].[CH2:3]([CH2:4][CH2:5][CH3:6])[N:7]1[CH2:8][CH2:9][N:10]([c:13]2[cH:14][cH:15][c:16]([O:19][CH3:20])[cH:17][cH:18]2)[CH2:11][CH2:12]1.[CH3:22][C:23](=[O:24])[OH:25].[ClH:1].[ClH:2]>>[BrH:21].[CH2:3]([CH2:4][CH2:5][CH3:6])[N:7]1[CH2:8][CH2:9][N:10]([c:13]2[cH:14][cH:15][c:16]([OH:19])[cH:17][cH:18]2)[CH2:11][CH2:12]1

SMILES/SMARTS of the MAPPET output:
[CH3:1][CH2:2][CH2:3][CH2:4][N:5]1[CH2:6][CH2:7][N:8]([CH2:9][CH2:10]1)[c:11]1[cH:12][cH:13][c:14]([O:15][CH3:16])[cH:17][cH:18]1>>[CH3:1][CH2:2][CH2:3][CH2:4][N:5]1[CH2:10][CH2:9][N:8]([CH2:7][CH2:6]1)[c:11]1[cH:18][cH:17][c:14]([OH:15])[cH:13][cH:12]1.[C:16]

Correctness of the mapping
MAPPET YES
USPTO YES

Reaction no 40 (1 bond changed)

SMILES/SMARTS of the USPTO output:
[C:31].[CH3:28][CH2:29][OH:30].[Pd:32].[c:1]1([CH:11]=[C:12]([C:13](=[O:14])[O:15][CH2:16][CH3:17])[CH2:18][CH2:19][CH2:20][O:21][CH:22]2[O:23][CH2:24][CH2:25][CH2:26][CH2:27]2)[cH:2][cH:3][cH:4][c:5]2[cH:6][cH:7][cH:8][cH:9][c:10]12>>[c:1]1([CH2:11][CH:12]([C:13](=[O:14])[O:15][CH2:16][CH3:17])[CH2:18][CH2:19][CH2:20][O:21][CH:22]2[O:23][CH2:24][CH2:25][CH2:26][CH2:27]2)[cH:2][cH:3][cH:4][c:5]2[cH:6][cH:7][cH:8][cH:9][c:10]12

SMILES/SMARTS of the MAPPET output:
[CH3:1][CH2:2][O:3][C:4](=[O:5])[C:6]([CH2:7][CH2:8][CH2:9][O:10][CH:11]1[CH2:12
][CH2:13][CH2:14][CH2:15][O:16]1)=[CH:17][c:18]1[cH:19][cH:20][cH:21][c:22]2[cH:
23][cH:24][cH:25][cH:26][c:27]12>>[CH3:1][CH2:2][O:3][C:4](=[O:5])[CH:6]([CH2:7]
[CH2:8][CH2:9][O:10][CH:11]1[CH2:12][CH2:13][CH2:14][CH2:15][O:16]1)[CH2:17][c:1
8]1[cH:19][cH:20][cH:21][c:22]2[cH:23][cH:24][cH:25][cH:26][c:27]12

Correctness of the mapping
MAPPET YES
USPTO YES

Reaction no 41 (1 bond changed)

SMILES/SMARTS of the USPTO output:
[C:1]([CH3:2])(=[O:3])[NH:4][c:5]1[c:6]([CH2:7][OH:8])[cH:9][cH:10][cH:11][cH:12
]1.[Cl:13][CH:14]([Cl:15])[Cl:16].[O:17]=[Mn:18]=[O:19]>>[C:1]([CH3:2])(=[O:3])[
NH:4][c:5]1[c:6]([CH:7]=[O:8])[cH:9][cH:10][cH:11][cH:12]1

SMILES/SMARTS of the MAPPET output:
[CH3:1][C:2](=[O:3])[NH:4][c:5]1[cH:6][cH:7][cH:8][cH:9][c:10]1[CH2:11][OH:12]>>
[CH3:1][C:2](=[O:3])[NH:4][c:5]1[cH:6][cH:7][cH:8][cH:9][c:10]1[CH:11]=[O:12]

Correctness of the mapping
MAPPET YES
USPTO YES

Reaction no 42 (1 bond changed)

SMILES/SMARTS of the USPTO output:
[CH2:36]([OH:37])[CH3:38].[CH3:1][C:2]1([CH3:32])[c:3]2[cH:4][cH:5][c:6]([CH:20]
=[CH:21][c:22]3[cH:23][cH:24][c:25]([C:26](=[O:27])[O:28][CH3:29])[cH:30][cH:31]
3)[cH:7][c:8]2[C:9]([CH:12]=[CH:13][c:14]2[cH:15][cH:16][cH:17][cH:18][cH:19]2)=
[CH:10][CH2:11]1.[ClH:35].[Na+:34].[O:39]1[CH2:40][CH2:41][CH2:42][CH2:43]1.[OH-
:33]>>[CH3:1][C:2]1([CH3:32])[c:3]2[cH:4][cH:5][c:6]([CH:20]=[CH:21][c:22]3[cH:2
3][cH:24][c:25]([C:26](=[O:27])[OH:28])[cH:30][cH:31]3)[cH:7][c:8]2[C:9]([CH:12]
=[CH:13][c:14]2[cH:15][cH:16][cH:17][cH:18][cH:19]2)=[CH:10][CH2:11]1

SMILES/SMARTS of the MAPPET output:
[CH3:2][O:3][C:4](=[O:5])[c:6]1[cH:7][cH:8][c:9]([CH:10]=[CH:11][c:12]2[cH:13][c
H:14][c:15]3[c:16]([cH:17]2)[C:18]([CH:19]=[CH:20][c:21]2[cH:22][cH:23][cH:24][c
H:25][cH:26]2)=[CH:27][CH2:28][C:29]3([CH3:30])[CH3:31])[cH:32][cH:33]1.[OH2:1]>
>[CH3:31][C:29]1([CH3:30])[CH2:28][CH:27]=[C:18]([CH:19]=[CH:20][c:21]2[cH:22][c
H:23][cH:24][cH:25][cH:26]2)[c:16]2[cH:17][c:12]([CH:11]=[CH:10][c:9]3[cH:8][cH:
7][c:6]([cH:33][cH:32]3)[C:4]([OH:1])=[O:5])[cH:13][cH:14][c:15]12.[CH3:2][OH:3]

Correct mapped SMILES/SMARTS of the reaction:

Correctness of the mapping
MAPPET YES
USPTO NO

Reaction no 43 (1 bond changed)

SMILES/SMARTS of the USPTO output:

[C:1](#[N:2])[c:3]1[cH:4][cH:5][c:6]([O:31][CH3:32])[c:7]([S:9](=[O:10])(=[O:11]
)[NH:12][CH2:13][CH2:14][c:15]2[c:16]([O:17][CH2:18][C:19](=[O:20])[O:21][CH2:22
][CH3:23])[cH:24][c:25]([CH:28]([CH3:29])[CH3:30])[cH:26][cH:27]2)[cH:8]1.[CH3:3
5][CH2:36][OH:37].[Na+:34].[OH-
:33]>>[C:1](#[N:2])[c:3]1[cH:4][cH:5][c:6]([O:31][CH3:32])[c:7]([S:9](=[O:10])(=
[O:11])[NH:12][CH2:13][CH2:14][c:15]2[c:16]([O:17][CH2:18][C:19](=[O:20])[OH:21]
)[cH:24][c:25]([CH:28]([CH3:29])[CH3:30])[cH:26][cH:27]2)[cH:8]1

SMILES/SMARTS of the MAPPET output:
[CH3:2][CH2:3][O:4][C:5](=[O:6])[CH2:7][O:8][c:9]1[cH:10][c:11]([cH:12][cH:13][c
:14]1[CH2:15][CH2:16][NH:17][S:18](=[O:19])(=[O:20])[c:21]1[cH:22][c:23]([cH:24]
[cH:25][c:26]1[O:27][CH3:28])[C:29]#[N:30])[CH:31]([CH3:32])[CH3:33].[OH2:1]>>[C
H3:28][O:27][c:26]1[cH:25][cH:24][c:23]([cH:22][c:21]1[S:18](=[O:20])(=[O:19])[N
H:17][CH2:16][CH2:15][c:14]1[cH:13][cH:12][c:11]([cH:10][c:9]1[O:8][CH2:7][C:5](
[OH:1])=[O:6])[CH:31]([CH3:32])[CH3:33])[C:29]#[N:30].[CH3:2][CH2:3][OH:4]

Correctness of the mapping
MAPPET YES
USPTO NO

Reaction no 44 (1 bond changed)

SMILES/SMARTS of the USPTO output:
[C:12]([CH3:13])([CH3:14])([CH3:15])[Si:16]([O:17][c:18]1[cH:19][c:20]([CH:21]([
OH:22])[c:23]2[cH:24][cH:25][c:26]([C:27](=[O:28])[N:29]([CH2:30][CH3:31])[CH2:3
2][CH3:33])[cH:34][cH:35]2)[cH:36][cH:37][cH:38]1)([CH3:39])[CH3:40].[Cl:41][CH2
:42][Cl:43].[O:1]=[Cr:2]([Cl:3])([O-
:4])=[O:5].[nH+:6]1[cH:7][cH:8][cH:9][cH:10][cH:11]1>>[C:12]([CH3:13])([CH3:14])
([CH3:15])[Si:16]([O:17][c:18]1[cH:19][c:20]([C:21](=[O:22])[c:23]2[cH:24][cH:25
][c:26]([C:27](=[O:28])[N:29]([CH2:30][CH3:31])[CH2:32][CH3:33])[cH:34][cH:35]2)
[cH:36][cH:37][cH:38]1)([CH3:39])[CH3:40]

SMILES/SMARTS of the MAPPET output:
[CH3:1][CH2:2][N:3]([CH2:4][CH3:5])[C:6](=[O:7])[c:8]1[cH:9][cH:10][c:11]([cH:12
][cH:13]1)[CH:14]([OH:15])[c:16]1[cH:17][cH:18][cH:19][c:20]([O:21][Si:22]([CH3:
23])([CH3:24])[C:25]([CH3:26])([CH3:27])[CH3:28])[cH:29]1>>[CH3:5][CH2:4][N:3]([
CH2:2][CH3:1])[C:6](=[O:7])[c:8]1[cH:13][cH:12][c:11]([cH:10][cH:9]1)[C:14](=[O:
15])[c:16]1[cH:17][cH:18][cH:19][c:20]([O:21][Si:22]([CH3:24])([CH3:23])[C:25]([
CH3:28])([CH3:27])[CH3:26])[cH:29]1

Correctness of the mapping
MAPPET YES
USPTO YES

Reaction no 45 (1 bond changed)

SMILES/SMARTS of the USPTO output:
[B:25]([Br:26])([Br:27])[Br:28].[CH2:30]([Cl:31])[Cl:32].[Cl:1][c:2]1[c:3]([I:24
])[c:4]([O:22][CH3:23])[c:5](-
[n:9]2[c:10](=[O:21])[n:11]([CH3:20])[c:12]([C:16]([F:17])([F:18])[F:19])[cH:13]
[c:14]2=[O:15])[c:6]([F:8])[cH:7]1.[OH2:29]>>[Cl:1][c:2]1[c:3]([I:24])[c:4]([OH:
22])[c:5](-
[n:9]2[c:10](=[O:21])[n:11]([CH3:20])[c:12]([C:16]([F:17])([F:18])[F:19])[cH:13]
[c:14]2=[O:15])[c:6]([F:8])[cH:7]1

SMILES/SMARTS of the MAPPET output:

[CH3:1][O:2][c:3]1[c:4]([I:5])[c:6]([Cl:7])[cH:8][c:9]([F:10])[c:11]1-[n:12]1[c:13](=[O:14])[cH:15][c:16]([n:17]([CH3:18])[c:19]1=[O:20])[C:21]([F:22])([F:23])[F:24]>>[CH3:18][n:17]1[c:16]([cH:15][c:13](=[O:14])[n:12](-[c:11]2[c:9]([F:10])[cH:8][c:6]([Cl:7])[c:4]([I:5])[c:3]2[OH:2])[c:19]1=[O:20])[C:21]([F:22])([F:23])[F:24].[C:1]

Correctness of the mapping
MAPPET YES
USPTO YES

Reaction no 46 (1 bond changed)

SMILES/SMARTS of the USPTO output:
[CH3:24][CH2:25][OH:26].[Na+:23].[OH-:22].[c:1]1([O:7][c:8]2[cH:9][c:10]([C:18]([F:19])([F:20])[F:21])[c:11]([C:12](=[O:13])[O:14][CH3:15])[cH:16][cH:17]2)[cH:2][cH:3][cH:4][cH:5][cH:6]1>>[c:1]1([O:7][c:8]2[cH:9][c:10]([C:18]([F:19])([F:20])[F:21])[c:11]([C:12](=[O:13])[OH:14])[cH:16][cH:17]2)[cH:2][cH:3][cH:4][cH:5][cH:6]1

SMILES/SMARTS of the MAPPET output:
[CH3:2][O:3][C:4](=[O:5])[c:6]1[cH:7][cH:8][c:9]([O:10][c:11]2[cH:12][cH:13][cH:14][cH:15][cH:16]2)[cH:17][c:18]1[C:19]([F:20])([F:21])[F:22].[OH2:1]>>[OH:1][C:4](=[O:5])[c:6]1[cH:7][cH:8][c:9]([O:10][c:11]2[cH:12][cH:13][cH:14][cH:15][cH:16]2)[cH:17][c:18]1[C:19]([F:20])([F:21])[F:22].[CH3:2][OH:3]

Correctness of the mapping
MAPPET YES
USPTO NO

Reaction no 47 (1 bond changed)

SMILES/SMARTS of the USPTO output:
[CH3:1][C:2]1=[C:3]([c:22]2[cH:23][cH:24][cH:25][cH:26][cH:27]2)[C:4](=[O:21])[N:5]([C:8]([CH:9]([OH:10])[c:11]2[cH:12][c:13]([CH:17]=[CH2:18])[cH:14][cH:15][cH:16]2)([CH3:19])[CH3:20])[CH2:6][O:7]1.[Cl:39][CH2:40][Cl:41].[O:28]=[Cr:29]([Cl:30])([O-:31])=[O:32].[nH+:33]1[cH:34][cH:35][cH:36][cH:37][cH:38]1>>[CH3:1][C:2]1=[C:3]([c:22]2[cH:23][cH:24][cH:25][cH:26][cH:27]2)[C:4](=[O:21])[N:5]([C:8]([C:9](=[O:10])[c:11]2[cH:12][c:13]([CH:17]=[CH2:18])[cH:14][cH:15][cH:16]2)([CH3:19])[CH3:20])[CH2:6][O:7]1

SMILES/SMARTS of the MAPPET output:
[CH3:1][C:2]1=[C:3]([C:4](=[O:5])[N:6]([CH2:7][O:8]1)[C:9]([CH3:10])([CH3:11])[CH:12]([OH:13])[c:14]1[cH:15][cH:16][cH:17][c:18]([CH:19]=[CH2:20])[cH:21]1)[c:22]1[cH:23][cH:24][cH:25][cH:26][cH:27]1>>[CH3:1][C:2]1=[C:3]([C:4](=[O:5])[N:6]([CH2:7][O:8]1)[C:9]([CH3:10])([CH3:11])[C:12](=[O:13])[c:14]1[cH:15][cH:16][cH:17][c:18]([CH:19]=[CH2:20])[cH:21]1)[c:22]1[cH:27][cH:26][cH:25][cH:24][cH:23]1

Correctness of the mapping
MAPPET YES
USPTO YES

Reaction no 48 (1 bond changed)

SMILES/SMARTS of the USPTO output:

[Al+3:13].[CH2:1]1[O:2][CH2:3][C:4](=[O:11])[N:5]2[CH:6]1[CH2:7][NH:8][CH2:9][CH2:10]2.[CH:18]([OH:19])([CH3:20])[CH3:21].[H-:12].[H-:15].[H-:16].[H-:17].[Li+:14].[Na+:22].[Na+:23].[O-:24][S:25]([O-:26])(=[O:27])=[O:28].[O:29]1[CH2:30][CH2:31][O:32][CH2:33][CH2:34]1>>[CH2:1]1[O:2][CH2:3][CH2:4][N:5]2[CH:6]1[CH2:7][NH:8][CH2:9][CH2:10]2

SMILES/SMARTS of the MAPPET output:
[O:1]=[C:2]1[CH2:3][O:4][CH2:5][CH:6]2[CH2:7][NH:8][CH2:9][CH2:10][N:11]12>>[CH2:9]1[CH2:10][N:11]2[CH2:2][CH2:3][O:4][CH2:5][CH:6]2[CH2:7][NH:8]1.[O:1]

Correctness of the mapping
MAPPET YES
USPTO YES

Reaction no 49 (1 bond changed)

SMILES/SMARTS of the USPTO output:
[CH3:1][O:2][c:3]1[cH:4][c:5]([CH:16]=[CH:17][CH:18]=[CH:19][C:20](=[O:21])[NH:22][CH2:23][CH2:24][N:25]2[CH2:26][CH2:27][CH:28]([O:31][CH:32]([c:33]3[cH:34][cH:35][cH:36][cH:37][cH:38]3)[c:39]3[cH:40][cH:41][cH:42][cH:43][cH:44]3)[CH2:29][CH2:30]2)[cH:6][cH:7][c:8]1[O:9][CH2:10][O:11][CH2:12][CH2:13][O:14][CH3:15].[CH3:64][OH:65].[Na+:58].[Na+:59].[O-:60][C:61](=[O:62])[O-:63].[OH2:45].[OH2:57].[c:46]1([CH3:47])[cH:48][cH:49][c:50]([S:51]([OH:52])(=[O:53])=[O:54])[cH:55][cH:56]1>>[CH3:1][O:2][c:3]1[cH:4][c:5]([CH:16]=[CH:17][CH:18]=[CH:19][C:20](=[O:21])[NH:22][CH2:23][CH2:24][N:25]2[CH2:26][CH2:27][CH:28]([O:31][CH:32]([c:33]3[cH:34][cH:35][cH:36][cH:37][cH:38]3)[c:39]3[cH:40][cH:41][cH:42][cH:43][cH:44]3)[CH2:29][CH2:30]2)[cH:6][cH:7][c:8]1[OH:9]

SMILES/SMARTS of the MAPPET output:
[CH3:1][O:2][CH2:3][CH2:4][O:5][CH2:6][O:7][c:8]1[cH:9][cH:10][c:11]([CH:12]=[CH:13][CH:14]=[CH:15][C:16](=[O:17])[NH:18][CH2:19][CH2:20][N:21]2[CH2:22][CH2:23][CH:24]([CH2:25][CH2:26]2)[O:27][CH:28]([c:29]2[cH:30][cH:31][cH:32][cH:33][cH:34]2)[c:35]2[cH:36][cH:37][cH:38][cH:39][cH:40]2)[cH:41][c:42]1[O:43][CH3:44]>>[CH3:44][O:43][c:42]1[cH:41][c:11]([CH:12]=[CH:13][CH:14]=[CH:15][C:16](=[O:17])[NH:18][CH2:19][CH2:20][N:21]2[CH2:26][CH2:25][CH:24]([CH2:23][CH2:22]2)[O:27][CH:28]([c:35]2[cH:40][cH:39][cH:38][cH:37][cH:36]2)[c:29]2[cH:34][cH:33][cH:32][cH:31][cH:30]2)[cH:10][cH:9][c:8]1[OH:7].[C:4].[C:3].[C:1].[C:6].[O:5].[O:2]

Correctness of the mapping
MAPPET YES
USPTO YES

Reaction no 50 (1 bond changed)

SMILES/SMARTS of the USPTO output:
[CH3:17][O-:18].[CH3:1][O:2][c:3]1[c:4]([CH:9]=[CH:10][c:11]2[cH:12][cH:13][cH:14][cH:15][cH:16]2)[cH:5][cH:6][cH:7][cH:8]1.[CH3:20][CH2:21][O-:22].[Na+:19].[Na+:23]>>[OH:2][c:3]1[c:4]([CH:9]=[CH:10][c:11]2[cH:12][cH:13][cH:14][cH:15][cH:16]2)[cH:5][cH:6][cH:7][cH:8]1

SMILES/SMARTS of the MAPPET output:
[CH3:1][O:2][c:3]1[cH:4][cH:5][cH:6][cH:7][c:8]1[CH:9]=[CH:10][c:11]1[cH:12][cH:13][cH:14][cH:15][cH:16]1>>[OH:2][c:3]1[cH:4][cH:5][cH:6][cH:7][c:8]1[CH:9]=[CH:10][c:11]1[cH:16][cH:15][cH:14][cH:13][cH:12]1.[C:1]

Correctness of the mapping

MAPPET YES
USPTO YES

Reaction no 51 (2 bonds changed)

SMILES/SMARTS of the USPTO output:
[CH3:1][C:2]1([CH3:22])[O:3][CH2:4][CH:5]([CH2:14][CH:15]2[CH2:16][O:17][CH2:18]
[CH2:19][CH2:20][CH2:21]2)[N:6]1[C:7](=[O:8])[O:9][C:10]([CH3:11])([CH3:12])[CH3
:13].[CH3:23][OH:24]>>[OH:3][CH2:4][CH:5]([NH:6][C:7](=[O:8])[O:9][C:10]([CH3:11
])([CH3:12])[CH3:13])[CH2:14][CH:15]1[CH2:16][O:17][CH2:18][CH2:19][CH2:20][CH2:
21]1

SMILES/SMARTS of the MAPPET output:
[OH2:1].[CH3:2][C:3]([CH3:4])([CH3:5])[O:6][C:7](=[O:8])[N:9]1[CH:10]([CH2:11][C
H:12]2[CH2:13][CH2:14][CH2:15][CH2:16][O:17][CH2:18]2)[CH2:19][O:20][C:21]1([CH3
:22])[CH3:23]>>[CH3:5][C:3]([CH3:4])([CH3:2])[O:6][C:7](=[O:8])[NH:9][CH:10]([CH
2:19][OH:20])[CH2:11][CH:12]1[CH2:13][CH2:14][CH2:15][CH2:16][O:17][CH2:18]1.[C:
22].[C:21].[C:23].[O:1]

Correct mapped SMILES/SMARTS of the reaction:
[OH2:1].[CH3:2][C:3]([CH3:4])([CH3:5])[O:6][C:7](=[O:8])[N:9]1[CH:10]([CH2:11][C
H:12]2[CH2:13][CH2:14][CH2:15][CH2:16][O:17][CH2:18]2)[CH2:19][O:20][C:21]1([CH3
:22])[CH3:23]>>[CH3:5][C:3]([CH3:4])([CH3:2])[O:6][C:7](=[O:8])[NH:9][CH:10]([CH
2:19][OH:1])[CH2:11][CH:12]1[CH2:13][CH2:14][CH2:15][CH2:16][O:17][CH2:18]1.[C:2
2].[C:21].[C:23].[O:20]

Correctness of the mapping
MAPPET NO
USPTO NO

Reaction no 52 (2 bonds changed)

SMILES/SMARTS of the USPTO output:
[CH3:34][CH2:35][N:36]([CH2:37][CH3:38])[CH2:39][CH3:40].[CH:7]([Cl:8])([Cl:9])[
Cl:10].[ClH:1].[F:11][C:12]([c:13]1[cH:14][cH:15][c:16]([O:17][c:18]2[cH:19][cH:
20][c:21]([O:22][CH:23]([C:24](=[O:25])[Cl:26])[CH3:27])[cH:28][cH:29]2)[cH:30][
cH:31]1)([F:32])[F:33].[O:2]1[NH:3][CH2:4][CH2:5][CH2:6]1.[OH2:41]>>[O:2]1[N:3](
[C:24]([CH:23]([O:22][c:21]2[cH:20][cH:19][c:18]([O:17][c:16]3[cH:15][cH:14][c:1
3]([C:12]([F:11])([F:32])[F:33])[cH:31][cH:30]3)[cH:29][cH:28]2)[CH3:27])=[O:25]
)[CH2:4][CH2:5][CH2:6]1

SMILES/SMARTS of the MAPPET output:
[CH2:1]1[CH2:2][NH:3][O:4][CH2:5]1.[CH3:6][CH:7]([O:8][c:9]1[cH:10][cH:11][c:12]
([O:13][c:14]2[cH:15][cH:16][c:17]([cH:18][cH:19]2)[C:20]([F:21])([F:22])[F:23])
[cH:24][cH:25]1)[C:26]([Cl:27])=[O:28]>>[CH3:6][CH:7]([O:8][c:9]1[cH:25][cH:24][
c:12]([O:13][c:14]2[cH:19][cH:18][c:17]([cH:16][cH:15]2)[C:20]([F:21])([F:22])[F
:23])[cH:11][cH:10]1)[C:26](=[O:28])[N:3]1[CH2:2][CH2:1][CH2:5][O:4]1.[Cl:27]

Correctness of the mapping
MAPPET YES
USPTO YES

Reaction no 53 (2 bonds changed)

SMILES/SMARTS of the USPTO output:

[C:18](=[O:19])([O-:20])[O-
:21].[CH3:15][NH:16][CH3:17].[CH3:1][O:2][C:3]([c:4]1[c:5]([C:11]#[N:12])[cH:6][
cH:7][c:8]([F:10])[cH:9]1)=[O:13].[ClH:14].[K+:22].[K+:23]>>[CH3:1][O:2][C:3]([c
:4]1[c:5]([C:11]#[N:12])[cH:6][cH:7][c:8]([N:16]([CH3:15])[CH3:17])[cH:9]1)=[O:1
3]

SMILES/SMARTS of the MAPPET output:
[CH3:1][O:2][C:3](=[O:4])[c:5]1[cH:6][c:7]([F:8])[cH:9][cH:10][c:11]1[C:12]#[N:1
3].[CH3:14][NH:15][CH3:16]>>[CH3:1][O:2][C:3](=[O:4])[c:5]1[cH:6][c:7]([cH:9][cH
:10][c:11]1[C:12]#[N:13])[N:15]([CH3:16])[CH3:14].[F:8]

Correctness of the mapping
MAPPET YES
USPTO YES

Reaction no 54 (2 bonds changed)

SMILES/SMARTS of the USPTO output:
[Br:1][c:2]1[cH:3][cH:4][cH:5][c:6]([CH2:8][OH:9])[n:7]1.[CH2:24]([P:25]([CH2:26
][CH2:27][CH2:28][CH3:29])[CH2:30][CH2:31][CH2:32][CH3:33])[CH2:34][CH2:35][CH3:
36].[CH3:60][CH2:61][O:62][CH2:63][CH3:64].[N:37]([C:38]([N:39]1[CH2:40][CH2:41]
[CH2:42][CH2:43][CH2:44]1)=[O:45])=[N:46][C:47]([N:48]1[CH2:49][CH2:50][CH2:51][
CH2:52][CH2:53]1)=[O:54].[O:55]1[CH2:56][CH2:57][CH2:58][CH2:59]1.[OH:10][c:11]1
[c:12]([C:21]([CH3:22])=[O:23])[cH:13][cH:14][c:15]([OH:20])[c:16]1[CH2:17][CH2:
18][CH3:19]>>[Br:1][c:2]1[cH:3][cH:4][cH:5][c:6]([CH2:8][O:9][c:15]2[cH:14][cH:1
3][c:12]([C:21]([CH3:22])=[O:23])[c:11]([OH:10])[c:16]2[CH2:17][CH2:18][CH3:19])
[n:7]1

SMILES/SMARTS of the MAPPET output:
[CH3:1][CH2:2][CH2:3][c:4]1[c:5]([OH:6])[cH:7][cH:8][c:9]([C:10]([CH3:11])=[O:12
])[c:13]1[OH:14].[OH:15][CH2:16][c:17]1[cH:18][cH:19][cH:20][c:21]([Br:22])[n:23
]1>>[OH2:6].[CH3:1][CH2:2][CH2:3][c:4]1[c:13]([OH:14])[c:9]([cH:8][cH:7][c:5]1[O
:15][CH2:16][c:17]1[cH:18][cH:19][cH:20][c:21]([Br:22])[n:23]1)[C:10]([CH3:11])=
[O:12]

Correct mapped SMILES/SMARTS of the reaction:
[CH3:1][CH2:2][CH2:3][c:4]1[c:5]([OH:6])[cH:7][cH:8][c:9]([C:10]([CH3:11])=[O:12
])[c:13]1[OH:14].[OH:15][CH2:16][c:17]1[cH:18][cH:19][cH:20][c:21]([Br:22])[n:23
]1>>[OH2:15].[CH3:1][CH2:2][CH2:3][c:4]1[c:13]([OH:14])[c:9]([cH:8][cH:7][c:5]1[
O:6][CH2:16][c:17]1[cH:18][cH:19][cH:20][c:21]([Br:22])[n:23]1)[C:10]([CH3:11])=
[O:12]

Correctness of the mapping
MAPPET NO
USPTO NO

Reaction no 55 (2 bonds changed)

SMILES/SMARTS of the USPTO output:
[CH3:33][N:34]([CH3:35])[CH:36]=[O:37].[Cl:26][CH:27]([C:28](=[O:29])[Cl:30])[CH
3:31].[H-
:1].[Na+:2].[OH2:32].[s:3]1[c:4]([CH2:12][O:13][c:14]2[cH:15][c:16]([CH3:25])[c:
17]([NH:20][C:21]([O:22][CH3:23])=[O:24])[cH:18][cH:19]2)[n:5][c:6]2[c:7]1[cH:8]
[cH:9][cH:10][cH:11]2>>[s:3]1[c:4]([CH2:12][O:13][c:14]2[cH:15][c:16]([CH3:25])[
c:17]([N:20]([C:21]([O:22][CH3:23])=[O:24])[C:28]([CH:27]([Cl:26])[CH3:31])=[O:2
9])[cH:18][cH:19]2)[n:5][c:6]2[c:7]1[cH:8][cH:9][cH:10][cH:11]2

SMILES/SMARTS of the MAPPET output:
[CH3:1][CH2:2][CH2:3][c:4]1[c:5]([OH:6])[cH:7][cH:8][c:9]([C:10]([CH3:11])=[O:12])[c:13]1[OH:14].[OH:15][CH2:16][c:17]1[cH:18][cH:19][cH:20][c:21]([Br:22])[n:23]1>>[OH2:6].[CH3:1][CH2:2][CH2:3][c:4]1[c:13]([OH:14])[c:9]([cH:8][cH:7][c:5]1[O:15][CH2:16][c:17]1[cH:18][cH:19][cH:20][c:21]([Br:22])[n:23]1)[C:10]([CH3:11])=[O:12]

Correctness of the mapping
MAPPET YES
USPTO YES

Reaction no 56 (2 bonds changed)

SMILES/SMARTS of the USPTO output:
[Br:1][c:2]1[cH:3][cH:4][c:5](-[c:8]2[n:9][n:10]([CH2:20][CH:21]3[O:22][CH2:23]3)[c:11]3[c:12]2[CH2:13][N:14]([C:17]([CH3:18])=[O:19])[CH2:15][CH2:16]3)[cH:6][cH:7]1.[CH3:24][c:25]1[c:26]([N:32]2[CH2:33][CH2:34][NH:35][CH2:36][CH2:37]2)[cH:27][c:28]([Cl:31])[cH:29][cH:30]1.[CH3:38][CH2:39][OH:40].[Cl:41][CH2:42][Cl:43]>>[Br:1][c:2]1[cH:3][cH:4][c:5](-[c:8]2[n:9][n:10]([CH2:20][CH:21]([OH:22])[CH2:23][N:35]3[CH2:34][CH2:33][N:32]([c:26]4[c:25]([CH3:24])[cH:30][cH:29][c:28]([Cl:31])[cH:27]4)[CH2:37][CH2:36]3)[c:11]3[c:12]2[CH2:13][N:14]([C:17]([CH3:18])=[O:19])[CH2:15][CH2:16]3)[cH:6][cH:7]1

SMILES/SMARTS of the MAPPET output:
[CH3:1][C:2](=[O:3])[N:4]1[CH2:5][CH2:6][c:7]2[c:8]([CH2:9]1)[c:10]([n:11][n:12]2[CH2:13][CH:14]1[CH2:15][O:16]1)-[c:17]1[cH:18][cH:19][c:20]([Br:21])[cH:22][cH:23]1.[CH3:24][c:25]1[cH:26][cH:27][c:28]([Cl:29])[cH:30][c:31]1[N:32]1[CH2:33][CH2:34][NH:35][CH2:36][CH2:37]1>>[CH3:1][C:2](=[O:3])[N:4]1[CH2:5][CH2:6][c:7]2[c:8]([CH2:9]1)[c:10]([n:11][n:12]2[CH2:13][CH:14]([OH:16])[CH2:15][N:35]1[CH2:34][CH2:33][N:32]([CH2:37][CH2:36]1)[c:31]1[cH:30][c:28]([Cl:29])[cH:27][cH:26][c:25]1[CH3:24])-[c:17]1[cH:23][cH:22][c:20]([Br:21])[cH:19][cH:18]1

Correctness of the mapping
MAPPET YES
USPTO YES

Reaction no 57 (2 bonds changed)

SMILES/SMARTS of the USPTO output:
[CH:20](=[O:21])[OH:22].[NH2:1][CH:2]1[c:3]2[c:4]([cH:13][cH:14][c:15]([C:17](=[O:18])[OH:19])[cH:16]2)[O:5][CH2:6][c:7]2[c:8]1[cH:9][cH:10][cH:11][cH:12]2>>[NH:1]([CH:2]1[c:3]2[c:4]([cH:13][cH:14][c:15]([C:17](=[O:18])[OH:19])[cH:16]2)[O:5][CH2:6][c:7]2[c:8]1[cH:9][cH:10][cH:11][cH:12]2)[CH:20]=[O:21]

SMILES/SMARTS of the MAPPET output:
[OH:20][CH:21]=[O:22].[NH2:1][CH:2]1[c:3]2[cH:4][cH:5][cH:6][cH:7][c:8]2[CH2:9][O:10][c:11]2[cH:12][cH:13][c:14]([cH:15][c:16]12)[C:17]([OH:18])=[O:19]>>[OH:18][C:17](=[O:19])[c:14]1[cH:13][cH:12][c:11]2[O:10][CH2:9][c:8]3[cH:7][cH:6][cH:5][cH:4][c:3]3[CH:2]([NH:1][CH:21]=[O:22])[c:16]2[cH:15]1.[OH2:20]

Correctness of the mapping
MAPPET YES
USPTO YES

Reaction no 58 (2 bonds changed)

SMILES/SMARTS of the USPTO output:
[Br:1][c:2]1[cH:3][s:4][c:5]2[c:6]1[n:7][c:8]([Cl:12])[n:9][c:10]2[Cl:11].[CH2:1
3]([CH:14]=[CH2:15])[NH2:16].[O:18]=[CH:19][N:20]([CH3:21])[CH3:22].[OH2:17]>>[B
r:1][c:2]1[cH:3][s:4][c:5]2[c:6]1[n:7][c:8]([Cl:12])[n:9][c:10]2[NH:16][CH2:13][
CH:14]=[CH2:15]

SMILES/SMARTS of the MAPPET output:
[Cl:5][c:6]1[n:7][c:8]([Cl:9])[c:10]2[s:11][cH:12][c:13]([Br:14])[c:15]2[n:16]1.
[NH2:1][CH2:2][CH:3]=[CH2:4]>>[Cl:5][c:6]1[n:7][c:8]([NH:1][CH2:2][CH:3]=[CH2:4]
)[c:10]2[s:11][cH:12][c:13]([Br:14])[c:15]2[n:16]1.[ClH:9]

Correctness of the mapping
MAPPET YES
USPTO YES

Reaction no 59 (2 bonds changed)

SMILES/SMARTS of the USPTO output:
[CH3:43][CH:44]([CH3:45])[CH2:46][C:47](=[O:48])[CH3:49].[Cl:1][CH:2]([CH3:3])[c
:4]1[cH:5][cH:6][c:7]([F:10])[cH:8][cH:9]1.[I-:42].[K+:41].[Na+:35].[Na+:36].[O-
:37][C:38](=[O:39])[O-
:40].[c:11]1([CH2:17][CH2:18][N:19]2[CH2:20][CH2:21][CH:22]([NH:25][c:26]3[n:27]
[c:28]4[c:29]([nH:30]3)[cH:31][cH:32][cH:33][cH:34]4)[CH2:23][CH2:24]2)[cH:12][c
H:13][cH:14][cH:15][cH:16]1>>[CH:2]([CH3:3])([c:4]1[cH:5][cH:6][c:7]([F:10])[cH:
8][cH:9]1)[n:27]1[c:26]([NH:25][CH:22]2[CH2:21][CH2:20][N:19]([CH2:18][CH2:17][c
:11]3[cH:12][cH:13][cH:14][cH:15][cH:16]3)[CH2:24][CH2:23]2)[n:30][c:29]2[c:28]1
[cH:34][cH:33][cH:32][cH:31]2

SMILES/SMARTS of the MAPPET output:
[CH3:25][CH:26]([Cl:27])[c:28]1[cH:29][cH:30][c:31]([F:32])[cH:33][cH:34]1.[CH2:
1]([CH2:2][c:3]1[cH:4][cH:5][cH:6][cH:7][cH:8]1)[N:9]1[CH2:10][CH2:11][CH:12]([C
H2:13][CH2:14]1)[NH:15][c:16]1[n:17][c:18]2[cH:19][cH:20][cH:21][cH:22][c:23]2[n
H:24]1>>[CH3:25][CH:26]([c:28]1[cH:34][cH:33][c:31]([F:32])[cH:30][cH:29]1)[n:24
]1[c:16]([NH:15][CH:12]2[CH2:11][CH2:10][N:9]([CH2:1][CH2:2][c:3]3[cH:4][cH:5][c
H:6][cH:7][cH:8]3)[CH2:14][CH2:13]2)[n:17][c:18]2[cH:19][cH:20][cH:21][cH:22][c:
23]12.[ClH:27]

Correctness of the mapping
MAPPET YES
USPTO YES

Reaction no 60 (2 bonds changed)

SMILES/SMARTS of the USPTO output:
[C:23](=[O:24])([O-:25])[O-
:26].[CH3:29][S:30]([CH3:31])=[O:32].[F:1][c:2]1[cH:3][cH:4][c:5]([C:12]#[N:13])
[c:6]2[c:7]1[cH:8][c:9]([CH3:11])[o:10]2.[K+:27].[K+:28].[OH2:33].[OH:14][CH2:15
][CH2:16][CH:17]1[CH2:18][CH2:19][NH:20][CH2:21][CH2:22]1>>[c:2]1([N:20]2[CH2:19
][CH2:18][CH:17]([CH2:16][CH2:15][OH:14])[CH2:22][CH2:21]2)[cH:3][cH:4][c:5]([C:
12]#[N:13])[c:6]2[c:7]1[cH:8][c:9]([CH3:11])[o:10]2

SMILES/SMARTS of the MAPPET output:

[OH:1][CH2:2][CH2:3][CH:4]1[CH2:5][CH2:6][NH:7][CH2:8][CH2:9]1.[CH3:10][c:11]1[cH:12][c:13]2[c:14]([F:15])[cH:16][cH:17][c:18]([C:19]#[N:20])[c:21]2[o:22]1>>[CH3:10][c:11]1[cH:12][c:13]2[c:14]([cH:16][cH:17][c:18]([C:19]#[N:20])[c:21]2[o:22]1)[N:7]1[CH2:6][CH2:5][CH:4]([CH2:3][CH2:2][OH:1])[CH2:9][CH2:8]1.[F:15]

Correctness of the mapping
MAPPET YES
USPTO YES

Reaction no 61 (2 bonds changed)

SMILES/SMARTS of the USPTO output:
[Br:20][c:21]1[cH:22][cH:23][c:24]([C:26]#[N:27])[s:25]1.[CH2:29]1[O:30][CH2:31][CH2:32][CH2:33]1.[CH3:10][Si:11]([N-:12][Si:13]([CH3:14])([CH3:15])[CH3:16])([CH3:17])[CH3:18].[NH2:1][c:2]1[cH:3][c:4]([Cl:5])[cH:6][cH:7][c:8]1[Cl:9].[Na+:19].[OH2:28]>>[NH:1]([c:2]1[cH:3][c:4]([Cl:5])[cH:6][cH:7][c:8]1[Cl:9])[C:26]([c:24]1[cH:23][cH:22][c:21]([Br:20])[s:25]1)=[NH:27]

SMILES/SMARTS of the MAPPET output:
[Br:1][c:2]1[cH:3][cH:4][c:5]([s:6]1)[C:7]#[N:8].[NH2:9][c:10]1[cH:11][c:12]([Cl:13])[cH:14][cH:15][c:16]1[Cl:17]>>[Cl:13][c:12]1[cH:14][cH:15][c:16]([Cl:17])[c:10]([NH:9][C:7](=[NH:8])[c:5]2[cH:4][cH:3][c:2]([Br:1])[s:6]2)[cH:11]1

Correctness of the mapping
MAPPET YES
USPTO YES

Reaction no 62 (2 bonds changed)

SMILES/SMARTS of the USPTO output:
[CH3:5][CH2:6][N:7]([CH2:8][CH3:9])[CH2:10][CH3:11].[CH:13]1([CH2:16][n:17]2[c:18]([CH2:35][C:36]([CH3:37])([CH3:38])[CH3:39])[n:19][c:20]3[c:21]2[cH:22][cH:23][c:24]([S:26](=[O:27])(=[O:28])[C:29]([C:30](=[O:31])[Cl:32])([CH3:33])[CH3:34])[cH:25]3)[CH2:14][CH2:15]1.[Cl:40][CH2:41][Cl:42].[ClH:12].[NH2:1][CH2:2][CH2:3][OH:4]>>[NH:1]([CH2:2][CH2:3][OH:4])[C:30]([C:29]([S:26]([c:24]1[cH:23][cH:22][c:21]2[n:17]([CH2:16][CH:13]3[CH2:14][CH2:15]3)[c:18]([CH2:35][C:36]([CH3:37])([CH3:38])[CH3:39])[n:19][c:20]2[cH:25]1)(=[O:27])=[O:28])([CH3:33])[CH3:34])=[O:31]

SMILES/SMARTS of the MAPPET output:
[NH2:1][CH2:2][CH2:3][OH:4].[CH3:5][C:6]([CH3:7])([CH3:8])[CH2:9][c:10]1[n:11][c:12]2[cH:13][c:14]([cH:15][cH:16][c:17]2[n:18]1[CH2:19][CH:20]1[CH2:21][CH2:22]1)[S:23](=[O:24])(=[O:25])[C:26]([CH3:27])([CH3:28])[C:29]([Cl:30])=[O:31]>>[CH3:8][C:6]([CH3:7])([CH3:5])[CH2:9][c:10]1[n:11][c:12]2[cH:13][c:14]([cH:15][cH:16][c:17]2[n:18]1[CH2:19][CH:20]1[CH2:22][CH2:21]1)[S:23](=[O:24])(=[O:25])[C:26]([CH3:28])([CH3:27])[C:29](=[O:31])[NH:1][CH2:2][CH2:3][OH:4].[Cl:30]

Correctness of the mapping
MAPPET YES
USPTO YES

Reaction no 63 (2 bonds changed)

SMILES/SMARTS of the USPTO output:

[C:1](=[O:2])([OH:3])[CH2:4][CH:5]1[CH2:6][O:7][c:8]2[c:9]1[c:10]([C:16](=[O:17])[NH:18][c:19]1[c:20]([Cl:26])[cH:21][n:22][cH:23][c:24]1[Cl:25])[cH:11][cH:12][c:13]2[O:14][CH3:15].[CH3:27][O:28][c:29]1[cH:30][cH:31][c:32]([CH2:33][NH2:34])[cH:35][cH:36]1>>[C:1](=[O:2])([CH2:4][CH:5]1[CH2:6][O:7][c:8]2[c:9]1[c:10]([C:16](=[O:17])[NH:18][c:19]1[c:20]([Cl:26])[cH:21][n:22][cH:23][c:24]1[Cl:25])[cH:11][cH:12][c:13]2[O:14][CH3:15])[NH:34][CH2:33][c:32]1[cH:31][cH:30][c:29]([O:28][CH3:27])[cH:36][cH:35]1

SMILES/SMARTS of the MAPPET output:
[CH3:11][O:12][c:13]1[cH:14][cH:15][c:16]([C:17](=[O:18])[NH:19][c:20]2[c:21]([Cl:22])[cH:23][n:24][cH:25][c:26]2[Cl:27])[c:28]2[CH:29]([CH2:30][C:31]([OH:32])=[O:33])[CH2:34][O:35][c:36]12.[CH3:1][O:2][c:3]1[cH:4][cH:5][c:6]([CH2:7][NH2:8])[cH:9][cH:10]1>>[CH3:1][O:2][c:3]1[cH:10][cH:9][c:6]([CH2:7][NH:8][C:31](=[O:33])[CH2:30][CH:29]2[CH2:34][O:35][c:36]3[c:28]2[c:16]([cH:15][cH:14][c:13]3[O:12][CH3:11])[C:17](=[O:18])[NH:19][c:20]2[c:21]([Cl:22])[cH:23][n:24][cH:25][c:26]2[Cl:27])[cH:5][cH:4]1.[OH2:32]

Correctness of the mapping
MAPPET YES
USPTO YES

Reaction no 64 (2 bonds changed)

SMILES/SMARTS of the USPTO output:
[CH2:26]1[O:27][CH2:28][CH2:29][CH2:30]1.[CH3:15][c:16]1[c:17]([NH2:18])[cH:19][cH:20][cH:21][cH:22]1.[CH3:23][CH2:24][OH:25].[Cl:1][c:2]1[c:3]([N+:12](=[O:13])[O-:14])[cH:4][n:5][c:6]2[cH:7][cH:8][cH:9][cH:10][c:11]12>>[c:2]1([NH:18][c:17]2[c:16]([CH3:15])[cH:22][cH:21][cH:20][cH:19]2)[c:3]([N+:12](=[O:13])[O-:14])[cH:4][n:5][c:6]2[cH:7][cH:8][cH:9][cH:10][c:11]12

SMILES/SMARTS of the MAPPET output:
[O-:9][N+:10](=[O:11])[c:12]1[cH:13][n:14][c:15]2[cH:16][cH:17][cH:18][cH:19][c:20]2[c:21]1[Cl:22].[CH3:1][c:2]1[cH:3][cH:4][cH:5][cH:6][c:7]1[NH2:8]>>[CH3:1][c:2]1[cH:3][cH:4][cH:5][cH:6][c:7]1[NH:8][c:21]1[c:12]([cH:13][n:14][c:15]2[cH:16][cH:17][cH:18][cH:19][c:20]12)[N+:10]([O-:9])=[O:11].[ClH:22]

Correctness of the mapping
MAPPET YES
USPTO YES

Reaction no 65 (2 bonds changed)

SMILES/SMARTS of the USPTO output:
[CH2:28]([CH3:29])[N:30]([CH2:31][CH2:32][CH2:33][Cl:34])[CH2:35][CH3:36].[Cl:1][c:2]1[cH:3][cH:4][c:5]([CH2:6][c:7]2[o:8][c:9]3[c:10]([c:11]2[S:12](=[O:13])(=[O:14])[c:15]2[cH:16][cH:17][c:18]([OH:21])[cH:19][cH:20]2)[cH:22][cH:23][cH:24][cH:25]3)[cH:26][cH:27]1>>[Cl:1][c:2]1[cH:3][cH:4][c:5]([CH2:6][c:7]2[o:8][c:9]3[c:10]([c:11]2[S:12](=[O:13])(=[O:14])[c:15]2[cH:16][cH:17][c:18]([O:21][CH2:33][CH2:32][CH2:31][N:30]([CH2:28][CH3:29])[CH2:35][CH3:36])[cH:19][cH:20]2)[cH:22][cH:23][cH:24][cH:25]3)[cH:26][cH:27]1

SMILES/SMARTS of the MAPPET output:
[OH:1][c:2]1[cH:3][cH:4][c:5]([cH:6][cH:7]1)[S:8](=[O:9])(=[O:10])[c:11]1[c:12]([CH2:13][c:14]2[cH:15][cH:16][c:17]([Cl:18])[cH:19][cH:20]2)[o:21][c:22]2[cH:23][cH:24][cH:25][cH:26][c:27]12.[CH3:28][CH2:29][N:30]([CH2:31][CH3:32])[CH2:33][C

H2:34][CH2:35][Cl:36]>>[CH3:28][CH2:29][N:30]([CH2:31][CH3:32])[CH2:33][CH2:34][
CH2:35][O:1][c:2]1[cH:7][cH:6][c:5]([cH:4][cH:3]1)[S:8](=[O:9])(=[O:10])[c:11]1[
c:12]([CH2:13][c:14]2[cH:20][cH:19][c:17]([Cl:18])[cH:16][cH:15]2)[o:21][c:22]2[
cH:23][cH:24][cH:25][cH:26][c:27]12.[Cl:36]

Correctness of the mapping
MAPPET YES
USPTO YES

Reaction no 66 (2 bonds changed)

SMILES/SMARTS of the USPTO output:
[CH3:21][CH:22]([CH2:23][S:24](=[O:25])(=[O:26])[Cl:27])[CH3:28].[NH2:1][c:2]1[n
:3][cH:4][c:5]([CH2:9][c:10]2[cH:11][c:12]([OH:20])[c:13]3[cH:14][cH:15][n:16]([
CH3:19])[c:17]3[cH:18]2)[c:6]([NH2:8])[n:7]1>>[NH2:1][c:2]1[n:3][cH:4][c:5]([CH2
:9][c:10]2[cH:11][c:12]([O:20][S:24]([CH2:23][CH:22]([CH3:21])[CH3:28])(=[O:25])
=[O:26])[c:13]3[cH:14][cH:15][n:16]([CH3:19])[c:17]3[cH:18]2)[c:6]([NH2:8])[n:7]
1

SMILES/SMARTS of the MAPPET output:
[CH3:1][n:2]1[cH:3][cH:4][c:5]2[c:6]([OH:7])[cH:8][c:9]([CH2:10][c:11]3[cH:12][n
:13][c:14]([NH2:15])[n:16][c:17]3[NH2:18])[cH:19][c:20]12.[CH3:21][CH:22]([CH3:2
3])[CH2:24][S:25]([Cl:26])(=[O:27])=[O:28]>>[CH3:21][CH:22]([CH3:23])[CH2:24][S:
25](=[O:28])(=[O:27])[O:7][c:6]1[cH:8][c:9]([CH2:10][c:11]2[cH:12][n:13][c:14]([
NH2:15])[n:16][c:17]2[NH2:18])[cH:19][c:20]2[n:2]([CH3:1])[cH:3][cH:4][c:5]12.[C
l:26]

Correctness of the mapping
MAPPET YES
USPTO YES

Reaction no 67 (2 bonds changed)

SMILES/SMARTS of the USPTO output:
[CH3:25][O:26][c:27]1[c:28]([C:29](=[O:30])[Cl:31])[cH:32][cH:33][c:34]([Cl:36])
[cH:35]1.[CH3:37][CH2:38][N:39]([CH2:40][CH3:41])[CH2:42][CH3:43].[Cl:44][CH2:45
][Cl:46].[NH2:1][c:2]1[cH:3][cH:4][c:5]([C:6](=[O:7])[N:8]2[c:9]3[c:10]([cH:19][
cH:20][cH:21][cH:22]3)[CH2:11][c:12]3[c:13]([cH:15][cH:16][cH:17][cH:18]3)[CH2:1
4]2)[cH:23][cH:24]1>>[NH:1]([c:2]1[cH:3][cH:4][c:5]([C:6](=[O:7])[N:8]2[c:9]3[c:
10]([cH:19][cH:20][cH:21][cH:22]3)[CH2:11][c:12]3[c:13]([cH:15][cH:16][cH:17][cH
:18]3)[CH2:14]2)[cH:23][cH:24]1)[C:29]([c:28]1[c:27]([O:26][CH3:25])[cH:35][c:34
]([Cl:36])[cH:33][cH:32]1)=[O:30]

SMILES/SMARTS of the MAPPET output:
[NH2:1][c:2]1[cH:3][cH:4][c:5]([cH:6][cH:7]1)[C:8](=[O:9])[N:10]1[CH2:11][c:12]2
[cH:13][cH:14][cH:15][cH:16][c:17]2[CH2:18][c:19]2[cH:20][cH:21][cH:22][cH:23][c
:24]12.[CH3:25][O:26][c:27]1[cH:28][c:29]([Cl:30])[cH:31][cH:32][c:33]1[C:34]([C
l:35])=[O:36]>>[CH3:25][O:26][c:27]1[cH:28][c:29]([Cl:30])[cH:31][cH:32][c:33]1[
C:34](=[O:36])[NH:1][c:2]1[cH:7][cH:6][c:5]([cH:4][cH:3]1)[C:8](=[O:9])[N:10]1[C
H2:11][c:12]2[cH:13][cH:14][cH:15][cH:16][c:17]2[CH2:18][c:19]2[cH:20][cH:21][cH
:22][cH:23][c:24]12.[Cl:35]

Correctness of the mapping
MAPPET YES
USPTO YES

Reaction no 68 (2 bonds changed)

SMILES/SMARTS of the USPTO output:
[Br:1][CH2:2][CH2:3][CH2:4][O:5][CH2:6][O:7][CH3:8].[CH3:28][c:29]1[cH:30][cH:31][cH:32][cH:33][cH:34]1.[c:9]1([P:15]([c:16]2[cH:17][cH:18][cH:19][cH:20][cH:21]2)[c:22]2[cH:23][cH:24][cH:25][cH:26][cH:27]2)[cH:10][cH:11][cH:12][cH:13][cH:14]1>>[Br-:1].[CH2:2]([CH2:3][CH2:4][O:5][CH2:6][O:7][CH3:8])[P+:15]([c:9]1[cH:10][cH:11][cH:12][cH:13][cH:14]1)([c:16]1[cH:17][cH:18][cH:19][cH:20][cH:21]1)[c:22]1[cH:23][cH:24][cH:25][cH:26][cH:27]1

SMILES/SMARTS of the MAPPET output:
[CH3:1][O:2][CH2:3][O:4][CH2:5][CH2:6][CH2:7][Br:8].[cH:9]1[cH:10][cH:11][c:12]([cH:13][cH:14]1)[P:15]([c:16]1[cH:17][cH:18][cH:19][cH:20][cH:21]1)[c:22]1[cH:23][cH:24][cH:25][cH:26][cH:27]1>>[Br-:8].[CH3:1][O:2][CH2:3][O:4][CH2:5][CH2:6][CH2:7][P+:15]([c:12]1[cH:13][cH:14][cH:9][cH:10][cH:11]1)([c:16]1[cH:21][cH:20][cH:19][cH:18][cH:17]1)[c:22]1[cH:27][cH:26][cH:25][cH:24][cH:23]1

Correctness of the mapping
MAPPET YES
USPTO YES

Reaction no 69 (2 bonds changed)

SMILES/SMARTS of the USPTO output:
[C:13]([O:14][CH2:15][CH3:16])(=[O:17])[CH3:18].[CH2:19]1[CH2:20][CH2:21][CH2:22][CH2:23][CH2:24]1.[CH3:11][NH2:12].[CH3:1][S:2][c:3]1[n:4][cH:5][c:6]([Cl:10])[c:7]([Cl:9])[n:8]1.[CH3:25][CH2:26][OH:27]>>[CH3:1][S:2][c:3]1[n:4][cH:5][c:6]([Cl:10])[c:7]([NH:12][CH3:11])[n:8]1

SMILES/SMARTS of the MAPPET output:
[CH3:3][S:4][c:5]1[n:6][cH:7][c:8]([Cl:9])[c:10]([Cl:11])[n:12]1.[CH3:1][NH2:2]>>[CH3:1][NH:2][c:10]1[n:12][c:5]([S:4][CH3:3])[n:6][cH:7][c:8]1[Cl:9].[ClH:11]

Correctness of the mapping
MAPPET YES
USPTO YES

Reaction no 70 (2 bond changed)

SMILES/SMARTS of the USPTO output:
[CH3:18][c:19]1[cH:20][cH:21][c:22]([CH2:25][Cl:26])[n:23][cH:24]1.[ClH:17].[Na+:16].[OH-:15].[OH2:27].[nH:1]1[c:2]([SH:14])[n:3][c:4]2[c:5]1[cH:6][c:7]1[c:8]([cH:9]2)[O:10][CH2:11][O:12][CH2:13]1>>[ClH:17].[ClH:26].[nH:1]1[c:2]([S:14][CH2:25][c:22]2[cH:21][cH:20][c:19]([CH3:18])[cH:24][n:23]2)[n:3][c:4]2[c:5]1[cH:6][c:7]1[c:8]([cH:9]2)[O:10][CH2:11][O:12][CH2:13]1

SMILES/SMARTS of the MAPPET output:
[SH:1][c:2]1[n:3][c:4]2[cH:5][c:6]3[O:7][CH2:8][O:9][CH2:10][c:11]3[cH:12][c:13]2[nH:14]1.[CH3:15][c:16]1[cH:17][cH:18][c:19]([CH2:20][Cl:21])[n:22][cH:23]1>>[CH3:15][c:16]1[cH:17][cH:18][c:19]([CH2:20][S:1][c:2]2[n:3][c:4]3[cH:5][c:6]4[O:7][CH2:8][O:9][CH2:10][c:11]4[cH:12][c:13]3[nH:14]2)[n:22][cH:23]1.[Cl:21]

Correctness of the mapping

MAPPET YES
USPTO YES

Reaction no 71 (2 bonds changed)

SMILES/SMARTS of the USPTO output:
[CH2:5]([CH3:6])[O:7][C:8]([NH:9][NH2:10])=[O:11].[Cl:1][C:2]([Cl:3])=[O:4]>>[Cl:1][C:2](=[O:4])[NH:10][NH:9][C:8]([O:7][CH2:5][CH3:6])=[O:11]

SMILES/SMARTS of the MAPPET output:
[Cl:1][C:2]([Cl:3])=[O:4].[CH3:5][CH2:6][O:7][C:8](=[O:9])[NH:10][NH2:11]>>[CH3:5][CH2:6][O:7][C:8](=[O:9])[NH:10][NH:11][C:2]([Cl:1])=[O:4].[Cl:3]

Correctness of the mapping
MAPPET YES
USPTO YES

Reaction no 72 (2 bonds changed)

SMILES/SMARTS of the USPTO output:
[C:44](=[O:45])([O-:46])[O-:47].[CH3:39][N:40]([CH3:41])[CH:42]=[O:43].[F:15][c:16]1[c:17]([N+:36](=[O:37])[O-:38])[cH:18][c:19]([F:35])[c:20](-[n:22]2[c:23](=[O:34])[n:24]([CH3:33])[c:25]([C:29]([F:30])([F:31])[F:32])[cH:26][c:27]2=[O:28])[cH:21]1.[K+:48].[K+:49].[OH2:50].[OH:1][c:2]1[c:3]([O:8][CH:9]([CH3:10])[C:11](=[O:12])[O:13][CH3:14])[n:4][cH:5][cH:6][cH:7]1>>[O:1]([c:2]1[c:3]([O:8][CH:9]([CH3:10])[C:11](=[O:12])[O:13][CH3:14])[n:4][cH:5][cH:6][cH:7]1)[c:16]1[c:17]([N+:36](=[O:37])[O-:38])[cH:18][c:19]([F:35])[c:20](-[n:22]2[c:23](=[O:34])[n:24]([CH3:33])[c:25]([C:29]([F:30])([F:31])[F:32])[cH:26][c:27]2=[O:28])[cH:21]1

SMILES/SMARTS of the MAPPET output:
[CH3:1][O:2][C:3](=[O:4])[CH:5]([CH3:6])[O:7][c:8]1[n:9][cH:10][cH:11][cH:12][c:13]1[OH:14].[CH3:15][n:16]1[c:17]([cH:18][c:19](=[O:20])[n:21](-[c:22]2[cH:23][c:24]([F:25])[c:26]([cH:27][c:28]2[F:29])[N+:30]([O-:31])=[O:32])[c:33]1=[O:34])[C:35]([F:36])([F:37])[F:38]>>[CH3:1][O:2][C:3](=[O:4])[CH:5]([CH3:6])[O:7][c:8]1[n:9][cH:10][cH:11][cH:12][c:13]1[O:14][c:24]1[cH:23][c:22]([c:28]([F:29])[cH:27][c:26]1[N+:30]([O-:31])=[O:32])-[n:21]1[c:19](=[O:20])[cH:18][c:17]([n:16]([CH3:15])[c:33]1=[O:34])[C:35]([F:36])([F:37])[F:38].[F:25]

Correctness of the mapping
MAPPET YES
USPTO YES

Reaction no 73 (2 bonds changed)

SMILES/SMARTS of the USPTO output:
[Br:39][CH2:40][CH2:41][CH2:42][O:43][c:44]1[c:45]([C:46]#[N:47])[cH:48][cH:49][cH:50][cH:51]1.[OH:1][c:2]1[cH:3][cH:4][c:5]([CH:8]2[CH:9]([O:21][CH2:22][c:23]3[cH:24][cH:25][c:26]4[c:31]([cH:32]3)[N:30]([CH2:33][CH2:34][CH2:35][O:36][CH3:37])[C:29](=[O:38])[CH2:28][CH2:27]4)[CH2:10][N:11]([C:14](=[O:15])[O:16][C:17]([CH3:18])([CH3:19])[CH3:20])[CH2:12][CH2:13]2)[cH:6][cH:7]1>>[O:1]([c:2]1[cH:3][cH:4][c:5]([CH:8]2[CH:9]([O:21][CH2:22][c:23]3[cH:24][cH:25][c:26]4[c:31]([cH:32]3)[N:30]([CH2:33][CH2:34][CH2:35][O:36][CH3:37])[C:29](=[O:38])[CH2:28][CH2:27]4

)[CH2:10][N:11]([C:14](=[O:15])[O:16][C:17]([CH3:18])([CH3:19])[CH3:20])[CH2:12][CH2:13]2)[cH:6][cH:7]1)[CH2:40][CH2:41][CH2:42][O:43][c:44]1[c:45]([C:46]#[N:47])[cH:48][cH:49][cH:50][cH:51]1

SMILES/SMARTS of the MAPPET output:
[CH3:1][O:2][CH2:3][CH2:4][CH2:5][N:6]1[C:7](=[O:8])[CH2:9][CH2:10][c:11]2[cH:12][cH:13][c:14]([CH2:15][O:16][CH:17]3[CH2:18][N:19]([CH2:20][CH2:21][CH:22]3[c:23]3[cH:24][cH:25][c:26]([OH:27])[cH:28][cH:29]3)[C:30](=[O:31])[O:32][C:33]([CH3:34])([CH3:35])[CH3:36])[cH:37][c:38]12.[Br:39][CH2:40][CH2:41][CH2:42][O:43][c:44]1[cH:45][cH:46][cH:47][cH:48][c:49]1[C:50]#[N:51]>>[CH3:1][O:2][CH2:3][CH2:4][CH2:5][N:6]1[C:7](=[O:8])[CH2:9][CH2:10][c:11]2[cH:12][cH:13][c:14]([CH2:15][O:16][CH:17]3[CH2:18][N:19]([CH2:20][CH2:21][CH:22]3[c:23]3[cH:29][cH:28][c:26]([O:27][CH2:40][CH2:41][CH2:42][O:43][c:44]4[cH:45][cH:46][cH:47][cH:48][c:49]4[C:50]#[N:51])[cH:25][cH:24]3)[C:30](=[O:31])[O:32][C:33]([CH3:36])([CH3:35])[CH3:34])[cH:37][c:38]12.[Br:39]

Correctness of the mapping
MAPPET YES
USPTO YES

Reaction no 74 (2 bonds changed)

SMILES/SMARTS of the USPTO output:
[Cl:12][CH2:13][S:14][CH3:15].[Cl:1][c:2]1[c:3]([OH:9])[cH:4][cH:5][c:6]([Cl:8])[cH:7]1.[H-:11].[Na+:10].[O:16]=[CH:17][N:18]([CH3:19])[CH3:20]>>[Cl:1][c:2]1[c:3]([O:9][CH2:13][S:14][CH3:15])[cH:4][cH:5][c:6]([Cl:8])[cH:7]1

SMILES/SMARTS of the MAPPET output:
[OH:1][c:2]1[cH:3][cH:4][c:5]([Cl:6])[cH:7][c:8]1[Cl:9].[CH3:10][S:11][CH2:12][Cl:13]>>[CH3:10][S:11][CH2:12][O:1][c:2]1[cH:3][cH:4][c:5]([Cl:6])[cH:7][c:8]1[Cl:9].[Cl:13]

Correctness of the mapping
MAPPET YES
USPTO YES

Reaction no 75 (2 bonds changed)

SMILES/SMARTS of the USPTO output:
[CH2:12]([c:13]1[cH:14][cH:15][cH:16][cH:17][cH:18]1)[c:19]1[c:20](=[O:28])[nH:21][c:22]([S:26][CH3:27])[n:23][c:24]1[CH3:25].[NH2:1][CH2:2][CH2:3][S:4][CH2:5][c:6]1[c:7]([CH3:11])[n:8][cH:9][nH:10]1>>[NH:1]([CH2:2][CH2:3][S:4][CH2:5][c:6]1[c:7]([CH3:11])[n:8][cH:9][nH:10]1)[c:22]1[nH:21][c:20](=[O:28])[c:19]([CH2:12][c:13]2[cH:14][cH:15][cH:16][cH:17][cH:18]2)[c:24]([CH3:25])[n:23]1

SMILES/SMARTS of the MAPPET output:
[CH3:1][c:2]1[n:3][cH:4][nH:5][c:6]1[CH2:7][S:8][CH2:9][CH2:10][NH2:11].[CH3:12][S:13][c:14]1[n:15][c:16]([CH3:17])[c:18]([CH2:19][c:20]2[cH:21][cH:22][cH:23][cH:24][cH:25]2)[c:26](=[O:27])[nH:28]1>>[CH3:1][c:2]1[n:3][cH:4][nH:5][c:6]1[CH2:7][S:8][CH2:9][CH2:10][NH:11][c:14]1[n:15][c:16]([CH3:17])[c:18]([CH2:19][c:20]2[cH:25][cH:24][cH:23][cH:22][cH:21]2)[c:26](=[O:27])[nH:28]1.[C:12].[S:13]

Correctness of the mapping
MAPPET YES
USPTO YES

Reaction no 76 (2 bonds changed)

SMILES/SMARTS of the USPTO output:
[C:19](=[O:20])([OH:21])[O-
:22].[CH3:15][C:16]([CH3:17])=[CH2:18].[CH:1]1([C:7](=[O:8])[OH:9])[CH:2]=[CH:3][CH2:4][CH:5]=[CH:6]1.[Cl:24][CH2:25][Cl:26].[Na+:23].[S:10](=[O:11])(=[O:12])([OH:13])[OH:14]>>[CH:1]1([C:7](=[O:8])[O:9][C:16]([CH3:15])([CH3:17])[CH3:18])[CH:2]=[CH:3][CH2:4][CH:5]=[CH:6]1

SMILES/SMARTS of the MAPPET output:
[CH3:1][C:2]([CH3:3])=[CH2:4].[OH:5][C:6](=[O:7])[CH:8]1[CH:9]=[CH:10][CH2:11][CH:12]=[CH:13]1>>[CH3:3][C:2]([CH3:4])([CH3:1])[O:5][C:6](=[O:7])[CH:8]1[CH:13]=[CH:12][CH2:11][CH:10]=[CH:9]1

Correctness of the mapping
MAPPET YES
USPTO YES

Reaction no 77 (2 bonds changed)

SMILES/SMARTS of the USPTO output:
[Cl:22][c:23]1[n:24][cH:25][c:26]([F:29])[cH:27][n:28]1.[F:1][c:2]1[cH:3][c:4]2[cH:5][cH:6][n:7]([CH2:11][CH:12]3[CH2:13][CH2:14][CH:15]4[N:16]([CH2:17][CH2:18][NH:19][CH2:20]4)[CH2:21]3)[c:8]2[cH:9][cH:10]1.[Na+:30].[Na+:31].[O-
:32][C:33](=[O:34])[O-
:35].[OH2:36]>>[F:1][c:2]1[cH:3][c:4]2[cH:5][cH:6][n:7]([CH2:11][CH:12]3[CH2:13][CH2:14][CH:15]4[N:16]([CH2:17][CH2:18][N:19]([c:23]5[n:24][cH:25][c:26]([F:29])[cH:27][n:28]5)[CH2:20]4)[CH2:21]3)[c:8]2[cH:9][cH:10]1

SMILES/SMARTS of the MAPPET output:
[F:22][c:23]1[cH:24][n:25][c:26]([Cl:27])[n:28][cH:29]1.[F:1][c:2]1[cH:3][cH:4][c:5]2[n:6]([CH2:7][CH:8]3[CH2:9][CH2:10][CH:11]4[CH2:12][NH:13][CH2:14][CH2:15][N:16]4[CH2:17]3)[cH:18][cH:19][c:20]2[cH:21]1>>[F:22][c:23]1[cH:29][n:28][c:26]([n:25][cH:24]1)[N:13]1[CH2:14][CH2:15][N:16]2[CH2:17][CH:8]([CH2:7][n:6]3[cH:18][cH:19][c:20]4[cH:21][c:2]([F:1])[cH:3][cH:4][c:5]34)[CH2:9][CH2:10][CH:11]2[CH2:12]1.[ClH:27]

Correctness of the mapping
MAPPET YES
USPTO YES

Reaction no 78 (2 bonds changed)

SMILES/SMARTS of the USPTO output:
[CH3:15][O:16][C:17](=[O:18])[O:19][CH3:20].[ClH:14].[H-
:1].[Na+:2].[O:3]=[C:4]1[CH2:5][c:6]2[cH:7][cH:8][cH:9][cH:10][c:11]2[CH2:12][CH2:13]1>>[O:3]=[C:4]1[CH:5]([C:17]([O:16][CH3:15])=[O:18])[c:6]2[cH:7][cH:8][cH:9][cH:10][c:11]2[CH2:12][CH2:13]1

SMILES/SMARTS of the MAPPET output:
[O:7]=[C:8]1[CH2:9][CH2:10][c:11]2[cH:12][cH:13][cH:14][cH:15][c:16]2[CH2:17]1.[CH3:1][O:2][C:3](=[O:4])[O:5][CH3:6]>>[CH3:1][O:2][C:3](=[O:4])[CH:17]1[C:8](=[O:7])[CH2:9][CH2:10][c:11]2[cH:12][cH:13][cH:14][cH:15][c:16]12.[CH3:6][OH:5]

Correctness of the mapping

MAPPET YES
USPTO YES

Reaction no 79 (2 bonds changed)

SMILES/SMARTS of the USPTO output:
[CH3:16][N:17]1[CH2:18][CH2:19][NH:20][CH2:21][CH2:22]1.[CH3:23][CH2:24][N:25]([CH2:26][CH3:27])[CH2:28][CH3:29].[CH3:30][C:31](=[O:32])[CH3:33].[Cl:1][S:2](=[O:3])(=[O:4])[c:5]1[cH:6][cH:7][c:8]([O:14][CH3:15])[c:9]([C:10](=[O:11])[OH:12])[cH:13]1>>[S:2](=[O:3])(=[O:4])([c:5]1[cH:6][cH:7][c:8]([O:14][CH3:15])[c:9]([C:10](=[O:11])[OH:12])[cH:13]1)[N:20]1[CH2:19][CH2:18][N:17]([CH3:16])[CH2:22][CH2:21]1

SMILES/SMARTS of the MAPPET output:
[CH3:1][O:2][c:3]1[cH:4][cH:5][c:6]([cH:7][c:8]1[C:9]([OH:10])=[O:11])[S:12]([Cl:13])(=[O:14])=[O:15].[CH3:16][N:17]1[CH2:18][CH2:19][NH:20][CH2:21][CH2:22]1>>[CH3:1][O:2][c:3]1[cH:4][cH:5][c:6]([cH:7][c:8]1[C:9]([OH:10])=[O:11])[S:12](=[O:15])(=[O:14])[N:20]1[CH2:19][CH2:18][N:17]([CH3:16])[CH2:22][CH2:21]1.[Cl:13]

Correctness of the mapping
MAPPET YES
USPTO YES

Reaction no 80 (2 bonds changed)

SMILES/SMARTS of the USPTO output:
[Br:1][C:2]1=[CH:3][C:4](=[O:10])[CH:5]2[CH2:6][CH2:7][CH:8]1[CH2:9]2.[CH3:11][O:12][CH2:13][CH2:14][O:15][CH2:16][c:17]1[c:18]([C:19](=[O:20])[OH:21])[cH:22][cH:23][c:24]([C:26]([F:27])([F:28])[F:29])[n:25]1.[CH:30]([N:31]([CH2:32][CH3:33])[CH:34]([CH3:35])[CH3:36])([CH3:37])[CH3:38].[Cl:39][c:40]1[cH:41][cH:42][cH:43][cH:44][cH:45]1>>[C:2]1([O:21][C:19]([c:18]2[c:17]([CH2:16][O:15][CH2:14][CH2:13][O:12][CH3:11])[n:25][c:24]([C:26]([F:27])([F:28])[F:29])[cH:23][cH:22]2)=[O:20])=[CH:3][C:4](=[O:10])[CH:5]2[CH2:6][CH2:7][CH:8]1[CH2:9]2

SMILES/SMARTS of the MAPPET output:
[CH3:1][O:2][CH2:3][CH2:4][O:5][CH2:6][c:7]1[n:8][c:9]([cH:10][cH:11][c:12]1[C:13]([OH:14])=[O:15])[C:16]([F:17])([F:18])[F:19].[Br:20][C:21]1=[CH:22][C:23](=[O:24])[CH:25]2[CH2:26][CH2:27][CH:28]1[CH2:29]2>>[CH3:1][O:2][CH2:3][CH2:4][O:5][CH2:6][c:7]1[n:8][c:9]([cH:10][cH:11][c:12]1[C:13](=[O:15])[O:14][C:21]1=[CH:22][C:23](=[O:24])[CH:25]2[CH2:26][CH2:27][CH:28]1[CH2:29]2)[C:16]([F:17])([F:18])[F:19].[Br:20]

Correctness of the mapping
MAPPET YES
USPTO YES

Reaction no 81 (2 bonds changed)

SMILES/SMARTS of the USPTO output:
[Cl:15][CH2:16][CH2:17][CH2:18][CH:19]([c:20]1[cH:21][cH:22][c:23]([F:26])[cH:24][cH:25]1)[c:27]1[cH:28][cH:29][c:30]([F:33])[cH:31][cH:32]1.[NH:1]1[CH2:2][CH2:3][C:4]2([O:5][CH2:6][c:7]3[cH:8][cH:9][cH:10][cH:11][c:12]32)[CH2:13][CH2:14]1>>[ClH:15].[N:1]1([CH2:16][CH2:17][CH2:18][CH:19]([c:20]2[cH:21][cH:22][c:23]([F:26])[cH:24][cH:25]2)[c:27]2[cH:28][cH:29][c:30]([F:33])[cH:31][cH:32]2)[CH2:2][C

H2:3][C:4]2([O:5][CH2:6][c:7]3[cH:8][cH:9][cH:10][cH:11][c:12]32)[CH2:13][CH2:14]1

SMILES/SMARTS of the MAPPET output:
[F:1][c:2]1[cH:3][cH:4][c:5]([cH:6][cH:7]1)[CH:8]([CH2:9][CH2:10][CH2:11][Cl:12])[c:13]1[cH:14][cH:15][c:16]([F:17])[cH:18][cH:19]1.[CH2:20]1[O:21][C:22]2([CH2:23][CH2:24][NH:25][CH2:26][CH2:27]2)[c:28]2[cH:29][cH:30][cH:31][cH:32][c:33]12>>[ClH:12].[F:1][c:2]1[cH:3][cH:4][c:5]([cH:6][cH:7]1)[CH:8]([CH2:9][CH2:10][CH2:11][N:25]1[CH2:26][CH2:27][C:22]2([CH2:23][CH2:24]1)[O:21][CH2:20][c:33]1[cH:32][cH:31][cH:30][cH:29][c:28]21)[c:13]1[cH:14][cH:15][c:16]([F:17])[cH:18][cH:19]1

Correctness of the mapping
MAPPET YES
USPTO YES

Reaction no 82 (2 bonds changed)

SMILES/SMARTS of the USPTO output:
[C:19]1(=[O:29])[c:20]2[c:21]([cH:25][cH:26][cH:27][cH:28]2)[C:22](=[O:24])[NH:23]1.[Cl:1][CH2:2][CH:3]([CH2:4][N:5]1[CH2:6][CH2:7][CH:8]([CH2:11][c:12]2[cH:13][cH:14][cH:15][cH:16][cH:17]2)[CH2:9][CH2:10]1)[OH:18].[K:30]>>[CH2:2]([CH:3]([CH2:4][N:5]1[CH2:6][CH2:7][CH:8]([CH2:11][c:12]2[cH:13][cH:14][cH:15][cH:16][cH:17]2)[CH2:9][CH2:10]1)[OH:18])[N:23]1[C:19](=[O:29])[c:20]2[c:21]([cH:25][cH:26][cH:27][cH:28]2)[C:22]1=[O:24]

SMILES/SMARTS of the MAPPET output:
[OH:12][CH:13]([CH2:14][Cl:15])[CH2:16][N:17]1[CH2:18][CH2:19][CH:20]([CH2:21][c:22]2[cH:23][cH:24][cH:25][cH:26][cH:27]2)[CH2:28][CH2:29]1.[O:1]=[C:2]1[NH:3][C:4](=[O:5])[c:6]2[cH:7][cH:8][cH:9][cH:10][c:11]12>>[OH:12][CH:13]([CH2:14][N:31[C:4](=[O:5])[c:6]2[cH:7][cH:8][cH:9][cH:10][c:11]2[C:2]1=[O:1])[CH2:16][N:17]1[CH2:18][CH2:19][CH:20]([CH2:21][c:22]2[cH:23][cH:24][cH:25][cH:26][cH:27]2)[CH2:28][CH2:29]1.[ClH:15]

Correctness of the mapping
MAPPET YES
USPTO YES

Reaction no 83 (2 bonds changed)

SMILES/SMARTS of the USPTO output:
[C:9](#[N:10])[CH2:11][C:12](=[O:13])[O:14][CH2:15][CH3:16].[F:17][c:18]1[cH:19][c:20]2[c:21](=[O:42])[c:22]([C:37](=[O:38])[O:39][CH2:40][CH3:41])[cH:23][n:24](-
[c:30]3[cH:31][cH:32][c:33]([F:36])[cH:34][cH:35]3)[c:25]2[c:26]([F:29])[c:27]1[F:28].[H-
:1].[Na+:2].[O:3]1[CH2:4][CH2:5][O:6][CH2:7][CH2:8]1.[OH2:43]>>[C:9](#[N:10])[CH:11]([C:12](=[O:13])[O:14][CH2:15][CH3:16])[c:27]1[c:18]([F:17])[cH:19][c:20]2[c:21](=[O:42])[c:22]([C:37](=[O:38])[O:39][CH2:40][CH3:41])[cH:23][n:24](-
[c:30]3[cH:31][cH:32][c:33]([F:36])[cH:34][cH:35]3)[c:25]2[c:26]1[F:29]

SMILES/SMARTS of the MAPPET output:
[CH3:1][CH2:2][O:3][C:4](=[O:5])[c:6]1[cH:7][n:8](-
[c:9]2[cH:10][cH:11][c:12]([F:13])[cH:14][cH:15]2)[c:16]2[c:17]([F:18])[c:19]([F:20])[c:21]([F:22])[cH:23][c:24]2[c:25]1=[O:26].[CH3:27][CH2:28][O:29][C:30](=[O:31])[CH2:32][C:33]#[N:34]>>[CH3:27][CH2:28][O:29][C:30](=[O:31])[CH:32]([C:33]#[N:34])[c:19]1[c:21]([F:22])[cH:23][c:24]2[c:16]([c:17]1[F:18])[n:8]([cH:7][c:6]

([C:4](=[O:5])[O:3][CH2:2][CH3:1])[c:25]2=[O:26])-
[c:9]1[cH:15][cH:14][c:12]([F:13])[cH:11][cH:10]1.[F:20]

Correctness of the mapping
MAPPET YES
USPTO YES

Reaction no 84 (2 bonds changed)

SMILES/SMARTS of the USPTO output:
[Br:1][c:2]1[c:3]([CH2:4][NH:5][CH2:6][CH3:7])[cH:8][c:9]([C:12]([F:13])([F:14])[F:15])[cH:10][cH:11]1.[Cl:16][c:17]1[cH:18][cH:19][c:20]([CH2:21][N:22]=[C:23]=[O:24])[cH:25][cH:26]1>>[Br:1][c:2]1[c:3]([CH2:4][N:5]([CH2:6][CH3:7])[C:23]([NH:22][CH2:21][c:20]2[cH:19][cH:18][c:17]([Cl:16])[cH:26][cH:25]2)=[O:24])[cH:8][c:9]([C:12]([F:13])([F:14])[F:15])[cH:10][cH:11]1

SMILES/SMARTS of the MAPPET output:
[CH3:1][CH2:2][NH:3][CH2:4][c:5]1[cH:6][c:7]([cH:8][cH:9][c:10]1[Br:11])[C:12]([F:13])([F:14])[F:15].[Cl:16][c:17]1[cH:18][cH:19][c:20]([CH2:21][N:22]=[C:23]=[O:24])[cH:25][cH:26]1>>[CH3:1][CH2:2][N:3]([CH2:4][c:5]1[cH:6][c:7]([cH:8][cH:9][c:10]1[Br:11])[C:12]([F:13])([F:14])[F:15])[C:23](=[O:24])[NH:22][CH2:21][c:20]1[cH:19][cH:18][c:17]([Cl:16])[cH:26][cH:25]1

Correctness of the mapping
MAPPET YES
USPTO YES

Reaction no 85 (2 bonds changed)

SMILES/SMARTS of the USPTO output:
[C:1]([Li:2])([CH3:3])([CH3:4])[CH3:5].[Cl:6][c:7]1[n:8][cH:9][c:10]([Cl:13])[cH:11][cH:12]1.[OH2:22].[n:14]1[cH:15][cH:16][c:17]([CH:20]=[O:21])[cH:18][cH:19]1>>[Cl:6][c:7]1[n:8][c:9]([CH:20]([c:17]2[cH:16][cH:15][n:14][cH:19][cH:18]2)[OH:21])[c:10]([Cl:13])[cH:11][cH:12]1

SMILES/SMARTS of the MAPPET output:
[O:1]=[CH:2][c:3]1[cH:4][cH:5][n:6][cH:7][cH:8]1.[Cl:9][c:10]1[cH:11][cH:12][c:13]([Cl:14])[n:15][cH:16]1>>[OH:1][CH:2]([c:3]1[cH:8][cH:7][n:6][cH:5][cH:4]1)[c:16]1[n:15][c:13]([Cl:14])[cH:12][cH:11][c:10]1[Cl:9]

Correctness of the mapping
MAPPET YES
USPTO YES

Reaction no 86 (2 bonds changed)

SMILES/SMARTS of the USPTO output:
[F:32][C:33]([c:34]1[cH:35][c:36]([CH2:37][Cl:38])[cH:39][cH:40][cH:41]1)([F:42])[F:43].[c:1]1([CH:7]([CH2:8][CH2:9][N:10]2[CH2:11][CH2:12][N:13]([c:16]3[cH:17][cH:18][c:19]4[c:23]([cH:24]3)[C:22](=[O:25])[NH:21][CH2:20]4)[CH2:14][CH2:15]2)[c:26]2[cH:27][cH:28][cH:29][cH:30][cH:31]2)[cH:2][cH:3][cH:4][cH:5][cH:6]1>>[c:1]1([CH:7]([CH2:8][CH2:9][N:10]2[CH2:11][CH2:12][N:13]([c:16]3[cH:17][cH:18][c:19]4[c:23]([cH:24]3)[C:22](=[O:25])[N:21]([CH2:37][c:36]3[cH:35][c:34]([C:33]([F:32])([F:42])[F:43])[cH:41][cH:40][cH:39]3)[CH2:20]4)[CH2:14][CH2:15]2)[c:26]2[cH:27][cH:28][cH:29][cH:30][cH:31]2)[cH:2][cH:3][cH:4][cH:5][cH:6]1

SMILES/SMARTS of the MAPPET output:
[F:32][C:33]([F:34])([F:35])[c:36]1[cH:37][cH:38][cH:39][c:40]([CH2:41][Cl:42])[cH:43]1.[O:1]=[C:2]1[NH:3][CH2:4][c:5]2[cH:6][cH:7][c:8]([cH:9][c:10]12)[N:11]1[CH2:12][CH2:13][N:14]([CH2:15][CH2:16][CH:17]([c:18]2[cH:19][cH:20][cH:21][cH:22][cH:23]2)[c:24]2[cH:25][cH:26][cH:27][cH:28][cH:29]2)[CH2:30][CH2:31]1>>[F:32][C:33]([F:34])([F:35])[c:36]1[cH:37][cH:38][cH:39][c:40]([CH2:41][N:3]2[CH2:4][c:5]3[cH:6][cH:7][c:8]([cH:9][c:10]3[C:2]2=[O:1])[N:11]2[CH2:12][CH2:13][N:14]([CH2:15][CH2:16][CH:17]([c:18]3[cH:19][cH:20][cH:21][cH:22][cH:23]3)[c:24]3[cH:25][cH:26][cH:27][cH:28][cH:29]3)[CH2:30][CH2:31]2)[cH:43]1.[ClH:42]

Correctness of the mapping
MAPPET YES
USPTO YES

Reaction no 87 (2 bonds changed)

SMILES/SMARTS of the USPTO output:
[CH3:29][CH2:30][N:31]([CH2:32][CH3:33])[CH2:34][CH3:35].[Cl:36][C:37](=[O:38])[O:39][CH3:40].[Cl:41][CH2:42][Cl:43].[NH2:1][CH2:2][CH2:3][CH2:4][C:5]1([c:23]2[cH:24][cH:25][cH:26][cH:27][cH:28]2)[N:6]([C:18](=[O:19])[N:20]([CH3:21])[CH3:22])[CH2:7][C:8]([c:10]2[c:11]([F:17])[cH:12][cH:13][c:14]([Cl:16])[cH:15]2)=[CH:9]1>>[NH:1]([CH2:2][CH2:3][CH2:4][C:5]1([c:23]2[cH:24][cH:25][cH:26][cH:27][cH:28]2)[N:6]([C:18](=[O:19])[N:20]([CH3:21])[CH3:22])[CH2:7][C:8]([c:10]2[c:11]([F:17])[cH:12][cH:13][c:14]([Cl:16])[cH:15]2)=[CH:9]1)[C:37](=[O:38])[O:39][CH3:40]

SMILES/SMARTS of the MAPPET output:
[CH3:1][N:2]([CH3:3])[C:4](=[O:5])[N:6]1[CH2:7][C:8](=[CH:9][C:10]1([CH2:11][CH2:12][CH2:13][NH2:14])[c:15]1[cH:16][cH:17][cH:18][cH:19][cH:20]1)[c:21]1[cH:22][c:23]([Cl:24])[cH:25][cH:26][c:27]1[F:28].[CH3:29][O:30][C:31]([Cl:32])=[O:33]>>[CH3:29][O:30][C:31](=[O:33])[NH:14][CH2:13][CH2:12][CH2:11][C:10]1([CH:9]=[C:8]([CH2:7][N:6]1[C:4](=[O:5])[N:2]([CH3:3])[CH3:1])[c:21]1[cH:22][c:23]([Cl:24])[cH:25][cH:26][c:27]1[F:28])[c:15]1[cH:20][cH:19][cH:18][cH:17][cH:16]1.[Cl:32]

Correctness of the mapping
MAPPET YES
USPTO YES

Reaction no 88 (2 bonds changed)

SMILES/SMARTS of the USPTO output:
[C:46](=[O:47])([O-:48])[O-:49].[CH3:52][CH2:53][O:54][C:55](=[O:56])[CH3:57].[CH3:58][N:59]([CH3:60])[CH:61]=[O:62].[ClH:37].[K+:50].[K+:51].[OH2:63].[OH:1][c:2]1[c:3]([O:35][CH3:36])[cH:4][c:5]2[c:6](-[c:23]3[cH:24][c:25]([O:33][CH3:34])[c:26]([O:31][CH3:32])[c:27]([O:29][CH3:30])[cH:28]3)[c:7]([C:19](=[O:20])[O:21][CH3:22])[n:8]([N:13]3[CH2:14][CH2:15][O:16][CH2:17][CH2:18]3)[c:9](=[O:12])[c:10]2[cH:11]1.[c:38]1([CH2:44][Cl:45])[cH:39][cH:40][cH:41][cH:42][n:43]1>>[ClH:45].[O:1]([c:2]1[c:3]([O:35][CH3:36])[cH:4][c:5]2[c:6](-[c:23]3[cH:24][c:25]([O:33][CH3:34])[c:26]([O:31][CH3:32])[c:27]([O:29][CH3:30])[cH:28]3)[c:7]([C:19](=[O:20])[O:21][CH3:22])[n:8]([N:13]3[CH2:14][CH2:15][O:16][CH2:17][CH2:18]3)[c:9](=[O:12])[c:10]2[cH:11]1)[CH2:44][c:38]1[cH:39][cH:40][cH:41][cH:42][n:43]1

SMILES/SMARTS of the MAPPET output:

[CH3:1][O:2][C:3](=[O:4])[c:5]1[c:6](-
[c:7]2[cH:8][c:9]([O:10][CH3:11])[c:12]([O:13][CH3:14])[c:15]([O:16][CH3:17])[cH
:18]2)[c:19]2[cH:20][c:21]([O:22][CH3:23])[c:24]([OH:25])[cH:26][c:27]2[c:28](=[
O:29])[n:30]1[N:31]1[CH2:32][CH2:33][O:34][CH2:35][CH2:36]1.[Cl:37][CH2:38][c:39
]1[cH:40][cH:41][cH:42][cH:43][n:44]1>>[ClH:37].[CH3:1][O:2][C:3](=[O:4])[c:5]1[
c:6](-
[c:7]2[cH:18][c:15]([O:16][CH3:17])[c:12]([O:13][CH3:14])[c:9]([O:10][CH3:11])[c
H:8]2)[c:19]2[cH:20][c:21]([O:22][CH3:23])[c:24]([O:25][CH2:38][c:39]3[cH:40][cH
:41][cH:42][cH:43][n:44]3)[cH:26][c:27]2[c:28](=[O:29])[n:30]1[N:31]1[CH2:36][CH
2:35][O:34][CH2:33][CH2:32]1

Correctness of the mapping
MAPPET YES
USPTO YES

Reaction no 89 (2 bonds changed)

SMILES/SMARTS of the USPTO output:
[BH4-
:28].[CH3:14][O:15][C:16]([CH:17]=[CH:18][c:19]1[cH:20][cH:21][c:22]([CH:25]=[O:
26])[cH:23][cH:24]1)=[O:27].[CH3:1][c:2]1[c:3]([CH2:4][CH2:5][NH2:6])[c:7]2[cH:8
][cH:9][cH:10][cH:11][c:12]2[nH:13]1.[CH3:33][OH:34].[ClH:30].[H:31][H:32].[Na+:
29].[OH2:35]>>[CH3:1][c:2]1[c:3]([CH2:4][CH2:5][NH:6][CH2:25][c:22]2[cH:21][cH:2
0][c:19]([CH:18]=[CH:17][C:16]([O:15][CH3:14])=[O:27])[cH:24][cH:23]2)[c:7]2[cH:
8][cH:9][cH:10][cH:11][c:12]2[nH:13]1.[ClH:30]

SMILES/SMARTS of the MAPPET output:
[CH3:1][c:2]1[nH:3][c:4]2[cH:5][cH:6][cH:7][cH:8][c:9]2[c:10]1[CH2:11][CH2:12][N
H2:13].[CH3:14][O:15][C:16](=[O:17])[CH:18]=[CH:19][c:20]1[cH:21][cH:22][c:23]([
CH:24]=[O:25])[cH:26][cH:27]1>>[CH3:14][O:15][C:16](=[O:17])[CH:18]=[CH:19][c:20
]1[cH:27][cH:26][c:23]([CH2:24][NH:13][CH2:12][CH2:11][c:10]2[c:2]([CH3:1])[nH:3
][c:4]3[cH:5][cH:6][cH:7][cH:8][c:9]23)[cH:22][cH:21]1.[O:25]

Correctness of the mapping
MAPPET YES
USPTO YES

Reaction no 90 (2 bonds changed)

SMILES/SMARTS of the USPTO output:
[Br:1][c:2]1[cH:3][cH:4][c:5]([C:7](=[O:8])[NH:9][CH:10]([CH2:11][C:12](=[O:13])
[O-
:14])[CH2:15][N+:16]([CH3:17])([CH3:18])[CH3:19])[o:6]1.[C:20](#[CH:21])[c:22]1[
cH:23][c:24]([O:28][CH2:29][CH2:30][CH2:31][CH2:32][CH2:33][CH3:34])[cH:25][cH:2
6][cH:27]1>>[c:2]1([C:21]#[C:20][c:22]2[cH:23][c:24]([O:28][CH2:29][CH2:30][CH2:
31][CH2:32][CH2:33][CH3:34])[cH:25][cH:26][cH:27]2)[cH:3][cH:4][c:5]([C:7](=[O:8
])[NH:9][CH:10]([CH2:11][C:12](=[O:13])[O-
:14])[CH2:15][N+:16]([CH3:17])([CH3:18])[CH3:19])[o:6]1

SMILES/SMARTS of the MAPPET output:
[CH3:1][CH2:2][CH2:3][CH2:4][CH2:5][CH2:6][O:7][c:8]1[cH:9][cH:10][cH:11][c:12](
[cH:13]1)[C:14]#[CH:15].[CH3:16][N+:17]([CH3:18])([CH3:19])[CH2:20][CH:21]([CH2:
22][C:23]([O-
:24])=[O:25])[NH:26][C:27](=[O:28])[c:29]1[cH:30][cH:31][c:32]([Br:33])[o:34]1>>
[CH3:1][CH2:2][CH2:3][CH2:4][CH2:5][CH2:6][O:7][c:8]1[cH:9][cH:10][cH:11][c:12](
[cH:13]1)[C:14]#[C:15][c:32]1[cH:31][cH:30][c:29]([o:34]1)[C:27](=[O:28])[NH:26]

[CH:21]([CH2:22][C:23]([O-
:24])=[O:25])[CH2:20][N+:17]([CH3:19])([CH3:18])[CH3:16].[Br:33]

Correctness of the mapping
MAPPET YES
USPTO YES

Reaction no 91 (2 bonds changed)

SMILES/SMARTS of the USPTO output:
[CH2:39]([N+:40]([CH2:41][CH3:42])([CH2:43][CH3:44])[CH2:45][CH3:46])[c:47]1[cH:
48][cH:49][cH:50][cH:51][cH:52]1.[CH3:2][N:3]([CH2:4][CH2:5][Cl:6])[CH3:7].[CH3:
8][c:9]1[cH:10][cH:11][cH:12][cH:13][cH:14]1.[Cl-
:38].[Cl:15][c:16]1[cH:17][cH:18][c:19]2[c:20]([cH:35]1)-
[c:21]1[c:22]([CH2:33][OH:34])[s:23][cH:24][c:25]1-
[c:26]1[c:27]([cH:29][cH:30][cH:31][cH:32]1)[O:28]2.[ClH:1].[Na+:37].[OH-
:36].[OH2:53]>>[CH3:2][N:3]([CH2:4][CH2:5][O:34][CH2:33][c:22]1[c:21]2[c:25]([cH
:24][s:23]1)-
[c:26]1[c:27]([cH:29][cH:30][cH:31][cH:32]1)[O:28][c:19]1[cH:18][cH:17][c:16]([C
l:15])[cH:35][c:20]1-2)[CH3:7]

SMILES/SMARTS of the MAPPET output:
[OH:1][CH2:2][c:3]1[s:4][cH:5][c:6]-2[c:7]1-
[c:8]1[cH:9][c:10]([Cl:11])[cH:12][cH:13][c:14]1[O:15][c:16]1[cH:17][cH:18][cH:1
9][cH:20][c:21]-
21.[CH3:22][N:23]([CH3:24])[CH2:25][CH2:26][Cl:27]>>[CH3:22][N:23]([CH3:24])[CH2
:25][CH2:26][O:1][CH2:2][c:3]1[s:4][cH:5][c:6]-2[c:7]1-
[c:8]1[cH:9][c:10]([Cl:11])[cH:12][cH:13][c:14]1[O:15][c:16]1[cH:17][cH:18][cH:1
9][cH:20][c:21]-21.[Cl:27]

Correctness of the mapping
MAPPET YES
USPTO YES

Reaction no 92 (2 bonds changed)

SMILES/SMARTS of the USPTO output:
[C:9](=[O:10])([O-:11])[O-:12].[Cl:15][c:16]1[c:17]([OH:43])[cH:18][c:19](-
[c:22]2[c:23](-
[c:33]3[c:34]([O:41][CH3:42])[cH:35][cH:36][cH:37][c:38]3[O:39][CH3:40])[cH:24][
cH:25][c:26]([C:28](=[O:29])[O:30][CH2:31][CH3:32])[n:27]2)[cH:20][cH:21]1.[Cl:2
][CH2:3][CH2:4][CH2:5][N:6]([CH3:7])[CH3:8].[ClH:1].[Cs+:13].[Cs+:14].[O:57]=[CH
:58][N:59]([CH3:60])[CH3:61].[OH:44][C:45]([CH2:46][C:47]([C:48](=[O:49])[OH:50]
)([CH2:51][C:52](=[O:53])[OH:54])[OH:55])=[O:56]>>[CH2:3]([CH2:4][CH2:5][N:6]([C
H3:7])[CH3:8])[O:43][c:17]1[c:16]([Cl:15])[cH:21][cH:20][c:19](-[c:22]2[c:23](-
[c:33]3[c:34]([O:41][CH3:42])[cH:35][cH:36][cH:37][c:38]3[O:39][CH3:40])[cH:24][
cH:25][c:26]([C:28](=[O:29])[O:30][CH2:31][CH3:32])[n:27]2)[cH:18]1

SMILES/SMARTS of the MAPPET output:
[CH3:1][N:2]([CH3:3])[CH2:4][CH2:5][CH2:6][Cl:7].[CH3:8][CH2:9][O:10][C:11](=[O:
12])[c:13]1[cH:14][cH:15][c:16]([c:17]([n:18]1)-
[c:19]1[cH:20][cH:21][c:22]([Cl:23])[c:24]([OH:25])[cH:26]1)-
[c:27]1[c:28]([O:29][CH3:30])[cH:31][cH:32][cH:33][c:34]1[O:35][CH3:36]>>[CH3:8]
[CH2:9][O:10][C:11](=[O:12])[c:13]1[cH:14][cH:15][c:16]([c:17]([n:18]1)-
[c:19]1[cH:20][cH:21][c:22]([Cl:23])[c:24]([O:25][CH2:6][CH2:5][CH2:4][N:2]([CH3
:1])[CH3:3])[cH:26]1)-
[c:27]1[c:34]([O:35][CH3:36])[cH:33][cH:32][cH:31][c:28]1[O:29][CH3:30].[Cl:7]

Correctness of the mapping
MAPPET YES
USPTO YES

Reaction no 93 (2 bonds changed)

SMILES/SMARTS of the USPTO output:
[Cl:15][c:16]1[cH:17][c:18]([CH2:23][SH:24])[cH:19][cH:20][c:21]1[Cl:22].[Cl:1][
CH2:2][c:3]1[c:4]2[c:9]([cH:10][cH:11][c:12]1[OH:13])[C:8](=[O:14])[CH2:7][CH2:6
][CH2:5]2>>[CH2:2]([c:3]1[c:4]2[c:9]([cH:10][cH:11][c:12]1[OH:13])[C:8](=[O:14])
[CH2:7][CH2:6][CH2:5]2)[S:24][CH2:23][c:18]1[cH:17][c:16]([Cl:15])[c:21]([Cl:22]
)[cH:20][cH:19]1

SMILES/SMARTS of the MAPPET output:
[OH:1][c:2]1[cH:3][cH:4][c:5]2[C:6](=[O:7])[CH2:8][CH2:9][CH2:10][c:11]2[c:12]1[
CH2:13][Cl:14].[SH:15][CH2:16][c:17]1[cH:18][cH:19][c:20]([Cl:21])[c:22]([Cl:23]
)[cH:24]1>>[OH:1][c:2]1[cH:3][cH:4][c:5]2[C:6](=[O:7])[CH2:8][CH2:9][CH2:10][c:1
1]2[c:12]1[CH2:13][S:15][CH2:16][c:17]1[cH:18][cH:19][c:20]([Cl:21])[c:22]([Cl:2
3])[cH:24]1.[Cl:14]

Correctness of the mapping
MAPPET YES
USPTO YES

Reaction no 94 (2 bonds changed)

SMILES/SMARTS of the USPTO output:
[CH3:13][CH2:14][OH:15].[CH3:1][CH2:2][CH2:3][CH2:4][NH2:5].[CH3:6][O:7][CH:8]([
CH:9]=[O:10])[O:11][CH3:12].[Cl:17][CH2:18][Cl:19].[OH2:16]>>[CH3:1][CH2:2][CH2:
3][CH2:4][NH:5][CH2:9][CH:8]([O:7][CH3:6])[O:11][CH3:12]

SMILES/SMARTS of the MAPPET output:
[CH3:1][O:2][CH:3]([O:4][CH3:5])[CH:6]=[O:7].[CH3:8][CH2:9][CH2:10][CH2:11][NH2:
12]>>[CH3:8][CH2:9][CH2:10][CH2:11][NH:12][CH2:6][CH:3]([O:2][CH3:1])[O:4][CH3:5
].[O:7]

Correctness of the mapping
MAPPET YES
USPTO YES

Reaction no 95 (2 bonds changed)

SMILES/SMARTS of the USPTO output:
[CH3:16][OH:17].[F:1][c:2]1[c:3]([N+:11]([O-
:12])=[O:13])[cH:4][c:5]([N:8]([CH3:9])[CH3:10])[cH:6][cH:7]1.[H:14][H:15]>>[F:1
][c:2]1[c:3]([NH2:11])[cH:4][c:5]([N:8]([CH3:9])[CH3:10])[cH:6][cH:7]1

SMILES/SMARTS of the MAPPET output:
[CH3:1][N:2]([CH3:3])[c:4]1[cH:5][cH:6][c:7]([F:8])[c:9]([cH:10]1)[N+:11]([O-
:12])=[O:13]>>[CH3:3][N:2]([CH3:1])[c:4]1[cH:5][cH:6][c:7]([F:8])[c:9]([NH2:11])
[cH:10]1.[O:12].[O:13]

Correctness of the mapping
MAPPET YES

USPTO YES

Reaction no 96 (2 bonds changed)

SMILES/SMARTS of the USPTO output:
[CH2:1]([CH3:2])[O:3][c:4]1[c:5]([C:6](=[O:7])[Cl:8])[cH:9][cH:10][cH:11][cH:12]1.[NH2:13][c:14]1[c:15]([C:22](=[O:23])[NH2:24])[n:16][cH:17][cH:18][c:19]1[C:20]#[N:21]>>[CH2:1]([CH3:2])[O:3][c:4]1[c:5]([C:6](=[O:7])[NH:13][c:14]2[c:15]([C:22](=[O:23])[NH2:24])[n:16][cH:17][cH:18][c:19]2[C:20]#[N:21])[cH:9][cH:10][cH:11][cH:12]1

SMILES/SMARTS of the MAPPET output:
[NH2:1][C:2](=[O:3])[c:4]1[n:5][cH:6][cH:7][c:8]([C:9]#[N:10])[c:11]1[NH2:12].[CH3:13][CH2:14][O:15][c:16]1[cH:17][cH:18][cH:19][cH:20][c:21]1[C:22]([Cl:23])=[O:24]>>[CH3:13][CH2:14][O:15][c:16]1[cH:17][cH:18][cH:19][cH:20][c:21]1[C:22](=[O:24])[NH:12][c:11]1[c:8]([cH:7][cH:6][n:5][c:4]1[C:2]([NH2:1])=[O:3])[C:9]#[N:10].[Cl:23]

Correctness of the mapping
MAPPET YES
USPTO YES

Reaction no 97 (2 bonds changed)

SMILES/SMARTS of the USPTO output:
[C:3]([c:4]1[cH:5][cH:6][cH:7][cH:8][cH:9]1)(=[O:10])[c:11]1[c:12]([C:24]([F:25])([F:26])[F:27])[o:13][c:14]2[c:15]1[c:16]([C:21](=[O:22])[OH:23])[c:17]([OH:20])[cH:18][cH:19]2.[CH3:28][O:29][c:30]1[n:31][c:32]([S:38]([CH3:39])(=[O:40])=[O:41])[n:33][c:34]([O:36][CH3:37])[cH:35]1.[CH3:43][N:44]([CH3:45])[CH:46]=[O:47].[H-:1].[Na+:2].[OH2:42]>>[C:3]([c:4]1[cH:5][cH:6][cH:7][cH:8][cH:9]1)(=[O:10])[c:11]1[c:12]([C:24]([F:25])([F:26])[F:27])[o:13][c:14]2[c:15]1[c:16]([C:21](=[O:22])[O-:23])[c:17]([O:20][c:32]1[n:31][c:30]([O:29][CH3:28])[cH:35][c:34]([O:36][CH3:37])[n:33]1)[cH:18][cH:19]2.[Na+:2]

SMILES/SMARTS of the MAPPET output:
[OH:1][C:2](=[O:3])[c:4]1[c:5]([OH:6])[cH:7][cH:8][c:9]2[o:10][c:11]([c:12]([C:13](=[O:14])[c:15]3[cH:16][cH:17][cH:18][cH:19][cH:20]3)[c:21]12)[C:22]([F:23])([F:24])[F:25].[CH3:26][O:27][c:28]1[cH:29][c:30]([O:31][CH3:32])[n:33][c:34]([n:35]1)[S:36]([CH3:37])(=[O:38])=[O:39]>>[CH3:26][O:27][c:28]1[cH:29][c:30]([O:31][CH3:32])[n:33][c:34]([O:6][c:5]2[cH:7][cH:8][c:9]3[o:10][c:11]([c:12]([C:13](=[O:14])[c:15]4[cH:20][cH:19][cH:18][cH:17][cH:16]4)[c:21]3[c:4]2[C:2]([O-:1])=[O:3])[C:22]([F:23])([F:24])[F:25])[n:35]1.[C:37].[O:38].[O:39].[S:36]

Correctness of the mapping
MAPPET YES
USPTO YES

Reaction no 98 (2 bonds changed)

SMILES/SMARTS of the USPTO output:
[CH:13]([N:14]([CH2:15][CH3:16])[CH:17]([CH3:18])[CH3:19])([CH3:20])[CH3:21].[Cl:38][CH2:39][Cl:40].[F:22][C:23]([F:24])([F:25])[S:26]([O:27][Si:28]([CH3:29])([CH3:30])[C:31]([CH3:32])([CH3:33])[CH3:34])(=[O:35])=[O:36].[NH2:1][c:2]1[s:3][c

H:4][c:5]([CH2:11][OH:12])[c:6]1[C:7](=[O:8])[O:9][CH3:10].[OH2:37]>>[NH2:1][c:2]1[s:3][cH:4][c:5]([CH2:11][O:12][Si:28]([CH3:29])([CH3:30])[C:31]([CH3:32])([CH3:33])[CH3:34])[c:6]1[C:7](=[O:8])[O:9][CH3:10]

SMILES/SMARTS of the MAPPET output:
[CH3:1][O:2][C:3](=[O:4])[c:5]1[c:6]([NH2:7])[s:8][cH:9][c:10]1[CH2:11][OH:12].[CH3:13][C:14]([CH3:15])([CH3:16])[Si:17]([CH3:18])([CH3:19])[O:20][S:21](=[O:22])(=[O:23])[C:24]([F:25])([F:26])[F:27]>>[CH3:1][O:2][C:3](=[O:4])[c:5]1[c:6]([NH2:7])[s:8][cH:9][c:10]1[CH2:11][O:12][Si:17]([CH3:18])([CH3:19])[C:14]([CH3:13])([CH3:16])[CH3:15].[C:24].[F:27].[F:25].[F:26].[O:22].[O:20].[O:23].[S:21]

Correctness of the mapping
MAPPET YES
USPTO YES

Reaction no 99 (2 bonds changed)

SMILES/SMARTS of the USPTO output:
[CH2:38]([CH3:39])[O:40][C:41]([CH2:42][NH2:43])=[O:44].[CH3:1][O:2][c:3]1[cH:4][c:5]([CH2:6][CH:7]2[N:8]([CH:22]([C:23](=[O:24])[OH:25])[c:26]3[cH:27][cH:28][cH:29][cH:30][cH:31]3)[CH2:9][CH2:10][CH2:11][c:12]3[c:13]2[cH:14][c:15]([O:20][CH3:21])[c:16]([O:18][CH3:19])[cH:17]3)[cH:32][cH:33][c:34]1[O:35][CH3:36].[ClH:37]>>[CH3:1][O:2][c:3]1[cH:4][c:5]([CH2:6][CH:7]2[N:8]([CH:22]([C:23](=[O:24])[NH:43][CH2:42][C:41]([O:40][CH2:38][CH3:39])=[O:44])[c:26]3[cH:27][cH:28][cH:29][cH:30][cH:31]3)[CH2:9][CH2:10][CH2:11][c:12]3[c:13]2[cH:14][c:15]([O:20][CH3:21])[c:16]([O:18][CH3:19])[cH:17]3)[cH:32][cH:33][c:34]1[O:35][CH3:36]

SMILES/SMARTS of the MAPPET output:
[CH3:8][O:9][c:10]1[cH:11][cH:12][c:13]([CH2:14][CH:15]2[N:16]([CH2:17][CH2:18][CH2:19][c:20]3[cH:21][c:22]([O:23][CH3:24])[c:25]([O:26][CH3:27])[cH:28][c:29]23)[CH:30]([C:31]([OH:32])=[O:33])[c:34]2[cH:35][cH:36][cH:37][cH:38][cH:39]2)[cH:40][c:41]1[O:42][CH3:43].[CH3:1][CH2:2][O:3][C:4](=[O:5])[CH2:6][NH2:7]>>[CH3:1][CH2:2][O:3][C:4](=[O:5])[CH2:6][NH:7][C:31](=[O:33])[CH:30]([N:16]1[CH2:17][CH2:18][CH2:19][c:20]2[cH:21][c:22]([O:23][CH3:24])[c:25]([O:26][CH3:27])[cH:28][c:29]2[CH:15]1[CH2:14][c:13]1[cH:12][cH:11][c:10]([O:9][CH3:8])[c:41]([O:42][CH3:43])[cH:40]1)[c:34]1[cH:35][cH:36][cH:37][cH:38][cH:39]1.[OH2:32]

Correctness of the mapping
MAPPET YES
USPTO YES

Reaction no 100 (2 bonds changed)

SMILES/SMARTS of the USPTO output:
[C:26](=[O:27])([O-:28])[O-:29].[CH2:1]([c:2]1[cH:3][cH:4][cH:5][cH:6][cH:7]1)[N:8]1[CH2:9][CH2:10][O:11][c:12]2[c:13]([n:15][cH:16][c:17]([Cl:19])[n:18]2)[CH2:14]1.[CH3:20][c:21]1[nH:22][cH:23][cH:24][n:25]1.[Cs+:30].[Cs+:31].[Cu:37]([I:38])[I:39].[O:32]=[CH:33][N:34]([CH3:35])[CH3:36]>>[CH2:1]([c:2]1[cH:3][cH:4][cH:5][cH:6][cH:7]1)[N:8]1[CH2:9][CH2:10][O:11][c:12]2[c:13]([n:15][cH:16][c:17](-[n:22]3[c:21]([CH3:20])[n:25][cH:24][cH:23]3)[n:18]2)[CH2:14]1

SMILES/SMARTS of the MAPPET output:
[Cl:7][c:8]1[cH:9][n:10][c:11]2[CH2:12][N:13]([CH2:14][c:15]3[cH:16][cH:17][cH:18][cH:19][cH:20]3)[CH2:21][CH2:22][O:23][c:24]2[n:25]1.[CH3:1][c:2]1[n:3][cH:4][cH:5][nH:6]1>>[CH3:1][c:2]1[n:3][cH:4][cH:5][n:6]1-

[c:8]1[cH:9][n:10][c:11]2[CH2:12][N:13]([CH2:14][c:15]3[cH:16][cH:17][cH:18][cH:19][cH:20]3)[CH2:21][CH2:22][O:23][c:24]2[n:25]1.[ClH:7]

Correctness of the mapping
MAPPET YES
USPTO YES

Reaction no 101 (3 bonds changed)

SMILES/SMARTS of the USPTO output:
[Cl:1][c:2]1[cH:3][cH:4][c:5](-[c:8]2[c:9](-[c:16]3[c:17]([F:23])[c:18]([F:22])[cH:19][cH:20][cH:21]3)[c:10](=[O:15])[nH:11][n:12][c:13]2[CH3:14])[cH:6][cH:7]1.[P:24]([Cl:25])([Cl:26])([Cl:27])=[O:28]>>[Cl:1][c:2]1[cH:3][cH:4][c:5](-[c:8]2[c:9](-[c:16]3[c:17]([F:23])[c:18]([F:22])[cH:19][cH:20][cH:21]3)[c:10]([Cl:26])[n:11][n:12][c:13]2[CH3:14])[cH:6][cH:7]1

SMILES/SMARTS of the MAPPET output:
[Cl:1][P:2]([Cl:3])([Cl:4])=[O:5].[CH3:6][c:7]1[n:8][nH:9][c:10](=[O:11])[c:12](-[c:13]2[cH:14][cH:15][cH:16][c:17]([F:18])[c:19]2[F:20])[c:21]1-[c:22]1[cH:23][cH:24][c:25]([Cl:26])[cH:27][cH:28]1>>[CH3:6][c:7]1[n:8][n:9][c:10]([Cl:4])[c:12](-[c:13]2[cH:14][cH:15][cH:16][c:17]([F:18])[c:19]2[F:20])[c:21]1-[c:22]1[cH:28][cH:27][c:25]([Cl:26])[cH:24][cH:23]1.[Cl:1].[Cl:3].[O:11].[O:5].[P:2]

Correctness of the mapping
MAPPET YES
USPTO YES

Reaction no 102 (3 bonds changed)

SMILES/SMARTS of the USPTO output:
[C:1]([CH3:2])([CH3:3])([CH3:4])[O:5][C:6](=[O:7])[NH:8][c:9]1[c:10]([CH:16]=[CH:17][C:18](=[O:19])[OH:20])[cH:11][c:12]([Cl:15])[cH:13][cH:14]1.[Cl:36][CH2:37][Cl:38].[F:21][c:22]1[cH:23][cH:24][c:25]([CH2:26][N:27]2[CH2:28][CH:29]([CH3:33])[NH:30][CH2:31][CH2:32]2)[cH:34][cH:35]1>>[C:1]([CH3:2])([CH3:3])([CH3:4])[O:5][C:6](=[O:7])[NH:8][c:9]1[c:10]([CH:16]=[CH:17][C:18](=[O:20])[N:30]2[CH:29]([CH3:33])[CH2:28][N:27]([CH2:26][c:25]3[cH:24][cH:23][c:22]([F:21])[cH:35][cH:34]3)[CH2:32][CH2:31]2)[cH:11][c:12]([Cl:15])[cH:13][cH:14]1

SMILES/SMARTS of the MAPPET output:
[CH3:16][C:17]([CH3:18])([CH3:19])[O:20][C:21](=[O:22])[NH:23][c:24]1[cH:25][cH:26][c:27]([Cl:28])[cH:29][c:30]1[CH:31]=[CH:32][C:33]([OH:34])=[O:35].[CH3:1][CH:2]1[CH2:3][N:4]([CH2:5][c:6]2[cH:7][cH:8][c:9]([F:10])[cH:11][cH:12]2)[CH2:13][CH2:14][NH:15]1>>[CH3:1][CH:2]1[CH2:3][N:4]([CH2:5][c:6]2[cH:12][cH:11][c:9]([F:10])[cH:8][cH:7]2)[CH2:13][CH2:14][N:15]1[C:33](=[O:35])[CH:32]=[CH:31][c:30]1[cH:29][c:27]([Cl:28])[cH:26][cH:25][c:24]1[NH:23][C:21](=[O:22])[O:20][C:17]([CH3:16])([CH3:19])[CH3:18].[OH2:34]

Correctness of the mapping
MAPPET YES
USPTO NO

Reaction no 103 (3 bonds changed)

SMILES/SMARTS of the USPTO output:
[CH3:16][c:17]1[cH:18][cH:19][cH:20][cH:21][cH:22]1.[CH3:5][c:6]1[cH:7][c:8]([C:9](=[O:10])[OH:11])[cH:12][c:13]([CH3:15])[cH:14]1.[S:1]([Cl:2])([Cl:3])=[O:4]>>[Cl:3][C:9]([c:8]1[cH:7][c:6]([CH3:5])[cH:14][c:13]([CH3:15])[cH:12]1)=[O:10]

SMILES/SMARTS of the MAPPET output:
[Cl:1][S:2]([Cl:3])=[O:4].[CH3:5][c:6]1[cH:7][c:8]([CH3:9])[cH:10][c:11]([cH:12]1)[C:13]([OH:14])=[O:15]>>[CH3:9][c:8]1[cH:7][c:6]([CH3:5])[cH:12][c:11]([cH:10]1)[C:13]([Cl:3])=[O:15].[Cl:1].[O:14].[O:4].[S:2]

Correctness of the mapping
MAPPET YES
USPTO YES

Reaction no 104 (3 bonds changed)

SMILES/SMARTS of the USPTO output:
[CH3:1][O:2][c:3]1[cH:4][c:5]([S:11][CH2:12][C:13](=[O:14])[OH:15])[cH:6][cH:7][c:8]1[O:9][CH3:10].[CH3:51][N:52]([CH3:53])[C:54](=[O:55])[CH3:56].[F:16][C:17]([F:18])([F:19])[C:20]([OH:21])=[O:22].[NH2:23][CH:24]1[CH:25]2[S:26][CH2:27][C:28]([CH:36]=[C:37]3[C:38](=[O:50])[N:39]([CH2:42][c:43]4[cH:44][c:45]([OH:49])[cH:46][cH:47][cH:48]4)[CH2:40][CH2:41]3)=[C:29]([C:33](=[O:34])[OH:35])[N:30]2[C:31]1=[O:32]>>[CH3:1][O:2][c:3]1[cH:4][c:5]([S:11][CH2:12][C:13](=[O:15])[NH:23][CH:24]2[CH:25]3[S:26][CH2:27][C:28]([CH:36]=[C:37]4[C:38](=[O:50])[N:39]([CH2:42][c:43]5[cH:44][c:45]([OH:49])[cH:46][cH:47][cH:48]5)[CH2:40][CH2:41]4)=[C:29]([C:33](=[O:34])[OH:35])[N:30]3[C:31]2=[O:32])[cH:6][cH:7][c:8]1[O:9][CH3:10]

SMILES/SMARTS of the MAPPET output:
[CH3:29][O:30][c:31]1[cH:32][cH:33][c:34]([S:35][CH2:36][C:37]([OH:38])=[O:39])[cH:40][c:41]1[O:42][CH3:43].[NH2:1][CH:2]1[CH:3]2[S:4][CH2:5][C:6]([CH:7]=[C:8]3[CH2:9][CH2:10][N:11]([CH2:12][c:13]4[cH:14][cH:15][cH:16][c:17]([OH:18])[cH:19]4)[C:20]3=[O:21])=[C:22]([N:23]2[C:24]1=[O:25])[C:26]([OH:27])=[O:28]>>[CH3:29][O:30][c:31]1[cH:32][cH:33][c:34]([S:35][CH2:36][C:37](=[O:39])[NH:1][CH:2]2[CH:3]3[S:4][CH2:5][C:6]([CH:7]=[C:8]4[CH2:9][CH2:10][N:11]([CH2:12][c:13]5[cH:14][cH:15][cH:16][c:17]([OH:18])[cH:19]5)[C:20]4=[O:21])=[C:22]([N:23]3[C:24]2=[O:25])[C:26]([OH:27])=[O:28])[cH:40][c:41]1[O:42][CH3:43].[OH2:38]

Correctness of the mapping
MAPPET YES
USPTO NO

Reaction no 105 (3 bonds changed)

SMILES/SMARTS of the USPTO output:
[CH3:50][N:51]([CH3:52])[CH:53]=[O:54].[CH:1]1([CH2:6][CH:7]([C:8](=[O:9])[OH:10])[N:11]2[C:12](=[O:18])[CH:13]=[C:14]([O:16][CH3:17])[CH2:15]2)[CH2:2][CH2:3][CH2:4][CH2:5]1.[CH:32]([N:33]([CH2:34][CH3:35])[CH:36]([CH3:37])[CH3:38])([CH3:39])[CH3:40].[Cl:19][C:20]([C:21]([Cl:22])=[O:23])=[O:24].[Cl:47][CH2:48][Cl:49].[cH:41]1[cH:42][cH:43][cH:44][cH:45][cH:46]1.[n:25]1[c:26]([NH2:31])[cH:27][n:28][cH:29][cH:30]1>>[CH:1]1([CH2:6][CH:7]([C:8](=[O:10])[NH:31][c:26]2[n:25][cH:30][cH:29][n:28][cH:27]2)[N:11]2[C:12](=[O:18])[CH:13]=[C:14]([O:16][CH3:17])[CH2:15]2)[CH2:2][CH2:3][CH2:4][CH2:5]1

SMILES/SMARTS of the MAPPET output:
[CH3:8][O:9][C:10]1=[CH:11][C:12](=[O:13])[N:14]([CH2:15]1)[CH:16]([CH2:17][CH:18]1[CH2:19][CH2:20][CH2:21][CH2:22]1)[C:23]([OH:24])=[O:25].[NH2:1][c:2]1[cH:3][n:4][cH:5][cH:6][n:7]1>>[CH3:8][O:9][C:10]1=[CH:11][C:12](=[O:13])[N:14]([CH2:15]1)[CH:16]([CH2:17][CH:18]1[CH2:19][CH2:20][CH2:21][CH2:22]1)[C:23](=[O:25])[NH:1][c:2]1[cH:3][n:4][cH:5][cH:6][n:7]1.[OH2:24]

Correctness of the mapping
MAPPET YES
USPTO NO

Reaction no 106 (3 bonds changed)

SMILES/SMARTS of the USPTO output:
[Br:1][CH2:2][c:3]1[c:4]([C:24]([F:25])([F:26])[F:27])[n:5][n:6](-[c:14]2[cH:15][cH:16][c:17]([S:20](=[O:21])(=[O:22])[NH2:23])[cH:18][cH:19]2)[c:7]1-[c:8]1[cH:9][cH:10][cH:11][cH:12][cH:13]1.[CH3:37][S:38]([CH3:39])=[O:40].[n:28]1[c:29]([CH3:30])[cH:31][c:32]([CH3:33])[cH:34][c:35]1[CH3:36]>>[CH:2]([c:3]1[c:4]([C:24]([F:25])([F:26])[F:27])[n:5][n:6](-[c:14]2[cH:15][cH:16][c:17]([S:20](=[O:21])(=[O:22])[NH2:23])[cH:18][cH:19]2)[c:7]1-[c:8]1[cH:9][cH:10][cH:11][cH:12][cH:13]1)=[O:40]

SMILES/SMARTS of the MAPPET output:
[CH3:1][S:2]([CH3:3])=[O:4].[NH2:5][S:6](=[O:7])(=[O:8])[c:9]1[cH:10][cH:11][c:12]([cH:13][cH:14]1)-[n:15]1[n:16][c:17]([c:18]([CH2:19][Br:20])[c:21]1-[c:22]1[cH:23][cH:24][cH:25][cH:26][cH:27]1)[C:28]([F:29])([F:30])[F:31]>>[NH2:5][S:6](=[O:7])(=[O:8])[c:9]1[cH:14][cH:13][c:12]([cH:11][cH:10]1)-[n:15]1[n:16][c:17]([c:18]([CH:19]=[O:4])[c:21]1-[c:22]1[cH:27][cH:26][cH:25][cH:24][cH:23]1)[C:28]([F:29])([F:30])[F:31].[Br:20].[C:3].[C:1].[S:2]

Correctness of the mapping
MAPPET YES
USPTO YES

Reaction no 107 (3 bonds changed)

SMILES/SMARTS of the USPTO output:
[CH3:1][C:2](=[CH:3][C:4](=[O:5])[OH:6])[CH2:7][CH2:8][CH:9]=[C:10]([CH2:11][CH2:12][CH:13]=[C:14]([CH2:15][CH2:16][CH:17]=[C:18]([CH3:19])[CH3:20])[CH3:21])[CH3:22].[OH:23][CH2:24][CH2:25][NH:26][CH2:27][CH2:28][OH:29]>>[CH3:1][C:2](=[CH:3][C:4](=[O:6])[N:26]([CH2:25][CH2:24][OH:23])[CH2:27][CH2:28][OH:29])[CH2:7][CH2:8][CH:9]=[C:10]([CH2:11][CH2:12][CH:13]=[C:14]([CH2:15][CH2:16][CH:17]=[C:18]([CH3:19])[CH3:20])[CH3:21])[CH3:22]

SMILES/SMARTS of the MAPPET output:
[CH3:8][C:9]([CH3:10])=[CH:11][CH2:12][CH2:13][C:14]([CH3:15])=[CH:16][CH2:17][CH2:18][C:19]([CH3:20])=[CH:21][CH2:22][CH2:23][C:24]([CH3:25])=[CH:26][C:27]([OH:28])=[O:29].[OH:1][CH2:2][CH2:3][NH:4][CH2:5][CH2:6][OH:7]>>[CH3:8][C:9]([CH3:10])=[CH:11][CH2:12][CH2:13][C:14]([CH3:15])=[CH:16][CH2:17][CH2:18][C:19]([CH3:20])=[CH:21][CH2:22][CH2:23][C:24]([CH3:25])=[CH:26][C:27](=[O:29])[N:4]([CH2:5][CH2:6][OH:7])[CH2:3][CH2:2][OH:1].[OH2:28]

Correctness of the mapping
MAPPET YES
USPTO NO

Reaction no 108 (3 bonds changed)

SMILES/SMARTS of the USPTO output:
[O:17]=[CH:18][N:19]([CH3:20])[CH3:21].[OH:1][c:2]1[c:3]([C:4](=[O:5])[OH:6])[cH:7][cH:8][c:9]([O:11][CH3:12])[cH:10]1.[S:13]([Cl:14])([Cl:15])=[O:16]>>[OH:1][c:2]1[c:3]([C:4](=[O:5])[Cl:15])[cH:7][cH:8][c:9]([O:11][CH3:12])[cH:10]1

SMILES/SMARTS of the MAPPET output:
[Cl:1][S:2]([Cl:3])=[O:4].[CH3:5][O:6][c:7]1[cH:8][cH:9][c:10]([C:11]([OH:12])=[O:13])[c:14]([OH:15])[cH:16]1>>[CH3:5][O:6][c:7]1[cH:8][cH:9][c:10]([C:11]([Cl:3])=[O:13])[c:14]([OH:15])[cH:16]1.[Cl:1].[O:12].[O:4].[S:2]

Correctness of the mapping
MAPPET YES
USPTO YES

Reaction no 109 (3 bonds changed)

SMILES/SMARTS of the USPTO output:
[Br:1][C:2]1=[CH:3][C:4]([CH2:16][F:17])([CH2:18][F:19])[O:5][c:6]2[c:7]1[cH:8][c:9]([C:12]([F:13])([F:14])[F:15])[cH:10][cH:11]2.[CH2:20]([Li:21])[CH2:22][CH2:23][CH3:24].[CH2:25]([CH3:26])[N:27]=[C:28]=[S:29].[CH3:30][CH2:31][O:32][CH2:33][CH3:34]>>[C:2]1([C:28]([NH:27][CH2:25][CH3:26])=[S:29])=[CH:3][C:4]([CH2:16][F:17])([CH2:18][F:19])[O:5][c:6]2[c:7]1[cH:8][c:9]([C:12]([F:13])([F:14])[F:15])[cH:10][cH:11]2

SMILES/SMARTS of the MAPPET output:
[CH3:1][CH2:2][N:3]=[C:4]=[S:5].[F:6][CH2:7][C:8]1([CH2:9][F:10])[O:11][c:12]2[cH:13][cH:14][c:15]([cH:16][c:17]2[C:18]([Br:19])=[CH:20]1)[C:21]([F:22])([F:23])[F:24]>>[CH3:1][CH2:2][NH:3][C:4](=[S:5])[C:18]1=[CH:20][C:8]([CH2:7][F:6])([CH2:9][F:10])[O:11][c:12]2[cH:13][cH:14][c:15]([cH:16][c:17]12)[C:21]([F:22])([F:23])[F:24].[Br:19]

Correctness of the mapping
MAPPET YES
USPTO YES

Reaction no 110 (3 bonds changed)

SMILES/SMARTS of the USPTO output:
[CH3:1][n:2]1[c:3]([CH3:11])[cH:4][c:5]2[cH:6][cH:7][cH:8][cH:9][c:10]12.[CH3:21][OH:22].[I-:20].[I:18].[K+:19].[NH:12]1[C:13](=[S:17])[NH:14][CH2:15][CH2:16]1.[OH2:23]>>[CH3:1][n:2]1[c:3]([CH3:11])[c:4]([S:17][C:13]2=[N:12][CH2:16][CH2:15][NH:14]2)[c:5]2[cH:6][cH:7][cH:8][cH:9][c:10]12.[IH:20]

SMILES/SMARTS of the MAPPET output:
[S:1]=[C:2]1[NH:3][CH2:4][CH2:5][NH:6]1.[CH3:7][c:8]1[cH:9][c:10]2[cH:11][cH:12][cH:13][cH:14][c:15]2[n:16]1[CH3:17]>>[CH3:7][c:8]1[c:9]([S:1][C:2]2=[N:6][CH2:5][CH2:4][NH:3]2)[c:10]2[cH:11][cH:12][cH:13][cH:14][c:15]2[n:16]1[CH3:17]

Correctness of the mapping
MAPPET YES
USPTO YES

Reaction no 111 (3 bonds changed)

SMILES/SMARTS of the USPTO output:
[CH2:38]1[O:39][CH2:40][CH2:41][CH2:42]1.[CH3:1][O:2][C:3]([CH:4]([CH2:5][c:6]1[cH:7][c:8]([Cl:13])[c:9]([Cl:12])[cH:10][cH:11]1)[NH:14][C:15]([c:16]1[c:17]([NH:23][S:24](=[O:25])(=[O:26])[c:27]2[cH:28][cH:29][cH:30][c:31]3[c:32]2[n:33][s:34][n:35]3)[cH:18][c:19]([Cl:22])[cH:20][cH:21]1)=[O:36])=[O:37].[ClH:45].[Li+:44].[OH-:43].[OH2:46]>>[O:2]=[C:3]([CH:4]([CH2:5][c:6]1[cH:7][c:8]([Cl:13])[c:9]([Cl:12])

)[cH:10][cH:11]1)[NH:14][C:15]([c:16]1[c:17]([NH:23][S:24](=[O:25])(=[O:26])[c:2
7]2[cH:28][cH:29][cH:30][c:31]3[c:32]2[n:33][s:34][n:35]3)[cH:18][c:19]([Cl:22])
[cH:20][cH:21]1)=[O:36])[OH:37]

SMILES/SMARTS of the MAPPET output:
[CH3:2][O:3][C:4](=[O:5])[CH:6]([CH2:7][c:8]1[cH:9][cH:10][c:11]([Cl:12])[c:13](
[Cl:14])[cH:15]1)[NH:16][C:17](=[O:18])[c:19]1[cH:20][cH:21][c:22]([Cl:23])[cH:2
4][c:25]1[NH:26][S:27](=[O:28])(=[O:29])[c:30]1[cH:31][cH:32][cH:33][c:34]2[n:35
][s:36][n:37][c:38]12.[OH2:1]>>[OH:1][C:4](=[O:5])[CH:6]([CH2:7][c:8]1[cH:9][cH:
10][c:11]([Cl:12])[c:13]([Cl:14])[cH:15]1)[NH:16][C:17](=[O:18])[c:19]1[cH:20][c
H:21][c:22]([Cl:23])[cH:24][c:25]1[NH:26][S:27](=[O:29])(=[O:28])[c:30]1[cH:31][
cH:32][cH:33][c:34]2[n:35][s:36][n:37][c:38]12.[CH3:2][OH:3]

Correctness of the mapping
MAPPET YES
USPTO NO

Reaction no 112 (3 bonds changed)

SMILES/SMARTS of the USPTO output:
[CH3:18][c:19]1[cH:20][cH:21][cH:22][cH:23][cH:24]1.[CH3:1][c:2]1[cH:3][c:4]([NH
2:5])[cH:6][c:7]([CH3:13])[c:8]1[C:9]([CH2:10][CH3:11])=[O:12].[Cl:14][C:15]([Cl
:16])=[O:17]>>[CH3:1][c:2]1[cH:3][c:4]([N:5]=[C:15]=[O:17])[cH:6][c:7]([CH3:13])
[c:8]1[C:9]([CH2:10][CH3:11])=[O:12]

SMILES/SMARTS of the MAPPET output:
[Cl:1][C:2]([Cl:3])=[O:4].[CH3:5][CH2:6][C:7](=[O:8])[c:9]1[c:10]([CH3:11])[cH:1
2][c:13]([NH2:14])[cH:15][c:16]1[CH3:17]>>[CH3:5][CH2:6][C:7](=[O:8])[c:9]1[c:16
]([CH3:17])[cH:15][c:13]([cH:12][c:10]1[CH3:11])[N:14]=[C:2]=[O:4].[Cl:3].[Cl:1]

Correctness of the mapping
MAPPET YES
USPTO YES

Reaction no 113 (3 bonds changed)

SMILES/SMARTS of the USPTO output:
[C:21](#[N:22])[c:23]1[cH:24][cH:25][c:26]([B:29]([OH:30])[OH:31])[cH:27][cH:28]
1.[CH3:1][C:2]1([CH3:20])[CH2:3][C:4](=[O:19])[c:5]2[cH:6][cH:7][c:8]([O:11][S:1
2]([C:13]([F:14])([F:15])[F:16])(=[O:17])=[O:18])[cH:9][c:10]21>>[CH3:1][C:2]1([
CH3:20])[CH2:3][C:4](=[O:19])[c:5]2[cH:6][cH:7][c:8](-
[c:26]3[cH:25][cH:24][c:23]([C:21]#[N:22])[cH:28][cH:27]3)[cH:9][c:10]21

SMILES/SMARTS of the MAPPET output:
[CH3:1][C:2]1([CH3:3])[CH2:4][C:5](=[O:6])[c:7]2[cH:8][cH:9][c:10]([O:11][S:12](
=[O:13])(=[O:14])[C:15]([F:16])([F:17])[F:18])[cH:19][c:20]12.[OH:21][B:22]([OH:
23])[c:24]1[cH:25][cH:26][c:27]([cH:28][cH:29]1)[C:30]#[N:31]>>[CH3:3][C:2]1([CH
3:1])[CH2:4][C:5](=[O:6])[c:7]2[cH:8][cH:9][c:10]([cH:19][c:20]12)-
[c:24]1[cH:25][cH:26][c:27]([cH:28][cH:29]1)[C:30]#[N:31].[B:22].[C:15].[F:18].[
F:16].[F:17].[O:23].[O:21].[O:11].[O:13].[O:14].[S:12]

Correctness of the mapping
MAPPET YES
USPTO YES

Reaction no 114 (3 bonds changed)

SMILES/SMARTS of the USPTO output:

[CH2:4]([CH3:5])[O:6][C:7]([CH2:8][c:9]1[n:10][c:11](-
[c:14]2[c:15]([CH3:44])[cH:16][c:17]([C:20]([CH2:21][CH3:22])([c:23]3[cH:24][c:2
5]([CH3:41])[c:26]([CH:29]=[CH:30][C:31]([C:32]([F:33])([F:34])[F:35])([C:36]([F
:37])([F:38])[F:39])[OH:40])[cH:27][cH:28]3)[CH2:42][CH3:43])[cH:18][cH:19]2)[s:
12][cH:13]1)=[O:45].[CH3:47][OH:48].[ClH:46].[Na+:2].[OH-
:1].[OH2:3]>>[O:6]=[C:7]([CH2:8][c:9]1[n:10][c:11](-
[c:14]2[c:15]([CH3:44])[cH:16][c:17]([C:20]([CH2:21][CH3:22])([c:23]3[cH:24][c:2
5]([CH3:41])[c:26]([CH:29]=[CH:30][C:31]([C:32]([F:33])([F:34])[F:35])([C:36]([F
:37])([F:38])[F:39])[OH:40])[cH:27][cH:28]3)[CH2:42][CH3:43])[cH:18][cH:19]2)[s:
12][cH:13]1)[OH:45]

SMILES/SMARTS of the MAPPET output:
[CH3:2][CH2:3][O:4][C:5](=[O:6])[CH2:7][c:8]1[cH:9][s:10][c:11]([n:12]1)-
[c:13]1[cH:14][cH:15][c:16]([cH:17][c:18]1[CH3:19])[C:20]([CH2:21][CH3:22])([CH2
:23][CH3:24])[c:25]1[cH:26][cH:27][c:28]([CH:29]=[CH:30][C:31]([OH:32])([C:33]([
F:34])([F:35])[F:36])[C:37]([F:38])([F:39])[F:40])[c:41]([CH3:42])[cH:43]1.[OH2:
1]>>[CH3:24][CH2:23][C:20]([CH2:21][CH3:22])([c:25]1[cH:26][cH:27][c:28]([CH:29]
=[CH:30][C:31]([OH:32])([C:33]([F:34])([F:35])[F:36])[C:37]([F:38])([F:39])[F:40
])[c:41]([CH3:42])[cH:43]1)[c:16]1[cH:15][cH:14][c:13](-
[c:11]2[n:12][c:8]([CH2:7][C:5]([OH:1])=[O:6])[cH:9][s:10]2)[c:18]([CH3:19])[cH:
17]1.[CH3:2][CH2:3][OH:4]

Correctness of the mapping
MAPPET YES
USPTO NO

Reaction no 115 (3 bonds changed)

SMILES/SMARTS of the USPTO output:
[C:19](=[O:20])([OH:21])[O-
:22].[CH3:36][CH2:37][O:38][C:39](=[O:40])[CH3:41].[Cu:24][C:25]#[N:26].[N:15]([
O-
:16])=[O:17].[NH2:1][c:2]1[c:3]2[c:4](=[O:14])[cH:5][c:6]([CH3:13])[o:7][c:8]2[c
:9]([CH3:12])[cH:10][cH:11]1.[Na+:18].[Na+:23].[Na:27][C:28]#[N:29].[OH2:35].[S:
30](=[O:31])(=[O:32])([OH:33])[OH:34]>>[c:2]1([C:25]#[N:26])[c:3]2[c:4](=[O:14])
[cH:5][c:6]([CH3:13])[o:7][c:8]2[c:9]([CH3:12])[cH:10][cH:11]1

SMILES/SMARTS of the MAPPET output:
[CH3:1][c:2]1[cH:3][c:4](=[O:5])[c:6]2[c:7]([NH2:8])[cH:9][cH:10][c:11]([CH3:12]
)[c:13]2[o:14]1.[O-
:15][N:16]=[O:17].[Cu:18][C:19]#[N:20]>>[CH3:1][c:2]1[cH:3][c:4](=[O:5])[c:6]2[c
:7]([cH:9][cH:10][c:11]([CH3:12])[c:13]2[o:14]1)[C:19]#[N:20].[Cu:18].[N:8].[N:1
6].[O:17].[O:15]

Correctness of the mapping
MAPPET YES
USPTO YES

Reaction no 116 (3 bonds changed)

SMILES/SMARTS of the USPTO output:
[CH3:47][OH:48].[N:1]([CH2:4][CH2:5][n:6]1[n:7][c:8]([C:23](=[O:24])[O:25][CH3:2
6])[c:9]([O:15][CH2:16][c:17]2[cH:18][cH:19][cH:20][cH:21][cH:22]2)[c:10]1[C:11]
([O:2][CH3:3])=[O:12])=[N+:13]=[N-
:14].[O:49]=[CH:50][N:51]([CH3:52])[CH3:53].[OH2:46].[c:27]1([P:28]([c:29]2[cH:3
0][cH:31][cH:32][cH:33][cH:34]2)[c:35]2[cH:36][cH:37][cH:38][cH:39][cH:40]2)[cH:
41][cH:42][cH:43][cH:44][cH:45]1>>[NH:1]1[CH2:4][CH2:5][n:6]2[n:7][c:8]([C:23](=
[O:24])[O:25][CH3:26])[c:9]([O:15][CH2:16][c:17]3[cH:18][cH:19][cH:20][cH:21][cH
:22]3)[c:10]2[C:11]1=[O:12]

SMILES/SMARTS of the MAPPET output:
[CH3:1][O:2][C:3](=[O:4])[c:5]1[n:6][n:7]([CH2:8][CH2:9][N:10]=[N+:11]=[N-
:12])[c:13]([C:14](=[O:15])[O:16][CH3:17])[c:18]1[O:19][CH2:20][c:21]1[cH:22][cH
:23][cH:24][cH:25][cH:26]1>>[CH3:1][O:2][C:3](=[O:4])[c:5]1[n:6][n:7]2[CH2:8][CH
2:9][NH:10][C:17](=[O:16])[c:13]2[c:18]1[O:19][CH2:20][c:21]1[cH:26][cH:25][cH:2
4][cH:23][cH:22]1.[C:14].[N:11].[N:12].[O:15]

Correct mapped SMILES/SMARTS of the reaction:

Correctness of the mapping
MAPPET NO
USPTO YES

Reaction no 117 (3 bonds changed)

SMILES/SMARTS of the USPTO output:
[C:40]([CH3:41])([CH3:42])([CH3:43])[O:44][C:45]([NH:46][CH2:47][c:48]1[cH:49][c
H:50][c:51]([CH2:54][NH2:55])[cH:52][cH:53]1)=[O:56].[CH3:29][N:30]([CH3:31])[CH
2:32][CH2:33][CH2:34][N:35]=[C:36]=[N:37][CH2:38][CH3:39].[CH3:57][N:58]1[CH2:59
][CH2:60][O:61][CH2:62][CH2:63]1.[CH3:64][N:65]([CH3:66])[CH:67]=[O:68].[ClH:28]
.[F:1][c:2]1[cH:3][cH:4][c:5]([O:6][c:7]2[c:8]([C:9](=[O:10])[OH:11])[cH:12][cH:
13][cH:14][n:15]2)[cH:16][cH:17]1.[OH:18][n:19]1[c:20]2[cH:21][cH:22][cH:23][cH:
24][c:25]2[n:26][n:27]1>>[F:1][c:2]1[cH:3][cH:4][c:5]([O:6][c:7]2[c:8]([C:9](=[O
:11])[NH:55][CH2:54][c:51]3[cH:50][cH:49][c:48]([CH2:47][NH:46][C:45]([O:44][C:4
0]([CH3:41])([CH3:42])[CH3:43])=[O:56])[cH:53][cH:52]3)[cH:12][cH:13][cH:14][n:1
5]2)[cH:16][cH:17]1

SMILES/SMARTS of the MAPPET output:
[OH:18][C:19](=[O:20])[c:21]1[cH:22][cH:23][cH:24][n:25][c:26]1[O:27][c:28]1[cH:
29][cH:30][c:31]([F:32])[cH:33][cH:34]1.[CH3:1][C:2]([CH3:3])([CH3:4])[O:5][C:6]
(=[O:7])[NH:8][CH2:9][c:10]1[cH:11][cH:12][c:13]([CH2:14][NH2:15])[cH:16][cH:17]
1>>[CH3:1][C:2]([CH3:4])([CH3:3])[O:5][C:6](=[O:7])[NH:8][CH2:9][c:10]1[cH:11][c
H:12][c:13]([CH2:14][NH:15][C:19](=[O:20])[c:21]2[cH:22][cH:23][cH:24][n:25][c:2
6]2[O:27][c:28]2[cH:34][cH:33][c:31]([F:32])[cH:30][cH:29]2)[cH:16][cH:17]1.[OH2
:18]

Correctness of the mapping
MAPPET YES
USPTO NO

Reaction no 118 (3 bonds changed)

SMILES/SMARTS of the USPTO output:
[CH3:23][c:24]1[cH:25][cH:26][cH:27][cH:28][cH:29]1.[OH:1][CH2:2][c:3]1[cH:4][c:
5]2[c:9]([cH:10][cH:11]1)[CH:8]([c:12]1[cH:13][cH:14][c:15]([F:18])[cH:16][cH:17
]1)[O:7][CH2:6]2.[S:19]([Br:20])([Br:21])=[O:22]>>[CH2:2]([c:3]1[cH:4][c:5]2[c:9
]([cH:10][cH:11]1)[CH:8]([c:12]1[cH:13][cH:14][c:15]([F:18])[cH:16][cH:17]1)[O:7
][CH2:6]2)[Br:21]

SMILES/SMARTS of the MAPPET output:
-

Correctness of the mapping
MAPPET NO
USPTO YES

Reaction no 119 (3 bonds changed)

SMILES/SMARTS of the USPTO output:
[C:27]([c:28]1[nH:29][cH:30][cH:31][n:32]1)([c:33]1[nH:34][cH:35][cH:36][n:37]1)=[O:38].[CH2:1]([c:2]1[cH:3][cH:4][cH:5][cH:6][cH:7]1)[O:8][c:9]1[c:10]([C:11](=[O:12])[OH:13])[cH:14][c:15]([Br:26])[c:16]([O:18][CH2:19][c:20]2[cH:21][cH:22][cH:23][cH:24][cH:25]2)[cH:17]1.[CH3:39][O:40][CH2:41][CH2:42][CH2:43][NH2:44].[O:45]=[CH:46][N:47]([CH3:48])[CH3:49].[OH2:50]>>[CH2:1]([c:2]1[cH:3][cH:4][cH:5][cH:6][cH:7]1)[O:8][c:9]1[c:10]([C:11](=[O:13])[NH:44][CH2:43][CH2:42][CH2:41][O:40][CH3:39])[cH:14][c:15]([Br:26])[c:16]([O:18][CH2:19][c:20]2[cH:21][cH:22][cH:23][cH:24][cH:25]2)[cH:17]1

SMILES/SMARTS of the MAPPET output:
[OH:7][C:8](=[O:9])[c:10]1[cH:11][c:12]([Br:13])[c:14]([O:15][CH2:16][c:17]2[cH:18][cH:19][cH:20][cH:21][cH:22]2)[cH:23][c:24]1[O:25][CH2:26][c:27]1[cH:28][cH:29][cH:30][cH:31][cH:32]1.[CH3:1][O:2][CH2:3][CH2:4][CH2:5][NH2:6]>>[CH3:1][O:2][CH2:3][CH2:4][CH2:5][NH:6][C:8](=[O:9])[c:10]1[cH:11][c:12]([Br:13])[c:14]([O:15][CH2:16][c:17]2[cH:18][cH:19][cH:20][cH:21][cH:22]2)[cH:23][c:24]1[O:25][CH2:26][c:27]1[cH:28][cH:29][cH:30][cH:31][cH:32]1.[OH2:7]

Correctness of the mapping
MAPPET YES
USPTO NO

Reaction no 120 (3 bonds changed)

SMILES/SMARTS of the USPTO output:
[Cl:36][CH2:37][Cl:38].[F:1][C:2]([c:3]1[cH:4][c:5]([CH:9]([CH2:10][CH2:11][CH2:12][CH:13]=[CH2:14])[NH:15][C:16](=[O:17])[c:18]2[cH:19][n:20][n:21](-[c:24]3[cH:25][cH:26][c:27]([Cl:30])[cH:28][cH:29]3)[c:22]2[CH3:23])[cH:6][cH:7][cH:8]1)([F:31])[F:32].[O-:33][O+:34]=[O:35]>>[F:1][C:2]([c:3]1[cH:4][c:5]([CH:9]([CH2:10][CH2:11][CH2:12][CH:13]=[O:33])[NH:15][C:16](=[O:17])[c:18]2[cH:19][n:20][n:21](-[c:24]3[cH:25][cH:26][c:27]([Cl:30])[cH:28][cH:29]3)[c:22]2[CH3:23])[cH:6][cH:7][cH:8]1)([F:31])[F:32]

SMILES/SMARTS of the MAPPET output:
[O-:1][O+:2]=[O:3].[CH3:4][c:5]1[c:6]([cH:7][n:8][n:9]1-[c:10]1[cH:11][cH:12][c:13]([Cl:14])[cH:15][cH:16]1)[C:17](=[O:18])[NH:19][CH:20]([CH2:21][CH2:22][CH2:23][CH:24]=[CH2:25])[c:26]1[cH:27][cH:28][cH:29][c:30]([cH:31]1)[C:32]([F:33])([F:34])[F:35]>>[CH3:4][c:5]1[c:6]([cH:7][n:8][n:9]1-[c:10]1[cH:16][cH:15][c:13]([Cl:14])[cH:12][cH:11]1)[C:17](=[O:18])[NH:19][CH:20]([CH2:21][CH2:22][CH2:23][CH:24]=[O:3])[c:26]1[cH:27][cH:28][cH:29][c:30]([cH:31]1)[C:32]([F:33])([F:34])[F:35].[C:25].[O:2].[O:1]

Correctness of the mapping
MAPPET YES
USPTO YES

Reaction no 121 (3 bonds changed)

SMILES/SMARTS of the USPTO output:
[CH3:1][O:2][C:3]([CH:4]([NH:5][C:6]([CH:7]([CH2:8][CH:9]([CH2:10][c:11]1[cH:12][cH:13][cH:14][cH:15][cH:16]1)[C:17](=[O:18])[NH:19][c:20]1[n:21][cH:22][cH:23][cH:24][cH:25]1)[CH2:26][c:27]1[cH:28][cH:29][cH:30][cH:31][cH:32]1)=[O:33])[CH2:34][S:35][CH2:36][c:37]1[cH:38][cH:39][cH:40][cH:41][cH:42]1)=[O:43].[CH3:47][OH:48].[ClH:46].[Na+:45].[OH-:44]>>[O:2]=[C:3]([CH:4]([NH:5][C:6]([CH:7]([CH2:8][CH:9]([CH2:10][c:11]1[cH:12][cH:13][cH:14][cH:15][cH:16]1)[C:17](=[O:18])[NH:19][c:20]1[n:21][cH:22][cH:23][

cH:24][cH:25]1)[CH2:26][c:27]1[cH:28][cH:29][cH:30][cH:31][cH:32]1)=[O:33])[CH2:34][S:35][CH2:36][c:37]1[cH:38][cH:39][cH:40][cH:41][cH:42]1)[OH:43]

SMILES/SMARTS of the MAPPET output:
[CH3:2][O:3][C:4](=[O:5])[CH:6]([CH2:7][S:8][CH2:9][c:10]1[cH:11][cH:12][cH:13][cH:14][cH:15]1)[NH:16][C:17](=[O:18])[CH:19]([CH2:20][CH:21]([CH2:22][c:23]1[cH:24][cH:25][cH:26][cH:27][cH:28]1)[C:29](=[O:30])[NH:31][c:32]1[cH:33][cH:34][cH:35][cH:36][n:37]1)[CH2:38][c:39]1[cH:40][cH:41][cH:42][cH:43][cH:44]1.[OH2:1]>>[OH:1][C:4](=[O:5])[CH:6]([CH2:7][S:8][CH2:9][c:10]1[cH:11][cH:12][cH:13][cH:14][cH:15]1)[NH:16][C:17](=[O:18])[CH:19]([CH2:20][CH:21]([CH2:22][c:23]1[cH:24][cH:25][cH:26][cH:27][cH:28]1)[C:29](=[O:30])[NH:31][c:32]1[cH:33][cH:34][cH:35][cH:36][n:37]1)[CH2:38][c:39]1[cH:40][cH:41][cH:42][cH:43][cH:44]1.[CH3:2][OH:3]

Correctness of the mapping
MAPPET YES
USPTO NO

Reaction no 122 (3 bonds changed)

SMILES/SMARTS of the USPTO output:
[Br:2][c:3]1[cH:4][cH:5][n:6][cH:7][cH:8]1.[C:24]([P:25]([C:26]([CH3:27])([CH3:28])[CH3:29])[C:30]([CH3:31])([CH3:32])[CH3:33])([CH3:34])([CH3:35])[CH3:36].[CH2:43]1[O:44][CH2:45][CH2:46][O:47][CH2:48]1.[Cl:49][CH2:50][Cl:51].[ClH:1].[F-:22].[F:11][c:12]1[c:13]([B:19]([OH:20])[OH:21])[c:14]([F:18])[cH:15][cH:16][cH:17]1.[K+:23].[Na+:10].[O:37]1[CH2:38][CH2:39][CH2:40][CH2:41]1.[O:54]=[C:55]([CH:56]=[CH:57][c:58]1[cH:59][cH:60][cH:61][cH:62][cH:63]1)[CH:64]=[CH:65][c:66]1[cH:67][cH:68][cH:69][cH:70][cH:71]1.[O:72]=[C:73]([CH:74]=[CH:75][c:76]1[cH:77][cH:78][cH:79][cH:80][cH:81]1)[CH:82]=[CH:83][c:84]1[cH:85][cH:86][cH:87][cH:88][cH:89]1.[O:90]=[C:91]([CH:92]=[CH:93][c:94]1[cH:95][cH:96][cH:97][cH:98][cH:99]1)[CH:100]=[CH:101][c:102]1[cH:103][cH:104][cH:105][cH:106][cH:107]1.[OH-:9].[OH2:42].[Pd:52].[Pd:53]>>[c:3]1(-[c:13]2[c:12]([F:11])[cH:17][cH:16][cH:15][c:14]2[F:18])[cH:4][cH:5][n:6][cH:7][cH:8]1

SMILES/SMARTS of the MAPPET output:
[OH:1][B:2]([OH:3])[c:4]1[c:5]([F:6])[cH:7][cH:8][cH:9][c:10]1[F:11].[Br:12][c:13]1[cH:14][cH:15][n:16][cH:17][cH:18]1>>[F:6][c:5]1[cH:7][cH:8][cH:9][c:10]([F:11])[c:4]1-[c:13]1[cH:14][cH:15][n:16][cH:17][cH:18]1.[B:2].[Br:12].[O:3].[O:1]

Correctness of the mapping
MAPPET YES
USPTO YES

Reaction no 123 (3 bonds changed)

SMILES/SMARTS of the USPTO output:
[Br:29][CH2:30][c:31]1[cH:32][cH:33][cH:34][cH:35][cH:36]1.[C:19](=[O:20])([O-:21])[OH:22].[CH3:24][N:25]([CH3:26])[CH:27]=[O:28].[CH3:37][CH2:38][O:39][C:40](=[O:41])[CH3:42].[K+:23].[NH2:1][c:2]1[n+:3]([O-:18])[c:4]([CH2:14][CH:15]([CH3:16])[CH3:17])[c:5](=[O:13])[nH:6][c:7]1[C:8](=[O:9])[O:10][CH2:11][CH3:12]>>[NH2:1][c:2]1[n+:3]([O-:18])[c:4]([CH2:14][CH:15]([CH3:16])[CH3:17])[c:5]([O:13][CH2:30][c:31]2[cH:32][cH:33][cH:34][cH:35][cH:36]2)[n:6][c:7]1[C:8](=[O:9])[O:10][CH2:11][CH3:12]

SMILES/SMARTS of the MAPPET output:
[CH3:1][CH2:2][O:3][C:4](=[O:5])[c:6]1[nH:7][c:8](=[O:9])[c:10]([CH2:11][CH:12]([CH3:13])[CH3:14])[n+:15]([O-:16])[c:17]1[NH2:18].[Br:19][CH2:20][c:21]1[cH:22][cH:23][cH:24][cH:25][cH:26]1>>[CH3:1][CH2:2][O:3][C:4](=[O:5])[c:6]1[n:7][c:8]([O:9][CH2:20][c:21]2[cH:22][cH

:23][cH:24][cH:25][cH:26]2)[c:10]([CH2:11][CH:12]([CH3:14])[CH3:13])[n+:15]([O-
:16])[c:17]1[NH2:18].[Br:19]

Correctness of the mapping
MAPPET YES
USPTO YES

Reaction no 124 (3 bonds changed)

SMILES/SMARTS of the USPTO output:
[CH:1](=[O:2])[c:3]1[cH:4][c:5]([CH2:24][CH3:25])[c:6]([N:9]=[C:10]2[N:11]([CH:1
9]3[CH2:20][CH2:21][CH2:22][CH2:23]3)[C:12]3([CH2:13][S:14]2)[CH2:15][CH2:16][CH
2:17][CH2:18]3)[cH:7][cH:8]1.[O:26]=[C:27]([CH2:28][P:29](=[O:30])([O:31][CH2:32
][CH3:33])[O:34][CH2:35][CH3:36])[CH3:37]>>[CH:1]([c:3]1[cH:4][c:5]([CH2:24][CH3
:25])[c:6]([N:9]=[C:10]2[N:11]([CH:19]3[CH2:20][CH2:21][CH2:22][CH2:23]3)[C:12]3
([CH2:13][S:14]2)[CH2:15][CH2:16][CH2:17][CH2:18]3)[cH:7][cH:8]1)=[CH:28][C:27](
=[O:26])[CH3:37]

SMILES/SMARTS of the MAPPET output:
[CH3:1][CH2:2][O:3][P:4](=[O:5])([CH2:6][C:7]([CH3:8])=[O:9])[O:10][CH2:11][CH3:
12].[CH3:13][CH2:14][c:15]1[cH:16][c:17]([CH:18]=[O:19])[cH:20][cH:21][c:22]1[N:
23]=[C:24]1[S:25][CH2:26][C:27]2([CH2:28][CH2:29][CH2:30][CH2:31]2)[N:32]1[CH:33
]1[CH2:34][CH2:35][CH2:36][CH2:37]1>>[CH3:13][CH2:14][c:15]1[cH:16][c:17]([CH:18
]=[CH:6][C:7]([CH3:8])=[O:9])[cH:20][cH:21][c:22]1[N:23]=[C:24]1[S:25][CH2:26][C
:27]2([CH2:31][CH2:30][CH2:29][CH2:28]2)[N:32]1[CH:33]1[CH2:37][CH2:36][CH2:35][
CH2:34]1.[C:12].[C:11].[C:1].[C:2].[O:10].[O:5].[O:3].[O:19].[P:4]

Correctness of the mapping
MAPPET YES
USPTO YES

Reaction no 125 (3 bonds changed)

SMILES/SMARTS of the USPTO output:
[BrH:1].[CH2:27]1[O:28][CH:29]1[CH3:30].[CH2:2]([CH3:3])[O:4][C:5]([CH:6]([CH2:7
][c:8]1[n:9][c:10]2[cH:11][cH:12][cH:13][cH:14][c:15]2[n:16][c:17]1[CH2:18][P:19
](=[O:20])([OH:21])[OH:22])[NH2:23])=[O:24].[K+:26].[OH-
:25].[OH2:31]>>[O:4]=[C:5]([CH:6]([CH2:7][c:8]1[n:9][c:10]2[cH:11][cH:12][cH:13]
[cH:14][c:15]2[n:16][c:17]1[CH2:18][P:19](=[O:20])([OH:21])[OH:22])[NH2:23])[OH:
24]

SMILES/SMARTS of the MAPPET output:
[CH3:2][CH2:3][O:4][C:5](=[O:6])[CH:7]([NH2:8])[CH2:9][c:10]1[n:11][c:12]2[cH:13
][cH:14][cH:15][cH:16][c:17]2[n:18][c:19]1[CH2:20][P:21]([OH:22])([OH:23])=[O:24
].[OH2:1]>>[NH2:8][CH:7]([CH2:9][c:10]1[n:11][c:12]2[cH:13][cH:14][cH:15][cH:16]
[c:17]2[n:18][c:19]1[CH2:20][P:21]([OH:23])([OH:22])=[O:24])[C:5]([OH:1])=[O:6].
[CH3:2][CH2:3][OH:4]

Correctness of the mapping
MAPPET YES
USPTO NO

Reaction no 126 (3 bonds changed)

SMILES/SMARTS of the USPTO output:
[F:1][c:2]1[cH:3][cH:4][c:5]([C:8]2([C:14](=[O:15])[OH:16])[CH2:9][CH2:10][CH2:1
1][CH2:12][CH2:13]2)[cH:6][cH:7]1.[NH2:17][CH2:18][CH2:19][CH2:20][N:21]1[CH2:22
][CH2:23][CH:24]([c:27]2[cH:28][c:29]([NH:33][C:34]([CH2:35][CH3:36])=[O:37])[cH

:30][cH:31][cH:32]2)[CH2:25][CH2:26]1>>[F:1][c:2]1[cH:3][cH:4][c:5]([C:8]2([C:14](=[O:16])[NH:17][CH2:18][CH2:19][CH2:20][N:21]3[CH2:22][CH2:23][CH:24]([c:27]4[cH:28][c:29]([NH:33][C:34]([CH2:35][CH3:36])=[O:37])[cH:30][cH:31][cH:32]4)[CH2:25][CH2:26]3)[CH2:9][CH2:10][CH2:11][CH2:12][CH2:13]2)[cH:6][cH:7]1

SMILES/SMARTS of the MAPPET output:
[OH:22][C:23](=[O:24])[C:25]1([CH2:26][CH2:27][CH2:28][CH2:29][CH2:30]1)[c:31]1[cH:32][cH:33][c:34]([F:35])[cH:36][cH:37]1.[CH3:1][CH2:2][C:3](=[O:4])[NH:5][c:6]1[cH:7][cH:8][cH:9][c:10]([cH:11]1)[CH:12]1[CH2:13][CH2:14][N:15]([CH2:16][CH2:17][CH2:18][NH2:19])[CH2:20][CH2:21]1>>[CH3:1][CH2:2][C:3](=[O:4])[NH:5][c:6]1[cH:7][cH:8][cH:9][c:10]([cH:11]1)[CH:12]1[CH2:13][CH2:14][N:15]([CH2:16][CH2:17][CH2:18][NH:19][C:23](=[O:24])[C:25]2([CH2:26][CH2:27][CH2:28][CH2:29][CH2:30]2)[c:31]2[cH:37][cH:36][c:34]([F:35])[cH:33][cH:32]2)[CH2:20][CH2:21]1.[OH2:22]

Correctness of the mapping
MAPPET YES
USPTO NO

Reaction no 127 (3 bonds changed)

SMILES/SMARTS of the USPTO output:
[Cl:1][C:2]([Cl:3])=[S:4].[NH2:5][c:6]1[cH:7][cH:8][cH:9][c:10]([F:11])[cH:12]1>>[C:2](=[S:4])=[N:5][c:6]1[cH:7][cH:8][cH:9][c:10]([F:11])[cH:12]1

SMILES/SMARTS of the MAPPET output:
[NH2:1][c:2]1[cH:3][cH:4][cH:5][c:6]([F:7])[cH:8]1.[Cl:9][C:10]([Cl:11])=[S:12]>>[F:7][c:6]1[cH:5][cH:4][cH:3][c:2]([cH:8]1)[N:1]=[C:10]=[S:12].[Cl:11].[Cl:9]

Correctness of the mapping
MAPPET YES
USPTO YES

Reaction no 128 (3 bonds changed)

SMILES/SMARTS of the USPTO output:
[NH2:1][c:2]1[c:3]([C:13](=[O:14])[NH:15][CH2:16][CH2:17][CH3:18])[n:4][n:5][c:6]2[c:7]([Br:12])[cH:8][cH:9][cH:10][c:11]12.[nH:19]1[cH:20][cH:21][c:22]2[cH:23][c:24]([B:28]([OH:29])[OH:30])[cH:25][cH:26][c:27]12>>[NH2:1][c:2]1[c:3]([C:13](=[O:14])[NH:15][CH2:16][CH2:17][CH3:18])[n:4][n:5][c:6]2[c:7](-[c:24]3[cH:23][c:22]4[cH:21][cH:20][nH:19][c:27]4[cH:26][cH:25]3)[cH:8][cH:9][cH:10][c:11]12

SMILES/SMARTS of the MAPPET output:
[OH:1][B:2]([OH:3])[c:4]1[cH:5][cH:6][c:7]2[nH:8][cH:9][cH:10][c:11]2[cH:12]1.[CH3:13][CH2:14][CH2:15][NH:16][C:17](=[O:18])[c:19]1[n:20][n:21][c:22]2[c:23]([Br:24])[cH:25][cH:26][cH:27][c:28]2[c:29]1[NH2:30]>>[CH3:13][CH2:14][CH2:15][NH:16][C:17](=[O:18])[c:19]1[n:20][n:21][c:22]2[c:23]([cH:25][cH:26][cH:27][c:28]2[c:29]1[NH2:30])-[c:4]1[cH:5][cH:6][c:7]2[nH:8][cH:9][cH:10][c:11]2[cH:12]1.[B:2].[Br:24].[O:3].[O:1]

Correctness of the mapping
MAPPET YES
USPTO YES

Reaction no 129 (3 bonds changed)

SMILES/SMARTS of the USPTO output:

[CH:1]([CH3:2])([CH3:3])[CH:4]([C:5](=[O:6])[OH:7])[c:8]1[cH:9][cH:10][c:11]([O:14][C:15]([F:16])([F:17])[F:18])[cH:12][cH:13]1.[S:19]([Cl:20])([Cl:21])=[O:22].[cH:23]1[cH:24][cH:25][cH:26][cH:27][cH:28]1>>[CH:1]([CH3:2])([CH3:3])[CH:4]([C:5](=[O:6])[Cl:21])[c:8]1[cH:9][cH:10][c:11]([O:14][C:15]([F:16])([F:17])[F:18])[cH:12][cH:13]1

SMILES/SMARTS of the MAPPET output:
[Cl:1][S:2]([Cl:3])=[O:4].[CH3:5][CH:6]([CH3:7])[CH:8]([C:9]([OH:10])=[O:11])[c:12]1[cH:13][cH:14][c:15]([O:16][C:17]([F:18])([F:19])[F:20])[cH:21][cH:22]1>>[CH3:7][CH:6]([CH3:5])[CH:8]([C:9]([Cl:3])=[O:11])[c:12]1[cH:22][cH:21][c:15]([O:16][C:17]([F:18])([F:19])[F:20])[cH:14][cH:13]1.[Cl:1].[O:10].[O:4].[S:2]

Correctness of the mapping
MAPPET YES
USPTO YES

Reaction no 130 (3 bonds changed)

SMILES/SMARTS of the USPTO output:
[CH3:22][O:23][C:24]([CH3:25])([CH3:26])[CH3:27].[F:3][c:4]1[c:5]([B:18]([OH:19])[OH:20])[cH:6][cH:7][c:8]2[c:16]1[CH2:15][c:14]1[c:9]-2[cH:10][cH:11][cH:12][c:13]1[F:17].[OH2:21].[OH:1][OH:2]>>[OH:1][c:5]1[c:4]([F:3])[c:16]2[c:8]([cH:7][cH:6]1)-[c:9]1[cH:10][cH:11][cH:12][c:13]([F:17])[c:14]1[CH2:15]2

SMILES/SMARTS of the MAPPET output:
[OH:1][OH:2].[OH:3][B:4]([OH:5])[c:6]1[cH:7][cH:8][c:9]-2[c:10]([CH2:11][c:12]3[c:13]-2[cH:14][cH:15][cH:16][c:17]3[F:18])[c:19]1[F:20]>>[OH:2][c:6]1[cH:7][cH:8][c:9]-2[c:10]([CH2:11][c:12]3[c:13]-2[cH:14][cH:15][cH:16][c:17]3[F:18])[c:19]1[F:20].[B:4].[O:5].[O:3].[O:1]

Correctness of the mapping
MAPPET YES
USPTO YES

Reaction no 131 (3 bonds changed)

SMILES/SMARTS of the USPTO output:
[ClH:25].[ClH:26].[ClH:27].[F:1][c:2]1[cH:3][c:4]2[c:5]([c:6]([CH2:9][O:10][c:11]3[c:12]4[cH:13][c:14]([C:20](=[O:21])[OH:22])[nH:15][c:16]4[cH:17][cH:18][cH:19]3)[cH:7][o:8]2)[cH:23][cH:24]1.[NH2:28][CH:29]1[CH2:30][CH2:31][N:32]([CH2:35][CH:36]([CH3:37])[N:38]2[CH2:39][CH:40]([CH3:45])[CH:41]([OH:44])[CH2:42][CH2:43]2)[CH2:33][CH2:34]1>>[F:1][c:2]1[cH:3][c:4]2[c:5]([c:6]([CH2:9][O:10][c:11]3[c:12]4[cH:13][c:14]([C:20](=[O:22])[NH:28][CH:29]5[CH2:30][CH2:31][N:32]([CH2:35][CH:36]([CH3:37])[N:38]6[CH2:39][CH:40]([CH3:45])[CH:41]([OH:44])[CH2:42][CH2:43]6)[CH2:33][CH2:34]5)[nH:15][c:16]4[cH:17][cH:18][cH:19]3)[cH:7][o:8]2)[cH:23][cH:24]1

SMILES/SMARTS of the MAPPET output:
[OH:19][C:20](=[O:21])[c:22]1[cH:23][c:24]2[c:25]([O:26][CH2:27][c:28]3[cH:29][o:30][c:31]4[cH:32][c:33]([F:34])[cH:35][cH:36][c:37]34)[cH:38][cH:39][cH:40][c:41]2[nH:42]1.[CH3:1][CH:2]([CH2:3][N:4]1[CH2:5][CH2:6][CH:7]([NH2:8])[CH2:9][CH2:10]1)[N:11]1[CH2:12][CH2:13][CH:14]([OH:15])[CH:16]([CH3:17])[CH2:18]1>>[CH3:1][CH:2]([CH2:3][N:4]1[CH2:10][CH2:9][CH:7]([CH2:6][CH2:5]1)[NH:8][C:20](=[O:21])[c:22]1[cH:23][c:24]2[c:25]([O:26][CH2:27][c:28]3[cH:29][o:30][c:31]4[cH:32][c:33]([F:34])[cH:35][cH:36][c:37]34)[cH:38][cH:39][cH:40][c:41]2[nH:42]1)[N:11]1[CH2:12][CH2:13][CH:14]([OH:15])[CH:16]([CH3:17])[CH2:18]1.[OH2:19]

Correctness of the mapping
MAPPET YES
USPTO NO

Reaction no 132 (3 bonds changed)

SMILES/SMARTS of the USPTO output:
[Cl:16][c:17]1[cH:18][cH:19][c:20]([CH2:21][NH2:22])[cH:23][cH:24]1.[Cl:25][P:26]([Cl:27])[Cl:28].[OH2:29].[OH:1][c:2]1[c:3]([C:13](=[O:14])[OH:15])[cH:4][n:5][c:6]2[n:7][c:8]([CH3:12])[cH:9][cH:10][c:11]12>>[OH:1][c:2]1[c:3]([C:13](=[O:15])[NH:22][CH2:21][c:20]2[cH:19][cH:18][c:17]([Cl:16])[cH:24][cH:23]2)[cH:4][n:5][c:6]2[n:7][c:8]([CH3:12])[cH:9][cH:10][c:11]12

SMILES/SMARTS of the MAPPET output:
[CH3:10][c:11]1[cH:12][cH:13][c:14]2[c:15]([OH:16])[c:17]([cH:18][n:19][c:20]2[n:21]1)[C:22]([OH:23])=[O:24].[NH2:1][CH2:2][c:3]1[cH:4][cH:5][c:6]([Cl:7])[cH:8][cH:9]1>>[CH3:10][c:11]1[cH:12][cH:13][c:14]2[c:15]([OH:16])[c:17]([cH:18][n:19][c:20]2[n:21]1)[C:22](=[O:24])[NH:1][CH2:2][c:3]1[cH:9][cH:8][c:6]([Cl:7])[cH:5][cH:4]1.[OH2:23]

Correctness of the mapping
MAPPET YES
USPTO NO

Reaction no 133 (3 bonds changed)

SMILES/SMARTS of the USPTO output:
[CH2:30]([OH:31])[CH3:32].[CH3:1][O:2][C:3]([c:4]1[cH:5][c:6]([O:12][CH2:13][CH2:14][CH2:15][CH2:16][CH2:17][CH2:18][CH2:19][CH2:20][CH2:21][CH2:22][CH2:23][CH2:24][CH2:25][CH3:26])[c:7]([O:10][CH3:11])[cH:8][cH:9]1)=[O:27].[CH3:28][OH:29].[K+:34].[OH-:33].[OH2:35]>>[O:2]=[C:3]([c:4]1[cH:5][c:6]([O:12][CH2:13][CH2:14][CH2:15][CH2:16][CH2:17][CH2:18][CH2:19][CH2:20][CH2:21][CH2:22][CH2:23][CH2:24][CH2:25][CH3:26])[c:7]([O:10][CH3:11])[cH:8][cH:9]1)[OH:27]

SMILES/SMARTS of the MAPPET output:
[CH3:2][CH2:3][CH2:4][CH2:5][CH2:6][CH2:7][CH2:8][CH2:9][CH2:10][CH2:11][CH2:12][CH2:13][CH2:14][CH2:15][O:16][c:17]1[cH:18][c:19]([cH:20][cH:21][c:22]1[O:23][CH3:24])[C:25](=[O:26])[O:27][CH3:28].[OH2:1]>>[CH3:2][CH2:3][CH2:4][CH2:5][CH2:6][CH2:7][CH2:8][CH2:9][CH2:10][CH2:11][CH2:12][CH2:13][CH2:14][CH2:15][O:16][c:17]1[cH:18][c:19]([cH:20][cH:21][c:22]1[O:23][CH3:24])[C:25]([OH:1])=[O:26].[CH3:28][OH:27]

Correctness of the mapping
MAPPET YES
USPTO NO

Reaction no 134 (3 bonds changed)

SMILES/SMARTS of the USPTO output:
[CH3:18][c:19]1[cH:20][cH:21][c:22]([NH2:23])[cH:24][cH:25]1.[CH3:26][C:27](=[O:28])[OH:29].[N+:1](=[O:2])([O-:3])[c:4]1[c:5]2[c:6]([cH:12][c:13]([N+:15](=[O:16])[O-:17])[cH:14]1)[C:7](=[O:8])[O:9][C:10]2=[O:11]>>[N+:1](=[O:2])([O-:3])[c:4]1[c:5]([C:10]([OH:9])=[O:11])[c:6]([C:7]([OH:8])=[N:23][c:22]2[cH:21][cH:20][c:19]([CH3:18])[cH:25][cH:24]2)[cH:12][c:13]([N+:15](=[O:16])[O-:17])[cH:14]1

SMILES/SMARTS of the MAPPET output:
[CH3:1][c:2]1[cH:3][cH:4][c:5]([NH2:6])[cH:7][cH:8]1.[O-
:9][N+:10](=[O:11])[c:12]1[cH:13][c:14]2[C:15](=[O:16])[O:17][C:18](=[O:19])[c:2
0]2[c:21]([cH:22]1)[N+:23]([O-
:24])=[O:25]>>[CH3:1][c:2]1[cH:3][cH:4][c:5]([cH:7][cH:8]1)[N:6]=[C:15]([OH:16])
[c:14]1[cH:13][c:12]([cH:22][c:21]([c:20]1[C:18]([OH:17])=[O:19])[N+:23]([O-
:24])=[O:25])[N+:10]([O-:9])=[O:11]

Correctness of the mapping
MAPPET YES
USPTO YES

Reaction no 135 (3 bonds changed)

SMILES/SMARTS of the USPTO output:
[C:1](=[O:2])([O:3][C:4]([CH3:5])([CH3:6])[CH3:7])[N:8]1[CH2:9][CH2:10][CH:11]([
C:12](=[O:13])[OH:14])[CH2:15][CH2:16]1.[CH3:17][O-
:18].[CH3:53][OH:54].[CH3:58][N:59]([c:60]1[cH:61][cH:62][n:63][cH:64][cH:65]1)[
CH3:66].[CH3:67][N:68]([CH3:69])[CH:70]=[O:71].[CH:44]([N:45]([CH2:46][CH3:47])[
CH:48]([CH3:49])[CH3:50])([CH3:51])[CH3:52].[Cl:20][C:21]([C:22]([Cl:23])=[O:24]
)=[O:25].[Cl:55][CH2:56][Cl:57].[NH2:26][c:27]1[c:28]([C:29](=[O:30])[NH:31][c:3
2]2[n:33][cH:34][c:35]([Cl:38])[cH:36][cH:37]2)[cH:39][c:40]([I:43])[cH:41][cH:4
2]1.[Na+:19]>>[C:1](=[O:2])([O:3][C:4]([CH3:5])([CH3:6])[CH3:7])[N:8]1[CH2:9][CH
2:10][CH:11]([C:12](=[O:14])[NH:26][c:27]2[c:28]([C:29](=[O:30])[NH:31][c:32]3[n
:33][cH:34][c:35]([Cl:38])[cH:36][cH:37]3)[cH:39][c:40]([I:43])[cH:41][cH:42]2)[
CH2:15][CH2:16]1

SMILES/SMARTS of the MAPPET output:
[CH3:19][C:20]([CH3:21])([CH3:22])[O:23][C:24](=[O:25])[N:26]1[CH2:27][CH2:28][C
H:29]([CH2:30][CH2:31]1)[C:32]([OH:33])=[O:34].[NH2:1][c:2]1[cH:3][cH:4][c:5]([I
:6])[cH:7][c:8]1[C:9](=[O:10])[NH:11][c:12]1[cH:13][cH:14][c:15]([Cl:16])[cH:17]
[n:18]1>>[CH3:22][C:20]([CH3:21])([CH3:19])[O:23][C:24](=[O:25])[N:26]1[CH2:31][
CH2:30][CH:29]([CH2:28][CH2:27]1)[C:32](=[O:34])[NH:1][c:2]1[cH:3][cH:4][c:5]([I
:6])[cH:7][c:8]1[C:9](=[O:10])[NH:11][c:12]1[cH:13][cH:14][c:15]([Cl:16])[cH:17]
[n:18]1.[OH2:33]

Correctness of the mapping
MAPPET YES
USPTO NO

Reaction no 136 (3 bonds changed)

SMILES/SMARTS of the USPTO output:
[CH:28]([N:29]([CH2:30][CH3:31])[CH:32]([CH3:33])[CH3:34])([CH3:35])[CH3:36].[CH
:37]12[CH2:38][CH:39]([OH:45])[CH2:40][CH:41]([CH2:42][CH2:43]1)[NH:44]2.[N:1](=
[N+:2]=[N-
:3])[CH2:4][CH2:5][n:6]1[cH:7][n:8][c:9]2[c:10]1[cH:11][cH:12][c:13]([C:15](=[O:
16])[OH:17])[cH:14]2.[O:46]=[CH:47][N:48]([CH3:49])[CH3:50].[OH2:51].[OH:18][n:1
9]1[c:20]2[c:21]([cH:22][cH:23][cH:24][cH:25]2)[n:26][n:27]1>>[N:1](=[N+:2]=[N-
:3])[CH2:4][CH2:5][n:6]1[cH:7][n:8][c:9]2[c:10]1[cH:11][cH:12][c:13]([C:15](=[O:
17])[N:44]1[CH:37]3[CH2:38][CH:39]([OH:45])[CH2:40][CH:41]1[CH2:42][CH2:43]3)[cH
:14]2

SMILES/SMARTS of the MAPPET output:
[OH:10][C:11](=[O:12])[c:13]1[cH:14][cH:15][c:16]2[n:17]([CH2:18][CH2:19][N:20]=
[N+:21]=[N-
:22])[cH:23][n:24][c:25]2[cH:26]1.[OH:1][CH:2]1[CH2:3][CH:4]2[CH2:5][CH2:6][CH:7
]([CH2:8]1)[NH:9]2>>[OH:1][CH:2]1[CH2:8][CH:7]2[CH2:6][CH2:5][CH:4]([CH2:3]1)[N:

9]2[C:11](=[O:12])[c:13]1[cH:14][cH:15][c:16]2[n:17]([CH2:18][CH2:19][N:20]=[N+:
21]=[N-:22])[cH:23][n:24][c:25]2[cH:26]1.[OH2:10]

Correctness of the mapping
MAPPET YES
USPTO NO

Reaction no 137 (3 bonds changed)

SMILES/SMARTS of the USPTO output:
[CH2:16]1[O:17][CH2:18][CH2:19][CH2:20]1.[CH2:1]([CH3:2])[O:3][C:4]([C:5](=[O:6]
)[c:7]1[s:8][c:9]([Br:12])[cH:10][cH:11]1)=[O:13].[CH3:21][OH:22].[Na+:15].[OH-
:14].[OH2:23]>>[O:3]=[C:4]([C:5](=[O:6])[c:7]1[s:8][c:9]([Br:12])[cH:10][cH:11]1
)[OH:13]

SMILES/SMARTS of the MAPPET output:
[CH3:2][CH2:3][O:4][C:5](=[O:6])[C:7](=[O:8])[c:9]1[cH:10][cH:11][c:12]([Br:13])
[s:14]1.[OH2:1]>>[OH:1][C:5](=[O:6])[C:7](=[O:8])[c:9]1[cH:10][cH:11][c:12]([Br:
13])[s:14]1.[CH3:2][CH2:3][OH:4]

Correctness of the mapping
MAPPET YES
USPTO NO

Reaction no 138 (3 bonds changed)

SMILES/SMARTS of the USPTO output:
[CH2:12]([O:13][C:14]([Cl:15])=[O:16])[CH3:17].[CH2:18]([c:19]1[cH:20][cH:21][cH
:22][cH:23][cH:24]1)[N:25]1[CH2:26][CH2:27][CH:28]([O:31][CH2:32][CH2:33][CH2:34
][NH2:35])[CH2:29][CH2:30]1.[CH3:36][c:37]1[cH:38][cH:39][cH:40][cH:41][cH:42]1.
[n:1]1[cH:2][c:3]([CH:7]=[CH:8][C:9](=[O:10])[OH:11])[cH:4][cH:5][cH:6]1>>[n:1]1
[cH:2][c:3]([CH:7]=[CH:8][C:9](=[O:11])[NH:35][CH2:34][CH2:33][CH2:32][O:31][CH:
28]2[CH2:27][CH2:26][N:25]([CH2:18][c:19]3[cH:20][cH:21][cH:22][cH:23][cH:24]3)[
CH2:30][CH2:29]2)[cH:4][cH:5][cH:6]1

SMILES/SMARTS of the MAPPET output:
[OH:19][C:20](=[O:21])[CH:22]=[CH:23][c:24]1[cH:25][cH:26][cH:27][n:28][cH:29]1.
[NH2:1][CH2:2][CH2:3][CH2:4][O:5][CH:6]1[CH2:7][CH2:8][N:9]([CH2:10][c:11]2[cH:1
2][cH:13][cH:14][cH:15][cH:16]2)[CH2:17][CH2:18]1>>[O:21]=[C:20]([NH:1][CH2:2][C
H2:3][CH2:4][O:5][CH:6]1[CH2:7][CH2:8][N:9]([CH2:10][c:11]2[cH:12][cH:13][cH:14]
[cH:15][cH:16]2)[CH2:17][CH2:18]1)[CH:22]=[CH:23][c:24]1[cH:25][cH:26][cH:27][n:
28][cH:29]1.[OH2:19]

Correctness of the mapping
MAPPET YES
USPTO NO

Reaction no 139 (3 bonds changed)

SMILES/SMARTS of the USPTO output:
[Cl:1][c:2]1[cH:3][cH:4][c:5]2[c:6]([cH:7][c:8]([C:10](=[O:11])[OH:12])[o:9]2)[c
H:13]1.[S:14]([Cl:15])([Cl:16])=[O:17]>>[Cl:1][c:2]1[cH:3][cH:4][c:5]2[c:6]([cH:
7][c:8]([C:10](=[O:11])[Cl:16])[o:9]2)[cH:13]1

SMILES/SMARTS of the MAPPET output:
[Cl:1][S:2]([Cl:3])=[O:4].[OH:5][C:6](=[O:7])[c:8]1[cH:9][c:10]2[cH:11][c:12]([C
l:13])[cH:14][cH:15][c:16]2[o:17]1>>[Cl:3][C:6](=[O:7])[c:8]1[cH:9][c:10]2[cH:11
][c:12]([Cl:13])[cH:14][cH:15][c:16]2[o:17]1.[Cl:1].[O:5].[O:4].[S:2]

Correctness of the mapping
MAPPET YES
USPTO YES

Reaction no 140 (3 bonds changed)

SMILES/SMARTS of the USPTO output:
[CH2:17]([CH3:18])[O:19][CH2:20][CH2:21][n:22]1[c:23]([N:31]2[CH2:32][CH2:33][N:34]([CH2:38][CH2:39][C:40]3([c:45]4[cH:46][cH:47][cH:48][cH:49][cH:50]4)[CH2:41][NH:42][CH2:43][CH2:44]3)[CH2:35][CH2:36][CH2:37]2)[n:24][c:25]2[c:26]1[cH:27][cH:28][cH:29][cH:30]2.[CH3:1][O:2][c:3]1[c:4]([C:5](=[O:6])[OH:7])[cH:8][c:9]([S:12](=[O:13])(=[O:14])[CH3:15])[cH:10][cH:11]1.[ClH:16]>>[CH3:1][O:2][c:3]1[c:4]([C:5](=[O:7])[N:42]2[CH2:41][C:40]([CH2:39][CH2:38][N:34]3[CH2:33][CH2:32][N:31]([c:23]4[n:22]([CH2:21][CH2:20][O:19][CH2:17][CH3:18])[c:26]5[c:25]([n:24]4)[cH:30][cH:29][cH:28][cH:27]5)[CH2:37][CH2:36][CH2:35]3)([c:45]3[cH:46][cH:47][cH:48][cH:49][cH:50]3)[CH2:44][CH2:43]2)[cH:8][c:9]([S:12](=[O:13])(=[O:14])[CH3:15])[cH:10][cH:11]1

SMILES/SMARTS of the MAPPET output:
[CH3:35][O:36][c:37]1[cH:38][cH:39][c:40]([cH:41][c:42]1[C:43]([OH:44])=[O:45])[S:46]([CH3:47])(=[O:48])=[O:49].[CH3:1][CH2:2][O:3][CH2:4][CH2:5][n:6]1[c:7]([n:8][c:9]2[cH:10][cH:11][cH:12][cH:13][c:14]12)[N:15]1[CH2:16][CH2:17][CH2:18][N:19]([CH2:20][CH2:21][C:22]2([CH2:23][CH2:24][NH:25][CH2:26]2)[c:27]2[cH:28][cH:29][cH:30][cH:31][cH:32]2)[CH2:33][CH2:34]1>>[CH3:1][CH2:2][O:3][CH2:4][CH2:5][n:6]1[c:7]([n:8][c:9]2[cH:10][cH:11][cH:12][cH:13][c:14]12)[N:15]1[CH2:16][CH2:17][CH2:18][N:19]([CH2:20][CH2:21][C:22]2([CH2:23][CH2:24][N:25]([CH2:26]2)[C:43](=[O:45])[c:42]2[cH:41][c:40]([cH:39][cH:38][c:37]2[O:36][CH3:35])[S:46]([CH3:47])(=[O:48])=[O:49])[c:27]2[cH:28][cH:29][cH:30][cH:31][cH:32]2)[CH2:33][CH2:34]1.[OH2:44]

Correctness of the mapping
MAPPET YES
USPTO NO

Reaction no 141 (3 bonds changed)

SMILES/SMARTS of the USPTO output:
[CH3:1][O:2][C:3]([CH2:4][CH2:5][CH2:6][CH2:7][CH2:8][CH2:9][CH2:10][n:11]1[c:12](=[O:23])[o:13][c:14]2[c:15]1[cH:16][c:17]([N+:20](=[O:21])[O-:22])[cH:18][cH:19]2)=[O:24].[Na+:26].[OH-:25]>>[O:2]=[C:3]([CH2:4][CH2:5][CH2:6][CH2:7][CH2:8][CH2:9][CH2:10][n:11]1[c:12](=[O:23])[o:13][c:14]2[c:15]1[cH:16][c:17]([N+:20](=[O:21])[O-:22])[cH:18][cH:19]2)[OH:24]

SMILES/SMARTS of the MAPPET output:
[CH3:2][O:3][C:4](=[O:5])[CH2:6][CH2:7][CH2:8][CH2:9][CH2:10][CH2:11][CH2:12][n:13]1[c:14]2[cH:15][c:16]([cH:17][cH:18][c:19]2[o:20][c:21]1=[O:22])[N+:23]([O-:24])=[O:25].[OH2:1]>>[OH:1][C:4](=[O:5])[CH2:6][CH2:7][CH2:8][CH2:9][CH2:10][CH2:11][CH2:12][n:13]1[c:14]2[cH:15][c:16]([cH:17][cH:18][c:19]2[o:20][c:21]1=[O:22])[N+:23]([O-:24])=[O:25].[CH3:2][OH:3]

Correctness of the mapping
MAPPET YES
USPTO NO

Reaction no 142 (3 bonds changed)

SMILES/SMARTS of the USPTO output:
[Br:1][c:2]1[n:3][cH:4][c:5]([CH3:26])[cH:6][c:7]1[N:8]([S:9](=[O:10])(=[O:11])[c:12]1[cH:13][c:14]([C:19]([F:20])([F:21])[F:22])[c:15]([Cl:18])[cH:16][cH:17]1)[CH2:23][O:24][CH3:25].[CH2:37]1[O:38][CH2:39][CH2:40][CH2:41]1.[CH3:32][N:33]([CH:34]=[O:35])[CH3:36].[CH:28]([Mg+:29])([CH3:30])[CH3:31].[Cl-:27]>>[c:2]1([CH:34]=[O:35])[n:3][cH:4][c:5]([CH3:26])[cH:6][c:7]1[N:8]([S:9](=[O:10])(=[O:11])[c:12]1[cH:13][c:14]([C:19]([F:20])([F:21])[F:22])[c:15]([Cl:18])[cH:16][cH:17]1)[CH2:23][O:24][CH3:25]

SMILES/SMARTS of the MAPPET output:
[Cl-:1].[CH3:2][CH:3]([CH3:4])[Mg+:5].[CH3:6][N:7]([CH3:8])[CH:9]=[O:10].[CH3:11][O:12][CH2:13][N:14]([c:15]1[cH:16][c:17]([CH3:18])[cH:19][n:20][c:21]1[Br:22])[S:23](=[O:24])(=[O:25])[c:26]1[cH:27][cH:28][c:29]([Cl:30])[c:31]([cH:32]1)[C:33]([F:34])([F:35])[F:36]>>[CH3:11][O:12][CH2:13][N:14]([c:15]1[cH:16][c:17]([CH3:18])[cH:19][n:20][c:21]1[CH:9]=[O:10])[S:23](=[O:24])(=[O:25])[c:26]1[cH:27][cH:28][c:29]([Cl:30])[c:31]([cH:32]1)[C:33]([F:34])([F:35])[F:36].[Br:22].[C:6].[C:4].[C:2].[C:3].[C:8].[Cl:1].[Mg:5].[N:7]

Correctness of the mapping
MAPPET YES
USPTO YES

Reaction no 143 (3 bonds changed)

SMILES/SMARTS of the USPTO output:
[Br:1][c:2]1[cH:3][cH:4][c:5]([C:8](=[O:9])[OH:10])[n:6][cH:7]1.[CH3:22][N:23]1[CH2:24][CH2:25][O:26][CH2:27][CH2:28]1.[Cl:11][c:12]1[n:13][c:14]([O:15][CH3:16])[n:17][c:18]([O:19][CH3:20])[n:21]1.[Cl:37][CH2:38][Cl:39].[N:29]1([CH2:34][CH2:35][NH2:36])[CH2:30][CH2:31][CH2:32][CH2:33]1>>[Br:1][c:2]1[cH:3][cH:4][c:5]([C:8](=[O:10])[NH:36][CH2:35][CH2:34][N:29]2[CH2:30][CH2:31][CH2:32][CH2:33]2)[n:6][cH:7]1

SMILES/SMARTS of the MAPPET output:
[OH:9][C:10](=[O:11])[c:12]1[cH:13][cH:14][c:15]([Br:16])[cH:17][n:18]1.[NH2:1][CH2:2][CH2:3][N:4]1[CH2:5][CH2:6][CH2:7][CH2:8]1>>[Br:16][c:15]1[cH:14][cH:13][c:12]([n:18][cH:17]1)[C:10](=[O:11])[NH:1][CH2:2][CH2:3][N:4]1[CH2:8][CH2:7][CH2:6][CH2:5]1.[OH2:9]

Correctness of the mapping
MAPPET YES
USPTO NO

Reaction no 144 (3 bonds changed)

SMILES/SMARTS of the USPTO output:
[Br:1][c:2]1[cH:3][cH:4][c:5]([S:8](=[O:9])(=[O:10])[NH:11][CH3:12])[cH:6][cH:7]1.[C:28]([CH3:29])([CH3:30])([CH3:31])[O:32][C:33](=[O:34])[N:35]1[CH2:36][CH2:37][CH:38]([CH:41]=[O:42])[CH2:39][CH2:40]1.[CH2:43]1[O:44][CH2:45][CH2:46][CH2:47]1.[CH3:13][Li:14].[CH3:15][CH2:16][CH2:17][CH2:18][Li:19].[CH3:20][N:21]([CH3:22])[CH2:23][CH2:24][N:25]([CH3:26])[CH3:27]>>[c:2]1([CH:41]([CH:38]2[CH2:37][CH2:36][N:35]([C:33]([O:32][C:28]([CH3:29])([CH3:30])[CH3:31])=[O:34])[CH2:40][CH2:39]2)[OH:42])[cH:3][cH:4][c:5]([S:8](=[O:9])(=[O:10])[NH:11][CH3:12])[cH:6][cH:7]1

SMILES/SMARTS of the MAPPET output:
[CH3:1][C:2]([CH3:3])([CH3:4])[O:5][C:6](=[O:7])[N:8]1[CH2:9][CH2:10][CH:11]([CH2:12][CH2:13]1)[CH:14]=[O:15].[CH3:16][NH:17][S:18](=[O:19])(=[O:20])[c:21]1[cH:22][cH:23][c:24]([Br:25])[cH:26][cH:27]1>>[CH3:16][NH:17][S:18](=[O:20])(=[O:19]

)[c:21]1[cH:22][cH:23][c:24]([cH:26][cH:27]1)[CH:14]([OH:15])[CH:11]1[CH2:12][CH2:13][N:8]([CH2:9][CH2:10]1)[C:6](=[O:7])[O:5][C:2]([CH3:4])([CH3:3])[CH3:1].[Br:25]

Correctness of the mapping
MAPPET YES
USPTO YES

Reaction no 145 (3 bonds changed)

SMILES/SMARTS of the USPTO output:
[Cl:1][c:2]1[n:3][n:4][cH:5][cH:6][cH:7]1.[Cl:8][c:9]1[cH:10][cH:11][c:12](-[n:15]2[c:16](-[c:31]3[cH:32][cH:33][c:34]([B:37]4[O:38][C:39]([CH3:40])([CH3:41])[C:42]([CH3:43])([CH3:44])[O:45]4)[cH:35][cH:36]3)[n:17][c:18]3[c:19]([c:20]2=[O:21])[cH:22][n:23][n:24]3-[c:25]2[cH:26][cH:27][cH:28][cH:29][cH:30]2)[cH:13][cH:14]1>>[c:2]1(-[c:34]2[cH:33][cH:32][c:31](-[c:16]3[n:15](-[c:12]4[cH:11][cH:10][c:9]([Cl:8])[cH:14][cH:13]4)[c:20](=[O:21])[c:19]4[c:18]([n:17]3)[n:24](-[c:25]3[cH:26][cH:27][cH:28][cH:29][cH:30]3)[n:23][cH:22]4)[cH:36][cH:35]2)[n:3][n:4][cH:5][cH:6][cH:7]1

SMILES/SMARTS of the MAPPET output:
-
Correctness of the mapping
MAPPET NO
USPTO YES

Reaction no 146 (3 bonds changed)

SMILES/SMARTS of the USPTO output:
[C:1]([CH3:2])([CH3:3])([CH3:4])[O:5][C:6](=[O:7])[N:8]([CH3:9])[CH:10]([C:11](=[O:12])[OH:13])[CH2:14][c:15]1[cH:16][c:17]2[cH:18][cH:19][cH:20][cH:21][c:22]2[cH:23][cH:24]1.[CH3:36][N:37]([CH3:38])[CH2:39][CH2:40][CH2:41][N:42]=[C:43]=[N:44][CH2:45][CH3:46].[CH3:47][NH:48][CH2:49][CH2:50][c:51]1[c:52]([O:53][CH2:54][CH2:55][CH2:56][OH:57])[cH:58][cH:59][cH:60][cH:61]1.[CH3:62][N:63]([CH3:64])[CH:65]=[O:66].[CH3:70][CH2:71][O:72][C:73](=[O:74])[CH3:75].[Cl:67][CH2:68][Cl:69].[ClH:35].[OH:25][n:26]1[c:27]2[n:28][cH:29][cH:30][cH:31][c:32]2[n:33][n:34]1>>[C:1]([CH3:2])([CH3:3])([CH3:4])[O:5][C:6](=[O:7])[N:8]([CH3:9])[CH:10]([C:11](=[O:13])[N:48]([CH3:47])[CH2:49][CH2:50][c:51]1[c:52]([O:53][CH2:54][CH2:55][CH2:56][OH:57])[cH:58][cH:59][cH:60][cH:61]1)[CH2:14][c:15]1[cH:16][c:17]2[cH:18][cH:19][cH:20][cH:21][c:22]2[cH:23][cH:24]1

SMILES/SMARTS of the MAPPET output:
[CH3:16][N:17]([CH:18]([CH2:19][c:20]1[cH:21][cH:22][c:23]2[cH:24][cH:25][cH:26][cH:27][c:28]2[cH:29]1)[C:30]([OH:31])=[O:32])[C:33](=[O:34])[O:35][C:36]([CH3:37])([CH3:38])[CH3:39].[CH3:1][NH:2][CH2:3][CH2:4][c:5]1[cH:6][cH:7][cH:8][cH:9][c:10]1[O:11][CH2:12][CH2:13][CH2:14][OH:15]>>[CH3:1][N:2]([CH2:3][CH2:4][c:5]1[cH:6][cH:7][cH:8][cH:9][c:10]1[O:11][CH2:12][CH2:13][CH2:14][OH:15])[C:30](=[O:32])[CH:18]([CH2:19][c:20]1[cH:21][cH:22][c:23]2[cH:24][cH:25][cH:26][cH:27][c:28]2[cH:29]1)[N:17]([CH3:16])[C:33](=[O:34])[O:35][C:36]([CH3:37])([CH3:39])[CH3:38].[OH2:31]

Correctness of the mapping
MAPPET YES
USPTO NO

Reaction no 147 (3 bonds changed)

SMILES/SMARTS of the USPTO output:
[C:18]([O:19][C:20](=[O:21])[CH3:22])(=[O:23])[CH3:24].[OH2:17].[P:1](=[O:2])([OH:3])([OH:4])[OH:5].[s:6]1[c:7]([CH2:11][CH2:12][CH2:13][C:14](=[O:15])[OH:16])[cH:8][cH:9][cH:10]1>>[s:6]1[c:7]2[c:8]([cH:9][cH:10]1)[C:14](=[O:16])[CH2:13][CH2:12][CH2:11]2

SMILES/SMARTS of the MAPPET output:
[OH:1][C:2](=[O:3])[CH2:4][CH2:5][CH2:6][c:7]1[cH:8][cH:9][cH:10][s:11]1>>[O:3]=[C:2]1[CH2:4][CH2:5][CH2:6][c:7]2[s:11][cH:10][cH:9][c:8]12.[O:1]

Correctness of the mapping
MAPPET YES
USPTO NO

Reaction no 148 (3 bonds changed)

SMILES/SMARTS of the USPTO output:
[CH3:19][OH:20].[CH:10](=[O:11])[c:12]1[cH:13][cH:14][cH:15][cH:16][c:17]1[OH:18].[NH2:1][CH:2]([C:3]([CH3:4])([CH3:5])[SH:6])[C:7](=[O:8])[OH:9]>>[NH:1]1[CH:2]([C:7](=[O:8])[OH:9])[C:3]([CH3:4])([CH3:5])[S:6][CH:10]1[c:12]1[cH:13][cH:14][cH:15][cH:16][c:17]1[OH:18]

SMILES/SMARTS of the MAPPET output:
[CH3:1][C:2]([CH3:3])([SH:4])[CH:5]([NH2:6])[C:7]([OH:8])=[O:9].[OH:10][c:11]1[cH:12][cH:13][cH:14][cH:15][c:16]1[CH:17]=[O:18]>>[OH2:18].[CH3:3][C:2]1([CH3:1])[S:4][CH:17]([NH:6][CH:5]1[C:7]([OH:8])=[O:9])[c:16]1[cH:15][cH:14][cH:13][cH:12][c:11]1[OH:10]

Correctness of the mapping
MAPPET YES
USPTO YES

Reaction no 149 (3 bonds changed)

SMILES/SMARTS of the USPTO output:
[CH3:1][O:2][C:3](=[O:4])[c:5]1[s:6][cH:7][c:8]([Br:12])[c:9]1[O:10][CH3:11].[Na+:14].[O:15]1[CH2:16][CH2:17][CH2:18][CH2:19]1.[OH-:13]>>[O:2]=[C:3]([OH:4])[c:5]1[s:6][cH:7][c:8]([Br:12])[c:9]1[O:10][CH3:11]

SMILES/SMARTS of the MAPPET output:
[CH3:2][O:3][C:4](=[O:5])[c:6]1[s:7][cH:8][c:9]([Br:10])[c:11]1[O:12][CH3:13].[OH2:1]>>[CH3:13][O:12][c:11]1[c:9]([Br:10])[cH:8][s:7][c:6]1[C:4]([OH:1])=[O:5].[CH3:2][OH:3]

Correctness of the mapping
MAPPET YES
USPTO NO

Reaction no 150 (3 bonds changed)

SMILES/SMARTS of the USPTO output:
[C:1]([CH3:2])([CH3:3])([CH3:4])[c:5]1[cH:6][c:7]([CH2:17][CH2:18][OH:19])[c:8]2[c:12]([cH:13]1)[CH:11]([O:14][CH3:15])[CH:10]([CH3:16])[CH2:9]2.[CH2:39]1[O:40][CH2:41][CH2:42][CH2:43]1.[O:44]=[C:45]1[N:46]([Br:51])[C:47](=[O:48])[CH2:49][CH2:50]1.[c:20]1([P:21]([c:22]2[cH:23][cH:24][cH:25][cH:26][cH:27]2)[c:28]2[cH:29][cH:30][cH:31][cH:32][cH:33]2)[cH:34][cH:35][cH:36][cH:37][cH:38]1>>[C:1]([CH3:

2])([CH3:3])([CH3:4])[c:5]1[cH:6][c:7]([CH2:17][CH2:18][Br:51])[c:8]2[c:12]([cH:13]1)[CH:11]([O:14][CH3:15])[CH:10]([CH3:16])[CH2:9]2

SMILES/SMARTS of the MAPPET output:
-

Correctness of the mapping
MAPPET NO
USPTO YES

Reaction no 151 (4 bonds changed)

SMILES/SMARTS of the USPTO output:
[C:1]([CH3:2])([CH3:3])([CH3:4])[Si:5]([O:6][CH2:7][CH:8]([O:9][c:10]1[c:11]([C:33]([O:35][CH3:34])=[O:36])[s:12][c:13](-
[n:15]2[cH:16][n:17][c:18]3[cH:19][n:20][c:21]([CH2:24][O:25][Si:26]([CH3:27])([CH3:28])[C:29]([CH3:30])([CH3:31])[CH3:32])[cH:22][c:23]23)[cH:14]1)[c:37]1[c:38]([Cl:43])[cH:39][cH:40][cH:41][cH:42]1)([CH3:44])[CH3:45].[CH3:47][OH:48].[NH3:46]>>[C:1]([CH3:2])([CH3:3])([CH3:4])[Si:5]([O:6][CH2:7][CH:8]([O:9][c:10]1[c:11]([C:33](=[O:35])[NH2:46])[s:12][c:13](-
[n:15]2[cH:16][n:17][c:18]3[cH:19][n:20][c:21]([CH2:24][O:25][Si:26]([CH3:27])([CH3:28])[C:29]([CH3:30])([CH3:31])[CH3:32])[cH:22][c:23]23)[cH:14]1)[c:37]1[c:38]([Cl:43])[cH:39][cH:40][cH:41][cH:42]1)([CH3:44])[CH3:45]

SMILES/SMARTS of the MAPPET output:
[CH3:2][O:3][C:4](=[O:5])[c:6]1[s:7][c:8]([cH:9][c:10]1[O:11][CH:12]([CH2:13][O:14][Si:15]([CH3:16])([CH3:17])[C:18]([CH3:19])([CH3:20])[CH3:21])[c:22]1[cH:23][cH:24][cH:25][cH:26][c:27]1[Cl:28])-
[n:29]1[cH:30][n:31][c:32]2[cH:33][n:34][c:35]([CH2:36][O:37][Si:38]([CH3:39])([CH3:40])[C:41]([CH3:42])([CH3:43])[CH3:44])[cH:45][c:46]12.[NH3:1]>>[CH3:19][C:18]([CH3:21])([CH3:20])[Si:15]([CH3:17])([CH3:16])[O:14][CH2:13][CH:12]([O:11][c:10]1[cH:9][c:8]([s:7][c:6]1[C:4]([NH2:1])=[O:5])-
[n:29]1[cH:30][n:31][c:32]2[cH:33][n:34][c:35]([CH2:36][O:37][Si:38]([CH3:40])([CH3:39])[C:41]([CH3:42])([CH3:44])[CH3:43])[cH:45][c:46]12)[c:22]1[cH:23][cH:24][cH:25][cH:26][c:27]1[Cl:28].[CH3:2][OH:3]

Correctness of the mapping
MAPPET YES
USPTO NO

Reaction no 152 (4 bonds changed)

SMILES/SMARTS of the USPTO output:
[CH3:15][OH:16].[CH3:1][O:2][c:3]1[n:4][cH:5][cH:6][cH:7][c:8]1[CH2:9][C:10]#[N:11].[ClH:14].[Na+:13].[OH-
:12]>>[CH3:1][O:2][c:3]1[n:4][cH:5][cH:6][cH:7][c:8]1[CH2:9][C:10](=[O:12])[OH:16]

SMILES/SMARTS of the MAPPET output:
[OH2:1].[OH2:2].[CH3:3][O:4][c:5]1[n:6][cH:7][cH:8][cH:9][c:10]1[CH2:11][C:12]#[N:13]>>[CH3:3][O:4][c:5]1[n:6][cH:7][cH:8][cH:9][c:10]1[CH2:11][C:12]([OH:2])=[O:1].[N:13]

Correctness of the mapping
MAPPET YES
USPTO NO

Reaction no 153 (4 bonds changed)

SMILES/SMARTS of the USPTO output:
[Cl:26][CH2:27][CH2:28][N:29]([CH3:30])[CH2:31][CH2:32][Cl:33].[K+:34].[K+:35].[
NH2:1][CH2:2][c:3]1[cH:4][c:5]2[c:9]([c:10]([CH3:12])[cH:11]1)[C:8](=[O:13])[N:7
]([CH2:14][c:15]1[cH:16][cH:17][c:18]([O:21][C:22]([F:23])([F:24])[F:25])[cH:19]
[cH:20]1)[CH2:6]2.[O-:36][C:37]([O-
:38])=[O:39].[O:40]=[CH:41][N:42]([CH3:43])[CH3:44]>>[N:1]1([CH2:2][c:3]2[cH:4][
c:5]3[c:9]([c:10]([CH3:12])[cH:11]2)[C:8](=[O:13])[N:7]([CH2:14][c:15]2[cH:16][c
H:17][c:18]([O:21][C:22]([F:23])([F:24])[F:25])[cH:19][cH:20]2)[CH2:6]3)[CH2:27]
[CH2:28][N:29]([CH3:30])[CH2:31][CH2:32]1

SMILES/SMARTS of the MAPPET output:
[CH3:1][c:2]1[cH:3][c:4]([CH2:5][NH2:6])[cH:7][c:8]2[CH2:9][N:10]([CH2:11][c:12]
3[cH:13][cH:14][c:15]([O:16][C:17]([F:18])([F:19])[F:20])[cH:21][cH:22]3)[C:23](
=[O:24])[c:25]12.[CH3:26][N:27]([CH2:28][CH2:29][Cl:30])[CH2:31][CH2:32][Cl:33]>
>[CH3:26][N:27]1[CH2:28][CH2:29][N:6]([CH2:5][c:4]2[cH:7][c:8]3[CH2:9][N:10]([CH
2:11][c:12]4[cH:22][cH:21][c:15]([O:16][C:17]([F:18])([F:19])[F:20])[cH:14][cH:1
3]4)[C:23](=[O:24])[c:25]3[c:2]([CH3:1])[cH:3]2)[CH2:32][CH2:31]1.[Cl:33].[Cl:30
]

Correctness of the mapping
MAPPET YES
USPTO YES

Reaction no 154 (4 bonds changed)

SMILES/SMARTS of the USPTO output:
[CH3:14][OH:15].[CH3:16][CH2:17][O:18][C:19]([CH3:20])=[O:21].[CH3:1][O:2][C:3](
=[O:4])[C:5]1([CH2:11][CH:12]=[CH2:13])[CH2:6][CH2:7][O:8][CH2:9][CH2:10]1.[CH:2
2]([OH:23])([CH3:24])[CH3:25].[Cl:27][CH2:28][Cl:29].[O:30]=[Os:31](=[O:32])(=[O
:33])=[O:34].[OH2:26]>>[CH3:1][O:2][C:3](=[O:4])[C:5]1([CH2:11][CH:12]=[O:18])[C
H2:6][CH2:7][O:8][CH2:9][CH2:10]1

SMILES/SMARTS of the MAPPET output:
[OH2:1].[O:2]=[Os:3](=[O:4])(=[O:5])=[O:6].[CH3:7][O:8][C:9](=[O:10])[C:11]1([CH
2:12][CH:13]=[CH2:14])[CH2:15][CH2:16][O:17][CH2:18][CH2:19]1>>[CH3:7][O:8][C:9]
(=[O:10])[C:11]1([CH2:12][CH:13]=[O:6])[CH2:15][CH2:16][O:17][CH2:18][CH2:19]1.[
C:14].[O:5].[O:2].[O:4].[O:1].[Os:3]

Correctness of the mapping
MAPPET YES
USPTO NO

Reaction no 155 (4 bonds changed)

SMILES/SMARTS of the USPTO output:
[CH3:17][CH2:18][N:19]([CH2:20][CH3:21])[CH2:22][CH3:23].[CH3:1][C:2]1([CH3:16])
[O:3][B:4]([c:9]2[cH:10][cH:11][c:12]([NH2:13])[cH:14][cH:15]2)[O:5][C:6]1([CH3:
7])[CH3:8].[CH3:35][CH2:36][O:37][C:38]([CH3:39])=[O:40].[Cl:24][CH2:25][CH2:26]
[CH2:27][S:28](=[O:29])(=[O:30])[Cl:31].[Cl:32][CH2:33][Cl:34].[OH2:41]>>[CH3:1]
[C:2]1([CH3:16])[O:3][B:4]([c:9]2[cH:10][cH:11][c:12]([N:13]3[CH2:25][CH2:26][CH
2:27][S:28]3(=[O:29])=[O:30])[cH:14][cH:15]2)[O:5][C:6]1([CH3:7])[CH3:8]

SMILES/SMARTS of the MAPPET output:
[Cl:1][CH2:2][CH2:3][CH2:4][S:5]([Cl:6])(=[O:7])=[O:8].[CH3:9][C:10]1([CH3:11])[
O:12][B:13]([O:14][C:15]1([CH3:16])[CH3:17])[c:18]1[cH:19][cH:20][c:21]([NH2:22]
)[cH:23][cH:24]1>>[CH3:9][C:10]1([CH3:11])[O:12][B:13]([O:14][C:15]1([CH3:17])[C
H3:16])[c:18]1[cH:24][cH:23][c:21]([cH:20][cH:19]1)[N:22]1[CH2:2][CH2:3][CH2:4][
S:5]1(=[O:7])=[O:8].[Cl:6].[Cl:1]

Correctness of the mapping
MAPPET YES
USPTO YES

Reaction no 156 (4 bonds changed)

SMILES/SMARTS of the USPTO output:
[Br:1][c:2]1[cH:3][cH:4][c:5]2[c:6]([cH:30]1)[O:7][CH2:8][CH2:9][c:10]1[c:11]-
2[s:12][c:13]([C:15](=[O:16])[NH:17][c:18]2[c:19]([Cl:29])[cH:20][c:21]([C:24]([
N:25]([CH3:26])[CH3:27])=[O:28])[cH:22][cH:23]2)[cH:14]1.[C:31](=[O:32])([O-
:33])[O-
:34].[CH3:37][I:38].[Cs+:35].[Cs+:36].[O:39]=[CH:40][N:41]([CH3:42])[CH3:43]>>[B
r:1][c:2]1[cH:3][cH:4][c:5]2[c:6]([cH:30]1)[O:7][CH2:8][CH2:9][c:10]1[c:11]-
2[s:12][c:13]([C:15](=[O:16])[N:17]([c:18]2[c:19]([Cl:29])[cH:20][c:21]([C:24]([
N:25]([CH3:26])[CH3:27])=[O:28])[cH:22][cH:23]2)[CH3:31])[cH:14]1

SMILES/SMARTS of the MAPPET output:
[CH3:1][I:2].[CH3:3][N:4]([CH3:5])[C:6](=[O:7])[c:8]1[cH:9][cH:10][c:11]([NH:12]
[C:13](=[O:14])[c:15]2[cH:16][c:17]3[CH2:18][CH2:19][O:20][c:21]4[cH:22][c:23]([
Br:24])[cH:25][cH:26][c:27]4-
[c:28]3[s:29]2)[c:30]([Cl:31])[cH:32]1>>[CH3:5][N:4]([CH3:3])[C:6](=[O:7])[c:8]1
[cH:9][cH:10][c:11]([N:12]([CH3:1])[C:13](=[O:14])[c:15]2[cH:16][c:17]3[CH2:18][
CH2:19][O:20][c:21]4[cH:22][c:23]([Br:24])[cH:25][cH:26][c:27]4-
[c:28]3[s:29]2)[c:30]([Cl:31])[cH:32]1.[I:2]

Correctness of the mapping
MAPPET YES
USPTO NO

Reaction no 157 (4 bonds changed)

SMILES/SMARTS of the USPTO output:
[CH2:36]1[O:37][CH2:38][CH2:39][CH2:40]1.[CH3:1][N:2]([c:3]1[c:4]([C:12](=[O:13]
)[NH:14][CH2:15][CH2:16][O:17][c:18]2[cH:19][cH:20][c:21]([C:22]([O:24][CH3:23])
=[O:25])[cH:26][cH:27]2)[o:5][c:6]2[c:7]1[cH:8][cH:9][cH:10][cH:11]2)[CH3:28].[C
H3:34][OH:35].[ClH:33].[NH2:29][OH:30].[Na+:32].[OH-
:31]>>[CH3:1][N:2]([c:3]1[c:4]([C:12](=[O:13])[NH:14][CH2:15][CH2:16][O:17][c:18
]2[cH:19][cH:20][c:21]([C:22](=[O:24])[NH:29][OH:30])[cH:26][cH:27]2)[o:5][c:6]2
[c:7]1[cH:8][cH:9][cH:10][cH:11]2)[CH3:28]

SMILES/SMARTS of the MAPPET output:
[CH3:3][O:4][C:5](=[O:6])[c:7]1[cH:8][cH:9][c:10]([O:11][CH2:12][CH2:13][NH:14][
C:15](=[O:16])[c:17]2[o:18][c:19]3[cH:20][cH:21][cH:22][cH:23][c:24]3[c:25]2[N:2
6]([CH3:27])[CH3:28])[cH:29][cH:30]1.[NH2:1][OH:2]>>[CH3:27][N:26]([CH3:28])[c:2
5]1[c:17]([o:18][c:19]2[cH:20][cH:21][cH:22][cH:23][c:24]12)[C:15](=[O:16])[NH:1
4][CH2:13][CH2:12][O:11][c:10]1[cH:9][cH:8][c:7]([cH:30][cH:29]1)[C:5](=[O:6])[N
H:1][OH:2].[CH3:3][OH:4]

Correctness of the mapping
MAPPET YES
USPTO NO

Reaction no 158 (4 bonds changed)

SMILES/SMARTS of the USPTO output:
[CH3:13][Al:14]([CH3:15])[CH3:16].[CH3:17][O:18][C:19]([c:20]1[c:21]([NH:26][CH2
:27][c:28]2[cH:29][c:30]([NH:34][C:35](=[O:36])[N:37]([CH3:38])[CH3:39])[n:31][c

H:32][cH:33]2)[cH:22][cH:23][cH:24][cH:25]1)=[O:40].[CH3:1][n:2]1[n:3][c:4]2[cH:5][c:6]([NH2:12])[cH:7][cH:8][c:9]2[c:10]1[CH3:11].[Cl:46][CH2:47][Cl:48].[Na+:41].[OH:42][C:43](=[O:44])[O-:45]>>[CH3:1][n:2]1[n:3][c:4]2[cH:5][c:6]([NH:12][C:19](=[O:18])[c:20]3[c:21]([NH:26][CH2:27][c:28]4[cH:29][c:30]([NH:34][C:35](=[O:36])[N:37]([CH3:38])[CH3:39])[n:31][cH:32][cH:33]4)[cH:22][cH:23][cH:24][cH:25]3)[cH:7][cH:8][c:9]2[c:10]1[CH3:11]

SMILES/SMARTS of the MAPPET output:
[CH3:13][O:14][C:15](=[O:16])[c:17]1[cH:18][cH:19][cH:20][cH:21][c:22]1[NH:23][CH2:24][c:25]1[cH:26][cH:27][n:28][c:29]([NH:30][C:31](=[O:32])[N:33]([CH3:34])[CH3:35])[cH:36]1.[CH3:1][c:2]1[n:3]([CH3:4])[n:5][c:6]2[cH:7][c:8]([NH2:9])[cH:10][cH:11][c:12]12>>[CH3:34][N:33]([CH3:35])[C:31](=[O:32])[NH:30][c:29]1[cH:36][c:25]([CH2:24][NH:23][c:22]2[cH:21][cH:20][cH:19][cH:18][c:17]2[C:15](=[O:16])[NH:9][c:8]2[cH:10][cH:11][c:12]3[c:2]([CH3:1])[n:3]([CH3:4])[n:5][c:6]3[cH:7]2)[cH:26][cH:27][n:28]1.[CH3:13][OH:14]

Correctness of the mapping
MAPPET YES
USPTO NO

Reaction no 159 (4 bonds changed)

SMILES/SMARTS of the USPTO output:
[C:21](=[O:22])([O-:23])[OH:24].[CH3:26][OH:27].[CH:1]([CH3:2])([CH3:3])[c:4]1[cH:5][cH:6][c:7]([C:8](=[O:9])[OH:10])[cH:11][cH:12]1.[NH2:19][NH2:20].[Na+:25].[OH2:18].[S:13](=[O:14])(=[O:15])([OH:16])[OH:17]>>[CH:1]([CH3:2])([CH3:3])[c:4]1[cH:5][cH:6][c:7]([C:8](=[O:9])[O:10][CH3:21])[cH:11][cH:12]1

SMILES/SMARTS of the MAPPET output:
[CH3:3][CH:4]([CH3:5])[c:6]1[cH:7][cH:8][c:9]([cH:10][cH:11]1)[C:12]([OH:13])=[O:14].[CH3:1][OH:2]>>[CH3:1][O:2][C:12](=[O:14])[c:9]1[cH:8][cH:7][c:6]([cH:11][cH:10]1)[CH:4]([CH3:3])[CH3:5].[OH2:13]

Correctness of the mapping
MAPPET YES
USPTO NO

Reaction no 160 (4 bonds changed)

SMILES/SMARTS of the USPTO output:
[C:1]([CH3:2])([CH3:3])([CH3:4])[c:5]1[c:6]([OH:15])[c:7]([C:11]([CH3:12])([CH3:13])[CH3:14])[cH:8][cH:9][cH:10]1.[CH2:19]([CH:20]=[CH2:21])[Br:22].[CH2:26]([CH2:27][O:28][CH3:29])[O:30][CH3:31].[ClH:18].[H-:16].[Na+:17].[OH2:32].[S:23]=[C:24]=[S:25]>>[C:1]([CH3:2])([CH3:3])([CH3:4])[c:5]1[c:6]([OH:15])[c:7]([C:11]([CH3:12])([CH3:13])[CH3:14])[cH:8][c:9]([C:24](=[S:23])[S:25][CH2:19][CH:20]=[CH2:21])[cH:10]1

SMILES/SMARTS of the MAPPET output:
[S:1]=[C:2]=[S:3].[Br:4][CH2:5][CH:6]=[CH2:7].[CH3:8][C:9]([CH3:10])([CH3:11])[c:12]1[cH:13][cH:14][cH:15][c:16]([c:17]1[OH:18])[C:19]([CH3:20])([CH3:21])[CH3:22]>>[CH3:11][C:9]([CH3:10])([CH3:8])[c:12]1[cH:13][c:14]([cH:15][c:16]([c:17]1[OH:18])[C:19]([CH3:22])([CH3:21])[CH3:20])[C:2](=[S:1])[S:3][CH2:5][CH:6]=[CH2:7].[Br:4]

Correctness of the mapping
MAPPET YES
USPTO YES

Reaction no 161 (4 bonds changed)

SMILES/SMARTS of the USPTO output:
[CH3:14][CH2:15][OH:16].[N+:1]([CH2:4][CH:5]([CH2:6][C:7]([O:2][CH3:3])=[O:8])[CH2:11][CH2:12][CH3:13])([O-:9])=[O:10].[Pt:17]=[O:18]>>[NH:1]1[CH2:4][CH:5]([CH2:11][CH2:12][CH3:13])[CH2:6][C:7]1=[O:8]

SMILES/SMARTS of the MAPPET output:
[CH3:1][CH2:2][CH2:3][CH:4]([CH2:5][C:6](=[O:7])[O:8][CH3:9])[CH2:10][N+:11]([O-:12])=[O:13]>>[CH3:1][CH2:2][CH2:3][CH:4]1[CH2:10][NH:11][C:9](=[O:8])[CH2:5]1.[C:6].[O:12].[O:7].[O:13]

Correctness of the mapping
MAPPET NO
USPTO YES

Reaction no 162 (4 bonds changed)

SMILES/SMARTS of the USPTO output:
[CH2:33]1[O:34][CH2:35][CH2:36][CH2:37]1.[OH:1][C:2]([CH:3]([c:4]1[cH:5][cH:6][cH:7][cH:8][cH:9]1)[NH:10][S:11](=[O:12])(=[O:13])[c:14]1[cH:15][cH:16][c:17]([CH3:20])[cH:18][cH:19]1)([CH3:21])[CH3:22].[S:23](=[O:24])([Cl:25])[Cl:26].[cH:27]1[cH:28][cH:29][n:30][cH:31][cH:32]1>>[O:1]1[C:2]([CH3:21])([CH3:22])[CH:3]([c:4]2[cH:5][cH:6][cH:7][cH:8][cH:9]2)[N:10]([S:11](=[O:12])(=[O:13])[c:14]2[cH:15][cH:16][c:17]([CH3:20])[cH:18][cH:19]2)[S:23]1=[O:24]

SMILES/SMARTS of the MAPPET output:
[Cl:1][S:2]([Cl:3])=[O:4].[CH3:5][c:6]1[cH:7][cH:8][c:9]([cH:10][cH:11]1)[S:12](=[O:13])(=[O:14])[NH:15][CH:16]([c:17]1[cH:18][cH:19][cH:20][cH:21][cH:22]1)[C:23]([CH3:24])([CH3:25])[OH:26]>>[CH3:5][c:6]1[cH:11][cH:10][c:9]([cH:8][cH:7]1)[S:12](=[O:13])(=[O:14])[N:15]1[CH:16]([c:17]2[cH:22][cH:21][cH:20][cH:19][cH:18]2)[C:23]([CH3:25])([CH3:24])[O:26][S:2]1=[O:4].[Cl:3].[Cl:1]

Correctness of the mapping
MAPPET YES
USPTO YES

Reaction no 163 (4 bonds changed)

SMILES/SMARTS of the USPTO output:
[CH3:38][N:39]([CH3:40])[CH:41]=[O:42].[H-:10].[N+:12]([c:13]1[cH:14][cH:15][c:16]([O:21][C:22](=[O:17])[c:24]2[c:25]3[c:26]([c:27]([O:30][CH3:31])[n:28][cH:29]2)[o:32][c:33]([C:35]([CH3:36])=[O:37])[cH:34]3)[cH:18][cH:19]1)([O-:20])=[O:23].[NH2:1][c:2]1[c:3]([Cl:9])[cH:4][n:5][cH:6][c:7]1[Cl:8].[Na+:11]>>[NH:1]([c:2]1[c:3]([Cl:9])[cH:4][n:5][cH:6][c:7]1[Cl:8])[C:22](=[O:21])[c:24]1[c:25]2[c:26]([c:27]([O:30][CH3:31])[n:28][cH:29]1)[o:32][c:33]([C:35]([CH3:36])=[O:37])[cH:34]2

SMILES/SMARTS of the MAPPET output:
[CH3:10][O:11][c:12]1[n:13][cH:14][c:15]([C:16](=[O:17])[O:18][c:19]2[cH:20][cH:21][c:22]([cH:23][cH:24]2)[N+:25]([O-:26])=[O:27])[c:28]2[cH:29][c:30]([o:31][c:32]12)[C:33]([CH3:34])=[O:35].[NH2:1][c:2]1[c:3]([Cl:4])[cH:5][n:6][cH:7][c:8]1[Cl:9]>>[CH3:10][O:11][c:12]1[n:13][cH:14][c:15]([C:16](=[O:17])[NH:1][c:2]2[c:3]([Cl:4])[cH:5][n:6][cH:7][c:8]2[Cl:9]

)[c:28]2[cH:29][c:30]([o:31][c:32]12)[C:33]([CH3:34])=[O:35].[OH:18][c:19]1[cH:24][cH:23][c:22]([cH:21][cH:20]1)[N+:25]([O-:26])=[O:27]

Correctness of the mapping
MAPPET YES
USPTO NO

Reaction no 164 (4 bonds changed)

SMILES/SMARTS of the USPTO output:
[C:11]([O-:12])(=[O:13])[OH:14].[CH2:2]([c:3]1[cH:4][cH:5][cH:6][cH:7][cH:8]1)[NH:9][OH:10].[CH3:24][O:25][C:26]([CH3:27])([CH3:28])[CH3:29].[CH:16]([O:17][CH2:18][C:19]([F:20])([F:21])[F:22])=[O:23].[ClH:1].[K+:15]>>[CH2:2]([c:3]1[cH:4][cH:5][cH:6][cH:7][cH:8]1)[N:9]([OH:10])[CH:11]=[O:12]

SMILES/SMARTS of the MAPPET output:
[F:10][C:11]([F:12])([F:13])[CH2:14][O:15][CH:16]=[O:17].[OH:1][NH:2][CH2:3][c:4]1[cH:5][cH:6][cH:7][cH:8][cH:9]1>>[OH:1][N:2]([CH2:3][c:4]1[cH:5][cH:6][cH:7][cH:8][cH:9]1)[CH:16]=[O:17].[OH:15][CH2:14][C:11]([F:10])([F:12])[F:13]

Correctness of the mapping
MAPPET YES
USPTO NO

Reaction no 165 (4 bonds changed)

SMILES/SMARTS of the USPTO output:
[C:3]([O:4][BH-:5]([O:6][C:7](=[O:8])[CH3:9])[O:10][C:11](=[O:12])[CH3:13])(=[O:14])[CH3:15].[C:49](=[O:50])([OH:51])[O-:52].[CH2:1]=[O:2].[Cl:17][c:18]1[cH:19][c:20]2[c:21]([cH:47][cH:48]1)-[n:22]1[c:23]([CH:35]3[CH2:36][CH2:37][N:38]([c:41]4[n:42][cH:43][cH:44][cH:45][cH:46]4)[CH2:39][CH2:40]3)[n:24][n:25][c:26]1[CH2:27][N:28]([CH:30]1[CH2:31][NH:32][CH2:33][CH2:34]1)[CH2:29]2.[Cl:54][CH2:55][Cl:56].[Na+:16].[Na+:53]>>[CH3:3][N:32]1[CH2:31][CH:30]([N:28]2[CH2:27][c:26]3[n:22]([c:23]([CH:35]4[CH2:36][CH2:37][N:38]([c:41]5[n:42][cH:43][cH:44][cH:45][cH:46]5)[CH2:39][CH2:40]4)[n:24][n:25]3)-[c:21]3[c:20]([cH:19][c:18]([Cl:17])[cH:48][cH:47]3)[CH2:29]2)[CH2:34][CH2:33]1

SMILES/SMARTS of the MAPPET output:
[Cl:1][c:2]1[cH:3][cH:4][c:5]-2[c:6]([CH2:7][N:8]([CH2:9][c:10]3[n:11][n:12][c:13]([CH:14]4[CH2:15][CH2:16][N:17]([CH2:18][CH2:19]4)[c:20]4[cH:21][cH:22][cH:23][cH:24][n:25]4)[n:26]-23)[CH:27]2[CH2:28][CH2:29][NH:30][CH2:31]2)[cH:32]1.[CH2:33]=[O:34]>>[CH3:33][N:30]1[CH2:29][CH2:28][CH:27]([CH2:31]1)[N:8]1[CH2:9][c:10]2[n:11][n:12][c:13]([CH:14]3[CH2:19][CH2:18][N:17]([CH2:16][CH2:15]3)[c:20]3[cH:21][cH:22][cH:23][cH:24][n:25]3)[n:26]2-[c:5]2[cH:4][cH:3][c:2]([Cl:1])[cH:32][c:6]2[CH2:7]1.[O:34]

Correctness of the mapping
MAPPET YES
USPTO NO

Reaction no 166 (4 bonds changed)

SMILES/SMARTS of the USPTO output:
[CH2:1]([CH:2]=[CH2:3])[c:4]1[c:5]([O:18][C:19]([CH3:20])=[O:21])[cH:6][cH:7][c:8]2[cH:9][n:10][n:11]([C:13]([NH:14][CH2:15][CH3:16])=[O:17])[c:12]12.[Cl:22][c:

23]1[cH:24][cH:25][cH:26][c:27]([C:28]([O:29][OH:31])=[O:30])[cH:32]1.[Cl:33][CH2:34][Cl:35]>>[CH2:1]([CH:2]1[CH2:3][O:30]1)[c:4]1[c:5]([O:18][C:19]([CH3:20])=[O:21])[cH:6][cH:7][c:8]2[cH:9][n:10][n:11]([C:13]([NH:14][CH2:15][CH3:16])=[O:17])[c:12]12

SMILES/SMARTS of the MAPPET output:
-

Correct mapped SMILES/SMARTS of the reaction:
[CH3:16][CH2:15][NH:14][C:13](=[O:17])[n:11]1[n:10][cH:9][c:8]2[cH:7][cH:6][c:5]([O:18][C:19]([CH3:20])=[O:21])[c:4]([CH2:1][CH:2]=[CH2:3])[c:12]12.[OH:31][O:29][C:28](=[O:30])[c:27]1[cH:26][cH:25][cH:24][c:23]([Cl:22])[cH:32]1>>[CH3:16][CH2:15][NH:14][C:13](=[O:17])[n:11]1[n:10][cH:9][c:8]2[cH:7][cH:6][c:5]([O:18][C:19]([CH3:20])=[O:21])[c:4]([CH2:1][CH:2]3[CH2:3][O:31]3)[c:12]12.[OH:29][C:28](=[O:30])[c:27]1[cH:26][cH:25][cH:24][c:23]([Cl:22])[cH:32]1

Correctness of the mapping
MAPPET NO
USPTO NO

Reaction no 167 (4 bonds changed)

SMILES/SMARTS of the USPTO output:
[CH2:51]([Cl:52])[Cl:53].[Cl:1][c:2]1[cH:3][c:4]([CH:12]([C:13](=[O:14])[NH:15][c:16]2[n:17][n:18]([CH2:21][c:22]3[cH:23][c:24]([C:25](=[O:26])[OH:27])[cH:28][cH:29][cH:30]3)[cH:19][cH:20]2)[CH2:31][CH:32]2[CH2:33][CH2:34][CH2:35][CH2:36]2)[cH:5][cH:6][c:7]1[S:8](=[O:9])(=[O:10])[CH3:11].[Cl:37][C:38]([C:39]([Cl:40])=[O:41])=[O:42].[n:43]1[c:44]([CH3:45])[cH:46][cH:47][cH:48][c:49]1[CH3:50]>>[Cl:1][c:2]1[cH:3][c:4]([CH:12]([C:13](=[O:14])[NH:15][c:16]2[n:17][n:18]([CH2:21][c:22]3[cH:23][c:24]([C:25](=[O:27])[Cl:37])[cH:28][cH:29][cH:30]3)[cH:19][cH:20]2)[CH2:31][CH:32]2[CH2:33][CH2:34][CH2:35][CH2:36]2)[cH:5][cH:6][c:7]1[S:8](=[O:9])(=[O:10])[CH3:11]

SMILES/SMARTS of the MAPPET output:
[Cl:1][C:2](=[O:3])[C:4]([Cl:5])=[O:6].[CH3:7][S:8](=[O:9])(=[O:10])[c:11]1[cH:12][cH:13][c:14]([cH:15][c:16]1[Cl:17])[CH:18]([CH2:19][CH:20]1[CH2:21][CH2:22][CH2:23][CH2:24]1)[C:25](=[O:26])[NH:27][c:28]1[cH:29][cH:30][n:31]([CH2:32][c:33]2[cH:34][cH:35][cH:36][c:37]([cH:38]2)[C:39]([OH:40])=[O:41])[n:42]1>>[CH3:7][S:8](=[O:9])(=[O:10])[c:11]1[cH:12][cH:13][c:14]([cH:15][c:16]1[Cl:17])[CH:18]([CH2:19][CH:20]1[CH2:24][CH2:23][CH2:22][CH2:21]1)[C:25](=[O:26])[NH:27][c:28]1[cH:29][cH:30][n:31]([CH2:32][c:33]2[cH:34][cH:35][cH:36][c:37]([cH:38]2)[C:2]([Cl:5])=[O:3])[n:42]1.[C:39].[C:4].[Cl:1].[O:40].[O:6].[O:41]

Correct mapped SMILES/SMARTS of the reaction:
[CH3:11][S:8](=[O:9])(=[O:10])[c:7]1[cH:6][cH:5][c:4]([cH:3][c:2]1[Cl:1])[CH:12]([CH2:31][CH:32]1[CH2:33][CH2:34][CH2:35][CH2:36]1)[C:13](=[O:14])[NH:15][c:16]1[cH:20][cH:19][n:18]([CH2:21][c:22]2[cH:30][cH:29][cH:28][c:24]([cH:23]2)[C:25]([OH:27])=[O:26])[n:17]1.[Cl:37][C:38](=[O:42])[C:39]([Cl:40])=[O:41]>>[CH3:11][S:8](=[O:9])(=[O:10])[c:7]1[cH:6][cH:5][c:4]([cH:3][c:2]1[Cl:1])[CH:12]([CH2:31][CH:32]1[CH2:33][CH2:34][CH2:35][CH2:36]1)[C:13](=[O:14])[NH:15][c:16]1[cH:20][cH:19][n:18]([CH2:21][c:22]2[cH:30][cH:29][cH:28][c:24]([cH:23]2)[C:25]([Cl:37])=[O:26])[n:17]1.[O:27]=[C:38]=[O:42].[C+:39]#[O-:41].[Cl-:40]

Correctness of the mapping
MAPPET NO
USPTO NO

Reaction no 168 (4 bonds changed)

SMILES/SMARTS of the USPTO output:
[C:1]([c:2]1[cH:3][n:4][cH:5][cH:6][cH:7]1)([O:9][CH2:8][CH3:10])=[O:11].[CH3:14][C:15]#[N:16].[CH3:17][c:18]1[cH:19][cH:20][cH:21][cH:22][cH:23]1.[H-:13].[Na+:12]>>[C:1]([c:2]1[cH:3][n:4][cH:5][cH:6][cH:7]1)(=[O:9])[CH2:14][C:15]#[N:16]

SMILES/SMARTS of the MAPPET output:
[CH3:1][C:2]#[N:3].[CH3:4][CH2:5][O:6][C:7](=[O:8])[c:9]1[cH:10][cH:11][cH:12][n:13][cH:14]1>>[O:6]=[C:5]([CH2:4][C:2]#[N:3])[c:9]1[cH:10][cH:11][cH:12][n:13][cH:14]1.[C:1].[C:7].[O:8]

Correct mapped SMILES/SMARTS of the reaction:
[CH3:1][C:2]#[N:3].[CH3:4][CH2:5][O:6][C:7](=[O:8])[c:9]1[cH:10][cH:11][cH:12][n:13][cH:14]1>>[O:8]=[C:7]([CH2:1][C:2]#[N:3])[c:9]1[cH:10][cH:11][cH:12][n:13][cH:14]1.[CH3:4][CH2:5][OH:6]

Correctness of the mapping
MAPPET NO
USPTO NO

Reaction no 169 (4 bonds changed)

SMILES/SMARTS of the USPTO output:
[Br:1][c:2]1[c:3]([CH2:20][C:21](=[O:22])[NH:23][N:24]([c:25]2[c:26]([CH3:37])[cH:27][cH:28][cH:29][c:30]2[N:31]2[CH2:32][CH2:33][CH2:34][CH2:35][CH2:36]2)[c:38]2[cH:39][cH:40][c:41]([O:44][CH3:45])[cH:42][cH:43]2)[cH:4][c:5]([O:18][CH3:19])[c:6]([C:7]([O:9][N:8]2[C:10](=[O:11])[CH2:12][CH2:13][C:14]2=[O:15])=[O:16])[cH:17]1.[CH3:46][N:47]1[CH2:48][CH2:49][NH:50][CH2:51][CH2:52]1.[Cl:53][CH2:54][Cl:55]>>[Br:1][c:2]1[c:3]([CH2:20][C:21](=[O:22])[NH:23][N:24]([c:25]2[c:26]([CH3:37])[cH:27][cH:28][cH:29][c:30]2[N:31]2[CH2:32][CH2:33][CH2:34][CH2:35][CH2:36]2)[c:38]2[cH:39][cH:40][c:41]([O:44][CH3:45])[cH:42][cH:43]2)[cH:4][c:5]([O:18][CH3:19])[c:6]([C:7](=[O:9])[N:50]2[CH2:49][CH2:48][N:47]([CH3:46])[CH2:52][CH2:51]2)[cH:17]1

SMILES/SMARTS of the MAPPET output:
[CH3:8][O:9][c:10]1[cH:11][cH:12][c:13]([cH:14][cH:15]1)[N:16]([NH:17][C:18](=[O:19])[CH2:20][c:21]1[cH:22][c:23]([O:24][CH3:25])[c:26]([cH:27][c:28]1[Br:29])[C:30](=[O:31])[O:32][N:33]1[C:34](=[O:35])[CH2:36][CH2:37][C:38]1=[O:39])[c:40]1[c:41]([CH3:42])[cH:43][cH:44][cH:45][c:46]1[N:47]1[CH2:48][CH2:49][CH2:50][CH2:51][CH2:52]1.[CH3:1][N:2]1[CH2:3][CH2:4][NH:5][CH2:6][CH2:7]1>>[CH3:8][O:9][c:10]1[cH:11][cH:12][c:13]([cH:14][cH:15]1)[N:16]([NH:17][C:18](=[O:19])[CH2:20][c:21]1[cH:22][c:23]([O:24][CH3:25])[c:26]([cH:27][c:28]1[Br:29])[C:30](=[O:31])[N:5]1[CH2:4][CH2:3][N:2]([CH3:1])[CH2:7][CH2:6]1)[c:40]1[c:41]([CH3:42])[cH:43][cH:44][cH:45][c:46]1[N:47]1[CH2:48][CH2:49][CH2:50][CH2:51][CH2:52]1.[OH:32][N:33]1[C:38](=[O:39])[CH2:37][CH2:36][C:34]1=[O:35]

Correctness of the mapping
MAPPET YES
USPTO NO

Reaction no 170 (4 bonds changed)

SMILES/SMARTS of the USPTO output:
[CH2:12]([C:13]#[CH:14])[Br:15].[CH3:16][CH2:17][O:18][C:19](=[O:20])[CH3:21].[CH3:22][N:23]([CH3:24])[CH:25]=[O:26].[CH3:2][S:3][c:4]1[n:5][c:6](=[O:11])[nH:7][cH:8][c:9]1[F:10].[K:1]>>[CH3:2][S:3][c:4]1[n:5][c:6](=[O:11])[n:7]([CH2:14][C:13]#[CH:12])[cH:8][c:9]1[F:10]

SMILES/SMARTS of the MAPPET output:

[Br:11][CH2:12][C:13]#[CH:14].[CH3:1][S:2][c:3]1[n:4][c:5](=[O:6])[nH:7][cH:8][c:9]1[F:10]>>[CH3:1][S:2][c:3]1[n:4][c:5](=[O:6])[n:7]([CH2:12][C:13]#[CH:14])[cH:8][c:9]1[F:10].[BrH:11]

Correctness of the mapping
MAPPET YES
USPTO NO

Reaction no 171 (4 bonds changed)

SMILES/SMARTS of the USPTO output:
[CH3:25][O-:26].[CH3:30][OH:31].[ClH:22].[ClH:28].[NH2:23][OH:24].[Na+:27].[OH2:29].[c:1]1(-[c:7]2[c:8]([CH2:16][C:17]([O:19][CH2:18][CH3:20])=[O:21])[o:9][c:10]3[cH:14][cH:13][cH:12][c:11]-3[cH:15]2)[cH:2][cH:3][cH:4][cH:5][cH:6]1>>[c:1]1(-[c:7]2[c:8]([CH2:16][C:17](=[O:19])[NH:23][OH:24])[o:9][c:10]3[cH:14][cH:13][cH:12][c:11]-3[cH:15]2)[cH:2][cH:3][cH:4][cH:5][cH:6]1

SMILES/SMARTS of the MAPPET output:
[CH3:3][CH2:4][O:5][C:6](=[O:7])[CH2:8][c:9]1[o:10][c:11]-2[cH:12][cH:13][cH:14][c:15]-2[cH:16][c:17]1-[c:18]1[cH:19][cH:20][cH:21][cH:22][cH:23]1.[NH2:1][OH:2]>>[OH:2][NH:1][C:6](=[O:7])[CH2:8][c:9]1[o:10][c:11]-2[cH:12][cH:13][cH:14][c:15]-2[cH:16][c:17]1-[c:18]1[cH:19][cH:20][cH:21][cH:22][cH:23]1.[CH3:3][CH2:4][OH:5]

Correctness of the mapping
MAPPET YES
USPTO NO

Reaction no 172 (4 bonds changed)

SMILES/SMARTS of the USPTO output:
[CH2:1]([c:2]1[cH:3][cH:4][cH:5][cH:6][cH:7]1)[O:8][c:9]1[cH:10][cH:11][c:12]([S:15](=[O:16])(=[O:17])[N:18]2[CH:19]3[C:20](=[O:26])[O:21][CH:22]([CH2:23]2)[CH2:24][CH2:25]3)[cH:13][cH:14]1.[CH2:27]1[CH2:30][CH2:29][CH2:28][O:31]1.[CH3:35][OH:36].[ClH:32].[Li+:34].[OH-:33]>>[CH2:1]([c:2]1[cH:3][cH:4][cH:5][cH:6][cH:7]1)[O:8][c:9]1[cH:10][cH:11][c:12]([S:15](=[O:16])(=[O:17])[N:18]2[CH:19]([C:20](=[O:26])[OH:31])[CH2:25][CH2:24][CH:22]([OH:21])[CH2:23]2)[cH:13][cH:14]1

SMILES/SMARTS of the MAPPET output:
[O:1]=[C:2]1[O:3][CH:4]2[CH2:5][CH2:6][CH:7]1[N:8]([CH2:9]2)[S:10](=[O:11])(=[O:12])[c:13]1[cH:14][cH:15][c:16]([O:17][CH2:18][c:19]2[cH:20][cH:21][cH:22][cH:23][cH:24]2)[cH:25][cH:26]1.[OH2:27]>>[OH:3][CH:4]1[CH2:5][CH2:6][CH:7]([N:8]([CH2:9]1)[S:10](=[O:11])(=[O:12])[c:13]1[cH:26][cH:25][c:16]([O:17][CH2:18][c:19]2[cH:24][cH:23][cH:22][cH:21][cH:20]2)[cH:15][cH:14]1)[C:2]([OH:27])=[O:1]

Correctness of the mapping
MAPPET YES
USPTO NO

Reaction no 173 (4 bonds changed)

SMILES/SMARTS of the USPTO output:
[C:10]([O:11][CH2:12][c:13]1[cH:14][cH:15][cH:16][cH:17][cH:18]1)([O:19][N:21]1[C:22](=[O:23])[CH2:24][CH2:25][C:26]1=[O:27])=[O:20].[C:28](=[O:29])([O-:30])[O-:31].[CH2:34]1[O:35][CH2:36][CH2:37][O:38][CH2:39]1.[NH:1]1[CH2:2][CH:3]([C:7](=

[O:8])[OH:9])[CH2:4][CH2:5][CH2:6]1.[Na+:32].[Na+:33].[OH2:40]>>[N:1]1([C:10]([O
:11][CH2:12][c:13]2[cH:14][cH:15][cH:16][cH:17][cH:18]2)=[O:19])[CH2:2][CH:3]([C
:7](=[O:8])[OH:9])[CH2:4][CH2:5][CH2:6]1

SMILES/SMARTS of the MAPPET output:
[O:10]=[C:11]([O:12][CH2:13][c:14]1[cH:15][cH:16][cH:17][cH:18][cH:19]1)[O:20][N
:21]1[C:22](=[O:23])[CH2:24][CH2:25][C:26]1=[O:27].[OH:1][C:2](=[O:3])[CH:4]1[CH
2:5][CH2:6][CH2:7][NH:8][CH2:9]1>>[OH:1][C:2](=[O:3])[CH:4]1[CH2:5][CH2:6][CH2:7
][N:8]([CH2:9]1)[C:11](=[O:10])[O:12][CH2:13][c:14]1[cH:19][cH:18][cH:17][cH:16]
[cH:15]1.[OH:20][N:21]1[C:22](=[O:23])[CH2:24][CH2:25][C:26]1=[O:27]

Correctness of the mapping
MAPPET YES
USPTO NO

Reaction no 174 (4 bonds changed)

SMILES/SMARTS of the USPTO output:
[CH:1]([Li:2])([CH2:3][CH3:4])[CH3:5].[F:6][c:7]1[cH:8][cH:9][c:10]([F:11])[cH:1
2][cH:13]1.[O:25]1[CH2:26][CH2:27][CH2:28][CH2:29]1.[n:14]1[n:15][cH:16][c:17]2[
c:18]([cH:19]1)[C:20](=[O:21])[O:22][C:23]2=[O:24]>>[F:6][c:7]1[c:8]([C:20]([c:1
8]2[c:17]([C:23](=[O:22])[OH:24])[cH:16][n:15][n:14][cH:19]2)=[O:21])[cH:9][c:10
]([F:11])[cH:12][cH:13]1

SMILES/SMARTS of the MAPPET output:
[O:1]=[C:2]1[O:3][C:4](=[O:5])[c:6]2[cH:7][n:8][n:9][cH:10][c:11]12.[F:12][c:13]
1[cH:14][cH:15][c:16]([F:17])[cH:18][cH:19]1>>[OH:3][C:4](=[O:5])[c:6]1[cH:7][n:
8][n:9][cH:10][c:11]1[C:2](=[O:1])[c:18]1[cH:19][c:13]([F:12])[cH:14][cH:15][c:1
6]1[F:17]

Correctness of the mapping
MAPPET YES
USPTO YES

Reaction no 175 (4 bonds changed)

SMILES/SMARTS of the USPTO output:
[CH2:6]([CH:7]([CH3:8])[CH3:9])[n:10]1[c:11]2[n:12][c:13](-
[c:32]3[cH:33][n:34][c:35]([NH2:38])[n:36][cH:37]3)[n:14][c:15]([N:25]3[CH:26]([
CH3:31])[CH2:27][O:28][CH2:29][CH2:30]3)[c:16]2[n:17][c:18]1[N:19]1[CH2:20][CH2:
21][NH:22][CH2:23][CH2:24]1.[CH:52]([Cl:53])([Cl:54])[Cl:55].[Na+:51].[O:1]1[CH2
:2][CH2:5][CH2:4][CH2:3]1.[OH-
:50].[n:39]1([CH:40]=[O:41])[c:42]2[cH:43][cH:44][cH:45][cH:46][c:47]2[n:48][n:4
9]1>>[O:1]=[CH:2][N:22]1[CH2:21][CH2:20][N:19]([c:18]2[n:10]([CH2:6][CH:7]([CH3:
8])[CH3:9])[c:11]3[n:12][c:13](-
[c:32]4[cH:33][n:34][c:35]([NH2:38])[n:36][cH:37]4)[n:14][c:15]([N:25]4[CH:26]([
CH3:31])[CH2:27][O:28][CH2:29][CH2:30]4)[c:16]3[n:17]2)[CH2:24][CH2:23]1

SMILES/SMARTS of the MAPPET output:
-

Correct mapped SMILES/SMARTS of the reaction:
[CH3:8][CH:7]([CH3:9])[CH2:6][n:10]1[c:18]([n:17][c:16]2[c:15]([n:14][c:13]([n:1
2][c:11]12)-
[c:32]1[cH:33][n:34][c:35]([NH2:38])[n:36][cH:37]1)[N:25]1[CH2:30][CH2:29][O:28]
[CH2:27][CH:26]1[CH3:31])[N:19]1[CH2:20][CH2:21][NH:22][CH2:23][CH2:24]1.[O:41]=
[CH:40][n:39]1[n:49][n:48][c:47]2[cH:46][cH:45][cH:44][cH:43][c:42]12>>[CH3:8][C
H:7]([CH3:9])[CH2:6][n:10]1[c:18]([n:17][c:16]2[c:15]([n:14][c:13]([n:12][c:11]1
2)-

[c:32]1[cH:33][n:34][c:35]([NH2:38])[n:36][cH:37]1)[N:25]1[CH2:30][CH2:29][O:28]
[CH2:27][CH:26]1[CH3:31])[N:19]1[CH2:20][CH2:21][N:22]([CH2:23][CH2:24]1)[CH:40]
=[O:41].[cH:45]1[cH:44][cH:43][c:42]2[nH:39][n:49][n:48][c:47]2[cH:46]1

Correctness of the mapping
MAPPET NO
USPTO NO

Reaction no 176 (4 bonds changed)

SMILES/SMARTS of the USPTO output:
[CH2:5]([CH3:6])[N:7]1[CH2:8][CH2:9][N:10]([c:14]2[n:15][cH:16][c:17]([C:20]([O:
22][CH3:21])=[O:23])[cH:18][n:19]2)[CH2:11][CH2:12][CH2:13]1.[CH3:1][Al:2]([CH3:
3])[CH3:4].[CH3:24][O:25][c:26]1[cH:27][c:28]([CH2:34][CH2:35][c:36]2[cH:37][c:3
8]([NH2:41])[nH:39][n:40]2)[cH:29][c:30]([O:32][CH3:33])[cH:31]1.[CH3:42][c:43]1
[cH:44][cH:45][cH:46][cH:47][cH:48]1>>[CH2:5]([CH3:6])[N:7]1[CH2:8][CH2:9][N:10]
([c:14]2[n:15][cH:16][c:17]([C:20](=[O:22])[NH:41][c:38]3[cH:37][c:36]([CH2:35][
CH2:34][c:28]4[cH:27][c:26]([O:25][CH3:24])[cH:31][c:30]([O:32][CH3:33])[cH:29]4
)[n:40][nH:39]3)[cH:18][n:19]2)[CH2:11][CH2:12][CH2:13]1

SMILES/SMARTS of the MAPPET output:
[CH3:19][CH2:20][N:21]1[CH2:22][CH2:23][CH2:24][N:25]([CH2:26][CH2:27]1)[c:28]1[
n:29][cH:30][c:31]([cH:32][n:33]1)[C:34](=[O:35])[O:36][CH3:37].[CH3:1][O:2][c:3
]1[cH:4][c:5]([CH2:6][CH2:7][c:8]2[cH:9][c:10]([NH2:11])[nH:12][n:13]2)[cH:14][c
:15]([O:16][CH3:17])[cH:18]1>>[CH3:19][CH2:20][N:21]1[CH2:22][CH2:23][CH2:24][N:
25]([CH2:26][CH2:27]1)[c:28]1[n:33][cH:32][c:31]([cH:30][n:29]1)[C:34](=[O:35])[
NH:11][c:10]1[cH:9][c:8]([CH2:7][CH2:6][c:5]2[cH:4][c:3]([O:2][CH3:1])[cH:18][c:
15]([O:16][CH3:17])[cH:14]2)[n:13][nH:12]1.[CH3:37][OH:36]

Correctness of the mapping
MAPPET YES
USPTO NO

Reaction no 177 (4 bonds changed)

SMILES/SMARTS of the USPTO output:
[Cl:11][c:12]1[c:13]([O:35][CH3:36])[cH:14][c:15]([O:33][CH3:34])[c:16]([CH2:18]
[CH2:19][C:20]2([CH:28]3[CH2:29][CH2:30][CH2:31][CH2:32]3)[CH2:21][C:22](=[O:27]
)[CH2:23][C:24](=[O:26])[O:25]2)[cH:17]1.[Cl:1][CH2:2][c:3]1[n:4][c:5]([CH3:10])
[cH:6][c:7](=[O:9])[nH:8]1.[OH2:37]>>[CH2:2]([c:3]1[n:4][c:5]([CH3:10])[cH:6][c:
7](=[O:9])[nH:8]1)[C:23]1=[C:22]([OH:27])[CH2:21][C:20]([CH2:19][CH2:18][c:16]2[
c:15]([O:33][CH3:34])[cH:14][c:13]([O:35][CH3:36])[c:12]([Cl:11])[cH:17]2)([CH:2
8]2[CH2:29][CH2:30][CH2:31][CH2:32]2)[O:25][C:24]1=[O:26]

SMILES/SMARTS of the MAPPET output:
[CH3:1][c:2]1[cH:3][c:4](=[O:5])[nH:6][c:7]([CH2:8][Cl:9])[n:10]1.[CH3:11][O:12]
[c:13]1[cH:14][c:15]([O:16][CH3:17])[c:18]([CH2:19][CH2:20][C:21]2([CH2:22][C:23
](=[O:24])[CH2:25][C:26](=[O:27])[O:28]2)[CH:29]2[CH2:30][CH2:31][CH2:32][CH2:33
]2)[cH:34][c:35]1[Cl:36]>>[CH3:11][O:12][c:13]1[cH:14][c:15]([O:16][CH3:17])[c:1
8]([CH2:19][CH2:20][C:21]2([CH2:22][C:23]([OH:24])=[C:25]([CH2:8][c:7]3[n:10][c:
2]([CH3:1])[cH:3][c:4](=[O:5])[nH:6]3)[C:26](=[O:27])[O:28]2)[CH:29]2[CH2:33][CH
2:32][CH2:31][CH2:30]2)[cH:34][c:35]1[Cl:36].[Cl:9]

Correctness of the mapping
MAPPET YES
USPTO YES

Reaction no 178 (4 bonds changed)

SMILES/SMARTS of the USPTO output:
[C:16]1([CH2:22][CH2:23][C:24](=[O:25])[CH:26]2[CH2:27][CH2:28][CH2:29][CH2:30]2)=[CH:17][CH2:18][CH2:19][CH2:20][CH2:21]1.[C:1]([CH2:2][C:3](=[O:4])[CH3:5])(=[O:6])[O:7][CH3:8].[C:31](=[O:32])([O-:33])[O-:34].[CH2:37]1[O:38][CH2:39][CH2:40][CH2:41]1.[CH3:11][CH2:12][CH2:13][CH2:14][Li:15].[H-:10].[K+:35].[K+:36].[Na+:9]>>[C:1]1(=[O:6])[CH2:2][C:3](=[O:4])[CH2:5][C:24]([CH2:23][CH2:22][C:16]2=[CH:17][CH2:18][CH2:19][CH2:20][CH2:21]2)([CH:26]2[CH2:27][CH2:28][CH2:29][CH2:30]2)[O:25]1

SMILES/SMARTS of the MAPPET output:
[CH3:1][O:2][C:3](=[O:4])[CH2:5][C:6]([CH3:7])=[O:8].[O:9]=[C:10]([CH2:11][CH2:12][C:13]1=[CH:14][CH2:15][CH2:16][CH2:17][CH2:18]1)[CH:19]1[CH2:20][CH2:21][CH2:22][CH2:23]1>>[O:8]=[C:6]1[CH2:5][C:3](=[O:4])[O:2][C:1]([CH2:11][CH2:12][C:13]2=[CH:14][CH2:15][CH2:16][CH2:17][CH2:18]2)([CH2:7]1)[CH:19]1[CH2:23][CH2:22][CH2:21][CH2:20]1.[C:10].[O:9]

Correctness of the mapping
MAPPET NO
USPTO YES

Reaction no 179 (4 bonds changed)

SMILES/SMARTS of the USPTO output:
[CH3:1][O:2][c:3]1[cH:4][c:5]2[c:6]([O:18][c:19]3[cH:20][c:21]([NH2:22])[cH:23][cH:24][cH:25]3)[n:7][cH:8][n:9][c:10]2[cH:11][c:12]1[O:13][CH2:14][CH2:15][O:16][CH3:17].[CH:26]([CH3:27])([CH3:28])[c:29]1[cH:30][c:31]([NH:34][C:35]([O:36][c:38]2[cH:39][cH:40][cH:41][cH:42][cH:43]2)=[O:37])[n:32][o:33]1>>[CH3:1][O:2][c:3]1[cH:4][c:5]2[c:6]([O:18][c:19]3[cH:20][c:21]([NH:22][C:35]([NH:34][c:31]4[cH:30][c:29]([CH:26]([CH3:27])[CH3:28])[o:33][n:32]4)=[O:36])[cH:23][cH:24][cH:25]3)[n:7][cH:8][n:9][c:10]2[cH:11][c:12]1[O:13][CH2:14][CH2:15][O:16][CH3:17]

SMILES/SMARTS of the MAPPET output:
[CH3:26][CH:27]([CH3:28])[c:29]1[cH:30][c:31]([NH:32][C:33](=[O:34])[O:35][c:36]2[cH:37][cH:38][cH:39][cH:40][cH:41]2)[n:42][o:43]1.[CH3:1][O:2][CH2:3][CH2:4][O:5][c:6]1[cH:7][c:8]2[n:9][cH:10][n:11][c:12]([O:13][c:14]3[cH:15][cH:16][cH:17][c:18]([NH2:19])[cH:20]3)[c:21]2[cH:22][c:23]1[O:24][CH3:25]>>[CH3:1][O:2][CH2:3][CH2:4][O:5][c:6]1[cH:7][c:8]2[n:9][cH:10][n:11][c:12]([O:13][c:14]3[cH:15][cH:16][cH:17][c:18]([NH:19][C:33](=[O:34])[NH:32][c:31]4[cH:30][c:29]([o:43][n:42]4)[CH:27]([CH3:28])[CH3:26])[cH:20]3)[c:21]2[cH:22][c:23]1[O:24][CH3:25].[OH:35][c:36]1[cH:37][cH:38][cH:39][cH:40][cH:41]1

Correctness of the mapping
MAPPET YES
USPTO NO

Reaction no 180 (4 bonds changed)

SMILES/SMARTS of the USPTO output:
[CH2:1]([CH2:2][CH2:3][CH2:4][CH3:5])[CH:6]1[CH2:7][CH2:8][CH:9]([c:12]2[cH:13][cH:14][c:15](-[c:18]3[cH:19][cH:20][c:21]([C:24](=[O:25])[OH:26])[cH:22][cH:23]3)[cH:16][cH:17]2)[CH2:10][CH2:11]1.[Cl-:31].[Cl:32][c:33]1[c:34]([CH:35]=[O:36])[cH:37][cH:38][c:39]([OH:41])[cH:40]1.[S:27]([Cl:28])([Cl:29])=[O:30]>>[CH2:1]([CH2:2][CH2:3][CH2:4][CH3:5])[CH:6]1[CH2:7][CH2:8][CH:9]([c:12]2[cH:13][cH:14][c:15](-[c:18]3[cH:19][cH:20][c:21]([C:24]([O:25][c:39]4[cH:38][cH:37][c:34]([CH:35]=[O:

36])[c:33]([Cl:32])[cH:40]4)=[O:26])[cH:22][cH:23]3)[cH:16][cH:17]2)[CH2:10][CH2:11]1

SMILES/SMARTS of the MAPPET output:
[CH3:11][CH2:12][CH2:13][CH2:14][CH2:15][CH:16]1[CH2:17][CH2:18][CH:19]([CH2:20][CH2:21]1)[c:22]1[cH:23][cH:24][c:25]([cH:26][cH:27]1)-[c:28]1[cH:29][cH:30][c:31]([cH:32][cH:33]1)[C:34]([OH:35])=[O:36].[OH:1][c:2]1[cH:3][cH:4][c:5]([CH:6]=[O:7])[c:8]([Cl:9])[cH:10]1>>[CH3:11][CH2:12][CH2:13][CH2:14][CH2:15][CH:16]1[CH2:17][CH2:18][CH:19]([CH2:20][CH2:21]1)[c:22]1[cH:23][cH:24][c:25]([cH:26][cH:27]1)-[c:28]1[cH:29][cH:30][c:31]([cH:32][cH:33]1)[C:34](=[O:36])[O:1][c:2]1[cH:3][cH:4][c:5]([CH:6]=[O:7])[c:8]([Cl:9])[cH:10]1.[OH2:35]

Correctness of the mapping
MAPPET YES
USPTO NO

Reaction no 181 (4 bonds changed)

SMILES/SMARTS of the USPTO output:
[C:1]([c:2]1[cH:3][cH:4][cH:5][cH:6][cH:7]1)(=[O:8])[S:9][CH2:10][CH:11]([C:12](=[O:13])[N:14]1[CH:15]([C:16](=[O:17])[OH:18])[CH2:19][CH:20]([O:22][c:23]2[cH:24][cH:25][c:26]([CH:29]=[CH:30][O:31][CH3:32])[cH:27][cH:28]2)[CH2:21]1)[CH3:33].[CH3:51][O:52][c:53]1[cH:54][cH:55][cH:56][cH:57][cH:58]1.[CH3:70][C:71]#[N:72].[NH2:34][c:35]1[c:36]([S:46](=[O:47])(=[O:48])[NH:49][CH3:50])[cH:37][c:38]([S:42](=[O:43])(=[O:44])[NH2:45])[c:39]([Cl:41])[cH:40]1.[c:59]1([CH3:60])[cH:61][cH:62][c:63]([S:64]([OH:65])(=[O:66])=[O:67])[cH:68][cH:69]1>>[C:1]([c:2]1[cH:3][cH:4][cH:5][cH:6][cH:7]1)(=[O:8])[S:9][CH2:10][CH:11]([C:12](=[O:13])[N:14]1[CH:15]([C:16](=[O:17])[OH:18])[CH2:19][CH:20]([O:22][c:23]2[cH:24][cH:25][c:26]([CH2:29][CH:30]3[NH:34][c:35]4[c:36]([cH:37][c:38]([S:42](=[O:43])(=[O:44])[NH2:45])[c:39]([Cl:41])[cH:40]4)[S:46](=[O:47])(=[O:48])[N:49]3[CH3:50])[cH:27][cH:28]2)[CH2:21]1)[CH3:33]

SMILES/SMARTS of the MAPPET output:
[CH3:1][NH:2][S:3](=[O:4])(=[O:5])[c:6]1[cH:7][c:8]([c:9]([Cl:10])[cH:11][c:12]1[NH2:13])[S:14]([NH2:15])(=[O:16])=[O:17].[CH3:18][O:19][CH:20]=[CH:21][c:22]1[cH:23][cH:24][c:25]([O:26][CH:27]2[CH2:28][CH:29]([N:30]([CH2:31]2)[C:32](=[O:33])[CH:34]([CH3:35])[CH2:36][S:37][C:38](=[O:39])[c:40]2[cH:41][cH:42][cH:43][cH:44][cH:45]2)[C:46]([OH:47])=[O:48])[cH:49][cH:50]1>>[CH3:35][CH:34]([CH2:36][S:37][C:38](=[O:39])[c:40]1[cH:45][cH:44][cH:43][cH:42][cH:41]1)[C:32](=[O:33])[N:30]1[CH2:31][CH:27]([CH2:28][CH:29]1[C:46]([OH:47])=[O:48])[O:26][c:25]1[cH:49][cH:50][c:22]([CH2:21][CH:20]2[NH:13][c:12]3[cH:11][c:9]([Cl:10])[c:8]([cH:7][c:6]3[S:3](=[O:5])(=[O:4])[N:2]2[CH3:1])[S:14]([NH2:15])(=[O:17])=[O:16])[cH:23][cH:24]1.[C:18].[O:19]

Correctness of the mapping
MAPPET YES
USPTO YES

Reaction no 182 (4 bonds changed)

SMILES/SMARTS of the USPTO output:
[C:22](=[O:23])([O-:24])[OH:25].[CH3:13][CH2:14][N:15]([CH2:16][CH3:17])[CH2:18][CH3:19].[CH3:20][I:21].[NH2:1][CH2:2][CH2:3][CH2:4][NH:5][C:6]([O:7][C:8]([CH3:9])([CH3:10])[CH3:11])=[O:12].[Na+:26].[O:30]1[CH2:31][CH2:32][CH2:33][CH2:34]1.[S:27]=[C:28]=[S:29]>>[NH:1]([CH2:2][CH2:3][CH2:4][NH:5][C:6]([O:7][C:8]([CH3:9])([CH3:10])[CH3:11])=[O:12])[C:28]([S:27][CH3:20])=[S:29]

SMILES/SMARTS of the MAPPET output:
[S:1]=[C:2]=[S:3].[CH3:4][C:5]([CH3:6])([CH3:7])[O:8][C:9](=[O:10])[NH:11][CH2:1
2][CH2:13][CH2:14][NH2:15].[CH3:16][I:17]>>[CH3:16][S:3][C:2](=[S:1])[NH:15][CH2
:14][CH2:13][CH2:12][NH:11][C:9](=[O:10])[O:8][C:5]([CH3:7])([CH3:6])[CH3:4].[I:
17]

Correctness of the mapping
MAPPET YES
USPTO YES

Reaction no 183 (4 bonds changed)

SMILES/SMARTS of the USPTO output:
[CH:1]([CH3:2])([CH3:3])[c:4]1[cH:5][c:6]2[cH:7][cH:8][cH:9][n:10][c:11]2[c:12](
-
[c:14]2[cH:15][c:16]([CH:20]=[CH2:21])[cH:17][cH:18][cH:19]2)[cH:13]1.[N+:22](=[
N-
:23])=[C:24]([c:25]1[cH:26][cH:27][c:28]([Cl:31])[cH:29][cH:30]1)[c:32]1[cH:33][
cH:34][c:35]([Cl:38])[cH:36][cH:37]1.[cH:39]1[cH:40][cH:41][cH:42][cH:43][cH:44]
1>>[CH:1]([CH3:2])([CH3:3])[c:4]1[cH:5][c:6]2[cH:7][cH:8][cH:9][n:10][c:11]2[c:1
2](-
[c:14]2[cH:15][c:16]([CH:20]3[CH2:21][C:24]3([c:25]3[cH:26][cH:27][c:28]([Cl:31]
)[cH:29][cH:30]3)[c:32]3[cH:33][cH:34][c:35]([Cl:38])[cH:36][cH:37]3)[cH:17][cH:
18][cH:19]2)[cH:13]1

SMILES/SMARTS of the MAPPET output:
[Cl:1][c:2]1[cH:3][cH:4][c:5]([cH:6][cH:7]1)[C:8](=[N+:9]=[N-
:10])[c:11]1[cH:12][cH:13][c:14]([Cl:15])[cH:16][cH:17]1.[CH3:18][CH:19]([CH3:20
])[c:21]1[cH:22][c:23](-
[c:24]2[cH:25][cH:26][cH:27][c:28]([CH:29]=[CH2:30])[cH:31]2)[c:32]2[n:33][cH:34
][cH:35][cH:36][c:37]2[cH:38]1>>[CH3:20][CH:19]([CH3:18])[c:21]1[cH:22][c:23](-
[c:24]2[cH:25][cH:26][cH:27][c:28]([cH:31]2)[CH:29]2[CH2:30][C:8]2([c:11]2[cH:12
][cH:13][c:14]([Cl:15])[cH:16][cH:17]2)[c:5]2[cH:4][cH:3][c:2]([Cl:1])[cH:7][cH:
6]2)[c:32]2[n:33][cH:34][cH:35][cH:36][c:37]2[cH:38]1.[N:10].[N:9]

Correctness of the mapping
MAPPET YES
USPTO YES

Reaction no 184 (4 bonds changed)

SMILES/SMARTS of the USPTO output:
[CH:23]([CH3:24])([CH3:25])[OH:26].[Cl:1][c:2]1[c:3]([O:4][c:5]2[cH:6][cH:7][c:8
]([O:9][CH:10]([C:11](=[O:12])[OH:13])[CH3:14])[cH:15][cH:16]2)[cH:17][cH:18][c:
19]([Cl:21])[cH:20]1.[ClH:22]>>[Cl:1][c:2]1[c:3]([O:4][c:5]2[cH:6][cH:7][c:8]([O
:9][CH:10]([C:11]([O:12][CH:23]([CH3:24])[CH3:25])=[O:13])[CH3:14])[cH:15][cH:16
]2)[cH:17][cH:18][c:19]([Cl:21])[cH:20]1

SMILES/SMARTS of the MAPPET output:
[CH3:5][CH:6]([O:7][c:8]1[cH:9][cH:10][c:11]([O:12][c:13]2[cH:14][cH:15][c:16]([
Cl:17])[cH:18][c:19]2[Cl:20])[cH:21][cH:22]1)[C:23]([OH:24])=[O:25].[CH3:1][CH:2
]([CH3:3])[OH:4]>>[CH3:3][CH:2]([CH3:1])[O:4][C:23](=[O:25])[CH:6]([CH3:5])[O:7]
[c:8]1[cH:9][cH:10][c:11]([O:12][c:13]2[cH:14][cH:15][c:16]([Cl:17])[cH:18][c:19
]2[Cl:20])[cH:21][cH:22]1.[OH2:24]

Correctness of the mapping
MAPPET YES
USPTO NO

Reaction no 185 (4 bonds changed)

SMILES/SMARTS of the USPTO output:
[CH2:23]([CH3:24])[O:25][CH:26]([CH2:27][NH2:28])[O:29][CH2:30][CH3:31].[CH3:19][C:20](=[O:21])[O-:22].[CH3:1][O:2][C:3]([c:4]1[c:5]([CH:10]=[C:11]([CH2:12][CH3:13])[N+:14](=[O:15])[O-:16])[cH:6][cH:7][cH:8][cH:9]1)=[O:17].[CH3:32][C:33]#[N:34].[Na+:18]>>[C:3]1(=[O:17])[c:4]2[c:5]([cH:6][cH:7][cH:8][cH:9]2)[CH:10]([CH:11]([CH2:12][CH3:13])[N+:14](=[O:15])[O-:16])[N:28]1[CH2:27][CH:26]([O:25][CH2:23][CH3:24])[O:29][CH2:30][CH3:31]

SMILES/SMARTS of the MAPPET output:
[CH3:1][CH2:2][C:3](=[CH:4][c:5]1[cH:6][cH:7][cH:8][cH:9][c:10]1[C:11](=[O:12])[O:13][CH3:14])[N+:15]([O-:16])=[O:17].[CH3:18][CH2:19][O:20][CH:21]([CH2:22][NH2:23])[O:24][CH2:25][CH3:26]>>[CH3:26][CH2:25][O:24][CH:21]([CH2:22][N:23]1[CH:4]([CH:3]([CH2:2][CH3:1])[N+:15]([O-:16])=[O:17])[c:5]2[cH:6][cH:7][cH:8][cH:9][c:10]2[C:14]1=[O:13])[O:20][CH2:19][CH3:18].[C:11].[O:12]

Correctness of the mapping
MAPPET NO
USPTO YES

Reaction no 186 (4 bonds changed)

SMILES/SMARTS of the USPTO output:
[C:1]([CH3:2])([CH3:3])([CH3:4])[O:5][C:6]([NH:7][c:8]1[c:9]([NH2:18])[cH:10][c:11]([C:14]([F:15])([F:16])[F:17])[cH:12][cH:13]1)=[O:19].[C:20]([CH3:22])([CH3:23])([O:24][C:25](=[O:21])[CH2:26][C:27](=[O:28])[c:29]1[cH:30][c:31](-[c:35]2[cH:36][n:37][c:38]([CH:41]3[CH2:42][CH2:43]3)[cH:39][cH:40]2)[cH:32][cH:33][cH:34]1)[CH3:44]>>[C:1]([CH3:2])([CH3:3])([CH3:4])[O:5][C:6]([NH:7][c:8]1[c:9]([NH:18][C:25](=[O:24])[CH2:26][C:27](=[O:28])[c:29]2[cH:30][c:31](-[c:35]3[cH:36][n:37][c:38]([CH:41]4[CH2:42][CH2:43]4)[cH:39][cH:40]3)[cH:32][cH:33][cH:34]2)[cH:10][c:11]([C:14]([F:15])([F:16])[F:17])[cH:12][cH:13]1)=[O:19]

SMILES/SMARTS of the MAPPET output:
[CH3:20][C:21]([CH3:22])([CH3:23])[O:24][C:25](=[O:26])[CH2:27][C:28](=[O:29])[c:30]1[cH:31][cH:32][cH:33][c:34]([cH:35]1)-[c:36]1[cH:37][cH:38][c:39]([n:40][cH:41]1)[CH:42]1[CH2:43][CH2:44]1.[CH3:1][C:2]([CH3:3])([CH3:4])[O:5][C:6](=[O:7])[NH:8][c:9]1[cH:10][cH:11][c:12]([cH:13][c:14]1[NH2:15])[C:16]([F:17])([F:18])[F:19]>>[CH3:4][C:2]([CH3:3])([CH3:1])[O:5][C:6](=[O:7])[NH:8][c:9]1[cH:10][cH:11][c:12]([cH:13][c:14]1[NH:15][C:25](=[O:26])[CH2:27][C:28](=[O:29])[c:30]1[cH:31][cH:32][cH:33][c:34]([cH:35]1)-[c:36]1[cH:37][cH:38][c:39]([n:40][cH:41]1)[CH:42]1[CH2:43][CH2:44]1)[C:16]([F:17])([F:18])[F:19].[CH3:20][C:21]([CH3:23])([CH3:22])[OH:24]

Correctness of the mapping
MAPPET YES
USPTO NO

Reaction no 187 (4 bonds changed)

SMILES/SMARTS of the USPTO output:
[C:31](=[O:32])([O-:33])[O-:34].[CH3:1][O:2][c:3]1[cH:4][cH:5][c:6](-[c:10]2[n:11][s:12][c:13]3[c:14]2[cH:15][c:16](-[n:19]2[c:20](=[O:30])[nH:21][c:22]([C:26]([F:27])([F:28])[F:29])[cH:23][c:24]2=[O:25])[cH:17][cH:18]3)[cH:7][c:8]1[CH3:9].[CH3:39][N:40]([CH3:41])[CH:42]=[O:43].[I:37][CH3:38].[K+:35].[K+:36]>>[CH3:1][O:2][c:3]1[cH:4][cH:5][c:6](-[c:10]2[n:11][s:12][c:13]3[c:14]2[cH:15][c:16](-[n:19]2[c:20](=[O:30])[n:21]([CH3:31])[c:22]([C:26]([F:27])([F:28])[F:29])[cH:23][c:24]2=[O:25])[cH:17][cH:18]3)[cH:7][c:8]1[CH3:9]

SMILES/SMARTS of the MAPPET output:
[CH3:1][I:2].[CH3:3][O:4][c:5]1[cH:6][cH:7][c:8]([cH:9][c:10]1[CH3:11])-[c:12]1[n:13][s:14][c:15]2[cH:16][cH:17][c:18]([cH:19][c:20]12)-[n:21]1[c:22](=[O:23])[cH:24][c:25]([nH:26][c:27]1=[O:28])[C:29]([F:30])([F:31])[F:32]>>[CH3:3][O:4][c:5]1[cH:6][cH:7][c:8]([cH:9][c:10]1[CH3:11])-[c:12]1[n:13][s:14][c:15]2[cH:16][cH:17][c:18]([cH:19][c:20]12)-[n:21]1[c:22](=[O:23])[cH:24][c:25]([n:26]([CH3:1])[c:27]1=[O:28])[C:29]([F:30])([F:31])[F:32].[I:2]

Correctness of the mapping
MAPPET YES
USPTO NO

Reaction no 188 (4 bonds changed)

SMILES/SMARTS of the USPTO output:
[NH2:19][c:20]1[c:21](=[O:28])[cH:22][c:23]([CH3:27])[cH:24][cH:25][cH:26]1.[OH:1][C:2]1=[C:3]([C:15]([O:17][CH3:16])=[O:18])[N:4]([CH3:14])[S:5](=[O:12])(=[O:13])[c:6]2[c:7]1[cH:8][cH:9][cH:10][cH:11]2.[c:29]1([CH3:30])[c:31]([CH3:32])[cH:33][cH:34][cH:35][cH:36]1>>[OH:1][C:2]1=[C:3]([C:15](=[O:17])[NH:19][c:20]2[c:21](=[O:28])[cH:22][c:23]([CH3:27])[cH:24][cH:25][cH:26]2)[N:4]([CH3:14])[S:5](=[O:12])(=[O:13])[c:6]2[c:7]1[cH:8][cH:9][cH:10][cH:11]2

SMILES/SMARTS of the MAPPET output:
[CH3:11][O:12][C:13](=[O:14])[C:15]1=[C:16]([OH:17])[c:18]2[cH:19][cH:20][cH:21][cH:22][c:23]2[S:24](=[O:25])(=[O:26])[N:27]1[CH3:28].[CH3:1][c:2]1[cH:3][cH:4][cH:5][c:6]([NH2:7])[c:8](=[O:9])[cH:10]1>>[CH3:28][N:27]1[C:15]([C:13](=[O:14])[NH:7][c:6]2[cH:5][cH:4][cH:3][c:2]([CH3:1])[cH:10][c:8]2=[O:9])=[C:16]([OH:17])[c:18]2[cH:19][cH:20][cH:21][cH:22][c:23]2[S:24]1(=[O:26])=[O:25].[CH3:11][OH:12]

Correctness of the mapping
MAPPET YES
USPTO NO

Reaction no 189 (4 bonds changed)

SMILES/SMARTS of the USPTO output:
[F:1][c:2]1[cH:3][cH:4][c:5]([N:8]2[CH2:9][CH2:10][CH:11]([C:14](=[O:15])[OH:16])[CH2:12][CH2:13]2)[cH:6][cH:7]1.[O:21]1[CH2:22][CH2:23][O:24][CH2:25][CH2:26]1.[S:17]([Cl:18])([Cl:19])=[O:20]>>[F:1][c:2]1[cH:3][cH:4][c:5]([N:8]2[CH2:9][CH2:10][CH:11]([C:14](=[O:16])[Cl:19])[CH2:12][CH2:13]2)[cH:6][cH:7]1

SMILES/SMARTS of the MAPPET output:
[Cl:1][S:2]([Cl:3])=[O:4].[OH:5][C:6](=[O:7])[CH:8]1[CH2:9][CH2:10][N:11]([CH2:12][CH2:13]1)[c:14]1[cH:15][cH:16][c:17]([F:18])[cH:19][cH:20]1>>[F:18][c:17]1[cH:19][cH:20][c:14]([cH:15][cH:16]1)[N:11]1[CH2:12][CH2:13][CH:8]([CH2:9][CH2:10]1)[C:6]([Cl:3])=[O:7].[Cl:1].[O:5].[O:4].[S:2]

Correctness of the mapping
MAPPET YES
USPTO NO

Reaction no 190 (4 bonds changed)

SMILES/SMARTS of the USPTO output:
[CH3:1][N:2]1[CH:3]([C:12]([O:14][CH2:13][CH3:15])=[O:16])[CH2:4][CH2:5][CH:6]1[C:7](=[O:8])[O:9][CH2:10][CH3:11].[NH2:17][CH2:18][c:19]1[cH:20][cH:21][cH:22][cH:23][cH:24]1.[c:25]1([CH3:26])[c:27]([CH3:28])[cH:29][cH:30][cH:31][cH:32]1>>[CH3:1][N:2]1[CH:3]([C:12](=[O:14])[NH:17][CH2:18][c:19]2[cH:20][cH:21][cH:22][cH:23][cH:24]2)[CH2:4][CH2:5][CH:6]1[C:7](=[O:8])[O:9][CH2:10][CH3:11]

SMILES/SMARTS of the MAPPET output:
[CH3:9][CH2:10][O:11][C:12](=[O:13])[CH:14]1[CH2:15][CH2:16][CH:17]([N:18]1[CH3:19])[C:20](=[O:21])[O:22][CH2:23][CH3:24].[NH2:1][CH2:2][c:3]1[cH:4][cH:5][cH:6][cH:7][cH:8]1>>[CH3:24][CH2:23][O:22][C:20](=[O:21])[CH:17]1[CH2:16][CH2:15][CH:14]([N:18]1[CH3:19])[C:12](=[O:13])[NH:1][CH2:2][c:3]1[cH:8][cH:7][cH:6][cH:5][cH:4]1.[CH3:9][CH2:10][OH:11]

Correctness of the mapping
MAPPET YES
USPTO NO

Reaction no 191 (4 bonds changed)

SMILES/SMARTS of the USPTO output:
[Br:1][CH2:2][C:3]#[CH:4].[Cl:5][c:6]1[cH:7][cH:8][c:9](-[c:12]2[cH:13][cH:14][c:15]([OH:18])[n:16][cH:17]2)[cH:10][cH:11]1.[K+:19].[K+:20].[O-:21][C:22]([O-:23])=[O:24].[O:25]=[CH:26][N:27]([CH3:28])[CH3:29]>>[CH:2]#[C:3][CH2:4][O:18][c:15]1[cH:14][cH:13][c:12](-[c:9]2[cH:8][cH:7][c:6]([Cl:5])[cH:11][cH:10]2)[cH:17][n:16]1

SMILES/SMARTS of the MAPPET output:
[OH:1][c:2]1[cH:3][cH:4][c:5]([cH:6][n:7]1)-[c:8]1[cH:9][cH:10][c:11]([Cl:12])[cH:13][cH:14]1.[Br:15][CH2:16][C:17]#[CH:18]>>[Cl:12][c:11]1[cH:13][cH:14][c:8]([cH:9][cH:10]1)-[c:5]1[cH:4][cH:3][c:2]([O:1][CH2:16][C:17]#[CH:18])[n:7][cH:6]1.[Br:15]

Correctness of the mapping
MAPPET YES
USPTO NO

Reaction no 192 (4 bonds changed)

SMILES/SMARTS of the USPTO output:
[CH3:16][I:17].[CH3:1][C:2]1([CH3:15])[CH2:3][N:4]([C:12]([CH3:13])=[O:14])[c:5]2[cH:6][cH:7][c:8]([OH:11])[cH:9][c:10]21.[K+:18].[K+:19].[O-:20][C:21]([O-:22])=[O:23].[O:24]=[CH:25][N:26]([CH3:27])[CH3:28].[OH2:29]>>[CH3:1][C:2]1([CH3:15])[CH2:3][N:4]([C:12]([CH3:13])=[O:14])[c:5]2[cH:6][cH:7][c:8]([O:11][CH3:21])[cH:9][c:10]21

SMILES/SMARTS of the MAPPET output:

[CH3:16][I:17].[CH3:1][C:2](=[O:3])[N:4]1[CH2:5][C:6]([CH3:7])([CH3:8])[c:9]2[cH
:10][c:11]([OH:12])[cH:13][cH:14][c:15]12>>[CH3:16][O:12][c:11]1[cH:13][cH:14][c
:15]2[N:4]([CH2:5][C:6]([CH3:8])([CH3:7])[c:9]2[cH:10]1)[C:2]([CH3:1])=[O:3].[IH
:17]

Correctness of the mapping
MAPPET YES
USPTO NO

Reaction no 193 (4 bonds changed)

SMILES/SMARTS of the USPTO output:
[CH3:23][CH2:24][OH:25].[H:21][H:22].[N+:1]([CH2:4][CH:5]([CH2:6][C:7]([O:2][CH2
:3][CH3:9])=[O:8])[c:12]1[o:13][c:14]2[c:15]([cH:16]1)[cH:17][cH:18][cH:19][cH:2
0]2)([O-
:10])=[O:11]>>[NH:1]1[CH2:4][CH:5]([c:12]2[o:13][c:14]3[c:15]([cH:16]2)[cH:17][c
H:18][cH:19][cH:20]3)[CH2:6][C:7]1=[O:8]

SMILES/SMARTS of the MAPPET output:
[CH3:1][CH2:2][O:3][C:4](=[O:5])[CH2:6][CH:7]([CH2:8][N+:9]([O-
:10])=[O:11])[c:12]1[cH:13][c:14]2[cH:15][cH:16][cH:17][cH:18][c:19]2[o:20]1>>[O
:3]=[C:2]1[CH2:6][CH:7]([CH2:8][NH:9]1)[c:12]1[cH:13][c:14]2[cH:15][cH:16][cH:17
][cH:18][c:19]2[o:20]1.[C:4].[C:1].[O:10].[O:5].[O:11]

Correctness of the mapping
MAPPET NO
USPTO YES

Reaction no 194 (4 bonds changed)

SMILES/SMARTS of the USPTO output:
[C:26](=[O:27])([O-:28])[O-
:29].[CH2:1]([CH:2]=[CH2:3])[n:4]1[c:5]([NH2:25])[n:6][c:7](-
[c:18]2[cH:19][c:20]([F:24])[cH:21][cH:22][cH:23]2)[c:8](-
[c:11]2[cH:12][nH:13][c:14](=[O:17])[cH:15][cH:16]2)[c:9]1=[O:10].[CH3:34][S:35]
(=[O:36])[CH3:37].[I:32][CH3:33].[K+:30].[K+:31]>>[CH2:1]([CH:2]=[CH2:3])[n:4]1[
c:5]([NH2:25])[n:6][c:7](-
[c:18]2[cH:19][c:20]([F:24])[cH:21][cH:22][cH:23]2)[c:8](-
[c:11]2[cH:12][n:13]([CH3:26])[c:14](=[O:17])[cH:15][cH:16]2)[c:9]1=[O:10]

SMILES/SMARTS of the MAPPET output:
[CH3:1][I:2].[NH2:3][c:4]1[n:5][c:6](-
[c:7]2[cH:8][cH:9][cH:10][c:11]([F:12])[cH:13]2)[c:14](-
[c:15]2[cH:16][cH:17][c:18](=[O:19])[nH:20][cH:21]2)[c:22](=[O:23])[n:24]1[CH2:2
5][CH:26]=[CH2:27]>>[CH3:1][n:20]1[cH:21][c:15]([cH:16][cH:17][c:18]1=[O:19])-
[c:14]1[c:6]([n:5][c:4]([NH2:3])[n:24]([CH2:25][CH:26]=[CH2:27])[c:22]1=[O:23])-
[c:7]1[cH:8][cH:9][cH:10][c:11]([F:12])[cH:13]1.[I:2]

Correctness of the mapping
MAPPET YES
USPTO NO

Reaction no 195 (4 bonds changed)

SMILES/SMARTS of the USPTO output:

[C:2]([CH3:3])(=[O:4])[O:5][c:6]1[c:7]([CH3:26])[n:8][c:9]([C:15]([NH:16][C:17](
[CH:18]([CH3:19])[CH3:20])([CH3:21])[C:22]([NH2:23])=[O:24])=[O:25])[c:10]([C:11
](=[O:12])[OH:13])[cH:14]1.[CH3:27][OH:28].[ClH:1]>>[C:2]([CH3:3])(=[O:4])[O:5][
c:6]1[c:7]([CH3:26])[n:8][c:9]([C:15]([NH:16][C:17]([CH:18]([CH3:19])[CH3:20])([
CH3:21])[C:22]([NH2:23])=[O:24])=[O:25])[c:10]([C:11]([O:12][CH3:27])=[O:13])[cH
:14]1

SMILES/SMARTS of the MAPPET output:
[CH3:3][CH:4]([CH3:5])[C:6]([CH3:7])([NH:8][C:9](=[O:10])[c:11]1[n:12][c:13]([CH
3:14])[c:15]([O:16][C:17]([CH3:18])=[O:19])[cH:20][c:21]1[C:22]([OH:23])=[O:24])
[C:25]([NH2:26])=[O:27].[CH3:1][OH:2]>>[CH3:1][O:2][C:22](=[O:24])[c:21]1[cH:20]
[c:15]([O:16][C:17]([CH3:18])=[O:19])[c:13]([CH3:14])[n:12][c:11]1[C:9](=[O:10])
[NH:8][C:6]([CH3:7])([CH:4]([CH3:3])[CH3:5])[C:25]([NH2:26])=[O:27].[OH2:23]

Correctness of the mapping
MAPPET YES
USPTO NO

Reaction no 196 (4 bonds changed)

SMILES/SMARTS of the USPTO output:
[C:20]([CH3:21])([CH3:22])([CH3:23])[O:24][C:25](=[O:26])[N:27]1[CH2:28][CH:29](
[OH:32])[CH2:30][CH2:31]1.[C:33]([CH3:34])(=[S:35])[OH:36].[CH2:37]1[O:38][CH2:3
9][CH2:40][CH2:41]1.[c:1]1([P:2]([c:3]2[cH:4][cH:5][cH:6][cH:7][cH:8]2)[c:9]2[cH
:10][cH:11][cH:12][cH:13][cH:14]2)[cH:15][cH:16][cH:17][cH:18][cH:19]1>>[C:20]([
CH3:21])([CH3:22])([CH3:23])[O:24][C:25](=[O:26])[N:27]1[CH2:28][CH:29]([S:35][C
:33]([CH3:34])=[O:36])[CH2:30][CH2:31]1

SMILES/SMARTS of the MAPPET output:
[CH3:1][C:2]([OH:3])=[S:4].[CH3:5][C:6]([CH3:7])([CH3:8])[O:9][C:10](=[O:11])[N:
12]1[CH2:13][CH2:14][CH:15]([OH:16])[CH2:17]1>>[OH2:16].[CH3:1][C:2](=[O:3])[S:4
][CH:15]1[CH2:14][CH2:13][N:12]([CH2:17]1)[C:10](=[O:11])[O:9][C:6]([CH3:8])([CH
3:7])[CH3:5]

Correctness of the mapping
MAPPET YES
USPTO YES

Reaction no 197 (4 bonds changed)

SMILES/SMARTS of the USPTO output:
[C:3]([CH3:4])([CH3:5])([CH3:6])[Si:7]([O:8][CH2:9][CH2:10][CH2:11][CH2:12][CH2:
13][CH2:14][CH2:15][CH2:16][CH2:17][N:18]([CH:19]1[CH:20]2[CH:21]([OH:26])[CH2:2
2][CH:23]1[CH2:24][CH2:25]2)[CH3:27])([CH3:28])[CH3:29].[CH2:30]([O:32][C:33](=[
O:31])[C:34]([c:35]1[s:36][cH:37][cH:38][cH:39]1)([c:40]1[s:41][cH:42][cH:43][cH
:44]1)[OH:45])[CH3:46].[CH3:49][c:50]1[cH:51][cH:52][cH:53][cH:54][cH:55]1.[Cl-
:47].[H-
:1].[NH4+:48].[Na+:2]>>[C:3]([CH3:4])([CH3:5])([CH3:6])[Si:7]([O:8][CH2:9][CH2:1
0][CH2:11][CH2:12][CH2:13][CH2:14][CH2:15][CH2:16][CH2:17][N:18]([CH:19]1[CH:20]
2[CH:21]([O:26][C:33](=[O:32])[C:34]([c:35]3[s:36][cH:37][cH:38][cH:39]3)([c:40]
3[s:41][cH:42][cH:43][cH:44]3)[OH:45])[CH2:22][CH:23]1[CH2:24][CH2:25]2)[CH3:27]
)([CH3:28])[CH3:29]

SMILES/SMARTS of the MAPPET output:
[CH3:28][CH2:29][O:30][C:31](=[O:32])[C:33]([OH:34])([c:35]1[cH:36][cH:37][cH:38
][s:39]1)[c:40]1[cH:41][cH:42][cH:43][s:44]1.[CH3:1][N:2]([CH2:3][CH2:4][CH2:5][
CH2:6][CH2:7][CH2:8][CH2:9][CH2:10][CH2:11][O:12][Si:13]([CH3:14])([CH3:15])[C:1

6]([CH3:17])([CH3:18])[CH3:19])[CH:20]1[CH:21]2[CH2:22][CH2:23][CH:24]1[CH:25]([OH:26])[CH2:27]2>>[CH3:1][N:2]([CH2:3][CH2:4][CH2:5][CH2:6][CH2:7][CH2:8][CH2:9][CH2:10][CH2:11][O:12][Si:13]([CH3:14])([CH3:15])[C:16]([CH3:17])([CH3:19])[CH3:18])[CH:20]1[CH:21]2[CH2:22][CH2:23][CH:24]1[CH:25]([CH2:27]2)[O:26][C:31](=[O:32])[C:33]([OH:34])([c:40]1[cH:41][cH:42][cH:43][s:44]1)[c:35]1[cH:36][cH:37][cH:38][s:39]1.[CH3:28][CH2:29][OH:30]

Correctness of the mapping
MAPPET YES
USPTO NO

Reaction no 198 (4 bonds changed)

SMILES/SMARTS of the USPTO output:
[Br:1][CH2:2][c:3]1[c:4]([C:13]([F:14])([F:15])[F:16])[n:5][n:6]([CH3:12])[c:7]1[O:8][CH:9]([F:10])[F:11].[CH3:21][CH2:22][OH:23].[NH2:17][C:18]([NH2:19])=[S:20]>>[BrH:1].[CH2:2]([c:3]1[c:4]([C:13]([F:14])([F:15])[F:16])[n:5][n:6]([CH3:12])[c:7]1[O:8][CH:9]([F:10])[F:11])[S:20][C:18](=[NH:17])[NH2:19]

SMILES/SMARTS of the MAPPET output:
[CH3:1][n:2]1[n:3][c:4]([c:5]([CH2:6][Br:7])[c:8]1[O:9][CH:10]([F:11])[F:12])[C:13]([F:14])([F:15])[F:16].[NH2:17][C:18]([NH2:19])=[S:20]>>[BrH:7].[CH3:1][n:2]1[n:3][c:4]([c:5]([CH2:6][S:20][C:18]([NH2:17])=[NH:19])[c:8]1[O:9][CH:10]([F:11])[F:12])[C:13]([F:14])([F:15])[F:16]

Correctness of the mapping
MAPPET YES
USPTO YES

Reaction no 199 (4 bonds changed)

SMILES/SMARTS of the USPTO output:
[C:24]([O-
:25])(=[O:26])[CH3:27].[CH3:14][O:15][CH2:16][C:17]([CH2:18][C:19](=[O:20])[O:21][CH3:22])=[O:23].[CH3:30][OH:31].[ClH:13].[N:1]([O-
:2])=[O:3].[NH2:5][c:6]1[cH:7][cH:8][cH:9][cH:10][c:11]1[F:12].[Na+:28].[Na+:4].[OH2:29]>>[N:1]([NH:5][c:6]1[cH:7][cH:8][cH:9][cH:10][c:11]1[F:12])=[C:18]([C:17]([CH2:16][O:15][CH3:14])=[O:23])[C:19](=[O:20])[O:21][CH3:22]

SMILES/SMARTS of the MAPPET output:
[NH2:1][c:2]1[cH:3][cH:4][cH:5][cH:6][c:7]1[F:8].[O-
:9][N:10]=[O:11].[CH3:12][O:13][CH2:14][C:15](=[O:16])[CH2:17][C:18](=[O:19])[O:20][CH3:21]>>[CH3:12][O:13][CH2:14][C:15](=[O:16])[C:17](=[N:10][NH:1][c:2]1[cH:3][cH:4][cH:5][cH:6][c:7]1[F:8])[C:18](=[O:19])[O:20][CH3:21].[O:9].[O:11]

Correctness of the mapping
MAPPET YES
USPTO YES

Reaction no 200 (4 bonds changed)

SMILES/SMARTS of the USPTO output:
[C:22](=[O:23])([O-:24])[O-
:25].[CH3:1][O:2][c:3]1[c:4]([N:9]2[S:10][c:11]3[c:12]([cH:16][cH:17][cH:18][cH:19]3)[CH2:13][C:14]2=[O:15])[cH:5][cH:6][cH:7][cH:8]1.[CH3:20][I:21].[CH3:28][C:

29](=[O:30])[CH3:31].[K+:26].[K+:27]>>[CH3:1][O:2][c:3]1[c:4]([N:9]2[S:10][c:11]3[c:12]([cH:16][cH:17][cH:18][cH:19]3)[CH:13]([CH3:22])[C:14]2=[O:15])[cH:5][cH:6][cH:7][cH:8]1

SMILES/SMARTS of the MAPPET output:
[CH3:1][O:2][c:3]1[cH:4][cH:5][cH:6][cH:7][c:8]1[N:9]1[S:10][c:11]2[cH:12][cH:13][cH:14][cH:15][c:16]2[CH2:17][C:18]1=[O:19].[CH3:20][I:21]>>[CH3:1][O:2][c:3]1[cH:4][cH:5][cH:6][cH:7][c:8]1[N:9]1[S:10][c:11]2[cH:12][cH:13][cH:14][cH:15][c:16]2[CH:17]([CH3:20])[C:18]1=[O:19].[I:21]

Correctness of the mapping
MAPPET YES
USPTO NO

Reaction no 201 (5 bonds changed)

SMILES/SMARTS of the USPTO output:
[C-:27]#[N:28].[C-:30]#[N:31].[CH3:16][N:17]([CH3:18])[CH:19]=[O:20].[CH3:21][CH2:22][O:23][C:24](=[O:25])[CH3:26].[Cl:13][CH2:14][Cl:15].[Cl:1][c:2]1[cH:3][c:4]([C:5](=[O:6])[O:7][CH3:8])[cH:9][c:10]([F:12])[cH:11]1.[Cl:32][Pd:33][Cl:34].[Zn+2:29]>>[Cl:1][c:2]1[cH:3][c:4]([C:5](=[O:6])[O:7][CH3:8])[cH:9][c:10]([C:16]#[N:17])[cH:11]1

SMILES/SMARTS of the MAPPET output:
[CH3:1][O:2][C:3](=[O:4])[c:5]1[cH:6][c:7]([F:8])[cH:9][c:10]([Cl:11])[cH:12]1.[C-:13]#[N:14]>>[CH3:1][O:2][C:3](=[O:4])[c:5]1[cH:12][c:10]([Cl:11])[cH:9][c:7]([cH:6]1)[C:13]#[N:14].[F:8]

Correctness of the mapping
MAPPET YES
USPTO NO

Reaction no 202 (5 bonds changed)

SMILES/SMARTS of the USPTO output:
[BrH:1].[CH2:56]1[O:57][CH2:58][CH2:59][CH2:60]1.[CH3:2][N:3]1[C:4](=[S:31])[CH:5]([NH:20][C:21]([O:22][CH2:24][c:25]2[cH:26][cH:27][cH:28][cH:29][cH:30]2)=[O:23])[N:6]=[C:7]([c:14]2[cH:15][cH:16][cH:17][cH:18][cH:19]2)[c:8]2[c:9]1[cH:10][cH:11][cH:12][cH:13]2.[CH3:32][CH2:33][N:34]([CH2:35][CH3:36])[CH2:37][CH3:38].[CH3:69][C:70](=[O:71])[OH:72].[CH:45]1([CH2:51][CH2:52][C:53]([OH:54])=[O:55])[CH2:46][CH2:47][CH2:48][CH2:49][CH2:50]1.[Cl:39][C:40]([C:41]([Cl:42])=[O:43])=[O:44].[Cl:66][CH2:67][Cl:68].[O:61]=[CH:62][N:63]([CH3:64])[CH3:65]>>[CH3:2][N:3]1[C:4](=[S:31])[CH:5]([NH:20][C:21](=[O:22])[CH2:52][CH2:51][CH:45]2[CH2:46][CH2:47][CH2:48][CH2:49][CH2:50]2)[N:6]=[C:7]([c:14]2[cH:15][cH:16][cH:17][cH:18][cH:19]2)[c:8]2[c:9]1[cH:10][cH:11][cH:12][cH:13]2

SMILES/SMARTS of the MAPPET output:
-

Correctness of the mapping
MAPPET NO
USPTO YES

Reaction no 203 (5 bonds changed)

SMILES/SMARTS of the USPTO output:
[Br:1][c:2]1[cH:3][c:4]([O:5][c:6]2[c:7]([C:13]([OH:14])=[O:15])[n:8][cH:9][cH:10][c:11]2[CH3:12])[cH:16][c:17]([Cl:19])[cH:18]1.[C:33]([OH:34])([CH3:35])([CH3:36])[CH3:37].[CH2:55]1[O:56][CH2:57][CH2:58][CH2:59]1.[CH3:20][CH2:21][N:22]([CH2:23][CH3:24])[CH2:25][CH3:26].[Cl:60][CH2:61][Cl:62].[c:38]1([P:39]([N:40]=[N+:41]=[N-:42])([c:43]2[cH:44][cH:45][cH:46][cH:47][cH:48]2)=[O:49])[cH:50][cH:51][cH:52][cH:53][cH:54]1.[cH:27]1[cH:28][cH:29][n:30][cH:31][cH:32]1>>[Br:1][c:2]1[cH:3][c:4]([O:5][c:6]2[c:7]([NH2:22])[n:8][cH:9][cH:10][c:11]2[CH3:12])[cH:16][c:17]([Cl:19])[cH:18]1

SMILES/SMARTS of the MAPPET output:
[N-:1]=[N+:2]=[N-:3].[CH3:4][c:5]1[cH:6][cH:7][n:8][c:9]([C:10]([OH:11])=[O:12])[c:13]1[O:14][c:15]1[cH:16][c:17]([Cl:18])[cH:19][c:20]([Br:21])[cH:22]1>>[CH3:4][c:5]1[cH:6][cH:7][n:8][c:9]([NH2:3])[c:13]1[O:14][c:15]1[cH:16][c:17]([Cl:18])[cH:19][c:20]([Br:21])[cH:22]1.[C:10].[N:2].[N:1].[O:11].[O:12]

Correctness of the mapping
MAPPET YES
USPTO NO

Reaction no 204 (5 bonds changed)

SMILES/SMARTS of the USPTO output:
[C:22](=[O:23])([O-:24])[OH:25].[CH3:20][Mg+:21].[CH:1]([c:2]1[cH:3][cH:4][cH:5][cH:6][cH:7]1)([c:8]1[cH:9][cH:10][cH:11][cH:12][cH:13]1)[N:14]1[CH2:15][C:16](=[O:18])[CH2:17]1.[I-:19].[Na+:26].[O:27]1[CH2:28][CH2:29][CH2:30][CH2:31]1>>[CH:1]([c:2]1[cH:3][cH:4][cH:5][cH:6][cH:7]1)([c:8]1[cH:9][cH:10][cH:11][cH:12][cH:13]1)[N:14]1[CH2:15][C:16]([OH:18])([CH3:22])[CH2:17]1

SMILES/SMARTS of the MAPPET output:
[O:1]=[C:2]1[CH2:3][N:4]([CH2:5]1)[CH:6]([c:7]1[cH:8][cH:9][cH:10][cH:11][cH:12]1)[c:13]1[cH:14][cH:15][cH:16][cH:17][cH:18]1.[CH3:19][Mg+:20]>>[CH3:19][C:2]1([OH:1])[CH2:3][N:4]([CH2:5]1)[CH:6]([c:13]1[cH:18][cH:17][cH:16][cH:15][cH:14]1)[c:7]1[cH:12][cH:11][cH:10][cH:9][cH:8]1.[Mg:20]

Correctness of the mapping
MAPPET YES
USPTO NO

Reaction no 205 (5 bonds changed)

SMILES/SMARTS of the USPTO output:
[CH3:1][O:2][c:3]1[cH:4][cH:5][c:6]([N:9]2[CH2:10][CH2:11][N:12]([C:15](=[O:16])[CH:17]([CH2:18][CH:19]([CH3:20])[CH3:21])[CH:22]3[C:23](=[O:29])[O:24][C:25]([CH3:27])([CH3:28])[O:26]3)[CH2:13][CH2:14]2)[cH:7][cH:8]1.[CH:32]([OH:33])([CH3:34])[CH3:35].[NH2:30][OH:31]>>[CH3:1][O:2][c:3]1[cH:4][cH:5][c:6]([N:9]2[CH2:10][CH2:11][N:12]([C:15](=[O:16])[CH:17]([CH2:18][CH:19]([CH3:20])[CH3:21])[CH:22]([C:23](=[O:24])[NH:30][OH:31])[OH:26])[CH2:13][CH2:14]2)[cH:7][cH:8]1

SMILES/SMARTS of the MAPPET output:
[NH2:1][OH:2].[CH3:3][O:4][c:5]1[cH:6][cH:7][c:8]([cH:9][cH:10]1)[N:11]1[CH2:12][CH2:13][N:14]([CH2:15][CH2:16]1)[C:17](=[O:18])[CH:19]([CH2:20][CH:21]([CH3:22])[CH3:23])[CH:24]1[O:25][C:26]([CH3:27])([CH3:28])[O:29][C:30]1=[O:31]>>[CH3:3][

O:4][c:5]1[cH:10][cH:9][c:8]([cH:7][cH:6]1)[N:11]1[CH2:16][CH2:15][N:14]([CH2:13][CH2:12]1)[C:17](=[O:18])[CH:19]([CH2:20][CH:21]([CH3:23])[CH3:22])[CH:24]([OH:25])[C:26](=[O:29])[NH:1][OH:2].[C:28].[C:27].[C:30].[O:31]

Correctness of the mapping
MAPPET NO
USPTO YES

Reaction no 206 (5 bonds changed)

SMILES/SMARTS of the USPTO output:
[Br:1][c:2]1[n:3][cH:4][c:5]([O:8][CH3:9])[cH:6][cH:7]1.[CH2:10]([Li:11])[CH2:12][CH2:13][CH3:14].[CH2:15]([c:16]1[cH:17][cH:18][cH:19][cH:20][cH:21]1)[O:22][c:23]1[cH:24][cH:25][c:26]([O:31][CH3:32])[c:27]([CH:28]=[O:29])[cH:30]1.[CH3:33][C:34](=[O:35])[O:36][C:37](=[O:38])[CH3:39].[CH3:46][OH:47].[cH:40]1[cH:41][cH:42][n:43][cH:44][cH:45]1>>[c:2]1([CH:28]([c:27]2[c:26]([O:31][CH3:32])[cH:25][cH:24][c:23]([O:22][CH2:15][c:16]3[cH:17][cH:18][cH:19][cH:20][cH:21]3)[cH:30]2)[O:29][C:34]([CH3:33])=[O:35])[n:3][cH:4][c:5]([O:8][CH3:9])[cH:6][cH:7]1

SMILES/SMARTS of the MAPPET output:
[CH3:1][C:2](=[O:3])[O:4][C:5]([CH3:6])=[O:7].[CH3:8][O:9][c:10]1[cH:11][cH:12][c:13]([O:14][CH2:15][c:16]2[cH:17][cH:18][cH:19][cH:20][cH:21]2)[cH:22][c:23]1[CH:24]=[O:25].[CH3:26][O:27][c:28]1[cH:29][cH:30][c:31]([Br:32])[n:33][cH:34]1>>[CH3:26][O:27][c:28]1[cH:29][cH:30][c:31]([n:33][cH:34]1)[CH:24]([O:25][C:5]([CH3:6])=[O:7])[c:23]1[cH:22][c:13]([O:14][CH2:15][c:16]2[cH:21][cH:20][cH:19][cH:18][cH:17]2)[cH:12][cH:11][c:10]1[O:9][CH3:8].[Br:32].[C:2].[C:1].[O:4].[O:3]

Correctness of the mapping
MAPPET YES
USPTO YES

Reaction no 207 (5 bonds changed)

SMILES/SMARTS of the USPTO output:
[Cl:1][c:2]1[n:3][c:4]([Cl:5])[n:6][c:7]([Cl:8])[n:9]1.[O:37]=[CH:38][N:39]([CH3:40])[CH3:41].[OH2:36].[OH:10][N:11]=[C:12]1[CH2:13][C:14]2([c:15]3[cH:16][cH:17][cH:18][cH:19][c:20]31)[CH2:21][CH2:22][N:23]([C:26](=[O:27])[O:28][CH2:29][c:30]1[cH:31][cH:32][cH:33][cH:34][cH:35]1)[CH2:24][CH2:25]2>>[NH:11]1[C:12](=[O:36])[CH2:13][C:14]2([c:15]3[cH:16][cH:17][cH:18][cH:19][c:20]31)[CH2:21][CH2:22][N:23]([C:26](=[O:27])[O:28][CH2:29][c:30]1[cH:31][cH:32][cH:33][cH:34][cH:35]1)[CH2:24][CH2:25]2

SMILES/SMARTS of the MAPPET output:
[OH:1][N:2]=[C:3]1[CH2:4][C:5]2([CH2:6][CH2:7][N:8]([CH2:9][CH2:10]2)[C:11](=[O:12])[O:13][CH2:14][c:15]2[cH:16][cH:17][cH:18][cH:19][cH:20]2)[c:21]2[cH:22][cH:23][cH:24][cH:25][c:26]12.[OH2:27]>>[OH2:27].[O:12]=[C:11]([O:13][CH2:14][c:15]1[cH:20][cH:19][cH:18][cH:17][cH:16]1)[N:8]1[CH2:7][CH2:6][C:5]2([CH2:10][CH2:9]1)[CH2:4][C:3](=[O:1])[NH:2][c:26]1[cH:25][cH:24][cH:23][cH:22][c:21]21

Correctness of the mapping
MAPPET NO
USPTO YES

Reaction no 208 (5 bonds changed)

SMILES/SMARTS of the USPTO output:
[C:13]([CH2:14][CH2:15][C:16]([CH3:18])=[O:20])([OH:17])=[O:19].[NH2:1][CH2:2][C
H2:3][c:4]1[cH:5][nH:6][c:7]2[cH:8][cH:9][cH:10][cH:11][c:12]12>>[N:1]12[CH2:2][
CH2:3][c:4]3[c:5]([nH:6][c:7]4[cH:8][cH:9][cH:10][cH:11][c:12]34)[C:16]1([CH3:18
])[CH2:15][CH2:14][C:13]2=[O:19]

SMILES/SMARTS of the MAPPET output:
[NH2:1][CH2:2][CH2:3][c:4]1[cH:5][nH:6][c:7]2[cH:8][cH:9][cH:10][cH:11][c:12]12.
[CH3:13][C:14](=[O:15])[CH2:16][CH2:17][C:18]([OH:19])=[O:20]>>[CH3:13][C:14]12[
CH2:16][CH2:17][C:18](=[O:20])[N:1]1[CH2:2][CH2:3][c:4]1[c:5]2[nH:6][c:7]2[cH:8]
[cH:9][cH:10][cH:11][c:12]12.[O:19].[O:15]

Correctness of the mapping
MAPPET YES
USPTO YES

Reaction no 209 (5 bonds changed)

SMILES/SMARTS of the USPTO output:
[C:24]([O:25][CH2:26][CH3:27])(=[O:28])[CH2:29][CH3:30].[C:4]1(=[O:5])[O:6][CH2:
7][c:8]2[cH:9][cH:10][cH:11][cH:12][c:13]21.[CH3:1][O-
:2].[CH3:31][OH:32].[CH:14](=[O:15])[c:16]1[cH:17][c:18]([C:19]#[N:20])[cH:21][c
H:22][cH:23]1.[Na+:3]>>[C:4]1(=[O:5])[c:13]2[c:8]([cH:9][cH:10][cH:11][cH:12]2)[
C:7](=[O:6])[CH:14]1[c:16]1[cH:17][c:18]([C:19]#[N:20])[cH:21][cH:22][cH:23]1

SMILES/SMARTS of the MAPPET output:
[O:1]=[CH:2][c:3]1[cH:4][cH:5][cH:6][c:7]([cH:8]1)[C:9]#[N:10].[O:11]=[C:12]1[O:
13][CH2:14][c:15]2[cH:16][cH:17][cH:18][cH:19][c:20]12>>[OH2:1].[O:13]=[C:14]1[C
H:2]([C:12](=[O:11])[c:20]2[cH:19][cH:18][cH:17][cH:16][c:15]12)[c:3]1[cH:4][cH:
5][cH:6][c:7]([cH:8]1)[C:9]#[N:10]

Correctness of the mapping
MAPPET YES
USPTO YES

Reaction no 210 (5 bonds changed)

SMILES/SMARTS of the USPTO output:
[CH3:18][CH2:19][N:20]([CH2:21][CH3:22])[CH2:23][CH3:24].[CH:25]([Cl:26])([Cl:27
])[Cl:28].[Cl:5][CH2:6][C:7](=[O:8])[C:9]([C:10](=[O:11])[O:12][CH2:13][CH3:14])
=[N:15][O:16][CH3:17].[SH:1][CH2:2][CH2:3][OH:4]>>[S:1]1[CH2:2][CH2:3][O:4][C:7]
([C:9]([C:10](=[O:11])[O:12][CH2:13][CH3:14])=[N:15][O:16][CH3:17])=[CH:6]1

SMILES/SMARTS of the MAPPET output:
[OH:1][CH2:2][CH2:3][SH:4].[CH3:5][CH2:6][O:7][C:8](=[O:9])[C:10](=[N:11][O:12][
CH3:13])[C:14](=[O:15])[CH2:16][Cl:17]>>[CH3:5][CH2:6][O:7][C:8](=[O:9])[C:10](=
[N:11][O:12][CH3:13])[C:14]1=[CH:16][S:4][CH2:3][CH2:2][O:1]1.[Cl:17].[O:15]

Correctness of the mapping
MAPPET YES
USPTO YES

Reaction no 211 (5 bonds changed)

SMILES/SMARTS of the USPTO output:

[C:10]([CH3:11])([CH3:12])([CH3:13])[O:14][C:15]([NH:16][CH2:17][CH:18]1[O:19][CH2:20]1)=[O:21].[F:1][c:2]1[cH:3][c:4]([NH2:5])[cH:6][cH:7][c:8]1[CH3:9].[O:22]=[CH:23][N:24]([CH3:25])[CH3:26]>>[F:1][c:2]1[cH:3][c:4]([N:5]2[CH2:20][CH:18]([CH2:17][NH:16][C:15]([O:14][C:10]([CH3:11])([CH3:12])[CH3:13])=[O:21])[O:19][C:23]2=[O:22])[cH:6][cH:7][c:8]1[CH3:9]

SMILES/SMARTS of the MAPPET output:
[CH3:1][N:2]([CH3:3])[CH:4]=[O:5].[CH3:6][c:7]1[cH:8][cH:9][c:10]([NH2:11])[cH:12][c:13]1[F:14].[CH3:15][C:16]([CH3:17])([CH3:18])[O:19][C:20](=[O:21])[NH:22][CH2:23][CH:24]1[CH2:25][O:26]1>>[CH3:6][c:7]1[cH:8][cH:9][c:10]([cH:12][c:13]1[F:14])[N:2]1[CH2:1][CH:24]([CH2:23][NH:22][C:20](=[O:21])[O:19][C:16]([CH3:18])([CH3:17])[CH3:15])[O:26][C:4]1=[O:5].[C:3].[C:25].[N:11]

Correctness of the mapping
MAPPET NO
USPTO YES

Reaction no 212 (5 bonds changed)

SMILES/SMARTS of the USPTO output:
[Br:1][c:2]1[cH:3][n:4][c:5]([Cl:11])[c:6]([C:7](=[O:8])[O-:9])[cH:10]1.[Cl:13][C:14]([C:15]([Cl:16])=[O:17])=[O:18].[Cl:25][CH2:26][Cl:27].[NH3:24].[Na+:12].[O:19]=[CH:20][N:21]([CH3:22])[CH3:23]>>[Br:1][c:2]1[cH:3][n:4][c:5]([Cl:11])[c:6]([C:7](=[O:8])[NH2:21])[cH:10]1

SMILES/SMARTS of the MAPPET output:
[NH3:1].[O-:2][C:3](=[O:4])[c:5]1[cH:6][c:7]([Br:8])[cH:9][n:10][c:11]1[Cl:12]>>[NH2:1][C:3](=[O:4])[c:5]1[cH:6][c:7]([Br:8])[cH:9][n:10][c:11]1[Cl:12].[O:2]

Correctness of the mapping
MAPPET YES
USPTO NO

Reaction no 213 (5 bonds changed)

SMILES/SMARTS of the USPTO output:
[Br:1][c:2]1[cH:3][c:4]([CH2:10][OH:11])[cH:5][c:6]([O:8][CH3:9])[cH:7]1.[C-:22]#[N:23].[C-:25]#[N:26].[CH3:12][N:13]([CH3:14])[CH:15]=[O:16].[CH3:17][CH2:18][O:19][CH2:20][CH3:21].[Zn+2:24].[cH:27]1[cH:28][cH:29][c:30]([P:31]([Pd:32]([P:33]([c:34]2[cH:35][cH:36][cH:37][cH:38][cH:39]2)([c:40]2[cH:41][cH:42][cH:43][cH:44][cH:45]2)[c:46]2[cH:47][cH:48][cH:49][cH:50][cH:51]2)([P:52]([c:53]2[cH:54][cH:55][cH:56][cH:57][cH:58]2)([c:59]2[cH:60][cH:61][cH:62][cH:63][cH:64]2)[c:65]2[cH:66][cH:67][cH:68][cH:69][cH:70]2)[P:71]([c:72]2[cH:73][cH:74][cH:75][cH:76][cH:77]2)([c:78]2[cH:79][cH:80][cH:81][cH:82][cH:83]2)[c:84]2[cH:85][cH:86][cH:87][cH:88][cH:89]2)([c:90]2[cH:91][cH:92][cH:93][cH:94][cH:95]2)[c:96]2[cH:97][cH:98][cH:99][cH:100][cH:101]2)[cH:102][cH:103]1>>[c:2]1([C:12]#[N:13])[cH:3][c:4]([CH2:10][OH:11])[cH:5][c:6]([O:8][CH3:9])[cH:7]1

SMILES/SMARTS of the MAPPET output:
[C-:1]#[N:2].[CH3:3][O:4][c:5]1[cH:6][c:7]([Br:8])[cH:9][c:10]([CH2:11][OH:12])[cH:13]1>>[CH3:3][O:4][c:5]1[cH:13][c:10]([CH2:11][OH:12])[cH:9][c:7]([cH:6]1)[C:1]#[N:2].[Br:8]

Correctness of the mapping

MAPPET YES
USPTO NO

Reaction no 214 (5 bonds changed)

SMILES/SMARTS of the USPTO output:
[CH2:1]([c:2]1[cH:3][cH:4][cH:5][cH:6][cH:7]1)[N:8]1[CH:9]([CH3:19])[CH2:10][N:1
1]([CH3:18])[CH2:12][CH:13]([N+:15]([O-
:16])=[O:17])[CH2:14]1.[CH3:20][C:21]([OH:22])=[O:23].[CH3:24][CH2:25][OH:26]>>[
CH2:1]([c:2]1[cH:3][cH:4][cH:5][cH:6][cH:7]1)[N:8]1[CH:9]([CH3:19])[CH2:10][N:11
]([CH3:18])[CH2:12][CH:13]([NH:15][C:21]([CH3:20])=[O:22])[CH2:14]1

SMILES/SMARTS of the MAPPET output:
[CH3:1][C:2]([OH:3])=[O:4].[CH3:5][CH:6]1[CH2:7][N:8]([CH3:9])[CH2:10][CH:11]([C
H2:12][N:13]1[CH2:14][c:15]1[cH:16][cH:17][cH:18][cH:19][cH:20]1)[N+:21]([O-
:22])=[O:23]>>[CH3:5][CH:6]1[CH2:7][N:8]([CH3:9])[CH2:10][CH:11]([CH2:12][N:13]1
[CH2:14][c:15]1[cH:20][cH:19][cH:18][cH:17][cH:16]1)[NH:21][C:2]([CH3:1])=[O:4].
[O:23].[O:22].[O:3]

Correctness of the mapping
MAPPET YES
USPTO YES

Reaction no 215 (5 bonds changed)

SMILES/SMARTS of the USPTO output:

SMILES/SMARTS of the MAPPET output:

Correct mapped SMILES/SMARTS of the reaction:

Correctness of the mapping
MAPPET YES
USPTO NO

Reaction no 216 (5 bonds changed)

SMILES/SMARTS of the USPTO output:
[CH2:18]([CH2:19][CH2:31][CH3:32])[Sn:20]([CH2:21][CH2:22][CH2:23][CH3:24])([CH2
:25][CH2:26][CH2:27][CH3:28])[CH:29]=[CH2:30].[CH3:38][CH2:39][O:40][C:41](=[O:4
2])[CH3:43].[Cl:1][c:2]1[n:3][cH:4][c:5]([S:8](=[O:9])(=[O:10])[c:11]2[c:12]([F:
17])[cH:13][cH:14][cH:15][cH:16]2)[cH:6][cH:7]1.[O:33]1[CH2:34][CH2:35][CH2:36][
CH2:37]1>>[c:2]1([CH:18]=[CH2:19])[n:3][cH:4][c:5]([S:8](=[O:9])(=[O:10])[c:11]2
[c:12]([F:17])[cH:13][cH:14][cH:15][cH:16]2)[cH:6][cH:7]1

SMILES/SMARTS of the MAPPET output:
-

Correct mapped SMILES/SMARTS of the reaction:
[CH3:32][CH2:31][CH2:19][CH2:18][Sn:20]([CH2:21][CH2:22][CH2:23][CH3:24])([CH2:2
5][CH2:26][CH2:27][CH3:28])[CH:29]=[CH2:30].[F:17][c:12]1[cH:13][cH:14][cH:15][c
H:16][c:11]1[S:8](=[O:9])(=[O:10])[c:5]1[cH:6][cH:7][c:2]([Cl:1])[n:3][cH:4]1>>[
F:17][c:12]1[cH:13][cH:14][cH:15][cH:16][c:11]1[S:8](=[O:9])(=[O:10])[c:5]1[cH:6

][cH:7][c:2]([CH:29]=[CH2:30])[n:3][cH:4]1.[CH3:28][CH2:27][CH2:26][CH2:25][Sn+:20]([CH2:21][CH2:22][CH2:23][CH3:24])[CH2:18][CH2:19][CH2:31][CH3:32].[Cl-:1]

Correctness of the mapping
MAPPET NO
USPTO NO

Reaction no 217 (5 bonds changed)

SMILES/SMARTS of the USPTO output:
[CH2:1]([CH3:2])[O:3][C:4]([C:5]([CH2:6][C:7](=[CH2:8])[c:9]1[c:10]([O:16][CH3:17])[cH:11][cH:12][c:13]([F:15])[cH:14]1)([C:18]([F:19])([F:20])[F:21])[OH:22])=[O:23].[CH3:28][CH2:29][O:30][C:31]([CH3:32])=[O:33].[Cu:34].[I:24].[I:25][CH2:26][I:27].[Zn:35]>>[CH2:1]([CH3:2])[O:3][C:4]([C:5]([CH2:6][C:7]1([c:9]2[c:10]([O:16][CH3:17])[cH:11][cH:12][c:13]([F:15])[cH:14]2)[CH2:8][CH2:26]1)([C:18]([F:19])([F:20])[F:21])[OH:22])=[O:23]

SMILES/SMARTS of the MAPPET output:
[I:1][CH2:2][I:3].[CH3:4][CH2:5][O:6][C:7](=[O:8])[C:9]([OH:10])([CH2:11][C:12](=[CH2:13])[c:14]1[cH:15][c:16]([F:17])[cH:18][cH:19][c:20]1[O:21][CH3:22])[C:23]([F:24])([F:25])[F:26]>>[CH3:4][CH2:5][O:6][C:7](=[O:8])[C:9]([OH:10])([CH2:11][C:12]1([CH2:13][CH2:2]1)[c:14]1[cH:15][c:16]([F:17])[cH:18][cH:19][c:20]1[O:21][CH3:22])[C:23]([F:24])([F:25])[F:26].[I:3].[I:1]

Correctness of the mapping
MAPPET YES
USPTO YES

Reaction no 218 (5 bonds changed)

SMILES/SMARTS of the USPTO output:
[C:1]([Br:2])([Br:3])([Br:4])[Br:5].[Cl:45][CH2:46][Cl:47].[OH:25][CH2:26][CH2:27][C:28]1([CH2:42][O:43][CH3:44])[CH2:29][C:30](=[O:41])[N:31]([CH:33]([CH3:34])[c:35]2[cH:36][cH:37][cH:38][cH:39][cH:40]2)[CH2:32]1.[c:6]1([P:7]([c:8]2[cH:9][cH:10][cH:11][cH:12][cH:13]2)[c:14]2[cH:15][cH:16][cH:17][cH:18][cH:19]2)[cH:20][cH:21][cH:22][cH:23][cH:24]1>>[CH2:1]([Br:5])[CH2:27][C:28]1([CH2:42][O:43][CH3:44])[CH2:29][C:30](=[O:41])[N:31]([CH:33]([CH3:34])[c:35]2[cH:36][cH:37][cH:38][cH:39][cH:40]2)[CH2:32]1

SMILES/SMARTS of the MAPPET output:
-

Correctness of the mapping
MAPPET NO
USPTO YES

Reaction no 219 (5 bonds changed)

SMILES/SMARTS of the USPTO output:
[Br:1][c:2]1[cH:3][cH:4][c:5](-[c:8]2[cH:9][cH:10][c:11](=[O:14])[nH:12][n:13]2)[cH:6][cH:7]1.[C-:15]#[N:16].[CH3:17][N:18]([CH3:19])[CH:20]=[O:21].[NH2:22][CH2:23][CH2:24][NH2:25].[OH2:26]>>[c:2]1([C:17]#[N:18])[cH:3][cH:4][c:5](-[c:8]2[cH:9][cH:10][c:11](=[O:14])[nH:12][n:13]2)[cH:6][cH:7]1

SMILES/SMARTS of the MAPPET output:
[C-:1]#[N:2].[Br:3][c:4]1[cH:5][cH:6][c:7]([cH:8][cH:9]1)-
[c:10]1[cH:11][cH:12][c:13](=[O:14])[nH:15][n:16]1>>[O:14]=[c:13]1[cH:12][cH:11]
[c:10]([n:16][nH:15]1)-[c:7]1[cH:8][cH:9][c:4]([cH:5][cH:6]1)[C:1]#[N:2].[Br:3]

Correctness of the mapping
MAPPET YES
USPTO NO

Reaction no 220 (5 bonds changed)

SMILES/SMARTS of the USPTO output:
[C-
:24]#[N:25].[CH3:1][N:2]1[CH2:3][CH2:4][C:5](=[C:8]2[c:9]3[c:10]([cH:20][cH:21][
cH:22][cH:23]3)[CH2:11][CH2:12][c:13]3[c:14]2[cH:15][c:16]([Br:19])[cH:17][cH:18
]3)[CH2:6][CH2:7]1.[CH3:26][N:27]([CH3:28])[CH:29]=[O:30].[CH:34]([Cl:35])([Cl:3
6])[Cl:37].[Na:31][C:32]#[N:33].[OH2:44].[cH:38]1[cH:39][cH:40][cH:41][cH:42][cH
:43]1>>[CH3:1][N:2]1[CH2:3][CH2:4][C:5](=[C:8]2[c:9]3[c:10]([cH:20][cH:21][cH:22
][cH:23]3)[CH2:11][CH2:12][c:13]3[c:14]2[cH:15][c:16]([C:26]#[N:27])[cH:17][cH:1
8]3)[CH2:6][CH2:7]1

SMILES/SMARTS of the MAPPET output:
[CH3:1][N:2]1[CH2:3][CH2:4][C:5]([CH2:6][CH2:7]1)=[C:8]1[c:9]2[cH:10][cH:11][cH:
12][cH:13][c:14]2[CH2:15][CH2:16][c:17]2[cH:18][cH:19][c:20]([Br:21])[cH:22][c:2
3]12.[C-
:24]#[N:25]>>[CH3:1][N:2]1[CH2:7][CH2:6][C:5]([CH2:4][CH2:3]1)=[C:8]1[c:9]2[cH:1
0][cH:11][cH:12][cH:13][c:14]2[CH2:15][CH2:16][c:17]2[cH:18][cH:19][c:20]([cH:22
][c:23]12)[C:24]#[N:25].[Br:21]

Correctness of the mapping
MAPPET YES
USPTO NO

Reaction no 221 (5 bonds changed)

SMILES/SMARTS of the USPTO output:
[CH:21](=[O:22])[O-
:23].[NH4+:24].[O:1]=[C:2]([CH2:3][c:4]1[cH:5][cH:6][c:7]([CH2:10][C:11](=[O:12]
)[O:13][CH3:14])[cH:8][cH:9]1)[c:15]1[cH:16][n:17][cH:18][cH:19][cH:20]1.[OH2:25
]>>[CH:2]([CH2:3][c:4]1[cH:5][cH:6][c:7]([CH2:10][C:11](=[O:12])[O:13][CH3:14])[
cH:8][cH:9]1)([c:15]1[cH:16][n:17][cH:18][cH:19][cH:20]1)[NH:24][CH:21]=[O:23]

SMILES/SMARTS of the MAPPET output:
[CH3:1][O:2][C:3](=[O:4])[CH2:5][c:6]1[cH:7][cH:8][c:9]([CH2:10][C:11](=[O:12])[
c:13]2[cH:14][cH:15][cH:16][n:17][cH:18]2)[cH:19][cH:20]1.[NH4+:21].[O-
:22][CH:23]=[O:24]>>[CH3:1][O:2][C:3](=[O:4])[CH2:5][c:6]1[cH:20][cH:19][c:9]([C
H2:10][CH:11]([NH:21][CH:23]=[O:24])[c:13]2[cH:14][cH:15][cH:16][n:17][cH:18]2)[
cH:8][cH:7]1.[O:12].[O:22]

Correctness of the mapping
MAPPET YES
USPTO YES

Reaction no 222 (5 bonds changed)

SMILES/SMARTS of the USPTO output:
[Br-
:6].[CH2:1]1[CH2:2][CH2:5][CH2:4][O:3]1.[CH2:7]([Mg+:8])[CH3:9].[CH3:32][CH:33]([CH3:34])[O-:35].[CH3:37][CH:38]([CH3:39])[O-:40].[CH3:41][CH:42]([CH3:43])[O-
:44].[CH3:45][CH:46]([CH3:47])[O-:48].[Cl-
:29].[NH4+:30].[OH2:31].[Ti+4:36].[c:10]1([CH2:16][N:17]2[CH:18]([C:22](=[O:23])[N:24]3[CH2:25][CH2:26][CH2:27][CH2:28]3)[CH2:19][CH2:20][CH2:21]2)[cH:11][cH:12][cH:13][cH:14][cH:15]1>>[CH2:1]1[CH2:2][C:22]1([CH:18]1[N:17]([CH2:16][c:10]2[cH:11][cH:12][cH:13][cH:14][cH:15]2)[CH2:21][CH2:20][CH2:19]1)[N:24]1[CH2:25][CH2:26][CH2:27][CH2:28]1

SMILES/SMARTS of the MAPPET output:
[O:1]=[C:2]([CH:3]1[CH2:4][CH2:5][CH2:6][N:7]1[CH2:8][c:9]1[cH:10][cH:11][cH:12][cH:13][cH:14]1)[N:15]1[CH2:16][CH2:17][CH2:18][CH2:19]1.[CH3:20][CH2:21][Mg+:22]>>[CH2:8]([N:7]1[CH2:6][CH2:5][CH2:4][CH:3]1[C:2]1([CH2:21][CH2:20]1)[N:15]1[CH2:19][CH2:18][CH2:17][CH2:16]1)[c:9]1[cH:14][cH:13][cH:12][cH:11][cH:10]1.[Mg:22].[O:1]

Correctness of the mapping
MAPPET YES
USPTO NO

Reaction no 223 (5 bonds changed)

SMILES/SMARTS of the USPTO output:
[CH3:25][c:26]1[cH:27][cH:28][cH:29][cH:30][cH:31]1.[NH2:1][CH2:2][CH2:3][c:4]1[cH:5][nH:6][c:7]2[cH:8][cH:9][cH:10][cH:11][c:12]12.[O:13]=[C:14]1[O:15][C:16](=[O:17])[c:18]2[cH:19][cH:20][cH:21][cH:22][c:23]21.[OH2:24]>>[N:1]1([CH2:2][CH2:3][c:4]2[cH:5][nH:6][c:7]3[cH:8][cH:9][cH:10][cH:11][c:12]23)[C:14](=[O:13])[c:23]2[c:18]([cH:19][cH:20][cH:21][cH:22]2)[C:16]1=[O:15]

SMILES/SMARTS of the MAPPET output:
[O:1]=[C:2]1[O:3][C:4](=[O:5])[c:6]2[cH:7][cH:8][cH:9][cH:10][c:11]12.[NH2:12][CH2:13][CH2:14][c:15]1[cH:16][nH:17][c:18]2[cH:19][cH:20][cH:21][cH:22][c:23]12>>[OH2:3].[O:1]=[C:2]1[N:12]([CH2:13][CH2:14][c:15]2[cH:16][nH:17][c:18]3[cH:19][cH:20][cH:21][cH:22][c:23]23)[C:4](=[O:5])[c:6]2[cH:7][cH:8][cH:9][cH:10][c:11]12

Correctness of the mapping
MAPPET YES
USPTO YES

Reaction no 224 (5 bonds changed)

SMILES/SMARTS of the USPTO output:
[CH3:1][c:2]1[c:3]([CH2:4][O:5][c:6]2[cH:7][c:8]([C:12]([CH2:13][CH2:14][C:15](=[O:16])[OH:17])=[O:18])[cH:9][cH:10][cH:11]2)[c:19]([CH3:23])[cH:20][cH:21][cH:22]1.[CH3:24][CH2:25][N:26]([CH2:27][CH3:28])[CH2:29][CH3:30].[NH3:31].[O:32]=[CH:33][N:34]([CH3:35])[CH3:36]>>[CH3:1][c:2]1[c:3]([CH2:4][O:5][c:6]2[cH:7][c:8]([C:12]([CH2:13][CH2:14][C:15](=[O:16])[NH2:26])=[O:18])[cH:9][cH:10][cH:11]2)[c:19]([CH3:23])[cH:20][cH:21][cH:22]1

SMILES/SMARTS of the MAPPET output:
[CH3:2][c:3]1[cH:4][cH:5][cH:6][c:7]([CH3:8])[c:9]1[CH2:10][O:11][c:12]1[cH:13][cH:14][cH:15][c:16]([cH:17]1)[C:18](=[O:19])[CH2:20][CH2:21][C:22]([OH:23])=[O:24].[NH3:1]>>[CH3:2][c:3]1[cH:4][cH:5][cH:6][c:7]([CH3:8])[c:9]1[CH2:10][O:11][c:12]1[cH:13][cH:14][cH:15][c:16]([cH:17]1)[C:18](=[O:19])[CH2:20][CH2:21][C:22]([NH2:1])=[O:24].[OH2:23]

Correctness of the mapping
MAPPET YES
USPTO NO

Reaction no 225 (5 bonds changed)

SMILES/SMARTS of the USPTO output:
[CH2:16]([CH3:17])[O:18][C:19]([CH:20]=[C:21]([O:22][CH2:23][CH3:24])[O:25][CH2:26][CH3:27])=[O:28].[CH3:29][CH2:30][N:31]([CH2:32][CH3:33])[CH2:34][CH3:35].[CH3:36][N:37]([CH3:38])[CH:39]=[O:40].[CH3:41][CH2:42][O:43][C:44](=[O:45])[CH3:46].[NH2:1][c:2]1[s:3][cH:4][c:5]([CH2:11][O:12][CH2:13][O:14][CH3:15])[c:6]1[S:7](=[O:8])(=[O:9])[NH2:10]>>[NH:1]1[c:2]2[s:3][cH:4][c:5]([CH2:11][O:12][CH2:13][O:14][CH3:15])[c:6]2[S:7](=[O:8])(=[O:9])[N:10]=[C:21]1[CH2:20][C:19]([O:18][CH2:16][CH3:17])=[O:28]

SMILES/SMARTS of the MAPPET output:
[CH3:1][O:2][CH2:3][O:4][CH2:5][c:6]1[cH:7][s:8][c:9]([NH2:10])[c:11]1[S:12]([NH2:13])(=[O:14])=[O:15].[CH3:16][CH2:17][O:18][C:19](=[O:20])[CH:21]=[C:22]([O:23][CH2:24][CH3:25])[O:26][CH2:27][CH3:28]>>[CH3:16][CH2:17][O:18][C:19](=[O:20])[CH2:21][C:22]1=[N:13][S:12](=[O:14])(=[O:15])[c:11]2[c:6]([CH2:5][O:4][CH2:3][O:2][CH3:1])[cH:7][s:8][c:9]2[NH:10]1.[C:28].[C:27].[C:24].[C:25].[O:23].[O:26]

Correctness of the mapping
MAPPET YES
USPTO YES

Reaction no 226 (5 bonds changed)

SMILES/SMARTS of the USPTO output:
[CH3:36][OH:37].[CH:28]1([NH2:31])[CH2:29][CH2:30]1.[Cl:1][S:2](=[O:3])(=[O:4])[OH:5].[NH:6]([c:7]1[cH:8][cH:9][cH:10][cH:11][cH:12]1)[c:13]1[n:14][cH:15][cH:16][c:17](-
[c:19]2[cH:20][n:21][c:22]([CH:25]([CH3:26])[CH3:27])[n:23]2[CH3:24])[n:18]1.[S:32]([Cl:33])([Cl:34])=[O:35]>>[S:2](=[O:3])(=[O:5])([c:10]1[cH:9][cH:8][c:7]([NH:6][c:13]2[n:14][cH:15][cH:16][c:17](-
[c:19]3[cH:20][n:21][c:22]([CH:25]([CH3:26])[CH3:27])[n:23]3[CH3:24])[n:18]2)[cH:12][cH:11]1)[NH:31][CH:28]1[CH2:29][CH2:30]1

SMILES/SMARTS of the MAPPET output:
[CH3:1][CH:2]([CH3:3])[c:4]1[n:5][cH:6][c:7](-
[c:8]2[cH:9][cH:10][n:11][c:12]([NH:13][c:14]3[cH:15][cH:16][cH:17][cH:18][cH:19]3)[n:20]2)[n:21]1[CH3:22].[OH:23][S:24]([Cl:25])(=[O:26])=[O:27].[NH2:28][CH:29]1[CH2:30][CH2:31]1>>[CH3:3][CH:2]([CH3:1])[c:4]1[n:5][cH:6][c:7](-
[c:8]2[cH:9][cH:10][n:11][c:12]([NH:13][c:14]3[cH:15][cH:16][c:17]([cH:18][cH:19]3)[S:24](=[O:27])(=[O:26])[NH:28][CH:29]3[CH2:30][CH2:31]3)[n:20]2)[n:21]1[CH3:22].[Cl:25].[O:23]

Correctness of the mapping
MAPPET YES
USPTO YES

Reaction no 227 (5 bonds changed)

SMILES/SMARTS of the USPTO output:

[C:1]([CH:2]([OH:3])[CH:4]([OH:5])[C:6](=[O:7])[OH:9])(=[O:8])[OH:10].[CH3:19][c:20]1[cH:21][c:22]([CH3:23])[cH:24][cH:25][cH:26]1.[NH2:11][CH2:12][c:13]1[cH:14][cH:15][cH:16][cH:17][cH:18]1.[OH2:27]>>[C:1]1(=[O:10])[CH:2]([OH:3])[CH:4]([OH:5])[C:6](=[O:7])[N:11]1[CH2:12][c:13]1[cH:14][cH:15][cH:16][cH:17][cH:18]1

SMILES/SMARTS of the MAPPET output:
[NH2:1][CH2:2][c:3]1[cH:4][cH:5][cH:6][cH:7][cH:8]1.[OH:9][CH:10]([CH:11]([OH:12])[C:13]([OH:14])=[O:15])[C:16]([OH:17])=[O:18]>>[OH2:17].[OH2:14].[OH:9][CH:10]1[CH:11]([OH:12])[C:13](=[O:15])[N:1]([CH2:2][c:3]2[cH:4][cH:5][cH:6][cH:7][cH:8]2)[C:16]1=[O:18]

Correctness of the mapping
MAPPET YES
USPTO YES

Reaction no 228 (5 bonds changed)

SMILES/SMARTS of the USPTO output:
[CH:12]([CH3:13])([CH3:14])[c:15]1[c:16]([NH2:17])[c:18]([CH:22]([CH3:23])[CH3:24])[cH:19][cH:20][cH:21]1.[O:1]=[C:2]1[O:3][C:4](=[O:5])[c:6]2[cH:7][cH:8][cH:9][cH:10][c:11]21>>[C:2]1(=[O:3])[c:11]2[c:6]([cH:7][cH:8][cH:9][cH:10]2)[C:4](=[O:5])[N:17]1[c:16]1[c:15]([CH:12]([CH3:13])[CH3:14])[cH:21][cH:20][cH:19][c:18]1[CH:22]([CH3:23])[CH3:24]

SMILES/SMARTS of the MAPPET output:
[O:1]=[C:2]1[O:3][C:4](=[O:5])[c:6]2[cH:7][cH:8][cH:9][cH:10][c:11]12.[CH3:12][CH:13]([CH3:14])[c:15]1[cH:16][cH:17][cH:18][c:19]([CH:20]([CH3:21])[CH3:22])[c:23]1[NH2:24]>>[OH2:3].[CH3:22][CH:20]([CH3:21])[c:19]1[cH:18][cH:17][cH:16][c:15]([CH:13]([CH3:14])[CH3:12])[c:23]1[N:24]1[C:2](=[O:1])[c:11]2[cH:10][cH:9][cH:8][cH:7][c:6]2[C:4]1=[O:5]

Correctness of the mapping
MAPPET YES
USPTO YES

Reaction no 229 (5 bonds changed)

SMILES/SMARTS of the USPTO output:
[C:6]([c:7]1[cH:8][cH:9][cH:10][cH:11][cH:12]1)(=[O:13])[Cl:14].[CH2:4]=[O:5].[K:1][C:2]#[N:3].[OH2:15]>>[C:2](#[N:3])[CH:4]([OH:5])[C:6]([c:7]1[cH:8][cH:9][cH:10][cH:11][cH:12]1)=[O:13]

SMILES/SMARTS of the MAPPET output:
[K:1][C:2]#[N:3].[CH2:4]=[O:5].[Cl:6][C:7](=[O:8])[c:9]1[cH:10][cH:11][cH:12][cH:13][cH:14]1>>[OH:5][CH:4]([C:2]#[N:3])[C:7](=[O:8])[c:9]1[cH:14][cH:13][cH:12][cH:11][cH:10]1.[Cl:6].[K:1]

Correctness of the mapping
MAPPET YES
USPTO YES

Reaction no 230 (5 bonds changed)

SMILES/SMARTS of the USPTO output:

[CH2:45]1[O:46][CH2:47][CH2:48][CH2:49]1.[CH3:28][CH2:29][N:30]([CH2:31][CH3:32])[CH2:33][CH3:34].[Cl-:43].[Cl:35][C:36]([O:37][CH2:38][CH3:39])=[O:40].[NH4+:42].[NH4+:44].[OH-:41].[OH:1][c:2]1[cH:3][cH:4][c:5]([C:8]([c:9]2[cH:10][cH:11][c:12]([C:13](=[O:14])[OH:15])[cH:16][cH:17]2)=[C:18]2[CH2:19][C:20]([CH3:26])([CH3:27])[CH2:21][C:22]([CH3:24])([CH3:25])[CH2:23]2)[cH:6][cH:7]1>>[OH:1][c:2]1[cH:3][cH:4][c:5]([C:8]([c:9]2[cH:10][cH:11][c:12]([C:13](=[O:14])[NH2:30])[cH:16][cH:17]2)=[C:18]2[CH2:19][C:20]([CH3:26])([CH3:27])[CH2:21][C:22]([CH3:24])([CH3:25])[CH2:23]2)[cH:6][cH:7]1

SMILES/SMARTS of the MAPPET output:
[CH3:1][C:2]1([CH3:3])[CH2:4][C:5]([CH2:6][C:7]([CH3:8])([CH3:9])[CH2:10]1)=[C:11]([c:12]1[cH:13][cH:14][c:15]([OH:16])[cH:17][cH:18]1)[c:19]1[cH:20][cH:21][c:22]([cH:23][cH:24]1)[C:25]([OH:26])=[O:27].[NH4+:28]>>[CH3:8][C:7]1([CH3:9])[CH2:6][C:5]([CH2:4][C:2]([CH3:1])([CH3:3])[CH2:10]1)=[C:11]([c:12]1[cH:18][cH:17][c:15]([OH:16])[cH:14][cH:13]1)[c:19]1[cH:24][cH:23][c:22]([cH:21][cH:20]1)[C:25]([NH2:28])=[O:27].[O:26]

Correctness of the mapping
MAPPET YES
USPTO NO

Reaction no 231 (5 bonds changed)

SMILES/SMARTS of the USPTO output:
[C:25]([O:26][CH2:27][CH3:28])(=[O:29])[CH2:30][CH3:31].[C:4]1(=[O:5])[O:6][CH2:7][c:8]2[cH:9][cH:10][cH:11][cH:12][c:13]21.[CH3:1][O-:2].[CH3:33][OH:34].[N+:14](=[O:15])([O-:16])[c:17]1[cH:18][c:19]([CH:20]=[O:21])[cH:22][cH:23][cH:24]1.[Na+:3].[OH2:32]>>[C:4]1(=[O:5])[c:13]2[c:8]([cH:9][cH:10][cH:11][cH:12]2)[C:7](=[O:6])[CH:20]1[c:19]1[cH:18][c:17]([N+:14](=[O:15])[O-:16])[cH:24][cH:23][cH:22]1

SMILES/SMARTS of the MAPPET output:
[O-:1][N+:2](=[O:3])[c:4]1[cH:5][cH:6][cH:7][c:8]([CH:9]=[O:10])[cH:11]1.[O:12]=[C:13]1[O:14][CH2:15][c:16]2[cH:17][cH:18][cH:19][cH:20][c:21]12>>[OH2:10].[O-:1][N+:2](=[O:3])[c:4]1[cH:5][cH:6][cH:7][c:8]([cH:11]1)[CH:9]1[C:13](=[O:12])[c:21]2[cH:20][cH:19][cH:18][cH:17][c:16]2[C:15]1=[O:14]

Correctness of the mapping
MAPPET YES
USPTO YES

Reaction no 232 (5 bonds changed)

SMILES/SMARTS of the USPTO output:
[Br:1][c:2]1[cH:3][n:4][n:5]([CH3:18])[c:6]1-[c:7]1[cH:8][c:9]([C:14](=[O:15])[O:16][CH3:17])[s:10][c:11]1[CH2:12][CH3:13].[C:19](=[O:20])([O-:21])[O-:22].[CH3:25][B:26]1[O:27][B:28]([CH3:29])[O:30][B:31]([CH3:32])[O:33]1.[CH3:34][N:35]([CH3:36])[CH:37]=[O:38].[K+:23].[K+:24]>>[c:2]1([CH3:19])[cH:3][n:4][n:5]([CH3:18])[c:6]1-[c:7]1[cH:8][c:9]([C:14](=[O:15])[O:16][CH3:17])[s:10][c:11]1[CH2:12][CH3:13]

SMILES/SMARTS of the MAPPET output:
[CH3:1][B:2]1[O:3][B:4]([CH3:5])[O:6][B:7]([CH3:8])[O:9]1.[CH3:10][CH2:11][c:12]1[s:13][c:14]([cH:15][c:16]1-

[c:17]1[c:18]([Br:19])[cH:20][n:21][n:22]1[CH3:23])[C:24](=[O:25])[O:26][CH3:27]
>>[CH3:10][CH2:11][c:12]1[s:13][c:14]([cH:15][c:16]1-
[c:17]1[c:18]([CH3:8])[cH:20][n:21][n:22]1[CH3:23])[C:24](=[O:25])[O:26][CH3:27]
.[B:4].[B:2].[B:7].[Br:19].[C:1].[C:5].[O:9].[O:6].[O:3]

Correctness of the mapping
MAPPET YES
USPTO NO

Reaction no 233 (5 bonds changed)

SMILES/SMARTS of the USPTO output:
[C:1]([CH3:2])([CH3:3])([CH3:4])[c:5]1[c:6]([NH2:14])[cH:7][c:8]([N+:11](=[O:12]
)[O-:13])[cH:9][cH:10]1.[ClH:25].[N:15]([O-
:16])=[O:17].[Na+:18].[Na+:23].[Na+:24].[OH2:26].[S:19](=[O:20])([O-:21])[O-
:22]>>[C:1]([CH3:2])([CH3:3])([CH3:4])[c:5]1[c:6]([S:19](=[O:20])(=[O:22])[Cl:25
])[cH:7][c:8]([N+:11](=[O:12])[O-:13])[cH:9][cH:10]1

SMILES/SMARTS of the MAPPET output:
[O-:1][S:2]([O-:3])=[O:4].[O-
:5][N:6]=[O:7].[ClH:8].[CH3:9][C:10]([CH3:11])([CH3:12])[c:13]1[cH:14][cH:15][c:
16]([cH:17][c:18]1[NH2:19])[N+:20]([O-
:21])=[O:22]>>[CH3:12][C:10]([CH3:11])([CH3:9])[c:13]1[cH:14][cH:15][c:16]([cH:1
7][c:18]1[S:2]([Cl:8])(=[O:4])=[O:1])[N+:20]([O-
:21])=[O:22].[N:6].[N:19].[O:7].[O:5].[O:3]

Correctness of the mapping
MAPPET YES
USPTO YES

Reaction no 234 (5 bonds changed)

SMILES/SMARTS of the USPTO output:
[Cl:1][c:2]1[c:3]([CH:9]([CH2:10][C:11](=[O:12])[OH:16])[CH2:14][C:15](=[O:13])[
OH:17])[c:4]([Cl:8])[cH:5][cH:6][cH:7]1.[NH4+:19].[OH-
:18]>>[Cl:1][c:2]1[c:3]([CH:9]2[CH2:10][C:11](=[O:12])[NH:19][C:15](=[O:17])[CH2
:14]2)[c:4]([Cl:8])[cH:5][cH:6][cH:7]1

SMILES/SMARTS of the MAPPET output:
[NH4+:1].[OH:2][C:3](=[O:4])[CH2:5][CH:6]([CH2:7][C:8]([OH:9])=[O:10])[c:11]1[c:
12]([Cl:13])[cH:14][cH:15][cH:16][c:17]1[Cl:18]>>[Cl:13][c:12]1[cH:14][cH:15][cH
:16][c:17]([Cl:18])[c:11]1[CH:6]1[CH2:7][C:8](=[O:10])[NH:1][C:3](=[O:4])[CH2:5]
1.[O:9].[O:2]

Correctness of the mapping
MAPPET YES
USPTO NO

Reaction no 235 (5 bonds changed)

SMILES/SMARTS of the USPTO output:
[Br:1][c:2]1[cH:3][cH:4][c:5]2[c:13]([cH:14]1)[CH2:12][c:11]1[c:6]-
2[cH:7][cH:8][c:9]([NH2:15])[cH:10]1.[CH3:21][C:22]([CH3:23])([O-
:24])[CH3:25].[I:16][CH2:17][CH2:18][CH2:19][CH3:20].[K+:26].[O:27]1[CH2:28][CH2
:29][CH2:30][CH2:31]1.[OH2:32]>>[Br:1][c:2]1[cH:3][cH:4][c:5]2[c:13]([cH:14]1)[C

:12]([CH2:17][CH2:18][CH2:19][CH3:20])([CH2:28][CH2:29][CH2:30][CH3:31])[c:11]1[c:6]-2[cH:7][cH:8][c:9]([NH2:15])[cH:10]1

SMILES/SMARTS of the MAPPET output:
[CH3:1][CH2:2][CH2:3][CH2:4][I:5].[NH2:6][c:7]1[cH:8][cH:9][c:10]-2[c:11]([CH2:12][c:13]3[cH:14][c:15]([Br:16])[cH:17][cH:18][c:19]-23)[cH:20]1.[C:21].[C:22].[C:23].[C:24]>>[CH3:22][CH2:23][CH2:21][CH2:24][C:12]1([CH2:4][CH2:3][CH2:2][CH3:1])[c:11]2[cH:20][c:7]([NH2:6])[cH:8][cH:9][c:10]2-[c:19]2[cH:18][cH:17][c:15]([Br:16])[cH:14][c:13]12.[I:5]

Correctness of the mapping
MAPPET YES
USPTO NO

Reaction no 236 (5 bonds changed)

SMILES/SMARTS of the USPTO output:
[Ag+:25].[Br:1][c:2]1[c:3]([C:4](=[O:5])[O:6][CH2:7][CH3:8])[cH:9][c:10]([C:13]([Br:14])([Br:15])[Br:16])[cH:11][n:12]1.[CH3:17][CH2:18][OH:19].[N+:21]([O-:22])([O-:23])=[O:24].[OH2:20]>>[Br:1][c:2]1[c:3]([C:4](=[O:5])[O:6][CH2:7][CH3:8])[cH:9][c:10]([C:13]([O:19][CH2:18][CH3:17])=[O:20])[cH:11][n:12]1

SMILES/SMARTS of the MAPPET output:
[OH2:1].[CH3:2][CH2:3][OH:4].[CH3:5][CH2:6][O:7][C:8](=[O:9])[c:10]1[cH:11][c:12]([cH:13][n:14][c:15]1[Br:16])[C:17]([Br:18])([Br:19])[Br:20]>>[CH3:2][CH2:3][O:4][C:17](=[O:1])[c:12]1[cH:13][n:14][c:15]([Br:16])[c:10]([cH:11]1)[C:8](=[O:9])[O:7][CH2:6][CH3:5].[Br:20].[Br:19].[Br:18]

Correctness of the mapping
MAPPET YES
USPTO YES

Reaction no 237 (5 bonds changed)

SMILES/SMARTS of the USPTO output:
[CH2:1]([CH3:2])[O:3][C:4]([CH2:5][NH:6][S:7](=[O:8])(=[O:9])[c:10]1[cH:11][n:12][c:13]([NH2:15])[s:14]1)=[O:16].[CH2:30]1[CH2:32][CH2:31][CH2:33][O:34]1.[CH3:35][N:36]([c:37]1[cH:38][cH:39][n:40][cH:41][cH:42]1)[CH3:43].[CH:17]1([NH:23][CH:24]2[CH2:25][CH2:26][CH2:27][CH2:28][CH2:29]2)[CH2:18][CH2:19][CH2:20][CH2:21][CH2:22]1>>[CH2:1]([CH3:2])[O:3][C:4]([CH2:5][NH:6][S:7](=[O:8])(=[O:9])[c:10]1[cH:11][n:12][c:13]([NH:15][C:33]([N:23]([CH:17]2[CH2:18][CH2:19][CH2:20][CH2:21][CH2:22]2)[CH:24]2[CH2:25][CH2:26][CH2:27][CH2:28][CH2:29]2)=[O:34])[s:14]1)=[O:16]

SMILES/SMARTS of the MAPPET output:
[CH2:1]1[CH2:2][CH2:3][CH:4]([CH2:5][CH2:6]1)[NH:7][CH:8]1[CH2:9][CH2:10][CH2:11][CH2:12][CH2:13]1.[CH3:14][CH2:15][O:16][C:17](=[O:18])[CH2:19][NH:20][S:21](=[O:22])(=[O:23])[c:24]1[cH:25][n:26][c:27]([NH2:28])[s:29]1.[C:30].[O:31]>>[CH3:14][CH2:15][O:16][C:17](=[O:18])[CH2:19][NH:20][S:21](=[O:22])(=[O:23])[c:24]1[cH:25][n:26][c:27]([NH:28][C:30](=[O:31])[N:7]([CH:8]2[CH2:9][CH2:10][CH2:11][CH2:12][CH2:13]2)[CH:4]2[CH2:3][CH2:2][CH2:1][CH2:6][CH2:5]2)[s:29]1

Correctness of the mapping
MAPPET YES
USPTO NO

Reaction no 238 (5 bonds changed)

SMILES/SMARTS of the USPTO output:
[CH2:1]1[CH2:2][CH2:3][C:4]2=[N:9][CH2:8][CH2:7][CH2:6][N:5]2[CH2:10][CH2:11]1.[CH2:36]([Cl:37])[Cl:38].[Cl:44][CH3:45].[NH2:12][c:13]1[cH:14][c:15](-[c:19]2[cH:20][cH:21][c:22]([C:25]([CH2:26][NH:27][S:28](=[O:29])(=[O:30])[CH:31]([CH3:32])[CH3:33])([CH3:34])[F:35])[cH:23][cH:24]2)[cH:16][cH:17][cH:18]1.[OH2:46].[S:39](=[O:40])(=[O:41])([Cl:42])[Cl:43]>>[NH:12]([c:13]1[cH:14][c:15](-[c:19]2[cH:20][cH:21][c:22]([C:25]([CH2:26][NH:27][S:28](=[O:29])(=[O:30])[CH:31]([CH3:32])[CH3:33])([CH3:34])[F:35])[cH:23][cH:24]2)[cH:16][cH:17][cH:18]1)[S:39](=[O:40])(=[O:41])[CH3:45]

SMILES/SMARTS of the MAPPET output:
[Cl:1][S:2]([Cl:3])(=[O:4])=[O:5].[CH3:6][CH:7]([CH3:8])[S:9](=[O:10])(=[O:11])[NH:12][CH2:13][C:14]([CH3:15])([F:16])[c:17]1[cH:18][cH:19][c:20]([cH:21][cH:22]1)-[c:23]1[cH:24][cH:25][cH:26][c:27]([NH2:28])[cH:29]1.[CH3:30][Cl:31]>>[CH3:8][CH:7]([CH3:6])[S:9](=[O:10])(=[O:11])[NH:12][CH2:13][C:14]([CH3:15])([F:16])[c:17]1[cH:22][cH:21][c:20]([cH:19][cH:18]1)-[c:23]1[cH:24][cH:25][cH:26][c:27]([NH:28][S:2]([CH3:30])(=[O:4])=[O:5])[cH:29]1.[Cl:3].[Cl:1].[Cl:31]

Correctness of the mapping
MAPPET YES
USPTO YES

Reaction no 239 (5 bonds changed)

SMILES/SMARTS of the USPTO output:
[C:1]([CH3:2])([CH3:3])([CH3:4])[OH:5].[CH3:22][C:23](=[O:24])[OH:25].[CH3:6][c:7]1[c:8]([C:13]#[N:14])[n:9][cH:10][cH:11][cH:12]1.[Na+:21].[OH-:20].[OH2:26].[S:15]([OH:16])(=[O:17])(=[O:18])[OH:19]>>[C:1]([CH3:2])([CH3:3])([CH3:4])[NH:14][C:13]([c:8]1[c:7]([CH3:6])[cH:12][cH:11][cH:10][n:9]1)=[O:16]

SMILES/SMARTS of the MAPPET output:
[OH2:1].[CH3:2][c:3]1[cH:4][cH:5][cH:6][n:7][c:8]1[C:9]#[N:10].[CH3:11][C:12]([CH3:13])([CH3:14])[OH:15]>>[OH2:1].[CH3:2][c:3]1[cH:4][cH:5][cH:6][n:7][c:8]1[C:9](=[O:15])[NH:10][C:12]([CH3:13])([CH3:11])[CH3:14]

Correctness of the mapping
MAPPET NO
USPTO YES

Reaction no 240 (5 bonds changed)

SMILES/SMARTS of the USPTO output:
[C:3]([CH2:4][CH2:5][C:6]([CH3:8])=[O:9])(=[O:7])[OH:10].[CH3:21][CH2:22][OH:23].[H:1][H:2].[NH2:11][c:12]1[cH:13][c:14]([NH2:15])[c:16]([CH3:20])[cH:17][c:18]1[CH3:19].[Pt:24]=[O:25]>>[C:3]1(=[O:10])[CH2:4][CH2:5][CH:6]([CH3:8])[N:15]1[c:14]1[cH:13][c:12]([NH2:11])[c:18]([CH3:19])[cH:17][c:16]1[CH3:20]

SMILES/SMARTS of the MAPPET output:
[CH3:1][c:2]1[cH:3][c:4]([CH3:5])[c:6]([NH2:7])[cH:8][c:9]1[NH2:10].[CH3:11][C:12](=[O:13])[CH2:14][CH2:15][C:16]([OH:17])=[O:18]>>[CH3:11][CH:12]1[CH2:14][CH2:

15][C:16](=[O:18])[N:7]1[c:6]1[cH:8][c:9]([NH2:10])[c:2]([CH3:1])[cH:3][c:4]1[CH3:5].[O:17].[O:13]

Correctness of the mapping
MAPPET YES
USPTO YES

Reaction no 241 (5 bonds changed)

SMILES/SMARTS of the USPTO output:
[C:12]([CH3:13])([O:14][CH2:15][CH3:16])([O:17][CH2:18][CH3:19])[O:20][CH2:21][CH3:22].[C:1]12([NH2:11])[CH2:2][CH:3]3[CH2:4][CH:5]([CH2:6][CH:7]([CH2:8]1)[CH2:9]3)[CH2:10]2.[CH:23]1([NH2:29])[CH2:24][CH2:25][CH2:26][CH2:27][CH2:28]1>>[C:1]12([NH:11][C:12]([CH3:13])=[N:29][CH:23]3[CH2:24][CH2:25][CH2:26][CH2:27][CH2:28]3)[CH2:2][CH:3]3[CH2:4][CH:5]([CH2:6][CH:7]([CH2:8]1)[CH2:9]3)[CH2:10]2

SMILES/SMARTS of the MAPPET output:
-

Correctness of the mapping
MAPPET NO
USPTO YES

Reaction no 242 (5 bonds changed)

SMILES/SMARTS of the USPTO output:
[CH2:54]1[O:55][CH2:56][CH2:57][CH2:58]1.[Cl:24][C:25](=[O:26])[Cl:27].[Cl:51][CH2:52][Cl:53].[N-:28]=[C:29]=[O:30].[NH2:1][c:2]1[c:3]([O:17][CH3:18])[c:4]([NH:12][S:13](=[O:14])(=[O:15])[CH3:16])[cH:5][c:6]([C:8]([CH3:9])([CH3:10])[CH3:11])[cH:7]1.[NH2:31][c:32]1[cH:33][cH:34][c:35](-[n:42]2[cH:43][cH:44][c:45]3[cH:46][n:47][cH:48][cH:49][c:50]23)[c:36]2[cH:37][cH:38][cH:39][cH:40][c:41]12.[Na+:23].[O-:19][C:20](=[O:21])[OH:22]>>[NH:1]([c:2]1[c:3]([O:17][CH3:18])[c:4]([NH:12][S:13](=[O:14])(=[O:15])[CH3:16])[cH:5][c:6]([C:8]([CH3:9])([CH3:10])[CH3:11])[cH:7]1)[C:20](=[O:22])[NH:31][c:32]1[cH:33][cH:34][c:35](-[n:42]2[cH:43][cH:44][c:45]3[cH:46][n:47][cH:48][cH:49][c:50]23)[c:36]2[cH:37][cH:38][cH:39][cH:40][c:41]12

SMILES/SMARTS of the MAPPET output:
[NH2:1][c:2]1[cH:3][cH:4][c:5](-[n:6]2[cH:7][cH:8][c:9]3[cH:10][n:11][cH:12][cH:13][c:14]23)[c:15]2[cH:16][cH:17][cH:18][cH:19][c:20]12.[CH3:21][O:22][c:23]1[c:24]([NH2:25])[cH:26][c:27]([cH:28][c:29]1[NH:30][S:31]([CH3:32])(=[O:33])=[O:34])[C:35]([CH3:36])([CH3:37])[CH3:38].[Cl:39][C:40]([Cl:41])=[O:42]>>[CH3:21][O:22][c:23]1[c:24]([NH:25][C:40](=[O:42])[NH:1][c:2]2[cH:3][cH:4][c:5](-[n:6]3[cH:7][cH:8][c:9]4[cH:10][n:11][cH:12][cH:13][c:14]34)[c:15]3[cH:16][cH:17][cH:18][cH:19][c:20]23)[cH:26][c:27]([cH:28][c:29]1[NH:30][S:31]([CH3:32])(=[O:34])=[O:33])[C:35]([CH3:36])([CH3:38])[CH3:37].[Cl:39].[Cl:41]

Correctness of the mapping
MAPPET YES
USPTO NO

Reaction no 243 (5 bonds changed)

SMILES/SMARTS of the USPTO output:
[CH3:21][C:22](=[O:23])[OH:24].[F:12][c:13]1[cH:14][n:15][cH:16][cH:17][c:18]1[NH:19][NH2:20].[O:1]=[C:2]1[O:3][C:4](=[O:5])[c:6]2[cH:7][cH:8][cH:9][cH:10][c:11]21>>[C:2]1(=[O:3])[c:11]2[c:6]([cH:7][cH:8][cH:9][cH:10]2)[C:4](=[O:5])[N:20]1[NH:19][c:18]1[c:13]([F:12])[cH:14][n:15][cH:16][cH:17]1

SMILES/SMARTS of the MAPPET output:
[O:1]=[C:2]1[O:3][C:4](=[O:5])[c:6]2[cH:7][cH:8][cH:9][cH:10][c:11]12.[NH2:12][NH:13][c:14]1[cH:15][cH:16][n:17][cH:18][c:19]1[F:20]>>[OH2:3].[F:20][c:19]1[cH:18][n:17][cH:16][cH:15][c:14]1[NH:13][N:12]1[C:4](=[O:5])[c:6]2[cH:7][cH:8][cH:9][cH:10][c:11]2[C:2]1=[O:1]

Correctness of the mapping
MAPPET YES
USPTO NO

Reaction no 244 (5 bonds changed)

SMILES/SMARTS of the USPTO output:
[CH3:14][N:15]1[CH2:16][CH2:17][O:18][CH2:19][CH2:20]1.[CH3:32][N:33]([CH3:34])[CH2:35][CH2:36][CH2:37][N:38]=[C:39]=[N:40][CH2:41][CH3:42].[CH3:50][CH2:51][O:52][C:53](=[O:54])[CH3:55].[ClH:31].[F:1][c:2]1[cH:3][c:4]([C:5](=[O:6])[OH:7])[cH:8][cH:9][c:10]1[N+:11](=[O:12])[O-:13].[NH4+:43].[O:45]1[CH2:46][CH2:47][CH2:48][CH2:49]1.[OH-:44].[OH:21][n:22]1[c:23]2[cH:24][cH:25][cH:26][cH:27][c:28]2[n:29][n:30]1>>[F:1][c:2]1[cH:3][c:4]([C:5](=[O:6])[NH2:15])[cH:8][cH:9][c:10]1[N+:11](=[O:12])[O-:13]

SMILES/SMARTS of the MAPPET output:
[NH4+:1].[OH:2][C:3](=[O:4])[c:5]1[cH:6][cH:7][c:8]([c:9]([F:10])[cH:11]1)[N+:12]([O-:13])=[O:14]>>[NH2:1][C:3](=[O:4])[c:5]1[cH:6][cH:7][c:8]([c:9]([F:10])[cH:11]1)[N+:12]([O-:13])=[O:14].[O:2]

Correctness of the mapping
MAPPET YES
USPTO NO

Reaction no 245 (5 bonds changed)

SMILES/SMARTS of the USPTO output:
[CH2:1]([c:2]1[cH:3][cH:4][cH:5][cH:6][cH:7]1)[O:8][C:9](=[O:10])[c:11]1[cH:12][c:13]([Br:34])[c:14]2[c:15]3[c:16]([nH:17][c:18]2[c:19]1[CH3:20])[C:21]([CH2:25][CH2:26][CH3:27])([CH2:28][C:29](=[O:30])[O:31][CH2:32][CH3:33])[O:22][CH2:23][CH2:24]3.[CH3:35][N:36]1[CH2:37][CH2:38][CH2:39][C:40]1=[O:41].[CH3:46][CH2:47][O:48][C:49]([CH3:50])=[O:51].[Cu:42][C:43]#[N:44].[OH2:45]>>[CH2:1]([c:2]1[cH:3][cH:4][cH:5][cH:6][cH:7]1)[O:8][C:9](=[O:10])[c:11]1[cH:12][c:13]([C:35]#[N:36])[c:14]2[c:15]3[c:16]([nH:17][c:18]2[c:19]1[CH3:20])[C:21]([CH2:25][CH2:26][CH3:27])([CH2:28][C:29](=[O:30])[O:31][CH2:32][CH3:33])[O:22][CH2:23][CH2:24]3

SMILES/SMARTS of the MAPPET output:
[Cu:1][C:2]#[N:3].[CH3:4][CH2:5][CH2:6][C:7]1([CH2:8][C:9](=[O:10])[O:11][CH2:12][CH3:13])[O:14][CH2:15][CH2:16][c:17]2[c:18]1[nH:19][c:20]1[c:21]([CH3:22])[c:23]([cH:24][c:25]([Br:26])[c:27]21)[C:28](=[O:29])[O:30][CH2:31][c:32]1[cH:33][cH:34][cH:35][cH:36][cH:37]1>>[CH3:4][CH2:5][CH2:6][C:7]1([CH2:8][C:9](=[O:10])[O:11][CH2:12][CH3:13])[O:14][CH2:15][CH2:16][c:17]2[c:18]1[nH:19][c:20]1[c:21]([CH

3:22])[c:23]([cH:24][c:25]([C:2]#[N:3])[c:27]21)[C:28](=[O:29])[O:30][CH2:31][c:
32]1[cH:37][cH:36][cH:35][cH:34][cH:33]1.[Br:26].[Cu:1]

Correctness of the mapping
MAPPET YES
USPTO NO

Reaction no 246 (5 bonds changed)

SMILES/SMARTS of the USPTO output:
[C:9]([CH:10]=[O:11])(=[O:12])[OH:13].[CH3:14][O:15][c:16]1[cH:17][c:18]([CH:19]
=[CH2:20])[cH:21][cH:22][c:23]1[O:24][CH3:25].[I:1][c:2]1[c:3]([NH2:4])[cH:5][cH
:6][cH:7][cH:8]1>>[I:1][c:2]1[c:3]2[c:5]([cH:6][cH:7][cH:8]1)[CH:19]([c:18]1[cH:
17][c:16]([O:15][CH3:14])[c:23]([O:24][CH3:25])[cH:22][cH:21]1)[CH2:20][CH:10]([
C:9](=[O:12])[OH:13])[NH:4]2

SMILES/SMARTS of the MAPPET output:
[NH2:1][c:2]1[cH:3][cH:4][cH:5][cH:6][c:7]1[I:8].[CH3:9][O:10][c:11]1[cH:12][cH:
13][c:14]([CH:15]=[CH2:16])[cH:17][c:18]1[O:19][CH3:20].[OH:21][C:22](=[O:23])[C
H:24]=[O:25]>>[CH3:9][O:10][c:11]1[cH:12][cH:13][c:14]([cH:17][c:18]1[O:19][CH3:
20])[CH:15]1[CH2:16][CH:24]([NH:1][c:2]2[c:7]([I:8])[cH:6][cH:5][cH:4][c:3]12)[C
:22]([OH:21])=[O:23].[O:25]

Correctness of the mapping
MAPPET YES
USPTO YES

Reaction no 247 (5 bonds changed)

SMILES/SMARTS of the USPTO output:
[CH2:14]([CH:15]=[CH2:16])[Cl:17].[CH3:18][Si:19]([CH3:20])([CH3:21])[C:22]#[N:2
3].[CH:80]([Cl:81])([Cl:82])[Cl:83].[Cl:1][c:2]1[cH:3][cH:4][c:5]([CH:8]=[C:9]([
C:10]#[N:11])[C:12]#[N:13])[cH:6][n:7]1.[O:26]=[C:27]([CH:28]=[CH:29][c:30]1[cH:
31][cH:32][cH:33][cH:34][cH:35]1)[CH:36]=[CH:37][c:38]1[cH:39][cH:40][cH:41][cH:
42][cH:43]1.[O:44]=[C:45]([CH:46]=[CH:47][c:48]1[cH:49][cH:50][cH:51][cH:52][cH:
53]1)[CH:54]=[CH:55][c:56]1[cH:57][cH:58][cH:59][cH:60][cH:61]1.[O:62]=[C:63]([C
H:64]=[CH:65][c:66]1[cH:67][cH:68][cH:69][cH:70][cH:71]1)[CH:72]=[CH:73][c:74]1[
cH:75][cH:76][cH:77][cH:78][cH:79]1.[O:84]1[CH2:85][CH2:86][CH2:87][CH2:88]1.[Pd
:24].[Pd:25]>>[Cl:1][c:2]1[cH:3][cH:4][c:5]([CH:8]([C:9]([C:10]#[N:11])([C:12]#[
N:13])[CH2:14][CH:15]=[CH2:16])[C:22]#[N:23])[cH:6][n:7]1

SMILES/SMARTS of the MAPPET output:
[Cl:1][c:2]1[cH:3][cH:4][c:5]([CH:6]=[C:7]([C:8]#[N:9])[C:10]#[N:11])[cH:12][n:1
3]1.[CH3:14][Si:15]([CH3:16])([CH3:17])[C:18]#[N:19].[Cl:20][CH2:21][CH:22]=[CH2
:23]>>[Cl:1][c:2]1[cH:3][cH:4][c:5]([cH:12][n:13]1)[CH:6]([C:8]#[N:9])[C:7]([CH2
:21][CH:22]=[CH2:23])([C:10]#[N:11])[C:18]#[N:19].[C:17].[C:16].[C:14].[Cl:20].[
Si:15]

Correctness of the mapping
MAPPET NO
USPTO YES

Reaction no 248 (5 bonds changed)

SMILES/SMARTS of the USPTO output:

[CH3:18][c:19]1[cH:20][c:21]2[c:22]([cH:28][cH:29]1)[C:23](=[O:24])[O:25][C:26]2=[O:27].[CH3:30][CH2:31][O:32][C:33](=[O:34])[CH3:35].[CH3:36][CH2:37][CH2:38][CH2:39][CH2:40][CH3:41].[CH3:42][C:43](=[O:44])[OH:45].[NH2:1][CH:2]([CH2:3][C:4](=[O:5])[OH:6])[c:7]1[cH:8][c:9]([O:15][CH2:16][CH3:17])[c:10]([O:13][CH3:14])[cH:11][cH:12]1.[OH2:46]>>[N:1]1([CH:2]([CH2:3][C:4](=[O:5])[OH:6])[c:7]2[cH:8][c:9]([O:15][CH2:16][CH3:17])[c:10]([O:13][CH3:14])[cH:11][cH:12]2)[C:23](=[O:24])[c:22]2[c:21]([cH:20][c:19]([CH3:18])[cH:29][cH:28]2)[C:26]1=[O:25]

SMILES/SMARTS of the MAPPET output:
[CH3:1][CH2:2][O:3][c:4]1[cH:5][c:6]([cH:7][cH:8][c:9]1[O:10][CH3:11])[CH:12]([NH2:13])[CH2:14][C:15]([OH:16])=[O:17].[CH3:18][c:19]1[cH:20][cH:21][c:22]2[C:23](=[O:24])[O:25][C:26](=[O:27])[c:28]2[cH:29]1>>[OH2:25].[CH3:1][CH2:2][O:3][c:4]1[cH:5][c:6]([cH:7][cH:8][c:9]1[O:10][CH3:11])[CH:12]([CH2:14][C:15]([OH:16])=[O:17])[N:13]1[C:23](=[O:24])[c:22]2[cH:21][cH:20][c:19]([CH3:18])[cH:29][c:28]2[C:26]1=[O:27]

Correctness of the mapping
MAPPET YES
USPTO NO

Reaction no 249 (5 bonds changed)

SMILES/SMARTS of the USPTO output:
[CH2:10]([N:11]=[C:12]=[N:13][CH2:14][CH2:15][CH2:16][N:17]([CH3:18])[CH3:19])[CH3:20].[CH3:52][C:53]#[N:54].[Cl:21][c:22]1[c:23](-[c:30]2[n:31][n:32]3[c:33]([c:34](=[O:40])[n:35]2[CH2:36][C:37](=[O:38])[OH:39])[c:41]([CH:45]([CH2:46][CH3:47])[CH2:48][CH3:49])[cH:42][c:43]3[CH3:44])[cH:24][cH:25][c:26]([O:28][CH3:29])[cH:27]1.[ClH:9].[NH3:50].[OH2:51].[OH:1][N:2]1[C:3](=[O:4])[CH2:5][CH2:6][C:7]1=[O:8]>>[NH2:2][C:37]([CH2:36][n:35]1[c:30](-[c:23]2[c:22]([Cl:21])[cH:27][c:26]([O:28][CH3:29])[cH:25][cH:24]2)[n:31][n:32]2[c:33]([c:34]1=[O:40])[c:41]([CH:45]([CH2:46][CH3:47])[CH2:48][CH3:49])[cH:42][c:43]2[CH3:44])=[O:38]

SMILES/SMARTS of the MAPPET output:
[CH3:2][CH2:3][CH:4]([CH2:5][CH3:6])[c:7]1[cH:8][c:9]([CH3:10])[n:11]2[n:12][c:13](-[c:14]3[cH:15][cH:16][c:17]([O:18][CH3:19])[cH:20][c:21]3[Cl:22])[n:23]([CH2:24][C:25]([OH:26])=[O:27])[c:28](=[O:29])[c:30]12.[NH3:1]>>[CH3:2][CH2:3][CH:4]([CH2:5][CH3:6])[c:7]1[cH:8][c:9]([CH3:10])[n:11]2[n:12][c:13](-[c:14]3[cH:15][cH:16][c:17]([O:18][CH3:19])[cH:20][c:21]3[Cl:22])[n:23]([CH2:24][C:25]([NH2:1])=[O:27])[c:28](=[O:29])[c:30]12.[OH2:26]

Correctness of the mapping
MAPPET YES
USPTO NO

Reaction no 250 (5 bonds changed)

SMILES/SMARTS of the USPTO output:
[CH3:20][N:21]([CH3:22])[CH:23]=[O:24].[CH3:31][NH2:32].[Cl:1][c:2]1[c:3]([C:17](=[O:18])[OH:19])[c:4]2[n:5][c:6]([O:15][CH3:16])[c:7]([O:13][CH3:14])[n:8][c:9]2[cH:10][c:11]1[Cl:12].[Cl:25][C:26]([C:27]([Cl:28])=[O:29])=[O:30].[Cl:33][CH2:34][Cl:35]>>[Cl:1][c:2]1[c:3]([C:17](=[O:19])[NH:21][CH3:20])[c:4]2[n:5][c:6]([O:15][CH3:16])[c:7]([O:13][CH3:14])[n:8][c:9]2[cH:10][c:11]1[Cl:12]

SMILES/SMARTS of the MAPPET output:

[CH3:3][O:4][c:5]1[n:6][c:7]2[cH:8][c:9]([Cl:10])[c:11]([Cl:12])[c:13]([C:14]([OH:15])=[O:16])[c:17]2[n:18][c:19]1[O:20][CH3:21].[CH3:1][NH2:2]>>[CH3:1][NH:2][C:14](=[O:16])[c:13]1[c:11]([Cl:12])[c:9]([Cl:10])[cH:8][c:7]2[n:6][c:5]([O:4][CH3:3])[c:19]([O:20][CH3:21])[n:18][c:17]12.[OH2:15]

Correctness of the mapping
MAPPET YES
USPTO NO

Reaction no 251 (6 bonds changed)

SMILES/SMARTS of the USPTO output:
[Br:2][c:3]1[c:4]([O:18][CH3:19])[c:5]2[c:6]([cH:7][cH:8]1)[CH:9]1[CH:10]([N:11]([CH3:15])[CH2:12][CH2:13][CH2:14]1)[CH2:16][O:17]2.[C:20]([Li:21])([CH3:22])([CH3:23])[CH3:24].[CH3:25][I:26].[CH3:32][CH2:33][CH2:34][CH2:35][CH2:36][CH3:37].[CH3:38][CH2:39][O:40][C:41](=[O:42])[CH3:43].[ClH:1].[O:27]1[CH2:28][CH2:29][CH2:30][CH2:31]1>>[ClH:1].[c:3]1([CH3:20])[c:4]([O:18][CH3:19])[c:5]2[c:6]([cH:7][cH:8]1)[CH:9]1[CH:10]([N:11]([CH3:15])[CH2:12][CH2:13][CH2:14]1)[CH2:16][O:17]2

SMILES/SMARTS of the MAPPET output:
[CH3:1][I:2].[CH3:3][O:4][c:5]1[c:6]([Br:7])[cH:8][cH:9][c:10]2[CH:11]3[CH2:12][CH2:13][CH2:14][N:15]([CH3:16])[CH:17]3[CH2:18][O:19][c:20]12>>[CH3:3][O:4][c:5]1[c:6]([CH3:1])[cH:8][cH:9][c:10]2[CH:11]3[CH2:12][CH2:13][CH2:14][N:15]([CH3:16])[CH:17]3[CH2:18][O:19][c:20]12.[Br:7].[I:2]

Correctness of the mapping
MAPPET YES
USPTO NO

Reaction no 252 (6 bonds changed)

SMILES/SMARTS of the USPTO output:
[Br:6][c:7]1[cH:8][n:9][cH:10][cH:11][cH:12]1.[CH2:1]([Li:2])[CH2:3][CH2:4][CH3:5].[CH3:31][CH2:32][CH2:33][CH2:34][CH2:35][CH3:36].[ClH:25].[F:13][C:14]([c:15]1[c:16]([C:17]#[N:18])[cH:19][cH:20][cH:21][cH:22]1)([F:23])[F:24].[O:26]([CH2:27][CH3:28])[CH2:29][CH3:30]>>[c:7]1([C:17]([c:16]2[c:15]([C:14]([F:13])([F:23])[F:24])[cH:22][cH:21][cH:20][cH:19]2)=[O:26])[cH:8][n:9][cH:10][cH:11][cH:12]1

SMILES/SMARTS of the MAPPET output:
[F:1][C:2]([F:3])([F:4])[c:5]1[cH:6][cH:7][cH:8][cH:9][c:10]1[C:11]#[N:12].[OH2:13].[Br:14][c:15]1[cH:16][cH:17][cH:18][n:19][cH:20]1>>[F:1][C:2]([F:3])([F:4])[c:5]1[cH:6][cH:7][cH:8][cH:9][c:10]1[C:11](=[O:13])[c:15]1[cH:16][cH:17][cH:18][n:19][cH:20]1.[Br:14].[N:12]

Correctness of the mapping
MAPPET YES
USPTO NO

Reaction no 253 (6 bonds changed)

SMILES/SMARTS of the USPTO output:
[CH3:25][c:26]1[cH:27][cH:28][cH:29][cH:30][cH:31]1.[CH3:32][C:33]([CH3:34])=[O:35].[N-:22]=[N+:23]=[N-:24].[Na+:21].[O:1]([c:2]1[cH:3][cH:4][cH:5][cH:6][cH:7]1)[CH2:8][CH2:9][CH2:10][CH2:11][CH2:12][CH2:13][CH2:14][CH2:15][CH2:16][CH2:17][C:18]([Cl:19])=[O:20].[

OH2:36]>>[O:1]([c:2]1[cH:3][cH:4][cH:5][cH:6][cH:7]1)[CH2:8][CH2:9][CH2:10][CH2:11][CH2:12][CH2:13][CH2:14][CH2:15][CH2:16][CH2:17][N:22]=[C:33]=[O:35]

SMILES/SMARTS of the MAPPET output:
[Cl:1][C:2](=[O:3])[CH2:4][CH2:5][CH2:6][CH2:7][CH2:8][CH2:9][CH2:10][CH2:11][CH2:12][CH2:13][O:14][c:15]1[cH:16][cH:17][cH:18][cH:19][cH:20]1.[N-:21]=[N+:22]=[N-:23]>>[O:3]=[C:2]=[N:23][CH2:4][CH2:5][CH2:6][CH2:7][CH2:8][CH2:9][CH2:10][CH2:11][CH2:12][CH2:13][O:14][c:15]1[cH:20][cH:19][cH:18][cH:17][cH:16]1.[Cl:1].[N:21].[N:22]

Correctness of the mapping
MAPPET YES
USPTO NO

Reaction no 254 (6 bonds changed)

SMILES/SMARTS of the USPTO output:
[CH2:2]([Br:3])[CH3:4].[CH3:5][N:6]1[CH2:7][CH2:8][CH:9]([Cl:12])[CH2:10][CH2:11]1.[Cl-:22].[ClH:24].[F:13][c:14]1[c:15]([C:16]#[N:17])[cH:18][cH:19][cH:20][cH:21]1.[Mg:1].[NH4+:23].[O:25]1[CH2:26][CH2:27][CH2:28][CH2:29]1.[OH2:30]>>[CH3:5][N:6]1[CH2:7][CH2:8][CH:9]([C:16]([c:15]2[c:14]([F:13])[cH:21][cH:20][cH:19][cH:18]2)=[O:25])[CH2:10][CH2:11]1.[ClH:12]

SMILES/SMARTS of the MAPPET output:
[OH2:1].[F:2][c:3]1[cH:4][cH:5][cH:6][cH:7][c:8]1[C:9]#[N:10].[CH3:11][N:12]1[CH2:13][CH2:14][CH:15]([Cl:16])[CH2:17][CH2:18]1>>[CH3:11][N:12]1[CH2:18][CH2:17][CH:15]([CH2:14][CH2:13]1)[C:9](=[O:1])[c:8]1[cH:7][cH:6][cH:5][cH:4][c:3]1[F:2].[Cl:16].[N:10]

Correctness of the mapping
MAPPET YES
USPTO NO

Reaction no 255 (6 bonds changed)

SMILES/SMARTS of the USPTO output:
[C:1]([CH3:2])([O:3][CH2:4][CH3:8])([O:6][CH2:7][CH3:5])[O:9][CH2:10][CH3:11].[CH3:21][c:22]1[cH:23][cH:24][c:25]([S:26]([OH:27])(=[O:28])=[O:29])[cH:30][cH:31]1.[CH3:32][O:33][CH2:34][CH2:35][O:36][CH2:37][CH2:38][O:39][CH3:40].[CH3:41][CH2:42][OH:43].[OH:12][CH2:13][C:14]([CH2:15][OH:16])([CH2:17][OH:18])[CH2:19][OH:20]>>[C:1]12([CH3:2])[O:3][CH2:4][C:11]([CH2:13][OH:12])([CH2:7][O:6]1)[CH2:10][O:9]2

SMILES/SMARTS of the MAPPET output:
-

Correct mapped SMILES/SMARTS of the reaction:
[CH3:8][CH2:4][O:3][C:1]([CH3:2])([O:6][CH2:7][CH3:5])[O:9][CH2:10][CH3:11].[OH:12][CH2:13][C:14]([CH2:15][OH:16])([CH2:17][OH:18])[CH2:19][OH:20]>>[CH3:2][C:1]12[O:18][CH2:17][C:14]([CH2:13][OH:12])([CH2:19][O:20]1)[CH2:15][O:16]2.[CH3:8][CH2:4][OH:3].[CH3:5][CH2:7][OH:6].[CH3:11][CH2:10][OH:9]

Correctness of the mapping
MAPPET NO
USPTO NO

Reaction no 256 (6 bonds changed)

SMILES/SMARTS of the USPTO output:
[C:1]([O-:2])([O-
:3])=[O:4].[C:7]([CH3:8])(=[O:9])[c:10]1[cH:11][cH:12][c:13]2[c:14]([cH:29]1)[C:
15]([c:22]1[c:23]([F:28])[cH:24][cH:25][cH:26][cH:27]1)=[N:16][CH:17]([CH3:21])[
C:18](=[O:20])[NH:19]2.[CH3:30][I:31].[CH3:32][C:33](=[O:34])[CH3:35].[K+:5].[K+
:6]>>[C:1]1(=[O:4])[CH:17]([CH3:21])[N:16]=[C:15]([c:22]2[c:23]([F:28])[cH:24][c
H:25][cH:26][cH:27]2)[c:14]2[c:13]([cH:12][cH:11][c:10]([C:7]([CH3:8])=[O:9])[cH
:29]2)[N:19]1[CH3:18]

SMILES/SMARTS of the MAPPET output:
[CH3:1][I:2].[CH3:3][CH:4]1[N:5]=[C:6]([c:7]2[cH:8][cH:9][cH:10][cH:11][c:12]2[F
:13])[c:14]2[cH:15][c:16]([cH:17][cH:18][c:19]2[NH:20][C:21]1=[O:22])[C:23]([CH3
:24])=[O:25]>>[CH3:3][CH:4]1[N:5]=[C:6]([c:7]2[cH:8][cH:9][cH:10][cH:11][c:12]2[
F:13])[c:14]2[cH:15][c:16]([cH:17][cH:18][c:19]2[N:20]([CH3:1])[C:21]1=[O:22])[C
:23]([CH3:24])=[O:25].[I:2]

Correctness of the mapping
MAPPET YES
USPTO NO

Reaction no 257 (6 bonds changed)

SMILES/SMARTS of the USPTO output:
[Cl:16][CH2:17][C:18]([CH2:19][C:20](=[O:21])[O:22][CH2:23][CH3:24])=[O:25].[Cl:
1][c:2]1[c:3]([CH:4]=[O:5])[cH:6][cH:7][cH:8][cH:9]1.[NH2:10][c:11]1[n:12][nH:13
][cH:14][cH:15]1>>[Cl:1][c:2]1[c:3]([CH:4]2[c:15]3[c:11]([n:12][nH:13][cH:14]3)[
NH:10][C:24]3=[C:19]2[C:20](=[O:21])[O:22][CH2:23]3)[cH:6][cH:7][cH:8][cH:9]1

SMILES/SMARTS of the MAPPET output:
[NH2:1][c:2]1[cH:3][cH:4][nH:5][n:6]1.[Cl:7][c:8]1[cH:9][cH:10][cH:11][cH:12][c:
13]1[CH:14]=[O:15].[CH3:16][CH2:17][O:18][C:19](=[O:20])[CH2:21][C:22](=[O:23])[
CH2:24][Cl:25]>>[Cl:7][c:8]1[cH:9][cH:10][cH:11][cH:12][c:13]1[CH:14]1[C:21]2=[C
:22]([CH2:17][O:18][C:19]2=[O:20])[NH:1][c:2]2[n:6][nH:5][cH:4][c:3]12.[C:24].[C
:16].[Cl:25].[O:15].[O:23]

Correct mapped SMILES/SMARTS of the reaction:
[O:30].[NH2:1][c:2]1[cH:3][cH:4][nH:5][n:6]1.[Cl:7][c:8]1[cH:9][cH:10][cH:11][cH
:12][c:13]1[CH:14]=[O:15].[CH3:16][CH2:17][O:18][C:19](=[O:20])[CH2:21][C:22](=[
O:23])[CH2:24][Cl:25]>>[Cl:7][c:8]1[cH:9][cH:10][cH:11][cH:12][c:13]1[CH:14]1[C:
21]2=[C:22]([CH2:24][O:30][C:19]2=[O:20])[NH:1][c:2]2[n:6][nH:5][cH:4][c:3]12.[C
H3:16][CH2:17][OH:18].[O:15].[Cl-:25]

Correctness of the mapping
MAPPET NO
USPTO NO

Reaction no 258 (6 bonds changed)

SMILES/SMARTS of the USPTO output:
[CH2:37]([Cl:38])[Cl:39].[CH3:28][CH2:29][NH2:30].[CH3:31][CH2:32][CH2:33][CH2:3
4][CH2:35][CH3:36].[CH3:9][CH2:10][N:11]([CH2:12][CH3:13])[CH2:14][CH3:15].[F:16
][C:17]1([F:27])[CH:18]([OH:26])[CH2:19][C:20]([F:24])([F:25])[C:21]1([F:22])[F:

23].[P:1](=[O:2])([S:3][CH2:4][CH2:5][CH3:6])([Cl:7])[Cl:8]>>[P:1](=[O:2])([S:3][CH2:4][CH2:5][CH3:6])([NH:11][CH2:14][CH3:15])[O:26][CH:18]1[C:17]([F:16])([F:27])[C:21]([F:22])([F:23])[C:20]([F:24])([F:25])[CH2:19]1

SMILES/SMARTS of the MAPPET output:
[CH3:1][CH2:2][CH2:3][S:4][P:5]([Cl:6])([Cl:7])=[O:8].[OH:9][CH:10]1[CH2:11][C:12]([F:13])([F:14])[C:15]([F:16])([F:17])[C:18]1([F:19])[F:20].[CH3:21][CH2:22][NH2:23]>>[CH3:1][CH2:2][CH2:3][S:4][P:5](=[O:8])([NH:23][CH2:22][CH3:21])[O:9][CH:10]1[CH2:11][C:12]([F:13])([F:14])[C:15]([F:16])([F:17])[C:18]1([F:19])[F:20].[Cl:6].[Cl:7]

Correctness of the mapping
MAPPET YES
USPTO NO

Reaction no 259 (6 bonds changed)

SMILES/SMARTS of the USPTO output:
[Br:1][c:2]1[cH:3][c:4]([C:9]([F:10])([F:11])[F:12])[c:5]([F:8])[cH:6][cH:7]1.[CH3:19][S:20]([CH3:21])=[O:22].[K:13][C:14]#[N:15].[Na+:18].[OH-:17].[OH2:16]>>[Br:1][c:2]1[cH:3][c:4]([C:9]([F:10])([F:11])[F:12])[c:5]([C:14](=[O:16])[OH:17])[cH:6][cH:7]1

SMILES/SMARTS of the MAPPET output:
[OH2:1].[OH2:2].[K:3][C:4]#[N:5].[F:6][c:7]1[cH:8][cH:9][c:10]([Br:11])[cH:12][c:13]1[C:14]([F:15])([F:16])[F:17]>>[OH:2][C:4](=[O:1])[c:7]1[cH:8][cH:9][c:10]([Br:11])[cH:12][c:13]1[C:14]([F:15])([F:16])[F:17].[F:6].[K:3].[N:5]

Correctness of the mapping
MAPPET YES
USPTO YES

Reaction no 260 (6 bonds changed)

SMILES/SMARTS of the USPTO output:
[Cl:1][c:2]1[cH:3][c:4]2[c:5]([nH:6][c:7]([C:9]([Cl:10])([Cl:11])[Cl:12])[n:8]2)[cH:13][cH:14]1.[NH2:15][CH2:16][c:17]1[cH:18][cH:19][cH:20][cH:21][cH:22]1.[Na+:27].[O-:23][C:24]([OH:25])=[O:26].[O:28]1[CH2:29][CH2:30][CH2:31][CH2:32]1.[OH2:33]>>[Cl:1][c:2]1[cH:3][c:4]2[c:5]([nH:6][c:7]([C:9]([NH:15][CH2:16][c:17]3[cH:18][cH:19][cH:20][cH:21][cH:22]3)=[O:23])[n:8]2)[cH:13][cH:14]1

SMILES/SMARTS of the MAPPET output:
[OH2:1].[NH2:2][CH2:3][c:4]1[cH:5][cH:6][cH:7][cH:8][cH:9]1.[Cl:10][c:11]1[cH:12][cH:13][c:14]2[nH:15][c:16]([n:17][c:18]2[cH:19]1)[C:20]([Cl:21])([Cl:22])[Cl:23]>>[Cl:10][c:11]1[cH:12][cH:13][c:14]2[nH:15][c:16]([n:17][c:18]2[cH:19]1)[C:20](=[O:1])[NH:2][CH2:3][c:4]1[cH:5][cH:6][cH:7][cH:8][cH:9]1.[Cl:23].[Cl:22].[Cl:21]

Correctness of the mapping
MAPPET YES
USPTO NO

Reaction no 261 (6 bonds changed)

SMILES/SMARTS of the USPTO output:

[C:5](#[N:6])[c:7]1[c:8]([NH2:9])[cH:10][cH:11][cH:12][cH:13]1.[CH2:15]1[CH2:18][CH2:17][CH2:16][O:19]1.[CH3:1][CH2:2][Mg:3][Cl:4].[ClH:14]>>[CH3:1][CH2:2][C:5]([c:7]1[c:8]([NH2:9])[cH:10][cH:11][cH:12][cH:13]1)=[O:19]

SMILES/SMARTS of the MAPPET output:
[CH3:1][CH2:2][Mg:3][Cl:4].[NH2:5][c:6]1[cH:7][cH:8][cH:9][cH:10][c:11]1[C:12]#[N:13].[O:14]>>[CH3:1][CH2:2][C:12](=[O:14])[c:11]1[cH:10][cH:9][cH:8][cH:7][c:6]1[NH2:5].[Cl:4].[Mg:3].[N:13]

Correctness of the mapping
MAPPET YES
USPTO NO

Reaction no 262 (6 bonds changed)

SMILES/SMARTS of the USPTO output:
[CH2:26]1[CH2:27][CH2:28][NH:29][CH2:30][CH2:31]1.[CH3:1][O:2][c:3]1[cH:4][c:5]2[c:9]([cH:10][cH:11]1)[N:8]([CH3:12])[C:7](=[O:13])[CH2:6]2.[CH3:35][S+:36]([CH3:37])([CH3:38])=[O:39].[CH3:45][OH:46].[H-:33].[I-:34].[I:14][c:15]1[n:16][nH:17][c:18]2[cH:19][c:20]([CH:24]=[O:25])[cH:21][cH:22][c:23]12.[Na+:32].[O:40]=[CH:41][N:42]([CH3:43])[CH3:44]>>[CH3:1][O:2][c:3]1[cH:4][c:5]2[c:9]([cH:10][cH:11]1)[N:8]([CH3:12])[C:7](=[O:13])[C:6]21[CH:24]([c:20]2[cH:19][c:18]3[nH:17][n:16][c:15]([I:14])[c:23]3[cH:22][cH:21]2)[CH2:26]1

SMILES/SMARTS of the MAPPET output:
[I:1][c:2]1[n:3][nH:4][c:5]2[cH:6][c:7]([CH:8]=[O:9])[cH:10][cH:11][c:12]12.[CH3:13][S+:14]([CH3:15])([CH3:16])=[O:17].[CH3:18][O:19][c:20]1[cH:21][cH:22][c:23]2[N:24]([CH3:25])[C:26](=[O:27])[CH2:28][c:29]2[cH:30]1>>[CH3:18][O:19][c:20]1[cH:21][cH:22][c:23]2[N:24]([CH3:25])[C:26](=[O:27])[C:28]3([CH2:16][CH:8]3[c:7]3[cH:10][cH:11][c:12]4[c:2]([I:1])[n:3][nH:4][c:5]4[cH:6]3)[c:29]2[cH:30]1.[C:15].[C:13].[O:9].[O:17].[S:14]

Correctness of the mapping
MAPPET YES
USPTO NO

Reaction no 263 (6 bonds changed)

SMILES/SMARTS of the USPTO output:
[Br:1][c:2]1[cH:3][cH:4][c:5]([C:6]#[N:7])[cH:8][cH:9]1.[CH3:17][CH2:18][O:19][CH2:20][CH3:21].[CH:11]([CH3:12])([CH3:13])[Mg+:14].[Cl-:10].[Cl-:15].[NH4+:16]>>[Br:1][c:2]1[cH:3][cH:4][c:5]([C:6]([CH:11]([CH3:12])[CH3:13])=[O:19])[cH:8][cH:9]1

SMILES/SMARTS of the MAPPET output:
[CH3:1][CH:2]([CH3:3])[Mg+:4].[Br:5][c:6]1[cH:7][cH:8][c:9]([cH:10][cH:11]1)[C:12]#[N:13].[O:14]>>[CH3:1][CH:2]([CH3:3])[C:12](=[O:14])[c:9]1[cH:10][cH:11][c:6]([Br:5])[cH:7][cH:8]1.[Mg:4].[N:13]

Correctness of the mapping
MAPPET YES
USPTO NO

Reaction no 264 (6 bonds changed)

SMILES/SMARTS of the USPTO output:
[CH2:1]([c:2]1[cH:3][cH:4][cH:5][cH:6][cH:7]1)[O:8][C:9](=[O:10])[NH:11][CH:12]1[C:13](=[O:18])[NH:14][CH2:15][CH2:16][CH2:17]1.[CH3:28][Si:29]([Cl:30])([CH3:31])[CH3:32].[CH3:42][c:43]1[cH:44][cH:45][cH:46][cH:47][cH:48]1.[CH:19]([N:20]([CH:21]([CH3:22])[CH3:23])[CH2:24][CH3:25])([CH3:26])[CH3:27].[Cl-:40].[NH3:39].[NH4+:41].[OH2:38].[P:33](=[O:34])([Cl:35])([Cl:36])[Cl:37]>>[CH2:1]([c:2]1[cH:3][cH:4][cH:5][cH:6][cH:7]1)[O:8][C:9](=[O:10])[NH:11][CH:12]1[C:13](=[O:18])[N:14]([P:33](=[O:34])([NH2:39])[NH2:41])[CH2:15][CH2:16][CH2:17]1

SMILES/SMARTS of the MAPPET output:
[Cl:1][P:2]([Cl:3])([Cl:4])=[O:5].[NH3:6].[NH3:7].[O:8]=[C:9]([NH:10][CH:11]1[CH2:12][CH2:13][CH2:14][NH:15][C:16]1=[O:17])[O:18][CH2:19][c:20]1[cH:21][cH:22][cH:23][cH:24][cH:25]1>>[NH2:6][P:2]([NH2:7])(=[O:5])[N:15]1[CH2:14][CH2:13][CH2:12][CH:11]([NH:10][C:9](=[O:8])[O:18][CH2:19][c:20]2[cH:25][cH:24][cH:23][cH:22][cH:21]2)[C:16]1=[O:17].[Cl:4].[Cl:3].[Cl:1]

Correctness of the mapping
MAPPET YES
USPTO YES

Reaction no 265 (6 bonds changed)

SMILES/SMARTS of the USPTO output:
[C:1](#[N:2])[c:3]1[cH:4][cH:5][c:6]([C:9]2([NH:13][C:14]([O:15][C:16]([CH3:17])([CH3:18])[CH3:19])=[O:20])[CH2:10][CH2:11][CH2:12]2)[cH:7][cH:8]1.[CH2:27]([c:28]1[cH:29][cH:30][cH:31][cH:32][cH:33]1)[Mg+:34].[CH2:35]1[CH2:38][CH2:37][CH2:36][O:39]1.[CH:22]([Mg+:23])([CH3:24])[CH3:25].[Cl-:21].[Cl-:26]>>[C:1]([c:3]1[cH:4][cH:5][c:6]([C:9]2([NH:13][C:14]([O:15][C:16]([CH3:17])([CH3:18])[CH3:19])=[O:20])[CH2:10][CH2:11][CH2:12]2)[cH:7][cH:8]1)([CH2:27][c:28]1[cH:29][cH:30][cH:31][cH:32][cH:33]1)=[O:39]

SMILES/SMARTS of the MAPPET output:
[Mg+:1][CH2:2][c:3]1[cH:4][cH:5][cH:6][cH:7][cH:8]1.[CH3:9][C:10]([CH3:11])([CH3:12])[O:13][C:14](=[O:15])[NH:16][C:17]1([CH2:18][CH2:19][CH2:20]1)[c:21]1[cH:22][cH:23][c:24]([cH:25][cH:26]1)[C:27]#[N:28].[O:29]>>[CH3:12][C:10]([CH3:11])([CH3:9])[O:13][C:14](=[O:15])[NH:16][C:17]1([CH2:20][CH2:19][CH2:18]1)[c:21]1[cH:26][cH:25][c:24]([cH:23][cH:22]1)[C:27](=[O:29])[CH2:2][c:3]1[cH:4][cH:5][cH:6][cH:7][cH:8]1.[Mg:1].[N:28]

Correctness of the mapping
MAPPET YES
USPTO NO

Reaction no 266 (6 bonds changed)

SMILES/SMARTS of the USPTO output:
[C:1]([O:2][CH2:5][c:6]1[cH:7][cH:8][c:9]([N+:12](=[O:13])[O-:14])[cH:10][cH:11]1)(=[S:3])[CH3:4].[OH2:15].[S:16](=[O:17])(=[O:18])([OH:19])[OH:20]>>[CH2:5]([c:6]1[cH:7][cH:8][c:9]([N+:12](=[O:13])[O-:14])[cH:10][cH:11]1)[SH:16]

SMILES/SMARTS of the MAPPET output:
[CH3:1][C:2](=[S:3])[O:4][CH2:5][c:6]1[cH:7][cH:8][c:9]([cH:10][cH:11]1)[N+:12]([O-:13])=[O:14]>>[O-:13][N+:12](=[O:14])[c:9]1[cH:10][cH:11][c:6]([CH2:5][SH:3])[cH:7][cH:8]1.[C:1].[C:2].[O:4]

Correctness of the mapping
MAPPET YES
USPTO NO

Reaction no 267 (6 bonds changed)

SMILES/SMARTS of the USPTO output:
[CH2:5]([c:6]1[cH:7][cH:8][cH:9][cH:10][cH:11]1)[O:12][CH2:13][CH:14]([CH2:15][CH2:16][CH:17]([CH2:18][O:19][CH2:20][c:21]1[cH:22][cH:23][cH:24][cH:25][cH:26]1)[OH:27])[OH:28].[Cl:36][C:37]([Cl:38])([Cl:39])[Cl:40].[I+3:29]([O-:30])([O-:31])([O-:32])[O-:33].[Na+:34].[OH2:35].[S:1](=[O:2])([Cl:3])[Cl:4]>>[S:1]1(=[O:2])(=[O:30])[O:27][CH:17]([CH2:18][O:19][CH2:20][c:21]2[cH:22][cH:23][cH:24][cH:25][cH:26]2)[CH2:16][CH2:15][CH:14]([CH2:13][O:12][CH2:5][c:6]2[cH:7][cH:8][cH:9][cH:10][cH:11]2)[O:28]1

SMILES/SMARTS of the MAPPET output:
[Cl:1][S:2]([Cl:3])=[O:4].[O-:5][I+3:6]([O-:7])([O-:8])[O-:9].[OH:10][CH:11]([CH2:12][CH2:13][CH:14]([OH:15])[CH2:16][O:17][CH2:18][c:19]1[cH:20][cH:21][cH:22][cH:23][cH:24]1)[CH2:25][O:26][CH2:27][c:28]1[cH:29][cH:30][cH:31][cH:32][cH:33]1>>[O:4]=[S:2]1(=[O:9])[O:10][CH:11]([CH2:25][O:26][CH2:27][c:28]2[cH:33][cH:32][cH:31][cH:30][cH:29]2)[CH2:12][CH2:13][CH:14]([CH2:16][O:17][CH2:18][c:19]2[cH:24][cH:23][cH:22][cH:21][cH:20]2)[O:15]1.[Cl:3].[Cl:1].[I:6].[O:8].[O:5].[O:7]

Correctness of the mapping
MAPPET YES
USPTO YES

Reaction no 268 (6 bonds changed)

SMILES/SMARTS of the USPTO output:
[C:25](=[O:26])([n:27]1[cH:28][cH:29][n:30][cH:31]1)[n:32]1[cH:33][cH:34][n:35][cH:36]1.[CH2:43]1[O:44][CH2:45][CH2:46][CH2:47]1.[CH3:37][CH2:38][O:39][C:40]([CH3:41])=[O:42].[N+:1]([O-:2])(=[O:3])[c:4]1[c:5]([CH2:6][NH:7][CH:8]2[CH2:9][CH2:10][N:11]([C:14](=[O:15])[O:16][C:17]([CH3:18])([CH3:19])[CH3:20])[CH2:12][CH2:13]2)[cH:21][cH:22][cH:23][cH:24]1>>[NH:1]1[c:4]2[c:5]([cH:21][cH:22][cH:23][cH:24]2)[CH2:6][N:7]([CH:8]2[CH2:9][CH2:10][N:11]([C:14](=[O:15])[O:16][C:17]([CH3:18])([CH3:19])[CH3:20])[CH2:12][CH2:13]2)[C:25]1=[O:26]

SMILES/SMARTS of the MAPPET output:
-

Correctness of the mapping
MAPPET NO
USPTO YES

Reaction no 269 (6 bonds changed)

SMILES/SMARTS of the USPTO output:
[Br:6][c:7]1[c:8]([O:46][CH3:47])[c:9]([O:44][CH3:45])[c:10]([O:42][CH3:43])[c:11]([O:40][CH3:41])[c:12]1[CH2:13][C:14]([C:15](=[O:16])[O:17][CH2:18][CH3:19])([CH2:20][CH2:21][CH2:22][CH2:23][O:24][c:25]1[cH:26][cH:27][c:28]([C:31](=[O:32])[O:33][CH3:34])[cH:29][cH:30]1)[C:35]([O:36][CH2:37][CH3:38])=[O:39].[C:52](=[O:

53])([OH:54])[O-
:55].[CH2:1]([Li:2])[CH2:3][CH2:4][CH3:5].[CH2:57]1[O:58][CH2:59][CH2:60][CH2:61
]1.[CH3:48][C:49]([OH:50])=[O:51].[CH3:62][CH2:63][CH2:64][CH2:65][CH2:66][CH3:6
7].[Na+:56]>>[c:7]12[c:8]([O:46][CH3:47])[c:9]([O:44][CH3:45])[c:10]([O:42][CH3:
43])[c:11]([O:40][CH3:41])[c:12]1[CH2:13][C:14]([C:15](=[O:16])[O:17][CH2:18][CH
3:19])([CH2:20][CH2:21][CH2:22][CH2:23][O:24][c:25]1[cH:26][cH:27][c:28]([C:31](
=[O:32])[O:33][CH3:34])[cH:29][cH:30]1)[C:35]2=[O:50]

SMILES/SMARTS of the MAPPET output:
[CH3:1][CH2:2][O:3][C:4](=[O:5])[C:6]([CH2:7][CH2:8][CH2:9][CH2:10][O:11][c:12]1
[cH:13][cH:14][c:15]([cH:16][cH:17]1)[C:18](=[O:19])[O:20][CH3:21])([CH2:22][c:2
3]1[c:24]([Br:25])[c:26]([O:27][CH3:28])[c:29]([O:30][CH3:31])[c:32]([O:33][CH3:
34])[c:35]1[O:36][CH3:37])[C:38](=[O:39])[O:40][CH2:41][CH3:42]>>[CH3:1][CH2:2][
O:3][C:4](=[O:5])[C:6]1([CH2:7][CH2:8][CH2:9][CH2:10][O:11][c:12]2[cH:17][cH:16]
[c:15]([cH:14][cH:13]2)[C:18](=[O:19])[O:20][CH3:21])[CH2:22][c:23]2[c:24]([C:41
]1=[O:40])[c:26]([O:27][CH3:28])[c:29]([O:30][CH3:31])[c:32]([O:33][CH3:34])[c:3
5]2[O:36][CH3:37].[Br:25].[C:38].[C:42].[O:39]

Correct mapped SMILES/SMARTS of the reaction:
[CH3:1][CH2:2][O:3][C:4](=[O:5])[C:6]([CH2:7][CH2:8][CH2:9][CH2:10][O:11][c:12]1
[cH:13][cH:14][c:15]([cH:16][cH:17]1)[C:18](=[O:19])[O:20][CH3:21])([CH2:22][c:2
3]1[c:24]([Br:25])[c:26]([O:27][CH3:28])[c:29]([O:30][CH3:31])[c:32]([O:33][CH3:
34])[c:35]1[O:36][CH3:37])[C:38](=[O:39])[O:40][CH2:41][CH3:42]>>[CH3:1][CH2:2][
O:3][C:4](=[O:5])[C:6]1([CH2:7][CH2:8][CH2:9][CH2:10][O:11][c:12]2[cH:17][cH:16]
[c:15]([cH:14][cH:13]2)[C:18](=[O:19])[O:20][CH3:21])[CH2:22][c:23]2[c:24]([C:38
]1=[O:39])[c:26]([O:27][CH3:28])[c:29]([O:30][CH3:31])[c:32]([O:33][CH3:34])[c:3
5]2[O:36][CH3:37].[Br:25].[CH3:42][CH2:41][OH:40]

Correctness of the mapping
MAPPET NO
USPTO NO

Reaction no 270 (6 bonds changed)

SMILES/SMARTS of the USPTO output:
[CH2:1]([CH3:2])[O:3][C:4](=[O:5])[c:6]1[n:7][c:8]([Br:13])[cH:9][cH:10][c:11]1[
NH2:12].[CH3:47][c:48]1[cH:49][cH:50][cH:51][cH:52][cH:53]1.[CH:22]1([P:23]([CH:
27]2[CH2:28][CH2:29][CH2:30][CH2:31][CH2:32]2)[CH:35]2[CH2:26][CH2:25][CH2:24][C
H2:39][CH2:40]2)[CH2:33][CH2:34][CH2:36][CH2:37][CH2:38]1.[CH:41]1([B:42]([OH:43
])[OH:44])[CH2:45][CH2:46]1.[K+:19].[K+:20].[K+:21].[O-
:55][C:56]([CH3:57])=[O:58].[O-:59][C:60]([CH3:61])=[O:62].[OH2:63].[P:14]([O-
:15])([O-:16])([O-
:17])=[O:18].[Pd+2:54]>>[CH2:1]([CH3:2])[O:3][C:4](=[O:5])[c:6]1[n:7][c:8]([CH:3
9]2[CH2:35][CH2:40]2)[cH:9][cH:10][c:11]1[NH2:12]

SMILES/SMARTS of the MAPPET output:
[OH:1][B:2]([OH:3])[CH:4]1[CH2:5][CH2:6]1.[CH3:7][CH2:8][O:9][C:10](=[O:11])[c:1
2]1[n:13][c:14]([Br:15])[cH:16][cH:17][c:18]1[NH2:19]>>[CH3:7][CH2:8][O:9][C:10]
(=[O:11])[c:12]1[n:13][c:14]([cH:16][cH:17][c:18]1[NH2:19])[CH:4]1[CH2:5][CH2:6]
1.[B:2].[Br:15].[O:3].[O:1]

Correctness of the mapping
MAPPET YES
USPTO NO

Reaction no 271 (6 bonds changed)

SMILES/SMARTS of the USPTO output:
[C:26]([O:27][BH-
:28]([O:29][C:30](=[O:31])[CH3:32])[O:33][C:34](=[O:35])[CH3:36])(=[O:37])[CH3:3
8].[C:42](=[O:43])([O-:44])[O-
:45].[CH2:40]=[O:41].[CH3:48][C:49]([CH3:50])=[O:51].[Cl:52][CH:53]([Cl:54])[CH3
:55].[NH2:1][CH:2]1[CH2:3][CH2:4][CH:5]([N:11]([CH2:12][c:13]2[cH:14][cH:15][cH:
16][cH:17][cH:18]2)[CH2:19][c:20]2[cH:21][cH:22][cH:23][cH:24][cH:25]2)[CH:6]([C
H2:8][C:9]#[N:10])[CH2:7]1.[Na+:39].[Na+:46].[Na+:47]>>[N:1]([CH:2]1[CH2:3][CH2:
4][CH:5]([N:11]([CH2:12][c:13]2[cH:14][cH:15][cH:16][cH:17][cH:18]2)[CH2:19][c:2
0]2[cH:21][cH:22][cH:23][cH:24][cH:25]2)[CH:6]([CH2:8][C:9]#[N:10])[CH2:7]1)([CH
3:26])[CH:49]([CH3:48])[CH3:50]

SMILES/SMARTS of the MAPPET output:
[NH2:1][CH:2]1[CH2:3][CH2:4][CH:5]([CH:6]([CH2:7][C:8]#[N:9])[CH2:10]1)[N:11]([C
H2:12][c:13]1[cH:14][cH:15][cH:16][cH:17][cH:18]1)[CH2:19][c:20]1[cH:21][cH:22][
cH:23][cH:24][cH:25]1.[CH3:26][C:27]([CH3:28])=[O:29].[CH2:30]=[O:31]>>[CH3:26][
CH:27]([CH3:28])[N:1]([CH3:30])[CH:2]1[CH2:3][CH2:4][CH:5]([CH:6]([CH2:7][C:8]#[
N:9])[CH2:10]1)[N:11]([CH2:19][c:20]1[cH:25][cH:24][cH:23][cH:22][cH:21]1)[CH2:1
2][c:13]1[cH:18][cH:17][cH:16][cH:15][cH:14]1.[O:31].[O:29]

Correctness of the mapping
MAPPET YES
USPTO NO

Reaction no 272 (6 bonds changed)

SMILES/SMARTS of the USPTO output:
[CH3:18][CH2:19][O:20][CH2:21][CH3:22].[CH3:23][CH2:24][O:25][C:26](=[O:27])[CH3
:28].[CH:11]1([C:14]#[N:15])[CH2:12][CH2:13]1.[Cl-
:16].[Cl:2][c:3]1[c:4]([CH2:5][Br:6])[cH:7][cH:8][cH:9][cH:10]1.[Mg:1].[NH4+:17]
>>[Cl:2][c:3]1[c:4]([CH2:5][C:14]([CH:11]2[CH2:12][CH2:13]2)=[O:20])[cH:7][cH:8]
[cH:9][cH:10]1

SMILES/SMARTS of the MAPPET output:
[Cl:1][c:2]1[cH:3][cH:4][cH:5][cH:6][c:7]1[CH2:8][Br:9].[N:10]#[C:11][CH:12]1[CH
2:13][CH2:14]1.[O:15]>>[Cl:1][c:2]1[cH:3][cH:4][cH:5][cH:6][c:7]1[CH2:8][C:11](=
[O:15])[CH:12]1[CH2:13][CH2:14]1.[Br:9].[N:10]

Correctness of the mapping
MAPPET YES
USPTO NO

Reaction no 273 (6 bonds changed)

SMILES/SMARTS of the USPTO output:
[Br:12][CH2:13][CH2:14][Br:15].[Br:1][c:2]1[cH:3][cH:4][c:5]([CH2:8][CH2:9][CH2:
10][CH3:11])[cH:6][cH:7]1.[CH3:35][OH:36].[ClH:29].[Mg:16].[O:30]1[CH2:31][CH2:3
2][CH2:33][CH2:34]1.[c:17]1([C:27]#[N:28])[n:18][cH:19][cH:20][c:21]2[cH:22][cH:
23][cH:24][cH:25][c:26]12>>[c:2]1([C:27]([c:17]2[n:18][cH:19][cH:20][c:21]3[cH:2
2][cH:23][cH:24][cH:25][c:26]23)=[O:30])[cH:3][cH:4][c:5]([CH2:8][CH2:9][CH2:10]
[CH3:11])[cH:6][cH:7]1

SMILES/SMARTS of the MAPPET output:
[N:1]#[C:2][c:3]1[n:4][cH:5][cH:6][c:7]2[cH:8][cH:9][cH:10][cH:11][c:12]12.[CH3:
13][CH2:14][CH2:15][CH2:16][c:17]1[cH:18][cH:19][c:20]([Br:21])[cH:22][cH:23]1.[
O:24]>>[CH3:13][CH2:14][CH2:15][CH2:16][c:17]1[cH:18][cH:19][c:20]([cH:22][cH:23

]1)[C:2](=[O:24])[c:3]1[n:4][cH:5][cH:6][c:7]2[cH:8][cH:9][cH:10][cH:11][c:12]12.[Br:21].[N:1]

Correctness of the mapping
MAPPET YES
USPTO NO

Reaction no 274 (6 bonds changed)

SMILES/SMARTS of the USPTO output:
[Br-:9].[CH2:13]1[CH2:16][CH2:15][CH2:14][O:17]1.[CH:10](=[CH2:11])[Mg+:12].[Cl:1][P:2]([CH:3]([CH3:4])[CH3:5])[CH:6]([CH3:7])[CH3:8]>>[P:2]([CH:3]([CH3:4])[CH3:5])([CH:6]([CH3:7])[CH3:8])([CH:10]=[CH2:11])=[O:17]

SMILES/SMARTS of the MAPPET output:
[CH3:1][CH:2]([CH3:3])[P:4]([Cl:5])[CH:6]([CH3:7])[CH3:8].[Mg+:9][CH:10]=[CH2:11].[O:12]>>[CH3:3][CH:2]([CH3:1])[P:4](=[O:12])([CH:10]=[CH2:11])[CH:6]([CH3:8])[CH3:7].[Cl:5].[Mg:9]

Correctness of the mapping
MAPPET YES
USPTO YES

Reaction no 275 (6 bonds changed)

SMILES/SMARTS of the USPTO output:
[Br:1][c:2]1[c:3]([C:4]#[N:5])[cH:6][cH:7][c:8]([CH2:10][O:11][CH:12]([C:13]([F:14])([F:15])[F:16])[c:17]2[c:18]([CH3:32])[n:19][c:20](-[c:22]3[cH:23][cH:24][c:25]([C:28]([F:29])([F:30])[F:31])[cH:26][cH:27]3)[s:21]2)[cH:9]1.[CH3:58][CH2:59][O:60][C:61](=[O:62])[CH3:63].[CH3:64][c:65]1[cH:66][cH:67][cH:68][cH:69][cH:70]1.[CH:33]1([P:34]([CH:38]2[CH2:39][CH2:40][CH2:41][CH2:42][CH2:43]2)[CH:46]2[CH2:37][CH2:36][CH2:35][CH2:50][CH2:51]2)[CH2:44][CH2:45][CH2:47][CH2:48][CH2:49]1.[CH:52]1([B:53]([OH:54])[OH:55])[CH2:56][CH2:57]1.[OH2:71]>>[c:2]1([CH:50]2[CH2:46][CH2:51]2)[c:3]([C:4]#[N:5])[cH:6][cH:7][c:8]([CH2:10][O:11][CH:12]([C:13]([F:14])([F:15])[F:16])[c:17]2[c:18]([CH3:32])[n:19][c:20](-[c:22]3[cH:23][cH:24][c:25]([C:28]([F:29])([F:30])[F:31])[cH:26][cH:27]3)[s:21]2)[cH:9]1

SMILES/SMARTS of the MAPPET output:
[OH:1][B:2]([OH:3])[CH:4]1[CH2:5][CH2:6]1.[CH3:7][c:8]1[n:9][c:10]([s:11][c:12]1[CH:13]([O:14][CH2:15][c:16]1[cH:17][cH:18][c:19]([C:20]#[N:21])[c:22]([Br:23])[cH:24]1)[C:25]([F:26])([F:27])[F:28])-[c:29]1[cH:30][cH:31][c:32]([cH:33][cH:34]1)[C:35]([F:36])([F:37])[F:38]>>[CH3:7][c:8]1[n:9][c:10]([s:11][c:12]1[CH:13]([O:14][CH2:15][c:16]1[cH:17][cH:18][c:19]([C:20]#[N:21])[c:22]([cH:24]1)[CH:4]1[CH2:5][CH2:6]1)[C:25]([F:26])([F:27])[F:28])-[c:29]1[cH:34][cH:33][c:32]([cH:31][cH:30]1)[C:35]([F:36])([F:37])[F:38].[B:2].[Br:23].[O:3].[O:1]

Correctness of the mapping
MAPPET YES
USPTO NO

Reaction no 276 (6 bonds changed)

SMILES/SMARTS of the USPTO output:
[Br:6][c:7]1[cH:8][cH:9][c:10](-
[c:13]2[cH:14][cH:15][cH:16][cH:17][cH:18]2)[cH:11][cH:12]1.[C:19](#[N:20])[CH:2
1]1[CH2:22][N:23]2[CH2:24][CH2:25][CH:26]1[CH2:27][CH2:28]2.[CH3:35][CH2:36][CH2
:37][CH2:38][CH2:39][CH3:40].[CH:1]([Li:2])([CH2:3][CH3:4])[CH3:5].[ClH:29].[O:3
0]1[CH2:31][CH2:32][CH2:33][CH2:34]1>>[ClH:29].[c:7]1([C:19]([CH:21]2[CH2:22][N:
23]3[CH2:24][CH2:25][CH:26]2[CH2:27][CH2:28]3)=[O:30])[cH:8][cH:9][c:10](-
[c:13]2[cH:14][cH:15][cH:16][cH:17][cH:18]2)[cH:11][cH:12]1

SMILES/SMARTS of the MAPPET output:
[N:1]#[C:2][CH:3]1[CH2:4][N:5]2[CH2:6][CH2:7][CH:8]1[CH2:9][CH2:10]2.[Br:11][c:1
2]1[cH:13][cH:14][c:15]([cH:16][cH:17]1)-
[c:18]1[cH:19][cH:20][cH:21][cH:22][cH:23]1.[O:24]>>[O:24]=[C:2]([CH:3]1[CH2:4][
N:5]2[CH2:6][CH2:7][CH:8]1[CH2:9][CH2:10]2)[c:12]1[cH:17][cH:16][c:15]([cH:14][c
H:13]1)-[c:18]1[cH:23][cH:22][cH:21][cH:20][cH:19]1.[Br:11].[N:1]

Correctness of the mapping
MAPPET YES
USPTO NO

Reaction no 277 (6 bonds changed)

SMILES/SMARTS of the USPTO output:
[Br:1][c:2]1[c:3]([NH:17][C:18]([C:19]([CH3:20])([CH3:21])[CH3:22])=[O:23])[cH:4
][c:5]2[c:6]([cH:16]1)[S:7](=[O:14])(=[O:15])[c:8]1[c:9]-
2[cH:10][cH:11][cH:12][cH:13]1.[C:24]([Li:25])([CH3:26])([CH3:27])[CH3:28].[CH2:
41]1[O:42][CH2:43][CH2:44][CH2:45]1.[CH3:29][CH2:30][CH2:31][CH2:32][CH3:33].[CH
3:37][C:38](=[O:39])[O-
:40].[Cl:46][CH2:47][Cl:48].[I:34][CH3:35].[NH4+:36]>>[c:2]1([CH3:24])[c:3]([NH:
17][C:18]([C:19]([CH3:20])([CH3:21])[CH3:22])=[O:23])[cH:4][c:5]2[c:6]([cH:16]1)
[S:7](=[O:14])(=[O:15])[c:8]1[c:9]-2[cH:10][cH:11][cH:12][cH:13]1

SMILES/SMARTS of the MAPPET output:
[CH3:1][I:2].[CH3:3][C:4]([CH3:5])([CH3:6])[C:7](=[O:8])[NH:9][c:10]1[cH:11][c:1
2]2-
[c:13]3[cH:14][cH:15][cH:16][cH:17][c:18]3[S:19](=[O:20])(=[O:21])[c:22]2[cH:23]
[c:24]1[Br:25]>>[CH3:1][c:24]1[cH:23][c:22]2[c:12]([cH:11][c:10]1[NH:9][C:7](=[O
:8])[C:4]([CH3:6])([CH3:5])[CH3:3])-
[c:13]1[cH:14][cH:15][cH:16][cH:17][c:18]1[S:19]2(=[O:20])=[O:21].[Br:25].[I:2]

Correctness of the mapping
MAPPET YES
USPTO NO

Reaction no 278 (6 bonds changed)

SMILES/SMARTS of the USPTO output:
[CH3:20][CH2:21][O:22][CH2:23][CH3:24].[ClH:19].[F:1][c:2]1[cH:3][cH:4][c:5]([CH
2:6][Br:7])[cH:8][cH:9]1.[Mg:10].[N:11]#[C:12][c:13]1[cH:14][cH:15][cH:16][cH:17
][cH:18]1>>[F:1][c:2]1[cH:3][cH:4][c:5]([CH2:6][C:12]([c:13]2[cH:14][cH:15][cH:1
6][cH:17][cH:18]2)=[O:22])[cH:8][cH:9]1

SMILES/SMARTS of the MAPPET output:
[N:1]#[C:2][c:3]1[cH:4][cH:5][cH:6][cH:7][cH:8]1.[F:9][c:10]1[cH:11][cH:12][c:13
]([CH2:14][Br:15])[cH:16][cH:17]1.[O:18]>>[F:9][c:10]1[cH:17][cH:16][c:13]([CH2:

14][C:2](=[O:18])[c:3]2[cH:4][cH:5][cH:6][cH:7][cH:8]2)[cH:12][cH:11]1.[Br:15].[N:1]

Correctness of the mapping
MAPPET YES
USPTO NO

Reaction no 279 (6 bonds changed)

SMILES/SMARTS of the USPTO output:
[C:1]([CH3:2])([CH3:3])([CH3:4])[O:5][C:6]([NH:7][CH:8]1[CH2:9][CH2:10][CH:11]([NH2:14])[CH2:12][CH2:13]1)=[O:15].[C:20]([O:21][BH-:22]([O:23][C:24](=[O:25])[CH3:26])[O:27][C:28](=[O:29])[CH3:30])(=[O:31])[CH3:32].[CH2:36]=[O:37].[CH2:38]([Cl:39])[Cl:40].[CH3:16][C:17]([CH3:18])=[O:19].[Na+:33].[Na+:35].[OH-:34]>>[C:1]([CH3:2])([CH3:3])([CH3:4])[O:5][C:6]([NH:7][CH:8]1[CH2:9][CH2:10][CH:11]([N:14]([CH:17]([CH3:16])[CH3:18])[CH3:20])[CH2:12][CH2:13]1)=[O:15]

SMILES/SMARTS of the MAPPET output:
[CH3:1][C:2]([CH3:3])=[O:4].[CH2:5]=[O:6].[CH3:7][C:8]([CH3:9])([CH3:10])[O:11][C:12](=[O:13])[NH:14][CH:15]1[CH2:16][CH2:17][CH:18]([NH2:19])[CH2:20][CH2:21]1>>[CH3:1][CH:2]([CH3:3])[N:19]([CH3:5])[CH:18]1[CH2:20][CH2:21][CH:15]([CH2:16][CH2:17]1)[NH:14][C:12](=[O:13])[O:11][C:8]([CH3:10])([CH3:9])[CH3:7].[O:6].[O:4]

Correctness of the mapping
MAPPET YES
USPTO NO

Reaction no 280 (6 bonds changed)

SMILES/SMARTS of the USPTO output:
[CH2:33]1[CH2:36][CH2:35][CH2:34][O:37]1.[CH:47]([Mg+:48])([CH3:49])[CH3:50].[Cl-:46].[Cl:2][c:3]1[cH:4][c:5]([S:10](=[O:11])(=[O:12])[NH:13][c:14]2[c:15]([C:21]#[N:22])[n:16][cH:17][c:18]([CH3:20])[cH:19]2)[cH:6][cH:7][c:8]1[Cl:9].[Cl:38][Mg:39][c:40]1[cH:41][cH:42][cH:43][cH:44][cH:45]1.[I:23][c:24]1[c:25]2[c:26]([n:27][cH:28][n:29]1)[nH:30][cH:31][cH:32]2.[Na:1]>>[Cl:2][c:3]1[cH:4][c:5]([S:10](=[O:11])(=[O:12])[NH:13][c:14]2[c:15]([C:21]([c:24]3[c:25]4[c:26]([n:27][cH:28][n:29]3)[nH:30][cH:31][cH:32]4)=[O:37])[n:16][cH:17][c:18]([CH3:20])[cH:19]2)[cH:6][cH:7][c:8]1[Cl:9]

SMILES/SMARTS of the MAPPET output:
[I:1][c:2]1[n:3][cH:4][n:5][c:6]2[nH:7][cH:8][cH:9][c:10]12.[CH3:11][c:12]1[cH:13][n:14][c:15]([C:16]#[N:17])[c:18]([NH:19][S:20](=[O:21])(=[O:22])[c:23]2[cH:24][cH:25][c:26]([Cl:27])[c:28]([Cl:29])[cH:30]2)[cH:31]1.[O:32]>>[CH3:11][c:12]1[cH:13][n:14][c:15]([C:16](=[O:32])[c:2]2[n:3][cH:4][n:5][c:6]3[nH:7][cH:8][cH:9][c:10]23)[c:18]([NH:19][S:20](=[O:21])(=[O:22])[c:23]2[cH:24][cH:25][c:26]([Cl:27])[c:28]([Cl:29])[cH:30]2)[cH:31]1.[I:1].[N:17]

Correctness of the mapping
MAPPET YES
USPTO NO

Reaction no 281 (6 bonds changed)

SMILES/SMARTS of the USPTO output:
[Br:1][c:2]1[cH:3][c:4]([Br:9])[cH:5][c:6]([Br:8])[cH:7]1.[C:15](#[N:16])[c:17]1[cH:18][n:19][cH:20][cH:21][cH:22]1.[CH2:23]([O:25][CH2:24][CH3:26])[CH3:27].[CH3:10][CH2:11][CH2:12][CH2:13][Li:14]>>[c:2]1([C:15]([c:17]2[cH:18][n:19][cH:20][cH:21][cH:22]2)=[O:25])[cH:3][c:4]([Br:9])[cH:5][c:6]([Br:8])[cH:7]1

SMILES/SMARTS of the MAPPET output:
[N:1]#[C:2][c:3]1[cH:4][cH:5][cH:6][n:7][cH:8]1.[Br:9][c:10]1[cH:11][c:12]([Br:13])[cH:14][c:15]([Br:16])[cH:17]1.[O:18]>>[Br:13][c:12]1[cH:14][c:15]([Br:16])[cH:17][c:10]([cH:11]1)[C:2](=[O:18])[c:3]1[cH:4][cH:5][cH:6][n:7][cH:8]1.[Br:9].[N:1]

Correctness of the mapping
MAPPET YES
USPTO NO

Supplementary Note 5. Test Set of 175 reactions with correct mappings, prepared and verified by external experts, compared to those of our software ("MAPPET")..pages 728-834

Anna Domżalska, Institute of Organic Chemistry PAS

Reaction no 1

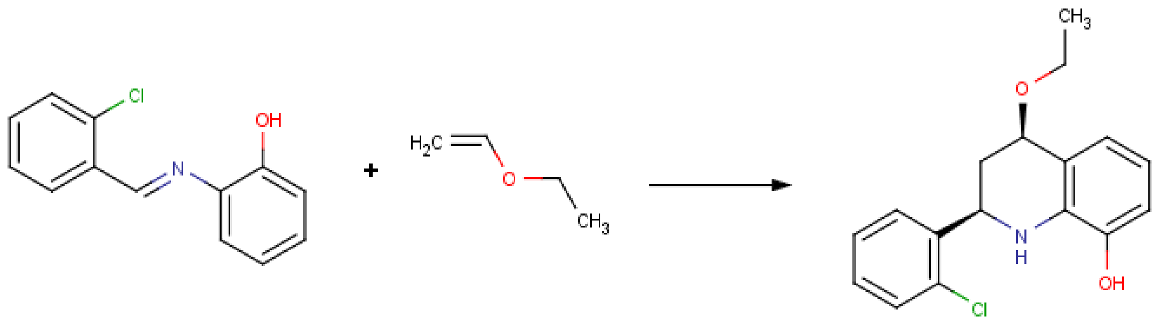

SMILES of the input:
OC1=CC=CC=C1\N=C\C1=CC=CC=C1Cl.CCOC=C>>CCO[C@@H]1C[C@@H](NC2=C1C=CC=C2O)C1=C(Cl)C=CC=C**1**

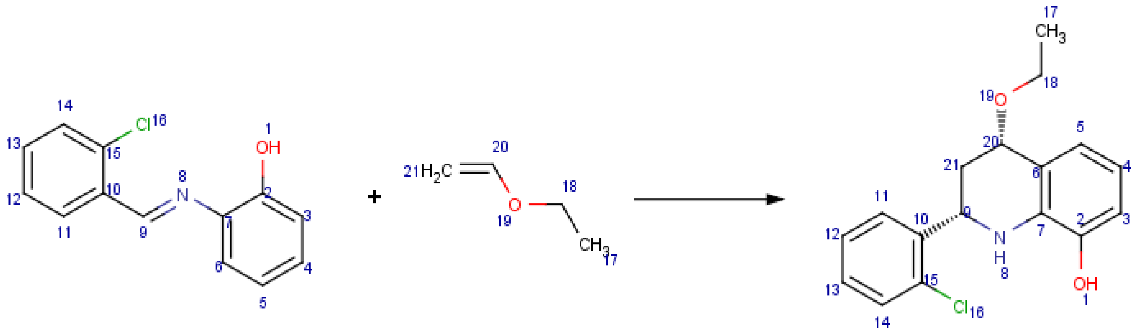

Correct mapped SMILES/SMARTS of the reaction:
[OH:1][C:2]1=[CH:3][CH:4]=[CH:5][CH:6]=[C:7]1\[N:8]=[CH:9]\[C:10]1=[CH:11][CH:12]=[CH:13][CH:14]=[C:15]1[Cl:16].[CH3:17][CH2:18][O:19][CH:20]=[CH2:21]>>[CH3:17][CH2:18][O:19][C@H:20]1[CH2:21][C@H:9]([NH:8][C:7]2=[C:6]1[CH:5]=[CH:4][CH:3]=[C:2]2[OH:1])[C:10]1=[C:15]([Cl:16])[CH:14]=[CH:13][CH:12]=[CH:11]1

Correctness of the mapping: YES

Reaction no 2

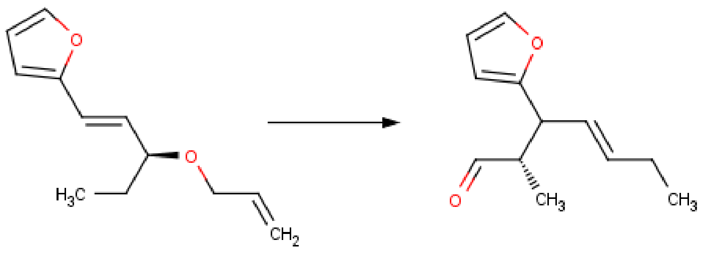

SMILES of the input:

CC[C@H](OCC=C)\C=C\C1=CC=CO1>>CC\C=C\C([C@H](C)C=O)C1=CC=CO1

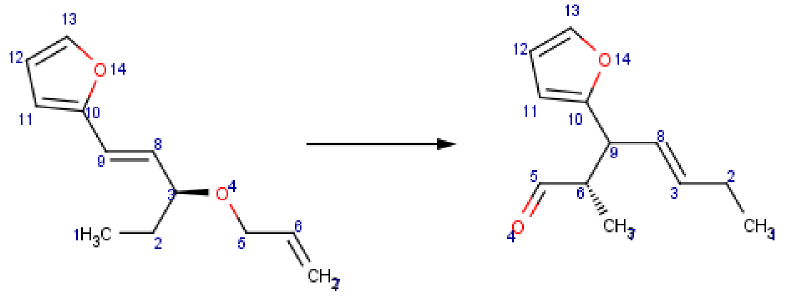

Correct mapped SMILES/SMARTS of the reaction:
[CH3:1][CH2:2][C@H:3]([O:4][CH2:5][CH:6]=[CH2:7])\[CH:8]=[CH:9]\[C:10]1=[CH:11][CH:12]=[CH:13][O:14]1>>[CH3:1][CH2:2]\[CH:3]=[CH:8]\[CH:9]([C@H:6]([CH3:7])[CH:5]=[O:4])[C:10]1=[CH:11][CH:12]=[CH:13][O:14]1

Correctness of the mapping: YES

Reaction no 3

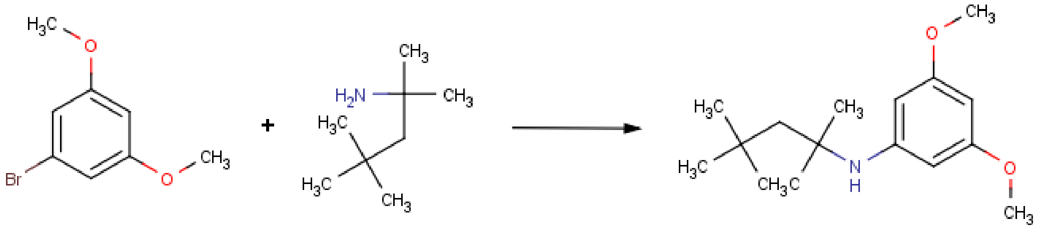

SMILES of the input:
COC1=CC(OC)=CC(Br)=C1.CC(C)(C)CC(C)(C)N>>COC1=CC(OC)=CC(NC(C)(C)CC(C)(C)C)=C1

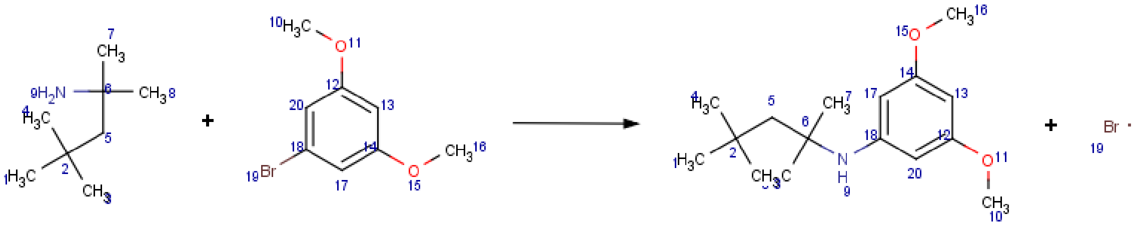

Correct mapped SMILES/SMARTS of the reaction:
[CH3:1][C:2]([CH3:3])([CH3:4])[CH2:5][C:6]([CH3:7])([CH3:8])[NH2:9].[CH3:10][O:11][C:12]1=[CH:13][C:14]([O:15][CH3:16])=[CH:17][C:18]([Br:19])=[CH:20]1>>[CH3:16][O:15][C:14]1=[CH:13][C:12]([O:11][CH3:10])=[CH:20][C:18]([NH:9][C:6]([CH3:7])([CH3:8])[CH2:5][C:2]([CH3:1])([CH3:4])[CH3:3])=[CH:17]1.[Br:19]

Correctness of the mapping: YES

Reaction no 4

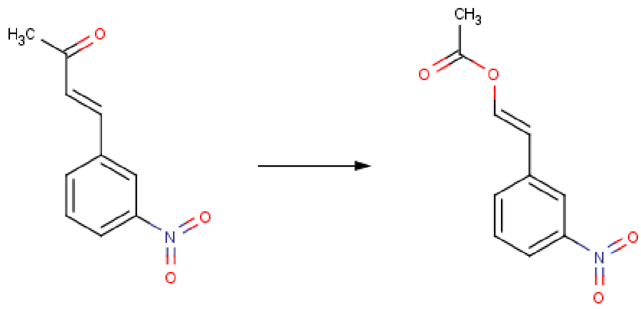

SMILES of the input:
CC(=O)\C=C\C1=CC(=CC=C1)N(=O)=O>>CC(=O)O\C=C\C1=CC(=CC=C1)N(=O)=O

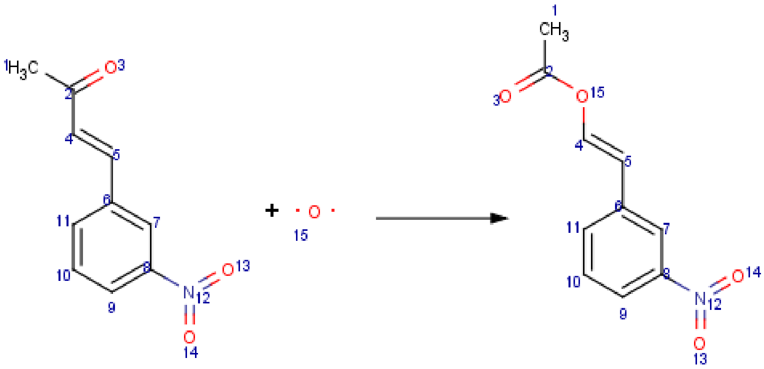

Correct mapped SMILES/SMARTS of the reaction:
[CH3:1][C:2](=[O:3])\[CH:4]=[CH:5]\[C:6]1=[CH:7][C:8](=[CH:9][CH:10]=[CH:11]1)[N:12](=[O:13])=[O:14].[O:15]>>[CH3:1][C:2](=[O:3])[O:15]\[CH:4]=[CH:5]\[C:6]1=[CH:7][C:8](=[CH:9][CH:10]=[CH:11]1)[N:12](=[O:14])=[O:13]

Correctness of the mapping: YES

Reaction no 5

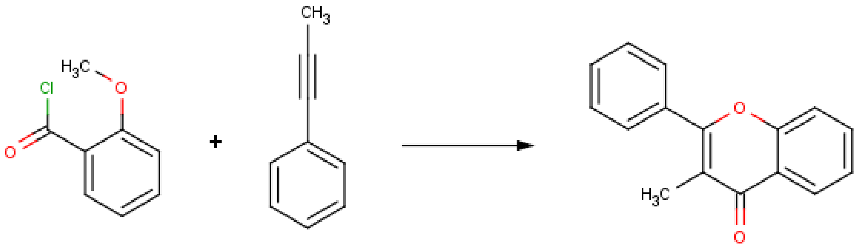

SMILES of the input:
COC1=CC=CC=C1C(Cl)=O.CC#CC1=CC=CC=C1>>CC1=C(OC2=CC=CC=C2C1=O)C1=CC=CC=C1

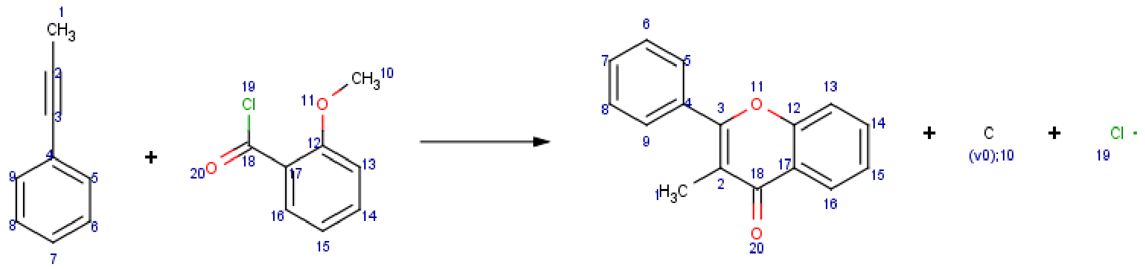

Correct mapped SMILES/SMARTS of the reaction:
[CH3:1][C:2]#[C:3][C:4]1=[CH:5][CH:6]=[CH:7][CH:8]=[CH:9]1.[CH3:10][O:11][C:12]1=[CH:13][CH:14]=[CH:15][CH:16]=[C:17]1[C:18]([Cl:19])=[O:20]>>[CH3:1][C:2]1=[C:3]([O:11][C:12]2=[CH:13][CH:14]=[CH:15][CH:16]=[C:17]2[C:18]1=[O:20])[C:4]1=[CH:9][CH:8]=[CH:7][CH:6]=[CH:5]1.[C:10].[Cl:19]

Correctness of the mapping: YES

Reaction no 6

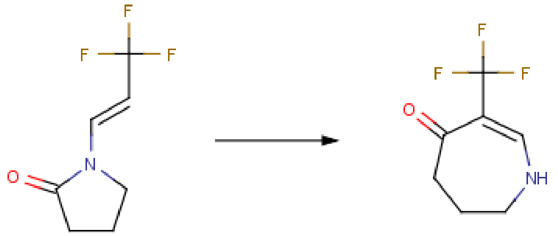

SMILES of the input:
FC(F)(F)\C=C\N1CCCC1=O>>FC(F)(F)C1=CNCCCC1=O

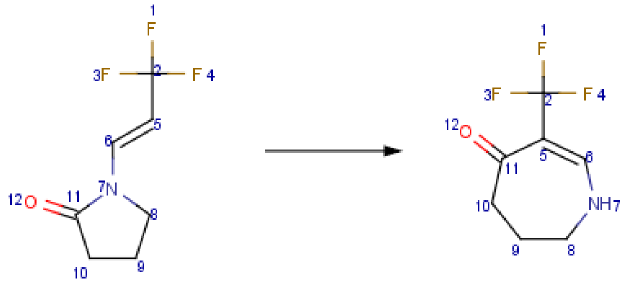

Correct mapped SMILES/SMARTS of the reaction:
[F:1][C:2]([F:3])([F:4])\[CH:5]=[CH:6]\[N:7]1[CH2:8][CH2:9][CH2:10][C:11]1=[O:12]>>[F:1][C:2]([F:3])([F:4])[C:5]1=[CH:6][NH:7][CH2:8][CH2:9][CH2:10][C:11]1=[O:12]

Correctness of the mapping: YES

Reaction no 7

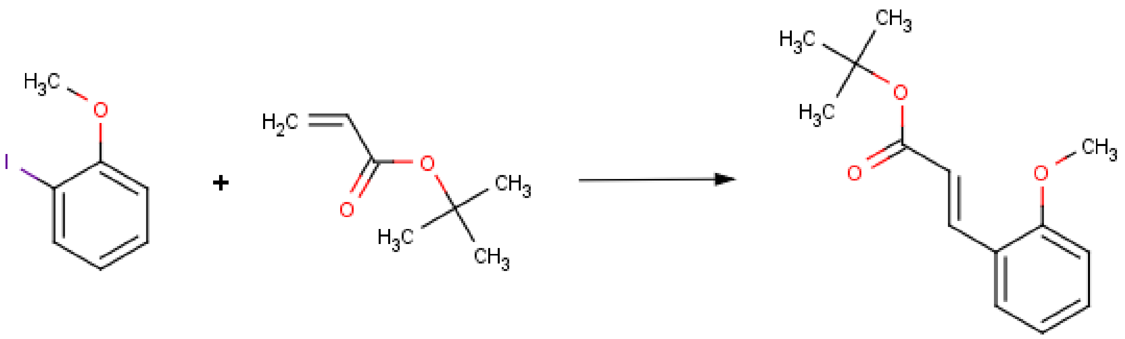

SMILES of the input:
COC1=CC=CC=C1I.CC(C)(C)OC(=O)C=C>>COC1=CC=CC=C1\C=C\C(=O)OC(C)(C)C

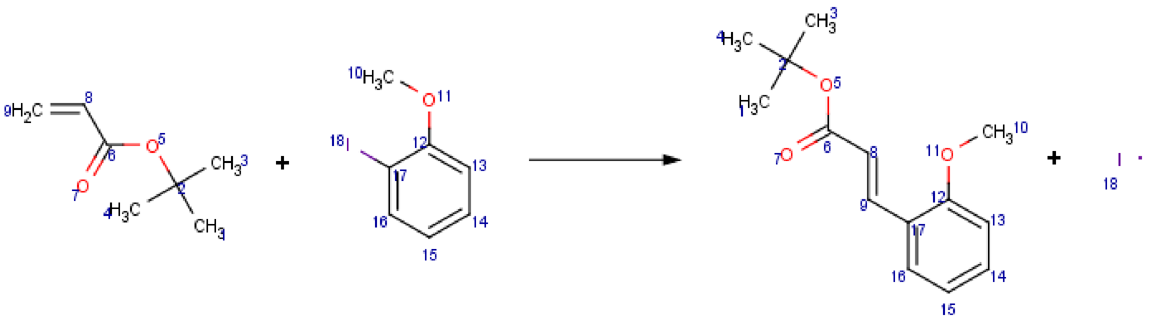

Correct mapped SMILES/SMARTS of the reaction:
[CH3:1][C:2]([CH3:3])([CH3:4])[O:5][C:6](=[O:7])[CH:8]=[CH2:9].[CH3:10][O:11][C:12]1=[CH:13][CH:14]=[CH:15][CH:16]=[C:17]1[I:18]>>[CH3:10][O:11][C:12]1=[CH:13][CH:14]=[CH:15][CH:16]=[C:17]1\[CH:9]=[CH:8]\[C:6](=[O:7])[O:5][C:2]([CH3:4])([CH3:3])[CH3:1].[I:18]

Correctness of the mapping: YES

Reaction no 8

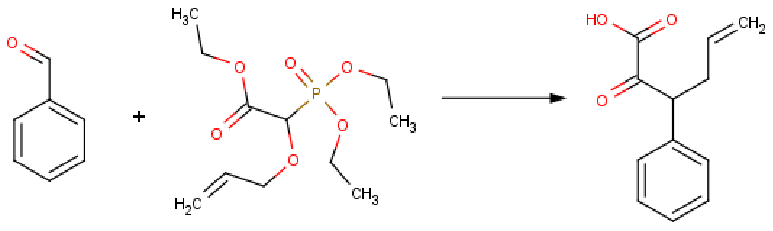

SMILES of the input:
O=CC1=CC=CC=C1.CCOC(=O)C(OCC=C)P(=O)(OCC)OCC>>OC(=O)C(=O)C(CC=C)C1=CC=CC=C1

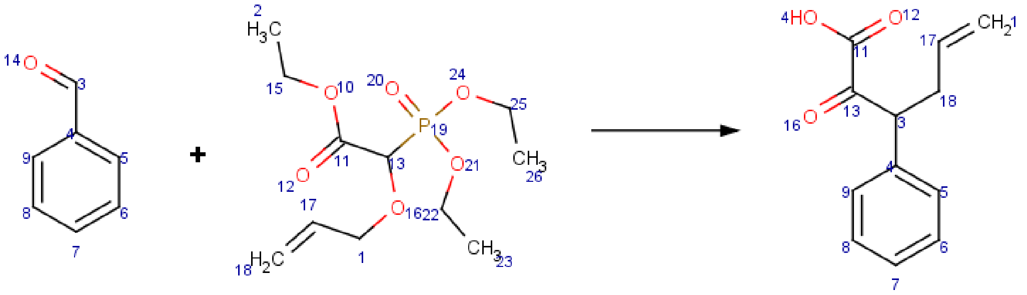

Correct mapped SMILES/SMARTS of the reaction:
[O:14]=[CH:3][C:4]1=[CH:5][CH:6]=[CH:7][CH:8]=[CH:9]1.[CH3:2][CH2:15][O:10][C:11](=[O:12])[CH:13]([O:16][CH2:1][CH:17]=[CH2:18])[P:19](=[O:20])([O:21][CH2:22][CH3:23])[O:24][CH2:25][CH3:26]>>[OH:4][C:11](=[O:12])[C:13](=[O:16])[CH:3]([CH2:18][CH:17]=[CH2:1])[C:4]1=[CH:5][CH:6]=[CH:7][CH:8]=[CH:9]1
Correctness of the mapping: NO

Reaction no 9

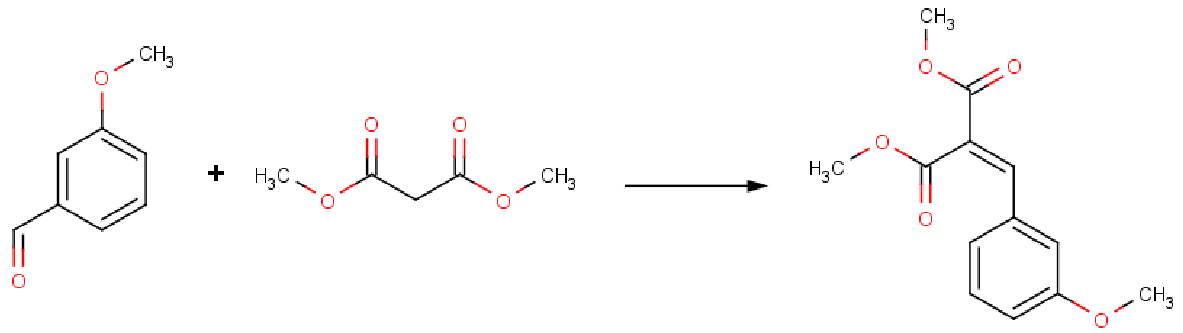

SMILES of the input:
COC1=CC=CC(C=O)=C1.COC(=O)CC(=O)OC>>COC(=O)C(=CC1=CC(OC)=CC=C1)C(=O)OC

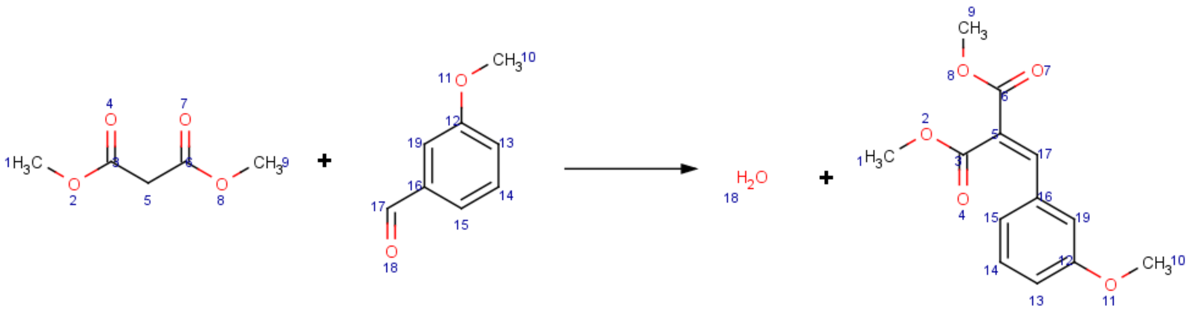

Correct mapped SMILES/SMARTS of the reaction:
[CH3:1][O:2][C:3](=[O:4])[CH2:5][C:6](=[O:7])[O:8][CH3:9].[CH3:10][O:11][C:12]1=[CH:13][CH:14]=[CH:15][C:16]([CH:17]=[O:18])=[CH:19]1>>[OH2:18].[CH3:1][O:2][C:3](=[O:4])[C:5](=[CH:17][C:16]1=[CH:19][C:12]([O:11][CH3:10])=[CH:13][CH:14]=[CH:15]1)[C:6](=[O:7])[O:8][CH3:9]

Correctness of the mapping: YES

Reaction no 10

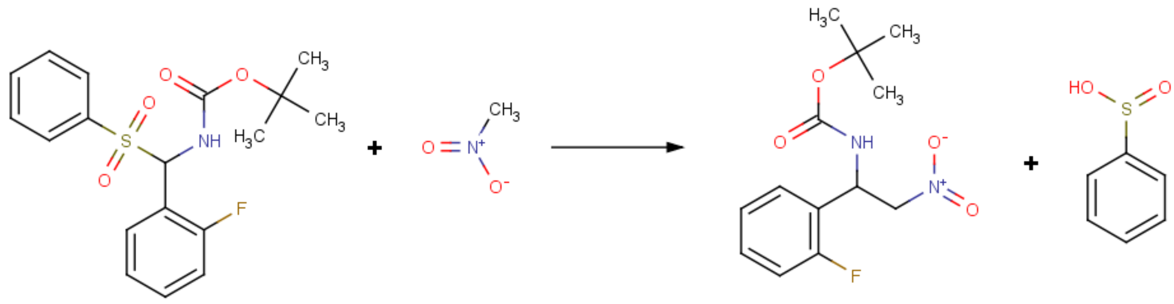

SMILES of the input:
CC(C)(C)OC(=O)NC(C1=C(F)C=CC=C1)S(=O)(=O)C1=CC=CC=C1.C[N+]([O-
])=O>>CC(C)(C)OC(=O)NC(C[N+]([O-])=O)C1=C(F)C=CC=C1.OS(=O)C1=CC=CC=C1

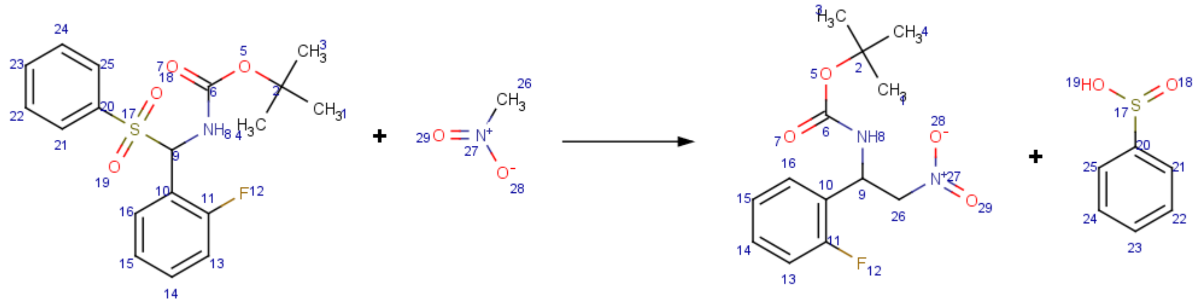

Correct mapped SMILES/SMARTS of the reaction:
[CH3:1][C:2]([CH3:3])([CH3:4])[O:5][C:6](=[O:7])[NH:8][CH:9]([C:10]1=[C:1
1]([F:12])[CH:13]=[CH:14][CH:15]=[CH:16]1)[S:17](=[O:18])(=[O:19])[C:20]1
=[CH:21][CH:22]=[CH:23][CH:24]=[CH:25]1.[CH3:26][N+:27]([O-
:28])=[O:29]>>[CH3:4][C:2]([CH3:3])([CH3:1])[O:5][C:6](=[O:7])[NH:8][CH:9
]([CH2:26][N+:27]([O-
:28])=[O:29])[C:10]1=[C:11]([F:12])[CH:13]=[CH:14][CH:15]=[CH:16]1.[OH:19
][S:17](=[O:18])[C:20]1=[CH:21][CH:22]=[CH:23][CH:24]=[CH:25]1

Correctness of the mapping: YES

Reaction no 11

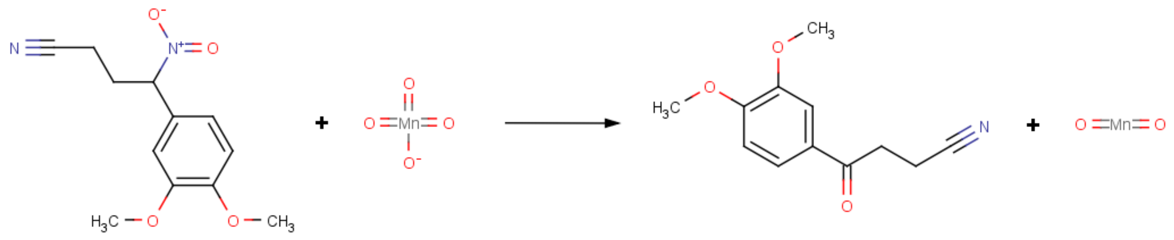

SMILES of the input:
COC1=C(OC)C=C(C=C1)C(CCC#N)[N+]([O-])=O.[O-
][Mn](=O)(=O)=O>>COC1=C(OC)C=C(C=C1)C(=O)CCC#N.O=[Mn]=O

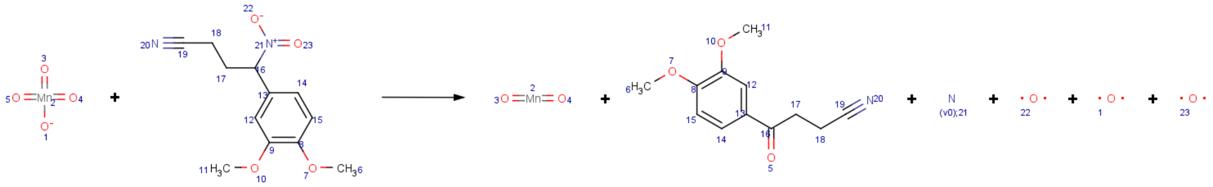

Correct mapped SMILES/SMARTS of the reaction:
[O-
:1][Mn:2](=[O:3])(=[O:4])=[O:5].[CH3:6][O:7][C:8]1=[C:9]([O:10][CH3:11])[
CH:12]=[C:13]([CH:14]=[CH:15]1)[CH:16]([CH2:17][CH2:18][C:19]#[N:20])[N+:
21]([O-
:22])=[O:23]>>[O:3]=[Mn:2]=[O:4].[CH3:6][O:7][C:8]1=[C:9]([O:10][CH3:11])
[CH:12]=[C:13]([CH:14]=[CH:15]1)[C:16](=[O:5])[CH2:17][CH2:18][C:19]#[N:2
0].[N:21].[O:22].[O:1].[O:23]

Correctness of the mapping: YES

Reaction no 12

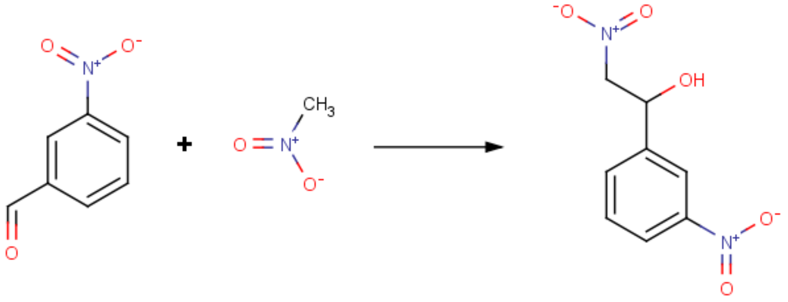

SMILES of the input:
[O-][N+](=O)C1=CC=CC(C=O)=C1.C[N+]([O-])=O>>OC(C[N+]([O-
])=O)C1=CC(=CC=C1)[N+]([O-])=O

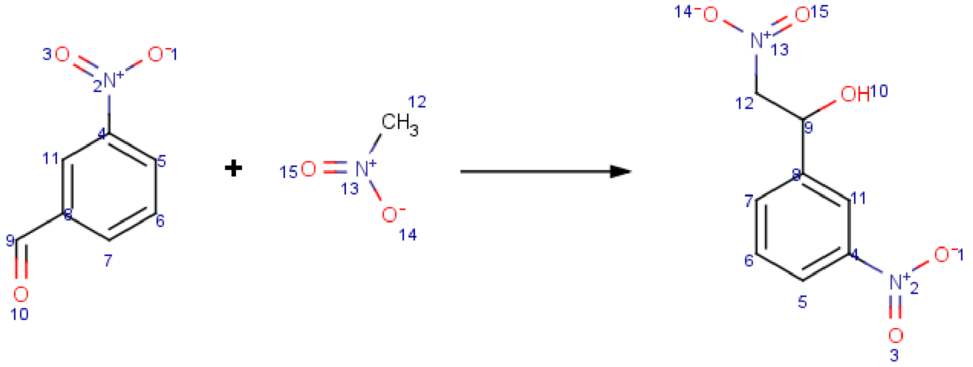

Correct mapped SMILES/SMARTS of the reaction:
[O-
:1][N+:2](=[O:3])[C:4]1=[CH:5][CH:6]=[CH:7][C:8]([CH:9]=[O:10])=[CH:11]1.
[CH3:12][N+:13]([O-:14])=[O:15]>>[OH:10][CH:9]([CH2:12][N+:13]([O-
:14])=[O:15])[C:8]1=[CH:11][C:4](=[CH:5][CH:6]=[CH:7]1)[N+:2]([O-
:1])=[O:3]

Correctness of the mapping: YES

Reaction no 13

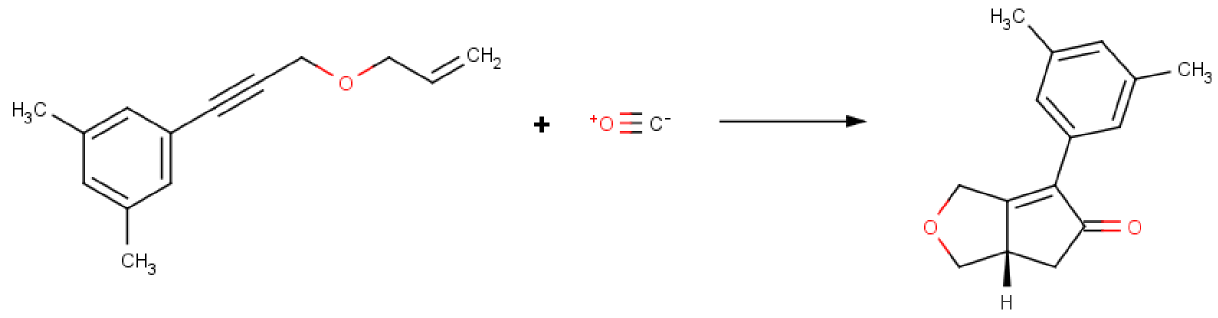

SMILES of the input:
CC1=CC(=CC(C)=C1)C#CCOCC=C.[C-
]#[O+]>>[H][C@@]12COCC1=C(C(=O)C2)C1=CC(C)=CC(C)=C1

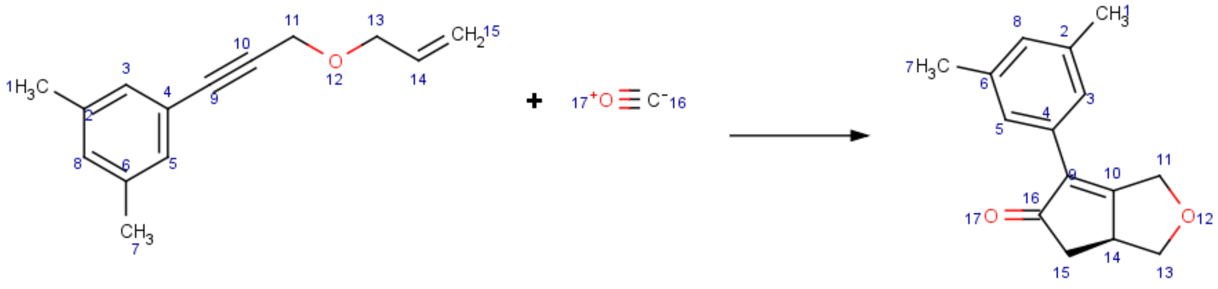

Correct mapped SMILES/SMARTS of the reaction:
[CH3:1][C:2]1=[CH:3][C:4](=[CH:5][C:6]([CH3:7])=[CH:8]1)[C:9]#[C:10][CH2:
11][O:12][CH2:13][CH:14]=[CH2:15].[C-
:16]#[O+:17]>>[CH3:1][C:2]1=[CH:3][C:4](=[CH:5][C:6]([CH3:7])=[CH:8]1)[C:
9]1=[C:10]2[CH2:11][O:12][CH2:13][C@H:14]2[CH2:15][C:16]1=[O:17]

Correctness of the mapping: YES

Reaction no 14

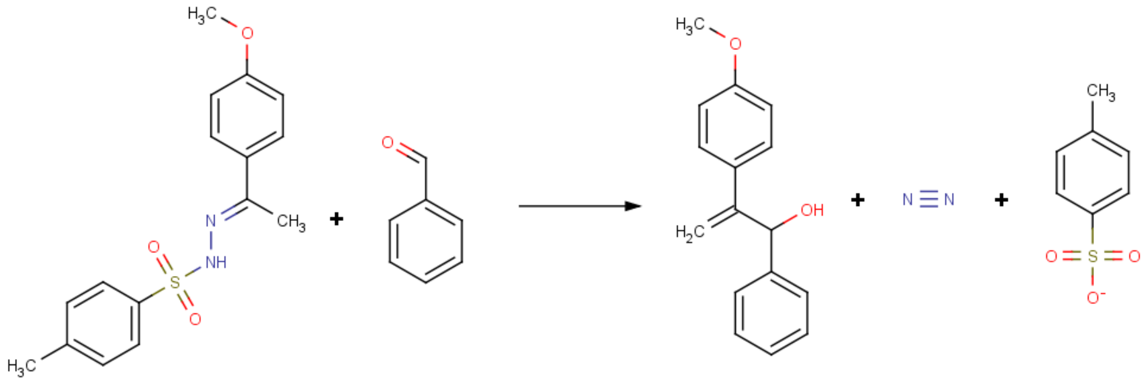

SMILES of the input:
COC1=CC=C(C=C1)C(\C)=N\NS(=O)(=O)C1=CC=C(C)C=C1.O=CC1=CC=CC=C1>>COC1=CC=C
(C=C1)C(=C)C(O)C1=CC=CC=C1.N#N.CC1=CC=C(C=C1)S([O-])(=O)=O

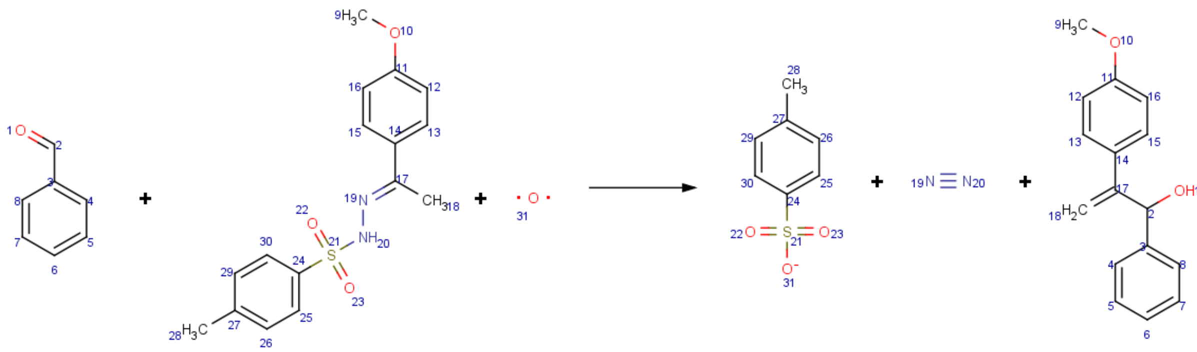

Correct mapped SMILES/SMARTS of the reaction:
[O:1]=[CH:2][C:3]1=[CH:4][CH:5]=[CH:6][CH:7]=[CH:8]1.[CH3:9][O:10][C:11]1=[CH:12][CH:13]=[C:14]([CH:15]=[CH:16]1)[C:17](\[CH3:18])=[N:19]\[NH:20][S:21](=[O:22])(=[O:23])[C:24]1=[CH:25][CH:26]=[C:27]([CH3:28])[CH:29]=[CH:30]1.[O:31]>>[CH3:28][C:27]1=[CH:26][CH:25]=[C:24]([CH:30]=[CH:29]1)[S:21]([O-:31])(=[O:22])=[O:23].[N:20]#[N:19].[CH3:9][O:10][C:11]1=[CH:16][CH:15]=[C:14]([CH:13]=[CH:12]1)[C:17](=[CH2:18])[CH:2]([OH:1])[C:3]1=[CH:8][CH:7]=[CH:6][CH:5]=[CH:4]1

Correctness of the mapping: YES

Reaction no 15

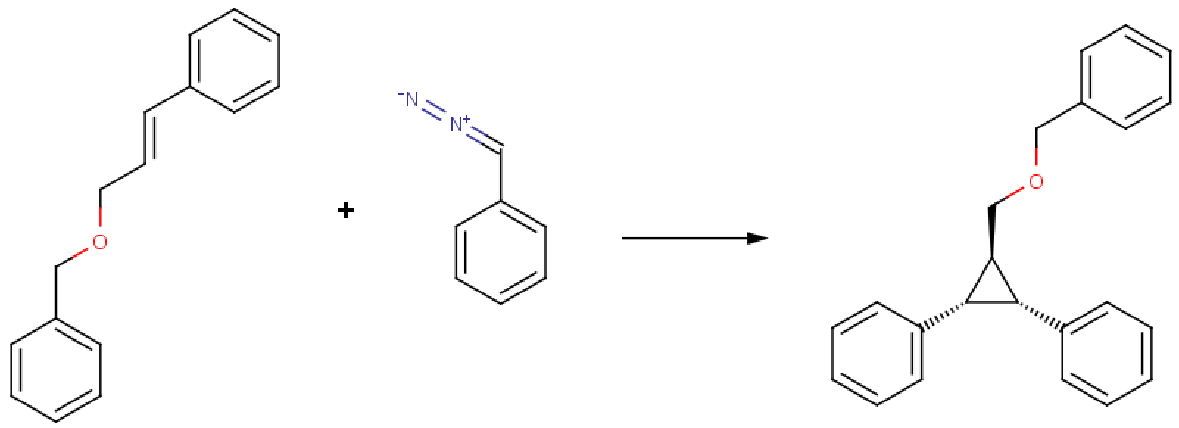

SMILES of the input:
C(OCC1=CC=CC=C1)\C=C\C1=CC=CC=C1.[N-]=[N+]=CC1=CC=CC=C1>>C(OCC1=CC=CC=C1)[C@H]1[C@@H]([C@@H]1C1=CC=CC=C1)C1=CC=CC=C1

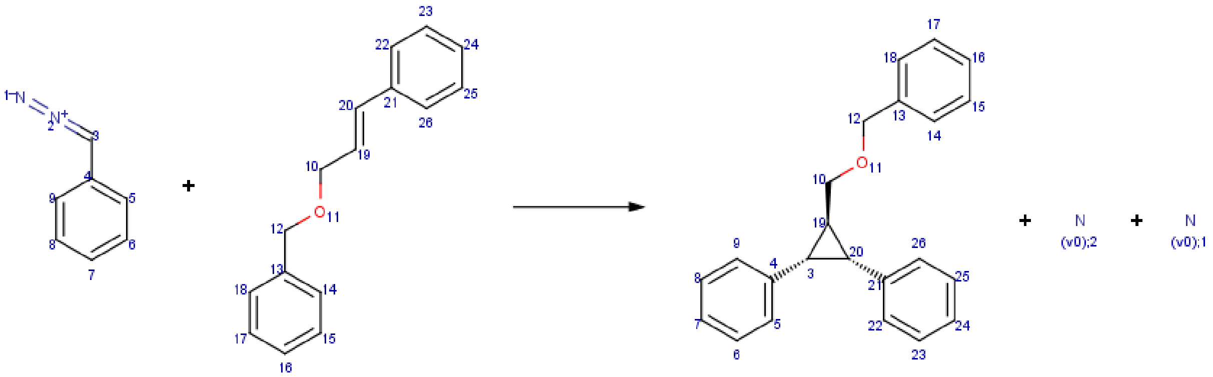

Correct mapped SMILES/SMARTS of the reaction:

[N-
:1]=[N+:2]=[CH:3][C:4]1=[CH:5][CH:6]=[CH:7][CH:8]=[CH:9]1.[CH2:10]([O:11]
[CH2:12][C:13]1=[CH:14][CH:15]=[CH:16][CH:17]=[CH:18]1)\[CH:19]=[CH:20]\[
C:21]1=[CH:22][CH:23]=[CH:24][CH:25]=[CH:26]1>>[CH2:10]([O:11][CH2:12][C:
13]1=[CH:18][CH:17]=[CH:16][CH:15]=[CH:14]1)[C@H:19]1[C@@H:20]([C@@H:3]1[
C:4]1=[CH:5][CH:6]=[CH:7][CH:8]=[CH:9]1)[C:21]1=[CH:26][CH:25]=[CH:24][CH
:23]=[CH:22]1.[N:2].[N:1]

Correctness of the mapping: YES

Reaction no 16

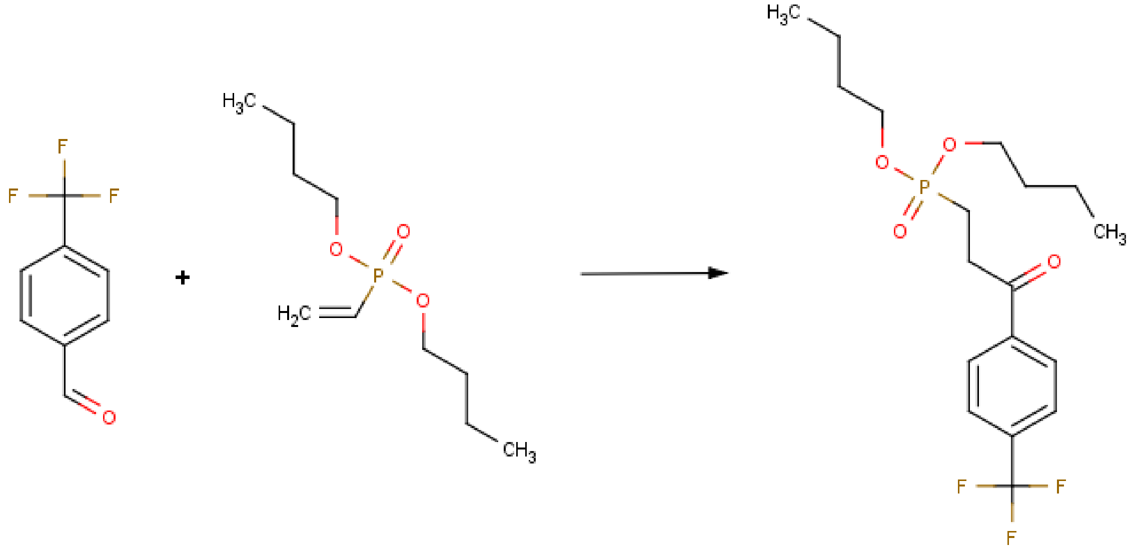

SMILES of the input:
FC(F)(F)C1=CC=C(C=O)C=C1.CCCCOP(=O)(OCCCC)C=C>>CCCCOP(=O)(CCC(=O)C1=CC=C(
C=C1)C(F)(F)F)OCCCC

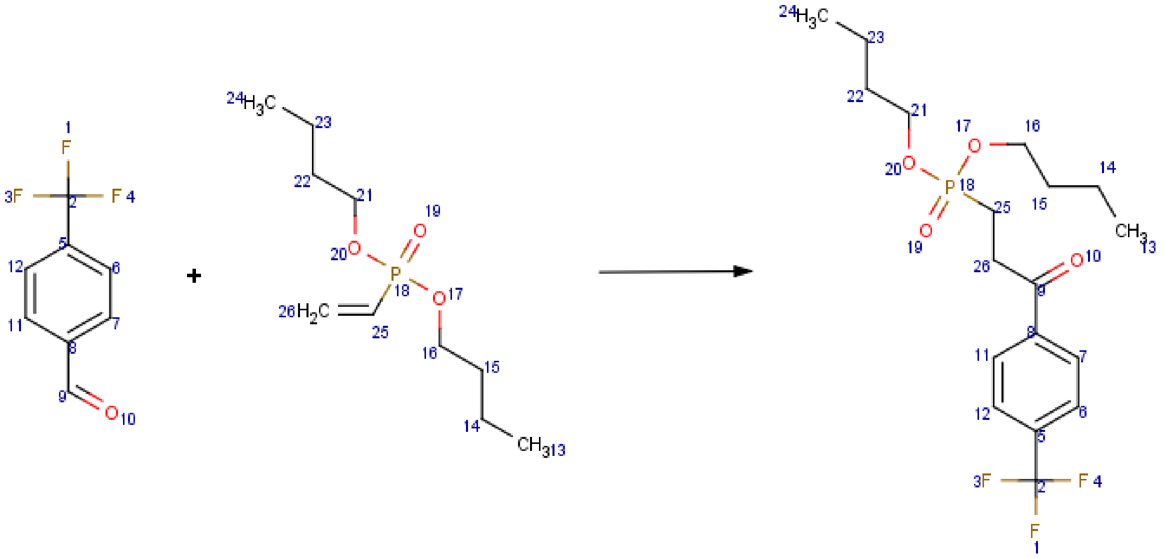

Correct mapped SMILES/SMARTS of the reaction:
[F:1][C:2]([F:3])([F:4])[C:5]1=[CH:6][CH:7]=[C:8]([CH:9]=[O:10])[CH:11]=[
CH:12]1.[CH3:13][CH2:14][CH2:15][CH2:16][O:17][P:18](=[O:19])([O:20][CH2:
21][CH2:22][CH2:23][CH3:24])[CH:25]=[CH2:26]>>[CH3:24][CH2:23][CH2:22][CH
2:21][O:20][P:18](=[O:19])([CH2:25][CH2:26][C:9](=[O:10])[C:8]1=[CH:7][CH
:6]=[C:5]([CH:12]=[CH:11]1)[C:2]([F:1])([F:3])[F:4])[O:17][CH2:16][CH2:15
][CH2:14][CH3:13]

Correctness of the mapping: YES

Reaction no 17

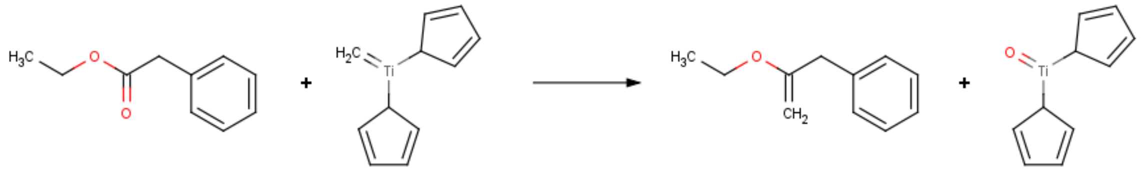

SMILES of the input:
CCOC(=O)CC1=CC=CC=C1.C=[Ti](C1C=CC=C1)C1C=CC=C1>>CCOC(=C)CC1=CC=CC=C1.O=[
Ti](C1C=CC=C1)C1C=CC=C1

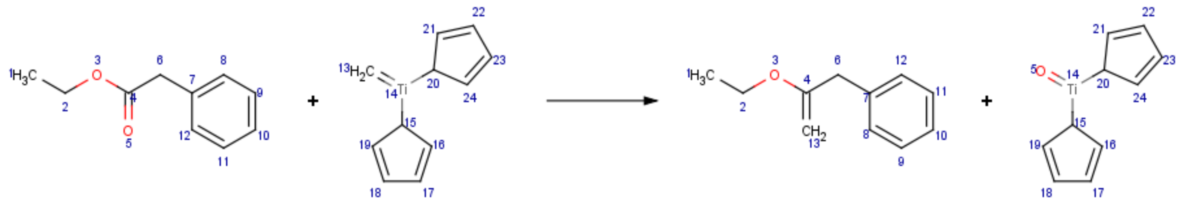

Correct mapped SMILES/SMARTS of the reaction:
[CH3:1][CH2:2][O:3][C:4](=[O:5])[CH2:6][C:7]1=[CH:8][CH:9]=[CH:10][CH:11]
=[CH:12]1.[CH2:13]=[Ti:14]([CH:15]1[CH:16]=[CH:17][CH:18]=[CH:19]1)[CH:20
]1[CH:21]=[CH:22][CH:23]=[CH:24]1>>[CH3:1][CH2:2][O:3][C:4](=[CH2:13])[CH
2:6][C:7]1=[CH:12][CH:11]=[CH:10][CH:9]=[CH:8]1.[O:5]=[Ti:14]([CH:15]1[CH
:16]=[CH:17][CH:18]=[CH:19]1)[CH:20]1[CH:21]=[CH:22][CH:23]=[CH:24]1

Correctness of the mapping: YES

Reaction no 18

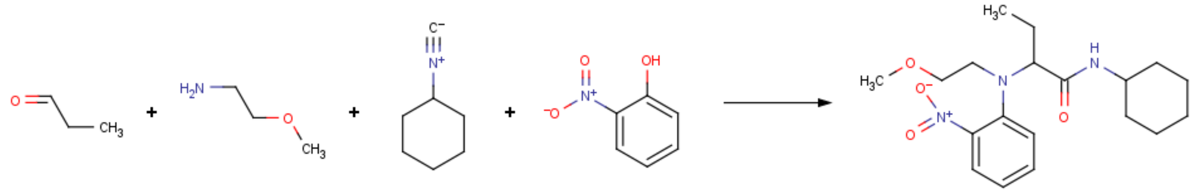

SMILES of the input:
CCC=O.COCCN.[C-]#[N+]C1CCCCC1.OC1=CC=CC=C1[N+]([O-
])=O>>CCC(N(CCOC)C1=CC=CC=C1[N+]([O-])=O)C(=O)NC1CCCCC1

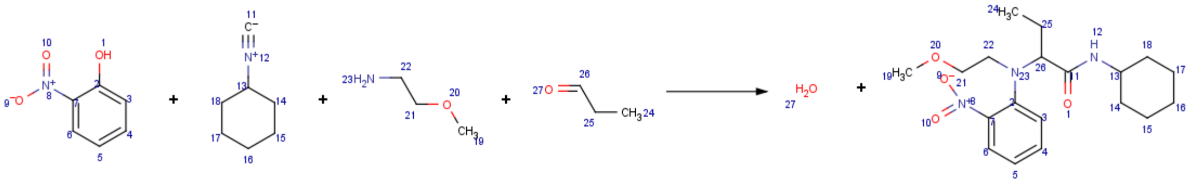

Correct mapped SMILES/SMARTS of the reaction:
[OH:1][C:2]1=[CH:3][CH:4]=[CH:5][CH:6]=[C:7]1[N+:8]([O-:9])=[O:10].[C-
:11]#[N+:12][CH:13]1[CH2:14][CH2:15][CH2:16][CH2:17][CH2:18]1.[CH3:19][O:
20][CH2:21][CH2:22][NH2:23].[CH3:24][CH2:25][CH:26]=[O:27]>>[OH2:27].[CH3
:24][CH2:25][CH:26]([N:23]([CH2:22][CH2:21][O:20][CH3:19])[C:2]1=[CH:3][C

H:4]=[CH:5][CH:6]=[C:7]1[N+:8]([O-
:9])=[O:10])[C:11](=[O:1])[NH:12][CH:13]1[CH2:18][CH2:17][CH2:16][CH2:15]
[CH2:14]1

Correctness of the mapping: YES

Reaction no 19

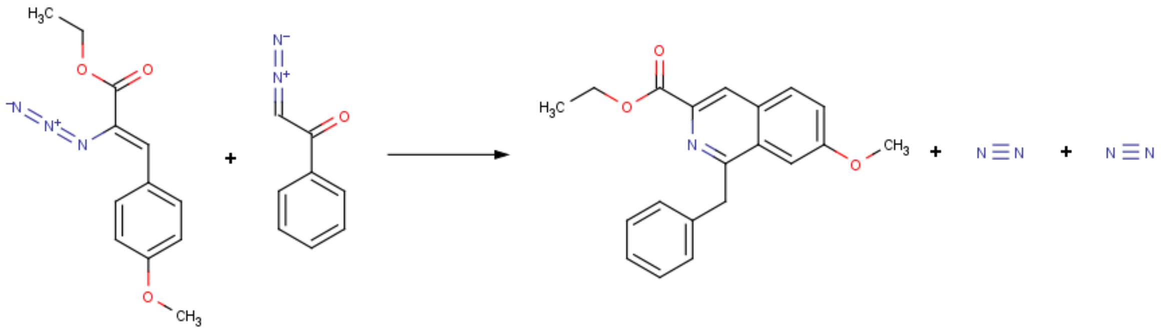

SMILES of the input:
CCOC(=O)C(=C\C1=CC=C(OC)C=C1)\N=[N+]=[N-].[N-
]=[N+]=CC(=O)C1=CC=CC=C1>>CCOC(=O)C1=CC2=CC=C(OC)C=C2C(CC2=CC=CC=C2)=N1.N
#N.N#N

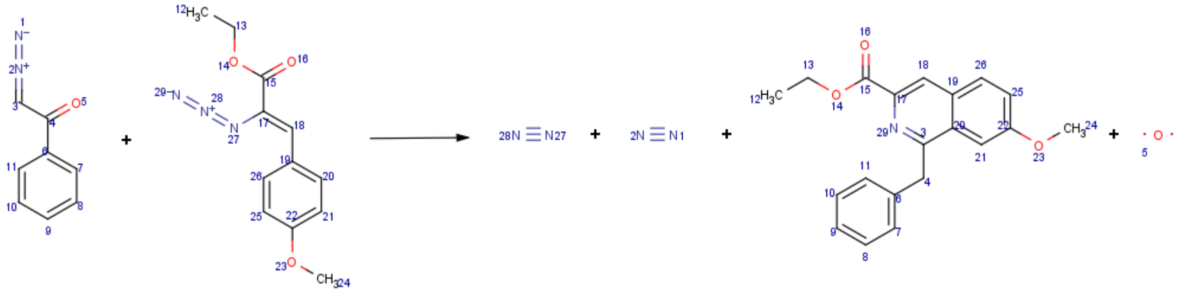

Correct mapped SMILES/SMARTS of the reaction:
[N-
:1]=[N+:2]=[CH:3][C:4](=[O:5])[C:6]1=[CH:7][CH:8]=[CH:9][CH:10]=[CH:11]1.
[CH3:12][CH2:13][O:14][C:15](=[O:16])[C:17](=[CH:18]\[C:19]1=[CH:20][CH:2
1]=[C:22]([O:23][CH3:24])[CH:25]=[CH:26]1)\[N:27]=[N+:28]=[N-
:29]>>[N:27]#[N:28].[N:1]#[N:2].[CH3:12][CH2:13][O:14][C:15](=[O:16])[C:1
7]1=[CH:18][C:19]2=[CH:26][CH:25]=[C:22]([O:23][CH3:24])[CH:21]=[C:20]2[C
:3]([CH2:4][C:6]2=[CH:7][CH:8]=[CH:9][CH:10]=[CH:11]2)=[N:29]1.[O:5]

Correctness of the mapping: NO

Reaction no 20

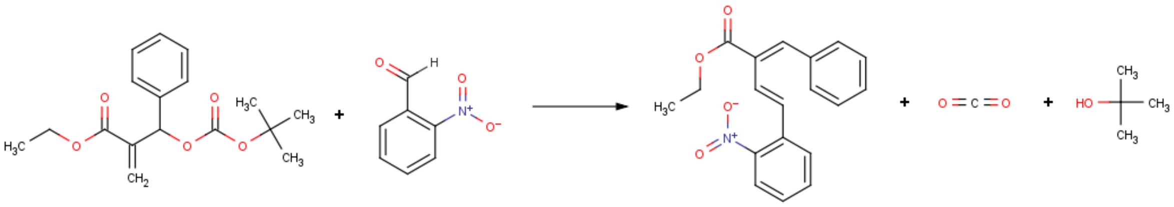

SMILES of the input:
CCOC(=O)C(=C)C(OC(=O)OC(C)(C)C)C1=CC=CC=C1.[H]C(=O)C1=C(C=CC=C1)[N+]([O-
])=O>>CCOC(=O)\C(\C=C\C1=C(C=CC=C1)[N+]([O-
])=O)=C\C1=CC=CC=C1.O=C=O.CC(C)(C)O

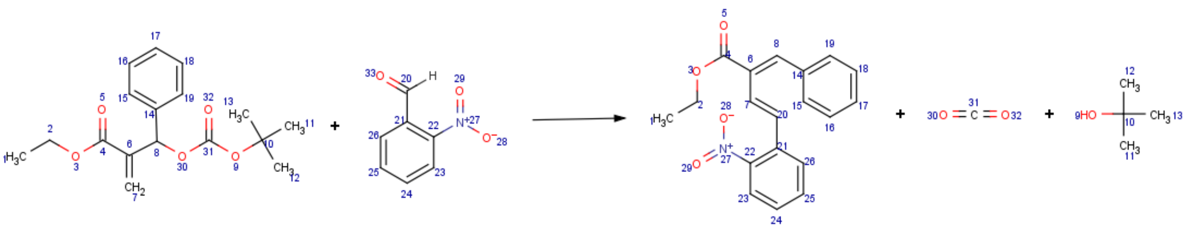

Correct mapped SMILES/SMARTS of the reaction:
[CH3:1][CH2:2][O:3][C:4](=[O:5])[C:6](=[CH2:7])[CH:8]([O:30][C:31](=[O:32
])[O:9][C:10]([CH3:11])([CH3:12])[CH3:13])[C:14]1=[CH:15][CH:16]=[CH:17][
CH:18]=[CH:19]1.[H][C:20](=[O:33])[C:21]1=[C:22]([CH:23]=[CH:24][CH:25]=[
CH:26]1)[N+:27]([O-
:28])=[O:29]>>[CH3:1][CH2:2][O:3][C:4](=[O:5])\[C:6](\[CH:7]=[CH:20]\[C:2
1]1=[C:22]([CH:23]=[CH:24][CH:25]=[CH:26]1)[N+:27]([O-
:28])=[O:29])=[CH:8]\[C:14]1=[CH:19][CH:18]=[CH:17][CH:16]=[CH:15]1.[O:30
]=[C:31]=[O:32].[CH3:11][C:10]([CH3:12])([CH3:13])[OH:9]

Correctness of the mapping: NO

Reaction no 21

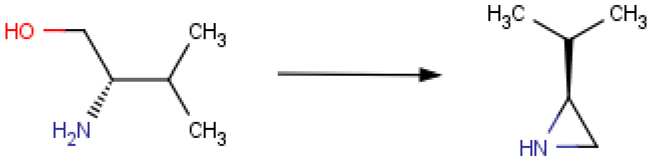

SMILES of the input:
CC(C)[C@H](N)CO>>CC(C)[C@H]1CN1

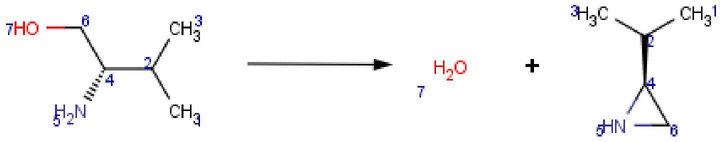

Correct mapped SMILES/SMARTS of the reaction:
[CH3:1][CH:2]([CH3:3])[C@H:4]([NH2:5])[CH2:6][OH:7]>>[OH2:7].[CH3:3][CH:2
]([CH3:1])[C@H:4]1[CH2:6][NH:5]1

Correctness of the mapping: YES

Reaction no 22

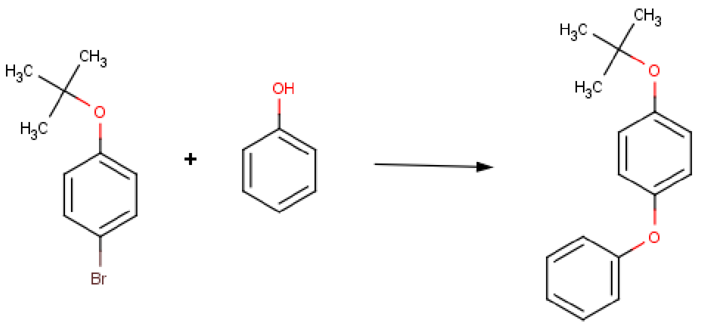

SMILES of the input:
CC(C)(C)OC1=CC=C(Br)C=C1.OC1=CC=CC=C1>>CC(C)(C)OC1=CC=C(OC2=CC=CC=C2)C=C1

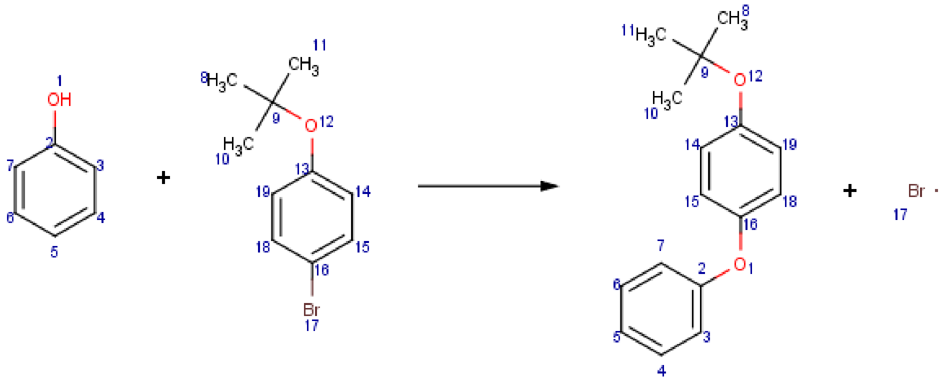

Correct mapped SMILES/SMARTS of the reaction:
[OH:1][C:2]1=[CH:3][CH:4]=[CH:5][CH:6]=[CH:7]1.[CH3:8][C:9]([CH3:10])([CH3:11])[O:12][C:13]1=[CH:14][CH:15]=[C:16]([Br:17])[CH:18]=[CH:19]1>>[CH3:11][C:9]([CH3:10])([CH3:8])[O:12][C:13]1=[CH:19][CH:18]=[C:16]([O:1][C:2]2=[CH:3][CH:4]=[CH:5][CH:6]=[CH:7]2)[CH:15]=[CH:14]1.[Br:17]

Correctness of the mapping: YES

Reaction no 23

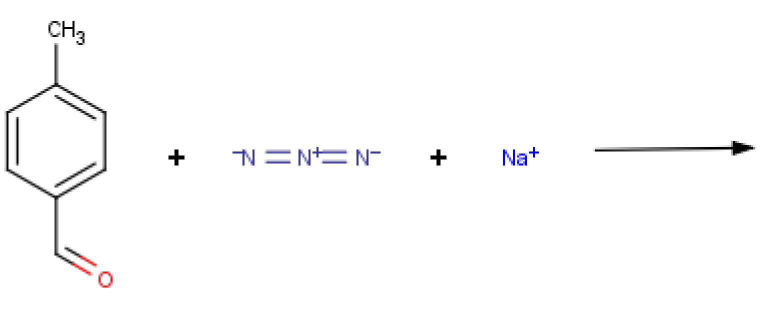

SMILES of the input:
CC1=CC=C(C=O)C=C1.[N-]=[N+]=[N-].[Na+]>>CC1=CC=C(C=C1)C#N

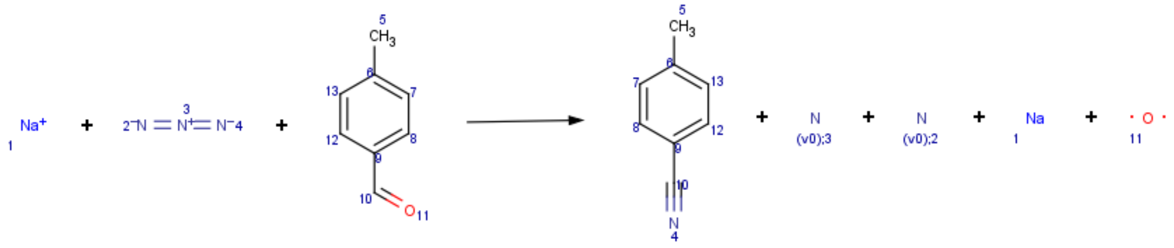

Correct mapped SMILES/SMARTS of the reaction:
[Na+:1].[N-:2]=[N+:3]=[N-
:4].[CH3:5][C:6]1=[CH:7][CH:8]=[C:9]([CH:10]=[O:11])[CH:12]=[CH:13]1>>[CH
3:5][C:6]1=[CH:13][CH:12]=[C:9]([CH:8]=[CH:7]1)[C:10]#[N:4].[N:3].[N:2].[
Na:1].[O:11]

Correctness of the mapping: YES

Reaction no 24

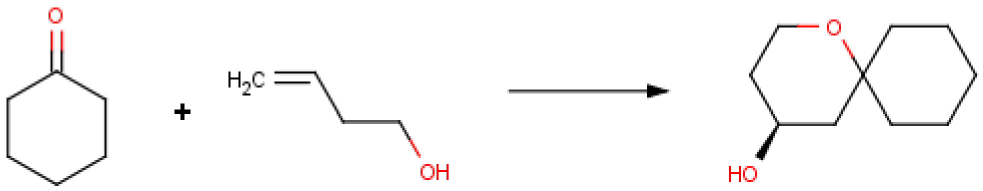

SMILES of the input:
O=C1CCCCC1.OCCC=C>>O[C@H]1CCOC2(CCCCC2)C1

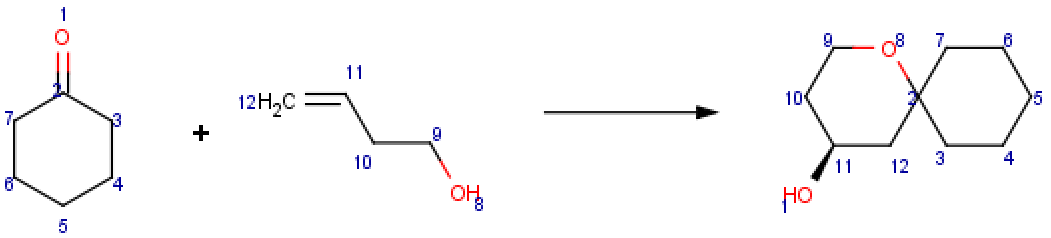

Correct mapped SMILES/SMARTS of the reaction:
[O:1]=[C:2]1[CH2:3][CH2:4][CH2:5][CH2:6][CH2:7]1.[OH:8][CH2:9][CH2:10][CH
:11]=[CH2:12]>>[OH:1][C@H:11]1[CH2:10][CH2:9][O:8][C:2]2([CH2:7][CH2:6][C
H2:5][CH2:4][CH2:3]2)[CH2:12]1

Correctness of the mapping: YES

Reaction no 25

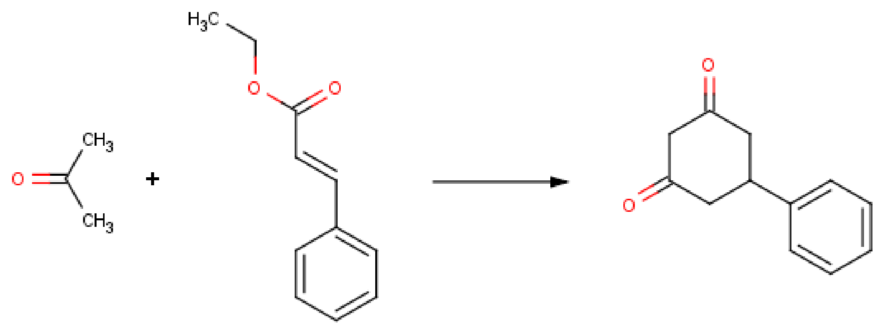

SMILES of the input:
CC(C)=O.CCOC(=O)\C=C\C1=CC=CC=C1>>O=C1CC(CC(=O)C1)C1=CC=CC=C1

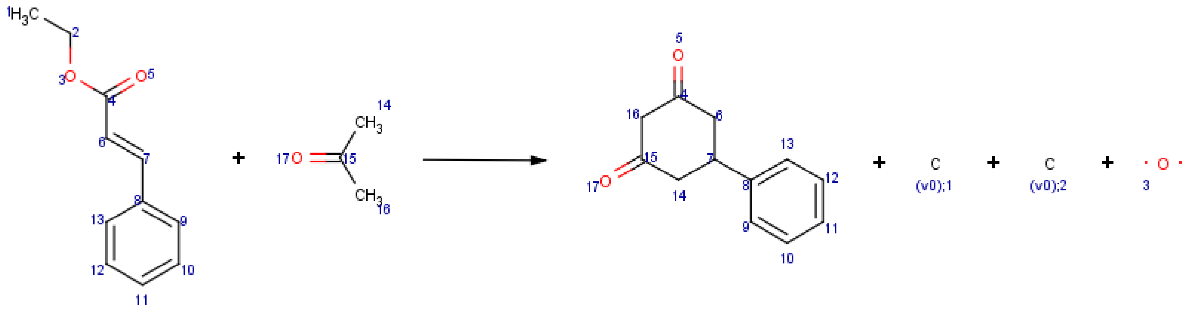

Correct mapped SMILES/SMARTS of the reaction:
[CH3:1][CH2:2][O:3][C:4](=[O:5])\[CH:6]=[CH:7]\[C:8]1=[CH:9][CH:10]=[CH:1
1][CH:12]=[CH:13]1.[CH3:14][C:15]([CH3:16])=[O:17]>>[O:5]=[C:4]1[CH2:6][C
H:7]([CH2:14][C:15](=[O:17])[CH2:16]1)[C:8]1=[CH:13][CH:12]=[CH:11][CH:10
]=[CH:9]1.[C:1].[C:2].[O:3]

Correctness of the mapping: NO

Michał Pieczykolan, PhD, Institute of Organic Chemistry PAS

Reaction no 1

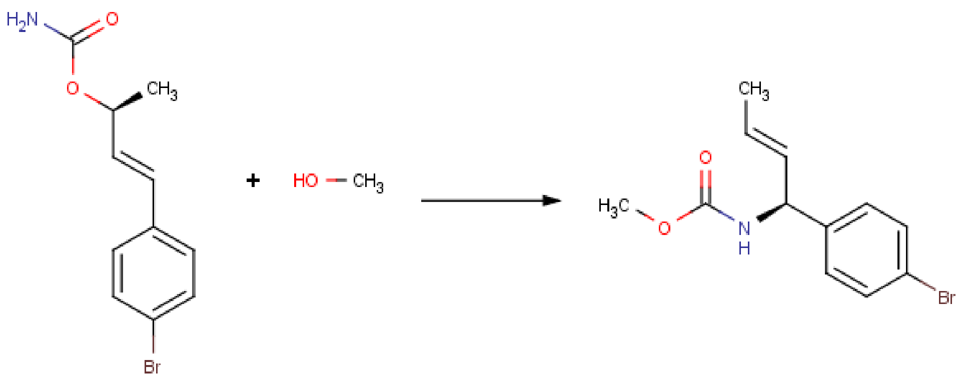

SMILES of the input:
C[C@H](OC(N)=O)\C=C\C1=CC=C(Br)C=C1.CO>>COC(=O)N[C@@H](\C=C\C)C1=CC=C(Br)
C=C1

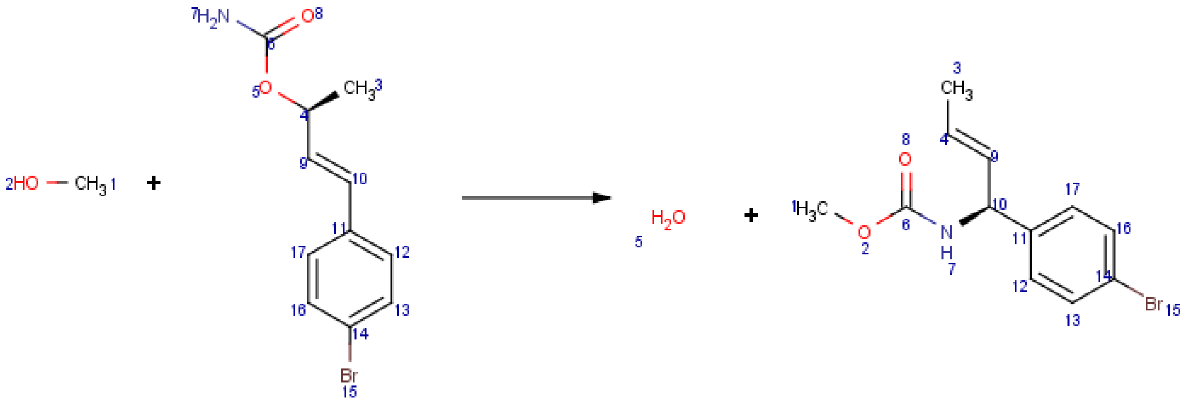

Correct mapped SMILES/SMARTS of the reaction:
[CH3:1][OH:2].[CH3:3][C@H:4]([O:5][C:6]([NH2:7])=[O:8])\[CH:9]=[CH:10]\[C:11]1=[CH:12][CH:13]=[C:14]([Br:15])[CH:16]=[CH:17]1>>[OH2:5].[CH3:1][O:2][C:6](=[O:8])[NH:7][C@@H:10](\[CH:9]=[CH:4]\[CH3:3])[C:11]1=[CH:17][CH:16]=[C:14]([Br:15])[CH:13]=[CH:12]1

Correctness of the mapping: NO

Reaction no 2

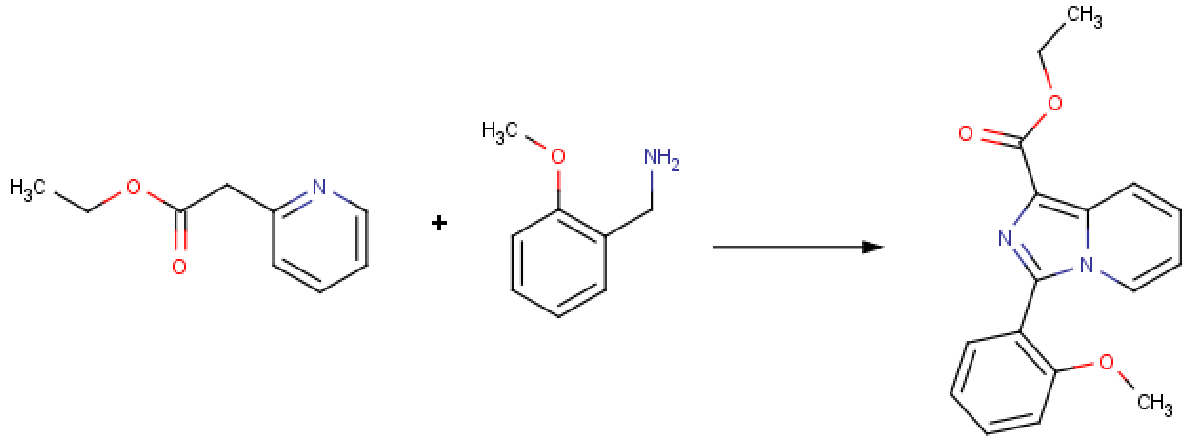

SMILES of the input:
CCOC(=O)CC1=NC=CC=C1.COC1=C(CN)C=CC=C1>>CCOC(=O)C1=C2C=CC=CN2C(=N1)C1=C(OC)C=CC=C1

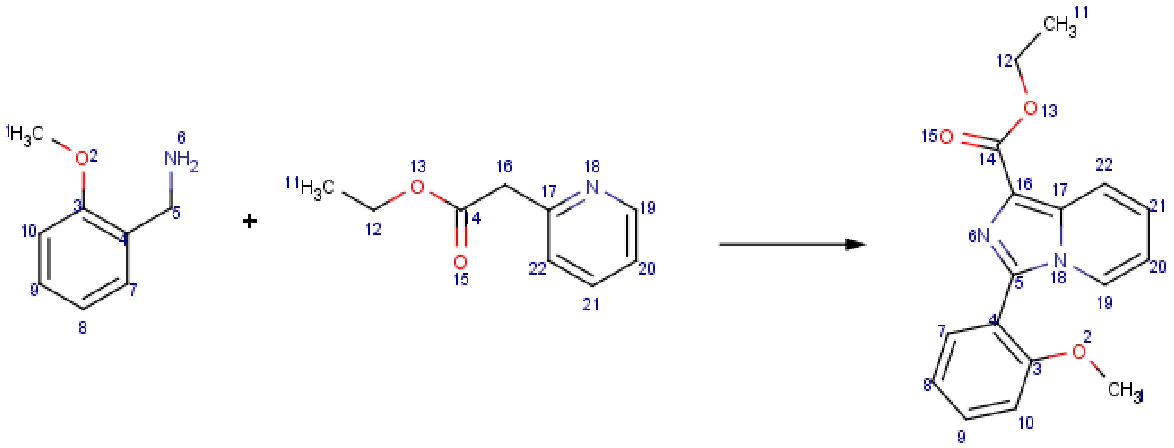

Correct mapped SMILES/SMARTS of the reaction:

[CH3:1][O:2][C:3]1=[C:4]([CH2:5][NH2:6])[CH:7]=[CH:8][CH:9]=[CH:10]1.[CH3
:11][CH2:12][O:13][C:14](=[O:15])[CH2:16][C:17]1=[N:18][CH:19]=[CH:20][CH
:21]=[CH:22]1>>[CH3:11][CH2:12][O:13][C:14](=[O:15])[C:16]1=[C:17]2[CH:22
]=[CH:21][CH:20]=[CH:19][N:18]2[C:5](=[N:6]1)[C:4]1=[C:3]([O:2][CH3:1])[C
H:10]=[CH:9][CH:8]=[CH:7]1

Correctness of the mapping: YES

Reaction no 3

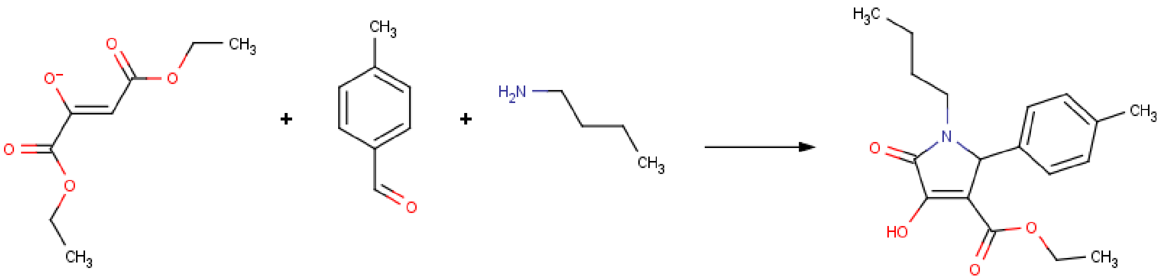

SMILES of the input:
CCOC(=O)\C=C(/[O-
])C(=O)OCC.CC1=CC=C(C=O)C=C1.CCCCN>>CCCCN1C(C(C(=O)OCC)=C(O)C1=O)C1=CC=C(
C)C=C1

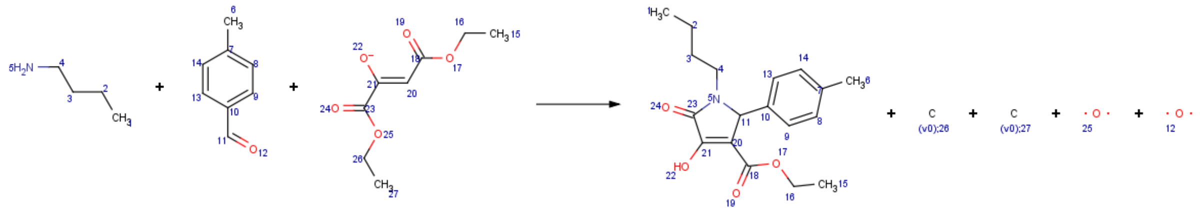

Correct mapped SMILES/SMARTS of the reaction:
[CH3:1][CH2:2][CH2:3][CH2:4][NH2:5].[CH3:6][C:7]1=[CH:8][CH:9]=[C:10]([CH
:11]=[O:12])[CH:13]=[CH:14]1.[CH3:15][CH2:16][O:17][C:18](=[O:19])\[CH:20
]=[C:21](/[O-
:22])[C:23](=[O:24])[O:25][CH2:26][CH3:27]>>[CH3:1][CH2:2][CH2:3][CH2:4][
N:5]1[CH:11]([C:20]([C:18](=[O:19])[O:17][CH2:16][CH3:15])=[C:21]([OH:22]
)[C:23]1=[O:24])[C:10]1=[CH:13][CH:14]=[C:7]([CH3:6])[CH:8]=[CH:9]1.[C:26
].[C:27].[O:25].[O:12]

Correctness of the mapping: NO

Reaction no 4

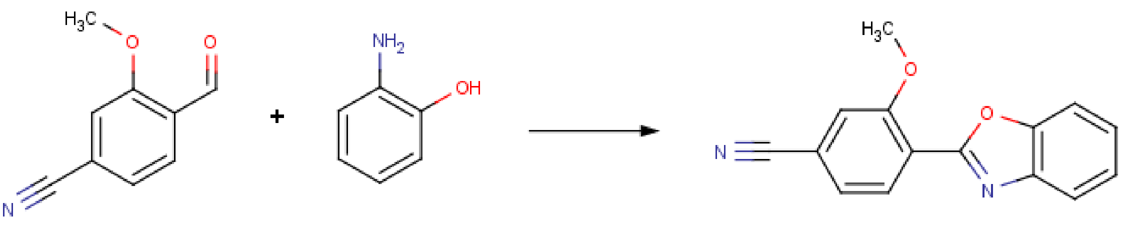

SMILES of the input:
COC1=C(C=O)C=CC(=C1)C#N.NC1=C(O)C=CC=C1>>COC1=C(C=CC(=C1)C#N)C1=NC2=C(O1)
C=CC=C2

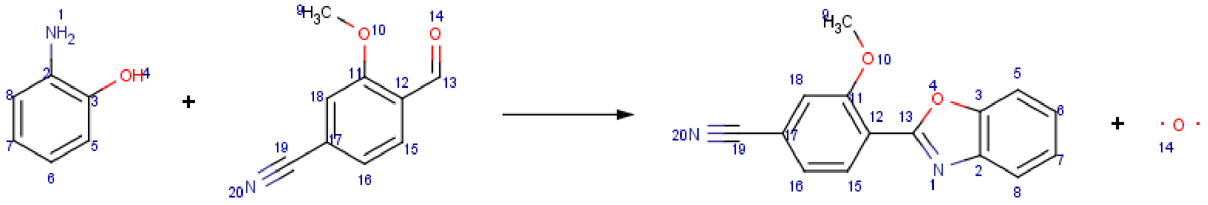

Correct mapped SMILES/SMARTS of the reaction:
[NH2:1][C:2]1=[C:3]([OH:4])[CH:5]=[CH:6][CH:7]=[CH:8]1.[CH3:9][O:10][C:11]1=[C:12]([CH:13]=[O:14])[CH:15]=[CH:16][C:17](=[CH:18]1)[C:19]#[N:20]>>[CH3:9][O:10][C:11]1=[C:12]([CH:15]=[CH:16][C:17](=[CH:18]1)[C:19]#[N:20])[C:13]1=[N:1][C:2]2=[C:3]([O:4]1)[CH:5]=[CH:6][CH:7]=[CH:8]2.[O:14]

Correctness of the mapping: YES

Reaction no 5

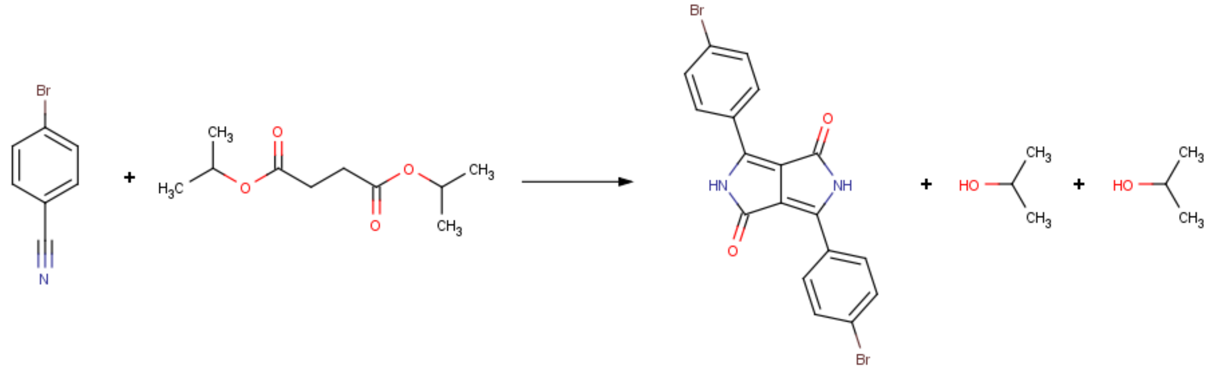

SMILES of the input:
BrC1=CC=C(C=C1)C#N.CC(C)OC(=O)CCC(=O)OC(C)C>>BrC1=CC=C(C=C1)C1=C2C(=O)NC(=C2C(=O)N1)C1=CC=C(Br)C=C1.CC(C)O.CC(C)O

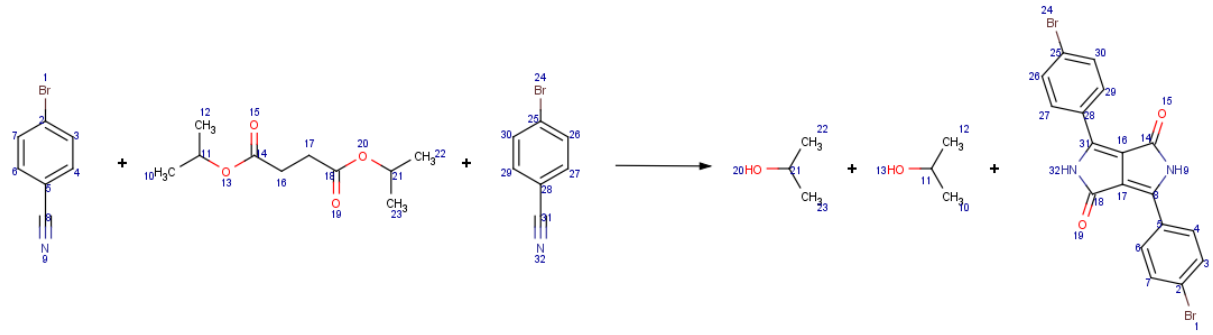

Correct mapped SMILES/SMARTS of the reaction:
[Br:1][C:2]1=[CH:3][CH:4]=[C:5]([CH:6]=[CH:7]1)[C:8]#[N:9].[CH3:10][CH:11]([CH3:12])[O:13][C:14](=[O:15])[CH2:16][CH2:17][C:18](=[O:19])[O:20][CH:21]([CH3:22])[CH3:23].[Br:24][C:25]1=[CH:26][CH:27]=[C:28]([CH:29]=[CH:30]1)[C:31]#[N:32]>>[CH3:22][CH:21]([CH3:23])[OH:20].[CH3:12][CH:11]([CH3:10])[OH:13].[Br:24][C:25]1=[CH:30][CH:29]=[C:28]([CH:27]=[CH:26]1)[C:31]1=[C:16]2[C:14](=[O:15])[NH:9][C:8](=[C:17]2[C:18](=[O:19])[NH:32]1)[C:5]1=[CH:4][CH:3]=[C:2]([Br:1])[CH:7]=[CH:6]1

Correctness of the mapping: YES

Reaction no 6

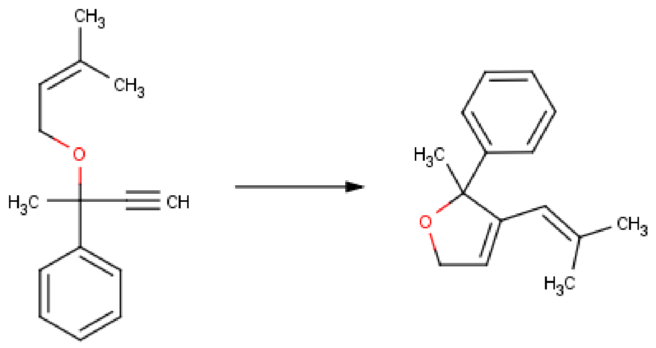

SMILES of the input:
CC(C)=CCOC(C)(C#C)C1=CC=CC=C1>>CC(C)=CC1=CCOC1(C)C1=CC=CC=C1

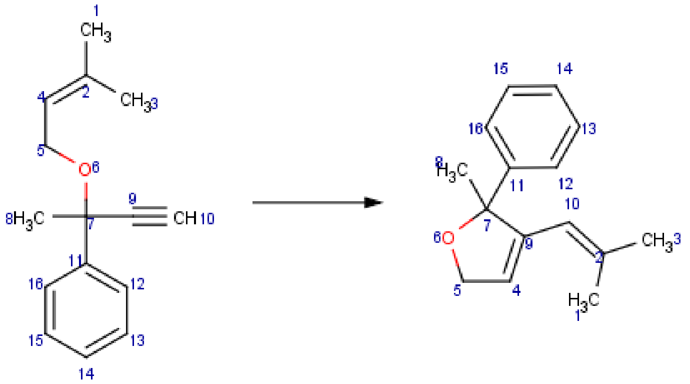

Correct mapped SMILES/SMARTS of the reaction:
[CH3:1][C:2]([CH3:3])=[CH:4][CH2:5][O:6][C:7]([CH3:8])([C:9]#[CH:10])[C:11]1=[CH:12][CH:13]=[CH:14][CH:15]=[CH:16]1>>[CH3:3][C:2]([CH3:1])=[CH:10][C:9]1=[CH:4][CH2:5][O:6][C:7]1([CH3:8])[C:11]1=[CH:16][CH:15]=[CH:14][CH:13]=[CH:12]1

Correctness of the mapping: NO

Reaction no 7

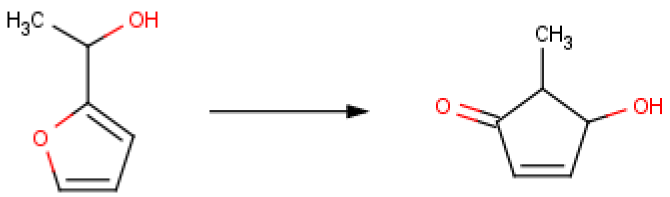

SMILES of the input:
CC(O)C1=CC=CO1>>CC1C(O)C=CC1=O

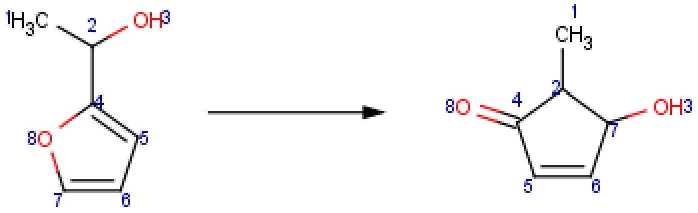

Correct mapped SMILES/SMARTS of the reaction:
[CH3:1][CH:2]([OH:3])[C:4]1=[CH:5][CH:6]=[CH:7][O:8]1>>[CH3:1][CH:2]1[CH:7]([OH:3])[CH:6]=[CH:5][C:4]1=[O:8]

Correctness of the mapping: YES

Reaction no 8

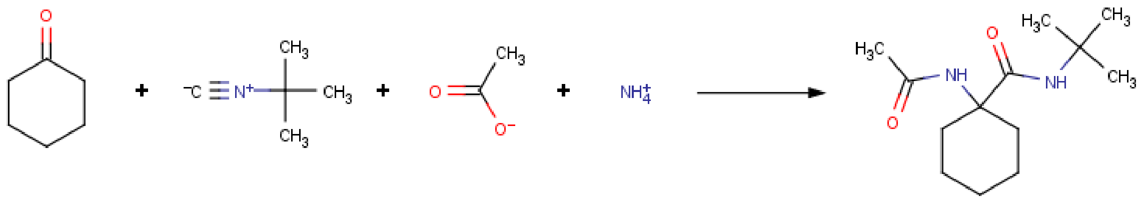

SMILES of the input:
O=C1CCCCC1.CC(C)(C)[N+]#[C-].CC([O-])=O.[NH4+]>>CC(=O)NC1(CCCCC1)C(=O)NC(C)(C)C

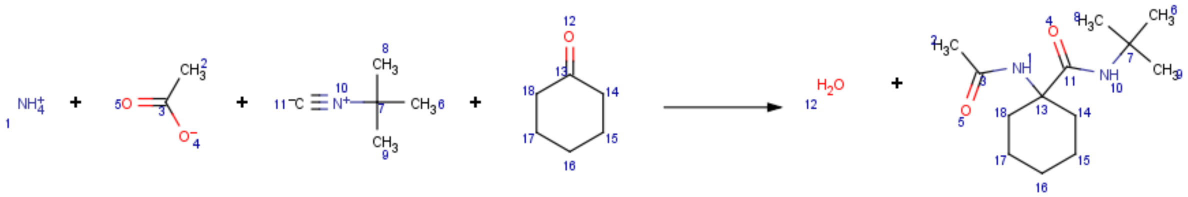

Correct mapped SMILES/SMARTS of the reaction:
[NH4+:1].[CH3:2][C:3]([O-:4])=[O:5].[CH3:6][C:7]([CH3:8])([CH3:9])[N+:10]#[C-:11].[O:12]=[C:13]1[CH2:14][CH2:15][CH2:16][CH2:17][CH2:18]1>>[OH2:12].[CH3:2][C:3](=[O:5])[NH:1][C:13]1([CH2:14][CH2:15][CH2:16][CH2:17][CH2:18]1)[C:11](=[O:4])[NH:10][C:7]([CH3:6])([CH3:9])[CH3:8]

Correctness of the mapping: YES

Reaction no 9

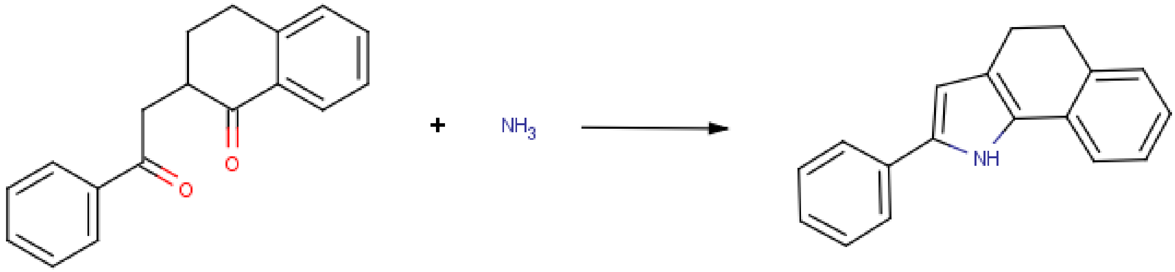

SMILES of the input:
O=C(CC1CCC2=CC=CC=C2C1=O)C1=CC=CC=C1.N>>C1CC2=CC=CC=C2C2=C1C=C(N2)C1=CC=CC=C1

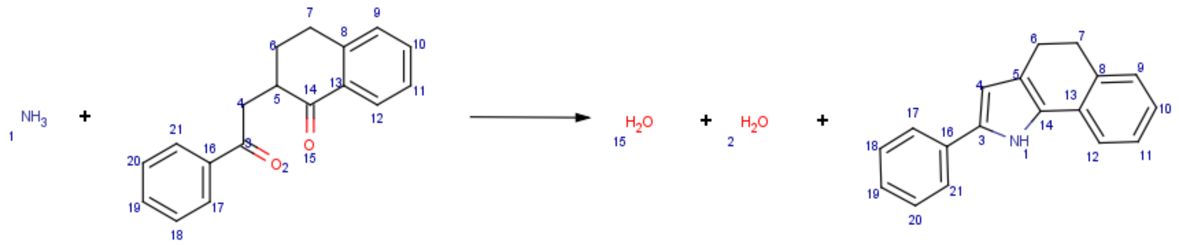

Correct mapped SMILES/SMARTS of the reaction:
[NH3:1].[O:2]=[C:3]([CH2:4][CH:5]1[CH2:6][CH2:7][C:8]2=[CH:9][CH:10]=[CH:11][CH:12]=[C:13]2[C:14]1=[O:15])[C:16]1=[CH:17][CH:18]=[CH:19][CH:20]=[CH:21]1>>[OH2:15].[OH2:2].[CH2:6]1[CH2:7][C:8]2=[CH:9][CH:10]=[CH:11][CH:12]=[C:13]2[C:14]2=[C:5]1[CH:4]=[C:3]([NH:1]2)[C:16]1=[CH:21][CH:20]=[CH:19][CH:18]=[CH:17]1

Correctness of the mapping: YES

Reaction no 10

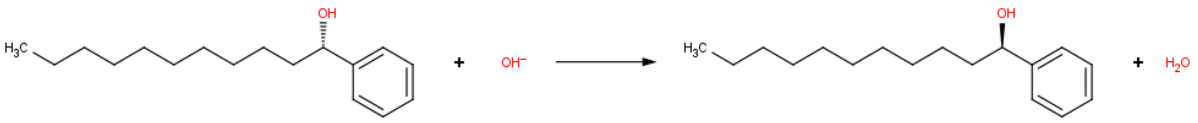

SMILES of the input:
CCCCCCCCCC[C@H](O)C1=CC=CC=C1.[OH-]>>CCCCCCCCCC[C@@H](O)C1=CC=CC=C1.O

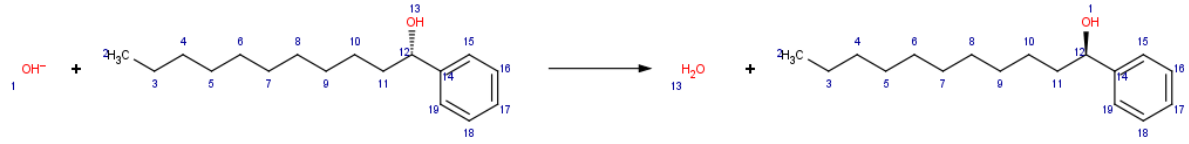

Correct mapped SMILES/SMARTS of the reaction:
[OH-:1].[CH3:2][CH2:3][CH2:4][CH2:5][CH2:6][CH2:7][CH2:8][CH2:9][CH2:10][CH2:11][C@H:12]([OH:13])[C:14]1=[CH:15][CH:16]=[CH:17][CH:18]=[CH:19]1>>[OH2:13].[CH3:2][CH2:3][CH2:4][CH2:5][CH2:6][CH2:7][CH2:8][CH2:9][CH2:10][CH2:11][C@@H:12]([OH:1])[C:14]1=[CH:15][CH:16]=[CH:17][CH:18]=[CH:19]1

Correctness of the mapping: NO

Reaction no 11

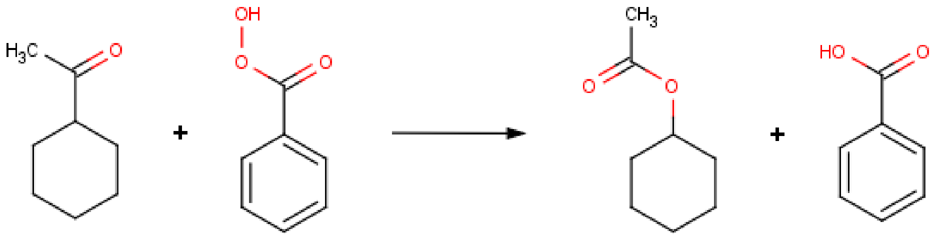

SMILES of the input:
CC(=O)C1CCCCC1.OOC(=O)C1=CC=CC=C1>>CC(=O)OC1CCCCC1.OC(=O)C1=CC=CC=C1

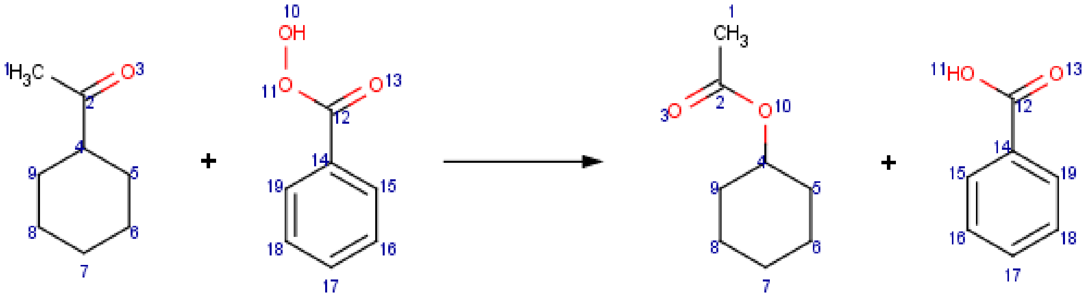

Correct mapped SMILES/SMARTS of the reaction:
[CH3:1][C:2](=[O:3])[CH:4]1[CH2:5][CH2:6][CH2:7][CH2:8][CH2:9]1.[OH:10][O:11][C:12](=[O:13])[C:14]1=[CH:15][CH:16]=[CH:17][CH:18]=[CH:19]1>>[CH3:1][C:2](=[O:3])[O:10][CH:4]1[CH2:5][CH2:6][CH2:7][CH2:8][CH2:9]1.[OH:11][C:12](=[O:13])[C:14]1=[CH:19][CH:18]=[CH:17][CH:16]=[CH:15]1

Correctness of the mapping: YES

Reaction no 12

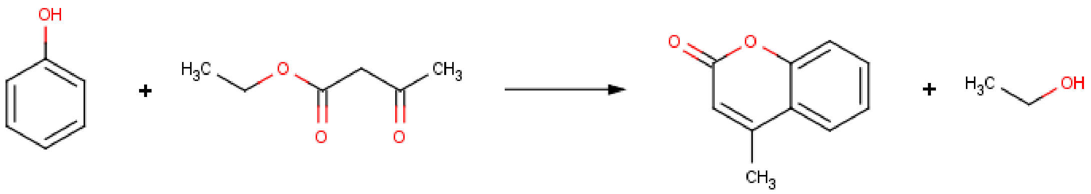

SMILES of the input:
OC1=CC=CC=C1.CCOC(=O)CC(C)=O>>CC1=CC(=O)OC2=CC=CC=C12.CCO

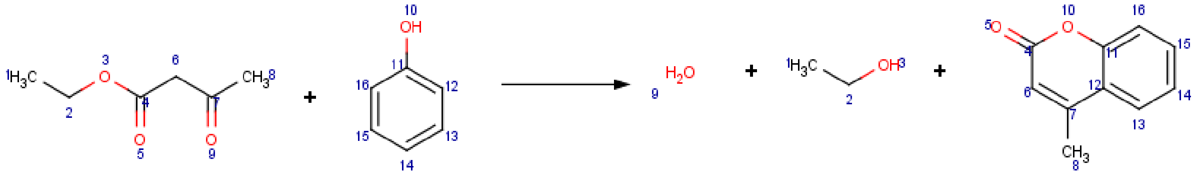

Correct mapped SMILES/SMARTS of the reaction:
[CH3:1][CH2:2][O:3][C:4](=[O:5])[CH2:6][C:7]([CH3:8])=[O:9].[OH:10][C:11]1=[CH:12][CH:13]=[CH:14][CH:15]=[CH:16]1>>[OH2:9].[CH3:1][CH2:2][OH:3].[CH3:8][C:7]1=[CH:6][C:4](=[O:5])[O:10][C:11]2=[CH:16][CH:15]=[CH:14][CH:13]=[C:12]12

Correctness of the mapping: YES

Reaction no 13

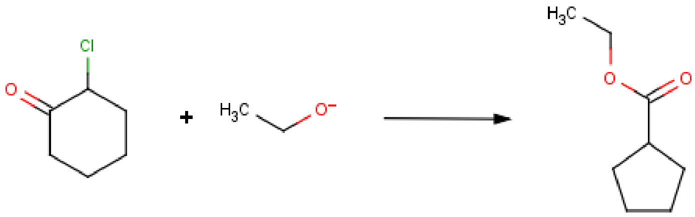

SMILES of the input:
ClC1CCCCC1=O.CC[O-]>>CCOC(=O)C1CCCC1

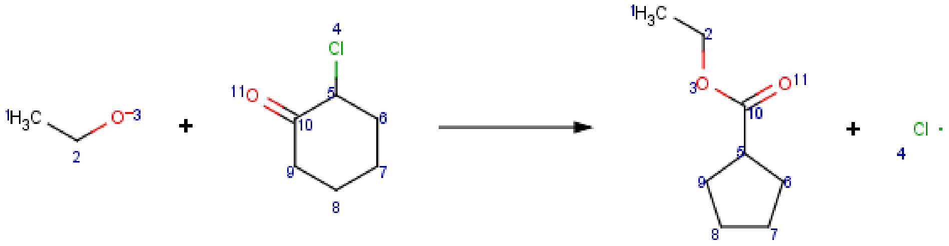

Correct mapped SMILES/SMARTS of the reaction:
[CH3:1][CH2:2][O-
:3].[Cl:4][CH:5]1[CH2:6][CH2:7][CH2:8][CH2:9][C:10]1=[O:11]>>[CH3:1][CH2:
2][O:3][C:10](=[O:11])[CH:5]1[CH2:6][CH2:7][CH2:8][CH2:9]1.[Cl:4]

Correctness of the mapping: YES

Reaction no 14

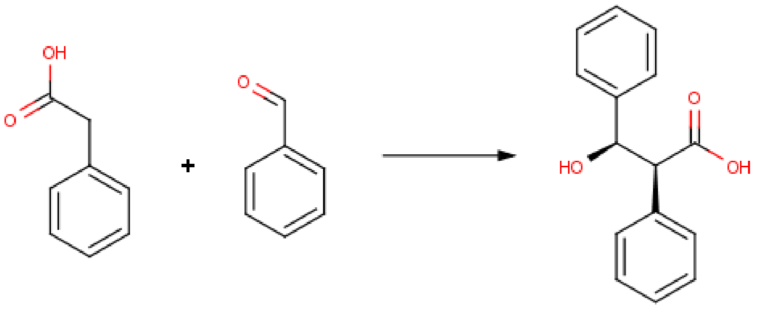

SMILES of the input:
OC(=O)CC1=CC=CC=C1.O=CC1=CC=CC=C1>>O[C@H]([C@@H](C(O)=O)C1=CC=CC=C1)C1=CC
=CC=C1

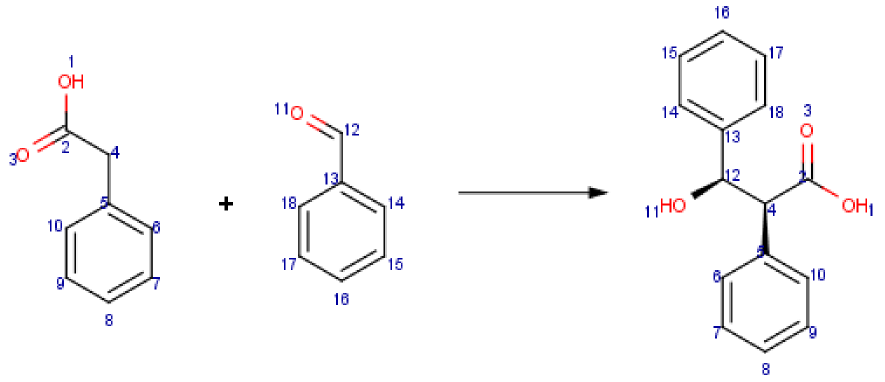

Correct mapped SMILES/SMARTS of the reaction:
[OH:1][C:2](=[O:3])[CH2:4][C:5]1=[CH:6][CH:7]=[CH:8][CH:9]=[CH:10]1.[O:11
]=[CH:12][C:13]1=[CH:14][CH:15]=[CH:16][CH:17]=[CH:18]1>>[OH:11][C@H:12](
[C@@H:4]([C:2]([OH:1])=[O:3])[C:5]1=[CH:10][CH:9]=[CH:8][CH:7]=[CH:6]1)[C
:13]1=[CH:14][CH:15]=[CH:16][CH:17]=[CH:18]1

Correctness of the mapping: YES

Reaction no 15

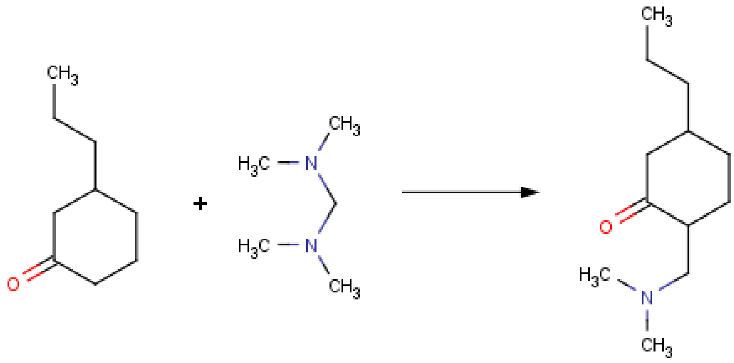

SMILES of the input:
CCCC1CCCC(=O)C1.CN(C)CN(C)C>>CCCC1CCC(CN(C)C)C(=O)C1

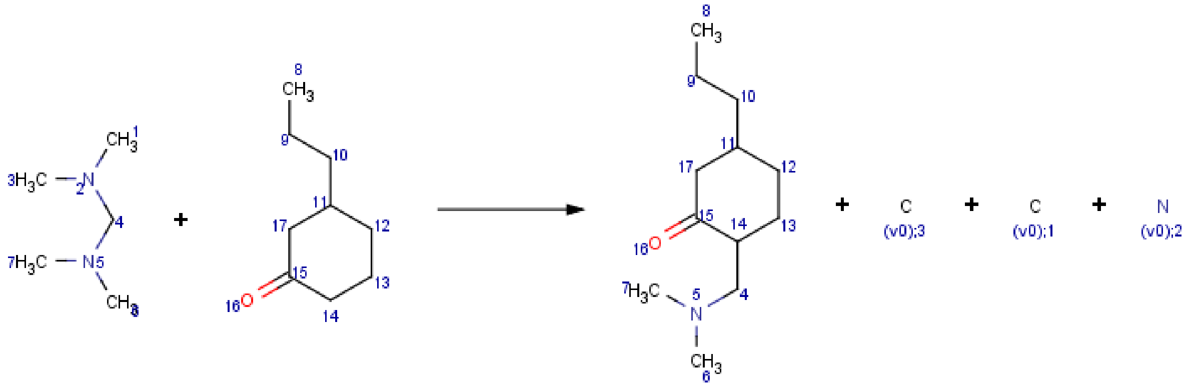

Correct mapped SMILES/SMARTS of the reaction:
[CH3:1][N:2]([CH3:3])[CH2:4][N:5]([CH3:6])[CH3:7].[CH3:8][CH2:9][CH2:10][
CH:11]1[CH2:12][CH2:13][CH2:14][C:15](=[O:16])[CH2:17]1>>[CH3:8][CH2:9][C
H2:10][CH:11]1[CH2:12][CH2:13][CH:14]([CH2:4][N:5]([CH3:6])[CH3:7])[C:15]
(=[O:16])[CH2:17]1.[C:3].[C:1].[N:2]

Correctness of the mapping: YES

Reaction no 16

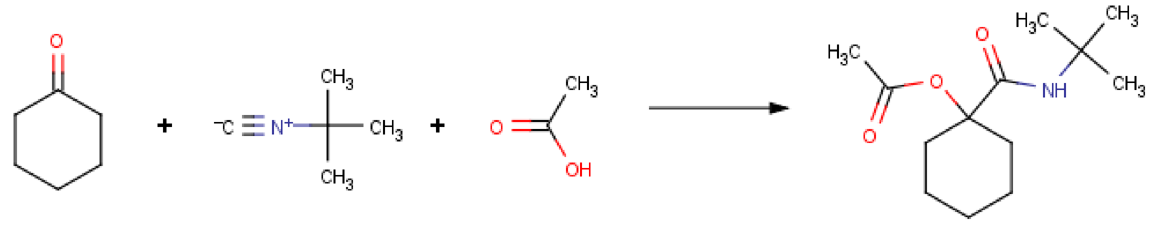

SMILES of the input:
O=C1CCCCC1.CC(C)(C)[N+]#[C-].CC(O)=O>>CC(=O)OC1(CCCCC1)C(=O)NC(C)(C)C

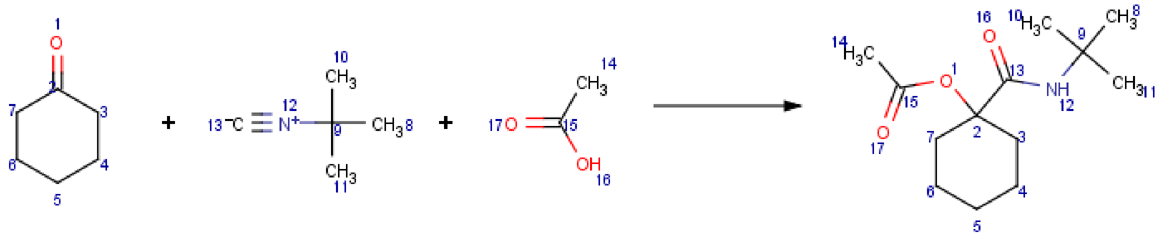

Correct mapped SMILES/SMARTS of the reaction:
[O:1]=[C:2]1[CH2:3][CH2:4][CH2:5][CH2:6][CH2:7]1.[CH3:8][C:9]([CH3:10])([CH3:11])[N+:12]#[C-:13].[CH3:14][C:15]([OH:16])=[O:17]>>[CH3:14][C:15](=[O:17])[O:1][C:2]1([CH2:3][CH2:4][CH2:5][CH2:6][CH2:7]1)[C:13](=[O:16])[NH:12][C:9]([CH3:8])([CH3:11])[CH3:10]

Correctness of the mapping: YES

Reaction no 17

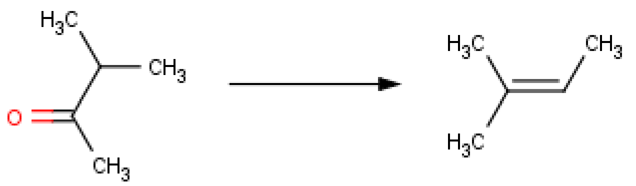

SMILES of the input:
CC(C)C(C)=O>>CC=C(C)C

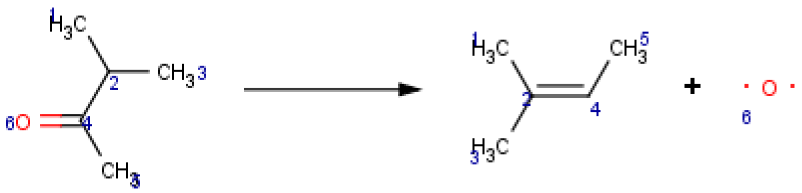

Correct mapped SMILES/SMARTS of the reaction:
[CH3:1][CH:2]([CH3:3])[C:4]([CH3:5])=[O:6]>>[CH3:5][CH:4]=[C:2]([CH3:3])[CH3:1].[O:6]

Correctness of the mapping: YES

Reaction no 18

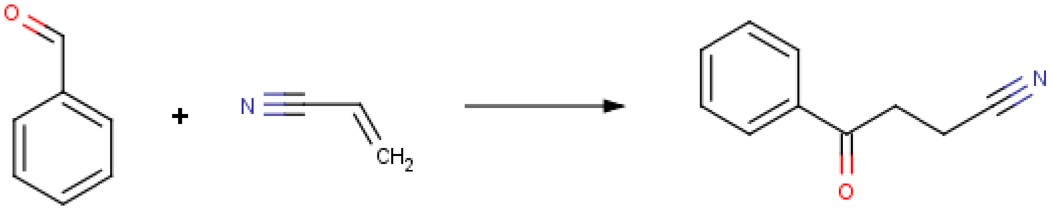

SMILES of the input:
O=CC1=CC=CC=C1.C=CC#N>>O=C(CCC#N)C1=CC=CC=C1

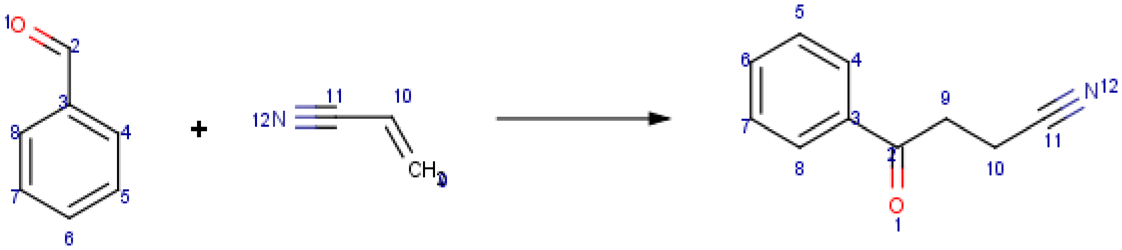

Correct mapped SMILES/SMARTS of the reaction:
[O:1]=[CH:2][C:3]1=[CH:4][CH:5]=[CH:6][CH:7]=[CH:8]1.[CH2:9]=[CH:10][C:11]#[N:12]>>[O:1]=[C:2]([CH2:9][CH2:10][C:11]#[N:12])[C:3]1=[CH:8][CH:7]=[CH:6][CH:5]=[CH:4]1

Correctness of the mapping: YES

Reaction no 19

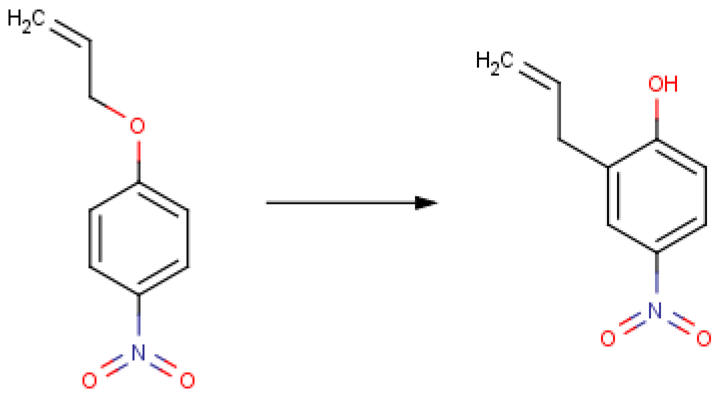

SMILES of the input:
C=CCOC1=CC=C(C=C1)N(=O)=O>>OC1=CC=C(C=C1CC=C)N(=O)=O

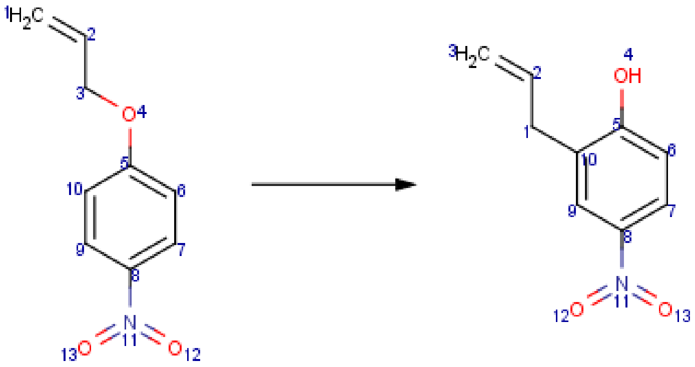

Correct mapped SMILES/SMARTS of the reaction:
[CH2:1]=[CH:2][CH2:3][O:4][C:5]1=[CH:6][CH:7]=[C:8]([CH:9]=[CH:10]1)[N:11](=[O:12])=[O:13]>>[OH:4][C:5]1=[CH:6][CH:7]=[C:8]([CH:9]=[C:10]1[CH2:1][CH:2]=[CH2:3])[N:11](=[O:13])=[O:12]

Correctness of the mapping: YES

Reaction no 20

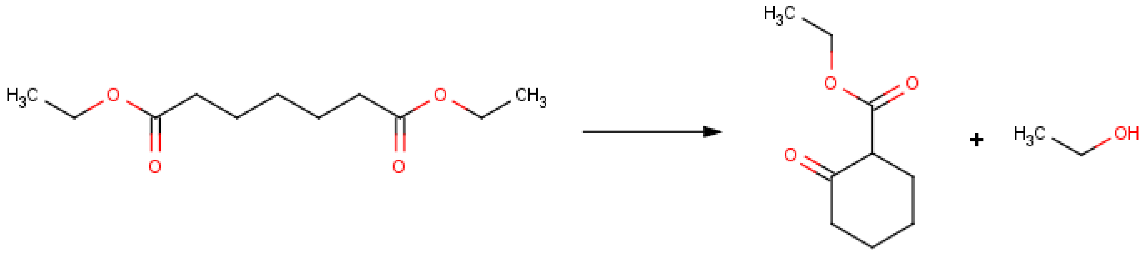

SMILES of the input:
CCOC(=O)CCCCCC(=O)OCC>>CCOC(=O)C1CCCCC1=O.CCO

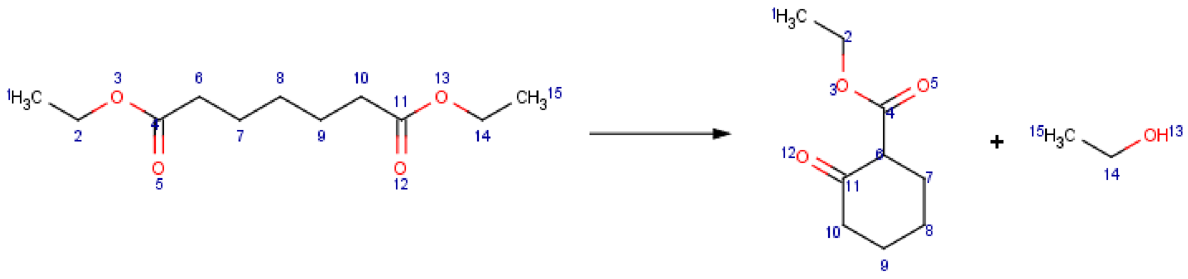

Correct mapped SMILES/SMARTS of the reaction:
[CH3:1][CH2:2][O:3][C:4](=[O:5])[CH2:6][CH2:7][CH2:8][CH2:9][CH2:10][C:11
](=[O:12])[O:13][CH2:14][CH3:15]>>[CH3:1][CH2:2][O:3][C:4](=[O:5])[CH:6]1
[CH2:7][CH2:8][CH2:9][CH2:10][C:11]1=[O:12].[CH3:15][CH2:14][OH:13]

Correctness of the mapping: YES

Reaction no 21

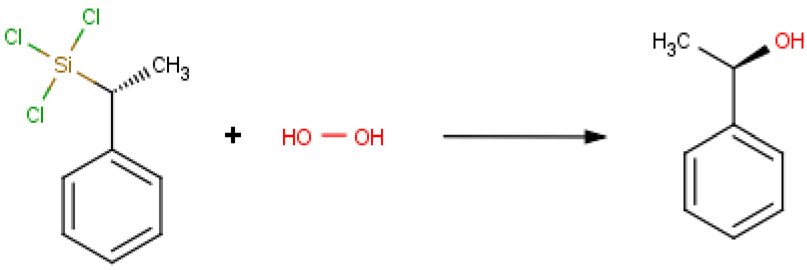

SMILES of the input:
C[C@H](C1=CC=CC=C1)[Si](Cl)(Cl)Cl.OO>>C[C@@H](O)C1=CC=CC=C1

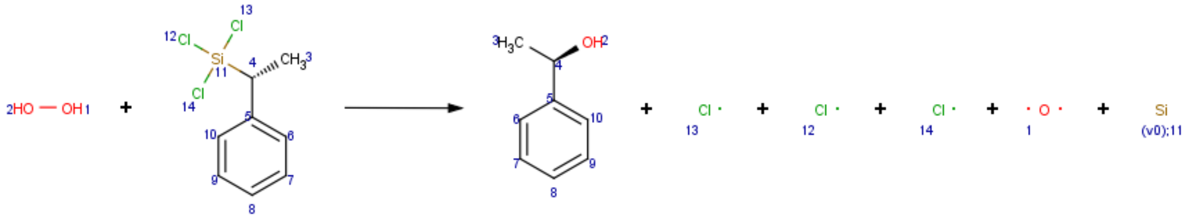

Correct mapped SMILES/SMARTS of the reaction:
[OH:1][OH:2].[CH3:3][C@H:4]([C:5]1=[CH:6][CH:7]=[CH:8][CH:9]=[CH:10]1)[Si
:11]([Cl:12])([Cl:13])[Cl:14]>>[CH3:3][C@@H:4]([OH:2])[C:5]1=[CH:10][CH:9
]=[CH:8][CH:7]=[CH:6]1.[Cl:13].[Cl:12].[Cl:14].[O:1].[Si:11]

Correctness of the mapping: YES

Reaction no 22

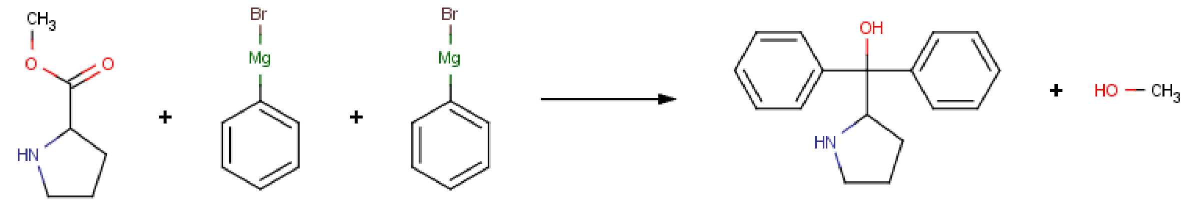

SMILES of the input:
COC(=O)C1CCCN1.Br[Mg]C1=CC=CC=C1.Br[Mg]C1=CC=CC=C1>>OC(C1CCCN1)(C1=CC=CC=C1)C1=CC=CC=C1.CO

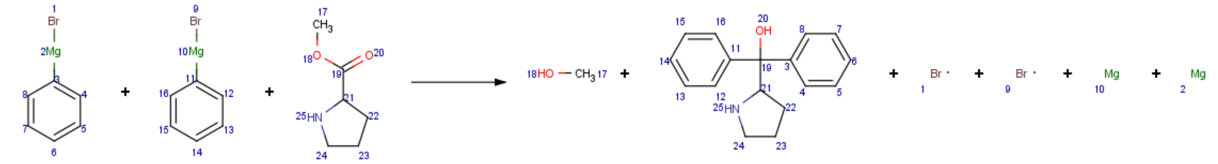

Correct mapped SMILES/SMARTS of the reaction:
[Br:1][Mg:2][C:3]1=[CH:4][CH:5]=[CH:6][CH:7]=[CH:8]1.[Br:9][Mg:10][C:11]1=[CH:12][CH:13]=[CH:14][CH:15]=[CH:16]1.[CH3:17][O:18][C:19](=[O:20])[CH:21]1[CH2:22][CH2:23][CH2:24][NH:25]1>>[CH3:17][OH:18].[OH:20][C:19]([CH:21]1[CH2:22][CH2:23][CH2:24][NH:25]1)([C:3]1=[CH:8][CH:7]=[CH:6][CH:5]=[CH:4]1)[C:11]1=[CH:12][CH:13]=[CH:14][CH:15]=[CH:16]1.[Br:1].[Br:9].[Mg:10].[Mg:2]

Correctness of the mapping: YES

Reaction no 23

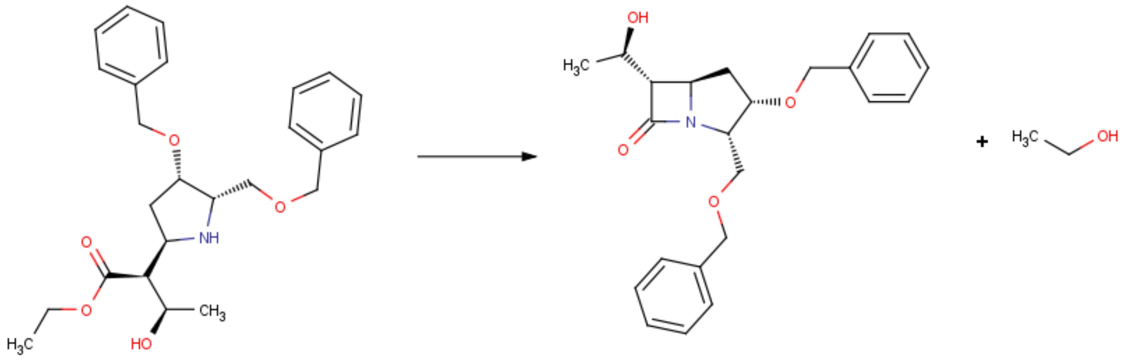

SMILES of the input:
CCOC(=O)[C@H]([C@@H](C)O)[C@H]1C[C@H](OCC2=CC=CC=C2)[C@H](COCC2=CC=CC=C2)N1>>C[C@@H](O)[C@@H]1[C@H]2C[C@H](OCC3=CC=CC=C3)[C@H](COCC3=CC=CC=C3)N2C1=O.CCO

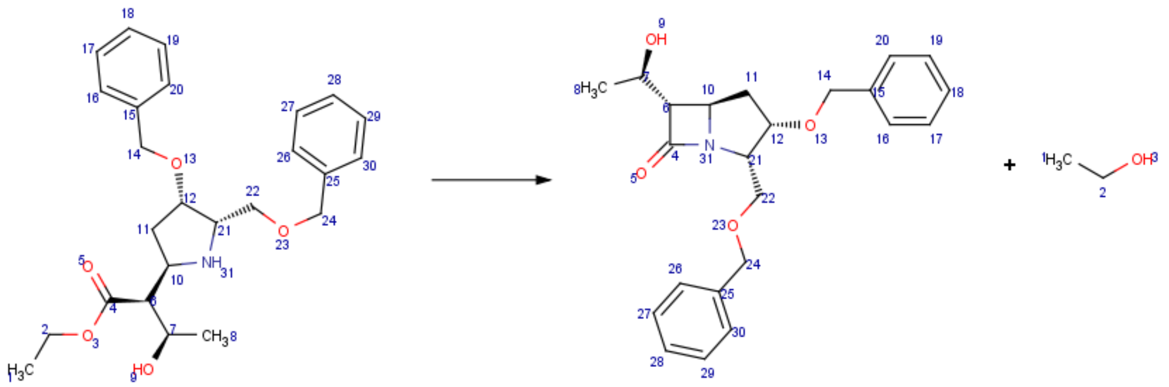

Correct mapped SMILES/SMARTS of the reaction:
[CH3:1][CH2:2][O:3][C:4](=[O:5])[C@H:6]([C@@H:7]([CH3:8])[OH:9])[C@H:10]1
[CH2:11][C@H:12]([O:13][CH2:14][C:15]2=[CH:16][CH:17]=[CH:18][CH:19]=[CH:
20]2)[C@H:21]([CH2:22][O:23][CH2:24][C:25]2=[CH:26][CH:27]=[CH:28][CH:29]
=[CH:30]2)[NH:31]1>>[CH3:8][C@@H:7]([OH:9])[C@@H:6]1[C@H:10]2[CH2:11][C@H
:12]([O:13][CH2:14][C:15]3=[CH:20][CH:19]=[CH:18][CH:17]=[CH:16]3)[C@H:21
]([CH2:22][O:23][CH2:24][C:25]3=[CH:30][CH:29]=[CH:28][CH:27]=[CH:26]3)[N
:31]2[C:4]1=[O:5].[CH3:1][CH2:2][OH:3]

Correctness of the mapping: YES

Reaction no 24

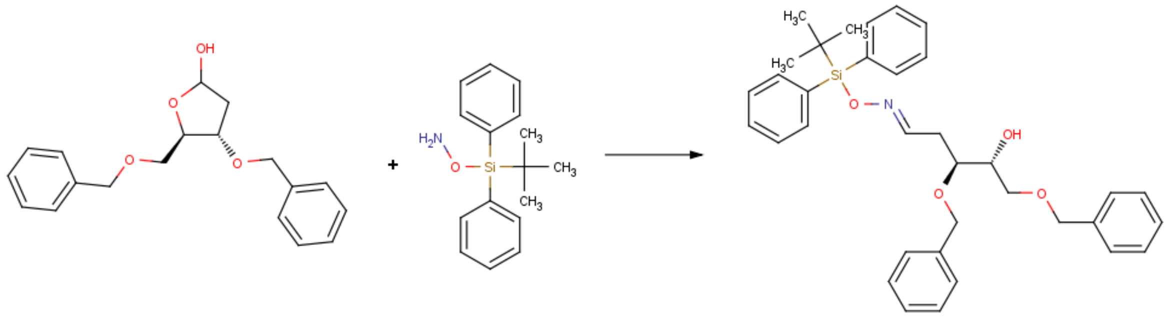

SMILES of the input:
OC1C[C@H](OCC2=CC=CC=C2)[C@@H](COCC2=CC=CC=C2)O1.CC(C)(C)[Si](ON)(C1=CC=C
C=C1)C1=CC=CC=C1>>CC(C)(C)[Si](O\N=C\C[C@H](OCC1=CC=CC=C1)[C@H](O)COCC1=C
C=CC=C1)(C1=CC=CC=C1)C1=CC=CC=C1

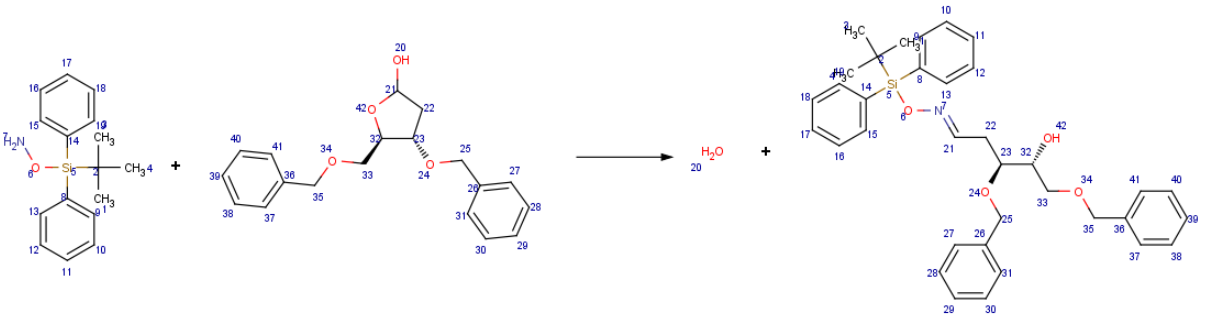

Correct mapped SMILES/SMARTS of the reaction:
[CH3:1][C:2]([CH3:3])([CH3:4])[Si:5]([O:6][NH2:7])([C:8]1=[CH:9][CH:10]=[
CH:11][CH:12]=[CH:13]1)[C:14]1=[CH:15][CH:16]=[CH:17][CH:18]=[CH:19]1.[OH
:20][CH:21]1[CH2:22][C@H:23]([O:24][CH2:25][C:26]2=[CH:27][CH:28]=[CH:29]
[CH:30]=[CH:31]2)[C@@H:32]([CH2:33][O:34][CH2:35][C:36]2=[CH:37][CH:38]=[
CH:39][CH:40]=[CH:41]2)[O:42]1>>[OH2:20].[CH3:1][C:2]([CH3:4])([CH3:3])[S
i:5]([O:6]\[N:7]=[CH:21]\[CH2:22][C@H:23]([O:24][CH2:25][C:26]1=[CH:31][C

H:30]=[CH:29][CH:28]=[CH:27]1)[C@H:32]([OH:42])[CH2:33][O:34][CH2:35][C:36]1=[CH:41][CH:40]=[CH:39][CH:38]=[CH:37]1)([C:8]1=[CH:9][CH:10]=[CH:11][CH:12]=[CH:13]1)[C:14]1=[CH:15][CH:16]=[CH:17][CH:18]=[CH:19]1

Correctness of the mapping: YES

Reaction no 25

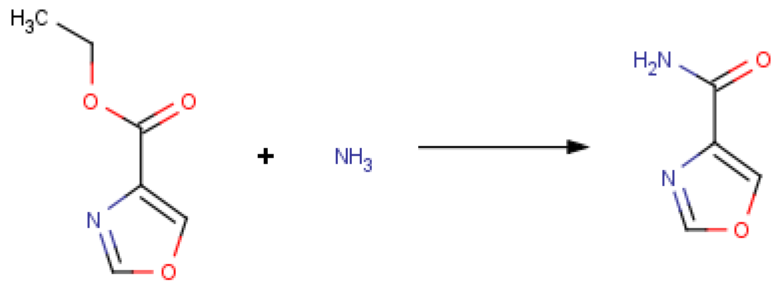

SMILES of the input:
CCOC(=O)C1=COC=N1.N>>NC(=O)C1=COC=N1

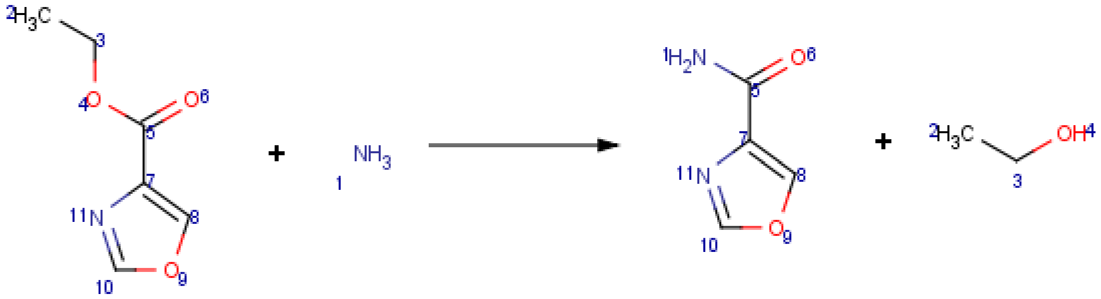

Correct mapped SMILES/SMARTS of the reaction:
[CH3:2][CH2:3][O:4][C:5](=[O:6])[C:7]1=[CH:8][O:9][CH:10]=[N:11]1.[NH3:1]>>[NH2:1][C:5](=[O:6])[C:7]1=[CH:8][O:9][CH:10]=[N:11]1.[CH3:2][CH2:3][OH:4]

Correctness of the mapping: YES

Dorota Jakubczyk, PhD, University of Lorraine

Reaction no 1

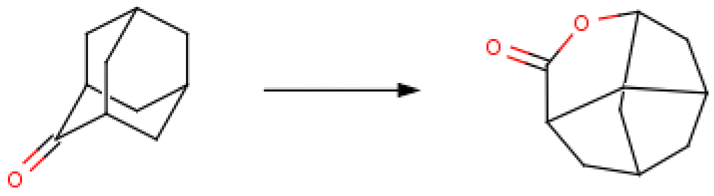

SMILES of the input:

O=C1C2CC3CC(C2)CC1C3>>O=C1OC2CC3CC(C2)CC1C3

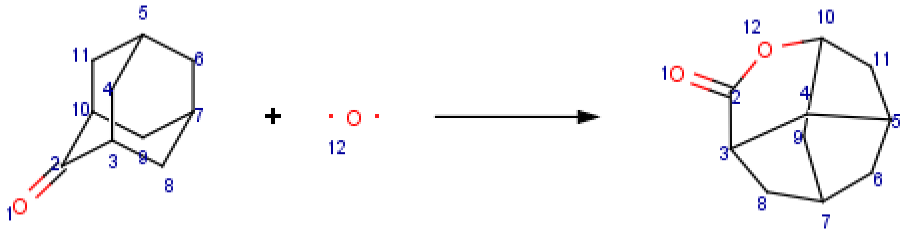

Correct mapped SMILES/SMARTS of the reaction:
[O:1]=[C:2]1[CH:3]2[CH2:4][CH:5]3[CH2:6][CH:7]([CH2:8]2)[CH2:9][CH:10]1[C
H2:11]3.[O:12]>>[O:1]=[C:2]1[O:12][CH:10]2[CH2:11][CH:5]3[CH2:6][CH:7]([C
H2:9]2)[CH2:8][CH:3]1[CH2:4]3

Correctness of the mapping: YES

Reaction no 2

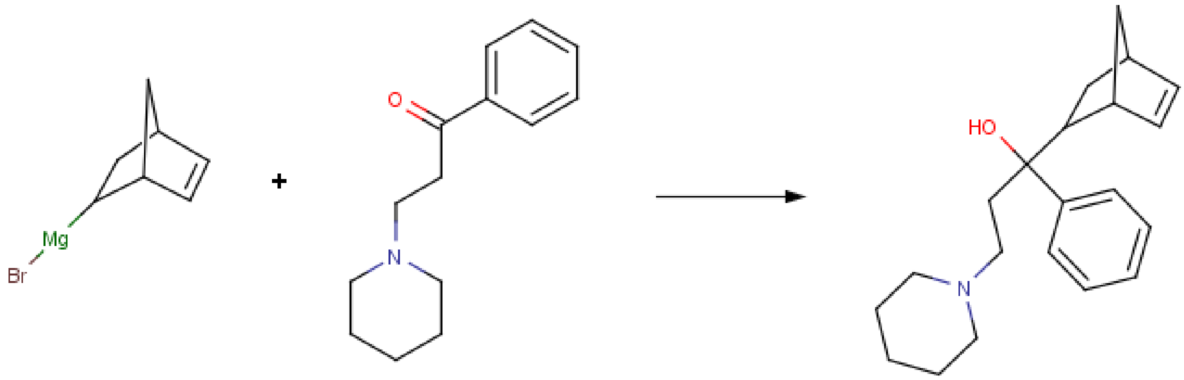

SMILES of the input:
Br[Mg]C1CC2CC1C=C2.O=C(CCN1CCCCC1)C1=CC=CC=C1>>OC(CCN1CCCCC1)(C1CC2CC1C=C
2)C1=CC=CC=C1

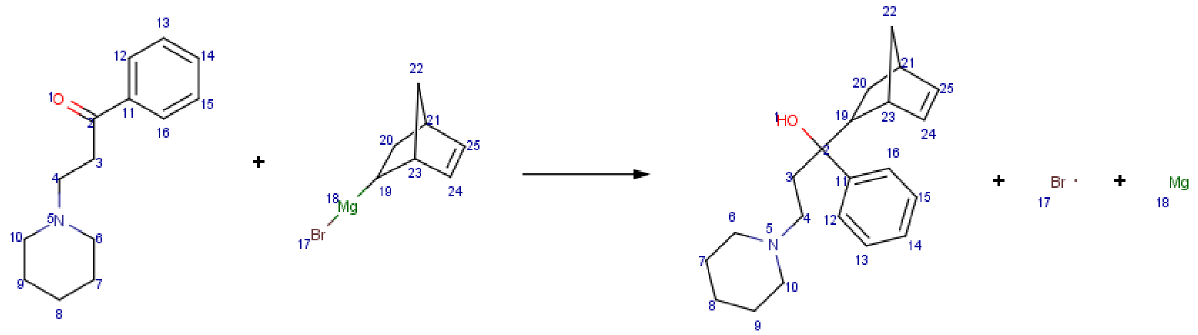

Correct mapped SMILES/SMARTS of the reaction:
[O:1]=[C:2]([CH2:3][CH2:4][N:5]1[CH2:6][CH2:7][CH2:8][CH2:9][CH2:10]1)[C:
11]1=[CH:12][CH:13]=[CH:14][CH:15]=[CH:16]1.[Br:17][Mg:18][CH:19]1[CH2:20
][CH:21]2[CH2:22][CH:23]1[CH:24]=[CH:25]2>>[OH:1][C:2]([CH2:3][CH2:4][N:5
]1[CH2:10][CH2:9][CH2:8][CH2:7][CH2:6]1)([CH:19]1[CH2:20][CH:21]2[CH2:22]
[CH:23]1[CH:24]=[CH:25]2)[C:11]1=[CH:16][CH:15]=[CH:14][CH:13]=[CH:12]1.[
Br:17].[Mg:18]

Correctness of the mapping: YES

Reaction no 3

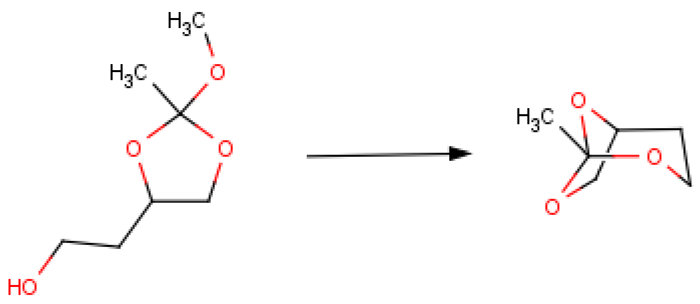

SMILES of the input:
COC1(C)OCC(CCO)O1>>CC12OCC(CCO1)O2

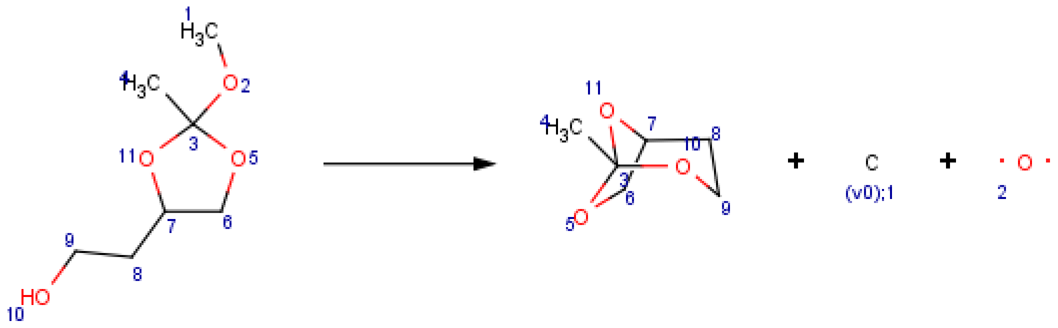

Correct mapped SMILES/SMARTS of the reaction:
[CH3:1][O:2][C:3]1([CH3:4])[O:5][CH2:6][CH:7]([CH2:8][CH2:9][OH:10])[O:11]1>>[CH3:4][C:3]12[O:5][CH2:6][CH:7]([CH2:8][CH2:9][O:10]1)[O:11]2.[C:1].[O:2]

Correctness of the mapping: YES

Reaction no 4

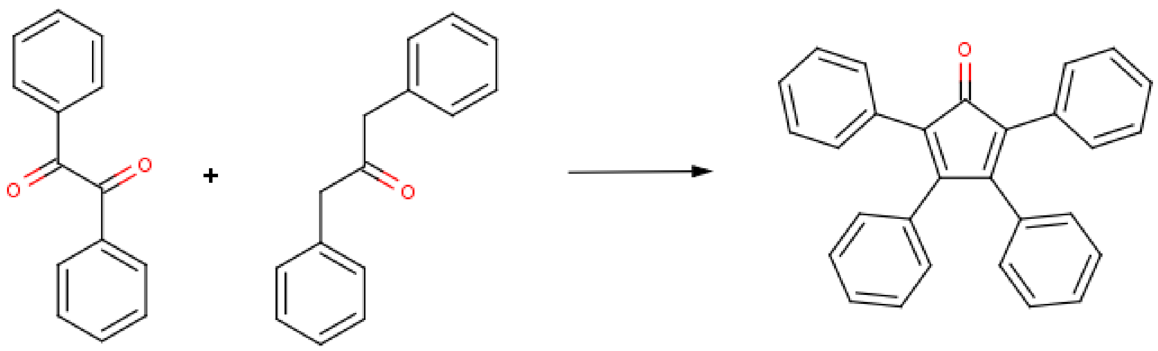

SMILES of the input:
O=C(C(=O)C1=CC=CC=C1)C1=CC=CC=C1.O=C(CC1=CC=CC=C1)CC1=CC=CC=C1>>O=C1C(=C(C(=C1C1=CC=CC=C1)C1=CC=CC=C1)C1=CC=CC=C1)C1=CC=CC=C1

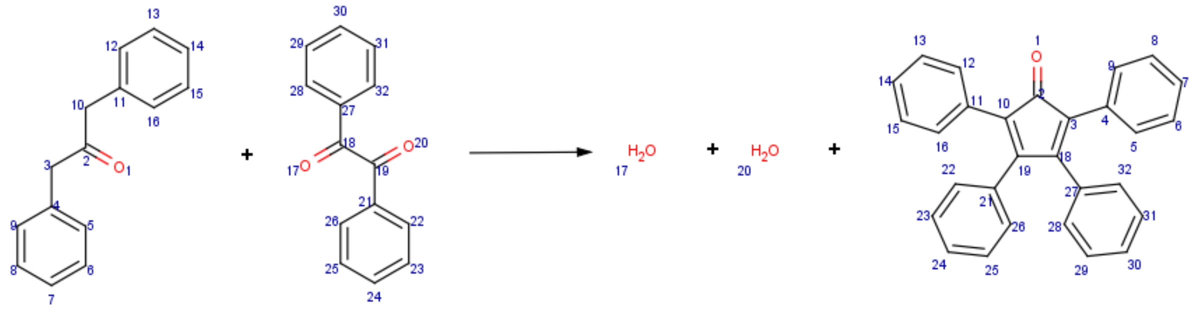

Correct mapped SMILES/SMARTS of the reaction:
[O:1]=[C:2]([CH2:3][C:4]1=[CH:5][CH:6]=[CH:7][CH:8]=[CH:9]1)[CH2:10][C:11
]1=[CH:12][CH:13]=[CH:14][CH:15]=[CH:16]1.[O:17]=[C:18]([C:19](=[O:20])[C
:21]1=[CH:22][CH:23]=[CH:24][CH:25]=[CH:26]1)[C:27]1=[CH:28][CH:29]=[CH:3
0][CH:31]=[CH:32]1>>[OH2:17].[OH2:20].[O:1]=[C:2]1[C:3](=[C:18]([C:19](=[
C:10]1[C:11]1=[CH:16][CH:15]=[CH:14][CH:13]=[CH:12]1)[C:21]1=[CH:26][CH:2
5]=[CH:24][CH:23]=[CH:22]1)[C:27]1=[CH:32][CH:31]=[CH:30][CH:29]=[CH:28]1
)[C:4]1=[CH:9][CH:8]=[CH:7][CH:6]=[CH:5]1

Correctness of the mapping: YES

Reaction no 5

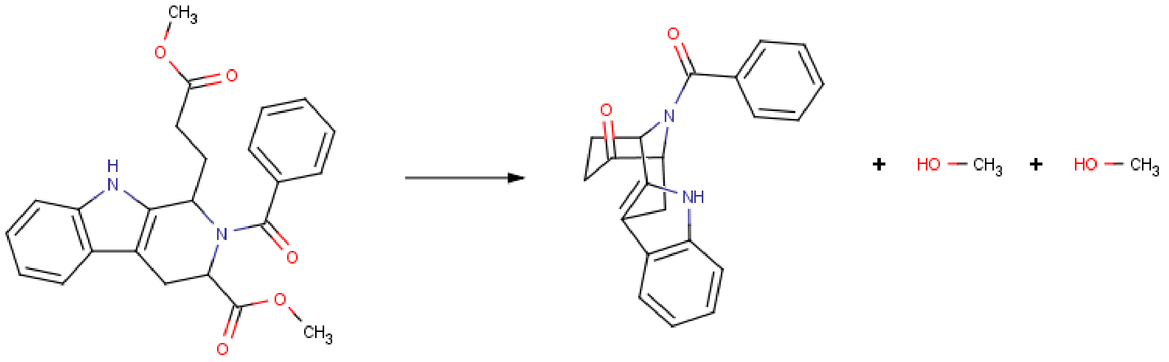

SMILES of the input:
COC(=O)CCC1N(C(CC2=C1NC1=C2C=CC=C1)C(=O)OC)C(=O)C1=CC=CC=C1>>O=C(N1C2CCC(
=O)C1CC1=C2NC2=C1C=CC=C2)C1=CC=CC=C1.CO.CO

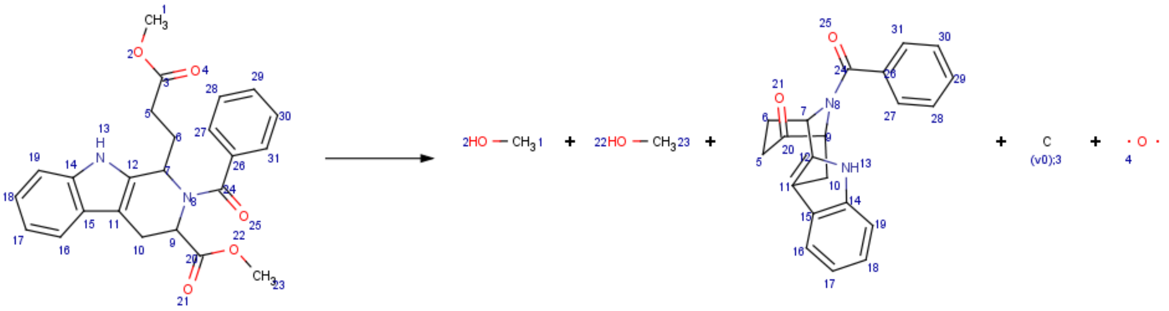

Correct mapped SMILES/SMARTS of the reaction:
[CH3:1][O:2][C:3](=[O:4])[CH2:5][CH2:6][CH:7]1[N:8]([CH:9]([CH2:10][C:11]
2=[C:12]1[NH:13][C:14]1=[C:15]2[CH:16]=[CH:17][CH:18]=[CH:19]1)[C:20](=[O
:21])[O:22][CH3:23])[C:24](=[O:25])[C:26]1=[CH:27][CH:28]=[CH:29][CH:30]=
[CH:31]1>>[CH3:1][OH:2].[CH3:23][OH:22].[O:25]=[C:24]([N:8]1[CH:7]2[CH2:6
][CH2:5][C:20](=[O:21])[CH:9]1[CH2:10][C:11]1=[C:12]2[NH:13][C:14]2=[C:15

]1[CH:16]=[CH:17][CH:18]=[CH:19]2)[C:26]1=[CH:31][CH:30]=[CH:29][CH:28]=[CH:27]1.[C:3].[O:4]

Correctness of the mapping: YES

Reaction no 6

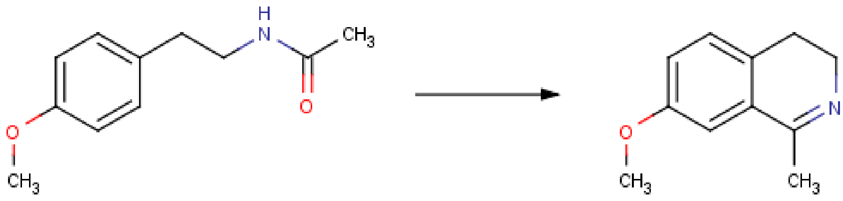

SMILES of the input:
COC1=CC=C(CCNC(C)=O)C=C1>>COC1=CC=C2CCN=C(C)C2=C1

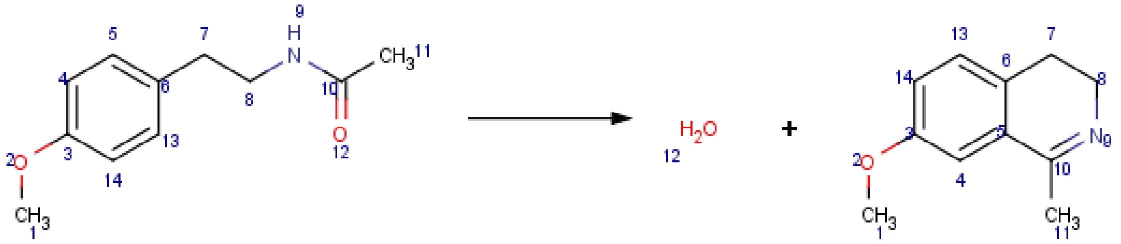

Correct mapped SMILES/SMARTS of the reaction:
[CH3:1][O:2][C:3]1=[CH:4][CH:5]=[C:6]([CH2:7][CH2:8][NH:9][C:10]([CH3:11])=[O:12])[CH:13]=[CH:14]1>>[OH2:12].[CH3:1][O:2][C:3]1=[CH:14][CH:13]=[C:6]2[CH2:7][CH2:8][N:9]=[C:10]([CH3:11])[C:5]2=[CH:4]1

Correctness of the mapping: YES

Reaction no 7

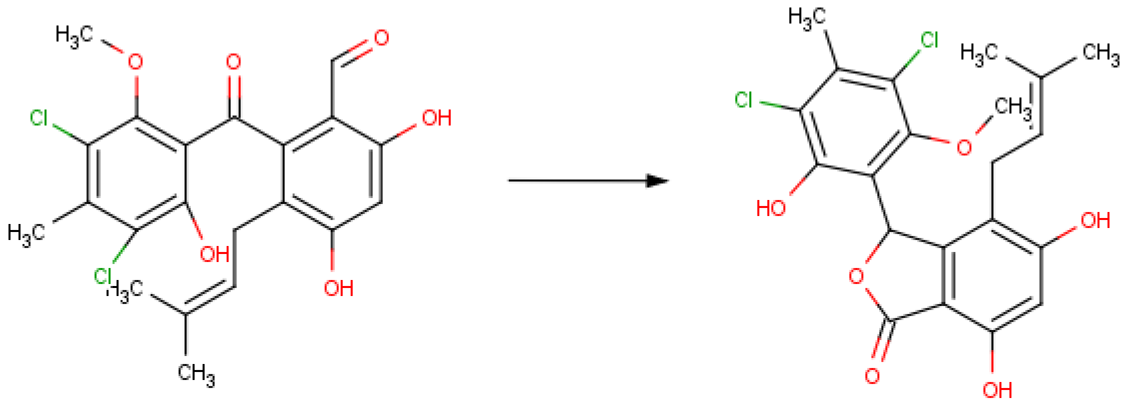

SMILES of the input:
COC1=C(C(=O)C2=C(C=O)C(O)=CC(O)=C2CC=C(C)C)C(O)=C(Cl)C(C)=C1Cl>>COC1=C(C2OC(=O)C3=C2C(CC=C(C)C)=C(O)C=C3O)C(O)=C(Cl)C(C)=C1Cl

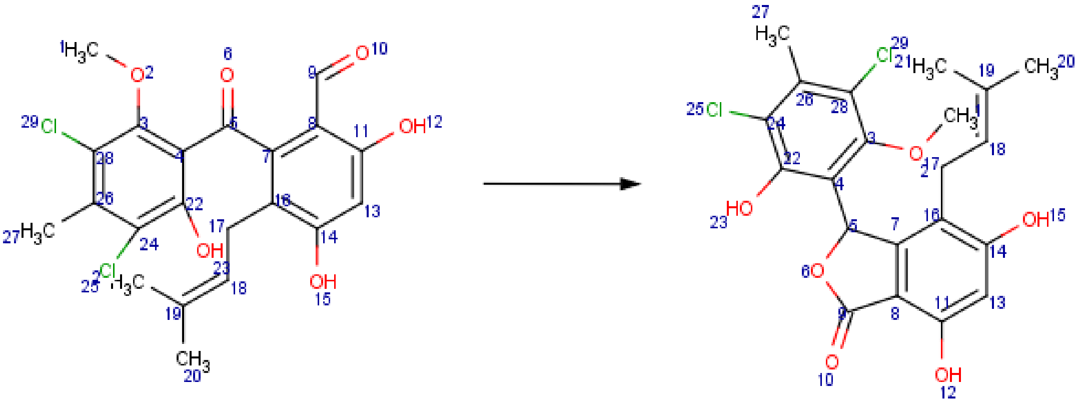

Correct mapped SMILES/SMARTS of the reaction:
[CH3:1][O:2][C:3]1=[C:4]([C:5](=[O:6])[C:7]2=[C:8]([CH:9]=[O:10])[C:11]([
OH:12])=[CH:13][C:14]([OH:15])=[C:16]2[CH2:17][CH:18]=[C:19]([CH3:20])[CH
3:21])[C:22]([OH:23])=[C:24]([Cl:25])[C:26]([CH3:27])=[C:28]1[Cl:29]>>[CH
3:1][O:2][C:3]1=[C:4]([CH:5]2[O:6][C:9](=[O:10])[C:8]3=[C:7]2[C:16]([CH2:
17][CH:18]=[C:19]([CH3:21])[CH3:20])=[C:14]([OH:15])[CH:13]=[C:11]3[OH:12
])[C:22]([OH:23])=[C:24]([Cl:25])[C:26]([CH3:27])=[C:28]1[Cl:29]

Correctness of the mapping: YES

Reaction no 8

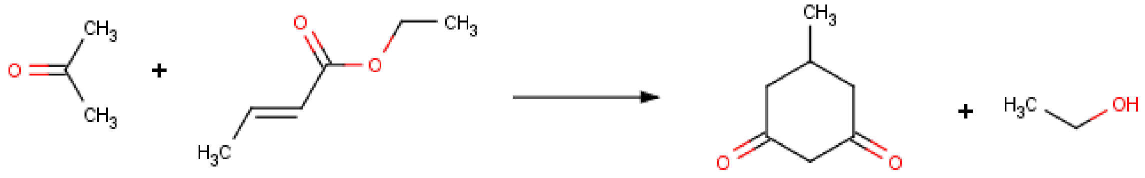

SMILES of the input:
CC(C)=O.CCOC(=O)\C=C\C>>CC1CC(=O)CC(=O)C1.CCO

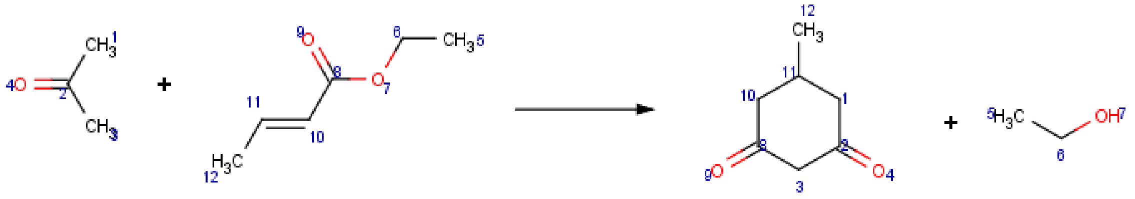

Correct mapped SMILES/SMARTS of the reaction:
[CH3:1][C:2]([CH3:3])=[O:4].[CH3:5][CH2:6][O:7][C:8](=[O:9])\[CH:10]=[CH:
11]\[CH3:12]>>[CH3:12][CH:11]1[CH2:1][C:2](=[O:4])[CH2:3][C:8](=[O:9])[CH
2:10]1.[CH3:5][CH2:6][OH:7]

Correctness of the mapping: YES

Reaction no 9

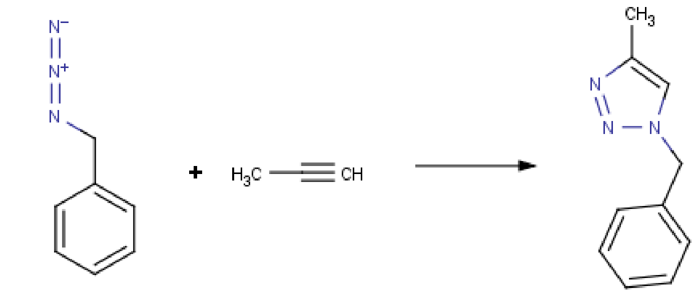

SMILES of the input:
[N-]=[N+]=NCC1=CC=CC=C1.CC#C>>CC1=CN(CC2=CC=CC=C2)N=N1

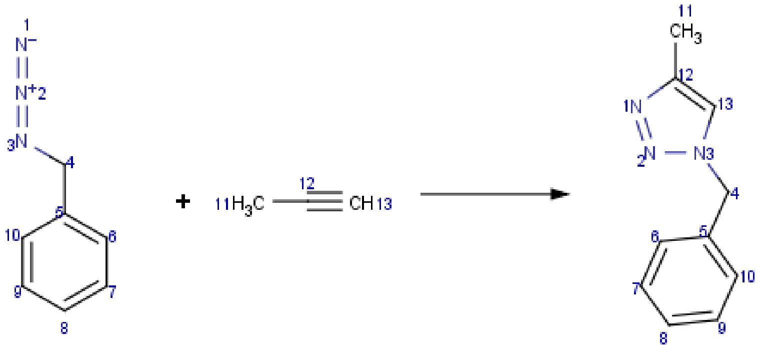

Correct mapped SMILES/SMARTS of the reaction:
[N-
:1]=[N+:2]=[N:3][CH2:4][C:5]1=[CH:6][CH:7]=[CH:8][CH:9]=[CH:10]1.[CH3:11]
[C:12]#[CH:13]>>[CH3:11][C:12]1=[CH:13][N:3]([CH2:4][C:5]2=[CH:10][CH:9]=
[CH:8][CH:7]=[CH:6]2)[N:2]=[N:1]1

Correctness of the mapping: YES

Reaction no 10

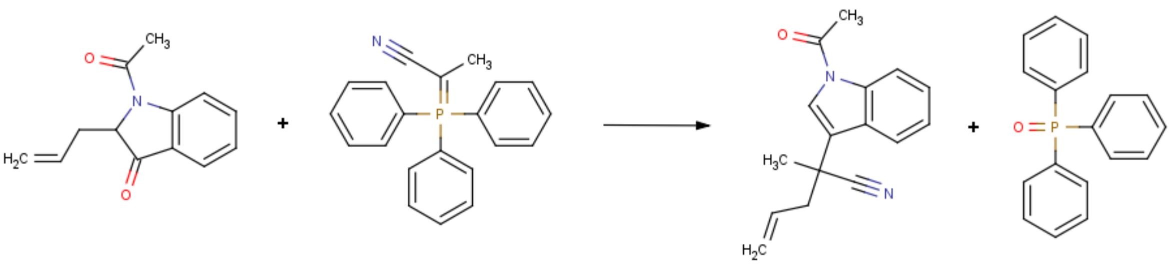

SMILES of the input:
CC(=O)N1C(CC=C)C(=O)C2=C1C=CC=C2.CC(C#N)=P(C1=CC=CC=C1)(C1=CC=CC=C1)C1=CC
=CC=C1>>CC(=O)N1C=C(C2=C1C=CC=C2)C(C)(CC=C)C#N.O=P(C1=CC=CC=C1)(C1=CC=CC=
C1)C1=CC=CC=C1

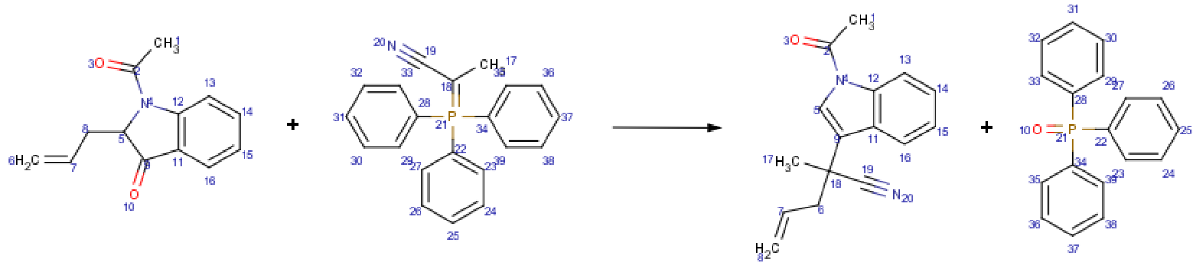

Correct mapped SMILES/SMARTS of the reaction:
[CH3:1][C:2](=[O:3])[N:4]1[CH:5]([CH2:8][CH:7]=[CH2:6])[C:9](=[O:10])[C:11]2=[C:12]1[CH:13]=[CH:14][CH:15]=[CH:16]2.[CH3:17][C:18]([C:19]#[N:20])=[P:21]([C:22]1=[CH:23][CH:24]=[CH:25][CH:26]=[CH:27]1)([C:28]1=[CH:29][CH:30]=[CH:31][CH:32]=[CH:33]1)[C:34]1=[CH:35][CH:36]=[CH:37][CH:38]=[CH:39]1>>[CH3:1][C:2](=[O:3])[N:4]1[CH:5]=[C:9]([C:11]2=[C:12]1[CH:13]=[CH:14][CH:15]=[CH:16]2)[C:18]([CH3:17])([CH2:6][CH:7]=[CH2:8])[C:19]#[N:20].[O:10]=[P:21]([C:34]1=[CH:39][CH:38]=[CH:37][CH:36]=[CH:35]1)([C:28]1=[CH:33][CH:32]=[CH:31][CH:30]=[CH:29]1)[C:22]1=[CH:27][CH:26]=[CH:25][CH:24]=[CH:23]1

Correctness of the mapping: NO

Reaction no 11

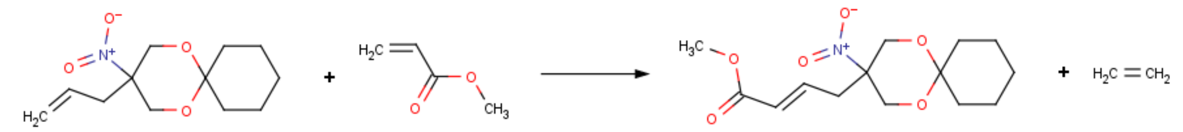

SMILES of the input:
[O-][N+](=O)C1(CC=C)COC2(CCCCC2)OC1.COC(=O)C=C>>COC(=O)\C=C\CC1(COC2(CCCCC2)OC1)[N+]([O-])=O.C=C

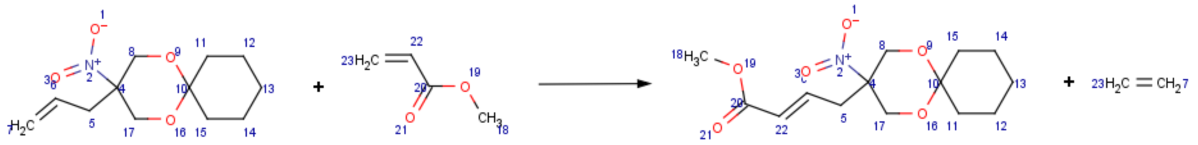

Correct mapped SMILES/SMARTS of the reaction:
[O-:1][N+:2](=[O:3])[C:4]1([CH2:5][CH:6]=[CH2:7])[CH2:8][O:9][C:10]2([CH2:11][CH2:12][CH2:13][CH2:14][CH2:15]2)[O:16][CH2:17]1.[CH3:18][O:19][C:20](=[O:21])[CH:22]=[CH2:23]>>[CH3:18][O:19][C:20](=[O:21])\[CH:22]=[CH:6]\[CH2:5][C:4]1([CH2:8][O:9][C:10]2([CH2:15][CH2:14][CH2:13][CH2:12][CH2:11]2)[O:16][CH2:17]1)[N+:2]([O-:1])=[O:3].[CH2:7]=[CH2:23]

Correctness of the mapping: YES

Reaction no 12

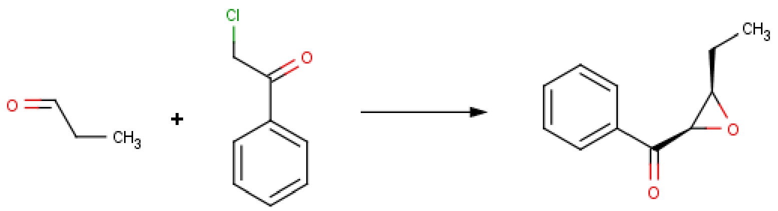

SMILES of the input:
CCC=O.ClCC(=O)C1=CC=CC=C1>>CC[C@H]1O[C@H]1C(=O)C1=CC=CC=C1

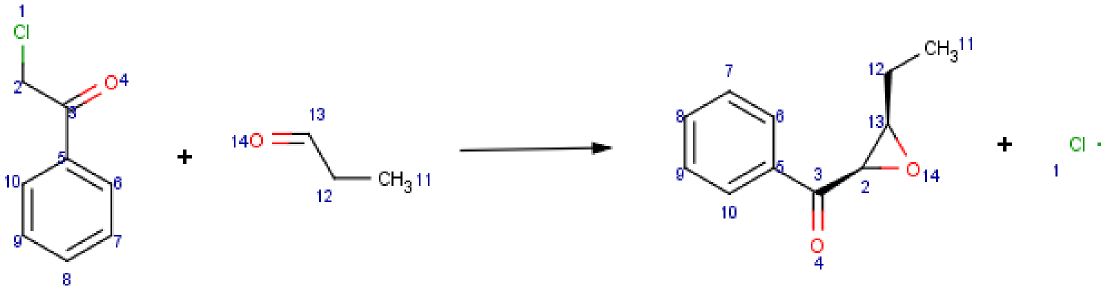

Correct mapped SMILES/SMARTS of the reaction:
[Cl:1][CH2:2][C:3](=[O:4])[C:5]1=[CH:6][CH:7]=[CH:8][CH:9]=[CH:10]1.[CH3:11][CH2:12][CH:13]=[O:14]>>[CH3:11][CH2:12][C@H:13]1[O:14][C@H:2]1[C:3](=[O:4])[C:5]1=[CH:10][CH:9]=[CH:8][CH:7]=[CH:6]1.[Cl:1]

Correctness of the mapping: YES

Reaction no 13

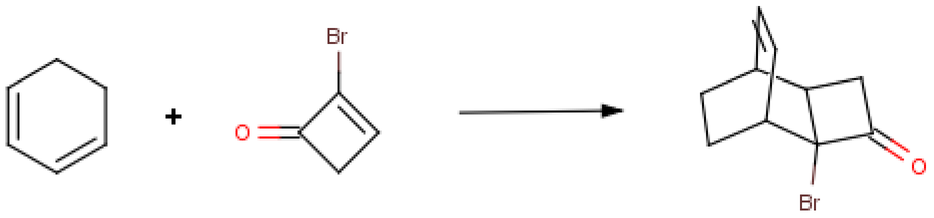

SMILES of the input:
C1CC=CC=C1.BrC1=CCC1=O>>BrC12C(CC1=O)C1CCC2C=C1

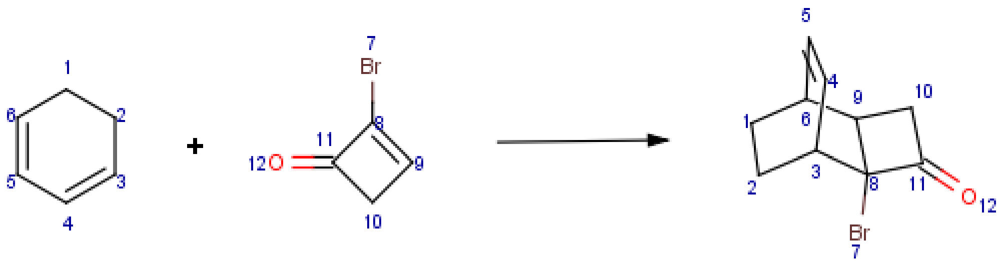

Correct mapped SMILES/SMARTS of the reaction:

[CH2:1]1[CH2:2][CH:3]=[CH:4][CH:5]=[CH:6]1.[Br:7][C:8]1=[CH:9][CH2:10][C:11]1=[O:12]>>[Br:7][C:8]12[CH:9]([CH2:10][C:11]1=[O:12])[CH:6]1[CH2:1][CH2:2][CH:3]2[CH:4]=[CH:5]1

Correctness of the mapping: YES

Reaction no 14

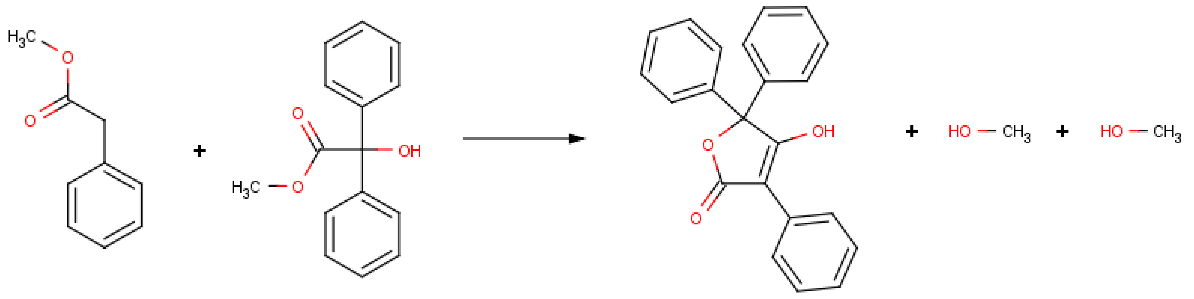

SMILES of the input:
COC(=O)CC1=CC=CC=C1.COC(=O)C(O)(C1=CC=CC=C1)C1=CC=CC=C1>>OC1=C(C(=O)OC1(C1=CC=CC=C1)C1=CC=CC=C1)C1=CC=CC=C1.CO.CO

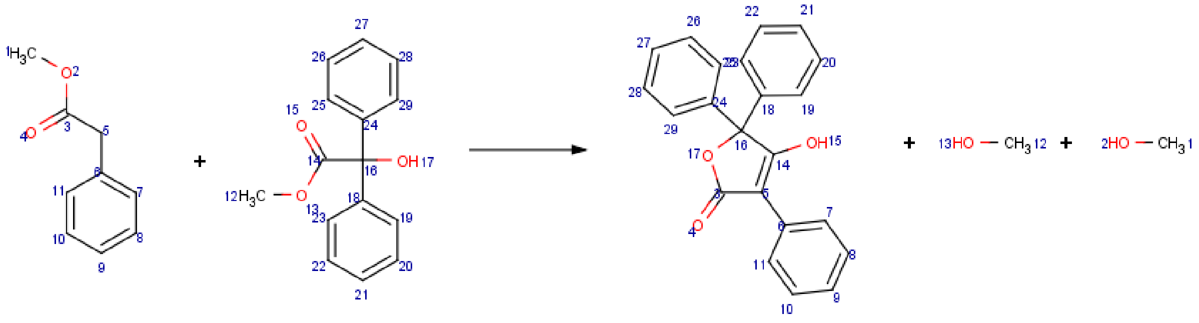

Correct mapped SMILES/SMARTS of the reaction:
[CH3:1][O:2][C:3](=[O:4])[CH2:5][C:6]1=[CH:7][CH:8]=[CH:9][CH:10]=[CH:11]1.[CH3:12][O:13][C:14](=[O:15])[C:16]([OH:17])([C:18]1=[CH:19][CH:20]=[CH:21][CH:22]=[CH:23]1)[C:24]1=[CH:25][CH:26]=[CH:27][CH:28]=[CH:29]1>>[OH:15][C:14]1=[C:5]([C:3](=[O:4])[O:17][C:16]1([C:24]1=[CH:29][CH:28]=[CH:27][CH:26]=[CH:25]1)[C:18]1=[CH:23][CH:22]=[CH:21][CH:20]=[CH:19]1)[C:6]1=[CH:7][CH:8]=[CH:9][CH:10]=[CH:11]1.[CH3:12][OH:13].[CH3:1][OH:2]

Correctness of the mapping: YES

Reaction no 15

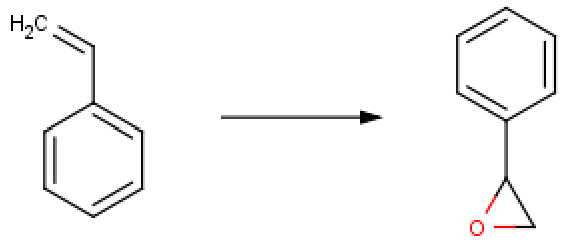

SMILES of the input:

C=CC1=CC=CC=C1>>C1OC1C1=CC=CC=C1

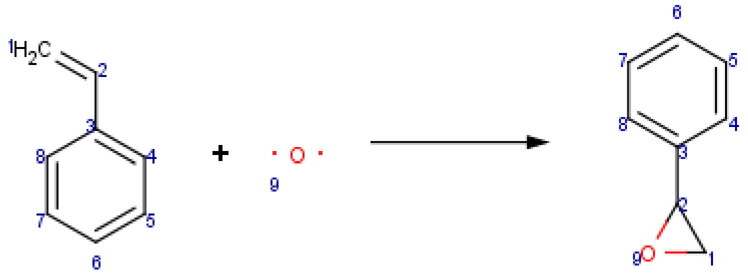

Correct mapped SMILES/SMARTS of the reaction:
[CH2:1]=[CH:2][C:3]1=[CH:4][CH:5]=[CH:6][CH:7]=[CH:8]1.[O:9]>>[CH2:1]1[O:9][CH:2]1[C:3]1=[CH:8][CH:7]=[CH:6][CH:5]=[CH:4]1

Correctness of the mapping: YES

Reaction no 16

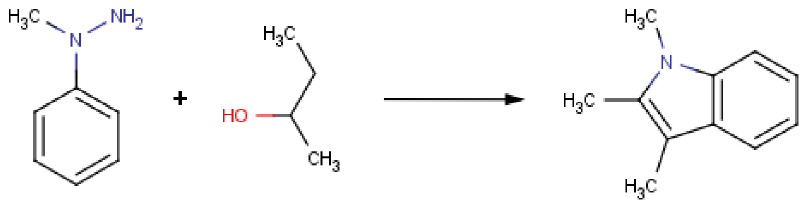

SMILES of the input:
CN(N)C1=CC=CC=C1.CCC(C)O>>CN1C(C)=C(C)C2=C1C=CC=C2

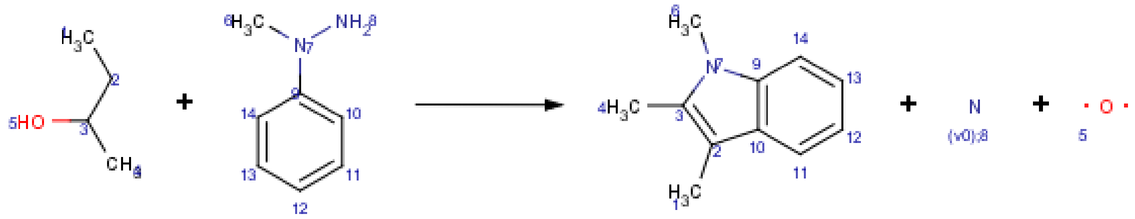

Correct mapped SMILES/SMARTS of the reaction:
[CH3:1][CH2:2][CH:3]([CH3:4])[OH:5].[CH3:6][N:7]([NH2:8])[C:9]1=[CH:10][CH:11]=[CH:12][CH:13]=[CH:14]1>>[CH3:6][N:7]1[C:3]([CH3:4])=[C:2]([CH3:1])[C:10]2=[C:9]1[CH:14]=[CH:13][CH:12]=[CH:11]2.[N:8].[O:5]

Correctness of the mapping: NO

Reaction no 17

SMILES

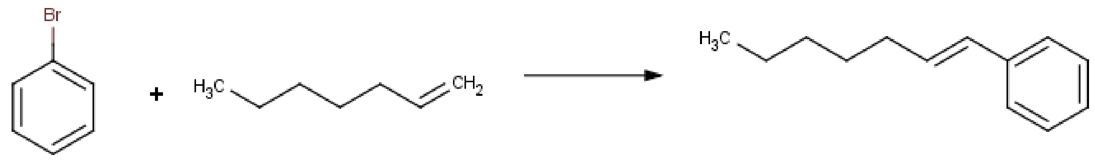

of the input:
BrC1=CC=CC=C1.CCCCCC=C>>CCCCC\C=C\C1=CC=CC=C1

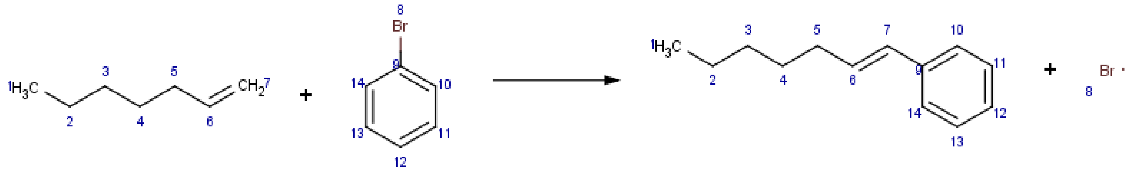

Correct mapped SMILES/SMARTS of the reaction:
[CH3:1][CH2:2][CH2:3][CH2:4][CH2:5][CH:6]=[CH2:7].[Br:8][C:9]1=[CH:10][CH:11]=[CH:12][CH:13]=[CH:14]1>>[CH3:1][CH2:2][CH2:3][CH2:4][CH2:5]\[CH:6]=[CH:7]\[C:9]1=[CH:10][CH:11]=[CH:12][CH:13]=[CH:14]1.[Br:8]

Correctness of the mapping: YES

Reaction no 18

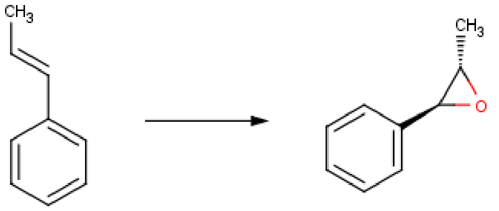

SMILES of the input:
C\C=C\C1=CC=CC=C1>>C[C@@H]1O[C@H]1C1=CC=CC=C1

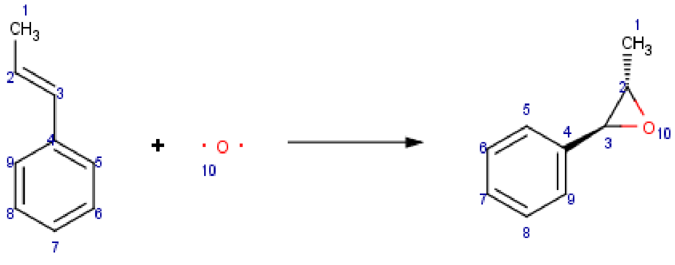

Correct mapped SMILES/SMARTS of the reaction:
[CH3:1]\[CH:2]=[CH:3]\[C:4]1=[CH:5][CH:6]=[CH:7][CH:8]=[CH:9]1.[O:10]>>[CH3:1][C@@H:2]1[O:10][C@H:3]1[C:4]1=[CH:9][CH:8]=[CH:7][CH:6]=[CH:5]1

Correctness of the mapping: YES

Reaction no 19

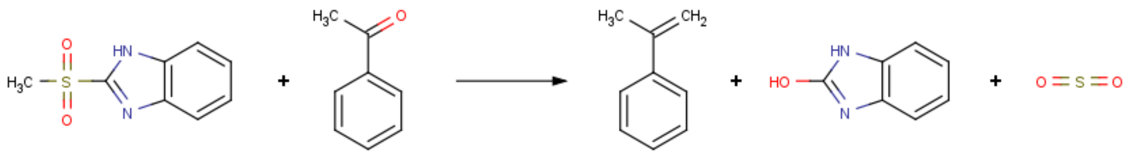

SMILES of the input:
CS(=O)(=O)C1=NC2=C(N1)C=CC=C2.CC(=O)C1=CC=CC=C1>>CC(=C)C1=CC=CC=C1.OC1=NC2=C(N1)C=CC=C2.O=S=O

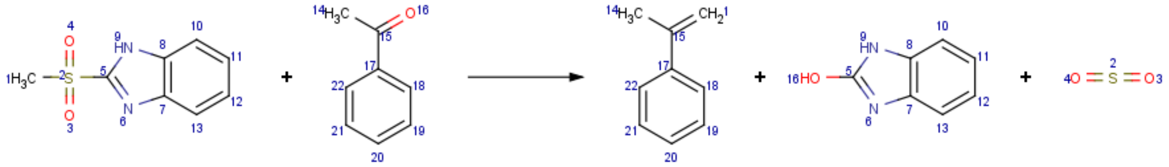

Correct mapped SMILES/SMARTS of the reaction:
[CH3:1][S:2](=[O:3])(=[O:4])[C:5]1=[N:6][C:7]2=[C:8]([NH:9]1)[CH:10]=[CH:11][CH:12]=[CH:13]2.[CH3:14][C:15](=[O:16])[C:17]1=[CH:18][CH:19]=[CH:20][CH:21]=[CH:22]1>>[CH3:14][C:15](=[CH2:1])[C:17]1=[CH:18][CH:19]=[CH:20][CH:21]=[CH:22]1.[OH:16][C:5]1=[N:6][C:7]2=[C:8]([NH:9]1)[CH:10]=[CH:11][CH:12]=[CH:13]2.[O:4]=[S:2]=[O:3]

Correctness of the mapping: YES

Reaction no 20

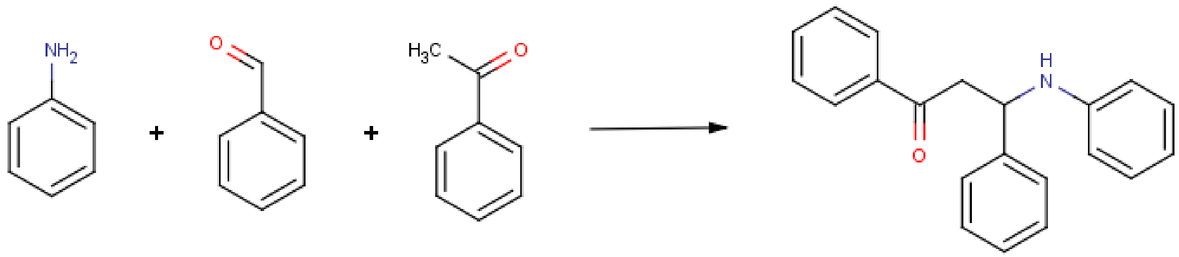

SMILES of the input:
NC1=CC=CC=C1.O=CC1=CC=CC=C1.CC(=O)C1=CC=CC=C1>>O=C(CC(NC1=CC=CC=C1)C1=CC=CC=C1)C1=CC=CC=C1

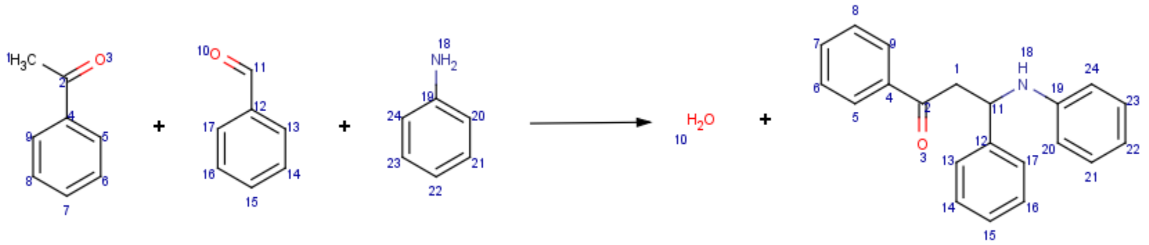

Correct mapped SMILES/SMARTS of the reaction:
[CH3:1][C:2](=[O:3])[C:4]1=[CH:5][CH:6]=[CH:7][CH:8]=[CH:9]1.[O:10]=[CH:11][C:12]1=[CH:13][CH:14]=[CH:15][CH:16]=[CH:17]1.[NH2:18][C:19]1=[CH:20][CH:21]=[CH:22][CH:23]=[CH:24]1>>[OH2:10].[O:3]=[C:2]([CH2:1][CH:11]([NH:18][C:19]1=[CH:24][CH:23]=[CH:22][CH:21]=[CH:20]1)[C:12]1=[CH:17][CH:16]=[CH:15][CH:14]=[CH:13]1)[C:4]1=[CH:5][CH:6]=[CH:7][CH:8]=[CH:9]1

Correctness of the mapping: YES

Reaction no 21

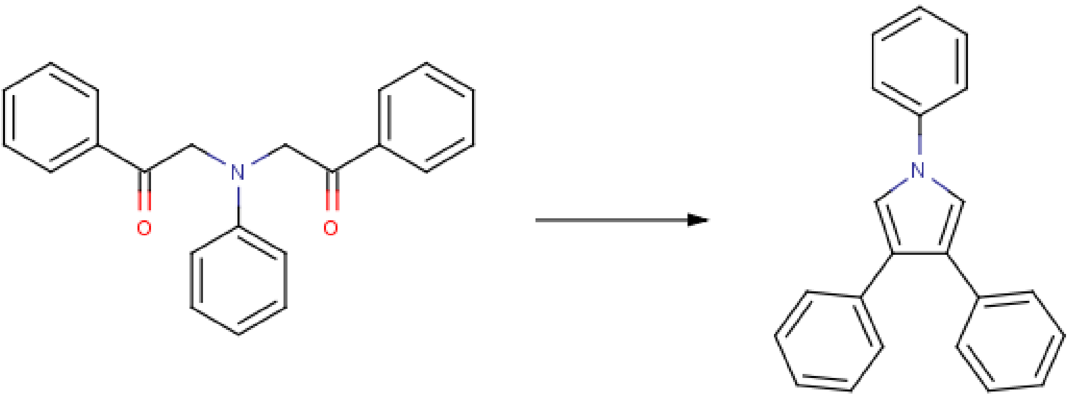

SMILES of the input:
O=C(CN(CC(=O)C1=CC=CC=C1)C1=CC=CC=C1)C1=CC=CC=C1>>C1=C(C(=CN1C1=CC=CC=C1)C1=CC=CC=C1)C1=CC=CC=C1

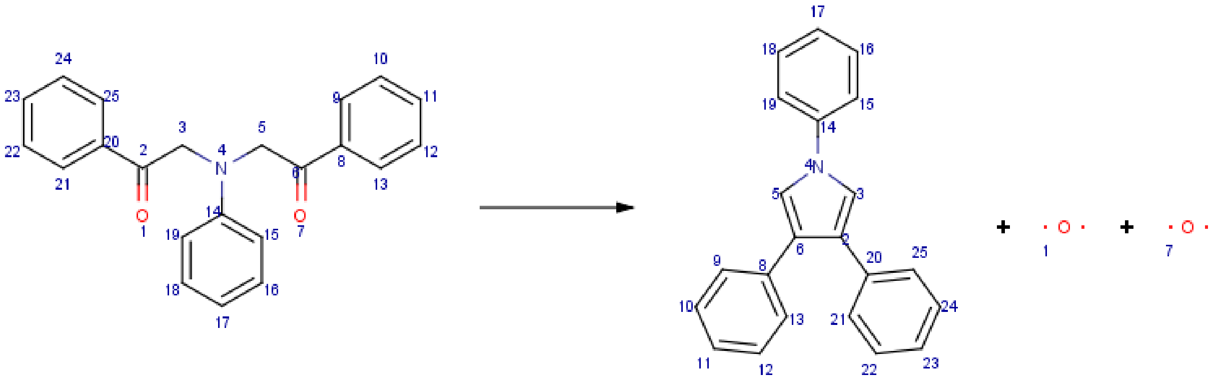

Correct mapped SMILES/SMARTS of the reaction:
[O:1]=[C:2]([CH2:3][N:4]([CH2:5][C:6](=[O:7])[C:8]1=[CH:9][CH:10]=[CH:11][CH:12]=[CH:13]1)[C:14]1=[CH:15][CH:16]=[CH:17][CH:18]=[CH:19]1)[C:20]1=[CH:21][CH:22]=[CH:23][CH:24]=[CH:25]1>>[CH:3]1=[C:2]([C:6](=[CH:5][N:4]1[C:14]1=[CH:19][CH:18]=[CH:17][CH:16]=[CH:15]1)[C:8]1=[CH:13][CH:12]=[CH:11][CH:10]=[CH:9]1)[C:20]1=[CH:25][CH:24]=[CH:23][CH:22]=[CH:21]1.[O:1].[O:7]

Correctness of the mapping: YES

Reaction no 22

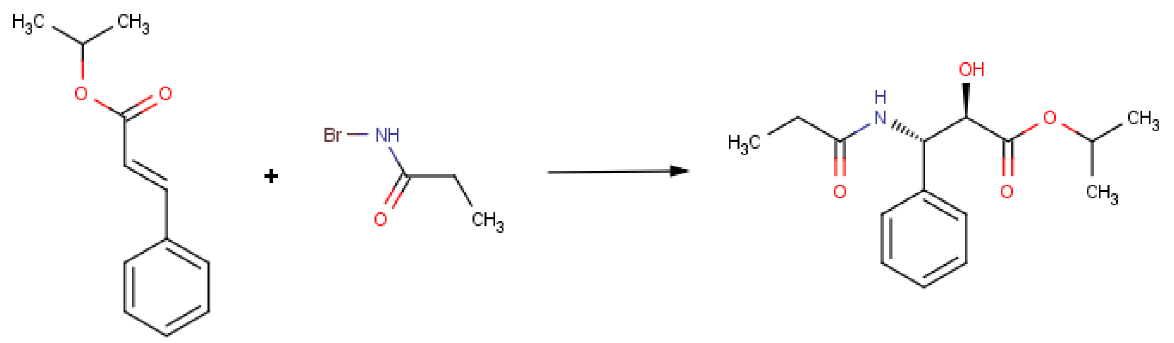

SMILES of the input:
CC(C)OC(=O)\C=C\C1=CC=CC=C1.CCC(=O)NBr>>CCC(=O)N[C@H]([C@@H](O)C(=O)OC(C)C)C1=CC=CC=C1

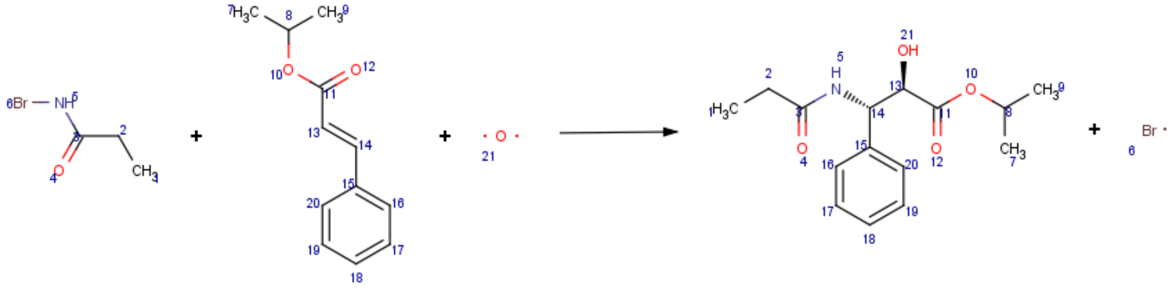

Correct mapped SMILES/SMARTS of the reaction:
[CH3:1][CH2:2][C:3](=[O:4])[NH:5][Br:6].[CH3:7][CH:8]([CH3:9])[O:10][C:11](=[O:12])\[CH:13]=[CH:14]\[C:15]1=[CH:16][CH:17]=[CH:18][CH:19]=[CH:20]1.[O:21]>>[CH3:1][CH2:2][C:3](=[O:4])[NH:5][C@H:14]([C@@H:13]([OH:21])[C:11](=[O:12])[O:10][CH:8]([CH3:9])[CH3:7])[C:15]1=[CH:20][CH:19]=[CH:18][CH:17]=[CH:16]1.[Br:6]

Correctness of the mapping: YES

Reaction no 23

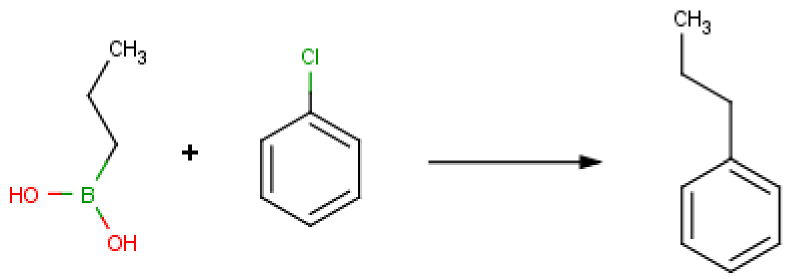

SMILES of the input:
CCCB(O)O.ClC1=CC=CC=C1>>CCCC1=CC=CC=C1

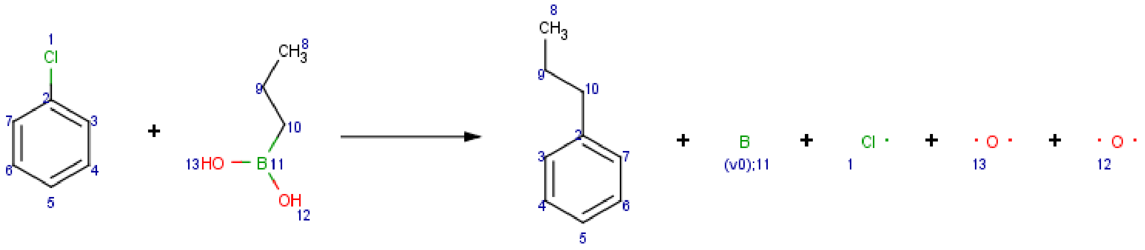

Correct mapped SMILES/SMARTS of the reaction:
[Cl:1][C:2]1=[CH:3][CH:4]=[CH:5][CH:6]=[CH:7]1.[CH3:8][CH2:9][CH2:10][B:11]([OH:12])[OH:13]>>[CH3:8][CH2:9][CH2:10][C:2]1=[CH:7][CH:6]=[CH:5][CH:4]=[CH:3]1.[B:11].[Cl:1].[O:13].[O:12]

Correctness of the mapping: YES

Reaction no 24

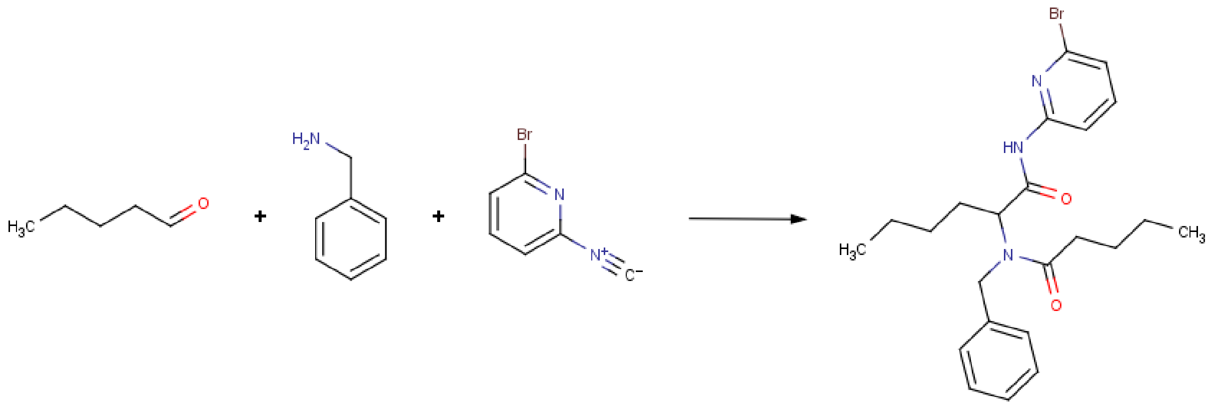

SMILES of the input:
CCCCC=O.NCC1=CC=CC=C1.BrC1=NC(=CC=C1)[N+]#[C-]>>CCCCC(N(CC1=CC=CC=C1)C(=O)CCCC)C(=O)NC1=NC(Br)=CC=C1

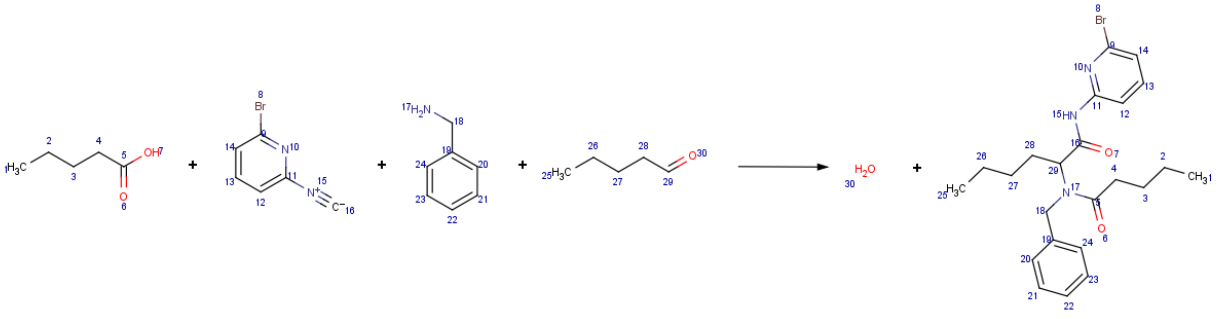

Correct mapped SMILES/SMARTS of the reaction:
[CH3:1][CH2:2][CH2:3][CH2:4][C:5]([OH:7])=[O:6].[Br:8][C:9]1=[N:10][C:11](=[CH:12][CH:13]=[CH:14]1)[N+:15]#[C-:16].[NH2:17][CH2:18][C:19]1=[CH:20][CH:21]=[CH:22][CH:23]=[CH:24]1.[CH3:25][CH2:26][CH2:27][CH2:28][CH:29]=[O:30]>>[OH2:30].[CH3:25][CH2:26][CH2:27][CH2:28][CH:29]([N:17]([CH2:18][C:19]1=[CH:24][CH:23]=[CH:22][CH:21]=[CH:20]1)[C:5](=[O:6])[CH2:4][CH2:3][CH2:2][CH3:1])[C:16](=[O:7])[NH:15][C:11]1=[N:10][C:9]([Br:8])=[CH:14][CH:13]=[CH:12]1

Correctness of the mapping: YES

Reaction no 25

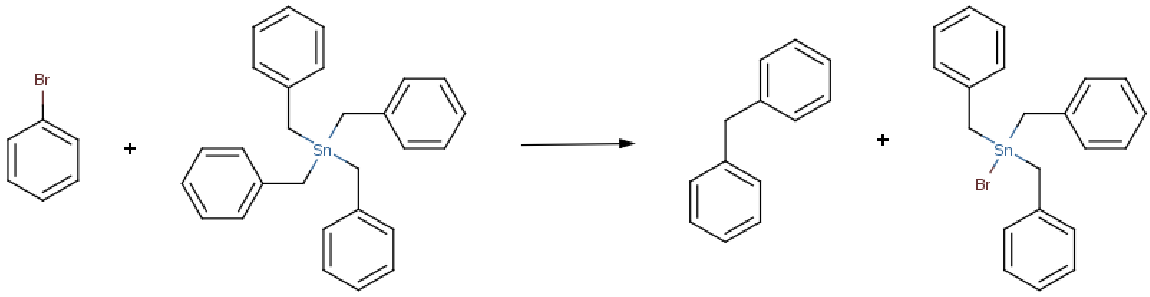

SMILES of the input:
BrC1=CC=CC=C1.C(C1=CC=CC=C1)[Sn](CC1=CC=CC=C1)(CC1=CC=CC=C1)CC1=CC=CC=C1>>C(C1=CC=CC=C1)C1=CC=CC=C1.Br[Sn](CC1=CC=CC=C1)(CC1=CC=CC=C1)CC1=CC=CC=C1

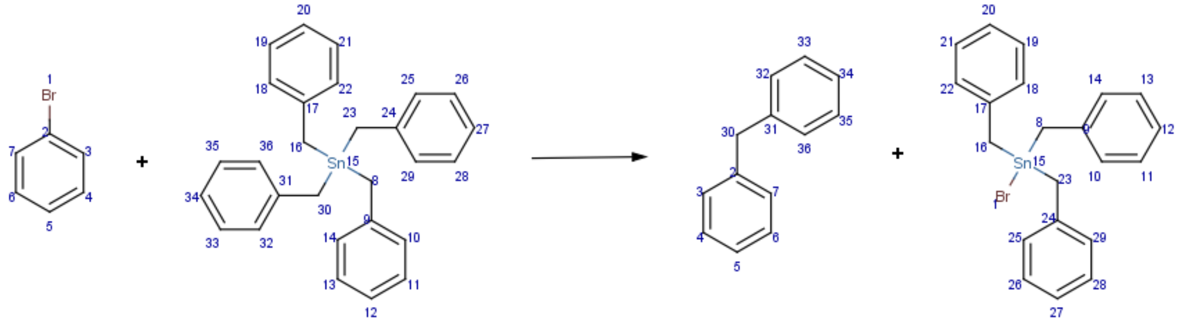

Correct mapped SMILES/SMARTS of the reaction:
[Br:1][C:2]1=[CH:3][CH:4]=[CH:5][CH:6]=[CH:7]1.[CH2:8]([C:9]1=[CH:10][CH:11]=[CH:12][CH:13]=[CH:14]1)[Sn:15]([CH2:16][C:17]1=[CH:18][CH:19]=[CH:20][CH:21]=[CH:22]1)([CH2:23][C:24]1=[CH:25][CH:26]=[CH:27][CH:28]=[CH:29]1)[CH2:30][C:31]1=[CH:32][CH:33]=[CH:34][CH:35]=[CH:36]1>>[CH2:30]([C:2]1=[CH:7][CH:6]=[CH:5][CH:4]=[CH:3]1)[C:31]1=[CH:32][CH:33]=[CH:34][CH:35]=[CH:36]1.[Br:1][Sn:15]([CH2:23][C:24]1=[CH:29][CH:28]=[CH:27][CH:26]=[CH:25]1)([CH2:16][C:17]1=[CH:22][CH:21]=[CH:20][CH:19]=[CH:18]1)[CH2:8][C:9]1=[CH:14][CH:13]=[CH:12][CH:11]=[CH:10]1

Correctness of the mapping: YES

Patryk Kasza, Jagiellonian University Medical College

Reaction no 1

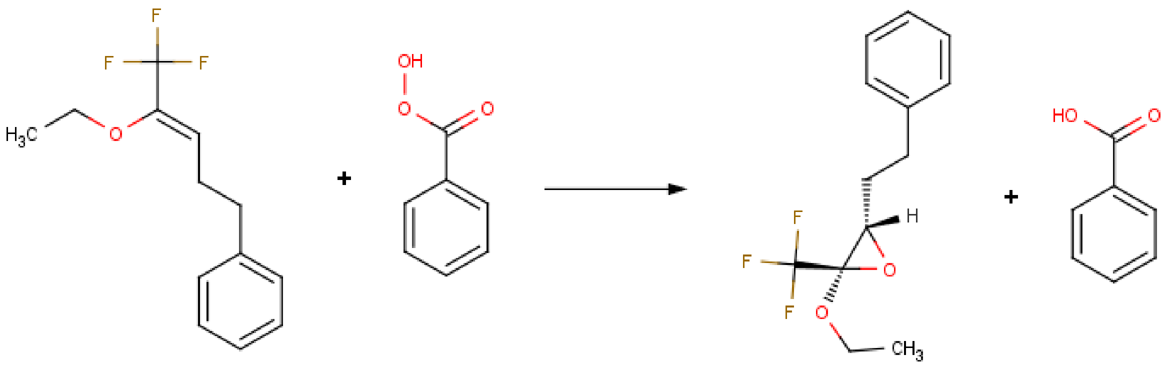

SMILES of the input:

CCO\C(=C/CCC1=CC=CC=C1)C(F)(F)F.OOC(=O)C1=CC=CC=C1>>[H][C@@]1(CCC2=CC=CC=C2)O[C@@]1(OCC)C(F)(F)F.OC(=O)C1=CC=CC=C1

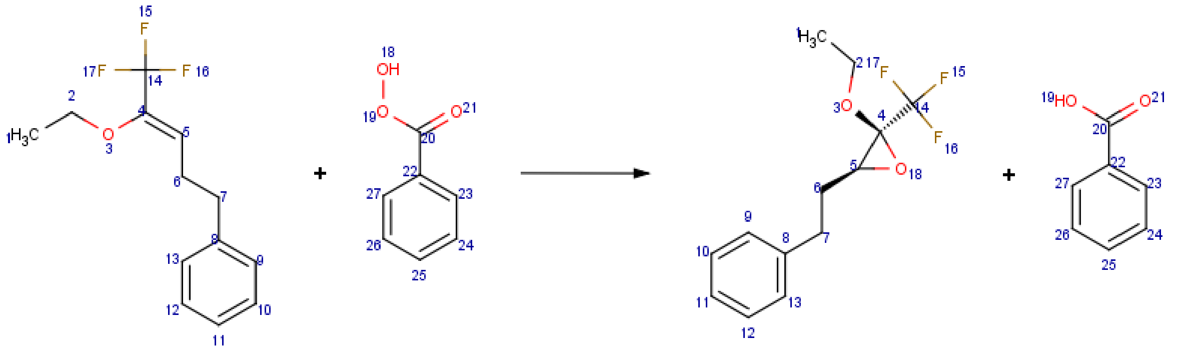

Correct mapped SMILES/SMARTS of the reaction:
[CH3:1][CH2:2][O:3]\[C:4](=[CH:5]/[CH2:6][CH2:7][C:8]1=[CH:9][CH:10]=[CH:11][CH:12]=[CH:13]1)[C:14]([F:15])([F:16])[F:17].[OH:18][O:19][C:20](=[O:21])[C:22]1=[CH:23][CH:24]=[CH:25][CH:26]=[CH:27]1>>[CH3:1][CH2:2][O:3][C@:4]1([O:18][C@H:5]1[CH2:6][CH2:7][C:8]1=[CH:13][CH:12]=[CH:11][CH:10]=[CH:9]1)[C:14]([F:15])([F:16])[F:17].[OH:19][C:20](=[O:21])[C:22]1=[CH:23][CH:24]=[CH:25][CH:26]=[CH:27]1

Correctness of the mapping: YES

Reaction no 2

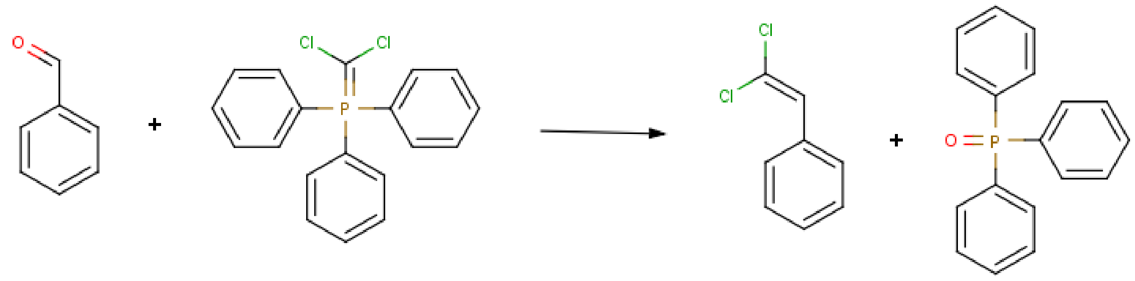

SMILES of the input:
O=CC1=CC=CC=C1.ClC(Cl)=P(C1=CC=CC=C1)(C1=CC=CC=C1)C1=CC=CC=C1>>ClC(Cl)=CC1=CC=CC=C1.O=P(C1=CC=CC=C1)(C1=CC=CC=C1)C1=CC=CC=C1

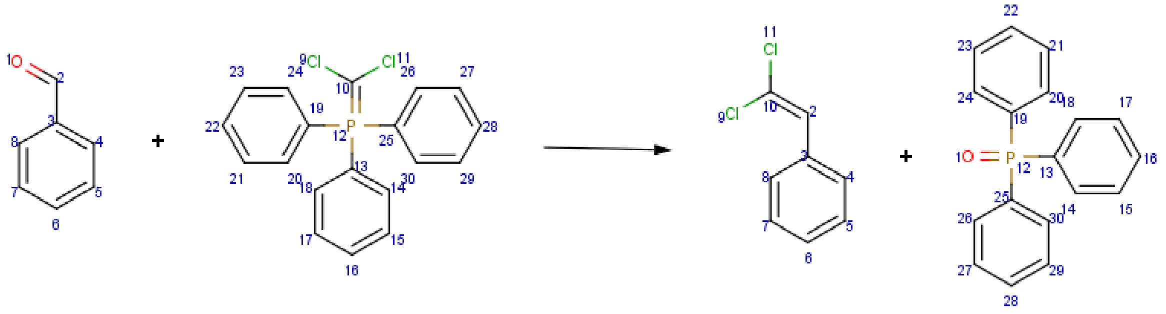

Correct mapped SMILES/SMARTS of the reaction:
[O:1]=[CH:2][C:3]1=[CH:4][CH:5]=[CH:6][CH:7]=[CH:8]1.[Cl:9][C:10]([Cl:11])=[P:12]([C:13]1=[CH:14][CH:15]=[CH:16][CH:17]=[CH:18]1)([C:19]1=[CH:20][CH:21]=[CH:22][CH:23]=[CH:24]1)[C:25]1=[CH:26][CH:27]=[CH:28][CH:29]=[CH:30]1>>[Cl:11][C:10]([Cl:9])=[CH:2][C:3]1=[CH:4][CH:5]=[CH:6][CH:7]=[CH:8]1.[O:1]=[P:12]([C:25]1=[CH:30][CH:29]=[CH:28][CH:27]=[CH:26]1)([C:19]1=[C

H:24][CH:23]=[CH:22][CH:21]=[CH:20]1)[C:13]1=[CH:18][CH:17]=[CH:16][CH:15]=[CH:14]1

Correctness of the mapping: YES

Reaction no 3

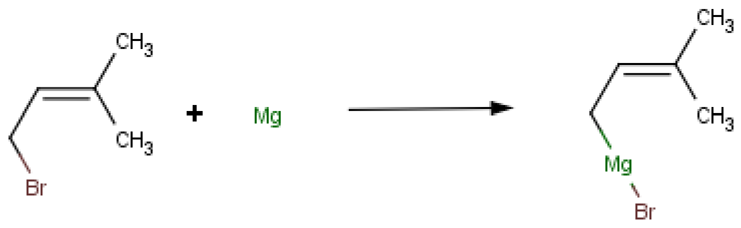

SMILES of the input:
CC(C)=CCBr.[Mg]>>CC(C)=CC[Mg]Br

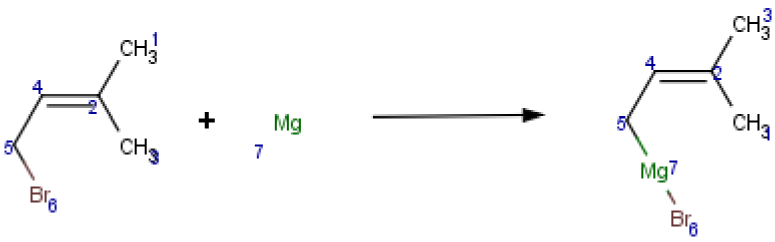

Correct mapped SMILES/SMARTS of the reaction:
[CH3:1][C:2]([CH3:3])=[CH:4][CH2:5][Br:6].[Mg:7]>>[CH3:3][C:2]([CH3:1])=[CH:4][CH2:5][Mg:7][Br:6]

Correctness of the mapping: YES

Reaction no 4

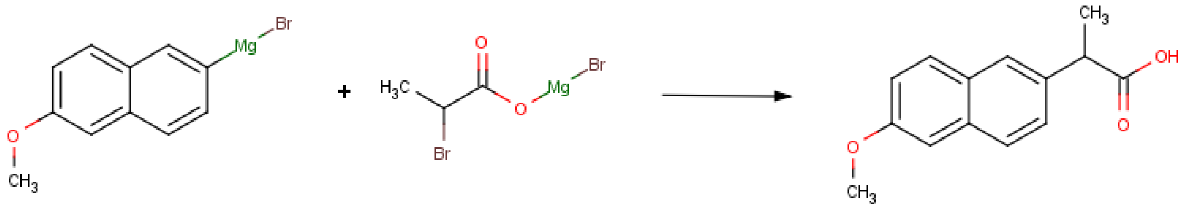

SMILES of the input:
COC1=CC=C2C=C([Mg]Br)C=CC2=C1.CC(Br)C(=O)O[Mg]Br>>COC1=CC=C2C=C(C=CC2=C1)C(C)C(O)=O

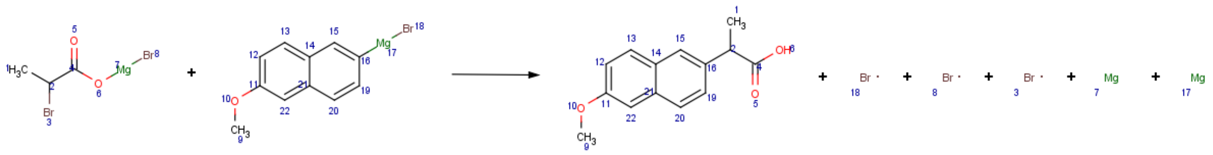

Correct mapped SMILES/SMARTS of the reaction:
[CH3:1][CH:2]([Br:3])[C:4](=[O:5])[O:6][Mg:7][Br:8].[CH3:9][O:10][C:11]1=[CH:12][CH:13]=[C:14]2[CH:15]=[C:16]([Mg:17][Br:18])[CH:19]=[CH:20][C:21]2=[CH:22]1>>[CH3:9][O:10][C:11]1=[CH:12][CH:13]=[C:14]2[CH:15]=[C:16]([CH:19]=[CH:20][C:21]2=[CH:22]1)[CH:2]([CH3:1])[C:4]([OH:6])=[O:5].[Br:18].[Br:8].[Br:3].[Mg:7].[Mg:17]

Correctness of the mapping: YES

Reaction no 5

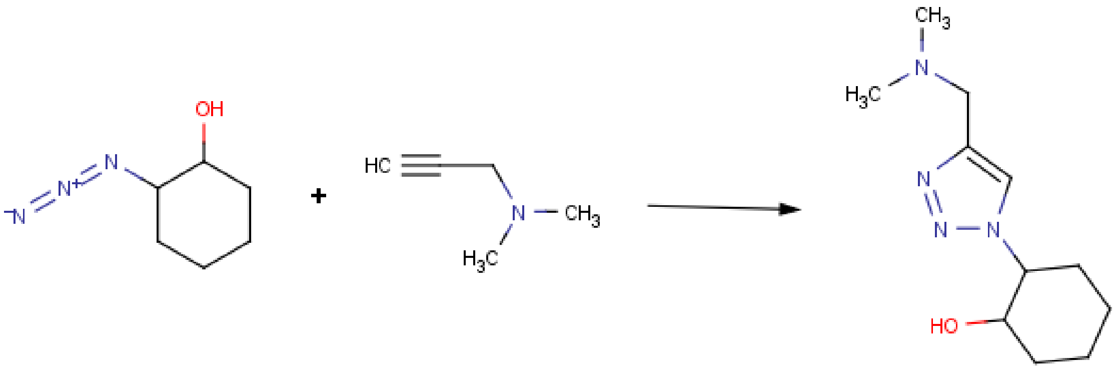

SMILES of the input:
OC1CCCCC1N=[N+]=[N-].CN(C)CC#C>>CN(C)CC1=CN(N=N1)C1CCCCC1O

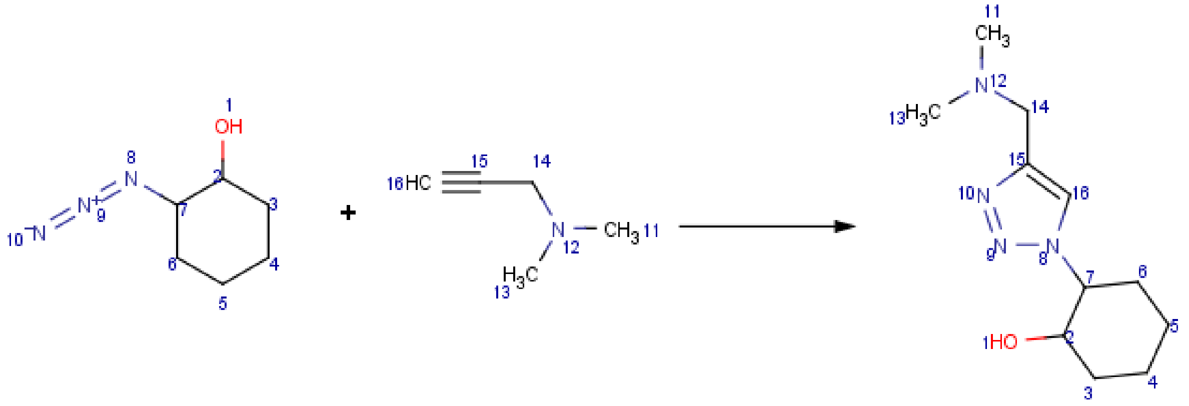

Correct mapped SMILES/SMARTS of the reaction:
[OH:1][CH:2]1[CH2:3][CH2:4][CH2:5][CH2:6][CH:7]1[N:8]=[N+:9]=[N-:10].[CH3:11][N:12]([CH3:13])[CH2:14][C:15]#[CH:16]>>[CH3:11][N:12]([CH3:13])[CH2:14][C:15]1=[CH:16][N:8]([N:9]=[N:10]1)[CH:7]1[CH2:6][CH2:5][CH2:4][CH2:3][CH:2]1[OH:1]

Correctness of the mapping: YES

Reaction no 6

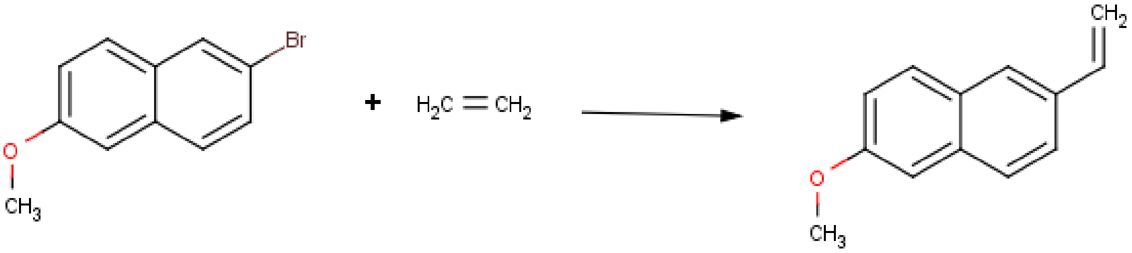

SMILES of the input:

COC1=CC=C2C=C(Br)C=CC2=C1.C=C>>COC1=CC=C2C=C(C=C)C=CC2=C1

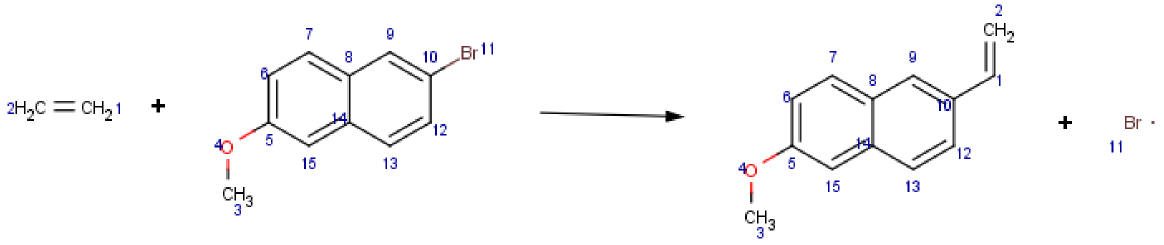

Correct mapped SMILES/SMARTS of the reaction:
[CH2:1]=[CH2:2].[CH3:3][O:4][C:5]1=[CH:6][CH:7]=[C:8]2[CH:9]=[C:10]([Br:11])[CH:12]=[CH:13][C:14]2=[CH:15]1>>[CH3:3][O:4][C:5]1=[CH:6][CH:7]=[C:8]2[CH:9]=[C:10]([CH:1]=[CH2:2])[CH:12]=[CH:13][C:14]2=[CH:15]1.[Br:11]

Correctness of the mapping: YES

Reaction no 7

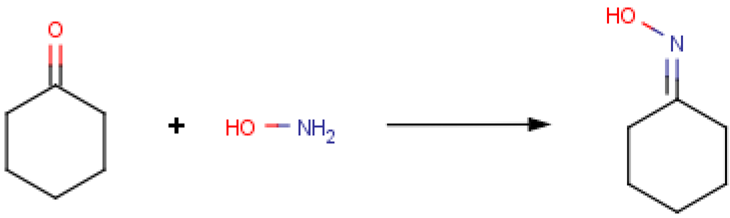

SMILES of the input:
O=C1CCCCC1.NO>>ON=C1CCCCC1

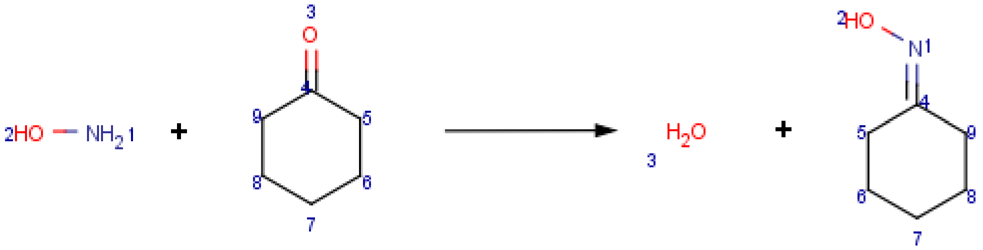

Correct mapped SMILES/SMARTS of the reaction:
[NH2:1][OH:2].[O:3]=[C:4]1[CH2:5][CH2:6][CH2:7][CH2:8][CH2:9]1>>[OH2:3].[OH:2][N:1]=[C:4]1[CH2:9][CH2:8][CH2:7][CH2:6][CH2:5]1

Correctness of the mapping: YES

Reaction no 8

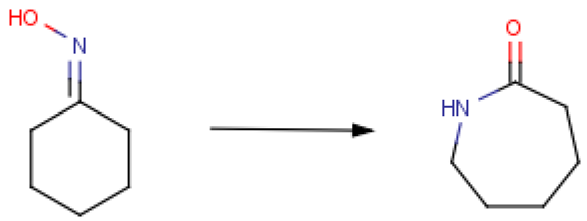

SMILES of the input:
ON=C1CCCCC1>>O=C1CCCCCN1

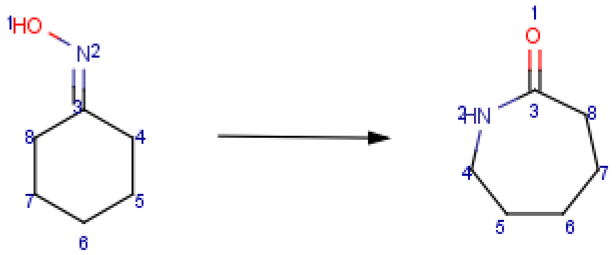

Correct mapped SMILES/SMARTS of the reaction:
[OH:1][N:2]=[C:3]1[CH2:4][CH2:5][CH2:6][CH2:7][CH2:8]1>>[O:1]=[C:3]1[CH2:8][CH2:7][CH2:6][CH2:5][CH2:4][NH:2]1

Correctness of the mapping: YES

Reaction no 9

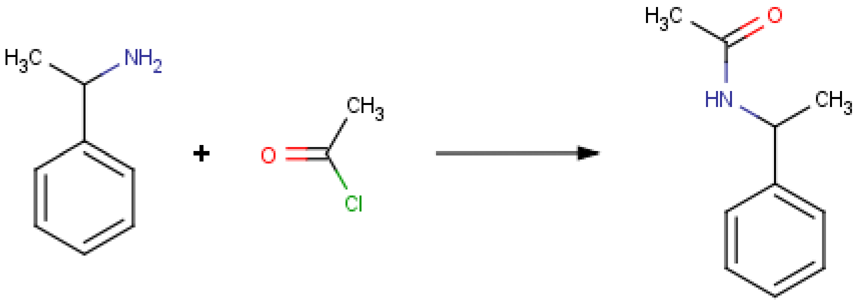

SMILES of the input:
CC(N)C1=CC=CC=C1.CC(Cl)=O>>CC(NC(C)=O)C1=CC=CC=C1

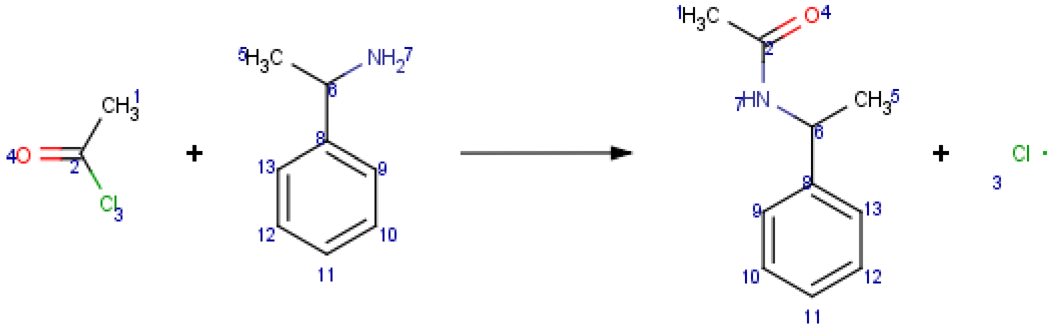

Correct mapped SMILES/SMARTS of the reaction:
[CH3:1][C:2]([Cl:3])=[O:4].[CH3:5][CH:6]([NH2:7])[C:8]1=[CH:9][CH:10]=[CH:11][CH:12]=[CH:13]1>>[CH3:5][CH:6]([NH:7][C:2]([CH3:1])=[O:4])[C:8]1=[CH:13][CH:12]=[CH:11][CH:10]=[CH:9]1.[Cl:3]

Correctness of the mapping: YES

Reaction no 10

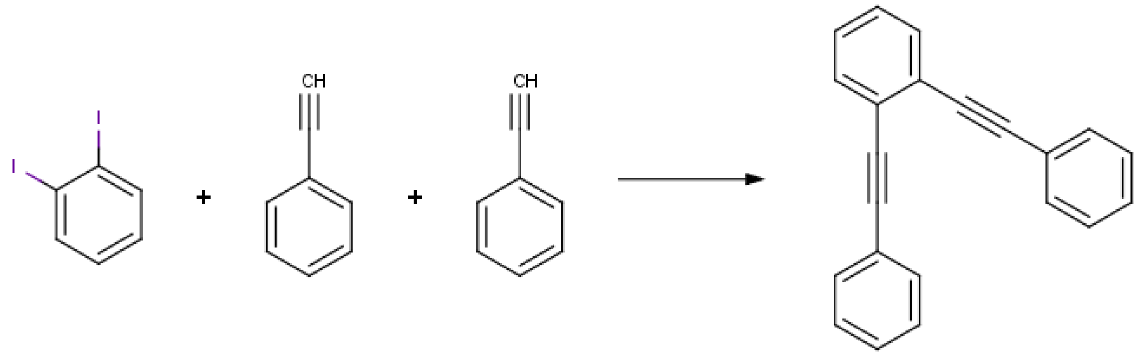

SMILES of the input:
IC1=CC=CC=C1I.C#CC1=CC=CC=C1.C#CC1=CC=CC=C1>>C1=CC=C(C=C1)C#CC1=CC=CC=C1C#CC1=CC=CC=C1

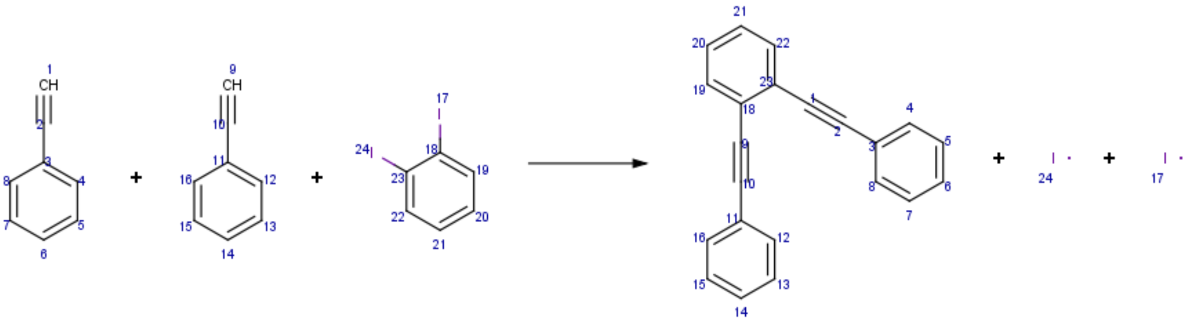

Correct mapped SMILES/SMARTS of the reaction:
[CH:1]#[C:2][C:3]1=[CH:4][CH:5]=[CH:6][CH:7]=[CH:8]1.[CH:9]#[C:10][C:11]1=[CH:12][CH:13]=[CH:14][CH:15]=[CH:16]1.[I:17][C:18]1=[CH:19][CH:20]=[CH:21][CH:22]=[C:23]1[I:24]>>[CH:14]1=[CH:15][CH:16]=[C:11]([CH:12]=[CH:13]1)[C:10]#[C:9][C:18]1=[CH:19][CH:20]=[CH:21][CH:22]=[C:23]1[C:1]#[C:2][C:3]1=[CH:4][CH:5]=[CH:6][CH:7]=[CH:8]1.[I:24].[I:17]

Correctness of the mapping: YES

Reaction no 11

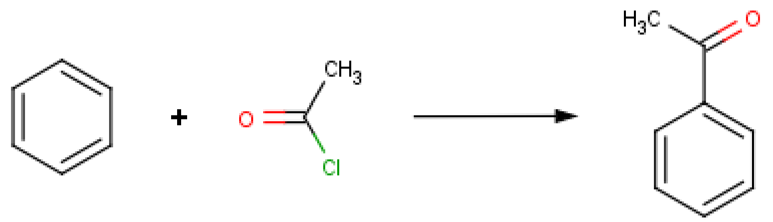

SMILES of the input:
C1=CC=CC=C1.CC(Cl)=O>>CC(=O)C1=CC=CC=C1

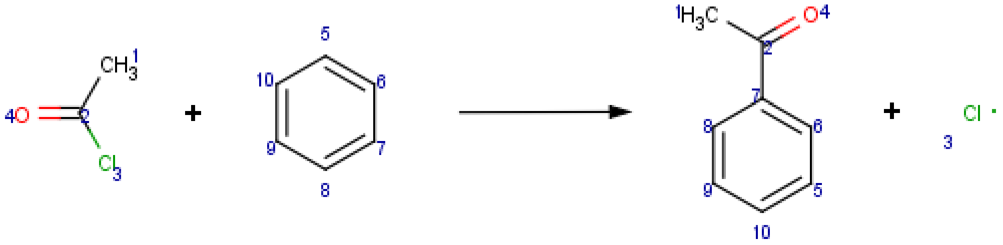

Correct mapped SMILES/SMARTS of the reaction:
[CH3:1][C:2]([Cl:3])=[O:4].[CH:5]1=[CH:6][CH:7]=[CH:8][CH:9]=[CH:10]1>>[CH3:1][C:2](=[O:4])[C:7]1=[CH:6][CH:5]=[CH:10][CH:9]=[CH:8]1.[Cl:3]

Correctness of the mapping: YES

Reaction no 12

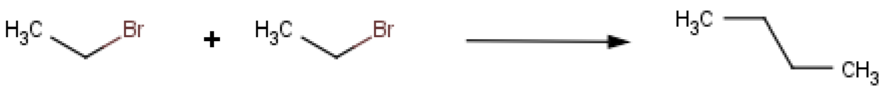

SMILES of the input:
CCBr.CCBr>>CCCC

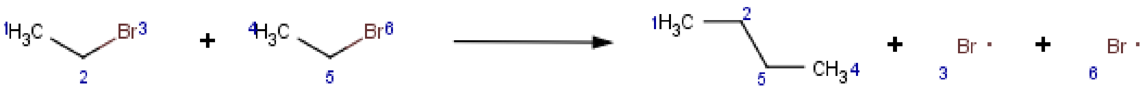

Correct mapped SMILES/SMARTS of the reaction:
[CH3:1][CH2:2][Br:3].[CH3:4][CH2:5][Br:6]>>[CH3:4][CH2:5][CH2:2][CH3:1].[Br:3].[Br:6]

Correctness of the mapping: YES

Reaction no 13

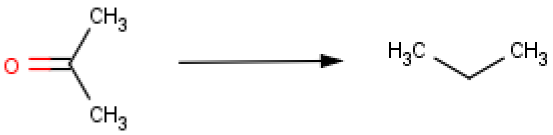

SMILES of the input:
CC(C)=O>>CCC

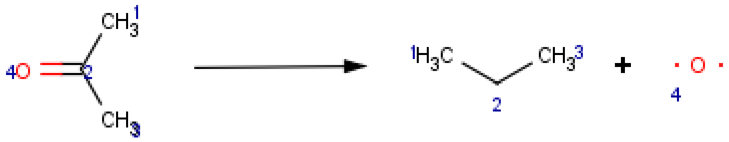

Correct mapped SMILES/SMARTS of the reaction:
[CH3:1][C:2]([CH3:3])=[O:4]>>[CH3:1][CH2:2][CH3:3].[O:4]

Correctness of the mapping: YES

Reaction no 14

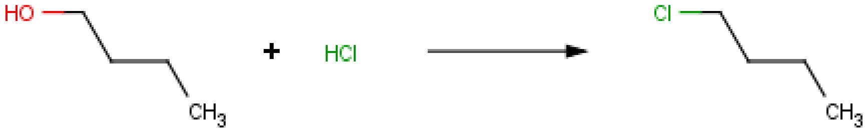

SMILES of the input:
CCCCO.Cl>>CCCCCl

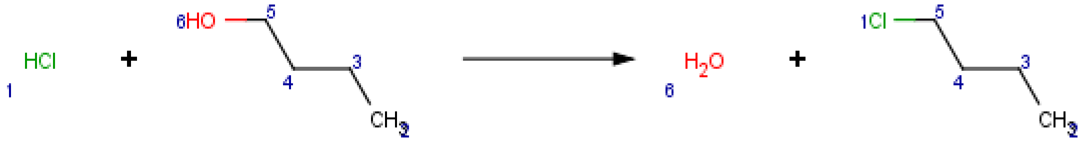

Correct mapped SMILES/SMARTS of the reaction:
[ClH:1].[CH3:2][CH2:3][CH2:4][CH2:5][OH:6]>>[OH2:6].[CH3:2][CH2:3][CH2:4][CH2:5][Cl:1]

Correctness of the mapping: YES

Reaction no 15

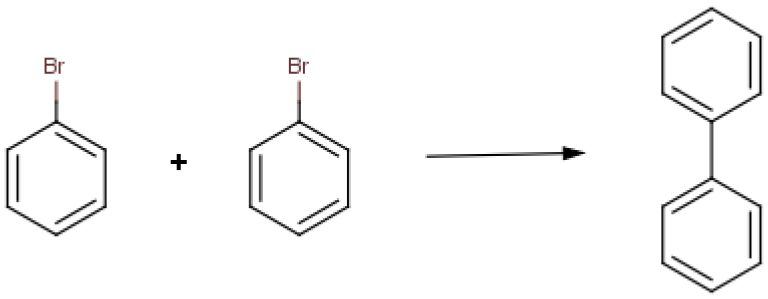

SMILES of the input:
BrC1=CC=CC=C1.BrC1=CC=CC=C1>>C1=CC=C(C=C1)C1=CC=CC=C1

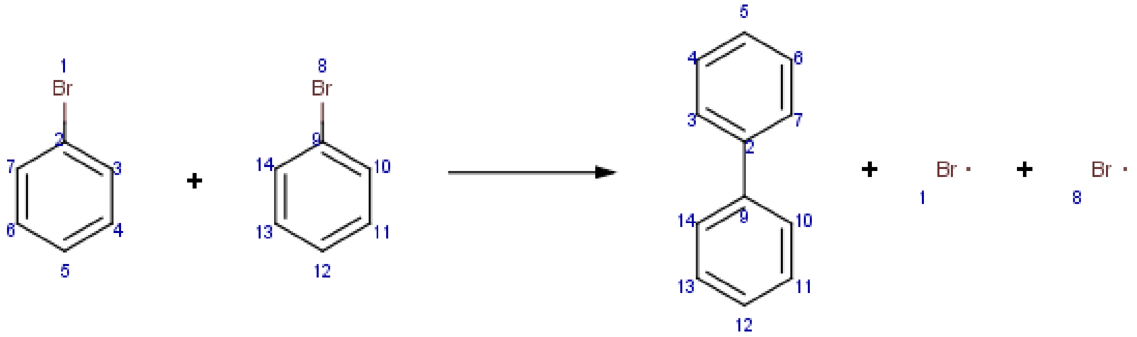

Correct mapped SMILES/SMARTS of the reaction:
[Br:1][C:2]1=[CH:3][CH:4]=[CH:5][CH:6]=[CH:7]1.[Br:8][C:9]1=[CH:10][CH:11
]=[CH:12][CH:13]=[CH:14]1>>[CH:12]1=[CH:13][CH:14]=[C:9]([CH:10]=[CH:11]1
)[C:2]1=[CH:3][CH:4]=[CH:5][CH:6]=[CH:7]1.[Br:1].[Br:8]

Correctness of the mapping: YES

Reaction no 16

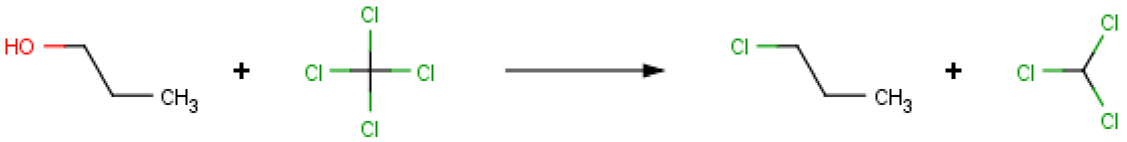

SMILES of the input:
CCCO.ClC(Cl)(Cl)Cl>>CCCCl.ClC(Cl)Cl

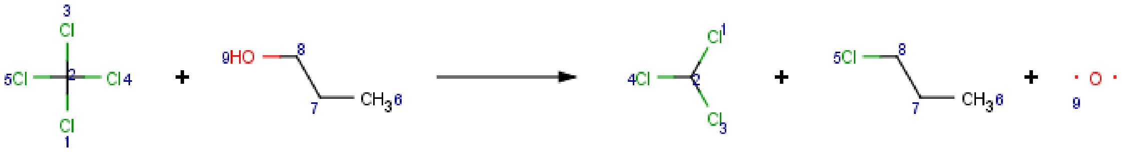

Correct mapped SMILES/SMARTS of the reaction:

[Cl:1][C:2]([Cl:3])([Cl:4])[Cl:5].[CH3:6][CH2:7][CH2:8][OH:9]>>[Cl:1][CH:2]([Cl:3])[Cl:4].[CH3:6][CH2:7][CH2:8][Cl:5].[O:9]

Correctness of the mapping: YES

Reaction no 17

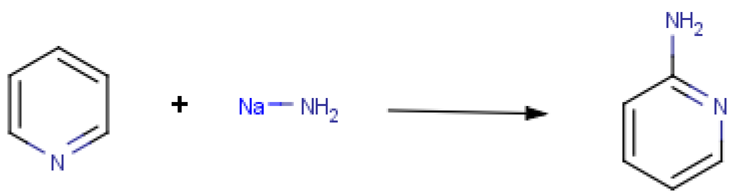

SMILES of the input:
C1=CC=NC=C1.N[Na]>>NC1=NC=CC=C1

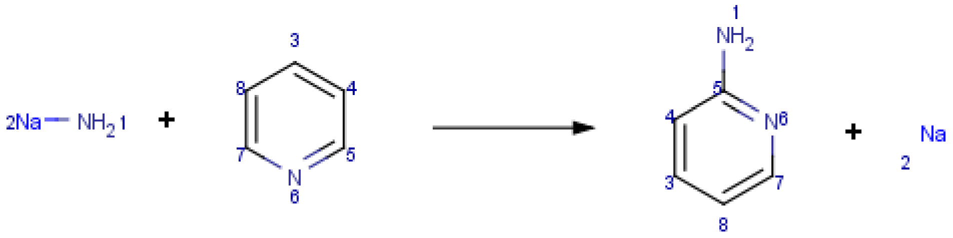

Correct mapped SMILES/SMARTS of the reaction:
[NH2:1][Na:2].[CH:3]1=[CH:4][CH:5]=[N:6][CH:7]=[CH:8]1>>[NH2:1][C:5]1=[N:6][CH:7]=[CH:8][CH:3]=[CH:4]1.[Na:2]

Correctness of the mapping: YES

Reaction no 18

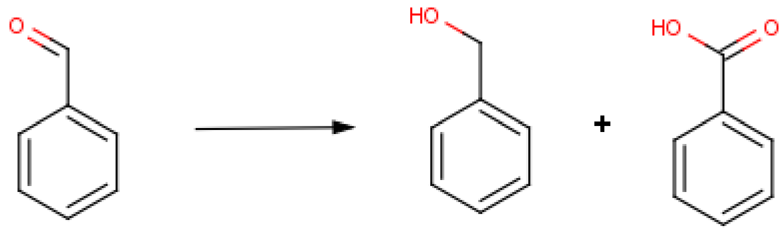

SMILES of the input:
O=CC1=CC=CC=C1>>OCC1=CC=CC=C1.OC(=O)C1=CC=CC=C1

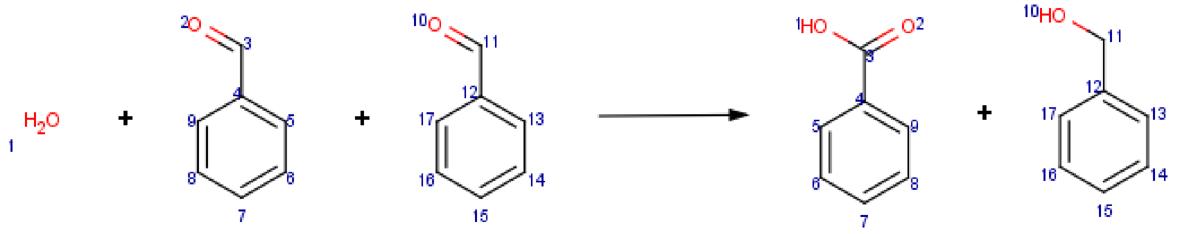

Correct mapped SMILES/SMARTS of the reaction:
[OH2:1].[O:2]=[CH:3][C:4]1=[CH:5][CH:6]=[CH:7][CH:8]=[CH:9]1.[O:10]=[CH:1
1][C:12]1=[CH:13][CH:14]=[CH:15][CH:16]=[CH:17]1>>[OH:1][C:3](=[O:2])[C:4
]1=[CH:9][CH:8]=[CH:7][CH:6]=[CH:5]1.[OH:10][CH2:11][C:12]1=[CH:13][CH:14
]=[CH:15][CH:16]=[CH:17]1

Correctness of the mapping: YES

Reaction no 19

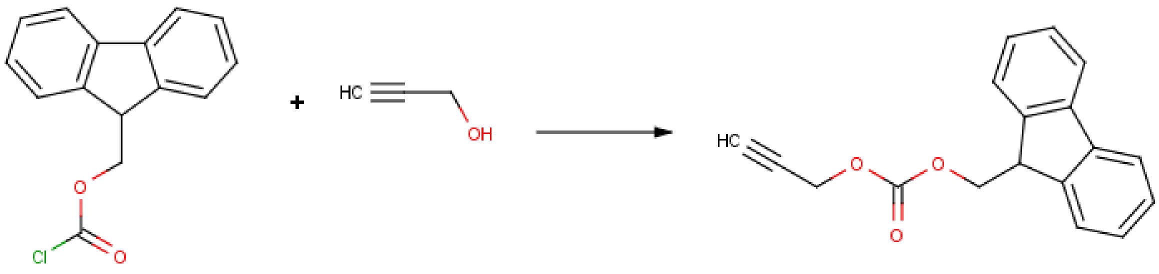

SMILES of the input:
ClC(=O)OCC1C2=CC=CC=C2C2=C1C=CC=C2.OCC#C>>O=C(OCC#C)OCC1C2=CC=CC=C2C2=C1C
=CC=C2

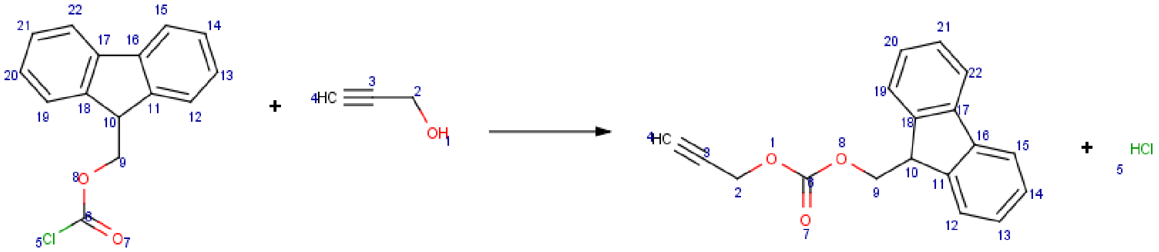

Correct mapped SMILES/SMARTS of the reaction:
[Cl:5][C:6](=[O:7])[O:8][CH2:9][CH:10]1[C:11]2=[CH:12][CH:13]=[CH:14][CH:
15]=[C:16]2[C:17]2=[C:18]1[CH:19]=[CH:20][CH:21]=[CH:22]2.[OH:1][CH2:2][C
:3]#[CH:4]>>[O:7]=[C:6]([O:1][CH2:2][C:3]#[CH:4])[O:8][CH2:9][CH:10]1[C:1
1]2=[CH:12][CH:13]=[CH:14][CH:15]=[C:16]2[C:17]2=[C:18]1[CH:19]=[CH:20][C
H:21]=[CH:22]2.[ClH:5]

Correctness of the mapping: YES

Reaction no 20

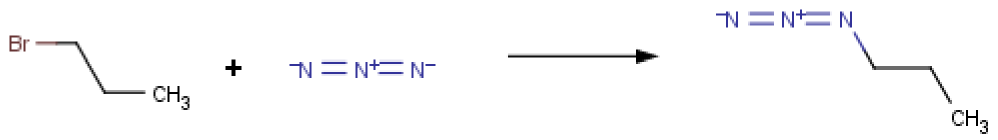

SMILES of the input:
CCCBr.[N-]=[N+]=[N-]>>CCCN=[N+]=[N-]

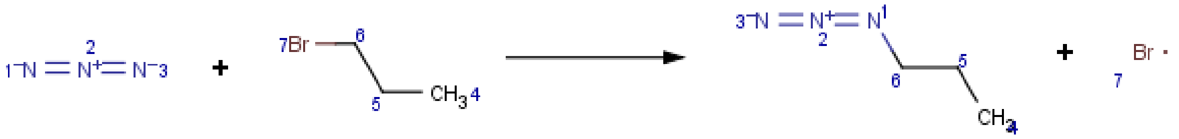

Correct mapped SMILES/SMARTS of the reaction:
[N-:1]=[N+:2]=[N-
:3].[CH3:4][CH2:5][CH2:6][Br:7]>>[CH3:4][CH2:5][CH2:6][N:1]=[N+:2]=[N-
:3].[Br:7]

Correctness of the mapping: YES

Reaction no 21

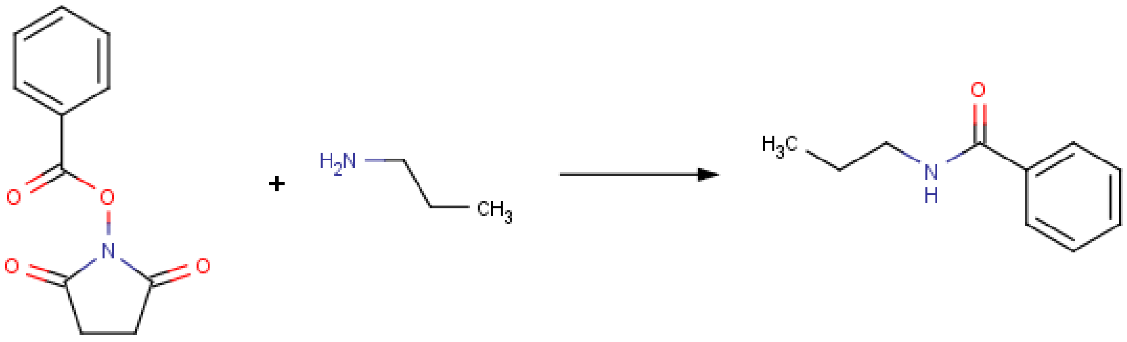

SMILES of the input:
O=C(ON1C(=O)CCC1=O)C1=CC=CC=C1.CCCN>>CCCNC(=O)C1=CC=CC=C1

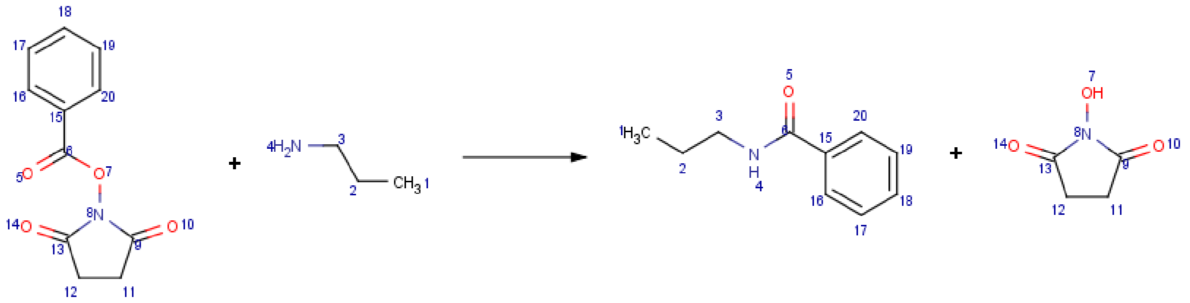

Correct mapped SMILES/SMARTS of the reaction:
[O:5]=[C:6]([O:7][N:8]1[C:9](=[O:10])[CH2:11][CH2:12][C:13]1=[O:14])[C:15
]1=[CH:16][CH:17]=[CH:18][CH:19]=[CH:20]1.[CH3:1][CH2:2][CH2:3][NH2:4]>>[
CH3:1][CH2:2][CH2:3][NH:4][C:6](=[O:5])[C:15]1=[CH:20][CH:19]=[CH:18][CH:
17]=[CH:16]1.[OH:7][N:8]1[C:9](=[O:10])[CH2:11][CH2:12][C:13]1=[O:14]

Correctness of the mapping: YES

Reaction no 22

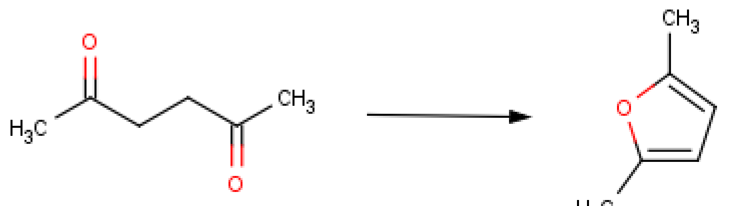

SMILES of the input:
CC(=O)CCC(C)=O>>CC1=CC=C(C)O1

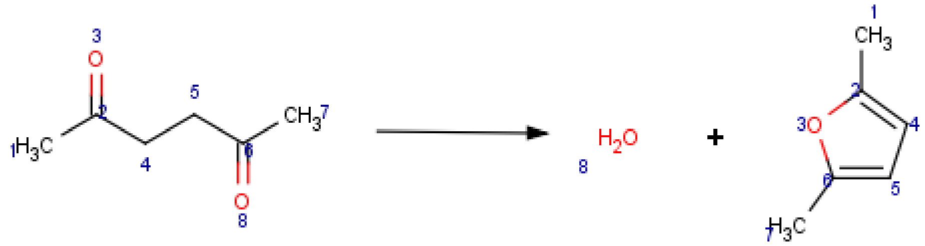

Correct mapped SMILES/SMARTS of the reaction:
[CH3:1][C:2](=[O:3])[CH2:4][CH2:5][C:6]([CH3:7])=[O:8]>>[OH2:8].[CH3:1][C:2]1=[CH:4][CH:5]=[C:6]([CH3:7])[O:3]1

Correctness of the mapping: YES

Reaction no 23

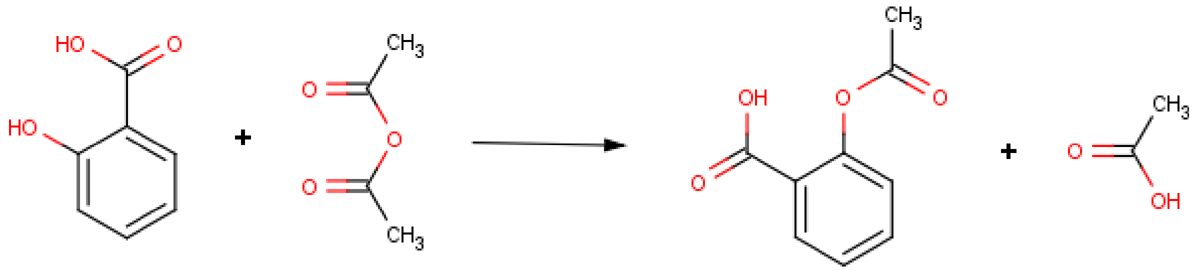

SMILES of the input:
OC(=O)C1=CC=CC=C1O.CC(=O)OC(C)=O>>CC(=O)OC1=CC=CC=C1C(O)=O.CC(O)=O

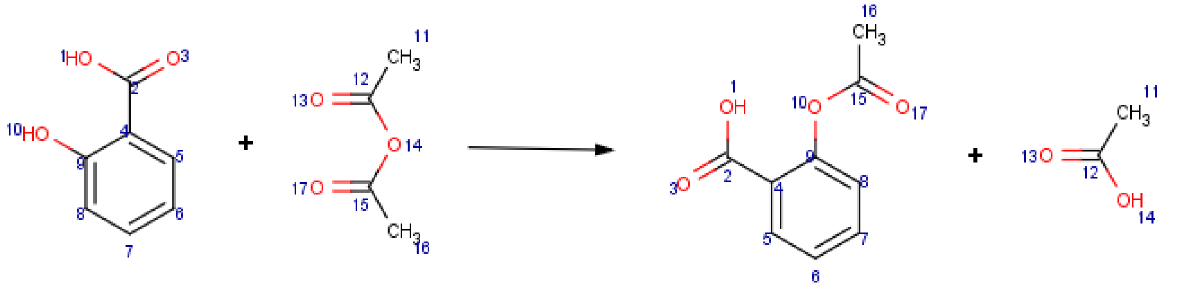

Correct mapped SMILES/SMARTS of the reaction:

[OH:1][C:2](=[O:3])[C:4]1=[CH:5][CH:6]=[CH:7][CH:8]=[C:9]1[OH:10].[CH3:11][C:12](=[O:13])[O:14][C:15]([CH3:16])=[O:17]>>[CH3:16][C:15](=[O:17])[O:10][C:9]1=[CH:8][CH:7]=[CH:6][CH:5]=[C:4]1[C:2]([OH:1])=[O:3].[CH3:11][C:12]([OH:14])=[O:13]

Correctness of the mapping: YES

Reaction no 24

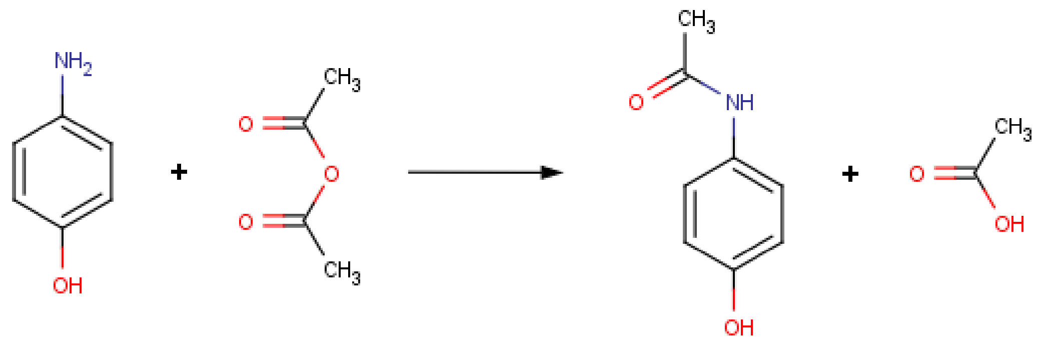

SMILES of the input:
NC1=CC=C(O)C=C1.CC(=O)OC(C)=O>>CC(=O)NC1=CC=C(O)C=C1.CC(O)=O

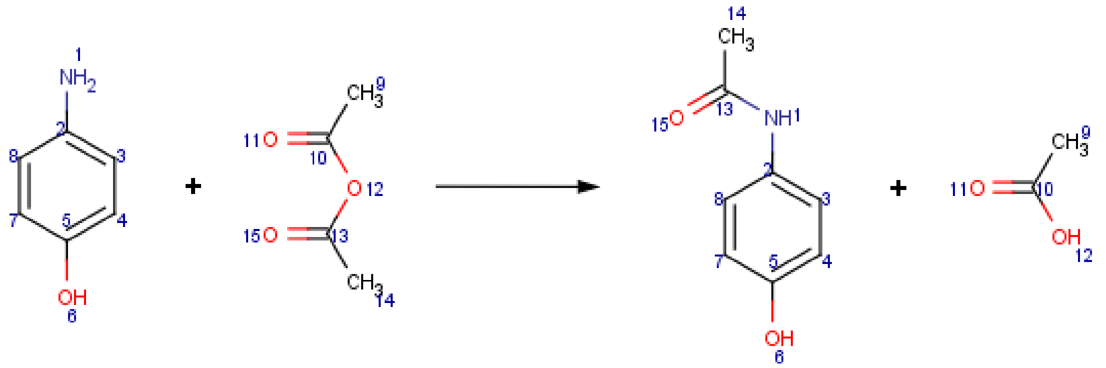

Correct mapped SMILES/SMARTS of the reaction:
[NH2:1][C:2]1=[CH:3][CH:4]=[C:5]([OH:6])[CH:7]=[CH:8]1.[CH3:9][C:10](=[O:11])[O:12][C:13]([CH3:14])=[O:15]>>[CH3:14][C:13](=[O:15])[NH:1][C:2]1=[CH:3][CH:4]=[C:5]([OH:6])[CH:7]=[CH:8]1.[CH3:9][C:10]([OH:12])=[O:11]

Correctness of the mapping: YES

Reaction no 25

SMILES of the input:

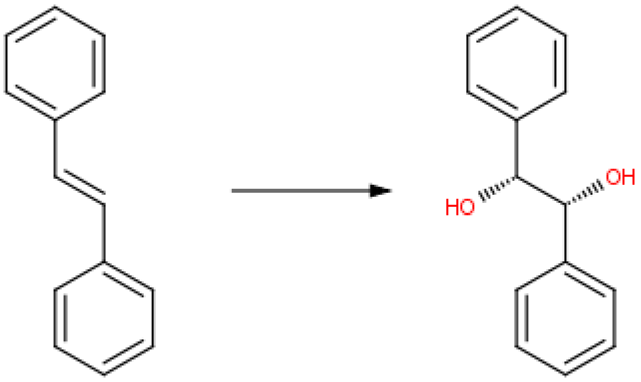

C(=C/C1=CC=CC=C1)\C1=CC=CC=C1>>O[C@@H]([C@H](O)C1=CC=CC=C1)C1=CC=CC=C1

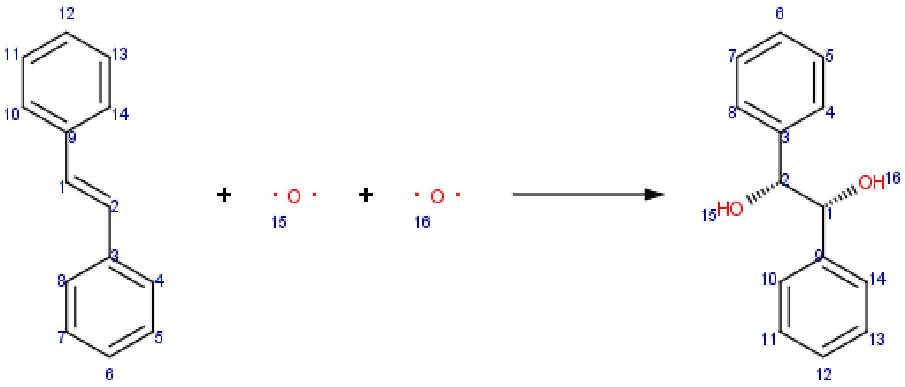

Correct mapped SMILES/SMARTS of the reaction:
[CH:1](=[CH:2]/[C:3]1=[CH:4][CH:5]=[CH:6][CH:7]=[CH:8]1)\[C:9]1=[CH:10][C
H:11]=[CH:12][CH:13]=[CH:14]1.[O:15].[O:16]>>[OH:15][C@@H:2]([C@H:1]([OH:
16])[C:9]1=[CH:14][CH:13]=[CH:12][CH:11]=[CH:10]1)[C:3]1=[CH:8][CH:7]=[CH
:6][CH:5]=[CH:4]1

Correctness of the mapping: YES

Students, Ulsan Institute of Science and Technology, Korea

Reaction no 1

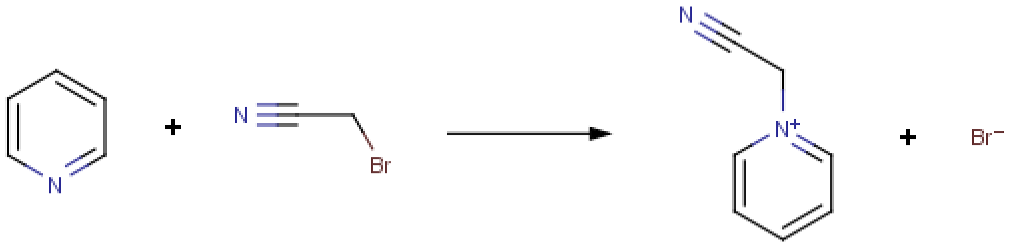

SMILES of the input:
C1=CC=NC=C1.BrCC#N>>N#CC[N+]1=CC=CC=C1.[Br-]

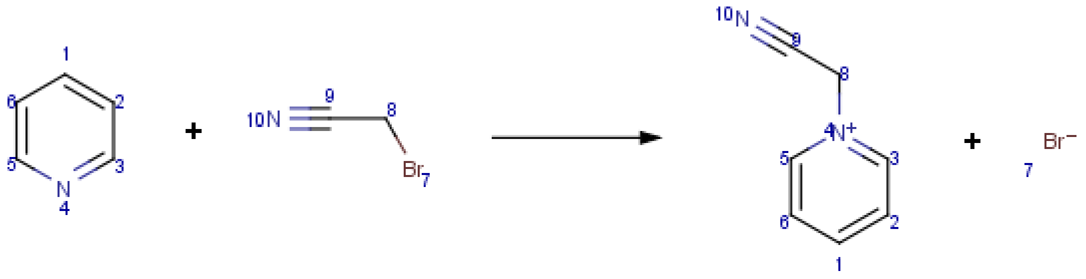

Correct mapped SMILES/SMARTS of the reaction:
[CH:1]1=[CH:2][CH:3]=[N:4][CH:5]=[CH:6]1.[Br:7][CH2:8][C:9]#[N:10]>>[N:10
]#[C:9][CH2:8][N+:4]1=[CH:3][CH:2]=[CH:1][CH:6]=[CH:5]1.[Br-:7]

Correctness of the mapping: YES

Reaction no 2

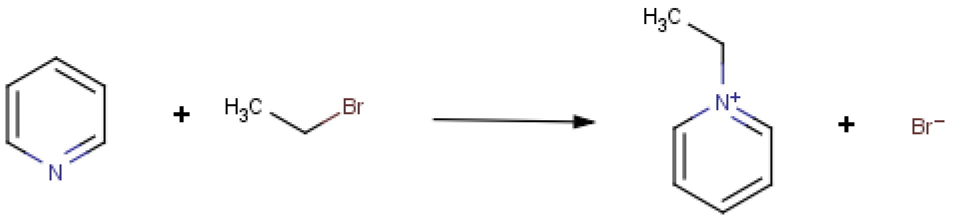

SMILES of the input:
C1=CC=NC=C1.CCBr>>CC[N+]1=CC=CC=C1.[Br-]

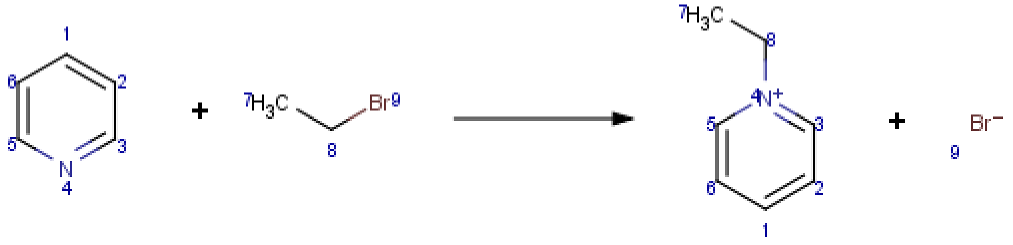

Correct mapped SMILES/SMARTS of the reaction:
[CH:1]1=[CH:2][CH:3]=[N:4][CH:5]=[CH:6]1.[CH3:7][CH2:8][Br:9]>>[CH3:7][CH
2:8][N+:4]1=[CH:3][CH:2]=[CH:1][CH:6]=[CH:5]1.[Br-:9]

Correctness of the mapping: YES

Reaction no 3

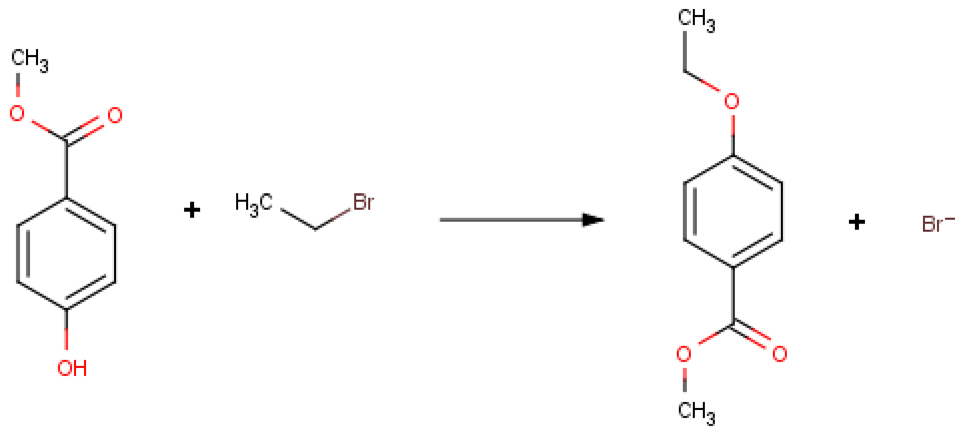

SMILES of the input:
COC(=O)C1=CC=C(O)C=C1.CCBr>>CCOC1=CC=C(C=C1)C(=O)OC.[Br-]

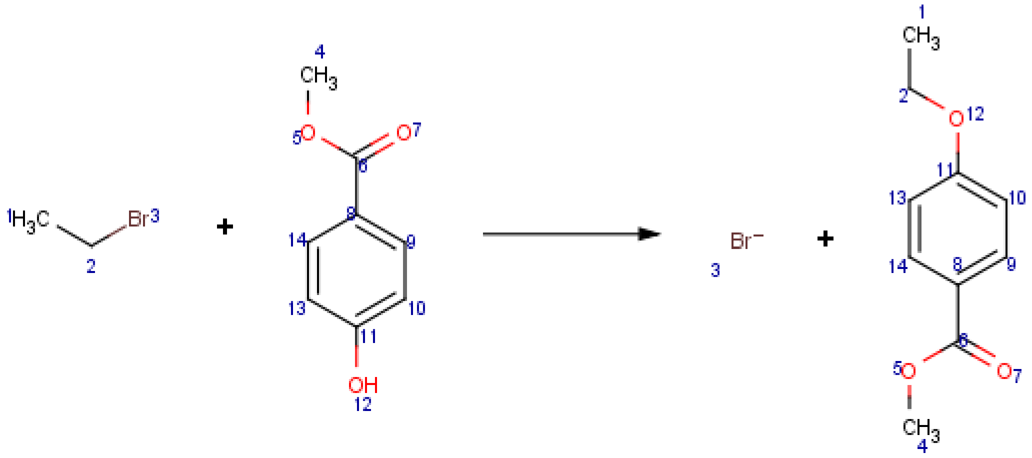

Correct mapped SMILES/SMARTS of the reaction:
[CH3:1][CH2:2][Br:3].[CH3:4][O:5][C:6](=[O:7])[C:8]1=[CH:9][CH:10]=[C:11]([OH:12])[CH:13]=[CH:14]1>>[Br-:3].[CH3:1][CH2:2][O:12][C:11]1=[CH:10][CH:9]=[C:8]([CH:14]=[CH:13]1)[C:6](=[O:7])[O:5][CH3:4]

Correctness of the mapping: YES

Reaction no 4

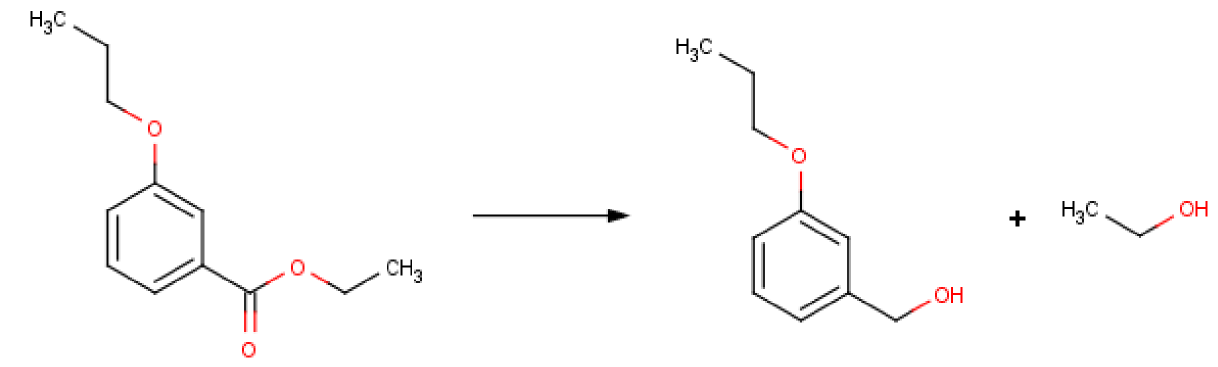

SMILES of the input:
CCCOC1=CC(=CC=C1)C(=O)OCC>>CCCOC1=CC(CO)=CC=C1.CCO

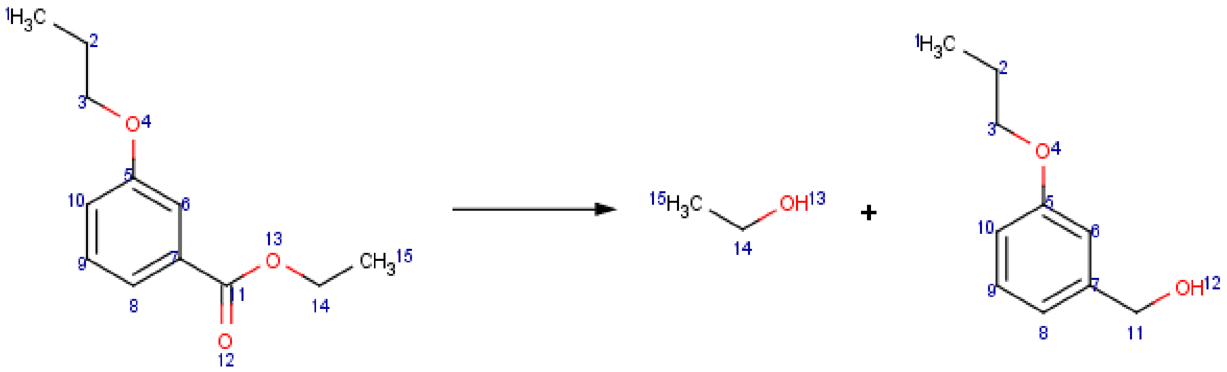

Correct mapped SMILES/SMARTS of the reaction:
[CH3:1][CH2:2][CH2:3][O:4][C:5]1=[CH:6][C:7](=[CH:8][CH:9]=[CH:10]1)[C:11](=[O:12])[O:13][CH2:14][CH3:15]>>[CH3:15][CH2:14][OH:13].[CH3:1][CH2:2][CH2:3][O:4][C:5]1=[CH:6][C:7]([CH2:11][OH:12])=[CH:8][CH:9]=[CH:10]1

Correctness of the mapping: YES

Reaction no 5

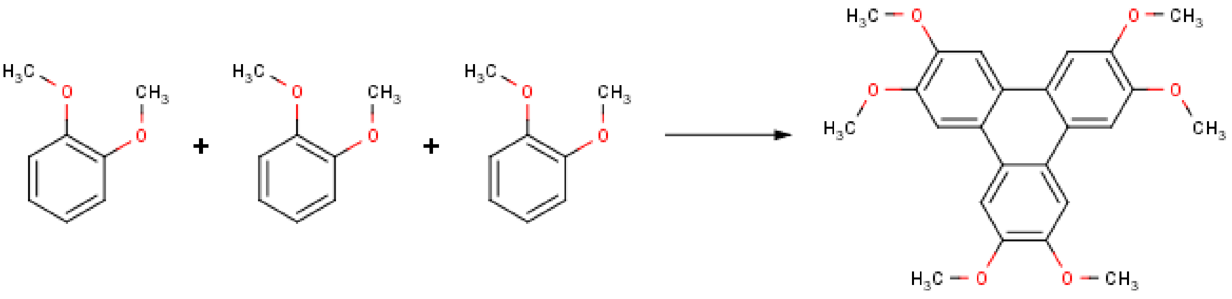

SMILES of the input:

COC1=C(OC)C=CC=C1.COC1=C(OC)C=CC=C1.COC1=C(OC)C=CC=C1>>COC1=C(OC)C=C2C(=C1)C1=CC(OC)=C(OC)C=C1C1=CC(OC)=C(OC)C=C21

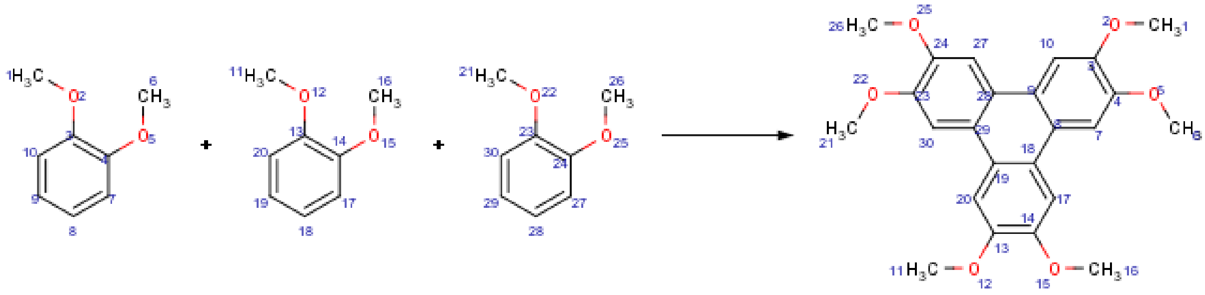

Correct mapped SMILES/SMARTS of the reaction:
[CH3:1][O:2][C:3]1=[C:4]([O:5][CH3:6])[CH:7]=[CH:8][CH:9]=[CH:10]1.[CH3:11][O:12][C:13]1=[C:14]([O:15][CH3:16])[CH:17]=[CH:18][CH:19]=[CH:20]1.[CH3:21][O:22][C:23]1=[C:24]([O:25][CH3:26])[CH:27]=[CH:28][CH:29]=[CH:30]1>>[CH3:26][O:25][C:24]1=[C:23]([O:22][CH3:21])[CH:30]=[C:29]2[C:28](=[CH:27]1)[C:9]1=[CH:10][C:3]([O:2][CH3:1])=[C:4]([O:5][CH3:6])[CH:7]=[C:8]1[C:18]1=[CH:17][C:14]([O:15][CH3:16])=[C:13]([O:12][CH3:11])[CH:20]=[C:19]21

Correctness of the mapping: YES

Reaction no 6

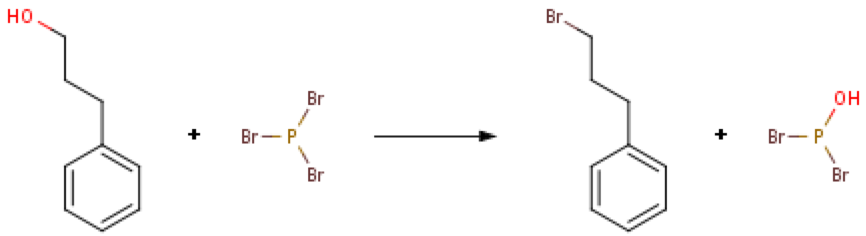

SMILES of the input:
OCCCC1=CC=CC=C1.BrP(Br)Br>>BrCCCC1=CC=CC=C1.OP(Br)Br

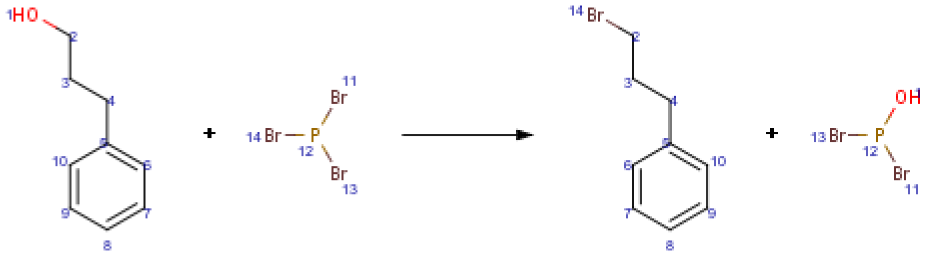

Correct mapped SMILES/SMARTS of the reaction:
[OH:1][CH2:2][CH2:3][CH2:4][C:5]1=[CH:6][CH:7]=[CH:8][CH:9]=[CH:10]1.[Br:11][P:12]([Br:13])[Br:14]>>[Br:14][CH2:2][CH2:3][CH2:4][C:5]1=[CH:10][CH:9]=[CH:8][CH:7]=[CH:6]1.[OH:1][P:12]([Br:11])[Br:13]

Correctness of the mapping: YES

Reaction no 7

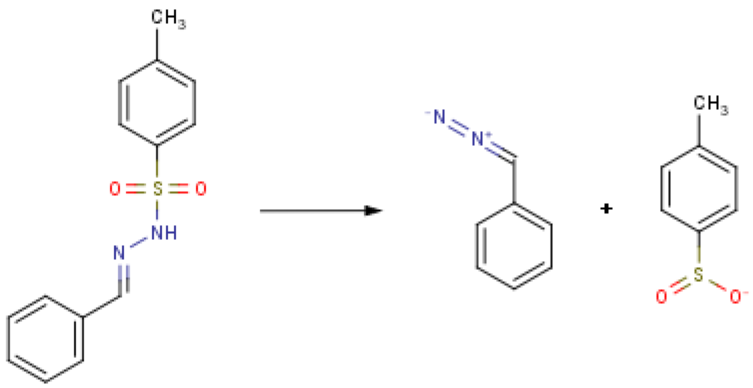

SMILES of the input:
CC1=CC=C(C=C1)S(=O)(=O)N\N=C\C1=CC=CC=C1>>[N-
]=[N+]=CC1=CC=CC=C1.CC1=CC=C(C=C1)S([O-])=O

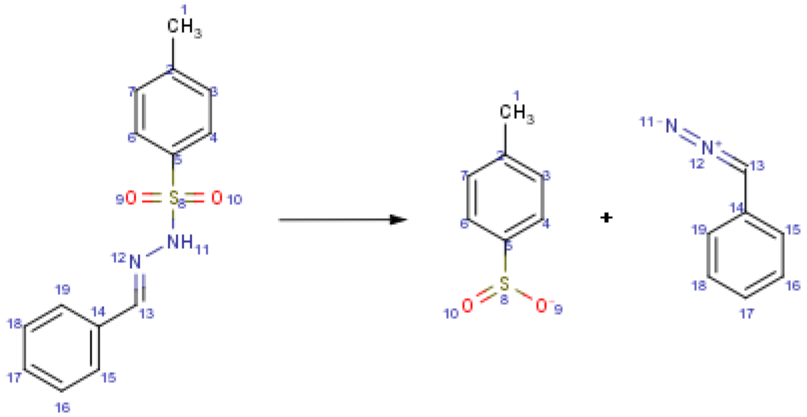

Correct mapped SMILES/SMARTS of the reaction:
[CH3:1][C:2]1=[CH:3][CH:4]=[C:5]([CH:6]=[CH:7]1)[S:8](=[O:9])(=[O:10])[NH
:11]\[N:12]=[CH:13]\[C:14]1=[CH:15][CH:16]=[CH:17][CH:18]=[CH:19]1>>[CH3:
1][C:2]1=[CH:3][CH:4]=[C:5]([CH:6]=[CH:7]1)[S:8]([O-:9])=[O:10].[N-
:11]=[N+:12]=[CH:13][C:14]1=[CH:15][CH:16]=[CH:17][CH:18]=[CH:19]1

Correctness of the mapping: YES

Reaction no 8

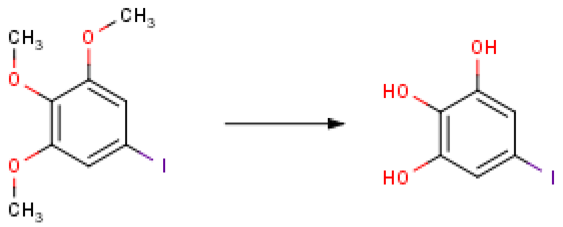

SMILES of the input:
COC1=CC(I)=CC(OC)=C1OC>>OC1=CC(I)=CC(O)=C1O

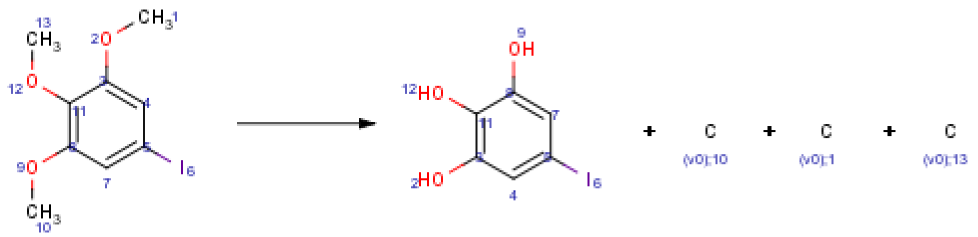

Correct mapped SMILES/SMARTS of the reaction:
[CH3:1][O:2][C:3]1=[CH:4][C:5]([I:6])=[CH:7][C:8]([O:9][CH3:10])=[C:11]1[O:12][CH3:13]>>[OH:9][C:8]1=[CH:7][C:5]([I:6])=[CH:4][C:3]([OH:2])=[C:11]1[OH:12].[C:10].[C:1].[C:13]

Correctness of the mapping: YES

Reaction no 9

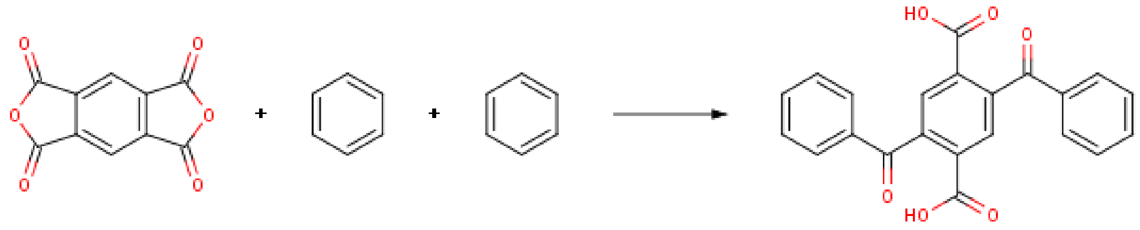

SMILES of the input:
O=C1OC(=O)C2=CC3=C(C=C12)C(=O)OC3=O.C1=CC=CC=C1.C1=CC=CC=C1>>OC(=O)C1=CC(C(=O)C2=CC=CC=C2)=C(C=C1C(=O)C1=CC=CC=C1)C(O)=O

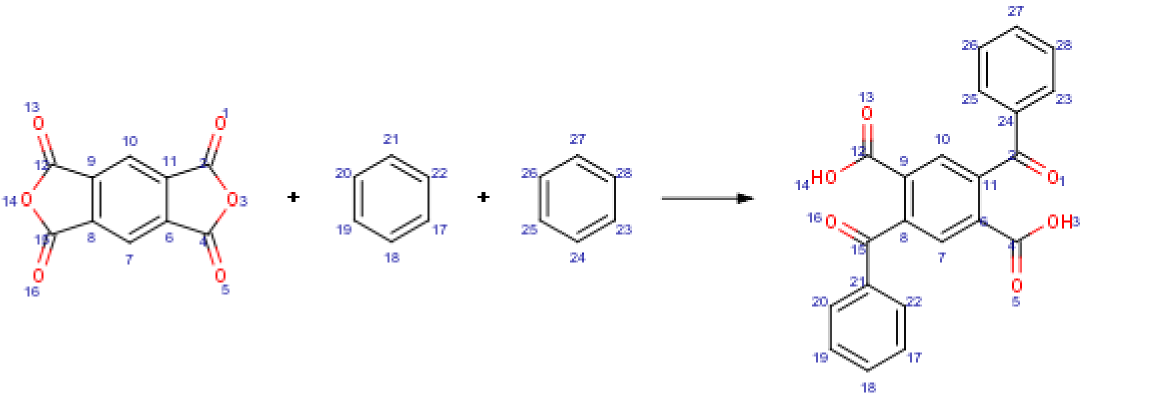

Correct mapped SMILES/SMARTS of the reaction:
[O:1]=[C:2]1[O:3][C:4](=[O:5])[C:6]2=[CH:7][C:8]3=[C:9]([CH:10]=[C:11]12)[C:12](=[O:13])[O:14][C:15]3=[O:16].[CH:17]1=[CH:18][CH:19]=[CH:20][CH:21]=[CH:22]1.[CH:23]1=[CH:24][CH:25]=[CH:26][CH:27]=[CH:28]1>>[OH:3][C:4](=[O:5])[C:6]1=[CH:7][C:8]([C:15](=[O:16])[C:21]2=[CH:20][CH:19]=[CH:18][CH:17]=[CH:22]2)=[C:9]([CH:10]=[C:11]1[C:2](=[O:1])[C:24]1=[CH:23][CH:28]=[CH:27][CH:26]=[CH:25]1)[C:12]([OH:14])=[O:13]

Correctness of the mapping: NO

Reaction no 10

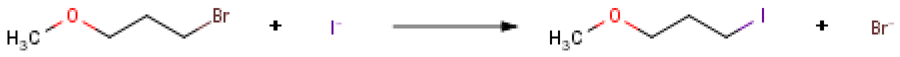

SMILES of the input:
COCCCBr.[I-]>>COCCCI.[Br-]

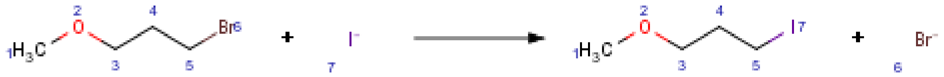

Correct mapped SMILES/SMARTS of the reaction:
[CH3:1][O:2][CH2:3][CH2:4][CH2:5][Br:6].[I-
:7]>>[CH3:1][O:2][CH2:3][CH2:4][CH2:5][I:7].[Br-:6]

Correctness of the mapping: YES

Reaction no 11

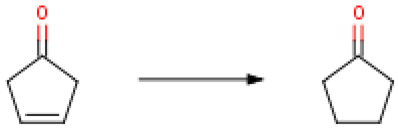

SMILES of the input:
O=C1CC=CC1>>O=C1CCCC1

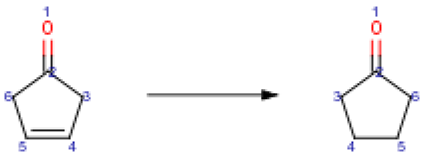

Correct mapped SMILES/SMARTS of the reaction:
[O:1]=[C:2]1[CH2:3][CH:4]=[CH:5][CH2:6]1>>[O:1]=[C:2]1[CH2:6][CH2:5][CH2:
4][CH2:3]1

Correctness of the mapping: YES

Reaction no 12

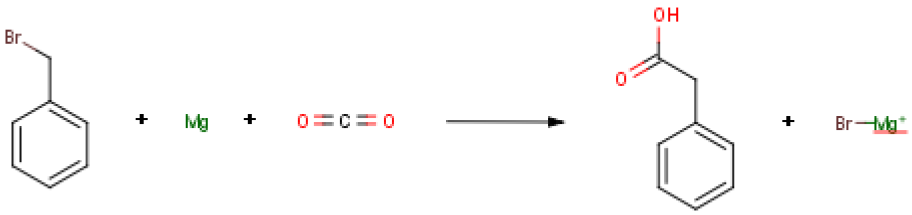

SMILES of the input:
BrCC1=CC=CC=C1.[Mg].O=C=O>>OC(=O)CC1=CC=CC=C1.[Mg+]Br

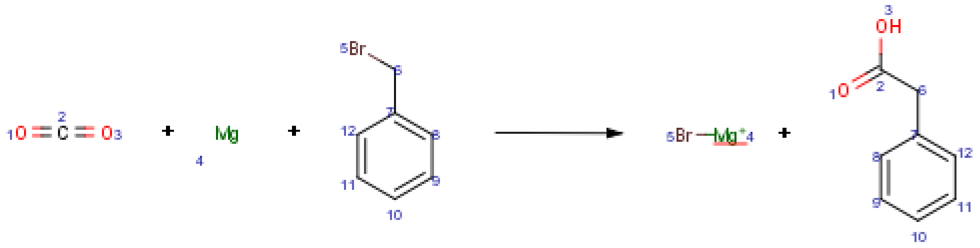

Correct mapped SMILES/SMARTS of the reaction:
[O:1]=[C:2]=[O:3].[Mg:4].[Br:5][CH2:6][C:7]1=[CH:8][CH:9]=[CH:10][CH:11]=[CH:12]1>>[Mg+:4][Br:5].[OH:3][C:2](=[O:1])[CH2:6][C:7]1=[CH:12][CH:11]=[CH:10][CH:9]=[CH:8]1

Correctness of the mapping: YES

Reaction no 13

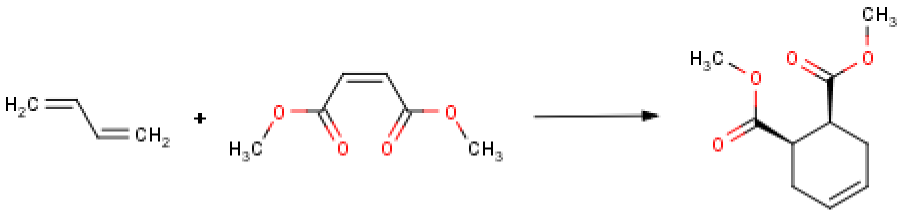

SMILES of the input:
C=CC=C.COC(=O)\C=C/C(=O)OC>>COC(=O)[C@H]1CC=CC[C@H]1C(=O)OC

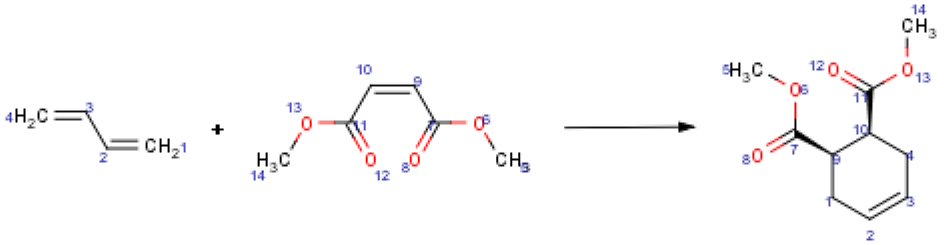

Correct mapped SMILES/SMARTS of the reaction:
[CH2:1]=[CH:2][CH:3]=[CH2:4].[CH3:5][O:6][C:7](=[O:8])\[CH:9]=[CH:10]/[C:11](=[O:12])[O:13][CH3:14]>>[CH3:14][O:13][C:11](=[O:12])[C@H:10]1[CH2:4][CH:3]=[CH:2][CH2:1][C@H:9]1[C:7](=[O:8])[O:6][CH3:5]

Correctness of the mapping: YES

Reaction no 14

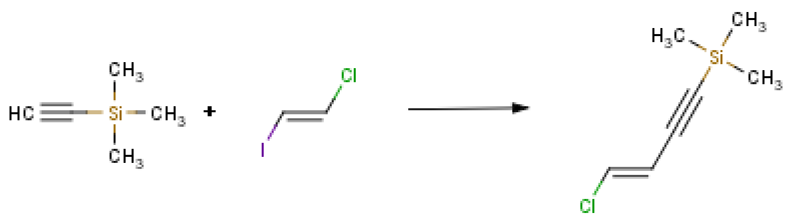

SMILES of the input:
C[Si](C)(C)C#C.Cl\C=C\I>>C[Si](C)(C)C#C\C=C\Cl

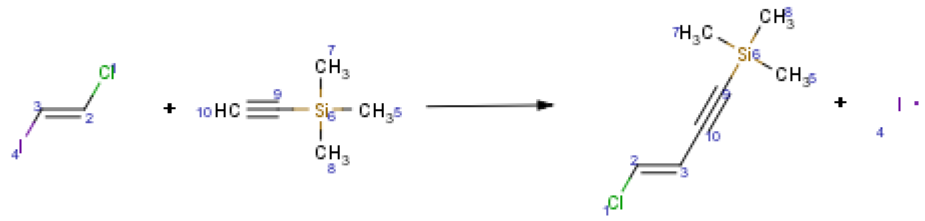

Correct mapped SMILES/SMARTS of the reaction:
[Cl:1]\[CH:2]=[CH:3]\[I:4].[CH3:5][Si:6]([CH3:7])([CH3:8])[C:9]#[CH:10]>>[CH3:8][Si:6]([CH3:7])([CH3:5])[C:9]#[C:10]\[CH:3]=[CH:2]\[Cl:1].[I:4]

Correctness of the mapping: YES

Reaction no 15

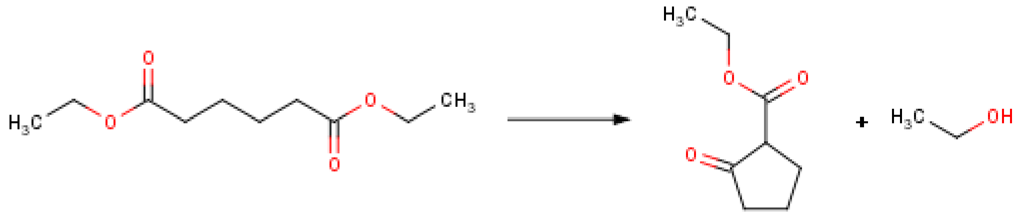

SMILES of the input:
CCOC(=O)CCCCC(=O)OCC>>CCOC(=O)C1CCCC1=O.CCO

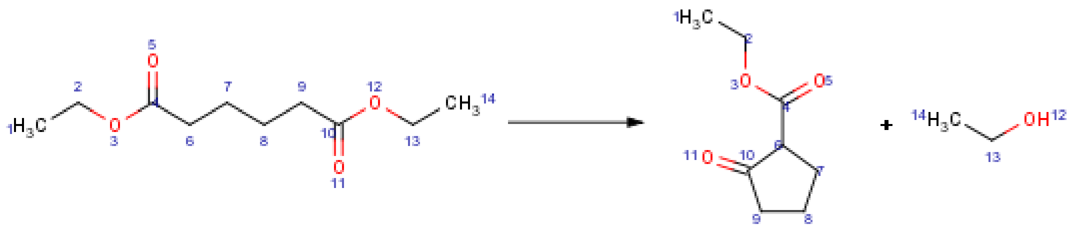

Correct mapped SMILES/SMARTS of the reaction:
[CH3:1][CH2:2][O:3][C:4](=[O:5])[CH2:6][CH2:7][CH2:8][CH2:9][C:10](=[O:11])[O:12][CH2:13][CH3:14]>>[CH3:1][CH2:2][O:3][C:4](=[O:5])[CH:6]1[CH2:7][CH2:8][CH2:9][C:10]1=[O:11].[CH3:14][CH2:13][OH:12]

Correctness of the mapping: YES

Reaction no 16

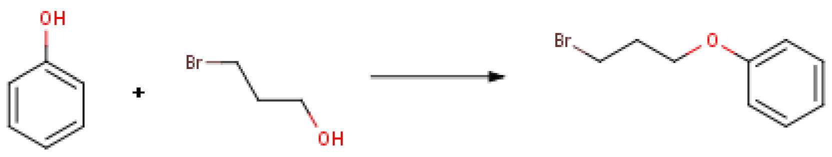

SMILES of the input:
OC1=CC=CC=C1.OCCCBr>>BrCCCOC1=CC=CC=C1

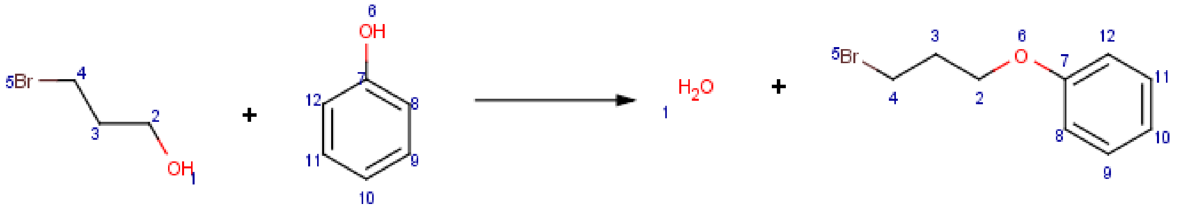

Correct mapped SMILES/SMARTS of the reaction:
[OH:1][CH2:2][CH2:3][CH2:4][Br:5].[OH:6][C:7]1=[CH:8][CH:9]=[CH:10][CH:11]=[CH:12]1>>[OH2:1].[Br:5][CH2:4][CH2:3][CH2:2][O:6][C:7]1=[CH:12][CH:11]=[CH:10][CH:9]=[CH:8]1

Correctness of the mapping: NO

Reaction no 17

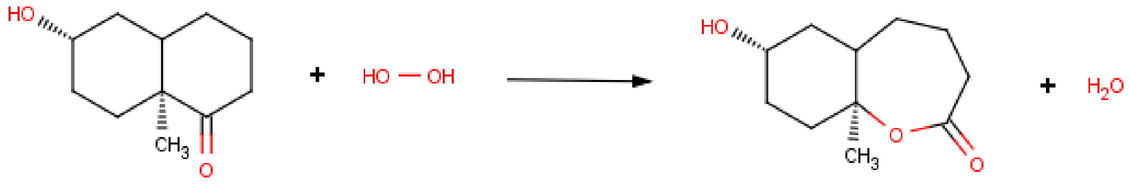

SMILES of the input:
C[C@]12CC[C@H](O)CC1CCCC2=O.OO>>C[C@]12CC[C@H](O)CC1CCCC(=O)O2.O

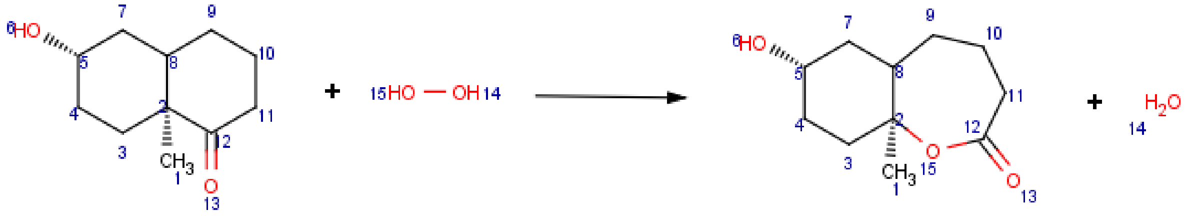

Correct mapped SMILES/SMARTS of the reaction:
[CH3:1][C@:2]12[CH2:3][CH2:4][C@H:5]([OH:6])[CH2:7][CH:8]1[CH2:9][CH2:10][CH2:11][C:12]2=[O:13].[OH:14][OH:15]>>[CH3:1][C@:2]12[CH2:3][CH2:4][C@H:5]([OH:6])[CH2:7][CH:8]1[CH2:9][CH2:10][CH2:11][C:12](=[O:13])[O:15]2.[OH2:14]

Correctness of the mapping: YES

Reaction no 18

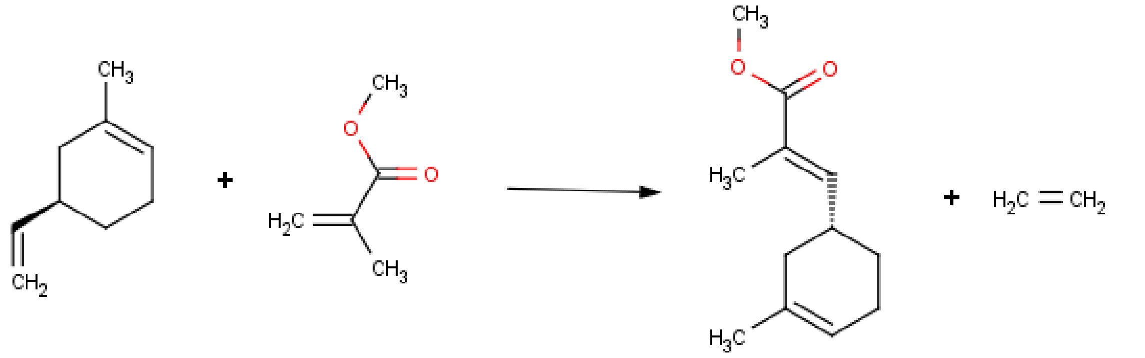

SMILES of the input:
CC1=CCC[C@H](C1)C=C.COC(=O)C(C)=C>>COC(=O)C(\C)=C\[C@@H]1CCC=C(C)C1.C=C

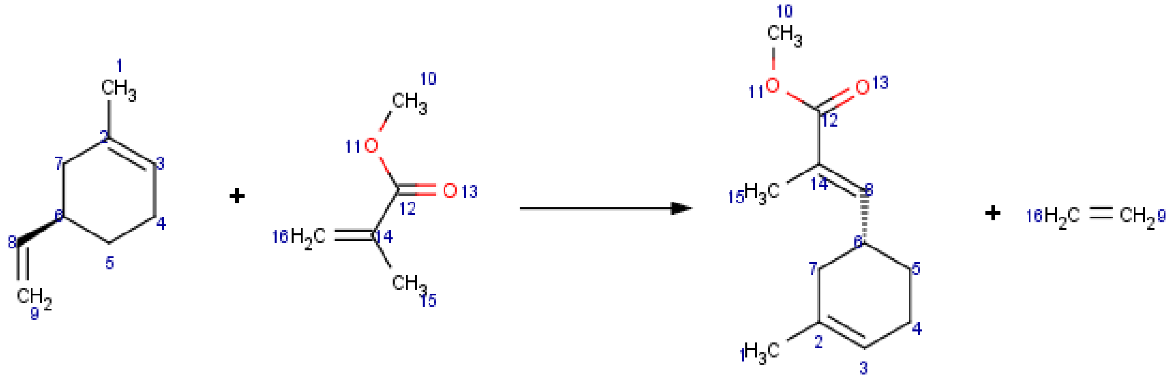

Correct mapped SMILES/SMARTS of the reaction:
[CH3:1][C:2]1=[CH:3][CH2:4][CH2:5][C@H:6]([CH2:7]1)[CH:8]=[CH2:9].[CH3:10][O:11][C:12](=[O:13])[C:14]([CH3:15])=[CH2:16]>>[CH3:10][O:11][C:12](=[O:13])[C:14](\[CH3:15])=[CH:8]\[C@@H:6]1[CH2:5][CH2:4][CH:3]=[C:2]([CH3:1])[CH2:7]1.[CH2:9]=[CH2:16]

Correctness of the mapping: YES

Reaction no 19

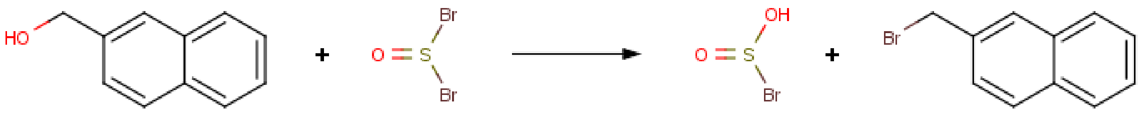

SMILES of the input:
OCC1=CC2=CC=CC=C2C=C1.BrS(Br)=O>>OS(Br)=O.BrCC1=CC2=CC=CC=C2C=C1

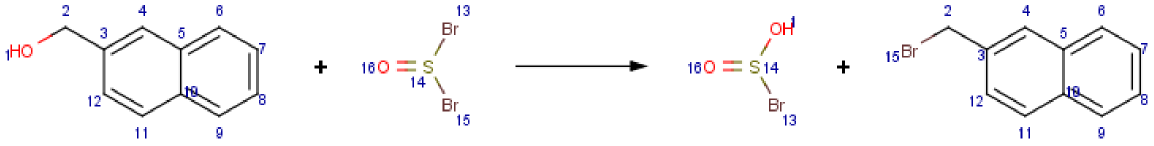

Correct mapped SMILES/SMARTS of the reaction:
[OH:1][CH2:2][C:3]1=[CH:4][C:5]2=[CH:6][CH:7]=[CH:8][CH:9]=[C:10]2[CH:11]=[CH:12]1.[Br:13][S:14]([Br:15])=[O:16]>>[OH:1][S:14]([Br:13])=[O:16].[Br

:15][CH2:2][C:3]1=[CH:4][C:5]2=[CH:6][CH:7]=[CH:8][CH:9]=[C:10]2[CH:11]=[CH:12]1

Correctness of the mapping: YES

Reaction no 20

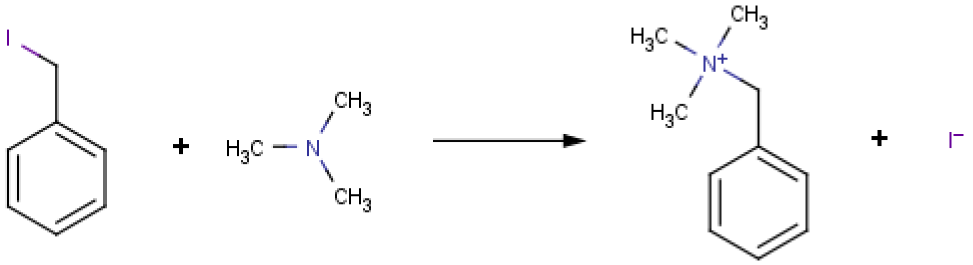

SMILES of the input:
ICC1=CC=CC=C1.CN(C)C>>C[N+](C)(C)CC1=CC=CC=C1.[I-]

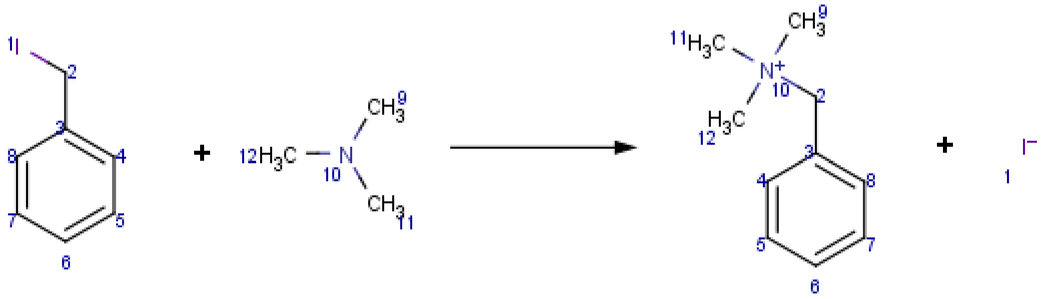

Correct mapped SMILES/SMARTS of the reaction:
[I:1][CH2:2][C:3]1=[CH:4][CH:5]=[CH:6][CH:7]=[CH:8]1.[CH3:9][N:10]([CH3:11])[CH3:12]>>[CH3:11][N+:10]([CH3:12])([CH3:9])[CH2:2][C:3]1=[CH:8][CH:7]=[CH:6][CH:5]=[CH:4]1.[I-:1]

Correctness of the mapping: YES

Reaction no 21

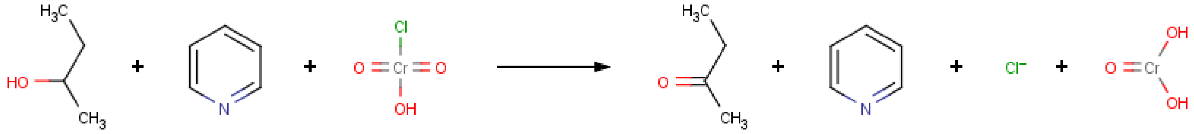

SMILES of the input:
CCC(C)O.C1=CC=NC=C1.O[Cr](Cl)(=O)=O>>CCC(C)=O.C1=CC=NC=C1.[Cl-].O[Cr](O)=O

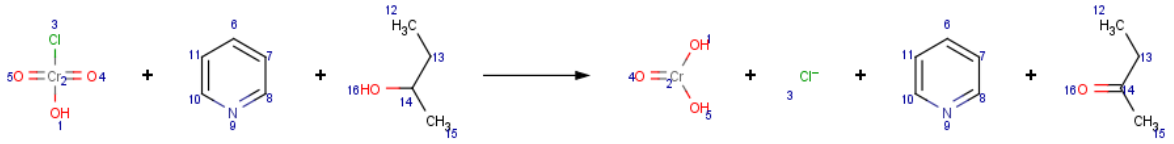

Correct mapped SMILES/SMARTS of the reaction:
[OH:1][Cr:2]([Cl:3])(=[O:4])=[O:5].[CH:6]1=[CH:7][CH:8]=[N:9][CH:10]=[CH:11]1.[CH3:12][CH2:13][CH:14]([CH3:15])[OH:16]>>[OH:1][Cr:2]([OH:5])=[O:4].[Cl-:3].[CH:6]1=[CH:7][CH:8]=[N:9][CH:10]=[CH:11]1.[CH3:12][CH2:13][C:14]([CH3:15])=[O:16]

Correctness of the mapping: YES

Reaction no 22

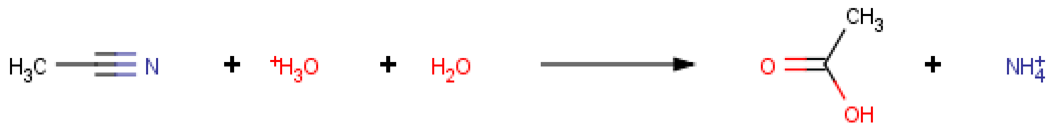

SMILES of the input:
CC#N.[OH3+].O>>CC(O)=O.[NH4+]

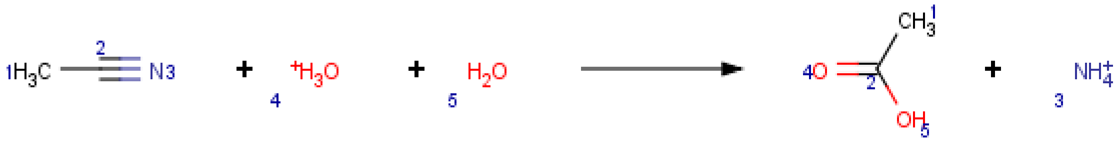

Correct mapped SMILES/SMARTS of the reaction:
[CH3:1][C:2]#[N:3].[OH3+:4].[OH2:5]>>[CH3:1][C:2]([OH:5])=[O:4].[NH4+:3]

Correctness of the mapping: YES

Reaction no 23

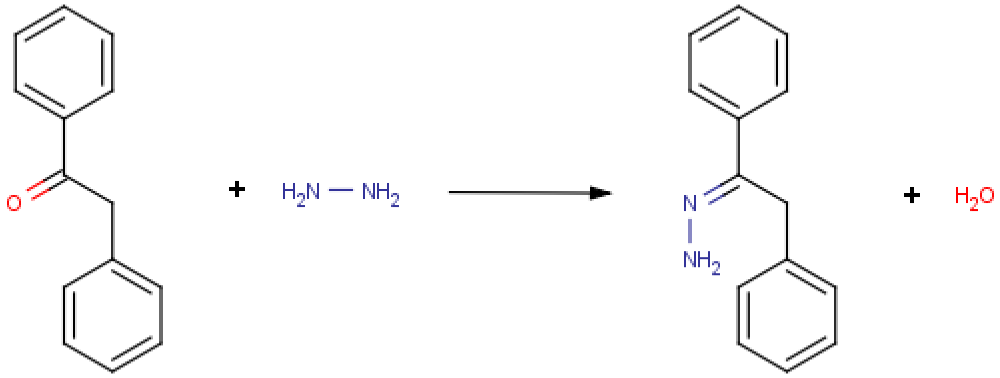

SMILES of the input:
O=C(CC1=CC=CC=C1)C1=CC=CC=C1.NN>>N\N=C(/CC1=CC=CC=C1)C1=CC=CC=C1.O

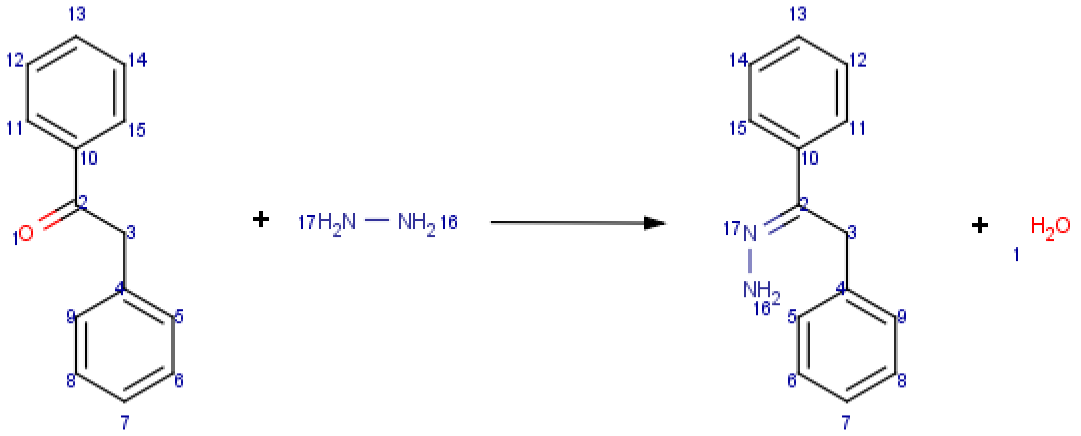

Correct mapped SMILES/SMARTS of the reaction:
[O:1]=[C:2]([CH2:3][C:4]1=[CH:5][CH:6]=[CH:7][CH:8]=[CH:9]1)[C:10]1=[CH:1
1][CH:12]=[CH:13][CH:14]=[CH:15]1.[NH2:16][NH2:17]>>[NH2:16]\[N:17]=[C:2]
(/[CH2:3][C:4]1=[CH:9][CH:8]=[CH:7][CH:6]=[CH:5]1)[C:10]1=[CH:15][CH:14]=
[CH:13][CH:12]=[CH:11]1.[OH2:1]

Correctness of the mapping: YES

Reaction no 24

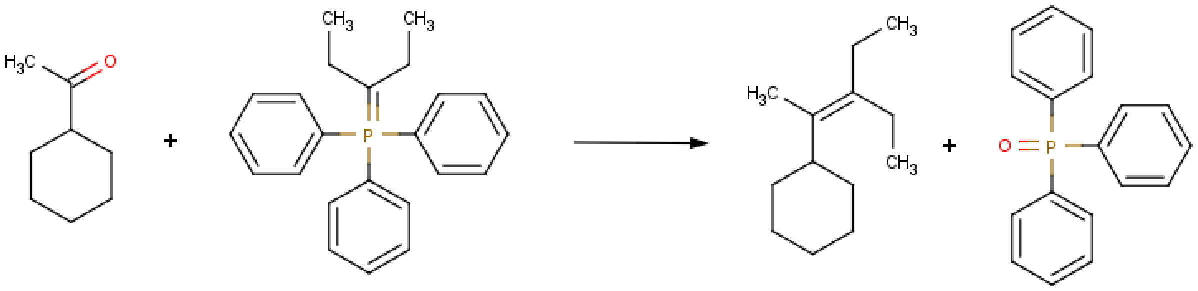

SMILES of the input:
CC(=O)C1CCCCC1.CCC(CC)=P(C1=CC=CC=C1)(C1=CC=CC=C1)C1=CC=CC=C1>>CCC(CC)=C(
C)C1CCCCC1.O=P(C1=CC=CC=C1)(C1=CC=CC=C1)C1=CC=CC=C1

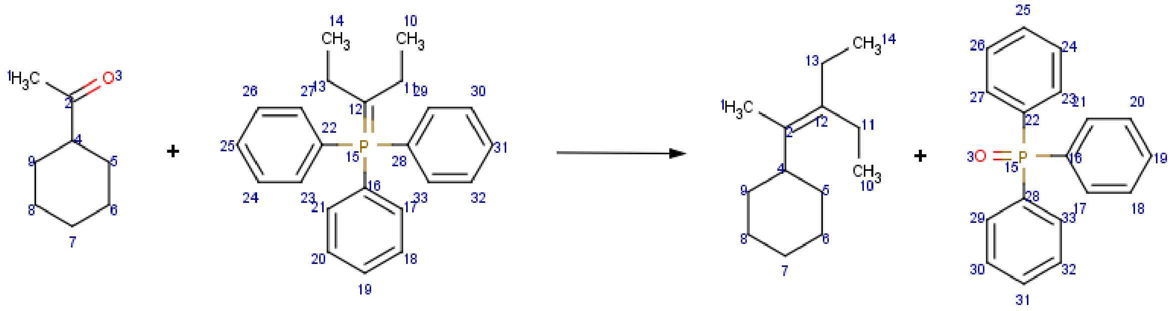

Correct mapped SMILES/SMARTS of the reaction:
[CH3:1][C:2](=[O:3])[CH:4]1[CH2:5][CH2:6][CH2:7][CH2:8][CH2:9]1.[CH3:10][
CH2:11][C:12]([CH2:13][CH3:14])=[P:15]([C:16]1=[CH:17][CH:18]=[CH:19][CH:
20]=[CH:21]1)([C:22]1=[CH:23][CH:24]=[CH:25][CH:26]=[CH:27]1)[C:28]1=[CH:
29][CH:30]=[CH:31][CH:32]=[CH:33]1>>[CH3:14][CH2:13][C:12]([CH2:11][CH3:1
0])=[C:2]([CH3:1])[CH:4]1[CH2:5][CH2:6][CH2:7][CH2:8][CH2:9]1.[O:3]=[P:15

]([C:28]1=[CH:33][CH:32]=[CH:31][CH:30]=[CH:29]1)([C:22]1=[CH:27][CH:26]=[CH:25][CH:24]=[CH:23]1)[C:16]1=[CH:21][CH:20]=[CH:19][CH:18]=[CH:17]1

Correctness of the mapping: YES

Reaction no 25

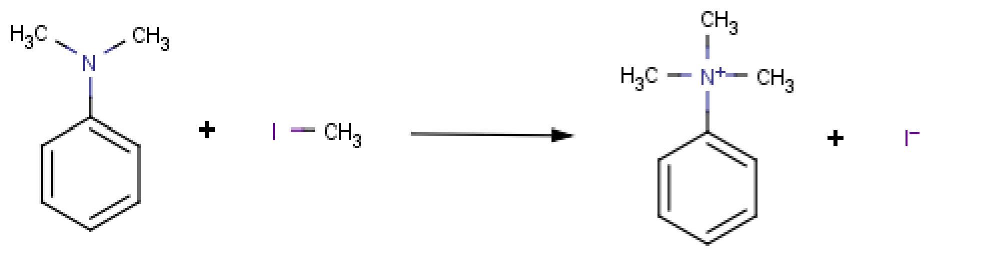

SMILES of the input:
CN(C)C1=CC=CC=C1.CI>>C[N+](C)(C)C1=CC=CC=C1.[I-]

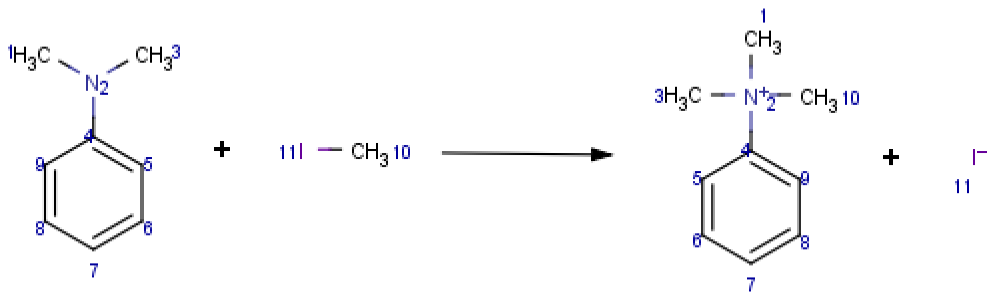

Correct mapped SMILES/SMARTS of the reaction:
[CH3:1][N:2]([CH3:3])[C:4]1=[CH:5][CH:6]=[CH:7][CH:8]=[CH:9]1.[CH3:10][I:11]>>[CH3:1][N+:2]([CH3:3])([CH3:10])[C:4]1=[CH:9][CH:8]=[CH:7][CH:6]=[CH:5]1.[I-:11]

Correctness of the mapping: YES

Reaction no 26

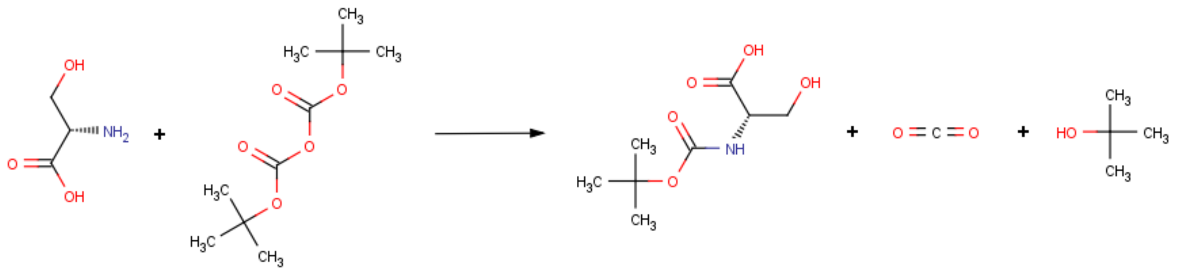

SMILES of the input:
N[C@@H](CO)C(O)=O.CC(C)(C)OC(=O)OC(=O)OC(C)(C)C>>CC(C)(C)OC(=O)N[C@@H](CO)C(O)=O.O=C=O.CC(C)(C)O

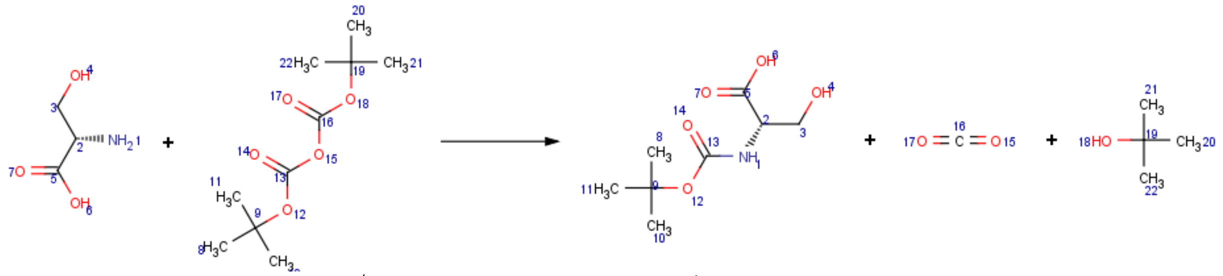

Correct mapped SMILES/SMARTS of the reaction:
[NH2:1][C@@H:2]([CH2:3][OH:4])[C:5]([OH:6])=[O:7].[CH3:8][C:9]([CH3:10])([CH3:11])[O:12][C:13](=[O:14])[O:15][C:16](=[O:17])[O:18][C:19]([CH3:20])([CH3:21])[CH3:22]>>[CH3:11][C:9]([CH3:10])([CH3:8])[O:12][C:13](=[O:14])[NH:1][C@@H:2]([CH2:3][OH:4])[C:5]([OH:6])=[O:7].[O:17]=[C:16]=[O:15].[CH3:22][C:19]([CH3:21])([CH3:20])[OH:18]

Correctness of the mapping: YES

Reaction no 27

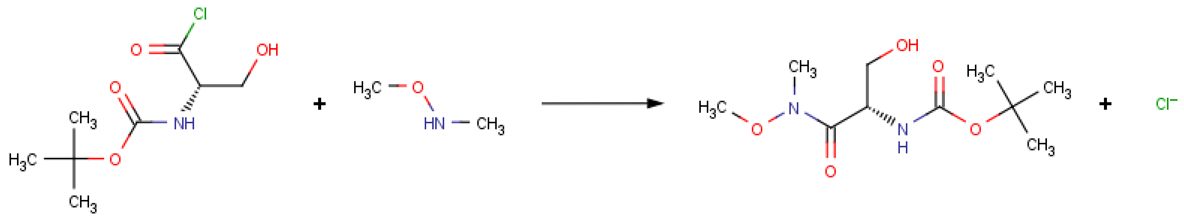

SMILES of the input:
CC(C)(C)OC(=O)N[C@@H](CO)C(Cl)=O.CNOC>>CON(C)C(=O)[C@H](CO)NC(=O)OC(C)(C)C.[Cl-]

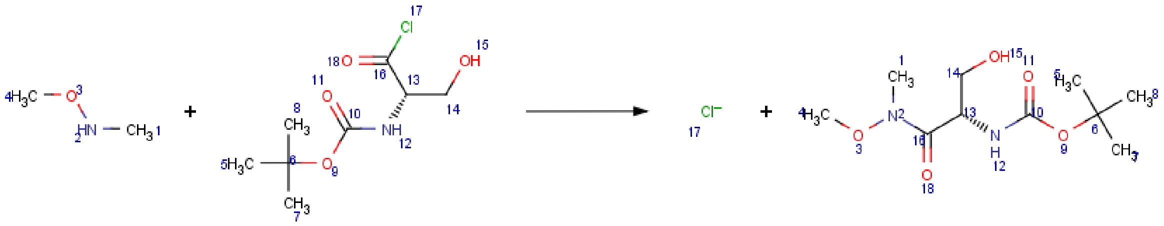

Correct mapped SMILES/SMARTS of the reaction:
[CH3:1][NH:2][O:3][CH3:4].[CH3:5][C:6]([CH3:7])([CH3:8])[O:9][C:10](=[O:11])[NH:12][C@@H:13]([CH2:14][OH:15])[C:16]([Cl:17])=[O:18]>>[Cl-:17].[CH3:4][O:3][N:2]([CH3:1])[C:16](=[O:18])[C@H:13]([CH2:14][OH:15])[NH:12][C:10](=[O:11])[O:9][C:6]([CH3:8])([CH3:7])[CH3:5]

Correctness of the mapping: YES

Reaction no 28

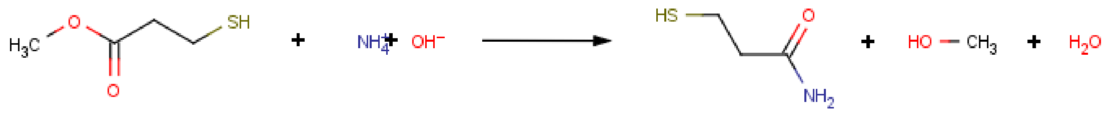

SMILES of the input:

COC(=O)CCS.[NH4+].[OH-]>>NC(=O)CCS.CO.O

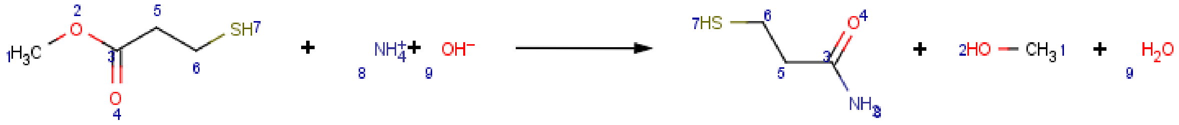

Correct mapped SMILES/SMARTS of the reaction:
[CH3:1][O:2][C:3](=[O:4])[CH2:5][CH2:6][SH:7].[NH4+:8].[OH-
:9]>>[NH2:8][C:3](=[O:4])[CH2:5][CH2:6][SH:7].[CH3:1][OH:2].[OH2:9]

Correctness of the mapping: YES

Reaction no 29

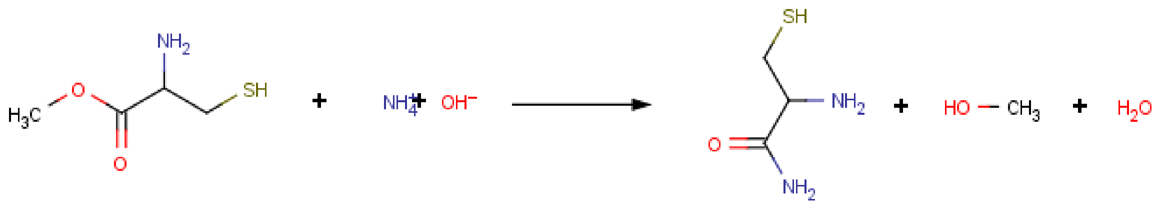

SMILES of the input:
COC(=O)C(N)CS.[NH4+].[OH-]>>NC(CS)C(N)=O.CO.O

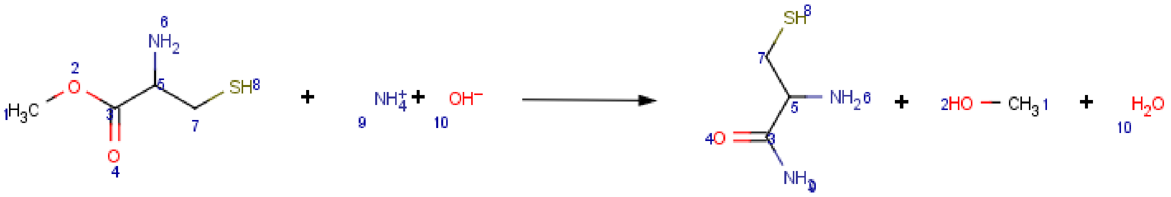

Correct mapped SMILES/SMARTS of the reaction:
[CH3:1][O:2][C:3](=[O:4])[CH:5]([NH2:6])[CH2:7][SH:8].[NH4+:9].[OH-
:10]>>[NH2:6][CH:5]([CH2:7][SH:8])[C:3]([NH2:9])=[O:4].[CH3:1][OH:2].[OH2
:10]

Correctness of the mapping: YES

Reaction no 30

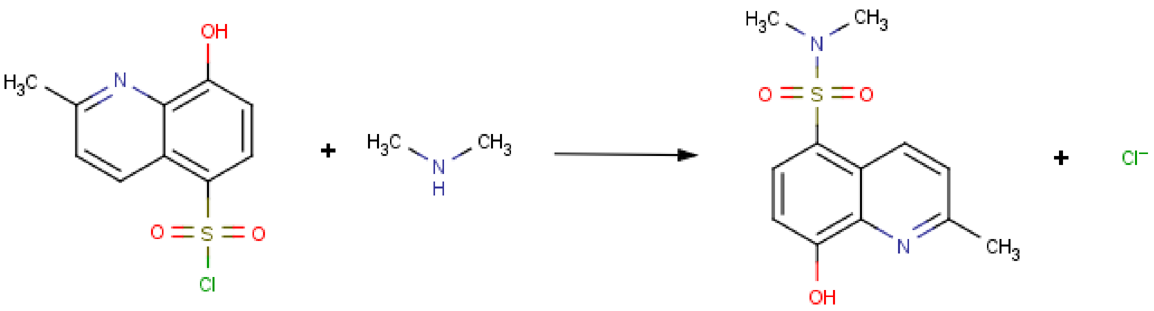

SMILES of the input:
CC1=NC2=C(O)C=CC(=C2C=C1)S(Cl)(=O)=O.CNC>>CN(C)S(=O)(=O)C1=C2C=CC(C)=NC2=
C(O)C=C1.[Cl-]

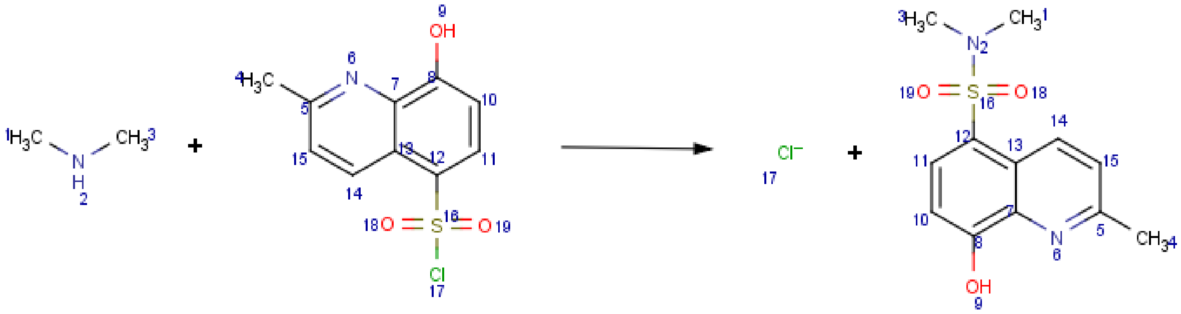

Correct mapped SMILES/SMARTS of the reaction:
[CH3:1][NH:2][CH3:3].[CH3:4][C:5]1=[N:6][C:7]2=[C:8]([OH:9])[CH:10]=[CH:11][C:12](=[C:13]2[CH:14]=[CH:15]1)[S:16]([Cl:17])(=[O:18])=[O:19]>>[Cl-:17].[CH3:3][N:2]([CH3:1])[S:16](=[O:19])(=[O:18])[C:12]1=[C:13]2[CH:14]=[CH:15][C:5]([CH3:4])=[N:6][C:7]2=[C:8]([OH:9])[CH:10]=[CH:11]1

Correctness of the mapping: YES

Reaction no 31

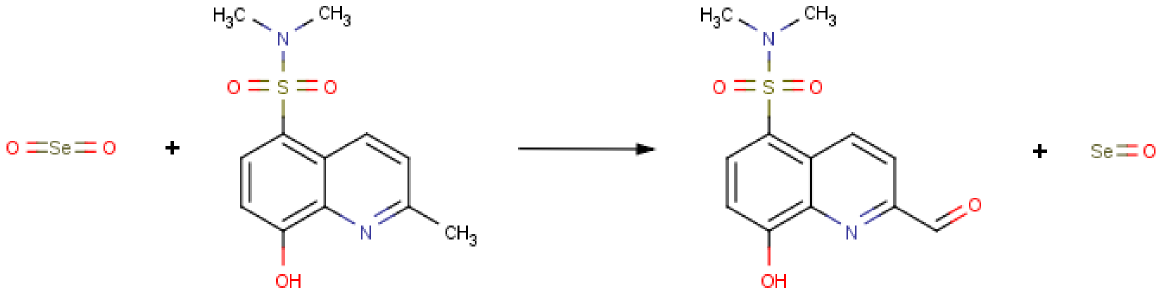

SMILES of the input:
O=[Se]=O.CN(C)S(=O)(=O)C1=C2C=CC(C)=NC2=C(O)C=C1>>CN(C)S(=O)(=O)C1=C2C=CC(C=O)=NC2=C(O)C=C1.O=[Se]

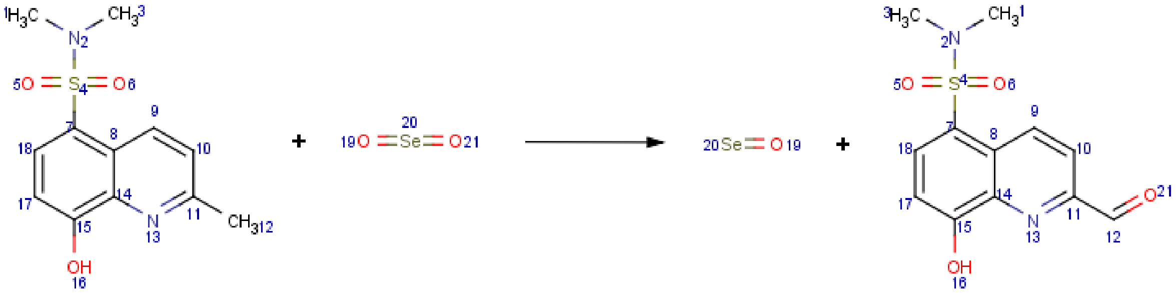

Correct mapped SMILES/SMARTS of the reaction:
[CH3:1][N:2]([CH3:3])[S:4](=[O:5])(=[O:6])[C:7]1=[C:8]2[CH:9]=[CH:10][C:11]([CH3:12])=[N:13][C:14]2=[C:15]([OH:16])[CH:17]=[CH:18]1.[O:19]=[Se:20]=[O:21]>>[O:19]=[Se:20].[CH3:3][N:2]([CH3:1])[S:4](=[O:5])(=[O:6])[C:7]1=[C:8]2[CH:9]=[CH:10][C:11]([CH:12]=[O:21])=[N:13][C:14]2=[C:15]([OH:16])[CH:17]=[CH:18]1

Correctness of the mapping: YES

Reaction no 32

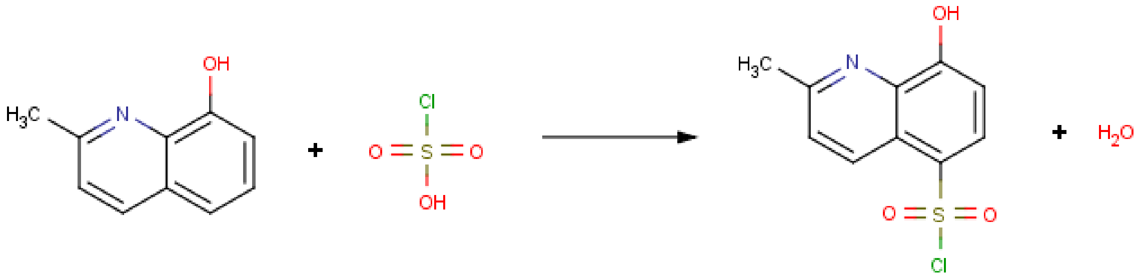

SMILES of the input:
CC1=NC2=C(O)C=CC=C2C=C1.OS(Cl)(=O)=O>>CC1=NC2=C(O)C=CC(=C2C=C1)S(Cl)(=O)=O.O

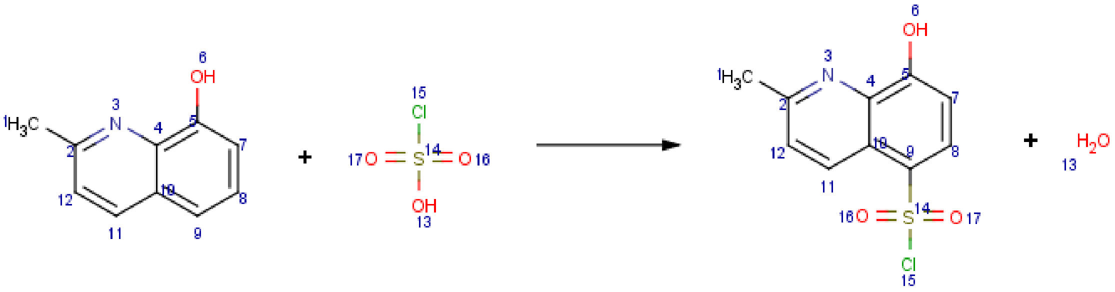

Correct mapped SMILES/SMARTS of the reaction:
[CH3:1][C:2]1=[N:3][C:4]2=[C:5]([OH:6])[CH:7]=[CH:8][CH:9]=[C:10]2[CH:11]=[CH:12]1.[OH:13][S:14]([Cl:15])(=[O:16])=[O:17]>>[CH3:1][C:2]1=[N:3][C:4]2=[C:5]([OH:6])[CH:7]=[CH:8][C:9](=[C:10]2[CH:11]=[CH:12]1)[S:14]([Cl:15])(=[O:16])=[O:17].[OH2:13]

Correctness of the mapping: YES

Reaction no 33

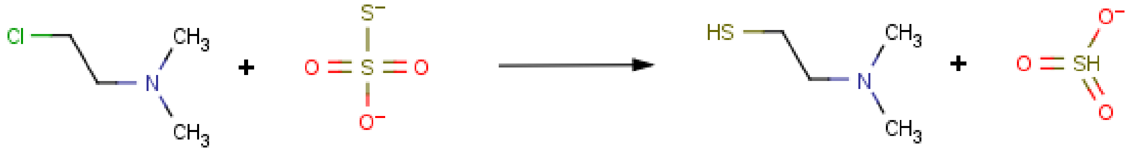

SMILES of the input:
CN(C)CCCl.[O-]S([S-])(=O)=O>>CN(C)CCS.[O-]S(=O)=O

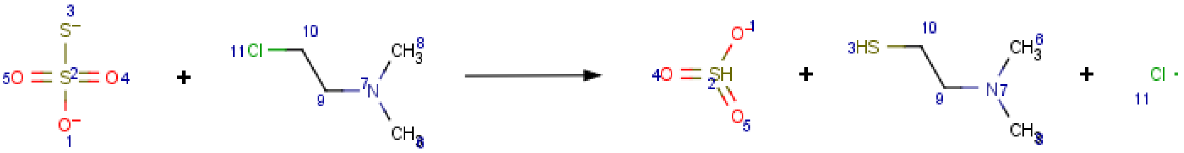

Correct mapped SMILES/SMARTS of the reaction:
[O-:1][S:2]([S-:3])(=[O:4])=[O:5].[CH3:6][N:7]([CH3:8])[CH2:9][CH2:10][Cl:11]>>[O-:1][SH:2](=[O:5])=[O:4].[CH3:8][N:7]([CH3:6])[CH2:9][CH2:10][SH:3].[Cl:11]

Correctness of the mapping: YES

Reaction no 34

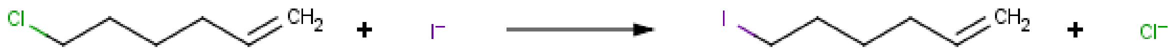

SMILES of the input:
ClCCCCC=C.[I-]>>ICCCCC=C.[Cl-]

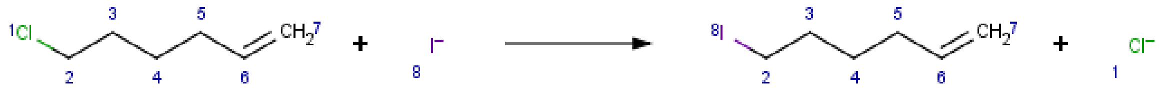

Correct mapped SMILES/SMARTS of the reaction:
[Cl:1][CH2:2][CH2:3][CH2:4][CH2:5][CH:6]=[CH2:7].[I-:8]>>[I:8][CH2:2][CH2:3][CH2:4][CH2:5][CH:6]=[CH2:7].[Cl-:1]

Correctness of the mapping: YES

Reaction no 35

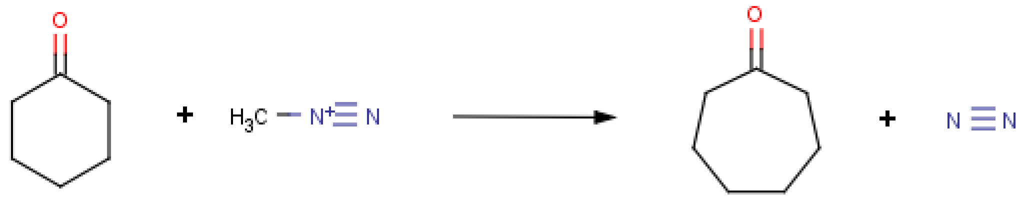

SMILES of the input:
O=C1CCCCC1.C[N+]#N>>O=C1CCCCCC1.N#N

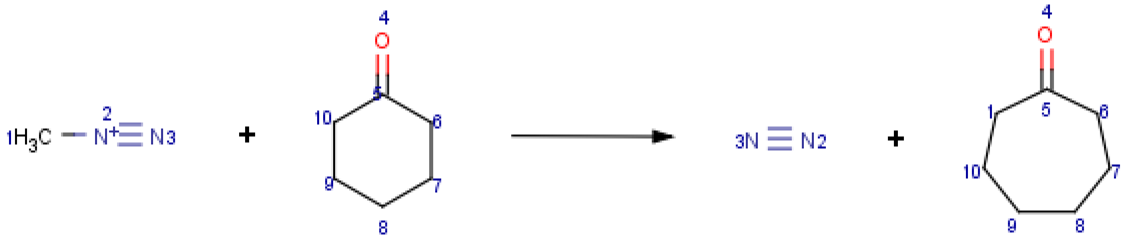

Correct mapped SMILES/SMARTS of the reaction:
[CH3:1][N+:2]#[N:3].[O:4]=[C:5]1[CH2:6][CH2:7][CH2:8][CH2:9][CH2:10]1>>[N:2]#[N:3].[O:4]=[C:5]1[CH2:6][CH2:7][CH2:8][CH2:9][CH2:10][CH2:1]1

Correctness of the mapping: NO

Reaction no 36

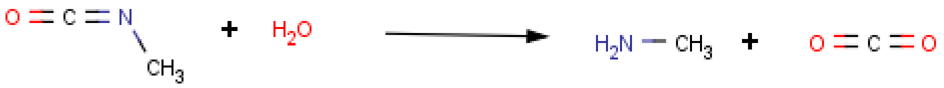

SMILES of the input:
CN=C=O.O>>CN.O=C=O

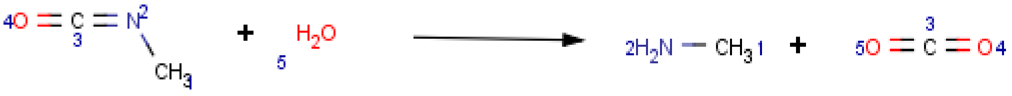

Correct mapped SMILES/SMARTS of the reaction:
[CH3:1][N:2]=[C:3]=[O:4].[OH2:5]>>[CH3:1][NH2:2].[O:5]=[C:3]=[O:4]

Correctness of the mapping: YES

Reaction no 37

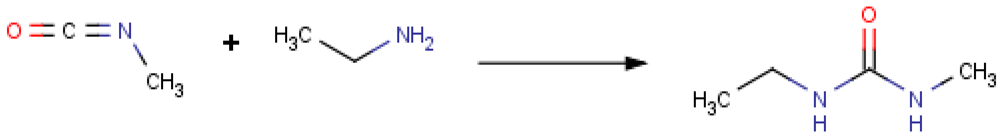

SMILES of the input:
CN=C=O.CCN>>CCNC(=O)NC

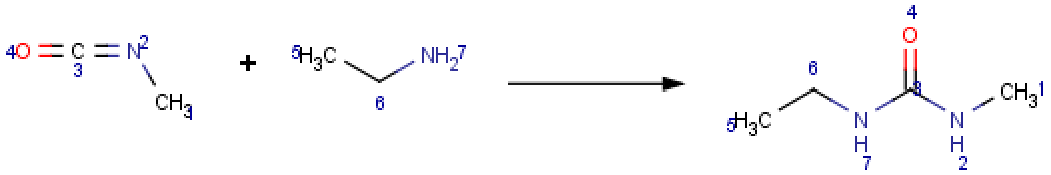

Correct mapped SMILES/SMARTS of the reaction:
[CH3:1][N:2]=[C:3]=[O:4].[CH3:5][CH2:6][NH2:7]>>[CH3:5][CH2:6][NH:7][C:3](=[O:4])[NH:2][CH3:1]

Correctness of the mapping: YES

Reaction no 38

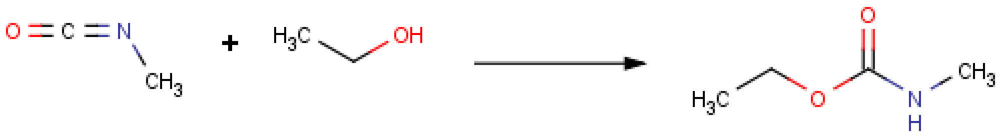

SMILES of the input:
CN=C=O.CCO>>CCOC(=O)NC

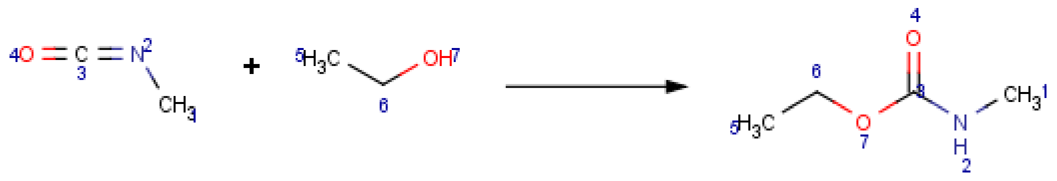

Correct mapped SMILES/SMARTS of the reaction:
[CH3:1][N:2]=[C:3]=[O:4].[CH3:5][CH2:6][OH:7]>>[CH3:5][CH2:6][O:7][C:3](=[O:4])[NH:2][CH3:1]

Correctness of the mapping: YES

Reaction no 39

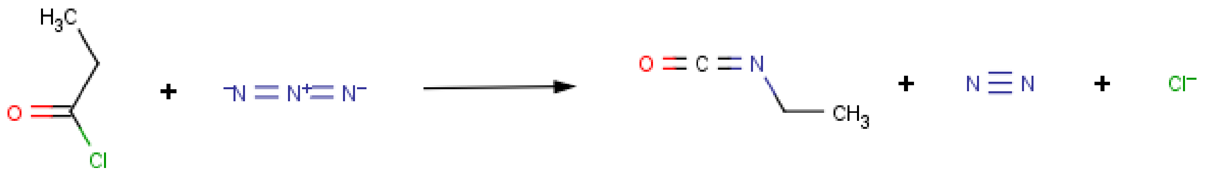

SMILES of the input:
CCC(Cl)=O.[N-]=[N+]=[N-]>>CCN=C=O.N#N.[Cl-]

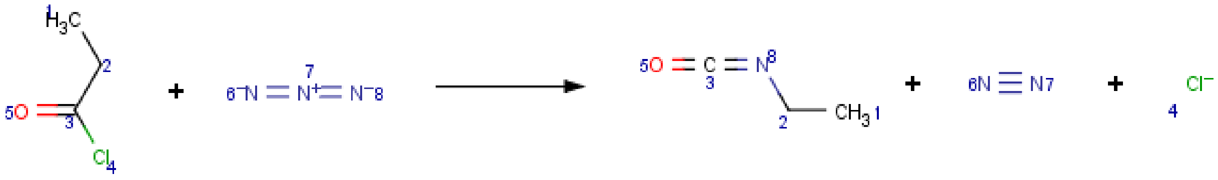

Correct mapped SMILES/SMARTS of the reaction:
[CH3:1][CH2:2][C:3]([Cl:4])=[O:5].[N-:6]=[N+:7]=[N-:8]>>[CH3:1][CH2:2][N:8]=[C:3]=[O:5].[N:7]#[N:6].[Cl-:4]

Correctness of the mapping: YES

Reaction no 40

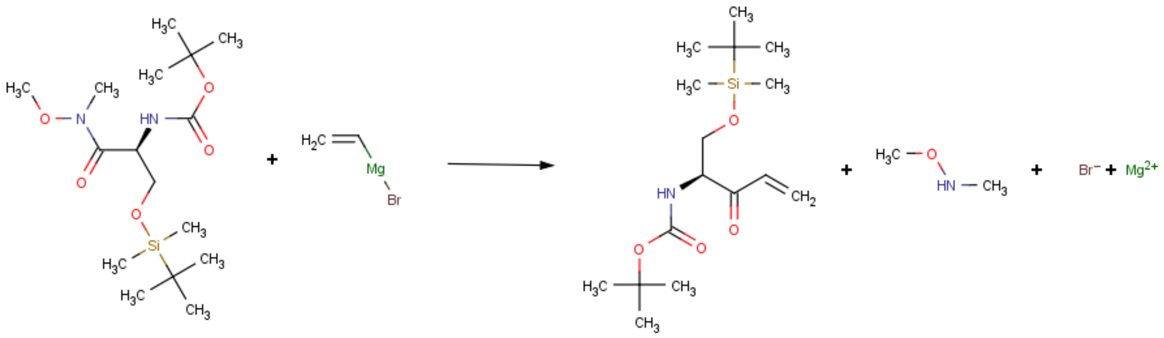

SMILES of the input:
CON(C)C(=O)[C@H](CO[Si](C)(C)C(C)(C)C)NC(=O)OC(C)(C)C.Br[Mg]C=C>>CC(C)(C)OC(=O)N[C@@H](CO[Si](C)(C)C(C)(C)C)C(=O)C=C.CNOC.[Br-].[Mg++]

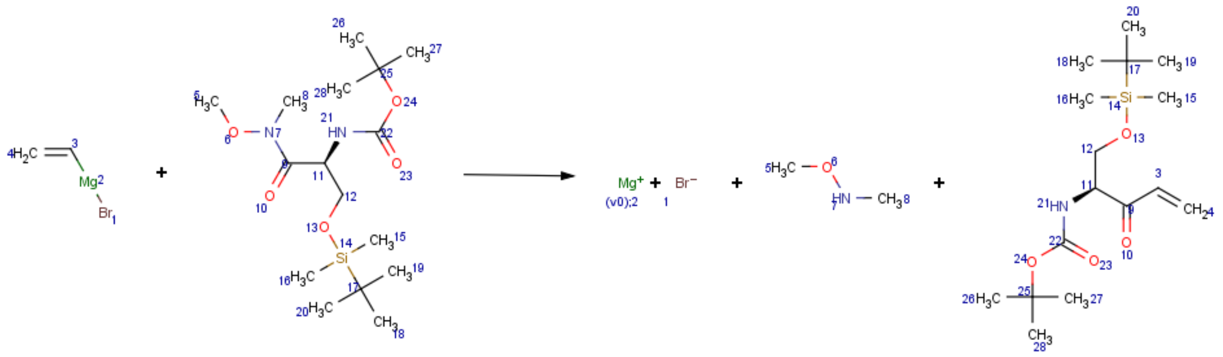

Correct mapped SMILES/SMARTS of the reaction:
[Br:1][Mg:2][CH:3]=[CH2:4].[CH3:5][O:6][N:7]([CH3:8])[C:9](=[O:10])[C@H:11]([CH2:12][O:13][Si:14]([CH3:15])([CH3:16])[C:17]([CH3:18])([CH3:19])[CH3:20])[NH:21][C:22](=[O:23])[O:24][C:25]([CH3:26])([CH3:27])[CH3:28]>>[Mg+:2].[Br-:1].[CH3:8][NH:7][O:6][CH3:5].[CH3:28][C:25]([CH3:27])([CH3:26])[O:24][C:22](=[O:23])[NH:21][C@@H:11]([CH2:12][O:13][Si:14]([CH3:15])([CH3:16])[C:17]([CH3:20])([CH3:19])[CH3:18])[C:9](=[O:10])[CH:3]=[CH2:4]

Correctness of the mapping: YES

Reaction no 41

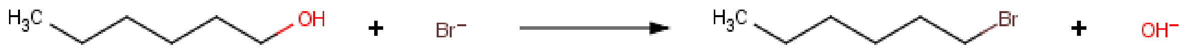

SMILES of the input:
CCCCCCO.[Br-]>>CCCCCCBr.[OH-]

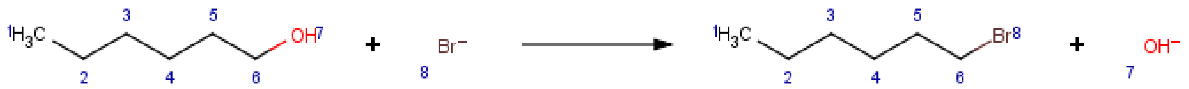

Correct mapped SMILES/SMARTS of the reaction:
[CH3:1][CH2:2][CH2:3][CH2:4][CH2:5][CH2:6][OH:7].[Br-:8]>>[CH3:1][CH2:2][CH2:3][CH2:4][CH2:5][CH2:6][Br:8].[OH-:7]

Correctness of the mapping: YES

Reaction no 42

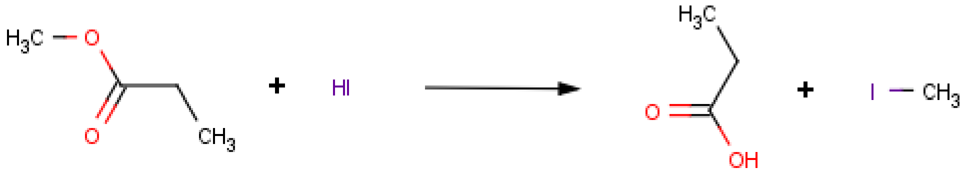

SMILES of the input:
CCC(=O)OC.I>>CCC(O)=O.CI

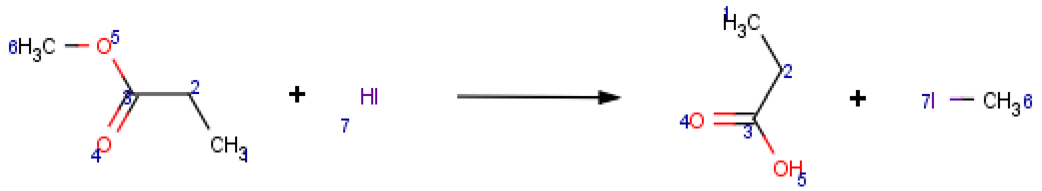

Correct mapped SMILES/SMARTS of the reaction:
[CH3:1][CH2:2][C:3](=[O:4])[O:5][CH3:6].[IH:7]>>[CH3:1][CH2:2][C:3]([OH:5
])=[O:4].[CH3:6][I:7]

Correctness of the mapping: YES

Reaction no 43

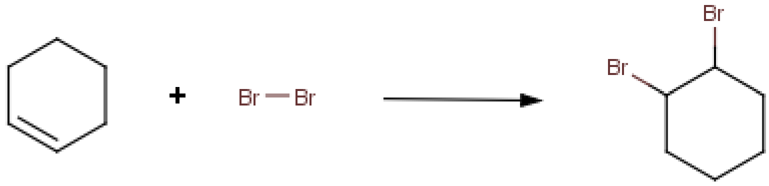

SMILES of the input:
C1CCC=CC1.BrBr>>BrC1CCCCC1Br

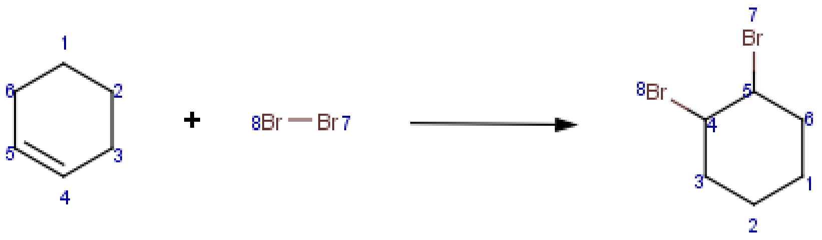

Correct mapped SMILES/SMARTS of the reaction:
[CH2:1]1[CH2:2][CH2:3][CH:4]=[CH:5][CH2:6]1.[Br:7][Br:8]>>[Br:7][CH:5]1[C
H2:6][CH2:1][CH2:2][CH2:3][CH:4]1[Br:8]

Correctness of the mapping: YES

Reaction no 44

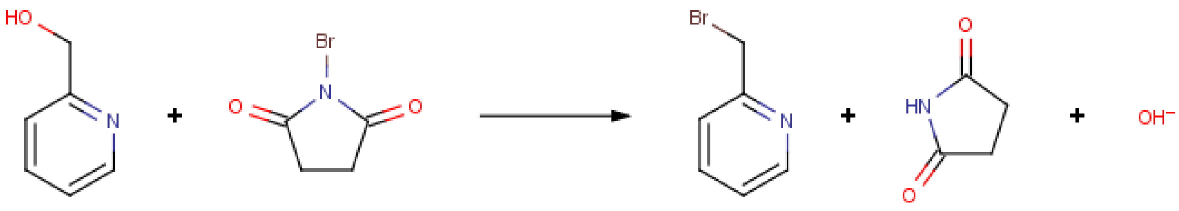

SMILES of the input:
OCC1=NC=CC=C1.BrN1C(=O)CCC1=O>>BrCC1=NC=CC=C1.O=C1CCC(=O)N1.[OH-]

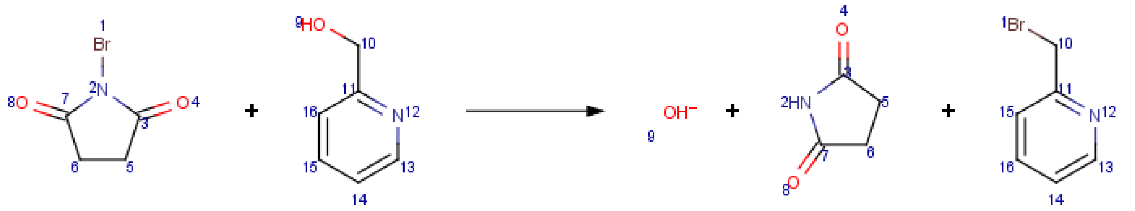

Correct mapped SMILES/SMARTS of the reaction:
[Br:1][N:2]1[C:3](=[O:4])[CH2:5][CH2:6][C:7]1=[O:8].[OH:9][CH2:10][C:11]1=[N:12][CH:13]=[CH:14][CH:15]=[CH:16]1>>[OH-:9].[O:4]=[C:3]1[CH2:5][CH2:6][C:7](=[O:8])[NH:2]1.[Br:1][CH2:10][C:11]1=[N:12][CH:13]=[CH:14][CH:16]=[CH:15]1

Correctness of the mapping: YES

Reaction no 45

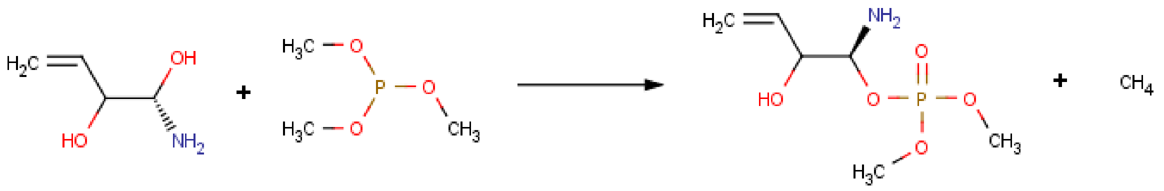

SMILES of the input:
N[C@@H](O)C(O)C=C.COP(OC)OC>>COP(=O)(OC)O[C@H](N)C(O)C=C.C

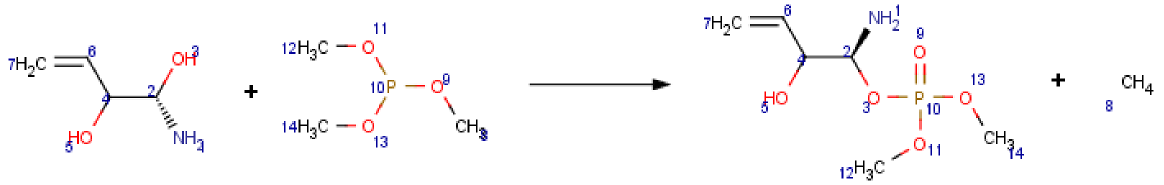

Correct mapped SMILES/SMARTS of the reaction:
[NH2:1][C@@H:2]([OH:3])[CH:4]([OH:5])[CH:6]=[CH2:7].[CH3:8][O:9][P:10]([O:11][CH3:12])[O:13][CH3:14]>>[CH3:14][O:13][P:10](=[O:9])([O:11][CH3:12])[O:3][C@H:2]([NH2:1])[CH:4]([OH:5])[CH:6]=[CH2:7].[CH4:8]

Correctness of the mapping: YES

Reaction no 46

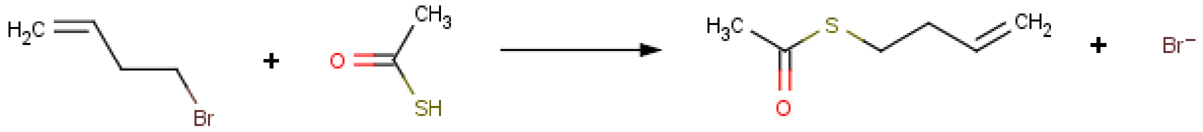

SMILES of the input:
BrCCC=C.CC(S)=O>>CC(=O)SCCC=C.[Br-]

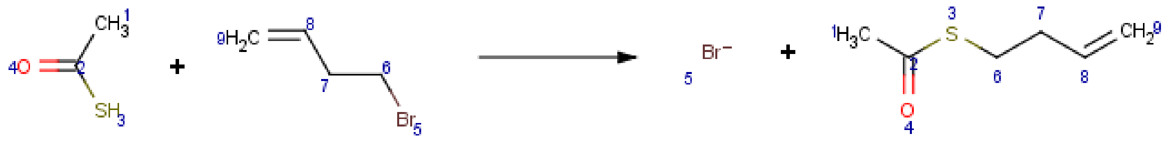

Correct mapped SMILES/SMARTS of the reaction:
[CH3:1][C:2]([SH:3])=[O:4].[Br:5][CH2:6][CH2:7][CH:8]=[CH2:9]>>[Br-:5].[CH3:1][C:2](=[O:4])[S:3][CH2:6][CH2:7][CH:8]=[CH2:9]

Correctness of the mapping: YES

Reaction no 47

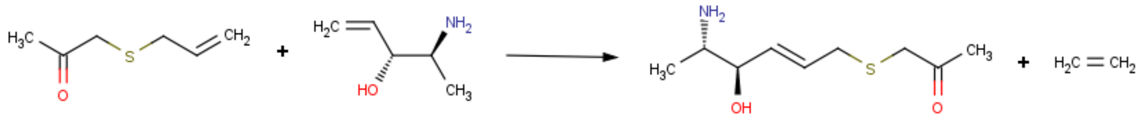

SMILES of the input:
CC(=O)CSCC=C.C[C@H](N)[C@H](O)C=C>>C[C@H](N)[C@H](O)\C=C\CSCC(C)=O.C=C

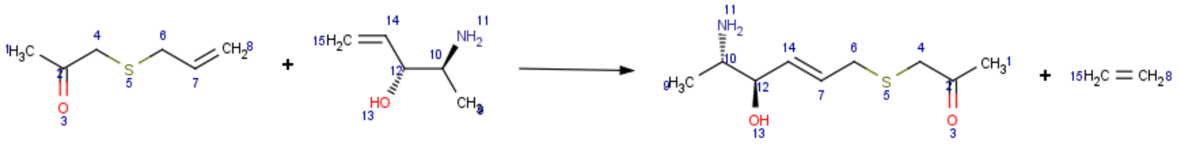

Correct mapped SMILES/SMARTS of the reaction:
[CH3:1][C:2](=[O:3])[CH2:4][S:5][CH2:6][CH:7]=[CH2:8].[CH3:9][C@H:10]([NH2:11])[C@H:12]([OH:13])[CH:14]=[CH2:15]>>[CH3:9][C@H:10]([NH2:11])[C@H:12]([OH:13])\[CH:14]=[CH:7]\[CH2:6][S:5][CH2:4][C:2]([CH3:1])=[O:3].[CH2:8]=[CH2:15]

Correctness of the mapping: YES

Reaction no 48

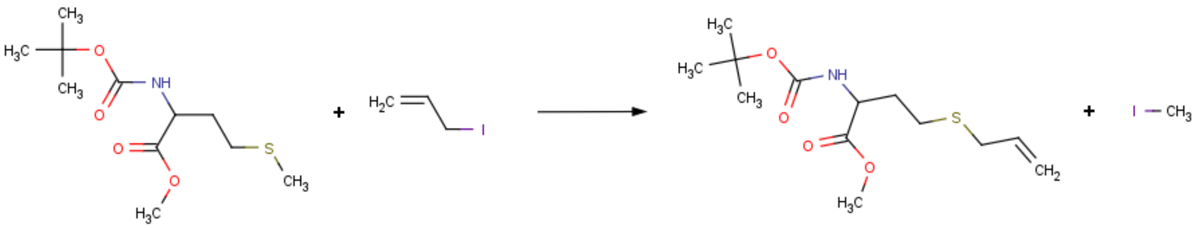

SMILES of the input:
COC(=O)C(CCSC)NC(=O)OC(C)(C)C.ICC=C>>COC(=O)C(CCSCC=C)NC(=O)OC(C)(C)C.CI

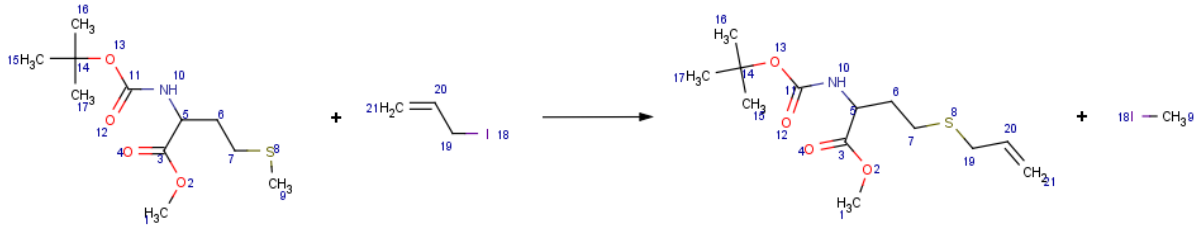

Correct mapped SMILES/SMARTS of the reaction:
[CH3:1][O:2][C:3](=[O:4])[CH:5]([CH2:6][CH2:7][S:8][CH3:9])[NH:10][C:11](
=[O:12])[O:13][C:14]([CH3:15])([CH3:16])[CH3:17].[I:18][CH2:19][CH:20]=[C
H2:21]>>[CH3:1][O:2][C:3](=[O:4])[CH:5]([CH2:6][CH2:7][S:8][CH2:19][CH:20
]=[CH2:21])[NH:10][C:11](=[O:12])[O:13][C:14]([CH3:17])([CH3:16])[CH3:15]
.[CH3:9][I:18]

Correctness of the mapping: NO

Reaction no 49

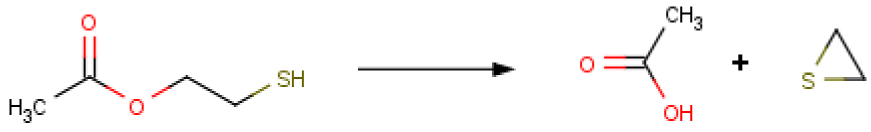

SMILES of the input:
CC(=O)OCCS>>CC(O)=O.C1CS1

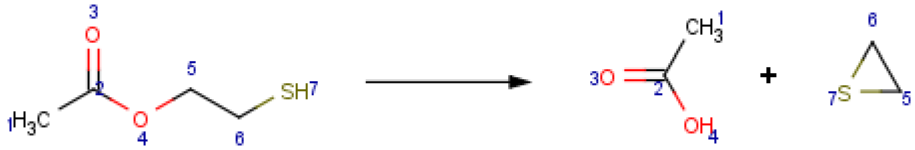

Correct mapped SMILES/SMARTS of the reaction:
[CH3:1][C:2](=[O:3])[O:4][CH2:5][CH2:6][SH:7]>>[CH3:1][C:2]([OH:4])=[O:3]
.[CH2:6]1[CH2:5][S:7]1

Correctness of the mapping: YES

Reaction no 50

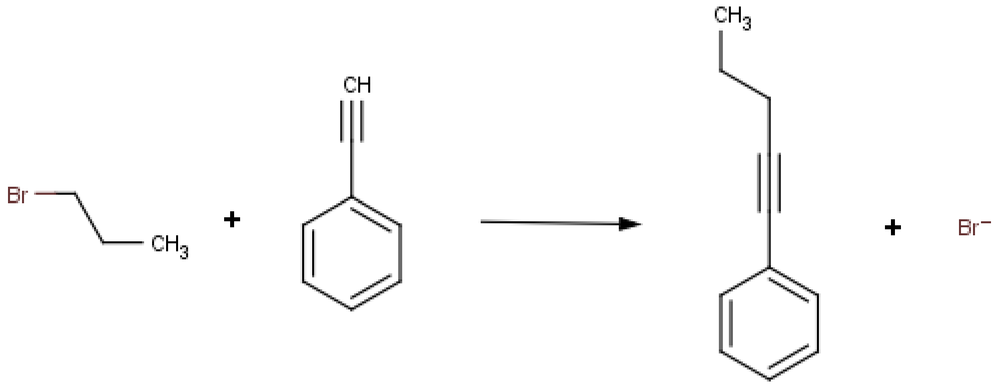

SMILES of the input:

CCCBr.C#CC1=CC=CC=C1>>CCCC#CC1=CC=CC=C1.[Br-]

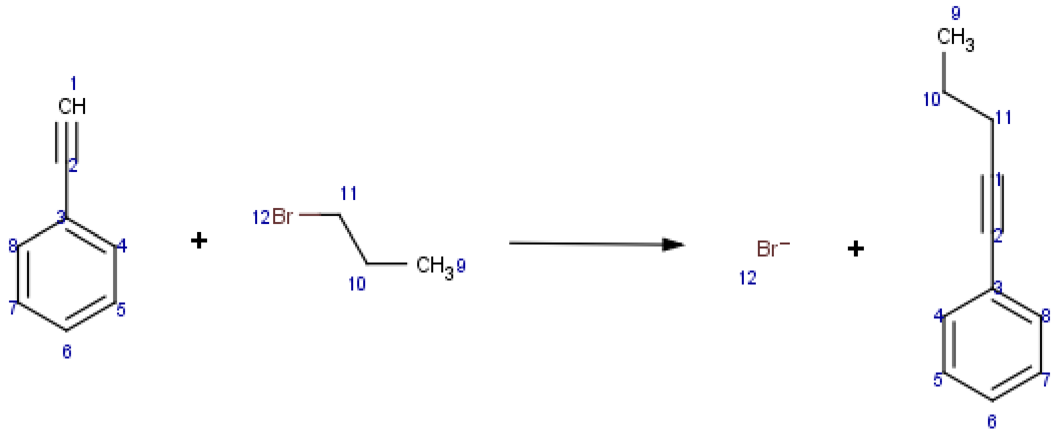

Correct mapped SMILES/SMARTS of the reaction:
[CH:1]#[C:2][C:3]1=[CH:4][CH:5]=[CH:6][CH:7]=[CH:8]1.[CH3:9][CH2:10][CH2:11][Br:12]>>[Br-:12].[CH3:9][CH2:10][CH2:11][C:1]#[C:2][C:3]1=[CH:8][CH:7]=[CH:6][CH:5]=[CH:4]1

Correctness of the mapping: YES

Reaction no 51

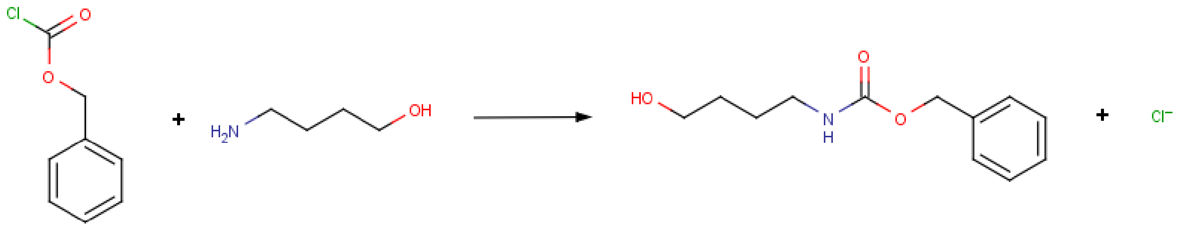

SMILES of the input:
ClC(=O)OCC1=CC=CC=C1.NCCCCO>>OCCCCNC(=O)OCC1=CC=CC=C1.[Cl-]

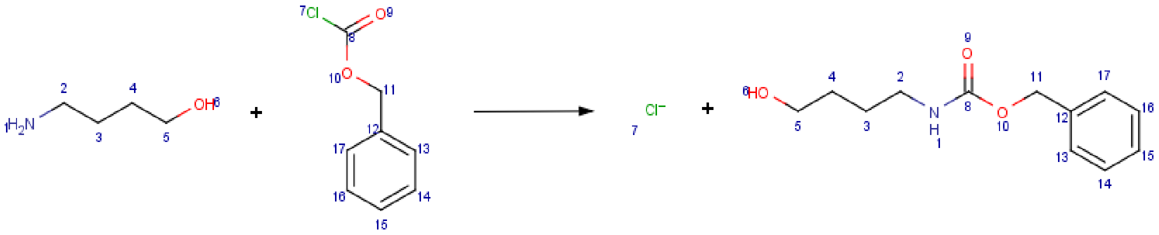

Correct mapped SMILES/SMARTS of the reaction:
[NH2:1][CH2:2][CH2:3][CH2:4][CH2:5][OH:6].[Cl:7][C:8](=[O:9])[O:10][CH2:11][C:12]1=[CH:13][CH:14]=[CH:15][CH:16]=[CH:17]1>>[Cl-:7].[OH:6][CH2:5][CH2:4][CH2:3][CH2:2][NH:1][C:8](=[O:9])[O:10][CH2:11][C:12]1=[CH:17][CH:16]=[CH:15][CH:14]=[CH:13]1

Correctness of the mapping: YES

Reaction no 52

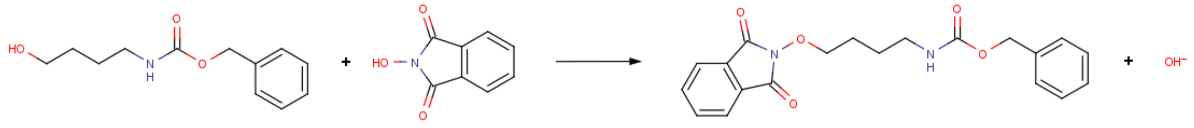

SMILES of the input:
OCCCCNC(=O)OCC1=CC=CC=C1.ON1C(=O)C2=C(C=CC=C2)C1=O>>O=C(NCCCCON1C(=O)C2=C(C=CC=C2)C1=O)OCC1=CC=CC=C1.[OH-]

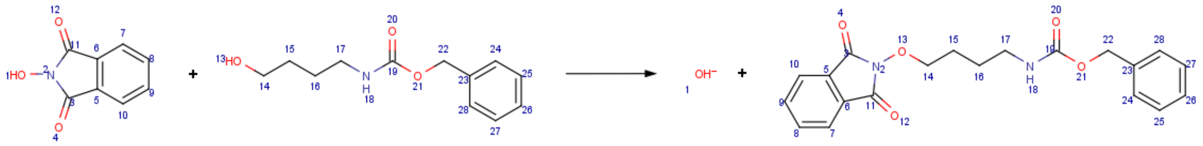

Correct mapped SMILES/SMARTS of the reaction:
[OH:1][N:2]1[C:3](=[O:4])[C:5]2=[C:6]([CH:7]=[CH:8][CH:9]=[CH:10]2)[C:11]1=[O:12].[OH:13][CH2:14][CH2:15][CH2:16][CH2:17][NH:18][C:19](=[O:20])[O:21][CH2:22][C:23]1=[CH:24][CH:25]=[CH:26][CH:27]=[CH:28]1>>[OH-:1].[O:20]=[C:19]([NH:18][CH2:17][CH2:16][CH2:15][CH2:14][O:13][N:2]1[C:3](=[O:4])[C:5]2=[C:6]([CH:7]=[CH:8][CH:9]=[CH:10]2)[C:11]1=[O:12])[O:21][CH2:22][C:23]1=[CH:28][CH:27]=[CH:26][CH:25]=[CH:24]1

Correctness of the mapping: YES

Reaction no 53

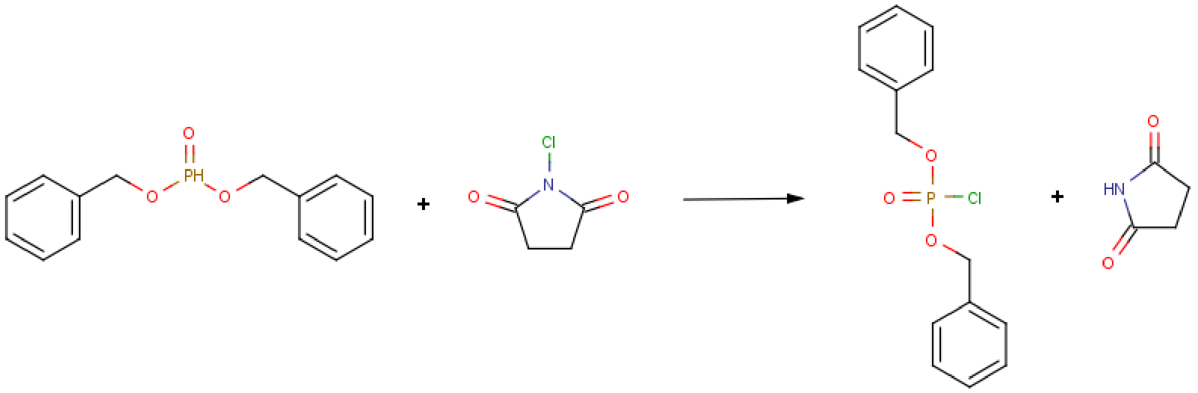

SMILES of the input:
O=P(OCC1=CC=CC=C1)OCC1=CC=CC=C1.ClN1C(=O)CCC1=O>>ClP(=O)(OCC1=CC=CC=C1)OCC1=CC=CC=C1.O=C1CCC(=O)N1

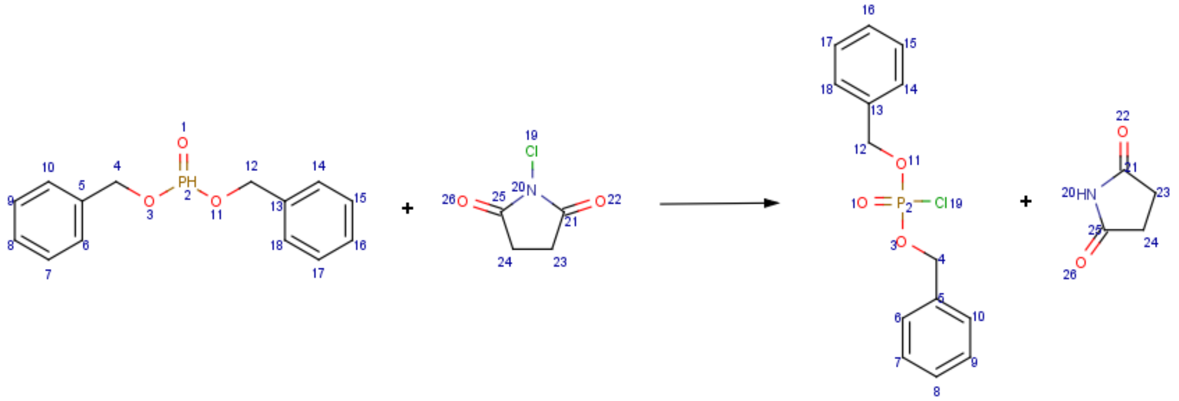

Correct mapped SMILES/SMARTS of the reaction:
[O:1]=[PH:2]([O:3][CH2:4][C:5]1=[CH:6][CH:7]=[CH:8][CH:9]=[CH:10]1)[O:11][CH2:12][C:13]1=[CH:14][CH:15]=[CH:16][CH:17]=[CH:18]1.[Cl:19][N:20]1[C:21](=[O:22])[CH2:23][CH2:24][C:25]1=[O:26]>>[Cl:19][P:2](=[O:1])([O:3][CH2:4][C:5]1=[CH:10][CH:9]=[CH:8][CH:7]=[CH:6]1)[O:11][CH2:12][C:13]1=[CH:18][CH:17]=[CH:16][CH:15]=[CH:14]1.[O:22]=[C:21]1[CH2:23][CH2:24][C:25](=[O:26])[NH:20]1

Correctness of the mapping: YES

Reaction no 54

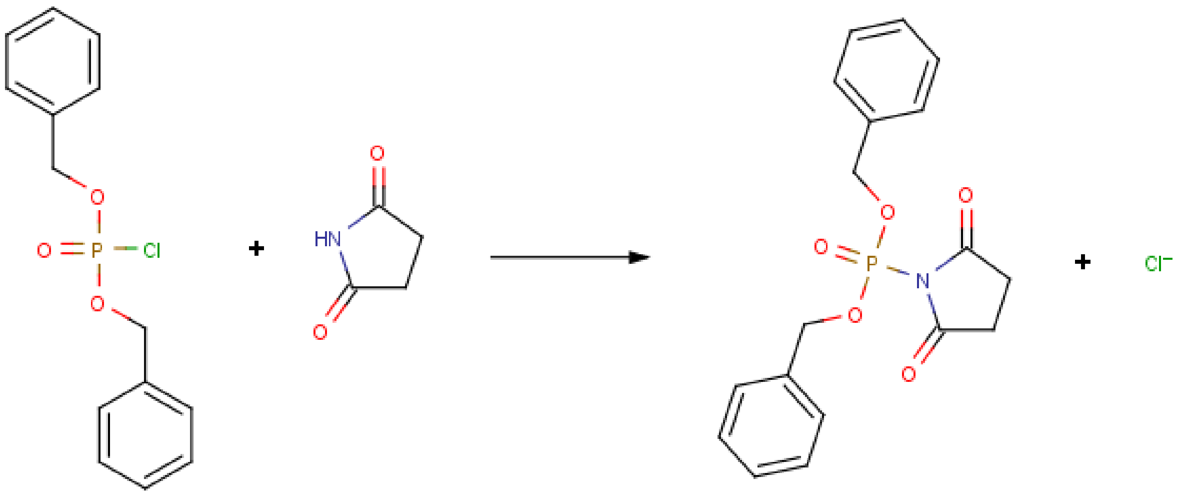

SMILES of the input:
ClP(=O)(OCC1=CC=CC=C1)OCC1=CC=CC=C1.O=C1CCC(=O)N1>>O=C1CCC(=O)N1P(=O)(OCC1=CC=CC=C1)OCC1=CC=CC=C1.[Cl-]

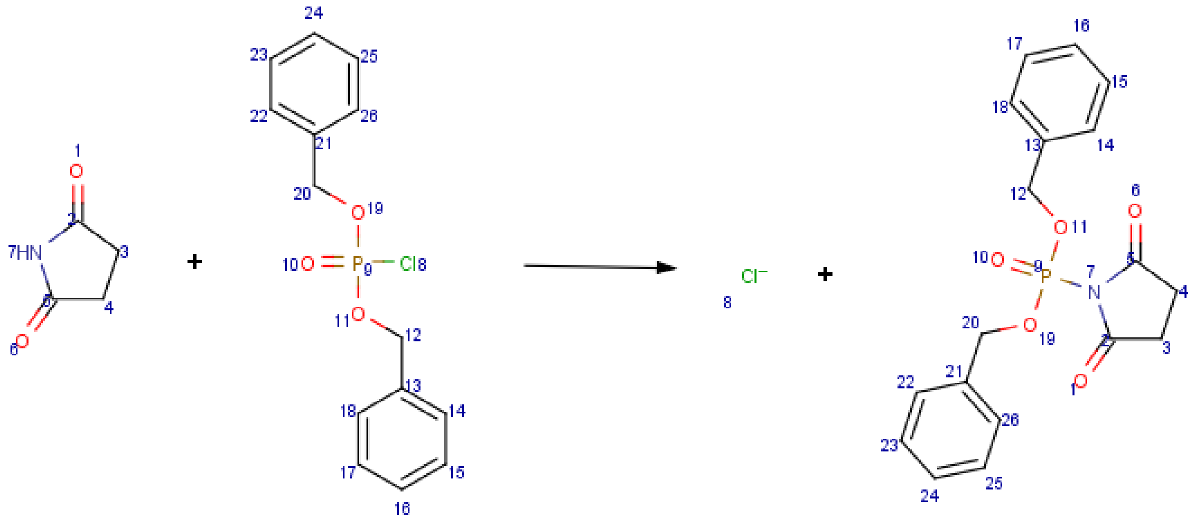

Correct mapped SMILES/SMARTS of the reaction:
[O:1]=[C:2]1[CH2:3][CH2:4][C:5](=[O:6])[NH:7]1.[Cl:8][P:9](=[O:10])([O:11][CH2:12][C:13]1=[CH:14][CH:15]=[CH:16][CH:17]=[CH:18]1)[O:19][CH2:20][C:21]1=[CH:22][CH:23]=[CH:24][CH:25]=[CH:26]1>>[Cl-:8].[O:6]=[C:5]1[CH2:4][CH2:3][C:2](=[O:1])[N:7]1[P:9](=[O:10])([O:19][CH2:20][C:21]1=[CH:26][CH:25]=[CH:24][CH:23]=[CH:22]1)[O:11][CH2:12][C:13]1=[CH:18][CH:17]=[CH:16][CH:15]=[CH:14]1

Correctness of the mapping: YES

Reaction no 55

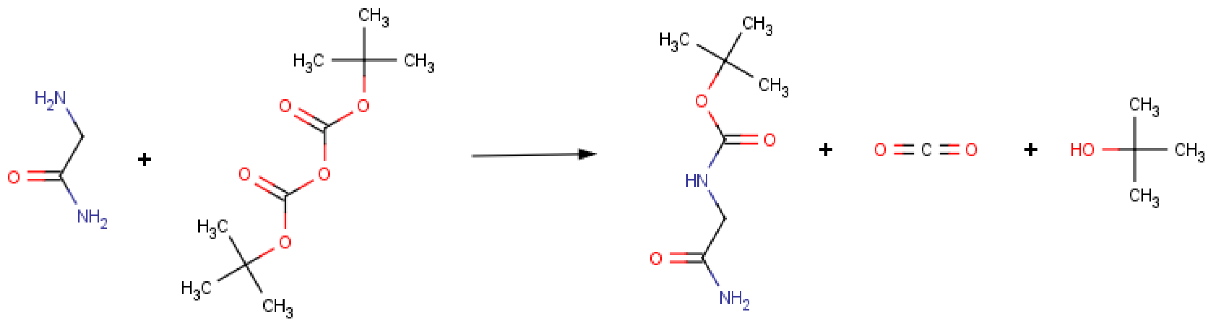

SMILES of the input:
NCC(N)=O.CC(C)(C)OC(=O)OC(=O)OC(C)(C)C>>CC(C)(C)OC(=O)NCC(N)=O.O=C=O.CC(C)(C)O

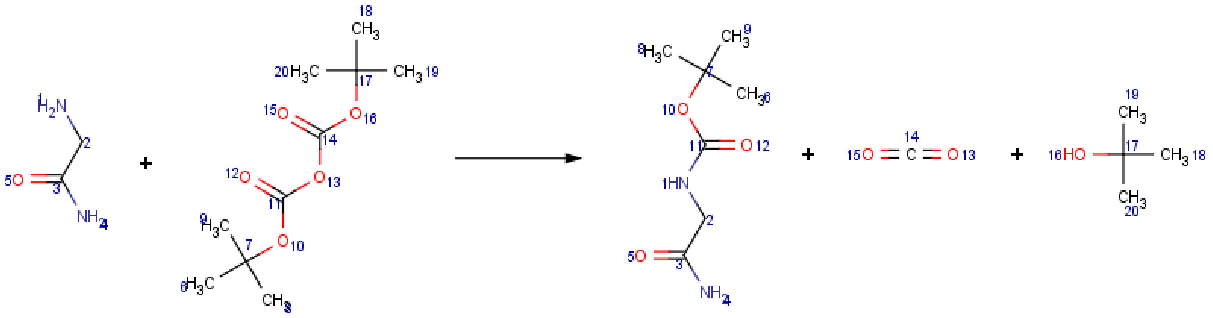

Correct mapped SMILES/SMARTS of the reaction:
[NH2:1][CH2:2][C:3]([NH2:4])=[O:5].[CH3:6][C:7]([CH3:8])([CH3:9])[O:10][C:11](=[O:12])[O:13][C:14](=[O:15])[O:16][C:17]([CH3:18])([CH3:19])[CH3:20]>>[CH3:9][C:7]([CH3:8])([CH3:6])[O:10][C:11](=[O:12])[NH:1][CH2:2][C:3]([NH2:4])=[O:5].[O:15]=[C:14]=[O:13].[CH3:20][C:17]([CH3:19])([CH3:18])[OH:16]

Correctness of the mapping: YES

Reaction no 56

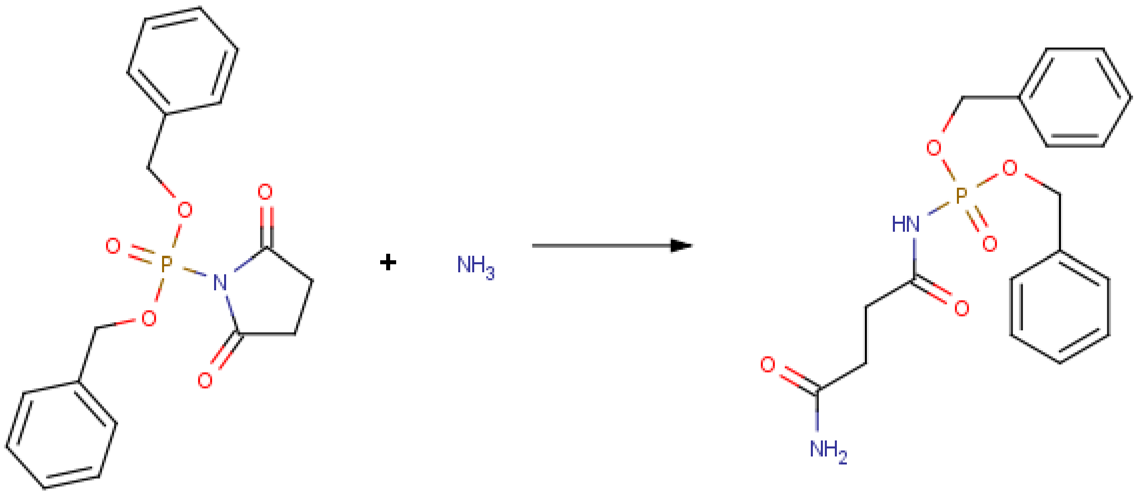

SMILES of the input:
O=C1CCC(=O)N1P(=O)(OCC1=CC=CC=C1)OCC1=CC=CC=C1.N>>NC(=O)CCC(=O)NP(=O)(OCC1=CC=CC=C1)OCC1=CC=CC=C1

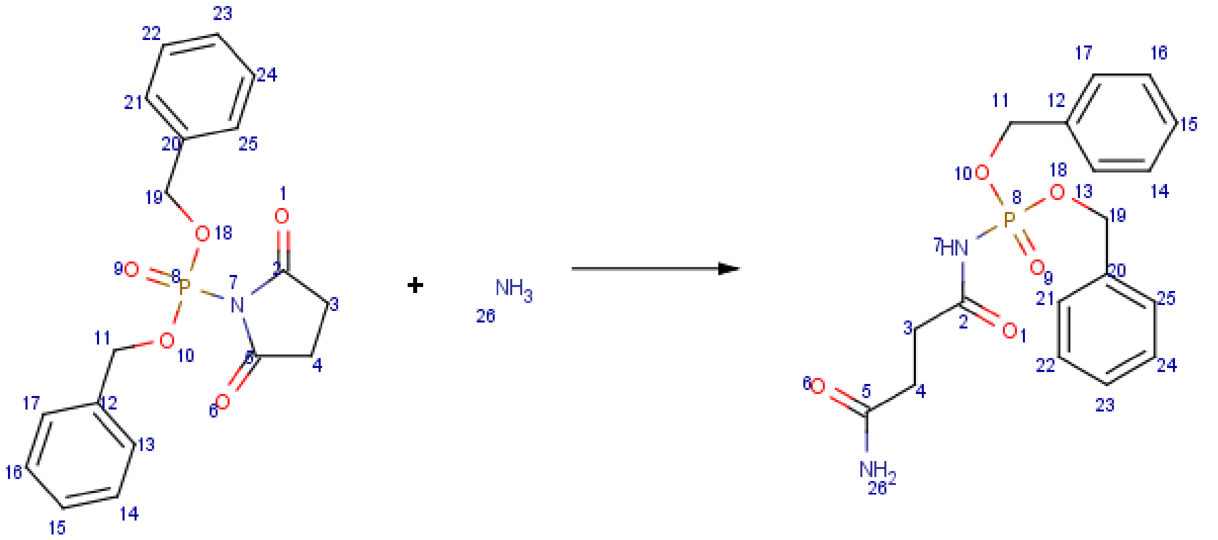

Correct mapped SMILES/SMARTS of the reaction:
[O:1]=[C:2]1[CH2:3][CH2:4][C:5](=[O:6])[N:7]1[P:8](=[O:9])([O:10][CH2:11][C:12]1=[CH:13][CH:14]=[CH:15][CH:16]=[CH:17]1)[O:18][CH2:19][C:20]1=[CH:21][CH:22]=[CH:23][CH:24]=[CH:25]1.[NH3:26]>>[NH2:26][C:5](=[O:6])[CH2:4][CH2:3][C:2](=[O:1])[NH:7][P:8](=[O:9])([O:18][CH2:19][C:20]1=[CH:25][CH:24]=[CH:23][CH:22]=[CH:21]1)[O:10][CH2:11][C:12]1=[CH:17][CH:16]=[CH:15][CH:14]=[CH:13]1

Correctness of the mapping: YES

Reaction no 57

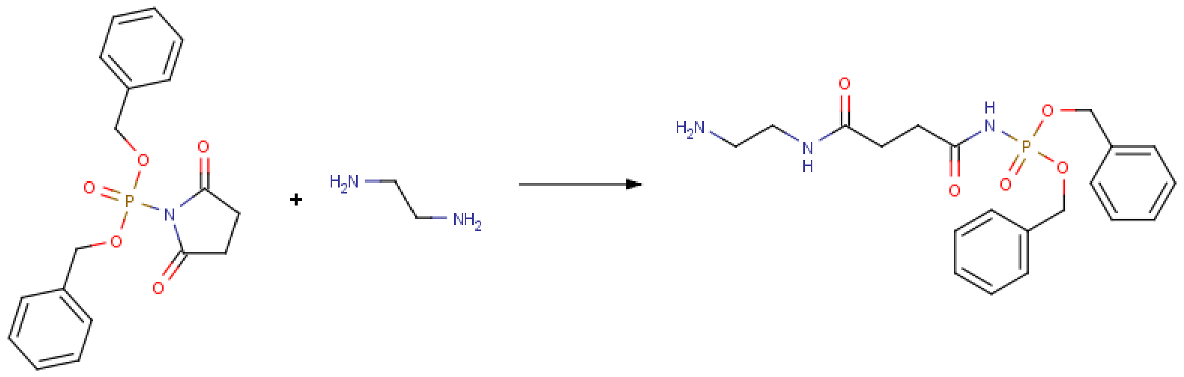

SMILES of the input:
O=C1CCC(=O)N1P(=O)(OCC1=CC=CC=C1)OCC1=CC=CC=C1.NCCN>>NCCNC(=O)CCC(=O)NP(=O)(OCC1=CC=CC=C1)OCC1=CC=CC=C1

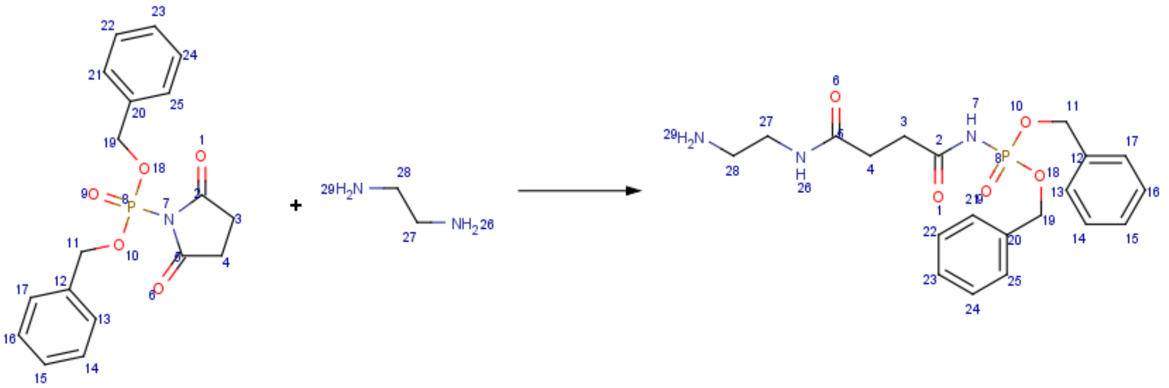

Correct mapped SMILES/SMARTS of the reaction:
[O:1]=[C:2]1[CH2:3][CH2:4][C:5](=[O:6])[N:7]1[P:8](=[O:9])([O:10][CH2:11][C:12]1=[CH:13][CH:14]=[CH:15][CH:16]=[CH:17]1)[O:18][CH2:19][C:20]1=[CH:21][CH:22]=[CH:23][CH:24]=[CH:25]1.[NH2:26][CH2:27][CH2:28][NH2:29]>>[NH2:29][CH2:28][CH2:27][NH:26][C:5](=[O:6])[CH2:4][CH2:3][C:2](=[O:1])[NH:7][P:8](=[O:9])([O:18][CH2:19][C:20]1=[CH:25][CH:24]=[CH:23][CH:22]=[CH:21]1)[O:10][CH2:11][C:12]1=[CH:17][CH:16]=[CH:15][CH:14]=[CH:13]1

Correctness of the mapping: YES

Reaction no 58

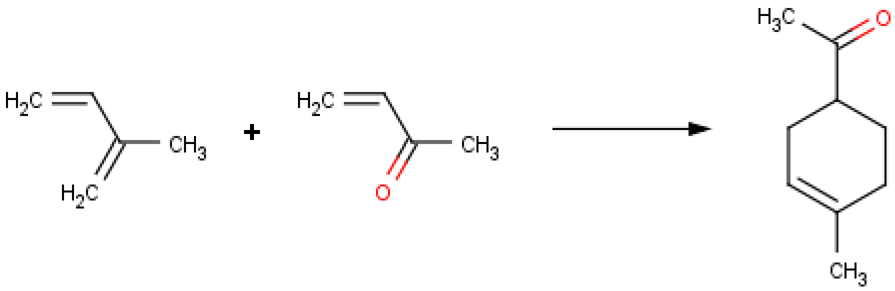

SMILES of the input:
CC(=C)C=C.CC(=O)C=C>>CC(=O)C1CCC(C)=CC1

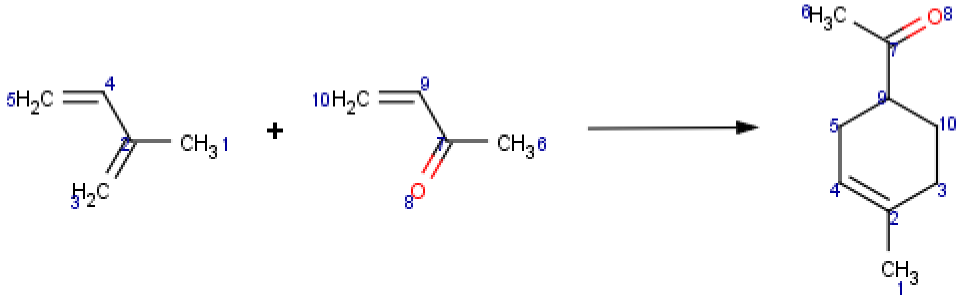

Correct mapped SMILES/SMARTS of the reaction:
[CH3:1][C:2](=[CH2:3])[CH:4]=[CH2:5].[CH3:6][C:7](=[O:8])[CH:9]=[CH2:10]>
>[CH3:6][C:7](=[O:8])[CH:9]1[CH2:10][CH2:3][C:2]([CH3:1])=[CH:4][CH2:5]1

Correctness of the mapping: YES

Reaction no 59

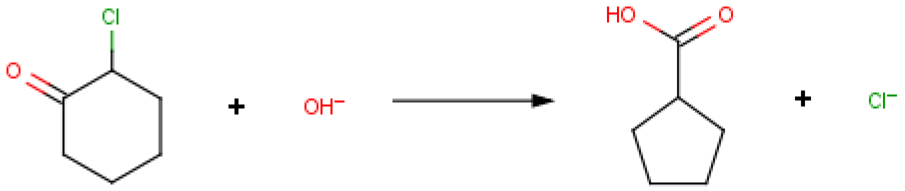

SMILES of the input:
ClC1CCCCC1=O.[OH-]>>OC(=O)C1CCCC1.[Cl-]

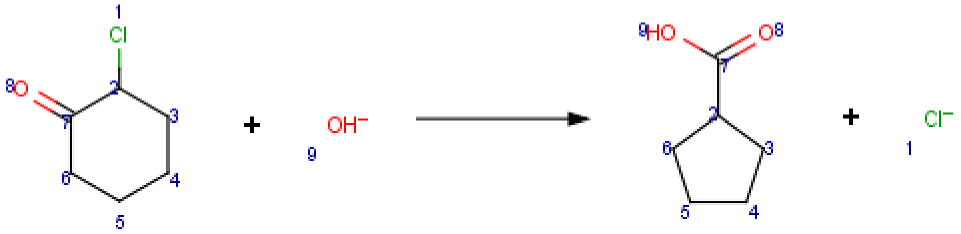

Correct mapped SMILES/SMARTS of the reaction:
[Cl:1][CH:2]1[CH2:3][CH2:4][CH2:5][CH2:6][C:7]1=[O:8].[OH-
:9]>>[OH:9][C:7](=[O:8])[CH:2]1[CH2:3][CH2:4][CH2:5][CH2:6]1.[Cl-:1]

Correctness of the mapping: YES

Reaction no 60

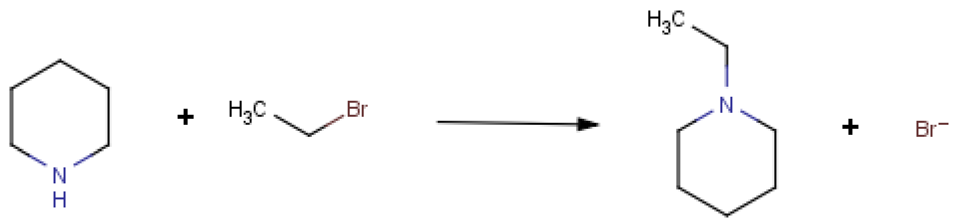

SMILES of the input:
C1CCNCC1.CCBr>>CCN1CCCCC1.[Br-]

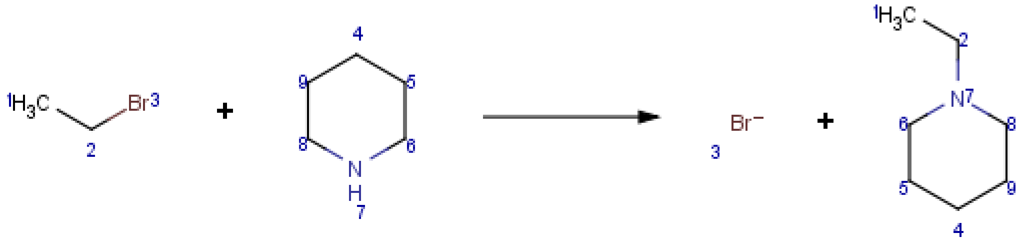

Correct mapped SMILES/SMARTS of the reaction:
[CH3:1][CH2:2][Br:3].[CH2:4]1[CH2:5][CH2:6][NH:7][CH2:8][CH2:9]1>>[Br-:3].[CH3:1][CH2:2][N:7]1[CH2:8][CH2:9][CH2:4][CH2:5][CH2:6]1

Correctness of the mapping: YES

Reaction no 61

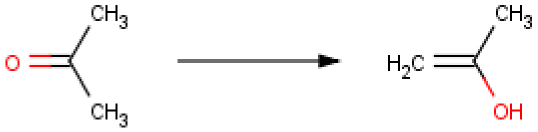

SMILES of the input:
CC(C)=O>>CC(O)=C

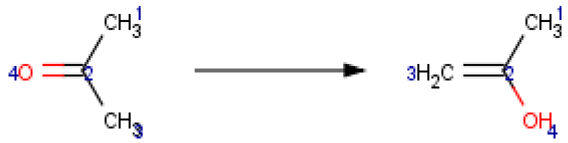

Correct mapped SMILES/SMARTS of the reaction:
[CH3:1][C:2]([CH3:3])=[O:4]>>[CH3:1][C:2]([OH:4])=[CH2:3]

Correctness of the mapping: YES

Reaction no 62

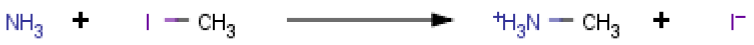

SMILES of the input:
N.CI>>C[NH3+].[I-]

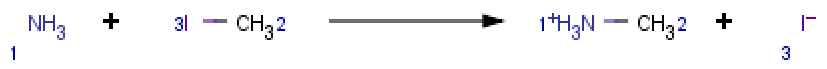

Correct mapped SMILES/SMARTS of the reaction:
[NH3:1].[CH3:2][I:3]>>[CH3:2][NH3+:1].[I-:3]

Correctness of the mapping: YES

Reaction no 63

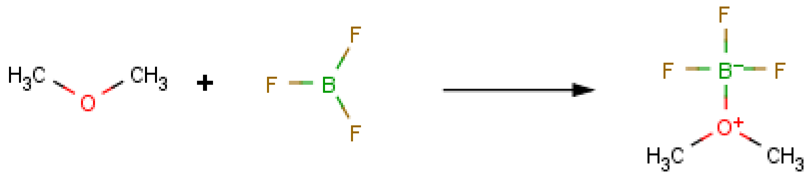

SMILES of the input:
COC.FB(F)F>>C[O+](C)[B-](F)(F)F

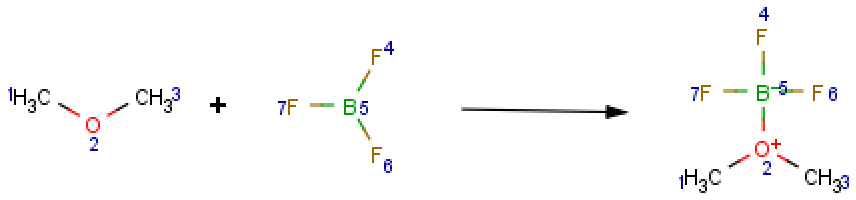

Correct mapped SMILES/SMARTS of the reaction:
[CH3:1][O:2][CH3:3].[F:4][B:5]([F:6])[F:7]>>[CH3:1][O+:2]([CH3:3])[B-:5]([F:4])([F:6])[F:7]

Correctness of the mapping: YES

Reaction no 64

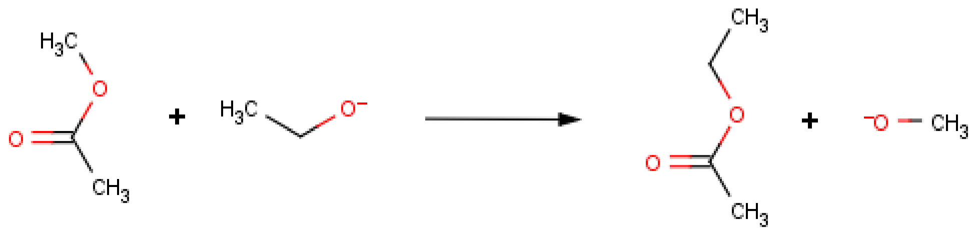

SMILES of the input:
COC(C)=O.CC[O-]>>CCOC(C)=O.C[O-]

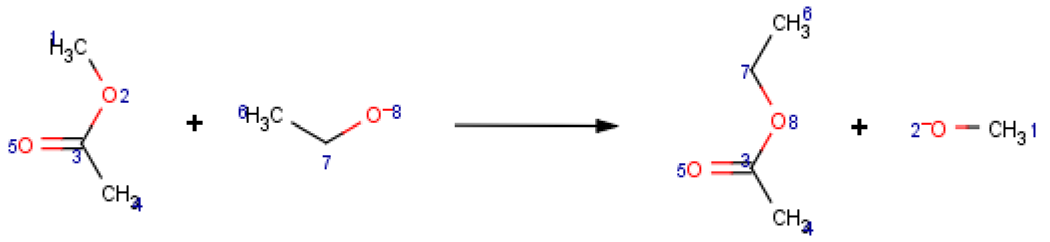

Correct mapped SMILES/SMARTS of the reaction:
[CH3:1][O:2][C:3]([CH3:4])=[O:5].[CH3:6][CH2:7][O-
:8]>>[CH3:6][CH2:7][O:8][C:3]([CH3:4])=[O:5].[CH3:1][O-:2]

Correctness of the mapping: YES

Reaction no 65

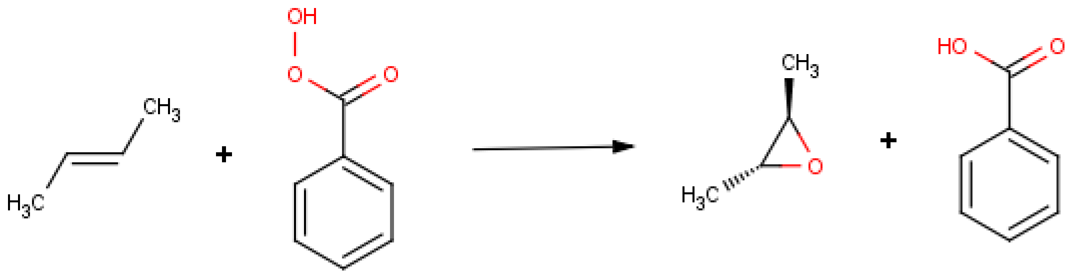

SMILES of the input:
C\C=C\C.OOC(=O)C1=CC=CC=C1>>C[C@H]1O[C@@H]1C.OC(=O)C1=CC=CC=C1

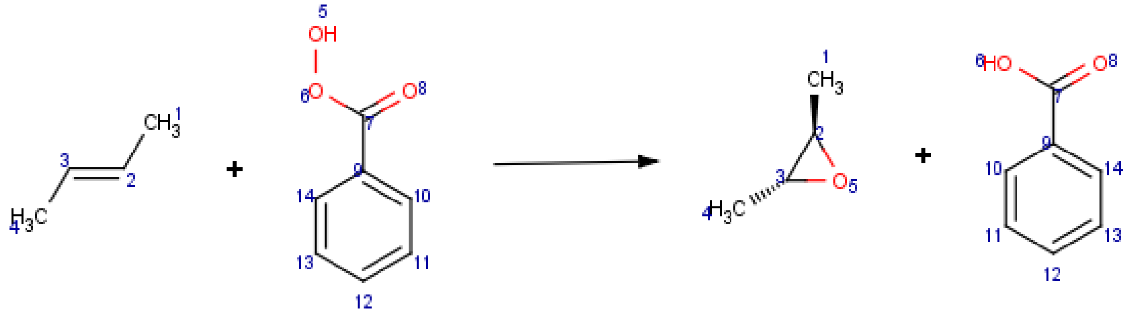

Correct mapped SMILES/SMARTS of the reaction:
[CH3:1]\[CH:2]=[CH:3]\[CH3:4].[OH:5][O:6][C:7](=[O:8])[C:9]1=[CH:10][CH:1
1]=[CH:12][CH:13]=[CH:14]1>>[CH3:1][C@H:2]1[O:5][C@@H:3]1[CH3:4].[OH:6][C
:7](=[O:8])[C:9]1=[CH:14][CH:13]=[CH:12][CH:11]=[CH:10]1

Correctness of the mapping: YES

Reaction no 66

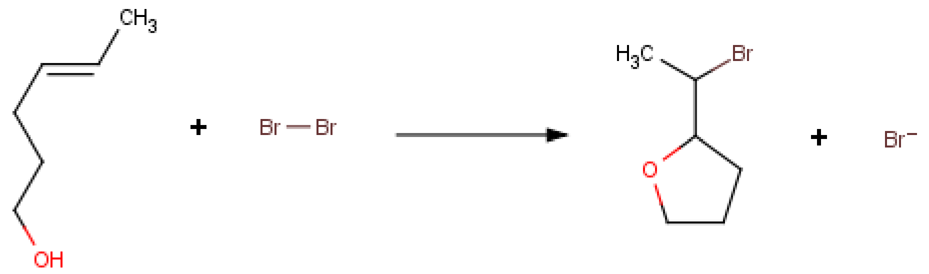

SMILES of the input:
C\C=C\CCCO.BrBr>>CC(Br)C1CCCO1.[Br-]

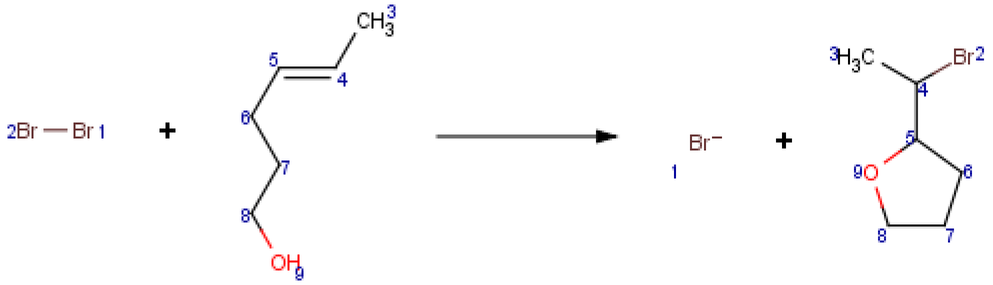

Correct mapped SMILES/SMARTS of the reaction:
[Br:1][Br:2].[CH3:3]\[CH:4]=[CH:5]\[CH2:6][CH2:7][CH2:8][OH:9]>>[Br-:1].[CH3:3][CH:4]([Br:2])[CH:5]1[CH2:6][CH2:7][CH2:8][O:9]1

Correctness of the mapping: YES

Reaction no 67

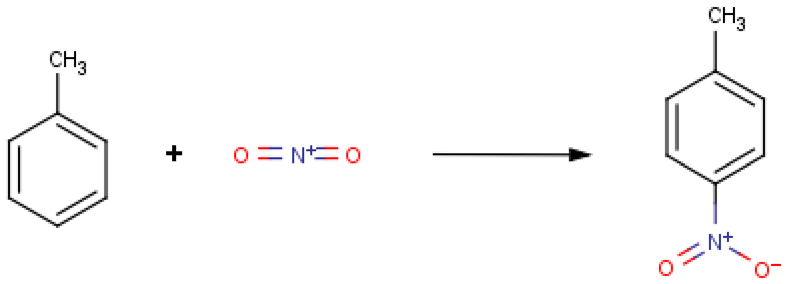

SMILES of the input:
CC1=CC=CC=C1.O=[N+]=O>>CC1=CC=C(C=C1)[N+]([O-])=O

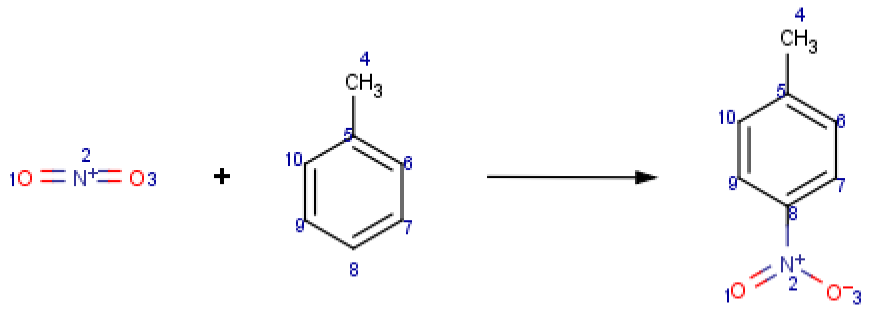

Correct mapped SMILES/SMARTS of the reaction:
[O:1]=[N+:2]=[O:3].[CH3:4][C:5]1=[CH:6][CH:7]=[CH:8][CH:9]=[CH:10]1>>[CH3:4][C:5]1=[CH:6][CH:7]=[C:8]([CH:9]=[CH:10]1)[N+:2]([O-:3])=[O:1]

Correctness of the mapping: YES

Reaction no 68

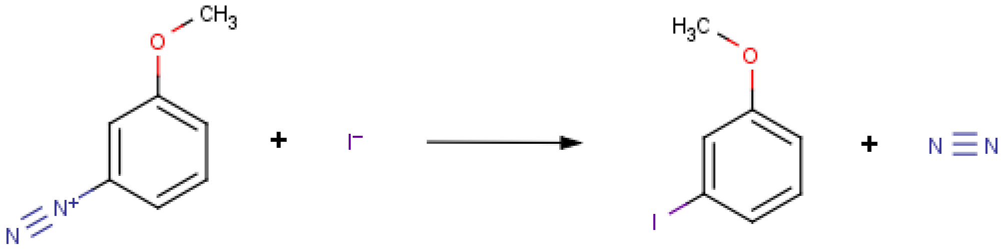

SMILES of the input:
COC1=CC=CC(=C1)[N+]#N.[I-]>>COC1=CC=CC(I)=C1.N#N

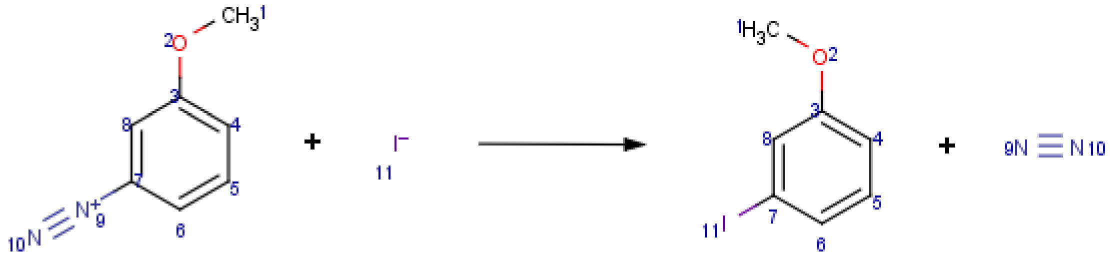

Correct mapped SMILES/SMARTS of the reaction:
[CH3:1][O:2][C:3]1=[CH:4][CH:5]=[CH:6][C:7](=[CH:8]1)[N+:9]#[N:10].[I-:11]>>[CH3:1][O:2][C:3]1=[CH:4][CH:5]=[CH:6][C:7]([I:11])=[CH:8]1.[N:10]#[N:9]

Correctness of the mapping: YES

Reaction no 69

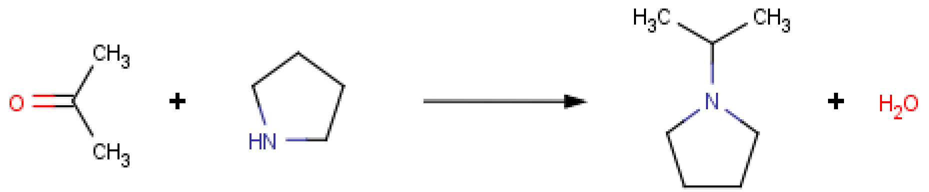

SMILES of the input:
CC(C)=O.C1CCNC1>>CC(C)N1CCCC1.O

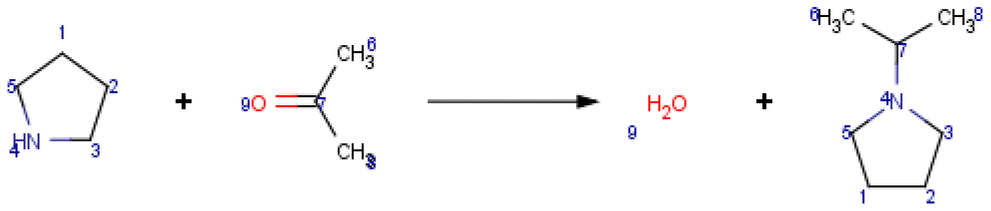

Correct mapped SMILES/SMARTS of the reaction:
[CH2:1]1[CH2:2][CH2:3][NH:4][CH2:5]1.[CH3:6][C:7]([CH3:8])=[O:9]>>[OH2:9]
.[CH3:6][CH:7]([CH3:8])[N:4]1[CH2:3][CH2:2][CH2:1][CH2:5]1

Correctness of the mapping: YES

Reaction no 70

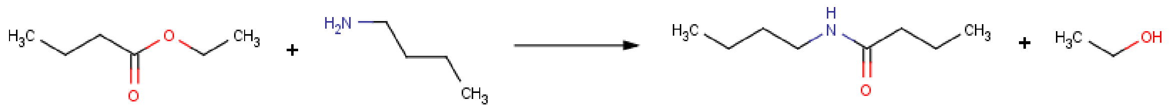

SMILES of the input:
CCCC(=O)OCC.CCCCN>>CCCCNC(=O)CCC.CCO

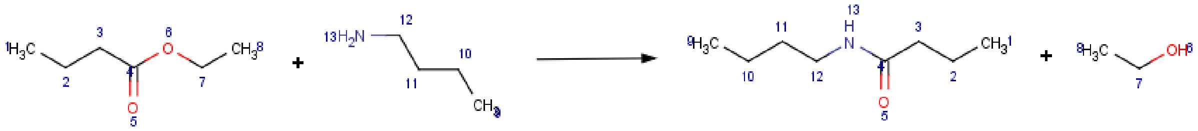

Correct mapped SMILES/SMARTS of the reaction:
[CH3:1][CH2:2][CH2:3][C:4](=[O:5])[O:6][CH2:7][CH3:8].[CH3:9][CH2:10][CH2
:11][CH2:12][NH2:13]>>[CH3:9][CH2:10][CH2:11][CH2:12][NH:13][C:4](=[O:5])
[CH2:3][CH2:2][CH3:1].[CH3:8][CH2:7][OH:6]

Correctness of the mapping: YES

Reaction no 71

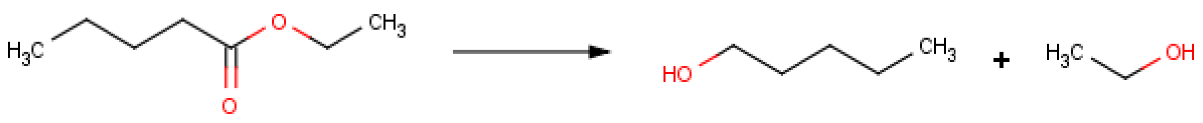

SMILES of the input:
CCCCC(=O)OCC>>C(O)CCCC.CCO

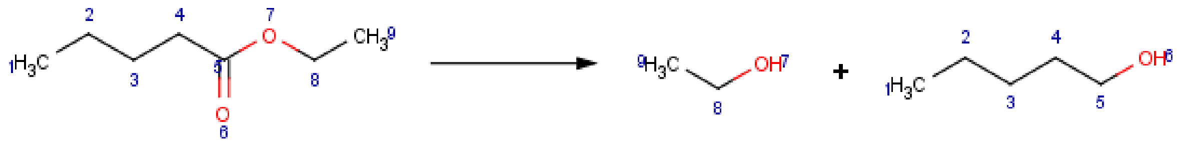

Correct mapped SMILES/SMARTS of the reaction:
[CH3:1][CH2:2][CH2:3][CH2:4][C:5](=[O:6])[O:7][CH2:8][CH3:9]>>[CH3:9][CH2:8][OH:7].[CH3:1][CH2:2][CH2:3][CH2:4][CH2:5][OH:6]

Correctness of the mapping: YES

Reaction no 72

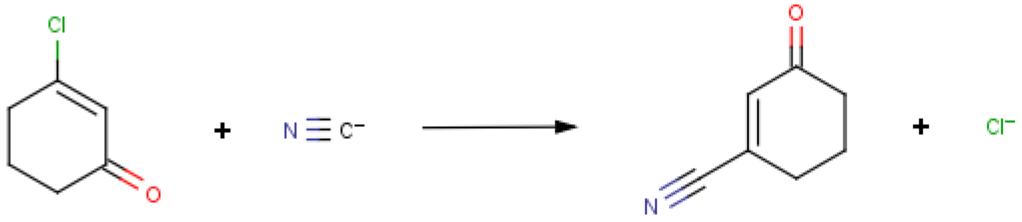

SMILES of the input:
ClC1=CC(=O)CCC1.[C-]#N>>O=C1CCCC(=C1)C#N.[Cl-]

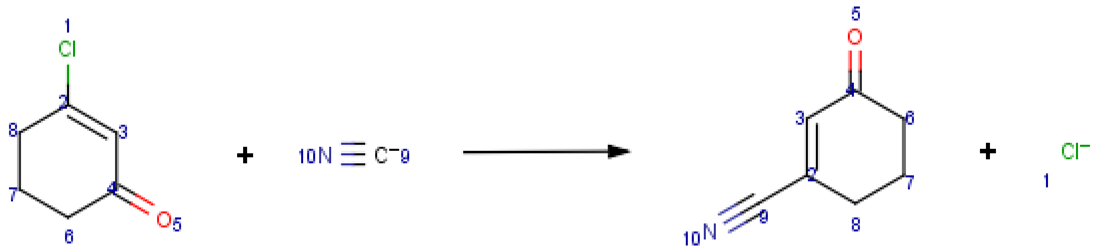

Correct mapped SMILES/SMARTS of the reaction:
[Cl:1][C:2]1=[CH:3][C:4](=[O:5])[CH2:6][CH2:7][CH2:8]1.[C-:9]#[N:10]>>[O:5]=[C:4]1[CH2:6][CH2:7][CH2:8][C:2](=[CH:3]1)[C:9]#[N:10].[Cl-:1]

Correctness of the mapping: YES

Reaction no 73

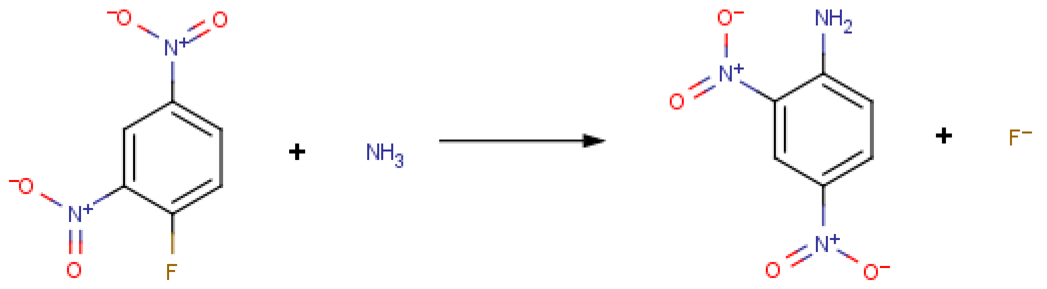

SMILES of the input:
[O-][N+](=O)C1=CC=C(F)C(=C1)[N+]([O-])=O.N>>NC1=CC=C(C=C1[N+]([O-
])=O)[N+]([O-])=O.[F-]

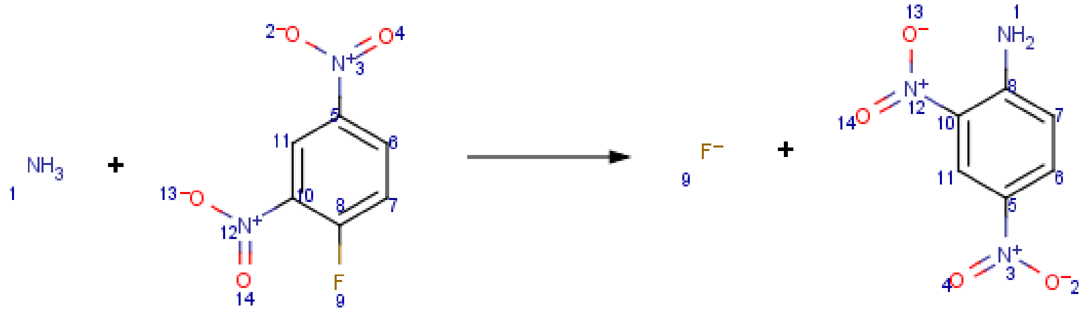

Correct mapped SMILES/SMARTS of the reaction:
[NH3:1].[O-
:2][N+:3](=[O:4])[C:5]1=[CH:6][CH:7]=[C:8]([F:9])[C:10](=[CH:11]1)[N+:12]
([O-:13])=[O:14]>>[F-
:9].[NH2:1][C:8]1=[CH:7][CH:6]=[C:5]([CH:11]=[C:10]1[N+:12]([O-
:13])=[O:14])[N+:3]([O-:2])=[O:4]

Correctness of the mapping: YES

Reaction no 74

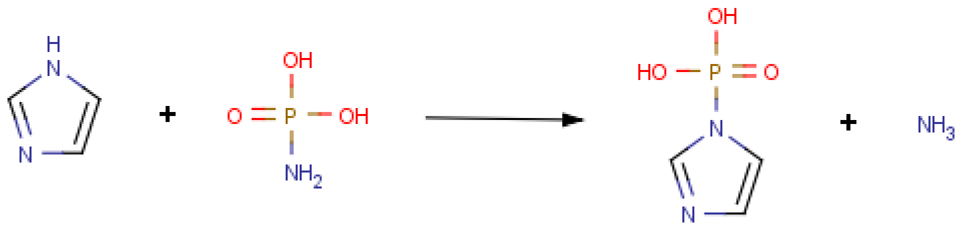

SMILES of the input:
N1C=CN=C1.NP(O)(O)=O>>OP(O)(=O)N1C=CN=C1.N

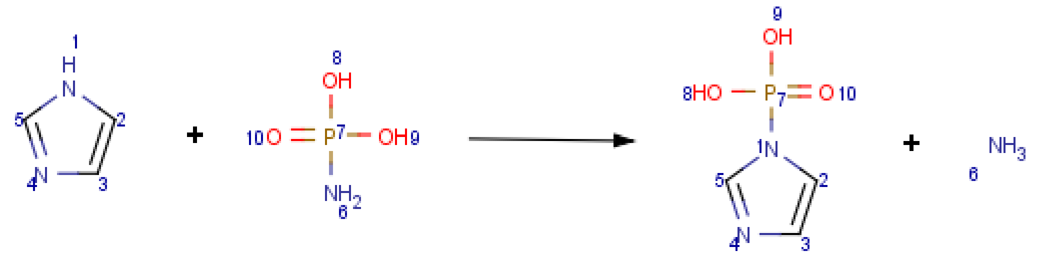

Correct mapped SMILES/SMARTS of the reaction:
[NH:1]1[CH:2]=[CH:3][N:4]=[CH:5]1.[NH2:6][P:7]([OH:8])([OH:9])=[O:10]>>[OH:9][P:7]([OH:8])(=[O:10])[N:1]1[CH:2]=[CH:3][N:4]=[CH:5]1.[NH3:6]

Correctness of the mapping: YES

Reaction no 75

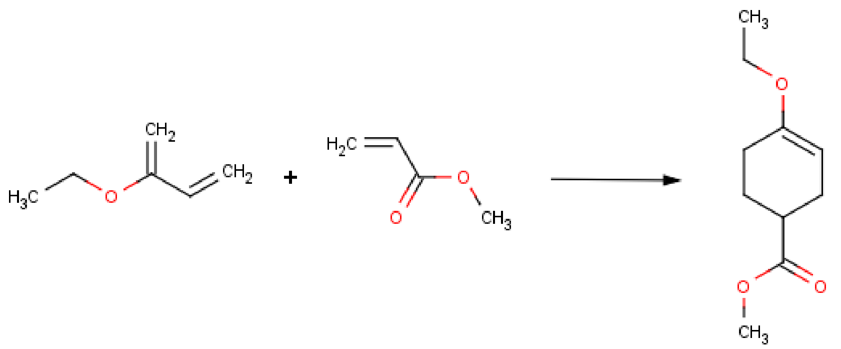

SMILES of the input:
CCOC(=C)C=C.COC(=O)C=C>>CCOC1=CCC(CC1)C(=O)OC

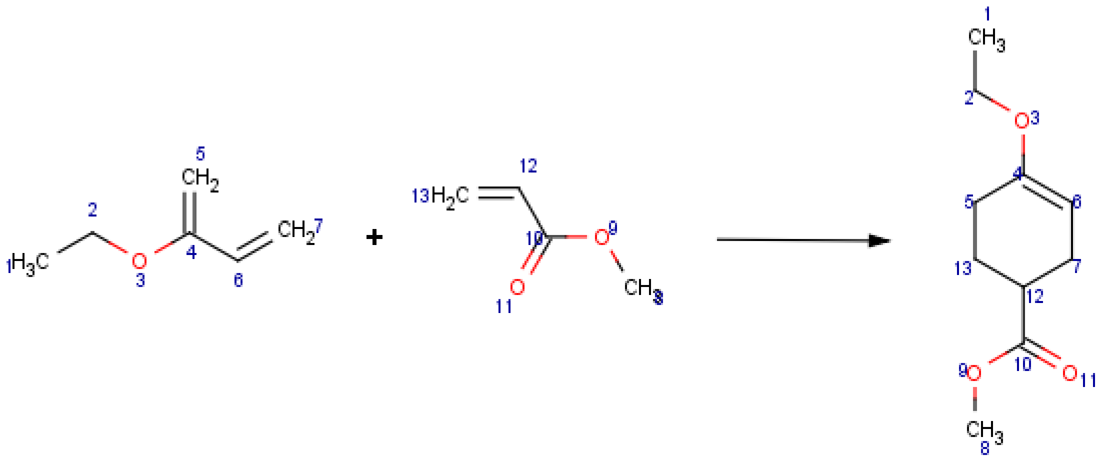

Correct mapped SMILES/SMARTS of the reaction:
[CH3:1][CH2:2][O:3][C:4](=[CH2:5])[CH:6]=[CH2:7].[CH3:8][O:9][C:10](=[O:11])[CH:12]=[CH2:13]>>[CH3:1][CH2:2][O:3][C:4]1=[CH:6][CH2:7][CH:12]([CH2:13][CH2:5]1)[C:10](=[O:11])[O:9][CH3:8]

Correctness of the mapping: YES

Supplementary Note 5. Consequences of incorrect atom mapping for the prediction of reaction outcomes.

5.1. An illustrative example of the correct outcome of a Diels-Alder reaction vs. top predictions (all incorrect) of the MIT's program.

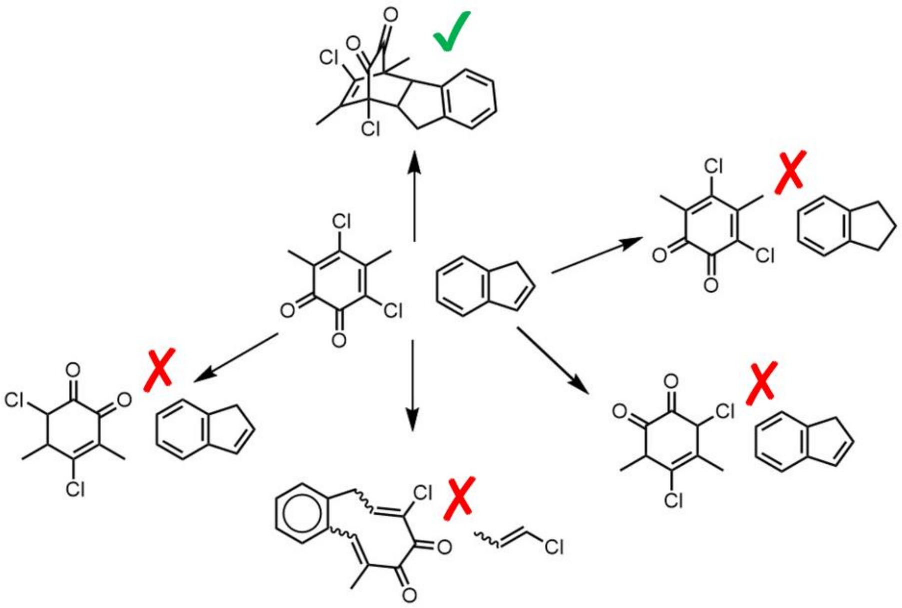

Supplementary Figure 10. The image shows correct Diels-Alder output for the reaction of the diene and a dienophile as well as four top-scoring but chemically incorrect predictions of the Weisfeiler-Lehman network described in ref. 11 and trained on 50,000 mapped reactions from patents. The large number of mapping errors (including those for the Diels-Alder reactions, see main-text Figure 6b) is one of the reasons why the network's predictions are chemically nonsensical.

S5.2. Examples of 10 pairs of reactions with *correct* mapping in the MIT training set.

The first reaction in each pair has correct mapping and was used with such a mapping to train the MIT's deep neural network for reaction prediction. The second reaction is a closely related transformation (with chemically correct product shown) with which we queried/tested the MIT's network. The question asked is whether the MIT network can or cannot predict the correct product based on reaction substrates. On this set of 10 examples, 90% of outputs are correct.

1.

MIT reaction:

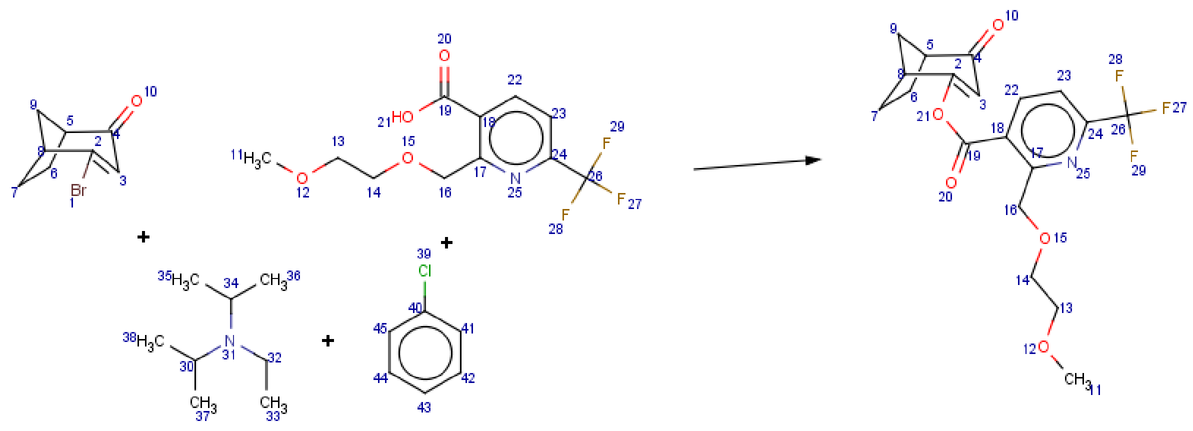

[Br:1][C:2]1=[CH:3][C:4](=[O:10])[CH:5]2[CH2:6][CH2:7][CH:8]1[CH2:9]2.[CH3:11][O:12][CH2:13][CH2:14][O:15][CH2:16][c:17]1[c:18]([C:19](=[O:20])[OH:21])[cH:22][cH:23][c:24]([C:26]([F:27])([F:28])[F:29])[n:25]1.[CH:30]([N:31]([CH2:32][CH3:33])[CH:34]([CH3:35])[CH3:36])([CH3:37])[CH3:38].[Cl:39][c:40]1[cH:41][cH:42][cH:43][cH:44][cH:45]1>>[C:2]1([O:21][C:19]([c:18]2[c:17]([CH2:16][O:15][CH2:14][CH2:13][O:12][CH3:11])[n:25][c:24]([C:26]([F:27])([F:28])[F:29])[cH:23][cH:22]2)=[O:20])=[CH:3][C:4](=[O:10])[CH:5]2[CH2:6][CH2:7][CH:8]1[CH2:9]2

Similar reaction:

Input:

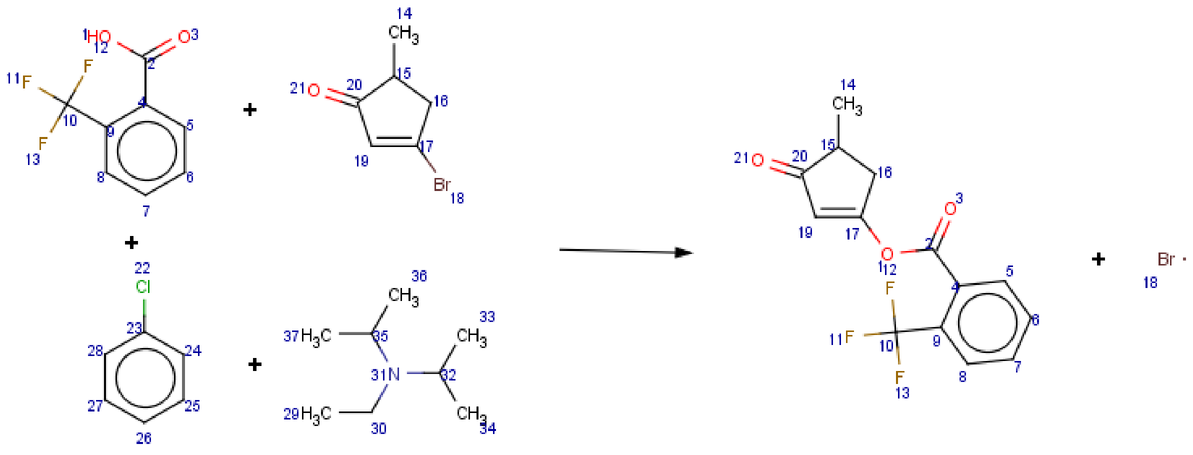

[OH:1][C:2](=[O:3])[c:4]1[cH:5][cH:6][cH:7][cH:8][c:9]1[C:10]([F:11])([F:12])[F:13].[CH3:14][CH:15]1[CH2:16][C:17]([Br:18])=[CH:19][C:20]1=[O:21].[Cl:22][c:23]1[cH:24][cH:25][cH:26][cH:27][cH:28]1.[CH3:29][CH2:30][N:31]([CH:32]([CH3:33])[CH3:34])[CH:35]([CH3:36])[CH3:37]>>[CH3:14][CH:15]1[CH2:16][C:17]([O:1][C:2](=[O:3])[c:4]2[cH:5][cH:6][cH:7][cH:8][c:9]2[C:10]([F:11])([F:12])[F:13])=[CH:19][C:20]1=[O:21].[Br:18]

Output (top prediction):

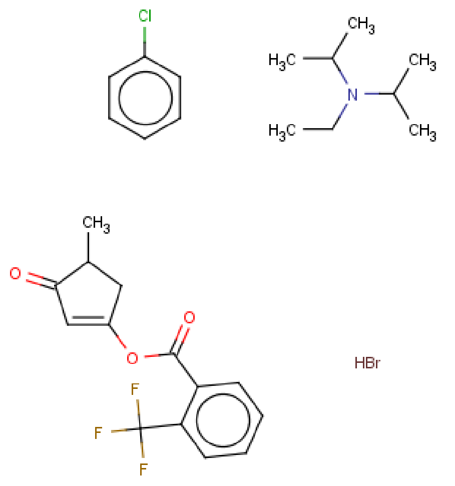

Clc1ccccc1.CCN(C(C)C)C(C)C.CC1CC(OC(=O)c2ccccc2C(F)(F)F)=CC1=O.Br

Correctness: CORRECT

2.

MIT reaction:

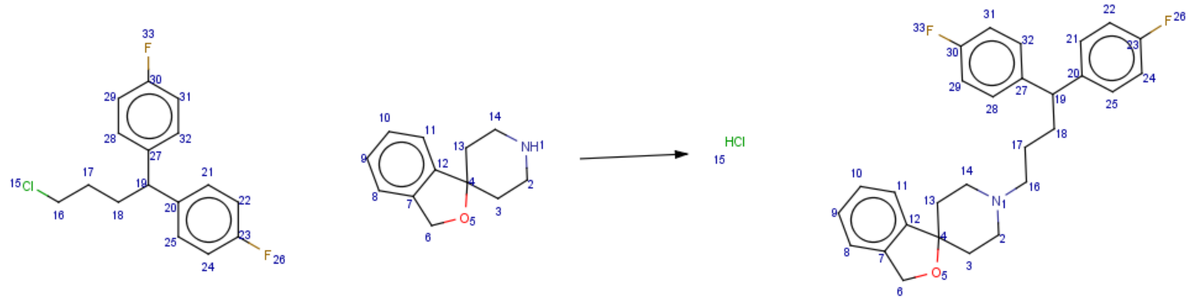

[Cl:15][CH2:16][CH2:17][CH2:18][CH:19]([c:20]1[cH:21][cH:22][c:23]([F:26])[cH:24][cH:
25]1)[c:27]1[cH:28][cH:29][c:30]([F:33])[cH:31][cH:32]1.[NH:1]1[CH2:2][CH2:3][C:4]2([
O:5][CH2:6][c:7]3[cH:8][cH:9][cH:10][cH:11][c:12]32)[CH2:13][CH2:14]1>>[ClH:15].[N:1
]1([CH2:16][CH2:17][CH2:18][CH:19]([c:20]2[cH:21][cH:22][c:23]([F:26])[cH:24][cH:25]2
)[c:27]2[cH:28][cH:29][c:30]([F:33])[cH:31][cH:32]2)[CH2:2][CH2:3][C:4]2([O:5][CH2:6][
c:7]3[cH:8][cH:9][cH:10][cH:11][c:12]32)[CH2:13][CH2:14]1

Similar reaction:

Input:

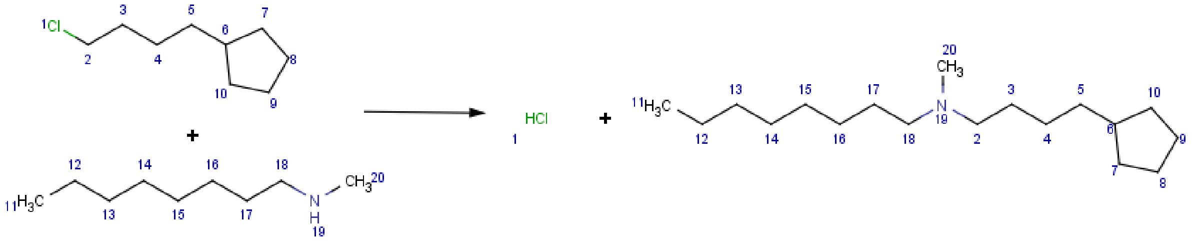

[Cl:1][CH2:2][CH2:3][CH2:4][CH2:5][CH:6]1[CH2:7][CH2:8][CH2:9][CH2:10]1.[CH3:11]
[CH2:12][CH2:13][CH2:14][CH2:15][CH2:16][CH2:17][CH2:18][NH:19][CH3:20]>>[ClH:
1].[CH3:11][CH2:12][CH2:13][CH2:14][CH2:15][CH2:16][CH2:17][CH2:18][N:19]([CH3:2
0])[CH2:2][CH2:3][CH2:4][CH2:5][CH:6]1[CH2:10][CH2:9][CH2:8][CH2:7]1

Output (top prediction):

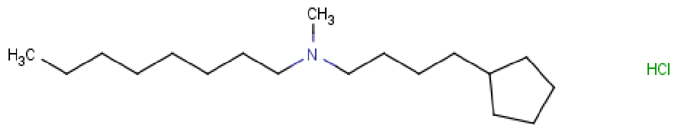

CCCCCCCCN(C)CCCCC1CCCC1.Cl

Correctness: CORRECT

3.

MIT reaction:

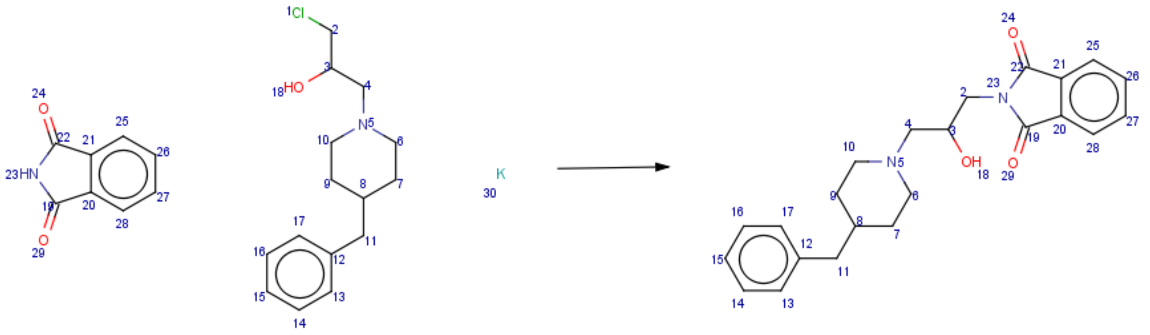

[C:19]1(=[O:29])[c:20]2[c:21]([cH:25][cH:26][cH:27][cH:28]2)[C:22](=[O:24])[NH:23]1.[Cl:1][CH2:2][CH:3]([CH2:4][N:5]1[CH2:6][CH2:7][CH:8]([CH2:11][c:12]2[cH:13][cH:14][cH:15][cH:16][cH:17]2)[CH2:9][CH2:10]1)[OH:18].[K:30]>>[CH2:2]([CH:3]([CH2:4][N:5]1[CH2:6][CH2:7][CH:8]([CH2:11][c:12]2[cH:13][cH:14][cH:15][cH:16][cH:17]2)[CH2:9][CH2:10]1)[OH:18])[N:23]1[C:19](=[O:29])[c:20]2[c:21]([cH:25][cH:26][cH:27][cH:28]2)[C:22]1=[O:24]

Similar reaction:

Input:

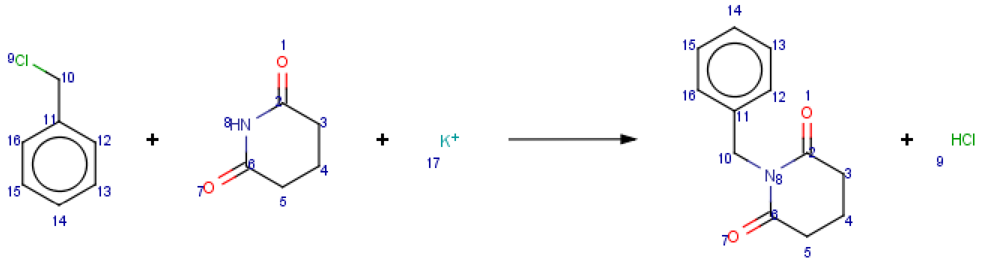

[Cl:9][CH2:10][c:11]1[cH:12][cH:13][cH:14][cH:15][cH:16]1.[O:1]=[C:2]1[CH2:3][CH2:4][CH2:5][C:6](=[O:7])[NH:8]1.[K+:17]>>[O:1]=[C:2]1[CH2:3][CH2:4][CH2:5][C:6](=[O:7])[N:8]1[CH2:10][c:11]1[cH:16][cH:15][cH:14][cH:13][cH:12]1.[ClH:9]

Output (top prediction):

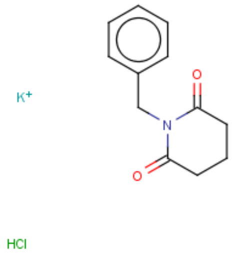

[K+].O=C1CCCC(=O)N1Cc1ccccc1.Cl

Correctness: CORRECT

4.

MIT reaction:

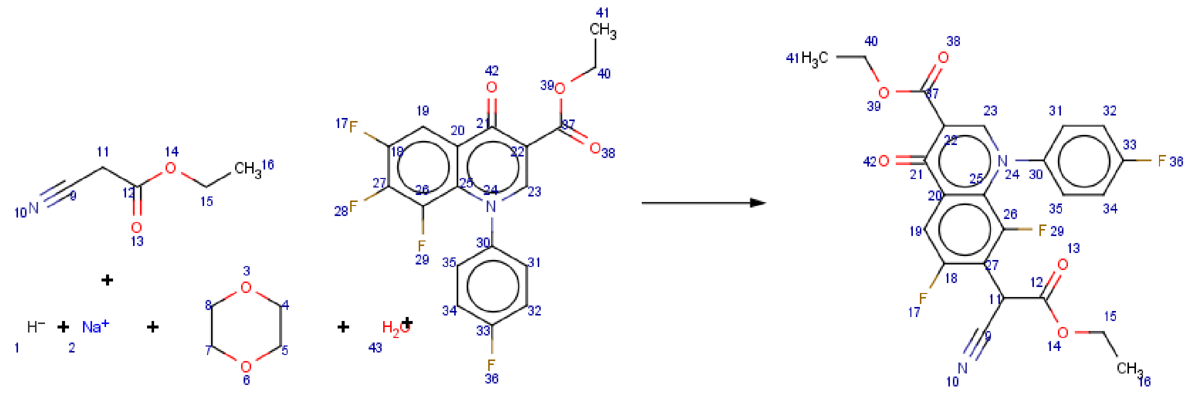

[C:9](#[N:10])[CH2:11][C:12](=[O:13])[O:14][CH2:15][CH3:16].[F:17][c:18]1[cH:19][c:20]2[c:21](=[O:42])[c:22]([C:37](=[O:38])[O:39][CH2:40][CH3:41])[cH:23][n:24](-[c:30]3[cH:31][cH:32][c:33]([F:36])[cH:34][cH:35]3)[c:25]2[c:26]([F:29])[c:27]1[F:28].[H-:1].[Na+:2].[O:3]1[CH2:4][CH2:5][O:6][CH2:7][CH2:8]1.[OH2:43]>>[C:9](#[N:10])[CH:11]([C:12](=[O:13])[O:14][CH2:15][CH3:16])[c:27]1[c:18]([F:17])[cH:19][c:20]2[c:21](=[O:42])[c:22]([C:37](=[O:38])[O:39][CH2:40][CH3:41])[cH:23][n:24](-[c:30]3[cH:31][cH:32][c:33]([F:36])[cH:34][cH:35]3)[c:25]2[c:26]1[F:29]

Similar reaction:

Input:

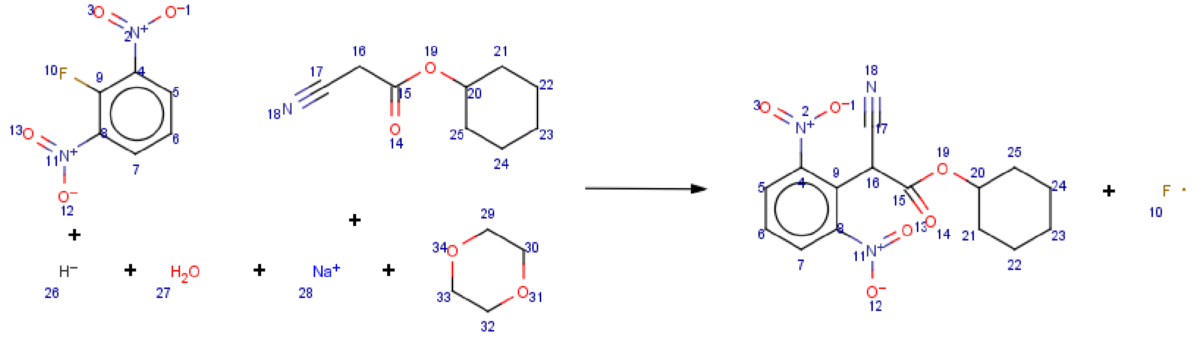

[O-:1][N+:2](=[O:3])[c:4]1[cH:5][cH:6][cH:7][c:8]([c:9]1[F:10])[N+:11]([O-:12])=[O:13].[O:14]=[C:15]([CH2:16][C:17]#[N:18])[O:19][CH:20]1[CH2:21][CH2:22][CH2:23][CH2:24][CH2:25]1.[H-:26].[OH2:27].[Na+:28].[CH2:29]1[CH2:30][O:31][CH2:32][CH2:33][O:34]1>>[O-:12][N+:11](=[O:13])[c:8]1[cH:7][cH:6][cH:5][c:4]([c:9]1[CH:16]([C:17]#[N:18])[C:15](=[O:14])[O:19][CH:20]1[CH2:25][CH2:24][CH2:23][CH2:22][CH2:21]1)[N+:2]([O-:1])=[O:3].[F:10]

Output (top prediction):

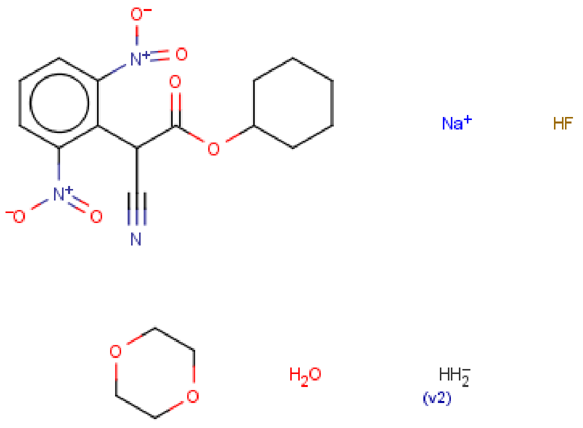

Correctness: CORRECT

5.

MIT reaction:

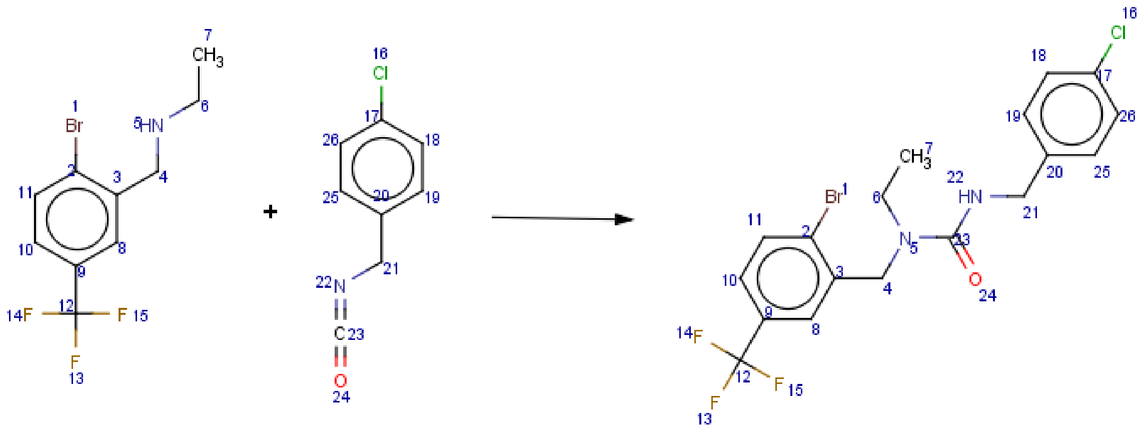

[Br:1][c:2]1[c:3]([CH2:4][NH:5][CH2:6][CH3:7])[cH:8][c:9]([C:12]([F:13])([F:14])[F:15])[cH:10][cH:11]1.[Cl:16][c:17]1[cH:18][cH:19][c:20]([CH2:21][N:22]=[C:23]=[O:24])[cH:25][cH:26]1>>[Br:1][c:2]1[c:3]([CH2:4][N:5]([CH2:6][CH3:7])[C:23]([NH:22][CH2:21][c:20]2[cH:19][cH:18][c:17]([Cl:16])[cH:26][cH:25]2)=[O:24])[cH:8][c:9]([C:12]([F:13])([F:14])[F:15])[cH:10][cH:11]1

Similar reaction:

Input:

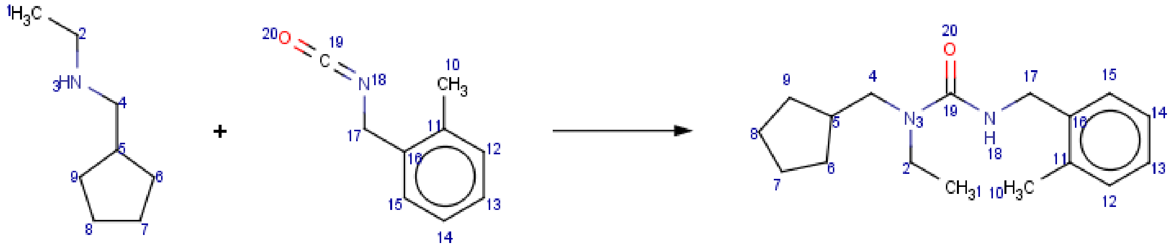

[CH3:1][CH2:2][NH:3][CH2:4][CH:5]1[CH2:6][CH2:7][CH2:8][CH2:9]1.[CH3:10][c:11]1[cH:12][cH:13][cH:14][cH:15][c:16]1[CH2:17][N:18]=[C:19]=[O:20]>>[CH3:1][CH2:2][N:3]([CH2:4][CH:5]1[CH2:6][CH2:7][CH2:8][CH2:9]1)[C:19](=[O:20])[NH:18][CH2:17][c:16]1[cH:15][cH:14][cH:13][cH:12][c:11]1[CH3:10]

Output (top prediction):

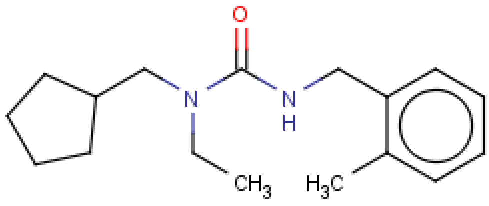

CCN(CC1CCCC1)C(=O)NCc1ccccc1C

Correctness: CORRECT

6.

MIT reaction:

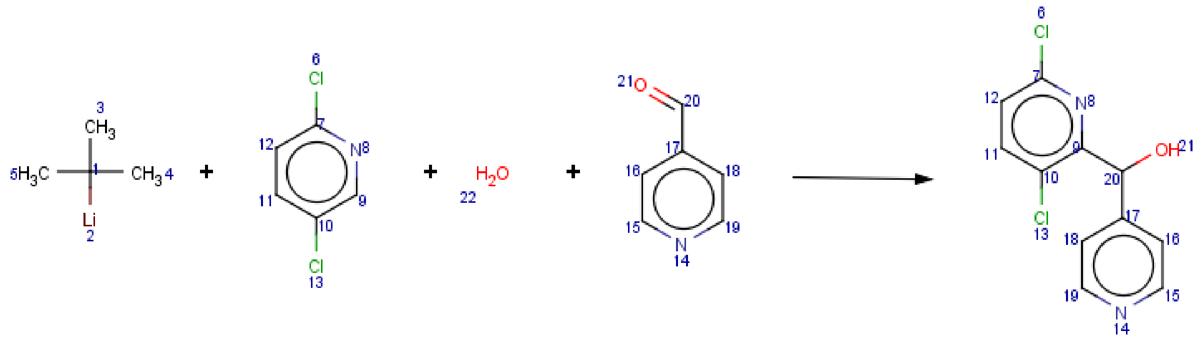

[C:1]([Li:2])([CH3:3])([CH3:4])[CH3:5].[Cl:6][c:7]1[n:8][cH:9][c:10]([Cl:13])[cH:11][cH:12]1.[OH2:22].[n:14]1[cH:15][cH:16][c:17]([CH:20]=[O:21])[cH:18][cH:19]1>>[Cl:6][c:7]1[n:8][c:9]([CH:20]([c:17]2[cH:16][cH:15][n:14][cH:19][cH:18]2)[OH:21])[c:10]([Cl:13])[cH:11][cH:12]1

Similar reaction:

Input:

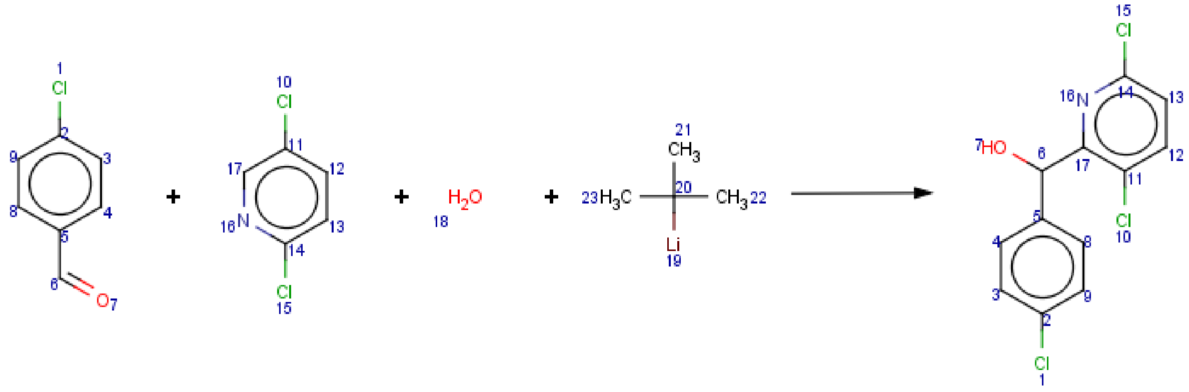

[Cl:1][c:2]1[cH:3][cH:4][c:5]([CH:6]=[O:7])[cH:8][cH:9]1.[Cl:10][c:11]1[cH:12][cH:13][c:14]([Cl:15])[n:16][cH:17]1.[OH2:18].[Li:19][C:20]([CH3:21])([CH3:22])[CH3:23]>>[OH:7][CH:6]([c:5]1[cH:8][cH:9][c:2]([Cl:1])[cH:3][cH:4]1)[c:17]1[n:16][c:14]([Cl:15])[cH:13][cH:12][c:11]1[Cl:10]

Output (top prediction):

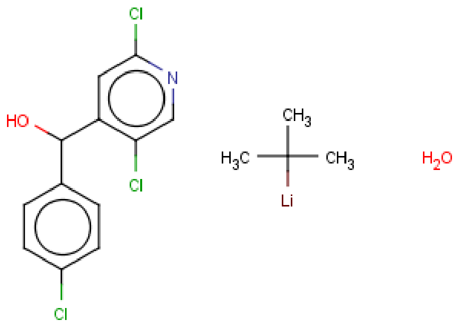

[Li]C(C)(C)C.O.OC(c1ccc(Cl)cc1)c1cc(Cl)ncc1Cl

Correctness: INCORRECT

(the wright answer was also generated, but ranked on the fifth place)

7.

MIT reaction:

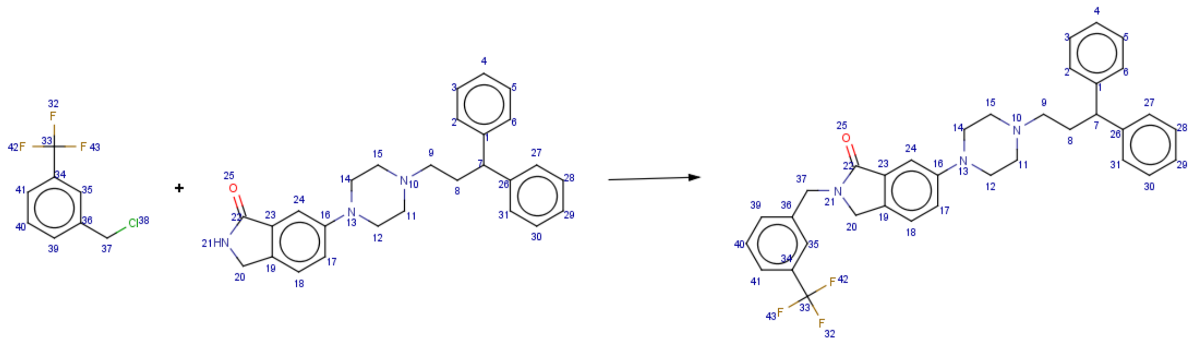

[F:32][C:33]([c:34]1[cH:35][c:36]([CH2:37][Cl:38])[cH:39][cH:40][cH:41]1)([F:42])[F:43].[c:1]1([CH:7]([CH2:8][CH2:9][N:10]2[CH2:11][CH2:12][N:13]([c:16]3[cH:17][cH:18][c:19]4[c:23]([cH:24]3)[C:22](=[O:25])[NH:21][CH2:20]4)[CH2:14][CH2:15]2)[c:26]2[cH:27][cH:28][cH:29][cH:30][cH:31]2)[cH:2][cH:3][cH:4][cH:5][cH:6]1>>[c:1]1([CH:7]([CH2:8][CH2:9][N:10]2[CH2:11][CH2:12][N:13]([c:16]3[cH:17][cH:18][c:19]4[c:23]([cH:24]3)[C:22](=[O:25])[N:21]([CH2:37][c:36]3[cH:35][c:34]([C:33]([F:32])([F:42])[F:43])[cH:41][cH:40][cH:39]3)[CH2:20]4)[CH2:14][CH2:15]2)[c:26]2[cH:27][cH:28][cH:29][cH:30][cH:31]2)[cH:2][cH:3][cH:4][cH:5][cH:6]1

Similar reaction:

Input:

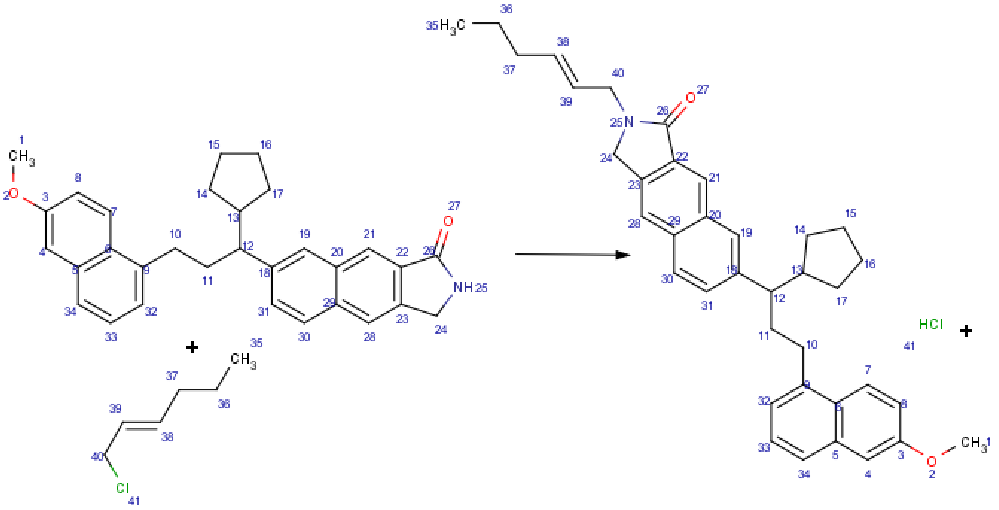

[CH3:35][CH2:36][CH2:37]\[CH:38]=[CH:39]\[CH2:40][Cl:41].[CH3:1][O:2][C:3]1=[CH:4][C:5]2=[C:6]([CH:7]=[CH:8]1)[C:9]([CH2:10][CH2:11][CH:12]([CH:13]1[CH2:14][CH2:15][CH2:16][CH2:17]1)[C:18]1=[CH:19][C:20]3=[CH:21][C:22]4=[C:23]([CH2:24][NH:25][C:26]4=[O:27])[CH:28]=[C:29]3[CH:30]=[CH:31]1)=[CH:32][CH:33]=[CH:34]2>>[CH3:35][CH2:36][CH2:37]\[CH:38]=[CH:39]\[CH2:40][N:25]1[CH2:24][C:23]2=[C:22]([CH:21]=[C:20]3[CH:19]=[C:18]([CH:31]=[CH:30][C:29]3=[CH:28]2)[CH:12]([CH2:11][CH2:10][C:9]2

=[CH:32][CH:33]=[CH:34][C:5]3=[C:6]2[CH:7]=[CH:8][C:3]([O:2][CH3:1])=[CH:4]3)[CH:13]2[CH2:14][CH2:15][CH2:16][CH2:17]2)[C:26]1=[O:27].[ClH:41]

Output (top prediction):

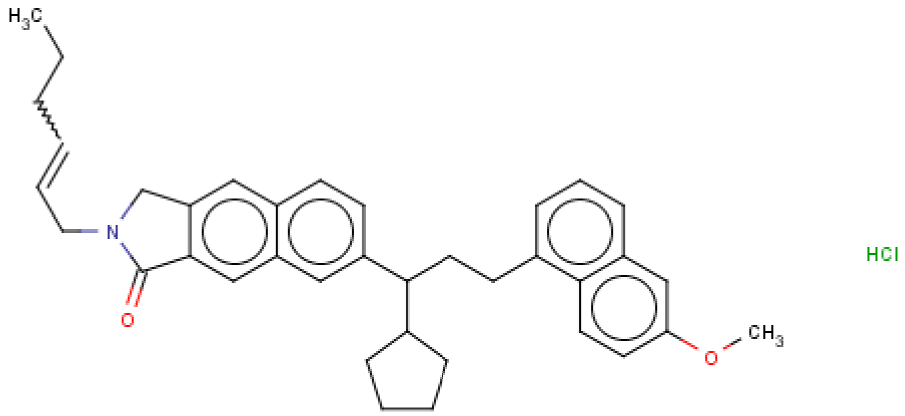

CCCC=CCN1Cc2cc3ccc(C(CCc4cccc5cc(OC)ccc45)C4CCCC4)cc3cc2C1=O.Cl

Correctness: CORRECT

8.

MIT reaction:

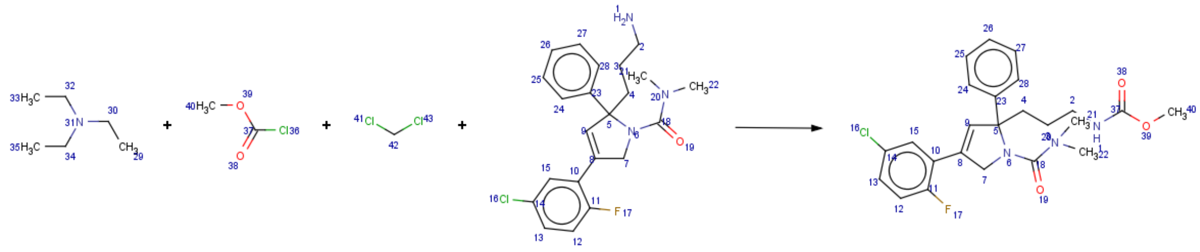

[CH3:29][CH2:30][N:31]([CH2:32][CH3:33])[CH2:34][CH3:35].[Cl:36][C:37](=[O:38])[O:39][CH3:40].[Cl:41][CH2:42][Cl:43].[NH2:1][CH2:2][CH2:3][CH2:4][C:5]1([c:23]2[cH:24][cH:25][cH:26][cH:27][cH:28]2)[N:6]([C:18](=[O:19])[N:20]([CH3:21])[CH3:22])[CH2:7][C:8]([c:10]2[c:11]([F:17])[cH:12][cH:13][c:14]([Cl:16])[cH:15]2)=[CH:9]1>>[NH:1]([CH2:2][CH2:3][CH2:4][C:5]1([c:23]2[cH:24][cH:25][cH:26][cH:27][cH:28]2)[N:6]([C:18](=[O:19])[N:20]([CH3:21])[CH3:22])[CH2:7][C:8]([c:10]2[c:11]([F:17])[cH:12][cH:13][c:14]([Cl:16])[cH:15]2)=[CH:9]1)[C:37](=[O:38])[O:39][CH3:40]

Similar reaction:

Input:

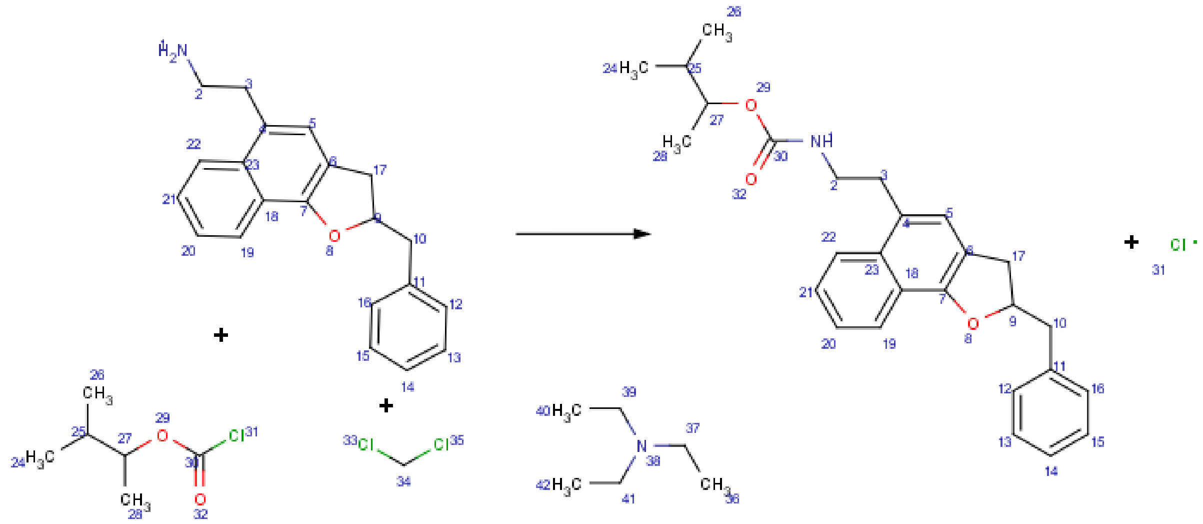

[NH2:1][CH2:2][CH2:3][C:4]1=[CH:5][C:6]2=[C:7]([O:8][CH:9]([CH2:10][C:11]3=[CH:12][CH:13]=[CH:14][CH:15]=[CH:16]3)[CH2:17]2)[C:18]2=[CH:19][CH:20]=[CH:21][CH:22]=[C:23]12.[CH3:24][CH:25]([CH3:26])[CH:27]([CH3:28])[O:29][C:30]([Cl:31])=[O:32].[Cl:33][CH2:34][Cl:35].[CH3:36][CH2:37][N:38]([CH2:39][CH3:40])[CH2:41][CH3:42]>>[CH3:24][CH:25]([CH3:26])[CH:27]([CH3:28])[O:29][C:30](=[O:32])[NH:1][CH2:2][CH2:3][C:4]1=[CH:5][C:6]2=[C:7]([O:8][CH:9]([CH2:10][C:11]3=[CH:16][CH:15]=[CH:14][CH:13]=[CH:12]3)[CH2:17]2)[C:18]2=[CH:19][CH:20]=[CH:21][CH:22]=[C:23]12.[Cl:31]

Output (top prediction):

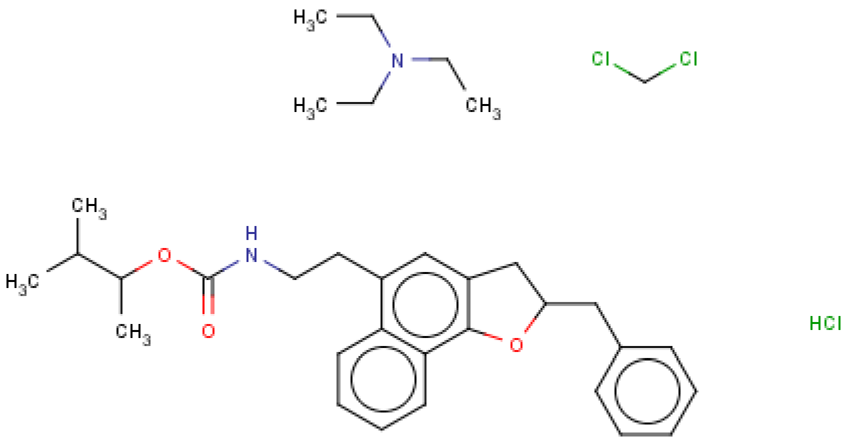

CCN(CC)CC.ClCCl.CC(C)C(C)OC(=O)NCCc1cc2c(c3ccccc13)OC(Cc1ccccc1)C2.Cl

Correctness: CORRECT

9.

MIT reaction:

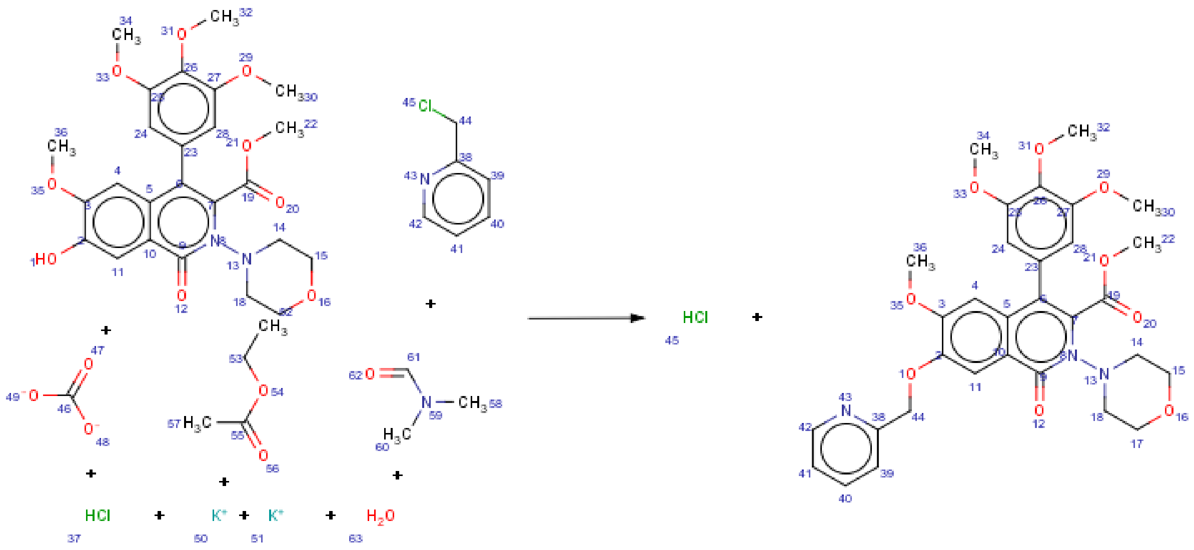

[C:46](=[O:47])([O-:48])[O-
:49].[CH3:52][CH2:53][O:54][C:55](=[O:56])[CH3:57].[CH3:58][N:59]([CH3:60])[CH:61]=
[O:62].[ClH:37].[K+:50].[K+:51].[OH2:63].[OH:1][c:2]1[c:3]([O:35][CH3:36])[cH:4][c:5]2[
c:6](-
[c:23]3[cH:24][c:25]([O:33][CH3:34])[c:26]([O:31][CH3:32])[c:27]([O:29][CH3:30])[cH:28]
3)[c:7]([C:19](=[O:20])[O:21][CH3:22])[n:8]([N:13]3[CH2:14][CH2:15][O:16][CH2:17][CH
2:18]3)[c:9](=[O:12])[c:10]2[cH:11]1.[c:38]1([CH2:44][Cl:45])[cH:39][cH:40][cH:41][cH:4
2][n:43]1>>[ClH:45].[O:1]([c:2]1[c:3]([O:35][CH3:36])[cH:4][c:5]2[c:6](-
[c:23]3[cH:24][c:25]([O:33][CH3:34])[c:26]([O:31][CH3:32])[c:27]([O:29][CH3:30])[cH:28]
3)[c:7]([C:19](=[O:20])[O:21][CH3:22])[n:8]([N:13]3[CH2:14][CH2:15][O:16][CH2:17][CH
2:18]3)[c:9](=[O:12])[c:10]2[cH:11]1)[CH2:44][c:38]1[cH:39][cH:40][cH:41][cH:42][n:43]1

Similar reaction:

Input:

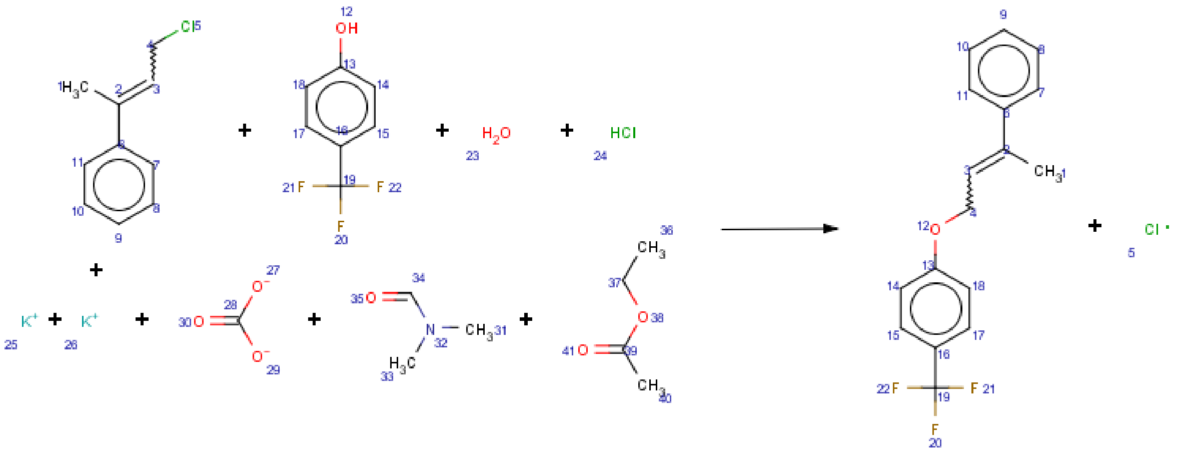

[CH3:1][C:2](=[CH:3][CH2:4][Cl:5])[c:6]1[cH:7][cH:8][cH:9][cH:10][cH:11]1.[OH:12][c:13]1[cH:14][cH:15][c:16]([cH:17][cH:18]1)[C:19]([F:20])([F:21])[F:22].[OH2:23].[ClH:24].[K+:25].[K+:26].[O-:27][C:28]([O-:29])=[O:30].[CH3:31][N:32]([CH3:33])[CH:34]=[O:35].[CH3:36][CH2:37][O:38][C:39]([CH3:40])=[O:41]>>[CH3:1][C:2](=[CH:3][CH2:4][O:12][c:13]1[cH:18][cH:17][c:16]([cH:15][cH:14]1)[C:19]([F:20])([F:21])[F:22])[c:6]1[cH:11][cH:10][cH:9][cH:8][cH:7]1.[Cl:5]

Output (top prediction):

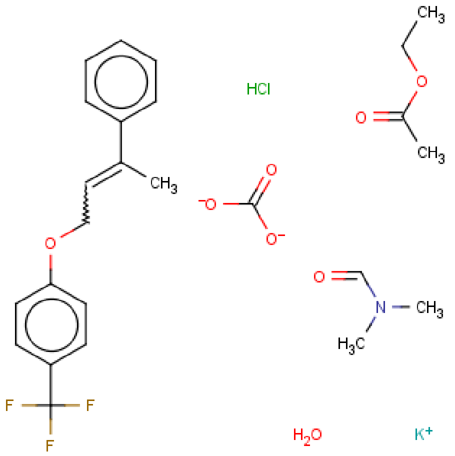

O=C([O-])[O-].Cl.CCOC(C)=O.CC(=CCOc1ccc(C(F)(F)F)cc1)c1ccccc1.O.[K+].CN(C)C=O

Correctness: CORRECT

10.

MIT reaction:

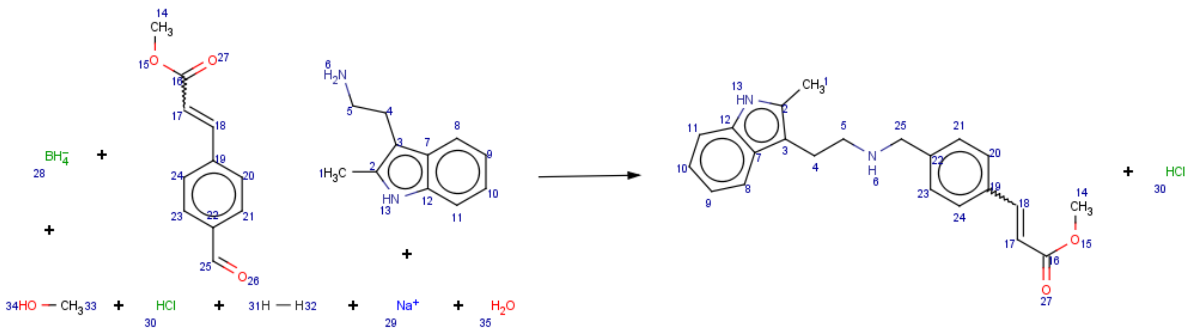

[BH4-:28].[CH3:14][O:15][C:16]([CH:17]=[CH:18][c:19]1[cH:20][cH:21][c:22]([CH:25]=[O:26])[cH:23][cH:24]1)=[O:27].[CH3:1][c:2]1[c:3]([CH2:4][CH2:5][NH2:6])[c:7]2[cH:8][cH:9][cH:10][cH:11][c:12]2[nH:13]1.[CH3:33][OH:34].[ClH:30].[H:31][H:32].[Na+:29].[OH2:35]>>[CH3:1][c:2]1[c:3]([CH2:4][CH2:5][NH:6][CH2:25][c:22]2[cH:21][cH:20][c:19]([CH:18]=[CH:17][C:16]([O:15][CH3:14])=[O:27])[cH:24][cH:23]2)[c:7]2[cH:8][cH:9][cH:10][cH:11][c:12]2[nH:13]1.[ClH:30]

Similar reaction:

Input:

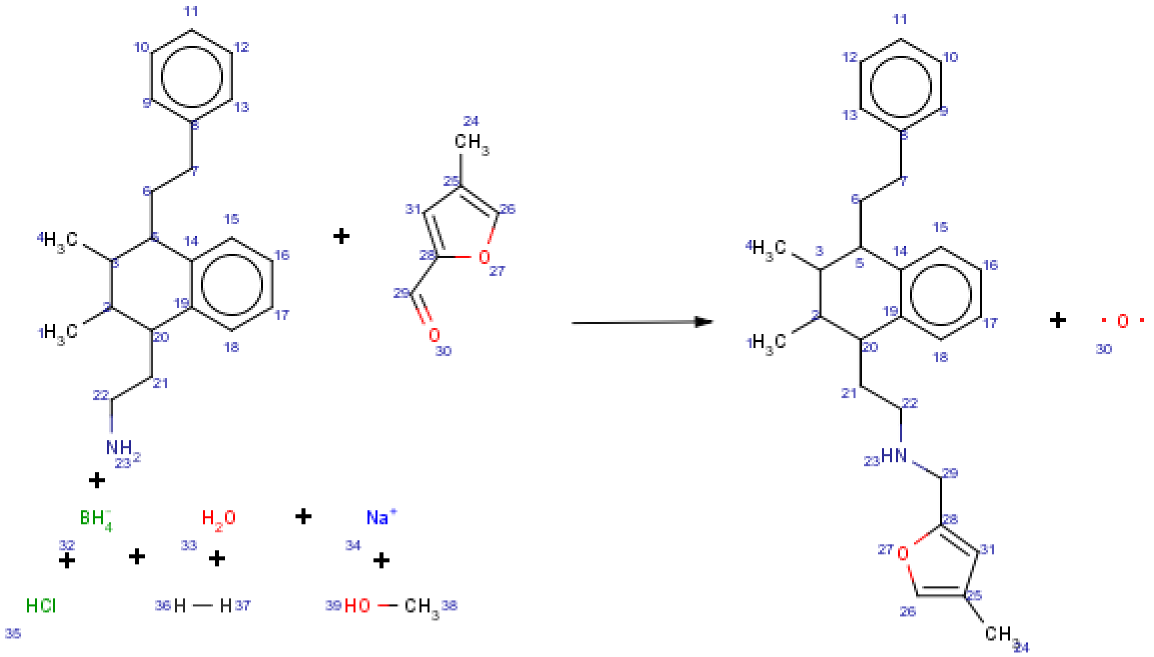

[CH3:1][CH:2]1[CH:3]([CH3:4])[CH:5]([CH2:6][CH2:7][c:8]2[cH:9][cH:10][cH:11][cH:12][cH:13]2)[c:14]2[cH:15][cH:16][cH:17][cH:18][c:19]2[CH:20]1[CH2:21][CH2:22][NH2:23].[CH3:24][C:25]1=[CH:26][O:27][C:28]([CH:29]=[O:30])=[CH:31]1.[BH4-:32].[OH2:33].[Na+:34].[ClH:35].[H:36][H:37].[CH3:38][OH:39]>>[CH3:1][CH:2]1[CH:3]([CH3:4])[CH:5]([CH2:6][CH2:7][c:8]2[cH:13][cH:12][cH:11][cH:10][cH:9]2)[c:14]2[cH:15][cH:16][cH:17][cH:18][c:19]2[CH:20]1[CH2:21][CH2:22][NH:23][CH2:29][C:28]1=[CH:31][C:25]([CH3:24])=[CH:26][O:27]1.[O:30]

Output (top prediction):

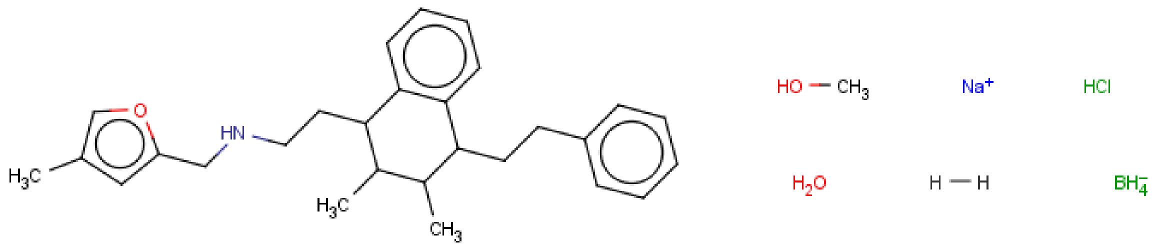

CO.[Na+].Cl.O.[H][H].[BH4-].Cc1coc(CNCCC2c3ccccc3C(CCc3ccccc3)C(C)C2C)c1

Correctness: CORRECT

S5.3. Examples of 10 pairs of reactions with *incorrect* mapping in the MIT training set.

The first reaction in each pair has incorrect mapping and was used with such a mapping to train the MIT's deep neural network for reaction prediction. The second reaction is a closely related transformation (with chemically correct product shown) with which we queried/tested the MIT's network. The question asked is whether the MIT network can or cannot predict the correct product based on reaction substrates. On this set of 10 examples, **only 20% of outputs are correct**.

1.

MIT reaction:

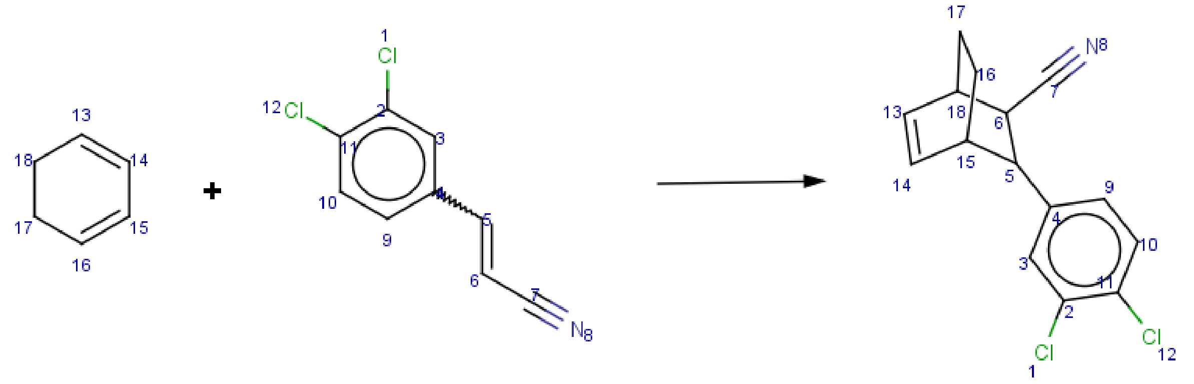

[CH:13]1=[CH:14][CH:15]=[CH:16][CH2:17][CH2:18]1.[Cl:1][c:2]1[cH:3][c:4]([CH:5]=[CH:6][C:7]#[N:8])[cH:9][cH:10][c:11]1[Cl:12]>>[Cl:1][c:2]1[cH:3][c:4]([CH:5]2[CH:6]([C:7]#[N:8])[CH:18]3[CH:13]=[CH:14][CH:15]2[CH2:16][CH2:17]3)[cH:9][cH:10][c:11]1[Cl:12]

Similar reaction:

Input:

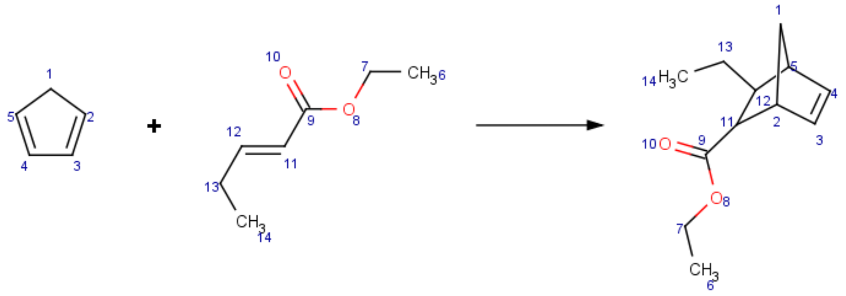

[CH2:1]1[CH:2]=[CH:3][CH:4]=[CH:5]1.[CH3:6][CH2:7][O:8][C:9](=[O:10])\[CH:11]=[CH:12]\[CH2:13][CH3:14]>>[CH3:6][CH2:7][O:8][C:9](=[O:10])[CH:11]1[CH:12]([CH2:13][CH3:14])[CH:5]2[CH2:1][CH:2]1[CH:3]=[CH:4]2

Output (top prediction):

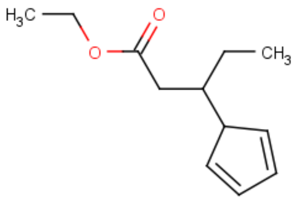

CCOC(=O)CC(CC)C1C=CC=C1

Correctness: INCORRECT

2.

MIT reaction:

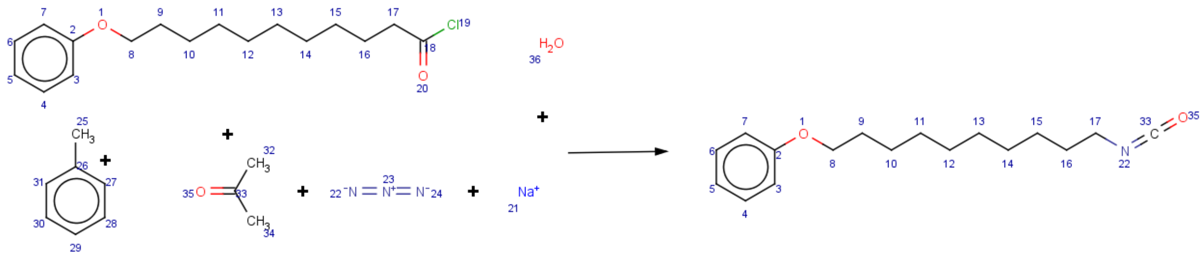

[CH3:25][c:26]1[cH:27][cH:28][cH:29][cH:30][cH:31]1.[CH3:32][C:33]([CH3:34])=[O:35].[N-:22]=[N+:23]=[N-:24].[Na+:21].[O:1]([c:2]1[cH:3][cH:4][cH:5][cH:6][cH:7]1)[CH2:8][CH2:9][CH2:10][CH2:11][CH2:12][CH2:13][CH2:14][CH2:15][CH2:16][CH2:17][C:18]([Cl:19])=[O:20].[OH2:36]>>[O:1]([c:2]1[cH:3][cH:4][cH:5][cH:6][cH:7]1)[CH2:8][CH2:9][CH2:10][CH2:11][CH2:12][CH2:13][CH2:14][CH2:15][CH2:16][CH2:17][N:22]=[C:33]=[O:35]

Similar reaction:

Input:

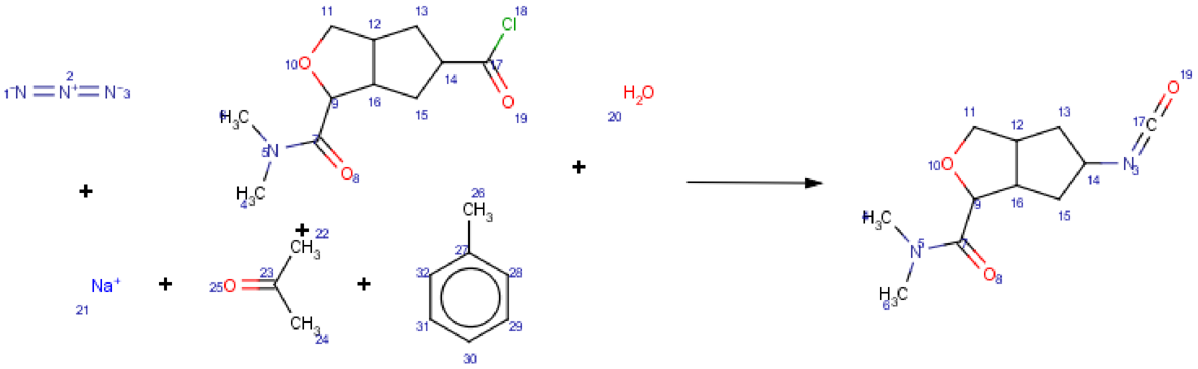

[N-:1]=[N+:2]=[N-:3].[CH3:4][N:5]([CH3:6])[C:7](=[O:8])[CH:9]1[O:10][CH2:11][CH:12]2[CH2:13][CH:14]([CH2:15][CH:16]12)[C:17]([Cl:18])=[O:19].[OH2:20].[Na+:21].[CH3:22][C:23]([CH3:24])=[O:25].[CH3:26][c:27]1[cH:28][cH:29][cH:30][cH:31][cH:32]1>>[CH3:6][N:5]([CH3:4])[C:7](=[O:8])[CH:9]1[O:10][CH2:11][CH:12]2[CH2:13][CH:14]([CH2:15][CH:16]12)[N:3]=[C:17]=[O:19]

Output (top prediction):

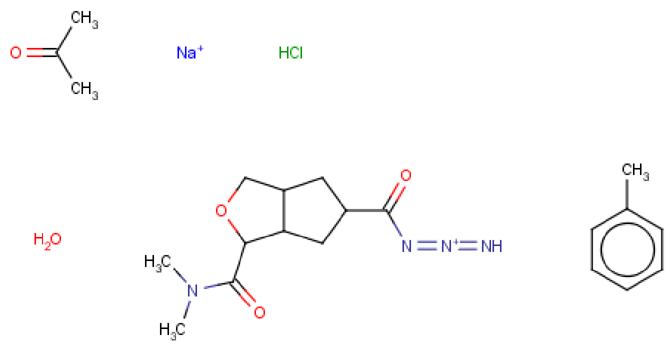

CC(C)=O.[Na+].Cl.O.CN(C)C(=O)C1OCC2CC(C(=O)N=[N+]=N)CC21.Cc1ccccc1

Correctness: INCORRECT

3.

MIT reaction:

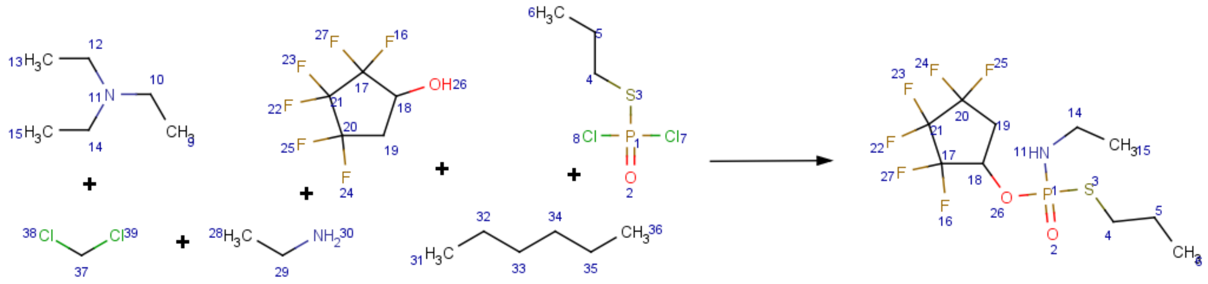

[CH2:37]([Cl:38])[Cl:39].[CH3:28][CH2:29][NH2:30].[CH3:31][CH2:32][CH2:33][CH2:34][CH2:35][CH3:36].[CH3:9][CH2:10][N:11]([CH2:12][CH3:13])[CH2:14][CH3:15].[F:16][C:17]1([F:27])[CH:18]([OH:26])[CH2:19][C:20]([F:24])([F:25])[C:21]1([F:22])[F:23].[P:1](=[O:2])([S:3][CH2:4][CH2:5][CH3:6])([Cl:7])[Cl:8]>>[P:1](=[O:2])([S:3][CH2:4][CH2:5][CH3:6])([NH:11][CH2:14][CH3:15])[O:26][CH:18]1[C:17]([F:16])([F:27])[C:21]([F:22])([F:23])[C:20]([F:24])([F:25])[CH2:19]1

Similar reaction:

Input:

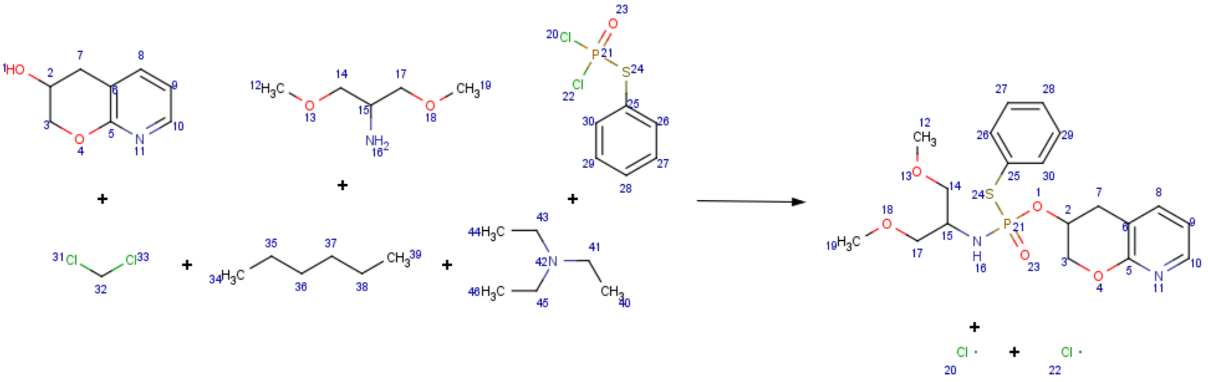

[OH:1][CH:2]1[CH2:3][O:4][C:5]2=[C:6]([CH2:7]1)[CH:8]=[CH:9][CH:10]=[N:11]2.[CH3:12][O:13][CH2:14][CH:15]([NH2:16])[CH2:17][O:18][CH3:19].[Cl:20][P:21]([Cl:22])(=[O:23])[S:24][C:25]1=[CH:26][CH:27]=[CH:28][CH:29]=[CH:30]1.[Cl:31][CH2:32][Cl:33].[CH3:34][CH2:35][CH2:36][CH2:37][CH2:38][CH3:39].[CH3:40][CH2:41][N:42]([CH2:43][CH3:44])[CH2:45][CH3:46]>>[CH3:19][O:18][CH2:17][CH:15]([CH2:14][O:13][CH3:12])[NH:16][P:21](=[O:23])([O:1][CH:2]1[CH2:3][O:4][C:5]2=[C:6]([CH2:7]1)[CH:8]=[CH:9][CH:10]=[N:11]2)[S:24][C:25]1=[CH:26][CH:27]=[CH:28][CH:29]=[CH:30]1.[Cl:20].[Cl:22]

Output (top prediction):

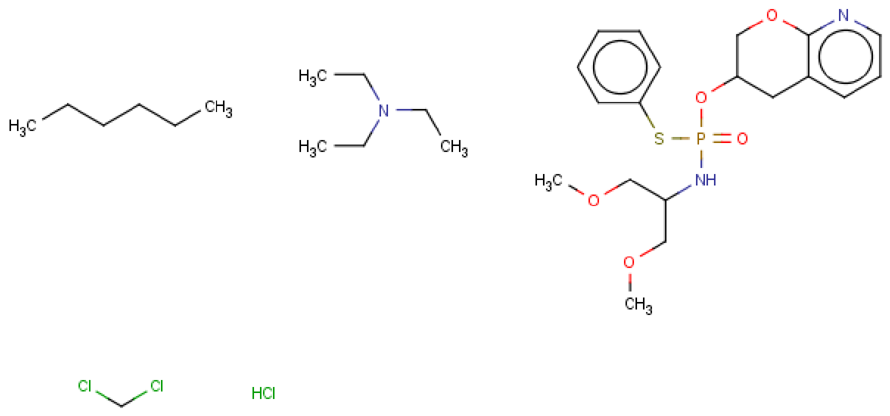

CCCCCC.CCN(CC)CC.COCC(COC)NP(=O)(OC1COc2ncccc2C1)Sc1ccccc1.ClCCl.Cl

Correctness: CORRECT

4.

MIT reaction:

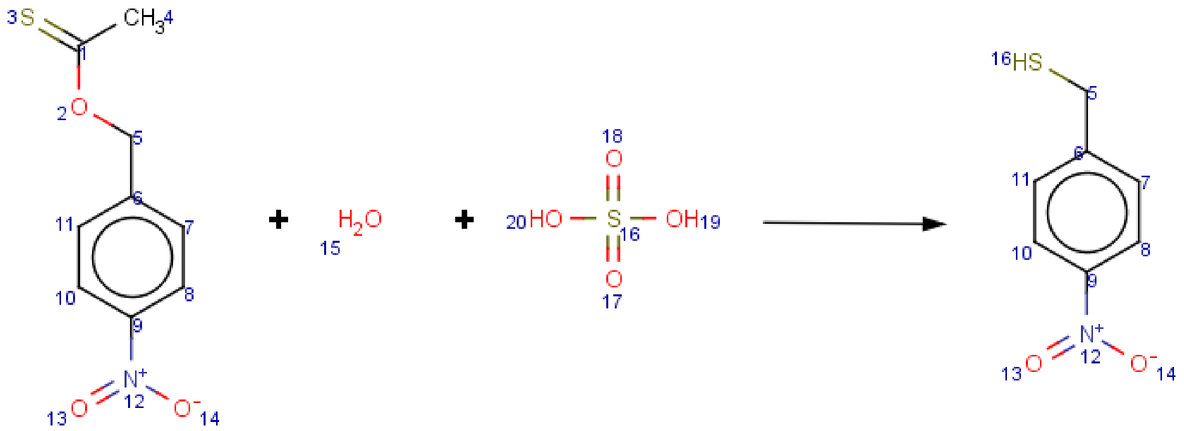

[C:1]([O:2][CH2:5][c:6]1[cH:7][cH:8][c:9]([N+:12](=[O:13])[O-:14])[cH:10][cH:11]1)(=[S:3])[CH3:4].[OH2:15].[S:16](=[O:17])(=[O:18])([OH:19])[OH:20]>>[CH2:5]([c:6]1[cH:7][cH:8][c:9]([N+:12](=[O:13])[O-:14])[cH:10][cH:11]1)[SH:16]

Similar reaction:

Input:

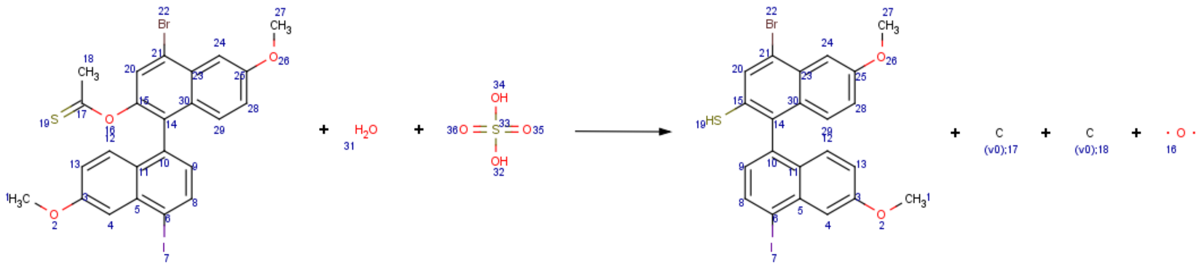

[CH3:1][O:2][C:3]1=[CH:4][C:5]2=[C:6]([I:7])[CH:8]=[CH:9][C:10](=[C:11]2[CH:12]=[CH:13]1)[C:14]1=[C:15]([O:16][C:17]([CH3:18])=[S:19])[CH:20]=[C:21]([Br:22])[C:23]2=[CH:24][C:25]([O:26][CH3:27])=[CH:28][CH:29]=[C:30]12.[OH2:31].[OH:32][S:33]([OH:34])(=[O:35])=[O:36]>>[CH3:1][O:2][C:3]1=[CH:4][C:5]2=[C:6]([I:7])[CH:8]=[CH:9][C:10](=[C:11]2[CH:12]=[CH:13]1)[C:14]1=[C:15]([SH:19])[CH:20]=[C:21]([Br:22])[C:23]2=[CH:24][C:25]([O:26][CH3:27])=[CH:28][CH:29]=[C:30]12.[C:17].[C:18].[O:16]

Output (top prediction):

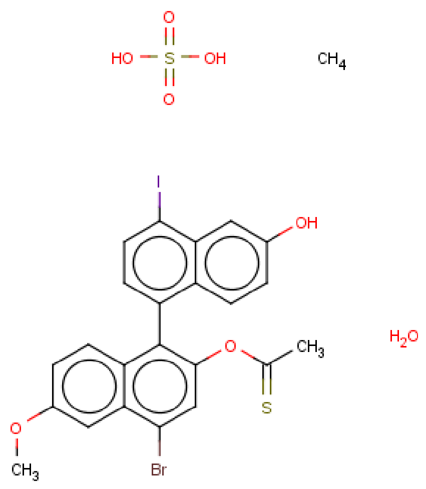

O=S(=O)(O)O.C.COc1ccc2c(-c3ccc(I)c4cc(O)ccc34)c(OC(C)=S)cc(Br)c2c1.O

Correctness: INCORRECT

5.

MIT reaction:

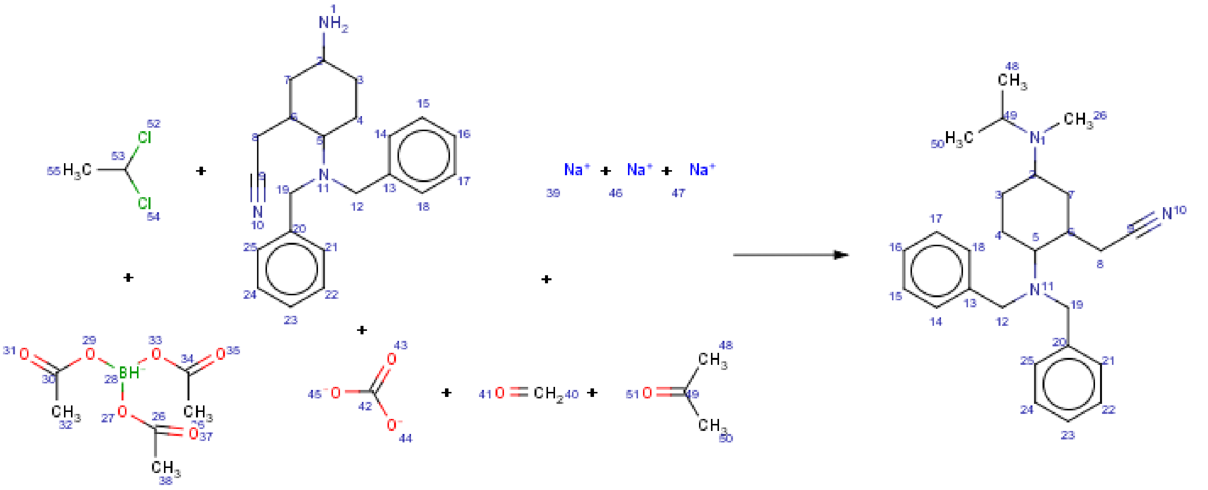

[C:26]([O:27][BH-:28]([O:29][C:30](=[O:31])[CH3:32])[O:33][C:34](=[O:35])[CH3:36])(=[O:37])[CH3:38].[C:42](=[O:43])([O-:44])[O-:45].[CH2:40]=[O:41].[CH3:48][C:49]([CH3:50])=[O:51].[Cl:52][CH:53]([Cl:54])[CH3:55].[NH2:1][CH:2]1[CH2:3][CH2:4][CH:5]([N:11]([CH2:12][c:13]2[cH:14][cH:15][cH:16][cH:17][cH:18]2)[CH2:19][c:20]2[cH:21][cH:22][cH:23][cH:24][cH:25]2)[CH:6]([CH2:8][C:9]#[N:10])[CH2:7]1.[Na+:39].[Na+:46].[Na+:47]>>[N:1]([CH:2]1[CH2:3][CH2:4][CH:5]([N:11]([CH2:12][c:13]2[cH:14][cH:15][cH:16][cH:17][cH:18]2)[CH2:19][c:20]2[cH:21][cH:22][cH:23][cH:24][cH:25]2)[CH:6]([CH2:8][C:9]#[N:10])[CH2:7]1)([CH3:26])[CH:49]([CH3:48])[CH3:50]

Similar reaction:

Input:

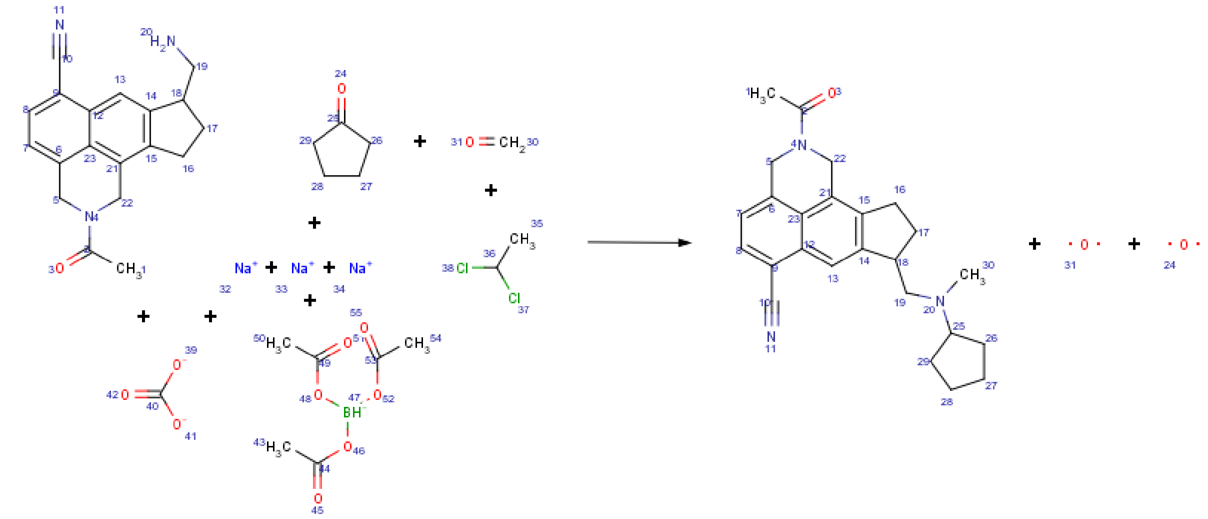

[CH3:1][C:2](=[O:3])[N:4]1[CH2:5][C:6]2=[CH:7][CH:8]=[C:9]([C:10]#[N:11])[C:12]3=[CH:13][C:14]4=[C:15]([CH2:16][CH2:17][CH:18]4[CH2:19][NH2:20])[C:21]([CH2:22]1)=[C

:23]23.[O:24]=[C:25]1[CH2:26][CH2:27][CH2:28][CH2:29]1.[CH2:30]=[O:31].[Na+:32].[Na+:33].[Na+:34].[CH3:35][CH:36]([Cl:37])[Cl:38].[O-:39][C:40]([O-:41])=[O:42].[CH3:43][C:44](=[O:45])[O:46][BH-:47]([O:48][C:49]([CH3:50])=[O:51])[O:52][C:53]([CH3:54])=[O:55]>>[CH3:30][N:20]([CH2:19][CH:18]1[CH2:17][CH2:16][C:15]2=[C:14]1[CH:13]=[C:12]1[C:9](=[CH:8][CH:7]=[C:6]3[CH2:5][N:4]([CH2:22][C:21]2=[C:23]13)[C:2]([CH3:1])=[O:3])[C:10]#[N:11])[CH:25]1[CH2:26][CH2:27][CH2:28][CH2:29]1.[O:31].[O:24]

Output (top prediction):

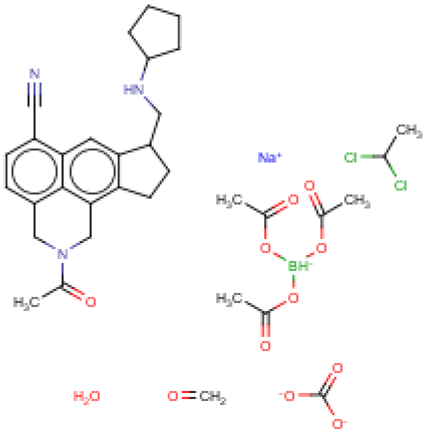

CC(=O)N1Cc2ccc(C#N)c3cc4c(c(c23)C1)CCC4CNC1CCCC1.[Na+].CC(Cl)Cl.O.C=O.O=C([O-])[O-].CC(=O)O[BH-](OC(C)=O)OC(C)=O

Correctness: INCORRECT

6.

MIT reaction:

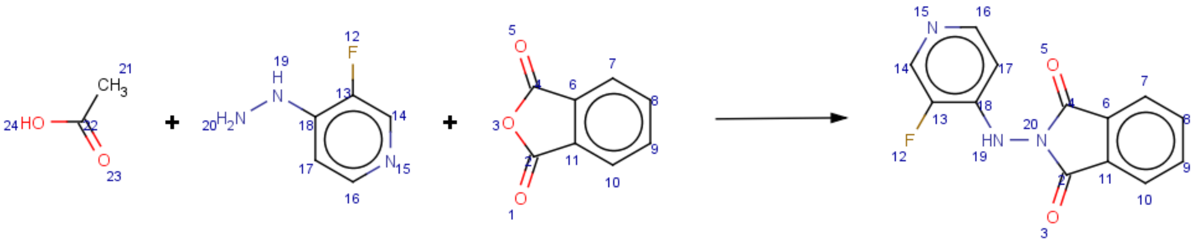

[CH3:21][C:22](=[O:23])[OH:24].[F:12][c:13]1[cH:14][n:15][cH:16][cH:17][c:18]1[NH:19][NH2:20].[O:1]=[C:2]1[O:3][C:4](=[O:5])[c:6]2[cH:7][cH:8][cH:9][cH:10][c:11]21>>[C:2]1(=[O:3])[c:11]2[c:6]([cH:7][cH:8][cH:9][cH:10]2)[C:4](=[O:5])[N:20]1[NH:19][c:18]1[c:13]([F:12])[cH:14][n:15][cH:16][cH:17]1

Similar reaction:

Input:

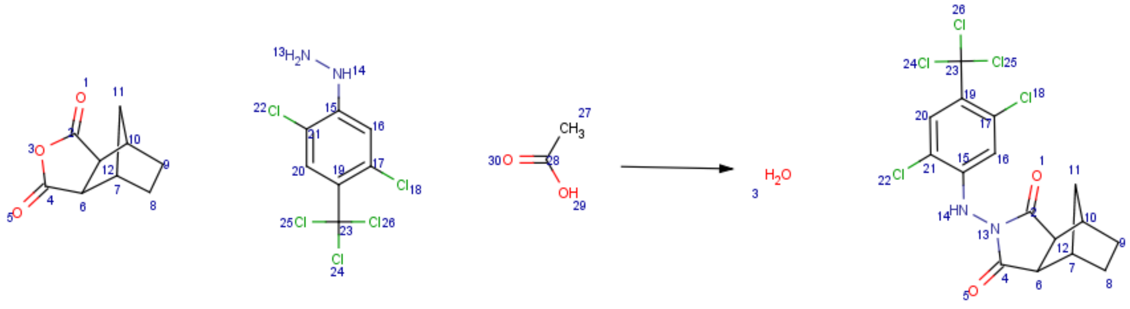

[O:1]=[C:2]1[O:3][C:4](=[O:5])[CH:6]2[CH:7]3[CH2:8][CH2:9][CH:10]([CH2:11]3)[CH:12]12.[NH2:13][NH:14][C:15]1=[CH:16][C:17]([Cl:18])=[C:19]([CH:20]=[C:21]1[Cl:22])[C:23]([Cl:24])([Cl:25])[Cl:26].[CH3:27][C:28]([OH:29])=[O:30]>>[OH2:3].[Cl:22][C:21]1=[CH:20][C:19](=[C:17]([Cl:18])[CH:16]=[C:15]1[NH:14][N:13]1[C:4](=[O:5])[CH:6]2[CH:7]3[CH2:8][CH2:9][CH:10]([CH2:11]3)[CH:12]2[C:2]1=[O:1])[C:23]([Cl:26])([Cl:25])[Cl:24]

Output (top prediction):

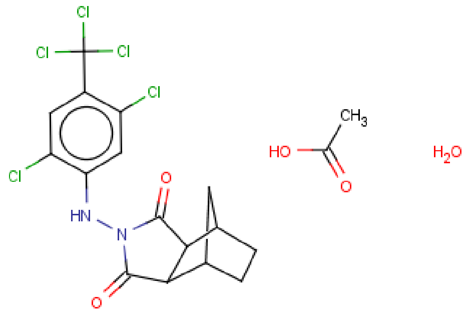

CC(=O)O.O.O=C1C2C3CCC(C3)C2C(=O)N1Nc1cc(Cl)c(C(Cl)(Cl)Cl)cc1Cl

Correctness: CORRECT

7.

MIT reaction:

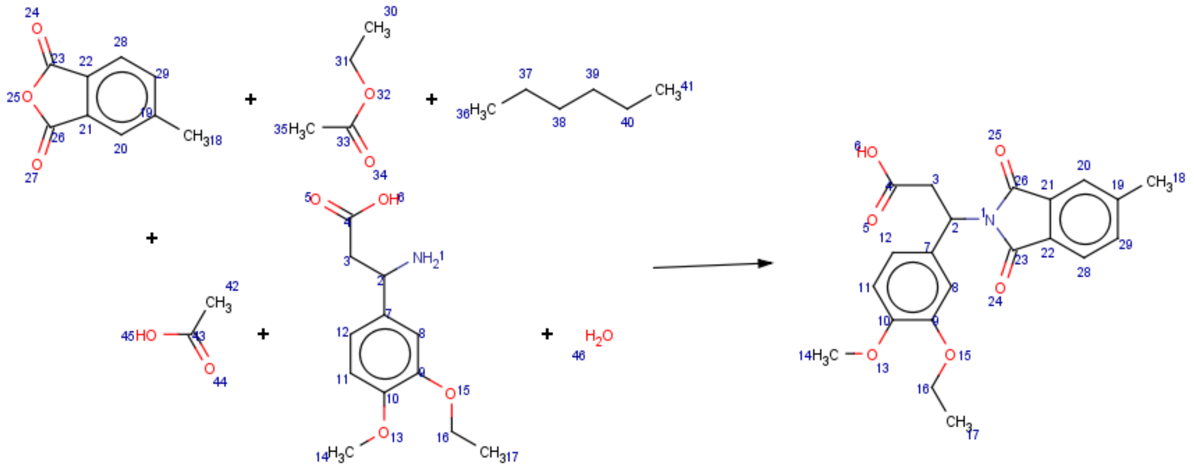

[CH3:18][c:19]1[cH:20][c:21]2[c:22]([cH:28][cH:29]1)[C:23](=[O:24])[O:25][C:26]2=[O:27].[CH3:30][CH2:31][O:32][C:33](=[O:34])[CH3:35].[CH3:36][CH2:37][CH2:38][CH2:39][CH2:40][CH3:41].[CH3:42][C:43](=[O:44])[OH:45].[NH2:1][CH:2]([CH2:3][C:4](=[O:5])[OH:6])[c:7]1[cH:8][c:9]([O:15][CH2:16][CH3:17])[c:10]([O:13][CH3:14])[cH:11][cH:12]1.[OH2:46]>>[N:1]1([CH:2]([CH2:3][C:4](=[O:5])[OH:6])[c:7]2[cH:8][c:9]([O:15][CH2:16][CH3:17])[c:10]([O:13][CH3:14])[cH:11][cH:12]2)[C:23](=[O:24])[c:22]2[c:21]([cH:20][c:19]([CH3:18])[cH:29][cH:28]2)[C:26]1=[O:25]

Similar reaction:

Input:

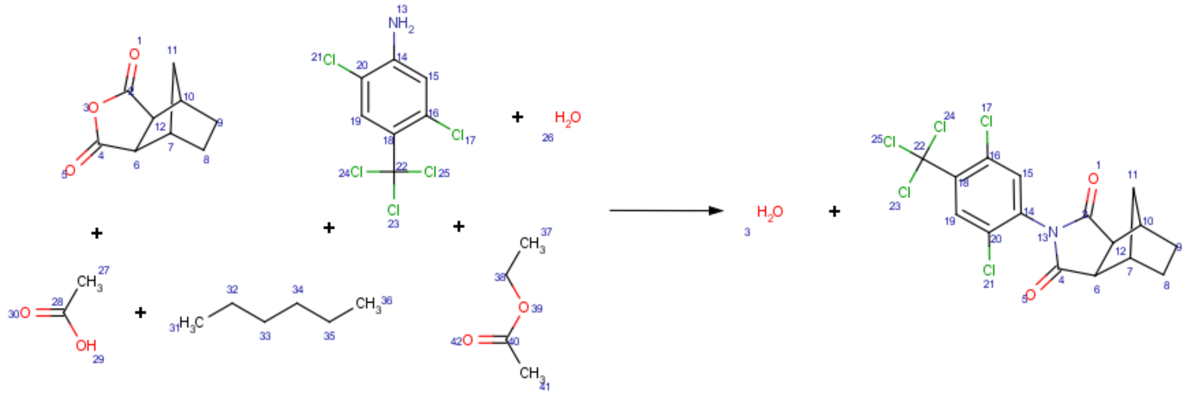

[O:1]=[C:2]1[O:3][C:4](=[O:5])[CH:6]2[CH:7]3[CH2:8][CH2:9][CH:10]([CH2:11]3)[CH:12]12.[NH2:13][C:14]1=[CH:15][C:16]([Cl:17])=[C:18]([CH:19]=[C:20]1[Cl:21])[C:22]([Cl:23])([Cl:24])[Cl:25].[OH2:26].[CH3:27][C:28]([OH:29])=[O:30].[CH3:31][CH2:32][CH2:33][CH2:34][CH2:35][CH3:36].[CH3:37][CH2:38][O:39][C:40]([CH3:41])=[O:42]>>[OH2:3].[Cl:21][C:20]1=[CH:19][C:18](=[C:16]([Cl:17])[CH:15]=[C:14]1[N:13]1[C:4](=[O:5])[CH:6]2[CH:7]3[CH2:8][CH2:9][CH:10]([CH2:11]3)[CH:12]2[C:2]1=[O:1])[C:22]([Cl:25])([Cl:24])[Cl:23]

Output (top prediction):

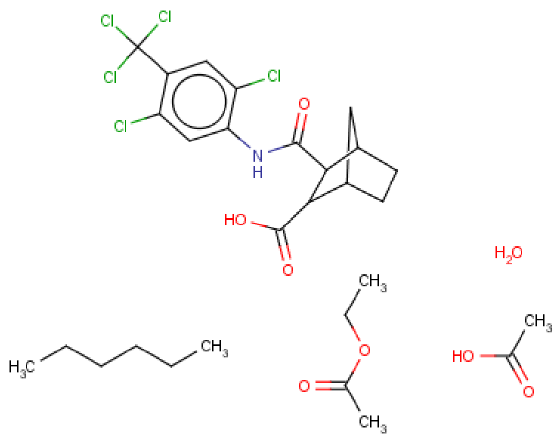

CCCCCC.CCOC(C)=O.CC(=O)O.O.O=C(O)C1C2CCC(C2)C1C(=O)Nc1cc(Cl)c(C(Cl)(Cl)Cl)cc1Cl

Correctness: INCORRECT

(the correct answer was also generated, but ranked on the third place)

8.

MIT reaction:

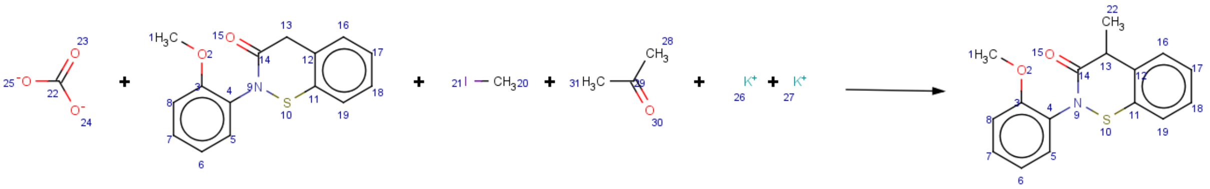

[C:22](=[O:23])([O-:24])[O-:25].[CH3:1][O:2][c:3]1[c:4]([N:9]2[S:10][c:11]3[c:12]([cH:16][cH:17][cH:18][cH:19]3)[CH2:13][C:14]2=[O:15])[cH:5][cH:6][cH:7][cH:8]1.[CH3:20][I:21].[CH3:28][C:29](=[O:30])[CH3:31].[K+:26].[K+:27]>>[CH3:1][O:2][c:3]1[c:4]([N:9]2[S:10][c:11]3[c:12]([cH:16][cH:17][cH:18][cH:19]3)[CH:13]([CH3:22])[C:14]2=[O:15])[cH:5][cH:6][cH:7][cH:8]1

Similar reaction:

Input:

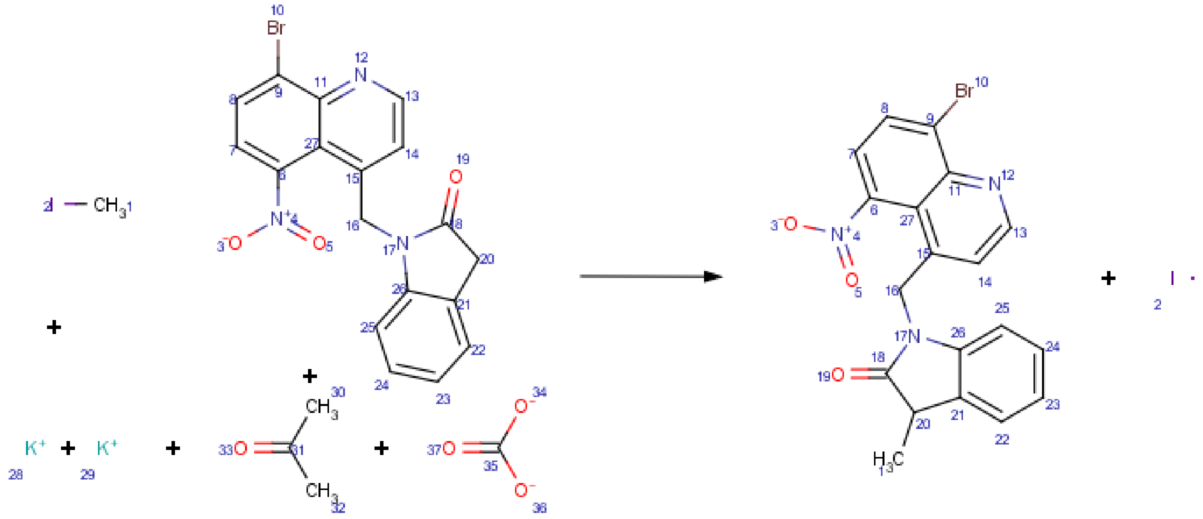

[CH3:1][I:2].[O-:3][N+:4](=[O:5])[C:6]1=[CH:7][CH:8]=[C:9]([Br:10])[C:11]2=[N:12][CH:13]=[CH:14][C:15]([CH2:16][N:17]3[C:18](=[O:19])[CH2:20][C:21]4=[CH:22][CH:23]=[CH:24][CH:25]=[C:26]34)=[C:27]12.[K+:28].[K+:29].[CH3:30][C:31]([CH3:32])=[O:33].[O-:34][C:35]([O-:36])=[O:37]>>[CH3:1][CH:20]1[C:18](=[O:19])[N:17]([CH2:16][C:15]2=[C:27]3[C:6](=[CH:7][CH:8]=[C:9]([Br:10])[C:11]3=[N:12][CH:13]=[CH:14]2)[N+:4]([O-:3])=[O:5])[C:26]2=[CH:25][CH:24]=[CH:23][CH:22]=[C:21]12.[I:2]

Output (top prediction):

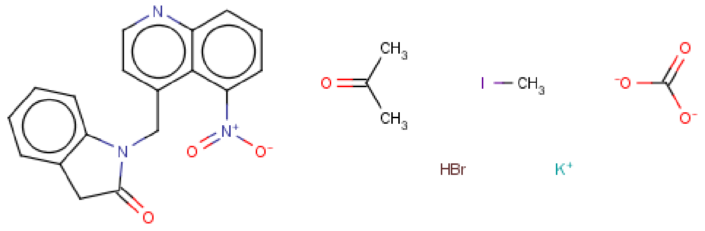

CC(C)=O.CI.O=C([O-])[O-].Br.[K+].O=C1Cc2ccccc2N1Cc1ccnc2cccc([N+](=O)[O-])c12

Correctness: INCORRECT

9.

MIT reaction:

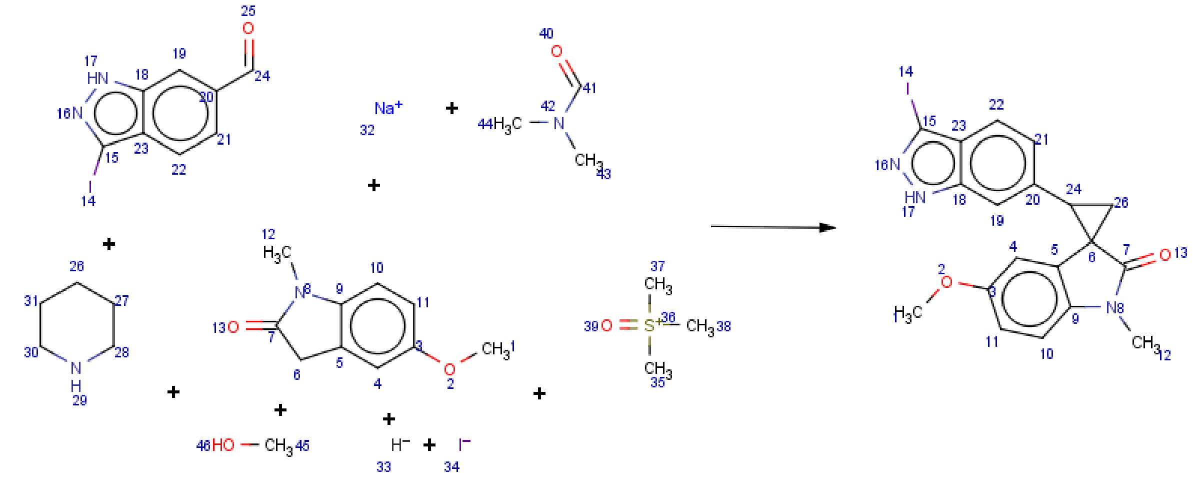

[CH2:26]1[CH2:27][CH2:28][NH:29][CH2:30][CH2:31]1.[CH3:1][O:2][c:3]1[cH:4][c:5]2[c:9]([cH:10][cH:11]1)[N:8]([CH3:12])[C:7](=[O:13])[CH2:6]2.[CH3:35][S+:36]([CH3:37])([CH3:38])=[O:39].[CH3:45][OH:46].[H-:33].[I-:34].[I:14][c:15]1[n:16][nH:17][c:18]2[cH:19][c:20]([CH:24]=[O:25])[cH:21][cH:22][c:23]12.[Na+:32].[O:40]=[CH:41][N:42]([CH3:43])[CH3:44]>>[CH3:1][O:2][c:3]1[cH:4][c:5]2[c:9]([cH:10][cH:11]1)[N:8]([CH3:12])[C:7](=[O:13])[C:6]21[CH:24]([c:20]2[cH:19][c:18]3[nH:17][n:16][c:15]([I:14])[c:23]3[cH:22][cH:21]2)[CH2:26]1

Similar reaction:

Input:

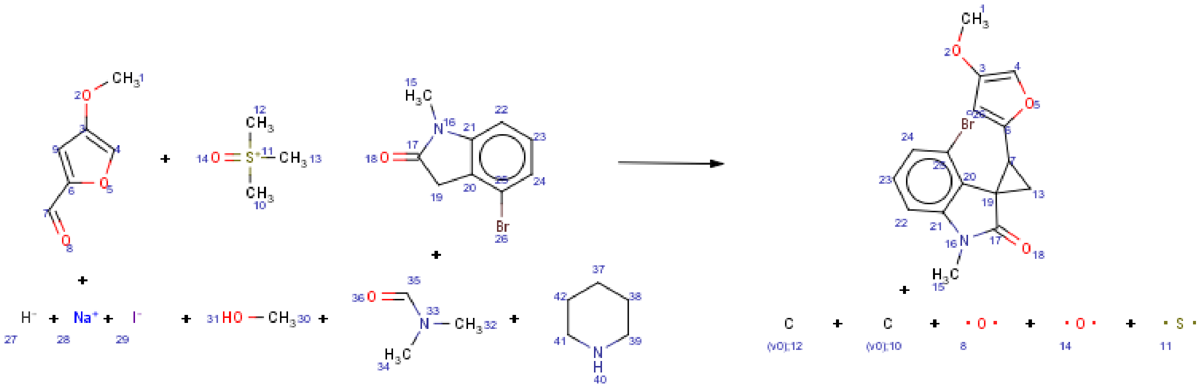

[CH3:1][O:2][C:3]1=[CH:4][O:5][C:6]([CH:7]=[O:8])=[CH:9]1.[CH3:10][S+:11]([CH3:12])([CH3:13])=[O:14].[CH3:15][N:16]1[C:17](=[O:18])[CH2:19][c:20]2[c:21]1[cH:22][cH:23][cH:24][c:25]2[Br:26].[H-:27].[Na+:28].[I-:29].[CH3:30][OH:31].[CH3:32][N:33]([CH3:34])[CH:35]=[O:36].[CH2:37]1[CH2:38][CH2:39][NH:40][CH2:41][CH2:42]1>>[CH3:1][O:2][C:3]1=[CH:4][O:5][C:6](=[CH:9]1)[CH:7]1[CH2:13][C:19]11[C:17](=[O:18])[N:16]([CH3:15])[c:21]2[cH:22][cH:23][cH:24][c:25]([Br:26])[c:20]12.[C:12].[C:10].[O:8].[O:14].[S:11]

Output (top prediction):

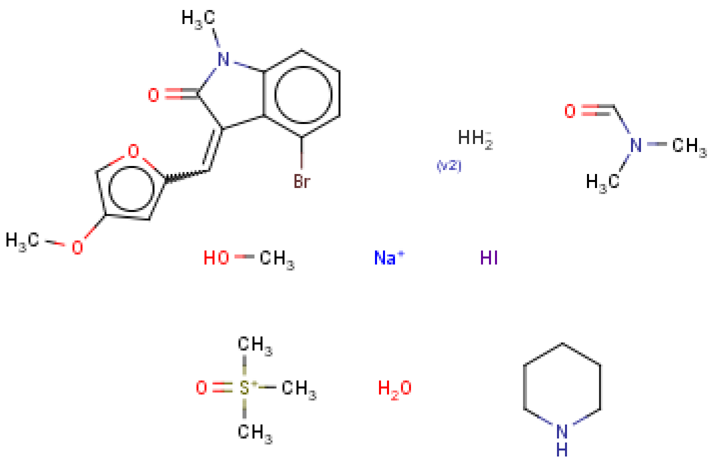

CO.[Na+].I.C[S+](C)(C)=O.O.C1CCNCC1.COc1coc(C=C2C(=O)N(C)c3cccc(Br)c32)c1.[HH2-].CN(C)C=O

Correctness: INCORRECT

10.

MIT reaction:

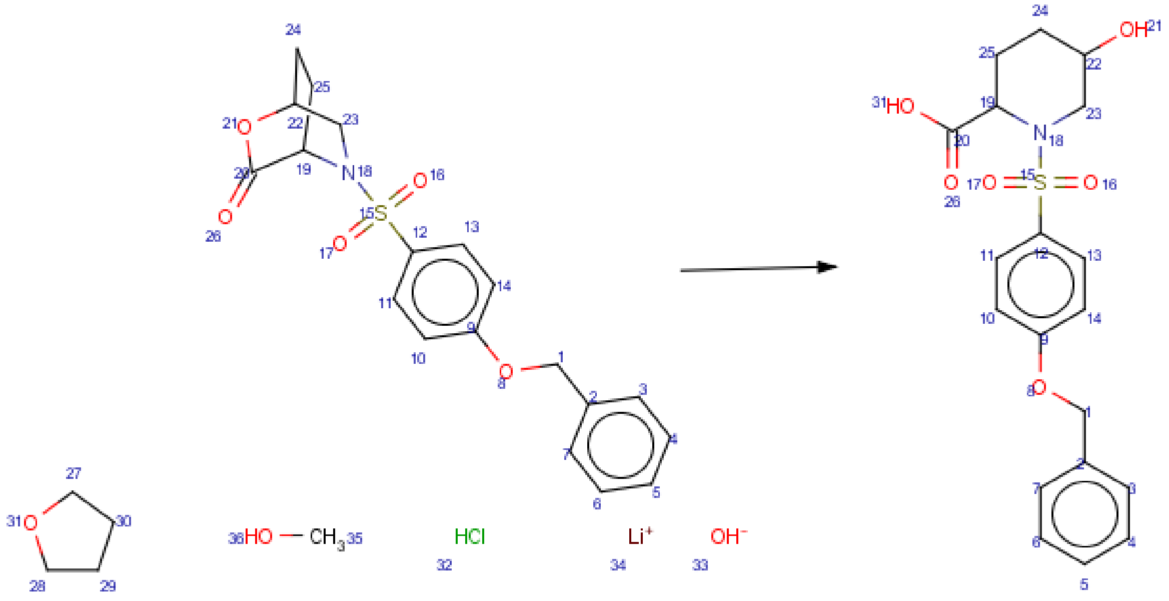

[CH2:1]([c:2]1[cH:3][cH:4][cH:5][cH:6][cH:7]1)[O:8][c:9]1[cH:10][cH:11][c:12]([S:15](=[O:16])(=[O:17])[N:18]2[CH:19]3[C:20](=[O:26])[O:21][CH:22]([CH2:23]2)[CH2:24][CH2:25]3)[cH:13][cH:14]1.[CH2:27]1[CH2:30][CH2:29][CH2:28][O:31]1.[CH3:35][OH:36].[ClH:32].[Li+:34].[OH-:33]>>[CH2:1]([c:2]1[cH:3][cH:4][cH:5][cH:6][cH:7]1)[O:8][c:9]1[cH:10][cH:11][c:12]([S:15](=[O:16])(=[O:17])[N:18]2[CH:19]([C:20](=[O:26])[OH:31])[CH2:25][CH2:24][CH:22]([OH:21])[CH2:23]2)[cH:13][cH:14]1

Similar reaction:

Input:

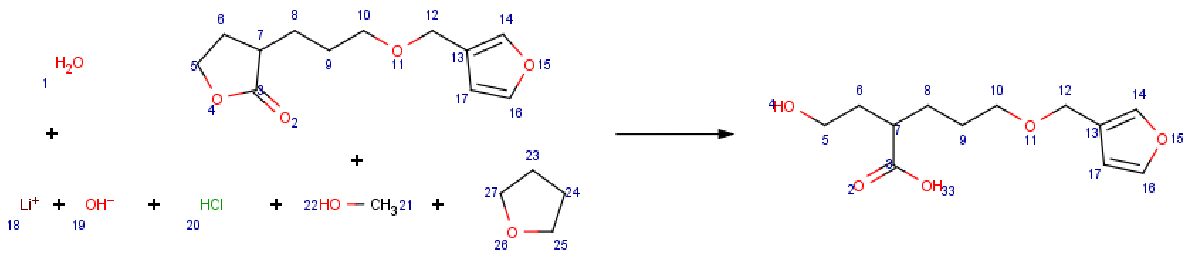

[OH2:1].[O:2]=[C:3]1[O:4][CH2:5][CH2:6][CH:7]1[CH2:8][CH2:9][CH2:10][O:11][CH2:12][C:13]1=[CH:14][O:15][CH:16]=[CH:17]1.[Li+:18].[OH-:19].[ClH:20].[CH3:21][OH:22].[CH2:23]1[CH2:24][CH2:25][O:26][CH2:27]1>>[OH:4][CH2:5][CH2:6][CH:7]([CH2:8][CH2:9][CH2:10][O:11][CH2:12][C:13]1=[CH:14][O:15][CH:16]=[CH:17]1)[C:3]([OH:33])=[O:2]

Output (top prediction):

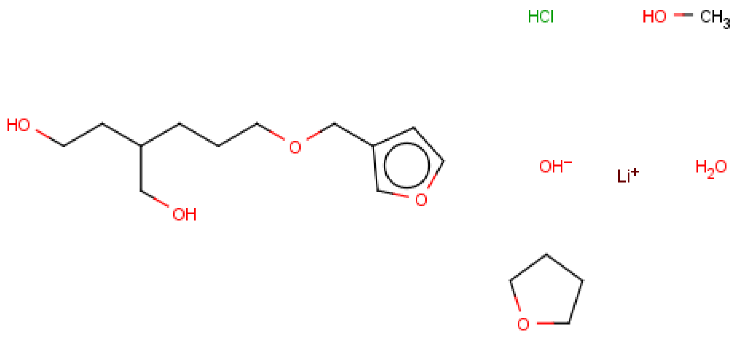

Cl.CO.OCCC(CO)CCCOCc1ccoc1.[OH-].[Li+].O.C1CCOC1

Correctness: INCORRECT
