# Supplementary material for: Automatic mapping of atoms across both simple and complex chemical reactions
Source: Nat Commun. 2019 Mar 29;10:1434. doi: 10.1038/s41467-019-09440-2 (PMC6441094; doi:10.1038/s41467-019-09440-2)
Supplement: Supplementary file 2 — Description of Additional Supplementary Files [file 41467_2019_9440_MOESM2_ESM.pdf]

Description of Additional Supplementary files

Fine name: Supplementary Data 1

Description: The benchmark set of reactions with correct mappings, used for developing and testing the algorithm
